# Life span‐associated ferroptosis‐related genes identification and validation for hepatocellular carcinoma patients as hepatitis B virus carriers

**DOI:** 10.1002/jcla.24930

**Published:** 2023-07-18

**Authors:** Weijie Weng, Defa Zhang, Shuang Li

**Affiliations:** ^1^ The third people's hospital health care group of Cixi Cixi China; ^2^ Tianjin Second People Hospital Tianjin China

**Keywords:** ferroptosis, hepatitis B virus carriers, hepatocellular carcinoma, life span, prognostic model

## Abstract

**Background:**

Hepatitis B virus (HBV)‐infected population accounts for approximately 50% of all hepatocellular carcinoma (HCC) cases and has a relatively poor prognosis. Although the significant role of ferroptosis in the development and therapeutic response of various cancers has been validated, the key ferroptosis‐related genes (FRGs) on the stratification of HBV‐associated HCC are still unclear.

**Methods:**

Through the random forest, GSVA and Cox regression analyses, we established a comprehensive prognostic system covering multiple FRGs to elevate the predictive accuracy for the survival rate of HBV‐related HCC using information obtained from public databases. The association between key FRGs and the immune microenvironment was evaluated, and the molecular mechanism was identified by GSEA and SNV analyses. Finally, the differential expression of key FRGs was validated by immunohistochemistry staining of patient tissue microarrays.

**Results:**

Within the top 10 key FRGs, EPAS1 and GABARAPL1 were taken as protective factors, and SQLE, RAD51AP1, RPL8, CAPG, RRM2, SLC1A5, SLC38A1, and SRC were the other eight dangerous markers. Cox regression analysis combined with clinicopathological features indicated the independent prognostic efficacy of GSVA complex score based on these FRGs. In addition, key FRGs were related to immune and metabolic‐related functions. Especially, the immunohistochemical analysis of SQLE in 50 clinical samples showed significantly higher expression in HBV+ HCC tissues.

**Conclusions:**

These results indicate that 10 FRGs may be potential biomarkers and therapeutic targets for long‐term survival in HBV‐related HCC.

## Graphical abstract

Hepatitis B virus (HBV)‐infected population accounts for approximately 50% of all hepatocellular carcinoma (HCC) cases and has a relatively poor prognosis. The significant role of ferroptosis in the development and therapeutic response of various cancers has been validated recently. In this study, we established a comprehensive prognostic system covering multiple ferroptosis‐related genes (FRGs) to elevate the predictive accuracy for the survival rate of HBV‐related HCC. Through survival, receiver‐operating characteristic (ROC) curve, random forest, GSVA, and a Cox regression model analysis, 10 key FRGs were identified that could be associated with survival time in HBV‐related HCC and then verified at protein levels. The molecular mechanism was identified by enrichment, GSEA and SNV analyses. In addition, key FRGs were related to immune and metabolic‐related functions. These results indicate that 10 FRGs may be potential biomarkers and therapeutic targets for long‐term survival in HBV‐related HCC.

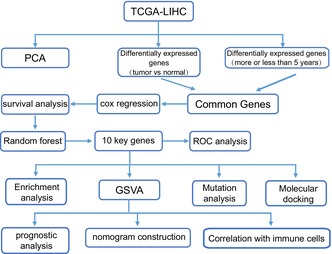

## 1 INTRODUCTION

Hepatocellular carcinoma (HCC) is one of the most common malignant tumors worldwide and is associated with an extremely poor prognosis.^1^, ^2^ Chronic Hepatitis B (HBV) infection, which causes about 50% of all instances of HCC, is a significant risk factor.^3^ The worst part is that the median survival for HBV‐related HCC is fewer than 16 months. After 1 year and after 5 years of diagnosis, the survival rates of HBV‐related HCC ranged from 36% to 67% and 15% to 26%, respectively.^4^ HBV infection should be treated as an independent clinic factor for OS of HCC patients, and there is an urgent need to predict the clinical outcomes for HCC patients in the context of HBV infection.

In contrast to normal apoptosis, autophagy, and planned necrosis, ferroptosis is a recently identified mechanism of regulated cell death characterized by iron‐dependent lipid peroxidation and buildup of reactive oxygen species (ROS).^5^ The significant role of ferroptosis inhibition in the development and therapeutic response of various cancers has been validated in recent studies.^6^ As the ferroptotic response is controlled by a complex signal network,^7^ targeting these pathways in tumor cells should be an emerging anticancer strategy, even though the extent to which ferroptosis affects tumor biology is still unknown. For example, two iron metabolic genes (FPN and LCN2) were selected to develop a cancer therapy based on gene interference‐enhanced ferroptosis.^8^ So far, there are many drugs for tumor ferroptosis, and most of them inhibit system xc− and GPX4 to promote ferroptosis.^9^ Besides, as ferroptosis could affect the efficacy of chemotherapy, radiotherapy, and immunotherapy, there have been increasing reports that have identified ferroptosis‐related genes (FRGs) of great prognostic importance for multiple cancer clinical practice.^10^, ^11^, ^12^, ^13^, ^14^ However, considering the important clinical characteristics, the development of a prognostic tool integrating FRGs on the stratification of HBV‐associated HCC is still urgently in need.

In this study, we utilized sequencing and clinical data of HBV+ HCC patients from the TCGA and GEO databases (GSE14520). The expression and mutation information of FRGs in patients with survival times longer or shorter than 5 years were analyzed. Then, the data were screened to identify genes and construct a prognostic model related to the overall survival rate of HBV+ HCC and the possible regulatory mechanisms.

## 2 MATERIALS AND METHODS

### 2.1 Data collection

The UCSC Xena^15^ (https://xenabrowser.net/) was used to obtain the TCGA‐LIHC transcriptome and patient clinicopathological data, and 368 HCC tumor samples and 50 paired normal samples with clinical annotation information were included first, and then 139 HBV+ tumor samples and 22 paired normal samples were further selected according to the “Hepatitis B Surface Antigen” annotation (Table S1). The “stage” information was retrieved from the “tumor_stage.diagnoses” column. Somatic mutation burden of prognostic genes for the HBV+ TCGA‐LIHC data set was downloaded through TCGAbiolinks^16^ and calculated using maftools.^17^

The gene expression data set of 225 HCC tumor samples and 220 paired normal samples for validating our conclusion were collected from the Gene Expression Omnibus (GEO) database (GSE14520, based on the GPL3921 platform) in the National Center for Biotechnology Information (http://www.ncbi.nlm.nih.gov/geo/). Within them, there were 212 HBV+ tumor samples and 13 HBV+ paired normal samples according to the “HBV viral status” information (Table S2).

The list of ferroptosis‐related genes (FRGs) was first obtained from related literature^18^, ^19^, ^20^, ^21^ and GeneCards, FerrDb, MsigDB, and KEGG database by browsing and searching the keyword “Ferroptosis.” After deleting the redundant information, 292 FRGs were preserved. Subsequently, through the correlation analysis between these FRGs and other genes in vst normalized TCGA‐LIHC gene expression data, 38 genes whose *p* < 1e‐10 and Pearson coefficients >0.9 were screened out as extra FRGs. Finally, 330 FRGs were kept for further analysis.

### 2.2 Identification and enrichment analysis of ferroptosis‐related genes

The RNA‐Seq data downloaded from TCGA were normalized and the differentially expressed genes between HCC tumoral and their paired normal samples were calculated using the DESeq2^22^ package in the R software. Similarly, we also used 5 years as the dividing line to construct the differential expression profiles. Benjamini and Hochberg method was then employed to adjust *p* values for multiple testing. Differentially expressed genes were screened with the following criteria: |log_2_(FoldChange)| > 0.58 and adjusted *p* value <0.05. To overview the expression of genes of TCGA‐LIHC data set, PCA was carried out with the “prcomp” function of the “stats” R package.

### 2.3 Construction of FRGs‐related prognostic model

To assess which differentially expressed FRGs could be taken as prognostic factors for HBV+ HCC patients, we utilized the univariate Cox regression model to identify significant predictors. The *p* value of <0.05 was considered statistically significant. As none of these FRGs obeys normal distribution, HBV+ HCC patients were divided into low‐expression and high‐expression groups according to the median value of gene expression value, respectively. Survival differences between the two groups were assessed by Kaplan–Meier and compared using log‐rank statistical methods.

Second, to pick out the most important factors associated with the HBV+ HCC progression, we subsequently constructed a random forest model of hub genes screened out by survival analysis. Packages “carat” and “randomForest” in R were used for parameter optimization and model construction. The model was built with 10‐fold cross‐validation. After that, genes whose MeanDecreaseGini ranked top 10 were taken as our FRGs‐related prognostic genes. The Human Protein Atlas (HPA) was used for validating the different expressions of selected FRGs on the protein level. ROC curves were used to study the accuracy of predictions of HBV+ HCC patients based on these prognostic genes, separately.

Third, gene set variation analysis (GSVA) as a nonparametric, unsupervised estimation was performed on log2 normalized counts of TCGA‐LIHC HBV+ expression data set with the GSVA package. GSVA scores were calculated nonparametrically using a Kolmogorov‐Smirnoff (KS)‐like random walk statistic and a negative value for a particular sample and gene set. Two protective factors and eight dangerous markers were taken as custom gene sets, separately. The GSVA score of samples based on eight dangerous factors minus that based on protective markers equals the final complex score of each sample.

Molecular stratification of HCC has been extensively described based on genetic, epigenetic, and transcriptomic profiles,^23^ for example, the 3 HCC subtypes (iC1‐3) identified by the so‐called iCluster algorithm. The prognostic assessment of complex scores in all HBV+ HCC samples and three subtypes was then provided.

At last, we employed univariate and multivariate Cox regression to check the effect of stage, gender, vascular, age, AFP, and complex score on the survival status of TCGA‐LIHC HBV+ patients. Nomogram was constructed based on these six factors using the “rms” and “survival” packages in R. Following that, calibration curves were drawn to assess the consistency between actual and predicted survival rates.

### 2.4 Analysis of immune cells composition from gene expression data

To assess the potential association between our selected prognostic signatures and tumor‐infiltrating immune cells in the HBV+ HCC tumor microenvironment, the TCGA and GSE14520 database was used to measure the abundance ratios of 22 types of immune cells through TIMER2 database (http://timer.comp‐genomics.org/) and CIBERSORT LM22 signature immune panel (http://cibersort.stanford.edu/). Benjamini and Hochberg's method was then employed to adjust *p* values for multiple testing.

### 2.5 Enrichment and GSEA analysis of key genes

Firstly, the enrichment analyses including Gene ontology (GO) functions and Kyoto Encyclopedia of Genes and Genomes (KEGG) pathways were performed on selected prognostic genes using clusterProfiler package.^24^ Second, the differentially expressed genes between HBV+ HCC patient groups with short or long survival time were highlighted. The gene set enrichment analysis (GSEA)^25^ was conducted to evaluate the enriched pathways with the GO Biological Process (BP) gene set. The overlap BP pathways highlighted by selected prognostic FRGs and differentially expressed genes between patients with different survival times were listed and the semantic similarity score between these pathways was computed through the GOSemSim package in R.^26^ Subsequently, similarity matrices were served as inputs for Euclidean distance hierarchical clustering using the R function hclust. The *p* < 0.05 and false discovery rate (FDR) *q*‐value of <0.2 were used as the screening criteria.

### 2.6 Molecular docking

Within compounds downloaded from the DrugBank (https://go.drugbank.com/), 5464 were obtained by applying Lipinski's rule^27^ (no more than five hydrogen bond donors, no more than 10 hydrogen bond acceptors, molecular mass between 180 and 480 Da, partition coefficient (log P) not greater than 5, no more than 10 rotatable bonds, and polar surface area equal to or less than 140). Within our FRGs prognostic markers, 3D structures files of SLC1A5 (PDB ID: 5LM4), SQLE (PDB ID: 6C6N), and SRC (PDB ID: 1Y57) were successfully downloaded from the PDB database. The docking grid box was defined according to the ligand in the original complex. The AutoDock Vina package was employed to generate the docking input files, PyMOL was used for 3D structure visualization, and Ligplus as the 2D structure–protein interaction visualizer to show the hydrogen bonds and hydrophobic contacts.

### 2.7 Immunohistochemistry staining of human liver tissue samples

For the purpose of detecting the key FRGs expression, tissue microarrays used for immunohistochemical (IHC) staining analysis were composed of 50 samples including 43 HBV+ HCC and 7 adjacent normal liver tissues, sampled from 11 patients with stage I, 12 patients with stage II, and 20 patients with stage III (Bioaitech, cat. no. D120Lv02) (Table S3). This study was authorized and supervised by the Ethics Committee of the Tianjin Second People Hospital. The tissues were fixed with 10% formalin, embedded by paraffin, and sectioned. Then we selected the optimal tissue sections for degreasing and Immunohistochemistry staining. Antibodies used in this study are as follows: SQLE (Affinity, DF12063). The results were randomly observed in 5–10 fields of view and then taken as the average. The expression grade was determined by the product of intensity score (grade 0–3) and frequency of positive cells (grade 0–4).

## 3 RESULTS

### 3.1 Differentially expressed FRGs of high and low survival time in HBV+ HCC patients

**FIGURE 1:**
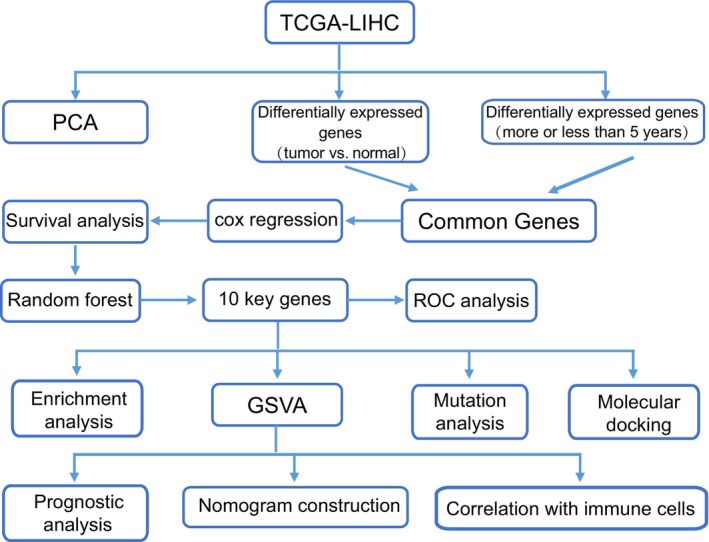
Study flowchart.

**FIGURE 2:**
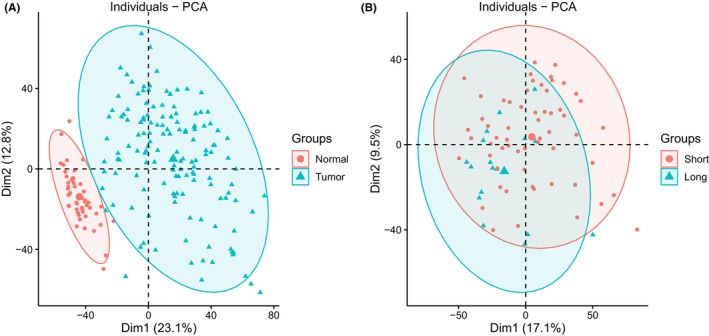
PCA plot of the TCGA‐LIHC cohort. (A) PCA supported the stratification into normal and tumor groups. (B) The distribution of patients with <5 years and ≥5 years survival time.

The study flowchart is presented in Figure 1. First, unsupervised principal component analysis (PCA) was utilized to provide an overview for the vst standardizing TCGA‐LIHC gene expression data set. Genes whose expression variation ranked top 5000 were selected for analysis. As shown in Figure 2A, tumor and normal samples were independent of each other, and there were also differences between the samples with survival time < 5 years and ≥5 years (Figure 2B).

**FIGURE 3:**
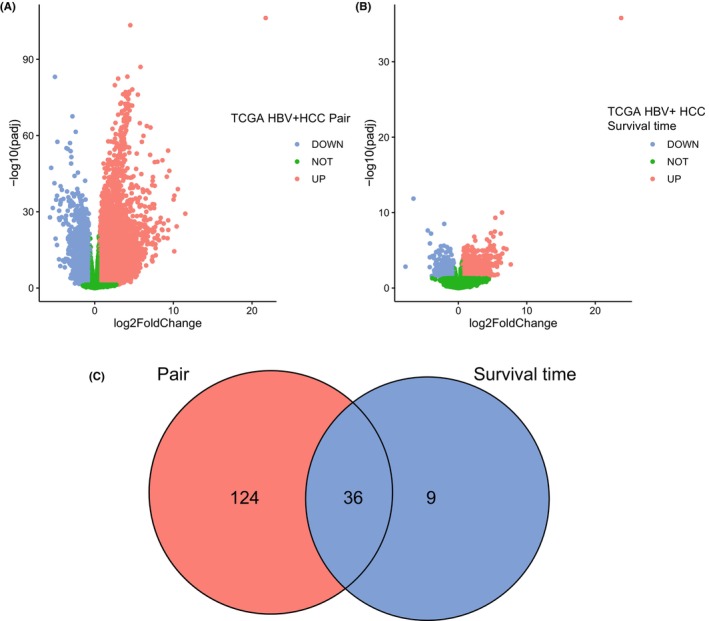
Identification of differentially expressed genes in HBV+ HCC patients. (A) Volcano map of differentially expressed genes in HBV+ HCC tumor tissues compared to adjacent normal tissues (A) and in patients with survival time <5 years compared to that ≥5 years (B), with red dots representing significantly upregulated genes, blue dots representing significantly downregulated genes, and green dots representing no differences gene. (C) Venn diagram of common differentially expressed FRGs in the two groups.

By comparing 139 HBV+ HCC tumor and 22 paired normal samples, considering the criteria mentioned above, we obtained 10,267 upregulated and 3347 downregulated genes (Figure 3A and Table S4). These genes may be related to the occurrence and development of HBV+ HCC. Compared with patients with a survival time ≥5 years, patients with a survival time < 5 years possessed 1496 upregulated and 979 downregulated genes (Figure 3B and Table S5). These genes may be related to the shorter survival time of HBV+ HCC. As shown in Figure 3C, 160 FRGs were detected as significantly differentially expressed between HCC tumor tissues and adjacent normal tissues, and 45 FRGs were found as significantly differentially expressed between patients with survival time < 5 years and that ≥5 years. As the intersection set, 36 common FRGs (Table S6) were screened out as prognostic candidates for subsequent analysis.

### 3.2 Construction of FRGs‐related prognostic model in TCGA HBV+ HCC data set

**FIGURE 4:**
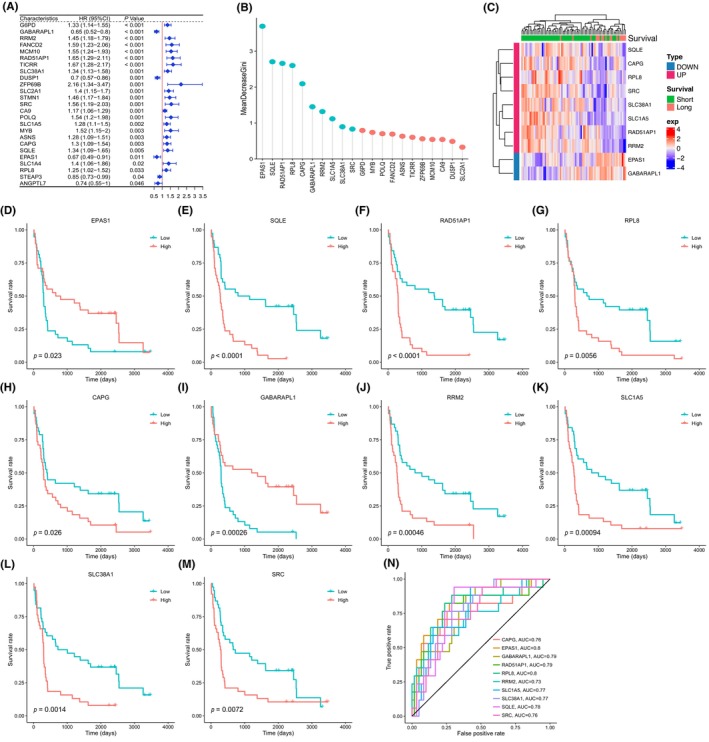
FRGs‐related prognostic markers were selected by univariate Cox, survival analysis, and random forest algorithm. (A) Results of the univariate Cox analysis in TCGA HBV+ HCC patients. (B) Random forest screening for the top 10 markers within 21 survival time‐related genes with a high Gini coefficient of average decline. The blue lollipops represented the markers we selected. (C) Expression of 8 dangerous and 2 protective genes within HBV+ HCC patients having <5 years or ≥5 years survival time. (D–M) Kaplan–Meier survival probability curve of TCGA HBV+ HCC patients associated with the expression level of 10 FRGs prognostic candidates. (N) Survival‐dependent receiver‐operating characteristic (ROC) curves validating the prognostic significance of 10 prognostic genes.

Following the method described above, within 36 FRGs prognostic candidates, a total of 25 genes were found significantly associated with the prognosis of TCGA HBV+ HCC patients after univariate Cox regression (Figure 4A and Table S7). Subsequently, according to the expression data of these genes, the median values were set as the thresholds to divide samples into high‐ or low‐expression groups, respectively. Survival differences between groups were assessed by Kaplan–Meier and compared using log‐rank statistical methods. For 21 of these genes, the survival probability between high‐ and low‐expression groups differed significantly. GABARAPL1, DUSP1, and EPAS1 should be considered protective factors for TNBC prognosis, whereas the other 18 genes (G6PD, RRM2, FANCD2, MCM10, RAD51AP1, TICRR, SLC38A1, ZFP69B, SLC2A1, SRC, CA9, POLQ, SLC1A5, MYB, ASNS, CAPG, SQLE, and RPL8) should be taken as dangerous factors (Figure S1). Furthermore, a random forest model based on these 21 genes was constructed to divide patients with <5 years or ≥5 years survival time. Genes whose MeanDecreaseGini ranked top 10 were considered our FRGs‐related prognostic markers, as they were evaluated as the most important features in this model (Figure 4B). Besides EPAS1 and GABARAPL1 as protective factors, SQLE, RAD51AP1, RPL8, CAPG, RRM2, SLC1A5, SLC38A1, and SRC, were the other eight dangerous markers. The expression of these 10 genes within TCGA HBV+ HCC patients was illustrated in Figure 4C. As expected, patients with shorter survival time had more possibilities to express eight dangerous markers, respectively. In contrast, patients with longer survival time had a disposition to express protective genes. Kaplan–Meier survival probability curves were used to study the accuracy of predictions of HBV+ HCC patients based on these prognostic genes, separately, as shown in Figure 4D–M. The AUC values of survival‐dependent receiver‐operating characteristic (ROC) curves ranged from 0.73 to 0.8 (Figure 4N).

**FIGURE 5:**
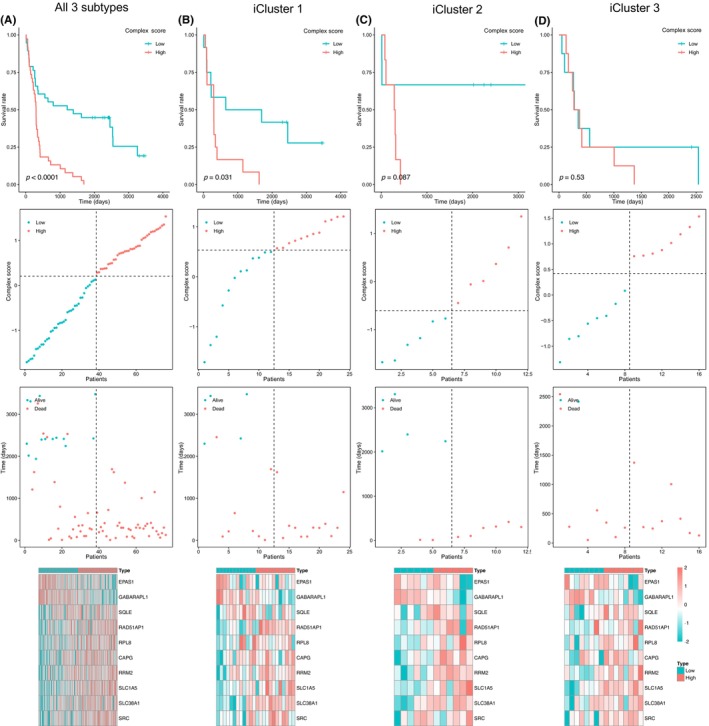
Establishment and assessment of the prognostic GSVA complex score model comprised of 10 ferroptosis‐related genes. The survival rate comparison between high‐ and low‐score patients, distribution of score of patients in different groups, survival status of patients in different groups, and the heatmap of expression profile of 10 ferroptosis‐related genes was provided for all HBV+ HCC patients (A), iCluster1 (B), iCluster2 (C), and iCluster3 (D) subtype samples, respectively.

To evaluate the role of 10 prognostic genes, we combined them into a complex score through the GSVA algorithm as described above. The median value was set as the threshold to divide each TCGA HBV+ HCC sample into high‐ or low‐complex score groups, respectively. In previous research,^23^ integrative clustering of TCGA data sets of DNA copy number, DNA methylation, mRNA expression, and miRNA expression could define three HCC subtypes (iCluster 1–3). As illustrated in Figure 5A–C, for all HBV+ HCC patients, iCluster1 and iCluster2 subtype samples, the survival rate in the high‐score group was significantly lower than that in the low‐score group, although for iCluster3 subtype, perhaps due to the lack of efficient samples, there was no statistically significant difference between two groups (Figure 5D). To further evaluate the predictive value of these 10 prognostic genes, we compared them with the other four genesets based on the multi‐Cox analysis of the TCGA‐LIHC HBV+ samples data set, and the comparison was provided in Table S8 and Figure S2.

As shown in the heatmap, in general, patients in the low‐score group have more possibilities to express two protective signatures, that is, EPAS1 and GABARAPL1.

We analyzed the protein expression patterns of these key FRGs in the HPA database (Figure S3). The results showed that apart from EPAS1 and RAD51AP1 that without immunohistochemistry data, SQLE, CAPG, RRM2, SLC1A5, and SRC as dangerous genes were higher expressed in cancer tissues.

**FIGURE 6:**
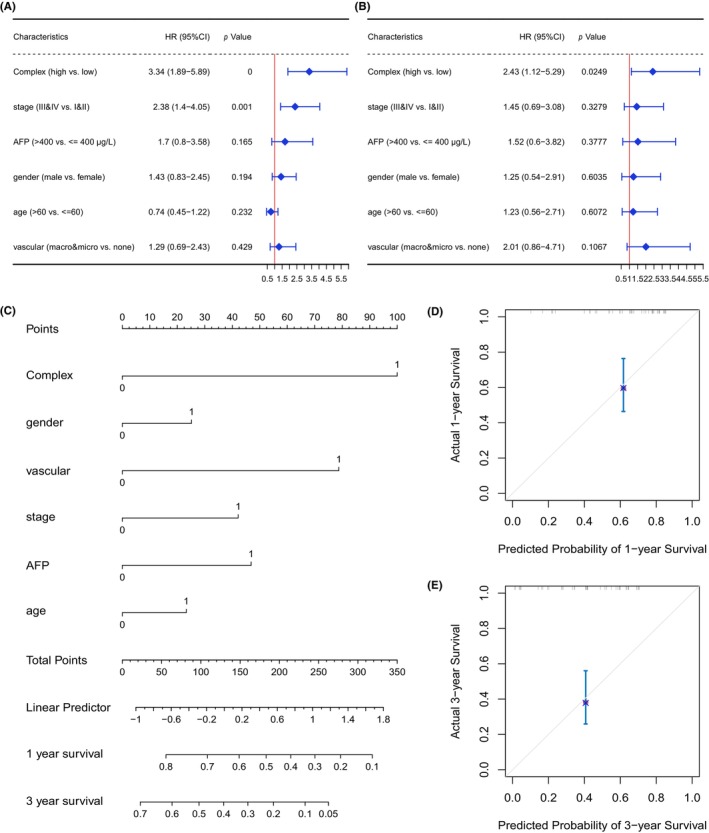
The effect of GSVA complex score on the prognosis of HBV+ HCC. Forest plot of prognostic features in univariate (A) and multivariate (B) Cox regression analysis. The construction of nomogram combining complex score and 5 clinical features (C), and the calibration curve of this nomogram for 1‐year (D) and 3‐year (E) prediction.

As the result of univariate and multivariate Cox regression analyses of variables such as stage, alpha‐fetoprotein (AFP), gender, age, vascular invasion, and GSVA complex score, the forest graph (Figure 6A,B) indicated the independent prognostic efficacy of GSVA complex score. Besides, the Chi‐square test showed no significant relationship between stage and vascular invasion status in HBV+ HCC patients (Figure S4). Subsequently, we generated a nomogram to predict the possibility of 1‐ and 3‐year overall survival based on these six factors (Figure 6C). The 1‐ and 3‐year calibration curves were, respectively, shown in Figure 6D,E, which indicated good agreements between the prediction and observation of overall survival.

**FIGURE 7:**
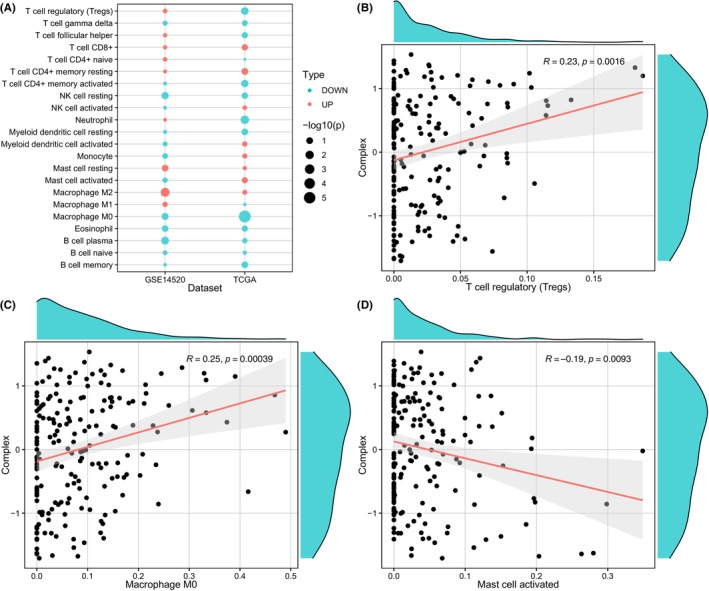
Immune cell subpopulation analysis. Relative fractions of 22 leukocyte subtypes (LM22 signature) evaluated by CIBERSORT in HBV+ HCC patients according to short or long survival time (A). The correlation curve between the GSVA complex score and T‐cell regulatory (B), Macrophage M0 (C), and Mast cell activated (D).

Furthermore, to understand the contribution of different immune cell subpopulations to our FRGs prognostic model for HBV+ HCC patients, we examined the correlation between the expression of 10 genes and tumor‐infiltrating immune cells by TIMER2, which included samples accessible in the TCGA‐LIHC HBV+ cohort and GSE14520 HBV+ cohort. Compared with patients with survival time ≥5 years, the upregulated and downregulated immune cell subpopulations of patients with survival time <5 years were illustrated in Figure 7A. Within these immune cell subpopulations varying between patients with different survival times, fraction as T‐cell regulatory (Tregs) was found higher (*R* = 0.23, *p* = 0.0016) in the tumor microenvironment in HBV+ HCC patients with higher complex scores, as shown in Figure 7B. Similarly, higher overall expression of Macrophage M0 (*R* = 0.25, *p* = 0.00039) was also observed in patients with higher complex scores (Figure 7C). On the other hand, there was a negative correlation between the complex score and the infiltration level of the Mast cell activated (*R* = −0.19, *p* = 0.0093) (Figure 7D). The relationship between the GSVA complex score and the proportion of other 19 immune cell subpopulations was provided Figure S5.

### 3.3 Comprehensive analysis of ferroptosis‐related prognostic signatures for HBV+ HCC patients

**FIGURE 8:**
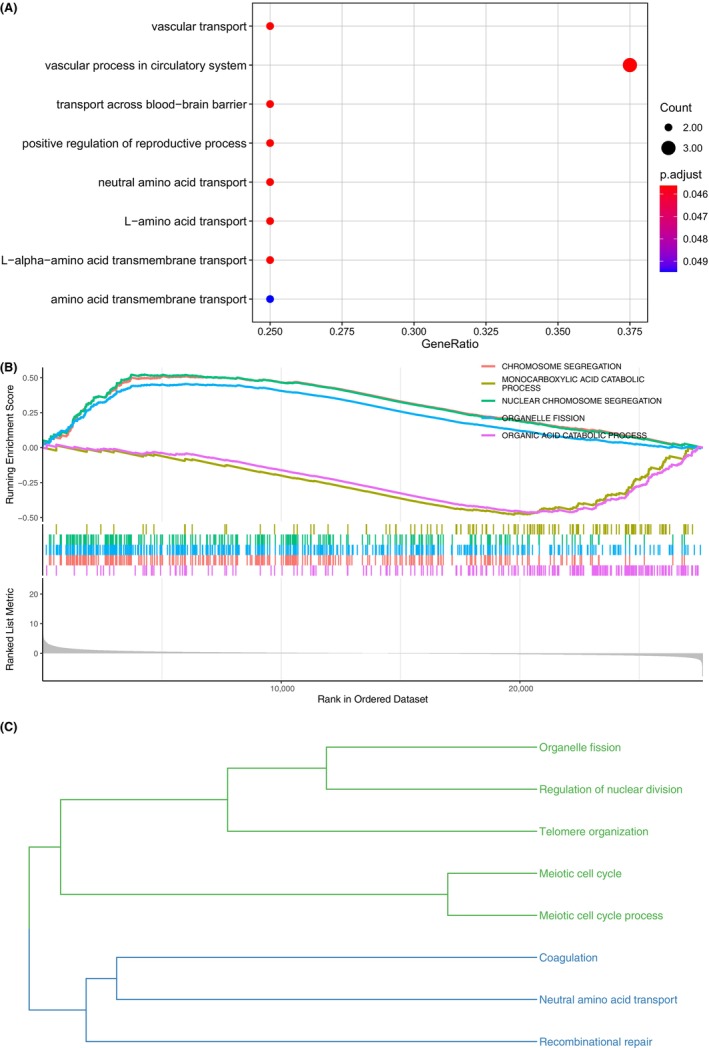
The biological function and signaling pathway of 10 key ferroptosis‐related prognostic signatures for HBV+ HCC. (A) Enriched GO BP and KEGG pathways of 10 ferroptosis‐related prognostic signatures. (B) GSEA enriched pathways of differentially expressed genes between HBV+ HCC patients with short or long survival time. (C) The overlap pathways were clustered into two types of biological functions based on GOSemSim semantic similarity.

Within our 10 key FRGs, EPAS1 and GABARAPL1 were taken as protective factors, and SQLE, RAD51AP1, RPL8, CAPG, RRM2, SLC1A5, SLC38A1, and SRC were the other eight dangerous markers. To explore the biological process involved in the influence of key genes on the survival time of HBV+ HCC patients, we analyzed the GO biological processes and KEGG pathway enrichment of these 10 genes (Tables S9 and S10). As shown in Figure 8A, ten key ferroptosis‐related prognostic signatures were mainly involved in pathways such as vascular transport, vascular process in the circulatory system, transport across blood–brain barrier, positive regulation of reproductive process, neutral amino acid transport, L‐amino acid transport, L‐alpha‐amino acid transmembrane transport, and amino acid transmembrane transport. The GSEA analysis further demonstrated the differential pathway enrichment in the two clusters, for the differentially expressed genes between patients with different survival times (Figure 8B and Table S11). The results showed that pathways such as chromosome segregation, nuclear chromosome segregation, and organelle fission enriched in one cluster, while the pathways involved in monocarboxylic acid catabolic process and organic acid catabolic process in the other cluster. As the semantic similarity analysis revealed in Figure 8C, the overlap biological processes shared by the above enrichment were divided into two branches. One branch included pathways such as organelle fission, regulation of nuclear division, telomere organization, meiotic cell cycle and meiotic cell cycle process, and the other included coagulation, neutral amino acid transport, and recombinational repair. In general, 10 key FRGs may affect the prognosis of HBV+ HCC through cell proliferation and repairment pathways.

**FIGURE 9:**
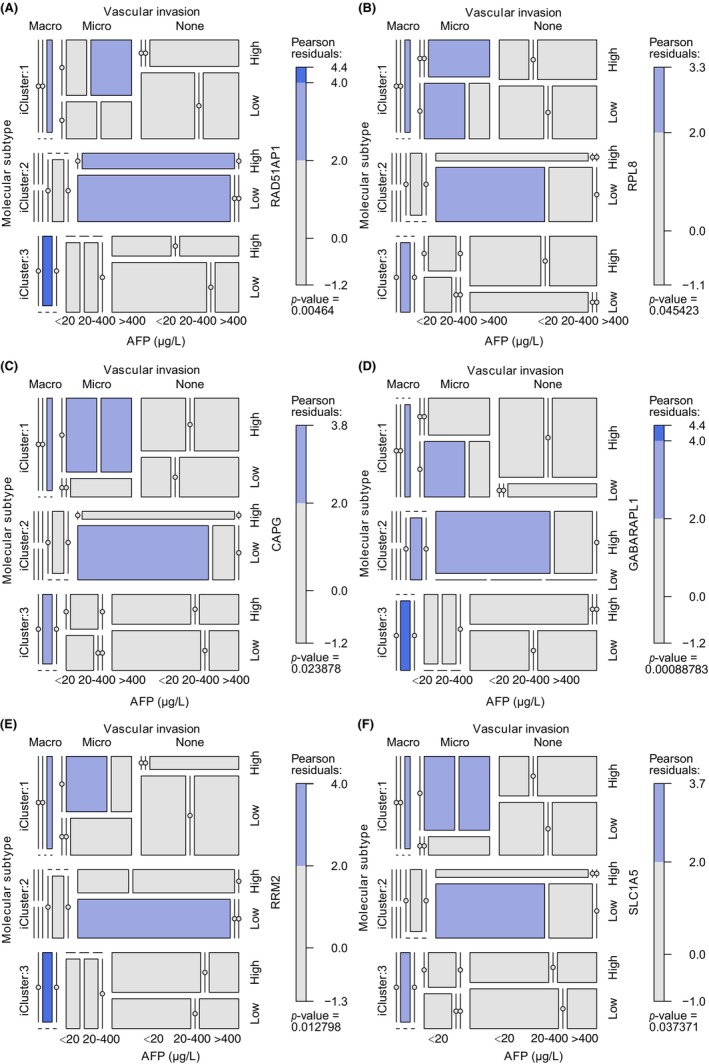
Mosaic plot showing cross‐link between molecular subtypes, AFP level, vascular invasion, and the expression of RAD51AP1 (A), RPL8 (B), CAPG (C), GABARAPL1 (D), RRM2 (E), and SLC1A5 (F) for HBV+ HCC patients, respectively.

Relationship was subsequently analyzed between our 10 ferroptosis‐related prognostic signatures and key clinical characteristics, including vascular invasion, molecular subtyping, and AFP level. Six key signatures found significant correlated with these clinical features were depicted in Figure 9, and the analysis of the other four genes was provided in Figure S6. As a protective factor, it was found that the expression of GABARAPL1 was negatively correlated with the ratio of iCluster 1 patients with macrovascular invasion and >400 μg/L AFP. The opposite phenomenon was observed for the other five dangerous markers. Similarly, for iCluster 2 subtype, patients with <20 μg/L AFP and without vascular invasion tended to have a low expression of RAD51AP1, CAPG, RRM2, RPL8, and SLC1A5, and a high expression of GABARAPL1. For the iCluster 3 subtype, patients with macrovascular invasion and 20–400 μg/L AFP tended to have a high expression of RAD51AP1, CAPG, RRM2, and SLC1A5 and a low expression of GABARAPL1 and RPL8. In general, the results of these analyses were consistent with the trend that these six genes could predict a long or short life span.

**FIGURE 10:**
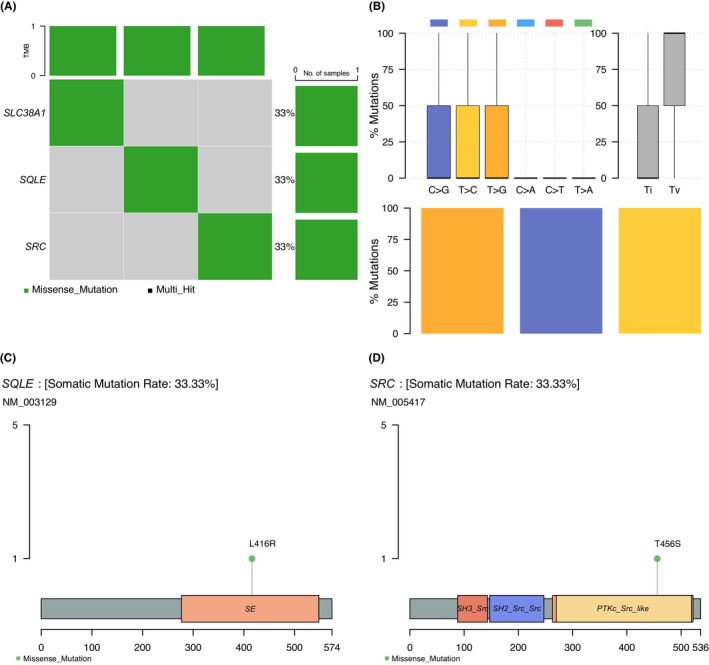
Three key ferroptosis‐related prognostic signatures with somatic mutation. (A) Oncoplot of somatic mutations of three FRGs in TCGA HBV+ HCC patients. (B) The graphs show the distribution of SNV in HBV+ HCC with transition and transversion events. The lollipop map shows the mutation distribution and protein domain of SQLE (C) and SRC (D).

In addition, exploring somatic mutations is helpful to understand the role of FRGs in the development of HBV+ HCC. As illustrated in Figure 10A, within 10 ferroptosis‐related genes, only three missense mutations on SLC38A1, SQLE, and SRC were found in three independent samples of TCGA HBV+ HCC patients. The SNV class of three somatic mutation sites was C > G transversion, T > C transition, and C > A transversion, respectively (Figure 10B). As SQLE and SRC were also taken as drug targets in the following section, the distribution of these two gene mutations was demonstrated in Figure 10C,D. The distribution of SLC38A1 mutation was uploaded as Figure S7.

To screen out drug candidates targeting our ferroptosis‐related prognostic genes, compounds information was downloaded from DrugBank and 3D structures of receptors were prepared as described previously. As shown in Table 1, two compounds DB09280 and DB14943 had a high amount of binding affinity (binding energy ≤ −9 kcal/mol from docking score) toward inhibition sites of three ferroptosis‐related proteins, as SLC1A5, SQLE, and SRC. The complete docking score was provided in Tables S12–S14. As illustrated in Figure S8, DB09280 could establish hydrogen bonds with the amino acid residues Phe‐369 of SLC1A5, Gln‐168 and Tyr‐335 of SQLE, and Glu‐310, Asp‐404, and Phe‐405 of SRC. As shown in Figure S9, DB14943 could establish hydrogen bonds with the amino acid residues Ile‐162, Phe‐166, and Met‐421 of SQLE and Ala‐390 of SRC. Furthermore, other lipophilic contacts are also detectable between DB09280, DB14943 and SLC1A5, SQLE, and SRC. As high‐expression levels of these three genes were correlated with poor prognoses, DB09280 and DB14943 could be taken as drug candidates aiming for improving the survival rate of HBV+ HCC patients.

**TABLE 1:** Docking score between drug compounds (DB09280, DB14943) and ferroptosis‐related prognostic receptors (SLC1A5, SQLE, and SRC).

DrugBank_ID	Generic name	Protein	Affinity (kcal/mol)
DB09280	Lumacaftor	SLC1A5	−9.2
		SQLE	−12.2
		SRC	−9.5
DB14943	LGH‐447	SLC1A5	−9.4
		SQLE	−10.2
		SRC	−9.2

**FIGURE 11:**
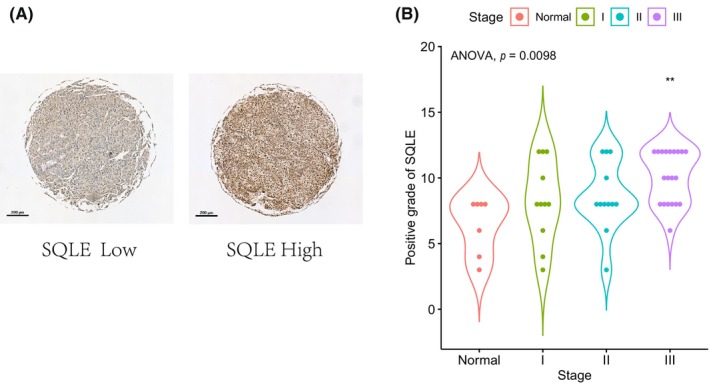
Experimental verification of SQLE. Protein expression (A) and positive grade (B) of SQLE in HBV+ HCC tissues and their adjacent normal tissue.

Considering SQLE as the most important dangerous FRGs and potential drug target discovered here, we used the SQLE gene to verify our model at last. The immunohistochemical analysis of 50 clinical samples showed that SQLE expression was significantly higher in HBV+ HCC tissues than in normal tissues, especially for the Stage III cases (Figure 11). It is worth noting that this is the first report of SQLE expression level in HBV+ HCC patients.

## 4 DISCUSSION

Chronic HBV infection was one of the major viral risk factors for HCC. In the core TCGA data set, 44 of 196 (22.4%) HCC patients displayed clinical and molecular evidence of HBV infection. In epidemiological terms, HBV infection was significantly associated with Asian ethnicity, younger age at initial diagnosis, and male gender.^23^ In biological terms, integrated viral DNA in the host genome could raise the possibility of cis‐activation or inactivation of cancer regulatory genes.^23^ One of the effects of HBV infection was the regulation of transcriptional levels of key oncogenes. To provide a useful prognostic tool, the stratification analysis focused on HBV‐related HCC based on high throughput sequencing data is required.

Ferroptosis is currently thought to be the combined effect of multiple metabolic pathways being disrupted and the failure of ferroptosis surveillance systems.^28^ However, the crosstalk between ferroptosis, HCC tumorigenesis mechanism, and the implication of HBV infection is still unclear. To our knowledge, this is the first research to screen key ferroptosis‐related genes and the related mechanism that is involved in the prognosis of HBV+ HCC. Unsurprisingly, compared with previous studies evaluating the prognostic values of FRGs in HCC,^13^, ^14^, ^29^, ^30^, ^31^ most of the key FRGs we highlighted were first reported as prognostic signatures for HBV+ HCC.

As our list of ferroptosis‐related genes was obtained from various databases, literature, and statistical analysis on TCGA transcriptome data, finally as many as 330 FRGs were kept for prognostic markers screening. First, 160 FRGs were detected as significantly differentially expressed between HCC tumor tissues and adjacent normal tissues. As in the clinic, no recurrence or metastasis 5 years after treatment means that the risk of cancer patients is greatly reduced, we subsequently screen out 36 FRGs differentially expressed in patients with long survival time.

Besides stratification of HBV+ HCC, relatively complete FRGs list, and long‐term survival‐associated genes screening, another characteristic of this research is the employment of machine‐learning algorithm (i.e., random forest in this article) to divide patients with <5 years or ≥5 years survival time. Compared to classic approaches, machine‐learning procedures allow researchers to reduce the complexity of extensive data into specific classifications, shorten the computational cost time, and improve accuracy. Considering other clinical factors such as older age, liver function impairment, vascular invasion, tumor aggressiveness, and elevated AFP are associated with HBV+ HCC survival,^4^ machine‐learning algorithm should be taken as a proper method for gene's prognostic contribution ranking.

As a result of univariate Cox regression analysis, our complex score was significantly associated with OS in the TCGA cohort (HR = 3.34, 95% CI = 1.89–5.89, *p* < 0.001). After correction for other confounding factors, the risk score still proved to be an independent predictor for OS in the multivariate Cox regression analysis (HR = 2.43, 95% CI = 1.12–5.29, *p* = 0.0249). Compared with the previous FRGs prognostic model for all HCC patients in TCGA,^13^, ^31^ our result provided a higher hazard ratio value.

Genes play a regulatory role through different biological functions and signaling pathway networks. The GO and KEGG enrichment analysis of our key FRGs focused on vascular and nutrient/metabolic transport pathways, while the combined analysis considering prognostic FRGs and survival time‐associated pathways highlighted the biological processes involved in the cell cycle and metabolic mechanism. This result was congruent with the opinion that numerous metabolic pathways contribute to ferroptosis through the generation of L‐ROS.^32^

It has been increasingly accepted that HBV is a noncytopathic virus and HBV pathogenesis lies mostly in immune‐mediated liver injury.^33^, ^34^ On the other hand, the role of ferroptosis in tumorigenesis depends on the release of damage‐associated molecular patterns and the activation of immune response triggered by ferroptosis damage within the tumor microenvironment.^6^ Regulatory T cells (Treg) suppress cytotoxic T‐cell antitumoral immune responses and thereby promote tumor progression. Macrophages M0 are nonactivated macrophages without any inflammatory or tumor‐associated function and can be transformed into classically activated M1 macrophages and alternatively activated M2 macrophages. As revealed in our immune infiltrates analysis, within immune cell subpopulations that varied between patients with different survival time, there was a positive correlation between fractions as Tregs and Macrophage M0 and FRGs complex score in HBV+ HCC patients. This result was consistent with previous reports^13^, ^29^ and may be that the mechanism of ferroptosis affects tumor cells by affecting immune cells. On the contrary, a lower infiltration level of Mast cell activated was in samples with higher complex scores. In general, our analysis indicated ferroptosis could affect HBV+ HCC tumor immune landscape, and the immune profile may guide the selection of immunotherapy in the future.

The prognostic model for HBV+ HCC proposed in the present study was composed of 10 ferroptosis‐related genes (EPAS1 and GABARAPL1 as protective factors and SQLE, RAD51AP1, RPL8, CAPG, RRM2, SLC1A5, SLC38A1, and SRC as dangerous markers). Within them, EPAS1 could regulate ferroptosis in kidney cancer^35^ and appears to promote ferroptosis in clear cell carcinoma cells through transcriptional upregulation of hypoxia‐inducible lipid droplet‐associated (HILPDA/HIG2), a regulator of enriched lipids that contain polyunsaturated fatty acyl side chains.^7^ GABARAPL1 as the other protective gene and ferroptosis driver has been proven as a potential positive regulator of ferroptosis via RNAi screening.^36^ For the dangerous markers, the SQLE gene encodes squalene epoxidase to catalyze the oxidation of squalene that could change the lipid profile of tumor cells and protect them from ferroptosis,^37^ and the inhibition of the flavoprotein SQLE with NB‐598 could enhance ferroptosis.^38^ RAD51AP1, as a DNA‐binding protein that stimulates RAD51 activity, could protect tumor cells against DNA damage and may be linked to cancer development and progression.^39^ RPL8 as a ferroptosis driver, which encodes RPL8/uL2, a protein of the 60S large ribosomal subunit, has an overexpression in HCC tumor as described previously.^40^ Conversely, CAPG, as a ferroptosis downregulated factor, is also upregulated in liver cancer tissues.^41^ RRM2 as a ferroptosis suppressor has been found a role in GSH synthesis in HCC.^42^ Besides, RRM2 is also found as a core gene in the p53 regulation pathway in hepatitis B virus‐related HCC.^43^ SLC1A5 is an essential transporter for glutamine uptake and ferroptosis regulator, the inhibition or knockdown of it also suppresses ferroptosis.^44^ SLC38A1 as an adverse prognostic marker is an essential mediator of glutamine uptake and metabolism in lipid peroxidation and SLC38A1 knockout can markedly block ferroptosis.^45^ SRC is an indispensable player in multiple physiological homeostatic pathways.^46^ It was also reported that SRC activation could contribute to ferroptosis resistance.^47^ In summary, two protective signatures are both ferroptosis driver genes, but adverse FRGs consist of both ferroptosis driver and suppressor genes. The role of these genes in HBV+ HCC tumorigenesis may deserve more attention.

Nevertheless, there is still a limitation in our study. As analyses and results are mainly based on the transcriptome data from public databases, besides SQLE, further effort in experiments to elaborate the specific mechanisms of other key FRGs is in need. However, as the first comprehensive research aiming to develop a prognostic tool based on the relationship between ferroptosis‐related genes and HBV+ HCC, our study demonstrates that the stratification of HBV+ HCC is necessary and the newfound biomarkers and related mechanism may be considered as drug targets for therapy in future.

## CONFLICT OF INTEREST STATEMENT

The authors declare that they have no known competing financial interests or personal relationships that could have appeared to influence the work reported in this paper.

## Supporting information


Figure S1

Figure S2

Figure S3

Figure S4

Figure S5

Figure S6

Figure S7

Figure S8

Figure S9

Tables S1–S14

## Data Availability

The data that support the findings of this study are available in the supporting information of this article.
