# Supplementary figures and images for: Life span‐associated ferroptosis‐related genes identification and validation for hepatocellular carcinoma patients as hepatitis B virus carriers
Source: J Clin Lab Anal. 2023 Jul 18;37(13-14):e24930. doi: 10.1002/jcla.24930 (PMC10492458; doi:10.1002/jcla.24930)

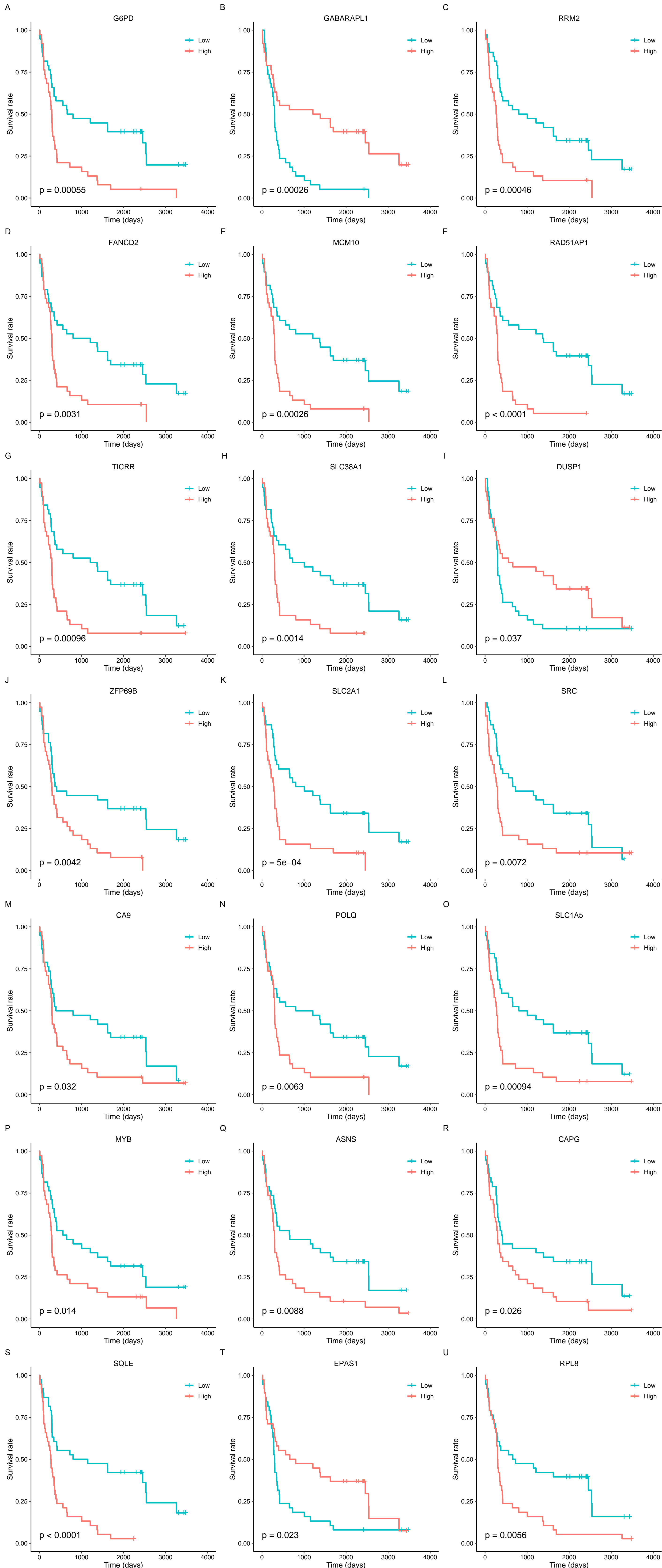

Supplement: Supplementary file 1 — Figure S1 [file JCLA-37-e24930-s008.pdf]

**A**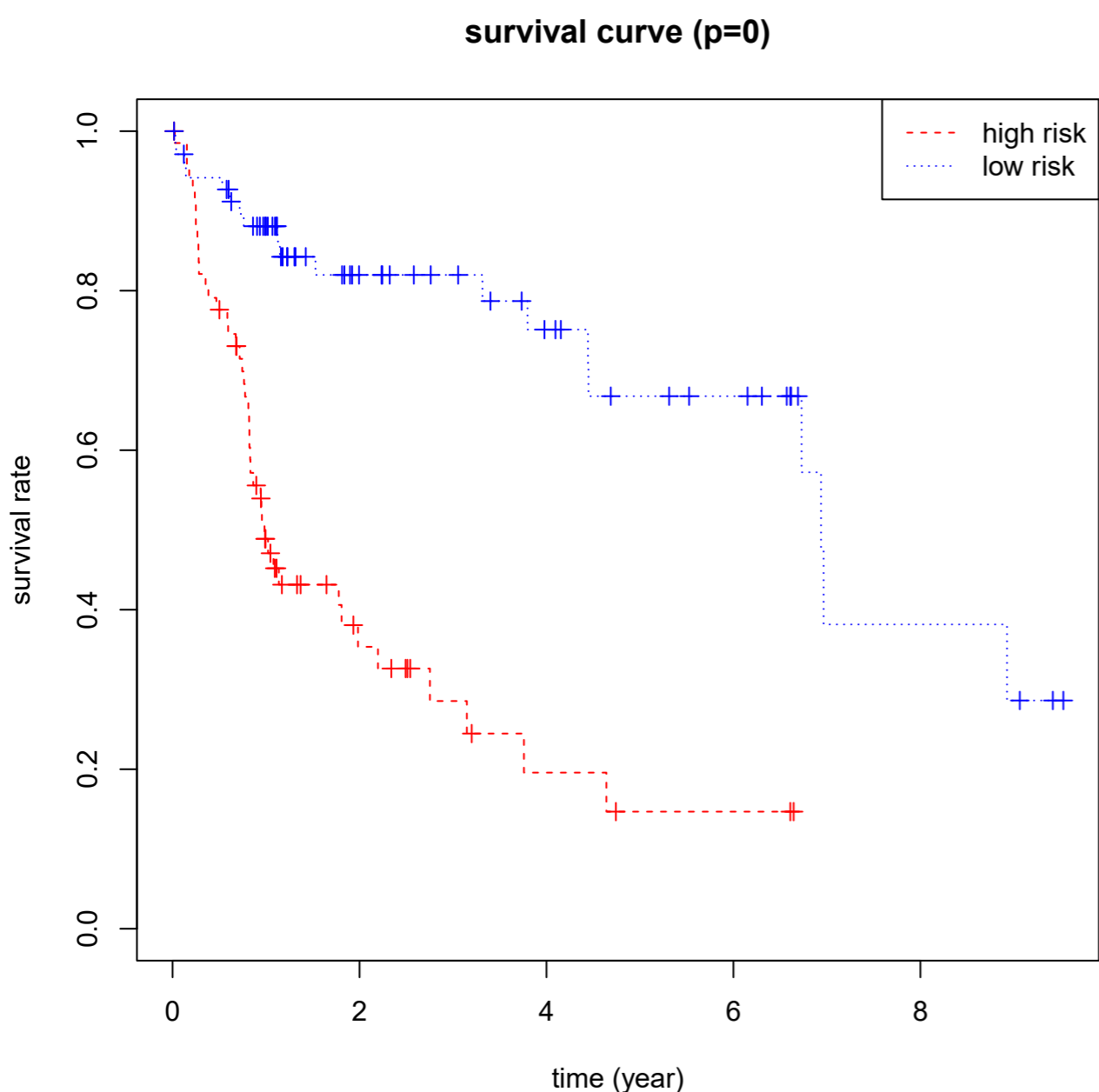**B**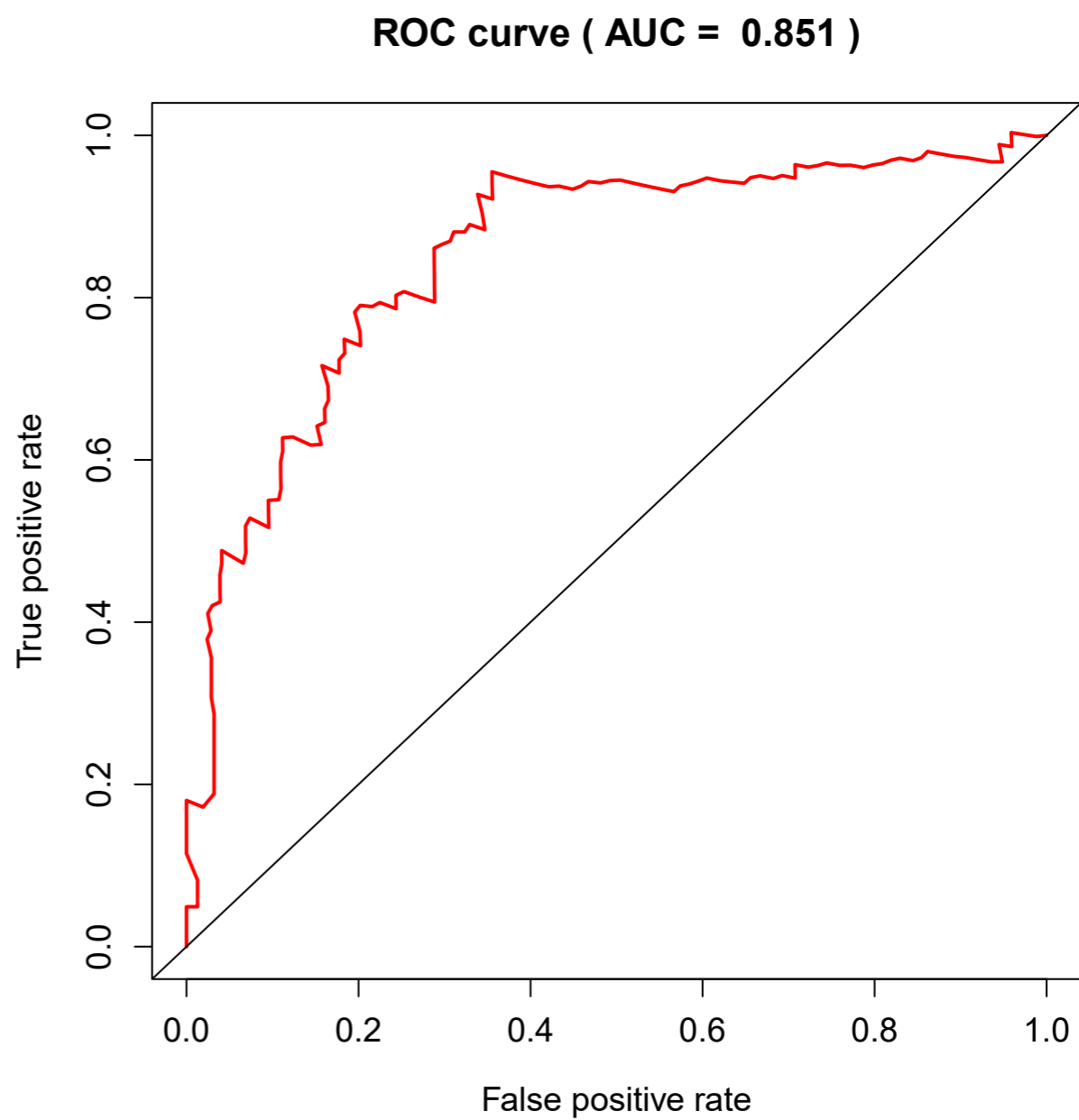**C**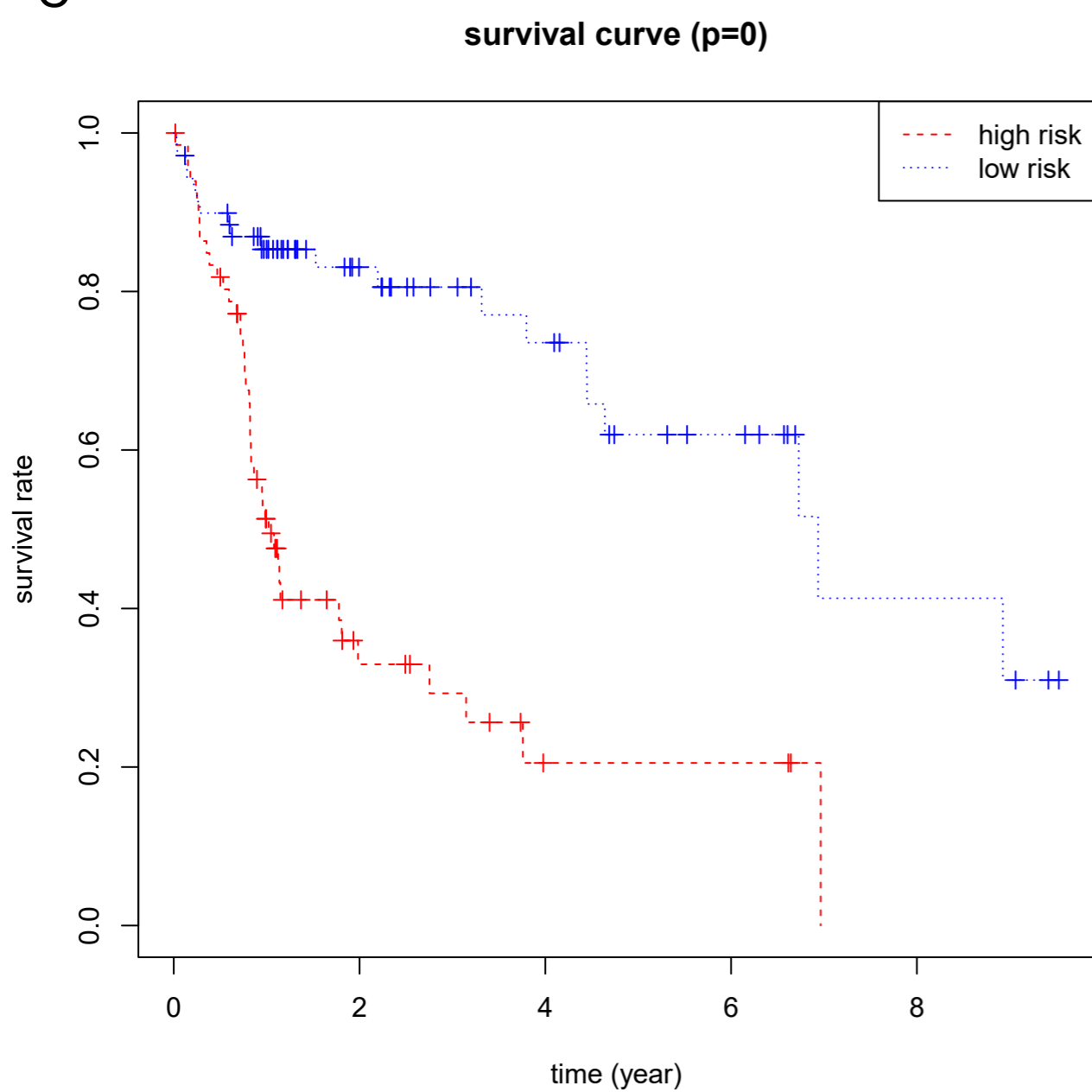**D**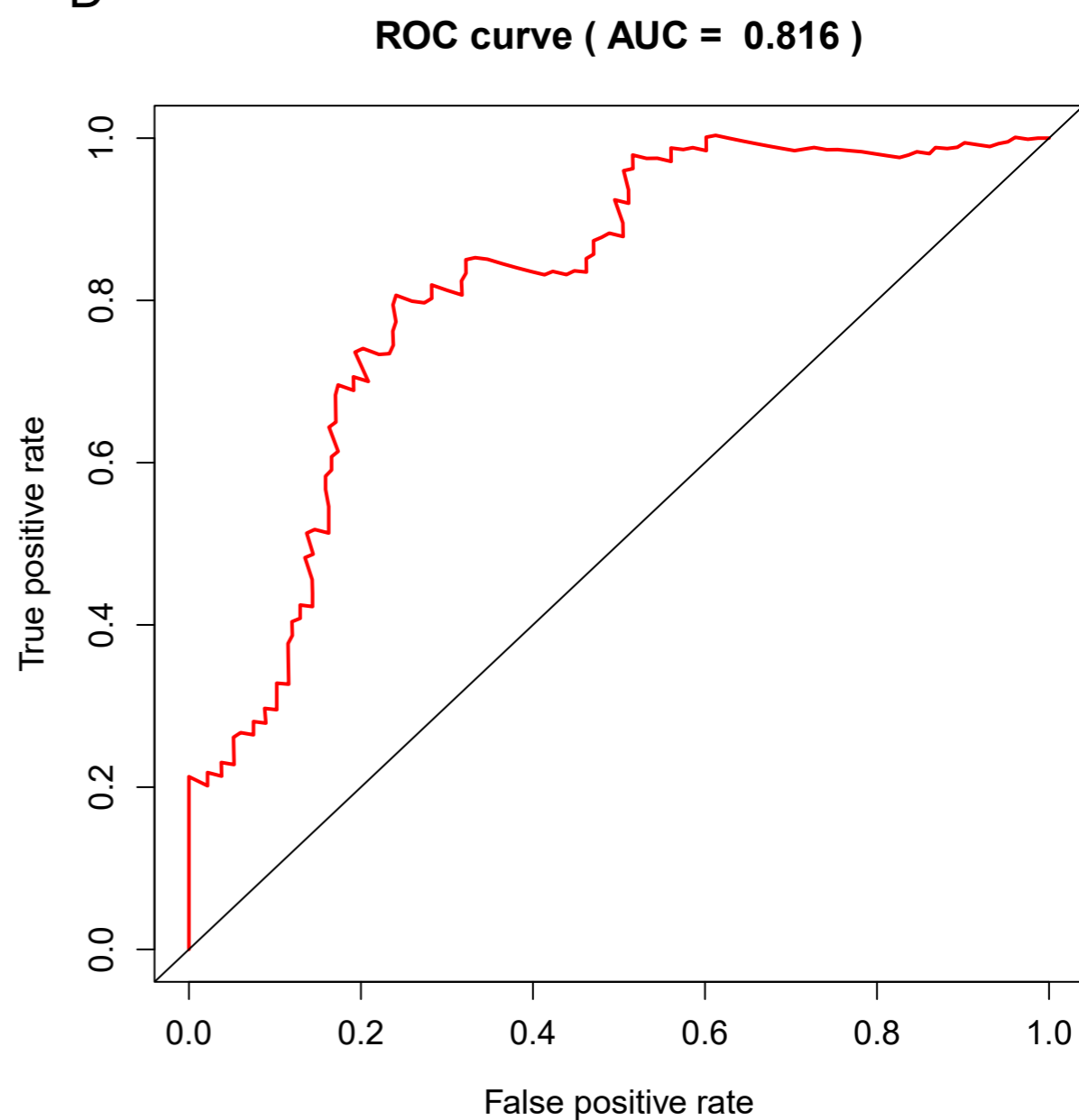**E**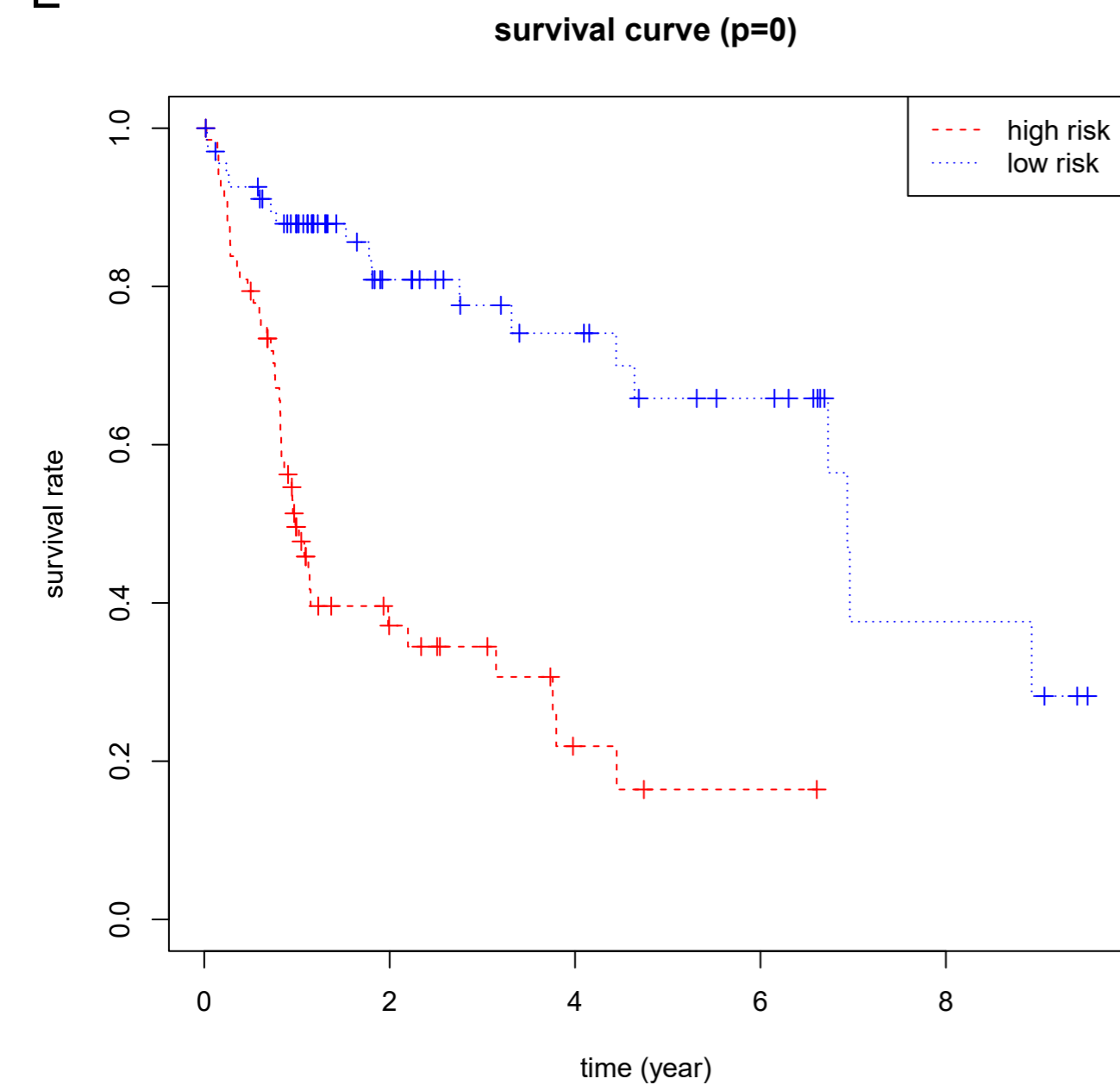**F**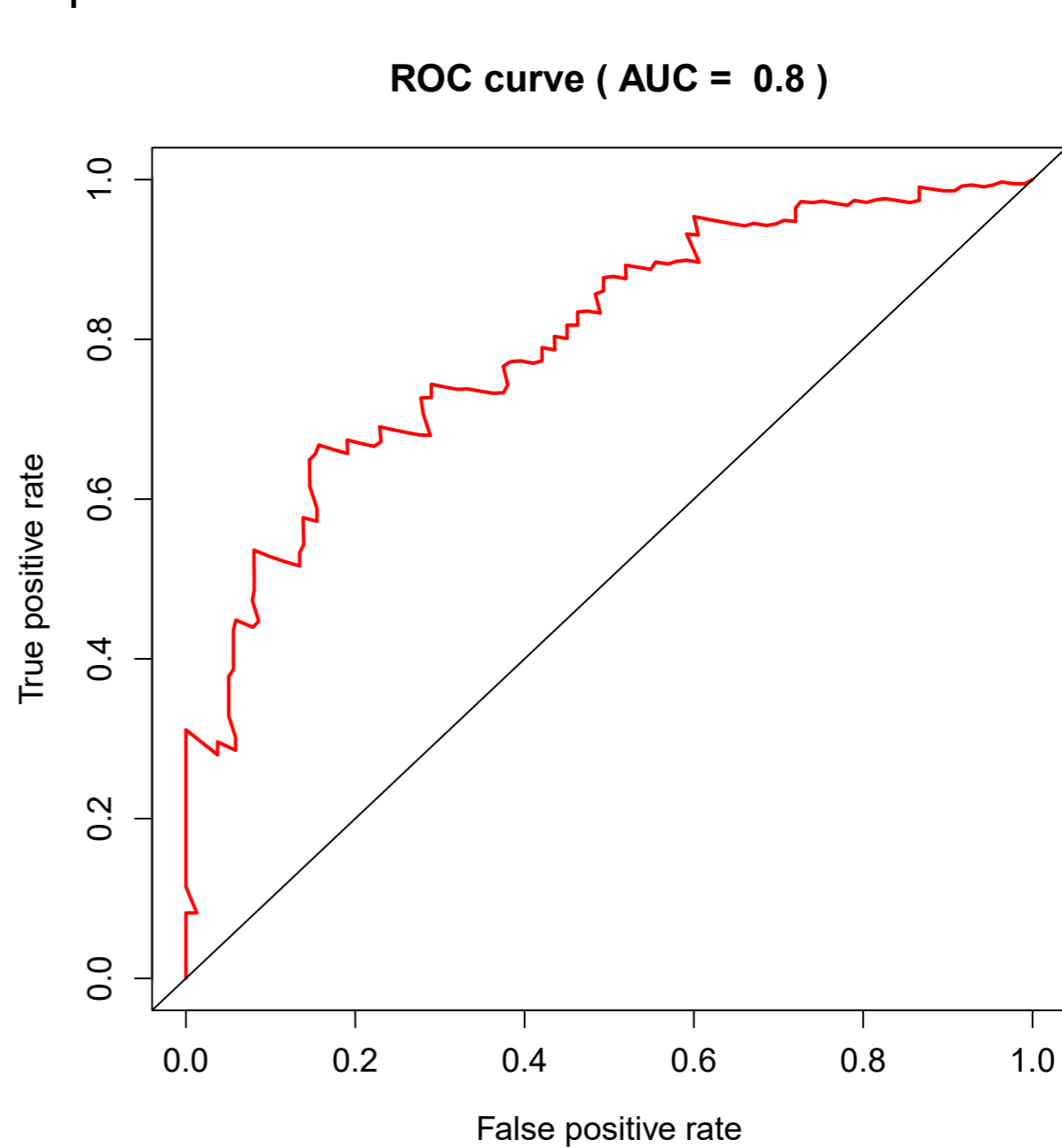**G**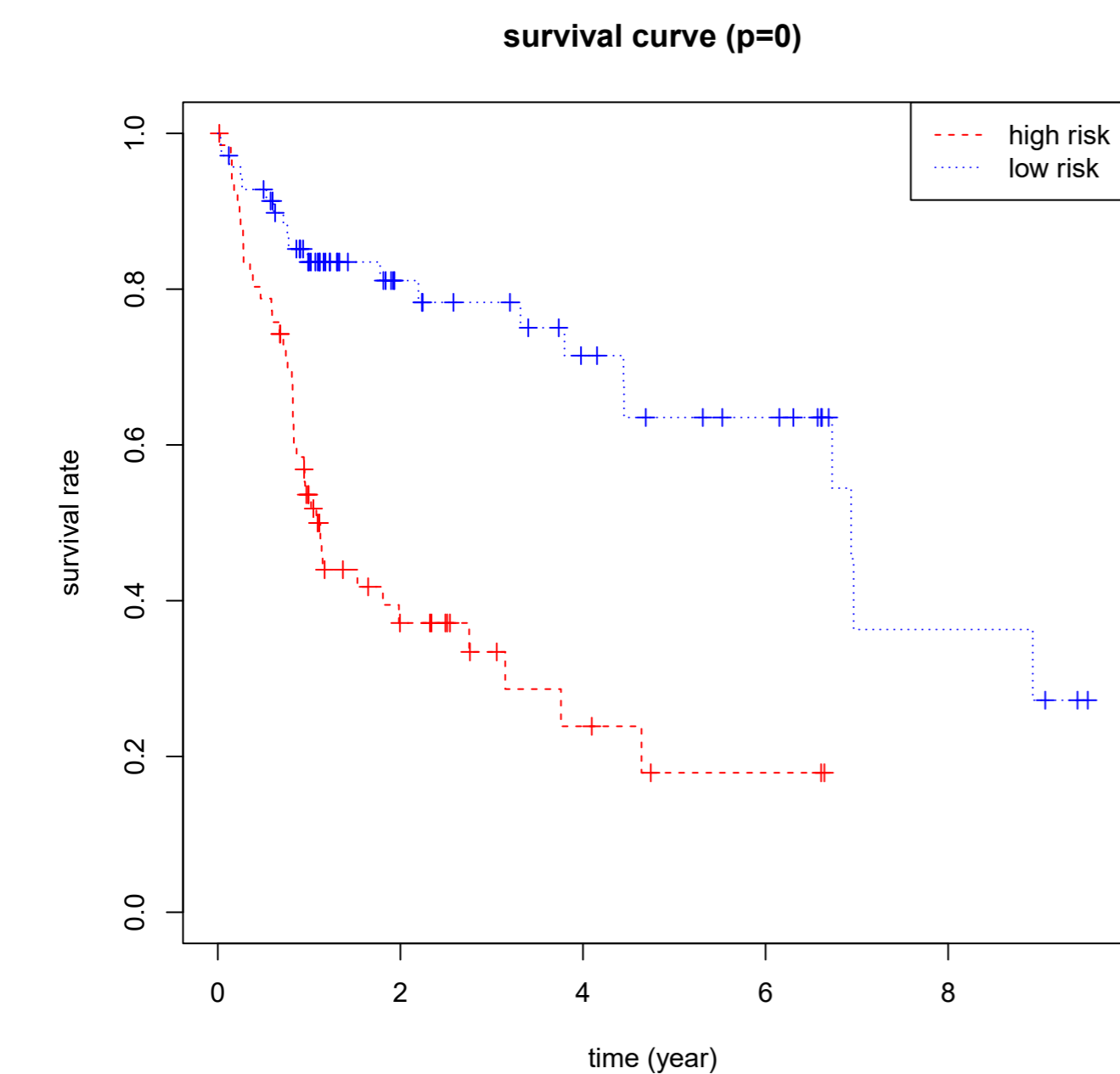**H**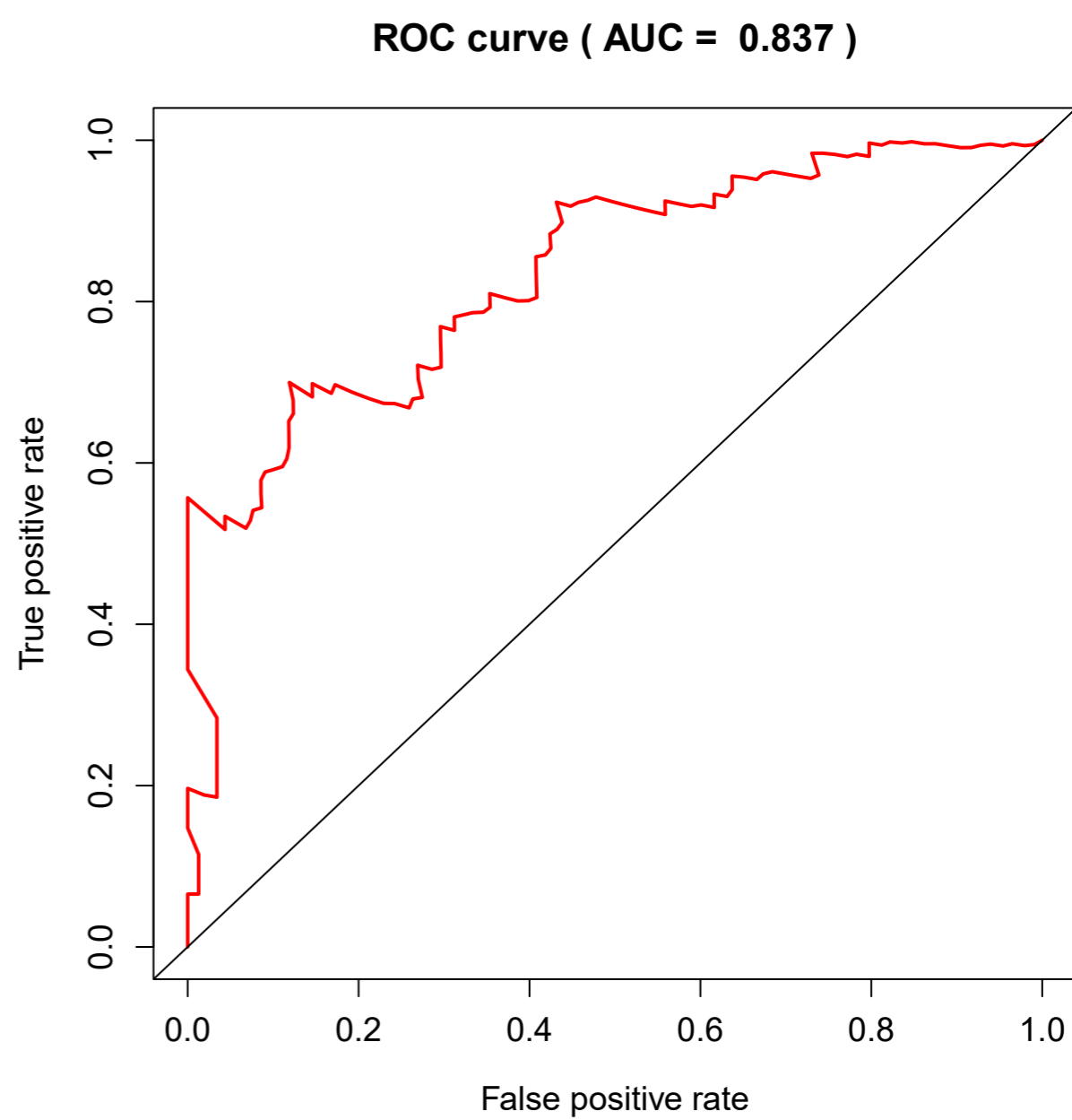**I**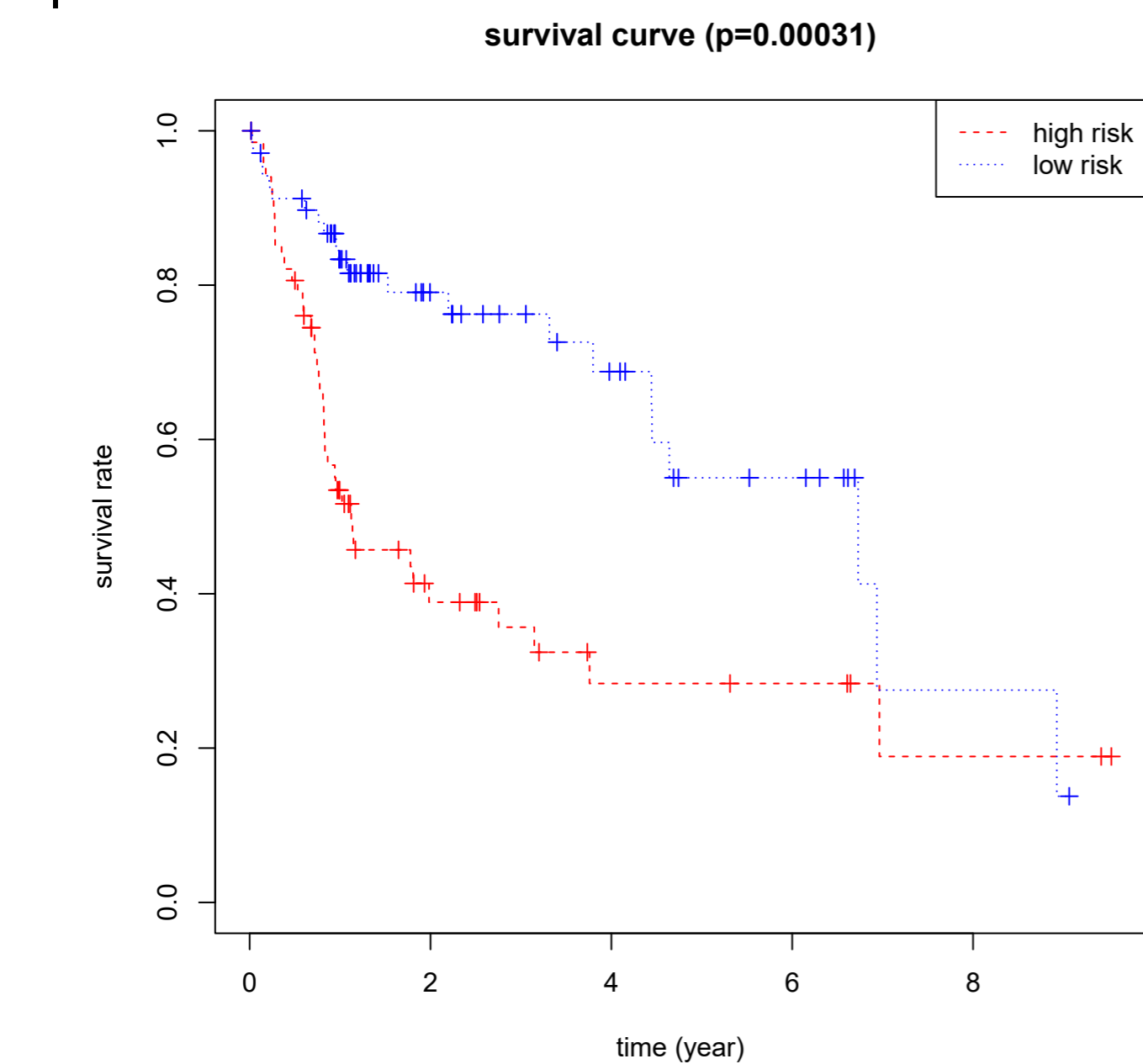**J**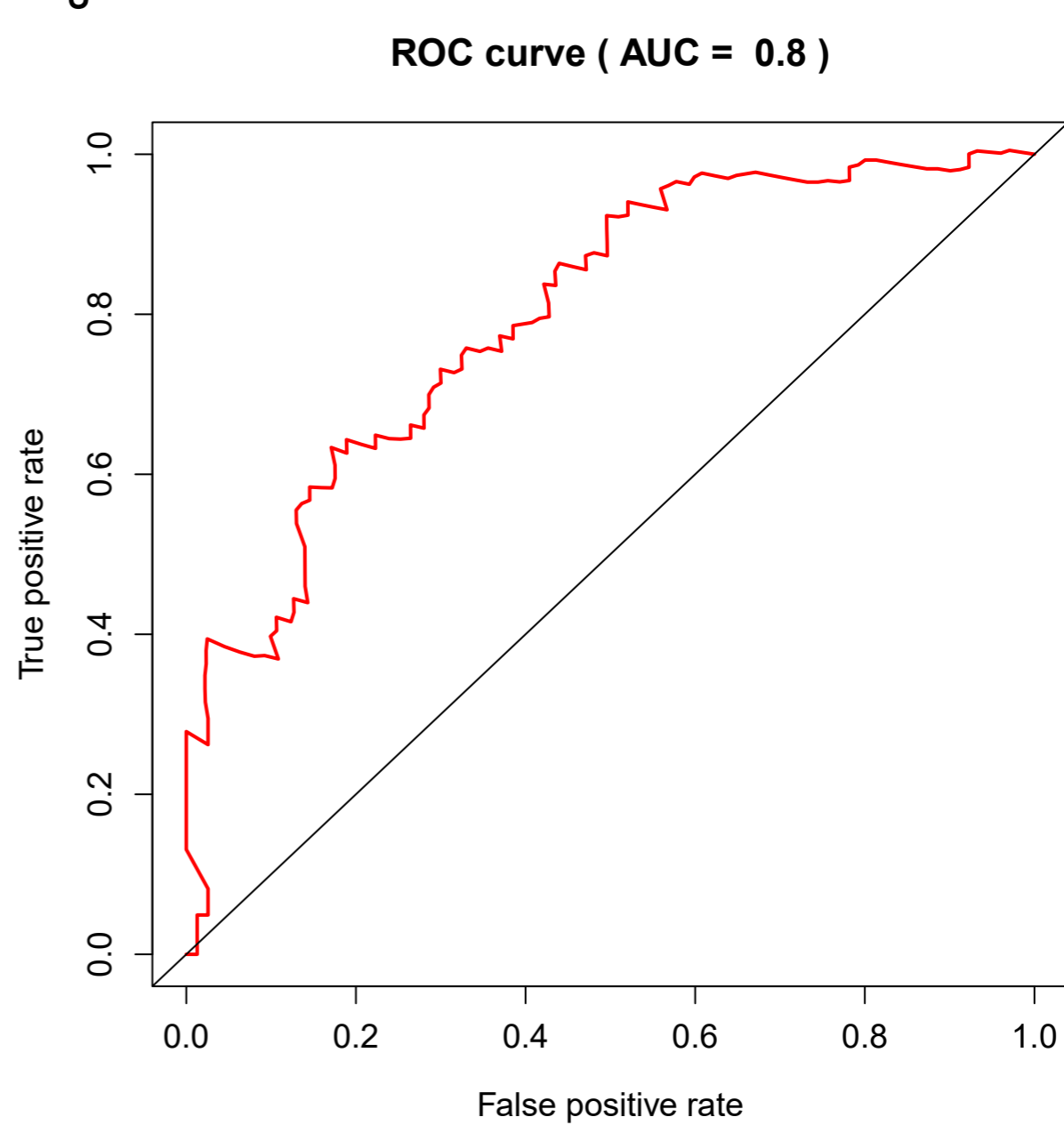

Supplement: Supplementary file 2 — Figure S2 [file JCLA-37-e24930-s002.pdf]

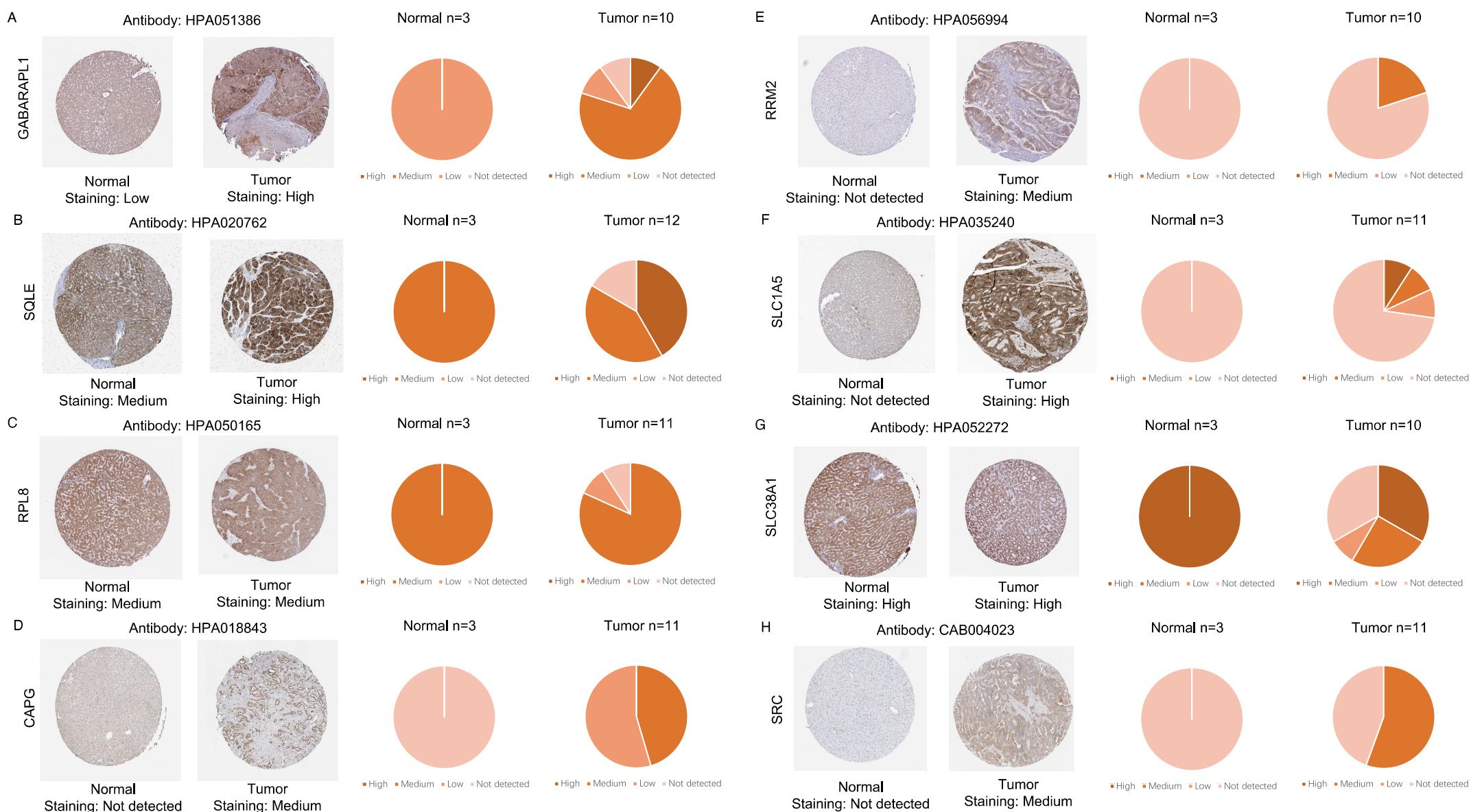

Supplement: Supplementary file 3 — Figure S3 [file JCLA-37-e24930-s007.pdf]

$\chi^2_{\text{pearson}}=14.15, p=0.068$

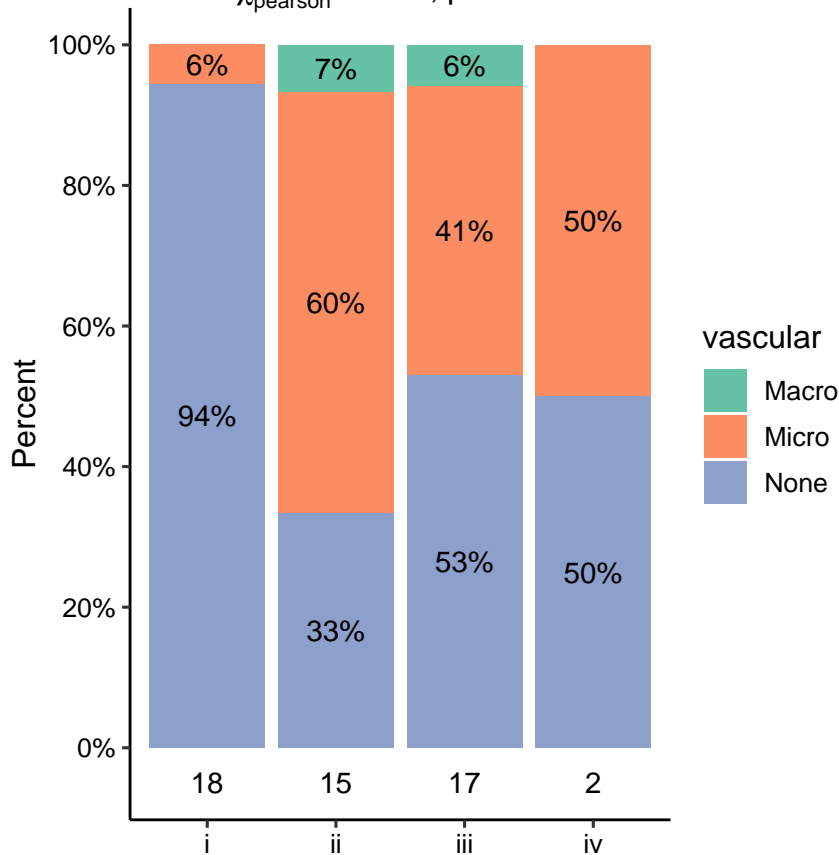

$\chi^2_{\text{pearson}}=1.03, p=0.704$

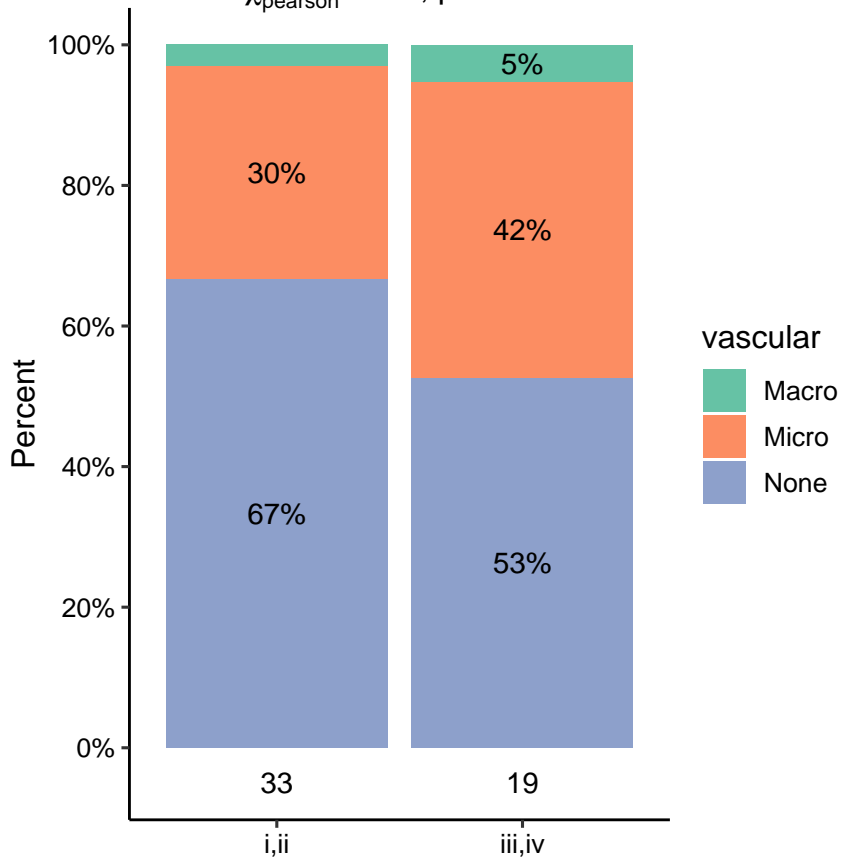

Supplement: Supplementary file 4 — Figure S4 [file JCLA-37-e24930-s003.pdf]

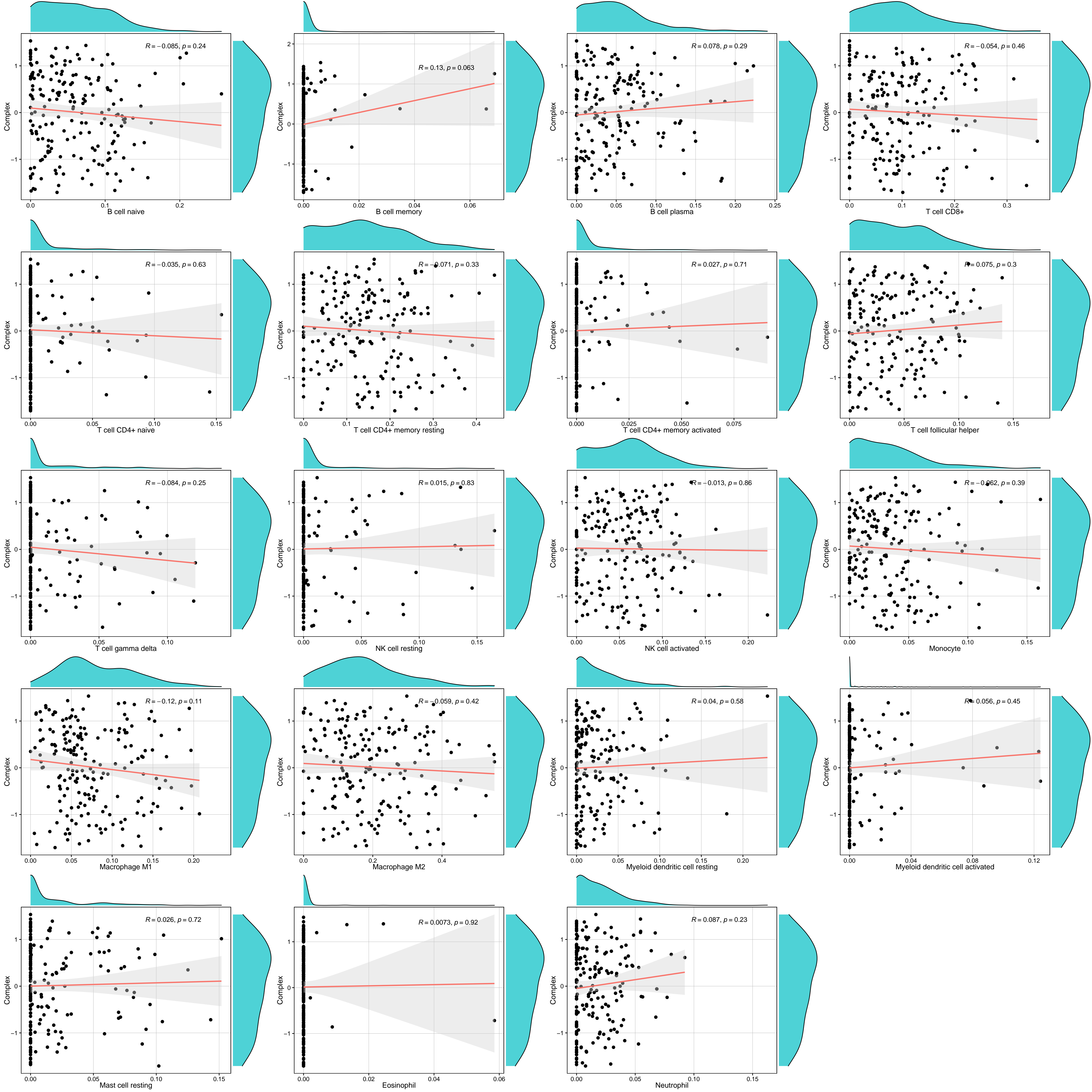

Supplement: Supplementary file 5 — Figure S5 [file JCLA-37-e24930-s004.pdf]

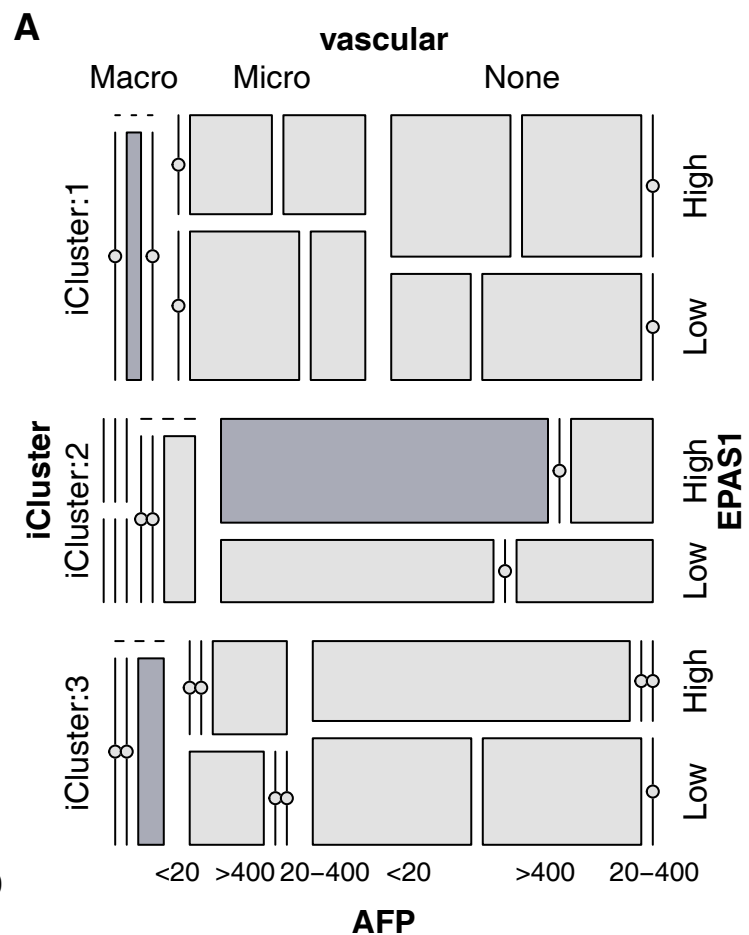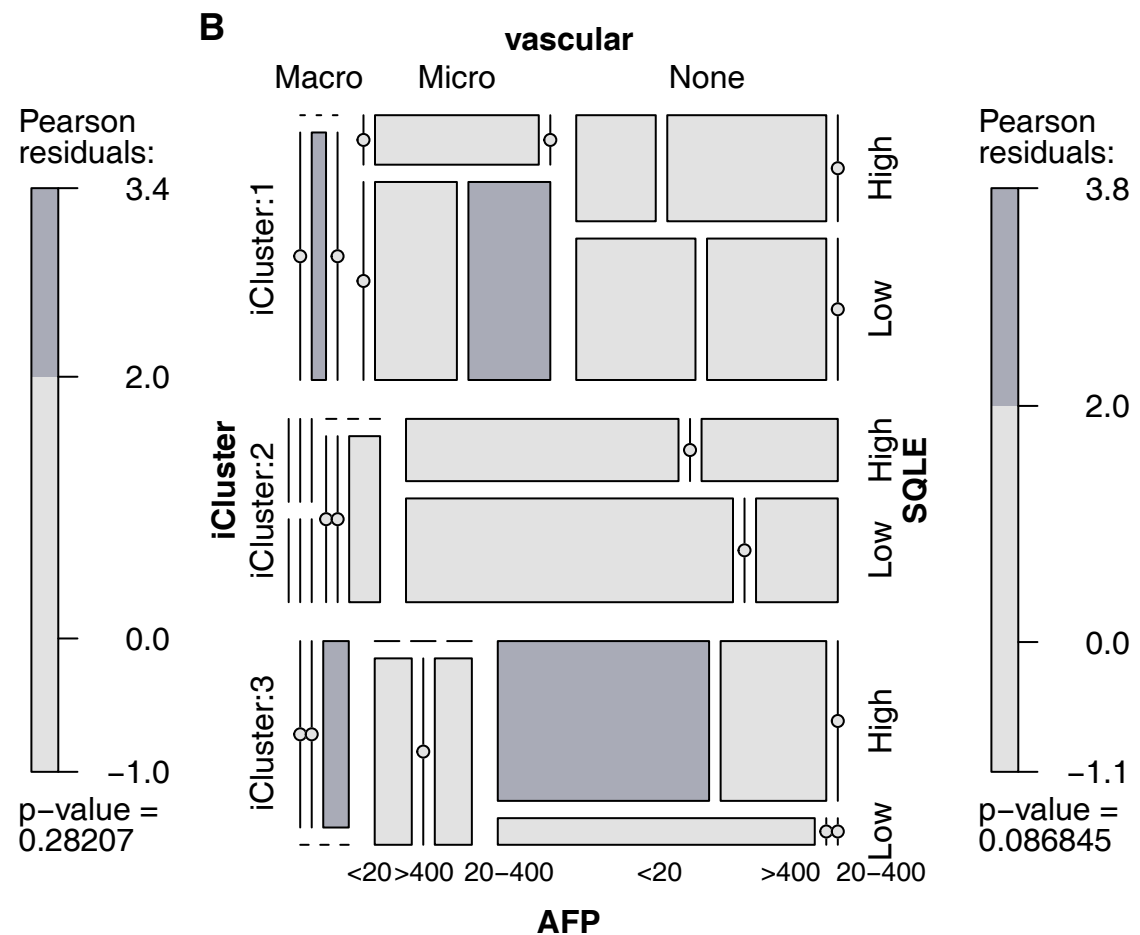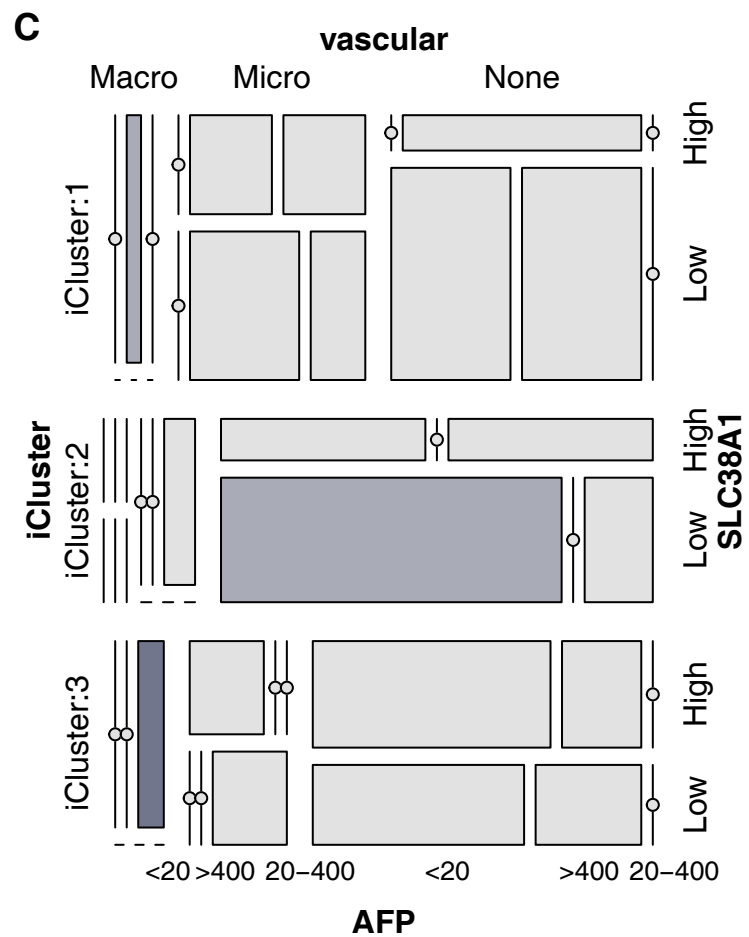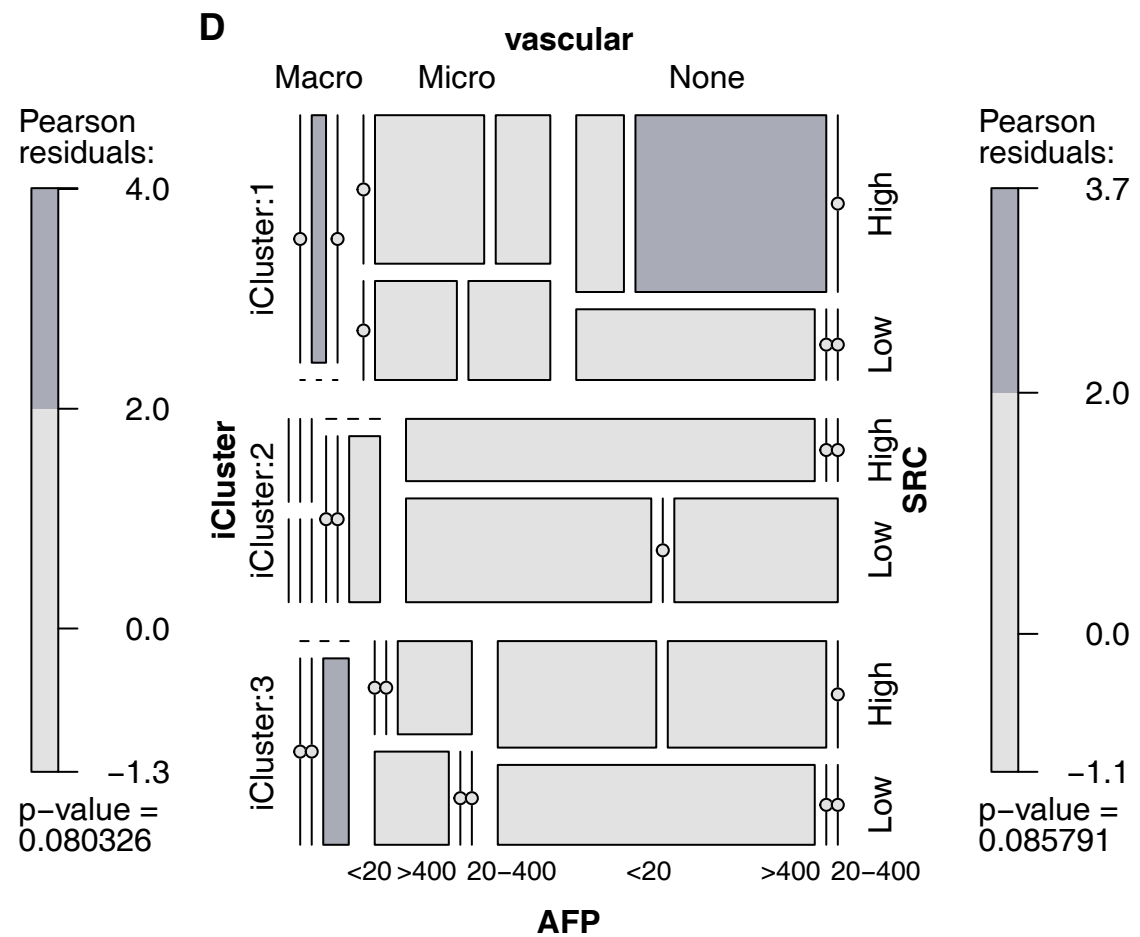

Supplement: Supplementary file 6 — Figure S6 [file JCLA-37-e24930-s005.pdf]

SLC38A1 : [Somatic Mutation Rate: 33.33%]

NM\_001077484

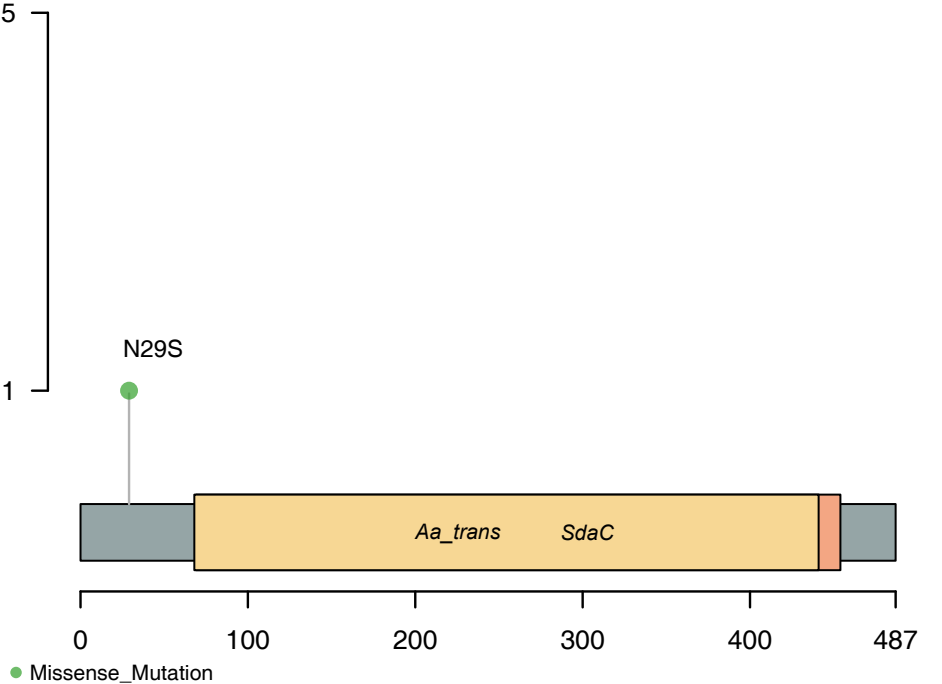

Supplement: Supplementary file 7 — Figure S7 [file JCLA-37-e24930-s010.pdf]

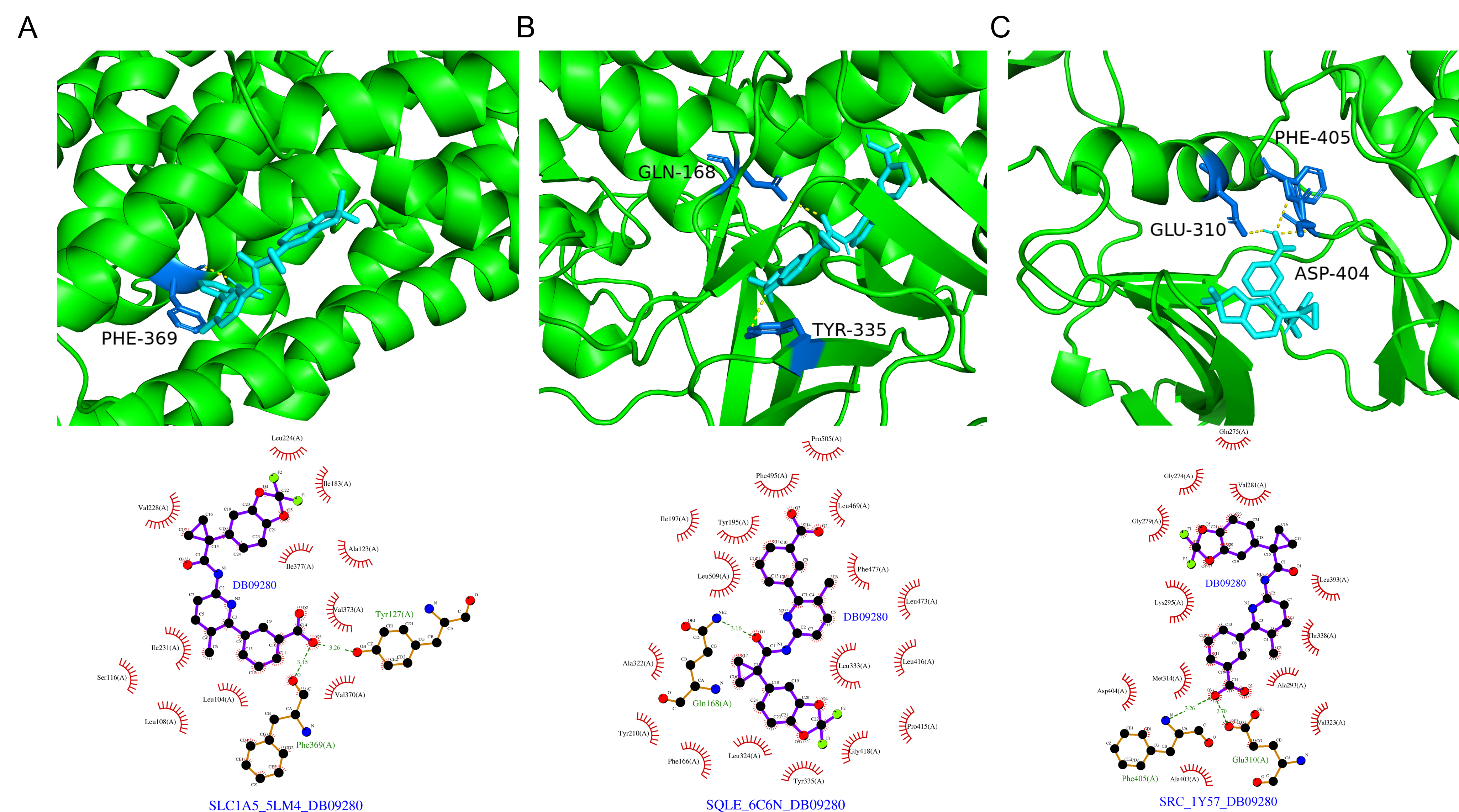

Supplement: Supplementary file 8 — Figure S8 [file JCLA-37-e24930-s001.tif]

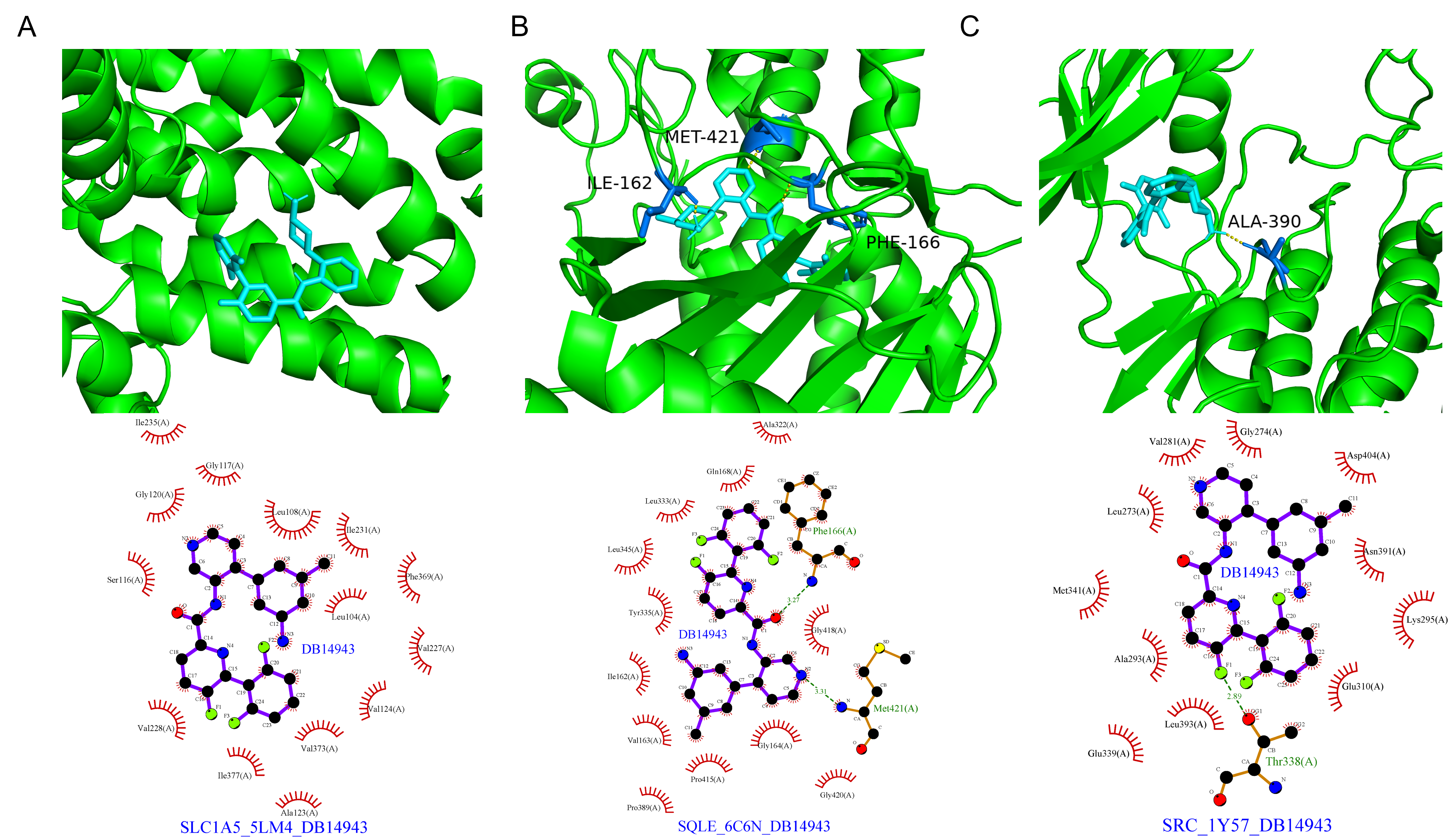

Supplement: Supplementary file 9 — Figure S9 [file JCLA-37-e24930-s006.tif]
