# Supplementary material for: Life span‐associated ferroptosis‐related genes identification and validation for hepatocellular carcinoma patients as hepatitis B virus carriers
Source: J Clin Lab Anal. 2023 Jul 18;37(13-14):e24930. doi: 10.1002/jcla.24930 (PMC10492458; doi:10.1002/jcla.24930)
Supplement: Supplementary file 10 — Tables S1–S14 [file JCLA-37-e24930-s009.zip › TableS1_TCGA_phen_HBV.docx]

TableS1_TCGA_phen_HBV

|  | submitter_id.samples | group | batch_number | age_at_index.demographic | gender | tumor_grade.diagnoses | vascular | fetoprotein_outcome_value | fibrosis_ishak_score | neoplasm_histologic_grade | tumor_stage.diagnoses | weight | alcohol_history.exposures | bmi.exposures | OS | OS.time | Survival | DSS | DSS.time | DFI | DFI.time | PFI | PFI.time | Redaction | iCluster | age | fibrosis | AFP | stage |
| --- | --- | --- | --- | --- | --- | --- | --- | --- | --- | --- | --- | --- | --- | --- | --- | --- | --- | --- | --- | --- | --- | --- | --- | --- | --- | --- | --- | --- | --- |
| 1 | TCGA-2Y-A9GS-01A | Tumor | 399.48.0 | 58 | male | not reported | NA | 74 | NA | G2 | not reported | 92 | Not Reported | 35.05563 | 1 | 724 | Low | 1 | 724 | 1 | 102 | 1 | 102 |  | NA | <=60 | NA | 20-400 | NA |
| 2 | TCGA-2Y-A9GT-01A | Tumor | 399.48.0 | 51 | male | not reported | None | 17 | NA | G2 | stage i | 122 | Not Reported | 36.8313 | 1 | 1624 | Low | 1 | 1624 | 1 | 1083 | 1 | 1083 |  | NA | <=60 | NA | <20 | i |
| 3 | TCGA-2Y-A9GU-01A | Tumor | 399.48.0 | 55 | female | not reported | NA | 304 | NA | G2 | stage i | 78 | Not Reported | 32.88919 | 0 | 1939 | High | 0 | 1939 | 0 | 1939 | 0 | 1939 |  | NA | <=60 | NA | 20-400 | i |
| 4 | TCGA-2Y-A9GV-01A | Tumor | 399.48.0 | 54 | female | not reported | NA | 6 | NA | G1 | stage i | 85 | Not Reported | 30.47797 | 1 | 2532 | High | 1 | 2532 | 0 | 2532 | 1 | 1745 |  | NA | <=60 | NA | <20 | i |
| 5 | TCGA-2Y-A9GX-01A | Tumor | 399.48.0 | 68 | male | not reported | NA | 1 | NA | G2 | stage i | 104 | Not Reported | 33.95918 | 0 | 2442 | High | 0 | 2442 | NA | NA | 1 | 2133 |  | NA | >60 | NA | <20 | i |
| 6 | TCGA-2Y-A9H2-01A | Tumor | 399.48.0 | 64 | female | not reported | None | 2 | NA | G3 | stage i | 55 | Not Reported | 17.75568 | 0 | 1731 | High | 0 | 1731 | 0 | 1731 | 0 | 1731 |  | NA | >60 | NA | <20 | i |
| 7 | TCGA-2Y-A9H3-01A | Tumor | 399.48.0 | 45 | male | not reported | Micro | 5640 | 1,2 - Portal Fibrosis | G1 | stage ii | 105 | Not Reported | 32.77051 | 0 | 1516 | High | 0 | 1516 | 1 | 22 | 1 | 22 |  | NA | <=60 | 1,2 | >400 | ii |
| 8 | TCGA-2Y-A9H4-01A | Tumor | 399.48.0 | 68 | male | not reported | None | 11 | NA | G2 | stage i | 61 | Not Reported | 21.87242 | 0 | 1452 | High | 0 | 1452 | 0 | 1452 | 0 | 1452 |  | NA | >60 | NA | <20 | i |
| 9 | TCGA-2Y-A9H7-01A | Tumor | 405.45.0 | 81 | female | not reported | None | 6 | 3,4 - Fibrous Speta | G2 | stage i | 104 | Not Reported | 42.19238 | 0 | 1168 | High | 0 | 1168 | 1 | 1117 | 1 | 1117 |  | NA | >60 | 3,4 | <20 | i |
| 10 | TCGA-4R-AA8I-01A | Tumor | 399.48.0 | 66 | male | not reported | Micro | 5 | 6 - Established Cirrhosis | G2 | stage ii | 103 | Not Reported | 35.22451 | 1 | 262 | Low | 1 | 262 | 1 | 158 | 1 | 158 |  | NA | >60 | 6 | <20 | ii |
| 11 | TCGA-5R-AA1C-01A | Tumor | 425.45.0 | 57 | male | not reported | Micro | 2 | 1,2 - Portal Fibrosis | G2 | stage ii | 90 | Not Reported | 29.72652 | 0 | 520 | High | 0 | 520 | 0 | 520 | 0 | 520 |  | NA | <=60 | 1,2 | <20 | ii |
| 12 | TCGA-5R-AA1D-01A | Tumor | 399.48.0 | 17 | female | not reported | Micro | 5 | 0 - No Fibrosis | G3 | stage iiia | 59 | Not Reported | 22.76147 | 0 | 449 | High | 0 | 449 | 0 | 449 | 0 | 449 |  | NA | <=60 | 0 | <20 | iii |
| 13 | TCGA-5R-AAAM-01A | Tumor | 425.45.0 | 65 | female | not reported | Micro | 32 | 6 - Established Cirrhosis | G2 | stage ii | 80 | Not Reported | 30.11028 | 1 | 46 | Low | 0 | 46 | 0 | 46 | 0 | 46 |  | NA | >60 | 6 | 20-400 | ii |
| 14 | TCGA-BC-A10W-01A | Tumor | 100.78.0 | 50 | male | not reported | NA | 79 | NA | G3 | not reported | 71 | Not Reported | NA | 1 | 91 | Low | 1 | 91 | NA | NA | 1 | 91 |  | iCluster:1 | <=60 | NA | 20-400 | NA |
| 15 | TCGA-BD-A2L6-01A | Tumor | 203.73.0 | 69 | male | not reported | Micro | 53 | 3,4 - Fibrous Speta | G2 | not reported | 110 | Not Reported | 38.97392 | 0 | 1363 | High | 0 | 1363 | 1 | 415 | 1 | 415 |  | iCluster:3 | >60 | 3,4 | 20-400 | NA |
| 16 | TCGA-BD-A3ER-01A | Tumor | 203.73.0 | 62 | male | not reported | None | 5 | 6 - Established Cirrhosis | G2 | stage ii | 111 | Not Reported | 31.40561 | 0 | 1115 | High | 0 | 1115 | 1 | 226 | 1 | 226 |  | NA | >60 | 6 | <20 | ii |
| 17 | TCGA-CC-5259-01A | Tumor | 203.73.0 | 60 | female | not reported | NA | NA | NA | G2 | stage iiic | 49 | Not Reported | 20.93212 | 0 | 250 | High | 0 | 250 | 1 | 180 | 1 | 180 |  | NA | <=60 | NA | NA | iii |
| 18 | TCGA-CC-5260-01A | Tumor | 100.78.0 | 61 | female | not reported | None | NA | NA | G1 | stage iiic | 46 | Not Reported | 19.65056 | 1 | 87 | Low | 0 | 87 | NA | NA | 0 | 87 |  | iCluster:1 | >60 | NA | NA | iii |
| 19 | TCGA-CC-5261-01A | Tumor | 100.78.0 | 44 | male | not reported | None | NA | NA | G2 | stage ii | 58 | Not Reported | 18.93878 | 1 | 97 | Low | 0 | 97 | NA | NA | 0 | 97 |  | iCluster:1 | <=60 | NA | NA | ii |
| 20 | TCGA-CC-5262-01A | Tumor | 100.78.0 | 67 | male | not reported | NA | NA | NA | G1 | stage iiic | 62 | Not Reported | 21.45329 | 1 | 103 | Low | 0 | 103 | NA | NA | 0 | 103 |  | iCluster:2 | >60 | NA | NA | iii |
| 21 | TCGA-CC-5263-01A | Tumor | 100.78.0 | 35 | male | not reported | None | NA | NA | G1 | stage iiia | 59 | Not Reported | 22.20633 | 1 | 129 | Low | 0 | 129 | NA | NA | 0 | 129 |  | iCluster:3 | <=60 | NA | NA | iii |
| 22 | TCGA-CC-5264-01A | Tumor | 100.78.0 | 71 | male | not reported | None | NA | NA | G2 | stage iiia | 52 | Not Reported | 20.3125 | 1 | 102 | Low | 0 | 102 | NA | NA | 0 | 102 |  | iCluster:3 | >60 | NA | NA | iii |
| 23 | TCGA-CC-A123-01A | Tumor | 100.78.0 | 24 | female | not reported | None | NA | NA | G1 | stage iiia | NA | Not Reported | NA | 0 | 219 | High | 0 | 219 | NA | NA | 1 | 145 |  | NA | <=60 | NA | NA | iii |
| 24 | TCGA-CC-A1HT-01A | Tumor | 100.78.0 | 50 | male | not reported | NA | NA | NA | G3 | stage iiia | 54 | Not Reported | 20.32444 | 1 | 101 | Low | 0 | 101 | NA | NA | 0 | 101 |  | iCluster:1 | <=60 | NA | NA | iii |
| 25 | TCGA-CC-A3M9-01A | Tumor | 203.73.0 | 45 | male | not reported | NA | NA | NA | G3 | stage iiia | 61 | Not Reported | 20.14797 | 1 | 300 | Low | 1 | 300 | 1 | 219 | 1 | 219 |  | iCluster:1 | <=60 | NA | NA | iii |
| 26 | TCGA-CC-A3MA-01A | Tumor | 203.73.0 | 61 | male | not reported | NA | NA | NA | G2 | stage iiia | 66 | Not Reported | 23.38435 | 1 | 303 | Low | 1 | 303 | 1 | 261 | 1 | 261 |  | iCluster:1 | >60 | NA | NA | iii |
| 27 | TCGA-CC-A3MB-01A | Tumor | 203.73.0 | 36 | male | not reported | NA | NA | NA | G1 | stage iiia | 58 | Not Reported | 18.72417 | 1 | 315 | Low | 1 | 315 | 0 | 315 | 1 | 260 |  | iCluster:2 | <=60 | NA | NA | iii |
| 28 | TCGA-CC-A3MC-01A | Tumor | 231.65.0 | 54 | male | not reported | NA | NA | NA | G2 | stage iiia | 52 | Not Reported | 19.57168 | 0 | 363 | High | 0 | 363 | 1 | 297 | 1 | 297 |  | iCluster:1 | <=60 | NA | NA | iii |
| 29 | TCGA-CC-A5UC-01A | Tumor | 303.59.0 | 63 | male | not reported | NA | NA | NA | G3 | stage iiia | 57 | Not Reported | 20.93664 | 1 | 347 | Low | 1 | 347 | 0 | 347 | 1 | 312 |  | iCluster:1 | >60 | NA | NA | iii |
| 30 | TCGA-CC-A5UD-01A | Tumor | 303.59.0 | 45 | male | not reported | NA | NA | NA | G2 | stage iiia | 62 | Not Reported | 21.45329 | 1 | 304 | Low | 1 | 304 | 1 | 182 | 1 | 182 |  | iCluster:2 | <=60 | NA | NA | iii |
| 31 | TCGA-CC-A5UE-01A | Tumor | 303.59.0 | 48 | male | not reported | NA | NA | NA | G2 | stage iiib | 65 | Not Reported | 21.22449 | 1 | 272 | Low | 1 | 272 | 1 | 175 | 1 | 175 |  | iCluster:3 | <=60 | NA | NA | iii |
| 32 | TCGA-CC-A7IE-01A | Tumor | 399.48.0 | 57 | male | not reported | NA | NA | NA | G2 | stage iiia | 62 | Not Reported | 20.95727 | 1 | 217 | Low | 1 | 217 | 0 | 217 | 1 | 169 |  | NA | <=60 | NA | NA | iii |
| 33 | TCGA-CC-A7IF-01A | Tumor | 341.53.0 | 59 | male | not reported | NA | NA | NA | G1 | stage iiia | 58 | Not Reported | 20.30741 | 1 | 649 | Low | 1 | 649 | 0 | 649 | 1 | 254 |  | iCluster:1 | <=60 | NA | NA | iii |
| 34 | TCGA-CC-A7IG-01A | Tumor | 341.53.0 | 47 | male | not reported | NA | NA | NA | G2 | stage ii | 62 | Not Reported | 20.47827 | 1 | 299 | Low | 1 | 299 | 1 | 195 | 1 | 195 |  | iCluster:1 | <=60 | NA | NA | ii |
| 35 | TCGA-CC-A7IH-01A | Tumor | 341.53.0 | 58 | male | not reported | NA | NA | NA | G1 | stage iiia | 56 | Not Reported | 18.28571 | 0 | 365 | High | 0 | 365 | 0 | 365 | 0 | 365 |  | iCluster:3 | <=60 | NA | NA | iii |
| 36 | TCGA-CC-A7II-01A | Tumor | 341.53.0 | 54 | male | not reported | NA | NA | NA | G3 | stage iiia | 52 | Not Reported | 18.42404 | 0 | 399 | High | 0 | 399 | 1 | 278 | 1 | 278 |  | iCluster:1 | <=60 | NA | NA | iii |
| 37 | TCGA-CC-A7IJ-01A | Tumor | 345.53.0 | 56 | male | not reported | NA | NA | NA | G3 | stage ii | 54 | Not Reported | 19.83471 | 0 | 382 | High | 0 | 382 | 0 | 382 | 0 | 382 |  | iCluster:1 | <=60 | NA | NA | ii |
| 38 | TCGA-CC-A7IK-01A | Tumor | 345.53.0 | 59 | male | not reported | NA | NA | NA | G3 | stage iiia | 57 | Not Reported | 20.19558 | 1 | 262 | Low | 1 | 262 | 1 | 150 | 1 | 150 |  | iCluster:3 | <=60 | NA | NA | iii |
| 39 | TCGA-CC-A7IL-01A | Tumor | 345.53.0 | 61 | male | not reported | NA | NA | NA | G1 | stage iiia | 63 | Not Reported | 20.80856 | 1 | 278 | Low | 1 | 278 | 1 | 179 | 1 | 179 |  | iCluster:3 | >60 | NA | NA | iii |
| 40 | TCGA-CC-A8HS-01A | Tumor | 377.51.0 | 18 | male | not reported | NA | NA | NA | G1 | stage iiic | 62 | Not Reported | 20.95727 | 1 | 300 | Low | 1 | 300 | 1 | 201 | 1 | 201 |  | NA | <=60 | NA | NA | iii |
| 41 | TCGA-CC-A8HT-01A | Tumor | 377.51.0 | 74 | male | not reported | NA | NA | NA | G2 | stage iiia | 48 | Not Reported | 18.98659 | 1 | 140 | Low | 1 | 140 | 1 | 83 | 1 | 83 |  | NA | >60 | NA | NA | iii |
| 42 | TCGA-CC-A8HU-01A | Tumor | 377.51.0 | 39 | female | not reported | NA | NA | NA | G3 | stage iiia | 42 | Not Reported | 15.8079 | 1 | 344 | Low | 1 | 344 | 1 | 301 | 1 | 301 |  | NA | <=60 | NA | NA | iii |
| 43 | TCGA-CC-A8HV-01A | Tumor | 377.51.0 | 51 | female | not reported | NA | NA | NA | G2 | stage ii | 51 | Not Reported | 20.17325 | 1 | 279 | Low | 1 | 279 | 1 | 149 | 1 | 149 |  | NA | <=60 | NA | NA | ii |
| 44 | TCGA-CC-A9FS-01A | Tumor | 384.50.0 | 55 | male | not reported | NA | NA | NA | G2 | stage ii | 72 | Not Reported | 23.78121 | 0 | 211 | High | 0 | 211 | 0 | 211 | 1 | 109 |  | NA | <=60 | NA | NA | ii |
| 45 | TCGA-CC-A9FW-01A | Tumor | 384.50.0 | 68 | male | not reported | NA | NA | NA | G2 | stage iiia | 67 | Not Reported | 21.87755 | 0 | 248 | High | 0 | 248 | 1 | 153 | 1 | 153 |  | NA | >60 | NA | NA | iii |
| 46 | TCGA-DD-A113-01A | Tumor | 100.78.0 | 55 | female | not reported | Micro | 133 | 0 - No Fibrosis | G3 | stage ii | 56 | Not Reported | 23.61275 | 0 | 2425 | High | 0 | 2425 | 1 | 1676 | 1 | 1676 |  | iCluster:1 | <=60 | 0 | 20-400 | ii |
| 47 | TCGA-DD-A114-01A | Tumor | 100.78.0 | 42 | male | not reported | Micro | 21 | 5 - Nodular Formation and Incomplete Cirrhosis | G3 | stage ii | 74 | Not Reported | 24.72518 | 1 | 1149 | Low | 0 | 1149 | 0 | 1149 | 0 | 1149 |  | iCluster:1 | <=60 | 5 | 20-400 | ii |
| 48 | TCGA-DD-A115-01A | Tumor | 100.78.0 | 53 | male | not reported | None | 10 | 0 - No Fibrosis | G2 | stage iiia | 79 | Not Reported | 27.99036 | 1 | 2542 | High | 1 | 2542 | 1 | 469 | 1 | 469 |  | iCluster:3 | <=60 | 0 | <20 | iii |
| 49 | TCGA-DD-A116-01A | Tumor | 100.78.0 | 68 | male | not reported | NA | 24 | 3,4 - Fibrous Speta | G3 | stage iiia | 73 | Not Reported | 23.83673 | 1 | 1622 | Low | 0 | 1622 | 1 | 828 | 1 | 828 | Redacted | iCluster:1 | >60 | 3,4 | 20-400 | iii |
| 50 | TCGA-DD-A118-01A | Tumor | 100.78.0 | 77 | female | not reported | None | 85150 | 0 - No Fibrosis | G2 | stage ii | 75 | Not Reported | 26.25958 | 0 | 3437 | High | 0 | 3437 | 1 | 658 | 1 | 658 |  | iCluster:1 | >60 | 0 | >400 | ii |
| 51 | TCGA-DD-A119-01A | Tumor | 100.78.0 | 40 | male | not reported | Micro | 1836 | 0 - No Fibrosis | G3 | stage iv | 58 | Not Reported | 22.65625 | 1 | 223 | Low | NA | 223 | NA | NA | 0 | 223 |  | iCluster:1 | <=60 | 0 | >400 | i |
| 52 | TCGA-DD-A11A-01A | Tumor | 100.78.0 | 67 | male | not reported | None | 223 | 0 - No Fibrosis | G3 | stage i | 67 | Not Reported | 27.53123 | 1 | 79 | Low | 0 | 79 | 0 | 79 | 0 | 79 |  | iCluster:2 | >60 | 0 | 20-400 | i |
| 53 | TCGA-DD-A11B-01A | Tumor | 100.78.0 | 73 | male | not reported | None | 15 | 6 - Established Cirrhosis | G2 | stage i | 134 | Not Reported | 40.90229 | 1 | 14 | Low | 0 | 14 | NA | NA | 0 | 14 |  | iCluster:2 | >60 | 6 | <20 | i |
| 54 | TCGA-DD-A11C-01A | Tumor | 100.78.0 | 69 | male | not reported | None | 11 | 0 - No Fibrosis | G3 | stage i | 123 | Not Reported | 35.55324 | 0 | 662 | High | 0 | 662 | NA | NA | 0 | 662 |  | iCluster:3 | >60 | 0 | <20 | i |
| 55 | TCGA-DD-A1EA-01A | Tumor | 100.78.0 | 68 | male | not reported | Micro | 3 | 1,2 - Portal Fibrosis | G2 | stage ii | 94 | Not Reported | 33.30499 | 0 | 2415 | High | 0 | 2415 | 1 | 754 | 1 | 754 |  | iCluster:3 | >60 | 1,2 | <20 | ii |
| 56 | TCGA-DD-A1EB-01A | Tumor | 100.78.0 | 72 | female | not reported | None | 7 | 0 - No Fibrosis | G2 | stage i | 57 | Not Reported | 21.19274 | 0 | 2017 | High | 0 | 2017 | 1 | 505 | 1 | 505 |  | iCluster:2 | >60 | 0 | <20 | i |
| 57 | TCGA-DD-A1ED-01A | Tumor | 131.77.0 | 68 | male | not reported | None | 3 | 0 - No Fibrosis | G1 | stage i | 85 | Not Reported | 30.11621 | 0 | 2301 | High | 0 | 2301 | 0 | 2301 | 0 | 2301 |  | iCluster:1 | >60 | 0 | <20 | i |
| 58 | TCGA-DD-A1EE-01A | Tumor | 100.78.0 | 73 | male | not reported | None | 3 | 6 - Established Cirrhosis | G3 | stage iiia | 77 | Not Reported | 25.14286 | 1 | 349 | Low | 1 | 349 | 1 | 44 | 1 | 44 |  | iCluster:3 | >60 | 6 | <20 | iii |
| 59 | TCGA-DD-A1EF-01A | Tumor | 100.78.0 | 57 | female | not reported | None | 16211 | 3,4 - Fibrous Speta | G3 | stage i | 81 | Not Reported | 30.116 | 1 | 394 | Low | 1 | 394 | NA | NA | 1 | 394 |  | iCluster:1 | <=60 | 3,4 | >400 | i |
| 60 | TCGA-DD-A1EG-01A | Tumor | 203.73.0 | 76 | male | not reported | None | 16 | 1,2 - Portal Fibrosis | G3 | stage i | 82 | Not Reported | 29.05329 | 1 | 1372 | Low | 1 | 1372 | 1 | 875 | 1 | 875 |  | iCluster:3 | >60 | 1,2 | <20 | i |
| 61 | TCGA-DD-A1EH-01A | Tumor | 100.78.0 | 23 | male | not reported | Macro | 94340 | 3,4 - Fibrous Speta | G3 | stage iii | 70 | Not Reported | 21.84701 | 0 | 1495 | High | 0 | 1495 | 1 | 120 | 1 | 120 |  | iCluster:1 | <=60 | 3,4 | >400 | iii |
| 62 | TCGA-DD-A1EI-01A | Tumor | 100.78.0 | 46 | male | not reported | None | 24 | 6 - Established Cirrhosis | G2 | stage i | 69 | Not Reported | 22.53061 | 0 | 183 | High | 0 | 183 | 0 | 183 | 0 | 183 |  | iCluster:1 | <=60 | 6 | 20-400 | i |
| 63 | TCGA-DD-A1EJ-01A | Tumor | 131.77.0 | 71 | female | not reported | None | 18840 | 0 - No Fibrosis | G2 | stage iiic | 64 | Not Reported | 26.986 | 1 | 1005 | Low | 1 | 1005 | 1 | 263 | 1 | 263 |  | iCluster:3 | >60 | 0 | >400 | iii |
| 64 | TCGA-DD-A1EK-01A | Tumor | 203.73.0 | 64 | female | not reported | None | 19 | 0 - No Fibrosis | G2 | stage ivb | 97 | Not Reported | 38.36874 | 1 | 558 | Low | 1 | 558 | NA | NA | 1 | 167 |  | iCluster:3 | >60 | 0 | <20 | i |
| 65 | TCGA-DD-A1EL-01A | Tumor | 131.77.0 | 23 | male | not reported | None | 5 | 0 - No Fibrosis | G3 | stage ii | 54 | Not Reported | 16.30238 | 1 | 415 | Low | 1 | 415 | NA | NA | 1 | 415 |  | iCluster:3 | <=60 | 0 | <20 | ii |
| 66 | TCGA-DD-A39X-01A | Tumor | 203.73.0 | 78 | female | not reported | None | 10 | 0 - No Fibrosis | G2 | stage i | 48 | Not Reported | 20.77562 | 1 | 1694 | Low | 1 | 1694 | 1 | 1032 | 1 | 1032 |  | iCluster:1 | >60 | 0 | <20 | i |
| 67 | TCGA-DD-A39Y-01A | Tumor | 203.73.0 | 67 | male | not reported | None | 5600 | 0 - No Fibrosis | G3 | stage i | 85 | Not Reported | 27.4406 | 1 | 171 | Low | 0 | 171 | 0 | 171 | 0 | 171 |  | iCluster:3 | >60 | 0 | >400 | i |
| 68 | TCGA-DD-A3A6-01A | Tumor | 231.65.0 | 72 | female | not reported | Micro | NA | 0 - No Fibrosis | G2 | stage ii | 52 | Not Reported | 16.97959 | 1 | 3258 | High | 0 | 3258 | 0 | 3258 | 0 | 3258 |  | NA | >60 | 0 | NA | ii |
| 69 | TCGA-DD-A3A7-01A | Tumor | 231.65.0 | 67 | male | not reported | Micro | 120 | 0 - No Fibrosis | G3 | stage iiib | 80 | Not Reported | 22.63468 | 1 | 419 | Low | 1 | 419 | 1 | 144 | 1 | 144 |  | iCluster:2 | >60 | 0 | 20-400 | iii |
| 70 | TCGA-DD-A3A8-01A | Tumor | 231.65.0 | 75 | male | not reported | None | 4 | 0 - No Fibrosis | G2 | stage ii | 99 | Not Reported | 31.60011 | 1 | 11 | Low | 0 | 11 | 0 | 11 | 0 | 11 |  | iCluster:2 | >60 | 0 | <20 | ii |
| 71 | TCGA-DD-A4NA-01A | Tumor | 275.62.0 | 67 | female | not reported | NA | 3 | 5 - Nodular Formation and Incomplete Cirrhosis | G3 | stage iiic | 93 | Not Reported | 33.74946 | 0 | 1008 | High | 0 | 1008 | 0 | 1008 | 0 | 1008 |  | iCluster:1 | >60 | 5 | <20 | iii |
| 72 | TCGA-DD-A4NE-01A | Tumor | 287.61.0 | 75 | female | not reported | None | 28 | 0 - No Fibrosis | G3 | stage iiia | 79 | Not Reported | 27.33564 | 1 | 660 | Low | 1 | 660 | 1 | 190 | 1 | 190 |  | NA | >60 | 0 | 20-400 | iii |
| 73 | TCGA-DD-A4NF-01A | Tumor | 287.61.0 | 72 | male | not reported | None | 6 | 6 - Established Cirrhosis | G2 | stage i | 86 | Not Reported | 28.73467 | 0 | 942 | High | 0 | 942 | 1 | 656 | 1 | 656 |  | iCluster:2 | >60 | 6 | <20 | i |
| 74 | TCGA-DD-A4NG-01A | Tumor | 287.61.0 | 77 | male | not reported | Micro | 3 | NA | G2 | stage iiia | 94 | Not Reported | 34.94943 | 1 | 802 | Low | 1 | 802 | 1 | 430 | 1 | 430 |  | NA | >60 | NA | <20 | iii |
| 75 | TCGA-DD-A4NH-01A | Tumor | 287.61.0 | 65 | female | not reported | Macro | 92889 | 1,2 - Portal Fibrosis | G3 | stage iiib | 81 | Not Reported | 32.86137 | 0 | 917 | High | 0 | 917 | 1 | 161 | 1 | 161 |  | iCluster:1 | >60 | 1,2 | >400 | iii |
| 76 | TCGA-DD-A4NI-01A | Tumor | 287.61.0 | 67 | male | not reported | None | 6 | 0 - No Fibrosis | G2 | stage ii | 99 | Not Reported | 33.46403 | 0 | 816 | High | 0 | 816 | 0 | 816 | 0 | 816 |  | iCluster:2 | >60 | 0 | <20 | ii |
| 77 | TCGA-DD-A4NJ-01A | Tumor | 287.61.0 | 54 | female | not reported | None | 5 | 1,2 - Portal Fibrosis | G2 | stage ii | 88 | Not Reported | 32.71862 | 0 | 928 | High | 0 | 928 | 1 | 549 | 1 | 549 |  | NA | <=60 | 1,2 | <20 | ii |
| 78 | TCGA-DD-A4NK-01A | Tumor | 303.59.0 | 80 | female | not reported | None | 3 | 0 - No Fibrosis | G2 | stage iiia | 84 | Not Reported | NA | 1 | 1210 | Low | 1 | 1210 | 1 | 89 | 1 | 89 |  | NA | >60 | 0 | <20 | iii |
| 79 | TCGA-DD-A4NL-01A | Tumor | 303.59.0 | 46 | male | not reported | None | 2 | 0 - No Fibrosis | G1 | stage i | 113 | Not Reported | 37.75602 | 0 | 1711 | High | 0 | 1711 | 0 | 1711 | 0 | 1711 |  | iCluster:1 | <=60 | 0 | <20 | i |
| 80 | TCGA-DD-A4NO-01A | Tumor | 303.59.0 | 65 | male | not reported | None | 3 | 0 - No Fibrosis | G1 | stage i | 109 | Not Reported | 35.18853 | 0 | 2245 | High | 0 | 2245 | 1 | 990 | 1 | 990 |  | iCluster:2 | >60 | 0 | <20 | i |
| 81 | TCGA-DD-A4NP-01A | Tumor | 303.59.0 | 32 | male | not reported | None | 2 | 0 - No Fibrosis | G3 | stage i | 79 | Not Reported | 25.21625 | 0 | 3308 | High | 0 | 3308 | 1 | 1286 | 1 | 1286 |  | iCluster:2 | <=60 | 0 | <20 | i |
| 82 | TCGA-DD-A4NQ-01A | Tumor | 303.59.0 | 60 | male | not reported | Macro | 141 | NA | G3 | stage ii | 75 | Not Reported | 29.66655 | 1 | 373 | Low | 1 | 373 | 1 | 126 | 1 | 126 |  | iCluster:3 | <=60 | NA | 20-400 | ii |
| 83 | TCGA-DD-A4NR-01A | Tumor | 314.59.0 | 85 | female | not reported | None | 40250 | 6 - Established Cirrhosis | G3 | stage i | 54 | Not Reported | 131.8359 | 1 | 9 | Low | 0 | 9 | 0 | 9 | 0 | 9 |  | iCluster:1 | >60 | 6 | >400 | i |
| 84 | TCGA-DD-A4NS-01A | Tumor | 314.59.0 | 61 | female | not reported | None | 2 | 0 - No Fibrosis | G2 | stage i | 58 | Not Reported | 20.79673 | 1 | 2456 | High | 1 | 2456 | 1 | 893 | 1 | 893 |  | iCluster:1 | >60 | 0 | <20 | i |
| 85 | TCGA-DD-A4NV-01A | Tumor | 314.59.0 | 61 | male | not reported | None | 3 | 0 - No Fibrosis | G1 | stage iiia | 129 | Not Reported | 40.26092 | 0 | 2398 | High | 0 | 2398 | 0 | 2398 | 0 | 2398 |  | iCluster:2 | >60 | 0 | <20 | iii |
| 86 | TCGA-DD-A73A-01A | Tumor | 327.54.0 | 71 | male | not reported | None | 4 | 3,4 - Fibrous Speta | G2 | stage i | 102 | Not Reported | 33.69005 | 0 | 728 | High | 0 | 728 | 0 | 728 | 0 | 728 |  | iCluster:3 | >60 | 3,4 | <20 | i |
| 87 | TCGA-DD-A73B-01A | Tumor | 327.54.0 | 72 | female | not reported | None | 30 | 6 - Established Cirrhosis | G2 | stage i | 74 | Not Reported | 31.20256 | 1 | 283 | Low | 1 | 283 | 1 | 240 | 1 | 240 |  | iCluster:2 | >60 | 6 | 20-400 | i |
| 88 | TCGA-DD-A73C-01A | Tumor | 341.53.0 | 65 | female | not reported | None | 3 | 0 - No Fibrosis | G1 | stage iiia | 49 | Not Reported | 19.38214 | 0 | 701 | High | 0 | 701 | 1 | 566 | 1 | 566 |  | iCluster:2 | >60 | 0 | <20 | iii |
| 89 | TCGA-DD-A73D-01A | Tumor | 327.54.0 | 68 | female | not reported | Micro | 4 | 6 - Established Cirrhosis | G1 | stage ii | 102 | Not Reported | 35.29412 | 0 | 693 | High | 0 | 693 | 1 | 592 | 1 | 592 |  | iCluster:3 | >60 | 6 | <20 | ii |
| 90 | TCGA-DD-A73E-01A | Tumor | 327.54.0 | 66 | male | not reported | None | 1 | 0 - No Fibrosis | G1 | stage i | 108 | Not Reported | 36.50622 | 0 | 44 | High | 0 | 44 | 0 | 44 | 0 | 44 |  | iCluster:3 | >60 | 0 | <20 | i |
| 91 | TCGA-DD-A73G-01A | Tumor | 327.54.0 | 73 | female | not reported | None | 2035400 | 0 - No Fibrosis | G3 | stage i | 44 | Not Reported | 18.31426 | 0 | 3478 | High | 0 | 3478 | 0 | 3478 | 0 | 3478 |  | iCluster:1 | >60 | 0 | >400 | i |
| 92 | TCGA-DD-AA3A-01A | Tumor | 384.50.0 | 81 | female | not reported | None | 1 | 0 - No Fibrosis | G4 | stage i | 60 | Not Reported | 26.31464 | 1 | 410 | Low | 0 | 410 | 0 | 410 | 0 | 410 |  | NA | >60 | 0 | <20 | i |
| 93 | TCGA-ED-A459-01A | Tumor | 275.62.0 | 47 | male | not reported | Micro | 267 | NA | G2 | stage ii | 51 | Not Reported | 18.28678 | 0 | 910 | High | 0 | 910 | 0 | 910 | 0 | 910 |  | iCluster:2 | <=60 | NA | 20-400 | ii |
| 94 | TCGA-ED-A4XI-01A | Tumor | 275.62.0 | 58 | male | not reported | Micro | 573 | NA | G3 | stage ii | 55 | Not Reported | 19.03114 | 0 | 819 | High | 0 | 819 | 0 | 819 | 0 | 819 |  | iCluster:1 | <=60 | NA | >400 | ii |
| 95 | TCGA-ED-A5KG-01A | Tumor | 287.61.0 | 60 | female | not reported | Micro | 3177 | NA | G2 | stage ii | 61 | Not Reported | 23.82813 | 0 | 854 | High | 0 | 854 | 0 | 854 | 1 | 110 |  | iCluster:1 | <=60 | NA | >400 | ii |
| 96 | TCGA-ED-A627-01A | Tumor | 314.59.0 | 74 | male | not reported | NA | NA | NA | G2 | stage i | 74 | Not Reported | 23.88946 | 0 | 423 | High | 0 | 423 | 0 | 423 | 0 | 423 |  | iCluster:1 | >60 | NA | NA | i |
| 97 | TCGA-ED-A66X-01A | Tumor | 314.59.0 | 35 | male | not reported | Micro | 5840 | NA | G3 | stage iiia | 61 | Not Reported | 21.10727 | 0 | 406 | High | 0 | 406 | 1 | 124 | 1 | 124 |  | iCluster:1 | <=60 | NA | >400 | iii |
| 98 | TCGA-ED-A66Y-01A | Tumor | 314.59.0 | 51 | female | not reported | Micro | 12100 | NA | G3 | stage iiia | 59 | Not Reported | 23.04688 | 1 | 296 | Low | 0 | 296 | 0 | 296 | 0 | 296 |  | iCluster:1 | <=60 | NA | >400 | iii |
| 99 | TCGA-ED-A7PX-01A | Tumor | 365.54.0 | 48 | female | not reported | Micro | 24 | NA | G3 | stage ii | 43 | Not Reported | 16.58887 | 0 | 6 | High | 0 | 6 | 0 | 6 | 0 | 6 |  | iCluster:1 | <=60 | NA | 20-400 | ii |
| 100 | TCGA-ED-A7PY-01A | Tumor | 345.53.0 | 20 | female | not reported | Micro | 2 | NA | G3 | stage ii | 40 | Not Reported | 16.43655 | 0 | 390 | High | 0 | 390 | 0 | 390 | 0 | 390 |  | iCluster:1 | <=60 | NA | <20 | ii |
| 101 | TCGA-ED-A7PZ-01A | Tumor | 345.53.0 | 61 | male | not reported | Micro | 2 | NA | G2 | stage ii | 50 | Not Reported | 19.7777 | 0 | 6 | High | 0 | 6 | 0 | 6 | 0 | 6 |  | iCluster:3 | >60 | NA | <20 | ii |
| 102 | TCGA-ED-A7XO-01A | Tumor | 365.54.0 | 29 | male | not reported | Micro | 4 | NA | G2 | stage iiia | 64 | Not Reported | 20.89796 | 0 | 427 | High | 0 | 427 | 1 | 262 | 1 | 262 |  | NA | <=60 | NA | <20 | iii |
| 103 | TCGA-ED-A7XP-01A | Tumor | 365.54.0 | 53 | female | not reported | Micro | 18 | NA | G3 | stage ii | 54 | Not Reported | 21.90758 | 0 | 400 | High | 0 | 400 | 1 | 187 | 1 | 187 |  | iCluster:1 | <=60 | NA | <20 | ii |
| 104 | TCGA-ED-A82E-01A | Tumor | 365.54.0 | 60 | female | not reported | Micro | 4 | NA | G2 | stage iiia | 44 | Not Reported | 19.55556 | 0 | 408 | High | 0 | 408 | 0 | 408 | 0 | 408 |  | iCluster:1 | <=60 | NA | <20 | iii |
| 105 | TCGA-ED-A8O5-01A | Tumor | 377.51.0 | 59 | female | not reported | Micro | 498 | NA | G3 | stage iiia | 63 | Not Reported | 23.1405 | 0 | 406 | High | 0 | 406 | 0 | 406 | 0 | 406 |  | NA | <=60 | NA | >400 | iii |
| 106 | TCGA-ED-A8O6-01A | Tumor | 377.51.0 | 50 | female | not reported | Micro | 3000 | NA | G3 | stage iiia | 45 | Not Reported | 16.52893 | 1 | 56 | Low | 0 | 56 | 0 | 56 | 0 | 56 |  | NA | <=60 | NA | >400 | iii |
| 107 | TCGA-ED-A97K-01A | Tumor | 399.48.0 | 54 | male | not reported | Micro | 3 | NA | G2 | stage iiia | 64 | Not Reported | 22.14533 | 0 | 6 | High | 0 | 6 | 0 | 6 | 0 | 6 |  | NA | <=60 | NA | <20 | iii |
| 108 | TCGA-FV-A3I1-01A | Tumor | 231.65.0 | 81 | female | not reported | Micro | NA | 0 - No Fibrosis | G2 | stage ii | NA | Not Reported | NA | 1 | 247 | Low | 0 | 247 | 0 | 247 | 0 | 247 |  | iCluster:3 | >60 | 0 | NA | ii |
| 109 | TCGA-FV-A3R2-01A | Tumor | 231.65.0 | 75 | male | not reported | NA | NA | NA | NA | stage i | 75 | Not Reported | NA | 1 | 194 | Low | 0 | 194 | 0 | 194 | 0 | 194 |  | NA | >60 | NA | NA | i |
| 110 | TCGA-G3-A6UC-01A | Tumor | 341.53.0 | 65 | male | not reported | Micro | 4 | 6 - Established Cirrhosis | G2 | stage iiib | 91 | Not Reported | 27.47253 | 0 | 671 | High | 0 | 671 | 1 | 639 | 1 | 639 |  | iCluster:3 | >60 | 6 | <20 | iii |
| 111 | TCGA-G3-A7M5-01A | Tumor | 345.53.0 | 76 | male | not reported | None | 6 | 0 - No Fibrosis | G2 | stage i | 68 | Not Reported | 25.28257 | 0 | 447 | High | 0 | 447 | 0 | 447 | 0 | 447 |  | iCluster:3 | >60 | 0 | <20 | i |
| 112 | TCGA-G3-A7M7-01A | Tumor | 365.54.0 | 65 | male | not reported | None | 4 | 3,4 - Fibrous Speta | G1 | stage i | 77 | Not Reported | 23.50356 | 0 | 361 | High | 0 | 361 | 0 | 361 | 0 | 361 |  | iCluster:2 | >60 | 3,4 | <20 | i |
| 113 | TCGA-G3-A7M8-01A | Tumor | 345.53.0 | 31 | male | not reported | None | 5 | 6 - Established Cirrhosis | G1 | stage i | NA | Not Reported | NA | 0 | 430 | High | 0 | 430 | NA | NA | 0 | 430 |  | iCluster:2 | <=60 | 6 | <20 | i |
| 114 | TCGA-G3-A7M9-01A | Tumor | 365.54.0 | 70 | male | not reported | Macro | 22868 | 6 - Established Cirrhosis | G2 | stage iiib | 73 | Not Reported | 25.25952 | 1 | 56 | Low | NA | 56 | 0 | 56 | 0 | 56 |  | iCluster:1 | >60 | 6 | >400 | iii |
| 115 | TCGA-G3-AAUZ-01A | Tumor | 399.48.0 | 48 | male | not reported | None | 3 | 1,2 - Portal Fibrosis | G2 | stage i | 77 | Not Reported | 30.07813 | 0 | 480 | High | 0 | 480 | 0 | 480 | 0 | 480 |  | NA | <=60 | 1,2 | <20 | i |
| 116 | TCGA-G3-AAV0-01A | Tumor | 384.50.0 | 58 | male | not reported | None | 4 | 0 - No Fibrosis | G2 | stage i | 58 | Not Reported | 23.23346 | 0 | 476 | High | 0 | 476 | 0 | 476 | 0 | 476 |  | NA | <=60 | 0 | <20 | i |
| 117 | TCGA-G3-AAV1-01A | Tumor | 399.48.0 | 51 | male | not reported | Micro | 8 | 6 - Established Cirrhosis | G3 | stage iiic | 99 | Not Reported | 30.21886 | 1 | 359 | Low | 1 | 359 | 1 | 49 | 1 | 49 |  | NA | <=60 | 6 | <20 | iii |
| 118 | TCGA-G3-AAV2-01A | Tumor | 384.50.0 | 50 | male | not reported | None | 4 | 6 - Established Cirrhosis | G1 | stage i | NA | Not Reported | NA | 0 | 372 | High | 0 | 372 | 0 | 372 | 0 | 372 |  | NA | <=60 | 6 | <20 | i |
| 119 | TCGA-G3-AAV5-01A | Tumor | 384.50.0 | 67 | male | not reported | Macro | NA | 6 - Established Cirrhosis | G2 | stage ii | 88 | Not Reported | 32.32323 | 0 | 354 | High | 0 | 354 | 1 | 117 | 1 | 117 |  | NA | >60 | 6 | NA | ii |
| 120 | TCGA-G3-AAV6-01A | Tumor | 384.50.0 | 53 | female | not reported | Micro | 57875 | 0 - No Fibrosis | G3 | stage iiia | 78 | Not Reported | 28.65014 | 1 | 65 | Low | 1 | 65 | 1 | 44 | 1 | 44 |  | NA | <=60 | 0 | >400 | iii |
| 121 | TCGA-G3-AAV7-01A | Tumor | 399.48.0 | 38 | male | not reported | Micro | 3 | 5 - Nodular Formation and Incomplete Cirrhosis | G2 | stage ii | 58 | Not Reported | 21.30395 | 0 | 361 | High | 0 | 361 | 0 | 361 | 0 | 361 |  | NA | <=60 | 5 | <20 | ii |
| 122 | TCGA-HP-A5MZ-01A | Tumor | 287.61.0 | 78 | male | not reported | None | NA | NA | G2 | stage i | 82 | Not Reported | 23.95909 | 1 | 91 | Low | 0 | 91 | 0 | 91 | 0 | 91 |  | iCluster:1 | >60 | NA | NA | i |
| 123 | TCGA-MR-A520-01A | Tumor | 275.62.0 | 58 | male | not reported | NA | NA | NA | G1 | stage i | 104 | Not Reported | 32.45841 | 0 | 229 | High | 0 | 229 | NA | NA | 0 | 229 |  | iCluster:2 | <=60 | NA | NA | i |
| 124 | TCGA-MR-A8JO-01A | Tumor | 377.51.0 | 34 | male | not reported | None | 2 | NA | G3 | stage i | 90 | Not Reported | 23.42774 | 0 | 330 | High | 0 | 330 | NA | NA | 0 | 330 |  | NA | <=60 | NA | <20 | i |
| 125 | TCGA-UB-A7MA-01A | Tumor | 345.53.0 | 62 | female | not reported | Micro | 2008 | 0 - No Fibrosis | G2 | stage ii | 85 | Not Reported | 31.2213 | 0 | 848 | High | 0 | 848 | NA | NA | 1 | 221 |  | NA | >60 | 0 | >400 | ii |
| 126 | TCGA-UB-A7MB-01A | Tumor | 345.53.0 | 24 | male | not reported | Micro | 1865 | 0 - No Fibrosis | G3 | stage ii | 62 | Not Reported | 19.1358 | 0 | 601 | High | 0 | 601 | 1 | 48 | 1 | 48 |  | iCluster:2 | <=60 | 0 | >400 | ii |
| 127 | TCGA-UB-A7MC-01A | Tumor | 345.53.0 | 59 | male | not reported | Micro | 126 | 1,2 - Portal Fibrosis | G3 | stage iiia | 78 | Not Reported | 25.18079 | 0 | 500 | High | 0 | 500 | 1 | 359 | 1 | 359 |  | iCluster:3 | <=60 | 1,2 | 20-400 | iii |
| 128 | TCGA-UB-A7MD-01A | Tumor | 365.54.0 | 67 | male | not reported | Micro | 23 | 6 - Established Cirrhosis | G3 | stage i | 57 | Not Reported | 18.61224 | 1 | 52 | Low | 0 | 52 | 0 | 52 | 0 | 52 |  | iCluster:3 | >60 | 6 | 20-400 | i |
| 129 | TCGA-UB-A7ME-01A | Tumor | 341.53.0 | 51 | male | not reported | None | 1388 | 1,2 - Portal Fibrosis | G2 | stage i | 75 | Not Reported | 27.54821 | 0 | 486 | High | 0 | 486 | 0 | 486 | 0 | 486 |  | iCluster:1 | <=60 | 1,2 | >400 | i |
| 130 | TCGA-UB-A7MF-01A | Tumor | 341.53.0 | 56 | male | not reported | Micro | 2505 | 6 - Established Cirrhosis | G2 | stage iiia | 95 | Not Reported | 24.98356 | 1 | 214 | Low | 1 | 214 | 1 | 133 | 1 | 133 |  | iCluster:1 | <=60 | 6 | >400 | iii |
| 131 | TCGA-UB-AA0U-01A | Tumor | 399.48.0 | 60 | male | not reported | None | 26 | 0 - No Fibrosis | G2 | stage ii | 93 | Not Reported | 29.35235 | 0 | 327 | High | 0 | 327 | NA | NA | 1 | 106 |  | NA | <=60 | 0 | 20-400 | ii |
| 132 | TCGA-UB-AA0V-01A | Tumor | 399.48.0 | 69 | female | not reported | None | 3 | 0 - No Fibrosis | G1 | stage i | 57 | Not Reported | 18.61224 | 0 | 314 | High | 0 | 314 | 0 | 314 | 0 | 314 |  | NA | >60 | 0 | <20 | i |
| 133 | TCGA-WJ-A86L-01A | Tumor | 405.45.0 | 68 | female | not reported | None | 16 | 0 - No Fibrosis | G2 | stage i | 56 | Not Reported | 22.15102 | 0 | 345 | High | 0 | 345 | 0 | 345 | 0 | 345 |  | NA | >60 | 0 | <20 | i |
| 134 | TCGA-ZP-A9CZ-01A | Tumor | 399.48.0 | 72 | male | not reported | None | 8 | 3,4 - Fibrous Speta | G1 | not reported | 73 | Not Reported | 26.17519 | 0 | 706 | High | 0 | 706 | 0 | 706 | 0 | 706 |  | NA | >60 | 3,4 | <20 | NA |
| 135 | TCGA-ZS-A9CD-01A | Tumor | 384.50.0 | 73 | male | not reported | None | NA | 5 - Nodular Formation and Incomplete Cirrhosis | G2 | stage ii | NA | Not Reported | NA | 1 | 1386 | Low | NA | 1386 | 1 | 371 | 1 | 371 |  | NA | >60 | 5 | NA | ii |
| 136 | TCGA-ZS-A9CE-01A | Tumor | 384.50.0 | 79 | female | not reported | Micro | NA | 0 - No Fibrosis | G1 | stage ii | NA | Not Reported | NA | 0 | 1241 | High | 0 | 1241 | 0 | 1241 | 1 | 892 |  | NA | >60 | 0 | NA | ii |
| 137 | TCGA-ZS-A9CF-01A | Tumor | 399.48.0 | 64 | male | not reported | Micro | NA | 0 - No Fibrosis | G2 | stage ii | NA | Not Reported | NA | 0 | 2412 | High | 0 | 2412 | 1 | 636 | 1 | 636 |  | NA | >60 | 0 | NA | ii |
| 138 | TCGA-ZS-A9CF-02A | Tumor | 399.48.0 | 64 | male | not reported | Micro | NA | 0 - No Fibrosis | G2 | stage ii | NA | Not Reported | NA | 0 | 2412 | High | 0 | 2412 | 1 | 636 | 1 | 636 |  | NA | >60 | 0 | NA | ii |
| 139 | TCGA-ZS-A9CG-01A | Tumor | 384.50.0 | 55 | male | not reported | Micro | NA | 0 - No Fibrosis | G2 | stage ii | 94 | Not Reported | 29.33741 | 0 | 341 | High | 0 | 341 | 0 | 341 | 0 | 341 |  | NA | <=60 | 0 | NA | ii |
