# Supplementary material for: Life span‐associated ferroptosis‐related genes identification and validation for hepatocellular carcinoma patients as hepatitis B virus carriers
Source: J Clin Lab Anal. 2023 Jul 18;37(13-14):e24930. doi: 10.1002/jcla.24930 (PMC10492458; doi:10.1002/jcla.24930)
Supplement: Supplementary file 10 — Tables S1–S14 [file JCLA-37-e24930-s009.zip › TableS10_KEGG.docx]

TableS10_KEGG

| ID | Description | BgRatio | pvalue | p.adjust | qvalue | geneID | Count |
| --- | --- | --- | --- | --- | --- | --- | --- |
| hsa00100 | Steroid biosynthesis | 19/5894 | 0.01602 | 0.108304 | 0.100592 | 6713 | 1 |
| hsa00480 | Glutathione metabolism | 50/5894 | 0.041716 | 0.108304 | 0.100592 | 6241 | 1 |
| hsa05120 | Epithelial cell signaling in Helicobacter pylori infection | 68/5894 | 0.056389 | 0.108304 | 0.100592 | 6714 | 1 |
| hsa04115 | p53 signaling pathway | 69/5894 | 0.057199 | 0.108304 | 0.100592 | 6241 | 1 |
| hsa05100 | Bacterial invasion of epithelial cells | 71/5894 | 0.058817 | 0.108304 | 0.100592 | 6714 | 1 |
| hsa04520 | Adherens junction | 73/5894 | 0.060432 | 0.108304 | 0.100592 | 6714 | 1 |
| hsa04370 | VEGF signaling pathway | 76/5894 | 0.062852 | 0.108304 | 0.100592 | 6714 | 1 |
| hsa04974 | Protein digestion and absorption | 81/5894 | 0.066873 | 0.108304 | 0.100592 | 6510 | 1 |
| hsa04012 | ErbB signaling pathway | 87/5894 | 0.071681 | 0.108304 | 0.100592 | 6714 | 1 |
| hsa04540 | Gap junction | 90/5894 | 0.074077 | 0.108304 | 0.100592 | 6714 | 1 |
| hsa03010 | Ribosome | 92/5894 | 0.075672 | 0.108304 | 0.100592 | 6132 | 1 |
| hsa00240 | Pyrimidine metabolism | 99/5894 | 0.081236 | 0.108304 | 0.100592 | 6241 | 1 |
| hsa04912 | GnRH signaling pathway | 101/5894 | 0.082821 | 0.108304 | 0.100592 | 6714 | 1 |
| hsa04530 | Tight junction | 133/5894 | 0.107883 | 0.131001 | 0.121673 | 6714 | 1 |
| hsa00230 | Purine metabolism | 162/5894 | 0.13012 | 0.147469 | 0.136968 | 6241 | 1 |
| hsa04510 | Focal adhesion | 200/5894 | 0.158584 | 0.160799 | 0.149349 | 6714 | 1 |
| hsa04144 | Endocytosis | 203/5894 | 0.160799 | 0.160799 | 0.149349 | 6714 | 1 |
