# Supplementary material for: Life span‐associated ferroptosis‐related genes identification and validation for hepatocellular carcinoma patients as hepatitis B virus carriers
Source: J Clin Lab Anal. 2023 Jul 18;37(13-14):e24930. doi: 10.1002/jcla.24930 (PMC10492458; doi:10.1002/jcla.24930)
Supplement: Supplementary file 10 — Tables S1–S14 [file JCLA-37-e24930-s009.zip › TableS11_GSEA.pdf]

ID	Description	setSize	enrichmentScore	NES	pvalue	p.adjust	qvalues	rank	leading_edge	core_enrichment
ORGANIC ACID CATABOLIC PROCESS	ORGANIC ACID CATABOLIC PROCESS	248	-0.465359	-2.17	1.00E-10	4.94E-07	4.62E-07	7255	tags=56%, list=26%, signal=42%	TST/CPT1C/ETFB/PCCB/PECR/FAH/HACL1/DLD/AUH/PLA2G15/MECR/FAAH/ALDH3A2/ACAA1/HMGCL/ACADM/MCCC1/ECI2/BCKDK/ACAA2/AKT2/TDO2/ACSF3/PCK2/QDPR/HADH/HSD17B4/ECHS1/AMDHD1/GLDC/ECI1/BDH2/PHYKPL/IRS2/ACADVL/PON3/ADHFE1/HIBADH/ARG1/ABCD2/NOS3/SDS/LONP2/ACAD8/ACADSB/MCCC2/LPIN1/SHMT1/CYP4F11/CPT2/HAO1/HGD/ALDH8A1/ACACB/ALDH1L2/MTHFS/GOT1/PHYH/ECH1/DBT/ILVBL/CEL/SARDH/SULT2A1/AFMID/PAH/MCEE/ACAD11/OGDH/DAO/ACADS/UROC1/HAAO/SORD/HPD/NUDT7/PLIN5/GLUL/BCKDHB/IVD/BCKDHA/LPIN2/ACMSD/HAL/MLYCD/CYP26A1/CRAT/ABHD2/CYP4F2/OTC/SCP2/CDO1/MAT1A/HOGA1/MMAA/CSAD/ECHDC2/HMGCLL1/ALDH4A1/ALDH7A1/CYP4F12/BCAT2/PON1/ABCB11/ACADL/ACOX1/EHHADH/GPT2/ATP2B4/ALDH6A1/KMO/GSTZ1/FTCD/ETFDH/GCDH/LDHD/ACAT1/HAO2/AGXT/GOT2/GPT/DCXR/PRODH/NOS2/ABAT/AASS/OAT/AADAT/CTH/GADL1/AGXT2/AKR1D1/CYP4F3/PCK1/GLS2/TAT/MFSD2A/HDC/PPM1K/ASPA

CHROMOSOME SEGREGATION	CHROMOSOME SEGREGATION	299	0.5117489	1.883	4.07E-09	1.00E-05	9.39E-06	5023	tags=32%, list=18%, signal=27%	TEX15/FMN2/SYCE1/MEI4/TRIP13/UBE2C/KIF2C/AURKB/CDC20/CDCA2/BUB1B/HJURP/TTK/KIF18B/DLGAP5/KIFC1/NCAPH/SMC1B/SPC25/NCAPG/ESCO2/CENPW/SKA3/MAEL/DSCC1/BANF2/KIF23/KIF4A/SKA1/NUF2/PSRC1/PLK1/KIF18A/NDC80/PTTG1/CDC6/BIRC5/BUB1/CDCA8/CCNB1/PRDM9/TOP2A/FBXO5/OIP5/CDT1/RACGAP1/FANCD2/TACC3/PRC1/NEK2/ECT2/EME1/RRS1/KNTC1/NSMCE2/CENPF/NCAPD2/CCNE1/CCNE2/MAPK15/MKI67/ZWINT/NCAPG2/ESPL1/NUSAP1/UBE2DNL/P3H4/CENPK/RCC2/RAN/BRCA1/FEN1/USP44/MSH4/CHMP4C/NUP37/CENPE/XRCC3/MAD2L2/RAD21/NCAPH2/TUBG1/FAM83D/CENPQ/SPDL1/CENPH/KNSTRN/CEP85/CDCA5/ANAPC7/SRPK1/RCC1/NEK11/INCENP/PCID2/RMI2

ORGANELLE FISSION	ORGANELLE FISSION	432	0.4565745	1.717	2.80E-08	4.61E-05	4.31E-05	5980	tags=34%, list=22%, signal=27%	TEX15/BRDT/FMN2/EREG/SYCE1/MYBL2/MEI4/CYP26B1/PSMA8/MISP/TEX19/TRIP13/UBE2C/SPHK1/KIF2C/AURKB/CDC20/DNM1/BUB1B/TTK/KIF18B/DLGAP5/KIFC1/NCAPH/SMC1B/MTFR2/NCAPG/RAD51/MAEL/RAD54L/DSCC1/KIF23/KIF4A/TPX2/NUF2/EDN3/HSF2BP/PSRC1/PLK1/FBXO43/KIF18A/CDK1/NDC80/PTTG1/CDC6/PSMC3IP/RAD51AP1/ANLN/CKS2/BUB1/CDCA8/CCNB1/PRDM9/TOP2A/FBXO5/CDT1/C11orf80/MTBP/PKMYT1/RACGAP1/CDC25C/GDAP1/CCNB2/FANCD2/TACC3/PRC1/BMP4/NEK2/GGNBP1/EME1/TUBB8/RRS1/KNTC1/NSMCE2/CENPF/NCAPD2/LIF/CCNE1/CHEK1/STRA8/TGFA/CCNE2/MAPK15/MKI67/ZWINT/NCAPG2/ESPL1/NUSAP1/UBE2DNL/P3H4/CENPK/RAN/MX2/HSPA1A/L3MBTL1/TDRD9/KIF11/USP44/WNT5A/MSH4/CHMP4C/CENPE/XRCC3/MAD2L2/RAD21/NCAPH2/HSPA1B/KIFC2/REEP4/CCDC8/TUBG1/CHEK2/IL1A/UBE2S/MZT1/SPDL1/CDC25B/RAD54B/BORA/VRK1/KNSTRN/CEP85/CDCA5/ANAPC7/RCC1/INCENP/CORO1C/PCID2/IGF2/NUP62/RANBP1/SPDYA/NDC1/SMC4/AURKA/HFM1/DDX4/PINX1/NME6/BTC/SPAG5/MND1/MAD2L1/MYO19/ANKRD53/TDRKH/DYNC1LI1

NUCLEAR CHROMOSOME SEGREGATION	NUCLEAR CHROMOSOME SEGREGATION	239	0.5231332	1.904	8.15E-08	9.04E-05	8.45E-05	4256	tags=31%, list=15%, signal=26%	TEX15/FMN2/SYCE1/MEI4/TRIP13/UBE2C/KIF2C/AURKB/CDC20/BUB1B/TTK/KIF18B/DLGAP5/KIFC1/NCAPH/SMC1B/NCAPG/ESCO2/MAEL/DSCC1/KIF23/KIF4A/NUF2/PSRC1/PLK1/KIF18A/NDC80/PTTG1/CDC6/BUB1/CDCA8/CCNB1/PRDM9/TOP2A/FBXO5/CDT1/RACGAP1/FANCD2/TACC3/PRC1/NEK2/ECT2/EME1/RRS1/KNTC1/NSMCE2/CENPF/NCAPD2/CCNE1/CCNE2/MAPK15/ZWINT/NCAPG2/ESPL1/NUSAP1/UBE2DNL/P3H4/CENPK/RCC2/RAN/FEN1/USP44/MSH4/CHMP4C/CENPE/XRCC3/MAD2L2/RAD21/NCAPH2/TUBG1/FAM83D/CENPQ/SPDL1

MONOCARBOXYLIC ACID CATABOLICPROCESS	MONOCARBOXYLIC ACID CATABOLICPROCESS	128	-0.479174	-2.05	9.16E-08	9.04E-05	8.45E-05	7910	tags=60%, list=29%, signal=43%	MTOR/CYP26C1/PPARA/IDNK/CPT1A/HADHB/CPT1C/ETFB/PCCB/PECR/FAH/HACL1/AUH/PLA2G15/MECR/FAAH/ALDH3A2/ACAA1/ACADM/ECI2/ACAA2/AKT2/PCK2/HADH/HSD17B4/ECHS1/ECI1/BDH2/IRS2/ACADVL/PON3/ABCD2/LONP2/LPIN1/CYP4F11/CPT2/HAO1/ACACB/PHYH/ECH1/ILVBL/CEL/SULT2A1/MCEE/ACAD11/ACADS/SORD/NUDT7/PLIN5/IVD/LPIN2/MLYCD/CYP26A1/CRAT/ABHD2/CYP4F2/SCP2/HOGA1/MMAA/ECHDC2/CYP4F12/ABCB11/ACADL/ACOX1/EHHADH/ETFDH/GCDH/LDHD/ACAT1/HAO2/AGXT/DCXR/AGXT2/AKR1D1/CYP4F3/PCK1/MFSD2A

MEIOTIC CELL CYCLE	MEIOTIC CELL CYCLE	209	0.5238168	1.883	1.47E-07	0.000119	0.00011	3718	tags=29%, list=13%, signal=25%	TEX15/BRDT/FMN2/EREG/SYCE1/DUSP13/MEI4/CYP26B1/PSMA8/FAM9A/TEX19/TRIP13/FKBP6/CDC20/BUB1B/TTK/SMC1B/RSPH1/RAD51/MAEL/RAD54L/XRCC2/NUF2/EXD1/CDC25A/HSF2BP/PLK1/FBXO43/KIF18A/PTTG1/PSMC3IP/RAD51AP1/CKS2/BUB1/PRDM9/TOP2A/FBXO5/PIWIL4/EXO1/C11orf80/PKMYT1/CDC25C/CCNB2/FANCD2/NEK2/EME1/TUBB8/NCAPD2/LIF/CCNE1/STRA8/CCNE2/MAPK15/HUS1B/ESPL1/P3H4/TDRD9/WNT5A/MSH4/RAD21/NCAPH2

CELLULAR AMINO ACID CATABOLICPROCESS	CELLULAR AMINO ACID CATABOLICPROCESS	99	-0.51454	-2.12	1.73E-07	0.000119	0.00011	5387	tags=56%, list=19%, signal=45%	AMDHD1/GLDC/PHYKPL/ADHFE1/HIBADH/ARG1/NOS3/SDS/ACAD8/MCCC2/SHMT1/HGD/ALDH8A1/GOT1/SARDH/AFMID/PAH/OGDH/DAO/UROC1/HAAO/HPD/GLUL/IVD/ACMSD/HAL/OTC/CDO1/MAT1A/HOGA1/CSAD/HMGCLL1/ALDH4A1/ALDH7A1/GPT2/ATP2B4/ALDH6A1/KMO/GSTZ1/FTCD/GCDH/AGXT/GOT2/GPT/PRODH/NOS2/ABAT/AASS/OAT/AADAT/AGXT2/GLS2/TAT/HDC/ASPA

FATTY ACID CATABOLIC PROCESS	FATTY ACID CATABOLIC PROCESS	103	-0.505864	-2.11	1.94E-07	0.000119	0.00011	7494	tags=56%, list=27%, signal=41%	CPT1A/HADHB/CPT1C/ETFB/PCCB/PECR/HACL1/AUH/PLA2G15/MECR/FAAH/ALDH3A2/ACAA1/ACADM/ECI2/ACAA2/AKT2/PCK2/HADH/HSD17B4/ECHS1/ECI1/BDH2/IRS2/ACADVL/ABCD2/LONP2/LPIN1/CPT2/HAO1/ACACB/PHYH/ECH1/ILVBL/CEL/MCEE/ACAD11/ACADS/NUDT7/PLIN5/IVD/LPIN2/MLYCD/CRAT/ABHD2/SCP2/MMAA/ECHDC2/ABCB11/ACADL/ACOX1/EHHADH/ETFDH/GCDH/ACAT1/HAO2/PCK1/MFSD2A

MITOTIC NUCLEAR DIVISION	MITOTIC NUCLEAR DIVISION	273	0.4943154	1.815	2.37E-07	0.000129	0.00012	5980	tags=37%, list=22%, signal=30%	EREG/MYBL2/MISP/TRIP13/UBE2C/SPHK1/KIF2C/AURKB/CDC20/BUB1B/TTK/KIF18B/DLGAP5/KIFC1/NCAPH/SMC1B/NCAPG/DSCC1/KIF23/KIF4A/TPX2/NUF2/EDN3/PSRC1/PLK1/FBXO43/KIF18A/CDK1/NDC80/PTTG1/CDC6/ANLN/BUB1/CDCA8/CCNB1/FBXO5/CDT1/MTBP/PKMYT1/RACGAP1/CDC25C/CCNB2/TACC3/PRC1/BMP4/NEK2/RRS1/KNTC1/NSMCE2/CENPF/NCAPD2/CHEK1/TGFA/MKI67/ZWINT/NCAPG2/ESPL1/NUSAP1/UBE2DNL/CENPK/RAN/HSPA1A/L3MBTL1/KIF11/USP44/CHMP4C/CENPE/XRCC3/MAD2L2/RAD21/NCAPH2/HSPA1B/KIFC2/REEP4/CCDC8/TUBG1/CHEK2/IL1A/UBE2S/MZT1/SPDL1/BORA/VRK1/KNSTRN/CEP85/CDCA5/ANAPC7/RCC1/INCENP/PCID2/IGF2/NUP62/RANBP1/SMC4/AURKA/PINX1/NME6/BTC/SPAG5/MAD2L1/ANKRD53/DYNC1LI1

SMALL MOLECULE CATABOLIC PROCESS	SMALL MOLECULE CATABOLIC PROCESS	407	-0.328665	-1.59	2.61E-07	0.000129	0.00012	6308	tags=39%, list=23%, signal=31%	MCCC1/ECI2/BCKDK/ACAA2/AKT2/CDADC1/TDO2/ACSF3/LRP5/PCK2/QDPR/SLC25A12/ADAL/UPP2/APOBEC3H/PTEN/HADH/PGM1/NUDT16/HSD17B4/GLYCTK/ECHS1/SRD5A3/AMDHD1/GLDC/ECI1/BDH2/PHYKPL/IRS2/ACADVL/PON3/ADHFE1/HIBADH/ARG1/ABCD2/NOS3/SDS/HAGH/LONP2/MGAT1/ACAD8/BDH1/ENTPD7/ACADSB/MCCC2/LPIN1/SHMT1/CYP4F11/CPT2/HAO1/HGD/ALDH8A1/ACACB/ALDH1L2/MTHFS/GOT1/PHYH/ECH1/DBT/ILVBL/CEL/SARDH/SULT2A1/AFMID/PAH/MCEE/PDXP/ACAD11/OGDH/DAO/ACADS/INPP5K/UROC1/HAAO/SORD/HPD/NUDT7/PLIN5/GLUL/BCKDHB/IVD/BCKDHA/GPI/LPIN2/ACMSD/HAL/MLYCD/CYP26A1/GK/CRAT/ABHD2/CYP4F2/OTC/SCP2/CDO1/ENTPD8/UPB1/MAT1A/HOGA1/MMAA/CSAD/APOE/ECHDC2/HMGCLL1/ALDH4A1/ALDH7A1/CYP4F12/BCAT2/PON1/ABCB11/ACADL/ACOX1/EHHADH/GPT2/ATP2B4/ALDH6A1/KMO/CYP39A1/HSD17B6/GSTZ1/FTCD/CYP7A1/ALDH2/ETFDH/GCDH/LDHD/ACAT1/HAO2/XDH/AGXT/GOT2/GPT/DCXR/PRODH/NOS2/CYP1A1/ABAT/AASS/FGF23/OAT/AADAT/CYP46A1/CTH/GCK/GADL1/AGXT2/AKR1D1/CYP4F3/PCK1/ALDOB/GLS2/TAT/

MITOTIC SISTER CHROMATIDSEGREGATION	MITOTIC SISTER CHROMATIDSEGREGATION	153	0.5686126	1.993	5.46E-07	0.000243	0.00023	5980	tags=42%, list=22%, signal=33%	TRIP13/UBE2C/KIF2C/AURKB/CDC20/BUB1B/TTK/KIF18B/DLGAP5/KIFC1/NCAPH/SMC1B/NCAPG/DSCC1/KIF23/KIF4A/NUF2/PSRC1/PLK1/KIF18A/NDC80/PTTG1/CDC6/BUB1/CDCA8/CCNB1/FBXO5/CDT1/RACGAP1/TACC3/PRC1/NEK2/RRS1/KNTC1/NSMCE2/CENPF/NCAPD2/ZWINT/NCAPG2/ESPL1/NUSAP1/UBE2DNL/CENPK/RAN/USP44/CHMP4C/CENPE/XRCC3/MAD2L2/RAD21/NCAPH2/TUBG1/SPDL1/KNSTRN/CDCA5/ANAPC7/INCENP/PCID2/NUP62/SMC4/PINX1/SPAG5/MAD2L1/ANKRD53/DYNC1LI1

ENERGY DERIVATION BY OXIDATIONOF ORGANIC COMPOUNDS	ENERGY DERIVATION BY OXIDATIONOF ORGANIC COMPOUNDS	256	-0.365815	-1.72	5.92E-07	0.000243	0.00023	7723	tags=43%, list=28%, signal=32%	UGP2/NDUFS1/CAT/NDUFA5/NDUFV2/NDUFA2/IDH3A/PDHA1/NDUFC1/COX15/NDUFA3/ETFB/PID1/NDUFS7/IREB2/UQCRC2/DLD/COQ10A/PHKA1/PIK3CA/COX8A/SURF1/NDUFB8/NDUFV1/SIRT3/NDUFA7/COX7B/AKT2/COX6B1/MT-ND4/MT-ND1/SUCLG2/NDUFA9/SLC25A23/COX5A/SLC25A12/NDUFB7/OGDHL/SDHD/PHKB/IDE/IL6ST/PGM1/SDHA/NDUFA4/NDUFAF1/EPM2AIP1/LYRM7/NDUFA8/FH/IRS2/ACADVL/SORBS1/NDUFV3/MT-CYB/PYGM/ACO2/BLOC1S1/NDUFS8/PHKG1/MT-CO3/LEPR/COX5B/UQCR11/NDUFB10/MT-CO2/MT-ND4L/PCDH12/GFPT2/IMMP2L/MT-ND5/PPP1R3B/MT-CO1/ACO1/OGDH/INSR/COQ9/INPP5K/PER2/MT-ND6/GPD1/KL/ENPP1/PINK1/COX4I1/UQCRFS1/NDUFB1/AGL/NNT/IDH2/CBFA2T3/PRKAG2/ETFDH/COX4I2/SLC25A25/POMC/NOS2/PPP1R1A/NDUFA13/ESRRB/GYG2/GCGR/GYS2/GCK/IGF1/CYP1A2/GNMT/PPARGC1A/UQCRFS1P1/G6PC/NR4A3

SISTER CHROMATID SEGREGATION	SISTER CHROMATID SEGREGATION	183	0.5238071	1.867	1.26E-06	0.000446	0.00042	5980	tags=38%, list=22%, signal=30%	TRIP13/UBE2C/KIF2C/AURKB/CDC20/BUB1B/TTK/KIF18B/DLGAP5/KIFC1/NCAPH/SMC1B/NCAPG/ESCO2/DSCC1/KIF23/KIF4A/NUF2/PSRC1/PLK1/KIF18A/NDC80/PTTG1/CDC6/BUB1/CDCA8/CCNB1/TOP2A/FBXO5/CDT1/RACGAP1/TACC3/PRC1/NEK2/RRS1/KNTC1/NSMCE2/CENPF/NCAPD2/MAPK15/ZWINT/NCAPG2/ESPL1/NUSAP1/UBE2DNL/CENPK/RAN/FEN1/USP44/CHMP4C/CENPE/XRCC3/MAD2L2/RAD21/NCAPH2/TUBG1/SPDL1/KNSTRN/CDCA5/ANAPC7/INCENP/PCID2/RMI2/NUP62/SMC4/PINX1/SPAG5/MAD2L1/ANKRD53/DYNC1LI1

FIBRINOLYSIS	FIBRINOLYSIS	25	-0.73752	-2.3	1.28E-06	0.000446	0.00042	3819	tags=64%, list=14%, signal=55%	APOH/SERPINF2/PLG/THBD/GP1BA/SERPING1/PLAT/THBS1/F12/FGG/HRG/FGB/FGA/KLKB1/F11/KRT1

EAR MORPHOGENESIS	EAR MORPHOGENESIS	98	0.6147325	2.061	1.36E-06	0.000446	0.00042	5021	tags=46%, list=18%, signal=38%	DLX6/COL2A1/MYO3A/WNT3A/USH1C/DLX5/GJB6/HMX3/POU3F4/NOG/MYO3B/USH1G/HMX2/EYA1/OSR2/COL11A1/CTHRC1/PTK7/ROR2/LHFPL5/TMIE/TPRN/SOX9/PAX8/GBX2/SIX4/FZD2/HOXA2/SIX1/HOXA1/TBX18/KCNQ4/WNT5A/SLC44A4/ATP8A2/TFAP2A/FZD6/OTX1/TWIST1/LRIG3/CHD7/PDZD7/OSR1/SIX2/DVL2

MEIOTIC CELL CYCLE PROCESS	MEIOTIC CELL CYCLE PROCESS	156	0.5490596	1.931	1.62E-06	0.000499	0.00047	4365	tags=35%, list=16%, signal=29%	TEX15/BRDT/FMN2/EREG/SYCE1/MEI4/CYP26B1/PSMA8/TEX19/TRIP13/CDC20/BUB1B/TTK/RAD51/MAEL/RAD54L/NUF2/CDC25A/HSF2BP/PLK1/FBXO43/KIF18A/PTTG1/PSMC3IP/RAD51AP1/CKS2/BUB1/PRDM9/TOP2A/FBXO5/C11orf80/CDC25C/CCNB2/FANCD2/EME1/TUBB8/NCAPD2/LIF/CCNE1/STRA8/CCNE2/MAPK15/HUS1B/ESPL1/P3H4/TDRD9/WNT5A/MSH4/RAD21/NCAPH2/TUBG1/OVOL1/CDC25B/RAD54B

EPIDERMIS DEVELOPMENT	EPIDERMIS DEVELOPMENT	317	0.4581912	1.692	1.91E-06	0.000555	0.00052	4783	tags=31%, list=17%, signal=26%	ESRP1/PITX2/S100A7/RBP2/KLK13/KRT20/KRTAP4-1/DSG3/GAL/KRT4/EREG/KRT79/CASP14/FOXN1/SFN/CALML5/USH1C/OVOL2/PKP3/KRT13/HOXB13/KLK7/CYP26B1/DSC3/KRT34/ABCA12/LIPM/EVPL/PTHLH/KRT19/KRT5/KRT23/TRIM16/KRTAP5-7/KRT2/POU3F2/KRTAP1-1/SPRR2D/CTSV/KRTAP5-5/KRT17/COL7A1/ETV4/FERMT1/SPRR1B/OVOL3/KRT80/MCOLN3/EZH2/LHFPL5/FABP5/ZBED2/KRT6C/DSC2/SOSTDC1/DSG2/FOXQ1/TPRN/SPRR2E/NME2/CD109/SOX9/VDR/BMP4/KRT39/FOXE1/KLK14/DSG4/FLG/POU3F1/ADAM9/LRP4/SPRR1A/TFDP1/WNT5A/SLC44A4/KRT15/CST6/SPRR3/KRT12/HDAC2/KRTAP5-1/SOX21/FZD6/GRHL2/AKR1C3/YBX1/OVOL1/HRNR/KRT8/GORAB/DSC1/KRT83/DKK4/PDZD7/EVPLL/SHARPIN

FATTY ACID METABOLIC PROCESS	FATTY ACID METABOLIC PROCESS	362	-0.32426	-1.59	2.58E-06	0.000674	0.00063	6942	tags=40%, list=25%, signal=31%	AASDH/PDP2/HACL1/PLA2G4C/DLD/AUH/PLA2G15/MECR/ACSL5/FAAH/ALDH3A2/ERLIN1/TNFRSF1A/ACAA1/WDTC1/ACADM/ACOT2/GSTA1/ECI2/PLP1/FASN/ACAA2/AKT2/ACSF3/PCK2/LIPC/ZADH2/ALOX15/PDK2/HADH/SIRT1/NCOR1/HSD17B4/ECHS1/ANGPTL3/ACSM2B/ECI1/BDH2/IRS2/ACADVL/PON3/ACSBG1/ABHD5/ABCD2/PDPR/SLC27A3/APOC2/ACSM5/LONP2/ACADSB/LPIN1/CYP4F11/CPT2/HAO1/ACSM3/MLXIPL/ACACB/LIPG/ACOT6/PHYH/ECH1/ACSF2/ILVBL/PTGS2/CEL/APOC1/MCEE/INSIG1/ACOT12/ACAD11/CD36/APOA1/ACADS/PTGIS/PER2/CYP2C8/ATP6V1B1/NUDT7/PLIN5/IVD/ACSM2A/LPIN2/PDK4/MLYCD/CRAT/ABHD2/CYP4F2/SCP2/BAAT/ALKBH7/ACSL1/MMAA/HPGDS/RGN/CYP2C18/ECHDC2/CYP4F12/EDN2/PON1/ABCB11/ACADL/ACOX1/FAAH2/NAAA/EHHADH/DGAT2/TH/APOC3/SLC27A5/CYP3A4/EPHX2/APOA5/GSTZ1/CYP7A1/PRKAG2/ETFDH/CYP2U1/GCDH/C3/ACAT1/SLC27A1/GSTM1/HAO2/CBR4/CYP1A1/GSTM2/TYRP1/CYP2A6/CYP2C9/CYP4F3/PCK1/CYP2E1/CYP4V2/GGT5/AVPR1A/CYP1A2/MFSD2A/PPARGC1A/TECRL/CYP2B6/ADH7/NR4A3/CYP2A7/ADH4/CYP2A13/CYP2G1P

MEIOSIS I CELL CYCLE PROCESS	MEIOSIS I CELL CYCLE PROCESS	102	0.5825043	1.962	2.59E-06	0.000674	0.00063	3698	tags=33%, list=13%, signal=29%	TEX15/BRDT/FMN2/SYCE1/MEI4/PSMA8/TEX19/TRIP13/RAD51/MAEL/RAD54L/CDC25A/HSF2BP/PLK1/PTTG1/PSMC3IP/RAD51AP1/CKS2/PRDM9/TOP2A/FBXO5/C11orf80/CDC25C/CCNB2/FANCD2/EME1/CCNE1/CCNE2/MAPK15/ESPL1/P3H4/TDRD9/MSH4/RAD21

INNER EAR MORPHOGENESIS	INNER EAR MORPHOGENESIS	78	0.6264678	2.03	2.84E-06	0.0007	0.00065	4742	tags=46%, list=17%, signal=38%	DLX6/COL2A1/MYO3A/WNT3A/USH1C/DLX5/HMX3/POU3F4/MYO3B/USH1G/HMX2/COL11A1/CTHRC1/PTK7/ROR2/LHFPL5/TMIE/TPRN/SOX9/PAX8/GBX2/SIX4/FZD2/SIX1/HOXA1/TBX18/KCNQ4/WNT5A/SLC44A4/ATP8A2/TFAP2A/FZD6/OTX1/LRIG3/CHD7/PDZD7

DOUBLE STRAND BREAK REPAIR	DOUBLE STRAND BREAK REPAIR	222	0.4846494	1.756	4.75E-06	0.001117	0.00104	7943	tags=42%, list=29%, signal=30%	TEX15/HMGA2/FMN2/DNTT/TRIP13/EYA1/ESCO2/RAD51/MCM2/AUNIP/RAD54L/XRCC2/RECQL4/HSF2BP/FOXM1/RAD51AP1/BLM/MCM3/MCM4/PRDM9/MCM6/BARD1/POLQ/CDC7/PARPBP/MMS22L/TONSL/FANCD2/MCM7/GINS4/EME1/RPA4/NSMCE2/CHEK1/MCM8/HUS1B/MCM5/FANCB/CDC45/BRCA1/FEN1/DCLRE1C/XRCC3/MAD2L2/RAD21/HMGB2/MSH2/CHEK2/TWIST1/RAD54B/PRKDC/OGG1/MCMDC2/CDCA5/POLA1/RMI2/TIMELESS/TDP2/PSMD14/SFR1/DEK/UBQLN4/DCLRE1B/NHEJ1/RBBP8/HSF1/TDP1/FIGNL1/RNF8/KDM1A/AP5Z1/XRCC4/XRCC5/UBE2V2/RAD51AP2/DDX1/RAD51B/NABP2/DNA2/SMARCAL1/RAD51D/UBR5/DDX11/ACTR5/MDC1/WRN/PAXIP1/GINS2/BRCA2/FUS/SFPQ/POLB/PRPF19

PROTEIN ACTIVATION CASCADE	PROTEIN ACTIVATION CASCADE	26	-0.714471	-2.22	6.08E-06	0.001337	0.00125	4178	tags=69%, list=15%, signal=59%	F7/SERPINC1/APOH/GP1BA/SERPING1/F10/F8/A2M/F12/F9/KNG1/F13A1/FGG/VWF/FGB/FGA/KLKB1/F11

OLEFINIC COMPOUND METABOLICPROCESS	OLEFINIC COMPOUND METABOLICPROCESS	112	-0.459481	-1.91	6.28E-06	0.001337	0.00125	2879	tags=29%, list=10%, signal=27%	CYP4F2/SCP2/ACSL1/HPGDS/CYP2C18/CYP4F12/ACOX1/FAAH2/RDH13/SRD5A2/EPHX2/SRD5A1/BCO2/CYP2U1/CYP17A1/ALDH1A2/BCO1/CYP1A1/GSTM2/CYP2A6/BMP5/CYP2C9/CYP46A1/CYP4F3/CYP2E1/CYP1A2/PPARGC1A/CYP2B6/CYP2A7/CACNA1H/WNT4/CYP2A13/CYP2G1P

MICROTUBULE CYTOSKELETONORGANIZATION INVOLVED IN MITOSIS	MICROTUBULE CYTOSKELETON ORGANIZATION INVOLVED IN MITOSIS	134	0.5337939	1.852	6.50E-06	0.001337	0.00125	4484	tags=31%, list=16%, signal=26%	EFHC2/MYBL2/MISP/SAPCD2/AURKB/CDC20/TTK/DLGAP5/KIFC1/CENPA/SPC25/KIF23/KIF4A/TPX2/NUF2/PSRC1/PLK1/NDC80/CCNB1/RACGAP1/WDR62/TACC3/PRC1/NEK2/STMN1/GPSM2/ESPL1/NUSAP1/RAN/HSPA1A/KIF11/CHMP4C/CENPE/HSPA1B/KIFC2/STIL/TUBG1/CHEK2/MZT1/SPDL1/CENPH/BORA

DNA REPLICATION	DNA REPLICATION	267	0.4641201	1.697	6.98E-06	0.001378	0.00129	7994	tags=44%, list=29%, signal=31%	MCIDAS/EREG/MCM10/GINS1/ESCO2/RAD51/MCM2/TICRR/CLSPN/HMGA1/TRAIP/DSCC1/RECQL4/ORC1/WDHD1/ORC6/CDK1/CDC6/AICDA/BLM/S100A11/MCM3/MCM4/FBXO5/MCM6/EXO1/CHAF1B/CDT1/BARD1/POLQ/CDC7/MMS22L/DBF4/RRM2/TOP1MT/TONSL/MCM7/GINS4/EME1/RPA4/RNASEH2A/CCNE1/CACYBP/CHEK1/STRA8/MCM8/CCNE2/DTL/MCM5/E2F7/RFC4/CDC45/BRCA1/FEN1/PCNA/PRIM1/E2F8/DTD1/GMNN/PRIM2/POLD1/DBF4B/CHEK2/EHMT2/NAP1L1/PIF1/CDK2/FAM111B/MCMDC2/ATAD5/POLA1/GINS3/RMI2/RHNO1/TIMELESS/CHAF1A/TIPIN/ENPP7/ALYREF/NASP/RBBP8/CDK2AP1/ING5/CHTF18/CCDC88A/SSRP1/UCN/RFC5/DNAJC2/GLI1/POLA2/GTPBP4/THOC1/DNA2/EGF/RRM1/SET/SMARCAL1/DONSON/DDX11/SSBP1/MGME1/RFC3/SLFN11/SUPT16H/WRN/POLE4/LIG1/POLG2/RFC2/KIN/TOPBP1/RAC1/GINS2/BRCA2/POLB/ORC4

ALPHA AMINO ACID CATABOLICPROCESS	ALPHA AMINO ACID CATABOLICPROCESS	82	-0.498948	-2	7.65E-06	0.001452	0.00136	5366	tags=55%, list=19%, signal=44%	GLDC/PHYKPL/ADHFE1/ARG1/NOS3/SDS/ACADSB/SHMT1/HGD/ALDH8A1/GOT1/SARDH/AFMID/PAH/OGDH/DAO/HAAO/HPD/GLUL/ACMSD/OTC/CDO1/MAT1A/HOGA1/CSAD/ALDH4A1/ALDH7A1/GPT2/ATP2B4/KMO/GSTZ1/GCDH/ACAT1/AGXT/GOT2/GPT/PRODH/NOS2/AASS/OAT/AADAT/AGXT2/GLS2/TAT/ASPA

RIBONUCLEOPROTEIN COMPLEX BIOGENESIS	RIBONUCLEOPROTEIN COMPLEXBIOGENESIS	424	0.4189931	1.575	1.00E-05	0.001831	0.00171	10706	tags=58%, list=39%, signal=36%	RPL10L/RNVU1-3/PWP2/BOP1/CELF3/FBLL1/DCAF13/CELF4/SRSF12/SMN2/RPLP0P6/EIF3E/NLE1/RRS1/EIF3H/NPM3/RAN/PAK1IP1/RRP9/VCX/PUF60/GEMIN2/RPP40/SNRPD1/NPM1/EXOSC4/RPS15/HSP90AB1/RIOK1/SNRPB/SNRPC/LYAR/RUVBL1/BRIX1/NIFK/MTERF3/PRKDC/NOP58/RPS7/NOP56/CHD7/AGO2/SNRPG/DKC1/SRPK1/RPL7/RPS8/RPF2/DHX37/RPL27/RPS9/BYSL/WDR12/TAF9/EIF3D/HSP90AA1/AATF/UTP23/MRTO4/WDR3/SF3A2/RPL23A/EXOSC3/SNRPD3/LTV1/METTL5/UTP18/PRPF3/SRSF9/SNRPF/PTBP2/NOP16/REXO4/CPSF6/WDR77/ERI1/WDR46/HEATR1/ZNHIT6/RPL14/ISG20L2/RPL5/EIF6/NSUN5/RRP12/LSM2/RRP15/RPSA/RPL6/NHP2/NOC2L/PIH1D2/WDR75/NVL/RPS6/PES1/CELF5/GAR1/XRCC5/EMG1/SNRPE/DDX27/UTP6/GCFC2/TRMT112/DENR/DDX1/POP5/RPS27/EIF3B/GTPBP4/PA2G4/MRPL10/RPL24/ESF1/ZNHIT3/DDX31/PTGES3/EIF2S2/TGS1/ISY1/RPF1/SMN1/GNL2/LSM4/UTP14A/NIP7/DDX52/GEMIN6/MAK16/RPL35A/RPS14/RRP36/NOL11/RPUSD3/RPL10A/DDX51/EIF3M/GTF3A/RRP1/PRPF39/NOC4L/CD2BP2/RPS10/EXOSC2/NOP2/PRPF19/RPS21/USP39/PRMT5/WDR43/NGDN/UTP

EMBRYONIC SKELETAL SYSTEMMORPHOGENESIS	EMBRYONIC SKELETAL SYSTEMMORPHOGENESIS	85	0.5883274	1.931	1.11E-05	0.001963	0.00184	4212	tags=41%, list=15%, signal=35%	COL2A1/HOXA11/DLX2/NOG/ALX1/HOXD10/HOXB9/OSR2/ALX3/SHOX2/COL11A1/HOXA6/HOXD4/FLVCR1/PAX5/DSCAML1/MDFI/HOXB7/BMP4/IRX5/SIX4/PDGFRA/HOXA2/MMP16/SIX1/HOXA1/MTHFD1L/HOXB8/TFAP2A/CHST11/GRHL2/MMP14/SLC39A1/HOXA9/TWIST1

REGULATION OF CHROMOSOMESEGREGATION	REGULATION OF CHROMOSOME SEGREGATION	81	0.5934673	1.94	1.25E-05	0.002132	0.00199	5023	tags=43%, list=18%, signal=35%	TRIP13/UBE2C/KIF2C/AURKB/CDC20/BUB1B/TTK/DLGAP5/PLK1/NDC80/PTTG1/CDC6/BUB1/CCNB1/FBXO5/CDT1/TACC3/KNTC1/NSMCE2/CENPF/MAPK15/MKI67/ZWINT/ESPL1/UBE2DNL/RCC2/USP44/CENPE/XRCC3/MAD2L2/RAD21/SPDL1/ANAPC7/PCID2/RMI2

EPOXYGENASE P450 PATHWAY	EPOXYGENASE P450 PATHWAY	19	-0.778218	-2.23	1.31E-05	0.002148	0.00201	3351	tags=74%, list=12%, signal=65%	CYP2C8/CYP4F2/CYP2C18/CYP4F12/EPHX2/CYP1A1/CYP2A6/CYP2C9/CYP2E1/CYP1A2/CYP2B6/CYP2A7/CYP2A13/CYP2G1P

SPINDLE ORGANIZATION	SPINDLE ORGANIZATION	170	0.5083511	1.8	1.44E-05	0.002292	0.00214	7066	tags=42%, list=26%, signal=31%	EFHC2/MYBL2/MISP/AURKB/CDC20/TTK/DLGAP5/KIFC1/SPC25/AUNIP/KIF23/KIF4A/TPX2/NUF2/PSRC1/PLK1/NDC80/CCNB1/FBXO5/RACGAP1/WDR62/CCNB2/TACC3/PRC1/NEK2/TUBB8/STMN1/GPSM2/MAPK15/ESPL1/RAN/HSPA1A/KIF11/CHMP4C/CENPE/HSPA1B/KIFC2/STIL/TRIM36/TUBG1/HAUS1/CHEK2/GOLGA2P5/MZT1/CENPH/BORA/KNSTRN/HAUS5/HAUS6/RCC1/INCENP/TUBB/EFHC1/NUP62/RGS14/AURKA/RAE1/SPAG5/ASPM/ANKRD53/LZTS2/NUDC/SAC3D1/CEP72/CENPJ/KPNB1/CKAP5/GOLGA8B/DRG1/CCSAP/HAUS8

DNA DEPENDENT DNA REPLICATION	DNA DEPENDENT DNA REPLICATION	150	0.5293124	1.85	2.12E-05	0.003273	0.00306	7994	tags=49%, list=29%, signal=35%	MCM10/GINS1/RAD51/MCM2/TICRR/HMGA1/TRAIP/DSCC1/RECQL4/ORC1/WDHD1/ORC6/CDC6/AICDA/BLM/MCM3/MCM4/FBXO5/MCM6/CDT1/POLQ/CDC7/MMS22L/DBF4/TONSL/MCM7/GINS4/EME1/RPA4/CCNE1/CCNE2/MCM5/E2F7/RFC4/CDC45/FEN1/PCNA/PRIM1/E2F8/GMNN/PRIM2/POLD1/DBF4B/CHEK2/MCMDC2/POLA1/GINS3/TIMELESS/TIPIN/ALYREF/CDK2AP1/CHTF18/RFC5/POLA2/THOC1/DNA2/SMARCAL1/DONSON/DDX11/SSBP1/MGME1/RFC3/SLFN11/WRN/POLE4/LIG1/POLG2/RFC2/TOPBP1/GINS2/BRCA2/POLB/ORC4

MITOTIC SPINDLE ORGANIZATION	MITOTIC SPINDLE ORGANIZATION	109	0.5649466	1.918	2.21E-05	0.003298	0.00308	5597	tags=39%, list=20%, signal=31%	EFHC2/MYBL2/MISP/AURKB/CDC20/TTK/DLGAP5/KIFC1/SPC25/KIF23/KIF4A/TPX2/NUF2/PSRC1/PLK1/NDC80/CCNB1/RACGAP1/WDR62/TACC3/PRC1/NEK2/STMN1/GPSM2/RAN/HSPA1A/KIF11/CHMP4C/CENPE/HSPA1B/KIFC2/STIL/TUBG1/CHEK2/MZT1/CENPH/BORA/RCC1/EFHC1/NUP62/AURKA/RAE1
REGULATION OF SYSTEMIC ARTERIAL BLOOD	REGULATION OF SYSTEMIC	16	-0.785724	-2.16	2.78E-05	0.003947	0.00369	470	tags=31%, list=2%, signal=31%	ENPEP/CPA3/REN/CTSG/CMA1

RIBOSOME BIOGENESIS	RIBOSOME BIOGENESIS	288	0.4420957	1.623	2.88E-05	0.003947	0.00369	10706	tags=60%, list=39%, signal=37%	RPL10L/PWP2/BOP1/FBLL1/DCAF13/RPLP0P6/NLE1/RRS1/NPM3/RAN/PAK1IP1/RRP9/VCX/RPP40/NPM1/EXOSC4/RPS15/RIOK1/LYAR/BRIX1/NIFK/MTERF3/PRKDC/NOP58/RPS7/NOP56/CHD7/DKC1/RPL7/RPS8/RPF2/DHX37/RPL27/RPS9/BYSL/WDR12/AATF/UTP23/MRTO4/WDR3/RPL23A/EXOSC3/LTV1/METTL5/UTP18/NOP16/REXO4/ERI1/WDR46/HEATR1/ZNHIT6/RPL14/ISG20L2/RPL5/EIF6/NSUN5/RRP12/RRP15/RPSA/RPL6/NHP2/NOC2L/PIH1D2/WDR75/NVL/RPS6/PES1/GAR1/XRCC5/EMG1/DDX27/UTP6/TRMT112/POP5/RPS27/GTPBP4/PA2G4/MRPL10/RPL24/ESF1/ZNHIT3/DDX31/RPF1/GNL2/UTP14A/NIP7/DDX52/MAK16/RPL35A/RPS14/RRP36/NOL11/RPUSD3/RPL10A/DDX51/GTF3A/RRP1/NOC4L/RPS10/EXOSC2/NOP2/RPS21/WDR43/NGDN/UTP20/RPP25/RPLP0/EXOSC5/FBL/DDX54/TRMT61B/CUL4A/NOP10/NSUN4/DDX10/ERAL1/EIF4A3/RPS24/NAT10/WDR74/XRN2/NOB1/RPL38/RPL10/ZCCHC4/MRPL1/RPP38/ERCC2/SURF6/ABT1/RSL1D1/RPP30/MRM1/PWP1/MYBBP1A/EXOSC1/RPL7L1/NOL6/EXOSC10/XPO1/SRFBP1/EBNA1BP2/LSG1/RRP7A/EXOSC7/URB2/RPL7A/SART1/DDX18/RPS5/C1QBP/RPS16/DROSHA/RPL26L1/ERI3/M

FATTY ACID BETA OXIDATION	FATTY ACID BETA OXIDATION	73	-0.497138	-1.96	2.94E-05	0.003947	0.00369	8358	tags=59%, list=30%, signal=41%	CROT/ACOX2/MTOR/PPARA/CPT1A/HADHB/CPT1C/ETFB/AUH/MECR/ACAA1/ACADM/ECI2/ACAA2/AKT2/HADH/HSD17B4/ECHS1/ECI1/BDH2/IRS2/ACADVL/ABCD2/LONP2/CPT2/ACACB/ECH1/ACAD11/ACADS/PLIN5/IVD/MLYCD/CRAT/SCP2/ECHDC2/ABCB11/ACADL/ACOX1/EHHADH/ETFDH/GCDH/ACAT1/MFSD2A

NCRNA METABOLIC PROCESS	NCRNA METABOLIC PROCESS	433	0.4127992	1.552	2.96E-05	0.003947	0.00369	10719	tags=54%, list=39%, signal=33%	CT45A10/LIN28B/LIN28A/ERN2/FKBP6/TRIM71/SLFN13/MAEL/PWP2/BOP1/EXD1/FBLL1/DCAF13/SAGE1/PIWIL4/EEF1E1/HENMT1/RRS1/WDR4/NPM3/RAN/XPO5/INTS8/TDRD9/RRP9/SSB/DTD1/RPP40/TRMT6/EXOSC4/RPS15/CSTF2/FARSB/RIOK1/LYAR/NIFK/PUS7/PRKDC/NOP58/RPS7/NOP56/PUS1/CHD7/AGO2/TSEN54/DKC1/POP1/METTL1/RPL7/RPS8/RPF2/TPRKB/DHX37/RPL27/RPS9/BYSL/TARBP1/WDR12/UTP23/MRTO4/WDR3/EXOSC3/TRMT13/METTL5/DALRD3/UTP18/DDX4/TDRD1/REXO4/ADAT2/ERI1/WDR46/HEATR1/ZNHIT6/RPL14/ISG20L2/RPL5/EIF6/TDRKH/NSUN5/RRP12/RRP15/TRMT61A/NHP2/PIH1D2/WDR75/LAGE3/NVL/METTL6/RPS6/PRKRA/PES1/TRMT12/GAR1/PARS2/EMG1/DDX27/CPSF1/UTP6/PNPT1/TRMT112/DDX1/POP5/RPS27/GTPBP4/TSEN15/PA2G4/TRDMT1/ESF1/TRMT10C/CPSF4/ZNHIT3/ZBTB8OS/MAPT/KTI12/RPF1/PIWIL1/THUMPD2/SRRT/TRMT1/UTP14A/DDX52/TYW3/MAK16/RPL35A/RPS14/RPP21/RRP36/NOL11/RPUSD3/RPL10A/DDX51/RRP1/TOE1/TRMU/NOC4L/METTL3/EXOSC2/NOP2/RPS21/WDR43/NGDN/UTP20/QRSL1/RPP25/CDKAL1/YARS2/EXOSC5/FBL/DDX54/TRMT61B/NOP10/HARS2/
REGULATION OF ANGIOTENSIN LEVELSIN [illegible]	REGULATION OF ANGIOTENSIN [illegible]	11	-0.841683	-2.04	3.10E-05	0.003952	0.0037	470	tags=45%, list=2%, signal=45%	ENPEP/CPA3/REN/CTSG/CMA1

EPIDERMAL CELL DIFFERENTIATION	EPIDERMAL CELL DIFFERENTIATION	219	0.4798784	1.733	3.14E-05	0.003952	0.0037	4783	tags=34%, list=17%, signal=28%	ESRP1/PITX2/S100A7/KLK13/KRT20/KRTAP4-1/DSG3/KRT4/EREG/KRT79/CASP14/FOXN1/SFN/USH1C/OVOL2/PKP3/KRT13/CYP26B1/DSC3/KRT34/ABCA12/LIPM/EVPL/KRT19/KRT5/KRT23/TRIM16/KRTAP5-7/KRT2/KRTAP1-1/SPRR2D/CTSV/KRTAP5-5/KRT17/ETV4/SPRR1B/OVOL3/KRT80/MCOLN3/EZH2/LHFPL5/ZBED2/KRT6C/DSC2/DSG2/TPRN/SPRR2E/NME2/CD109/VDR/BMP4/KRT39/KLK14/DSG4/FLG/POU3F1/ADAM9/SPRR1A/WNT5A/SLC44A4/KRT15/SPRR3/KRT12/HDAC2/KRTAP5-1/GRHL2/AKR1C3/OVOL1/HRNR/KRT8/DSC1/KRT83/PDZD7/SHARPIN

NCRNA PROCESSING	NCRNA PROCESSING	361	0.4262967	1.584	3.20E-05	0.003952	0.0037	10719	tags=56%, list=39%, signal=35%	CT45A10/LIN28B/LIN28A/PWP2/BOP1/FBLL1/DCAF13/SAGE1/RRS1/WDR4/NPM3/INTS8/RRP9/SSB/RPP40/TRMT6/EXOSC4/RPS15/CSTF2/RIOK1/LYAR/NIFK/PUS7/PRKDC/NOP58/RPS7/NOP56/PUS1/CHD7/AGO2/TSEN54/DKC1/POP1/METTL1/RPL7/RPS8/RPF2/TPRKB/DHX37/RPL27/RPS9/BYSL/TARBP1/WDR12/UTP23/MRTO4/WDR3/EXOSC3/TRMT13/METTL5/UTP18/REXO4/ADAT2/ERI1/WDR46/HEATR1/ZNHIT6/RPL14/ISG20L2/RPL5/EIF6/NSUN5/RRP12/RRP15/TRMT61A/NHP2/PIH1D2/WDR75/LAGE3/NVL/METTL6/RPS6/PRKRA/PES1/TRMT12/GAR1/EMG1/DDX27/CPSF1/UTP6/TRMT112/DDX1/POP5/RPS27/GTPBP4/TSEN15/PA2G4/TRDMT1/ESF1/TRMT10C/CPSF4/ZNHIT3/ZBTB8OS/KTI12/RPF1/THUMPD2/SRRT/TRMT1/UTP14A/DDX52/TYW3/MAK16/RPL35A/RPS14/RPP21/RRP36/NOL11/RPUSD3/RPL10A/DDX51/RRP1/TOE1/TRMU/NOC4L/METTL3/EXOSC2/NOP2/RPS21/WDR43/NGDN/UTP20/RPP25/CDKAL1/EXOSC5/FBL/DDX54/TRMT61B/NOP10/OSGEPL1/RNF113A/NSUN4/DDX10/EIF4A3/RPS24/NAT10/WDR74/XRN2/NOB1/TSEN2/ELP3/HNRNPA2B1/ZCCHC4/MRPL1/RPP38/ERCC2/TSEN34/ABT1/RSL1D1/RPP30/MRM1/FAM98B/EX

REGULATION OF CHROMOSOMESEPARATION	REGULATION OF CHROMOSOME SEPARATION	68	0.6101019	1.934	3.84E-05	0.00462	0.00432	3698	tags=41%, list=13%, signal=36%	TRIP13/UBE2C/AURKB/CDC20/BUB1B/TTK/DLGAP5/PLK1/NDC80/PTTG1/CDC6/BUB1/CCNB1/FBXO5/CDT1/TACC3/KNTC1/NSMCE2/CENPF/MAPK15/ZWINT/ESPL1/UBE2DNL/USP44/CENPE/XRCC3/MAD2L2/RAD21

EMBRYONIC ORGAN MORPHOGENESIS	EMBRYONIC ORGAN MORPHOGENESIS	254	0.4645051	1.695	4.16E-05	0.00489	0.00457	4298	tags=30%, list=16%, signal=25%	DLX6/COL2A1/SIX3/MYO3A/WNT3A/USH1C/OVOL2/DLX5/HOXA11/STRA6/GJB6/HMX3/POU3F4/DLX2/NOG/ALX1/MYO3B/HOXD10/USH1G/HOXB9/HMX2/NKX2-5/EYA1/MFAP2/OSR2/ALX3/SHOX2/COL11A1/CTHRC1/HOXA6/PTK7/ROR2/LHFPL5/TMIE/FOLR1/HOXD4/FLVCR1/TPRN/PAX5/FBN2/DSCAML1/MDFI/SOX9/HOXB7/PAX8/GBX2/BMP4/FOXE1/IRX5/SIX4/FZD2/PDGFRA/HOXA2/MMP16/SIX1/HOXA1/TBX18/KCNQ4/WNT5A/SLC44A4/MTHFD1L/ATP8A2/STIL/HOXB8/TFAP2A/MESP1/CHST11/FZD6/GRHL2/MMP14/SLC39A1/OTX1/HOXA9/TWIST1/LRIG3

ALPHA AMINO ACID METABOLICPROCESS	ALPHA AMINO ACID METABOLICPROCESS	174	-0.37807	-1.67	4.62E-05	0.005158	0.00482	5483	tags=40%, list=20%, signal=32%	MRI1/AMDHD1/GLDC/PHYKPL/ADHFE1/ARG1/NOS3/SDS/ACADSB/SHMT1/HGD/ALDH8A1/MTHFS/GOT1/GFPT2/ILVBL/SARDH/SLC39A8/AFMID/PAH/OGDH/DAO/ASL/UROC1/SEPSECS/HAAO/HPD/ACCS/GLUL/ACMSD/ART4/HAL/OTC/SERINC5/CDO1/BAAT/MAT1A/HOGA1/CSAD/ASS1/MTHFD1/ALDH4A1/ALDH7A1/GPT2/ATP2B4/TH/KMO/IYD/GSTZ1/FTCD/GCDH/ACAT1/AGXT/CPS1/GOT2/GPT/PRODH/NOS2/AASS/GLYATL1/OAT/AADAT/CTH/AGXT2/GLS2/TAT/GNMT/ASPA/TTC36

SKIN DEVELOPMENT	SKIN DEVELOPMENT	279	0.4508625	1.655	4.69E-05	0.005158	0.00482	4662	tags=29%, list=17%, signal=24%	S100A7/KLK13/KRT20/KRTAP4-1/DSG3/GAL/KRT4/EREG/KRT79/FRAS1/CASP14/FOXN1/SFN/OVOL2/PKP3/KRT13/CYP26B1/DSC3/KRT34/ABCA12/LIPM/EVPL/KRT19/KRT5/CLDN4/KRT23/TRIM16/KRTAP5-7/GJB3/KRT2/KRTAP1-1/SPRR2D/ALOX12B/CTSV/KRTAP5-5/KRT17/ETV4/FERMT1/SPRR1B/KRT80/ITGB4/ZBED2/KRT6C/DSC2/SOSTDC1/DSG2/FOXQ1/SPRR2E/NME2/CD109/SOX9/VDR/KRT39/FOXE1/STMN1/KLK14/DSG4/FLG/POU3F1/ADAM9/LRP4/SPRR1A/WNT5A/ATP8A2/KRT15/SPRR3/KRT12/HDAC2/KRTAP5-1/SOX21/FZD6/GRHL2/AKR1C3/OVOL1/HRNR/KRT8/GORAB/DSC1/KRT83/DKK4

KERATINOCYTE DIFFERENTIATION	KERATINOCYTE DIFFERENTIATION	169	0.5020341	1.778	4.72E-05	0.005158	0.00482	4143	tags=33%, list=15%, signal=28%	S100A7/KLK13/KRT20/KRTAP4-1/DSG3/KRT4/EREG/KRT79/CASP14/FOXN1/SFN/OVOL2/PKP3/KRT13/CYP26B1/DSC3/KRT34/ABCA12/LIPM/EVPL/KRT19/KRT5/KRT23/TRIM16/KRTAP5-7/KRT2/KRTAP1-1/SPRR2D/CTSV/KRTAP5-5/KRT17/ETV4/SPRR1B/KRT80/ZBED2/KRT6C/DSC2/DSG2/SPRR2E/NME2/CD109/VDR/KRT39/KLK14/DSG4/FLG/POU3F1/ADAM9/SPRR1A/WNT5A/KRT15/SPRR3/KRT12/KRTAP5-1/GRHL2/AKR1C3

DNA CONFORMATION CHANGE	DNA CONFORMATION CHANGE	255	0.4549682	1.661	4.82E-05	0.005158	0.00482	7738	tags=40%, list=28%, signal=29%	DQX1/HMGA2/TNP1/ERN2/CHD5/HJURP/NCAPH/CENPA/NCAPG/ERCC6L/CENPW/RAD51/MCM2/RAD54L/HMGA1/DSCC1/BANF2/RECQL4/CENPM/AICDA/BLM/CCNB1/MCM3/MCM4/PRDM9/TOP2A/MCM6/OIP5/CENPI/CHAF1B/ASF1B/POLQ/BEND3/SOX9/TOP1MT/MCM7/NCAPD2/MCM8/MCM5/NCAPG2/NUSAP1/RFC4/CENPK/TET1/CENPL/NCAPH2/NPM1/HMGB2/CDKN2A/RUVBL1/CENPQ/SMYD3/CENPH/RAD54B/NAP1L1/CENPO/PIF1/CHD7/MCMDC2/CDCA5/ITGB3BP/DNMT1/SRPK1/INCENP/CHAF1A/DNMT3A/HAT1/SMC4/NASP/HFM1/CHD1L/IPO4/CHTF18/RNF8/DAXX/NOC2L/ANP32B/XRCC5/TRIM28/MIS18A/RFC5/DDX1/BANF1/CENPV/ASF1A/DNA2/SET/PPHLN1/SMC2/RPS27A/SMARCAL1/DDX11/DDX12P/SSBP1/PIWIL1/RFC3/MNAT1/WRN/GTF2H1/RBX1/RFC2

COMPLEMENT ACTIVATION LECTINPATHWAY	COMPLEMENT ACTIVATION LECTIN PATHWAY	12	-0.836052	-2.13	5.01E-05	0.005158	0.00482	4170	tags=83%, list=15%, signal=71%	MBL2/MASP2/SERPING1/MASP1/A2M/MFAP4/COLEC10/FCN3/KRT1/FCN2

APPENDAGE DEVELOPMENT	APPENDAGE DEVELOPMENT	154	0.5136755	1.802	5.02E-05	0.005158	0.00482	5040	tags=34%, list=18%, signal=28%	DLX6/PITX2/HOXD13/COL2A1/SP8/FRAS1/FOXN1/TBX4/DLX5/HOXA11/CYP26B1/SALL4/NOG/HOXD10/KREMEN2/OSR2/ALX3/SHOX2/SOX4/ROR2/SCX/ITGB4/FMN1/FLVCR1/AFF3/FBN2/GPC3/SOX9/BMP4/SLC7A11/GDF5/LRP4/WNT5A/LEF1/TFAP2A/HDAC2/BAK1/IFT80/CHST11/FZD6/GRHL2/SLC39A1/SFRP2/HOXA9/TWIST1/CHD7/MEOX2/OSR1/IFT52/TBX5/PITX1/WNT7A

REGULATION OF TRIGLYCERIDEBIOSYNTHETIC PROCESS	REGULATION OF TRIGLYCERIDE BIOSYNTHETIC PROCESS	18	-0.741264	-2.1	5.16E-05	0.005202	0.00486	4880	tags=61%, list=18%, signal=50%	THRSP/FBXW7/LDLR/PLIN5/RGN/DGAT2/C3/SLC27A1/GPLD1/MFSD2A/SIK1

COMPLEMENT ACTIVATION	COMPLEMENT ACTIVATION	157	-0.387945	-1.72	5.28E-05	0.005213	0.00488	4170	tags=39%, list=15%, signal=33%	MBL2/IGHV4-4/IGHV3-23/IGHV3-11/IGHV3-64/IGKV5-2/IGLV1-44/CLU/IGHV1-45/IGKV1D-12/APCS/IGHV3-74/VTN/C1RL/IGHV3-49/IGLV3-19/MASP2/IGHV3-21/SERPING1/CFH/IGLC7/CFHR2/MASP1/RGCC/C5/C4BPB/CD5L/IGHG1/C4A/A2M/CFHR1/IGHA2/IGLV2-14/CFP/CFB/IGLV1-51/C1S/C3/C8B/IGLV1-40/MFAP4/CFI/IGHV2-70/IGKV2D-30/IGLC2/C4BPA/C1R/IGHA1/C7/CFHR5/IGKV1-12/C9/IGKV1D-39/C8A/IGLC6/C6/CFHR4/COLEC10/FCN3/KRT1/FCN2

DNA RECOMBINATION	DNA RECOMBINATION	251	0.4508828	1.645	5.85E-05	0.00566	0.00529	6723	tags=35%, list=24%, signal=27%	TEX15/MEI4/TEX19/TRIP13/RAD51/MCM2/AUNIP/RAD54L/XRCC2/BATF/RECQL4/HSF2BP/EID3/PSMC3IP/RAD51AP1/BLM/MCM3/MCM4/PRDM9/TOP2A/MCM6/EXO1/KPNA2/C11orf80/POLQ/CDC7/PARPBP/TNFSF4/MMS22L/TONSL/FANCD2/TGFB1/MCM7/GINS4/EME1/RPA4/NSMCE2/CHEK1/MCM8/HUS1B/MCM5/FANCB/CDC45/BRCA1/FEN1/TCF3/DCLRE1C/MSH4/XRCC3/MAD2L2/RAD21/LEF1/HMGB2/ACTL6A/RUVBL1/MSH2/INO80C/HSPD1/RAD54B/PRKDC/PIF1/MCMDC2/UNG/TPRKB/RMI2/RHNO1/TIMELESS/PSMD14/SFR1/ALYREF/EXOSC3/UBQLN4/NONO/HFM1/CLCF1/RBBP8/MND1/FIGNL1/IL27RA/RNF8/KDM1A/AP5Z1/PRDM7/XRCC4/XRCC5/TCF7/RAD51AP2/RAD51B/NABP2

BLOOD COAGULATION INTRINSIC PATHWAY	BLOOD COAGULATION INTRINSICPATHWAY	16	-0.772088	-2.13	6.03E-05	0.005721	0.00535	3879	tags=75%, list=14%, signal=65%	SERPINC1/APOH/GP1BA/SERPING1/F8/A2M/F12/F9/KNG1/VWF/KLKB1/F11
METAPHASE ANAPHASE TRANSITION OFCELL CYCLE	METAPHASE ANAPHASE TRANSITION OFCELL CYCLE	63	0.6204431	1.941	6.37E-05	0.005933	0.00555	3698	tags=43%, list=13%, signal=37%	TRIP13/UBE2C/AURKB/CDC20/BUB1B/TTK/DLGAP5/PLK1/NDC80/CDC6/BUB1/CCNB1/FBXO5/CDT1/TACC3/KNTC1/NSMCE2/CENPF/MAPK15/ZWINT/ESPL1/UBE2DNL/USP44/CENPE/XRCC3/MAD2L2/RAD21

LIPID OXIDATION	LIPID OXIDATION	108	-0.426291	-1.77	6.95E-05	0.006192	0.00579	7494	tags=53%, list=27%, signal=39%	CPT1A/ADIPOR2/HADHB/CPT1C/ETFB/PECR/HACL1/AUH/MECR/ALDH3A2/ACAA1/ACADM/ECI2/ACAA2/AKT2/ALOX15/HADH/HSD17B4/ECHS1/ECI1/BDH2/IRS2/ACADVL/ABCD2/LONP2/CPT2/HAO1/ACACB/PHYH/ECH1/ILVBL/APOD/ACAD11/ACADS/PLIN5/IVD/PDK4/MLYCD/CRAT/SCP2/ECHDC2/ABCB11/ACADL/ACOX1/EHHADH/DGAT2/PRKAG2/ETFDH/GCDH/ACAT1/HAO2/CYP4V2/MFSD2A/PPARGC1A/ADH7/NR4A3/ADH4

MALE GAMETE GENERATION	MALE GAMETE GENERATION	430	0.4067587	1.53	6.98E-05	0.006192	0.00579	6218	tags=28%, list=22%, signal=22%	TEX15/RNF17/NR0B1/SPATA31D1/BRDT/PAEP/TNP1/TDRD5/GTSF1/RPL10L/SSTR3/DUSP13/FOXJ1/ADAM18/MEI4/HOXA11/SLC26A3/CYP26B1/PSMA8/FAM9A/TEX19/SPATA31C2/TRIP13/CHD5/FKBP6/PLA2G3/PTCHD3/GGT3P/SSTR2/CABYR/EIF5A2/KIFC1/GJB3/SOHLH2/GGT1/RIMBP3C/RPL39L/RSPH1/MAEL/DPY19L2P2/SPOCD1/HSF2BP/ROPN1/KIF18A/OSBP2/PTTG1/CLDN11/GGT2/OCA2/PMFBP1/CCNB1/PRDM9/PIWIL4/ASF1B/PAX5/RACGAP1/CDC25C/SPINK1/NEURL1/SOX9/ETV5/GGNBP1/SOHLH1/ACSBG2/STRA8/MCM8/ZFP41/TAF4B/WIPF3/TTC26/SLC26A6/ZNF296/TDRD9/ROPN1L/MSH4/SPAG4/VCX/HMGB2/GAL3ST1/RUVBL1/E2F1/INSL3/HOXA9/OVOL1/FANCG/TDRD6/SPATA24/RGS2/SRPK1/DEFB1/TYRO3/ELL3/NPHP1/MORN2/SPATC1L/SNRPA1/HSF2/DPY19L2/PYGO2/NUP62/SPDYA/TTLL1/SOX30/DNMT3A/NDC1/SPEF2/DDX4/NANOS3/TDRD1/MORC1/CCDC136/CEP131/CNR1/DYNLL1/TDRKH/HSPB11/FIGNL1/RNF8/NDRG3/MAST2/ATAT1

REGULATION OF LIPASE ACTIVITY	REGULATION OF LIPASE ACTIVITY	90	-0.455712	-1.85	7.02E-05	0.006192	0.00579	6055	tags=44%, list=22%, signal=35%	PDGFRB/TXK/LIPC/RASGRP4/CCL5/ANGPTL3/GNAQ/EDNRA/ABHD5/FLT1/APOC2/NTRK2/ARHGAP6/APOC1/APOH/APOA1/LMF1/PLIN5/ITK/FURIN/NTRK3/ANG/MIR590/APOC3/APOA5/ANXA8/EGFR/ANGPTL4/PCSK6/PNLIP/GPIHBP1/HTR2A/KIT/SELE/NMUR1/NTF3/AVPR1A/ADRA1A/ESR1/HTR2B
REGULATION OF SYSTEMIC ARTERIAL [illegible]	REGULATION OF SYSTEMIC [illegible]	20	-0.716613	-2.09	7.36E-05	0.006375	0.00596	470	tags=25%, list=2%, signal=25%	ENPEP/CPA3/REN/CTSG/CMA1

CHROMOSOME SEPARATION	CHROMOSOME SEPARATION	89	0.5564705	1.835	7.72E-05	0.006571	0.00615	3718	tags=36%, list=13%, signal=31%	TRIP13/UBE2C/AURKB/CDC20/BUB1B/TTK/DLGAP5/PLK1/NDC80/PTTG1/CDC6/BUB1/CCNB1/TOP2A/FBXO5/CDT1/TACC3/EME1/KNTC1/NSMCE2/CENPF/MAPK15/ZWINT/ESPL1/UBE2DNL/USP44/MSH4/CENPE/XRCC3/MAD2L2/RAD21/NCAPH2

EMBRYONIC ORGAN DEVELOPMENT	EMBRYONIC ORGAN DEVELOPMENT	384	0.4109253	1.535	9.42E-05	0.007881	0.00737	4212	tags=24%, list=15%, signal=21%	DLX6/PITX2/COL2A1/SIX3/MYO3A/WNT3A/USH1C/TBX4/OVOL2/DLX5/SPINT2/HOXA11/CDX2/STRA6/GJB6/HMX3/POU3F4/DLX2/NOG/ALX1/MYO3B/SPINT1/HOXD10/USH1G/HOXB9/HMX2/PIFO/NKX2-5/KRT19/EYA1/MFAP2/OSR2/ALX3/SHOX2/COL11A1/PHLDA2/CTHRC1/HOXA6/PTK7/ROR2/LHFPL5/TMIE/FOLR1/HOXD4/FLVCR1/ASCL2/TPRN/PAX5/FBN2/DSCAML1/MDFI/PLCD3/SOX9/HOXB7/PAX8/TGFB1/GBX2/BMP4/FOXE1/IRX5/SIX4/FZD2/PDGFRA/LIF/HOXA2/MMP16/E2F7/SIX1/HOXA1/CXCL8/TBX18/KCNQ4/WNT5A/PPP1R13L/SLC44A4/E2F8/MTHFD1L/LEF1/ATP8A2/STIL/HOXB8/TFAP2A/MESP1/TUBB2B/CHST11/FZD6/GRHL2/MMP14/SLC39A1/OTX1/HOXA9/TWIST1

DNA REPLICATION INITIATION	DNA REPLICATION INITIATION	40	0.6718035	1.953	9.85E-05	0.008104	0.00758	4957	tags=52%, list=18%, signal=43%	MCM10/MCM2/TICRR/ORC1/ORC6/CDC6/MCM3/MCM4/MCM6/CDT1/MCM7/RPA4/CCNE1/CCNE2/MCM5/CDC45/PRIM1/PRIM2/MCMDC2/POLA1/GINS3

NEGATIVE REGULATION OFVASCULATURE DEVELOPMENT	NEGATIVE REGULATION OFVASCULATURE DEVELOPMENT	88	-0.455515	-1.84	0.0001102	0.008883	0.00831	4808	tags=34%, list=17%, signal=28%	COL4A2/STAB1/SYNJ2BP/ECSCR/THBS2/PTN/APOH/TIE1/HSPG2/KLF2/SEMA3E/GPR4/SERPINF1/RGCC/THBS1/ADAMTS1/ATP2B4/DCN/THBS4/TEK/GADD45A/NPR1/SPINK5/HRG/CD160/ANGPT4/CLDN5/WNT4/GDF2/ANGPTL7

EMBRYONIC SKELETAL SYSTEMDEVELOPMENT	EMBRYONIC SKELETAL SYSTEMDEVELOPMENT	111	0.5430453	1.848	0.0001116	0.008883	0.00831	4212	tags=36%, list=15%, signal=31%	COL2A1/DMRT2/HOXA11/DLX2/NOG/ALX1/HOXD1/HOXD10/HOXB9/DLX1/OSR2/ALX3/SHOX2/COL11A1/HOXA6/SCX/HOXD4/FLVCR1/PAX5/DSCAML1/MDFI/HOXB7/BMP4/IRX5/SIX4/PDGFRA/HOXA2/MMP16/SIX1/HOXA1/WNT5A/MTHFD1L/HOXB8/TFAP2A/CHST11/GRHL2/MMP14/SLC39A1/HOXA9/TWIST1

LONG CHAIN FATTY ACID METABOLICPROCESS	LONG CHAIN FATTY ACID METABOLICPROCESS	115	-0.406448	-1.7	0.000137	0.010732	0.01004	4836	tags=41%, list=17%, signal=34%	HACL1/PLA2G4C/ACSL5/FAAH/ACAA1/ACOT2/GSTA1/PLP1/ALOX15/HSD17B4/ACSM2B/ACSBG1/SLC27A3/ACSM5/CYP4F11/CPT2/ACSM3/PTGS2/PTGIS/CYP2C8/ACSM2A/CYP4F2/SCP2/ACSL1/HPGDS/CYP2C18/CYP4F12/ACADL/ACOX1/FAAH2/SLC27A5/CYP3A4/EPHX2/CYP2U1/SLC27A1/GSTM1/CYP1A1/GSTM2/CYP2A6/CYP2C9/CYP4F3/CYP2E1/CYP1A2/CYP2B6/CYP2A7/CYP2A13/CYP2G1P

CELLULAR PROCESS INVOLVED INREPRODUCTION IN MULTICELLULARORGANISM	CELLULAR PROCESS INVOLVED INREPRODUCTION IN MULTICELLULARORGANISM	274	0.4325726	1.587	0.0001561	0.012036	0.01126	6099	tags=33%, list=22%, signal=26%	TEX15/SPESP1/BRDT/PAEP/TNP1/TDRD5/FMN2/EREG/MEI4/SLC26A3/LIN28A/CYP26B1/FAM9A/TEX19/TRIP13/CHD5/FKBP6/PLA2G3/PTCHD3/CABYR/TTK/SOHLH2/RIMBP3C/RSPH1/PAQR8/MAEL/DPY19L2P2/HSF2BP/ROPN1/PLK1/KIF18A/OSBP2/OCA2/FOLR1/CCNB1/ROBO2/TOP2A/FBXO5/PIWIL4/SRC/CELF4/SPINK1/NEURL1/RETN/CTCFL/NOX5/ETV5/CCNB2/BMP4/TUBB8/SOHLH1/STRA8/TAF4B/TTC26/SLC26A6/TDRD9/ROPN1L/NCAPH2/HMGB2/MSH2/FANCG/CDC25B/PABPC1L/SRPK1/DEFB1/NPHP1/DPY19L2/PYGO2/SPDYA/IZUMO1/TTLL1/SOX30/RNF2/DNMT3A/SPEF2/AURKA/IZUMO1R/IHH/DDX4/NANOS3/TDRD1/WDR77/MORC1/CCDC136/CEP131/DYNLL1/TDRKH/FIGNL1/PAQR7/RNF8

ENDOTHELIUM DEVELOPMENT	ENDOTHELIUM DEVELOPMENT	123	-0.394764	-1.67	0.0001675	0.012722	0.0119	6798	tags=40%, list=25%, signal=30%	S1PR1/PROX1/EZR/TNFRSF1A/NRG1/MET/FASN/KDM6B/GJA1/RDX/RAP2C/STARD13/PECAM1/GJA5/HAPLN2/ETV2/S1PR3/GJA4/STC1/ACVRL1/SOX17/CD34/MARVELD2/PPP1R16B/RHOB/PTN/COL18A1/NOTCH4/TIE1/SOX18/HOXA13/CDH5/ADAMTS12/RAPGEF2/ATOH8/COL15A1/DLL1/HEY2/KDR/RAPGEF3/XDH/ENG/APOLD1/ARHGEF26/ROBO4/PTPRS/PDE2A/CLDN5/GDF2

CELLULAR RESPIRATION	CELLULAR RESPIRATION	178	-0.364017	-1.62	0.0001835	0.013725	0.01283	7700	tags=43%, list=28%, signal=31%	NDUFS1/CAT/NDUFA5/NDUFV2/NDUFA2/IDH3A/PDHA1/NDUFC1/COX15/NDUFA3/ETFB/NDUFS7/IREB2/UQCRC2/DLD/COQ10A/PIK3CA/COX8A/SURF1/NDUFB8/NDUFV1/SIRT3/NDUFA7/COX7B/COX6B1/MT-ND4/MT-ND1/SUCLG2/NDUFA9/SLC25A23/COX5A/SLC25A12/NDUFB7/OGDHL/SDHD/IDE/SDHA/NDUFA4/NDUFAF1/LYRM7/NDUFA8/FH/NDUFV3/MT-CYB/ACO2/BLOC1S1/NDUFS8/MT-CO3/COX5B/UQCR11/NDUFB10/MT-CO2/MT-ND4L/IMMP2L/MT-ND5/MT-CO1/ACO1/OGDH/COQ9/MT-ND6/GPD1/PINK1/COX4I1/UQCRFS1/NDUFB1/NNT/IDH2/CBFA2T3/ETFDH/COX4I2/SLC25A25/NOS2/NDUFA13/CYP1A2/PPARGC1A/UQCRFS1P1/NR4A3

MITOTIC CELL CYCLE CHECKPOINT	MITOTIC CELL CYCLE CHECKPOINT	151	0.4931681	1.726	0.0001933	0.01424	0.01332	4543	tags=32%, list=16%, signal=27%	HMGA2/SFN/MUC1/TRIP13/AURKB/CDC20/BUB1B/GTSE1/TTK/TICRR/CLSPN/SOX4/ARID3A/ORC1/PLK1/CDK1/NDC80/CDC6/BUB1/BLM/CCNB1/CDT1/CDC25C/FANCD2/EME1/KNTC1/CENPF/CHEK1/HUS1B/ZWINT/E2F7/BRSK1/BRCA1/TFDP1/PCNA/USP44/CHMP4C/XRCC3/MAD2L2/E2F8/CNOT11/MSH2/E2F1/CHEK2/CASP2/SPDL1/PRKDC/PLAGL1/CDK2

CORNIFICATION	CORNIFICATION	76	0.5538732	1.79	0.0002046	0.014852	0.01389	3916	tags=43%, list=14%, signal=37%	KLK13/KRT20/DSG3/KRT4/KRT79/CASP14/PKP3/KRT13/CYP26B1/DSC3/KRT34/LIPM/EVPL/KRT19/KRT5/KRT23/KRT2/SPRR2D/KRT17/SPRR1B/KRT80/KRT6C/DSC2/DSG2/SPRR2E/KRT39/KLK14/DSG4/FLG/SPRR1A/KRT15/SPRR3/KRT12

APPENDAGE MORPHOGENESIS	APPENDAGE MORPHOGENESIS	124	0.5162057	1.78	0.0002195	0.015702	0.01468	5040	tags=35%, list=18%, signal=28%	DLX6/HOXD13/COL2A1/SP8/FRAS1/TBX4/DLX5/HOXA11/CYP26B1/SALL4/NOG/HOXD10/OSR2/ALX3/SHOX2/SOX4/ROR2/FMN1/FLVCR1/AFF3/FBN2/GPC3/SOX9/BMP4/GDF5/LRP4/WNT5A/LEF1/TFAP2A/HDAC2/BAK1/CHST11/FZD6/GRHL2/SFRP2/HOXA9/TWIST1/CHD7/OSR1/IFT52/TBX5/PITX1/WNT7A

NEGATIVE REGULATION OFENDOTHELIAL CELL APOPTOTICPROCESS	NEGATIVE REGULATION OFENDOTHELIAL CELL APOPTOTICPROCESS	30	-0.616478	-2.01	0.0002489	0.017454	0.01632	3255	tags=40%, list=12%, signal=35%	CDH5/GAS6/RAMP2/MIR590/NDNF/KDR/TEK/ANGPTL4/FGG/FGB/FGA/MIR126
SENSORY PERCEPTION OF CHEMICALSTIMULUS	SENSORY PERCEPTION OF CHEMICALSTIMULUS	84	0.5448453	1.788	0.0002511	0.017454	0.01632	1868	tags=27%, list=7%, signal=26%	OR56A3/CST1/GFY/OR8A1/SLC6A3/OR8G5/OR2H1/CST4/OR13A1/OR8G3P/OR12D2/CALHM3/CST2/LPO/TRPM5/SCNN1G/ASIC1/TAS2R60/OR1N2/SCNN1B/REEP2/UGT2A1/ITPR3

GLYOXYLATE METABOLIC PROCESS	GLYOXYLATE METABOLIC PROCESS	10	-0.835777	-1.96	0.0002581	0.017609	0.01647	2628	tags=60%, list=9%, signal=54%	HOGA1/ALDH4A1/IDH2/AGXT/GOT2/AGXT2
ETHANOL OXIDATION	ETHANOL OXIDATION	12	-0.803054	-2.04	0.000263	0.017609	0.01647	2718	tags=58%, list=10%, signal=53%	ADH6/ALDH2/ADH1C/ADH1B/ADH1A/ADH7/ADH4
NEUTRAL LIPID BIOSYNTHETICPROCESS	NEUTRAL LIPID BIOSYNTHETIC PROCESS	44	-0.541648	-1.92	0.000264	0.017609	0.01647	4880	tags=45%, list=18%, signal=37%	THRSP/LPIN1/FBXW7/LDLR/PLIN5/LPIN2/GK/ACSL1/RGN/ANG/DGAT2/C3/SLC27A1/MOGAT1/MOGAT2/AVIL/PCK1/GPLD1/MFSD2A/SIK1

REGULATION OF MITOTIC NUCLEAR DIVISION	REGULATION OF MITOTIC NUCLEARDIVISION	104	0.5197806	1.748	0.0002782	0.01831	0.01712	5980	tags=47%, list=22%, signal=37%	EREG/TRIP13/SPHK1/AURKB/CDC20/BUB1B/TTK/DLGAP5/EDN3/PLK1/FBXO43/NDC80/BUB1/CCNB1/FBXO5/CDT1/MTBP/PKMYT1/CDC25C/BMP4/NEK2/KNTC1/NSMCE2/CENPF/CHEK1/TGFA/MKI67/ZWINT/ESPL1/NUSAP1/L3MBTL1/USP44/XRCC3/MAD2L2/RAD21/CCDC8/IL1A/SPDL1/BORA/ANAPC7/RCC1/PCID2/IGF2/NUP62/AURKA/NME6/BTC/MAD2L1/DYNC1LI1

CELLULAR GLUCAN METABOLICPROCESS	CELLULAR GLUCAN METABOLICPROCESS	67	-0.488653	-1.89	0.000298	0.018994	0.01776	5749	tags=40%, list=21%, signal=32%	PHKB/IL6ST/PGM1/EPM2AIP1/IRS2/SORBS1/PYGM/PHKG1/LEPR/PCDH12/PPP1R3B/INSR/INPP5K/PER2/ENPP1/AGL/PRKAG2/POMC/PPP1R1A/ESRRB/GYG2/GCGR/GYS2/GCK/IGF1/GNMT/G6PC

RESPIRATORY ELECTRON TRANSPORTCHAIN	RESPIRATORY ELECTRON TRANSPORTCHAIN	113	-0.398259	-1.67	0.0002993	0.018994	0.01776	8878	tags=54%, list=32%, signal=37%	SLC25A13/GHITM/UQCRQ/NDUFAB1/SDHB/NDUFA1/NDUFB2/NDUFS3/COX10/NDUFS1/NDUFA5/NDUFV2/NDUFA2/NDUFC1/COX15/NDUFA3/ETFB/NDUFS7/UQCRC2/DLD/COX8A/NDUFB8/NDUFV1/NDUFA7/COX7B/COX6B1/MT-ND4/MT-ND1/NDUFA9/COX5A/SLC25A12/NDUFB7/SDHD/SDHA/NDUFA4/NDUFAF1/NDUFA8/NDUFV3/MT-CYB/NDUFS8/MT-CO3/COX5B/UQCR11/NDUFB10/MT-CO2/MT-ND4L/IMMP2L/MT-ND5/MT-CO1/COQ9/MT-ND6/GPD1/PINK1/COX4I1/UQCRFS1/NDUFB1/ETFDH/COX4I2/NDUFA13/PPARGC1A/UQCRFS1P1

COAGULATION	COAGULATION	307	-0.300754	-1.44	0.0003002	0.018994	0.01776	5000	tags=29%, list=18%, signal=24%	NOS3/GGCX/ARRB1/CPB2/MPL/JMJD1C/PLCG2/C1QTNF1/CD34/CYP4F11/PRKCH/AK3/PRKACA/ADAMTS13/GNG2/F7/SH2B3/EHD3/SERPINC1/PEAR1/APOH/PRDX2/SERPINF2/CD36/PLG/THBD/PRKG1/VTN/FYN/PRTN3/IL6/SERPIND1/COMP/NFE2/F2RL3/DGKI/GAS6/GP1BA/SERPING1/ZFPM1/ZFPM2/PRKCE/SERPINA10/CYP4F2/F10/PLAT/STXBP1/ITPR2/SLC4A1/TBXA2R/SELP/F5/F8/C4BPB/APOE/PIK3R1/SERPINA5/THBS1/A2M/F13B/HPS5/ANXA8/GATA1/F12/ITGB3/GP6/P2RX3/F9/KNG1/F13A1/FGG/PROZ/HRG/VWF/FGB/FGA/HBG2/P2RX1/GNA14/KLKB1/SAA1/HGFAC/HBB/F11/HBE1/KRT1/CLEC1B/P2RX2

KERATINIZATION	KERATINIZATION	97	0.5285699	1.767	0.000308	0.019148	0.01791	4783	tags=46%, list=17%, signal=39%	KLK13/KRT20/KRTAP4-1/DSG3/KRT4/KRT79/CASP14/SFN/PKP3/KRT13/CYP26B1/DSC3/KRT34/ABCA12/LIPM/EVPL/KRT19/KRT5/KRT23/KRTAP5-7/KRT2/KRTAP1-1/SPRR2D/KRTAP5-5/KRT17/SPRR1B/KRT80/KRT6C/DSC2/DSG2/SPRR2E/KRT39/KLK14/DSG4/FLG/SPRR1A/KRT15/SPRR3/KRT12/KRTAP5-1/HRNR/KRT8/DSC1/KRT83/SHARPIN

TRIGLYCERIDE BIOSYNTHETICPROCESS	TRIGLYCERIDE BIOSYNTHETIC PROCESS	37	-0.571744	-1.96	0.0003162	0.019148	0.01791	4880	tags=49%, list=18%, signal=40%	THRSP/LPIN1/FBXW7/LDLR/PLIN5/LPIN2/GK/ACSL1/RGN/DGAT2/C3/SLC27A1/MOGAT1/MOGAT2/PCK1/GPLD1/MFSD2A/SIK1
POSITIVE REGULATION OF ORGANGROWTH	POSITIVE REGULATION OF ORGAN GROWTH	44	-0.535553	-1.9	0.0003179	0.019148	0.01791	4968	tags=36%, list=18%, signal=30%	HLX/SMO/AKAP6/ACACB/MEF2C/ZFPM2/MIR590/HEY2/TGFBR3/TBX20/WT1/IGF1/BASP1/ARX/HAMP/BMP10

REGULATION OF CHROMOSOMEORGANIZATION	REGULATION OF CHROMOSOME ORGANIZATION	252	0.4216369	1.538	0.0003223	0.019148	0.01791	8239	tags=40%, list=30%, signal=28%	PKIB/MUC1/TRIP13/UBE2C/SDR16C5/AURKB/CDC20/BUB1B/TTK/MYB/DLGAP5/MCM2/DNMT3B/PLK1/NDC80/PTTG1/CDC6/AICDA/BUB1/CCNB1/PRDM9/TOP2A/FBXO5/CDT1/SRC/GCG/TACC3/NEK2/KNTC1/NSMCE2/CENPF/LIF/MYC/MAPK15/PHF19/ZWINT/ESPL1/UBE2DNL/BRCA1/FEN1/CCT3/TET1/USP44/CENPE/CCT5/XRCC3/MAD2L2/RAD21/SMG5/CCT2/SPDL1/PIF1/DKC1/CDCA5/CCT6A/DNMT1/ANAPC7/NOS1/PCID2/MTF2/RMI2/CCT4/GNL3/NELFE/KAT2A/PINX1/SMARCB1/MAD2L1/CCT7/DYNC1LI1/KDM1A/ACD/XRCC5/TRIM28/NABP2/CENPV/TCP1/CHFR/MAD2L1BP/PPHLN1/MAPKAPK5/CCT8/DDX11/SSBP1/MAPT/MAPK3/CDC16/MNAT1/BUB3/PAXIP1/ZNF207/IL1B/HNRNPA1/SFPQ/HNRNPC/HNRNPU/PHF1/CDK5RAP2/CHTOP/ANAPC1/SIRT6

REGULATION OF MITOTIC SISTERCHROMATID SEGREGATION	REGULATION OF MITOTIC SISTER CHROMATID SEGREGATION	43	0.6253137	1.836	0.0003286	0.019148	0.01791	4256	tags=47%, list=15%, signal=39%	TRIP13/AURKB/CDC20/BUB1B/TTK/PLK1/NDC80/PTTG1/BUB1/CCNB1/FBXO5/CDT1/KNTC1/CENPF/ZWINT/USP44/XRCC3/MAD2L2/RAD21/SPDL1

MITOCHONDRIAL ELECTRON TRANSPORTNADH TO UBIQUINONE	MITOCHONDRIAL ELECTRON TRANSPORTNADH TO UBIQUINONE	54	-0.498608	-1.83	0.0003317	0.019148	0.01791	8675	tags=59%, list=31%, signal=41%	NDUFAB1/NDUFA1/NDUFB2/NDUFS3/NDUFS1/NDUFA5/NDUFV2/NDUFA2/NDUFC1/NDUFA3/NDUFS7/DLD/NDUFB8/NDUFV1/NDUFA7/MT-ND4/MT-ND1/NDUFA9/NDUFB7/NDUFA4/NDUFAF1/NDUFA8/NDUFV3/NDUFS8/NDUFB10/MT-ND4L/MT-ND5/COQ9/MT-ND6/PINK1/NDUFB1/NDUFA13

RRNA METABOLIC PROCESS	RRNA METABOLIC PROCESS	223	0.4467514	1.618	0.000333	0.019148	0.01791	10932	tags=62%, list=40%, signal=38%	ERN2/SLFN13/PWP2/BOP1/FBLL1/DCAF13/RRS1/NPM3/RRP9/RPP40/EXOSC4/RPS15/RIOK1/LYAR/NIFK/PRKDC/NOP58/RPS7/NOP56/CHD7/DKC1/RPL7/RPS8/RPF2/DHX37/RPL27/RPS9/BYSL/WDR12/UTP23/MRTO4/WDR3/EXOSC3/METTL5/UTP18/REXO4/ERI1/WDR46/HEATR1/ZNHIT6/RPL14/ISG20L2/RPL5/EIF6/NSUN5/RRP12/RRP15/NHP2/PIH1D2/WDR75/NVL/RPS6/PES1/GAR1/EMG1/DDX27/UTP6/TRMT112/POP5/RPS27/GTPBP4/PA2G4/ESF1/ZNHIT3/MAPT/RPF1/UTP14A/DDX52/MAK16/RPL35A/RPS14/RRP36/NOL11/RPUSD3/RPL10A/DDX51/RRP1/NOC4L/EXOSC2/NOP2/RPS21/WDR43/NGDN/UTP20/RPP25/EXOSC5/FBL/DDX54/TRMT61B/NOP10/NSUN4/DDX10/EIF4A3/RPS24/NAT10/WDR74/XRN2/NOB1/ZCCHC4/MRPL1/RPP38/ERCC2/ABT1/RSL1D1/RPP30/MRM1/EXOSC1/RPL7L1/NOL6/EXOSC10/SRFBP1/EBNA1BP2/RRP7A/EXOSC7/RPL7A/SART1/DDX18/RPS16/DROSHA/ERI3/DDX56/NOL10/PDCD11/FCF1/DIMT1/POP7/IMP4/SUV39H1/RPS17/LAS1L/TEX10/RPS25/RPL35/RPS2/RPL11/NOL9/SENP3/C1D/MPHOSPH6

POSITIVE REGULATION OF MUSCLEHYPERTROPHY	POSITIVE REGULATION OF MUSCLEHYPERTROPHY	27	-0.621249	-1.95	0.0003336	0.019148	0.01791	716	tags=59%, list=3%, signal=58%	CDK9/ROCK1/MTOR/PIN1/TRPC3/PPP3CA/MEF2A/CAMK2D/HAND2/IL6ST/AKAP6/IGF1/ADRA1A/NR4A3/HAMP/BMP10
GAMMA AMINOBUTYRIC ACIDSIGNALING PATHWAY	GAMMA AMINOBUTYRIC ACIDSIGNALIN[illegible]	21	0.733475	1.863	0.0003472	0.019697	0.01842	1025	tags=29%, list=4%, signal=28%	GABRA2/GABRA3/GABRB1/GABRR1/GABRR3/GABRA5

ENDOTHELIAL CELL APOPTOTICPROCESS	ENDOTHELIAL CELL APOPTOTICPROCESS	54	-0.495566	-1.82	0.0003801	0.021157	0.01978	3255	tags=37%, list=12%, signal=33%	ITGA4/CD40LG/GATA2/ECSCR/CDH5/GAS6/RGCC/RAMP2/THBS1/MIR590/NDNF/KDR/MAP3K5/TEK/ANGPTL4/FGG/FGB/FGA/CD160/MIR126

ARACHIDONIC ACID METABOLICPROCESS	ARACHIDONIC ACID METABOLICPROCESS	56	-0.499023	-1.86	0.0003815	0.021157	0.01978	3481	tags=34%, list=13%, signal=30%	PTGIS/CYP2C8/CYP4F2/HPGDS/CYP2C18/CYP4F12/FAAH2/EPHX2/CYP2U1/CYP1A1/CYP2A6/CYP2C9/CYP4F3/CYP2E1/CYP1A2/CYP2B6/CYP2A7/CYP2A13/CYP2G1P

TELOMERE ORGANIZATION	TELOMERE ORGANIZATION	140	0.4917764	1.71	0.0003991	0.021836	0.02042	9517	tags=57%, list=34%, signal=38%	PKIB/AURKB/RAD51/RECQL4/BLM/EXO1/SRC/NEK2/NSMCE2/CCNE1/MYC/CCNE2/MAPK15/HUS1B/RFC4/FEN1/CCT3/DCLRE1C/PCNA/PRIM1/CCT5/XRCC3/SMG5/PRIM2/CCT2/POLD1/HSP90AB1/PRKDC/PIF1/DKC1/POLA1/CCT6A/TPRKB/HSP90AA1/CCT4/GNL3/DCLRE1B/PINX1/CCT7/NHP2/ACD/GAR1/XRCC5/RFC5/NABP2/POLA2/TCP1/DNA2/MAPKAPK5/APEX1/SMARCAL1/RAD51D/CCT8/PTGES3/MAPK3/RFC3/WRN/DOT1L/POLE4/RFC2/BRCA2/HNRNPA1/HNRNPC/HNRNPU/XRCC6/RAD51C/SIRT6/NOP10/NAT10/WRAP53/HNRNPA2B1/PARP1/TERF1/MAP3K4/TEN1/RIF1/EXOSC10/HNRNPD/POLE2/NBN

REGULATION OF HUMORAL IMMUNERESPONSE	REGULATION OF HUMORAL IMMUNERESPONSE	126	-0.37923	-1.61	0.0004055	0.021836	0.02042	3975	tags=37%, list=14%, signal=32%	IGHV3-23/IGHV3-11/IGKV5-2/IGLV1-44/CLU/IGKV1D-12/VTN/IGLV3-19/CCR7/SERPING1/CFH/IGLC7/CFHR2/C5/C4BPB/SPNS2/CD5L/IGHG1/C4A/A2M/CFHR1/IGLV2-14/CFP/CFB/IGLV1-51/C1S/C3/C8B/IGLV1-40/CFI/IGHV2-70/IGKV2D-30/IGLC2/C4BPA/SPINK5/C1R/C7/CFHR5/IGKV1-12/C9/IGKV1D-39/C8A/HPX/IGLC6/C6/CFHR4/KLK5

EMBRYONIC APPENDAGEMORPHOGENESIS	EMBRYONIC APPENDAGEMORPHOGENESIS	105	0.516902	1.743	0.000407	0.021836	0.02042	5040	tags=36%, list=18%, signal=30%	DLX6/HOXD13/SP8/FRAS1/TBX4/DLX5/HOXA11/CYP26B1/SALL4/NOG/HOXD10/OSR2/ALX3/SHOX2/ROR2/FLVCR1/AFF3/FBN2/GPC3/BMP4/GDF5/LRP4/WNT5A/LEF1/TFAP2A/HDAC2/CHST11/FZD6/GRHL2/SFRP2/HOXA9/TWIST1/CHD7/OSR1/IFT52/TBX5/PITX1/WNT7A

HOMOPHILIC CELL ADHESIONVIA PLASMA MEMBRANE ADHESIONMOLECULES	HOMOPHILIC CELL ADHESIONVIA PLASMA MEMBRANE ADHESIONMOLECULES	159	-0.350441	-1.55	0.0004406	0.023383	0.02187	3844	tags=36%, list=14%, signal=32%	CDH19/PCDHB3/PCDH15/PCDHB7/DSG1/CDHR5/CNTN2/PCDHB18P/RET/PCDHGA7/CDH5/TRO/CDH1/PCDHGB1/PCDHGA6/CDH23/CDH7/PCDHGA9/PCDHB2/PCDHGB3/PCDHB12/PCDHGB7/CNTN6/PCDHB14/PCDHA1/PCDHA10/CADM1/PCDHGA12/PCDHB4/EMB/PCDHB16/PCDHB13/CDH12/PCDHGC5/PCDHGC3/PCDHB6/PCDH10/IGSF9/ROBO4/PCDHA8/PCDHGA1/PCDHGA2/PCDHGA5/SDK1/IGFN1/PCDHB15/PCDHB8/PCDHGA3/PCDHB11/PCDHA4/FAT4/PCDHGC4/SDK2/CNTN4/PCDHGB2/PCDHA12/CDH8/PCDH20

COTRANSLATIONAL PROTEINTARGETING TO MEMBRANE	COTRANSLATIONAL PROTEINTARGETING TO MEMBRANE	102	0.508049	1.711	0.0004534	0.0234	0.02188	10826	tags=72%, list=39%, signal=44%	RPL36A/BHLHE40-AS1/RPL8/RPL30/RPS15/RPL36/RPL17/RPS7/RPS18/RPL23/RPL7/RPS8/RPL27/RPS9/TRAM1/RPL37/RPL29/RPL23A/RPS23/RPS20/RPL14/RPL5/RPSA/RPL37A/RPL6/RPL31/RPS6/RPL19/RPS27/RPS27A/TRAM1L1/RPL24/RPL32/RPS12/RPL35A/RPS4Y1/RPS14/RPL10A/RPL39/RPS10/RPS21/RPL12/RPLP0/SSR2/RPL4/ZFAND2B/RPS24/SRP14/SRP9/RPS15A/RPL15/RPS3/RPL38/RPS13/RPL10/RPL27A/RPL22/SSR1/RPL18A/RPL7A/RPS29/RPS5/SSR3/RPS16/SNAP25-AS1/ARL6IP1/RPS17/RPLP2/RPS25/RPL35/RPS2/RPL11/RPL21

REGULATION OF VASCULATUREDEVELOPMENT	REGULATION OF VASCULATURE DEVELOPMENT	253	-0.315853	-1.48	0.0004546	0.0234	0.02188	5808	tags=31%, list=21%, signal=25%	CCL24/SPARC/ANGPT2/HSPB6/PTK2B/SIRT1/ANGPTL3/SMOC2/GATA2/ERBB2/GAB1/NOS3/FLT1/COL4A2/HYAL1/STAB1/CD34/RHOJ/SYNJ2BP/PPP1R16B/ECSCR/NODAL/THBS2/RHOB/PTN/PLXND1/APOH/TIE1/PTGIS/SMAD1/HSPG2/ETS1/IL6/CDH5/KLF2/GLUL/SEMA3E/ECM1/PROK1/SASH1/GPR4/HIPK2/TBXA2R/SERPINF1/RGCC/DLL1/TLR3/C5/RAMP2/APLNR/THBS1/ADAMTS1/ATP2B4/DCN/KDR/CCBE1/C3/RAPGEF3/THBS4/TEK/GADD45A/ENG/ANGPTL4/NPR1/SPINK5/HRG/TMIGD2/CD160/ANGPT4/MIR126/CLDN5/SFRP1/BMPER/WNT4/KRT1/GDF2/CMA1/ANGPTL7

DNA PACKAGING	DNA PACKAGING	147	0.4867923	1.7	0.0004551	0.0234	0.02188	7015	tags=42%, list=25%, signal=32%	HMGA2/TNP1/ERN2/CHD5/HJURP/NCAPH/CENPA/NCAPG/CENPW/MCM2/HMGA1/BANF2/CENPM/AICDA/CCNB1/PRDM9/TOP2A/OIP5/CENPI/CHAF1B/ASF1B/BEND3/SOX9/NCAPD2/NCAPG2/NUSAP1/CENPK/TET1/CENPL/NCAPH2/NPM1/HMGB2/CDKN2A/RUVBL1/CENPQ/SMYD3/CENPH/NAP1L1/CENPO/CDCA5/ITGB3BP/DNMT1/SRPK1/INCENP/CHAF1A/DNMT3A/HAT1/SMC4/NASP/IPO4/RNF8/DAXX/NOC2L/ANP32B/TRIM28/MIS18A/BANF1/CENPV/ASF1A/SET/PPHLN1/SMC2

REGULATION OF COAGULATION	REGULATION OF COAGULATION	70	-0.460762	-1.8	0.0004675	0.023791	0.02225	5000	tags=43%, list=18%, signal=35%	NOS3/CPB2/C1QTNF1/CD34/F7/SH2B3/SERPINC1/APOH/PRDX2/SERPINF2/CD36/PLG/THBD/PRKG1/VTN/GP1BA/SERPING1/PLAT/TBXA2R/APOE/THBS1/F12/KNG1/FGG/HRG/FGB/FGA/KLKB1/F11/KRT1

RNA 3 END PROCESSING	RNA 3 END PROCESSING	135	0.46945	1.626	0.0004733	0.023837	0.02229	10178	tags=52%, list=37%, signal=33%	CT45A10/LIN28B/LIN28A/FBLL1/SAGE1/CCNB1/PABPC1/BARD1/CPEB1/CPSF4L/INTS8/SSB/THOC7/EXOSC4/CSTF2/PABPC1L/DKC1/MAGOH/UPF3B/NELFE/NCBP2/ALYREF/EXOSC3/SRSF9/HSF1/CPSF6/ZC3H3/ERI1/SLBP/CPSF1/PNPT1/THOC5/THOC1/TRMT10C/CPSF4/SRSF3/SRSF7/RNPS1/THOC3/SARNP/TOE1/THOC2/EXOSC2/RPS21/CHTOP/EXOSC5/FBL/LSM11/U2AF2/CPSF3/POLDIP3/EIF4A3/LEO1/POLR2D/ZNF473/FIP1L1/RBM8A/SRSF2/SNRPA/PTCD1/PAPOLA/EXOSC10/SRSF1/EXOSC7/MTPAP/ERI3/TRNT1/INTS7/CSTF3/DDX39A

REGULATION OF COMPLEMENTACTIVATION	REGULATION OF COMPLEMENT ACTIVATION	107	-0.403732	-1.68	0.0004945	0.024653	0.02305	3975	tags=39%, list=14%, signal=34%	IGHV3-23/IGHV3-11/IGKV5-2/IGLV1-44/CLU/IGKV1D-12/VTN/IGLV3-19/SERPING1/CFH/IGLC7/CFHR2/C5/C4BPB/CD5L/IGHG1/C4A/A2M/CFHR1/IGLV2-14/CFP/CFB/IGLV1-51/C1S/C3/C8B/IGLV1-40/CFI/IGHV2-70/IGKV2D-30/IGLC2/C4BPA/C1R/C7/CFHR5/IGKV1-12/C9/IGKV1D-39/C8A/IGLC6/C6/CFHR4

DRUG CATABOLIC PROCESS	DRUG CATABOLIC PROCESS	24	-0.655475	-2.02	0.0005047	0.024912	0.0233	3400	tags=58%, list=12%, signal=51%	CYP3A5/CYP2C8/FMO4/CYP2C18/CYP3A4/CYP2U1/CYP2A6/CYP2C9/CYP2E1/CYP1A2/CYP2B6/CYP2A7/CYP2A13/CYP2G1P

DRUG METABOLIC PROCESS	DRUG METABOLIC PROCESS	44	-0.523876	-1.86	0.0005228	0.025552	0.02389	3520	tags=50%, list=13%, signal=44%	FMO5/CYP3A5/CYP2C8/CYP4F2/FMO4/CYP2C18/CYP4F12/ABCB11/AOX1/CYP3A4/CYP2U1/FMO2/CYP1A1/CYP2A6/CYP2C9/CYP2E1/ADH1A/CYP1A2/CYP2B6/CYP2A7/CYP2A13/CYP2G1P
REGULATION OF FIBRINOLYSIS	REGULATION OF FIBRINOLYSIS	14	-0.735447	-1.94	0.0005338	0.025833	0.02416	4775	tags=71%, list=17%, signal=59%	CPB2/APOH/SERPINF2/PLG/THBD/THBS1/F12/HRG/KLKB1/F11

MEIOTIC CHROMOSOME SEGREGATION	MEIOTIC CHROMOSOME SEGREGATION	67	0.5902652	1.863	0.0005626	0.02696	0.02521	3718	tags=36%, list=13%, signal=31%	TEX15/FMN2/SYCE1/MEI4/TRIP13/BUB1B/TTK/MAEL/NUF2/PLK1/PTTG1/BUB1/PRDM9/TOP2A/FANCD2/EME1/CCNE1/CCNE2/MAPK15/ESPL1/P3H4/MSH4/RAD21/NCAPH2

SENSORY PERCEPTION	SENSORY PERCEPTION	462	0.3781086	1.426	0.0006215	0.029445	0.02753	4060	tags=22%, list=15%, signal=19%	OR56A3/CST1/COL2A1/OTOG/GJA10/GFY/OR8A1/SLC6A3/SIX3/MYO3A/OR8G5/LHFPL4/OR2H1/GIP/SFRP5/ADORA1/CST4/OR13A1/OR8G3P/OPRK1/USH1C/OR12D2/ATP6V0A4/CLDN19/CALHM3/CNGB3/CST2/GJB6/POU3F4/LPO/LHFPL3/MYO3B/HOXD1/SRRM4/TRPM5/USH1G/NDP/SLC52A3/GABRA5/SCNN1G/ASIC1/PDE6B/SERPINE2/NR2E1/TAS2R60/CHRNA7/RDH8/OR1N2/EYA1/GRIN2D/ZIC2/TULP1/COL11A1/PAX3/SCNN1B/KCNJ10/GRM1/NDN/SLC17A8/LRP2/REEP2/UGT2A1/LHFPL5/ITPR3/CCK/FABP5/BIRC5/TMIE/CRYGN/TPRN/OTOGL/MYO1A/PIGR/TRPA1/IRX5/COCH/PPEF1/POU4F1/FZD2/P2RX4/SCN1A/VSX1/PTGES/DCDC2/PKHD1L1/SIX1/HOXA1/GJA3/RDH12/KCNQ4/RS1/CABP4/LEF1/ATP8A2/TIMM13/HOXB8/TFAP2A/KRT12/CACNA2D4/TACSTD2/FAM161A/OPRL1
ANDROGEN BIOSYNTHETIC PROCESS	ANDROGEN BIOSYNTHETIC PROCESS	11	-0.766396	-1.85	0.0006313	0.029445	0.02753	2102	tags=55%, list=8%, signal=50%	SRD5A3/SRD5A2/HSD17B6/SRD5A1/CYP17A1/WNT4

POSITIVE REGULATION OFVASOCONSTRICTION	POSITIVE REGULATION OFVASOCONSTRICTION	29	-0.59851	-1.92	0.0006323	0.029445	0.02753	1435	tags=38%, list=5%, signal=36%	TBXA2R/EGFR/ADRA1B/FGG/FGB/AVPR2/FGA/HTR2A/AVPR1A/DBH/ADRA1A
HISTONE EXCHANGE	HISTONE EXCHANGE	39	0.632484	1.832	0.0006418	0.029605	0.02768	6534	tags=56%, list=24%, signal=43%	TNP1/CHD5/HJURP/CENPA/CENPW/CENPM/OIP5/CENPI/CENPK/VPS72/CENPL/NPM1/RUVBL1/CENPQ/CENPH/CENPO/ITGB3BP/ANP32E/NASP/RNF8/ACTR6/MIS18A

CELLULAR AMINO ACID METABOLICPROCESS	CELLULAR AMINO ACID METABOLICPROCESS	293	-0.294855	-1.41	0.0006583	0.030085	0.02813	5483	tags=29%, list=20%, signal=24%	MRI1/PPA2/AMDHD1/GLDC/PHYKPL/ADHFE1/HIBADH/ARG1/NOS3/FOLH1/SDS/ACAD8/ACADSB/MCCC2/SHMT1/HGD/ALDH8A1/MTHFS/GOT1/GFPT2/ILVBL/SARDH/SLC39A8/AFMID/PAH/OGDH/DAO/ASL/UROC1/SEPSECS/HAAO/HPD/ACCS/GLUL/IVD/ACMSD/ART4/HAL/OTC/SERINC5/CDO1/BAAT/UPB1/MAT1A/HOGA1/CSAD/RIMKLB/ASS1/MTHFD1/HMGCLL1/ALDH4A1/ALDH7A1/BCAT2/GPT2/ATP2B4/ALDH6A1/TH/KMO/IYD/GSTZ1/FTCD/GCDH/ACAT1/AGXT/CPS1/GOT2/GPT/PRODH/NOS2/ABAT/AASS/GLYATL1/ASPG/OAT/AADAT/CTH/GADL1/AGXT2/GGT5/GLS2/TAT/GNMT/HDC/ASPA/TTC36

PROTEIN LOCALIZATION TOCHROMOSOME	PROTEIN LOCALIZATION TOCHROMOSOME	79	0.5328473	1.735	0.0006798	0.030783	0.02879	7164	tags=46%, list=26%, signal=34%	AURKB/BUB1B/TTK/CENPA/ESCO2/EZH2/PLK1/CDK1/NDC80/CDT1/MTBP/MCM8/RCC2/CCT3/CCT5/RAD21/CCT2/MSH2/CENPQ/SPDL1/DKC1/CDCA5/CCT6A/CCT4/GNL3/PINX1/CCT7/BOD1/ACD/XRCC4/XRCC5/NABP2/TCP1/LEMD2/PPHLN1/CCT8

CELL CYCLE DNA REPLICATION	CELL CYCLE DNA REPLICATION	63	0.5739973	1.795	0.0006992	0.031373	0.02934	7867	tags=60%, list=28%, signal=43%	GINS1/RAD51/MCM2/AICDA/MCM3/MCM4/FBXO5/MCM6/CDT1/CDC7/DBF4/MCM7/MCM5/E2F7/RFC4/CDC45/FEN1/PCNA/PRIM1/E2F8/GMNN/PRIM2/POLD1/DBF4B/CHEK2/MCMDC2/POLA1/GINS3/TIPIN/RFC5/POLA2/DNA2/DONSON/RFC3/POLE4/LIG1/RFC2/BRCA2

NEUTRAL AMINO ACID TRANSPORT	NEUTRAL AMINO ACID TRANSPORT	40	0.6260417	1.82	0.0007347	0.032673	0.03055	5299	tags=42%, list=19%, signal=34%	SLC7A10/SLC6A19/SLC6A14/SLC6A20/SLC6A15/SLC1A5/SLC38A1/ACE2/SLC6A9/SLC3A1/SLC1A4/SLC7A8/LEP/RGS2/SLC3A2/SLC38A5/NFKBIE

REGULATION OF CELL CYCLE G2 MPHASE TRANSITION	REGULATION OF CELL CYCLE G2 MPHASE TRANSITION	207	0.4405566	1.582	0.0007435	0.032767	0.03064	9122	tags=47%, list=33%, signal=32%	HMGA2/TUBB4A/PSMA8/PKIA/AURKB/GTSE1/KCNH5/TICRR/CLSPN/TPX2/CDC25A/ORC1/PLK1/CDK1/CDC6/BLM/CCNB1/FBXO5/CDC7/CDC25C/NEK2/CENPF/CHEK1/HUS1B/DTL/CDK4/RCC2/BRSK1/BRCA1/STOX1/CHMP4C/RAD21/NPM1/NEK10/SFI1/TUBA4A/DBF4B/HMMR/TUBG1/HAUS1/PSMB9/CDC25B/CDK2/ATAD5/HAUS5/HAUS6/TUBB/HSP90AA1/PSMD14/AURKA/PINX1/CEP131/DYNLL1/MTA3/PSMD4/CEP72/CENPJ/PSMD10/RAD51B/NABP2/CKAP5/PRKAR2B/HAUS8/DONSON/DYNC1I2/NEDD1/CEP41/KIF14/CEP152/RBX1/PAXIP1/CEP78/PSMB3/TOPBP1/MAPRE1/PSME2/CCND1/HAUS2/CDK5RAP2/RAD51C/PLK4/PSMD6/WNT10B/CSNK1E/PSMB8/PSMA5/CETN2/UIMC1/MIIP/CEP135/CEP250/NAE1/PSMB5/NDE1/PSMD1/ZFYVE19/PSMB4

ATP SYNTHESIS COUPLED ELECTRONTRANSPORT	ATP SYNTHESIS COUPLED ELECTRONTRANSPORT	97	-0.399279	-1.65	0.000788	0.034423	0.03219	8801	tags=55%, list=32%, signal=37%	GHITM/UQCRQ/NDUFAB1/NDUFA1/NDUFB2/NDUFS3/COX10/NDUFS1/NDUFA5/NDUFV2/NDUFA2/NDUFC1/COX15/NDUFA3/NDUFS7/UQCRC2/DLD/COX8A/NDUFB8/NDUFV1/NDUFA7/COX7B/COX6B1/MT-ND4/MT-ND1/NDUFA9/COX5A/NDUFB7/SDHD/SDHA/NDUFA4/NDUFAF1/NDUFA8/NDUFV3/MT-CYB/NDUFS8/MT-CO3/COX5B/UQCR11/NDUFB10/MT-CO2/MT-ND4L/MT-ND5/MT-CO1/COQ9/MT-ND6/PINK1/COX4I1/UQCRFS1/NDUFB1/COX4I2/NDUFA13/UQCRFS1P1

REGULATION OF CELL CYCLE PHASETRANSITION	REGULATION OF CELL CYCLE PHASETRANSITION	426	0.3808383	1.432	0.0008475	0.036359	0.034	5152	tags=24%, list=19%, signal=20%	TFDP3/HMGA2/SIX3/TUBB4A/SFN/MUC1/PSMA8/TRIP13/PKIA/UBE2C/AURKB/CDC20/BUB1B/GTSE1/TTK/DLGAP5/KCNH5/TICRR/CLSPN/SOX4/ARID3A/TPX2/CDC25A/ORC1/EZH2/PLK1/CDK1/NDC80/UBD/CDC6/ANLN/BUB1/BLM/CCNB1/FBXO5/CDT1/MTBP/CDC7/CDC25C/DBF4/NEK2/KNTC1/NSMCE2/CENPF/CHEK1/MAPK15/HUS1B/DTL/ZWINT/ESPL1/CDK4/E2F7/UBE2DNL/RCC2/BRSK1/BRCA1/STOX1/TFDP1/PCNA/USP44/RBL1/CHMP4C/CENPE/XRCC3/MAD2L2/RAD21/E2F8/NPM1/CNOT11/CDKN2A/NEK10/SFI1/TUBA4A/DBF4B/HMMR/TUBG1/HAUS1/FAM83D/PSMB9/E2F1/CHEK2/CASP2/OVOL1/SPDL1/CDC25B/PRKDC/PLAGL1/BID/CDK2/TMEM14B/ATAD5/CDCA5/HAUS5/ANAPC7/TFAP4/HAUS6/NEK11/UBE2E1/PCID2/ZNF16/TUBB/HSP90AA1

NEGATIVE REGULATION OF METAPHASEANAPHASE TRANSITION OF CELLCYCLE	NEGATIVE REGULATION OF METAPHASEANAPHASE TRANSITION OF CELLCYCLE	40	0.6223195	1.81	0.0008518	0.036359	0.034	4256	tags=48%, list=15%, signal=40%	TRIP13/AURKB/CDC20/BUB1B/TTK/PLK1/NDC80/BUB1/CCNB1/FBXO5/CDT1/KNTC1/CENPF/ZWINT/USP44/XRCC3/MAD2L2/RAD21/SPDL1

RECOMBINATIONAL REPAIR	RECOMBINATIONAL REPAIR	126	0.4906009	1.693	0.0008545	0.036359	0.034	6723	tags=37%, list=24%, signal=28%	TEX15/RAD51/MCM2/AUNIP/RAD54L/XRCC2/RECQL4/RAD51AP1/BLM/MCM3/MCM4/MCM6/POLQ/CDC7/PARPBP/MMS22L/TONSL/MCM7/GINS4/RPA4/NSMCE2/CHEK1/MCM8/HUS1B/MCM5/FANCB/CDC45/BRCA1/FEN1/XRCC3/MAD2L2/RAD21/RAD54B/MCMDC2/RMI2/RHNO1/TIMELESS/PSMD14/SFR1/UBQLN4/RBBP8/FIGNL1/KDM1A/AP5Z1/RAD51AP2/RAD51B/NABP2

RIBOSOMAL LARGE SUBUNITBIOGENESIS	RIBOSOMAL LARGE SUBUNITBIOGENESIS	69	0.5495194	1.741	0.0008949	0.037754	0.0353	9897	tags=68%, list=36%, signal=44%	RPL10L/BOP1/RPLP0P6/NLE1/RRS1/PAK1IP1/NPM1/BRIX1/NIFK/RPL7/RPF2/WDR12/MRTO4/RPL23A/NOP16/ZNHIT6/RPL14/RPL5/EIF6/RRP15/RPL6/NHP2/NOC2L/NVL/PES1/GTPBP4/RPL24/ZNHIT3/RPF1/NIP7/MAK16/RPL35A/RPL10A/GTF3A/NOP2/RPLP0/WDR74/RPL38/RPL10/MRPL1/SURF6/RSL1D1/RPL7L1/EBNA1BP2/RPL7A/DDX18/RPL26L1
POSITIVE REGULATION OFTRIGLYCERIDE BIOSYNTHETICPRO	POSITIVE REGULATION OFTRIGLYCERIDE	13	-0.75282	-1.96	0.0009294	0.038877	0.03636	3951	tags=54%, list=14%, signal=46%	LDLR/PLIN5/RGN/DGAT2/SLC27A1/GPLD1/MFSD2A

CELL CYCLE CHECKPOINT	CELL CYCLE CHECK POINT	197	0.4381923	1.57	0.0009394	0.038965	0.03644	5499	tags=31%, list=20%, signal=25%	HMGA2/SFN/MUC1/TRIP13/AURKB/CDC20/BUB1B/GTSE1/TTK/TICRR/CLSPN/SOX4/ARID3A/ORC1/PLK1/CDK1/NDC80/CDC6/BUB1/BLM/CCNB1/CDT1/WDR76/CDC25C/FANCD2/EME1/RPA4/KNTC1/CENPF/CHEK1/HUS1B/DTL/ZWINT/E2F7/BRSK1/CDC45/BRCA1/TFDP1/PCNA/USP44/CHMP4C/XRCC3/MAD2L2/E2F8/CNOT11/MSH2/E2F1/CHEK2/CASP2/SPDL1/FBXO6/PRKDC/PLAGL1/CDK2/NEK11/PCID2/RHNO1/TIMELESS/TIPIN/DCLRE1B/AURKA

EAR DEVELOPMENT	EAR DEVELOPMENT	180	0.449642	1.603	0.0009529	0.039194	0.03665	5282	tags=33%, list=19%, signal=27%	DLX6/ESRP1/COL2A1/MYO3A/WNT3A/SOX2/USH1C/DLX5/STRA6/GJB6/HMX3/POU3F4/NOG/MYO3B/USH1G/HMX2/GABRA5/EYA1/OSR2/COL11A1/MCM2/CTHRC1/PTK7/MCOLN3/ROR2/SLC17A8/LHFPL5/TMIE/TPRN/SOX9/PAX8/GBX2/BMP4/SIX4/FZD2/HOXA2/SIX1/HOXA1/TBX18/KCNQ4/WNT5A/DDR1/SLC44A4/HPCA/ATP8A2/TFAP2A/MPV17/FZD6/OTX1/TWIST1/LRIG3/CHD7/PDZD7/OSR1/SIX2/SLC4A7/DVL2/KCNK2/FGF20/PAX2

CENTROMERE COMPLEX ASSEMBLY	CENTROMERE COMPLEX ASSEMBLY	37	0.6245234	1.785	0.001009	0.040973	0.03831	4755	tags=46%, list=17%, signal=38%	HJURP/DLGAP5/CENPA/CENPW/CENPM/OIP5/CENPI/CENPF/CENPK/CENPE/CENPL/NPM1/RUVBL1/CENPQ/CENPH/CENPO/ITGB3BP
NEGATIVE REGULATION OFCOAGULATION	NEGATIVE REGULATION OFCOAGULATION	51	-0.484052	-1.74	0.0010127	0.040973	0.03831	5000	tags=49%, list=18%, signal=40%	NOS3/CPB2/C1QTNF1/CD34/SH2B3/APOH/SERPINF2/PLG/THBD/PRKG1/VTN/GP1BA/SERPING1/PLAT/APOE/THBS1/F12/KNG1/FGG/HRG/FGB/FGA/KLKB1/F11/KRT1

DNA GEOMETRIC CHANGE	DNA GEOMETRIC CHANGE	110	0.4815341	1.638	0.0010637	0.042685	0.03992	8982	tags=47%, list=32%, signal=32%	DQX1/CHD5/ERCC6L/RAD51/MCM2/RAD54L/HMGA1/DSCC1/RECQL4/BLM/MCM3/MCM4/TOP2A/MCM6/POLQ/MCM7/MCM8/MCM5/RFC4/HMGB2/RUVBL1/RAD54B/PIF1/CHD7/MCMDC2/HFM1/CHD1L/CHTF18/XRCC5/RFC5/DDX1/DNA2/RPS27A/SMARCAL1/DDX11/DDX12P/SSBP1/RFC3/MNAT1/WRN/GTF2H1/RBX1/RFC2/XRCC6/DHX9/WRNIP1/CUL4A/CETN2/RUVBL2/HNRNPA2B1/ERCC2/PARP1

HOMOLOGOUS CHROMOSOMESEGREGATION	HOMOLOGOUS CHROMOSOME SEGREGATION	42	0.6286316	1.835	0.0011182	0.044512	0.04162	3698	tags=38%, list=13%, signal=33%	TEX15/FMN2/SYCE1/MEI4/TRIP13/MAEL/PLK1/PTTG1/PRDM9/FANCD2/CCNE1/CCNE2/ESPL1/P3H4/MSH4/RAD21

NUCLEAR TRANSCRIBED MRNACATABOLIC PROCESS NONSENSEMEDIATED DECAY	NUCLEAR TRANSCRIBED MRNACATABOLIC PROCESS NONSENSEMEDIATED DECAY	119	0.4835328	1.657	0.0011653	0.046015	0.04303	11722	tags=74%, list=42%, signal=43%	PABPC1/RPL36A/EIF3E/RPL8/RPL30/SMG5/RPS15/RPL36/RPL17/RPS7/RPS18/RPL23/RPL7/RPS8/MAGOH/RPL27/RPS9/UPF3B/RPL37/RPL29/NCBP2/RPL23A/RPS23/DHX34/RPS20/RPL14/RPL5/RPSA/RPL37A/RPL6/RPL31/RPS6/RPL19/RPS27/RPS27A/RPL24/RPL32/RPS12/UPF3A/RNPS1/UPF2/RPL35A/RPS4Y1/RPS14/RPL10A/RPL39/RPS10/RPS21/RPL12/RPLP0/RPL4/EIF4A3/RPS24/RPS15A/RPL15/RPS3/RPL38/RPS13/RPL10/RPL27A/RBM8A/RPL22/EXOSC10/RPL18A/RPL7A/RPS29/RPS5/RPS16/RPS17/RPLP2/CASC3/RPS25/RPL35/RPS2/RPL11/RPL21/RPL13A/RPS4X/RPS11/DCP2/DCP1A/RPL26/PPP2R2A/SMG9/RPL18/MAGOHB/PPP2CA/SMG8
NEGATIVE REGULATION OF EXECUTIONPHASE OF	NEGATIVE REGULATION OF EXECUT	16	-0.709084	-1.95	0.0012007	0.047037	0.04399	597	tags=25%, list=2%, signal=24%	MTRNR2L10/MTRNR2L3/MTRNR2L1/CIDEA

REGULATION OF NUCLEAR DIVISION	REGULATION OF NUCLEAR DIVISION	127	0.4862007	1.675	0.0012263	0.047661	0.04457	5980	tags=43%, list=22%, signal=34%	EREG/PSMA8/TRIP13/SPHK1/AURKB/CDC20/BUB1B/TTK/DLGAP5/EDN3/PLK1/FBXO43/NDC80/RAD51AP1/BUB1/CCNB1/PRDM9/FBXO5/CDT1/MTBP/PKMYT1/CDC25C/BMP4/NEK2/KNTC1/NSMCE2/CENPF/LIF/CHEK1/STRA8/TGFA/MKI67/ZWINT/ESPL1/NUSAP1/L3MBTL1/USP44/WNT5A/XRCC3/MAD2L2/RAD21/CCDC8/IL1A/SPDL1/BORA/ANAPC7/RCC1/PCID2/IGF2/NUP62/AURKA/NME6/BTC/MAD2L1/DYNC1LI1

BRANCHED CHAIN AMINO ACIDMETABOLIC PROCESS	BRANCHED CHAIN AMINO ACIDMETABOLIC PROCESS	23	-0.628409	-1.92	0.0012387	0.047709	0.04461	6868	tags=78%, list=25%, signal=59%	DLD/AUH/MCCC1/BCKDK/HIBADH/SDS/ACAD8/ACADSB/MCCC2/DBT/ILVBL/BCKDHB/IVD/BCKDHA/BCAT2/ALDH6A1/ACAT1/PPM1K
CYTOLYSIS	CYTOLYSIS	30	-0.567335	-1.85	0.0012618	0.047709	0.04461	2723	tags=40%, list=10%, signal=36%	CFHR2/C5/CFHR1/C8B/HRG/C7/CFHR5/C9/PLA2G2A/C8A/C6/REG3G

NEGATIVE REGULATION OFCHROMOSOME ORGANIZATION	NEGATIVE REGULATION OFCHROMOSOME ORGANIZATION	81	0.5224889	1.708	0.0012648	0.047709	0.04461	4480	tags=33%, list=16%, signal=28%	TRIP13/AURKB/CDC20/BUB1B/TTK/MCM2/PLK1/NDC80/PTTG1/AICDA/BUB1/CCNB1/TOP2A/FBXO5/CDT1/SRC/KNTC1/CENPF/ZWINT/ESPL1/TET1/USP44/XRCC3/MAD2L2/RAD21/SPDL1/PIF1

CHROMOSOME CONDENSATION	CHROMOSOME CONDENSATION	28	0.6613116	1.784	0.0012662	0.047709	0.04461	5490	tags=50%, list=20%, signal=40%	HMGA2/ERN2/NCAPH/NCAPG/BANF2/CCNB1/TOP2A/NCAPD2/NCAPG2/NUSAP1/NCAPH2/CDCA5/INCENP/SMC4
PROTEIN LOCALIZATION TOCHROMOSOME CENTROMERIC REGION	PROTEIN LOCALIZATION TOCHROMOSOME CENTROMERIC	23	0.6959666	1.795	0.0012827	0.047718	0.04462	4256	tags=48%, list=15%, signal=41%	AURKB/BUB1B/TTK/CENPA/CDK1/NDC80/CDT1/MTBP/RCC2/CENPQ/SPDL1
TYROSINE METABOLIC PROCESS	TYROSINE METABOLIC PROCESS	12	-0.76385	-1.95	0.0012858	0.047718	0.04462	4398	tags=67%, list=16%, signal=56%	HGD/PAH/HPD/TH/IYD/GSTZ1/TAT/TTC36

REGULATION OF SYSTEMIC ARTERIAL BLOOD PRESSURE MEDIATED BY ACHEMICAL	REGULATION OF SYSTEMIC ARTERIALBLOOD PRESSURE	40	-0.52674	-1.83	0.0013132	0.048371	0.04523	2392	tags=28%, list=9%, signal=25%	HSD11B2/EDN2/ADRA1B/AVPR2/AVPR1A/ENPEP/ADRA1A/CPA3/REN/CTSG/CMA1
