# Supplementary material for: Life span‐associated ferroptosis‐related genes identification and validation for hepatocellular carcinoma patients as hepatitis B virus carriers
Source: J Clin Lab Anal. 2023 Jul 18;37(13-14):e24930. doi: 10.1002/jcla.24930 (PMC10492458; doi:10.1002/jcla.24930)
Supplement: Supplementary file 10 — Tables S1–S14 [file JCLA-37-e24930-s009.zip › TableS14_SRC_1Y57_Docking.pdf]

DrugBank_ID	Hydrogen_Acceptors	Hydrogen_Donors	Rotatable_Bonds	LogP	Molecular_Weight	TPSA	Affinity(kcal/mol)
DB08674	5	1	0	2.8	435.5	83.2	-11.1
DB08676	5	1	0	2.5	453.5	83.2	-11.1
DB08683	3	1	0	3.8	393.4	65.3	-11
DB01395	3	0	0	3.5	366.5	43.4	-10.7
DB15054	5	2	3	2.5	424.5	86.2	-10.6
DB06469	4	3	1	2.2	439.5	88.6	-10.5
DB08006	4	1	2	4.6	325.4	55.1	-10.5
DB07430	5	2	1	4	374.5	95.2	-10.4
DB12185	7	2	1	0.4	435.4	106	-10.4
DB12368	7	1	4	3.2	431.4	77	-10.4
DB04281	3	2	1	4.8	366.8	57.5	-10.3
DB07435	3	3	0	3	304.3	60.7	-10.3
DB02152	5	2	2	2.8	467.5	94.7	-10.2
DB03373	3	3	6	3.8	455.6	106	-10.2
DB06888	6	2	0	2.2	403.4	86.3	-10.2
DB07241	3	5	1	3.8	355.3	105	-10.2
DB08036	1	3	0	3.7	311.3	60.7	-10.2
DB12640	3	1	5	4	480	58.4	-10.2
DB05423	4	1	2	3.5	430.5	55.8	-10.1
DB06666	3	1	3	4.8	473.9	54.3	-10.1
DB06852	4	3	3	3.5	419.9	73	-10.1
DB07307	3	1	4	4.9	393.5	54.9	-10.1
DB08553	5	2	3	2.5	373.5	75.3	-10.1
DB04608	3	3	1	3.5	328.3	82.2	-10
DB07226	3	3	2	3.6	389.8	91.1	-10
DB12200	2	2	2	2.9	369.4	66.9	-10
DB13950	4	0	4	4.4	426.5	43.7	-10
DB15308	4	2	3	3.9	388.4	83.1	-10
DB06732	2	0	1	4.4	272.3	26.3	-9.9
DB06925	7	2	3	4.9	422.4	80.9	-9.9
DB07189	5	2	6	4	464.5	88.5	-9.9
DB07193	7	1	2	1.9	395.4	86.4	-9.9
DB08975	3	1	4	3.6	302.3	54.4	-9.9
DB11911	4	1	3	2.4	369.4	86.7	-9.9
DB12108	6	1	5	1.8	460.5	119	-9.9
DB12306	3	3	0	3.9	390.2	56.9	-9.9
DB14878	4	1	2	3.7	366.5	55	-9.9
DB14982	5	2	4	3.8	432.5	95.6	-9.9
DB15029	8	1	5	3	457.5	71.1	-9.9
DB15408	5	2	3	4.4	349.8	75.1	-9.9
DB07396	8	3	4	4	455.8	98.7	-9.8
DB07969	8	1	2	3.6	388.4	52.5	-9.8
DB08358	3	2	2	2.6	340.4	70.7	-9.8
DB14896	6	1	0	2	380.4	75.4	-9.8
DB02010	4	2	2	3.2	466.5	69.4	-9.7
DB04982	5	1	1	2.7	337.4	77.2	-9.7
DB05812	2	1	1	4.6	349.5	33.1	-9.7
DB07213	5	4	5	2.5	427.5	110	-9.7
DB07830	7	1	4	2.4	392.4	64.8	-9.7
DB07861	5	3	6	4.2	420.5	104	-9.7
DB08738	4	0	5	2.5	444.5	73.3	-9.7
DB14773	8	2	3	3.7	478.4	89.1	-9.7
DB15305	6	1	2	0.5	401.5	78.1	-9.7
DB05608	4	1	2	2.6	400.4	102	-9.6
DB06159	7	1	1	0.8	393.3	126	-9.6
DB06870	2	1	2	3	312.4	37.3	-9.6
DB06959	5	1	6	3	394.5	73.4	-9.6
DB07072	10	1	2	2	419.4	60	-9.6
DB07265	5	3	4	2.8	400.4	109	-9.6
DB07514	5	2	2	3.7	397.5	84.1	-9.6
DB07528	7	1	2	3.7	396.4	72.1	-9.6
DB08166	3	2	0	1.6	328.8	69.8	-9.6
DB08191	3	2	3	4.1	314.3	66	-9.6
DB09199	5	1	5	4.9	381.4	80.7	-9.6
DB11562	7	0	1	2.6	367.4	66.5	-9.6
DB11697	7	1	4	3.8	472.6	68.7	-9.6
DB12012	8	1	4	3.3	455.4	80.2	-9.6

DB14974	8	3	4	3.8	399.4	104	-9.6
DB15446	5	2	5	3.4	355.4	75.6	-9.6
DB03476	1	2	3	4.3	286.4	49.9	-9.5
DB04064	7	2	2	3	378.3	118	-9.5
DB04240	7	4	7	3.5	430.5	124	-9.5
DB04289	2	1	2	4.2	393.5	39.3	-9.5
DB07453	2	0	1	4.8	272.3	26.3	-9.5
DB07608	4	4	7	3.6	447.9	113	-9.5
DB07794	5	2	2	1.9	327.3	97.8	-9.5
DB08044	10	1	2	1.4	445.4	77	-9.5
DB08349	6	2	5	3.5	449.5	79.3	-9.5
DB08512	4	4	3	2.7	356.4	108	-9.5
DB08703	3	1	4	4.3	424.5	63.8	-9.5
DB08731	6	2	6	3.2	403.4	112	-9.5
DB09070	2	1	1	2.4	312.4	37.3	-9.5
DB09280	8	2	5	4.4	452.4	97.8	-9.5
DB09371	2	1	1	2.1	298.4	37.3	-9.5
DB11687	7	4	2	3.3	389.4	116	-9.5
DB12121	6	2	4	3.6	411.5	83.4	-9.5
DB12558	5	2	7	4.6	440.6	60.1	-9.5
DB12897	4	1	3	4	399.5	65.8	-9.5
DB13685	2	1	3	4.7	366.5	29.5	-9.5
DB14584	3	1	1	2.3	328.4	54.4	-9.5
DB14882	5	1	3	3.1	418.5	68.9	-9.5
DB15294	8	1	4	2.9	462.5	81.9	-9.5
DB15345	8	1	5	1.1	451.5	79.8	-9.5
DB04690	5	1	1	1	348.4	79.7	-9.4
DB04886	5	1	2	3.8	370.4	65	-9.4
DB06718	2	2	0	4.5	328.5	48.9	-9.4
DB07029	7	0	5	3.9	388.4	80.5	-9.4
DB07064	2	1	1	3.1	324.4	43.8	-9.4
DB07266	2	2	1	3.6	348.4	64.8	-9.4
DB07320	4	3	5	2.2	401.5	78	-9.4
DB07336	4	3	3	4.1	356.4	86.8	-9.4
DB07507	3	1	2	4.1	329.3	57.6	-9.4
DB07994	5	3	7	4.2	421.5	88.8	-9.4
DB08418	7	1	4	2.3	469.6	128	-9.4
DB08450	5	2	3	4.1	352.4	79.4	-9.4
DB09095	6	2	2	2.3	394.5	74.6	-9.4
DB09304	1	0	0	3.3	261.4	3.2	-9.4
DB11450	4	2	3	2.7	334.4	79.4	-9.4
DB11737	7	1	3	3.1	391.4	74.7	-9.4
DB11963	7	2	7	4.4	469.9	79.4	-9.4
DB12221	3	0	0	2.7	340.5	43.4	-9.4
DB12341	4	1	6	5	418.5	55.6	-9.4
DB12369	6	2	3	2.6	438.5	94.2	-9.4
DB12465	5	1	5	2.6	395.4	69.7	-9.4
DB12515	6	2	1	0.3	363.4	106	-9.4
DB12611	5	4	4	1.5	419.5	130	-9.4
DB13029	5	1	5	2.5	422.4	87.8	-9.4
DB13113	7	0	3	2.1	377.3	73.8	-9.4
DB13953	3	1	3	4.5	376.5	46.5	-9.4
DB14935	6	1	2	2.3	363.4	98.9	-9.4
DB15047	8	2	6	3.1	468.5	132	-9.4
DB15114	4	2	2	2.7	356.5	74.6	-9.4
DB00396	2	0	1	3.9	314.5	34.1	-9.3
DB00967	2	1	0	4.5	310.8	24.9	-9.3
DB01357	2	1	2	4	310.4	29.5	-9.3
DB01514	4	1	0	4	330.5	59.2	-9.3
DB01564	2	1	0	3.6	316.5	37.3	-9.3
DB01573	4	1	3	2.6	375.5	41.9	-9.3
DB01940	7	4	7	4.1	474.5	125	-9.3
DB02840	7	4	4	3.3	368.3	125	-9.3
DB03496	6	3	2	3.3	401.8	90.2	-9.3
DB03583	7	4	3	1.8	441.5	135	-9.3
DB05039	4	4	6	3.3	392.5	81.6	-9.3
DB06075	5	2	3	4.2	421.5	89.3	-9.3
DB06680	5	1	5	3.2	449.6	68.1	-9.3

DB06721	7	1	4	2	447.5	101	-9.3
DB06933	6	2	6	4.6	432.5	92.4	-9.3
DB06977	5	2	6	2.1	383.4	89.2	-9.3
DB07000	8	2	6	2.7	442.4	113	-9.3
DB07006	5	3	5	3	416.4	101	-9.3
DB07142	5	2	4	4.9	386.5	87	-9.3
DB07181	4	2	5	3.5	352.4	69.1	-9.3
DB07220	6	2	5	2.9	453.6	107	-9.3
DB07236	4	2	1	3.9	277.27	66.5	-9.3
DB07334	7	1	7	4	435.5	103	-9.3
DB07458	3	2	5	3.7	438.5	70.1	-9.3
DB07557	2	0	1	4.4	316.5	34.1	-9.3
DB07605	6	1	3	2.5	477	136	-9.3
DB07728	3	2	2	2.4	307.3	57.8	-9.3
DB07827	4	1	5	4.1	424.4	77.9	-9.3
DB08701	4	2	4	3.7	411.2	69.6	-9.3
DB08705	3	2	2	3.3	367.2	73.1	-9.3
DB09074	5	1	4	1.9	434.5	82.1	-9.3
DB09215	7	0	1	1.2	357.3	105	-9.3
DB09383	5	2	2	1.9	372.5	91.7	-9.3
DB11529	3	1	1	2.5	354.5	54.4	-9.3
DB11791	6	1	4	2.9	412.4	85.1	-9.3
DB11829	4	1	2	2.7	468.5	68.5	-9.3
DB11925	8	1	4	2.8	462.5	92.7	-9.3
DB11984	5	2	5	1.9	480	115	-9.3
DB12168	6	1	3	2.6	406.4	86.1	-9.3
DB12194	2	0	0	2.9	298.4	34.1	-9.3
DB12504	5	2	4	4.3	461.6	104	-9.3
DB12690	9	1	3	3.6	445.4	75.5	-9.3
DB12854	5	3	4	3.9	462.9	108	-9.3
DB13035	6	3	5	3.7	418.4	82.3	-9.3
DB13072	7	2	5	1.4	452.5	91.8	-9.3
DB13927	3	1	2	4	343.17	47.1	-9.3
DB14652	6	1	4	3.1	452.9	80.7	-9.3
DB14675	7	1	5	1.7	473.5	126	-9.3
DB15157	9	3	3	4.1	469.8	90.2	-9.3
DB15282	6	0	3	3.3	427.5	65.7	-9.3
DB15442	7	2	3	2.6	446.5	91.2	-9.3
DB00223	7	3	2	1.9	410.5	94.8	-9.2
DB00693	5	2	0	3.4	332.3	76	-9.2
DB00838	5	2	2	2.5	410.9	74.6	-9.2
DB00894	3	0	0	3	300.4	43.4	-9.2
DB00977	2	2	1	3.7	296.4	40.5	-9.2
DB01405	9	2	3	0.6	417.4	72.9	-9.2
DB01536	2	0	0	2.7	286.4	34.1	-9.2
DB01977	2	3	3	3	289.3	79	-9.2
DB02698	9	2	3	4.7	415.3	87.1	-9.2
DB03515	2	1	0	3.5	266.3	37.3	-9.2
DB03623	3	1	1	3.6	272.3	46	-9.2
DB04154	4	0	3	2.4	343.4	63.4	-9.2
DB04424	5	4	10	2.9	458.6	131	-9.2
DB04583	3	2	4	3.6	317.3	80.4	-9.2
DB04698	8	2	3	1.4	343.32	135	-9.2
DB05482	6	2	2	1.4	392.4	100	-9.2
DB05498	9	2	2	2.6	419.4	137	-9.2
DB05804	5	1	2	2.8	368.5	89	-9.2
DB06229	6	0	4	3.3	420.5	61.9	-9.2
DB06412	3	2	0	4.4	332.5	57.5	-9.2
DB07025	3	3	4	2.8	386.5	74.2	-9.2
DB07145	5	2	6	3.2	433.5	91.8	-9.2
DB07247	3	4	4	2.5	358.4	104	-9.2
DB07393	2	0	3	3.7	368.4	25.8	-9.2
DB07570	4	1	5	5	446.5	71.8	-9.2
DB07604	3	2	0	2.9	336.4	73.4	-9.2
DB07618	5	3	5	3.2	421.5	104	-9.2
DB07622	7	3	3	2.5	437.5	135	-9.2
DB07666	9	1	4	2.5	448.5	97.6	-9.2
DB07700	3	1	1	4.9	395.5	69.4	-9.2

DB07764	5	3	2	3.7	334.3	87	-9.2
DB07786	5	2	7	3.4	424.9	107	-9.2
DB07970	7	3	5	3.4	414.4	97.1	-9.2
DB08073	4	3	6	3.7	397.5	92.6	-9.2
DB08091	5	2	6	3.9	444.5	70.2	-9.2
DB08197	7	2	2	2.7	363.4	97.3	-9.2
DB08339	5	2	4	4	427.3	78.4	-9.2
DB08444	5	1	4	4.1	420.3	87.5	-9.2
DB08905	3	1	0	2.6	302.4	54.4	-9.2
DB09124	2	0	1	4.1	340.5	34.1	-9.2
DB11429	2	1	0	3.2	302.5	37.3	-9.2
DB11734	3	1	4	3.2	462.6	73	-9.2
DB11799	9	2	3	2.7	449.4	99.2	-9.2
DB12015	8	2	4	3.2	441.5	129	-9.2
DB12081	4	2	2	2	344.4	74.6	-9.2
DB12101	6	1	6	1.1	449.5	88.2	-9.2
DB12339	7	3	8	1.1	438.5	112	-9.2
DB12385	6	2	1	0.6	364.4	100	-9.2
DB12387	7	3	4	2	406.4	131	-9.2
DB12812	6	1	6	4	406.4	74.2	-9.2
DB12886	5	2	5	4.9	402.4	53.6	-9.2
DB13158	5	1	2	2.6	408.9	71.4	-9.2
DB13230	3	1	1	2.6	316.4	54.4	-9.2
DB14035	4	1	4	4.4	353.4	80.7	-9.2
DB14664	7	1	5	4.2	478.6	80.7	-9.2
DB14723	7	2	3	1.7	428.4	86	-9.2
DB14845	6	1	5	1.4	425.5	105	-9.2
DB14887	7	2	4	3.3	396.4	119	-9.2
DB14943	7	2	4	3.9	440.5	80.9	-9.2
DB00377	2	0	1	2.8	296.4	23.6	-9.1
DB00443	6	3	2	1.9	392.5	94.8	-9.1
DB00547	5	2	2	2.3	376.5	74.6	-9.1
DB00596	6	2	2	2.7	428.9	74.6	-9.1
DB00973	5	2	6	4	409.4	60.8	-9.1
DB01452	6	0	4	1.5	369.4	65.1	-9.1
DB01471	2	1	0	3.6	316.5	37.3	-9.1
DB01668	6	1	0	1	300.26	89.9	-9.1
DB02998	2	1	0	2.1	284.4	37.3	-9.1
DB03037	6	3	2	2.2	298.25	112	-9.1
DB03383	4	3	5	3.8	443.9	85.4	-9.1
DB04011	5	2	4	4.4	451.6	67.1	-9.1
DB04405	6	1	10	4.1	471.5	101	-9.1
DB04574	5	1	2	2.5	350.4	89	-9.1
DB04764	3	1	6	3.6	399.5	59.2	-9.1
DB06306	5	2	4	3.1	409.5	67.2	-9.1
DB06435	3	1	3	4.6	448.5	66.7	-9.1
DB06780	4	0	4	3.1	372.5	60.4	-9.1
DB06871	2	2	0	4	282.4	40.5	-9.1
DB06875	4	2	1	3.9	279.26	64.2	-9.1
DB06896	6	2	5	4.1	458.4	87.3	-9.1
DB06932	6	2	2	3	334.4	96.3	-9.1
DB06985	6	2	3	4.3	392.4	69.6	-9.1
DB06992	3	0	2	4.4	326.4	33.2	-9.1
DB07020	5	3	4	3.2	370.4	113	-9.1
DB07024	8	3	2	2	403.4	133	-9.1
DB07078	3	3	3	3	332.4	74.4	-9.1
DB07141	5	2	4	4.5	372.5	87	-9.1
DB07144	5	2	4	4.9	386.5	87	-9.1
DB07151	2	1	2	3.3	278.3	62	-9.1
DB07450	6	1	2	2.1	449.2	83.6	-9.1
DB07594	5	3	3	3.7	352.4	87.6	-9.1
DB08025	6	2	6	4	420.5	76.1	-9.1
DB08039	4	2	3	2.1	371.5	90.6	-9.1
DB08246	3	0	2	3	328.4	23.6	-9.1
DB08366	7	4	5	2.2	443.5	133	-9.1
DB08386	4	1	5	4.2	378.4	63.7	-9.1
DB11371	3	1	1	3.2	332.5	54.4	-9.1
DB11372	2	1	2	2.8	310.4	37.3	-9.1

DB11636	3	1	1	2	328.4	54.4	-9.1
DB11751	8	2	3	2.1	405.4	99.2	-9.1
DB11795	8	1	4	4	433.4	67.6	-9.1
DB11832	6	1	5	3.7	443.5	78.4	-9.1
DB12047	4	3	3	3.1	364.4	90.1	-9.1
DB12066	3	2	2	1.3	307.3	67.2	-9.1
DB12289	6	3	6	3.5	474.6	94.1	-9.1
DB12523	5	1	5	3.2	432.5	65.8	-9.1
DB12571	5	1	5	3.4	394.4	76.9	-9.1
DB12787	2	1	1	3.3	302.5	37.3	-9.1
DB13223	6	2	2	2.8	390.4	91.7	-9.1
DB13936	10	2	2	3	364.25	77.3	-9.1
DB14625	6	1	4	1.6	414.5	97.7	-9.1
DB14660	3	0	2	2.5	312.4	43.4	-9.1
DB14795	8	1	5	4.1	459.9	79.8	-9.1
DB15035	5	2	6	3.5	471.5	103	-9.1
DB15192	4	0	3	3	372.5	39.2	-9.1
DB15265	6	1	5	3	422.6	64.6	-9.1
DB15295	7	2	5	4.2	415.4	92.8	-9.1
DB15310	6	2	5	3.2	399.4	83.8	-9.1
DB15579	3	0	0	3.8	296.4	43.4	-9.1
DB00685	10	2	3	0.3	416.4	99.8	-9
DB00757	4	1	3	3.4	324.4	62.4	-9
DB00858	2	1	0	4.2	304.5	37.3	-9
DB00972	3	0	3	4.4	381.9	35.9	-9
DB00990	2	0	0	3.1	296.4	34.1	-9
DB01267	7	1	4	2.2	426.5	82.2	-9
DB01392	4	2	2	2.9	354.4	65.6	-9
DB01420	3	0	3	4.4	344.5	43.4	-9
DB01434	2	0	0	2.5	272.4	34.1	-9
DB01456	2	0	0	2.9	286.4	34.1	-9
DB01561	2	0	0	3.6	288.4	34.1	-9
DB01765	3	2	2	2.4	292.29	71.3	-9
DB02187	2	1	0	2.9	268.3	37.3	-9
DB02551	3	4	4	3.1	372.5	91	-9
DB02705	3	4	4	3.7	386.5	91	-9
DB03571	5	3	4	2.8	430.2	127	-9
DB03777	3	2	6	3.1	412.5	70.1	-9
DB03944	6	1	4	3.8	425.5	106	-9
DB04434	5	2	2	1.6	289.33	62.7	-9
DB04573	3	3	0	2.5	288.4	60.7	-9
DB04709	4	0	1	4.1	350.4	83.2	-9
DB04850	10	2	7	0.5	465.4	126	-9
DB06166	4	2	5	3	413.8	109	-9
DB06347	8	3	6	3.2	451.5	99.4	-9
DB06446	4	0	8	4.9	442.6	24.9	-9
DB06589	8	2	5	3.1	437.5	127	-9
DB06922	6	2	5	3.3	389.4	112	-9
DB06938	8	3	5	4.9	461.8	91.9	-9
DB06997	6	3	5	4	422.4	96.1	-9
DB07019	7	1	2	3.3	459.6	111	-9
DB07021	6	1	1	1.5	318.3	56.7	-9
DB07031	5	3	4	4.9	399.5	86.6	-9
DB07034	8	3	6	2.4	343.29	126	-9
DB07075	4	3	4	2.7	384.5	97.5	-9
DB07229	3	4	4	3.9	357.4	95.1	-9
DB07294	5	3	4	4.9	399.5	86.6	-9
DB07354	4	1	2	3.7	322.3	51.1	-9
DB07373	2	0	0	3	284.4	34.1	-9
DB07409	7	5	6	1.2	434.3	135	-9
DB07456	3	2	5	3.7	438.5	70.1	-9
DB07545	8	3	6	4.5	413.4	78.9	-9
DB07586	4	3	5	3	417.5	98.7	-9
DB07691	6	2	5	3.5	436.5	112	-9
DB07716	8	3	1	0.1	422.5	107	-9
DB07833	5	1	4	4.4	394.4	91.8	-9
DB07847	6	1	5	2	466	104	-9
DB07975	9	2	5	4.7	427.3	75.6	-9

DB07976	6	2	5	4	371.4	104	-9
DB08079	6	0	5	3.1	383.4	74.4	-9
DB08111	4	1	3	3	302.3	63.3	-9
DB08124	6	2	3	2.2	393.4	117	-9
DB08159	4	1	4	2.2	333.3	74.7	-9
DB08164	9	1	4	2.9	462.5	97.6	-9
DB08218	5	0	4	2.7	373.4	114	-9
DB08293	5	2	0	3.4	324.75	83.8	-9
DB08338	3	3	2	2.9	363.4	95.7	-9
DB08446	6	1	4	4.4	458.7	87.5	-9
DB08454	4	2	3	4.4	301.3	66.5	-9
DB08497	6	2	5	1.9	366.3	119	-9
DB08707	5	1	2	3.6	322.7	77.8	-9
DB08956	3	1	2	3.8	332.5	54.4	-9
DB08971	5	2	2	2.4	376.5	74.6	-9
DB09015	4	2	3	1.9	358.5	74.6	-9
DB11443	9	2	3	0.9	395.4	72.9	-9
DB11511	8	1	3	1.6	399.4	64.1	-9
DB11622	5	1	4	2.6	402.5	88.5	-9
DB11650	3	1	6	4.9	393.5	47.6	-9
DB11769	8	0	2	3.5	429.3	61.1	-9
DB11793	3	2	3	2.2	320.4	72.9	-9
DB11838	8	1	3	4.8	440.2	88.2	-9
DB12158	6	0	5	3.9	420.5	68.2	-9
DB12644	6	0	5	3	381.8	78.9	-9
DB12742	7	1	3	3.5	447.5	108	-9
DB12998	5	1	7	4.2	430.5	57.7	-9
DB13208	5	3	2	1.6	372.5	94.8	-9
DB13958	3	0	2	3.4	330.5	43.4	-9
DB14029	4	1	2	3	293.3	48.7	-9
DB14570	3	1	1	3.2	330.5	54.4	-9
DB14717	5	0	1	1.3	319.32	88.9	-9
DB14814	4	1	3	3	412.5	72.9	-9
DB14914	3	1	1	3.3	262.27	41.6	-9
DB15033	3	1	1	3.3	263.27	41.6	-9
DB15145	8	2	4	1.8	439.5	119	-9
DB15173	7	1	3	2.6	410.8	66.9	-9
DB15219	6	3	2	1.3	340.4	113	-9
DB15358	9	0	2	3	440.8	76.8	-9
DB15399	8	3	6	2.9	461.5	116	-9
DB15585	3	0	2	4.5	445.6	29.5	-9
DB00294	2	1	2	3.3	324.5	37.3	-8.9
DB00365	7	2	3	-0.2	359.4	72.9	-8.9
DB00624	2	1	0	3.3	288.4	37.3	-8.9
DB01485	3	2	0	3.2	304.4	57.5	-8.9
DB01572	2	1	0	4.1	302.5	37.3	-8.9
DB01609	6	3	4	3.8	373.4	109	-8.9
DB01619	1	0	1	3.1	261.4	3.2	-8.9
DB01708	2	1	0	3.2	288.4	37.3	-8.9
DB02019	5	3	2	2.7	284.26	94.8	-8.9
DB02081	3	4	6	3.4	376.5	117	-8.9
DB02360	4	2	4	4	376.3	74.6	-8.9
DB02932	9	2	5	2.3	430.4	116	-8.9
DB03159	2	4	3	3.9	327.4	85.9	-8.9
DB03367	5	2	7	4.4	428.5	108	-8.9
DB03865	1	4	3	4.6	362.8	87.6	-8.9
DB04014	3	2	0	2.4	293.28	90.7	-8.9
DB04452	5	4	2	2.5	372.4	119	-8.9
DB04591	9	2	7	3.9	439.5	93.7	-8.9
DB04652	4	2	2	1.9	346.5	74.6	-8.9
DB04693	2	0	0	3.2	274.4	34.1	-8.9
DB04739	7	2	7	3.2	479.6	86.3	-8.9
DB04794	1	0	4	4.8	310.4	17.8	-8.9
DB04888	4	1	4	4.2	385.5	44.8	-8.9
DB05016	6	1	3	3.1	284.24	76.2	-8.9
DB05171	4	0	5	4.3	419.5	47.4	-8.9
DB05234	9	2	2	3.4	394.31	52.6	-8.9
DB05520	4	2	3	2.6	410.9	70.9	-8.9

DB05830	2	1	0	2.9	288.4	37.3	-8.9
DB06730	2	1	2	2.9	310.4	37.3	-8.9
DB06876	5	2	4	2.6	412.5	77.2	-8.9
DB07008	7	0	5	3.5	374.3	80.5	-8.9
DB07180	2	3	2	2.4	329.8	74	-8.9
DB07264	4	4	6	4.1	459.3	93.8	-8.9
DB07270	7	2	5	4.8	464.3	116	-8.9
DB07337	6	3	4	3.9	375.8	116	-8.9
DB07444	3	2	3	2.2	321.4	77.1	-8.9
DB07479	3	3	0	2.4	280.3	60.7	-8.9
DB07486	10	1	4	4.6	476.4	80.9	-8.9
DB07493	3	2	1	3.4	341.16	65.4	-8.9
DB07544	8	2	4	4.3	418.8	96.8	-8.9
DB07768	2	1	0	3.3	288.4	37.3	-8.9
DB07832	6	2	4	4.2	361.4	103	-8.9
DB07856	4	2	3	4.2	389.9	66.5	-8.9
DB07891	5	2	5	4	372.5	83.8	-8.9
DB07993	3	3	4	3.7	314.4	66.7	-8.9
DB08057	5	2	3	5	406.9	57.5	-8.9
DB08058	5	2	3	1.9	380.4	73.8	-8.9
DB08092	4	2	3	2.7	339.4	57.4	-8.9
DB08174	4	2	5	4.2	441.9	107	-8.9
DB08280	4	2	6	2.4	386.5	90.6	-8.9
DB08365	4	3	4	2.7	476.7	100	-8.9
DB08498	6	2	5	2.6	400.8	119	-8.9
DB08504	6	1	5	1	424.5	96.8	-8.9
DB08569	4	2	6	4.3	437.3	76.8	-8.9
DB08588	4	1	3	1.7	359.4	85.7	-8.9
DB08730	5	1	6	3.5	444.5	59.4	-8.9
DB08739	6	0	4	1.2	405.4	78	-8.9
DB08907	7	4	5	3.2	444.5	118	-8.9
DB08970	6	3	2	1.5	390.4	94.8	-8.9
DB08976	9	3	7	3.7	406.4	91.7	-8.9
DB09034	6	0	3	4.9	450.9	80.3	-8.9
DB09054	7	2	5	3.7	415.4	99.2	-8.9
DB11551	2	1	0	1.9	270.4	37.3	-8.9
DB11750	5	2	2	2.5	410.9	74.6	-8.9
DB11800	7	2	6	4	454.9	108	-8.9
DB11859	2	1	1	4.9	318.5	37.3	-8.9
DB11968	8	2	6	1.3	466.5	99.5	-8.9
DB12024	6	1	6	3.5	409.4	76.1	-8.9
DB12130	7	1	0	1.5	406.4	110	-8.9
DB12235	4	4	0	1.5	304.4	80.9	-8.9
DB12260	5	2	3	3	397.4	87.7	-8.9
DB12303	3	1	7	3.8	458.6	79	-8.9
DB12408	4	2	9	4.8	444.6	75.8	-8.9
DB12447	7	2	2	2.9	360.4	81.1	-8.9
DB12494	8	1	5	4.3	405.4	54.7	-8.9
DB12764	2	1	4	4.7	380.9	42	-8.9
DB12848	7	1	5	0.7	372.4	107	-8.9
DB12877	3	1	7	4.1	426.6	38.8	-8.9
DB12924	6	2	4	4.1	363.4	82.5	-8.9
DB12949	6	2	4	2.5	433.5	82.1	-8.9
DB12972	2	1	1	4.9	318.5	37.3	-8.9
DB13040	7	2	6	3.8	469.9	83.4	-8.9
DB13059	9	3	6	3	474.5	99.2	-8.9
DB13174	6	3	1	2.2	284.22	112	-8.9
DB13418	3	2	2	3	326.4	49.7	-8.9
DB13533	2	1	0	2.8	288.4	37.3	-8.9
DB13563	2	1	1	1.8	294.4	37.3	-8.9
DB13710	2	1	0	3.9	302.5	37.3	-8.9
DB13793	2	0	1	3	294.4	25.2	-8.9
DB13806	3	0	5	4.1	391.5	46.1	-8.9
DB14093	2	1	0	3.3	296.5	37.3	-8.9
DB14221	6	2	5	2	465	101	-8.9
DB14681	5	2	2	1.5	360.4	91.7	-8.9
DB14716	5	0	1	1.6	353.8	88.9	-8.9
DB14770	9	2	5	1.4	443.5	110	-8.9

DB14844	3	0	4	2.3	386.5	39.2	-8.9
DB14918	6	3	4	2.7	393.5	96	-8.9
DB14980	7	2	5	2	408.4	94.5	-8.9
DB15407	7	2	4	3.2	401.4	99.2	-8.9
DB15419	8	3	5	1	394.4	103	-8.9
DB15490	4	1	3	4.5	409.6	78.9	-8.9
DB00247	3	2	4	2.3	353.5	57.5	-8.8
DB00304	1	1	2	4.9	310.5	20.2	-8.8
DB00398	7	3	5	4.1	464.8	92.4	-8.8
DB00628	4	2	2	3.3	314.72	78.8	-8.8
DB00635	5	2	2	1.5	358.4	91.7	-8.8
DB00655	2	1	0	3.1	270.4	37.3	-8.8
DB00683	3	0	1	2.5	325.8	30.2	-8.8
DB00687	6	3	2	1.7	380.4	94.8	-8.8
DB00734	6	0	4	2.7	410.5	61.9	-8.8
DB01030	7	2	3	0.5	421.4	103	-8.8
DB01165	8	1	2	-0.4	361.4	73.3	-8.8
DB01179	8	1	4	2	414.4	92.7	-8.8
DB01208	9	3	3	0.1	392.4	98.9	-8.8
DB01216	2	2	2	3	372.5	58.2	-8.8
DB01406	3	1	1	3.8	337.5	46.3	-8.8
DB01451	2	0	0	3.6	286.4	34.1	-8.8
DB01496	7	2	3	3.1	368.3	103	-8.8
DB01540	3	2	0	2.8	304.4	57.5	-8.8
DB01946	3	2	5	2.1	398.5	82	-8.8
DB02194	6	3	5	2.9	439.5	106	-8.8
DB02519	6	3	2	1.5	342.3	128	-8.8
DB02656	4	0	2	3.1	307.3	38.8	-8.8
DB02901	2	1	0	3.7	290.4	37.3	-8.8
DB03034	8	1	2	-0.4	361.4	73.3	-8.8
DB03207	5	1	4	3.9	393.5	83.1	-8.8
DB03555	5	4	4	4.1	382.4	108	-8.8
DB04049	5	3	6	3.8	411.5	95.3	-8.8
DB04115	4	0	2	3.6	336.4	40.8	-8.8
DB04432	6	4	6	3.1	398.4	133	-8.8
DB04716	3	2	1	4	309.3	57.8	-8.8
DB04823	3	3	2	3.2	317.3	69.6	-8.8
DB04824	4	2	2	3.6	318.3	66.8	-8.8
DB04839	4	0	3	3.6	416.9	60.4	-8.8
DB05713	4	2	5	3	459.6	71.7	-8.8
DB05738	3	2	1	1	296.37	72.9	-8.8
DB05959	2	1	2	4.3	311.4	52.3	-8.8
DB06144	3	1	5	4.1	440.9	40.5	-8.8
DB06212	3	2	3	4.8	448.9	69.6	-8.8
DB06472	5	4	7	1.5	367.4	126	-8.8
DB06518	8	1	4	2.8	388.4	76.6	-8.8
DB06597	5	2	2	1.9	351.5	98	-8.8
DB06605	5	1	5	2.2	459.5	111	-8.8
DB06710	2	1	0	3.4	302.5	37.3	-8.8
DB06834	3	3	4	2.3	362.4	82.9	-8.8
DB06957	7	3	5	2.5	463	118	-8.8
DB06961	7	3	6	3	457.9	108	-8.8
DB06962	4	1	4	3.7	412.5	68.7	-8.8
DB06972	3	0	3	4.2	408.3	67.9	-8.8
DB07041	7	3	6	3.8	428.5	129	-8.8
DB07140	5	2	4	4.1	358.4	87	-8.8
DB07255	8	2	5	4.1	425.9	80.8	-8.8
DB07297	6	2	6	3.9	399.5	66.2	-8.8
DB07326	9	2	4	4.2	433.8	92.9	-8.8
DB07369	4	1	5	3.8	456	91	-8.8
DB07404	7	5	6	1.2	434.3	135	-8.8
DB07474	5	3	5	1.7	397.4	101	-8.8
DB07494	1	0	2	4.8	348.5	9.2	-8.8
DB07508	5	3	6	2.9	419.5	122	-8.8
DB07519	3	1	5	2.7	351.4	67.9	-8.8
DB07596	6	1	4	4.2	404.5	111	-8.8
DB07630	5	3	4	3.9	472	118	-8.8
DB07715	5	3	0	2.7	270.24	94.8	-8.8

DB07778	5	1	5	5	374.4	67.9	-8.8
DB07966	6	3	6	4.1	381.4	102	-8.8
DB07974	3	2	6	4.6	453	66.4	-8.8
DB08011	7	1	2	3.2	346.28	73	-8.8
DB08035	4	1	3	3.4	309.4	69.6	-8.8
DB08080	6	2	1	2.9	395.5	110	-8.8
DB08261	6	3	4	2.6	330.3	96.2	-8.8
DB08299	6	1	3	3.8	371.3	109	-8.8
DB08362	7	3	5	2.7	384.4	120	-8.8
DB08384	4	1	6	4.5	392.5	63.7	-8.8
DB08387	4	0	5	4.2	392.5	52.8	-8.8
DB08459	5	1	5	4.8	411.2	83.8	-8.8
DB08460	6	2	5	4.5	426.3	110	-8.8
DB08489	4	4	6	2.5	456.6	113	-8.8
DB08608	7	1	2	1.9	383.4	84.6	-8.8
DB08619	5	1	5	3.3	388.5	80.7	-8.8
DB08656	2	2	3	4	304.4	55.1	-8.8
DB08749	3	1	6	2.9	418.5	79	-8.8
DB08772	9	1	4	3.5	419.4	111	-8.8
DB08865	6	2	5	3.7	450.3	78	-8.8
DB11453	8	2	3	0.4	396.4	96.7	-8.8
DB11877	8	0	5	1.9	445.5	111	-8.8
DB11923	10	1	3	4.4	447.3	69.1	-8.8
DB11962	5	1	2	3	333.4	97.2	-8.8
DB12072	3	3	4	2.2	310.3	82.2	-8.8
DB12134	7	1	5	0.1	448.5	90.4	-8.8
DB12183	8	2	7	4	473.9	88.6	-8.8
DB12206	4	2	7	2.8	414.5	103	-8.8
DB12305	1	1	1	4.3	292.2	26	-8.8
DB12379	3	2	1	2.7	262.26	65.4	-8.8
DB12522	5	2	6	4.4	392.5	69.7	-8.8
DB12535	6	3	4	3.4	349.4	86.7	-8.8
DB12720	8	2	5	2.1	415.4	110	-8.8
DB12725	5	2	7	3.4	455.6	90.6	-8.8
DB12888	4	0	3	4.3	337.4	68.6	-8.8
DB12903	4	3	3	3.9	356.4	105	-8.8
DB12978	7	2	5	4.5	417.8	66.5	-8.8
DB12999	5	1	4	4.6	435.3	92.4	-8.8
DB13093	6	2	6	2.4	445.5	115	-8.8
DB13169	2	1	0	2.6	274.4	37.3	-8.8
DB13175	5	3	1	2.7	270.24	94.8	-8.8
DB13347	3	0	4	3.7	340.4	51.2	-8.8
DB13528	3	1	1	3	362.9	54.4	-8.8
DB13587	2	1	0	4.1	304.5	37.3	-8.8
DB13610	6	1	1	3.9	398.5	54.4	-8.8
DB13708	5	3	2	1.8	337.8	110	-8.8
DB13951	3	0	2	4.2	332.5	43.4	-8.8
DB13991	2	1	4	5	316.4	24.9	-8.8
DB14583	4	0	3	2.9	370.5	60.4	-8.8
DB14769	4	2	0	1.6	298.31	60.5	-8.8
DB14790	7	2	5	4.1	406.8	80.9	-8.8
DB15028	7	1	7	2.7	461.9	99.1	-8.8
DB15099	4	2	2	3.3	368.8	69.6	-8.8
DB15207	7	2	6	2.9	466.5	96.8	-8.8
DB00246	5	1	4	4	412.9	76.7	-8.7
DB00253	3	1	1	2.7	344.5	54.4	-8.7
DB00351	4	0	3	3.1	384.5	60.4	-8.7
DB00404	3	0	1	2.1	308.8	43.1	-8.7
DB00421	5	0	2	2.9	416.6	85.7	-8.7
DB00434	1	0	0	4.7	287.4	3.2	-8.7
DB00603	4	0	3	4.1	386.5	60.4	-8.7
DB00621	3	1	0	3.7	306.4	46.5	-8.7
DB00700	6	0	2	1.4	414.5	82.2	-8.7
DB00741	5	3	2	1.6	362.5	94.8	-8.7
DB00783	2	2	0	4	272.4	40.5	-8.7
DB00820	4	1	1	2.3	389.4	74.9	-8.7
DB01025	6	2	2	3.1	298.29	103	-8.7
DB01067	6	3	7	1.9	445.5	139	-8.7

DB01349	6	1	4	3	411.5	101	-8.7
DB01443	2	0	0	2.1	272.4	34.1	-8.7
DB01500	3	2	0	2.5	290.4	57.5	-8.7
DB01513	2	2	0	4.3	306.5	40.5	-8.7
DB01541	2	1	0	3.5	286.4	37.3	-8.7
DB01544	5	0	1	2.1	313.28	78.5	-8.7
DB01554	2	2	0	2.7	276.4	40.5	-8.7
DB02118	5	0	3	1.1	372.4	75.3	-8.7
DB02342	3	2	1	4	302.4	49.7	-8.7
DB02473	3	3	4	3.7	384.5	91.3	-8.7
DB02621	6	2	1	3.7	421.6	110	-8.7
DB02789	2	1	1	4.2	316.5	37.3	-8.7
DB02830	4	4	9	1.1	446.5	127	-8.7
DB03351	7	3	3	2.2	331.4	116	-8.7
DB03782	2	4	4	2	341.5	106	-8.7
DB03926	2	2	0	4.2	292.5	40.5	-8.7
DB04030	3	2	3	3.9	398.5	65.7	-8.7
DB04582	5	2	3	2.5	311.33	91.6	-8.7
DB04847	6	3	5	3.4	352.3	109	-8.7
DB05107	2	1	0	4.6	369.3	37.3	-8.7
DB05838	3	1	5	4.8	454	52.6	-8.7
DB06169	3	1	5	3.9	389.8	64	-8.7
DB06532	5	2	6	3.2	364.4	108	-8.7
DB06626	4	2	5	4.2	386.5	96	-8.7
DB06678	3	0	0	3.3	265.35	19.4	-8.7
DB06847	5	2	4	4.1	358.4	87	-8.7
DB06860	3	0	1	2.7	272.68	50.3	-8.7
DB06963	4	2	7	3.9	399.5	85.8	-8.7
DB06974	7	2	6	3.3	468.5	129	-8.7
DB07010	2	3	5	4	376.8	73.6	-8.7
DB07026	5	3	4	1	383.4	100	-8.7
DB07036	6	2	3	3.6	362.4	58.9	-8.7
DB07076	3	3	5	4.2	373.4	88.2	-8.7
DB07081	10	1	4	1.7	421.34	77	-8.7
DB07162	5	3	4	2.8	344.4	109	-8.7
DB07245	3	1	5	4.4	304.4	48.1	-8.7
DB07257	3	2	3	2.6	354.8	71.3	-8.7
DB07267	5	1	3	3.4	313.4	63.6	-8.7
DB07274	5	1	6	4.7	414.5	69.7	-8.7
DB07343	7	2	4	4.3	373.2	110	-8.7
DB07457	3	3	5	2.1	384.4	92.9	-8.7
DB07535	2	3	3	1.1	254.29	83.3	-8.7
DB07546	6	2	6	2.4	376.4	97.9	-8.7
DB07561	4	2	5	4.4	322.4	82.4	-8.7
DB07612	7	2	3	4.7	360.4	63.8	-8.7
DB07706	3	3	0	3.7	288.4	60.7	-8.7
DB07874	3	1	3	2	323.4	67.9	-8.7
DB08204	6	2	2	4.9	413.4	113	-8.7
DB08219	6	1	3	2.3	368.5	117	-8.7
DB08221	8	2	5	4.8	449.4	79.8	-8.7
DB08244	4	1	4	2.9	354.4	46.3	-8.7
DB08268	3	3	3	2.3	319.4	96.2	-8.7
DB08423	6	2	5	3.6	380.4	82.2	-8.7
DB08445	9	1	4	2.5	401.4	77.2	-8.7
DB08521	5	1	4	3.2	377.4	77.8	-8.7
DB08639	5	2	4	4.8	329.4	73.6	-8.7
DB08708	7	1	3	4.4	361.4	55.1	-8.7
DB08709	2	2	3	4.9	313.3	53.1	-8.7
DB08864	6	2	5	4.5	366.4	97.4	-8.7
DB09123	3	1	1	1.8	311.4	61.1	-8.7
DB09195	7	0	4	3.3	405.5	37.2	-8.7
DB09378	6	3	2	1.4	378.4	94.8	-8.7
DB11457	6	0	3	4.1	394.4	63.2	-8.7
DB11491	8	2	3	0.3	385.4	72.9	-8.7
DB11645	4	1	5	3	393.5	95.4	-8.7
DB11730	7	2	5	2.2	434.5	91.2	-8.7
DB11830	6	3	6	2.8	396.4	106	-8.7
DB11952	5	2	4	4.1	416.9	86.8	-8.7

DB12096	4	1	2	2.7	405.5	83.9	-8.7
DB12131	3	0	4	4.1	350.5	34.5	-8.7
DB12167	5	1	5	1.7	406.5	78.8	-8.7
DB12332	3	3	3	2.5	323.4	56.9	-8.7
DB12467	4	0	0	2.4	315.3	54.4	-8.7
DB12562	4	1	3	4.1	402.4	62.5	-8.7
DB12579	9	1	4	3.4	372.34	41	-8.7
DB12658	4	1	4	2.5	375.4	75.4	-8.7
DB12659	8	1	4	3.4	468.4	86.1	-8.7
DB12663	5	1	3	3.5	391.2	85.6	-8.7
DB12680	5	1	3	2.5	353.4	70.7	-8.7
DB12774	9	1	5	2.6	465.5	93.1	-8.7
DB12776	8	2	8	4.2	472.5	112	-8.7
DB12804	3	1	0	1.4	249.27	58.7	-8.7
DB13386	3	2	1	2.8	302.4	49.7	-8.7
DB13602	2	0	2	3.3	326.5	34.1	-8.7
DB13643	7	0	2	2.9	464.9	97.3	-8.7
DB13931	5	2	8	4.6	453.5	94.3	-8.7
DB13935	3	1	1	3.5	278.3	47.3	-8.7
DB13952	3	1	2	2.8	314.4	46.5	-8.7
DB13993	8	0	5	3	397.4	83	-8.7
DB14034	6	1	7	4.1	420.5	115	-8.7
DB14569	8	1	4	1.4	370.3	106	-8.7
DB14649	7	2	4	2.8	434.5	101	-8.7
DB14917	7	2	4	2.3	412.5	116	-8.7
DB15197	4	3	3	3.3	388.4	82.6	-8.7
DB15292	7	1	5	3.7	421.5	95.4	-8.7
DB15346	4	0	4	2.4	378.4	60.4	-8.7
DB15414	7	3	5	4.1	467.8	92.4	-8.7
DB00209	3	1	5	4.4	392.5	46.5	-8.6
DB00367	2	1	2	3.3	312.4	37.3	-8.6
DB00408	3	0	1	3.1	327.8	28.1	-8.6
DB00620	7	4	2	1.2	394.4	115	-8.6
DB00714	3	2	0	2.3	267.32	43.7	-8.6
DB00836	3	1	7	5	477	43.8	-8.6
DB00860	5	3	2	1.6	360.4	94.8	-8.6
DB00922	6	2	3	2.3	280.28	113	-8.6
DB01095	5	3	8	3.5	411.5	82.7	-8.6
DB01128	9	2	5	2.3	430.4	116	-8.6
DB01134	4	0	5	4.5	414.6	60.4	-8.6
DB01185	4	2	0	2.4	336.4	57.5	-8.6
DB01380	6	1	4	2.1	402.5	97.7	-8.6
DB01455	2	2	0	2.8	276.4	40.5	-8.6
DB01501	4	1	7	2.7	424.5	64.3	-8.6
DB01567	3	0	1	2.8	302.73	32.7	-8.6
DB01888	8	3	7	3.2	471.6	126	-8.6
DB02052	3	3	1	3.2	277.28	81.2	-8.6
DB02366	3	4	3	4	362.8	98.8	-8.6
DB03878	6	2	5	2.8	382.4	92.7	-8.6
DB03960	7	4	6	4.7	449.5	115	-8.6
DB04216	7	5	1	1.5	302.23	127	-8.6
DB04270	4	1	9	5	403.5	60.7	-8.6
DB04750	9	3	3	2.4	412.3	121	-8.6
DB04879	4	1	4	4.5	346.8	50.7	-8.6
DB04908	6	1	4	3.3	390.4	38.8	-8.6
DB05265	5	2	3	4.3	380.5	100	-8.6
DB05884	7	2	6	3.1	420.5	106	-8.6
DB05944	8	2	7	4.2	466.9	122	-8.6
DB05992	3	3	3	2.8	336.4	86.9	-8.6
DB06077	5	0	5	3.8	393.5	26.8	-8.6
DB06160	8	2	5	2.1	426.4	78.9	-8.6
DB06604	6	2	6	3	432.5	129	-8.6
DB06684	5	2	7	4	441.5	102	-8.6
DB06740	3	1	2	4.8	312.4	54.4	-8.6
DB06879	7	1	2	0.9	412.4	98.1	-8.6
DB06908	5	1	8	4.8	438.9	77.5	-8.6
DB06916	7	1	6	2.8	420.5	94.4	-8.6
DB07009	4	2	1	4	277.27	66.5	-8.6

DB07074	5	4	5	2.8	377.4	126	-8.6
DB07175	6	2	5	3.1	354.4	92.4	-8.6
DB07202	3	1	2	3.7	336.8	55.1	-8.6
DB07207	4	5	3	1.7	318.33	128	-8.6
DB07212	3	4	3	3	319.4	99.2	-8.6
DB07230	3	2	1	3.3	317.13	57.5	-8.6
DB07238	7	2	7	2.7	446.5	108	-8.6
DB07240	4	1	4	3.7	267.28	58.9	-8.6
DB07253	8	2	5	3.6	418.9	111	-8.6
DB07311	5	2	0	2.4	348.78	85.4	-8.6
DB07340	7	3	5	3.9	393.5	91	-8.6
DB07375	2	0	0	3.6	288.4	34.1	-8.6
DB07403	8	2	5	2.7	478.9	112	-8.6
DB07419	8	2	5	3.7	416.8	82.4	-8.6
DB07512	4	0	4	3.2	314.7	52.6	-8.6
DB07536	6	4	8	1.3	437.5	111	-8.6
DB07539	6	3	3	2.6	314.3	129	-8.6
DB07629	5	3	4	3.9	472	118	-8.6
DB07638	5	2	1	4	318.3	49.7	-8.6
DB07642	7	2	5	3.2	381.4	90.3	-8.6
DB07675	6	1	9	5	419.5	68.2	-8.6
DB07676	6	4	6	2.4	365.4	110	-8.6
DB07698	4	2	3	3.8	335.8	68.2	-8.6
DB07702	3	3	0	2.5	288.4	60.7	-8.6
DB07788	7	3	1	2.6	362.4	113	-8.6
DB07795	6	4	1	2	286.24	107	-8.6
DB07811	4	1	4	4	347.4	68	-8.6
DB07929	4	1	3	4.3	396.5	67.9	-8.6
DB07936	7	1	6	2.8	451.5	79.2	-8.6
DB08097	8	1	5	4.8	399.4	64.9	-8.6
DB08173	4	2	6	3.2	401.9	107	-8.6
DB08230	7	5	1	1	302.23	127	-8.6
DB08242	5	2	4	3.4	358.4	84.7	-8.6
DB08300	4	1	1	2.7	275.31	69.6	-8.6
DB08350	4	1	4	2.9	372.4	71.1	-8.6
DB08351	6	2	7	3.3	431.5	79.4	-8.6
DB08407	7	4	5	2.3	441.5	133	-8.6
DB08439	5	1	5	3.3	370.4	97.6	-8.6
DB08500	7	5	2	0.4	347.4	111	-8.6
DB08549	7	3	6	1	419.5	133	-8.6
DB08568	4	2	6	3.6	358.4	76.8	-8.6
DB08583	4	2	4	2.5	374.4	92.8	-8.6
DB08597	5	0	6	3.4	399.5	55.6	-8.6
DB09078	5	3	6	2.8	426.9	116	-8.6
DB11522	6	3	2	1.5	378.4	94.8	-8.6
DB11732	7	1	4	2.8	377.4	62.3	-8.6
DB11743	6	2	6	2.5	458	81.6	-8.6
DB11763	7	2	6	2.6	414.5	103	-8.6
DB11870	8	3	5	4.6	469.4	87.3	-8.6
DB11953	7	1	4	0	395.5	97.5	-8.6
DB11978	4	3	3	2.4	374.4	96.8	-8.6
DB11992	8	1	3	0.3	398.4	98.8	-8.6
DB11994	8	1	5	1.9	368.3	124	-8.6
DB12009	5	0	5	3.5	393.5	81.9	-8.6
DB12055	8	2	6	3.1	422.4	93.7	-8.6
DB12078	8	2	5	2.7	389.3	106	-8.6
DB12082	5	1	4	2.1	395.5	71.1	-8.6
DB12124	8	2	5	0.1	434.4	127	-8.6
DB12140	9	3	6	3	456.4	98.6	-8.6
DB12275	9	2	7	4.7	399.3	80.3	-8.6
DB12286	6	2	3	2.3	280.28	113	-8.6
DB12327	8	0	5	2.5	432.5	109	-8.6
DB12388	4	2	6	2.1	374.5	52.6	-8.6
DB12394	8	0	4	3.7	415.4	64.6	-8.6
DB12492	4	1	7	3.7	430.6	73.4	-8.6
DB12573	4	1	5	3.2	374.4	79.9	-8.6
DB12590	5	0	4	2.3	376.4	95.8	-8.6
DB12781	6	1	5	2.7	395.4	113	-8.6

DB12802	8	1	4	2	414.4	92.7	-8.6
DB12890	3	3	0	2.5	267.32	52.5	-8.6
DB12964	3	1	3	2.8	292.4	33.1	-8.6
DB13042	6	0	4	4.2	459.6	70.6	-8.6
DB13466	2	0	2	4.8	333.5	12.5	-8.6
DB13552	7	1	6	3.5	409.4	40.5	-8.6
DB13579	4	0	3	3.6	323.4	38.8	-8.6
DB13866	2	2	1	3.1	300.4	40.5	-8.6
DB14539	6	2	4	2.2	404.5	101	-8.6
DB14624	5	1	2	2.2	354.5	89	-8.6
DB14626	3	0	3	4.8	358.5	43.4	-8.6
DB14634	7	2	4	2.3	432.5	101	-8.6
DB14646	6	1	4	1.5	400.5	97.7	-8.6
DB14671	4	0	3	2.8	342.8	59	-8.6
DB14672	4	1	3	3	328.7	67.8	-8.6
DB14774	7	0	5	3.8	428.4	77.5	-8.6
DB14821	8	0	3	4.7	456.5	107	-8.6
DB14823	5	0	3	3.4	409.9	56.1	-8.6
DB14895	5	3	6	1.8	444.5	94	-8.6
DB14916	6	1	6	2.7	445.5	90.5	-8.6
DB14995	6	1	4	4.7	399.4	60.1	-8.6
DB15048	7	4	5	1.9	416.5	109	-8.6
DB15156	6	2	7	3.6	469.6	113	-8.6
DB15418	6	2	3	1.1	399.4	97	-8.6
DB15460	5	3	10	4.7	476.5	95.5	-8.6
DB15465	5	0	4	4.1	403.5	48	-8.6
DB00301	8	2	4	2.6	453.9	138	-8.5
DB00378	2	0	1	3.8	312.4	34.1	-8.5
DB00522	5	4	7	3.3	404.4	116	-8.5
DB00543	3	1	1	2.6	313.8	36.9	-8.5
DB00717	2	1	1	3	298.4	37.3	-8.5
DB00719	2	0	0	3.6	290.4	16.1	-8.5
DB00731	3	2	6	3.2	317.4	66.4	-8.5
DB00823	4	0	5	3.9	384.5	52.6	-8.5
DB00834	3	1	3	3.8	429.6	40.5	-8.5
DB00842	3	2	1	2.2	286.71	61.7	-8.5
DB00875	7	1	5	4.5	434.5	52	-8.5
DB00889	3	1	2	2.8	312.4	50.2	-8.5
DB00969	2	1	2	1.6	294.35	53.9	-8.5
DB01108	4	2	0	2.9	329.4	76.8	-8.5
DB01137	8	1	2	-0.4	361.4	73.3	-8.5
DB01155	10	2	5	-0.7	389.4	121	-8.5
DB01184	3	2	5	3.9	425.9	67.9	-8.5
DB01474	2	2	0	3.4	304.5	40.5	-8.5
DB01481	2	1	0	4	288.4	37.3	-8.5
DB01524	2	2	0	3.5	290.4	40.5	-8.5
DB01530	2	2	0	4.2	292.5	40.5	-8.5
DB01569	4	2	1	2.2	344.4	74.6	-8.5
DB01595	4	1	1	2.2	281.27	87.3	-8.5
DB01623	6	0	5	3.8	443.6	77.5	-8.5
DB01689	6	3	7	2.3	390.5	121	-8.5
DB01691	3	1	4	1.8	374.4	67.2	-8.5
DB01772	6	4	6	2	409.4	104	-8.5
DB01793	5	3	4	3.5	377.2	95.5	-8.5
DB02224	7	5	1	1.5	304.25	127	-8.5
DB02241	7	3	6	3.5	455.5	115	-8.5
DB02300	3	3	5	4.3	412.6	60.7	-8.5
DB02799	6	1	6	1.7	383.4	96	-8.5
DB03035	6	2	0	2.5	285.21	120	-8.5
DB03444	3	3	1	3.9	356.17	81.2	-8.5
DB03446	7	5	6	-0.1	389.4	129	-8.5
DB03534	3	1	5	1.6	378.5	71.6	-8.5
DB03595	7	4	3	2.9	336.27	108	-8.5
DB03672	2	1	3	4.9	288.4	24.9	-8.5
DB03882	2	2	0	4.2	292.5	40.5	-8.5
DB04177	4	2	1	2.6	374.5	66.8	-8.5
DB04430	6	3	5	3.1	303.32	102	-8.5
DB04607	6	2	5	4.8	461.3	115	-8.5

DB04797	5	0	3	4.2	322.3	56.2	-8.5
DB04816	4	2	0	3.2	240.21	74.6	-8.5
DB04891	2	2	3	4.7	344.5	40.5	-8.5
DB04971	6	1	7	4	392.4	90.7	-8.5
DB05087	2	1	1	5	332.5	37.3	-8.5
DB05288	5	1	4	2.1	386.5	80.7	-8.5
DB05316	4	1	8	4.5	427.6	44.8	-8.5
DB06228	6	1	5	2.5	435.9	116	-8.5
DB06246	5	1	4	3.5	372.4	79.8	-8.5
DB06454	4	1	5	4.1	348.4	34.2	-8.5
DB06777	4	3	4	4.9	392.6	77.8	-8.5
DB06858	4	4	7	2.5	413.6	111	-8.5
DB06923	1	3	2	3	249.31	65.7	-8.5
DB06971	4	2	5	2.1	311.33	85.6	-8.5
DB06994	7	1	7	3.7	454	108	-8.5
DB07084	7	1	1	1.4	417.5	84.5	-8.5
DB07092	7	1	5	1.9	448.5	96.8	-8.5
DB07119	2	2	1	4.7	270.71	40.5	-8.5
DB07123	1	0	3	4.4	293.4	20.3	-8.5
DB07201	4	1	4	3.7	281.3	58.9	-8.5
DB07322	5	2	4	3.9	331.4	91.8	-8.5
DB07323	7	2	7	2.2	432.5	95.1	-8.5
DB07460	8	3	7	4.1	468.9	101	-8.5
DB07469	3	1	1	2.4	292.3	52.9	-8.5
DB07584	5	3	3	2.7	291.31	95.2	-8.5
DB07606	5	3	4	4.6	465.7	99.7	-8.5
DB07639	6	3	9	3.1	433.5	99.6	-8.5
DB07647	4	3	5	4	344.4	73.8	-8.5
DB07683	6	2	5	4.2	363.5	120	-8.5
DB07707	3	2	2	3.6	316.4	49.7	-8.5
DB07793	5	2	4	1.3	402.9	130	-8.5
DB07831	6	3	7	4.8	429.4	70.2	-8.5
DB07840	5	1	4	4.5	364.3	55	-8.5
DB07859	2	2	3	4.3	337.8	40.7	-8.5
DB07909	4	3	7	3.3	381.5	86.6	-8.5
DB07941	5	1	5	4.2	477.3	58.6	-8.5
DB07943	5	2	4	1.9	397.9	95	-8.5
DB07949	6	1	3	1.7	335.3	101	-8.5
DB07986	6	2	6	2.8	374.5	95.1	-8.5
DB08030	4	2	5	2	282.29	82.4	-8.5
DB08034	5	3	4	3.3	336.3	91.2	-8.5
DB08052	5	2	2	2.1	319.4	98.3	-8.5
DB08059	8	0	4	1.2	428.4	109	-8.5
DB08068	6	1	6	3	445.9	63.7	-8.5
DB08088	7	1	1	3.2	359.73	68.8	-8.5
DB08141	6	4	4	1.5	349.34	98.9	-8.5
DB08169	3	2	4	4.2	304.3	73.1	-8.5
DB08304	7	1	3	1.8	413.6	104	-8.5
DB08332	4	1	3	2.5	296.3	55.8	-8.5
DB08354	6	2	5	4.1	375.8	90.9	-8.5
DB08355	6	2	3	2.2	321.33	92.9	-8.5
DB08429	5	2	8	2.1	478	118	-8.5
DB08490	6	2	6	2.3	425.9	110	-8.5
DB08510	5	1	6	4.9	418.6	80.7	-8.5
DB08541	5	2	2	2.1	286.28	83.8	-8.5
DB08546	7	3	4	0.4	448.5	129	-8.5
DB08547	6	1	6	2.4	430.5	97.7	-8.5
DB08584	6	0	3	2.4	359.4	99.1	-8.5
DB08609	8	2	6	3.2	430.4	95.1	-8.5
DB08743	6	1	3	1	393.4	112	-8.5
DB08883	3	0	3	3.4	349.4	57	-8.5
DB08899	8	1	3	3.6	464.4	109	-8.5
DB08930	8	2	3	2.4	419.4	99.2	-8.5
DB09073	8	2	5	1.8	447.5	103	-8.5
DB11219	1	0	1	4.5	254.4	17.1	-8.5
DB11440	4	0	3	3.1	372.5	60.4	-8.5
DB11455	7	2	5	4.1	327.27	49.3	-8.5
DB11633	8	1	6	3.5	437.5	116	-8.5

DB11686	2	1	6	4.7	348.5	32.3	-8.5
DB11698	7	4	4	2.5	404.5	118	-8.5
DB11741	4	2	6	2.5	410.5	68.4	-8.5
DB11881	7	3	7	4.1	479.6	108	-8.5
DB11950	6	1	4	2.4	426.6	81.9	-8.5
DB11958	6	2	5	3	370.4	84.7	-8.5
DB12020	6	1	1	2.6	376.3	66.5	-8.5
DB12048	6	0	3	1.5	345.4	91.6	-8.5
DB12073	7	1	5	2.5	431.8	83.6	-8.5
DB12135	7	4	4	2.5	463.3	119	-8.5
DB12218	6	4	6	1.7	428.9	120	-8.5
DB12229	5	1	7	4.3	409.5	83.7	-8.5
DB12247	6	3	8	3.9	463.6	91.5	-8.5
DB12270	4	2	6	3.8	383.5	71.1	-8.5
DB12308	2	1	1	4.9	318.5	37.3	-8.5
DB12404	5	0	5	3.4	439.3	69.4	-8.5
DB12432	5	2	6	3.8	388.5	82.7	-8.5
DB12436	9	2	6	3.1	432.4	76.9	-8.5
DB12446	4	3	3	3.3	308.4	73.8	-8.5
DB12468	5	2	4	2.5	357.8	95.1	-8.5
DB12474	1	1	1	4.5	284.4	20.2	-8.5
DB12545	3	1	4	3.1	295.3	57.6	-8.5
DB12561	6	1	5	1.8	396.4	93.6	-8.5
DB12899	7	0	4	2.7	395.5	70.9	-8.5
DB12952	5	2	2	1.8	372.5	91.7	-8.5
DB13019	6	3	5	1.1	468.5	97.9	-8.5
DB13094	4	1	4	3.1	376.4	63.1	-8.5
DB13101	5	2	6	2.4	447.5	120	-8.5
DB13129	3	1	2	2.6	342.5	54.4	-8.5
DB13212	5	2	2	3	354.4	92.2	-8.5
DB13246	2	0	1	4.5	304.4	6.5	-8.5
DB13252	2	0	0	4.8	333.5	28.5	-8.5
DB13463	3	0	3	4.1	319.4	25.6	-8.5
DB13586	2	1	0	3.6	300.4	37.3	-8.5
DB13618	3	0	3	4	280.3	35.5	-8.5
DB13720	0	0	2	4.4	278.4	0	-8.5
DB13857	2	0	1	2.8	312.4	34.1	-8.5
DB13981	4	0	3	2.6	370.5	60.4	-8.5
DB14637	7	2	4	2.1	420.5	101	-8.5
DB14775	4	2	2	3.7	299.71	70.4	-8.5
DB14802	3	1	2	4.1	398.9	45.3	-8.5
DB14966	6	1	5	3.5	389.5	82.7	-8.5
DB15009	8	1	5	1.8	432.4	123	-8.5
DB15055	7	1	4	3.1	455.9	78.1	-8.5
DB15092	5	1	4	0.6	379.5	65.4	-8.5
DB15208	6	1	5	3.5	420.5	69.8	-8.5
DB15221	6	1	1	1.6	302.35	94.5	-8.5
DB15227	6	1	4	3	454.5	105	-8.5
DB15275	8	2	5	1.5	407.4	112	-8.5
DB15377	4	1	6	4.2	365.4	93.6	-8.5
DB15431	8	2	6	3.9	449.9	84.1	-8.5
DB15448	5	2	5	3.4	385.4	114	-8.5
DB15584	6	4	1	1.4	286.24	107	-8.5
DB00240	5	3	2	2.2	408.9	94.8	-8.4
DB00276	6	2	5	4	393.5	88.7	-8.4
DB00348	8	0	2	2.3	329.23	97	-8.4
DB00358	9	2	2	3.6	378.31	45.2	-8.4
DB00450	4	1	6	3.5	379.4	52.6	-8.4
DB00540	1	1	3	4.5	263.4	12	-8.4
DB00590	9	1	4	2.5	451.5	112	-8.4
DB00591	8	2	2	2.5	452.5	93.1	-8.4
DB00605	5	1	4	3.4	356.4	73.6	-8.4
DB00808	5	2	3	2.9	365.8	101	-8.4
DB00829	2	0	1	3	284.74	32.7	-8.4
DB00962	4	0	3	1	305.33	74.3	-8.4
DB00978	8	2	3	-0.8	351.35	72.9	-8.4
DB01148	5	0	6	4.3	391.5	55.8	-8.4
DB01222	6	2	4	2.5	430.5	93.1	-8.4

DB01543	2	1	1	3	288.4	37.3	-8.4
DB01608	5	1	4	3.5	365.5	75.8	-8.4
DB01725	3	4	3	3.4	328.4	98.8	-8.4
DB01771	3	4	4	4.2	369.8	95.1	-8.4
DB01782	2	1	0	2.7	220.23	45.8	-8.4
DB01852	6	4	1	1.9	286.24	107	-8.4
DB01944	3	1	1	2.4	292.3	52.9	-8.4
DB02195	4	1	2	3.4	305.3	50.9	-8.4
DB02310	1	0	0	4	251.35	16.8	-8.4
DB02491	6	2	7	3.7	452	76.3	-8.4
DB03072	4	1	5	3.4	377.5	44.7	-8.4
DB03220	3	2	6	2.5	323.4	81.1	-8.4
DB03336	10	2	5	3.7	439.4	110	-8.4
DB03421	2	1	3	2.6	266.29	49.4	-8.4
DB03453	4	3	10	3.2	446.5	109	-8.4
DB03490	2	1	1	2.5	233.27	41.6	-8.4
DB03671	5	3	1	1.1	390.5	87	-8.4
DB03744	4	3	6	3.1	425.9	103	-8.4
DB03758	6	2	0	3.4	364.8	96.4	-8.4
DB04246	3	5	5	2.7	372.4	126	-8.4
DB04563	4	4	3	3.1	387.2	126	-8.4
DB04610	5	4	4	0.5	313.35	112	-8.4
DB04632	8	3	8	3.7	471.5	99.3	-8.4
DB04669	4	2	4	3.9	382.2	70.6	-8.4
DB04760	6	2	6	3.3	410.4	84	-8.4
DB04812	4	1	3	4.1	301.72	63.3	-8.4
DB04862	7	3	8	2.1	452.5	124	-8.4
DB04863	6	2	9	2.7	439.5	129	-8.4
DB04885	2	0	2	2.6	319.4	39.8	-8.4
DB04946	7	0	8	4.1	426.5	64.8	-8.4
DB05137	3	1	6	3.8	337.5	40.5	-8.4
DB05586	3	2	2	1.8	270.33	61	-8.4
DB06235	4	1	2	3.2	282.29	63.6	-8.4
DB06274	5	3	8	1.7	424.5	89.9	-8.4
DB06334	5	3	6	2.3	390.4	97.1	-8.4
DB06622	3	2	0	2.3	306.4	57.5	-8.4
DB06635	5	3	9	2	446.5	131	-8.4
DB06831	7	1	5	3.8	450.9	122	-8.4
DB06886	5	3	10	3	467.6	108	-8.4
DB06942	3	3	7	1.9	378.5	99.3	-8.4
DB06945	9	2	5	2.6	445.4	110	-8.4
DB06953	5	0	6	4.5	474.3	87.5	-8.4
DB07011	5	0	5	3.5	377.4	48.8	-8.4
DB07065	6	1	5	4.5	427.3	104	-8.4
DB07117	8	2	5	2.2	458.5	117	-8.4
DB07124	7	1	6	0.3	384.4	97.6	-8.4
DB07131	3	3	7	3.1	384.5	99.3	-8.4
DB07146	5	2	6	4.2	398.5	53.3	-8.4
DB07147	6	2	8	2.9	420.5	97.8	-8.4
DB07150	3	2	2	4.2	263.29	52.8	-8.4
DB07179	4	1	4	2.8	410.3	67.7	-8.4
DB07186	6	2	5	2.6	448.5	115	-8.4
DB07187	5	1	2	2.2	324.74	97.1	-8.4
DB07198	4	2	1	3	251.24	77.4	-8.4
DB07217	4	1	2	4.4	300.4	81.1	-8.4
DB07242	3	1	0	2.7	377.3	61.1	-8.4
DB07352	5	3	1	1.7	270.24	87	-8.4
DB07421	10	3	6	3.1	411.3	131	-8.4
DB07443	3	2	3	4	278.3	73.1	-8.4
DB07468	3	1	1	2.4	292.3	52.9	-8.4
DB07655	4	3	5	3.6	330.4	73.8	-8.4
DB07663	7	3	5	1.7	355.3	132	-8.4
DB07692	10	0	4	1.6	460.5	110	-8.4
DB07704	4	3	4	1.9	335.4	105	-8.4
DB07717	5	1	5	5	446.6	46.5	-8.4
DB07741	7	2	5	2.2	444.5	75.6	-8.4
DB07766	6	4	6	2.4	365.4	110	-8.4
DB07775	6	4	1	4.1	444.03	107	-8.4

DB07806	6	4	8	0.1	441.5	133	-8.4
DB07813	4	3	5	1.2	368.4	105	-8.4
DB07853	6	2	5	3.5	381.4	103	-8.4
DB07863	3	1	2	3.3	276.67	74.9	-8.4
DB07872	6	2	6	3.4	474.9	110	-8.4
DB07968	5	3	3	4.1	338.72	87.7	-8.4
DB07982	7	2	6	1.7	435.5	99.4	-8.4
DB08029	6	3	7	0.9	350.4	115	-8.4
DB08054	5	1	2	2.5	304.3	82.5	-8.4
DB08067	4	5	5	0.5	382.4	112	-8.4
DB08087	4	1	1	1.4	307.3	84.6	-8.4
DB08172	4	2	4	4.6	342.8	82.4	-8.4
DB08239	4	1	3	3.2	397.5	35.6	-8.4
DB08251	7	3	7	0.3	342.3	134	-8.4
DB08252	5	2	3	4.1	292.29	82.2	-8.4
DB08267	3	3	3	1.9	305.33	96.2	-8.4
DB08305	7	1	3	2.1	427.6	104	-8.4
DB08348	3	2	3	1.7	295.34	61.4	-8.4
DB08448	8	3	2	2	385.4	127	-8.4
DB08491	6	2	6	1.7	391.4	110	-8.4
DB08499	7	3	8	0.8	427.4	131	-8.4
DB08542	4	2	3	2.5	316.4	74.6	-8.4
DB08602	5	0	2	3.6	304.32	58	-8.4
DB08801	2	0	5	2.7	292.4	16.1	-8.4
DB08846	8	4	0	1.1	302.19	134	-8.4
DB08860	6	3	8	3.5	421.5	90.6	-8.4
DB08882	7	1	4	1.9	472.5	114	-8.4
DB08931	9	2	5	1.6	422.4	138	-8.4
DB08951	3	1	3	2.8	281.3	57.6	-8.4
DB09047	9	2	3	-0.7	398.4	106	-8.4
DB09128	5	1	7	4.7	433.6	73	-8.4
DB09181	6	0	8	4.1	466.6	71.8	-8.4
DB11426	9	1	2	-0.5	362.4	76.6	-8.4
DB11619	2	1	2	2.2	308.4	37.3	-8.4
DB11679	7	1	5	3.4	393.4	95.7	-8.4
DB11706	4	2	5	2.4	314.4	64.4	-8.4
DB11708	4	4	3	1.9	326.4	104	-8.4
DB11725	2	0	3	3.5	319.4	21.1	-8.4
DB11778	6	2	6	1.8	474.6	93.8	-8.4
DB11794	8	2	7	2.3	463.6	132	-8.4
DB11809	6	0	5	4.6	469.6	49.4	-8.4
DB11867	9	1	6	2.4	416.4	94.4	-8.4
DB12021	6	2	7	1.9	396.5	91.2	-8.4
DB12238	7	0	6	3.4	395.4	83	-8.4
DB12285	7	2	3	0.6	409.4	126	-8.4
DB12382	8	2	4	1.3	413.5	124	-8.4
DB12399	6	1	3	2.6	361.4	94.8	-8.4
DB12459	6	2	5	1.6	433.5	91.8	-8.4
DB12479	10	2	4	-0.6	401.4	107	-8.4
DB12505	5	2	3	2.4	336.4	118	-8.4
DB12553	8	4	2	1.1	412.4	115	-8.4
DB12597	8	3	6	3	449.8	103	-8.4
DB12627	8	0	7	4	456.6	107	-8.4
DB12637	4	2	5	3.2	449.6	60.8	-8.4
DB12666	4	0	6	2.6	401.5	69.6	-8.4
DB12782	4	1	4	4.8	440.4	49.2	-8.4
DB12792	2	1	3	4.9	343.2	42	-8.4
DB12910	5	1	4	2.9	404.5	75.5	-8.4
DB12966	8	2	4	3.4	387.8	78.9	-8.4
DB13062	4	3	2	4.4	348.4	69.9	-8.4
DB13102	4	0	4	2.7	373.8	66.6	-8.4
DB13292	2	0	0	3.9	293.4	28.5	-8.4
DB13808	1	0	2	3.5	276.4	8.2	-8.4
DB13856	6	3	2	2.6	447.3	94.8	-8.4
DB13865	6	1	7	3.7	478.6	52.2	-8.4
DB13872	3	1	1	2.4	335.2	52.9	-8.4
DB13939	7	2	5	3.1	393.8	119	-8.4
DB14627	5	1	6	3.5	401.4	81.7	-8.4

DB14715	6	2	6	2.5	465.7	105	-8.4
DB14876	5	1	6	3.1	454.5	109	-8.4
DB14985	6	3	6	1.6	340.33	111	-8.4
DB15120	4	0	4	3.4	377.5	45.7	-8.4
DB15138	6	2	5	2.9	442.5	56.3	-8.4
DB15247	4	3	3	1.6	439.5	97.5	-8.4
DB15256	7	2	6	1.5	384.4	98.7	-8.4
DB15348	5	1	4	3.8	321.3	21.3	-8.4
DB15393	7	2	6	3.1	409.8	102	-8.4
DB15450	5	1	6	4.7	428.5	70.5	-8.4
DB15566	6	2	4	2.4	402.5	101	-8.4
DB00180	7	2	2	2.5	434.5	93.1	-8.3
DB00216	3	1	6	4.1	382.5	61.6	-8.3
DB00275	7	3	8	3.2	446.5	130	-8.3
DB00340	2	0	2	4.9	309.5	28.5	-8.3
DB00353	3	3	4	2.3	339.4	68.4	-8.3
DB00713	7	2	4	2.4	401.4	138	-8.3
DB00715	5	1	4	3.5	329.4	39.7	-8.3
DB00817	5	1	3	0.6	294.3	70.5	-8.3
DB00861	5	2	2	4.4	250.2	57.5	-8.3
DB00896	3	1	2	3.5	370.5	54.4	-8.3
DB00910	3	3	5	5	416.6	60.7	-8.3
DB00920	3	0	0	3.2	309.4	48.6	-8.3
DB01029	5	1	7	4.1	428.5	87.1	-8.3
DB01044	8	2	4	-0.7	375.4	82.1	-8.3
DB01238	4	1	7	4.6	448.4	44.8	-8.3
DB01261	10	1	4	0.7	407.31	77	-8.3
DB01503	2	2	0	4	290.4	40.5	-8.3
DB01586	4	3	4	4.9	392.6	77.8	-8.3
DB01657	6	3	5	-1.6	328.4	113	-8.3
DB02058	3	1	3	2.2	335.4	52.6	-8.3
DB02205	4	1	3	3.9	314.4	55.8	-8.3
DB02659	5	4	4	3.6	408.6	98	-8.3
DB02882	4	1	5	3.7	364.4	64.2	-8.3
DB03181	5	2	4	3.9	367.4	91.2	-8.3
DB03996	3	1	2	3	314.8	52.9	-8.3
DB04114	2	1	1	4.1	298.8	38.9	-8.3
DB04392	6	0	2	2.9	336.3	86.7	-8.3
DB04480	3	0	2	3.3	293.3	30.7	-8.3
DB04759	4	2	6	3.1	374.4	84	-8.3
DB04833	2	0	1	2.5	250.29	32.7	-8.3
DB04859	3	1	6	4.9	376.5	32.3	-8.3
DB04861	7	3	6	3	405.4	71	-8.3
DB05212	3	3	1	2.2	330.5	60.7	-8.3
DB05524	7	2	8	4.5	467.9	90.3	-8.3
DB05590	6	4	9	1.8	428.5	102	-8.3
DB06137	5	1	9	2.9	431.5	63.7	-8.3
DB06157	5	1	3	1.5	360.5	81.8	-8.3
DB06307	2	2	1	4.1	316.5	40.5	-8.3
DB06395	4	2	5	4.3	378.5	86.8	-8.3
DB06436	1	2	1	2.5	238.28	44.9	-8.3
DB06457	4	2	4	3.6	324.4	41.9	-8.3
DB06499	3	1	3	4	266.29	50.4	-8.3
DB06533	6	1	9	5	419.5	68.2	-8.3
DB06771	7	2	3	1.1	393.8	86.9	-8.3
DB06850	4	4	7	1.5	385.5	111	-8.3
DB06907	4	1	2	4.2	308.1	63.3	-8.3
DB06964	6	3	5	3.5	388.8	105	-8.3
DB06986	3	3	2	3.6	332.8	93.4	-8.3
DB07148	5	0	4	2.9	389.8	62.2	-8.3
DB07152	6	2	3	1.6	362.4	96.4	-8.3
DB07156	4	2	4	3.2	426.3	64.9	-8.3
DB07183	5	2	7	4.5	396.4	76.1	-8.3
DB07237	4	1	6	4.7	363.5	66.6	-8.3
DB07252	9	3	5	3	419.8	137	-8.3
DB07254	9	3	6	3.4	433.9	123	-8.3
DB07256	7	3	5	3.3	383.8	111	-8.3
DB07275	5	2	4	2.9	411.5	107	-8.3

DB07284	4	2	4	2.5	276.34	63.8	-8.3
DB07447	2	1	0	3.7	290.4	37.3	-8.3
DB07495	5	4	5	3.4	387.8	108	-8.3
DB07513	4	1	5	2.9	329.8	47.6	-8.3
DB07549	6	4	8	1.2	448.4	102	-8.3
DB07685	8	2	6	3.2	402.5	133	-8.3
DB07729	5	2	3	2.1	283.26	83.6	-8.3
DB07756	9	3	5	3.4	426.4	105	-8.3
DB07763	6	1	3	3.7	318.27	58.6	-8.3
DB07809	4	3	6	3.3	344.4	91.9	-8.3
DB07917	5	1	8	3.8	377.5	66.9	-8.3
DB07945	3	2	4	3.8	362.4	85.3	-8.3
DB08000	7	2	3	1	335.29	137	-8.3
DB08023	4	0	3	4	307.4	46.3	-8.3
DB08024	3	2	5	2	323.4	72.4	-8.3
DB08047	6	2	3	4.2	334.29	58.3	-8.3
DB08051	6	1	5	3.2	419.4	66.6	-8.3
DB08147	5	1	9	4.3	387.4	74.7	-8.3
DB08150	3	2	3	3.1	342.8	72.4	-8.3
DB08241	6	3	6	3.5	366.4	119	-8.3
DB08278	6	2	4	2.8	427.5	107	-8.3
DB08390	5	1	6	4.9	362.4	55.8	-8.3
DB08391	8	0	5	3.4	403.4	82.5	-8.3
DB08393	5	2	5	3.9	331.4	71.2	-8.3
DB08395	9	1	7	4	423.4	94	-8.3
DB08400	7	1	4	4.2	380.4	62.7	-8.3
DB08513	7	4	7	3.2	377.4	130	-8.3
DB08531	4	1	4	4	370.2	55.1	-8.3
DB08867	4	1	3	2.9	433.6	57.6	-8.3
DB08950	2	2	5	4.1	347.5	48.1	-8.3
DB08954	3	2	5	3.9	325.4	43.7	-8.3
DB09053	6	1	5	3.6	440.5	99.2	-8.3
DB09091	5	3	2	2	378.5	75.6	-8.3
DB11157	3	2	0	3.2	226.23	57.5	-8.3
DB11393	7	1	3	-0.3	357.4	64.1	-8.3
DB11452	9	1	4	3.1	457.4	122	-8.3
DB11518	7	2	3	4.1	296.24	62.2	-8.3
DB11656	4	3	5	2.4	370.8	95.5	-8.3
DB11665	7	3	5	0.7	368.4	101	-8.3
DB11703	6	2	4	3	465.5	119	-8.3
DB11789	4	3	4	4.9	392.6	77.8	-8.3
DB11798	10	3	5	3.1	448.4	97.1	-8.3
DB11845	6	2	7	3.6	443.5	95.7	-8.3
DB11921	7	1	4	2	441.5	102	-8.3
DB11939	8	4	6	1.8	454.9	109	-8.3
DB12155	4	0	1	2.3	240.21	64.4	-8.3
DB12191	3	2	2	3.3	317.4	49.4	-8.3
DB12204	5	3	10	4.2	405.5	110	-8.3
DB12547	7	2	3	1.5	390.4	115	-8.3
DB12567	5	2	6	3.3	424.6	47.6	-8.3
DB12612	7	2	7	3.1	404.5	104	-8.3
DB12744	4	3	5	3.4	466.3	94.2	-8.3
DB12867	4	1	6	3.7	381.4	52.6	-8.3
DB12879	2	0	3	3.4	275.3	12.5	-8.3
DB12941	5	3	6	1.8	398.8	120	-8.3
DB12963	6	1	5	2.5	451.9	92.2	-8.3
DB13036	6	2	6	2.9	416.5	96.8	-8.3
DB13078	7	2	2	4.2	473.6	110	-8.3
DB13213	5	0	7	5	409.6	52.1	-8.3
DB13317	5	1	3	3.6	285.27	63.3	-8.3
DB13471	6	2	5	1	476.6	86.4	-8.3
DB13520	3	1	5	3.8	403.5	46.5	-8.3
DB13651	5	1	4	3	402.9	53	-8.3
DB13752	4	0	5	4.7	331.4	63.7	-8.3
DB13937	4	1	2	4	342.74	34	-8.3
DB13978	1	2	1	2.5	248.71	58.9	-8.3
DB14055	4	1	4	2.7	308.3	63.6	-8.3
DB14127	5	3	10	3	383.4	104	-8.3

DB14632	6	2	6	4.1	458.6	101	-8.3
DB14633	8	3	7	1.8	460.5	138	-8.3
DB14640	7	2	4	1.5	420.5	101	-8.3
DB14659	4	0	3	3.1	396.5	60.4	-8.3
DB14719	3	1	1	3.7	296.4	69.7	-8.3
DB14765	5	2	6	4.1	397.5	90.7	-8.3
DB14799	5	1	6	2.1	467.3	77.1	-8.3
DB14969	5	2	3	4	341.3	60.8	-8.3
DB14970	6	2	5	3.2	437.5	101	-8.3
DB14979	3	2	6	3.9	354.5	52.6	-8.3
DB15052	4	1	7	4.7	381.5	49.8	-8.3
DB15307	6	2	3	2	367.4	130	-8.3
DB15347	4	3	3	2.8	370.4	91.2	-8.3
DB15590	7	0	4	2.9	396.4	72.4	-8.3
DB00231	3	1	1	2.2	300.74	52.9	-8.2
DB00321	1	0	3	5	277.4	3.2	-8.2
DB00324	5	2	1	2	376.5	74.6	-8.2
DB00436	10	3	3	1.2	421.4	135	-8.2
DB00468	4	1	4	2.9	324.4	45.6	-8.2
DB00482	7	1	3	3.4	381.4	86.4	-8.2
DB00496	3	1	7	4.6	426.5	55.6	-8.2
DB00546	4	0	3	1.6	351.8	46.3	-8.2
DB00564	1	1	0	2.5	236.27	46.3	-8.2
DB00663	7	3	2	1.9	410.5	94.8	-8.2
DB00768	4	1	5	1.5	337.4	49.8	-8.2
DB00846	7	2	2	1.4	436.5	93.1	-8.2
DB00873	5	2	3	2.6	394.9	83.8	-8.2
DB00897	3	0	1	2.4	343.2	43.1	-8.2
DB00991	4	1	5	4.2	293.3	63.3	-8.2
DB01104	1	1	2	4.8	306.2	12	-8.2
DB01113	5	0	6	3.9	339.4	49.8	-8.2
DB01147	7	2	4	2.4	435.9	138	-8.2
DB01196	3	1	6	4	440.4	49.8	-8.2
DB01219	6	1	3	1.7	314.25	121	-8.2
DB01228	3	1	6	4.3	352.5	41.6	-8.2
DB01234	6	3	2	1.9	392.5	94.8	-8.2
DB01253	3	3	3	1.8	325.4	68.4	-8.2
DB01469	6	1	6	3.2	453.6	68.2	-8.2
DB01479	2	2	0	4.3	306.5	40.5	-8.2
DB01489	4	0	3	2.8	371.8	62.2	-8.2
DB01526	2	2	0	3.4	290.4	40.5	-8.2
DB01645	5	3	1	2.7	270.24	87	-8.2
DB02014	9	2	8	4.1	413.4	119	-8.2
DB02021	5	3	1	-0.3	279.22	111	-8.2
DB02049	6	2	6	2.3	425.9	110	-8.2
DB02123	5	4	6	4.3	449.6	107	-8.2
DB02166	2	2	7	4.9	414.6	55.9	-8.2
DB02217	8	3	3	-0.9	410.3	135	-8.2
DB02390	2	4	4	3	389.3	73.7	-8.2
DB02398	3	4	4	1.8	318.4	105	-8.2
DB02848	8	2	8	3.9	422.5	118	-8.2
DB02854	2	1	0	3.7	290.4	37.3	-8.2
DB02917	7	2	5	2.1	389.5	136	-8.2
DB03118	5	3	3	2.3	289.68	108	-8.2
DB03176	4	2	5	4.9	355.2	66.8	-8.2
DB03288	4	4	7	2.4	365.9	88.6	-8.2
DB03365	6	2	4	3	297.31	76.5	-8.2
DB03368	4	2	3	2.4	310.3	84.5	-8.2
DB03467	5	3	1	2.4	272.25	87	-8.2
DB03585	3	1	5	2.7	324.4	60.8	-8.2
DB03596	4	3	6	2.5	371.5	113	-8.2
DB04140	7	2	6	0.7	419.5	125	-8.2
DB04200	6	2	6	3.3	358.4	85.2	-8.2
DB04288	6	3	6	3.7	405.5	93.7	-8.2
DB04685	5	1	7	4.7	472.5	67.9	-8.2
DB04829	2	1	3	3	323.4	39.3	-8.2
DB04836	3	2	8	2.1	337.5	49.3	-8.2
DB05104	3	1	7	4.1	414.5	43.8	-8.2

DB05105	9	0	6	4.6	381.3	74.2	-8.2
DB06257	3	3	0	2.4	306.4	60.7	-8.2
DB06358	7	2	5	2.5	354.4	106	-8.2
DB06414	7	2	4	4.5	435.3	121	-8.2
DB06578	5	2	3	2.9	391.8	75.6	-8.2
DB06693	5	1	7	3.9	390.5	72.8	-8.2
DB06713	3	2	2	3.6	327.5	52.8	-8.2
DB06739	4	1	8	4.4	354.4	71.4	-8.2
DB06766	3	0	1	1.7	307.4	38.1	-8.2
DB06786	6	1	2	3.6	455	72.8	-8.2
DB06837	5	5	6	0.8	370.4	119	-8.2
DB06853	4	4	7	2	399.5	111	-8.2
DB06856	4	4	4	4.6	381.4	95.1	-8.2
DB06889	6	1	2	1.2	288.33	115	-8.2
DB06927	4	2	2	2.9	265.26	77.4	-8.2
DB06930	1	2	4	3.1	318.4	86.4	-8.2
DB06976	4	4	3	0.6	366.4	102	-8.2
DB06995	7	3	5	3.2	398.4	106	-8.2
DB07210	4	1	4	3.4	380.2	55.1	-8.2
DB07216	6	1	3	2.9	473	73.9	-8.2
DB07227	4	0	5	2.7	405.9	65.2	-8.2
DB07246	9	3	6	1.6	404.4	127	-8.2
DB07319	5	2	1	2.9	316.16	90.7	-8.2
DB07441	5	2	4	1.9	335.4	93.4	-8.2
DB07446	5	2	7	3.8	347.4	91.8	-8.2
DB07455	6	2	9	2.9	424.6	109	-8.2
DB07503	7	1	1	2.8	285.23	89.9	-8.2
DB07511	3	0	1	2.5	262.26	61.2	-8.2
DB07537	4	3	6	3.5	366.5	97.1	-8.2
DB07563	5	1	5	3.1	418.6	63.2	-8.2
DB07583	6	2	7	2.9	431.9	88.7	-8.2
DB07588	4	5	4	3.9	385.4	122	-8.2
DB07616	7	3	4	4.7	344.4	111	-8.2
DB07619	6	1	5	4.3	432.5	53.4	-8.2
DB07643	6	2	5	4.3	432.3	90.3	-8.2
DB07653	6	2	5	4.1	345.4	88.2	-8.2
DB07697	9	0	5	1.4	454.5	119	-8.2
DB07812	4	3	5	2.9	362.4	112	-8.2
DB07834	4	1	5	3.8	347.4	68	-8.2
DB07938	7	2	5	2.5	354.4	106	-8.2
DB08005	4	3	4	3.2	398.9	85.9	-8.2
DB08015	6	1	3	4.7	404.4	73	-8.2
DB08020	4	2	3	3.7	326.4	58.9	-8.2
DB08037	6	1	4	2.8	459.5	47	-8.2
DB08042	8	2	7	4	420.5	97.4	-8.2
DB08043	6	2	4	3.8	373.3	63.2	-8.2
DB08055	3	1	3	4.9	320.8	42.2	-8.2
DB08056	3	1	3	5	314.4	42.2	-8.2
DB08184	1	3	2	3	249.31	65.7	-8.2
DB08205	3	1	1	3.8	239.27	46.3	-8.2
DB08207	2	0	1	4.2	223.27	26	-8.2
DB08282	4	1	5	3.4	360.8	90.7	-8.2
DB08303	8	2	3	2	428.6	116	-8.2
DB08353	6	2	5	4.4	347.4	90.9	-8.2
DB08441	7	3	0	2.2	384.25	104	-8.2
DB08486	4	2	6	3.6	341.4	75.6	-8.2
DB08493	4	3	6	3.2	441.6	100	-8.2
DB08517	5	2	2	2.7	286.28	76	-8.2
DB08530	4	1	3	1	355.4	78.3	-8.2
DB08564	5	2	6	3.6	426.3	70.2	-8.2
DB08715	5	0	6	4.3	326.4	61.3	-8.2
DB08745	6	0	6	2.9	475	82.2	-8.2
DB08807	4	2	9	4.7	380.5	63.4	-8.2
DB08922	6	0	6	3.9	426.6	85	-8.2
DB09068	3	1	3	4.2	298.4	40.6	-8.2
DB09193	1	1	2	3.7	237.34	12	-8.2
DB09488	4	1	6	1.6	348.4	53.4	-8.2
DB11811	7	1	8	4.3	415.8	64.6	-8.2

DB11885	6	2	6	3.7	407.4	82.4	-8.2
DB11903	8	2	5	4.1	449.3	76.1	-8.2
DB11948	3	0	0	2.2	242.27	43.4	-8.2
DB12058	8	1	7	2.7	386.4	101	-8.2
DB12068	10	3	5	1.5	411.31	119	-8.2
DB12129	3	0	3	4.3	334.4	65.9	-8.2
DB12149	6	3	8	5	464.6	136	-8.2
DB12268	6	1	4	0.9	377.5	68	-8.2
DB12291	3	3	3	1.3	269.3	84.2	-8.2
DB12350	4	3	1	3.3	374.5	73.8	-8.2
DB12357	4	2	1	2.2	289.71	66.6	-8.2
DB12364	5	3	7	3.6	451.9	107	-8.2
DB12570	7	2	4	1.1	397.5	101	-8.2
DB12582	3	0	3	3.5	285.34	38.8	-8.2
DB12591	5	1	6	2.9	432.5	85.8	-8.2
DB12675	8	1	7	2.4	432.5	86.4	-8.2
DB12685	4	3	7	2.2	399.5	90.9	-8.2
DB12687	7	2	8	2.7	410.5	96.6	-8.2
DB12739	4	1	6	3	324.4	64.4	-8.2
DB12746	5	3	3	2	302.33	92.5	-8.2
DB12784	7	2	6	3.2	389.4	80.6	-8.2
DB12796	10	1	5	2	408.3	87.9	-8.2
DB12830	3	1	2	4.3	378.5	28.3	-8.2
DB12840	3	0	2	3.7	300.4	35.5	-8.2
DB12852	6	1	6	4.7	442.9	79.8	-8.2
DB12914	7	2	4	2.7	435.2	136	-8.2
DB13016	6	3	9	4.9	434.5	104	-8.2
DB13022	5	2	7	3.5	444.3	88.8	-8.2
DB13080	4	0	5	3.2	366.4	40.6	-8.2
DB13358	1	1	3	2.7	262.3	24.4	-8.2
DB13374	4	1	3	2.9	354.4	54.7	-8.2
DB13397	4	0	7	5	361.5	42.2	-8.2
DB13718	4	1	4	3.1	326.4	45.6	-8.2
DB13728	7	3	2	2.6	444.9	94.8	-8.2
DB13772	8	1	2	-0.3	363.4	89.4	-8.2
DB13843	5	3	2	1.9	392.9	94.8	-8.2
DB13867	8	2	3	3.2	444.5	99.9	-8.2
DB13919	7	2	7	4.1	440.5	119	-8.2
DB14008	6	3	2	1.7	300.26	96.2	-8.2
DB14065	7	0	8	4.9	468.5	34.2	-8.2
DB14210	8	3	10	0.5	470.5	125	-8.2
DB14662	8	1	4	3.2	476.5	99.1	-8.2
DB14736	2	1	2	5	282.4	29.5	-8.2
DB14810	9	1	9	1.3	478.5	129	-8.2
DB14899	7	1	3	3.3	391.3	71.5	-8.2
DB15078	8	2	6	1.7	466.6	135	-8.2
DB15143	6	2	6	2	361.4	104	-8.2
DB15170	6	2	7	4.2	429.5	93.4	-8.2
DB15191	8	2	5	3.6	438.5	105	-8.2
DB15250	5	1	6	4.6	444.9	97.1	-8.2
DB15281	7	2	6	2	440.9	96.2	-8.2
DB15323	6	1	3	0.7	357.4	93.7	-8.2
DB15493	3	3	3	4.1	360.4	93.4	-8.2
DB00218	8	2	4	0.6	401.4	82.1	-8.1
DB00227	5	1	7	4.3	404.5	72.8	-8.1
DB00245	2	0	4	4.5	307.4	12.5	-8.1
DB00248	4	2	8	3.4	451.6	71.7	-8.1
DB00317	8	1	8	4.1	446.9	68.7	-8.1
DB00363	3	1	1	3.1	326.8	30.9	-8.1
DB00448	8	1	5	2.8	369.4	87.1	-8.1
DB00487	7	1	3	0.3	333.36	64.1	-8.1
DB00641	5	1	7	4.7	418.6	72.8	-8.1
DB00705	7	3	6	2.4	456.6	119	-8.1
DB00821	2	2	2	4	273.71	53.1	-8.1
DB00957	4	1	4	4.1	369.5	58.9	-8.1
DB00959	5	3	2	1.9	374.5	94.8	-8.1
DB01094	6	3	2	2.4	302.28	96.2	-8.1
DB01157	8	3	6	2.5	369.4	118	-8.1

DB01260	6	2	2	2.7	416.5	93.1	-8.1
DB01320	6	3	5	0.6	362.27	116	-8.1
DB01384	6	3	2	2	392.5	94.8	-8.1
DB01521	2	1	0	4.2	322.9	37.3	-8.1
DB01591	3	0	3	4	362.5	32.8	-8.1
DB01685	5	1	2	1.2	248.24	91.1	-8.1
DB01838	4	2	1	4.6	426.05	66.8	-8.1
DB01967	5	1	6	2.5	403.5	79.8	-8.1
DB02008	5	2	8	3.6	463.5	98.4	-8.1
DB02062	6	4	9	-0.9	418.4	130	-8.1
DB02101	5	3	1	-0.3	279.22	111	-8.1
DB02177	7	0	6	4.4	478.6	117	-8.1
DB02191	2	1	0	2.4	240.3	36.1	-8.1
DB02354	4	3	6	3.4	423.5	97.5	-8.1
DB02455	6	4	4	2.4	434.5	140	-8.1
DB02526	4	4	4	3	336.4	108	-8.1
DB02636	8	2	2	3.2	357.3	131	-8.1
DB02639	8	4	3	-0.8	338.31	126	-8.1
DB02691	6	4	5	3.6	464.6	130	-8.1
DB02699	2	1	5	4.6	300.4	37.3	-8.1
DB02929	4	0	6	4.1	424.6	58.1	-8.1
DB03154	10	4	4	0.6	388.14	115	-8.1
DB03173	3	4	4	3.6	335.4	95.1	-8.1
DB03509	3	1	2	2.4	283.71	68.9	-8.1
DB03619	4	3	4	4.9	392.6	77.8	-8.1
DB03849	5	1	5	3.4	343.4	79.6	-8.1
DB03916	7	2	6	3	423.9	83.2	-8.1
DB03924	6	4	0	1.9	270.24	127	-8.1
DB03950	4	2	6	2.3	371.5	103	-8.1
DB04202	4	1	2	2.8	268.26	55.8	-8.1
DB04215	2	4	2	3.4	344.21	85.9	-8.1
DB04463	5	2	2	2	283.33	89.8	-8.1
DB04552	7	2	3	3.7	282.22	62.2	-8.1
DB04576	9	1	4	-0.1	369.34	64.1	-8.1
DB04630	5	2	3	1.1	360.4	91.7	-8.1
DB04715	4	1	5	2.7	306.4	59.3	-8.1
DB04787	3	1	1	2.7	297.4	82.1	-8.1
DB04790	6	4	6	1	382.4	137	-8.1
DB04791	6	4	6	1	382.4	137	-8.1
DB04867	5	2	5	4.5	411.9	113	-8.1
DB04892	4	1	3	2.3	337.4	44.8	-8.1
DB04903	4	0	6	4.6	407.9	63.2	-8.1
DB05575	5	3	5	3.9	373.5	78.9	-8.1
DB05611	8	1	8	3.8	418.5	84.8	-8.1
DB05861	7	1	3	3.5	406.4	70.1	-8.1
DB05928	6	3	2	1.6	392.4	90.3	-8.1
DB05936	7	1	6	4.2	446.4	87.1	-8.1
DB06148	2	0	0	3.4	264.4	6.5	-8.1
DB06153	2	0	0	3.8	295.4	31.5	-8.1
DB06203	5	1	3	0.6	339.4	93.7	-8.1
DB06280	3	1	4	4.1	323.3	43.1	-8.1
DB06603	3	4	7	3	349.4	77.2	-8.1
DB06620	4	2	4	1.6	310.3	59.6	-8.1
DB06803	4	2	2	4	327.12	95.2	-8.1
DB06909	5	2	6	2.8	379.5	81.1	-8.1
DB06918	1	3	2	3	249.31	65.7	-8.1
DB06940	6	3	6	2	382.4	110	-8.1
DB06944	2	2	4	3.1	291.3	57.8	-8.1
DB06999	7	2	6	3.3	413.8	100	-8.1
DB07014	8	0	5	3.6	360.4	91.9	-8.1
DB07039	5	2	5	2.4	447.2	106	-8.1
DB07049	6	0	4	4.6	417.5	94.8	-8.1
DB07134	5	1	2	2.4	328.8	103	-8.1
DB07137	3	1	4	2	337.4	86.5	-8.1
DB07204	5	2	6	2.9	395.5	89.7	-8.1
DB07222	2	1	3	3.1	334.8	49.4	-8.1
DB07280	7	1	5	2.7	400.5	80.4	-8.1
DB07283	2	1	3	4.4	305.4	42.2	-8.1

DB07333	8	2	9	4.1	476.5	115	-8.1
DB07414	5	1	4	3	396.5	95.4	-8.1
DB07470	3	1	1	2	278.3	52.9	-8.1
DB07522	5	3	7	2.6	370.5	101	-8.1
DB07550	7	2	8	1.2	467.8	99.3	-8.1
DB07578	4	0	3	3.9	372.4	76.3	-8.1
DB07601	7	3	5	2.9	389.8	91	-8.1
DB07648	5	4	6	3.5	360.4	94.1	-8.1
DB07654	5	1	5	4	345.4	62.4	-8.1
DB07662	4	2	4	3.9	369.2	66.9	-8.1
DB07680	5	3	4	4.4	431.8	115	-8.1
DB07703	4	1	1	3.3	296.4	63.6	-8.1
DB07770	7	2	5	4.7	391.4	120	-8.1
DB07772	7	3	7	2.2	399.4	121	-8.1
DB07776	2	0	1	3.6	222.24	26.3	-8.1
DB07814	6	3	1	0.2	346.4	104	-8.1
DB07855	4	2	3	2.3	301.3	80.5	-8.1
DB07905	8	3	1	1.4	378.4	126	-8.1
DB07933	3	2	1	4	282.3	49.7	-8.1
DB07935	5	1	2	3	292.27	85.5	-8.1
DB07942	4	1	2	3	257.24	41.6	-8.1
DB07946	7	4	10	2.9	443.8	115	-8.1
DB07959	2	2	1	2.8	234.26	57.4	-8.1
DB08063	4	1	6	4.3	384.4	58.6	-8.1
DB08064	4	3	6	3.8	399.9	91.9	-8.1
DB08192	5	2	3	0.7	302.4	90.5	-8.1
DB08272	5	3	6	-0.3	287.31	101	-8.1
DB08315	4	1	5	2.8	307.3	72.4	-8.1
DB08317	4	2	1	2.8	250.3	77.8	-8.1
DB08340	5	2	4	3.8	302.33	67.1	-8.1
DB08346	5	2	0	3.9	338.8	83.8	-8.1
DB08360	7	1	4	2.3	348.4	85.4	-8.1
DB08397	7	3	3	1.4	302.17	94.8	-8.1
DB08402	6	2	5	4.2	404.2	101	-8.1
DB08406	7	2	2	2.8	367.4	107	-8.1
DB08535	4	1	4	3.4	380.2	55.1	-8.1
DB08538	8	2	5	2.7	381.4	94	-8.1
DB08539	4	1	5	3.6	341.4	55.1	-8.1
DB08591	5	1	3	2	318.3	84.1	-8.1
DB08660	6	4	0	2.5	272.21	115	-8.1
DB08699	4	1	3	3	295.4	69.6	-8.1
DB08713	6	0	6	3.7	327.4	78.9	-8.1
DB08714	6	0	6	4.1	327.4	78.9	-8.1
DB08746	6	0	6	3.4	461	65.1	-8.1
DB08972	5	1	1	2.9	261.25	57.6	-8.1
DB08974	5	2	4	2.9	313.28	84.1	-8.1
DB09038	7	4	6	2	450.9	109	-8.1
DB09177	3	0	6	4.2	354.5	23.6	-8.1
DB09200	6	1	6	3.1	397.4	108	-8.1
DB09209	6	1	4	0.8	398.5	54.4	-8.1
DB11259	6	3	2	1.7	300.26	96.2	-8.1
DB11404	7	1	4	-0.2	359.4	64.1	-8.1
DB11699	3	1	3	3.8	284.35	45.3	-8.1
DB11726	3	1	2	3.8	320.8	60.6	-8.1
DB11827	7	4	6	1.7	436.9	109	-8.1
DB11873	5	1	4	4.1	348.4	99.3	-8.1
DB11951	7	1	6	3.2	410.4	77	-8.1
DB11974	8	2	6	1.6	425.5	124	-8.1
DB12013	5	3	9	2.6	457.6	107	-8.1
DB12016	6	2	8	4.6	461	108	-8.1
DB12188	2	0	2	4.7	354.9	6.5	-8.1
DB12233	7	2	5	1.4	374.4	119	-8.1
DB12239	5	2	7	2.8	411.5	88.5	-8.1
DB12242	5	4	4	2.3	362.4	125	-8.1
DB12255	5	3	4	2.5	306.7	99.5	-8.1
DB12273	2	1	0	4.4	313.8	23.5	-8.1
DB12463	4	3	5	1.3	361.4	98.7	-8.1
DB12512	7	1	6	4.5	406.5	90.4	-8.1

DB12517	5	1	4	4.5	470.6	43.8	-8.1
DB12574	4	2	3	3.9	387.2	92.9	-8.1
DB12645	5	3	9	3.6	421.5	90.9	-8.1
DB12705	8	2	9	4.2	453.5	111	-8.1
DB12707	3	3	5	2.8	312.4	78.4	-8.1
DB12712	2	1	3	2.7	272.4	32.3	-8.1
DB12740	7	2	3	0.7	336.35	113	-8.1
DB12868	4	3	5	4.3	348.4	64.2	-8.1
DB12904	10	0	4	2.3	417.4	81.4	-8.1
DB13039	5	3	3	3.6	309.3	92.8	-8.1
DB13143	3	1	5	4.7	286.4	46.5	-8.1
DB13333	5	0	6	4.2	324.3	61.8	-8.1
DB13351	3	0	6	4.4	323.4	29.5	-8.1
DB13371	2	1	4	3.8	288.3	42	-8.1
DB13425	3	0	4	4.6	346.4	17.8	-8.1
DB13491	6	3	2	1.9	392.5	94.8	-8.1
DB13506	7	2	7	3	454.5	138	-8.1
DB13514	4	1	2	2.7	255.27	59.4	-8.1
DB13566	2	1	3	4.1	321.5	23.5	-8.1
DB13657	5	1	6	2.2	313.3	81.7	-8.1
DB13713	2	1	3	3.4	293.4	23.5	-8.1
DB13790	5	0	5	3.2	388.8	51.2	-8.1
DB14086	6	5	1	0.4	290.27	110	-8.1
DB14673	8	1	4	3	478.5	99.1	-8.1
DB14875	6	2	8	4.4	419.6	108	-8.1
DB14934	6	2	7	4.7	461.5	62.2	-8.1
DB14949	5	2	5	3.2	367.4	106	-8.1
DB14961	7	0	3	2.8	407.4	80	-8.1
DB15003	8	1	4	1.5	389.4	79.2	-8.1
DB15031	5	1	4	4.1	450.9	88.9	-8.1
DB15123	6	3	10	1.6	462.9	117	-8.1
DB15124	6	1	6	3.7	436.9	112	-8.1
DB15186	9	1	2	-0.3	382.4	117	-8.1
DB15190	8	2	6	2.5	458.5	118	-8.1
DB15391	9	2	4	1.4	437.4	128	-8.1
DB15581	4	2	1	2.1	254.24	66.8	-8.1
DB00252	2	2	2	2.5	252.27	58.2	-8
DB00349	2	0	1	2.1	300.74	40.6	-8
DB00374	5	3	10	4.5	390.5	87	-8
DB00475	3	1	1	2.4	299.75	48.2	-8
DB00554	6	2	2	3.1	331.3	108	-8
DB00580	5	1	3	2.6	314.4	94.6	-8
DB00607	6	2	5	2.9	414.5	121	-8
DB00625	5	1	1	4	315.67	38.3	-8
DB00716	8	2	5	2.2	371.3	121	-8
DB00749	3	2	4	2.8	287.35	62.3	-8
DB00751	1	1	0	3.5	249.31	41.6	-8
DB00769	7	2	8	3.1	475.6	104	-8
DB00776	2	1	0	1.7	252.27	63.4	-8
DB00801	5	0	2	4	352.7	32.7	-8
DB00812	2	0	5	3.2	308.4	40.6	-8
DB00831	7	0	4	5	407.5	35	-8
DB00908	4	1	4	2.9	324.4	45.6	-8
DB01068	4	1	1	2.4	315.71	87.3	-8
DB01106	5	1	4	2.5	420.5	64.3	-8
DB01136	5	3	10	4.2	406.5	75.7	-8
DB01138	4	0	6	2.3	404.5	76.9	-8
DB01162	8	1	4	1.4	387.4	103	-8
DB01205	5	0	3	1	303.29	64.4	-8
DB01215	3	0	1	1.7	294.74	43.1	-8
DB01511	2	1	1	3.1	305.2	41.5	-8
DB01656	6	1	7	4.6	403.2	60.4	-8
DB01659	5	2	3	-0.8	267.28	89.1	-8
DB01906	6	3	2	0.3	272.22	136	-8
DB02020	4	1	2	1.7	255.22	74.7	-8
DB02170	6	2	0	2.6	273.2	113	-8
DB02400	6	0	4	2.4	356.3	103	-8
DB02479	4	2	6	2.3	371.5	103	-8

DB02504	5	3	9	1.3	416.4	115	-8
DB02538	4	1	2	1.7	267.29	72.2	-8
DB02611	6	4	5	2.2	370.4	108	-8
DB02834	10	2	6	3.2	414.3	117	-8
DB02875	4	4	2	1.9	270.26	98.8	-8
DB03682	5	2	2	2.7	256.21	87.7	-8
DB04186	3	2	2	1.3	308.33	74.3	-8
DB04239	4	3	4	1	312.4	119	-8
DB04338	6	2	3	1.4	338.4	81.6	-8
DB04429	3	1	1	2.8	240.25	46.5	-8
DB04549	7	2	4	1.6	344.31	97.6	-8
DB04578	4	2	5	1.3	355.4	89.4	-8
DB04707	5	2	2	0.2	307.37	86.9	-8
DB04726	3	1	0	1.1	256.26	74.1	-8
DB04792	6	4	6	1	382.4	137	-8
DB04821	2	1	1	2.6	238.33	29.3	-8
DB04915	3	2	1	2.7	240.25	49.7	-8
DB05327	5	1	2	1.3	420.2	88.5	-8
DB05470	7	2	4	2.5	404.3	102	-8
DB05532	6	1	5	1.5	422.4	105	-8
DB06040	7	1	6	3.3	420.5	96.5	-8
DB06090	4	1	4	3.5	361.4	58.4	-8
DB06190	5	4	9	1.8	410.9	81.6	-8
DB06525	5	1	4	4.7	381.5	54	-8
DB06529	5	0	3	1.5	301.3	73	-8
DB06609	7	3	2	0.1	324.4	122	-8
DB06625	7	2	3	1.4	321.34	122	-8
DB06849	3	1	3	2.7	348.4	55.6	-8
DB06874	7	2	5	3.9	450.3	115	-8
DB06898	4	2	1	1.9	240.26	77	-8
DB06956	5	4	4	2.3	371.8	115	-8
DB06970	6	1	6	4.2	438	127	-8
DB07035	3	1	4	3	406.22	62.1	-8
DB07040	6	2	1	1.4	273.22	108	-8
DB07058	5	1	3	3.2	324.4	92.8	-8
DB07116	6	3	4	0.1	327.27	101	-8
DB07161	2	2	1	2.8	209.25	54.7	-8
DB07206	2	2	3	2.9	237.3	54.7	-8
DB07235	6	3	7	3.8	436.4	121	-8
DB07244	7	3	5	3.7	355.4	89.8	-8
DB07271	9	2	5	2	411.34	75.4	-8
DB07303	5	2	5	2.5	306.4	73.1	-8
DB07348	4	2	0	2	280.36	66.8	-8
DB07382	6	4	5	2.7	332.4	107	-8
DB07394	8	1	8	2.2	460.5	101	-8
DB07395	10	3	4	4.5	438.69	105	-8
DB07415	7	2	6	3.3	386.4	119	-8
DB07471	6	1	7	4.8	457.9	117	-8
DB07472	2	0	1	3.4	281.4	45.6	-8
DB07502	5	3	1	3	322.11	86.7	-8
DB07595	3	2	4	4.1	381.2	57.7	-8
DB07686	8	3	5	2.7	387.5	136	-8
DB07688	8	2	5	2.7	388.4	133	-8
DB07713	7	3	7	2.2	399.4	121	-8
DB07743	8	1	5	4.6	363.3	97.1	-8
DB07747	5	3	4	1.9	352.8	86.8	-8
DB07751	9	3	9	3.5	415.4	82.5	-8
DB07755	7	3	9	4.6	448.3	82.5	-8
DB07769	9	2	5	3.1	402.3	104	-8
DB07779	4	2	7	2.4	383.5	75.4	-8
DB07783	4	3	10	1.6	445.5	115	-8
DB07787	4	1	6	3.3	393.5	52.6	-8
DB07846	4	1	4	4.1	381.5	59.6	-8
DB07848	6	2	5	1.2	454.9	120	-8
DB07851	4	1	4	3.4	326.4	47.7	-8
DB07867	2	1	4	4.8	319.8	38.3	-8
DB07871	2	1	3	4.6	319.8	38.3	-8
DB07963	6	3	5	3.4	447.17	86.6	-8

DB07967	7	1	8	3.2	428.5	107	-8
DB07971	5	2	3	3.6	361.4	115	-8
DB07981	4	2	7	4.3	428.9	80.6	-8
DB08049	4	2	1	2.4	254.24	66.8	-8
DB08066	3	3	3	2.3	303.32	86.5	-8
DB08096	7	2	5	4.9	400.8	67.7	-8
DB08099	6	2	7	4.2	417.5	90.3	-8
DB08122	5	3	4	2.3	329.4	103	-8
DB08138	6	3	4	3.1	360.29	86.9	-8
DB08213	6	0	5	2.3	400.4	92.4	-8
DB08233	6	3	5	4.3	339.4	96	-8
DB08236	3	1	2	4.3	384.7	65.8	-8
DB08385	3	1	2	2.7	270.33	53.4	-8
DB08470	3	1	2	3.4	239.25	41.6	-8
DB08485	6	4	4	1.7	358.4	123	-8
DB08534	5	1	4	2.8	319.3	55.1	-8
DB08536	4	1	4	3.5	380.2	55.1	-8
DB08543	5	1	7	4.7	395.5	48.8	-8
DB08557	4	2	6	3.1	377.4	86.4	-8
DB08560	7	1	5	4.3	417.5	101	-8
DB08561	5	0	6	2.9	392.4	92.2	-8
DB08562	3	2	7	3.2	309.4	66.4	-8
DB08577	3	3	4	1.8	296.32	82.2	-8
DB08613	8	2	3	1.9	423.4	107	-8
DB08647	3	1	5	4.5	316.4	54.4	-8
DB08702	5	2	4	3.4	308.3	87.7	-8
DB08737	3	2	1	4	282.3	49.7	-8
DB08742	3	3	3	3.4	394.5	60.7	-8
DB08828	4	1	4	3.8	421.3	84.5	-8
DB08927	4	1	7	4.1	401.5	35.6	-8
DB08940	3	0	5	1.2	322.4	57.7	-8
DB08997	3	1	4	3.1	362.5	49.4	-8
DB09020	5	0	7	3.8	361.4	65.5	-8
DB09175	5	0	6	3.1	376.5	62.5	-8
DB09226	5	1	7	4.4	450.4	54	-8
DB09290	2	1	2	2.2	279.34	50.7	-8
DB11115	4	2	2	2	274.3	91.4	-8
DB11466	4	1	6	3.4	385.8	67.6	-8
DB11478	5	3	0	4.3	322.4	87	-8
DB11739	5	1	5	2	345.4	72.4	-8
DB11774	7	2	2	-0.8	318.3	92.9	-8
DB11817	7	1	5	-0.5	371.4	129	-8
DB11824	6	4	4	1.5	386.4	99.4	-8
DB11833	3	0	3	4.5	325.8	30.7	-8
DB11841	5	3	7	2	376.4	106	-8
DB11899	5	2	2	0.8	376.25	86.1	-8
DB11901	9	1	3	3	477.4	121	-8
DB11910	7	0	6	4.2	431.4	45.1	-8
DB11922	6	4	4	3.1	441.9	116	-8
DB11969	6	2	6	3.4	429.2	86.1	-8
DB11987	7	1	8	4	440.9	103	-8
DB11990	8	1	6	3.5	455.6	46.6	-8
DB12022	7	1	10	4.2	468.6	105	-8
DB12086	4	1	6	1.7	332.4	59.1	-8
DB12147	7	1	9	3.2	446.5	77.3	-8
DB12251	3	0	5	4.8	307.4	31.4	-8
DB12354	3	0	5	3.1	369.5	62.8	-8
DB12389	5	2	6	3.2	401.5	68.6	-8
DB12433	4	2	4	1.6	276.29	84.5	-8
DB12543	5	3	3	1.1	370.4	104	-8
DB12565	6	3	8	2.2	397.4	104	-8
DB12621	1	0	0	1.9	236.27	32.7	-8
DB12623	8	1	10	2.5	478.5	123	-8
DB12672	6	3	4	4.8	368.4	96.2	-8
DB12696	5	3	7	3	406.5	100	-8
DB12731	3	1	8	3.7	391.5	62.3	-8
DB12930	4	1	6	3.6	363.5	30	-8
DB12988	5	2	9	3.6	409.5	91.2	-8

DB13024	10	1	6	3.4	467.2	119	-8
DB13119	6	3	5	1.8	371.4	103	-8
DB13225	3	0	3	2.7	295.4	26.8	-8
DB13276	5	1	7	3.1	423.5	71.1	-8
DB13308	4	2	4	3.5	372.8	80.6	-8
DB13445	2	0	3	3.2	271.4	20.3	-8
DB13458	5	0	3	2.4	341.4	48	-8
DB13511	4	2	5	3.2	373.9	67.6	-8
DB13531	2	1	5	3.6	302.4	42	-8
DB13554	4	1	6	3	355.4	40.5	-8
DB13591	6	1	6	3.1	445.6	117	-8
DB13596	4	2	2	3.6	265.26	70.4	-8
DB13759	1	1	6	3.5	337.5	43.1	-8
DB13841	4	1	5	4.3	401	52	-8
DB13983	7	3	3	2.6	330.29	105	-8
DB14059	7	1	3	3.7	401.8	86.4	-8
DB14071	1	1	1	4.3	292.2	26	-8
DB14232	3	2	3	3.6	277.3	53.4	-8
DB14657	7	2	4	2.5	434.5	101	-8
DB14718	4	0	1	1.4	354.2	56	-8
DB14791	5	1	3	2.6	321.4	77.8	-8
DB14801	6	1	6	4.5	434.9	126	-8
DB14841	1	1	1	3	209.29	12	-8
DB14854	5	1	4	3	357.4	45.2	-8
DB14987	8	1	10	4.8	446.4	82.8	-8
DB15038	2	1	3	2.9	241.33	21.3	-8
DB15229	8	0	3	2.6	427.4	79	-8
DB15440	6	3	10	2.9	403.5	101	-8
DB15464	4	2	0	2.3	228.2	66.8	-8
DB00425	2	0	3	2.5	307.4	37.6	-7.9
DB00533	4	0	3	2.3	314.4	68.8	-7.9
DB00537	7	2	3	-1.1	331.34	72.9	-7.9
DB00601	6	1	4	0.7	337.35	71.1	-7.9
DB00643	4	2	4	2.8	295.29	84.1	-7.9
DB00674	4	1	1	1.8	287.35	41.9	-7.9
DB00771	3	1	5	3.3	352.4	46.5	-7.9
DB00782	3	0	7	4.2	368.5	35.5	-7.9
DB00904	2	0	2	2.3	293.4	39.8	-7.9
DB01006	4	0	3	2.7	285.3	78.3	-7.9
DB01053	5	2	4	1.8	334.4	112	-7.9
DB01058	2	0	1	2.7	312.4	40.6	-7.9
DB01059	7	2	3	-1	319.33	72.9	-7.9
DB01071	3	0	2	4.6	322.5	31.8	-7.9
DB01149	5	0	10	4.3	470	51.6	-7.9
DB01166	5	1	7	3.1	369.5	81.9	-7.9
DB01186	2	1	4	4.2	314.5	44.3	-7.9
DB01250	8	4	4	3.1	302.24	140	-7.9
DB01399	5	2	4	3	258.23	83.8	-7.9
DB01532	2	0	5	4	336.5	23.6	-7.9
DB01588	2	0	3	3.6	324.8	32.7	-7.9
DB01621	7	1	8	3.5	475.7	97.8	-7.9
DB01628	4	0	3	3.3	358.8	68.3	-7.9
DB01905	3	4	3	1.7	283.3	110	-7.9
DB02030	8	4	4	-1.1	358.28	134	-7.9
DB02154	8	2	7	2.8	386.4	112	-7.9
DB02197	7	2	4	2.2	366.4	124	-7.9
DB02427	7	2	5	2.4	339.4	99.5	-7.9
DB02610	9	2	4	1.8	380.29	97.6	-7.9
DB02842	4	2	5	2.5	322.4	71.2	-7.9
DB02861	7	2	4	1.6	344.31	97.6	-7.9
DB02936	4	2	9	2.6	380.4	94.6	-7.9
DB03572	3	2	6	2.1	309.4	81.1	-7.9
DB03601	4	2	1	2.2	256.25	66.8	-7.9
DB03622	5	3	4	0.9	331.4	98.7	-7.9
DB03695	7	2	4	2.1	325.4	109	-7.9
DB03737	6	5	5	0.2	385.4	121	-7.9
DB03753	3	0	4	4.5	258.29	26.3	-7.9
DB03804	6	3	3	0.8	389.22	127	-7.9

DB03987	8	2	5	1.7	340.4	112	-7.9
DB04293	5	3	4	-1.7	349.77	113	-7.9
DB04345	4	2	0	1.1	242.23	84	-7.9
DB04442	2	4	2	0.4	254.27	109	-7.9
DB04509	4	2	2	1.5	342.4	66.8	-7.9
DB04518	6	2	3	3.3	298.4	99.2	-7.9
DB04571	3	0	0	3	228.24	39.4	-7.9
DB04644	5	3	7	4.7	439.3	105	-7.9
DB04793	6	4	6	1	382.4	137	-7.9
DB04957	6	0	8	3.2	458	72.6	-7.9
DB05015	5	3	5	1.7	318.3	104	-7.9
DB05294	7	1	6	4.9	475.4	59.5	-7.9
DB05395	5	2	9	3.9	403.9	67.8	-7.9
DB06140	5	0	5	3.5	478.4	63.2	-7.9
DB06209	6	0	6	3.6	373.4	74.8	-7.9
DB06230	4	2	2	2.7	339.4	52.9	-7.9
DB06272	4	3	6	3.5	418.6	69.9	-7.9
DB06370	6	3	4	1.8	385.8	139	-7.9
DB06440	8	1	6	3.5	437.5	116	-7.9
DB06741	3	3	4	4.4	375.2	82.2	-7.9
DB06832	5	2	2	3.6	271.24	66.5	-7.9
DB06848	5	1	3	4.3	424.6	109	-7.9
DB06948	5	2	5	4.7	323.4	75.7	-7.9
DB06967	5	2	6	3.5	375.5	92	-7.9
DB06983	5	2	5	1.8	331.4	75.3	-7.9
DB07032	3	2	1	3.3	226.23	53.6	-7.9
DB07033	2	2	2	1.4	240.26	65.4	-7.9
DB07043	9	2	4	2.3	407.5	140	-7.9
DB07066	3	3	2	3.6	345.8	102	-7.9
DB07090	2	1	3	3.1	334.8	49.4	-7.9
DB07095	3	3	7	2.5	370.5	99.3	-7.9
DB07168	7	4	7	3.1	360.4	114	-7.9
DB07250	9	2	7	4.3	430.8	96	-7.9
DB07268	6	4	5	3	321.33	113	-7.9
DB07288	3	2	5	4.3	337.8	54	-7.9
DB07315	3	4	4	2.3	330.8	85.4	-7.9
DB07324	6	2	5	1.6	321.33	93.4	-7.9
DB07385	4	1	3	1.8	322.4	62.3	-7.9
DB07405	7	2	1	1.2	382.4	107	-7.9
DB07423	9	3	6	2.2	441.4	134	-7.9
DB07615	5	2	6	3.2	327.3	84.9	-7.9
DB07628	4	0	2	3.1	327.4	65.8	-7.9
DB07644	5	2	3	3.3	314.77	87	-7.9
DB07714	4	1	6	3	286.32	71.4	-7.9
DB07742	6	2	4	1.5	326.32	97.6	-7.9
DB07785	4	2	7	2.2	339.4	90.4	-7.9
DB07816	4	3	8	3.7	384.4	97.5	-7.9
DB07826	3	1	6	4.8	365.8	55.4	-7.9
DB07835	2	2	6	3.8	348.4	58.2	-7.9
DB07843	7	1	5	2.1	472	133	-7.9
DB07882	6	3	8	1.2	459.5	126	-7.9
DB07932	7	2	6	1.7	394.4	102	-7.9
DB07972	2	1	1	2.8	223.27	43.8	-7.9
DB08008	7	1	2	3.3	293.25	55.1	-7.9
DB08061	2	2	2	2.7	261.75	40.7	-7.9
DB08077	4	3	5	3.8	383.2	87.7	-7.9
DB08093	3	1	3	3.2	250.29	48.1	-7.9
DB08095	6	3	7	4.1	455.9	99.6	-7.9
DB08142	4	4	4	2.6	382.2	98.9	-7.9
DB08146	5	1	2	2.8	289.33	67.9	-7.9
DB08208	7	3	7	3.2	350.29	70.6	-7.9
DB08216	5	2	4	4.5	298.34	82.2	-7.9
DB08240	5	3	4	3	377.7	95.9	-7.9
DB08274	3	1	3	4.2	298.4	46.5	-7.9
DB08277	5	1	5	3.8	444	80.8	-7.9
DB08308	5	1	5	2.6	374.5	80.7	-7.9
DB08361	6	4	5	2.6	340.4	119	-7.9
DB08379	4	1	4	3.5	330.7	50.7	-7.9

DB08392	7	2	7	3.8	391.4	89.6	-7.9
DB08424	7	3	7	2.2	371.4	111	-7.9
DB08467	2	1	4	5	300.4	37.3	-7.9
DB08514	5	4	3	1.2	312.35	136	-7.9
DB08532	5	1	4	3.2	319.3	55.1	-7.9
DB08537	4	1	4	4.2	380.2	55.1	-7.9
DB08555	2	0	2	4.6	350.64	21.6	-7.9
DB08659	5	4	3	1.1	330.4	139	-7.9
DB08666	7	3	2	3.4	268.2	96.8	-7.9
DB08668	6	4	3	2.1	244.21	134	-7.9
DB08687	5	3	3	2.5	280.66	99.5	-7.9
DB08694	7	3	1	1.1	293.28	124	-7.9
DB08710	8	2	5	3.3	398.4	134	-7.9
DB08750	5	3	2	1	367.4	96.7	-7.9
DB08787	5	1	3	4.5	344.2	76.5	-7.9
DB08953	1	2	3	3.3	228.33	27.8	-7.9
DB08981	3	1	5	3.2	254.28	54.4	-7.9
DB08998	3	1	4	4	278.3	33.6	-7.9
DB09022	6	1	8	4.6	351.4	38.3	-7.9
DB09080	6	4	7	1.8	386.4	100	-7.9
DB09166	4	0	2	2.6	342.8	71.3	-7.9
DB09218	4	2	3	3.8	262.69	62.2	-7.9
DB09244	1	1	0	2.3	226.32	17	-7.9
DB09285	9	1	7	3.7	395.4	63.7	-7.9
DB11591	5	1	10	2.3	463.6	67.6	-7.9
DB11674	3	2	1	3	242.27	49.7	-7.9
DB11675	6	2	7	2.8	421.9	76.8	-7.9
DB11749	2	0	1	2.7	312.4	40.6	-7.9
DB11760	7	2	2	2.3	380.4	84.2	-7.9
DB11816	6	2	5	1.4	401.4	86.8	-7.9
DB11843	7	1	6	2.9	392.5	125	-7.9
DB11883	6	2	3	3.8	429.2	57.3	-7.9
DB11900	4	1	4	4.4	348.4	55.1	-7.9
DB11909	5	2	8	2.8	380.4	112	-7.9
DB11957	7	2	9	5	398.4	37	-7.9
DB11964	5	1	5	2.3	366.4	92	-7.9
DB12007	2	0	1	3.2	222.24	26.3	-7.9
DB12011	8	1	5	2.8	426.4	97.6	-7.9
DB12025	6	1	1	0.2	360.4	84.1	-7.9
DB12092	5	1	7	4.1	392.5	45.2	-7.9
DB12220	4	1	6	1.6	339.4	49.8	-7.9
DB12236	7	4	9	2.4	464.9	109	-7.9
DB12301	8	1	4	2.1	425.7	98	-7.9
DB12302	7	2	9	4.3	469.5	98.3	-7.9
DB12345	9	0	7	3.6	448.5	123	-7.9
DB12417	6	2	6	0	383.4	115	-7.9
DB12542	9	1	6	2.8	439.5	132	-7.9
DB12569	5	2	8	4.6	416.5	107	-7.9
DB12686	4	2	4	1	361.5	81.3	-7.9
DB12717	6	2	10	3.4	438.6	109	-7.9
DB12779	4	4	2	2.2	271.31	72.7	-7.9
DB12869	3	1	5	4.5	347.9	23.5	-7.9
DB12979	4	2	2	1.4	295.35	73.4	-7.9
DB13066	2	1	3	3.5	308.8	46.5	-7.9
DB13077	6	1	3	0.6	308.31	68.6	-7.9
DB13114	1	0	3	4.5	293.4	18.1	-7.9
DB13182	4	2	1	2.5	254.24	66.8	-7.9
DB13327	5	2	7	1.3	376.4	93.2	-7.9
DB13364	2	0	4	3.1	320.4	40.6	-7.9
DB13396	5	3	7	-0.6	367.4	113	-7.9
DB13468	2	0	5	4.8	321.5	12.5	-7.9
DB13649	1	0	2	3	278.3	32.7	-7.9
DB13656	1	0	1	4.9	329.2	17.8	-7.9
DB13767	4	0	3	3.1	324.77	61.4	-7.9
DB13851	5	0	2	3.4	312.4	46.2	-7.9
DB13873	4	1	5	3.9	318.7	63.6	-7.9
DB13957	6	0	4	4.7	350.3	41.9	-7.9
DB14025	7	2	3	0.4	365.8	86.9	-7.9

DB14750	2	0	3	4.3	279.4	12.5	-7.9
DB14857	5	2	1	0	286.29	105	-7.9
DB14867	7	2	5	2.4	432.9	108	-7.9
DB14924	4	2	3	2.1	285.34	73.9	-7.9
DB14938	5	0	7	4.9	330.3	52.6	-7.9
DB14977	10	0	6	2.5	432.8	88	-7.9
DB15021	6	2	7	2.5	372.4	114	-7.9
DB15096	4	1	5	2.9	368.5	54.6	-7.9
DB15117	6	0	5	5	371.2	26.3	-7.9
DB15149	7	1	6	2	418.4	108	-7.9
DB15241	4	3	3	1.1	385.5	101	-7.9
DB15300	4	1	4	3.1	326.4	45.6	-7.9
DB15550	8	1	6	1.1	423.8	114	-7.9
DB15551	6	3	4	1.1	299.7	99.4	-7.9
DB00186	3	2	1	2.4	321.2	61.7	-7.8
DB00203	8	1	7	1.5	474.6	118	-7.8
DB00238	4	1	1	2	266.3	58.1	-7.8
DB00370	3	0	0	3.3	265.35	19.4	-7.8
DB00417	6	2	5	2.1	350.4	121	-7.8
DB00497	5	1	1	1.2	315.4	59	-7.8
DB00542	6	2	10	1.3	424.5	95.9	-7.8
DB00606	7	3	2	2	389.9	135	-7.8
DB00680	6	1	6	3	427.5	96.4	-7.8
DB00799	4	0	5	4.9	351.5	64.5	-7.8
DB00814	7	2	2	3	351.4	136	-7.8
DB00843	4	0	6	4.3	379.5	38.8	-7.8
DB00844	5	3	2	0.2	357.4	73.2	-7.8
DB00906	5	1	6	2.7	375.6	97	-7.8
DB00933	5	0	4	4.5	386.6	68.1	-7.8
DB00934	1	1	4	4.6	277.4	12	-7.8
DB00952	4	2	5	2	335.5	73.6	-7.8
DB00956	4	0	1	2.2	299.4	38.8	-7.8
DB01013	6	1	5	3.8	467	80.7	-7.8
DB01116	1	0	4	3.6	365.5	24.6	-7.8
DB01283	4	2	4	4.2	293.72	49.3	-7.8
DB01426	4	2	1	1.8	326.4	46.9	-7.8
DB01568	4	1	1	0.6	315.4	56.8	-7.8
DB01767	3	4	3	2	290.32	107	-7.8
DB01865	3	1	4	2	320.4	64.2	-7.8
DB02288	3	4	2	2.8	345.19	98.8	-7.8
DB02292	6	1	2	2.3	309.34	104	-7.8
DB02383	7	1	4	3.7	357.3	81.9	-7.8
DB02463	1	4	2	2.3	252.29	87.6	-7.8
DB02559	5	4	2	1	307.5	79.3	-7.8
DB02583	8	2	5	1.7	340.4	112	-7.8
DB02915	8	1	3	4.5	350.4	78.9	-7.8
DB02973	6	1	3	1.9	393.22	123	-7.8
DB02989	4	4	5	3.7	358.8	108	-7.8
DB03039	6	2	4	1.5	326.32	97.6	-7.8
DB03046	5	2	4	1.7	344.4	119	-7.8
DB03083	4	1	4	2.8	311.3	56.8	-7.8
DB03650	3	2	1	2.3	238.24	61.7	-7.8
DB03895	1	0	3	4.8	329.5	6.2	-7.8
DB04010	3	2	3	2	267.28	81	-7.8
DB04069	3	1	0	1.5	197.24	51.8	-7.8
DB04204	10	4	9	2.6	470.3	115	-7.8
DB04232	8	2	7	1.1	449.5	134	-7.8
DB04254	7	1	5	3.1	343.36	88.1	-7.8
DB04274	3	1	1	2.5	240.25	46.5	-7.8
DB04470	4	4	5	3.1	324.4	108	-7.8
DB04796	3	3	4	4.4	400.6	60.7	-7.8
DB04843	3	1	5	3.2	340.4	46.5	-7.8
DB04866	5	2	4	1.4	414.7	82	-7.8
DB04876	4	2	3	0.9	303.4	76.4	-7.8
DB06083	2	2	3	4.6	254.32	40.5	-7.8
DB06266	3	1	3	4.3	321.2	55.1	-7.8
DB06471	4	1	5	2.8	343.4	78.6	-7.8
DB06582	6	0	6	3.2	382.5	61.6	-7.8

DB06685	3	1	3	3.7	356.8	60.8	-7.8
DB06697	5	0	1	3.1	298.37	46.2	-7.8
DB06845	4	4	7	0.9	371.5	111	-7.8
DB06865	5	5	8	2.4	382.5	128	-7.8
DB06866	5	4	9	2.7	396.5	117	-7.8
DB06883	3	3	4	3.8	349.4	85	-7.8
DB06913	10	3	4	3.5	357.28	79.9	-7.8
DB06919	3	2	6	2.8	385.9	75.4	-7.8
DB06954	6	1	4	3.7	371.5	120	-7.8
DB07027	4	2	6	2.3	369.4	75.4	-7.8
DB07061	2	2	5	4.3	340.5	37.2	-7.8
DB07063	7	1	4	4.4	376.4	83.4	-7.8
DB07067	7	1	5	0.8	378.4	128	-7.8
DB07088	4	3	7	1.2	372.5	109	-7.8
DB07112	5	3	3	2.2	372.11	99.5	-7.8
DB07132	2	3	3	0.3	282.3	100	-7.8
DB07153	7	2	6	3	356.4	106	-7.8
DB07154	6	2	4	0.9	343.34	75.4	-7.8
DB07185	4	2	5	4.2	305.71	75.6	-7.8
DB07194	7	3	6	3.3	397.5	128	-7.8
DB07218	4	1	0	2.3	263.68	58.4	-7.8
DB07243	4	1	3	2.4	285.34	58.2	-7.8
DB07300	7	1	6	1.9	365.4	97.3	-7.8
DB07335	5	2	2	1.8	269.3	89.8	-7.8
DB07362	10	3	6	3.1	462.5	138	-7.8
DB07701	4	0	6	4.3	379.5	38.8	-7.8
DB07708	3	2	1	3.5	260.67	58.3	-7.8
DB07712	3	2	2	3.4	254.28	58.3	-7.8
DB07739	6	1	3	1.3	330.4	83.1	-7.8
DB07750	7	3	9	4.6	448.3	82.5	-7.8
DB07815	5	2	1	1.7	332.4	83.8	-7.8
DB07879	5	2	3	1.5	368.4	116	-7.8
DB07883	2	1	3	3.6	291.4	43.1	-7.8
DB07889	7	2	8	3.4	418.5	87.8	-7.8
DB07920	5	1	5	1.8	304.32	101	-7.8
DB07973	3	1	6	4.6	405.5	46.5	-7.8
DB07983	4	1	4	4.3	449.2	68.5	-7.8
DB07984	4	2	7	4.3	428.9	80.6	-7.8
DB07985	4	4	9	2.2	352.4	126	-7.8
DB08009	3	3	7	3.2	414.9	77.2	-7.8
DB08018	5	4	8	2.1	373.9	75	-7.8
DB08100	1	1	2	4.6	224.3	20.2	-7.8
DB08133	5	3	3	1.3	316.34	126	-7.8
DB08137	4	3	4	2.9	324.31	86.9	-7.8
DB08177	6	1	5	3.9	393.9	128	-7.8
DB08292	5	2	0	3.4	324.75	83.8	-7.8
DB08331	5	5	6	3.5	331.4	93.4	-7.8
DB08345	7	3	6	2.1	345.4	120	-7.8
DB08371	4	2	5	0.2	269.29	80.4	-7.8
DB08449	8	1	4	3.7	377.3	96.2	-7.8
DB08481	6	1	3	3.5	362.4	122	-7.8
DB08488	6	1	5	2.1	436.9	123	-7.8
DB08506	6	4	10	-1	418.4	130	-7.8
DB08516	4	1	6	2.6	300.33	64.7	-7.8
DB08519	8	3	7	3.9	406.4	106	-7.8
DB08528	4	1	2	3	286.4	92.3	-7.8
DB08631	4	2	3	2.5	322.8	66.6	-7.8
DB08697	4	2	6	4.1	401.9	67.6	-7.8
DB08736	4	2	4	2.6	310.3	66.8	-7.8
DB08747	4	2	1	4.5	445.3	69.6	-7.8
DB08755	5	2	9	3	456.6	104	-7.8
DB08795	7	2	5	2.8	375.4	126	-7.8
DB08811	6	0	6	3.2	382.5	61.6	-7.8
DB08893	6	4	9	2.1	396.5	129	-7.8
DB08910	5	2	1	0.2	273.24	110	-7.8
DB08979	3	1	3	2.1	260.33	41.6	-7.8
DB08994	5	2	7	2.7	324.4	69.7	-7.8
DB09076	2	1	8	4.7	428.6	29.5	-7.8

DB09171	2	0	6	4.4	350.5	23.6	-7.8
DB09173	2	0	7	4.4	350.5	23.6	-7.8
DB09189	2	1	5	4.3	280.4	15.3	-7.8
DB09214	3	1	4	3.1	254.28	54.4	-7.8
DB09355	4	2	3	0.7	276.31	97.6	-7.8
DB11064	3	1	3	5	262.34	46.5	-7.8
DB11433	5	1	8	5	365.4	64.6	-7.8
DB11521	3	4	4	0.4	348.4	89.9	-7.8
DB11560	5	1	5	4.7	404.3	93.3	-7.8
DB11629	5	1	5	3.5	435.9	84.8	-7.8
DB11644	4	1	2	4.2	308.1	63.3	-7.8
DB11765	8	2	6	3.3	396.4	97.1	-7.8
DB11836	7	2	2	1.7	309.33	122	-7.8
DB11865	8	2	8	3.1	441.5	117	-7.8
DB11905	6	1	4	1.5	353.4	87.2	-7.8
DB12000	6	2	6	2.3	370.4	89.4	-7.8
DB12050	8	3	7	1.8	464.4	112	-7.8
DB12063	6	1	6	3.1	358.4	67.4	-7.8
DB12123	6	0	7	1.5	417.5	71.6	-7.8
DB12161	4	0	4	2.9	323.5	38.8	-7.8
DB12173	1	0	2	3.3	206.24	17.1	-7.8
DB12230	7	1	5	2.2	373.4	93.8	-7.8
DB12361	4	0	6	3.6	409.9	45.7	-7.8
DB12378	4	1	5	3.2	356.4	82.7	-7.8
DB12402	5	2	2	1.7	303.4	89.7	-7.8
DB12549	6	1	5	3	367.4	88.1	-7.8
DB12780	4	0	4	3.8	354.2	59.8	-7.8
DB12857	7	1	5	2.9	432.6	78.3	-7.8
DB12900	4	1	6	2.5	313.4	53.9	-7.8
DB12925	7	3	3	2.6	417.3	130	-7.8
DB12929	5	0	4	3.4	369.4	45.7	-7.8
DB12985	6	3	6	1.3	394.5	95.3	-7.8
DB13177	8	4	3	-0.8	338.31	126	-7.8
DB13349	3	0	5	2.8	307.4	35.9	-7.8
DB13482	4	0	4	3.6	323.4	38.8	-7.8
DB13551	3	1	2	3.4	249.26	50.2	-7.8
DB13555	3	2	3	2.7	369.5	43.7	-7.8
DB13727	1	0	2	3.6	235.32	3.2	-7.8
DB13783	6	1	7	4.2	415.8	94.8	-7.8
DB13803	5	3	3	2.9	354.8	118	-7.8
DB13930	4	4	7	4.1	433.3	90	-7.8
DB13934	9	1	2	3.7	338.25	47.3	-7.8
DB14125	6	3	8	0.6	396.4	107	-7.8
DB14545	8	3	7	1.8	462.5	138	-7.8
DB14575	2	2	0	1.4	254.28	66.6	-7.8
DB14643	7	1	7	3.8	472.6	107	-7.8
DB14654	6	1	6	3.6	417.4	90.9	-7.8
DB14753	3	5	4	1.8	280.32	120	-7.8
DB14888	3	1	5	3.4	340.4	66.5	-7.8
DB14975	5	1	6	2.7	337.4	77.2	-7.8
DB15317	4	1	3	2.3	337.4	44.8	-7.8
DB15439	4	2	3	2.1	316.4	58.3	-7.8
DB15498	8	2	8	4.3	446.5	103	-7.8
DB00243	6	2	9	2.7	427.5	74.3	-7.7
DB00298	4	0	4	2.4	325.5	37.2	-7.7
DB00323	5	2	2	3.3	273.24	103	-7.7
DB00328	4	1	4	4.3	357.8	68.5	-7.7
DB00344	1	1	4	4.4	263.4	12	-7.7
DB00457	8	1	4	2	383.4	107	-7.7
DB00467	8	2	3	-0.2	320.32	85.8	-7.7
DB00485	7	2	4	2.9	470.3	138	-7.7
DB00524	5	2	2	4.1	365.8	101	-7.7
DB00579	2	1	1	2.2	284.74	35.8	-7.7
DB00623	8	1	6	4.4	437.5	55.2	-7.7
DB00670	5	1	2	0.1	351.4	68.8	-7.7
DB00682	4	1	4	2.7	308.3	63.6	-7.7
DB00764	4	2	2	2.7	427.4	74.6	-7.7
DB00813	2	0	6	4	336.5	23.6	-7.7

DB00839	4	2	3	1.5	311.4	86.9	-7.7
DB00854	2	1	0	3.1	257.37	23.5	-7.7
DB00998	2	3	2	1.2	243.3	70.9	-7.7
DB01038	6	1	8	3.1	425.6	72.3	-7.7
DB01041	4	1	1	0.3	258.23	83.6	-7.7
DB01120	4	2	3	1.5	323.4	86.9	-7.7
DB01466	4	1	2	1.5	313.4	41.9	-7.7
DB01538	5	0	3	2.7	343.4	48	-7.7
DB01622	7	0	6	3.7	446.6	80.8	-7.7
DB01644	5	3	3	2.4	286.28	87	-7.7
DB01674	7	5	6	-1.7	362.4	137	-7.7
DB01712	7	1	9	3.5	476.6	127	-7.7
DB01734	5	3	3	2.4	259.21	104	-7.7
DB01809	4	1	2	2.8	281.36	69.6	-7.7
DB01938	3	3	4	1.6	280.32	83.8	-7.7
DB01958	5	2	3	4.6	324.4	103	-7.7
DB02069	5	2	4	1	308.33	97.6	-7.7
DB02139	4	0	8	5	444.3	30.3	-7.7
DB02287	2	4	2	1.8	253.28	101	-7.7
DB02402	6	2	3	2.3	282.3	96.3	-7.7
DB02628	4	3	7	3	442.6	90.5	-7.7
DB02819	8	4	4	-1.5	344.26	134	-7.7
DB03062	6	1	9	3.1	404.5	82.2	-7.7
DB03086	4	4	6	3.9	305.4	87.4	-7.7
DB03133	6	5	2	-0.6	294.3	119	-7.7
DB03267	4	2	8	2.6	395.5	98.4	-7.7
DB03285	4	3	3	3.2	256.25	77.8	-7.7
DB03643	3	4	2	1.8	252.27	98.8	-7.7
DB03841	5	1	5	2.8	299.32	88.1	-7.7
DB03844	6	2	4	1.5	326.32	97.6	-7.7
DB03921	3	1	2	2.7	272.3	54.5	-7.7
DB04059	4	3	3	2.3	263.3	87.7	-7.7
DB04180	6	2	4	1.5	326.32	97.6	-7.7
DB04769	5	1	1	1.5	257.27	97.2	-7.7
DB04806	6	5	3	1.2	408.63	115	-7.7
DB04873	3	1	6	3.7	369.5	46.5	-7.7
DB04975	8	4	8	3.3	444.5	135	-7.7
DB05154	9	0	4	2.8	359.26	91.3	-7.7
DB05422	4	0	6	3.8	413.9	36	-7.7
DB05708	4	0	4	3	308.4	43.7	-7.7
DB05767	5	3	3	2.2	350.4	87	-7.7
DB06292	6	4	6	2.3	408.9	99.4	-7.7
DB06579	6	0	5	2.3	351.4	73.8	-7.7
DB06654	4	2	7	2.2	302.34	64.4	-7.7
DB06736	5	2	7	4.3	354.2	75.6	-7.7
DB06855	5	4	5	3.2	342.4	108	-7.7
DB06857	3	4	3	3.6	429.64	99.2	-7.7
DB06935	4	1	2	3.3	234.2	37.3	-7.7
DB06937	5	3	1	2.3	243.21	86.7	-7.7
DB06949	4	3	3	4.8	464.1	81.9	-7.7
DB06958	6	5	4	1.2	365.81	114	-7.7
DB06990	8	3	8	0.5	346.29	139	-7.7
DB07007	5	2	3	1.6	329.4	97.1	-7.7
DB07030	6	2	6	2.7	364.73	121	-7.7
DB07107	4	2	7	3.2	370.4	76.8	-7.7
DB07125	6	3	6	2.1	326.35	99.8	-7.7
DB07133	3	2	6	2.5	365.5	75.4	-7.7
DB07143	3	2	6	2.2	351.4	75.4	-7.7
DB07188	2	1	3	3.3	355.3	49.4	-7.7
DB07190	3	2	6	3.9	391.9	75.4	-7.7
DB07290	7	3	5	0.8	346.38	113	-7.7
DB07312	5	1	3	4.5	377.6	80.6	-7.7
DB07316	7	2	7	2.2	453.5	86.9	-7.7
DB07350	3	2	5	1.2	284.31	71.3	-7.7
DB07379	7	4	7	3.6	403.9	114	-7.7
DB07389	4	2	4	1.9	280.32	91.8	-7.7
DB07429	6	2	7	3.4	455.3	112	-7.7
DB07459	3	1	5	3.2	304.3	51.2	-7.7

DB07488	7	2	6	4.7	355.4	115	-7.7
DB07490	5	1	2	4.9	322.7	112	-7.7
DB07598	4	0	0	1.5	247.25	48	-7.7
DB07649	6	3	6	1.5	369.5	116	-7.7
DB07695	3	3	2	4.2	415.08	69.6	-7.7
DB07753	4	1	2	3.3	234.2	37.3	-7.7
DB07761	9	3	9	3.5	415.4	82.5	-7.7
DB07820	3	1	6	3.3	330.4	58.6	-7.7
DB07829	3	1	2	2.5	239.25	41.6	-7.7
DB07869	3	1	4	3.9	351.9	65.4	-7.7
DB07876	5	1	2	1.7	319.4	70.7	-7.7
DB07944	5	3	8	3.6	384.5	90	-7.7
DB07964	7	2	5	1.6	398.5	130	-7.7
DB08004	5	3	5	2.3	311.4	88.5	-7.7
DB08046	5	3	2	0.9	338.8	118	-7.7
DB08048	4	4	1	2.1	242.23	89.4	-7.7
DB08162	5	1	2	1	291.37	70.7	-7.7
DB08187	4	3	7	1.8	386.5	87.5	-7.7
DB08235	2	2	4	3.7	284.4	73.1	-7.7
DB08250	5	2	6	2.7	386.4	70.7	-7.7
DB08284	4	1	5	3.4	452.3	90.7	-7.7
DB08330	3	0	6	4.2	294.3	35.5	-7.7
DB08388	7	2	8	2.7	402.4	103	-7.7
DB08461	5	2	3	2.3	297.35	89.8	-7.7
DB08462	4	2	4	3.2	290.32	66.9	-7.7
DB08471	6	0	4	2.9	359.5	116	-7.7
DB08496	4	1	4	2.7	308.3	63.6	-7.7
DB08646	5	5	6	2.4	310.35	103	-7.7
DB08655	2	0	0	2.3	227.26	39.1	-7.7
DB08664	6	3	8	3.5	452.5	114	-7.7
DB08671	7	3	2	3.4	268.2	96.8	-7.7
DB08686	3	1	3	4.8	312.4	46.5	-7.7
DB08741	7	2	5	2.5	354.4	106	-7.7
DB08752	5	2	5	1.8	405.5	97.7	-7.7
DB08753	5	1	9	2.9	367.4	81.7	-7.7
DB08754	4	4	5	2.4	299.32	89.8	-7.7
DB08757	3	2	2	2.1	236.65	68.3	-7.7
DB08758	3	2	1	1.9	226.23	72.4	-7.7
DB08770	8	3	5	2.2	337.34	127	-7.7
DB08774	5	2	3	1.3	309.4	80.1	-7.7
DB08789	5	2	3	4.1	367.3	109	-7.7
DB08810	6	2	6	3.6	402.5	113	-7.7
DB08942	7	2	2	3	335.34	121	-7.7
DB08968	4	1	6	2.4	401.9	61.9	-7.7
DB09017	4	0	1	2.8	393.7	71.3	-7.7
DB09083	6	0	10	2.4	468.6	60.5	-7.7
DB11395	8	1	5	2.8	397.4	95.6	-7.7
DB11398	4	1	3	4.1	407.6	85.6	-7.7
DB11464	6	2	3	1.7	300.34	106	-7.7
DB11638	5	1	0	2.5	284.35	57.2	-7.7
DB11649	5	1	6	2.1	310.35	94.9	-7.7
DB11658	5	1	5	2	358.4	97.6	-7.7
DB11681	4	0	3	2.8	339.5	76.8	-7.7
DB11757	5	0	6	2.5	384.4	76.9	-7.7
DB11875	0	2	2	4.3	246.31	31.6	-7.7
DB11915	6	1	8	4.3	418.6	74	-7.7
DB11933	8	4	5	1.1	335.36	126	-7.7
DB11982	6	3	6	3.7	456.21	87.9	-7.7
DB12027	3	3	6	4.6	328.4	52.7	-7.7
DB12057	3	0	2	3.3	285.4	25.6	-7.7
DB12106	3	3	4	1.5	274.36	97.8	-7.7
DB12196	5	2	6	3.3	426.6	78	-7.7
DB12214	7	4	7	2.9	434.5	125	-7.7
DB12367	6	2	4	1	377.4	122	-7.7
DB12393	10	3	3	-0.5	409.25	119	-7.7
DB12395	4	1	6	3	313.4	39.7	-7.7
DB12445	7	0	7	2.9	331.28	108	-7.7
DB12461	7	1	3	2.9	389.3	84.6	-7.7

DB12679	3	1	4	3.4	356.2	65.8	-7.7
DB12704	3	0	4	4.3	425.4	32.8	-7.7
DB12713	6	3	6	3.2	424.9	104	-7.7
DB12721	3	0	4	3.3	438	37.7	-7.7
DB12745	9	3	7	0.3	425.5	137	-7.7
DB12752	4	3	8	3.3	363.5	81.1	-7.7
DB12919	3	1	2	5	291.8	71.3	-7.7
DB12921	6	2	3	2.3	334.8	106	-7.7
DB13027	5	2	5	2.6	272.25	83.8	-7.7
DB13132	5	0	0	2.8	282.33	54	-7.7
DB13136	3	0	1	3	240.23	34.1	-7.7
DB13407	4	1	3	2	308.33	65.5	-7.7
DB13432	3	1	4	3.6	312.7	55.1	-7.7
DB13501	4	1	5	3.1	282.29	64.4	-7.7
DB13545	2	0	1	3.6	301.13	34.1	-7.7
DB13680	3	2	1	2	215.21	88	-7.7
DB13686	6	2	5	2.7	433.3	121	-7.7
DB13696	2	2	2	4.6	326.57	49.3	-7.7
DB13784	6	1	9	3.7	427.6	64.5	-7.7
DB14028	2	1	1	2.9	270.71	41.5	-7.7
DB14971	4	0	3	1.5	411.19	64.4	-7.7
DB14998	5	2	5	0.3	357.4	106	-7.7
DB15261	9	0	8	3.5	474.8	93.2	-7.7
DB15333	4	1	5	4.7	371.4	46.9	-7.7
DB15553	6	3	4	0.4	265.26	99.4	-7.7
DB00179	4	4	5	4.3	302.4	80.9	-7.6
DB00310	5	3	2	0.9	338.8	118	-7.6
DB00315	3	2	5	2.2	287.36	57.4	-7.6
DB00318	4	1	1	1.1	299.4	41.9	-7.6
DB00327	4	1	0	1.8	285.34	49.8	-7.6
DB00334	4	1	1	2.9	312.4	59.1	-7.6
DB00338	6	1	5	2.2	345.4	96.3	-7.6
DB00432	8	3	2	-0.5	296.2	99.1	-7.6
DB00480	4	2	1	-0.5	259.26	92.5	-7.6
DB00502	4	1	6	3.2	375.9	40.5	-7.6
DB00587	5	2	9	4.6	412.5	108	-7.6
DB00613	4	2	6	2.6	355.9	48.4	-7.6
DB00704	5	2	2	1.9	341.4	70	-7.6
DB00712	3	1	3	4.2	244.26	37.3	-7.6
DB00736	6	1	5	2.2	345.4	96.3	-7.6
DB00881	6	2	10	1.2	438.5	95.9	-7.6
DB00946	3	1	3	3.6	280.3	46.5	-7.6
DB00986	3	1	5	3.1	318.4	46.5	-7.6
DB01146	2	0	4	3.9	281.4	12.5	-7.6
DB01183	5	2	2	2.1	327.4	70	-7.6
DB01192	5	2	0	0.8	301.34	70	-7.6
DB01207	8	1	8	3.9	366.3	71.8	-7.6
DB01252	3	1	5	3	315.4	57.6	-7.6
DB01268	4	3	7	2.6	398.5	77.2	-7.6
DB01340	7	2	9	0.6	417.5	99.2	-7.6
DB01453	3	1	6	2.9	352.5	43.8	-7.6
DB01480	5	2	4	3	423.5	62.2	-7.6
DB01497	5	2	4	3.1	411.5	62.2	-7.6
DB01531	3	1	0	2.8	271.35	32.7	-7.6
DB01535	4	0	8	3.8	394.5	49.8	-7.6
DB01562	3	0	6	3.9	323.4	29.5	-7.6
DB01587	3	0	1	3	368.8	49.8	-7.6
DB01589	6	0	2	4	386.8	47.7	-7.6
DB01953	1	1	3	3.4	222.28	28.7	-7.6
DB01976	1	1	0	3.7	193.24	26	-7.6
DB02140	5	2	10	1.3	410.5	109	-7.6
DB02586	2	0	0	3.2	208.26	25.8	-7.6
DB02587	7	3	3	1	410.5	113	-7.6
DB02712	4	2	0	0.7	236.2	67.4	-7.6
DB02911	6	4	1	1.1	242.28	102	-7.6
DB02984	6	1	5	3.9	327.4	81.6	-7.6
DB03060	7	2	4	2.5	337.4	109	-7.6
DB03272	7	1	2	3.8	401.54	55.1	-7.6

DB03369	1	1	0	3.7	193.24	26	-7.6
DB03370	3	2	6	2.5	342.2	81.1	-7.6
DB03477	7	1	3	3.7	446.2	86.4	-7.6
DB03608	5	5	5	1.9	281.32	137	-7.6
DB03851	2	1	4	3.1	253.29	42.2	-7.6
DB04416	7	2	8	2.5	403.5	110	-7.6
DB04520	3	2	2	1	260.29	69.6	-7.6
DB04600	8	1	6	2.3	449.3	136	-7.6
DB04645	5	3	8	4.7	439.3	105	-7.6
DB04751	6	3	7	4.3	388.9	87.9	-7.6
DB04763	3	1	4	2.6	305.4	72.4	-7.6
DB04849	7	1	8	4.9	450.5	72.5	-7.6
DB04854	6	1	5	3.9	316.4	111	-7.6
DB04890	5	1	8	1	388.9	62.7	-7.6
DB05289	3	1	3	4.2	244.26	37.3	-7.6
DB05421	7	2	6	4.1	380.4	42.5	-7.6
DB05737	5	1	9	3.1	394.4	101	-7.6
DB05772	4	1	5	3.9	409.9	63	-7.6
DB06282	5	1	8	1.7	388.9	53	-7.6
DB06313	10	2	8	2.3	452.3	134	-7.6
DB06354	6	3	4	-0.8	279.34	104	-7.6
DB06506	5	1	7	3	400.5	84.1	-7.6
DB06588	4	2	3	4.4	348.4	58.9	-7.6
DB06600	7	2	4	0.4	371.4	96.1	-7.6
DB06814	6	4	6	2.1	331.4	91.2	-7.6
DB06868	2	1	6	3.3	336.9	49.4	-7.6
DB06873	5	2	9	1.9	382.4	88.1	-7.6
DB06877	2	2	3	2.1	280.32	64.8	-7.6
DB06993	9	3	7	1.2	458.5	122	-7.6
DB06996	4	4	7	0.7	359.5	125	-7.6
DB07005	3	4	7	1.1	394.5	127	-7.6
DB07055	5	1	3	0.4	243.22	91.8	-7.6
DB07070	8	2	8	4.3	427.4	75.6	-7.6
DB07087	3	2	2	3	276.4	49.7	-7.6
DB07135	6	1	5	1.3	430.5	96.8	-7.6
DB07163	4	4	4	1.7	310.33	82.9	-7.6
DB07167	5	2	4	2.7	339.4	89.5	-7.6
DB07192	2	1	3	2.8	365.3	49.4	-7.6
DB07195	3	2	2	2.4	288.4	49.7	-7.6
DB07232	3	3	2	0.5	244.29	83.8	-7.6
DB07233	6	3	8	3.6	426.5	117	-7.6
DB07249	8	1	8	5	470.9	78	-7.6
DB07318	6	3	4	1.2	320.29	92.6	-7.6
DB07425	4	2	6	4.9	328.4	66.8	-7.6
DB07504	7	3	9	4	458.4	82.5	-7.6
DB07543	3	3	6	3.6	298.4	57.3	-7.6
DB07548	8	2	8	0.7	432.4	99.6	-7.6
DB07562	6	1	4	3.7	325.4	82.2	-7.6
DB07564	6	4	4	-0.3	329.36	121	-7.6
DB07667	5	2	3	3.6	270.28	82.2	-7.6
DB07679	5	3	2	2.2	362.4	105	-7.6
DB07694	2	2	2	5	439.9	49.3	-7.6
DB07791	7	2	6	1.7	384.5	110	-7.6
DB07808	5	2	3	0.9	241.19	66.4	-7.6
DB07844	7	1	5	2.1	472	133	-7.6
DB07864	3	1	3	3.7	285.31	38.3	-7.6
DB07934	6	4	10	2.6	466.6	130	-7.6
DB08017	5	0	3	2.6	284.36	41.5	-7.6
DB08022	7	1	6	3.3	372.4	116	-7.6
DB08026	6	2	5	2	365.4	87.4	-7.6
DB08149	4	2	4	3.4	355.9	70.8	-7.6
DB08211	4	2	3	1.6	372.24	105	-7.6
DB08313	5	2	4	2.8	301.32	112	-7.6
DB08325	6	3	6	2.9	346.81	87.9	-7.6
DB08334	5	1	4	3.6	265.28	58.9	-7.6
DB08356	4	3	3	3.2	296.32	78.4	-7.6
DB08363	1	1	3	3	238.33	17	-7.6
DB08369	1	0	3	3.2	234.29	17.8	-7.6

DB08373	4	0	3	4.4	448.2	85	-7.6
DB08383	4	1	5	4.7	362.4	75.4	-7.6
DB08463	7	3	9	3.8	431.5	101	-7.6
DB08502	5	1	8	4.5	451.4	108	-7.6
DB08503	7	5	2	-0.4	311.33	111	-7.6
DB08559	6	4	4	1.4	352.4	108	-7.6
DB08600	2	1	2	3.5	321.9	50.6	-7.6
DB08663	4	1	4	3.6	310.3	55.8	-7.6
DB08672	4	2	8	2.9	328.4	72.2	-7.6
DB08698	4	1	3	3.2	373.5	76.4	-7.6
DB08706	7	2	5	3.8	374.4	117	-7.6
DB08728	5	0	6	3.9	355.2	56.8	-7.6
DB08773	3	2	1	3.9	242.29	68.7	-7.6
DB08877	4	1	4	2.1	306.4	83.2	-7.6
DB08990	4	0	9	3.7	380.5	32.8	-7.6
DB09021	1	1	2	3.7	249.3	12	-7.6
DB09040	6	1	5	2	348.4	54.2	-7.6
DB09167	2	0	3	4.5	295.4	28.5	-7.6
DB09197	3	1	4	3.2	304.82	35.2	-7.6
DB09201	4	1	5	4.8	333.4	80.7	-7.6
DB09219	4	3	2	3.3	333.3	78.8	-7.6
DB09225	3	0	4	4.8	331.9	37.8	-7.6
DB09262	2	1	6	2.7	319.4	60.9	-7.6
DB11446	5	2	4	2.3	315.3	103	-7.6
DB11530	6	1	5	2.5	428.5	73	-7.6
DB11672	6	2	8	3.2	368.4	93.1	-7.6
DB11682	6	2	5	2.5	393.4	124	-7.6
DB11702	3	2	3	0.4	246.26	75.3	-7.6
DB11880	3	1	4	3.3	293.4	45.2	-7.6
DB11912	10	3	8	2	479.5	123	-7.6
DB11960	5	2	5	3.9	424.7	117	-7.6
DB11993	4	0	5	2.4	376.9	41.4	-7.6
DB11999	10	2	5	-0.3	401.4	136	-7.6
DB12039	6	5	1	0.4	290.27	110	-7.6
DB12080	6	4	9	0.7	373.4	99	-7.6
DB12093	5	0	4	3.2	355.4	40.2	-7.6
DB12150	3	1	3	3	258.31	54.4	-7.6
DB12203	7	2	3	1.7	406.5	115	-7.6
DB12264	5	2	5	3	379.5	73.5	-7.6
DB12288	4	2	5	1.7	312.32	80.4	-7.6
DB12293	2	0	1	2.9	253.34	12.5	-7.6
DB12398	5	0	7	2.5	336.4	78	-7.6
DB12566	8	3	6	3.1	392.4	95.6	-7.6
DB12833	6	0	6	1.9	383.5	69.6	-7.6
DB13065	6	1	2	2.1	320.4	89.5	-7.6
DB13165	6	1	2	1.5	323.4	70.7	-7.6
DB13166	6	1	8	4.4	429.6	125	-7.6
DB13300	6	3	4	-1.3	351.4	138	-7.6
DB13329	0	0	1	4.8	198.3	0	-7.6
DB13335	2	0	2	3.1	308.8	32.7	-7.6
DB13403	4	1	6	4.3	379.5	40.7	-7.6
DB13457	5	0	2	3.2	273.29	12.5	-7.6
DB13481	4	2	2	3.8	320.8	114	-7.6
DB13496	1	0	4	4.6	284.4	8.2	-7.6
DB13557	6	0	8	4.8	446	61.3	-7.6
DB13606	2	1	3	3.8	321.5	23.5	-7.6
DB13617	4	1	2	2	328.8	88.8	-7.6
DB13653	2	0	5	4.6	370.9	23.6	-7.6
DB13782	2	0	4	4.8	296.4	21.3	-7.6
DB13837	5	2	3	1.6	348.8	73.1	-7.6
DB13844	3	1	6	3.6	354.5	46.5	-7.6
DB13876	3	1	2	3.1	310.19	34.4	-7.6
DB13994	6	2	5	3.3	354.4	90.1	-7.6
DB14209	6	3	8	1.2	402.5	107	-7.6
DB14538	7	1	7	3.3	460.6	107	-7.6
DB14540	6	2	6	3.2	432.5	101	-7.6
DB14644	8	3	7	2.2	474.5	138	-7.6
DB14826	7	1	4	0.9	363.4	114	-7.6

DB14849	5	2	3	2.9	273.24	103	-7.6
DB15091	6	2	3	2.7	380.4	78.3	-7.6
DB15209	7	0	7	4.9	469.6	73.2	-7.6
DB15239	7	2	5	1.4	354.4	114	-7.6
DB15360	4	0	8	3.8	393.5	49.8	-7.6
DB15565	7	3	7	-2.4	389.4	110	-7.6
DB00192	2	2	6	3	308.4	55.1	-7.5
DB00295	4	2	0	0.8	285.34	52.9	-7.5
DB00400	6	0	3	2.2	352.8	71.1	-7.5
DB00406	2	0	4	4.9	372.5	9.5	-7.5
DB00427	2	0	4	3.9	278.4	16.1	-7.5
DB00447	5	3	4	-1.7	349.77	113	-7.5
DB00461	2	0	4	3.1	228.29	26.3	-7.5
DB00476	3	1	6	4.3	297.4	49.5	-7.5
DB00490	6	0	6	2.6	385.5	69.6	-7.5
DB00589	2	2	3	2.7	338.4	51.4	-7.5
DB00656	4	0	5	2.8	371.9	42.4	-7.5
DB00678	5	2	8	4.3	422.9	92.5	-7.5
DB00688	8	1	10	3.2	433.5	94.5	-7.5
DB00802	6	0	9	2.2	416.5	81	-7.5
DB00837	4	2	6	3	334.8	75.7	-7.5
DB00876	6	2	10	4.5	424.5	121	-7.5
DB00885	5	1	1	-0.3	228.21	87.1	-7.5
DB00886	6	3	5	2.8	408.5	113	-7.5
DB00913	4	1	7	3.7	352.5	55.6	-7.5
DB00921	5	2	5	5	467.6	62.2	-7.5
DB01063	6	1	7	2.6	411.6	72.3	-7.5
DB01097	6	1	2	2.5	270.21	55.1	-7.5
DB01132	5	1	7	3.8	356.4	93.6	-7.5
DB01217	4	0	4	2.1	293.4	78.3	-7.5
DB01231	2	1	6	4.2	309.4	23.5	-7.5
DB01342	6	1	10	4.9	416.5	98.1	-7.5
DB01393	4	2	7	3.8	361.8	75.6	-7.5
DB01548	5	2	4	3.6	425.6	62.2	-7.5
DB01553	3	1	1	3.2	349.2	41.6	-7.5
DB01558	3	1	1	1.7	316.15	54.4	-7.5
DB01571	2	0	6	4.5	350.5	23.6	-7.5
DB01594	5	1	3	2	357.8	76.7	-7.5
DB01625	1	1	8	4.3	353.5	43.1	-7.5
DB01748	4	2	4	0.8	290.34	97.6	-7.5
DB01791	4	1	5	4.2	381.2	60.4	-7.5
DB02004	6	2	5	3.8	405.3	96.3	-7.5
DB02047	2	1	3	3.5	226.27	37.3	-7.5
DB02221	7	2	4	1.6	344.31	97.6	-7.5
DB02429	5	2	4	0.9	308.33	97.6	-7.5
DB02669	5	3	7	3	412.5	87.7	-7.5
DB02733	8	4	8	3.8	432.9	125	-7.5
DB02850	6	1	6	3.7	442.5	101	-7.5
DB03023	3	2	2	3.7	302.78	71.5	-7.5
DB03121	3	1	5	3.7	309.4	51.5	-7.5
DB03337	3	4	5	3	346.4	100	-7.5
DB03626	4	0	4	2.6	311.3	57.5	-7.5
DB04474	4	2	3	3.5	299.3	74.8	-7.5
DB04567	7	4	5	-2.7	319.31	136	-7.5
DB04601	8	1	6	1.6	370.4	136	-7.5
DB04643	5	3	7	4.6	445.7	105	-7.5
DB04712	6	5	4	-0.9	305.33	111	-7.5
DB05022	4	1	3	1.5	283.32	66.6	-7.5
DB05271	3	1	6	4.9	315.5	51.7	-7.5
DB05298	7	1	7	3.8	370.4	110	-7.5
DB05501	4	2	7	2.4	349.5	70.8	-7.5
DB05562	6	2	10	3.2	450.6	90.1	-7.5
DB05585	4	0	3	3.9	279.34	38.2	-7.5
DB05676	7	1	8	1.8	460.5	128	-7.5
DB05695	4	2	8	5	408.9	65.3	-7.5
DB05786	3	2	1	-0.2	246.3	57.5	-7.5
DB06016	3	1	5	4.3	427.4	38.8	-7.5
DB06174	8	0	4	2.7	413.4	75.7	-7.5

DB06345	2	2	2	2.1	319.4	61.4	-7.5
DB06458	2	2	5	2.3	268.35	55.1	-7.5
DB06538	4	1	4	2.8	318.4	55.6	-7.5
DB06725	7	2	2	2.1	371.8	136	-7.5
DB06800	4	2	2	1.6	356.4	66.8	-7.5
DB06884	3	3	5	3.2	284.35	61.4	-7.5
DB06936	3	3	7	1.7	344.5	99.3	-7.5
DB06947	4	4	6	-0.1	331.4	125	-7.5
DB07028	5	2	6	3.7	416.6	75.6	-7.5
DB07091	4	3	7	1.7	386.5	109	-7.5
DB07108	3	1	2	3.7	216.21	37.3	-7.5
DB07113	9	2	7	3.2	469.5	117	-7.5
DB07120	2	3	3	1.5	269.3	96.7	-7.5
DB07128	5	2	6	4.9	344.8	67.1	-7.5
DB07155	2	1	3	2.1	286.37	49.4	-7.5
DB07174	7	2	6	1.9	385.5	117	-7.5
DB07182	5	4	9	0.9	389.4	139	-7.5
DB07286	4	1	1	1.1	305.71	84.6	-7.5
DB07289	7	3	8	4.9	462.3	124	-7.5
DB07330	3	2	4	1.9	286.37	75	-7.5
DB07401	8	0	8	3.7	403.4	104	-7.5
DB07439	6	3	9	1.7	398.4	108	-7.5
DB07462	5	2	2	2.9	259.21	103	-7.5
DB07467	5	2	3	2.9	365.8	101	-7.5
DB07501	7	3	9	4	458.4	82.5	-7.5
DB07556	7	2	8	1.7	393.5	117	-7.5
DB07624	9	2	6	1	449.5	112	-7.5
DB07724	6	1	7	2.9	389.4	103	-7.5
DB07790	8	2	9	1.8	430.5	119	-7.5
DB07796	4	2	4	1.7	407.5	92.2	-7.5
DB07857	2	2	4	3.2	297.8	54.7	-7.5
DB07858	2	2	4	3.2	297.8	54.7	-7.5
DB07901	4	1	5	4.1	324.8	50.7	-7.5
DB07965	7	1	5	2	368.5	85.9	-7.5
DB07995	5	2	8	3.5	446.4	79.5	-7.5
DB08019	5	4	8	2.1	373.9	75	-7.5
DB08040	5	4	7	0.2	294.3	116	-7.5
DB08053	6	1	4	2.3	325.4	88.1	-7.5
DB08152	5	1	9	3.1	410.9	84.9	-7.5
DB08181	5	2	3	3.2	256.26	82.2	-7.5
DB08196	7	2	5	2.8	302.28	101	-7.5
DB08247	5	2	4	3.6	289.38	89.7	-7.5
DB08307	8	1	1	2.8	329.23	100	-7.5
DB08396	6	2	6	1.7	274.27	95.9	-7.5
DB08398	3	1	1	2.2	224.26	56.7	-7.5
DB08399	4	4	2	2.9	244.24	80.9	-7.5
DB08440	3	1	1	1.6	237.26	54.9	-7.5
DB08458	3	0	8	4.8	400.3	29.5	-7.5
DB08574	6	1	2	3.5	291.3	86.5	-7.5
DB08579	2	1	1	3.7	311.22	46.3	-7.5
DB08590	7	2	8	3.2	440.4	139	-7.5
DB08635	5	1	8	4.4	354.4	86.4	-7.5
DB08680	4	1	5	4.5	350.9	78.8	-7.5
DB08734	3	2	6	2.8	394.7	98.5	-7.5
DB08761	4	4	4	2	326.3	98.7	-7.5
DB08775	6	3	10	2	402.4	105	-7.5
DB08796	6	0	7	3.4	399.5	80.2	-7.5
DB08926	6	3	5	1	306.34	118	-7.5
DB08962	4	3	3	3.2	366.5	104	-7.5
DB09172	7	0	8	2.7	420.5	81	-7.5
DB09182	2	0	5	3.6	322.4	23.6	-7.5
DB09286	5	1	7	2	375.5	66.6	-7.5
DB09306	2	0	1	1.4	255.31	29.8	-7.5
DB11376	5	0	6	3.3	327.4	36.4	-7.5
DB11584	2	2	3	3	267.4	32.3	-7.5
DB11662	6	3	5	1.4	436.3	97.4	-7.5
DB11721	6	1	7	3.5	370.4	111	-7.5
DB11781	6	4	10	2.3	406.5	125	-7.5

DB11821	5	3	4	-2.7	291.3	113	-7.5
DB11961	7	2	5	0.6	402.9	126	-7.5
DB12049	7	2	9	4.9	462	102	-7.5
DB12254	7	3	6	2.8	371.4	109	-7.5
DB12401	4	1	6	3.3	420.3	40.5	-7.5
DB12475	4	0	5	3.9	270.28	52.6	-7.5
DB12478	6	0	3	1.8	298.34	50.7	-7.5
DB12555	5	2	4	3.3	437.2	68.2	-7.5
DB12593	5	2	4	-0.5	288.3	82.5	-7.5
DB12594	3	3	10	4.6	419	61.4	-7.5
DB12670	3	1	5	4.9	356.5	69.3	-7.5
DB12938	8	0	4	2.4	359.3	85.6	-7.5
DB13291	2	1	2	3.8	258.7	33.4	-7.5
DB13324	2	0	1	3.2	288.77	32.7	-7.5
DB13399	2	2	3	2.5	340.5	51.4	-7.5
DB13532	7	3	3	1.3	379.9	135	-7.5
DB13542	3	1	6	3.6	354.5	46.5	-7.5
DB13564	5	1	9	2.2	384.5	45.2	-7.5
DB13593	3	0	2	2.7	272.32	59.8	-7.5
DB13665	5	0	7	3.6	356.4	32.8	-7.5
DB13692	6	3	5	2.5	345.4	80.2	-7.5
DB13792	5	2	3	2.3	345.8	101	-7.5
DB13810	1	0	0	3.8	255.4	3.2	-7.5
DB13820	4	0	4	3.4	330.4	49	-7.5
DB14060	6	1	4	2.9	314.36	110	-7.5
DB14885	9	0	3	2.2	414.4	77	-7.5
DB15024	6	1	3	2.3	364.4	84.1	-7.5
DB15334	5	2	2	3	284.26	76	-7.5
DB15335	4	1	2	2.8	268.26	55.8	-7.5
DB15425	7	3	8	3.3	397.5	101	-7.5
DB00214	5	3	5	2.7	348.4	109	-7.4
DB00382	2	1	0	2.7	198.26	38.9	-7.4
DB00393	8	1	9	3.1	418.4	120	-7.4
DB00402	7	0	3	0.5	388.8	91.8	-7.4
DB00415	6	3	4	-1.1	349.4	138	-7.4
DB00465	3	1	3	1.9	255.27	59.3	-7.4
DB00495	6	2	3	0	267.24	93.2	-7.4
DB00519	6	2	10	2	430.5	95.9	-7.4
DB00561	3	0	6	3.3	378.5	32.8	-7.4
DB00573	3	1	4	3.3	242.27	46.5	-7.4
DB00805	5	1	5	2	298.4	50.3	-7.4
DB00810	2	1	5	4.3	311.5	23.5	-7.4
DB01009	3	1	4	3.1	254.28	54.4	-7.4
DB01123	3	2	0	1.8	209.25	64.9	-7.4
DB01176	2	0	3	3.6	266.4	6.5	-7.4
DB01198	7	0	3	0.5	388.8	91.8	-7.4
DB01209	2	2	0	3.7	245.36	46.2	-7.4
DB01210	4	2	6	2.4	291.4	58.6	-7.4
DB01475	4	0	8	3.6	353.5	38.8	-7.4
DB01491	2	0	7	4.8	349.5	20.3	-7.4
DB01545	5	1	4	2.9	360.8	67.8	-7.4
DB02001	7	2	3	2.7	353.4	116	-7.4
DB02255	4	5	9	0.9	388.5	123	-7.4
DB02558	5	4	9	2.6	400.5	96.5	-7.4
DB02603	4	2	3	2.7	246.31	76.8	-7.4
DB02709	3	3	2	3.1	228.24	60.7	-7.4
DB02862	5	4	2	-0.8	276.29	98.7	-7.4
DB03016	4	4	2	0.7	253.26	112	-7.4
DB03093	7	2	4	2.1	303.29	98.9	-7.4
DB03137	7	1	7	2.9	369.4	88.1	-7.4
DB03451	4	3	9	4.9	460.7	69.9	-7.4
DB03666	9	3	5	-1	347.22	140	-7.4
DB03890	6	1	8	1	399.6	95.2	-7.4
DB03980	5	1	4	2.1	309.34	69.6	-7.4
DB03982	5	2	4	3.6	335.3	94.9	-7.4
DB04054	7	1	7	3.2	359.4	88.1	-7.4
DB04070	6	3	1	3.1	386.5	104	-7.4
DB04336	2	4	3	2.1	288.73	91	-7.4

DB04367	3	4	0	-1.2	245.24	112	-7.4
DB04590	8	4	9	2.6	417.4	127	-7.4
DB04656	6	4	6	0.9	310.34	107	-7.4
DB04743	6	1	4	2.6	308.31	110	-7.4
DB04761	6	2	6	0.3	348.4	110	-7.4
DB04770	6	2	8	3.1	355.4	85.1	-7.4
DB04917	4	2	3	2.1	323.82	67.6	-7.4
DB05223	4	2	10	3.1	358.5	70.4	-7.4
DB06193	7	4	6	1	325.4	123	-7.4
DB06504	5	1	1	0.3	242.23	76.8	-7.4
DB06530	5	2	5	1.3	314.4	86.2	-7.4
DB06585	2	1	2	3.1	230.28	28.7	-7.4
DB06618	4	0	9	3.9	368.5	24.9	-7.4
DB06737	4	1	2	3.5	298.4	79.7	-7.4
DB06802	3	2	4	1.9	254.28	86.2	-7.4
DB06902	1	1	2	3.7	212.29	20.2	-7.4
DB06929	3	3	6	0.9	316.4	99.3	-7.4
DB06988	6	4	6	-0.9	314.34	116	-7.4
DB06991	5	3	6	3.4	346.4	111	-7.4
DB07056	5	1	7	2.9	395.9	87.8	-7.4
DB07059	7	3	5	1	398.5	137	-7.4
DB07096	3	2	0	1.3	212.2	72.2	-7.4
DB07100	5	4	2	1.3	294.74	84.4	-7.4
DB07158	2	1	1	1.7	227.26	49	-7.4
DB07260	3	2	6	3.5	282.4	33.3	-7.4
DB07295	6	3	4	3.2	351.3	115	-7.4
DB07305	5	1	2	3	267.62	96.3	-7.4
DB07310	7	1	2	3.7	320.31	66.8	-7.4
DB07364	3	2	4	3.5	267.33	61.8	-7.4
DB07402	3	0	3	3.7	298.34	56.2	-7.4
DB07498	7	1	5	1.8	277.23	122	-7.4
DB07505	5	1	10	3.6	416.6	67	-7.4
DB07527	6	3	8	2.4	392.5	110	-7.4
DB07613	3	1	2	2	211.22	54.7	-7.4
DB07626	5	2	8	4.5	446	76.8	-7.4
DB07652	8	3	4	0	324.21	96.2	-7.4
DB07732	7	3	6	0.4	331.28	121	-7.4
DB07757	2	1	3	4.6	335.2	37.3	-7.4
DB07759	6	1	2	3.4	256.18	50.4	-7.4
DB07862	4	2	3	2.7	269.34	82.8	-7.4
DB07866	3	1	3	4.5	282.4	66.8	-7.4
DB07926	5	4	10	1.3	379.5	117	-7.4
DB07937	9	1	10	3.4	446.5	77.3	-7.4
DB07962	6	2	6	3.7	461.2	75.6	-7.4
DB07996	5	1	2	1	291.37	70.7	-7.4
DB08001	9	3	9	3.2	425.4	139	-7.4
DB08021	4	1	4	3.9	422.7	48.7	-7.4
DB08045	5	4	6	2.8	359.8	104	-7.4
DB08119	6	2	8	3.5	372.4	75.3	-7.4
DB08130	9	3	6	3.3	475.21	89	-7.4
DB08167	3	0	1	2.2	284.4	43.9	-7.4
DB08227	4	1	1	0.5	254.33	60.8	-7.4
DB08234	6	2	5	2.6	312.37	96.3	-7.4
DB08253	2	2	3	1.5	214.26	69.1	-7.4
DB08333	4	1	4	3.5	247.29	58.9	-7.4
DB08382	8	1	3	0.6	366.4	64.6	-7.4
DB08422	5	3	8	1.3	381.5	100	-7.4
DB08473	5	3	2	1.1	319.14	87.7	-7.4
DB08478	4	3	2	1.4	279.65	103	-7.4
DB08665	5	0	1	2.2	298.3	82.3	-7.4
DB08682	5	1	5	3.4	355.8	89.9	-7.4
DB08735	4	2	4	2.6	310.3	66.8	-7.4
DB08880	6	2	2	3.3	270.21	73.1	-7.4
DB08895	5	1	3	1.5	312.37	88.9	-7.4
DB08986	2	1	2	4.1	300.8	33.6	-7.4
DB09012	5	3	1	0	236.23	112	-7.4
DB09179	3	0	8	4.2	380.5	32.8	-7.4
DB09194	4	0	7	3	377.9	42.4	-7.4

DB09196	4	1	3	2.1	251.3	30.5	-7.4
DB09246	2	2	4	2.9	240.3	41.1	-7.4
DB09289	6	2	8	1.2	437	95.1	-7.4
DB09305	2	0	0	1.8	253.34	11.4	-7.4
DB09495	3	0	6	4.8	310.4	43.4	-7.4
DB11366	3	1	2	2.7	308.3	60.8	-7.4
DB11410	4	2	4	3.6	299.3	92.3	-7.4
DB11490	4	2	2	1.9	311.4	52.9	-7.4
DB11648	5	2	6	4	427.3	101	-7.4
DB11654	5	0	6	2.3	368.4	76.2	-7.4
DB11689	6	3	6	3.6	457.7	88.4	-7.4
DB11764	8	3	10	3.6	423.4	97.4	-7.4
DB11766	8	1	9	2	434.5	87.1	-7.4
DB12210	6	2	6	3.1	366.4	99.4	-7.4
DB12271	6	3	10	1.4	428.3	104	-7.4
DB12359	6	1	3	1.9	318.76	91.7	-7.4
DB12499	5	1	5	3.9	402.5	80.7	-7.4
DB12572	6	1	5	3.8	340.4	77.2	-7.4
DB12641	2	1	3	4.5	283.4	37.3	-7.4
DB12766	3	1	1	2.4	261.7	42.4	-7.4
DB12818	6	2	3	2.1	264.23	93.1	-7.4
DB12853	6	2	8	1.7	406.9	98.3	-7.4
DB12889	8	2	5	2.6	407.5	96.5	-7.4
DB12974	10	3	7	4.5	430.4	117	-7.4
DB13111	2	1	0	3.6	220.31	29.5	-7.4
DB13256	3	0	1	3.6	343.9	44.1	-7.4
DB13275	2	0	1	3.5	256.68	34.1	-7.4
DB13286	4	2	8	4.9	326.4	69.6	-7.4
DB13318	6	1	5	1.4	371.4	66	-7.4
DB13437	2	0	1	4.4	270.75	15.6	-7.4
DB13611	1	0	2	4.1	256.73	17.8	-7.4
DB13725	1	1	6	5	281.4	12	-7.4
DB13805	3	2	2	1.7	262.3	75.3	-7.4
DB13825	5	0	7	2.4	347.4	48	-7.4
DB14033	6	1	3	1.3	309.34	115	-7.4
DB14064	2	0	9	3.9	334.5	27	-7.4
DB14083	4	0	8	4	340.4	43.5	-7.4
DB14881	5	1	7	3.5	386.6	71.6	-7.4
DB14945	5	1	7	3.9	364.5	41.9	-7.4
DB15411	5	0	7	2.9	314.34	70.4	-7.4
DB15489	3	1	1	3.7	363.2	41.6	-7.4
DB15534	6	2	4	1.2	385.4	94.1	-7.4
DB00257	1	0	4	5	344.8	17.8	-7.3
DB00283	2	0	6	5	343.9	12.5	-7.3
DB00341	5	1	8	1.7	388.9	53	-7.3
DB00346	8	2	8	1.7	389.4	112	-7.3
DB00383	4	1	6	2.9	344.4	62.1	-7.3
DB00412	6	1	7	3.1	357.4	96.8	-7.3
DB00498	2	0	1	2.9	222.24	34.1	-7.3
DB00567	6	3	4	0.6	347.4	138	-7.3
DB00571	3	2	6	3	259.34	41.5	-7.3
DB00572	4	1	5	1.8	289.4	49.8	-7.3
DB00604	7	2	9	3.4	465.9	86	-7.3
DB00622	8	1	10	3.8	479.5	114	-7.3
DB00652	2	1	2	3.3	285.4	23.5	-7.3
DB00692	3	2	4	2.6	281.35	47.9	-7.3
DB00767	6	0	7	2.1	404.5	68.3	-7.3
DB00777	4	0	5	4.8	340.5	48.8	-7.3
DB00800	4	4	1	2.4	305.75	72.7	-7.3
DB00827	7	1	2	2.1	262.22	88.4	-7.3
DB00850	5	1	6	4.2	404	55.2	-7.3
DB00940	3	0	7	3.3	340.4	35.5	-7.3
DB00980	2	1	4	2.7	259.34	38.3	-7.3
DB01000	6	3	3	1.3	341.4	138	-7.3
DB01079	3	3	8	2.6	301.39	87.8	-7.3
DB01151	2	1	4	4.9	266.4	15.3	-7.3
DB01174	3	2	2	1.5	232.23	75.3	-7.3
DB01188	2	1	1	2	207.27	40.5	-7.3

DB01322	3	0	3	2.3	232.27	35.5	-7.3
DB01354	3	2	2	2	250.29	75.3	-7.3
DB01425	5	2	6	1.6	315.37	83.1	-7.3
DB01437	2	1	2	1.9	217.26	46.2	-7.3
DB01450	5	2	4	3.8	413.5	62.2	-7.3
DB01463	1	1	3	3.3	215.33	12	-7.3
DB01470	3	0	6	4.4	356.5	51.8	-7.3
DB01529	3	0	6	3.8	392.5	32.8	-7.3
DB01551	4	1	1	2.2	301.4	41.9	-7.3
DB01557	2	0	6	4.5	350.5	23.6	-7.3
DB01559	3	0	2	3.5	318.8	60.9	-7.3
DB01565	4	2	0	1.8	287.35	52.9	-7.3
DB01570	3	1	6	3.6	366.5	43.8	-7.3
DB01614	4	0	5	4.2	326.5	48.8	-7.3
DB01618	3	1	3	1.7	276.37	45.3	-7.3
DB01634	8	5	6	-2	304.3	138	-7.3
DB01731	2	1	4	4.1	426.1	28.4	-7.3
DB01951	5	0	9	3.1	360.4	76.6	-7.3
DB01959	5	0	4	2.7	289.29	89.9	-7.3
DB02132	5	1	4	3.3	441.6	77.9	-7.3
DB02161	4	1	4	1.9	275.34	49.8	-7.3
DB02593	8	3	2	1	322.27	123	-7.3
DB02647	2	2	3	2	227.26	57.8	-7.3
DB02918	6	1	4	2.2	268.22	59.9	-7.3
DB02919	9	2	6	1.6	370.4	122	-7.3
DB02963	5	1	3	2.4	322.77	84.7	-7.3
DB03079	8	4	4	-1.8	330.23	134	-7.3
DB03250	7	4	2	0.3	311.36	131	-7.3
DB03428	3	2	2	1	241.24	67	-7.3
DB03456	5	2	8	3	358.4	94.5	-7.3
DB03749	4	4	3	1.8	270.29	97.8	-7.3
DB03914	7	4	5	-2.6	342.35	134	-7.3
DB03984	5	2	10	2.2	455.6	113	-7.3
DB04163	5	2	2	2.9	268.34	103	-7.3
DB04297	4	2	6	2.7	302.37	69.6	-7.3
DB04306	5	2	2	3.3	282.4	103	-7.3
DB04639	3	2	4	0.1	241.28	63.3	-7.3
DB04662	7	4	8	2.8	370.4	108	-7.3
DB04857	3	0	3	4.1	327.2	24.8	-7.3
DB05095	6	1	4	2.9	381.8	95.6	-7.3
DB05659	9	1	6	0.9	397.4	137	-7.3
DB05943	6	1	6	3	361.8	80.8	-7.3
DB06711	1	1	2	2.1	210.27	24.4	-7.3
DB06743	9	2	1	0.6	408.4	129	-7.3
DB06869	5	3	9	2.9	479	114	-7.3
DB06973	2	2	2	3.3	228.29	40.5	-7.3
DB06989	8	2	10	2.4	407.4	127	-7.3
DB07002	5	1	4	2.6	336.4	78.2	-7.3
DB07017	5	2	3	3.1	310.4	87	-7.3
DB07018	3	1	7	3	328.4	41.6	-7.3
DB07085	6	3	6	3.9	364.8	95.5	-7.3
DB07099	3	2	5	1.7	256.3	64.4	-7.3
DB07122	2	2	4	1.7	253.3	81.5	-7.3
DB07149	5	3	3	0.2	275.28	96.7	-7.3
DB07173	6	3	1	-0.2	376.15	106	-7.3
DB07231	6	3	9	1.5	431.5	128	-7.3
DB07248	7	1	6	3.3	377.4	83.3	-7.3
DB07262	5	3	4	2	276.28	114	-7.3
DB07313	5	2	4	2.9	291.32	91.8	-7.3
DB07328	6	4	7	0.1	403.5	126	-7.3
DB07338	8	1	3	3	361.65	92.4	-7.3
DB07420	7	3	8	1.6	386.4	130	-7.3
DB07431	5	3	2	1.4	277.34	105	-7.3
DB07515	9	5	8	0.7	457.9	111	-7.3
DB07525	2	1	3	3.2	238.28	37.9	-7.3
DB07582	4	3	8	1.9	446.6	108	-7.3
DB07693	2	2	2	4.5	399.08	49.3	-7.3
DB07746	6	2	5	3.8	337.4	91.8	-7.3

DB07758	3	1	2	3.8	257.07	50.4	-7.3
DB07825	4	1	3	0.2	247.25	74.7	-7.3
DB07852	4	1	2	3.2	272.08	68	-7.3
DB07877	8	2	7	3.3	465.4	125	-7.3
DB07878	4	1	8	3.3	368.5	76.9	-7.3
DB07956	4	2	7	3.8	354.8	84.5	-7.3
DB07961	2	2	3	3.8	363.3	106	-7.3
DB08014	6	0	10	4.6	397.5	64.6	-7.3
DB08041	5	3	8	2.4	329.3	95.9	-7.3
DB08136	4	3	3	1.2	262.24	86.9	-7.3
DB08144	5	2	1	3.1	395.05	90.7	-7.3
DB08151	7	4	2	-0.5	295.29	112	-7.3
DB08198	3	2	3	2.9	338.4	84.7	-7.3
DB08285	6	3	8	3.6	354.4	87.4	-7.3
DB08302	5	1	5	3.6	301.29	96.3	-7.3
DB08320	7	2	8	2.2	424.4	102	-7.3
DB08403	4	4	9	0.8	349.4	108	-7.3
DB08472	5	1	6	4	309.33	21.3	-7.3
DB08522	3	0	4	3	293.3	30.7	-7.3
DB08524	4	1	6	2.3	285.29	66.8	-7.3
DB08595	2	1	1	3.1	258.35	29.5	-7.3
DB08596	5	2	3	-0.2	309.36	82.1	-7.3
DB08615	3	1	2	3.9	285.4	55.6	-7.3
DB08642	7	3	6	1	375.5	107	-7.3
DB08724	5	0	8	3.7	328.4	56.8	-7.3
DB08794	8	2	5	2.5	408.4	119	-7.3
DB08936	2	0	3	4.5	300.8	6.5	-7.3
DB08984	8	2	9	4.7	369.3	67.8	-7.3
DB09170	4	1	6	2.9	358.5	72	-7.3
DB09188	2	1	5	3.2	294.4	32.3	-7.3
DB09212	3	1	4	2.4	246.3	54.4	-7.3
DB09271	4	1	7	2.1	358.5	59.1	-7.3
DB09274	8	1	5	2.5	384.4	101	-7.3
DB09477	6	3	8	-0.7	348.4	107	-7.3
DB11380	5	2	4	2.9	302.35	108	-7.3
DB11390	6	0	6	4.5	362.8	86.1	-7.3
DB11463	6	2	4	2	335.34	130	-7.3
DB11561	2	1	0	2.8	194.23	38.9	-7.3
DB11783	7	2	10	-0.7	405.4	116	-7.3
DB12008	8	3	8	1.4	365.4	135	-7.3
DB12071	2	1	0	2.5	228.33	15.3	-7.3
DB12201	4	1	5	2.5	322.4	32.3	-7.3
DB12252	7	2	1	-0.9	376.4	104	-7.3
DB12299	4	0	4	1.2	329.4	36	-7.3
DB12311	2	2	3	2	234.29	58.2	-7.3
DB12347	4	1	6	2.9	340.4	83.8	-7.3
DB12360	4	1	6	3.4	350.4	32.3	-7.3
DB12448	4	2	8	4.8	342.4	66.8	-7.3
DB12588	8	3	5	2.4	348.76	104	-7.3
DB12758	4	2	4	2.7	318.4	94.8	-7.3
DB12927	7	4	6	-0.8	375.4	131	-7.3
DB12931	2	2	1	1.2	266.68	73.8	-7.3
DB12946	3	1	5	4	304.39	69.3	-7.3
DB13001	5	1	5	3.4	316.4	83.8	-7.3
DB13004	3	1	3	2.9	313.4	49.8	-7.3
DB13012	3	3	3	3	321.5	66.5	-7.3
DB13063	3	0	0	2.3	248.32	38.8	-7.3
DB13064	1	2	2	2	215.29	36.4	-7.3
DB13118	3	1	4	3.8	350.4	60.8	-7.3
DB13283	6	2	3	1.2	278.33	106	-7.3
DB13435	5	2	2	0.4	269.3	84.1	-7.3
DB13465	4	2	4	1.6	259.26	84.1	-7.3
DB13489	2	0	4	4.1	280.4	6.5	-7.3
DB13635	4	1	5	3.1	367.4	61.9	-7.3
DB13672	3	0	2	3.5	298.4	48.8	-7.3
DB13674	4	1	2	2.7	253.25	57.6	-7.3
DB13687	5	1	6	2.6	356.4	48.5	-7.3
DB13739	7	1	8	2.1	406.5	127	-7.3

DB13823	8	2	3	-2.1	303.32	98.7	-7.3
DB13836	6	2	5	3	361.4	124	-7.3
DB14207	5	2	9	3.8	435.5	94.9	-7.3
DB14768	7	2	7	1.8	431.9	110	-7.3
DB15161	4	2	2	3.9	255.33	73.4	-7.3
DB15316	10	1	4	1.6	359.29	107	-7.3
DB00177	6	2	10	4.4	435.5	112	-7.2
DB00234	4	1	6	3	313.4	39.7	-7.2
DB00261	2	1	0	1.6	256.08	44.7	-7.2
DB00266	6	2	2	2.6	336.3	93.1	-7.2
DB00357	3	2	2	1.2	232.28	72.2	-7.2
DB00384	7	3	1	1	253.26	130	-7.2
DB00433	4	0	4	4.9	373.9	35	-7.2
DB00504	2	1	2	3.5	283.4	23.5	-7.2
DB00557	4	1	8	3.7	374.9	35.9	-7.2
DB00582	8	1	5	1.5	349.31	76.7	-7.2
DB00586	3	2	4	4.4	296.1	49.3	-7.2
DB00611	3	2	2	3.8	327.5	43.7	-7.2
DB00690	4	0	6	3	387.9	35.9	-7.2
DB00788	3	1	3	3.3	230.26	46.5	-7.2
DB00833	6	3	4	-1.8	367.8	138	-7.2
DB00907	5	0	5	2.3	303.35	55.8	-7.2
DB01054	7	1	6	2.9	360.4	110	-7.2
DB01125	3	0	2	2.9	252.26	43.4	-7.2
DB01288	5	5	6	2	303.35	93	-7.2
DB01333	6	3	4	0.4	349.4	138	-7.2
DB01394	6	1	5	1	399.4	83.1	-7.2
DB01512	5	3	0	-1.1	303.35	73.2	-7.2
DB01615	4	0	4	4.3	326.5	48.8	-7.2
DB01925	2	2	1	3.3	220.65	40.5	-7.2
DB01954	3	1	4	2.2	275.34	47.6	-7.2
DB02104	9	2	6	2	384.4	122	-7.2
DB02660	5	1	6	2.6	292.33	83.1	-7.2
DB02880	3	2	3	4.1	338.17	49.3	-7.2
DB02909	3	1	2	3.1	222.62	50.4	-7.2
DB02921	4	3	2	-0.5	252.27	89.9	-7.2
DB03535	4	0	5	1.7	330.4	66.9	-7.2
DB03537	7	4	5	-3.1	305.29	136	-7.2
DB03575	1	0	2	3.6	243.4	3.2	-7.2
DB03781	4	1	5	5	327.2	55.8	-7.2
DB03809	5	1	6	2.8	311.4	78.8	-7.2
DB04037	4	1	5	4.2	334.2	57.7	-7.2
DB04175	4	3	4	2.9	302.11	90.4	-7.2
DB04407	7	3	4	3.2	313.4	111	-7.2
DB04513	4	2	8	3.3	340.9	80.6	-7.2
DB04627	5	2	1	-2.5	226.19	91.6	-7.2
DB04817	6	1	4	0	311.36	89.5	-7.2
DB04828	3	1	4	2.8	291.73	59.3	-7.2
DB04953	5	3	6	2.8	303.33	76.4	-7.2
DB04954	9	4	4	-0.3	337.33	135	-7.2
DB05013	6	3	4	2	430.5	104	-7.2
DB05351	8	1	5	2.8	369.4	87.1	-7.2
DB05444	4	1	3	0.8	260.33	49.8	-7.2
DB05596	5	2	6	3.5	377.5	101	-7.2
DB06234	6	4	4	2.2	376.2	99.8	-7.2
DB06299	2	1	1	1.5	201.22	40.5	-7.2
DB06335	4	2	2	0.7	315.4	90.4	-7.2
DB06465	4	1	2	0.8	265.28	52.6	-7.2
DB06649	4	2	7	2.2	302.34	64.4	-7.2
DB06726	3	2	5	3.5	261.36	45.4	-7.2
DB06731	5	1	5	3.5	295.3	35.2	-7.2
DB06787	2	1	4	3.1	317.5	23.5	-7.2
DB06878	3	2	5	1.7	323.82	75.4	-7.2
DB06885	7	1	5	2.3	261.2	58.9	-7.2
DB06887	7	3	5	0.5	385.4	133	-7.2
DB06892	7	2	5	2.8	452.17	70.9	-7.2
DB06903	2	1	1	4	234.33	37.3	-7.2
DB07048	4	2	7	3	338.5	97.6	-7.2

DB07083	3	2	7	3	379.5	75.4	-7.2
DB07102	5	3	8	-0.7	328.4	102	-7.2
DB07139	5	1	4	2.9	277.3	111	-7.2
DB07211	7	1	6	1.9	462	133	-7.2
DB07251	9	2	8	4.2	417.8	99.6	-7.2
DB07272	7	2	7	2.9	435.3	120	-7.2
DB07365	3	2	3	-0.1	215.25	63.3	-7.2
DB07451	5	3	5	3.3	422.2	91.3	-7.2
DB07465	4	2	2	0.7	315.4	90.4	-7.2
DB07473	4	0	3	2.9	325.4	71.9	-7.2
DB07510	6	2	4	1.9	254.19	74.6	-7.2
DB07516	4	2	4	2.3	252.65	74.6	-7.2
DB07560	3	2	5	2.8	303.4	71.1	-7.2
DB07719	7	3	5	0.5	385.4	133	-7.2
DB07730	4	2	1	0.3	225.22	91.8	-7.2
DB07773	5	3	5	1.5	273.2	82.6	-7.2
DB07798	8	2	5	2.2	340.34	66.6	-7.2
DB07842	3	1	6	4.3	270.32	46.5	-7.2
DB07918	3	1	6	4.5	259.34	40.5	-7.2
DB07958	3	3	6	1.7	274.31	96.2	-7.2
DB08118	7	2	5	3	360.5	138	-7.2
DB08140	6	3	4	1.9	313.4	104	-7.2
DB08148	4	2	3	3.1	341.8	70.8	-7.2
DB08170	8	3	5	1.2	408.4	134	-7.2
DB08209	5	1	4	3.3	269.3	65.3	-7.2
DB08212	6	3	5	2.3	382.4	139	-7.2
DB08224	4	2	3	1.9	306.4	66.8	-7.2
DB08229	6	3	9	-1.9	385.4	124	-7.2
DB08281	4	1	5	3.5	405.3	90.7	-7.2
DB08312	7	2	3	2.1	251.29	117	-7.2
DB08414	6	1	3	2.9	306.77	103	-7.2
DB08436	6	1	5	2.7	325.4	88.1	-7.2
DB08456	5	1	4	1.5	341.5	91.3	-7.2
DB08466	3	3	3	3.1	230.26	60.7	-7.2
DB08545	4	1	6	1.4	332.31	78.5	-7.2
DB08581	4	2	5	3	411.3	86.7	-7.2
DB08598	2	1	2	3.5	321.9	50.6	-7.2
DB08604	2	1	2	5	289.5	29.5	-7.2
DB08610	3	2	8	3.4	360.9	64.4	-7.2
DB08611	5	1	4	3.1	268.23	29.5	-7.2
DB08634	3	1	7	3.7	364.4	58.6	-7.2
DB08667	5	3	2	2.6	218.19	96.8	-7.2
DB08691	4	1	4	3.1	338.2	81	-7.2
DB08723	5	0	8	4.6	383.3	56.8	-7.2
DB08784	4	2	3	3.4	248.66	62.2	-7.2
DB08918	2	1	5	1.4	246.35	46.3	-7.2
DB08967	6	0	5	3.8	391.6	77.5	-7.2
DB08992	2	0	5	3.8	259.4	20.3	-7.2
DB09000	4	0	4	4.4	323.5	55.6	-7.2
DB09002	2	0	6	4.8	329.9	12.5	-7.2
DB09084	3	0	7	3.9	309.4	30.3	-7.2
DB09119	3	1	2	2	296.32	72.6	-7.2
DB09180	3	0	6	3.8	342.5	51.8	-7.2
DB09206	10	2	7	1.2	435.5	133	-7.2
DB11181	4	1	4	1.9	275.34	49.8	-7.2
DB11185	6	2	4	2.2	308.31	109	-7.2
DB11315	4	1	5	1.3	318.4	59.1	-7.2
DB11481	1	1	2	3	212.29	28.7	-7.2
DB11540	4	0	6	4.7	340.5	48.8	-7.2
DB11683	4	2	4	1	325.77	117	-7.2
DB11711	6	2	8	3.6	410.5	99.8	-7.2
DB11785	5	2	5	0.9	305.4	70	-7.2
DB11837	3	0	1	1.9	227.24	41.6	-7.2
DB11947	3	0	4	2.6	281.4	45.8	-7.2
DB12462	5	1	9	5	431.9	76.1	-7.2
DB12464	7	2	9	1.1	386.4	109	-7.2
DB12625	7	2	7	1	401.4	84.7	-7.2
DB12714	3	0	4	4.4	283.34	21.1	-7.2

DB12733	3	0	3	3.5	265.28	30.2	-7.2
DB12846	7	4	7	-0.5	389.4	131	-7.2
DB12954	6	2	4	-0.2	302.29	101	-7.2
DB13028	6	1	3	1.4	350.4	128	-7.2
DB13203	5	1	8	1.2	385.5	81.9	-7.2
DB13337	6	2	5	2.2	364.4	121	-7.2
DB13355	7	0	6	3.4	388.4	88.1	-7.2
DB13395	4	1	8	2.7	354.5	35.9	-7.2
DB13439	1	0	5	4.9	279.4	3.2	-7.2
DB13479	2	0	6	4.1	280.4	6.5	-7.2
DB13507	3	1	6	3.2	340.4	46.5	-7.2
DB13546	5	0	7	4.1	412.9	84	-7.2
DB13605	4	1	8	3.7	367.5	49.8	-7.2
DB13627	6	1	2	-0.2	261.23	76.1	-7.2
DB13630	1	0	7	3.6	284.4	9.2	-7.2
DB13671	3	0	4	4.3	314.5	63.9	-7.2
DB13709	3	0	3	3.8	247.33	39.2	-7.2
DB13729	6	2	9	1.1	398.4	137	-7.2
DB13835	4	0	8	3.2	365.5	45.1	-7.2
DB13855	6	2	3	2.1	275.22	121	-7.2
DB13869	3	1	3	3	241.28	41.8	-7.2
DB13938	5	2	3	2.3	293.36	81.3	-7.2
DB13988	5	1	5	2.9	352.5	69.2	-7.2
DB14061	5	2	7	2.7	356.5	77.9	-7.2
DB14191	1	2	3	2.4	211.26	50.4	-7.2
DB14217	6	3	8	0.5	410.5	107	-7.2
DB14596	7	1	7	3.9	466.9	99.1	-7.2
DB14701	5	2	7	2.1	298.34	71.4	-7.2
DB14941	4	4	6	2.8	454	102	-7.2
DB15288	5	2	3	1.2	311.35	82.6	-7.2
DB15380	3	0	6	3.2	345.4	34.5	-7.2
DB15578	3	2	8	4.4	341.5	43.7	-7.2
DB00270	8	1	6	4.3	371.4	104	-7.1
DB00380	6	2	3	-1.4	268.27	98.8	-7.1
DB00414	4	2	4	2.4	324.4	101	-7.1
DB00449	6	2	9	2.9	351.4	84.9	-7.1
DB00472	5	1	6	4	309.33	21.3	-7.1
DB00514	2	0	1	3.4	271.4	12.5	-7.1
DB00633	1	1	2	3.1	200.28	28.7	-7.1
DB00665	7	1	1	2	317.22	95.2	-7.1
DB00695	7	3	5	2	330.74	131	-7.1
DB00744	3	2	2	1.6	236.29	94.8	-7.1
DB00745	3	1	5	1.7	273.4	79.4	-7.1
DB00841	4	4	7	3.4	301.4	72.7	-7.1
DB00887	7	3	8	2.8	364.4	127	-7.1
DB00918	4	1	6	1.6	335.5	64.8	-7.1
DB00942	2	1	5	3.9	287.4	23.5	-7.1
DB00961	2	1	2	1.9	246.35	32.3	-7.1
DB00963	4	2	4	3.3	334.16	80.4	-7.1
DB01024	6	2	6	3.2	320.3	93.1	-7.1
DB01048	6	3	4	0.9	286.33	102	-7.1
DB01195	10	2	7	3.8	414.34	59.6	-7.1
DB01214	5	2	8	2.7	309.4	67.8	-7.1
DB01224	5	1	6	2.1	383.5	73.6	-7.1
DB01418	6	1	4	2.5	353.3	109	-7.1
DB01428	3	1	3	3.6	228.24	46.5	-7.1
DB01441	2	1	6	3.1	274.4	28.3	-7.1
DB01482	4	1	6	1.4	341.4	70.5	-7.1
DB01515	5	1	4	-0.3	289.33	66.8	-7.1
DB01523	4	0	7	4.6	386.9	66.9	-7.1
DB01547	5	2	2	1.6	333.4	62.2	-7.1
DB01600	4	1	4	3.3	260.31	82.6	-7.1
DB01766	3	2	3	0.3	215.25	63.3	-7.1
DB02036	4	3	2	0.2	238.27	104	-7.1
DB02277	2	2	3	3.5	306.79	59	-7.1
DB02359	6	1	7	2.8	341.4	88.1	-7.1
DB02450	6	2	7	3	342.3	93.1	-7.1
DB02550	8	1	8	3.5	433.9	97.3	-7.1

DB02568	3	3	2	0.1	241.25	96.2	-7.1
DB02706	8	2	9	3.2	429.5	140	-7.1
DB02752	5	1	3	1.6	269.32	83.1	-7.1
DB02869	3	1	6	2.7	301.4	68.5	-7.1
DB02923	2	2	1	2.7	186.21	40.5	-7.1
DB02946	1	1	4	4.5	334.7	29.1	-7.1
DB03009	7	1	3	1.5	321.32	87.1	-7.1
DB03183	4	1	4	2.2	259.3	70.1	-7.1
DB03348	2	2	0	0.6	256.34	41.1	-7.1
DB03606	3	1	4	2.2	275.34	47.6	-7.1
DB03752	4	1	4	4	386.21	82.6	-7.1
DB04108	4	3	8	2.6	346.5	101	-7.1
DB04149	3	1	4	2.2	275.34	47.6	-7.1
DB04458	3	1	4	3	413.2	65.4	-7.1
DB04640	6	2	2	-0.6	288.3	126	-7.1
DB04676	7	3	9	-0.1	379.5	121	-7.1
DB04776	6	2	8	3.3	368.5	79.1	-7.1
DB04826	3	0	4	3.8	286.4	34.7	-7.1
DB04838	3	1	4	4.2	276.4	46.5	-7.1
DB04880	3	2	3	1.6	248.3	83.5	-7.1
DB04951	1	0	1	1.9	185.22	20.3	-7.1
DB04967	4	1	6	4	340.5	57.6	-7.1
DB05266	2	0	3	3	230.31	34.4	-7.1
DB05431	5	2	5	1.4	322.33	85	-7.1
DB05719	4	2	4	2.9	400.5	129	-7.1
DB05805	2	0	3	4.1	314.2	12.5	-7.1
DB05864	6	2	9	2.4	424.5	116	-7.1
DB05871	3	1	5	4.9	335.8	66.5	-7.1
DB06061	6	3	6	2.2	461.23	90.9	-7.1
DB06127	6	1	6	1.3	351.4	82.4	-7.1
DB06247	3	0	1	1.8	241.29	46.1	-7.1
DB06264	2	0	4	3.4	245.36	20.3	-7.1
DB06397	4	2	5	0.5	284.31	84	-7.1
DB06422	5	3	4	0.8	313.78	85.6	-7.1
DB06468	3	2	3	1.2	283.35	124	-7.1
DB06480	5	2	6	2	367.9	76.8	-7.1
DB06594	2	1	4	2.7	243.3	38.3	-7.1
DB06623	6	3	6	2.4	304.32	89.3	-7.1
DB06712	8	1	6	2.9	385.4	134	-7.1
DB06729	5	2	4	1.5	314.4	98.4	-7.1
DB06738	3	1	3	1	247.33	40.5	-7.1
DB06793	3	0	9	4.2	339.5	29.5	-7.1
DB06950	4	3	3	4.1	448.49	81.9	-7.1
DB06978	5	3	4	3.5	444.07	91.2	-7.1
DB07015	5	1	2	0.4	339.4	92.4	-7.1
DB07057	4	2	2	0.2	221.21	77.8	-7.1
DB07093	6	1	4	1.6	351.8	119	-7.1
DB07115	6	1	5	1.8	374.9	114	-7.1
DB07199	7	2	7	4.5	458.6	136	-7.1
DB07200	9	2	6	4.3	469.5	123	-7.1
DB07228	6	2	7	2.1	379.8	88.6	-7.1
DB07239	3	1	2	2.3	296.75	67.6	-7.1
DB07273	6	2	3	2.1	318.78	109	-7.1
DB07278	7	1	6	1.2	448	133	-7.1
DB07279	4	1	8	3.9	355.5	54.5	-7.1
DB07309	5	2	4	3.8	390.64	91.8	-7.1
DB07332	3	2	5	4.4	351.2	72.2	-7.1
DB07408	5	1	2	2.3	233.18	96.3	-7.1
DB07412	1	1	2	2.4	183.25	26	-7.1
DB07445	5	3	3	2.4	415.04	94.8	-7.1
DB07448	5	3	9	-0.4	361.4	101	-7.1
DB07477	2	1	3	3	212.24	37.3	-7.1
DB07481	4	4	4	-0.3	279.3	122	-7.1
DB07484	6	3	6	0.5	269.25	124	-7.1
DB07592	3	1	10	4.7	305.4	55.4	-7.1
DB07625	5	2	9	3.2	372.5	82.8	-7.1
DB07670	4	0	4	3.3	327.4	63.6	-7.1
DB07854	4	2	3	1.1	239.28	66.5	-7.1

DB07903	5	2	4	2.5	304.37	112	-7.1
DB07908	4	2	2	1.1	220.22	66.8	-7.1
DB07952	3	3	4	0.8	232.23	82.2	-7.1
DB08012	6	0	8	3.7	369.5	64.6	-7.1
DB08179	6	3	4	3.5	304.29	96.2	-7.1
DB08188	3	1	6	2.6	302.37	58.6	-7.1
DB08206	3	1	1	4.5	369.01	46.3	-7.1
DB08262	4	2	2	2.8	216.19	74.6	-7.1
DB08289	4	2	8	1.5	334.4	84.5	-7.1
DB08465	8	3	8	3.4	397.8	121	-7.1
DB08507	4	2	8	3.2	374.4	69.6	-7.1
DB08727	5	0	10	4.8	356.5	56.8	-7.1
DB08756	3	2	3	0.9	247.34	68	-7.1
DB08933	4	0	2	4	354.3	92.2	-7.1
DB08988	3	0	4	2.8	261.36	29.5	-7.1
DB09151	5	2	2	3.8	273.32	73.4	-7.1
DB09191	2	1	5	4.3	311.5	37.3	-7.1
DB09192	2	0	4	3.4	302.5	33.5	-7.1
DB09217	5	0	5	2.2	336.4	78	-7.1
DB09300	4	1	8	2.6	360.5	59.1	-7.1
DB11221	4	2	3	3.3	244.24	66.8	-7.1
DB11419	6	0	8	3.3	415.6	71.1	-7.1
DB11428	1	1	2	3.1	200.28	28.7	-7.1
DB11436	5	1	3	1.1	246.22	92.1	-7.1
DB11555	4	0	1	1.7	286.3	50.5	-7.1
DB11872	8	3	6	1.3	437.4	124	-7.1
DB11946	4	2	5	4	388.9	96.6	-7.1
DB12059	2	0	5	4.6	344.9	23.6	-7.1
DB12074	4	2	3	-0.7	248.23	78.9	-7.1
DB12177	5	1	7	4.1	328.4	45.1	-7.1
DB12184	6	0	6	1.8	359.5	69.6	-7.1
DB12244	4	1	0	2.4	227.22	12	-7.1
DB12392	5	2	6	0.9	349.4	100	-7.1
DB12400	5	2	2	0.5	270.29	101	-7.1
DB12476	6	2	7	1.9	361.5	111	-7.1
DB12585	6	2	8	2	373.4	86.5	-7.1
DB12596	6	2	7	2.6	334.4	77.4	-7.1
DB12657	6	0	5	1.2	353.4	64.8	-7.1
DB12710	4	0	4	4.1	339.5	35	-7.1
DB12715	6	2	6	2.1	292.34	75.6	-7.1
DB12884	6	2	9	2.2	359.5	75.4	-7.1
DB12926	5	2	6	0.3	357.4	90.7	-7.1
DB12986	8	1	3	0.8	354.4	108	-7.1
DB13048	5	2	8	3.3	392.5	68.2	-7.1
DB13051	10	1	3	-0.6	377.4	136	-7.1
DB13356	2	0	0	4.9	244.4	50.6	-7.1
DB13398	4	2	8	2.4	313.4	58.6	-7.1
DB13469	4	0	0	1.1	210.19	59.9	-7.1
DB13478	2	1	2	3.4	233.35	23.5	-7.1
DB13609	5	1	8	4.4	477.4	80	-7.1
DB13642	2	1	5	3.8	295.4	23.5	-7.1
DB13645	5	1	8	4.1	413.5	87.2	-7.1
DB13675	4	3	3	2.2	311.4	110	-7.1
DB13737	3	2	2	1.8	236.27	75.3	-7.1
DB13744	7	1	3	1.3	288.3	86.6	-7.1
DB13750	5	2	6	1.1	288.3	82.2	-7.1
DB13804	2	2	2	1.6	218.28	73.2	-7.1
DB14013	6	0	8	4.4	437.9	107	-7.1
DB14122	4	2	4	3.5	270.28	66.8	-7.1
DB14208	6	3	8	0.7	388.5	107	-7.1
DB14682	2	1	0	3.1	257.37	23.5	-7.1
DB14921	3	2	4	2.1	273.33	61.4	-7.1
DB15058	5	2	2	3.8	274.32	73.4	-7.1
DB15279	3	1	3	3.7	242.32	48.1	-7.1
DB15324	6	0	5	2.2	354.4	47.1	-7.1
DB15491	3	1	1	3	328.8	41.6	-7.1
DB15597	2	1	6	3.1	296.4	46.3	-7.1
DB00205	4	2	2	2.7	248.71	77.8	-7

DB00280	3	1	8	3.2	339.5	59.2	-7
DB00289	2	1	6	3.7	255.35	21.3	-7
DB00376	2	1	5	4.5	301.5	23.5	-7
DB00442	5	4	2	-1.3	277.28	126	-7
DB00458	2	0	4	4.8	280.4	6.5	-7
DB00469	7	2	2	1.1	337.4	136	-7
DB00499	6	1	2	3.3	276.21	74.9	-7
DB00598	4	4	8	3.1	328.4	95.6	-7
DB00708	4	0	8	4	386.6	61	-7
DB00724	3	1	2	2.6	240.3	56.7	-7
DB00739	6	2	3	-0.6	389.5	115	-7
DB00760	7	3	5	-2.4	383.5	136	-7
DB00925	2	0	8	4.4	303.8	12.5	-7
DB01023	5	1	6	3.9	384.2	64.6	-7
DB01084	4	0	5	2.2	302.4	33.5	-7
DB01129	6	1	8	1.9	359.4	96.3	-7
DB01247	4	2	4	1.5	231.25	67.2	-7
DB01265	5	3	2	-1.2	242.23	99.1	-7
DB01298	4	2	4	0.1	294.33	113	-7
DB01462	5	0	9	4.3	396.5	76.1	-7
DB01478	3	0	4	2.5	247.33	29.5	-7
DB01518	4	0	9	3.8	367.5	38.8	-7
DB01605	7	0	8	3.1	439.6	114	-7
DB02096	3	2	6	0.8	259.3	81.1	-7
DB02116	6	3	6	1.5	298.34	87.9	-7
DB02754	7	1	8	2.8	371.4	97.3	-7
DB02925	7	2	5	2.2	362.4	118	-7
DB03171	4	3	5	1	255.21	82.6	-7
DB03195	8	3	4	-1.4	324.2	125	-7
DB03242	7	5	3	-1	271.27	125	-7
DB03322	3	2	6	3	259.34	41.5	-7
DB03449	3	2	7	4.6	369.9	78.6	-7
DB03475	6	4	8	1.2	350.4	110	-7
DB03551	5	5	3	-1.9	267.3	98.4	-7
DB03609	6	4	2	-1.5	267.24	135	-7
DB03807	3	0	4	3.5	278.73	44.1	-7
DB03928	6	1	4	2.9	270.69	102	-7
DB03952	7	3	2	0.1	280.28	114	-7
DB04168	2	2	1	1.2	266.09	67.5	-7
DB04376	7	4	3	-0.3	406.5	124	-7
DB04604	7	4	2	-0.7	392.15	127	-7
DB04641	4	3	1	2.5	204.18	77.8	-7
DB04655	4	2	1	2.8	269.13	77.8	-7
DB04800	5	2	3	0.7	253.28	92.6	-7
DB04820	4	3	7	0.9	298.34	83.1	-7
DB04832	2	0	4	3.9	317.22	16.1	-7
DB05969	7	2	6	3	380.5	134	-7
DB06133	6	1	9	4.6	396.4	74.2	-7
DB06152	3	3	7	3.5	299.4	52.5	-7
DB06195	6	3	8	3.2	354.4	87.9	-7
DB06217	5	1	7	2.9	349.5	51.2	-7
DB06288	6	2	7	1.5	369.5	110	-7
DB06403	6	1	7	3.8	378.4	81.5	-7
DB06413	3	1	5	1.7	273.4	79.4	-7
DB06478	8	2	4	-0.4	348.35	128	-7
DB06481	6	2	4	3.7	308.25	73.1	-7
DB06742	3	3	3	2.6	378.1	58.3	-7
DB06807	4	2	3	3.2	229.23	72.6	-7
DB06859	4	2	10	3.2	394.9	103	-7
DB06911	3	2	6	2.4	351.9	75.4	-7
DB07013	7	3	8	1.8	465.6	133	-7
DB07086	3	2	2	1.7	260.33	49.7	-7
DB07164	5	1	3	1.4	252.27	68	-7
DB07184	4	1	6	3.2	326.5	83.9	-7
DB07197	7	3	5	3	373.18	132	-7
DB07234	5	2	5	2.3	293.32	71.1	-7
DB07276	6	1	7	2.3	341.4	93.5	-7
DB07304	7	1	3	3.7	272.18	59.7	-7

DB07327	3	2	5	4.5	440.1	72.2	-7
DB07449	3	3	7	-0.1	276.33	111	-7
DB07454	7	2	3	2.6	371.11	95.2	-7
DB07476	5	3	3	1.8	308.4	98.6	-7
DB07478	2	2	1	2.6	186.21	40.5	-7
DB07524	2	2	2	2.9	209.25	40.7	-7
DB07614	3	1	2	2.6	227.29	69.8	-7
DB07669	2	0	0	1.7	186.21	34.1	-7
DB07684	4	1	2	2.2	251.3	66	-7
DB07762	4	3	6	1.6	292.33	95.5	-7
DB07870	8	1	5	4.2	361.7	68.6	-7
DB07880	5	2	3	2.9	242.23	82.2	-7
DB07892	5	2	6	1.1	308.35	104	-7
DB08033	4	1	5	5	376.5	106	-7
DB08070	2	2	3	1.6	201.27	54.7	-7
DB08072	4	2	7	3.8	383.3	73.6	-7
DB08076	4	1	3	2.9	334.2	69.6	-7
DB08113	2	1	1	2.1	195.22	41.6	-7
DB08127	0	0	2	5	204.35	0	-7
DB08128	4	1	0	2.3	227.22	26	-7
DB08153	5	2	0	4.5	352.8	83.8	-7
DB08186	1	0	2	2.1	199.25	22	-7
DB08286	3	1	3	2.8	202.21	46.5	-7
DB08344	3	0	9	4.8	415	32.8	-7
DB08434	7	4	5	-1.1	315.22	133	-7
DB08442	3	2	2	3.6	297.3	60.8	-7
DB08469	6	0	4	3	335.4	96.7	-7
DB08540	7	0	5	3.2	388.5	112	-7
DB08544	5	1	6	4	309.33	21.3	-7
DB08573	6	2	4	2.6	317.8	120	-7
DB08645	7	3	2	0.6	380.7	135	-7
DB08661	5	2	3	-0.5	295.33	82.1	-7
DB08719	5	0	8	4	348.8	56.8	-7
DB08824	4	0	6	4	427.3	29.5	-7
DB08952	3	2	6	2.4	247.33	41.5	-7
DB08987	2	1	7	3.7	276.4	32.3	-7
DB09097	6	2	7	3.1	395.6	81.3	-7
DB09118	3	1	3	3.6	234.29	38.7	-7
DB09144	9	1	8	-0.5	370.31	138	-7
DB09184	6	2	5	1.3	339.4	60	-7
DB09229	8	1	7	2	388.4	128	-7
DB11479	3	3	2	0.5	261.32	64.6	-7
DB11537	7	5	6	1.7	411	136	-7
DB11609	2	0	7	3.5	295.4	20.3	-7
DB11967	7	3	6	3.1	441.2	88.4	-7
DB12145	3	0	0	1.6	216.28	25.4	-7
DB12187	8	3	7	0.8	344.37	128	-7
DB12300	2	0	1	3.8	199.2	45.8	-7
DB12614	3	1	5	2.9	283.39	71.6	-7
DB12912	4	2	2	1.5	284.34	106	-7
DB13032	5	0	8	4.6	357.5	38.2	-7
DB13082	2	1	3	1.4	246.3	49.4	-7
DB13087	6	2	7	0	340.37	115	-7
DB13279	6	0	10	3.1	361.4	65.1	-7
DB13361	1	0	5	4.5	253.4	3.2	-7
DB13369	3	1	7	3.6	354.5	46.5	-7
DB13421	5	3	3	-0.7	256.25	99.1	-7
DB13448	6	1	5	4.5	405.6	106	-7
DB13475	6	1	3	0.5	264.19	121	-7
DB13505	0	0	6	4.8	282.4	0	-7
DB13581	4	1	8	4.8	339.5	49.8	-7
DB13652	2	0	9	4.7	326.5	23.6	-7
DB13660	6	2	6	2.7	378.4	121	-7
DB13794	5	0	7	3.3	358.5	67.3	-7
DB13815	4	1	8	4.4	313.4	49.8	-7
DB13833	3	1	5	1.8	304.4	46.5	-7
DB13840	3	1	5	4.1	329.5	48.8	-7
DB14120	2	2	2	3.5	214.26	40.5	-7

DB14203	8	1	6	3.4	335.38	135	-7
DB14650	8	4	4	0.2	334.16	134	-7
DB14904	6	4	6	1.7	431.2	94.5	-7
DB14933	5	4	3	-1.8	255.25	117	-7
DB15412	5	1	2	-3	268.31	70.1	-7
DB15457	4	1	2	4.3	257.32	53.2	-7
DB15496	7	3	6	2.2	477.5	121	-7
DB00168	6	3	8	-2.7	294.3	119	-6.9
DB00193	3	1	4	2.6	263.37	32.7	-6.9
DB00208	2	0	2	3.6	263.8	31.5	-6.9
DB00213	9	1	7	2.4	383.4	106	-6.9
DB00215	4	0	5	3.2	324.4	36.3	-6.9
DB00235	3	1	1	0	211.22	65.8	-6.9
DB00250	4	2	2	1	248.3	94.6	-6.9
DB00333	2	0	7	3.9	309.4	20.3	-6.9
DB00392	3	0	5	4.8	312.5	31.8	-6.9
DB00401	7	1	7	3.3	388.4	110	-6.9
DB00500	3	1	4	2.8	257.28	59.3	-6.9
DB00600	2	1	3	3.4	200.23	29.5	-6.9
DB00849	3	1	2	1.8	246.26	66.5	-6.9
DB00953	3	1	5	1.7	269.34	49.7	-6.9
DB00981	4	1	2	0.7	275.35	44.8	-6.9
DB00983	5	4	8	1.8	344.4	90.8	-6.9
DB01021	7	3	2	0.6	380.7	135	-6.9
DB01062	4	1	8	4.2	357.5	49.8	-6.9
DB01088	4	3	8	2.8	360.5	77.8	-6.9
DB01175	4	0	5	3.2	324.4	36.3	-6.9
DB01273	3	1	0	0.8	211.26	37.8	-6.9
DB01274	5	4	8	1.8	344.4	90.8	-6.9
DB01295	5	2	10	3	345.4	60	-6.9
DB01359	3	2	7	4.2	291.4	41.5	-6.9
DB01409	6	1	5	2.3	392.5	116	-6.9
DB01438	5	2	2	1.9	213.24	89.6	-6.9
DB01476	4	1	1	2.8	377.2	41.6	-6.9
DB01502	2	0	8	4.2	324.5	23.6	-6.9
DB01549	1	0	2	3.3	229.36	3.2	-6.9
DB01850	4	2	9	1.4	331.4	72.6	-6.9
DB01931	4	2	1	2.5	258.05	66.4	-6.9
DB02495	4	3	6	0.8	299.33	91.5	-6.9
DB02740	2	2	4	2.3	203.24	53.1	-6.9
DB03150	7	3	4	-2.8	306.21	125	-6.9
DB03217	4	3	2	0.6	238.18	77.8	-6.9
DB03342	6	5	4	-3.3	238.24	131	-6.9
DB03372	2	2	2	1.6	260.36	37.1	-6.9
DB03504	7	1	8	3.4	405.9	97.3	-6.9
DB03679	4	4	3	-1.3	220.22	99.3	-6.9
DB03693	5	2	4	0.8	285.75	93.5	-6.9
DB03767	5	2	9	1.4	402.5	104	-6.9
DB03818	5	2	5	1.6	328.5	75.9	-6.9
DB03899	5	1	6	2.8	311.4	78.8	-6.9
DB03998	6	3	4	-2.4	275.26	116	-6.9
DB04001	5	4	3	1.3	248.19	120	-6.9
DB04060	7	4	4	-2.4	305.22	125	-6.9
DB04101	6	2	3	1	249.29	112	-6.9
DB04143	6	5	5	-1.2	287.21	123	-6.9
DB04469	4	0	5	2.9	274.31	53.4	-6.9
DB04485	5	3	2	-1.2	242.23	99.1	-6.9
DB04844	4	0	4	2.9	317.4	38.8	-6.9
DB04864	2	2	0	0	242.32	55.1	-6.9
DB04924	5	1	9	2.3	358.4	60	-6.9
DB05246	2	0	1	1.2	203.24	37.4	-6.9
DB05252	6	0	7	4.1	361.8	70.8	-6.9
DB05284	6	2	7	2.6	334.4	77.4	-6.9
DB05377	3	0	3	4.3	303.2	51.8	-6.9
DB06156	2	0	4	4.5	328.3	12.5	-6.9
DB06198	5	2	2	-0.3	244.22	78.9	-6.9
DB06200	2	0	4	3.9	288.5	6.5	-6.9
DB06682	6	0	9	4.1	347.4	90.6	-6.9

DB06905	4	2	9	1.4	331.4	72.6	-6.9
DB06914	3	0	5	2.3	292.76	36.3	-6.9
DB07104	3	2	7	1.9	284.35	64.4	-6.9
DB07277	7	1	7	1.2	450	133	-6.9
DB07345	2	2	3	3.4	219.32	46.2	-6.9
DB07383	6	3	4	1.3	277.24	69.6	-6.9
DB07480	8	4	6	0.5	360.19	134	-6.9
DB07496	1	2	2	3	212.25	41.1	-6.9
DB07569	4	2	7	2.7	349.5	96.4	-6.9
DB07585	4	2	1	1.5	274.71	73.5	-6.9
DB07635	3	2	2	2.7	214.22	57.5	-6.9
DB07731	6	4	2	0.9	218.22	126	-6.9
DB07805	4	1	3	3.4	279.68	29.1	-6.9
DB07899	3	2	8	1.6	324.8	75.3	-6.9
DB07919	2	1	1	3.6	212.25	37.9	-6.9
DB07951	5	4	6	0.3	290.27	120	-6.9
DB07953	3	3	5	2.1	274.31	82.2	-6.9
DB08084	6	2	6	3.8	416.2	90.6	-6.9
DB08115	3	1	5	2.1	229.27	52.3	-6.9
DB08203	6	2	5	0.6	301.34	101	-6.9
DB08210	7	2	4	2.6	349.4	139	-6.9
DB08238	4	2	1	-0.1	223.25	88.8	-6.9
DB08378	6	3	6	-0.2	292.29	107	-6.9
DB08511	3	3	0	0.1	215.21	96.2	-6.9
DB08662	3	1	3	4.6	373.2	46.5	-6.9
DB08669	6	4	10	1.3	393.5	131	-6.9
DB08690	4	0	7	4.6	318.4	52.6	-6.9
DB08760	3	1	5	4.1	276.71	46.5	-6.9
DB08777	3	1	0	1.9	206.27	69.7	-6.9
DB08781	5	2	2	0.4	312.17	80.1	-6.9
DB08799	2	1	5	2.6	265.35	27.6	-6.9
DB08941	4	3	7	2.8	301.4	61.7	-6.9
DB08955	2	2	4	2.8	221.29	49.3	-6.9
DB09007	2	0	6	4.1	303.8	12.5	-6.9
DB09190	2	1	5	3.5	295.4	21.3	-6.9
DB11378	6	1	9	4.6	361.4	73.9	-6.9
DB11396	2	2	2	4.3	269.12	40.5	-6.9
DB11461	6	2	3	1	284.72	106	-6.9
DB11770	4	4	8	2.6	363.5	82.6	-6.9
DB11919	5	2	3	1.2	241.25	89.7	-6.9
DB12045	7	1	8	1.3	399.4	121	-6.9
DB12179	6	4	9	2.5	362.4	99.4	-6.9
DB12216	4	0	1	2.3	216.19	48.7	-6.9
DB12278	4	0	8	4.7	367.5	38.8	-6.9
DB12606	6	4	3	-1.2	282.25	119	-6.9
DB12661	6	1	7	1.5	387.5	68.4	-6.9
DB12763	3	3	5	0.9	236.35	58.3	-6.9
DB12878	5	3	4	-1.6	242.66	106	-6.9
DB12882	7	3	9	2.2	402.4	112	-6.9
DB13056	5	2	1	2	256.09	90.7	-6.9
DB13258	5	0	9	4.1	427.3	84.6	-6.9
DB13273	5	1	7	1.6	354.5	84.1	-6.9
DB13284	5	1	1	0.5	275.3	111	-6.9
DB13342	7	0	9	1.8	392.4	77.5	-6.9
DB13367	6	0	10	3.8	395.9	65.1	-6.9
DB13420	2	0	3	4.8	299.5	28.5	-6.9
DB13430	7	3	4	2.4	381.9	135	-6.9
DB13512	5	1	7	3.2	399.2	95.6	-6.9
DB13515	2	0	7	3.9	309.4	20.3	-6.9
DB13548	4	4	8	3.2	470.2	118	-6.9
DB13550	2	1	8	4.1	269.4	21.3	-6.9
DB13558	6	0	8	3.5	405.9	57.2	-6.9
DB13629	2	1	4	2.7	232.28	49.4	-6.9
DB13668	2	1	0	2.1	188.27	38.9	-6.9
DB13670	2	1	2	1.5	216.28	35.8	-6.9
DB13677	7	1	7	-0.2	321.33	102	-6.9
DB13773	7	2	4	0.3	280.31	116	-6.9
DB13827	1	2	2	1.4	201.27	36.4	-6.9

DB13842	6	0	5	0	329.31	97.6	-6.9
DB14020	2	1	3	2.1	212.24	37.3	-6.9
DB14109	7	5	3	-0.7	272.25	120	-6.9
DB14195	2	2	4	4	226.32	24.1	-6.9
DB14763	1	2	1	1	251.71	80	-6.9
DB14835	5	1	6	2.3	373.5	54	-6.9
DB14973	6	2	6	1.7	323.42	99.4	-6.9
DB15012	4	0	1	1.5	231.25	59.2	-6.9
DB15230	10	0	7	4.8	401.4	97.6	-6.9
DB15235	4	1	4	4	353.5	85.5	-6.9
DB15293	5	1	4	3.8	319.4	115	-6.9
DB15338	6	2	5	1	366.4	129	-6.9
DB00178	6	2	10	1.4	416.5	95.9	-6.8
DB00332	3	1	6	2.6	332.5	46.5	-6.8
DB00343	6	0	7	3.1	414.5	84.4	-6.8
DB00387	2	1	5	4.1	287.4	23.5	-6.8
DB00441	6	3	2	-1.5	263.2	108	-6.8
DB00454	3	0	4	2.5	247.33	29.5	-6.8
DB00521	4	3	6	1	292.37	70.6	-6.8
DB00555	5	2	1	1.4	256.09	90.7	-6.8
DB00584	6	2	10	-0.1	376.4	95.9	-6.8
DB00631	8	3	2	0.9	303.68	119	-6.8
DB00647	3	0	9	4.2	339.5	29.5	-6.8
DB00662	6	1	10	2.3	388.5	69.3	-6.8
DB00698	6	1	2	-0.5	238.16	121	-6.8
DB00738	4	4	10	2.6	340.4	118	-6.8
DB00747	5	1	5	0.9	303.35	62.3	-6.8
DB00774	10	3	1	0.4	331.3	135	-6.8
DB00779	5	1	2	1.4	232.23	70.5	-6.8
DB00794	2	2	2	0.9	218.25	58.2	-6.8
DB00867	4	4	6	2.3	287.35	72.7	-6.8
DB00870	4	1	4	3.3	260.31	82.6	-6.8
DB00914	1	3	4	-0.8	205.26	103	-6.8
DB00960	3	3	6	1.8	248.32	57.3	-6.8
DB01015	6	2	3	0.9	253.28	107	-6.8
DB01065	2	2	4	0.8	232.28	54.1	-6.8
DB01096	5	3	4	2.2	279.33	90.1	-6.8
DB01102	5	5	8	0.9	317.4	93	-6.8
DB01168	3	3	5	0.1	221.3	53.2	-6.8
DB01403	4	0	5	4.7	328.5	41	-6.8
DB01439	3	0	6	4.5	356.5	51.8	-6.8
DB01461	4	0	9	3.6	327.4	38.8	-6.8
DB01611	4	2	9	3.6	335.9	48.4	-6.8
DB01629	7	4	2	-1.7	262.19	119	-6.8
DB01647	4	1	5	2.3	265.3	56.8	-6.8
DB01877	8	3	6	-0.3	345.37	126	-6.8
DB01950	6	2	4	2.6	308.32	137	-6.8
DB01994	5	2	3	1.4	229.24	75.3	-6.8
DB02029	1	2	2	3.6	344.19	41.1	-6.8
DB02365	2	0	0	1.8	180.2	25.8	-6.8
DB02395	5	2	5	-0.3	290.31	82.5	-6.8
DB02407	5	2	3	2.1	247.3	89.7	-6.8
DB02472	8	5	2	-1.8	270.24	127	-6.8
DB02565	4	3	9	1.7	307.39	81.7	-6.8
DB02796	6	5	2	-2	267.24	127	-6.8
DB02896	8	3	3	0.1	298.32	139	-6.8
DB02947	8	3	2	0.3	269.23	119	-6.8
DB03030	2	0	3	3.4	254.4	46.1	-6.8
DB03078	5	1	6	1.8	292.22	72.8	-6.8
DB03114	5	1	6	2.6	298.27	72.8	-6.8
DB03249	8	3	5	-1.6	350.26	135	-6.8
DB03307	7	3	3	0.3	265.29	132	-6.8
DB03314	4	3	3	-0.7	222.22	79.1	-6.8
DB04012	7	2	7	2.6	414.5	138	-6.8
DB04033	5	2	6	1.7	320.34	95.9	-6.8
DB04152	5	3	3	-2.6	238.24	102	-6.8
DB04275	2	3	3	0.5	218.25	65.1	-6.8
DB04307	2	2	2	1.5	187.24	32.3	-6.8

DB04672	7	3	4	-1.9	304.19	125	-6.8
DB04920	7	1	10	4.3	456.3	90.9	-6.8
DB05409	6	1	7	0.9	282.25	110	-6.8
DB05592	2	0	4	3.5	281.4	12.5	-6.8
DB05824	3	1	5	4.2	351.9	92.2	-6.8
DB06119	5	1	5	1.5	267.67	95.9	-6.8
DB06700	3	2	4	2.6	263.37	43.7	-6.8
DB06762	3	2	5	2.7	245.32	73.1	-6.8
DB06764	1	1	1	1.8	200.28	24.4	-6.8
DB06774	3	2	9	3.6	305.4	58.6	-6.8
DB06828	1	1	4	2.4	226.27	30	-6.8
DB06910	3	2	8	3.6	406.5	64	-6.8
DB06951	7	3	7	1.3	315.34	129	-6.8
DB06980	2	2	5	3.5	231.29	53.1	-6.8
DB07012	3	3	0	-0.3	201.18	96.2	-6.8
DB07038	3	2	3	3.8	219.28	49.3	-6.8
DB07130	6	2	5	3.4	357.18	112	-6.8
DB07308	3	1	3	3.4	236.65	50.4	-6.8
DB07314	6	2	4	1.3	333.73	109	-6.8
DB07380	5	1	4	2.4	259.22	46.2	-6.8
DB07428	3	0	4	3.2	229.27	31.4	-6.8
DB07437	4	2	6	0.4	276.29	78.9	-6.8
DB07452	4	4	0	-0.7	216.2	122	-6.8
DB07483	3	1	2	2.7	218.27	56.5	-6.8
DB07487	3	0	2	1.3	225.2	49.4	-6.8
DB07575	3	3	6	1.8	270.37	72.3	-6.8
DB07577	4	2	2	2.1	214.27	77.8	-6.8
DB07593	3	1	9	4.3	317.4	55.4	-6.8
DB07760	8	3	8	0.5	346.29	139	-6.8
DB07802	3	1	0	3.1	333.96	46.5	-6.8
DB07810	5	4	4	2.6	274.27	98	-6.8
DB07893	6	3	10	1.5	343.31	113	-6.8
DB07902	6	2	5	2.5	320.37	121	-6.8
DB07947	6	2	10	2.6	419.9	88.7	-6.8
DB08027	4	2	8	3	381.9	82.8	-6.8
DB08032	4	1	5	4.8	362.5	106	-6.8
DB08038	4	4	7	0.6	307.39	104	-6.8
DB08074	2	2	2	1.3	203.24	68.1	-6.8
DB08102	1	1	1	4.6	328	20.2	-6.8
DB08104	2	2	2	3.8	371.02	49.3	-6.8
DB08109	5	2	8	2.1	309.4	75.6	-6.8
DB08117	6	1	5	1	399.4	83.1	-6.8
DB08145	7	2	3	1.7	297.31	109	-6.8
DB08254	3	1	1	0.6	208.24	62.8	-6.8
DB08314	4	1	2	2.4	267.08	69.1	-6.8
DB08515	4	2	3	0.1	279.33	75.6	-6.8
DB08575	2	3	4	2.9	270.4	43.8	-6.8
DB08638	5	3	4	-1.4	236.22	97.7	-6.8
DB08640	6	2	8	1.1	313.37	109	-6.8
DB08744	4	0	1	1	233.22	48	-6.8
DB09207	5	2	5	2.1	399.3	56.8	-6.8
DB09253	4	2	4	1.8	208.26	56.5	-6.8
DB11071	3	1	3	3.8	214.22	46.5	-6.8
DB11160	2	0	6	3.9	255.35	12.5	-6.8
DB11217	7	5	3	-0.7	272.25	120	-6.8
DB11462	7	2	5	0.7	294.33	116	-6.8
DB11547	6	2	3	1	357.23	106	-6.8
DB11790	3	2	1	1.9	255.74	41.5	-6.8
DB11820	7	0	2	1.3	287.29	117	-6.8
DB11941	4	1	3	4.6	415.1	99.9	-6.8
DB12061	4	1	1	1.1	233.27	44.8	-6.8
DB12143	1	2	2	2.7	232.35	56.2	-6.8
DB12827	3	2	3	-1.3	218.25	68.2	-6.8
DB12906	7	3	8	1.7	389.4	112	-6.8
DB13216	4	0	6	2.9	245.32	42.2	-6.8
DB13228	5	0	1	1.1	239.27	56.6	-6.8
DB13305	1	0	5	3.8	253.4	3.2	-6.8
DB13320	6	2	3	0.3	264.31	106	-6.8

DB13380	4	1	7	3.3	327.4	49.8	-6.8
DB13431	4	2	10	2.4	334.4	86.7	-6.8
DB13523	7	2	8	0.8	383.5	119	-6.8
DB13614	2	1	2	2.2	230.33	52.6	-6.8
DB13762	6	1	8	1.9	359.4	96.3	-6.8
DB13845	3	0	10	4.2	325.4	29.5	-6.8
DB13875	2	1	1	2.1	214.26	37.4	-6.8
DB14176	3	1	4	3.6	228.24	46.5	-6.8
DB14200	4	1	3	4.4	264.4	78.5	-6.8
DB14638	4	0	5	3.4	328.1	59.8	-6.8
DB14755	2	0	0	2.5	234.38	6.5	-6.8
DB14930	5	2	2	-0.3	243.22	78.9	-6.8
DB15152	8	1	9	2.3	422.4	135	-6.8
DB15198	2	0	7	3.9	309.4	20.3	-6.8
DB00150	3	3	3	-1.1	204.22	79.1	-6.7
DB00219	3	1	9	4.1	348.5	46.5	-6.7
DB00232	7	2	2	1.4	360.2	126	-6.7
DB00249	5	3	2	-1	354.1	99.1	-6.7
DB00263	6	2	3	1	267.31	107	-6.7
DB00296	2	1	4	2.9	274.4	32.3	-6.7
DB00297	2	1	5	3.4	288.4	32.3	-6.7
DB00322	6	3	2	-1.2	246.19	99.1	-6.7
DB00391	6	2	6	0.6	341.4	110	-6.7
DB00505	1	1	8	4.8	318.5	20.2	-6.7
DB00527	4	1	10	4.4	343.5	54.5	-6.7
DB00574	4	1	4	3.4	231.26	12	-6.7
DB00608	3	1	8	4.6	319.9	28.2	-6.7
DB00660	3	1	3	2.2	221.25	47.6	-6.7
DB00676	2	0	4	4	212.24	26.3	-6.7
DB00750	2	2	5	2.1	220.31	41.1	-6.7
DB00754	2	1	2	1	204.22	49.4	-6.7
DB00899	6	0	9	1.9	376.4	76.2	-6.7
DB00900	5	2	2	-1.2	236.23	88.7	-6.7
DB00903	4	1	6	3.8	303.13	63.6	-6.7
DB00949	4	2	7	0.6	238.24	105	-6.7
DB01002	2	1	5	3.4	288.4	32.3	-6.7
DB01069	3	0	3	4.8	284.4	31.8	-6.7
DB01171	3	1	4	1.5	268.74	41.6	-6.7
DB01173	2	0	6	3.8	269.4	12.5	-6.7
DB01191	4	1	4	3.4	231.26	12	-6.7
DB01221	2	1	2	2.2	237.72	29.1	-6.7
DB01240	5	3	10	2.9	352.5	87	-6.7
DB01241	3	1	6	3.8	250.33	46.5	-6.7
DB01297	4	3	7	0.8	266.34	70.6	-6.7
DB01325	5	3	2	1.2	289.74	110	-6.7
DB01351	3	2	4	2.1	226.27	75.3	-6.7
DB01355	3	1	1	1.5	236.27	66.5	-6.7
DB01366	4	4	5	1.5	290.36	81.6	-6.7
DB01750	2	1	2	2.7	186.21	37.3	-6.7
DB01935	4	3	6	1.5	253.32	67.4	-6.7
DB02275	5	1	5	-0.9	261.28	92.8	-6.7
DB02380	6	3	2	-1.7	252.23	109	-6.7
DB02566	5	3	2	0.2	235.24	90.6	-6.7
DB02728	5	1	3	2	234.2	72.8	-6.7
DB02866	4	1	2	2	250.32	71.8	-6.7
DB02934	7	3	2	0.1	280.28	114	-6.7
DB02988	3	3	3	1.4	202.21	76.9	-6.7
DB03172	7	4	2	-1.3	266.25	127	-6.7
DB03259	2	2	1	4.1	255.09	40.5	-6.7
DB03273	8	3	2	-1.2	265.23	136	-6.7
DB03312	5	3	3	-0.4	333.13	99.1	-6.7
DB03332	5	3	0	-1.5	223.19	109	-6.7
DB03386	4	3	3	-1.1	222.22	79.1	-6.7
DB03528	8	4	2	-1.1	267.24	140	-6.7
DB03577	4	3	5	-1.2	277.25	69.6	-6.7
DB03667	4	2	3	1.4	195.17	75.6	-6.7
DB04055	6	2	7	4	406.8	105	-6.7
DB04316	4	4	4	-0.1	224.21	98.7	-6.7

DB04335	7	4	2	-1.3	268.23	129	-6.7
DB04537	2	3	3	0.6	203.24	84.9	-6.7
DB04831	5	1	5	4.1	331.2	91.8	-6.7
DB04846	5	3	10	1.9	379.5	90.9	-6.7
DB04883	8	1	9	3.6	410.4	100	-6.7
DB04947	4	0	5	3.9	429.3	29.5	-6.7
DB05047	5	0	1	0.3	233.22	68.5	-6.7
DB05680	4	1	2	2	255.32	69.6	-6.7
DB06201	5	1	3	0.7	238.19	73.8	-6.7
DB06236	4	2	2	-1.3	227.19	88.2	-6.7
DB06683	6	3	2	-0.9	260.22	99.1	-6.7
DB06981	2	2	4	3.2	217.26	53.1	-6.7
DB07073	5	0	1	2.3	266.36	70.7	-6.7
DB07407	4	1	3	2.5	218.2	59.7	-6.7
DB07461	4	1	3	0.7	273.33	66.6	-6.7
DB07620	9	0	2	4.9	459.2	134	-6.7
DB07627	4	4	6	2.1	351.5	115	-6.7
DB07632	6	1	2	1.6	275.7	123	-6.7
DB07803	3	2	2	1.6	188.18	66	-6.7
DB07807	4	3	5	1.2	265.35	63.9	-6.7
DB08013	6	0	9	4	383.5	64.6	-6.7
DB08028	3	1	4	3.9	279.79	62	-6.7
DB08083	6	2	2	1	280.3	138	-6.7
DB08134	6	2	3	1.2	284.72	106	-6.7
DB08139	4	1	2	2.1	235.67	66	-6.7
DB08182	5	2	4	1.8	269.3	89.7	-6.7
DB08287	5	2	7	1.3	354.5	107	-6.7
DB08297	7	3	2	-0.7	285.25	125	-6.7
DB08335	4	1	5	3.1	235.28	58.9	-6.7
DB08347	5	2	6	1.1	287.36	77.6	-6.7
DB08372	5	2	6	2.5	334.4	91.2	-6.7
DB08618	6	1	5	-1.1	339.32	76.1	-6.7
DB08677	4	1	4	2.1	261.34	83.1	-6.7
DB08790	3	1	2	1.4	188.18	55.1	-6.7
DB08983	6	0	9	3.5	363.8	74.7	-6.7
DB09023	4	1	9	2.6	327.4	49.8	-6.7
DB09064	3	1	4	3.4	289.15	46.5	-6.7
DB09071	2	1	4	2.2	245.32	38.3	-6.7
DB09187	3	1	0	1.8	292.76	41.6	-6.7
DB09234	8	1	7	4.2	371.4	104	-6.7
DB09237	7	2	10	3	408.9	99.9	-6.7
DB09245	3	1	2	1.2	207.23	49.8	-6.7
DB09254	3	1	2	0.2	206.2	72.6	-6.7
DB11102	4	3	4	-0.2	223.22	86.6	-6.7
DB11541	4	4	7	2.7	301.4	72.7	-6.7
DB11823	2	1	2	2.2	237.72	29.1	-6.7
DB12028	5	3	2	-0.3	307.1	99.1	-6.7
DB12190	6	2	3	0.3	285.32	112	-6.7
DB13181	3	1	9	3.4	342.5	46.5	-6.7
DB13296	4	4	8	1.8	312.37	118	-6.7
DB13316	5	1	9	2.9	307.4	64.6	-6.7
DB13353	2	1	9	4.8	362.9	28.4	-6.7
DB13414	3	2	4	2	214.26	45.2	-6.7
DB13459	3	1	7	2.9	291.4	57.6	-6.7
DB13485	7	2	4	0.6	294.33	116	-6.7
DB13541	5	3	2	0.8	264.28	112	-6.7
DB13683	3	0	7	4.2	272.4	25.4	-6.7
DB13822	4	1	5	3.4	329.5	58	-6.7
DB13871	5	2	5	1.4	281.33	103	-6.7
DB13918	4	0	6	4.8	341.2	44.8	-6.7
DB14030	4	3	8	3.1	361.5	92.8	-6.7
DB14544	6	2	7	3.8	446.6	101	-6.7
DB14913	6	3	2	-0.9	259.22	99.1	-6.7
DB15144	4	0	5	3.9	425.3	29.5	-6.7
DB15203	6	2	3	0.8	278.71	99	-6.7
DB00176	7	1	9	2.6	318.33	56.8	-6.6
DB00196	7	1	5	0.4	306.27	81.6	-6.6
DB00242	7	3	2	0.8	285.69	119	-6.6

DB00268	2	1	7	2.7	260.37	32.3	-6.6
DB00285	3	1	5	2.9	277.4	32.7	-6.6
DB00335	4	3	8	0.2	266.34	84.6	-6.6
DB00350	3	3	1	1.2	209.25	88.9	-6.6
DB00366	3	0	6	2.5	270.37	25.4	-6.6
DB00420	3	0	4	4.5	284.4	31.8	-6.6
DB00424	4	1	5	1.8	289.4	49.8	-6.6
DB00553	4	0	1	1.9	216.19	48.7	-6.6
DB00649	4	2	2	-0.8	224.21	78.9	-6.6
DB00758	4	0	4	3.8	321.8	57.8	-6.6
DB00880	6	2	1	-0.2	295.7	135	-6.6
DB00935	2	2	3	2.9	260.37	44.6	-6.6
DB01101	7	3	7	0.6	359.35	121	-6.6
DB01163	5	1	3	2.1	325.4	98.5	-6.6
DB01190	7	4	7	2.2	425	128	-6.6
DB01193	5	3	10	1.7	336.4	87.7	-6.6
DB01246	3	0	4	4.7	298.4	31.8	-6.6
DB01498	2	1	7	3.7	311.5	23.5	-6.6
DB01539	2	0	1	2.5	192.3	27	-6.6
DB01581	6	2	3	0.1	264.31	106	-6.6
DB01603	7	2	5	1.2	380.4	131	-6.6
DB02066	8	5	3	-1.6	281.27	136	-6.6
DB02103	6	2	2	0.8	269.69	99.1	-6.6
DB02286	4	4	3	-1.9	219.24	105	-6.6
DB02391	6	5	6	-2.9	268.27	138	-6.6
DB02416	9	5	2	-1.1	395.15	137	-6.6
DB02710	6	5	4	0	241.2	127	-6.6
DB02758	2	2	3	1.8	189.21	53.1	-6.6
DB02762	6	2	2	0.6	250.15	103	-6.6
DB02852	7	4	7	-1.3	311.33	124	-6.6
DB02959	4	4	3	-1.2	220.22	99.3	-6.6
DB03216	6	3	1	-0.9	233.23	110	-6.6
DB03225	3	3	3	-1.1	204.22	79.1	-6.6
DB03294	6	1	1	0.6	244.3	114	-6.6
DB03424	5	4	8	-1	308.37	113	-6.6
DB03468	4	2	1	-0.1	212.27	80.6	-6.6
DB03588	2	1	3	3.1	212.24	37.3	-6.6
DB03735	6	2	2	0.2	250.25	93.3	-6.6
DB04007	3	2	6	1.6	370.24	98.5	-6.6
DB04061	3	2	2	-1.1	191.23	63.3	-6.6
DB04159	4	4	3	-2.4	220.22	99.3	-6.6
DB04198	7	5	2	-2.5	268.23	140	-6.6
DB04393	2	1	2	4.4	255.09	29.5	-6.6
DB04400	6	5	2	-2.2	239.23	132	-6.6
DB04441	9	4	2	-0.6	285.23	140	-6.6
DB04565	5	4	4	-0.9	252.22	116	-6.6
DB04905	2	0	8	4.6	283.4	12.5	-6.6
DB05644	7	4	10	1.5	372.42	133	-6.6
DB05964	1	1	1	3	228.11	12	-6.6
DB06106	5	2	7	-1.4	332.36	109	-6.6
DB06479	4	0	7	0.7	306.36	75.5	-6.6
DB06512	2	0	5	4.4	301.5	12.5	-6.6
DB06694	1	1	3	3.2	244.37	24.4	-6.6
DB06727	2	0	0	2.5	234.38	6.5	-6.6
DB06890	2	1	4	2.6	240.3	42	-6.6
DB06917	3	0	2	1.8	201.2	30	-6.6
DB06928	6	3	8	0.4	426.14	112	-6.6
DB06987	4	3	8	0.2	266.34	84.6	-6.6
DB07051	2	1	4	3.6	245.3	46	-6.6
DB07060	3	3	3	1.5	205.21	73.3	-6.6
DB07176	4	2	1	1.4	223.25	88.8	-6.6
DB07223	6	2	10	1.2	397.5	117	-6.6
DB07258	4	1	6	2.2	298.4	45.6	-6.6
DB07281	3	2	3	2	199.25	50.9	-6.6
DB07342	2	0	3	2.4	244.33	34.9	-6.6
DB07376	4	1	2	2.2	251.3	66	-6.6
DB07388	4	1	4	2.4	263.34	54.5	-6.6
DB07427	3	0	4	2.7	229.27	31.4	-6.6

DB07542	4	3	8	1.1	285.42	83.6	-6.6
DB07553	5	1	8	3.9	301.3	46.2	-6.6
DB07723	3	2	4	1.7	219.24	62.3	-6.6
DB07749	3	2	8	2.1	304.4	75.3	-6.6
DB07818	2	2	2	2.2	232.06	49.3	-6.6
DB07824	5	2	2	0.5	194.19	86.8	-6.6
DB07860	1	1	3	3.3	231.72	26	-6.6
DB07884	4	1	3	2.5	280.29	58.6	-6.6
DB07888	5	1	4	1.3	235.24	68.1	-6.6
DB08007	4	4	5	-0.2	238.24	98.7	-6.6
DB08090	6	4	7	0.9	316.39	106	-6.6
DB08103	2	1	2	4.5	344	29.5	-6.6
DB08110	5	2	4	0.6	265.3	75.6	-6.6
DB08132	4	2	1	1.5	223.25	88.8	-6.6
DB08178	5	2	2	0.9	241.25	89.7	-6.6
DB08190	2	2	4	2.3	358.17	54.1	-6.6
DB08263	5	3	5	1.1	237.21	104	-6.6
DB08295	6	2	7	2.4	309.29	135	-6.6
DB08319	4	2	2	2.4	234.27	76.7	-6.6
DB08326	5	1	1	2.4	250.3	116	-6.6
DB08626	4	3	6	1.3	253.32	67.4	-6.6
DB08633	6	1	7	2.5	401.9	89.7	-6.6
DB08648	6	3	2	-0.6	228.2	104	-6.6
DB08689	4	0	4	2.8	250.29	52.6	-6.6
DB08695	3	1	1	1.7	189.17	74.5	-6.6
DB08717	4	2	2	0.5	234.25	69.6	-6.6
DB08726	5	0	10	4.4	342.4	56.8	-6.6
DB08778	5	2	4	4.1	275.37	96.2	-6.6
DB08802	4	0	3	3.5	285.4	44.7	-6.6
DB09010	4	2	5	2.6	257.26	78.5	-6.6
DB09120	3	2	9	3.6	305.4	58.6	-6.6
DB09174	4	0	8	4.2	408.5	49.8	-6.6
DB09186	3	1	7	3.1	271.35	30.5	-6.6
DB11421	4	1	2	1.2	255.66	86.3	-6.6
DB11556	1	1	2	2.7	186.25	28.7	-6.6
DB11570	3	0	9	5	277.4	29.5	-6.6
DB11719	6	1	6	2.4	352.5	106	-6.6
DB11989	4	1	4	0.9	260.25	92.7	-6.6
DB11998	6	4	3	-0.9	349.13	119	-6.6
DB12017	3	1	4	1.6	228.25	55.1	-6.6
DB12209	3	1	4	1.4	230.27	58.1	-6.6
DB12353	5	4	3	-1.1	264.28	101	-6.6
DB12497	2	0	3	3.4	232.32	49.2	-6.6
DB12531	5	4	3	-1.9	265.27	126	-6.6
DB12577	8	2	8	2.4	396.3	104	-6.6
DB12608	3	2	6	2.8	263.37	55.5	-6.6
DB12664	2	2	3	0.8	190.24	55.1	-6.6
DB12894	5	2	2	0.2	264.64	78.9	-6.6
DB12958	4	0	4	3.4	285.4	44.7	-6.6
DB13021	3	0	4	3.3	356.16	44.1	-6.6
DB13153	1	1	4	3.8	222.37	20.2	-6.6
DB13211	3	2	2	0.2	207.23	82.9	-6.6
DB13219	3	0	6	3.8	257.33	21.7	-6.6
DB13341	3	1	2	1.6	204.22	50.7	-6.6
DB13377	3	2	3	1.7	224.26	75.3	-6.6
DB13405	7	1	5	0.9	382.9	124	-6.6
DB13410	3	3	3	1.6	247.08	83	-6.6
DB13419	5	2	5	1.2	294.35	68.8	-6.6
DB13524	2	0	2	1.7	230.31	23.6	-6.6
DB13530	3	3	6	2.3	262.35	57.3	-6.6
DB13538	5	0	6	2.9	286.28	61.8	-6.6
DB13623	5	1	6	2.3	307.4	75.7	-6.6
DB13641	3	2	1	2	234.28	108	-6.6
DB13795	6	2	4	2	339.19	96.3	-6.6
DB13819	4	0	8	3.8	287.4	42.2	-6.6
DB13948	5	2	7	3.1	312.4	74.5	-6.6
DB15492	4	2	6	3.1	340.8	61.8	-6.6
DB15596	4	0	5	4.8	327.5	35	-6.6

DB00273	9	1	3	-0.8	339.36	124	-6.5
DB00440	7	2	5	0.9	290.32	106	-6.5
DB00494	6	2	4	2.1	305.29	130	-6.5
DB00576	7	2	3	0.5	270.3	135	-6.5
DB00645	3	0	8	3.6	289.4	29.5	-6.5
DB00806	4	0	5	0.3	278.31	75.5	-6.5
DB00809	3	1	6	1.5	284.35	53.4	-6.5
DB00832	2	0	1	0.8	189.21	37.4	-6.5
DB00919	9	5	2	-3.1	332.35	130	-6.5
DB01011	3	0	3	2	226.27	42.8	-6.5
DB01032	5	1	7	3.2	285.36	83.1	-6.5
DB01075	2	0	6	3.3	255.35	12.5	-6.5
DB01087	4	2	6	2.2	259.35	60.2	-6.5
DB01114	2	0	5	3.4	274.79	16.1	-6.5
DB01156	2	1	4	3.2	239.74	29.1	-6.5
DB01227	3	0	9	4.3	353.5	29.5	-6.5
DB01408	6	2	8	1.2	367.4	91.3	-6.5
DB01433	3	0	9	4.3	353.5	29.5	-6.5
DB01460	1	1	5	3.3	216.32	19	-6.5
DB01487	3	2	9	2.7	293.4	58.6	-6.5
DB01522	3	0	9	4.3	353.5	29.5	-6.5
DB01582	6	2	3	0.3	278.33	106	-6.5
DB01754	3	2	0	0.7	191.18	60.8	-6.5
DB01878	1	0	2	3.4	182.22	17.1	-6.5
DB02071	7	2	7	2.2	427.5	117	-6.5
DB02352	3	1	3	2	200.24	48.1	-6.5
DB02414	3	2	2	-0.4	234.25	78.1	-6.5
DB02521	5	3	0	0.7	206.15	94.8	-6.5
DB02529	6	3	5	-3.1	301.34	122	-6.5
DB02546	3	3	8	1.9	264.32	78.4	-6.5
DB02617	2	1	8	4.7	341.3	38	-6.5
DB02898	6	3	3	-0.5	248.24	119	-6.5
DB03065	4	2	1	0.8	205.17	114	-6.5
DB03185	5	4	2	-1.1	242.23	102	-6.5
DB03240	5	3	3	-3.7	239.23	113	-6.5
DB03257	6	2	7	0.8	301.34	102	-6.5
DB03299	5	3	6	0.3	251.23	104	-6.5
DB03562	5	3	2	-1.5	230.22	99.1	-6.5
DB03717	7	5	2	-3.1	263.24	129	-6.5
DB03763	5	3	2	-1.6	242.23	99.1	-6.5
DB03886	6	4	2	-2.4	237.22	134	-6.5
DB03992	4	3	4	-2.4	208.21	92.4	-6.5
DB04169	4	4	0	-0.4	192.17	110	-6.5
DB04206	4	3	3	-2	205.21	92	-6.5
DB04213	1	2	5	3.5	260.37	41.1	-6.5
DB04218	7	4	2	-0.7	266.25	127	-6.5
DB04273	1	1	0	2.9	216.1	12	-6.5
DB04505	7	1	8	3.1	415.9	97.3	-6.5
DB04546	7	4	2	-1.6	266.25	127	-6.5
DB04837	2	1	5	3.6	289.8	23.5	-6.5
DB05565	3	1	2	3	271.14	36.4	-6.5
DB05650	4	1	3	1.9	241.33	49.8	-6.5
DB05938	4	1	7	2.6	291.4	60.9	-6.5
DB06218	3	2	6	0.3	250.29	67.4	-6.5
DB06258	5	1	6	2.2	297.78	58	-6.5
DB06433	5	3	2	-2.3	257.22	108	-6.5
DB06477	2	2	1	0.4	203.24	44.4	-6.5
DB06619	8	3	2	-1.2	252.23	134	-6.5
DB06656	5	4	3	-2.7	267.24	129	-6.5
DB06691	4	0	7	3.3	285.4	28.6	-6.5
DB06701	3	1	4	0.2	233.31	38.3	-6.5
DB06769	4	1	9	2.9	358.3	58.4	-6.5
DB06821	7	2	4	0.4	280.31	116	-6.5
DB06939	4	1	4	1.2	327.4	66.6	-6.5
DB06952	4	2	1	1.8	192.17	66.8	-6.5
DB07110	2	1	2	2.6	193.26	12	-6.5
DB07170	7	2	2	-0.1	253.23	99.1	-6.5
DB07285	7	1	6	1.5	294.27	89	-6.5

DB07298	7	2	4	1.6	253.23	125	-6.5
DB07306	4	1	4	2.6	283.75	54.5	-6.5
DB07317	3	1	2	1.9	189.21	38.3	-6.5
DB07366	5	3	9	1	334.4	96.7	-6.5
DB07367	6	4	7	0.9	316.39	106	-6.5
DB07370	6	4	7	1.4	332.5	122	-6.5
DB07374	5	2	5	0.9	265.3	67.8	-6.5
DB07377	5	4	7	1.6	359.9	130	-6.5
DB07398	3	2	3	2.2	264.35	100	-6.5
DB07463	6	3	7	1.2	335.5	116	-6.5
DB07506	4	2	5	1.2	208.21	74.6	-6.5
DB07526	6	3	10	2.7	409.6	87.9	-6.5
DB07572	6	2	8	1.8	349.4	106	-6.5
DB07590	5	2	7	-0.1	322.2	106	-6.5
DB07636	5	1	6	3.8	279.36	95.5	-6.5
DB07745	9	3	6	0.6	329.17	105	-6.5
DB07823	4	1	2	0.9	237.25	74.7	-6.5
DB08065	2	2	1	1.5	184.2	57.4	-6.5
DB08260	5	4	7	1.3	332.5	117	-6.5
DB08271	6	2	7	1.2	316.38	104	-6.5
DB08273	6	2	7	3	435.19	135	-6.5
DB08310	5	1	7	2.6	359.4	86.9	-6.5
DB08329	6	1	2	0.1	290.4	114	-6.5
DB08443	4	3	1	1.7	219.19	82.7	-6.5
DB08447	2	2	5	1.8	232.32	39.3	-6.5
DB08480	2	1	2	2.2	187.24	32.6	-6.5
DB08533	4	1	3	1.8	239.28	55.1	-6.5
DB08603	5	2	9	1.8	332.8	97.6	-6.5
DB08606	6	3	4	0	293.39	116	-6.5
DB08617	6	2	4	-0.1	247.21	63.3	-6.5
DB08627	3	1	2	3	224.32	62.6	-6.5
DB08636	4	2	3	1.4	192.17	74.6	-6.5
DB08673	6	2	4	2.6	297.4	122	-6.5
DB08720	5	0	8	3.3	314.4	56.8	-6.5
DB08771	4	1	2	3.4	252.31	66.8	-6.5
DB08798	6	2	3	1.3	267.31	107	-6.5
DB08800	3	0	6	3.9	289.8	19.4	-6.5
DB09013	5	2	7	2	291.34	71.7	-6.5
DB09202	2	1	4	1.8	216.28	33.6	-6.5
DB09343	3	3	2	-0.7	242.66	85.3	-6.5
DB11447	2	1	0	4.2	199.27	37.3	-6.5
DB11448	6	0	5	2.8	317.3	113	-6.5
DB11504	3	0	8	3.9	289.4	29.5	-6.5
DB11889	2	1	3	1.7	198.26	38.9	-6.5
DB11932	2	0	1	2.2	184.24	25.8	-6.5
DB11937	4	2	1	1.3	189.17	66.4	-6.5
DB12125	3	0	0	1.4	216.28	25.4	-6.5
DB12156	7	3	2	-1.2	251.24	119	-6.5
DB12314	1	3	4	3	288.17	88.8	-6.5
DB12377	7	3	4	-3.6	348.38	137	-6.5
DB12383	4	3	2	-0.9	261.66	108	-6.5
DB12928	5	1	6	2.1	275.27	38.3	-6.5
DB12957	5	3	2	-1.5	245.21	108	-6.5
DB13030	6	3	2	-1	246.19	99.1	-6.5
DB13096	2	0	2	4.4	263.4	31.5	-6.5
DB13160	3	0	4	2.8	261.36	29.5	-6.5
DB13214	4	2	3	0.5	254.31	97.6	-6.5
DB13297	2	2	4	0.9	191.23	49.3	-6.5
DB13306	2	1	0	3.5	228.07	33.1	-6.5
DB13350	6	1	2	-0.8	246.23	115	-6.5
DB13413	3	1	6	2.1	288.4	49.4	-6.5
DB13436	4	1	8	1.6	305.4	49.8	-6.5
DB13438	1	0	5	4	217.35	3.2	-6.5
DB13460	6	0	10	3.9	377.8	74.7	-6.5
DB13462	1	0	6	3.4	256.36	9.2	-6.5
DB13559	4	4	2	0.8	223.27	72.7	-6.5
DB13625	4	5	5	-0.1	267.32	108	-6.5
DB13730	7	1	3	-0.3	268.18	132	-6.5

DB13736	2	1	4	2	218.29	33.6	-6.5
DB13754	4	1	4	1.4	223.22	56.8	-6.5
DB13775	4	2	6	2.7	295.4	66.8	-6.5
DB13776	4	3	2	-0.7	353.11	108	-6.5
DB13990	2	2	7	3.8	260.37	39.3	-6.5
DB14126	8	3	5	-1.6	287.21	136	-6.5
DB14213	6	3	7	0.2	340.4	107	-6.5
DB15580	3	0	5	3.7	292.76	44.1	-6.5
DB00194	8	4	2	-1.1	267.24	140	-6.4
DB00221	4	4	5	1.7	239.31	72.7	-6.4
DB00292	3	0	5	3	244.29	44.1	-6.4
DB00359	6	2	3	-0.1	250.28	106	-6.4
DB00381	7	2	10	3	408.9	99.9	-6.4
DB00405	2	0	5	3.5	319.24	16.1	-6.4
DB00418	3	2	5	2	238.28	75.3	-6.4
DB00422	3	1	4	0.2	233.31	38.3	-6.4
DB00484	3	2	2	0.6	292.13	62.2	-6.4
DB00489	5	3	6	0.2	272.37	86.8	-6.4
DB00518	4	2	5	2.9	265.33	92.3	-6.4
DB00532	2	1	2	1.5	218.25	49.4	-6.4
DB00640	8	4	2	-1.1	267.24	140	-6.4
DB00664	7	2	4	0.7	280.31	116	-6.4
DB00669	4	2	6	0.9	295.4	73.6	-6.4
DB00790	6	2	9	0.9	368.5	95.9	-6.4
DB00792	3	0	6	3.3	255.36	19.4	-6.4
DB00835	2	0	5	3.5	319.24	16.1	-6.4
DB00848	2	0	1	1.8	204.29	40.9	-6.4
DB00891	5	2	3	0	249.29	93.5	-6.4
DB01037	1	0	4	2.8	187.28	3.2	-6.4
DB01073	9	4	2	-0.6	285.23	140	-6.4
DB01115	7	1	5	2.2	346.3	110	-6.4
DB01119	3	1	0	1.2	230.67	66.9	-6.4
DB01203	5	4	6	0.7	309.4	82	-6.4
DB01353	3	2	4	1.7	212.25	75.3	-6.4
DB01424	3	0	2	1	231.29	26.8	-6.4
DB01620	2	0	5	2.8	240.34	16.1	-6.4
DB01980	3	3	3	0.8	306.1	75.4	-6.4
DB02070	5	3	4	-2.2	208.21	106	-6.4
DB02185	5	2	1	0	211.17	79.2	-6.4
DB02256	5	3	2	-1.6	228.2	99.1	-6.4
DB02441	3	3	3	1	208.22	86.9	-6.4
DB02462	4	0	5	2.5	263.36	54.8	-6.4
DB02514	5	0	2	1	225.11	83.5	-6.4
DB02552	7	3	8	0.7	314.21	113	-6.4
DB02608	5	3	4	0	254.24	115	-6.4
DB02642	5	3	5	-0.3	245.17	95.9	-6.4
DB02745	6	4	2	-2	244.2	119	-6.4
DB02781	6	0	7	1.8	331.4	115	-6.4
DB02829	4	4	4	-3.4	252.25	123	-6.4
DB02883	6	3	4	-3.3	291.2	135	-6.4
DB02933	7	3	3	-0.6	296.35	132	-6.4
DB03012	6	3	5	0.2	244.27	118	-6.4
DB04058	3	3	4	0	208.21	92.4	-6.4
DB04085	5	0	4	-1.2	236.18	84	-6.4
DB04292	4	3	6	-1.4	236.31	75.4	-6.4
DB04331	4	3	4	1.6	292.36	103	-6.4
DB04385	6	4	2	-2.1	242.23	116	-6.4
DB04433	3	3	4	-1.4	253.3	114	-6.4
DB04459	2	0	0	3.1	215.03	26.3	-6.4
DB04478	8	1	4	-1.2	301.37	98.2	-6.4
DB04523	3	1	5	2.9	241.33	55.4	-6.4
DB04597	8	4	1	-2.4	250.2	126	-6.4
DB04599	3	0	2	1.6	219.24	46.6	-6.4
DB04646	2	0	1	3.3	321.99	34.1	-6.4
DB04910	4	2	5	2.4	249.27	76.2	-6.4
DB04970	5	0	6	1.8	320.82	50.1	-6.4
DB05025	5	1	6	1.2	313.78	70.5	-6.4
DB06112	3	1	4	3	221.29	30.5	-6.4

DB06150	8	2	5	1.6	310.33	125	-6.4
DB06231	5	2	2	-1.8	256.32	78	-6.4
DB06485	4	2	2	-0.5	338.1	82.4	-6.4
DB06842	3	2	7	2.4	278.35	59.6	-6.4
DB06864	6	3	5	-0.3	240.21	112	-6.4
DB06872	5	2	6	0.8	294.33	104	-6.4
DB06984	5	4	4	0	269.36	98	-6.4
DB07004	3	3	4	2.8	279.36	98.4	-6.4
DB07037	6	3	4	1	262.26	117	-6.4
DB07224	7	3	10	0.6	344.36	134	-6.4
DB07517	5	2	6	1.9	240.25	87.7	-6.4
DB07574	7	2	6	1.3	431.5	84.1	-6.4
DB07720	2	1	1	2.2	208.69	24.9	-6.4
DB07781	6	2	6	1.9	345.4	115	-6.4
DB07797	5	2	6	2.8	354.8	91.2	-6.4
DB07906	4	2	1	0.5	208.21	78.1	-6.4
DB07911	4	0	4	2.1	217.2	74.3	-6.4
DB07921	6	1	4	0.3	246.22	101	-6.4
DB07979	5	4	3	0.6	195.17	102	-6.4
DB07997	5	2	5	0.6	265.33	79.5	-6.4
DB08129	5	2	2	1	205.18	46.2	-6.4
DB08155	4	2	4	0.1	242.3	97.6	-6.4
DB08165	3	1	1	1.3	197.26	68.5	-6.4
DB08228	3	0	2	1.4	233.26	38.8	-6.4
DB08318	7	2	5	1.2	247.25	109	-6.4
DB08321	5	5	9	0.8	287.39	93	-6.4
DB08476	2	2	0	2.6	212.33	55.1	-6.4
DB08508	3	2	3	1.2	193.2	66.4	-6.4
DB08570	6	3	2	0.6	240.17	132	-6.4
DB08593	4	1	1	0.4	212.23	74.9	-6.4
DB08599	6	2	5	0.9	259.28	101	-6.4
DB08651	8	4	4	-1.4	338.28	126	-6.4
DB08718	3	2	2	3.4	220.29	68.7	-6.4
DB08766	5	2	5	2.6	325.5	83.9	-6.4
DB08776	4	2	1	0.5	225.27	99.3	-6.4
DB08803	2	1	4	2.4	232.32	33.6	-6.4
DB08982	5	0	4	1.6	284.38	75.2	-6.4
DB08991	5	0	3	1.3	234.25	62.1	-6.4
DB09089	6	0	10	4	387.5	57.2	-6.4
DB09213	2	1	4	3.5	206.28	37.3	-6.4
DB09252	2	2	4	2.2	206.28	41.1	-6.4
DB11285	4	1	5	2.2	222.24	55.8	-6.4
DB11327	4	0	3	0.6	252.3	102	-6.4
DB11336	5	2	3	1	215.21	79.6	-6.4
DB11472	5	1	5	3.2	266.32	88.7	-6.4
DB11667	5	3	2	-2.4	252.23	132	-6.4
DB12248	2	2	4	2.3	227.73	32.3	-6.4
DB12328	4	0	0	0.6	196.2	52.6	-6.4
DB12472	4	2	4	0.6	236.31	46.9	-6.4
DB12518	4	2	5	2.9	347.2	61.8	-6.4
DB12551	3	1	1	0.7	204.22	42.8	-6.4
DB12650	1	0	4	2.8	188.29	3.2	-6.4
DB12749	2	0	3	2.8	190.24	26.3	-6.4
DB12803	2	2	4	3.3	248.15	32.3	-6.4
DB12829	3	3	3	1	236.3	112	-6.4
DB12883	4	1	1	1.1	220.27	33.7	-6.4
DB12947	6	3	1	-1.7	246.19	99.1	-6.4
DB13222	2	1	0	3.1	238.08	33.1	-6.4
DB13362	2	2	3	1.8	206.24	72.2	-6.4
DB13488	2	0	7	4.6	289.5	12.5	-6.4
DB13619	3	0	9	4.7	329.5	29.5	-6.4
DB13679	2	0	5	3.4	274.79	16.1	-6.4
DB13770	3	2	4	2	224.26	75.3	-6.4
DB13801	2	1	2	2	272.13	58.7	-6.4
DB13850	3	0	3	3.3	320.5	65.7	-6.4
DB14130	4	0	7	3.2	250.29	52.6	-6.4
DB14136	4	1	6	3.7	258.35	55.8	-6.4
DB14808	7	1	9	1.5	433.9	121	-6.4

DB15167	5	1	10	3.3	364.5	50.8	-6.4
DB15447	2	1	6	4	206.28	37.3	-6.4
DB00152	5	2	4	1	265.36	104	-6.3
DB00187	5	2	10	1.9	295.37	67.8	-6.3
DB00264	4	2	9	1.9	267.36	50.7	-6.3
DB00281	2	1	5	2.3	234.34	32.3	-6.3
DB00306	3	2	4	1.4	224.26	75.3	-6.3
DB00311	6	1	3	2	258.3	119	-6.3
DB00312	3	2	4	2.1	226.27	75.3	-6.3
DB00409	4	1	6	2.1	371.27	50.8	-6.3
DB00426	8	1	9	0	321.33	122	-6.3
DB00473	3	1	6	3.6	261.36	38.3	-6.3
DB00740	7	1	1	3.6	234.2	76.4	-6.3
DB00748	3	0	6	2.9	290.79	25.4	-6.3
DB00865	1	0	5	4.1	239.35	3.2	-6.3
DB00999	7	3	1	-0.1	297.7	135	-6.3
DB01001	4	4	5	0.3	239.31	72.7	-6.3
DB01050	2	1	4	3.5	206.28	37.3	-6.3
DB01124	3	2	5	2.3	270.35	83.6	-6.3
DB01235	5	4	3	-2.7	197.19	104	-6.3
DB01255	3	3	8	1.2	263.38	81.1	-6.3
DB01299	8	2	5	0.7	310.33	125	-6.3
DB01352	3	2	3	1.1	210.23	75.3	-6.3
DB01427	3	2	1	-0.2	187.2	68	-6.3
DB01435	2	0	1	0.4	188.23	23.6	-6.3
DB01464	5	0	9	2.7	361.5	48	-6.3
DB01499	3	0	5	3.2	275.4	29.5	-6.3
DB01533	3	0	6	4.9	291.5	59.7	-6.3
DB01542	3	0	6	3	287.4	29.5	-6.3
DB01546	1	2	3	2.5	188.27	41.8	-6.3
DB01566	3	1	4	2.5	207.27	30.5	-6.3
DB01579	2	0	1	1.9	191.27	12.5	-6.3
DB01606	7	1	3	-2	300.29	131	-6.3
DB01662	3	2	3	2	180.2	57.5	-6.3
DB01973	6	2	6	-2.4	259.28	115	-6.3
DB01997	3	1	0	2.4	242.03	74.5	-6.3
DB02099	0	1	4	3.7	208.36	4.4	-6.3
DB02278	4	4	0	-1.3	209.21	109	-6.3
DB02350	7	2	4	1.3	374.5	130	-6.3
DB02594	4	3	2	-1.8	227.22	108	-6.3
DB02599	4	3	4	0.8	264.35	119	-6.3
DB02600	5	3	6	-1.9	284.35	102	-6.3
DB02652	4	4	4	-0.1	224.21	98.7	-6.3
DB02662	5	3	3	1.8	335.05	104	-6.3
DB02816	7	2	3	-0.6	266.28	122	-6.3
DB02855	6	4	10	0.4	385.5	136	-6.3
DB02950	3	2	1	-0.6	324.13	109	-6.3
DB03090	4	2	7	0.2	304.4	69.6	-6.3
DB03112	4	3	6	0.9	258.34	104	-6.3
DB03196	7	3	3	0.2	271.22	125	-6.3
DB03302	2	0	0	3.2	271.9	26.3	-6.3
DB03716	8	3	2	-0.6	269.23	119	-6.3
DB03881	5	5	3	-1.5	297.36	140	-6.3
DB03985	0	1	4	3.7	208.36	4.4	-6.3
DB04236	2	3	3	0.9	190.24	62	-6.3
DB04440	7	3	2	-0.7	252.23	114	-6.3
DB04631	5	2	8	2.8	366.2	84.9	-6.3
DB04634	4	2	5	1.4	223.22	75.6	-6.3
DB04779	6	1	6	2.3	275.3	110	-6.3
DB06421	3	2	6	1.7	269.77	58.4	-6.3
DB06851	2	1	3	2.3	184.24	24.9	-6.3
DB06943	5	1	4	2.2	311.4	56	-6.3
DB07069	4	3	3	0	195.17	86.6	-6.3
DB07109	4	2	3	1.5	194.18	66.8	-6.3
DB07293	3	1	5	1.2	207.23	55.4	-6.3
DB07573	4	1	5	1.7	265.35	41.9	-6.3
DB07581	4	3	7	0.8	271.4	83.6	-6.3
DB07734	3	2	6	2.2	292.4	33.3	-6.3

DB07738	5	2	8	2.8	431.4	51.8	-6.3
DB07819	2	2	2	1.8	197.62	49.3	-6.3
DB07885	5	1	5	2.7	340.5	108	-6.3
DB07922	5	1	4	0.2	228.23	101	-6.3
DB07960	6	3	5	0.2	273.28	105	-6.3
DB08016	6	4	6	-0.9	298.62	125	-6.3
DB08060	4	2	4	1.1	193.2	80.4	-6.3
DB08062	3	1	2	3.1	225.7	66.9	-6.3
DB08069	3	2	1	1.3	206.27	85.7	-6.3
DB08085	1	0	7	4.9	216.32	17.1	-6.3
DB08135	2	2	2	1.4	187.2	57.8	-6.3
DB08161	3	2	6	2.9	224.32	38.3	-6.3
DB08201	3	1	8	4.7	304.4	46.5	-6.3
DB08259	3	3	8	2.3	270.37	78.4	-6.3
DB08316	4	1	0	1.4	191.23	68.9	-6.3
DB08336	5	1	5	1.7	241.29	77.2	-6.3
DB08359	4	1	3	3.2	232.3	70.2	-6.3
DB08427	6	3	4	-0.5	226.18	112	-6.3
DB08479	3	3	4	-0.2	237.26	121	-6.3
DB08751	7	0	4	0.6	293.28	108	-6.3
DB08806	5	1	10	2.1	348.4	67.9	-6.3
DB09019	2	1	3	4.3	376.13	29.3	-6.3
DB09088	3	0	6	3	235.32	29.5	-6.3
DB09092	6	2	6	-1.8	311.34	102	-6.3
DB09096	4	0	5	3.5	242.23	52.6	-6.3
DB09205	4	0	7	3.2	279.37	38.8	-6.3
DB09224	3	0	5	3.3	263.35	20.3	-6.3
DB09247	3	2	5	2	242.7	50.4	-6.3
DB09273	5	0	2	-0.9	266.25	76.9	-6.3
DB09284	4	1	6	2.5	260.33	51.9	-6.3
DB11207	3	0	7	3.9	248.32	35.5	-6.3
DB11323	4	2	4	1.9	182.17	66.8	-6.3
DB11324	3	2	10	4.2	293.4	58.6	-6.3
DB11413	5	2	6	0.8	358.2	91.8	-6.3
DB11449	4	1	6	2.5	246.26	63.6	-6.3
DB12337	3	1	2	1.4	233.67	71.6	-6.3
DB12470	4	2	2	-1.3	227.19	88.2	-6.3
DB12527	3	1	1	2	244.12	28.2	-6.3
DB12968	5	3	3	0.2	265.69	102	-6.3
DB13069	6	2	4	0.4	272.69	114	-6.3
DB13139	4	4	5	0.3	239.31	72.7	-6.3
DB13233	3	1	5	2.8	255.74	52.3	-6.3
DB13288	3	1	6	2.1	280.41	41.6	-6.3
DB13289	3	0	3	0.5	251.3	49.6	-6.3
DB13328	2	2	6	2.1	254.75	41.1	-6.3
DB13634	3	0	5	2.3	264.32	58.4	-6.3
DB13662	2	1	3	1.7	181.23	52.3	-6.3
DB13724	7	0	4	0.7	285.28	126	-6.3
DB13726	4	3	2	-0.7	214.25	133	-6.3
DB13757	6	4	10	0.9	369.4	115	-6.3
DB13785	4	2	4	0.6	236.31	46.9	-6.3
DB13858	5	0	7	4	293.4	56.8	-6.3
DB13921	5	4	2	-2.4	257.24	129	-6.3
DB15027	6	3	5	1.1	262.35	79.1	-6.3
DB15427	6	3	2	-0.9	372.09	99.1	-6.3
DB00121	4	3	5	0.3	244.31	104	-6.2
DB00198	5	2	8	1.1	312.4	90.6	-6.2
DB00226	3	2	2	0	213.28	82.9	-6.2
DB00237	3	2	3	1.6	212.25	75.3	-6.2
DB00291	3	1	9	1.7	304.2	40.5	-6.2
DB00336	5	2	2	0.2	198.14	126	-6.2
DB00446	5	3	5	1.1	323.13	115	-6.2
DB00552	6	4	2	-2.1	268.27	112	-6.2
DB00614	6	0	2	-0.1	225.16	101	-6.2
DB00629	2	2	2	1.7	231.08	76.8	-6.2
DB00697	4	2	2	1.1	253.71	90.4	-6.2
DB00786	5	5	8	0.5	331.41	128	-6.2
DB00807	5	1	10	2.7	294.39	64.8	-6.2

DB00879	5	2	2	-0.6	247.25	113	-6.2
DB00909	5	1	2	0.2	212.23	94.6	-6.2
DB01035	3	2	6	0.9	235.33	58.4	-6.2
DB01133	7	4	4	-0.3	318.61	140	-6.2
DB01154	3	2	5	3.2	254.35	90.3	-6.2
DB01178	3	0	1	1.1	273.74	62.8	-6.2
DB01237	2	0	6	4	334.2	12.5	-6.2
DB01243	2	1	0	3.5	214.04	33.1	-6.2
DB01423	6	2	6	1.7	273.3	137	-6.2
DB01445	2	2	3	1.2	204.27	39.3	-6.2
DB01473	3	0	4	2.8	261.36	29.5	-6.2
DB01505	5	1	9	1.6	321.4	59	-6.2
DB01552	3	0	5	3.2	275.4	29.5	-6.2
DB01555	3	0	9	4.3	353.5	29.5	-6.2
DB02097	5	4	2	-2.1	243.22	129	-6.2
DB02126	4	2	3	1.3	192.17	74.6	-6.2
DB02251	5	2	5	0.2	222.19	91.7	-6.2
DB02384	4	1	2	2.5	181.14	60.4	-6.2
DB03052	4	1	5	1.4	232.23	64.4	-6.2
DB03125	6	3	5	1.1	291.33	107	-6.2
DB03354	8	4	2	-2.3	246.22	129	-6.2
DB03359	5	2	2	1.3	250.24	40.5	-6.2
DB03384	3	3	3	2	238.28	45.9	-6.2
DB03628	4	3	3	0.4	229.17	86.6	-6.2
DB04004	5	3	5	-0.1	293.39	122	-6.2
DB04129	5	2	4	-1.7	199.16	102	-6.2
DB04394	6	1	2	-0.3	285.28	135	-6.2
DB04411	4	4	5	-0.3	293.14	95.6	-6.2
DB04650	4	3	5	0.3	244.31	104	-6.2
DB04657	3	1	2	2.1	235.3	63.6	-6.2
DB04677	1	0	5	3.3	189.3	3.2	-6.2
DB04896	2	1	5	1.4	246.35	46.3	-6.2
DB06904	7	1	9	1.4	299.34	107	-6.2
DB06915	5	5	0	1.9	208.17	101	-6.2
DB06924	4	1	4	1.5	209.2	83.1	-6.2
DB06941	4	1	3	1.8	277.39	89.3	-6.2
DB07617	3	1	3	1	188.23	38.1	-6.2
DB07668	4	2	5	2.7	249.3	60.8	-6.2
DB07737	5	3	6	1.2	311.4	45.4	-6.2
DB07990	3	0	6	2.8	262.67	35.5	-6.2
DB08050	3	1	5	2.3	192.21	54.4	-6.2
DB08175	3	1	9	4.4	268.39	46.5	-6.2
DB08200	3	1	8	4.7	304.4	46.5	-6.2
DB08258	3	3	7	1.7	256.34	78.4	-6.2
DB08296	7	1	7	0.7	302.2	133	-6.2
DB08370	4	2	5	-0.5	290.18	88.6	-6.2
DB08620	4	0	2	2.2	252.72	77.6	-6.2
DB08621	5	3	6	-0.3	356.2	112	-6.2
DB08644	7	2	10	1.6	433.6	130	-6.2
DB08681	4	1	6	4.2	315.8	79.6	-6.2
DB08740	3	3	6	2.9	243.39	66.5	-6.2
DB08814	7	1	3	2.1	248.15	63.6	-6.2
DB08872	6	2	9	3.1	329.39	102	-6.2
DB09028	2	1	0	0.2	190.24	32.3	-6.2
DB09185	4	1	5	1.5	237.29	39.7	-6.2
DB09242	4	2	3	0.6	241.68	71.4	-6.2
DB09288	4	1	7	1.1	264.32	58.6	-6.2
DB09324	5	1	1	-1	233.24	100	-6.2
DB11282	1	0	3	2	191.27	20.3	-6.2
DB11291	2	0	8	4.5	234.33	26.3	-6.2
DB11664	5	3	5	-1.6	284.25	85.8	-6.2
DB11825	3	2	4	-1.7	209.28	63.3	-6.2
DB12162	2	1	6	4.4	236.35	29.5	-6.2
DB12265	5	0	4	2.5	279.32	98.2	-6.2
DB12406	4	1	5	0.7	280.32	78.7	-6.2
DB13009	3	2	2	1.5	191.19	67	-6.2
DB13202	5	0	5	2	298.4	75.2	-6.2
DB13278	3	2	5	1.8	223.27	58.6	-6.2

DB13294	7	3	6	1	295.25	130	-6.2
DB13311	6	1	10	2.1	310.34	82.1	-6.2
DB13406	4	3	5	1	271.34	110	-6.2
DB13443	4	3	8	0.2	266.34	84.6	-6.2
DB13449	4	1	2	-0.8	238.24	78.7	-6.2
DB13495	6	0	6	2	275.19	90.6	-6.2
DB13497	2	0	10	4.4	306.5	26.3	-6.2
DB13508	3	2	6	3.1	292.2	41.5	-6.2
DB13561	1	1	5	4.3	259.77	12	-6.2
DB13603	2	1	1	4.4	257.07	61.6	-6.2
DB13722	3	1	3	2.9	251.71	40.5	-6.2
DB13781	6	4	8	-0.6	339.39	103	-6.2
DB13787	3	0	5	3.1	273.37	29.5	-6.2
DB13849	4	0	8	2.8	327.8	55.8	-6.2
DB13853	4	0	2	2.8	240.4	91.9	-6.2
DB13868	8	3	5	-2	273.19	136	-6.2
DB14202	5	0	2	2.7	252.4	78.9	-6.2
DB14680	5	1	6	3.7	316.3	57.2	-6.2
DB14754	3	2	5	0.6	194.23	78.3	-6.2
DB14940	4	3	2	-1.4	186.17	95.5	-6.2
DB15122	4	1	5	0.7	285.35	78.7	-6.2
DB15467	4	0	8	2.4	250.29	44.8	-6.2
DB15610	5	2	2	2	220.15	64.4	-6.2
DB00190	6	5	4	-2.2	226.23	116	-6.1
DB00345	4	3	3	-0.9	194.19	92.4	-6.1
DB00474	3	1	3	2.3	262.3	66.5	-6.1
DB00599	3	2	4	2.9	242.34	90.3	-6.1
DB00634	4	2	2	-1	214.24	97.6	-6.1
DB00730	3	1	1	2.5	201.25	69.8	-6.1
DB00866	3	2	8	3.1	249.35	41.5	-6.1
DB00937	2	0	5	2.8	205.3	20.3	-6.1
DB00979	4	1	7	2.7	291.4	49.8	-6.1
DB00989	3	0	5	2.3	250.34	32.8	-6.1
DB01018	1	2	2	2	246.09	81.5	-6.1
DB01400	2	0	3	1.5	223.29	29.5	-6.1
DB01407	3	3	4	2.2	277.19	58.3	-6.1
DB01488	1	1	3	2.5	188.27	19	-6.1
DB01520	2	0	2	3.9	249.4	31.5	-6.1
DB01800	7	2	2	0.4	235.17	138	-6.1
DB01880	4	3	2	1.2	180.16	77.8	-6.1
DB02018	4	3	4	0.2	206.2	104	-6.1
DB02215	4	2	5	1.9	225.24	79.5	-6.1
DB02488	6	3	6	-1.1	271.31	116	-6.1
DB02509	1	1	7	4.8	222.37	20.2	-6.1
DB02562	5	4	2	-2.6	239.23	133	-6.1
DB02622	5	3	3	1.8	209.16	104	-6.1
DB02674	4	3	4	0	230.29	104	-6.1
DB02991	5	1	3	3.2	248.27	63.7	-6.1
DB03019	4	1	1	3.1	246.12	80	-6.1
DB03068	5	3	2	-1.5	228.2	103	-6.1
DB03132	6	1	5	1.6	289.4	129	-6.1
DB03629	7	3	4	-2.1	263.14	130	-6.1
DB03697	5	3	6	-0.4	271.34	124	-6.1
DB03833	5	1	3	1.6	223.27	68	-6.1
DB03867	6	3	3	-2	226.19	129	-6.1
DB04126	5	4	10	1.2	316.39	116	-6.1
DB04436	5	3	3	-2.3	199.18	83.6	-6.1
DB04472	5	1	3	1.8	208.17	80.4	-6.1
DB04638	2	2	2	4.6	222.32	40.5	-6.1
DB04702	7	3	8	1.1	330.28	129	-6.1
DB04719	2	1	1	4.7	476.79	31.9	-6.1
DB04795	6	0	3	2.2	222.19	62.4	-6.1
DB04815	2	1	0	3.5	305.5	33.1	-6.1
DB04961	4	2	2	-1.7	213.19	97.4	-6.1
DB05885	4	1	4	0.3	232.23	63.4	-6.1
DB06147	6	2	3	0.1	255.3	122	-6.1
DB06965	3	1	10	4	298.36	46.5	-6.1
DB07291	3	1	1	2.4	218.64	67.6	-6.1

DB07292	4	2	1	2.2	192.24	87.4	-6.1
DB07356	3	2	3	0.4	249.35	72.4	-6.1
DB07492	5	3	5	1.4	412.03	115	-6.1
DB07529	4	2	1	1.3	205.24	91.1	-6.1
DB07718	4	2	3	0.9	180.16	74.6	-6.1
DB07733	7	2	3	2.4	309.3	76.2	-6.1
DB07736	5	3	5	0.9	297.39	45.4	-6.1
DB07894	6	3	5	1	278.24	103	-6.1
DB07957	7	2	6	0.6	267.23	116	-6.1
DB07992	4	2	2	1.3	213.21	87.8	-6.1
DB08156	5	2	4	0.1	229.26	106	-6.1
DB08202	6	3	3	0.1	329.4	131	-6.1
DB08255	6	3	3	-0.3	247.31	108	-6.1
DB08283	5	4	9	2.1	289.41	84.2	-6.1
DB08419	3	0	6	2.8	262.67	35.5	-6.1
DB08768	4	2	3	2.1	203.24	66.5	-6.1
DB08924	1	0	3	4.1	264.6	12.4	-6.1
DB08944	2	0	5	3.6	244.37	27	-6.1
DB09069	5	1	5	1	266.34	43	-6.1
DB09282	7	0	4	2.2	242.23	87	-6.1
DB09345	4	0	9	3.1	293.4	30.9	-6.1
DB11235	5	0	7	2.6	286.37	41.5	-6.1
DB11477	2	1	2	2.8	220.34	49.7	-6.1
DB11480	5	1	1	0.7	225.16	135	-6.1
DB12484	6	5	2	-2.4	248.23	123	-6.1
DB12847	4	3	8	0.9	265.31	91.3	-6.1
DB12901	5	3	2	-0.7	371.1	108	-6.1
DB13025	5	1	8	0.9	328.4	84.1	-6.1
DB13314	3	2	5	3.2	219.28	49.3	-6.1
DB13346	3	2	6	1.9	223.27	58.6	-6.1
DB13357	3	2	4	0.4	181.19	72.6	-6.1
DB13429	1	2	2	1.8	209.67	36.4	-6.1
DB13452	7	3	8	1	283.33	99.6	-6.1
DB13474	3	0	2	3.7	275.4	59.7	-6.1
DB13510	5	0	8	2.3	307.4	48	-6.1
DB13536	2	1	0	3.2	302.95	33.1	-6.1
DB13573	5	1	2	-0.8	238.2	95.7	-6.1
DB13578	4	2	7	2	236.31	64.4	-6.1
DB13592	4	0	5	0.3	279.34	61.7	-6.1
DB13758	4	0	7	2.5	257.71	38.8	-6.1
DB14010	2	1	4	1.5	218.29	28.3	-6.1
DB14074	3	1	5	2	229.32	49.8	-6.1
DB15445	3	2	3	1	305.07	66.4	-6.1
DB15589	3	1	4	0.8	194.23	46.5	-6.1
DB00181	3	2	4	-1	213.66	63.3	-6
DB00241	3	2	4	1.7	224.26	75.3	-6
DB00265	1	0	3	2.8	203.28	20.3	-6
DB00299	5	4	5	-1.9	253.26	126	-6
DB00501	4	3	7	0.4	252.34	114	-6
DB00651	5	2	3	-1.8	254.24	98.9	-6
DB00672	3	2	4	2.3	276.74	83.6	-6
DB00709	4	2	2	-0.9	229.26	113	-6
DB00853	5	1	1	-1.1	194.15	106	-6
DB00943	3	2	2	-1.3	211.22	88.2	-6
DB00987	5	4	2	-2.1	243.22	129	-6
DB01085	3	0	3	1.1	208.26	44.1	-6
DB01131	1	3	4	1.5	253.73	88.8	-6
DB01161	4	1	7	2.9	270.75	55.6	-6
DB01262	4	3	2	-1.2	228.21	121	-6
DB01422	4	1	0	2.1	190.16	78.9	-6
DB01442	4	1	3	1.6	209.24	53.7	-6
DB01454	3	1	3	2.2	193.24	30.5	-6
DB01468	3	0	5	4.5	277.5	59.7	-6
DB01483	3	2	2	0.6	184.19	75.3	-6
DB01651	8	3	2	-1.8	264.23	115	-6
DB01758	4	3	3	-1.1	307.08	83.6	-6
DB02119	5	4	1	-2	195.18	112	-6
DB02211	1	0	2	2.3	185.26	3.2	-6

DB02272	5	4	6	-3.5	226.23	116	-6
DB02908	6	4	3	-1.8	252.1	115	-6
DB02960	3	3	6	-2.6	209.26	77	-6
DB03152	6	4	9	1.4	292.37	99.4	-6
DB03223	5	4	8	-3.5	298.36	135	-6
DB03228	6	1	6	1.8	351.14	138	-6
DB03274	4	2	1	-1.1	212.2	78.9	-6
DB03455	5	4	6	-0.7	266.32	75.4	-6
DB03479	7	5	1	-3.2	262.22	140	-6
DB03525	5	3	2	0.6	229.17	78.8	-6
DB03631	6	4	3	-4	210.19	125	-6
DB03649	8	5	4	-1.6	302.54	140	-6
DB03663	6	2	4	0.5	235.24	107	-6
DB04002	6	4	8	0.5	317.4	111	-6
DB04080	4	2	3	1.2	180.16	74.6	-6
DB04123	3	2	4	0.5	339.07	66.4	-6
DB04256	5	3	3	-2	209.2	101	-6
DB04324	5	2	4	0.7	298.37	79.3	-6
DB04386	8	4	1	-2.4	250.2	126	-6
DB04449	5	4	3	0.4	212.2	90.2	-6
DB04531	4	2	5	-1.2	211.28	88.6	-6
DB04871	1	1	0	2.7	195.69	12	-6
DB06204	2	1	5	3.5	221.34	23.5	-6
DB06716	5	2	6	2.5	288.28	76	-6
DB06861	3	3	7	0.4	226.32	87.2	-6
DB06966	3	1	10	4	298.36	46.5	-6
DB07378	6	4	9	1.1	291.38	105	-6
DB07440	3	1	2	2.3	214.28	62.8	-6
DB07711	5	2	7	2.6	277.37	89.8	-6
DB07721	5	0	7	2.2	260.22	54	-6
DB07767	4	2	3	1.5	194.18	66.8	-6
DB07915	4	2	4	1.8	218.2	74.6	-6
DB07924	6	4	9	1.4	292.37	99.4	-6
DB07925	6	3	6	0.1	278.26	114	-6
DB07954	3	1	2	1.5	222.24	69.3	-6
DB08082	2	2	3	0.7	199.63	49.3	-6
DB08288	4	2	6	2.5	243.34	72.6	-6
DB08451	4	1	2	1.6	222.27	67.4	-6
DB08523	3	2	5	1.5	181.23	43.7	-6
DB08578	4	2	4	0.5	273.08	79.3	-6
DB08587	5	2	4	1.5	224.21	76	-6
DB08632	6	3	3	0.5	210.14	112	-6
DB08678	4	2	3	1.2	201.25	74.8	-6
DB08765	6	2	1	1.1	230.3	130	-6
DB08891	3	2	4	-1	213.66	63.3	-6
DB09004	2	1	5	3.2	255.78	23.5	-6
DB09011	1	1	4	1.7	197.66	29.1	-6
DB11156	2	0	2	1.6	206.31	43.8	-6
DB11337	5	3	4	0.7	219.24	86.7	-6
DB11438	5	1	5	2.4	270.71	74	-6
DB11642	2	0	8	4.3	295.8	12.5	-6
DB11762	4	2	4	1.8	313.77	75.6	-6
DB12032	3	2	3	-1.3	185.26	63.3	-6
DB12110	2	1	1	1.7	196.67	15.3	-6
DB12365	7	3	3	-1.6	260.18	107	-6
DB12418	3	1	0	0.9	183.19	71.6	-6
DB12450	5	3	4	1.8	212.2	87	-6
DB12485	5	1	4	1	254.29	87.1	-6
DB12809	4	1	2	0.8	193.2	61.6	-6
DB12945	6	4	2	0.2	190.21	102	-6
DB13206	3	3	6	0.6	209.28	52.5	-6
DB13313	5	3	4	-0.2	211.21	92.8	-6
DB13632	7	2	8	4.2	288.32	121	-6
DB13663	6	2	2	-0.7	270.7	137	-6
DB13848	6	4	3	-2	214.18	104	-6
DB13877	3	1	1	1.3	292.03	88.9	-6
DB14174	4	0	3	3.4	320.6	121	-6
DB14175	1	0	6	4.2	202.29	17.1	-6

DB14656	4	2	6	1.2	245.66	81.8	-6
DB14665	2	0	2	3.3	196.29	26.3	-6
DB15296	5	2	5	-0.7	212.21	72.6	-6
DB00636	3	0	5	3.3	242.7	35.5	-5.9
DB00721	4	1	7	1.9	236.31	55.6	-5.9
DB00968	5	4	3	-1.9	211.21	104	-5.9
DB01034	3	1	7	0.8	223.27	72.7	-5.9
DB01042	4	2	8	-0.5	305.2	66.6	-5.9
DB01064	4	4	4	-0.6	211.26	72.7	-5.9
DB01233	4	2	7	2.6	299.79	67.6	-5.9
DB01291	5	4	5	-0.1	240.3	85.6	-5.9
DB01382	7	1	7	0.9	309.34	98.8	-5.9
DB01550	2	1	5	1.9	188.27	35.8	-5.9
DB01639	6	3	4	-0.9	262.18	108	-5.9
DB01676	6	0	0	1.6	227.13	138	-5.9
DB01704	4	3	2	1.2	180.16	77.8	-5.9
DB01962	7	4	5	-2.8	261.17	130	-5.9
DB02055	3	1	5	2.1	213.3	54.6	-5.9
DB02138	3	0	6	2.1	242.25	35.5	-5.9
DB02268	4	3	2	-0.1	194.19	92.4	-5.9
DB02369	8	0	8	-3.2	314.19	125	-5.9
DB02420	4	3	3	-2.4	194.19	92.4	-5.9
DB02451	6	4	10	1.9	306.39	99.4	-5.9
DB02500	4	4	4	-1.4	214.22	98.7	-5.9
DB02793	6	1	2	2.1	224.17	110	-5.9
DB02891	3	0	0	1.7	189.24	58.4	-5.9
DB02953	3	2	4	2	196.27	38.3	-5.9
DB03064	3	2	9	4.3	253.34	66.4	-5.9
DB03136	1	2	1	2.9	303.14	79.8	-5.9
DB03353	4	3	5	-0.2	243.33	113	-5.9
DB03374	4	3	3	-0.5	432.98	83.6	-5.9
DB03380	3	3	3	-0.6	180.2	89.3	-5.9
DB03441	2	1	4	2.8	290.1	37.3	-5.9
DB03660	3	2	3	-0.9	291.09	63.3	-5.9
DB03747	6	3	1	-1.1	219.26	108	-5.9
DB03757	4	3	6	-0.6	259.3	111	-5.9
DB03772	5	3	7	1.3	248.32	79.2	-5.9
DB03775	3	3	6	0.8	214.26	78.4	-5.9
DB03828	6	2	4	-0.4	244.14	101	-5.9
DB03839	4	3	3	-2.3	181.19	83.6	-5.9
DB04208	4	1	5	1.2	210.23	55.8	-5.9
DB04302	3	1	2	2.8	210.29	62.6	-5.9
DB04404	5	3	2	-1.4	216.23	85.5	-5.9
DB04462	2	1	0	3.8	434.71	41.6	-5.9
DB04560	6	2	9	-1.6	230.21	109	-5.9
DB04723	4	4	4	0.2	239.3	103	-5.9
DB04819	4	0	6	2.6	261.39	47.6	-5.9
DB04948	2	1	3	2.6	259.13	33.6	-5.9
DB05458	3	1	3	1.3	192.26	34.2	-5.9
DB05541	2	1	5	1	212.29	63.4	-5.9
DB05814	4	1	4	1.5	241.28	74.7	-5.9
DB06535	5	3	5	0.3	225.24	92.8	-5.9
DB06580	6	3	4	-0.8	259.3	90.2	-5.9
DB06862	7	4	5	1.8	277.3	113	-5.9
DB06880	3	2	3	1	263.38	72.4	-5.9
DB07259	2	1	2	2.6	189.28	54.3	-5.9
DB07282	5	1	6	1.4	223.22	68.1	-5.9
DB07353	4	4	5	0.2	210.27	92.5	-5.9
DB07551	6	2	5	2.7	254.72	86.5	-5.9
DB07591	4	2	2	1.4	239.3	99.3	-5.9
DB07609	3	0	3	1.5	202.25	29.3	-5.9
DB07634	3	1	3	2.6	180.2	46.5	-5.9
DB07637	4	3	3	-0.4	338.98	83.6	-5.9
DB07890	5	3	5	0.9	260.25	103	-5.9
DB08081	4	1	7	1.9	241.28	72.5	-5.9
DB08114	3	1	2	2.6	190.27	67.2	-5.9
DB08157	5	1	6	1.3	257.31	94.8	-5.9
DB08193	4	1	2	1.5	181.15	83.1	-5.9

DB08337	5	1	5	1.7	241.29	77.2	-5.9
DB08374	2	1	4	1.7	197.66	43.1	-5.9
DB08474	2	2	7	1.9	242.36	72.2	-5.9
DB08475	5	1	8	1	278.28	65	-5.9
DB08509	3	0	9	3.6	308.8	36.3	-5.9
DB08525	3	1	4	0.9	194.23	53.4	-5.9
DB08548	5	1	8	1	278.28	65	-5.9
DB08571	5	4	2	-0.4	210.19	113	-5.9
DB08629	3	1	4	1.9	225.37	32.5	-5.9
DB08637	4	1	4	1.7	206.19	63.6	-5.9
DB08808	3	2	6	2.8	271.78	41.5	-5.9
DB09018	4	2	7	2.8	344.25	67.6	-5.9
DB09085	4	1	9	3.7	264.36	41.6	-5.9
DB09115	2	1	0	3.1	396.95	33.1	-5.9
DB09220	5	1	4	0.8	211.17	97	-5.9
DB09480	1	1	4	3.6	299.18	12	-5.9
DB11148	3	1	5	2.9	193.24	52.3	-5.9
DB11174	2	1	4	2.9	200.32	29.5	-5.9
DB11254	2	2	5	3.5	194.27	40.5	-5.9
DB11412	5	0	5	4.1	278.3	85.1	-5.9
DB11543	2	2	2	1.4	258.09	36.4	-5.9
DB11768	4	1	5	4.3	314.17	62.6	-5.9
DB12192	4	4	6	1.1	284.4	60.2	-5.9
DB12338	3	2	4	1	215.63	72.6	-5.9
DB12440	3	2	4	1	253.32	89.4	-5.9
DB13167	3	1	5	2.5	226.65	46.5	-5.9
DB13221	2	2	4	1.7	184.24	72.2	-5.9
DB13229	3	1	3	2.3	303.15	66.5	-5.9
DB13699	4	3	2	-0.7	231.3	139	-5.9
DB13779	3	3	5	2.1	263.12	85.7	-5.9
DB13802	3	2	0	0.2	186.25	49.7	-5.9
DB13992	4	0	5	1.9	259.76	71.4	-5.9
DB14084	3	1	5	3.6	194.23	46.5	-5.9
DB15326	6	2	3	-0.4	251.3	117	-5.9
DB00114	7	3	4	-1.1	247.14	117	-5.8
DB00765	4	3	3	-1.6	195.21	83.6	-5.8
DB00787	5	3	4	-1.9	225.2	115	-5.8
DB00816	4	4	4	0.7	211.26	72.7	-5.8
DB00871	4	4	4	0.9	225.28	72.7	-5.8
DB01004	6	4	5	-2.5	255.23	135	-5.8
DB01056	2	2	2	0.8	192.26	55.1	-5.8
DB01144	6	2	2	0.3	305.2	137	-5.8
DB01444	3	0	4	4.1	263.4	59.7	-5.8
DB01580	4	2	9	2.1	265.35	50.7	-5.8
DB01827	6	1	2	1.2	223.12	52.3	-5.8
DB01862	6	4	3	-0.5	238.3	115	-5.8
DB02091	5	1	1	1.4	206.27	92.9	-5.8
DB02105	6	2	0	0.7	200.11	132	-5.8
DB02368	4	4	6	-2.3	217.22	122	-5.8
DB02742	6	5	1	-2.8	232.19	130	-5.8
DB02773	3	1	5	2.5	228.67	46.5	-5.8
DB02906	3	2	2	0.8	186.25	57.5	-5.8
DB02941	5	3	9	-1.5	231.29	101	-5.8
DB03338	6	4	8	0.8	278.34	99.4	-5.8
DB03552	4	3	3	-2.4	181.19	83.6	-5.8
DB03739	7	5	1	-1.6	205.17	131	-5.8
DB04141	6	4	7	0.1	264.31	99.4	-5.8
DB04253	6	1	2	0	252.18	138	-5.8
DB04334	6	3	3	-4.1	211.17	118	-5.8
DB04368	4	2	9	1.8	329.43	90	-5.8
DB04450	6	4	8	1.6	294.41	115	-5.8
DB06704	1	2	2	1	275.09	64.4	-5.8
DB06931	7	5	9	-0.1	304.21	135	-5.8
DB07103	3	0	4	0.8	199.16	49.4	-5.8
DB07196	2	1	3	2.6	203.31	40.3	-5.8
DB07357	6	4	7	0	263.33	105	-5.8
DB07368	3	2	2	0.1	198.24	92.4	-5.8
DB07497	3	2	5	0.3	228.31	83.5	-5.8

DB07532	4	3	8	0.8	217.26	86.6	-5.8
DB07735	5	2	8	2.1	352.5	51.8	-5.8
DB07955	4	1	6	1.3	286.12	69.4	-5.8
DB08199	6	4	8	0.7	312.34	106	-5.8
DB08256	3	3	3	0.8	200.23	78.4	-5.8
DB08257	3	3	5	1	228.29	78.4	-5.8
DB08294	5	2	2	1.5	197.14	103	-5.8
DB08550	1	1	0	2.6	202.08	12	-5.8
DB08554	5	3	7	-0.1	217.22	104	-5.8
DB08628	3	1	2	2.2	196.27	62.6	-5.8
DB08989	3	1	4	2.1	223.27	49.8	-5.8
DB09009	5	2	7	2.8	284.38	95.7	-5.8
DB11408	7	0	6	2.2	325.3	106	-5.8
DB11587	2	1	4	2.1	193.28	23.5	-5.8
DB12098	3	2	4	-1	213.66	63.3	-5.8
DB12438	5	4	3	-2.3	263.25	126	-5.8
DB12819	5	2	7	3.3	311.9	103	-5.8
DB12855	4	2	2	-1.2	229.26	113	-5.8
DB13204	4	0	4	1	210.23	52.6	-5.8
DB13516	3	1	6	4.7	347.7	74.7	-5.8
DB13577	3	2	4	1	208.21	75.3	-5.8
DB13777	4	3	6	1.4	225.28	61.7	-5.8
DB13839	6	1	5	0.9	238.19	89.9	-5.8
DB14018	3	1	0	1	259.06	69.3	-5.8
DB14132	3	1	0	0.9	214.61	69.3	-5.8
DB14177	3	1	4	3	180.2	46.5	-5.8
DB14628	3	0	0	1.4	201.63	54.9	-5.8
DB15220	5	2	4	1.3	235.33	67.1	-5.8
DB15357	5	0	7	3.3	281.42	66.5	-5.8
DB15405	5	2	6	-0.8	226.23	72.6	-5.8
DB00135	4	3	3	-2.3	181.19	83.6	-5.7
DB00211	5	3	6	-0.6	254.28	93.8	-5.7
DB00413	4	2	3	1.9	211.33	79.2	-5.7
DB00463	3	1	2	1.2	198.22	66.5	-5.7
DB00575	1	2	2	1.6	230.09	36.4	-5.7
DB00710	8	5	9	-4.1	319.23	139	-5.7
DB00766	5	2	2	-1.2	199.16	87.1	-5.7
DB00824	3	2	2	0.3	194.19	78.1	-5.7
DB00856	3	2	4	1.2	202.63	49.7	-5.7
DB01055	6	3	3	-3.5	198.18	104	-5.7
DB01170	2	2	3	0.5	198.31	67.6	-5.7
DB01412	3	1	0	-0.8	180.16	67.2	-5.7
DB01467	3	1	5	2.8	223.31	44.5	-5.7
DB01525	4	2	1	-1.8	185.22	60.8	-5.7
DB01778	4	2	0	-0.4	195.18	95.3	-5.7
DB01783	5	4	6	-1.1	219.23	107	-5.7
DB02148	3	1	5	0.4	193.24	52.3	-5.7
DB02528	6	2	4	-1.5	207.19	115	-5.7
DB03008	7	4	1	-1.3	200.14	90.2	-5.7
DB03197	5	3	1	-2.2	193.16	114	-5.7
DB03293	3	3	0	-1.1	182.14	90.5	-5.7
DB03738	5	5	7	-1	276.35	99.7	-5.7
DB03824	1	1	0	2.2	259.09	12	-5.7
DB04017	2	0	6	4.2	272.17	12.5	-5.7
DB04090	5	1	3	0.1	234.25	60.4	-5.7
DB04214	6	2	2	0.3	219.09	113	-5.7
DB06262	6	5	3	-3.2	213.19	124	-5.7
DB06797	4	2	8	1.4	232.28	105	-5.7
DB06946	5	3	3	0.1	200.16	77.8	-5.7
DB07160	6	2	8	0.9	273.28	105	-5.7
DB07347	4	1	3	1.7	203.24	68.5	-5.7
DB07355	3	1	8	4.4	236.31	46.5	-5.7
DB07391	5	1	3	1	197.23	82	-5.7
DB07552	6	2	5	2.7	254.72	86.5	-5.7
DB07677	3	1	0	-0.2	182.25	66.8	-5.7
DB07696	5	1	7	0.5	245.27	81.7	-5.7
DB07722	4	1	4	1.9	197.19	75.3	-5.7
DB07923	6	4	9	1.4	292.37	99.4	-5.7

DB07928	3	1	7	2.6	227.3	55.4	-5.7
DB08217	5	1	3	0.3	265.4	91.3	-5.7
DB08484	5	4	4	0	275.4	111	-5.7
DB08558	6	4	9	2.2	308.44	115	-5.7
DB08685	3	1	1	0.8	191.27	32.5	-5.7
DB08759	5	3	8	-0.2	266.27	94.8	-5.7
DB08780	5	1	1	0.3	205.22	66.9	-5.7
DB09060	6	2	3	-1.8	265.25	139	-5.7
DB09256	4	1	1	-0.3	200.17	58.6	-5.7
DB11299	3	1	6	1.8	210.27	38.7	-5.7
DB11364	5	2	2	-0.7	244.27	112	-5.7
DB12473	8	2	2	-1.7	284.4	116	-5.7
DB12716	6	3	8	-0.2	264.33	102	-5.7
DB13247	3	1	7	0.8	269.38	52.6	-5.7
DB13253	4	3	4	0.2	226.23	95.5	-5.7
DB13319	6	0	5	0.4	267.24	107	-5.7
DB13417	5	1	3	-0.8	222.24	67.4	-5.7
DB13583	3	2	4	1.4	182.22	49.7	-5.7
DB13695	4	1	9	3.3	340.5	74.8	-5.7
DB13716	4	0	10	4.7	278.34	52.6	-5.7
DB13852	1	1	6	3.1	211.73	12	-5.7
DB13940	4	1	6	2.5	255.38	69.8	-5.7
DB14708	3	1	2	1.7	205.66	54.6	-5.7
DB14783	6	0	7	-0.9	255.22	90	-5.7
DB15098	6	1	4	-0.3	270.29	96.3	-5.7
DB15134	7	2	2	-0.7	246.18	113	-5.7
DB15601	2	1	2	0.3	194.23	49.4	-5.7
DB00371	4	2	8	0.7	218.25	105	-5.6
DB00419	5	4	4	-0.6	219.28	84.2	-5.6
DB00883	8	0	2	1.3	236.14	129	-5.6
DB00964	2	3	2	1.3	245.11	62.4	-5.6
DB01528	3	1	4	2.2	209.28	44.5	-5.6
DB01711	6	1	1	1.5	198.09	20.2	-5.6
DB01866	5	2	3	0.4	216.21	100	-5.6
DB02219	4	2	5	-1.4	221.32	74.8	-5.6
DB02248	6	3	2	-2.9	209.22	112	-5.6
DB02283	4	2	4	0.9	201.25	74.8	-5.6
DB02320	6	5	2	-1.5	221.21	119	-5.6
DB02695	6	4	6	-0.4	250.29	99.4	-5.6
DB02725	4	3	4	-2.3	196.25	89.3	-5.6
DB02813	6	4	2	-2	219.19	116	-5.6
DB02928	6	5	3	-2.8	212.2	130	-5.6
DB02966	6	3	3	-4.4	217.15	113	-5.6
DB02985	3	3	0	-0.4	277.02	96.2	-5.6
DB03244	5	2	5	-2.3	212.25	94	-5.6
DB03319	6	2	4	-0.6	227.24	112	-5.6
DB03539	5	3	2	0.2	200.19	94.6	-5.6
DB03624	5	4	8	-2	232.28	113	-5.6
DB03705	5	3	1	-0.5	185.14	125	-5.6
DB03762	3	2	5	2.8	280.17	39.3	-5.6
DB03868	6	4	1	-2.4	190.15	115	-5.6
DB03898	4	3	2	-1.4	201.61	83.6	-5.6
DB03972	4	2	0	0.9	183.23	80.8	-5.6
DB04021	3	1	9	2.6	270.41	41.6	-5.6
DB04106	6	1	8	0.9	315.69	97.3	-5.6
DB04139	3	3	3	-0.7	184.19	78.4	-5.6
DB04528	5	1	0	1.7	184.11	112	-5.6
DB04559	2	3	2	0.9	185.61	70.6	-5.6
DB04636	4	2	6	-0.6	202.25	95.4	-5.6
DB04713	3	2	3	-0.9	291.09	63.3	-5.6
DB05817	6	1	5	-0.8	307.8	121	-5.6
DB06331	3	1	1	3.6	311.4	38.3	-5.6
DB06707	4	4	2	-0.8	183.2	86.7	-5.6
DB06830	7	5	7	-1.2	276.16	135	-5.6
DB07079	4	1	6	2.4	284.4	52.8	-5.6
DB07114	5	2	3	0.7	215.23	91.8	-5.6
DB07850	3	3	2	0.9	227.29	106	-5.6
DB08413	5	1	2	0.6	217.12	92.4	-5.6

DB08421	2	2	2	0.7	184.28	41.1	-5.6
DB08792	2	1	2	2	234.08	40.5	-5.6
DB08985	3	3	4	0.1	181.23	52.5	-5.6
DB09342	5	1	10	3.1	294.39	64.8	-5.6
DB09366	4	0	5	2.4	447.01	46.6	-5.6
DB11392	7	1	5	1.6	297.3	128	-5.6
DB11610	3	3	3	0.6	181.23	52.5	-5.6
DB11738	2	1	4	1.2	180.25	33.6	-5.6
DB11831	4	0	0	2.3	202.55	91.6	-5.6
DB11876	5	4	6	-4.4	217.22	136	-5.6
DB12099	4	2	8	-1.8	239.31	81.1	-5.6
DB12111	4	1	1	0.5	198.65	41	-5.6
DB12821	10	0	1	3.8	238.03	0	-5.6
DB13304	5	0	3	-0.1	231.25	43.2	-5.6
DB13604	2	3	2	0.1	184.28	76.4	-5.6
DB13613	2	1	0	1.9	185.63	54.4	-5.6
DB14630	5	2	4	-2	217.24	98	-5.6
DB15130	4	2	3	1.9	211.33	79.2	-5.6
DB00185	3	0	0	1.5	199.32	37.8	-5.5
DB00236	2	0	4	0.4	356.05	40.6	-5.5
DB00373	8	2	7	1.8	316.42	108	-5.5
DB00395	4	2	9	1.9	260.33	90.6	-5.5
DB00703	6	1	1	0.1	236.3	139	-5.5
DB00723	4	2	4	0.5	211.26	64.7	-5.5
DB00945	4	1	3	1.2	180.16	63.6	-5.5
DB01107	2	1	2	0.8	183.25	46.2	-5.5
DB01458	4	1	7	2.6	255.38	69.8	-5.5
DB01465	3	1	4	1.7	195.26	44.5	-5.5
DB01556	1	1	2	2.6	183.68	26	-5.5
DB01985	4	4	6	-1.7	216.24	131	-5.5
DB02260	5	2	9	0.1	216.23	91.7	-5.5
DB02475	4	3	6	-1.8	228.25	122	-5.5
DB02570	3	2	3	2.7	308.14	38.3	-5.5
DB02604	8	5	3	-3.3	244.14	137	-5.5
DB02645	7	4	5	-2	250.25	112	-5.5
DB02786	6	4	1	-1.7	192.14	98	-5.5
DB02818	5	3	3	-3.7	325.06	113	-5.5
DB02892	5	3	6	-2.2	187.19	101	-5.5
DB02945	7	5	1	-2.3	194.14	127	-5.5
DB03109	6	5	2	-1.7	221.21	119	-5.5
DB03392	9	5	5	-2.9	287.2	138	-5.5
DB04000	5	3	3	-3.5	278.06	113	-5.5
DB04075	5	3	5	-1.8	189.17	104	-5.5
DB04340	5	0	1	1	199.08	83.5	-5.5
DB04406	6	3	3	-1.3	217.12	108	-5.5
DB04415	2	2	4	1.3	199.68	46.2	-5.5
DB04598	4	3	1	1.6	187.58	83.6	-5.5
DB04674	3	2	1	4.6	389.91	57.5	-5.5
DB04688	4	1	2	0.6	199.25	49.8	-5.5
DB06657	2	2	7	1.3	200.28	72.2	-5.5
DB07001	3	1	2	1.8	210.3	75.8	-5.5
DB07331	4	1	2	1.4	181.15	83.1	-5.5
DB07433	4	3	6	0.5	259.3	96.5	-5.5
DB07900	4	3	5	-0.4	189.21	86.6	-5.5
DB08291	4	2	4	1.6	195.22	83.9	-5.5
DB08433	3	0	3	1.7	184.21	51.8	-5.5
DB08589	5	1	4	1.4	212.2	65	-5.5
DB08782	4	2	3	-0.2	200.26	94.6	-5.5
DB09283	4	0	3	1.7	205.26	46.3	-5.5
DB11379	5	0	7	2.2	327.5	61.8	-5.5
DB11549	3	1	3	2	223.34	57.3	-5.5
DB11940	4	3	2	-0.2	195.25	121	-5.5
DB12493	3	1	4	1.1	197.27	52.3	-5.5
DB12911	4	0	8	2.1	223.27	48.4	-5.5
DB13272	7	2	7	1.9	253.35	83.2	-5.5
DB13394	4	3	3	0.5	181.19	69.6	-5.5
DB13661	5	1	1	0.9	214.2	119	-5.5
DB14107	5	2	1	-3.1	214.24	109	-5.5

DB14872	5	4	4	-0.6	219.28	84.2	-5.5
DB00201	3	0	0	-0.1	194.19	58.4	-5.4
DB00277	3	1	0	0	180.16	69.3	-5.4
DB00668	4	4	3	-1.4	183.2	72.7	-5.4
DB00793	1	0	2	4.6	361.4	9.2	-5.4
DB01020	6	1	1	-0.4	191.14	93.7	-5.4
DB01206	3	1	3	2.8	233.69	61.8	-5.4
DB01516	4	1	5	1.2	225.28	53.7	-5.4
DB01785	7	3	5	-1.1	246.09	113	-5.4
DB02057	5	3	3	-3.2	186.17	102	-5.4
DB02142	7	4	4	-4.4	248.17	126	-5.4
DB02190	7	3	5	-1.1	190.11	129	-5.4
DB02270	7	3	6	-0.6	264.18	129	-5.4
DB02765	5	2	2	-0.4	193.21	89.8	-5.4
DB02878	6	4	9	-6	232.28	127	-5.4
DB02940	5	2	2	0.5	186.23	101	-5.4
DB03270	5	1	1	0.7	193.17	68.5	-5.4
DB03309	4	2	4	-1.2	207.29	74.8	-5.4
DB03652	7	5	1	-2.3	194.14	127	-5.4
DB03801	5	4	6	-2.7	190.2	113	-5.4
DB04167	4	3	5	-1.4	188.18	110	-5.4
DB04426	6	4	3	-2.1	235.23	108	-5.4
DB04568	6	4	2	-1.9	215.21	114	-5.4
DB04714	7	3	6	-0.9	246.09	113	-5.4
DB05103	4	0	7	2.3	202.25	52.6	-5.4
DB05152	3	1	1	1.4	214.33	57.6	-5.4
DB05492	4	2	4	1.7	209.24	83.9	-5.4
DB06091	7	2	9	0.6	449.04	114	-5.4
DB06255	7	5	4	-3.5	287.19	127	-5.4
DB06775	5	4	5	-2.4	190.15	130	-5.4
DB07392	5	2	4	2.6	215.68	62.7	-5.4
DB07623	4	0	3	1.9	220.3	76.4	-5.4
DB08075	6	2	1	0.2	193.23	119	-5.4
DB08576	5	1	4	1.1	197.23	68	-5.4
DB08711	4	1	3	1.8	182.17	55.8	-5.4
DB08779	4	2	3	2.6	226.3	102	-5.4
DB09357	4	4	6	-0.9	205.25	89.8	-5.4
DB11944	4	3	7	-0.7	225.24	95.1	-5.4
DB12060	5	0	1	0	268.02	112	-5.4
DB12172	5	0	3	-0.4	226.23	76.1	-5.4
DB12223	4	0	8	2.4	226.27	52.6	-5.4
DB12727	6	2	6	0.3	293.08	71	-5.4
DB12902	4	0	7	1.8	323.6	32.8	-5.4
DB13389	4	2	3	0.3	420.97	60.8	-5.4
DB13494	5	3	5	0.2	227.26	71	-5.4
DB14044	5	4	7	-0.7	233.26	107	-5.4
DB15041	6	3	7	-2.4	206.2	101	-5.4
DB15264	3	2	7	-0.5	187.28	63.3	-5.4
DB00545	2	0	2	0.7	181.21	33.4	-5.3
DB00711	2	0	2	0.3	199.29	26.8	-5.3
DB00874	4	2	5	1.4	198.22	58.9	-5.3
DB00911	5	0	4	-0.4	247.27	106	-5.3
DB01090	0	0	6	2.4	240.43	0	-5.3
DB01484	3	1	4	2.6	274.15	44.5	-5.3
DB01799	8	4	7	-2.2	264.11	134	-5.3
DB01805	7	3	6	-3.8	227.15	119	-5.3
DB01816	5	4	0	-2.2	189.21	84.2	-5.3
DB01947	5	2	3	0.1	202.1	83.8	-5.3
DB01978	4	4	0	-1.6	184.22	79.1	-5.3
DB01981	8	3	2	-2.5	242.21	131	-5.3
DB02087	5	1	1	0.7	193.17	68.5	-5.3
DB02209	7	4	4	-1.9	249.16	120	-5.3
DB02302	4	4	7	-3.7	202.25	99.7	-5.3
DB02370	6	4	8	-5	218.25	113	-5.3
DB02471	7	4	1	-2.9	202.17	125	-5.3
DB02707	7	3	7	-0.9	248.11	113	-5.3
DB02720	6	5	2	-2.5	207.18	133	-5.3
DB03000	5	2	2	-0.4	193.21	89.8	-5.3

DB03071	5	3	3	-2.9	199.21	101	-5.3
DB03094	6	4	4	-4.3	216.24	122	-5.3
DB03306	6	2	4	-0.3	232.13	93.1	-5.3
DB03511	7	5	1	-2.3	194.14	127	-5.3
DB03567	6	5	2	-1.7	221.21	119	-5.3
DB03582	6	4	8	-3.9	232.23	130	-5.3
DB03657	7	5	3	-1.1	237.21	129	-5.3
DB03740	6	5	2	-1.7	221.21	119	-5.3
DB03847	7	4	5	-3.7	191.14	138	-5.3
DB03861	5	5	2	-2.1	204.22	102	-5.3
DB03941	1	1	6	4.8	192.3	20.2	-5.3
DB04072	7	4	5	-1.6	206.15	132	-5.3
DB04228	5	4	2	-2.4	183.16	104	-5.3
DB04282	6	4	1	-1.6	182.15	90.2	-5.3
DB04303	7	4	2	-2.4	208.17	116	-5.3
DB04798	5	2	4	0	244.31	129	-5.3
DB05057	6	2	5	-0.1	249.3	134	-5.3
DB05343	2	1	8	3.7	186.29	37.3	-5.3
DB06795	4	2	2	-0.7	186.23	94.6	-5.3
DB06801	4	2	10	2	218.29	66.8	-5.3
DB06906	4	2	3	-0.9	183.16	93.8	-5.3
DB07003	3	1	0	0.9	182.25	75.8	-5.3
DB07491	5	3	2	2.4	417.83	101	-5.3
DB07681	3	0	10	4.6	249.39	65.6	-5.3
DB07989	4	1	5	0.9	203.24	66.8	-5.3
DB11695	5	4	6	-4	226.23	121	-5.3
DB12736	5	2	4	-1.3	214.18	113	-5.3
DB12951	3	2	5	0.8	187.24	66.4	-5.3
DB12987	3	0	2	4	325.4	31.4	-5.3
DB13336	4	0	4	1.6	194.18	52.6	-5.3
DB13408	6	0	6	2.9	222.2	110	-5.3
DB13817	2	2	3	1.5	237.09	72.2	-5.3
DB14031	6	0	3	0.7	204.23	47.7	-5.3
DB15086	3	0	9	4.1	209.28	50.1	-5.3
DB00423	5	2	7	0.6	241.24	91	-5.2
DB00548	4	2	8	1.6	188.22	74.6	-5.2
DB01527	1	1	2	2.5	183.68	26	-5.2
DB01537	3	1	4	2.1	260.13	44.5	-5.2
DB01686	4	3	6	-3.6	202.25	105	-5.2
DB01715	4	3	7	-2.3	188.27	89.3	-5.2
DB01797	6	5	3	-5.1	204.16	136	-5.2
DB01835	5	3	6	-3.2	205.23	102	-5.2
DB01854	2	1	1	0.4	201.02	56	-5.2
DB02228	6	4	1	-1.6	182.15	90.2	-5.2
DB02274	4	2	7	-2.2	187.24	80.4	-5.2
DB02318	6	3	1	-0.5	184.14	69.9	-5.2
DB02364	7	2	8	-3.3	241.18	108	-5.2
DB02419	3	0	3	1.2	185.22	46.6	-5.2
DB02508	7	3	6	-1	248.11	113	-5.2
DB02644	3	5	8	-3.3	217.29	115	-5.2
DB02763	5	2	1	1.7	199.19	84.1	-5.2
DB03340	7	4	6	-4.6	239.16	138	-5.2
DB03390	5	4	5	-1.2	191.18	107	-5.2
DB03569	7	5	1	-1.8	192.12	127	-5.2
DB03892	3	5	8	-3.2	215.27	115	-5.2
DB04046	6	4	2	-2.2	194.18	99.4	-5.2
DB04173	6	5	2	-2.3	180.16	110	-5.2
DB04272	7	4	5	-1.7	192.12	132	-5.2
DB04283	3	2	0	0.6	215.01	70.1	-5.2
DB06261	4	1	10	1.1	215.29	69.4	-5.2
DB06881	6	3	5	-0.8	210.12	104	-5.2
DB07171	3	3	10	1.3	204.31	60.7	-5.2
DB07518	4	1	7	2.3	210.29	72	-5.2
DB07603	4	3	8	1.7	233.33	67.4	-5.2
DB07645	4	2	9	2.1	202.25	74.6	-5.2
DB07930	3	2	10	3.6	216.32	57.5	-5.2
DB08194	4	1	0	0.8	181.26	77.1	-5.2
DB08215	6	2	5	3.7	241.36	88	-5.2

DB11684	4	1	3	-0.1	199.25	49.8	-5.2
DB11815	4	4	6	-4.1	188.23	114	-5.2
DB12040	3	0	8	3.9	209.28	50.1	-5.2
DB12539	5	0	1	1.1	226.3	109	-5.2
DB12667	2	1	3	1.5	180.27	71	-5.2
DB13370	2	2	2	1.1	223.07	72.2	-5.2
DB13493	3	2	3	2.1	186.25	57.5	-5.2
DB13513	1	1	0	4	344.83	20.2	-5.2
DB13522	2	0	0	-0.9	198.22	47.1	-5.2
DB00141	6	5	6	-3.4	221.21	127	-5.1
DB00166	4	1	5	1.7	206.3	87.9	-5.1
DB01706	3	1	0	1.8	233.45	54.5	-5.1
DB01727	7	4	5	-1.8	192.12	132	-5.1
DB01832	5	2	4	-1.7	180.1	91.7	-5.1
DB01882	5	3	5	-0.6	228.31	105	-5.1
DB01982	7	5	1	-2.3	194.14	127	-5.1
DB02100	6	4	2	-2.2	194.18	99.4	-5.1
DB02294	7	4	1	-2.9	202.17	125	-5.1
DB02408	4	4	7	0.4	202.29	78.5	-5.1
DB02625	4	2	6	0.8	189.21	77.8	-5.1
DB02722	7	4	2	-2.4	208.17	116	-5.1
DB02999	6	3	3	-3.9	189.13	122	-5.1
DB03145	6	2	4	-0.3	223.19	108	-5.1
DB03236	5	4	6	-4.7	189.21	118	-5.1
DB03408	7	5	7	-3.8	250.27	131	-5.1
DB03653	4	1	3	0.5	180.25	55	-5.1
DB03683	4	2	5	0.5	189.21	77.8	-5.1
DB03707	2	1	3	2.4	180.27	63.7	-5.1
DB03710	3	3	7	-2.8	202.27	103	-5.1
DB03997	6	4	5	-4.6	221.15	122	-5.1
DB04084	6	4	1	-1.6	182.15	90.2	-5.1
DB04483	6	4	1	-1.6	182.15	90.2	-5.1
DB06839	4	2	6	1.6	203.24	75.6	-5.1
DB06893	3	1	9	3.6	222.35	62.8	-5.1
DB07094	3	1	2	1.9	195.3	82.5	-5.1
DB07387	4	1	6	0.4	194.25	79.8	-5.1
DB07912	4	2	6	0.1	194.17	74.6	-5.1
DB08939	7	2	8	-1	279.4	126	-5.1
DB11688	4	1	4	-1	222.63	96.1	-5.1
DB12083	8	2	9	0.4	401.3	130	-5.1
DB13191	6	4	4	-2.6	211.11	136	-5.1
DB13529	5	3	1	-0.1	190.18	103	-5.1
DB14557	3	2	9	1.9	204.31	49.7	-5.1
DB00431	0	0	0	3.8	290.8	0	-5
DB00491	6	5	3	-2.6	207.22	104	-5
DB00585	8	2	9	1.6	331.5	140	-5
DB00791	3	2	5	0.5	252.09	61.4	-5
DB00851	5	2	3	-0.6	182.18	99.7	-5
DB00863	7	2	9	0.3	314.41	112	-5
DB01979	6	4	2	-2.2	194.18	99.4	-5
DB02257	5	2	4	-1.3	262	87.1	-5
DB02337	6	3	9	-1.7	235.3	126	-5
DB02480	8	4	7	-2	343	134	-5
DB02613	1	0	9	4.2	201.35	18.1	-5
DB03101	8	5	3	-3.1	230.11	137	-5
DB03144	5	5	6	-4.3	190.2	134	-5
DB03156	7	5	1	-2.3	194.14	127	-5
DB03237	7	2	3	-0.8	190.11	138	-5
DB03303	6	5	5	-2.4	180.16	118	-5
DB03339	1	1	1	2.3	234.03	20.2	-5
DB03799	7	2	4	-0.7	203.19	88.6	-5
DB03856	6	3	5	-2.9	182.17	89.3	-5
DB03974	4	4	6	-3.7	188.23	128	-5
DB03989	6	5	1	-2.6	180.16	110	-5
DB04127	8	5	3	-3.6	230.11	137	-5
DB04170	8	4	7	-2	343	134	-5
DB04199	7	3	10	-0.4	270.22	113	-5
DB06968	2	0	7	1.1	218.3	35.6	-5

DB07641	1	0	9	3.9	218.32	17.1	-5
DB08171	3	2	10	4	218.36	38.3	-5
DB08786	5	1	4	0.4	183.21	70.3	-5
DB12834	4	1	2	0.2	185.18	83.9	-5
DB12970	6	5	5	-2.9	180.16	118	-5
DB13121	5	4	2	-1.8	192.21	90.2	-5
DB00330	4	4	9	-0.1	204.31	64.5	-4.9
DB01158	0	0	3	2.8	243.16	0	-4.9
DB01181	4	1	5	0.9	261.08	41.6	-4.9
DB01197	4	2	3	0.3	217.29	58.6	-4.9
DB01236	8	0	2	2.8	200.05	9.2	-4.9
DB01534	3	2	4	2.1	265.6	49.7	-4.9
DB01642	6	4	2	-2.2	194.18	99.4	-4.9
DB02032	4	2	3	0.3	217.29	58.6	-4.9
DB02415	3	1	9	2	206.35	56.5	-4.9
DB02807	6	5	5	-2.4	180.16	118	-4.9
DB02810	6	3	6	-3.9	190.15	121	-4.9
DB04317	5	3	3	-0.5	234.96	94.8	-4.9
DB04321	3	1	7	1.6	194.21	46.5	-4.9
DB04508	6	3	2	-1.8	208.23	111	-4.9
DB04511	6	3	8	-0.4	249.29	129	-4.9
DB04660	6	2	8	-2.3	257.22	99	-4.9
DB04936	6	5	5	-3.2	180.16	118	-4.9
DB06243	6	3	5	-2.9	182.17	89.3	-4.9
DB07646	1	0	10	4.8	215.38	18.1	-4.9
DB08842	4	0	5	0.4	203.24	66.4	-4.9
DB08969	7	0	2	2.3	182.06	9.2	-4.9
DB09502	6	4	5	-2.4	181.15	98	-4.9
DB11117	2	1	9	3.9	184.27	37.3	-4.9
DB11497	7	0	10	3.9	322.3	110	-4.9
DB11716	5	1	4	-0.4	201.18	93.1	-4.9
DB12197	4	1	2	-1.9	222.09	66	-4.9
DB12237	5	3	7	-3.3	219.31	127	-4.9
DB12536	6	5	6	-3.4	221.21	127	-4.9
DB12870	5	3	7	-0.6	222.31	113	-4.9
DB13026	4	1	3	0.6	219.62	83.9	-4.9
DB15087	6	5	5	-3.2	180.16	118	-4.9
DB00531	4	1	5	0.6	261.08	41.6	-4.8
DB00556	8	0	0	3.1	188.02	0	-4.8
DB00753	6	0	2	2.1	184.49	9.2	-4.8
DB01695	5	2	3	-2.6	181.08	113	-4.8
DB01914	6	5	5	-2.9	180.16	118	-4.8
DB02168	3	1	0	1.3	199.01	54.5	-4.8
DB02303	2	2	0	-0.4	240	58.2	-4.8
DB02379	6	5	1	-2.6	180.16	110	-4.8
DB02438	6	4	6	-2.7	194.18	107	-4.8
DB02730	6	4	2	-1.8	210.25	115	-4.8
DB03017	2	1	10	4.2	200.32	37.3	-4.8
DB03584	6	5	1	-2.2	196.22	91.2	-4.8
DB03816	6	2	5	-0.9	185.19	88.6	-4.8
DB03936	7	4	3	-3.1	214.11	116	-4.8
DB04082	2	1	10	4	188.31	29.5	-4.8
DB04510	7	4	4	-2.6	186.06	124	-4.8
DB04731	7	3	9	-5	258.19	130	-4.8
DB11473	4	1	3	0.5	257.43	55.8	-4.8
DB12969	6	5	1	-3.2	194.18	110	-4.8
DB14103	5	3	4	-1.8	180.23	113	-4.8
DB14830	5	1	3	-0.5	188.15	83.9	-4.8
DB00228	6	0	3	2.1	184.49	9.2	-4.7
DB00488	6	0	3	2.7	210.28	48.4	-4.7
DB00720	6	4	2	-2.1	244.89	115	-4.7
DB01077	7	5	2	-3.7	206.03	135	-4.7
DB01648	6	4	2	-1.6	210.25	80.2	-4.7
DB01679	7	3	5	-1.8	220.05	113	-4.7
DB02417	1	1	0	4.4	330.8	20.2	-4.7
DB02482	7	4	4	-4.7	199.1	130	-4.7
DB02561	6	5	1	-2.8	180.16	110	-4.7
DB02822	1	1	1	2	187.03	20.2	-4.7

DB02922	8	2	0	1.1	184.04	40.5	-4.7
DB03002	1	1	0	2.9	220.01	20.2	-4.7
DB03040	7	3	6	-3.2	191.14	115	-4.7
DB03165	8	3	6	-4.9	249.1	117	-4.7
DB03248	6	3	4	-1	184.08	104	-4.7
DB03406	6	4	2	-1.6	210.25	80.2	-4.7
DB03434	5	1	4	-3.1	209.27	75.2	-4.7
DB03554	2	2	0	0	237.98	58.2	-4.7
DB03790	5	2	4	-3.1	181.21	106	-4.7
DB03977	3	2	6	-2.1	189.28	63.3	-4.7
DB04352	8	5	3	-3.6	230.11	137	-4.7
DB08805	3	3	5	0.5	244.4	110	-4.7
DB00262	3	1	4	1.5	214.05	61.8	-4.6
DB00389	3	0	2	0.7	186.23	64.9	-4.6
DB00677	4	0	4	1.2	184.15	35.5	-4.6
DB01057	4	0	8	1	256.33	60.8	-4.6
DB01646	4	2	5	0.1	191.25	91.7	-4.6
DB01870	6	5	1	-1.2	212.3	71.9	-4.6
DB02371	7	5	7	-5.8	229.25	136	-4.6
DB02899	5	3	5	0.1	193.22	112	-4.6
DB03364	4	3	5	-0.5	192.24	118	-4.6
DB03522	7	3	6	-3.9	191.14	127	-4.6
DB03859	6	5	1	-1.6	196.22	91.2	-4.6
DB03953	4	4	5	-3.7	191.25	134	-4.6
DB07650	2	0	10	4.6	186.29	26.3	-4.6
DB08120	3	3	7	1.1	207.4	45.1	-4.6
DB08160	6	3	5	-0.5	234.3	133	-4.6
DB08501	4	2	8	0.8	218.25	76.7	-4.6
DB11735	6	5	5	-2.9	180.16	118	-4.6
DB14199	2	0	3	1.4	265.93	47.6	-4.6
DB00822	4	0	7	3.9	296.5	121	-4.5
DB01709	7	4	4	-2.6	186.06	124	-4.5
DB02127	3	0	4	0.6	180.18	35.5	-4.5
DB02160	5	2	6	-2.1	191.25	106	-4.5
DB02496	7	4	5	-3.1	214.11	124	-4.5
DB02663	5	3	4	-5	181.13	101	-4.5
DB03113	7	3	4	-1.6	188.05	104	-4.5
DB03414	6	5	1	-2	195.24	132	-4.5
DB03497	7	3	4	-4.4	185.16	135	-4.5
DB03590	6	4	6	-5.9	190.2	127	-4.5
DB03814	5	1	3	-3.5	195.24	75.2	-4.5
DB03836	0	0	0	4.2	181.4	0	-4.5
DB03937	7	4	5	-3.3	200.08	124	-4.5
DB04087	7	5	6	-3.1	216.13	127	-4.5
DB04522	7	4	4	-5.1	185.07	130	-4.5
DB05448	3	1	4	2.3	188.3	79.3	-4.5
DB12160	5	4	4	0.4	223.3	68.4	-4.5
DB12916	4	4	5	-0.3	307.96	80.9	-4.5
DB13071	5	4	5	-2.5	197.13	120	-4.5
DB13768	3	1	2	0.4	244.03	38.7	-4.5
DB01008	6	0	7	-0.5	246.3	104	-4.4
DB01798	7	3	4	-2.3	206.03	113	-4.4
DB03484	7	4	7	-5.5	215.14	122	-4.4
DB03630	1	1	1	3	250.1	1	-4.4
DB03760	4	3	7	1.8	208.3	39.3	-4.4
DB04530	6	3	6	-3.4	197.3	134	-4.4
DB06405	6	2	4	-1.6	204.2	126	-4.4
DB06753	4	2	2	0.3	229.38	66.8	-4.4
DB08431	4	2	5	-0.6	194.17	74.6	-4.4
DB08960	0	0	7	1.8	202.38	0	-4.4
DB11397	4	0	4	1.4	220.97	44.8	-4.4
DB13960	4	2	2	-0.6	199.99	86.3	-4.4
DB00659	4	2	4	-1.2	181.21	91.8	-4.3
DB04188	2	2	9	0.6	192.3	24.1	-4.3
DB04250	2	0	6	1.5	190.33	42.4	-4.3
DB08658	4	1	5	0.3	181.17	49.8	-4.3
DB13543	4	4	5	-0.3	307.96	80.9	-4.3
DB14159	7	5	2	-3.9	192	135	-4.3

DB02618	3	0	4	-0.2	195.28	65.6	-4.2
DB03347	4	0	6	0.8	182.15	44.8	-4.2
DB03857	6	5	1	-1.2	212.3	71.9	-4.2
DB12907	6	5	5	-2.9	180.16	118	-4.2
DB02252	0	0	0	3.2	204.01	0	-4.1
DB02948	5	3	4	-2.2	183.1	98.1	-4.1
DB03163	5	1	5	-0.5	198.3	105	-4.1
DB05668	4	3	6	-0.4	221.02	61.4	-4.1
DB14089	6	4	3	0.1	182.2	76.6	-4.1
DB00566	6	4	3	0.1	182.2	76.6	-4
DB01143	6	4	7	-4.5	214.23	121	-4
DB02151	5	3	4	-3.7	185.18	109	-4
DB03945	4	2	4	-1.5	184.15	66.8	-4
DB13712	6	0	7	3.2	242.4	133	-4
DB04637	1	1	5	1.7	181.07	20.2	-3.9
DB13245	4	0	3	1.7	240.4	121	-3.9
DB01159	3	0	0	2.3	197.38	0	-3.7
DB04572	4	0	3	0.5	189.22	41.1	-3.7
DB14178	3	0	2	1.7	208.4	96	-3.5
DB02721	1	1	0	1.7	193.97	28.7	-3.3
DB13321	3	1	1	-0.1	222	62.8	-3.3
DB13383	1	1	2	1.8	311.89	20.2	-3
DB13813	0	0	0	2.7	393.732	0	-2.5
DB03054	0	0	0	2.8	252.73	0	-2.4
DB05382	0	0	0	1.7	253.8089	0	-1.6
