# Supplementary material for: Life span‐associated ferroptosis‐related genes identification and validation for hepatocellular carcinoma patients as hepatitis B virus carriers
Source: J Clin Lab Anal. 2023 Jul 18;37(13-14):e24930. doi: 10.1002/jcla.24930 (PMC10492458; doi:10.1002/jcla.24930)
Supplement: Supplementary file 10 — Tables S1–S14 [file JCLA-37-e24930-s009.zip › TableS2_GSE14520_pheno_HBV.docx]

TableS2_GSE14520_pheno_HBV

| LCS ID | ID | Affy_GSM | Tissue Type | Predicted risk Metastasis Signature | Agilent_GSM | CGH_survival_group | Gender | Age | HBV viral status | ALT(>/<=50U/L) | Main Tumor Size (>/<=5 cm) | Multinodular | Cirrhosis | TNM staging | BCLC staging | CLIP staging | AFP (>/<=300ng/ml) | Survival status | Survival months | Recurr status | Recurr months |
| --- | --- | --- | --- | --- | --- | --- | --- | --- | --- | --- | --- | --- | --- | --- | --- | --- | --- | --- | --- | --- | --- |
| LCS_193A | 03-195 | GSM363205 | Tumor | low | NA | NA | M | 70 | CC | low | small | N | N | I | 0 | 0 | high | 0 | 58 | 0 | 58 |
| LCS_094A | 02-304 | GSM363115 | Tumor | low | NA | NA | M | 74 | CC | low | small | N | Y | I | A | 0 | low | 0 | 66.6 | 0 | 66.6 |
| LCS_085A | 02-286 | GSM362970 | Tumor | low | NA | NA | M | 39 | CC | low | small | N | N | I | 0 | 0 | low | 0 | 67.3 | 0 | 67.3 |
| LCS_021A | 03-077 | GSM363209 | Tumor | low | NA | NA | F | 45 | CC | low | small | N | Y | I | A | 1 | high | 0 | 60.8 | 0 | 60.8 |
| LCS_137A | 02-448 | GSM363344 | Tumor | low | NA | NA | F | 52 | CC | low | small | N | Y | II | A | 0 | low | 0 | 38.7 | 0 | 38.7 |
| LCS_268A | 03-419 | GSM363271 | Tumor | high | NA | NA | M | 52 | CC | low | large | N | Y | IIIC | C | 0 | low | 0 | 28.7 | 0 | 28.7 |
| LCS_046A | 03-311 | GSM363404 | Tumor | low | NA | NA | M | 46 | CC | low | large | N | Y | IIIA | C | 4 | high | 1 | 12 | 1 | 12 |
| LCS_250A | 03-342 | GSM363217 | Tumor | high | NA | NA | M | 58 | CC | low | small | Y | N | IIIC | C | 1 | low | 0 | 54.5 | 0 | 54.5 |
| LCS_211A | 03-234 | GSM363053 | Tumor | low | NA | NA | F | 57 | CC | low | small | N | Y | I | A | 0 | low | 0 | 57 | 0 | 57 |
| LCS_048A | 03-321 | GSM363295 | Tumor | low | GSM358056 | G2 | M | 63 | CC | low | large | N | Y | I | C | 3 | high | 0 | 54.9 | 0 | 54.9 |
| LCS_222A | 03-262 | GSM363272 | Tumor | low | NA | NA | M | 65 | CC | low | large | Y | Y | II | A | 2 | high | 0 | 56.3 | 0 | 56.3 |
| LCS_047A | 03-309 | GSM363164 | Tumor | low | GSM358055 | G1 | F | 27 | CC | low | small | N | N | I | A | 1 | high | 0 | 55.2 | 0 | 55.2 |
| LCS_095A | 02-307 | GSM362965 | Tumor | high | NA | NA | M | 57 | CC | low | large | N | N | II | A | 0 | low | 0 | 66.6 | 0 | 66.6 |
| LCS_179A | 03-138 | GSM363126 | Tumor | high | NA | NA | M | 65 | CC | low | large | N | Y | IIIB | A | 1 | high | 0 | 59.5 | 1 | 59.5 |
| LCS_210A | 03-233 | GSM363082 | Tumor | low | NA | NA | M | 64 | CC | low | small | N | Y | II | 0 | 0 | low | 0 | 57 | 0 | 57 |
| LCS_143A | 02-458 | GSM363176 | Tumor | low | NA | NA | F | 65 | CC | low | small | N | Y | I | A | 0 | low | 0 | 50.7 | 0 | 50.7 |
| LCS_033A | 03-150 | GSM363099 | Tumor | high | GSM358045 | G2 | M | 50 | CC | low | large | Y | Y | III | B | 2 | high | 1 | 30.9 | 1 | 30.9 |
| LCS_056A | 03-333 | GSM363316 | Tumor | low | GSM358062 | G1 | F | 35 | AVR-CC | low | small | N | Y | I | A | 1 | high | 0 | 54.7 | 0 | 54.7 |
| LCS_199A | 03-218 | GSM363178 | Tumor | high | NA | NA | M | 42 | CC | low | small | N | N | II | A | 0 | low | 0 | 57.3 | 1 | 41.6 |
| LCS_116A | 02-359 | GSM363343 | Tumor | low | NA | NA | F | 30 | CC | low | small | N | N | I | A | 1 | high | 0 | 65.1 | 0 | 65.1 |
| LCS_150A | 03-017 | GSM363033 | Tumor | high | NA | NA | M | 63 | AVR-CC | low | small | N | Y | I | A | 1 | high | 1 | 36.4 | 1 | 36 |
| LCS_230A | 03-274 | GSM363055 | Tumor | high | NA | NA | F | 55 | CC | low | large | N | Y | I | A | 1 | high | 0 | 7.3 | 0 | 7.3 |
| LCS_164A | 03-061 | GSM363100 | Tumor | high | NA | NA | M | 56 | CC | low | large | N | Y | IIIB | C | 4 | high | 1 | 3.4 | 1 | 1.5 |
| LCS_182A | 03-151 | GSM363249 | Tumor | high | NA | NA | F | 60 | CC | low | small | N | Y | II | A | 1 | high | 0 | 59.2 | 0 | 59.2 |
| LCS_078A | 02-269 | GSM363130 | Tumor | low | NA | NA | M | 72 | CC | low | large | N | Y | II | A | 0 | low | 0 | 61.8 | 0 | 61.8 |
| LCS_156A | 03-042 | GSM363149 | Tumor | low | NA | NA | M | 53 | CC | low | large | N | N | I | A | 0 | low | 0 | 14.4 | 0 | 14.4 |
| LCS_106A | 02-339 | GSM363337 | Tumor | high | NA | NA | F | 53 | CC | low | small | N | Y | I | A | 1 | high | 0 | 65.5 | 0 | 65.5 |
| LCS_274A | 03-438 | GSM363031 | Tumor | low | NA | NA | M | 58 | CC | low | small | N | Y | II | A | 0 | low | 0 | 52.8 | 1 | 20.8 |
| LCS_075A | 03-381 | GSM363166 | Tumor | low | GSM358079 | G2 | M | 59 | CC | low | large | N | Y | IIIB | A | 1 | high | 0 | 53.8 | 0 | 53.8 |
| LCS_152A | 03-024 | GSM363049 | Tumor | high | NA | NA | M | 45 | AVR-CC | low | . | N | Y | I | A | 0 | high | 0 | 62.4 | 0 | 62.4 |
| LCS_159A | 03-049 | GSM363032 | Tumor | high | NA | NA | M | 52 | CC | low | large | N | Y | IIIB | A | 1 | high | 0 | 24.9 | 0 | 24.9 |
| LCS_183A | 03-162 | GSM363072 | Tumor | low | NA | NA | M | 49 | CC | low | small | N | N | I | A | 0 | low | 0 | 10.4 | 0 | 10.4 |
| LCS_066A | 03-354 | GSM363289 | Tumor | low | GSM358072 | G1 | M | 46 | CC | low | small | N | Y | I | A | 1 | high | 0 | 54.2 | 0 | 54.2 |
| LCS_012A | 03-196 | GSM363296 | Tumor | high | GSM358033 | G2 | F | 52 | AVR-CC | low | small | N | Y | II | A | 2 | high | 1 | 27.5 | 1 | 27.5 |
| LCS_206A | 03-227 | GSM363235 | Tumor | high | NA | NA | M | 41 | CC | low | large | Y | Y | III | B | 2 | high | 0 | 32.8 | 0 | 32.8 |
| LCS_079A | 02-271 | GSM362958 | Tumor | low | NA | NA | M | 56 | CC | low | large | N | Y | II | A | 0 | low | 1 | 28.2 | 1 | 28.2 |
| LCS_289A | 03-457 | GSM363366 | Tumor | low | NA | NA | M | 73 | CC | low | large | N | Y | I | A | 0 | low | 0 | 52.3 | 0 | 52.3 |
| LCS_051A | 03-292 | GSM363311 | Tumor | low | GSM358058 | G2 | M | 77 | CC | low | large | N | Y | II | A | 0 | low | 0 | 19 | 0 | 19 |
| LCS_180A | 03-139 | GSM363071 | Tumor | low | NA | NA | M | 35 | CC | low | large | Y | Y | IIIB | C | 3 | high | 1 | 13.8 | 1 | 13.8 |
| LCS_266A | 03-416 | GSM363329 | Tumor | low | NA | NA | M | 56 | AVR-CC | low | small | N | Y | I | A | 0 | low | 0 | 53.2 | 0 | 53.2 |
| LCS_044A | 03-241 | GSM363200 | Tumor | low | GSM358053 | G2 | M | 26 | CC | low | small | N | Y | II | A | 1 | high | 1 | 23.9 | 1 | 23.9 |
| LCS_130A | 02-429 | GSM362984 | Tumor | low | NA | NA | M | 49 | CC | low | small | N | Y | II | A | 0 | low | 0 | 51.4 | 0 | 51.4 |
| LCS_162A | 03-057 | GSM363037 | Tumor | high | NA | NA | M | 54 | AVR-CC | low | small | N | Y | I | A | 0 | low | 0 | 61.2 | 0 | 61.2 |
| LCS_269A | 03-424 | GSM363362 | Tumor | low | NA | NA | M | 53 | CC | low | small | N | Y | II | A | 0 | low | 1 | 5.8 | 1 | 5.8 |
| LCS_251A | 03-343 | GSM363357 | Tumor | high | NA | NA | F | 47 | AVR-CC | low | large | N | Y | II | A | 0 | low | 1 | 8.8 | 1 | 8.8 |
| LCS_069A | 03-364 | GSM363080 | Tumor | high | GSM358075 | G2 | M | 49 | CC | low | large | N | Y | IIIB | C | 5 | high | 1 | 2.3 | 1 | 2.3 |
| LCS_160A | 03-054 | GSM363125 | Tumor | high | NA | NA | M | 43 | AVR-CC | low | large | N | Y | II | A | 1 | high | 1 | 16.5 | 1 | 16.5 |
| LCS_135A | 02-445 | GSM362988 | Tumor | low | NA | NA | F | 52 | AVR-CC | low | small | N | Y | I | 0 | 1 | high | 0 | 51.1 | 0 | 51.1 |
| LCS_249A | 03-336 | GSM363107 | Tumor | low | NA | NA | F | 71 | AVR-CC | low | small | N | Y | I | A | 1 | high | 1 | 7 | 1 | 7 |
| LCS_231A | 03-277 | GSM363355 | Tumor | high | NA | NA | M | 53 | CC | low | small | N | Y | II | A | 0 | low | 1 | 9.1 | 1 | 6.2 |
| LCS_228A | 03-272 | GSM363268 | Tumor | high | NA | NA | M | 56 | AVR-CC | low | small | N | Y | I | A | 1 | high | 0 | 56 | 0 | 56 |
| LCS_177A | 03-131 | GSM363015 | Tumor | high | NA | NA | M | 39 | CC | low | large | N | N | I | A | 1 | high | 0 | 59.7 | 0 | 59.7 |
| LCS_023A | 03-082 | GSM363014 | Tumor | low | GSM358039 | G1 | M | 61 | CC | low | small | N | Y | I | A | 0 | low | 0 | 60.7 | 1 | 32.7 |
| LCS_273A | 03-435 | GSM363364 | Tumor | low | NA | NA | M | 49 | CC | low | small | N | Y | I | A | 0 | low | 1 | 27.1 | 1 | 10.2 |
| LCS_014A | 03-062 | GSM363034 | Tumor | low | NA | NA | F | 30 | CC | low | small | N | N | I | A | 0 | low | 1 | 45.9 | 1 | 45.9 |
| LCS_015A | 03-071 | GSM363078 | Tumor | high | GSM358034 | G1 | M | 47 | CC | low | small | N | Y | IIIB | A | 0 | low | 1 | 57.9 | 1 | 57.9 |
| LCS_174A | 03-112 | GSM363202 | Tumor | low | NA | NA | M | 33 | CC | low | small | N | Y | I | A | 0 | low | 0 | 60 | 1 | 6.7 |
| LCS_032A | 03-148 | GSM363332 | Tumor | high | NA | NA | M | 54 | AVR-CC | low | large | N | Y | I | A | 1 | high | 1 | 12.6 | 1 | 2.5 |
| LCS_165A | 03-073 | GSM363313 | Tumor | low | NA | NA | M | 71 | CC | low | large | N | Y | II | A | 0 | low | 0 | 36.6 | 1 | 36.6 |
| LCS_035A | 03-186 | GSM363017 | Tumor | high | NA | NA | M | 50 | AVR-CC | low | large | Y | Y | IIIB | B | 2 | low | 1 | 12.7 | 1 | 8.4 |
| LCS_148A | 03-012 | GSM363294 | Tumor | high | NA | NA | M | 25 | AVR-CC | low | small | Y | Y | II | A | 2 | high | 0 | 62.6 | 0 | 62.6 |
| LCS_167A | 03-087 | GSM363330 | Tumor | high | NA | NA | M | 50 | CC | low | large | N | Y | I | A | 0 | low | 1 | 12.6 | 1 | 12.6 |
| LCS_263A | 03-404 | GSM363360 | Tumor | low | NA | NA | M | 71 | CC | low | small | N | Y | I | A | 0 | high | 0 | 53.3 | 0 | 53.3 |
| LCS_188A | 03-180 | GSM363008 | Tumor | high | NA | NA | F | 50 | CC | low | small | Y | Y | II | B | 1 | low | 0 | 58.4 | 1 | 23.6 |
| LCS_253A | 03-350 | GSM363143 | Tumor | high | NA | NA | F | 48 | CC | low | small | N | Y | II | 0 | 0 | low | 1 | 32.6 | 1 | 32.6 |
| LCS_134A | 02-437 | GSM363127 | Tumor | high | NA | NA | M | 55 | CC | low | small | N | Y | II | A | 1 | high | 1 | 22.2 | 1 | 14.6 |
| LCS_245A | 03-314 | GSM363102 | Tumor | low | NA | NA | M | 49 | CC | low | large | N | Y | II | A | 0 | low | 1 | 37.9 | 1 | 37.9 |
| LCS_090A | 02-295 | GSM363151 | Tumor | low | NA | NA | F | 70 | CC | low | small | Y | Y | II | A | 1 | low | 0 | 67.1 | 0 | 67.1 |
| LCS_074A | 03-398 | GSM363124 | Tumor | low | NA | NA | M | 67 | CC | low | small | N | Y | I | A | 0 | . | 0 | 53.5 | 0 | 53.5 |
| LCS_190A | 03-187 | GSM712542 | Tumor | high | NA | NA | M | 61 | CC | low | small | N | Y | IIIA | C | 2 | high | 1 | 7.5 | 1 | 4.4 |
| LCS_031A | 03-141 | GSM363335 | Tumor | low | NA | NA | M | 45 | CC | low | small | N | Y | I | A | 0 | low | 0 | 59.4 | 0 | 59.4 |
| LCS_142A | 02-457 | GSM363350 | Tumor | high | NA | NA | M | 43 | CC | low | large | N | Y | I | A | 1 | high | 0 | 62.9 | 0 | 62.9 |
| LCS_197A | 03-210 | GSM363386 | Tumor | high | NA | NA | M | 61 | CC | low | large | N | Y | IIIA | C | 2 | high | 1 | 10 | 1 | 1.9 |
| LCS_215A | 03-243 | GSM363207 | Tumor | high | NA | NA | M | 53 | CC | low | large | N | Y | I | A | 1 | high | 1 | 53.3 | 1 | 53.3 |
| LCS_123A | 02-384 | GSM362986 | Tumor | high | NA | NA | M | 55 | AVR-CC | low | small | N | Y | II | 0 | 0 | low | 0 | 64.5 | 0 | 64.5 |
| LCS_103A | 02-325 | GSM362972 | Tumor | low | NA | NA | M | 45 | CC | low | small | N | Y | I | 0 | 0 | low | 0 | 66 | 0 | 66 |
| LCS_184A | 03-164 | GSM363186 | Tumor | low | NA | NA | M | 45 | AVR-CC | low | small | N | Y | II | A | 0 | low | 1 | 7.6 | 1 | 7.6 |
| LCS_025A | 03-101 | GSM363288 | Tumor | low | GSM358041 | G2 | M | 56 | CC | low | small | N | Y | I | A | 1 | high | 0 | 60.3 | 1 | 35.2 |
| LCS_140A | 02-455 | GSM363016 | Tumor | low | NA | NA | M | 44 | AVR-CC | low | large | Y | Y | IIIA | B | 1 | low | 0 | 2 | 0 | 2 |
| LCS_109A | 02-346 | GSM363341 | Tumor | high | NA | NA | M | 32 | CC | low | large | N | Y | II | A | 1 | high | 0 | 65.4 | 0 | 65.4 |
| LCS_236A | 03-282 | GSM363243 | Tumor | low | NA | NA | M | 69 | CC | low | small | N | Y | I | A | 1 | low | 0 | 55.8 | 0 | 55.8 |
| LCS_259A | 03-383 | GSM363168 | Tumor | low | NA | NA | M | 51 | CC | low | large | Y | Y | IIIA | B | 1 | low | 0 | 53.8 | 0 | 53.8 |
| LCS_209A | 03-231 | GSM363051 | Tumor | high | NA | NA | M | 43 | CC | low | large | Y | Y | IIIA | B | 2 | high | 0 | 20.6 | 0 | 20.6 |
| LCS_213A | 03-237 | GSM363098 | Tumor | high | NA | NA | M | 60 | AVR-CC | low | small | N | Y | II | 0 | 1 | high | 0 | 56.9 | 1 | 21.3 |
| LCS_208A | 03-230 | GSM363314 | Tumor | low | NA | NA | F | 48 | AVR-CC | low | small | Y | N | II | A | 2 | high | 0 | 57.1 | 0 | 57.1 |
| LCS_264A | 03-405 | GSM363170 | Tumor | low | NA | NA | M | 59 | CC | low | small | N | Y | I | 0 | 0 | low | 0 | 53.3 | 0 | 53.3 |
| LCS_132A | 02-431 | GSM363029 | Tumor | high | NA | NA | M | 48 | CC | low | small | N | Y | I | A | 0 | low | 0 | 51.3 | 0 | 51.3 |
| LCS_260A | 03-387 | GSM363298 | Tumor | high | NA | NA | M | 52 | CC | low | small | N | N | I | A | 1 | high | 0 | 53.6 | 1 | 40.1 |
| LCS_117A | 02-361 | GSM363265 | Tumor | high | NA | NA | M | 41 | CC | low | small | Y | Y | II | A | 1 | low | 0 | 65.1 | 0 | 65.1 |
| LCS_104A | 02-335 | GSM363038 | Tumor | low | NA | NA | M | 41 | CC | low | small | N | Y | II | A | 0 | low | 1 | 15.1 | 1 | 5.8 |
| LCS_247A | 03-316 | GSM363309 | Tumor | high | NA | NA | M | 34 | CC | low | small | N | Y | II | 0 | 0 | low | 1 | 53 | 1 | 53 |
| LCS_061A | 03-361 | GSM363104 | Tumor | low | GSM358067 | G2 | M | 59 | CC | low | small | N | Y | I | A | 0 | low | 1 | 35.9 | 1 | 29.9 |
| LCS_216A | 03-244 | GSM363393 | Tumor | high | NA | NA | M | 46 | AVR-CC | low | small | Y | Y | IIIA | C | 3 | high | 1 | 4 | 1 | 4 |
| LCS_120A | 02-370 | GSM363011 | Tumor | high | NA | NA | M | 57 | CC | low | small | Y | Y | II | B | 2 | high | 1 | 17.6 | 1 | 9.1 |
| LCS_034A | 03-149 | GSM363180 | Tumor | low | GSM358046 | G2 | M | 52 | CC | low | large | Y | Y | IIIA | B | 2 | high | 1 | 47.1 | 1 | 3.2 |
| LCS_237A | 03-284 | GSM363048 | Tumor | low | NA | NA | M | 50 | AVR-CC | low | large | Y | Y | IIIA | B | 2 | high | 1 | 20.3 | 1 | 20.3 |
| LCS_275A | 03-439 | GSM363077 | Tumor | low | NA | NA | M | 40 | CC | low | small | N | Y | I | 0 | 1 | high | 0 | 52.7 | 0 | 52.7 |
| LCS_016A | 03-064 | GSM363336 | Tumor | low | GSM358035 | G2 | M | 63 | CC | low | small | N | Y | I | A | 0 | low | 1 | 8 | 1 | 8 |
| LCS_153A | 03-025 | GSM363073 | Tumor | high | NA | NA | M | 41 | CC | low | small | N | Y | II | A | 1 | high | 1 | 26.5 | 1 | 21.8 |
| LCS_099A | 02-315 | GSM362977 | Tumor | low | NA | NA | M | 41 | CC | low | large | N | Y | I | A | 0 | low | 0 | 66.3 | 0 | 66.3 |
| LCS_286A | 03-422 | GSM363012 | Tumor | low | NA | NA | M | 61 | CC | low | large | N | Y | I | A | 0 | low | 1 | 4.5 | 1 | 4.5 |
| LCS_240A | 03-291 | GSM363328 | Tumor | low | NA | NA | M | 63 | AVR-CC | low | large | N | Y | I | A | 0 | high | 1 | 12 | 1 | 12 |
| LCS_084A | 02-285 | GSM363215 | Tumor | low | NA | NA | M | 48 | CC | low | small | N | Y | I | 0 | 1 | high | 0 | 18.6 | 0 | 18.6 |
| LCS_238A | 03-285 | GSM363400 | Tumor | low | NA | NA | M | 58 | CC | low | small | N | Y | II | A | 0 | low | 0 | 55.6 | 1 | 19.1 |
| LCS_072A | 03-375 | GSM363358 | Tumor | low | GSM358077 | G1 | M | 56 | CC | low | small | N | Y | II | A | 0 | low | 0 | 53.9 | 1 | 9.4 |
| LCS_201A | 03-220 | GSM363391 | Tumor | low | NA | NA | M | 55 | CC | low | small | N | Y | II | 0 | 0 | low | 0 | 57.3 | 1 | 21.5 |
| LCS_050A | 03-319 | GSM363333 | Tumor | high | NA | NA | M | 48 | CC | low | large | N | Y | II | A | 1 | high | 1 | 13.5 | 1 | 13.5 |
| LCS_265A | 03-407 | GSM363083 | Tumor | high | NA | NA | M | 45 | AVR-CC | low | small | N | N | I | 0 | 0 | low | 0 | 53.3 | 0 | 53.3 |
| LCS_011A | 02-461 | GSM363218 | Tumor | high | NA | NA | M | 48 | CC | low | large | N | Y | IIIB | A | 3 | high | 1 | 15.2 | 1 | 3.9 |
| LCS_169A | 03-093 | GSM363086 | Tumor | low | NA | NA | M | 44 | AVR-CC | low | small | Y | Y | II | A | 2 | high | 0 | 60.5 | 0 | 60.5 |
| LCS_100A | 02-318 | GSM363267 | Tumor | low | NA | NA | M | 54 | CC | low | small | Y | Y | II | A | 1 | low | 1 | 47.9 | 1 | 13.4 |
| LCS_136A | 02-447 | GSM362994 | Tumor | low | NA | NA | M | 49 | CC | low | small | N | Y | II | A | 1 | high | 0 | 54.8 | 1 | 54.8 |
| LCS_194A | 03-199 | GSM363224 | Tumor | low | NA | NA | M | 70 | CC | low | small | N | Y | I | A | 1 | high | 0 | 57.9 | 1 | 13.1 |
| LCS_054A | 03-322 | GSM363269 | Tumor | low | GSM358060 | G2 | M | 55 | AVR-CC | low | small | N | Y | I | A | 0 | low | 1 | 52.7 | 1 | 12.4 |
| LCS_278A | 03-444 | GSM363182 | Tumor | low | NA | NA | M | 50 | CC | low | small | N | Y | I | A | 0 | low | 0 | 52.7 | 0 | 52.7 |
| LCS_045A | 03-306 | GSM363109 | Tumor | high | GSM358054 | G2 | M | 50 | CC | low | small | N | Y | II | A | 0 | low | 0 | 55.2 | 0 | 55.2 |
| LCS_073A | 03-384 | GSM363054 | Tumor | high | GSM358078 | G1 | M | 51 | CC | low | large | N | Y | II | A | 0 | low | 0 | 53.8 | 0 | 53.8 |
| LCS_018A | 03-051 | GSM363075 | Tumor | low | NA | NA | F | 54 | CC | low | large | N | Y | IIIB | C | 1 | low | 1 | 18 | 1 | 18 |
| LCS_224A | 03-265 | GSM363129 | Tumor | high | NA | NA | M | 34 | CC | low | large | Y | Y | IIIA | C | 3 | high | 1 | 3.5 | 1 | 3.2 |
| LCS_223A | 03-263 | GSM363101 | Tumor | high | NA | NA | M | 50 | CC | low | small | N | Y | I | A | 1 | high | 0 | 56.3 | 0 | 56.3 |
| LCS_161A | 03-055 | GSM363378 | Tumor | high | NA | NA | M | 47 | AVR-CC | low | small | N | Y | I | A | 0 | low | 1 | 31.9 | 1 | 13.3 |
| LCS_195A | 03-200 | GSM363232 | Tumor | low | NA | NA | M | 49 | AVR-CC | low | small | N | N | II | A | 0 | low | 0 | 57.9 | 1 | 30.7 |
| LCS_127A | 02-423 | GSM362993 | Tumor | low | NA | NA | M | 50 | CC | low | small | N | Y | I | A | 1 | high | 0 | 3.8 | 1 | 3.8 |
| LCS_282A | 03-456 | GSM363150 | Tumor | high | NA | NA | M | 68 | CC | low | large | N | Y | II | A | 1 | high | 1 | 3.3 | 1 | 3.3 |
| LCS_093A | 02-302 | GSM363142 | Tumor | low | NA | NA | M | 28 | CC | high | small | Y | Y | II | B | 2 | high | 1 | 46.1 | 1 | 46.1 |
| LCS_086A | 02-287 | GSM363013 | Tumor | high | NA | NA | M | 58 | CC | high | small | N | Y | II | A | 1 | high | 1 | 7.7 | 1 | 7.7 |
| LCS_028A | 03-121 | GSM363331 | Tumor | low | GSM358043 | G2 | F | 54 | AVR-CC | high | large | N | Y | I | A | 1 | high | 1 | 36.5 | 1 | 28.4 |
| LCS_009A | 02-409 | GSM363128 | Tumor | high | GSM358032 | G2 | M | 36 | AVR-CC | high | small | N | Y | II | A | 0 | low | 1 | 30.1 | 1 | 10 |
| LCS_110A | 02-347 | GSM363315 | Tumor | low | NA | NA | M | 43 | CC | high | small | N | Y | II | A | 1 | high | 0 | 65.4 | 0 | 65.4 |
| LCS_172A | 03-105 | GSM363384 | Tumor | high | NA | NA | M | 43 | AVR-CC | high | small | N | Y | II | A | 0 | low | 1 | 51.6 | 1 | 51.6 |
| LCS_291A | AN | GSM363147 | Tumor | high | NA | NA | M | 44 | AVR-CC | high | small | Y | Y | II | B | 1 | low | 1 | 28.7 | 1 | 28.7 |
| LCS_234A | 03-280 | GSM363081 | Tumor | high | NA | NA | M | 34 | CC | high | large | N | Y | IIIA | C | 1 | low | 1 | 5.2 | 1 | 0.1 |
| LCS_065A | 03-363 | GSM363052 | Tumor | high | GSM358071 | G2 | M | 46 | CC | high | large | Y | Y | IIIA | C | 2 | low | 1 | 14.3 | 1 | 14.3 |
| LCS_102A | 02-323 | GSM362966 | Tumor | low | NA | NA | M | 58 | AVR-CC | high | small | N | Y | I | A | 0 | low | 0 | 66.1 | 0 | 66.1 |
| LCS_010A | 02-424 | GSM363169 | Tumor | high | NA | NA | M | 45 | AVR-CC | high | large | Y | Y | IIIA | B | 2 | high | 0 | 39.4 | 0 | 39.4 |
| LCS_040A | 03-215 | GSM363085 | Tumor | high | NA | NA | M | 49 | CC | high | large | Y | Y | IIIA | B | 2 | high | 1 | 54.8 | 1 | 46.3 |
| LCS_200A | 03-219 | GSM363388 | Tumor | high | NA | NA | F | 58 | CC | high | small | N | Y | I | 0 | 0 | low | 0 | 57.3 | 1 | 11.6 |
| LCS_043A | 03-208 | GSM363196 | Tumor | low | NA | NA | M | 42 | CC | high | small | N | Y | I | A | 0 | low | 0 | 57.7 | 1 | 51.1 |
| LCS_267A | 03-418 | GSM363266 | Tumor | low | NA | NA | M | 35 | CC | high | small | N | Y | IIIB | C | 1 | low | 1 | 2.5 | 1 | 1.5 |
| LCS_147A | 02-466 | GSM363326 | Tumor | high | NA | NA | M | 37 | CC | high | small | N | Y | I | A | 2 | high | 1 | 37.2 | 0 | 37.2 |
| LCS_261A | 03-394 | GSM363230 | Tumor | low | NA | NA | M | 40 | CC | high | large | N | Y | II | A | 0 | low | 0 | 4.8 | 0 | 4.8 |
| LCS_036A | 03-214 | GSM363237 | Tumor | high | GSM358047 | G2 | M | 69 | CC | high | large | N | Y | I | A | 1 | high | 0 | 57.5 | 1 | 4.7 |
| LCS_166A | 03-081 | GSM363204 | Tumor | high | NA | NA | M | 53 | CC | high | large | N | Y | IIIA | C | 2 | high | 1 | 14.1 | 1 | 8.4 |
| LCS_125A | 02-396 | GSM362992 | Tumor | low | NA | NA | F | 41 | CC | high | large | N | Y | I | A | 1 | . | 0 | 64.3 | 0 | 64.3 |
| LCS_277A | 03-443 | GSM363264 | Tumor | low | NA | NA | M | 43 | CC | high | large | Y | Y | III | B | 1 | low | 1 | 17.8 | 1 | 17.8 |
| LCS_243A | 03-303 | GSM363312 | Tumor | low | NA | NA | F | 53 | CC | high | small | N | Y | II | A | 0 | low | 0 | 55.3 | 0 | 55.3 |
| LCS_057A | 03-326 | GSM363123 | Tumor | low | GSM358063 | G1 | M | 48 | AVR-CC | high | large | N | Y | II | A | 0 | low | 0 | 42.7 | 0 | 42.7 |
| LCS_038A | 03-213 | GSM363245 | Tumor | high | GSM358049 | G2 | M | 47 | CC | high | large | Y | Y | IIIA | C | 3 | high | 0 | 57.5 | 0 | 57.5 |
| LCS_284A | 03-467 | GSM363192 | Tumor | low | NA | NA | M | 50 | CC | high | small | N | Y | I | 0 | 0 | low | 0 | 52.2 | 0 | 52.2 |
| LCS_121A | 02-375 | GSM363291 | Tumor | low | NA | NA | M | 54 | CC | high | small | N | Y | I | A | 0 | low | 1 | 50.1 | 1 | 43.2 |
| LCS_192A | 03-192 | GSM363273 | Tumor | low | NA | NA | M | 56 | CC | high | small | N | Y | I | A | 0 | low | 0 | 58 | 0 | 58 |
| LCS_281A | 03-453 | GSM363148 | Tumor | high | NA | NA | M | 64 | CC | high | small | N | Y | II | A | 2 | high | 0 | 52.4 | 1 | 11.8 |
| LCS_189A | 03-182 | GSM363263 | Tumor | high | NA | NA | M | 39 | AVR-CC | high | large | N | Y | IIIA | C | 2 | high | 1 | 3 | 1 | 3 |
| LCS_105A | 02-337 | GSM362978 | Tumor | low | NA | NA | M | 61 | AVR-CC | high | small | N | Y | I | A | 1 | low | 1 | 28.8 | 1 | 28.8 |
| LCS_262A | 03-397 | GSM363056 | Tumor | high | NA | NA | F | 59 | AVR-CC | high | small | Y | Y | II | B | 2 | high | 1 | 19.2 | 1 | 19.2 |
| LCS_088A | 02-290 | GSM363310 | Tumor | high | NA | NA | M | 41 | AVR-CC | high | small | N | Y | II | A | 0 | low | 1 | 23.5 | 1 | 23.5 |
| LCS_175A | 03-125 | GSM363275 | Tumor | low | NA | NA | F | 67 | CC | high | small | Y | Y | IIIB | A | 1 | low | 0 | 59.8 | 0 | 59.8 |
| LCS_196A | 03-205 | GSM363352 | Tumor | high | GSM358081 | G2 | M | 51 | CC | high | small | N | Y | II | A | 1 | high | 1 | 16.2 | 1 | 5.1 |
| LCS_157A | 03-043 | GSM363293 | Tumor | low | NA | NA | M | 54 | CC | high | small | N | Y | I | A | 0 | low | 0 | 61.5 | 1 | 27.9 |
| LCS_027A | 03-119 | GSM363292 | Tumor | high | GSM358042 | G2 | M | 53 | CC | high | small | N | Y | I | A | 0 | low | 0 | 59.9 | 0 | 59.9 |
| LCS_129A | 02-428 | GSM363211 | Tumor | high | NA | NA | M | 61 | CC | high | large | Y | Y | IIIA | C | 3 | high | 1 | 8.9 | 1 | 8.9 |
| LCS_107A | 02-344 | GSM363339 | Tumor | high | NA | NA | F | 49 | AVR-CC | high | small | N | Y | II | 0 | 1 | high | 0 | 65.4 | 0 | 65.4 |
| LCS_063A | 03-346 | GSM363270 | Tumor | low | GSM358069 | G2 | M | 58 | CC | high | small | Y | Y | II | B | 1 | low | 1 | 21.3 | 1 | 21.3 |
| LCS_097A | 02-313 | GSM363371 | Tumor | low | NA | NA | F | 69 | CC | high | small | N | Y | I | A | 0 | low | 0 | 66.3 | 0 | 66.3 |
| LCS_185A | 03-165 | GSM363009 | Tumor | high | NA | NA | M | 50 | CC | high | large | N | Y | II | A | 1 | high | 0 | 58.9 | 0 | 58.9 |
| LCS_122A | 02-380 | GSM362982 | Tumor | high | NA | NA | M | 39 | CC | high | small | N | Y | I | A | 0 | low | 0 | 64.6 | 0 | 64.6 |
| LCS_203A | 03-223 | GSM363188 | Tumor | low | NA | NA | M | 35 | AVR-CC | high | small | N | Y | II | A | 0 | low | 0 | 57.3 | 1 | 46.3 |
| LCS_002A | 02-395 | GSM363251 | Tumor | low | GSM358026 | G2 | M | 47 | CC | high | small | Y | Y | IIIA | C | 2 | low | 1 | 19.6 | 1 | 19.6 |
| LCS_144A | 02-459 | GSM363057 | Tumor | high | NA | NA | M | 63 | CC | high | small | Y | Y | II | A | 1 | low | 1 | 60.5 | 1 | 22.8 |
| LCS_205A | 03-226 | GSM363030 | Tumor | high | NA | NA | M | 58 | CC | high | small | N | Y | IIIA | A | 2 | high | 1 | 4.5 | 1 | 4.5 |
| LCS_022A | 03-099 | GSM363274 | Tumor | high | GSM358038 | G2 | M | 53 | CC | high | small | Y | Y | II | B | 2 | high | 1 | 26.9 | 1 | 26.9 |
| LCS_049A | 03-317 | GSM363146 | Tumor | high | GSM358057 | G2 | M | 50 | CC | high | large | Y | Y | IIIA | C | 2 | low | 0 | 54.9 | 0 | 54.9 |
| LCS_004A | 02-404 | GSM362983 | Tumor | low | GSM358028 | G1 | M | 32 | CC | high | small | N | Y | I | A | 1 | high | 0 | 64.2 | 0 | 64.2 |
| LCS_219A | 03-255 | GSM363222 | Tumor | low | NA | NA | M | 59 | AVR-CC | high | small | N | Y | I | A | 0 | low | 0 | 48.4 | 1 | 49.1 |
| LCS_042A | 03-206 | GSM363074 | Tumor | high | GSM358052 | G1 | M | 41 | CC | high | small | N | Y | II | A | 0 | low | 0 | 57.7 | 0 | 57.7 |
| LCS_248A | 03-334 | GSM363290 | Tumor | high | NA | NA | F | 63 | CC | high | small | N | Y | I | 0 | 0 | low | 0 | 54.7 | 0 | 54.7 |
| LCS_007A | 02-421 | GSM362987 | Tumor | low | GSM358031 | G2 | M | 60 | AVR-CC | high | small | N | Y | II | A | 0 | low | 0 | 60.4 | 1 | 57.7 |
| LCS_227A | 03-270 | GSM363035 | Tumor | low | NA | NA | M | 40 | CC | high | large | N | Y | I | A | 0 | low | 0 | 56.1 | 1 | 24.6 |
| LCS_283A | 03-460 | GSM363036 | Tumor | low | NA | NA | M | 65 | CC | high | small | N | Y | II | A | 1 | low | 1 | 32.7 | 1 | 4.6 |
| LCS_118A | 02-363 | GSM363079 | Tumor | high | NA | NA | M | 53 | CC | high | large | N | Y | IIIA | C | 2 | high | 1 | 7.8 | 1 | 5.2 |
| LCS_146A | 02-462 | GSM363317 | Tumor | high | NA | NA | M | 58 | CC | high | small | N | Y | I | 0 | 1 | high | 0 | 62.8 | 1 | 8 |
| LCS_163A | 03-058 | GSM363172 | Tumor | high | NA | NA | M | 67 | CC | high | small | N | Y | II | A | 0 | low | 1 | 6.7 | 1 | 6.7 |
| LCS_149A | 03-015 | GSM363084 | Tumor | low | NA | NA | M | 67 | CC | high | large | N | Y | I | A | 0 | low | 0 | 62.6 | 1 | 48 |
| LCS_145A | 02-460 | GSM363239 | Tumor | high | NA | NA | M | 54 | AVR-CC | high | small | Y | Y | II | A | 1 | low | 1 | 42.2 | 1 | 6.8 |
| LCS_131A | 02-430 | GSM363121 | Tumor | high | NA | NA | M | 39 | CC | high | small | Y | Y | IIIC | C | 3 | high | 0 | 51.2 | 0 | 51.2 |
| LCS_020A | 03-070 | GSM363108 | Tumor | low | GSM358037 | G1 | M | 49 | CC | high | large | N | Y | II | A | 0 | low | 0 | 48.8 | 0 | 48.8 |
| LCS_151A | 03-023 | GSM363152 | Tumor | low | NA | NA | M | 39 | CC | high | large | Y | Y | IIIA | C | 4 | high | 1 | 8.3 | 1 | 8.3 |
| LCS_191A | 03-188 | GSM363105 | Tumor | high | NA | NA | M | 36 | AVR-CC | high | small | N | Y | II | A | 1 | low | 0 | 9.5 | 1 | 9.5 |
| LCS_173A | 03-108 | GSM363327 | Tumor | low | NA | NA | M | 52 | AVR-CC | high | small | N | Y | I | A | 0 | low | 0 | 60.1 | 0 | 60.1 |
| LCS_108A | 02-345 | GSM363194 | Tumor | high | NA | NA | M | 44 | AVR-CC | high | small | Y | Y | II | B | 2 | high | 0 | 4.5 | 0 | 4.5 |
| LCS_138A | 02-452 | GSM363346 | Tumor | low | NA | NA | M | 51 | AVR-CC | high | large | N | Y | IIIB | A | 0 | low | 1 | 14.3 | 1 | 7.3 |
| LCS_089A | 02-291 | GSM363174 | Tumor | high | NA | NA | M | 48 | CC | high | small | Y | Y | II | A | 2 | high | 0 | 67.1 | 0 | 67.1 |
| LCS_091A | 02-298 | GSM362976 | Tumor | low | NA | NA | M | 50 | CC | high | small | N | Y | I | A | 0 | low | 0 | 67 | 1 | 51.1 |
| LCS_178A | 03-133 | GSM363190 | Tumor | high | NA | NA | M | 41 | AVR-CC | high | small | N | Y | II | A | 1 | low | 0 | 4.8 | 1 | 2.7 |
| LCS_024A | 03-088 | GSM363069 | Tumor | high | GSM358040 | G2 | M | 34 | CC | high | large | N | Y | IIIA | C | 2 | high | 1 | 13.6 | 1 | 1.7 |
| LCS_270A | 03-425 | GSM363247 | Tumor | low | NA | NA | M | 44 | CC | high | small | N | N | I | A | 0 | low | 0 | 52.9 | 0 | 52.9 |
| LCS_062A | 03-345 | GSM363076 | Tumor | high | GSM358068 | G2 | M | 35 | CC | high | large | Y | Y | IIIB | C | 3 | high | 1 | 23 | 1 | 23 |
| LCS_158A | 03-047 | GSM363376 | Tumor | low | NA | NA | M | 62 | CC | high | small | N | Y | I | 0 | 0 | low | 0 | 61.5 | 0 | 61.5 |
| LCS_092A | 02-301 | GSM362959 | Tumor | high | NA | NA | M | 45 | AVR-CC | high | small | N | Y | I | A | 1 | high | 1 | 9.5 | 1 | 9.5 |
| LCS_170A | 03-098 | GSM363226 | Tumor | low | NA | NA | M | 54 | AVR-CC | high | large | N | Y | IIIC | C | 0 | low | 0 | 60.4 | 1 | 19.4 |
| LCS_154A | 03-033 | GSM363010 | Tumor | high | NA | NA | M | 47 | CC | high | large | N | Y | I | A | 1 | . | 0 | 62 | 0 | 62 |
| LCS_071A | 03-370 | GSM363220 | Tumor | high | GSM358076 | G2 | M | 59 | AVR-CC | high | large | Y | Y | IIIA | B | 2 | high | 0 | 54.2 | 1 | 29.9 |
| LCS_083A | 02-284 | GSM362964 | Tumor | low | NA | NA | M | 50 | CC | high | small | N | Y | I | A | 0 | low | 0 | 67.4 | 0 | 67.4 |
| LCS_139A | 02-454 | GSM363348 | Tumor | high | NA | NA | M | 41 | CC | high | small | N | Y | I | A | 0 | low | 0 | 14.3 | 1 | 4.2 |
| LCS_041A | 03-197 | GSM363198 | Tumor | high | GSM358051 | G2 | M | 41 | AVR-CC | high | small | N | Y | I | A | 0 | low | 0 | 57.9 | 0 | 57.9 |
| LCS_119A | 02-366 | GSM363184 | Tumor | high | NA | NA | M | 34 | AVR-CC | high | large | Y | Y | IIIA | B | 1 | low | 0 | 16.3 | 0 | 16.3 |
| LCS_101A | 02-321 | GSM362960 | Tumor | high | NA | NA | M | 21 | CC | high | small | N | Y | II | A | 1 | high | 0 | 66.1 | 0 | 66.1 |
| LCS_008A | 02-416 | GSM363145 | Tumor | high | NA | NA | M | 43 | CC | high | small | N | N | I | A | 0 | low | 1 | 33 | 1 | 26.4 |
| LCS_067A | 03-365 | GSM363050 | Tumor | low | GSM358073 | G1 | M | 32 | AVR-CC | high | small | Y | Y | II | B | 2 | high | 0 | 54.2 | 1 | 2.8 |
| LCS_064A | 03-360 | GSM363241 | Tumor | low | GSM358070 | G1 | M | 55 | CC | high | large | N | Y | I | A | 1 | high | 0 | 54.2 | 0 | 54.2 |
| LCS_241A | 03-295 | GSM363122 | Tumor | low | NA | NA | M | 49 | AVR-CC | high | small | N | Y | I | A | 0 | low | 0 | 55.4 | 0 | 55.4 |
| LCS_096A | 02-309 | GSM362971 | Tumor | low | NA | NA | M | 52 | AVR-CC | high | small | N | Y | I | A | 2 | high | 1 | 48.1 | 1 | 40.4 |
