# Supplementary material for: Life span‐associated ferroptosis‐related genes identification and validation for hepatocellular carcinoma patients as hepatitis B virus carriers
Source: J Clin Lab Anal. 2023 Jul 18;37(13-14):e24930. doi: 10.1002/jcla.24930 (PMC10492458; doi:10.1002/jcla.24930)
Supplement: Supplementary file 10 — Tables S1–S14 [file JCLA-37-e24930-s009.zip › TableS3_Immunohistochemistry_samples_pheno.docx]

TableS3_Immunohistochemistry_samples_pheno

| ID | age | gender | Grade | TNM | Stage | group | Primary/Metastasis | tumor size | IHC |
| --- | --- | --- | --- | --- | --- | --- | --- | --- | --- |
| 1 | 55 | male | - | - | - | adjacent tissue | - | - | - |
| 2 | 58 | male | - | - | - | adjacent tissue | - | - | - |
| 3 | 65 | female | - | - | - | adjacent tissue | - | - | - |
| 4 | 50 | male | - | - | - | adjacent tissue | - | - | - |
| 5 | 37 | male | - | - | - | adjacent tissue | - | - | - |
| 6 | 47 | female | - | - | - | adjacent tissue | - | - | - |
| 7 | 73 | male | - | - | - | adjacent tissue | - | - | - |
| 8 | 68 | male | 3 | T1N0M0 | I | Tumor | Primary | 3.6x3.2x3.2 | HBsAg（+） |
| 9 | 63 | male | 2--3 | T1aN0M0 | IA | Tumor | Primary | 1.5 in diameter | HBsAg（+） |
| 10 | 53 | male | 2 | T1bN0M0 | IB | Tumor | Primary | 3.5*3 | HBsAg（+） |
| 11 | 46 | female | 2 | T1bN0M0 | IB | Tumor | Primary | 2.8*2.5 | HBsAg（+） |
| 12 | 47 | female | 2 | T1bN0M0 | IB | Tumor | Primary | 3*2.5 | HBsAg（+） |
| 13 | 42 | female | 2 | T1bN0M0 | IB | Tumor | Primary | 4.5*3 | HBsAg（+） |
| 14 | 58 | male | 2 | T1bN0M0 | IB | Tumor | Primary | 2.5*2.5 | HBsAg（+） |
| 15 | 27 | male | 2 | T1bN0M0 | IB | Tumor | Primary | 3*3*4 | HBsAg（+） |
| 16 | 61 | male | 2 | T1bN0M0 | IB | Tumor | Primary | 3.5 in diameter | HBsAg（+） |
| 17 | 57 | female | 3 | T1bN0M0 | IB | Tumor | Primary | 3.5*3 | HBsAg（+） |
| 18 | 65 | male | 3 | T1bN0M0 | IB | Tumor | Primary | 4x3.6 | HBsAg（+） |
| 19 | 55 | male | 1 | T2N0M0 | II | Tumor | Primary | 5*3.5 | HBsAg（+） |
| 20 | 57 | female | 2 | T2N0M0 | II | Tumor | Primary | 4*3.5 | HBsAg（+） |
| 21 | 68 | male | 2 | T2N0M0 | II | Tumor | Primary | 2.7 in diameter | HBsAg（+） |
| 22 | 50 | male | 2 | T2N0M0 | II | Tumor | Primary | 5 in diameter | HBsAg（+） |
| 23 | 35 | male | 2 | T2N0M0 | II | Tumor | Primary | 2.5*2.5*2.2 | HBsAg（+） |
| 24 | 65 | male | 2 | T2N0M0 | II | Tumor | Primary | 6*5.5*5.5 | HBsAg（+） |
| 25 | 49 | male | 2 | T2N0M0 | II | Tumor | Primary | 1.5 in diameter | HBsAg（+） |
| 26 | 37 | male | 2 | T2N0M0 | II | Tumor | Primary | 2.5*2*2 | HBsAg（+） |
| 27 | 47 | female | 2 | T2N0M0 | II | Tumor | Primary | 14*12*7 | HBsAg（+） |
| 28 | 60 | male | 2--3 | T2N0M0 | II | Tumor | Primary | 4.5*4 | HBsAg（+） |
| 29 | 57 | female | 2--3 | T2N0M0 | II | Tumor | Primary | 1.3 in diameter | HBsAg（+） |
| 30 | 80 | male | 3 | T2N0M0 | II | Tumor | Primary | 4x3 | HBsAg（+） |
| 31 | 70 | male | 2 | T3N0M0 | IIIA | Tumor | Primary | 5*4.5*4.5 | HBsAg（+） |
| 32 | 54 | male | 2 | T3N0M0 | IIIA | Tumor | Primary | 10.5*9 | HBsAg（+） |
| 33 | 83 | male | 2 | T3N0M0 | IIIA | Tumor | Primary | 7*4 | HBsAg（+） |
| 34 | 64 | male | 2 | T3N0M0 | IIIA | Tumor | Primary | 3.5*3*3 | HBsAg（+） |
| 35 | 33 | male | 2 | T3N0M0 | IIIA | Tumor | Primary | 6*6*5 | HBsAg（+） |
| 36 | 55 | male | 2 | T3N0M0 | IIIA | Tumor | Primary | 7*6 | HBsAg（+） |
| 37 | 65 | female | 2 | T3N0M0 | IIIA | Tumor | Primary | 9.6x6 | HBsAg（+） |
| 38 | 73 | male | 2 | T3N0M0 | IIIA | Tumor | Primary | 5.5x5.5 | HBsAg（+） |
| 39 | 67 | female | 2 | T3N0M0 | IIIA | Tumor | Primary | 10*6 | HBsAg（+） |
| 40 | 53 | male | 2 | T3N0M0 | IIIA | Tumor | Primary | Diffuse | HBsAg（+） |
| 41 | 56 | male | 2 | T3N0M0 | IIIA | Tumor | Primary | 5*4 | HBsAg（+） |
| 42 | 50 | male | 2 | T3N0M0 | IIIA | Tumor | Primary | 9*3 | HBsAg（+） |
| 43 | 41 | male | 2--3 | T3N0M0 | IIIA | Tumor | Primary | 10*9 | HBsAg（+） |
| 44 | 51 | male | 2--3 | T3N0M0 | IIIA | Tumor | Primary | 7*5.5 | HBsAg（+） |
| 45 | 57 | female | 3 | T3N0M0 | IIIA | Tumor | Primary | 28*20*9 | HBsAg（+） |
| 46 | 55 | male | 3 | T3N0M0 | IIIA | Tumor | Primary | 9*7 | HBsAg（+） |
| 47 | 71 | male | 3 | T3N0M0 | IIIA | Tumor | Primary | 4*3 | HBsAg（+） |
| 48 | 54 | female | 1--2 | T4N0M0 | IIIB | Tumor | Primary | 4*3 | HBsAg（+） |
| 49 | 46 | female | 2 | T4N0M0 | IIIB | Tumor | Primary | Diffuse | HBsAg（+） |
| 50 | 77 | female | 2 | T4N0M0 | IIIB | Tumor | Primary | 8*4*4 | HBsAg（+） |
