# Supplementary material for: Life span‐associated ferroptosis‐related genes identification and validation for hepatocellular carcinoma patients as hepatitis B virus carriers
Source: J Clin Lab Anal. 2023 Jul 18;37(13-14):e24930. doi: 10.1002/jcla.24930 (PMC10492458; doi:10.1002/jcla.24930)
Supplement: Supplementary file 10 — Tables S1–S14 [file JCLA-37-e24930-s009.zip › TableS4_DEG_tumor_normal.pdf]

Gene	baseMean	log2FoldCh	lfcSE	stat	pvalue	padj	reg
RP11-402I	17.0105	21.73762	0.96917	22.42911	2.05E-111	5.66E-107	UP
GABRD	141.0271	4.528462	0.204871	22.10396	2.90E-108	4.01E-104	UP
THBS4	755.9968	5.825642	0.286876	20.30717	1.11E-91	1.02E-87	UP
CENPF	1011.792	4.150965	0.209059	19.85551	9.88E-88	6.83E-84	UP
ASS1P2	47.01119	-5.056	0.2549	-19.8352	1.48E-87	8.18E-84	DOWN
PLVAP	4021.709	2.983317	0.151092	19.74501	8.85E-87	4.08E-83	UP
SLC26A6	839.001	2.562794	0.131874	19.43365	4.01E-84	1.58E-80	UP
SKA1	232.015	4.78238	0.248687	19.23053	2.05E-82	7.11E-79	UP
NUF2	262.5849	4.386721	0.229489	19.11521	1.89E-81	5.80E-78	UP
KIFC1	611.3231	3.931717	0.205918	19.09361	2.85E-81	7.90E-78	UP
TROAP	357.1217	4.3479	0.228726	19.00923	1.43E-80	3.60E-77	UP
CDC25C	158.2461	4.273629	0.224977	18.99582	1.85E-80	4.26E-77	UP
CDCA5	509.6219	3.605678	0.190023	18.97499	2.75E-80	5.84E-77	UP
EBF2	50.38334	5.485459	0.289426	18.95292	4.18E-80	8.26E-77	UP
CDKN3	331.6947	3.985568	0.211328	18.85963	2.45E-79	4.52E-76	UP
KIF4A	463.8174	4.142906	0.220249	18.81008	6.24E-79	1.08E-75	UP
BIRC5	990.5596	4.271624	0.228155	18.72246	3.25E-78	5.29E-75	UP
PRC1	742.1352	3.388124	0.182628	18.5521	7.84E-77	1.21E-73	UP
UBE2T	331.3061	3.257877	0.176207	18.48897	2.53E-76	3.36E-73	UP
ASPM	793.1094	3.891794	0.210495	18.4888	2.54E-76	3.36E-73	UP
TOP2A	1902.397	4.206254	0.227505	18.48863	2.55E-76	3.36E-73	UP
COL15A1	938.6975	3.894453	0.210937	18.46264	4.13E-76	5.19E-73	UP
HJURP	324.6766	4.076798	0.22093	18.45289	4.94E-76	5.95E-73	UP
CDC20	804.6415	4.52805	0.245958	18.40985	1.10E-75	1.26E-72	UP
MYBL2	934.9169	4.766911	0.259183	18.39206	1.52E-75	1.68E-72	UP
MELK	285.3867	4.002822	0.217886	18.37113	2.24E-75	2.38E-72	UP
KIF18B	276.1628	4.347349	0.236769	18.36116	2.69E-75	2.75E-72	UP
KIF2C	426.2493	4.189354	0.229181	18.27967	1.20E-74	1.19E-71	UP
CCNB2	391.9821	3.843074	0.211399	18.17925	7.54E-74	7.19E-71	UP
CENPA	142.2683	4.440745	0.244729	18.14555	1.39E-73	1.28E-70	UP
SPC24	415.6454	3.468772	0.191661	18.09845	3.28E-73	2.93E-70	UP
CCNB1	875.3959	3.435806	0.190853	18.00235	1.87E-72	1.61E-69	UP
NEK2	327.7607	4.125781	0.229753	17.95748	4.19E-72	3.52E-69	UP
KIF20A	429.3391	3.947573	0.22009	17.93621	6.15E-72	5.01E-69	UP
ARHGEF39	193.6536	2.98042	0.166296	17.92233	7.90E-72	6.24E-69	UP
APLN	311.2238	3.921374	0.219593	17.85749	2.53E-71	1.94E-68	UP
FOXM1	734.2329	3.884673	0.217801	17.83585	3.72E-71	2.78E-68	UP
ADAMTS1	775.6336	-2.82659	0.158503	-17.833	3.92E-71	2.85E-68	DOWN
NCAPG	408.9285	4.042564	0.226857	17.81988	4.95E-71	3.51E-68	UP
UBE2C	668.7269	4.394266	0.247709	17.73961	2.07E-70	1.43E-67	UP
BUB1	327.5491	3.702723	0.209273	17.69327	4.73E-70	3.19E-67	UP
ESM1	448.5765	4.379772	0.248169	17.64831	1.05E-69	6.91E-67	UP
PLK1	497.6263	3.827221	0.216966	17.63977	1.22E-69	7.85E-67	UP
DLGAP5	250.6864	3.932399	0.22356	17.58991	2.94E-69	1.85E-66	UP
EXO1	192.076	3.789672	0.215656	17.57276	3.98E-69	2.45E-66	UP
CDK1	621.6293	3.696051	0.210381	17.56838	4.30E-69	2.59E-66	UP
MXD3	370.191	2.640001	0.150972	17.48674	1.81E-68	1.06E-65	UP
GPC3	21640.8	5.737095	0.328357	17.47212	2.34E-68	1.35E-65	UP
FBXO43	51.87882	4.094641	0.234388	17.46953	2.45E-68	1.38E-65	UP
ANLN	541.6088	4.001419	0.230914	17.32864	2.86E-67	1.58E-64	UP
ZIC2	192.0852	6.62358	0.382427	17.31985	3.33E-67	1.81E-64	UP

PTTG1	628.8485	3.756405	0.217089	17.30349	4.43E-67	2.36E-64	UP
E2F1	754.7886	3.664171	0.211939	17.28884	5.71E-67	2.98E-64	UP
IGF2BP1	565.4665	7.096987	0.411698	17.23832	1.37E-66	7.02E-64	UP
CDCA3	294.5364	3.401695	0.197348	17.23701	1.40E-66	7.05E-64	UP
NDC80	320.4617	3.501134	0.203292	17.22218	1.81E-66	8.94E-64	UP
CDC6	569.3394	3.461726	0.201261	17.20017	2.65E-66	1.29E-63	UP
CDCA8	358.8049	3.408883	0.198274	17.19282	3.01E-66	1.43E-63	UP
CXorf36	391.5541	2.263989	0.131898	17.16463	4.89E-66	2.29E-63	UP
PKMYT1	239.6861	3.275412	0.191642	17.09132	1.72E-65	7.94E-63	UP
TTK	215.3126	4.002752	0.234271	17.08596	1.89E-65	8.56E-63	UP
EME1	90.09904	3.31214	0.194551	17.0245	5.41E-65	2.41E-62	UP
CSRNP1	2633.947	-2.41423	0.141995	-17.0022	7.92E-65	3.48E-62	DOWN
GTSE1	234.2154	3.874898	0.228366	16.96796	1.42E-64	6.13E-62	UP
RACGAP1	653.9012	2.672331	0.158326	16.87865	6.46E-64	2.75E-61	UP
TRIP13	246.3104	3.744913	0.222226	16.85181	1.02E-63	4.27E-61	UP
HIGD1B	42.77664	3.163712	0.187893	16.8378	1.29E-63	5.32E-61	UP
SKA3	169.2471	3.690348	0.219285	16.82904	1.49E-63	6.08E-61	UP
HAGLR	214.9142	6.198198	0.369346	16.78156	3.33E-63	1.34E-60	UP
HMMR	346.0466	3.36084	0.200848	16.73321	7.51E-63	2.97E-60	UP
CDT1	460.7212	3.410847	0.204835	16.65169	2.94E-62	1.15E-59	UP
BUB1B	254.5941	3.712433	0.223155	16.63611	3.82E-62	1.47E-59	UP
MKI67	1360.173	3.624773	0.217976	16.62926	4.28E-62	1.62E-59	UP
CENPL	186.8163	2.580581	0.155241	16.62311	4.74E-62	1.77E-59	UP
KIF23	289.8599	3.618961	0.218486	16.56378	1.27E-61	4.70E-59	UP
CELSR3	240.9454	3.427907	0.207818	16.49478	4.00E-61	1.46E-58	UP
DTL	436.0228	3.38006	0.204955	16.49168	4.21E-61	1.51E-58	UP
FAM189B	1196.147	1.722002	0.104523	16.47484	5.56E-61	1.97E-58	UP
CENPE	228.6539	3.417143	0.207499	16.46824	6.21E-61	2.17E-58	UP
AURKB	345.2329	3.745191	0.22747	16.46457	6.59E-61	2.28E-58	UP
CD34	1642.03	2.07635	0.126121	16.46312	6.75E-61	2.31E-58	UP
STAB2	490.3816	-4.74568	0.288548	-16.4468	8.85E-61	2.98E-58	DOWN
NXPH4	273.8387	5.996021	0.364631	16.44409	9.25E-61	3.08E-58	UP
CDH13	374.3093	2.432178	0.148104	16.42206	1.33E-60	4.38E-58	UP
CCNA2	485.2394	3.521731	0.214603	16.41043	1.61E-60	5.24E-58	UP
OIT3	1777.77	-3.13941	0.191696	-16.377	2.79E-60	8.98E-58	DOWN
AC092171	393.3792	2.76314	0.168735	16.37564	2.85E-60	9.08E-58	UP
TICRR	103.851	3.816649	0.233391	16.35305	4.14E-60	1.30E-57	UP
TRAIP	146.5243	2.881345	0.176419	16.33242	5.80E-60	1.80E-57	UP
CKAP2L	207.1344	3.454245	0.212282	16.27193	1.56E-59	4.80E-57	UP
NCAPH	251.8687	3.241357	0.199719	16.22957	3.12E-59	9.47E-57	UP
CENPM	252.9026	3.547842	0.219285	16.17913	7.08E-59	2.13E-56	UP
PARPBP	134.2758	2.845226	0.175947	16.17097	8.08E-59	2.40E-56	UP
AURKA	728.4274	2.75142	0.170318	16.15463	1.05E-58	3.10E-56	UP
DEPDC1	219.0945	4.029949	0.249488	16.15287	1.08E-58	3.16E-56	UP
CDKN2A	616.2275	4.122437	0.255243	16.15104	1.12E-58	3.22E-56	UP
NEIL3	78.21602	4.376132	0.271405	16.12396	1.73E-58	4.94E-56	UP
KIF14	198.6673	3.374976	0.209333	16.12254	1.77E-58	5.00E-56	UP
CENPI	91.7199	3.371943	0.20944	16.09982	2.56E-58	7.15E-56	UP
CFP	677.2638	-3.58718	0.223022	-16.0844	3.28E-58	9.08E-56	DOWN
PTH1R	455.4301	-3.41816	0.213127	-16.0382	6.92E-58	1.90E-55	DOWN
SHCBP1	184.7169	3.699975	0.230744	16.035	7.28E-58	1.97E-55	UP
RRM2	1144.431	3.212052	0.200364	16.03104	7.76E-58	2.08E-55	UP

KIF18A	115.1651	3.621976	0.225981	16.02781	8.17E-58	2.17E-55	UP
EZH2	488.3956	2.529779	0.158084	16.00272	1.22E-57	3.22E-55	UP
CPLX2	2450.962	9.331526	0.585422	15.93983	3.35E-57	8.75E-55	UP
SGOL1	124.1183	3.252014	0.20415	15.92949	3.96E-57	1.02E-54	UP
CCL23	37.24804	-3.05773	0.192193	-15.9097	5.43E-57	1.39E-54	DOWN
B4GALNT1	320.5426	4.398289	0.277923	15.82558	2.07E-56	5.26E-54	UP
SPDL1	270.1929	1.913022	0.120963	15.81488	2.46E-56	6.18E-54	UP
JRK	370.6129	1.902224	0.120432	15.79507	3.36E-56	8.39E-54	UP
SPC25	122.2351	3.220379	0.20398	15.78774	3.78E-56	9.33E-54	UP
DUSP9	1211.758	5.348233	0.339161	15.76903	5.08E-56	1.24E-53	UP
ASF1B	395.5967	3.200623	0.203517	15.72658	9.94E-56	2.41E-53	UP
E2F8	134.6943	3.81229	0.242769	15.70334	1.43E-55	3.45E-53	UP
HOXA13	242.6455	7.046596	0.449045	15.6924	1.70E-55	4.07E-53	UP
C5orf34	62.36989	2.482389	0.15846	15.66573	2.59E-55	6.13E-53	UP
PLXDC1	287.5318	2.981016	0.190435	15.65374	3.13E-55	7.35E-53	UP
ERCC6L	67.57176	3.489575	0.223422	15.61879	5.42E-55	1.26E-52	UP
ECM1	1151.362	-2.96477	0.190479	-15.5648	1.26E-54	2.91E-52	DOWN
TCF19	751.3127	2.960962	0.190579	15.53667	1.96E-54	4.48E-52	UP
OLFML2B	392.5179	3.24209	0.208747	15.53116	2.13E-54	4.84E-52	UP
KIF15	153.2181	3.576769	0.230523	15.51588	2.71E-54	6.09E-52	UP
MSTO1	502.3473	1.676337	0.108861	15.39892	1.66E-53	3.71E-51	UP
TBC1D16	1956.901	1.875077	0.121788	15.39627	1.73E-53	3.84E-51	UP
E2F7	89.2201	3.853057	0.250463	15.38373	2.10E-53	4.62E-51	UP
ARHGAP11	335.0515	2.702338	0.175676	15.38252	2.14E-53	4.67E-51	UP
TRIM71	296.6536	8.61752	0.560595	15.3721	2.52E-53	5.44E-51	UP
RASD2	58.51078	2.770577	0.180315	15.36522	2.80E-53	6.01E-51	UP
CEP131	562.0864	1.800855	0.117361	15.34455	3.85E-53	8.20E-51	UP
TERT	125.5738	7.981616	0.522207	15.28439	9.72E-53	2.05E-50	UP
PBK	256.5047	3.664341	0.239848	15.27778	1.08E-52	2.25E-50	UP
FLVCR1	614.3034	2.329195	0.152575	15.26585	1.29E-52	2.69E-50	UP
AFAP1-AS	365.3304	7.627136	0.499764	15.26147	1.38E-52	2.85E-50	UP
XRCC2	101.5041	3.096745	0.203028	15.25278	1.58E-52	3.23E-50	UP
DIAPH3	154.0102	3.673442	0.240924	15.24729	1.72E-52	3.49E-50	UP
STIL	191.4282	2.595666	0.170718	15.20437	3.31E-52	6.68E-50	UP
TONSL	655.9892	2.051623	0.134978	15.19969	3.55E-52	7.12E-50	UP
VPS45	1161.393	1.116368	0.073572	15.17375	5.28E-52	1.05E-49	UP
ANGPTL6	427.754	-2.99912	0.197657	-15.1733	5.31E-52	1.05E-49	DOWN
POLQ	121.6769	3.236858	0.213344	15.172	5.42E-52	1.06E-49	UP
FANCI	504.7225	2.36724	0.156038	15.17096	5.51E-52	1.07E-49	UP
SPATS2	699.387	1.683935	0.11113	15.15291	7.25E-52	1.40E-49	UP
WDR62	302.7524	2.804208	0.185095	15.15008	7.57E-52	1.45E-49	UP
NOX4	56.36608	2.9591	0.195615	15.12716	1.07E-51	2.05E-49	UP
GJC1	217.8237	2.700461	0.178598	15.12037	1.19E-51	2.25E-49	UP
GINS1	369.6683	2.936342	0.194389	15.1055	1.49E-51	2.80E-49	UP
RECQL4	819.1786	2.718096	0.180046	15.09671	1.70E-51	3.18E-49	UP
GMNN	1504.848	2.208177	0.146525	15.0703	2.54E-51	4.72E-49	UP
UHRF1	271.0544	3.523533	0.234348	15.03547	4.30E-51	7.93E-49	UP
ANKRD52	1427.238	1.642709	0.109334	15.02474	5.06E-51	9.26E-49	UP
CLEC4G	1084.321	-5.53443	0.371175	-14.9106	2.81E-50	5.12E-48	DOWN
MMP11	849.2018	4.160117	0.279523	14.88294	4.25E-50	7.69E-48	UP
TMEM120	398.6053	1.273306	0.085581	14.87831	4.56E-50	8.19E-48	UP
CDC45	233.4528	3.158082	0.212732	14.84538	7.45E-50	1.33E-47	UP

ORC1	159.5029	3.07678	0.207267	14.84456	7.55E-50	1.34E-47	UP
TGM3	693.0182	5.265834	0.354759	14.8434	7.68E-50	1.35E-47	UP
TOMM40L	1317.509	1.711985	0.115445	14.82949	9.44E-50	1.65E-47	UP
SGOL2	187.408	2.626539	0.177122	14.82895	9.52E-50	1.66E-47	UP
THY1	2024.585	2.647877	0.178603	14.82549	1.00E-49	1.73E-47	UP
CCNF	357.5986	2.356125	0.158948	14.82327	1.04E-49	1.78E-47	UP
LRRC14	1127.797	1.511128	0.101994	14.81583	1.16E-49	1.98E-47	UP
OLFML2A	425.8473	2.496473	0.168542	14.81218	1.22E-49	2.07E-47	UP
FAM83D	533.4966	2.643369	0.178485	14.81	1.26E-49	2.13E-47	UP
TARBP1	1208.299	1.815546	0.122634	14.80463	1.37E-49	2.29E-47	UP
RAD54L	104.6535	3.612166	0.244964	14.74572	3.28E-49	5.46E-47	UP
PGC	1179.727	9.48965	0.64458	14.72222	4.64E-49	7.69E-47	UP
CCNE1	257.2284	3.865278	0.262629	14.71765	4.97E-49	8.18E-47	UP
ORC6	142.1946	3.129277	0.212876	14.7	6.45E-49	1.06E-46	UP
IQGAP3	666.5811	3.491921	0.237954	14.67477	9.35E-49	1.52E-46	UP
SEMA5B	188.2679	2.454982	0.167302	14.67393	9.47E-49	1.53E-46	UP
CEP55	192.2079	3.465043	0.236217	14.66889	1.02E-48	1.64E-46	UP
ECT2	579.0477	2.679886	0.182699	14.66831	1.03E-48	1.65E-46	UP
TRIB1	7512.29	-2.13465	0.146107	-14.6101	2.42E-48	3.85E-46	DOWN
E2F2	108.9326	3.129371	0.214372	14.59785	2.90E-48	4.58E-46	UP
TTC13	626.3806	1.434855	0.098319	14.59386	3.07E-48	4.83E-46	UP
C16orf59	187.8629	2.828661	0.1941	14.57321	4.16E-48	6.50E-46	UP
NUSAP1	889.0133	2.352219	0.161774	14.54016	6.74E-48	1.05E-45	UP
CDCA2	108.0342	3.496908	0.240831	14.52017	9.03E-48	1.40E-45	UP
RP11-443I	190.6652	5.925494	0.408169	14.51724	9.42E-48	1.45E-45	UP
RP5-890E1	21.22081	2.710134	0.187004	14.4924	1.35E-47	2.07E-45	UP
MDK	6101.378	3.796298	0.263402	14.41258	4.31E-47	6.56E-45	UP
MCM10	169.2261	3.33617	0.231579	14.4062	4.73E-47	7.15E-45	UP
RND3	3373.555	-2.56293	0.177985	-14.3997	5.19E-47	7.81E-45	DOWN
ZWINT	709.5681	2.642476	0.18356	14.39573	5.50E-47	8.23E-45	UP
C21orf58	154.1562	2.334447	0.162412	14.3736	7.58E-47	1.13E-44	UP
HOXD9	88.05504	4.919762	0.342314	14.37209	7.75E-47	1.15E-44	UP
CENPK	107.9758	2.779535	0.193624	14.35532	9.87E-47	1.45E-44	UP
MAGEA1	294.8482	9.169083	0.638978	14.34961	1.07E-46	1.57E-44	UP
ZFP41	408.0496	1.95003	0.136083	14.32968	1.43E-46	2.08E-44	UP
MYO18B	148.9035	7.405512	0.516879	14.32735	1.48E-46	2.14E-44	UP
MTBP	109.7403	2.040866	0.142574	14.31441	1.78E-46	2.56E-44	UP
CTHRC1	315.0917	4.168939	0.291434	14.30492	2.04E-46	2.92E-44	UP
LINC00176	172.6034	4.717108	0.329759	14.30473	2.04E-46	2.92E-44	UP
FANCD2	301.646	2.310002	0.161517	14.3019	2.13E-46	3.02E-44	UP
PYGO2	2145.857	1.239221	0.086683	14.29604	2.32E-46	3.27E-44	UP
SOCS7	426.6194	1.796709	0.125695	14.29422	2.38E-46	3.34E-44	UP
OTX1	51.36733	4.546907	0.31837	14.28181	2.84E-46	3.97E-44	UP
MND1	90.15561	2.846775	0.199542	14.26656	3.54E-46	4.92E-44	UP
GNAZ	451.1888	3.681126	0.258634	14.23294	5.72E-46	7.92E-44	UP
CDH24	145.965	2.399617	0.169321	14.17202	1.37E-45	1.88E-43	UP
DSCC1	157.8326	2.181171	0.154729	14.09668	3.98E-45	5.45E-43	UP
MESP2	56.55165	3.61604	0.256553	14.09473	4.09E-45	5.58E-43	UP
MAP2K1	2429.055	-1.21793	0.086465	-14.0858	4.64E-45	6.30E-43	DOWN
ISX	304.5061	7.368045	0.523318	14.07947	5.08E-45	6.85E-43	UP
UCK2	1331.627	1.76863	0.125793	14.05983	6.70E-45	9.01E-43	UP
NDUFA4L2	1149.742	3.019338	0.215268	14.02593	1.08E-44	1.45E-42	UP

XRCC3	306.0373	1.698916	0.121231	14.01391	1.28E-44	1.70E-42	UP
FEN1	1179.639	1.683257	0.120184	14.00564	1.44E-44	1.91E-42	UP
KIAA0101	356.0193	2.538116	0.18125	14.00341	1.49E-44	1.96E-42	UP
NT5DC2	1277.765	3.060986	0.219136	13.9684	2.43E-44	3.19E-42	UP
MCM2	1337.394	2.520171	0.180459	13.96535	2.54E-44	3.31E-42	UP
MTFR2	61.62729	3.118593	0.223365	13.96186	2.66E-44	3.46E-42	UP
MAD2L1	353.5674	2.390754	0.171584	13.93346	3.97E-44	5.13E-42	UP
KIF11	373.3252	2.47934	0.178029	13.92659	4.37E-44	5.62E-42	UP
DBF4B	199.9859	1.724848	0.123863	13.9255	4.43E-44	5.68E-42	UP
RD3L	3.51909	-5.11528	0.367347	-13.9249	4.47E-44	5.70E-42	DOWN
RP11-196(	239.992	2.264569	0.163223	13.87407	9.10E-44	1.15E-41	UP
COCH	251.251	4.679807	0.337878	13.85056	1.26E-43	1.59E-41	UP
RFC4	618.9839	1.784199	0.129273	13.80174	2.49E-43	3.13E-41	UP
HOTTIP	53.04259	7.309238	0.529866	13.7945	2.75E-43	3.44E-41	UP
PIF1	112.0982	2.859262	0.20754	13.77692	3.51E-43	4.37E-41	UP
SFTA1P	9.650768	5.297163	0.384503	13.77665	3.52E-43	4.37E-41	UP
CTD-2371	38.12641	2.344896	0.170253	13.77304	3.70E-43	4.57E-41	UP
DEPDC1B	215.3743	3.32958	0.242033	13.75671	4.64E-43	5.71E-41	UP
ADCY6	1353.544	1.34305	0.097646	13.75425	4.80E-43	5.88E-41	UP
RP11-495I	48.06319	7.632124	0.55495	13.75282	4.90E-43	5.97E-41	UP
CHAF1B	262.6252	2.736235	0.198969	13.75207	4.95E-43	6.01E-41	UP
BCAN	123.77	4.662641	0.33962	13.729	6.81E-43	8.22E-41	UP
BMPER	188.2461	-4.33705	0.316059	-13.7223	7.47E-43	8.98E-41	DOWN
EHMT2	2151.261	1.338224	0.097598	13.71158	8.66E-43	1.04E-40	UP
STMN1	3000.978	2.153606	0.157325	13.68886	1.18E-42	1.41E-40	UP
CSPG4	518.4844	2.359035	0.17237	13.6859	1.23E-42	1.46E-40	UP
PRR11	268.0669	2.758862	0.201752	13.67455	1.44E-42	1.70E-40	UP
CAPN10-A	45.54281	2.366778	0.173091	13.67364	1.46E-42	1.72E-40	UP
USP49	190.6273	1.415946	0.103621	13.66462	1.65E-42	1.94E-40	UP
CENPW	230.3655	2.600902	0.190471	13.65514	1.88E-42	2.20E-40	UP
EPS8L3	642.1495	4.904181	0.359259	13.65082	2.00E-42	2.32E-40	UP
HOXA10	85.45564	5.516593	0.404345	13.64328	2.21E-42	2.56E-40	UP
IGF2BP3	138.575	4.863863	0.357108	13.62016	3.04E-42	3.50E-40	UP
RASL12	148.5707	2.396679	0.176028	13.61534	3.25E-42	3.73E-40	UP
ABCC10	587.3074	1.452213	0.106774	13.60085	3.96E-42	4.53E-40	UP
SLC7A11	326.3759	4.508221	0.331717	13.59055	4.56E-42	5.19E-40	UP
USP21	645.2229	1.208898	0.089138	13.5621	6.72E-42	7.62E-40	UP
ZFP36	10224.22	-2.21864	0.163824	-13.5428	8.73E-42	9.86E-40	DOWN
KAT2A	2158.653	1.482036	0.109469	13.53841	9.28E-42	1.04E-39	UP
OR51E1	21.70772	3.129284	0.231272	13.53078	1.03E-41	1.15E-39	UP
LINC0141(	182.682	10.57298	0.781702	13.52559	1.10E-41	1.23E-39	UP
PLK4	115.8306	2.307654	0.170686	13.51984	1.19E-41	1.33E-39	UP
PITX1	254.9081	6.106362	0.452187	13.50407	1.48E-41	1.64E-39	UP
FCN3	2383.334	-4.17388	0.309758	-13.4746	2.21E-41	2.43E-39	DOWN
HHIPL2	143.4806	6.157879	0.457337	13.46464	2.53E-41	2.77E-39	UP
CDKN2C	710.2957	2.369459	0.176093	13.45572	2.85E-41	3.12E-39	UP
LIN9	178.718	1.698898	0.126354	13.44553	3.27E-41	3.56E-39	UP
SIX2	49.24456	6.680269	0.497143	13.43733	3.65E-41	3.96E-39	UP
TK1	1336.528	2.332956	0.173785	13.42439	4.35E-41	4.70E-39	UP
RP11-175I	11.9213	3.651327	0.272088	13.41964	4.64E-41	5.00E-39	UP
CBX2	240.2185	3.009587	0.224516	13.40481	5.67E-41	6.08E-39	UP
FOXK1	911.5571	1.390582	0.103798	13.39696	6.30E-41	6.73E-39	UP

MAPT	300.5945	3.072524	0.229436	13.39162	6.77E-41	7.20E-39	UP
TRIM45	162.2219	2.399702	0.179456	13.3721	8.80E-41	9.33E-39	UP
CHTF18	408.3222	1.719743	0.12865	13.36765	9.35E-41	9.87E-39	UP
VIPR1	538.2199	-3.51726	0.263166	-13.3652	9.66E-41	1.02E-38	DOWN
HELLS	330.6773	2.662393	0.199368	13.35418	1.12E-40	1.17E-38	UP
TACC3	1207.214	2.306906	0.173083	13.32834	1.58E-40	1.65E-38	UP
AFP	13438.92	6.158274	0.462062	13.32782	1.59E-40	1.66E-38	UP
CTC-526N	38.54214	-2.10921	0.158702	-13.2904	2.63E-40	2.73E-38	DOWN
ZIC5	86.30594	6.941254	0.522522	13.28415	2.86E-40	2.95E-38	UP
NVL	533.1017	1.007066	0.075872	13.2733	3.31E-40	3.40E-38	UP
NOTCH3	2061.365	2.309669	0.174315	13.24993	4.52E-40	4.63E-38	UP
CABYR	338.6444	4.696349	0.354574	13.24504	4.82E-40	4.92E-38	UP
RAD51	125.8525	2.366102	0.178675	13.24248	4.99E-40	5.07E-38	UP
CAD	1471.211	1.454632	0.109878	13.23856	5.25E-40	5.33E-38	UP
KIAA1841	180.0021	1.719876	0.129937	13.2362	5.42E-40	5.48E-38	UP
CBFA2T2	771.0467	1.271825	0.096109	13.23309	5.65E-40	5.69E-38	UP
LRAT	222.465	-2.82982	0.213959	-13.226	6.21E-40	6.22E-38	DOWN
RUSC1	942.2209	1.518752	0.114941	13.21333	7.35E-40	7.34E-38	UP
RCAN1	4933.98	-2.30699	0.174623	-13.2112	7.56E-40	7.52E-38	DOWN
TNFRSF4	146.6204	2.428589	0.184034	13.19642	9.20E-40	9.12E-38	UP
GSG2	36.19559	3.03674	0.230177	13.19305	9.62E-40	9.51E-38	UP
COX4I2	78.89457	2.814988	0.213694	13.17301	1.25E-39	1.24E-37	UP
KIAA0907	1137.929	1.429734	0.108548	13.17145	1.28E-39	1.26E-37	UP
CDKN2B-/	27.07415	3.527605	0.26796	13.16466	1.40E-39	1.37E-37	UP
MSH2	711.8003	1.427036	0.108641	13.13537	2.06E-39	2.01E-37	UP
RDM1	33.34525	3.529885	0.268944	13.12497	2.37E-39	2.30E-37	UP
CEP250	1026.01	1.132271	0.086411	13.10327	3.15E-39	3.05E-37	UP
WDHD1	185.3511	2.197928	0.167965	13.08566	3.98E-39	3.83E-37	UP
SPRY2	781.651	-1.56956	0.119968	-13.0832	4.11E-39	3.95E-37	DOWN
RP11-774I	46.41653	10.13255	0.775001	13.07424	4.62E-39	4.42E-37	UP
ZFP1	500.1453	-1.69229	0.129491	-13.0688	4.96E-39	4.73E-37	DOWN
MARCO	1550.737	-4.79009	0.366575	-13.0672	5.07E-39	4.82E-37	DOWN
HMGXB3	1407.919	0.886218	0.067822	13.06688	5.09E-39	4.82E-37	UP
COL9A1	24.38635	3.999885	0.306116	13.06658	5.11E-39	4.83E-37	UP
DDX12P	61.91775	2.170113	0.166307	13.04884	6.45E-39	6.07E-37	UP
DNAJC6	202.4082	2.745416	0.210447	13.04563	6.73E-39	6.31E-37	UP
SMG5	5594.476	1.356508	0.10415	13.02461	8.87E-39	8.29E-37	UP
LIFR	1402.616	-2.6242	0.201542	-13.0206	9.34E-39	8.70E-37	DOWN
C17orf82	26.83137	3.23633	0.248797	13.00793	1.10E-38	1.02E-36	UP
KIFC2	500.1829	2.020441	0.155358	13.00504	1.15E-38	1.06E-36	UP
RNASEH2/	776.2773	1.866935	0.143668	12.99477	1.31E-38	1.21E-36	UP
ZNF517	608.1551	1.687816	0.129892	12.99399	1.32E-38	1.22E-36	UP
FOXO1	1898.622	-1.9377	0.149249	-12.983	1.53E-38	1.40E-36	DOWN
WDR76	265.129	2.35447	0.181443	12.97637	1.67E-38	1.52E-36	UP
NTF3	103.768	-3.34793	0.258351	-12.9588	2.09E-38	1.90E-36	DOWN
C22orf29	614.8624	1.226309	0.094632	12.95865	2.10E-38	1.90E-36	UP
MYO19	1238.746	1.332919	0.102911	12.95218	2.28E-38	2.06E-36	UP
DDX11-AS	17.49834	2.894684	0.223679	12.94123	2.63E-38	2.37E-36	UP
S100P	2126.003	5.721408	0.442135	12.9404	2.66E-38	2.39E-36	UP
RAD51AP1	164.7127	2.435808	0.188266	12.9381	2.74E-38	2.46E-36	UP
TP73	126.024	3.516048	0.271869	12.93286	2.94E-38	2.62E-36	UP
TMEM206	179.8087	1.393061	0.107888	12.91207	3.85E-38	3.42E-36	UP

EMC3-AS1	40.61741	2.312127	0.179268	12.8976	4.64E-38	4.12E-36	UP
CENPH	166.3972	1.846048	0.143182	12.89302	4.93E-38	4.36E-36	UP
TMEM79	265.0015	1.116583	0.086613	12.89162	5.02E-38	4.42E-36	UP
MCM3	3005.669	1.686445	0.130836	12.88981	5.14E-38	4.51E-36	UP
CENPU	386.5486	2.267067	0.175953	12.88447	5.51E-38	4.82E-36	UP
DENND4B	1586.747	1.122498	0.087219	12.8699	6.65E-38	5.80E-36	UP
MUC13	5135.907	5.199853	0.404115	12.86726	6.88E-38	5.99E-36	UP
DQX1	103.3229	6.47338	0.503121	12.86646	6.95E-38	6.03E-36	UP
DUSP6	4554.658	-1.83598	0.142753	-12.8612	7.44E-38	6.43E-36	DOWN
COLEC10	459.676	-3.98263	0.31019	-12.8393	9.87E-38	8.49E-36	DOWN
SPATC1L	220.4728	2.975519	0.231752	12.83925	9.88E-38	8.49E-36	UP
CAP2	1139.126	2.249034	0.17536	12.82522	1.18E-37	1.01E-35	UP
MAFG-AS1	101.1843	3.01913	0.235407	12.82517	1.19E-37	1.01E-35	UP
KCTD7	276.437	1.449816	0.113224	12.80481	1.54E-37	1.31E-35	UP
CLSPN	87.24548	3.163954	0.247229	12.79765	1.69E-37	1.43E-35	UP
DCUN1D3	559.2126	-1.16364	0.090974	-12.7909	1.84E-37	1.56E-35	DOWN
REG3A	4058.145	10.02861	0.784144	12.78925	1.88E-37	1.59E-35	UP
CRNDE	118.7389	3.287964	0.257107	12.7883	1.91E-37	1.60E-35	UP
ETS2	12124.56	-1.7923	0.140186	-12.7852	1.98E-37	1.66E-35	DOWN
FATE1	16.14851	4.54709	0.355765	12.78117	2.09E-37	1.75E-35	UP
RP11-556I	82.6393	5.506375	0.430934	12.77777	2.18E-37	1.82E-35	UP
FCN2	940.0927	-4.90628	0.384019	-12.7762	2.23E-37	1.85E-35	DOWN
NQO1	6868.238	4.821047	0.377383	12.77494	2.26E-37	1.87E-35	UP
ZNF692	796.3649	1.644273	0.128792	12.7669	2.51E-37	2.07E-35	UP
C9orf172	213.5814	1.963633	0.153807	12.76687	2.51E-37	2.07E-35	UP
KIAA1524	154.8514	2.510442	0.196646	12.76627	2.53E-37	2.08E-35	UP
SLC2A5	374.5769	4.44134	0.348183	12.75576	2.90E-37	2.37E-35	UP
OIP5	85.42329	2.339573	0.183548	12.74637	3.27E-37	2.67E-35	UP
KNTC1	528.5854	2.09758	0.164617	12.7422	3.45E-37	2.80E-35	UP
C17orf53	125.9643	2.031823	0.159462	12.74176	3.46E-37	2.81E-35	UP
BLM	130.0377	2.501044	0.196292	12.74147	3.48E-37	2.81E-35	UP
SIX1	32.32966	4.531642	0.355698	12.74012	3.54E-37	2.85E-35	UP
CRHBP	1237.783	-4.3072	0.338103	-12.7393	3.58E-37	2.88E-35	DOWN
DNASE1L3	2040.621	-3.08023	0.24199	-12.7287	4.09E-37	3.28E-35	DOWN
FANCG	412.4993	1.521814	0.119682	12.71552	4.85E-37	3.88E-35	UP
CTD-2227	54.7928	2.834807	0.223048	12.70938	5.24E-37	4.18E-35	UP
CDC7	189.8341	2.304872	0.181454	12.70227	5.74E-37	4.57E-35	UP
SEMA3F	853.849	1.298806	0.102268	12.70004	5.91E-37	4.68E-35	UP
HDAC11	884.51	1.696337	0.13357	12.69995	5.92E-37	4.68E-35	UP
LYVE1	655.724	-3.0553	0.24059	-12.6992	5.97E-37	4.71E-35	DOWN
MSTO2P	66.97961	2.16123	0.170233	12.69572	6.25E-37	4.91E-35	UP
CDC37L1	1869.676	-1.59261	0.125476	-12.6926	6.50E-37	5.10E-35	DOWN
LHX4	38.13509	2.72635	0.214814	12.69166	6.58E-37	5.14E-35	UP
MSH5	83.8605	2.278824	0.179648	12.68496	7.17E-37	5.58E-35	UP
PRIM2	338.3242	1.231274	0.09707	12.68435	7.22E-37	5.61E-35	UP
ST8SIA6-A	121.9649	5.478378	0.431936	12.68332	7.32E-37	5.67E-35	UP
S100A12	17.47945	-3.28974	0.259409	-12.6816	7.47E-37	5.78E-35	DOWN
RP11-147I	189.5244	1.696407	0.133851	12.67382	8.26E-37	6.36E-35	UP
GPRIN1	162.8354	2.727669	0.215318	12.66809	8.88E-37	6.83E-35	UP
TMEM145	45.53516	4.175402	0.329615	12.6675	8.95E-37	6.86E-35	UP
RP11-547I	7.303283	4.308561	0.340422	12.65651	1.03E-36	7.87E-35	UP
DBH	732.5897	-3.21002	0.253715	-12.6521	1.09E-36	8.30E-35	DOWN

TUBE1	750.9309	-1.77446	0.140334	-12.6445	1.20E-36	9.12E-35	DOWN
CHEK1	324.8841	1.752041	0.138668	12.63479	1.36E-36	1.03E-34	UP
LY6H	27.58187	4.619695	0.366011	12.62172	1.60E-36	1.21E-34	UP
RNFT2	76.92657	2.77995	0.220555	12.60435	2.00E-36	1.51E-34	UP
RFPL1	2.264835	-4.32609	0.343449	-12.596	2.22E-36	1.67E-34	DOWN
TRIM50	244.864	5.44729	0.43253	12.594	2.28E-36	1.71E-34	UP
IP6K1	1756.823	0.900712	0.071547	12.58917	2.42E-36	1.81E-34	UP
CETP	704.6255	-2.84965	0.226526	-12.5798	2.73E-36	2.03E-34	DOWN
EDIL3	285.3666	2.955407	0.235009	12.57572	2.87E-36	2.14E-34	UP
HAGLROS	6.840881	4.627817	0.368069	12.57324	2.96E-36	2.20E-34	UP
POLD1	886.4742	1.344481	0.106945	12.57174	3.02E-36	2.23E-34	UP
SCX	36.17144	3.163272	0.251644	12.57043	3.07E-36	2.27E-34	UP
MCM6	1255.112	1.823083	0.145163	12.55889	3.55E-36	2.61E-34	UP
SOGA1	813.6939	1.790809	0.142758	12.54435	4.27E-36	3.13E-34	UP
SCRIB	4075.737	1.379274	0.110074	12.53043	5.09E-36	3.72E-34	UP
TBC1D31	242.9242	1.422584	0.113571	12.52599	5.38E-36	3.93E-34	UP
SLCO1C1	10.46756	4.114661	0.328719	12.51727	6.01E-36	4.37E-34	UP
MAPK8IP2	364.9406	3.206059	0.256215	12.51317	6.33E-36	4.59E-34	UP
CCDC93	884.9696	1.109069	0.088715	12.50153	7.32E-36	5.30E-34	UP
LYPD1	254.149	4.097661	0.328054	12.49081	8.38E-36	6.05E-34	UP
EGR1	11181.29	-2.69506	0.216043	-12.4747	1.03E-35	7.40E-34	DOWN
B4GALNT2	162.5713	7.374874	0.592244	12.45243	1.36E-35	9.75E-34	UP
FOXS1	112.2329	3.163228	0.254114	12.44808	1.43E-35	1.03E-33	UP
CTD-2547	42.71299	1.969385	0.158234	12.44606	1.47E-35	1.05E-33	UP
RFPL4AP6	1.58763	-4.37936	0.352188	-12.4347	1.69E-35	1.21E-33	DOWN
ILF3	5879.683	0.915898	0.073682	12.43044	1.79E-35	1.27E-33	UP
C1RL	8924.372	-1.70762	0.137378	-12.4301	1.79E-35	1.27E-33	DOWN
TM4SF20	290.8795	7.570585	0.609179	12.42752	1.85E-35	1.31E-33	UP
CDCA7	187.6181	3.99207	0.321743	12.40763	2.38E-35	1.68E-33	UP
MTX1P1	99.78355	2.194739	0.176888	12.40751	2.38E-35	1.68E-33	UP
FSTL4	90.3321	4.51479	0.363941	12.40529	2.45E-35	1.72E-33	UP
RP11-124(	61.79952	2.249317	0.18135	12.40321	2.51E-35	1.76E-33	UP
HOXD8	44.77894	3.352236	0.270589	12.38869	3.01E-35	2.10E-33	UP
FAM72D	15.59394	3.74648	0.302729	12.37569	3.54E-35	2.47E-33	UP
MEP1A	210.4771	5.953836	0.481222	12.37233	3.69E-35	2.56E-33	UP
PCNXL3	2250.746	1.028405	0.083137	12.37004	3.80E-35	2.63E-33	UP
COX6B1P4	8.918843	-3.10386	0.250928	-12.3695	3.82E-35	2.64E-33	DOWN
GNG4	497.6156	5.723519	0.462718	12.36933	3.83E-35	2.64E-33	UP
ASB16	39.75759	2.314685	0.18729	12.35882	4.36E-35	3.00E-33	UP
ZBTB12	134.5115	2.038654	0.165001	12.35543	4.55E-35	3.13E-33	UP
SUV420H2	355.1304	1.564541	0.126643	12.35393	4.64E-35	3.18E-33	UP
G6PD	1837.757	2.790413	0.225913	12.35169	4.77E-35	3.26E-33	UP
AADAT	926.2748	-2.72743	0.220823	-12.3512	4.80E-35	3.27E-33	DOWN
TIGD5	559.8359	1.653926	0.134072	12.33607	5.79E-35	3.94E-33	UP
TTC39A	510.5028	3.380343	0.274214	12.32739	6.45E-35	4.37E-33	UP
EPHA2	1948.239	-2.18826	0.177698	-12.3145	7.57E-35	5.12E-33	DOWN
FAM111B	314.0695	2.806427	0.227974	12.31027	7.98E-35	5.38E-33	UP
AP5Z1	1536.194	1.144628	0.093109	12.29341	9.83E-35	6.62E-33	UP
MAP3K9	271.6341	1.490513	0.121316	12.2862	1.07E-34	7.21E-33	UP
CHAF1A	688.9874	1.504133	0.122522	12.27646	1.21E-34	8.12E-33	UP
RP11-328I	22.07003	6.391305	0.520643	12.27579	1.22E-34	8.17E-33	UP
COL6A6	35.64005	-3.11184	0.253748	-12.2635	1.42E-34	9.48E-33	DOWN

BMS1P8	104.3653	5.709141	0.465667	12.26013	1.48E-34	9.86E-33	UP
UBAP2L	4479.111	1.049801	0.085711	12.24813	1.72E-34	1.14E-32	UP
COL4A1	9099.581	1.942044	0.158598	12.24507	1.79E-34	1.18E-32	UP
TBX4	42.47346	6.937381	0.566817	12.2392	1.92E-34	1.27E-32	UP
PTP4A3	1125.047	2.634264	0.215239	12.23879	1.93E-34	1.27E-32	UP
RAD54B	71.56801	1.70939	0.139698	12.2363	1.99E-34	1.31E-32	UP
RP5-821D	105.7478	1.652587	0.135063	12.2357	2.00E-34	1.31E-32	UP
ZNF251	525.8165	1.294152	0.105855	12.22568	2.27E-34	1.48E-32	UP
ARHGAP11	77.44235	2.295526	0.187841	12.22055	2.41E-34	1.58E-32	UP
MATN3	85.72107	4.541195	0.371769	12.21511	2.58E-34	1.68E-32	UP
ATAD5	131.3999	1.804051	0.147879	12.1995	3.13E-34	2.03E-32	UP
PNRC1	5880.956	-1.57132	0.128875	-12.1927	3.40E-34	2.20E-32	DOWN
ROBO1	2231.423	2.706058	0.222068	12.18571	3.70E-34	2.39E-32	UP
EBF1	139.6211	2.068884	0.169884	12.17821	4.06E-34	2.62E-32	UP
RP11-46A	26.25711	2.266167	0.186168	12.17269	4.35E-34	2.80E-32	UP
RP11-739I	14.56612	3.307221	0.271785	12.1685	4.57E-34	2.94E-32	UP
TRIM65	637.5778	1.14081	0.093761	12.16718	4.65E-34	2.98E-32	UP
CCDC34	443.3478	1.676032	0.137828	12.16028	5.06E-34	3.23E-32	UP
CLEC1B	303.5108	-5.32686	0.438084	-12.1594	5.11E-34	3.26E-32	DOWN
FAM64A	77.31815	3.478868	0.286108	12.15926	5.12E-34	3.26E-32	UP
COL2A1	232.2447	7.555672	0.62168	12.15363	5.49E-34	3.48E-32	UP
SIX4	52.2238	3.501323	0.288302	12.14466	6.12E-34	3.88E-32	UP
TMC7	96.41063	2.304905	0.190044	12.12828	7.48E-34	4.73E-32	UP
ZC2HC1C	228.7941	-1.54966	0.127786	-12.1271	7.59E-34	4.79E-32	DOWN
RP11-495I	31.93773	7.099448	0.585556	12.12428	7.85E-34	4.94E-32	UP
COLCA2	122.0737	2.999296	0.247385	12.12398	7.88E-34	4.95E-32	UP
FANCE	157.137	1.757445	0.145156	12.10725	9.67E-34	6.05E-32	UP
FBXL18	150.3215	1.521827	0.125736	12.10339	1.01E-33	6.33E-32	UP
RFX8	17.26141	2.834867	0.234488	12.08958	1.20E-33	7.47E-32	UP
IGSF3	684.3852	2.868977	0.237316	12.08929	1.20E-33	7.48E-32	UP
NRBF2	1326.217	-1.08452	0.089774	-12.0805	1.34E-33	8.31E-32	DOWN
SCAMP3	4136.525	1.211766	0.100328	12.07806	1.38E-33	8.54E-32	UP
TPX2	1277.656	2.331857	0.193207	12.06923	1.54E-33	9.48E-32	UP
MRAP2	137.3797	4.01002	0.332622	12.05579	1.81E-33	1.11E-31	UP
LILRA5	99.26954	-2.33218	0.193466	-12.0547	1.83E-33	1.13E-31	DOWN
PEA15	4758.497	1.206627	0.100214	12.04052	2.18E-33	1.33E-31	UP
TBXA2R	237.5158	-1.78868	0.148557	-12.0403	2.18E-33	1.34E-31	DOWN
PXDNL	23.69342	2.868088	0.238275	12.03691	2.27E-33	1.39E-31	UP
NAA40	507.8545	1.223607	0.101704	12.03108	2.44E-33	1.49E-31	UP
RP11-600I	30.74528	2.182535	0.181411	12.03087	2.45E-33	1.49E-31	UP
TRIM16	624.2873	2.887821	0.240244	12.02037	2.78E-33	1.69E-31	UP
CTSV	131.6048	4.006758	0.333421	12.01713	2.89E-33	1.75E-31	UP
MSRA	1674.157	-1.62387	0.135205	-12.0105	3.13E-33	1.89E-31	DOWN
POLA2	567.2011	1.30498	0.108659	12.00992	3.15E-33	1.90E-31	UP
TRPC6	35.53645	2.174394	0.181091	12.0072	3.26E-33	1.96E-31	UP
ARHGEF11	2271.985	1.225612	0.102156	11.99744	3.66E-33	2.20E-31	UP
NPLOC4	5228.104	0.889727	0.074163	11.99685	3.69E-33	2.21E-31	UP
TMEM201	433.8637	1.358835	0.11327	11.99647	3.71E-33	2.22E-31	UP
MAP2K3	3432.344	-1.35896	0.113307	-11.9937	3.84E-33	2.29E-31	DOWN
MYD88	2618.664	-1.07943	0.090061	-11.9855	4.23E-33	2.52E-31	DOWN
SOCS2	1418.979	-2.42562	0.202563	-11.9746	4.83E-33	2.87E-31	DOWN
TRIM11	873.2916	1.138387	0.095086	11.97215	4.97E-33	2.95E-31	UP

DSN1	703.9063	1.293248	0.10807	11.96671	5.31E-33	3.14E-31	UP
ERLIN1	4142.718	-1.31267	0.109713	-11.9645	5.45E-33	3.22E-31	DOWN
GABRQ	15.95635	4.867179	0.406832	11.9636	5.51E-33	3.24E-31	UP
DACH1	56.83264	-2.43729	0.203777	-11.9606	5.72E-33	3.36E-31	DOWN
PPAP2B	6848.856	-1.68314	0.140747	-11.9586	5.86E-33	3.43E-31	DOWN
PZP	1869.509	-4.5159	0.377917	-11.9495	6.53E-33	3.82E-31	DOWN
GPR182	116.3558	-3.42994	0.287102	-11.9468	6.75E-33	3.94E-31	DOWN
IQCC	81.70565	1.462345	0.122446	11.94281	7.08E-33	4.12E-31	UP
3-Sep	64.41779	3.354306	0.280874	11.94241	7.11E-33	4.13E-31	UP
RAB3B	503.4224	3.780727	0.316696	11.93802	7.50E-33	4.35E-31	UP
RP5-1074I	102.4876	2.01646	0.169234	11.9152	9.86E-33	5.71E-31	UP
FAM132B	66.4011	2.953794	0.24792	11.9143	9.97E-33	5.76E-31	UP
ZNF335	820.8639	0.773516	0.064978	11.90428	1.12E-32	6.48E-31	UP
KIAA1522	3142.223	1.629644	0.136923	11.90187	1.16E-32	6.66E-31	UP
DHX34	982.1235	1.17314	0.098637	11.8935	1.28E-32	7.34E-31	UP
CPEB3	1045.456	-2.16644	0.18222	-11.8892	1.35E-32	7.72E-31	DOWN
SPP1	28491.79	4.464936	0.375611	11.88714	1.38E-32	7.89E-31	UP
PLSCR4	1397.581	-1.85876	0.156405	-11.8842	1.43E-32	8.16E-31	DOWN
SPOCK1	138.8364	4.169308	0.350835	11.88397	1.43E-32	8.16E-31	UP
LOXL2	707.1451	1.931472	0.162627	11.87671	1.56E-32	8.89E-31	UP
NPSR1-AS	39.69554	6.563126	0.552961	11.86907	1.71E-32	9.72E-31	UP
CENPO	292.7417	1.791858	0.151138	11.85574	2.01E-32	1.14E-30	UP
SAPCD2	141.1552	3.014793	0.254337	11.85356	2.06E-32	1.16E-30	UP
KIF24	105.5883	2.150979	0.181496	11.85136	2.12E-32	1.19E-30	UP
KDSR	3294.881	-0.95994	0.081081	-11.8393	2.45E-32	1.38E-30	DOWN
N4BP3	86.66132	2.21999	0.187842	11.81841	3.14E-32	1.76E-30	UP
ABCC5	676.0028	1.185362	0.100306	11.81749	3.17E-32	1.78E-30	UP
COG2	1020.934	1.049112	0.088793	11.81522	3.26E-32	1.82E-30	UP
PMFBP1	43.29796	2.94087	0.249113	11.80538	3.66E-32	2.04E-30	UP
CTD-2649	18.35058	2.436036	0.206441	11.80016	3.90E-32	2.17E-30	UP
GBAP1	65.50964	1.65801	0.14058	11.79407	4.19E-32	2.33E-30	UP
SOCS6	1580.258	-1.10916	0.094107	-11.7862	4.60E-32	2.55E-30	DOWN
PIGC	1288.265	1.031291	0.087506	11.7854	4.64E-32	2.57E-30	UP
NUDT17	69.27316	1.770006	0.150253	11.78014	4.94E-32	2.73E-30	UP
UBE2S	669.7608	2.086845	0.177329	11.7682	5.69E-32	3.14E-30	UP
SERTAD1	1020.377	-1.73238	0.147275	-11.7629	6.06E-32	3.34E-30	DOWN
ZNF696	308.7335	1.207148	0.102671	11.7574	6.47E-32	3.55E-30	UP
SSUH2	225.5267	3.992273	0.339606	11.75562	6.61E-32	3.62E-30	UP
CUL7	1599.474	1.182119	0.1006	11.7507	7.00E-32	3.83E-30	UP
NETO2	146.017	2.610302	0.222143	11.75055	7.02E-32	3.83E-30	UP
ACLY	4096.073	1.118647	0.095229	11.74691	7.32E-32	3.99E-30	UP
SP6	48.16988	3.030458	0.258022	11.74495	7.50E-32	4.07E-30	UP
SEC62	7088.027	-0.9289	0.079093	-11.7444	7.54E-32	4.09E-30	DOWN
NSUN6	1245.563	-1.34792	0.114798	-11.7416	7.80E-32	4.22E-30	DOWN
PHLDA1	6695.706	-2.45541	0.209237	-11.7351	8.43E-32	4.55E-30	DOWN
CTAG2	218.5796	11.51867	0.982946	11.71851	1.02E-31	5.53E-30	UP
CNBP	11910.27	-0.75257	0.064238	-11.7154	1.06E-31	5.72E-30	DOWN
C1orf35	755.4673	1.244189	0.106218	11.71354	1.09E-31	5.84E-30	UP
TRIM59	94.91677	2.169153	0.185245	11.70964	1.14E-31	6.10E-30	UP
CSMD2	77.14457	3.480343	0.297604	11.69455	1.36E-31	7.27E-30	UP
SNHG1	854.8735	1.665015	0.142445	11.68883	1.45E-31	7.76E-30	UP
CDC25A	176.5072	2.304626	0.197194	11.68709	1.48E-31	7.91E-30	UP

IER2	3840.264	-1.47686	0.126599	-11.6657	1.91E-31	1.02E-29	DOWN
ADRA2B	176.9581	-2.16651	0.185873	-11.6559	2.14E-31	1.14E-29	DOWN
IRAK1	4773.946	1.30766	0.112217	11.65298	2.22E-31	1.17E-29	UP
ZNF707	348.5045	0.978611	0.084001	11.65001	2.29E-31	1.21E-29	UP
ARHGAP39	195.7375	1.785115	0.153312	11.64371	2.47E-31	1.30E-29	UP
POLR1A	1525.002	0.894255	0.076859	11.63501	2.74E-31	1.44E-29	UP
MCM4	1957.07	1.716021	0.147541	11.6308	2.87E-31	1.51E-29	UP
CCNE2	103.0544	2.417181	0.207838	11.63013	2.90E-31	1.52E-29	UP
EHD3	511.9265	-1.97111	0.169495	-11.6293	2.93E-31	1.53E-29	DOWN
CTD-3065	73.79039	2.010244	0.172921	11.62519	3.07E-31	1.61E-29	UP
MPC1	4968.067	-1.69457	0.145804	-11.6223	3.18E-31	1.66E-29	DOWN
GIT1	1376.466	1.118629	0.096273	11.61934	3.29E-31	1.71E-29	UP
RP1-63M2	15.83039	2.729313	0.234925	11.61782	3.35E-31	1.74E-29	UP
FOS	9888.097	-2.93374	0.252616	-11.6134	3.52E-31	1.83E-29	DOWN
IL1RAP	2591.787	-2.00883	0.173106	-11.6046	3.90E-31	2.02E-29	DOWN
LAMA4	1107.948	1.694801	0.146098	11.60047	4.10E-31	2.12E-29	UP
TIGD1	124.4728	1.883148	0.162334	11.60044	4.10E-31	2.12E-29	UP
BAIAP2L2	487.8572	2.977857	0.257095	11.58272	5.04E-31	2.60E-29	UP
CTA-204B	61.20786	1.519598	0.131197	11.5826	5.05E-31	2.60E-29	UP
TOB2P1	24.05876	2.527227	0.218221	11.58106	5.14E-31	2.64E-29	UP
YEATS2	709.9196	1.208378	0.104421	11.57215	5.70E-31	2.92E-29	UP
CTD-2510	26.09037	3.004924	0.259691	11.57114	5.77E-31	2.95E-29	UP
SEZ6	114.5553	5.884409	0.50858	11.57028	5.83E-31	2.98E-29	UP
MYLK-AS1	26.41206	2.271266	0.196335	11.56834	5.96E-31	3.04E-29	UP
MCL1	19231.42	-1.29566	0.112061	-11.5621	6.42E-31	3.26E-29	DOWN
IL1RL1	131.8012	-3.6286	0.314021	-11.5553	6.94E-31	3.52E-29	DOWN
ZBTB45	422.5064	0.880108	0.076231	11.54524	7.80E-31	3.95E-29	UP
RASGRF2	231.9605	1.746446	0.151301	11.54283	8.02E-31	4.05E-29	UP
DNMT3A	1103.828	1.489384	0.129031	11.54283	8.02E-31	4.05E-29	UP
RHNO1	495.4675	1.225752	0.106209	11.54094	8.20E-31	4.13E-29	UP
UPK3A	267.7747	5.369127	0.465842	11.52563	9.80E-31	4.93E-29	UP
COL22A1	169.5702	4.833758	0.419402	11.52537	9.83E-31	4.94E-29	UP
LENG8-AS	40.82957	1.569091	0.136179	11.52227	1.02E-30	5.11E-29	UP
PPM1F	1246.871	1.107654	0.096194	11.51475	1.11E-30	5.56E-29	UP
ACAN	83.05233	3.971996	0.345361	11.50099	1.30E-30	6.51E-29	UP
SFN	1248.3	3.912774	0.340407	11.49441	1.41E-30	7.02E-29	UP
NUDT1	397.0511	1.76049	0.153427	11.47448	1.77E-30	8.82E-29	UP
RTKN2	69.95929	3.04827	0.265703	11.47248	1.81E-30	9.01E-29	UP
RIBC2	55.66803	3.233646	0.281937	11.4694	1.88E-30	9.32E-29	UP
CHGA	50.36707	6.376307	0.556319	11.4616	2.06E-30	1.02E-28	UP
PAQR4	307.9591	2.1917	0.191372	11.45257	2.28E-30	1.13E-28	UP
RASGEF1B	1309.134	-1.98802	0.173624	-11.4502	2.35E-30	1.16E-28	DOWN
CD4	3722.401	-1.96276	0.171445	-11.4483	2.40E-30	1.18E-28	DOWN
IL1RN	4180.332	-2.48705	0.217342	-11.443	2.55E-30	1.25E-28	DOWN
LRRC37A6	34.09436	3.364957	0.294131	11.44035	2.63E-30	1.29E-28	UP
CYP26A1	443.6391	-4.19646	0.366974	-11.4353	2.79E-30	1.36E-28	DOWN
ZNF219	1194.659	1.378928	0.120641	11.43001	2.96E-30	1.45E-28	UP
GBA	3156.103	1.339433	0.117207	11.42794	3.03E-30	1.48E-28	UP
LINC01311	23.36002	2.127852	0.186226	11.4262	3.09E-30	1.51E-28	UP
C21orf91	462.7344	-1.5684	0.137299	-11.4232	3.20E-30	1.56E-28	DOWN
CLEC4M	447.874	-5.6836	0.497668	-11.4205	3.30E-30	1.60E-28	DOWN
C8orf4	3304.985	-2.26911	0.198699	-11.4198	3.33E-30	1.61E-28	DOWN

GPSM2	233.3776	1.860096	0.163021	11.41014	3.72E-30	1.80E-28	UP
EDC3	872.0521	0.716395	0.062788	11.40977	3.74E-30	1.80E-28	UP
CLK2	1278.507	1.053182	0.092309	11.40932	3.76E-30	1.81E-28	UP
SLC38A2	14650.34	-1.70646	0.149627	-11.4047	3.96E-30	1.91E-28	DOWN
RNF157	746.5134	2.765975	0.24256	11.40327	4.03E-30	1.93E-28	UP
COL4A2	10363.62	1.811041	0.159002	11.39002	4.69E-30	2.25E-28	UP
NANOS1	76.20024	3.067363	0.269348	11.38812	4.79E-30	2.29E-28	UP
MAGEC2	246.2884	8.39091	0.737036	11.38466	4.99E-30	2.38E-28	UP
RP11-533I	14.19914	2.473954	0.217314	11.38423	5.01E-30	2.39E-28	UP
INCENP	399.4995	1.449389	0.127395	11.37714	5.44E-30	2.59E-28	UP
MRO	207.8411	-2.23077	0.196138	-11.3735	5.67E-30	2.69E-28	DOWN
ILF2	5165.701	0.977642	0.085964	11.37263	5.72E-30	2.72E-28	UP
MAST2	1253.896	1.148301	0.101043	11.36453	6.28E-30	2.97E-28	UP
RP11-486(	78.62633	1.373939	0.120898	11.36443	6.29E-30	2.97E-28	UP
STRIP2	91.57044	2.739492	0.241098	11.36259	6.42E-30	3.03E-28	UP
NDRG2	11853.17	-1.62532	0.143057	-11.3614	6.51E-30	3.07E-28	DOWN
FBXL19	385.1321	1.243599	0.109462	11.36098	6.54E-30	3.08E-28	UP
BCKDHB	1894.954	-1.61904	0.14254	-11.3585	6.73E-30	3.16E-28	DOWN
HAMP	10063.33	-4.53667	0.39943	-11.3578	6.78E-30	3.18E-28	DOWN
LINC0108(	192.7901	1.6902	0.148829	11.35662	6.88E-30	3.22E-28	UP
RP11-127!	10.70785	2.590308	0.228163	11.35286	7.18E-30	3.35E-28	UP
ADAM15	3306.467	1.294684	0.114197	11.33731	8.57E-30	4.00E-28	UP
C19orf40	101.4401	1.492374	0.131693	11.33219	9.09E-30	4.23E-28	UP
JAG2	541.8767	1.908111	0.168441	11.3281	9.52E-30	4.43E-28	UP
CCDC28B	222.1707	2.382368	0.210331	11.32674	9.67E-30	4.49E-28	UP
NDOR1	353.9029	1.070517	0.094538	11.32365	1.00E-29	4.64E-28	UP
SIGLEC11	65.44822	-2.48303	0.219385	-11.3181	1.07E-29	4.94E-28	DOWN
NCAPD2	1317.422	1.644907	0.145354	11.31653	1.09E-29	5.02E-28	UP
GABRE	715.4597	2.846456	0.251569	11.31483	1.11E-29	5.11E-28	UP
ENAH	3285.774	1.758038	0.155421	11.31146	1.15E-29	5.30E-28	UP
ANAPC7	1390.117	0.923877	0.081687	11.30996	1.17E-29	5.38E-28	UP
PSMC3IP	78.38739	1.755154	0.155195	11.30938	1.18E-29	5.41E-28	UP
PXDC1	6332.327	-1.44951	0.128179	-11.3085	1.19E-29	5.46E-28	DOWN
CCHCR1	1210.979	1.356794	0.120002	11.30639	1.22E-29	5.58E-28	UP
PDGFA	932.0161	1.95357	0.172827	11.3036	1.26E-29	5.75E-28	UP
CHIC2	444.1283	-0.9434	0.083479	-11.301	1.30E-29	5.91E-28	DOWN
ZBED8	124.2173	1.310515	0.11597	11.30042	1.31E-29	5.94E-28	UP
RP11-250I	68.90419	-1.68095	0.148766	-11.2992	1.32E-29	6.01E-28	DOWN
C8orf33	2178.145	1.271324	0.112529	11.2977	1.35E-29	6.11E-28	UP
ASIC1	121.9958	3.427325	0.303404	11.29624	1.37E-29	6.20E-28	UP
ZSCAN9	285.3039	0.991888	0.087824	11.29404	1.40E-29	6.35E-28	UP
SPSB2	372.0521	1.418759	0.125667	11.28979	1.47E-29	6.65E-28	UP
PRAME	406.1043	7.081291	0.627245	11.28952	1.48E-29	6.66E-28	UP
OSR2	60.86007	4.244087	0.375944	11.28913	1.48E-29	6.68E-28	UP
CSPG5	61.91844	2.68015	0.237424	11.28845	1.50E-29	6.72E-28	UP
EFNA4	386.9332	1.518036	0.134484	11.28785	1.51E-29	6.76E-28	UP
METTL6	277.8725	0.833823	0.073877	11.28669	1.53E-29	6.84E-28	UP
KCP	58.37867	3.106421	0.27533	11.28254	1.60E-29	7.15E-28	UP
IQCD	57.00636	2.803225	0.248475	11.2817	1.62E-29	7.21E-28	UP
MCM7	3265.163	1.415865	0.125562	11.27623	1.72E-29	7.66E-28	UP
GLI4	602.2234	1.574993	0.139703	11.27384	1.77E-29	7.86E-28	UP
DUXAP8	62.04033	3.216319	0.285299	11.27348	1.77E-29	7.88E-28	UP

MMAA	1036.17	-1.24474	0.110436	-11.2711	1.82E-29	8.08E-28	DOWN
RAB11FIP4	1523.705	1.887651	0.16752	11.26819	1.88E-29	8.34E-28	UP
TBCE	1346.775	1.044539	0.092703	11.26756	1.90E-29	8.39E-28	UP
TMEM106	2600.107	1.482078	0.131543	11.26686	1.91E-29	8.44E-28	UP
ZNF646	548.5113	0.939462	0.083407	11.26361	1.98E-29	8.74E-28	UP
ALDH2	52492.13	-1.87753	0.166716	-11.2619	2.02E-29	8.90E-28	DOWN
JUN	9504.659	-1.64952	0.146474	-11.2615	2.03E-29	8.93E-28	DOWN
TIMD4	127.8855	-3.69688	0.328296	-11.2608	2.05E-29	8.98E-28	DOWN
CCDC71L	1625.438	-1.92955	0.171563	-11.2469	2.40E-29	1.05E-27	DOWN
CDCA4	278.2676	1.528522	0.135908	11.24676	2.40E-29	1.05E-27	UP
PPOX	728.777	1.066313	0.094943	11.2311	2.87E-29	1.25E-27	UP
WHSC1	1517.323	1.292912	0.115143	11.22872	2.95E-29	1.28E-27	UP
OTUB2	105.8726	1.706453	0.152056	11.22252	3.16E-29	1.38E-27	UP
SNORD104	79.38631	2.663523	0.237362	11.22137	3.20E-29	1.39E-27	UP
ADRA2C	230.0474	3.401292	0.303162	11.21937	3.28E-29	1.42E-27	UP
4-Mar	8.271648	4.062891	0.362249	11.21574	3.41E-29	1.48E-27	UP
KLHL15	749.9661	-1.31271	0.117074	-11.2127	3.53E-29	1.53E-27	DOWN
KCNK9	37.29206	6.265079	0.558817	11.21133	3.59E-29	1.55E-27	UP
RP11-152I	24.98911	1.816986	0.162091	11.20964	3.66E-29	1.58E-27	UP
RP11-172I	17.94978	-4.45992	0.39809	-11.2033	3.93E-29	1.69E-27	DOWN
LINC0066!	372.4035	3.018026	0.269608	11.19413	4.36E-29	1.87E-27	UP
LEF1	236.4487	2.876411	0.257017	11.19152	4.49E-29	1.92E-27	UP
ZNF330	1220.473	-1.02637	0.091715	-11.1909	4.52E-29	1.94E-27	DOWN
HAGHL	101.0266	3.030841	0.270898	11.18811	4.66E-29	1.99E-27	UP
MAGEB17	56.39655	6.657871	0.595205	11.18585	4.78E-29	2.04E-27	UP
IQCE	636.1374	1.257266	0.112421	11.18357	4.91E-29	2.09E-27	UP
DDX39A	2299.909	1.52583	0.13647	11.18067	5.07E-29	2.16E-27	UP
ZC3H3	1402.752	1.075861	0.096258	11.17681	5.30E-29	2.25E-27	UP
ZNF605	410.4197	1.224594	0.109577	11.17565	5.37E-29	2.28E-27	UP
DCAF8L2	22.24044	9.342357	0.836143	11.17315	5.52E-29	2.34E-27	UP
NPY1R	240.3879	-2.64377	0.236737	-11.1675	5.88E-29	2.49E-27	DOWN
VPS72	1570.11	1.012677	0.090698	11.16538	6.02E-29	2.54E-27	UP
KIAA1462	1003.19	1.976066	0.177058	11.16053	6.36E-29	2.68E-27	UP
SMARCA4	3339.629	1.048122	0.093934	11.15803	6.54E-29	2.75E-27	UP
RP5-1120I	44.83883	3.896784	0.349241	11.15788	6.55E-29	2.75E-27	UP
RP11-480/	20.58097	2.262517	0.202773	11.15786	6.55E-29	2.75E-27	UP
CAPN11	22.13074	1.985021	0.177904	11.15781	6.56E-29	2.75E-27	UP
SH3BP5L	1507.278	0.868576	0.077892	11.15102	7.08E-29	2.96E-27	UP
COL7A1	707.4617	3.339198	0.299632	11.14433	7.63E-29	3.19E-27	UP
LAMC1	6432.737	1.581932	0.142011	11.13953	8.05E-29	3.36E-27	UP
NSMCE2	834.7564	1.309884	0.117612	11.13732	8.26E-29	3.44E-27	UP
TAOK2	2134.312	0.77062	0.0692	11.13606	8.37E-29	3.48E-27	UP
ATAD2	1718.255	1.572561	0.141222	11.13539	8.44E-29	3.51E-27	UP
CBX1	1351.982	0.942503	0.084644	11.13489	8.49E-29	3.52E-27	UP
RP11-572(	15.70297	2.564157	0.230311	11.13347	8.62E-29	3.57E-27	UP
KCNMB2-/	38.06582	4.607324	0.414349	11.11942	1.01E-28	4.17E-27	UP
CD200	137.5788	2.0231	0.18195	11.11901	1.01E-28	4.19E-27	UP
RP11-175I	8.513102	2.700198	0.243048	11.10971	1.13E-28	4.64E-27	UP
MTL5	98.87888	2.334953	0.210283	11.10388	1.20E-28	4.95E-27	UP
WNK4	278.7335	3.762587	0.339142	11.09444	1.33E-28	5.49E-27	UP
EPPK1	457.6599	3.592456	0.323882	11.09188	1.37E-28	5.64E-27	UP
NR2C2AP	662.1015	1.297811	0.117018	11.09074	1.39E-28	5.70E-27	UP

ARHGAP33	241.9576	1.629393	0.146919	11.09041	1.40E-28	5.72E-27	UP
LINC00205	72.55958	1.639794	0.147919	11.08578	1.47E-28	6.01E-27	UP
SIGLEC7	95.42537	-2.04009	0.184136	-11.0793	1.58E-28	6.45E-27	DOWN
CERS1	47.21345	5.053817	0.456283	11.07606	1.64E-28	6.68E-27	UP
DYNC1I1	209.7015	3.770033	0.340671	11.0665	1.82E-28	7.42E-27	UP
SLC6A8	1509.581	3.332193	0.301303	11.05928	1.98E-28	8.03E-27	UP
MMADHC	3610.531	-0.84029	0.076031	-11.0519	2.15E-28	8.71E-27	DOWN
RUSC1-AS	209.2986	1.815561	0.164418	11.04238	2.39E-28	9.67E-27	UP
LINC01287	237.161	8.291158	0.750982	11.04042	2.44E-28	9.87E-27	UP
5-Sep	346.655	2.187538	0.198194	11.03738	2.52E-28	1.02E-26	UP
GPR19	21.96535	2.790149	0.252824	11.03593	2.56E-28	1.03E-26	UP
KLC2	589.3101	1.183003	0.107212	11.03424	2.61E-28	1.05E-26	UP
LILRA2	79.3651	-1.9524	0.176944	-11.034	2.62E-28	1.05E-26	DOWN
PI4KB	2588.392	0.865495	0.078441	11.03376	2.63E-28	1.05E-26	UP
C5orf46	26.59458	4.013206	0.363864	11.02942	2.76E-28	1.11E-26	UP
CYP17A1	3740.631	4.643594	0.421036	11.02898	2.77E-28	1.11E-26	UP
PRND	35.474	4.203432	0.381318	11.02343	2.95E-28	1.18E-26	UP
ZBTB40	632.2311	1.02752	0.093234	11.02092	3.03E-28	1.21E-26	UP
PIGU	1109.235	1.10766	0.100516	11.01974	3.07E-28	1.22E-26	UP
SH3PXD2E	616.8317	1.502754	0.13644	11.01403	3.27E-28	1.30E-26	UP
ZNF142	504.1819	0.934408	0.084872	11.00964	3.43E-28	1.36E-26	UP
DGKI	21.13255	2.37685	0.215888	11.00962	3.43E-28	1.36E-26	UP
BRSK2	55.09205	3.929748	0.357045	11.00632	3.56E-28	1.41E-26	UP
IGFBP3	14306.1	-1.9818	0.180093	-11.0043	3.64E-28	1.44E-26	DOWN
LIG1	1224.896	1.153906	0.104868	11.00342	3.68E-28	1.45E-26	UP
RP11-498(	12.63438	2.426192	0.220502	11.00302	3.70E-28	1.46E-26	UP
DDIAS	97.55387	1.774993	0.161341	11.00147	3.76E-28	1.48E-26	UP
RP11-284I	476.8004	3.743357	0.340261	11.00143	3.76E-28	1.48E-26	UP
MT2P1	158.4577	-3.61923	0.328985	-11.0012	3.77E-28	1.48E-26	DOWN
EGFL6	15.82484	4.435056	0.403213	10.99929	3.85E-28	1.51E-26	UP
GAD1	32.26593	4.720174	0.429142	10.99909	3.86E-28	1.51E-26	UP
TEX19	9.162653	4.884093	0.444367	10.99113	4.22E-28	1.65E-26	UP
SLC7A6	151.459	1.82111	0.165696	10.99069	4.24E-28	1.66E-26	UP
SAPCD1	15.58783	2.716115	0.247142	10.99009	4.26E-28	1.66E-26	UP
FANCB	18.4653	2.400957	0.218654	10.98063	4.74E-28	1.85E-26	UP
ALG1L	238.8435	3.288644	0.299655	10.97478	5.05E-28	1.97E-26	UP
TYMS	877.5566	1.99343	0.181664	10.97315	5.14E-28	2.00E-26	UP
OSBPL3	358.9534	1.898542	0.173083	10.96899	5.39E-28	2.09E-26	UP
TTL	1190.409	0.935236	0.08532	10.96148	5.85E-28	2.27E-26	UP
MROH1	1675.249	1.349055	0.123086	10.96029	5.93E-28	2.29E-26	UP
FAM222A	626.8297	1.883132	0.171814	10.96029	5.93E-28	2.29E-26	UP
FERMT2	3076.228	-1.0975	0.100138	-10.9598	5.96E-28	2.30E-26	DOWN
NSUN5P1	292.7149	1.482395	0.135338	10.95328	6.41E-28	2.47E-26	UP
REG1A	911.82	7.914837	0.722749	10.95102	6.57E-28	2.53E-26	UP
BCL9	875.7094	1.4742	0.134623	10.9506	6.60E-28	2.54E-26	UP
ACAA2	19694.63	-1.70877	0.156055	-10.9497	6.66E-28	2.56E-26	DOWN
FAM171A2	69.42217	2.531733	0.231255	10.94778	6.81E-28	2.61E-26	UP
BNIP2	1737.244	-0.77754	0.07105	-10.9437	7.13E-28	2.72E-26	DOWN
N4BP2L1	1482.195	-1.58427	0.144767	-10.9436	7.13E-28	2.72E-26	DOWN
RP11-309I	27.33747	2.42648	0.221729	10.94345	7.14E-28	2.73E-26	UP
LINC01296	13.38128	4.203317	0.3841	10.94328	7.16E-28	2.73E-26	UP
FAM57B	10.00525	4.111457	0.375969	10.93562	7.79E-28	2.96E-26	UP

SMC1B	36.76259	3.600748	0.329585	10.92508	8.75E-28	3.32E-26	UP
SRRM3	155.0165	3.628755	0.332171	10.92434	8.82E-28	3.35E-26	UP
FLJ44511	43.03847	3.186371	0.29169	10.92382	8.87E-28	3.36E-26	UP
CRAMP1L	325.9577	1.031131	0.094393	10.9238	8.87E-28	3.36E-26	UP
UNC119B	652.1311	1.372907	0.125737	10.91886	9.37E-28	3.54E-26	UP
ACADS	7021.333	-1.75742	0.160957	-10.9185	9.40E-28	3.55E-26	DOWN
MCM8	353.0272	1.491521	0.136613	10.91783	9.47E-28	3.57E-26	UP
DHX57	608.0619	0.821169	0.075218	10.91721	9.54E-28	3.59E-26	UP
PPP2R2C	194.0566	5.232149	0.479305	10.91612	9.65E-28	3.63E-26	UP
TLDC2	104.0664	2.350731	0.215386	10.91406	9.87E-28	3.71E-26	UP
FAM46A	1569.947	-1.62633	0.149021	-10.9134	9.95E-28	3.73E-26	DOWN
TSSK6	81.54679	1.469889	0.134741	10.90898	1.04E-27	3.91E-26	UP
GINS3	157.1749	1.573053	0.1442	10.90885	1.05E-27	3.91E-26	UP
GAREML	124.484	2.551728	0.234026	10.9036	1.11E-27	4.14E-26	UP
SSX1	303.7221	8.773092	0.804821	10.90068	1.14E-27	4.27E-26	UP
FEZF1-AS1	39.22374	5.615337	0.515148	10.90043	1.15E-27	4.27E-26	UP
CD109	587.9504	2.703846	0.248121	10.8973	1.19E-27	4.42E-26	UP
PVT1	182.5674	2.326675	0.213593	10.89302	1.24E-27	4.62E-26	UP
FOXD2	98.14079	2.116397	0.194323	10.89114	1.27E-27	4.71E-26	UP
U73166.2	20.29629	2.371745	0.217881	10.8855	1.35E-27	5.01E-26	UP
DUXAP10	11.9745	3.915962	0.359884	10.88117	1.42E-27	5.24E-26	UP
LIN7C	1715.523	-0.82241	0.075618	-10.8759	1.50E-27	5.55E-26	DOWN
NCAPD2P1	20.38483	5.76155	0.529793	10.8751	1.51E-27	5.59E-26	UP
TUBG1	1522.234	1.139606	0.104816	10.87243	1.56E-27	5.75E-26	UP
CYP2C19	211.1347	-3.98237	0.366341	-10.8707	1.59E-27	5.85E-26	DOWN
SNHG4	34.60122	2.723182	0.250572	10.86786	1.64E-27	6.03E-26	UP
RP11-34F2	7.392765	2.740588	0.252294	10.86269	1.74E-27	6.37E-26	UP
H2AFZ	4102.752	1.228957	0.113218	10.85482	1.89E-27	6.93E-26	UP
TCOF1	1610.739	1.076492	0.099195	10.85231	1.94E-27	7.12E-26	UP
NAT9	1053.177	1.117666	0.103033	10.84761	2.05E-27	7.48E-26	UP
BACE1-AS	86.28941	1.310307	0.120819	10.84523	2.10E-27	7.67E-26	UP
ATXN7L3	1877.95	0.78476	0.072369	10.84393	2.13E-27	7.77E-26	UP
HCFC1	2630.356	0.785659	0.072454	10.84359	2.14E-27	7.79E-26	UP
SLC9A3	104.1786	3.760208	0.34682	10.84197	2.18E-27	7.92E-26	UP
EVX1	13.33144	5.95527	0.549352	10.84053	2.21E-27	8.03E-26	UP
MFI2-AS1	41.21573	2.531938	0.233693	10.83447	2.36E-27	8.57E-26	UP
PEX13	1788.588	-0.76059	0.07021	-10.8331	2.40E-27	8.69E-26	DOWN
NDST3	25.66399	-3.99423	0.368932	-10.8265	2.58E-27	9.33E-26	DOWN
AC079466	463.14	5.777059	0.533616	10.82625	2.59E-27	9.34E-26	UP
HCG25	21.62245	1.721126	0.158995	10.82503	2.62E-27	9.45E-26	UP
XPO5	1527.764	0.972406	0.089852	10.82229	2.70E-27	9.72E-26	UP
ETFDH	3891.454	-1.63553	0.151134	-10.8217	2.72E-27	9.78E-26	DOWN
CSMD1	83.57203	6.375696	0.589239	10.82022	2.76E-27	9.92E-26	UP
ZNF74	320.8033	1.025157	0.094747	10.81991	2.77E-27	9.94E-26	UP
MT1XP1	42.99499	-3.34915	0.309546	-10.8195	2.78E-27	9.97E-26	DOWN
KLF10	3242.751	-1.50386	0.139017	-10.8178	2.83E-27	1.01E-25	DOWN
NECAB3	1611.264	1.321561	0.122179	10.81656	2.87E-27	1.03E-25	UP
SF3B4	3201.71	1.069932	0.098926	10.81546	2.91E-27	1.04E-25	UP
FRMD4B	1167.53	-1.31871	0.121956	-10.813	2.99E-27	1.06E-25	DOWN
RNF152	2365.187	-1.80858	0.167297	-10.8106	3.07E-27	1.09E-25	DOWN
HIST1H4H	54.31361	2.886836	0.267248	10.8021	3.36E-27	1.20E-25	UP
SULT1C2	658.0092	3.589143	0.33237	10.79865	3.49E-27	1.24E-25	UP

DNMT3B	122.9507	1.832334	0.169702	10.79738	3.54E-27	1.26E-25	UP
CCDC142	253.4222	0.988928	0.091634	10.79218	3.75E-27	1.33E-25	UP
ZFP62	481.7914	0.975026	0.090394	10.78644	3.99E-27	1.41E-25	UP
DUSP12	417.231	0.914258	0.084794	10.78215	4.18E-27	1.48E-25	UP
ZNF740	971.0363	0.936019	0.086863	10.77584	4.48E-27	1.58E-25	UP
RP11-57H	65.46625	1.737804	0.161311	10.77298	4.62E-27	1.63E-25	UP
AC006277	14.42308	2.029989	0.188565	10.76544	5.01E-27	1.76E-25	UP
ZADH2	1367.059	-1.03023	0.095729	-10.762	5.20E-27	1.83E-25	DOWN
LINC0142€	116.1924	3.123536	0.29028	10.76042	5.29E-27	1.86E-25	UP
PSPH	1015.436	1.630834	0.151629	10.75542	5.59E-27	1.96E-25	UP
HCN2	66.21986	3.255099	0.302695	10.75373	5.69E-27	1.99E-25	UP
IFNAR1	4405.617	-0.85146	0.07925	-10.7441	6.32E-27	2.21E-25	DOWN
SLC26A2	393.5273	1.315012	0.122395	10.744	6.32E-27	2.21E-25	UP
TMEM184	1683.604	1.137419	0.105909	10.73957	6.64E-27	2.31E-25	UP
FAM220A	509.184	0.815154	0.07591	10.73844	6.72E-27	2.34E-25	UP
GPAA1	9148.511	1.365229	0.127151	10.73707	6.82E-27	2.37E-25	UP
FOXD2-AS	132.0139	2.012756	0.187488	10.73537	6.94E-27	2.41E-25	UP
CCDC64	528.7394	2.401634	0.223739	10.73411	7.04E-27	2.44E-25	UP
LINC0094z	63.94771	6.29664	0.586646	10.73328	7.10E-27	2.46E-25	UP
FAM219A	555.7984	0.83102	0.07746	10.72834	7.49E-27	2.59E-25	UP
HSF2BP	15.71643	2.231605	0.208108	10.72329	7.91E-27	2.74E-25	UP
RBM7	804.4541	-0.7816	0.072943	-10.7152	8.64E-27	2.98E-25	DOWN
PRCC	2901.855	0.883221	0.082442	10.7133	8.82E-27	3.04E-25	UP
MIOX	51.51127	4.402485	0.411102	10.70898	9.24E-27	3.18E-25	UP
C4orf46	163.3621	1.36225	0.127231	10.70687	9.45E-27	3.25E-25	UP
RP11-148I	26.23107	1.727068	0.161408	10.7	1.02E-26	3.50E-25	UP
FIBCD1	21.42581	5.86822	0.548478	10.69911	1.03E-26	3.53E-25	UP
CENPJ	221.3832	1.359547	0.127133	10.69393	1.09E-26	3.73E-25	UP
PANK1	3158.274	-1.59639	0.149381	-10.6867	1.17E-26	4.02E-25	DOWN
MYCN	108.883	3.904161	0.365331	10.68663	1.18E-26	4.02E-25	UP
RP13-143(	63.73781	4.292094	0.401783	10.68261	1.23E-26	4.19E-25	UP
MCM5	2297.491	1.39321	0.130518	10.67447	1.34E-26	4.57E-25	UP
NSUN5P2	40.09424	1.573269	0.14752	10.66481	1.49E-26	5.07E-25	UP
LINC0123	82.27079	6.164753	0.578111	10.66361	1.51E-26	5.13E-25	UP
ZNF775	643.8024	1.505596	0.141227	10.66081	1.55E-26	5.28E-25	UP
SPTAN1	9966.505	0.695027	0.065204	10.65922	1.58E-26	5.36E-25	UP
PDIA2	50.9941	4.796324	0.450059	10.65711	1.62E-26	5.48E-25	UP
KPNA2	2463.788	1.540003	0.144517	10.65617	1.63E-26	5.53E-25	UP
LIN28B	54.94455	10.41845	0.978335	10.64916	1.76E-26	5.95E-25	UP
SUCO	1587.381	1.235238	0.115999	10.64873	1.77E-26	5.97E-25	UP
ZKSCAN3	307.536	1.440543	0.135281	10.64856	1.77E-26	5.98E-25	UP
AC011294	27.86704	4.096051	0.384665	10.64837	1.77E-26	5.98E-25	UP
PODXL	1640.686	1.699093	0.159565	10.64828	1.78E-26	5.98E-25	UP
PHF19	509.4361	1.531246	0.143834	10.64594	1.82E-26	6.12E-25	UP
DDX19B	663.6996	-0.97383	0.091509	-10.642	1.90E-26	6.38E-25	DOWN
ZFP36L1	13010.18	-1.08291	0.101778	-10.6399	1.94E-26	6.52E-25	DOWN
ZGRF1	92.48096	1.46988	0.138192	10.63652	2.02E-26	6.75E-25	UP
DNM3	73.02586	1.720628	0.161845	10.63133	2.13E-26	7.13E-25	UP
RP11-726(	15.86621	2.697293	0.253745	10.62991	2.16E-26	7.23E-25	UP
C1R	82374.14	-1.86646	0.175605	-10.6288	2.19E-26	7.31E-25	DOWN
MYO1A	114.9455	2.91407	0.274297	10.62376	2.31E-26	7.70E-25	UP
DNAH14	269.5862	1.266028	0.119186	10.62232	2.35E-26	7.81E-25	UP

SDC4	22905.57	-1.49836	0.141088	-10.62	2.40E-26	7.99E-25	DOWN
RP11-529I	20.66092	2.475112	0.233061	10.62001	2.41E-26	7.99E-25	UP
PNCK	50.68784	5.758011	0.542278	10.61819	2.45E-26	8.14E-25	UP
ASB16-AS	261.3172	1.02999	0.097006	10.61776	2.46E-26	8.16E-25	UP
HSP90AB1	42322.32	0.97432	0.091779	10.61599	2.51E-26	8.31E-25	UP
BCO2	912.403	-2.95638	0.278572	-10.6126	2.60E-26	8.61E-25	DOWN
SMG9	959.3053	1.29693	0.122243	10.60941	2.69E-26	8.90E-25	UP
RP11-81H	56.40393	7.689336	0.725006	10.60589	2.80E-26	9.23E-25	UP
VMO1	177.33	-1.62255	0.153004	-10.6046	2.84E-26	9.33E-25	DOWN
LPL	143.6745	2.104116	0.198417	10.6045	2.84E-26	9.33E-25	UP
RP6-65G2	25.64453	2.088263	0.196923	10.60449	2.84E-26	9.33E-25	UP
SPRYD4	3480.277	-1.42655	0.134528	-10.6041	2.85E-26	9.36E-25	DOWN
MAFG	862.5099	1.394693	0.13154	10.60277	2.89E-26	9.48E-25	UP
TINAG	123.7166	6.541815	0.617036	10.602	2.92E-26	9.55E-25	UP
NELFE	2682.149	1.23117	0.116167	10.59825	3.04E-26	9.93E-25	UP
CCT3	14010.74	1.135922	0.107206	10.59573	3.12E-26	1.02E-24	UP
DKK1	301.6848	4.767597	0.450003	10.59459	3.16E-26	1.03E-24	UP
KLHL12	1409.749	0.828379	0.078224	10.58987	3.32E-26	1.08E-24	UP
CSPG4P8	41.42033	1.903866	0.179804	10.58857	3.37E-26	1.10E-24	UP
MESP1	114.4335	2.026876	0.191427	10.58822	3.38E-26	1.10E-24	UP
CTC-471F	24.53466	2.239017	0.211483	10.58723	3.42E-26	1.11E-24	UP
TMEM132	735.707	2.919649	0.2758	10.58613	3.46E-26	1.12E-24	UP
RP4-616B	12.52719	3.075154	0.290537	10.58439	3.52E-26	1.14E-24	UP
GSTZ1	3364.518	-2.07759	0.19631	-10.5832	3.56E-26	1.15E-24	DOWN
LINC01451	120.4411	2.962965	0.279994	10.58226	3.60E-26	1.16E-24	UP
TMEM26	65.70125	-1.9638	0.185579	-10.582	3.61E-26	1.16E-24	DOWN
DOCK6	1356.893	0.893134	0.084401	10.582	3.61E-26	1.16E-24	UP
CENPP	122.7491	1.203051	0.113792	10.57241	4.00E-26	1.29E-24	UP
RP11-466I	11.84403	2.139678	0.202414	10.57079	4.07E-26	1.31E-24	UP
FGD1	317.9259	1.489909	0.140954	10.5702	4.10E-26	1.32E-24	UP
ZNF7	675.8983	0.891202	0.084317	10.56962	4.12E-26	1.32E-24	UP
DLG5	783.955	1.973962	0.186803	10.5671	4.23E-26	1.36E-24	UP
RP11-138	16.74043	6.3998	0.606151	10.55809	4.66E-26	1.49E-24	UP
LHX4-AS1	1187.148	0.979647	0.092792	10.55749	4.69E-26	1.50E-24	UP
PACS2	1412.911	0.834937	0.079089	10.55691	4.72E-26	1.51E-24	UP
PYGB	3412.947	1.556707	0.147461	10.55671	4.73E-26	1.51E-24	UP
SCNM1	994.5729	1.290254	0.122268	10.55267	4.94E-26	1.57E-24	UP
STIP1	4955.521	1.005754	0.095335	10.5497	5.10E-26	1.62E-24	UP
DNA2	170.0087	1.569277	0.148802	10.54609	5.30E-26	1.68E-24	UP
RP11-242	141.8743	4.738603	0.449505	10.54182	5.54E-26	1.76E-24	UP
SCN4A	76.40663	2.252857	0.213767	10.53885	5.72E-26	1.81E-24	UP
KIAA1244	394.2447	3.285979	0.311797	10.53884	5.72E-26	1.81E-24	UP
TRIM17	40.02901	4.111882	0.390187	10.53823	5.76E-26	1.82E-24	UP
CMB9-22I	90.32698	2.869963	0.272347	10.5379	5.78E-26	1.83E-24	UP
SBF1	2212.512	1.182323	0.112257	10.53228	6.13E-26	1.93E-24	UP
KIAA0100	3209.731	1.043289	0.099056	10.53227	6.13E-26	1.93E-24	UP
KIAA1614	88.92539	2.067399	0.196299	10.53187	6.16E-26	1.94E-24	UP
FAM72B	20.5304	2.536416	0.240937	10.52728	6.47E-26	2.04E-24	UP
PI15	77.32789	4.033879	0.383278	10.52467	6.65E-26	2.09E-24	UP
TAPT1	1849.584	-1.05261	0.100029	-10.523	6.77E-26	2.13E-24	DOWN
HOXD10	24.47499	5.141043	0.48864	10.52112	6.90E-26	2.17E-24	UP
VAX2	28.66028	4.086113	0.388427	10.51965	7.01E-26	2.20E-24	UP

SLC7A10	51.49331	5.370031	0.51052	10.51875	7.08E-26	2.22E-24	UP
MIR4435-:	636.7248	1.821575	0.173177	10.51859	7.09E-26	2.22E-24	UP
ATF3	2729.944	-1.8991	0.180579	-10.5167	7.24E-26	2.26E-24	DOWN
CD302	1813.621	-1.69187	0.160915	-10.5141	7.44E-26	2.32E-24	DOWN
PAGE4	340.3501	7.498744	0.713908	10.50379	8.30E-26	2.59E-24	UP
RP1-170O	10.09871	5.722925	0.54509	10.49905	8.73E-26	2.72E-24	UP
HMGA1	3396.273	1.784182	0.169961	10.49757	8.86E-26	2.76E-24	UP
PRPF3	1388.877	1.015477	0.096833	10.48694	9.92E-26	3.08E-24	UP
BRSK1	101.5255	1.816486	0.17329	10.48236	1.04E-25	3.23E-24	UP
DLX5	16.86304	5.481005	0.522978	10.48037	1.06E-25	3.29E-24	UP
FBF1	119.1576	1.470858	0.140346	10.48021	1.07E-25	3.30E-24	UP
FUT2	120.5595	3.277016	0.312715	10.47924	1.08E-25	3.32E-24	UP
CCT6A	5916.12	0.887101	0.084654	10.47919	1.08E-25	3.32E-24	UP
MAPK12	434.8399	2.263849	0.216043	10.47867	1.08E-25	3.34E-24	UP
AP001469.	35.42388	1.963251	0.187375	10.47768	1.09E-25	3.37E-24	UP
ALPI	81.49074	6.73589	0.642891	10.4775	1.10E-25	3.37E-24	UP
TCF3	1443.388	1.106115	0.105607	10.47393	1.14E-25	3.50E-24	UP
ZFPM2-AS	92.39318	3.844469	0.367196	10.46981	1.19E-25	3.65E-24	UP
C6orf223	253.2718	4.628083	0.442467	10.45974	1.32E-25	4.06E-24	UP
CTD-2561	19.50112	1.798755	0.172012	10.45713	1.36E-25	4.16E-24	UP
ANKRD13E	102.0905	1.871162	0.178954	10.45613	1.37E-25	4.20E-24	UP
LINGO1	319.8452	2.695913	0.257843	10.45564	1.38E-25	4.22E-24	UP
RP11-424(	11.11854	3.870475	0.370197	10.45517	1.39E-25	4.24E-24	UP
LINC00511	139.6799	3.161299	0.302446	10.45245	1.43E-25	4.36E-24	UP
EFNA3	129.6132	2.22476	0.212912	10.44922	1.48E-25	4.50E-24	UP
GATSL2	41.7461	1.720818	0.164684	10.4492	1.48E-25	4.50E-24	UP
ANTXR2	1754.887	-1.3056	0.124968	-10.4474	1.51E-25	4.58E-24	DOWN
GGA3	1080.84	0.733805	0.07024	10.44718	1.51E-25	4.58E-24	UP
ITPKA	308.338	2.493221	0.238672	10.44623	1.52E-25	4.63E-24	UP
CHML	561.6563	1.817098	0.173996	10.44335	1.57E-25	4.76E-24	UP
DLK2	78.64302	2.271326	0.217507	10.44252	1.59E-25	4.80E-24	UP
HIST1H2A	12.61022	4.2497	0.406972	10.44225	1.59E-25	4.81E-24	UP
POLG2	273.357	0.982357	0.094084	10.4413	1.61E-25	4.85E-24	UP
HSF4	362.4827	1.677126	0.160634	10.44068	1.62E-25	4.88E-24	UP
GATS	280.9177	1.541249	0.147621	10.44061	1.62E-25	4.88E-24	UP
TCF15	26.0797	2.266627	0.217157	10.43774	1.67E-25	5.02E-24	UP
RP11-520I	8.738232	3.253897	0.311846	10.43431	1.73E-25	5.20E-24	UP
RHBDL3	64.28944	3.892551	0.373161	10.43128	1.78E-25	5.36E-24	UP
EGR2	308.2039	-2.42087	0.232179	-10.4267	1.87E-25	5.62E-24	DOWN
SEZ6L2	1310.26	3.397442	0.326034	10.42051	2.00E-25	5.99E-24	UP
SUZ12P1	145.1907	1.322857	0.126963	10.41922	2.03E-25	6.07E-24	UP
CXCL12	5230.172	-2.44549	0.234815	-10.4146	2.13E-25	6.36E-24	DOWN
FLNC	1187.299	3.786136	0.363983	10.40195	2.43E-25	7.26E-24	UP
CYP19A1	32.97891	5.302929	0.509884	10.40027	2.47E-25	7.38E-24	UP
FAM72A	23.4526	2.06825	0.19889	10.39895	2.51E-25	7.47E-24	UP
GLDN	160.2631	3.674749	0.353401	10.39824	2.53E-25	7.52E-24	UP
NAAA	1363.464	-1.52869	0.147021	-10.3978	2.54E-25	7.55E-24	DOWN
PEG10	6873.292	4.581524	0.440688	10.3963	2.58E-25	7.66E-24	UP
UNC13A	40.72862	3.725321	0.358418	10.3938	2.65E-25	7.86E-24	UP
COL24A1	34.69432	3.835433	0.369083	10.39178	2.70E-25	8.01E-24	UP
LDLR	6314.327	-1.57672	0.151755	-10.39	2.75E-25	8.16E-24	DOWN
CLPX	3423.481	-0.95093	0.091551	-10.387	2.84E-25	8.41E-24	DOWN

LCAT	5426.076	-2.49664	0.240436	-10.3838	2.94E-25	8.69E-24	DOWN
C1QL1	276.8826	3.76789	0.362941	10.38154	3.01E-25	8.88E-24	UP
MAGEC1	131.6177	8.540888	0.822976	10.37805	3.12E-25	9.21E-24	UP
RP11-105!	10.07467	3.687241	0.355433	10.37394	3.26E-25	9.60E-24	UP
TP53I3	1228.502	1.815735	0.175075	10.37118	3.35E-25	9.87E-24	UP
CACYBP	2369.808	1.143318	0.11025	10.37023	3.39E-25	9.96E-24	UP
H2AFX	1170.499	1.568488	0.15128	10.36811	3.46E-25	1.02E-23	UP
LMNB2	1280.014	1.609352	0.155265	10.36519	3.57E-25	1.05E-23	UP
CD244	66.4653	-1.93682	0.186911	-10.3622	3.68E-25	1.08E-23	DOWN
TMEM164	687.0923	1.507592	0.145499	10.36155	3.71E-25	1.09E-23	UP
CCDC97	1071.321	0.786789	0.075955	10.35863	3.82E-25	1.12E-23	UP
CEP72	230.6408	1.409081	0.136033	10.35839	3.83E-25	1.12E-23	UP
NEB	888.1332	3.087262	0.298274	10.35044	4.17E-25	1.22E-23	UP
APOF	8017.438	-3.18194	0.3075	-10.3478	4.28E-25	1.25E-23	DOWN
MRPL53	121.4596	1.005027	0.097126	10.34769	4.29E-25	1.25E-23	UP
NDRG3	1086.091	1.128061	0.109065	10.34303	4.50E-25	1.31E-23	UP
LCN2	4713.306	3.844127	0.371798	10.3393	4.68E-25	1.36E-23	UP
MAFA	34.89378	5.088103	0.49213	10.33894	4.70E-25	1.36E-23	UP
RP11-89K2	29.5114	7.10075	0.686814	10.33868	4.71E-25	1.37E-23	UP
NMB	149.1016	1.863215	0.180258	10.3364	4.82E-25	1.40E-23	UP
ADM2	743.2978	2.379045	0.230199	10.33475	4.91E-25	1.42E-23	UP
DCAF4L2	300.245	9.353598	0.905213	10.33304	5.00E-25	1.44E-23	UP
RFX5	1403.064	1.185806	0.114764	10.33252	5.02E-25	1.45E-23	UP
RECQL5	1142.891	0.861611	0.083421	10.32845	5.24E-25	1.51E-23	UP
PTP4A2P2	5.689634	3.059162	0.296196	10.32815	5.26E-25	1.51E-23	UP
COX7B2	126.8486	8.613071	0.834557	10.32053	5.69E-25	1.64E-23	UP
PPID	1956.173	-0.89245	0.086484	-10.3192	5.77E-25	1.66E-23	DOWN
HOXD4	6.39081	4.777659	0.462998	10.31897	5.78E-25	1.66E-23	UP
EMX1	64.18556	3.832164	0.371578	10.31323	6.14E-25	1.76E-23	UP
STK39	500.1008	2.191925	0.212695	10.30548	6.66E-25	1.91E-23	UP
FXN	751.5697	-1.18128	0.11463	-10.3051	6.68E-25	1.91E-23	DOWN
ZWILCH	332.6922	0.965149	0.09367	10.30376	6.78E-25	1.94E-23	UP
RAB6B	199.0801	1.846218	0.17928	10.29798	7.20E-25	2.06E-23	UP
CKS1B	952.9822	1.166266	0.113304	10.29321	7.56E-25	2.16E-23	UP
CYB561D1	238.8529	1.37297	0.13339	10.29288	7.59E-25	2.16E-23	UP
RP11-77P(	29.74517	1.569062	0.152443	10.29278	7.59E-25	2.16E-23	UP
FAM72C	11.34947	3.397484	0.330098	10.29237	7.63E-25	2.17E-23	UP
CASKIN1	16.61933	4.232212	0.4112	10.29235	7.63E-25	2.17E-23	UP
REEP2	37.37918	2.780587	0.270218	10.29016	7.80E-25	2.22E-23	UP
P4HA2	1466.512	1.517054	0.147447	10.28882	7.91E-25	2.25E-23	UP
FAM133A	125.4028	5.412539	0.526234	10.28542	8.20E-25	2.32E-23	UP
POLE2	180.8508	1.659278	0.161347	10.28393	8.33E-25	2.36E-23	UP
EBF3	39.76656	2.405765	0.234159	10.27407	9.22E-25	2.61E-23	UP
KRT8P12	204.3207	1.272855	0.123945	10.26948	9.67E-25	2.73E-23	UP
GOLPH3L	1037.546	0.980625	0.095492	10.26915	9.71E-25	2.74E-23	UP
DNMT1	1762.719	1.379585	0.134355	10.26823	9.80E-25	2.76E-23	UP
ZNF789	224.6224	1.020504	0.099389	10.26778	9.84E-25	2.77E-23	UP
TRAF2	1091.772	1.140244	0.111078	10.26522	1.01E-24	2.85E-23	UP
MTHFD1L	487.1565	1.581951	0.154151	10.26237	1.04E-24	2.93E-23	UP
B3GNTL1	223.9022	1.367904	0.133296	10.26216	1.04E-24	2.93E-23	UP
POLR1E	874.2724	-0.9619	0.093753	-10.26	1.07E-24	2.99E-23	DOWN
SNCG	647.5654	2.923208	0.284991	10.2572	1.10E-24	3.08E-23	UP

RP4-717I2	188.5846	1.427819	0.13922	10.25583	1.11E-24	3.12E-23	UP
KCNN2	335.0086	-3.22468	0.314527	-10.2525	1.15E-24	3.23E-23	DOWN
MED22	784.8841	1.012136	0.098741	10.2504	1.18E-24	3.29E-23	UP
RP11-863I	51.24616	-2.41481	0.235717	-10.2445	1.25E-24	3.50E-23	DOWN
RP5-1011(	15.49857	4.94951	0.483251	10.2421	1.28E-24	3.58E-23	UP
C8orf44	89.57246	1.235007	0.120602	10.24033	1.31E-24	3.64E-23	UP
SGMS2	1039.71	-1.70662	0.166659	-10.2402	1.31E-24	3.64E-23	DOWN
DSCR8	36.96581	7.577699	0.740029	10.23973	1.32E-24	3.66E-23	UP
JUNB	6610.544	-1.59421	0.155698	-10.2391	1.32E-24	3.68E-23	DOWN
NRCAM	611.264	3.781769	0.369375	10.23829	1.34E-24	3.71E-23	UP
GAS5	4342.406	1.776659	0.173578	10.23549	1.37E-24	3.81E-23	UP
GPR35	240.0825	3.264131	0.318995	10.23253	1.42E-24	3.93E-23	UP
LEMD2	1738.036	0.750036	0.073306	10.23162	1.43E-24	3.96E-23	UP
PNMA3	249.5057	3.868543	0.37811	10.23127	1.44E-24	3.97E-23	UP
ZNF252P-/	13.23174	2.181206	0.213196	10.231	1.44E-24	3.98E-23	UP
PGM1	10099.16	-1.33074	0.130073	-10.2307	1.44E-24	3.99E-23	DOWN
INTS8	945.2243	0.957961	0.093733	10.22005	1.61E-24	4.44E-23	UP
RP11-284I	67.58973	3.311056	0.324092	10.2164	1.67E-24	4.61E-23	UP
PLK3	483.3215	-1.69232	0.165659	-10.2157	1.69E-24	4.64E-23	DOWN
ELFN2	243.6512	3.621258	0.354516	10.21465	1.70E-24	4.68E-23	UP
AGO2	1249.503	1.14944	0.112574	10.21055	1.78E-24	4.88E-23	UP
P2RY13	141.0011	-1.96631	0.192578	-10.2105	1.78E-24	4.88E-23	DOWN
SFSWAP	1153.231	0.694075	0.067979	10.2102	1.79E-24	4.89E-23	UP
RP5-908M	13.91835	2.412462	0.236308	10.20898	1.81E-24	4.95E-23	UP
CASC3	2490.578	0.678409	0.06646	10.2078	1.83E-24	5.00E-23	UP
GLP1R	23.79623	6.500395	0.636836	10.20734	1.84E-24	5.02E-23	UP
CEP152	106.0594	1.41491	0.138678	10.20285	1.93E-24	5.25E-23	UP
HAUS5	573.1837	1.024294	0.100414	10.20071	1.97E-24	5.37E-23	UP
TNFRSF1A	7412.549	-0.92394	0.09059	-10.1991	2.00E-24	5.45E-23	DOWN
UBE2O	1192.399	0.753148	0.073855	10.19765	2.03E-24	5.53E-23	UP
RP11-620J	94.94717	1.656923	0.162583	10.19126	2.17E-24	5.90E-23	UP
DTYMK	1052.375	1.292618	0.126847	10.19037	2.19E-24	5.94E-23	UP
CUL9	1184.648	0.878078	0.086173	10.18972	2.20E-24	5.98E-23	UP
NCAPG2	544.1467	1.403841	0.137784	10.18869	2.23E-24	6.04E-23	UP
PDLIM5	4807.404	-1.05648	0.103758	-10.1822	2.38E-24	6.45E-23	DOWN
CCDC163I	93.43553	1.510282	0.148329	10.182	2.39E-24	6.45E-23	UP
RAD18	421.7919	0.824434	0.081046	10.17246	2.63E-24	7.11E-23	UP
MS4A6A	1536.731	-1.84295	0.181179	-10.172	2.64E-24	7.13E-23	DOWN
KNOP1	424.1045	0.849932	0.083556	10.17199	2.64E-24	7.13E-23	UP
MAMSTR	60.63073	2.221292	0.218457	10.16812	2.75E-24	7.41E-23	UP
ARID3A	734.2486	2.617186	0.257436	10.16634	2.80E-24	7.54E-23	UP
PGLYRP1	3.321364	-2.28883	0.225223	-10.1625	2.91E-24	7.84E-23	DOWN
GSDMC	17.43882	3.62976	0.357226	10.16097	2.96E-24	7.95E-23	UP
RP11-328J	24.04287	6.805663	0.669795	10.16081	2.97E-24	7.95E-23	UP
KIF19	37.69182	-2.31109	0.227452	-10.1608	2.97E-24	7.95E-23	DOWN
CNIH4	1423.314	0.999235	0.098348	10.16024	2.98E-24	7.99E-23	UP
DVL3	2759.776	0.742323	0.073073	10.15871	3.03E-24	8.11E-23	UP
RP11-388(	12.73662	2.107984	0.207599	10.15413	3.18E-24	8.49E-23	UP
AHSA2	858.8746	1.381006	0.13601	10.15373	3.19E-24	8.52E-23	UP
LARS	2710.404	0.692498	0.068204	10.15326	3.20E-24	8.55E-23	UP
GPR158	111.2938	3.292141	0.324311	10.1512	3.27E-24	8.72E-23	UP
PTK7	736.3069	2.685124	0.264549	10.14981	3.32E-24	8.84E-23	UP

GADD45B	9768.913	-2.04908	0.201887	-10.1496	3.33E-24	8.85E-23	DOWN
PRRG3	12.29001	2.464687	0.242849	10.14907	3.35E-24	8.89E-23	UP
TMPO-AS:	139.6365	1.614279	0.159077	10.14778	3.39E-24	9.00E-23	UP
CEL	39.7644	2.975472	0.293308	10.14452	3.50E-24	9.30E-23	UP
PLXNA1	990.7468	1.595556	0.157299	10.14345	3.54E-24	9.39E-23	UP
ZNF296	60.74784	2.422931	0.238874	10.14314	3.55E-24	9.41E-23	UP
SIPA1L3	1215.592	1.381333	0.136222	10.14029	3.66E-24	9.68E-23	UP
RP11-560J	13.97646	2.178111	0.214869	10.13692	3.79E-24	1.00E-22	UP
AC098973	50.25335	7.394346	0.729782	10.13226	3.97E-24	1.05E-22	UP
LINC01532	9.781792	5.449111	0.537918	10.13	4.07E-24	1.07E-22	UP
GINS4	127.4782	1.950977	0.192689	10.12503	4.28E-24	1.13E-22	UP
SAE1	2882.822	0.876808	0.086605	10.12418	4.32E-24	1.14E-22	UP
ALPK3	801.6504	2.30718	0.227913	10.12305	4.37E-24	1.15E-22	UP
CDK16	2139.558	0.991938	0.097988	10.12305	4.37E-24	1.15E-22	UP
RPS6KL1	204.0243	1.981785	0.195775	10.12277	4.38E-24	1.15E-22	UP
ZNF623	888.9952	0.91443	0.090335	10.12261	4.39E-24	1.15E-22	UP
SLC30A3	33.20992	3.68881	0.364531	10.11934	4.53E-24	1.19E-22	UP
PROSC	2550.141	-1.13092	0.111768	-10.1185	4.58E-24	1.20E-22	DOWN
PTPN3	2472.102	-1.07434	0.10618	-10.1181	4.59E-24	1.20E-22	DOWN
RP11-618I	42.23056	6.072551	0.600221	10.1172	4.63E-24	1.21E-22	UP
AC018890	29.94148	4.985584	0.492895	10.11489	4.75E-24	1.24E-22	UP
FIGNL1	321.3592	1.287387	0.127361	10.10816	5.08E-24	1.33E-22	UP
NPFFR2	41.81322	6.083997	0.601907	10.10787	5.10E-24	1.33E-22	UP
SETDB1	1374.217	0.786531	0.077818	10.10732	5.13E-24	1.33E-22	UP
ADK	2803.326	-1.16994	0.115781	-10.1047	5.26E-24	1.37E-22	DOWN
GINS2	313.9374	1.706082	0.168844	10.10447	5.28E-24	1.37E-22	UP
DVL2	831.0258	1.063443	0.105274	10.10171	5.43E-24	1.41E-22	UP
SFRP4	189.1083	2.928406	0.289989	10.09835	5.62E-24	1.46E-22	UP
RP11-284I	104.5337	3.870748	0.383321	10.09792	5.64E-24	1.46E-22	UP
TSEN54	1085.443	0.991866	0.098265	10.09376	5.89E-24	1.52E-22	UP
MTHFD2L	510.7705	-1.34003	0.13277	-10.0928	5.94E-24	1.54E-22	DOWN
AP4M1	396.8749	0.87456	0.08667	10.09069	6.07E-24	1.57E-22	UP
MCRS1	1731.405	0.786696	0.077972	10.08951	6.15E-24	1.59E-22	UP
GPM6A	190.2736	-3.05536	0.302868	-10.0881	6.24E-24	1.61E-22	DOWN
CBX8	435.961	1.120702	0.111118	10.08571	6.39E-24	1.65E-22	UP
ALDH3A1	3597.606	4.756251	0.471619	10.08495	6.44E-24	1.66E-22	UP
RIPK4	1416.548	-1.4126	0.140149	-10.0793	6.82E-24	1.75E-22	DOWN
AC139100	24.52655	2.158448	0.214186	10.07743	6.95E-24	1.79E-22	UP
DBT	2004.002	-1.1102	0.110171	-10.077	6.98E-24	1.79E-22	DOWN
CCDC137	900.9167	0.977517	0.097008	10.07664	7.01E-24	1.80E-22	UP
ACADSB	16146.47	-1.76136	0.174866	-10.0726	7.30E-24	1.87E-22	DOWN
NXF3	21.30744	-2.98768	0.296631	-10.072	7.34E-24	1.88E-22	DOWN
DSTYK	533.8754	1.221972	0.121379	10.06741	7.70E-24	1.97E-22	UP
FPR2	25.82126	-2.81835	0.280017	-10.0649	7.90E-24	2.02E-22	DOWN
MAVS	3146.076	0.934803	0.092907	10.06166	8.16E-24	2.08E-22	UP
UXS1	1302.093	0.915226	0.090962	10.06161	8.17E-24	2.08E-22	UP
SLC39A10	447.0949	1.420393	0.141182	10.06073	8.24E-24	2.10E-22	UP
ASIC3	60.22153	1.997004	0.198519	10.05952	8.34E-24	2.12E-22	UP
HGS	2387.775	0.9293	0.092383	10.0592	8.37E-24	2.13E-22	UP
CACNB4	36.0497	3.061368	0.304549	10.05214	8.99E-24	2.28E-22	UP
MT1DP	97.31467	-3.01603	0.300059	-10.0514	9.05E-24	2.30E-22	DOWN
UBXN8	802.2145	-1.12321	0.111756	-10.0505	9.14E-24	2.32E-22	DOWN

TFAP2A-A	15.69246	4.167208	0.41469	10.04896	9.28E-24	2.35E-22	UP
RPLP0P2	24.00681	2.955137	0.294085	10.04859	9.32E-24	2.36E-22	UP
VASH2	95.29906	2.472347	0.246075	10.04715	9.46E-24	2.39E-22	UP
AC016999	5.156621	-2.81032	0.279832	-10.0429	9.87E-24	2.50E-22	DOWN
ITGA6	2404.216	1.302447	0.129696	10.04227	9.94E-24	2.51E-22	UP
DUOX1	159.5432	2.478904	0.246848	10.04224	9.94E-24	2.51E-22	UP
PLOD3	5128.859	0.941462	0.093751	10.04216	9.95E-24	2.51E-22	UP
SPINK5	60.66843	3.102163	0.30893	10.04162	1.00E-23	2.52E-22	UP
SAMD4B	2752.859	0.69763	0.069484	10.04016	1.02E-23	2.55E-22	UP
ZNF341	222.5826	1.021089	0.101715	10.03877	1.03E-23	2.59E-22	UP
MEX3A	283.3103	2.07247	0.206458	10.03823	1.04E-23	2.60E-22	UP
C2orf44	357.9915	0.791479	0.078856	10.03696	1.05E-23	2.63E-22	UP
TSSK5P	13.16722	2.619611	0.261234	10.02782	1.15E-23	2.88E-22	UP
SARAF	10315.51	-0.93914	0.093654	-10.0277	1.15E-23	2.88E-22	DOWN
RP11-25H	10.38015	5.062815	0.504899	10.02737	1.16E-23	2.89E-22	UP
SLC22A31	589.6218	5.152793	0.513895	10.02693	1.16E-23	2.90E-22	UP
RAD9A	537.314	0.964547	0.096203	10.02612	1.17E-23	2.92E-22	UP
ZNF687	1537.283	0.958059	0.095565	10.02523	1.18E-23	2.95E-22	UP
PGS1	575.6659	0.736188	0.07344	10.02438	1.19E-23	2.97E-22	UP
RP11-363I	7.513826	2.810469	0.28041	10.02271	1.21E-23	3.02E-22	UP
LRRC1	410.5447	2.382276	0.237935	10.01228	1.35E-23	3.35E-22	UP
TUBG2	386.7628	0.940303	0.093928	10.01091	1.36E-23	3.39E-22	UP
NOL12	153.1597	1.130492	0.112929	10.01061	1.37E-23	3.40E-22	UP
PRRX1	134.2264	2.565375	0.256319	10.00853	1.40E-23	3.47E-22	UP
TSC22D2	1360.964	-1.0349	0.103406	-10.0081	1.40E-23	3.48E-22	DOWN
MT-ATP6	323269.7	-1.43546	0.143499	-10.0032	1.48E-23	3.65E-22	DOWN
MSH5-SAI	31.7046	1.692234	0.169346	9.992757	1.64E-23	4.06E-22	UP
DHODH	3837.649	-1.87798	0.187994	-9.98959	1.69E-23	4.19E-22	DOWN
RABIF	654.1861	0.864165	0.08655	9.984526	1.78E-23	4.40E-22	UP
RP11-110(	14.75187	2.150663	0.21546	9.98173	1.83E-23	4.52E-22	UP
AC009005	43.93771	2.210013	0.22141	9.981526	1.84E-23	4.53E-22	UP
TBC1D15	1642.553	-0.6477	0.064895	-9.98074	1.85E-23	4.56E-22	DOWN
IGDCC4	67.72853	3.597528	0.360505	9.979135	1.88E-23	4.63E-22	UP
RGMA	45.98649	2.112347	0.2117	9.978001	1.90E-23	4.68E-22	UP
RHPN1	321.0376	2.041586	0.20462	9.977428	1.91E-23	4.70E-22	UP
IRGQ	1018.604	0.972767	0.097532	9.973834	1.98E-23	4.87E-22	UP
KRT20	25.40907	6.137455	0.615376	9.973505	1.99E-23	4.88E-22	UP
SLC4A11	96.55039	3.180122	0.318941	9.970865	2.04E-23	5.01E-22	UP
IVD	9592.524	-1.2924	0.129708	-9.9639	2.19E-23	5.37E-22	DOWN
C11orf84	384.8259	1.403543	0.140893	9.961769	2.24E-23	5.48E-22	UP
ZNF512B	1615.952	0.993008	0.09969	9.960951	2.26E-23	5.52E-22	UP
SNHG20	153.8413	1.169819	0.117447	9.960393	2.27E-23	5.55E-22	UP
HOXA3	109.7975	2.870906	0.288249	9.959804	2.29E-23	5.58E-22	UP
RNF139-A	43.39864	1.537015	0.154324	9.959663	2.29E-23	5.58E-22	UP
TEX41	79.29373	3.254751	0.326807	9.959238	2.30E-23	5.59E-22	UP
RMDN2	562.4686	-1.24805	0.125316	-9.95923	2.30E-23	5.59E-22	DOWN
RAB24	357.6413	1.033827	0.103825	9.957433	2.34E-23	5.69E-22	UP
MUT	7010.251	-1.3768	0.138372	-9.95001	2.52E-23	6.13E-22	DOWN
RBL1	248.0703	1.437604	0.144506	9.948403	2.56E-23	6.22E-22	UP
FBXW10	49.05869	4.126548	0.414826	9.947652	2.58E-23	6.26E-22	UP
MSX1	99.28405	2.171586	0.218517	9.937858	2.85E-23	6.90E-22	UP
AP004782.	3.649303	-2.91287	0.293171	-9.93574	2.91E-23	7.04E-22	DOWN

CREG2	11.01769	3.454743	0.347737	9.93494	2.93E-23	7.09E-22	UP
TSC1	863.9262	0.946384	0.095264	9.934319	2.95E-23	7.13E-22	UP
IBSP	14.85203	5.759984	0.579846	9.933645	2.97E-23	7.17E-22	UP
CTSL	7774.212	-0.90367	0.090996	-9.93087	3.06E-23	7.37E-22	DOWN
ACYP1	109.377	1.151972	0.116007	9.930168	3.08E-23	7.42E-22	UP
ATAT1	287.3158	1.12385	0.113213	9.926886	3.18E-23	7.66E-22	UP
FAHD2A	1877.494	-1.15309	0.116236	-9.92023	3.40E-23	8.18E-22	DOWN
GHR	5466.664	-2.36618	0.238547	-9.91916	3.44E-23	8.26E-22	DOWN
STX1A	89.27907	1.552098	0.156577	9.912685	3.67E-23	8.81E-22	UP
PIAS3	509.7041	1.139548	0.114966	9.912075	3.69E-23	8.85E-22	UP
MPP3	96.73687	2.023921	0.204277	9.907714	3.85E-23	9.24E-22	UP
SCIMP	161.3713	-1.73528	0.175336	-9.89689	4.29E-23	1.03E-21	DOWN
FAM101A	44.05969	3.385145	0.342051	9.896616	4.31E-23	1.03E-21	UP
DLG5-AS1	22.53099	2.151217	0.217395	9.895415	4.36E-23	1.04E-21	UP
MCAM	2430.333	1.544204	0.156075	9.89398	4.42E-23	1.06E-21	UP
PTGES2-A	6.971672	3.629393	0.366999	9.889383	4.63E-23	1.10E-21	UP
KDM8	1946.702	-2.34285	0.236925	-9.88855	4.67E-23	1.11E-21	DOWN
DNAJC25	1230.145	-1.24918	0.126484	-9.87618	5.28E-23	1.26E-21	DOWN
NR4A1	2170.323	-2.16471	0.219226	-9.87433	5.38E-23	1.28E-21	DOWN
APLP1	176.2302	3.083894	0.312362	9.872836	5.46E-23	1.30E-21	UP
ID2	10109.7	-1.39442	0.141248	-9.87209	5.50E-23	1.31E-21	DOWN
NPEPL1	402.9846	1.192371	0.120799	9.870724	5.58E-23	1.32E-21	UP
MSI1	270.4226	3.458414	0.350442	9.868706	5.69E-23	1.35E-21	UP
L3MBTL1	104.3614	1.465959	0.148567	9.867318	5.77E-23	1.37E-21	UP
SLC52A2	1267.897	1.527584	0.154826	9.866482	5.82E-23	1.38E-21	UP
CTBS	1416.579	-1.05994	0.107431	-9.86629	5.83E-23	1.38E-21	DOWN
RGS5	4425.286	1.602839	0.162457	9.866254	5.83E-23	1.38E-21	UP
DNAJC5	2581.44	0.839338	0.085079	9.865436	5.88E-23	1.39E-21	UP
CTD-2589	34.53479	2.485348	0.251977	9.863379	6.00E-23	1.42E-21	UP
LINC0113	148.7787	1.48945	0.151037	9.861482	6.11E-23	1.44E-21	UP
FER1L6	64.99956	5.790061	0.587142	9.861435	6.12E-23	1.44E-21	UP
GCH1	2450.443	-1.76102	0.17858	-9.86125	6.13E-23	1.44E-21	DOWN
MT2A	50679.18	-2.8812	0.292184	-9.8609	6.15E-23	1.45E-21	DOWN
TAZ	1001.892	0.965868	0.09799	9.856813	6.41E-23	1.51E-21	UP
LA16c-312	33.69035	4.18453	0.424554	9.856299	6.44E-23	1.51E-21	UP
PABPC1L	858.314	1.702475	0.172739	9.855744	6.47E-23	1.52E-21	UP
TM4SF19-	11.70181	1.946329	0.197558	9.85196	6.72E-23	1.58E-21	UP
LINC0153	15.48792	3.771366	0.3829	9.849468	6.89E-23	1.61E-21	UP
RP11-431	37.08735	4.450423	0.451847	9.849402	6.90E-23	1.61E-21	UP
CCDC25	1770.345	-1.04109	0.105701	-9.8494	6.90E-23	1.61E-21	DOWN
RP11-92C	3.756369	3.927458	0.398811	9.847924	7.00E-23	1.63E-21	UP
FAM83H	4088.982	1.449073	0.147147	9.847786	7.01E-23	1.63E-21	UP
HOXC9	31.85887	6.106167	0.620055	9.847776	7.01E-23	1.63E-21	UP
ID1	1841.164	-2.04391	0.207661	-9.8425	7.39E-23	1.72E-21	DOWN
SMYD3	276.6764	1.508217	0.153259	9.840939	7.50E-23	1.75E-21	UP
MAGEA12	205.0907	8.016558	0.814658	9.840392	7.54E-23	1.75E-21	UP
ACAT1	18139.93	-1.50887	0.153374	-9.83785	7.73E-23	1.80E-21	DOWN
UPF3B	438.0388	0.960847	0.09775	9.829624	8.39E-23	1.95E-21	UP
NKD1	1279.597	3.872446	0.394035	9.827672	8.56E-23	1.99E-21	UP
PRIM1	343.72	1.321459	0.134464	9.827585	8.56E-23	1.99E-21	UP
LILRB5	475.1417	-1.91029	0.194393	-9.82693	8.62E-23	2.00E-21	DOWN
PKN3	438.0068	1.494912	0.152133	9.826333	8.67E-23	2.01E-21	UP

ITGA9	883.7034	-1.74792	0.177922	-9.8241	8.87E-23	2.05E-21	DOWN
TRIM54	52.1477	4.10517	0.417879	9.823822	8.89E-23	2.05E-21	UP
ZBTB21	855.0924	-1.33669	0.136078	-9.82302	8.96E-23	2.07E-21	DOWN
HOXD1	8.261275	5.14951	0.524284	9.821979	9.05E-23	2.09E-21	UP
SLC6A9	200.2434	2.088516	0.212653	9.821257	9.12E-23	2.10E-21	UP
LEF1-AS1	6.063211	4.098832	0.417417	9.819508	9.28E-23	2.14E-21	UP
LRRC4	54.16315	-1.75204	0.178461	-9.8175	9.47E-23	2.18E-21	DOWN
HDAC4	443.3935	1.14557	0.116732	9.813701	9.83E-23	2.26E-21	UP
SASS6	158.0534	1.226244	0.124974	9.812031	9.99E-23	2.30E-21	UP
RHOB	32373.25	-1.63975	0.167121	-9.81172	1.00E-22	2.30E-21	DOWN
LINC0154(	42.48812	3.35533	0.341974	9.811667	1.00E-22	2.30E-21	UP
CABIN1	1597.524	0.80691	0.082242	9.811377	1.01E-22	2.31E-21	UP
PCNA	2774.064	1.103147	0.112439	9.81108	1.01E-22	2.31E-21	UP
PCDH17	304.8895	1.589485	0.162018	9.810533	1.01E-22	2.32E-21	UP
SSR2	10523.89	1.107443	0.112896	9.809366	1.03E-22	2.35E-21	UP
NAT1	291.86	-1.28674	0.131181	-9.80888	1.03E-22	2.36E-21	DOWN
RP3-508I1	33.02646	1.660434	0.169285	9.808519	1.03E-22	2.36E-21	UP
CST1	68.57685	6.569103	0.669813	9.807368	1.05E-22	2.39E-21	UP
LILRA1	44.44192	-1.65282	0.168579	-9.80446	1.08E-22	2.45E-21	DOWN
GMEB2	786.7653	0.639109	0.065201	9.80217	1.10E-22	2.51E-21	UP
DCST1	20.0768	2.420928	0.247078	9.79824	1.15E-22	2.61E-21	UP
TMCC1-A!	52.25588	1.786506	0.182342	9.797528	1.15E-22	2.62E-21	UP
RP11-295(	480.5991	2.099503	0.214353	9.794619	1.19E-22	2.70E-21	UP
RMI2	262.0573	1.921209	0.19615	9.79457	1.19E-22	2.70E-21	UP
RP5-882C:	40.18227	1.608278	0.164302	9.788568	1.26E-22	2.86E-21	UP
RTBDN	20.54946	5.315021	0.542995	9.788333	1.26E-22	2.86E-21	UP
NTM	98.39618	3.3281	0.340057	9.786894	1.28E-22	2.90E-21	UP
PRMT3	425.6229	0.865235	0.08841	9.786624	1.29E-22	2.91E-21	UP
LINC0111(	26.32929	3.150535	0.321955	9.785633	1.30E-22	2.93E-21	UP
CTB-50L1'	22.78317	1.708485	0.174597	9.785333	1.30E-22	2.94E-21	UP
FOSB	3313.192	-3.09149	0.315973	-9.78401	1.32E-22	2.98E-21	DOWN
PRKRIP1	817.8825	0.748885	0.076554	9.782412	1.34E-22	3.02E-21	UP
TCHP	525.5424	0.757939	0.077483	9.782001	1.35E-22	3.03E-21	UP
DIO2	99.60798	3.993673	0.408272	9.781888	1.35E-22	3.03E-21	UP
DLC1	1975.985	-1.28722	0.131633	-9.77884	1.39E-22	3.12E-21	DOWN
ZNF512	595.8602	0.948216	0.096967	9.778716	1.39E-22	3.12E-21	UP
LINC0123!	23.64237	3.450773	0.353156	9.771249	1.50E-22	3.36E-21	UP
LYPD8	37.68456	4.618184	0.47264	9.771031	1.50E-22	3.36E-21	UP
CYR61	4244.051	-1.89437	0.193881	-9.77076	1.50E-22	3.37E-21	DOWN
MTA3	844.9641	0.848698	0.086898	9.766654	1.57E-22	3.51E-21	UP
IGDCC3	24.13006	5.158154	0.528249	9.764635	1.60E-22	3.57E-21	UP
AARS2	877.4278	0.831846	0.085199	9.763609	1.61E-22	3.61E-21	UP
RP11-395I	69.28942	1.149256	0.117735	9.76136	1.65E-22	3.69E-21	UP
ANKRD55	50.19602	-2.34284	0.240041	-9.76018	1.67E-22	3.73E-21	DOWN
CPXM1	135.8289	2.577795	0.264141	9.759156	1.69E-22	3.76E-21	UP
EPHX4	12.35036	2.773904	0.284251	9.758631	1.69E-22	3.78E-21	UP
RSPO3	108.7953	-3.24347	0.332442	-9.7565	1.73E-22	3.85E-21	DOWN
HMGB2	1833.391	1.420789	0.145662	9.75403	1.77E-22	3.95E-21	UP
HOXA11-/	7.809776	5.177952	0.531106	9.749382	1.86E-22	4.13E-21	UP
SNHG3	538.063	1.852256	0.19	9.748717	1.87E-22	4.15E-21	UP
SLX4	257.8971	0.907074	0.093049	9.748317	1.88E-22	4.16E-21	UP
ZNF207	3304.608	0.523598	0.053727	9.745573	1.93E-22	4.28E-21	NOT

CD160	37.04148	-1.71606	0.176235	-9.73735	2.09E-22	4.63E-21	DOWN
TJAP1	1178.355	0.676584	0.069494	9.735882	2.12E-22	4.70E-21	UP
BLOC1S3	623.5037	0.969777	0.099618	9.73496	2.14E-22	4.73E-21	UP
NAP1L4P1	29.02444	2.937719	0.301822	9.73329	2.17E-22	4.81E-21	UP
PAEP	83.88875	6.768007	0.695534	9.730665	2.23E-22	4.93E-21	UP
PLXNC1	699.5418	1.947184	0.200118	9.730186	2.24E-22	4.95E-21	UP
SLC22A11	701.5445	4.217799	0.433491	9.729831	2.25E-22	4.96E-21	UP
CMTM4	624.3728	1.628349	0.167359	9.729663	2.25E-22	4.97E-21	UP
ARL16	847.5223	1.164556	0.119734	9.726164	2.33E-22	5.14E-21	UP
PVRL1	753.553	1.350468	0.138867	9.724878	2.36E-22	5.20E-21	UP
ZSCAN2	346.3512	0.982363	0.101053	9.721282	2.45E-22	5.38E-21	UP
NFIL3	2955.654	-1.41277	0.145391	-9.71706	2.55E-22	5.60E-21	DOWN
8-Sep	1338.981	0.879551	0.090521	9.716578	2.56E-22	5.63E-21	UP
TPM2	1600.352	1.720286	0.177094	9.713986	2.63E-22	5.77E-21	UP
GNAL	252.1544	2.185223	0.225065	9.709278	2.75E-22	6.03E-21	UP
BOP1	3211.141	1.607565	0.165593	9.707947	2.79E-22	6.11E-21	UP
ETV4	758.2185	2.899446	0.298744	9.705457	2.86E-22	6.26E-21	UP
PAFAH1B3	809.7628	2.187189	0.225392	9.70392	2.90E-22	6.35E-21	UP
DPF2	1068.547	0.623432	0.064269	9.700305	3.01E-22	6.57E-21	UP
EGF	52.67456	4.944505	0.510015	9.694828	3.17E-22	6.93E-21	UP
CCL14	223.7459	-1.94795	0.200954	-9.69354	3.21E-22	7.00E-21	DOWN
PHEX	39.45076	3.407921	0.351567	9.693513	3.21E-22	7.00E-21	UP
CCRN4L	417.5228	-1.88768	0.194742	-9.6932	3.22E-22	7.02E-21	DOWN
DFNB31	307.174	1.377138	0.142094	9.691766	3.27E-22	7.11E-21	UP
RP4-800G	58.85558	1.94444	0.200688	9.688877	3.36E-22	7.31E-21	UP
QRICH2	209.4447	1.557881	0.160817	9.687295	3.41E-22	7.42E-21	UP
LPPR4	23.96916	2.985733	0.30823	9.686717	3.43E-22	7.46E-21	UP
ENGASE	880.6099	0.98917	0.102122	9.686209	3.45E-22	7.48E-21	UP
SAP130	893.7225	0.670047	0.069175	9.686194	3.45E-22	7.48E-21	UP
GRAMD1A	2032.466	1.62051	0.167312	9.68557	3.47E-22	7.52E-21	UP
COA6	1246.465	1.12597	0.116293	9.682145	3.59E-22	7.77E-21	UP
ACAA1	13362.3	-1.56751	0.161916	-9.68101	3.63E-22	7.86E-21	DOWN
RP11-396	28.86398	2.269387	0.234446	9.679798	3.67E-22	7.94E-21	UP
FAM81A	53.31091	2.442781	0.252523	9.673515	3.91E-22	8.44E-21	UP
SNRPE	2071.566	1.011438	0.104569	9.672454	3.95E-22	8.52E-21	UP
YJEFN3	80.67375	1.901564	0.196631	9.670725	4.02E-22	8.66E-21	UP
TMEM262	30.01196	1.164134	0.120432	9.666344	4.19E-22	9.03E-21	UP
PLCG1	1554.825	1.069935	0.110694	9.665712	4.22E-22	9.08E-21	UP
TIGD3	37.69392	2.23409	0.23122	9.662196	4.36E-22	9.39E-21	UP
AP000439.	5.185377	-2.69347	0.278805	-9.66076	4.43E-22	9.51E-21	DOWN
MCM3AP-	58.63815	1.187673	0.122985	9.657059	4.59E-22	9.86E-21	UP
GPSM1	495.9227	1.932733	0.200158	9.656051	4.63E-22	9.95E-21	UP
CEACAM2	76.25935	4.709157	0.48787	9.652488	4.80E-22	1.03E-20	UP
AF131217.	17.32128	-2.295	0.237786	-9.65151	4.84E-22	1.04E-20	DOWN
AC005154	450.1277	1.094465	0.113449	9.647213	5.05E-22	1.08E-20	UP
RP11-511	9.726885	2.161694	0.224177	9.642786	5.27E-22	1.13E-20	UP
ITGA2	426.4602	2.268892	0.235338	9.640975	5.37E-22	1.15E-20	UP
CTC-260E	28.34583	3.29461	0.341741	9.640653	5.38E-22	1.15E-20	UP
ZNF572	78.11958	1.404257	0.14566	9.640639	5.39E-22	1.15E-20	UP
ENTHD2	455.5827	0.824671	0.085552	9.63944	5.45E-22	1.16E-20	UP
GUSBP11	45.15967	1.755557	0.182133	9.638858	5.48E-22	1.17E-20	UP
CLCN2	290.221	1.032238	0.107095	9.638561	5.50E-22	1.17E-20	UP

HMGCL	9221.909	-1.43941	0.149353	-9.63764	5.54E-22	1.18E-20	DOWN
RP11-488I	307.8053	1.664906	0.172862	9.631438	5.89E-22	1.25E-20	UP
MAP2K4	1042.702	-0.81191	0.084307	-9.63048	5.94E-22	1.26E-20	DOWN
EME2	294.1703	1.231231	0.127857	9.629722	5.99E-22	1.27E-20	UP
RP11-704I	98.39091	2.252692	0.233936	9.629529	6.00E-22	1.27E-20	UP
RP11-126(	19.61384	-1.90729	0.198191	-9.62346	6.37E-22	1.35E-20	DOWN
DBN1	1332.077	2.112088	0.219473	9.623438	6.37E-22	1.35E-20	UP
UBE2Q1	3927.453	0.721296	0.074961	9.622225	6.44E-22	1.36E-20	UP
GNPNAT1	2336.263	-1.10822	0.115199	-9.62006	6.58E-22	1.39E-20	DOWN
EML6	93.84547	1.880106	0.195474	9.61817	6.70E-22	1.42E-20	UP
EPHB1	67.44873	-2.08414	0.216723	-9.61661	6.80E-22	1.44E-20	DOWN
SETD4	345.638	0.763047	0.079352	9.615933	6.85E-22	1.45E-20	UP
PRDM15	311.9722	1.171903	0.121925	9.611686	7.14E-22	1.51E-20	UP
AC009014	179.6152	4.658327	0.484677	9.611192	7.17E-22	1.51E-20	UP
SLC41A3	1398.819	0.77621	0.080765	9.61078	7.20E-22	1.52E-20	UP
BHLHE40	8923.281	-1.46815	0.152767	-9.61042	7.23E-22	1.52E-20	DOWN
CRIP3	283.9244	2.06315	0.214702	9.609378	7.30E-22	1.53E-20	UP
LARP1B	1549.848	-1.0942	0.113877	-9.60864	7.35E-22	1.54E-20	DOWN
GS1-309P	59.06607	8.761868	0.911899	9.608378	7.37E-22	1.55E-20	UP
SLC39A14	23159.92	-1.54754	0.16108	-9.60731	7.45E-22	1.56E-20	DOWN
STXBP4	330.4341	1.304954	0.135841	9.606466	7.51E-22	1.57E-20	UP
FBXL19-AS	53.02894	1.668442	0.173682	9.606294	7.52E-22	1.58E-20	UP
INMT	890.3298	-2.47637	0.257789	-9.60618	7.53E-22	1.58E-20	DOWN
FAM183DI	2.04145	-3.39677	0.353774	-9.60154	7.88E-22	1.65E-20	DOWN
TPBGL	41.89331	2.963134	0.30867	9.599696	8.02E-22	1.68E-20	UP
LINC00853	49.15139	2.249111	0.234381	9.595979	8.31E-22	1.74E-20	UP
ANKRD27	795.3492	1.111442	0.115904	9.589293	8.87E-22	1.85E-20	UP
FLAD1	2151.33	0.948632	0.098942	9.58777	9.00E-22	1.88E-20	UP
THUMPD3	357.2319	1.131001	0.117973	9.58694	9.07E-22	1.89E-20	UP
RIC8A	3248.064	0.531267	0.055422	9.585818	9.17E-22	1.91E-20	NOT
RP11-277I	8.291248	2.440076	0.254568	9.585174	9.23E-22	1.92E-20	UP
DIRAS3	158.3395	-2.77087	0.289145	-9.58296	9.43E-22	1.96E-20	DOWN
LUCAT1	64.53595	4.29859	0.448575	9.582762	9.45E-22	1.96E-20	UP
DCTN2	3169.543	0.681808	0.071154	9.582161	9.50E-22	1.97E-20	UP
ODAM	100.4037	6.454701	0.674239	9.573308	1.04E-21	2.15E-20	UP
RP11-742I	11.38341	5.250948	0.548702	9.569755	1.07E-21	2.22E-20	UP
THEM5	130.1606	1.699141	0.177579	9.568371	1.09E-21	2.25E-20	UP
NRM	580.381	1.615579	0.168901	9.565267	1.12E-21	2.32E-20	UP
DIRAS1	68.75988	3.82944	0.400353	9.56515	1.12E-21	2.32E-20	UP
ECHS1	31299.69	-1.39467	0.145816	-9.56461	1.13E-21	2.33E-20	DOWN
MLYCD	1656.782	-1.33296	0.139387	-9.56301	1.14E-21	2.36E-20	DOWN
STX6	809.4575	0.868621	0.090839	9.562217	1.15E-21	2.38E-20	UP
CMTM6	4689.686	-1.01497	0.10617	-9.5598	1.18E-21	2.43E-20	DOWN
SNRPB	5626.594	1.102931	0.115391	9.558227	1.20E-21	2.47E-20	UP
TMEM104	1397.159	0.913301	0.095557	9.55761	1.21E-21	2.48E-20	UP
LINC00221	87.5486	7.616725	0.797011	9.556614	1.22E-21	2.50E-20	UP
HSPB1	19420.21	1.456431	0.152429	9.554828	1.24E-21	2.54E-20	UP
P3H4	540.183	1.524899	0.159649	9.551578	1.28E-21	2.62E-20	UP
MAGEB2	60.9294	9.259353	0.96961	9.549561	1.30E-21	2.67E-20	UP
PRKAR2A-	50.80015	1.443246	0.151159	9.547849	1.32E-21	2.72E-20	UP
RP11-128I	46.64998	2.221895	0.232759	9.545892	1.35E-21	2.77E-20	UP
AC025171	47.65094	1.543687	0.161756	9.543289	1.38E-21	2.83E-20	UP

MIR4664	17.14133	2.421749	0.253871	9.539299	1.44E-21	2.94E-20	UP
TRIM7	41.13671	2.309183	0.242152	9.53608	1.48E-21	3.03E-20	UP
LINC01224	28.52676	4.83728	0.507419	9.533108	1.53E-21	3.12E-20	UP
HOXC6	27.46359	5.302291	0.556397	9.529692	1.58E-21	3.22E-20	UP
UBD	2664.926	2.441299	0.256207	9.528606	1.59E-21	3.25E-20	UP
ERRFI1	16107.8	-1.70358	0.178873	-9.52393	1.67E-21	3.40E-20	DOWN
UBE2D3	8488.073	-0.55508	0.058295	-9.52196	1.70E-21	3.46E-20	NOT
NAB2	1034.919	-0.94709	0.099472	-9.52113	1.71E-21	3.49E-20	DOWN
RNF130	5837.892	-0.82141	0.086284	-9.51984	1.73E-21	3.53E-20	DOWN
LZTS1	224.4705	1.583804	0.166413	9.517324	1.78E-21	3.61E-20	UP
DONSON	582.3402	1.016658	0.106825	9.517077	1.78E-21	3.62E-20	UP
CTD-2529	5.375147	3.516702	0.369534	9.516583	1.79E-21	3.63E-20	UP
LIN37	140.9969	0.937653	0.098557	9.513859	1.84E-21	3.73E-20	UP
CKAP2	512.4605	1.421013	0.149375	9.513084	1.85E-21	3.75E-20	UP
AC006960	4.119965	-4.97755	0.523331	-9.51128	1.88E-21	3.81E-20	DOWN
CCBE1	178.9945	-3.15981	0.332273	-9.5097	1.91E-21	3.87E-20	DOWN
NKIRAS1	581.4298	-1.00648	0.105841	-9.50936	1.92E-21	3.88E-20	DOWN
TMBIM6	74052.87	-0.89515	0.094138	-9.50894	1.93E-21	3.89E-20	DOWN
CANT1	1972.417	0.739833	0.077808	9.508431	1.94E-21	3.91E-20	UP
NABP2	1150.52	0.884338	0.093017	9.50726	1.96E-21	3.95E-20	UP
FAM212B	223.9422	1.391842	0.146416	9.5061	1.98E-21	3.99E-20	UP
ADRA1A	818.9331	-3.08354	0.324377	-9.50605	1.98E-21	3.99E-20	DOWN
ATG2A	2681.95	-0.83463	0.087816	-9.50428	2.01E-21	4.06E-20	DOWN
HAVCR1	30.48091	4.370993	0.459962	9.502952	2.04E-21	4.11E-20	UP
RFPL4B	37.26322	7.963505	0.838062	9.502281	2.05E-21	4.13E-20	UP
LINC0068	13.03437	2.105067	0.221571	9.500631	2.09E-21	4.19E-20	UP
SPARCL1	4858.012	2.472846	0.260299	9.500009	2.10E-21	4.21E-20	UP
PPT2-EGFI	33.0006	1.165266	0.122673	9.498989	2.12E-21	4.25E-20	UP
R3HDM1	807.1624	0.749547	0.078917	9.497945	2.14E-21	4.29E-20	UP
ZNF174	297.1299	0.648586	0.068292	9.497211	2.16E-21	4.32E-20	UP
TPD52L2	3184.599	0.820732	0.086421	9.496884	2.16E-21	4.33E-20	UP
CHTOP	2762.605	0.578547	0.06094	9.493743	2.23E-21	4.46E-20	NOT
SLC9B2	1560.39	-1.50219	0.158252	-9.4924	2.26E-21	4.51E-20	DOWN
CTA-228A	26.10332	1.998331	0.210532	9.491796	2.27E-21	4.54E-20	UP
RP4-565E(	32.35442	3.516705	0.370595	9.489358	2.32E-21	4.64E-20	UP
SPOCD1	27.17789	2.772035	0.292132	9.48897	2.33E-21	4.65E-20	UP
MKRN3	24.8746	4.030235	0.425029	9.482251	2.49E-21	4.96E-20	UP
DLX6-AS1	26.26348	6.093537	0.64281	9.479524	2.55E-21	5.09E-20	UP
RP11-649/	30.52827	1.539117	0.16237	9.479052	2.57E-21	5.11E-20	UP
UBQLN4	1834.178	0.913275	0.09635	9.478751	2.57E-21	5.12E-20	UP
STAM-AS:	7.375698	2.454174	0.259006	9.475338	2.66E-21	5.28E-20	UP
GON4L	973.7866	0.792686	0.083678	9.47309	2.72E-21	5.40E-20	UP
CTD-2574	53.89459	1.095203	0.115615	9.472823	2.72E-21	5.41E-20	UP
CROCCP3	47.54692	1.256317	0.132691	9.467988	2.85E-21	5.66E-20	UP
NUP155	1027.315	0.807181	0.085267	9.46656	2.89E-21	5.73E-20	UP
EDNRB	1386.183	-1.45964	0.154239	-9.46355	2.98E-21	5.89E-20	DOWN
HOXD3	4.400779	3.748198	0.396381	9.456042	3.20E-21	6.33E-20	UP
ANKS3	447.3797	1.01968	0.107838	9.455673	3.21E-21	6.35E-20	UP
EPHA10	60.17858	3.902626	0.412957	9.450444	3.37E-21	6.67E-20	UP
GLUD1P3	26.6918	1.262829	0.133641	9.449443	3.41E-21	6.73E-20	UP
GABBR2	116.4279	4.519764	0.478331	9.449025	3.42E-21	6.75E-20	UP
RP11-2N1	3.121353	-3.39029	0.358824	-9.44833	3.44E-21	6.79E-20	DOWN

MIS18A	326.7128	1.011938	0.107115	9.447171	3.48E-21	6.86E-20	UP
CCDC78	38.61567	2.586632	0.27382	9.446483	3.50E-21	6.90E-20	UP
C9orf117	38.94544	1.078	0.114128	9.445508	3.54E-21	6.96E-20	UP
TRAM1L1	39.44109	3.19379	0.33815	9.444889	3.56E-21	7.00E-20	UP
SYNE1	1437.344	-1.24444	0.131847	-9.43855	3.78E-21	7.43E-20	DOWN
GCDH	3976.742	-1.52662	0.161764	-9.43736	3.82E-21	7.51E-20	DOWN
IPO9	2919.381	0.774209	0.082042	9.436701	3.85E-21	7.55E-20	UP
CTD-2228	585.3974	2.451804	0.25991	9.433287	3.97E-21	7.79E-20	UP
ANO2	35.39373	2.770004	0.293643	9.433248	3.98E-21	7.79E-20	UP
AGL	2366.594	-1.42944	0.151541	-9.4327	4.00E-21	7.82E-20	DOWN
AFF2	26.64291	3.930205	0.416658	9.432695	4.00E-21	7.82E-20	UP
RCN2	1044.014	0.807959	0.085748	9.422442	4.41E-21	8.62E-20	UP
BFSP1	50.69589	1.55067	0.164663	9.417245	4.63E-21	9.05E-20	UP
GNE	5300.883	-1.53722	0.163255	-9.41604	4.68E-21	9.15E-20	DOWN
PHACTR3	12.84421	-2.60411	0.276658	-9.41274	4.83E-21	9.43E-20	DOWN
SNX25P1	17.6313	2.153292	0.228788	9.411751	4.88E-21	9.51E-20	UP
PVALB	28.60894	-3.08552	0.327945	-9.40866	5.03E-21	9.79E-20	DOWN
IRF8	1066.818	-1.61169	0.171329	-9.407	5.11E-21	9.94E-20	DOWN
OACYLP	3.182454	3.510437	0.373194	9.40646	5.13E-21	9.98E-20	UP
AKIP1	613.3496	0.739625	0.07864	9.405206	5.19E-21	1.01E-19	UP
KB-1460A	179.5146	1.501202	0.159635	9.403968	5.25E-21	1.02E-19	UP
RP11-501(	5.934982	4.547984	0.483825	9.400064	5.45E-21	1.06E-19	UP
RP11-348	7.189158	4.222174	0.449303	9.397166	5.61E-21	1.09E-19	UP
CFL2	3920.95	-1.12715	0.119985	-9.39402	5.78E-21	1.12E-19	DOWN
PMS2P3	61.91941	1.062848	0.113211	9.388218	6.10E-21	1.18E-19	UP
NUP37	459.4727	0.826351	0.088025	9.387711	6.13E-21	1.19E-19	UP
FITM1	165.0021	-2.22372	0.236904	-9.38659	6.20E-21	1.20E-19	DOWN
EPHX2	7639.349	-1.66995	0.178005	-9.3815	6.50E-21	1.26E-19	DOWN
SLC36A1	381.1424	1.140307	0.121559	9.38072	6.55E-21	1.27E-19	UP
C15orf27	60.99283	2.179769	0.232404	9.379232	6.65E-21	1.28E-19	UP
UCHL1	194.5366	3.324346	0.354505	9.377421	6.76E-21	1.30E-19	UP
IL6ST	11825.06	-0.98173	0.104725	-9.37444	6.95E-21	1.34E-19	DOWN
TRAF5	285.3139	1.563748	0.166852	9.372067	7.11E-21	1.37E-19	UP
CCDC77	218.8658	0.966735	0.103172	9.370138	7.24E-21	1.39E-19	UP
RP11-723(	23.15433	2.104655	0.224644	9.368845	7.33E-21	1.41E-19	UP
RP11-651	19.40381	1.765986	0.188546	9.366331	7.51E-21	1.44E-19	UP
RP3-406P	14.45864	1.882891	0.201032	9.366133	7.52E-21	1.45E-19	UP
C12orf75	620.8464	2.528509	0.270013	9.364404	7.65E-21	1.47E-19	UP
GTPBP2	1462.216	1.031619	0.110174	9.363522	7.71E-21	1.48E-19	UP
GRPEL2	564.9744	0.671073	0.07167	9.363426	7.72E-21	1.48E-19	UP
LINC0015	526.5872	1.935339	0.206706	9.362763	7.77E-21	1.49E-19	UP
EPRS	4244.967	0.837579	0.089467	9.361898	7.83E-21	1.50E-19	UP
ZNF618	670.1508	1.214008	0.129705	9.359753	7.99E-21	1.53E-19	UP
GPR128	403.9915	-2.63624	0.281683	-9.35889	8.06E-21	1.54E-19	DOWN
MAGEA6	286.0917	8.17776	0.87393	9.357451	8.17E-21	1.56E-19	UP
DDX11	489.0541	1.32272	0.141382	9.355639	8.31E-21	1.59E-19	UP
PYCR2	2124.081	0.746426	0.079802	9.353498	8.48E-21	1.62E-19	UP
ZNF767P	194.0515	1.047794	0.112069	9.349504	8.81E-21	1.68E-19	UP
SLC35D1	4719.15	-1.27169	0.13605	-9.34722	9.00E-21	1.71E-19	DOWN
METTL3	846.661	0.704114	0.075343	9.345419	9.15E-21	1.74E-19	UP
KLF6	7877.105	-1.38338	0.148078	-9.34221	9.43E-21	1.80E-19	DOWN
ELOVL1	2224.533	0.789078	0.084483	9.340091	9.63E-21	1.83E-19	UP

TOPBP1	988.9284	0.926421	0.099217	9.337311	9.88E-21	1.88E-19	UP
ADAM11	33.30229	2.334641	0.25009	9.335198	1.01E-20	1.91E-19	UP
ITGB1BP1	1117.805	0.927801	0.099388	9.335107	1.01E-20	1.91E-19	UP
SNX22	588.7466	2.168045	0.232266	9.334329	1.02E-20	1.93E-19	UP
LINC01121	8.193515	4.552393	0.487729	9.333865	1.02E-20	1.93E-19	UP
IL3RA	263.1321	1.22901	0.131675	9.333663	1.02E-20	1.94E-19	UP
KLF11	1889.287	-1.135	0.121667	-9.32869	1.07E-20	2.03E-19	DOWN
ATP6V1C1	3070.128	1.018879	0.109252	9.325947	1.10E-20	2.08E-19	UP
LINC0100	34.66116	1.3447	0.144194	9.325644	1.10E-20	2.08E-19	UP
RHBDL1	105.5973	1.812361	0.194375	9.32405	1.12E-20	2.11E-19	UP
RAP2A	1513.182	1.179035	0.126487	9.32141	1.15E-20	2.17E-19	UP
OSBP2	188.9228	1.860323	0.199578	9.321262	1.15E-20	2.17E-19	UP
BX322557.	32.76315	1.338181	0.143575	9.320417	1.16E-20	2.18E-19	UP
MDFI	157.8841	2.728034	0.292697	9.320345	1.16E-20	2.18E-19	UP
ZNF883	46.65326	3.032927	0.325411	9.320295	1.16E-20	2.18E-19	UP
LPPR2	1138.116	1.146706	0.123055	9.318638	1.18E-20	2.22E-19	UP
TLCD1	483.819	1.531958	0.164432	9.316662	1.20E-20	2.26E-19	UP
OCRL	1350.39	0.920381	0.098793	9.316281	1.20E-20	2.26E-19	UP
PPP2R3B	381.7121	1.02644	0.110186	9.315526	1.21E-20	2.28E-19	UP
PKM	8622.019	2.179992	0.234037	9.314721	1.22E-20	2.29E-19	UP
C14orf93	338.3092	0.748024	0.080306	9.314643	1.22E-20	2.29E-19	UP
MRGBP	759.0357	0.81797	0.087874	9.30842	1.30E-20	2.43E-19	UP
SBF2-AS1	122.7341	1.094373	0.11759	9.306701	1.32E-20	2.47E-19	UP
FAM212A	60.08712	1.462419	0.157139	9.306505	1.32E-20	2.47E-19	UP
SNX9	3045.001	-0.82033	0.088172	-9.30375	1.36E-20	2.53E-19	DOWN
ZSCAN16	145.7003	1.0572	0.113702	9.297978	1.43E-20	2.67E-19	UP
KCNQ3	24.73308	2.326652	0.250238	9.297748	1.43E-20	2.68E-19	UP
RP4-773N	147.7164	1.451203	0.156173	9.292253	1.51E-20	2.82E-19	UP
SCG2	12.91762	3.589226	0.386335	9.290455	1.54E-20	2.86E-19	UP
UNKL	365.4245	1.150801	0.123872	9.290248	1.54E-20	2.87E-19	UP
TMEM178	100.833	2.995631	0.322493	9.288976	1.56E-20	2.90E-19	UP
DUSP14	786.1484	-0.9518	0.102501	-9.28574	1.61E-20	2.99E-19	DOWN
PAMR1	290.7102	-1.9018	0.20481	-9.28566	1.61E-20	2.99E-19	DOWN
FAR2P1	37.36778	6.688803	0.720346	9.285548	1.61E-20	2.99E-19	UP
KCNN3	144.566	1.617926	0.174298	9.282552	1.65E-20	3.07E-19	UP
C20orf96	209.0242	1.038397	0.111876	9.281673	1.67E-20	3.10E-19	UP
EPB41L4B	3455.048	-1.58652	0.170942	-9.28104	1.68E-20	3.11E-19	DOWN
RBM28	803.7243	0.635133	0.068438	9.280432	1.69E-20	3.13E-19	UP
DYDC2	67.95193	3.234256	0.348517	9.280051	1.69E-20	3.14E-19	UP
NR4A3	308.0888	-2.33073	0.251195	-9.27857	1.72E-20	3.18E-19	DOWN
SMARCD1	1540.029	0.7346	0.079175	9.278144	1.72E-20	3.19E-19	UP
HPS5	2062.247	-1.49802	0.161476	-9.27706	1.74E-20	3.22E-19	DOWN
SCGB3A1	32.70824	-2.31484	0.249579	-9.275	1.78E-20	3.28E-19	DOWN
C12orf56	23.35118	4.622932	0.49846	9.274432	1.79E-20	3.30E-19	UP
NFE2L2	4389.673	-0.92183	0.099396	-9.27425	1.79E-20	3.30E-19	DOWN
ZNF681	71.15442	2.311849	0.249285	9.273928	1.79E-20	3.31E-19	UP
RP11-102	6.600609	2.514475	0.271164	9.272891	1.81E-20	3.34E-19	UP
GTF2IRD1	559.6835	1.141235	0.123118	9.269435	1.87E-20	3.44E-19	UP
ZSWIM1	348.8607	0.753995	0.081347	9.268915	1.88E-20	3.46E-19	UP
NUDT10	15.44554	-3.11604	0.336183	-9.26887	1.88E-20	3.46E-19	DOWN
CDK5R1	86.08859	1.334001	0.143948	9.267239	1.91E-20	3.51E-19	UP
GNA14	149.0861	-1.78805	0.192978	-9.26561	1.94E-20	3.56E-19	DOWN

DCST2	62.57769	1.828011	0.197299	9.26518	1.95E-20	3.57E-19	UP
NEU1	4851.989	1.088895	0.117553	9.263018	1.99E-20	3.64E-19	UP
SPRED3	23.78589	1.97425	0.213168	9.26149	2.02E-20	3.69E-19	UP
FHAD1	36.44473	2.371535	0.256067	9.261369	2.02E-20	3.70E-19	UP
PDE4C	68.42753	1.942873	0.209784	9.261293	2.02E-20	3.70E-19	UP
CDH8	9.480496	3.990058	0.430857	9.260745	2.03E-20	3.71E-19	UP
STRA6	32.59157	3.767189	0.406845	9.259512	2.05E-20	3.75E-19	UP
ACPT	15.88701	3.973471	0.429179	9.258302	2.08E-20	3.79E-19	UP
ZNF668	100.8447	0.879406	0.095014	9.255545	2.13E-20	3.89E-19	UP
LLGL1	496.5325	1.104197	0.119315	9.254505	2.15E-20	3.93E-19	UP
SOX12	1240.84	1.589721	0.171856	9.250327	2.24E-20	4.08E-19	UP
POPDC3	32.77131	6.178082	0.667909	9.249887	2.25E-20	4.09E-19	UP
DUSP5P1	5.99567	4.804791	0.519513	9.248637	2.27E-20	4.14E-19	UP
SUV39H2	285.6067	0.857495	0.092724	9.247795	2.29E-20	4.17E-19	UP
AGAP1	768.0141	1.046817	0.113211	9.246576	2.32E-20	4.21E-19	UP
RP11-398(	24.92593	1.501194	0.162368	9.24564	2.34E-20	4.25E-19	UP
GOLGA3	2453.679	0.673157	0.072811	9.245228	2.35E-20	4.26E-19	UP
RP11-105!	85.49508	2.120665	0.229395	9.244594	2.36E-20	4.28E-19	UP
KCTD9P4	9.967481	-2.82914	0.306055	-9.24389	2.38E-20	4.31E-19	DOWN
TMEM147	134.5121	1.344962	0.145499	9.2438	2.38E-20	4.31E-19	UP
RP11-329I	45.74241	2.122169	0.229604	9.242726	2.40E-20	4.35E-19	UP
RP11-5O1	48.60375	1.425375	0.154235	9.241603	2.43E-20	4.39E-19	UP
GOLGA8B	728.4544	1.572922	0.170262	9.238242	2.51E-20	4.53E-19	UP
POGZ	2429.004	0.835802	0.090482	9.237177	2.53E-20	4.57E-19	UP
RP11-197I	12.85468	1.505047	0.162985	9.234282	2.60E-20	4.70E-19	UP
LINC0035!	13.76121	6.117361	0.662532	9.233312	2.62E-20	4.74E-19	UP
EEF1A2	3235.069	3.874859	0.41972	9.232019	2.66E-20	4.79E-19	UP
PRAM1	76.82348	-1.51835	0.164501	-9.22999	2.71E-20	4.88E-19	DOWN
RP11-298I	6.440538	1.92331	0.208393	9.229257	2.73E-20	4.91E-19	UP
FLVCR1-A!	130.5479	1.604975	0.174	9.223984	2.86E-20	5.15E-19	UP
TMCO3	1806.12	1.425674	0.154595	9.221964	2.92E-20	5.25E-19	UP
RP11-806I	4.027409	3.447376	0.373837	9.221605	2.93E-20	5.26E-19	UP
ARL5B	2595.771	-1.03452	0.11219	-9.22113	2.94E-20	5.28E-19	DOWN
ZNF526	401.5455	0.682324	0.074045	9.214944	3.11E-20	5.59E-19	UP
HMGCLL1	13.48815	-2.64743	0.287314	-9.21439	3.13E-20	5.62E-19	DOWN
MPV17	1123.914	1.040394	0.112957	9.210535	3.25E-20	5.82E-19	UP
TPPP2	115.8521	-2.37689	0.258127	-9.20823	3.32E-20	5.94E-19	DOWN
TCAM1P	22.43724	5.229727	0.568029	9.206801	3.36E-20	6.02E-19	UP
DNAJB11	4435.527	0.785742	0.085354	9.205725	3.39E-20	6.07E-19	UP
FAM155B	75.88344	2.955661	0.321086	9.205192	3.41E-20	6.10E-19	UP
DNASE1L2	31.27019	2.443389	0.26561	9.199169	3.61E-20	6.45E-19	UP
NKX3-2	9.69402	4.790574	0.52078	9.198851	3.62E-20	6.46E-19	UP
TEX22	16.02548	2.059107	0.223884	9.197189	3.67E-20	6.56E-19	UP
RP11-139:	24.2901	1.794943	0.195179	9.196413	3.70E-20	6.60E-19	UP
B4GALT1-,	82.59979	-1.56424	0.170138	-9.19389	3.79E-20	6.75E-19	DOWN
KNSTRN	455.5542	0.934978	0.101714	9.192241	3.85E-20	6.85E-19	UP
PCDHB2	55.84645	3.027801	0.329434	9.190932	3.89E-20	6.93E-19	UP
PLEKHG2	639.7876	1.257809	0.136914	9.186848	4.05E-20	7.20E-19	UP
KCNC1	7.227143	4.422139	0.481452	9.184995	4.12E-20	7.32E-19	UP
S100A3	19.58925	2.819494	0.306996	9.184133	4.15E-20	7.37E-19	UP
CAPN10	490.839	0.83565	0.091016	9.18137	4.26E-20	7.56E-19	UP
B4GALNT4	159.7968	4.087848	0.445237	9.181284	4.26E-20	7.56E-19	UP

RP11-404(	2.565263	3.354629	0.365398	9.180753	4.28E-20	7.59E-19	UP
BANF2	7.391589	4.740476	0.516353	9.180683	4.28E-20	7.59E-19	UP
TAF6	814.6778	1.001519	0.10913	9.177307	4.42E-20	7.83E-19	UP
UNK	947.4795	0.717744	0.07821	9.177147	4.43E-20	7.84E-19	UP
MMS22L	191.6745	1.466507	0.159815	9.176256	4.46E-20	7.90E-19	UP
TRIM16L	1004.009	2.457533	0.267846	9.175164	4.51E-20	7.97E-19	UP
ACTN2	148.0446	3.269501	0.356435	9.172782	4.61E-20	8.14E-19	UP
POC5	212.9958	0.713689	0.07782	9.171063	4.68E-20	8.27E-19	UP
SLC45A4	451.0957	2.186306	0.238402	9.170672	4.70E-20	8.29E-19	UP
CAMK2N2	98.81802	2.50138	0.272803	9.169168	4.77E-20	8.41E-19	UP
SERPINI1	233.4913	1.668675	0.182044	9.166333	4.89E-20	8.62E-19	UP
LARP1	6857	0.664238	0.072515	9.160013	5.19E-20	9.14E-19	UP
AIM1L	104.8625	2.271016	0.248	9.157331	5.32E-20	9.36E-19	UP
STRN4	2313.742	0.628545	0.068671	9.152938	5.54E-20	9.75E-19	UP
AC092155	5.532242	-3.22435	0.352375	-9.15033	5.68E-20	9.98E-19	DOWN
USF1	2061.053	0.697989	0.076288	9.149377	5.73E-20	1.01E-18	UP
ALDH6A1	15161.68	-1.8017	0.196936	-9.14863	5.77E-20	1.01E-18	DOWN
SLC31A1	6252.728	-1.03423	0.113081	-9.14588	5.91E-20	1.04E-18	DOWN
CLEC12A	54.22632	-1.88318	0.205912	-9.14554	5.93E-20	1.04E-18	DOWN
CD163	2247.844	-1.94789	0.21305	-9.14291	6.08E-20	1.06E-18	DOWN
HIST1H3B	12.57185	3.283084	0.359086	9.142903	6.08E-20	1.06E-18	UP
PLEKHG4	150.8362	2.677623	0.292882	9.142312	6.11E-20	1.07E-18	UP
POU5F1	72.55771	1.927809	0.210884	9.141584	6.15E-20	1.08E-18	UP
VKORC1	3565.764	-1.222	0.13368	-9.14125	6.17E-20	1.08E-18	DOWN
MARK4	1819.359	0.89298	0.097697	9.140322	6.23E-20	1.09E-18	UP
KLRF1	24.22516	-1.81415	0.198484	-9.14001	6.24E-20	1.09E-18	DOWN
RP11-445I	8.406583	3.377825	0.369668	9.137469	6.39E-20	1.12E-18	UP
LRRC37BP1	177.619	0.918763	0.100563	9.136169	6.47E-20	1.13E-18	UP
PRRT2	27.82662	1.80199	0.197266	9.13484	6.55E-20	1.14E-18	UP
CUEDC1	805.5526	1.109827	0.12151	9.133598	6.63E-20	1.15E-18	UP
ZNF8	172.3192	0.994151	0.108853	9.13293	6.67E-20	1.16E-18	UP
ZKSCAN5	486.6534	0.616146	0.067471	9.132063	6.72E-20	1.17E-18	UP
PRR36	91.43988	2.575499	0.282048	9.131415	6.76E-20	1.17E-18	UP
AKAP8L	1420.304	0.740776	0.081128	9.130915	6.79E-20	1.18E-18	UP
C1orf64	55.70134	5.397086	0.591272	9.127931	6.98E-20	1.21E-18	UP
SULT4A1	117.8387	4.748105	0.520231	9.126918	7.05E-20	1.22E-18	UP
KB-1460A1	7.817114	4.226332	0.46313	9.125585	7.13E-20	1.24E-18	UP
HIST2H2BI	28.4074	2.387123	0.261601	9.125064	7.17E-20	1.24E-18	UP
AQP8	115.6358	3.837785	0.420731	9.121707	7.40E-20	1.28E-18	UP
LOXHD1	8.062984	3.10034	0.339922	9.120744	7.46E-20	1.29E-18	UP
FRS3	186.7558	0.792404	0.08688	9.120699	7.46E-20	1.29E-18	UP
SUGP2	1169.821	0.910466	0.099834	9.119801	7.53E-20	1.30E-18	UP
ANKFN1	82.22717	7.36849	0.807971	9.119744	7.53E-20	1.30E-18	UP
ZNF300	99.07839	2.082755	0.228402	9.118804	7.60E-20	1.31E-18	UP
RBP7	413.1549	1.86916	0.205022	9.116884	7.73E-20	1.33E-18	UP
C21orf62	9.384135	-2.49485	0.273662	-9.11655	7.76E-20	1.34E-18	DOWN
PLEKHH1	166.7649	1.371252	0.150427	9.115703	7.82E-20	1.35E-18	UP
RP11-351I	10.36801	1.755508	0.192586	9.115443	7.83E-20	1.35E-18	UP
HS6ST2	150.1259	4.500912	0.493803	9.114803	7.88E-20	1.36E-18	UP
ALKBH6	112.3213	1.19382	0.131052	9.109521	8.27E-20	1.42E-18	UP
SAC3D1	634.2117	1.236026	0.135688	9.109312	8.29E-20	1.42E-18	UP
AL589743.	9.930301	3.224064	0.35399	9.107789	8.41E-20	1.44E-18	UP

PYCR1	883.4126	2.67682	0.293934	9.106889	8.48E-20	1.46E-18	UP
MBNL2	3319.891	-1.35411	0.148694	-9.1067	8.49E-20	1.46E-18	DOWN
HIST1H2A	9.364864	2.694087	0.295844	9.106445	8.51E-20	1.46E-18	UP
NPM1P25	84.98918	-1.45912	0.160271	-9.10408	8.70E-20	1.49E-18	DOWN
DSTNP2	123.0816	1.093932	0.120233	9.098395	9.17E-20	1.57E-18	UP
FHDC1	63.3147	2.230872	0.245239	9.096744	9.31E-20	1.59E-18	UP
RP11-15E1	4.946429	2.398921	0.263772	9.094683	9.49E-20	1.62E-18	UP
LHFPL4	44.30142	4.700243	0.516818	9.094576	9.50E-20	1.62E-18	UP
MASP1	7468.868	-1.70832	0.187902	-9.09157	9.76E-20	1.67E-18	DOWN
RP11-154J	8.899603	2.270038	0.249769	9.088548	1.00E-19	1.71E-18	UP
HIST1H3D	28.45407	2.351162	0.25879	9.085209	1.03E-19	1.77E-18	UP
RP11-598I	7.697167	-2.7233	0.299773	-9.08452	1.04E-19	1.78E-18	DOWN
ASS1P1	80.78009	-1.92225	0.211609	-9.08397	1.05E-19	1.78E-18	DOWN
UBALD1	1494.585	-1.05329	0.115954	-9.08369	1.05E-19	1.79E-18	DOWN
FSD1L	96.80834	1.39233	0.15331	9.081804	1.07E-19	1.82E-18	UP
AKR1C3	13616.14	1.48006	0.162973	9.081602	1.07E-19	1.82E-18	UP
CACNG4	201.1237	3.999415	0.440628	9.07662	1.12E-19	1.90E-18	UP
TBC1D22B	398.3308	0.824125	0.09081	9.075292	1.13E-19	1.93E-18	UP
BARD1	165.9387	1.429357	0.157532	9.073416	1.15E-19	1.96E-18	UP
WEE1	1871.18	-1.2431	0.137014	-9.07282	1.16E-19	1.97E-18	DOWN
ABCB5	20.72012	5.252855	0.579004	9.072225	1.17E-19	1.98E-18	UP
RP11-800A	10.42363	5.129489	0.565438	9.071716	1.17E-19	1.98E-18	UP
INPP5J	54.97596	2.334036	0.257314	9.070756	1.18E-19	2.00E-18	UP
PDLIM7	895.1523	1.335974	0.147311	9.069082	1.20E-19	2.03E-18	UP
DOCK3	17.50998	2.152621	0.237387	9.067985	1.21E-19	2.05E-18	UP
CTD-2349	15.60627	1.623312	0.179038	9.066841	1.23E-19	2.07E-18	UP
UBE2A	1859.383	0.678196	0.074823	9.064041	1.26E-19	2.12E-18	UP
MT1X	18800.43	-2.97269	0.328064	-9.06132	1.29E-19	2.18E-18	DOWN
DNAH12	43.2298	2.953542	0.325987	9.060293	1.30E-19	2.19E-18	UP
TEX11	41.60608	3.854982	0.425514	9.059596	1.31E-19	2.21E-18	UP
FER1L4	102.8129	2.722112	0.300511	9.058275	1.33E-19	2.23E-18	UP
LDLRAD1	82.46452	3.636491	0.401463	9.058108	1.33E-19	2.24E-18	UP
ZMIZ2	2576.64	0.793485	0.087611	9.056873	1.34E-19	2.26E-18	UP
TIGD7	63.21149	1.156621	0.127739	9.054561	1.37E-19	2.31E-18	UP
C19orf81	15.12871	5.131946	0.566872	9.053092	1.39E-19	2.34E-18	UP
CTB-92J24	13.16818	4.813886	0.531781	9.052392	1.40E-19	2.35E-18	UP
PEMT	3536.462	-1.53749	0.169866	-9.05122	1.41E-19	2.37E-18	DOWN
RP11-399J	37.48469	2.76967	0.306007	9.050992	1.42E-19	2.38E-18	UP
CSTF2	408.021	1.002593	0.110779	9.050414	1.42E-19	2.39E-18	UP
SSTR5-AS1	125.2424	5.796125	0.640461	9.049927	1.43E-19	2.40E-18	UP
BRD8	1290.326	0.652197	0.07208	9.048225	1.45E-19	2.43E-18	UP
RP11-18H	19.62118	1.417691	0.156706	9.046792	1.47E-19	2.46E-18	UP
RP11-105!	41.38647	1.997109	0.22077	9.046109	1.48E-19	2.48E-18	UP
ZNF282	1212.853	0.72783	0.080532	9.037802	1.60E-19	2.67E-18	UP
RBM24	198.1804	2.356565	0.260982	9.029595	1.72E-19	2.88E-18	UP
LL0XNC01	35.33495	1.444829	0.160069	9.026283	1.78E-19	2.97E-18	UP
BCAS4	160.5083	1.57723	0.17475	9.02562	1.79E-19	2.98E-18	UP
GRPR	37.13705	3.977642	0.440757	9.02457	1.80E-19	3.01E-18	UP
CKS2	747.2869	1.449692	0.160652	9.023785	1.82E-19	3.03E-18	UP
RAD1	695.6011	0.619971	0.06872	9.021646	1.85E-19	3.09E-18	UP
RP11-909I	10.63584	5.743411	0.63668	9.020869	1.87E-19	3.11E-18	UP
ATXN7L2	157.001	0.928145	0.102906	9.019319	1.89E-19	3.15E-18	UP

TEAD2	889.6021	1.551994	0.172099	9.018005	1.92E-19	3.18E-18	UP
CAT	23103.75	-1.45383	0.161219	-9.01777	1.92E-19	3.19E-18	DOWN
AC108463	10.65181	2.104809	0.233435	9.016682	1.94E-19	3.22E-18	UP
SYN3	26.79997	2.651093	0.294049	9.015806	1.95E-19	3.24E-18	UP
CSAG1	183.8666	5.878878	0.652145	9.01468	1.97E-19	3.28E-18	UP
SNHG7	1029.697	1.478678	0.164037	9.014279	1.98E-19	3.29E-18	UP
PIK3C2B	938.1013	1.061311	0.11776	9.012455	2.01E-19	3.34E-18	UP
ZNF99	18.14417	3.991	0.442875	9.011571	2.03E-19	3.36E-18	UP
ADAMTS1	107.8649	3.374974	0.374572	9.01021	2.06E-19	3.40E-18	UP
LAPTM4B	5133.642	1.696237	0.188401	9.003334	2.19E-19	3.62E-18	UP
PTPDC1	137.4178	0.984875	0.109403	9.002299	2.21E-19	3.65E-18	UP
TTLL4	948.2055	1.493981	0.165966	9.001749	2.22E-19	3.67E-18	UP
TSLP	173.2555	-2.45063	0.272247	-9.00152	2.23E-19	3.68E-18	DOWN
MAP7D2	122.9801	3.507081	0.389629	9.001069	2.24E-19	3.69E-18	UP
ZNF449	136.158	0.845387	0.093947	8.998571	2.29E-19	3.77E-18	UP
ASXL1	2332.187	0.714721	0.079435	8.99753	2.31E-19	3.80E-18	UP
RP11-523I	19.75915	1.727395	0.192033	8.995307	2.36E-19	3.88E-18	UP
RNF215	576.3109	0.743203	0.082643	8.992929	2.41E-19	3.96E-18	UP
ACTL8	23.80822	5.968	0.663709	8.991893	2.43E-19	4.00E-18	UP
MAGEA3	313.3392	7.05585	0.784875	8.989773	2.48E-19	4.07E-18	UP
ALDH1B1	8069.953	-1.49118	0.165912	-8.9878	2.52E-19	4.14E-18	DOWN
DPY19L1P	52.62207	1.012787	0.112692	8.987193	2.54E-19	4.16E-18	UP
CNTNAP1	214.8609	1.9976	0.222273	8.987152	2.54E-19	4.16E-18	UP
RPL32P3	154.7839	1.099666	0.122366	8.986714	2.55E-19	4.18E-18	UP
NCOR1	3426.953	-1.00852	0.112245	-8.98493	2.59E-19	4.24E-18	DOWN
BRCA2	132.7385	1.405508	0.156485	8.981728	2.67E-19	4.37E-18	UP
LINC0063	3.871116	3.570261	0.397647	8.97846	2.75E-19	4.50E-18	UP
DRP2	5.023078	3.793989	0.422655	8.976567	2.79E-19	4.57E-18	UP
RP11-106I	36.79559	1.193908	0.133007	8.976298	2.80E-19	4.58E-18	UP
NFRKB	1030.004	0.653732	0.072829	8.976275	2.80E-19	4.58E-18	UP
CCL3	246.8298	-1.78113	0.198437	-8.97581	2.81E-19	4.59E-18	DOWN
RP11-167I	12.59288	4.89452	0.545418	8.973882	2.86E-19	4.67E-18	UP
RP11-93H	15.76069	1.739151	0.193822	8.972919	2.89E-19	4.71E-18	UP
LA16c-60I	5.922291	3.345721	0.37292	8.971675	2.92E-19	4.76E-18	UP
ZFAT	359.3911	0.804535	0.089677	8.971468	2.93E-19	4.77E-18	UP
ZNF785	208.8974	0.869851	0.096965	8.970742	2.95E-19	4.80E-18	UP
SHPK	352.582	-1.12919	0.125876	-8.97063	2.95E-19	4.80E-18	DOWN
PRRT3	71.6085	1.094305	0.121989	8.970494	2.95E-19	4.80E-18	UP
ATP5G1P4	7.593753	3.013824	0.336016	8.969275	2.98E-19	4.85E-18	UP
LRRC37A3	127.1652	1.518981	0.169358	8.969064	2.99E-19	4.86E-18	UP
PTGES	256.2355	3.100867	0.345752	8.96848	3.01E-19	4.88E-18	UP
MIR600HG	98.93404	1.568838	0.17494	8.967852	3.02E-19	4.91E-18	UP
SERINC1	8785.26	-0.85114	0.094948	-8.96429	3.12E-19	5.06E-18	DOWN
HRCT1	263.3181	2.421942	0.270227	8.962627	3.17E-19	5.14E-18	UP
HERC2P2	519.5223	1.423001	0.158774	8.96242	3.18E-19	5.15E-18	UP
USP38	1447.807	-0.95148	0.106177	-8.96123	3.21E-19	5.20E-18	DOWN
YKT6	2008.885	0.848161	0.094663	8.959833	3.25E-19	5.26E-18	UP
RP11-383	8.243227	5.203325	0.580794	8.958993	3.28E-19	5.30E-18	UP
TANGO6	977.6189	-1.03761	0.115827	-8.95829	3.30E-19	5.33E-18	DOWN
SCML2	147.6209	1.480801	0.165303	8.958114	3.30E-19	5.33E-18	UP
CLVS1	23.75293	2.649264	0.295955	8.951572	3.50E-19	5.66E-18	UP
DPY19L2P	22.38562	2.324627	0.259717	8.950617	3.54E-19	5.70E-18	UP

IGFALS	4011.795	-3.05155	0.340962	-8.94981	3.56E-19	5.74E-18	DOWN
SNHG12	233.9163	1.313838	0.146807	8.949415	3.57E-19	5.76E-18	UP
STEAP1B	8.6542	2.999155	0.335157	8.948495	3.60E-19	5.80E-18	UP
AC004381	204.3298	1.372899	0.153428	8.948143	3.62E-19	5.82E-18	UP
MNX1-AS:	23.68479	7.721959	0.862977	8.948049	3.62E-19	5.82E-18	UP
SNX27	1879.823	0.826758	0.092409	8.946755	3.66E-19	5.89E-18	UP
NR0B1	24.22524	6.680825	0.746787	8.946095	3.68E-19	5.92E-18	UP
C18orf54	65.63275	1.637772	0.183118	8.94381	3.76E-19	6.04E-18	UP
C1QTNF6	837.1288	1.34254	0.150124	8.942843	3.79E-19	6.09E-18	UP
RP11-98G	22.56978	2.394122	0.267799	8.939986	3.89E-19	6.24E-18	UP
ZNF695	10.63982	4.528126	0.506513	8.939795	3.90E-19	6.25E-18	UP
LINC0089	46.50609	1.612441	0.180426	8.936861	4.00E-19	6.41E-18	UP
POLM	674.2982	0.729171	0.08161	8.934849	4.08E-19	6.53E-18	UP
C1QL4	15.47764	4.203854	0.470551	8.933895	4.11E-19	6.58E-18	UP
RP11-506I	6.155337	2.429191	0.271995	8.931001	4.22E-19	6.75E-18	UP
PLA2G6	610.9426	1.353706	0.151613	8.928683	4.31E-19	6.89E-18	UP
SLC22A5	532.107	0.811486	0.090889	8.928282	4.33E-19	6.91E-18	UP
MMS19	1494.311	0.563326	0.063108	8.926397	4.40E-19	7.03E-18	NOT
SPAG5	1212.68	1.438641	0.161194	8.924877	4.46E-19	7.12E-18	UP
RP11-587I	29.18462	7.366669	0.825522	8.923654	4.51E-19	7.19E-18	UP
C19orf48	2216.927	1.383008	0.155004	8.922394	4.56E-19	7.27E-18	UP
VSIG1	72.6708	3.734517	0.418588	8.921696	4.59E-19	7.31E-18	UP
KRTCAP2	1187.187	1.025003	0.11496	8.91617	4.83E-19	7.68E-18	UP
CYP2C8	32213.84	-2.73173	0.306466	-8.91364	4.94E-19	7.86E-18	DOWN
CPSF6	1757.284	0.622679	0.06987	8.912013	5.01E-19	7.97E-18	UP
RNF125	821.2148	-1.78549	0.200425	-8.90853	5.17E-19	8.22E-18	DOWN
SMC4	844.613	1.54167	0.173122	8.90511	5.33E-19	8.47E-18	UP
PIM3	3017.479	-1.08705	0.122146	-8.89962	5.60E-19	8.90E-18	DOWN
TCERG1	1018.989	0.73284	0.082363	8.897647	5.70E-19	9.05E-18	UP
CEP89	482.7882	0.865901	0.097325	8.896978	5.74E-19	9.10E-18	UP
HOXB9	8.154407	4.954025	0.556922	8.895366	5.82E-19	9.23E-18	UP
EFNB3	57.82703	-1.94053	0.218193	-8.89367	5.91E-19	9.36E-18	DOWN
AP003774.	3.158097	-1.85347	0.208406	-8.89355	5.92E-19	9.37E-18	DOWN
DCLRE1B	233.3214	0.889361	0.100006	8.893083	5.94E-19	9.40E-18	UP
ISOC1	2636.332	-0.99235	0.11164	-8.88891	6.17E-19	9.76E-18	DOWN
C4orf29	492.6464	-0.78957	0.088841	-8.88746	6.25E-19	9.88E-18	DOWN
LINC0052	50.09542	1.638221	0.184383	8.884903	6.40E-19	1.01E-17	UP
SERPINB8	632.3857	-1.65519	0.18633	-8.88309	6.50E-19	1.03E-17	DOWN
MT-CYB	456193.1	-1.31957	0.14858	-8.88122	6.61E-19	1.04E-17	DOWN
ZNF213	315.5683	1.157863	0.130374	8.881082	6.62E-19	1.04E-17	UP
CEP295	243.847	0.998919	0.112479	8.880969	6.63E-19	1.04E-17	UP
ZCCHC24	2237.214	-1.06804	0.120266	-8.88064	6.65E-19	1.05E-17	DOWN
EIF5	9196.493	-0.74417	0.083818	-8.87839	6.78E-19	1.07E-17	DOWN
PFKFB4	185.3003	1.82747	0.205865	8.877036	6.87E-19	1.08E-17	UP
TNNC1	101.1996	2.939997	0.331259	8.875233	6.98E-19	1.10E-17	UP
RP5-1157I	6.633875	2.218607	0.249993	8.874693	7.01E-19	1.10E-17	UP
DGKQ	802.8674	0.800734	0.090243	8.873111	7.11E-19	1.12E-17	UP
LINC0048	153.6856	1.688523	0.190324	8.871855	7.19E-19	1.13E-17	UP
GALNT10	886.8749	1.167241	0.131584	8.870719	7.27E-19	1.14E-17	UP
SLC38A6	348.7127	1.134197	0.127903	8.86766	7.47E-19	1.17E-17	UP
PON3	8642.762	-1.79803	0.202771	-8.86731	7.49E-19	1.17E-17	DOWN
RP11-440I	44.60295	1.081189	0.121938	8.866684	7.54E-19	1.18E-17	UP

PPP2CB	2634.544	-0.84066	0.094815	-8.86632	7.56E-19	1.18E-17	DOWN
CTD-2297	20.15761	5.587127	0.630246	8.864991	7.65E-19	1.20E-17	UP
AC006538	16.03172	2.629016	0.296591	8.864122	7.71E-19	1.21E-17	UP
DNER	33.55887	5.130369	0.578842	8.863158	7.78E-19	1.22E-17	UP
PIP4K2B	2063.191	0.637194	0.071895	8.862816	7.80E-19	1.22E-17	UP
PPP1R3B	5856.366	-1.67009	0.188474	-8.86112	7.92E-19	1.24E-17	DOWN
BMP10	22.22829	-4.79326	0.540934	-8.86107	7.92E-19	1.24E-17	DOWN
NNMT	40778.8	-2.62725	0.29653	-8.86	8.00E-19	1.25E-17	DOWN
LAMA3	1136.871	2.343512	0.264536	8.85895	8.08E-19	1.26E-17	UP
HSP90AB3	30.44496	1.536063	0.173434	8.856764	8.24E-19	1.28E-17	UP
TAF1A	108.4526	1.036486	0.117028	8.85675	8.24E-19	1.28E-17	UP
CCDC154	40.5719	2.097087	0.236786	8.85646	8.26E-19	1.28E-17	UP
NUPL2	757.2891	0.597625	0.067489	8.855158	8.36E-19	1.30E-17	UP
SRSF8	1374.452	-0.867	0.097913	-8.85481	8.38E-19	1.30E-17	DOWN
NES	1545.565	1.299406	0.146756	8.854219	8.43E-19	1.31E-17	UP
OSTF1	1299.198	-0.65572	0.074061	-8.85376	8.46E-19	1.31E-17	DOWN
SFI1	606.32	1.292419	0.145974	8.853735	8.46E-19	1.31E-17	UP
YAF2	607.5479	-0.63163	0.07137	-8.85007	8.75E-19	1.36E-17	DOWN
ATXN2L	3149.474	0.667929	0.075486	8.848382	8.88E-19	1.38E-17	UP
ANKRD65	296.0183	2.527729	0.285681	8.848071	8.90E-19	1.38E-17	UP
SHC1	6035.074	0.82354	0.093092	8.846541	9.03E-19	1.40E-17	UP
PSRC1	225.2235	1.735573	0.196216	8.845202	9.14E-19	1.41E-17	UP
AVPI1	1316.533	-1.30371	0.147393	-8.84515	9.14E-19	1.41E-17	DOWN
ZNF473	339.0439	0.734576	0.083097	8.839953	9.58E-19	1.48E-17	UP
CNOT3	1373.355	0.602834	0.06821	8.837914	9.75E-19	1.51E-17	UP
RP11-496I	12.10259	2.587353	0.292767	8.837571	9.78E-19	1.51E-17	UP
RP11-802I	36.64932	1.58505	0.179391	8.835716	9.95E-19	1.53E-17	UP
RP3-329A!	15.95656	1.900765	0.215164	8.83401	1.01E-18	1.56E-17	UP
KLHL17	180.5998	0.928312	0.105087	8.833773	1.01E-18	1.56E-17	UP
FAP	109.6599	2.596528	0.293953	8.833139	1.02E-18	1.57E-17	UP
MMRN1	237.4858	-1.88424	0.213351	-8.83164	1.03E-18	1.59E-17	DOWN
VPS11	1417.889	0.561817	0.063654	8.826094	1.08E-18	1.67E-17	NOT
LPCAT1	1744.174	1.491294	0.16898	8.825258	1.09E-18	1.68E-17	UP
INPPL1	2796.578	0.731931	0.082937	8.825132	1.09E-18	1.68E-17	UP
STK40	2617.475	-0.81432	0.092273	-8.82511	1.09E-18	1.68E-17	DOWN
INHA	41.10122	3.693499	0.418632	8.822774	1.12E-18	1.71E-17	UP
ADAMTS1	14.85119	3.545508	0.401937	8.821054	1.13E-18	1.74E-17	UP
SOWAHC	2341.259	-1.13671	0.128865	-8.82092	1.14E-18	1.74E-17	DOWN
RP11-134(	13.78677	1.440037	0.163302	8.818243	1.16E-18	1.78E-17	UP
RP11-733(	14.68085	-1.70645	0.193521	-8.8179	1.17E-18	1.79E-17	DOWN
RCL1	2144.125	-1.54258	0.174939	-8.81785	1.17E-18	1.79E-17	DOWN
DGKZ	1444.883	0.731913	0.083012	8.81698	1.18E-18	1.80E-17	UP
CA4	57.10264	3.330939	0.377959	8.812958	1.22E-18	1.86E-17	UP
PARP2	700.7115	0.665121	0.0755	8.809538	1.26E-18	1.92E-17	UP
FIRRE	9.9713	3.309538	0.3757	8.809001	1.26E-18	1.93E-17	UP
CTD-2231	9.479979	2.123129	0.241048	8.807915	1.27E-18	1.95E-17	UP
CYP2R1	417.4634	0.667492	0.075785	8.807686	1.28E-18	1.95E-17	UP
RP11-421I	5.421605	2.230486	0.253266	8.806894	1.29E-18	1.96E-17	UP
FLJ31104	14.43466	2.009909	0.228258	8.805424	1.30E-18	1.99E-17	UP
INTS10	1802.747	-0.84018	0.095418	-8.80529	1.31E-18	1.99E-17	DOWN
GAREM	660.753	-1.41486	0.160733	-8.80255	1.34E-18	2.04E-17	DOWN
C1QTNF3	365.0834	2.572177	0.292215	8.802351	1.34E-18	2.04E-17	UP

CTD-2562	6.38504	2.110344	0.239751	8.802245	1.34E-18	2.04E-17	UP
GRHPR	18666.2	-1.365	0.155079	-8.80196	1.34E-18	2.04E-17	DOWN
CPT2	3673.045	-1.22079	0.138707	-8.80124	1.35E-18	2.05E-17	DOWN
CPSF1	3707.005	0.849949	0.096577	8.800746	1.36E-18	2.06E-17	UP
ZNF337-A	100.5385	1.08729	0.123548	8.800566	1.36E-18	2.06E-17	UP
FLJ39080	7.751165	4.819996	0.547747	8.799673	1.37E-18	2.08E-17	UP
EPM2A	291.9866	-1.02103	0.116041	-8.79891	1.38E-18	2.09E-17	DOWN
TSPO2	28.06536	2.083903	0.236859	8.798073	1.39E-18	2.11E-17	UP
MYO15B	2133.344	1.428592	0.162378	8.797927	1.39E-18	2.11E-17	UP
SOCS3	3451.555	-2.15464	0.244915	-8.79751	1.40E-18	2.12E-17	DOWN
MT-ND4	822855.8	-1.29328	0.147033	-8.79583	1.42E-18	2.15E-17	DOWN
SOX11	12.11092	4.228448	0.480801	8.794585	1.44E-18	2.17E-17	UP
RPL18P13	10.28424	4.875207	0.554516	8.79182	1.47E-18	2.22E-17	UP
RFC3	346.8421	1.074317	0.122201	8.791404	1.48E-18	2.23E-17	UP
NUP107	1008.042	0.733569	0.083452	8.790272	1.49E-18	2.25E-17	UP
NR4A2	654.6468	-2.05055	0.233372	-8.78662	1.54E-18	2.32E-17	DOWN
PROCA1	58.20447	1.504706	0.171272	8.785485	1.56E-18	2.35E-17	UP
THOC5	990.3095	0.812561	0.092495	8.784934	1.56E-18	2.36E-17	UP
KCNH4	20.27404	2.413939	0.274789	8.784699	1.57E-18	2.36E-17	UP
NAT2	925.5319	-2.88528	0.328509	-8.78294	1.59E-18	2.40E-17	DOWN
RP11-465I	44.01849	1.444418	0.164458	8.782915	1.59E-18	2.40E-17	UP
PLIN2	23666.38	-1.64315	0.187148	-8.77991	1.64E-18	2.46E-17	DOWN
C1orf198	2155.936	1.137275	0.129563	8.777804	1.67E-18	2.50E-17	UP
PRR7-AS1	5.914882	2.757288	0.314134	8.777428	1.67E-18	2.51E-17	UP
KIAA0195	2586.786	0.660668	0.075297	8.774129	1.72E-18	2.58E-17	UP
SLC50A1	3101.365	1.127488	0.128522	8.772747	1.74E-18	2.61E-17	UP
H2AFY	4445.239	0.716723	0.081702	8.772425	1.75E-18	2.62E-17	UP
ANKZF1	1125.839	0.762134	0.086883	8.771932	1.76E-18	2.63E-17	UP
HIST3H2A	121.1863	3.164724	0.360818	8.770973	1.77E-18	2.65E-17	UP
GPC2	16.90938	2.382755	0.271676	8.770562	1.78E-18	2.66E-17	UP
PRRC2A	6848.452	0.690662	0.078793	8.765471	1.86E-18	2.78E-17	UP
MT1E	13368.89	-3.06263	0.349432	-8.76458	1.87E-18	2.80E-17	DOWN
ZNF385D	125.1236	3.314186	0.378186	8.763377	1.89E-18	2.83E-17	UP
TMPOP2	3.167236	3.168559	0.361662	8.761097	1.93E-18	2.89E-17	UP
ZNF607	107.1534	1.664351	0.189996	8.759914	1.95E-18	2.92E-17	UP
IL17D	64.4766	2.620205	0.299126	8.759545	1.96E-18	2.92E-17	UP
BAI2	41.13907	2.60125	0.297046	8.757061	2.00E-18	2.99E-17	UP
HIST1H2B	40.22546	2.415736	0.27588	8.756463	2.01E-18	3.00E-17	UP
AGBL5	949.2197	0.852915	0.097431	8.754045	2.06E-18	3.07E-17	UP
BRD9	940.4455	0.675823	0.077202	8.753954	2.06E-18	3.07E-17	UP
BDKRB1	25.23159	2.449655	0.279958	8.750074	2.13E-18	3.17E-17	UP
C15orf39	930.6907	1.06037	0.121192	8.749532	2.14E-18	3.19E-17	UP
SERTAD4-	22.53334	2.941396	0.336181	8.749447	2.14E-18	3.19E-17	UP
MTMR11	495.6478	1.90456	0.217744	8.746775	2.20E-18	3.26E-17	UP
TSPYL1	3041.084	-0.90155	0.103082	-8.74595	2.21E-18	3.28E-17	DOWN
SERPINE1	12632.42	-2.2494	0.257216	-8.74516	2.23E-18	3.30E-17	DOWN
CD1D	395.6091	-1.81561	0.207617	-8.74501	2.23E-18	3.31E-17	DOWN
SMPD4	1696.357	0.606421	0.069347	8.74469	2.24E-18	3.31E-17	UP
TLX1	103.9888	2.448667	0.28002	8.744623	2.24E-18	3.31E-17	UP
FAM53B	660.742	1.042015	0.119237	8.739004	2.35E-18	3.48E-17	UP
MYBL1	173.634	1.293208	0.148056	8.734602	2.45E-18	3.62E-17	UP
RP11-968/	12.7064	2.122228	0.243012	8.733002	2.48E-18	3.67E-17	UP

CH17-264	25.60732	1.836659	0.210333	8.73217	2.50E-18	3.69E-17	UP
MCC	1056.903	-1.61032	0.18444	-8.73084	2.53E-18	3.73E-17	DOWN
HID1-AS1	6.616275	-1.61438	0.184915	-8.73039	2.54E-18	3.75E-17	DOWN
RP11-347I	24.88412	1.717819	0.196787	8.729341	2.56E-18	3.78E-17	UP
RP11-143I	5.849716	1.943586	0.222675	8.728367	2.58E-18	3.81E-17	UP
SYP	99.31055	1.838268	0.210651	8.72662	2.62E-18	3.87E-17	UP
RP11-158I	18.79259	1.864916	0.213822	8.72183	2.74E-18	4.03E-17	UP
MAFA-AS1	8.838933	5.475499	0.627917	8.720106	2.78E-18	4.09E-17	UP
HEATR6	377.7537	0.716299	0.082145	8.719968	2.78E-18	4.10E-17	UP
CTC-351M	52.89366	1.262753	0.144814	8.719819	2.79E-18	4.10E-17	UP
MUS81	931.271	0.497787	0.057102	8.717459	2.85E-18	4.18E-17	NOT
AACS	675.1736	1.110739	0.127442	8.71567	2.89E-18	4.25E-17	UP
SAMD1	1363.443	0.915714	0.105066	8.715592	2.89E-18	4.25E-17	UP
MCCD1	40.15179	5.280593	0.605889	8.715447	2.90E-18	4.25E-17	UP
RP11-108I	6.107192	2.724679	0.312638	8.715114	2.90E-18	4.26E-17	UP
NGFR	852.644	-2.12964	0.244366	-8.71496	2.91E-18	4.26E-17	DOWN
TPT1	108546.6	-0.95435	0.109515	-8.7143	2.93E-18	4.29E-17	DOWN
IFT52	486.6168	0.873413	0.100237	8.713516	2.95E-18	4.31E-17	UP
CYP2B6	12726.64	-2.74918	0.315513	-8.71335	2.95E-18	4.32E-17	DOWN
RP11-37C7	8.229839	2.177492	0.249937	8.712159	2.98E-18	4.36E-17	UP
RCOR3	1028.105	0.746348	0.085671	8.711838	2.99E-18	4.37E-17	UP
EPS15L1	900.44	0.768764	0.088253	8.710864	3.02E-18	4.41E-17	UP
MED24	2715.443	0.582712	0.066916	8.708144	3.09E-18	4.51E-17	NOT
AC004447	371.1566	1.059308	0.121681	8.705586	3.16E-18	4.61E-17	UP
CPNE7	116.2223	2.688941	0.308883	8.705376	3.17E-18	4.62E-17	UP
LRRC55	40.04203	-2.42138	0.278344	-8.69922	3.34E-18	4.87E-17	DOWN
AC005229	26.86525	-1.17498	0.135076	-8.69862	3.36E-18	4.90E-17	DOWN
CLCN7	2719.044	0.854861	0.098279	8.698347	3.37E-18	4.91E-17	UP
JRKL	254.9698	1.003342	0.115353	8.697982	3.38E-18	4.92E-17	UP
HES2	25.49784	2.751284	0.316386	8.695973	3.44E-18	5.00E-17	UP
C6orf48	2395.473	1.138022	0.130923	8.692335	3.55E-18	5.16E-17	UP
PCBP4	1000.04	1.035141	0.119093	8.691841	3.57E-18	5.18E-17	UP
EZH1	873.3959	0.672804	0.077407	8.691726	3.57E-18	5.18E-17	UP
RAB14	4779.234	-0.61253	0.070473	-8.69172	3.57E-18	5.18E-17	DOWN
CSE1L	3033.756	0.694607	0.079926	8.690682	3.60E-18	5.23E-17	UP
SIRT7	817.8794	0.914838	0.10527	8.690435	3.61E-18	5.24E-17	UP
DXO	726.9229	0.808766	0.093072	8.68966	3.64E-18	5.27E-17	UP
GTSF1	202.1225	4.167815	0.479675	8.688826	3.66E-18	5.31E-17	UP
LPHN1	544.6517	2.198307	0.253021	8.688233	3.68E-18	5.33E-17	UP
TMEM136	132.2303	1.544931	0.177843	8.687036	3.72E-18	5.39E-17	UP
DAND5	6.523783	3.1529	0.362977	8.686221	3.75E-18	5.42E-17	UP
CCDC180	23.10247	1.845331	0.21247	8.68515	3.78E-18	5.47E-17	UP
STEAP3	7922.378	-1.7247	0.198595	-8.68453	3.80E-18	5.50E-17	DOWN
TRIM25	1600.119	0.959832	0.110524	8.684345	3.81E-18	5.50E-17	UP
PIGV	1145.911	-1.0085	0.116158	-8.68209	3.89E-18	5.61E-17	DOWN
GLA	927.6874	1.16759	0.134514	8.680095	3.95E-18	5.71E-17	UP
UGT1A10	44.4872	5.004082	0.576612	8.678419	4.01E-18	5.79E-17	UP
LOX	454.1805	2.032971	0.234263	8.678141	4.02E-18	5.80E-17	UP
ADAM23	110.5592	2.905699	0.334844	8.677769	4.04E-18	5.82E-17	UP
NCOR2	3788.378	0.679284	0.07828	8.677585	4.04E-18	5.82E-17	UP
GNPAT	2933.802	0.824261	0.094998	8.676627	4.08E-18	5.87E-17	UP
CTC-435M	8.529583	2.039823	0.23525	8.67086	4.29E-18	6.17E-17	UP

SYT3	43.09546	3.773403	0.435195	8.670596	4.30E-18	6.18E-17	UP
SLC7A2	12918.39	-1.82144	0.210082	-8.67015	4.32E-18	6.20E-17	DOWN
CBR4	1793.791	-1.16053	0.133941	-8.66448	4.54E-18	6.52E-17	DOWN
HIST1H3H	158.8455	2.205069	0.254497	8.664415	4.54E-18	6.52E-17	UP
PCDHA1	19.42659	4.430612	0.511366	8.66427	4.54E-18	6.52E-17	UP
ADCY10P1	108.5409	1.386991	0.160115	8.662488	4.62E-18	6.62E-17	UP
RP11-268(	7.365844	4.624467	0.533884	8.661927	4.64E-18	6.65E-17	UP
CD5L	1613.245	-3.19625	0.369049	-8.66077	4.69E-18	6.71E-17	DOWN
CRYGS	65.37192	1.208678	0.139578	8.659547	4.74E-18	6.78E-17	UP
RP11-98D	12.39634	1.676001	0.193555	8.659021	4.76E-18	6.81E-17	UP
CTB-31O2	80.08764	1.131031	0.130696	8.653907	4.98E-18	7.12E-17	UP
AC024560	298.0628	0.968492	0.111945	8.651533	5.08E-18	7.27E-17	UP
USP15	1497	-0.51599	0.059654	-8.64973	5.16E-18	7.38E-17	NOT
NSD1	1758.351	0.726489	0.083992	8.649531	5.17E-18	7.39E-17	UP
ASB9P1	9.932009	3.176601	0.367279	8.649016	5.19E-18	7.42E-17	UP
KDM6B	1392.782	-0.95752	0.110742	-8.64641	5.31E-18	7.58E-17	DOWN
TRMU	983.2686	0.953636	0.110303	8.645612	5.35E-18	7.63E-17	UP
SNX15	23.27512	1.209554	0.13991	8.645201	5.37E-18	7.66E-17	UP
TMEM150	395.4736	2.161977	0.250092	8.644732	5.39E-18	7.68E-17	UP
MTATP6P1	41167.27	-1.39918	0.161884	-8.64313	5.47E-18	7.79E-17	DOWN
PDE2A	580.7536	-1.51963	0.175821	-8.64306	5.47E-18	7.79E-17	DOWN
SMC2	586.9326	1.07371	0.124259	8.640873	5.58E-18	7.94E-17	UP
MFGE8	1129.904	1.32689	0.153567	8.640474	5.60E-18	7.96E-17	UP
CLCNKA	41.59821	3.007	0.348043	8.639738	5.63E-18	8.01E-17	UP
CDAN1	507.6573	0.631824	0.073137	8.638963	5.67E-18	8.06E-17	UP
STXBP6	447.7146	1.562318	0.180861	8.638243	5.71E-18	8.10E-17	UP
ANKS1B	51.64055	2.600904	0.301112	8.637673	5.74E-18	8.14E-17	UP
AKR1B15	268.9295	4.084526	0.472911	8.636987	5.77E-18	8.18E-17	UP
ACSL1	40973.63	-1.78646	0.206876	-8.63541	5.85E-18	8.29E-17	DOWN
DNASE1	288.552	1.219381	0.141238	8.633487	5.95E-18	8.43E-17	UP
IDNK	876.3292	-1.29053	0.149504	-8.6321	6.02E-18	8.53E-17	DOWN
MPZL1	2922.214	0.946146	0.109632	8.630159	6.13E-18	8.67E-17	UP
VPS33A	832.3794	0.711352	0.082428	8.629949	6.14E-18	8.68E-17	UP
CELSR3-A:	6.646872	2.265293	0.262497	8.629775	6.15E-18	8.69E-17	UP
MIP	24.55829	-1.87066	0.216778	-8.62939	6.17E-18	8.72E-17	DOWN
RP11-334(	143.217	1.378675	0.159779	8.628621	6.21E-18	8.77E-17	UP
C11orf96	1274.041	-2.0489	0.237466	-8.62818	6.23E-18	8.80E-17	DOWN
STK11IP	486.0748	0.818722	0.094898	8.627386	6.28E-18	8.86E-17	UP
DARS2	1377.795	0.920151	0.106656	8.627302	6.28E-18	8.86E-17	UP
CCDC14	761.678	1.06215	0.123139	8.62563	6.37E-18	8.98E-17	UP
JDP2	637.2949	-1.14896	0.133204	-8.62557	6.38E-18	8.98E-17	DOWN
POLR3F	359.0056	0.514422	0.059641	8.625337	6.39E-18	9.00E-17	NOT
PSMD10	1249.985	0.738161	0.085584	8.624986	6.41E-18	9.02E-17	UP
ARPC5	6467.139	0.684683	0.079399	8.623374	6.50E-18	9.14E-17	UP
FAM24B	19.08735	1.940874	0.225101	8.622249	6.57E-18	9.23E-17	UP
KCNF1	74.01647	3.684668	0.427521	8.618688	6.77E-18	9.52E-17	UP
AC022007	51.0807	1.252476	0.145327	8.618337	6.79E-18	9.54E-17	UP
CYP4A11	28187.01	-2.33191	0.270585	-8.61803	6.81E-18	9.56E-17	DOWN
PBLD	6977.805	-1.71836	0.199429	-8.61641	6.91E-18	9.69E-17	DOWN
RHO	4.986635	3.760697	0.436473	8.616096	6.93E-18	9.71E-17	UP
BEND3	163.4059	1.266821	0.147042	8.61536	6.97E-18	9.77E-17	UP
MAPK3	1415.347	0.699695	0.081241	8.612545	7.15E-18	1.00E-16	UP

RP5-994D	22.16625	-0.83749	0.097256	-8.61112	7.23E-18	1.01E-16	DOWN
WFS1	1805.293	1.101403	0.127917	8.610323	7.29E-18	1.02E-16	UP
HEY1	250.0584	1.398704	0.162493	8.607769	7.45E-18	1.04E-16	UP
SLC35E4	89.68625	1.57895	0.18348	8.60557	7.59E-18	1.06E-16	UP
RP11-46F1	6.84987	2.105198	0.244698	8.603253	7.75E-18	1.08E-16	UP
ILDR2	337.2119	2.623837	0.304993	8.602932	7.77E-18	1.09E-16	UP
GNA12	1759.082	0.774252	0.090006	8.602203	7.82E-18	1.09E-16	UP
CCDC170	328.9482	2.501254	0.290778	8.601937	7.84E-18	1.09E-16	UP
KCNH6	17.51913	3.93822	0.457852	8.601506	7.87E-18	1.10E-16	UP
FBXL22	25.0983	1.234148	0.143483	8.60134	7.88E-18	1.10E-16	UP
KBTBD11	532.7467	-2.20793	0.256769	-8.59891	8.05E-18	1.12E-16	DOWN
RP11-173I	5.800428	2.547318	0.296238	8.598887	8.05E-18	1.12E-16	UP
DROSHA	1344.803	0.649675	0.075556	8.598583	8.07E-18	1.12E-16	UP
AMH	31.68037	2.86388	0.333067	8.598509	8.08E-18	1.12E-16	UP
PABPC4L	21.18029	1.66696	0.193882	8.597796	8.13E-18	1.13E-16	UP
RP13-516I	5.691309	2.497423	0.290507	8.596761	8.20E-18	1.14E-16	UP
SDHD	5061.549	-0.87438	0.101719	-8.59608	8.25E-18	1.15E-16	DOWN
IDUA	630.0153	1.08004	0.125698	8.592353	8.52E-18	1.18E-16	UP
RXFP1	29.28138	-1.70666	0.198688	-8.58967	8.72E-18	1.21E-16	DOWN
CCNO	51.92417	2.914436	0.339389	8.587307	8.90E-18	1.23E-16	UP
SDHB	5841.266	-0.98263	0.114432	-8.58698	8.93E-18	1.24E-16	DOWN
SARDH	6728.177	-1.63059	0.189999	-8.58208	9.32E-18	1.29E-16	DOWN
PORCN	179.3144	0.978593	0.114067	8.579073	9.56E-18	1.32E-16	UP
DMD	2049.773	-1.31469	0.153251	-8.57868	9.60E-18	1.33E-16	DOWN
C20orf144	4.852835	2.435666	0.28393	8.578414	9.62E-18	1.33E-16	UP
RBMXL1	698.2323	-0.8856	0.103247	-8.57746	9.70E-18	1.34E-16	DOWN
FAM21A	1199.325	0.714525	0.083306	8.577145	9.73E-18	1.34E-16	UP
ZNF200	322.7568	0.663843	0.077405	8.576207	9.81E-18	1.35E-16	UP
NF2	1227.313	0.763593	0.089042	8.575645	9.85E-18	1.36E-16	UP
CTC-246B	19.31148	2.645982	0.308553	8.575455	9.87E-18	1.36E-16	UP
MRPS23	1514.266	0.760384	0.088678	8.57469	9.94E-18	1.37E-16	UP
MAZ	1672.628	0.868056	0.101242	8.574106	9.99E-18	1.38E-16	UP
PDLIM2	884.1515	-0.96054	0.112028	-8.57407	9.99E-18	1.38E-16	DOWN
MPZ	477.8824	2.687813	0.313533	8.572664	1.01E-17	1.39E-16	UP
PFKFB2	548.2357	1.212615	0.141506	8.569369	1.04E-17	1.43E-16	UP
TTF2	290.0029	1.164088	0.13585	8.568913	1.04E-17	1.44E-16	UP
CLEC2L	19.78194	5.662215	0.660801	8.568719	1.05E-17	1.44E-16	UP
KMO	1616.379	-1.99285	0.232656	-8.56564	1.07E-17	1.48E-16	DOWN
DLL4	606.549	1.170513	0.136682	8.563796	1.09E-17	1.50E-16	UP
PPFIA4	37.09584	2.877114	0.336032	8.562025	1.11E-17	1.52E-16	UP
SLC39A13	1344.023	0.686064	0.080135	8.561348	1.12E-17	1.53E-16	UP
MYEF2	150.2391	2.674559	0.312404	8.561219	1.12E-17	1.53E-16	UP
TRIM60P1	23.90807	1.712866	0.20008	8.560893	1.12E-17	1.54E-16	UP
ZNRD1-AS	102.7285	1.22254	0.142864	8.557393	1.15E-17	1.58E-16	UP
SPDYA	14.80827	1.483123	0.173318	8.557257	1.16E-17	1.58E-16	UP
KLRD1	99.16016	-1.5656	0.182958	-8.55715	1.16E-17	1.58E-16	DOWN
BEST4	15.33929	2.224688	0.259985	8.557002	1.16E-17	1.59E-16	UP
CPSF4	927.0638	0.762033	0.089061	8.556309	1.17E-17	1.59E-16	UP
RP11-631I	19.9246	1.732393	0.202471	8.556244	1.17E-17	1.59E-16	UP
WDYHV1	354.0673	0.996316	0.11646	8.554975	1.18E-17	1.61E-16	UP
EED	491.4415	0.595173	0.069582	8.553555	1.19E-17	1.63E-16	UP
COLCA1	133.6036	2.109338	0.246616	8.553123	1.20E-17	1.64E-16	UP

MSC	832.8515	3.007763	0.351672	8.552746	1.20E-17	1.64E-16	UP
ZMIZ1-AS	37.05011	1.801478	0.21066	8.551601	1.21E-17	1.66E-16	UP
LIMK1	770.2594	1.377194	0.161055	8.551061	1.22E-17	1.66E-16	UP
ALB	3892493	-1.96456	0.229799	-8.54903	1.24E-17	1.69E-16	DOWN
SNAPIN	1198.249	0.627943	0.07346	8.54805	1.25E-17	1.70E-16	UP
MCF2L2	31.56503	2.537796	0.2969	8.547647	1.26E-17	1.71E-16	UP
ORM1	181990.6	-2.08915	0.244504	-8.54446	1.29E-17	1.76E-16	DOWN
RP5-1068I	16.84028	1.573503	0.184165	8.544006	1.30E-17	1.76E-16	UP
LINC0133(	7.798405	2.615479	0.306156	8.542957	1.31E-17	1.78E-16	UP
GIGYF1	2044.375	0.678509	0.079428	8.542403	1.31E-17	1.79E-16	UP
CEND1	10.72645	2.514468	0.294388	8.54135	1.33E-17	1.80E-16	UP
WDR4	365.4822	0.963854	0.112873	8.539298	1.35E-17	1.83E-16	UP
TUBB3	16.79031	3.071101	0.359644	8.539271	1.35E-17	1.83E-16	UP
LYRM5	1448.732	-1.04802	0.122732	-8.53909	1.35E-17	1.83E-16	DOWN
C1S	120006.2	-1.42538	0.166944	-8.53808	1.36E-17	1.85E-16	DOWN
TNFSF4	141.5464	1.71248	0.200595	8.537	1.38E-17	1.87E-16	UP
REPS1	1069.472	-0.79871	0.09357	-8.53603	1.39E-17	1.88E-16	DOWN
PCDHGA1	29.92586	2.480029	0.290538	8.535977	1.39E-17	1.88E-16	UP
GAS2L3	267.2049	1.435455	0.168165	8.535967	1.39E-17	1.88E-16	UP
SLC26A11	737.9051	0.756633	0.08865	8.535047	1.40E-17	1.89E-16	UP
CBR3	63.15215	1.974154	0.23133	8.533932	1.41E-17	1.91E-16	UP
C2orf47	1331.603	-0.69673	0.081653	-8.53288	1.43E-17	1.93E-16	DOWN
PLEKHG4B	56.42249	3.590266	0.420807	8.531858	1.44E-17	1.94E-16	UP
ARIH2OS	47.3233	0.935181	0.109612	8.531764	1.44E-17	1.94E-16	UP
PCAT6	70.95275	1.637063	0.191924	8.529734	1.47E-17	1.98E-16	UP
KCNE5	28.81962	2.886845	0.338497	8.528425	1.48E-17	2.00E-16	UP
HIST2H4A	15.90319	2.147382	0.251793	8.528357	1.48E-17	2.00E-16	UP
AGTR1	2280.937	-1.59341	0.186838	-8.52829	1.49E-17	2.00E-16	DOWN
LIG3	839.1343	0.639763	0.07502	8.527868	1.49E-17	2.01E-16	UP
NSUN5	1044.745	0.809708	0.094955	8.527309	1.50E-17	2.01E-16	UP
DNAJC9	435.2015	0.940893	0.110345	8.526862	1.50E-17	2.02E-16	UP
BRICD5	87.61667	1.47505	0.173006	8.525995	1.52E-17	2.04E-16	UP
RP11-564I	6.643363	3.151572	0.36966	8.525591	1.52E-17	2.04E-16	UP
RP11-152I	31.26047	1.55082	0.181938	8.523897	1.54E-17	2.07E-16	UP
CTD-2199	4.594995	2.866217	0.336284	8.523201	1.55E-17	2.08E-16	UP
MGC3958	35.75891	7.740586	0.908647	8.518801	1.61E-17	2.16E-16	UP
AC013275	72.8307	2.708439	0.317961	8.518141	1.62E-17	2.17E-16	UP
ZNF37BP	217.0185	1.118064	0.131263	8.51772	1.63E-17	2.18E-16	UP
AKR1B10	21213.4	3.561241	0.418117	8.517324	1.63E-17	2.19E-16	UP
DCAF4L1	14.44105	2.046053	0.240239	8.516757	1.64E-17	2.20E-16	UP
GRK6	1138.763	0.765053	0.089831	8.516565	1.64E-17	2.20E-16	UP
CASP2	738.4567	0.903799	0.106124	8.516411	1.65E-17	2.20E-16	UP
KIAA0556	506.1679	0.91732	0.107722	8.515657	1.66E-17	2.21E-16	UP
BBOX1-AS	15.57863	5.127623	0.602361	8.512547	1.70E-17	2.27E-16	UP
SUV39H1	630.1669	0.814014	0.095646	8.510671	1.73E-17	2.31E-16	UP
FAM122A	775.0051	-0.68335	0.080337	-8.50605	1.80E-17	2.40E-16	DOWN
SEC24B	2131.027	-1.03646	0.121851	-8.50596	1.80E-17	2.40E-16	DOWN
ZNF16	360.8595	0.803608	0.09448	8.505591	1.81E-17	2.41E-16	UP
BLNK	1189.417	-1.13856	0.133882	-8.50424	1.83E-17	2.44E-16	DOWN
TMEM81	134.763	0.967323	0.113747	8.504126	1.83E-17	2.44E-16	UP
TMEM220	2584.88	-1.55593	0.182966	-8.50391	1.83E-17	2.44E-16	DOWN
KIF20B	254.1658	1.181102	0.138893	8.503671	1.84E-17	2.44E-16	UP

F2RL3	141.8738	2.112665	0.248489	8.502051	1.86E-17	2.48E-16	UP
CTD-2336	391.6533	-1.08153	0.12725	-8.49926	1.91E-17	2.54E-16	DOWN
PLXNA3	653.8829	1.55956	0.183495	8.499199	1.91E-17	2.54E-16	UP
RFWD2	2072.149	0.662846	0.07799	8.499081	1.91E-17	2.54E-16	UP
TMEM27	513.8924	-2.34633	0.276084	-8.49862	1.92E-17	2.55E-16	DOWN
DMBX1	7.552625	4.665123	0.549	8.497495	1.94E-17	2.57E-16	UP
INO80E	1305.187	0.688551	0.081046	8.495857	1.96E-17	2.61E-16	UP
DNAJC16	1460.519	-0.75503	0.088877	-8.49522	1.98E-17	2.62E-16	DOWN
TSPEAR-A	91.20035	2.588206	0.304695	8.494424	1.99E-17	2.63E-16	UP
PPM1G	4092.036	0.602082	0.07088	8.49441	1.99E-17	2.63E-16	UP
LMAN1	9745.668	-0.82922	0.097623	-8.49404	2.00E-17	2.64E-16	DOWN
ABCD3	5394.39	-0.84367	0.099329	-8.49372	2.00E-17	2.65E-16	DOWN
CYP2W1	14.31538	2.661668	0.313371	8.493655	2.00E-17	2.65E-16	UP
HOXA11	9.90397	5.229804	0.615843	8.492105	2.03E-17	2.68E-16	UP
PDLIM1P4	16.75573	-1.62743	0.191653	-8.49152	2.04E-17	2.69E-16	DOWN
KB-1208A	109.9008	1.276462	0.15035	8.489923	2.07E-17	2.73E-16	UP
COL25A1	30.67957	-2.47608	0.291651	-8.48988	2.07E-17	2.73E-16	DOWN
SERPINH1	4687.166	1.326756	0.156283	8.489453	2.08E-17	2.74E-16	UP
CTNNA2	154.5874	4.870932	0.573772	8.48932	2.08E-17	2.74E-16	UP
PPP1R14D	23.29671	3.640966	0.428932	8.488442	2.09E-17	2.76E-16	UP
STK24-AS	10.7078	1.747584	0.205906	8.487288	2.12E-17	2.79E-16	UP
ZEB1-AS1	126.0263	0.930278	0.109618	8.486577	2.13E-17	2.80E-16	UP
GABRR3	31.43973	4.200927	0.495018	8.486407	2.13E-17	2.81E-16	UP
RP11-334	17.42824	1.607515	0.18946	8.484705	2.16E-17	2.85E-16	UP
B4GALT3	1521.792	0.719278	0.084806	8.481416	2.22E-17	2.93E-16	UP
METTL18	260.2514	0.807015	0.095167	8.47995	2.25E-17	2.96E-16	UP
RNF44	1357.53	0.833152	0.098288	8.476611	2.32E-17	3.05E-16	UP
DLX6	16.99382	6.013767	0.709487	8.476213	2.33E-17	3.05E-16	UP
IRX3	215.9292	2.658495	0.313691	8.474883	2.35E-17	3.09E-16	UP
ATP6AP1	5656.235	0.695433	0.082061	8.474589	2.36E-17	3.09E-16	UP
LSM11	233.1139	0.7715	0.091048	8.473579	2.38E-17	3.12E-16	UP
DDX3X	8363.197	-0.69179	0.08166	-8.47156	2.42E-17	3.17E-16	DOWN
CEP68	617.4762	0.761483	0.0899	8.470317	2.45E-17	3.21E-16	UP
RP11-31F	20.45547	-1.35824	0.160354	-8.47026	2.45E-17	3.21E-16	DOWN
BDH2	1502.103	-1.25787	0.148506	-8.47018	2.45E-17	3.21E-16	DOWN
MIER1	1377.863	-0.73779	0.087129	-8.46781	2.50E-17	3.27E-16	DOWN
RP11-286	7.449974	2.578734	0.304644	8.464755	2.57E-17	3.36E-16	UP
TUBB4A	348.1472	3.053075	0.360695	8.46442	2.57E-17	3.36E-16	UP
DAPK2	469.8336	1.545523	0.182591	8.464378	2.58E-17	3.36E-16	UP
CMPK1	8862.284	-0.65972	0.077943	-8.46419	2.58E-17	3.37E-16	DOWN
MNS1	163.7963	2.293494	0.271026	8.462258	2.62E-17	3.42E-16	UP
AP000593	12.00083	4.099939	0.484606	8.460354	2.67E-17	3.48E-16	UP
RPTOR	1552.298	0.661088	0.078144	8.45987	2.68E-17	3.49E-16	UP
BGLAP	23.79746	1.613315	0.190711	8.459462	2.69E-17	3.50E-16	UP
ESCO2	103.8671	1.90157	0.224823	8.458068	2.72E-17	3.54E-16	UP
LINC0109	649.5642	-3.2164	0.380302	-8.45748	2.73E-17	3.56E-16	DOWN
RP13-516	86.81577	1.19419	0.141205	8.457154	2.74E-17	3.57E-16	UP
KITLG	377.5893	1.503805	0.17782	8.456886	2.75E-17	3.57E-16	UP
TMEM237	325.4544	0.996523	0.11784	8.456552	2.75E-17	3.58E-16	UP
TNFRSF25	252.8666	1.540836	0.182331	8.450769	2.89E-17	3.76E-16	UP
CPED1	1071.971	-1.93211	0.228634	-8.45065	2.90E-17	3.76E-16	DOWN
RP5-967N	39.88625	1.785518	0.211298	8.450225	2.91E-17	3.77E-16	UP

ST8SIA6	13.87044	-2.39518	0.283468	-8.44956	2.92E-17	3.79E-16	DOWN
MAPK11	321.5879	1.279841	0.151505	8.447535	2.98E-17	3.86E-16	UP
UNC5A	26.08317	2.501929	0.29622	8.446174	3.01E-17	3.90E-16	UP
MMP12	72.61749	3.732691	0.442045	8.444143	3.06E-17	3.97E-16	UP
XRCC1	1108.05	0.801194	0.094894	8.443017	3.09E-17	4.01E-16	UP
CHKA	1635.979	1.024289	0.121386	8.438253	3.22E-17	4.17E-16	UP
GAL3ST1	734.7013	2.677275	0.317282	8.438148	3.22E-17	4.17E-16	UP
RP3-323A	14.44729	3.334553	0.395177	8.438131	3.22E-17	4.17E-16	UP
P2RY12	24.60269	-2.28212	0.270491	-8.43695	3.26E-17	4.21E-16	DOWN
GCN1L1	3824.632	0.607996	0.072073	8.43589	3.29E-17	4.25E-16	UP
ABCF2	1520.426	0.682915	0.080956	8.435676	3.29E-17	4.25E-16	UP
AP000347	23.12435	1.58753	0.18822	8.434428	3.33E-17	4.30E-16	UP
PAFAH2	1039.246	-0.85768	0.101699	-8.43351	3.35E-17	4.33E-16	DOWN
RP11-492	25.92248	3.173561	0.376352	8.432437	3.39E-17	4.37E-16	UP
BNIPL	29.5351	1.557949	0.184778	8.431452	3.41E-17	4.40E-16	UP
HIST1H3E	29.42463	1.889883	0.22421	8.429089	3.48E-17	4.49E-16	UP
RP13-516	5.51102	2.495464	0.296115	8.427352	3.54E-17	4.55E-16	UP
ACTBP11	8.793296	2.085012	0.247442	8.426258	3.57E-17	4.59E-16	UP
FOCAD	679.6244	0.756363	0.089799	8.422856	3.67E-17	4.73E-16	UP
HHIP	331.8783	-3.31028	0.393058	-8.42187	3.71E-17	4.77E-16	DOWN
ATP13A2	822.7629	1.028843	0.122183	8.42054	3.75E-17	4.82E-16	UP
REPIN1	5445.203	0.721273	0.085669	8.419305	3.79E-17	4.87E-16	UP
LINC0095	29.4453	1.491792	0.17721	8.418231	3.82E-17	4.91E-16	UP
DEAF1	890.7917	0.795801	0.094544	8.417264	3.85E-17	4.95E-16	UP
WASF2	2599.952	0.795156	0.094483	8.415844	3.90E-17	5.01E-16	UP
NDFIP2	1591.47	-0.85279	0.101342	-8.415	3.93E-17	5.04E-16	DOWN
SEMA6C	749.8379	1.445481	0.171802	8.413655	3.97E-17	5.10E-16	UP
UGT2B11	800.7165	2.646474	0.314572	8.412941	4.00E-17	5.12E-16	UP
TUBA1B	4055.041	1.11635	0.1327	8.412611	4.01E-17	5.13E-16	UP
CTD-2201	22.86991	1.643981	0.195419	8.412601	4.01E-17	5.13E-16	UP
TMEM53	1525.152	-1.00342	0.119348	-8.40755	4.19E-17	5.36E-16	DOWN
DUSP10	2068.866	-1.28569	0.152942	-8.40638	4.23E-17	5.41E-16	DOWN
RP1-257A	19.35316	1.579358	0.187881	8.406144	4.24E-17	5.42E-16	UP
KCNU1	57.2525	6.454555	0.768001	8.404363	4.30E-17	5.50E-16	UP
ST3GAL4-	76.64297	1.405736	0.167289	8.403017	4.35E-17	5.56E-16	UP
TDRKH	464.9483	1.244485	0.148129	8.401349	4.41E-17	5.64E-16	UP
LRRN3	32.6303	-2.24484	0.267207	-8.4011	4.42E-17	5.64E-16	DOWN
PDE1C	22.72545	1.818683	0.216518	8.399704	4.48E-17	5.71E-16	UP
PLGLB2	231.4813	-2.00017	0.238128	-8.39954	4.48E-17	5.71E-16	DOWN
RNF8	610.8374	0.708936	0.084403	8.399366	4.49E-17	5.72E-16	UP
DUSP28	113.6014	0.626249	0.074571	8.39802	4.54E-17	5.78E-16	UP
CCDC102	146.7155	1.466547	0.174639	8.39758	4.56E-17	5.80E-16	UP
GOLGA6L9	54.94182	1.204771	0.143469	8.397403	4.56E-17	5.81E-16	UP
CRIPAK	314.6753	0.941703	0.112178	8.394732	4.67E-17	5.94E-16	UP
RP11-104	66.4167	1.392418	0.165869	8.394698	4.67E-17	5.94E-16	UP
DEDD	1496.11	0.548655	0.065367	8.393429	4.72E-17	6.00E-16	NOT
TMEM192	2294.736	-0.92826	0.110609	-8.3923	4.77E-17	6.06E-16	DOWN
RP11-159	192.0036	1.413222	0.168399	8.392084	4.78E-17	6.06E-16	UP
BRIP1	205.0368	1.482203	0.176667	8.389826	4.87E-17	6.18E-16	UP
DPH7	518.6251	0.908901	0.108345	8.388942	4.91E-17	6.22E-16	UP
RP11-521	6.314969	-1.45697	0.173679	-8.38889	4.91E-17	6.22E-16	DOWN
COX19	458.7702	0.881201	0.105046	8.38871	4.92E-17	6.23E-16	UP

RP11-488I	187.5746	1.35704	0.161798	8.38723	4.98E-17	6.31E-16	UP
CCSAP	342.3164	1.042483	0.124311	8.386081	5.03E-17	6.36E-16	UP
HIC2	321.0192	1.451155	0.173059	8.38533	5.06E-17	6.40E-16	UP
MYBPHL	11.43327	4.717693	0.562637	8.384964	5.07E-17	6.42E-16	UP
AL450992.	91.16485	1.560995	0.186271	8.38024	5.28E-17	6.68E-16	UP
FGF13	291.1176	2.162358	0.25807	8.378944	5.34E-17	6.75E-16	UP
RP11-55K	5.183448	2.269662	0.270936	8.377115	5.42E-17	6.85E-16	UP
RP11-16E	61.18409	2.442071	0.291542	8.376387	5.46E-17	6.89E-16	UP
RCOR2	42.83532	2.761626	0.329731	8.375388	5.50E-17	6.95E-16	UP
EPHB2	414.8997	2.480574	0.296278	8.372459	5.64E-17	7.12E-16	UP
GLMN	174.2006	0.840678	0.100413	8.372191	5.66E-17	7.13E-16	UP
SUN2	8466.54	-0.92781	0.110824	-8.37188	5.67E-17	7.15E-16	DOWN
PLP2	1698.835	1.67606	0.200286	8.368336	5.84E-17	7.36E-16	UP
CPA6	7.787847	4.025034	0.481052	8.367142	5.90E-17	7.43E-16	UP
MAT1A	63860.77	-1.85416	0.221623	-8.3663	5.95E-17	7.48E-16	DOWN
C2orf48	11.35656	2.530375	0.302455	8.366129	5.95E-17	7.49E-16	UP
RBM3	4676.114	0.833109	0.099591	8.365314	6.00E-17	7.54E-16	UP
PRRX2	18.55316	3.717912	0.444467	8.36488	6.02E-17	7.56E-16	UP
PDGFRB	3042.577	1.316177	0.157381	8.363009	6.11E-17	7.68E-16	UP
AP3M2	330.9789	1.019288	0.121895	8.362043	6.16E-17	7.74E-16	UP
PART1	25.50544	6.117666	0.731609	8.361933	6.17E-17	7.75E-16	UP
NRIP2	121.6082	1.200614	0.143589	8.36149	6.19E-17	7.77E-16	UP
VRK1	287.5632	0.895233	0.107066	8.361475	6.19E-17	7.77E-16	UP
PIM1	2449.038	-1.47782	0.176758	-8.36068	6.24E-17	7.82E-16	DOWN
TTLL1	144.7104	0.950675	0.113728	8.3592	6.31E-17	7.91E-16	UP
CHP1	10974.29	-0.89134	0.106659	-8.35692	6.44E-17	8.06E-16	DOWN
PSME3	3045.943	0.546868	0.065445	8.356137	6.48E-17	8.11E-16	NOT
SNRK	1701.773	-0.83831	0.100352	-8.35367	6.62E-17	8.28E-16	DOWN
PPP6R1	2928.894	0.627227	0.075096	8.352315	6.69E-17	8.37E-16	UP
MSS51	32.61937	1.294343	0.154975	8.35192	6.72E-17	8.40E-16	UP
FABP5	260.6254	1.71198	0.205004	8.350948	6.77E-17	8.46E-16	UP
ALDH8A1	6551.805	-1.90124	0.227725	-8.34884	6.89E-17	8.61E-16	DOWN
EFTUD2	3379.64	0.573254	0.068701	8.344133	7.17E-17	8.96E-16	NOT
CTD-3065	15.55003	1.800659	0.215889	8.340689	7.39E-17	9.22E-16	UP
SHB	1200.141	-1.05356	0.126319	-8.34048	7.40E-17	9.23E-16	DOWN
RP11-749I	4.21743	2.892785	0.34685	8.340163	7.42E-17	9.25E-16	UP
KIAA1875	79.62976	1.521164	0.182391	8.340145	7.42E-17	9.25E-16	UP
CTC-425C	36.19443	1.523203	0.182636	8.340115	7.42E-17	9.25E-16	UP
RP11-629	31.62732	1.634795	0.196037	8.339204	7.48E-17	9.31E-16	UP
SERPING1	139819.7	-1.39249	0.167018	-8.33739	7.59E-17	9.45E-16	DOWN
UBR5	2419.081	0.752002	0.090216	8.335607	7.71E-17	9.59E-16	UP
PROS1	10953.39	-1.25046	0.150025	-8.33498	7.75E-17	9.64E-16	DOWN
AP3B1	1485.486	0.580958	0.06971	8.333973	7.82E-17	9.72E-16	NOT
TBC1D7	707.2097	0.803952	0.096477	8.333091	7.88E-17	9.78E-16	UP
CCL26	15.3974	3.248545	0.38997	8.330244	8.07E-17	1.00E-15	UP
COMP	120.8599	3.578528	0.429586	8.330171	8.07E-17	1.00E-15	UP
VWF	3502.479	1.660248	0.19931	8.329979	8.09E-17	1.00E-15	UP
AC069513	5.957403	2.602123	0.312402	8.329417	8.12E-17	1.01E-15	UP
HN1	1976.484	1.290799	0.155023	8.326491	8.33E-17	1.03E-15	UP
TMC5	505.5904	3.412091	0.409792	8.326397	8.33E-17	1.03E-15	UP
LL22NC03	74.97047	0.871106	0.104625	8.326003	8.36E-17	1.04E-15	UP
TSPAN17	1516.432	0.773181	0.092876	8.324883	8.44E-17	1.04E-15	UP

PACSIN1	127.9656	2.584471	0.310726	8.317527	8.98E-17	1.11E-15	UP
NMRK1	1195.596	-0.91503	0.110061	-8.31385	9.26E-17	1.15E-15	DOWN
HS3ST3B1	1994.394	-1.70705	0.205345	-8.31309	9.32E-17	1.15E-15	DOWN
UTP3	1095.201	-0.59659	0.071779	-8.31148	9.45E-17	1.17E-15	DOWN
MT1L	494.2506	-2.64852	0.318667	-8.31123	9.47E-17	1.17E-15	DOWN
TBC1D13	1367.749	0.857813	0.103214	8.311025	9.49E-17	1.17E-15	UP
CYP4V2	6021.422	-1.37943	0.165982	-8.31067	9.52E-17	1.17E-15	DOWN
HES6	297.0402	1.281317	0.154197	8.309617	9.60E-17	1.18E-15	UP
RP11-336I	8.961033	2.691115	0.323856	8.309591	9.60E-17	1.18E-15	UP
ASMTL-AS	137.557	1.513705	0.182165	8.309534	9.61E-17	1.18E-15	UP
GMPS	1920.119	0.627064	0.075467	8.309115	9.64E-17	1.19E-15	UP
RP11-498I	17.6051	2.489706	0.299668	8.308214	9.72E-17	1.20E-15	UP
LY6E	11250.93	-2.09768	0.252514	-8.30721	9.80E-17	1.21E-15	DOWN
RP11-259(	11.29979	2.153133	0.259235	8.305705	9.92E-17	1.22E-15	UP
LRRN2	184.1215	2.120196	0.255273	8.3056	9.93E-17	1.22E-15	UP
HEATR1	1054.607	0.969912	0.116789	8.304858	9.99E-17	1.23E-15	UP
KB-1440D	4.429284	3.277434	0.394709	8.303418	1.01E-16	1.24E-15	UP
RP11-97O	18.04933	1.427167	0.171877	8.303398	1.01E-16	1.24E-15	UP
AP006285.	14.41028	2.59977	0.313101	8.303304	1.01E-16	1.24E-15	UP
SP5	379.2528	2.414924	0.290881	8.302089	1.02E-16	1.26E-15	UP
RP11-394I	7.72773	2.221719	0.267635	8.301306	1.03E-16	1.26E-15	UP
CD81	28548.79	-1.10928	0.133628	-8.30121	1.03E-16	1.26E-15	DOWN
RP11-599J	10.55868	2.612627	0.314766	8.300221	1.04E-16	1.27E-15	UP
RP4-563E1	15.27108	1.628515	0.196291	8.296424	1.07E-16	1.31E-15	UP
LINC00893	39.661	1.534569	0.184981	8.295809	1.08E-16	1.32E-15	UP
VSIG10L	368.5494	2.108516	0.254181	8.295343	1.08E-16	1.32E-15	UP
HIF1AN	1243.151	0.67365	0.081208	8.295315	1.08E-16	1.32E-15	UP
ALAS1	15845.46	-1.49018	0.179653	-8.29476	1.09E-16	1.33E-15	DOWN
BTNL8	182.1525	3.45757	0.416842	8.294679	1.09E-16	1.33E-15	UP
ZNF382	80.49822	1.638692	0.197564	8.29449	1.09E-16	1.33E-15	UP
ZNF581	520.6527	1.167458	0.140754	8.294299	1.09E-16	1.33E-15	UP
RP11-29H	11.33128	1.437709	0.173343	8.294016	1.09E-16	1.34E-15	UP
ADAM22	77.8291	2.085954	0.251516	8.293513	1.10E-16	1.34E-15	UP
RP11-776I	11.46306	5.094816	0.614324	8.293366	1.10E-16	1.34E-15	UP
RP13-582(	181.0938	1.430954	0.172547	8.293133	1.10E-16	1.34E-15	UP
PLCD3	353.0608	1.757927	0.211975	8.293084	1.10E-16	1.34E-15	UP
C9orf78	2707.303	-0.65426	0.078893	-8.29296	1.10E-16	1.35E-15	DOWN
LINC01126	11.35454	1.953928	0.235643	8.291885	1.11E-16	1.36E-15	UP
PPP2R2A	1089.562	-0.78274	0.094402	-8.29153	1.12E-16	1.36E-15	DOWN
FBLIM1	1149.338	1.530745	0.184648	8.290088	1.13E-16	1.38E-15	UP
TEX26	4.497494	-3.35399	0.404605	-8.28954	1.14E-16	1.38E-15	DOWN
LINC00628	4.457915	2.817695	0.339914	8.289433	1.14E-16	1.38E-15	UP
HERC5	559.2455	-1.71238	0.206574	-8.28942	1.14E-16	1.38E-15	DOWN
RP11-90L1	69.52087	-2.16549	0.261236	-8.28941	1.14E-16	1.38E-15	DOWN
PIDD1	688.4416	0.810594	0.0978	8.288297	1.15E-16	1.39E-15	UP
RP11-474(	6.193288	1.973768	0.238152	8.287832	1.15E-16	1.40E-15	UP
RP11-257(	171.233	0.860536	0.10384	8.287121	1.16E-16	1.41E-15	UP
CFI	24509.77	-1.36908	0.165206	-8.28711	1.16E-16	1.41E-15	DOWN
AC016747	369.3767	0.741475	0.089481	8.286361	1.17E-16	1.41E-15	UP
SERPINB9	811.7433	-1.46228	0.176473	-8.28613	1.17E-16	1.42E-15	DOWN
DLX1	6.009486	3.744758	0.451984	8.285161	1.18E-16	1.43E-15	UP
SERPINA1	1091793	-1.42966	0.172563	-8.28483	1.18E-16	1.43E-15	DOWN

RP11-504I	72.92738	1.222135	0.147523	8.284361	1.19E-16	1.44E-15	UP
COLEC12	172.1167	2.480867	0.299497	8.283445	1.20E-16	1.45E-15	UP
FAM13A	1515.312	-1.73553	0.209529	-8.28299	1.20E-16	1.45E-15	DOWN
RP11-449J	27.0131	2.664807	0.321724	8.2829	1.20E-16	1.45E-15	UP
RP11-57A1	10.81783	3.995412	0.482536	8.280031	1.23E-16	1.49E-15	UP
LINC00346	118.027	2.074046	0.250498	8.279701	1.23E-16	1.49E-15	UP
BPIFA2	10.21034	5.508091	0.665403	8.27783	1.25E-16	1.51E-15	UP
MORC2	1570.086	0.555982	0.06717	8.277179	1.26E-16	1.52E-15	NOT
SLC39A1	6505.117	0.762173	0.092085	8.27683	1.26E-16	1.52E-15	UP
CCL25	75.17954	3.189263	0.385343	8.276423	1.27E-16	1.53E-15	UP
FO538757.	80.71075	1.089825	0.1317	8.275043	1.28E-16	1.55E-15	UP
FBXO32	479.3802	1.566913	0.189375	8.274116	1.29E-16	1.56E-15	UP
USP39	1411.398	0.49299	0.059583	8.273966	1.30E-16	1.56E-15	NOT
RNF24	360.7703	1.180424	0.142684	8.273007	1.31E-16	1.57E-15	UP
ENTPD6	2882.893	0.781755	0.094517	8.271055	1.33E-16	1.60E-15	UP
ARL6IP1	10298.35	-0.80389	0.097198	-8.27071	1.33E-16	1.60E-15	DOWN
POLA1	407.3238	0.948773	0.114728	8.269773	1.34E-16	1.61E-15	UP
SHOX2	15.83774	3.089325	0.37358	8.269522	1.34E-16	1.61E-15	UP
C17orf80	665.6044	0.643035	0.07776	8.269496	1.35E-16	1.61E-15	UP
ANXA2R	90.67431	1.520221	0.183857	8.268514	1.36E-16	1.63E-15	UP
ZNF580	443.1427	1.173789	0.142028	8.264509	1.40E-16	1.68E-15	UP
POC1A	417.4488	1.037185	0.125508	8.263864	1.41E-16	1.69E-15	UP
AC068858	3.283992	3.321283	0.401932	8.2633	1.42E-16	1.70E-15	UP
C11orf54	5461.199	-1.14771	0.138898	-8.26298	1.42E-16	1.70E-15	DOWN
MYBPC1	12.29804	2.794825	0.338249	8.262621	1.43E-16	1.71E-15	UP
ANKRD29	351.5609	1.779519	0.215382	8.262171	1.43E-16	1.71E-15	UP
RNY4P10	6.220953	1.980318	0.239755	8.259767	1.46E-16	1.75E-15	UP
CEMIP	195.9602	2.410108	0.291814	8.259049	1.47E-16	1.75E-15	UP
UCN2	4.757215	4.107226	0.497363	8.258012	1.48E-16	1.77E-15	UP
MAPKBP1	368.5675	0.674466	0.081678	8.257624	1.49E-16	1.77E-15	UP
INTS4	571.3376	0.490527	0.059406	8.257135	1.49E-16	1.78E-15	NOT
BACH2	193.2411	-1.74498	0.211338	-8.25679	1.50E-16	1.79E-15	DOWN
MED20	646.7011	0.676936	0.081992	8.256139	1.50E-16	1.79E-15	UP
CYP39A1	1540.918	-2.50259	0.303163	-8.25494	1.52E-16	1.81E-15	DOWN
RBMS3	254.5792	-1.40338	0.170006	-8.25489	1.52E-16	1.81E-15	DOWN
FAM162B	33.01957	1.53664	0.186164	8.254228	1.53E-16	1.82E-15	UP
LINC01572	7.405946	2.314947	0.28048	8.253524	1.54E-16	1.83E-15	UP
SLC25A29	793.9238	0.905394	0.109706	8.252889	1.55E-16	1.84E-15	UP
DBF4	250.0502	1.015675	0.123075	8.252472	1.55E-16	1.85E-15	UP
ISL2	12.26002	3.644249	0.441739	8.249773	1.59E-16	1.89E-15	UP
DNAH17	57.43541	1.507881	0.18282	8.247922	1.61E-16	1.92E-15	UP
RP1-86C1	13.40053	2.460735	0.298411	8.246134	1.64E-16	1.94E-15	UP
CD300E	66.06955	-1.67801	0.203565	-8.24312	1.68E-16	1.99E-15	DOWN
AC090587	15.61458	1.646869	0.199956	8.236174	1.78E-16	2.11E-15	UP
RNU6-415	4.059603	-1.28387	0.155904	-8.23496	1.80E-16	2.13E-15	DOWN
KAZALD1	133.5847	1.901015	0.230856	8.234648	1.80E-16	2.14E-15	UP
FAM86JP	81.84647	1.144355	0.138994	8.233125	1.82E-16	2.16E-15	UP
LENG8	2775.889	0.886238	0.107648	8.232771	1.83E-16	2.17E-15	UP
PDRG1	783.1339	0.761184	0.092461	8.23253	1.83E-16	2.17E-15	UP
LINC01355	88.60554	1.361989	0.165446	8.232228	1.84E-16	2.18E-15	UP
NDEL1	1217.503	-0.8577	0.104207	-8.23066	1.86E-16	2.20E-15	DOWN
KPNA7	50.70519	2.124831	0.258172	8.230292	1.87E-16	2.21E-15	UP

RP13-735I	12.26925	2.23132	0.27113	8.229718	1.88E-16	2.22E-15	UP
PPP1R1B	24.9852	3.488189	0.423955	8.227739	1.91E-16	2.25E-15	UP
RP11-20I2	82.36586	1.778662	0.216193	8.227203	1.92E-16	2.26E-15	UP
TULP3	548.1934	0.810086	0.098481	8.225845	1.94E-16	2.29E-15	UP
MZT1	360.3333	0.830276	0.100945	8.225076	1.95E-16	2.30E-15	UP
ZNF738	60.14035	1.799401	0.218818	8.223268	1.98E-16	2.34E-15	UP
MCMDC2	67.77354	1.269629	0.154395	8.223256	1.98E-16	2.34E-15	UP
AL161668.	122.2745	-2.11529	0.257265	-8.22221	2.00E-16	2.36E-15	DOWN
MAST3	1115.2	0.968656	0.11784	8.220133	2.03E-16	2.40E-15	UP
CKAP4	5204.566	1.09751	0.13355	8.217969	2.07E-16	2.44E-15	UP
RYBP	1624.013	-0.68544	0.083416	-8.21709	2.08E-16	2.45E-15	DOWN
RP11-313.	37.37739	2.552824	0.310722	8.215787	2.11E-16	2.48E-15	UP
HIVEP1	990.9612	-1.05834	0.12882	-8.21563	2.11E-16	2.48E-15	DOWN
TRMT1	1304.877	0.855052	0.104116	8.212517	2.17E-16	2.55E-15	UP
MAPKAPK!	370.3702	0.909347	0.110728	8.212409	2.17E-16	2.55E-15	UP
RP11-448/	79.21492	1.001222	0.121927	8.211679	2.18E-16	2.56E-15	UP
GOLGA2P1	58.53784	1.342936	0.163577	8.209803	2.22E-16	2.60E-15	UP
PLEKHA8P	103.8609	0.901761	0.10985	8.209004	2.23E-16	2.62E-15	UP
TNRC18	4093.35	0.68424	0.083359	8.208315	2.24E-16	2.63E-15	UP
HIST1H2A	16.47141	2.255377	0.274772	8.208172	2.25E-16	2.63E-15	UP
FBP1	22185.52	-2.00575	0.244361	-8.20813	2.25E-16	2.63E-15	DOWN
RCCD1	502.8348	0.679499	0.082785	8.207958	2.25E-16	2.64E-15	UP
AC012146	115.4898	1.498915	0.182655	8.20628	2.28E-16	2.67E-15	UP
CFL1P1	11.2405	1.433929	0.174771	8.204609	2.31E-16	2.71E-15	UP
RP11-344I	21.09597	1.264686	0.154148	8.204349	2.32E-16	2.71E-15	UP
SNRNP70	5827.924	0.7985	0.097327	8.204322	2.32E-16	2.71E-15	UP
BSDC1	4227.679	-0.6794	0.082818	-8.20362	2.33E-16	2.73E-15	DOWN
RSAD2	379.5583	-1.9731	0.240551	-8.20243	2.36E-16	2.75E-15	DOWN
HOXD-AS:	6.618369	3.776997	0.460522	8.201548	2.37E-16	2.77E-15	UP
STMND1	36.00681	3.095197	0.377504	8.199101	2.42E-16	2.83E-15	UP
CECR7	27.49592	4.013276	0.489688	8.195579	2.49E-16	2.91E-15	UP
ST6GALNA	2490.966	-0.94214	0.114958	-8.19548	2.50E-16	2.91E-15	DOWN
ZNF169	41.5364	1.071107	0.130719	8.193972	2.53E-16	2.95E-15	UP
ZNF736	190.7147	1.239995	0.151332	8.193864	2.53E-16	2.95E-15	UP
PSMD4	9954.347	0.936275	0.114274	8.193264	2.54E-16	2.96E-15	UP
AC139100	25.89907	1.871846	0.228486	8.192391	2.56E-16	2.98E-15	UP
TNPO2	1839.873	0.625782	0.076389	8.192029	2.57E-16	2.99E-15	UP
LINC01011	46.70598	1.068261	0.130411	8.191497	2.58E-16	3.00E-15	UP
MMP14	4325.067	1.678149	0.20493	8.18887	2.64E-16	3.07E-15	UP
RP11-708.	19.73928	1.280994	0.156459	8.187402	2.67E-16	3.10E-15	UP
DUSP1	16333.33	-1.5704	0.191869	-8.18475	2.73E-16	3.17E-15	DOWN
RP11-783I	18.26568	1.365338	0.166817	8.184629	2.73E-16	3.17E-15	UP
PFN4	23.92635	1.638626	0.200222	8.184038	2.74E-16	3.19E-15	UP
TESC	866.7861	2.429505	0.296884	8.183351	2.76E-16	3.21E-15	UP
ZSWIM5	274.6506	1.894082	0.231503	8.181663	2.80E-16	3.25E-15	UP
PTPN14	534.0494	1.529614	0.186966	8.181238	2.81E-16	3.26E-15	UP
AP001412.	4.860502	1.978967	0.241898	8.181006	2.81E-16	3.26E-15	UP
HOGA1	1541.923	-1.8474	0.22582	-8.18082	2.82E-16	3.27E-15	DOWN
NAPSB	263.4086	-1.74647	0.2135	-8.18021	2.83E-16	3.28E-15	DOWN
RBMX	3364.792	0.504362	0.061659	8.179805	2.84E-16	3.29E-15	NOT
KLF13	2780.957	1.199743	0.146708	8.177762	2.89E-16	3.35E-15	UP
TNFSF15	115.1304	2.235249	0.27339	8.176033	2.93E-16	3.39E-15	UP

CENPQ	187.863	1.068892	0.130748	8.175183	2.95E-16	3.42E-15	UP
OR11Q1P	17.328	5.866481	0.717723	8.173744	2.99E-16	3.46E-15	UP
PRUNE	1232.863	0.855068	0.10462	8.17307	3.01E-16	3.47E-15	UP
DHX37	919.1209	0.699835	0.085635	8.172292	3.03E-16	3.50E-15	UP
HTR1D	65.00432	3.573272	0.43727	8.171768	3.04E-16	3.51E-15	UP
TCEANC2	308.4231	0.677918	0.082969	8.170707	3.07E-16	3.54E-15	UP
RELL2	51.41006	1.303551	0.159547	8.1703	3.08E-16	3.55E-15	UP
TBX19	75.10829	0.965177	0.118135	8.170148	3.08E-16	3.55E-15	UP
DPF1	7.850994	2.612344	0.319748	8.170011	3.08E-16	3.55E-15	UP
ZHX1-C8o	43.39235	1.294026	0.158407	8.169013	3.11E-16	3.58E-15	UP
LGALS14	22.14884	10.1143	1.238154	8.168853	3.11E-16	3.59E-15	UP
TPR	3759.49	0.749556	0.091764	8.1683	3.13E-16	3.60E-15	UP
LMNB1	1022.249	1.355538	0.165985	8.166652	3.17E-16	3.65E-15	UP
GPR107	2468.657	0.692222	0.084804	8.162628	3.28E-16	3.77E-15	UP
MT1F	4613.664	-3.10042	0.379878	-8.16161	3.31E-16	3.80E-15	DOWN
RP5-890O	41.09399	0.991882	0.121562	8.159502	3.36E-16	3.87E-15	UP
SPECC1	446.7143	2.005657	0.245853	8.157937	3.41E-16	3.92E-15	UP
NAMPT	10492.96	-1.57329	0.19287	-8.15724	3.43E-16	3.94E-15	DOWN
EGR3	116.5493	-1.84886	0.22668	-8.15624	3.46E-16	3.97E-15	DOWN
EXTL1	12.10366	2.717891	0.333243	8.155893	3.47E-16	3.98E-15	UP
LYSMD1	421.6465	0.74488	0.091331	8.15579	3.47E-16	3.98E-15	UP
TOR1A	1785.424	-0.57683	0.070745	-8.15357	3.53E-16	4.05E-15	NOT
MANBAL	1660.186	0.647383	0.079401	8.153286	3.54E-16	4.06E-15	UP
RNPC3	278.5151	1.068779	0.131087	8.15319	3.54E-16	4.06E-15	UP
GRIN2A	48.24976	3.413091	0.41865	8.152605	3.56E-16	4.08E-15	UP
CTSB	53144.99	-0.93488	0.114677	-8.15226	3.57E-16	4.09E-15	DOWN
RP11-440(	4.372136	4.283083	0.525418	8.151764	3.59E-16	4.10E-15	UP
ACVR1C	108.256	-1.63922	0.201116	-8.15061	3.62E-16	4.14E-15	DOWN
TMEM65	806.3503	1.160836	0.142443	8.149456	3.66E-16	4.18E-15	UP
RASGEF1A	119.4099	2.784808	0.341805	8.147352	3.72E-16	4.25E-15	UP
LAMB4	9.446919	1.968881	0.241682	8.146575	3.74E-16	4.28E-15	UP
PDIA3P1	220.0302	0.917696	0.112661	8.145646	3.77E-16	4.31E-15	UP
CTC-573N	13.03444	4.453954	0.546852	8.144721	3.80E-16	4.34E-15	UP
SPIRE2	661.7775	1.143596	0.140429	8.143604	3.84E-16	4.38E-15	UP
RALY	4636.534	0.755547	0.092788	8.142752	3.86E-16	4.41E-15	UP
SLC25A19	225.2577	0.953605	0.117135	8.141076	3.92E-16	4.47E-15	UP
FENDRR	22.56549	-2.43514	0.299155	-8.14007	3.95E-16	4.50E-15	DOWN
WNT3A	7.264282	4.380931	0.538225	8.139589	3.97E-16	4.52E-15	UP
CALM1	9528.924	-0.59582	0.073211	-8.1385	4.00E-16	4.56E-15	DOWN
RP11-214I	13.5594	1.457988	0.179151	8.138304	4.01E-16	4.56E-15	UP
BAK1	694.6321	1.075719	0.132198	8.137201	4.05E-16	4.60E-15	UP
CTA-984G	8.784049	1.670098	0.205249	8.136918	4.05E-16	4.61E-15	UP
PRPF40B	181.6906	0.877038	0.107787	8.136797	4.06E-16	4.61E-15	UP
MAN1C1	1364.954	-1.69106	0.207846	-8.1361	4.08E-16	4.64E-15	DOWN
PTK2	3232.634	0.731456	0.089914	8.135092	4.12E-16	4.68E-15	UP
RP11-216I	35.17556	2.135273	0.262482	8.134916	4.12E-16	4.68E-15	UP
CACNA1E	15.21891	3.534765	0.434602	8.133343	4.18E-16	4.74E-15	UP
RAD51D	385.6447	0.697454	0.085762	8.13243	4.21E-16	4.77E-15	UP
SHISA2	19.78023	2.758262	0.339284	8.129663	4.30E-16	4.88E-15	UP
SPSB4	13.52449	-2.53634	0.311987	-8.12962	4.31E-16	4.88E-15	DOWN
BICD1	161.6716	1.42514	0.175327	8.128471	4.35E-16	4.93E-15	UP
ZNF500	355.7311	0.584346	0.071908	8.126312	4.43E-16	5.01E-15	NOT

COPS7B	1004.837	0.619692	0.076283	8.123641	4.52E-16	5.12E-15	UP
DDX39B	1862.67	1.053479	0.129728	8.120694	4.64E-16	5.25E-15	UP
KB-1572G	17.64547	1.711195	0.210722	8.120628	4.64E-16	5.25E-15	UP
CTC-297N	113.4994	-1.57648	0.194152	-8.11982	4.67E-16	5.28E-15	DOWN
SIN3B	1104.552	0.590714	0.072756	8.119146	4.69E-16	5.31E-15	UP
RP11-326I	32.10133	-1.08725	0.133944	-8.11722	4.77E-16	5.39E-15	DOWN
HGF	745.2292	-2.18489	0.269206	-8.11607	4.82E-16	5.44E-15	DOWN
MT-ND1	292970.9	-1.1665	0.143743	-8.11517	4.85E-16	5.48E-15	DOWN
ZFYVE26	886.2647	0.686064	0.084556	8.113764	4.91E-16	5.54E-15	UP
CIZ1	1786.865	0.708828	0.087363	8.113585	4.91E-16	5.54E-15	UP
ATP2A1-A	10.28251	2.306443	0.284295	8.112849	4.94E-16	5.57E-15	UP
LINC0023	23.98194	1.607283	0.198137	8.111993	4.98E-16	5.61E-15	UP
DMBT1	30.24158	3.937577	0.485528	8.109885	5.07E-16	5.71E-15	UP
RP11-382I	4.415052	3.053988	0.37659	8.109575	5.08E-16	5.72E-15	UP
MT-CO1	980412.3	-1.15201	0.142083	-8.10803	5.14E-16	5.79E-15	DOWN
GRPEL1	2586.683	-0.7534	0.092929	-8.10724	5.18E-16	5.83E-15	DOWN
GJA5	307.5442	1.514054	0.186769	8.106566	5.21E-16	5.86E-15	UP
NIT2	4023.666	-0.77516	0.095633	-8.10554	5.25E-16	5.90E-15	DOWN
MRPL39	856.9322	-0.6285	0.077543	-8.10518	5.27E-16	5.92E-15	DOWN
KIF5A	9.775846	3.283387	0.405251	8.102115	5.40E-16	6.07E-15	UP
RP11-480I	208.6401	1.754632	0.216583	8.101425	5.43E-16	6.10E-15	UP
RP11-392(	11.86364	1.727466	0.213292	8.099072	5.54E-16	6.21E-15	UP
RNF144A	309.6259	1.480237	0.182766	8.099067	5.54E-16	6.21E-15	UP
RP11-546I	136.4125	0.886695	0.109491	8.098316	5.57E-16	6.25E-15	UP
DCAF13	1533.684	1.060695	0.130998	8.097048	5.63E-16	6.31E-15	UP
FOXF2	23.08069	2.469584	0.305031	8.096167	5.67E-16	6.36E-15	UP
PRR12	932.7947	0.790189	0.097621	8.094481	5.75E-16	6.44E-15	UP
RP11-608(	5.632918	4.764574	0.588633	8.094308	5.76E-16	6.45E-15	UP
LAMA5	3254.39	1.293079	0.159756	8.094094	5.77E-16	6.46E-15	UP
AQP3	4046.992	-1.46582	0.181101	-8.09394	5.78E-16	6.46E-15	DOWN
CHP2	13.698	6.36443	0.786392	8.093201	5.81E-16	6.50E-15	UP
C1orf106	308.1723	2.664234	0.329222	8.092526	5.84E-16	6.53E-15	UP
TYRO3	480.6094	2.006364	0.247969	8.091187	5.91E-16	6.60E-15	UP
DNAAF5	1073.621	0.606702	0.074986	8.090888	5.92E-16	6.61E-15	UP
RP5-1112I	48.59036	1.343868	0.166097	8.090885	5.92E-16	6.61E-15	UP
IARS	2734.546	0.761984	0.094185	8.090271	5.95E-16	6.64E-15	UP
PDE7B	207.7757	-1.55769	0.192547	-8.0899	5.97E-16	6.66E-15	DOWN
TTC36	1708.937	-2.92645	0.36176	-8.08949	5.99E-16	6.68E-15	DOWN
KDM7A	866.2722	-0.90867	0.112334	-8.089	6.02E-16	6.71E-15	DOWN
RP1-267D	42.30742	1.070485	0.132362	8.087529	6.09E-16	6.78E-15	UP
SUCLG2	7728.176	-1.06284	0.131417	-8.0875	6.09E-16	6.78E-15	DOWN
PPEF1	8.03915	2.611674	0.322973	8.086362	6.15E-16	6.84E-15	UP
RP11-521I	47.83031	1.291836	0.15977	8.085591	6.19E-16	6.88E-15	UP
SERPINA1	11856.35	-2.26009	0.279553	-8.08468	6.23E-16	6.93E-15	DOWN
MNX1	36.63858	5.355917	0.662521	8.084141	6.26E-16	6.96E-15	UP
PSCA	11.46714	3.244519	0.401389	8.083226	6.31E-16	7.01E-15	UP
CTD-2619	11.04492	1.737678	0.215015	8.081669	6.39E-16	7.10E-15	UP
E2F5	231.7302	1.255222	0.155338	8.080575	6.45E-16	7.16E-15	UP
C19orf57	107.2968	1.444656	0.178783	8.080512	6.45E-16	7.16E-15	UP
RPS6KC1	794.2	0.689542	0.085335	8.080407	6.46E-16	7.16E-15	UP
RHPN1-AS	34.83596	1.546125	0.191343	8.080364	6.46E-16	7.16E-15	UP
C8A	16827.21	-1.92627	0.23843	-8.07898	6.53E-16	7.24E-15	DOWN

AC018766	20.57202	1.328943	0.164499	8.078708	6.55E-16	7.26E-15	UP
COL8A1	300.0513	1.845998	0.228509	8.078463	6.56E-16	7.27E-15	UP
KIF3A	220.415	0.775898	0.096049	8.078143	6.58E-16	7.28E-15	UP
DAP3	4829.699	0.647778	0.08019	8.078062	6.58E-16	7.29E-15	UP
PCED1A	1185.722	0.772481	0.095628	8.078002	6.58E-16	7.29E-15	UP
CREM	1206.687	-0.79197	0.098047	-8.07749	6.61E-16	7.31E-15	DOWN
CTD-3222	16.12584	1.324087	0.163924	8.077454	6.61E-16	7.31E-15	UP
SCP2	25820.04	-1.35993	0.168381	-8.07648	6.67E-16	7.37E-15	DOWN
CDKN2B	353.0865	1.475282	0.182676	8.075948	6.70E-16	7.40E-15	UP
LRP8	93.16995	2.105274	0.260709	8.075178	6.74E-16	7.44E-15	UP
GOLGA2P7	11.40531	1.796541	0.222478	8.075125	6.74E-16	7.44E-15	UP
LINC01108	13.91678	3.08828	0.382456	8.074866	6.76E-16	7.45E-15	UP
MDM4	1083.979	0.827621	0.1025	8.074368	6.78E-16	7.48E-15	UP
C12orf73	316.4372	0.810337	0.100362	8.074135	6.80E-16	7.49E-15	UP
RP11-903I	69.31672	-1.36256	0.168759	-8.07401	6.80E-16	7.50E-15	DOWN
HIST1H4E	9.202523	2.834315	0.351051	8.073797	6.81E-16	7.51E-15	UP
MIR148A	2.902311	3.007156	0.37249	8.073126	6.85E-16	7.55E-15	UP
RP11-495I	15.7168	6.073563	0.75244	8.071824	6.93E-16	7.62E-15	UP
NSF	1345.312	0.586267	0.072637	8.071162	6.96E-16	7.66E-15	UP
ZNF192P1	19.63529	1.762568	0.218413	8.069881	7.04E-16	7.74E-15	UP
GRIK4	34.61224	2.780991	0.344629	8.069527	7.06E-16	7.76E-15	UP
CTD-2270	22.67937	1.542084	0.191134	8.068088	7.14E-16	7.85E-15	UP
ORM2	48487.94	-1.85755	0.230269	-8.06689	7.21E-16	7.92E-15	DOWN
LRRC69	25.37322	1.702042	0.211005	8.066374	7.24E-16	7.95E-15	UP
DLK1	1361.303	5.611838	0.695741	8.065993	7.26E-16	7.98E-15	UP
CS	3704.038	0.748066	0.092747	8.06565	7.28E-16	7.99E-15	UP
LPA	2494.341	-2.34058	0.290228	-8.06462	7.35E-16	8.06E-15	DOWN
POLR2K	1988.93	0.804527	0.099761	8.064546	7.35E-16	8.06E-15	UP
PARP1	6066.828	0.717191	0.088935	8.06421	7.37E-16	8.08E-15	UP
STC2	420.6975	2.068059	0.25645	8.064189	7.37E-16	8.08E-15	UP
ARHGAP22	75.62733	1.583616	0.196448	8.061244	7.55E-16	8.27E-15	UP
PFN1P11	40.50523	3.485336	0.432359	8.061206	7.55E-16	8.27E-15	UP
AC027601	31.05614	1.127459	0.139898	8.059126	7.68E-16	8.41E-15	UP
CTB-176F2	23.13261	1.880242	0.233318	8.058704	7.71E-16	8.44E-15	UP
CNIH2	24.6957	1.81263	0.224941	8.058244	7.74E-16	8.46E-15	UP
CTD-2561	23.17727	2.537856	0.314943	8.058138	7.75E-16	8.47E-15	UP
RP4-794I6	13.91356	2.680255	0.33268	8.056566	7.85E-16	8.57E-15	UP
SSTR5	27.42642	5.528841	0.686565	8.0529	8.09E-16	8.83E-15	UP
ZNF792	154.9672	0.987919	0.122689	8.05225	8.13E-16	8.88E-15	UP
TJP2	3652.555	-0.93354	0.11595	-8.05125	8.20E-16	8.94E-15	DOWN
VPS37C	665.365	0.610567	0.075852	8.04941	8.32E-16	9.08E-15	UP
MYO1B	13841.35	-1.13993	0.141618	-8.04936	8.32E-16	9.08E-15	DOWN
PCGF2	739.4665	1.100493	0.136765	8.04661	8.51E-16	9.28E-15	UP
RNF216	1597.799	0.560485	0.069663	8.045699	8.58E-16	9.34E-15	NOT
SCIN	47.84381	2.397373	0.297975	8.045564	8.58E-16	9.35E-15	UP
FAM122B	772.5887	0.860001	0.106893	8.045408	8.60E-16	9.36E-15	UP
CXCR2	29.75455	-2.15138	0.267449	-8.04407	8.69E-16	9.46E-15	DOWN
LHX2	79.30226	-1.91178	0.237732	-8.04175	8.86E-16	9.64E-15	DOWN
EI24	8313.203	-0.79205	0.098494	-8.04163	8.86E-16	9.64E-15	DOWN
DPH2	877.3196	0.788783	0.098094	8.041107	8.90E-16	9.68E-15	UP
SCARA3	935.4819	1.771302	0.220297	8.040526	8.95E-16	9.72E-15	UP
CREB3L1	298.8603	2.429575	0.302213	8.039282	9.04E-16	9.82E-15	UP

PLEC	11492.65	0.8497	0.105701	8.038743	9.08E-16	9.86E-15	UP
LINC0118(	14.74434	2.503779	0.311477	8.038403	9.10E-16	9.88E-15	UP
PTS	905.3779	-0.76794	0.095537	-8.03809	9.12E-16	9.90E-15	DOWN
UGP2	16615.91	-1.15428	0.143603	-8.03803	9.13E-16	9.90E-15	DOWN
ATP2A1	36.36106	1.599431	0.198992	8.037671	9.16E-16	9.92E-15	UP
PDSS1	273.8582	0.912293	0.113502	8.037659	9.16E-16	9.92E-15	UP
UPK1A	17.90197	2.863019	0.356296	8.035497	9.32E-16	1.01E-14	UP
RP11-497(	15.24936	3.490774	0.434449	8.034948	9.36E-16	1.01E-14	UP
CTD-2325	13.87771	2.362122	0.294009	8.03419	9.42E-16	1.02E-14	UP
C4orf48	86.58098	2.200607	0.273915	8.033899	9.44E-16	1.02E-14	UP
PTH2R	227.6326	3.780195	0.470633	8.032144	9.58E-16	1.04E-14	UP
TPM3	9270.452	0.613299	0.076365	8.031178	9.65E-16	1.04E-14	UP
PRR3	461.1714	0.751321	0.093559	8.030463	9.71E-16	1.05E-14	UP
STAU2-AS	6.0998	2.383296	0.296793	8.030154	9.74E-16	1.05E-14	UP
C15orf41	326.0308	0.803482	0.100068	8.029381	9.80E-16	1.06E-14	UP
SH3D21	82.62296	1.39423	0.173665	8.028266	9.89E-16	1.07E-14	UP
CGREF1	955.2786	1.832922	0.228387	8.025508	1.01E-15	1.09E-14	UP
LONP2	6903.972	-0.97644	0.12167	-8.02532	1.01E-15	1.09E-14	DOWN
SLC41A2	3135.123	-1.34032	0.167038	-8.02399	1.02E-15	1.10E-14	DOWN
ZIC4	87.82703	3.539645	0.441254	8.021777	1.04E-15	1.12E-14	UP
ZNF250	272.1072	0.693791	0.086494	8.021262	1.05E-15	1.13E-14	UP
CXCL17	33.92019	2.966185	0.369915	8.018569	1.07E-15	1.15E-14	UP
ANKRD33	25.31346	4.848451	0.604814	8.016433	1.09E-15	1.17E-14	UP
HIST1H1B	3.511236	3.3528	0.418241	8.016431	1.09E-15	1.17E-14	UP
ADAMTS1	1780.795	-1.52196	0.189865	-8.01602	1.09E-15	1.17E-14	DOWN
GTF3C2	1283.024	0.561144	0.070003	8.015967	1.09E-15	1.17E-14	NOT
GLYATL1	7053.075	-2.13697	0.26659	-8.01595	1.09E-15	1.17E-14	DOWN
C11orf80	215.3627	1.259494	0.15715	8.01458	1.11E-15	1.19E-14	UP
PRDM7	6.574915	3.207463	0.400212	8.014408	1.11E-15	1.19E-14	UP
RP11-546]	8.682791	1.800975	0.224728	8.014019	1.11E-15	1.19E-14	UP
C5orf30	155.7843	1.875148	0.23414	8.008669	1.16E-15	1.24E-14	UP
HIBCH	2796.403	-1.00784	0.125849	-8.00835	1.16E-15	1.25E-14	DOWN
PCCA	2910.835	-1.03597	0.129377	-8.0074	1.17E-15	1.26E-14	DOWN
JMJD4	862.974	0.854019	0.106666	8.006482	1.18E-15	1.27E-14	UP
SOWAHA	472.9994	1.766808	0.220723	8.004639	1.20E-15	1.28E-14	UP
NR6A1	285.6424	1.317003	0.164542	8.004056	1.20E-15	1.29E-14	UP
RP11-829I	5.213585	3.428913	0.428404	8.003929	1.21E-15	1.29E-14	UP
PALM2	79.14787	-1.74379	0.217872	-8.00377	1.21E-15	1.29E-14	DOWN
CROCC	535.6133	0.991473	0.12389	8.002838	1.22E-15	1.30E-14	UP
RP11-498(	77.66656	1.240555	0.155021	8.002499	1.22E-15	1.30E-14	UP
RP11-355(	31.80677	1.189058	0.148593	8.002122	1.22E-15	1.31E-14	UP
RP4-584D	2.319632	2.808769	0.351006	8.002046	1.22E-15	1.31E-14	UP
DMC1	17.2006	2.314551	0.289249	8.001942	1.22E-15	1.31E-14	UP
CTD-3001	13.97442	1.74765	0.21841	8.001683	1.23E-15	1.31E-14	UP
RP11-573I	12.07919	1.360764	0.17006	8.001674	1.23E-15	1.31E-14	UP
RP11-307(	61.8436	1.491183	0.186363	8.00152	1.23E-15	1.31E-14	UP
MS4A7	943.1932	-1.4611	0.182613	-8.00106	1.23E-15	1.32E-14	DOWN
CD300A	319.8384	-1.34031	0.167522	-8.00083	1.24E-15	1.32E-14	DOWN
ADAMTS7	179.3827	1.078683	0.134884	7.997086	1.27E-15	1.36E-14	UP
TBCC	716.7007	0.674042	0.084292	7.996545	1.28E-15	1.36E-14	UP
STK19	405.8381	0.656935	0.082162	7.995588	1.29E-15	1.37E-14	UP
BORA	73.37531	1.201236	0.150244	7.995206	1.29E-15	1.38E-14	UP

PRKDC	4477.788	0.936078	0.117121	7.992382	1.32E-15	1.41E-14	UP
NUP133	1430.664	0.574596	0.071908	7.990758	1.34E-15	1.43E-14	NOT
RP11-465I	86.25776	1.62424	0.203312	7.988913	1.36E-15	1.45E-14	UP
DYNC1H1	7867.174	0.639833	0.080108	7.987084	1.38E-15	1.47E-14	UP
AC005785	22.31216	1.62733	0.203794	7.985176	1.40E-15	1.49E-14	UP
RRN3	1656.973	-0.67343	0.084348	-7.98401	1.42E-15	1.50E-14	DOWN
RP11-127!	88.52761	1.273106	0.159459	7.9839	1.42E-15	1.50E-14	UP
CHST1	222.7353	1.814569	0.227279	7.983888	1.42E-15	1.50E-14	UP
BCAT1	266.3029	1.649	0.206564	7.982997	1.43E-15	1.52E-14	UP
RP5-1103I	23.56264	-1.76284	0.220833	-7.98268	1.43E-15	1.52E-14	DOWN
PIP5K1C	1081.454	0.795631	0.099673	7.982427	1.43E-15	1.52E-14	UP
NOVA1-A!	10.14987	5.556332	0.69616	7.981404	1.45E-15	1.53E-14	UP
KCTD17	562.2813	1.868941	0.234203	7.980016	1.46E-15	1.55E-14	UP
TAL2	6.491381	2.772783	0.347497	7.979289	1.47E-15	1.56E-14	UP
DESI2	1337.872	0.746941	0.093626	7.977944	1.49E-15	1.57E-14	UP
CDK5	705.883	0.777768	0.097502	7.976956	1.50E-15	1.59E-14	UP
RP11-295I	28.02718	1.210463	0.151794	7.97438	1.53E-15	1.62E-14	UP
FER1L5	9.528241	2.513783	0.315267	7.973507	1.54E-15	1.63E-14	UP
KCNH2	76.23535	2.981818	0.373969	7.973427	1.54E-15	1.63E-14	UP
AC011290	7.812609	1.734955	0.217677	7.970327	1.58E-15	1.67E-14	UP
ACTG2	248.4099	1.888708	0.236969	7.970265	1.58E-15	1.67E-14	UP
RP11-723(	16.60292	1.689004	0.211936	7.969412	1.59E-15	1.68E-14	UP
CHRNB4	11.23257	2.608138	0.327306	7.968498	1.61E-15	1.69E-14	UP
DPF3	128.5183	-1.78111	0.223556	-7.96717	1.62E-15	1.71E-14	DOWN
PDZRN4	18.87717	-2.59269	0.325523	-7.96468	1.66E-15	1.75E-14	DOWN
SLC34A3	5.454369	3.123568	0.392214	7.963946	1.67E-15	1.76E-14	UP
RAB3D	333.4423	1.940698	0.243726	7.962633	1.68E-15	1.77E-14	UP
LINC0066{	21.71063	5.68556	0.714251	7.960174	1.72E-15	1.81E-14	UP
CTD-2555	7.559844	4.014852	0.504371	7.96011	1.72E-15	1.81E-14	UP
CEP41	292.505	0.821177	0.103164	7.959889	1.72E-15	1.81E-14	UP
RANBP3L	148.2238	-2.48545	0.31226	-7.95955	1.73E-15	1.82E-14	DOWN
CDRT1	15.28563	3.103118	0.38987	7.959358	1.73E-15	1.82E-14	UP
NAV3	260.8342	2.458695	0.308979	7.957495	1.76E-15	1.85E-14	UP
GS1-124K!	167.964	0.819247	0.102992	7.954459	1.80E-15	1.89E-14	UP
LZTS2	1445.368	1.016995	0.127897	7.951672	1.84E-15	1.93E-14	UP
DNM1P35	18.66008	1.847352	0.232334	7.951286	1.85E-15	1.94E-14	UP
CH507-51	30.09337	-2.09415	0.263387	-7.95085	1.85E-15	1.94E-14	DOWN
RP11-235I	19.02907	1.852309	0.233055	7.94794	1.90E-15	1.99E-14	UP
PTP4A1	30675.51	-0.97738	0.122973	-7.94791	1.90E-15	1.99E-14	DOWN
SMYD5	993.8918	0.739362	0.093031	7.94746	1.90E-15	2.00E-14	UP
UBL4A	2000.839	0.724943	0.091225	7.946785	1.91E-15	2.01E-14	UP
CITF22-1A	23.6922	1.127668	0.141912	7.946248	1.92E-15	2.01E-14	UP
ZNF385C	54.37243	1.918543	0.241452	7.945843	1.93E-15	2.02E-14	UP
MAPK8IP3	1037.037	1.002324	0.126205	7.942035	1.99E-15	2.08E-14	UP
MIR647	13.9264	1.660649	0.209134	7.940584	2.01E-15	2.11E-14	UP
NIPAL1	513.3699	-1.65882	0.208956	-7.93864	2.04E-15	2.14E-14	DOWN
EXOG	168.4762	0.804589	0.101357	7.938173	2.05E-15	2.15E-14	UP
HMGA2	25.90004	3.758943	0.473609	7.936802	2.07E-15	2.17E-14	UP
PDGFRL	81.2663	1.828841	0.230427	7.936755	2.08E-15	2.17E-14	UP
DDX53	18.34854	6.472921	0.815649	7.935914	2.09E-15	2.18E-14	UP
SLC22A12	307.8477	4.442945	0.560019	7.93356	2.13E-15	2.22E-14	UP
CHST6	10.33778	2.816816	0.355061	7.933332	2.13E-15	2.23E-14	UP

PDIA5	3073.793	-0.81785	0.103103	-7.93236	2.15E-15	2.24E-14	DOWN
UBAP2	964.5704	0.737158	0.092936	7.931928	2.16E-15	2.25E-14	UP
COL1A1	20934.92	2.142589	0.270128	7.931755	2.16E-15	2.25E-14	UP
THBS1	7528.882	-1.73868	0.219264	-7.92963	2.20E-15	2.29E-14	DOWN
RP11-923I	131.6691	1.999438	0.252158	7.929312	2.20E-15	2.30E-14	UP
HIST1H1PS	6.364824	3.068345	0.386967	7.929215	2.21E-15	2.30E-14	UP
ZCCHC2	1469.834	-0.77211	0.097382	-7.9287	2.21E-15	2.31E-14	DOWN
TMEM220	302.4246	-1.53944	0.194206	-7.92684	2.25E-15	2.34E-14	DOWN
PP7080	449.5629	1.31853	0.16634	7.926711	2.25E-15	2.34E-14	UP
ZNF514	292.46	0.9695	0.12232	7.925962	2.26E-15	2.35E-14	UP
CDIPT-AS	7.996179	2.827914	0.356811	7.925523	2.27E-15	2.36E-14	UP
IL18R1	275.4955	-1.52311	0.19219	-7.92503	2.28E-15	2.37E-14	DOWN
RFXANK	1249.787	1.079168	0.136173	7.924997	2.28E-15	2.37E-14	UP
CBX3	4232.153	0.570363	0.071978	7.924078	2.30E-15	2.39E-14	NOT
DCAF11	7754.68	-1.02001	0.128742	-7.92289	2.32E-15	2.41E-14	DOWN
PLA2G4C	907.468	1.248161	0.15756	7.92183	2.34E-15	2.43E-14	UP
NARF	1774.662	0.904531	0.114183	7.921755	2.34E-15	2.43E-14	UP
RP1-228H	70.07616	1.105972	0.139625	7.921036	2.36E-15	2.44E-14	UP
AGPAT1	3061.745	0.666129	0.084097	7.920983	2.36E-15	2.44E-14	UP
HPDL	22.24797	2.561507	0.32343	7.919826	2.38E-15	2.46E-14	UP
SMIM14	10687.92	-1.10414	0.139444	-7.91812	2.41E-15	2.50E-14	DOWN
CEP290	329.4922	0.838452	0.105903	7.917166	2.43E-15	2.52E-14	UP
AC007773	26.69901	1.729906	0.218577	7.914402	2.48E-15	2.57E-14	UP
PGP	897.1123	1.080068	0.136473	7.914162	2.49E-15	2.57E-14	UP
ELMSAN1	995.6508	-0.90827	0.114766	-7.91407	2.49E-15	2.58E-14	DOWN
MARS	3042.998	0.675835	0.085407	7.913095	2.51E-15	2.59E-14	UP
AP000347.	16.55421	1.968281	0.248799	7.911114	2.55E-15	2.64E-14	UP
CDKN2AIP	869.944	0.85837	0.108508	7.910669	2.56E-15	2.64E-14	UP
ARHGEF2	1274.53	1.188138	0.150243	7.908096	2.61E-15	2.70E-14	UP
C1orf162	434.0115	-1.28029	0.161901	-7.90785	2.62E-15	2.70E-14	DOWN
SLC39A7	7974.948	0.757157	0.09577	7.906023	2.66E-15	2.74E-14	UP
LINC0143(	17.17195	-2.21915	0.280712	-7.90543	2.67E-15	2.75E-14	DOWN
CCT5	5097.988	0.765712	0.096859	7.905423	2.67E-15	2.75E-14	UP
FAM50A	3442.37	1.002344	0.126811	7.90421	2.70E-15	2.78E-14	UP
RP11-540/	16.78514	1.641007	0.207615	7.904085	2.70E-15	2.78E-14	UP
RRM1	1787.909	0.737074	0.093263	7.903163	2.72E-15	2.80E-14	UP
RP11-186I	16.77351	1.32284	0.167391	7.902697	2.73E-15	2.81E-14	UP
RP11-563I	6.404023	1.92222	0.243258	7.901966	2.75E-15	2.82E-14	UP
FGB	445638.6	-1.82488	0.230943	-7.90185	2.75E-15	2.83E-14	DOWN
ABCA3	445.3704	1.733845	0.219447	7.900991	2.77E-15	2.84E-14	UP
PIEZO2	1308.257	1.372465	0.173732	7.899878	2.79E-15	2.87E-14	UP
FZD10	12.84643	2.79805	0.354208	7.899462	2.80E-15	2.88E-14	UP
RIT1	626.995	0.737609	0.093389	7.898278	2.83E-15	2.90E-14	UP
SLC22A4	90.18473	1.852683	0.234601	7.897152	2.85E-15	2.93E-14	UP
SIGLEC9	110.3205	-1.3823	0.175061	-7.89608	2.88E-15	2.95E-14	DOWN
C14orf180	55.35454	-3.35217	0.424598	-7.89492	2.90E-15	2.98E-14	DOWN
SRSF5	5965.371	-0.56815	0.071968	-7.89448	2.92E-15	2.99E-14	NOT
PLCE1	363.6947	1.035263	0.131153	7.893567	2.94E-15	3.01E-14	UP
CCDC138	137.671	1.069496	0.135516	7.892018	2.97E-15	3.05E-14	UP
PPP1R15A	2186.087	-1.00751	0.127665	-7.89183	2.98E-15	3.05E-14	DOWN
C8B	21311.82	-1.70174	0.215642	-7.8915	2.99E-15	3.06E-14	DOWN
PIGT	5780.09	0.672231	0.085201	7.889975	3.02E-15	3.09E-14	UP

CNTNAP4	47.86393	4.369625	0.553854	7.889485	3.03E-15	3.10E-14	UP
OXLD1	820.4274	0.943388	0.119585	7.888878	3.05E-15	3.12E-14	UP
RTEL1-TNI	55.21948	1.202498	0.152443	7.888176	3.07E-15	3.14E-14	UP
RP11-6O2	18.43975	2.390705	0.30308	7.888023	3.07E-15	3.14E-14	UP
ZKSCAN4	163.0219	0.779684	0.098845	7.887917	3.07E-15	3.14E-14	UP
RP11-109.	11.10805	4.759891	0.603515	7.886945	3.10E-15	3.16E-14	UP
LINC0113(	8.233153	2.489501	0.315655	7.886773	3.10E-15	3.17E-14	UP
SRGAP2	896.6399	0.925927	0.117416	7.885845	3.12E-15	3.19E-14	UP
RP11-128/	4.306326	3.124321	0.396274	7.884251	3.16E-15	3.23E-14	UP
GUCY2C	205.9791	3.047831	0.386643	7.882814	3.20E-15	3.26E-14	UP
CISH	1998.316	-1.40756	0.178615	-7.88043	3.26E-15	3.33E-14	DOWN
XPO1	4323.191	0.492494	0.062505	7.879229	3.29E-15	3.36E-14	NOT
CXCL14	753.1727	-3.17544	0.403036	-7.8788	3.31E-15	3.37E-14	DOWN
CDKN2A-/	2.086645	3.402853	0.431906	7.878694	3.31E-15	3.37E-14	UP
GLOD4	2186.154	-0.67987	0.086297	-7.87825	3.32E-15	3.38E-14	DOWN
CTC-261N	4.848337	4.672898	0.593207	7.877345	3.34E-15	3.40E-14	UP
CCDC90B	1250.43	-0.56412	0.071635	-7.87493	3.41E-15	3.47E-14	NOT
LY6G5B	46.68136	1.453423	0.184609	7.872992	3.46E-15	3.52E-14	UP
FAM171B	91.67023	1.638671	0.208174	7.871655	3.50E-15	3.56E-14	UP
CLDN15	1753.88	1.737119	0.220692	7.871251	3.51E-15	3.57E-14	UP
MYCBPAP	9.761192	1.983611	0.252027	7.87062	3.53E-15	3.58E-14	UP
U47924.6	54.78568	1.057042	0.134344	7.868159	3.60E-15	3.65E-14	UP
FBXL3	1361.967	-0.7818	0.099364	-7.86798	3.60E-15	3.66E-14	DOWN
ATP6V1C2	66.07121	1.162425	0.147757	7.867134	3.63E-15	3.68E-14	UP
LINC0062:	17.73529	1.891953	0.240529	7.865801	3.67E-15	3.72E-14	UP
FBN3	66.75548	2.968052	0.37735	7.865506	3.68E-15	3.73E-14	UP
LINC00261	8531.95	-1.40334	0.17849	-7.86229	3.77E-15	3.82E-14	DOWN
SKAP1	533.8478	-1.7634	0.224317	-7.86117	3.81E-15	3.86E-14	DOWN
CFAP53	27.10006	1.459429	0.185651	7.861124	3.81E-15	3.86E-14	UP
AC005253	22.35198	1.237964	0.157483	7.860951	3.81E-15	3.86E-14	UP
TSEN15	894.2248	0.614162	0.078155	7.858218	3.90E-15	3.94E-14	UP
MAP4K2	571.6196	0.787331	0.1002	7.857619	3.92E-15	3.96E-14	UP
TLR4	514.6719	-1.34047	0.170643	-7.85541	3.98E-15	4.03E-14	DOWN
KDM4A-A	32.52532	1.228625	0.15641	7.855158	3.99E-15	4.04E-14	UP
ETFA	9246.717	-0.8058	0.102599	-7.85393	4.03E-15	4.07E-14	DOWN
RP11-190(	3.417295	2.685302	0.341962	7.852631	4.07E-15	4.12E-14	UP
ATP6V0D2	41.71812	2.82984	0.360427	7.851349	4.12E-15	4.16E-14	UP
ZFP64	542.617	0.550859	0.070165	7.85095	4.13E-15	4.17E-14	NOT
PPIP5K1	326.7791	0.74472	0.094859	7.850768	4.13E-15	4.17E-14	UP
LYSMD4	319.5051	0.901063	0.114779	7.850442	4.15E-15	4.18E-14	UP
TXNRD1	7400.144	1.455894	0.185465	7.849962	4.16E-15	4.20E-14	UP
PLCB1	617.041	1.53598	0.195678	7.849538	4.18E-15	4.21E-14	UP
SMG7	3331.058	0.558686	0.071176	7.849322	4.18E-15	4.21E-14	NOT
SEPT7P2	258.8006	0.856811	0.109167	7.848646	4.21E-15	4.24E-14	UP
GPR137C	76.15295	1.274602	0.162406	7.848242	4.22E-15	4.25E-14	UP
CD14	23178.45	-1.62176	0.206674	-7.84695	4.26E-15	4.29E-14	DOWN
ESR1	902.6496	-2.31076	0.294503	-7.84632	4.28E-15	4.31E-14	DOWN
CNNM1	252.968	2.718273	0.34646	7.845857	4.30E-15	4.32E-14	UP
NUAK1	742.5466	1.00656	0.128306	7.845001	4.33E-15	4.35E-14	UP
TKT	11740.09	1.430857	0.182414	7.844024	4.36E-15	4.39E-14	UP
RP11-285I	133.7094	1.148642	0.14645	7.84324	4.39E-15	4.41E-14	UP
RP3-439F{	11.11769	2.236088	0.285103	7.843074	4.40E-15	4.42E-14	UP

RGS9BP	3.655869	2.927488	0.373271	7.842789	4.41E-15	4.42E-14	UP
AC074117	186.7546	0.857794	0.109375	7.842694	4.41E-15	4.43E-14	UP
IGF2BP2	565.515	2.06756	0.263667	7.841549	4.45E-15	4.46E-14	UP
FRRS1L	22.525	3.729468	0.475622	7.841248	4.46E-15	4.47E-14	UP
GK5	449.2684	0.821296	0.104763	7.839595	4.52E-15	4.53E-14	UP
ZNF133	372.4908	0.655082	0.083583	7.837519	4.60E-15	4.60E-14	UP
MED12	1222.748	0.691538	0.08824	7.836995	4.61E-15	4.62E-14	UP
CYB5RL	97.89221	1.013919	0.129395	7.835831	4.66E-15	4.66E-14	UP
FAM20B	2565.06	0.667891	0.085244	7.835063	4.69E-15	4.69E-14	UP
RP11-875(	16.53278	2.390503	0.305108	7.834951	4.69E-15	4.69E-14	UP
CKB	2928.773	2.420238	0.308943	7.833918	4.73E-15	4.73E-14	UP
PRSS3P2	12.66938	5.059019	0.645837	7.83328	4.75E-15	4.75E-14	UP
PAQR5	560.8852	2.151984	0.274725	7.833232	4.75E-15	4.75E-14	UP
DUSP13	4.891168	3.840377	0.490318	7.832415	4.79E-15	4.78E-14	UP
U8	5.70546	3.86762	0.493908	7.830655	4.85E-15	4.85E-14	UP
BROX	1709.169	0.63066	0.08054	7.830358	4.86E-15	4.86E-14	UP
TRIM3	378.0744	0.57635	0.073608	7.830031	4.88E-15	4.87E-14	NOT
HARS2	1115.986	0.540304	0.069021	7.828081	4.95E-15	4.94E-14	NOT
FCGRT	14201.35	-1.00062	0.127829	-7.82782	4.96E-15	4.95E-14	DOWN
RP11-68I1	24.59556	1.815143	0.231939	7.825954	5.04E-15	5.02E-14	UP
CRHR2	13.87667	1.767874	0.225901	7.825883	5.04E-15	5.02E-14	UP
ST3GAL6	1605.726	-1.4542	0.185834	-7.82527	5.07E-15	5.05E-14	DOWN
RP11-38M	33.79806	2.546808	0.325607	7.821713	5.21E-15	5.19E-14	UP
TRIM52	287.9046	0.757528	0.096851	7.821543	5.22E-15	5.19E-14	UP
EN2	11.94773	4.043705	0.51701	7.821326	5.23E-15	5.20E-14	UP
TIMELESS	1572.159	0.838987	0.10727	7.821275	5.23E-15	5.20E-14	UP
GCNT3	364.1237	2.919536	0.373283	7.821245	5.23E-15	5.20E-14	UP
CAMK2G	950.9188	0.633576	0.081009	7.821048	5.24E-15	5.21E-14	UP
RP11-495I	28.09207	1.411211	0.180438	7.821007	5.24E-15	5.21E-14	UP
TATDN2	821.6804	0.688792	0.088072	7.820781	5.25E-15	5.22E-14	UP
AC007277	11.03316	3.730407	0.477014	7.820326	5.27E-15	5.23E-14	UP
PADI3	13.22126	5.231008	0.668906	7.820249	5.27E-15	5.23E-14	UP
RP11-14C:	13.6756	2.640883	0.33779	7.81811	5.36E-15	5.32E-14	UP
SPATC1	15.628	1.731709	0.221546	7.81646	5.43E-15	5.39E-14	UP
ABCC4	536.623	1.721754	0.220293	7.815746	5.46E-15	5.42E-14	UP
AL163953.	8.139527	3.621682	0.46348	7.814112	5.54E-15	5.49E-14	UP
DAXX	2179.55	0.578393	0.074031	7.812822	5.59E-15	5.54E-14	NOT
MAN1A1	9353.968	-1.11816	0.143128	-7.81232	5.61E-15	5.56E-14	DOWN
IFT80	263.0756	1.153721	0.147702	7.811136	5.67E-15	5.61E-14	UP
NADK2	10719.36	-1.20191	0.153875	-7.81091	5.68E-15	5.62E-14	DOWN
METTL12	87.95713	1.099202	0.140728	7.810799	5.68E-15	5.62E-14	UP
NRG2	48.48587	2.216441	0.283768	7.810738	5.69E-15	5.62E-14	UP
AC012358	28.1859	1.051128	0.134593	7.809674	5.73E-15	5.67E-14	UP
LRG1	38893.98	-1.81749	0.232727	-7.80955	5.74E-15	5.67E-14	DOWN
BCAS1	144.386	2.709175	0.346987	7.807707	5.82E-15	5.75E-14	UP
VSIG4	680.2893	-1.82427	0.233686	-7.80652	5.88E-15	5.81E-14	DOWN
HEBP1	3323.05	-0.79158	0.101406	-7.8061	5.90E-15	5.82E-14	DOWN
HIPK3	2705.976	-0.72116	0.092387	-7.8059	5.91E-15	5.83E-14	DOWN
GPATCH1	411.2504	0.601051	0.077001	7.805783	5.91E-15	5.83E-14	UP
ZNF248	283.2153	0.701134	0.089859	7.802641	6.06E-15	5.98E-14	UP
OR2B6	10.06442	2.347107	0.30084	7.801852	6.10E-15	6.02E-14	UP
RBBP7	3350.06	0.555064	0.071161	7.800101	6.19E-15	6.10E-14	NOT

ZNF555	155.2236	0.8849	0.11345	7.799926	6.19E-15	6.10E-14	UP
MAFF	1504.358	-1.37494	0.1763	-7.79886	6.25E-15	6.15E-14	DOWN
MORF4L2-	11.73473	1.362659	0.174727	7.798799	6.25E-15	6.15E-14	UP
ASS1	65147.14	-1.75098	0.224565	-7.79724	6.33E-15	6.23E-14	DOWN
KCNJ6	22.08807	3.856019	0.49454	7.797178	6.33E-15	6.23E-14	UP
ZNF716	18.20002	5.996079	0.769106	7.796166	6.38E-15	6.28E-14	UP
AC145343	19.49692	1.757567	0.225492	7.794358	6.47E-15	6.36E-14	UP
RNF144A-	16.04208	2.699373	0.346539	7.789525	6.73E-15	6.61E-14	UP
ZNF532	497.555	1.260702	0.161851	7.789268	6.74E-15	6.62E-14	UP
RP11-353I	12.18422	3.315278	0.425653	7.788685	6.77E-15	6.65E-14	UP
RP11-137I	2.667549	-1.51616	0.194699	-7.7872	6.85E-15	6.73E-14	DOWN
PDZK1IP1	2232.951	2.921889	0.375517	7.780986	7.20E-15	7.06E-14	UP
RRP7B	113.303	0.967655	0.124362	7.780938	7.20E-15	7.06E-14	UP
PRR19	66.6983	2.131517	0.273982	7.77977	7.27E-15	7.13E-14	UP
COMMD5	1316.682	0.878245	0.112892	7.779522	7.28E-15	7.14E-14	UP
KREMEN2	15.52677	2.320882	0.298387	7.778103	7.36E-15	7.22E-14	UP
AC092667	10.42189	2.435052	0.313099	7.777251	7.41E-15	7.26E-14	UP
CPA2	21.79266	5.829574	0.749574	7.777181	7.42E-15	7.26E-14	UP
TELO2	1329.279	0.6695	0.086092	7.776562	7.45E-15	7.30E-14	UP
DHRS1	3612.028	-1.29523	0.16662	-7.77357	7.63E-15	7.47E-14	DOWN
RP11-388I	7.051654	5.032859	0.647598	7.771586	7.75E-15	7.58E-14	UP
ZNF530	86.54194	1.181987	0.152093	7.771464	7.76E-15	7.59E-14	UP
RP11-261I	2.372049	2.836758	0.365086	7.770099	7.84E-15	7.67E-14	UP
RP11-187I	5.865752	3.453335	0.444446	7.76997	7.85E-15	7.67E-14	UP
EIF2D	2396.95	0.59765	0.076926	7.769113	7.90E-15	7.72E-14	UP
OR2I1P	2573.245	2.232857	0.287462	7.767489	8.01E-15	7.82E-14	UP
LINC0094	28.87668	2.861707	0.368422	7.767464	8.01E-15	7.82E-14	UP
CA12	990.8593	2.932068	0.3775	7.767071	8.03E-15	7.84E-14	UP
CLN6	1893.546	0.748524	0.096372	7.766992	8.04E-15	7.84E-14	UP
POP1	233.0019	0.787936	0.101452	7.766609	8.06E-15	7.86E-14	UP
ACSM3	2281.037	-1.82797	0.235362	-7.7666	8.06E-15	7.86E-14	DOWN
AJ003147.	6.340828	1.655509	0.213188	7.765493	8.13E-15	7.93E-14	UP
SMIM19	1695.6	-1.02045	0.131409	-7.76546	8.14E-15	7.93E-14	DOWN
PROSER3	164.3921	0.947273	0.122025	7.762932	8.30E-15	8.08E-14	UP
PLCD4	90.74761	0.976781	0.125856	7.761078	8.42E-15	8.20E-14	UP
OGT	3040.716	0.736673	0.094983	7.755871	8.77E-15	8.54E-14	UP
SH2D1B	21.31532	-1.48228	0.191204	-7.75233	9.02E-15	8.78E-14	DOWN
RP11-932(	5.319416	2.435834	0.314213	7.752178	9.03E-15	8.78E-14	UP
SMKR1	10.81897	2.581229	0.333042	7.750454	9.16E-15	8.90E-14	UP
AIG1	6727.003	-0.99665	0.12862	-7.74881	9.28E-15	9.01E-14	DOWN
STARD5	340.2119	-1.48405	0.19154	-7.74801	9.33E-15	9.07E-14	DOWN
KCNN1	13.33067	2.452223	0.316533	7.747124	9.40E-15	9.13E-14	UP
CCER2	19.96138	1.794964	0.231745	7.745418	9.53E-15	9.25E-14	UP
AC074212	69.62276	1.509024	0.19483	7.745343	9.53E-15	9.25E-14	UP
AC004076	14.70947	1.550235	0.200202	7.74337	9.68E-15	9.39E-14	UP
RP11-480I	50.51734	1.845701	0.238362	7.743265	9.69E-15	9.40E-14	UP
RAD51C	461.786	0.685981	0.088601	7.742395	9.76E-15	9.46E-14	UP
ACADM	6625.03	-1.18884	0.153556	-7.74206	9.78E-15	9.48E-14	DOWN
HAUS3	370.436	0.564887	0.072974	7.740987	9.86E-15	9.56E-14	NOT
RP11-727/	42.05041	0.973196	0.12572	7.740945	9.87E-15	9.56E-14	UP
FAM219B	202.7416	0.684901	0.088496	7.739381	9.99E-15	9.67E-14	UP
HOXB8	5.057537	3.833904	0.495379	7.739336	9.99E-15	9.67E-14	UP

CDH19	104.676	-2.60548	0.336683	-7.73868	1.00E-14	9.72E-14	DOWN
C22orf23	20.83862	1.430727	0.184887	7.738371	1.01E-14	9.74E-14	UP
CTA-315H	33.24794	1.756618	0.227019	7.737767	1.01E-14	9.78E-14	UP
STRA8	7.553474	3.900191	0.504069	7.737421	1.01E-14	9.80E-14	UP
HINFP	469.7421	0.487396	0.062994	7.737227	1.02E-14	9.82E-14	NOT
ALDOA	18622.4	1.273306	0.164581	7.736648	1.02E-14	9.86E-14	UP
CLN3	1349.661	0.935582	0.120935	7.736248	1.02E-14	9.88E-14	UP
RP11-85I2	5.523567	4.383257	0.566764	7.733835	1.04E-14	1.01E-13	UP
RP11-14N	8.756715	2.421187	0.313145	7.731846	1.06E-14	1.02E-13	UP
NKX3-1	136.4917	-1.56167	0.202006	-7.7308	1.07E-14	1.03E-13	DOWN
HIST1H2B	91.62956	1.582479	0.204762	7.728385	1.09E-14	1.05E-13	UP
STX16	1757.22	0.552799	0.071531	7.728148	1.09E-14	1.05E-13	NOT
SESTD1	1016.453	0.882932	0.114258	7.727545	1.10E-14	1.06E-13	UP
RP13-104	68.32885	1.067566	0.138165	7.726751	1.10E-14	1.06E-13	UP
CD164	10304.33	-0.63638	0.082363	-7.7265	1.11E-14	1.06E-13	DOWN
FAXDC2	4379.547	-1.38889	0.179768	-7.72603	1.11E-14	1.07E-13	DOWN
RP1-95L4.	197.4931	-1.22707	0.158826	-7.72587	1.11E-14	1.07E-13	DOWN
RP11-465	13.92771	2.355466	0.304902	7.725317	1.12E-14	1.07E-13	UP
PAQR6	87.81602	1.472429	0.190604	7.725078	1.12E-14	1.07E-13	UP
LAP3	6642.324	-1.02078	0.132143	-7.72485	1.12E-14	1.08E-13	DOWN
HPX	169719.2	-1.83236	0.237225	-7.72411	1.13E-14	1.08E-13	DOWN
CENPT	829.8355	0.603741	0.078187	7.721762	1.15E-14	1.10E-13	UP
TLX2	7.052551	4.504001	0.583295	7.721654	1.15E-14	1.10E-13	UP
CHRND	6.591582	4.224277	0.547123	7.720884	1.16E-14	1.11E-13	UP
CHRNA5	20.69393	2.091228	0.270888	7.719903	1.16E-14	1.12E-13	UP
CAPN9	17.30192	2.42096	0.313694	7.717589	1.19E-14	1.14E-13	UP
LGI3	30.78634	3.163663	0.409935	7.717481	1.19E-14	1.14E-13	UP
FANCM	127.2353	0.769165	0.099668	7.717258	1.19E-14	1.14E-13	UP
PMPCA	4190.645	-0.83662	0.108409	-7.71723	1.19E-14	1.14E-13	DOWN
AP1M2	601.9871	2.914444	0.377746	7.715359	1.21E-14	1.16E-13	UP
CREBL2	2779.6	-0.75954	0.098448	-7.71507	1.21E-14	1.16E-13	DOWN
DGCR9	22.1139	2.413745	0.312897	7.714185	1.22E-14	1.17E-13	UP
LINC0010	40.09195	1.622235	0.210295	7.714082	1.22E-14	1.17E-13	UP
HNRNPA1	23.28153	1.407553	0.182479	7.713497	1.22E-14	1.17E-13	UP
AFM	11565.46	-2.12673	0.275741	-7.71277	1.23E-14	1.18E-13	DOWN
TMEM56	6946.447	-1.39506	0.180905	-7.7116	1.24E-14	1.19E-13	DOWN
RAD21	5958.426	0.689501	0.089414	7.711302	1.25E-14	1.19E-13	UP
LINC0060	47.10259	2.401828	0.311472	7.711213	1.25E-14	1.19E-13	UP
CYP4A22	7549.641	-2.22099	0.288056	-7.71028	1.26E-14	1.20E-13	DOWN
CREB3L4	667.082	0.886204	0.114951	7.709419	1.26E-14	1.21E-13	UP
EPAS1	10872.16	-0.82413	0.106922	-7.70775	1.28E-14	1.22E-13	DOWN
SNHG10	117.8952	1.088533	0.141227	7.707698	1.28E-14	1.22E-13	UP
ZP3	82.64072	1.570996	0.203827	7.707491	1.28E-14	1.22E-13	UP
XXYLT1	277.3351	1.023134	0.132747	7.707385	1.28E-14	1.22E-13	UP
YY1AP1	1895.319	0.573033	0.074358	7.706394	1.29E-14	1.23E-13	NOT
NME1-NM	2048.891	0.81237	0.105433	7.705096	1.31E-14	1.25E-13	UP
ENOX1	48.28335	2.261284	0.293491	7.704781	1.31E-14	1.25E-13	UP
AC006486	12.21002	1.407439	0.182688	7.704055	1.32E-14	1.25E-13	UP
ADORA3	162.089	-1.45894	0.189374	-7.70401	1.32E-14	1.25E-13	DOWN
RP11-466	11.19075	3.21464	0.417415	7.701305	1.35E-14	1.28E-13	UP
STX10	1492.046	0.735944	0.095575	7.70017	1.36E-14	1.29E-13	UP
PRTFDC1	133.8093	1.509169	0.196011	7.699417	1.37E-14	1.30E-13	UP

C9orf163	13.20815	1.503183	0.195236	7.699314	1.37E-14	1.30E-13	UP
RP11-715J	5.393026	2.19926	0.28565	7.699129	1.37E-14	1.30E-13	UP
CITED2	2123.211	-1.12482	0.146108	-7.69854	1.38E-14	1.31E-13	DOWN
CDC25B	2319.628	1.124267	0.146038	7.698449	1.38E-14	1.31E-13	UP
RP4-655J1	7.10146	2.437035	0.316631	7.696777	1.40E-14	1.32E-13	UP
ERVK3-1	401.1918	0.658975	0.085626	7.696009	1.40E-14	1.33E-13	UP
NAA11	12.17286	7.758892	1.008274	7.695224	1.41E-14	1.34E-13	UP
FAM78B	109.1573	1.305667	0.169695	7.69421	1.42E-14	1.35E-13	UP
FAM200A	219.896	0.598147	0.07774	7.694179	1.42E-14	1.35E-13	UP
RP11-295I	39.69627	-2.09554	0.272411	-7.69258	1.44E-14	1.37E-13	DOWN
PHPT1	3272.933	1.254638	0.163105	7.69223	1.45E-14	1.37E-13	UP
ABHD6	1914.225	-1.13877	0.148053	-7.69165	1.45E-14	1.37E-13	DOWN
SHC3	45.12357	1.792213	0.233009	7.691592	1.45E-14	1.37E-13	UP
TSACC	49.61114	1.483837	0.192943	7.690527	1.47E-14	1.39E-13	UP
ZNF589	186.0573	0.803953	0.104542	7.690231	1.47E-14	1.39E-13	UP
XX-C2158	4.535685	4.093333	0.532295	7.689977	1.47E-14	1.39E-13	UP
RP11-106I	11.6843	2.28478	0.297118	7.689809	1.47E-14	1.39E-13	UP
IFIT1	2240.658	-1.68527	0.21916	-7.68968	1.47E-14	1.39E-13	DOWN
LRRC10B	19.42165	1.719605	0.223625	7.68967	1.48E-14	1.39E-13	UP
RP11-401I	146.716	2.753069	0.358049	7.689094	1.48E-14	1.40E-13	UP
SLC12A7	5570.458	0.743619	0.096713	7.68895	1.48E-14	1.40E-13	UP
KRI1	1034.896	0.550757	0.07164	7.687814	1.50E-14	1.41E-13	NOT
IRF2BPL	1358.164	-0.8722	0.113485	-7.6856	1.52E-14	1.44E-13	DOWN
AMDHD1	4158.1	-1.62866	0.211912	-7.68556	1.52E-14	1.44E-13	DOWN
NPC1	1587.389	0.811962	0.105657	7.684885	1.53E-14	1.44E-13	UP
RGP1	1080.876	0.656173	0.085391	7.684322	1.54E-14	1.45E-13	UP
XXbac-BP(	175.7688	1.103855	0.143658	7.683898	1.54E-14	1.45E-13	UP
USP22	4579.075	0.778413	0.101318	7.68285	1.56E-14	1.46E-13	UP
ZNF57	140.7471	1.147196	0.14933	7.682287	1.56E-14	1.47E-13	UP
DCN	6811.01	-2.20639	0.287237	-7.68144	1.57E-14	1.48E-13	DOWN
UFC1	3675.5	0.669458	0.087153	7.681437	1.57E-14	1.48E-13	UP
RP11-264I	47.71161	-0.97435	0.126851	-7.68109	1.58E-14	1.48E-13	DOWN
KIAA0196	1788.113	0.777236	0.1012	7.680192	1.59E-14	1.49E-13	UP
PTGS2	110.5188	-2.07337	0.269965	-7.68014	1.59E-14	1.49E-13	DOWN
GABRA3	30.89569	5.9859	0.779449	7.679656	1.60E-14	1.50E-13	UP
ZNF865	616.4289	0.748062	0.097417	7.678943	1.60E-14	1.51E-13	UP
ARFGAP1	2754.794	0.660483	0.086024	7.677886	1.62E-14	1.52E-13	UP
RP11-109I	13.13594	5.930341	0.772468	7.677139	1.63E-14	1.53E-13	UP
RP11-147I	4.821186	2.014911	0.262461	7.676991	1.63E-14	1.53E-13	UP
ABL2	914.285	0.724284	0.09436	7.675739	1.64E-14	1.54E-13	UP
PCDHB8	24.80628	2.733208	0.356085	7.675707	1.65E-14	1.54E-13	UP
KLHL2	1058.829	-0.9455	0.123195	-7.67485	1.66E-14	1.55E-13	DOWN
LINC0066	8.458986	2.945798	0.383851	7.674317	1.66E-14	1.56E-13	UP
RBM12B	346.7119	0.772165	0.100622	7.673935	1.67E-14	1.56E-13	UP
ESYT3	46.46597	2.142213	0.279162	7.673728	1.67E-14	1.56E-13	UP
TMEM26-	2.311157	-1.87754	0.24468	-7.67346	1.67E-14	1.57E-13	DOWN
MURC	18.16474	2.010602	0.262025	7.673308	1.68E-14	1.57E-13	UP
MECOM	311.1651	1.472887	0.191979	7.672139	1.69E-14	1.58E-13	UP
GNB1L	227.6883	0.985168	0.128418	7.671562	1.70E-14	1.59E-13	UP
SPARC	18574.65	1.09186	0.142337	7.670958	1.71E-14	1.60E-13	UP
NAT8L	38.80359	2.513473	0.327665	7.670856	1.71E-14	1.60E-13	UP
RP11-676J	58.49994	-2.79272	0.364143	-7.66929	1.73E-14	1.61E-13	DOWN

SLC4A3	111.3094	2.785253	0.363176	7.669147	1.73E-14	1.62E-13	UP
PTGDR2	133.2775	2.118181	0.276263	7.667258	1.76E-14	1.64E-13	UP
RP11-517I	70.2926	-1.01669	0.132627	-7.66577	1.78E-14	1.66E-13	DOWN
NCAPH2	1751.803	0.819265	0.10688	7.66527	1.78E-14	1.66E-13	UP
VARS	4568.748	0.721942	0.094187	7.665004	1.79E-14	1.67E-13	UP
CALY	8.921675	2.971619	0.387747	7.663813	1.80E-14	1.68E-13	UP
ISG20L2	1513.099	0.605992	0.079075	7.663536	1.81E-14	1.69E-13	UP
VWA5B2	17.93698	2.224931	0.290506	7.658817	1.88E-14	1.75E-13	UP
RP11-191(	15.74158	-1.43369	0.1872	-7.65859	1.88E-14	1.75E-13	DOWN
TBKBP1	315.9448	1.128177	0.147346	7.656673	1.91E-14	1.78E-13	UP
CTD-2201	3.786069	2.712499	0.354298	7.655982	1.92E-14	1.78E-13	UP
WASH3P	189.9485	0.736866	0.096257	7.655187	1.93E-14	1.80E-13	UP
HOMER3	566.8721	1.474713	0.192647	7.655009	1.93E-14	1.80E-13	UP
KB-431C1.	170.7574	0.730403	0.095441	7.652961	1.96E-14	1.83E-13	UP
RP5-965G:	8.682106	1.984685	0.259378	7.651709	1.98E-14	1.84E-13	UP
RN7SL521I	4.152894	2.355486	0.307853	7.651346	1.99E-14	1.85E-13	UP
RASGRF1	37.68079	2.37832	0.310854	7.650911	2.00E-14	1.85E-13	UP
ZMYND10	31.96848	1.472172	0.192441	7.649996	2.01E-14	1.87E-13	UP
OBSCN	482.5668	2.244862	0.293494	7.648738	2.03E-14	1.88E-13	UP
OAS2	1817.477	-1.66031	0.217074	-7.64858	2.03E-14	1.88E-13	DOWN
HBB	1536.355	-2.07129	0.270829	-7.64793	2.04E-14	1.89E-13	DOWN
GCM1	4.947596	3.583757	0.468628	7.647339	2.05E-14	1.90E-13	UP
NHLRC2	1179.589	-0.60854	0.079586	-7.64632	2.07E-14	1.92E-13	DOWN
NOP56	2898.934	0.916391	0.119854	7.645873	2.08E-14	1.92E-13	UP
PADI4	8.491773	-2.25308	0.294689	-7.64562	2.08E-14	1.93E-13	DOWN
RP11-22B:	116.9877	1.179049	0.154216	7.645426	2.08E-14	1.93E-13	UP
CTB-129O	15.1121	1.721019	0.22511	7.645226	2.09E-14	1.93E-13	UP
CTB-52I2.4	13.67307	1.456202	0.190487	7.644616	2.10E-14	1.94E-13	UP
CTD-2095	8.771753	1.533756	0.200636	7.644454	2.10E-14	1.94E-13	UP
CDC20B	12.51522	3.685265	0.48214	7.643553	2.11E-14	1.95E-13	UP
TTLL3	243.4286	1.090344	0.142719	7.639787	2.18E-14	2.01E-13	UP
GLMP	4175.905	0.981159	0.128446	7.638678	2.19E-14	2.03E-13	UP
NUDCD1	590.7066	0.879826	0.115194	7.637768	2.21E-14	2.04E-13	UP
ARTN	19.84829	1.994854	0.261197	7.637345	2.22E-14	2.05E-13	UP
GDPD1	102.3952	1.18568	0.155254	7.637028	2.22E-14	2.05E-13	UP
BAI3	18.18215	-2.08214	0.272684	-7.63575	2.25E-14	2.07E-13	DOWN
RP11-264I	8.001972	1.347145	0.17643	7.635597	2.25E-14	2.07E-13	UP
MAGI1	1574.416	-0.71037	0.093035	-7.63549	2.25E-14	2.07E-13	DOWN
FLYWCH1	1009.917	0.697248	0.091329	7.634434	2.27E-14	2.09E-13	UP
OTUD6B	392.7757	0.817697	0.107156	7.630899	2.33E-14	2.15E-13	UP
QDPR	6644.059	-1.1464	0.150262	-7.62938	2.36E-14	2.17E-13	DOWN
BMP5	63.99979	-2.64839	0.347133	-7.62933	2.36E-14	2.17E-13	DOWN
APOL6	6220.54	-1.13724	0.149095	-7.62762	2.39E-14	2.20E-13	DOWN
ACACA	2130.055	0.871336	0.114244	7.626977	2.40E-14	2.21E-13	UP
FLJ12825	6.336608	3.022835	0.396427	7.6252	2.44E-14	2.24E-13	UP
C19orf68	345.0377	0.69697	0.091411	7.624563	2.45E-14	2.25E-13	UP
DUOX2	518.9553	3.441264	0.451385	7.623788	2.46E-14	2.26E-13	UP
LONRF1	452.3449	-0.97673	0.12816	-7.62112	2.51E-14	2.31E-13	DOWN
SAMD10	279.0525	0.800396	0.105047	7.619444	2.55E-14	2.34E-13	UP
CPSF3	1298.638	0.524751	0.068872	7.619269	2.55E-14	2.34E-13	NOT
PTCD1	339.2777	0.70821	0.09296	7.618474	2.57E-14	2.36E-13	UP
MAGED2	5077.005	0.714518	0.09379	7.618287	2.57E-14	2.36E-13	UP

SAA4	6848.43	-2.17874	0.286017	-7.61753	2.59E-14	2.37E-13	DOWN
E4F1	1024.78	0.715702	0.093959	7.617162	2.59E-14	2.38E-13	UP
PNMA1	580.3283	1.110086	0.145742	7.616791	2.60E-14	2.38E-13	UP
NUP93	810.7508	0.778175	0.102176	7.616046	2.62E-14	2.40E-13	UP
TMC2	2.364935	2.858533	0.375342	7.615813	2.62E-14	2.40E-13	UP
RP11-800/	113.4222	1.700999	0.223396	7.61428	2.65E-14	2.43E-13	UP
TSPAN5	365.6552	2.202185	0.289268	7.612962	2.68E-14	2.45E-13	UP
PPIAP29	85.8909	2.053258	0.269734	7.612157	2.70E-14	2.47E-13	UP
ANAPC4	713.312	0.620154	0.081472	7.611874	2.70E-14	2.47E-13	UP
ABAT	19624.63	-1.63038	0.214238	-7.61012	2.74E-14	2.50E-13	DOWN
KCNJ5	180.4447	1.81954	0.239172	7.607651	2.79E-14	2.55E-13	UP
HOXB13	20.31243	5.424458	0.713046	7.607443	2.80E-14	2.56E-13	UP
RP11-321I	5.684898	1.786402	0.234976	7.60248	2.91E-14	2.65E-13	UP
STAG3L5P	161.6408	1.216024	0.159958	7.602127	2.91E-14	2.66E-13	UP
ZNF724P	17.73437	1.645682	0.216507	7.601061	2.94E-14	2.68E-13	UP
TIPARP	1233.174	-0.94498	0.124336	-7.60024	2.96E-14	2.70E-13	DOWN
C5orf28	360.8701	0.711786	0.093662	7.59951	2.97E-14	2.71E-13	UP
RP11-923I	5.677254	2.311712	0.304207	7.599141	2.98E-14	2.72E-13	UP
FABP4	357.704	2.221438	0.292371	7.598022	3.01E-14	2.74E-13	UP
KCNJ11	132.8054	1.872995	0.246518	7.597805	3.01E-14	2.75E-13	UP
NCOA6	1661.582	0.60182	0.079231	7.595749	3.06E-14	2.79E-13	UP
SOX13	1901.57	0.703069	0.092561	7.595699	3.06E-14	2.79E-13	UP
EID2B	43.75735	1.061915	0.139818	7.594964	3.08E-14	2.80E-13	UP
APC2	114.3538	0.967044	0.12735	7.593584	3.11E-14	2.83E-13	UP
PLA2G1B	62.65017	2.226573	0.293225	7.593402	3.12E-14	2.84E-13	UP
LNX2	1154.509	-0.90035	0.118573	-7.59316	3.12E-14	2.84E-13	DOWN
MGC5072:	96.75646	3.641518	0.479585	7.593067	3.12E-14	2.84E-13	UP
CTD-2292	15.67966	1.764489	0.232424	7.591699	3.16E-14	2.87E-13	UP
HAAO	8724.438	-1.5071	0.198661	-7.58625	3.29E-14	2.99E-13	DOWN
CABLES2	361.5015	0.935152	0.123286	7.585223	3.32E-14	3.02E-13	UP
TFAP2A	112.2109	2.601438	0.342964	7.585153	3.32E-14	3.02E-13	UP
LINC00941	12.88035	2.681527	0.35356	7.584366	3.34E-14	3.03E-13	UP
LAGE3	981.9176	1.076384	0.141934	7.583704	3.36E-14	3.05E-13	UP
MYH4	998.283	5.386179	0.710231	7.583696	3.36E-14	3.05E-13	UP
MFAP3L	1520.642	-1.84356	0.243109	-7.58328	3.37E-14	3.06E-13	DOWN
SQLE	3739.217	1.582205	0.208683	7.581862	3.41E-14	3.09E-13	UP
INPP5B	565.139	-0.66316	0.087474	-7.58124	3.42E-14	3.10E-13	DOWN
DDX49	2104.668	0.821461	0.108367	7.580386	3.45E-14	3.12E-13	UP
RNU6-8	13.83056	1.983485	0.261729	7.578382	3.50E-14	3.17E-13	UP
SNHG21	27.16095	1.097606	0.144841	7.578021	3.51E-14	3.18E-13	UP
ZNF431	172.1218	1.323484	0.174653	7.577796	3.51E-14	3.18E-13	UP
KIAA0895L	244.9227	0.950046	0.125383	7.577133	3.53E-14	3.20E-13	UP
LSM4	4198.407	0.954948	0.126031	7.577087	3.53E-14	3.20E-13	UP
AQP10	23.50029	4.373607	0.577372	7.575023	3.59E-14	3.25E-13	UP
LINC00173	8.59939	2.560803	0.338064	7.574913	3.59E-14	3.25E-13	UP
VBP1	1242.134	0.589949	0.077906	7.572569	3.66E-14	3.31E-13	UP
BFSP2	8.310716	2.795432	0.369196	7.571678	3.68E-14	3.33E-13	UP
MSH4	6.378284	2.590045	0.342178	7.569293	3.75E-14	3.39E-13	UP
SRD5A1	3083.974	-1.35438	0.178931	-7.56925	3.75E-14	3.39E-13	DOWN
CXCR1	30.56135	-2.17948	0.287978	-7.56824	3.78E-14	3.42E-13	DOWN
RP11-435(	32.49514	1.269098	0.1677	7.567688	3.80E-14	3.43E-13	UP
SLC5A11	109.4168	2.447334	0.323401	7.567488	3.81E-14	3.43E-13	UP

LINC0144€	22.93976	4.79625	0.633843	7.566936	3.82E-14	3.45E-13	UP
FAH	10184.96	-1.11088	0.14686	-7.56424	3.90E-14	3.52E-13	DOWN
UCKL1-AS	13.50348	1.632119	0.215794	7.56331	3.93E-14	3.54E-13	UP
CTD-2574	23.63911	1.395586	0.184524	7.563168	3.93E-14	3.54E-13	UP
RPH3AL	669.892	-0.91767	0.121356	-7.56183	3.97E-14	3.58E-13	DOWN
AP006621.	15.80823	1.977152	0.261466	7.561803	3.98E-14	3.58E-13	UP
CTSA	12345.68	0.808449	0.106936	7.560108	4.03E-14	3.62E-13	UP
SP8	12.90859	5.676012	0.750863	7.559313	4.05E-14	3.65E-13	UP
RP11-16N	20.5667	1.480314	0.195855	7.558231	4.09E-14	3.68E-13	UP
RP11-285.	4.871814	2.052798	0.27161	7.557894	4.10E-14	3.68E-13	UP
MT-ND6	82229.66	-1.43521	0.189896	-7.55788	4.10E-14	3.68E-13	DOWN
DACH2	3.315905	3.752428	0.496493	7.55787	4.10E-14	3.68E-13	UP
AATF	2218.735	0.603649	0.079873	7.557611	4.11E-14	3.69E-13	UP
MT-ND2	317067	-1.11187	0.147132	-7.55691	4.13E-14	3.71E-13	DOWN
S100PBP	479.7834	0.615993	0.08153	7.555447	4.17E-14	3.75E-13	UP
CASC15	55.55918	2.25135	0.298001	7.554846	4.19E-14	3.76E-13	UP
AC141928	24.23224	2.724699	0.360657	7.55483	4.19E-14	3.76E-13	UP
GCHFR	885.6917	-1.17473	0.155499	-7.55456	4.20E-14	3.77E-13	DOWN
PDSS2	1295.009	-0.79064	0.104661	-7.55431	4.21E-14	3.78E-13	DOWN
ABLIM2	231.6941	1.607318	0.212773	7.554144	4.22E-14	3.78E-13	UP
SNRPC	3384.807	0.801212	0.106086	7.552456	4.27E-14	3.83E-13	UP
HIST2H2B(	5.529639	1.870567	0.24768	7.552342	4.28E-14	3.83E-13	UP
CDC23	1226.507	0.488826	0.064727	7.552176	4.28E-14	3.83E-13	NOT
TMEM101	1481.268	0.784994	0.103968	7.550326	4.34E-14	3.89E-13	UP
NUP210	2513.316	0.947596	0.125551	7.547517	4.44E-14	3.97E-13	UP
RP11-95M	12.3543	3.14303	0.4165	7.54629	4.48E-14	4.01E-13	UP
PPP1R37	1381.875	0.803386	0.106462	7.546233	4.48E-14	4.01E-13	UP
SIGLEC16	35.94818	-1.64657	0.218205	-7.54597	4.49E-14	4.01E-13	DOWN
PPP1R35	635.3984	0.905069	0.119966	7.544353	4.55E-14	4.06E-13	UP
FPR1	186.3888	-1.87718	0.248821	-7.54429	4.55E-14	4.06E-13	DOWN
RP3-337H	11.79752	1.556082	0.206302	7.542752	4.60E-14	4.11E-13	UP
BUD13	559.0235	0.426932	0.056603	7.542565	4.61E-14	4.11E-13	NOT
PSKH1	1175.973	0.807219	0.107038	7.541414	4.65E-14	4.15E-13	UP
GOT1	15831.68	-1.26787	0.168147	-7.54023	4.69E-14	4.19E-13	DOWN
PER1	3306.316	-1.13275	0.150234	-7.53989	4.70E-14	4.20E-13	DOWN
ACAD11	147.532	-1.51466	0.200922	-7.53856	4.75E-14	4.24E-13	DOWN
FUS	6343.982	0.672807	0.089255	7.538064	4.77E-14	4.25E-13	UP
PNPLA7	706.2118	-1.31646	0.174642	-7.53806	4.77E-14	4.25E-13	DOWN
BPGM	592.7821	0.617232	0.081886	7.537708	4.78E-14	4.26E-13	UP
SCN4B	126.1737	1.440926	0.191168	7.5375	4.79E-14	4.27E-13	UP
LINC0048{	15.57607	3.799922	0.504183	7.536794	4.82E-14	4.29E-13	UP
RHBDF2	943.7267	1.112283	0.147589	7.536348	4.83E-14	4.30E-13	UP
LYRM1	1998.786	-0.79649	0.105696	-7.53565	4.86E-14	4.32E-13	DOWN
STK35	1095.972	0.738416	0.098	7.53484	4.89E-14	4.35E-13	UP
SF3A2	2289.225	1.011058	0.134194	7.534301	4.91E-14	4.36E-13	UP
TRIM46	26.63482	1.982363	0.263171	7.532611	4.97E-14	4.42E-13	UP
CCDC28A	1244.49	-0.82847	0.110001	-7.53144	5.02E-14	4.46E-13	DOWN
AC109829	4.553701	2.151235	0.285672	7.530443	5.06E-14	4.49E-13	UP
RMST	12.7271	3.612189	0.47968	7.53041	5.06E-14	4.49E-13	UP
CSNK1E	2123.059	0.769683	0.102216	7.529975	5.08E-14	4.50E-13	UP
HP	659664.3	-2.14519	0.284901	-7.52961	5.09E-14	4.52E-13	DOWN
DUSP5	1327.189	-1.58159	0.210051	-7.52956	5.09E-14	4.52E-13	DOWN

AC006547	8.678807	1.457569	0.193587	7.529263	5.10E-14	4.52E-13	UP
OR51E2	4.611852	3.072583	0.408174	7.527632	5.17E-14	4.58E-13	UP
BZW1	4664.626	-0.60105	0.079856	-7.52666	5.21E-14	4.61E-13	DOWN
REG1B	26.10936	7.372853	0.979568	7.526637	5.21E-14	4.61E-13	UP
FAM134B	1455.077	-2.21237	0.293966	-7.52592	5.23E-14	4.64E-13	DOWN
TET1	66.67897	1.597592	0.212303	7.525038	5.27E-14	4.67E-13	UP
ARID3C	224.8925	-1.78183	0.236801	-7.52459	5.29E-14	4.68E-13	DOWN
GDPD3	67.7892	1.656195	0.22011	7.52439	5.30E-14	4.69E-13	UP
ARSD	3417.585	-1.098	0.145928	-7.52426	5.30E-14	4.69E-13	DOWN
GGN	9.488086	1.863981	0.247736	7.524061	5.31E-14	4.70E-13	UP
ACTBP9	9.88669	-1.48817	0.197816	-7.52301	5.35E-14	4.73E-13	DOWN
POGK	2117.776	0.834779	0.110992	7.521071	5.43E-14	4.80E-13	UP
VAMP1	238.7678	1.025209	0.136336	7.519751	5.49E-14	4.85E-13	UP
ZNF2	141.8997	0.538186	0.071579	7.518822	5.53E-14	4.88E-13	NOT
MTMR7	218.6831	2.184104	0.290509	7.518202	5.55E-14	4.90E-13	UP
GCSH	503.3178	-0.94494	0.125692	-7.51794	5.56E-14	4.91E-13	DOWN
STRC	6.753566	3.233583	0.430192	7.516596	5.62E-14	4.96E-13	UP
RP11-256I	31.16226	-2.11041	0.280768	-7.51658	5.62E-14	4.96E-13	DOWN
PMS1	362.396	0.552744	0.073547	7.51552	5.67E-14	5.00E-13	NOT
FANCA	336.3527	0.997165	0.13271	7.513865	5.74E-14	5.06E-13	UP
RP11-284(	9.86673	5.753361	0.765764	7.513227	5.77E-14	5.08E-13	UP
TMEM30A	5431.596	-0.57062	0.075978	-7.51033	5.90E-14	5.20E-13	NOT
CTD-2537	33.37132	1.431012	0.190573	7.509005	5.96E-14	5.25E-13	UP
MFSD6	484.6219	1.310592	0.174542	7.508745	5.97E-14	5.25E-13	UP
RP13-582(	23.42918	1.562855	0.208184	7.507098	6.05E-14	5.32E-13	UP
NT5M	130.377	1.408283	0.187601	7.506786	6.06E-14	5.33E-13	UP
RP11-94C:	2.06899	2.930549	0.39042	7.506153	6.09E-14	5.35E-13	UP
PHF12	1186.263	0.514453	0.068543	7.505544	6.12E-14	5.38E-13	NOT
SLC25A39	7349.176	0.724262	0.096514	7.504228	6.18E-14	5.43E-13	UP
MFSD10	1167.789	1.276097	0.17008	7.502934	6.24E-14	5.48E-13	UP
AC006077	15.27535	1.658972	0.22115	7.501585	6.31E-14	5.54E-13	UP
FAM193B	1119.578	0.772551	0.102997	7.500712	6.35E-14	5.57E-13	UP
SMPX	36.44157	3.088842	0.411808	7.500686	6.35E-14	5.57E-13	UP
ATP6V0E2	40.6413	1.423877	0.189834	7.500628	6.35E-14	5.57E-13	UP
MT-ND3	119422.4	-1.23224	0.164295	-7.50016	6.37E-14	5.59E-13	DOWN
KLKB1	6512.913	-1.58347	0.211161	-7.49885	6.44E-14	5.65E-13	DOWN
RDH5	535.27	-1.57088	0.209539	-7.49681	6.54E-14	5.73E-13	DOWN
AAK1	1065.201	0.780114	0.104067	7.496301	6.56E-14	5.75E-13	UP
TBX20	3.799846	-2.53006	0.337512	-7.49621	6.57E-14	5.75E-13	DOWN
CAMK4	94.93465	-1.51091	0.201569	-7.49575	6.59E-14	5.77E-13	DOWN
ASNSP1	20.90636	5.895954	0.786643	7.495078	6.63E-14	5.80E-13	UP
PHYHIPL	385.6411	2.04537	0.272919	7.494421	6.66E-14	5.83E-13	UP
RP11-796I	6.528001	4.440564	0.592572	7.493711	6.70E-14	5.86E-13	UP
CXCR2P1	184.4464	-2.04118	0.272435	-7.49236	6.76E-14	5.92E-13	DOWN
DNAJB6	2090.509	0.510737	0.068178	7.491255	6.82E-14	5.97E-13	NOT
CSPG4P12	8.772729	1.731446	0.231139	7.490916	6.84E-14	5.98E-13	UP
SNIP1	654.1959	-0.55836	0.07455	-7.48969	6.90E-14	6.03E-13	NOT
KIF21B	457.6941	1.609538	0.214935	7.488494	6.97E-14	6.09E-13	UP
GHRHR	45.71488	3.194445	0.426702	7.486369	7.08E-14	6.18E-13	UP
RP11-429J	10.84604	1.435338	0.191728	7.486325	7.08E-14	6.18E-13	UP
FCN1	193.8387	-1.54387	0.206234	-7.48604	7.10E-14	6.20E-13	DOWN
RP11-216I	2.225198	2.888077	0.385807	7.485813	7.11E-14	6.20E-13	UP

DKK4	84.48219	3.746828	0.500693	7.48329	7.25E-14	6.32E-13	UP
RP11-527I	14.13279	1.896041	0.253387	7.482786	7.28E-14	6.34E-13	UP
FTSJ3	2251.093	0.534107	0.07138	7.482624	7.29E-14	6.35E-13	NOT
CCDC66	243.3093	0.630085	0.084215	7.4819	7.33E-14	6.38E-13	UP
TIPIN	163.0364	0.734323	0.098159	7.480934	7.38E-14	6.43E-13	UP
C6	21168.47	-1.92708	0.257643	-7.47964	7.45E-14	6.49E-13	DOWN
DRGX	7.972999	4.622297	0.618023	7.47917	7.48E-14	6.51E-13	UP
CTD-2116	2.753748	3.010093	0.402565	7.477287	7.59E-14	6.60E-13	UP
RP11-439I	3.2421	3.469861	0.464085	7.476774	7.62E-14	6.63E-13	UP
XYLT2	1310.644	0.613063	0.082	7.476396	7.64E-14	6.64E-13	UP
MAP3K10	387.9803	0.769238	0.102898	7.475761	7.68E-14	6.67E-13	UP
CUZD1	12.34095	1.579641	0.211304	7.47567	7.68E-14	6.68E-13	UP
CD226	97.57573	-1.49306	0.199724	-7.4756	7.69E-14	6.68E-13	DOWN
FAM180A	174.6583	-2.43067	0.325153	-7.47545	7.69E-14	6.68E-13	DOWN
FGL2	991.7655	-1.31399	0.175825	-7.47328	7.82E-14	6.79E-13	DOWN
PKN1	4350.02	0.954669	0.127749	7.473026	7.84E-14	6.80E-13	UP
RCC2	2096.418	0.849268	0.113649	7.472695	7.86E-14	6.82E-13	UP
DHTKD1	8409.786	-1.14431	0.153137	-7.4725	7.87E-14	6.82E-13	DOWN
TNNT1	36.81284	2.999642	0.401425	7.472492	7.87E-14	6.82E-13	UP
RNF122	208.866	-0.91201	0.12206	-7.47184	7.91E-14	6.86E-13	DOWN
RP11-973I	5.174008	3.357759	0.44939	7.471807	7.91E-14	6.86E-13	UP
CATSPER2	57.05721	1.148638	0.153741	7.471245	7.94E-14	6.88E-13	UP
CTC-1337	7.798881	2.124714	0.284408	7.470641	7.98E-14	6.91E-13	UP
STRADB	2269.429	-0.7201	0.096391	-7.47057	7.98E-14	6.91E-13	DOWN
RP11-127I	10.00369	1.41557	0.189489	7.470453	7.99E-14	6.92E-13	UP
C11orf24	2822.218	-0.74335	0.099509	-7.47025	8.00E-14	6.93E-13	DOWN
FAM168B	3738.341	0.625308	0.083708	7.470091	8.01E-14	6.93E-13	UP
HIST1H2B	9.229275	2.202658	0.294911	7.468897	8.09E-14	6.99E-13	UP
SH2B1	1597.365	0.5806	0.077762	7.466393	8.24E-14	7.13E-13	NOT
AC005618	9.744865	2.215904	0.296786	7.466328	8.25E-14	7.13E-13	UP
CRLF1	58.45623	1.654949	0.221656	7.466303	8.25E-14	7.13E-13	UP
ASPHD1	199.0563	2.341313	0.313587	7.466229	8.25E-14	7.13E-13	UP
PAGE1	21.97311	6.291411	0.842765	7.465201	8.32E-14	7.18E-13	UP
TRAPPC6B	1006.574	-0.51341	0.068774	-7.4651	8.32E-14	7.18E-13	NOT
RP11-112	8.396929	1.810903	0.242584	7.465068	8.33E-14	7.18E-13	UP
NKIRAS2	1763.303	0.513458	0.068789	7.46422	8.38E-14	7.23E-13	NOT
RP11-832	3.635839	2.967425	0.397588	7.463563	8.42E-14	7.26E-13	UP
PMS2P4	41.76833	0.831034	0.111346	7.463536	8.42E-14	7.26E-13	UP
IFT172	485.2287	0.74199	0.099419	7.463243	8.44E-14	7.28E-13	UP
NDUFB2-A	24.29956	0.857492	0.114914	7.462017	8.52E-14	7.34E-13	UP
RP11-61J1	75.274	1.193504	0.159945	7.461977	8.52E-14	7.34E-13	UP
DGKD	820.935	0.771021	0.103332	7.461616	8.55E-14	7.36E-13	UP
ELL3	16.56571	1.255687	0.168302	7.460926	8.59E-14	7.40E-13	UP
FAM188A	534.6278	-0.61411	0.08233	-7.45913	8.71E-14	7.50E-13	DOWN
PRKAR2B	120.216	-1.72295	0.230991	-7.45897	8.72E-14	7.50E-13	DOWN
RP1-34B2	14.81144	2.275077	0.305089	7.457103	8.84E-14	7.61E-13	UP
RSRP1	970.4717	0.888827	0.119196	7.456864	8.86E-14	7.62E-13	UP
EVI5L	517.9554	0.66796	0.089578	7.456769	8.87E-14	7.62E-13	UP
CTNNA3	159.2199	-2.37795	0.318904	-7.45662	8.88E-14	7.63E-13	DOWN
AC098820	19.30212	1.472533	0.197528	7.454802	9.00E-14	7.73E-13	UP
SLBP	1441.191	0.629211	0.084413	7.453992	9.06E-14	7.78E-13	UP
ZNF273	76.78882	1.080131	0.144907	7.453951	9.06E-14	7.78E-13	UP

MDGA1	238.6518	2.108087	0.28282	7.453824	9.07E-14	7.78E-13	UP
TPSP2	64.44675	2.092508	0.280847	7.450693	9.29E-14	7.97E-13	UP
OAF	11896.11	-1.02122	0.137066	-7.45058	9.29E-14	7.97E-13	DOWN
LPIN2	10005.04	-1.17138	0.157233	-7.44999	9.33E-14	8.00E-13	DOWN
CTD-2269	2.884866	3.312745	0.444673	7.44984	9.35E-14	8.01E-13	UP
AKR1B10P:	100.2466	3.746989	0.502986	7.449495	9.37E-14	8.03E-13	UP
KLHL23	643.1396	1.067122	0.143263	7.448678	9.43E-14	8.08E-13	UP
FAM49B	1474.693	0.87582	0.117589	7.448146	9.47E-14	8.11E-13	UP
CTC-338M	47.5617	0.965917	0.129687	7.448073	9.47E-14	8.11E-13	UP
TMPRSS2	2195.234	-1.41756	0.190329	-7.44795	9.48E-14	8.11E-13	DOWN
SELM	1585.838	1.76804	0.237398	7.447569	9.51E-14	8.13E-13	UP
PSORS1C1	35.89438	2.386713	0.320517	7.44645	9.59E-14	8.20E-13	UP
CACNG1	6.236641	4.374865	0.587532	7.446176	9.61E-14	8.22E-13	UP
TMEM170	706.0834	-0.50996	0.068511	-7.44356	9.80E-14	8.38E-13	NOT
PRDM9	4.219504	3.805755	0.511286	7.443488	9.81E-14	8.38E-13	UP
BTN2A3P	60.99533	1.054879	0.14172	7.443383	9.81E-14	8.38E-13	UP
GRAMD1C	702.9782	-1.4478	0.194509	-7.44334	9.82E-14	8.38E-13	DOWN
VCX	5.828718	4.697553	0.631264	7.4415	9.95E-14	8.50E-13	UP
KLK4	26.05903	5.817988	0.781918	7.440665	1.00E-13	8.55E-13	UP
RHEBL1	32.11672	1.126828	0.151507	7.43746	1.03E-13	8.76E-13	UP
TMEM198	252.6574	1.378967	0.185409	7.43744	1.03E-13	8.76E-13	UP
RP1-90L14	7.986461	5.387727	0.724461	7.436873	1.03E-13	8.79E-13	UP
LA16c-390	8.566062	1.921177	0.258351	7.436296	1.04E-13	8.83E-13	UP
RP11-643I	3.256657	2.50415	0.336805	7.43501	1.05E-13	8.91E-13	UP
SLC22A10	1999.463	-2.30382	0.309907	-7.43389	1.05E-13	8.98E-13	DOWN
RP11-245I	4.552766	3.31245	0.445595	7.433768	1.06E-13	8.99E-13	UP
CLYBL	1811.482	-1.10882	0.149171	-7.43324	1.06E-13	9.02E-13	DOWN
STRADA	206.4406	0.642763	0.086474	7.433026	1.06E-13	9.03E-13	UP
CBFA2T3	276.1719	-1.62661	0.218851	-7.43247	1.07E-13	9.07E-13	DOWN
PODNL1	23.85896	1.814106	0.244132	7.430844	1.08E-13	9.18E-13	UP
C8orf76	389.5047	0.887183	0.119398	7.430479	1.08E-13	9.20E-13	UP
B4GALT1	8347.798	-0.7486	0.100751	-7.43022	1.08E-13	9.22E-13	DOWN
RPL23AP82	168.0423	0.786544	0.105877	7.428868	1.10E-13	9.31E-13	UP
ANKRD23	24.05674	1.088304	0.146528	7.42728	1.11E-13	9.42E-13	UP
AQP7	637.8924	-1.34295	0.180859	-7.42541	1.12E-13	9.55E-13	DOWN
SRXN1	168.5035	1.554778	0.209387	7.425396	1.12E-13	9.55E-13	UP
HIST3H2BI	14.11628	3.788843	0.510288	7.424917	1.13E-13	9.58E-13	UP
HTR2A-AS	2.630108	-2.262	0.304683	-7.42413	1.14E-13	9.63E-13	DOWN
CTD-2207	3.483572	3.539163	0.476715	7.424069	1.14E-13	9.63E-13	UP
CCDC162I	115.7253	2.703714	0.364257	7.422548	1.15E-13	9.74E-13	UP
SLC16A3	1000.632	1.737596	0.234123	7.421733	1.16E-13	9.80E-13	UP
RP11-139I	4.255608	3.637452	0.490112	7.421672	1.16E-13	9.80E-13	UP
RNU6-850	4.236799	2.264848	0.305172	7.421559	1.16E-13	9.80E-13	UP
RP4-713B5	3.543325	2.330137	0.31397	7.421533	1.16E-13	9.80E-13	UP
LRR1	218.8934	0.75446	0.101669	7.420772	1.16E-13	9.86E-13	UP
CTD-2591	7.40187	5.043066	0.679593	7.420716	1.16E-13	9.86E-13	UP
MRPL9	1597.647	0.611247	0.08238	7.419851	1.17E-13	9.92E-13	UP
SSFA2	4425.585	-0.8233	0.110961	-7.41978	1.17E-13	9.92E-13	DOWN
SORL1	5850.524	-1.34653	0.181514	-7.41834	1.19E-13	1.00E-12	DOWN
MDC1	1342.555	0.735872	0.099198	7.418178	1.19E-13	1.00E-12	UP
POMT2	509.7414	0.655715	0.088402	7.417381	1.19E-13	1.01E-12	UP
FAM182B	9.553761	2.059405	0.27765	7.417267	1.20E-13	1.01E-12	UP

FERMT1	195.6213	2.272286	0.306398	7.416126	1.21E-13	1.02E-12	UP
CTC-251D	6.429385	1.72474	0.232567	7.416109	1.21E-13	1.02E-12	UP
GINM1	1601.638	-0.60116	0.081066	-7.41568	1.21E-13	1.02E-12	DOWN
HSDL1	551.1458	0.690305	0.0931	7.414657	1.22E-13	1.03E-12	UP
CNFN	44.10722	2.213098	0.298614	7.411226	1.25E-13	1.06E-12	UP
RHOF	56.43042	1.577109	0.212853	7.409385	1.27E-13	1.07E-12	UP
ZNF529-A	69.99583	1.024351	0.138291	7.407218	1.29E-13	1.09E-12	UP
CCNH	893.1001	-0.5984	0.0808	-7.40596	1.30E-13	1.10E-12	DOWN
MKL1	1134.983	0.565102	0.076314	7.404957	1.31E-13	1.11E-12	NOT
PTOV1-AS	60.89489	1.118644	0.151076	7.404523	1.32E-13	1.11E-12	UP
DMTF1	810.9512	0.735942	0.099396	7.404124	1.32E-13	1.11E-12	UP
PDGFB	687.3223	1.069034	0.144388	7.403876	1.32E-13	1.11E-12	UP
CCDC40	135.0463	1.105029	0.149257	7.403514	1.33E-13	1.12E-12	UP
APOBEC3A	19.47287	-1.82691	0.246766	-7.4034	1.33E-13	1.12E-12	DOWN
C9orf173-	50.02272	1.840611	0.24867	7.401821	1.34E-13	1.13E-12	UP
PPCDC	371.1115	0.762003	0.102954	7.401404	1.35E-13	1.13E-12	UP
ANXA2	13104.6	1.16578	0.157511	7.401264	1.35E-13	1.13E-12	UP
RP11-169I	74.24823	4.758069	0.642977	7.40006	1.36E-13	1.14E-12	UP
TMEM151	1.733101	2.727365	0.368584	7.399578	1.37E-13	1.15E-12	UP
MED25	1385.428	0.58756	0.079422	7.397913	1.38E-13	1.16E-12	UP
CST2	16.55315	3.220818	0.435418	7.397072	1.39E-13	1.17E-12	UP
HK3	215.3505	-1.46982	0.198729	-7.39609	1.40E-13	1.18E-12	DOWN
TCF21	69.57942	-1.55012	0.209608	-7.39532	1.41E-13	1.18E-12	DOWN
NR3C2	464.1521	-1.30283	0.176213	-7.39349	1.43E-13	1.20E-12	DOWN
CTD-3162	5.55131	2.643393	0.35762	7.391634	1.45E-13	1.22E-12	UP
RP11-348J	2.914912	-2.603	0.352195	-7.39078	1.46E-13	1.22E-12	DOWN
CSNK1G1	399.0325	0.627203	0.084875	7.389716	1.47E-13	1.23E-12	UP
FGF12	188.4101	2.146046	0.290422	7.389398	1.47E-13	1.24E-12	UP
RP11-468I	5.509541	-1.63982	0.22196	-7.38791	1.49E-13	1.25E-12	DOWN
GOLGA2P5	592.1986	1.281472	0.173458	7.387775	1.49E-13	1.25E-12	UP
CAPN15	1469.416	0.580913	0.078639	7.38712	1.50E-13	1.26E-12	NOT
ATP13A4	17.25645	-2.27543	0.308034	-7.38692	1.50E-13	1.26E-12	DOWN
C1orf131	540.6145	0.741551	0.100392	7.386578	1.51E-13	1.26E-12	UP
RNF126P1	6.887245	2.55562	0.345988	7.386451	1.51E-13	1.26E-12	UP
SMARCAL1	723.5174	0.517124	0.070021	7.385214	1.52E-13	1.27E-12	NOT
IMPG2	10.03214	1.349232	0.18271	7.384547	1.53E-13	1.28E-12	UP
LLNLR-268	5.142648	2.161983	0.29283	7.383064	1.55E-13	1.29E-12	UP
KLHL31	46.87284	1.372583	0.185967	7.380782	1.57E-13	1.31E-12	UP
CDK9	2608.586	-0.66855	0.090589	-7.38006	1.58E-13	1.32E-12	DOWN
VSIG10	1260.857	0.761384	0.103177	7.379422	1.59E-13	1.33E-12	UP
RP1-276N	14.75245	3.57549	0.484568	7.378719	1.60E-13	1.33E-12	UP
PHF6	620.6151	0.718488	0.097373	7.378686	1.60E-13	1.33E-12	UP
CACNB3	141.4156	1.35202	0.183235	7.3786	1.60E-13	1.33E-12	UP
XXbac-B47	53.93025	-0.94743	0.128405	-7.37845	1.60E-13	1.33E-12	DOWN
MICB	190.1419	1.344406	0.182214	7.378176	1.60E-13	1.34E-12	UP
FAM213B	890.5492	1.018784	0.138088	7.377797	1.61E-13	1.34E-12	UP
SHARPIN	3310.628	0.877126	0.118888	7.377754	1.61E-13	1.34E-12	UP
DDX55	608.9092	0.57743	0.078267	7.377722	1.61E-13	1.34E-12	NOT
MLEC	10365.87	0.748289	0.101426	7.377711	1.61E-13	1.34E-12	UP
THNSL1	884.5575	-0.89036	0.120691	-7.37714	1.62E-13	1.35E-12	DOWN
RP11-449J	39.98062	1.542048	0.209033	7.377038	1.62E-13	1.35E-12	UP
AC093724	15.65402	1.717288	0.232859	7.374784	1.65E-13	1.37E-12	UP

RP11-199I	20.32666	1.282527	0.173932	7.373728	1.66E-13	1.38E-12	UP
ZNF862	349.3831	0.990285	0.134314	7.372919	1.67E-13	1.39E-12	UP
RAD51-AS	69.84369	0.973872	0.132103	7.372079	1.68E-13	1.40E-12	UP
DHDH	21.91008	2.085323	0.282876	7.371875	1.68E-13	1.40E-12	UP
MFI2	522.6216	2.020946	0.274161	7.371387	1.69E-13	1.40E-12	UP
BAX	2158.564	0.843433	0.114445	7.369756	1.71E-13	1.42E-12	UP
MAGEA8	9.744474	5.034556	0.683202	7.369063	1.72E-13	1.43E-12	UP
CDYL	508.7395	0.532387	0.072248	7.368876	1.72E-13	1.43E-12	NOT
PSMG3	1031.677	0.939119	0.127452	7.368385	1.73E-13	1.43E-12	UP
IFT81	283.3816	0.855134	0.116057	7.36822	1.73E-13	1.43E-12	UP
HIST1H3J	1.595763	2.889971	0.39224	7.367862	1.73E-13	1.44E-12	UP
IZUMO1	8.974239	1.889374	0.256497	7.366062	1.76E-13	1.46E-12	UP
FUCA1	3689.87	-0.79394	0.107784	-7.36606	1.76E-13	1.46E-12	DOWN
RP5-1198(	9.107668	1.905916	0.258756	7.365677	1.76E-13	1.46E-12	UP
DLX4	14.33659	2.982325	0.404953	7.364624	1.78E-13	1.47E-12	UP
RP11-264I	16.93571	1.20957	0.164288	7.362497	1.81E-13	1.49E-12	UP
PFDN6	2173.049	0.855622	0.116225	7.361761	1.82E-13	1.50E-12	UP
RP11-498(	8.948872	2.639281	0.358521	7.361577	1.82E-13	1.50E-12	UP
TRMT6	563.5664	0.614978	0.083544	7.361091	1.82E-13	1.51E-12	UP
RP11-19E1	11.35226	5.133686	0.697441	7.360745	1.83E-13	1.51E-12	UP
GGT5	1638.487	-1.54586	0.210022	-7.36046	1.83E-13	1.51E-12	DOWN
SBSPON	98.1675	1.955729	0.265737	7.359644	1.84E-13	1.52E-12	UP
NUP205	1173.999	0.733876	0.09972	7.359348	1.85E-13	1.53E-12	UP
ATP13A1	2860.376	0.629147	0.085499	7.358524	1.86E-13	1.54E-12	UP
CLDN19	41.05935	2.771459	0.376678	7.357642	1.87E-13	1.55E-12	UP
ZBTB41	894.4202	0.939524	0.127701	7.357209	1.88E-13	1.55E-12	UP
ZNF71	170.5345	1.106933	0.150465	7.356741	1.88E-13	1.55E-12	UP
RP1-80N2	38.27364	1.338309	0.181928	7.356245	1.89E-13	1.56E-12	UP
AKIRIN1	2028.734	-0.67226	0.091402	-7.35504	1.91E-13	1.57E-12	DOWN
CLUL1	11.85097	2.326143	0.316292	7.354411	1.92E-13	1.58E-12	UP
FAM149A	1076.698	-1.25377	0.170519	-7.35269	1.94E-13	1.60E-12	DOWN
RP11-332I	41.33974	0.91195	0.12403	7.352679	1.94E-13	1.60E-12	UP
PCSK1N	120.5983	3.431606	0.466729	7.352461	1.95E-13	1.60E-12	UP
ABLIM3	3647.589	-1.14668	0.155963	-7.35224	1.95E-13	1.60E-12	DOWN
AARSD1	151.4367	0.672869	0.091526	7.351676	1.96E-13	1.61E-12	UP
LINC00907	30.38504	-2.65136	0.360648	-7.35165	1.96E-13	1.61E-12	DOWN
UGT2B7	21130.49	-2.02262	0.275166	-7.35055	1.97E-13	1.62E-12	DOWN
RP11-268(	5.224666	3.938772	0.535897	7.349861	1.98E-13	1.63E-12	UP
NFIA	3109.79	-0.84609	0.115128	-7.34911	2.00E-13	1.64E-12	DOWN
ABCD1	1427.894	0.977454	0.133023	7.348014	2.01E-13	1.65E-12	UP
PDXK	3872.981	0.728059	0.099103	7.346502	2.03E-13	1.67E-12	UP
ZBED4	660.4014	0.720695	0.098122	7.344888	2.06E-13	1.69E-12	UP
EGFL8	35.94066	1.313612	0.178875	7.343734	2.08E-13	1.71E-12	UP
ZNF85	57.59806	1.483707	0.202051	7.343219	2.09E-13	1.71E-12	UP
NME6	482.1514	0.545224	0.07425	7.343068	2.09E-13	1.71E-12	NOT
FKBPL	295.346	0.822822	0.112061	7.342608	2.09E-13	1.72E-12	UP
SSPO	140.6727	1.819006	0.247748	7.342147	2.10E-13	1.72E-12	UP
MMP17	118.5347	2.466556	0.336017	7.340568	2.13E-13	1.74E-12	UP
PRDX3	11563.93	-0.75844	0.103358	-7.33802	2.17E-13	1.78E-12	DOWN
MGME1	698.3039	0.535589	0.073	7.336866	2.19E-13	1.79E-12	NOT
NOL7	1876.514	0.621713	0.084739	7.336775	2.19E-13	1.79E-12	UP
KLHL30	26.88304	2.181318	0.297378	7.335183	2.21E-13	1.81E-12	UP

B3GALNT1	136.4248	1.295375	0.176607	7.334776	2.22E-13	1.82E-12	UP
PTPN23	2116.369	0.507069	0.069133	7.33473	2.22E-13	1.82E-12	NOT
RP11-396I	217.2248	1.047252	0.142794	7.333995	2.23E-13	1.83E-12	UP
MLF2	10743.38	-0.60309	0.082236	-7.33363	2.24E-13	1.83E-12	DOWN
RP11-70D	60.36911	-1.32987	0.181346	-7.33331	2.25E-13	1.84E-12	DOWN
CTAGE7P	8.703836	1.611927	0.219823	7.332849	2.25E-13	1.84E-12	UP
RPP21	281.226	0.839772	0.114538	7.331843	2.27E-13	1.85E-12	UP
CSNK1D	4708.154	0.438511	0.059823	7.330105	2.30E-13	1.88E-12	NOT
C1orf61	16.76053	2.42553	0.33095	7.328994	2.32E-13	1.89E-12	UP
RDH8	5.447295	4.241367	0.57875	7.328493	2.33E-13	1.90E-12	UP
RP11-63A	2.179382	2.7521	0.375562	7.327956	2.34E-13	1.91E-12	UP
LGI2	81.11101	1.82122	0.248546	7.327483	2.35E-13	1.91E-12	UP
BSG	19101.48	1.050151	0.143327	7.326978	2.35E-13	1.92E-12	UP
SFXN1	5015.243	-0.84262	0.115006	-7.32668	2.36E-13	1.92E-12	DOWN
RP11-525I	27.33478	2.640866	0.360453	7.326528	2.36E-13	1.93E-12	UP
MEP1B	39.25617	-2.67317	0.364871	-7.32634	2.37E-13	1.93E-12	DOWN
SRC	1369.339	1.266972	0.172943	7.32596	2.37E-13	1.93E-12	UP
RP11-327I	46.97715	-0.83405	0.113856	-7.32555	2.38E-13	1.94E-12	DOWN
TRAM2	1617.229	-0.79063	0.107936	-7.32492	2.39E-13	1.95E-12	DOWN
ADCK5	606.812	0.90767	0.123928	7.324166	2.40E-13	1.96E-12	UP
WDR66	69.49997	-1.36326	0.18615	-7.32345	2.42E-13	1.97E-12	DOWN
GTF2E1	383.7962	0.648622	0.088573	7.323001	2.42E-13	1.97E-12	UP
CTD-2287	38.7703	1.059772	0.144728	7.322506	2.43E-13	1.98E-12	UP
RP11-247(	3.992882	3.491652	0.476909	7.321424	2.45E-13	1.99E-12	UP
LRRC26	7.299377	4.074855	0.556595	7.321036	2.46E-13	2.00E-12	UP
FAM210A	806.8107	-0.57378	0.078377	-7.32077	2.47E-13	2.00E-12	NOT
EP400	1328.717	0.55844	0.076292	7.31978	2.48E-13	2.02E-12	NOT
CTB-50L1	4.823598	1.859567	0.254051	7.319667	2.49E-13	2.02E-12	UP
RP11-756/	38.97552	2.936573	0.401376	7.316273	2.55E-13	2.07E-12	UP
RNF224	4.275385	2.94515	0.402582	7.315655	2.56E-13	2.08E-12	UP
ITGB4	945.4108	1.783059	0.243734	7.315601	2.56E-13	2.08E-12	UP
BPIFB4	5.601416	3.724847	0.509232	7.314632	2.58E-13	2.09E-12	UP
CMB9-55F	84.8287	1.538656	0.21036	7.314379	2.59E-13	2.10E-12	UP
RP11-79N	4.739458	2.880317	0.393805	7.314061	2.59E-13	2.10E-12	UP
OTUD3	150.1199	0.908987	0.124281	7.313981	2.59E-13	2.10E-12	UP
AD000684	11.331	1.483291	0.202805	7.313886	2.60E-13	2.10E-12	UP
COL11A1	57.63374	2.95521	0.404083	7.313376	2.61E-13	2.11E-12	UP
HEXA-AS1	10.90425	1.705502	0.233247	7.311998	2.63E-13	2.13E-12	UP
COMT	7456.354	-1.03944	0.142225	-7.30839	2.70E-13	2.19E-12	DOWN
ALDH9A1	7925.272	-0.90624	0.124012	-7.30769	2.72E-13	2.20E-12	DOWN
AC016682	6.939927	-2.5905	0.354491	-7.30765	2.72E-13	2.20E-12	DOWN
TNFRSF19	505.1272	2.47144	0.338213	7.307337	2.72E-13	2.20E-12	UP
ABCA1	3939.024	-0.97572	0.133527	-7.30727	2.73E-13	2.20E-12	DOWN
RAB12	1263.347	-0.5197	0.071124	-7.30694	2.73E-13	2.21E-12	NOT
RP11-946I	15.64189	5.835512	0.798639	7.306823	2.74E-13	2.21E-12	UP
RP5-1039I	80.44471	0.935982	0.128099	7.306703	2.74E-13	2.21E-12	UP
C1orf74	163.6531	0.787148	0.107734	7.306426	2.74E-13	2.22E-12	UP
RGS20	7.679299	2.717461	0.371986	7.30528	2.77E-13	2.23E-12	UP
GC	153263.5	-1.34819	0.184563	-7.30474	2.78E-13	2.24E-12	DOWN
YPEL2	1501.539	-0.9756	0.133581	-7.30347	2.80E-13	2.26E-12	DOWN
RP11-174(	45.8273	1.411179	0.193246	7.302491	2.82E-13	2.28E-12	UP
CTC-429P	150.0498	1.009565	0.138308	7.299416	2.89E-13	2.33E-12	UP

FMO1	240.3061	2.538411	0.347767	7.299164	2.90E-13	2.33E-12	UP
TLL2	16.08562	1.996174	0.273513	7.298283	2.91E-13	2.35E-12	UP
SNPH	68.13076	1.607137	0.22021	7.298207	2.92E-13	2.35E-12	UP
GLTSCR1	520.2813	0.574191	0.078679	7.297851	2.92E-13	2.36E-12	NOT
RP1-12G1	10.66486	-1.08248	0.14833	-7.29779	2.93E-13	2.36E-12	DOWN
HPS4	1046.102	0.597762	0.081918	7.297054	2.94E-13	2.37E-12	UP
Z97634.3	17.70995	1.312484	0.179871	7.296814	2.95E-13	2.37E-12	UP
DCAF16	570.192	0.896325	0.122847	7.296288	2.96E-13	2.38E-12	UP
CYTH3	518.0193	0.907514	0.124402	7.295027	2.99E-13	2.40E-12	UP
HGH1	1962.105	0.965313	0.132334	7.29451	3.00E-13	2.41E-12	UP
AZGP1	55172.8	-1.76663	0.242189	-7.29441	3.00E-13	2.41E-12	DOWN
RP11-108	2.942018	-1.79147	0.245597	-7.29434	3.00E-13	2.41E-12	DOWN
DTNA	664.7804	1.646444	0.225754	7.293093	3.03E-13	2.43E-12	UP
PKD1	1552.215	0.73982	0.101453	7.29227	3.05E-13	2.45E-12	UP
ZMYM3	1358.596	0.620472	0.085109	7.290368	3.09E-13	2.48E-12	UP
MEFV	22.50784	-1.21336	0.166446	-7.28979	3.10E-13	2.49E-12	DOWN
ARHGEF37	363.6608	1.434194	0.196794	7.287783	3.15E-13	2.53E-12	UP
BSND	6.173279	4.18696	0.574617	7.286519	3.18E-13	2.55E-12	UP
SMYD2	2378.012	0.994292	0.136457	7.28649	3.18E-13	2.55E-12	UP
LINC0113	52.80374	1.24892	0.171405	7.28635	3.18E-13	2.55E-12	UP
STYX	1457.66	-0.64873	0.08904	-7.28587	3.20E-13	2.56E-12	DOWN
SPHK1	814.6868	2.17496	0.298534	7.285473	3.21E-13	2.57E-12	UP
C19orf67	3.995814	2.646203	0.363277	7.284253	3.23E-13	2.59E-12	UP
IRX6	6.149617	4.424986	0.607533	7.283536	3.25E-13	2.60E-12	UP
SHBG	2141.994	-2.18918	0.300586	-7.28305	3.26E-13	2.61E-12	DOWN
RCBTB2	329.5099	-0.77046	0.105801	-7.28215	3.29E-13	2.63E-12	DOWN
PPME1	1177.976	0.50386	0.069221	7.279035	3.36E-13	2.69E-12	NOT
LRRC16B	94.86956	-1.40039	0.1924	-7.27853	3.37E-13	2.70E-12	DOWN
MEA1	2648.047	0.668927	0.091911	7.277956	3.39E-13	2.71E-12	UP
RP11-314	9.731524	5.499648	0.755744	7.277129	3.41E-13	2.73E-12	UP
MAML1	985.0615	0.553591	0.076077	7.276749	3.42E-13	2.73E-12	NOT
RP11-66N	75.47408	1.219399	0.167586	7.276256	3.43E-13	2.74E-12	UP
AC022154	4.854516	1.687042	0.231864	7.276013	3.44E-13	2.75E-12	UP
RALGAPB	1553.318	0.528788	0.072677	7.275835	3.44E-13	2.75E-12	NOT
CTD-3064	10.1598	2.012693	0.27663	7.275772	3.44E-13	2.75E-12	UP
FBXL6	1168.992	0.981365	0.134893	7.275156	3.46E-13	2.76E-12	UP
FTSJ1	824.2477	0.524859	0.072147	7.274843	3.47E-13	2.77E-12	NOT
SMIM12	1800.058	-0.67061	0.092184	-7.27472	3.47E-13	2.77E-12	DOWN
HIST1H2A	2.345119	3.124725	0.429537	7.274626	3.47E-13	2.77E-12	UP
SNRPEP4	9.144752	1.460902	0.200853	7.273494	3.50E-13	2.79E-12	UP
LYG1	32.29939	1.159269	0.159388	7.273246	3.51E-13	2.80E-12	UP
SEPSECS	1813.618	-0.89085	0.122503	-7.27203	3.54E-13	2.82E-12	DOWN
KIRREL2	7.216945	3.642305	0.500868	7.27199	3.54E-13	2.82E-12	UP
ALS2CL	1205.294	1.357828	0.186748	7.270909	3.57E-13	2.84E-12	UP
BAGE2	13.42026	6.022826	0.828374	7.270665	3.58E-13	2.85E-12	UP
CACNA1S	5.356952	2.838982	0.390481	7.270474	3.58E-13	2.85E-12	UP
S100A10	5404.484	1.153483	0.158703	7.268202	3.64E-13	2.90E-12	UP
MYCT1	346.5603	-0.97659	0.13438	-7.26734	3.67E-13	2.92E-12	DOWN
RP11-386	6.765199	2.121672	0.292043	7.26493	3.73E-13	2.97E-12	UP
SH2D4B	2.988471	2.808401	0.386658	7.263276	3.78E-13	3.00E-12	UP
SPATA3-A	4.839925	2.799007	0.385374	7.263085	3.78E-13	3.01E-12	UP
CEP83-AS	7.994961	1.5414	0.212242	7.262466	3.80E-13	3.02E-12	UP

LINC01537	17.23458	-1.45628	0.200552	-7.26134	3.83E-13	3.04E-12	DOWN
PTGES3L	9.279274	1.800324	0.247937	7.261201	3.84E-13	3.05E-12	UP
MCOLN3	104.8057	2.957272	0.407285	7.260934	3.84E-13	3.05E-12	UP
RP11-188I	4.417588	1.824292	0.251263	7.260489	3.86E-13	3.06E-12	UP
ACACB	5452.431	-1.15298	0.158819	-7.25975	3.88E-13	3.08E-12	DOWN
KIF28P	20.52777	1.725132	0.23765	7.259135	3.90E-13	3.09E-12	UP
CYHR1	2811.128	0.82717	0.113967	7.257964	3.93E-13	3.12E-12	UP
GOLT1B	1469.329	0.498126	0.068638	7.25728	3.95E-13	3.13E-12	NOT
MRPL32	1826.811	-0.56048	0.077232	-7.25714	3.95E-13	3.13E-12	NOT
KIAA1211L	101.8309	1.308187	0.180264	7.257072	3.96E-13	3.13E-12	UP
OTOG	8.533862	4.5088	0.621322	7.256779	3.96E-13	3.14E-12	UP
TREML3P	17.13231	4.099574	0.564972	7.256244	3.98E-13	3.15E-12	UP
RP11-432J	10.75042	1.379645	0.19014	7.255953	3.99E-13	3.16E-12	UP
RP11-250I	9.608286	-1.40294	0.19337	-7.25525	4.01E-13	3.17E-12	DOWN
RP4-533D	3.318866	2.812483	0.387713	7.254027	4.05E-13	3.20E-12	UP
AK3	6864.431	-0.89689	0.123653	-7.25331	4.07E-13	3.22E-12	DOWN
AFF4	4466.644	-0.73191	0.100914	-7.25281	4.08E-13	3.23E-12	DOWN
AP006222.	10.16225	1.969024	0.271639	7.248687	4.21E-13	3.33E-12	UP
MT1P3	11.48577	-3.69657	0.509971	-7.24859	4.21E-13	3.33E-12	DOWN
HTR3A	13.4192	3.906171	0.53889	7.248553	4.21E-13	3.33E-12	UP
RP11-97C.	51.96256	0.93234	0.128625	7.248509	4.21E-13	3.33E-12	UP
CCDC15	91.67742	0.804897	0.111047	7.248222	4.22E-13	3.33E-12	UP
ACOT2	1937.443	-1.06172	0.146482	-7.24808	4.23E-13	3.34E-12	DOWN
ZNF706	2727.249	0.745297	0.10283	7.247859	4.23E-13	3.34E-12	UP
SUOX	2534.32	-0.81498	0.112445	-7.24784	4.23E-13	3.34E-12	DOWN
RP11-147I	34.72846	1.510588	0.208427	7.247561	4.24E-13	3.35E-12	UP
TTLL6	17.4617	2.249206	0.310357	7.247157	4.26E-13	3.35E-12	UP
GPHN	1695.376	-1.19052	0.164311	-7.24553	4.31E-13	3.39E-12	DOWN
CCR1	321.5216	-1.41482	0.1953	-7.24438	4.34E-13	3.42E-12	DOWN
PRKAA2	567.2933	1.95711	0.270204	7.243079	4.39E-13	3.45E-12	UP
RUNDC3A	20.84155	1.987894	0.274521	7.24131	4.44E-13	3.50E-12	UP
CTD-2033	4.684455	3.59521	0.496598	7.239683	4.50E-13	3.54E-12	UP
U2AF2	4378.503	0.4265	0.058913	7.239472	4.50E-13	3.54E-12	NOT
UBN1	1603.996	0.552091	0.076274	7.238284	4.54E-13	3.57E-12	NOT
AC006547	56.19117	0.875066	0.120909	7.2374	4.57E-13	3.60E-12	UP
RP11-420A	53.1472	1.273736	0.176007	7.236852	4.59E-13	3.61E-12	UP
RP11-572C	3.612629	2.276157	0.31453	7.236688	4.60E-13	3.61E-12	UP
FOXH1	4.969182	2.661804	0.367838	7.236354	4.61E-13	3.62E-12	UP
POU3F2	6.078429	3.160017	0.436718	7.235829	4.63E-13	3.63E-12	UP
GNG12-AS	27.15783	-1.31577	0.18188	-7.23425	4.68E-13	3.68E-12	DOWN
CEP164	618.6913	0.585987	0.081019	7.232693	4.74E-13	3.72E-12	UP
WSCD1	198.1457	1.584967	0.219145	7.232508	4.74E-13	3.72E-12	UP
RP11-758I	15.98796	5.925024	0.81966	7.228637	4.88E-13	3.83E-12	UP
TTC9C	504.4315	0.431649	0.059732	7.226415	4.96E-13	3.89E-12	NOT
CYP27B1	46.69311	1.718644	0.237883	7.22475	5.02E-13	3.94E-12	UP
HIST2H2BI	3.229336	2.308442	0.319525	7.224614	5.03E-13	3.94E-12	UP
HIST1H4K	8.31852	2.099609	0.290643	7.224024	5.05E-13	3.96E-12	UP
HCG18	529.5946	0.805828	0.11156	7.223253	5.08E-13	3.98E-12	UP
CYP1A2	11098.48	-3.476	0.481246	-7.22291	5.09E-13	3.99E-12	DOWN
TRIM28	8050.946	0.777614	0.107661	7.222827	5.09E-13	3.99E-12	UP
RAET1E	5.470906	1.89473	0.262469	7.218867	5.24E-13	4.10E-12	UP
ZFPM2	111.3163	-1.32278	0.183248	-7.21854	5.25E-13	4.11E-12	DOWN

GOT2	15106.68	-1.06799	0.147958	-7.21822	5.27E-13	4.12E-12	DOWN
GPATCH4	1439.792	0.75743	0.10494	7.217767	5.28E-13	4.13E-12	UP
RPUSD1	734.6443	0.675543	0.093596	7.217685	5.29E-13	4.14E-12	UP
ECE1	5947.798	-0.67563	0.093609	-7.21756	5.29E-13	4.14E-12	DOWN
C5orf58	28.76353	2.774609	0.384426	7.217537	5.29E-13	4.14E-12	UP
TTYH3	3364.677	1.164107	0.161291	7.217426	5.30E-13	4.14E-12	UP
RAE1	1026.739	0.53625	0.074318	7.215606	5.37E-13	4.19E-12	NOT
CEBPD	5838.983	-1.23681	0.17141	-7.21551	5.37E-13	4.20E-12	DOWN
TESK2	297.0247	-0.91393	0.126677	-7.21464	5.41E-13	4.22E-12	DOWN
WFDC21P	60.75285	2.489052	0.345095	7.212652	5.49E-13	4.28E-12	UP
MCCC2	6555.148	-0.76696	0.10634	-7.21236	5.50E-13	4.29E-12	DOWN
PRPF39	540.5451	0.618485	0.085755	7.21223	5.50E-13	4.29E-12	UP
MGAT5	1000.241	0.983961	0.136432	7.212113	5.51E-13	4.30E-12	UP
ZSCAN31	555.667	1.475571	0.204603	7.211878	5.52E-13	4.30E-12	UP
RP11-428I	2.662347	2.850963	0.395369	7.210898	5.56E-13	4.33E-12	UP
TSPEAR	34.03677	2.04642	0.2838	7.210791	5.56E-13	4.33E-12	UP
STAG3L2	88.19136	0.841155	0.116661	7.210239	5.59E-13	4.35E-12	UP
METTL13	1546.067	0.523745	0.072641	7.210066	5.59E-13	4.35E-12	NOT
TTC39B	531.6606	-1.17702	0.163272	-7.20898	5.64E-13	4.39E-12	DOWN
CRTC2	2095.203	0.530894	0.073657	7.207632	5.69E-13	4.43E-12	NOT
ESPL1	507.9969	1.629519	0.22612	7.206435	5.74E-13	4.47E-12	UP
KIF26B	238.3813	2.178638	0.302324	7.206293	5.75E-13	4.47E-12	UP
RP11-146I	2.785866	3.520538	0.488624	7.20501	5.80E-13	4.51E-12	UP
RP11-567I	107.4668	0.946637	0.131408	7.203777	5.86E-13	4.55E-12	UP
CHRNB2	8.727328	2.357061	0.327218	7.203339	5.88E-13	4.57E-12	UP
RP11-89B1	1.997541	-2.10917	0.292842	-7.2024	5.92E-13	4.60E-12	DOWN
ECEL1	41.03468	3.153581	0.437859	7.202283	5.92E-13	4.60E-12	UP
TTC9	891.653	1.831856	0.254368	7.201594	5.95E-13	4.62E-12	UP
RP11-492I	16.00397	2.930455	0.406973	7.20062	5.99E-13	4.65E-12	UP
UTP14A	715.88	0.59542	0.082704	7.199453	6.05E-13	4.69E-12	UP
TBX2	553.9978	1.021118	0.141842	7.198975	6.07E-13	4.71E-12	UP
CNR1	36.47145	2.500381	0.347347	7.198502	6.09E-13	4.72E-12	UP
ATF6B	3254.245	0.550655	0.076504	7.197752	6.12E-13	4.75E-12	NOT
GLOD5	101.4596	-1.66357	0.231172	-7.19626	6.19E-13	4.80E-12	DOWN
GLUD1	27737.99	-1.19982	0.166761	-7.19485	6.25E-13	4.85E-12	DOWN
CTC-471J1	29.8607	1.312174	0.182392	7.194236	6.28E-13	4.87E-12	UP
AXL	763.4451	-1.23831	0.172184	-7.1918	6.39E-13	4.95E-12	DOWN
TBX2-AS1	40.1287	1.725211	0.239887	7.19176	6.40E-13	4.95E-12	UP
NAB1	1587.543	-0.59069	0.082155	-7.18999	6.48E-13	5.02E-12	DOWN
GDF2	278.1312	-4.53936	0.631408	-7.18927	6.51E-13	5.04E-12	DOWN
ZNF783	209.6902	1.042948	0.145109	7.187323	6.61E-13	5.11E-12	UP
CRK	3034.843	-0.60399	0.084038	-7.1871	6.62E-13	5.12E-12	DOWN
CLDN18	10.12791	2.86699	0.398959	7.18617	6.66E-13	5.15E-12	UP
FAM229A	90.82962	1.064675	0.148156	7.186151	6.66E-13	5.15E-12	UP
ADAM12	112.9961	2.018257	0.280866	7.18583	6.68E-13	5.16E-12	UP
FAM65C	247.1101	-1.91699	0.266778	-7.18572	6.69E-13	5.17E-12	DOWN
ASPA	270.587	-1.61253	0.224423	-7.18522	6.71E-13	5.18E-12	DOWN
STAT3	9295.086	-0.75516	0.105104	-7.1849	6.73E-13	5.19E-12	DOWN
ASNS	444.1512	1.902721	0.26488	7.183334	6.80E-13	5.25E-12	UP
HCG27	36.71154	1.130533	0.157396	7.182746	6.83E-13	5.27E-12	UP
CTA-390C	21.47296	1.450567	0.201996	7.181182	6.91E-13	5.33E-12	UP
CTD-2162	5.929951	2.743116	0.382006	7.18082	6.93E-13	5.34E-12	UP

LSM2	1147.759	0.810621	0.112899	7.180079	6.97E-13	5.37E-12	UP
RP11-715J	13.1936	1.568536	0.218462	7.179893	6.98E-13	5.38E-12	UP
DEF8	1101.239	0.582099	0.081074	7.179887	6.98E-13	5.38E-12	NOT
NCOA4	13114.3	-0.68546	0.095472	-7.17968	6.99E-13	5.38E-12	DOWN
DUOXA2	136.9655	3.740855	0.521065	7.179251	7.01E-13	5.40E-12	UP
LINC01389	14.49934	1.65166	0.230077	7.178727	7.04E-13	5.42E-12	UP
RPIA	436.9441	0.607919	0.084706	7.176829	7.13E-13	5.49E-12	UP
HHIP-AS1	23.22171	-2.1001	0.292662	-7.17587	7.18E-13	5.53E-12	DOWN
MAP1A	135.8601	1.613227	0.224827	7.175428	7.21E-13	5.55E-12	UP
SPATA25	39.49809	1.198946	0.167103	7.174891	7.24E-13	5.57E-12	UP
TET3	645.2513	0.799518	0.111497	7.170746	7.46E-13	5.74E-12	UP
LINC01269	4.880647	3.557011	0.496065	7.170447	7.48E-13	5.75E-12	UP
GAP43	9.492453	2.946495	0.410942	7.170101	7.49E-13	5.76E-12	UP
UAP1L1	476.3842	1.835159	0.255975	7.16929	7.54E-13	5.79E-12	UP
PDZD2	132.4083	1.373505	0.191599	7.168631	7.58E-13	5.82E-12	UP
RP11-4O3	1.69965	-3.40728	0.475315	-7.16846	7.58E-13	5.82E-12	DOWN
TNNT2	34.24312	2.612042	0.36448	7.166494	7.69E-13	5.91E-12	UP
LDHD	5222.486	-1.45676	0.203312	-7.16514	7.77E-13	5.96E-12	DOWN
AGBL4	21.16759	-2.35601	0.328822	-7.16501	7.78E-13	5.97E-12	DOWN
MT-ND5	196700.6	-1.29683	0.181009	-7.16444	7.81E-13	5.99E-12	DOWN
PFKP	1018.805	1.987177	0.277374	7.164248	7.82E-13	6.00E-12	UP
TMEM72	38.68808	2.807793	0.391922	7.164161	7.83E-13	6.00E-12	UP
ENTPD2	187.4232	1.716623	0.239616	7.164055	7.83E-13	6.00E-12	UP
CTBP1-AS	583.9567	0.622895	0.086948	7.163961	7.84E-13	6.01E-12	UP
CEP83	242.7235	0.590922	0.082488	7.163737	7.85E-13	6.01E-12	UP
PCTP	3161.373	-0.9113	0.127213	-7.16355	7.86E-13	6.02E-12	DOWN
ACOX1	13227.49	-0.99251	0.138566	-7.16271	7.91E-13	6.06E-12	DOWN
UPK1A-AS	10.51498	3.676769	0.513372	7.162	7.95E-13	6.09E-12	UP
PTPRG-AS	11.71116	2.034873	0.284151	7.161238	8.00E-13	6.12E-12	UP
ZC3H8	272.514	0.678934	0.094812	7.160856	8.02E-13	6.13E-12	UP
LINC01194	13.28732	6.066774	0.847343	7.159761	8.08E-13	6.18E-12	UP
CD248	549.3881	1.152686	0.160998	7.159617	8.09E-13	6.18E-12	UP
RP11-772(	10.49151	-2.20412	0.307856	-7.15959	8.09E-13	6.18E-12	DOWN
ARL2	946.9428	1.203709	0.168135	7.159185	8.12E-13	6.20E-12	UP
RP11-446J	2.932123	3.467689	0.484379	7.15904	8.12E-13	6.21E-12	UP
VCAN	1600.464	2.154026	0.300914	7.15827	8.17E-13	6.24E-12	UP
RPAP1	1133.702	0.497221	0.069476	7.156764	8.26E-13	6.31E-12	NOT
FGG	371466.1	-1.57263	0.219747	-7.15655	8.27E-13	6.31E-12	DOWN
SENP1	423.2004	0.56187	0.078511	7.156549	8.27E-13	6.31E-12	NOT
DCLK3	11.63378	1.743249	0.243588	7.156544	8.27E-13	6.31E-12	UP
CNTD1	29.1275	0.923816	0.129106	7.155511	8.34E-13	6.36E-12	UP
SEMA4F	204.1077	1.321641	0.184703	7.155491	8.34E-13	6.36E-12	UP
ADH1A	29672.23	-2.02133	0.282489	-7.15543	8.34E-13	6.36E-12	DOWN
GRAMD3	786.9443	-0.64463	0.090091	-7.15528	8.35E-13	6.36E-12	DOWN
MT-CO2	425431	-1.00898	0.141045	-7.15362	8.45E-13	6.44E-12	DOWN
PLEKHA8	338.6751	0.74764	0.104526	7.15269	8.51E-13	6.48E-12	UP
BOLA2B	18.10364	1.475255	0.206313	7.150567	8.64E-13	6.58E-12	UP
ANO10	1107.351	0.842574	0.117835	7.150475	8.65E-13	6.58E-12	UP
PRR16	32.08103	1.507432	0.21086	7.148968	8.74E-13	6.65E-12	UP
NME1	2321.226	1.055938	0.147705	7.148954	8.74E-13	6.65E-12	UP
RP11-142I	29.16912	1.324273	0.185261	7.148134	8.80E-13	6.69E-12	UP
AP001046.	28.13246	-1.37441	0.192278	-7.148	8.81E-13	6.69E-12	DOWN

RASGRF2-/	1.618345	2.630752	0.368075	7.14732	8.85E-13	6.73E-12	UP
LINC01372	11.05598	1.317514	0.184341	7.147173	8.86E-13	6.73E-12	UP
FGA	662079.7	-1.55575	0.217683	-7.14687	8.88E-13	6.74E-12	DOWN
LKAAEAR1	3.81397	4.074736	0.570225	7.145845	8.94E-13	6.79E-12	UP
CIB2	120.6563	1.728146	0.24187	7.144943	9.00E-13	6.84E-12	UP
LZTR1	1866.778	0.496033	0.069426	7.144787	9.01E-13	6.84E-12	NOT
RP11-7F18	4.878041	1.961215	0.274519	7.144198	9.05E-13	6.87E-12	UP
AC024937	6.221785	2.139437	0.299497	7.143445	9.10E-13	6.91E-12	UP
TK2	1782.844	-0.66411	0.092974	-7.14293	9.14E-13	6.93E-12	DOWN
BCORL1	643.9328	0.945084	0.132346	7.141028	9.26E-13	7.02E-12	UP
TMEM165	1220.647	0.791462	0.110851	7.139853	9.34E-13	7.08E-12	UP
CTXN1	28.35666	2.029338	0.284232	7.139725	9.35E-13	7.09E-12	UP
ANKRD34/	25.8191	1.074261	0.150478	7.139004	9.40E-13	7.12E-12	UP
RBCK1	4793.403	0.757056	0.106049	7.13872	9.42E-13	7.13E-12	UP
NFYA	977.7518	0.702545	0.098413	7.138705	9.42E-13	7.13E-12	UP
RP11-458I	46.4668	1.49263	0.20909	7.138695	9.42E-13	7.13E-12	UP
HOXC-AS2	5.109052	4.149171	0.581243	7.138449	9.44E-13	7.14E-12	UP
TAGLN2	16693.7	0.876788	0.122827	7.138401	9.44E-13	7.14E-12	UP
MAP1B	756.8002	1.446138	0.202591	7.138217	9.45E-13	7.15E-12	UP
KAZN	174.5968	-1.5305	0.214441	-7.13718	9.53E-13	7.20E-12	DOWN
IBA57-AS1	3.636017	2.178034	0.305176	7.136983	9.54E-13	7.21E-12	UP
RP11-229I	6.973713	1.697093	0.237822	7.135971	9.61E-13	7.26E-12	UP
FRMD3	202.4462	1.787966	0.250568	7.135658	9.63E-13	7.28E-12	UP
MT1A	681.2652	-2.23787	0.31363	-7.13539	9.65E-13	7.29E-12	DOWN
ITGB3BP	314.5676	0.792756	0.111104	7.135279	9.66E-13	7.29E-12	UP
CCDC183	63.57567	1.529103	0.214308	7.135067	9.67E-13	7.30E-12	UP
TRIL	105.5454	0.998251	0.139927	7.134081	9.74E-13	7.35E-12	UP
RP11-158I	25.34903	1.336492	0.187344	7.133899	9.76E-13	7.36E-12	UP
CR1	79.43601	-1.9666	0.275672	-7.13386	9.76E-13	7.36E-12	DOWN
HOXA-AS3	3.229382	3.657101	0.512656	7.13364	9.77E-13	7.37E-12	UP
CORO1B	3483.661	0.592091	0.083003	7.133326	9.80E-13	7.39E-12	UP
CCDC114	14.45754	1.728884	0.242409	7.132091	9.89E-13	7.45E-12	UP
RP11-80P2	8.924589	1.683317	0.236022	7.132021	9.89E-13	7.45E-12	UP
SGPP1	1143.98	-0.75679	0.10614	-7.13011	1.00E-12	7.55E-12	DOWN
WIZ	1852.201	0.574407	0.080568	7.129483	1.01E-12	7.59E-12	NOT
GXYLT2	44.11201	2.245427	0.314962	7.129206	1.01E-12	7.60E-12	UP
DRC1	7.080614	2.373327	0.332976	7.127625	1.02E-12	7.69E-12	UP
NPTX1	10.42132	3.130446	0.439218	7.127313	1.02E-12	7.70E-12	UP
SMARCD3	387.2318	1.569274	0.220211	7.126228	1.03E-12	7.76E-12	UP
U91328.22	4.647583	2.014077	0.282638	7.125985	1.03E-12	7.77E-12	UP
XPOT	3143.565	0.712598	0.100002	7.125837	1.03E-12	7.78E-12	UP
DYRK2	783.3063	0.830425	0.116537	7.125824	1.03E-12	7.78E-12	UP
BAALC-AS	25.25785	1.698701	0.238398	7.125478	1.04E-12	7.79E-12	UP
KCNMB3	58.10945	1.38709	0.194698	7.124333	1.05E-12	7.86E-12	UP
IGFBP4	52263.95	-1.11051	0.155887	-7.1238	1.05E-12	7.88E-12	DOWN
RP3-375P9	13.75327	1.764085	0.247664	7.122901	1.06E-12	7.93E-12	UP
PRORSD1F	41.43269	0.932662	0.130985	7.120349	1.08E-12	8.08E-12	UP
RP11-119I	44.22958	1.306042	0.183435	7.1199	1.08E-12	8.10E-12	UP
RWDD2B	855.2275	-0.6317	0.088728	-7.1195	1.08E-12	8.13E-12	DOWN
COMMD2	885.6876	0.503843	0.070772	7.119254	1.09E-12	8.14E-12	NOT
ZBED1	3738.987	-0.60287	0.084696	-7.11807	1.09E-12	8.21E-12	DOWN
CNOT6	732.3761	0.644473	0.090541	7.118029	1.09E-12	8.21E-12	UP

HSPA14	646.947	0.577063	0.081071	7.118014	1.09E-12	8.21E-12	NOT
EVPL	168.0087	2.848919	0.400255	7.11776	1.10E-12	8.22E-12	UP
LINC01436	40.82398	3.078037	0.432497	7.116907	1.10E-12	8.27E-12	UP
BRINP3	8.839861	5.125058	0.720259	7.115579	1.11E-12	8.34E-12	UP
CTD-3138	129.2243	0.955992	0.134363	7.115002	1.12E-12	8.38E-12	UP
BEX2	265.3963	2.45621	0.345239	7.114521	1.12E-12	8.40E-12	UP
IL33	674.134	-1.55612	0.218759	-7.11339	1.13E-12	8.47E-12	DOWN
TRAF7	2825.27	0.565219	0.079459	7.113329	1.13E-12	8.47E-12	NOT
LCMT1	619.5181	0.657553	0.092448	7.112673	1.14E-12	8.51E-12	UP
ADH4	59696.75	-2.49355	0.350653	-7.11116	1.15E-12	8.60E-12	DOWN
RP11-206I	15.13001	1.307984	0.183959	7.11019	1.16E-12	8.66E-12	UP
SCAMP5	823.0373	1.557183	0.219034	7.109333	1.17E-12	8.71E-12	UP
NIN	1040.497	0.890965	0.125349	7.107865	1.18E-12	8.80E-12	UP
MIER2	485.0745	0.826532	0.116284	7.107845	1.18E-12	8.80E-12	UP
RP11-135/	10.93156	1.862456	0.262032	7.107731	1.18E-12	8.81E-12	UP
RP11-295I	225.3549	0.83414	0.117363	7.107373	1.18E-12	8.83E-12	UP
RP11-111I	8.06737	1.887588	0.26567	7.105002	1.20E-12	8.98E-12	UP
CTD-2035	12.64773	1.646386	0.231738	7.1045	1.21E-12	9.01E-12	UP
RPS6KA4	1041.04	0.551874	0.077691	7.103407	1.22E-12	9.08E-12	NOT
RP11-685I	9.605978	-3.83476	0.539916	-7.10252	1.23E-12	9.13E-12	DOWN
FGF17	10.69389	2.407035	0.338943	7.101587	1.23E-12	9.19E-12	UP
LINC01101	5.113999	3.795137	0.534419	7.101423	1.23E-12	9.20E-12	UP
RP11-337(	61.87515	1.174501	0.165409	7.100588	1.24E-12	9.25E-12	UP
THBS3	641.8638	0.845636	0.119095	7.100547	1.24E-12	9.25E-12	UP
PROK2	6.319725	-2.52036	0.354966	-7.10027	1.25E-12	9.27E-12	DOWN
CYP4A22-	4.524825	2.020141	0.284562	7.099111	1.26E-12	9.35E-12	UP
DRD4	44.81013	1.692661	0.238444	7.098771	1.26E-12	9.37E-12	UP
DDX41	2769.962	0.570524	0.080377	7.098106	1.26E-12	9.41E-12	NOT
TECPR1	1175.436	0.730085	0.102863	7.097618	1.27E-12	9.44E-12	UP
RP11-173I	129.7867	-1.26407	0.178126	-7.09651	1.28E-12	9.51E-12	DOWN
CDPF1	362.5903	0.757788	0.106784	7.096449	1.28E-12	9.51E-12	UP
MCTP1	317.5825	1.642335	0.231506	7.094124	1.30E-12	9.67E-12	UP
ZFP69B	38.99409	0.975256	0.137479	7.093834	1.30E-12	9.69E-12	UP
PTOV1-AS	210.9469	0.707767	0.099773	7.093802	1.30E-12	9.69E-12	UP
RP11-809(	21.97995	2.210096	0.311562	7.093592	1.31E-12	9.70E-12	UP
RARRES2P	11.99756	7.27365	1.025386	7.093569	1.31E-12	9.70E-12	UP
SHMT1	15874.49	-1.36472	0.192392	-7.09345	1.31E-12	9.71E-12	DOWN
RP11-245I	12.00368	1.576832	0.222322	7.092571	1.32E-12	9.77E-12	UP
SLC41A1	664.0302	1.207595	0.170291	7.091371	1.33E-12	9.85E-12	UP
NELFA	931.3753	0.520748	0.073441	7.09073	1.33E-12	9.89E-12	NOT
PHF21A	776.0795	0.665976	0.093925	7.090534	1.34E-12	9.90E-12	UP
SSC4D	304.0406	1.766821	0.249185	7.09041	1.34E-12	9.91E-12	UP
ST6GALNA	15.24452	3.03517	0.428078	7.090233	1.34E-12	9.92E-12	UP
RP11-430(	29.88108	1.616915	0.228067	7.089647	1.34E-12	9.96E-12	UP
CTB-43E1	6.441097	-2.6327	0.371385	-7.08887	1.35E-12	1.00E-11	DOWN
RP11-127	58.90634	1.263809	0.178327	7.087042	1.37E-12	1.01E-11	UP
CTB-58E1	14.31564	1.423435	0.200858	7.086757	1.37E-12	1.02E-11	UP
LINC0154	35.75794	2.765919	0.390322	7.086246	1.38E-12	1.02E-11	UP
CCNI	5922.345	-0.6086	0.085887	-7.08603	1.38E-12	1.02E-11	DOWN
CEP85	421.7947	0.929943	0.131247	7.085427	1.39E-12	1.03E-11	UP
EIF2B1	1805.744	0.387783	0.054736	7.084571	1.39E-12	1.03E-11	NOT
C19orf25	1064.422	0.826642	0.116687	7.084246	1.40E-12	1.03E-11	UP

ACSM5	6770.59	-1.9432	0.27433	-7.08345	1.41E-12	1.04E-11	DOWN
ZNF467	521.7843	1.336265	0.188648	7.083379	1.41E-12	1.04E-11	UP
LUC7L3	2541.064	0.574994	0.081186	7.082402	1.42E-12	1.05E-11	NOT
RP5-1112I	57.87075	0.99967	0.141152	7.082249	1.42E-12	1.05E-11	UP
CTD-2171	8.723732	3.100488	0.437824	7.081593	1.43E-12	1.05E-11	UP
AL133493.	23.35676	2.983121	0.421277	7.081131	1.43E-12	1.06E-11	UP
AP001432.	4.367292	2.082946	0.294175	7.080628	1.44E-12	1.06E-11	UP
FAM178A	882.9251	0.706652	0.09981	7.079942	1.44E-12	1.06E-11	UP
LINC00921	38.48454	0.92561	0.130786	7.077292	1.47E-12	1.08E-11	UP
ARHGAP1(	415.5735	-1.33856	0.189146	-7.07684	1.47E-12	1.09E-11	DOWN
NCDN	719.6328	0.842303	0.119027	7.076584	1.48E-12	1.09E-11	UP
RP11-126	19.79177	2.671467	0.377531	7.076157	1.48E-12	1.09E-11	UP
LYG2	5.919605	2.543249	0.359473	7.07493	1.50E-12	1.10E-11	UP
PCGF1	581.7676	0.527314	0.074541	7.074133	1.50E-12	1.11E-11	NOT
SORT1	2188.176	1.256722	0.177652	7.074081	1.50E-12	1.11E-11	UP
CAPN8	47.52561	2.066353	0.29215	7.07291	1.52E-12	1.12E-11	UP
FIGNL2	30.00105	1.784423	0.25231	7.072347	1.52E-12	1.12E-11	UP
EFHC1	212.4039	0.839923	0.118776	7.071497	1.53E-12	1.13E-11	UP
HBA1	10.84016	-2.20012	0.311184	-7.07017	1.55E-12	1.14E-11	DOWN
MCCC1-A	9.050009	1.631341	0.230737	7.070149	1.55E-12	1.14E-11	UP
BLVRA	804.0063	1.187449	0.167954	7.070086	1.55E-12	1.14E-11	UP
PRR18	225.6433	-1.69845	0.240239	-7.06983	1.55E-12	1.14E-11	DOWN
TRIM39	493.1897	0.543826	0.076924	7.069688	1.55E-12	1.14E-11	NOT
GOLGA8A	246.8223	1.661654	0.235101	7.06782	1.57E-12	1.16E-11	UP
RP11-324I	44.97987	1.095143	0.154955	7.067491	1.58E-12	1.16E-11	UP
PDZD11	1189.597	0.62174	0.087976	7.067119	1.58E-12	1.16E-11	UP
AC069277	6.362546	4.647643	0.657643	7.067118	1.58E-12	1.16E-11	UP
FBXL16	118.391	2.312151	0.327237	7.065675	1.60E-12	1.17E-11	UP
DAGLA	214.8203	1.610738	0.228002	7.064576	1.61E-12	1.18E-11	UP
TERF2IP	2032.619	-0.54796	0.077571	-7.06398	1.62E-12	1.19E-11	NOT
LINC01424	3.238646	2.274187	0.321979	7.063157	1.63E-12	1.19E-11	UP
DLGAP4	2633.827	0.655811	0.092869	7.061645	1.65E-12	1.21E-11	UP
ZNF280C	104.0024	0.773055	0.109494	7.060235	1.66E-12	1.22E-11	UP
KIAA1429	2146.901	0.55939	0.079232	7.060172	1.66E-12	1.22E-11	NOT
COX18	887.2483	-0.66687	0.094456	-7.06011	1.66E-12	1.22E-11	DOWN
NFKBIA	5897.2	-0.81517	0.11548	-7.05897	1.68E-12	1.23E-11	DOWN
MGAT3	56.88296	1.725935	0.244538	7.05794	1.69E-12	1.24E-11	UP
FABP6	10.92707	4.011144	0.568326	7.057819	1.69E-12	1.24E-11	UP
RP11-31F1	2.743726	-2.16209	0.306345	-7.05769	1.69E-12	1.24E-11	DOWN
CTTN	5868.733	0.612064	0.086724	7.057589	1.69E-12	1.24E-11	UP
IFT46	436.1144	-0.84047	0.119112	-7.05611	1.71E-12	1.25E-11	DOWN
KLF3	3055.556	-0.58708	0.083205	-7.05575	1.72E-12	1.25E-11	DOWN
SULT1A1	5003.317	-1.31882	0.186992	-7.05283	1.75E-12	1.28E-11	DOWN
TPCN1	2415.511	0.778573	0.110402	7.05216	1.76E-12	1.29E-11	UP
PCK1	66493.82	-2.29702	0.325759	-7.05129	1.77E-12	1.29E-11	DOWN
PLEKHG5	203.7328	0.962469	0.136505	7.050815	1.78E-12	1.30E-11	UP
RP4-710M	356.7872	-1.73223	0.24571	-7.04989	1.79E-12	1.31E-11	DOWN
ATP6V1B1	32.57137	2.469187	0.350253	7.049724	1.79E-12	1.31E-11	UP
ZNF316	1097.374	0.52461	0.074425	7.04889	1.80E-12	1.32E-11	NOT
FAM71F2	34.20567	1.514359	0.214839	7.048808	1.80E-12	1.32E-11	UP
PUS10	542.6107	-0.70624	0.100202	-7.04816	1.81E-12	1.32E-11	DOWN
RP11-87H	53.35375	0.970352	0.137714	7.046148	1.84E-12	1.34E-11	UP

RP11-150(	32.75705	3.031579	0.430381	7.043938	1.87E-12	1.36E-11	UP
FGF23	14.62374	-4.05808	0.576166	-7.04325	1.88E-12	1.37E-11	DOWN
LINC0086	20.1628	1.762273	0.250239	7.042356	1.89E-12	1.38E-11	UP
AKT1	4401.936	-0.55793	0.079229	-7.04199	1.90E-12	1.38E-11	NOT
ICAM5	22.55593	2.67594	0.380037	7.041256	1.91E-12	1.39E-11	UP
STOM	16491.82	-0.95063	0.135012	-7.04113	1.91E-12	1.39E-11	DOWN
LL22NC03	26.91552	1.285159	0.182522	7.04112	1.91E-12	1.39E-11	UP
GTF3C3	783.4122	0.474079	0.067335	7.040653	1.91E-12	1.39E-11	NOT
GOSR2	780.2508	0.502475	0.07137	7.040383	1.92E-12	1.39E-11	NOT
FAM46C	626.0227	-1.32953	0.188846	-7.04027	1.92E-12	1.40E-11	DOWN
RP5-858B(	4.005347	3.255662	0.462523	7.03892	1.94E-12	1.41E-11	UP
CLN5	532.5679	-0.63404	0.09008	-7.03863	1.94E-12	1.41E-11	DOWN
STRN3	1141.873	-0.62108	0.088242	-7.03843	1.94E-12	1.41E-11	DOWN
TAF11	1116.11	0.526266	0.074802	7.035457	1.99E-12	1.44E-11	NOT
AC025335	55.89906	1.211745	0.172252	7.034724	2.00E-12	1.45E-11	UP
TDO2	14643.7	-1.99908	0.284181	-7.03452	2.00E-12	1.45E-11	DOWN
C3	594454.3	-1.13381	0.16118	-7.03441	2.00E-12	1.45E-11	DOWN
SIGLEC1	695.04	-1.35372	0.192451	-7.03412	2.01E-12	1.45E-11	DOWN
C15orf48	271.7905	2.314772	0.329102	7.033596	2.01E-12	1.46E-11	UP
NFATC4	300.1841	1.465795	0.208424	7.032771	2.02E-12	1.47E-11	UP
RP11-666/	10.19726	1.264418	0.1798	7.032357	2.03E-12	1.47E-11	UP
DCC	15.27142	3.1283	0.444873	7.0319	2.04E-12	1.48E-11	UP
TMEM57	2735.516	-0.63824	0.090766	-7.03173	2.04E-12	1.48E-11	DOWN
RP11-701I	7.350856	-1.77765	0.252838	-7.03078	2.05E-12	1.49E-11	DOWN
INTS3	2026.621	0.828491	0.117855	7.029776	2.07E-12	1.50E-11	UP
KCNIP3	183.1556	1.906974	0.271278	7.029579	2.07E-12	1.50E-11	UP
CTD-2015	7.469735	3.453228	0.491277	7.02908	2.08E-12	1.50E-11	UP
GBP2	3340.073	1.24576	0.177235	7.028843	2.08E-12	1.51E-11	UP
CEP95	713.419	0.617687	0.087886	7.028247	2.09E-12	1.51E-11	UP
PLCB3	812.6192	0.628031	0.089367	7.027553	2.10E-12	1.52E-11	UP
SOX2	15.8313	3.723404	0.529863	7.027112	2.11E-12	1.52E-11	UP
GPR146	105.9731	-1.18488	0.168631	-7.02645	2.12E-12	1.53E-11	DOWN
TUG1	3306.745	0.516617	0.073528	7.026167	2.12E-12	1.53E-11	NOT
TSPEAR-A	70.99632	2.019634	0.287495	7.024937	2.14E-12	1.55E-11	UP
ADM	1203.259	-1.36784	0.194717	-7.02478	2.14E-12	1.55E-11	DOWN
HOXC4	20.17859	2.368298	0.337145	7.024563	2.15E-12	1.55E-11	UP
MYO9B	2712.278	0.561657	0.07996	7.024197	2.15E-12	1.55E-11	NOT
RP11-369I	1.750478	-2.89607	0.412383	-7.02278	2.18E-12	1.57E-11	DOWN
ZNF43	181.0336	1.528425	0.217663	7.021979	2.19E-12	1.58E-11	UP
NFAM1	281.3933	-1.29225	0.184054	-7.02104	2.20E-12	1.59E-11	DOWN
KCNJ14	44.87605	1.19516	0.170242	7.020358	2.21E-12	1.60E-11	UP
CTD-3037	4.091904	4.128892	0.588161	7.019998	2.22E-12	1.60E-11	UP
BCL2L13	2510.596	-0.46722	0.066564	-7.01904	2.23E-12	1.61E-11	NOT
SPRTN	337.9089	0.476885	0.067942	7.019027	2.23E-12	1.61E-11	NOT
SLC8A1	200.7366	-1.21385	0.172947	-7.01863	2.24E-12	1.61E-11	DOWN
RP11-122I	2.540015	2.991334	0.426275	7.017382	2.26E-12	1.63E-11	UP
ATP8A2	15.06876	2.126243	0.303112	7.014715	2.30E-12	1.66E-11	UP
MAG	11.10755	-2.77562	0.395688	-7.01465	2.31E-12	1.66E-11	DOWN
MFSD5	1111.878	0.688197	0.098113	7.014365	2.31E-12	1.66E-11	UP
CNDP1	1349.034	-2.86187	0.408001	-7.01436	2.31E-12	1.66E-11	DOWN
AC012314	29.67785	1.143297	0.163	7.014074	2.31E-12	1.67E-11	UP
COL1A2	12490.45	1.76491	0.251629	7.013931	2.32E-12	1.67E-11	UP

BUB3	2417.204	0.470784	0.067123	7.013781	2.32E-12	1.67E-11	NOT
AC010524	61.21426	2.306208	0.328906	7.011746	2.35E-12	1.69E-11	UP
PUF60	8300.815	0.821471	0.117165	7.011236	2.36E-12	1.70E-11	UP
PSAT1	5211.148	-1.36593	0.194829	-7.01092	2.37E-12	1.70E-11	DOWN
KLHL38	5.712169	2.852626	0.406895	7.010723	2.37E-12	1.70E-11	UP
CHRM2	26.55005	-3.34982	0.477824	-7.01058	2.37E-12	1.70E-11	DOWN
CTD-2196	12.12374	1.256721	0.179282	7.009729	2.39E-12	1.71E-11	UP
UVSSA	266.8548	0.847393	0.120898	7.00917	2.40E-12	1.72E-11	UP
KIAA0040	1150.232	-1.09307	0.155952	-7.00899	2.40E-12	1.72E-11	DOWN
RP11-434I	31.40819	2.628466	0.375019	7.008896	2.40E-12	1.72E-11	UP
DZIP3	444.8662	0.723486	0.103227	7.008686	2.41E-12	1.72E-11	UP
RP11-278I	14.74359	1.765087	0.251844	7.008666	2.41E-12	1.72E-11	UP
SMPD1	3898.8	-0.72807	0.103889	-7.00813	2.42E-12	1.73E-11	DOWN
RP11-844I	62.37787	2.142309	0.30576	7.006494	2.44E-12	1.75E-11	UP
PLG	90824.76	-1.7485	0.249554	-7.00649	2.44E-12	1.75E-11	DOWN
MC1R	77.90891	1.188863	0.169683	7.006361	2.45E-12	1.75E-11	UP
ST7	1666.964	-0.61358	0.087575	-7.00629	2.45E-12	1.75E-11	DOWN
RP11-631I	53.72553	1.146606	0.16366	7.006031	2.45E-12	1.76E-11	UP
RP11-715J	3.720151	2.406589	0.343523	7.005613	2.46E-12	1.76E-11	UP
PTPRG	1576.26	0.860068	0.122793	7.004215	2.48E-12	1.78E-11	UP
RP11-179I	10.0749	1.384008	0.197619	7.003408	2.50E-12	1.79E-11	UP
MMP24	179.4823	1.83333	0.261805	7.002656	2.51E-12	1.80E-11	UP
TMEM133	252.962	-0.78923	0.112713	-7.00215	2.52E-12	1.80E-11	DOWN
CTD-2555	5.885964	1.519614	0.217026	7.002002	2.52E-12	1.80E-11	UP
HOXB7	72.52687	1.90962	0.272734	7.001758	2.53E-12	1.81E-11	UP
RP11-66N	25.13506	1.234312	0.176307	7.000926	2.54E-12	1.82E-11	UP
SLC22A1	20333.63	-2.54212	0.363163	-6.99994	2.56E-12	1.83E-11	DOWN
GLI1	57.79764	1.680908	0.240148	6.999482	2.57E-12	1.83E-11	UP
RP11-693I	81.09589	1.155757	0.165142	6.998569	2.59E-12	1.85E-11	UP
PARD6G	244.122	0.77149	0.110236	6.998545	2.59E-12	1.85E-11	UP
IQCB1	372.7581	0.663959	0.094874	6.998351	2.59E-12	1.85E-11	UP
PRKCDBP	419.4908	1.505878	0.215192	6.997839	2.60E-12	1.85E-11	UP
CLIP2	877.8574	1.39497	0.199349	6.997618	2.60E-12	1.86E-11	UP
C1orf159	316.3512	0.670698	0.095854	6.997093	2.61E-12	1.86E-11	UP
PRDM12	7.523475	1.906498	0.272519	6.995824	2.64E-12	1.88E-11	UP
RPGRIP1L	107.6061	0.849279	0.121402	6.99559	2.64E-12	1.88E-11	UP
RP4-758J1	64.94509	0.906703	0.129614	6.99541	2.64E-12	1.88E-11	UP
C19orf45	9.557394	2.044908	0.292331	6.995171	2.65E-12	1.89E-11	UP
MUC15	36.13385	4.680739	0.669161	6.994934	2.65E-12	1.89E-11	UP
MYPOP	317.1978	0.716316	0.10243	6.993232	2.69E-12	1.91E-11	UP
AKR1C7P	13.32979	2.013862	0.287975	6.993181	2.69E-12	1.91E-11	UP
RP11-252I	29.06785	-2.39127	0.341963	-6.99276	2.70E-12	1.92E-11	DOWN
FBXO8	1135.331	-0.81078	0.115963	-6.99175	2.71E-12	1.93E-11	DOWN
ITCH	3022.045	-0.66991	0.095828	-6.99075	2.73E-12	1.94E-11	DOWN
RP11-304I	23.33863	1.152564	0.164892	6.989789	2.75E-12	1.96E-11	UP
COL9A2	174.6601	1.850206	0.264753	6.98841	2.78E-12	1.98E-11	UP
NUP43	905.9067	0.598053	0.085591	6.987354	2.80E-12	1.99E-11	UP
RP11-893I	5.270943	1.729454	0.247521	6.987106	2.81E-12	1.99E-11	UP
PRX	201.0894	0.985288	0.141019	6.986912	2.81E-12	2.00E-11	UP
TPTEP1	16.96408	-1.69533	0.242672	-6.98612	2.83E-12	2.01E-11	DOWN
HES1	2241.319	-0.9978	0.142872	-6.98385	2.87E-12	2.04E-11	DOWN
TFAP2E	17.00145	1.693249	0.242489	6.982789	2.89E-12	2.05E-11	UP

RP11-972I	3.584851	1.878188	0.268983	6.982548	2.90E-12	2.06E-11	UP
AC008746	12.55023	1.401008	0.200713	6.980143	2.95E-12	2.09E-11	UP
PITPNM3	132.8154	-2.09959	0.300803	-6.97996	2.95E-12	2.09E-11	DOWN
USP12	1398.963	-0.75451	0.108101	-6.97966	2.96E-12	2.10E-11	DOWN
RP5-943J3	12.4374	1.462788	0.209582	6.979559	2.96E-12	2.10E-11	UP
DEFA3	2.885208	-2.9564	0.423672	-6.97805	2.99E-12	2.12E-11	DOWN
CCL4	290.7748	-1.49708	0.214558	-6.9775	3.00E-12	2.13E-11	DOWN
EIF4HP2	9.348055	1.662352	0.238253	6.977269	3.01E-12	2.13E-11	UP
CDX2	5.517143	3.699641	0.530275	6.97683	3.02E-12	2.14E-11	UP
RP1-278C	5.216672	1.937816	0.277774	6.976234	3.03E-12	2.15E-11	UP
SRPR	10172.77	-0.57563	0.082517	-6.97586	3.04E-12	2.15E-11	NOT
SH3GL1P2	3.602025	1.982846	0.284257	6.975541	3.05E-12	2.16E-11	UP
MAF	2414.309	-0.79433	0.113887	-6.9747	3.07E-12	2.17E-11	DOWN
CDH11	366.4572	1.81137	0.259721	6.97428	3.07E-12	2.17E-11	UP
CTD-2013	31.31871	1.181182	0.169392	6.973073	3.10E-12	2.19E-11	UP
RUVBL1	1290.221	0.616455	0.088415	6.972273	3.12E-12	2.20E-11	UP
C1QTNF1-	22.79128	2.690092	0.385903	6.970897	3.15E-12	2.22E-11	UP
TNFRSF18	55.03497	1.624165	0.233034	6.969635	3.18E-12	2.24E-11	UP
ATHL1	2355.381	1.617727	0.232113	6.969574	3.18E-12	2.24E-11	UP
PCNXL2	180.2938	1.623446	0.232983	6.968082	3.21E-12	2.27E-11	UP
ZNF320	319.8688	1.57709	0.226448	6.964461	3.30E-12	2.33E-11	UP
AL023806.	4.519662	1.867852	0.268248	6.963163	3.33E-12	2.35E-11	UP
ZNF826P	87.58746	2.130184	0.305942	6.9627	3.34E-12	2.35E-11	UP
C12orf49	973.2946	0.960402	0.137943	6.962302	3.35E-12	2.36E-11	UP
TPGS2	1375.511	0.803133	0.115362	6.961874	3.36E-12	2.37E-11	UP
MRAS	694.0023	1.180036	0.169505	6.96165	3.36E-12	2.37E-11	UP
HERC6	406.9213	-1.29045	0.185387	-6.96088	3.38E-12	2.38E-11	DOWN
UQCC2	1715.669	0.896668	0.128819	6.96068	3.39E-12	2.39E-11	UP
FAM21C	1031.062	0.513673	0.073812	6.959217	3.42E-12	2.41E-11	NOT
ORAOV1	417.9138	1.012927	0.145581	6.957824	3.46E-12	2.43E-11	UP
MOB3C	518.2715	-0.57431	0.082549	-6.95723	3.47E-12	2.44E-11	NOT
PPP1CC	3701.556	0.555611	0.079869	6.956499	3.49E-12	2.46E-11	NOT
NRSN2	664.2173	1.665039	0.239356	6.956339	3.49E-12	2.46E-11	UP
PPAPDC2	1073.568	-0.7981	0.114731	-6.95633	3.49E-12	2.46E-11	DOWN
CTD-2527	38.18551	2.027759	0.291537	6.95542	3.52E-12	2.47E-11	UP
GPR1	8.001822	2.821157	0.405633	6.954953	3.53E-12	2.48E-11	UP
RP11-286I	9.431853	-1.55494	0.22358	-6.95473	3.53E-12	2.48E-11	DOWN
STOX1	55.25687	1.892271	0.272091	6.954564	3.54E-12	2.49E-11	UP
RNF213	7953.977	0.871906	0.125399	6.953046	3.57E-12	2.51E-11	UP
CYB5A	23210.41	-1.10757	0.159306	-6.95246	3.59E-12	2.52E-11	DOWN
RP11-680/	18.1734	1.182644	0.170131	6.951379	3.62E-12	2.54E-11	UP
CTD-2265	6.003261	1.729369	0.2488	6.950848	3.63E-12	2.55E-11	UP
SRCAP	3120.023	0.541939	0.077968	6.950787	3.63E-12	2.55E-11	NOT
CKLF	246.4639	0.912385	0.131297	6.94899	3.68E-12	2.58E-11	UP
AC108463	4.132374	1.834234	0.263985	6.948242	3.70E-12	2.59E-11	UP
RP13-415(	1.968864	2.691796	0.387412	6.948152	3.70E-12	2.60E-11	UP
NISCH	2394.884	0.474488	0.068293	6.947868	3.71E-12	2.60E-11	NOT
PCK2	24940.81	-1.39552	0.200863	-6.9476	3.72E-12	2.60E-11	DOWN
DEPDC7	1449.113	-1.34975	0.194287	-6.94724	3.73E-12	2.61E-11	DOWN
UHRF1BP1	833.8844	0.748875	0.1078	6.946872	3.73E-12	2.62E-11	UP
LMNA	16357.54	0.755982	0.108834	6.946216	3.75E-12	2.63E-11	UP
CTD-3234	3.875726	2.220029	0.319638	6.945449	3.77E-12	2.64E-11	UP

PGBD1	203.3688	1.069356	0.154022	6.942866	3.84E-12	2.69E-11	UP
HMOX1	4412.844	-1.38242	0.199131	-6.94226	3.86E-12	2.70E-11	DOWN
NLRC4	73.19475	-0.79671	0.114767	-6.94196	3.87E-12	2.71E-11	DOWN
ACTL6A	1020.57	0.624473	0.089958	6.941825	3.87E-12	2.71E-11	UP
KRBA1	192.7576	1.210945	0.174448	6.941595	3.88E-12	2.71E-11	UP
RBMS3-AS	3.085372	-1.76444	0.254185	-6.94154	3.88E-12	2.71E-11	DOWN
ALX3	8.714971	5.399331	0.778111	6.939027	3.95E-12	2.76E-11	UP
RP11-500(	3.824161	1.844638	0.26586	6.938393	3.97E-12	2.77E-11	UP
ZNF841	228.4826	0.990045	0.1427	6.93795	3.98E-12	2.78E-11	UP
HSPD1P6	5.254298	1.601123	0.230779	6.937915	3.98E-12	2.78E-11	UP
CTD-2267	27.23935	1.173325	0.169142	6.936904	4.01E-12	2.80E-11	UP
IPO13	1495.459	0.545966	0.078707	6.936652	4.02E-12	2.80E-11	NOT
ADIPOR2	6391.959	-0.72121	0.103992	-6.93518	4.06E-12	2.83E-11	DOWN
CTB-193M	104.7993	1.072428	0.154645	6.934764	4.07E-12	2.84E-11	UP
SLC25A20	3721.535	-0.95342	0.137493	-6.93431	4.08E-12	2.85E-11	DOWN
DUSP15	55.05637	2.274858	0.328117	6.933073	4.12E-12	2.87E-11	UP
C11orf1	498.3888	-0.90816	0.130996	-6.93274	4.13E-12	2.88E-11	DOWN
NDST1	5422.463	0.818688	0.118112	6.931456	4.17E-12	2.90E-11	UP
ELAVL2	3.602312	2.405672	0.347087	6.93104	4.18E-12	2.91E-11	UP
TRIM37	729.5824	0.586711	0.084653	6.930774	4.19E-12	2.92E-11	UP
RNASEH1	512.8163	0.53246	0.076836	6.929865	4.21E-12	2.93E-11	NOT
ZNF891	107.8558	0.814269	0.117525	6.9285	4.25E-12	2.96E-11	UP
RPS18P9	51.30937	-0.67661	0.097657	-6.92838	4.26E-12	2.96E-11	DOWN
RP11-191I	4.815178	4.541278	0.655542	6.927516	4.28E-12	2.98E-11	UP
RABL6	1443.072	0.891236	0.128657	6.927236	4.29E-12	2.99E-11	UP
SLC25A47	13073.19	-2.55923	0.369455	-6.92704	4.30E-12	2.99E-11	DOWN
KIAA1549	264.1939	1.79574	0.259238	6.926989	4.30E-12	2.99E-11	UP
RP11-109/	4.202908	-2.09699	0.302733	-6.92686	4.30E-12	2.99E-11	DOWN
AC133644	17.72174	1.601742	0.231243	6.926656	4.31E-12	3.00E-11	UP
RP3-337H	12.82622	1.072301	0.154837	6.925349	4.35E-12	3.02E-11	UP
C14orf169	405.5545	0.510292	0.073687	6.925137	4.36E-12	3.03E-11	NOT
ZFC3H1	705.9784	0.688513	0.099434	6.924309	4.38E-12	3.04E-11	UP
GTPBP3	631.3645	0.592323	0.085553	6.923465	4.41E-12	3.06E-11	UP
NAT14	327.9081	1.418429	0.20488	6.92323	4.41E-12	3.06E-11	UP
PEX3	720.4796	-0.8553	0.123545	-6.923	4.42E-12	3.07E-11	DOWN
RTN4	12955.89	-0.68938	0.099589	-6.9222	4.45E-12	3.09E-11	DOWN
DBIL5P	6.628591	1.30327	0.188302	6.921185	4.48E-12	3.11E-11	UP
CACNA1C	139.0308	1.361526	0.196728	6.920842	4.49E-12	3.11E-11	UP
RBM45	292.4865	0.381572	0.055135	6.920665	4.50E-12	3.12E-11	NOT
TMEM74B	194.5322	1.537855	0.222234	6.919987	4.52E-12	3.13E-11	UP
IFI6	6649.641	-1.68584	0.243619	-6.91996	4.52E-12	3.13E-11	DOWN
CKMT1A	15.30737	3.66746	0.530006	6.91966	4.53E-12	3.14E-11	UP
STK36	530.5681	0.721537	0.104291	6.91851	4.56E-12	3.16E-11	UP
CCDC112	109.56	1.169032	0.168985	6.917957	4.58E-12	3.17E-11	UP
TMEM74	70.22863	1.672041	0.241699	6.917879	4.58E-12	3.17E-11	UP
GATAD2B	1205.355	0.553473	0.080009	6.917667	4.59E-12	3.18E-11	NOT
WDR53	259.0376	0.446121	0.064492	6.917469	4.60E-12	3.18E-11	NOT
XPR1	1175.4	0.786426	0.113691	6.917214	4.61E-12	3.19E-11	UP
JUND	10261.49	-1.08604	0.157006	-6.91721	4.61E-12	3.19E-11	DOWN
MIR25	8.235394	1.555465	0.224926	6.915447	4.66E-12	3.23E-11	UP
INTS7	1161.904	0.581832	0.084148	6.914415	4.70E-12	3.25E-11	NOT
RP11-161I	3.895471	3.880372	0.561213	6.914259	4.70E-12	3.25E-11	UP

C4orf3	4477.618	-0.68022	0.098382	-6.91408	4.71E-12	3.25E-11	DOWN
AGPAT4	196.1714	1.484865	0.214781	6.913395	4.73E-12	3.27E-11	UP
PRKCA	1758.585	0.829711	0.120026	6.912762	4.75E-12	3.28E-11	UP
ZNF550	213.7415	1.036267	0.149926	6.911837	4.78E-12	3.30E-11	UP
RP11-180I	9.8914	3.084834	0.446417	6.910211	4.84E-12	3.34E-11	UP
LRRC37B	117.5714	0.767524	0.111075	6.909985	4.85E-12	3.35E-11	UP
RP11-115:	93.06633	-1.39018	0.201191	-6.90977	4.85E-12	3.35E-11	DOWN
FAM110A	261.842	0.944141	0.136641	6.909623	4.86E-12	3.35E-11	UP
STAB1	3336.53	-1.04421	0.151143	-6.90879	4.89E-12	3.37E-11	DOWN
MEGF8	1909.794	0.912376	0.132062	6.908703	4.89E-12	3.37E-11	UP
FAM3B	648.8507	2.786095	0.403315	6.907991	4.92E-12	3.39E-11	UP
LINC0101§	14.29453	7.349517	1.063987	6.907522	4.93E-12	3.40E-11	UP
GLS	1645.069	1.226638	0.177582	6.907439	4.93E-12	3.40E-11	UP
RP11-212I	163.0605	0.767314	0.111101	6.906467	4.97E-12	3.42E-11	UP
ACE	293.598	1.071361	0.155131	6.906151	4.98E-12	3.43E-11	UP
WDR7	736.9832	-0.60947	0.088253	-6.90599	4.99E-12	3.43E-11	DOWN
LINC0067:	20.50839	1.362594	0.197308	6.90591	4.99E-12	3.43E-11	UP
ARHGEF26	1461.109	-1.32093	0.191309	-6.9047	5.03E-12	3.46E-11	DOWN
HSF1	3893.899	0.797436	0.115496	6.904425	5.04E-12	3.47E-11	UP
TPMT	3260.093	-0.7911	0.114587	-6.90391	5.06E-12	3.48E-11	DOWN
ZNF676	26.83111	2.920794	0.423086	6.903542	5.07E-12	3.49E-11	UP
GBA3	2854.973	-2.12301	0.307542	-6.90316	5.09E-12	3.50E-11	DOWN
ATP11C	1718.579	-1.13945	0.16507	-6.90283	5.10E-12	3.50E-11	DOWN
FAM178B	18.25118	3.40585	0.493415	6.902604	5.11E-12	3.51E-11	UP
DUS4L	261.9824	0.551559	0.079925	6.900972	5.16E-12	3.55E-11	NOT
RP11-529I	10.71633	1.360898	0.197218	6.900458	5.18E-12	3.56E-11	UP
RP5-901A	6.340591	1.685329	0.244267	6.899543	5.22E-12	3.58E-11	UP
FAM167B	303.8977	-1.05548	0.152986	-6.89921	5.23E-12	3.59E-11	DOWN
KMT2B	2193.332	0.910058	0.131932	6.897939	5.28E-12	3.62E-11	UP
RP11-385I	34.72037	1.121716	0.162641	6.896871	5.32E-12	3.65E-11	UP
TCAP	91.67219	1.862686	0.270082	6.896734	5.32E-12	3.65E-11	UP
LINC0090(	2.859945	3.837265	0.55644	6.896101	5.34E-12	3.66E-11	UP
PRAMEF4	24.48989	4.708239	0.682922	6.894253	5.41E-12	3.71E-11	UP
JAKMIP3	25.17287	1.997668	0.289772	6.893934	5.43E-12	3.72E-11	UP
AC024995	1.511902	2.624332	0.380704	6.893366	5.45E-12	3.73E-11	UP
MLLT6	2427.103	0.763198	0.11072	6.893073	5.46E-12	3.74E-11	UP
RP1-302G:	8.201729	2.197055	0.318737	6.893	5.46E-12	3.74E-11	UP
GBAS	822.2243	0.777933	0.112876	6.891905	5.51E-12	3.77E-11	UP
SLC35F6	3285.417	0.698429	0.101342	6.891775	5.51E-12	3.77E-11	UP
RP11-369I	2.254347	-2.98845	0.43369	-6.89075	5.55E-12	3.80E-11	DOWN
RP1-63G5.	2.643251	3.105621	0.450694	6.890747	5.55E-12	3.80E-11	UP
RP11-304I	25.54419	1.300156	0.188709	6.889745	5.59E-12	3.82E-11	UP
SNX8	1338.458	0.750141	0.108884	6.889345	5.60E-12	3.83E-11	UP
KPNB1	6399.253	0.522913	0.075906	6.888944	5.62E-12	3.84E-11	NOT
RP11-799I	15.02652	1.826897	0.265209	6.888525	5.64E-12	3.85E-11	UP
PABPC1	35308.31	0.903671	0.1312	6.887728	5.67E-12	3.87E-11	UP
PTGFR	331.6885	2.391271	0.347196	6.887384	5.68E-12	3.88E-11	UP
GUCY2D	32.78704	2.110386	0.306441	6.886751	5.71E-12	3.90E-11	UP
CLU	226498.7	-1.03757	0.150669	-6.88642	5.72E-12	3.91E-11	DOWN
RP11-168(	2.320125	2.99301	0.434714	6.885003	5.78E-12	3.94E-11	UP
RP4-789D	3.957088	2.1544	0.312976	6.883584	5.84E-12	3.98E-11	UP
PTMS	29351.43	-0.98776	0.143513	-6.88274	5.87E-12	4.01E-11	DOWN

RP11-476I	24.15349	3.219651	0.467821	6.882231	5.89E-12	4.02E-11	UP
SCUBE2	226.9192	2.318797	0.336981	6.881087	5.94E-12	4.05E-11	UP
PLXND1	3576.402	0.890178	0.129404	6.879081	6.02E-12	4.11E-11	UP
QARS	6039.052	0.552763	0.080356	6.878915	6.03E-12	4.11E-11	NOT
NUP85	1445.338	0.54387	0.079065	6.878747	6.04E-12	4.11E-11	NOT
NAPB	365.0694	0.494391	0.071881	6.877878	6.08E-12	4.14E-11	NOT
LINC00648	13.63354	5.04852	0.734284	6.875431	6.18E-12	4.21E-11	UP
RP11-268J	29.13487	1.282383	0.186538	6.874646	6.21E-12	4.23E-11	UP
CD24	4918.902	2.030343	0.295372	6.873852	6.25E-12	4.25E-11	UP
ZNF815P	42.38984	0.882002	0.128347	6.872024	6.33E-12	4.31E-11	UP
C9	32608.2	-2.68907	0.391314	-6.87191	6.33E-12	4.31E-11	DOWN
CLTCL1	585.6542	1.179772	0.171687	6.871663	6.35E-12	4.32E-11	UP
TRAPPC3L	4.42246	-1.67584	0.243944	-6.86974	6.43E-12	4.37E-11	DOWN
RP11-385I	4.877482	3.146835	0.458172	6.868235	6.50E-12	4.42E-11	UP
SNORA60	4.392339	1.698311	0.247273	6.868171	6.50E-12	4.42E-11	UP
GPR126	3664.102	-1.33224	0.194009	-6.86692	6.56E-12	4.46E-11	DOWN
RP11-574I	27.24373	0.917968	0.133697	6.866048	6.60E-12	4.48E-11	UP
RP11-325I	2.503234	2.516473	0.366513	6.865988	6.60E-12	4.49E-11	UP
COQ10B	1427.481	-0.5451	0.079398	-6.86546	6.63E-12	4.50E-11	NOT
USP32P3	3.856003	2.949853	0.429677	6.865273	6.64E-12	4.51E-11	UP
ZNF143	439.9738	0.435846	0.063486	6.865192	6.64E-12	4.51E-11	NOT
CDK4	1892.137	0.769962	0.112157	6.865025	6.65E-12	4.51E-11	UP
C1orf233	138.3361	1.885981	0.274727	6.864937	6.65E-12	4.51E-11	UP
ZNF544	673.8909	0.830728	0.121014	6.864757	6.66E-12	4.52E-11	UP
HIST1H2BI	4.277859	2.700685	0.393432	6.864433	6.68E-12	4.53E-11	UP
PDZD7	15.97217	1.462773	0.213107	6.864022	6.69E-12	4.54E-11	UP
GADD45G	4399.857	-1.55143	0.22607	-6.86263	6.76E-12	4.58E-11	DOWN
CLEC4D	3.084337	-2.14113	0.31203	-6.86195	6.79E-12	4.60E-11	DOWN
HAPLN1	10.32411	3.634557	0.529685	6.861729	6.80E-12	4.61E-11	UP
ANKRD13D	632.5107	0.835616	0.12179	6.861123	6.83E-12	4.63E-11	UP
CDK5RAP2	2489.591	0.627853	0.091514	6.860694	6.85E-12	4.64E-11	UP
SIRT3	1308.827	-0.58652	0.085491	-6.8606	6.86E-12	4.64E-11	DOWN
ZSCAN12F	32.71472	1.144487	0.166846	6.859525	6.91E-12	4.68E-11	UP
AP001350.	14.30964	1.174337	0.171203	6.859343	6.92E-12	4.68E-11	UP
C10orf95	7.172463	1.443441	0.210451	6.85879	6.94E-12	4.70E-11	UP
RP11-666(	17.0291	1.286234	0.187541	6.858401	6.96E-12	4.71E-11	UP
RP11-479(	184.8769	1.09624	0.159852	6.857827	6.99E-12	4.73E-11	UP
MT-CO3	513088	-0.96164	0.140227	-6.85774	7.00E-12	4.73E-11	DOWN
PLAU	384.9767	1.205834	0.175839	6.857612	7.00E-12	4.73E-11	UP
RCVRN	1.92288	-1.78741	0.260656	-6.85735	7.01E-12	4.74E-11	DOWN
ABHD14B	7063.237	-0.88647	0.129293	-6.85633	7.07E-12	4.77E-11	DOWN
MFAP2	133.8811	2.167561	0.316191	6.855221	7.12E-12	4.81E-11	UP
ARID4A	915.5862	-0.75383	0.10997	-6.85492	7.14E-12	4.82E-11	DOWN
ANKS6	512.3263	1.504434	0.219474	6.854721	7.15E-12	4.82E-11	UP
CTD-3037	3.350677	3.71794	0.542432	6.8542	7.17E-12	4.84E-11	UP
U91324.1	79.88932	-1.57065	0.229208	-6.8525	7.26E-12	4.90E-11	DOWN
RP5-1009I	6.293277	2.174758	0.317373	6.852373	7.26E-12	4.90E-11	UP
TPH1	14.42229	1.996437	0.291355	6.852249	7.27E-12	4.90E-11	UP
DTX1	1369.022	-1.84903	0.269856	-6.85192	7.29E-12	4.91E-11	DOWN
USP18	316.2505	-1.09622	0.159996	-6.8515	7.31E-12	4.93E-11	DOWN
SNRPA	1859.558	0.732614	0.106939	6.850771	7.35E-12	4.95E-11	UP
RP11-7O1	7.651448	1.411589	0.206057	6.850462	7.36E-12	4.96E-11	UP

PNPLA4	1022.49	-0.77798	0.113584	-6.84937	7.42E-12	5.00E-11	DOWN
ADH6	15212.78	-1.60523	0.234378	-6.84887	7.44E-12	5.01E-11	DOWN
RP11-813I	4.397886	-2.59248	0.378542	-6.8486	7.46E-12	5.02E-11	DOWN
WDR88	23.53273	1.05098	0.153459	6.848592	7.46E-12	5.02E-11	UP
PWWP2B	667.3131	1.185326	0.173083	6.848291	7.47E-12	5.03E-11	UP
RP5-908M	75.31027	1.187887	0.173498	6.846697	7.56E-12	5.08E-11	UP
INS-IGF2	150.1141	-3.84345	0.561404	-6.84614	7.59E-12	5.10E-11	DOWN
RP6-74O6	9.802794	1.543487	0.225473	6.845555	7.62E-12	5.12E-11	UP
NPNT	765.6073	1.938176	0.283144	6.845195	7.64E-12	5.13E-11	UP
GRIN1	6.523189	3.023371	0.441735	6.844314	7.68E-12	5.16E-11	UP
PKDCC	831.5429	1.361614	0.198945	6.844184	7.69E-12	5.17E-11	UP
TM2D2	1387.197	-0.64782	0.094653	-6.84417	7.69E-12	5.17E-11	DOWN
PKD1L2	70.59737	2.123918	0.310351	6.843608	7.72E-12	5.19E-11	UP
AC002116	10.38702	1.379996	0.201667	6.842943	7.76E-12	5.21E-11	UP
TSNARE1	480.7604	0.780123	0.114069	6.839055	7.97E-12	5.35E-11	UP
TRIM47	1452.712	1.111891	0.162604	6.838049	8.03E-12	5.39E-11	UP
RGL2	1484.604	0.700661	0.102474	6.83746	8.06E-12	5.41E-11	UP
SERPINF2	69012.03	-1.43825	0.210389	-6.83613	8.14E-12	5.46E-11	DOWN
LINC0122	5.873652	2.677825	0.391741	6.835704	8.16E-12	5.47E-11	UP
ARMC4P1	2.606698	3.285132	0.480616	6.835251	8.19E-12	5.49E-11	UP
SORBS3	4336.672	-0.79571	0.116419	-6.8349	8.21E-12	5.50E-11	DOWN
FNDC4	2664.927	-1.32469	0.193844	-6.83379	8.27E-12	5.54E-11	DOWN
ZNF793-A	29.19678	1.927893	0.282116	6.833684	8.28E-12	5.54E-11	UP
RP11-410I	1.161179	2.407552	0.352313	6.833554	8.28E-12	5.55E-11	UP
AC005042	5.117227	1.777254	0.260092	6.833174	8.31E-12	5.56E-11	UP
AP001187.	64.94097	1.327309	0.194258	6.832704	8.33E-12	5.58E-11	UP
SRPK3	26.85568	1.65062	0.24163	6.831195	8.42E-12	5.64E-11	UP
PGK1P2	3.906066	1.697236	0.248472	6.83068	8.45E-12	5.65E-11	UP
PAGE2B	8.013599	4.508314	0.660063	6.830124	8.48E-12	5.68E-11	UP
RBM19	1119.334	0.496554	0.072702	6.83	8.49E-12	5.68E-11	NOT
RP11-443I	26.80036	1.52788	0.223756	6.828324	8.59E-12	5.74E-11	UP
KIR2DL1	2.508094	-1.97703	0.289539	-6.82818	8.60E-12	5.75E-11	DOWN
IL17B	2.762465	2.782926	0.407609	6.827435	8.64E-12	5.78E-11	UP
COLGALT1	3357.121	0.602452	0.088242	6.827266	8.65E-12	5.78E-11	UP
HSD17B1C	6159.003	-0.79467	0.116399	-6.82707	8.67E-12	5.79E-11	DOWN
CDK5RAP1	958.2148	0.401289	0.05878	6.826976	8.67E-12	5.79E-11	NOT
RARRES1	547.0898	1.990124	0.291539	6.826258	8.72E-12	5.82E-11	UP
FOXD4	9.731717	1.999755	0.292977	6.825645	8.75E-12	5.84E-11	UP
SYDE2	129.7608	-1.12044	0.164154	-6.82552	8.76E-12	5.85E-11	DOWN
PRPF38AP1	2.690377	-3.30307	0.48393	-6.82551	8.76E-12	5.85E-11	DOWN
VTI1B	2582.376	-0.47109	0.06903	-6.82447	8.83E-12	5.89E-11	NOT
PKI55	116.3092	1.051292	0.154074	6.823297	8.90E-12	5.93E-11	UP
ASAP1	1195.895	0.932718	0.136701	6.823029	8.91E-12	5.94E-11	UP
SLC9A9	244.3455	-1.17417	0.17209	-6.823	8.92E-12	5.94E-11	DOWN
TOMM40	3052.956	0.968584	0.141983	6.821853	8.99E-12	5.99E-11	UP
RNF165	108.1983	-1.57911	0.231506	-6.82103	9.04E-12	6.02E-11	DOWN
FAM118A	603.0767	0.950294	0.13932	6.820922	9.05E-12	6.02E-11	UP
AL022393.	6.587627	2.090363	0.306476	6.820646	9.06E-12	6.03E-11	UP
METTL7A	29399.5	-1.05669	0.154956	-6.81933	9.15E-12	6.09E-11	DOWN
MYOM2	149.17	-1.84286	0.270251	-6.81907	9.16E-12	6.10E-11	DOWN
RP11-258(	133.5374	0.867617	0.127234	6.819053	9.16E-12	6.10E-11	UP
RP1-29C1	16.56804	2.585629	0.37919	6.818823	9.18E-12	6.11E-11	UP

SRGN	1865.023	-1.1785	0.172846	-6.8182	9.22E-12	6.13E-11	DOWN
TMEM108	22.5258	1.665293	0.244251	6.817971	9.23E-12	6.14E-11	UP
MZF1-AS1	78.44731	0.928362	0.136166	6.817865	9.24E-12	6.14E-11	UP
THAP8	347.8261	0.797108	0.116921	6.817485	9.26E-12	6.16E-11	UP
CCDC185	5.141227	4.103229	0.601879	6.817371	9.27E-12	6.16E-11	UP
FAM220CI	3.625077	1.958568	0.287304	6.817049	9.29E-12	6.17E-11	UP
RP11-263I	14.53646	1.318601	0.193529	6.813447	9.53E-12	6.33E-11	UP
RP11-178I	32.96127	1.122203	0.164716	6.812958	9.56E-12	6.35E-11	UP
AGBL3	141.2185	0.89257	0.131016	6.812653	9.58E-12	6.36E-11	UP
AC087294	3.922091	1.76108	0.258505	6.812565	9.59E-12	6.36E-11	UP
PPIAP22	900.7825	0.597459	0.087708	6.811922	9.63E-12	6.39E-11	UP
PAQR8	306.7817	1.54239	0.22643	6.811766	9.64E-12	6.39E-11	UP
ACVR2A	468.7825	-0.56594	0.083085	-6.8116	9.65E-12	6.40E-11	NOT
DCAF4	364.7053	0.586847	0.08616	6.811167	9.68E-12	6.42E-11	UP
STX11	145.1546	-1.31746	0.193459	-6.81003	9.76E-12	6.47E-11	DOWN
ANGPT2	336.8435	1.256381	0.184502	6.809576	9.79E-12	6.49E-11	UP
VLDLR	464.6351	2.140562	0.314363	6.809213	9.81E-12	6.50E-11	UP
PPIA	12856.87	0.610817	0.089706	6.809077	9.82E-12	6.51E-11	UP
RP11-962(	8.921538	5.550476	0.815194	6.808777	9.84E-12	6.52E-11	UP
DCK	505.9017	0.802633	0.117897	6.807897	9.90E-12	6.56E-11	UP
FNIP2	2700.881	-1.18734	0.174418	-6.80745	9.93E-12	6.58E-11	DOWN
SLC25A5P	7.215578	1.48208	0.217737	6.806733	9.98E-12	6.61E-11	UP
MBIP	602.2907	-0.60949	0.089545	-6.80654	1.00E-11	6.61E-11	DOWN
GRAPL	1.931815	-1.6708	0.24547	-6.80653	1.00E-11	6.61E-11	DOWN
NDRG1	10803.97	1.381667	0.202993	6.806473	1.00E-11	6.61E-11	UP
RP11-415	1.771552	2.446982	0.359521	6.806222	1.00E-11	6.62E-11	UP
RP11-455(	3.588672	1.901625	0.27944	6.805123	1.01E-11	6.67E-11	UP
LHX3	33.53909	3.705122	0.544484	6.804828	1.01E-11	6.69E-11	UP
CYP2C58P	2.146747	-2.53406	0.372408	-6.80454	1.01E-11	6.70E-11	DOWN
PRC1-AS1	3.480262	1.894202	0.27838	6.804382	1.01E-11	6.70E-11	UP
UBE2Q2	717.6102	0.952194	0.139941	6.80424	1.02E-11	6.71E-11	UP
CACNB1	58.31892	1.229515	0.180725	6.803236	1.02E-11	6.75E-11	UP
ALMS1	585.9872	0.615094	0.090434	6.801587	1.03E-11	6.83E-11	UP
RP11-141(	1.891346	2.547706	0.374577	6.801553	1.03E-11	6.83E-11	UP
CABP1	12.35208	1.759646	0.258733	6.801014	1.04E-11	6.85E-11	UP
MICE	24.65432	1.411453	0.207551	6.800519	1.04E-11	6.88E-11	UP
PYCRL	1198.203	0.958305	0.140937	6.799524	1.05E-11	6.92E-11	UP
ZNF587	355.0629	0.765547	0.112593	6.79921	1.05E-11	6.93E-11	UP
CERS5	774.2293	0.58068	0.085405	6.799163	1.05E-11	6.94E-11	NOT
DUSP8P5	19.05364	1.008395	0.148315	6.798986	1.05E-11	6.94E-11	UP
GPR161	137.9779	1.394977	0.205219	6.797504	1.06E-11	7.01E-11	UP
ACTR5	354.7367	0.664691	0.097786	6.797376	1.07E-11	7.02E-11	UP
IGSF1	293.1589	2.146108	0.315772	6.796396	1.07E-11	7.06E-11	UP
GNPDA1	1181.434	0.782676	0.115164	6.79617	1.07E-11	7.07E-11	UP
LRCH1	729.6255	-0.72535	0.106732	-6.79599	1.08E-11	7.08E-11	DOWN
LINC0112!	61.26847	1.081652	0.159196	6.794457	1.09E-11	7.15E-11	UP
ZCCHC12	8.306684	2.964075	0.436273	6.794089	1.09E-11	7.17E-11	UP
ANXA2P2	99.59163	1.220314	0.179635	6.793291	1.10E-11	7.21E-11	UP
MIR4292	5.090846	1.753661	0.258218	6.791393	1.11E-11	7.30E-11	UP
SPINK4	5.264369	4.372435	0.643891	6.790645	1.12E-11	7.34E-11	UP
RP11-227I	3.891722	3.185676	0.469141	6.790444	1.12E-11	7.35E-11	UP
SLC25A35	148.2263	0.877739	0.129281	6.789404	1.13E-11	7.40E-11	UP

ATPAF1	3014.39	-0.60382	0.088939	-6.7891	1.13E-11	7.41E-11	DOWN
TDRD5	14.20338	4.085661	0.601798	6.789094	1.13E-11	7.41E-11	UP
RP11-333I	5.657445	1.835352	0.270356	6.788653	1.13E-11	7.43E-11	UP
LINC01091	21.2222	1.845557	0.271869	6.78841	1.13E-11	7.44E-11	UP
PIK3R2	59.85484	1.353278	0.199372	6.787713	1.14E-11	7.48E-11	UP
ERVMER34	31.82573	2.474014	0.364523	6.786992	1.14E-11	7.51E-11	UP
USH1G	5.060293	3.767872	0.555509	6.782736	1.18E-11	7.73E-11	UP
HIST1H2B.	61.6154	1.8144	0.267519	6.782327	1.18E-11	7.75E-11	UP
TAS2R4	7.13129	1.618118	0.23858	6.78228	1.18E-11	7.76E-11	UP
RP11-567(	10.08753	3.206387	0.472807	6.781596	1.19E-11	7.79E-11	UP
ANO4	14.49891	2.277426	0.335906	6.779947	1.20E-11	7.88E-11	UP
FOXJ1	73.60841	2.625285	0.387238	6.779506	1.21E-11	7.90E-11	UP
SLC4A2	5243.926	0.702989	0.103702	6.778951	1.21E-11	7.93E-11	UP
STAMBPL1	187.3328	1.147682	0.169316	6.778346	1.22E-11	7.96E-11	UP
CMPK2	379.1179	-1.38766	0.204722	-6.77824	1.22E-11	7.96E-11	DOWN
MAOB	12660.73	-0.98279	0.144992	-6.7782	1.22E-11	7.96E-11	DOWN
CYTL1	11.43556	1.788193	0.263836	6.777666	1.22E-11	7.99E-11	UP
CLIC1	7571.715	0.928112	0.136942	6.777401	1.22E-11	8.00E-11	UP
RRP15	848.5437	0.571552	0.084335	6.777175	1.23E-11	8.02E-11	NOT
FGFR1OP2	892.6761	-0.41933	0.061875	-6.77697	1.23E-11	8.03E-11	NOT
PI3	110.0129	2.788572	0.411488	6.776807	1.23E-11	8.03E-11	UP
ZNF730	5.93443	3.074454	0.453682	6.776667	1.23E-11	8.04E-11	UP
SLC19A3	1027.186	-1.39074	0.205234	-6.77636	1.23E-11	8.05E-11	DOWN
SULT1C2P1	5.025775	2.925581	0.431793	6.775429	1.24E-11	8.10E-11	UP
KLHL35	13.859	2.050582	0.302658	6.775244	1.24E-11	8.11E-11	UP
CTA-963H	29.88601	1.023348	0.151047	6.775046	1.24E-11	8.12E-11	UP
C11orf65	28.63129	-0.88868	0.13117	-6.77501	1.24E-11	8.12E-11	DOWN
PEX11G	484.8984	-1.19218	0.175971	-6.77489	1.24E-11	8.13E-11	DOWN
PDCD5	2014.601	0.783689	0.115703	6.773272	1.26E-11	8.22E-11	UP
EDARADD	53.35752	2.017995	0.297954	6.772829	1.26E-11	8.24E-11	UP
CA8	20.33813	2.213183	0.326778	6.772751	1.26E-11	8.24E-11	UP
BMP7	17.5825	3.579	0.528517	6.77178	1.27E-11	8.29E-11	UP
NLE1	632.4613	0.764619	0.112929	6.770769	1.28E-11	8.35E-11	UP
HIST1H2BI	4.856235	1.740196	0.257035	6.770263	1.29E-11	8.38E-11	UP
TMEM132	16.18482	2.03144	0.300092	6.769392	1.29E-11	8.43E-11	UP
DUT	1535.495	0.69084	0.102058	6.769095	1.30E-11	8.44E-11	UP
C5orf45	493.0752	0.889783	0.131463	6.768322	1.30E-11	8.49E-11	UP
LARP4B	1924.875	0.516969	0.076382	6.768202	1.30E-11	8.49E-11	NOT
UBL3	2413.85	-0.70708	0.10448	-6.76759	1.31E-11	8.52E-11	DOWN
LINC01341	84.27709	1.458311	0.215488	6.767484	1.31E-11	8.53E-11	UP
AOX3P	20.32109	2.294442	0.339164	6.764999	1.33E-11	8.67E-11	UP
AC010148	11.88406	1.847262	0.273089	6.764335	1.34E-11	8.71E-11	UP
C5AR1	471.1121	-1.1287	0.166874	-6.76381	1.34E-11	8.74E-11	DOWN
RP11-6N1	2.793717	3.930859	0.581186	6.763515	1.35E-11	8.76E-11	UP
GSTA4	1173.174	1.134659	0.167769	6.763215	1.35E-11	8.77E-11	UP
CHD3	1450.839	1.243856	0.183922	6.762945	1.35E-11	8.79E-11	UP
RP11-1094	211.4466	0.913981	0.135148	6.762813	1.35E-11	8.79E-11	UP
AC068831	6.525804	1.324668	0.195955	6.760051	1.38E-11	8.96E-11	UP
IQGAP2	8162.176	-0.93606	0.138484	-6.75935	1.39E-11	9.00E-11	DOWN
HAO2	5096.484	-2.33983	0.346191	-6.75879	1.39E-11	9.03E-11	DOWN
GAL3ST4	123.2726	1.486664	0.219965	6.758625	1.39E-11	9.04E-11	UP
ZNF793	70.29954	1.523463	0.225419	6.75837	1.40E-11	9.06E-11	UP

C1orf220	31.80929	1.452071	0.214857	6.758309	1.40E-11	9.06E-11	UP
PLGLA	196.7233	-2.25313	0.333461	-6.75682	1.41E-11	9.15E-11	DOWN
RP11-356(	19.75804	2.073503	0.306878	6.756764	1.41E-11	9.15E-11	UP
HSD3BP5	4.863642	2.021874	0.299268	6.756073	1.42E-11	9.19E-11	UP
GRM4	4.946407	3.230575	0.47824	6.755138	1.43E-11	9.25E-11	UP
TMEM59	12825.74	-0.46644	0.069055	-6.75463	1.43E-11	9.28E-11	NOT
DNAJC10	2129.406	0.656829	0.097249	6.754094	1.44E-11	9.31E-11	UP
DKKL1	11.88865	2.070492	0.306568	6.753782	1.44E-11	9.33E-11	UP
CTD-2154	2.36579	-1.5234	0.225578	-6.7533	1.45E-11	9.36E-11	DOWN
CITF22-92	68.88215	0.897725	0.132961	6.751769	1.46E-11	9.46E-11	UP
RP11-365(	11.10612	-1.7122	0.253603	-6.7515	1.46E-11	9.47E-11	DOWN
F13A1	450.521	1.875325	0.277766	6.751446	1.46E-11	9.47E-11	UP
MFSD2B	26.9399	1.832154	0.271372	6.751443	1.46E-11	9.47E-11	UP
ELMOD3	616.1532	0.485089	0.07185	6.751371	1.46E-11	9.47E-11	NOT
MTHFD1	14889.2	-1.23213	0.182502	-6.75131	1.47E-11	9.48E-11	DOWN
USP14	2402.743	0.490523	0.072667	6.750327	1.48E-11	9.54E-11	NOT
RPL10L	5.784194	4.150588	0.614921	6.749796	1.48E-11	9.57E-11	UP
DUSP16	4147.977	-0.8847	0.131084	-6.74915	1.49E-11	9.61E-11	DOWN
NTN4	901.142	-1.18618	0.175765	-6.74869	1.49E-11	9.64E-11	DOWN
KIF7	133.805	1.295581	0.191998	6.747905	1.50E-11	9.69E-11	UP
TMEM253	6.346042	1.6212	0.24026	6.747681	1.50E-11	9.70E-11	UP
KCNQ1OT:	105.1286	1.321807	0.195901	6.747327	1.51E-11	9.72E-11	UP
2-Mar	5484.226	-0.97018	0.143794	-6.74701	1.51E-11	9.74E-11	DOWN
FAM87A	5.46667	2.896603	0.429336	6.746701	1.51E-11	9.76E-11	UP
HIST1H2A	151.6029	2.162942	0.320614	6.746255	1.52E-11	9.79E-11	UP
RP11-260I	17.30424	1.107761	0.16422	6.745578	1.52E-11	9.83E-11	UP
ZNF205	531.0305	0.675267	0.100105	6.74557	1.52E-11	9.83E-11	UP
TMEM163	79.72408	2.256487	0.334553	6.744781	1.53E-11	9.88E-11	UP
ALDH4A1	16561.05	-1.18172	0.175246	-6.7432	1.55E-11	9.99E-11	DOWN
DCLRE1C	175.7685	0.948159	0.140614	6.742995	1.55E-11	1.00E-10	UP
UCN	48.88072	1.25834	0.186625	6.7426	1.56E-11	1.00E-10	UP
ZNF26	340.0381	0.606241	0.089917	6.742204	1.56E-11	1.00E-10	UP
CPEB4	3080.357	-0.89667	0.133026	-6.74056	1.58E-11	1.02E-10	DOWN
RIMS3	90.59645	1.396275	0.20716	6.740078	1.58E-11	1.02E-10	UP
TAGLN2P1	8.675298	1.340176	0.198852	6.739582	1.59E-11	1.02E-10	UP
ACRV1	5.189278	2.071951	0.307441	6.739346	1.59E-11	1.02E-10	UP
PRSS1	11.78877	5.231061	0.776209	6.739246	1.59E-11	1.02E-10	UP
AVPR2	11.90752	1.794338	0.26627	6.738799	1.60E-11	1.03E-10	UP
TPK1	165.3161	-0.87753	0.130228	-6.73845	1.60E-11	1.03E-10	DOWN
CTD-2026	61.27658	0.85116	0.126328	6.737677	1.61E-11	1.03E-10	UP
PIGZ	242.0792	1.180804	0.175282	6.736597	1.62E-11	1.04E-10	UP
TRPM8	1354.164	-1.77979	0.264209	-6.73629	1.62E-11	1.04E-10	DOWN
DNM1L	1589.528	0.55592	0.082528	6.736138	1.63E-11	1.05E-10	NOT
B3GALT1	11.15015	3.411203	0.506452	6.735495	1.63E-11	1.05E-10	UP
ASNA1	2118.724	0.534995	0.079431	6.735318	1.64E-11	1.05E-10	NOT
COL4A5	418.1632	2.175045	0.322953	6.734858	1.64E-11	1.05E-10	UP
MTND6P4	41.51984	-1.76133	0.261557	-6.73401	1.65E-11	1.06E-10	DOWN
PRICKLE4	254.7512	0.778691	0.115643	6.733602	1.66E-11	1.06E-10	UP
MROH6	485.365	1.497145	0.222373	6.732577	1.67E-11	1.07E-10	UP
CPLX1	440.2449	1.564129	0.232373	6.731111	1.68E-11	1.08E-10	UP
NDUFS1	5876.235	-0.56482	0.083913	-6.73106	1.68E-11	1.08E-10	NOT
LL22NC03	5.190846	2.57622	0.3828	6.729941	1.70E-11	1.09E-10	UP

CMTM5	1.950542	-1.99676	0.296741	-6.72897	1.71E-11	1.10E-10	DOWN
FNDC3A	3591.175	-0.86597	0.128703	-6.72843	1.72E-11	1.10E-10	DOWN
TRIM31	371.6952	2.177761	0.32367	6.728341	1.72E-11	1.10E-10	UP
ZBED5	671.2893	0.677983	0.100773	6.727843	1.72E-11	1.10E-10	UP
ULBP1	15.2044	2.23738	0.332571	6.727516	1.73E-11	1.11E-10	UP
SUPT7L	1314.174	0.36812	0.054723	6.726977	1.73E-11	1.11E-10	NOT
AC003090	13.44982	3.084092	0.458469	6.726942	1.73E-11	1.11E-10	UP
PNMA5	12.34344	3.362133	0.49998	6.724534	1.76E-11	1.13E-10	UP
C10orf91	10.269	3.920121	0.583031	6.723693	1.77E-11	1.13E-10	UP
FOXN4	164.7508	2.727808	0.40574	6.723046	1.78E-11	1.14E-10	UP
ZNF492	8.166807	2.812777	0.418396	6.722758	1.78E-11	1.14E-10	UP
RP11-44N	3.260752	3.967439	0.590171	6.722527	1.79E-11	1.14E-10	UP
RP11-343I	15.86343	1.192837	0.1775	6.720207	1.81E-11	1.16E-10	UP
COL4A6	38.56281	2.467972	0.367265	6.71986	1.82E-11	1.16E-10	UP
SFRP1	155.9275	-2.07113	0.308277	-6.71842	1.84E-11	1.17E-10	DOWN
SNHG6	1960.906	1.084205	0.16138	6.718342	1.84E-11	1.17E-10	UP
ZNF436	303.7576	0.574865	0.08558	6.717273	1.85E-11	1.18E-10	NOT
GRIN2C	22.85626	1.138305	0.169538	6.714176	1.89E-11	1.21E-10	UP
FEZ1	303.8178	-1.25743	0.18728	-6.71414	1.89E-11	1.21E-10	DOWN
ABHD13	742.7321	-0.59212	0.088202	-6.71323	1.90E-11	1.21E-10	DOWN
SPIC	13.12844	-1.84388	0.274665	-6.71322	1.90E-11	1.21E-10	DOWN
LASP1	11245.48	0.593643	0.088434	6.712872	1.91E-11	1.22E-10	UP
GATA6	765.3637	-0.94509	0.14079	-6.71275	1.91E-11	1.22E-10	DOWN
MPEG1	1413.187	-1.15689	0.172342	-6.71271	1.91E-11	1.22E-10	DOWN
AP000442.	21.49259	-0.76404	0.113833	-6.71195	1.92E-11	1.22E-10	DOWN
RP1-92O1	9.243744	1.303976	0.194311	6.710758	1.94E-11	1.23E-10	UP
KIAA1614-	3.369912	2.324197	0.346354	6.71046	1.94E-11	1.24E-10	UP
RP11-118.	32.7305	-1.90748	0.284268	-6.71017	1.94E-11	1.24E-10	DOWN
SLC52A3	93.33071	1.903361	0.283731	6.70833	1.97E-11	1.25E-10	UP
RBP2	17.77964	3.186975	0.475108	6.707901	1.97E-11	1.26E-10	UP
PINK1	2057.075	-1.00982	0.150555	-6.70731	1.98E-11	1.26E-10	DOWN
RP11-468I	15.59039	1.018896	0.151914	6.707055	1.99E-11	1.26E-10	UP
LINC0070.	25.10925	1.355697	0.20214	6.706735	1.99E-11	1.27E-10	UP
PSMD2	6660.381	0.507554	0.075687	6.70596	2.00E-11	1.27E-10	NOT
WDR27	255.3609	0.869267	0.129636	6.705433	2.01E-11	1.28E-10	UP
RNF187	7025.321	0.743888	0.110984	6.702675	2.05E-11	1.30E-10	UP
RP11-265I	2.997014	2.113818	0.315387	6.702293	2.05E-11	1.30E-10	UP
NAGPA	849.0978	0.653125	0.097459	6.701523	2.06E-11	1.31E-10	UP
AC003973	26.92502	2.198153	0.328015	6.701376	2.06E-11	1.31E-10	UP
AQPEP	7.05957	2.356719	0.351687	6.701174	2.07E-11	1.31E-10	UP
POLD3	481.0151	0.678694	0.101297	6.700013	2.08E-11	1.32E-10	UP
ZNF76	1264.558	0.544472	0.081268	6.699736	2.09E-11	1.33E-10	NOT
GIPC2	826.9834	-1.27646	0.19055	-6.69882	2.10E-11	1.33E-10	DOWN
DENND5A	1599.541	0.548264	0.081867	6.69702	2.13E-11	1.35E-10	NOT
HIST1H2BI	3.376588	2.771519	0.413864	6.696692	2.13E-11	1.35E-10	UP
LINC0089(	44.66421	1.859614	0.277701	6.69645	2.14E-11	1.35E-10	UP
FAM227A	19.8389	2.015131	0.300933	6.696282	2.14E-11	1.36E-10	UP
RP4-694B.	31.47436	1.10362	0.16486	6.694302	2.17E-11	1.37E-10	UP
SUB1	5999.856	0.59596	0.089029	6.693972	2.17E-11	1.38E-10	UP
CCDC80	1283.348	1.919691	0.286801	6.69345	2.18E-11	1.38E-10	UP
MRPL45P1	2.872291	3.856479	0.576176	6.693231	2.18E-11	1.38E-10	UP
VN1R1	28.15512	1.533278	0.229097	6.692718	2.19E-11	1.39E-10	UP

FCAR	7.910372	-1.94962	0.291313	-6.69254	2.19E-11	1.39E-10	DOWN
MMP10	26.33351	2.794211	0.417598	6.691145	2.21E-11	1.40E-10	UP
GYS2	3833.451	-2.21332	0.33081	-6.6906	2.22E-11	1.41E-10	DOWN
CACNA1G	7.586945	2.853624	0.426558	6.689877	2.23E-11	1.41E-10	UP
FBXO45	665.2999	0.532587	0.079612	6.689771	2.24E-11	1.41E-10	NOT
CFAP44	178.3744	0.961536	0.143769	6.688046	2.26E-11	1.43E-10	UP
HIGD1A	3720.735	-0.80894	0.120961	-6.6876	2.27E-11	1.43E-10	DOWN
AC068580	8.737221	1.517573	0.226925	6.68756	2.27E-11	1.44E-10	UP
GRIN2B	73.16779	-2.329	0.34829	-6.68696	2.28E-11	1.44E-10	DOWN
CP	50953.23	-1.57397	0.235398	-6.68644	2.29E-11	1.45E-10	DOWN
GHITM	11098.45	-0.62049	0.092806	-6.68589	2.30E-11	1.45E-10	DOWN
CCDC42	4.946972	-1.40567	0.210271	-6.68506	2.31E-11	1.46E-10	DOWN
CACYBPP2	5.608349	1.47601	0.220819	6.684257	2.32E-11	1.47E-10	UP
RP11-421I	3.041934	-3.15401	0.47186	-6.6842	2.32E-11	1.47E-10	DOWN
SNX33	2086.717	-0.54483	0.081513	-6.68397	2.33E-11	1.47E-10	NOT
SDHA	11603.47	-0.74695	0.111753	-6.68394	2.33E-11	1.47E-10	DOWN
CTB-179K	8.829165	1.221434	0.182768	6.682962	2.34E-11	1.48E-10	UP
SCRT1	2.37587	2.768257	0.414259	6.682438	2.35E-11	1.48E-10	UP
INTS1	4964.529	0.586896	0.087831	6.682074	2.36E-11	1.49E-10	UP
AKAP3	36.82729	-1.34092	0.200697	-6.68134	2.37E-11	1.49E-10	DOWN
PXMP2	3838.288	-1.15393	0.172722	-6.68085	2.38E-11	1.50E-10	DOWN
FLRT1	17.61631	1.618585	0.242277	6.680726	2.38E-11	1.50E-10	UP
SNORD60	4.048759	2.057436	0.307974	6.680552	2.38E-11	1.50E-10	UP
TMEM61	16.2863	2.502449	0.374611	6.680123	2.39E-11	1.50E-10	UP
CKAP5	2910.009	0.503771	0.075414	6.680085	2.39E-11	1.50E-10	NOT
RP13-103	117.941	0.880743	0.131868	6.678972	2.41E-11	1.52E-10	UP
RIPPLY3	34.4828	2.101179	0.314644	6.677951	2.42E-11	1.53E-10	UP
CTD-3025	41.93672	1.002038	0.150069	6.677186	2.44E-11	1.53E-10	UP
PSMD5-A	543.4133	0.849084	0.127163	6.677152	2.44E-11	1.53E-10	UP
KRTAP5-A	18.97425	2.227224	0.333583	6.676662	2.44E-11	1.54E-10	UP
DPYD	2746.767	-1.00503	0.150546	-6.67586	2.46E-11	1.55E-10	DOWN
ABCA4	123.3109	1.497554	0.224358	6.674856	2.47E-11	1.56E-10	UP
RP11-295I	22.35184	1.353266	0.202741	6.674847	2.47E-11	1.56E-10	UP
MTX1	808.6716	0.588712	0.088232	6.672339	2.52E-11	1.58E-10	UP
BCYRN1	21.38551	1.765977	0.264704	6.671519	2.53E-11	1.59E-10	UP
PCBD1	8702.118	-0.81602	0.122321	-6.67118	2.54E-11	1.59E-10	DOWN
IL1B	96.89061	-1.58516	0.237616	-6.67109	2.54E-11	1.60E-10	DOWN
AC005307	4.469868	4.194007	0.628732	6.670579	2.55E-11	1.60E-10	UP
RP11-379I	35.84366	1.028752	0.154246	6.669539	2.57E-11	1.61E-10	UP
TMEM80	525.8103	0.591013	0.088636	6.667857	2.60E-11	1.63E-10	UP
TCTE3	52.4136	0.88155	0.132223	6.667162	2.61E-11	1.64E-10	UP
TMEM55B	1011.905	0.48051	0.072082	6.666166	2.63E-11	1.65E-10	NOT
CYP2C9	29234.41	-1.92955	0.289509	-6.66488	2.65E-11	1.66E-10	DOWN
THOC2	1452.396	0.567032	0.085081	6.664573	2.65E-11	1.66E-10	NOT
MIR325HG	16.88575	2.845972	0.427034	6.664517	2.66E-11	1.67E-10	UP
ADCK1	356.0704	0.621746	0.093294	6.664399	2.66E-11	1.67E-10	UP
ZNF436-A	53.4516	0.865588	0.129891	6.663953	2.67E-11	1.67E-10	UP
NHLRC3	1074.597	-0.66078	0.099168	-6.66322	2.68E-11	1.68E-10	DOWN
RP11-305I	60.08116	0.790149	0.118587	6.663056	2.68E-11	1.68E-10	UP
FEZF1	5.131918	4.150762	0.623008	6.662458	2.69E-11	1.69E-10	UP
ABCB9	132.6144	1.079353	0.162006	6.662429	2.69E-11	1.69E-10	UP
WDR46	2001.154	0.621626	0.093304	6.662358	2.69E-11	1.69E-10	UP

GATA2-AS	38.72638	1.846628	0.277185	6.662081	2.70E-11	1.69E-10	UP
RP11-299I	20.53324	2.639346	0.39619	6.661827	2.70E-11	1.69E-10	UP
RP11-328I	333.1931	-2.08536	0.313119	-6.65995	2.74E-11	1.71E-10	DOWN
CCDC88A	980.4309	0.820667	0.123232	6.659524	2.75E-11	1.72E-10	UP
DKC1	1741.92	0.624716	0.093816	6.65895	2.76E-11	1.72E-10	UP
CHST10	143.9568	1.451103	0.217918	6.658931	2.76E-11	1.72E-10	UP
ZNF107	168.2717	1.145806	0.172106	6.657556	2.78E-11	1.74E-10	UP
ANKAR	68.7836	0.687844	0.103319	6.657489	2.79E-11	1.74E-10	UP
CTD-2374	3.867106	4.344573	0.652587	6.657459	2.79E-11	1.74E-10	UP
ZNF675	114.5955	1.049422	0.157636	6.657231	2.79E-11	1.74E-10	UP
RP11-121I	4.655077	1.706469	0.256375	6.65614	2.81E-11	1.76E-10	UP
RP11-188I	6.93412	-1.26272	0.18973	-6.65533	2.83E-11	1.76E-10	DOWN
TCEB3	2265.465	-0.55506	0.083404	-6.65507	2.83E-11	1.77E-10	NOT
DSCAM	7.163332	-2.00542	0.30134	-6.65499	2.83E-11	1.77E-10	DOWN
CWH43	18.73557	-2.84904	0.428119	-6.65479	2.84E-11	1.77E-10	DOWN
XXbac-B13	22.36144	1.510282	0.226954	6.654587	2.84E-11	1.77E-10	UP
ABHD12	2946.047	0.657817	0.098855	6.654335	2.85E-11	1.77E-10	UP
RP11-27N	13.48202	1.641955	0.24678	6.653519	2.86E-11	1.78E-10	UP
DGKH	259.9857	0.917742	0.137952	6.65262	2.88E-11	1.79E-10	UP
ENTPD1	927.2865	0.648449	0.097473	6.652611	2.88E-11	1.79E-10	UP
ADH1B	117838.3	-1.96342	0.295207	-6.65099	2.91E-11	1.81E-10	DOWN
TIPRL	1883.304	0.48714	0.073245	6.650857	2.91E-11	1.82E-10	NOT
RP11-120I	8.088989	1.46119	0.219748	6.649397	2.94E-11	1.83E-10	UP
OXCT1	223.0764	1.783024	0.268204	6.648011	2.97E-11	1.85E-10	UP
PTCRA	8.415128	-1.52363	0.2292	-6.64761	2.98E-11	1.85E-10	DOWN
TSPAN15	798.1734	1.486597	0.223669	6.646406	3.00E-11	1.87E-10	UP
TRIO	1579.308	0.66229	0.099652	6.646028	3.01E-11	1.87E-10	UP
HSPA4	5305.053	0.580173	0.087319	6.644289	3.05E-11	1.90E-10	NOT
LIMK2	1347.04	1.039531	0.156476	6.643383	3.07E-11	1.91E-10	UP
TH	10.20329	-2.3018	0.346484	-6.64332	3.07E-11	1.91E-10	DOWN
CTD-2621	11.682	1.565139	0.235597	6.643282	3.07E-11	1.91E-10	UP
RASSF1	824.7207	0.557445	0.083919	6.642669	3.08E-11	1.91E-10	NOT
HRSP12	17153.33	-1.33322	0.200728	-6.64192	3.10E-11	1.92E-10	DOWN
RP11-114I	7.969529	5.268126	0.793188	6.64171	3.10E-11	1.93E-10	UP
MICA	594.0887	0.790934	0.119092	6.641396	3.11E-11	1.93E-10	UP
H1FX-AS1	64.86778	1.091318	0.164371	6.639345	3.15E-11	1.96E-10	UP
ZBTB22	829.4808	0.519978	0.078319	6.639253	3.15E-11	1.96E-10	NOT
MFN2	5735.381	-0.59746	0.090015	-6.63732	3.19E-11	1.98E-10	DOWN
YWHAEP1	4.604542	3.323574	0.500779	6.636808	3.21E-11	1.99E-10	UP
CCPG1	1204.797	-0.66877	0.100784	-6.63566	3.23E-11	2.00E-10	DOWN
AKR7A3	4363.13	-1.74242	0.262625	-6.63462	3.25E-11	2.02E-10	DOWN
SERPINA1(	13967.85	-1.27805	0.192641	-6.63433	3.26E-11	2.02E-10	DOWN
CAPN12	580.3045	1.535974	0.231529	6.634037	3.27E-11	2.03E-10	UP
C9orf40	226.4314	0.862167	0.129992	6.632452	3.30E-11	2.05E-10	UP
SLC27A2	9210.221	-1.48314	0.223636	-6.63193	3.31E-11	2.05E-10	DOWN
RP11-347(	10.91748	1.578201	0.23798	6.631665	3.32E-11	2.06E-10	UP
TREM2	210.187	1.591818	0.240063	6.630848	3.34E-11	2.07E-10	UP
RP11-404I	63.75245	2.159604	0.325813	6.628364	3.39E-11	2.10E-10	UP
NCOA7-A:	5.08864	-2.26562	0.341809	-6.62833	3.40E-11	2.10E-10	DOWN
LINC01021	59.77993	2.330438	0.351614	6.627827	3.41E-11	2.11E-10	UP
FCRL6	43.04041	-1.33308	0.201137	-6.62772	3.41E-11	2.11E-10	DOWN
OR1F1	2.971746	3.659942	0.552259	6.627225	3.42E-11	2.12E-10	UP

TCL6	15.44157	2.963033	0.447133	6.626735	3.43E-11	2.12E-10	UP
HADH	6301.481	-0.90496	0.136564	-6.62663	3.43E-11	2.12E-10	DOWN
UROC1	4117.243	-2.43392	0.367297	-6.62658	3.44E-11	2.12E-10	DOWN
MSL3P1	22.34787	1.383963	0.208887	6.62543	3.46E-11	2.14E-10	UP
YES1	2680.905	-0.554	0.083661	-6.62193	3.55E-11	2.19E-10	NOT
AC010761	26.70434	1.118965	0.168991	6.621447	3.56E-11	2.20E-10	UP
RP11-16P(	20.88247	1.183044	0.178677	6.621136	3.56E-11	2.20E-10	UP
PDCD6	2765.165	0.457679	0.069133	6.620241	3.59E-11	2.22E-10	NOT
MRPS21	3024.99	0.782783	0.118253	6.619588	3.60E-11	2.22E-10	UP
MPDU1	3939.653	-0.71579	0.108136	-6.61934	3.61E-11	2.23E-10	DOWN
RP11-121(	75.30013	0.856324	0.12937	6.619211	3.61E-11	2.23E-10	UP
TMEM9	4490.225	0.628206	0.09492	6.618239	3.64E-11	2.24E-10	UP
LRRC37A1	77.4535	0.947054	0.143105	6.61789	3.64E-11	2.25E-10	UP
SMUG1P1	64.84472	-3.16193	0.477831	-6.61726	3.66E-11	2.26E-10	DOWN
UFD1L	2497.282	0.547383	0.082721	6.617195	3.66E-11	2.26E-10	NOT
NACC1	2351.63	0.610969	0.092337	6.616727	3.67E-11	2.26E-10	UP
CALN1	16.11394	-2.70786	0.40925	-6.61663	3.67E-11	2.27E-10	DOWN
ARL17A	16.12599	0.985358	0.148934	6.616067	3.69E-11	2.27E-10	UP
RP11-279(	2.250199	3.256039	0.492276	6.614252	3.73E-11	2.30E-10	UP
ALG1L6P	14.6953	0.992238	0.150016	6.614209	3.74E-11	2.30E-10	UP
PPIL4	630.5735	-0.57428	0.086839	-6.61318	3.76E-11	2.32E-10	NOT
REEP4	654.0426	0.921886	0.13941	6.612783	3.77E-11	2.32E-10	UP
POLR2G	1608.454	0.546876	0.082712	6.611808	3.80E-11	2.34E-10	NOT
CIC	2698.86	0.542828	0.0821	6.611778	3.80E-11	2.34E-10	NOT
KIF2A	357.2973	0.726636	0.109905	6.611501	3.80E-11	2.34E-10	UP
AC090154	3.546188	1.936371	0.292884	6.611403	3.81E-11	2.34E-10	UP
TRAF3	681.7917	0.647561	0.097965	6.610132	3.84E-11	2.36E-10	UP
PDCD11	1672.016	0.54539	0.082509	6.610051	3.84E-11	2.36E-10	NOT
CH507-39	1.642507	-2.37015	0.358612	-6.60922	3.86E-11	2.38E-10	DOWN
INTS6	1236.576	-0.64819	0.098077	-6.60898	3.87E-11	2.38E-10	DOWN
MRPL40P1	2.836516	2.692285	0.407373	6.608892	3.87E-11	2.38E-10	UP
NOA1	737.8859	0.50795	0.076859	6.60881	3.87E-11	2.38E-10	NOT
TMEM154	219.5692	-1.85471	0.280654	-6.60854	3.88E-11	2.38E-10	DOWN
KIF1A	237.1697	2.717211	0.411176	6.608383	3.89E-11	2.39E-10	UP
CYTH2	1786.432	0.564356	0.085417	6.607063	3.92E-11	2.41E-10	NOT
RP11-122	7.865766	-2.25484	0.341305	-6.60653	3.93E-11	2.42E-10	DOWN
RP11-326I	7.157923	1.41246	0.213801	6.606433	3.94E-11	2.42E-10	UP
MOGAT2	2691.878	-2.2238	0.336619	-6.60627	3.94E-11	2.42E-10	DOWN
CTD-2006	61.22182	1.022124	0.15475	6.605005	3.98E-11	2.44E-10	UP
RP11-573I	6.934747	1.806343	0.273542	6.603535	4.01E-11	2.46E-10	UP
RMI1	395.7894	0.721417	0.109268	6.602268	4.05E-11	2.48E-10	UP
A4GALT	324.2674	1.23961	0.187758	6.602154	4.05E-11	2.48E-10	UP
WDR90	575.3876	0.763404	0.115631	6.602065	4.05E-11	2.49E-10	UP
AC005624	1.477354	2.60815	0.395059	6.601919	4.06E-11	2.49E-10	UP
GULP1	178.0943	2.184362	0.330885	6.601567	4.07E-11	2.49E-10	UP
KATNB1	713.7061	0.590291	0.089434	6.600332	4.10E-11	2.51E-10	UP
PAX8	144.9291	1.178918	0.178622	6.600059	4.11E-11	2.52E-10	UP
UBAP1	2538.941	-0.42266	0.064048	-6.59909	4.14E-11	2.53E-10	NOT
POPDC2	52.34553	1.065723	0.161513	6.598391	4.16E-11	2.54E-10	UP
PUS7	498.6459	0.6958	0.10547	6.597139	4.19E-11	2.57E-10	UP
RP5-1061I	37.38732	1.039234	0.157567	6.595491	4.24E-11	2.59E-10	UP
MANF	3864.479	0.885569	0.134291	6.594415	4.27E-11	2.61E-10	UP

PDE8A	1613.962	-0.55325	0.083909	-6.59342	4.30E-11	2.63E-10	NOT
BX842568.	70.65608	-1.79051	0.271562	-6.59337	4.30E-11	2.63E-10	DOWN
CLASRP	1275.354	0.696228	0.105597	6.593252	4.30E-11	2.63E-10	UP
MYL3	16.96812	-1.42716	0.2165	-6.59199	4.34E-11	2.65E-10	DOWN
TNNI3	9.607755	3.545752	0.537891	6.591956	4.34E-11	2.65E-10	UP
SLC29A2	686.0639	0.985588	0.149515	6.591886	4.34E-11	2.65E-10	UP
AGRN	7186.017	0.978459	0.148444	6.59144	4.36E-11	2.66E-10	UP
MCIDAS	3.969842	3.671069	0.556977	6.591057	4.37E-11	2.67E-10	UP
RP11-417I	8.54419	1.452681	0.220414	6.590693	4.38E-11	2.67E-10	UP
AIFM3	88.42434	1.311862	0.1991	6.588948	4.43E-11	2.70E-10	UP
SPERT	5.288496	4.610564	0.699777	6.588618	4.44E-11	2.71E-10	UP
FBXO15	22.5847	-1.14974	0.174508	-6.58847	4.44E-11	2.71E-10	DOWN
ZMYND19	617.2154	0.633776	0.096213	6.587192	4.48E-11	2.73E-10	UP
AC009133	137.9051	0.644972	0.097924	6.586429	4.51E-11	2.75E-10	UP
MYL6B	1059.696	1.008079	0.153055	6.586385	4.51E-11	2.75E-10	UP
RP11-111I	122.5516	1.055315	0.16023	6.586265	4.51E-11	2.75E-10	UP
USP54	296.5226	1.196103	0.181625	6.585571	4.53E-11	2.76E-10	UP
PSMC4	4364.156	0.627129	0.095232	6.58525	4.54E-11	2.77E-10	UP
CCDC65	43.46615	1.02829	0.156159	6.584896	4.55E-11	2.77E-10	UP
EIF5A2	303.4746	1.595752	0.242339	6.584797	4.56E-11	2.77E-10	UP
HNRNPH1	6352.186	0.509111	0.077317	6.584701	4.56E-11	2.78E-10	NOT
CLIC5	148.473	1.579054	0.239814	6.584503	4.56E-11	2.78E-10	UP
RP11-33E1	80.92316	-0.99174	0.150618	-6.58445	4.57E-11	2.78E-10	DOWN
TFRC	3538.417	0.940488	0.142839	6.584243	4.57E-11	2.78E-10	UP
CAPRIN1	5135.377	0.434669	0.066019	6.584034	4.58E-11	2.79E-10	NOT
MT1H	3121.164	-3.18041	0.483054	-6.58397	4.58E-11	2.79E-10	DOWN
SWAP70	1171.798	0.70472	0.107038	6.583814	4.59E-11	2.79E-10	UP
HAND2-A	91.74722	-2.24004	0.340243	-6.58363	4.59E-11	2.79E-10	DOWN
AGAP4	39.76789	0.908763	0.13805	6.582838	4.62E-11	2.81E-10	UP
ZBED6CL	862.7838	1.103451	0.16763	6.582677	4.62E-11	2.81E-10	UP
RHEB	2319.836	0.509419	0.077398	6.581812	4.65E-11	2.82E-10	NOT
ARMC9	133.0901	1.122455	0.170541	6.581716	4.65E-11	2.82E-10	UP
TMEM147	2601.025	0.84954	0.129079	6.58153	4.66E-11	2.83E-10	UP
CTA-941F9	18.05491	1.177742	0.17895	6.58139	4.66E-11	2.83E-10	UP
RP11-352I	7.071035	2.720229	0.413329	6.581274	4.66E-11	2.83E-10	UP
SLC35B2	3033.677	0.58051	0.088213	6.580773	4.68E-11	2.84E-10	NOT
AC007128	3.724253	4.230917	0.642957	6.580399	4.69E-11	2.85E-10	UP
SALL2	218.5592	1.740403	0.264507	6.579812	4.71E-11	2.86E-10	UP
MAEL	33.88013	2.249024	0.341815	6.579644	4.72E-11	2.86E-10	UP
DMPK	614.4746	0.971337	0.147646	6.578837	4.74E-11	2.87E-10	UP
RP11-44N	33.53144	1.947705	0.296105	6.577745	4.78E-11	2.90E-10	UP
SMAD2	1964.412	0.452868	0.068851	6.577487	4.78E-11	2.90E-10	NOT
ZNF543	109.9964	0.689525	0.10484	6.576912	4.80E-11	2.91E-10	UP
ETHE1	1255.815	-0.77181	0.117355	-6.57676	4.81E-11	2.91E-10	DOWN
RAB27A	1500.214	-0.87625	0.133235	-6.57669	4.81E-11	2.91E-10	DOWN
LA16c-358	2.742339	2.02666	0.308181	6.57621	4.83E-11	2.92E-10	UP
BIK	63.58277	1.816011	0.276205	6.574861	4.87E-11	2.95E-10	UP
PNP	1888.388	-0.83703	0.12733	-6.5737	4.91E-11	2.97E-10	DOWN
MYRF	2488.598	1.12201	0.170694	6.573207	4.92E-11	2.98E-10	UP
U47924.32	2.982557	2.213132	0.336701	6.572994	4.93E-11	2.98E-10	UP
APOL1	12079.53	-1.10886	0.168703	-6.57284	4.94E-11	2.99E-10	DOWN
WRAP73	545.7675	0.476923	0.072568	6.572059	4.96E-11	3.00E-10	NOT

CCDC24	262.3975	0.919452	0.139914	6.571538	4.98E-11	3.01E-10	UP
GNAO1	884.0153	-1.92715	0.293301	-6.57054	5.01E-11	3.03E-10	DOWN
RP4-612B1	1.684985	2.544975	0.387362	6.57002	5.03E-11	3.04E-10	UP
HES4	165.3081	1.386032	0.210972	6.569736	5.04E-11	3.05E-10	UP
C1QTNF1	2001.676	-1.59397	0.242644	-6.56917	5.06E-11	3.06E-10	DOWN
RP3-453C1	20.287	-1.23076	0.187367	-6.56869	5.08E-11	3.07E-10	DOWN
SETD3	2319.831	-0.42419	0.06459	-6.56739	5.12E-11	3.09E-10	NOT
FAIM	339.0032	0.758086	0.115442	6.566827	5.14E-11	3.10E-10	UP
BRINP2	7.083138	-1.94534	0.296247	-6.56662	5.15E-11	3.11E-10	DOWN
TYRP1	17.60927	3.297412	0.502158	6.566485	5.15E-11	3.11E-10	UP
RP13-104I	86.16116	0.936639	0.142644	6.566269	5.16E-11	3.11E-10	UP
AIF1L	408.527	1.331958	0.202849	6.566262	5.16E-11	3.11E-10	UP
EYA1	6.020579	3.17044	0.482855	6.566033	5.17E-11	3.12E-10	UP
PABPC3	15.32807	1.315187	0.200315	6.565603	5.18E-11	3.12E-10	UP
ENPP6	37.0697	2.476134	0.377138	6.5656	5.18E-11	3.12E-10	UP
MT-ATP8	31773.78	-1.13939	0.173548	-6.56528	5.19E-11	3.13E-10	DOWN
SEMA3B	732.4082	1.724037	0.262601	6.565226	5.20E-11	3.13E-10	UP
AC000067	1.340814	2.554981	0.38919	6.564871	5.21E-11	3.14E-10	UP
RPL22L1	1593.32	1.267765	0.193118	6.564726	5.21E-11	3.14E-10	UP
MIR8071-1	3.502529	-2.4295	0.370165	-6.56329	5.26E-11	3.17E-10	DOWN
BAHCC1	571.9888	0.83719	0.127585	6.561799	5.32E-11	3.20E-10	UP
RP11-317I	11.60918	5.753914	0.876908	6.561595	5.32E-11	3.20E-10	UP
BOLA3	654.7577	0.801783	0.122193	6.561592	5.32E-11	3.20E-10	UP
LINC0062	19.29119	1.593359	0.242856	6.560927	5.35E-11	3.22E-10	UP
DMGDH	6482.64	-1.5131	0.230732	-6.55782	5.46E-11	3.28E-10	DOWN
FBLN1	2039.833	1.626696	0.248067	6.557475	5.47E-11	3.29E-10	UP
MASTL	375.7879	0.698799	0.10657	6.557179	5.48E-11	3.30E-10	UP
KCTD1	216.7155	1.026088	0.156501	6.556418	5.51E-11	3.31E-10	UP
HADHB	10651.77	-0.65524	0.099957	-6.55526	5.55E-11	3.34E-10	DOWN
SRPK1	1747.466	0.636137	0.097049	6.554798	5.57E-11	3.35E-10	UP
RGAG1	43.18008	2.276758	0.347371	6.554263	5.59E-11	3.36E-10	UP
PLAC8L1	13.10099	1.523915	0.232527	6.553703	5.61E-11	3.37E-10	UP
ACSL4	9461.999	1.934551	0.295195	6.553462	5.62E-11	3.38E-10	UP
RP11-417I	4.236806	2.559389	0.390541	6.553443	5.62E-11	3.38E-10	UP
IFI27L1	397.4283	0.97941	0.149453	6.553311	5.63E-11	3.38E-10	UP
LL09NC01	4.756568	1.584117	0.241767	6.552251	5.67E-11	3.40E-10	UP
ADAMDEC	53.16537	2.268825	0.346272	6.552147	5.67E-11	3.40E-10	UP
MRM1	371.0959	0.665951	0.101647	6.551593	5.69E-11	3.41E-10	UP
PLAG1	80.3034	1.507099	0.230041	6.551441	5.70E-11	3.42E-10	UP
HSD17B12	3814.007	-0.71843	0.10966	-6.55143	5.70E-11	3.42E-10	DOWN
TEK	391.1052	-1.30478	0.199203	-6.55	5.75E-11	3.45E-10	DOWN
RRP36	1451.295	0.524246	0.080053	6.548732	5.80E-11	3.48E-10	NOT
RP11-422I	91.92864	-2.35352	0.359412	-6.54824	5.82E-11	3.49E-10	DOWN
RP11-501(	3.480211	3.573049	0.54567	6.547999	5.83E-11	3.49E-10	UP
TTR	142201.3	-1.76712	0.269901	-6.54726	5.86E-11	3.51E-10	DOWN
RP11-94I2	5.932146	2.735517	0.417834	6.546901	5.87E-11	3.52E-10	UP
AP001062.	56.36417	1.01438	0.154949	6.546546	5.89E-11	3.52E-10	UP
RP11-533I	14.98104	1.057591	0.161562	6.546024	5.91E-11	3.54E-10	UP
ELOVL3	11.98094	2.476906	0.378393	6.545863	5.92E-11	3.54E-10	UP
MRE11A	523.2451	0.535284	0.08178	6.545387	5.93E-11	3.55E-10	NOT
ATP6V1F	2485.684	0.673447	0.102903	6.544472	5.97E-11	3.57E-10	UP
KDM5B	1358.041	0.837798	0.128019	6.544328	5.98E-11	3.57E-10	UP

MIR217HG	8.964342	3.812167	0.582583	6.543556	6.01E-11	3.59E-10	UP
TP53I11	1450.953	0.89255	0.136411	6.543087	6.03E-11	3.60E-10	UP
MAP4K4	4631.512	0.594734	0.090907	6.542247	6.06E-11	3.62E-10	UP
C2CD4D	12.89029	1.511105	0.230996	6.541703	6.08E-11	3.63E-10	UP
CELF3	1.813047	2.780137	0.425071	6.5404	6.14E-11	3.66E-10	UP
RP11-337(	118.6692	0.938246	0.143541	6.536443	6.30E-11	3.76E-10	UP
U2SURP	2207.764	0.533462	0.081618	6.53611	6.31E-11	3.77E-10	NOT
TANK	1432.248	-0.5635	0.086238	-6.53431	6.39E-11	3.81E-10	NOT
SEMA3G	444.2807	1.183305	0.181121	6.533243	6.44E-11	3.84E-10	UP
ADAMTS1	53.68217	1.608004	0.246144	6.532771	6.46E-11	3.85E-10	UP
FCGR2B	403.0159	-1.85336	0.283712	-6.53255	6.47E-11	3.86E-10	DOWN
ZBTB11-A:	78.97088	0.684188	0.104738	6.532401	6.47E-11	3.86E-10	UP
RNF167	4477.872	-0.456	0.069813	-6.53171	6.50E-11	3.88E-10	NOT
FZD2	68.25122	1.757375	0.269056	6.531645	6.51E-11	3.88E-10	UP
RP5-965G:	5.821957	1.710428	0.261911	6.530582	6.55E-11	3.90E-10	UP
ZNF101	267.3274	0.624636	0.09565	6.530452	6.56E-11	3.91E-10	UP
ZNF714	66.52507	1.594815	0.244213	6.530415	6.56E-11	3.91E-10	UP
CPT1B	65.48308	1.148058	0.175819	6.529766	6.59E-11	3.92E-10	UP
B3GNT5	395.1877	1.605212	0.245835	6.529642	6.59E-11	3.93E-10	UP
RP11-283I	21.54147	2.244968	0.343868	6.528572	6.64E-11	3.95E-10	UP
INSC	22.93252	2.023952	0.3101	6.526777	6.72E-11	4.00E-10	UP
XPO6	2482.187	0.441125	0.067607	6.52488	6.81E-11	4.05E-10	NOT
LRP4-AS1	3.271942	2.243636	0.343868	6.524697	6.81E-11	4.05E-10	UP
RP11-431I	11.00524	-1.84671	0.283039	-6.52456	6.82E-11	4.06E-10	DOWN
GLUL	115783.7	1.895865	0.290575	6.524519	6.82E-11	4.06E-10	UP
FGF14-AS2	106.8344	-1.23607	0.189456	-6.52433	6.83E-11	4.06E-10	DOWN
LRRFIP1	3527.874	-0.56388	0.08643	-6.52409	6.84E-11	4.07E-10	NOT
NCBP2	1924.777	0.465724	0.07139	6.52363	6.86E-11	4.08E-10	NOT
WBP4	489.0863	-0.46649	0.071511	-6.52332	6.88E-11	4.09E-10	NOT
RASGRP4	63.84661	-0.95658	0.146641	-6.5233	6.88E-11	4.09E-10	DOWN
ITGB1BP2	33.83816	1.277175	0.195788	6.523259	6.88E-11	4.09E-10	UP
GOLM1	4060.045	1.416891	0.217215	6.522983	6.89E-11	4.09E-10	UP
COBLL1	4494.345	-1.1064	0.16967	-6.52089	6.99E-11	4.15E-10	DOWN
RP1-179N	18.22655	1.330828	0.204163	6.518443	7.10E-11	4.22E-10	UP
UBB	31176.93	-0.61526	0.094407	-6.51707	7.17E-11	4.25E-10	DOWN
RP3-449O	56.90419	0.896359	0.137541	6.517021	7.17E-11	4.26E-10	UP
PFAS	752.0331	0.62142	0.095357	6.516775	7.18E-11	4.26E-10	UP
RP11-110!	3.184308	2.13498	0.327691	6.515223	7.26E-11	4.30E-10	UP
SPAG4	229.439	1.269561	0.194879	6.514622	7.29E-11	4.32E-10	UP
SFT2D2	2526.158	0.573474	0.088036	6.514109	7.31E-11	4.33E-10	NOT
HAUS1	369.392	0.710436	0.109071	6.513542	7.34E-11	4.35E-10	UP
ZBTB26	172.7135	0.733774	0.112655	6.513477	7.34E-11	4.35E-10	UP
JADE2	833.8048	0.709438	0.108935	6.512473	7.39E-11	4.38E-10	UP
FOXL1	30.91569	1.66358	0.255476	6.511684	7.43E-11	4.40E-10	UP
TGFBR3	1426.867	-1.14794	0.176298	-6.51134	7.45E-11	4.41E-10	DOWN
PHBP11	14.4302	1.603074	0.246206	6.511112	7.46E-11	4.42E-10	UP
GBX2	2.271511	3.321378	0.510118	6.510999	7.47E-11	4.42E-10	UP
HRAS	1004.235	0.890834	0.136835	6.510277	7.50E-11	4.44E-10	UP
NPHS1	6.427915	3.145764	0.483259	6.509482	7.54E-11	4.46E-10	UP
PPFIA3	389.9204	0.89467	0.137444	6.509358	7.55E-11	4.46E-10	UP
HAO1	11667.4	-1.58556	0.243602	-6.50884	7.57E-11	4.48E-10	DOWN
ANKRD45	14.82697	2.02285	0.310791	6.508705	7.58E-11	4.48E-10	UP

BOLA1	834.2477	0.714442	0.109769	6.508595	7.59E-11	4.48E-10	UP
LPAL2	234.7773	-1.30141	0.199957	-6.50844	7.59E-11	4.49E-10	DOWN
CTC-459F	83.6963	0.816158	0.1254	6.508416	7.59E-11	4.49E-10	UP
LLNLR-47(	11.80032	-1.89529	0.291265	-6.50711	7.66E-11	4.53E-10	DOWN
AVL9	1505.246	0.54134	0.083198	6.506627	7.69E-11	4.54E-10	NOT
RPP14	954.2946	-0.49637	0.076288	-6.50655	7.69E-11	4.54E-10	NOT
HSD17B1F	15.65688	1.162256	0.178637	6.506255	7.70E-11	4.55E-10	UP
CYB5D2	1221.349	-0.9128	0.140299	-6.50612	7.71E-11	4.55E-10	DOWN
CDHR2	2432.042	-2.11507	0.325127	-6.50538	7.75E-11	4.57E-10	DOWN
CASC9	127.0004	3.401049	0.522813	6.50529	7.75E-11	4.58E-10	UP
SYAP1	3976.488	-0.57326	0.088127	-6.50486	7.78E-11	4.59E-10	NOT
RP11-341I	8.612748	1.835674	0.282246	6.503804	7.83E-11	4.62E-10	UP
MEF2D	2385.98	0.554955	0.085334	6.503359	7.85E-11	4.63E-10	NOT
RP11-496I	2.534945	2.828013	0.43491	6.502532	7.90E-11	4.66E-10	UP
CASC20	7.533061	5.070786	0.779849	6.502265	7.91E-11	4.66E-10	UP
HSP90AB2	26.36889	1.218815	0.187447	6.502168	7.92E-11	4.67E-10	UP
OGG1	938.1468	0.675083	0.103838	6.501331	7.96E-11	4.69E-10	UP
PASK	242.3337	0.875381	0.134654	6.500985	7.98E-11	4.70E-10	UP
CTC-487M	19.41113	1.056373	0.16251	6.50035	8.01E-11	4.72E-10	UP
CCAR1	1700.8	0.489324	0.075279	6.500152	8.02E-11	4.72E-10	NOT
RP11-9E1	81.21401	1.036891	0.159519	6.500106	8.03E-11	4.72E-10	UP
TYMSOS	113.6459	1.27971	0.196882	6.499892	8.04E-11	4.73E-10	UP
YWHAEP7	8.52512	3.241538	0.498736	6.499513	8.06E-11	4.74E-10	UP
LINC01315	30.5884	1.316752	0.202594	6.49945	8.06E-11	4.74E-10	UP
CRAT	7787.723	-0.75308	0.115887	-6.4984	8.12E-11	4.77E-10	DOWN
MICU1	3931.268	-0.59717	0.091897	-6.49824	8.13E-11	4.78E-10	DOWN
AC093616	12.08888	1.335638	0.205546	6.497997	8.14E-11	4.79E-10	UP
MPP2	37.29614	1.799453	0.277025	6.495643	8.27E-11	4.86E-10	UP
TMEM254	1782.22	-0.65824	0.101344	-6.49509	8.30E-11	4.88E-10	DOWN
UBTFL6	1.716595	2.657491	0.409211	6.49418	8.35E-11	4.90E-10	UP
GOLGA6L1	10.39341	1.235658	0.190272	6.494157	8.35E-11	4.90E-10	UP
PPP1R16A	4144.582	1.075566	0.165634	6.493639	8.38E-11	4.92E-10	UP
TBC1D10B	1196.372	0.538883	0.082989	6.493443	8.39E-11	4.93E-10	NOT
ZNF32-AS	10.76933	1.369892	0.210975	6.493147	8.41E-11	4.93E-10	UP
SLC9A3R2	10232.98	-0.95816	0.147569	-6.493	8.41E-11	4.94E-10	DOWN
AC068580	82.8751	1.094682	0.168597	6.492903	8.42E-11	4.94E-10	UP
KIAA0922	1411.137	-0.83057	0.127922	-6.49281	8.42E-11	4.94E-10	DOWN
TIA1	1587.514	0.684428	0.105423	6.492214	8.46E-11	4.96E-10	UP
ZNF30	142.1277	0.663024	0.102127	6.492131	8.46E-11	4.96E-10	UP
AC159540	5.170706	2.088665	0.321734	6.491898	8.48E-11	4.97E-10	UP
ZNF318	1188.662	0.663087	0.10215	6.49133	8.51E-11	4.99E-10	UP
AC004540	49.31747	-2.17065	0.334439	-6.49043	8.56E-11	5.02E-10	DOWN
TMEM180	428.1813	0.746964	0.115139	6.487487	8.73E-11	5.11E-10	UP
PPM1K	560.0252	-1.20299	0.185471	-6.48614	8.81E-11	5.16E-10	DOWN
MCF2L	665.3312	0.868958	0.133983	6.485603	8.84E-11	5.18E-10	UP
CAMSAP2	998.5254	0.772272	0.119088	6.484883	8.88E-11	5.20E-10	UP
SPATA22	2.262724	-2.71724	0.419034	-6.48454	8.90E-11	5.21E-10	DOWN
CSF3	3.840244	-2.76349	0.426221	-6.4837	8.95E-11	5.24E-10	DOWN
HDGFRP2	2332.016	0.63355	0.097717	6.483541	8.96E-11	5.24E-10	UP
ME3	266.4684	1.106626	0.170696	6.483005	8.99E-11	5.26E-10	UP
TOMM20	8686.206	0.605533	0.093404	6.48297	8.99E-11	5.26E-10	UP
NAA20	2826.834	0.552847	0.085291	6.481866	9.06E-11	5.30E-10	NOT

WBP2NL	26.84655	0.954308	0.147235	6.481545	9.08E-11	5.31E-10	UP
RP11-42I1	14.62856	1.052763	0.162441	6.480909	9.12E-11	5.33E-10	UP
RP11-129!	5.608589	3.009566	0.46442	6.480261	9.16E-11	5.35E-10	UP
PRRT4	32.68707	2.042402	0.315178	6.480163	9.16E-11	5.35E-10	UP
HIST1H2A	102.1897	1.656103	0.255584	6.479694	9.19E-11	5.37E-10	UP
COPG2	805.1504	0.618737	0.095492	6.479458	9.21E-11	5.38E-10	UP
RP11-531/	19.21745	1.341721	0.207081	6.479203	9.22E-11	5.38E-10	UP
CXCL2	2563.969	-1.5614	0.241016	-6.4784	9.27E-11	5.41E-10	DOWN
FAM83H-/	68.72224	1.558931	0.240654	6.477885	9.30E-11	5.43E-10	UP
HSPH1	2620.819	0.74557	0.115096	6.477783	9.31E-11	5.43E-10	UP
KB-1742H	7.109729	2.131855	0.329103	6.477769	9.31E-11	5.43E-10	UP
DENND4A	1489.364	-0.70744	0.109214	-6.4776	9.32E-11	5.44E-10	DOWN
HKDC1	1821.32	1.891414	0.291996	6.477524	9.32E-11	5.44E-10	UP
TSEN2	257.7236	0.565266	0.087271	6.477145	9.35E-11	5.45E-10	NOT
DDN	7.066136	2.536043	0.391543	6.477041	9.35E-11	5.45E-10	UP
NAA10	1539.911	0.814632	0.125785	6.476394	9.39E-11	5.48E-10	UP
C6orf1	750.28	0.825119	0.127414	6.475907	9.42E-11	5.49E-10	UP
MITD1	456.1773	0.515381	0.079599	6.474707	9.50E-11	5.53E-10	NOT
RP11-104I	9.081003	1.573295	0.243017	6.47401	9.54E-11	5.56E-10	UP
MAPT-IT1	5.392401	3.099971	0.478848	6.473808	9.56E-11	5.57E-10	UP
AC144449	23.94456	-1.25313	0.193584	-6.47331	9.59E-11	5.58E-10	DOWN
CNIH1	5120.992	-0.61749	0.095413	-6.47175	9.69E-11	5.64E-10	DOWN
PSMB4	11856.9	0.606358	0.093694	6.471692	9.69E-11	5.64E-10	UP
RP11-694I	25.88558	1.454892	0.224827	6.471155	9.73E-11	5.66E-10	UP
RAB20	1561.01	-1.01217	0.156434	-6.47027	9.78E-11	5.69E-10	DOWN
THRA	958.7418	0.690077	0.10666	6.469897	9.81E-11	5.70E-10	UP
RBM12B-/	7.842819	1.257378	0.194343	6.469892	9.81E-11	5.70E-10	UP
C8orf59	1139.485	0.767332	0.118605	6.46966	9.82E-11	5.71E-10	UP
ZNF83	566.742	1.511057	0.23357	6.469388	9.84E-11	5.72E-10	UP
RAN	6080.684	0.576008	0.089045	6.468761	9.88E-11	5.74E-10	NOT
SCUBE3	20.63607	1.219819	0.188573	6.468686	9.89E-11	5.74E-10	UP
TFAP4	376.3319	0.621226	0.096058	6.467185	9.98E-11	5.80E-10	UP
RP11-475I	3.361505	2.046019	0.316423	6.466087	1.01E-10	5.84E-10	UP
SERHL	13.25484	1.212385	0.187504	6.465917	1.01E-10	5.85E-10	UP
ZFPL1	280.5936	0.464906	0.071901	6.465898	1.01E-10	5.85E-10	NOT
RP11-295I	18.71744	1.411586	0.218346	6.464917	1.01E-10	5.88E-10	UP
MYADML2	1.845806	2.659226	0.411362	6.464445	1.02E-10	5.90E-10	UP
AC006128	68.34799	1.277014	0.197574	6.463482	1.02E-10	5.94E-10	UP
SIRT1	812.2332	-0.66791	0.103342	-6.46308	1.03E-10	5.95E-10	DOWN
RP5-874C:	2.596976	2.861215	0.442739	6.462538	1.03E-10	5.97E-10	UP
RP11-314I	9.753156	4.913232	0.760292	6.462295	1.03E-10	5.98E-10	UP
MIS18A-A	3.622976	1.770685	0.274009	6.462141	1.03E-10	5.98E-10	UP
BAZ2A	2352.449	0.576603	0.089235	6.461598	1.04E-10	6.00E-10	NOT
CTD-2527	6.86932	1.927429	0.298323	6.46087	1.04E-10	6.03E-10	UP
RP11-325I	4.469012	1.967409	0.30452	6.460679	1.04E-10	6.04E-10	UP
FKBP9	1953.355	0.620448	0.096051	6.459546	1.05E-10	6.08E-10	UP
RP11-635I	134.6712	-1.06725	0.165241	-6.45873	1.06E-10	6.11E-10	DOWN
NAALADL1	164.6967	1.593518	0.24678	6.457239	1.07E-10	6.17E-10	UP
ADRA1B	261.8253	-1.45411	0.225193	-6.45716	1.07E-10	6.18E-10	DOWN
ROCK1P1	16.58252	1.417859	0.219582	6.457085	1.07E-10	6.18E-10	UP
PKD1P6	71.86166	0.966141	0.149625	6.457061	1.07E-10	6.18E-10	UP
RP11-496I	67.38353	1.22982	0.190486	6.456213	1.07E-10	6.21E-10	UP

DYNLL1-A	165.2828	0.705566	0.109289	6.455988	1.08E-10	6.22E-10	UP
EMR1	67.63676	-1.79863	0.278632	-6.45521	1.08E-10	6.25E-10	DOWN
KBTBD11-	6.151347	-2.05657	0.318623	-6.45456	1.09E-10	6.27E-10	DOWN
RP11-285(	4.685192	1.716693	0.265992	6.453929	1.09E-10	6.30E-10	UP
DDX60L	599.2635	-0.94392	0.14626	-6.45374	1.09E-10	6.31E-10	DOWN
TMEM88B	3.125669	3.733379	0.57857	6.45277	1.10E-10	6.34E-10	UP
AXDND1	7.276815	1.928919	0.299003	6.451159	1.11E-10	6.41E-10	UP
CDA	1096.806	-1.51685	0.235148	-6.45063	1.11E-10	6.43E-10	DOWN
GABARAPL	5332.724	-1.12149	0.173876	-6.44993	1.12E-10	6.46E-10	DOWN
POLN	87.03529	1.398708	0.21689	6.448938	1.13E-10	6.50E-10	UP
CCDC3	873.0378	-1.18699	0.184083	-6.44816	1.13E-10	6.53E-10	DOWN
CASS4	95.56094	-1.08011	0.167575	-6.44553	1.15E-10	6.65E-10	DOWN
MTERF3	758.5184	0.62063	0.096289	6.445513	1.15E-10	6.65E-10	UP
BPIFB2	197.6592	3.150394	0.488784	6.445376	1.15E-10	6.65E-10	UP
ASS1P11	21.39354	-2.35646	0.365606	-6.44534	1.15E-10	6.65E-10	DOWN
HOXA6	2.959502	3.604091	0.559193	6.445159	1.15E-10	6.66E-10	UP
GCNT1P3	1.818745	-2.16005	0.335149	-6.44504	1.16E-10	6.66E-10	DOWN
ZNF552	220.647	0.755169	0.117179	6.444587	1.16E-10	6.68E-10	UP
ARHGEF12	8178.995	-0.65953	0.102354	-6.44363	1.17E-10	6.72E-10	DOWN
RGN	8073.843	-1.31167	0.203574	-6.44324	1.17E-10	6.74E-10	DOWN
SLC38A8	6.63327	3.986932	0.618782	6.443197	1.17E-10	6.74E-10	UP
PHLDB2	1808.948	-0.82553	0.128126	-6.44311	1.17E-10	6.74E-10	DOWN
RAB33B	604.1319	-0.78634	0.122044	-6.44307	1.17E-10	6.74E-10	DOWN
RNVU1-6	3.944552	2.162084	0.3356	6.44244	1.18E-10	6.77E-10	UP
CHRNA3	5.488363	2.890533	0.448727	6.441636	1.18E-10	6.80E-10	UP
RP11-972I	13.46089	1.271887	0.197463	6.441154	1.19E-10	6.82E-10	UP
PDE6G	57.35969	-1.12035	0.17394	-6.44101	1.19E-10	6.83E-10	DOWN
MAP4	7430.272	0.479176	0.0744	6.440541	1.19E-10	6.85E-10	NOT
CNOT11	1963.655	0.596122	0.092564	6.440095	1.19E-10	6.86E-10	UP
R3HCC1	1240.553	-0.56211	0.087288	-6.43971	1.20E-10	6.88E-10	NOT
C16orf87	655.5296	-0.77605	0.120513	-6.43954	1.20E-10	6.89E-10	DOWN
GOLPH3	6371.055	-0.59946	0.093096	-6.43915	1.20E-10	6.90E-10	DOWN
IL4R	3563.861	-0.88246	0.137059	-6.43856	1.21E-10	6.93E-10	DOWN
ASS1P9	8.630381	-1.64499	0.255532	-6.4375	1.21E-10	6.98E-10	DOWN
HDAC7	1324.453	0.859488	0.133519	6.437175	1.22E-10	6.99E-10	UP
DPYSL4	40.46291	2.130029	0.330902	6.437047	1.22E-10	6.99E-10	UP
CACNA2D	34.5849	1.889017	0.293551	6.435061	1.23E-10	7.08E-10	UP
RTN2	352.5781	1.293733	0.201048	6.434934	1.24E-10	7.09E-10	UP
AC007405	14.03538	1.334653	0.207408	6.434928	1.24E-10	7.09E-10	UP
ADAT2	176.8034	0.787005	0.122303	6.434874	1.24E-10	7.09E-10	UP
RBM33	1368.082	0.412849	0.064162	6.434522	1.24E-10	7.10E-10	NOT
ARMC1	1509.123	0.530433	0.082436	6.434511	1.24E-10	7.10E-10	NOT
ANKRD9	734.5568	0.915723	0.142334	6.43363	1.25E-10	7.14E-10	UP
SCGN	534.6279	2.152893	0.334654	6.433187	1.25E-10	7.16E-10	UP
ABCC1	804.9924	1.368162	0.212693	6.432568	1.25E-10	7.19E-10	UP
PITPNB	2825.241	-0.49262	0.076584	-6.43234	1.26E-10	7.20E-10	NOT
ARHGAP4	1277.207	1.136537	0.176711	6.431628	1.26E-10	7.23E-10	UP
LARP7	1251.845	-0.44911	0.069831	-6.43141	1.26E-10	7.24E-10	NOT
RP11-156I	11.07188	1.361649	0.211727	6.43116	1.27E-10	7.25E-10	UP
G6PC3	1096.689	0.83539	0.129913	6.43037	1.27E-10	7.29E-10	UP
SUN1	3069.823	0.569536	0.088571	6.430269	1.27E-10	7.29E-10	NOT
IMPDH2	3912.253	0.751376	0.11685	6.430241	1.27E-10	7.29E-10	UP

ZNF778	181.1982	0.611325	0.095114	6.427296	1.30E-10	7.43E-10	UP
AMBP	319827.7	-1.27902	0.198999	-6.42727	1.30E-10	7.43E-10	DOWN
ATN1	2793.98	0.613394	0.095442	6.426887	1.30E-10	7.45E-10	UP
LEAP2	5921.974	-1.42893	0.222341	-6.42677	1.30E-10	7.45E-10	DOWN
BMP4	457.021	1.715786	0.267004	6.426072	1.31E-10	7.49E-10	UP
GABRP	84.0828	-2.32837	0.362374	-6.42532	1.32E-10	7.52E-10	DOWN
EVA1A	2834.736	-1.2314	0.19165	-6.42526	1.32E-10	7.52E-10	DOWN
DLL3	3.271874	3.376315	0.525494	6.425035	1.32E-10	7.53E-10	UP
PRRG4	1494.006	-0.98892	0.153947	-6.42376	1.33E-10	7.59E-10	DOWN
SRRM5	20.58631	1.067315	0.166153	6.423669	1.33E-10	7.60E-10	UP
NPM1	12925.17	0.653472	0.101737	6.423155	1.33E-10	7.62E-10	UP
TNNI2	41.86379	1.616266	0.251679	6.421928	1.35E-10	7.68E-10	UP
ITLN1	64.3828	-2.52118	0.39259	-6.42192	1.35E-10	7.68E-10	DOWN
VPS8	710.3081	0.365035	0.056848	6.421221	1.35E-10	7.71E-10	NOT
C1orf112	329.1689	1.122228	0.174772	6.421104	1.35E-10	7.72E-10	UP
AC104534	13.33684	1.672437	0.260493	6.420266	1.36E-10	7.76E-10	UP
FUT1	157.7384	1.215437	0.189319	6.420037	1.36E-10	7.77E-10	UP
ADAMTS1	503.3634	1.212697	0.188915	6.419263	1.37E-10	7.81E-10	UP
CYP2J2	4365.929	-1.2377	0.19284	-6.41828	1.38E-10	7.86E-10	DOWN
YEATS2-A	11.15645	1.223158	0.190587	6.417861	1.38E-10	7.88E-10	UP
GATM	56759.58	-1.24745	0.194418	-6.41634	1.40E-10	7.95E-10	DOWN
NKPD1	4.93544	2.310644	0.360212	6.414682	1.41E-10	8.04E-10	UP
HNRNPA1I	10.43966	1.253384	0.195399	6.414485	1.41E-10	8.05E-10	UP
MIR4653	2.653536	2.279709	0.355442	6.413734	1.42E-10	8.09E-10	UP
UBE2Q2P2	22.66826	0.959833	0.149654	6.413701	1.42E-10	8.09E-10	UP
TTC27	634.2404	0.460984	0.071879	6.413378	1.42E-10	8.10E-10	NOT
PLIN1	654.2939	-1.7017	0.265337	-6.41338	1.42E-10	8.10E-10	DOWN
RP4-758J1	5.073066	2.063202	0.321715	6.413129	1.43E-10	8.11E-10	UP
ABHD15	1501.407	-0.93665	0.146065	-6.41259	1.43E-10	8.14E-10	DOWN
C1orf229	22.09383	1.607889	0.250757	6.412147	1.43E-10	8.16E-10	UP
F8	889.1523	-1.11087	0.173252	-6.41191	1.44E-10	8.17E-10	DOWN
ADNP	2697.493	0.465624	0.072632	6.410722	1.45E-10	8.23E-10	NOT
SPTY2D1-	18.18547	1.09295	0.170502	6.410181	1.45E-10	8.26E-10	UP
PPP1R13L	640.364	0.910024	0.14199	6.409085	1.46E-10	8.32E-10	UP
GRM2	18.38933	1.760712	0.274725	6.408986	1.46E-10	8.32E-10	UP
MKLN1-AS	53.47868	0.927524	0.144726	6.408822	1.47E-10	8.33E-10	UP
KLHDC8A	22.15904	2.425938	0.378623	6.407273	1.48E-10	8.41E-10	UP
ZNF93	76.87338	1.516701	0.236724	6.407044	1.48E-10	8.42E-10	UP
PAXIP1	509.9981	0.54809	0.085545	6.407029	1.48E-10	8.42E-10	NOT
TOR3A	2209.261	0.872161	0.136169	6.40497	1.50E-10	8.54E-10	UP
ABHD4	1772.167	0.766104	0.119616	6.4047	1.51E-10	8.55E-10	UP
HYI-AS1	10.25504	1.417959	0.221394	6.404681	1.51E-10	8.55E-10	UP
AGXT2	3176.817	-1.69572	0.264768	-6.40457	1.51E-10	8.55E-10	DOWN
SNORA11	5.519157	1.347293	0.210375	6.404257	1.51E-10	8.57E-10	UP
UBE2L6	3734.018	-0.9116	0.142365	-6.40328	1.52E-10	8.62E-10	DOWN
CBX5	2503.157	0.687289	0.107365	6.40145	1.54E-10	8.72E-10	UP
RP1-118J2	3.272634	2.976724	0.465008	6.401444	1.54E-10	8.72E-10	UP
RP11-267(	6.291691	4.624947	0.722528	6.401059	1.54E-10	8.74E-10	UP
ZNF195	473.2708	0.579195	0.090486	6.400924	1.54E-10	8.75E-10	NOT
XXbac-B56	10.52396	1.481928	0.231524	6.400757	1.55E-10	8.76E-10	UP
MAPK13	764.2256	1.713492	0.267738	6.399893	1.55E-10	8.80E-10	UP
CORIN	30.07755	1.65037	0.257909	6.399032	1.56E-10	8.85E-10	UP

PTHLH	68.84676	2.125318	0.33222	6.397315	1.58E-10	8.95E-10	UP
DSE	487.5614	-1.13434	0.17733	-6.39677	1.59E-10	8.98E-10	DOWN
ABCC9	1780.564	-1.45192	0.226992	-6.39633	1.59E-10	9.00E-10	DOWN
DHX35	456.0015	0.454787	0.071106	6.395883	1.60E-10	9.03E-10	NOT
CKMT1B	16.05126	3.518681	0.550156	6.395782	1.60E-10	9.03E-10	UP
RP11-126(	54.59583	-1.47409	0.230486	-6.39558	1.60E-10	9.04E-10	DOWN
CACNA1I	34.02373	2.621298	0.409944	6.394291	1.61E-10	9.12E-10	UP
AIFM2	1980.767	0.776308	0.121407	6.394269	1.61E-10	9.12E-10	UP
UTP6	1433.839	0.433183	0.067764	6.39252	1.63E-10	9.22E-10	NOT
ZNF547	56.11894	0.956738	0.149707	6.390731	1.65E-10	9.33E-10	UP
NKAIN2	45.86793	2.538843	0.397349	6.389458	1.66E-10	9.40E-10	UP
MMACHC	1280.135	-0.68689	0.107522	-6.38835	1.68E-10	9.47E-10	DOWN
NOV	127.8567	1.366392	0.213899	6.388011	1.68E-10	9.49E-10	UP
UGT2B10	13253.51	-1.88851	0.295638	-6.38791	1.68E-10	9.49E-10	DOWN
ZNF324B	116.9448	0.512934	0.0803	6.387682	1.68E-10	9.50E-10	NOT
KMT2D	1986.546	0.65726	0.102895	6.387673	1.68E-10	9.50E-10	UP
KISS1R	8.983303	3.684422	0.576876	6.386848	1.69E-10	9.55E-10	UP
RNF183	8.044717	2.503988	0.392142	6.385418	1.71E-10	9.64E-10	UP
RNF207	388.2377	0.838175	0.131264	6.385416	1.71E-10	9.64E-10	UP
RP11-59H	3.477385	2.138026	0.334837	6.385265	1.71E-10	9.65E-10	UP
TIGD6	241.7762	0.488936	0.076583	6.384387	1.72E-10	9.70E-10	NOT
CTC-548K	1.931316	2.90245	0.454637	6.384107	1.72E-10	9.72E-10	UP
RIMKLBP2	6.329159	1.543625	0.241823	6.383279	1.73E-10	9.77E-10	UP
MT-RNR2	525073.3	-1.03474	0.162125	-6.38238	1.74E-10	9.82E-10	DOWN
PSD4	3299.11	-0.69487	0.108884	-6.38179	1.75E-10	9.86E-10	DOWN
KRT86	42.72941	2.025899	0.317506	6.380654	1.76E-10	9.93E-10	UP
SLC6A14	12.55793	4.192104	0.657047	6.380217	1.77E-10	9.96E-10	UP
RRP9	810.9056	0.68998	0.108149	6.379905	1.77E-10	9.97E-10	UP
NCOA7	2146.946	-1.07517	0.168555	-6.37875	1.79E-10	1.00E-09	DOWN
AC142472	45.71309	0.896398	0.140538	6.378351	1.79E-10	1.01E-09	UP
RP11-783I	26.89075	1.288807	0.202078	6.377777	1.80E-10	1.01E-09	UP
BX842568.	25.49261	-2.09932	0.3292	-6.37703	1.81E-10	1.02E-09	DOWN
RP11-32D	1.4343	2.544914	0.3991	6.376636	1.81E-10	1.02E-09	UP
SRPX	321.0485	-1.81553	0.28472	-6.37653	1.81E-10	1.02E-09	DOWN
STK25	2986.736	0.457553	0.071759	6.376213	1.82E-10	1.02E-09	NOT
C7	6534.216	-2.09945	0.329264	-6.37621	1.82E-10	1.02E-09	DOWN
INSRR	4.494784	2.453201	0.384761	6.375909	1.82E-10	1.02E-09	UP
DNAH3	8.934677	2.157415	0.338497	6.373506	1.85E-10	1.04E-09	UP
PGF	328.9256	1.052172	0.165102	6.372867	1.86E-10	1.04E-09	UP
AZGP1P2	17.18047	-1.8437	0.289309	-6.37278	1.86E-10	1.04E-09	DOWN
TMIE	85.18801	1.836178	0.288155	6.37218	1.86E-10	1.05E-09	UP
RP11-81H	190.0256	2.435645	0.382246	6.371923	1.87E-10	1.05E-09	UP
NEDD4L	2351.169	0.768151	0.120557	6.371673	1.87E-10	1.05E-09	UP
ACSBG2	3.406059	2.393674	0.37571	6.371072	1.88E-10	1.05E-09	UP
TRIM27	3207.308	0.496902	0.078025	6.368498	1.91E-10	1.07E-09	NOT
ULK3	1265.337	0.568036	0.089196	6.368377	1.91E-10	1.07E-09	NOT
SNX4	2118.759	-0.47735	0.074974	-6.36693	1.93E-10	1.08E-09	NOT
FDCSP	49.03535	3.900115	0.612596	6.366542	1.93E-10	1.08E-09	UP
PRKD2	1545.283	0.497475	0.078141	6.366409	1.94E-10	1.08E-09	NOT
RNASE4	1110.886	-1.22416	0.192287	-6.36631	1.94E-10	1.08E-09	DOWN
MTMR9	317.407	-0.61098	0.095971	-6.3663	1.94E-10	1.08E-09	DOWN
SLC22A15	148.0062	1.806087	0.283702	6.366145	1.94E-10	1.09E-09	UP

POLR3A	931.6673	0.429142	0.067416	6.365536	1.95E-10	1.09E-09	NOT
GGPS1	984.8501	0.512823	0.080568	6.3651	1.95E-10	1.09E-09	NOT
NHP2	1692.699	0.64013	0.100572	6.364872	1.95E-10	1.09E-09	UP
TLR3	472.9093	-1.12597	0.176923	-6.36418	1.96E-10	1.10E-09	DOWN
ACRC	32.21598	0.910118	0.143008	6.364123	1.96E-10	1.10E-09	UP
TMPRSS15	9.893737	5.172732	0.812901	6.363302	1.97E-10	1.10E-09	UP
IMPDH1P6	7.093085	1.703857	0.267775	6.363024	1.98E-10	1.11E-09	UP
RP5-894A	42.72958	0.876356	0.137731	6.362819	1.98E-10	1.11E-09	UP
NAT10	1590.788	0.409593	0.064383	6.361789	1.99E-10	1.12E-09	NOT
RP11-455I	3.865151	1.744067	0.274163	6.361414	2.00E-10	1.12E-09	UP
RP11-826I	5.311305	1.578527	0.24816	6.360922	2.01E-10	1.12E-09	UP
TTC39A-A	5.646791	2.500336	0.39309	6.360714	2.01E-10	1.12E-09	UP
GSC	3.99396	3.238118	0.509119	6.360238	2.01E-10	1.13E-09	UP
ANO9	218.3668	1.847413	0.290477	6.359918	2.02E-10	1.13E-09	UP
NCKIPSD	1229.268	0.558311	0.087791	6.35957	2.02E-10	1.13E-09	NOT
CTD-2566	4.619385	3.944329	0.620221	6.359555	2.02E-10	1.13E-09	UP
SDHAP1	215.8058	0.58221	0.091563	6.358552	2.04E-10	1.14E-09	NOT
HMGN1	3091.852	0.61603	0.096887	6.358232	2.04E-10	1.14E-09	UP
ZPR1	1675.461	0.518474	0.081565	6.356572	2.06E-10	1.15E-09	NOT
KRTAP5-1	4.92885	2.48739	0.391318	6.356443	2.06E-10	1.15E-09	UP
CFHR3	7314.652	-2.05722	0.323655	-6.35621	2.07E-10	1.15E-09	DOWN
RGSL1	87.10779	3.096322	0.487158	6.355885	2.07E-10	1.16E-09	UP
ZEB2P1	3.536917	3.559392	0.560015	6.355884	2.07E-10	1.16E-09	UP
CRLF2	13.23823	2.801545	0.440807	6.355489	2.08E-10	1.16E-09	UP
NTS	413.2175	3.178196	0.500208	6.353745	2.10E-10	1.17E-09	UP
KAT7	1110.883	0.444126	0.069915	6.352343	2.12E-10	1.18E-09	NOT
CTB-129P	5.099228	1.684963	0.265272	6.35184	2.13E-10	1.19E-09	UP
OLA1	2305.628	0.550222	0.086625	6.351741	2.13E-10	1.19E-09	NOT
DENR	1943.501	0.396561	0.062436	6.351524	2.13E-10	1.19E-09	NOT
GPR65	112.0303	-1.12374	0.176932	-6.35125	2.14E-10	1.19E-09	DOWN
SLC38A4	18719.1	-1.47845	0.232789	-6.35103	2.14E-10	1.19E-09	DOWN
CALML6	18.8563	1.664749	0.26213	6.35086	2.14E-10	1.19E-09	UP
LRRC45	1479.716	0.854648	0.134574	6.35077	2.14E-10	1.19E-09	UP
TOP3B	13.98629	0.987445	0.155495	6.350344	2.15E-10	1.20E-09	UP
AGMAT	3770.004	-1.06879	0.168312	-6.35002	2.15E-10	1.20E-09	DOWN
CTC-444N	714.039	0.647725	0.102012	6.349519	2.16E-10	1.20E-09	UP
DGUOK-A	13.86273	1.20782	0.190239	6.348967	2.17E-10	1.21E-09	UP
NFE2L3	362.8457	1.290097	0.203201	6.348857	2.17E-10	1.21E-09	UP
KSR1	320.8523	0.845582	0.133189	6.348731	2.17E-10	1.21E-09	UP
LTBP2	1300.511	1.35875	0.214025	6.348557	2.17E-10	1.21E-09	UP
STRCP1	10.88983	2.055812	0.323828	6.348471	2.17E-10	1.21E-09	UP
LBHD1	120.418	0.782353	0.123238	6.34832	2.18E-10	1.21E-09	UP
ZNF92	189.5779	0.705328	0.111118	6.347553	2.19E-10	1.21E-09	UP
C2orf27A	46.01113	1.463931	0.230633	6.347436	2.19E-10	1.22E-09	UP
RGS17	17.10412	1.386916	0.218506	6.347274	2.19E-10	1.22E-09	UP
RP11-28H	1.566269	2.482143	0.39107	6.347051	2.19E-10	1.22E-09	UP
OSR1	59.47692	1.946785	0.306746	6.346569	2.20E-10	1.22E-09	UP
SPEF2	168.3182	1.080389	0.170238	6.346337	2.21E-10	1.22E-09	UP
RP5-1039I	36.81618	1.008374	0.158901	6.34592	2.21E-10	1.23E-09	UP
CIDECP	296.4832	0.702395	0.110685	6.345899	2.21E-10	1.23E-09	UP
TAB2	2803.224	-0.68205	0.107498	-6.34477	2.23E-10	1.24E-09	DOWN
ERC2	7.542105	2.965113	0.467362	6.344364	2.23E-10	1.24E-09	UP

SPNS1	105.9013	0.773858	0.121996	6.34332	2.25E-10	1.25E-09	UP
CTD-2292	17.2336	1.260929	0.198797	6.342794	2.26E-10	1.25E-09	UP
RP11-485(	1.787087	2.760861	0.435279	6.342738	2.26E-10	1.25E-09	UP
LRP12	219.1714	1.367127	0.215543	6.342709	2.26E-10	1.25E-09	UP
PITX2	8.994351	3.833841	0.60457	6.341429	2.28E-10	1.26E-09	UP
MAGI2-AS	681.5492	-1.58052	0.249239	-6.34136	2.28E-10	1.26E-09	DOWN
LPAR4	2.477717	2.416869	0.381145	6.341069	2.28E-10	1.26E-09	UP
DENND6B	210.2646	0.830101	0.130921	6.340498	2.29E-10	1.27E-09	UP
RP11-347I	10.6912	1.188751	0.187507	6.339757	2.30E-10	1.27E-09	UP
PAPSS1	809.2897	0.677237	0.106826	6.339595	2.30E-10	1.27E-09	UP
RP11-20J1	3.74342	3.877338	0.611609	6.339575	2.30E-10	1.27E-09	UP
FMNL3	665.1486	0.872758	0.137672	6.33942	2.31E-10	1.28E-09	UP
CTC-459F	18.58658	1.239166	0.195477	6.33919	2.31E-10	1.28E-09	UP
FRMD6	584.935	-1.05214	0.166003	-6.33807	2.33E-10	1.29E-09	DOWN
SLC16A4	271.1745	-1.55225	0.244917	-6.33786	2.33E-10	1.29E-09	DOWN
RP11-96B	8.314092	2.565211	0.404797	6.337024	2.34E-10	1.29E-09	UP
BRI3BP	1030.037	0.650888	0.102749	6.334731	2.38E-10	1.31E-09	UP
DKFZP434I	64.7862	0.746489	0.117842	6.334653	2.38E-10	1.31E-09	UP
9-Sep	9741.891	0.489766	0.077317	6.334488	2.38E-10	1.32E-09	NOT
CDK5RAP3	4247.063	0.57292	0.090456	6.333703	2.39E-10	1.32E-09	NOT
CAPS	508.6334	0.972552	0.153557	6.33347	2.40E-10	1.32E-09	UP
LRRK2	378.345	-1.16653	0.184198	-6.33306	2.40E-10	1.33E-09	DOWN
LINC01524	3.170682	3.758243	0.593524	6.332086	2.42E-10	1.33E-09	UP
OAS1	2077.816	-1.08878	0.171955	-6.33174	2.42E-10	1.34E-09	DOWN
RP11-287I	4.912602	2.336014	0.368987	6.330876	2.44E-10	1.34E-09	UP
GBA2	2096.854	0.504924	0.079759	6.330623	2.44E-10	1.35E-09	NOT
WIPF2	1538.082	0.444329	0.070189	6.330475	2.44E-10	1.35E-09	NOT
RP11-536I	59.71444	0.979029	0.154659	6.330256	2.45E-10	1.35E-09	UP
DECR1	10480.81	-0.85621	0.135263	-6.32999	2.45E-10	1.35E-09	DOWN
MST1R	161.2431	2.08175	0.328882	6.329772	2.46E-10	1.35E-09	UP
RP11-348A	1.775491	2.911477	0.459973	6.329662	2.46E-10	1.35E-09	UP
CDC37	5258.576	0.44097	0.069673	6.329165	2.46E-10	1.36E-09	NOT
IGHA2	767.2147	-2.07472	0.327813	-6.32897	2.47E-10	1.36E-09	DOWN
FANCF	573.5512	0.479653	0.075805	6.327484	2.49E-10	1.37E-09	NOT
XAGE5	10.89483	6.541752	1.033908	6.32721	2.50E-10	1.37E-09	UP
RFWD3	656.989	0.577349	0.091268	6.325866	2.52E-10	1.39E-09	NOT
HNRNPA1	12500.78	0.538323	0.085112	6.324879	2.53E-10	1.39E-09	NOT
PODXL2	387.092	2.103509	0.3326	6.32444	2.54E-10	1.40E-09	UP
MSL1	2310.643	0.401126	0.063425	6.324366	2.54E-10	1.40E-09	NOT
LINC01573	33.04501	1.265937	0.200173	6.324213	2.55E-10	1.40E-09	UP
MEOX2	49.84634	1.677665	0.265291	6.323862	2.55E-10	1.40E-09	UP
LDHA	27660.2	-0.69542	0.109975	-6.32345	2.56E-10	1.41E-09	DOWN
CAMK2D	1668.508	-0.5844	0.09242	-6.32333	2.56E-10	1.41E-09	NOT
AC093850	2.937564	3.0675	0.485283	6.32105	2.60E-10	1.43E-09	UP
RP11-10O	6.347798	1.257456	0.198937	6.320867	2.60E-10	1.43E-09	UP
RP11-551I	6.073428	2.702104	0.427505	6.320639	2.60E-10	1.43E-09	UP
AAGAB	1158.255	0.405499	0.064158	6.32031	2.61E-10	1.43E-09	NOT
EDEM1	3977.549	-0.73241	0.115885	-6.32011	2.61E-10	1.44E-09	DOWN
CTSO	3336.812	-0.95184	0.15062	-6.31949	2.62E-10	1.44E-09	DOWN
ZC3H10	348.6564	0.434596	0.068776	6.319029	2.63E-10	1.44E-09	NOT
LYRM4	908.9114	0.708356	0.1121	6.318953	2.63E-10	1.45E-09	UP
RP11-552I	10.33223	1.092594	0.172909	6.31889	2.63E-10	1.45E-09	UP

DUSP7	520.5301	0.541325	0.085669	6.318805	2.64E-10	1.45E-09	NOT
MBOAT7	2303.723	0.588972	0.093221	6.318031	2.65E-10	1.45E-09	UP
SGK494	48.39263	1.09337	0.173064	6.31771	2.65E-10	1.46E-09	UP
STK32B	28.89552	1.56933	0.248421	6.317233	2.66E-10	1.46E-09	UP
RP11-98D	2.443533	2.218697	0.351336	6.315025	2.70E-10	1.48E-09	UP
PCSK2	5.162657	-2.98685	0.473072	-6.31375	2.72E-10	1.49E-09	DOWN
CMTR2	687.3567	-0.62581	0.099155	-6.31146	2.76E-10	1.51E-09	DOWN
bP-2189O	4.599663	3.620146	0.573636	6.310881	2.77E-10	1.52E-09	UP
AXIN2	415.2143	1.964875	0.31136	6.310612	2.78E-10	1.52E-09	UP
CRBN	1244.042	-0.44723	0.070871	-6.31051	2.78E-10	1.52E-09	NOT
FOXRED2	1706.032	0.827549	0.131142	6.310321	2.78E-10	1.52E-09	UP
RP11-264I	12.74193	1.366737	0.216589	6.310281	2.79E-10	1.52E-09	UP
STOML1	768.3329	0.621961	0.09857	6.309839	2.79E-10	1.53E-09	UP
SNHG11	562.8378	0.88927	0.140953	6.308973	2.81E-10	1.54E-09	UP
CTC-537E	47.76372	-2.18779	0.346829	-6.30798	2.83E-10	1.55E-09	DOWN
ZNF800	633.4908	-0.53917	0.085475	-6.30794	2.83E-10	1.55E-09	NOT
FAM186A	15.72035	1.485802	0.235547	6.307893	2.83E-10	1.55E-09	UP
MT-ND4L	46814.51	-1.19091	0.188806	-6.30762	2.83E-10	1.55E-09	DOWN
RP11-508I	6.599198	1.506178	0.238792	6.307489	2.84E-10	1.55E-09	UP
LINC0083	24.77382	1.776076	0.281604	6.306995	2.85E-10	1.55E-09	UP
NLRP12	8.508147	-1.18993	0.18867	-6.30694	2.85E-10	1.55E-09	DOWN
GADD45A	3602.223	-1.0161	0.161111	-6.30683	2.85E-10	1.56E-09	DOWN
COL4A3BP	1431.84	-0.49308	0.078188	-6.30629	2.86E-10	1.56E-09	NOT
IYD	1405.217	-1.69127	0.268201	-6.30599	2.86E-10	1.56E-09	DOWN
RP11-557I	21.97001	-1.58563	0.251465	-6.30557	2.87E-10	1.57E-09	DOWN
ELP2	2901.818	-0.44864	0.071155	-6.30514	2.88E-10	1.57E-09	NOT
RP11-219I	1.853418	2.463268	0.390684	6.305014	2.88E-10	1.57E-09	UP
ZNF432	246.9065	0.870843	0.138147	6.303722	2.91E-10	1.59E-09	UP
FYN	1190.691	-0.90848	0.144132	-6.3031	2.92E-10	1.59E-09	DOWN
RP5-1031I	6.530333	1.861702	0.295383	6.302661	2.93E-10	1.60E-09	UP
SPATA6L	151.5469	-1.04338	0.165552	-6.30244	2.93E-10	1.60E-09	DOWN
ZNF764	340.29	0.566586	0.089902	6.302265	2.93E-10	1.60E-09	NOT
SALL1	2404.748	-0.98884	0.156903	-6.30222	2.93E-10	1.60E-09	DOWN
ABCA7	506.058	0.95445	0.151474	6.301095	2.96E-10	1.61E-09	UP
CTNNA1	10553.06	0.433691	0.068832	6.300762	2.96E-10	1.61E-09	NOT
AC009120	73.49069	0.953833	0.151414	6.299524	2.99E-10	1.63E-09	UP
PIGG	1131.648	0.493641	0.078364	6.299313	2.99E-10	1.63E-09	NOT
CTD-2547	18.27608	-1.22641	0.194691	-6.29926	2.99E-10	1.63E-09	DOWN
DLGAP1-A	52.7544	1.218562	0.193476	6.298272	3.01E-10	1.64E-09	UP
AC096574	5.132678	1.955034	0.310445	6.297524	3.02E-10	1.65E-09	UP
PCDHB10	30.91816	1.524215	0.242048	6.297149	3.03E-10	1.65E-09	UP
SNRPGP15	4.290631	1.943698	0.308701	6.29637	3.05E-10	1.66E-09	UP
CTA-292E	536.4608	-0.87983	0.139787	-6.29409	3.09E-10	1.68E-09	DOWN
ZBTB7B	3167.251	0.699897	0.111205	6.293758	3.10E-10	1.69E-09	UP
HDAC6	4379.712	-0.73554	0.116884	-6.29296	3.11E-10	1.69E-09	DOWN
RP11-15B	10.9037	2.670645	0.424435	6.292241	3.13E-10	1.70E-09	UP
C16orf70	1676.509	-0.78363	0.124539	-6.29223	3.13E-10	1.70E-09	DOWN
ZNF337	55.31693	0.959402	0.152485	6.291766	3.14E-10	1.71E-09	UP
INPP5E	445.7603	0.564863	0.089781	6.291548	3.14E-10	1.71E-09	NOT
RP11-132	70.38929	-1.79322	0.285196	-6.28769	3.22E-10	1.75E-09	DOWN
FAM212B-	3.042731	2.404026	0.382506	6.284935	3.28E-10	1.78E-09	UP
GTPBP4	1608.383	0.554201	0.088185	6.284551	3.29E-10	1.79E-09	NOT

SOSTDC1	15.18258	2.230086	0.354859	6.284423	3.29E-10	1.79E-09	UP
SLC44A5	171.7051	2.249145	0.357916	6.28401	3.30E-10	1.79E-09	UP
CTB-61M7	7.31975	-2.06081	0.327953	-6.28386	3.30E-10	1.79E-09	DOWN
BZW2	1252.911	0.678375	0.107958	6.283686	3.31E-10	1.79E-09	UP
SLC25A15	10.19064	3.581742	0.570009	6.283655	3.31E-10	1.79E-09	UP
CTA-796E	6.85074	3.974043	0.632543	6.282649	3.33E-10	1.81E-09	UP
KISS1	88.84519	1.696069	0.269971	6.282421	3.33E-10	1.81E-09	UP
OMG	12.96302	-1.43845	0.228983	-6.2819	3.34E-10	1.81E-09	DOWN
SATB2-AS	8.937689	1.659839	0.264228	6.281852	3.35E-10	1.81E-09	UP
NT5E	2421.854	-1.07297	0.170824	-6.28115	3.36E-10	1.82E-09	DOWN
TREX1	7.497936	0.925779	0.147391	6.281124	3.36E-10	1.82E-09	UP
B3GNT4	15.94689	1.317405	0.20977	6.280232	3.38E-10	1.83E-09	UP
RP11-379	3.968684	1.737102	0.27662	6.279748	3.39E-10	1.84E-09	UP
ABCA17P	10.27186	1.576483	0.251047	6.279636	3.39E-10	1.84E-09	UP
GPX8	219.4753	1.294118	0.206093	6.279306	3.40E-10	1.84E-09	UP
KSR2	19.40798	2.268976	0.361358	6.279024	3.41E-10	1.84E-09	UP
COLEC11	2577.083	-1.50323	0.239407	-6.27899	3.41E-10	1.84E-09	DOWN
RP11-169	2.75401	2.707877	0.431289	6.27856	3.42E-10	1.85E-09	UP
PDCD1LG2	74.66472	-1.30278	0.207506	-6.27828	3.42E-10	1.85E-09	DOWN
RP11-105	4.382676	2.03813	0.324649	6.277958	3.43E-10	1.86E-09	UP
AC012485	5.134849	1.782984	0.28403	6.277445	3.44E-10	1.86E-09	UP
RP11-485	3.788225	2.003511	0.31919	6.276858	3.45E-10	1.87E-09	UP
P3H1	1724.789	0.594975	0.094794	6.276536	3.46E-10	1.87E-09	UP
TMEM119	196.6809	1.850802	0.294888	6.276295	3.47E-10	1.87E-09	UP
LINC0113	32.6986	3.492901	0.556589	6.275545	3.48E-10	1.88E-09	UP
AP000997	3.767793	3.154567	0.502756	6.274554	3.51E-10	1.89E-09	UP
RP13-39P	5.920477	1.81652	0.289543	6.273751	3.52E-10	1.90E-09	UP
SUCNR1	197.4669	-1.28233	0.204401	-6.27359	3.53E-10	1.91E-09	DOWN
5-Mar	1501.132	-0.3995	0.063681	-6.27351	3.53E-10	1.91E-09	NOT
SH2D5	12.30971	2.291432	0.36529	6.272917	3.54E-10	1.91E-09	UP
CA5B	165.3632	1.143197	0.182244	6.272885	3.54E-10	1.91E-09	UP
ZNF233	39.05955	1.455014	0.231956	6.27279	3.55E-10	1.91E-09	UP
C22orf46	606.8538	0.560158	0.089309	6.272117	3.56E-10	1.92E-09	NOT
STARD9	156.2749	0.89787	0.143173	6.271244	3.58E-10	1.93E-09	UP
TTC19	1316.027	-0.6437	0.102647	-6.27098	3.59E-10	1.93E-09	DOWN
RFC5	587.8874	0.606035	0.096644	6.270782	3.59E-10	1.94E-09	UP
RP3-523E	2.164536	2.560629	0.408379	6.270226	3.61E-10	1.94E-09	UP
SHKBP1	1683.539	0.676711	0.107941	6.269266	3.63E-10	1.96E-09	UP
PDCL3	520.6644	0.522261	0.083327	6.267581	3.67E-10	1.98E-09	NOT
ST6GAL1	35374.33	-0.92098	0.146958	-6.26698	3.68E-10	1.98E-09	DOWN
RP11-383	3.712865	1.751515	0.279501	6.266575	3.69E-10	1.99E-09	UP
RPL39L	243.2638	1.627474	0.259761	6.265263	3.72E-10	2.00E-09	UP
RP11-398	8.703063	1.666608	0.266028	6.264772	3.73E-10	2.01E-09	UP
CBLN2	3.602714	-2.59559	0.414366	-6.26401	3.75E-10	2.02E-09	DOWN
RP11-879	6.592191	1.62435	0.259355	6.263036	3.78E-10	2.03E-09	UP
RP4-614O	36.22208	0.910556	0.145396	6.262577	3.79E-10	2.04E-09	UP
SNRPD1	1331.13	0.695685	0.111113	6.261078	3.82E-10	2.06E-09	UP
ACAT2	3487.12	-0.9269	0.148058	-6.26036	3.84E-10	2.07E-09	DOWN
TMEM82	1211.552	-1.90918	0.304981	-6.26001	3.85E-10	2.07E-09	DOWN
AC084219	4.396197	2.007642	0.320713	6.259927	3.85E-10	2.07E-09	UP
GNGT1	8.329231	4.741533	0.757489	6.259539	3.86E-10	2.08E-09	UP
ACVRL1	561.0662	0.756336	0.120833	6.259365	3.87E-10	2.08E-09	UP

PPP2R5D	1970.482	0.505919	0.080826	6.259355	3.87E-10	2.08E-09	NOT
ZBED3-AS	44.72147	1.26092	0.201451	6.259189	3.87E-10	2.08E-09	UP
RP11-315(	42.97574	1.172298	0.187294	6.259139	3.87E-10	2.08E-09	UP
GNRHR2	30.78978	0.817973	0.130693	6.258716	3.88E-10	2.08E-09	UP
IGHA1	8013.032	-2.14926	0.343431	-6.25821	3.89E-10	2.09E-09	DOWN
DPEP2	95.10546	-0.94295	0.150679	-6.258	3.90E-10	2.09E-09	DOWN
RP11-278/	11.29436	1.232538	0.196963	6.2577	3.91E-10	2.10E-09	UP
RP11-89H	19.83094	1.586235	0.253491	6.257553	3.91E-10	2.10E-09	UP
MYO5C	625.4333	0.848169	0.135584	6.255669	3.96E-10	2.12E-09	UP
TRANK1	1350.814	-0.92828	0.148399	-6.25532	3.97E-10	2.13E-09	DOWN
MAGEE1	61.61928	1.138627	0.182041	6.254778	3.98E-10	2.14E-09	UP
RDH16	14633.19	-1.93836	0.309969	-6.2534	4.02E-10	2.15E-09	DOWN
FLYWCH2	570.4871	0.810453	0.129603	6.253357	4.02E-10	2.15E-09	UP
C1orf95	41.47346	1.584449	0.253443	6.251687	4.06E-10	2.18E-09	UP
NOS2	64.18092	1.411811	0.225858	6.250866	4.08E-10	2.19E-09	UP
SLC46A3	2853.874	-1.44487	0.231157	-6.25061	4.09E-10	2.19E-09	DOWN
GTF2H4	99.7733	0.626473	0.100233	6.250137	4.10E-10	2.20E-09	UP
RP1-28H2	20.29402	-1.08048	0.172889	-6.24952	4.12E-10	2.21E-09	DOWN
CAPG	1300.189	1.388895	0.222252	6.249202	4.13E-10	2.21E-09	UP
RP11-146I	30.87088	0.919939	0.14721	6.249167	4.13E-10	2.21E-09	UP
PAIP1P1	8.399337	1.324244	0.21191	6.249091	4.13E-10	2.21E-09	UP
TUFT1	598.795	0.896507	0.143463	6.24906	4.13E-10	2.21E-09	UP
CYP27A1	28743.24	-1.15835	0.185368	-6.24895	4.13E-10	2.21E-09	DOWN
CTD-2311	6.0029	2.056411	0.329084	6.248886	4.13E-10	2.21E-09	UP
CASK	1555.723	0.629056	0.100669	6.248722	4.14E-10	2.21E-09	UP
SPP2	5680.912	-2.06246	0.33008	-6.24837	4.15E-10	2.22E-09	DOWN
UNC45A	2431.739	0.495426	0.079291	6.248171	4.15E-10	2.22E-09	NOT
ARPC1A	4476.051	0.472823	0.07568	6.247664	4.17E-10	2.23E-09	NOT
SPSB1	2507.843	-1.08776	0.174114	-6.24742	4.17E-10	2.23E-09	DOWN
BCL6B	484.5696	0.821001	0.131423	6.247002	4.18E-10	2.24E-09	UP
KRT16P1	8.984204	-4.37715	0.70076	-6.24629	4.20E-10	2.25E-09	DOWN
IGF2BP2-A	5.148595	2.839444	0.454608	6.245914	4.21E-10	2.25E-09	UP
KLK2	6.72841	4.846248	0.775974	6.245376	4.23E-10	2.26E-09	UP
BBS4	552.4821	0.562152	0.090028	6.244156	4.26E-10	2.28E-09	NOT
NMNAT2	22.28875	1.708219	0.273601	6.243469	4.28E-10	2.28E-09	UP
GLB1L3	13.77245	3.2205	0.515968	6.241661	4.33E-10	2.31E-09	UP
APOBEC3E	142.228	1.751381	0.280596	6.241656	4.33E-10	2.31E-09	UP
SLC22A8	5.25186	4.247895	0.680665	6.240804	4.35E-10	2.32E-09	UP
HIST1H3G	9.733786	3.191498	0.511515	6.239306	4.40E-10	2.34E-09	UP
NMD3	2649.2	-0.51514	0.082574	-6.23857	4.42E-10	2.36E-09	NOT
SEC23A	3562.546	-0.62939	0.100894	-6.23809	4.43E-10	2.36E-09	DOWN
CPB2	28400.37	-1.23858	0.198588	-6.23692	4.46E-10	2.38E-09	DOWN
RP11-3J1.:	3.38853	3.201393	0.513329	6.236529	4.47E-10	2.38E-09	UP
TRIM6	91.64821	1.4127	0.226531	6.236231	4.48E-10	2.39E-09	UP
RP11-574I	124.002	0.947998	0.15202	6.236012	4.49E-10	2.39E-09	UP
CDNF	138.6233	-0.969	0.155397	-6.23566	4.50E-10	2.40E-09	DOWN
RP11-793I	38.79795	1.019947	0.163589	6.234811	4.52E-10	2.41E-09	UP
AP000487.	13.75495	0.917328	0.147149	6.234021	4.55E-10	2.42E-09	UP
MCEE	727.4336	-0.76952	0.123442	-6.23382	4.55E-10	2.42E-09	DOWN
FAM21EP	12.3443	1.140005	0.182922	6.232196	4.60E-10	2.45E-09	UP
RPRML	5.69547	2.730705	0.438178	6.231949	4.61E-10	2.45E-09	UP
ALG6	578.9745	0.490367	0.078688	6.231802	4.61E-10	2.45E-09	NOT

TUBGCP6	1331.426	0.585934	0.094024	6.231761	4.61E-10	2.45E-09	UP
ADAMTSL:	1578.425	-1.47606	0.23687	-6.23149	4.62E-10	2.46E-09	DOWN
RP11-817I	5.721547	1.604778	0.257595	6.229853	4.67E-10	2.48E-09	UP
WDR17	7.860188	-1.54596	0.248162	-6.22963	4.68E-10	2.49E-09	DOWN
STEAP4	1214.304	-1.70182	0.273231	-6.22851	4.71E-10	2.50E-09	DOWN
RP11-165/	7.707758	2.059668	0.330696	6.228291	4.72E-10	2.51E-09	UP
PPIL1	1104.526	0.627258	0.100717	6.227942	4.73E-10	2.51E-09	UP
HNRNPU-/	191.3618	1.042809	0.167444	6.227814	4.73E-10	2.51E-09	UP
GLP2R	37.48803	-1.99101	0.319718	-6.2274	4.74E-10	2.52E-09	DOWN
C9orf41	831.7362	-0.5167	0.082976	-6.22704	4.75E-10	2.52E-09	NOT
CEACAM4	12.80506	-1.27211	0.204292	-6.2269	4.76E-10	2.53E-09	DOWN
OLFML3	709.5356	-1.39034	0.223287	-6.2267	4.76E-10	2.53E-09	DOWN
CIPC	759.1815	-0.6363	0.102212	-6.22533	4.81E-10	2.55E-09	DOWN
MFSD2A	4441.087	-2.25246	0.361834	-6.22512	4.81E-10	2.55E-09	DOWN
FAM53A	94.30934	0.993295	0.159563	6.225109	4.81E-10	2.55E-09	UP
TMCO1	6785.086	0.513173	0.082444	6.224501	4.83E-10	2.56E-09	NOT
SLC16A1-/	104.646	0.902758	0.145035	6.224428	4.83E-10	2.56E-09	UP
ZNF24	2615.825	-0.38853	0.062449	-6.22162	4.92E-10	2.61E-09	NOT
PON1	18362.72	-1.61166	0.259094	-6.2204	4.96E-10	2.63E-09	DOWN
LINC0048!	2.408643	2.674491	0.430012	6.219577	4.98E-10	2.64E-09	UP
RUFY1	1775.017	0.438874	0.070567	6.219228	5.00E-10	2.65E-09	NOT
PLA2G5	126.46	-1.14317	0.183835	-6.21845	5.02E-10	2.66E-09	DOWN
KCNG1	30.58406	2.211247	0.355598	6.218389	5.02E-10	2.66E-09	UP
RP11-626(	2.064782	2.301243	0.370086	6.218123	5.03E-10	2.67E-09	UP
DYNLL1	5253.504	0.5667	0.091142	6.217777	5.04E-10	2.67E-09	NOT
RP11-172I	7.201181	2.192755	0.352676	6.217474	5.05E-10	2.68E-09	UP
RP11-20D	57.04975	3.127346	0.503008	6.217289	5.06E-10	2.68E-09	UP
ENTPD3	12.15221	2.482009	0.399243	6.216783	5.07E-10	2.69E-09	UP
RP11-79O	1.902424	2.196394	0.353317	6.216488	5.08E-10	2.69E-09	UP
RP5-965G:	19.29916	1.36803	0.220093	6.215684	5.11E-10	2.70E-09	UP
RP11-249(	5.588343	-2.84165	0.457309	-6.21385	5.17E-10	2.74E-09	DOWN
RP11-936I	15.21911	1.96602	0.316462	6.212498	5.21E-10	2.76E-09	UP
RP11-403I	12.10235	1.154538	0.185853	6.212098	5.23E-10	2.76E-09	UP
HMGN4	825.0324	0.71341	0.11485	6.211652	5.24E-10	2.77E-09	UP
DPT	473.9026	-2.17304	0.349861	-6.21115	5.26E-10	2.78E-09	DOWN
ST13	11363.09	-0.45506	0.073267	-6.21095	5.27E-10	2.78E-09	NOT
TMEM249	5.934542	1.636117	0.263435	6.210706	5.27E-10	2.79E-09	UP
CTC-260E	9.030602	1.801799	0.290197	6.208888	5.34E-10	2.82E-09	UP
RP5-1056I	7.620702	2.510534	0.404359	6.208678	5.34E-10	2.82E-09	UP
RP4-545L1	8.380187	1.149106	0.185087	6.208472	5.35E-10	2.83E-09	UP
RP11-327.	50.07858	-1.60258	0.258131	-6.20838	5.35E-10	2.83E-09	DOWN
DBNDD1	1211.078	1.355308	0.218321	6.207854	5.37E-10	2.84E-09	UP
COL18A1	47204.71	-0.83324	0.134228	-6.20764	5.38E-10	2.84E-09	DOWN
RP1-59D1	13.63978	1.289426	0.207726	6.20735	5.39E-10	2.84E-09	UP
MX1	2944.92	-1.32403	0.213311	-6.20705	5.40E-10	2.85E-09	DOWN
E2F4	1364.157	0.515768	0.083116	6.205369	5.46E-10	2.88E-09	NOT
LINC0115:	53.94051	1.991094	0.32098	6.203163	5.53E-10	2.92E-09	UP
FDX1	2446.456	-0.74637	0.120334	-6.20251	5.56E-10	2.93E-09	DOWN
ADA	295.3802	0.832543	0.134241	6.201867	5.58E-10	2.94E-09	UP
C12orf66	415.569	-0.5666	0.091364	-6.20152	5.59E-10	2.95E-09	NOT
ACOX2	6192.909	-1.1824	0.190669	-6.20129	5.60E-10	2.95E-09	DOWN
PTGES3	10497.35	0.38776	0.06253	6.20114	5.61E-10	2.95E-09	NOT

LINC0068(	14.86238	3.217236	0.518828	6.20097	5.61E-10	2.96E-09	UP
C16orf72	1849.353	-0.46225	0.074555	-6.20006	5.64E-10	2.97E-09	NOT
RP11-35N	704.5177	1.52748	0.246376	6.199786	5.65E-10	2.98E-09	UP
SAAL1	374.0406	0.558171	0.090041	6.199055	5.68E-10	2.99E-09	NOT
RP11-273(	59.87898	-1.79618	0.289757	-6.19891	5.69E-10	2.99E-09	DOWN
LINC0049/	25.05276	3.076033	0.496349	6.197321	5.74E-10	3.02E-09	UP
CD58	301.503	0.710113	0.114592	6.19686	5.76E-10	3.03E-09	UP
MPP4	3.400415	1.901147	0.306795	6.196803	5.76E-10	3.03E-09	UP
ATAD3B	596.4784	0.775737	0.125203	6.195838	5.80E-10	3.05E-09	UP
SMARCC1	2628.656	0.501668	0.080973	6.195478	5.81E-10	3.06E-09	NOT
RP11-91K(	1.973763	2.51816	0.406541	6.19411	5.86E-10	3.08E-09	UP
RP11-381I	3.277035	2.115696	0.341569	6.194057	5.86E-10	3.08E-09	UP
SLCO6A1	6.699685	4.959159	0.800649	6.193925	5.87E-10	3.08E-09	UP
ELL2	5898.484	-0.91993	0.148525	-6.1938	5.87E-10	3.09E-09	DOWN
PHGDH	5489.021	-1.3441	0.217021	-6.19339	5.89E-10	3.09E-09	DOWN
RP11-10N	14.45672	1.055537	0.170467	6.192048	5.94E-10	3.12E-09	UP
PLA2G12A	1619.516	-0.64789	0.104646	-6.19121	5.97E-10	3.14E-09	DOWN
SDK1	200.1679	1.879217	0.303537	6.191066	5.98E-10	3.14E-09	UP
CYP27C1	30.62676	1.767379	0.28548	6.190902	5.98E-10	3.14E-09	UP
RCHY1	536.7945	-0.49793	0.080442	-6.18999	6.02E-10	3.16E-09	NOT
PRKAG2	1337.606	-0.85733	0.138524	-6.18909	6.05E-10	3.18E-09	DOWN
ATG5	1113.489	-0.42985	0.069466	-6.18798	6.09E-10	3.20E-09	NOT
WDR5	1885.666	0.524286	0.084727	6.187958	6.09E-10	3.20E-09	NOT
TMEM71	38.62279	-1.04288	0.168537	-6.18785	6.10E-10	3.20E-09	DOWN
ST6GALNA	331.2354	1.018469	0.164596	6.187691	6.11E-10	3.20E-09	UP
ZNF703	647.4192	1.303773	0.210716	6.187356	6.12E-10	3.21E-09	UP
ITGA11	211.8963	1.275566	0.206172	6.1869	6.14E-10	3.22E-09	UP
DHX16	1847.991	0.417148	0.067435	6.185942	6.17E-10	3.24E-09	NOT
RP11-380I	34.33639	1.318368	0.213125	6.185895	6.18E-10	3.24E-09	UP
TBXAS1	391.4145	-0.95034	0.153632	-6.18583	6.18E-10	3.24E-09	DOWN
PRR7	115.8103	1.245335	0.20133	6.185528	6.19E-10	3.24E-09	UP
PHTF2	507.8335	0.616788	0.099718	6.185317	6.20E-10	3.25E-09	UP
ACBD7	8.137594	1.691783	0.27354	6.184784	6.22E-10	3.26E-09	UP
THBD	683.4966	-1.17483	0.189965	-6.18444	6.23E-10	3.26E-09	DOWN
PAPLN	739.3424	1.784729	0.288598	6.184128	6.24E-10	3.27E-09	UP
SHISA4	600.3823	1.320296	0.213504	6.183932	6.25E-10	3.27E-09	UP
CORO6	33.21459	1.399224	0.226276	6.183706	6.26E-10	3.28E-09	UP
HAO2-IT1	8.666958	-2.18868	0.353966	-6.18331	6.28E-10	3.28E-09	DOWN
TBX15	1231.266	-1.74009	0.281429	-6.18305	6.29E-10	3.29E-09	DOWN
CTD-2318	4.079407	2.366888	0.382814	6.182863	6.29E-10	3.29E-09	UP
RP11-227I	2.583561	2.253541	0.364494	6.182659	6.30E-10	3.30E-09	UP
ARR3	5.639617	1.324242	0.214196	6.18239	6.31E-10	3.30E-09	UP
CTD-2523	3.585282	2.24803	0.363626	6.182267	6.32E-10	3.30E-09	UP
PDCD2L	233.2079	0.769929	0.124549	6.181747	6.34E-10	3.31E-09	UP
C20orf196	98.67249	0.781154	0.12637	6.181465	6.35E-10	3.32E-09	UP
FAM96AP2	6.465558	1.907449	0.308578	6.181423	6.35E-10	3.32E-09	UP
PCDHB11	46.05001	1.657131	0.268111	6.180754	6.38E-10	3.33E-09	UP
CDKL3	22.22983	0.663023	0.107274	6.180646	6.38E-10	3.33E-09	UP
SLC16A8	17.46417	1.49039	0.241165	6.179952	6.41E-10	3.35E-09	UP
DNAJB13	7.203464	2.259645	0.365643	6.179923	6.41E-10	3.35E-09	UP
IFFO1	727.5857	0.547309	0.088572	6.179272	6.44E-10	3.36E-09	NOT
AC005387	2.422148	1.899283	0.307404	6.178451	6.47E-10	3.38E-09	UP

PID1	2355.664	-0.97361	0.157602	-6.17766	6.51E-10	3.39E-09	DOWN
TTC38	8790.731	-0.90222	0.146054	-6.17727	6.52E-10	3.40E-09	DOWN
PSMD14	2228.114	0.544169	0.088102	6.176572	6.55E-10	3.42E-09	NOT
ZNF654	422.6747	-0.5189	0.084015	-6.17631	6.56E-10	3.42E-09	NOT
FGL1	84441.53	-1.58658	0.256896	-6.17595	6.58E-10	3.43E-09	DOWN
LYZ	9842.09	1.930838	0.312644	6.175832	6.58E-10	3.43E-09	UP
RP11-106(	9.407153	-1.17845	0.190863	-6.17434	6.64E-10	3.46E-09	DOWN
CTD-3138	5.312735	1.492235	0.241758	6.172439	6.72E-10	3.50E-09	UP
SULF1	813.9094	1.650498	0.267429	6.171731	6.75E-10	3.52E-09	UP
FAM163B	72.20512	-2.34373	0.37979	-6.17113	6.78E-10	3.53E-09	DOWN
PLK5	6.147074	2.266036	0.367208	6.17099	6.79E-10	3.53E-09	UP
ZNF669	108.4792	0.88657	0.143676	6.170605	6.80E-10	3.54E-09	UP
USP6NL	1123.427	-0.59357	0.096201	-6.17004	6.83E-10	3.55E-09	DOWN
RP11-359(	1.541994	2.188926	0.354772	6.169946	6.83E-10	3.55E-09	UP
PYY2	12.04976	1.477724	0.239529	6.169296	6.86E-10	3.57E-09	UP
CTB-25B1:	296.2857	0.790744	0.12818	6.169024	6.87E-10	3.57E-09	UP
PPBP	12.87498	-2.3326	0.37813	-6.16879	6.88E-10	3.58E-09	DOWN
STARD7-A	91.85592	0.53637	0.086953	6.168539	6.89E-10	3.58E-09	NOT
ELFN1-AS:	19.10168	2.871406	0.465516	6.168215	6.91E-10	3.59E-09	UP
RNF34	756.3747	0.462598	0.075	6.167975	6.92E-10	3.60E-09	NOT
BBC3	442.0086	0.818731	0.132769	6.166603	6.98E-10	3.63E-09	UP
CTD-2616	3.081282	2.62904	0.426341	6.166512	6.98E-10	3.63E-09	UP
EFHC2	10.09794	2.837082	0.46016	6.165425	7.03E-10	3.65E-09	UP
ZSCAN20	39.59589	0.917383	0.148797	6.165342	7.03E-10	3.65E-09	UP
RBM15B	1967.219	0.356887	0.057888	6.165079	7.04E-10	3.66E-09	NOT
RP11-475I	1.909638	-2.37958	0.385984	-6.16498	7.05E-10	3.66E-09	DOWN
RND1	3060.369	-1.28401	0.208288	-6.1646	7.07E-10	3.67E-09	DOWN
KRT17	124.5243	2.078296	0.337136	6.164565	7.07E-10	3.67E-09	UP
PPAP2C	932.8805	2.142293	0.347562	6.163771	7.10E-10	3.69E-09	UP
PRSS56	7.158099	4.39573	0.713227	6.163155	7.13E-10	3.70E-09	UP
PBX2	2045.65	0.594467	0.096459	6.162926	7.14E-10	3.70E-09	UP
ERICH4	2.356005	3.134608	0.508643	6.162682	7.15E-10	3.71E-09	UP
RP11-166(	10.24875	1.214033	0.197026	6.16178	7.19E-10	3.73E-09	UP
ZNF391	45.74823	1.634235	0.265239	6.161369	7.21E-10	3.74E-09	UP
CPE	2405.149	1.292117	0.209717	6.161253	7.22E-10	3.74E-09	UP
TATDN1	747.1841	0.670409	0.108818	6.160857	7.24E-10	3.75E-09	UP
SLC6A17	5.925177	1.865444	0.30279	6.16084	7.24E-10	3.75E-09	UP
NFYC-AS1	25.40016	1.003621	0.162905	6.160755	7.24E-10	3.75E-09	UP
RP11-37B:	108.5369	0.8189	0.132936	6.160105	7.27E-10	3.77E-09	UP
EEF2KMT	347.6422	0.583802	0.094775	6.159891	7.28E-10	3.77E-09	NOT
RP11-519I	3.208541	4.130947	0.670644	6.159669	7.29E-10	3.77E-09	UP
THEM4	745.3264	0.608997	0.098892	6.158211	7.36E-10	3.81E-09	UP
BOD1	1242.034	0.499886	0.081178	6.157891	7.37E-10	3.82E-09	NOT
RP11-141(	3.884282	2.051102	0.333132	6.157028	7.41E-10	3.84E-09	UP
SORBS1	2639.209	-0.76437	0.124156	-6.15654	7.44E-10	3.85E-09	DOWN
RP11-736I	40.55402	1.24882	0.20286	6.156065	7.46E-10	3.86E-09	UP
RP11-60A:	20.49439	2.357158	0.382902	6.156043	7.46E-10	3.86E-09	UP
RP11-764I	29.78217	1.347621	0.218911	6.156028	7.46E-10	3.86E-09	UP
DPEP3	5.196106	-1.53015	0.248584	-6.15545	7.49E-10	3.87E-09	DOWN
RP11-677I	10.14733	1.246153	0.202459	6.155101	7.50E-10	3.88E-09	UP
SLC5A10	39.85866	1.84166	0.299283	6.153564	7.58E-10	3.91E-09	UP
VSTM5	5.80933	1.952062	0.317231	6.153438	7.58E-10	3.92E-09	UP

ZG16	874.2735	-1.89008	0.30716	-6.15342	7.58E-10	3.92E-09	DOWN
U52111.14	4.090127	1.839513	0.298985	6.152531	7.63E-10	3.94E-09	UP
IP6K2	1661.675	0.545445	0.088662	6.151973	7.65E-10	3.95E-09	NOT
NXF1	2302.657	0.376739	0.061243	6.151522	7.67E-10	3.96E-09	NOT
RP5-1132I	15.93343	-1.63404	0.265648	-6.15115	7.69E-10	3.97E-09	DOWN
USP46	347.7784	0.566493	0.092102	6.150716	7.71E-10	3.98E-09	NOT
ING5	624.1609	0.575664	0.093599	6.150324	7.73E-10	3.99E-09	NOT
ZNF92P3	3.738947	2.40893	0.391683	6.150205	7.74E-10	3.99E-09	UP
CTA-223H	3.763267	1.73697	0.282447	6.149709	7.76E-10	4.00E-09	UP
VAT1	6495.174	0.818008	0.13303	6.149044	7.80E-10	4.02E-09	UP
AC007292	28.19348	1.059399	0.172299	6.148594	7.82E-10	4.03E-09	UP
A1CF	10648.97	-0.9988	0.162458	-6.14804	7.84E-10	4.04E-09	DOWN
SPIN3	91.55227	0.615124	0.10006	6.147557	7.87E-10	4.06E-09	UP
ZNF84	607.1348	0.52557	0.085504	6.146754	7.91E-10	4.08E-09	NOT
FAHD2CP	127.1446	0.869716	0.141502	6.146313	7.93E-10	4.09E-09	UP
RP5-849H	458.3745	-1.39614	0.227171	-6.14575	7.96E-10	4.10E-09	DOWN
MBL2	6845.066	-1.78038	0.289728	-6.14502	8.00E-10	4.12E-09	DOWN
POLR3C	828.9517	0.589412	0.095924	6.144598	8.02E-10	4.13E-09	UP
SMCR8	794.5657	0.663538	0.10799	6.144452	8.02E-10	4.13E-09	UP
JPH1	73.82365	2.016011	0.328124	6.144051	8.04E-10	4.14E-09	UP
MXI1	2017.923	-0.67062	0.109153	-6.14385	8.05E-10	4.15E-09	DOWN
TMEM191	10.31628	1.366858	0.222491	6.143435	8.08E-10	4.16E-09	UP
RP11-382I	7.803531	-1.36851	0.222769	-6.14319	8.09E-10	4.16E-09	DOWN
TDGF1	277.3117	2.133134	0.347237	6.143156	8.09E-10	4.16E-09	UP
ASH1L-AS	39.21523	0.776244	0.126367	6.142788	8.11E-10	4.17E-09	UP
RP11-106I	5.343025	2.122833	0.345638	6.141779	8.16E-10	4.20E-09	UP
PSORS1C3	17.44069	1.726504	0.281113	6.141679	8.17E-10	4.20E-09	UP
TM4SF19	11.51112	2.063938	0.336071	6.141368	8.18E-10	4.20E-09	UP
USH1C	558.7684	2.730391	0.444647	6.140588	8.22E-10	4.22E-09	UP
HOOK1	1808.093	-0.83837	0.136549	-6.13971	8.27E-10	4.25E-09	DOWN
PHOSPHO	14.31708	-1.0371	0.168938	-6.13894	8.31E-10	4.27E-09	DOWN
HECTD2	240.8716	0.764065	0.124489	6.137629	8.38E-10	4.30E-09	UP
S100A1	180.1301	1.437414	0.234219	6.137042	8.41E-10	4.32E-09	UP
PLEKHN1	38.38629	1.65315	0.26944	6.135513	8.49E-10	4.36E-09	UP
CSPP1	597.6409	0.877205	0.142992	6.13464	8.54E-10	4.38E-09	UP
NPM1P27	66.39838	0.960322	0.15655	6.134298	8.55E-10	4.39E-09	UP
RP11-84N	3.884237	4.408486	0.718683	6.134122	8.56E-10	4.39E-09	UP
RP11-365I	5.040077	1.585114	0.258424	6.133762	8.58E-10	4.40E-09	UP
MSI2	1276.538	0.703829	0.11475	6.133562	8.59E-10	4.41E-09	UP
GNRH1	20.27265	1.033103	0.168452	6.132932	8.63E-10	4.42E-09	UP
TPRG1L	4227.874	-0.73081	0.119171	-6.13248	8.65E-10	4.44E-09	DOWN
CABP7	12.89687	1.139792	0.185928	6.130301	8.77E-10	4.50E-09	UP
TPM3P9	174.8138	1.094026	0.178486	6.129464	8.82E-10	4.52E-09	UP
RP11-173I	2.907105	2.597301	0.423786	6.128807	8.85E-10	4.54E-09	UP
TPRXL	8.120213	2.776088	0.453	6.128233	8.89E-10	4.55E-09	UP
SH3RF3	292.3421	1.451918	0.236934	6.127944	8.90E-10	4.56E-09	UP
PLXNA2	1104.968	0.654725	0.106844	6.127875	8.91E-10	4.56E-09	UP
AP002954.	5.922737	-1.60839	0.262489	-6.12746	8.93E-10	4.57E-09	DOWN
SPIDR	1714.501	-0.6714	0.109581	-6.12696	8.96E-10	4.59E-09	DOWN
FAM21FP	14.24181	0.895957	0.146233	6.126923	8.96E-10	4.59E-09	UP
RP11-192I	35.28123	0.931678	0.152071	6.126583	8.98E-10	4.60E-09	UP
NTPCR	1760.105	0.659106	0.107614	6.124729	9.08E-10	4.65E-09	UP

WASH6P	207.6419	0.668222	0.109114	6.12408	9.12E-10	4.67E-09	UP
GPR37L1	27.36971	1.515058	0.247403	6.123836	9.13E-10	4.67E-09	UP
HSF2	285.3093	0.599673	0.097934	6.123218	9.17E-10	4.69E-09	UP
KCNG2	6.358702	1.692443	0.276431	6.12248	9.21E-10	4.71E-09	UP
AHCTF1	1746.091	0.570399	0.093187	6.121005	9.30E-10	4.75E-09	NOT
RP11-115I	4.458021	3.125107	0.510564	6.120895	9.31E-10	4.76E-09	UP
SLC30A2	101.3773	2.525586	0.41265	6.120407	9.33E-10	4.77E-09	UP
CTC-559E	37.00816	1.008315	0.164759	6.119935	9.36E-10	4.78E-09	UP
RP11-338I	4.382513	3.39732	0.555195	6.119148	9.41E-10	4.81E-09	UP
C3orf62	296.455	0.592862	0.096891	6.118873	9.42E-10	4.81E-09	UP
NRARP	161.8365	1.034131	0.169017	6.11849	9.45E-10	4.82E-09	UP
ZNF788	68.87268	1.275799	0.208522	6.118281	9.46E-10	4.83E-09	UP
RNF144B	971.5973	-0.94746	0.154879	-6.11742	9.51E-10	4.85E-09	DOWN
SEPN1	2585.57	0.610498	0.099818	6.116129	9.59E-10	4.89E-09	UP
IL18RAP	44.43958	-1.1697	0.191277	-6.11522	9.64E-10	4.92E-09	DOWN
AC005519	11.45684	1.233065	0.201648	6.114941	9.66E-10	4.93E-09	UP
ZNF354A	202.0268	0.865104	0.141483	6.114527	9.68E-10	4.94E-09	UP
CWC27	560.9996	0.444065	0.072632	6.113904	9.72E-10	4.96E-09	NOT
HM13-AS:	12.53526	-1.08402	0.177316	-6.11349	9.75E-10	4.97E-09	DOWN
SQSTM1	31326.81	1.139414	0.18641	6.11242	9.81E-10	5.00E-09	UP
ARHGAP42	471.8855	-0.83326	0.136351	-6.11115	9.89E-10	5.04E-09	DOWN
PALD1	301.3918	0.843358	0.138006	6.111022	9.90E-10	5.04E-09	UP
RP13-476I	2.826529	2.316969	0.379148	6.11098	9.90E-10	5.05E-09	UP
FOLR1	59.0323	2.322793	0.380104	6.110938	9.90E-10	5.05E-09	UP
RP11-351J	31.67392	1.46022	0.239002	6.109647	9.99E-10	5.09E-09	UP
RSPH14	17.06579	1.699188	0.278124	6.109462	1.00E-09	5.09E-09	UP
B4GALT7	1242.04	0.647653	0.106036	6.107883	1.01E-09	5.14E-09	UP
BAIAP2-AS	903.2448	0.76761	0.125688	6.107239	1.01E-09	5.16E-09	UP
A2M	143636	-1.32341	0.21671	-6.10683	1.02E-09	5.17E-09	DOWN
TP53TG5	10.10898	0.978534	0.16024	6.106692	1.02E-09	5.18E-09	UP
AKAP14	2.377438	3.067368	0.502332	6.106252	1.02E-09	5.19E-09	UP
CD69	152.0233	-1.41784	0.232202	-6.10604	1.02E-09	5.20E-09	DOWN
DSCR9	3.632563	1.747396	0.286182	6.105886	1.02E-09	5.20E-09	UP
SNRPA1	900.0394	0.581076	0.095196	6.104005	1.03E-09	5.26E-09	NOT
C9orf69	1511.513	0.578691	0.094809	6.103742	1.04E-09	5.27E-09	NOT
RP11-159I	26.48476	0.804642	0.13184	6.103187	1.04E-09	5.29E-09	UP
SRRM2-AS	56.50001	0.970574	0.159041	6.102676	1.04E-09	5.30E-09	UP
FALEC	2.956148	1.805777	0.295932	6.101993	1.05E-09	5.32E-09	UP
AC093609	13.93412	-1.52516	0.249945	-6.10197	1.05E-09	5.32E-09	DOWN
ASRGL1	314.8479	1.327263	0.217558	6.100722	1.06E-09	5.36E-09	UP
OTUD1	514.906	-0.62649	0.102714	-6.09933	1.07E-09	5.41E-09	DOWN
ARF6	4237.084	-0.43448	0.071253	-6.09775	1.08E-09	5.46E-09	NOT
ZNF75A	496.7064	0.560326	0.091892	6.097621	1.08E-09	5.47E-09	NOT
SIK3	1754.923	-0.52333	0.085835	-6.0969	1.08E-09	5.49E-09	NOT
ANG	25280.21	-1.41758	0.232509	-6.09687	1.08E-09	5.49E-09	DOWN
NPTX2	551.5173	2.595414	0.425701	6.096801	1.08E-09	5.49E-09	UP
METTL20	295.455	-0.71624	0.117489	-6.09622	1.09E-09	5.51E-09	DOWN
GUCA2A	26.3962	2.996498	0.491543	6.096109	1.09E-09	5.51E-09	UP
SNORA26	4.011239	1.683262	0.276219	6.093945	1.10E-09	5.59E-09	UP
GPR83	9.993063	-1.57162	0.257912	-6.09364	1.10E-09	5.60E-09	DOWN
RP11-795I	32.21295	1.129243	0.185329	6.093189	1.11E-09	5.61E-09	UP
AC009237	56.78565	1.048811	0.172147	6.092549	1.11E-09	5.63E-09	UP

GIPR	47.39785	1.854255	0.30435	6.092516	1.11E-09	5.63E-09	UP
SBSN	5.24023	3.462637	0.568361	6.092315	1.11E-09	5.64E-09	UP
PARP9	3526.021	-0.69889	0.114727	-6.09171	1.12E-09	5.66E-09	DOWN
CLEC18A	3.655851	1.79999	0.295524	6.090839	1.12E-09	5.69E-09	UP
VMAC	149.7362	0.56727	0.093145	6.090203	1.13E-09	5.71E-09	NOT
NT5DC1	1787.888	-0.77319	0.126971	-6.08952	1.13E-09	5.73E-09	DOWN
DNAH10C	57.57242	1.190293	0.195497	6.088544	1.14E-09	5.77E-09	UP
RP11-103:	7.906331	5.318342	0.873515	6.088435	1.14E-09	5.77E-09	UP
RHBDF1	525.8424	0.839472	0.137899	6.087588	1.15E-09	5.80E-09	UP
RP5-858L1	14.78145	1.538175	0.252678	6.087495	1.15E-09	5.80E-09	UP
EXTL3-AS:	13.93088	1.225054	0.20126	6.086916	1.15E-09	5.82E-09	UP
VWA8	1712.605	-0.80408	0.132107	-6.08658	1.15E-09	5.83E-09	DOWN
ATP5J2-PT	4.744716	1.452041	0.238588	6.085979	1.16E-09	5.85E-09	UP
SLC16A2	4227.117	-1.20173	0.197459	-6.08597	1.16E-09	5.85E-09	DOWN
LINC0068(	144.1777	0.743029	0.122091	6.085881	1.16E-09	5.86E-09	UP
NDE1	380.768	0.718405	0.118054	6.085379	1.16E-09	5.87E-09	UP
RHBG	1710.246	2.554415	0.419873	6.083776	1.17E-09	5.93E-09	UP
RP11-583I	3.135174	2.181766	0.358661	6.083081	1.18E-09	5.96E-09	UP
HLTF	1556.771	0.658996	0.108357	6.081721	1.19E-09	6.01E-09	UP
TRAF6	345.7783	-0.43543	0.071599	-6.08159	1.19E-09	6.01E-09	NOT
CIART	260.5594	1.253504	0.206122	6.081356	1.19E-09	6.02E-09	UP
CTC-378H	31.30782	1.994962	0.328059	6.081103	1.19E-09	6.02E-09	UP
FARSB	1402.35	0.525048	0.086358	6.079917	1.20E-09	6.07E-09	NOT
RP11-167I	2.886285	2.132384	0.350727	6.079901	1.20E-09	6.07E-09	UP
TEAD3	941.6029	0.578858	0.095231	6.078487	1.21E-09	6.12E-09	NOT
AC007038	12.19071	1.118053	0.183939	6.07838	1.21E-09	6.12E-09	UP
GDF10	31.06946	2.799796	0.460762	6.076452	1.23E-09	6.20E-09	UP
RP11-430I	25.93277	1.645946	0.270895	6.075952	1.23E-09	6.21E-09	UP
AGAP6	167.0161	0.762111	0.125449	6.075079	1.24E-09	6.25E-09	UP
HAPLN3	138.8736	1.481092	0.243817	6.074614	1.24E-09	6.26E-09	UP
RHOBTB2	396.2669	0.765709	0.12606	6.074187	1.25E-09	6.28E-09	UP
VDAC1P8	64.45862	0.816234	0.134378	6.074148	1.25E-09	6.28E-09	UP
PITPNA	2275.54	-0.51331	0.084511	-6.07388	1.25E-09	6.29E-09	NOT
LINC0113:	24.48628	1.201228	0.197774	6.073755	1.25E-09	6.29E-09	UP
CTD-2035	7.252409	1.557724	0.256513	6.072703	1.26E-09	6.33E-09	UP
CTD-2006	46.62228	0.825759	0.136033	6.070272	1.28E-09	6.43E-09	UP
RN7SL2	1149.761	-1.21279	0.199794	-6.07019	1.28E-09	6.43E-09	DOWN
OSBP	4787.464	-0.50251	0.082787	-6.06986	1.28E-09	6.44E-09	NOT
FOXO6	60.83012	2.06187	0.339774	6.068358	1.29E-09	6.50E-09	UP
MAP3K15	18.00744	1.878292	0.309543	6.067951	1.30E-09	6.52E-09	UP
GOLGA7B	185.1757	1.988267	0.32767	6.067901	1.30E-09	6.52E-09	UP
ZNF781	30.61578	1.446599	0.238451	6.066649	1.31E-09	6.57E-09	UP
KCNK17	48.23889	-1.68828	0.278306	-6.06629	1.31E-09	6.58E-09	DOWN
MTCH1	6124.101	0.477715	0.078753	6.066026	1.31E-09	6.59E-09	NOT
GTF3C2-A	5.015203	1.351627	0.222829	6.065773	1.31E-09	6.60E-09	UP
LUZP2	12.88464	2.670852	0.44034	6.06543	1.32E-09	6.61E-09	UP
KALRN	1355.347	-0.77731	0.128158	-6.06523	1.32E-09	6.62E-09	DOWN
RP11-43N	2.436332	2.643523	0.435895	6.064579	1.32E-09	6.65E-09	UP
RP11-545I	66.79175	0.840521	0.138613	6.06378	1.33E-09	6.68E-09	UP
RP11-972I	5.201165	1.945569	0.320877	6.063282	1.33E-09	6.70E-09	UP
CLEC3B	468.6628	-1.21146	0.199804	-6.06327	1.33E-09	6.70E-09	DOWN
TCF20	1454.508	0.521855	0.086071	6.063053	1.34E-09	6.71E-09	NOT

RP3-395M	9.030079	1.497448	0.246986	6.062895	1.34E-09	6.71E-09	UP
RP11-874J	3.86763	4.104133	0.67702	6.062059	1.34E-09	6.74E-09	UP
SLC7A11-/	2.693321	3.096075	0.51078	6.061464	1.35E-09	6.77E-09	UP
LRRC19	48.7114	-2.12895	0.351244	-6.06115	1.35E-09	6.78E-09	DOWN
ZNF346	338.3777	0.465932	0.076883	6.060248	1.36E-09	6.82E-09	NOT
CACNG8	9.836557	1.5307	0.252595	6.059888	1.36E-09	6.83E-09	UP
PRKCD	854.2829	0.764185	0.126135	6.058482	1.37E-09	6.89E-09	UP
MYADM	3502.955	-0.84408	0.139325	-6.05839	1.37E-09	6.89E-09	DOWN
GLRX3	1639.186	0.501071	0.082707	6.058362	1.38E-09	6.89E-09	NOT
ADRB2	367.9636	-1.37994	0.227819	-6.05719	1.39E-09	6.94E-09	DOWN
B9D1	367.8877	0.856253	0.141378	6.056459	1.39E-09	6.97E-09	UP
PLA2G16	3079.298	-1.0914	0.180208	-6.05631	1.39E-09	6.98E-09	DOWN
MLH3	715.5366	0.506874	0.083703	6.055657	1.40E-09	7.00E-09	NOT
AC020594	5.989464	1.466132	0.242111	6.055628	1.40E-09	7.00E-09	UP
KANSL1	1265.433	0.479243	0.079168	6.053535	1.42E-09	7.09E-09	NOT
RNF185	2146.569	-0.4716	0.077915	-6.05279	1.42E-09	7.13E-09	NOT
PPP1R14C	10.45758	2.629471	0.434496	6.051775	1.43E-09	7.17E-09	UP
RP11-73M	178.4255	0.741143	0.122478	6.051256	1.44E-09	7.19E-09	UP
FBXO33	480.1341	-0.5054	0.08354	-6.04982	1.45E-09	7.25E-09	NOT
ITPR3	624.7861	1.725095	0.28515	6.049779	1.45E-09	7.26E-09	UP
LINC0088(	14.64585	1.940134	0.320739	6.04896	1.46E-09	7.29E-09	UP
EHF	349.4088	1.714695	0.283499	6.048318	1.46E-09	7.32E-09	UP
TNFSF11	62.44905	-1.76916	0.292536	-6.04766	1.47E-09	7.35E-09	DOWN
NDC1	904.3717	0.559044	0.092442	6.047509	1.47E-09	7.35E-09	NOT
RBBP4	3456.199	0.451286	0.074624	6.047427	1.47E-09	7.36E-09	NOT
RAB34	820.9004	1.458506	0.241182	6.047323	1.47E-09	7.36E-09	UP
RNY3P8	5.614848	2.296892	0.379828	6.047191	1.47E-09	7.36E-09	UP
EXOSC9	738.2656	0.478608	0.079146	6.047166	1.47E-09	7.36E-09	NOT
KRT16P2	7.788409	-3.79657	0.627829	-6.04713	1.47E-09	7.36E-09	DOWN
PUS7L	526.7792	0.508208	0.084044	6.046952	1.48E-09	7.37E-09	NOT
STAG3L4	212.8148	0.794758	0.131446	6.04629	1.48E-09	7.40E-09	UP
TMEM151	57.32641	1.878174	0.310646	6.046033	1.48E-09	7.41E-09	UP
MYRFL	12.86258	1.799344	0.29761	6.045984	1.49E-09	7.41E-09	UP
MAF1	4123.664	0.678148	0.112185	6.044937	1.49E-09	7.46E-09	UP
SYNM	588.1428	0.958688	0.158635	6.043354	1.51E-09	7.53E-09	UP
ZNF628	281.5975	0.585625	0.096907	6.043181	1.51E-09	7.54E-09	UP
PTPRB	1044.196	-0.9561	0.158221	-6.04281	1.51E-09	7.55E-09	DOWN
TBC1D20	1480.18	0.406597	0.067287	6.042702	1.52E-09	7.56E-09	NOT
AC135048	30.922	-1.0843	0.179458	-6.0421	1.52E-09	7.58E-09	DOWN
PIPSL	19.13097	0.862775	0.1428	6.041856	1.52E-09	7.59E-09	UP
CTB-161C	2.665804	2.538376	0.420177	6.041207	1.53E-09	7.62E-09	UP
RPL8	57583.98	0.963258	0.159454	6.040991	1.53E-09	7.63E-09	UP
TMEM263	2079.592	-0.53812	0.089085	-6.0406	1.54E-09	7.65E-09	NOT
RP11-497(	3.311624	3.757762	0.622102	6.040431	1.54E-09	7.66E-09	UP
HK2	521.6928	1.835173	0.303867	6.039389	1.55E-09	7.70E-09	UP
RP11-214(	7.190185	-1.78041	0.294811	-6.03915	1.55E-09	7.71E-09	DOWN
OTUD4	1266.625	-0.61698	0.102164	-6.03911	1.55E-09	7.71E-09	DOWN
AC253576	3.909447	1.800922	0.298219	6.038925	1.55E-09	7.72E-09	UP
RP11-693I	2.553307	-1.46618	0.242799	-6.03865	1.55E-09	7.73E-09	DOWN
KLHL34	21.28101	2.657644	0.440135	6.038251	1.56E-09	7.75E-09	UP
OCA2	60.75232	2.322591	0.384753	6.03658	1.57E-09	7.83E-09	UP
TRIM66	353.3062	0.813084	0.134714	6.035619	1.58E-09	7.88E-09	UP

GJC3	38.51192	-1.30971	0.217011	-6.03523	1.59E-09	7.89E-09	DOWN
AC005071	1.556503	2.33807	0.38741	6.035128	1.59E-09	7.90E-09	UP
MRPL16	2146.694	-0.45229	0.074948	-6.03476	1.59E-09	7.91E-09	NOT
NRG1	404.9541	-2.13639	0.354029	-6.0345	1.59E-09	7.92E-09	DOWN
RNF11	2866.073	-0.53256	0.08826	-6.034	1.60E-09	7.95E-09	NOT
GABRA2	16.19251	4.032278	0.668287	6.033749	1.60E-09	7.96E-09	UP
RP11-29B2	3.712621	1.591695	0.263799	6.033743	1.60E-09	7.96E-09	UP
ARFRP1	1870.326	0.58353	0.096713	6.033642	1.60E-09	7.96E-09	NOT
AC099850	15.41939	0.95199	0.157781	6.033609	1.60E-09	7.96E-09	UP
HOXA9	4.693415	2.241863	0.371631	6.032506	1.61E-09	8.01E-09	UP
AUNIP	38.83562	1.120168	0.185696	6.03227	1.62E-09	8.02E-09	UP
ERLIN2	2681.475	-0.65936	0.109323	-6.03133	1.63E-09	8.07E-09	DOWN
TAX1BP3	559.7564	1.024389	0.169855	6.030977	1.63E-09	8.09E-09	UP
GLULP4	7.541677	2.373554	0.393589	6.030535	1.63E-09	8.11E-09	UP
CTD-2561	4.848981	1.594593	0.26443	6.030295	1.64E-09	8.12E-09	UP
ZNF556	8.211354	2.301705	0.381724	6.029761	1.64E-09	8.14E-09	UP
NONO	11572.44	0.47987	0.079585	6.029626	1.64E-09	8.15E-09	NOT
BNIP3P17	3.550341	2.855699	0.473621	6.029498	1.64E-09	8.15E-09	UP
UPK2	4.070635	2.385165	0.395593	6.029339	1.65E-09	8.16E-09	UP
ADAM9	1681.423	1.107831	0.183763	6.028599	1.65E-09	8.20E-09	UP
RP11-110	10.87397	1.1504	0.190854	6.027641	1.66E-09	8.24E-09	UP
PALM2-AK	29.00992	-1.43177	0.237538	-6.02755	1.66E-09	8.25E-09	DOWN
ZFP14	233.1587	0.677603	0.112433	6.026699	1.67E-09	8.29E-09	UP
RP11-78L1	4.474485	4.437864	0.736448	6.026035	1.68E-09	8.32E-09	UP
RP1-81D8	10.49874	-1.65122	0.274056	-6.0251	1.69E-09	8.37E-09	DOWN
PROKR1	7.917325	2.806416	0.465788	6.025095	1.69E-09	8.37E-09	UP
TXNDC11	3887.616	-0.54382	0.09026	-6.02504	1.69E-09	8.37E-09	NOT
MISP	258.1171	2.805103	0.465634	6.024271	1.70E-09	8.41E-09	UP
RP11-267	3.674183	-1.64247	0.272653	-6.02403	1.70E-09	8.42E-09	DOWN
MALAT1	2238.953	0.931628	0.154654	6.023967	1.70E-09	8.42E-09	UP
RP4-635E	4.218079	1.41766	0.235346	6.023731	1.70E-09	8.43E-09	UP
RP5-899E	10.09963	-1.08552	0.180211	-6.02362	1.71E-09	8.43E-09	DOWN
CD2BP2	2042.987	0.532866	0.088468	6.023255	1.71E-09	8.45E-09	NOT
LYSMD2	552.9276	-0.61461	0.102068	-6.02155	1.73E-09	8.54E-09	DOWN
SP3P	4.596194	4.059004	0.674091	6.021447	1.73E-09	8.54E-09	UP
ANXA10	1293.041	-1.76186	0.292628	-6.02083	1.74E-09	8.57E-09	DOWN
VAMP2	1669.47	-0.58481	0.097131	-6.02082	1.74E-09	8.57E-09	NOT
STK19B	31.52577	1.683859	0.27968	6.020671	1.74E-09	8.58E-09	UP
RP11-471	3.494021	3.593296	0.596854	6.020397	1.74E-09	8.59E-09	UP
STC1	640.0436	1.311299	0.217832	6.019769	1.75E-09	8.62E-09	UP
DMKN	512.3801	2.377028	0.394941	6.018687	1.76E-09	8.68E-09	UP
UFSP2	650.9366	-0.48324	0.080298	-6.01811	1.76E-09	8.71E-09	NOT
HBA2	578.4027	-1.75079	0.290966	-6.01717	1.77E-09	8.76E-09	DOWN
SYT5	10.46541	2.23464	0.371385	6.017038	1.78E-09	8.77E-09	UP
PEBP1	67446.96	-0.83485	0.13877	-6.01603	1.79E-09	8.82E-09	DOWN
ZRANB2	1670.115	0.483929	0.08044	6.016023	1.79E-09	8.82E-09	NOT
RP4-763G	388.5358	-2.0177	0.335417	-6.01551	1.79E-09	8.84E-09	DOWN
PLGLB1	149.7375	-1.35169	0.224702	-6.01546	1.79E-09	8.84E-09	DOWN
RNF168	756.2211	-0.5153	0.085673	-6.01468	1.80E-09	8.89E-09	NOT
SLC13A4	30.56928	1.212995	0.20168	6.014447	1.81E-09	8.90E-09	UP
GPANK1	1121.619	0.641278	0.106627	6.014241	1.81E-09	8.91E-09	UP
PTDSS2	1132.813	0.768832	0.12784	6.01403	1.81E-09	8.92E-09	UP

KTN1-AS1	56.29255	0.755705	0.125666	6.013603	1.81E-09	8.94E-09	UP
MST1	13194.53	-1.14501	0.190406	-6.0135	1.82E-09	8.94E-09	DOWN
RP11-640I	8.703302	1.039431	0.17285	6.013494	1.82E-09	8.94E-09	UP
MAP2K2	5247.823	0.699332	0.116294	6.013478	1.82E-09	8.94E-09	UP
RHBDD3	1140.494	0.632181	0.105137	6.012936	1.82E-09	8.97E-09	UP
SCAF1	3167.019	0.466269	0.077568	6.011116	1.84E-09	9.07E-09	NOT
AC087793	3.738998	1.591174	0.264714	6.010913	1.84E-09	9.08E-09	UP
RASAL2	703.3196	0.847881	0.141064	6.010593	1.85E-09	9.10E-09	UP
AC002550	6.314085	1.106598	0.184112	6.010453	1.85E-09	9.10E-09	UP
RP11-445I	7.206877	1.590923	0.26473	6.009603	1.86E-09	9.15E-09	UP
RP11-180(	7.216873	5.185132	0.862863	6.00922	1.86E-09	9.17E-09	UP
DBNDD2	41.41179	1.112965	0.185238	6.008298	1.87E-09	9.22E-09	UP
NRF1	459.1224	0.376675	0.062693	6.008226	1.88E-09	9.22E-09	NOT
FTH1P23	41.00456	0.916022	0.152462	6.008209	1.88E-09	9.22E-09	UP
ZNF213-A	170.5525	0.659927	0.109842	6.007969	1.88E-09	9.23E-09	UP
FAM229B	282.9897	-1.00339	0.167021	-6.0076	1.88E-09	9.25E-09	DOWN
SRGAP1	298.3326	1.174246	0.195482	6.006918	1.89E-09	9.29E-09	UP
EGFR-AS1	90.11964	2.5322	0.421593	6.006264	1.90E-09	9.32E-09	UP
RP11-242I	34.29433	0.986937	0.164324	6.006055	1.90E-09	9.33E-09	UP
TMEM47	991.1012	-1.03949	0.17308	-6.00583	1.90E-09	9.35E-09	DOWN
ZSWIM6	410.1397	-0.76122	0.126754	-6.00551	1.91E-09	9.36E-09	DOWN
C15orf59	42.48973	1.685653	0.280755	6.004008	1.93E-09	9.45E-09	UP
LINC01431	16.12662	1.155592	0.192481	6.003656	1.93E-09	9.47E-09	UP
CNBD2	12.99468	1.015971	0.16923	6.003482	1.93E-09	9.48E-09	UP
NFKBIL1	1113.901	0.658336	0.10966	6.003423	1.93E-09	9.48E-09	UP
CTD-2036	3.452788	1.440819	0.240014	6.003052	1.94E-09	9.50E-09	UP
C9orf173	45.81749	1.511649	0.25182	6.002894	1.94E-09	9.50E-09	UP
ADI1	24255.82	-0.98046	0.16334	-6.00257	1.94E-09	9.52E-09	DOWN
CLCN1	12.85763	2.143156	0.357056	6.002297	1.95E-09	9.54E-09	UP
YBX2	183.9461	1.792918	0.298715	6.0021	1.95E-09	9.55E-09	UP
RP11-104:	10.20988	5.803058	0.966916	6.001618	1.95E-09	9.57E-09	UP
RP11-411I	1.586145	2.455333	0.409132	6.00132	1.96E-09	9.59E-09	UP
RP11-157I	44.47849	1.105003	0.184159	6.00028	1.97E-09	9.65E-09	UP
ESD	4393.959	-0.55903	0.093171	-6.00004	1.97E-09	9.66E-09	NOT
KANSL3	1886.715	0.448573	0.074762	6.000037	1.97E-09	9.66E-09	NOT
UBE2G1	1591.67	-0.47435	0.07907	-5.99908	1.98E-09	9.72E-09	NOT
SLC12A5	46.45224	1.435751	0.239344	5.998688	1.99E-09	9.73E-09	UP
TNFRSF1B	2555.533	-0.86408	0.144046	-5.99867	1.99E-09	9.73E-09	DOWN
MPPED2	56.95	1.46556	0.244314	5.998669	1.99E-09	9.73E-09	UP
RP11-454I	3.330444	-1.93634	0.322817	-5.99827	1.99E-09	9.76E-09	DOWN
C3orf67-A	2.800871	2.832594	0.472261	5.99794	2.00E-09	9.78E-09	UP
C1orf27	1613.275	0.556729	0.092833	5.99712	2.01E-09	9.82E-09	NOT
CMSS1	613.1858	0.607107	0.101248	5.996205	2.02E-09	9.88E-09	UP
MYC	3186.352	-1.23999	0.206804	-5.99597	2.02E-09	9.89E-09	DOWN
DGCR2	4141.185	0.456981	0.076221	5.995445	2.03E-09	9.92E-09	NOT
IKBKE	275.1747	1.191772	0.198782	5.995361	2.03E-09	9.92E-09	UP
MICALL1	945.5833	0.631296	0.105299	5.995253	2.03E-09	9.93E-09	UP
AL109763.	9.405837	5.567246	0.928738	5.99442	2.04E-09	9.98E-09	UP
MED15	2148.97	0.449032	0.074917	5.99377	2.05E-09	1.00E-08	NOT
MYH16	4.129463	1.565438	0.261214	5.992924	2.06E-09	1.01E-08	UP
METTL14	901.922	-0.46346	0.077339	-5.99262	2.06E-09	1.01E-08	NOT
LINC01117	2.368123	2.911582	0.485877	5.992431	2.07E-09	1.01E-08	UP

RCE1	495.643	0.531564	0.088722	5.99131	2.08E-09	1.02E-08	NOT
SGSM3	2036.777	0.444795	0.074249	5.99059	2.09E-09	1.02E-08	NOT
COIL	752.5191	0.409398	0.068352	5.989564	2.10E-09	1.03E-08	NOT
MTMR3	184.0733	0.604433	0.100916	5.98949	2.11E-09	1.03E-08	UP
MZF1	520.4433	0.579385	0.096737	5.989274	2.11E-09	1.03E-08	NOT
RP13-467I	9.306241	1.718359	0.286911	5.98917	2.11E-09	1.03E-08	UP
NRIP1	1652.323	-0.78554	0.131162	-5.98911	2.11E-09	1.03E-08	DOWN
C3orf70	61.43785	0.899003	0.15012	5.98856	2.12E-09	1.03E-08	UP
CCDC130	843.618	0.68335	0.114116	5.9882	2.12E-09	1.03E-08	UP
ZNF234	202.3575	0.640158	0.106979	5.983978	2.18E-09	1.06E-08	UP
RNF220	1858.885	0.416399	0.069586	5.983928	2.18E-09	1.06E-08	NOT
RETSAT	7978.147	-0.76612	0.12803	-5.9839	2.18E-09	1.06E-08	DOWN
ASPG	2588.912	-2.09916	0.350815	-5.98368	2.18E-09	1.06E-08	DOWN
CARD18	4.199499	4.34198	0.725676	5.983361	2.19E-09	1.06E-08	UP
HSPB8	895.5601	1.720156	0.287527	5.982596	2.20E-09	1.07E-08	UP
PHF14	1364.857	0.396429	0.066266	5.982367	2.20E-09	1.07E-08	NOT
C10orf88	248.5916	0.423119	0.070729	5.982242	2.20E-09	1.07E-08	NOT
ACAP3	1264.463	0.605319	0.101192	5.981869	2.21E-09	1.07E-08	UP
LINC01057	30.30277	1.436563	0.240158	5.981747	2.21E-09	1.07E-08	UP
CTD-3088	64.71904	1.305753	0.218291	5.981709	2.21E-09	1.07E-08	UP
ZNF239	70.24525	1.598667	0.267297	5.980861	2.22E-09	1.08E-08	UP
C2CD3	507.3946	0.473264	0.079132	5.980676	2.22E-09	1.08E-08	NOT
RP3-467N	64.12817	1.10394	0.184612	5.979774	2.23E-09	1.09E-08	UP
ASPSCR1	3825.68	1.135416	0.189885	5.979492	2.24E-09	1.09E-08	UP
RP3-395C	2.759291	2.103066	0.35177	5.978522	2.25E-09	1.09E-08	UP
VPS13B	955.6425	0.583928	0.097684	5.977742	2.26E-09	1.10E-08	NOT
LINC00491	5.278462	4.792296	0.801723	5.977493	2.27E-09	1.10E-08	UP
RP11-332	5.157638	4.702082	0.786641	5.977418	2.27E-09	1.10E-08	UP
TUBAP2	9.23737	1.197777	0.200426	5.976151	2.28E-09	1.11E-08	UP
MMP1	62.60076	2.015965	0.33735	5.97589	2.29E-09	1.11E-08	UP
RP11-322I	7.969246	1.492279	0.249722	5.97576	2.29E-09	1.11E-08	UP
RP11-165I	2.469986	2.595723	0.43438	5.975702	2.29E-09	1.11E-08	UP
ALYREF	1692.903	0.727187	0.121692	5.975638	2.29E-09	1.11E-08	UP
FSTL5	51.88482	3.520571	0.589204	5.975136	2.30E-09	1.12E-08	UP
FAM111A	919.3065	0.58764	0.098355	5.974673	2.31E-09	1.12E-08	UP
ITSN1	1869.807	-0.6314	0.105691	-5.974	2.32E-09	1.12E-08	DOWN
DDOST	7768.355	0.445029	0.074497	5.973821	2.32E-09	1.12E-08	NOT
AC105760	14.03542	0.968701	0.16216	5.973721	2.32E-09	1.12E-08	UP
STRA13	2294.841	0.92914	0.155551	5.973231	2.33E-09	1.13E-08	UP
BTG3	584.7282	0.644792	0.107948	5.973168	2.33E-09	1.13E-08	UP
B3GALNT2	217.5678	0.769062	0.128759	5.972858	2.33E-09	1.13E-08	UP
CTD-3099	17.97555	1.793707	0.300314	5.972776	2.33E-09	1.13E-08	UP
OGDHL	6067.9	-1.43219	0.239807	-5.97229	2.34E-09	1.13E-08	DOWN
GREB1	653.1643	1.560854	0.261387	5.971431	2.35E-09	1.14E-08	UP
RP11-554I	3.615005	1.565159	0.26211	5.971376	2.35E-09	1.14E-08	UP
AC005264	2.594925	1.908484	0.319641	5.970716	2.36E-09	1.14E-08	UP
EMC2	1550.7	0.52798	0.088444	5.969662	2.38E-09	1.15E-08	NOT
C10orf2	457.5306	0.65589	0.10989	5.968624	2.39E-09	1.16E-08	UP
HTRA3	363.6229	1.570878	0.263263	5.966961	2.42E-09	1.17E-08	UP
MTG1	344.5524	0.666215	0.111656	5.966688	2.42E-09	1.17E-08	UP
NPIPA1	99.69217	0.80984	0.135734	5.966387	2.43E-09	1.17E-08	UP
C10orf35	185.0244	1.148573	0.192535	5.965537	2.44E-09	1.18E-08	UP

RP11-435I	2.360794	2.125942	0.356399	5.965065	2.45E-09	1.18E-08	UP
FRMD8	1259.812	0.455606	0.076382	5.964873	2.45E-09	1.18E-08	NOT
ADAMTS6	32.87185	1.297995	0.21762	5.964504	2.45E-09	1.19E-08	UP
ADCK2	1234.283	0.564761	0.094696	5.963907	2.46E-09	1.19E-08	NOT
VKORC1L1	2133.205	-0.42776	0.071725	-5.96384	2.46E-09	1.19E-08	NOT
HERC3	624.4885	-0.75975	0.127399	-5.96355	2.47E-09	1.19E-08	DOWN
CTD-2291	3.549575	3.249948	0.545025	5.96293	2.48E-09	1.20E-08	UP
CYCSP6	3.863419	4.039533	0.677548	5.961986	2.49E-09	1.20E-08	UP
CCT2	4008.799	0.51187	0.085865	5.961315	2.50E-09	1.21E-08	NOT
TRNP1	819.6053	1.744392	0.292647	5.960745	2.51E-09	1.21E-08	UP
TMEM209	813.786	0.526385	0.088316	5.960245	2.52E-09	1.22E-08	NOT
RMDN3	2409.035	-0.48587	0.081526	-5.95972	2.53E-09	1.22E-08	NOT
RP11-505I	29.30074	-1.13734	0.190904	-5.95764	2.56E-09	1.24E-08	DOWN
WDR91	924.5291	0.826437	0.13872	5.957608	2.56E-09	1.24E-08	UP
FGFR1OP	759.9734	-0.58097	0.097526	-5.95706	2.57E-09	1.24E-08	NOT
LINC-ROR	3.451182	3.264515	0.548031	5.956805	2.57E-09	1.24E-08	UP
RP11-395I	29.34082	1.287083	0.216077	5.956604	2.58E-09	1.24E-08	UP
ZNF746	738.2172	0.589038	0.098891	5.956465	2.58E-09	1.24E-08	UP
ZNF519	53.80063	1.063133	0.17849	5.956259	2.58E-09	1.24E-08	UP
SAT1	15592.27	-0.75186	0.126239	-5.95584	2.59E-09	1.25E-08	DOWN
OSGEPL1	321.4316	0.46034	0.077294	5.955739	2.59E-09	1.25E-08	NOT
LRRC40	629.0674	-0.61539	0.103329	-5.95566	2.59E-09	1.25E-08	DOWN
RCC1	1170.018	0.649951	0.109138	5.955294	2.60E-09	1.25E-08	UP
FZD10-AS	11.19	1.859723	0.312316	5.954628	2.61E-09	1.26E-08	UP
CASC8	4.55801	2.871903	0.482305	5.954534	2.61E-09	1.26E-08	UP
RP11-148I	1.981431	2.14515	0.360358	5.952826	2.64E-09	1.27E-08	UP
C1orf204	13.41254	1.484971	0.249539	5.950867	2.67E-09	1.28E-08	UP
C20orf27	1201.112	0.72335	0.121586	5.949277	2.69E-09	1.30E-08	UP
RP5-890E1	13.44721	0.840628	0.141309	5.948873	2.70E-09	1.30E-08	UP
GDI1	3752.193	0.489225	0.082246	5.948323	2.71E-09	1.30E-08	NOT
FASTK	3638.477	0.563681	0.094773	5.947693	2.72E-09	1.31E-08	NOT
MEF2BNB	511.306	0.609432	0.102479	5.946876	2.73E-09	1.31E-08	UP
FUOM	4219.87	-1.00021	0.1682	-5.94656	2.74E-09	1.32E-08	DOWN
RP11-864I	123.816	0.621711	0.104555	5.946272	2.74E-09	1.32E-08	UP
BAG2	743.0118	0.801141	0.134745	5.945616	2.75E-09	1.32E-08	UP
SLC39A4	942.8296	1.895502	0.318812	5.945525	2.76E-09	1.32E-08	UP
EPB41L5	1884.039	-0.59644	0.100335	-5.94449	2.77E-09	1.33E-08	DOWN
RAB21	2371.742	-0.37156	0.062511	-5.94398	2.78E-09	1.34E-08	NOT
SBK3	8.362955	2.378999	0.400251	5.943769	2.79E-09	1.34E-08	UP
RGS18	58.0619	-1.14679	0.19301	-5.9416	2.82E-09	1.36E-08	DOWN
CTC-429L1	3.950495	1.549056	0.260725	5.941346	2.83E-09	1.36E-08	UP
SLC5A4	10.68693	1.612311	0.271371	5.941345	2.83E-09	1.36E-08	UP
PI4KAP2	143.8436	0.720297	0.121258	5.940209	2.85E-09	1.37E-08	UP
RP11-95M	2.30286	2.676385	0.450599	5.939622	2.86E-09	1.37E-08	UP
LRRC4C	28.48316	-1.54924	0.260853	-5.93913	2.87E-09	1.38E-08	DOWN
CTC-524C	61.93711	0.973375	0.163897	5.938931	2.87E-09	1.38E-08	UP
RP4-798A1	13.47632	0.876753	0.147637	5.93857	2.88E-09	1.38E-08	UP
TMEM38B	1100.615	0.635178	0.106967	5.938088	2.88E-09	1.38E-08	UP
HNRNPA1I	4.387947	1.670752	0.281365	5.938032	2.88E-09	1.38E-08	UP
ACD	485.2694	0.533032	0.089767	5.937979	2.89E-09	1.38E-08	NOT
MYO1H	4.831346	1.865983	0.314249	5.937917	2.89E-09	1.38E-08	UP
TMSB10	18802.61	1.189308	0.200315	5.937204	2.90E-09	1.39E-08	UP

BCHE	3561.773	-1.80823	0.304568	-5.93704	2.90E-09	1.39E-08	DOWN
GARS	4120.669	0.620888	0.104582	5.936877	2.91E-09	1.39E-08	UP
HDAC5	2466.39	0.544118	0.091653	5.936699	2.91E-09	1.39E-08	NOT
TPRKB	622.8156	0.477925	0.080509	5.93626	2.92E-09	1.40E-08	NOT
GRIN2D	69.03898	1.472122	0.247995	5.936101	2.92E-09	1.40E-08	UP
AF127936.	170.7897	-0.85512	0.144056	-5.93604	2.92E-09	1.40E-08	DOWN
RP11-574I	21.17909	0.89027	0.149982	5.935825	2.92E-09	1.40E-08	UP
CHEK2	424.5058	0.916466	0.154399	5.935679	2.93E-09	1.40E-08	UP
ASPDH	3146.453	-1.70932	0.287986	-5.93542	2.93E-09	1.40E-08	DOWN
GIPC1	3416.688	0.777261	0.130957	5.935255	2.93E-09	1.40E-08	UP
RP11-399I	18.07276	1.02262	0.172303	5.935011	2.94E-09	1.41E-08	UP
EHD2	1790.004	0.957725	0.161379	5.934648	2.94E-09	1.41E-08	UP
YAE1D1	248.6416	0.50281	0.08473	5.934241	2.95E-09	1.41E-08	NOT
C19orf33	72.63988	2.128722	0.358736	5.933947	2.96E-09	1.41E-08	UP
GPR63	8.576663	1.890143	0.318549	5.933606	2.96E-09	1.42E-08	UP
RP11-134{	23.60649	0.809855	0.136505	5.932799	2.98E-09	1.42E-08	UP
UGGT1	4360.518	0.638265	0.107584	5.932701	2.98E-09	1.42E-08	UP
HID1	692.4517	1.277548	0.215347	5.932497	2.98E-09	1.43E-08	UP
TTYH2	203.4004	1.156684	0.194983	5.932224	2.99E-09	1.43E-08	UP
CTC-429P	13.52629	1.222077	0.206017	5.931917	2.99E-09	1.43E-08	UP
EHHADH	13645.2	-1.27316	0.214671	-5.93074	3.02E-09	1.44E-08	DOWN
RP11-166I	6.633867	1.75119	0.295283	5.930551	3.02E-09	1.44E-08	UP
RP4-671G	5.790488	1.363849	0.229972	5.930502	3.02E-09	1.44E-08	UP
RP11-113I	5.847955	1.624724	0.273972	5.93025	3.02E-09	1.44E-08	UP
CENPN	359.9181	0.703069	0.118564	5.929844	3.03E-09	1.45E-08	UP
RBP4	276858.6	-1.24657	0.210222	-5.92979	3.03E-09	1.45E-08	DOWN
WBP1L	2708.472	-0.50335	0.084889	-5.92958	3.04E-09	1.45E-08	NOT
CRACR2B	314.4533	1.369533	0.230969	5.929496	3.04E-09	1.45E-08	UP
RP11-884I	141.569	0.515487	0.086937	5.929401	3.04E-09	1.45E-08	NOT
CTD-2587	19.89675	1.161409	0.195883	5.929096	3.05E-09	1.45E-08	UP
RP11-278I	5.856259	3.189068	0.537875	5.929015	3.05E-09	1.45E-08	UP
CC2D1B	1505.818	0.407523	0.06874	5.928476	3.06E-09	1.46E-08	NOT
RP13-20L1	7.670379	1.540879	0.259952	5.92755	3.07E-09	1.47E-08	UP
SNRPD2	4504.865	0.812418	0.137082	5.926524	3.09E-09	1.47E-08	UP
TCEB1	2032.257	0.686623	0.115863	5.926153	3.10E-09	1.48E-08	UP
ERV3-1	152.4458	0.898231	0.15158	5.925771	3.11E-09	1.48E-08	UP
BLZF1	782.5189	0.545719	0.092105	5.924952	3.12E-09	1.49E-08	NOT
RN7SL751I	1.866818	2.806516	0.473745	5.924108	3.14E-09	1.50E-08	UP
RPLP0P6	179.7868	1.054827	0.17807	5.923675	3.15E-09	1.50E-08	UP
LA16c-38(	5.85963	1.2128	0.204738	5.923671	3.15E-09	1.50E-08	UP
LRP11	1149.991	0.638677	0.107831	5.922945	3.16E-09	1.51E-08	UP
RP5-1112I	15.9638	1.269304	0.214311	5.922734	3.17E-09	1.51E-08	UP
SLC20A1	2216.798	-0.71491	0.120722	-5.92195	3.18E-09	1.51E-08	DOWN
DGCR14	629.6978	0.446929	0.075477	5.921425	3.19E-09	1.52E-08	NOT
RP11-486/	57.79161	1.281056	0.216351	5.921197	3.20E-09	1.52E-08	UP
MSANTD1	10.4609	1.125271	0.190046	5.92105	3.20E-09	1.52E-08	UP
CTD-3220	3.17958	2.512807	0.4244	5.92085	3.20E-09	1.52E-08	UP
CTD-2517	9.11494	1.303739	0.220245	5.919493	3.23E-09	1.54E-08	UP
EIF3B	8345.045	0.592181	0.10004	5.919462	3.23E-09	1.54E-08	UP
IL17RC	2749.269	-0.70829	0.119656	-5.9194	3.23E-09	1.54E-08	DOWN
OASL	1289.417	-1.52747	0.258072	-5.91876	3.24E-09	1.54E-08	DOWN
LINC0011	3.816487	2.822267	0.47691	5.917813	3.26E-09	1.55E-08	UP

LSM14B	1256.564	0.479974	0.081107	5.91778	3.26E-09	1.55E-08	NOT
CTC-480C	3.933795	4.248818	0.718018	5.917429	3.27E-09	1.55E-08	UP
MTFP1	260.2739	0.860616	0.145456	5.916664	3.29E-09	1.56E-08	UP
VPS39	1844.415	0.393673	0.066551	5.91533	3.31E-09	1.57E-08	NOT
RP11-11N	17.47875	1.07406	0.181581	5.915032	3.32E-09	1.57E-08	UP
ASPH	8438.677	1.037998	0.175485	5.915027	3.32E-09	1.57E-08	UP
SARM1	157.914	0.940815	0.159087	5.913847	3.34E-09	1.59E-08	UP
HIST1H4D	3.277373	1.977316	0.334366	5.913621	3.35E-09	1.59E-08	UP
FXYD1	1505.539	-1.88227	0.318316	-5.9132	3.36E-09	1.59E-08	DOWN
RP13-890I	15.72446	1.282715	0.21695	5.912482	3.37E-09	1.60E-08	UP
RP11-395I	2.714987	2.682402	0.453754	5.911571	3.39E-09	1.61E-08	UP
VWA7	197.169	1.073403	0.181581	5.911431	3.39E-09	1.61E-08	UP
SGK1	3785.545	-1.14274	0.193311	-5.91141	3.39E-09	1.61E-08	DOWN
C5	27962.72	-0.9792	0.165647	-5.91137	3.39E-09	1.61E-08	DOWN
RP11-115I	13.41511	3.141149	0.53141	5.910969	3.40E-09	1.61E-08	UP
NAP1L1	7683.326	0.64536	0.109186	5.910638	3.41E-09	1.61E-08	UP
PROZ	1632.463	-1.5161	0.256559	-5.90938	3.43E-09	1.63E-08	DOWN
RNF180	191.6704	-1.09219	0.184836	-5.90899	3.44E-09	1.63E-08	DOWN
CLN8	685.1532	-0.72849	0.123292	-5.90866	3.45E-09	1.63E-08	DOWN
NIPSNAP3	1096.101	-0.69675	0.117953	-5.90705	3.48E-09	1.65E-08	DOWN
TYK2	2839.524	0.406199	0.068766	5.90695	3.48E-09	1.65E-08	NOT
MRC1	1571.241	-1.49093	0.252461	-5.90557	3.51E-09	1.66E-08	DOWN
AC139887	6.91673	1.293423	0.219024	5.905383	3.52E-09	1.67E-08	UP
KIZ	632.4015	0.805619	0.136438	5.904652	3.53E-09	1.67E-08	UP
ZNF461	103.876	0.602798	0.102093	5.904425	3.54E-09	1.67E-08	UP
MAP2	829.8794	1.454111	0.246286	5.904156	3.54E-09	1.68E-08	UP
PPP1R14BI	3.705231	2.054515	0.348039	5.903112	3.57E-09	1.69E-08	UP
RP11-313I	2.417434	1.878509	0.318256	5.902504	3.58E-09	1.69E-08	UP
SEMA3B-/	22.29853	1.508329	0.255554	5.902202	3.59E-09	1.70E-08	UP
NNAT	12.34886	1.533932	0.25991	5.901784	3.60E-09	1.70E-08	UP
BCAM	8120.404	1.09161	0.184981	5.901205	3.61E-09	1.71E-08	UP
RP11-67L3	11.61237	-1.17061	0.19837	-5.90114	3.61E-09	1.71E-08	DOWN
RP11-154I	4.281245	1.771341	0.300227	5.900001	3.63E-09	1.72E-08	UP
KRTAP5-1	6.823591	2.022939	0.342894	5.89961	3.64E-09	1.72E-08	UP
RP11-435	2.630516	2.33754	0.39623	5.899447	3.65E-09	1.72E-08	UP
IGKV1-8	32.29444	-2.57394	0.436356	-5.89871	3.66E-09	1.73E-08	DOWN
SGSM2	1069.119	0.709663	0.120328	5.897753	3.68E-09	1.74E-08	UP
RPS10L	5.683864	1.512622	0.256509	5.89696	3.70E-09	1.75E-08	UP
DHRS4L2	1463.128	-0.85634	0.145218	-5.8969	3.70E-09	1.75E-08	DOWN
USP1	1058.026	0.626671	0.106275	5.896695	3.71E-09	1.75E-08	UP
APOA1BP	5143.139	0.661703	0.112243	5.895259	3.74E-09	1.77E-08	UP
LINC0068	6.344908	-2.18207	0.370153	-5.89505	3.75E-09	1.77E-08	DOWN
RP11-290I	2.059797	-1.85341	0.314404	-5.89501	3.75E-09	1.77E-08	DOWN
ZNF761	344.5731	0.908719	0.154153	5.894926	3.75E-09	1.77E-08	UP
MAU2	1449.855	0.501647	0.085099	5.894833	3.75E-09	1.77E-08	NOT
FAM69A	954.4617	-0.74763	0.126845	-5.89408	3.77E-09	1.78E-08	DOWN
RPN2	16103.54	0.450906	0.076502	5.894003	3.77E-09	1.78E-08	NOT
RP11-365	6.821419	1.37243	0.232858	5.893848	3.77E-09	1.78E-08	UP
DHRS12	758.8558	-0.81006	0.137451	-5.89347	3.78E-09	1.78E-08	DOWN
CTSZ	14949.18	-0.64519	0.109481	-5.89312	3.79E-09	1.79E-08	DOWN
TPST1	1169.75	-0.93207	0.158167	-5.89299	3.79E-09	1.79E-08	DOWN
PECAM1	3719.385	0.698084	0.118472	5.892377	3.81E-09	1.79E-08	UP

CASC22	6.315508	3.47877	0.59039	5.892324	3.81E-09	1.79E-08	UP
SSR1	7951.135	0.45706	0.077575	5.891833	3.82E-09	1.80E-08	NOT
MYLK2	7.519252	1.738012	0.294987	5.891821	3.82E-09	1.80E-08	UP
RP11-83N	13.24998	1.442142	0.244792	5.891301	3.83E-09	1.80E-08	UP
ATP6V1E2	143.0337	0.769891	0.130687	5.891097	3.84E-09	1.81E-08	UP
SLC6A3	6.192867	2.950781	0.500909	5.890856	3.84E-09	1.81E-08	UP
bP-21264(	3.202371	2.374866	0.403158	5.89066	3.85E-09	1.81E-08	UP
SGSM1	66.00644	1.472648	0.249998	5.890644	3.85E-09	1.81E-08	UP
YWHAZ	13948.42	0.685602	0.116406	5.889758	3.87E-09	1.82E-08	UP
SLC7A8	479.3239	-1.23206	0.209218	-5.88889	3.89E-09	1.83E-08	DOWN
C6orf222	8.162336	3.064791	0.520486	5.888328	3.90E-09	1.83E-08	UP
AC093375	11.04905	1.870646	0.317695	5.88818	3.90E-09	1.84E-08	UP
MUTYH	422.4704	0.567322	0.09635	5.888147	3.91E-09	1.84E-08	NOT
TRIB2	1295.277	1.075058	0.182584	5.888008	3.91E-09	1.84E-08	UP
WWC3	866.2007	0.6712	0.114001	5.887664	3.92E-09	1.84E-08	UP
AC137932	22.47072	0.878469	0.149215	5.887287	3.93E-09	1.84E-08	UP
SYNDIG1	18.67646	2.625697	0.446006	5.887135	3.93E-09	1.85E-08	UP
RP11-631I	2.450204	-1.61858	0.274944	-5.88694	3.93E-09	1.85E-08	DOWN
SNORA2	7.810896	-0.82453	0.140065	-5.8868	3.94E-09	1.85E-08	DOWN
SAYSD1	778.4211	0.598399	0.101669	5.885746	3.96E-09	1.86E-08	UP
ATP8B4	146.763	-0.82228	0.139712	-5.88553	3.97E-09	1.86E-08	DOWN
EFR3B	35.44356	1.017467	0.172882	5.885328	3.97E-09	1.86E-08	UP
OPN4	1.881278	3.348107	0.569038	5.883801	4.01E-09	1.88E-08	UP
SCN7A	46.57646	-1.84428	0.313461	-5.8836	4.01E-09	1.88E-08	DOWN
PRKAA1	2832.73	-0.53564	0.09104	-5.88355	4.02E-09	1.88E-08	NOT
SLC12A9	1453.765	0.55817	0.094874	5.88325	4.02E-09	1.89E-08	NOT
PLAC8	240.1398	-1.78428	0.303284	-5.8832	4.02E-09	1.89E-08	DOWN
BRMS1	1836.806	0.643551	0.109395	5.882792	4.03E-09	1.89E-08	UP
CCNI2	11.6349	1.726648	0.29351	5.882763	4.03E-09	1.89E-08	UP
CHCHD3	1561.888	0.501729	0.085288	5.882757	4.03E-09	1.89E-08	NOT
RP11-616I	50.93753	1.229321	0.208971	5.882746	4.04E-09	1.89E-08	UP
OXTR	48.07246	1.457288	0.247769	5.881641	4.06E-09	1.90E-08	UP
PPFIBP2	1749.38	-0.61945	0.10532	-5.88155	4.06E-09	1.90E-08	DOWN
SLC22A23	1657.537	0.730106	0.124139	5.881362	4.07E-09	1.91E-08	UP
PLCH2	369.6163	1.906323	0.32415	5.880994	4.08E-09	1.91E-08	UP
HNRNPU	11759.29	0.340126	0.057838	5.880681	4.09E-09	1.91E-08	NOT
AHNAK2	222.7601	1.894725	0.322205	5.880488	4.09E-09	1.91E-08	UP
RP1-30M3	27.19258	-0.79321	0.134909	-5.87957	4.11E-09	1.92E-08	DOWN
BCAP31	12783.01	0.699284	0.11894	5.879285	4.12E-09	1.93E-08	UP
ANKRD18E	91.37806	1.202497	0.204532	5.879267	4.12E-09	1.93E-08	UP
RP11-276I	11.30429	3.526689	0.600023	5.877591	4.16E-09	1.95E-08	UP
RHBDL2	28.45722	1.381692	0.235086	5.877382	4.17E-09	1.95E-08	UP
PTEN	3688.72	-0.51317	0.087333	-5.87602	4.20E-09	1.96E-08	NOT
RP11-849I	38.30317	0.847994	0.144316	5.875964	4.20E-09	1.97E-08	UP
ZNF138	206.5206	0.502747	0.085584	5.874315	4.25E-09	1.98E-08	NOT
PGLYRP2	9946.255	-1.68061	0.286101	-5.87418	4.25E-09	1.99E-08	DOWN
SNRPEP2	22.94335	0.73496	0.125117	5.874163	4.25E-09	1.99E-08	UP
DKK2	36.64651	1.615018	0.274953	5.873788	4.26E-09	1.99E-08	UP
SLC16A9	349.0294	2.289793	0.389843	5.873621	4.26E-09	1.99E-08	UP
NPM2	135.5753	1.628994	0.277346	5.873518	4.27E-09	1.99E-08	UP
RP11-762I	6.720903	0.93472	0.159144	5.873406	4.27E-09	1.99E-08	UP
VSX1	11.09294	2.007018	0.341758	5.872632	4.29E-09	2.00E-08	UP

RRAGD	1336.958	1.017182	0.173241	5.8715	4.32E-09	2.02E-08	UP
LILRB1	210.0762	-1.13052	0.192582	-5.87034	4.35E-09	2.03E-08	DOWN
RP11-471I	18.22386	-0.92751	0.158029	-5.86927	4.38E-09	2.04E-08	DOWN
PHF20L1	1277.51	0.49584	0.084487	5.868829	4.39E-09	2.05E-08	NOT
ZNF579	428.9623	0.880807	0.150085	5.86871	4.39E-09	2.05E-08	UP
MIR589	1.408232	2.171544	0.370048	5.868278	4.40E-09	2.05E-08	UP
CAPN2	4288.882	0.772185	0.131587	5.868255	4.40E-09	2.05E-08	UP
VPS16	1397.442	0.440619	0.075093	5.867655	4.42E-09	2.06E-08	NOT
RP1-8B1.4	10.57624	1.589525	0.270924	5.867054	4.44E-09	2.07E-08	UP
TSPAN7	744.1336	-0.99767	0.170064	-5.86643	4.45E-09	2.08E-08	DOWN
SCARNA15	8.701347	1.20755	0.20586	5.86588	4.47E-09	2.08E-08	UP
KHDC1	9.351544	2.000193	0.340997	5.865722	4.47E-09	2.08E-08	UP
ZNF300P1	38.49581	1.928475	0.328785	5.865464	4.48E-09	2.09E-08	UP
MYH13	4.66338	3.772453	0.643316	5.864076	4.52E-09	2.10E-08	UP
FTCD	20654.49	-1.50422	0.256516	-5.86404	4.52E-09	2.10E-08	DOWN
PRRG1	419.6792	-0.7183	0.122503	-5.86356	4.53E-09	2.11E-08	DOWN
SHMT2	9995.257	-0.5919	0.100954	-5.86309	4.54E-09	2.11E-08	DOWN
RAB10	4816.97	0.516312	0.088063	5.86297	4.55E-09	2.12E-08	NOT
CTC-510F	14.08675	0.844841	0.144098	5.862953	4.55E-09	2.12E-08	UP
SMAGP	422.8691	0.830879	0.141728	5.862478	4.56E-09	2.12E-08	UP
CCR3	9.007473	2.679605	0.457162	5.861389	4.59E-09	2.14E-08	UP
AP000648.	55.97629	0.921419	0.157212	5.860987	4.60E-09	2.14E-08	UP
COPA	10654.99	0.548633	0.093611	5.860793	4.61E-09	2.14E-08	NOT
TSNAX	1465.581	0.445347	0.075989	5.860684	4.61E-09	2.14E-08	NOT
COL5A3	2543.37	1.382763	0.235951	5.860393	4.62E-09	2.15E-08	UP
SPIN4	127.4106	0.878096	0.149849	5.859891	4.63E-09	2.15E-08	UP
FAM35DP	63.21303	-1.26207	0.215389	-5.85948	4.64E-09	2.16E-08	DOWN
ZSCAN29	572.7434	0.438058	0.074769	5.858842	4.66E-09	2.17E-08	NOT
NCOA5	1066.581	0.437605	0.074693	5.858738	4.66E-09	2.17E-08	NOT
TSPAN9	4206.857	-0.67565	0.115324	-5.8587	4.66E-09	2.17E-08	DOWN
MEF2B	13.1689	1.157978	0.19767	5.858142	4.68E-09	2.17E-08	UP
FBRS	2050.395	0.345588	0.058995	5.85794	4.69E-09	2.18E-08	NOT
AD000092	9.682528	1.006458	0.171834	5.857144	4.71E-09	2.19E-08	UP
SLC37A4	7965.78	-0.84738	0.144679	-5.85699	4.71E-09	2.19E-08	DOWN
SORD	11674.16	-1.13522	0.193824	-5.85694	4.71E-09	2.19E-08	DOWN
RP11-103I	5.102455	2.20009	0.375653	5.856709	4.72E-09	2.19E-08	UP
DCXR	34973.39	-1.35598	0.231543	-5.85625	4.73E-09	2.20E-08	DOWN
RP11-133I	1.293678	2.72772	0.465833	5.855573	4.75E-09	2.20E-08	UP
OFD1	660.8379	0.551122	0.094121	5.855452	4.76E-09	2.21E-08	NOT
AIRE	3.768561	2.81143	0.48015	5.855317	4.76E-09	2.21E-08	UP
HCG20	6.878261	1.964391	0.335491	5.855269	4.76E-09	2.21E-08	UP
HEIH	855.5825	0.542938	0.092737	5.854597	4.78E-09	2.22E-08	NOT
CTNND2	403.2184	2.575161	0.439884	5.854182	4.79E-09	2.22E-08	UP
TRMT112F	16.26298	1.566496	0.267601	5.853859	4.80E-09	2.23E-08	UP
HEY2	314.1588	-0.98199	0.167758	-5.85359	4.81E-09	2.23E-08	DOWN
ZNF852	53.94208	0.55557	0.094917	5.853205	4.82E-09	2.23E-08	NOT
RP11-3D4	13.15301	1.086333	0.185597	5.853197	4.82E-09	2.23E-08	UP
MRPL46	1104.125	-0.67807	0.115859	-5.85253	4.84E-09	2.24E-08	DOWN
RNU1-75P	3.680789	-1.70827	0.291897	-5.85229	4.85E-09	2.24E-08	DOWN
HCAR1	8.68628	1.857428	0.317409	5.851837	4.86E-09	2.25E-08	UP
RP11-436I	3.11907	2.997017	0.512162	5.851698	4.87E-09	2.25E-08	UP
FOLH1	782.3158	-1.22925	0.210109	-5.85053	4.90E-09	2.27E-08	DOWN

RP11-475(	18.47538	-1.6836	0.287781	-5.85027	4.91E-09	2.27E-08	DOWN
KB-1125A:	4.269417	1.476121	0.252369	5.849063	4.94E-09	2.29E-08	UP
PRF1	319.0177	-1.04921	0.179382	-5.84904	4.94E-09	2.29E-08	DOWN
SLC27A5	30762.39	-1.59186	0.27221	-5.84793	4.98E-09	2.30E-08	DOWN
PNPLA8	1445.075	-0.60089	0.10277	-5.84697	5.01E-09	2.31E-08	DOWN
RBKS	1083.097	-0.73277	0.125328	-5.84682	5.01E-09	2.32E-08	DOWN
CLRN1-AS	8.965733	-2.35597	0.402955	-5.84672	5.01E-09	2.32E-08	DOWN
RP11-184I	2.840536	2.497983	0.42726	5.846516	5.02E-09	2.32E-08	UP
BCL2L12	742.9207	0.71861	0.122918	5.846258	5.03E-09	2.32E-08	UP
ZNF648	54.65566	2.202861	0.376808	5.846107	5.03E-09	2.32E-08	UP
RP11-173I	3.915044	1.606937	0.274894	5.845661	5.05E-09	2.33E-08	UP
DIP2A	725.1285	0.498411	0.085273	5.84489	5.07E-09	2.34E-08	NOT
AVIL	173.3662	1.002726	0.171569	5.844461	5.08E-09	2.35E-08	UP
PHYKPL	1846.629	-0.60977	0.104342	-5.84401	5.10E-09	2.35E-08	DOWN
TAF1B	282.5456	0.490016	0.083849	5.843996	5.10E-09	2.35E-08	NOT
SIGLEC14	69.90096	-1.8567	0.317734	-5.84357	5.11E-09	2.36E-08	DOWN
ZNF257	31.46052	1.655208	0.283253	5.843571	5.11E-09	2.36E-08	UP
ZNF571-A	11.38825	1.63502	0.279799	5.843559	5.11E-09	2.36E-08	UP
RP11-53O	13.79465	1.218556	0.208531	5.843511	5.11E-09	2.36E-08	UP
RP11-687I	2.676649	1.738506	0.297536	5.843017	5.13E-09	2.36E-08	UP
LINC00501	3.190622	2.957127	0.506145	5.842448	5.14E-09	2.37E-08	UP
RP11-280(	4.439445	-1.23327	0.2111	-5.84213	5.15E-09	2.38E-08	DOWN
CTA-228A	6.403263	1.310099	0.224256	5.84198	5.16E-09	2.38E-08	UP
SAT2	6955.633	-0.88108	0.150822	-5.84186	5.16E-09	2.38E-08	DOWN
HIST1H1C	5445.791	1.260493	0.215787	5.841372	5.18E-09	2.39E-08	UP
VAC14-AS	49.86653	1.777702	0.304338	5.841215	5.18E-09	2.39E-08	UP
PAIP2B	954.7145	-1.3809	0.236457	-5.83997	5.22E-09	2.41E-08	DOWN
SNAP47	1538.164	0.523647	0.089672	5.839603	5.23E-09	2.41E-08	NOT
NPM3	550.4831	0.949087	0.162532	5.839374	5.24E-09	2.41E-08	UP
DISP2	94.51857	1.382021	0.236692	5.838908	5.25E-09	2.42E-08	UP
RFC1	2517.081	0.49745	0.0852	5.838615	5.26E-09	2.42E-08	NOT
E2F3	607.4831	0.816233	0.139802	5.838509	5.27E-09	2.42E-08	UP
ANKRD46	1300.992	-0.68065	0.116586	-5.83817	5.28E-09	2.43E-08	DOWN
RP11-452I	227.4504	0.80805	0.138426	5.837406	5.30E-09	2.44E-08	UP
CTD-3195	5.810066	1.312261	0.224833	5.836608	5.33E-09	2.45E-08	UP
CEP128	84.92647	0.801101	0.137281	5.835488	5.36E-09	2.47E-08	UP
BTNL10	3.294009	2.095775	0.359175	5.834977	5.38E-09	2.47E-08	UP
UNC5B	724.4317	1.064274	0.182412	5.834457	5.40E-09	2.48E-08	UP
NASP	2737.384	0.564741	0.096801	5.83407	5.41E-09	2.49E-08	NOT
FTCDNL1	240.1016	-0.88943	0.152458	-5.83394	5.41E-09	2.49E-08	DOWN
RP11-876I	31.46504	0.946499	0.162247	5.83369	5.42E-09	2.49E-08	UP
OSBPL11	1090.738	-0.66183	0.113463	-5.83303	5.44E-09	2.50E-08	DOWN
PHYH	15934.75	-1.02796	0.176232	-5.83297	5.44E-09	2.50E-08	DOWN
NPM1P9	2.181181	1.882313	0.322779	5.831592	5.49E-09	2.52E-08	UP
AC006042	13.17451	1.411803	0.242104	5.831395	5.50E-09	2.52E-08	UP
RP11-54O	38.2683	-1.81436	0.311152	-5.8311	5.51E-09	2.53E-08	DOWN
ANKRD1	128.7999	1.999849	0.342984	5.83074	5.52E-09	2.53E-08	UP
GBP1	2904.223	-1.12628	0.193168	-5.83059	5.52E-09	2.54E-08	DOWN
LINC0051(	1.828794	2.641272	0.453168	5.828461	5.59E-09	2.57E-08	UP
RP11-42O	248.0009	-1.44568	0.248048	-5.82824	5.60E-09	2.57E-08	DOWN
KCTD2	1152.863	0.5071	0.087036	5.826314	5.67E-09	2.60E-08	NOT
FAM168A	1944.301	0.481619	0.082663	5.826284	5.67E-09	2.60E-08	NOT

COL21A1	124.6025	1.297324	0.222677	5.826044	5.68E-09	2.60E-08	UP
RP11-378.	38.35558	0.713047	0.122395	5.82578	5.68E-09	2.61E-08	UP
HIST4H4	17.22288	1.000476	0.17174	5.825541	5.69E-09	2.61E-08	UP
AMPD1	5.755652	-2.21309	0.37993	-5.82498	5.71E-09	2.62E-08	DOWN
NEDD1	373.7147	0.508831	0.087354	5.82492	5.71E-09	2.62E-08	NOT
RP11-627(	4.583358	-1.20423	0.206744	-5.82475	5.72E-09	2.62E-08	DOWN
ACYP2	635.7014	-0.67523	0.115931	-5.82438	5.73E-09	2.63E-08	DOWN
PEX14	1293.813	-0.61858	0.106212	-5.824	5.75E-09	2.63E-08	DOWN
GCKR	2904.22	-1.19396	0.205021	-5.82363	5.76E-09	2.64E-08	DOWN
C6orf62	6782.607	0.465417	0.079938	5.822222	5.81E-09	2.66E-08	NOT
OAZ3	70.61438	0.877161	0.150663	5.82202	5.81E-09	2.66E-08	UP
HES5	6.841719	1.712156	0.294153	5.820627	5.86E-09	2.68E-08	UP
FXYD6	628.9627	-0.82564	0.141853	-5.82042	5.87E-09	2.69E-08	DOWN
CXorf23	269.3751	0.523599	0.089972	5.819603	5.90E-09	2.70E-08	NOT
RP3-420J1	55.14133	-2.30289	0.395738	-5.81923	5.91E-09	2.71E-08	DOWN
DCTN6	647.311	-0.51575	0.088645	-5.8181	5.95E-09	2.72E-08	NOT
GREB1L	362.9159	0.934413	0.160605	5.818085	5.95E-09	2.72E-08	UP
GABPB2	488.6133	0.560344	0.096319	5.817578	5.97E-09	2.73E-08	NOT
AATK	140.1404	1.454247	0.249996	5.817074	5.99E-09	2.74E-08	UP
GLYAT	6154.638	-2.03664	0.350118	-5.81701	5.99E-09	2.74E-08	DOWN
KIAA0226	541.7126	0.436052	0.074968	5.816535	6.01E-09	2.75E-08	NOT
HSD17B1	84.96408	0.861349	0.14809	5.816405	6.01E-09	2.75E-08	UP
RP1-16A9.	1.907458	2.383879	0.409954	5.814992	6.06E-09	2.77E-08	UP
AP3D1	6336.584	0.466892	0.080305	5.813998	6.10E-09	2.79E-08	NOT
GPR125	3293.256	-1.03936	0.178842	-5.81162	6.19E-09	2.83E-08	DOWN
SPATA17	23.20196	2.04401	0.351716	5.81153	6.19E-09	2.83E-08	UP
DICER1	1899.318	-0.58446	0.100578	-5.81103	6.21E-09	2.84E-08	NOT
ANGPTL3	19258.65	-1.32219	0.227532	-5.81099	6.21E-09	2.84E-08	DOWN
STX17	546.9732	-0.59462	0.10233	-5.81081	6.22E-09	2.84E-08	DOWN
DHRS4	1467.517	-0.79654	0.137088	-5.81044	6.23E-09	2.84E-08	DOWN
ITPRIP	927.5391	-0.86186	0.14833	-5.81039	6.23E-09	2.84E-08	DOWN
TRPC1	89.98974	1.169507	0.201297	5.809869	6.25E-09	2.85E-08	UP
ABCC8	16.38117	2.068125	0.355999	5.809352	6.27E-09	2.86E-08	UP
CTD-2349	3.701338	1.407648	0.24231	5.809295	6.27E-09	2.86E-08	UP
ACADVL	23898.09	-0.7223	0.124347	-5.8088	6.29E-09	2.87E-08	DOWN
RP3-446N	6.71128	3.476637	0.598525	5.808679	6.30E-09	2.87E-08	UP
UIMC1	579.9198	0.381366	0.06566	5.808209	6.31E-09	2.88E-08	NOT
CTC-260E	1.849913	2.983351	0.513735	5.807182	6.35E-09	2.90E-08	UP
WASH7P	279.6331	0.89573	0.154253	5.806883	6.36E-09	2.90E-08	UP
FOXI2	2.839133	2.334227	0.401993	5.806629	6.37E-09	2.90E-08	UP
SLC6A7	5.144029	2.492994	0.429377	5.806069	6.40E-09	2.91E-08	UP
SLCO2A1	576.6348	1.382284	0.23812	5.804985	6.44E-09	2.93E-08	UP
ASLP1	7.198483	1.451555	0.250059	5.804846	6.44E-09	2.93E-08	UP
SLC25A25	4686.753	-1.13827	0.196098	-5.80457	6.45E-09	2.94E-08	DOWN
KCNE1	13.79094	-1.48674	0.256169	-5.80374	6.49E-09	2.95E-08	DOWN
ANKLE1	16.69921	1.254393	0.21617	5.802807	6.52E-09	2.97E-08	UP
DMWD	648.464	0.462996	0.079792	5.802533	6.53E-09	2.97E-08	NOT
IMPDH1	644.4151	1.128099	0.194434	5.801972	6.55E-09	2.98E-08	UP
GGA2	2658.395	0.539525	0.092991	5.801905	6.56E-09	2.98E-08	NOT
GLIS2	547.3247	1.253665	0.216081	5.801843	6.56E-09	2.98E-08	UP
RP13-131I	5.856639	1.622082	0.279583	5.801799	6.56E-09	2.98E-08	UP
RNPEPL1	4119.17	0.544483	0.093853	5.801422	6.58E-09	2.99E-08	NOT

RP11-785I	20.99258	2.941897	0.507127	5.8011	6.59E-09	3.00E-08	UP
POLR3B	706.1729	-0.41642	0.071794	-5.80026	6.62E-09	3.01E-08	NOT
RAB3GAP2	1414.129	0.4638	0.079964	5.800098	6.63E-09	3.01E-08	NOT
KRT12	4.475241	3.653085	0.629903	5.799438	6.65E-09	3.02E-08	UP
CBX4	1505.227	0.645947	0.111391	5.798939	6.67E-09	3.03E-08	UP
USP35	470.6618	0.799293	0.137846	5.798435	6.69E-09	3.04E-08	UP
CLEC4GP1	4.039399	-2.20276	0.379981	-5.79703	6.75E-09	3.07E-08	DOWN
C17orf99	2.760196	2.826547	0.487613	5.796698	6.76E-09	3.07E-08	UP
RP11-30J2	27.74705	3.128641	0.539833	5.795571	6.81E-09	3.09E-08	UP
HOXC8	5.456775	3.798441	0.65559	5.793924	6.88E-09	3.12E-08	UP
SNORA51	2.389268	2.260805	0.390277	5.792826	6.92E-09	3.14E-08	UP
PTBP2	418.3461	0.671699	0.115958	5.7926	6.93E-09	3.15E-08	UP
IL6	54.43633	-1.90523	0.32893	-5.79221	6.95E-09	3.15E-08	DOWN
RP11-146I	2.921736	1.912771	0.330233	5.792192	6.95E-09	3.15E-08	UP
C1QB	6621.942	-1.20648	0.208314	-5.79164	6.97E-09	3.16E-08	DOWN
CTC-236F	5.699091	1.987845	0.343298	5.790428	7.02E-09	3.18E-08	UP
RP13-152(	5.292907	1.680169	0.290199	5.789707	7.05E-09	3.20E-08	UP
COL5A2	2250.991	1.098856	0.189807	5.789345	7.07E-09	3.20E-08	UP
AC015849	34.66387	1.640002	0.283287	5.789193	7.07E-09	3.21E-08	UP
SFR1	130.3832	0.551712	0.095303	5.789055	7.08E-09	3.21E-08	NOT
MYO1F	703.159	-0.83846	0.144841	-5.78883	7.09E-09	3.21E-08	DOWN
TMEM168	734.7732	0.527003	0.091042	5.788586	7.10E-09	3.22E-08	NOT
CHN1	125.8646	0.931511	0.160934	5.788139	7.12E-09	3.22E-08	UP
AL590431.	5.363739	1.233369	0.213087	5.788103	7.12E-09	3.22E-08	UP
CYP4F2	7157.618	-1.62587	0.280903	-5.78801	7.12E-09	3.23E-08	DOWN
FAM199X	1397.54	0.478466	0.082674	5.787358	7.15E-09	3.24E-08	NOT
DTNB	436.7442	0.475249	0.082126	5.786817	7.17E-09	3.25E-08	NOT
HIST1H4I	579.3647	0.967236	0.167176	5.785745	7.22E-09	3.27E-08	UP
CH17-262	4.914546	1.507417	0.260561	5.785276	7.24E-09	3.28E-08	UP
GFOD2	1436.697	-0.64589	0.111681	-5.78333	7.32E-09	3.31E-08	DOWN
AC080008	8.146052	-1.44442	0.249757	-5.7833	7.32E-09	3.31E-08	DOWN
EFCAB7	108.0244	0.693818	0.120002	5.781735	7.39E-09	3.35E-08	UP
KCNK12	3.256353	2.44855	0.423526	5.781339	7.41E-09	3.35E-08	UP
ARL6IP6	281.7222	0.65107	0.11262	5.781133	7.42E-09	3.36E-08	UP
NR2C2	795.0417	0.66795	0.115545	5.78087	7.43E-09	3.36E-08	UP
RP11-49C(	5.113781	1.673302	0.28947	5.780564	7.45E-09	3.37E-08	UP
ZNF726	100.4447	1.364654	0.236088	5.780275	7.46E-09	3.37E-08	UP
EGFEM1P	7.396478	2.832455	0.490024	5.780235	7.46E-09	3.37E-08	UP
ACIN1	3525.817	0.319119	0.05521	5.780085	7.47E-09	3.37E-08	NOT
STT3A	6553.718	0.474245	0.082051	5.779917	7.47E-09	3.38E-08	NOT
LZTFL1	467.7291	-0.75734	0.131032	-5.77981	7.48E-09	3.38E-08	DOWN
COLGALT2	45.96685	1.641469	0.284038	5.779043	7.51E-09	3.39E-08	UP
PDDC1	2000.894	0.52849	0.091452	5.778861	7.52E-09	3.40E-08	NOT
GDAP1	216.8101	1.038701	0.179775	5.777793	7.57E-09	3.42E-08	UP
SYT2	16.47506	1.567604	0.271317	5.777753	7.57E-09	3.42E-08	UP
AC073063	8.623939	0.865783	0.149875	5.776698	7.62E-09	3.44E-08	UP
RP11-181I	3.064927	2.200464	0.381017	5.77524	7.68E-09	3.47E-08	UP
SAMD4A	1056.01	-0.75835	0.131333	-5.77427	7.73E-09	3.49E-08	DOWN
TMEM98	1702.863	1.067098	0.18481	5.774016	7.74E-09	3.49E-08	UP
RP1-40E1(	4.046551	4.22551	0.731856	5.773694	7.76E-09	3.50E-08	UP
GJB2	1650.649	-1.14519	0.198382	-5.77262	7.80E-09	3.52E-08	DOWN
RP11-755I	3.647235	1.633857	0.283047	5.772387	7.82E-09	3.52E-08	UP

PCMT1	2072.01	-0.4985	0.086367	-5.77193	7.84E-09	3.53E-08	NOT
MT3	13.20946	2.828661	0.490101	5.771582	7.85E-09	3.54E-08	UP
OCIAD1	5660.556	-0.40025	0.069349	-5.77154	7.85E-09	3.54E-08	NOT
ABL1	1852.091	0.526311	0.091204	5.770671	7.90E-09	3.56E-08	NOT
GPR137	1421.98	0.574552	0.099577	5.769957	7.93E-09	3.57E-08	NOT
RP11-796I	18.65168	1.378386	0.238905	5.769591	7.95E-09	3.58E-08	UP
RP11-900I	2.842241	2.431037	0.421382	5.769195	7.97E-09	3.59E-08	UP
RP11-44N	26.43559	1.220603	0.211595	5.768577	7.99E-09	3.60E-08	UP
TRIP10	938.3385	0.767688	0.133086	5.768367	8.00E-09	3.60E-08	UP
EIF4E3	604.3862	-1.07763	0.186824	-5.76818	8.01E-09	3.61E-08	DOWN
FBXW8	435.0308	0.427312	0.074093	5.767205	8.06E-09	3.63E-08	NOT
SH3GLB2	1648.162	0.579676	0.100519	5.766836	8.08E-09	3.64E-08	NOT
CTD-2583	95.4114	0.866789	0.150349	5.765165	8.16E-09	3.67E-08	UP
XX-FW835	36.6906	0.716797	0.124348	5.76443	8.19E-09	3.69E-08	UP
RP11-528I	1.881638	2.096664	0.36374	5.764181	8.21E-09	3.69E-08	UP
RP11-545I	2.436572	-1.14901	0.199341	-5.76401	8.21E-09	3.69E-08	DOWN
COG1	1187.791	0.400012	0.0694	5.763844	8.22E-09	3.70E-08	NOT
RP11-540(	5.744951	1.445342	0.250764	5.763759	8.23E-09	3.70E-08	UP
SLC25A26	928.7873	-0.5218	0.090541	-5.76315	8.26E-09	3.71E-08	NOT
FAM198A	309.5306	-1.75302	0.304179	-5.76312	8.26E-09	3.71E-08	DOWN
FZD6	469.9437	1.027442	0.178282	5.762999	8.26E-09	3.71E-08	UP
RP11-729I	5.580324	-1.48873	0.258329	-5.76291	8.27E-09	3.72E-08	DOWN
GGA1	2023.403	0.538564	0.093455	5.762819	8.27E-09	3.72E-08	NOT
HOXC13	4.219603	4.16273	0.722377	5.762547	8.29E-09	3.72E-08	UP
CASP7	1029.54	-0.57663	0.100074	-5.76201	8.31E-09	3.73E-08	NOT
RP11-468I	114.5435	-1.76537	0.306425	-5.76119	8.35E-09	3.75E-08	DOWN
PLEKHA3	702.8242	-0.42283	0.0734	-5.76065	8.38E-09	3.76E-08	NOT
SCGB1D2	5.917408	4.121707	0.715498	5.760613	8.38E-09	3.76E-08	UP
PCDHB6	23.62681	1.623413	0.281828	5.760306	8.40E-09	3.77E-08	UP
RP4-622L5	3.517989	1.736659	0.301501	5.760037	8.41E-09	3.77E-08	UP
F9	16950.54	-1.80844	0.314017	-5.75905	8.46E-09	3.80E-08	DOWN
CTD-2201	79.05921	1.043415	0.181187	5.758764	8.47E-09	3.80E-08	UP
WASF1	424.6437	0.808392	0.140377	5.75873	8.47E-09	3.80E-08	UP
CREB3L2	2283.888	0.594046	0.103158	5.758584	8.48E-09	3.80E-08	UP
ULK4	308.0342	1.235692	0.214615	5.757717	8.53E-09	3.82E-08	UP
TRIM32	373.3173	0.555575	0.096493	5.757647	8.53E-09	3.82E-08	NOT
ENSA	7096.798	0.535119	0.092962	5.756341	8.60E-09	3.85E-08	NOT
HMCES	2549.714	-0.42255	0.07341	-5.75607	8.61E-09	3.86E-08	NOT
SKIV2L	2720.626	0.463738	0.080568	5.755878	8.62E-09	3.86E-08	NOT
RP11-135I	441.3021	-0.48223	0.083782	-5.75584	8.62E-09	3.86E-08	NOT
KIAA1407	121.1638	0.772418	0.134228	5.754512	8.69E-09	3.89E-08	UP
SOX4	1586.297	1.371185	0.23828	5.754506	8.69E-09	3.89E-08	UP
DDAH1	4955.122	-0.83777	0.1456	-5.75395	8.72E-09	3.90E-08	DOWN
ELOVL7	376.1435	1.769804	0.307583	5.753905	8.72E-09	3.90E-08	UP
RNU6-762	1.861058	1.947319	0.338443	5.753751	8.73E-09	3.91E-08	UP
DOK7	138.2432	1.583976	0.275295	5.753732	8.73E-09	3.91E-08	UP
FKBP7	287.4903	-0.68103	0.118365	-5.75365	8.73E-09	3.91E-08	DOWN
RP9P	98.01562	0.83881	0.145793	5.753435	8.74E-09	3.91E-08	UP
RP11-399I	2.172636	2.346659	0.407985	5.751823	8.83E-09	3.95E-08	UP
MYCNOS	4.978363	2.910529	0.506085	5.751064	8.87E-09	3.97E-08	UP
GPBP1	2627.522	0.31668	0.055073	5.750182	8.91E-09	3.99E-08	NOT
WDR36	1244.759	-0.42182	0.073361	-5.74996	8.93E-09	3.99E-08	NOT

ANGEL2	887.1433	0.460297	0.080057	5.749625	8.94E-09	4.00E-08	NOT
RP11-548I	29.35666	1.082104	0.188236	5.748667	8.99E-09	4.02E-08	UP
RP11-634I	14.82907	1.117017	0.19432	5.748346	9.01E-09	4.03E-08	UP
IL2RB	440.4617	-1.15479	0.200891	-5.74835	9.01E-09	4.03E-08	DOWN
CHD1L	2473.306	0.62976	0.109565	5.747837	9.04E-09	4.04E-08	UP
ATXN2	1330.571	0.350951	0.061058	5.747792	9.04E-09	4.04E-08	NOT
RP11-261I	9.217385	1.348086	0.234556	5.74739	9.06E-09	4.05E-08	UP
DRC7	38.79463	1.296276	0.225561	5.746902	9.09E-09	4.06E-08	UP
MAP7	1287.365	-0.78044	0.135821	-5.74606	9.13E-09	4.08E-08	DOWN
AC083843	45.59192	1.184786	0.206213	5.745458	9.17E-09	4.09E-08	UP
SPATA21	64.35263	2.130161	0.370758	5.745428	9.17E-09	4.09E-08	UP
AVPR1A	2473.279	-2.24923	0.391485	-5.74538	9.17E-09	4.09E-08	DOWN
MSANTD2	168.1134	0.594975	0.103566	5.744886	9.20E-09	4.10E-08	UP
RAET1K	4.053681	1.922033	0.334577	5.744672	9.21E-09	4.11E-08	UP
CTD-2165	18.49881	1.090251	0.189799	5.744231	9.23E-09	4.12E-08	UP
RP1-45C1	5.662048	2.140436	0.372629	5.744155	9.24E-09	4.12E-08	UP
HIST1H2BI	3.985412	2.424317	0.422086	5.743658	9.27E-09	4.13E-08	UP
RBM8A	3372.155	0.428812	0.07466	5.743496	9.27E-09	4.14E-08	NOT
PEX5L	5.409521	1.644617	0.286361	5.743159	9.29E-09	4.14E-08	UP
SLCO1B1	7548.093	-1.42828	0.248705	-5.74287	9.31E-09	4.15E-08	DOWN
ZFAND6	2900.753	-0.34952	0.060862	-5.74285	9.31E-09	4.15E-08	NOT
SOX9-AS1	242.9808	1.544121	0.268877	5.742842	9.31E-09	4.15E-08	UP
SLMO1	23.29289	1.403223	0.244343	5.742835	9.31E-09	4.15E-08	UP
CTB-31N1	10.50979	-0.81319	0.141606	-5.74261	9.32E-09	4.15E-08	DOWN
FOXF1	130.9619	-1.16442	0.202807	-5.74153	9.38E-09	4.18E-08	DOWN
RP11-350I	6.115879	2.327788	0.405434	5.741466	9.39E-09	4.18E-08	UP
ZNF711	96.33006	1.638829	0.285459	5.74104	9.41E-09	4.19E-08	UP
MTX3	563.3691	0.459745	0.080082	5.740916	9.42E-09	4.19E-08	NOT
ACO1	8294.408	-0.7552	0.131568	-5.74002	9.47E-09	4.21E-08	DOWN
CCT4	5059.516	0.460621	0.080249	5.739869	9.47E-09	4.22E-08	NOT
RP1-209A	2.758169	3.733238	0.650439	5.739564	9.49E-09	4.22E-08	UP
PDCL	426.1122	0.434475	0.075699	5.739536	9.49E-09	4.22E-08	NOT
STX16-NP	26.03069	1.137092	0.198135	5.738987	9.52E-09	4.24E-08	UP
RP11-69L1	2.691433	-1.19459	0.208156	-5.73894	9.53E-09	4.24E-08	DOWN
SFXN2	1121.818	-0.83211	0.145	-5.73873	9.54E-09	4.24E-08	DOWN
RP4-803J1	2.404438	1.823093	0.317683	5.738726	9.54E-09	4.24E-08	UP
TMEM161	207.0915	0.851274	0.148345	5.73849	9.55E-09	4.25E-08	UP
RP11-59H	3.892158	1.82089	0.317352	5.737757	9.59E-09	4.26E-08	UP
DGKZP1	14.60288	0.797906	0.139063	5.737711	9.60E-09	4.26E-08	UP
ERCC3	1252.654	0.346631	0.060413	5.737668	9.60E-09	4.26E-08	NOT
LL09NC01	7.975633	1.290965	0.225001	5.737603	9.60E-09	4.27E-08	UP
FBXL4	1062.32	-0.52109	0.090824	-5.73735	9.62E-09	4.27E-08	NOT
CCDC84	397.9089	0.673509	0.117403	5.73673	9.65E-09	4.29E-08	UP
SLC6A12	2891.896	-1.35616	0.236444	-5.73562	9.72E-09	4.31E-08	DOWN
LLNLR-304	2.175967	2.085102	0.363545	5.735478	9.72E-09	4.32E-08	UP
RPL39P36	3.889074	1.172504	0.204432	5.735421	9.73E-09	4.32E-08	UP
MICAL1	602.2551	0.994458	0.173404	5.734909	9.76E-09	4.33E-08	UP
HIVEP2	691.3389	-0.68494	0.119438	-5.73467	9.77E-09	4.34E-08	DOWN
NEIL2	542.5181	-0.58281	0.101647	-5.73367	9.83E-09	4.36E-08	NOT
RP11-173I	4.762326	1.741877	0.303803	5.733581	9.83E-09	4.36E-08	UP
CYP2C18	2808.794	-1.40513	0.245335	-5.72739	1.02E-08	4.52E-08	DOWN
ZNF354B	160.192	0.596678	0.10418	5.727382	1.02E-08	4.52E-08	UP

ARHGAP19	316.555	0.486709	0.084981	5.727261	1.02E-08	4.53E-08	NOT
AC005154	12.85364	1.04311	0.182145	5.726796	1.02E-08	4.54E-08	UP
TECTA	48.13029	-1.00293	0.175136	-5.72659	1.02E-08	4.54E-08	DOWN
RP3-414A	77.58348	-1.0376	0.181236	-5.72514	1.03E-08	4.58E-08	DOWN
SEMA3A	61.64183	1.817308	0.317431	5.725054	1.03E-08	4.58E-08	UP
LTK	86.49694	-1.41739	0.247595	-5.72463	1.04E-08	4.59E-08	DOWN
CDX1	4.502209	1.796409	0.313827	5.724196	1.04E-08	4.60E-08	UP
UAP1	4899.552	-0.7208	0.125923	-5.72418	1.04E-08	4.60E-08	DOWN
ABHD2	13445.43	-0.86864	0.151756	-5.72395	1.04E-08	4.61E-08	DOWN
AP001258	74.43376	0.706391	0.12341	5.723921	1.04E-08	4.61E-08	UP
CHODL	13.54462	2.389478	0.417538	5.722782	1.05E-08	4.64E-08	UP
HOXC-AS	3.755706	4.360015	0.761926	5.722363	1.05E-08	4.65E-08	UP
BCAT2	1614.649	0.767191	0.134074	5.72216	1.05E-08	4.65E-08	UP
ZNF77	96.89088	0.614517	0.107401	5.721728	1.05E-08	4.67E-08	UP
AC004985	8.019038	1.025436	0.179226	5.721465	1.06E-08	4.67E-08	UP
CUL4B	2417.805	0.433348	0.075742	5.721336	1.06E-08	4.67E-08	NOT
FHL3	512.2382	0.767043	0.134069	5.72127	1.06E-08	4.68E-08	UP
TMEM8B	471.0886	0.564213	0.09862	5.721066	1.06E-08	4.68E-08	NOT
AK8	26.11737	1.699265	0.297036	5.720745	1.06E-08	4.69E-08	UP
TCTEX1D2	54.83999	0.988681	0.172828	5.720596	1.06E-08	4.69E-08	UP
PPP2R5B	610.8612	0.45533	0.079627	5.718319	1.08E-08	4.75E-08	NOT
SCUBE1	845.2885	2.046377	0.35807	5.715013	1.10E-08	4.85E-08	UP
AZGP1P1	623.3069	-1.47946	0.258903	-5.71436	1.10E-08	4.87E-08	DOWN
MAST1	87.41494	1.425213	0.24941	5.714339	1.10E-08	4.87E-08	UP
RP11-649	4.515216	4.450921	0.779044	5.713312	1.11E-08	4.89E-08	UP
IGJ	1331.765	-1.97161	0.345092	-5.71327	1.11E-08	4.89E-08	DOWN
C9orf152	123.1054	1.928385	0.337538	5.713096	1.11E-08	4.90E-08	UP
ENPP1	3780.922	-0.86666	0.151718	-5.7123	1.11E-08	4.92E-08	DOWN
HSPA12A	144.5063	1.107939	0.193961	5.712172	1.12E-08	4.92E-08	UP
RP11-126	6.741268	1.522625	0.266572	5.711866	1.12E-08	4.93E-08	UP
DCTPP1	1007.505	0.630137	0.110365	5.709584	1.13E-08	5.00E-08	UP
COL6A3	4149.63	1.28369	0.224832	5.709543	1.13E-08	5.00E-08	UP
ADAM32	8.169662	1.864021	0.326483	5.709402	1.13E-08	5.00E-08	UP
CCR10	19.93511	1.122741	0.196659	5.709067	1.14E-08	5.01E-08	UP
RP11-848	4.259728	1.814387	0.317827	5.70872	1.14E-08	5.02E-08	UP
CTD-2184	14.13837	1.950705	0.341711	5.70864	1.14E-08	5.02E-08	UP
RP11-4O1	89.77707	0.880441	0.154232	5.708547	1.14E-08	5.02E-08	UP
KLF9	5758.794	-1.0682	0.187169	-5.7071	1.15E-08	5.07E-08	DOWN
bP-2171C	3.435087	2.041061	0.35766	5.706704	1.15E-08	5.08E-08	UP
NAP1L5	183.2076	-0.96781	0.169597	-5.7065	1.15E-08	5.08E-08	DOWN
CILP	78.29482	-1.5023	0.263263	-5.70647	1.15E-08	5.08E-08	DOWN
CPNE1	3306.077	0.678421	0.118895	5.70606	1.16E-08	5.09E-08	UP
PIK3R6	60.48412	0.98313	0.172309	5.705611	1.16E-08	5.11E-08	UP
RP11-274	8.560562	1.753765	0.307404	5.705078	1.16E-08	5.12E-08	UP
RP5-1136	157.9266	0.725869	0.127236	5.704894	1.16E-08	5.13E-08	UP
CTD-2587	43.22721	0.82866	0.145261	5.704614	1.17E-08	5.13E-08	UP
RCC2P7	3.108624	-1.71934	0.301395	-5.70459	1.17E-08	5.13E-08	DOWN
TACC2	1447.721	0.655819	0.114966	5.704462	1.17E-08	5.14E-08	UP
ITGA3	1028.158	1.511452	0.264969	5.704255	1.17E-08	5.14E-08	UP
NR0B2	3496.592	-1.32425	0.23216	-5.70405	1.17E-08	5.15E-08	DOWN
RP11-218	9.964939	1.082391	0.189775	5.703563	1.17E-08	5.16E-08	UP
ZC3H12C	385.7202	-0.63892	0.112028	-5.70318	1.18E-08	5.17E-08	DOWN

AC093838	423.696	0.62451	0.109509	5.702806	1.18E-08	5.18E-08	UP
CTD-2368	71.15557	0.979551	0.171778	5.702428	1.18E-08	5.19E-08	UP
ALOX12-A	99.47291	0.611585	0.10725	5.702422	1.18E-08	5.19E-08	UP
RP11-795I	16.49543	1.309965	0.229725	5.702326	1.18E-08	5.19E-08	UP
KDM3B	1968.473	0.438229	0.076854	5.702093	1.18E-08	5.20E-08	NOT
SOAT2	418.0075	2.126201	0.372886	5.702017	1.18E-08	5.20E-08	UP
HNRNPUL1	6659.976	0.390062	0.068408	5.701981	1.18E-08	5.20E-08	NOT
FAM104A	479.8677	0.396832	0.069605	5.70118	1.19E-08	5.23E-08	NOT
SPAG8	20.33911	0.828208	0.14527	5.701155	1.19E-08	5.23E-08	UP
ZNF208	13.93416	1.749684	0.306946	5.700299	1.20E-08	5.25E-08	UP
CEP350	1660.397	0.566158	0.099326	5.700005	1.20E-08	5.26E-08	NOT
ZNF34	229.4173	0.538149	0.09443	5.698906	1.21E-08	5.29E-08	NOT
HMGB2P1	3.475397	2.261954	0.39693	5.698617	1.21E-08	5.30E-08	UP
SATB1	636.8721	-1.0163	0.17836	-5.69803	1.21E-08	5.32E-08	DOWN
AC097724	18.05959	1.08006	0.189572	5.697361	1.22E-08	5.34E-08	UP
UBXN11	525.0464	0.681866	0.119683	5.697264	1.22E-08	5.34E-08	UP
OXNAD1	731.8059	-0.57847	0.101541	-5.69691	1.22E-08	5.35E-08	NOT
RP11-255I	19.70135	1.828771	0.321068	5.695907	1.23E-08	5.38E-08	UP
AF196972.	7.546884	1.262146	0.221593	5.695776	1.23E-08	5.38E-08	UP
TNN	75.62503	-1.15258	0.202411	-5.69426	1.24E-08	5.43E-08	DOWN
TAF1D	1508.089	0.638984	0.112216	5.694235	1.24E-08	5.43E-08	UP
VCX3A	4.201774	4.228058	0.742517	5.694222	1.24E-08	5.43E-08	UP
GPD1L	370.3588	0.915472	0.160773	5.694195	1.24E-08	5.43E-08	UP
ZSCAN26	316.7055	0.537037	0.094321	5.69374	1.24E-08	5.45E-08	NOT
TSPAN12	1028.374	-0.91147	0.16009	-5.69351	1.24E-08	5.45E-08	DOWN
PTGIR	166.2687	-1.07791	0.189352	-5.69262	1.25E-08	5.48E-08	DOWN
RP11-131I	6.196489	1.243839	0.21853	5.69185	1.26E-08	5.50E-08	UP
RP11-68L1	2.463316	1.997689	0.350991	5.691565	1.26E-08	5.51E-08	UP
G0S2	6010.792	-1.5806	0.277732	-5.69111	1.26E-08	5.53E-08	DOWN
CTD-2021	6.315681	3.011881	0.529246	5.690889	1.26E-08	5.53E-08	UP
C9orf72	458.1752	-0.84839	0.149086	-5.69065	1.27E-08	5.54E-08	DOWN
RP11-295I	6.359048	1.297108	0.227943	5.690488	1.27E-08	5.54E-08	UP
AC226118	2.817331	2.756733	0.48448	5.690089	1.27E-08	5.55E-08	UP
RP11-305I	78.59653	1.069597	0.187978	5.690011	1.27E-08	5.56E-08	UP
RP11-651I	10.06114	0.786758	0.138272	5.689935	1.27E-08	5.56E-08	UP
HDHD2	889.893	-0.52202	0.091745	-5.68989	1.27E-08	5.56E-08	NOT
PRCP	4100.236	-0.67141	0.118006	-5.68959	1.27E-08	5.57E-08	DOWN
CBWD5	173.3119	-0.63939	0.112379	-5.68956	1.27E-08	5.57E-08	DOWN
RP11-255I	4.086114	3.1868	0.560246	5.688212	1.28E-08	5.61E-08	UP
ARHGEF18	251.5781	0.734826	0.12919	5.687954	1.29E-08	5.62E-08	UP
B3GNT3	596.652	1.885983	0.331587	5.687755	1.29E-08	5.62E-08	UP
SAFB2	1933.466	0.364538	0.064093	5.687644	1.29E-08	5.63E-08	NOT
C11orf71	632.5	-0.71873	0.126379	-5.68711	1.29E-08	5.64E-08	DOWN
RAVER2	279.7289	1.204671	0.211829	5.686996	1.29E-08	5.65E-08	UP
IRF3	2340.472	0.601	0.10568	5.686962	1.29E-08	5.65E-08	UP
PRMT9	270.3587	-0.456	0.080194	-5.6862	1.30E-08	5.67E-08	NOT
RP11-1114	25.67937	0.809126	0.142302	5.685979	1.30E-08	5.68E-08	UP
ASH1L	1868.417	0.651657	0.114614	5.685666	1.30E-08	5.69E-08	UP
POSTN	1024.776	1.759317	0.309461	5.685096	1.31E-08	5.71E-08	UP
ITGAV	3264.419	0.942082	0.165728	5.684509	1.31E-08	5.72E-08	UP
GPD1	5704.625	-1.54625	0.272029	-5.68415	1.31E-08	5.74E-08	DOWN
AP000695.	6.416217	1.375576	0.242009	5.683981	1.32E-08	5.74E-08	UP

MAN2B2	3263.522	-0.54585	0.096036	-5.68383	1.32E-08	5.74E-08	NOT
PCAT18	5.163334	-1.84465	0.32458	-5.6832	1.32E-08	5.76E-08	DOWN
ATP1B3	1277.731	0.964419	0.1697	5.683067	1.32E-08	5.77E-08	UP
CD200R1	49.37572	-1.10921	0.195196	-5.68256	1.33E-08	5.78E-08	DOWN
PIK3R4	1349.301	-0.53578	0.094318	-5.6806	1.34E-08	5.85E-08	NOT
ZEB2	810.4081	-0.93746	0.16503	-5.68051	1.34E-08	5.85E-08	DOWN
RP11-108I	2.604197	2.010714	0.353991	5.68013	1.35E-08	5.86E-08	UP
SPON2	7337.276	0.932377	0.164159	5.679723	1.35E-08	5.88E-08	UP
RP11-111I	1.36907	2.279729	0.401422	5.679135	1.35E-08	5.90E-08	UP
FAM86EP	115.7478	0.62432	0.109935	5.678988	1.35E-08	5.90E-08	UP
GPR150	14.15289	1.6376	0.2884	5.678229	1.36E-08	5.93E-08	UP
RP11-573I	2.195978	2.005449	0.353229	5.677479	1.37E-08	5.95E-08	UP
PXMP4	1186.694	0.637512	0.112292	5.677292	1.37E-08	5.96E-08	UP
TMEM256	1794.088	-0.84091	0.148122	-5.67717	1.37E-08	5.96E-08	DOWN
ALPL	3422.769	-1.52554	0.268758	-5.67625	1.38E-08	5.99E-08	DOWN
CTD-2207	10.46386	4.585027	0.807834	5.675707	1.38E-08	6.01E-08	UP
RP11-412I	64.53226	0.74769	0.131743	5.67536	1.38E-08	6.02E-08	UP
SYTL5	128.4343	-1.49827	0.264018	-5.67487	1.39E-08	6.04E-08	DOWN
LA16c-316	3.116263	1.669633	0.294235	5.674489	1.39E-08	6.05E-08	UP
KIAA1217	1806.81	-0.58035	0.102283	-5.67402	1.39E-08	6.06E-08	NOT
KPTN	454.254	0.652193	0.114993	5.671608	1.41E-08	6.15E-08	UP
RP11-104(	2.912458	1.922739	0.339054	5.67089	1.42E-08	6.17E-08	UP
SOHLH2	11.77578	4.491092	0.792067	5.670088	1.43E-08	6.20E-08	UP
ABCA8	1673.39	-1.52136	0.268317	-5.67003	1.43E-08	6.20E-08	DOWN
RP11-443I	8.098619	2.058954	0.36314	5.66986	1.43E-08	6.21E-08	UP
LINC00535	42.23199	1.632135	0.287944	5.668246	1.44E-08	6.27E-08	UP
TRIM35	1017.796	-0.84989	0.149967	-5.66722	1.45E-08	6.30E-08	DOWN
RP11-66N	51.32937	1.214635	0.214351	5.666559	1.46E-08	6.33E-08	UP
RP11-298(	1.405576	2.151755	0.37991	5.663857	1.48E-08	6.43E-08	UP
RAB3A	151.087	0.911219	0.160884	5.663816	1.48E-08	6.43E-08	UP
RP1-90J4.1	6.301959	-1.92937	0.340665	-5.66354	1.48E-08	6.44E-08	DOWN
RP11-98D	18.79801	1.097523	0.193818	5.662652	1.49E-08	6.47E-08	UP
ERMP1	1311.477	0.716045	0.126454	5.662487	1.49E-08	6.47E-08	UP
WDR70	798.8433	0.398363	0.07036	5.661747	1.50E-08	6.50E-08	NOT
SMOC1	4734.771	-0.95697	0.169026	-5.6617	1.50E-08	6.50E-08	DOWN
ARHGEF26	31.26287	-1.17429	0.207423	-5.66131	1.50E-08	6.52E-08	DOWN
MIS18BP1	323.5635	0.700055	0.123662	5.661033	1.50E-08	6.52E-08	UP
CRELD2	2184.424	0.656877	0.116039	5.66081	1.51E-08	6.53E-08	UP
HCLS1	1317.009	-0.88698	0.156702	-5.66029	1.51E-08	6.55E-08	DOWN
BRAT1	2440.925	0.509128	0.089952	5.659987	1.51E-08	6.56E-08	NOT
BLK	31.81966	-1.62857	0.287764	-5.6594	1.52E-08	6.58E-08	DOWN
RP11-46H	6.221482	1.551176	0.274123	5.658691	1.53E-08	6.61E-08	UP
CTD-2203	16.80307	0.949568	0.167825	5.658096	1.53E-08	6.63E-08	UP
RP2	583.2766	-0.62692	0.110806	-5.65782	1.53E-08	6.64E-08	DOWN
SMARCE1	2015.214	0.375643	0.066401	5.657183	1.54E-08	6.66E-08	NOT
FAM86GP	5.647626	1.516268	0.268032	5.657042	1.54E-08	6.67E-08	UP
LINC00202	15.44396	1.408457	0.249006	5.656315	1.55E-08	6.70E-08	UP
RP13-39P1	2.997347	1.899839	0.33596	5.654956	1.56E-08	6.75E-08	UP
CMBL	9590.545	-1.12636	0.19921	-5.65411	1.57E-08	6.78E-08	DOWN
FGR	273.1662	-0.8786	0.15541	-5.65343	1.57E-08	6.81E-08	DOWN
RP11-552(	2.577631	3.765021	0.665978	5.653369	1.57E-08	6.81E-08	UP
LINC00294	274.2689	0.532161	0.094133	5.653266	1.57E-08	6.81E-08	NOT

CBS	429.7794	-1.16209	0.205566	-5.65312	1.58E-08	6.82E-08	DOWN
EIF2AK4	2267.13	-0.49432	0.087468	-5.6514	1.59E-08	6.88E-08	NOT
AFAP1L1	438.3985	0.652888	0.115527	5.65139	1.59E-08	6.88E-08	UP
HOMER2P	6.484708	4.854289	0.859116	5.65033	1.60E-08	6.92E-08	UP
FOXC2	19.77925	1.310179	0.23191	5.649519	1.61E-08	6.96E-08	UP
TRPM5	3.252052	2.263133	0.400604	5.649297	1.61E-08	6.96E-08	UP
UBE2Z	3261.894	0.427687	0.075715	5.648642	1.62E-08	6.99E-08	NOT
RP11-196(	5.171237	-1.32081	0.233871	-5.64762	1.63E-08	7.03E-08	DOWN
POU2F1	733.7967	0.468435	0.082945	5.647549	1.63E-08	7.03E-08	NOT
RPL17	1028.573	0.613357	0.108612	5.647225	1.63E-08	7.04E-08	UP
PHLDA3	866.4426	1.359346	0.240723	5.646932	1.63E-08	7.05E-08	UP
RAPSN	15.84534	1.291738	0.228758	5.646751	1.64E-08	7.06E-08	UP
FAXC	16.71685	2.351471	0.416433	5.646699	1.64E-08	7.06E-08	UP
UBE2M	2460.903	0.63409	0.112298	5.646477	1.64E-08	7.07E-08	UP
RP11-345.	123.609	0.740737	0.131199	5.645902	1.64E-08	7.09E-08	UP
TSPAN10	103.4845	0.988601	0.175109	5.645619	1.65E-08	7.10E-08	UP
RP11-307I	2.391433	2.358007	0.417676	5.64554	1.65E-08	7.11E-08	UP
KRTAP5-5	3.258308	4.02939	0.71375	5.645382	1.65E-08	7.11E-08	UP
GORAB	451.9502	0.777867	0.137789	5.64534	1.65E-08	7.11E-08	UP
KIR2DS4	4.092274	-1.4951	0.264866	-5.64474	1.65E-08	7.13E-08	DOWN
FIBP	1649.165	0.471172	0.083471	5.644717	1.65E-08	7.13E-08	NOT
RP11-568I	4.124195	2.116195	0.374906	5.644604	1.66E-08	7.14E-08	UP
PTPRR	11.43398	1.608997	0.285053	5.644558	1.66E-08	7.14E-08	UP
RP11-358I	6.094312	1.398942	0.247843	5.644462	1.66E-08	7.14E-08	UP
RALA	1482.658	0.365397	0.064741	5.643949	1.66E-08	7.16E-08	NOT
HAND2	155.1357	-1.80084	0.319103	-5.64345	1.67E-08	7.18E-08	DOWN
SPEF1	8.562823	1.523716	0.270036	5.642637	1.67E-08	7.21E-08	UP
AP001437.	4.671149	1.484989	0.263176	5.642573	1.68E-08	7.22E-08	UP
MID1IP1-/	15.98152	1.108553	0.196485	5.641931	1.68E-08	7.24E-08	UP
NAALADL2	194.7922	-1.22265	0.216727	-5.64146	1.69E-08	7.26E-08	DOWN
IL1RAPL2	28.75002	-1.91545	0.339589	-5.64049	1.70E-08	7.30E-08	DOWN
SNORA16	3.669944	-1.42823	0.25321	-5.64048	1.70E-08	7.30E-08	DOWN
RP1-267L1	6.207328	1.094592	0.194073	5.640108	1.70E-08	7.31E-08	UP
GABARAP	2154.078	-0.47474	0.08418	-5.63958	1.70E-08	7.34E-08	NOT
KCND3	1166.82	-1.63232	0.289459	-5.6392	1.71E-08	7.35E-08	DOWN
RP11-583I	3.129813	1.856403	0.329229	5.63864	1.71E-08	7.37E-08	UP
RP11-434I	1.909319	3.018963	0.535409	5.638612	1.71E-08	7.37E-08	UP
ROS1	15.36946	2.27526	0.403516	5.638589	1.71E-08	7.37E-08	UP
NR5A1	3.382218	2.726514	0.483552	5.638511	1.72E-08	7.38E-08	UP
CA9	388.7148	2.473663	0.438793	5.637426	1.73E-08	7.42E-08	UP
HMGCS2	73432.14	-1.27272	0.22582	-5.63601	1.74E-08	7.48E-08	DOWN
RAB41	16.56353	0.949681	0.168529	5.635129	1.75E-08	7.52E-08	UP
LAMP5	48.37469	2.168692	0.384866	5.63493	1.75E-08	7.53E-08	UP
F2	75361.59	-1.18705	0.210667	-5.63469	1.75E-08	7.54E-08	DOWN
MRPS12	1983.588	0.788436	0.139928	5.634579	1.75E-08	7.54E-08	UP
C6orf183	31.92913	1.896553	0.336619	5.63413	1.76E-08	7.56E-08	UP
MIF4GD	811.2401	0.540302	0.095899	5.634065	1.76E-08	7.56E-08	NOT
DHX9	4351.825	0.393237	0.069797	5.633997	1.76E-08	7.56E-08	NOT
UCKL1	1425.707	0.481369	0.085461	5.632588	1.78E-08	7.62E-08	NOT
TMSB15A	5.454092	1.65792	0.294345	5.632568	1.78E-08	7.62E-08	UP
NAPEPLD	688.0184	-0.5267	0.09351	-5.6325	1.78E-08	7.62E-08	NOT
ZIM2-AS1	7.26946	2.336457	0.414824	5.632406	1.78E-08	7.63E-08	UP

RP11-15A:	7.930783	2.062542	0.366196	5.632341	1.78E-08	7.63E-08	UP
KIAA0513	322.1775	0.706968	0.125522	5.632201	1.78E-08	7.63E-08	UP
RP11-707(	7.631701	1.411826	0.250678	5.632029	1.78E-08	7.64E-08	UP
RP11-297I	16.08051	0.798558	0.141792	5.631897	1.78E-08	7.64E-08	UP
STX4	1752.442	0.438046	0.077783	5.631644	1.79E-08	7.65E-08	NOT
FAM107B	5397.061	-0.5997	0.106503	-5.6308	1.79E-08	7.69E-08	DOWN
RP11-613I	7.463352	-1.44469	0.256598	-5.63016	1.80E-08	7.72E-08	DOWN
SPG20	555.1485	-1.16563	0.207043	-5.62991	1.80E-08	7.73E-08	DOWN
GAB2	697.3191	0.654101	0.116193	5.629442	1.81E-08	7.75E-08	UP
CTA-445C	83.66285	0.919767	0.163385	5.629436	1.81E-08	7.75E-08	UP
EIF5A	14448.65	-0.5668	0.100696	-5.62882	1.81E-08	7.77E-08	NOT
FYTTD1	1746.15	-0.367	0.065213	-5.62772	1.83E-08	7.82E-08	NOT
LMTK3	102.8132	1.834445	0.325971	5.627633	1.83E-08	7.82E-08	UP
REEP5	7165.545	-0.47895	0.085122	-5.62662	1.84E-08	7.87E-08	NOT
CTB-129P(	160.4677	1.170172	0.20799	5.626107	1.84E-08	7.89E-08	UP
LURAP1L	1509.064	-1.07306	0.190731	-5.62606	1.84E-08	7.89E-08	DOWN
PREP	1166.809	-0.42898	0.076258	-5.62536	1.85E-08	7.92E-08	NOT
HIST1H2BI	1386.556	0.975213	0.173375	5.624888	1.86E-08	7.94E-08	UP
NAT8B	129.6589	1.704411	0.303024	5.624678	1.86E-08	7.95E-08	UP
PIP5KL1	13.03794	1.423798	0.253166	5.623967	1.87E-08	7.98E-08	UP
TVP23B	711.6157	-0.56104	0.099777	-5.62296	1.88E-08	8.03E-08	NOT
SLC2A6	430.7995	1.16548	0.207281	5.622713	1.88E-08	8.04E-08	UP
SIRT6	662.1409	0.592322	0.105346	5.622646	1.88E-08	8.04E-08	UP
EFCAB2	399.8878	0.643531	0.11449	5.620831	1.90E-08	8.13E-08	UP
CPT1C	81.96298	1.040437	0.185106	5.620774	1.90E-08	8.13E-08	UP
FBXW9	262.1009	0.577072	0.102681	5.620037	1.91E-08	8.16E-08	NOT
RP11-475(	33.11979	-1.14077	0.202986	-5.61997	1.91E-08	8.16E-08	DOWN
ITIH1	91491.65	-1.26068	0.224341	-5.61949	1.92E-08	8.18E-08	DOWN
DYDC1	2.687968	2.734349	0.48659	5.619415	1.92E-08	8.18E-08	UP
CNGB3	4.509757	2.412345	0.429288	5.61941	1.92E-08	8.18E-08	UP
PLXNB3	113.316	1.63796	0.291483	5.619408	1.92E-08	8.18E-08	UP
C17orf75	618.9407	0.39486	0.070268	5.619329	1.92E-08	8.19E-08	NOT
AF011889.	6.983858	1.606095	0.285823	5.619203	1.92E-08	8.19E-08	UP
RILPL2	558.0562	-0.58339	0.103828	-5.61878	1.92E-08	8.21E-08	NOT
BEND4	7.224843	-1.76513	0.314178	-5.61823	1.93E-08	8.23E-08	DOWN
MT1M	2814.003	-2.52013	0.448623	-5.61749	1.94E-08	8.27E-08	DOWN
SC5D	6433.816	-0.97546	0.173689	-5.61615	1.95E-08	8.33E-08	DOWN
KLC3	34.60274	2.096683	0.373438	5.614535	1.97E-08	8.41E-08	UP
RP5-827C:	77.39077	1.001236	0.178346	5.614001	1.98E-08	8.43E-08	UP
MROH3P	3.874669	2.754727	0.49071	5.613756	1.98E-08	8.44E-08	UP
IFITM1	2248.712	-1.25751	0.224008	-5.6137	1.98E-08	8.45E-08	DOWN
RP11-890I	144.5078	0.747735	0.133208	5.613301	1.99E-08	8.46E-08	UP
RP11-159I	268.5571	0.781809	0.139297	5.612518	1.99E-08	8.50E-08	UP
RP11-672I	24.68173	1.071446	0.190911	5.612279	2.00E-08	8.51E-08	UP
RP4-806M	2.519157	2.85283	0.508355	5.611886	2.00E-08	8.53E-08	UP
PDPR	811.4043	0.647236	0.115348	5.611173	2.01E-08	8.56E-08	UP
BMS1P4	11.7705	0.851353	0.15173	5.610954	2.01E-08	8.57E-08	UP
RP11-475J	4.05552	-1.5883	0.283088	-5.61061	2.02E-08	8.59E-08	DOWN
GTSE1-AS	6.793861	1.135483	0.202399	5.610132	2.02E-08	8.61E-08	UP
FAM69B	538.1663	0.982118	0.175079	5.609564	2.03E-08	8.64E-08	UP
RP11-364I	4.774997	2.488074	0.443553	5.609421	2.03E-08	8.64E-08	UP
FAM193A	934.5995	0.386666	0.068935	5.609101	2.03E-08	8.66E-08	NOT

MAP7D1	1676.411	0.624541	0.111352	5.608705	2.04E-08	8.68E-08	UP
BHLHE40-	31.65637	1.407252	0.250931	5.608129	2.05E-08	8.70E-08	UP
PLEKHS1	15.71895	2.253658	0.401867	5.607965	2.05E-08	8.71E-08	UP
LINC0096(	595.4173	0.680021	0.121273	5.60737	2.05E-08	8.74E-08	UP
LINC0047(	9.558052	1.671982	0.298207	5.606788	2.06E-08	8.77E-08	UP
CCDC107	368.7108	0.64995	0.115938	5.606009	2.07E-08	8.81E-08	UP
LARP4	3760.331	-0.51073	0.09111	-5.60559	2.08E-08	8.83E-08	NOT
RP11-658I	142.9274	0.667286	0.119057	5.604783	2.09E-08	8.87E-08	UP
EBI3	127.1778	-1.05168	0.187657	-5.60424	2.09E-08	8.89E-08	DOWN
STRBP	799.7995	0.490278	0.087497	5.603381	2.10E-08	8.94E-08	NOT
HNRNPA1I	2.168691	2.335621	0.416859	5.602908	2.11E-08	8.96E-08	UP
RP11-485I	5.368861	4.756097	0.848865	5.602892	2.11E-08	8.96E-08	UP
A1BG	5611.081	-1.47351	0.263036	-5.60195	2.12E-08	9.01E-08	DOWN
OR13A1	2.583982	3.100084	0.553401	5.601879	2.12E-08	9.01E-08	UP
SEMA7A	376.1226	1.238042	0.221026	5.601332	2.13E-08	9.03E-08	UP
ATG101	979.7103	-0.44701	0.079828	-5.59969	2.15E-08	9.12E-08	NOT
AC004538	69.10617	-1.92265	0.343363	-5.59947	2.15E-08	9.13E-08	DOWN
CPD	7450.243	0.753344	0.134541	5.599372	2.15E-08	9.13E-08	UP
AC017002	2.956956	1.874124	0.334705	5.599327	2.15E-08	9.13E-08	UP
HSD17B6	27338.36	-1.42819	0.255086	-5.59886	2.16E-08	9.16E-08	DOWN
CTC-453G	1.66877	2.019925	0.360775	5.598845	2.16E-08	9.16E-08	UP
C15orf52	448.6993	1.187995	0.212206	5.598312	2.16E-08	9.18E-08	UP
ATP8B3	100.8314	1.026698	0.183447	5.596715	2.18E-08	9.27E-08	UP
VPS28	6626.808	0.768565	0.137327	5.596587	2.19E-08	9.27E-08	UP
RP11-611I	69.29689	0.752255	0.134426	5.596041	2.19E-08	9.30E-08	UP
LRFN1	111.8881	1.279575	0.228658	5.596026	2.19E-08	9.30E-08	UP
MMP9	529.7092	1.588017	0.28381	5.595342	2.20E-08	9.33E-08	UP
RP11-280I	8.702145	-1.47533	0.263715	-5.5944	2.21E-08	9.38E-08	DOWN
SLC30A1	6208.626	-0.9115	0.162957	-5.59347	2.23E-08	9.43E-08	DOWN
RP4-584D	20.58678	0.95124	0.170067	5.593332	2.23E-08	9.44E-08	UP
SLC35E2	29.67253	0.834564	0.149263	5.591217	2.25E-08	9.55E-08	UP
WARS2-IT	5.635201	-1.68161	0.300766	-5.5911	2.26E-08	9.56E-08	DOWN
ACTR3C	286.9414	-0.732	0.130931	-5.59076	2.26E-08	9.58E-08	DOWN
PCCB	5919.359	-0.75737	0.135485	-5.59009	2.27E-08	9.61E-08	DOWN
STAM	585.9121	0.420927	0.075299	5.590072	2.27E-08	9.61E-08	NOT
FSCN2	7.179328	1.655758	0.296202	5.589964	2.27E-08	9.62E-08	UP
RP11-757(	4.083518	3.089386	0.552699	5.589632	2.28E-08	9.63E-08	UP
ZNF117	286.6499	0.994213	0.177919	5.588016	2.30E-08	9.72E-08	UP
MRPS31	743.4746	-0.53047	0.094937	-5.58755	2.30E-08	9.75E-08	NOT
RP11-136I	6.989945	1.843895	0.330035	5.586976	2.31E-08	9.78E-08	UP
CHTF8	2951.069	-0.36926	0.066094	-5.58692	2.31E-08	9.78E-08	NOT
IL10	19.07859	-1.35742	0.242993	-5.58623	2.32E-08	9.82E-08	DOWN
MB	75.57804	1.738558	0.31123	5.586081	2.32E-08	9.82E-08	UP
AC007743	7.007284	1.470918	0.263324	5.58597	2.32E-08	9.83E-08	UP
AACSP1	5.306001	4.090816	0.732341	5.585944	2.32E-08	9.83E-08	UP
RP1-92O1	13.26806	1.147201	0.205376	5.58586	2.33E-08	9.83E-08	UP
PA2G4P4	7.117491	1.076914	0.19281	5.585357	2.33E-08	9.86E-08	UP
PPIH	522.1034	0.586132	0.10499	5.582758	2.37E-08	1.00E-07	UP
UBR3	2081.569	-0.51522	0.0923	-5.58203	2.38E-08	1.00E-07	NOT
PUS1	1104.571	0.617349	0.110604	5.581636	2.38E-08	1.01E-07	UP
L2HGDH	535.1454	-0.70894	0.127017	-5.58144	2.39E-08	1.01E-07	DOWN
ALDH5A1	6784.989	-0.89674	0.160665	-5.58142	2.39E-08	1.01E-07	DOWN

ABCA11P	31.03269	0.862184	0.154484	5.581036	2.39E-08	1.01E-07	UP
GNAT1	27.77433	1.410062	0.252706	5.579859	2.41E-08	1.02E-07	UP
RP5-1021I	1.893028	-1.89788	0.340162	-5.57935	2.41E-08	1.02E-07	DOWN
DAK	9477.959	-1.18319	0.212068	-5.57931	2.41E-08	1.02E-07	DOWN
LAMB1	3950.604	0.966849	0.173331	5.578037	2.43E-08	1.03E-07	UP
XXbac-BP(	3.820274	1.433245	0.256954	5.577826	2.44E-08	1.03E-07	UP
PIK3R3	551.5129	0.757636	0.135832	5.577731	2.44E-08	1.03E-07	UP
CTA-363E	8.580734	1.017589	0.18244	5.577656	2.44E-08	1.03E-07	UP
WDSUB1	287.535	0.535164	0.095966	5.576619	2.45E-08	1.03E-07	NOT
AC008746	3.895795	1.528591	0.274119	5.576367	2.46E-08	1.04E-07	UP
AIM1	834.3492	-1.03303	0.185292	-5.57517	2.47E-08	1.04E-07	DOWN
ZAN	2.897326	-1.75641	0.315064	-5.57477	2.48E-08	1.04E-07	DOWN
MTMR2	512.2388	0.662161	0.11879	5.574205	2.49E-08	1.05E-07	UP
AC011322	2.299635	2.477905	0.444642	5.572811	2.51E-08	1.06E-07	UP
FTSJ2	1089.045	0.352743	0.063302	5.572393	2.51E-08	1.06E-07	NOT
RBM6	1820.091	0.443037	0.079506	5.572373	2.51E-08	1.06E-07	NOT
UQCRHL	88.61263	0.731827	0.13134	5.572014	2.52E-08	1.06E-07	UP
GGCX	9272.557	-0.61353	0.110116	-5.57164	2.52E-08	1.06E-07	DOWN
RP11-661/	7.288695	1.683367	0.302133	5.571615	2.52E-08	1.06E-07	UP
ATOH8	1150.354	-1.52563	0.273832	-5.57141	2.53E-08	1.06E-07	DOWN
RP11-326(	7.873604	1.272243	0.228405	5.570131	2.55E-08	1.07E-07	UP
COPS5	2621.399	0.482223	0.086578	5.569823	2.55E-08	1.07E-07	NOT
CSF1R	1613.903	-1.00288	0.180066	-5.56951	2.55E-08	1.08E-07	DOWN
RP11-670I	19.68772	1.33054	0.238905	5.569327	2.56E-08	1.08E-07	UP
RP11-813I	195.6009	-1.63146	0.292945	-5.56917	2.56E-08	1.08E-07	DOWN
KCNE3	298.536	0.928253	0.166681	5.569042	2.56E-08	1.08E-07	UP
C1QC	5326.263	-1.12026	0.201159	-5.56903	2.56E-08	1.08E-07	DOWN
SERTAD4	36.56482	1.795478	0.322409	5.568953	2.56E-08	1.08E-07	UP
FBXO28	1395.752	-0.4288	0.077008	-5.56825	2.57E-08	1.08E-07	NOT
NOVA1	98.77957	1.541623	0.276862	5.568209	2.57E-08	1.08E-07	UP
PRDM16	89.77856	1.565666	0.281196	5.56789	2.58E-08	1.08E-07	UP
DDR1	1835.94	1.395989	0.250725	5.567802	2.58E-08	1.08E-07	UP
BRCA1	357.0858	0.863755	0.155137	5.567708	2.58E-08	1.08E-07	UP
PC	13372.95	-1.02771	0.184586	-5.56765	2.58E-08	1.08E-07	DOWN
CCDC155	8.822898	2.445623	0.439285	5.567281	2.59E-08	1.09E-07	UP
RP3-412A!	12.84433	1.127116	0.202461	5.567078	2.59E-08	1.09E-07	UP
RP11-420I	4.175795	3.794825	0.681665	5.566992	2.59E-08	1.09E-07	UP
AC068134	6.019854	1.384783	0.248765	5.566625	2.60E-08	1.09E-07	UP
HECA	951.1301	-0.60443	0.108583	-5.56649	2.60E-08	1.09E-07	DOWN
PIK3AP1	5302.442	-0.80417	0.144482	-5.56586	2.61E-08	1.10E-07	DOWN
RP11-392(	1.527925	2.465835	0.44304	5.565712	2.61E-08	1.10E-07	UP
PLEKHA4	1073.052	-0.98013	0.176105	-5.56559	2.61E-08	1.10E-07	DOWN
RP11-818I	1.497267	2.91624	0.524037	5.564954	2.62E-08	1.10E-07	UP
YDJC	606.2621	0.636583	0.114394	5.564848	2.62E-08	1.10E-07	UP
PTPRVP	7.870263	1.229137	0.220914	5.563879	2.64E-08	1.11E-07	UP
CTB-178M	4.273668	1.812889	0.325836	5.563812	2.64E-08	1.11E-07	UP
C2orf40	19.9979	-1.45872	0.262197	-5.56345	2.64E-08	1.11E-07	DOWN
NUP62	1901.908	0.500129	0.089897	5.563372	2.65E-08	1.11E-07	NOT
MTR	1569.776	0.771932	0.138768	5.562736	2.66E-08	1.11E-07	UP
TREX2	14.73714	1.098308	0.197492	5.561272	2.68E-08	1.12E-07	UP
ZNF610	35.13183	1.43247	0.257591	5.561024	2.68E-08	1.12E-07	UP
FHL2	730.0445	-1.16047	0.20868	-5.56101	2.68E-08	1.12E-07	DOWN

PPP1R14B	2113.133	0.768425	0.138214	5.559686	2.70E-08	1.13E-07	UP
ZNF468	347.4606	1.170105	0.210471	5.55946	2.71E-08	1.13E-07	UP
CBR3-AS1	37.70322	0.853282	0.153495	5.55904	2.71E-08	1.14E-07	UP
IGFBP1	43871.46	-1.53013	0.275275	-5.55855	2.72E-08	1.14E-07	DOWN
NBEAL2	899.7387	0.734576	0.132155	5.558433	2.72E-08	1.14E-07	UP
CEP112	190.8187	0.61901	0.11137	5.558154	2.73E-08	1.14E-07	UP
SLC25A16	1412.401	-0.58665	0.105562	-5.55736	2.74E-08	1.15E-07	DOWN
SLC5A2	18.8352	1.397295	0.251443	5.55711	2.74E-08	1.15E-07	UP
RXRB	1895.67	0.470386	0.084654	5.556551	2.75E-08	1.15E-07	NOT
GSTM5	77.05764	-1.72728	0.310872	-5.55623	2.76E-08	1.15E-07	DOWN
MIR3131	1.801411	2.681704	0.482702	5.555614	2.77E-08	1.16E-07	UP
HSPA7	132.6431	1.516833	0.273038	5.555383	2.77E-08	1.16E-07	UP
AC072062	5.026787	1.822023	0.328016	5.554668	2.78E-08	1.16E-07	UP
RP11-464I	9.014436	1.308166	0.235509	5.554642	2.78E-08	1.16E-07	UP
C15orf40	617.944	0.420256	0.07566	5.554526	2.78E-08	1.16E-07	NOT
APIP	832.2797	0.47827	0.086109	5.554228	2.79E-08	1.17E-07	NOT
RP11-153I	10.54624	1.117398	0.201186	5.554063	2.79E-08	1.17E-07	UP
RP11-727I	11.53095	0.987473	0.1778	5.553845	2.79E-08	1.17E-07	UP
PDE7A	451.2424	0.789151	0.142099	5.553531	2.80E-08	1.17E-07	UP
AL035610.	8.260363	3.862332	0.695504	5.553284	2.80E-08	1.17E-07	UP
TMEM155	4.840784	2.461392	0.443243	5.553138	2.81E-08	1.17E-07	UP
FAM126B	599.4159	-0.51196	0.092195	-5.553	2.81E-08	1.17E-07	NOT
DCAF7	2830.983	0.405407	0.073011	5.552698	2.81E-08	1.17E-07	NOT
IGLV4-60	18.09026	-2.46635	0.444182	-5.55256	2.82E-08	1.18E-07	DOWN
IKBIP	601.1161	0.639229	0.11513	5.552257	2.82E-08	1.18E-07	UP
THRAP3	4207.083	-0.29459	0.053059	-5.55223	2.82E-08	1.18E-07	NOT
DNMBP	1039.063	-0.57502	0.103593	-5.55077	2.84E-08	1.19E-07	NOT
SNORD7	2.130554	1.923615	0.346581	5.550261	2.85E-08	1.19E-07	UP
RP11-834(	1.72264	2.703313	0.487093	5.549886	2.86E-08	1.19E-07	UP
PDXDC1	6801.151	-0.5301	0.095517	-5.54982	2.86E-08	1.19E-07	NOT
RBM39	5567.88	0.358782	0.064651	5.549488	2.87E-08	1.19E-07	NOT
FAM13C	88.19831	1.09113	0.196629	5.549174	2.87E-08	1.20E-07	UP
RP11-603J	1.505726	2.09313	0.377217	5.548872	2.88E-08	1.20E-07	UP
ADORA1	143.7611	1.800866	0.324581	5.548273	2.89E-08	1.20E-07	UP
ERBB3	9099.59	0.766847	0.138232	5.547554	2.90E-08	1.21E-07	UP
SULT1A2	897.8611	-1.28829	0.232228	-5.5475	2.90E-08	1.21E-07	DOWN
TMEM234	261.5016	0.559247	0.100817	5.547153	2.90E-08	1.21E-07	NOT
LPHN2	2218.867	-0.82615	0.148954	-5.54636	2.92E-08	1.22E-07	DOWN
CDC42SE1	2180.059	0.525903	0.094821	5.546297	2.92E-08	1.22E-07	NOT
RNF103-C	3.25962	1.978378	0.356709	5.546196	2.92E-08	1.22E-07	UP
ADHFE1	1573.816	-1.04859	0.189093	-5.54538	2.93E-08	1.22E-07	DOWN
HM13	8802.831	0.532322	0.096026	5.543493	2.96E-08	1.23E-07	NOT
CLEC16A	1046.645	0.496945	0.089649	5.543228	2.97E-08	1.24E-07	NOT
RNF138	705.0795	-0.46609	0.084094	-5.54253	2.98E-08	1.24E-07	NOT
SNAPC2	488.3286	0.507315	0.091535	5.542324	2.98E-08	1.24E-07	NOT
LGI1	16.30415	-2.24149	0.404471	-5.54178	2.99E-08	1.25E-07	DOWN
PPP3CA	1046.225	-0.46646	0.084177	-5.54141	3.00E-08	1.25E-07	NOT
HIST2H3D	1.954778	2.191625	0.395555	5.540626	3.01E-08	1.25E-07	UP
RP5-836J3	3.781413	-1.74365	0.314729	-5.54017	3.02E-08	1.26E-07	DOWN
AGXT	71456.24	-1.41571	0.255561	-5.53962	3.03E-08	1.26E-07	DOWN
HIST1H4P	1.891356	2.736818	0.494122	5.538745	3.05E-08	1.27E-07	UP
ADAMTS9	589.9382	1.017699	0.18376	5.538187	3.06E-08	1.27E-07	UP

EIF4ENIF1	754.5487	0.426617	0.077032	5.53817	3.06E-08	1.27E-07	NOT
DUSP2	197.9624	-1.2147	0.219341	-5.53796	3.06E-08	1.27E-07	DOWN
SELL	233.8474	-0.98048	0.17706	-5.53755	3.07E-08	1.27E-07	DOWN
ACOX3	1122.505	-0.56591	0.102201	-5.53723	3.07E-08	1.28E-07	NOT
FCGBP	537.1918	1.546574	0.279328	5.536757	3.08E-08	1.28E-07	UP
CTC-453G	2.081857	1.7502	0.316136	5.536225	3.09E-08	1.28E-07	UP
WNT2B	52.40534	0.949517	0.171516	5.53603	3.09E-08	1.28E-07	UP
BCL3	3677.124	-0.85093	0.153716	-5.53569	3.10E-08	1.29E-07	DOWN
CAMKV	2.930786	3.392187	0.61284	5.535196	3.11E-08	1.29E-07	UP
CDH17	17.52442	2.305139	0.416516	5.534338	3.12E-08	1.30E-07	UP
ZNF541	45.63383	1.559889	0.281877	5.533938	3.13E-08	1.30E-07	UP
GS1-124K	9.230537	0.970328	0.175344	5.533853	3.13E-08	1.30E-07	UP
RP11-807I	2.513468	-1.23076	0.222409	-5.53379	3.13E-08	1.30E-07	DOWN
ECHDC1	2554.485	-0.66106	0.119479	-5.53282	3.15E-08	1.31E-07	DOWN
VANGL2	163.0329	1.844188	0.333318	5.532815	3.15E-08	1.31E-07	UP
C19orf44	259.2061	0.527782	0.095393	5.532723	3.15E-08	1.31E-07	NOT
ZNF485	75.33649	0.610712	0.110385	5.532542	3.16E-08	1.31E-07	UP
RP5-1142/	29.38945	1.044946	0.188929	5.530892	3.19E-08	1.32E-07	UP
CTB-147N	5.503528	1.631392	0.294977	5.53058	3.19E-08	1.32E-07	UP
RP11-504(	2.047052	1.805226	0.326516	5.528745	3.23E-08	1.34E-07	UP
INVS	493.0437	-0.48191	0.087171	-5.52827	3.23E-08	1.34E-07	NOT
C2orf15	34.18748	1.090758	0.197323	5.527768	3.24E-08	1.34E-07	UP
ITFG2	504.5581	0.435952	0.078866	5.527753	3.24E-08	1.34E-07	NOT
MT1JP	17.46222	-2.76186	0.499642	-5.52768	3.24E-08	1.34E-07	DOWN
KIAA0319	15.44243	1.403338	0.253904	5.527044	3.26E-08	1.35E-07	UP
LA16c-313	10.72018	1.060674	0.191926	5.526481	3.27E-08	1.35E-07	UP
SIRPB1	47.40237	-1.19141	0.215596	-5.52613	3.27E-08	1.36E-07	DOWN
NAP1L6	8.905441	3.041151	0.550353	5.525822	3.28E-08	1.36E-07	UP
CELF4	17.00403	1.80849	0.327295	5.525571	3.28E-08	1.36E-07	UP
AC016995	7.304455	1.584541	0.286768	5.525521	3.29E-08	1.36E-07	UP
TRMT10B	210.8994	0.420175	0.076047	5.525224	3.29E-08	1.36E-07	NOT
CTD-2265	1.912846	2.980833	0.539514	5.525033	3.29E-08	1.36E-07	UP
ZNF704	1044.616	0.817129	0.147897	5.524979	3.30E-08	1.36E-07	UP
ASS1P10	3.567089	-1.3616	0.246457	-5.52468	3.30E-08	1.37E-07	DOWN
PCDHGA4	53.93244	1.574839	0.285082	5.524171	3.31E-08	1.37E-07	UP
RP11-511I	4.131123	3.588521	0.649697	5.523374	3.33E-08	1.38E-07	UP
WSB2	2818.674	0.474354	0.085883	5.523273	3.33E-08	1.38E-07	NOT
RP11-439I	11.24285	0.978552	0.177172	5.523174	3.33E-08	1.38E-07	UP
ASCL5	2.715359	2.250061	0.40739	5.523107	3.33E-08	1.38E-07	UP
ACTL10	32.02973	1.313269	0.237787	5.522875	3.33E-08	1.38E-07	UP
RP11-6B4.	105.8242	-2.24433	0.406383	-5.52269	3.34E-08	1.38E-07	DOWN
RP11-519(	6.347408	3.03177	0.549023	5.522118	3.35E-08	1.38E-07	UP
SRGAP2D	6.455853	1.530415	0.277173	5.521521	3.36E-08	1.39E-07	UP
ATIC	2984.323	0.556147	0.100724	5.52149	3.36E-08	1.39E-07	NOT
RP11-122(	4.600067	1.201713	0.21765	5.521307	3.36E-08	1.39E-07	UP
CCNT2-AS	17.24908	0.808439	0.146432	5.520918	3.37E-08	1.39E-07	UP
TYSND1	1163.408	0.63851	0.115654	5.520872	3.37E-08	1.39E-07	UP
RP11-106	217.6554	-1.43914	0.260682	-5.52067	3.38E-08	1.39E-07	DOWN
PQLC2L	6.846696	2.877603	0.52126	5.520472	3.38E-08	1.40E-07	UP
CEP162	185.8821	0.551015	0.099825	5.519783	3.39E-08	1.40E-07	NOT
RP11-150(	4.0991	2.158268	0.391033	5.519404	3.40E-08	1.40E-07	UP
SPPL2B	1553.907	0.547038	0.099124	5.518733	3.41E-08	1.41E-07	NOT

RYR2	55.90652	1.891568	0.342754	5.51873	3.41E-08	1.41E-07	UP
COMMD4	3076.653	0.580314	0.105164	5.518185	3.43E-08	1.41E-07	NOT
PES1P1	3.991516	4.450491	0.80655	5.517937	3.43E-08	1.41E-07	UP
YIF1B	2151.428	0.664286	0.120389	5.517828	3.43E-08	1.41E-07	UP
TSC2	2614.568	0.486968	0.088256	5.517704	3.43E-08	1.42E-07	NOT
RP11-636(	3.041164	3.879014	0.703016	5.517676	3.44E-08	1.42E-07	UP
HTR1B	2.216651	2.461502	0.446156	5.51713	3.45E-08	1.42E-07	UP
DGKK	9.19386	3.246858	0.588625	5.516	3.47E-08	1.43E-07	UP
CTD-2270	185.2283	0.777524	0.140976	5.515281	3.48E-08	1.43E-07	UP
EXOSC6	1060.182	-0.46826	0.084907	-5.51505	3.49E-08	1.44E-07	NOT
DNAH11	114.0813	1.668824	0.302609	5.514797	3.49E-08	1.44E-07	UP
ZNF480	487.3342	0.575953	0.104458	5.513735	3.51E-08	1.45E-07	NOT
HTT	3155.995	0.446655	0.081025	5.512534	3.54E-08	1.46E-07	NOT
DYNLRB1	3610.763	0.61817	0.112152	5.511915	3.55E-08	1.46E-07	UP
RP11-323I	2.61582	1.766381	0.320488	5.511536	3.56E-08	1.46E-07	UP
CXorf40B	515.1798	0.437664	0.079418	5.510901	3.57E-08	1.47E-07	NOT
C9orf57	5.565066	2.777797	0.504073	5.51071	3.57E-08	1.47E-07	UP
INHBC	2960.18	-1.35913	0.246666	-5.51	3.59E-08	1.48E-07	DOWN
RP11-382I	3.483278	4.192176	0.760864	5.509758	3.59E-08	1.48E-07	UP
LINC0031(	9.409861	-1.07879	0.195808	-5.50944	3.60E-08	1.48E-07	DOWN
PUSL1	472.9842	0.758199	0.137626	5.509136	3.61E-08	1.48E-07	UP
PER2	845.3399	-0.66961	0.121547	-5.50906	3.61E-08	1.48E-07	DOWN
FKBP10	1281.487	1.335125	0.242354	5.508994	3.61E-08	1.48E-07	UP
RP11-116I	2625.318	-1.23456	0.22411	-5.50874	3.61E-08	1.49E-07	DOWN
RP11-640I	2.910317	3.125912	0.567481	5.508399	3.62E-08	1.49E-07	UP
CCDC86	825.6852	0.585328	0.10628	5.507425	3.64E-08	1.50E-07	UP
NFATC2IP	1015.426	0.430368	0.078147	5.507165	3.65E-08	1.50E-07	NOT
AC083899	42.5596	0.561655	0.10199	5.506947	3.65E-08	1.50E-07	NOT
SUPT5H	5233.836	0.427527	0.077653	5.505584	3.68E-08	1.51E-07	NOT
RP11-90P1	5.881105	1.784706	0.32417	5.505469	3.68E-08	1.51E-07	UP
BTN2A1	674.2029	0.475607	0.086397	5.504879	3.69E-08	1.52E-07	NOT
ZBTB37	662.6974	0.527835	0.09589	5.504561	3.70E-08	1.52E-07	NOT
LINC0018(	20.88458	1.87843	0.341259	5.504417	3.70E-08	1.52E-07	UP
MT1G	27574.17	-2.53963	0.461388	-5.50433	3.71E-08	1.52E-07	DOWN
PALB2	383.7125	0.478465	0.086956	5.502357	3.75E-08	1.54E-07	NOT
RNF2P1	8.244939	1.229258	0.223447	5.501331	3.77E-08	1.55E-07	UP
DDX20	442.037	0.403241	0.073304	5.500962	3.78E-08	1.55E-07	NOT
ISYNA1	1515.242	1.465918	0.266494	5.500756	3.78E-08	1.55E-07	UP
CTC-458A	4.952143	2.391413	0.434765	5.500475	3.79E-08	1.55E-07	UP
FXYD3	185.5236	2.07434	0.377141	5.500169	3.79E-08	1.56E-07	UP
KLHDC8B	903.5587	0.729235	0.132593	5.499779	3.80E-08	1.56E-07	UP
SHISA8	2.820774	2.775751	0.504722	5.499568	3.81E-08	1.56E-07	UP
EEF1E1	326.0842	0.684008	0.12438	5.499345	3.81E-08	1.56E-07	UP
ZNF728	6.090991	3.354085	0.609912	5.499289	3.81E-08	1.56E-07	UP
DEGS1	1898.459	0.579132	0.105314	5.499118	3.82E-08	1.56E-07	NOT
RP5-942I1	1.618222	2.353542	0.427995	5.498989	3.82E-08	1.56E-07	UP
RANP1	5.184631	1.165639	0.211976	5.498917	3.82E-08	1.57E-07	UP
CNOT10	976.5495	0.42427	0.077165	5.498242	3.84E-08	1.57E-07	NOT
TRMT61A	945.449	0.551793	0.100372	5.497501	3.85E-08	1.58E-07	NOT
NAF1	237.5059	-0.41077	0.07473	-5.49674	3.87E-08	1.58E-07	NOT
GSTCD	222.9193	0.635654	0.115647	5.496484	3.87E-08	1.59E-07	UP
GHRLOS	14.84074	1.035439	0.188383	5.496444	3.88E-08	1.59E-07	UP

C19orf71	38.83555	0.841792	0.153156	5.496285	3.88E-08	1.59E-07	UP
EXOC3	2045.573	0.415096	0.075524	5.496231	3.88E-08	1.59E-07	NOT
HOXA-AS2	80.74335	1.275071	0.232037	5.495118	3.90E-08	1.60E-07	UP
LINC01314	1019.667	-1.23406	0.22459	-5.4947	3.91E-08	1.60E-07	DOWN
RP11-110:	5.940626	4.606137	0.838378	5.494104	3.93E-08	1.61E-07	UP
RP1-152L7	627.5797	1.203242	0.219074	5.4924	3.97E-08	1.62E-07	UP
ALLC	13.34452	-1.78397	0.324851	-5.49166	3.98E-08	1.63E-07	DOWN
RP11-646I	4.733415	1.255293	0.228585	5.49157	3.98E-08	1.63E-07	UP
SRM	2488.231	0.71291	0.129835	5.490908	4.00E-08	1.63E-07	UP
ZNHIT3	931.1569	0.391792	0.071356	5.490673	4.00E-08	1.64E-07	NOT
RP11-151/	7.38387	1.462831	0.266431	5.490458	4.01E-08	1.64E-07	UP
ADAM21	8.297867	1.662007	0.302712	5.490391	4.01E-08	1.64E-07	UP
PARM1	313.0097	1.226785	0.223451	5.490186	4.02E-08	1.64E-07	UP
TRMT11	523.849	-0.62722	0.114258	-5.48948	4.03E-08	1.65E-07	DOWN
CDK7	598.2775	0.456595	0.083182	5.489129	4.04E-08	1.65E-07	NOT
ASAP2	234.6973	1.185052	0.215923	5.488315	4.06E-08	1.66E-07	UP
FCHSD2	991.6307	-0.47146	0.085902	-5.48827	4.06E-08	1.66E-07	NOT
AC006946	3.139574	3.086176	0.562371	5.487795	4.07E-08	1.66E-07	UP
ARCN1	5566.179	-0.35693	0.065048	-5.48717	4.08E-08	1.67E-07	NOT
ENO3	2585.061	-1.52898	0.278651	-5.48706	4.09E-08	1.67E-07	DOWN
AL132988.	44.61947	-1.26546	0.230635	-5.48684	4.09E-08	1.67E-07	DOWN
OPHN1	80.03686	0.964579	0.175806	5.486602	4.10E-08	1.67E-07	UP
GPATCH2	415.7511	0.490113	0.089329	5.48659	4.10E-08	1.67E-07	NOT
BBS1	37.23874	0.636482	0.116009	5.486466	4.10E-08	1.67E-07	UP
RTEL1	28.64532	0.847391	0.154454	5.486349	4.10E-08	1.67E-07	UP
PDE3B	1617.584	-0.79208	0.144394	-5.48553	4.12E-08	1.68E-07	DOWN
CTD-2527	1.480978	2.340973	0.426786	5.485122	4.13E-08	1.68E-07	UP
STX18-AS:	34.32869	0.744822	0.13579	5.485105	4.13E-08	1.68E-07	UP
ZFAS1	1686.128	0.882069	0.160817	5.484919	4.14E-08	1.69E-07	UP
RP11-488I	5.148939	1.764181	0.321666	5.48452	4.15E-08	1.69E-07	UP
NFATC3	782.416	-0.61606	0.112339	-5.48393	4.16E-08	1.69E-07	DOWN
RP11-320I	1.730923	2.397271	0.437145	5.48392	4.16E-08	1.69E-07	UP
RP11-269I	5.445323	1.152384	0.210199	5.482343	4.20E-08	1.71E-07	UP
RP11-91H	3.896584	3.17366	0.578919	5.48205	4.20E-08	1.71E-07	UP
RAB18	2736.952	-0.37874	0.069092	-5.48162	4.21E-08	1.72E-07	NOT
AFG3L1P	236.4264	0.500507	0.091308	5.48153	4.22E-08	1.72E-07	NOT
XXbac-B44	8.175488	1.101491	0.200977	5.480682	4.24E-08	1.72E-07	UP
SIGLEC15	127.4575	1.745453	0.318477	5.480616	4.24E-08	1.73E-07	UP
RUFY2	319.913	0.456904	0.083374	5.480156	4.25E-08	1.73E-07	NOT
TSGA10IP	4.200664	1.694075	0.30913	5.480144	4.25E-08	1.73E-07	UP
CFAP20	1097.717	-0.44972	0.082072	-5.47962	4.26E-08	1.73E-07	NOT
CFAP61	3.822591	2.098658	0.383111	5.477931	4.30E-08	1.75E-07	UP
TRNAU1AI	473.3463	0.522723	0.09543	5.477559	4.31E-08	1.75E-07	NOT
AQP7P4	3.248907	-1.9639	0.358547	-5.4774	4.32E-08	1.76E-07	DOWN
INPP1	919.9434	-0.69682	0.127226	-5.47704	4.32E-08	1.76E-07	DOWN
FAM166A	10.96168	1.606281	0.293312	5.476359	4.34E-08	1.77E-07	UP
C17orf58	632.1549	0.592493	0.108191	5.476342	4.34E-08	1.77E-07	UP
ZNF732	3.927722	1.883589	0.343957	5.476238	4.34E-08	1.77E-07	UP
RP11-43N	35.61556	0.813598	0.14857	5.476188	4.35E-08	1.77E-07	UP
CTD-2008	7.016797	4.324891	0.789896	5.475264	4.37E-08	1.78E-07	UP
ZNF713	54.02079	0.631836	0.115412	5.474629	4.38E-08	1.78E-07	UP
GABRG2	8.595034	5.259819	0.960872	5.474008	4.40E-08	1.79E-07	UP

RP5-874C:	2.58615	2.355917	0.430439	5.473295	4.42E-08	1.79E-07	UP
ACTBP8	8.999434	2.456116	0.448805	5.472564	4.44E-08	1.80E-07	UP
VN1R81P	4.756958	1.964656	0.359053	5.471769	4.46E-08	1.81E-07	UP
FHIT	498.9063	0.813	0.148582	5.471728	4.46E-08	1.81E-07	UP
RP11-101:	3.950146	2.52103	0.460766	5.471386	4.47E-08	1.81E-07	UP
GAS2L1	991.348	0.551489	0.100808	5.470702	4.48E-08	1.82E-07	NOT
RP11-266I	23.9935	0.719639	0.131567	5.46974	4.51E-08	1.83E-07	UP
PI4KAP1	99.78263	1.063531	0.194473	5.468781	4.53E-08	1.84E-07	UP
F11	3873.689	-1.17353	0.214603	-5.46835	4.54E-08	1.84E-07	DOWN
THEMIS2	579.1567	-0.99196	0.181421	-5.46773	4.56E-08	1.85E-07	DOWN
LRFN2	7.607216	2.463274	0.450569	5.467029	4.58E-08	1.86E-07	UP
DAZAP2	8881.632	-0.36456	0.066685	-5.46689	4.58E-08	1.86E-07	NOT
CLEC9A	17.16255	-1.37402	0.251338	-5.46682	4.58E-08	1.86E-07	DOWN
MPDZ	3087.961	-1.03327	0.189044	-5.46578	4.61E-08	1.87E-07	DOWN
DCTD	2535.04	-0.49127	0.089887	-5.46543	4.62E-08	1.87E-07	NOT
FAM132A	28.79247	1.092722	0.199946	5.465072	4.63E-08	1.88E-07	UP
DEPDC1-/	1.254858	2.599716	0.475741	5.464566	4.64E-08	1.88E-07	UP
SNORA40	3.80047	1.463824	0.267913	5.463793	4.66E-08	1.89E-07	UP
DCDC2	1460.35	1.851197	0.33882	5.463668	4.66E-08	1.89E-07	UP
ZNF836	115.8898	0.56743	0.10386	5.463416	4.67E-08	1.89E-07	NOT
RP11-57H	2.905898	1.6607	0.303969	5.463394	4.67E-08	1.89E-07	UP
TMEM194	624.1335	0.692483	0.126763	5.46283	4.69E-08	1.90E-07	UP
RP11-849I	11.67881	0.997826	0.182667	5.462553	4.69E-08	1.90E-07	UP
IFI44L	573.0318	-1.53112	0.280308	-5.46227	4.70E-08	1.90E-07	DOWN
RNF214	378.6051	0.386318	0.070725	5.462252	4.70E-08	1.90E-07	NOT
SLC25A37	875.4692	-0.63636	0.116504	-5.4621	4.71E-08	1.90E-07	DOWN
RP11-341(	23.36936	1.205679	0.220748	5.461791	4.71E-08	1.91E-07	UP
GATM-AS:	6.000886	1.447425	0.265016	5.461654	4.72E-08	1.91E-07	UP
RP11-426(	6.102325	1.466222	0.26849	5.461	4.73E-08	1.92E-07	UP
CSTB	5929.837	0.687669	0.125925	5.460956	4.74E-08	1.92E-07	UP
CTBP1-AS	33.339	0.925032	0.169402	5.46058	4.75E-08	1.92E-07	UP
ALS2CR11	8.570306	1.530758	0.280342	5.460334	4.75E-08	1.92E-07	UP
DNHD1	358.1811	0.796792	0.145943	5.459622	4.77E-08	1.93E-07	UP
RP11-318/	5.632033	1.142432	0.209275	5.458998	4.79E-08	1.94E-07	UP
ZNF182	227.3714	0.449654	0.082373	5.458726	4.80E-08	1.94E-07	NOT
NCSTN	5501.463	0.469649	0.086037	5.45867	4.80E-08	1.94E-07	NOT
LNP1	162.8382	-0.82981	0.152038	-5.45795	4.82E-08	1.95E-07	DOWN
NOL10	1168.519	0.351505	0.064403	5.457869	4.82E-08	1.95E-07	NOT
PPARGC1A	3182.581	-1.29847	0.23793	-5.45737	4.83E-08	1.95E-07	DOWN
SMAD6	375.6004	-0.86088	0.15776	-5.45687	4.85E-08	1.96E-07	DOWN
NADK	3498.955	-0.5468	0.100205	-5.45684	4.85E-08	1.96E-07	NOT
RP11-21L:	18.19414	1.545004	0.283135	5.456775	4.85E-08	1.96E-07	UP
ENTPD5	10985.05	-0.9591	0.175765	-5.4567	4.85E-08	1.96E-07	DOWN
PRRC2C	4976.511	0.557469	0.102168	5.456408	4.86E-08	1.96E-07	NOT
TLX1NB	1.690812	2.787013	0.510795	5.456228	4.86E-08	1.96E-07	UP
ARF4P2	2.729047	2.572141	0.471479	5.455474	4.88E-08	1.97E-07	UP
RP11-565/	3.632401	4.249923	0.779057	5.455211	4.89E-08	1.97E-07	UP
RP11-379I	21.15745	1.102813	0.202173	5.454787	4.90E-08	1.98E-07	UP
CD320	1202.446	0.794306	0.145622	5.454579	4.91E-08	1.98E-07	UP
SERPINA4	13864.26	-1.36082	0.249506	-5.45407	4.92E-08	1.99E-07	DOWN
TSPAN8	3151.1	1.600913	0.293535	5.453908	4.93E-08	1.99E-07	UP
PTGER2	58.03814	-1.23755	0.226952	-5.45292	4.95E-08	2.00E-07	DOWN

TBX6	47.32394	0.838699	0.153817	5.452586	4.96E-08	2.00E-07	UP
CHKB	138.0087	0.75113	0.137763	5.452336	4.97E-08	2.00E-07	UP
STEAP3-A	10.9189	-1.21241	0.222375	-5.45209	4.98E-08	2.01E-07	DOWN
CTC-523E	51.15012	0.806876	0.148011	5.451462	5.00E-08	2.01E-07	UP
GPR82	17.40765	-1.19798	0.219754	-5.45145	5.00E-08	2.01E-07	DOWN
ARRDC1-A	361.1994	0.548842	0.100686	5.45102	5.01E-08	2.02E-07	NOT
CASQ2	46.82347	1.289539	0.236572	5.450941	5.01E-08	2.02E-07	UP
NOL3	855.5235	0.803135	0.14734	5.450879	5.01E-08	2.02E-07	UP
LINC00982	45.52774	1.812537	0.332584	5.449864	5.04E-08	2.03E-07	UP
ADH1C	32301.61	-1.72165	0.315926	-5.44952	5.05E-08	2.03E-07	DOWN
ABCD4	1688.201	-0.44867	0.082333	-5.44947	5.05E-08	2.03E-07	NOT
SUPT3H	181.7847	0.750991	0.137831	5.44862	5.08E-08	2.04E-07	UP
COG5	675.178	0.496319	0.091093	5.448478	5.08E-08	2.04E-07	NOT
CD300C	41.9452	-1.00511	0.18448	-5.44831	5.08E-08	2.05E-07	DOWN
RP11-394I	2.980812	1.569513	0.288081	5.448161	5.09E-08	2.05E-07	UP
GLIDR	23.80574	-0.89309	0.163937	-5.44775	5.10E-08	2.05E-07	DOWN
AC012074	4.446008	1.546553	0.283892	5.447677	5.10E-08	2.05E-07	UP
LA16c-385	1.66531	1.924131	0.353215	5.447478	5.11E-08	2.05E-07	UP
TCTEX1D1	102.1364	-1.79581	0.329709	-5.44664	5.13E-08	2.06E-07	DOWN
RP11-889I	17.98022	1.050899	0.192948	5.446548	5.14E-08	2.06E-07	UP
SPRYD3	1754.974	0.39083	0.071764	5.446048	5.15E-08	2.07E-07	NOT
AJ006998.1	75.91428	2.090811	0.383934	5.445761	5.16E-08	2.07E-07	UP
ZBTB34	303.7362	0.562374	0.10327	5.445691	5.16E-08	2.07E-07	NOT
C2CD4B	61.68838	-1.45764	0.267681	-5.44542	5.17E-08	2.08E-07	DOWN
CEBPA-AS	401.6009	0.976796	0.17938	5.445415	5.17E-08	2.08E-07	UP
CCDC43	470.0926	0.442729	0.081313	5.444773	5.19E-08	2.08E-07	NOT
TMEM132	43.639	-1.40062	0.257266	-5.44424	5.20E-08	2.09E-07	DOWN
HSPB11	809.869	0.48817	0.089677	5.443623	5.22E-08	2.10E-07	NOT
CRYL1	7502.831	-0.938	0.172314	-5.44352	5.22E-08	2.10E-07	DOWN
UGT1A1	4424.675	-1.64383	0.301993	-5.44326	5.23E-08	2.10E-07	DOWN
CTD-3088	1.554924	2.229694	0.40963	5.443191	5.23E-08	2.10E-07	UP
PPIF	6744.488	-0.7071	0.129914	-5.44282	5.24E-08	2.10E-07	DOWN
CMB9-94E	4.685424	1.47878	0.271715	5.442387	5.26E-08	2.11E-07	UP
SGSH	1049.664	0.455959	0.083784	5.442072	5.27E-08	2.11E-07	NOT
EGLN3	415.8093	1.509662	0.277406	5.442065	5.27E-08	2.11E-07	UP
CAHM	15.95613	1.060329	0.194842	5.442004	5.27E-08	2.11E-07	UP
APOB	259223	-0.97104	0.17844	-5.44184	5.27E-08	2.11E-07	DOWN
ARHGAP28	56.43253	1.284512	0.236103	5.440466	5.31E-08	2.13E-07	UP
CCDC134	104.1903	0.601293	0.110525	5.440316	5.32E-08	2.13E-07	UP
RP11-817I	12.63073	1.156586	0.2126	5.4402	5.32E-08	2.13E-07	UP
VPS33B	503.5234	0.339906	0.062481	5.440127	5.32E-08	2.13E-07	NOT
NUBP1	657.7583	-0.38595	0.07095	-5.43977	5.33E-08	2.14E-07	NOT
GHET1	10.75729	1.040554	0.191287	5.439767	5.34E-08	2.14E-07	UP
RP4-595K1	3.385486	3.67161	0.675048	5.439033	5.36E-08	2.15E-07	UP
CDH12	20.45736	3.792597	0.697302	5.438956	5.36E-08	2.15E-07	UP
FRAS1	439.7744	1.948769	0.358307	5.438822	5.36E-08	2.15E-07	UP
ASGR2	29061.77	-1.0787	0.198344	-5.43851	5.37E-08	2.15E-07	DOWN
C12orf29	577.7428	-0.4291	0.078904	-5.43834	5.38E-08	2.15E-07	NOT
TM4SF1-A	9.86912	1.462773	0.268997	5.437889	5.39E-08	2.16E-07	UP
QSOX2	721.504	0.525379	0.096617	5.437772	5.40E-08	2.16E-07	NOT
TRAPPC8	1748.722	-0.52022	0.095676	-5.43729	5.41E-08	2.16E-07	NOT
RP3-395M	5.603928	1.661257	0.305542	5.437081	5.42E-08	2.17E-07	UP

ZNF710	490.677	0.710692	0.130713	5.437025	5.42E-08	2.17E-07	UP
ARMC2	48.47408	0.590503	0.108614	5.436718	5.43E-08	2.17E-07	UP
BTN2A2	463.2945	0.724455	0.133258	5.436498	5.43E-08	2.17E-07	UP
BCL2L2-PA	8.490515	1.020976	0.187802	5.43646	5.43E-08	2.17E-07	UP
EXOC7	1883.974	0.420104	0.077287	5.435613	5.46E-08	2.18E-07	NOT
PLA2G4F	9.728242	2.743081	0.504657	5.435539	5.46E-08	2.18E-07	UP
ZNF621	540.0235	0.479821	0.088276	5.435476	5.47E-08	2.18E-07	NOT
KLRAP1	31.36522	0.952976	0.175345	5.434875	5.48E-08	2.19E-07	UP
FCGR3A	1860.047	-1.13649	0.209133	-5.43427	5.50E-08	2.20E-07	DOWN
TRIM15	735.4452	-1.21847	0.224219	-5.43425	5.50E-08	2.20E-07	DOWN
MLLT11	235.9356	1.218942	0.224316	5.434033	5.51E-08	2.20E-07	UP
REXO4	1166.409	0.489615	0.090107	5.433733	5.52E-08	2.20E-07	NOT
AC005754	4.678036	2.101379	0.38676	5.433291	5.53E-08	2.21E-07	UP
NEURL3	237.813	1.485986	0.273517	5.432876	5.55E-08	2.21E-07	UP
SLC45A1	32.78338	0.835218	0.153767	5.431707	5.58E-08	2.23E-07	UP
LINC0085(	2.618901	2.98895	0.550289	5.431604	5.58E-08	2.23E-07	UP
C1orf194	1.52346	2.079085	0.382808	5.43114	5.60E-08	2.23E-07	UP
FAM53C	1020.943	0.391509	0.072089	5.43092	5.61E-08	2.24E-07	NOT
DGAT2L7P	6.375258	1.450452	0.267077	5.43084	5.61E-08	2.24E-07	UP
TAF9	1603.204	0.504637	0.092933	5.430126	5.63E-08	2.25E-07	NOT
APTR	343.1173	0.562012	0.103505	5.429821	5.64E-08	2.25E-07	NOT
ZNF560	4.170737	4.034286	0.743015	5.429619	5.65E-08	2.25E-07	UP
REPS2	833.3518	-0.83384	0.153589	-5.42904	5.67E-08	2.26E-07	DOWN
BOC	88.57814	1.283424	0.236421	5.428557	5.68E-08	2.27E-07	UP
PXYLP1	352.6535	0.817556	0.15062	5.427942	5.70E-08	2.27E-07	UP
CRMP1	263.6503	1.20755	0.222478	5.427732	5.71E-08	2.28E-07	UP
NANOGP1	5.859974	2.380341	0.438663	5.426355	5.75E-08	2.29E-07	UP
ALDOB	266923	-1.53954	0.283855	-5.42369	5.84E-08	2.33E-07	DOWN
RP11-156I	335.2939	0.404399	0.074572	5.422928	5.86E-08	2.34E-07	NOT
RFX3-AS1	14.38836	1.321125	0.24362	5.422896	5.86E-08	2.34E-07	UP
AC012613	2.854025	-1.77531	0.327394	-5.42254	5.88E-08	2.34E-07	DOWN
BACE2	1901.789	1.678201	0.309493	5.422428	5.88E-08	2.34E-07	UP
HSPB1P1	56.74402	1.117883	0.206176	5.421989	5.89E-08	2.35E-07	UP
ALOX15B	33.31226	1.679794	0.309831	5.421645	5.91E-08	2.35E-07	UP
ARHGEF38	50.02265	1.675443	0.30903	5.421612	5.91E-08	2.35E-07	UP
ANKRD61	9.243365	0.887441	0.163706	5.420962	5.93E-08	2.36E-07	UP
PLCB4	50.05889	1.187567	0.219074	5.420843	5.93E-08	2.36E-07	UP
RP11-806(	20.20596	1.437902	0.265268	5.420565	5.94E-08	2.36E-07	UP
NRSN2-AS	35.52681	0.837646	0.154533	5.420503	5.94E-08	2.36E-07	UP
DDA1	1338.901	0.462941	0.085416	5.41985	5.96E-08	2.37E-07	NOT
FITM2	785.5982	0.626732	0.115637	5.419835	5.97E-08	2.37E-07	UP
RP11-274I	16.29684	1.120413	0.206726	5.419806	5.97E-08	2.37E-07	UP
ANAPC1	407.905	0.455219	0.084	5.419305	5.98E-08	2.38E-07	NOT
USP9X	5960.983	-0.45936	0.084768	-5.41899	5.99E-08	2.38E-07	NOT
AMHR2	19.13222	-2.12741	0.392608	-5.41866	6.00E-08	2.39E-07	DOWN
RABEP1	1858.596	-0.48608	0.089709	-5.4184	6.01E-08	2.39E-07	NOT
NFKB2	2727.821	0.638078	0.117765	5.41825	6.02E-08	2.39E-07	UP
AC005562	38.90984	0.650046	0.119984	5.417779	6.03E-08	2.40E-07	UP
C1D	640.5776	-0.39288	0.072519	-5.41763	6.04E-08	2.40E-07	NOT
EXTL3	1270.735	0.739547	0.136529	5.416765	6.07E-08	2.41E-07	UP
TCFL5	410.5669	0.540374	0.09978	5.415661	6.11E-08	2.43E-07	NOT
RP11-705(	70.17054	0.837722	0.154694	5.415365	6.12E-08	2.43E-07	UP

SGPP2	151.5742	1.960046	0.361954	5.415182	6.12E-08	2.43E-07	UP
C4orf33	251.1269	-0.77832	0.143752	-5.41436	6.15E-08	2.44E-07	DOWN
SGIP1	63.89641	1.250236	0.230934	5.413831	6.17E-08	2.45E-07	UP
PAPSS2	4069.388	-0.85646	0.158205	-5.41358	6.18E-08	2.45E-07	DOWN
GPR56	1147.214	1.195141	0.220797	5.412852	6.20E-08	2.46E-07	UP
IL27	317.0048	-1.20206	0.222076	-5.41283	6.20E-08	2.46E-07	DOWN
RP11-114I	3.433859	2.693935	0.497788	5.411808	6.24E-08	2.48E-07	UP
ZNF563	326.1609	0.769909	0.142274	5.411462	6.25E-08	2.48E-07	UP
C19orf35	11.92982	-0.92308	0.17058	-5.41138	6.25E-08	2.48E-07	DOWN
DPPA4	12.7189	-1.78229	0.329402	-5.41067	6.28E-08	2.49E-07	DOWN
UBE2U	12.69052	3.990221	0.737507	5.410415	6.29E-08	2.49E-07	UP
ATP6V1H	1444.591	0.502481	0.092874	5.410352	6.29E-08	2.49E-07	NOT
NETO1	2.706324	2.673233	0.49413	5.409978	6.30E-08	2.50E-07	UP
MANEA	1156.553	-0.75994	0.140482	-5.40951	6.32E-08	2.50E-07	DOWN
FAM159A	30.20088	1.726178	0.319132	5.408978	6.34E-08	2.51E-07	UP
CYB5R1	3093.655	0.559665	0.103497	5.407557	6.39E-08	2.53E-07	NOT
ROR1	92.42011	1.495224	0.276527	5.407146	6.40E-08	2.54E-07	UP
TRIP4	695.9399	0.30522	0.056451	5.406848	6.41E-08	2.54E-07	NOT
RNA5SP18	1.545022	3.031234	0.560653	5.406609	6.42E-08	2.54E-07	UP
SART3	1451.4	0.304521	0.056337	5.405379	6.47E-08	2.56E-07	NOT
UBE2Q1-A	3.492764	1.479831	0.273772	5.405335	6.47E-08	2.56E-07	UP
LAS1L	1851.408	0.411961	0.07622	5.40491	6.48E-08	2.57E-07	NOT
UTP23	566.4099	0.45544	0.084274	5.404262	6.51E-08	2.58E-07	NOT
BDH1	8807.626	-1.0824	0.200292	-5.40414	6.51E-08	2.58E-07	DOWN
FNBP4	1243.368	0.48086	0.088997	5.403097	6.55E-08	2.59E-07	NOT
FBXL2	61.24001	1.085649	0.200932	5.403069	6.55E-08	2.59E-07	UP
TAS2R5	6.101399	1.422787	0.263341	5.402841	6.56E-08	2.59E-07	UP
SVEP1	415.9294	-1.42661	0.264053	-5.40273	6.56E-08	2.60E-07	DOWN
ANKRD54	925.5218	0.522536	0.096719	5.402632	6.57E-08	2.60E-07	NOT
SOD1	31153.82	-0.7873	0.145727	-5.40252	6.57E-08	2.60E-07	DOWN
NPEPPS	1978.593	0.52563	0.097298	5.402275	6.58E-08	2.60E-07	NOT
RP11-728I	10.39804	2.921457	0.540836	5.401741	6.60E-08	2.61E-07	UP
RP11-324I	11.94613	-0.84928	0.157227	-5.40163	6.60E-08	2.61E-07	DOWN
RP3-402G	52.78529	0.81398	0.150696	5.401466	6.61E-08	2.61E-07	UP
HERPUD1	6406.378	-0.57823	0.107071	-5.40039	6.65E-08	2.63E-07	NOT
SOX18	422.4161	0.861675	0.159563	5.400204	6.66E-08	2.63E-07	UP
RP11-45P	11.82959	0.973633	0.180299	5.400114	6.66E-08	2.63E-07	UP
SH3D19	3314.466	-0.652	0.120752	-5.39949	6.68E-08	2.64E-07	DOWN
TMEM183	1197.794	0.353208	0.065419	5.399195	6.69E-08	2.64E-07	NOT
RP5-857K	7390.039	-1.00339	0.18585	-5.39894	6.70E-08	2.65E-07	DOWN
XXbac-B4	6.311131	1.268946	0.235046	5.398711	6.71E-08	2.65E-07	UP
RP11-577I	10.0539	1.011742	0.187408	5.398589	6.72E-08	2.65E-07	UP
RP11-230I	9.994071	1.231363	0.228098	5.398388	6.72E-08	2.65E-07	UP
LRRC39	24.52816	0.821032	0.152109	5.397675	6.75E-08	2.66E-07	UP
AC018641	3.064099	3.342647	0.61928	5.397631	6.75E-08	2.66E-07	UP
CTD-2517	31.2877	0.826307	0.153091	5.397495	6.76E-08	2.67E-07	UP
MT-TM	2.563541	2.615561	0.48461	5.397248	6.77E-08	2.67E-07	UP
ERG	414.4666	-0.72373	0.134121	-5.39608	6.81E-08	2.69E-07	DOWN
UBE2SP1	10.88601	1.32788	0.246085	5.396016	6.81E-08	2.69E-07	UP
LPCAT4	321.6084	0.902816	0.167336	5.39522	6.84E-08	2.70E-07	UP
RP6-109B	4.062582	1.495897	0.27727	5.395099	6.85E-08	2.70E-07	UP
RP11-521I	16.43256	0.944568	0.175087	5.394854	6.86E-08	2.70E-07	UP

FBL	4600.538	0.870746	0.161408	5.394686	6.86E-08	2.71E-07	UP
ATR	711.2721	0.446932	0.082857	5.394007	6.89E-08	2.72E-07	NOT
RRP1	1324.8	0.531154	0.09849	5.39296	6.93E-08	2.73E-07	NOT
AC007191	42.89966	1.007531	0.186852	5.392126	6.96E-08	2.74E-07	UP
MARK1	65.0439	1.332999	0.247221	5.391932	6.97E-08	2.75E-07	UP
IFIT5	864.5189	-0.5468	0.101425	-5.39123	7.00E-08	2.76E-07	NOT
HADHA	15330.28	-0.4537	0.084155	-5.39119	7.00E-08	2.76E-07	NOT
NOL8	789.6959	0.386705	0.071729	5.391165	7.00E-08	2.76E-07	NOT
RP11-430I	4.604745	-1.16472	0.216078	-5.39028	7.03E-08	2.77E-07	DOWN
RP11-29H	2.204875	1.724708	0.319968	5.390256	7.04E-08	2.77E-07	UP
CAMP	2.341169	-1.53558	0.284891	-5.39005	7.04E-08	2.77E-07	DOWN
SIRT5	1630.891	-0.57317	0.106343	-5.38979	7.05E-08	2.78E-07	NOT
SEPT7P9	3.831762	1.358593	0.25213	5.388469	7.11E-08	2.80E-07	UP
KXD1	2143.759	0.420133	0.077972	5.388242	7.12E-08	2.80E-07	NOT
SLC25A13	5831.325	-0.68185	0.126548	-5.38808	7.12E-08	2.80E-07	DOWN
NAMPTP1	301.1891	-1.42938	0.265293	-5.38793	7.13E-08	2.80E-07	DOWN
CTC-505C	534.0235	-1.84317	0.342097	-5.38786	7.13E-08	2.80E-07	DOWN
CPTP	2633.992	-0.67906	0.126039	-5.38772	7.14E-08	2.81E-07	DOWN
BSN	151.3277	1.069983	0.198623	5.387015	7.16E-08	2.82E-07	UP
CNN2P1	9.759324	1.311515	0.243508	5.385917	7.21E-08	2.83E-07	UP
ZFHX2	50.90966	0.956176	0.177539	5.385723	7.22E-08	2.84E-07	UP
RP11-393I	6.597516	1.366744	0.253775	5.38565	7.22E-08	2.84E-07	UP
LRRC25	287.2603	-0.89702	0.166565	-5.3854	7.23E-08	2.84E-07	DOWN
MEOX1	28.10249	1.791534	0.332693	5.38494	7.25E-08	2.85E-07	UP
CLHC1	45.66199	0.756108	0.140417	5.384717	7.26E-08	2.85E-07	UP
AOX1	49631.36	-1.405	0.260926	-5.38467	7.26E-08	2.85E-07	DOWN
SMPD3	202.2599	-1.42464	0.264597	-5.38419	7.28E-08	2.86E-07	DOWN
AC023590	4.730616	-1.06495	0.197795	-5.3841	7.28E-08	2.86E-07	DOWN
NIPSNAP1	11387.31	-0.62501	0.116096	-5.38356	7.30E-08	2.87E-07	DOWN
RP11-495I	1.747653	2.260818	0.419953	5.3835	7.31E-08	2.87E-07	UP
GJA1	796.7422	0.97546	0.181195	5.38347	7.31E-08	2.87E-07	UP
FBXO46	738.7487	0.546159	0.10147	5.38244	7.35E-08	2.88E-07	NOT
SLC1A4	1430.458	0.738917	0.137288	5.382249	7.36E-08	2.89E-07	UP
PI4K2B	1599.434	-0.58359	0.108433	-5.38207	7.36E-08	2.89E-07	NOT
XXbac-BP(	25.97128	0.827806	0.153859	5.38028	7.44E-08	2.92E-07	UP
PAGE2	8.48805	4.832316	0.898154	5.380273	7.44E-08	2.92E-07	UP
GRN	14382.45	0.52344	0.097292	5.380115	7.44E-08	2.92E-07	NOT
RP11-139!	5.993945	2.119188	0.393979	5.378939	7.49E-08	2.94E-07	UP
SCMH1	716.3671	0.523631	0.097353	5.378707	7.50E-08	2.94E-07	NOT
ZNF384	446.5315	0.577467	0.10737	5.378282	7.52E-08	2.95E-07	NOT
LSM8	718.9075	0.486366	0.090439	5.377848	7.54E-08	2.95E-07	NOT
RNF17	6.538841	3.983553	0.740764	5.377625	7.55E-08	2.96E-07	UP
UQCRC2	7410.78	-0.54265	0.100928	-5.37659	7.59E-08	2.97E-07	NOT
MN1	146.4675	1.278272	0.237749	5.376553	7.59E-08	2.97E-07	UP
RP4-751H	3.665015	1.651253	0.307128	5.376438	7.60E-08	2.98E-07	UP
RP11-161I	1.541345	2.009688	0.37383	5.37594	7.62E-08	2.98E-07	UP
C4orf19	862.2882	-0.82655	0.153756	-5.37575	7.63E-08	2.99E-07	DOWN
TBCB	1738.534	0.693432	0.128994	5.375686	7.63E-08	2.99E-07	UP
LRRC59	5728.711	0.421033	0.078322	5.375667	7.63E-08	2.99E-07	NOT
UTP20	705.2042	0.446838	0.083127	5.37537	7.64E-08	2.99E-07	NOT
ZP1	2.205986	2.421498	0.4505	5.375131	7.65E-08	2.99E-07	UP
PA2G4	4624.584	0.472373	0.087905	5.373646	7.72E-08	3.02E-07	NOT

ST13P20	1.602926	-2.21014	0.411338	-5.37304	7.74E-08	3.03E-07	DOWN
ARPC1B	4889.688	0.675542	0.125729	5.373012	7.74E-08	3.03E-07	UP
SNF8	2763.064	0.517569	0.096336	5.37252	7.76E-08	3.04E-07	NOT
AGAP2	137.1686	0.774189	0.144113	5.372103	7.78E-08	3.04E-07	UP
RASAL2-A	24.04427	0.82119	0.152899	5.370811	7.84E-08	3.06E-07	UP
BSN-AS2	6.657393	1.639384	0.305247	5.370679	7.84E-08	3.07E-07	UP
RP13-672I	11.3557	-1.08941	0.20285	-5.37052	7.85E-08	3.07E-07	DOWN
CTA-941F	5.734793	2.426754	0.451869	5.370475	7.85E-08	3.07E-07	UP
AFAP1	433.1213	0.954117	0.177671	5.37014	7.87E-08	3.07E-07	UP
TNXB	559.0364	-1.26385	0.235385	-5.36931	7.90E-08	3.09E-07	DOWN
BCKDK	3487.105	-0.55088	0.102604	-5.369	7.92E-08	3.09E-07	NOT
APOC3	227693	-1.37811	0.256681	-5.36895	7.92E-08	3.09E-07	DOWN
IL4I1	145.7575	1.299604	0.242064	5.368843	7.92E-08	3.09E-07	UP
RP11-229I	4.753143	1.25034	0.232912	5.368303	7.95E-08	3.10E-07	UP
RP13-923(	14.68268	0.95553	0.177998	5.368204	7.95E-08	3.10E-07	UP
NCS1	454.6048	1.123014	0.209198	5.36819	7.95E-08	3.10E-07	UP
MRPL13	1988.037	0.637605	0.118777	5.368102	7.96E-08	3.11E-07	UP
SLC47A2	22.39782	1.11372	0.207484	5.367738	7.97E-08	3.11E-07	UP
RP11-96D	96.75378	-1.51862	0.282943	-5.36724	8.00E-08	3.12E-07	DOWN
C1orf109	388.0823	0.480053	0.089448	5.366859	8.01E-08	3.13E-07	NOT
LRGUK	12.84351	1.337919	0.249303	5.366646	8.02E-08	3.13E-07	UP
DNAH8	8.3736	1.848941	0.34456	5.366092	8.05E-08	3.14E-07	UP
ZNF32-AS	3.965268	1.35324	0.252186	5.366037	8.05E-08	3.14E-07	UP
PKIB	327.6322	1.523299	0.283901	5.365609	8.07E-08	3.15E-07	UP
CTD-2035	2.367271	2.053152	0.382701	5.364904	8.10E-08	3.16E-07	UP
RNF13	2204.267	-0.41382	0.077135	-5.36488	8.10E-08	3.16E-07	NOT
MRPS17P1	1.876112	2.368048	0.441405	5.364795	8.10E-08	3.16E-07	UP
CDK3	22.61014	0.997366	0.185923	5.364393	8.12E-08	3.17E-07	UP
TRPC4	22.57792	1.367377	0.254954	5.363234	8.17E-08	3.19E-07	UP
RP11-254I	3.974531	-1.35996	0.253575	-5.36315	8.18E-08	3.19E-07	DOWN
ZYG11B	1888.66	-0.5841	0.108912	-5.36306	8.18E-08	3.19E-07	NOT
SYNGR3	25.2636	1.614961	0.301165	5.362383	8.21E-08	3.20E-07	UP
RP11-521I	26.03951	0.824381	0.153736	5.362322	8.22E-08	3.20E-07	UP
AC007036	2.533634	2.51395	0.46882	5.362288	8.22E-08	3.20E-07	UP
NCR1	10.24567	-1.31067	0.244446	-5.3618	8.24E-08	3.21E-07	DOWN
PDK4	9441.242	-1.35695	0.253086	-5.36162	8.25E-08	3.21E-07	DOWN
ZNF821	76.34802	0.585126	0.109134	5.361538	8.25E-08	3.21E-07	UP
RRAGA	2897.974	-0.37464	0.069884	-5.36089	8.28E-08	3.22E-07	NOT
ZNF398	496.3556	0.434691	0.081093	5.360436	8.30E-08	3.23E-07	NOT
ECH1	16625.69	-0.78531	0.146501	-5.3604	8.30E-08	3.23E-07	DOWN
CYP21A1P	220.2512	1.138272	0.21235	5.360358	8.31E-08	3.23E-07	UP
TRMT2A	1640.969	0.428665	0.079988	5.359097	8.36E-08	3.25E-07	NOT
RASD1	2870.957	-1.42588	0.266107	-5.35832	8.40E-08	3.27E-07	DOWN
RRP12	1341.096	0.665141	0.124135	5.358223	8.40E-08	3.27E-07	UP
RP11-432	5.637704	2.34611	0.437875	5.357941	8.42E-08	3.27E-07	UP
SCHIP1	17.4343	0.874151	0.163154	5.357828	8.42E-08	3.27E-07	UP
SLC45A2	41.85336	1.615901	0.301612	5.35755	8.44E-08	3.28E-07	UP
HINT2	2805.778	-0.72775	0.135845	-5.35722	8.45E-08	3.28E-07	DOWN
PGRMC1	19897.9	-0.76153	0.142153	-5.35712	8.46E-08	3.29E-07	DOWN
NUP188	1298.718	0.505803	0.09443	5.356392	8.49E-08	3.30E-07	NOT
CTC-429P	19.97117	1.059336	0.197804	5.355487	8.53E-08	3.31E-07	UP
AGMO	3155.984	-1.02081	0.190621	-5.35522	8.55E-08	3.32E-07	DOWN

DGAT2	9643.786	-1.10051	0.205513	-5.35493	8.56E-08	3.32E-07	DOWN
QKI	3163.887	-0.47806	0.08928	-5.35462	8.57E-08	3.33E-07	NOT
MSH6	2005.537	0.459621	0.085847	5.353945	8.61E-08	3.34E-07	NOT
TNFSF9	35.12411	1.490891	0.278495	5.35338	8.63E-08	3.35E-07	UP
RNU6-529	9.229789	-1.08467	0.202629	-5.35297	8.65E-08	3.36E-07	DOWN
ERICH5	1522.893	1.534517	0.286692	5.352486	8.68E-08	3.37E-07	UP
AC007099	5.569151	2.721347	0.508474	5.351994	8.70E-08	3.38E-07	UP
AAAS	1534.07	0.368487	0.068851	5.351982	8.70E-08	3.38E-07	NOT
RP11-390I	120.7381	0.667199	0.124665	5.35192	8.70E-08	3.38E-07	UP
SH2B2	115.6295	0.847725	0.158402	5.351731	8.71E-08	3.38E-07	UP
CNTD2	173.5105	1.620077	0.302726	5.351633	8.72E-08	3.38E-07	UP
CTC-534A	81.46849	0.524872	0.09808	5.35149	8.72E-08	3.38E-07	NOT
DNAJC12	1046.16	-1.35995	0.25418	-5.35034	8.78E-08	3.40E-07	DOWN
HNRNPA1I	14.70023	1.521969	0.284484	5.349925	8.80E-08	3.41E-07	UP
VSTM1	1.616849	-1.68869	0.31566	-5.34972	8.81E-08	3.41E-07	DOWN
SPA17	128.8035	0.811315	0.15167	5.34922	8.83E-08	3.42E-07	UP
WDR83	282.0884	0.550279	0.102879	5.348798	8.85E-08	3.43E-07	NOT
REL	183.7232	-0.67713	0.126599	-5.34861	8.86E-08	3.43E-07	DOWN
BTBD19	187.5616	-0.78705	0.147159	-5.34829	8.88E-08	3.44E-07	DOWN
GDAP1L1	3.388619	2.247075	0.42018	5.347893	8.90E-08	3.45E-07	UP
RP11-794(	4.773018	1.882946	0.3521	5.347763	8.90E-08	3.45E-07	UP
PIGS	963.7261	0.60886	0.113854	5.347719	8.91E-08	3.45E-07	UP
ABHD10	1539.886	-0.43612	0.081555	-5.34756	8.91E-08	3.45E-07	NOT
DDIT3	1326.455	0.835705	0.156291	5.347122	8.94E-08	3.46E-07	UP
HNRNPA1I	4.777624	1.372038	0.256598	5.347026	8.94E-08	3.46E-07	UP
RP11-410I	34.34916	0.909582	0.170128	5.346467	8.97E-08	3.47E-07	UP
AC093162	3.652854	1.378539	0.257884	5.34557	9.01E-08	3.49E-07	UP
IL9RP3	3.758577	1.674065	0.313173	5.345499	9.02E-08	3.49E-07	UP
C2orf76	185.7445	0.484136	0.090588	5.344368	9.07E-08	3.51E-07	NOT
NAV1	383.4428	0.839836	0.157162	5.343759	9.10E-08	3.52E-07	UP
FGFBP2	23.05657	-1.13272	0.211973	-5.34368	9.11E-08	3.52E-07	DOWN
CTD-2033	9.66534	0.993119	0.185859	5.34339	9.12E-08	3.53E-07	UP
ALOX12	30.81419	0.769603	0.14403	5.343352	9.12E-08	3.53E-07	UP
VNN2	600.3078	1.5802	0.295746	5.343094	9.14E-08	3.53E-07	UP
WASIR2	2.229627	2.438123	0.456355	5.342598	9.16E-08	3.54E-07	UP
DLG2	86.97908	-1.10286	0.206456	-5.34187	9.20E-08	3.56E-07	DOWN
SEC24B-A	15.10228	0.732327	0.137093	5.341826	9.20E-08	3.56E-07	UP
ST8SIA5	9.521205	1.672512	0.313111	5.341587	9.21E-08	3.56E-07	UP
CTD-2331	21.87018	1.586882	0.297096	5.341316	9.23E-08	3.56E-07	UP
HSD17B13	12992.85	-2.18217	0.408602	-5.34056	9.27E-08	3.58E-07	DOWN
AC016738	9.708485	1.371988	0.256955	5.339414	9.32E-08	3.60E-07	UP
BRPF1	577.3886	0.354984	0.066488	5.339097	9.34E-08	3.61E-07	NOT
DCAF8L1	9.450506	5.573725	1.043988	5.338876	9.35E-08	3.61E-07	UP
AKR1D1	5527.808	-1.7554	0.32885	-5.33801	9.40E-08	3.63E-07	DOWN
SSBP4	1135.06	0.595084	0.111486	5.337728	9.41E-08	3.63E-07	UP
CASP12	6.008851	1.414945	0.265095	5.337498	9.42E-08	3.64E-07	UP
CTAGE5	429.4464	-0.54391	0.101906	-5.33738	9.43E-08	3.64E-07	NOT
FKBP1A	5354.508	0.486699	0.091192	5.337076	9.45E-08	3.64E-07	NOT
RP11-440I	4.480407	1.392433	0.260898	5.33707	9.45E-08	3.64E-07	UP
RP11-111I	3.262684	1.334505	0.250117	5.335517	9.53E-08	3.67E-07	UP
LINC01226	7.758756	1.799123	0.33725	5.334685	9.57E-08	3.69E-07	UP
SOX6	530.5518	-1.21032	0.22689	-5.33438	9.59E-08	3.70E-07	DOWN

RP11-60E8	1.78911	2.346803	0.439964	5.334077	9.60E-08	3.70E-07	UP
ZBED2	11.11657	2.086844	0.391272	5.333481	9.63E-08	3.71E-07	UP
UTP18	1127.434	0.44661	0.083758	5.332154	9.71E-08	3.74E-07	NOT
EHBP1	2746.079	-0.59767	0.11214	-5.32967	9.84E-08	3.79E-07	DOWN
RXFP4	1.677837	2.696456	0.506061	5.32832	9.91E-08	3.82E-07	UP
CXXC1	1442.677	0.410176	0.076982	5.328172	9.92E-08	3.82E-07	NOT
LIPA	5149.244	-0.58194	0.109227	-5.32785	9.94E-08	3.83E-07	NOT
LA16c-429	12.22826	-1.14268	0.214517	-5.32678	1.00E-07	3.85E-07	DOWN
PLEKHM2	1930.49	0.432893	0.081273	5.326379	1.00E-07	3.86E-07	NOT
TAC3	5.877107	2.200924	0.413229	5.326165	1.00E-07	3.86E-07	UP
RP11-156I	55.7097	-0.71674	0.134595	-5.32515	1.01E-07	3.88E-07	DOWN
GS1-393G	5.79616	1.234209	0.231777	5.324988	1.01E-07	3.89E-07	UP
RP13-644I	3.894872	2.00425	0.376394	5.324874	1.01E-07	3.89E-07	UP
FANCC	1027.661	-0.83329	0.156493	-5.32479	1.01E-07	3.89E-07	DOWN
CEPT1	917.2857	-0.5415	0.101696	-5.32473	1.01E-07	3.89E-07	NOT
SLC45A3	710.0692	-0.92351	0.173439	-5.32468	1.01E-07	3.89E-07	DOWN
FBXO44	1009.864	0.66174	0.124281	5.32456	1.01E-07	3.89E-07	UP
RP11-81H	15.22835	2.276082	0.427481	5.324402	1.01E-07	3.90E-07	UP
CCNL2	3152.122	0.634799	0.119229	5.324207	1.01E-07	3.90E-07	UP
DERA	2375.405	-0.57517	0.108032	-5.32403	1.01E-07	3.90E-07	NOT
RAMP3	763.0798	-1.08119	0.203084	-5.32383	1.02E-07	3.91E-07	DOWN
MED14OS	22.20831	-0.72177	0.13558	-5.32361	1.02E-07	3.91E-07	DOWN
LRRC47	2318.989	-0.3648	0.068525	-5.32357	1.02E-07	3.91E-07	NOT
RASSF4	2704.885	0.677167	0.127208	5.323286	1.02E-07	3.92E-07	UP
CX3CR1	80.06437	-0.89032	0.167264	-5.32285	1.02E-07	3.93E-07	DOWN
ECHDC2	9140.333	-0.84997	0.159686	-5.32274	1.02E-07	3.93E-07	DOWN
ENOPH1	998.0556	0.402192	0.075565	5.32244	1.02E-07	3.93E-07	NOT
UBBP4	319.8543	-0.596	0.111979	-5.3224	1.02E-07	3.93E-07	DOWN
RNU1-106	2.776666	1.893445	0.355753	5.322364	1.02E-07	3.93E-07	UP
FAM195CI	1.546908	2.32705	0.437249	5.322023	1.03E-07	3.94E-07	UP
KB-68A7.1	105.0999	-1.28849	0.242114	-5.32182	1.03E-07	3.94E-07	DOWN
SOCS2-AS	107.2027	-1.15129	0.216384	-5.32057	1.03E-07	3.97E-07	DOWN
S100A11	3221.209	1.159716	0.217977	5.32037	1.04E-07	3.97E-07	UP
RP11-308I	12.98331	1.228829	0.230974	5.320208	1.04E-07	3.98E-07	UP
LZTS3	814.8418	0.694211	0.1305	5.319631	1.04E-07	3.99E-07	UP
SLC39A8	1726.363	-0.78961	0.148453	-5.31895	1.04E-07	4.00E-07	DOWN
BPESC1	1.617972	3.031236	0.569901	5.318885	1.04E-07	4.01E-07	UP
U91328.19	216.0072	0.760819	0.143054	5.318395	1.05E-07	4.02E-07	UP
RP11-24M	3.72416	1.336283	0.251264	5.31825	1.05E-07	4.02E-07	UP
STMN3	329.9241	1.416539	0.266363	5.31808	1.05E-07	4.02E-07	UP
PPM1A	2880.669	-0.44726	0.084102	-5.31807	1.05E-07	4.02E-07	NOT
CLGN	581.9148	1.551159	0.291701	5.317623	1.05E-07	4.03E-07	UP
AC104777	1.650223	-1.73973	0.327202	-5.317	1.05E-07	4.04E-07	DOWN
PTCHD2	6.238362	1.860758	0.350026	5.316057	1.06E-07	4.06E-07	UP
ANK3	456.5621	-1.21812	0.229161	-5.31557	1.06E-07	4.07E-07	DOWN
ANGPTL1	397.0205	-1.47154	0.27684	-5.3155	1.06E-07	4.08E-07	DOWN
RP11-370I	3.576852	1.965372	0.369864	5.313771	1.07E-07	4.11E-07	UP
PTK2B	848.1591	-0.65279	0.122851	-5.31369	1.07E-07	4.11E-07	DOWN
UBA52P6	4.438704	1.263613	0.237806	5.31362	1.07E-07	4.12E-07	UP
HIST1H3C	4.936977	1.591406	0.299509	5.313379	1.08E-07	4.12E-07	UP
RP11-87C	4.214293	1.231952	0.231859	5.31336	1.08E-07	4.12E-07	UP
PQLC1	7454.129	-0.70478	0.132657	-5.31284	1.08E-07	4.13E-07	DOWN

RILPL1	256.0969	0.498167	0.093769	5.312695	1.08E-07	4.13E-07	NOT
RALGDS	1353.679	-0.80491	0.151513	-5.31245	1.08E-07	4.14E-07	DOWN
RP11-598I	27.74785	1.037079	0.195223	5.312272	1.08E-07	4.14E-07	UP
RP4-564F2	247.8776	-1.35266	0.254638	-5.31206	1.08E-07	4.15E-07	DOWN
MPV17L2	703.5196	0.592344	0.111522	5.31145	1.09E-07	4.16E-07	UP
AC104655	3.86543	1.405796	0.264686	5.311191	1.09E-07	4.17E-07	UP
RP5-858B(	5.0768	1.305016	0.245811	5.309027	1.10E-07	4.22E-07	UP
MGST1	32743.48	-0.94921	0.178795	-5.30896	1.10E-07	4.22E-07	DOWN
PLEK2	563.4895	-1.45654	0.274373	-5.30862	1.10E-07	4.22E-07	DOWN
NDUFAF1	1082.359	-0.5475	0.103143	-5.30815	1.11E-07	4.23E-07	NOT
RP11-15A	22.4459	1.192076	0.22463	5.306845	1.12E-07	4.26E-07	UP
TAF4	764.3878	0.44402	0.083671	5.306741	1.12E-07	4.27E-07	NOT
DNAH6	185.8199	-1.41722	0.26708	-5.30635	1.12E-07	4.27E-07	DOWN
CTB-75G1	4.859473	0.967237	0.182286	5.306163	1.12E-07	4.28E-07	UP
KCNG3	2.274355	3.39873	0.640536	5.306072	1.12E-07	4.28E-07	UP
CPSF7	2540.279	0.340228	0.064124	5.305803	1.12E-07	4.29E-07	NOT
ZNF232	244.3859	0.500177	0.094276	5.305454	1.12E-07	4.29E-07	NOT
RP11-262I	2.373259	2.890259	0.544798	5.305189	1.13E-07	4.30E-07	UP
ARNT2	269.9521	1.625145	0.306349	5.304877	1.13E-07	4.30E-07	UP
Z98750.1	1.637052	1.883891	0.355126	5.304856	1.13E-07	4.30E-07	UP
AP000253.	10.40374	-1.17338	0.221194	-5.30476	1.13E-07	4.31E-07	DOWN
CELA3A	2.324262	3.234199	0.609778	5.303897	1.13E-07	4.33E-07	UP
WASH1	198.3298	0.698159	0.131634	5.303798	1.13E-07	4.33E-07	UP
DOT1L	1303.089	0.618023	0.11653	5.303551	1.14E-07	4.33E-07	UP
ATP6V1E1	3647.877	0.393259	0.07415	5.303542	1.14E-07	4.33E-07	NOT
PDPK2P	9.518709	1.19081	0.224534	5.303478	1.14E-07	4.33E-07	UP
ZCCHC17	719.9652	0.427111	0.080538	5.303248	1.14E-07	4.34E-07	NOT
CD83	343.6717	-0.83261	0.157005	-5.30308	1.14E-07	4.34E-07	DOWN
DUSP18	65.24455	0.652455	0.123034	5.303028	1.14E-07	4.34E-07	UP
PECR	5241.962	-0.83013	0.15654	-5.30298	1.14E-07	4.34E-07	DOWN
RAPGEF3	251.482	0.748916	0.141248	5.302124	1.14E-07	4.36E-07	UP
RP11-432	3.446126	3.185627	0.60098	5.300718	1.15E-07	4.40E-07	UP
ANKS4B	1340.996	-1.03583	0.195418	-5.30058	1.15E-07	4.40E-07	DOWN
GPATCH8	1128.375	0.480762	0.090704	5.300342	1.16E-07	4.40E-07	NOT
RP11-46B	2.06399	3.061132	0.577587	5.299858	1.16E-07	4.42E-07	UP
PCDH9	128.1244	-1.59612	0.301167	-5.29978	1.16E-07	4.42E-07	DOWN
GSK3A	2028.957	0.395052	0.074547	5.299366	1.16E-07	4.43E-07	NOT
CHD8	1715.45	0.373134	0.070423	5.298438	1.17E-07	4.45E-07	NOT
KLHL7	648.6421	0.37401	0.070591	5.298282	1.17E-07	4.45E-07	NOT
RPL34-AS	6.431365	-0.98233	0.185409	-5.29815	1.17E-07	4.45E-07	DOWN
CTD-2008	8.06425	2.702606	0.510108	5.29811	1.17E-07	4.45E-07	UP
GAST	3.581637	4.050013	0.764514	5.297497	1.17E-07	4.47E-07	UP
RP11-490I	49.22577	1.408379	0.26586	5.297455	1.17E-07	4.47E-07	UP
RP11-552I	2.140225	1.772158	0.334568	5.296849	1.18E-07	4.48E-07	UP
RP3-508I1	2.003772	1.725419	0.325749	5.296777	1.18E-07	4.48E-07	UP
SLC25A51	266.3146	0.501993	0.094776	5.296595	1.18E-07	4.49E-07	NOT
ZNF48	378.7834	0.485935	0.091749	5.296339	1.18E-07	4.49E-07	NOT
CACNA1B	3.744507	2.86316	0.540602	5.296245	1.18E-07	4.50E-07	UP
MEIS3P2	2.908588	-1.33631	0.252313	-5.29623	1.18E-07	4.50E-07	DOWN
EVI5	973.8783	-0.51215	0.096719	-5.29525	1.19E-07	4.52E-07	NOT
MIDN	2956.904	-0.68831	0.130006	-5.29443	1.19E-07	4.54E-07	DOWN
BMP8A	9.843004	1.235895	0.233528	5.292272	1.21E-07	4.59E-07	UP

FAM110C	920.0226	-1.22706	0.231863	-5.29217	1.21E-07	4.59E-07	DOWN
SERPINA1:	60.20211	1.627541	0.307538	5.292167	1.21E-07	4.59E-07	UP
IFI44	817.1374	-1.1516	0.217604	-5.29216	1.21E-07	4.59E-07	DOWN
TEDDM1	2.327449	3.237987	0.611923	5.291495	1.21E-07	4.61E-07	UP
EPB41L1	2783.762	1.488937	0.281395	5.291261	1.21E-07	4.61E-07	UP
KCTD10	1242.137	0.430758	0.081412	5.291102	1.22E-07	4.62E-07	NOT
MXD4	2677.61	0.600174	0.113432	5.291025	1.22E-07	4.62E-07	UP
RP11-849I	9.62713	0.804205	0.151996	5.290953	1.22E-07	4.62E-07	UP
LRP4	145.0837	1.446427	0.273429	5.28995	1.22E-07	4.64E-07	UP
CEBPB	5467.237	-0.73526	0.139014	-5.28908	1.23E-07	4.67E-07	DOWN
SIX5	415.1337	0.573698	0.108474	5.288814	1.23E-07	4.67E-07	NOT
ARAF	3768.666	-0.45526	0.086096	-5.2878	1.24E-07	4.70E-07	NOT
IK	3565.462	0.303157	0.057337	5.287302	1.24E-07	4.71E-07	NOT
RP1-232P:	60.49028	-2.1598	0.408493	-5.28723	1.24E-07	4.71E-07	DOWN
CBLN1	97.21037	1.527101	0.28883	5.287204	1.24E-07	4.71E-07	UP
SYNGR4	7.495404	1.4687	0.277799	5.28691	1.24E-07	4.72E-07	UP
NBL1	310.274	1.305606	0.246954	5.286843	1.24E-07	4.72E-07	UP
FTH1P8	60.6156	0.856067	0.161936	5.286461	1.25E-07	4.73E-07	UP
C1QA	6244.54	-1.05921	0.200367	-5.28632	1.25E-07	4.73E-07	DOWN
ELF1	1375.374	-0.61116	0.115613	-5.28624	1.25E-07	4.73E-07	DOWN
WIF1	7.480165	3.736096	0.706828	5.285724	1.25E-07	4.75E-07	UP
PICALM	5551.08	-0.33463	0.063311	-5.2855	1.25E-07	4.75E-07	NOT
BMP8B	130.5114	1.469236	0.277998	5.285053	1.26E-07	4.76E-07	UP
RAP1A	2512.645	-0.36235	0.068566	-5.28466	1.26E-07	4.77E-07	NOT
DDAH2	2822.472	0.742913	0.140579	5.284651	1.26E-07	4.77E-07	UP
LGMN	4818.393	-0.56818	0.107517	-5.28456	1.26E-07	4.77E-07	NOT
H1F0	14212.65	0.616174	0.116609	5.28412	1.26E-07	4.78E-07	UP
MAP3K12	151.7958	0.678734	0.128461	5.283566	1.27E-07	4.80E-07	UP
C3P1	6249.256	-1.59291	0.301512	-5.28308	1.27E-07	4.81E-07	DOWN
GRAP	40.78809	-0.86516	0.163772	-5.28275	1.27E-07	4.82E-07	DOWN
RP5-940J5	2.351119	1.799181	0.34066	5.281465	1.28E-07	4.85E-07	UP
RP5-1014I	62.29804	0.604415	0.11445	5.281044	1.28E-07	4.86E-07	UP
ZNF91	486.8384	0.592683	0.112229	5.281022	1.28E-07	4.86E-07	UP
LILRB2	297.4339	-0.93999	0.178001	-5.28083	1.29E-07	4.87E-07	DOWN
THYN1	1203.947	-0.48755	0.092331	-5.28046	1.29E-07	4.87E-07	NOT
RP11-400I	3.569621	4.228021	0.800705	5.280375	1.29E-07	4.88E-07	UP
RP11-334(	8.91638	1.237238	0.234338	5.27972	1.29E-07	4.89E-07	UP
ASB15	3.076168	2.743097	0.519603	5.279216	1.30E-07	4.91E-07	UP
RP11-188I	8.678222	1.253227	0.237427	5.278375	1.30E-07	4.93E-07	UP
DHRS4-AS	1311.131	-0.79834	0.151247	-5.27837	1.30E-07	4.93E-07	DOWN
PPHLN1	1210.24	0.287455	0.054464	5.27789	1.31E-07	4.94E-07	NOT
ANAPC16	4081.627	-0.36907	0.069931	-5.27764	1.31E-07	4.95E-07	NOT
C6orf136	930.1278	0.518289	0.098209	5.27741	1.31E-07	4.95E-07	NOT
CTC-537E	5.7	-1.97905	0.375098	-5.27609	1.32E-07	4.99E-07	DOWN
RP11-451(	7.361302	3.795299	0.719374	5.275836	1.32E-07	4.99E-07	UP
BGN	14716.04	-1.11297	0.210973	-5.27541	1.32E-07	5.00E-07	DOWN
CDCA7L	303.7029	1.036416	0.196481	5.274896	1.33E-07	5.02E-07	UP
ZNF90	25.93301	1.403177	0.266017	5.27477	1.33E-07	5.02E-07	UP
CTB-58E1	4.471808	1.237822	0.234674	5.274641	1.33E-07	5.02E-07	UP
MIPEP	574.7667	-0.59304	0.112441	-5.27419	1.33E-07	5.03E-07	DOWN
RP11-295I	34.0641	1.228472	0.232941	5.273741	1.34E-07	5.05E-07	UP
RP11-474I	4.391714	-1.31325	0.249037	-5.27332	1.34E-07	5.06E-07	DOWN

SELP	142.1246	-1.38866	0.263346	-5.27313	1.34E-07	5.06E-07	DOWN
CTD-2328	46.52395	-1.68865	0.320257	-5.27281	1.34E-07	5.07E-07	DOWN
SERPINA3	1256.869	-1.26165	0.23929	-5.27247	1.35E-07	5.08E-07	DOWN
PPIL2	1753.456	0.360009	0.068282	5.272416	1.35E-07	5.08E-07	NOT
CTD-2015	8.443935	2.27639	0.43177	5.272224	1.35E-07	5.08E-07	UP
GAPDHP6(	5.199332	1.221971	0.231786	5.271979	1.35E-07	5.09E-07	UP
RP11-318(	5.555582	1.375633	0.260939	5.271854	1.35E-07	5.09E-07	UP
PXN-AS1	183.03	0.667784	0.126673	5.2717	1.35E-07	5.10E-07	UP
IGLV7-46	38.3313	-2.0129	0.381932	-5.27031	1.36E-07	5.13E-07	DOWN
KCNK6	295.097	-0.85266	0.161792	-5.27009	1.36E-07	5.14E-07	DOWN
CFL1	20943.8	0.445759	0.084586	5.269879	1.37E-07	5.14E-07	NOT
NCK1-AS1	83.73575	0.500344	0.094954	5.269338	1.37E-07	5.16E-07	NOT
SS18	2377.917	-0.37184	0.070584	-5.26807	1.38E-07	5.19E-07	NOT
AC007750	3.149601	2.078178	0.394488	5.268037	1.38E-07	5.19E-07	UP
APOA5	16755.6	-1.46031	0.277206	-5.26797	1.38E-07	5.20E-07	DOWN
PCDHB9	27.87683	1.506748	0.286044	5.267541	1.38E-07	5.21E-07	UP
GPR180	653.9959	-0.85664	0.162627	-5.2675	1.38E-07	5.21E-07	DOWN
NAA25	610.9909	0.402446	0.076402	5.267494	1.38E-07	5.21E-07	NOT
ORMDL2	1202.679	0.506855	0.09623	5.267129	1.39E-07	5.22E-07	NOT
SLC16A11	1115.062	1.617673	0.30714	5.266887	1.39E-07	5.22E-07	UP
RP11-10J2	1.408091	2.37492	0.450974	5.266204	1.39E-07	5.24E-07	UP
HUNK	260.8326	1.648515	0.313038	5.266188	1.39E-07	5.24E-07	UP
GDNF-AS1	23.62619	2.137557	0.405931	5.265816	1.40E-07	5.25E-07	UP
HYAL1	6825.106	-0.83691	0.158936	-5.26571	1.40E-07	5.25E-07	DOWN
RP11-77H	13.34586	-1.6354	0.310582	-5.2656	1.40E-07	5.26E-07	DOWN
ACTN4	13815.06	0.44197	0.083946	5.264924	1.40E-07	5.27E-07	NOT
PRODH2	4502.089	-1.30519	0.247945	-5.26403	1.41E-07	5.30E-07	DOWN
PM20D2	515.8536	0.708946	0.134679	5.263974	1.41E-07	5.30E-07	UP
SBK1	77.94432	1.354399	0.257306	5.263768	1.41E-07	5.31E-07	UP
RP11-92K2	20.99055	0.89236	0.169547	5.263218	1.42E-07	5.32E-07	UP
FOXK2	1524.525	0.499014	0.094818	5.262883	1.42E-07	5.33E-07	NOT
AC104088	2.944845	3.584848	0.681214	5.262438	1.42E-07	5.34E-07	UP
AC004076	1.185047	2.098675	0.398855	5.261749	1.43E-07	5.36E-07	UP
HIBADH	5679.99	-0.8018	0.152401	-5.26114	1.43E-07	5.38E-07	DOWN
RP11-276I	3.667055	2.464553	0.468454	5.261035	1.43E-07	5.38E-07	UP
AC084125	15.87243	0.86735	0.164866	5.260929	1.43E-07	5.38E-07	UP
SOX5	476.5505	-0.90125	0.17132	-5.2606	1.44E-07	5.39E-07	DOWN
PDE6A	5.291871	1.612253	0.306511	5.260014	1.44E-07	5.41E-07	UP
ASGR1	26316.38	-1.00413	0.190905	-5.25983	1.44E-07	5.41E-07	DOWN
ATP6V1B2	2459.311	-0.47423	0.090163	-5.25968	1.44E-07	5.42E-07	NOT
CHAMP1	850.1715	-0.59958	0.114002	-5.25942	1.45E-07	5.42E-07	DOWN
SLC25A14	172.1508	0.48589	0.092386	5.259322	1.45E-07	5.43E-07	NOT
ZC3HAV1L	34.81195	1.321546	0.251281	5.259246	1.45E-07	5.43E-07	UP
RP11-430(	1.829789	2.184802	0.41545	5.258877	1.45E-07	5.44E-07	UP
AC024937	1.647522	2.489044	0.473381	5.258018	1.46E-07	5.46E-07	UP
RP11-92K1	13.51461	1.13591	0.216049	5.257646	1.46E-07	5.47E-07	UP
RP11-574I	14.16086	-1.12897	0.214741	-5.25734	1.46E-07	5.48E-07	DOWN
FEM1C	1314.336	-0.49601	0.094353	-5.25693	1.46E-07	5.49E-07	NOT
IGKV1-6	75.56073	-2.11585	0.402487	-5.25693	1.46E-07	5.49E-07	DOWN
CELF5	70.46257	1.88391	0.358373	5.256845	1.47E-07	5.49E-07	UP
RP11-473I	14.00791	1.079252	0.205307	5.256764	1.47E-07	5.49E-07	UP
LMO1	2.810545	3.634257	0.691365	5.256642	1.47E-07	5.50E-07	UP

RP11-368I	2.643176	3.480841	0.662208	5.25642	1.47E-07	5.50E-07	UP
SDK2	127.4792	1.776024	0.337927	5.25564	1.48E-07	5.53E-07	UP
FAM83C-/	3.480517	1.434262	0.272903	5.255582	1.48E-07	5.53E-07	UP
PCA3	1.534091	-2.76537	0.526273	-5.25463	1.48E-07	5.56E-07	DOWN
CD3EAP	172.8491	0.736505	0.140165	5.254554	1.48E-07	5.56E-07	UP
RP11-34P1	13.25338	1.446704	0.275333	5.254387	1.49E-07	5.56E-07	UP
GJA10	7.56792	5.19496	0.988741	5.254114	1.49E-07	5.57E-07	UP
SLC2A1	467.3054	1.203689	0.229105	5.253882	1.49E-07	5.57E-07	UP
TUBA1C	1862.516	0.732921	0.139513	5.253428	1.49E-07	5.59E-07	UP
COL10A1	80.61688	2.186177	0.416151	5.253322	1.49E-07	5.59E-07	UP
QRFP	5.199695	1.344544	0.255996	5.252202	1.50E-07	5.62E-07	UP
SLC1A7	406.3848	1.881238	0.358193	5.252021	1.50E-07	5.63E-07	UP
RQCD1	1542.144	0.37547	0.071492	5.251911	1.51E-07	5.63E-07	NOT
DTNBP1	470.6801	0.615245	0.117165	5.251107	1.51E-07	5.65E-07	UP
EXPH5	401.0327	-1.44915	0.275999	-5.25056	1.52E-07	5.67E-07	DOWN
ZNF343	323.6425	0.428317	0.081585	5.249913	1.52E-07	5.69E-07	NOT
PRR34-AS1	135.1474	1.041319	0.198363	5.249569	1.52E-07	5.70E-07	UP
PLSCR1	1512.198	-0.81889	0.156023	-5.24852	1.53E-07	5.73E-07	DOWN
OSBPL7	166.6038	0.983982	0.18748	5.248463	1.53E-07	5.73E-07	UP
CTA-989H	16.23131	0.84823	0.16163	5.247987	1.54E-07	5.75E-07	UP
LRFN4	133.6834	1.276055	0.24318	5.247368	1.54E-07	5.77E-07	UP
TUBB	22500.36	0.550323	0.104899	5.246217	1.55E-07	5.80E-07	NOT
NEU3	419.054	0.555151	0.105848	5.244779	1.56E-07	5.85E-07	NOT
SNX18	1423.862	-0.4324	0.082452	-5.24427	1.57E-07	5.86E-07	NOT
PUS3	930.0647	-0.49874	0.095105	-5.2441	1.57E-07	5.87E-07	NOT
KBTBD7	399.896	-0.53369	0.101778	-5.24363	1.57E-07	5.88E-07	NOT
MYOM3	14.09154	1.239545	0.236419	5.243012	1.58E-07	5.90E-07	UP
RP11-96D	7.271188	1.421345	0.271095	5.242976	1.58E-07	5.90E-07	UP
MYO16	144.9675	-1.1881	0.22662	-5.24268	1.58E-07	5.91E-07	DOWN
AC016722	7.181846	1.205771	0.230023	5.24196	1.59E-07	5.93E-07	UP
RP11-496I	2.698466	1.518685	0.289722	5.241876	1.59E-07	5.93E-07	UP
FAM118B	363.262	0.369945	0.070581	5.241437	1.59E-07	5.94E-07	NOT
RP11-135I	18.28986	0.924299	0.176351	5.241238	1.60E-07	5.95E-07	UP
PCDHA2	2.96414	2.916914	0.556579	5.24079	1.60E-07	5.96E-07	UP
RP3-340N	68.90167	1.341705	0.256017	5.240698	1.60E-07	5.97E-07	UP
KIAA1024	11.92067	1.441906	0.275156	5.240312	1.60E-07	5.98E-07	UP
RP11-276I	2.490602	1.537393	0.293386	5.240175	1.60E-07	5.98E-07	UP
PLCXD3	122.4256	-1.6637	0.317505	-5.2399	1.61E-07	5.99E-07	DOWN
ISG15	2865.625	-1.12664	0.215016	-5.23979	1.61E-07	5.99E-07	DOWN
PJA2	4333.326	-0.46768	0.089261	-5.23949	1.61E-07	6.00E-07	NOT
RP11-258I	161.6039	-1.77932	0.339682	-5.2382	1.62E-07	6.04E-07	DOWN
RP11-661/	4.511976	1.48222	0.282979	5.237922	1.62E-07	6.05E-07	UP
FNBP1L	1374.558	0.609897	0.116443	5.23772	1.63E-07	6.06E-07	UP
RP11-504I	3.230815	1.622553	0.309825	5.237005	1.63E-07	6.08E-07	UP
SLC38A1	1803.809	1.237558	0.236314	5.236919	1.63E-07	6.08E-07	UP
RP5-1092/	59.91506	0.961212	0.183557	5.236588	1.64E-07	6.09E-07	UP
ST6GAL2	105.7676	-1.69428	0.323584	-5.23599	1.64E-07	6.11E-07	DOWN
RHOT2	2520.197	0.410055	0.078316	5.235895	1.64E-07	6.11E-07	NOT
CTD-2319	6.785548	1.097953	0.209698	5.235885	1.64E-07	6.11E-07	UP
AC013439	2.691469	2.183833	0.417096	5.2358	1.64E-07	6.11E-07	UP
PCDHB16	108.7245	1.36895	0.261541	5.234167	1.66E-07	6.17E-07	UP
RPSA	16352.08	0.737537	0.140911	5.234055	1.66E-07	6.17E-07	UP

HSPB1P2	7.085614	1.070442	0.20454	5.233411	1.66E-07	6.19E-07	UP
QRSL1P1	2.825807	2.415497	0.461594	5.232942	1.67E-07	6.21E-07	UP
GLS2	683.6984	-1.86544	0.356527	-5.23226	1.67E-07	6.23E-07	DOWN
PTPRN	3.503565	2.042141	0.390342	5.231667	1.68E-07	6.25E-07	UP
CTD-2192	21.91305	0.737326	0.140938	5.23157	1.68E-07	6.25E-07	UP
RIMS2	9.741772	1.918806	0.366797	5.231246	1.68E-07	6.26E-07	UP
SMPDL3A	1933.199	-0.72673	0.138926	-5.23107	1.69E-07	6.27E-07	DOWN
TATDN2P2	10.14503	1.276715	0.244084	5.230628	1.69E-07	6.28E-07	UP
C2orf42	470.9151	-0.42782	0.081792	-5.23062	1.69E-07	6.28E-07	NOT
SIPA1L2	1264.907	1.05819	0.202331	5.22999	1.70E-07	6.30E-07	UP
CETN2	1326.081	0.527148	0.100803	5.229509	1.70E-07	6.32E-07	NOT
FOLH1B	256.8293	-1.89352	0.362114	-5.22906	1.70E-07	6.33E-07	DOWN
LINC00342	116.0025	0.925365	0.176968	5.228995	1.70E-07	6.33E-07	UP
SALL4	75.7631	1.879839	0.359612	5.227409	1.72E-07	6.38E-07	UP
TUBA3C	10.36975	3.91563	0.749082	5.22724	1.72E-07	6.39E-07	UP
FZD9	25.13019	1.852384	0.354391	5.226955	1.72E-07	6.40E-07	UP
GOLGA2P8	1.867437	2.235715	0.427824	5.22578	1.73E-07	6.44E-07	UP
ZNF446	258.6604	0.501939	0.096053	5.225663	1.74E-07	6.44E-07	NOT
RP13-942I	34.69211	0.834476	0.159688	5.225655	1.74E-07	6.44E-07	UP
RP11-137S	7.430159	1.066697	0.204136	5.225431	1.74E-07	6.45E-07	UP
TSSC1	839.2835	0.476125	0.091119	5.225314	1.74E-07	6.45E-07	NOT
CSNK1A1F	8.699046	-1.1652	0.223059	-5.22373	1.75E-07	6.51E-07	DOWN
RP11-336I	4.383618	-1.31458	0.251671	-5.22342	1.76E-07	6.52E-07	DOWN
SETX	2284.254	-0.43563	0.083408	-5.22294	1.76E-07	6.53E-07	NOT
WASH2P	105.3199	0.607463	0.116309	5.222842	1.76E-07	6.53E-07	UP
RP1-140K8	54.81385	1.61722	0.309681	5.22221	1.77E-07	6.56E-07	UP
RP5-966M	3.259632	1.465209	0.280578	5.222116	1.77E-07	6.56E-07	UP
RP11-803I	3.325026	1.696996	0.324977	5.221893	1.77E-07	6.57E-07	UP
HTRA4	9.147849	1.879032	0.359859	5.221572	1.77E-07	6.58E-07	UP
AKAP17A	1455.117	0.446063	0.085429	5.221449	1.78E-07	6.58E-07	NOT
EXOSC4	1563.415	0.867076	0.166064	5.221332	1.78E-07	6.58E-07	UP
F11-AS1	403.7553	-1.10759	0.212144	-5.22094	1.78E-07	6.60E-07	DOWN
NXPH3	30.74322	1.049423	0.20103	5.220223	1.79E-07	6.62E-07	UP
CTB-92J24	34.38484	1.227641	0.235202	5.219521	1.79E-07	6.64E-07	UP
RP11-699A	5.187286	1.655782	0.317254	5.219112	1.80E-07	6.66E-07	UP
RUNDC3B	424.7321	-1.15295	0.220959	-5.21793	1.81E-07	6.70E-07	DOWN
DPCD	273.999	0.691536	0.132546	5.217334	1.82E-07	6.72E-07	UP
COL6A4P1	2.707628	2.272352	0.43557	5.21696	1.82E-07	6.73E-07	UP
GPBP1L1	4248.133	-0.38215	0.073261	-5.21627	1.83E-07	6.76E-07	NOT
RORA	1933.953	-0.89529	0.17164	-5.2161	1.83E-07	6.76E-07	DOWN
IGBP1	1496.9	0.452972	0.086864	5.214745	1.84E-07	6.81E-07	NOT
GS1-304P7	2.630584	1.979782	0.379692	5.214184	1.85E-07	6.83E-07	UP
MIR4635	3.276431	1.711754	0.328289	5.214177	1.85E-07	6.83E-07	UP
CTB-175E5	2.540266	1.499607	0.287608	5.214067	1.85E-07	6.83E-07	UP
CTC-471J1	1.75662	1.77735	0.340944	5.21303	1.86E-07	6.87E-07	UP
STK3	913.9458	0.460459	0.088328	5.213027	1.86E-07	6.87E-07	NOT
LINC01521	23.08088	0.886065	0.170007	5.211936	1.87E-07	6.91E-07	UP
AC007319	33.7436	-0.93616	0.179624	-5.2118	1.87E-07	6.91E-07	DOWN
SKA2	915.6316	0.505617	0.097014	5.21177	1.87E-07	6.91E-07	NOT
ERCC2	1268.162	0.58494	0.112241	5.211454	1.87E-07	6.93E-07	NOT
ENPEP	2618.782	-1.01825	0.195397	-5.2112	1.88E-07	6.93E-07	DOWN
CDC123	1977.616	0.437401	0.083946	5.21052	1.88E-07	6.96E-07	NOT

ELDR	1.557938	2.350575	0.451151	5.210172	1.89E-07	6.97E-07	UP
ERP27	46.35442	1.371778	0.26329	5.210129	1.89E-07	6.97E-07	UP
PANO1	14.17347	0.960038	0.184269	5.20997	1.89E-07	6.98E-07	UP
EN1	3.012617	3.617289	0.694405	5.209191	1.90E-07	7.01E-07	UP
C17orf59	345.2932	0.566838	0.108818	5.209037	1.90E-07	7.01E-07	NOT
TRIM56	2073.46	0.439505	0.084375	5.208935	1.90E-07	7.01E-07	NOT
GLYCTK	8233.267	-0.8783	0.16862	-5.20876	1.90E-07	7.02E-07	DOWN
PLAGL2	490.2601	0.641505	0.123166	5.208458	1.90E-07	7.03E-07	UP
CDC37P1	1.79225	2.463862	0.473068	5.208266	1.91E-07	7.04E-07	UP
MTTP	9704.45	-1.12494	0.215997	-5.20811	1.91E-07	7.04E-07	DOWN
DBNL	3458.034	0.367754	0.070634	5.206462	1.92E-07	7.10E-07	NOT
RBM34	60.57246	0.561752	0.107902	5.20612	1.93E-07	7.11E-07	NOT
MT1P1	5.217864	-1.25089	0.240298	-5.20559	1.93E-07	7.13E-07	DOWN
NPPB	2.348146	2.75144	0.528587	5.205271	1.94E-07	7.14E-07	UP
GLRX	2710.745	-0.85549	0.164351	-5.20526	1.94E-07	7.14E-07	DOWN
CTD-2012	2.810832	1.669768	0.320797	5.205066	1.94E-07	7.15E-07	UP
RP11-181(	97.26471	-1.10372	0.21205	-5.205	1.94E-07	7.15E-07	DOWN
TCHH	21.79095	-1.3533	0.260027	-5.20444	1.95E-07	7.17E-07	DOWN
CKMT2	119.4186	1.602412	0.307955	5.203395	1.96E-07	7.21E-07	UP
CCT8	4926.522	0.351379	0.067529	5.203363	1.96E-07	7.21E-07	NOT
THUMPD2	250.3437	0.480451	0.092335	5.203338	1.96E-07	7.21E-07	NOT
RP11-175I	2.970442	1.475875	0.283698	5.202275	1.97E-07	7.25E-07	UP
IL5RA	4.377374	-1.27756	0.245579	-5.20225	1.97E-07	7.25E-07	DOWN
TBCEL	949.9562	-0.52132	0.100228	-5.20131	1.98E-07	7.29E-07	NOT
YWHAE	12311.13	-0.38801	0.074607	-5.20072	1.99E-07	7.31E-07	NOT
CCDC85C	1043.248	0.67441	0.129679	5.200622	1.99E-07	7.31E-07	UP
REN	79.82443	1.704689	0.327801	5.200379	1.99E-07	7.32E-07	UP
GRP	3.122412	3.611623	0.694584	5.199695	2.00E-07	7.35E-07	UP
CTD-3131	27.76498	0.784668	0.150911	5.199558	2.00E-07	7.35E-07	UP
IFIT3	1478.178	-0.97686	0.187927	-5.19809	2.01E-07	7.41E-07	DOWN
ZNF184	192.2879	0.559269	0.107596	5.197874	2.02E-07	7.42E-07	NOT
HHAT	400.1342	0.861124	0.165681	5.19748	2.02E-07	7.43E-07	UP
NOTUM	3862.232	2.147294	0.413143	5.197461	2.02E-07	7.43E-07	UP
RP3-393E:	14.50673	1.396986	0.268786	5.197388	2.02E-07	7.43E-07	UP
NRBP2	4403.126	0.876262	0.168605	5.197115	2.02E-07	7.44E-07	UP
PRRT3-AS:	40.01482	1.196341	0.230207	5.196807	2.03E-07	7.46E-07	UP
RP11-114ℓ	118.2489	0.61341	0.118041	5.196563	2.03E-07	7.46E-07	UP
RP1-228P:	8.151117	1.299678	0.250125	5.196117	2.03E-07	7.48E-07	UP
ZNF124	102.9804	0.79791	0.153568	5.195814	2.04E-07	7.49E-07	UP
FBXO21	1540.962	-0.58305	0.112216	-5.19573	2.04E-07	7.50E-07	NOT
HMGN3-A	45.12909	0.773682	0.148915	5.195469	2.04E-07	7.50E-07	UP
ERP29	9904.136	-0.44049	0.084787	-5.19518	2.05E-07	7.52E-07	NOT
STAG3L3	39.86671	0.674154	0.129769	5.195037	2.05E-07	7.52E-07	UP
AC106786	4.105913	2.065061	0.397519	5.194872	2.05E-07	7.53E-07	UP
RP3-354N	2.05177	-1.26715	0.243968	-5.19391	2.06E-07	7.56E-07	DOWN
C19orf54	502.2068	0.602606	0.116032	5.193456	2.06E-07	7.58E-07	UP
IL13RA1	7960.381	-0.57797	0.111304	-5.19272	2.07E-07	7.61E-07	NOT
DUTP6	14.05713	0.985316	0.189761	5.192396	2.08E-07	7.62E-07	UP
FAM187B2	2.881946	-1.1515	0.221777	-5.19213	2.08E-07	7.63E-07	DOWN
DDX39B-A	3.782292	1.471917	0.283493	5.192081	2.08E-07	7.63E-07	UP
AC102953	4.720528	1.377289	0.265281	5.191812	2.08E-07	7.64E-07	UP
UPF3A	799.76	0.494274	0.095203	5.191763	2.08E-07	7.64E-07	NOT

LILRA6	52.15388	-0.91637	0.176509	-5.19162	2.08E-07	7.65E-07	DOWN
SMC2-AS:	4.121625	1.322097	0.254673	5.191352	2.09E-07	7.66E-07	UP
TULP4	718.5045	0.453418	0.087354	5.190603	2.10E-07	7.69E-07	NOT
RP13-554I	44.77144	1.0721	0.206562	5.190207	2.10E-07	7.70E-07	UP
BRPF3	2008.373	0.595035	0.11466	5.189563	2.11E-07	7.73E-07	UP
APBB1	572.0806	0.634247	0.122219	5.189431	2.11E-07	7.73E-07	UP
MBLAC1	103.4354	0.731137	0.140895	5.189235	2.11E-07	7.74E-07	UP
RP11-302I	10.15018	1.287583	0.248151	5.188711	2.12E-07	7.76E-07	UP
LL22NC03	19.95336	2.004026	0.386247	5.188453	2.12E-07	7.77E-07	UP
KIAA1671	3605.748	-0.64188	0.123718	-5.18822	2.12E-07	7.78E-07	DOWN
DDX60	991.9674	-0.97674	0.188275	-5.18784	2.13E-07	7.80E-07	DOWN
DEFB132	54.81087	2.361451	0.455199	5.187731	2.13E-07	7.80E-07	UP
C10orf55	3.948057	1.619424	0.312172	5.187605	2.13E-07	7.80E-07	UP
BMF	726.2415	1.051682	0.202738	5.187394	2.13E-07	7.81E-07	UP
SWT1	228.6708	0.498139	0.09603	5.187335	2.13E-07	7.81E-07	NOT
NODAL	3.220989	1.563919	0.3015	5.187132	2.14E-07	7.82E-07	UP
IRF4	96.62237	-1.58362	0.305311	-5.1869	2.14E-07	7.83E-07	DOWN
ICK	1421.421	0.887095	0.171042	5.186427	2.14E-07	7.85E-07	UP
AC104532	6.24313	1.013917	0.195508	5.186061	2.15E-07	7.86E-07	UP
NDUFA5	2622.587	-0.46937	0.090506	-5.18606	2.15E-07	7.86E-07	NOT
LPGAT1	7463.465	0.693989	0.133826	5.185737	2.15E-07	7.87E-07	UP
WISP3	21.54881	-1.12964	0.217848	-5.18542	2.16E-07	7.89E-07	DOWN
C1orf145	12.6209	1.556747	0.300218	5.185397	2.16E-07	7.89E-07	UP
ZMYM1	255.848	0.51166	0.09869	5.184529	2.17E-07	7.92E-07	NOT
SPRED2	1430.891	-0.5139	0.099137	-5.18376	2.17E-07	7.95E-07	NOT
SLAIN1	179.6778	-1.37451	0.265167	-5.18355	2.18E-07	7.96E-07	DOWN
KHK	11996.75	-1.01953	0.196687	-5.18349	2.18E-07	7.96E-07	DOWN
RP5-991G:	64.69704	0.793488	0.153097	5.182906	2.18E-07	7.99E-07	UP
PARD6G-/	89.66431	0.810035	0.156315	5.182067	2.19E-07	8.02E-07	UP
SEC61A1	17135.9	0.426679	0.082346	5.181509	2.20E-07	8.05E-07	NOT
AC138969	8.763247	1.081932	0.208843	5.18061	2.21E-07	8.08E-07	UP
RP11-96C:	2.381346	2.223351	0.429168	5.180604	2.21E-07	8.08E-07	UP
SERPINE2	1137.759	1.352359	0.261053	5.180397	2.21E-07	8.09E-07	UP
DHFRL1	357.7881	-0.52378	0.101109	-5.18031	2.22E-07	8.09E-07	NOT
FAM86C1	180.9356	0.60841	0.117448	5.180244	2.22E-07	8.09E-07	UP
LGALS8-A:	11.0796	1.074509	0.207449	5.17964	2.22E-07	8.12E-07	UP
PSMG3-A:	253.242	0.767825	0.148266	5.178701	2.23E-07	8.16E-07	UP
RP11-923I	2.131182	2.152943	0.415813	5.177666	2.25E-07	8.20E-07	UP
CSAD	1710.629	-0.92675	0.178998	-5.17745	2.25E-07	8.21E-07	DOWN
CASQ1	6.860961	1.311813	0.253423	5.176369	2.26E-07	8.26E-07	UP
HNRNPUL:	1824.974	0.349362	0.067497	5.175988	2.27E-07	8.27E-07	NOT
RP11-486(	34.2739	0.933843	0.180422	5.17589	2.27E-07	8.28E-07	UP
C10orf111	5.684757	0.95092	0.183726	5.175757	2.27E-07	8.28E-07	UP
HMGXB4	603.0103	0.440681	0.085149	5.175386	2.27E-07	8.30E-07	NOT
SLITRK6	19.42456	-2.15405	0.416212	-5.17537	2.27E-07	8.30E-07	DOWN
SSSCA1-A	41.98091	0.507681	0.098103	5.174971	2.28E-07	8.31E-07	NOT
CD180	124.0357	-1.02199	0.197503	-5.17456	2.28E-07	8.33E-07	DOWN
C19orf66	3458.32	-0.64892	0.125412	-5.17431	2.29E-07	8.34E-07	DOWN
RP11-536(	3.921202	1.592882	0.30788	5.173716	2.29E-07	8.37E-07	UP
STARD3	2022.838	0.396259	0.076592	5.173619	2.30E-07	8.37E-07	NOT
TMEM40	19.03553	1.850914	0.357779	5.17335	2.30E-07	8.38E-07	UP
LINC0100:	154.0877	0.645202	0.124725	5.173015	2.30E-07	8.40E-07	UP

SPTBN2	2080.544	-1.165	0.225211	-5.17291	2.30E-07	8.40E-07	DOWN
RP11-455I	5.576746	1.419409	0.274422	5.172353	2.31E-07	8.42E-07	UP
LRIG2	387.2765	0.429148	0.082973	5.17215	2.31E-07	8.43E-07	NOT
RP3-425C	253.1765	-0.61884	0.119651	-5.17202	2.32E-07	8.44E-07	DOWN
MPHOSPH	1365.497	-0.35083	0.06784	-5.17148	2.32E-07	8.46E-07	NOT
RP11-506I	7.527508	1.263713	0.24437	5.171312	2.32E-07	8.47E-07	UP
CCL4L1	152.5665	-1.2227	0.236464	-5.17075	2.33E-07	8.49E-07	DOWN
DNM1P51	2.97805	1.681746	0.325255	5.170545	2.33E-07	8.50E-07	UP
RP11-418J	96.88015	0.696405	0.134691	5.170402	2.34E-07	8.50E-07	UP
CYP20A1	1124.762	-0.35398	0.068464	-5.17037	2.34E-07	8.50E-07	NOT
RP11-707I	12.169	1.021927	0.197665	5.170002	2.34E-07	8.52E-07	UP
SAA1	91809.75	-2.29714	0.444347	-5.16971	2.34E-07	8.53E-07	DOWN
HIST1H2A	1.07636	2.161156	0.418095	5.169051	2.35E-07	8.56E-07	UP
KRTAP5-6	20.88782	1.469445	0.28429	5.168815	2.36E-07	8.57E-07	UP
LATS2	865.8923	-0.63534	0.122922	-5.16869	2.36E-07	8.57E-07	DOWN
HCG9	4.729512	1.674565	0.323983	5.168686	2.36E-07	8.57E-07	UP
LINC01353	4.902677	1.669764	0.323067	5.16848	2.36E-07	8.58E-07	UP
C10orf25	176.9302	-0.59002	0.114163	-5.16821	2.36E-07	8.59E-07	DOWN
RAD52	206.0834	0.462299	0.089463	5.1675	2.37E-07	8.63E-07	NOT
LAMTOR1	3102.683	0.410164	0.079378	5.167247	2.38E-07	8.64E-07	NOT
FRZB	318.8596	0.954368	0.184759	5.165482	2.40E-07	8.72E-07	UP
SLC26A9	19.52139	1.95947	0.379357	5.165246	2.40E-07	8.73E-07	UP
CNKSR3	1956.817	-0.67834	0.131331	-5.16513	2.40E-07	8.73E-07	DOWN
LINC01301	18.43413	0.962794	0.186404	5.165085	2.40E-07	8.73E-07	UP
RP11-561(	1.278788	1.936664	0.374997	5.16448	2.41E-07	8.76E-07	UP
FSIP2	21.33544	1.449834	0.28075	5.164143	2.42E-07	8.77E-07	UP
FBRSL1	1997.137	0.423813	0.082069	5.164087	2.42E-07	8.77E-07	NOT
RP11-109I	6.630146	1.034158	0.200262	5.16403	2.42E-07	8.78E-07	UP
CYP7A1	3382.357	1.902458	0.368426	5.163744	2.42E-07	8.79E-07	UP
MIR126	3.895135	1.678116	0.325023	5.163074	2.43E-07	8.82E-07	UP
TNS2	4072.02	-0.6143	0.119006	-5.16193	2.44E-07	8.87E-07	DOWN
IHH	301.543	1.472028	0.285189	5.161587	2.45E-07	8.89E-07	UP
CEP19	49.02413	0.683822	0.132489	5.161353	2.45E-07	8.90E-07	UP
RP13-638(	1.55982	1.942448	0.376356	5.161206	2.45E-07	8.90E-07	UP
RP11-67L3	18.99137	-0.91968	0.178199	-5.16094	2.46E-07	8.91E-07	DOWN
NPIPP1	83.38031	0.734248	0.142273	5.160827	2.46E-07	8.92E-07	UP
IMP3	2148.766	-0.5037	0.09761	-5.16031	2.47E-07	8.94E-07	NOT
RP11-383I	170.3252	1.4641	0.283793	5.159046	2.48E-07	9.00E-07	UP
IKBKG	410.3723	0.811532	0.157325	5.158322	2.49E-07	9.04E-07	UP
PDE6D	531.6123	0.351557	0.068155	5.158229	2.49E-07	9.04E-07	NOT
MDGA2	25.43421	2.925562	0.567175	5.15813	2.49E-07	9.04E-07	UP
GATAD1	1331.662	0.423757	0.082154	5.158081	2.49E-07	9.04E-07	NOT
RP11-494I	1.202343	2.252003	0.436605	5.157987	2.50E-07	9.05E-07	UP
RP11-538I	32.68846	1.833899	0.35557	5.157627	2.50E-07	9.06E-07	UP
FOXRED1	2292.467	-0.49632	0.096239	-5.15722	2.51E-07	9.08E-07	NOT
bP-21264(	10.99706	1.046273	0.202879	5.157121	2.51E-07	9.08E-07	UP
SSBP3	2870.763	-0.51227	0.099364	-5.15552	2.53E-07	9.16E-07	NOT
CYP2E1	173107.7	-2.00238	0.388409	-5.15533	2.53E-07	9.17E-07	DOWN
CTD-2278	2.600175	1.431777	0.277728	5.155325	2.53E-07	9.17E-07	UP
NSMAF	504.0972	0.573877	0.11133	5.154717	2.54E-07	9.20E-07	NOT
NFXL1	361.9373	0.578456	0.112234	5.15402	2.55E-07	9.23E-07	NOT
RP11-499(	22.06491	1.126902	0.218661	5.153642	2.55E-07	9.25E-07	UP

RP11-192I	12.1517	1.011073	0.196206	5.153128	2.56E-07	9.27E-07	UP
SLC4A1	9.86853	-1.79846	0.349012	-5.15302	2.56E-07	9.28E-07	DOWN
SLC4A1AP	987.6361	0.28223	0.054776	5.152442	2.57E-07	9.30E-07	NOT
ACTRT3	99.52104	-0.72298	0.140346	-5.1514	2.59E-07	9.35E-07	DOWN
RP11-65J2	5.021624	2.797929	0.543198	5.150848	2.59E-07	9.38E-07	UP
ZNF445	658.7274	0.406799	0.07898	5.150684	2.60E-07	9.39E-07	NOT
CES3	2139.817	-1.20081	0.233165	-5.15005	2.60E-07	9.42E-07	DOWN
P2RY8	319.8879	1.057276	0.205307	5.149729	2.61E-07	9.43E-07	UP
SLC16A6P	2.052174	1.952879	0.379275	5.148985	2.62E-07	9.47E-07	UP
KCNC2	33.96262	2.856774	0.554885	5.148406	2.63E-07	9.50E-07	UP
RP11-731	2.099601	2.350877	0.456626	5.148369	2.63E-07	9.50E-07	UP
PDCD6IPP	13.31775	1.335504	0.259406	5.148319	2.63E-07	9.50E-07	UP
RPRD2	2026.822	0.446358	0.086702	5.148172	2.63E-07	9.50E-07	NOT
DDX59	726.2256	0.498622	0.096854	5.148172	2.63E-07	9.50E-07	NOT
CANX	34699.58	0.434987	0.084494	5.148146	2.63E-07	9.50E-07	NOT
RAB23	130.9976	0.789786	0.153429	5.147573	2.64E-07	9.53E-07	UP
CAPN14	3.940922	1.877471	0.364781	5.146839	2.65E-07	9.57E-07	UP
PAK1	1250.438	0.574075	0.11154	5.146827	2.65E-07	9.57E-07	NOT
RP3-368A	63.47905	0.80285	0.156007	5.146255	2.66E-07	9.60E-07	UP
RP11-52L5	2.853489	3.417992	0.664197	5.146049	2.66E-07	9.60E-07	UP
RP11-713I	536.8692	1.035283	0.20118	5.14604	2.66E-07	9.60E-07	UP
PTGDR	20.71345	-1.1076	0.215258	-5.14543	2.67E-07	9.63E-07	DOWN
DCAF15	825.0536	0.553308	0.107534	5.145423	2.67E-07	9.63E-07	NOT
IRF5	495.5519	0.578586	0.112467	5.144475	2.68E-07	9.68E-07	NOT
TCAIM	2074.175	-0.49137	0.095519	-5.14429	2.69E-07	9.69E-07	NOT
GPRC5A	82.02497	-1.50154	0.291912	-5.14381	2.69E-07	9.71E-07	DOWN
SURF4	15537.35	-0.3593	0.069868	-5.14258	2.71E-07	9.77E-07	NOT
PCGF5	2792.383	-0.44935	0.087396	-5.14147	2.73E-07	9.83E-07	NOT
ZNF511	644.2908	-0.59016	0.114816	-5.14004	2.75E-07	9.91E-07	DOWN
OXSM	670.8885	-0.41071	0.079911	-5.13966	2.75E-07	9.92E-07	NOT
GIMAP8	551.0968	-0.73182	0.142395	-5.13938	2.76E-07	9.94E-07	DOWN
OVOL1	47.10288	2.235321	0.435036	5.138244	2.77E-07	1.00E-06	UP
P2RX2	7.543045	2.833065	0.55145	5.137481	2.78E-07	1.00E-06	UP
SOX9	1441.911	1.304514	0.253927	5.137353	2.79E-07	1.00E-06	UP
CDC40	604.7249	-0.50378	0.09807	-5.13688	2.79E-07	1.01E-06	NOT
RP11-411I	4.895644	1.867612	0.363574	5.136809	2.79E-07	1.01E-06	UP
HPCA	2.715717	1.931086	0.375951	5.136532	2.80E-07	1.01E-06	UP
RPL36A	2128.918	0.844087	0.164352	5.135834	2.81E-07	1.01E-06	UP
IGKV6D-2	5.195616	-2.20342	0.42906	-5.13547	2.81E-07	1.01E-06	DOWN
C3orf36	8.043734	1.575054	0.306719	5.135176	2.82E-07	1.02E-06	UP
CHRFAM7	3.249779	2.357381	0.459087	5.134928	2.82E-07	1.02E-06	UP
CCDC150I	9.291584	1.294126	0.252027	5.134878	2.82E-07	1.02E-06	UP
CTD-2349	3.506284	1.383739	0.269495	5.134566	2.83E-07	1.02E-06	UP
PIGX	610.3887	0.393238	0.076588	5.134471	2.83E-07	1.02E-06	NOT
UBL4B	2.443904	3.246683	0.632336	5.134426	2.83E-07	1.02E-06	UP
ALG3	3189.164	0.467542	0.091066	5.134078	2.84E-07	1.02E-06	NOT
XXbac-BP	7.45636	1.121124	0.218371	5.134023	2.84E-07	1.02E-06	UP
CXorf57	128.0584	1.001167	0.195028	5.133448	2.84E-07	1.02E-06	UP
KIAA0232	2073.771	0.43133	0.084043	5.132281	2.86E-07	1.03E-06	NOT
PTGES3P1	108.5455	0.676333	0.131793	5.131783	2.87E-07	1.03E-06	UP
RP1-142L7	7.368802	-1.556	0.303216	-5.13164	2.87E-07	1.03E-06	DOWN
EDA2R	84.1313	1.453807	0.283343	5.130919	2.88E-07	1.04E-06	UP

RALY-AS1	86.24992	0.713525	0.13907	5.130678	2.89E-07	1.04E-06	UP
AC005592	5.243534	1.817266	0.354242	5.130007	2.90E-07	1.04E-06	UP
CHGB	9.857919	2.280236	0.444499	5.129906	2.90E-07	1.04E-06	UP
AC015849	11.48543	1.042953	0.203328	5.129407	2.91E-07	1.04E-06	UP
KLHL21	1865.525	0.705663	0.137573	5.129376	2.91E-07	1.04E-06	UP
ZFP82	86.53611	1.02462	0.199756	5.129365	2.91E-07	1.04E-06	UP
RP11-750I	24.986	-1.06727	0.208145	-5.12753	2.94E-07	1.05E-06	DOWN
CDH10	7.268276	4.258439	0.830589	5.127014	2.94E-07	1.06E-06	UP
ODF3L1	38.07039	-1.35849	0.264972	-5.12692	2.95E-07	1.06E-06	DOWN
DMRTA1	550.762	-1.05271	0.205332	-5.12689	2.95E-07	1.06E-06	DOWN
AK2	5133.59	-0.41575	0.081098	-5.12656	2.95E-07	1.06E-06	NOT
TMEM56-	60.88646	-0.74403	0.145139	-5.12631	2.95E-07	1.06E-06	DOWN
RP11-131I	8.211585	1.053433	0.205505	5.126076	2.96E-07	1.06E-06	UP
RP4-607I7	3.179021	2.373561	0.463096	5.125418	2.97E-07	1.07E-06	UP
FAM99A	1007.71	-2.00868	0.391929	-5.12511	2.97E-07	1.07E-06	DOWN
SNCAIP	121.2795	1.266048	0.247049	5.124689	2.98E-07	1.07E-06	UP
RP11-383I	5.908202	-1.24604	0.243188	-5.12378	2.99E-07	1.07E-06	DOWN
LINC00471	8.508934	0.943066	0.18406	5.123685	3.00E-07	1.07E-06	UP
RP11-663I	1.030843	2.403485	0.469109	5.123507	3.00E-07	1.08E-06	UP
LL22NC03	1.314126	2.748903	0.536551	5.123281	3.00E-07	1.08E-06	UP
GAMT	16224.73	-0.9351	0.182524	-5.12316	3.00E-07	1.08E-06	DOWN
RAP1B	1213.386	-0.34204	0.066765	-5.12303	3.01E-07	1.08E-06	NOT
FBXO9	2177.505	0.500817	0.097781	5.121842	3.03E-07	1.08E-06	NOT
PACS1	1940.382	0.633243	0.123638	5.121749	3.03E-07	1.09E-06	UP
PTGFRN	1834.729	0.802221	0.156634	5.121625	3.03E-07	1.09E-06	UP
HSD17B2	6000.07	-1.14692	0.223942	-5.12151	3.03E-07	1.09E-06	DOWN
LRRN4CL	10.94529	1.723068	0.336458	5.121196	3.04E-07	1.09E-06	UP
AKR7L	335.9055	-0.9761	0.190602	-5.12113	3.04E-07	1.09E-06	DOWN
PIPOX	17869.94	-1.07833	0.210573	-5.12093	3.04E-07	1.09E-06	DOWN
CTD-2535	2.132451	3.553886	0.694166	5.119648	3.06E-07	1.10E-06	UP
AP001628.	2.152143	1.720459	0.336066	5.119401	3.07E-07	1.10E-06	UP
ZNF723P	4.301897	3.900729	0.761982	5.119188	3.07E-07	1.10E-06	UP
ELF2	831.0321	-0.35806	0.069955	-5.11849	3.08E-07	1.10E-06	NOT
RP11-30K!	7.908812	0.943246	0.184288	5.118325	3.08E-07	1.10E-06	UP
RP11-13P!	1.721671	2.363947	0.461891	5.117981	3.09E-07	1.11E-06	UP
JAGN1	2317.51	-0.46061	0.090002	-5.11779	3.09E-07	1.11E-06	NOT
GALNT13	10.88705	1.820679	0.355763	5.11767	3.09E-07	1.11E-06	UP
ACSF3	1224.513	-0.60342	0.11791	-5.11765	3.09E-07	1.11E-06	DOWN
RP11-755I	25.19357	0.726539	0.14197	5.117538	3.10E-07	1.11E-06	UP
AC005534	2.380451	1.669592	0.326271	5.117191	3.10E-07	1.11E-06	UP
C16orf96	16.4016	-1.06668	0.208462	-5.11689	3.11E-07	1.11E-06	DOWN
PSMA2	1032.77	-0.54351	0.106222	-5.11672	3.11E-07	1.11E-06	NOT
TRUB2	2469.142	-0.48013	0.093843	-5.11627	3.12E-07	1.11E-06	NOT
TTI1	1088.19	0.319102	0.062376	5.115808	3.12E-07	1.12E-06	NOT
DNALI1	682.8753	-1.1116	0.217306	-5.11538	3.13E-07	1.12E-06	DOWN
AATBC	16.92495	1.009295	0.197313	5.115195	3.13E-07	1.12E-06	UP
CBLL1	875.3217	0.402552	0.0787	5.114997	3.14E-07	1.12E-06	NOT
PNMAL2	44.21245	-0.90938	0.177789	-5.11494	3.14E-07	1.12E-06	DOWN
CHMP3	1333.673	0.444843	0.086973	5.114709	3.14E-07	1.12E-06	NOT
IFIH1	965.6905	-0.68053	0.133066	-5.11423	3.15E-07	1.13E-06	DOWN
CTC-487M	15.66375	0.875442	0.171203	5.113483	3.16E-07	1.13E-06	UP
APOBEC2	4.974824	1.290553	0.252422	5.112678	3.18E-07	1.13E-06	UP

RP4-597N	4.321872	1.442696	0.282191	5.112481	3.18E-07	1.14E-06	UP
FETUB	5287.228	-1.57851	0.30877	-5.11227	3.18E-07	1.14E-06	DOWN
CTC-429P	10.8467	0.880346	0.172207	5.112143	3.19E-07	1.14E-06	UP
AMOTL2	1634.914	-0.81896	0.160248	-5.11059	3.21E-07	1.15E-06	DOWN
FP236383.:	2.265485	-2.33554	0.457128	-5.10916	3.24E-07	1.15E-06	DOWN
RWDD4	305.7566	-0.40294	0.078866	-5.1091	3.24E-07	1.16E-06	NOT
RP11-103ℰ	9.077767	3.612558	0.707097	5.109001	3.24E-07	1.16E-06	UP
KLF4	694.3155	-1.06147	0.207783	-5.10857	3.25E-07	1.16E-06	DOWN
RP11-399I	43.28769	1.184627	0.231898	5.108402	3.25E-07	1.16E-06	UP
ANGPT1	134.4821	0.978017	0.191457	5.108286	3.25E-07	1.16E-06	UP
MARK2	1246.297	0.391408	0.076635	5.107448	3.27E-07	1.16E-06	NOT
KIR3DL1	3.623951	-1.55462	0.304388	-5.10737	3.27E-07	1.16E-06	DOWN
CTD-2537	6.713261	1.035286	0.202724	5.106869	3.28E-07	1.17E-06	UP
ZSCAN25	400.1707	0.374662	0.073371	5.106434	3.28E-07	1.17E-06	NOT
AC006262	2.680929	3.082791	0.603766	5.105938	3.29E-07	1.17E-06	UP
RP11-522I	1.665528	-2.54981	0.499399	-5.10576	3.29E-07	1.17E-06	DOWN
LMOD1	547.8371	1.321824	0.258928	5.104981	3.31E-07	1.18E-06	UP
BDKRB2	228.0679	1.22629	0.240237	5.104503	3.32E-07	1.18E-06	UP
COL5A1	2663.033	1.261911	0.247292	5.102922	3.34E-07	1.19E-06	UP
ZNF221	23.0583	0.999994	0.195975	5.102662	3.35E-07	1.19E-06	UP
MEX3B	64.74065	0.973851	0.190862	5.102371	3.35E-07	1.19E-06	UP
KIF22	1788.931	0.620503	0.121611	5.102359	3.35E-07	1.19E-06	UP
GMPPB	1203.687	0.468504	0.091823	5.102258	3.36E-07	1.20E-06	NOT
RP11-347(	6.060087	1.031469	0.202164	5.102137	3.36E-07	1.20E-06	UP
C12orf65	682.1058	0.361577	0.070868	5.10211	3.36E-07	1.20E-06	NOT
DOC2GP	14.37974	1.104345	0.216453	5.102017	3.36E-07	1.20E-06	UP
CH17-13I:	5.544728	1.892105	0.37091	5.101251	3.37E-07	1.20E-06	UP
SLC8A2	5.289745	1.799016	0.352666	5.101183	3.38E-07	1.20E-06	UP
SAP30	209.5495	0.622003	0.121935	5.101092	3.38E-07	1.20E-06	UP
C6orf47	973.015	0.496055	0.097252	5.10069	3.38E-07	1.20E-06	NOT
ARHGAP2(	79.41326	-1.13449	0.22243	-5.10044	3.39E-07	1.21E-06	DOWN
LDB2	659.4476	-0.78899	0.154703	-5.10006	3.40E-07	1.21E-06	DOWN
GPR1-AS	7.422548	4.191977	0.822017	5.099623	3.40E-07	1.21E-06	UP
CTD-2256	1.443189	1.811217	0.355229	5.098726	3.42E-07	1.22E-06	UP
CTD-2384	4.148389	4.025533	0.789536	5.098605	3.42E-07	1.22E-06	UP
ISM2	3.553161	3.549109	0.696115	5.09845	3.42E-07	1.22E-06	UP
SLC4A4	1825.256	-1.12318	0.220299	-5.09841	3.43E-07	1.22E-06	DOWN
KB-226F1.:	41.33872	1.300818	0.255159	5.098068	3.43E-07	1.22E-06	UP
RP11-214I	2.398141	1.61365	0.316549	5.097623	3.44E-07	1.22E-06	UP
AP4B1	432.6083	0.408291	0.080095	5.097598	3.44E-07	1.22E-06	NOT
C3orf35	16.97021	0.824705	0.161799	5.097109	3.45E-07	1.23E-06	UP
HTR4	14.71515	1.88637	0.370087	5.097094	3.45E-07	1.23E-06	UP
AQP5	5.900886	1.608021	0.315485	5.096974	3.45E-07	1.23E-06	UP
CEACAMP	3.797727	3.043974	0.597227	5.096849	3.45E-07	1.23E-06	UP
RP1-239B:	83.69008	1.451613	0.284886	5.095423	3.48E-07	1.24E-06	UP
HDAC11-/	1.248517	1.877951	0.368565	5.095311	3.48E-07	1.24E-06	UP
DNM3OS	50.52233	-1.09378	0.214679	-5.09495	3.49E-07	1.24E-06	DOWN
OTC	5624.477	-1.38328	0.271523	-5.09454	3.50E-07	1.24E-06	DOWN
SLC25A12	234.935	0.915056	0.179633	5.094043	3.51E-07	1.24E-06	UP
RET	186.939	-1.80317	0.353981	-5.09396	3.51E-07	1.24E-06	DOWN
FAT4	388.4038	-1.08956	0.213911	-5.09353	3.51E-07	1.25E-06	DOWN
CD274	118.5054	-1.06142	0.208406	-5.09304	3.52E-07	1.25E-06	DOWN

RP11-834(	5.632001	-1.18442	0.232566	-5.09285	3.53E-07	1.25E-06	DOWN
ARHGAP4(	12.28942	2.514976	0.49383	5.092801	3.53E-07	1.25E-06	UP
DDIT4L	24.90352	1.32849	0.260905	5.091851	3.55E-07	1.26E-06	UP
RP11-475(	3.347951	-1.56318	0.30701	-5.09164	3.55E-07	1.26E-06	DOWN
JPH2	28.35798	1.099467	0.215956	5.091173	3.56E-07	1.26E-06	UP
KIAA1586	244.8595	0.475114	0.093324	5.090996	3.56E-07	1.26E-06	NOT
CEACAM3	8.063241	-1.50401	0.295431	-5.09092	3.56E-07	1.26E-06	DOWN
IRF2	1366.004	-0.43408	0.085279	-5.09014	3.58E-07	1.27E-06	NOT
RP1-39G2:	138.6977	0.588933	0.115702	5.090081	3.58E-07	1.27E-06	UP
AC005537	5.846162	3.069956	0.60315	5.089871	3.58E-07	1.27E-06	UP
LGR5	476.7912	2.227481	0.437638	5.089777	3.58E-07	1.27E-06	UP
KLHL22	805.3666	0.441584	0.086763	5.08954	3.59E-07	1.27E-06	NOT
FAM135B	36.12881	1.720645	0.338078	5.089493	3.59E-07	1.27E-06	UP
RP11-179(	12.9054	0.985909	0.193718	5.089409	3.59E-07	1.27E-06	UP
RP11-219(	8.269042	2.242194	0.440764	5.087067	3.64E-07	1.29E-06	UP
ABHD17C	419.9644	0.718322	0.141228	5.086253	3.65E-07	1.29E-06	UP
ITGAE	327.9344	0.806396	0.158545	5.086218	3.65E-07	1.29E-06	UP
RGS12	1466.677	0.510558	0.100383	5.086117	3.65E-07	1.29E-06	NOT
UNC119	859.6747	0.663375	0.130438	5.085761	3.66E-07	1.30E-06	UP
PPP1R14BI	375.0078	0.783491	0.154065	5.085458	3.67E-07	1.30E-06	UP
RP11-311I	2.761026	3.794608	0.746174	5.085419	3.67E-07	1.30E-06	UP
MAPK8	1151.414	-0.35156	0.069132	-5.0854	3.67E-07	1.30E-06	NOT
CTC-205M	16.3714	1.018766	0.200368	5.084469	3.69E-07	1.30E-06	UP
GAS2L1P2	2.885505	2.851194	0.560805	5.084107	3.69E-07	1.31E-06	UP
MIR1254-:	2.76218	1.567914	0.308397	5.084081	3.69E-07	1.31E-06	UP
SEC22C	911.7223	0.314555	0.061874	5.083753	3.70E-07	1.31E-06	NOT
CTH	3736.4	-1.33249	0.262107	-5.08375	3.70E-07	1.31E-06	DOWN
EPSTI1	540.8995	-1.13915	0.224112	-5.08296	3.72E-07	1.31E-06	DOWN
AC000095	2.473542	2.150288	0.423048	5.082844	3.72E-07	1.31E-06	UP
TF	414297.4	-1.1342	0.223156	-5.08253	3.72E-07	1.32E-06	DOWN
TLK2	839.3376	0.328342	0.064609	5.081999	3.73E-07	1.32E-06	NOT
TOM1L2	1097.705	0.523024	0.102918	5.08193	3.74E-07	1.32E-06	NOT
3-Mar	116.9192	0.957138	0.188343	5.081893	3.74E-07	1.32E-06	UP
RP11-407/	3.997064	3.84784	0.757173	5.08185	3.74E-07	1.32E-06	UP
RP11-119I	11.63112	0.849476	0.167174	5.081395	3.75E-07	1.32E-06	UP
TRIOBP	1000.676	0.552343	0.108703	5.08123	3.75E-07	1.32E-06	NOT
GPAT2	69.53154	1.161986	0.228685	5.081167	3.75E-07	1.32E-06	UP
RP11-455(	2.03536	1.720954	0.338699	5.081079	3.75E-07	1.32E-06	UP
SPAG7	1820.294	-0.546	0.107462	-5.08082	3.76E-07	1.33E-06	NOT
CSTL1	3.327942	2.537071	0.499351	5.080732	3.76E-07	1.33E-06	UP
CRADD	917.2767	-0.59948	0.117992	-5.08069	3.76E-07	1.33E-06	DOWN
RP11-102:	44.63645	0.82014	0.161426	5.080584	3.76E-07	1.33E-06	UP
ATP10A	142.503	1.286601	0.253246	5.080433	3.77E-07	1.33E-06	UP
MICU3	245.0744	-0.94172	0.185373	-5.08015	3.77E-07	1.33E-06	DOWN
UNC13B	2510.279	0.759245	0.149456	5.080043	3.77E-07	1.33E-06	UP
CTB-13F3.	12.54954	1.032904	0.203341	5.079667	3.78E-07	1.33E-06	UP
FOXP4	3117.06	0.505724	0.099562	5.079511	3.78E-07	1.33E-06	NOT
EEF1E1P1	3.925975	1.382487	0.272188	5.079162	3.79E-07	1.34E-06	UP
MGMT	4706.473	-0.82828	0.163099	-5.07843	3.81E-07	1.34E-06	DOWN
RP5-915N	1.741547	1.750458	0.344689	5.078363	3.81E-07	1.34E-06	UP
DHRS3	12351.17	-0.64579	0.127165	-5.07835	3.81E-07	1.34E-06	DOWN
SUGP1	1100.346	0.371375	0.073129	5.078322	3.81E-07	1.34E-06	NOT

SLC2A14	49.22695	1.833431	0.361046	5.078107	3.81E-07	1.34E-06	UP
FAM109B	209.212	1.104678	0.217544	5.077948	3.82E-07	1.34E-06	UP
CCDC132	592.6885	0.414948	0.08173	5.077081	3.83E-07	1.35E-06	NOT
ZBTB43	514.0857	-0.51647	0.101733	-5.07675	3.84E-07	1.35E-06	NOT
TRIM60P1	1.536741	2.330874	0.459139	5.076624	3.84E-07	1.35E-06	UP
ANKRD39	549.4982	0.491519	0.096837	5.075718	3.86E-07	1.36E-06	NOT
CD82	2490.773	-0.68757	0.135482	-5.07497	3.88E-07	1.36E-06	DOWN
HIST1H2A	1.262326	2.28353	0.449968	5.074878	3.88E-07	1.37E-06	UP
DPYS	14428.76	-1.31112	0.258396	-5.07407	3.89E-07	1.37E-06	DOWN
RP11-624I	5.013509	1.3624	0.268533	5.073495	3.91E-07	1.37E-06	UP
FAM127A	2177.708	0.766473	0.151095	5.072774	3.92E-07	1.38E-06	UP
OPA3	1387.511	0.476763	0.093986	5.072719	3.92E-07	1.38E-06	NOT
PTMA	15800.31	0.387367	0.076371	5.072166	3.93E-07	1.38E-06	NOT
RPSAP15	5.217318	1.120895	0.220997	5.072002	3.94E-07	1.38E-06	UP
RP11-34P1	36.83621	1.354213	0.26706	5.070818	3.96E-07	1.39E-06	UP
RP5-902P8	19.04871	0.954673	0.188281	5.070466	3.97E-07	1.40E-06	UP
SLC44A3	721.8567	1.062139	0.209506	5.069736	3.98E-07	1.40E-06	UP
DNAH1	685.8891	0.841026	0.165909	5.069202	3.99E-07	1.40E-06	UP
TFR2	43071.72	-1.0683	0.210751	-5.069	4.00E-07	1.41E-06	DOWN
RP5-1158I	1.241332	2.43676	0.480754	5.068625	4.01E-07	1.41E-06	UP
NOTCH4	925.8662	0.60642	0.119648	5.068363	4.01E-07	1.41E-06	UP
WNT6	9.469523	1.376147	0.271539	5.067947	4.02E-07	1.41E-06	UP
AC010518	1.390396	-1.96356	0.387485	-5.06746	4.03E-07	1.42E-06	DOWN
RP11-234I	3.850983	1.764645	0.348238	5.06735	4.03E-07	1.42E-06	UP
EIF4A3	2530.818	0.425342	0.083944	5.067005	4.04E-07	1.42E-06	NOT
RP11-16K1	3.281781	1.962712	0.387371	5.066751	4.05E-07	1.42E-06	UP
RP11-433A	4.866256	1.627225	0.321179	5.066406	4.05E-07	1.42E-06	UP
IL1R1	4834.887	-0.6977	0.137717	-5.06618	4.06E-07	1.43E-06	DOWN
FLNA	10391.05	1.008973	0.199162	5.066083	4.06E-07	1.43E-06	UP
RP1-315G1	5.630225	1.355893	0.267643	5.066062	4.06E-07	1.43E-06	UP
ZNF503-A	148.0486	0.698203	0.137821	5.066023	4.06E-07	1.43E-06	UP
TUBB8P7	3.59072	2.084218	0.411422	5.065895	4.06E-07	1.43E-06	UP
CXCL5	164.4705	2.349632	0.463854	5.065454	4.07E-07	1.43E-06	UP
MRPL44	1470.852	-0.36749	0.072552	-5.06523	4.08E-07	1.43E-06	NOT
TMPRSS5	14.54929	1.038462	0.205021	5.065153	4.08E-07	1.43E-06	UP
RP11-460I	1.366003	1.943132	0.383655	5.064783	4.09E-07	1.43E-06	UP
TAS2R6P	1.632062	2.038307	0.402518	5.063894	4.11E-07	1.44E-06	UP
NR1H4	3510.481	-0.73183	0.144525	-5.06373	4.11E-07	1.44E-06	DOWN
ASAH2B	218.4801	-0.5189	0.102481	-5.06345	4.12E-07	1.44E-06	NOT
NMUR1	43.16721	-0.93498	0.184665	-5.06313	4.12E-07	1.45E-06	DOWN
CPN2	13719.27	-1.16826	0.230745	-5.06297	4.13E-07	1.45E-06	DOWN
MGC27382	38.02762	-1.13153	0.223514	-5.06244	4.14E-07	1.45E-06	DOWN
RP11-82L1	17.18769	1.369965	0.270735	5.060166	4.19E-07	1.47E-06	UP
ZNF260	568.5408	0.476812	0.094232	5.060007	4.19E-07	1.47E-06	NOT
KCNJ9	1.826694	2.340341	0.46253	5.059864	4.20E-07	1.47E-06	UP
LRRC37A2	67.32402	0.724294	0.143148	5.059771	4.20E-07	1.47E-06	UP
RP11-158I	6.589862	1.347719	0.266381	5.05937	4.21E-07	1.47E-06	UP
XKRX	6.719287	1.67523	0.331117	5.059323	4.21E-07	1.47E-06	UP
ANKRD2	14.95229	1.617136	0.319641	5.059227	4.21E-07	1.47E-06	UP
TMUB2	1510.253	0.335389	0.066298	5.058818	4.22E-07	1.48E-06	NOT
NAT16	5.22704	1.610348	0.318338	5.058618	4.22E-07	1.48E-06	UP
SLC1A5	1253.285	1.206789	0.238565	5.05853	4.23E-07	1.48E-06	UP

MIR17HG	36.25269	1.171325	0.231569	5.058217	4.23E-07	1.48E-06	UP
RP11-484I	2.225038	1.650709	0.326369	5.057807	4.24E-07	1.48E-06	UP
AC003003	1.856658	2.095591	0.414332	5.057764	4.24E-07	1.48E-06	UP
C2orf16	90.31059	0.915933	0.181099	5.057646	4.24E-07	1.49E-06	UP
ABHD17A	986.7692	0.543668	0.107498	5.057481	4.25E-07	1.49E-06	NOT
SUCLG2P2	22.14626	-0.94864	0.187595	-5.05686	4.26E-07	1.49E-06	DOWN
SETD1A	1292.564	0.333755	0.066013	5.055897	4.28E-07	1.50E-06	NOT
NOL11	1694.176	0.378565	0.074877	5.055852	4.28E-07	1.50E-06	NOT
RP11-114(	52.07549	2.055965	0.4067	5.055232	4.30E-07	1.50E-06	UP
RP11-1C8.	4.463812	1.243818	0.24613	5.053504	4.34E-07	1.52E-06	UP
SAP30L	1491.692	0.430508	0.085197	5.053081	4.35E-07	1.52E-06	NOT
CYSTM1	3107.679	0.637235	0.126119	5.052662	4.36E-07	1.52E-06	UP
NOX5	7.157417	1.68469	0.333477	5.051891	4.37E-07	1.53E-06	UP
LST1	377.0554	-0.86256	0.170762	-5.05122	4.39E-07	1.53E-06	DOWN
HIST1H1E	9.784916	1.224935	0.24252	5.050853	4.40E-07	1.54E-06	UP
ITGA5	4891.944	0.678193	0.134277	5.050707	4.40E-07	1.54E-06	UP
RP13-225(	3.367443	2.03055	0.402078	5.050137	4.41E-07	1.54E-06	UP
ACTA1	10.89423	1.171643	0.232003	5.050123	4.42E-07	1.54E-06	UP
RP11-802I	3.070217	1.390767	0.275394	5.050105	4.42E-07	1.54E-06	UP
MSMO1	13391.05	-0.87799	0.173857	-5.05009	4.42E-07	1.54E-06	DOWN
FAM185A	226.4731	-0.45356	0.089822	-5.04957	4.43E-07	1.55E-06	NOT
RP11-410I	2.961616	-1.33144	0.263689	-5.0493	4.43E-07	1.55E-06	DOWN
ARPIN	1031.811	0.494593	0.097954	5.049264	4.44E-07	1.55E-06	NOT
FBXL7	270.7895	0.915836	0.181383	5.049175	4.44E-07	1.55E-06	UP
RP11-57A:	3.413492	1.969491	0.390175	5.047714	4.47E-07	1.56E-06	UP
MIR663AH	4.686536	4.44404	0.88042	5.047633	4.47E-07	1.56E-06	UP
CNTNAP3	57.59433	1.525571	0.302235	5.047627	4.47E-07	1.56E-06	UP
TMED10	11156.42	-0.37264	0.073826	-5.04754	4.48E-07	1.56E-06	NOT
SCGB2A1	8.104331	2.149897	0.425945	5.047353	4.48E-07	1.56E-06	UP
ADH5	9817.962	-0.50139	0.099369	-5.04568	4.52E-07	1.58E-06	NOT
RP11-513(	118.5057	-1.43918	0.285235	-5.04561	4.52E-07	1.58E-06	DOWN
NUPR1	11347.24	0.998762	0.197958	5.045331	4.53E-07	1.58E-06	UP
CD68	93.41392	-0.96412	0.191118	-5.04462	4.54E-07	1.58E-06	DOWN
KB-1471A	4.3464	1.244437	0.246706	5.04421	4.55E-07	1.59E-06	UP
RP11-107(	2.828711	3.773876	0.748222	5.043794	4.56E-07	1.59E-06	UP
S100A6	3918.004	1.301021	0.257954	5.043616	4.57E-07	1.59E-06	UP
RP11-767I	10.38907	-1.71191	0.339434	-5.04343	4.57E-07	1.59E-06	DOWN
ANKRD49	328.823	0.370599	0.073485	5.043165	4.58E-07	1.60E-06	NOT
UQCRH	4135.489	0.59009	0.117032	5.042113	4.60E-07	1.60E-06	UP
RP11-49I1	14.58289	1.036443	0.205579	5.041582	4.62E-07	1.61E-06	UP
RAC3	675.4705	0.9415	0.186749	5.04153	4.62E-07	1.61E-06	UP
LMBRD1	2796.774	-0.50528	0.100229	-5.04122	4.63E-07	1.61E-06	NOT
KRT85	17.23495	2.268658	0.450026	5.041173	4.63E-07	1.61E-06	UP
RP11-285(	1.737532	2.200337	0.436491	5.040968	4.63E-07	1.61E-06	UP
CTB-40H1	2.87561	1.735848	0.344357	5.040846	4.63E-07	1.61E-06	UP
PRAMEF8	3.765755	3.09956	0.614999	5.039943	4.66E-07	1.62E-06	UP
TFPI2	221.8424	-1.49953	0.297534	-5.03987	4.66E-07	1.62E-06	DOWN
CTD-2537	3.759858	1.332065	0.264351	5.039003	4.68E-07	1.63E-06	UP
NWD2	26.17108	2.344425	0.465298	5.038549	4.69E-07	1.63E-06	UP
RP11-107(	2.999058	3.556369	0.705883	5.038188	4.70E-07	1.63E-06	UP
C19orf38	76.08924	-0.70698	0.140335	-5.0378	4.71E-07	1.64E-06	DOWN
RP11-336/	6.288933	1.565567	0.310773	5.037662	4.71E-07	1.64E-06	UP

RRS1	1113.529	0.763803	0.151632	5.037206	4.72E-07	1.64E-06	UP
CTD-2553	2.625211	1.987951	0.394665	5.037055	4.73E-07	1.64E-06	UP
MIR3685	8.040924	1.145719	0.227465	5.036903	4.73E-07	1.64E-06	UP
MAP3K2	2303.828	-0.42051	0.083489	-5.03672	4.74E-07	1.65E-06	NOT
BDH2P1	4.025006	-1.58021	0.313776	-5.0361	4.75E-07	1.65E-06	DOWN
RP4-742C:	18.90335	1.077139	0.213891	5.035915	4.76E-07	1.65E-06	UP
RP11-944(	10.80381	1.181622	0.234645	5.035789	4.76E-07	1.65E-06	UP
MAPKAPK2	4838.656	0.481403	0.09561	5.035058	4.78E-07	1.66E-06	NOT
RP11-346(	13.62699	0.807791	0.160434	5.03504	4.78E-07	1.66E-06	UP
TPD52L1	1316.815	-0.95156	0.18899	-5.03497	4.78E-07	1.66E-06	DOWN
RP11-290I	34.36804	1.116575	0.221794	5.03428	4.80E-07	1.67E-06	UP
TIAM2	188.9626	0.812953	0.161495	5.033904	4.81E-07	1.67E-06	UP
EIF3H	7133.156	0.544135	0.108101	5.033574	4.81E-07	1.67E-06	NOT
HSDL2	6867.598	-0.63066	0.125292	-5.03351	4.82E-07	1.67E-06	DOWN
PRR14	1064.295	0.315457	0.062672	5.033478	4.82E-07	1.67E-06	NOT
SLC2A2	22796.78	-1.2196	0.242308	-5.03327	4.82E-07	1.67E-06	DOWN
SNORA31	7.283187	-0.94164	0.187086	-5.03322	4.82E-07	1.67E-06	DOWN
SDCCAG3I	1.975769	-1.39096	0.276355	-5.03322	4.82E-07	1.67E-06	DOWN
PRMT1	2763.215	0.580278	0.115306	5.032507	4.84E-07	1.68E-06	NOT
SNAP25	177.1076	1.531541	0.304378	5.031715	4.86E-07	1.69E-06	UP
TMEM25	458.5877	-1.03572	0.205844	-5.03157	4.86E-07	1.69E-06	DOWN
CTD-3131	3.188109	1.432444	0.284699	5.031429	4.87E-07	1.69E-06	UP
AC096559	7.318555	1.954832	0.388589	5.030584	4.89E-07	1.70E-06	UP
IGF1	533.3176	-1.37947	0.27425	-5.02998	4.91E-07	1.70E-06	DOWN
MRPL19	1974.665	-0.35864	0.071307	-5.02952	4.92E-07	1.70E-06	NOT
NRAS	2341.048	0.437042	0.086906	5.028889	4.93E-07	1.71E-06	NOT
RP5-850E!	5.01942	1.273294	0.253223	5.028356	4.95E-07	1.71E-06	UP
CTC-344H	1.363738	1.836421	0.365216	5.028312	4.95E-07	1.71E-06	UP
RP11-737(	5.636916	-1.19639	0.237945	-5.02802	4.96E-07	1.72E-06	DOWN
ZKSCAN8	760.3204	0.547789	0.108957	5.027578	4.97E-07	1.72E-06	NOT
LINC0144E	4.893496	4.198512	0.835107	5.027515	4.97E-07	1.72E-06	UP
RP11-927I	5.391523	1.137198	0.226218	5.026995	4.98E-07	1.73E-06	UP
GRIN3B	6.678456	1.421317	0.282783	5.026182	5.00E-07	1.73E-06	UP
SMARCB1	2130.847	0.451689	0.089872	5.025922	5.01E-07	1.74E-06	NOT
PSMC1P1(	1.980086	2.443146	0.486146	5.025538	5.02E-07	1.74E-06	UP
SCAMP1-/	169.5568	0.498314	0.099158	5.025435	5.02E-07	1.74E-06	NOT
RBM20	31.49893	1.373107	0.273286	5.024438	5.05E-07	1.75E-06	UP
SAMSN1	185.5762	-1.0244	0.203883	-5.02442	5.05E-07	1.75E-06	DOWN
RP11-155(	3.746069	1.246917	0.248184	5.024159	5.06E-07	1.75E-06	UP
FGD6	594.7759	0.776728	0.154601	5.024088	5.06E-07	1.75E-06	UP
ZNF3	1057.855	0.347335	0.06914	5.023652	5.07E-07	1.75E-06	NOT
METTL4	254.7914	0.444746	0.088535	5.023412	5.08E-07	1.76E-06	NOT
RBM14	1480.902	0.280182	0.055778	5.023185	5.08E-07	1.76E-06	NOT
RP11-732/	5.36726	1.231699	0.245208	5.023087	5.08E-07	1.76E-06	UP
VPS54	1359.437	0.399233	0.079496	5.022066	5.11E-07	1.77E-06	NOT
PYDC1	3.872713	3.303622	0.65783	5.022001	5.11E-07	1.77E-06	UP
MEX3D	562.6694	0.539923	0.10752	5.021585	5.12E-07	1.77E-06	NOT
SBDS	3420.436	-0.38806	0.077281	-5.02144	5.13E-07	1.77E-06	NOT
DDX27	1838.192	0.379946	0.07568	5.020407	5.16E-07	1.78E-06	NOT
THOC1	691.1381	0.429609	0.085576	5.020196	5.16E-07	1.78E-06	NOT
TCIRG1	3549.316	0.606231	0.120777	5.019413	5.18E-07	1.79E-06	UP
SLC1A3	301.0161	1.147264	0.228578	5.019143	5.19E-07	1.79E-06	UP

NUS1	1115.463	-0.41958	0.083624	-5.01739	5.24E-07	1.81E-06	NOT
MAP10	195.7268	0.814217	0.162292	5.016972	5.25E-07	1.81E-06	UP
C1orf216	442.6732	0.539387	0.107517	5.01676	5.26E-07	1.82E-06	NOT
CCDC157	55.05974	0.566634	0.112951	5.016633	5.26E-07	1.82E-06	NOT
LINC0148(	13.45555	1.480213	0.295075	5.016392	5.27E-07	1.82E-06	UP
MKRN2OS	16.47314	1.352193	0.269563	5.01625	5.27E-07	1.82E-06	UP
CPNE5	86.91406	0.956693	0.19072	5.016209	5.27E-07	1.82E-06	UP
PRMT5-AS	5.704369	0.933776	0.186168	5.015766	5.28E-07	1.82E-06	UP
ELAC1	298.1014	-0.36818	0.073408	-5.01553	5.29E-07	1.83E-06	NOT
TMEM44	258.887	0.682276	0.136079	5.013817	5.34E-07	1.84E-06	UP
ARAP2	503.8311	-0.83086	0.165731	-5.01331	5.35E-07	1.85E-06	DOWN
C3orf22	2.012892	1.838313	0.366755	5.012377	5.38E-07	1.85E-06	UP
HLA-F-AS	80.53601	0.782704	0.15616	5.01218	5.38E-07	1.86E-06	UP
RNF20	1517.779	0.403798	0.080564	5.012142	5.38E-07	1.86E-06	NOT
RP11-131I	12.48265	0.941629	0.187879	5.011896	5.39E-07	1.86E-06	UP
MKKS	2659.428	0.485348	0.096842	5.01175	5.39E-07	1.86E-06	NOT
USP27X-A	36.72507	0.962595	0.192096	5.011022	5.41E-07	1.87E-06	UP
DAB2IP	1752.914	0.674852	0.134674	5.011016	5.41E-07	1.87E-06	UP
HPSE2	2.805739	2.094123	0.417912	5.010918	5.42E-07	1.87E-06	UP
DNAL4	443.4424	0.467598	0.093337	5.009802	5.45E-07	1.88E-06	NOT
TFCP2	833.5683	0.375997	0.075053	5.009746	5.45E-07	1.88E-06	NOT
RP11-480I	2.168478	1.737193	0.346763	5.009737	5.45E-07	1.88E-06	UP
GFY	1.748547	3.086319	0.616194	5.008679	5.48E-07	1.89E-06	UP
RP11-253I	1.854654	1.556507	0.310773	5.00851	5.49E-07	1.89E-06	UP
CTD-2118	5.477358	3.61028	0.720901	5.008008	5.50E-07	1.89E-06	UP
APOD	109.9073	1.353254	0.270251	5.00739	5.52E-07	1.90E-06	UP
NUGGC	930.7549	-1.30569	0.260824	-5.00603	5.56E-07	1.91E-06	DOWN
LCP2	679.6089	-0.841	0.168005	-5.00582	5.56E-07	1.92E-06	DOWN
HTR1F	1.676856	2.270849	0.453643	5.005809	5.56E-07	1.92E-06	UP
UCP3	23.84411	0.716015	0.143044	5.005554	5.57E-07	1.92E-06	UP
RP11-462(	1.928207	3.08336	0.615996	5.005488	5.57E-07	1.92E-06	UP
RP11-256I	4.447437	-1.45863	0.291417	-5.0053	5.58E-07	1.92E-06	DOWN
QPCT	133.0797	1.199358	0.239629	5.005062	5.58E-07	1.92E-06	UP
SMC6	1209.403	0.493137	0.098531	5.004917	5.59E-07	1.92E-06	NOT
FTLP12	6.058642	1.557655	0.311308	5.003576	5.63E-07	1.94E-06	UP
EDRF1	313.9894	0.437833	0.087512	5.003125	5.64E-07	1.94E-06	NOT
MTNR1B	5.301926	4.558248	0.911254	5.002171	5.67E-07	1.95E-06	UP
HCG4P11	6.674697	1.312331	0.262397	5.00132	5.69E-07	1.96E-06	UP
LINC0118:	6.158483	1.434111	0.286773	5.00085	5.71E-07	1.96E-06	UP
RPL7P1	50.00819	0.880166	0.176062	4.999189	5.76E-07	1.98E-06	UP
CSPG4P13	1.897276	1.958063	0.391677	4.999177	5.76E-07	1.98E-06	UP
RP11-705(	34.51388	0.877542	0.175547	4.9989	5.77E-07	1.98E-06	UP
AC104654	8.434782	2.036974	0.407508	4.998616	5.77E-07	1.98E-06	UP
AC097721	12.81038	0.775353	0.155119	4.998444	5.78E-07	1.99E-06	UP
MAP3K7CI	240.3897	0.56042	0.112131	4.99789	5.80E-07	1.99E-06	NOT
FADS3	975.9137	0.618573	0.123771	4.997739	5.80E-07	1.99E-06	UP
KCNJ10	74.08088	-1.53355	0.306863	-4.99751	5.81E-07	1.99E-06	DOWN
CCDC144(	1.297346	2.088549	0.417953	4.997095	5.82E-07	2.00E-06	UP
KRT18P5	3.872118	1.372761	0.274746	4.996465	5.84E-07	2.01E-06	UP
LINC0063:	5.301617	2.92488	0.585488	4.995624	5.86E-07	2.01E-06	UP
AC012512	1.825263	3.018119	0.604232	4.994967	5.88E-07	2.02E-06	UP
MACROD1	1391.911	-0.88069	0.176316	-4.99493	5.89E-07	2.02E-06	DOWN

RFC2	1141.856	0.473988	0.094895	4.994848	5.89E-07	2.02E-06	NOT
CSNK2A1	2472.36	0.376715	0.075421	4.994824	5.89E-07	2.02E-06	NOT
RP11-54O	6.516504	1.676952	0.335743	4.994751	5.89E-07	2.02E-06	UP
AC024560	7.266906	1.659002	0.332161	4.99458	5.90E-07	2.02E-06	UP
PITPNA-A:	99.35371	0.729653	0.1461	4.994216	5.91E-07	2.03E-06	UP
RPL7P26	1.862691	1.63341	0.327065	4.994144	5.91E-07	2.03E-06	UP
LINC0117(	39.54619	0.802573	0.160705	4.994063	5.91E-07	2.03E-06	UP
CHD4	6456.258	0.32925	0.065932	4.993804	5.92E-07	2.03E-06	NOT
CPA5	5.533159	1.969259	0.394364	4.993502	5.93E-07	2.03E-06	UP
RP5-984P	1.299115	2.647915	0.530318	4.993074	5.94E-07	2.04E-06	UP
RP11-429	3.316701	2.251952	0.451116	4.991961	5.98E-07	2.05E-06	UP
ALDH1L2	51.33187	1.060091	0.212397	4.991089	6.00E-07	2.06E-06	UP
CLEC4E	47.06627	-1.21212	0.242891	-4.99039	6.03E-07	2.07E-06	DOWN
PRR5	797.4091	-0.68735	0.137738	-4.99025	6.03E-07	2.07E-06	DOWN
ADSL	2014.773	0.476774	0.095544	4.990097	6.03E-07	2.07E-06	NOT
PHKG1	29.74013	0.683393	0.136964	4.989582	6.05E-07	2.07E-06	UP
TMTC3	768.1693	-0.43721	0.087628	-4.98938	6.06E-07	2.08E-06	NOT
LIPG	2466.746	-1.04602	0.209662	-4.98911	6.07E-07	2.08E-06	DOWN
RP3-510O	3.111182	1.543472	0.309395	4.988671	6.08E-07	2.08E-06	UP
AREL1	1048.925	0.400578	0.080301	4.988444	6.09E-07	2.08E-06	NOT
RP11-114:	71.50995	1.520795	0.304869	4.988356	6.09E-07	2.09E-06	UP
RP11-278(	25.76062	0.609508	0.122202	4.98772	6.11E-07	2.09E-06	UP
CCDC124	2425.673	0.563587	0.112999	4.987528	6.12E-07	2.09E-06	NOT
AC093495	32.38903	0.646547	0.129635	4.98745	6.12E-07	2.09E-06	UP
RRAGB	298.2035	0.466478	0.093537	4.987081	6.13E-07	2.10E-06	NOT
FKBP11	2344.645	0.773773	0.155167	4.986696	6.14E-07	2.10E-06	UP
JAG1	1671.49	0.871577	0.174785	4.986567	6.15E-07	2.10E-06	UP
MTCH2	7184.664	-0.40517	0.081258	-4.98627	6.16E-07	2.11E-06	NOT
RIOK1	620.0957	0.480606	0.096388	4.98619	6.16E-07	2.11E-06	NOT
RP11-589I	39.47965	0.757446	0.151925	4.985671	6.17E-07	2.11E-06	UP
ZNF606	286.6067	0.666239	0.133632	4.985627	6.18E-07	2.11E-06	UP
PHLPP1	1724.896	-0.61636	0.12363	-4.98556	6.18E-07	2.11E-06	DOWN
NFKB1	1669.255	-0.44258	0.088777	-4.98536	6.18E-07	2.11E-06	NOT
CDO1	9382.418	-1.16366	0.233467	-4.98425	6.22E-07	2.13E-06	DOWN
RP11-92G	7.518702	1.689093	0.338935	4.983528	6.24E-07	2.13E-06	UP
COA1	945.9889	0.388238	0.077908	4.983286	6.25E-07	2.14E-06	NOT
HEXDC-IT	3.730926	1.332382	0.267378	4.983129	6.26E-07	2.14E-06	UP
CTD-2532	2.047306	1.823285	0.365913	4.982842	6.27E-07	2.14E-06	UP
RP11-485I	4.020524	1.462972	0.293613	4.982657	6.27E-07	2.14E-06	UP
CCDC36	7.519779	1.423893	0.285775	4.982561	6.27E-07	2.14E-06	UP
RAB5A	1834.023	-0.31862	0.063948	-4.9825	6.28E-07	2.14E-06	NOT
UGGT2	608.1795	0.440432	0.088401	4.982193	6.29E-07	2.15E-06	NOT
RP11-64C	2.824409	2.367523	0.475222	4.981925	6.30E-07	2.15E-06	UP
RP1-257I2	5.530603	0.956467	0.191993	4.981788	6.30E-07	2.15E-06	UP
RP11-344I	66.77291	-1.10284	0.221413	-4.98091	6.33E-07	2.16E-06	DOWN
RP11-867(	4.305633	-1.28556	0.25812	-4.98048	6.34E-07	2.17E-06	DOWN
CCDC108	9.844497	1.494859	0.300163	4.980157	6.35E-07	2.17E-06	UP
KCNA3	17.25365	-1.35987	0.273072	-4.97992	6.36E-07	2.17E-06	DOWN
CTA-221G	97.24905	-0.87625	0.175998	-4.97874	6.40E-07	2.18E-06	DOWN
CTD-2531	3.423493	2.289399	0.459836	4.978726	6.40E-07	2.18E-06	UP
CCDC74B	9.242794	1.727227	0.346952	4.978289	6.41E-07	2.19E-06	UP
AL590226.	2.688612	2.358107	0.47373	4.977748	6.43E-07	2.19E-06	UP

PRR26	314.0179	-1.11678	0.224382	-4.97713	6.45E-07	2.20E-06	DOWN
NBAS	1633.647	0.415887	0.083567	4.97668	6.47E-07	2.21E-06	NOT
RP11-574I	5.080776	1.131609	0.227392	4.976478	6.48E-07	2.21E-06	UP
DHX58	907.2829	-0.70399	0.141464	-4.97645	6.48E-07	2.21E-06	DOWN
ITGA10	55.73091	0.911618	0.183188	4.976419	6.48E-07	2.21E-06	UP
SREBF2	7010.999	0.561944	0.112925	4.976268	6.48E-07	2.21E-06	NOT
RP11-571I	4.666404	1.10244	0.221545	4.976155	6.49E-07	2.21E-06	UP
NOP14	1978.715	-0.35809	0.07198	-4.97489	6.53E-07	2.22E-06	NOT
FOXE3	1.626391	2.132275	0.428636	4.974564	6.54E-07	2.23E-06	UP
CDHR4	3.226708	1.833757	0.368638	4.974416	6.54E-07	2.23E-06	UP
CRTC1	555.8645	0.512423	0.103017	4.974176	6.55E-07	2.23E-06	NOT
SYNGR1	621.0315	1.200556	0.241382	4.97368	6.57E-07	2.24E-06	UP
AC002398	15.83647	0.912142	0.183409	4.973258	6.58E-07	2.24E-06	UP
MT1CP	12.90166	-2.18804	0.440017	-4.97263	6.61E-07	2.25E-06	DOWN
CNTNAP3I	77.58244	1.54573	0.31085	4.972598	6.61E-07	2.25E-06	UP
ZSCAN12	139.4464	0.557921	0.1122	4.972558	6.61E-07	2.25E-06	NOT
RP4-545L1	1.707144	2.198494	0.442144	4.972349	6.61E-07	2.25E-06	UP
APOA1	469728.7	-1.42591	0.286777	-4.97219	6.62E-07	2.25E-06	DOWN
EIF3D	6184.895	0.452949	0.091105	4.971745	6.64E-07	2.26E-06	NOT
SSRP1	4231.759	0.361663	0.072748	4.97148	6.64E-07	2.26E-06	NOT
AP001331.	5.586092	3.017117	0.606913	4.971252	6.65E-07	2.26E-06	UP
RP3-434P1	64.37079	1.719943	0.345993	4.971031	6.66E-07	2.27E-06	UP
TMC1	4.507561	1.546698	0.311155	4.970827	6.67E-07	2.27E-06	UP
ZNF397	647.4287	0.360299	0.072492	4.97016	6.69E-07	2.28E-06	NOT
TRIM72	7.364332	1.587297	0.319397	4.969666	6.71E-07	2.28E-06	UP
DCX	8.761087	1.919793	0.386327	4.969344	6.72E-07	2.28E-06	UP
RP11-180(	1.326557	2.10011	0.422654	4.968859	6.73E-07	2.29E-06	UP
URB1	1142.743	0.432436	0.087031	4.968764	6.74E-07	2.29E-06	NOT
MUC5B	925.7995	2.604963	0.524334	4.96814	6.76E-07	2.30E-06	UP
AMDHD2	876.6135	0.569076	0.114565	4.967259	6.79E-07	2.31E-06	NOT
P2RX3	30.81959	-1.24694	0.251034	-4.96722	6.79E-07	2.31E-06	DOWN
ASAP3	828.3434	0.731591	0.147288	4.967085	6.80E-07	2.31E-06	UP
PRNP	2573.194	-0.86592	0.174331	-4.96708	6.80E-07	2.31E-06	DOWN
AC005740	5.016623	1.154188	0.232379	4.966844	6.81E-07	2.31E-06	UP
KBTBD12	6.193078	2.637722	0.53118	4.965776	6.84E-07	2.32E-06	UP
RBM17	2176.475	0.377354	0.075999	4.965218	6.86E-07	2.33E-06	NOT
CTD-2126	3.072897	3.463535	0.697574	4.965118	6.87E-07	2.33E-06	UP
SMARCA2	3195.069	-0.70822	0.142643	-4.96499	6.87E-07	2.33E-06	DOWN
LAYN	80.68849	0.842465	0.169708	4.964191	6.90E-07	2.34E-06	UP
PFDN4	503.945	0.604467	0.12177	4.964004	6.91E-07	2.34E-06	UP
RP11-553/	8.951818	1.504032	0.303003	4.963753	6.91E-07	2.35E-06	UP
CTD-2270	4.332358	1.27386	0.256638	4.963644	6.92E-07	2.35E-06	UP
UBE3A	2373.629	-0.33747	0.067991	-4.96344	6.93E-07	2.35E-06	NOT
CDADC1	409.7057	-0.58082	0.117026	-4.96314	6.94E-07	2.35E-06	NOT
PRR5L	265.2687	1.200671	0.241924	4.963012	6.94E-07	2.35E-06	UP
SLC24A4	15.11068	-0.8465	0.170563	-4.96297	6.94E-07	2.35E-06	DOWN
SLC44A1	3658.343	-0.46077	0.092853	-4.96235	6.96E-07	2.36E-06	NOT
RP4-747G.	2.694057	1.429482	0.288079	4.962124	6.97E-07	2.36E-06	UP
SQRDL	2111.907	-0.53227	0.107268	-4.96204	6.98E-07	2.37E-06	NOT
PTPN5	5.13373	1.866222	0.376129	4.961658	6.99E-07	2.37E-06	UP
PCDHA3	10.83256	2.070091	0.417281	4.960904	7.02E-07	2.38E-06	UP
CPOX	1090.77	-0.48041	0.096849	-4.96042	7.03E-07	2.38E-06	NOT

METTL7B	7264.928	-0.90111	0.181683	-4.95977	7.06E-07	2.39E-06	DOWN
RBM4B	468.5551	0.3475	0.070075	4.958993	7.09E-07	2.40E-06	NOT
LINC01012	3.359728	2.386543	0.481263	4.958915	7.09E-07	2.40E-06	UP
CSNK2B-L	3.618454	1.094833	0.220833	4.957742	7.13E-07	2.42E-06	UP
FAM210B	5021.685	-0.56528	0.114023	-4.9576	7.14E-07	2.42E-06	NOT
SEC24D	2638.04	-0.61625	0.124322	-4.95692	7.16E-07	2.43E-06	DOWN
ITPA	1686.185	0.539416	0.108824	4.956781	7.17E-07	2.43E-06	NOT
BNIP3P11	9.266458	1.254218	0.253034	4.956713	7.17E-07	2.43E-06	UP
SOCS5P4	3.462599	1.288766	0.260051	4.955815	7.20E-07	2.44E-06	UP
CTD-2517	73.12315	0.93543	0.188772	4.955348	7.22E-07	2.44E-06	UP
CTC-542B	6.405409	1.090625	0.220102	4.95508	7.23E-07	2.45E-06	UP
RBM4	278.3147	0.40643	0.082031	4.954611	7.25E-07	2.45E-06	NOT
TGFB2-AS	6.877827	1.758764	0.354989	4.954423	7.25E-07	2.45E-06	UP
PKIA	28.88504	1.53835	0.310538	4.953828	7.28E-07	2.46E-06	UP
TMEM52B	9.403514	1.858188	0.375123	4.953548	7.29E-07	2.47E-06	UP
RDH13	503.4184	0.743526	0.15011	4.953219	7.30E-07	2.47E-06	UP
ZNF727	10.0591	1.867231	0.37701	4.952743	7.32E-07	2.47E-06	UP
PMPCAP1	2.832729	3.285278	0.663352	4.95254	7.33E-07	2.48E-06	UP
TCF24	2.437706	3.182714	0.642728	4.951882	7.35E-07	2.49E-06	UP
RP5-839B	2.526051	2.642701	0.53378	4.950922	7.39E-07	2.50E-06	UP
IFRD1	990.2214	0.535467	0.108181	4.949741	7.43E-07	2.51E-06	NOT
PXN	2384.673	0.302192	0.061057	4.949363	7.45E-07	2.52E-06	NOT
RP11-472	12.75904	-0.78636	0.158912	-4.94839	7.48E-07	2.53E-06	DOWN
ACSM2A	12147.67	-1.31083	0.264907	-4.94826	7.49E-07	2.53E-06	DOWN
GATA4	1851.752	-0.70845	0.143176	-4.94811	7.49E-07	2.53E-06	DOWN
TMLHE	437.6429	-0.46219	0.093418	-4.94757	7.51E-07	2.54E-06	NOT
SIRPA	3338.708	-0.68799	0.139072	-4.947	7.54E-07	2.55E-06	DOWN
RP11-338	2.868651	2.701108	0.546063	4.946514	7.56E-07	2.55E-06	UP
GPD2	537.1495	0.590477	0.119386	4.945935	7.58E-07	2.56E-06	UP
TBC1D12	332.0672	0.44357	0.089685	4.945885	7.58E-07	2.56E-06	NOT
SSR3	6810.617	0.376698	0.076168	4.945612	7.59E-07	2.56E-06	NOT
HECTD4	1307.905	0.474001	0.095843	4.945609	7.59E-07	2.56E-06	NOT
ALG1L9P	9.946435	0.716029	0.144787	4.945401	7.60E-07	2.57E-06	UP
MIA2	78.5486	-0.79804	0.161373	-4.9453	7.60E-07	2.57E-06	DOWN
RPL9P29	13.52745	0.960485	0.194222	4.945288	7.60E-07	2.57E-06	UP
AANAT	3.932854	1.567547	0.317015	4.944711	7.63E-07	2.57E-06	UP
BTBD10	523.0728	0.372772	0.07539	4.944595	7.63E-07	2.57E-06	NOT
AC006273	6.404507	1.540326	0.311524	4.944488	7.63E-07	2.58E-06	UP
THOC3	326.7726	0.481765	0.097454	4.943525	7.67E-07	2.59E-06	NOT
CYTIP	219.8424	-1.02554	0.207468	-4.94309	7.69E-07	2.59E-06	DOWN
UBL7	1444.605	0.401828	0.081294	4.942875	7.70E-07	2.60E-06	NOT
HBEGF	384.1466	-0.75663	0.153095	-4.94222	7.72E-07	2.60E-06	DOWN
API5	2897.479	-0.29638	0.059969	-4.9422	7.72E-07	2.60E-06	NOT
LINC01197	58.46007	-0.88644	0.179376	-4.94181	7.74E-07	2.61E-06	DOWN
SLCO5A1	21.10669	1.445869	0.2926	4.941452	7.75E-07	2.61E-06	UP
CTD-2540	6.635076	1.303772	0.263847	4.941394	7.76E-07	2.61E-06	UP
ERICD	25.9085	0.884482	0.179007	4.941042	7.77E-07	2.62E-06	UP
C2orf82	882.1638	1.240992	0.251164	4.940964	7.77E-07	2.62E-06	UP
ACBD4	2907.015	-0.82689	0.167361	-4.94077	7.78E-07	2.62E-06	DOWN
GNMT	5616.113	-1.61298	0.326479	-4.94051	7.79E-07	2.62E-06	DOWN
PPAPDC3	26.97635	1.166228	0.236059	4.940415	7.80E-07	2.63E-06	UP
APOH	234611.1	-1.15374	0.233564	-4.93973	7.82E-07	2.63E-06	DOWN

MTHFR	882.8955	0.543195	0.109966	4.93968	7.83E-07	2.64E-06	NOT
RP11-147I	83.45398	0.6877	0.139222	4.939607	7.83E-07	2.64E-06	UP
AC005534	6.286814	1.233537	0.249738	4.93932	7.84E-07	2.64E-06	UP
ALAS2	12.03881	-1.54055	0.311913	-4.93902	7.85E-07	2.64E-06	DOWN
RBP5	5879.533	-1.11079	0.224929	-4.9384	7.88E-07	2.65E-06	DOWN
RP11-116I	10.85738	-1.27687	0.258569	-4.93822	7.88E-07	2.65E-06	DOWN
FAM225B	3.436765	1.975107	0.400014	4.937598	7.91E-07	2.66E-06	UP
ANKFY1	1571.35	-0.51308	0.103931	-4.93678	7.94E-07	2.67E-06	NOT
RP11-95O	4.368304	1.395001	0.282601	4.936298	7.96E-07	2.68E-06	UP
TTC23L	9.750362	0.888118	0.179923	4.936093	7.97E-07	2.68E-06	UP
PRAF2	519.5778	0.611961	0.123982	4.935886	7.98E-07	2.68E-06	UP
SURF1	2857.862	-0.54756	0.110938	-4.93572	7.99E-07	2.69E-06	NOT
ANKRD32	162.9143	0.469763	0.095179	4.935585	7.99E-07	2.69E-06	NOT
RP11-64B1	12.58143	1.251529	0.25358	4.935438	8.00E-07	2.69E-06	UP
SGCA	88.52454	-1.25896	0.255099	-4.93519	8.01E-07	2.69E-06	DOWN
DPY19L2P3	22.99357	0.732528	0.148448	4.934582	8.03E-07	2.70E-06	UP
IL31RA	5.070329	2.030643	0.411555	4.934071	8.05E-07	2.71E-06	UP
NTNG2	26.6002	1.265495	0.256495	4.933791	8.06E-07	2.71E-06	UP
ZNF678	239.3498	0.6223	0.126133	4.933682	8.07E-07	2.71E-06	UP
FAIM3	142.542	-0.89734	0.181889	-4.93344	8.08E-07	2.71E-06	DOWN
RASA4	10.03879	1.037719	0.210384	4.932509	8.12E-07	2.73E-06	UP
RP4-536B2	1.515034	2.085583	0.422874	4.931926	8.14E-07	2.73E-06	UP
IGKV2-24	51.16844	-2.04945	0.415552	-4.93187	8.14E-07	2.74E-06	DOWN
HGFAC	6414.582	-1.82977	0.371012	-4.93183	8.15E-07	2.74E-06	DOWN
FAAH	1664.607	-0.74183	0.150425	-4.93158	8.16E-07	2.74E-06	DOWN
REEP6	15623.92	-1.05628	0.214189	-4.93152	8.16E-07	2.74E-06	DOWN
NOMO1	1603.325	0.454903	0.092259	4.930739	8.19E-07	2.75E-06	NOT
HTR3B	1.791488	3.042493	0.617122	4.930134	8.22E-07	2.76E-06	UP
RP11-244(	71.51997	0.845376	0.171505	4.929159	8.26E-07	2.77E-06	UP
HNRNPA1I	69.29075	0.620019	0.125823	4.927728	8.32E-07	2.79E-06	UP
C7orf50	2526.184	0.653512	0.13262	4.927694	8.32E-07	2.79E-06	UP
CTD-3051	4.187211	1.180974	0.239688	4.927137	8.34E-07	2.80E-06	UP
RP11-723(	5.72688	1.004338	0.203854	4.926756	8.36E-07	2.80E-06	UP
ANKRD36E	16.08613	1.048015	0.212766	4.925679	8.41E-07	2.82E-06	UP
NLGN1	7.51147	2.055019	0.417348	4.923992	8.48E-07	2.84E-06	UP
ROR2	129.867	1.493049	0.303232	4.923777	8.49E-07	2.85E-06	UP
SLITRK4	17.71998	1.522785	0.30928	4.923652	8.49E-07	2.85E-06	UP
FAM183A	13.83494	2.504046	0.508576	4.923644	8.49E-07	2.85E-06	UP
PCDH12	665.6609	0.586197	0.119059	4.923602	8.50E-07	2.85E-06	UP
ABRACL	645.3322	-0.81059	0.164641	-4.92339	8.51E-07	2.85E-06	DOWN
NIF3L1	697.3703	0.295988	0.060119	4.923367	8.51E-07	2.85E-06	NOT
FIGF	75.57374	1.340008	0.272193	4.923003	8.52E-07	2.86E-06	UP
AC124789	15.14361	-0.93795	0.190528	-4.92288	8.53E-07	2.86E-06	DOWN
BNIP3P9	2.116521	3.10662	0.631084	4.922674	8.54E-07	2.86E-06	UP
AC005355	152.3244	1.190248	0.241798	4.922489	8.55E-07	2.86E-06	UP
CTD-2020	8.777492	1.349443	0.274153	4.922219	8.56E-07	2.87E-06	UP
LRRC66	15.83065	1.374624	0.279318	4.921355	8.59E-07	2.88E-06	UP
SLC35A3	1659.85	-0.5996	0.121836	-4.92134	8.60E-07	2.88E-06	DOWN
NKTR	1326.774	0.592928	0.120506	4.920342	8.64E-07	2.89E-06	UP
TMEM246	400.4723	1.243974	0.252826	4.920268	8.64E-07	2.89E-06	UP
ZDHHC13	220.9881	0.932498	0.189535	4.919915	8.66E-07	2.90E-06	UP
RP11-407I	31.63857	-0.94888	0.192886	-4.91939	8.68E-07	2.91E-06	DOWN

PFKM	703.5605	0.809138	0.164481	4.919342	8.68E-07	2.91E-06	UP
CNPY4	306.6883	0.511644	0.104011	4.919147	8.69E-07	2.91E-06	NOT
CTD-2503	3.530988	1.520474	0.3091	4.919029	8.70E-07	2.91E-06	UP
FBLN5	1386.153	-1.03671	0.210773	-4.91861	8.72E-07	2.92E-06	DOWN
SLC17A8	14.88086	-1.83413	0.372899	-4.91859	8.72E-07	2.92E-06	DOWN
SRSF12	44.11144	1.154215	0.23467	4.918454	8.72E-07	2.92E-06	UP
DPYSL5	1.81047	2.87208	0.583947	4.918392	8.73E-07	2.92E-06	UP
AP1G1	3151.07	-0.38554	0.078396	-4.91785	8.75E-07	2.93E-06	NOT
CTC-327F	1.541025	2.530676	0.514596	4.917787	8.75E-07	2.93E-06	UP
IPO8	1856.808	-0.31273	0.063591	-4.91777	8.75E-07	2.93E-06	NOT
LIX1	3.227556	3.603049	0.732693	4.917543	8.76E-07	2.93E-06	UP
ECHDC3	4388.227	-0.99032	0.201413	-4.91686	8.79E-07	2.94E-06	DOWN
BAG6	10931.12	0.33962	0.069074	4.916723	8.80E-07	2.94E-06	NOT
CNOT7	1988.254	-0.45621	0.092796	-4.91623	8.82E-07	2.95E-06	NOT
CTD-2012	189.1346	-0.90551	0.18423	-4.9151	8.87E-07	2.96E-06	DOWN
SKP2	900.3738	0.705545	0.143547	4.915089	8.87E-07	2.96E-06	UP
CTD-2521	6.991683	0.927389	0.188712	4.91431	8.91E-07	2.98E-06	UP
NCKAP5	176.8624	-1.00484	0.204478	-4.91415	8.92E-07	2.98E-06	DOWN
RP11-150	2.532006	2.389851	0.486357	4.913782	8.93E-07	2.98E-06	UP
PPP4R1	1732.327	0.36671	0.074629	4.913748	8.94E-07	2.98E-06	NOT
RP5-1092	7.732452	2.373487	0.48306	4.913439	8.95E-07	2.99E-06	UP
H2AFZP3	2.29206	1.40379	0.285707	4.913385	8.95E-07	2.99E-06	UP
PPP1R15B	4106.779	-0.45429	0.092468	-4.91297	8.97E-07	2.99E-06	NOT
MGLL	7092.739	-0.79469	0.161779	-4.91218	9.01E-07	3.01E-06	DOWN
ZNF528-A	63.87073	1.025147	0.208695	4.912176	9.01E-07	3.01E-06	UP
MRPS36P4	1.136016	-2.16588	0.44093	-4.91206	9.01E-07	3.01E-06	DOWN
AC090945	2.570183	1.813787	0.369275	4.911755	9.03E-07	3.01E-06	UP
GTPBP10	882.7941	-0.40431	0.082315	-4.9117	9.03E-07	3.01E-06	NOT
DDT	6569.798	-0.84634	0.172332	-4.91109	9.06E-07	3.02E-06	DOWN
TTC7A	1460.153	0.603385	0.122882	4.910285	9.09E-07	3.03E-06	UP
PPAT	434.4752	0.452253	0.092109	4.909964	9.11E-07	3.04E-06	NOT
CUBN	27.85678	0.855705	0.174286	4.909789	9.12E-07	3.04E-06	UP
ZNF285	54.34988	1.167361	0.237791	4.909194	9.15E-07	3.05E-06	UP
CPA4	13.27369	1.788763	0.364419	4.90854	9.18E-07	3.06E-06	UP
UBTF	4194.127	0.270293	0.055068	4.908389	9.18E-07	3.06E-06	NOT
AC016831	37.51703	1.226949	0.249986	4.908072	9.20E-07	3.06E-06	UP
RP11-111	4.161497	1.227232	0.250044	4.908069	9.20E-07	3.06E-06	UP
ANKRD10-	79.16256	0.911296	0.185708	4.90714	9.24E-07	3.08E-06	UP
RP11-316	11.85173	0.909768	0.18541	4.906789	9.26E-07	3.08E-06	UP
LINC0063	42.86201	0.856067	0.174478	4.906434	9.27E-07	3.09E-06	UP
RP11-392	137.5039	0.587668	0.119783	4.906088	9.29E-07	3.09E-06	UP
QSER1	840.8911	0.470897	0.095983	4.906058	9.29E-07	3.09E-06	NOT
RP3-368A	138.4339	0.702007	0.1431	4.905727	9.31E-07	3.10E-06	UP
SDC3	2746.96	-0.71764	0.146289	-4.90562	9.31E-07	3.10E-06	DOWN
ARHGEF15	387.7182	0.652326	0.132994	4.904932	9.35E-07	3.11E-06	UP
RP11-195	5.783877	1.040378	0.212121	4.90464	9.36E-07	3.12E-06	UP
SLC35E2B	983.1611	0.59482	0.121279	4.90456	9.36E-07	3.12E-06	UP
CLTA	4789.131	0.466186	0.095061	4.90408	9.39E-07	3.12E-06	NOT
RP4-680D	50.98245	-0.98616	0.201095	-4.90395	9.39E-07	3.12E-06	DOWN
TRAPPC13	12.35652	2.077452	0.423631	4.903922	9.39E-07	3.13E-06	UP
PLCXD1	1398.122	0.956741	0.1951	4.903842	9.40E-07	3.13E-06	UP
RP11-497	3.376042	1.489399	0.303754	4.903304	9.42E-07	3.13E-06	UP

RP11-15A:	7.191838	1.171143	0.238877	4.902711	9.45E-07	3.14E-06	UP
HSD17B3	105.842	1.196624	0.244123	4.901729	9.50E-07	3.16E-06	UP
RP11-797I	5.500996	1.47155	0.300219	4.901587	9.51E-07	3.16E-06	UP
AC004980	54.24633	0.50567	0.103171	4.901281	9.52E-07	3.17E-06	NOT
GPS2	100.6884	0.596273	0.121687	4.900076	9.58E-07	3.18E-06	UP
SELO	3249.704	-0.61139	0.124782	-4.89966	9.60E-07	3.19E-06	DOWN
RPP40	308.3146	0.601864	0.12286	4.898771	9.64E-07	3.20E-06	UP
CICP14	74.63402	0.919027	0.187614	4.898509	9.66E-07	3.21E-06	UP
MARCKS	3843.527	0.752686	0.153666	4.898185	9.67E-07	3.21E-06	UP
ULBP2	30.84657	1.257305	0.256778	4.89647	9.76E-07	3.24E-06	UP
GLB1L	322.8093	0.750806	0.153341	4.896307	9.77E-07	3.24E-06	UP
ANKMY1	366.4984	0.438404	0.089568	4.894678	9.85E-07	3.27E-06	NOT
C6orf163	11.37635	1.051358	0.214807	4.894427	9.86E-07	3.27E-06	UP
ANKRD18(	3.070476	1.90666	0.38957	4.894267	9.87E-07	3.28E-06	UP
SNAI2	532.3618	1.159851	0.236993	4.894039	9.88E-07	3.28E-06	UP
RP11-883/	2.210432	2.591019	0.529448	4.893809	9.89E-07	3.28E-06	UP
RNF39	32.07369	1.113098	0.227488	4.892991	9.93E-07	3.30E-06	UP
COX6B2	4.838735	1.814258	0.370851	4.892151	9.97E-07	3.31E-06	UP
SLC6A6	621.2494	1.26966	0.259554	4.891691	1.00E-06	3.32E-06	UP
NDUFV2-/	42.75703	0.493473	0.100883	4.891554	1.00E-06	3.32E-06	NOT
RP11-115(	57.90479	0.590267	0.120672	4.891479	1.00E-06	3.32E-06	UP
MYOT	6.116728	-1.44694	0.295811	-4.89143	1.00E-06	3.32E-06	DOWN
DIXDC1	508.5519	-0.72146	0.147503	-4.89113	1.00E-06	3.33E-06	DOWN
DKK3	1266.845	1.195269	0.244388	4.89087	1.00E-06	3.33E-06	UP
H1FNT	2.090778	2.899291	0.592973	4.889414	1.01E-06	3.35E-06	UP
SLC10A1	9744.623	-1.65868	0.339256	-4.88915	1.01E-06	3.36E-06	DOWN
SSX5	4.391622	4.464899	0.913277	4.888875	1.01E-06	3.36E-06	UP
RP1-80N2	11.84535	1.016778	0.207986	4.888682	1.02E-06	3.36E-06	UP
ZNF454	9.909359	1.312648	0.26852	4.888448	1.02E-06	3.37E-06	UP
ZNF460	36.9885	-0.73917	0.151213	-4.88828	1.02E-06	3.37E-06	DOWN
WFDC1	106.7337	-1.07468	0.219853	-4.88819	1.02E-06	3.37E-06	DOWN
RP11-522I	1.219085	-1.90678	0.390158	-4.88719	1.02E-06	3.39E-06	DOWN
RP11-408(	1.684969	1.545883	0.316346	4.886678	1.03E-06	3.40E-06	UP
C17orf61-	3.921368	-1.0061	0.20592	-4.88585	1.03E-06	3.41E-06	DOWN
ANKRD7	2.273465	2.901161	0.593927	4.884712	1.04E-06	3.43E-06	UP
APCS	49521.67	-1.2803	0.262136	-4.88412	1.04E-06	3.44E-06	DOWN
SNORD12I	1.710776	1.751946	0.358781	4.883054	1.04E-06	3.46E-06	UP
RP11-115:	208.4227	-1.50765	0.308753	-4.88302	1.04E-06	3.46E-06	DOWN
TMEM199	660.112	0.37991	0.077828	4.88139	1.05E-06	3.49E-06	NOT
ZMYND8	1054.872	0.458802	0.09399	4.881373	1.05E-06	3.49E-06	NOT
RP5-836N	1.823114	1.767556	0.362145	4.880802	1.06E-06	3.50E-06	UP
POLH	429.2799	0.518111	0.106163	4.880338	1.06E-06	3.50E-06	NOT
CD151	10351.99	0.70126	0.143692	4.880305	1.06E-06	3.50E-06	UP
RP11-746I	1.04288	2.544833	0.521454	4.880259	1.06E-06	3.50E-06	UP
RHOC	7044.865	0.572442	0.117307	4.879869	1.06E-06	3.51E-06	NOT
WBSCR28	8.990939	1.729891	0.354561	4.878966	1.07E-06	3.53E-06	UP
RP11-438I	13.25323	0.788103	0.161537	4.878789	1.07E-06	3.53E-06	UP
CTC-559E	41.19506	0.929519	0.190524	4.878758	1.07E-06	3.53E-06	UP
GAPDHP6:	9.950657	1.077775	0.220912	4.878748	1.07E-06	3.53E-06	UP
SPEG	69.34603	1.395205	0.28598	4.87868	1.07E-06	3.53E-06	UP
TMED3	1391.165	1.168807	0.23962	4.877752	1.07E-06	3.55E-06	UP
GDF5	1.397771	2.094731	0.429476	4.877411	1.07E-06	3.55E-06	UP

OAZ2	3427.813	-0.35671	0.073136	-4.87736	1.08E-06	3.55E-06	NOT
FAT1	5245.485	0.831951	0.170584	4.877089	1.08E-06	3.56E-06	UP
UNC5B-AS	2.403216	2.085658	0.427715	4.876283	1.08E-06	3.57E-06	UP
TGFB2	169.1816	1.248846	0.256165	4.87517	1.09E-06	3.59E-06	UP
CTD-2033	4.736214	-1.4832	0.304287	-4.87436	1.09E-06	3.61E-06	DOWN
HRG	125809.7	-1.44908	0.297292	-4.87426	1.09E-06	3.61E-06	DOWN
LIPJ	3.948061	-1.21898	0.250138	-4.87322	1.10E-06	3.63E-06	DOWN
LIPC	6539.306	-1.04679	0.214819	-4.8729	1.10E-06	3.63E-06	DOWN
CCL2	1004.552	-1.21077	0.248481	-4.8727	1.10E-06	3.63E-06	DOWN
MEGF9	2657.966	-0.59192	0.121483	-4.87243	1.10E-06	3.64E-06	DOWN
KLRB1	161.7593	-0.9716	0.199414	-4.87226	1.10E-06	3.64E-06	DOWN
RP11-494	2.909544	1.681641	0.345184	4.871721	1.11E-06	3.65E-06	UP
PRR14L	1079.44	0.421812	0.086587	4.871536	1.11E-06	3.65E-06	NOT
NELFB	2314.045	0.352619	0.072392	4.870955	1.11E-06	3.66E-06	NOT
NECAP1	940.6268	-0.36344	0.074616	-4.87083	1.11E-06	3.67E-06	NOT
PMS2	241.5843	0.561518	0.115287	4.870619	1.11E-06	3.67E-06	NOT
PERM1	27.87339	1.323753	0.271859	4.869262	1.12E-06	3.69E-06	UP
PAFAH1B1	2860.474	-0.32237	0.06621	-4.86891	1.12E-06	3.70E-06	NOT
FBXO41	96.74672	1.164551	0.239186	4.868808	1.12E-06	3.70E-06	UP
MTHFSD	438.8323	0.379001	0.077847	4.868526	1.12E-06	3.71E-06	NOT
RP11-131	11.0834	-1.08767	0.223441	-4.86781	1.13E-06	3.72E-06	DOWN
NR1D1	1707.148	-0.74811	0.153746	-4.86588	1.14E-06	3.76E-06	DOWN
IGSF10	31.2585	-1.32931	0.273215	-4.86544	1.14E-06	3.76E-06	DOWN
SUZ12	1401.921	0.429583	0.088294	4.865383	1.14E-06	3.76E-06	NOT
GDPD2	2.231934	1.604663	0.32984	4.86497	1.14E-06	3.77E-06	UP
RN7SL541	1.076965	2.005174	0.412179	4.864817	1.15E-06	3.77E-06	UP
ARIH2	2067.098	0.283905	0.05836	4.864751	1.15E-06	3.78E-06	NOT
BCL9L	1009.393	0.765418	0.157341	4.864699	1.15E-06	3.78E-06	UP
PPP1R3E	375.992	0.524249	0.107768	4.864629	1.15E-06	3.78E-06	NOT
PDE5A	226.6866	0.887408	0.182437	4.86419	1.15E-06	3.78E-06	UP
RP5-857K	185.4585	-0.82214	0.169024	-4.86403	1.15E-06	3.79E-06	DOWN
RP11-177	5.842838	1.113289	0.228888	4.863898	1.15E-06	3.79E-06	UP
FUBP1	1638.177	0.459792	0.094536	4.863684	1.15E-06	3.79E-06	NOT
C2orf68	1251.488	0.385039	0.079177	4.862999	1.16E-06	3.81E-06	NOT
AC005753	1.615776	1.84313	0.379018	4.862912	1.16E-06	3.81E-06	UP
JARID2	575.764	0.438813	0.090244	4.862507	1.16E-06	3.81E-06	NOT
RP11-894	7.93537	0.727562	0.149637	4.862192	1.16E-06	3.82E-06	UP
RP11-977	6.881928	1.063793	0.218794	4.862077	1.16E-06	3.82E-06	UP
SLC5A5	5.19239	1.861974	0.382969	4.86195	1.16E-06	3.82E-06	UP
AC007405	94.46694	0.727177	0.149565	4.861942	1.16E-06	3.82E-06	UP
TTC9B	5.739821	1.246202	0.256327	4.861769	1.16E-06	3.83E-06	UP
BEND6	11.0801	1.663513	0.342188	4.861403	1.17E-06	3.83E-06	UP
RP11-297	9.007391	2.860782	0.588518	4.860995	1.17E-06	3.84E-06	UP
RP11-133	6.936494	1.251958	0.257571	4.860622	1.17E-06	3.85E-06	UP
MANEAL	703.6318	0.763205	0.157022	4.860506	1.17E-06	3.85E-06	UP
LRRC46	46.89418	0.677872	0.139472	4.860279	1.17E-06	3.85E-06	UP
HNRNPA3	7107.693	0.305068	0.062779	4.859367	1.18E-06	3.87E-06	NOT
MRPS2	2449.364	-0.48954	0.100747	-4.85915	1.18E-06	3.87E-06	NOT
ADAMTSL	825.0038	-1.00781	0.207414	-4.85892	1.18E-06	3.88E-06	DOWN
PIGM	869.72	0.433088	0.089137	4.858675	1.18E-06	3.88E-06	NOT
MFF	2065.088	0.344545	0.070916	4.858469	1.18E-06	3.89E-06	NOT
TARDBP	3786.508	-0.2187	0.045016	-4.85843	1.18E-06	3.89E-06	NOT

CTD-3064	1.974987	2.869256	0.590626	4.857993	1.19E-06	3.89E-06	UP
ZNF333	221.4919	0.442163	0.091029	4.857395	1.19E-06	3.91E-06	NOT
RP11-652I	6.157362	-1.16713	0.240296	-4.85707	1.19E-06	3.91E-06	DOWN
RP11-509/	4.40863	2.161435	0.445061	4.856495	1.19E-06	3.92E-06	UP
RP11-430(	2.996236	1.571352	0.323557	4.856486	1.19E-06	3.92E-06	UP
AKAP11	1118.066	-0.5226	0.107611	-4.85637	1.20E-06	3.92E-06	NOT
BMX	32.17096	-0.95036	0.195717	-4.85577	1.20E-06	3.94E-06	DOWN
DANCR	1165.497	0.806182	0.166033	4.855554	1.20E-06	3.94E-06	UP
PRDX4	6267.821	-0.56435	0.11623	-4.85543	1.20E-06	3.94E-06	NOT
FARP2	796.3598	-0.42523	0.087617	-4.85328	1.21E-06	3.98E-06	NOT
CTD-3203	8.077735	0.940505	0.1938	4.852961	1.22E-06	3.99E-06	UP
NDST2	59.80986	0.392299	0.080851	4.852117	1.22E-06	4.01E-06	NOT
RP3-324O	1.885382	1.556597	0.320824	4.851871	1.22E-06	4.01E-06	UP
SNORA5C	4.572329	1.071538	0.220852	4.851837	1.22E-06	4.01E-06	UP
RP11-752(	2.174073	1.523841	0.31408	4.851764	1.22E-06	4.01E-06	UP
RP11-458I	1.887216	1.562387	0.322027	4.85173	1.22E-06	4.01E-06	UP
FMR1-IT1	4.593004	1.301038	0.268175	4.851452	1.23E-06	4.02E-06	UP
PKD1L1	18.78122	0.872318	0.179826	4.850899	1.23E-06	4.03E-06	UP
CTD-3184	141.1769	0.682216	0.140657	4.850217	1.23E-06	4.04E-06	UP
CTD-2623	10.86384	0.747952	0.154213	4.850118	1.23E-06	4.04E-06	UP
TMPRSS9	325.0002	1.308433	0.269782	4.849959	1.23E-06	4.05E-06	UP
MIR22HG	549.9672	-0.67569	0.139328	-4.84965	1.24E-06	4.05E-06	DOWN
RP11-394I	9.191904	-1.01529	0.209356	-4.84959	1.24E-06	4.05E-06	DOWN
CELSR1	684.0744	0.959788	0.19796	4.84839	1.24E-06	4.08E-06	UP
SNX32	15.90974	0.827845	0.170753	4.848189	1.25E-06	4.08E-06	UP
NUMB	2269.85	-0.36182	0.074634	-4.84784	1.25E-06	4.09E-06	NOT
CNNM4	468.7541	0.492634	0.101623	4.847675	1.25E-06	4.09E-06	NOT
DDX58	774.0057	-0.58281	0.120226	-4.84764	1.25E-06	4.09E-06	NOT
CLEC4C	1.530083	-1.87472	0.386734	-4.84756	1.25E-06	4.09E-06	DOWN
RP11-163I	5.271095	1.029464	0.21237	4.847504	1.25E-06	4.09E-06	UP
RP11-177I	3.488757	1.42176	0.293365	4.846392	1.26E-06	4.11E-06	UP
TSPYL5	120.9706	-1.07507	0.221861	-4.8457	1.26E-06	4.13E-06	DOWN
SPG7	2798.807	-0.41058	0.084732	-4.84563	1.26E-06	4.13E-06	NOT
ZNF286B	19.49826	0.812262	0.167636	4.845385	1.26E-06	4.13E-06	UP
DPM2	1805.079	0.576782	0.11904	4.845278	1.26E-06	4.14E-06	NOT
SLC29A1	4924.001	0.719854	0.148572	4.845158	1.27E-06	4.14E-06	UP
VPS36	1474.939	-0.47551	0.098145	-4.84496	1.27E-06	4.14E-06	NOT
RP11-321(	2.073657	2.478881	0.511663	4.844754	1.27E-06	4.15E-06	UP
TAC4	4.159665	1.253441	0.258729	4.844607	1.27E-06	4.15E-06	UP
ARHGEF3	533.2342	0.845761	0.174588	4.844323	1.27E-06	4.15E-06	UP
CACTIN	705.7347	0.340086	0.070203	4.844316	1.27E-06	4.15E-06	NOT
JMJD1C	1852.408	-0.51878	0.107095	-4.84409	1.27E-06	4.16E-06	NOT
WDR38	1.178998	2.093757	0.432231	4.844073	1.27E-06	4.16E-06	UP
FAM208A	1682.8	0.379824	0.078414	4.843837	1.27E-06	4.16E-06	NOT
FGD4	1236.674	-0.7404	0.152858	-4.84369	1.27E-06	4.16E-06	DOWN
METTL5	958.1085	0.434079	0.089621	4.843508	1.28E-06	4.17E-06	NOT
C17orf62	2406.53	0.443717	0.091611	4.843466	1.28E-06	4.17E-06	NOT
ARFIP2	1870.464	0.391914	0.080916	4.84345	1.28E-06	4.17E-06	NOT
RP1-60O1	1340.719	-0.89139	0.184046	-4.84333	1.28E-06	4.17E-06	DOWN
AL022476.	4.064355	1.048913	0.216575	4.843174	1.28E-06	4.17E-06	UP
ADAMTS2	2.640795	3.122928	0.644835	4.842991	1.28E-06	4.18E-06	UP
CEP192	535.8572	0.527808	0.108995	4.842501	1.28E-06	4.19E-06	NOT

HNMT	5160.936	-0.62624	0.129324	-4.84241	1.28E-06	4.19E-06	DOWN
CCDC167	1050.752	0.673812	0.139149	4.842375	1.28E-06	4.19E-06	UP
DDX51	794.0178	0.407733	0.084203	4.842234	1.28E-06	4.19E-06	NOT
POLDIP3	2363.247	0.37369	0.077176	4.842061	1.28E-06	4.19E-06	NOT
RP11-338I	2.814925	1.513906	0.312726	4.841	1.29E-06	4.21E-06	UP
BHMT	25302.44	-1.55545	0.321366	-4.84012	1.30E-06	4.23E-06	DOWN
CRISPLD2	1021.529	-1.11387	0.230138	-4.84002	1.30E-06	4.23E-06	DOWN
IL10RA	770.4438	-0.87582	0.180955	-4.83997	1.30E-06	4.24E-06	DOWN
KIF3C	229.6135	1.092566	0.225772	4.839244	1.30E-06	4.25E-06	UP
RP11-363I	1.769463	1.918067	0.39652	4.837252	1.32E-06	4.29E-06	UP
NR1I2	2116.391	-1.39086	0.287557	-4.83681	1.32E-06	4.30E-06	DOWN
CNDP2	10097.46	-0.48774	0.100839	-4.83678	1.32E-06	4.30E-06	NOT
HSPA12B	215.2347	0.689862	0.14263	4.836724	1.32E-06	4.30E-06	UP
RP11-523I	6.275642	0.900758	0.186317	4.834554	1.33E-06	4.35E-06	UP
RP11-134(	2.991274	1.149958	0.237864	4.834524	1.33E-06	4.35E-06	UP
USP36	1605.333	0.40214	0.083186	4.834241	1.34E-06	4.35E-06	NOT
HAGH	8557.046	-0.78979	0.163381	-4.83406	1.34E-06	4.36E-06	DOWN
RP11-622(	2.661797	1.68232	0.348021	4.833959	1.34E-06	4.36E-06	UP
XXyac-YX1	3.555057	2.169786	0.448879	4.833793	1.34E-06	4.36E-06	UP
HMCN2	463.9899	1.566328	0.324037	4.833791	1.34E-06	4.36E-06	UP
CNEP1R1	355.0697	-0.35664	0.073784	-4.8336	1.34E-06	4.37E-06	NOT
RP11-20I2	12.49801	0.918904	0.19011	4.833529	1.34E-06	4.37E-06	UP
PCDHB3	23.67755	1.368711	0.28318	4.833363	1.34E-06	4.37E-06	UP
RAB11FIP3	1664.185	0.434211	0.089842	4.833036	1.34E-06	4.38E-06	NOT
KANTR	14.83131	0.944558	0.195455	4.832612	1.35E-06	4.39E-06	UP
RP11-360I	17.22104	0.82616	0.170955	4.832607	1.35E-06	4.39E-06	UP
USP3	1017.701	0.319839	0.066185	4.832499	1.35E-06	4.39E-06	NOT
APEX1	4640.017	0.356058	0.073698	4.831341	1.36E-06	4.41E-06	NOT
IGFBPL1	19.13847	1.93347	0.400246	4.830709	1.36E-06	4.43E-06	UP
AC079613	2.979249	3.165676	0.655391	4.83021	1.36E-06	4.44E-06	UP
ZNF503-A	14.22608	-0.86118	0.178316	-4.8295	1.37E-06	4.45E-06	DOWN
TEAD4	256.8316	0.964059	0.19963	4.82924	1.37E-06	4.46E-06	UP
RBM42	3824.175	0.612534	0.126841	4.829148	1.37E-06	4.46E-06	UP
SCRN1	617.694	1.217749	0.252186	4.828781	1.37E-06	4.47E-06	UP
RP11-711I	1.346037	1.900312	0.393565	4.828461	1.38E-06	4.47E-06	UP
RP11-640I	2.628529	1.678875	0.347774	4.827481	1.38E-06	4.49E-06	UP
RP11-626(	3.029794	1.515209	0.313874	4.827452	1.38E-06	4.49E-06	UP
SDF2L1	2198.228	0.736849	0.152641	4.827326	1.38E-06	4.50E-06	UP
ORAI3	1269.272	-0.56362	0.116771	-4.82668	1.39E-06	4.51E-06	NOT
DERL3	389.4631	1.232772	0.255421	4.826433	1.39E-06	4.52E-06	UP
AGAP1-IT1	45.40523	-1.35423	0.280592	-4.82633	1.39E-06	4.52E-06	DOWN
RP11-472(	2.224196	2.140207	0.443508	4.825631	1.40E-06	4.53E-06	UP
GALM	3601.175	-0.59639	0.123595	-4.82539	1.40E-06	4.54E-06	DOWN
TRAF1	418.2187	0.693999	0.143824	4.82533	1.40E-06	4.54E-06	UP
ARHGAP15	265.4957	-0.85126	0.17643	-4.82491	1.40E-06	4.55E-06	DOWN
SPATA45	3.558932	1.39159	0.288446	4.824437	1.40E-06	4.56E-06	UP
PDE6C	15.14911	0.914012	0.189461	4.824286	1.41E-06	4.56E-06	UP
CPN1	2783.058	-1.07995	0.223878	-4.82382	1.41E-06	4.57E-06	DOWN
PRPF4B	1900.44	0.391895	0.081244	4.82366	1.41E-06	4.57E-06	NOT
ZNF350-A	3.700944	1.489805	0.308934	4.822409	1.42E-06	4.60E-06	UP
MOSPD1	416.4204	0.641539	0.133033	4.822402	1.42E-06	4.60E-06	UP
ZDHHC24	811.8415	0.462459	0.095906	4.822016	1.42E-06	4.61E-06	NOT

NIPAL2	1155.945	0.753067	0.156175	4.821952	1.42E-06	4.61E-06	UP
PNPO	3219.917	-0.71262	0.147787	-4.82192	1.42E-06	4.61E-06	DOWN
C7orf55-LU	1094.104	0.242838	0.050363	4.821773	1.42E-06	4.62E-06	NOT
PRR29	47.38719	0.620004	0.128589	4.821601	1.42E-06	4.62E-06	UP
LANCL1	1859.529	0.42534	0.088223	4.821212	1.43E-06	4.63E-06	NOT
CTC-542B	1.540363	2.371141	0.491843	4.820932	1.43E-06	4.63E-06	UP
KHSRP	5556.599	0.353344	0.073294	4.820891	1.43E-06	4.63E-06	NOT
RP11-363I	1.997263	1.579031	0.327553	4.820691	1.43E-06	4.64E-06	UP
CTC-327F	1.146656	2.439168	0.506022	4.820282	1.43E-06	4.65E-06	UP
C11orf16	2.248546	1.311511	0.272085	4.820232	1.43E-06	4.65E-06	UP
PRPF6	5288.891	0.40448	0.083914	4.820202	1.43E-06	4.65E-06	NOT
SIGLEC5	11.48486	-1.09991	0.22819	-4.82013	1.43E-06	4.65E-06	DOWN
DYM	1272.044	0.319147	0.066213	4.819995	1.44E-06	4.65E-06	NOT
CYP2U1	391.937	-0.69888	0.144999	-4.81991	1.44E-06	4.65E-06	DOWN
RP11-490I	3.383841	1.210163	0.251098	4.819485	1.44E-06	4.66E-06	UP
C3orf18	266.2359	0.671228	0.139304	4.818451	1.45E-06	4.69E-06	UP
CTD-3006	2.387379	3.648798	0.757273	4.818339	1.45E-06	4.69E-06	UP
FMR1-AS1	3.974997	1.403475	0.291278	4.818331	1.45E-06	4.69E-06	UP
RUNDC1	485.9893	0.392223	0.081416	4.817542	1.45E-06	4.71E-06	NOT
IPO9-AS1	5.368878	1.017742	0.211283	4.816962	1.46E-06	4.72E-06	UP
IFITM2	9134.787	-0.76298	0.158403	-4.81668	1.46E-06	4.72E-06	DOWN
PROC	16877.46	-0.94472	0.196135	-4.81667	1.46E-06	4.72E-06	DOWN
TPSG1	21.79541	1.783206	0.370219	4.81663	1.46E-06	4.72E-06	UP
BNIP3P10	1.323898	1.810797	0.375994	4.816025	1.46E-06	4.74E-06	UP
VNN3	866.9301	-1.25178	0.259924	-4.81597	1.46E-06	4.74E-06	DOWN
TRPC7-AS	1.588257	2.788013	0.578962	4.815537	1.47E-06	4.75E-06	UP
RP3-438O	1.219623	2.498883	0.518981	4.81498	1.47E-06	4.76E-06	UP
IGHMBP2	786.4782	0.529891	0.110051	4.81497	1.47E-06	4.76E-06	NOT
TCEAL7	25.20571	-0.78905	0.163884	-4.81469	1.47E-06	4.77E-06	DOWN
LUC7L	799.5743	0.484643	0.100663	4.814484	1.48E-06	4.77E-06	NOT
AC006273	28.73911	1.268557	0.263493	4.814379	1.48E-06	4.77E-06	UP
RP11-102(	4.364039	1.330951	0.276514	4.813327	1.48E-06	4.80E-06	UP
MAP1S	1187.641	0.442501	0.091934	4.81325	1.48E-06	4.80E-06	NOT
AMN1	102.6392	0.495373	0.102933	4.8126	1.49E-06	4.81E-06	NOT
BTBD3	838.0515	0.62145	0.12916	4.81147	1.50E-06	4.84E-06	UP
RP11-552I	1.297751	1.925754	0.400382	4.809786	1.51E-06	4.88E-06	UP
CTC-550B	13.38771	0.994453	0.206764	4.809597	1.51E-06	4.89E-06	UP
RASAL1	58.89861	1.58073	0.328667	4.80952	1.51E-06	4.89E-06	UP
RP11-326(	22.45521	0.75331	0.15664	4.809192	1.52E-06	4.89E-06	UP
TLE6	123.7622	1.07049	0.222604	4.808946	1.52E-06	4.90E-06	UP
RP11-727I	4.075871	1.192576	0.248036	4.808081	1.52E-06	4.92E-06	UP
NR3C1	3103.751	-0.46063	0.095809	-4.80782	1.53E-06	4.93E-06	NOT
RP4-756G	16.04451	0.872329	0.18145	4.807551	1.53E-06	4.93E-06	UP
RP11-96A	17.24465	1.936601	0.402847	4.80729	1.53E-06	4.94E-06	UP
CDH22	16.89726	2.746636	0.571365	4.807149	1.53E-06	4.94E-06	UP
APBB3	751.5453	0.65955	0.137206	4.807015	1.53E-06	4.94E-06	UP
CCDC176	145.6497	-0.51367	0.106859	-4.80697	1.53E-06	4.94E-06	NOT
AC007966	4.142005	1.771771	0.368601	4.806741	1.53E-06	4.95E-06	UP
NKAP	906.1597	0.387681	0.080658	4.806477	1.54E-06	4.96E-06	NOT
bP-21201I	2.821625	2.272448	0.472874	4.805611	1.54E-06	4.98E-06	UP
RP11-734I	49.15964	3.091317	0.64329	4.80548	1.54E-06	4.98E-06	UP
MRPL23-A	13.13438	2.319152	0.4827	4.804538	1.55E-06	5.00E-06	UP

HNRNPA3I	28.53245	0.575941	0.119883	4.804206	1.55E-06	5.01E-06	NOT
RP11-435I	11.3071	0.880926	0.183402	4.803263	1.56E-06	5.03E-06	UP
SHANK3	1723.676	0.542447	0.112938	4.803073	1.56E-06	5.04E-06	NOT
RP11-961/	5.905313	-1.10251	0.229547	-4.80299	1.56E-06	5.04E-06	DOWN
RPS18P12	14.25346	1.011828	0.210699	4.80225	1.57E-06	5.06E-06	UP
SVIL	1640.807	0.645403	0.134404	4.801961	1.57E-06	5.06E-06	UP
SIM2	77.98611	1.066134	0.222026	4.801842	1.57E-06	5.07E-06	UP
BAK1P1	4.079605	1.180942	0.245946	4.801636	1.57E-06	5.07E-06	UP
RP11-86H	12.58144	1.350638	0.281299	4.801434	1.58E-06	5.08E-06	UP
RP11-295I	5.271374	1.02554	0.213598	4.801272	1.58E-06	5.08E-06	UP
MGST2	5065.734	-0.61583	0.128265	-4.80123	1.58E-06	5.08E-06	DOWN
NIPAL3	457.4309	0.513045	0.106864	4.800901	1.58E-06	5.09E-06	NOT
MS4A4E	12.33506	-0.8861	0.184595	-4.80024	1.58E-06	5.10E-06	DOWN
POP4	1287.352	0.398723	0.083069	4.799883	1.59E-06	5.11E-06	NOT
IFT140	362.2079	0.606611	0.126384	4.799746	1.59E-06	5.11E-06	UP
MCCC1	2391.063	-0.5907	0.123079	-4.79936	1.59E-06	5.12E-06	DOWN
ZBTB12P1	3.415759	1.321136	0.275281	4.799226	1.59E-06	5.13E-06	UP
AL713999.	1.014429	1.941754	0.404622	4.798936	1.60E-06	5.13E-06	UP
YWHAH	4053.642	0.435823	0.090831	4.798164	1.60E-06	5.15E-06	NOT
IRF2BP2	7178.539	0.424635	0.088512	4.797501	1.61E-06	5.17E-06	NOT
NNT	7871.816	-0.66261	0.138127	-4.79711	1.61E-06	5.18E-06	DOWN
TUBD1	246.7604	0.433809	0.090432	4.797095	1.61E-06	5.18E-06	NOT
RP11-110(	60.26751	0.584809	0.121909	4.797079	1.61E-06	5.18E-06	NOT
AC091729	229.1116	0.629661	0.131261	4.797017	1.61E-06	5.18E-06	UP
GM2A	2966.572	0.463423	0.096609	4.79689	1.61E-06	5.18E-06	NOT
RP5-1096J	2.615485	3.665234	0.764092	4.796849	1.61E-06	5.18E-06	UP
RP11-20J1	5.222076	-1.52276	0.317497	-4.79614	1.62E-06	5.20E-06	DOWN
RP11-110I	9.957966	0.995733	0.207612	4.796118	1.62E-06	5.20E-06	UP
PRKCB	239.3116	-1.04337	0.217549	-4.79601	1.62E-06	5.20E-06	DOWN
DOC2B	151.8425	1.002728	0.209097	4.795527	1.62E-06	5.21E-06	UP
SLC9A6	635.758	0.430692	0.089819	4.795136	1.63E-06	5.22E-06	NOT
RP11-353I	31.80768	-1.46894	0.306371	-4.79464	1.63E-06	5.24E-06	DOWN
NARS	4805.227	0.355455	0.07415	4.793737	1.64E-06	5.26E-06	NOT
DLST	4669.765	-0.4217	0.08797	-4.79367	1.64E-06	5.26E-06	NOT
FAM35BP	29.8549	-1.05304	0.219694	-4.79319	1.64E-06	5.27E-06	DOWN
NME3	1360.152	0.608153	0.126892	4.792698	1.65E-06	5.28E-06	UP
RP11-426(	6.184905	2.022928	0.422103	4.792494	1.65E-06	5.29E-06	UP
AC005150	4.966785	4.646437	0.969694	4.791656	1.65E-06	5.31E-06	UP
ORMDL3	11638.72	-0.60248	0.125736	-4.79161	1.65E-06	5.31E-06	DOWN
ZNF506	266.5638	0.849114	0.177249	4.790522	1.66E-06	5.34E-06	UP
FLJ22763	126.8116	-1.76429	0.368289	-4.7905	1.66E-06	5.34E-06	DOWN
RP11-101I	410.9649	0.426383	0.089012	4.79017	1.67E-06	5.35E-06	NOT
TRMT12	421.5401	0.449923	0.093935	4.7897	1.67E-06	5.36E-06	NOT
ENDOG	454.3463	-0.67287	0.140483	-4.78966	1.67E-06	5.36E-06	DOWN
SH3BGRL2	3623.721	-0.73313	0.153075	-4.78939	1.67E-06	5.37E-06	DOWN
MED28P8	1.502933	-2.44781	0.511158	-4.78876	1.68E-06	5.38E-06	DOWN
MOB3A	963.9496	0.487941	0.101894	4.788693	1.68E-06	5.38E-06	NOT
SLCO1B3	1730.792	-2.06658	0.431567	-4.78854	1.68E-06	5.39E-06	DOWN
ASIC4	2.201361	2.020709	0.422023	4.788146	1.68E-06	5.40E-06	UP
NAA30	1123.726	-0.349	0.072897	-4.78758	1.69E-06	5.41E-06	NOT
FHL1	1182.248	-0.88855	0.185606	-4.78728	1.69E-06	5.42E-06	DOWN
RP11-407I	16.6383	0.741259	0.154841	4.787241	1.69E-06	5.42E-06	UP

CTD-3199	14.50505	0.743293	0.155269	4.787139	1.69E-06	5.42E-06	UP
ZCCHC14	2050.217	-0.45334	0.094708	-4.78668	1.70E-06	5.43E-06	NOT
ASS1P7	2.387018	-1.44256	0.301415	-4.78596	1.70E-06	5.45E-06	DOWN
AC144530	61.03618	0.703113	0.146919	4.785725	1.70E-06	5.46E-06	UP
PPAPDC1A	29.59257	-1.74582	0.364805	-4.78562	1.70E-06	5.46E-06	DOWN
TFDP3	8.657208	5.211272	1.08907	4.785067	1.71E-06	5.48E-06	UP
DIS3L2	858.132	0.295496	0.061758	4.784711	1.71E-06	5.48E-06	NOT
RP1-20C7.	9.87488	0.824589	0.172353	4.784312	1.72E-06	5.49E-06	UP
RP4-585I1	1.959063	1.657069	0.346367	4.784148	1.72E-06	5.50E-06	UP
PTGER3	62.81568	1.28948	0.269552	4.783782	1.72E-06	5.51E-06	UP
RPL5P23	5.523297	0.886351	0.185307	4.783155	1.73E-06	5.52E-06	UP
SSH2	649.167	0.40431	0.08453	4.783054	1.73E-06	5.53E-06	NOT
SNHG17	650.5803	0.711454	0.148779	4.781952	1.74E-06	5.56E-06	UP
AMT	2391.579	-0.66263	0.138578	-4.78164	1.74E-06	5.56E-06	DOWN
PCDH9-AS	12.54977	-2.97004	0.621137	-4.78163	1.74E-06	5.56E-06	DOWN
HNRNPUL2	19.76411	0.730756	0.152828	4.781559	1.74E-06	5.57E-06	UP
RP11-308I	3.21561	4.078212	0.852969	4.781198	1.74E-06	5.57E-06	UP
FXYD2	889.3587	2.056834	0.4302	4.78111	1.74E-06	5.58E-06	UP
FAAH2	433.3995	-0.87966	0.184016	-4.78037	1.75E-06	5.60E-06	DOWN
LRRC42	1223.343	0.482792	0.100995	4.780354	1.75E-06	5.60E-06	NOT
HIST2H2A	37.89813	0.957054	0.200208	4.780293	1.75E-06	5.60E-06	UP
WASH4P	23.74582	0.66734	0.139603	4.780282	1.75E-06	5.60E-06	UP
CDC14B	1852.905	-0.78935	0.165126	-4.78026	1.75E-06	5.60E-06	DOWN
MYO5A	519.3832	0.757258	0.158438	4.779519	1.76E-06	5.62E-06	UP
CDKN2AIP	624.9364	-0.40957	0.0857	-4.77912	1.76E-06	5.63E-06	NOT
FAM19A4	11.18522	2.935511	0.614244	4.779059	1.76E-06	5.63E-06	UP
CFAP69	100.9235	-0.71371	0.149348	-4.77884	1.76E-06	5.63E-06	DOWN
ZNF766	454.0614	0.375517	0.078582	4.778686	1.76E-06	5.64E-06	NOT
LINC00578	3.805311	2.063664	0.431897	4.778141	1.77E-06	5.65E-06	UP
CCK	1.812865	-1.99079	0.416688	-4.77764	1.77E-06	5.67E-06	DOWN
WDR45B	3069.799	0.352542	0.073817	4.77589	1.79E-06	5.71E-06	NOT
C1orf186	152.5207	1.588182	0.332602	4.775017	1.80E-06	5.74E-06	UP
WI2-8903	1.786593	1.887808	0.395462	4.773672	1.81E-06	5.78E-06	UP
KCTD13	389.1917	0.442926	0.092785	4.773671	1.81E-06	5.78E-06	NOT
POLD2P1	2.462052	2.362523	0.494916	4.773581	1.81E-06	5.78E-06	UP
RP11-304I	1.472122	2.253873	0.472209	4.77304	1.81E-06	5.79E-06	UP
BMPR2	2055.279	-0.50375	0.105553	-4.77249	1.82E-06	5.81E-06	NOT
HNRNPA3I	6.630947	0.889789	0.186472	4.771691	1.83E-06	5.83E-06	UP
ZNF691	409.6646	0.416071	0.087205	4.771206	1.83E-06	5.84E-06	NOT
ANAPC5	4052.166	0.274843	0.057606	4.771102	1.83E-06	5.85E-06	NOT
AGER	110.7007	0.652021	0.136673	4.770666	1.84E-06	5.86E-06	UP
DDB1	8230.932	0.256352	0.053739	4.770357	1.84E-06	5.87E-06	NOT
PDPN	78.05981	1.758003	0.368594	4.769486	1.85E-06	5.89E-06	UP
LRCOL1	386.4482	-1.66691	0.349505	-4.76934	1.85E-06	5.89E-06	DOWN
TPM4	6109.468	0.735054	0.154133	4.768953	1.85E-06	5.91E-06	UP
BCKDHA	309.1399	-0.72846	0.152778	-4.76806	1.86E-06	5.93E-06	DOWN
EOGT	349.4079	0.477897	0.100231	4.767944	1.86E-06	5.93E-06	NOT
RP11-203I	15.6857	0.659367	0.138293	4.767888	1.86E-06	5.93E-06	UP
RP11-196(	4.035868	1.768541	0.370931	4.76784	1.86E-06	5.94E-06	UP
CAGE1	3.119049	1.846731	0.387343	4.767683	1.86E-06	5.94E-06	UP
TNFRSF10I	2554.518	-0.48223	0.101162	-4.76691	1.87E-06	5.96E-06	NOT
SLC25A5P	3.872313	1.758537	0.368935	4.766523	1.87E-06	5.97E-06	UP

BLOC1S4	513.7122	0.396995	0.083292	4.766318	1.88E-06	5.98E-06	NOT
GPR160	187.4557	1.010296	0.211969	4.766237	1.88E-06	5.98E-06	UP
RBMX2	620.749	0.388529	0.081518	4.766178	1.88E-06	5.98E-06	NOT
AP006216.	38.35077	-1.41256	0.296387	-4.76593	1.88E-06	5.99E-06	DOWN
RP11-15A	4.643664	1.392735	0.292248	4.765589	1.88E-06	6.00E-06	UP
CTD-2515	13.07116	1.105182	0.231915	4.765452	1.88E-06	6.00E-06	UP
TBL2	1473.32	0.343361	0.072053	4.765415	1.88E-06	6.00E-06	NOT
PCYOX1L	125.3196	0.737005	0.1547	4.764102	1.90E-06	6.04E-06	UP
EML2	902.9897	0.55341	0.116175	4.763608	1.90E-06	6.05E-06	NOT
PLCL1	76.80897	0.848303	0.178085	4.763476	1.90E-06	6.06E-06	UP
SUPT16H	2737.732	0.335484	0.070433	4.7632	1.91E-06	6.06E-06	NOT
RP11-326(	39.28622	1.356792	0.284875	4.762763	1.91E-06	6.08E-06	UP
MUL1	1502.654	-0.37243	0.078198	-4.76264	1.91E-06	6.08E-06	NOT
CNGA1	787.1874	-1.25232	0.262956	-4.76246	1.91E-06	6.08E-06	DOWN
AL365181.	7.174287	0.829061	0.174114	4.761592	1.92E-06	6.11E-06	UP
RP11-425I	3.379277	1.335628	0.280523	4.761211	1.92E-06	6.12E-06	UP
RP11-774(	143.1006	0.727942	0.152898	4.760952	1.93E-06	6.13E-06	UP
SRSF6	4926.832	-0.28438	0.059735	-4.76065	1.93E-06	6.14E-06	NOT
RP5-968D	51.75776	1.177695	0.247382	4.760624	1.93E-06	6.14E-06	UP
KIR2DL3	2.729196	-1.39383	0.292868	-4.75923	1.94E-06	6.18E-06	DOWN
TMEM200	62.24884	1.221295	0.256648	4.758634	1.95E-06	6.20E-06	UP
ART4	1240.029	-1.04018	0.218597	-4.75844	1.95E-06	6.20E-06	DOWN
AGPAT2	7835.307	-0.86772	0.182359	-4.75832	1.95E-06	6.20E-06	DOWN
GUCY1B2	27.48631	1.984376	0.417035	4.758297	1.95E-06	6.20E-06	UP
PELI2	146.0499	-1.12653	0.236788	-4.75755	1.96E-06	6.23E-06	DOWN
SOWAHB	750.9237	-0.88504	0.186033	-4.75744	1.96E-06	6.23E-06	DOWN
TAP1	2718.657	0.724593	0.152317	4.757153	1.96E-06	6.24E-06	UP
TDP1	406.5212	0.418644	0.088009	4.756823	1.97E-06	6.25E-06	NOT
CHRM3	156.6497	1.293167	0.271857	4.756795	1.97E-06	6.25E-06	UP
AC004932	2.598421	1.2858	0.270313	4.756715	1.97E-06	6.25E-06	UP
RP11-830I	65.7639	-1.82045	0.382736	-4.75641	1.97E-06	6.26E-06	DOWN
RP11-384J	1.69859	2.957282	0.621807	4.755948	1.98E-06	6.27E-06	UP
SPATA33	151.902	0.477984	0.100512	4.755501	1.98E-06	6.28E-06	NOT
FAM157C	8.948012	1.293032	0.271908	4.755405	1.98E-06	6.29E-06	UP
RP11-113I	2.717199	1.498997	0.315253	4.754899	1.99E-06	6.30E-06	UP
SYTL3	71.02423	-0.87271	0.183574	-4.75399	1.99E-06	6.33E-06	DOWN
PRR15L	263.8615	1.522689	0.320338	4.753378	2.00E-06	6.35E-06	UP
ATP1A1	21280.58	0.669637	0.140879	4.753278	2.00E-06	6.35E-06	UP
SRD5A2	1997.112	-1.61708	0.340308	-4.75181	2.02E-06	6.39E-06	DOWN
CLCN5	1747.07	-0.6495	0.136693	-4.7515	2.02E-06	6.40E-06	DOWN
HIP1R	2526.869	0.534453	0.112494	4.75093	2.02E-06	6.42E-06	NOT
TM6SF2	957.2493	-1.13797	0.239527	-4.75088	2.03E-06	6.42E-06	DOWN
RIIAD1	2.178666	1.906262	0.401245	4.750874	2.03E-06	6.42E-06	UP
SYT9	140.497	-2.32034	0.488407	-4.75083	2.03E-06	6.42E-06	DOWN
PREB	4669.086	0.584351	0.123002	4.75074	2.03E-06	6.42E-06	NOT
TAGAP	141.5124	-1.03539	0.217945	-4.75067	2.03E-06	6.43E-06	DOWN
AP000472.	2.240379	-1.7418	0.366657	-4.75049	2.03E-06	6.43E-06	DOWN
SLC31A2	68.6684	-0.83014	0.174765	-4.75001	2.03E-06	6.45E-06	DOWN
SYCP2L	7.866662	1.441932	0.303567	4.749962	2.03E-06	6.45E-06	UP
ARMC6	2677.614	-0.7214	0.151889	-4.74949	2.04E-06	6.46E-06	DOWN
IFITM5	3.991493	2.639664	0.555796	4.749343	2.04E-06	6.46E-06	UP
RP11-583I	2.23608	1.545574	0.325457	4.748934	2.04E-06	6.48E-06	UP

NUDT11	7.531227	1.775432	0.373883	4.748629	2.05E-06	6.49E-06	UP
ZNF813	155.3814	1.074544	0.226292	4.748485	2.05E-06	6.49E-06	UP
ERGIC1	12041.6	-0.49273	0.103768	-4.74837	2.05E-06	6.49E-06	NOT
KIF5C	71.89267	1.276222	0.268773	4.748321	2.05E-06	6.49E-06	UP
ITFG1-AS1	1.862099	1.529625	0.322169	4.747896	2.06E-06	6.51E-06	UP
CTD-3193	2.97447	1.23473	0.260076	4.747577	2.06E-06	6.52E-06	UP
ACSS1	775.2635	0.923666	0.194559	4.747491	2.06E-06	6.52E-06	UP
RP11-43F1	284.21	0.756561	0.159361	4.747473	2.06E-06	6.52E-06	UP
SLC10A4	2.88604	1.886861	0.397453	4.747376	2.06E-06	6.52E-06	UP
ARRDC3	3252.459	-0.77528	0.163312	-4.74724	2.06E-06	6.52E-06	DOWN
BCL7A	803.3676	0.463018	0.097536	4.747143	2.06E-06	6.53E-06	NOT
HCG23	2.606112	-1.25674	0.264804	-4.74594	2.08E-06	6.56E-06	DOWN
RP11-390I	7.576216	1.115602	0.235072	4.745794	2.08E-06	6.57E-06	UP
ZFAND5	11093.64	-0.79768	0.168085	-4.74573	2.08E-06	6.57E-06	DOWN
LINC0063(	23.62907	0.772639	0.162813	4.745572	2.08E-06	6.57E-06	UP
RP11-448(	33.54841	0.931863	0.196391	4.74494	2.09E-06	6.59E-06	UP
SULT1A3	1.764044	1.445331	0.30461	4.744855	2.09E-06	6.60E-06	UP
DGCR11	60.80179	0.61217	0.129032	4.744309	2.09E-06	6.61E-06	UP
CCDC22	917.1219	0.37274	0.078567	4.744228	2.09E-06	6.62E-06	NOT
RP5-991G:	3.181758	1.284702	0.270822	4.743706	2.10E-06	6.63E-06	UP
IRF1	2645.129	-0.68354	0.144102	-4.74346	2.10E-06	6.64E-06	DOWN
CITF22-49	8.49316	1.392276	0.293517	4.743422	2.10E-06	6.64E-06	UP
RP4-760C!	2.429622	1.639012	0.345558	4.743087	2.10E-06	6.65E-06	UP
RP11-34F1	1.282047	2.283746	0.481544	4.74255	2.11E-06	6.67E-06	UP
RP11-72M	11.04164	-0.96786	0.204137	-4.74122	2.12E-06	6.71E-06	DOWN
ACY3	1053.658	-0.99141	0.209109	-4.74112	2.13E-06	6.71E-06	DOWN
IL12A	9.869666	1.224969	0.258409	4.740427	2.13E-06	6.73E-06	UP
RP11-181(	25.37952	0.744737	0.157121	4.739891	2.14E-06	6.75E-06	UP
SRRM4	1.743028	2.369772	0.499988	4.73966	2.14E-06	6.76E-06	UP
SPAG6	2.745466	2.069601	0.436663	4.739584	2.14E-06	6.76E-06	UP
RP11-483I	7.4521	-0.93181	0.196643	-4.73859	2.15E-06	6.79E-06	DOWN
CTA-38K2	1.591947	1.670431	0.352581	4.737728	2.16E-06	6.82E-06	UP
KHNYN	1273.589	0.543389	0.114698	4.737569	2.16E-06	6.83E-06	NOT
SLC25A43	782.4354	0.519691	0.109697	4.737516	2.16E-06	6.83E-06	NOT
SLC35F2	93.76672	1.248528	0.263548	4.737381	2.16E-06	6.83E-06	UP
ELAVL3	1.777501	1.666119	0.351708	4.737225	2.17E-06	6.83E-06	UP
SLC7A1	455.0214	1.158972	0.244661	4.737061	2.17E-06	6.84E-06	UP
NUDCD3	3053.807	0.323001	0.068189	4.736856	2.17E-06	6.85E-06	NOT
CTU1	153.3781	0.682916	0.144175	4.736715	2.17E-06	6.85E-06	UP
MTND2P2	12419.45	-0.98884	0.208777	-4.73632	2.18E-06	6.86E-06	DOWN
RP11-428:	53.45236	0.695892	0.146929	4.73626	2.18E-06	6.86E-06	UP
CTA-313A	1.061583	1.956875	0.413203	4.735864	2.18E-06	6.88E-06	UP
MAOA	8201.103	-0.63613	0.134327	-4.73568	2.18E-06	6.88E-06	DOWN
TUBB1	10.63463	-0.92224	0.194761	-4.73525	2.19E-06	6.89E-06	DOWN
AP001372.	129.5482	0.484564	0.102349	4.734414	2.20E-06	6.92E-06	NOT
ZNF786	228.3709	0.37624	0.079469	4.734414	2.20E-06	6.92E-06	NOT
RP11-474(	19.14159	-0.78198	0.165171	-4.73438	2.20E-06	6.92E-06	DOWN
AP001469.	2.189287	1.425307	0.301091	4.733802	2.20E-06	6.94E-06	UP
SH2D3A	196.7719	1.101887	0.232775	4.733702	2.20E-06	6.94E-06	UP
CAPN13	42.50715	2.286365	0.483009	4.733591	2.21E-06	6.95E-06	UP
SLC12A1	81.33509	2.308601	0.487718	4.733479	2.21E-06	6.95E-06	UP
ZMYND12	144.2978	-0.99487	0.210202	-4.73294	2.21E-06	6.97E-06	DOWN

MRPL34	3122.969	-0.52957	0.111895	-4.73274	2.22E-06	6.97E-06	NOT
IGLV3-25	174.2962	-1.78889	0.377999	-4.73253	2.22E-06	6.98E-06	DOWN
RP11-319(	4.535017	1.506824	0.318415	4.732267	2.22E-06	6.99E-06	UP
NIFK-AS1	122.9958	0.482147	0.101896	4.731737	2.23E-06	7.01E-06	NOT
RP11-674I	2.271508	2.021134	0.427162	4.731539	2.23E-06	7.01E-06	UP
WWC2	1234.487	-0.61503	0.12999	-4.73133	2.23E-06	7.02E-06	DOWN
CA2	4239.069	-1.07196	0.226578	-4.73108	2.23E-06	7.03E-06	DOWN
ISPD	169.1291	-0.72304	0.152842	-4.73064	2.24E-06	7.04E-06	DOWN
VNN1	9029.229	-1.39798	0.295532	-4.73039	2.24E-06	7.05E-06	DOWN
BAG4	843.7776	-0.50999	0.107822	-4.72988	2.25E-06	7.07E-06	NOT
HELQ	335.5488	-0.34211	0.07233	-4.72984	2.25E-06	7.07E-06	NOT
CTD-3116	4.460743	-1.11792	0.236361	-4.7297	2.25E-06	7.07E-06	DOWN
GLDC	4228.031	-1.00802	0.213129	-4.72964	2.25E-06	7.07E-06	DOWN
PIGF	358.828	0.364704	0.077113	4.729455	2.25E-06	7.08E-06	NOT
RNF43	1283.753	1.071589	0.226582	4.729356	2.25E-06	7.08E-06	UP
METTL2A	583.6414	0.352317	0.074496	4.729323	2.25E-06	7.08E-06	NOT
RP11-571I	44.15538	-0.67458	0.142643	-4.72913	2.25E-06	7.09E-06	DOWN
XDH	4086.383	-1.19653	0.253039	-4.72862	2.26E-06	7.10E-06	DOWN
RALGPS1	286.5218	1.041514	0.220265	4.728462	2.26E-06	7.11E-06	UP
PRKAB2	3280.093	0.67198	0.142122	4.728189	2.27E-06	7.12E-06	UP
AP001505.	66.88082	0.844258	0.178581	4.727587	2.27E-06	7.14E-06	UP
CATIP	15.43313	0.928161	0.196334	4.727447	2.27E-06	7.14E-06	UP
JMJD7-PL.	79.76115	0.858395	0.181598	4.726903	2.28E-06	7.16E-06	UP
RP11-417I	15.91776	1.570721	0.332301	4.726804	2.28E-06	7.16E-06	UP
PHC2	5178.865	-0.46426	0.09822	-4.7267	2.28E-06	7.16E-06	NOT
C12orf4	370.5286	0.358454	0.075843	4.726253	2.29E-06	7.18E-06	NOT
CDKAL1	572.2839	0.354348	0.074979	4.725988	2.29E-06	7.19E-06	NOT
GEMIN7	654.189	0.628838	0.133075	4.725444	2.30E-06	7.21E-06	UP
MMP19	290.5134	-0.90707	0.191954	-4.72544	2.30E-06	7.21E-06	DOWN
ZNF578	7.465826	1.468095	0.310702	4.725083	2.30E-06	7.22E-06	UP
PRSS16	71.17739	2.16432	0.458082	4.724746	2.30E-06	7.23E-06	UP
KIAA2013	3768.76	-0.4827	0.102166	-4.7247	2.30E-06	7.23E-06	NOT
TAF5	195.2381	0.395777	0.083769	4.724646	2.31E-06	7.23E-06	NOT
AP000925.	3.738891	-1.02585	0.217133	-4.72451	2.31E-06	7.23E-06	DOWN
DPCR1	14.8701	-2.52715	0.535001	-4.72365	2.32E-06	7.26E-06	DOWN
C7orf60	180.3239	0.51309	0.108627	4.723419	2.32E-06	7.27E-06	NOT
SMURF1	1491.037	0.444017	0.094006	4.72329	2.32E-06	7.28E-06	NOT
ATF5	39294.07	-1.31041	0.277459	-4.72291	2.32E-06	7.29E-06	DOWN
RP11-789(	6.963721	1.3392	0.283576	4.722551	2.33E-06	7.30E-06	UP
SNURF	7.088404	1.233346	0.261175	4.722297	2.33E-06	7.31E-06	UP
RP5-1024I	5.081014	1.091832	0.231269	4.721055	2.35E-06	7.35E-06	UP
COQ9	3035.672	-0.5275	0.111759	-4.71998	2.36E-06	7.39E-06	NOT
LINC01207	27.54725	2.903646	0.61519	4.71992	2.36E-06	7.39E-06	UP
AGAP5	6.845545	0.990502	0.209861	4.719804	2.36E-06	7.40E-06	UP
ZNF665	31.96821	1.088767	0.23076	4.71817	2.38E-06	7.45E-06	UP
AP1S1	2042.598	0.432837	0.091742	4.718004	2.38E-06	7.46E-06	NOT
RP13-16H	1.800846	1.572933	0.333406	4.717765	2.38E-06	7.47E-06	UP
PADI1	71.20401	2.543325	0.539101	4.717711	2.39E-06	7.47E-06	UP
RP11-757(	118.2316	1.679617	0.356027	4.717668	2.39E-06	7.47E-06	UP
RP3-467L1	7.639829	0.863444	0.183025	4.71764	2.39E-06	7.47E-06	UP
SLC9A5	18.26535	0.991262	0.21015	4.716928	2.39E-06	7.50E-06	UP
TRAF3IP2-	47.83466	0.473697	0.100426	4.716878	2.39E-06	7.50E-06	NOT

RP11-241I	2.12788	3.164835	0.671065	4.71614	2.40E-06	7.52E-06	UP
ZNF189	910.9571	0.568923	0.120639	4.715923	2.41E-06	7.53E-06	NOT
KB-431C1.	9.2315	0.974237	0.206592	4.715743	2.41E-06	7.54E-06	UP
ZNF274	958.2612	-0.41911	0.088877	-4.71555	2.41E-06	7.54E-06	NOT
DLX2	2.000253	2.514549	0.533295	4.715121	2.42E-06	7.56E-06	UP
VPS4B	1500.252	-0.33698	0.07147	-4.71502	2.42E-06	7.56E-06	NOT
PPIAP9	4.674161	1.276314	0.270768	4.713686	2.43E-06	7.61E-06	UP
TRIM67	17.66593	1.143534	0.242615	4.713362	2.44E-06	7.62E-06	UP
KLHDC7B	83.72987	1.124529	0.238619	4.712659	2.45E-06	7.65E-06	UP
CTD-2026	1.691332	1.590456	0.337529	4.712064	2.45E-06	7.67E-06	UP
IPCEF1	64.14217	-0.94559	0.200682	-4.71188	2.45E-06	7.67E-06	DOWN
NTRK3	24.81239	-1.2348	0.2621	-4.71116	2.46E-06	7.70E-06	DOWN
CD46	9025.729	0.472542	0.100314	4.710642	2.47E-06	7.72E-06	NOT
IGKV1D-4	2.2475	-2.15003	0.45643	-4.71054	2.47E-06	7.72E-06	DOWN
GPR176	311.9026	0.834042	0.177071	4.710223	2.47E-06	7.73E-06	UP
GYS1	789.6599	0.51623	0.109606	4.709878	2.48E-06	7.74E-06	NOT
CFD	323.448	-0.97258	0.206519	-4.70942	2.48E-06	7.76E-06	DOWN
C1QTNF7	43.87166	-1.13213	0.240407	-4.70924	2.49E-06	7.77E-06	DOWN
RP5-994D	2.074337	1.614492	0.342842	4.709144	2.49E-06	7.77E-06	UP
ARMC12	21.45451	0.937652	0.19914	4.70851	2.50E-06	7.79E-06	UP
RP11-48B	39.97989	0.623772	0.13248	4.708407	2.50E-06	7.80E-06	UP
VN1R51P	1.761279	2.282849	0.484867	4.708195	2.50E-06	7.80E-06	UP
GGCT	1111.69	0.466301	0.09905	4.707734	2.50E-06	7.82E-06	NOT
GALNT18	804.0313	0.77531	0.164705	4.707254	2.51E-06	7.84E-06	UP
LTBP4	2116.112	-0.85461	0.181554	-4.70717	2.51E-06	7.84E-06	DOWN
SBNO1	1279.108	0.314188	0.06675	4.706903	2.52E-06	7.85E-06	NOT
CTD-2147	3.304938	3.388855	0.71998	4.706874	2.52E-06	7.85E-06	UP
AC112497	46.3183	0.607175	0.129003	4.70666	2.52E-06	7.86E-06	UP
RP11-279	6.75042	1.211968	0.257537	4.706003	2.53E-06	7.88E-06	UP
CHMP7	1249.508	-0.3381	0.071848	-4.70579	2.53E-06	7.89E-06	NOT
SOS1-IT1	30.67025	0.587789	0.124928	4.705022	2.54E-06	7.92E-06	UP
NELFCD	3479.095	0.345721	0.07348	4.704951	2.54E-06	7.92E-06	NOT
AP000569.	1.79433	2.293512	0.487498	4.704659	2.54E-06	7.93E-06	UP
CCDC7	77.92323	0.570835	0.121341	4.704396	2.55E-06	7.94E-06	NOT
FKTN	492.2725	0.45103	0.095879	4.704146	2.55E-06	7.95E-06	NOT
CTD-2224	5.255399	1.750416	0.372106	4.704085	2.55E-06	7.95E-06	UP
COX6C	10185.66	0.760278	0.161629	4.70385	2.55E-06	7.96E-06	UP
PTP4A2P1	18.4031	0.827942	0.176029	4.703452	2.56E-06	7.97E-06	UP
RP11-867	9.536005	0.77624	0.165081	4.702191	2.57E-06	8.02E-06	UP
CHKB-AS1	32.3811	0.695955	0.148023	4.701669	2.58E-06	8.04E-06	UP
SLC6A11	430.0964	2.419787	0.514677	4.701564	2.58E-06	8.04E-06	UP
CTDSP1	6752.71	-0.3451	0.073401	-4.70155	2.58E-06	8.04E-06	NOT
FGF22	4.618789	1.437128	0.305692	4.701226	2.59E-06	8.06E-06	UP
RP11-618I	1.481927	2.28272	0.485578	4.701041	2.59E-06	8.06E-06	UP
KCNJ15	115.0104	-1.06499	0.22655	-4.70088	2.59E-06	8.07E-06	DOWN
CNTN4	114.9693	-1.06848	0.227293	-4.70086	2.59E-06	8.07E-06	DOWN
TMEM132	5.212253	-2.17926	0.46359	-4.70083	2.59E-06	8.07E-06	DOWN
GS1-279B	5.799182	1.038148	0.220846	4.700771	2.59E-06	8.07E-06	UP
AC016739	80.47696	0.73941	0.157299	4.700673	2.59E-06	8.07E-06	UP
RP11-401I	17.04041	1.404345	0.298782	4.700238	2.60E-06	8.09E-06	UP
SAP30L-A	6.636729	0.791803	0.168471	4.699929	2.60E-06	8.10E-06	UP
DCTN1	3768.152	0.332975	0.070847	4.699906	2.60E-06	8.10E-06	NOT

RP11-73M	5.720175	1.749742	0.372308	4.699711	2.61E-06	8.11E-06	UP
FCGR2C	66.32819	1.352278	0.287759	4.699334	2.61E-06	8.12E-06	UP
ZNF525	137.4791	1.010009	0.214937	4.699081	2.61E-06	8.13E-06	UP
SAMD8	687.8369	-0.43179	0.091894	-4.69875	2.62E-06	8.14E-06	NOT
CCDC38	63.7571	-1.02407	0.217968	-4.69824	2.62E-06	8.16E-06	DOWN
LPAR2	225.2344	1.172313	0.24953	4.698076	2.63E-06	8.17E-06	UP
PRKG1-AS	2.349171	2.698912	0.574503	4.697822	2.63E-06	8.18E-06	UP
IL11	9.462685	1.815668	0.386521	4.69746	2.63E-06	8.19E-06	UP
NUP88	1610.636	-0.43246	0.092067	-4.69729	2.64E-06	8.20E-06	NOT
RBFOX2	2225.481	0.459193	0.097763	4.696993	2.64E-06	8.21E-06	NOT
PVRIG	2.664329	1.286799	0.273989	4.696542	2.65E-06	8.22E-06	UP
RP11-169I	44.69365	1.346667	0.286737	4.696515	2.65E-06	8.22E-06	UP
UTS2R	2.19174	2.726834	0.580634	4.696305	2.65E-06	8.23E-06	UP
ZNF70	93.7918	0.637363	0.135718	4.696229	2.65E-06	8.23E-06	UP
GPX7	317.9953	1.037415	0.220912	4.696056	2.65E-06	8.24E-06	UP
MAGEA11	3.762271	4.126641	0.878785	4.695847	2.66E-06	8.25E-06	UP
RP11-440I	14.54811	0.860727	0.183299	4.695749	2.66E-06	8.25E-06	UP
MAPKAPK5	1007.972	0.264207	0.056266	4.695696	2.66E-06	8.25E-06	NOT
ZNF28	274.1859	1.070505	0.227979	4.695627	2.66E-06	8.25E-06	UP
MCPH1	427.971	-0.41488	0.088357	-4.6955	2.66E-06	8.26E-06	NOT
SLA	446.0984	-0.91895	0.19571	-4.69549	2.66E-06	8.26E-06	DOWN
RP11-422I	9.825929	0.793223	0.168933	4.695494	2.66E-06	8.26E-06	UP
AC004221	1.305376	2.286519	0.487013	4.694983	2.67E-06	8.28E-06	UP
RP11-680I	11.15476	0.762415	0.162397	4.694752	2.67E-06	8.28E-06	UP
ZNF790-A	22.33647	1.067017	0.227302	4.694273	2.68E-06	8.30E-06	UP
PCDHA4	6.282079	1.702059	0.362589	4.694187	2.68E-06	8.31E-06	UP
MIR7111	1.859811	1.769479	0.376972	4.693922	2.68E-06	8.31E-06	UP
CCT6B	109.9562	-0.88183	0.187873	-4.69377	2.68E-06	8.32E-06	DOWN
RP11-739I	43.06102	-0.78825	0.16795	-4.69335	2.69E-06	8.34E-06	DOWN
SOCS5	610.7122	0.368891	0.078602	4.693131	2.69E-06	8.34E-06	NOT
WDR54	187.5353	0.80773	0.172122	4.692784	2.70E-06	8.36E-06	UP
IQCG	188.1987	0.627996	0.133838	4.692222	2.70E-06	8.38E-06	UP
CYC1	8819.625	0.630102	0.134288	4.692181	2.70E-06	8.38E-06	UP
RP11-96K1	7.190514	1.034334	0.220445	4.692021	2.71E-06	8.39E-06	UP
CTA-384D	134.1469	0.660825	0.140847	4.691789	2.71E-06	8.39E-06	UP
RP11-180I	7.870497	0.968393	0.206402	4.69178	2.71E-06	8.39E-06	UP
RP11-186I	1.506405	2.473303	0.527162	4.691736	2.71E-06	8.39E-06	UP
FASN	29197.76	0.911368	0.194266	4.691352	2.71E-06	8.41E-06	UP
ME1	1213.476	1.130159	0.240956	4.690303	2.73E-06	8.45E-06	UP
ABHD5	588.1891	-0.50825	0.108371	-4.68995	2.73E-06	8.47E-06	NOT
RP11-110I	5.620725	0.993857	0.211925	4.689659	2.74E-06	8.48E-06	UP
TFAM	1423.542	-0.33375	0.071171	-4.68943	2.74E-06	8.49E-06	NOT
ITM2B	19765.17	-0.43559	0.092891	-4.6893	2.74E-06	8.49E-06	NOT
CCR9	3.119192	-1.56231	0.333172	-4.68921	2.74E-06	8.49E-06	DOWN
SYNGR2	5412.743	0.474102	0.101111	4.688948	2.75E-06	8.50E-06	NOT
MEGF10	19.87887	-1.51469	0.323043	-4.68881	2.75E-06	8.51E-06	DOWN
STPG1	163.4719	0.555753	0.118528	4.688794	2.75E-06	8.51E-06	NOT
SLC4A9	2.504022	1.483772	0.316469	4.688517	2.75E-06	8.52E-06	UP
PWWP2A	468.3812	0.294878	0.062897	4.688272	2.76E-06	8.53E-06	NOT
AC003988	1.584642	-2.11837	0.451854	-4.68818	2.76E-06	8.53E-06	DOWN
SPRN	233.356	0.784275	0.167296	4.687945	2.76E-06	8.54E-06	UP
CEP97	231.3322	0.541616	0.115541	4.687666	2.76E-06	8.55E-06	NOT

CWF19L1	731.6128	0.345521	0.073717	4.687131	2.77E-06	8.57E-06	NOT
ZNF319	382.7819	0.372577	0.079505	4.686194	2.78E-06	8.61E-06	NOT
SAA2	39617.03	-2.22933	0.475725	-4.68618	2.78E-06	8.61E-06	DOWN
HERC2P4	6.428897	1.772763	0.378327	4.685802	2.79E-06	8.62E-06	UP
RNU1-122	1.260287	1.954493	0.417113	4.685767	2.79E-06	8.62E-06	UP
IGF2-AS	77.06067	2.216344	0.473019	4.685527	2.79E-06	8.63E-06	UP
RP3-460G:	15.84942	1.772931	0.378546	4.683526	2.82E-06	8.72E-06	UP
AC104532	2.70388	-1.0385	0.221736	-4.68351	2.82E-06	8.72E-06	DOWN
PCAT19	130.663	-0.68006	0.145205	-4.68344	2.82E-06	8.72E-06	DOWN
RP11-488I	2.466831	1.810812	0.386656	4.683257	2.82E-06	8.73E-06	UP
HSPBAP1	245.5823	0.421724	0.09005	4.683217	2.82E-06	8.73E-06	NOT
COQ6	629.1539	-0.42919	0.091649	-4.683	2.83E-06	8.73E-06	NOT
UTP14C	861.4055	-0.51722	0.110454	-4.68272	2.83E-06	8.75E-06	NOT
ZNF518A	702.4108	0.552499	0.117991	4.682534	2.83E-06	8.75E-06	NOT
SCARA5	102.8687	-1.75705	0.375252	-4.68231	2.84E-06	8.76E-06	DOWN
TNR	50.72644	-1.9221	0.410506	-4.68226	2.84E-06	8.76E-06	DOWN
EXO5	219.6773	0.429136	0.091661	4.681765	2.84E-06	8.78E-06	NOT
HMGN2P5	16.2748	0.741723	0.158443	4.681335	2.85E-06	8.80E-06	UP
SLC22A20	12.95941	1.103675	0.23579	4.680757	2.86E-06	8.82E-06	UP
SIGIRR	2835.817	-0.84304	0.18011	-4.6807	2.86E-06	8.83E-06	DOWN
TUSC1	439.7678	-0.72059	0.153972	-4.68	2.87E-06	8.85E-06	DOWN
MT-TP	2402.344	-0.72551	0.155028	-4.67983	2.87E-06	8.86E-06	DOWN
DZANK1	47.66243	0.649252	0.138739	4.67968	2.87E-06	8.87E-06	UP
PIEZO1	3056.611	0.490736	0.104868	4.679582	2.87E-06	8.87E-06	NOT
IKZF4	141.2208	0.449066	0.095973	4.679067	2.88E-06	8.89E-06	NOT
EIF4E	739.1776	-0.31334	0.066968	-4.67889	2.88E-06	8.90E-06	NOT
AP000892.	65.66656	-0.97791	0.209019	-4.6786	2.89E-06	8.91E-06	DOWN
GPR18	14.92939	-0.9578	0.204729	-4.6784	2.89E-06	8.92E-06	DOWN
AGPAT9	875.5583	-1.12589	0.240712	-4.67735	2.91E-06	8.96E-06	DOWN
ZC2HC1A	116.8703	0.854193	0.182636	4.67702	2.91E-06	8.97E-06	UP
RP11-230I	2.247419	1.669153	0.356938	4.676305	2.92E-06	9.01E-06	UP
MYL4	12.42029	0.969902	0.207416	4.676106	2.92E-06	9.01E-06	UP
CSNK1G2	1845.203	0.39383	0.084224	4.676005	2.93E-06	9.02E-06	NOT
IER5	924.0855	0.552602	0.118183	4.675802	2.93E-06	9.02E-06	NOT
AC098820	4.131867	1.237875	0.264754	4.675569	2.93E-06	9.03E-06	UP
SEZ6L	6.305209	-1.33766	0.286118	-4.6752	2.94E-06	9.05E-06	DOWN
SYNJ2BP	1835.004	-0.43875	0.093848	-4.67518	2.94E-06	9.05E-06	NOT
DNAJC3-A	137.8633	0.68458	0.146434	4.675017	2.94E-06	9.05E-06	UP
DNAJB2	3704.161	0.468356	0.100187	4.674802	2.94E-06	9.06E-06	NOT
RP11-67L2	156.1846	0.604839	0.129384	4.674776	2.94E-06	9.06E-06	UP
ZNF404	62.39679	0.960059	0.205421	4.673613	2.96E-06	9.11E-06	UP
RP11-54O	39.50481	1.233699	0.264015	4.672831	2.97E-06	9.15E-06	UP
CTD-2015	133.0143	0.576982	0.123481	4.672657	2.97E-06	9.15E-06	NOT
RARS	2158.22	0.327464	0.070083	4.672535	2.98E-06	9.16E-06	NOT
RP3-417G:	20.27244	0.933645	0.199818	4.672488	2.98E-06	9.16E-06	UP
ZFYVE21	1081.124	-0.44323	0.094863	-4.67228	2.98E-06	9.17E-06	NOT
CCL13	13.0055	1.397434	0.2991	4.672136	2.98E-06	9.17E-06	UP
ZBTB10	1579.87	-0.55593	0.118991	-4.67203	2.98E-06	9.18E-06	NOT
SMAP2	3702.908	-0.38768	0.08298	-4.67192	2.98E-06	9.18E-06	NOT
HSP90AA2	11.84603	0.941847	0.201631	4.671148	3.00E-06	9.21E-06	UP
SMPD2	303.1655	0.459222	0.098317	4.670848	3.00E-06	9.23E-06	NOT
CHST7	381.7607	-0.9108	0.195017	-4.67036	3.01E-06	9.25E-06	DOWN

RPS21	11691.63	0.721558	0.154505	4.670134	3.01E-06	9.26E-06	UP
RP11-50C:	51.8256	0.603428	0.12921	4.670129	3.01E-06	9.26E-06	UP
CFAP74	35.26831	-1.40273	0.300422	-4.66918	3.02E-06	9.30E-06	DOWN
MED27	407.7136	0.493672	0.105749	4.668315	3.04E-06	9.34E-06	NOT
HNF4A-AS	364.8544	-1.31513	0.281743	-4.66783	3.04E-06	9.36E-06	DOWN
NSL1	1047.202	0.372896	0.079896	4.667275	3.05E-06	9.38E-06	NOT
FMO3	20562.55	-1.2308	0.263735	-4.66681	3.06E-06	9.40E-06	DOWN
RP5-940J5	1.515846	1.587835	0.340254	4.666618	3.06E-06	9.41E-06	UP
CNOT6L	1288.091	-0.46256	0.09914	-4.66571	3.08E-06	9.45E-06	NOT
ZNF202	267.4625	0.396446	0.084971	4.665687	3.08E-06	9.45E-06	NOT
RP11-195I	45.25689	-0.79912	0.171277	-4.66568	3.08E-06	9.45E-06	DOWN
RP5-1052I	4.384793	-1.29296	0.277122	-4.66566	3.08E-06	9.45E-06	DOWN
SLC29A4	1099.682	1.509819	0.323614	4.6655	3.08E-06	9.46E-06	UP
CASC5	574.5485	0.688242	0.14752	4.665428	3.08E-06	9.46E-06	UP
RP11-680I	431.6419	0.996799	0.213679	4.664946	3.09E-06	9.48E-06	UP
MED28P7	1.529512	1.903867	0.408138	4.664762	3.09E-06	9.49E-06	UP
RP11-656I	2.215683	1.686726	0.361595	4.664681	3.09E-06	9.49E-06	UP
IFIT2	1124.127	-0.9476	0.203154	-4.66447	3.09E-06	9.50E-06	DOWN
AKAP8	982.8519	0.299898	0.0643	4.664044	3.10E-06	9.52E-06	NOT
C20orf202	11.58214	1.006684	0.215857	4.663663	3.11E-06	9.53E-06	UP
RP11-114I	1.98242	1.507445	0.323292	4.662801	3.12E-06	9.57E-06	UP
TOLLIP	6775.823	-0.42083	0.090255	-4.66269	3.12E-06	9.58E-06	NOT
RP11-525(	4.714901	1.289832	0.276638	4.662523	3.12E-06	9.58E-06	UP
CTD-2554	4.375862	1.178375	0.252737	4.662448	3.12E-06	9.59E-06	UP
EXOSC5	1006.728	0.576177	0.123587	4.662108	3.13E-06	9.60E-06	NOT
RAB11FIP1	18.3001	1.032336	0.221441	4.661902	3.13E-06	9.61E-06	UP
CTD-2206	11.51881	1.010187	0.216695	4.661789	3.13E-06	9.61E-06	UP
C6orf89	4529.203	0.35208	0.075527	4.661657	3.14E-06	9.62E-06	NOT
P2RY4	1.679756	2.264708	0.485863	4.661211	3.14E-06	9.64E-06	UP
TMEM189	1606.688	0.535527	0.114892	4.66112	3.14E-06	9.64E-06	NOT
ACBD5	3334.711	-0.52397	0.112422	-4.66076	3.15E-06	9.66E-06	NOT
AC004854	2.137393	1.826181	0.391836	4.660574	3.15E-06	9.66E-06	UP
PEAK1	811.4725	0.502239	0.107787	4.659546	3.17E-06	9.71E-06	NOT
WDR75	1064.691	0.355958	0.076394	4.659482	3.17E-06	9.71E-06	NOT
TRAPPC12	15.90351	0.918791	0.197195	4.659298	3.17E-06	9.72E-06	UP
CDKL1	29.72439	0.955209	0.205038	4.658693	3.18E-06	9.75E-06	UP
FTH1P12	12.27272	0.919093	0.197306	4.658202	3.19E-06	9.77E-06	UP
DNAJC15	1466.917	-0.6735	0.144636	-4.65651	3.22E-06	9.85E-06	DOWN
FPGS	2902.884	-0.48713	0.104629	-4.65577	3.23E-06	9.89E-06	NOT
INMT-FAN	1.363084	-1.74156	0.374096	-4.65537	3.23E-06	9.90E-06	DOWN
MROH8	85.79146	-0.69034	0.148323	-4.65433	3.25E-06	9.95E-06	DOWN
ZNRF2P2	2.871406	1.299411	0.279184	4.654316	3.25E-06	9.95E-06	UP
TGDS	679.0036	-0.6742	0.144857	-4.65421	3.25E-06	9.96E-06	DOWN
OR7E29P	8.3964	-1.49283	0.320766	-4.65395	3.26E-06	9.97E-06	DOWN
ANO1	3921.186	-1.19048	0.25582	-4.65359	3.26E-06	9.98E-06	DOWN
AC005329	20.25922	1.228182	0.263954	4.65302	3.27E-06	1.00E-05	UP
MT-TE	2.206745	-1.31233	0.282042	-4.65296	3.27E-06	1.00E-05	DOWN
CLSTN1	2500.363	0.716965	0.154094	4.652771	3.28E-06	1.00E-05	UP
LINC0148:	1.679565	2.436295	0.523628	4.652724	3.28E-06	1.00E-05	UP
RP11-12M	4.693737	1.172403	0.251992	4.652542	3.28E-06	1.00E-05	UP
ESRP2	2628.661	-0.72344	0.155496	-4.65245	3.28E-06	1.00E-05	DOWN
TIMM8AP:	7.785252	1.230515	0.264498	4.652265	3.28E-06	1.00E-05	UP

RP11-85I1	2.022459	1.738858	0.373777	4.652122	3.29E-06	1.00E-05	UP
VIPR1-AS1	4.254645	-1.26721	0.272397	-4.65206	3.29E-06	1.00E-05	DOWN
GUSBP1	129.6346	0.489457	0.105218	4.651852	3.29E-06	1.01E-05	NOT
DOPEY1	433.37	-0.54294	0.116727	-4.65136	3.30E-06	1.01E-05	NOT
SCARNA5	2.61591	-1.72351	0.370614	-4.65041	3.31E-06	1.01E-05	DOWN
ZBTB2	513.6478	-0.38614	0.083036	-4.65025	3.32E-06	1.01E-05	NOT
C7orf49	1042.892	0.409582	0.088083	4.649978	3.32E-06	1.01E-05	NOT
NOP58	2383.798	0.42995	0.092465	4.649889	3.32E-06	1.01E-05	NOT
HABP2	20962.59	-1.11301	0.239369	-4.64978	3.32E-06	1.02E-05	DOWN
MGP	1720.406	1.073605	0.230894	4.649766	3.32E-06	1.02E-05	UP
CDC37L1-	45.6403	-0.82626	0.177702	-4.64971	3.32E-06	1.02E-05	DOWN
ZSCAN5D	1.349814	1.82478	0.39246	4.649595	3.33E-06	1.02E-05	UP
RP11-380I	2.54249	1.504485	0.323607	4.649118	3.33E-06	1.02E-05	UP
FGD2	238.2142	-0.81298	0.174906	-4.6481	3.35E-06	1.02E-05	DOWN
LAMB3	1198.941	1.048777	0.225645	4.647902	3.35E-06	1.02E-05	UP
DCP2	997.2004	0.351281	0.075579	4.647845	3.35E-06	1.02E-05	NOT
CTD-2267	1.869649	1.472735	0.316882	4.647586	3.36E-06	1.03E-05	UP
RP11-244I	9.676689	0.9709	0.208919	4.64726	3.36E-06	1.03E-05	UP
EBPL	2765.177	-0.7258	0.156186	-4.64702	3.37E-06	1.03E-05	DOWN
SMPDL3B	89.20403	1.435399	0.308918	4.646537	3.38E-06	1.03E-05	UP
PGRMC2	4880.847	-0.40418	0.086988	-4.64638	3.38E-06	1.03E-05	NOT
PTF1A	4.084889	4.062234	0.874291	4.646321	3.38E-06	1.03E-05	UP
GPATCH2L	565.9781	0.390481	0.084047	4.646002	3.38E-06	1.03E-05	NOT
RP11-417I	5.982069	1.226866	0.264111	4.645261	3.40E-06	1.04E-05	UP
DAZAP1	3426.155	0.313899	0.067576	4.645102	3.40E-06	1.04E-05	NOT
RP5-827C	2.612822	2.087074	0.449312	4.645047	3.40E-06	1.04E-05	UP
REEP3	1812.014	-0.39061	0.084093	-4.64496	3.40E-06	1.04E-05	NOT
WTAPP1	5.968164	1.905223	0.410182	4.644829	3.40E-06	1.04E-05	UP
RP5-1074I	26.39144	0.614361	0.132271	4.644724	3.41E-06	1.04E-05	UP
RP11-454I	2.615592	1.286048	0.276895	4.644536	3.41E-06	1.04E-05	UP
RP11-686	10.05919	0.791739	0.170523	4.643001	3.43E-06	1.05E-05	UP
RP11-958I	45.81203	0.951834	0.205027	4.642481	3.44E-06	1.05E-05	UP
CTD-2008	2.900137	3.565818	0.768114	4.642305	3.45E-06	1.05E-05	UP
CRISP2	6.497468	4.438495	0.956108	4.642251	3.45E-06	1.05E-05	UP
RP1-228P	9.708639	0.796982	0.171698	4.641758	3.45E-06	1.05E-05	UP
LAPTM4A	12055.88	-0.31489	0.06784	-4.64157	3.46E-06	1.05E-05	NOT
SLC4A5	12.32454	0.911962	0.196478	4.641552	3.46E-06	1.05E-05	UP
PLK2	1730.084	-0.67049	0.144459	-4.6414	3.46E-06	1.05E-05	DOWN
LLGL2	3555.8	0.541835	0.116746	4.641122	3.47E-06	1.06E-05	NOT
ATG4B	1818.518	0.382692	0.082458	4.641036	3.47E-06	1.06E-05	NOT
RP11-766I	16.56463	-0.83154	0.179178	-4.64084	3.47E-06	1.06E-05	DOWN
UBE2I	2354.95	0.367041	0.079095	4.640528	3.48E-06	1.06E-05	NOT
RP11-864I	3.421254	1.290917	0.278214	4.640016	3.48E-06	1.06E-05	UP
FBLN7	537.1333	0.809562	0.174477	4.639939	3.49E-06	1.06E-05	UP
ANKRD34E	3.971921	2.540298	0.547548	4.639407	3.49E-06	1.06E-05	UP
RP11-700	2.065889	1.541713	0.332342	4.638931	3.50E-06	1.07E-05	UP
AC145110	2.124964	1.643539	0.354308	4.638732	3.51E-06	1.07E-05	UP
RP11-748I	1.264226	2.079673	0.448385	4.638145	3.52E-06	1.07E-05	UP
EMILIN2	310.3435	0.904839	0.19514	4.636861	3.54E-06	1.08E-05	UP
SH3GL1P1	7.113944	0.913919	0.197099	4.636857	3.54E-06	1.08E-05	UP
ST6GALNA	186.3918	1.053625	0.227237	4.636681	3.54E-06	1.08E-05	UP
EFCAB12	200.9545	1.169515	0.252311	4.635217	3.57E-06	1.08E-05	UP

CFAP43	30.47649	0.901939	0.194587	4.635146	3.57E-06	1.08E-05	UP
RP11-20G	4.541549	1.612358	0.347856	4.635131	3.57E-06	1.08E-05	UP
AHSP	2.901312	-1.85761	0.400795	-4.63481	3.57E-06	1.09E-05	DOWN
FILIP1L	776.898	-0.81902	0.176721	-4.63453	3.58E-06	1.09E-05	DOWN
RP1-140A	17.36173	1.031195	0.222527	4.634027	3.59E-06	1.09E-05	UP
PPP2R1A	9098.839	0.483935	0.104432	4.633963	3.59E-06	1.09E-05	NOT
RP11-329I	27.80605	-0.75073	0.162015	-4.63369	3.59E-06	1.09E-05	DOWN
MAK16	452.4368	-0.47067	0.10158	-4.63347	3.60E-06	1.09E-05	NOT
PRKD1	173.5435	1.009152	0.217798	4.633433	3.60E-06	1.09E-05	UP
AC130469	2.937125	1.623739	0.35048	4.632906	3.61E-06	1.10E-05	UP
RP11-510I	1.931567	1.386015	0.299198	4.632429	3.61E-06	1.10E-05	UP
RP11-203I	4.199767	1.238466	0.267369	4.632045	3.62E-06	1.10E-05	UP
RP11-129	55.82553	-0.83128	0.17952	-4.63057	3.65E-06	1.11E-05	DOWN
RP1-102D	1.088644	2.330372	0.503298	4.630203	3.65E-06	1.11E-05	UP
GPS1	5535.21	0.464444	0.100309	4.630122	3.65E-06	1.11E-05	NOT
RP11-552I	2.123738	3.541961	0.764992	4.630061	3.66E-06	1.11E-05	UP
CTC-273B	1.941465	1.815238	0.392091	4.62964	3.66E-06	1.11E-05	UP
SORBS2	4771.271	-0.72439	0.156521	-4.62804	3.69E-06	1.12E-05	DOWN
SLC1A1	2694.533	-1.23236	0.266284	-4.628	3.69E-06	1.12E-05	DOWN
DDX11L2	26.27204	1.222658	0.264206	4.627672	3.70E-06	1.12E-05	UP
EFCAB11	249.7	0.481763	0.104113	4.6273	3.70E-06	1.12E-05	NOT
CTC-543D	11.00598	1.016504	0.219694	4.626901	3.71E-06	1.13E-05	UP
HYDIN2	5.179598	1.764946	0.38148	4.62657	3.72E-06	1.13E-05	UP
CTD-2583	82.48167	0.575322	0.124353	4.626511	3.72E-06	1.13E-05	NOT
HMGN1P3	8.150878	0.783782	0.169418	4.626315	3.72E-06	1.13E-05	UP
SCTR	135.1528	1.475674	0.318985	4.626158	3.73E-06	1.13E-05	UP
RP11-554I	2.861651	-1.10196	0.238305	-4.62415	3.76E-06	1.14E-05	DOWN
PRTG	26.42145	1.367375	0.29573	4.623722	3.77E-06	1.14E-05	UP
GAPDHP3	1.550889	1.792374	0.38768	4.623329	3.78E-06	1.14E-05	UP
RP5-905G	2.065001	2.812495	0.608387	4.62287	3.78E-06	1.15E-05	UP
RP11-789	31.02049	-2.66139	0.575722	-4.62271	3.79E-06	1.15E-05	DOWN
FLJ21408	10.6317	0.809517	0.175122	4.622575	3.79E-06	1.15E-05	UP
USP3-AS1	40.78462	0.534803	0.115694	4.622564	3.79E-06	1.15E-05	NOT
RP11-96D	12.45923	-0.66357	0.143558	-4.62227	3.80E-06	1.15E-05	DOWN
PYCARD	669.8733	1.036345	0.224244	4.621511	3.81E-06	1.15E-05	UP
TXNL4A	2060.23	0.470046	0.101708	4.621509	3.81E-06	1.15E-05	NOT
CBX3P4	1.279214	1.651264	0.357345	4.620919	3.82E-06	1.16E-05	UP
CTD-2587	16.58271	0.900573	0.194913	4.620379	3.83E-06	1.16E-05	UP
HYOU1	9515.937	0.563206	0.121902	4.620163	3.83E-06	1.16E-05	NOT
FAAHP1	9.809219	1.160208	0.251122	4.620105	3.84E-06	1.16E-05	UP
OCLM	5.379864	1.128275	0.244214	4.62003	3.84E-06	1.16E-05	UP
UGT3A2	70.00998	1.563976	0.33854	4.619768	3.84E-06	1.16E-05	UP
TIMM50	2659.613	0.518936	0.112347	4.619066	3.85E-06	1.17E-05	NOT
DSCR4	8.033813	5.339587	1.156012	4.618971	3.86E-06	1.17E-05	UP
RP11-707I	10.47065	1.050714	0.227482	4.618899	3.86E-06	1.17E-05	UP
MRPL37	4747.863	-0.4402	0.09531	-4.61857	3.86E-06	1.17E-05	NOT
AP003068.	230.8468	0.847302	0.183464	4.618354	3.87E-06	1.17E-05	UP
ATP5J	6046.268	-0.49214	0.106565	-4.61819	3.87E-06	1.17E-05	NOT
RP11-26J	4.847045	0.966382	0.209258	4.618139	3.87E-06	1.17E-05	UP
RP3-476K	2.626363	1.144859	0.247909	4.61806	3.87E-06	1.17E-05	UP
TBC1D22A	1470.465	0.378345	0.081939	4.617399	3.89E-06	1.17E-05	NOT
MIR6835	0.954028	2.138431	0.463142	4.617226	3.89E-06	1.18E-05	UP

RHOBTB1	1109.208	1.269794	0.275025	4.61702	3.89E-06	1.18E-05	UP
CELF1	4664.896	-0.24866	0.053861	-4.61671	3.90E-06	1.18E-05	NOT
ICE1	1116.674	0.393948	0.085332	4.616652	3.90E-06	1.18E-05	NOT
SEC14L5	20.46723	1.184329	0.25655	4.616372	3.91E-06	1.18E-05	UP
DNM2	4657.752	0.325595	0.070534	4.616129	3.91E-06	1.18E-05	NOT
BCDIN3D-	23.13464	0.599365	0.129853	4.615738	3.92E-06	1.18E-05	UP
AC006539	1.379274	2.220079	0.480989	4.615658	3.92E-06	1.18E-05	UP
PDGFC	800.4742	-0.69915	0.151478	-4.61556	3.92E-06	1.18E-05	DOWN
LA16c-361	2.192756	1.439927	0.312054	4.614354	3.94E-06	1.19E-05	UP
LECT2	4584.568	-1.48149	0.321085	-4.61403	3.95E-06	1.19E-05	DOWN
AC019186	7.376708	-0.68855	0.149239	-4.61375	3.95E-06	1.19E-05	DOWN
RP11-352(	4.304596	1.017146	0.22046	4.613744	3.95E-06	1.19E-05	UP
SAMD5	548.1737	-1.33824	0.290079	-4.61336	3.96E-06	1.20E-05	DOWN
STX12	1312.413	-0.315	0.068283	-4.61318	3.97E-06	1.20E-05	NOT
NPPA-AS1	20.97547	0.955826	0.207199	4.613076	3.97E-06	1.20E-05	UP
AC091654	4.612023	1.103731	0.239316	4.612019	3.99E-06	1.20E-05	UP
CNR2	7.528649	-1.1178	0.24238	-4.61176	3.99E-06	1.20E-05	DOWN
SNORD56	1.894025	1.364478	0.295871	4.61173	3.99E-06	1.20E-05	UP
GBP7	1616.13	-1.3595	0.294821	-4.61127	4.00E-06	1.21E-05	DOWN
RNU1-70P	1227.388	-1.09762	0.238052	-4.61084	4.01E-06	1.21E-05	DOWN
MIR135A1	80.57233	-0.9019	0.195626	-4.61031	4.02E-06	1.21E-05	DOWN
RP11-757(	2.941155	2.81387	0.610384	4.609998	4.03E-06	1.21E-05	UP
TOPORS	809.937	-0.38883	0.08435	-4.60975	4.03E-06	1.22E-05	NOT
GCLC	5196.894	-0.61951	0.134401	-4.60941	4.04E-06	1.22E-05	DOWN
GPATCH3	668.1906	0.427452	0.092737	4.609294	4.04E-06	1.22E-05	NOT
LSMEM1	55.88754	0.70208	0.152322	4.60918	4.04E-06	1.22E-05	UP
UBTFL10	3.398997	-1.99987	0.433948	-4.60855	4.05E-06	1.22E-05	DOWN
GNL3LP1	1.402959	1.657486	0.359667	4.608388	4.06E-06	1.22E-05	UP
NOP2	1332.997	0.464974	0.100905	4.608038	4.06E-06	1.23E-05	NOT
PARG	603.5988	0.316593	0.068708	4.607766	4.07E-06	1.23E-05	NOT
WBP5	1045.42	0.875833	0.190103	4.607145	4.08E-06	1.23E-05	UP
CH17-340	223.8338	0.724149	0.157188	4.606905	4.09E-06	1.23E-05	UP
SLC35A1	452.3649	-0.42349	0.091925	-4.6069	4.09E-06	1.23E-05	NOT
ZNF414	423.9255	0.419588	0.091087	4.606442	4.10E-06	1.23E-05	NOT
FHOD1	403.5119	0.656208	0.142458	4.606318	4.10E-06	1.23E-05	UP
RAB17	3988.7	-0.80595	0.174967	-4.60629	4.10E-06	1.23E-05	DOWN
AC091849	3.428221	1.520719	0.330156	4.606067	4.10E-06	1.24E-05	UP
CTD-2630	35.4869	0.603701	0.131089	4.605291	4.12E-06	1.24E-05	UP
MVB12A	1178.45	0.548766	0.119163	4.605185	4.12E-06	1.24E-05	NOT
NOXRED1	13.32232	0.706421	0.153401	4.605073	4.12E-06	1.24E-05	UP
METTL21A	533.24	0.364802	0.079219	4.604976	4.13E-06	1.24E-05	NOT
GP6	6.059276	-1.1985	0.260265	-4.60493	4.13E-06	1.24E-05	DOWN
TSC22D1-	6.592245	1.121734	0.243635	4.604155	4.14E-06	1.25E-05	UP
PRRC1	2478.741	0.331747	0.07206	4.603759	4.15E-06	1.25E-05	NOT
AF127936.	7.25294	-0.82351	0.178901	-4.60318	4.16E-06	1.25E-05	DOWN
CTD-2114	99.35849	-1.19335	0.259245	-4.60317	4.16E-06	1.25E-05	DOWN
CTD-3064	2.374554	2.275338	0.494328	4.602891	4.17E-06	1.25E-05	UP
CHFR	744.2633	0.467721	0.101618	4.602721	4.17E-06	1.25E-05	NOT
GAPDH	83377.78	0.57351	0.124613	4.602324	4.18E-06	1.26E-05	NOT
HCK	476.6104	-0.82108	0.178406	-4.60229	4.18E-06	1.26E-05	DOWN
PTMAP2	21.55881	0.749221	0.162799	4.602123	4.18E-06	1.26E-05	UP
DDX46	1695.708	0.293205	0.063716	4.601763	4.19E-06	1.26E-05	NOT

RP1-178F1	15.55476	0.815486	0.177214	4.601694	4.19E-06	1.26E-05	UP
CDKL4	2.151294	2.028601	0.440862	4.601443	4.20E-06	1.26E-05	UP
C1orf115	7133.494	-0.58775	0.127758	-4.60051	4.21E-06	1.27E-05	DOWN
MAPRE2	1982.469	-0.53088	0.1154	-4.6003	4.22E-06	1.27E-05	NOT
AC010149	2.163138	1.841425	0.400342	4.599635	4.23E-06	1.27E-05	UP
CCNL1	2469.063	-0.45022	0.097893	-4.59909	4.24E-06	1.27E-05	NOT
DIEXF	1111.062	0.444685	0.096692	4.598985	4.25E-06	1.28E-05	NOT
RP11-407(	3.091928	1.451127	0.31554	4.598862	4.25E-06	1.28E-05	UP
SLC35F3	18.48792	1.86231	0.404971	4.59863	4.25E-06	1.28E-05	UP
MAPK7	403.8282	0.442666	0.096274	4.597979	4.27E-06	1.28E-05	NOT
NPY5R	13.67502	-1.48247	0.32243	-4.59781	4.27E-06	1.28E-05	DOWN
AC078883	4.699747	-0.98004	0.21316	-4.59769	4.27E-06	1.28E-05	DOWN
CDH16	60.43127	1.821278	0.396143	4.597531	4.28E-06	1.28E-05	UP
FAM192A	1823.01	0.31173	0.067806	4.597371	4.28E-06	1.28E-05	NOT
AC002117	3.039884	1.145956	0.249283	4.597009	4.29E-06	1.29E-05	UP
CCND2	448.8607	0.898698	0.195589	4.594835	4.33E-06	1.30E-05	UP
TRIM31-A	2.126403	2.052212	0.446675	4.594424	4.34E-06	1.30E-05	UP
NEFL	5.957102	2.041547	0.444363	4.594325	4.34E-06	1.30E-05	UP
PHF13	854.6529	-0.4233	0.092145	-4.59391	4.35E-06	1.31E-05	NOT
NOP14-AS	339.0878	0.446429	0.097184	4.593671	4.36E-06	1.31E-05	NOT
ARG1	20431.88	-1.21352	0.26418	-4.59353	4.36E-06	1.31E-05	DOWN
SSX2IP	1047.157	0.80411	0.175053	4.593524	4.36E-06	1.31E-05	UP
AC009120	8.164889	0.98722	0.214918	4.59348	4.36E-06	1.31E-05	UP
CLDN1	16160.78	-0.67698	0.147385	-4.59326	4.36E-06	1.31E-05	DOWN
SEPP1	41076.82	-0.85244	0.185643	-4.59181	4.39E-06	1.32E-05	DOWN
LBR	3584.971	0.478902	0.104303	4.591472	4.40E-06	1.32E-05	NOT
MTND6P3	3.447162	-1.4152	0.308231	-4.59135	4.40E-06	1.32E-05	DOWN
BBOX1	1232.299	-1.49748	0.326163	-4.59119	4.41E-06	1.32E-05	DOWN
OVOL1-AS	13.65052	2.30751	0.50261	4.591059	4.41E-06	1.32E-05	UP
ABCF1	4088.322	0.341082	0.074293	4.591046	4.41E-06	1.32E-05	NOT
MIR210HG	131.4733	0.999122	0.217632	4.59089	4.41E-06	1.32E-05	UP
ACOT12	2207.615	-1.23719	0.269489	-4.59087	4.41E-06	1.32E-05	DOWN
PRSS27	25.87566	0.79089	0.172287	4.59055	4.42E-06	1.32E-05	UP
SECISBP2L	1510.989	-0.46384	0.101045	-4.59043	4.42E-06	1.32E-05	NOT
AHR	4149.353	-0.68062	0.148271	-4.59039	4.42E-06	1.33E-05	DOWN
POF1B	191.0836	1.778508	0.387489	4.589823	4.44E-06	1.33E-05	UP
LCNL1	11.20147	1.910423	0.416302	4.589034	4.45E-06	1.33E-05	UP
RP11-359I	21.48451	1.200779	0.26167	4.58891	4.46E-06	1.33E-05	UP
TMEM68	613.0838	0.422052	0.091973	4.588888	4.46E-06	1.33E-05	NOT
MKS1	349.5846	0.392355	0.085503	4.588773	4.46E-06	1.33E-05	NOT
CTD-2281	1.147286	2.247767	0.489878	4.588424	4.47E-06	1.34E-05	UP
ESRP1	320.3576	2.263649	0.493344	4.588379	4.47E-06	1.34E-05	UP
ARL4A	627.6655	0.752142	0.163926	4.588304	4.47E-06	1.34E-05	UP
RP11-477I	10.26706	1.153926	0.25153	4.587628	4.48E-06	1.34E-05	UP
HEYL	539.049	0.883522	0.192594	4.587488	4.49E-06	1.34E-05	UP
MDN1	1799.545	-0.54121	0.117974	-4.58749	4.49E-06	1.34E-05	NOT
LINC01561	2.2206	-1.75576	0.382753	-4.58719	4.49E-06	1.34E-05	DOWN
GSDMD	6540.792	0.55099	0.120119	4.58705	4.50E-06	1.34E-05	NOT
EVI2B	305.0453	-0.8593	0.187374	-4.586	4.52E-06	1.35E-05	DOWN
THEM6	3467.108	0.627758	0.136891	4.585807	4.52E-06	1.35E-05	UP
MRPL24	5103.057	0.584258	0.127425	4.585132	4.54E-06	1.36E-05	NOT
RAB7A	9452.826	-0.25885	0.056456	-4.58506	4.54E-06	1.36E-05	NOT

CREB3L3	14208.44	-1.04446	0.227824	-4.58449	4.55E-06	1.36E-05	DOWN
RP3-510D	35.045	0.775287	0.169142	4.583656	4.57E-06	1.37E-05	UP
PAK4	1917.392	0.478759	0.104453	4.583485	4.57E-06	1.37E-05	NOT
RP11-145(	3.083515	3.891175	0.849384	4.581174	4.62E-06	1.38E-05	UP
AC092301	3.51588	1.200712	0.262113	4.580895	4.63E-06	1.38E-05	UP
ACP6	841.4535	0.533226	0.116428	4.579855	4.65E-06	1.39E-05	NOT
FRMPD1	131.9567	1.362866	0.297613	4.579324	4.66E-06	1.39E-05	UP
AP000240.	11.8725	0.961891	0.21006	4.579116	4.67E-06	1.39E-05	UP
TUBA3D	21.1197	1.383719	0.30221	4.578669	4.68E-06	1.40E-05	UP
AC009961	16.15242	0.806694	0.176187	4.578616	4.68E-06	1.40E-05	UP
IGLV8-61	75.18916	-1.93024	0.421601	-4.57835	4.69E-06	1.40E-05	DOWN
ZBTB17	782.5747	0.34583	0.07554	4.578083	4.69E-06	1.40E-05	NOT
TRIM62	146.2236	0.488549	0.106735	4.577206	4.71E-06	1.41E-05	NOT
RP11-83M	15.68278	2.055728	0.449169	4.576735	4.72E-06	1.41E-05	UP
RP11-114(	53.63932	0.690901	0.15096	4.576715	4.72E-06	1.41E-05	UP
ASL	8631.816	-0.83137	0.181672	-4.5762	4.73E-06	1.41E-05	DOWN
PPCS	2036.79	-0.31609	0.069087	-4.57529	4.76E-06	1.42E-05	NOT
SAA2-SAA	4022.684	-2.13108	0.46583	-4.57479	4.77E-06	1.42E-05	DOWN
PLCG1-AS	3.934625	0.997223	0.217998	4.574449	4.77E-06	1.42E-05	UP
SI	2.719996	3.592044	0.785279	4.574228	4.78E-06	1.43E-05	UP
RP11-182.	2.051038	1.190865	0.260389	4.573407	4.80E-06	1.43E-05	UP
CLEC10A	220.7318	-0.82282	0.179914	-4.5734	4.80E-06	1.43E-05	DOWN
KB-1615E	5.673349	1.770347	0.387118	4.573145	4.80E-06	1.43E-05	UP
UFL1	1546.199	-0.45284	0.099024	-4.57306	4.81E-06	1.43E-05	NOT
C16orf74	34.75997	1.050053	0.229645	4.572504	4.82E-06	1.44E-05	UP
AC097523	4.995995	0.800567	0.175128	4.571328	4.85E-06	1.44E-05	UP
PHBP9	6.624759	0.955557	0.209033	4.571323	4.85E-06	1.44E-05	UP
RP11-295I	2.778273	1.256349	0.274836	4.571275	4.85E-06	1.44E-05	UP
RP11-396I	44.18277	-0.78793	0.172375	-4.571	4.85E-06	1.45E-05	DOWN
RP11-73M	17.4849	0.616556	0.134895	4.570642	4.86E-06	1.45E-05	UP
CMTM1	9.980951	0.899873	0.196914	4.569871	4.88E-06	1.45E-05	UP
AP001107.	1.641266	1.674182	0.366437	4.568815	4.90E-06	1.46E-05	UP
PAXBP1-A	36.10545	0.513206	0.112339	4.568361	4.92E-06	1.46E-05	NOT
RP11-401I	23.64829	1.004366	0.219872	4.567947	4.93E-06	1.47E-05	UP
RASEF	409.2058	1.290639	0.282552	4.567797	4.93E-06	1.47E-05	UP
FAM230C	4.811695	4.598918	1.006932	4.567257	4.94E-06	1.47E-05	UP
ARHGEF1	2677.044	0.35872	0.078552	4.566662	4.96E-06	1.48E-05	NOT
RP11-504(	2.029828	1.364885	0.298958	4.565478	4.98E-06	1.48E-05	UP
KIFAP3	1233.62	0.474791	0.104012	4.564794	5.00E-06	1.49E-05	NOT
ABCA6	4072.834	-1.03542	0.226885	-4.56364	5.03E-06	1.50E-05	DOWN
LINC01271	27.28475	0.871191	0.190899	4.563624	5.03E-06	1.50E-05	UP
RP11-524(	2.063506	1.374172	0.301125	4.563455	5.03E-06	1.50E-05	UP
RP11-276I	9.22802	-0.88742	0.194471	-4.56324	5.04E-06	1.50E-05	DOWN
PAGR1	278.2168	0.416844	0.09135	4.563168	5.04E-06	1.50E-05	NOT
CDH9	5.838908	4.818361	1.055998	4.562849	5.05E-06	1.50E-05	UP
ROM1	126.5266	-0.63801	0.139832	-4.56268	5.05E-06	1.50E-05	DOWN
HIATL1	1100.039	0.412334	0.090393	4.561581	5.08E-06	1.51E-05	NOT
RP11-465(	1.475223	3.003015	0.658332	4.561549	5.08E-06	1.51E-05	UP
PACSIN2	3694.41	0.431666	0.09464	4.561143	5.09E-06	1.51E-05	NOT
AC005077	13.73449	-1.85511	0.406734	-4.561	5.09E-06	1.51E-05	DOWN
CTD-2047	14.74011	0.78105	0.171248	4.56092	5.09E-06	1.51E-05	UP
ABCA12	7.525063	1.684152	0.3693	4.560383	5.11E-06	1.52E-05	UP

MCHR1	190.5406	1.289962	0.282874	4.560202	5.11E-06	1.52E-05	UP
F12	28161.61	-1.10501	0.242332	-4.55988	5.12E-06	1.52E-05	DOWN
PTPN9	1218.421	-0.38211	0.083831	-4.5581	5.16E-06	1.53E-05	NOT
TATDN3	617.272	0.403887	0.088615	4.557759	5.17E-06	1.54E-05	NOT
MYH7B	129.7971	1.114366	0.244505	4.557642	5.17E-06	1.54E-05	UP
ZGLP1	41.17888	0.767959	0.168502	4.557576	5.17E-06	1.54E-05	UP
TBX21	44.06938	-0.8479	0.186054	-4.55729	5.18E-06	1.54E-05	DOWN
AC093627	20.02687	0.650662	0.142778	4.557174	5.18E-06	1.54E-05	UP
TIAM1	302.5981	-0.8938	0.196135	-4.55703	5.19E-06	1.54E-05	DOWN
C9orf64	1032.251	-0.43349	0.095139	-4.55645	5.20E-06	1.54E-05	NOT
LIX1L	1401.669	0.464261	0.101907	4.55574	5.22E-06	1.55E-05	NOT
H2AFY2	747.6214	1.093273	0.239994	4.555421	5.23E-06	1.55E-05	UP
C1orf50	367.183	-0.47892	0.105141	-4.55508	5.24E-06	1.55E-05	NOT
AC073043	1.953266	1.629109	0.357716	4.554203	5.26E-06	1.56E-05	UP
AP001596.	2.075238	2.004172	0.440112	4.553777	5.27E-06	1.56E-05	UP
RP11-407I	128.6967	-0.99829	0.219226	-4.55372	5.27E-06	1.56E-05	DOWN
RP11-434I	22.96632	1.881389	0.413218	4.553014	5.29E-06	1.57E-05	UP
TRIM14	1778.209	-0.48713	0.106991	-4.55299	5.29E-06	1.57E-05	NOT
TAF10	2465.718	0.466717	0.10251	4.552916	5.29E-06	1.57E-05	NOT
MMP25-A	190.2173	0.736689	0.161814	4.55269	5.30E-06	1.57E-05	UP
CTD-2314	1.818848	3.321138	0.729666	4.55159	5.32E-06	1.58E-05	UP
ANKRD16	310.4298	0.411066	0.090323	4.551075	5.34E-06	1.58E-05	NOT
PPFIBP1	1199.57	-0.49412	0.108625	-4.54889	5.39E-06	1.60E-05	NOT
GOLIM4	3668.821	-0.55399	0.121786	-4.54885	5.39E-06	1.60E-05	NOT
ITGA1	5583.065	-0.4684	0.102972	-4.54883	5.39E-06	1.60E-05	NOT
NFKBIZ	983.2248	-0.71085	0.156274	-4.54872	5.40E-06	1.60E-05	DOWN
TMEM176	53242.25	-0.7988	0.175625	-4.54835	5.41E-06	1.60E-05	DOWN
DBR1	406.8873	0.339417	0.074641	4.547323	5.43E-06	1.61E-05	NOT
RP11-122(	32.44053	2.063039	0.453683	4.547311	5.43E-06	1.61E-05	UP
RP11-88E1	179.1494	0.731466	0.160884	4.546545	5.45E-06	1.62E-05	UP
RP11-363I	1.649416	1.83362	0.40331	4.546432	5.46E-06	1.62E-05	UP
WDR12	1011.232	0.360693	0.079338	4.546282	5.46E-06	1.62E-05	NOT
RP11-81A	80.33461	0.59646	0.131209	4.545894	5.47E-06	1.62E-05	UP
AP003068.	33.10625	0.766125	0.168544	4.54555	5.48E-06	1.62E-05	UP
AC004156	5.664167	1.03131	0.226903	4.545152	5.49E-06	1.63E-05	UP
ZNF782	105.6385	0.460518	0.101321	4.54513	5.49E-06	1.63E-05	NOT
RP11-137(	6.071079	0.997433	0.219463	4.544883	5.50E-06	1.63E-05	UP
KCNV2	1.055356	1.669091	0.367257	4.544752	5.50E-06	1.63E-05	UP
IL34	225.7579	1.009635	0.222157	4.54468	5.50E-06	1.63E-05	UP
LPAR3	15.38792	2.310039	0.508298	4.544658	5.50E-06	1.63E-05	UP
RP11-139I	2.921763	-1.34158	0.29521	-4.54449	5.51E-06	1.63E-05	DOWN
RP1-199J3	4.549586	0.988327	0.217524	4.543526	5.53E-06	1.64E-05	UP
ZNF286A	69.56757	0.593595	0.130658	4.54311	5.54E-06	1.64E-05	UP
ZNF701	97.17269	0.896256	0.197284	4.542972	5.55E-06	1.64E-05	UP
MIR4783	4.568744	1.40182	0.308588	4.542691	5.55E-06	1.64E-05	UP
ARHGEF16	669.6166	1.084915	0.238851	4.542232	5.57E-06	1.65E-05	UP
MT-TY	26.10568	-1.02611	0.225943	-4.54146	5.59E-06	1.65E-05	DOWN
RP11-416I	3.897274	1.627156	0.358305	4.541261	5.59E-06	1.65E-05	UP
AC005104	7.025694	0.999945	0.220205	4.540981	5.60E-06	1.66E-05	UP
GOLGA2P1	2.522533	2.416035	0.532115	4.54044	5.61E-06	1.66E-05	UP
CYP3A43	268.5061	-1.49392	0.329093	-4.53951	5.64E-06	1.67E-05	DOWN
GTF3C5	1896.191	0.431523	0.095063	4.539339	5.64E-06	1.67E-05	NOT

ZNF702P	45.18929	1.152921	0.253991	4.539214	5.65E-06	1.67E-05	UP
RP13-638(	3.552577	1.055011	0.232452	4.53861	5.66E-06	1.67E-05	UP
BMPR1B	27.46359	-1.51853	0.334649	-4.53768	5.69E-06	1.68E-05	DOWN
CCZ1	184.4291	0.461859	0.101784	4.537649	5.69E-06	1.68E-05	NOT
RP11-290I	19.42274	0.757951	0.167037	4.537631	5.69E-06	1.68E-05	UP
HSD11B2	356.3167	0.764677	0.168536	4.537188	5.70E-06	1.68E-05	UP
TTC7B	733.4533	-0.63843	0.140727	-4.53666	5.72E-06	1.69E-05	DOWN
NAE1	1091.192	0.321184	0.070798	4.536608	5.72E-06	1.69E-05	NOT
TMEM67	101.1885	0.665605	0.146735	4.536093	5.73E-06	1.69E-05	UP
RP11-76C:	2.868079	3.791002	0.835761	4.535989	5.73E-06	1.69E-05	UP
CRTAP	5071.931	0.501003	0.110458	4.535682	5.74E-06	1.70E-05	NOT
UBXN6	5578.844	-0.39149	0.086316	-4.5355	5.75E-06	1.70E-05	NOT
CXorf67	4.490663	4.202512	0.926654	4.535148	5.76E-06	1.70E-05	UP
RP5-1101(	3.132351	1.364035	0.300777	4.535037	5.76E-06	1.70E-05	UP
NXT2	528.6516	0.543012	0.119739	4.534958	5.76E-06	1.70E-05	NOT
MECP2	1280.365	0.387838	0.085524	4.534835	5.76E-06	1.70E-05	NOT
RP11-347(	2.242034	2.015411	0.444528	4.53382	5.79E-06	1.71E-05	UP
PPIAP31	8.865401	0.875986	0.193212	4.533803	5.79E-06	1.71E-05	UP
LCTL	7.978662	0.919607	0.20284	4.533653	5.80E-06	1.71E-05	UP
FERP1	2.187876	1.324601	0.292191	4.533343	5.81E-06	1.71E-05	UP
VLDLR-AS	9.031715	1.582346	0.349142	4.532099	5.84E-06	1.72E-05	UP
RP13-516I	8.327984	0.914793	0.201855	4.531933	5.84E-06	1.72E-05	UP
AP000997.	2.007136	2.063987	0.455444	4.531817	5.85E-06	1.72E-05	UP
PLEKHM1F	232.0264	0.511536	0.112882	4.53158	5.85E-06	1.73E-05	NOT
RPL7L1P3	7.530654	-0.91896	0.202804	-4.53125	5.86E-06	1.73E-05	DOWN
RP11-325I	4.555949	-1.23145	0.271774	-4.53116	5.87E-06	1.73E-05	DOWN
ERVFRD-1	8.065612	-1.55609	0.343439	-4.53092	5.87E-06	1.73E-05	DOWN
RPRD1B	2430.382	-0.56527	0.124766	-4.53067	5.88E-06	1.73E-05	NOT
RP11-739I	1.856367	1.399715	0.308967	4.530307	5.89E-06	1.74E-05	UP
GPR27	32.30396	1.772682	0.391311	4.530103	5.90E-06	1.74E-05	UP
TRIM24	2536.531	0.4899	0.10815	4.529813	5.90E-06	1.74E-05	NOT
CTD-2622	2.833055	1.385583	0.305906	4.529441	5.91E-06	1.74E-05	UP
GRIK2	12.97387	1.647328	0.363722	4.529079	5.92E-06	1.75E-05	UP
AC007163	2.969629	3.714181	0.82011	4.528882	5.93E-06	1.75E-05	UP
LAT	13.26647	0.870538	0.19222	4.528864	5.93E-06	1.75E-05	UP
MNX1-AS:	3.09113	3.285671	0.725559	4.528465	5.94E-06	1.75E-05	UP
CEACAM1	323.7084	0.764622	0.168864	4.528032	5.95E-06	1.75E-05	UP
ASMTL	1990.739	-0.53475	0.118104	-4.5278	5.96E-06	1.75E-05	NOT
RP11-432I	1.782883	2.193638	0.484495	4.527678	5.96E-06	1.76E-05	UP
SLC5A7	1.045409	-2.45028	0.541191	-4.52756	5.97E-06	1.76E-05	DOWN
USP10	2142.575	-0.30084	0.066452	-4.52717	5.98E-06	1.76E-05	NOT
RP11-266I	6.051058	1.167236	0.257849	4.526814	5.99E-06	1.76E-05	UP
MAGEB1	6.491075	5.030778	1.111332	4.526802	5.99E-06	1.76E-05	UP
TGIF2LX	2.886639	3.738825	0.825931	4.526801	5.99E-06	1.76E-05	UP
CMYA5	228.4146	-0.96329	0.212805	-4.52663	5.99E-06	1.76E-05	DOWN
TTC34	12.85412	1.154099	0.254964	4.526524	6.00E-06	1.76E-05	UP
IDH2	14319.06	-0.65293	0.144246	-4.5265	6.00E-06	1.76E-05	DOWN
LINC0112ε	407.5451	-0.63208	0.139646	-4.52631	6.00E-06	1.77E-05	DOWN
RP11-126(	21.55577	-1.01331	0.223873	-4.52628	6.00E-06	1.77E-05	DOWN
SPINK1	7306.169	1.95671	0.432312	4.526149	6.01E-06	1.77E-05	UP
AC010980	4.122526	2.059801	0.455111	4.525928	6.01E-06	1.77E-05	UP
CTD-2231	2.742804	2.994431	0.661686	4.525454	6.03E-06	1.77E-05	UP

AC002310	1.365749	1.753578	0.387504	4.525317	6.03E-06	1.77E-05	UP
RP11-182J	7.085349	-0.66535	0.147063	-4.52427	6.06E-06	1.78E-05	DOWN
THOC6	882.819	0.4363	0.096441	4.523991	6.07E-06	1.78E-05	NOT
GPR173	20.28431	1.114295	0.246346	4.523289	6.09E-06	1.79E-05	UP
GUCY2EP	10.95231	1.723917	0.381125	4.523236	6.09E-06	1.79E-05	UP
MIR99AHG	170.3923	-0.94887	0.209777	-4.52323	6.09E-06	1.79E-05	DOWN
ATP8B2	643.7516	0.778186	0.172044	4.523172	6.09E-06	1.79E-05	UP
BYSL	869.5786	0.491324	0.10863	4.522899	6.10E-06	1.79E-05	NOT
CALCB	8.146802	1.250826	0.276607	4.52204	6.12E-06	1.80E-05	UP
AC092614	6.408333	0.903929	0.1999	4.521906	6.13E-06	1.80E-05	UP
TP53BP1	493.7974	0.60559	0.133933	4.52159	6.14E-06	1.80E-05	UP
RAVER1	354.279	0.690941	0.152809	4.521584	6.14E-06	1.80E-05	UP
RP11-10L7	1.248216	-1.66493	0.368226	-4.52149	6.14E-06	1.80E-05	DOWN
RP4-564F2	8.867177	1.070498	0.236796	4.520759	6.16E-06	1.81E-05	UP
RP11-766I	6.887143	2.070329	0.457962	4.520741	6.16E-06	1.81E-05	UP
MS4A10	5.483795	2.81444	0.622579	4.520616	6.17E-06	1.81E-05	UP
ZNF529	339.6935	0.474624	0.104997	4.520346	6.17E-06	1.81E-05	NOT
MYOF	742.7341	0.956142	0.211545	4.519799	6.19E-06	1.82E-05	UP
SHF	1076.116	-0.85057	0.188191	-4.51974	6.19E-06	1.82E-05	DOWN
NT5C1B	13.39356	-0.78956	0.174723	-4.51891	6.22E-06	1.82E-05	DOWN
RP11-3P17	72.03981	0.808702	0.178962	4.518845	6.22E-06	1.82E-05	UP
GMFB	1743.32	0.370801	0.08206	4.518679	6.22E-06	1.83E-05	NOT
MAEA	1864.078	0.307462	0.068045	4.518519	6.23E-06	1.83E-05	NOT
AP000525.	1.024101	2.298696	0.508766	4.518178	6.24E-06	1.83E-05	UP
KLHL8	751.0873	-0.55084	0.121939	-4.5173	6.26E-06	1.84E-05	NOT
ZNF682	123.9967	0.976035	0.21607	4.517223	6.27E-06	1.84E-05	UP
DHRS13	163.2972	0.748043	0.165629	4.51639	6.29E-06	1.84E-05	UP
SGK223	603.1442	0.892546	0.197635	4.516131	6.30E-06	1.85E-05	UP
AC008991	2.090613	3.515394	0.77846	4.515832	6.31E-06	1.85E-05	UP
ZNF14	92.67633	0.94094	0.208384	4.515407	6.32E-06	1.85E-05	UP
SLN	2.076371	3.007055	0.666035	4.514859	6.34E-06	1.86E-05	UP
RN7SKP26	1.657241	1.527666	0.338394	4.514462	6.35E-06	1.86E-05	UP
WDR55	1323.388	0.324475	0.071883	4.513905	6.36E-06	1.86E-05	NOT
RP11-76C1	4.330372	3.58013	0.793171	4.513692	6.37E-06	1.87E-05	UP
CMTM3	755.5429	0.792356	0.175549	4.51359	6.37E-06	1.87E-05	UP
AKR1A1	9809.487	-0.51093	0.1132	-4.51352	6.38E-06	1.87E-05	NOT
RP11-205I	29.39078	-1.79423	0.397563	-4.51308	6.39E-06	1.87E-05	DOWN
HEBP2	2764.122	-0.52205	0.115706	-4.51187	6.43E-06	1.88E-05	NOT
RP11-385I	6.277651	0.953801	0.211424	4.511327	6.44E-06	1.89E-05	UP
CHST11	559.2626	0.967507	0.214464	4.511281	6.44E-06	1.89E-05	UP
DHX8	1621.028	0.296041	0.065625	4.51107	6.45E-06	1.89E-05	NOT
AC012360	2.332782	1.398988	0.310133	4.510923	6.45E-06	1.89E-05	UP
UGT1A5	9.023425	2.004913	0.44447	4.5108	6.46E-06	1.89E-05	UP
CRYBA2	2.06618	2.504543	0.55525	4.510658	6.46E-06	1.89E-05	UP
TNFRSF9	73.58852	1.317183	0.292054	4.510074	6.48E-06	1.90E-05	UP
PRDM4	648.259	0.324692	0.072001	4.509521	6.50E-06	1.90E-05	NOT
RPS5	19873.25	0.667906	0.148111	4.509497	6.50E-06	1.90E-05	UP
GS1-600G	2.710775	2.978448	0.660577	4.508858	6.52E-06	1.91E-05	UP
HPN	23533.3	-0.79888	0.17719	-4.5086	6.53E-06	1.91E-05	DOWN
RP11-498I	8.728554	0.879223	0.195045	4.507792	6.55E-06	1.92E-05	UP
RP11-372I	6.215595	0.973976	0.216078	4.507528	6.56E-06	1.92E-05	UP
MT1HL1	1.868021	-2.47184	0.548414	-4.50724	6.57E-06	1.92E-05	DOWN

RP11-274I	3.314745	3.939805	0.874156	4.50698	6.58E-06	1.92E-05	UP
SSTR3	6.968478	2.070785	0.459465	4.506948	6.58E-06	1.92E-05	UP
SEMA4B	2029.941	-0.62504	0.138689	-4.50676	6.58E-06	1.92E-05	DOWN
PCYOX1	7706.072	-0.53278	0.118219	-4.50673	6.58E-06	1.92E-05	NOT
TMEM240	15.43265	0.937854	0.208103	4.506686	6.58E-06	1.92E-05	UP
RP11-848I	27.12623	0.851828	0.189018	4.506586	6.59E-06	1.92E-05	UP
SNX29	531.5308	0.53094	0.117824	4.506205	6.60E-06	1.93E-05	NOT
MAP2K4P1	4.634312	-0.89202	0.197955	-4.50615	6.60E-06	1.93E-05	DOWN
ADRA1D	28.42463	1.758841	0.390326	4.506083	6.60E-06	1.93E-05	UP
CTRC	2.075815	1.707291	0.378887	4.506064	6.60E-06	1.93E-05	UP
METTL11B	1.720339	3.103838	0.688827	4.505975	6.61E-06	1.93E-05	UP
ZSCAN32	48.94052	0.437832	0.097206	4.504156	6.66E-06	1.95E-05	NOT
GTF2H2C	113.0295	0.534109	0.1186	4.503437	6.69E-06	1.95E-05	NOT
CH507-42	964.4377	-1.09973	0.24422	-4.50303	6.70E-06	1.96E-05	DOWN
ZSWIM7	561.8347	-0.4848	0.107666	-4.50281	6.71E-06	1.96E-05	NOT
AKAP5	65.10074	0.799035	0.177462	4.502571	6.71E-06	1.96E-05	UP
SH3PXD2A	1535.969	0.53474	0.118765	4.502503	6.72E-06	1.96E-05	NOT
STAR	9.334007	1.519773	0.33759	4.501828	6.74E-06	1.97E-05	UP
CALU	4070.596	0.480909	0.106835	4.501401	6.75E-06	1.97E-05	NOT
DENND5B	1285.195	-0.47983	0.106618	-4.5005	6.78E-06	1.98E-05	NOT
MTHFS	1100.238	-0.70794	0.157306	-4.50042	6.78E-06	1.98E-05	DOWN
RP11-21B2	3.923432	1.256684	0.279237	4.500415	6.78E-06	1.98E-05	UP
CACNA1A	19.16035	1.08972	0.242171	4.499789	6.80E-06	1.98E-05	UP
ZC3H12A	696.9284	-0.76897	0.170914	-4.49918	6.82E-06	1.99E-05	DOWN
AC064836	1.562951	1.654629	0.367785	4.498902	6.83E-06	1.99E-05	UP
ARAP1	3417.713	0.354793	0.078867	4.498625	6.84E-06	1.99E-05	NOT
PHYHD1	1318.407	-1.20106	0.267	-4.49836	6.85E-06	2.00E-05	DOWN
LINC00887	12.25766	1.490842	0.33142	4.498351	6.85E-06	2.00E-05	UP
CCM2	3359.288	-0.46782	0.104004	-4.49807	6.86E-06	2.00E-05	NOT
RP11-54A	3.315145	1.819516	0.40452	4.497959	6.86E-06	2.00E-05	UP
LYPD3	49.40959	0.858711	0.190927	4.497592	6.87E-06	2.00E-05	UP
AHSA1	3223.577	0.380086	0.084512	4.497436	6.88E-06	2.00E-05	NOT
RP11-701I	6.764128	1.526792	0.339511	4.497036	6.89E-06	2.01E-05	UP
NRXN1	7.28803	-2.08449	0.46353	-4.497	6.89E-06	2.01E-05	DOWN
SULT2A1	30788.72	-1.21633	0.270491	-4.49675	6.90E-06	2.01E-05	DOWN
DCTN5	1411.269	0.367694	0.081777	4.496301	6.91E-06	2.01E-05	NOT
FAM228B	128.2036	0.64191	0.142768	4.496184	6.92E-06	2.01E-05	UP
NRSN1	2.472888	2.778099	0.617902	4.496017	6.92E-06	2.02E-05	UP
NARF-IT1	4.056359	1.142432	0.254121	4.495629	6.94E-06	2.02E-05	UP
UCHL5	1687.682	0.405496	0.090198	4.495613	6.94E-06	2.02E-05	NOT
AC141586	41.8363	0.514463	0.114444	4.495341	6.95E-06	2.02E-05	NOT
AP1AR	856.354	-0.42824	0.095266	-4.49521	6.95E-06	2.02E-05	NOT
CTB-152G	12.25916	0.662901	0.147499	4.494263	6.98E-06	2.03E-05	UP
TRPV4	476.4948	-1.12138	0.24954	-4.49377	7.00E-06	2.04E-05	DOWN
IFT22	455.5427	0.452215	0.10066	4.492478	7.04E-06	2.05E-05	NOT
NFYAP1	1.598683	3.074619	0.684399	4.492439	7.04E-06	2.05E-05	UP
SLC30A6	1093.434	0.334037	0.074363	4.491987	7.06E-06	2.05E-05	NOT
ADRBK2	699.1404	1.022125	0.227558	4.491716	7.07E-06	2.06E-05	UP
RP1-81D8	8.539306	-2.0005	0.445406	-4.49141	7.08E-06	2.06E-05	DOWN
RP11-382	13.68489	0.69814	0.15545	4.491083	7.09E-06	2.06E-05	UP
NUMBL	315.3807	0.67451	0.150191	4.491029	7.09E-06	2.06E-05	UP
NUP98	3025.336	-0.29344	0.065348	-4.49048	7.11E-06	2.07E-05	NOT

TACR1	12.26027	1.351157	0.300908	4.490269	7.11E-06	2.07E-05	UP
RP11-128(	7.564443	1.059823	0.236056	4.489717	7.13E-06	2.07E-05	UP
DOK2	329.3519	-0.79012	0.176008	-4.48908	7.15E-06	2.08E-05	DOWN
KCNQ1	376.7558	1.046703	0.233169	4.489032	7.15E-06	2.08E-05	UP
RP11-387I	2.679385	1.158007	0.257975	4.488834	7.16E-06	2.08E-05	UP
AC016710	5.296996	4.797747	1.06896	4.488239	7.18E-06	2.09E-05	UP
HSPG2	4070.933	0.875371	0.195097	4.486851	7.23E-06	2.10E-05	UP
PCIF1	1342.402	0.264505	0.05896	4.486182	7.25E-06	2.11E-05	NOT
ZNF283	97.07096	0.651367	0.14521	4.485693	7.27E-06	2.11E-05	UP
GNAI2	9982.631	-0.32216	0.071835	-4.48468	7.30E-06	2.12E-05	NOT
RP5-1092/	1.431555	1.713187	0.382066	4.484013	7.33E-06	2.13E-05	UP
TSKU	11648.43	-0.98579	0.219867	-4.48357	7.34E-06	2.13E-05	DOWN
TAB1	1282.883	0.307488	0.068582	4.48348	7.34E-06	2.13E-05	NOT
AC017002	1.785059	2.141689	0.477687	4.483456	7.34E-06	2.13E-05	UP
SCO1	1541.064	-0.40537	0.090419	-4.4832	7.35E-06	2.13E-05	NOT
SLC35A4	3478.22	0.29297	0.065351	4.483011	7.36E-06	2.14E-05	NOT
P2RX4	1197.716	0.580468	0.129485	4.482897	7.36E-06	2.14E-05	NOT
SYT10	5.028958	-2.36119	0.526779	-4.48232	7.38E-06	2.14E-05	DOWN
RP11-91I8	1.860925	3.238346	0.722484	4.482236	7.39E-06	2.14E-05	UP
MCU	707.8355	0.410374	0.09156	4.482003	7.39E-06	2.15E-05	NOT
FAM180B	2.040641	1.943007	0.433518	4.48195	7.40E-06	2.15E-05	UP
UBXN10-A	10.71007	1.408005	0.314178	4.481549	7.41E-06	2.15E-05	UP
RP11-542I	1.120709	2.245997	0.50119	4.481333	7.42E-06	2.15E-05	UP
CASP3	1089.388	0.40892	0.091257	4.48095	7.43E-06	2.16E-05	NOT
RP11-317I	1.828621	2.946918	0.65767	4.480844	7.43E-06	2.16E-05	UP
TXNIP	13420.65	-0.83919	0.18732	-4.48	7.46E-06	2.16E-05	DOWN
ATP9A	2160.865	0.650805	0.145292	4.4793	7.49E-06	2.17E-05	UP
ARF1	19873.92	0.299169	0.066794	4.478975	7.50E-06	2.17E-05	NOT
RP11-182I	42.86776	-0.55689	0.124341	-4.47875	7.51E-06	2.18E-05	NOT
RBL2	2381.902	-0.68388	0.152709	-4.47834	7.52E-06	2.18E-05	DOWN
CALR4P	3.094559	-1.19095	0.265951	-4.47808	7.53E-06	2.18E-05	DOWN
NKX2-3	6.261111	2.051806	0.458219	4.477788	7.54E-06	2.19E-05	UP
TRIM73	10.16266	0.955285	0.21334	4.477765	7.54E-06	2.19E-05	UP
CTD-3051	2.227242	1.555416	0.347366	4.47774	7.54E-06	2.19E-05	UP
RP11-426/	5.444728	0.950025	0.21219	4.47723	7.56E-06	2.19E-05	UP
CTB-41I6.2	4.656248	-1.02515	0.228969	-4.47723	7.56E-06	2.19E-05	DOWN
IDSP1	1.540731	1.783564	0.398403	4.476789	7.58E-06	2.19E-05	UP
ARHGEF9	518.7358	0.479668	0.107147	4.47674	7.58E-06	2.20E-05	NOT
ATMIN	1536.647	-0.33625	0.075118	-4.47624	7.60E-06	2.20E-05	NOT
RAB6A	4008.077	-0.36262	0.081016	-4.47593	7.61E-06	2.20E-05	NOT
CRTC3-AS	6.940639	0.742031	0.165787	4.47582	7.61E-06	2.20E-05	UP
RP11-354I	2.936202	1.963116	0.438671	4.475139	7.64E-06	2.21E-05	UP
RP11-407I	1.936573	2.233658	0.499131	4.47509	7.64E-06	2.21E-05	UP
ALG1L7P	1.587477	1.750597	0.391199	4.474954	7.64E-06	2.21E-05	UP
ELOVL4	15.94582	1.23692	0.276439	4.474481	7.66E-06	2.22E-05	UP
YIPF4	2551.368	-0.33702	0.075322	-4.47433	7.67E-06	2.22E-05	NOT
RP11-314/	7.761912	1.055834	0.236017	4.473558	7.69E-06	2.23E-05	UP
CLIC3	52.34546	1.212087	0.270952	4.473431	7.70E-06	2.23E-05	UP
CSAG4	6.153091	4.479766	1.001524	4.47295	7.71E-06	2.23E-05	UP
RAD23B	9556.155	-0.33144	0.074103	-4.47265	7.73E-06	2.23E-05	NOT
TNFRSF10I	656.3527	-0.68776	0.153775	-4.47253	7.73E-06	2.24E-05	DOWN
RP11-817(	126.6688	-0.6955	0.155515	-4.47221	7.74E-06	2.24E-05	DOWN

STXBP1	429.8213	0.764054	0.170847	4.472146	7.74E-06	2.24E-05	UP
SETBP1	474.9736	-0.61707	0.137983	-4.47211	7.74E-06	2.24E-05	DOWN
SLCO2B1	13266.26	-0.83904	0.187636	-4.47166	7.76E-06	2.24E-05	DOWN
RP11-690I	2.72828	1.383387	0.30938	4.471483	7.77E-06	2.25E-05	UP
C1orf158	2.420495	3.053486	0.682895	4.471381	7.77E-06	2.25E-05	UP
KDELR3	874.1794	0.806133	0.180297	4.471148	7.78E-06	2.25E-05	UP
C1orf100	2.572161	1.513321	0.338486	4.470858	7.79E-06	2.25E-05	UP
KCNH3	41.71078	1.375933	0.307765	4.47073	7.80E-06	2.25E-05	UP
EHD4-AS1	2.780904	1.38497	0.309814	4.470333	7.81E-06	2.26E-05	UP
RP11-430I	6.920196	-1.51047	0.33789	-4.47029	7.81E-06	2.26E-05	DOWN
RP11-439I	68.88333	0.790539	0.176845	4.470229	7.81E-06	2.26E-05	UP
EFHD1	1361.752	-1.19543	0.267445	-4.46981	7.83E-06	2.26E-05	DOWN
SERINC3	6546.312	-0.30064	0.067267	-4.46936	7.85E-06	2.27E-05	NOT
RP11-349I	6.315121	0.733711	0.16417	4.469204	7.85E-06	2.27E-05	UP
CLDN4	1660.025	1.591181	0.356056	4.468912	7.86E-06	2.27E-05	UP
ALKBH5	3701.853	-0.35713	0.079922	-4.46851	7.88E-06	2.27E-05	NOT
DZIP1L	77.81305	1.021551	0.228633	4.468088	7.89E-06	2.28E-05	UP
IGKV1-9	93.0591	-1.77215	0.396635	-4.46797	7.90E-06	2.28E-05	DOWN
CES5A	155.5365	-1.34895	0.301918	-4.46793	7.90E-06	2.28E-05	DOWN
ADCY4	252.693	0.640437	0.143346	4.467773	7.90E-06	2.28E-05	UP
AC073321	6.908279	-1.77718	0.397796	-4.46755	7.91E-06	2.28E-05	DOWN
CTD-2008	1.553817	2.135276	0.477963	4.467452	7.92E-06	2.28E-05	UP
SHISA6	6.278745	1.65661	0.37084	4.467179	7.93E-06	2.29E-05	UP
MVP	7296.554	0.608717	0.136291	4.466301	7.96E-06	2.30E-05	UP
RPS19	29843.7	0.723581	0.162015	4.46615	7.96E-06	2.30E-05	UP
RP11-41O	127.3674	-0.76307	0.170856	-4.46615	7.96E-06	2.30E-05	DOWN
PSMD11	3808.644	0.284014	0.063605	4.465276	8.00E-06	2.31E-05	NOT
ATXN7	803.7651	-0.361	0.080845	-4.46527	8.00E-06	2.31E-05	NOT
RP11-44F2	5.875669	2.724145	0.610119	4.46494	8.01E-06	2.31E-05	UP
RP3-428L1	18.69504	0.864352	0.193601	4.464596	8.02E-06	2.31E-05	UP
RP1-310O	3.417331	1.162284	0.26035	4.46432	8.03E-06	2.32E-05	UP
PPIAP3	2.167932	1.340553	0.300349	4.463313	8.07E-06	2.33E-05	UP
NKAIN4	2.945595	1.831905	0.410529	4.462301	8.11E-06	2.34E-05	UP
RFX6	4.770999	2.604526	0.583684	4.46222	8.11E-06	2.34E-05	UP
CCS	2902.703	-0.5699	0.127719	-4.46214	8.11E-06	2.34E-05	NOT
LINC01124	284.8152	1.189872	0.266683	4.461752	8.13E-06	2.34E-05	UP
CTC-205M	43.88366	0.711551	0.159479	4.461715	8.13E-06	2.34E-05	UP
HCG15	11.19863	1.048698	0.235054	4.461519	8.14E-06	2.34E-05	UP
PCDHA5	3.395634	2.032165	0.455544	4.460959	8.16E-06	2.35E-05	UP
NLRP3	77.20252	-0.83834	0.187956	-4.46028	8.19E-06	2.36E-05	DOWN
TMEM120	3923.708	-0.50616	0.113486	-4.46011	8.19E-06	2.36E-05	NOT
URB2	406.9153	0.496905	0.111433	4.459226	8.23E-06	2.37E-05	NOT
CTA-243E	3.16067	1.105462	0.247912	4.459085	8.23E-06	2.37E-05	UP
ATP5J2	4946.268	0.537695	0.120589	4.458917	8.24E-06	2.37E-05	NOT
MPPED1	689.9868	-1.42562	0.319733	-4.45878	8.24E-06	2.37E-05	DOWN
SRP9P1	6.814808	0.901053	0.202086	4.458763	8.24E-06	2.37E-05	UP
MRPS18C	530.0302	-0.3414	0.07657	-4.45861	8.25E-06	2.37E-05	NOT
ZNF534	3.609414	2.175937	0.488045	4.458478	8.25E-06	2.38E-05	UP
PMF1	1547.512	0.546408	0.122573	4.457804	8.28E-06	2.38E-05	NOT
MID1	734.3572	0.698736	0.156752	4.457588	8.29E-06	2.38E-05	UP
ARID3B	195.9496	0.516042	0.115768	4.457542	8.29E-06	2.38E-05	NOT
CKLF-CMT	3.99651	1.113591	0.249829	4.457403	8.30E-06	2.39E-05	UP

HSP90AA1	32418.51	0.420627	0.094367	4.457369	8.30E-06	2.39E-05	NOT
AC010969	26.46707	-1.90712	0.427961	-4.45629	8.34E-06	2.40E-05	DOWN
RP11-247I	1.384637	1.73217	0.388703	4.456278	8.34E-06	2.40E-05	UP
RP3-402G	15.96558	0.712172	0.159821	4.456065	8.35E-06	2.40E-05	UP
SMURF2	530.102	0.499068	0.112007	4.455684	8.36E-06	2.40E-05	NOT
ARV1	874.7663	0.370206	0.083096	4.455191	8.38E-06	2.41E-05	NOT
GPC5	93.11647	1.639077	0.367932	4.454832	8.40E-06	2.41E-05	UP
HCG16	5.465719	1.169942	0.26263	4.454714	8.40E-06	2.41E-05	UP
RP11-460I	1.404336	1.760327	0.395163	4.454691	8.40E-06	2.41E-05	UP
RP11-169I	46.42027	1.179078	0.264684	4.454657	8.40E-06	2.41E-05	UP
SLC25A40	529.9269	0.413902	0.092923	4.454259	8.42E-06	2.42E-05	NOT
RP11-159I	2.106834	2.003757	0.449901	4.45377	8.44E-06	2.42E-05	UP
ZNF491	41.654	0.693976	0.155821	4.453681	8.44E-06	2.42E-05	UP
CTD-3157	31.17044	-0.87265	0.195946	-4.45352	8.45E-06	2.43E-05	DOWN
SH2D3C	538.9116	0.541208	0.121527	4.453414	8.45E-06	2.43E-05	NOT
RP11-798I	2.928811	1.174232	0.263684	4.453177	8.46E-06	2.43E-05	UP
IQSEC1	4219.797	0.456507	0.102519	4.452893	8.47E-06	2.43E-05	NOT
HNRNPR	3138.862	0.293177	0.065847	4.452423	8.49E-06	2.44E-05	NOT
RP11-227(	7.759898	1.10299	0.247744	4.452138	8.50E-06	2.44E-05	UP
ALOX15	10.37656	1.226851	0.275579	4.451906	8.51E-06	2.44E-05	UP
LINC0023E	53.88117	-1.85125	0.415882	-4.45137	8.53E-06	2.45E-05	DOWN
GNB3	8.598622	1.108373	0.249001	4.451273	8.54E-06	2.45E-05	UP
CALD1	12487.23	-0.41408	0.093036	-4.45078	8.56E-06	2.46E-05	NOT
MLIP	856.6807	-1.20491	0.270724	-4.45069	8.56E-06	2.46E-05	DOWN
RP5-1125/	46.63269	0.630042	0.141564	4.450563	8.56E-06	2.46E-05	UP
KLHL29	462.3329	1.043845	0.234559	4.450245	8.58E-06	2.46E-05	UP
BANF1	4196.896	0.412944	0.092804	4.449622	8.60E-06	2.47E-05	NOT
HMBS	1102.429	0.417564	0.093844	4.449566	8.60E-06	2.47E-05	NOT
CDK17	817.2355	-0.37848	0.085064	-4.44939	8.61E-06	2.47E-05	NOT
MIR635	14.14182	0.709578	0.159479	4.449349	8.61E-06	2.47E-05	UP
IFI27L2	316.9146	0.952988	0.214202	4.449004	8.63E-06	2.47E-05	UP
FAM160A1	29.09613	-1.06435	0.23929	-4.44796	8.67E-06	2.49E-05	DOWN
GYLTL1B	291.1288	1.184174	0.266253	4.447551	8.69E-06	2.49E-05	UP
NCBP2-AS	2.71904	1.237201	0.278233	4.446637	8.72E-06	2.50E-05	UP
NPM1P39	8.001632	0.846764	0.190428	4.446627	8.72E-06	2.50E-05	UP
STXBP5L	2.316272	2.857459	0.642711	4.445947	8.75E-06	2.51E-05	UP
FAM90A1	11.7375	1.424879	0.320532	4.445354	8.77E-06	2.51E-05	UP
RP11-46H	4.577375	1.058788	0.2382	4.444959	8.79E-06	2.52E-05	UP
HTATSF1	2386.11	0.3379	0.076026	4.44453	8.81E-06	2.52E-05	NOT
RP11-855/	2.858489	1.196944	0.269308	4.444512	8.81E-06	2.52E-05	UP
PGBD4P3	2.641832	-1.04123	0.234295	-4.44411	8.83E-06	2.53E-05	DOWN
AP1G2	557.1778	0.740443	0.166613	4.444088	8.83E-06	2.53E-05	UP
CTC-559E	25.71769	0.867747	0.195308	4.442959	8.87E-06	2.54E-05	UP
CCNJL	61.0928	1.16781	0.262847	4.442923	8.87E-06	2.54E-05	UP
DYRK1B	642.161	0.660225	0.148601	4.442923	8.87E-06	2.54E-05	UP
RP11-318(	3.331408	1.013174	0.22809	4.441985	8.91E-06	2.55E-05	UP
RP11-582	25.05847	-0.55998	0.126084	-4.44129	8.94E-06	2.56E-05	NOT
MYLK	4469.674	-0.68906	0.155162	-4.44092	8.96E-06	2.56E-05	DOWN
INHBA	754.6544	-0.93333	0.21018	-4.44062	8.97E-06	2.57E-05	DOWN
MOB3B	428.4062	0.915834	0.206246	4.440483	8.98E-06	2.57E-05	UP
RGL3	614.5283	0.694186	0.156333	4.440433	8.98E-06	2.57E-05	UP
FOLR2	748.4737	-0.87171	0.196316	-4.44036	8.98E-06	2.57E-05	DOWN

RP11-493I	3.484143	1.271925	0.286449	4.440318	8.98E-06	2.57E-05	UP
RP11-239I	1.031709	1.876214	0.422554	4.44018	8.99E-06	2.57E-05	UP
SIAH1	489.7112	-0.33432	0.075294	-4.44016	8.99E-06	2.57E-05	NOT
GNS	7835.497	0.42684	0.096136	4.43997	9.00E-06	2.57E-05	NOT
SH3GL3	5.824416	2.789234	0.62823	4.439828	9.00E-06	2.57E-05	UP
POLR3K	431.3439	0.425833	0.095924	4.439285	9.03E-06	2.58E-05	NOT
HCG4B	43.02518	0.887902	0.200026	4.438939	9.04E-06	2.58E-05	UP
RP11-100(	6.439552	-1.0889	0.245311	-4.43884	9.04E-06	2.59E-05	DOWN
MKRN9P	2.621899	2.982609	0.672005	4.438372	9.06E-06	2.59E-05	UP
LGALS1	5398.849	0.804155	0.181187	4.438257	9.07E-06	2.59E-05	UP
ARMC10	937.9341	-0.28035	0.063167	-4.43825	9.07E-06	2.59E-05	NOT
PIP4K2C	1266.684	0.341405	0.076925	4.438178	9.07E-06	2.59E-05	NOT
PVRL3	957.937	-0.68071	0.153407	-4.43726	9.11E-06	2.60E-05	DOWN
RANBP17	119.1395	1.182706	0.266566	4.43682	9.13E-06	2.61E-05	UP
C9orf84	5.974895	1.239578	0.279385	4.436808	9.13E-06	2.61E-05	UP
AC011330	2.813771	1.16054	0.261586	4.436555	9.14E-06	2.61E-05	UP
CPNE8	601.1288	-0.53739	0.121151	-4.43576	9.17E-06	2.62E-05	NOT
LHFPL3-AS	27.62664	1.942778	0.438046	4.435102	9.20E-06	2.63E-05	UP
HYPK	60.04815	0.474825	0.107067	4.434833	9.21E-06	2.63E-05	NOT
RNU2-11P	1.302251	1.643824	0.370674	4.434684	9.22E-06	2.63E-05	UP
ALX4	1.795328	2.036394	0.459223	4.434439	9.23E-06	2.64E-05	UP
RP11-459I	3.502903	0.948402	0.213918	4.43348	9.27E-06	2.65E-05	UP
AC104134	2.483167	2.405649	0.542613	4.433455	9.27E-06	2.65E-05	UP
TSHR	6.936779	-0.9708	0.218975	-4.43337	9.28E-06	2.65E-05	DOWN
FAM227B	100.8981	-0.59989	0.135315	-4.43326	9.28E-06	2.65E-05	DOWN
RP11-145/	9.362296	1.505834	0.339693	4.43293	9.30E-06	2.65E-05	UP
KCNC4	209.4133	0.785888	0.177286	4.432895	9.30E-06	2.65E-05	UP
RNVU1-3	2.204961	1.652908	0.37292	4.432337	9.32E-06	2.66E-05	UP
RGPD2	20.45051	-1.04079	0.234826	-4.43218	9.33E-06	2.66E-05	DOWN
SLC26A3	92.9857	1.692517	0.381899	4.431845	9.34E-06	2.66E-05	UP
CNIH3	108.4075	0.825777	0.186328	4.431836	9.34E-06	2.66E-05	UP
CH17-302	5.911276	1.112438	0.251044	4.431242	9.37E-06	2.67E-05	UP
RP4-673M	20.83585	0.776598	0.175272	4.430806	9.39E-06	2.68E-05	UP
ENG	4568.933	-0.66728	0.150629	-4.42992	9.43E-06	2.69E-05	DOWN
RP11-478(	3.002931	1.177013	0.265714	4.42962	9.44E-06	2.69E-05	UP
RP11-217I	8.435165	0.880177	0.198717	4.429307	9.45E-06	2.69E-05	UP
MTFR1L	2294.655	-0.38481	0.086885	-4.429	9.47E-06	2.70E-05	NOT
TGM4	2.217821	1.656449	0.374048	4.428443	9.49E-06	2.70E-05	UP
PRG4	9861.479	-1.27111	0.287044	-4.42828	9.50E-06	2.71E-05	DOWN
GAL	6.346422	2.170179	0.490084	4.428176	9.50E-06	2.71E-05	UP
PLEKHF2	764.3079	0.410903	0.092814	4.427168	9.55E-06	2.72E-05	NOT
CTD-2536	7.371297	-1.08427	0.244943	-4.4266	9.57E-06	2.73E-05	DOWN
SYMPK	4384.075	0.475806	0.107505	4.425875	9.61E-06	2.74E-05	NOT
PILRB	174.6904	0.925978	0.209227	4.425714	9.61E-06	2.74E-05	UP
TANC2	292.1216	1.016945	0.22979	4.425547	9.62E-06	2.74E-05	UP
RP11-192I	0.98874	1.7292	0.390735	4.425502	9.62E-06	2.74E-05	UP
RPS10	5440.208	0.631935	0.142798	4.425371	9.63E-06	2.74E-05	UP
AOC1	261.703	-1.76084	0.397913	-4.42518	9.64E-06	2.74E-05	DOWN
NAGLU	3710.402	-0.50016	0.113026	-4.42515	9.64E-06	2.74E-05	NOT
RP11-572I	175.0743	-0.98795	0.223259	-4.42512	9.64E-06	2.74E-05	DOWN
RP11-138I	5.600574	1.091414	0.246645	4.425034	9.64E-06	2.74E-05	UP
FAM161B	133.7551	0.418491	0.094576	4.424921	9.65E-06	2.75E-05	NOT

KLHL28	381.9695	-0.42274	0.095536	-4.42488	9.65E-06	2.75E-05	NOT
U2AF1L4	298.185	0.58424	0.132052	4.424306	9.68E-06	2.75E-05	NOT
NOVA2	138.2338	0.588078	0.132922	4.424217	9.68E-06	2.75E-05	UP
ARHGAP44	201.7357	1.131669	0.255848	4.42321	9.72E-06	2.77E-05	UP
RP11-397(	3.40939	2.085326	0.47147	4.423028	9.73E-06	2.77E-05	UP
LINC00635	2.114502	3.166451	0.715917	4.42293	9.74E-06	2.77E-05	UP
BRDTP1	1.877358	2.798276	0.632698	4.422765	9.74E-06	2.77E-05	UP
RP11-122I	22.23916	0.738913	0.167077	4.422604	9.75E-06	2.77E-05	UP
CTD-2538	22.88083	0.733818	0.165929	4.422482	9.76E-06	2.77E-05	UP
RP11-347(	10.81773	0.754579	0.170631	4.422289	9.77E-06	2.78E-05	UP
FNBP1P1	10.81627	1.140185	0.257843	4.422018	9.78E-06	2.78E-05	UP
TEX38	2.092022	1.394525	0.315401	4.421431	9.80E-06	2.79E-05	UP
GAPDHP1	444.553	1.277589	0.288963	4.421294	9.81E-06	2.79E-05	UP
DNAH10	22.29333	0.949406	0.214735	4.421289	9.81E-06	2.79E-05	UP
ANXA2P1	3.157997	1.108181	0.250658	4.421095	9.82E-06	2.79E-05	UP
FAM175A	146.9734	0.484806	0.109668	4.420677	9.84E-06	2.80E-05	NOT
DNAJB1P1	1.7772	2.520797	0.570264	4.420405	9.85E-06	2.80E-05	UP
ZMAT5	662.1536	0.418931	0.094774	4.420317	9.86E-06	2.80E-05	NOT
CTD-3035	1.811167	2.078921	0.47037	4.419762	9.88E-06	2.81E-05	UP
RP11-552I	23.72867	0.812579	0.183923	4.418033	9.96E-06	2.83E-05	UP
USP2-AS1	60.86116	1.095726	0.248033	4.41766	9.98E-06	2.83E-05	UP
TTPA	3205.81	-1.04456	0.236454	-4.41759	9.98E-06	2.83E-05	DOWN
FTH1P7	75.20948	0.583612	0.132116	4.417434	9.99E-06	2.84E-05	NOT
CTD-2325	1.353806	2.297253	0.520056	4.417314	9.99E-06	2.84E-05	UP
RP11-77H	40.47134	0.532423	0.120544	4.416843	1.00E-05	2.84E-05	NOT
RAMP2-AS	38.93326	1.014145	0.229619	4.416646	1.00E-05	2.84E-05	UP
AC010458	1.095803	1.836358	0.415823	4.4162	1.00E-05	2.85E-05	UP
EXOC4	1905.021	0.302897	0.068594	4.415775	1.01E-05	2.86E-05	NOT
HOXC10	16.79935	3.618584	0.81949	4.415652	1.01E-05	2.86E-05	UP
LINC00399	4.859944	-1.45363	0.329224	-4.41533	1.01E-05	2.86E-05	DOWN
CBX3P2	13.18064	0.713192	0.161527	4.415327	1.01E-05	2.86E-05	UP
SREK1	1603.269	0.363021	0.082242	4.414071	1.01E-05	2.88E-05	NOT
DALRD3	473.3176	0.383224	0.086826	4.413697	1.02E-05	2.88E-05	NOT
RP11-390I	257.8038	-1.00425	0.227536	-4.41359	1.02E-05	2.88E-05	DOWN
RP11-372I	8.046435	0.988121	0.223892	4.41338	1.02E-05	2.89E-05	UP
ZNF624	70.63768	0.567644	0.128633	4.412914	1.02E-05	2.89E-05	NOT
ZNF888	6.841656	1.374659	0.311523	4.412712	1.02E-05	2.89E-05	UP
RP11-279I	6.392972	1.778342	0.403012	4.412632	1.02E-05	2.89E-05	UP
PGGT1B	903.0024	-0.28373	0.064301	-4.41255	1.02E-05	2.90E-05	NOT
MKRN4P	3.515614	2.76418	0.626497	4.412122	1.02E-05	2.90E-05	UP
RMND5B	1424.155	0.3314	0.075114	4.411982	1.02E-05	2.90E-05	NOT
AC010970	23.33887	-2.30432	0.522291	-4.41196	1.02E-05	2.90E-05	DOWN
PLD1	1575.574	-0.70555	0.159923	-4.41182	1.03E-05	2.90E-05	DOWN
PIP5K1A	1689.611	0.408462	0.092592	4.411442	1.03E-05	2.91E-05	NOT
RP11-481	2.184999	1.321323	0.299535	4.41125	1.03E-05	2.91E-05	UP
RPS10P3	7.976041	1.012333	0.229522	4.410604	1.03E-05	2.92E-05	UP
ZCWPW1	243.3405	-0.58348	0.132294	-4.41052	1.03E-05	2.92E-05	NOT
MARCKSL1	3102.691	0.754051	0.170998	4.409698	1.04E-05	2.93E-05	UP
PKNOX1	570.871	0.273987	0.062133	4.409653	1.04E-05	2.93E-05	NOT
SAMM50	2548.841	0.514776	0.116739	4.409622	1.04E-05	2.93E-05	NOT
ASB6	697.4728	0.370521	0.08403	4.409372	1.04E-05	2.93E-05	NOT
LAIR2	10.1637	1.416461	0.321246	4.409277	1.04E-05	2.94E-05	UP

FAM58A	648.292	0.490843	0.111323	4.409164	1.04E-05	2.94E-05	NOT
IGFL2	2.365884	2.454618	0.556719	4.409082	1.04E-05	2.94E-05	UP
XRCC6	9170.385	0.324112	0.073511	4.409051	1.04E-05	2.94E-05	NOT
CTD-2192	3.061114	1.2198	0.276709	4.408243	1.04E-05	2.95E-05	UP
MTUS2	54.81979	-1.69692	0.384971	-4.40791	1.04E-05	2.95E-05	DOWN
HMGN2P1	15.0741	1.100402	0.249643	4.4079	1.04E-05	2.95E-05	UP
PIK3R5	216.9717	-0.74361	0.168706	-4.40776	1.04E-05	2.95E-05	DOWN
BMP2K	212.5599	-0.49935	0.113291	-4.40762	1.05E-05	2.96E-05	NOT
AC005281	1.389052	2.494777	0.566042	4.407409	1.05E-05	2.96E-05	UP
SLC22A6	2.492908	3.204884	0.727401	4.40594	1.05E-05	2.98E-05	UP
RP11-408I	126.1046	0.508844	0.115493	4.40585	1.05E-05	2.98E-05	NOT
XRN1	1498.855	-0.44605	0.101246	-4.40562	1.05E-05	2.98E-05	NOT
XXbac-BP(	7.334328	0.803716	0.182443	4.405307	1.06E-05	2.99E-05	UP
SMU1	2119.018	-0.26164	0.059395	-4.40516	1.06E-05	2.99E-05	NOT
ZSWIM3	152.5862	0.339907	0.077173	4.404462	1.06E-05	3.00E-05	NOT
RP3-462C:	2.744446	2.412673	0.547805	4.404256	1.06E-05	3.00E-05	UP
IGHV3-43	19.89337	-1.7362	0.3943	-4.40324	1.07E-05	3.01E-05	DOWN
ENPP7P13	2.132648	3.365514	0.76438	4.402931	1.07E-05	3.02E-05	UP
ERCC6L2	328.1668	-0.40673	0.092377	-4.40288	1.07E-05	3.02E-05	NOT
RP11-778I	21.3948	0.817174	0.18561	4.402634	1.07E-05	3.02E-05	UP
MYT1	17.61356	1.487415	0.337865	4.402393	1.07E-05	3.02E-05	UP
KDM5C	2887.775	0.350053	0.079515	4.40236	1.07E-05	3.02E-05	NOT
UPB1	9945.089	-1.13301	0.257366	-4.40234	1.07E-05	3.02E-05	DOWN
DRAP1	3219.442	0.474804	0.107863	4.401934	1.07E-05	3.03E-05	NOT
CAPNS1	10299.83	0.486692	0.110569	4.401684	1.07E-05	3.03E-05	NOT
MBD1	1812.78	0.249093	0.056593	4.401464	1.08E-05	3.04E-05	NOT
STAU1	8635.57	-0.33822	0.076846	-4.40125	1.08E-05	3.04E-05	NOT
RP11-75C:	3.809614	1.170081	0.265867	4.400995	1.08E-05	3.04E-05	UP
ACAD8	1250.965	-0.41049	0.093272	-4.40096	1.08E-05	3.04E-05	NOT
ENTPD8	634.0139	-0.99452	0.225985	-4.40085	1.08E-05	3.04E-05	DOWN
RP11-296I	2.497949	1.707093	0.387905	4.400807	1.08E-05	3.04E-05	UP
ITLN2	35.12799	1.481548	0.336665	4.400663	1.08E-05	3.04E-05	UP
RP11-95P:	1.981552	2.302353	0.523301	4.399672	1.08E-05	3.06E-05	UP
XXyac-YX6	1.200345	2.459768	0.559167	4.398986	1.09E-05	3.07E-05	UP
UBOX5	455.7566	0.373266	0.084854	4.398923	1.09E-05	3.07E-05	NOT
RP11-266I	5.109369	0.965237	0.219429	4.398849	1.09E-05	3.07E-05	UP
RGL1	809.8072	-0.66357	0.150853	-4.39879	1.09E-05	3.07E-05	DOWN
SYCP3	8.258196	0.788707	0.179316	4.398416	1.09E-05	3.07E-05	UP
LDB1	900.4652	0.394659	0.089739	4.397869	1.09E-05	3.08E-05	NOT
CTB-75G1	5.390562	1.201906	0.273333	4.397226	1.10E-05	3.09E-05	UP
RPL39P3	110.3158	0.77155	0.175491	4.396527	1.10E-05	3.10E-05	UP
FBXO3	1576.205	-0.30302	0.068929	-4.39615	1.10E-05	3.11E-05	NOT
RP11-4B1(	3.297123	-1.28831	0.293123	-4.39512	1.11E-05	3.12E-05	DOWN
PPIAP6	4.526202	1.012618	0.230403	4.394989	1.11E-05	3.12E-05	UP
AC064875	3.538156	1.266123	0.288099	4.394752	1.11E-05	3.12E-05	UP
TTC26	115.1708	0.627771	0.142847	4.394693	1.11E-05	3.13E-05	UP
ST7-OT4	3.002963	1.161209	0.264266	4.394085	1.11E-05	3.13E-05	UP
AC010900	2.239116	1.43863	0.327425	4.393773	1.11E-05	3.14E-05	UP
CLDND2	88.28424	0.939529	0.213873	4.392932	1.12E-05	3.15E-05	UP
SLC25A25	477.0751	0.752225	0.171251	4.392536	1.12E-05	3.15E-05	UP
DNAJB5-A	2.670931	1.783722	0.4061	4.392322	1.12E-05	3.16E-05	UP
TMEM178	98.76682	1.046329	0.238234	4.392025	1.12E-05	3.16E-05	UP

NAP1L4	3885.626	0.267038	0.060811	4.391283	1.13E-05	3.17E-05	NOT
GPR61	5.246282	1.044342	0.237836	4.391009	1.13E-05	3.18E-05	UP
CTA-246H	1.351679	2.700192	0.614987	4.390648	1.13E-05	3.18E-05	UP
USP47	2990.123	-0.2891	0.065852	-4.39015	1.13E-05	3.19E-05	NOT
RABEP2	719.0901	0.511378	0.116494	4.389751	1.13E-05	3.19E-05	NOT
CLRN3	809.8536	-1.45596	0.331676	-4.38969	1.14E-05	3.19E-05	DOWN
FRMPD2	2.828226	2.2479	0.512107	4.389517	1.14E-05	3.20E-05	UP
RP11-168I	2.977626	1.795518	0.409091	4.389043	1.14E-05	3.20E-05	UP
WBP1	403.1211	0.413742	0.094271	4.388854	1.14E-05	3.21E-05	NOT
AL031587.	6.570972	0.926113	0.211015	4.388838	1.14E-05	3.21E-05	UP
RP11-834(	108.7027	1.210582	0.275834	4.388802	1.14E-05	3.21E-05	UP
ALDH7A1	7989.164	-0.62769	0.143031	-4.38852	1.14E-05	3.21E-05	DOWN
RNU6-481	4.105158	1.042981	0.237664	4.388465	1.14E-05	3.21E-05	UP
MED10	797.6414	0.372547	0.084903	4.387892	1.14E-05	3.22E-05	NOT
SLC35A5	951.8192	-0.34721	0.079151	-4.3867	1.15E-05	3.24E-05	NOT
AC093668	3.648492	1.232405	0.280959	4.386421	1.15E-05	3.24E-05	UP
RP11-269(	1.436311	2.334173	0.532213	4.385783	1.16E-05	3.25E-05	UP
CTD-2319	13.4384	0.86059	0.196232	4.385568	1.16E-05	3.25E-05	UP
HCP5	1013.421	0.786778	0.179459	4.384178	1.16E-05	3.27E-05	UP
PIR	1171.515	0.755395	0.172304	4.384094	1.16E-05	3.27E-05	UP
SAP30BP	2021.226	0.349874	0.079814	4.383603	1.17E-05	3.28E-05	NOT
COQ2	435.656	-0.44386	0.10127	-4.38293	1.17E-05	3.29E-05	NOT
AC115617	17.64891	-0.80249	0.183097	-4.38289	1.17E-05	3.29E-05	DOWN
RP11-49O	7.879834	1.274767	0.29089	4.382294	1.17E-05	3.30E-05	UP
CTNNAP1	1.51625	1.571882	0.358697	4.3822	1.17E-05	3.30E-05	UP
CENPB	3719.802	0.359155	0.081961	4.382004	1.18E-05	3.30E-05	NOT
KCNJ4	143.4164	1.388031	0.316807	4.381321	1.18E-05	3.31E-05	UP
GTF2E2	823.4048	-0.42415	0.096832	-4.3803	1.19E-05	3.33E-05	NOT
RP11-883(	27.56389	-1.57802	0.360257	-4.38026	1.19E-05	3.33E-05	DOWN
DNAJA1	9444.366	-0.38778	0.088535	-4.37999	1.19E-05	3.33E-05	NOT
FBXL13	9.242163	0.900103	0.205558	4.378839	1.19E-05	3.35E-05	UP
ALKBH4	426.1667	0.322151	0.073576	4.378496	1.20E-05	3.35E-05	NOT
SECTM1	820.3931	0.898736	0.205271	4.37829	1.20E-05	3.36E-05	UP
TEFM	212.6713	0.309839	0.070771	4.378044	1.20E-05	3.36E-05	NOT
LA16c-83F	2.061781	3.372161	0.770259	4.377956	1.20E-05	3.36E-05	UP
CENPBD1F	549.55	0.324312	0.074083	4.377709	1.20E-05	3.36E-05	NOT
RP11-107	83.65652	0.763244	0.174356	4.377506	1.20E-05	3.37E-05	UP
GABRG3	12.51149	1.906785	0.43561	4.377275	1.20E-05	3.37E-05	UP
PIK3C2G	567.2042	-1.18894	0.271638	-4.37694	1.20E-05	3.38E-05	DOWN
TG	33.18063	1.158131	0.26461	4.376742	1.20E-05	3.38E-05	UP
CTD-2619	193.5153	0.463017	0.105807	4.376046	1.21E-05	3.39E-05	NOT
RP1-40E1(	19.2397	0.832434	0.190234	4.37584	1.21E-05	3.39E-05	UP
KRT81	17.35702	1.619818	0.370258	4.374831	1.22E-05	3.41E-05	UP
RP11-460I	6.196418	0.891433	0.203778	4.374542	1.22E-05	3.41E-05	UP
LOXL1-AS	27.65836	1.257634	0.287522	4.37404	1.22E-05	3.42E-05	UP
RP11-95D	169.6482	0.565444	0.129277	4.373881	1.22E-05	3.42E-05	NOT
ATP5G1	4761.381	0.611097	0.139716	4.373859	1.22E-05	3.42E-05	UP
RP11-216I	14.91862	0.492928	0.112705	4.373603	1.22E-05	3.42E-05	NOT
RP11-843I	2.098435	1.769621	0.404642	4.373297	1.22E-05	3.43E-05	UP
PERP	7371.921	-0.58395	0.133542	-4.37278	1.23E-05	3.44E-05	NOT
PHIP	984.2489	0.421976	0.096504	4.372628	1.23E-05	3.44E-05	NOT
CTB-119C	12.83213	0.863073	0.197391	4.372411	1.23E-05	3.44E-05	UP

ATG2B	1486.621	-0.43961	0.100542	-4.37235	1.23E-05	3.44E-05	NOT
AC092171	1.535595	1.490316	0.340866	4.372148	1.23E-05	3.44E-05	UP
AC112198	3.363355	1.604747	0.367052	4.371984	1.23E-05	3.45E-05	UP
RP11-326(	42.73773	0.894964	0.204714	4.371785	1.23E-05	3.45E-05	UP
EIF2B4	1473.938	0.346712	0.079307	4.371785	1.23E-05	3.45E-05	NOT
BCL2L15	16.65571	1.179928	0.269901	4.371711	1.23E-05	3.45E-05	UP
NBPF11	292.4214	0.552485	0.12638	4.371615	1.23E-05	3.45E-05	NOT
RP11-474I	1.605925	2.452733	0.56108	4.371446	1.23E-05	3.45E-05	UP
CLCNKB	3.839648	1.844962	0.422056	4.371366	1.23E-05	3.45E-05	UP
RP11-378J	4.894115	1.130503	0.258618	4.371329	1.23E-05	3.46E-05	UP
FECH	1461.093	-0.46724	0.106897	-4.37093	1.24E-05	3.46E-05	NOT
FLJ31356	4.321775	1.533578	0.3509	4.370411	1.24E-05	3.47E-05	UP
PPP3R1	2108.437	-0.28734	0.065749	-4.37026	1.24E-05	3.47E-05	NOT
PRRC2B	2486.126	0.514559	0.117755	4.369737	1.24E-05	3.48E-05	NOT
CCDC144N	49.14807	1.15967	0.265389	4.36969	1.24E-05	3.48E-05	UP
TPTE2P1	3.404432	1.457819	0.33363	4.369566	1.24E-05	3.48E-05	UP
ATG14	627.1075	0.334654	0.0766	4.368836	1.25E-05	3.49E-05	NOT
TANGO2	731.5426	0.482172	0.110375	4.368502	1.25E-05	3.50E-05	NOT
FCF1P2	125.8112	0.326355	0.074714	4.368063	1.25E-05	3.50E-05	NOT
MEPE	2.517125	2.038329	0.466665	4.367866	1.25E-05	3.51E-05	UP
BRMS1L	244.7503	-0.30748	0.070402	-4.36749	1.26E-05	3.51E-05	NOT
PDPK1	975.7178	0.459487	0.105212	4.367246	1.26E-05	3.52E-05	NOT
ATP2A2	9931.995	0.391941	0.08975	4.367036	1.26E-05	3.52E-05	NOT
NECAB1	53.37935	1.145557	0.262334	4.366794	1.26E-05	3.52E-05	UP
RP5-894A	103.2888	0.598409	0.137045	4.366525	1.26E-05	3.53E-05	UP
MIR181A2	6.281452	1.358388	0.311156	4.365614	1.27E-05	3.54E-05	UP
CATSPER1	8.653829	0.999665	0.228988	4.365576	1.27E-05	3.54E-05	UP
KB-1572G	1.966182	1.570879	0.359845	4.365433	1.27E-05	3.54E-05	UP
BEGAIN	44.21665	1.417588	0.324771	4.364887	1.27E-05	3.55E-05	UP
KYNU	2098.607	-0.89803	0.205753	-4.36459	1.27E-05	3.56E-05	DOWN
PTBP1	8534.006	0.242678	0.055605	4.364347	1.28E-05	3.56E-05	NOT
HKR1	703.4373	0.536532	0.122943	4.364085	1.28E-05	3.56E-05	NOT
RP11-314N	2.825353	3.829378	0.877543	4.363749	1.28E-05	3.57E-05	UP
RP11-443I	2.781071	1.306541	0.299409	4.363731	1.28E-05	3.57E-05	UP
RP11-434I	315.6357	-1.53643	0.352098	-4.36364	1.28E-05	3.57E-05	DOWN
RP11-94B	1.862263	3.105522	0.711744	4.363255	1.28E-05	3.58E-05	UP
CDH26	13.04371	1.104598	0.25316	4.363247	1.28E-05	3.58E-05	UP
FAT2	11.1438	1.245184	0.285386	4.363158	1.28E-05	3.58E-05	UP
RBMXP4	3.696991	-0.91315	0.209326	-4.36231	1.29E-05	3.59E-05	DOWN
PTBP3	2272.753	0.417597	0.09575	4.361343	1.29E-05	3.61E-05	NOT
SEMA3F-A	27.17505	0.630527	0.144592	4.36072	1.30E-05	3.62E-05	UP
DAPL1	7.65062	2.694051	0.617843	4.360414	1.30E-05	3.62E-05	UP
COPE	6038.448	0.505254	0.115877	4.360247	1.30E-05	3.62E-05	NOT
CTB-31O2	31.15519	0.836684	0.19191	4.359773	1.30E-05	3.63E-05	UP
SLC5A1	166.4001	-1.91456	0.439149	-4.35971	1.30E-05	3.63E-05	DOWN
FAM99B	152.9687	-1.58032	0.362629	-4.35796	1.31E-05	3.66E-05	DOWN
YTHDC1	1909.684	-0.25088	0.057568	-4.35795	1.31E-05	3.66E-05	NOT
LA16c-38(	8.358669	0.954094	0.218937	4.357839	1.31E-05	3.66E-05	UP
RP11-434I	101.5969	-0.73311	0.168231	-4.35778	1.31E-05	3.66E-05	DOWN
BEND3P1	28.83494	1.084398	0.248844	4.357749	1.31E-05	3.66E-05	UP
CTB-113D	8.534565	1.221553	0.280444	4.355781	1.33E-05	3.69E-05	UP
RP1-191J1	41.89777	0.588019	0.134999	4.355739	1.33E-05	3.69E-05	UP

ANKDD1A	94.77958	0.690577	0.158554	4.355469	1.33E-05	3.70E-05	UP
FOXD1	3.715816	2.290937	0.525996	4.355428	1.33E-05	3.70E-05	UP
SAR1B	7166.401	-0.53432	0.12268	-4.3554	1.33E-05	3.70E-05	NOT
TXN2	4780.031	-0.43069	0.098894	-4.35511	1.33E-05	3.70E-05	NOT
RP11-398I	13.20187	1.112599	0.25548	4.354938	1.33E-05	3.71E-05	UP
ADRB1	35.37419	-1.32709	0.304749	-4.35468	1.33E-05	3.71E-05	DOWN
PI16	19.29054	-1.62874	0.374021	-4.35467	1.33E-05	3.71E-05	DOWN
HOPX	107.7026	0.955715	0.219473	4.354596	1.33E-05	3.71E-05	UP
CCT7P2	2.291796	3.526376	0.809839	4.354417	1.33E-05	3.71E-05	UP
WI2-2118(	1.854223	3.219971	0.739479	4.354375	1.33E-05	3.71E-05	UP
SUSD4	684.1215	1.432021	0.328875	4.354302	1.33E-05	3.71E-05	UP
RPL30	25621.79	0.54876	0.126032	4.354123	1.34E-05	3.72E-05	NOT
PJA1	563.5028	0.488102	0.112107	4.353911	1.34E-05	3.72E-05	NOT
CTB-191K	1.827295	1.395269	0.320479	4.353702	1.34E-05	3.72E-05	UP
CTD-2135	2.474845	1.510763	0.347016	4.353579	1.34E-05	3.73E-05	UP
PIK3R1	5022.689	-0.68774	0.157981	-4.35331	1.34E-05	3.73E-05	DOWN
RN7SL333I	3.064157	1.437482	0.330206	4.353293	1.34E-05	3.73E-05	UP
RP11-156I	8.944318	-1.09732	0.252071	-4.3532	1.34E-05	3.73E-05	DOWN
FABP5P7	2.341403	1.488099	0.341853	4.353036	1.34E-05	3.73E-05	UP
ETV2	95.19227	0.759028	0.174377	4.352799	1.34E-05	3.74E-05	UP
FAM103A1	412.7315	0.328362	0.075444	4.352365	1.35E-05	3.74E-05	NOT
MAGEA10	3.929086	3.823487	0.878491	4.352332	1.35E-05	3.74E-05	UP
ACOT13	2897.958	-0.51876	0.119196	-4.35214	1.35E-05	3.75E-05	NOT
RP11-214I	2.033446	1.483226	0.340828	4.351836	1.35E-05	3.75E-05	UP
MED4	1123.371	-0.32898	0.075603	-4.35141	1.35E-05	3.76E-05	NOT
RP11-131I	4.267933	1.086608	0.249735	4.351044	1.35E-05	3.76E-05	UP
THEM7P	1.094989	2.310063	0.530985	4.350523	1.36E-05	3.77E-05	UP
EPCAM	1920.104	1.857963	0.427096	4.350226	1.36E-05	3.78E-05	UP
GSPT2	448.7288	-1.02548	0.235735	-4.35014	1.36E-05	3.78E-05	DOWN
TM2D3	1686.049	-0.32772	0.075348	-4.34937	1.37E-05	3.79E-05	NOT
ANAPC10	278.9706	-0.29205	0.067152	-4.34901	1.37E-05	3.80E-05	NOT
RP5-907D	1.198072	2.54167	0.584495	4.348487	1.37E-05	3.81E-05	UP
ZNF737	60.72626	1.032104	0.237349	4.348459	1.37E-05	3.81E-05	UP
MALL	24.11505	1.089833	0.250635	4.348285	1.37E-05	3.81E-05	UP
RP5-1085I	176.1142	0.658345	0.151408	4.348165	1.37E-05	3.81E-05	UP
FNTB	340.7498	0.311344	0.07161	4.347795	1.38E-05	3.82E-05	NOT
NUDT2	746.4698	0.487463	0.112132	4.347231	1.38E-05	3.83E-05	NOT
LINC00641	106.319	0.647853	0.14904	4.346834	1.38E-05	3.83E-05	UP
STAU2	1378.225	0.361359	0.083134	4.346702	1.38E-05	3.84E-05	NOT
AC083884	1.924176	1.480187	0.340538	4.346608	1.38E-05	3.84E-05	UP
RP11-4B1(	10.01272	0.842624	0.193861	4.346538	1.38E-05	3.84E-05	UP
PRKAG1	1780.203	0.23434	0.053918	4.346206	1.39E-05	3.84E-05	NOT
AASDHPPT	1357.968	-0.32876	0.075647	-4.34592	1.39E-05	3.85E-05	NOT
RP11-574I	12.37006	0.649334	0.149419	4.345714	1.39E-05	3.85E-05	UP
TSSC2	17.52594	1.222916	0.28143	4.345371	1.39E-05	3.86E-05	UP
CTB-55O6	3.893542	1.220581	0.280934	4.344729	1.39E-05	3.87E-05	UP
RASSF3	1150.217	0.674509	0.155249	4.344683	1.39E-05	3.87E-05	UP
SLAIN2	1744.702	-0.29271	0.067381	-4.34407	1.40E-05	3.88E-05	NOT
HIST1H2A	1.325212	1.677391	0.38614	4.343998	1.40E-05	3.88E-05	UP
CCDC127	655.743	0.336097	0.077371	4.343983	1.40E-05	3.88E-05	NOT
SLC46A1	1351.191	0.542534	0.124894	4.343954	1.40E-05	3.88E-05	NOT
SLC25A28	1478.271	-0.41699	0.095998	-4.34373	1.40E-05	3.88E-05	NOT

RP11-872J	39.46845	0.69734	0.160557	4.343263	1.40E-05	3.89E-05	UP
RPS15AP3(	7.053	1.007943	0.232095	4.3428	1.41E-05	3.90E-05	UP
RP11-94A1	6.275293	1.729526	0.398265	4.342656	1.41E-05	3.90E-05	UP
RP11-452(	82.8833	0.745504	0.171676	4.342499	1.41E-05	3.90E-05	UP
CECR6	19.1744	0.960541	0.221207	4.342274	1.41E-05	3.91E-05	UP
TSSK3	8.370579	0.847664	0.195216	4.342184	1.41E-05	3.91E-05	UP
BACH1	1712.731	-0.46506	0.107113	-4.34179	1.41E-05	3.91E-05	NOT
TMEM241	203.3348	0.47482	0.109361	4.341772	1.41E-05	3.91E-05	NOT
CUTA	7665.594	0.532602	0.122678	4.341467	1.42E-05	3.92E-05	NOT
RP11-10A1	28.65397	1.043826	0.240441	4.341294	1.42E-05	3.92E-05	UP
TBC1D23	1214.97	-0.34448	0.079353	-4.34111	1.42E-05	3.92E-05	NOT
AP001626.	5.479933	1.992607	0.459022	4.340988	1.42E-05	3.93E-05	UP
AC109642	5.139804	1.012788	0.233316	4.34084	1.42E-05	3.93E-05	UP
NDUFAF2	534.2089	0.527686	0.121572	4.340524	1.42E-05	3.93E-05	NOT
RP11-464I	12.63067	0.792755	0.182646	4.340403	1.42E-05	3.94E-05	UP
ZNF345	84.67059	0.454426	0.104697	4.34039	1.42E-05	3.94E-05	NOT
TES	991.9115	0.701157	0.161544	4.340361	1.42E-05	3.94E-05	UP
AP3B2	11.19662	1.699505	0.391581	4.340112	1.42E-05	3.94E-05	UP
RP11-61L1	19.09168	0.634145	0.146114	4.340067	1.42E-05	3.94E-05	UP
ERCC8	195.566	0.300634	0.069274	4.339792	1.43E-05	3.94E-05	NOT
GNB5	1709.765	0.572032	0.131815	4.339661	1.43E-05	3.95E-05	NOT
FBXO7	5471.144	-0.42349	0.097585	-4.33964	1.43E-05	3.95E-05	NOT
CAST	6276.985	-0.35489	0.081784	-4.33942	1.43E-05	3.95E-05	NOT
ZNF814	367.8875	0.61413	0.141525	4.339388	1.43E-05	3.95E-05	UP
PPP2R2D	1030.595	-0.32526	0.074957	-4.33929	1.43E-05	3.95E-05	NOT
XRCC4	209.6014	0.45123	0.103989	4.339197	1.43E-05	3.95E-05	NOT
HELB	182.8301	-0.48359	0.111449	-4.3391	1.43E-05	3.95E-05	NOT
RP11-582I	236.619	-0.42536	0.098033	-4.33897	1.43E-05	3.96E-05	NOT
HBD	6.121584	-1.87691	0.432595	-4.33873	1.43E-05	3.96E-05	DOWN
UFM1	2390.252	-0.36005	0.082992	-4.33839	1.44E-05	3.97E-05	NOT
SLC38A7	1397.3	0.421114	0.097069	4.338292	1.44E-05	3.97E-05	NOT
RP11-156I	248.3596	-1.14458	0.263835	-4.33825	1.44E-05	3.97E-05	DOWN
CEP170B	1561.848	0.432347	0.099663	4.338074	1.44E-05	3.97E-05	NOT
ZNF395	1317.357	-0.51716	0.119224	-4.33773	1.44E-05	3.98E-05	NOT
PNKP	1126.275	0.39801	0.091779	4.33662	1.45E-05	4.00E-05	NOT
RP11-723J	3.771283	0.883415	0.203717	4.336485	1.45E-05	4.00E-05	UP
CASC4	3308.076	-0.4003	0.092331	-4.33546	1.45E-05	4.02E-05	NOT
RP11-596(	4.09103	0.964065	0.222372	4.335368	1.46E-05	4.02E-05	UP
ARL5A	1468.168	-0.35128	0.081039	-4.33463	1.46E-05	4.03E-05	NOT
TRIM22	1363.182	-0.90742	0.209346	-4.33453	1.46E-05	4.03E-05	DOWN
KNOP1P5	2.658216	3.74555	0.864142	4.334413	1.46E-05	4.03E-05	UP
RP11-968/	1.268486	1.662856	0.383653	4.334274	1.46E-05	4.04E-05	UP
NUTF2	2800.416	0.48433	0.111767	4.333373	1.47E-05	4.05E-05	NOT
RP4-756H	31.13558	-0.69073	0.1594	-4.3333	1.47E-05	4.05E-05	DOWN
IL1RAPL1	6.814914	1.883398	0.434694	4.332699	1.47E-05	4.06E-05	UP
SYNPO2	615.6352	-0.89764	0.207183	-4.3326	1.47E-05	4.07E-05	DOWN
NRBP1	4407.748	0.324978	0.075009	4.33252	1.47E-05	4.07E-05	NOT
NKX2-5	2.604108	3.082866	0.711568	4.332495	1.47E-05	4.07E-05	UP
GNPDA2	215.2229	0.520695	0.120185	4.332463	1.47E-05	4.07E-05	NOT
RP1-151B1	1.857069	1.661594	0.383541	4.332242	1.48E-05	4.07E-05	UP
MATR3	96.25864	0.524664	0.121108	4.332195	1.48E-05	4.07E-05	NOT
LINC00707	3.243566	-1.91267	0.441534	-4.33188	1.48E-05	4.08E-05	DOWN

RP11-15N	9.451011	0.823182	0.190042	4.33157	1.48E-05	4.08E-05	UP
AC118754	8.628584	-1.14386	0.264082	-4.33146	1.48E-05	4.08E-05	DOWN
CTC-359D	37.71976	0.769759	0.177725	4.331172	1.48E-05	4.09E-05	UP
RP11-875(	11.57718	-0.6296	0.145372	-4.33097	1.48E-05	4.09E-05	DOWN
GZMH	95.49611	-0.90267	0.208426	-4.3309	1.48E-05	4.09E-05	DOWN
LRRC37A	15.10879	0.906412	0.209323	4.330213	1.49E-05	4.10E-05	UP
AC112229	3.801872	1.554077	0.358916	4.329922	1.49E-05	4.11E-05	UP
OVOL3	1.709832	1.752242	0.404682	4.329919	1.49E-05	4.11E-05	UP
KCNAB1	99.73868	-0.593	0.136983	-4.32905	1.50E-05	4.13E-05	DOWN
RP11-179I	8.062458	-1.47428	0.340573	-4.32883	1.50E-05	4.13E-05	DOWN
AF146191.	3.609423	1.18706	0.274231	4.328681	1.50E-05	4.13E-05	UP
RP11-528/	7.543564	1.509795	0.348838	4.328074	1.50E-05	4.14E-05	UP
RP13-49I1	1.785141	1.847574	0.426916	4.327721	1.51E-05	4.15E-05	UP
RP5-827C	15.4004	0.854058	0.197353	4.327559	1.51E-05	4.15E-05	UP
HAX1	4348.817	0.417359	0.096466	4.326476	1.52E-05	4.17E-05	NOT
SATB2	475.3811	0.637417	0.147357	4.325679	1.52E-05	4.19E-05	UP
RP11-47P	3.947724	3.144747	0.727012	4.325578	1.52E-05	4.19E-05	UP
PTCHD3P2	5.152838	1.751561	0.404954	4.325329	1.52E-05	4.19E-05	UP
RPRD1A	1601.568	0.340249	0.078668	4.325102	1.52E-05	4.20E-05	NOT
ZNF718	133.6921	0.600203	0.138772	4.325094	1.52E-05	4.20E-05	UP
SUMO2	4086.165	0.340762	0.078801	4.324321	1.53E-05	4.21E-05	NOT
GP2	43.56907	2.440194	0.564349	4.323911	1.53E-05	4.22E-05	UP
C9orf50	2.712826	1.578837	0.365215	4.323039	1.54E-05	4.23E-05	UP
LRP1	27892.84	-0.52892	0.122356	-4.32276	1.54E-05	4.24E-05	NOT
RP4-680D	2.124109	1.280282	0.296198	4.322384	1.54E-05	4.25E-05	UP
ADPRHL1	162.9956	1.008191	0.233255	4.322277	1.54E-05	4.25E-05	UP
ZNF324	247.4772	0.324827	0.075161	4.321764	1.55E-05	4.26E-05	NOT
AC104809	295.8522	-2.15057	0.497638	-4.32156	1.55E-05	4.26E-05	DOWN
PROX1	7868.616	-0.67488	0.156173	-4.32135	1.55E-05	4.26E-05	DOWN
HSBP1P2	5.366504	-1.5751	0.364498	-4.32129	1.55E-05	4.26E-05	DOWN
ZCCHC6	1907.698	-0.60556	0.14018	-4.31988	1.56E-05	4.29E-05	DOWN
PLCXD2	291.125	-0.79562	0.184184	-4.3197	1.56E-05	4.29E-05	DOWN
ADIG	4.208417	2.213029	0.512328	4.319551	1.56E-05	4.30E-05	UP
NCR3	26.03477	-0.94565	0.218942	-4.31918	1.57E-05	4.30E-05	DOWN
LHB	2.7177	1.865592	0.431996	4.318537	1.57E-05	4.32E-05	UP
RP11-311I	6.650856	0.822983	0.19057	4.318524	1.57E-05	4.32E-05	UP
FAM47E-S	730.6109	-0.64438	0.149224	-4.31823	1.57E-05	4.32E-05	DOWN
ZNF252P	1185.503	0.387059	0.089644	4.317752	1.58E-05	4.33E-05	NOT
MTMR6	942.338	-0.38856	0.089996	-4.31757	1.58E-05	4.33E-05	NOT
DPY19L2	15.35213	1.293975	0.299706	4.317483	1.58E-05	4.33E-05	UP
RP11-626I	1.582809	3.055122	0.707628	4.317412	1.58E-05	4.33E-05	UP
FBXW7	578.6249	-0.35571	0.082389	-4.31741	1.58E-05	4.33E-05	NOT
SNRPGP10	6.786607	0.766214	0.177502	4.316653	1.58E-05	4.35E-05	UP
AIF1	791.6146	-0.72508	0.167979	-4.31648	1.59E-05	4.35E-05	DOWN
CSAG3	30.42114	2.965398	0.687009	4.316391	1.59E-05	4.35E-05	UP
NDUFV2	1047.889	-0.53892	0.124857	-4.31631	1.59E-05	4.35E-05	NOT
CST7	187.8902	-0.88966	0.206126	-4.31611	1.59E-05	4.36E-05	DOWN
ATP13A3	7111.385	-0.44832	0.103884	-4.31557	1.59E-05	4.37E-05	NOT
TMEM69	820.4595	0.369597	0.085661	4.314669	1.60E-05	4.39E-05	NOT
PMS2P1	306.6468	0.366065	0.084847	4.314433	1.60E-05	4.39E-05	NOT
CTB-131B	17.74811	0.884295	0.204969	4.314277	1.60E-05	4.39E-05	UP
PML	1716.473	0.446374	0.103477	4.313747	1.61E-05	4.40E-05	NOT

CUL2	1319.162	0.307872	0.071373	4.313593	1.61E-05	4.41E-05	NOT
RP11-138/	3.93045	1.468588	0.340577	4.312059	1.62E-05	4.44E-05	UP
SLC5A3	785.1902	-0.64122	0.148711	-4.31184	1.62E-05	4.44E-05	DOWN
AC005682	33.51186	0.870152	0.201813	4.311672	1.62E-05	4.44E-05	UP
RP11-227I	1.430327	2.070857	0.480328	4.311344	1.62E-05	4.45E-05	UP
RP11-93B1	4.55214	-1.95758	0.454116	-4.31075	1.63E-05	4.46E-05	DOWN
AC012354	1.298649	1.574462	0.365252	4.310616	1.63E-05	4.46E-05	UP
IGSF6	458.5685	-0.70836	0.164336	-4.31043	1.63E-05	4.47E-05	DOWN
SMO	2694.411	0.666971	0.15474	4.310285	1.63E-05	4.47E-05	UP
RP11-504I	203.0309	0.623934	0.144772	4.309775	1.63E-05	4.48E-05	UP
AR	5176.576	-1.11201	0.258023	-4.30974	1.63E-05	4.48E-05	DOWN
FAM127B	1053.105	0.666512	0.15468	4.308967	1.64E-05	4.49E-05	UP
CCDC152	736.5469	-0.74095	0.171979	-4.30838	1.64E-05	4.50E-05	DOWN
TMEM54	527.9609	0.950601	0.220673	4.307745	1.65E-05	4.52E-05	UP
BNIP3	6320.933	-0.51788	0.120222	-4.30772	1.65E-05	4.52E-05	NOT
CTD-2263	2.610755	2.262705	0.525279	4.307621	1.65E-05	4.52E-05	UP
LRRC36	8.547947	1.414257	0.328398	4.306534	1.66E-05	4.54E-05	UP
JAM3	401.4697	0.584648	0.135771	4.306144	1.66E-05	4.55E-05	NOT
BSPRY	152.7398	1.287389	0.29901	4.305508	1.67E-05	4.56E-05	UP
BAP1	3268.752	0.368286	0.085541	4.30536	1.67E-05	4.56E-05	NOT
AGR2	204.14	2.195951	0.510054	4.305332	1.67E-05	4.56E-05	UP
WDR92	49.79179	0.467198	0.108519	4.305228	1.67E-05	4.57E-05	NOT
DEK	3734.34	0.471735	0.109578	4.30502	1.67E-05	4.57E-05	NOT
RP11-146I	4.417975	1.039507	0.241535	4.30375	1.68E-05	4.59E-05	UP
C18orf8	985.9723	-0.34087	0.079204	-4.30374	1.68E-05	4.59E-05	NOT
RP11-341(	1.341931	2.628302	0.610769	4.303266	1.68E-05	4.60E-05	UP
RNPS1	2442.165	0.328425	0.076324	4.303055	1.68E-05	4.61E-05	NOT
RP11-114I	1.809285	3.2301	0.750661	4.303006	1.68E-05	4.61E-05	UP
ANAPC11	4297.03	0.597362	0.138828	4.302907	1.69E-05	4.61E-05	UP
RP11-434I	10.03337	1.093336	0.254113	4.302565	1.69E-05	4.62E-05	UP
SPDEF	37.1177	1.808834	0.420425	4.302397	1.69E-05	4.62E-05	UP
MEOX2-A:	3.91881	2.304219	0.535573	4.302342	1.69E-05	4.62E-05	UP
CTC-459F	9.520796	0.934485	0.217245	4.301534	1.70E-05	4.64E-05	UP
RPL38	14011.91	0.65993	0.153436	4.301016	1.70E-05	4.65E-05	UP
CRHR1-IT1	147.8267	0.83901	0.195078	4.300891	1.70E-05	4.65E-05	UP
TDRD12	4.991823	1.267403	0.294697	4.300699	1.70E-05	4.65E-05	UP
RP11-455I	46.19323	0.696069	0.161852	4.30066	1.70E-05	4.65E-05	UP
RP11-603J	4.483381	1.031808	0.23992	4.300627	1.70E-05	4.65E-05	UP
HERC2P9	127.6622	0.582151	0.13538	4.300137	1.71E-05	4.66E-05	NOT
RP11-348I	19.59592	0.809454	0.188251	4.299878	1.71E-05	4.67E-05	UP
NTN5	11.19035	0.973863	0.226494	4.299739	1.71E-05	4.67E-05	UP
YIPF3	6281.29	0.377319	0.087762	4.299344	1.71E-05	4.68E-05	NOT
TMEM14C	4049.107	0.366315	0.085203	4.299309	1.71E-05	4.68E-05	NOT
LAGE3P1	3.374577	0.998799	0.232328	4.299081	1.72E-05	4.68E-05	UP
FGF14-IT1	1.499018	-1.9699	0.458271	-4.29855	1.72E-05	4.69E-05	DOWN
RPS27P25	1.421048	2.234301	0.519823	4.298193	1.72E-05	4.70E-05	UP
SNORA33	21.06536	0.848359	0.197385	4.297985	1.72E-05	4.71E-05	UP
C10orf131	2.657442	1.206243	0.280691	4.297405	1.73E-05	4.72E-05	UP
AP001462.	6.048944	0.750372	0.174615	4.297293	1.73E-05	4.72E-05	UP
AC006460	2.437364	1.719829	0.400223	4.297172	1.73E-05	4.72E-05	UP
SLC13A5	16227.32	-1.20232	0.279829	-4.29663	1.73E-05	4.73E-05	DOWN
PLEKHA1	1963.044	-0.36496	0.084942	-4.29656	1.73E-05	4.73E-05	NOT

KRTAP5-7	4.576289	2.17071	0.505249	4.296315	1.74E-05	4.74E-05	UP
RP11-638I	1.407446	2.497501	0.581351	4.296032	1.74E-05	4.74E-05	UP
SNX11	848.6058	0.325982	0.075886	4.295693	1.74E-05	4.75E-05	NOT
RP11-495I	4.659536	1.151036	0.267962	4.295513	1.74E-05	4.75E-05	UP
RP11-63G	1.865099	1.277945	0.297537	4.295076	1.75E-05	4.76E-05	UP
RP11-10G	15.39401	0.715411	0.166601	4.294163	1.75E-05	4.78E-05	UP
CTA-217C	5.322282	0.947824	0.220733	4.293992	1.75E-05	4.79E-05	UP
PDK2	1873.165	-0.54125	0.126067	-4.29338	1.76E-05	4.80E-05	NOT
ICA1L	42.45399	0.761968	0.17748	4.293249	1.76E-05	4.80E-05	UP
RP11-83M	5.563505	1.750741	0.407807	4.293067	1.76E-05	4.80E-05	UP
GOLGA8IP	3.120083	1.688056	0.393232	4.29277	1.76E-05	4.81E-05	UP
NSMF	1537.794	0.553905	0.129053	4.29206	1.77E-05	4.82E-05	NOT
C14orf132	277.2056	1.106353	0.257771	4.291995	1.77E-05	4.83E-05	UP
TRABD2A	211.829	1.282922	0.298993	4.290811	1.78E-05	4.85E-05	UP
PPP1R12C	2445.956	0.308255	0.071842	4.290752	1.78E-05	4.85E-05	NOT
BEST1	89.553	0.805531	0.187742	4.290618	1.78E-05	4.85E-05	UP
DNAJA4	233.3025	0.979811	0.228372	4.290412	1.78E-05	4.86E-05	UP
FOSL1	139.3571	-1.28709	0.300002	-4.29028	1.78E-05	4.86E-05	DOWN
GRSF1	2758.583	-0.28368	0.066123	-4.2901	1.79E-05	4.86E-05	NOT
RP11-161I	19.62999	0.971198	0.226409	4.289573	1.79E-05	4.88E-05	UP
AMBRA1	1216.663	0.252108	0.058787	4.288527	1.80E-05	4.90E-05	NOT
HIATL2	60.76827	0.397934	0.092791	4.288485	1.80E-05	4.90E-05	NOT
F7	8601.323	-0.85948	0.200418	-4.28844	1.80E-05	4.90E-05	DOWN
PSMD3	5681.321	0.315902	0.073665	4.288364	1.80E-05	4.90E-05	NOT
RBBP5	1099.468	0.3289	0.076704	4.287925	1.80E-05	4.91E-05	NOT
RP11-452I	142.8788	0.66658	0.155459	4.287829	1.80E-05	4.91E-05	UP
ATP1A4	4.756773	1.64851	0.384486	4.287569	1.81E-05	4.92E-05	UP
MIIP	950.0861	0.510142	0.118987	4.287386	1.81E-05	4.92E-05	NOT
REG4	12.87352	1.301128	0.30348	4.287359	1.81E-05	4.92E-05	UP
RP5-1056I	25.70966	0.705929	0.16466	4.287179	1.81E-05	4.92E-05	UP
LINC0109	21.14239	1.069361	0.249435	4.287135	1.81E-05	4.92E-05	UP
SETMAR	406.7094	0.442374	0.103187	4.287131	1.81E-05	4.92E-05	NOT
POLR3G	109.2528	0.580004	0.135301	4.286771	1.81E-05	4.93E-05	NOT
PCDHGA2	105.2415	1.185918	0.276663	4.28651	1.82E-05	4.94E-05	UP
BUD31	1435.713	0.357572	0.083424	4.286217	1.82E-05	4.94E-05	NOT
RP1-46F2.	1.617761	2.846217	0.664098	4.285841	1.82E-05	4.95E-05	UP
RGAG4	315.5109	-0.91695	0.213967	-4.28547	1.82E-05	4.96E-05	DOWN
RFPL1S	18.2981	1.343057	0.313409	4.285319	1.82E-05	4.96E-05	UP
HMOX2	2862.643	-0.48479	0.113128	-4.28528	1.83E-05	4.96E-05	NOT
RP4-755D	17.95048	1.29376	0.301942	4.284801	1.83E-05	4.97E-05	UP
PDAP1	3654.867	0.275693	0.064343	4.284719	1.83E-05	4.97E-05	NOT
RP11-388	2.24353	1.238017	0.288961	4.284373	1.83E-05	4.98E-05	UP
RP11-574I	4.331718	0.905407	0.211349	4.283952	1.84E-05	4.99E-05	UP
ATP7A	260.9864	0.521966	0.121862	4.283249	1.84E-05	5.00E-05	NOT
LINC0024	1.710164	1.710884	0.39957	4.281814	1.85E-05	5.04E-05	UP
PAK3	42.09403	1.548277	0.361597	4.281771	1.85E-05	5.04E-05	UP
ZNF664	4040.904	0.370542	0.086539	4.28177	1.85E-05	5.04E-05	NOT
PLEKHJ1	2163.398	0.579486	0.135359	4.281111	1.86E-05	5.05E-05	NOT
RP11-230	2.402284	1.275494	0.297943	4.280994	1.86E-05	5.05E-05	UP
INSIG2	2002.09	-0.54254	0.126741	-4.28071	1.86E-05	5.06E-05	NOT
REG3G	6.941508	5.187433	1.211873	4.28051	1.86E-05	5.06E-05	UP
ADAP2	411.4664	-0.61048	0.142661	-4.27922	1.88E-05	5.09E-05	DOWN

RP11-479(	1.850242	1.424265	0.332843	4.279093	1.88E-05	5.09E-05	UP
CTD-2311	6.076786	1.159495	0.270973	4.278997	1.88E-05	5.10E-05	UP
LINC0018/	1.26488	1.787285	0.4177	4.278878	1.88E-05	5.10E-05	UP
SLC2A9	946.7763	-1.04373	0.24395	-4.27845	1.88E-05	5.11E-05	DOWN
HSPA8P15	2.531575	1.188726	0.277893	4.277634	1.89E-05	5.13E-05	UP
C4B	12900.21	-0.80339	0.187818	-4.27752	1.89E-05	5.13E-05	DOWN
PCDHB14	134.4651	0.8807	0.205926	4.276772	1.90E-05	5.14E-05	UP
bP-2189O	2.780123	1.654399	0.386914	4.275882	1.90E-05	5.16E-05	UP
IFFO2	581.058	0.603423	0.141128	4.275723	1.91E-05	5.17E-05	UP
LRRC3B	4.802453	1.362848	0.318743	4.2757	1.91E-05	5.17E-05	UP
TNFRSF21	1630.435	0.902576	0.21111	4.275381	1.91E-05	5.17E-05	UP
ZNF620	84.83574	0.469151	0.109739	4.275144	1.91E-05	5.18E-05	NOT
FAM214A	1302.913	-0.47501	0.111117	-4.27488	1.91E-05	5.19E-05	NOT
RP11-4C2(	2.469524	1.596988	0.373652	4.273999	1.92E-05	5.21E-05	UP
PPIAP19	1.611851	1.442597	0.337534	4.273926	1.92E-05	5.21E-05	UP
RAI1	745.0628	0.567543	0.132797	4.273755	1.92E-05	5.21E-05	NOT
LRRC75A	35.45429	0.801917	0.18764	4.273705	1.92E-05	5.21E-05	UP
DIDO1	2076.131	0.305875	0.071576	4.273465	1.92E-05	5.22E-05	NOT
MS4A4A	420.4908	-0.8204	0.191986	-4.27322	1.93E-05	5.22E-05	DOWN
ZNF749	104.3135	0.533253	0.124812	4.272456	1.93E-05	5.24E-05	NOT
MSR1	609.2639	-0.78959	0.184812	-4.27239	1.93E-05	5.24E-05	DOWN
ANKRD36	35.19632	0.768134	0.179803	4.272082	1.94E-05	5.25E-05	UP
EGLN2	1097.58	0.654948	0.153318	4.271838	1.94E-05	5.25E-05	UP
RP11-308I	2.281089	2.239986	0.524417	4.271383	1.94E-05	5.26E-05	UP
HCFC1R1	1297.251	0.561604	0.131481	4.271375	1.94E-05	5.26E-05	NOT
LINC0029(	19.73254	1.09774	0.25703	4.270869	1.95E-05	5.27E-05	UP
LL22NC03	170.5612	0.446049	0.10444	4.27085	1.95E-05	5.27E-05	NOT
CPNE9	3.811519	1.316214	0.308194	4.270724	1.95E-05	5.28E-05	UP
MRVI1	224.0837	0.747284	0.174982	4.270636	1.95E-05	5.28E-05	UP
EIF1P5	4.199812	-1.06682	0.24981	-4.27051	1.95E-05	5.28E-05	DOWN
CTA-384D	2.763976	1.776355	0.416054	4.269527	1.96E-05	5.30E-05	UP
RP11-326(	187.8891	1.42372	0.333483	4.269243	1.96E-05	5.31E-05	UP
RASL10B	523.5682	1.546408	0.362228	4.269163	1.96E-05	5.31E-05	UP
FIZ1	551.7167	0.369533	0.086563	4.268965	1.96E-05	5.31E-05	NOT
FBLL1	81.27691	1.623895	0.380441	4.26846	1.97E-05	5.33E-05	UP
TMEM117	181.4847	0.525534	0.123129	4.268163	1.97E-05	5.33E-05	NOT
SNX14	2308.193	-0.3443	0.080667	-4.26816	1.97E-05	5.33E-05	NOT
RP11-251(	8.844312	1.217249	0.285207	4.267949	1.97E-05	5.34E-05	UP
RN7SKP80	2.882998	1.369055	0.320797	4.267665	1.98E-05	5.34E-05	UP
DRICH1	6.575937	1.033946	0.242283	4.267509	1.98E-05	5.35E-05	UP
SMCO2	7.898839	1.341495	0.314375	4.267186	1.98E-05	5.35E-05	UP
NANOGNE	5.032354	1.027434	0.240776	4.267182	1.98E-05	5.35E-05	UP
ZIK1	64.12756	0.985744	0.231013	4.267054	1.98E-05	5.35E-05	UP
HIP1	1881.657	0.520471	0.121975	4.267046	1.98E-05	5.35E-05	NOT
RP11-408I	3.958088	3.024819	0.708884	4.267018	1.98E-05	5.35E-05	UP
UGT8	20.23155	2.056153	0.481879	4.266949	1.98E-05	5.36E-05	UP
SMIM22	52.60476	1.71551	0.402052	4.266888	1.98E-05	5.36E-05	UP
PLCZ1	5.247334	-1.18276	0.277208	-4.26669	1.98E-05	5.36E-05	DOWN
RNF146	851.684	-0.30878	0.072375	-4.26633	1.99E-05	5.37E-05	NOT
RP11-215I	6.897183	0.720216	0.16882	4.266179	1.99E-05	5.37E-05	UP
ACADL	749.1441	-1.26675	0.296948	-4.26589	1.99E-05	5.38E-05	DOWN
RP13-991I	1.532616	1.731342	0.405876	4.265688	1.99E-05	5.38E-05	UP

RP11-817.	36.85762	-2.06163	0.483311	-4.26565	1.99E-05	5.38E-05	DOWN
RP11-156I	10.42467	1.699953	0.39853	4.265554	1.99E-05	5.39E-05	UP
PAX8-AS1	322.3524	1.194308	0.280028	4.264958	2.00E-05	5.40E-05	UP
RP11-11N	1.824937	2.686731	0.63001	4.264585	2.00E-05	5.41E-05	UP
AC079145	4.167772	1.128721	0.264687	4.264365	2.00E-05	5.41E-05	UP
KCNQ4	40.63281	1.010297	0.236968	4.263438	2.01E-05	5.43E-05	UP
CYB561	1522.207	0.738214	0.173157	4.263265	2.01E-05	5.44E-05	UP
PDCD7	553.5123	0.230442	0.054054	4.263159	2.02E-05	5.44E-05	NOT
AC005517	3.964741	0.94633	0.221996	4.262828	2.02E-05	5.45E-05	UP
RP3-325F2	20.54027	-1.06353	0.249499	-4.26264	2.02E-05	5.45E-05	DOWN
MATN1-A	38.71503	0.558834	0.131105	4.262476	2.02E-05	5.46E-05	NOT
RUFY4	18.26684	1.314284	0.308355	4.262238	2.02E-05	5.46E-05	UP
ATP1A2	92.54785	1.158522	0.271824	4.262038	2.03E-05	5.46E-05	UP
FAM65B	184.2655	-0.79769	0.187162	-4.26201	2.03E-05	5.46E-05	DOWN
GALNT16	149.8614	-0.88532	0.207728	-4.26193	2.03E-05	5.47E-05	DOWN
OGFOD2	79.98028	0.355802	0.083489	4.261672	2.03E-05	5.47E-05	NOT
SLC10A3	602.7733	0.576956	0.135386	4.261577	2.03E-05	5.47E-05	NOT
CCDC81	13.1209	0.717295	0.168345	4.260859	2.04E-05	5.49E-05	UP
SNHG9	224.0503	0.894882	0.21003	4.260744	2.04E-05	5.49E-05	UP
RPL35P2	12.39125	0.82	0.192456	4.260716	2.04E-05	5.49E-05	UP
MTUS1	3838.411	-0.62028	0.145591	-4.26042	2.04E-05	5.50E-05	DOWN
RPS7	17691.15	0.506961	0.119032	4.259043	2.05E-05	5.53E-05	NOT
TFG	5453.836	0.272682	0.064029	4.25871	2.06E-05	5.54E-05	NOT
AC062029	73.15061	0.552327	0.129709	4.25819	2.06E-05	5.55E-05	NOT
KRT23	927.0987	1.886074	0.442988	4.257624	2.07E-05	5.57E-05	UP
TRPM2	196.4732	0.836901	0.196582	4.257269	2.07E-05	5.58E-05	UP
RP11-282I	1.051673	2.201947	0.517222	4.257255	2.07E-05	5.58E-05	UP
RP11-352I	1.665635	1.922311	0.451558	4.257068	2.07E-05	5.58E-05	UP
RNMTL1	777.8606	-0.46955	0.1103	-4.25698	2.07E-05	5.58E-05	NOT
MRPL33	1163.013	0.437486	0.102786	4.25628	2.08E-05	5.60E-05	NOT
ZBTB32	12.57744	0.962153	0.226063	4.256123	2.08E-05	5.60E-05	UP
RP11-159I	5.007702	3.26539	0.76725	4.255969	2.08E-05	5.60E-05	UP
NT5C	777.078	0.544273	0.127893	4.255692	2.08E-05	5.61E-05	NOT
PALM	834.9585	1.020903	0.239893	4.255657	2.08E-05	5.61E-05	UP
FZD7	210.9689	1.060072	0.249106	4.255501	2.09E-05	5.61E-05	UP
ADAR	11900.48	0.356386	0.083747	4.255492	2.09E-05	5.61E-05	NOT
DOCK9-AS	15.32616	0.808665	0.190085	4.254236	2.10E-05	5.65E-05	UP
C1orf105	21.06758	0.988849	0.232461	4.25382	2.10E-05	5.66E-05	UP
RP11-367I	2.405157	1.756842	0.413005	4.253809	2.10E-05	5.66E-05	UP
E2F6	335.0103	0.352919	0.082983	4.252891	2.11E-05	5.68E-05	NOT
RMND5A	4231.245	-0.44244	0.10404	-4.25263	2.11E-05	5.68E-05	NOT
RNMT	1011.618	0.315391	0.074168	4.252369	2.12E-05	5.69E-05	NOT
STAC2	3.21433	2.350111	0.55267	4.25229	2.12E-05	5.69E-05	UP
RP11-467I	1.816057	1.413675	0.332483	4.251874	2.12E-05	5.70E-05	UP
RP11-254I	9.282224	0.87208	0.205141	4.251124	2.13E-05	5.72E-05	UP
WTIP	198.5791	0.910757	0.214253	4.25085	2.13E-05	5.73E-05	UP
MTA1	1258.706	0.32852	0.07729	4.250495	2.13E-05	5.74E-05	NOT
IRGM	5.023325	1.898809	0.446734	4.25042	2.13E-05	5.74E-05	UP
SEC22B	1830.757	-0.355	0.083543	-4.24927	2.14E-05	5.76E-05	NOT
SNORA5A	1.917325	1.306656	0.307501	4.249269	2.14E-05	5.76E-05	UP
BICC1	1092.794	1.340305	0.315437	4.249039	2.15E-05	5.77E-05	UP
WDTC1	2432.883	-0.34805	0.081921	-4.24858	2.15E-05	5.78E-05	NOT

HIST1H2BI	1.247105	2.103263	0.495095	4.248203	2.15E-05	5.79E-05	UP
RIMBP3	3.208132	1.294923	0.30483	4.248016	2.16E-05	5.79E-05	UP
PCDHGC5	4.765065	1.701538	0.400594	4.247532	2.16E-05	5.81E-05	UP
GABRR1	1.03856	2.017311	0.474942	4.247489	2.16E-05	5.81E-05	UP
RP11-314I	9.253767	0.763576	0.179777	4.247358	2.16E-05	5.81E-05	UP
SLC6A1-A	12.38013	1.163547	0.273964	4.247076	2.17E-05	5.82E-05	UP
FAM131B	16.40939	0.764388	0.179985	4.246959	2.17E-05	5.82E-05	UP
RP11-108I	36.12194	-1.4218	0.334791	-4.24683	2.17E-05	5.82E-05	DOWN
RP11-38L1	11.85399	1.09319	0.257439	4.246405	2.17E-05	5.83E-05	UP
PTCHD3P3	1.26521	2.521245	0.593754	4.246279	2.17E-05	5.84E-05	UP
CTC-246B	3.220335	1.560993	0.367684	4.24548	2.18E-05	5.86E-05	UP
RP11-63K(	4.286148	4.190928	0.987334	4.244692	2.19E-05	5.88E-05	UP
UBOX5-AS	5.750869	0.75037	0.176804	4.244084	2.19E-05	5.89E-05	UP
LINC0135S	8.289623	0.869147	0.2048	4.243889	2.20E-05	5.90E-05	UP
ANKRD20A	1.890115	2.563577	0.604064	4.243885	2.20E-05	5.90E-05	UP
TRIM74	5.790395	1.177281	0.27744	4.243372	2.20E-05	5.91E-05	UP
KLC4	4865.642	-0.54632	0.12875	-4.24324	2.20E-05	5.91E-05	NOT
TXNDC9	818.944	0.316482	0.074601	4.24233	2.21E-05	5.93E-05	NOT
RP11-286I	8.693138	0.734673	0.173178	4.242315	2.21E-05	5.93E-05	UP
HECTD1	4880.384	-0.41091	0.096868	-4.242	2.22E-05	5.94E-05	NOT
RFT1	830.0463	0.284524	0.067075	4.241859	2.22E-05	5.95E-05	NOT
CD177	13.84278	1.584345	0.373522	4.241638	2.22E-05	5.95E-05	UP
TMEM45A	2336.991	-1.15438	0.272164	-4.24148	2.22E-05	5.95E-05	DOWN
RCBTB1	690.9997	0.528032	0.124513	4.240787	2.23E-05	5.97E-05	NOT
LINC01272	97.41061	-0.99471	0.234561	-4.24073	2.23E-05	5.97E-05	DOWN
MED12L	12.25721	-0.96008	0.226409	-4.24044	2.23E-05	5.98E-05	DOWN
ZNF835	31.33604	1.0243	0.241584	4.23994	2.24E-05	5.99E-05	UP
AC137932	4.275557	1.075724	0.253719	4.239828	2.24E-05	6.00E-05	UP
OPRL1	49.43571	0.817964	0.192937	4.239531	2.24E-05	6.00E-05	UP
RP11-419(	8.728383	0.858668	0.202542	4.239453	2.24E-05	6.00E-05	UP
RPS7P10	26.26535	0.669142	0.157837	4.239438	2.24E-05	6.00E-05	UP
EFNA1	12857.01	0.678409	0.160025	4.239404	2.24E-05	6.00E-05	UP
GLULP3	2.431685	2.181081	0.514486	4.23934	2.24E-05	6.01E-05	UP
AL022341.	4.82032	1.047675	0.247157	4.238903	2.25E-05	6.02E-05	UP
TSN	2826.676	0.320141	0.075531	4.238554	2.25E-05	6.03E-05	NOT
IGLV3-16	2.755047	-1.91682	0.452237	-4.23853	2.25E-05	6.03E-05	DOWN
BNIP3L	1763.686	-0.47879	0.112965	-4.2384	2.25E-05	6.03E-05	NOT
WNT4	309.2006	1.431037	0.337675	4.237915	2.26E-05	6.04E-05	UP
ZNF81	84.92567	0.483239	0.114051	4.237046	2.26E-05	6.06E-05	NOT
MIR331	2.402825	1.289539	0.304361	4.236869	2.27E-05	6.07E-05	UP
AC234917	1.343763	2.752182	0.649592	4.236783	2.27E-05	6.07E-05	UP
ADSS	1496.401	0.369308	0.087174	4.23643	2.27E-05	6.08E-05	NOT
RP11-264I	3.735495	-1.39638	0.329642	-4.23604	2.27E-05	6.09E-05	DOWN
DRAM2	1385.999	0.344159	0.081246	4.236022	2.28E-05	6.09E-05	NOT
TCTN2	187.4183	0.700125	0.165288	4.235795	2.28E-05	6.09E-05	UP
CSAG2	15.60377	3.415892	0.806463	4.235647	2.28E-05	6.10E-05	UP
HS1BP3-IT	170.8915	-1.11397	0.263039	-4.23499	2.29E-05	6.11E-05	DOWN
ARPC3	6060.326	0.38058	0.089868	4.234892	2.29E-05	6.12E-05	NOT
RP1-149A	2.15497	1.464141	0.345737	4.234838	2.29E-05	6.12E-05	UP
MIRLET7BI	25.81479	0.833672	0.196865	4.234749	2.29E-05	6.12E-05	UP
BECN1P1	1.035663	2.391856	0.56486	4.234425	2.29E-05	6.13E-05	UP
RN7SL268I	2.662568	1.156965	0.273236	4.234301	2.29E-05	6.13E-05	UP

FAM217B	242.678	0.716985	0.16935	4.233741	2.30E-05	6.15E-05	UP
IQCH	72.61588	1.003576	0.237059	4.233448	2.30E-05	6.15E-05	UP
SOHLH1	1.662582	2.556726	0.603992	4.233047	2.31E-05	6.16E-05	UP
RP11-649/	1.46026	1.566789	0.370148	4.232867	2.31E-05	6.17E-05	UP
RP11-713(	1.241501	2.172469	0.513273	4.232577	2.31E-05	6.17E-05	UP
TST	17812.05	-0.70114	0.165664	-4.2323	2.31E-05	6.18E-05	DOWN
DIS3	1226.27	-0.36549	0.086358	-4.23224	2.31E-05	6.18E-05	NOT
AC079325	3.483556	-1.08713	0.256912	-4.23154	2.32E-05	6.20E-05	DOWN
RP11-669I	2.581477	3.571167	0.843996	4.231263	2.32E-05	6.21E-05	UP
ACTN1-AS	2.398576	1.331483	0.314704	4.230909	2.33E-05	6.22E-05	UP
SLX1A-SUI	2.381463	1.116907	0.264027	4.230272	2.33E-05	6.23E-05	UP
PDX1	213.4501	2.017526	0.476948	4.230075	2.34E-05	6.24E-05	UP
CLINT1	4126.137	-0.36508	0.086314	-4.22972	2.34E-05	6.25E-05	NOT
CTC-559E	1.478461	1.628799	0.385106	4.229479	2.34E-05	6.25E-05	UP
C7orf25	23.69801	0.484967	0.114665	4.229438	2.34E-05	6.26E-05	NOT
RP11-190/	7.283726	1.229945	0.290834	4.229029	2.35E-05	6.27E-05	UP
RN7SL138I	10.90347	-0.79213	0.187333	-4.22847	2.35E-05	6.28E-05	DOWN
RP5-1148/	214.93	0.746361	0.176509	4.228462	2.35E-05	6.28E-05	UP
SNRNP20(	7376.954	0.309217	0.073154	4.226926	2.37E-05	6.32E-05	NOT
RPS3AP5	21.0399	0.975666	0.230845	4.226491	2.37E-05	6.33E-05	UP
RP11-26J3	1.734196	1.631731	0.386098	4.226209	2.38E-05	6.34E-05	UP
ZC3H13	2016.696	-0.50598	0.119741	-4.22562	2.38E-05	6.36E-05	NOT
XKR3	1.760074	2.691904	0.637158	4.224858	2.39E-05	6.38E-05	UP
TRMT13	308.6669	0.398599	0.094346	4.22485	2.39E-05	6.38E-05	NOT
RP11-168/	3.080551	1.456124	0.344719	4.224093	2.40E-05	6.40E-05	UP
SACM1L	1425.205	-0.27533	0.065187	-4.2237	2.40E-05	6.41E-05	NOT
DDX39BP2	1.377654	2.377565	0.562928	4.223569	2.40E-05	6.41E-05	UP
RASSF5	793.8505	-0.63524	0.150407	-4.2235	2.41E-05	6.41E-05	DOWN
CTD-2528	1.42138	1.809986	0.428554	4.223471	2.41E-05	6.41E-05	UP
B3GAT3	2087.69	0.490571	0.116163	4.223128	2.41E-05	6.42E-05	NOT
CLEC18B	6.759908	1.232585	0.291879	4.222935	2.41E-05	6.43E-05	UP
ASIC5	3.817163	1.415103	0.335103	4.222896	2.41E-05	6.43E-05	UP
IFT27	922.1832	0.425102	0.100678	4.222369	2.42E-05	6.44E-05	NOT
ENPP7P4	1.830396	1.455152	0.34464	4.222242	2.42E-05	6.45E-05	UP
RP11-17G	1.881465	1.401466	0.331938	4.222069	2.42E-05	6.45E-05	UP
HMGN5	299.2898	-0.91556	0.216878	-4.22154	2.43E-05	6.47E-05	DOWN
AGTRAP	932.0797	0.511874	0.121263	4.22117	2.43E-05	6.48E-05	NOT
ERMAP	686.3908	-0.471	0.111609	-4.22007	2.44E-05	6.51E-05	NOT
S100A14	1072.64	1.2699	0.300949	4.219657	2.45E-05	6.52E-05	UP
FTH1P5	16.79518	0.712522	0.168872	4.219308	2.45E-05	6.53E-05	UP
PER3	1019.531	-0.6525	0.154655	-4.21905	2.45E-05	6.53E-05	DOWN
CASP8	864.0438	0.372568	0.088319	4.218447	2.46E-05	6.55E-05	NOT
SYT8	62.8622	1.640063	0.38883	4.217942	2.47E-05	6.57E-05	UP
IGLV1-41	4.289894	-2.09059	0.495713	-4.21733	2.47E-05	6.58E-05	DOWN
AC073621	1.712807	-1.54311	0.365946	-4.21677	2.48E-05	6.60E-05	DOWN
AADAC	13958.32	-0.94404	0.223891	-4.21653	2.48E-05	6.60E-05	DOWN
TMPRSS7	1.561474	2.275448	0.539666	4.216403	2.48E-05	6.61E-05	UP
FLRT3	755.8848	-0.93083	0.220793	-4.21585	2.49E-05	6.62E-05	DOWN
WDFY3-AS	48.70091	-0.78946	0.187264	-4.21576	2.49E-05	6.62E-05	DOWN
EEF1D	5489.967	0.447721	0.106209	4.215456	2.49E-05	6.63E-05	NOT
VPS35	2786.594	0.364041	0.086368	4.214981	2.50E-05	6.65E-05	NOT
CPQ	3722.656	0.477242	0.113232	4.21473	2.50E-05	6.65E-05	NOT

VASH1	485.2483	0.6133	0.145517	4.214644	2.50E-05	6.66E-05	UP
CTD-3193	24.89073	-1.09397	0.259572	-4.21452	2.50E-05	6.66E-05	DOWN
TMED9	10024.93	0.371263	0.088098	4.214195	2.51E-05	6.67E-05	NOT
TUBA4B	28.66383	1.157412	0.274654	4.214071	2.51E-05	6.67E-05	UP
WNT11	317.1958	-1.23845	0.293896	-4.21392	2.51E-05	6.67E-05	DOWN
YIPF5	1606.718	-0.25506	0.060528	-4.21389	2.51E-05	6.67E-05	NOT
MEN1	1614.436	0.287715	0.068285	4.213422	2.52E-05	6.69E-05	NOT
RP11-21G	1.462644	1.557079	0.369575	4.213167	2.52E-05	6.69E-05	UP
EEF1DP4	3.452708	1.706853	0.405142	4.21297	2.52E-05	6.70E-05	UP
P3H2	292.4338	-1.00356	0.238233	-4.21254	2.53E-05	6.71E-05	DOWN
SMTN	1309.482	0.463307	0.11	4.211884	2.53E-05	6.73E-05	NOT
HCN4	14.06228	2.00072	0.475061	4.211499	2.54E-05	6.74E-05	UP
KLRG1	50.2707	-0.69363	0.164703	-4.21142	2.54E-05	6.74E-05	DOWN
PLP1	10.46455	-1.99452	0.473615	-4.21126	2.54E-05	6.75E-05	DOWN
KPNA1	1923.43	-0.28535	0.067761	-4.21113	2.54E-05	6.75E-05	NOT
BHLHA9	3.529563	-2.21164	0.525198	-4.21105	2.54E-05	6.75E-05	DOWN
CFAP45	17.54721	1.134944	0.269519	4.211001	2.54E-05	6.75E-05	UP
MAPRE1	2120.743	0.460469	0.109352	4.210898	2.54E-05	6.76E-05	NOT
AP001610	2.301061	-1.39095	0.330323	-4.21087	2.54E-05	6.76E-05	DOWN
RP5-902P8	5.669494	0.97203	0.230852	4.210627	2.55E-05	6.76E-05	UP
RN7SKP97	1.673342	1.625736	0.386124	4.210397	2.55E-05	6.77E-05	UP
TRMT10A	295.4936	-0.39843	0.094634	-4.21027	2.55E-05	6.77E-05	NOT
RP11-616I	5.909283	1.929136	0.458247	4.209816	2.56E-05	6.78E-05	UP
AOC3	963.4775	-0.58546	0.139071	-4.20979	2.56E-05	6.78E-05	DOWN
SPTB	61.08313	0.969662	0.230343	4.209638	2.56E-05	6.79E-05	UP
LINC01348	885.0465	-1.17144	0.278282	-4.20954	2.56E-05	6.79E-05	DOWN
RP11-730I	3.008134	-1.08921	0.258756	-4.2094	2.56E-05	6.79E-05	DOWN
AC018712	3.28959	2.300246	0.546463	4.209335	2.56E-05	6.80E-05	UP
CYP4X1	204.1387	-0.92232	0.219152	-4.2086	2.57E-05	6.82E-05	DOWN
PPP1R1A	4414.905	-1.19878	0.284864	-4.20827	2.57E-05	6.83E-05	DOWN
SNORA45E	2.212614	1.483027	0.35242	4.208127	2.57E-05	6.83E-05	UP
RP11-278(	32.2856	0.623152	0.148084	4.208093	2.58E-05	6.83E-05	UP
XRCC6P2	6.882642	0.822354	0.195451	4.20746	2.58E-05	6.85E-05	UP
GAPDHP6	2.746315	1.23635	0.293899	4.206713	2.59E-05	6.87E-05	UP
RHOV	33.17158	1.538551	0.365839	4.205541	2.60E-05	6.91E-05	UP
MTO1	1152.46	-0.38358	0.091213	-4.20534	2.61E-05	6.91E-05	NOT
CTD-2033	9.369316	0.67176	0.159741	4.205317	2.61E-05	6.91E-05	UP
HAS3	70.02756	0.687735	0.163546	4.205135	2.61E-05	6.92E-05	UP
PEX7	319.1393	-0.41304	0.098233	-4.20471	2.61E-05	6.93E-05	NOT
ITGB1P1	15.0876	0.777663	0.184958	4.20453	2.62E-05	6.93E-05	UP
AC005387	4.748996	1.031389	0.245309	4.204445	2.62E-05	6.94E-05	UP
KLHL7-AS	16.37075	0.924402	0.219931	4.203146	2.63E-05	6.97E-05	UP
APMAP	14508.78	-0.54231	0.129025	-4.20314	2.63E-05	6.97E-05	NOT
FAR2P4	3.707803	2.988274	0.711022	4.202789	2.64E-05	6.98E-05	UP
RP6-109B	1.300321	1.531749	0.364542	4.201841	2.65E-05	7.01E-05	UP
FTH1P2	31.38387	0.671738	0.159871	4.201753	2.65E-05	7.02E-05	UP
RP11-599I	2.001092	2.829725	0.673508	4.201473	2.65E-05	7.02E-05	UP
AP4S1	241.7218	-0.43179	0.102774	-4.20129	2.65E-05	7.03E-05	NOT
SERPINF1	40010.63	-0.72674	0.173004	-4.2007	2.66E-05	7.05E-05	DOWN
HARBI1	127.6166	0.294597	0.070139	4.200195	2.67E-05	7.06E-05	NOT
RP11-3B7	4.061109	1.506331	0.358636	4.200168	2.67E-05	7.06E-05	UP
RP11-161I	0.921785	1.826698	0.435012	4.199188	2.68E-05	7.09E-05	UP

LINC0070	9.529942	-1.36049	0.324024	-4.19874	2.68E-05	7.10E-05	DOWN
NUDT7	589.0804	-0.68363	0.16282	-4.19866	2.69E-05	7.11E-05	DOWN
LOXL1	232.7427	1.098023	0.261521	4.198599	2.69E-05	7.11E-05	UP
SSB	2493.046	0.361127	0.086018	4.198293	2.69E-05	7.12E-05	NOT
RPL23AP6	7.823814	0.969775	0.231049	4.197272	2.70E-05	7.15E-05	UP
IGFN1	37.72073	1.979842	0.471744	4.196859	2.71E-05	7.16E-05	UP
AC004870	2.307274	3.600109	0.857975	4.196056	2.72E-05	7.19E-05	UP
KRT8P5	2.084364	-1.05809	0.252183	-4.19571	2.72E-05	7.20E-05	DOWN
RP11-384I	3.373693	0.997522	0.237755	4.195586	2.72E-05	7.20E-05	UP
PDX1-AS1	3.083229	2.210177	0.526803	4.195449	2.72E-05	7.20E-05	UP
ITPR1-AS1	7.596523	0.781298	0.186225	4.195442	2.72E-05	7.20E-05	UP
CYP3A5	11425.7	-0.97954	0.23348	-4.19541	2.72E-05	7.20E-05	DOWN
EEF1A1	116339.1	-0.35902	0.085575	-4.19533	2.72E-05	7.20E-05	NOT
LINC0031	1.264892	2.421557	0.577256	4.194946	2.73E-05	7.22E-05	UP
TMEM63C	28.24861	1.459053	0.347846	4.194542	2.73E-05	7.23E-05	UP
RP11-423I	3.984274	-1.09396	0.260815	-4.19437	2.74E-05	7.23E-05	DOWN
ZNF224	258.7568	0.453471	0.108118	4.194229	2.74E-05	7.24E-05	NOT
GJB6	9.014185	2.001039	0.477119	4.194005	2.74E-05	7.24E-05	UP
LFNG	290.3569	0.820667	0.195696	4.193582	2.75E-05	7.26E-05	UP
VN1R85P	3.508765	2.536402	0.604851	4.193433	2.75E-05	7.26E-05	UP
RP11-100	1.521935	1.723141	0.410929	4.193284	2.75E-05	7.26E-05	UP
RP11-120I	1.68822	2.442301	0.582554	4.192407	2.76E-05	7.29E-05	UP
RP11-241I	1.567886	2.585151	0.616629	4.192394	2.76E-05	7.29E-05	UP
TEKT5	15.80885	1.128394	0.269191	4.191797	2.77E-05	7.31E-05	UP
GAS8	517.2035	0.479111	0.114306	4.19147	2.77E-05	7.32E-05	NOT
TMEM161	2301.883	0.45516	0.108593	4.191438	2.77E-05	7.32E-05	NOT
TUB	131.05	1.08168	0.258093	4.191043	2.78E-05	7.33E-05	UP
ZNF584	303.6375	-0.36881	0.08801	-4.19059	2.78E-05	7.35E-05	NOT
GPT2	10743.53	-0.87395	0.208618	-4.18924	2.80E-05	7.39E-05	DOWN
ZFR2	6.744284	1.465969	0.349946	4.189132	2.80E-05	7.39E-05	UP
ACY1	895.7138	-0.56686	0.13532	-4.18902	2.80E-05	7.39E-05	NOT
AC133528	158.2506	0.5318	0.126955	4.1889	2.80E-05	7.40E-05	NOT
SLC39A3	925.8302	0.44222	0.105571	4.188858	2.80E-05	7.40E-05	NOT
LINC0108	1.399512	2.497234	0.596166	4.188819	2.80E-05	7.40E-05	UP
MATN1	4.101366	0.919178	0.219474	4.188093	2.81E-05	7.42E-05	UP
THAP1	319.5886	-0.31396	0.074967	-4.18805	2.81E-05	7.42E-05	NOT
THRB	1523.111	-0.51312	0.122531	-4.18766	2.82E-05	7.43E-05	NOT
LY6K	8.629568	1.307435	0.312257	4.187055	2.83E-05	7.45E-05	UP
KAAG1	20.34064	1.322763	0.315924	4.186967	2.83E-05	7.46E-05	UP
RP11-667I	16.50205	-0.56654	0.135328	-4.18641	2.83E-05	7.47E-05	NOT
RFPL3S	5.509422	0.939064	0.224315	4.186363	2.83E-05	7.47E-05	UP
RP1-265C	2.791477	1.104852	0.263942	4.185972	2.84E-05	7.49E-05	UP
RPP30	721.6454	0.356191	0.085093	4.185915	2.84E-05	7.49E-05	NOT
SENP8	76.8654	-0.51274	0.122499	-4.18564	2.84E-05	7.50E-05	NOT
SLC2A3	805.1703	-0.89867	0.214718	-4.18535	2.85E-05	7.51E-05	DOWN
PBDC1	767.2731	-0.37356	0.089258	-4.18516	2.85E-05	7.51E-05	NOT
KRT16P3	1.25379	-2.68034	0.640448	-4.18511	2.85E-05	7.51E-05	DOWN
MYHAS	1.528713	2.439572	0.582921	4.185082	2.85E-05	7.51E-05	UP
CCND2P1	398.9521	-1.33908	0.319992	-4.18474	2.85E-05	7.52E-05	DOWN
RPL23AP3	1.779065	1.461778	0.349368	4.184067	2.86E-05	7.54E-05	UP
HNF1A-AS	512.6355	0.697247	0.166653	4.183827	2.87E-05	7.55E-05	UP
KDELC1	414.624	0.613857	0.146723	4.183777	2.87E-05	7.55E-05	UP

PDGFRA	936.2356	-1.29265	0.308975	-4.18367	2.87E-05	7.56E-05	DOWN
RP1-182D	18.57115	1.035297	0.24749	4.183194	2.87E-05	7.57E-05	UP
AHSG	168752.9	-1.10603	0.264402	-4.18313	2.88E-05	7.57E-05	DOWN
EIF2B2	899.1503	0.299963	0.071709	4.183074	2.88E-05	7.57E-05	NOT
IDH1	14967.57	-0.48399	0.115714	-4.18264	2.88E-05	7.59E-05	NOT
FLOT1	7014.616	0.402077	0.096146	4.181928	2.89E-05	7.61E-05	NOT
SYNJ2	971.2418	0.577036	0.137988	4.181781	2.89E-05	7.61E-05	NOT
PTPN21	638.6959	-0.47736	0.114158	-4.1816	2.89E-05	7.62E-05	NOT
AC004593	12.40137	1.475079	0.352757	4.181578	2.89E-05	7.62E-05	UP
SYT6	4.693756	1.371706	0.328063	4.181221	2.90E-05	7.63E-05	UP
TNFRSF10(	105.429	0.919989	0.220067	4.180503	2.91E-05	7.65E-05	UP
GPIHBP1	160.8318	0.838516	0.200579	4.18048	2.91E-05	7.65E-05	UP
RP11-126I	44.35498	0.631978	0.151202	4.179685	2.92E-05	7.68E-05	UP
ACMSD	3505.177	-0.96295	0.230392	-4.17961	2.92E-05	7.68E-05	DOWN
RP11-338I	1.513747	2.415329	0.577976	4.178946	2.93E-05	7.70E-05	UP
G2E3	333.7082	0.388522	0.092973	4.178842	2.93E-05	7.71E-05	NOT
ACOT1	557.0088	-0.82369	0.197114	-4.17877	2.93E-05	7.71E-05	DOWN
GIP	1.798799	3.106453	0.743453	4.178412	2.94E-05	7.72E-05	UP
ASH2L	1159.746	-0.38236	0.091535	-4.17724	2.95E-05	7.76E-05	NOT
TMEM190	0.97575	1.966048	0.470668	4.177141	2.95E-05	7.76E-05	UP
FMO2	140.3586	-1.04144	0.249322	-4.17707	2.95E-05	7.76E-05	DOWN
CTD-2012	2.354177	1.161659	0.27811	4.176973	2.95E-05	7.77E-05	UP
AC007387	3.372852	1.126405	0.269677	4.17687	2.96E-05	7.77E-05	UP
DAO	3187.083	-1.09493	0.262149	-4.17674	2.96E-05	7.77E-05	DOWN
RP11-813I	6.474607	1.415735	0.338967	4.176619	2.96E-05	7.78E-05	UP
NOX1	42.44801	0.757615	0.181412	4.176223	2.96E-05	7.79E-05	UP
GRIP2	34.63579	1.212261	0.290288	4.176057	2.97E-05	7.79E-05	UP
CCDC68	342.267	-0.77514	0.185619	-4.17597	2.97E-05	7.80E-05	DOWN
EIF5AP4	4.286916	-0.79816	0.191143	-4.1757	2.97E-05	7.80E-05	DOWN
TOMM34	1268.199	0.421912	0.101043	4.175558	2.97E-05	7.81E-05	NOT
MIR8071-:	2.36315	-1.90957	0.457397	-4.17486	2.98E-05	7.83E-05	DOWN
RP11-305I	3.711431	1.283528	0.307455	4.174692	2.98E-05	7.84E-05	UP
KCNJ13	4.204787	-1.9329	0.463032	-4.17445	2.99E-05	7.84E-05	DOWN
PPIAP2	2.671803	1.17054	0.280409	4.174402	2.99E-05	7.84E-05	UP
ABCA9	346.5663	-0.9354	0.224096	-4.17411	2.99E-05	7.85E-05	DOWN
DLEU2	93.36136	0.589828	0.141319	4.173742	3.00E-05	7.87E-05	UP
AC099684	47.65774	-1.16393	0.278874	-4.17369	3.00E-05	7.87E-05	DOWN
SNORD99	5.801651	1.047462	0.250974	4.173592	3.00E-05	7.87E-05	UP
ZNF684	364.9669	-0.56898	0.136335	-4.17341	3.00E-05	7.88E-05	NOT
GFM1	2669.364	-0.3449	0.082645	-4.17328	3.00E-05	7.88E-05	NOT
CTD-2589	2.197736	3.017991	0.723246	4.17284	3.01E-05	7.89E-05	UP
C4BPA	59642.4	-1.01935	0.244324	-4.17214	3.02E-05	7.92E-05	DOWN
FABP1	68187.86	-1.27709	0.306123	-4.17182	3.02E-05	7.93E-05	DOWN
GTF2B	999.6041	-0.31484	0.075472	-4.17162	3.02E-05	7.93E-05	NOT
KIAA2018	1326.68	-0.44554	0.106804	-4.17152	3.03E-05	7.94E-05	NOT
GLDCP1	23.57847	-0.99527	0.238596	-4.17135	3.03E-05	7.94E-05	DOWN
SPDYE2	2.414039	1.388256	0.332817	4.171234	3.03E-05	7.94E-05	UP
RP11-119I	403.6448	-1.36064	0.326228	-4.17081	3.04E-05	7.96E-05	DOWN
ARRDC2	1934.105	0.716605	0.17182	4.170671	3.04E-05	7.96E-05	UP
RP11-118I	1.033244	2.038944	0.488958	4.169975	3.05E-05	7.99E-05	UP
DUS1L	5788.627	0.430187	0.103163	4.169954	3.05E-05	7.99E-05	NOT
KLF2P1	2.56744	3.754197	0.900301	4.169935	3.05E-05	7.99E-05	UP

C1orf147	2.197642	1.477029	0.354218	4.169832	3.05E-05	7.99E-05	UP
RP11-426(	20.5359	-1.46378	0.351067	-4.16951	3.05E-05	8.00E-05	DOWN
SERPINA6	24603.2	-0.81912	0.196462	-4.16933	3.05E-05	8.01E-05	DOWN
PLCH1	34.0703	1.105282	0.265101	4.169281	3.06E-05	8.01E-05	UP
SAMD11	121.1133	-1.19918	0.287716	-4.16795	3.07E-05	8.05E-05	DOWN
ASXL2	1078.97	0.42723	0.102504	4.167927	3.07E-05	8.05E-05	NOT
RP11-109!	5.265389	1.369293	0.328584	4.167258	3.08E-05	8.08E-05	UP
TMEM140	2685.987	-0.51087	0.122596	-4.16714	3.08E-05	8.08E-05	NOT
GTF2IRD1I	5.520565	1.074504	0.257861	4.166994	3.09E-05	8.08E-05	UP
LANCL1-A	4.647667	1.01725	0.244133	4.166782	3.09E-05	8.09E-05	UP
BRWD1	1927.054	-0.35379	0.084922	-4.166	3.10E-05	8.12E-05	NOT
TMEM261	1036.567	-0.58875	0.141326	-4.16589	3.10E-05	8.12E-05	DOWN
SCN11A	13.44324	-1.05715	0.253771	-4.16575	3.10E-05	8.12E-05	DOWN
RP11-474I	5.339572	0.822111	0.197352	4.165715	3.10E-05	8.12E-05	UP
RP11-244I	6.058345	-0.62068	0.149002	-4.16562	3.11E-05	8.13E-05	DOWN
DNAJC5G	1.973497	1.764476	0.423621	4.165219	3.11E-05	8.14E-05	UP
C1orf168	547.6187	-0.85586	0.205513	-4.16449	3.12E-05	8.17E-05	DOWN
MTFMT	421.9608	-0.25061	0.060182	-4.16425	3.12E-05	8.17E-05	NOT
SRRT	3292.892	0.276784	0.066478	4.163546	3.13E-05	8.20E-05	NOT
WDR64	4.942834	-0.95898	0.230345	-4.16325	3.14E-05	8.21E-05	DOWN
RAPGEF4	1038.162	-0.6619	0.159002	-4.16285	3.14E-05	8.22E-05	DOWN
AC114812	0.912622	1.979354	0.475481	4.162848	3.14E-05	8.22E-05	UP
IMPG1	4.572763	1.09276	0.262554	4.162038	3.15E-05	8.25E-05	UP
AC002398	2.731792	-1.45738	0.350169	-4.16194	3.16E-05	8.25E-05	DOWN
CCDC18	128.0099	0.491285	0.118055	4.161492	3.16E-05	8.27E-05	NOT
CACNA1F	9.528068	1.194947	0.287171	4.161104	3.17E-05	8.28E-05	UP
RP11-73M	2.409659	1.217981	0.29275	4.160487	3.18E-05	8.30E-05	UP
GJB3	60.19976	-1.4635	0.351815	-4.15986	3.18E-05	8.32E-05	DOWN
VDR	210.7338	0.970533	0.233315	4.159755	3.19E-05	8.33E-05	UP
RP11-452I	9.885623	0.803347	0.193171	4.15874	3.20E-05	8.36E-05	UP
CTC-241N	43.59164	0.504591	0.121336	4.158632	3.20E-05	8.37E-05	NOT
CTC-529I1	11.24385	-0.80658	0.193957	-4.15854	3.20E-05	8.37E-05	DOWN
TAP2	1140.441	0.587136	0.14119	4.158494	3.20E-05	8.37E-05	UP
RP11-102!	2.725812	1.132836	0.272418	4.158453	3.20E-05	8.37E-05	UP
MVD	2687.272	0.738319	0.177566	4.157998	3.21E-05	8.39E-05	UP
RP11-181I	3.442694	1.232691	0.296531	4.157035	3.22E-05	8.42E-05	UP
RP11-474I	4.737403	-0.95814	0.230509	-4.15663	3.23E-05	8.44E-05	DOWN
PIGP	785.5611	-0.39623	0.095326	-4.15658	3.23E-05	8.44E-05	NOT
CCDC61	448.3038	0.451947	0.108742	4.156126	3.24E-05	8.45E-05	NOT
C10orf10	10251.99	-0.90325	0.217339	-4.15596	3.24E-05	8.46E-05	DOWN
AL163953.	8.48914	1.027473	0.247278	4.155129	3.25E-05	8.49E-05	UP
RP11-439(	12.59341	-1.51527	0.36468	-4.15507	3.25E-05	8.49E-05	DOWN
RP11-637/	11.27211	0.725587	0.174629	4.155025	3.25E-05	8.49E-05	UP
SAMD13	21.14297	0.886726	0.213413	4.154973	3.25E-05	8.49E-05	UP
SIGLEC8	44.61806	-1.13349	0.272831	-4.15454	3.26E-05	8.51E-05	DOWN
ZNF765	219.0551	0.443725	0.106807	4.154465	3.26E-05	8.51E-05	NOT
TFCP2L1	65.82809	1.247846	0.30038	4.154227	3.26E-05	8.52E-05	UP
RP11-753I	3.628839	-1.33221	0.320713	-4.15389	3.27E-05	8.53E-05	DOWN
FKBP14	339.9169	0.471406	0.113495	4.153552	3.27E-05	8.54E-05	NOT
AFF1	2334.385	-0.47805	0.1151	-4.1534	3.28E-05	8.55E-05	NOT
CHD7	809.3701	0.477515	0.114984	4.152897	3.28E-05	8.56E-05	NOT
NPIPB3	17.33441	0.734677	0.176925	4.152472	3.29E-05	8.58E-05	UP

ZNF816-ZI	24.3761	0.962848	0.231881	4.152333	3.29E-05	8.58E-05	UP
RP11-757(	3.438273	1.12438	0.270784	4.152316	3.29E-05	8.58E-05	UP
R3HCC1L	430.9753	0.304342	0.073303	4.151827	3.30E-05	8.60E-05	NOT
THG1L	438.7464	-0.33746	0.08128	-4.1518	3.30E-05	8.60E-05	NOT
RP11-367(	5.446984	-1.09475	0.263693	-4.15162	3.30E-05	8.61E-05	DOWN
BNIP3P27	1.023199	2.001241	0.482041	4.1516	3.30E-05	8.61E-05	UP
GALNT7	198.4641	1.016738	0.244919	4.151333	3.31E-05	8.62E-05	UP
RP11-513I	12.90443	0.709748	0.170973	4.151226	3.31E-05	8.62E-05	UP
ETS1	2279.986	-0.61191	0.147432	-4.15049	3.32E-05	8.65E-05	DOWN
VGF	30.47338	1.517902	0.36573	4.150339	3.32E-05	8.65E-05	UP
RP11-641]	1.792653	1.381145	0.332842	4.14955	3.33E-05	8.68E-05	UP
RP11-468I	10.52132	0.684847	0.165069	4.148852	3.34E-05	8.71E-05	UP
DTD1	730.2938	0.63525	0.153134	4.148319	3.35E-05	8.73E-05	UP
RP11-162(	3.28014	1.190256	0.286934	4.148181	3.35E-05	8.73E-05	UP
ERI3	1899.372	0.382659	0.092252	4.147997	3.35E-05	8.74E-05	NOT
RP11-478(	3.595364	1.218971	0.293923	4.147251	3.36E-05	8.76E-05	UP
PKP4	2568.777	0.398376	0.096072	4.146649	3.37E-05	8.79E-05	NOT
WNT8B	2.742809	1.922638	0.463674	4.146528	3.38E-05	8.79E-05	UP
POR	30576.84	-0.62158	0.149907	-4.14647	3.38E-05	8.79E-05	DOWN
ZNF365	4.615006	1.327939	0.320285	4.146115	3.38E-05	8.80E-05	UP
CTC-308K	13.35395	0.584569	0.140992	4.146105	3.38E-05	8.80E-05	NOT
KIR3DL2	1.643374	-1.31757	0.317816	-4.14569	3.39E-05	8.82E-05	DOWN
ZNF212	542.5674	0.316713	0.0764	4.14545	3.39E-05	8.83E-05	NOT
LMO3	22.62044	1.062374	0.256277	4.14542	3.39E-05	8.83E-05	UP
AC004540	23.70495	1.247616	0.30098	4.145177	3.40E-05	8.84E-05	UP
RP3-510D	3.560927	1.028179	0.248081	4.144532	3.41E-05	8.86E-05	UP
RP11-483(	1.225906	2.15876	0.520909	4.144214	3.41E-05	8.87E-05	UP
RP11-342I	12.8196	0.784911	0.189406	4.14406	3.41E-05	8.88E-05	UP
SPCS3	3954.278	-0.34261	0.082681	-4.14373	3.42E-05	8.89E-05	NOT
SCARNA1(	2.671017	-1.47753	0.356605	-4.14331	3.42E-05	8.90E-05	DOWN
RP11-395I	4.274255	1.555103	0.375378	4.142763	3.43E-05	8.92E-05	UP
ITGAD	89.62321	-1.1848	0.286029	-4.14224	3.44E-05	8.94E-05	DOWN
EMR4P	15.64466	-0.925	0.22333	-4.14183	3.45E-05	8.96E-05	DOWN
PFDN2	2274.468	0.44617	0.10774	4.14116	3.46E-05	8.98E-05	NOT
PNRC2	1331.084	-0.29665	0.071636	-4.14104	3.46E-05	8.99E-05	NOT
SNHG14	929.472	0.955149	0.230656	4.141005	3.46E-05	8.99E-05	UP
CLUH	11317.93	-0.42772	0.103301	-4.1405	3.47E-05	9.01E-05	NOT
TMEM59L	8.69884	1.096442	0.264834	4.14011	3.47E-05	9.02E-05	UP
FAM3D	6.543607	-1.09897	0.265472	-4.13969	3.48E-05	9.04E-05	DOWN
TPT1P12	4.104223	-1.26765	0.306254	-4.13921	3.48E-05	9.06E-05	DOWN
RP11-650I	5.133341	1.37219	0.331541	4.138825	3.49E-05	9.07E-05	UP
RP3-395M	1.960494	2.195407	0.530444	4.138814	3.49E-05	9.07E-05	UP
KIAA1257	9.340302	1.10521	0.267038	4.138767	3.49E-05	9.07E-05	UP
NDUFB9	10213.29	0.614414	0.148472	4.138246	3.50E-05	9.09E-05	UP
CATSPERB	20.943	1.293517	0.312596	4.137986	3.50E-05	9.10E-05	UP
CPA1	5.336185	2.107771	0.509439	4.137437	3.51E-05	9.12E-05	UP
L3MBTL4	501.6238	-0.49703	0.120136	-4.13726	3.51E-05	9.13E-05	NOT
S1PR1	1076.357	-0.59982	0.14499	-4.13695	3.52E-05	9.14E-05	DOWN
C18orf21	451.5174	0.32571	0.07875	4.135979	3.53E-05	9.18E-05	NOT
RP4-738P1	2.315102	2.467524	0.596612	4.135895	3.54E-05	9.18E-05	UP
HAUS6	528.7185	0.455344	0.110097	4.135841	3.54E-05	9.18E-05	NOT
MTM1	770.5428	-0.49079	0.11867	-4.1358	3.54E-05	9.18E-05	NOT

EAPP	1296.485	-0.29286	0.070812	-4.13574	3.54E-05	9.18E-05	NOT
LA16c-380	33.27646	0.950154	0.229761	4.135395	3.54E-05	9.20E-05	UP
ZNF419	218.1063	0.531951	0.128634	4.135378	3.54E-05	9.20E-05	NOT
AP006285.	15.78785	-1.47417	0.356506	-4.13505	3.55E-05	9.21E-05	DOWN
RP11-494(	1.499894	1.489783	0.360293	4.134915	3.55E-05	9.21E-05	UP
NAPSA	24.70082	0.635061	0.153585	4.134913	3.55E-05	9.21E-05	UP
AC007879	3.396761	1.117827	0.270358	4.134616	3.56E-05	9.22E-05	UP
AC027612	47.47103	0.857334	0.20737	4.134323	3.56E-05	9.23E-05	UP
AKT1S1	3016.539	0.441148	0.106709	4.13411	3.56E-05	9.24E-05	NOT
PSPC1	801.2505	0.370775	0.089689	4.133985	3.57E-05	9.25E-05	NOT
TTLL10	2.669627	1.248927	0.302124	4.133827	3.57E-05	9.25E-05	UP
TRAPPC4	1289.797	0.371253	0.08981	4.133768	3.57E-05	9.25E-05	NOT
MIR616	2.289113	1.442749	0.349035	4.133542	3.57E-05	9.26E-05	UP
CRNKL1	1214.471	0.252386	0.061061	4.133333	3.58E-05	9.27E-05	NOT
RP11-526I	34.43244	0.893829	0.216316	4.132054	3.60E-05	9.32E-05	UP
PHOSPHO	72.86699	0.487868	0.118078	4.13173	3.60E-05	9.33E-05	NOT
RP4-593H	1.406098	1.390624	0.336591	4.131495	3.60E-05	9.34E-05	UP
DRAM1	657.656	0.526793	0.127517	4.13115	3.61E-05	9.35E-05	NOT
TRMT2B	515.6705	0.401759	0.097256	4.130965	3.61E-05	9.36E-05	NOT
RP11-18C:	8.487823	-0.67012	0.162227	-4.13076	3.62E-05	9.37E-05	DOWN
CTGLF12P	11.39033	1.198941	0.290255	4.130651	3.62E-05	9.37E-05	UP
PLCE1-AS:	0.900571	1.691708	0.409559	4.13056	3.62E-05	9.37E-05	UP
GALR2	5.400491	1.049708	0.254141	4.130415	3.62E-05	9.38E-05	UP
EAF1-AS1	7.880184	0.774896	0.187612	4.130311	3.62E-05	9.38E-05	UP
ATP8B1	1758.478	0.586043	0.141903	4.129892	3.63E-05	9.40E-05	UP
RP11-422I	54.05247	0.530336	0.128415	4.129863	3.63E-05	9.40E-05	NOT
RP5-855D	6.425728	0.930352	0.225316	4.1291	3.64E-05	9.43E-05	UP
IGLV5-37	3.228786	-1.99389	0.482911	-4.1289	3.65E-05	9.44E-05	DOWN
POTEF	2.493132	1.704574	0.412938	4.127917	3.66E-05	9.48E-05	UP
RP11-700.	3.957881	0.960298	0.232659	4.127489	3.67E-05	9.49E-05	UP
RP11-388I	2.660934	1.2066	0.292498	4.125152	3.70E-05	9.59E-05	UP
FTH1P4	8.118961	0.880419	0.213435	4.125004	3.71E-05	9.59E-05	UP
C6orf25	6.693459	-0.99616	0.241521	-4.12451	3.72E-05	9.61E-05	DOWN
RP5-1147/	1.259131	1.607608	0.38978	4.124404	3.72E-05	9.62E-05	UP
BMI1	1768.22	0.348345	0.084461	4.124349	3.72E-05	9.62E-05	NOT
EDRF1-AS	2.478898	1.163609	0.282135	4.124302	3.72E-05	9.62E-05	UP
AC002454	2.965111	1.627159	0.39453	4.124296	3.72E-05	9.62E-05	UP
RP11-718I	1.721262	-1.1111	0.269411	-4.1242	3.72E-05	9.62E-05	DOWN
CUL3	1886.685	-0.27202	0.065963	-4.12379	3.73E-05	9.64E-05	NOT
AC007879	3.344427	1.136421	0.2756	4.123448	3.73E-05	9.65E-05	UP
RP11-15I1	1.3392	1.876949	0.455203	4.12332	3.73E-05	9.66E-05	UP
FPR3	447.1556	-0.7818	0.189607	-4.12326	3.74E-05	9.66E-05	DOWN
RP11-22P(	3.232858	0.948316	0.230009	4.122949	3.74E-05	9.67E-05	UP
GPR183	213.5957	-0.87812	0.213013	-4.1224	3.75E-05	9.69E-05	DOWN
GDI2	10314.7	-0.27864	0.067592	-4.12239	3.75E-05	9.69E-05	NOT
GDF6	19.80356	-1.16607	0.282916	-4.12163	3.76E-05	9.72E-05	DOWN
CCDC102/	175.7121	0.682185	0.165581	4.119959	3.79E-05	9.79E-05	UP
RP1-35C2:	2.024336	-1.03146	0.250397	-4.11928	3.80E-05	9.82E-05	DOWN
RP11-315I	20.75631	0.595275	0.14457	4.117563	3.83E-05	9.89E-05	UP
RNF26	1758.081	0.29602	0.071894	4.117455	3.83E-05	9.90E-05	NOT
BMS1P20	55.61806	0.46855	0.113799	4.117346	3.83E-05	9.90E-05	NOT
LINC0119:	1.75081	2.943877	0.714997	4.117327	3.83E-05	9.90E-05	UP

RP11-31H	10.87823	0.662633	0.160941	4.117233	3.83E-05	9.90E-05	UP
ERLEC1	2432.42	0.277393	0.067384	4.116613	3.84E-05	9.93E-05	NOT
SLC25A5P:	1.375414	1.443797	0.350731	4.116532	3.85E-05	9.93E-05	UP
FTH1P3	32.82053	0.641905	0.155934	4.116517	3.85E-05	9.93E-05	UP
UQCRB	10481.59	0.549692	0.133535	4.116466	3.85E-05	9.93E-05	NOT
RGPD3	10.81727	-0.85665	0.208135	-4.11583	3.86E-05	9.96E-05	DOWN
NBPF13P	221.3342	-1.16828	0.283857	-4.11574	3.86E-05	9.96E-05	DOWN
RP11-423I	26.6659	0.487201	0.118378	4.115637	3.86E-05	9.97E-05	NOT
LINC0133(	5.750901	1.112076	0.270212	4.115574	3.86E-05	9.97E-05	UP
MAGEB6	1.971273	3.430492	0.83367	4.114929	3.87E-05	9.99E-05	UP
OSER1	1220.542	0.310142	0.075392	4.113724	3.89E-05	0.0001005	NOT
P2RY6	117.3972	0.998375	0.242748	4.112805	3.91E-05	0.0001009	UP
CD86	247.7722	-0.72995	0.177485	-4.11273	3.91E-05	0.0001009	DOWN
PSAPL1	5.128408	2.400549	0.583715	4.112538	3.91E-05	0.000101	UP
FICD	432.8218	0.526972	0.128141	4.11242	3.92E-05	0.000101	NOT
AP000344.	4.799145	1.914613	0.465572	4.112386	3.92E-05	0.000101	UP
FTH1P16	38.40546	0.674163	0.163947	4.112082	3.92E-05	0.0001011	UP
C9orf106	9.811259	1.272017	0.309352	4.111876	3.92E-05	0.0001012	UP
CNTNAP5	1.598868	2.95491	0.718697	4.111484	3.93E-05	0.0001014	UP
RP11-298I	1.884523	-1.67887	0.408513	-4.10972	3.96E-05	0.0001021	DOWN
RP5-856G.	1.670331	2.252997	0.548283	4.109187	3.97E-05	0.0001024	UP
DRAXIN	8.188783	0.926284	0.225419	4.109158	3.97E-05	0.0001024	UP
COX17P1	8.103028	-0.71343	0.173633	-4.10884	3.98E-05	0.0001025	DOWN
POU6F2	29.33392	-1.78499	0.434438	-4.10874	3.98E-05	0.0001025	DOWN
CTD-3099	15.11437	0.886238	0.215697	4.108712	3.98E-05	0.0001025	UP
SIX3	3.014348	2.913088	0.709003	4.10871	3.98E-05	0.0001025	UP
ST3GAL6-,	24.81562	-0.75796	0.184479	-4.10866	3.98E-05	0.0001025	DOWN
PAGE5	40.47034	1.720213	0.418686	4.108601	3.98E-05	0.0001026	UP
RP11-763I	2.625136	-1.18594	0.288651	-4.10855	3.98E-05	0.0001026	DOWN
NHS	40.49979	0.920944	0.224163	4.108369	3.98E-05	0.0001026	UP
BRIX1	868.788	0.387279	0.094268	4.108258	3.99E-05	0.0001027	NOT
ALAD	10489.93	-0.64275	0.156453	-4.10825	3.99E-05	0.0001027	DOWN
RILP	1052.021	-0.58322	0.141965	-4.10816	3.99E-05	0.0001027	NOT
PCDHGB2	61.62247	1.117981	0.272137	4.108158	3.99E-05	0.0001027	UP
QRFPR	3.871577	1.854611	0.451465	4.107986	3.99E-05	0.0001028	UP
CEP170	514.5015	0.493338	0.120102	4.107663	4.00E-05	0.0001029	NOT
ABI1	1703.112	0.289113	0.070388	4.107395	4.00E-05	0.000103	NOT
RP11-241I	1.651141	2.521911	0.61416	4.106279	4.02E-05	0.0001035	UP
SUCLA2	835.5579	-0.3823	0.093101	-4.10623	4.02E-05	0.0001035	NOT
ZNF98	3.983092	2.068695	0.503823	4.105994	4.03E-05	0.0001036	UP
ZBTB39	270.1317	0.460153	0.112075	4.105754	4.03E-05	0.0001037	NOT
TXNRD2	3110.244	-0.54376	0.132443	-4.1056	4.03E-05	0.0001038	NOT
SRGAP2B	44.487	0.628467	0.153077	4.105559	4.03E-05	0.0001038	UP
C5orf42	296.7839	0.540222	0.131583	4.105553	4.03E-05	0.0001038	NOT
RP11-747I	43.66611	1.282097	0.312305	4.105275	4.04E-05	0.0001039	UP
CHRNA7	3.086487	1.79023	0.436093	4.105153	4.04E-05	0.0001039	UP
TMOD2	237.4801	0.632233	0.154049	4.104092	4.06E-05	0.0001044	UP
MTRNR2L8	31.7635	-0.8349	0.20345	-4.10372	4.07E-05	0.0001045	DOWN
RP11-42O	4.723037	1.061122	0.258593	4.103439	4.07E-05	0.0001047	UP
RP11-579I	27.34242	0.849639	0.207061	4.103327	4.07E-05	0.0001047	UP
ZNF440	187.0671	0.401907	0.097949	4.103227	4.07E-05	0.0001047	NOT
GPR114	307.6732	1.274866	0.310753	4.102511	4.09E-05	0.0001051	UP

RP11-465I	72.93534	1.055421	0.257278	4.102256	4.09E-05	0.0001052	UP
FUNDC1	339.7238	0.371953	0.090672	4.102194	4.09E-05	0.0001052	NOT
RP11-545/	2.813044	2.016456	0.491716	4.100852	4.12E-05	0.0001058	UP
BAI1	27.04546	1.11459	0.271812	4.100594	4.12E-05	0.0001059	UP
POM121L4	1.295482	1.999575	0.487666	4.100296	4.13E-05	0.000106	UP
TMC6	990.7792	0.891246	0.217373	4.100083	4.13E-05	0.0001061	UP
RP11-73M	12.75652	0.59166	0.144323	4.099553	4.14E-05	0.0001063	UP
AC108676	9.540398	1.019313	0.248685	4.098809	4.15E-05	0.0001067	UP
LRRC20	1913.128	0.58218	0.142047	4.098493	4.16E-05	0.0001068	NOT
RP11-353I	7.673978	1.239688	0.302523	4.097832	4.17E-05	0.0001071	UP
GBF1	2946.478	0.296339	0.072329	4.097126	4.18E-05	0.0001074	NOT
CLPSL2	1.845831	2.756845	0.672945	4.096686	4.19E-05	0.0001076	UP
C5orf51	1088.514	0.317953	0.077615	4.096531	4.19E-05	0.0001077	NOT
RBM47	3440.093	-0.4757	0.11614	-4.09591	4.21E-05	0.000108	NOT
RP11-1379	7.90108	0.778732	0.19013	4.09578	4.21E-05	0.000108	UP
C8orf37-A	5.085157	1.196175	0.292063	4.095602	4.21E-05	0.0001081	UP
OTOP3	2.219623	3.171456	0.774364	4.095562	4.21E-05	0.0001081	UP
FAM222B	953.2036	0.350052	0.085478	4.095222	4.22E-05	0.0001082	NOT
TIMM9	830.7129	0.360821	0.088131	4.094153	4.24E-05	0.0001087	NOT
RP5-855D	5.324381	0.895284	0.218697	4.093711	4.25E-05	0.0001089	UP
EIF1B	1574.916	-0.30319	0.074076	-4.09293	4.26E-05	0.0001093	NOT
IGKV1D-8	10.09844	-1.74097	0.425388	-4.09266	4.26E-05	0.0001094	DOWN
HERC2P3	56.10361	1.123967	0.274635	4.092581	4.27E-05	0.0001094	UP
RP11-552I	1.636025	1.325067	0.323788	4.092396	4.27E-05	0.0001095	UP
CEP135	137.7049	0.545296	0.133255	4.092121	4.27E-05	0.0001096	NOT
AC005355	2.002146	1.529653	0.373869	4.09142	4.29E-05	0.0001099	UP
MED7	382.4647	0.312537	0.07639	4.091348	4.29E-05	0.00011	NOT
L1CAM	25.84814	-1.14242	0.279252	-4.091	4.30E-05	0.0001101	DOWN
IL27RA	305.4423	0.65141	0.159231	4.090977	4.30E-05	0.0001101	UP
GATA3	98.95857	-1.03326	0.252579	-4.09084	4.30E-05	0.0001102	DOWN
IGHM	4447.09	-1.50407	0.367727	-4.09018	4.31E-05	0.0001105	DOWN
RP11-337I	19.86577	0.567611	0.13878	4.090001	4.31E-05	0.0001106	NOT
SLC30A8	2.422597	2.504947	0.612475	4.08988	4.32E-05	0.0001106	UP
TGM2	16248.42	-0.72571	0.177458	-4.08948	4.32E-05	0.0001108	DOWN
RPL7AP10	1.699807	1.299869	0.317911	4.088785	4.34E-05	0.0001111	UP
AC105053	1.673736	-1.44856	0.354282	-4.08874	4.34E-05	0.0001111	DOWN
RP11-556I	4.999075	-3.07851	0.75294	-4.08865	4.34E-05	0.0001112	DOWN
ZNF165	140.401	0.720869	0.176313	4.088574	4.34E-05	0.0001112	UP
RP5-1142/	7.654007	0.903997	0.221121	4.08824	4.35E-05	0.0001113	UP
SMG7-AS1	15.8438	0.605948	0.148224	4.088067	4.35E-05	0.0001114	UP
IGKV2D-24	3.583371	-1.98509	0.485617	-4.08777	4.36E-05	0.0001115	DOWN
PCDHGB9I	4.911386	1.308826	0.320214	4.087344	4.36E-05	0.0001117	UP
C11orf98	120.9564	0.396318	0.096975	4.086815	4.37E-05	0.000112	NOT
GRID1	233.9047	1.40456	0.343699	4.086596	4.38E-05	0.0001121	UP
SCARB2	11417.39	-0.3432	0.083982	-4.08654	4.38E-05	0.0001121	NOT
LINC01511	1.845512	3.155287	0.772186	4.086173	4.39E-05	0.0001123	UP
TEX29	12.2115	1.100629	0.269362	4.086064	4.39E-05	0.0001123	UP
EIF3E	9810.368	0.520751	0.127446	4.086047	4.39E-05	0.0001123	NOT
DDX4	1.725831	1.94773	0.476725	4.085644	4.40E-05	0.0001125	UP
CYP4F8	1.77659	2.386834	0.584224	4.085479	4.40E-05	0.0001125	UP
PRKRIRP3	1.122255	2.293247	0.56136	4.085166	4.40E-05	0.0001127	UP
BCL11A	54.04737	1.17725	0.288187	4.085025	4.41E-05	0.0001127	UP

VAC14	1906.459	0.35293	0.086403	4.084692	4.41E-05	0.0001129	NOT
GAPDHP6!	24.37167	0.818135	0.2003	4.08455	4.42E-05	0.000113	UP
RP11-105!	1.558448	1.697778	0.415683	4.084314	4.42E-05	0.0001131	UP
PTGIS	492.6819	-1.25077	0.306271	-4.08387	4.43E-05	0.0001133	DOWN
MIOS	675.465	-0.31585	0.077344	-4.0837	4.43E-05	0.0001133	NOT
AC025016	2.526394	3.541374	0.867322	4.083113	4.44E-05	0.0001136	UP
AP000997.	2.256317	1.664834	0.407751	4.082973	4.45E-05	0.0001137	UP
ZNF599	146.9439	0.48078	0.117756	4.082839	4.45E-05	0.0001137	NOT
CTD-2334	1.845611	1.461811	0.358073	4.082435	4.46E-05	0.0001139	UP
SRGAP3	173.717	0.795539	0.194873	4.082348	4.46E-05	0.0001139	UP
CTD-2323	10.44475	0.600223	0.147065	4.081344	4.48E-05	0.0001144	UP
RPS4XP6	5.118125	0.838662	0.205497	4.081132	4.48E-05	0.0001145	UP
CPAMD8	79.5449	-0.72332	0.177256	-4.08065	4.49E-05	0.0001148	DOWN
HERC2P5	1.623839	2.34838	0.575506	4.080547	4.49E-05	0.0001148	UP
SPDYC	98.33278	-0.9999	0.245048	-4.08042	4.50E-05	0.0001148	DOWN
RP11-738I	1.079024	2.376355	0.58243	4.080068	4.50E-05	0.000115	UP
DNAJC2	1038.478	0.338701	0.083026	4.079429	4.51E-05	0.0001153	NOT
AC078941	2.229024	2.283282	0.559739	4.079191	4.52E-05	0.0001154	UP
UNQ6494	29.0455	-1.03139	0.252882	-4.07855	4.53E-05	0.0001157	DOWN
AC009120	1.960066	1.240605	0.304191	4.07837	4.54E-05	0.0001158	UP
CTD-2005	29.30576	1.59205	0.390435	4.07763	4.55E-05	0.0001162	UP
LMO7-AS:	6.842063	1.269461	0.311323	4.077629	4.55E-05	0.0001162	UP
MLXIP	2138.744	0.405542	0.099464	4.077257	4.56E-05	0.0001163	NOT
RP3-395M	259.5229	0.841004	0.206293	4.076752	4.57E-05	0.0001166	UP
LINC0106(	2.192919	2.817643	0.691157	4.076704	4.57E-05	0.0001166	UP
IGSF21	68.76047	0.770876	0.189093	4.076702	4.57E-05	0.0001166	UP
NEURL4	484.6559	0.311385	0.076391	4.07617	4.58E-05	0.0001168	NOT
RP11-426I	6.678549	0.909911	0.223254	4.075678	4.59E-05	0.0001171	UP
RAPH1	321.185	-0.7865	0.192981	-4.07555	4.59E-05	0.0001171	DOWN
LACTB	1546.191	-0.36424	0.089375	-4.0754	4.59E-05	0.0001172	NOT
MYEOV2	1210.634	0.577376	0.141696	4.074761	4.61E-05	0.0001175	NOT
C5orf66	26.25693	0.840645	0.206313	4.074612	4.61E-05	0.0001176	UP
PCDHGA5	24.59424	0.907782	0.222824	4.073991	4.62E-05	0.0001179	UP
MIR497HG	34.50517	-0.682	0.167412	-4.07376	4.63E-05	0.000118	DOWN
RUNDC3A	3.107974	1.297092	0.318422	4.073505	4.63E-05	0.0001181	UP
AC004160	10.23982	-1.66163	0.407913	-4.07349	4.63E-05	0.0001181	DOWN
OR2AT1P	1.832868	3.250545	0.797981	4.073463	4.63E-05	0.0001181	UP
WNK2	669.035	1.510334	0.370799	4.073185	4.64E-05	0.0001182	UP
RP11-510I	30.16346	-2.08046	0.510775	-4.07315	4.64E-05	0.0001182	DOWN
RPS10P2	8.209241	0.869866	0.213586	4.072669	4.65E-05	0.0001185	UP
RP11-536I	2.156483	1.395511	0.342656	4.072626	4.65E-05	0.0001185	UP
RP11-30L1	5.840523	0.987932	0.242586	4.072497	4.65E-05	0.0001185	UP
RP3-388M	5.796582	0.900463	0.22112	4.072279	4.66E-05	0.0001186	UP
CPSF4L	4.48212	1.304455	0.320337	4.072139	4.66E-05	0.0001187	UP
ZNF876P	15.42709	0.686666	0.16865	4.071543	4.67E-05	0.000119	UP
RP9	239.9258	0.418166	0.10271	4.071337	4.67E-05	0.0001191	NOT
HIST2H2BI	678.1042	0.746211	0.183294	4.071111	4.68E-05	0.0001192	UP
BTBD9	528.7314	0.383289	0.094153	4.070896	4.68E-05	0.0001193	NOT
RP11-101	3.607773	0.896668	0.220264	4.070887	4.68E-05	0.0001193	UP
FBXO27	539.4804	0.884308	0.217264	4.070206	4.70E-05	0.0001196	UP
NHLRC1	160.045	0.608858	0.149595	4.070037	4.70E-05	0.0001197	UP
C11orf68	1286.884	0.315297	0.077496	4.068551	4.73E-05	0.0001204	NOT

RP11-537I	2.189305	1.205603	0.296349	4.068181	4.74E-05	0.0001206	UP
SMCO3	39.52816	-0.77515	0.19057	-4.06753	4.75E-05	0.0001209	DOWN
XBP1P1	3.329449	1.034566	0.254348	4.067524	4.75E-05	0.0001209	UP
ACP2	4305.469	-0.39141	0.096228	-4.06748	4.75E-05	0.000121	NOT
METTL23	1071.277	0.373424	0.09181	4.067359	4.75E-05	0.000121	NOT
RP11-399I	1.765197	2.168221	0.533126	4.066995	4.76E-05	0.0001212	UP
FLJ46284	15.29074	0.617899	0.151942	4.066663	4.77E-05	0.0001213	UP
PDZK1	2785.056	0.705024	0.173387	4.066188	4.78E-05	0.0001216	UP
RAP1GAP	2374.894	0.864958	0.212734	4.065919	4.78E-05	0.0001217	UP
ELL	789.8356	-0.33482	0.082353	-4.06566	4.79E-05	0.0001218	NOT
SULT2B1	37.20931	1.405505	0.345729	4.065341	4.80E-05	0.000122	UP
RP11-481I	21.05551	2.883912	0.709394	4.065318	4.80E-05	0.000122	UP
MLST8	1693.668	0.375182	0.09229	4.065238	4.80E-05	0.000122	NOT
ZNF266	632.3308	0.557032	0.137027	4.065122	4.80E-05	0.0001221	NOT
RP11-6N1	1.40386	1.550453	0.38142	4.064954	4.80E-05	0.0001221	UP
SLC6A16	212.0338	-0.69409	0.170774	-4.06439	4.82E-05	0.0001224	DOWN
CFB	21021.16	-0.78002	0.191917	-4.06433	4.82E-05	0.0001224	DOWN
RTL1	2.17754	2.517346	0.61942	4.064041	4.82E-05	0.0001226	UP
RP11-338I	2.858583	0.973192	0.239477	4.063822	4.83E-05	0.0001227	UP
COMMD3	999.2763	0.376652	0.092704	4.06294	4.85E-05	0.0001231	NOT
SLC39A2	2.307642	-1.32977	0.327299	-4.06286	4.85E-05	0.0001232	DOWN
AF230666.	11.24597	0.649246	0.159803	4.062794	4.85E-05	0.0001232	UP
TLR8	65.80287	-0.9781	0.240757	-4.0626	4.85E-05	0.0001233	DOWN
UBQLN4P1	4.072106	0.923772	0.227387	4.062548	4.85E-05	0.0001233	UP
LINC00582	1.527889	-1.53739	0.378435	-4.0625	4.86E-05	0.0001233	DOWN
RP11-91P2	2.331181	1.123419	0.276551	4.062247	4.86E-05	0.0001234	UP
CTD-3035	18.48128	1.003412	0.247013	4.062176	4.86E-05	0.0001235	UP
STRIP1	784.4054	0.362685	0.089287	4.061995	4.87E-05	0.0001236	NOT
SLC20A2	2660.576	-0.56196	0.138346	-4.06197	4.87E-05	0.0001236	NOT
SCARNA21	1.878274	1.651988	0.406699	4.061946	4.87E-05	0.0001236	UP
SACS	267.9618	0.72627	0.178803	4.061847	4.87E-05	0.0001236	UP
RP11-415I	2.615865	1.249842	0.307728	4.061515	4.88E-05	0.0001238	UP
STARD6	1.256813	2.138344	0.526515	4.06132	4.88E-05	0.0001239	UP
TUBA1A	1379.033	0.775244	0.190889	4.061234	4.88E-05	0.0001239	UP
GCGR	2479.278	-1.50366	0.370302	-4.06062	4.89E-05	0.0001242	DOWN
LSM7	1328.566	0.567168	0.139676	4.060598	4.89E-05	0.0001242	NOT
RP11-554I	2.522829	-0.97132	0.239217	-4.06041	4.90E-05	0.0001243	DOWN
COQ5	1836.041	-0.34854	0.085842	-4.06025	4.90E-05	0.0001244	NOT
CYSRT1	45.92683	0.78481	0.193294	4.060193	4.90E-05	0.0001244	UP
RP11-91K9	194.3697	-0.8587	0.211516	-4.05972	4.91E-05	0.0001246	DOWN
CTA-363E	3.93444	2.133631	0.525614	4.059315	4.92E-05	0.0001248	UP
P4HA3	50.23174	-0.6903	0.170063	-4.05911	4.93E-05	0.0001249	DOWN
RIMKLA	30.3867	1.085337	0.267404	4.058799	4.93E-05	0.0001251	UP
TCF7	786.0632	0.81813	0.201586	4.058474	4.94E-05	0.0001252	UP
NRXN3	122.8093	1.301381	0.320662	4.058421	4.94E-05	0.0001253	UP
RP4-798A	4.210686	1.221698	0.30104	4.058252	4.94E-05	0.0001253	UP
RP11-361I	3.904026	-0.86602	0.2134	-4.05818	4.95E-05	0.0001254	DOWN
DCLK1	50.39495	1.031054	0.254107	4.057557	4.96E-05	0.0001257	UP
LINC01338	1.741909	2.995611	0.738281	4.05755	4.96E-05	0.0001257	UP
GXYLT1P6	7.119214	-1.69352	0.417483	-4.0565	4.98E-05	0.0001262	DOWN
TMEM169	115.5119	0.686812	0.169312	4.056488	4.98E-05	0.0001262	UP
MPP7	288.6302	0.769232	0.189644	4.056187	4.99E-05	0.0001264	UP

SART1	2898.881	0.320097	0.078918	4.05605	4.99E-05	0.0001265	NOT
USPL1	556.4242	-0.33705	0.08311	-4.0555	5.00E-05	0.0001267	NOT
CA10	1.440851	2.083956	0.513887	4.055281	5.01E-05	0.0001268	UP
RP11-47I2	6.187564	1.494803	0.368625	4.055077	5.01E-05	0.0001269	UP
AC114730	6.76208	0.889261	0.219298	4.055034	5.01E-05	0.000127	UP
ZNF497	76.57491	0.439862	0.108491	4.054356	5.03E-05	0.0001273	NOT
RP11-305I	19.36248	0.855913	0.211133	4.053899	5.04E-05	0.0001276	UP
DHX30	3000.919	0.231853	0.057203	4.053151	5.05E-05	0.0001279	NOT
RP11-690I	2.047	1.45424	0.358824	4.052799	5.06E-05	0.0001281	UP
AC138035	6.894478	0.913254	0.225339	4.052797	5.06E-05	0.0001281	UP
CDH3	21.77528	1.206481	0.297706	4.052597	5.07E-05	0.0001282	UP
VPS52	2100.171	0.35338	0.0872	4.052513	5.07E-05	0.0001283	NOT
RAPGEFL1	317.8063	0.638269	0.1575	4.052488	5.07E-05	0.0001283	UP
TIMM17B	1756.412	0.394606	0.097386	4.051961	5.08E-05	0.0001285	NOT
LINC0030	3.82529	1.610766	0.397572	4.05151	5.09E-05	0.0001288	UP
SEPSECS-	123.7677	0.56042	0.138324	4.051493	5.09E-05	0.0001288	NOT
THAP3	454.0674	0.420561	0.103811	4.051224	5.10E-05	0.0001289	NOT
CRYBB2P1	279.9882	0.521373	0.128697	4.051163	5.10E-05	0.0001289	NOT
FASTKD3	382.1582	0.268356	0.066248	4.050755	5.11E-05	0.0001291	NOT
RP11-629I	8.397583	-0.76846	0.18972	-4.05049	5.11E-05	0.0001293	DOWN
HRNR	5.971822	1.403828	0.346633	4.049893	5.12E-05	0.0001296	UP
MIR3125	1.359556	2.623014	0.647685	4.049828	5.13E-05	0.0001296	UP
CYBB	964.801	-0.87701	0.216563	-4.04966	5.13E-05	0.0001297	DOWN
PALLD	2500.276	0.756197	0.186743	4.049399	5.13E-05	0.0001298	UP
PSD3	1684.914	-0.74359	0.183632	-4.04936	5.14E-05	0.0001298	DOWN
ZNF263	970.2244	0.244715	0.060435	4.049239	5.14E-05	0.0001299	NOT
RP11-295I	3.856058	1.022521	0.252527	4.049148	5.14E-05	0.0001299	UP
DDX42	3389.031	0.222371	0.05492	4.048968	5.14E-05	0.00013	NOT
FAM195B	3499.113	0.629933	0.155582	4.048883	5.15E-05	0.0001301	UP
CHCHD3P	6.213512	0.703326	0.173739	4.04818	5.16E-05	0.0001304	UP
FOCAD-A	1.570209	1.407356	0.34766	4.048082	5.16E-05	0.0001305	UP
SYNGAP1	224.2936	0.555997	0.137351	4.048003	5.17E-05	0.0001305	NOT
RWDD4P2	30.58603	-0.40776	0.10074	-4.04771	5.17E-05	0.0001307	NOT
KLF12	1255.709	-0.61001	0.150733	-4.04694	5.19E-05	0.0001311	DOWN
LINC0038	2.264795	3.510842	0.867545	4.04687	5.19E-05	0.0001311	UP
CREBRF	924.2664	-0.40342	0.099694	-4.04655	5.20E-05	0.0001313	NOT
AMZ2P1	221.2464	0.385765	0.095339	4.046256	5.20E-05	0.0001314	NOT
PLCB2	428.0911	-0.6641	0.164137	-4.046	5.21E-05	0.0001316	DOWN
BCRP2	1.368597	2.333861	0.576877	4.045681	5.22E-05	0.0001317	UP
ZNF302	818.6201	0.402693	0.099539	4.045571	5.22E-05	0.0001318	NOT
IGFL4	1.334218	1.753445	0.433445	4.045368	5.22E-05	0.0001319	UP
GNB4	373.8315	-0.61178	0.151237	-4.04515	5.23E-05	0.000132	DOWN
NDUFAF6	816.5141	0.455743	0.112668	4.045009	5.23E-05	0.0001321	NOT
SPACA6P	59.37923	0.968414	0.239427	4.044718	5.24E-05	0.0001322	UP
SLC35E3	806.2016	0.29541	0.073043	4.044336	5.25E-05	0.0001324	NOT
RP11-529I	7.332655	0.698597	0.172744	4.044115	5.25E-05	0.0001325	UP
CYP7B1	420.7611	-0.76028	0.188026	-4.04348	5.27E-05	0.0001329	DOWN
SEC23B	2415.531	0.328746	0.081317	4.042794	5.28E-05	0.0001332	NOT
CTA-384D	18.25667	0.936172	0.231575	4.042626	5.29E-05	0.0001333	UP
CHRAC1	1224.518	0.394721	0.097646	4.042346	5.29E-05	0.0001335	NOT
LILRA4	15.09319	-1.05482	0.260964	-4.04201	5.30E-05	0.0001337	DOWN
RP5-1050I	3.899818	0.985918	0.243929	4.041827	5.30E-05	0.0001337	UP

MIF	2182.281	0.656501	0.162434	4.041637	5.31E-05	0.0001338	UP
ZNF284	60.71194	0.560326	0.13864	4.041588	5.31E-05	0.0001339	NOT
KANSL2	646.1618	0.312338	0.077289	4.041155	5.32E-05	0.0001341	NOT
SMARCE1I	2.75398	1.592478	0.394073	4.04107	5.32E-05	0.0001341	UP
SLC39A5	6896.401	-1.13312	0.280431	-4.04063	5.33E-05	0.0001344	DOWN
DIAPH1	9500.415	-0.3911	0.096813	-4.03977	5.35E-05	0.0001349	NOT
RGS19	382.6917	0.550784	0.13636	4.039206	5.36E-05	0.0001352	NOT
SPINT1	1289.112	1.447373	0.358331	4.039203	5.36E-05	0.0001352	UP
RP1-159M	2.324944	1.032192	0.255576	4.038685	5.38E-05	0.0001354	UP
TMEM191	6.462222	1.318583	0.326498	4.038561	5.38E-05	0.0001355	UP
EXOC6B	590.8634	0.424727	0.105179	4.038129	5.39E-05	0.0001357	NOT
RP11-251I	14.27866	-0.83556	0.206924	-4.03798	5.39E-05	0.0001358	DOWN
SENP3	573.1625	0.35717	0.088457	4.037792	5.40E-05	0.0001359	NOT
ZNRD1	663.7225	0.476886	0.118123	4.037217	5.41E-05	0.0001362	NOT
ANO8	443.3013	0.505017	0.125091	4.037189	5.41E-05	0.0001362	NOT
CCSER2	1220.989	-0.34666	0.085869	-4.03711	5.41E-05	0.0001363	NOT
CTD-3014	10.63015	0.81157	0.20104	4.036853	5.42E-05	0.0001364	UP
AC084219	4.126774	1.145188	0.283684	4.03684	5.42E-05	0.0001364	UP
SAV1	828.7223	-0.48014	0.118946	-4.03668	5.42E-05	0.0001365	NOT
KCNJ2	110.2569	0.659733	0.163461	4.036018	5.44E-05	0.0001369	UP
FASTKD2	1169.593	-0.26975	0.066839	-4.03578	5.44E-05	0.000137	NOT
MPO	8.733398	-1.10153	0.272987	-4.0351	5.46E-05	0.0001374	DOWN
RP11-982I	1.645403	1.819982	0.451039	4.035092	5.46E-05	0.0001374	UP
RPPH1	2.202285	-1.73063	0.428899	-4.03505	5.46E-05	0.0001374	DOWN
B4GALT6	164.2811	0.663562	0.164454	4.034942	5.46E-05	0.0001374	UP
RP4-734P1	1.786503	1.255997	0.311281	4.034933	5.46E-05	0.0001374	UP
CALB2	8.628333	1.588929	0.393795	4.034919	5.46E-05	0.0001374	UP
AC091729	8.88879	-2.17043	0.537934	-4.03475	5.47E-05	0.0001375	DOWN
IL13RA2	128.7108	-1.4826	0.367464	-4.03468	5.47E-05	0.0001375	DOWN
TSC22D4	1513.747	0.408954	0.101361	4.034628	5.47E-05	0.0001375	NOT
RAP1GAP2	227.7204	0.969257	0.240236	4.034596	5.47E-05	0.0001375	UP
RP11-569A	4.77003	1.085959	0.269171	4.034452	5.47E-05	0.0001376	UP
SRPK2	935.7027	0.389447	0.096534	4.034292	5.48E-05	0.0001377	NOT
CDK2	887.8501	0.448149	0.111091	4.034072	5.48E-05	0.0001378	NOT
CTD-2012	2.288611	1.350975	0.334934	4.033561	5.49E-05	0.0001381	UP
NFIA-AS2	2.923992	1.636619	0.405761	4.033456	5.50E-05	0.0001382	UP
RP11-110I	6.953956	0.885674	0.21963	4.03257	5.52E-05	0.0001387	UP
IDH3G	2204.562	0.405972	0.100674	4.032556	5.52E-05	0.0001387	NOT
CD24P4	1.649111	1.812623	0.449509	4.032449	5.52E-05	0.0001387	UP
SERPINA5	25669.32	-0.88886	0.22043	-4.03239	5.52E-05	0.0001387	DOWN
RP11-513(	2.562335	1.476316	0.366175	4.031717	5.54E-05	0.0001391	UP
RNU6-611	1.115428	1.520072	0.37707	4.031274	5.55E-05	0.0001394	UP
RP11-289I	5.677974	-2.33858	0.580157	-4.03095	5.56E-05	0.0001395	DOWN
HS3ST3A1	25.81088	-1.09402	0.271411	-4.03085	5.56E-05	0.0001396	DOWN
CYP8B1	24096.14	-1.44301	0.358027	-4.03046	5.57E-05	0.0001398	DOWN
RP5-1142	1.397167	1.97212	0.489307	4.030432	5.57E-05	0.0001398	UP
RP11-99L1	1.324611	2.347442	0.582433	4.030408	5.57E-05	0.0001398	UP
TMEM91	176.3418	0.750366	0.186189	4.03013	5.57E-05	0.00014	UP
ZNF488	6.737566	1.494805	0.370912	4.030084	5.58E-05	0.00014	UP
NREP	1764.361	0.712668	0.176847	4.029861	5.58E-05	0.0001401	UP
RNA5SP20	1.120575	1.527417	0.37904	4.0297	5.58E-05	0.0001402	UP
JOSD2	895.081	0.551106	0.136763	4.029641	5.59E-05	0.0001402	NOT

GBE1	3606.723	-0.59488	0.147627	-4.02962	5.59E-05	0.0001402	DOWN
LINC0157(	2.919611	2.870027	0.712239	4.029582	5.59E-05	0.0001402	UP
C12orf79	19.07247	1.017328	0.252467	4.029545	5.59E-05	0.0001402	UP
AAED1	421.7957	-0.42526	0.105537	-4.02947	5.59E-05	0.0001403	NOT
OLFM4	18.32676	2.188501	0.543234	4.028653	5.61E-05	0.0001407	UP
AC018766	2.425976	1.13376	0.281463	4.028095	5.62E-05	0.000141	UP
RP11-108I	55.6595	0.739104	0.183487	4.028088	5.62E-05	0.000141	UP
ZNF396	69.21593	0.445204	0.110525	4.028085	5.62E-05	0.000141	NOT
ZNF326	824.9241	-0.30176	0.074918	-4.0279	5.63E-05	0.0001411	NOT
PRPH2	12.83435	0.958268	0.237935	4.027433	5.64E-05	0.0001414	UP
PATL1	1973.731	-0.34522	0.085719	-4.02739	5.64E-05	0.0001414	NOT
PRR15	65.0261	1.624683	0.403412	4.027352	5.64E-05	0.0001414	UP
RP11-33N	4.974942	0.942413	0.234007	4.027295	5.64E-05	0.0001414	UP
WDR73	105.8546	0.37534	0.0932	4.027266	5.64E-05	0.0001415	NOT
GLYATL3	5.43355	-1.58445	0.393442	-4.02715	5.65E-05	0.0001415	DOWN
PRKCQ-AS	33.30209	-0.85676	0.212765	-4.02681	5.65E-05	0.0001417	DOWN
SERHL2	59.35841	0.883078	0.219306	4.026687	5.66E-05	0.0001418	UP
NCLP1	1.781495	1.321057	0.328107	4.026297	5.67E-05	0.000142	UP
CTD-2619	1.250716	1.98544	0.49316	4.025951	5.67E-05	0.0001422	UP
CLEC1A	76.55914	-0.69302	0.172151	-4.02562	5.68E-05	0.0001424	DOWN
BPNT1	1496.726	0.325708	0.080911	4.025497	5.69E-05	0.0001424	NOT
SLC25A15	1973.637	-0.8912	0.221399	-4.02533	5.69E-05	0.0001425	DOWN
VTA1	1438.597	-0.33155	0.08237	-4.02517	5.69E-05	0.0001426	NOT
APLP2	28405.99	-0.33846	0.084088	-4.02503	5.70E-05	0.0001427	NOT
SMC5	1139.546	-0.38541	0.095766	-4.02452	5.71E-05	0.000143	NOT
C11orf49	401.0849	0.598474	0.148712	4.024389	5.71E-05	0.000143	UP
CLCN3P1	2.819706	2.110696	0.524526	4.024004	5.72E-05	0.0001433	UP
RAB9B	14.56959	1.047421	0.260318	4.023624	5.73E-05	0.0001435	UP
S100A2	44.60432	1.382978	0.343727	4.023479	5.73E-05	0.0001436	UP
ARX	2.129759	2.601473	0.646592	4.023362	5.74E-05	0.0001436	UP
SESN1	1112.894	-0.51593	0.128242	-4.02309	5.74E-05	0.0001438	NOT
NCKAP1L	657.4852	-0.73614	0.182986	-4.02292	5.75E-05	0.0001439	DOWN
CTC-325J:	1.130088	1.494598	0.371525	4.022877	5.75E-05	0.0001439	UP
NDUFS6	4001.875	0.56544	0.140562	4.022695	5.75E-05	0.000144	NOT
ROBO3	166.0042	0.747093	0.185724	4.022599	5.76E-05	0.000144	UP
EBAG9P1	2.367062	1.183869	0.294313	4.022484	5.76E-05	0.0001441	UP
C9orf16	1553.423	0.660833	0.164298	4.022156	5.77E-05	0.0001443	UP
CXorf21	43.1691	-0.76703	0.190727	-4.02163	5.78E-05	0.0001446	DOWN
ECI2	9305.28	-0.52774	0.131228	-4.02151	5.78E-05	0.0001446	NOT
ZNF37A	608.0886	0.398682	0.099146	4.021183	5.79E-05	0.0001448	NOT
DNAJC18	95.04791	0.537363	0.133633	4.021179	5.79E-05	0.0001448	NOT
ZSCAN21	258.4513	0.348792	0.086745	4.020878	5.80E-05	0.000145	NOT
PRKCI	728.8566	0.370942	0.092261	4.020578	5.81E-05	0.0001451	NOT
RP11-345I	14.60317	0.959026	0.238535	4.020485	5.81E-05	0.0001452	UP
CTD-2054	8.70806	1.373535	0.341662	4.020163	5.82E-05	0.0001454	UP
RP11-127	17.35617	-0.77127	0.191898	-4.01915	5.84E-05	0.000146	DOWN
RP11-437I	2.578649	-1.63508	0.406846	-4.01893	5.85E-05	0.0001461	DOWN
RP3-414A	48.07498	-0.73879	0.183843	-4.01858	5.85E-05	0.0001463	DOWN
RP11-63E(	1.572592	2.545349	0.633516	4.01781	5.87E-05	0.0001468	UP
RP11-56M	5.754497	-0.75453	0.187797	-4.01778	5.88E-05	0.0001468	DOWN
CH17-260	1.50467	1.349717	0.335947	4.017652	5.88E-05	0.0001469	UP
PWP1	1611.183	0.239173	0.059534	4.017413	5.88E-05	0.000147	NOT

TDGF1P3	1.603067	2.49757	0.621717	4.017211	5.89E-05	0.0001471	UP
TMIGD2	15.11348	-0.83428	0.207679	-4.01716	5.89E-05	0.0001471	DOWN
PLEKHH3	974.073	0.469043	0.116765	4.017	5.89E-05	0.0001472	NOT
AC012487	10.28084	0.905636	0.225468	4.016695	5.90E-05	0.0001474	UP
CTA-280A	1.174916	2.638301	0.656942	4.016033	5.92E-05	0.0001478	UP
VOPP1	1148.286	0.441367	0.109911	4.015669	5.93E-05	0.000148	NOT
RP3-327A	2.455637	1.166675	0.290552	4.015372	5.94E-05	0.0001482	UP
IGHD	137.44	-1.57917	0.393293	-4.01526	5.94E-05	0.0001482	DOWN
RP3-508I1	23.6437	0.749826	0.186754	4.015043	5.94E-05	0.0001484	UP
KRCC1	1212.857	-0.40523	0.100935	-4.01477	5.95E-05	0.0001485	NOT
AC008592	14.89475	-1.26043	0.313962	-4.01461	5.95E-05	0.0001486	DOWN
NLRC5	953.5208	0.674533	0.168023	4.014542	5.96E-05	0.0001486	UP
KIF5B	3604.961	0.329787	0.082162	4.013854	5.97E-05	0.0001491	NOT
CLVS2	12.24714	2.927859	0.729451	4.013786	5.98E-05	0.0001491	UP
MSRB1	6171.002	-0.53654	0.133691	-4.01332	5.99E-05	0.0001494	NOT
RP11-760I	2.233366	2.753632	0.686177	4.013004	6.00E-05	0.0001496	UP
GFRA2	108.5766	-0.78052	0.194503	-4.01292	6.00E-05	0.0001496	DOWN
RTN4RL2	1078.747	0.74104	0.184669	4.0128	6.00E-05	0.0001497	UP
CHRNE	108.872	-0.76502	0.190647	-4.01278	6.00E-05	0.0001497	DOWN
BHMT2	14041.34	-0.77439	0.193021	-4.01194	6.02E-05	0.0001502	DOWN
TMEM39A	898.2957	0.283369	0.070643	4.011265	6.04E-05	0.0001506	NOT
ACBD3	2038.165	0.3395	0.084643	4.010948	6.05E-05	0.0001508	NOT
RP11-472I	2.82479	1.99025	0.496206	4.010939	6.05E-05	0.0001508	UP
PIAS4	960.1036	0.327315	0.081609	4.010781	6.05E-05	0.0001509	NOT
MRPL17	1497.723	0.410241	0.102286	4.010723	6.05E-05	0.0001509	NOT
RP11-302I	3.847676	1.038011	0.25881	4.010709	6.05E-05	0.0001509	UP
GAGE2A	3.110604	4.029998	1.004964	4.010091	6.07E-05	0.0001513	UP
TLR9	1.772514	1.308419	0.326289	4.010005	6.07E-05	0.0001513	UP
C2orf81	52.25895	0.712541	0.177704	4.009712	6.08E-05	0.0001515	UP
ERVMER61	2.78199	3.559804	0.887797	4.009706	6.08E-05	0.0001515	UP
SLC17A9	3295.117	0.738278	0.184131	4.009523	6.08E-05	0.0001516	UP
CTSK	410.2199	0.759385	0.189449	4.008377	6.11E-05	0.0001523	UP
AKAP6	230.135	-0.74147	0.184979	-4.00838	6.11E-05	0.0001523	DOWN
SMOC2	422.6849	0.973519	0.242896	4.007972	6.12E-05	0.0001525	UP
LINC00327	3.396312	1.517924	0.378762	4.007597	6.13E-05	0.0001528	UP
ISLR2	31.73883	0.941826	0.235012	4.007574	6.13E-05	0.0001528	UP
RP13-941I	3.939009	0.977328	0.243885	4.007322	6.14E-05	0.0001529	UP
FGFR4	8008.591	0.668918	0.166931	4.007161	6.15E-05	0.000153	UP
LINC00526	344.4976	-0.67231	0.167795	-4.0067	6.16E-05	0.0001533	DOWN
SLC16A13	1584.317	-0.72073	0.179891	-4.00649	6.16E-05	0.0001534	DOWN
XPA	540.3786	-0.30516	0.076166	-4.00644	6.16E-05	0.0001534	NOT
RP11-752(	12.35773	0.750327	0.187291	4.006206	6.17E-05	0.0001536	UP
RAPGEF2	1791.114	-0.49209	0.122835	-4.00613	6.17E-05	0.0001536	NOT
VGLL4	627.3255	0.52607	0.131318	4.006089	6.17E-05	0.0001536	NOT
IMPA1P	1.461007	1.78378	0.44528	4.005978	6.18E-05	0.0001537	UP
CTD-3220	8.103117	0.834826	0.208453	4.004866	6.21E-05	0.0001544	UP
ACAD10	2333.838	-0.39023	0.097449	-4.00444	6.22E-05	0.0001546	NOT
KMT2E-AS	181.6977	0.602492	0.15046	4.004346	6.22E-05	0.0001547	UP
N4BP2	728.6062	-0.51668	0.129034	-4.00424	6.22E-05	0.0001547	NOT
ATP1B1	12983.85	0.610554	0.152486	4.003996	6.23E-05	0.0001549	UP
LHPP	2477.403	-0.67154	0.16772	-4.00396	6.23E-05	0.0001549	DOWN
VTN	285649.9	-0.73957	0.184731	-4.00352	6.24E-05	0.0001552	DOWN

AMZ2	1344.195	0.304051	0.075947	4.003463	6.24E-05	0.0001552	NOT
CCDC87	22.96066	0.792415	0.197934	4.003425	6.24E-05	0.0001552	UP
ELAVL1	2556.183	0.272615	0.068102	4.003055	6.25E-05	0.0001554	NOT
SLC43A2	859.0394	0.657409	0.164244	4.002648	6.26E-05	0.0001557	UP
RP11-307(	26.75343	0.574118	0.143446	4.002324	6.27E-05	0.0001559	NOT
WDR89	466.9238	-0.28581	0.071413	-4.00221	6.28E-05	0.0001559	NOT
TTPAL	1893.479	-0.71141	0.177759	-4.00212	6.28E-05	0.000156	DOWN
RPL7AP66	15.32791	0.737051	0.184172	4.001974	6.28E-05	0.0001561	UP
BRDT	1.934386	2.340862	0.584994	4.001516	6.29E-05	0.0001564	UP
AK4	5705.623	-0.67096	0.167677	-4.00151	6.29E-05	0.0001564	DOWN
RP11-113(	1.801202	-1.34739	0.336728	-4.00141	6.30E-05	0.0001564	DOWN
ENO2	269.7562	0.992105	0.247986	4.000643	6.32E-05	0.0001569	UP
PVRL4	31.64529	1.221211	0.30532	3.999773	6.34E-05	0.0001575	UP
CTD-2280	9.140589	1.588629	0.397218	3.999391	6.35E-05	0.0001577	UP
ZNF527	152.0206	0.375102	0.093792	3.999296	6.35E-05	0.0001578	NOT
RP11-445I	6.763439	0.983023	0.245818	3.998993	6.36E-05	0.0001579	UP
SLC9A1	498.1118	0.6258	0.156514	3.998361	6.38E-05	0.0001584	UP
MFSD3	2189.036	0.676406	0.169215	3.997308	6.41E-05	0.000159	UP
MIEN1	1966.346	0.424043	0.106087	3.997109	6.41E-05	0.0001592	NOT
AC008069	3.85482	1.669944	0.417789	3.997105	6.41E-05	0.0001592	UP
TMEM51-	24.84478	1.310634	0.327945	3.996506	6.43E-05	0.0001595	UP
RP11-536(	2.053908	1.561979	0.390863	3.996227	6.44E-05	0.0001597	UP
SLX4IP	335.6966	0.357506	0.089466	3.996	6.44E-05	0.0001599	NOT
BCS1L	988.2963	0.396405	0.099203	3.995909	6.44E-05	0.0001599	NOT
ZNF616	274.3145	0.351764	0.088061	3.994559	6.48E-05	0.0001608	NOT
KCNMB1	45.30339	0.705656	0.176665	3.994318	6.49E-05	0.0001609	UP
BAATP1	30.46404	1.113272	0.27872	3.994236	6.49E-05	0.000161	UP
PCNPP1	4.372997	0.772548	0.193435	3.993843	6.50E-05	0.0001612	UP
SLC9C2	8.361779	1.364329	0.341687	3.992917	6.53E-05	0.0001619	UP
RP11-206I	3.947107	-2.91801	0.730824	-3.99277	6.53E-05	0.0001619	DOWN
AC011738	4.008463	1.538368	0.385293	3.992727	6.53E-05	0.000162	UP
PCSK9	2371.797	0.801225	0.20068	3.992558	6.54E-05	0.0001621	UP
MS4A8	12.21261	2.553597	0.639738	3.991629	6.56E-05	0.0001627	UP
OCIAD2	2794.023	-0.69327	0.173683	-3.99156	6.56E-05	0.0001627	DOWN
RP11-128I	1.463439	1.514348	0.379441	3.990995	6.58E-05	0.0001631	UP
CTC-455F.	3.004446	1.521798	0.38131	3.99097	6.58E-05	0.0001631	UP
LINC0085i	99.47669	0.767665	0.192363	3.990704	6.59E-05	0.0001633	UP
AC019129	2.67647	1.932996	0.484385	3.990617	6.59E-05	0.0001633	UP
IRAK3	159.1027	-0.74476	0.186659	-3.98996	6.61E-05	0.0001637	DOWN
ZNF501	55.58468	0.715822	0.179419	3.989668	6.62E-05	0.0001639	UP
NAT6	396.8893	0.505182	0.126624	3.989628	6.62E-05	0.0001639	NOT
CTD-2574	12.03951	0.836371	0.209647	3.989417	6.62E-05	0.0001641	UP
RP11-127	52.88828	0.517251	0.129662	3.989222	6.63E-05	0.0001642	NOT
UBA2	2603.888	0.352435	0.088348	3.989175	6.63E-05	0.0001642	NOT
LL22NC03	3.234566	1.206472	0.302447	3.989036	6.63E-05	0.0001643	UP
B3GNT2	833.5053	-0.4022	0.100835	-3.98874	6.64E-05	0.0001645	NOT
ECD	1029.898	0.233736	0.058601	3.988596	6.65E-05	0.0001646	NOT
AC147651	24.85324	0.822922	0.206323	3.988503	6.65E-05	0.0001646	UP
NPAS2	850.2371	0.635573	0.159357	3.988365	6.65E-05	0.0001647	UP
DAPK1	2708.549	-0.56005	0.140425	-3.98827	6.66E-05	0.0001648	NOT
EPHA1-AS	11.89444	1.342988	0.336746	3.988129	6.66E-05	0.0001648	UP
MICALL2	970.7422	0.61387	0.153933	3.987892	6.67E-05	0.000165	UP

GK	1774.903	-0.71002	0.178054	-3.98765	6.67E-05	0.0001651	DOWN
POLRMT	3099.131	0.422366	0.105927	3.987331	6.68E-05	0.0001653	NOT
CTD-3216	5.426048	0.828434	0.207767	3.987325	6.68E-05	0.0001653	UP
METTL1	616.2168	0.459746	0.115302	3.987323	6.68E-05	0.0001653	NOT
SYN2	7.168661	1.241298	0.311345	3.986895	6.69E-05	0.0001656	UP
SNAP25-A	79.68149	1.129108	0.283207	3.986871	6.70E-05	0.0001656	UP
C16orf93	11.26291	0.774777	0.194352	3.98647	6.71E-05	0.0001659	UP
AIMP1	1737.03	0.309933	0.077751	3.986242	6.71E-05	0.000166	NOT
AC000068	15.78808	0.666977	0.16732	3.986238	6.71E-05	0.000166	UP
TRAPPC9	1606.507	0.395175	0.099143	3.985904	6.72E-05	0.0001662	NOT
NCK2	1020.441	0.733391	0.183998	3.985852	6.72E-05	0.0001662	UP
ZNF137P	25.08354	0.933808	0.234291	3.985684	6.73E-05	0.0001663	UP
ZNF425	76.5644	0.591445	0.148394	3.985629	6.73E-05	0.0001664	UP
RNF216P1	572.7662	0.364011	0.091334	3.985482	6.73E-05	0.0001665	NOT
SV2A	92.27566	0.980698	0.246078	3.985312	6.74E-05	0.0001666	UP
FGD5P1	2.972179	2.236207	0.561116	3.985286	6.74E-05	0.0001666	UP
CA13	185.3295	-0.7316	0.183576	-3.98528	6.74E-05	0.0001666	DOWN
IGHV3-11	95.76626	-1.61832	0.406079	-3.98524	6.74E-05	0.0001666	DOWN
PWAR6	174.4475	1.056164	0.26502	3.985228	6.74E-05	0.0001666	UP
DBI	9272.856	-0.44617	0.11196	-3.98505	6.75E-05	0.0001667	NOT
AC005540	1.86086	1.493121	0.374684	3.985013	6.75E-05	0.0001667	UP
AC009120	4.781566	0.956102	0.23993	3.984922	6.75E-05	0.0001667	UP
KCNH5	2.755099	3.303032	0.828978	3.984464	6.76E-05	0.000167	UP
KIAA1549L	19.6751	1.254564	0.314874	3.984333	6.77E-05	0.0001671	UP
CRYAB	599.308	1.089444	0.27345	3.984075	6.77E-05	0.0001673	UP
GSDMB	809.7075	0.684531	0.171824	3.983902	6.78E-05	0.0001674	UP
MAPK9	1425.952	0.274016	0.068789	3.98345	6.79E-05	0.0001677	NOT
ACTL6B	5.795605	1.150765	0.288926	3.982902	6.81E-05	0.0001681	UP
RAPGEF4-/	2.512434	1.529686	0.384199	3.981498	6.85E-05	0.000169	UP
CYP2D7	877.934	-1.10245	0.276908	-3.98127	6.85E-05	0.0001692	DOWN
NPIPB5	32.23742	0.825211	0.207286	3.981029	6.86E-05	0.0001694	UP
WT1-AS	6.794792	1.841869	0.46268	3.980871	6.87E-05	0.0001694	UP
PAH	40669.19	-0.89826	0.225647	-3.98081	6.87E-05	0.0001695	DOWN
ACSM5P1	48.65958	-1.11803	0.280879	-3.98047	6.88E-05	0.0001697	DOWN
BOK	4992.293	-0.67356	0.169222	-3.98033	6.88E-05	0.0001698	DOWN
TAT	58954.66	-1.35191	0.339659	-3.9802	6.89E-05	0.0001699	DOWN
ALDH18A1	2590.967	0.446969	0.112303	3.980021	6.89E-05	0.00017	NOT
WDR60	323.3739	0.43384	0.10901	3.979813	6.90E-05	0.0001701	NOT
RP11-809(	1.451671	-1.64317	0.413042	-3.97822	6.94E-05	0.0001712	DOWN
RP11-501(	3.175402	1.139874	0.286536	3.978116	6.95E-05	0.0001713	UP
TTLL5	455.4118	0.333991	0.083958	3.978075	6.95E-05	0.0001713	NOT
AC106869	9.296856	1.687955	0.424318	3.978043	6.95E-05	0.0001713	UP
MAP4K3	1100.697	0.434081	0.109124	3.977859	6.95E-05	0.0001714	NOT
WRNIP1	1900.312	0.299579	0.075318	3.977514	6.96E-05	0.0001717	NOT
DDX31	429.1012	0.29418	0.073972	3.976923	6.98E-05	0.0001721	NOT
RHOXF1	4.358425	1.430499	0.359706	3.976855	6.98E-05	0.0001721	UP
GAS6-AS2	23.78639	0.853553	0.214648	3.97652	6.99E-05	0.0001723	UP
RP11-602I	1.583365	1.402185	0.352643	3.976216	7.00E-05	0.0001726	UP
FCER1G	973.3182	-0.72385	0.182068	-3.97573	7.02E-05	0.0001729	DOWN
LINC01073	2.604319	-1.18157	0.297196	-3.97571	7.02E-05	0.0001729	DOWN
MBLAC2	320.0423	-0.43565	0.109579	-3.97569	7.02E-05	0.0001729	NOT
PTGES2	2066.086	0.452461	0.113817	3.975357	7.03E-05	0.0001731	NOT

DCAF5	1944.695	-0.25923	0.065219	-3.97471	7.05E-05	0.0001736	NOT
TMCC2	45.84763	0.855277	0.2152	3.974327	7.06E-05	0.0001738	UP
CYP2A6	47629.37	-1.62478	0.408857	-3.97396	7.07E-05	0.0001741	DOWN
CTD-2550	2.919437	1.148901	0.289125	3.973721	7.08E-05	0.0001742	UP
AC003088	1.688825	2.059555	0.518303	3.973648	7.08E-05	0.0001743	UP
CORO2A	492.6272	0.748415	0.188355	3.973425	7.08E-05	0.0001744	UP
UEVLD	635.2168	0.283132	0.071261	3.973148	7.09E-05	0.0001746	NOT
PCGF3	1302.447	0.324355	0.081641	3.97295	7.10E-05	0.0001747	NOT
ARRDC4	1393.389	-0.89756	0.225919	-3.97294	7.10E-05	0.0001747	DOWN
HINT3	1444.195	-0.32018	0.080593	-3.97285	7.10E-05	0.0001748	NOT
RP11-444I	8.713428	-1.04035	0.26188	-3.97264	7.11E-05	0.0001749	DOWN
RP1-148H	3.829399	1.356958	0.341635	3.971947	7.13E-05	0.0001754	UP
TMEM35	4.258265	1.330165	0.334901	3.971819	7.13E-05	0.0001755	UP
PRDM10	286.5033	0.326465	0.082196	3.971781	7.13E-05	0.0001755	NOT
CCDC141	25.85009	-1.12625	0.283565	-3.97176	7.13E-05	0.0001755	DOWN
MOSPD2	486.8163	0.398686	0.100406	3.970753	7.16E-05	0.0001762	NOT
AC074286	42.95792	0.442223	0.111371	3.970698	7.17E-05	0.0001763	NOT
RP11-849I	2.929157	1.31875	0.332153	3.970308	7.18E-05	0.0001765	UP
RP11-74E2	1.923048	1.896994	0.477868	3.969699	7.20E-05	0.000177	UP
CTB-89H1	296.873	0.447815	0.112815	3.969482	7.20E-05	0.0001771	NOT
GRB2	5489.234	0.209389	0.052753	3.969208	7.21E-05	0.0001773	NOT
ZNF486	63.72361	0.973929	0.245372	3.969195	7.21E-05	0.0001773	UP
PPP2R5E	1352.707	-0.27213	0.068565	-3.96898	7.22E-05	0.0001775	NOT
ANKLE2	1838.117	0.287324	0.072405	3.968283	7.24E-05	0.000178	NOT
FUBP3	2226.07	-0.29619	0.074641	-3.96816	7.24E-05	0.000178	NOT
SOX21-AS	1.73565	2.035708	0.513109	3.967401	7.27E-05	0.0001786	UP
POP5	990.358	0.392704	0.098994	3.966958	7.28E-05	0.0001789	NOT
CSNK1G3	969.2638	-0.29803	0.075129	-3.96695	7.28E-05	0.0001789	NOT
SNX12	1577.071	0.318026	0.080175	3.966627	7.29E-05	0.0001791	NOT
MIAT	55.56686	1.052623	0.265419	3.965891	7.31E-05	0.0001797	UP
NELL1	2.329617	2.92829	0.738382	3.965817	7.31E-05	0.0001797	UP
TMEM123	16777.83	-0.45563	0.114893	-3.96573	7.32E-05	0.0001797	NOT
RP11-475(	166.9983	0.678054	0.170983	3.96562	7.32E-05	0.0001798	UP
CD33	111.2588	-0.67446	0.170082	-3.96552	7.32E-05	0.0001799	DOWN
RP4-753F5	1.21002	1.633957	0.412044	3.96549	7.32E-05	0.0001799	UP
RP11-126(	14.82621	-0.68553	0.172874	-3.96548	7.32E-05	0.0001799	DOWN
DHRS9	70.56089	-0.76122	0.191963	-3.96544	7.33E-05	0.0001799	DOWN
RBMS1P1	4.626605	-0.80279	0.20246	-3.96518	7.33E-05	0.0001801	DOWN
CTB-55O6	2.233249	2.45028	0.618038	3.964608	7.35E-05	0.0001805	UP
MPP6	903.4253	0.508423	0.128256	3.964119	7.37E-05	0.0001808	NOT
RPL39P38	2.944513	1.110578	0.280236	3.963003	7.40E-05	0.0001817	UP
XXbac-BP(	1.695992	1.649972	0.416351	3.962929	7.40E-05	0.0001817	UP
PMVK	3902.437	0.502837	0.126908	3.962207	7.43E-05	0.0001822	NOT
ILDR1	92.37087	1.360523	0.343383	3.962119	7.43E-05	0.0001823	UP
AADACP1	206.0455	-0.93798	0.236754	-3.96184	7.44E-05	0.0001825	DOWN
LMO7	2912.549	-0.52364	0.132172	-3.9618	7.44E-05	0.0001825	NOT
RP11-299I	2.91896	1.257985	0.317558	3.961432	7.45E-05	0.0001828	UP
CAMLG	1258.572	0.331151	0.083611	3.960624	7.48E-05	0.0001834	NOT
DYNLL2	5770.09	-0.31115	0.078567	-3.96037	7.48E-05	0.0001835	NOT
RP11-148I	2.209463	1.776266	0.448541	3.960099	7.49E-05	0.0001837	UP
HS1BP3	2090.228	0.386205	0.097525	3.960082	7.49E-05	0.0001837	NOT
DTX2	562.4087	0.369276	0.093259	3.959692	7.50E-05	0.000184	NOT

RP11-427I	1.182524	2.106549	0.5321	3.958932	7.53E-05	0.0001846	UP
RP11-463(	6.718076	1.027074	0.259469	3.958369	7.55E-05	0.000185	UP
FAM57A	211.5713	0.675555	0.170673	3.958175	7.55E-05	0.0001851	UP
RP11-318(	2.517545	2.553152	0.645075	3.957916	7.56E-05	0.0001853	UP
C16orf52	564.7743	-0.40777	0.103027	-3.9579	7.56E-05	0.0001853	NOT
RANBP2	2671.128	-0.28547	0.072129	-3.9577	7.57E-05	0.0001855	NOT
LETM1	2116.033	0.32309	0.081642	3.957391	7.58E-05	0.0001857	NOT
SRPK2P	1.201343	1.964209	0.496375	3.957108	7.59E-05	0.0001859	UP
INHBA-AS	12.27748	0.911603	0.23038	3.956946	7.59E-05	0.000186	UP
RP11-99E:	1.220086	1.442211	0.364487	3.956827	7.60E-05	0.0001861	UP
SIGLEC22F	2.194158	-1.03649	0.26204	-3.95549	7.64E-05	0.0001871	DOWN
CASP1P2	2.037147	1.620528	0.40976	3.954825	7.66E-05	0.0001876	UP
BANF1P3	6.197337	0.806162	0.20386	3.954491	7.67E-05	0.0001879	UP
SAPCD1-A	5.207754	0.899387	0.227437	3.954443	7.67E-05	0.0001879	UP
FHL5	17.60294	1.04482	0.264285	3.953383	7.71E-05	0.0001887	UP
ZBTB3	159.4274	0.341581	0.086405	3.953265	7.71E-05	0.0001888	NOT
RP11-98F1	3.493355	1.19259	0.301676	3.95322	7.71E-05	0.0001888	UP
ELP3	1306.95	-0.35939	0.090919	-3.95284	7.72E-05	0.0001891	NOT
SLCO4C1	306.8203	-1.38729	0.350977	-3.95267	7.73E-05	0.0001892	DOWN
GSTO2	347.3856	0.838462	0.212126	3.952667	7.73E-05	0.0001892	UP
HAUS7	90.95522	0.649689	0.1644	3.951875	7.75E-05	0.0001898	UP
NOD1	189.4042	0.366635	0.092777	3.95179	7.76E-05	0.0001898	NOT
TGS1	553.6718	0.349816	0.088528	3.951454	7.77E-05	0.0001901	NOT
AC016768	103.1479	-1.08078	0.27353	-3.95124	7.77E-05	0.0001902	DOWN
NDUFC1	1853.955	-0.39632	0.100308	-3.95103	7.78E-05	0.0001904	NOT
C9orf116	69.73786	0.694366	0.175751	3.950859	7.79E-05	0.0001905	UP
RP1-283E:	69.71344	0.715162	0.181019	3.950766	7.79E-05	0.0001906	UP
ZCCHC7	544.9296	0.280144	0.070912	3.950585	7.80E-05	0.0001907	NOT
DUSP27	1.858613	1.689476	0.427666	3.950454	7.80E-05	0.0001908	UP
RP5-1125/	8.917986	0.684092	0.17318	3.950182	7.81E-05	0.000191	UP
NUDT6	229.9031	-0.69404	0.175705	-3.95001	7.81E-05	0.0001911	DOWN
FNDC1	231.0023	1.256808	0.318195	3.94981	7.82E-05	0.0001912	UP
THRSP	3837.636	-1.67812	0.424871	-3.94972	7.82E-05	0.0001913	DOWN
KLHDC9	269.1247	0.702027	0.177756	3.949393	7.83E-05	0.0001915	UP
IGHV3-72	29.50789	-1.57919	0.399866	-3.94928	7.84E-05	0.0001916	DOWN
RP11-543(	1.202067	2.222732	0.562842	3.949123	7.84E-05	0.0001917	UP
OR7E128P	2.811665	1.182626	0.299487	3.948842	7.85E-05	0.0001919	UP
AC000123	19.11936	0.523351	0.132534	3.948809	7.85E-05	0.0001919	NOT
CYP4F3	10974.25	-0.84837	0.214853	-3.94862	7.86E-05	0.0001921	DOWN
BLMH	1171.943	0.692168	0.175305	3.948364	7.87E-05	0.0001923	UP
DTD2	466.9782	-0.30728	0.077826	-3.94832	7.87E-05	0.0001923	NOT
RNF128	5003.989	-0.54588	0.138267	-3.94804	7.88E-05	0.0001925	NOT
SMAD3	2179.287	0.410069	0.103897	3.946866	7.92E-05	0.0001934	NOT
ETV3	757.8298	0.359912	0.09121	3.945966	7.95E-05	0.0001941	NOT
INTU	117.1523	0.656452	0.166371	3.945708	7.96E-05	0.0001943	UP
GPRC5D	12.47791	0.894987	0.226847	3.945324	7.97E-05	0.0001946	UP
CHRNA1	7.503587	1.634903	0.414419	3.94505	7.98E-05	0.0001948	UP
AC007461	2.410737	1.447785	0.366997	3.944948	7.98E-05	0.0001949	UP
ZNF229	96.5219	1.077223	0.273077	3.944764	7.99E-05	0.000195	UP
CTC-448F:	2.564206	1.29098	0.327266	3.944738	7.99E-05	0.000195	UP
TBC1D3L	23.60979	0.916311	0.232296	3.944578	7.99E-05	0.0001951	UP
CSTF3-AS:	2.085305	1.266921	0.321203	3.944299	8.00E-05	0.0001953	UP

DGCR6L	3090.54	-0.54069	0.137081	-3.94427	8.00E-05	0.0001954	NOT
GEMIN2	186.8652	0.368659	0.093468	3.944225	8.01E-05	0.0001954	NOT
VPS9D1-A	58.08624	0.896024	0.227202	3.943738	8.02E-05	0.0001958	UP
RP1-286D	20.05634	0.628252	0.159318	3.943378	8.03E-05	0.000196	UP
VN1R20P	1.450027	1.438922	0.364899	3.943341	8.04E-05	0.000196	UP
RP11-333I	1.551915	1.256493	0.318647	3.943216	8.04E-05	0.0001961	UP
RP11-655I	6.499522	1.291235	0.327513	3.94255	8.06E-05	0.0001967	UP
RP11-761I	14.37991	0.886671	0.224908	3.942375	8.07E-05	0.0001968	UP
LINC0153	37.63339	0.594411	0.150795	3.941851	8.09E-05	0.0001972	UP
AC023347	1.976454	2.753968	0.698719	3.941453	8.10E-05	0.0001975	UP
RNASEH1-	150.2279	0.47369	0.120186	3.941316	8.10E-05	0.0001976	NOT
CYP4F12	2038.873	-0.8877	0.225254	-3.9409	8.12E-05	0.0001979	DOWN
FDPS	8712.363	0.642657	0.16308	3.940743	8.12E-05	0.000198	UP
SLC25A22	1785.39	-0.47718	0.121097	-3.9405	8.13E-05	0.0001982	NOT
FAM102A	3212.221	-0.58709	0.149	-3.94016	8.14E-05	0.0001985	DOWN
RPS3AP54	4.343994	1.034796	0.262686	3.939291	8.17E-05	0.0001992	UP
RP13-379I	1.523685	2.096327	0.532197	3.939007	8.18E-05	0.0001994	UP
RP11-356I	38.9159	1.122635	0.285011	3.938924	8.18E-05	0.0001995	UP
XXbac-B4	6.382714	1.041344	0.264424	3.938163	8.21E-05	0.0002001	UP
ZNF256	94.63294	0.665501	0.168989	3.938133	8.21E-05	0.0002001	UP
FRMD1	35.9659	1.590044	0.403808	3.93762	8.23E-05	0.0002005	UP
KLHL30-A	1.576895	2.011218	0.510796	3.937418	8.24E-05	0.0002006	UP
RPS23P1	1.977274	1.318522	0.334893	3.937148	8.25E-05	0.0002009	UP
LL0XNC01	9.695842	0.782998	0.198878	3.937073	8.25E-05	0.0002009	UP
IDO2	145.5547	-1.52321	0.386915	-3.93681	8.26E-05	0.0002011	DOWN
TNKS1BP1	5574.668	-0.33456	0.084994	-3.9363	8.27E-05	0.0002015	NOT
CTB-12O2	1.647775	1.904065	0.483742	3.936113	8.28E-05	0.0002017	UP
RP11-110	9.352542	-0.94868	0.24107	-3.93528	8.31E-05	0.0002023	DOWN
NDFIP1	8185.913	-0.32281	0.082031	-3.93518	8.31E-05	0.0002024	NOT
DAPK3	1644.215	0.395376	0.100484	3.934702	8.33E-05	0.0002028	NOT
GDF9	25.84385	0.591257	0.150268	3.934683	8.33E-05	0.0002028	UP
FAM63A	1297.951	0.457047	0.116159	3.934667	8.33E-05	0.0002028	NOT
RP11-215	18.2263	0.769622	0.195643	3.93382	8.36E-05	0.0002035	UP
AC007566	16.35722	0.722662	0.18371	3.933704	8.36E-05	0.0002036	UP
CLIC1P1	1.47089	1.34324	0.341606	3.932137	8.42E-05	0.0002049	UP
PQBP1	2435.367	0.437408	0.11125	3.931746	8.43E-05	0.0002052	NOT
MIR646HG	15.82309	1.331505	0.338658	3.931706	8.43E-05	0.0002052	UP
BHLHA15	60.96796	1.013295	0.257765	3.931078	8.46E-05	0.0002057	UP
VPS25	2285.766	0.319175	0.081203	3.930596	8.47E-05	0.0002061	NOT
MCM3AP	2050.988	0.28921	0.073587	3.9302	8.49E-05	0.0002064	NOT
GRM7	10.44827	1.628055	0.414255	3.930082	8.49E-05	0.0002065	UP
STXBP5-A	14.39738	0.768312	0.195501	3.929962	8.50E-05	0.0002066	UP
CTD-2015	1.614719	2.112332	0.537496	3.929947	8.50E-05	0.0002066	UP
CTD-2270	15.63538	0.740555	0.188439	3.929937	8.50E-05	0.0002066	UP
AATK-AS1	2.138687	2.860373	0.727857	3.929856	8.50E-05	0.0002066	UP
LRCH2	31.39547	0.914992	0.232848	3.929572	8.51E-05	0.0002069	UP
CLEC2B	283.4855	-0.67101	0.170783	-3.92905	8.53E-05	0.0002073	DOWN
RP5-1154I	96.69575	1.518681	0.386528	3.929034	8.53E-05	0.0002073	UP
RP11-112	4.823077	1.05355	0.268165	3.928746	8.54E-05	0.0002075	UP
POLR2H	1604.255	0.364103	0.092679	3.928645	8.54E-05	0.0002076	NOT
CFLAR	3816.865	-0.27013	0.068763	-3.92839	8.55E-05	0.0002078	NOT
CTC-332L	14.95319	-0.57032	0.145197	-3.92787	8.57E-05	0.0002082	NOT

RP11-34P1	3.084159	2.083104	0.530403	3.927401	8.59E-05	0.0002086	UP
ARG2	150.3523	1.001938	0.255139	3.927027	8.60E-05	0.0002089	UP
AP1S3	70.38004	0.6121	0.155884	3.926645	8.61E-05	0.0002092	UP
RNF141	1217.41	-0.28547	0.072723	-3.9254	8.66E-05	0.0002103	NOT
RP1-111B2	2.56898	1.270447	0.323682	3.924978	8.67E-05	0.0002107	UP
EMID1	465.6354	0.890126	0.226817	3.924429	8.69E-05	0.0002111	UP
SLC25A18	2676.368	-0.88683	0.225985	-3.92429	8.70E-05	0.0002112	DOWN
RP11-383(	1.657136	1.357152	0.345859	3.924009	8.71E-05	0.0002114	UP
RP11-532I	1.966241	1.395268	0.355589	3.923828	8.72E-05	0.0002116	UP
BIN2	230.2999	-0.67419	0.171826	-3.92365	8.72E-05	0.0002117	DOWN
C16orf45	870.4915	-0.73939	0.188483	-3.92283	8.75E-05	0.0002124	DOWN
RP11-426I	1.483675	1.368057	0.348743	3.922827	8.75E-05	0.0002124	UP
SLC28A1	2636.002	-1.13545	0.289466	-3.92256	8.76E-05	0.0002126	DOWN
AC074183	2.595776	1.511331	0.385314	3.922333	8.77E-05	0.0002128	UP
RP11-290I	500.1123	-1.12843	0.287695	-3.92231	8.77E-05	0.0002128	DOWN
XCR1	33.4466	-1.19	0.303404	-3.92215	8.78E-05	0.0002129	DOWN
SMIM5	17.89375	0.953587	0.243156	3.921705	8.79E-05	0.0002133	UP
SHROOM4	164.1154	0.613548	0.156452	3.921635	8.80E-05	0.0002134	UP
AP001626.	45.7563	1.278352	0.325985	3.92151	8.80E-05	0.0002134	UP
ASB14	20.86873	0.506366	0.129126	3.921482	8.80E-05	0.0002135	NOT
CH17-408	1.796598	2.241734	0.57166	3.921443	8.80E-05	0.0002135	UP
CYCSP10	1.619072	1.328562	0.338808	3.921281	8.81E-05	0.0002136	UP
SMTNL2	5.193668	1.101199	0.280848	3.920982	8.82E-05	0.0002138	UP
PCDHB7	54.7853	1.056376	0.26944	3.920632	8.83E-05	0.0002141	UP
HOXB3	147.0085	0.813737	0.207565	3.920392	8.84E-05	0.0002143	UP
SSNA1	1979.602	0.487069	0.124249	3.920103	8.85E-05	0.0002146	NOT
PPP4R1-A!	2.35213	1.416675	0.361485	3.919044	8.89E-05	0.0002155	UP
TEDDM2P	3.035448	3.379614	0.862436	3.918682	8.90E-05	0.0002158	UP
RP11-417I	4.35916	1.717996	0.438443	3.9184	8.91E-05	0.000216	UP
LY86	162.2864	-0.69298	0.176856	-3.91833	8.92E-05	0.0002161	DOWN
CTD-2639	17.83168	0.653633	0.166824	3.918108	8.92E-05	0.0002163	UP
MKRN3-A!	0.938509	2.236737	0.570912	3.917833	8.93E-05	0.0002165	UP
LINC01509	1.091927	2.469045	0.63021	3.917812	8.94E-05	0.0002165	UP
CXCR6	132.8332	-0.89191	0.22766	-3.91774	8.94E-05	0.0002165	DOWN
HMGB3P24	3.780956	0.84329	0.215303	3.916767	8.97E-05	0.0002174	UP
NUDT14	776.6295	0.686216	0.175208	3.916568	8.98E-05	0.0002175	UP
RP11-22B2	1.834949	1.560524	0.398459	3.916395	8.99E-05	0.0002177	UP
FDPSP1	1.619856	1.391186	0.355226	3.916341	8.99E-05	0.0002177	UP
RP11-370/	52.99502	-0.75617	0.193086	-3.91624	8.99E-05	0.0002178	DOWN
DNAJA3	3505.744	-0.34079	0.08702	-3.91622	8.99E-05	0.0002178	NOT
RP11-121I	17.40239	-0.60932	0.15559	-3.9162	9.00E-05	0.0002178	DOWN
CTB-41I6.1	2.249689	-0.99107	0.253115	-3.9155	9.02E-05	0.0002184	DOWN
GMCL1P1	3.274224	2.357414	0.602214	3.914582	9.06E-05	0.0002192	UP
LGR4	4253.428	-0.51822	0.132383	-3.91455	9.06E-05	0.0002192	NOT
MRPL55	2746.732	0.572668	0.146299	3.914356	9.06E-05	0.0002194	NOT
RP11-69M	28.36001	0.61741	0.157755	3.913727	9.09E-05	0.0002199	UP
RP11-481	0.926423	1.966006	0.502365	3.913498	9.10E-05	0.0002201	UP
FLT3	30.07305	-1.02166	0.261068	-3.91338	9.10E-05	0.0002202	DOWN
RS1	2.558961	1.273709	0.325489	3.913215	9.11E-05	0.0002203	UP
TMEM242	618.2741	-0.39067	0.099839	-3.91297	9.12E-05	0.0002205	NOT
MFAP4	1240.134	-1.26184	0.322502	-3.91265	9.13E-05	0.0002208	DOWN
RP5-1086I	29.75203	-0.76753	0.196186	-3.91224	9.14E-05	0.0002212	DOWN

RUVBL2	3301.851	0.440098	0.112509	3.91167	9.17E-05	0.0002217	NOT
LOXL4	1252.501	1.230156	0.314492	3.911563	9.17E-05	0.0002218	UP
RP11-127I	2.751891	1.075777	0.27507	3.910913	9.19E-05	0.0002223	UP
SF1	6286.15	-0.17864	0.045679	-3.91067	9.20E-05	0.0002225	NOT
RNASE1	2127.976	0.660423	0.168889	3.910409	9.21E-05	0.0002228	UP
TAS2R10	1.499199	1.353067	0.346043	3.910112	9.23E-05	0.000223	UP
CYLD	1449.908	-0.35742	0.091419	-3.90968	9.24E-05	0.0002234	NOT
SYCP2	30.93969	1.00242	0.256408	3.90947	9.25E-05	0.0002236	UP
SLC25A6	10642.86	0.462818	0.118393	3.909176	9.26E-05	0.0002238	NOT
LRRC52	4.712415	2.519214	0.644482	3.9089	9.27E-05	0.0002241	UP
AGBL2	125.3483	0.876542	0.224264	3.908524	9.29E-05	0.0002244	UP
NR2E1	3.892483	1.624017	0.415521	3.908383	9.29E-05	0.0002245	UP
RECQL	460.4868	0.527989	0.135094	3.908317	9.29E-05	0.0002245	NOT
RP11-23J9	5.377892	0.971881	0.248705	3.907763	9.32E-05	0.000225	UP
RBM5	1903.351	0.260052	0.066549	3.907709	9.32E-05	0.0002251	NOT
UPF1	4639.406	0.305623	0.078219	3.907267	9.33E-05	0.0002255	NOT
STAG3L5P	103.0067	0.531489	0.13603	3.907157	9.34E-05	0.0002255	NOT
AF131216.	5.065489	-0.74306	0.190195	-3.90682	9.35E-05	0.0002258	DOWN
RP5-1057I	1.36229	1.553803	0.397735	3.90663	9.36E-05	0.000226	UP
CNN1	222.8993	0.897417	0.229719	3.906581	9.36E-05	0.000226	UP
BNIP3P30	2.02094	2.341526	0.599404	3.906421	9.37E-05	0.0002261	UP
CTC-203F	11.59983	0.634527	0.162436	3.906327	9.37E-05	0.0002262	UP
TNFRSF13I	8.107817	-1.16851	0.299145	-3.90616	9.38E-05	0.0002264	DOWN
DLEU7	12.91253	-0.83365	0.21343	-3.90596	9.39E-05	0.0002265	DOWN
SCYL1	4536.581	-0.28642	0.073353	-3.90466	9.44E-05	0.0002277	NOT
SEC61G	2293.486	0.475872	0.121879	3.90445	9.44E-05	0.0002279	NOT
RP11-320(	3.70262	2.589498	0.663262	3.904186	9.45E-05	0.0002281	UP
AC097639	1.954202	1.188881	0.304529	3.903999	9.46E-05	0.0002283	UP
AC002075	4.915195	0.945699	0.242246	3.903871	9.47E-05	0.0002284	UP
EPS15	2077.297	-0.26919	0.068981	-3.90231	9.53E-05	0.0002298	NOT
MPRIP	2991.731	0.361444	0.092626	3.902188	9.53E-05	0.0002299	NOT
RP11-179I	1.385205	1.836376	0.470605	3.902161	9.53E-05	0.0002299	UP
SPATA24	106.0574	0.500529	0.128274	3.902039	9.54E-05	0.00023	NOT
SIAE	2550.945	-0.57514	0.147397	-3.90197	9.54E-05	0.0002301	NOT
RP11-386I	3.579984	1.432005	0.367004	3.901879	9.54E-05	0.0002302	UP
NUDT12	998.2298	-0.5862	0.150239	-3.90182	9.55E-05	0.0002302	DOWN
SLITRK3	242.6407	-2.07861	0.532784	-3.90141	9.56E-05	0.0002306	DOWN
CYCSP34	5.922053	1.02581	0.262937	3.901357	9.57E-05	0.0002306	UP
PKDREJ	15.71383	0.839948	0.215324	3.900849	9.59E-05	0.0002311	UP
HES7	1.348492	2.066327	0.52974	3.900647	9.59E-05	0.0002312	UP
XKR5	1.617699	1.885958	0.483503	3.900614	9.59E-05	0.0002312	UP
AC068535	124.0127	-1.63837	0.420038	-3.90054	9.60E-05	0.0002313	DOWN
RP11-36C	39.55654	0.672595	0.172449	3.900251	9.61E-05	0.0002315	UP
PLA2G4E	4.949884	1.581035	0.405378	3.900148	9.61E-05	0.0002316	UP
RP11-615I	1.187919	1.876483	0.481133	3.900132	9.61E-05	0.0002316	UP
AC004980	1.40235	1.540393	0.395003	3.899699	9.63E-05	0.000232	UP
RP11-314	16.14891	0.719602	0.184528	3.899684	9.63E-05	0.000232	UP
SCN5A	4.362172	1.238254	0.317547	3.899437	9.64E-05	0.0002322	UP
RP11-578I	1.686106	2.573194	0.659966	3.898981	9.66E-05	0.0002326	UP
MEGF11	4.657846	1.060431	0.271982	3.898906	9.66E-05	0.0002327	UP
LINC00847	447.9412	0.372265	0.095489	3.898506	9.68E-05	0.0002331	NOT
RP4-781K	4.556423	1.764847	0.452734	3.898197	9.69E-05	0.0002333	UP

APBA3	709.1023	0.30276	0.07767	3.898032	9.70E-05	0.0002335	NOT
NKX1-2	3.020713	2.880424	0.738987	3.897801	9.71E-05	0.0002337	UP
ZNF516	742.1877	0.344236	0.088316	3.897777	9.71E-05	0.0002337	NOT
CIB3	1.439149	2.018012	0.517783	3.89741	9.72E-05	0.000234	UP
ABCE1	1893.321	-0.3005	0.077104	-3.89738	9.72E-05	0.000234	NOT
LINC01237	8.471018	0.853333	0.21898	3.896861	9.74E-05	0.0002345	UP
RP11-401I	1.873066	2.358148	0.605146	3.896824	9.75E-05	0.0002345	UP
LYPD6	45.41173	1.264003	0.324382	3.896656	9.75E-05	0.0002347	UP
KB-208E9.	7.602757	1.074084	0.275684	3.896069	9.78E-05	0.0002352	UP
PNMT	10.04579	1.636745	0.420123	3.895869	9.78E-05	0.0002354	UP
TRPC2	5.575262	-0.82321	0.211339	-3.89523	9.81E-05	0.000236	DOWN
ABCC6	7211.567	-0.66455	0.170609	-3.89514	9.81E-05	0.0002361	DOWN
AP000936.	5.039703	0.871763	0.22381	3.895093	9.82E-05	0.0002361	UP
AP4B1-AS	6.787639	0.863343	0.221651	3.895051	9.82E-05	0.0002361	UP
RP11-74C.	8.876203	1.224392	0.314409	3.89427	9.85E-05	0.0002368	UP
RSPH9	30.60002	0.394721	0.101364	3.894082	9.86E-05	0.000237	NOT
C4A	10570.37	-0.76273	0.195871	-3.89406	9.86E-05	0.000237	DOWN
LYPD2	14.78716	-1.76858	0.454175	-3.89405	9.86E-05	0.000237	DOWN
RP11-162.	1.089243	1.795668	0.461144	3.893946	9.86E-05	0.0002371	UP
EWSAT1	17.6268	1.124189	0.28871	3.893835	9.87E-05	0.0002372	UP
WAC-AS1	759.2703	0.362466	0.093093	3.893591	9.88E-05	0.0002374	NOT
RP11-59D	50.16906	1.14086	0.293057	3.892963	9.90E-05	0.000238	UP
UBE2SP2	1.685549	1.471047	0.377883	3.89286	9.91E-05	0.0002381	UP
PARS2	260.3025	0.368013	0.094537	3.892813	9.91E-05	0.0002381	NOT
CCDC183-	48.43011	0.584855	0.150243	3.892722	9.91E-05	0.0002381	NOT
CTD-2091	1.464809	2.543378	0.653444	3.892269	9.93E-05	0.0002386	UP
CYB561A3	1559.538	0.333156	0.085597	3.892137	9.94E-05	0.0002387	NOT
SLC17A5	1786.805	0.475967	0.122303	3.89169	9.95E-05	0.0002391	NOT
DNAJB5	259.6661	0.428518	0.110116	3.891507	9.96E-05	0.0002393	NOT
PGK1	12814.67	0.453547	0.116551	3.891391	9.97E-05	0.0002394	NOT
ZNF470	209.4543	-0.71723	0.184321	-3.89118	9.98E-05	0.0002395	DOWN
TADA2A	399.3973	0.290098	0.074556	3.890989	9.98E-05	0.0002397	NOT
RP11-278(	13.5607	0.58391	0.150072	3.890857	9.99E-05	0.0002398	NOT
CTPS2	784.7784	0.350303	0.090038	3.890615	1.00E-04	0.00024	NOT
CTSE	69.76835	1.725097	0.443434	3.89031	0.000100116	0.0002403	UP
SIK1	285.7702	-1.60717	0.41314	-3.89013	0.000100191	0.0002405	DOWN
1-Mar	2630.338	-0.6852	0.176147	-3.88995	0.000100265	0.0002406	DOWN
RP11-184/	1.446941	2.160294	0.555356	3.889929	0.000100273	0.0002406	UP
SCAMP4	2111.888	0.320562	0.082419	3.889407	0.000100489	0.0002411	NOT
PPIL3	548.284	0.329258	0.084657	3.889322	0.000100525	0.0002412	NOT
C6orf226	248.1703	0.540073	0.138867	3.889129	0.000100604	0.0002414	NOT
TIMM44	1679.483	0.372595	0.095811	3.888848	0.000100721	0.0002416	NOT
SLC18B1	473.3035	0.477367	0.122761	3.888587	0.00010083	0.0002419	NOT
MVB12B	611.2154	0.408263	0.10499	3.888579	0.000100833	0.0002419	NOT
RP11-416I	1.270876	1.72265	0.443006	3.888543	0.000100848	0.0002419	UP
LINC0095E	3.862388	2.331504	0.599586	3.888524	0.000100856	0.0002419	UP
PPP1R1C	164.7828	-1.09263	0.281014	-3.88817	0.000101004	0.0002422	DOWN
CMKLR1	372.6845	-0.69894	0.179761	-3.88814	0.000101014	0.0002422	DOWN
RP11-299I	1.641783	1.961835	0.50469	3.887204	0.000101405	0.0002431	UP
URGCP	1732.25	-0.33481	0.086156	-3.88606	0.000101884	0.0002442	NOT
IGBP1-AS1	2.453887	0.972789	0.250335	3.885944	0.000101933	0.0002443	UP
COX10-AS	112.6305	-0.42554	0.109509	-3.88591	0.000101948	0.0002444	NOT

CCDC85A	8.059543	1.02914	0.264852	3.885717	0.000102028	0.0002445	UP
IL17RD	48.97542	0.959396	0.246944	3.885075	0.000102298	0.0002452	UP
PPP6C	1960.363	-0.23216	0.059764	-3.88468	0.000102466	0.0002455	NOT
NR2C1	677.0085	0.288466	0.074259	3.884571	0.000102511	0.0002456	NOT
YBX1P6	3.079574	0.948417	0.244152	3.884542	0.000102523	0.0002456	UP
RPL14P1	118.9584	0.524242	0.134967	3.884212	0.000102662	0.0002459	NOT
AC098617	4.656585	1.843063	0.474531	3.883965	0.000102767	0.0002462	UP
RP11-400I	12.0535	1.29737	0.334062	3.883624	0.000102911	0.0002465	UP
DYNC2LI1	401.2521	0.322787	0.08312	3.883387	0.000103011	0.0002467	NOT
RP5-1057I	2.075399	1.111494	0.286231	3.88321	0.000103087	0.0002469	UP
RP11-123I	1.019914	2.219443	0.571598	3.882877	0.000103228	0.0002472	UP
CHN2	1740.78	-0.60017	0.154571	-3.88279	0.000103263	0.0002473	DOWN
AC008079	17.46005	0.626852	0.161472	3.88212	0.00010355	0.0002479	UP
RP4-640H	11.9218	1.679968	0.43276	3.881987	0.000103606	0.000248	UP
RP11-790I	4.385339	-1.54973	0.399226	-3.88184	0.000103668	0.0002482	DOWN
ZNF554	202.9352	0.419601	0.108105	3.881437	0.000103841	0.0002485	NOT
ELOVL6	2747.714	-0.70606	0.181916	-3.88125	0.000103922	0.0002487	DOWN
RP11-767I	4.957115	0.868165	0.223686	3.881175	0.000103953	0.0002488	UP
SUCLG2-A	47.60366	-0.61798	0.159243	-3.88075	0.000104135	0.0002492	DOWN
AOC2	56.83181	0.554044	0.142772	3.880617	0.000104192	0.0002493	NOT
NT5C3A	614.9878	0.474409	0.122263	3.88022	0.000104362	0.0002497	NOT
EIF3EP1	11.02398	0.806014	0.20773	3.880098	0.000104414	0.0002498	UP
UNC79	15.73658	-0.59702	0.153899	-3.87931	0.000104753	0.0002506	DOWN
SNORA14E	2.34232	1.074915	0.277096	3.879218	0.000104793	0.0002506	UP
ARL10	136.651	0.5701	0.146963	3.879206	0.000104798	0.0002506	NOT
EOMES	61.80204	-0.92743	0.239123	-3.87846	0.00010512	0.0002514	DOWN
TEN1-CDK	63.34609	0.641465	0.165425	3.877685	0.000105455	0.0002522	UP
PARP4P2	3.719516	1.285345	0.331498	3.877388	0.000105584	0.0002525	UP
NPAS4	1.534208	-1.22406	0.315712	-3.87715	0.000105686	0.0002527	DOWN
MLLT3	192.6213	0.878495	0.226614	3.876611	0.000105922	0.0002532	UP
CCM2L	122.0922	0.540814	0.139513	3.876429	0.000106001	0.0002534	NOT
RP11-458I	222.5157	0.505389	0.130375	3.876417	0.000106006	0.0002534	NOT
RP11-482I	2.257082	1.311871	0.338428	3.876366	0.000106028	0.0002534	UP
BCR	2252.542	0.349188	0.090093	3.875865	0.000106247	0.0002539	NOT
OR8G5	2.176615	3.45296	0.890888	3.875863	0.000106247	0.0002539	UP
SNAI1	156.2961	-0.80051	0.206573	-3.87518	0.000106547	0.0002546	DOWN
APEX2	1152.018	0.298769	0.077101	3.875053	0.000106601	0.0002547	NOT
FAM3C	999.8114	-0.40719	0.105081	-3.87503	0.000106612	0.0002547	NOT
PARVB	1292.466	0.725782	0.187308	3.874808	0.000106709	0.0002549	UP
RP11-131I	4.450074	-0.74472	0.192199	-3.87472	0.000106749	0.000255	DOWN
FOXC1	179.7034	0.894016	0.230739	3.874581	0.000106808	0.0002551	UP
RP11-423I	33.3508	-1.19841	0.309376	-3.87365	0.000107218	0.0002561	DOWN
CHST4	266.6334	-1.83511	0.473753	-3.87355	0.00010726	0.0002561	DOWN
ZNF774	64.13363	0.483517	0.124833	3.873312	0.000107366	0.0002564	NOT
RP11-100I	9.689015	-0.69061	0.178304	-3.87324	0.0001074	0.0002564	DOWN
LINC00315	3.777905	0.887659	0.229183	3.873139	0.000107443	0.0002565	UP
PCDHA6	7.270962	1.82278	0.47066	3.872819	0.000107584	0.0002568	UP
OTOGL	3.816401	1.281311	0.330897	3.872231	0.000107844	0.0002574	UP
10-Sep	1859.002	-0.39587	0.102236	-3.87213	0.000107886	0.0002575	NOT
USO1	3446.053	-0.38457	0.099321	-3.87199	0.00010795	0.0002576	NOT
H2AFV	4617.053	0.278855	0.07202	3.871913	0.000107985	0.0002577	NOT
TAOK1	1760.912	0.34849	0.090023	3.871133	0.000108331	0.0002585	NOT

ZDHHC23	393.5603	0.604772	0.156234	3.870938	0.000108417	0.0002587	UP
UQCRBP1	5.121771	0.903704	0.233503	3.870196	0.000108748	0.0002594	UP
STK31	14.94263	1.154499	0.298338	3.86977	0.000108938	0.0002599	UP
RP11-503I	2.304882	1.239968	0.320426	3.869749	0.000108947	0.0002599	UP
RP11-187(	2.660838	1.031241	0.266519	3.8693	0.000109148	0.0002603	UP
TXN	11695.65	0.577458	0.149247	3.869155	0.000109213	0.0002605	NOT
PPDPF	5333.086	0.64431	0.166537	3.868862	0.000109345	0.0002608	UP
MUC2	5.569698	2.154118	0.556792	3.868802	0.000109371	0.0002608	UP
RYR3	27.30272	0.61094	0.157945	3.868045	0.000109711	0.0002616	UP
VN1R48P	4.871006	1.510972	0.390644	3.867895	0.000109779	0.0002617	UP
RP6-201G.	3.519185	1.082247	0.279816	3.867714	0.00010986	0.0002619	UP
RP5-1042I	2.083749	1.216832	0.314624	3.867574	0.000109924	0.000262	UP
TNFAIP8L1	3194.888	-0.51957	0.134349	-3.86735	0.000110023	0.0002622	NOT
CTD-2152	11.54496	0.71358	0.184551	3.866584	0.000110371	0.000263	UP
AP003900.	1.900964	3.383473	0.875112	3.866331	0.000110485	0.0002633	UP
LINC0121.	1.232484	2.629333	0.680097	3.866113	0.000110584	0.0002635	UP
ABCG4	6.52934	-0.83147	0.215071	-3.86604	0.000110619	0.0002636	DOWN
RP11-91I8	1.257597	2.322367	0.600713	3.866019	0.000110626	0.0002636	UP
MYLIP	351.2105	0.536684	0.138825	3.865906	0.000110678	0.0002637	NOT
KRT10	866.8313	0.436198	0.112839	3.865682	0.000110779	0.0002639	NOT
OTP	1.546492	2.436506	0.630314	3.865545	0.000110841	0.000264	UP
ZNF559-ZI	6.273601	0.95177	0.246234	3.865308	0.000110949	0.0002642	UP
RAB29	1707.34	0.375187	0.097067	3.86525	0.000110976	0.0002643	NOT
GAPDHP6.	1.779989	1.122713	0.290466	3.865206	0.000110995	0.0002643	UP
SMC3	1636.579	0.400872	0.103715	3.865114	0.000111037	0.0002644	NOT
CTB-39G8	7.51963	0.975807	0.252496	3.864647	0.00011125	0.0002649	UP
PIP	1.453159	2.408706	0.62327	3.864625	0.00011126	0.0002649	UP
IL21-AS1	4.322721	2.465525	0.637978	3.864595	0.000111274	0.0002649	UP
RPL7	20451.81	0.535811	0.138652	3.864435	0.000111346	0.000265	NOT
CTD-2044	2.619221	1.135513	0.293847	3.864303	0.000111407	0.0002651	UP
ACHE	337.6794	1.207848	0.312577	3.864157	0.000111473	0.0002653	UP
PCDHGC4	2.841978	1.683422	0.435675	3.863944	0.000111571	0.0002655	UP
LINC0023(	9.298335	1.247651	0.322954	3.863249	0.000111889	0.0002662	UP
SMIM4	625.5662	0.59588	0.154249	3.863107	0.000111954	0.0002664	UP
RP11-19D	84.1012	-1.17039	0.303058	-3.86195	0.000112488	0.0002676	DOWN
SPTA1	11.69147	1.39581	0.361441	3.861793	0.000112558	0.0002677	UP
ATP5A1	21231.31	-0.31987	0.082838	-3.8614	0.00011274	0.0002682	NOT
ZNF362	829.0346	0.337137	0.087314	3.86122	0.000112822	0.0002683	NOT
TRIM36	35.653	0.848364	0.219714	3.861214	0.000112825	0.0002683	UP
VANGL1	523.4536	0.443449	0.114859	3.860804	0.000113015	0.0002687	NOT
EFCAB5	4.892892	0.740579	0.19183	3.8606	0.000113109	0.0002689	UP
RP11-37N	1.489197	1.588611	0.411512	3.860419	0.000113193	0.0002691	UP
SMOX	497.36	0.766145	0.198479	3.860091	0.000113345	0.0002695	UP
UTS2	12.19735	1.554588	0.402879	3.858695	0.000113994	0.000271	UP
BRF2	190.7544	0.436727	0.113182	3.858635	0.000114022	0.000271	NOT
RP11-120I	15.43843	-0.66673	0.172792	-3.85859	0.000114044	0.000271	DOWN
TMEM258	2793.64	0.406183	0.10527	3.858499	0.000114086	0.0002711	NOT
POFUT2	824.8607	0.327172	0.084797	3.858287	0.000114185	0.0002713	NOT
PHACTR2	747.6672	-0.53789	0.139453	-3.85712	0.000114732	0.0002726	NOT
RAB40A	7.031673	0.835317	0.21657	3.857037	0.00011477	0.0002727	UP
PCNX	1755.14	-0.33797	0.087632	-3.85672	0.000114918	0.000273	NOT
FBXL8	169.6155	0.555093	0.143935	3.856552	0.000114997	0.0002732	NOT

C3orf33	151.2625	0.345043	0.08947	3.85652	0.000115013	0.0002732	NOT
DOC2A	16.51251	1.107166	0.287094	3.856465	0.000115039	0.0002732	UP
ACSM4	2.945217	1.257578	0.326136	3.855992	0.000115261	0.0002737	UP
GRB7	637.9172	0.706301	0.183177	3.855845	0.000115331	0.0002739	UP
LINC0065	47.64424	0.854343	0.22159	3.855522	0.000115483	0.0002742	UP
ATP11A	1962.521	0.54073	0.14026	3.855189	0.00011564	0.0002746	NOT
ALKBH1	331.4509	-0.25368	0.065803	-3.85512	0.000115675	0.0002746	NOT
RP11-319	59.30383	0.383835	0.099568	3.855025	0.000115718	0.0002747	NOT
RP11-522	27.4882	-0.75689	0.196344	-3.85489	0.00011578	0.0002748	DOWN
RP11-226	278.1462	0.443542	0.115081	3.854173	0.000116121	0.0002756	NOT
MMP28	44.3624	0.936929	0.243099	3.854112	0.000116151	0.0002757	UP
ZNF66	13.71568	1.041549	0.270246	3.854079	0.000116166	0.0002757	UP
SEL1L3	1888.336	1.039669	0.26978	3.853772	0.000116312	0.000276	UP
GFAP	29.51762	1.05566	0.273954	3.853421	0.000116479	0.0002764	UP
FOXD4L1	2.688912	1.284119	0.333258	3.853228	0.000116571	0.0002766	UP
RNU6-817	2.402016	1.314734	0.341212	3.853134	0.000116616	0.0002766	UP
TYROBP	1593.853	-0.6292	0.163312	-3.85275	0.000116797	0.000277	DOWN
TNFSF13	162.4506	-0.60038	0.155834	-3.85266	0.000116843	0.0002771	DOWN
AC114498	16.15918	-0.93652	0.243085	-3.85264	0.00011685	0.0002771	DOWN
PINK1-AS	131.4186	-0.40369	0.104788	-3.85249	0.000116925	0.0002773	NOT
HDAC10	380.4688	0.445655	0.115688	3.852217	0.000117053	0.0002776	NOT
RP11-134	22.3415	-0.7396	0.19201	-3.8519	0.000117203	0.0002779	DOWN
NCEH1	642.7643	0.943243	0.244911	3.851362	0.000117463	0.0002785	UP
ADAMTS4	919.6668	-0.75973	0.197266	-3.85127	0.000117506	0.0002786	DOWN
TNFRSF11	150.5055	0.911072	0.236581	3.850987	0.000117643	0.0002789	UP
RCAN3	306.4143	0.632058	0.164132	3.850918	0.000117676	0.0002789	UP
QPCTL	769.0454	0.497103	0.129091	3.850795	0.000117735	0.000279	NOT
GPSM3	563.0383	-0.60347	0.156723	-3.85054	0.000117858	0.0002793	DOWN
NOL4L	612.6656	0.463355	0.120341	3.850333	0.000117957	0.0002795	NOT
APOC1P1	3281.725	-1.25983	0.327206	-3.85027	0.000117989	0.0002796	DOWN
TRAM2-A	407.965	-0.5229	0.135809	-3.85024	0.000118003	0.0002796	NOT
SRGAP3-A	1.417576	2.702406	0.701956	3.849823	0.000118203	0.00028	UP
THPO	808.6295	-0.6105	0.158583	-3.84973	0.000118248	0.0002801	DOWN
C19orf12	2227.512	-0.42386	0.110106	-3.84956	0.000118332	0.0002803	NOT
STAG3L1	7.499786	0.701337	0.182196	3.84935	0.000118432	0.0002805	UP
ATG13	3178.333	0.244462	0.063511	3.849155	0.000118526	0.0002807	NOT
LINC0150	2.688562	-2.27789	0.591836	-3.84886	0.000118671	0.000281	DOWN
C6orf132	179.7619	1.3353	0.34697	3.848463	0.000118861	0.0002814	UP
AC008060	1.541166	2.94384	0.764986	3.848229	0.000118975	0.0002817	UP
DLX3	4.78379	2.107054	0.547561	3.848071	0.000119052	0.0002818	UP
RPL14P3	4.038522	0.833378	0.21658	3.847896	0.000119137	0.000282	UP
DCUN1D5	526.3554	0.385417	0.100164	3.84787	0.000119149	0.000282	NOT
NR1H3	2949.388	-0.39916	0.103736	-3.8478	0.000119181	0.0002821	NOT
RP11-71E	21.51765	0.950399	0.247035	3.847216	0.000119468	0.0002827	UP
RAB32	1181.647	-0.4611	0.119866	-3.8468	0.000119671	0.0002832	NOT
JAK1	5832.572	-0.35458	0.092187	-3.84634	0.000119896	0.0002837	NOT
KCNK13	59.19176	1.121451	0.291566	3.846295	0.000119917	0.0002837	UP
FAM92A1	6.578119	-0.735	0.191111	-3.84592	0.000120101	0.0002841	DOWN
TLR2	359.0484	-0.82483	0.214481	-3.84572	0.000120197	0.0002843	DOWN
CEP78	559.6097	0.405531	0.105451	3.845684	0.000120217	0.0002843	NOT
RPS2P4	3.615976	1.014244	0.263738	3.845656	0.00012023	0.0002844	UP
RP11-275	1.837512	1.09957	0.285936	3.845512	0.000120301	0.0002845	UP

EIF2S3L	10.44229	0.926536	0.240966	3.845089	0.000120509	0.000285	UP
RP3-394A:	198.0883	0.797245	0.207358	3.844782	0.00012066	0.0002853	UP
NEK6	3459.231	-0.37731	0.098147	-3.84437	0.000120864	0.0002858	NOT
PRPF31	2259.967	0.396888	0.103258	3.843663	0.000121212	0.0002866	NOT
ATG10	314.0801	0.369678	0.096186	3.843385	0.000121349	0.0002869	NOT
RP11-212I	14.35001	0.819039	0.213121	3.843073	0.000121503	0.0002872	UP
AZIN2	79.34129	0.554918	0.14442	3.842383	0.000121846	0.000288	NOT
COQ4	2223.706	-0.38518	0.100256	-3.84195	0.00012206	0.0002885	NOT
IGLV10-54	26.4381	-1.68672	0.439052	-3.84173	0.000122168	0.0002887	DOWN
ZFR	2556.511	0.258915	0.067396	3.841715	0.000122178	0.0002887	NOT
FAF1	1848.103	0.288381	0.075067	3.841638	0.000122216	0.0002888	NOT
RP11-521I	4.368627	0.944686	0.245917	3.841487	0.000122291	0.0002889	UP
ELL2P1	6.705681	-0.81081	0.21107	-3.84144	0.000122312	0.0002889	DOWN
HOXC11	2.048848	3.233103	0.841639	3.841439	0.000122315	0.0002889	UP
EFCAB8	3.864217	1.073701	0.279513	3.841328	0.000122371	0.000289	UP
AC007792	1.209808	1.770172	0.460842	3.841169	0.00012245	0.0002892	UP
CTD-2588	2.647525	-0.95431	0.248503	-3.84024	0.000122912	0.0002903	DOWN
LINC0120E	1.678438	1.955585	0.509246	3.840158	0.000122955	0.0002903	UP
NT5DC3	525.349	0.475624	0.123863	3.839935	0.000123067	0.0002906	NOT
MAPKAP1	2736.05	0.251747	0.065567	3.839522	0.000123274	0.000291	NOT
TRPM6	16.17137	1.195339	0.311328	3.839485	0.000123293	0.0002911	UP
CDKL2	2.351498	-1.16401	0.303174	-3.8394	0.000123337	0.0002911	DOWN
RPS2P7	16.54004	0.67226	0.175104	3.839211	0.00012343	0.0002913	UP
HSD17B4	15286.36	-0.5124	0.133467	-3.83913	0.000123471	0.0002914	NOT
PCDHGB8I	3.599022	1.420935	0.37012	3.839117	0.000123478	0.0002914	UP
RASSF7	2223.792	0.5433	0.14155	3.83821	0.000123934	0.0002924	NOT
KDM1A	1651.068	0.317272	0.082664	3.838089	0.000123995	0.0002926	NOT
RP3-486I3	5.356838	-0.72223	0.188206	-3.83746	0.000124313	0.0002933	DOWN
GIT2	607.0378	0.353978	0.092256	3.83693	0.000124582	0.0002939	NOT
SPAG9	2594.629	-0.31646	0.082477	-3.83689	0.000124602	0.0002939	NOT
TSSK4	14.5673	0.708589	0.184696	3.836516	0.000124792	0.0002943	UP
RP11-218(	8.342239	0.733202	0.191126	3.836232	0.000124936	0.0002947	UP
AGFG1	2173.214	0.305009	0.079514	3.83592	0.000125095	0.000295	NOT
NUP210P3	1.755151	1.477318	0.385135	3.835845	0.000125133	0.0002951	UP
KIAA1328	134.9231	0.303695	0.079179	3.835565	0.000125276	0.0002954	NOT
TRIM58	3.862112	-0.97338	0.253798	-3.83525	0.000125435	0.0002957	DOWN
MAFIP	9.349043	1.058606	0.276022	3.835227	0.000125448	0.0002957	UP
SNX29P2	17.31828	-0.85787	0.223682	-3.83521	0.000125457	0.0002957	DOWN
CTB-186H	8.509085	0.872879	0.227609	3.834994	0.000125567	0.000296	UP
RP11-442(	19.80354	-1.09531	0.285623	-3.8348	0.000125668	0.0002962	DOWN
MORN2	325.109	0.423366	0.110411	3.834443	0.000125849	0.0002966	NOT
ASPN	831.4305	-0.7878	0.205484	-3.83388	0.000126136	0.0002972	DOWN
CSTF3	1001.579	0.222014	0.057909	3.83385	0.000126153	0.0002972	NOT
TROVE2	1105.355	0.376932	0.098323	3.833626	0.000126268	0.0002975	NOT
PHF1	1728.312	0.32137	0.083829	3.833612	0.000126275	0.0002975	NOT
SSH1	1341.707	0.29698	0.077468	3.833583	0.00012629	0.0002975	NOT
LUZP1	570.088	0.364203	0.095003	3.833574	0.000126295	0.0002975	NOT
TXK	28.16971	-0.68594	0.178973	-3.83264	0.000126777	0.0002986	DOWN
RPSAP14	3.487017	0.816089	0.212937	3.832544	0.000126825	0.0002987	UP
DPP3	1670.021	0.334538	0.087289	3.832532	0.000126831	0.0002987	NOT
CTC-487M	4.079893	0.813529	0.212273	3.832464	0.000126866	0.0002987	UP
UBE2E1	1586.196	0.281248	0.073397	3.831875	0.00012717	0.0002994	NOT

PDCL3P4	23.96836	0.717284	0.187212	3.831404	0.000127414	0.0003	UP
RP11-159I	6.313749	0.612949	0.160009	3.830725	0.000127766	0.0003008	UP
CPEB2-AS	3.199785	1.00998	0.263658	3.830646	0.000127807	0.0003008	UP
TBX18	34.37863	1.296805	0.338606	3.829833	0.00012823	0.0003018	UP
RP11-470I	2.692673	-1.4739	0.38485	-3.8298	0.000128247	0.0003018	DOWN
RP11-493I	2.212053	1.126858	0.294262	3.829431	0.00012844	0.0003022	UP
RP11-165I	0.809473	1.912105	0.499354	3.829161	0.000128581	0.0003026	UP
SLC35G2	180.2093	0.712909	0.186182	3.829102	0.000128612	0.0003026	UP
SRD5A3-A	19.66554	0.515909	0.134736	3.829034	0.000128647	0.0003027	NOT
ONECUT3	2.038478	1.611618	0.420911	3.828883	0.000128726	0.0003028	UP
DOLK	908.4188	0.3016	0.078784	3.828199	0.000129084	0.0003036	NOT
GP5	6.994919	-1.17583	0.307165	-3.828	0.000129191	0.0003039	DOWN
CHURC1	1308.989	-0.34694	0.09065	-3.82719	0.000129615	0.0003048	NOT
LINC0114(	12.03558	0.830737	0.217069	3.827058	0.000129684	0.000305	UP
RP11-175I	18.52066	0.88321	0.230791	3.826881	0.000129777	0.0003052	UP
PES1	3289.946	0.371479	0.097076	3.826686	0.00012988	0.0003054	NOT
ZC3H14	1491.596	-0.31916	0.083408	-3.82649	0.000129983	0.0003056	NOT
TMCO2	1.222956	1.642717	0.429302	3.826487	0.000129985	0.0003056	UP
N4BP2L2-I	38.74179	0.629581	0.164557	3.825915	0.000130287	0.0003063	UP
MTCL1	293.9934	0.929913	0.243065	3.825772	0.000130363	0.0003064	UP
KLHL42	684.7201	0.382255	0.099921	3.825555	0.000130478	0.0003067	NOT
RP1-193H	199.9939	0.855945	0.223801	3.824577	0.000130997	0.0003078	UP
GAL3ST2	10.37724	1.481777	0.387447	3.824465	0.000131056	0.000308	UP
RP11-54D	3.72769	1.593431	0.416673	3.824179	0.000131209	0.0003083	UP
GAPDHP1	11.9181	1.342604	0.351141	3.823542	0.000131548	0.0003091	UP
CTC-513N	3.754666	1.934567	0.505973	3.823461	0.000131591	0.0003091	UP
RP1-197B	11.64044	-0.73579	0.192452	-3.82324	0.00013171	0.0003094	DOWN
ADAMTS9	17.70444	-0.84689	0.221511	-3.82324	0.000131711	0.0003094	DOWN
RELB	1102.987	0.584879	0.152985	3.823113	0.000131777	0.0003095	NOT
CICP27	1.283212	1.584644	0.414536	3.822694	0.000132002	0.00031	UP
GPR115	5.287621	2.001747	0.523686	3.822415	0.000132151	0.0003103	UP
RPL31P52	1.437141	1.292732	0.338235	3.821996	0.000132376	0.0003108	UP
MTMR9LP	132.7668	0.530517	0.138809	3.821934	0.000132409	0.0003109	NOT
ADORA2B	55.66109	0.987728	0.25844	3.821886	0.000132435	0.0003109	UP
CTD-2501	1.632684	1.222302	0.319843	3.82157	0.000132605	0.0003113	UP
FAM208B	1291.737	0.38702	0.101273	3.821541	0.00013262	0.0003113	NOT
EPT1	2718.226	-0.38258	0.100112	-3.82147	0.00013266	0.0003114	NOT
SEC24A	2666.704	-0.4291	0.112294	-3.8212	0.000132802	0.0003117	NOT
LINC0146(	0.974354	1.674091	0.438112	3.821152	0.00013283	0.0003117	UP
COPS6	3724.412	0.316878	0.082927	3.821144	0.000132834	0.0003117	NOT
DCTN4	2241.895	0.251072	0.065708	3.821043	0.000132888	0.0003118	NOT
PAK1IP1	672.2984	0.350713	0.091795	3.820613	0.00013312	0.0003123	NOT
AASDH	329.944	-0.29429	0.077036	-3.8201	0.000133398	0.0003129	NOT
AC109826	7.508627	1.064209	0.278583	3.820079	0.000133409	0.0003129	UP
RP4-669K	16.73993	0.658996	0.172519	3.819856	0.00013353	0.0003132	UP
NCAM1	173.6363	-1.20949	0.316648	-3.81968	0.000133625	0.0003134	DOWN
RP11-146I	2.904349	-1.09561	0.286913	-3.81863	0.000134197	0.0003147	DOWN
SAP18	4325.217	-0.31283	0.081925	-3.81846	0.000134286	0.0003149	NOT
RP11-863I	2.686225	-1.42015	0.371947	-3.81816	0.000134453	0.0003152	DOWN
WDR72	1703.883	-1.0446	0.273641	-3.81743	0.000134849	0.0003161	DOWN
TTC23	679.2602	0.398381	0.104364	3.817238	0.000134954	0.0003164	NOT
CFH	62992.13	-0.68829	0.180314	-3.81716	0.000134998	0.0003164	DOWN

MB21D1	101.223	0.758774	0.19882	3.816379	0.000135424	0.0003174	UP
MIR483	2.314527	3.29214	0.862651	3.816308	0.000135464	0.0003175	UP
SH3RF3-A	18.13471	1.065929	0.279342	3.815859	0.00013571	0.000318	UP
AC099552	1.763519	3.278075	0.859118	3.815629	0.000135837	0.0003183	UP
UBE2MP1	13.66631	0.529366	0.138742	3.815465	0.000135927	0.0003185	NOT
IPO11	632.8431	0.248147	0.065038	3.815403	0.000135961	0.0003185	NOT
RP11-728I	3.096755	1.117401	0.29288	3.815216	0.000136064	0.0003188	UP
ST5	1558.34	0.464099	0.121652	3.814973	0.000136198	0.000319	NOT
CPNE4	3.37503	1.673157	0.438586	3.814888	0.000136245	0.0003191	UP
MFN1	961.9248	0.259282	0.067969	3.81472	0.000136337	0.0003193	NOT
ENO1	40033.81	0.543812	0.142557	3.814693	0.000136352	0.0003193	NOT
YWHAZP6	2.308842	1.024333	0.268526	3.814649	0.000136376	0.0003194	UP
RP11-700I	1.694096	1.218808	0.31958	3.813776	0.000136859	0.0003205	UP
C11orf88	1.099493	1.616397	0.423867	3.813457	0.000137036	0.0003208	UP
GUCD1	9054.984	-0.42176	0.110602	-3.81332	0.000137114	0.000321	NOT
MMEL1	12.90915	1.12556	0.29522	3.812617	0.000137503	0.0003219	UP
CLEC17A	4.68353	-1.22675	0.321764	-3.81258	0.000137522	0.0003219	DOWN
MTRR	1291.392	-0.31405	0.082373	-3.8125	0.00013757	0.000322	NOT
RP11-809I	15.69029	-0.82516	0.216446	-3.81232	0.000137667	0.0003222	DOWN
RP11-512I	5.439179	0.739295	0.193969	3.811413	0.000138175	0.0003233	UP
CIT	372.7655	0.698531	0.183305	3.810764	0.000138538	0.0003242	UP
KDM3A	1269.288	0.348146	0.09136	3.810696	0.000138576	0.0003242	NOT
ADAMTS1	196.2104	0.762277	0.200077	3.809918	0.000139013	0.0003252	UP
CTD-2666	3.136566	0.984474	0.258403	3.809843	0.000139055	0.0003253	UP
RP11-549I	2.761441	2.479048	0.650768	3.809417	0.000139295	0.0003258	UP
CTD-2540	0.95915	1.931607	0.507236	3.808101	0.000140038	0.0003275	UP
LINC0108	3.019913	1.381374	0.362777	3.807772	0.000140224	0.0003279	UP
FTH1P10	16.44536	0.768965	0.201952	3.807653	0.000140292	0.0003281	UP
GPRC5C	7261.911	-0.44938	0.118024	-3.8075	0.000140377	0.0003282	NOT
CHRNA6	2.530939	1.658014	0.435519	3.806986	0.00014067	0.0003289	UP
LA16c-35	3.346591	-0.87985	0.23114	-3.80657	0.000140906	0.0003294	DOWN
RP11-715	97.87309	0.889864	0.233783	3.806365	0.000141024	0.0003297	UP
ZNF135	68.97797	0.939447	0.246821	3.806188	0.000141125	0.0003299	UP
RP11-656I	18.92747	0.771464	0.202691	3.806106	0.000141172	0.00033	UP
RP11-83M	1.563469	2.090171	0.549172	3.806043	0.000141208	0.00033	UP
IGLVI-70	3.062941	-2.49529	0.655618	-3.80602	0.000141224	0.00033	DOWN
CIAPIN1	1417.262	0.320392	0.084183	3.805922	0.000141277	0.0003301	NOT
SETD9	308.2108	-0.43667	0.114737	-3.80585	0.00014132	0.0003302	NOT
ITFG1	2709.768	-0.41927	0.110178	-3.80538	0.000141587	0.0003308	NOT
ZNF416	122.1223	0.404698	0.106354	3.805188	0.000141697	0.000331	NOT
IGHV1OR2	1.283761	-1.78358	0.468785	-3.80469	0.000141981	0.0003317	DOWN
PNMAL1	83.75389	1.410016	0.370671	3.803958	0.000142403	0.0003326	UP
CYP2AB1P	1.36799	2.641701	0.69452	3.803634	0.000142589	0.000333	UP
VWA9	1179.959	-0.19841	0.052172	-3.80299	0.000142958	0.0003339	NOT
ARHGEF17	820.9567	0.581069	0.152823	3.802241	0.000143393	0.0003348	NOT
RP11-973I	1.519952	1.343915	0.353495	3.80179	0.000143655	0.0003354	UP
CTC-378H	2.842539	-0.93974	0.247191	-3.80167	0.000143725	0.0003356	DOWN
KCNC3	267.4469	0.613946	0.161532	3.800781	0.000144241	0.0003367	UP
CA5A	912.2851	-0.97696	0.257049	-3.80067	0.000144304	0.0003369	DOWN
PSMF1	6854.984	-0.28055	0.073818	-3.80059	0.000144354	0.0003369	NOT
CPT1A	8724.519	-0.54064	0.142252	-3.80054	0.000144383	0.000337	NOT
TMOD1	587.94	-0.90705	0.23867	-3.80045	0.000144433	0.0003371	DOWN

AC005154	2.022547	1.24461	0.327504	3.800286	0.000144529	0.0003373	UP
RP11-532I	1.5347	1.715872	0.451526	3.800159	0.000144603	0.0003374	UP
JMJD6	1015.584	0.419903	0.110497	3.800142	0.000144613	0.0003374	NOT
HDC	49.89683	-0.95304	0.250812	-3.79984	0.000144788	0.0003378	DOWN
SDF4	7523.484	-0.31995	0.084206	-3.79961	0.000144923	0.0003381	NOT
CCDC186	425.6776	-0.42135	0.110895	-3.7995	0.000144989	0.0003382	NOT
FAM129C	16.68087	-0.91293	0.240294	-3.79923	0.000145145	0.0003385	DOWN
COL3A1	17527.85	1.006628	0.26499	3.798736	0.000145436	0.0003392	UP
RP11-666/	4.365875	0.933803	0.245885	3.797718	0.000146034	0.0003406	UP
LINC0115Z	24.3495	1.328404	0.349796	3.797658	0.00014607	0.0003406	UP
LCE2D	1.612446	-1.91157	0.503362	-3.79761	0.000146097	0.0003406	DOWN
TAF2	906.1041	0.408002	0.107449	3.797185	0.000146349	0.0003412	NOT
CYP4Z1	8.663269	-1.10302	0.290527	-3.79663	0.000146677	0.0003419	DOWN
ZNF114	6.64988	0.884391	0.232972	3.796132	0.000146971	0.0003426	UP
CTD-3128	5.877971	0.965611	0.254378	3.795961	0.000147073	0.0003428	UP
RP11-895I	1.007362	1.804099	0.475323	3.795527	0.00014733	0.0003434	UP
AP005901.	1.620526	3.086264	0.81317	3.795349	0.000147436	0.0003436	UP
METAP2	2962.476	-0.23469	0.061837	-3.7953	0.000147465	0.0003436	NOT
RP11-752(	2.576302	1.14441	0.301618	3.794234	0.0001481	0.0003451	UP
ALS2	1481.959	-0.30453	0.080267	-3.79395	0.000148268	0.0003454	NOT
RP5-1186I	1.571027	1.934269	0.509831	3.793937	0.000148277	0.0003454	UP
GGNBP2	2326.626	0.205215	0.05409	3.793933	0.00014828	0.0003454	NOT
SEC63	4690.782	-0.30531	0.080472	-3.79393	0.000148283	0.0003454	NOT
RP11-352(	2.679167	1.245531	0.328317	3.793687	0.000148427	0.0003457	UP
LINC00365	41.70607	-1.01846	0.268497	-3.79318	0.000148728	0.0003464	DOWN
WDR5B	311.2062	0.32396	0.085407	3.79311	0.000148772	0.0003464	NOT
CDK5R2	1.478677	2.705471	0.71327	3.793051	0.000148808	0.0003465	UP
RP11-254I	212.9504	0.47027	0.123996	3.792614	0.00014907	0.0003471	NOT
LA16c-36(	9.433127	0.620843	0.163719	3.792124	0.000149365	0.0003477	UP
KLHDC3	5198.625	0.382027	0.100746	3.791985	0.000149448	0.0003479	NOT
MYCBP2	1124.864	-0.43246	0.114048	-3.79186	0.000149523	0.000348	NOT
CASP14	1.927509	2.707277	0.714032	3.791533	0.00014972	0.0003485	UP
RP11-815I	10.97334	0.641675	0.169242	3.791456	0.000149766	0.0003486	UP
RP11-377I	5.352788	0.764327	0.201596	3.791389	0.000149807	0.0003486	UP
FLJ26850	4.593022	0.993696	0.262103	3.791239	0.000149898	0.0003488	UP
HBM	1.206286	-1.93682	0.510934	-3.79074	0.000150197	0.0003495	DOWN
GUSBP2	19.66311	0.685961	0.180965	3.790578	0.000150297	0.0003497	UP
ARL4D	1914.338	-0.78934	0.208249	-3.79035	0.000150435	0.00035	DOWN
ITIH3	79645.67	-0.82687	0.21817	-3.79003	0.000150632	0.0003504	DOWN
AF277315.	4.895931	4.622297	1.219609	3.789982	0.000150658	0.0003504	UP
SLC35F1	9.398905	0.914278	0.241248	3.789786	0.000150777	0.0003507	UP
ETAA1	409.3192	0.311376	0.082163	3.789739	0.000150806	0.0003507	NOT
H2AFJ	3112.868	0.623195	0.164449	3.789587	0.000150898	0.0003509	UP
RP11-29H	3.186113	0.983598	0.25959	3.78904	0.00015123	0.0003516	UP
DPEP1	48.43599	1.324247	0.349496	3.789024	0.00015124	0.0003516	UP
RP11-394(	85.97996	0.880419	0.232368	3.788907	0.000151312	0.0003518	UP
ZFP92	81.79736	1.010917	0.266857	3.788237	0.00015172	0.0003527	UP
CCT7	7737.068	0.278816	0.073609	3.787819	0.000151975	0.0003532	NOT
RARG	293.7476	0.539849	0.142523	3.787787	0.000151995	0.0003533	NOT
PACRGL	178.3075	0.314624	0.083083	3.786886	0.000152547	0.0003545	NOT
WDR13	4906.428	0.523266	0.13818	3.786834	0.000152579	0.0003546	NOT
METTL9	1096.156	0.383048	0.101159	3.786604	0.00015272	0.0003549	NOT

VMP1	5984.922	0.335708	0.088662	3.786353	0.000152874	0.0003552	NOT
RHBDD1	1175.756	-0.24842	0.065613	-3.78609	0.000153035	0.0003555	NOT
FAM96A	3829.73	-0.38508	0.101745	-3.78477	0.000153851	0.0003574	NOT
ACTA2	4255.05	0.626021	0.165419	3.784452	0.000154048	0.0003578	UP
AC011899	56.40788	-0.76649	0.20254	-3.78438	0.000154091	0.0003579	DOWN
RP11-644I	233.114	0.399188	0.105492	3.784072	0.000154283	0.0003583	NOT
RP11-580I	15.85819	0.533827	0.141073	3.784056	0.000154293	0.0003583	NOT
PWAR1	1.840925	1.577977	0.417075	3.783435	0.000154679	0.0003592	UP
PFKFB1	831.3033	-0.95366	0.252112	-3.78268	0.000155151	0.0003602	DOWN
RP11-380I	14.58331	-0.8603	0.227436	-3.78262	0.000155189	0.0003603	DOWN
RNF219	206.8225	0.38951	0.102976	3.782535	0.000155239	0.0003604	NOT
XAGE3	7.278494	-1.03988	0.274919	-3.78252	0.000155251	0.0003604	DOWN
DRG1	2019.57	0.320547	0.084749	3.78229	0.000155392	0.0003607	NOT
AC068282	122.0551	0.690836	0.182654	3.782209	0.000155443	0.0003608	UP
TAS2R20	6.677822	0.786367	0.207959	3.781358	0.000155975	0.000362	UP
PRKAB1	1419.015	0.295519	0.078156	3.781151	0.000156105	0.0003622	NOT
RPS4XP1	3.318404	0.851929	0.225336	3.780712	0.00015638	0.0003628	UP
GHc-857G	5.519849	0.761581	0.201448	3.780526	0.000156497	0.0003631	UP
RP11-114I	1.109086	1.994203	0.527518	3.780353	0.000156606	0.0003633	UP
MRPL35P2	6.476914	-0.89745	0.237422	-3.77999	0.000156832	0.0003638	DOWN
RP11-107(	19.87563	-0.94846	0.250967	-3.7792	0.000157332	0.0003649	DOWN
DTX3	319.484	0.721462	0.190926	3.778743	0.000157622	0.0003656	UP
CAB39L	244.1589	-0.40546	0.107301	-3.77868	0.000157662	0.0003656	NOT
NPIPB11	18.13194	0.793424	0.20998	3.778566	0.000157734	0.0003658	UP
RPL7AP11	8.119912	0.7587	0.20084	3.777628	0.000158329	0.0003671	UP
IFT88	619.7634	-0.43295	0.11461	-3.77757	0.000158364	0.0003672	NOT
TPM3P6	10.59013	0.829744	0.219655	3.777494	0.000158414	0.0003673	UP
CDHR3	196.8486	0.548106	0.145102	3.777377	0.000158489	0.0003674	NOT
XK	161.9746	1.0448	0.276618	3.777052	0.000158696	0.0003678	UP
TUBAL3	2.231064	2.303044	0.609749	3.777036	0.000158706	0.0003678	UP
RP11-160(	79.93518	0.719287	0.190464	3.776506	0.000159044	0.0003686	UP
SMAD1	503.1925	-0.59947	0.158765	-3.77586	0.000159457	0.0003695	DOWN
RP11-97O	4.721098	0.791445	0.209639	3.775268	0.000159836	0.0003704	UP
CTD-2083	9.720547	0.668803	0.177158	3.775176	0.000159895	0.0003705	UP
MYH3	130.3965	0.863599	0.228768	3.775005	0.000160005	0.0003707	UP
EEF1A1P3(	3.778223	0.849328	0.224988	3.775	0.000160007	0.0003707	UP
CLDND1	1365.69	-0.23458	0.062143	-3.77483	0.000160115	0.0003709	NOT
FKBP6	2.15847	1.603777	0.424874	3.774714	0.000160191	0.000371	UP
NEXN-AS1	3.381824	1.160701	0.307507	3.774552	0.000160295	0.0003712	UP
XXbac-BP(	1.689624	1.483465	0.393018	3.774552	0.000160296	0.0003712	UP
TUBB2B	118.474	0.793264	0.210164	3.774506	0.000160325	0.0003713	UP
CALCOCO:	4764.288	-0.28825	0.07637	-3.77431	0.00016045	0.0003715	NOT
RP11-284I	9.297069	-1.01092	0.267845	-3.77429	0.000160465	0.0003715	DOWN
NIPA1	790.5405	0.500976	0.132745	3.773979	0.000160664	0.0003719	NOT
PRSS50	30.31951	1.156544	0.306472	3.773739	0.000160819	0.0003723	UP
EFNA5	130.5828	1.370684	0.363228	3.773615	0.000160899	0.0003724	UP
LRRFIP2	2358.987	-0.26121	0.06922	-3.77357	0.000160926	0.0003725	NOT
LAD1	2937.648	0.960907	0.254661	3.773273	0.00016112	0.0003729	UP
LINC0089(	62.85798	0.563707	0.149399	3.773163	0.000161191	0.000373	NOT
SARNP	71.82391	0.457929	0.121368	3.773055	0.000161261	0.0003731	NOT
CAMK2A	22.30561	1.266983	0.335807	3.772957	0.000161324	0.0003733	UP
RP11-565I	33.97729	-0.49217	0.130449	-3.77288	0.000161371	0.0003733	NOT

DDX19A	1393.565	-0.23918	0.063396	-3.77284	0.000161398	0.0003734	NOT
RP11-480(	1.618606	1.523313	0.403775	3.772677	0.000161506	0.0003736	UP
RP11-387I	16.1076	-0.78152	0.207194	-3.77193	0.000161991	0.0003747	DOWN
RP11-356(	2.892169	-1.35345	0.358831	-3.77184	0.00016205	0.0003748	DOWN
CDC26	254.8916	-0.31474	0.083451	-3.77161	0.000162198	0.0003751	NOT
AC061992	4.218238	1.395729	0.370067	3.771554	0.000162234	0.0003751	UP
EHBP1L1	1374.429	0.405275	0.107457	3.771519	0.000162257	0.0003752	NOT
CCR8	4.522937	1.45411	0.385572	3.771308	0.000162394	0.0003754	UP
HBS1L	1757.407	-0.30492	0.08086	-3.77093	0.000162641	0.000376	NOT
CCDC159	699.789	0.439626	0.116588	3.770771	0.000162744	0.0003762	NOT
IPO4	227.8231	0.493222	0.130804	3.770682	0.000162802	0.0003763	NOT
PANK3	3959.779	-0.38168	0.101224	-3.77062	0.00016284	0.0003764	NOT
HCG17	7.928822	0.915951	0.242978	3.769685	0.000163454	0.0003777	UP
CHD1	1125.225	-0.33457	0.088753	-3.76964	0.000163485	0.0003778	NOT
RP5-1057I	1.176777	1.664002	0.441427	3.769596	0.000163512	0.0003778	UP
DHRS2	3393.427	1.550187	0.411234	3.769593	0.000163514	0.0003778	UP
FAM20C	4562.169	-0.42475	0.112693	-3.76914	0.000163809	0.0003784	NOT
MIR570	14.07041	0.656144	0.174085	3.769102	0.000163836	0.0003785	UP
C3orf14	112.2624	0.974128	0.258479	3.768691	0.000164106	0.0003791	UP
SMPD4P1	1.837752	2.129718	0.565257	3.767699	0.000164759	0.0003805	UP
NANOS3	3.542985	1.189086	0.31562	3.767462	0.000164916	0.0003809	UP
CTC-366B	18.48868	0.521235	0.138353	3.767433	0.000164935	0.0003809	NOT
RP11-573(	2.468758	1.049215	0.278511	3.767225	0.000165073	0.0003812	UP
MAN1B1	3204.074	0.335812	0.089144	3.767054	0.000165185	0.0003814	NOT
RBM41	328.0953	0.384565	0.102093	3.766804	0.000165351	0.0003817	NOT
RP11-2C2	3.1619	1.135048	0.301348	3.766571	0.000165505	0.0003821	UP
CMB9-55/	1.63443	1.309525	0.347679	3.766474	0.00016557	0.0003822	UP
GPR4	301.9048	0.513105	0.136233	3.766375	0.000165635	0.0003823	NOT
ADM5	21.4431	0.75373	0.200123	3.766332	0.000165664	0.0003823	UP
TFPT	529.5374	0.477233	0.126727	3.765817	0.000166005	0.0003831	NOT
PDHX	1397.563	-0.30132	0.080014	-3.76581	0.000166011	0.0003831	NOT
RP11-647I	12.74321	0.638877	0.169677	3.765263	0.000166374	0.0003839	UP
AMFR	7234.834	-0.3939	0.104615	-3.76523	0.000166394	0.0003839	NOT
EIF3J-AS1	138.9084	0.411984	0.10942	3.765179	0.00016643	0.0003839	NOT
ZCCHC9	1102.915	0.340027	0.090316	3.764876	0.000166632	0.0003844	NOT
APPL2	1082.546	0.326181	0.086641	3.764726	0.000166732	0.0003846	NOT
NME7	328.0123	0.38108	0.101226	3.764633	0.000166794	0.0003847	NOT
ORMDL1	1079.783	0.248102	0.065904	3.764582	0.000166828	0.0003847	NOT
LINC0064(	2.189432	1.549373	0.411589	3.764372	0.000166968	0.000385	UP
BIRC2	1984.169	-0.25731	0.068356	-3.76428	0.000167029	0.0003851	NOT
RP11-326I	2.821213	1.373553	0.364894	3.764256	0.000167046	0.0003851	UP
MYH8	1.542056	2.570457	0.682876	3.764163	0.000167108	0.0003852	UP
RPL19P21	5.577816	0.80031	0.212631	3.763844	0.000167321	0.0003857	UP
RP11-16F1	12.7424	0.831645	0.22097	3.763607	0.00016748	0.000386	UP
TTF1	295.4227	0.310742	0.082567	3.763508	0.000167546	0.0003862	NOT
TNFAIP8L2	149.8605	-0.64001	0.170072	-3.76317	0.000167775	0.0003867	DOWN
CAND2	125.2578	-0.9761	0.259406	-3.76282	0.000168005	0.0003872	DOWN
RP11-58E2	9.342405	0.86035	0.228661	3.762561	0.000168182	0.0003875	UP
B2M	102639.8	-0.50294	0.133678	-3.76229	0.000168364	0.0003879	NOT
RP11-49K2	6.688686	0.924499	0.24574	3.762094	0.000168497	0.0003882	UP
LIN7B	134.4186	0.528186	0.140401	3.761976	0.000168576	0.0003883	NOT
CAMK2B	334.9205	-1.23474	0.32826	-3.76148	0.00016891	0.0003891	DOWN

RP11-255I	3.099638	1.04258	0.277186	3.761298	0.000169034	0.0003893	UP
AZU1	4.380922	-0.99296	0.264003	-3.76117	0.000169119	0.0003895	DOWN
CTB-63M2	115.4004	0.896624	0.238404	3.76094	0.000169276	0.0003898	UP
PRAMEF9	8.874903	2.431593	0.646583	3.760682	0.00016945	0.0003902	UP
NDUFA1	5297.213	0.469995	0.124984	3.760442	0.000169614	0.0003905	NOT
FKBP9P1	26.05388	0.902598	0.240048	3.760078	0.00016986	0.0003911	UP
GUSB	7310.581	-0.4044	0.107568	-3.75951	0.000170245	0.0003919	NOT
DLGAP2	14.08123	-1.38112	0.367374	-3.75945	0.000170284	0.000392	DOWN
AP000688.	3.651395	1.912096	0.50865	3.759159	0.000170485	0.0003924	UP
ATAD3C	212.0873	-1.12953	0.300488	-3.75898	0.000170606	0.0003927	DOWN
APH1A	9519.42	0.257716	0.068564	3.758757	0.00017076	0.000393	NOT
DFNA5	415.7527	0.793712	0.211184	3.758395	0.000171007	0.0003935	UP
RP11-203I	3.800149	0.963461	0.256388	3.757829	0.000171394	0.0003944	UP
LINC0151(	3.756626	1.782712	0.474423	3.757641	0.000171523	0.0003946	UP
C8orf88	18.95132	1.157045	0.307981	3.756867	0.000172054	0.0003958	UP
EXOC1	666.7727	0.301953	0.080374	3.756851	0.000172065	0.0003958	NOT
ALDH3B1	688.5802	0.628094	0.167198	3.756598	0.000172239	0.0003962	UP
ANP32E	2182.748	0.356341	0.094858	3.756579	0.000172252	0.0003962	NOT
INTS2	376.3692	0.419575	0.111702	3.756187	0.000172522	0.0003968	NOT
RP11-6O2	14.32463	0.763271	0.203211	3.756041	0.000172622	0.000397	UP
RN7SL221I	2.425862	1.256744	0.334597	3.75599	0.000172657	0.000397	UP
PCDHB13	79.27619	0.873555	0.232582	3.755904	0.000172717	0.0003971	UP
RP1-102E2	7.117039	0.775826	0.206572	3.755723	0.000172842	0.0003974	UP
RP11-242I	1.402731	1.241087	0.330483	3.755368	0.000173087	0.0003979	UP
FAM83G	1968.461	0.585599	0.155944	3.755196	0.000173206	0.0003981	UP
FMO4	1341.563	-0.73786	0.196571	-3.75364	0.000174282	0.0004006	DOWN
INTS6-AS1	47.52011	0.527693	0.140582	3.753631	0.000174292	0.0004006	NOT
RP11-214I	1.690056	1.390094	0.370372	3.753242	0.000174563	0.0004012	UP
AC007879	1.408195	1.326594	0.353459	3.753178	0.000174607	0.0004012	UP
LINC00282	7.566548	0.955603	0.254622	3.753026	0.000174713	0.0004014	UP
DMRTA2	0.977982	2.212753	0.589602	3.752961	0.000174758	0.0004015	UP
RP11-365I	6.676239	0.793972	0.211573	3.752706	0.000174936	0.0004019	UP
WBSCR27	161.7193	0.724007	0.19295	3.752307	0.000175214	0.0004025	UP
PYURF	104.817	-0.46914	0.125035	-3.75212	0.000175347	0.0004028	NOT
MRRF	978.7452	0.294135	0.078405	3.751506	0.000175776	0.0004037	NOT
LINC01063	4.509707	1.032634	0.275259	3.751498	0.000175781	0.0004037	UP
UGT1A7	14.12808	1.300583	0.346709	3.75122	0.000175976	0.0004041	UP
AC000123	20.92752	0.495678	0.132145	3.750999	0.000176131	0.0004044	NOT
RP1-34H1	1.598478	2.034601	0.542433	3.750878	0.000176216	0.0004046	UP
RP13-516I	15.77405	0.641795	0.171111	3.750752	0.000176305	0.0004047	UP
SFXN3	641.2369	0.604243	0.161099	3.750744	0.000176311	0.0004047	UP
RP11-129I	13.4287	2.056712	0.548352	3.750713	0.000176333	0.0004048	UP
AC004237	1.553454	1.202677	0.320696	3.750211	0.000176686	0.0004055	UP
RP11-452I	3.261697	1.529925	0.407964	3.750145	0.000176732	0.0004056	UP
PLCG2	1586.037	-0.55834	0.148886	-3.75011	0.000176754	0.0004056	NOT
C9orf9	164.657	0.534051	0.142411	3.750074	0.000176783	0.0004057	NOT
CTC-463A	53.26672	-0.52108	0.138954	-3.75001	0.000176827	0.0004057	NOT
RP11-102I	1.213086	1.531664	0.408448	3.749958	0.000176864	0.0004058	UP
JSRP1	28.63508	0.955432	0.254829	3.749315	0.000177318	0.0004068	UP
CCDC174	618.9798	0.227634	0.060719	3.748969	0.000177563	0.0004073	NOT
BAZ2B	737.0884	-0.42925	0.114498	-3.74893	0.000177588	0.0004073	NOT
CNTLN	689.6301	-0.46994	0.125359	-3.74877	0.000177703	0.0004076	NOT

CDH18	3.351109	2.564791	0.684176	3.748731	0.000177732	0.0004076	UP
PRELID2	82.81287	0.637317	0.170014	3.748608	0.000177819	0.0004078	UP
MED19	370.5047	0.29947	0.079894	3.748359	0.000177996	0.0004081	NOT
RP11-481I	4.294698	1.328801	0.354506	3.748316	0.000178026	0.0004082	UP
HDAC1	3275.609	0.318277	0.084916	3.748148	0.000178145	0.0004084	NOT
WDR26	4191.319	0.291288	0.077721	3.747849	0.000178357	0.0004089	NOT
LINC00336	1.331866	2.003608	0.534634	3.747627	0.000178515	0.0004092	UP
PHB2	9019.384	-0.34617	0.092382	-3.74723	0.000178801	0.0004098	NOT
RP11-108	6.242446	0.915409	0.244355	3.74623	0.000179512	0.0004114	UP
CCKBR	1.568629	2.725845	0.727686	3.745908	0.000179742	0.0004119	UP
LINC00115	21.76239	0.52447	0.140012	3.745899	0.000179749	0.0004119	NOT
RP11-542I	1.067881	1.979541	0.528589	3.74495	0.00018043	0.0004134	UP
RP11-505I	7.128658	-0.75108	0.20058	-3.74452	0.000180735	0.0004141	DOWN
TAX1BP1	5022.397	0.299059	0.079877	3.744002	0.000181112	0.0004149	NOT
RP11-662	2.310981	2.590876	0.692076	3.743631	0.00018138	0.0004155	UP
SEC13	4559.868	0.325702	0.087007	3.743388	0.000181556	0.0004159	NOT
TUBA3E	6.147083	1.658275	0.443004	3.743254	0.000181653	0.000416	UP
RHOQ	904.2022	0.483552	0.129184	3.743116	0.000181752	0.0004162	NOT
RP11-2E1	1.718649	1.330977	0.355592	3.742991	0.000181843	0.0004164	UP
RP11-173I	3.134803	1.18011	0.315286	3.742985	0.000181847	0.0004164	UP
PRICKLE3	216.0414	0.278079	0.074293	3.742982	0.00018185	0.0004164	NOT
LPIN3	696.5782	0.471889	0.126079	3.742805	0.000181977	0.0004166	NOT
LRP5	8158.878	-0.39838	0.10644	-3.74275	0.000182015	0.0004167	NOT
GPC4	448.4024	1.137635	0.304016	3.742024	0.000182544	0.0004178	UP
SLC6A2	186.2052	2.167132	0.579205	3.741565	0.000182878	0.0004186	UP
RSPRY1	1130.874	-0.27854	0.074444	-3.74154	0.000182893	0.0004186	NOT
RP11-527	12.70723	0.554583	0.148224	3.74151	0.000182918	0.0004186	NOT
PCNT	1074.644	0.320915	0.085774	3.741399	0.000182999	0.0004187	NOT
NWD1	5.330372	1.418263	0.379094	3.741191	0.00018315	0.0004191	UP
RP4-575N	4.97328	-0.73228	0.195741	-3.74106	0.000183243	0.0004192	DOWN
PSMB5	4497.166	0.295579	0.079021	3.740508	0.000183649	0.0004201	NOT
MFRP	1.979382	1.375893	0.36785	3.740361	0.000183756	0.0004203	UP
PRDM6	35.95012	0.853948	0.228329	3.73998	0.000184035	0.0004209	UP
CTC-444N	3.078557	1.125344	0.300915	3.739741	0.00018421	0.0004213	UP
PPIAP21	5.175821	0.978038	0.261527	3.739723	0.000184223	0.0004213	UP
MINPP1	1442.243	-0.41642	0.11135	-3.73968	0.000184252	0.0004213	NOT
RN7SL812	0.862578	1.5147	0.40511	3.738983	0.000184766	0.0004225	UP
LINC0016	1.226535	2.337735	0.625258	3.738835	0.000184875	0.0004227	UP
MAP1LC3I	2234.541	-0.38627	0.103319	-3.73866	0.000185004	0.0004229	NOT
EIF2S2	3211.554	0.304601	0.081475	3.738561	0.000185077	0.0004231	NOT
RNF31	468.0616	0.417328	0.111634	3.738372	0.000185216	0.0004234	NOT
WNT5A	426.8602	0.990534	0.264996	3.737917	0.000185551	0.0004241	UP
SNAI3	38.35765	-0.57109	0.152788	-3.73781	0.000185627	0.0004242	NOT
CPEB2	1229.817	-0.49723	0.133031	-3.73768	0.000185725	0.0004244	NOT
LINC00323	3.331976	1.614422	0.431936	3.737644	0.000185753	0.0004244	UP
TPM1	5196.782	0.534791	0.143093	3.737365	0.000185959	0.0004249	NOT
NPIPB4	31.58485	0.779257	0.208509	3.737292	0.000186013	0.000425	UP
CDKN1A	8326.845	-0.64403	0.172333	-3.7371	0.000186157	0.0004253	DOWN
TEC	129.1276	-0.598	0.160021	-3.73701	0.000186218	0.0004254	DOWN
RP11-754I	1.318696	1.330561	0.356074	3.736755	0.000186411	0.0004258	UP
CTD-2331	2.322266	1.500361	0.401553	3.736401	0.000186673	0.0004263	UP
PRAMEF7	2.252278	2.361531	0.632047	3.73632	0.000186733	0.0004264	UP

AC114730	5.021264	1.738413	0.46532	3.735947	0.00018701	0.000427	UP
RP11-797/	1.520768	1.434215	0.383904	3.735867	0.00018707	0.0004271	UP
CHPF2	1916.616	0.312361	0.083612	3.735862	0.000187073	0.0004271	NOT
RP4-545C:	9.258781	0.888418	0.237824	3.735606	0.000187264	0.0004275	UP
PDE4B	453.7479	-0.74336	0.199021	-3.73509	0.000187645	0.0004283	DOWN
YWHAZP3	4.903412	0.874047	0.234014	3.735017	0.000187702	0.0004284	UP
ZNF496	747.8057	0.603844	0.161675	3.734926	0.00018777	0.0004286	UP
CBLN3	176.9994	-0.89821	0.240501	-3.73476	0.000187896	0.0004288	DOWN
IGLV2-11	164.0838	-1.43934	0.385424	-3.73445	0.000188127	0.0004293	DOWN
AC156455	18.51443	0.863221	0.231172	3.734113	0.000188378	0.0004298	UP
ESCO1	663.1124	0.292064	0.078217	3.734001	0.000188462	0.00043	NOT
ERP44	3987.88	-0.27318	0.073165	-3.73373	0.000188667	0.0004304	NOT
MMP13	1.501738	2.344439	0.627941	3.733534	0.000188811	0.0004307	UP
RP11-131I	5.152746	0.731242	0.195875	3.73321	0.000189055	0.0004312	UP
RP11-10E:	6.415729	-0.80565	0.215833	-3.73273	0.000189414	0.000432	DOWN
RP1-197B:	14.60772	-0.64533	0.172903	-3.7323	0.000189736	0.0004327	DOWN
RP11-445I	1.27108	2.536325	0.679574	3.732229	0.000189793	0.0004328	UP
MTRF1L	271.1395	-0.31008	0.083086	-3.73202	0.000189952	0.0004332	NOT
KLHL33	2.129271	-1.07061	0.286876	-3.73197	0.000189991	0.0004332	DOWN
CCT6P3	77.57014	0.426512	0.114291	3.731789	0.000190125	0.0004335	NOT
ANXA11	5758.765	0.323482	0.08669	3.731489	0.000190351	0.000434	NOT
WNT2	39.25194	-1.51651	0.406419	-3.7314	0.000190419	0.0004341	DOWN
RP11-819(	55.22794	-0.39798	0.106659	-3.73135	0.000190459	0.0004341	NOT
CTC-338M	1.121048	2.371935	0.635768	3.730817	0.00019086	0.000435	UP
CSMD3	5.69053	2.242783	0.601162	3.730744	0.000190915	0.0004351	UP
ZNF790	236.0034	-0.36565	0.09801	-3.73072	0.000190935	0.0004351	NOT
LINC0120:	2.210745	3.595442	0.963861	3.73025	0.00019129	0.0004359	UP
IVL	1.875222	2.723311	0.730162	3.729736	0.000191681	0.0004367	UP
MASP2	9583.091	-0.94305	0.25285	-3.72969	0.000191718	0.0004368	DOWN
MIR103A2	4.395354	-0.57767	0.154912	-3.72904	0.00019221	0.0004379	NOT
WFIKKN1	35.62627	0.86219	0.231219	3.728888	0.000192326	0.0004381	UP
PRAC2	1.92459	3.201244	0.858609	3.728408	0.000192693	0.0004389	UP
NBR1	7150.151	-0.32716	0.087757	-3.72798	0.000193023	0.0004396	NOT
DPH1	656.2299	-0.35826	0.096106	-3.72781	0.000193154	0.0004399	NOT
SIM1	49.2959	1.805001	0.484217	3.72767	0.000193259	0.0004401	UP
NAV2	2058.982	-0.55253	0.148268	-3.72658	0.000194097	0.0004419	NOT
IGHV3-20	10.68615	-1.84954	0.496317	-3.72652	0.000194141	0.000442	DOWN
C16orf54	78.81222	-0.83279	0.223478	-3.72649	0.000194164	0.000442	DOWN
INPP5F	270.0987	0.417458	0.112043	3.725867	0.000194645	0.0004431	NOT
RP11-159:	81.0431	1.091398	0.292948	3.725569	0.000194875	0.0004436	UP
PMP2	3.205603	-1.96413	0.52725	-3.72524	0.000195126	0.0004441	DOWN
SLC4A1AP	1.778599	1.131448	0.303761	3.724793	0.000195475	0.0004449	UP
SEMA6B	481.5566	0.53687	0.144147	3.724457	0.000195736	0.0004454	NOT
TSGA10	71.44802	0.412447	0.11075	3.724125	0.000195994	0.000446	NOT
GLRX3P2	1.869672	1.131877	0.303939	3.724027	0.00019607	0.0004461	UP
AC010883	18.07668	0.686802	0.18443	3.723913	0.000196159	0.0004463	UP
CTB-49A3	1.0326	2.426188	0.651602	3.723418	0.000196544	0.0004471	UP
RP4-800G	1.634018	1.197736	0.321735	3.722747	0.000197067	0.0004483	UP
DNAJB9	4230.08	-0.49459	0.132857	-3.7227	0.000197102	0.0004483	NOT
LINC0033:	1.655633	2.32176	0.623741	3.722317	0.000197403	0.000449	UP
CCDC144I	2.137384	2.99798	0.805422	3.722246	0.000197458	0.000449	UP
HN1L	3747.889	0.357867	0.096152	3.721904	0.000197727	0.0004496	NOT

OSBPL9	4003.759	-0.37885	0.101791	-3.72182	0.000197796	0.0004497	NOT
GAS5-AS1	37.24686	0.435397	0.116992	3.721581	0.000197979	0.0004501	NOT
THSD7A	206.3798	0.684708	0.184007	3.721098	0.000198359	0.0004509	UP
ZNF503	786.7306	0.696954	0.187313	3.720806	0.000198588	0.0004514	UP
PRPH	2.891298	1.653572	0.444419	3.720752	0.000198631	0.0004515	UP
PFN1P3	2.658299	-1.29119	0.347026	-3.72072	0.000198656	0.0004515	DOWN
RP4-730K3	7.929818	0.746268	0.200573	3.72068	0.000198687	0.0004515	UP
FBXW11P1	23.22787	-0.862	0.23168	-3.72067	0.000198696	0.0004515	DOWN
RAP2CP1	4.745515	1.535216	0.412657	3.72032	0.000198971	0.0004521	UP
DHRS4L1	55.67808	-0.71525	0.192256	-3.72029	0.000198997	0.0004521	DOWN
RARRES3	2215.71	-0.69676	0.187292	-3.72018	0.000199084	0.0004523	DOWN
AC005785	4.019658	0.931713	0.250458	3.720038	0.000199193	0.0004525	UP
PGBD5	623.5423	0.816131	0.219404	3.719766	0.000199407	0.000453	UP
ALKBH2	933.2275	0.513036	0.137924	3.719694	0.000199464	0.000453	NOT
ARHGAP24	404.1867	-0.63052	0.169518	-3.7195	0.000199621	0.0004534	DOWN
APOO	536.7768	0.383902	0.103214	3.719479	0.000199634	0.0004534	NOT
GNG12	2556.409	-0.44786	0.120413	-3.71939	0.000199703	0.0004535	NOT
CHRM4	2.837325	1.406404	0.378135	3.719317	0.000199762	0.0004536	UP
ENSAP2	2.753675	0.932024	0.250595	3.719243	0.000199821	0.0004537	UP
CYP3A4	87578.88	-1.69755	0.456455	-3.71898	0.00020003	0.0004541	DOWN
CTB-55O6	43.3257	0.46706	0.125596	3.718767	0.000200197	0.0004545	NOT
CSNK2B	2826.038	0.454545	0.122231	3.71873	0.000200227	0.0004545	NOT
TGOLN2	13487.5	-0.35419	0.095273	-3.71769	0.000201054	0.0004563	NOT
MRPL54	2038.618	-0.52139	0.140272	-3.71703	0.000201579	0.0004575	NOT
AP2B1	4625.821	0.290585	0.078181	3.716799	0.000201763	0.0004579	NOT
RP11-69L1	10.06252	0.860433	0.231521	3.71644	0.00020205	0.0004585	UP
TMSB10P1	2.53609	1.325669	0.356712	3.716355	0.000202118	0.0004586	UP
NEK8	297.9045	0.443712	0.119396	3.716296	0.000202165	0.0004587	NOT
VEGFB	2063.139	0.752016	0.202366	3.716127	0.0002023	0.0004589	UP
RP11-250I	17.53789	-0.65691	0.176809	-3.71538	0.0002029	0.0004602	DOWN
FGFR3	3390.851	0.754825	0.203178	3.715097	0.000203126	0.0004607	UP
CTD-3030	1.053542	2.06106	0.554861	3.714555	0.000203562	0.0004617	UP
ALG12	767.0376	0.401701	0.108171	3.713578	0.00020435	0.0004634	NOT
FAM124B	101.5722	0.905759	0.243955	3.712807	0.000204973	0.0004648	UP
TBCD	3377.202	0.300446	0.080925	3.712666	0.000205088	0.000465	NOT
RNU4-2	16.74696	-1.3333	0.359195	-3.71191	0.000205702	0.0004664	DOWN
ZIM2	2.088129	1.942278	0.523367	3.711123	0.000206342	0.0004678	UP
AC009236	0.820176	2.051153	0.552774	3.710655	0.000206724	0.0004686	UP
RP11-33O	5.523755	0.989434	0.266666	3.710389	0.000206941	0.0004691	UP
KB-1980E6	1.655285	2.219469	0.598212	3.71017	0.00020712	0.0004694	UP
RP13-392I	37.88082	1.060147	0.285772	3.709766	0.000207451	0.0004701	UP
TIGD2	877.2456	-0.62924	0.169626	-3.70956	0.000207623	0.0004705	DOWN
RPLP0	47703.65	0.447288	0.120646	3.707451	0.000209356	0.0004744	NOT
DMXL2	703.8628	0.426039	0.11494	3.706622	0.000210042	0.0004759	NOT
NADSYN1	1842.606	0.324204	0.087478	3.706103	0.000210473	0.0004768	NOT
LINC00593	2.971898	1.808465	0.48799	3.705948	0.000210601	0.0004771	UP
AC083843	187.322	0.621945	0.167826	3.705885	0.000210654	0.0004772	UP
FRG1B	239.8665	0.493458	0.133159	3.705778	0.000210743	0.0004773	NOT
RP11-248I	2.27313	1.818127	0.490652	3.70553	0.00021095	0.0004778	UP
RP4-539M	29.14499	-0.87724	0.23677	-3.70504	0.000211355	0.0004786	DOWN
GPT	6531.24	-0.88998	0.240234	-3.70463	0.0002117	0.0004794	DOWN
RP11-2B6.	6.418763	0.826271	0.223043	3.704532	0.000211782	0.0004795	UP

KAT2B	1859.516	-0.54961	0.148362	-3.70452	0.000211794	0.0004795	NOT
HNRNPA1I	4.91526	0.733034	0.1979	3.704052	0.000212183	0.0004804	UP
RP11-315I	1.882855	1.549771	0.418499	3.703166	0.000212925	0.000482	UP
TRRAP	1945.343	0.338014	0.091294	3.702479	0.000213503	0.0004833	NOT
RP11-496I	0.978693	2.229289	0.602162	3.702143	0.000213786	0.0004839	UP
HMGA1P2	2.157285	1.059326	0.286153	3.701958	0.000213942	0.0004842	UP
CTD-3076	3.253979	1.421074	0.383923	3.701458	0.000214364	0.0004851	UP
AF001548.	26.08795	0.60849	0.164402	3.701225	0.000214561	0.0004855	UP
RPL23A	13868.79	0.49816	0.134608	3.700813	0.00021491	0.0004863	NOT
BIN3	313.4692	-0.37175	0.100468	-3.70014	0.000215484	0.0004875	NOT
PDE11A	391.7351	-0.96613	0.261106	-3.70012	0.000215497	0.0004875	DOWN
DIRAS2	48.48527	1.423715	0.384808	3.699808	0.000215762	0.0004881	UP
RP11-814I	1.613267	1.214684	0.328347	3.699397	0.000216112	0.0004888	UP
ITIH5	500.7387	1.124752	0.304052	3.699209	0.000216273	0.0004891	UP
COX15	1814.906	-0.26162	0.070724	-3.69908	0.000216381	0.0004893	NOT
RP11-175(	236.0422	0.361237	0.097662	3.698866	0.000216565	0.0004897	NOT
NDUFA10	4578.064	-0.29236	0.079045	-3.69859	0.000216802	0.0004902	NOT
CTD-2026	9.117438	0.797005	0.21549	3.698564	0.000216822	0.0004902	UP
RP13-714.	5.516054	1.557352	0.421077	3.6985	0.000216878	0.0004903	UP
PF4	8.574242	-1.35053	0.365201	-3.69804	0.00021727	0.0004912	DOWN
SPIN2B	51.08378	0.336532	0.09101	3.697749	0.00021752	0.0004917	NOT
HAL	6160.888	-1.09018	0.294831	-3.69762	0.000217627	0.0004919	DOWN
SLC39A6	1291.07	0.466231	0.126091	3.69758	0.000217665	0.0004919	NOT
CBX6	1114.595	0.704819	0.190621	3.697488	0.000217743	0.000492	UP
ADRA2A	152.555	1.097951	0.296946	3.697477	0.000217753	0.000492	UP
GALNT11	1337.229	0.311703	0.084302	3.697442	0.000217783	0.0004921	NOT
IGLV7-43	33.72959	-1.46295	0.39567	-3.69741	0.000217811	0.0004921	DOWN
TNIP2	1235.936	0.315477	0.085329	3.697187	0.000218002	0.0004925	NOT
RP11-148I	1.882863	3.123667	0.845037	3.696484	0.000218606	0.0004938	UP
OGFR-AS1	19.42478	-0.67786	0.183403	-3.69599	0.00021903	0.0004947	DOWN
POMGNT2	634.3257	0.341558	0.092423	3.695615	0.000219355	0.0004954	NOT
SUMO1	4623.257	-0.24666	0.066745	-3.69561	0.000219361	0.0004954	NOT
ZSCAN30	332.6011	0.326901	0.088457	3.695605	0.000219364	0.0004954	NOT
FAM221B	7.534191	0.845058	0.228679	3.695391	0.000219549	0.0004957	UP
BATF2	223.4281	-0.48695	0.131793	-3.69477	0.000220083	0.0004969	NOT
AUH	807.0836	-0.38928	0.105363	-3.69467	0.000220174	0.0004971	NOT
PCDHB5	119.0361	1.063158	0.287759	3.694607	0.000220227	0.0004971	UP
VMA21	1283.282	0.319618	0.086516	3.694328	0.000220469	0.0004977	NOT
YWHAQ	5387.494	0.29176	0.078977	3.694225	0.000220559	0.0004978	NOT
AP004372.	2.32719	3.224616	0.872914	3.694082	0.000220683	0.0004981	UP
RP11-260I	11.43933	-1.33451	0.361282	-3.69382	0.000220907	0.0004985	DOWN
AGGF1P2	1.01967	1.777684	0.481309	3.693434	0.000221246	0.0004992	UP
ALMS1P	9.403549	-0.70528	0.190954	-3.69343	0.00022125	0.0004992	DOWN
AC090954	1.152086	2.591178	0.701585	3.693318	0.000221347	0.0004994	UP
RP11-278.	11.67694	0.789485	0.213842	3.691915	0.000222572	0.0005021	UP
RP11-799I	34.72424	0.637873	0.172779	3.691835	0.000222642	0.0005022	UP
RENBP	633.1608	0.954424	0.258526	3.691796	0.000222676	0.0005023	UP
GADL1	2.750601	2.204063	0.59707	3.691463	0.000222967	0.0005029	UP
PRIMPOL	299.2017	0.330902	0.089646	3.691204	0.000223195	0.0005034	NOT
RP4-621N	1.419215	1.393405	0.377572	3.690435	0.000223871	0.0005048	UP
RP11-234/	137.7686	0.626814	0.169857	3.690245	0.000224039	0.0005052	UP
PPP1R3F	245.0055	0.603964	0.163675	3.690029	0.000224229	0.0005055	UP

AC092159	1.938769	1.079382	0.292515	3.690008	0.000224247	0.0005055	UP
RP11-79P!	16.6166	0.714827	0.19372	3.689996	0.000224258	0.0005055	UP
BNIP3P28	3.268906	-1.42364	0.385833	-3.68979	0.000224443	0.0005059	DOWN
RP1-313L4	3.504154	0.816441	0.221303	3.689248	0.000224918	0.0005069	UP
VSTM4	763.5222	0.748044	0.202788	3.688802	0.000225312	0.0005078	UP
PCAT29	17.17933	1.137143	0.308304	3.688388	0.00022568	0.0005086	UP
RP11-797/	3.886508	1.150196	0.31185	3.688294	0.000225763	0.0005087	UP
RP11-375I	6.67533	-1.03143	0.279665	-3.68811	0.000225928	0.0005091	DOWN
FOXQ1	223.1057	1.231773	0.334037	3.687538	0.000226435	0.0005102	UP
IL16	382.5736	-0.60997	0.165425	-3.68729	0.000226656	0.0005106	DOWN
ALDH1A3	151.6994	-0.92325	0.250391	-3.68723	0.000226713	0.0005107	DOWN
FMO5	14200.11	-0.75515	0.204811	-3.68707	0.000226849	0.000511	DOWN
SLC9A2	6.521898	1.220313	0.331014	3.686593	0.000227276	0.0005119	UP
CERK	2494.122	0.42684	0.115782	3.68658	0.000227288	0.0005119	NOT
SUPT20H	674.8842	0.35175	0.095422	3.686257	0.000227576	0.0005125	NOT
INTS4P1	6.975883	0.978205	0.265367	3.686233	0.000227598	0.0005125	UP
PCDHGB7	49.30055	-0.8186	0.222072	-3.68621	0.000227619	0.0005125	DOWN
TMIGD1	1.10599	2.211932	0.600119	3.685822	0.000227966	0.0005132	UP
DDO	525.9626	-0.52282	0.141848	-3.68579	0.000227995	0.0005133	NOT
GAPDHP69	2.765139	1.215984	0.329914	3.685765	0.000228016	0.0005133	UP
PPM1M	517.0219	0.462377	0.125495	3.684441	0.000229205	0.0005159	NOT
RP5-1171I	37.24914	0.986698	0.26781	3.684327	0.000229308	0.0005161	UP
ATG16L2	561.2021	0.426982	0.115894	3.684259	0.000229369	0.0005162	NOT
RP11-333I	5.681053	1.307632	0.355076	3.682686	0.000230789	0.0005193	UP
HECW1	27.89978	1.203465	0.326806	3.682508	0.00023095	0.0005197	UP
GNGT2	44.4159	-0.59745	0.162265	-3.68195	0.000231458	0.0005208	DOWN
RP11-594I	1.080577	2.067148	0.561482	3.681591	0.000231783	0.0005214	UP
SNRK-AS1	1.647404	1.480318	0.402169	3.680835	0.000232472	0.000523	UP
RP11-100(	2.350097	0.981018	0.266557	3.680335	0.000232927	0.0005239	UP
LGALS9C	23.89998	1.355539	0.368387	3.679661	0.000233544	0.0005253	UP
MOGS	3153.335	0.223154	0.060648	3.679475	0.000233715	0.0005256	NOT
GALR3	13.63183	1.306844	0.355182	3.679369	0.000233812	0.0005258	UP
GBGT1	112.8351	0.693608	0.188515	3.679323	0.000233854	0.0005258	UP
LINC00265	32.58466	0.679583	0.184722	3.678958	0.000234189	0.0005266	UP
HNRNPCP	13.28318	0.701048	0.190607	3.677975	0.000235093	0.0005285	UP
HAUS4	818.2813	0.524653	0.142662	3.677596	0.000235442	0.0005293	NOT
CPNE6	14.86338	-1.25623	0.341598	-3.6775	0.000235528	0.0005294	DOWN
DNAJC30	815.0931	-0.38049	0.103466	-3.67739	0.000235637	0.0005296	NOT
EDNRA	197.9518	0.642737	0.174782	3.677367	0.000235654	0.0005296	UP
FDX1L	100.0487	0.472899	0.128606	3.677116	0.000235886	0.0005301	NOT
PIFO	16.78529	1.10163	0.299597	3.677039	0.000235957	0.0005302	UP
IGFLR1	119.7658	-0.65394	0.177847	-3.67698	0.000236015	0.0005303	DOWN
SREBF1	8983.518	-0.615	0.167277	-3.67655	0.000236411	0.0005312	DOWN
AKR1B1	1299.483	0.855843	0.232823	3.675931	0.000236984	0.0005324	UP
RP11-121(	4.825352	0.867947	0.236142	3.675527	0.000237359	0.0005332	UP
CTB-60B1	1.230903	1.971389	0.536361	3.675491	0.000237393	0.0005332	UP
MAGOHB	490.6705	0.315641	0.085881	3.675316	0.000237555	0.0005336	NOT
TCP10	6.65248	1.844424	0.501874	3.675077	0.000237778	0.000534	UP
GPN1	1081.515	0.248948	0.067745	3.674769	0.000238064	0.0005346	NOT
RP11-575I	4.133374	1.891609	0.514883	3.673861	0.000238912	0.0005365	UP
LINC01146	727.0827	-0.97501	0.265397	-3.67377	0.000238997	0.0005366	DOWN
C1orf116	232.3013	1.24737	0.33958	3.673274	0.000239463	0.0005376	UP

RP11-426.	5.986713	1.158761	0.315481	3.672998	0.000239721	0.0005382	UP
CTD-2104	8.84762	0.888425	0.24189	3.67284	0.000239869	0.0005385	UP
C7orf61	5.66637	0.798107	0.217339	3.672168	0.000240501	0.0005398	UP
PITPNM1	945.5754	0.484741	0.132026	3.671554	0.00024108	0.0005411	NOT
EWSR1	6798.162	0.197371	0.053758	3.671496	0.000241135	0.0005412	NOT
CYP1B1-A	23.74432	0.726876	0.197985	3.67137	0.000241253	0.0005414	UP
OCM	2.205479	1.135715	0.309373	3.671025	0.00024158	0.0005421	UP
AMBN	1.452942	2.714905	0.739666	3.670447	0.000242126	0.0005433	UP
CTD-2310	17.21895	-0.52228	0.142312	-3.66996	0.000242584	0.0005442	NOT
COG4	2080.544	0.281648	0.076747	3.669841	0.000242701	0.0005445	NOT
RP11-367I	9.504598	0.912228	0.248579	3.669779	0.00024276	0.0005445	UP
ABHD11	692.6995	0.467555	0.127413	3.669607	0.000242924	0.0005449	NOT
CCDC17	53.10407	0.595508	0.162282	3.669578	0.000242951	0.0005449	UP
POLL	928.3281	0.296898	0.080913	3.669376	0.000243143	0.0005453	NOT
MOGAT3	959.8363	0.93827	0.255717	3.669169	0.00024334	0.0005456	UP
RP11-114I	9.26161	-0.66266	0.180603	-3.66916	0.000243348	0.0005456	DOWN
CTC-479C	31.77166	0.428491	0.116783	3.669121	0.000243386	0.0005457	NOT
SRRM1	1976.194	-0.24109	0.065708	-3.66906	0.00024344	0.0005458	NOT
KCNK2	17.18598	1.908728	0.520237	3.668957	0.000243542	0.0005459	UP
MIR621	466.6019	-0.85531	0.233201	-3.6677	0.000244743	0.0005486	DOWN
AC020571	7.04056	1.30766	0.356536	3.667682	0.000244759	0.0005486	UP
GAPDHP7.	5.249362	0.911753	0.248606	3.667468	0.000244964	0.000549	UP
MCUR1	1635.055	0.319757	0.087189	3.667421	0.000245009	0.0005491	NOT
NUDT9	1671.255	-0.35255	0.096133	-3.66734	0.000245089	0.0005492	NOT
COQ3	408.7741	-0.39063	0.106522	-3.66719	0.000245232	0.0005495	NOT
RP11-766I	1.066142	2.031791	0.554072	3.667019	0.000245394	0.0005498	UP
ZNF493	159.2991	0.527001	0.143722	3.666797	0.000245608	0.0005502	NOT
PTPN4	541.1323	-0.34345	0.093672	-3.66655	0.000245843	0.0005507	NOT
POMGNT1	2801.863	0.284466	0.077591	3.666224	0.000246158	0.0005514	NOT
ZNF131	642.6429	0.247714	0.067571	3.665965	0.000246407	0.0005519	NOT
DIAPH2	1190.004	-0.35866	0.097847	-3.66549	0.000246865	0.0005529	NOT
AP2M1	9804.453	0.342832	0.093544	3.664937	0.0002474	0.000554	NOT
ATP2B4	2896.043	0.583519	0.159226	3.664714	0.000247615	0.0005544	NOT
AC084117	4.191966	-0.83918	0.22901	-3.6644	0.000247923	0.0005551	DOWN
LA16c-431	3.546412	1.166443	0.31835	3.664028	0.00024828	0.0005558	UP
PSIP1	1487.928	0.422386	0.115279	3.664028	0.00024828	0.0005558	NOT
RCSD1	353.8652	-0.61284	0.167299	-3.66316	0.000249126	0.0005577	DOWN
ACSL6	512.3417	1.097319	0.299566	3.663031	0.000249248	0.0005579	UP
LTB	475.9135	0.963798	0.263143	3.662636	0.000249633	0.0005587	UP
CTB-1I21.:	2.226401	3.291969	0.898813	3.662574	0.000249694	0.0005588	UP
NDUFV2P1	85.89907	-0.59792	0.163258	-3.66244	0.000249822	0.000559	DOWN
RPS18	39678.99	0.48072	0.131269	3.662112	0.000250144	0.0005597	NOT
DAB2	2376.412	0.682065	0.18625	3.662102	0.000250154	0.0005597	UP
HSD17B11	8112.723	-0.47454	0.129594	-3.66177	0.000250483	0.0005604	NOT
HULC	4721.196	0.98605	0.269288	3.661688	0.000250559	0.0005605	UP
CERCAM	384.6963	0.820302	0.224024	3.661673	0.000250574	0.0005605	UP
VWFP1	4.426838	1.382701	0.377616	3.66166	0.000250587	0.0005605	UP
RP4-635A.	1.446053	1.264098	0.345245	3.661449	0.000250793	0.0005609	UP
ZNF23	28.10035	0.565324	0.154402	3.661368	0.000250873	0.000561	NOT
DMP1	0.855338	1.873827	0.511819	3.661113	0.000251122	0.0005615	UP
RP11-481(	31.64432	0.410267	0.112063	3.661036	0.000251198	0.0005617	NOT
MSMB	10.09077	1.447524	0.395393	3.660978	0.000251255	0.0005617	UP

REM1	30.03915	0.730948	0.199661	3.660947	0.000251285	0.0005618	UP
RP11-609I	10.31174	0.923275	0.252206	3.660799	0.00025143	0.000562	UP
STXBP5	403.9546	0.475359	0.129857	3.660623	0.000251602	0.0005624	NOT
GID8	2875.402	0.270646	0.073937	3.660472	0.000251751	0.0005627	NOT
LINC01096	1.472131	2.951263	0.806264	3.660416	0.000251806	0.0005627	UP
AC009487	14.37242	0.735209	0.200878	3.65998	0.000252235	0.0005636	UP
C16orf47	6.896018	-1.04418	0.285297	-3.65997	0.000252246	0.0005636	DOWN
NAT8	2204.458	-0.91163	0.249141	-3.6591	0.000253101	0.0005655	DOWN
MUC12	22.14745	1.112889	0.304147	3.659047	0.000253154	0.0005656	UP
AHCYP2	7.797603	-0.92744	0.253494	-3.65861	0.00025359	0.0005665	DOWN
RP11-164(	15.88679	-0.85149	0.232787	-3.6578	0.000254385	0.0005682	DOWN
MRPS22	1565.719	-0.23984	0.065589	-3.65666	0.000255525	0.0005707	NOT
ANKS1A	980.9087	-0.34321	0.093863	-3.65645	0.000255732	0.0005711	NOT
RP11-715I	25.64406	-1.55017	0.423977	-3.65625	0.000255928	0.0005715	DOWN
KCNJ2-AS	8.207815	0.680349	0.186106	3.6557	0.000256481	0.0005727	UP
TSFM	1599.562	-0.29336	0.08025	-3.65562	0.000256558	0.0005728	NOT
RP11-40C(	20.70186	0.723374	0.197883	3.655571	0.00025661	0.0005729	UP
MICU2	1133.89	-0.3192	0.087352	-3.65414	0.000258045	0.0005761	NOT
KRTAP10-4	1.009985	2.117724	0.579543	3.654129	0.000258056	0.0005761	UP
FOXO3	1665.518	-0.37533	0.102714	-3.65408	0.000258105	0.0005761	NOT
LINC00677	1.748639	1.29339	0.354005	3.653594	0.000258595	0.0005772	UP
GCSAML	7.425274	1.57586	0.431397	3.652919	0.000259276	0.0005786	UP
RP4-568B1	9.388093	0.994736	0.272314	3.652893	0.000259303	0.0005787	UP
NT5DC4	5.241882	1.320628	0.361573	3.652457	0.000259744	0.0005796	UP
PURA	1152.862	-0.31606	0.086537	-3.65229	0.000259916	0.0005799	NOT
PILRA	168.2818	-0.52883	0.144813	-3.65184	0.000260371	0.0005809	NOT
BRE	1277.213	0.322207	0.088234	3.651722	0.000260488	0.0005811	NOT
RP11-671I	1.561107	1.181643	0.323607	3.651481	0.000260733	0.0005816	UP
ZMIZ1	2326.428	0.432697	0.11851	3.651143	0.000261076	0.0005823	NOT
RP11-355I	3.423357	-0.78797	0.215816	-3.65112	0.000261097	0.0005823	DOWN
PTPRM	1637.122	0.590267	0.161676	3.65092	0.000261303	0.0005827	UP
RAB8A	1898.25	-0.29761	0.081517	-3.6509	0.000261328	0.0005827	NOT
CARM1	1272.735	0.323676	0.08866	3.650765	0.00026146	0.000583	NOT
SRRM2	12368.73	0.335008	0.091764	3.650741	0.000261485	0.000583	NOT
RAB30-AS	253.5518	0.415647	0.113874	3.650055	0.000262184	0.0005845	NOT
LARS2	1004.017	-0.34206	0.093722	-3.64972	0.000262524	0.0005852	NOT
RANP4	3.63547	0.884065	0.242238	3.649568	0.000262681	0.0005855	UP
UGT2A1	131.9715	-1.52593	0.418196	-3.64884	0.000263431	0.0005871	DOWN
RP11-242I	1.835062	1.10754	0.303534	3.648819	0.000263449	0.0005871	UP
RASGRP3	524.5717	0.504268	0.138236	3.647878	0.000264415	0.0005893	NOT
RP11-192(	3.691636	1.101285	0.301954	3.647199	0.000265115	0.0005908	UP
RP11-130I	213.6185	-0.97313	0.266824	-3.64711	0.000265211	0.0005909	DOWN
PRSS30P	10.49185	1.028	0.281943	3.646124	0.000266226	0.0005931	UP
AC007041	3.054306	0.855293	0.234589	3.645922	0.000266435	0.0005936	UP
AC011524	1.439891	2.841123	0.779335	3.645575	0.000266794	0.0005943	UP
OVAAL	1.236397	2.44088	0.669619	3.645177	0.000267208	0.0005952	UP
GLT1D1	1338.497	-0.99063	0.271811	-3.64455	0.00026786	0.0005966	DOWN
RP11-502I	1.606561	2.486015	0.682127	3.644503	0.000267909	0.0005967	UP
RP11-259I	20.34472	1.164718	0.319615	3.644131	0.000268296	0.0005975	UP
RP11-462I	0.880148	1.869851	0.513124	3.644055	0.000268376	0.0005976	UP
C2orf66	1.667075	1.572535	0.431559	3.643846	0.000268595	0.000598	UP
ARFGEF1	1831.232	0.321814	0.088328	3.643388	0.000269072	0.0005991	NOT

MEIS3P1	16.13925	-0.88568	0.243102	-3.64324	0.000269224	0.0005993	DOWN
XXbac-BP(	8.733676	-0.85883	0.235742	-3.6431	0.000269374	0.0005996	DOWN
RP11-26J3	54.26106	-0.51692	0.141893	-3.64304	0.000269438	0.0005997	NOT
NOL4	34.49439	-1.62963	0.447386	-3.64256	0.000269945	0.0006008	DOWN
FHOD3	137.6216	1.20652	0.33123	3.642545	0.000269956	0.0006008	UP
MAFK	1112.728	-0.57301	0.157314	-3.64243	0.000270075	0.000601	NOT
PTRH2	1110.113	0.406224	0.111533	3.642176	0.000270343	0.0006015	NOT
IGLON5	54.24058	-1.00693	0.276478	-3.64201	0.000270522	0.0006019	DOWN
SH3PXD2A	3.101274	0.999002	0.274312	3.641847	0.000270689	0.0006022	UP
COX5BP6	3.590741	0.866428	0.237909	3.641847	0.000270689	0.0006022	UP
MRPL1	819.6727	-0.31929	0.087686	-3.6413	0.000271262	0.0006034	NOT
CTC-457E	1.522852	2.79791	0.768445	3.641002	0.000271579	0.0006041	UP
CTD-2349	2.759846	1.060939	0.29141	3.640704	0.000271894	0.0006047	UP
EIF4H	6845.262	-0.22696	0.062341	-3.64067	0.000271926	0.0006047	NOT
AP000344.	4.03503	2.183099	0.599755	3.639982	0.000272657	0.0006063	UP
LINC0089	1.368119	2.649765	0.728055	3.639512	0.000273156	0.0006073	UP
RP11-467I	25.7065	0.492568	0.135339	3.639506	0.000273162	0.0006073	NOT
ZNF429	242.1532	-0.45882	0.126071	-3.63936	0.000273314	0.0006076	NOT
RP5-864K1	2.59442	0.933048	0.256422	3.638716	0.000274001	0.0006091	UP
RP1-265C	3.920822	0.966796	0.265703	3.638639	0.000274083	0.0006092	UP
FAM103A2	2.487367	1.411275	0.387872	3.63851	0.00027422	0.0006095	UP
ANKRD11	1986.79	0.271327	0.07459	3.637592	0.000275199	0.0006116	NOT
RP11-378(	3.064721	1.070373	0.294264	3.637457	0.000275343	0.0006119	UP
WNT5A-A	12.7942	1.099285	0.302228	3.637275	0.000275538	0.0006123	UP
TSPYL4	465.6606	-0.44324	0.121864	-3.63717	0.000275651	0.0006125	NOT
SLITRK5	8.040616	1.322541	0.363647	3.636884	0.000275956	0.0006131	UP
H3F3AP6	3.823453	0.729461	0.200577	3.636815	0.00027603	0.0006132	UP
HNRNPA2I	21508.92	0.189002	0.051974	3.636495	0.000276373	0.0006139	NOT
LINC00601	1.95319	3.095199	0.851158	3.636455	0.000276416	0.000614	UP
EXOSC2	687.2993	0.309766	0.085187	3.636295	0.000276588	0.0006143	NOT
LNPEP	1232.563	0.475662	0.130817	3.63609	0.000276807	0.0006147	NOT
ATP4A	3.009384	2.195279	0.603805	3.635743	0.000277181	0.0006155	UP
RP5-826L7	1.867	-1.00555	0.276612	-3.63523	0.000277731	0.0006167	DOWN
AMER1	387.9504	0.645227	0.177502	3.635035	0.000277943	0.0006171	UP
RP11-418J	24.12574	-0.95023	0.261411	-3.635	0.00027798	0.0006171	DOWN
GPR89A	218.9521	0.379535	0.10442	3.63469	0.000278315	0.0006178	NOT
RP11-546I	1.976291	1.163661	0.320156	3.634674	0.000278333	0.0006178	UP
AC092625	2.054984	3.493499	0.961159	3.634673	0.000278333	0.0006178	UP
RFX3	158.0804	0.461262	0.126917	3.634361	0.00027867	0.0006185	NOT
KCNH7	10.3518	-1.25633	0.3457	-3.63416	0.000278885	0.0006189	DOWN
RP11-324I	3.442286	1.120954	0.30848	3.633799	0.000279279	0.0006197	UP
YWHAB	7476.772	0.214732	0.059096	3.633587	0.000279509	0.0006202	NOT
RP11-699(	4.924712	0.908542	0.250059	3.633305	0.000279814	0.0006208	UP
RP11-823I	13.21078	-0.71397	0.196507	-3.63329	0.00027983	0.0006208	DOWN
SCML4	87.95466	-0.81501	0.224318	-3.63329	0.000279834	0.0006208	DOWN
RPL28	24690.13	0.566248	0.155858	3.633099	0.000280038	0.0006212	NOT
FAM154A	3.697109	1.010786	0.27822	3.633049	0.000280092	0.0006212	UP
OTUD6B-A	854.2249	0.358232	0.098615	3.632633	0.000280544	0.0006222	NOT
EIF4G3	1881.38	0.321339	0.08846	3.632608	0.000280571	0.0006222	NOT
ZNF833P	17.20692	0.60885	0.167607	3.632605	0.000280574	0.0006222	UP
UBXN2B	1471.521	0.430775	0.118587	3.632561	0.000280622	0.0006222	NOT
C16orf46	37.78271	-0.49467	0.136185	-3.63234	0.000280867	0.0006227	NOT

TMEM45B	2910.721	0.940116	0.258825	3.632239	0.000280973	0.0006229	UP
MYH10	2603.66	-0.50746	0.139741	-3.63144	0.000281842	0.0006248	NOT
PSEN2	1070.215	0.390513	0.107545	3.631162	0.000282148	0.0006254	NOT
RP11-456I	49.99425	-0.92948	0.255982	-3.63102	0.000282302	0.0006257	DOWN
AC009410	5.373707	1.866123	0.513949	3.630949	0.000282381	0.0006258	UP
RP11-104I	14.65345	0.675819	0.18614	3.63071	0.000282642	0.0006263	UP
RP11-106(	1.901765	1.064765	0.293317	3.630081	0.000283332	0.0006278	UP
RP1-27K1:	11.06132	0.932044	0.256779	3.629755	0.00028369	0.0006286	UP
RUNX3	268.4969	-0.719	0.198095	-3.62956	0.00028391	0.000629	DOWN
PCDHA7	5.292826	1.358679	0.37434	3.629537	0.00028393	0.000629	UP
GRAMD1B	380.9859	1.01701	0.280243	3.629035	0.000284483	0.0006301	UP
GSTO1	12675.62	-0.51302	0.141366	-3.62903	0.000284492	0.0006301	NOT
IGKV1-17	68.50614	-1.50183	0.41389	-3.62858	0.000284985	0.0006312	DOWN
USP2	516.4703	-0.7586	0.209084	-3.62821	0.000285392	0.000632	DOWN
KARSP2	44.60571	0.777314	0.214258	3.62794	0.000285691	0.0006326	UP
PPIC	1606.064	0.387884	0.106923	3.6277	0.000285957	0.0006332	NOT
AC004988	3.371932	1.402775	0.386716	3.627404	0.000286286	0.0006339	UP
PFDN1	1221.312	0.268517	0.074031	3.627073	0.000286652	0.0006346	NOT
RP11-816:	4.438303	0.867571	0.239207	3.626872	0.000286876	0.0006351	UP
CHST5	1.63491	1.954022	0.538989	3.625345	0.000288576	0.0006388	UP
DOHH	582.9456	0.365338	0.100785	3.62493	0.00028904	0.0006397	NOT
RPL17P11	13.36017	-1.19036	0.328416	-3.62456	0.00028945	0.0006406	DOWN
TCEA3	6463.243	-0.52999	0.146225	-3.6245	0.000289526	0.0006407	NOT
RP11-363I	139.6713	-0.49494	0.136563	-3.62429	0.000289761	0.0006412	NOT
TRGC2	40.61725	-0.86799	0.239501	-3.62416	0.000289904	0.0006415	DOWN
PARPG1	31.67033	0.568981	0.157013	3.623785	0.000290323	0.0006423	NOT
RP11-89C:	3.907104	1.33926	0.369621	3.623336	0.000290828	0.0006434	UP
CTD-2017	8.51175	0.73262	0.202212	3.623038	0.000291163	0.0006441	UP
CTD-3187	3.658598	-1.04563	0.288612	-3.62295	0.000291261	0.0006443	DOWN
ZNRF2	1240.228	-0.3732	0.103019	-3.6226	0.000291659	0.0006451	NOT
RP11-540I	127.8397	0.35527	0.098076	3.622401	0.000291881	0.0006455	NOT
PFN1P7	5.864604	1.187053	0.327758	3.621736	0.000292632	0.0006471	UP
TLR5	176.5219	0.658375	0.181788	3.621666	0.000292712	0.0006473	UP
WDR59	2044.361	-0.34552	0.095421	-3.62104	0.000293425	0.0006488	NOT
FREM2	331.6262	-1.70869	0.471973	-3.62032	0.000294235	0.0006505	DOWN
RNU6-116	2.082792	1.317357	0.363889	3.62022	0.000294353	0.0006507	UP
RN7SL336I	1.924956	-0.89956	0.248493	-3.62005	0.000294544	0.0006511	DOWN
SPINK13	2.072924	1.697578	0.468954	3.619925	0.000294689	0.0006514	UP
RP11-90M	1.907124	1.168094	0.322711	3.619632	0.000295022	0.0006521	UP
14-Sep	1.982811	2.761684	0.763108	3.618993	0.000295752	0.0006536	UP
EXOSC1	757.0416	0.301396	0.083282	3.618988	0.000295757	0.0006536	NOT
TEX2	2931.463	-0.36551	0.101019	-3.61828	0.000296568	0.0006553	NOT
RP1-97D1	1.618092	1.792857	0.495582	3.617681	0.000297255	0.0006568	UP
RP11-104I	15.17575	0.771556	0.213292	3.617377	0.000297603	0.0006575	UP
RAMP1	3713.468	0.850018	0.234985	3.617324	0.000297664	0.0006576	UP
TMEM176	35719.64	-0.6669	0.184377	-3.61702	0.000298015	0.0006583	DOWN
SNORA47	1.5934	1.321731	0.365442	3.616805	0.000298262	0.0006587	UP
RP11-467:	2.229435	1.361201	0.376355	3.616804	0.000298264	0.0006587	UP
CNTROB	844.6374	0.327112	0.090449	3.616542	0.000298565	0.0006594	NOT
RP11-834(	1.550615	1.590966	0.44001	3.615747	0.000299483	0.0006613	UP
CELF6	1.476576	1.473856	0.407746	3.614646	0.000300758	0.0006641	UP
PITHD1	1510.585	-0.31144	0.086162	-3.61457	0.000300844	0.0006642	NOT

CTD-2262	4.870631	0.942789	0.260838	3.614466	0.000300968	0.0006644	UP
MIR503HG	45.31411	-0.71923	0.199003	-3.61415	0.000301338	0.0006652	DOWN
IDI2-AS1	3.750854	0.908859	0.251479	3.61405	0.000301451	0.0006654	UP
RP11-310I	1.754514	1.311589	0.362919	3.613996	0.000301514	0.0006655	UP
CMC2	1201.959	-0.32397	0.089645	-3.61391	0.000301617	0.0006657	NOT
PEX2	1541.518	0.29796	0.082471	3.612921	0.000302767	0.0006682	NOT
CTD-2561	6.845788	1.046599	0.289689	3.612832	0.000302871	0.0006683	UP
SCLY	225.814	0.465712	0.128912	3.612634	0.000303102	0.0006687	NOT
PRMT2	1234.101	0.346815	0.096001	3.612633	0.000303103	0.0006687	NOT
CH507-42	2.993436	1.494282	0.413629	3.612619	0.00030312	0.0006687	UP
NEURL1B	1006.979	0.535844	0.148332	3.612471	0.000303293	0.000669	NOT
RP11-173I	2.498181	1.08986	0.301722	3.612128	0.000303695	0.0006699	UP
PSMD1	4078.766	0.223627	0.061929	3.611036	0.000304976	0.0006727	NOT
RP11-329/	14.33231	-0.63298	0.175296	-3.61091	0.00030513	0.0006729	DOWN
CCDC136	16.5145	0.853569	0.236404	3.610642	0.00030544	0.0006736	UP
AOAH	298.2651	-0.72001	0.199446	-3.61004	0.000306147	0.0006751	DOWN
PKD2L1	54.46464	0.906304	0.251056	3.609966	0.000306238	0.0006752	UP
AC116366	34.79865	0.670388	0.185726	3.609553	0.000306725	0.0006762	UP
ROMO1	3145.603	0.552237	0.152995	3.60951	0.000306776	0.0006763	NOT
RNF4	1694.433	0.276273	0.076543	3.60939	0.000306917	0.0006766	NOT
TBRG4	2793.308	0.290871	0.080594	3.609108	0.000307251	0.0006772	NOT
ARHGAP1	3333.286	0.308912	0.085604	3.60864	0.000307806	0.0006784	NOT
TRPM3	39.10941	1.133503	0.314112	3.608599	0.000307855	0.0006785	UP
SCARNA6	2.253786	-1.01443	0.28118	-3.60775	0.000308861	0.0006806	DOWN
RP1-13D1	1.278844	1.666043	0.461816	3.607592	0.000309052	0.000681	UP
BTBD7P1	3.921904	1.214269	0.336603	3.607424	0.000309253	0.0006814	UP
FAM173B	453.0912	0.287329	0.079667	3.606635	0.000310194	0.0006834	NOT
CLEC7A	159.0472	-0.73933	0.204993	-3.6066	0.000310237	0.0006834	DOWN
RP1-89D4	5.156468	0.808388	0.22416	3.606296	0.000310599	0.0006842	UP
ZNF600	215.262	0.606424	0.168182	3.605753	0.00031125	0.0006856	UP
NLRP1	494.8287	0.601083	0.166731	3.605097	0.000312036	0.0006872	UP
SNORD14/	12.4561	0.655418	0.181813	3.604895	0.00031228	0.0006877	UP
CEACAM7	23.90804	2.830606	0.785238	3.604775	0.000312423	0.000688	UP
LINC01559	7.005888	1.812619	0.502845	3.604728	0.00031248	0.0006881	UP
RP11-493I	1.188095	2.111906	0.585883	3.604653	0.000312571	0.0006882	UP
PRELP	1238.678	-1.00843	0.279807	-3.60404	0.000313312	0.0006897	DOWN
SDC4P	1.659976	-1.29873	0.360355	-3.60403	0.000313319	0.0006897	DOWN
SYCE3	25.00955	0.89256	0.24766	3.603971	0.000313392	0.0006898	UP
DUSP23	3820.065	0.539909	0.149815	3.603845	0.000313544	0.0006901	NOT
CH507-15	1.593502	1.326578	0.368216	3.602715	0.000314911	0.0006931	UP
CLEC12B	2.271384	-1.0687	0.296656	-3.60249	0.000315182	0.0006936	DOWN
RP11-96H	2.903183	2.71585	0.75397	3.602064	0.0003157	0.0006947	UP
RPL24P4	88.42528	0.562406	0.156156	3.60156	0.000316313	0.000696	NOT
RP11-160I	3.899012	-0.81523	0.226365	-3.6014	0.000316513	0.0006964	DOWN
SEC31B	192.3504	0.610185	0.169447	3.60105	0.000316935	0.0006973	UP
RP11-672/	9.415707	1.427291	0.396386	3.60076	0.000317288	0.000698	UP
RP11-680I	1.607361	1.227512	0.340914	3.600653	0.000317419	0.0006982	UP
LINC00885	66.3517	-1.4401	0.40004	-3.59988	0.000318359	0.0007002	DOWN
SMARCE1I	2.172616	0.927135	0.257546	3.599876	0.000318369	0.0007002	UP
GORASP2	3602.432	0.22876	0.063547	3.599862	0.000318386	0.0007002	NOT
RP11-285/	1.691107	1.199709	0.333279	3.599715	0.000318566	0.0007005	UP
AMD1	1869.095	-0.33648	0.093496	-3.59891	0.000319558	0.0007026	NOT

RP11-101	64.9271	0.578351	0.160707	3.598801	0.000319688	0.0007029	NOT
TPTE	2.325179	3.486509	0.968981	3.598119	0.000320527	0.0007046	UP
U91319.1	91.63825	-1.23686	0.343753	-3.59811	0.000320534	0.0007046	DOWN
GGNBP1	1.11533	1.299727	0.361236	3.597995	0.00032068	0.0007049	UP
CEACAM1	3.462857	1.245222	0.346093	3.597939	0.000320748	0.000705	UP
UHRF1BP1	1328.914	-0.36766	0.102191	-3.59775	0.000320987	0.0007054	NOT
YWHAZP4	11.9986	0.657113	0.182649	3.597689	0.000321058	0.0007055	UP
RP11-134	6.920103	-0.68835	0.191335	-3.5976	0.000321171	0.0007057	DOWN
TBX10	36.90631	0.886678	0.246467	3.59756	0.000321216	0.0007058	UP
USH2A	652.7733	-1.04508	0.290513	-3.59736	0.000321463	0.0007063	DOWN
RP11-13N	2.753685	1.068332	0.296987	3.597241	0.00032161	0.0007065	UP
TRPC7	1.064128	2.171416	0.603683	3.596949	0.000321972	0.0007073	UP
RP4-761J1	11.93176	0.691654	0.192335	3.596099	0.000323025	0.0007095	UP
MRPL10	1619.586	0.271941	0.075624	3.595978	0.000323175	0.0007098	NOT
RPS21P1	1.485038	1.275267	0.354683	3.595516	0.00032375	0.000711	UP
RP11-440	204.5629	0.501616	0.139512	3.595507	0.000323761	0.000711	NOT
RPLP1	42466.64	0.457697	0.127298	3.595479	0.000323795	0.000711	NOT
NKX2-2	1.724476	2.92959	0.814833	3.595328	0.000323984	0.0007113	UP
GNL1	2365.961	0.294287	0.081854	3.59527	0.000324055	0.0007114	NOT
GDE1	1669.913	-0.28916	0.080439	-3.59478	0.000324667	0.0007127	NOT
MT1B	10.97664	-2.25864	0.628391	-3.59433	0.000325229	0.0007139	DOWN
NRBF2P5	2.976401	0.954353	0.265523	3.594235	0.000325346	0.0007141	UP
TBX1	26.53611	1.234571	0.343516	3.593927	0.000325731	0.0007149	UP
CTD-2012	3.050136	0.855327	0.238004	3.593758	0.000325943	0.0007153	UP
HOXA7	1.873185	1.728557	0.48099	3.593751	0.000325951	0.0007153	UP
RTN4RL1	302.8875	0.844912	0.235118	3.593563	0.000326187	0.0007157	UP
TMX2P1	39.82027	0.548852	0.152746	3.593238	0.000326594	0.0007166	NOT
RP11-692	11.91313	0.531847	0.148019	3.593094	0.000326774	0.0007169	NOT
RTN3	6240.424	0.283609	0.078937	3.592845	0.000327087	0.0007175	NOT
RP11-397	2.391233	3.529582	0.982403	3.592807	0.000327135	0.0007176	UP
RP11-789	4.137945	1.926613	0.536336	3.592174	0.000327931	0.0007193	UP
LINC0092	22.70131	1.100655	0.306456	3.591556	0.000328709	0.0007209	UP
SLC35B1	3195.555	0.297652	0.082892	3.590827	0.00032963	0.0007229	NOT
LINC0125	1.611266	2.818783	0.784998	3.590816	0.000329644	0.0007229	UP
IGKV1D-1	8.98863	-1.53772	0.428317	-3.59015	0.000330489	0.0007246	DOWN
RP13-36G	50.87443	-0.48872	0.136135	-3.59	0.000330684	0.000725	NOT
RP11-424	1.691144	1.216068	0.338754	3.589823	0.000330902	0.0007254	UP
SLC40A1	11847.61	-0.48405	0.134841	-3.5898	0.000330936	0.0007255	NOT
FAM198B	691.1903	0.553609	0.154239	3.589286	0.000331585	0.0007268	NOT
RP11-96K	21.97413	0.56541	0.157532	3.589186	0.000331712	0.000727	NOT
RP3-465N	5.492144	0.806538	0.22477	3.588288	0.000332857	0.0007295	UP
RP11-428	9.893348	-0.55262	0.15401	-3.58821	0.000332958	0.0007297	NOT
PPP1R27	7.311704	1.005067	0.280131	3.587851	0.000333414	0.0007306	UP
ANKHD1-	148.4018	0.369594	0.103015	3.587762	0.000333529	0.0007308	NOT
HSP90B3P	3.231277	0.867175	0.241706	3.587732	0.000333567	0.0007308	UP
PHLDB1	729.9781	0.632739	0.176371	3.587541	0.000333812	0.0007313	UP
OR7E14P	87.46808	-0.52179	0.145458	-3.58725	0.000334186	0.0007321	NOT
RP5-966M	1522.692	-0.68339	0.190506	-3.58721	0.000334233	0.0007321	DOWN
RP11-65I1	20.84493	1.17099	0.326467	3.586857	0.000334688	0.000733	UP
AC007349	0.742266	1.763385	0.491666	3.586554	0.000335077	0.0007338	UP
ABHD17B	649.0952	-0.29454	0.082123	-3.58655	0.000335078	0.0007338	NOT
TRAPPC11	1020.813	-0.30576	0.085258	-3.58631	0.000335388	0.0007344	NOT

UBR2	2179.27	-0.28948	0.080724	-3.5861	0.000335666	0.000735	NOT
RIPK2	668.0022	0.494397	0.137867	3.586038	0.00033574	0.0007351	NOT
RP11-568/	2.83072	1.921416	0.535821	3.585927	0.000335882	0.0007353	UP
LINC0146{	0.99592	2.436603	0.679515	3.585795	0.000336053	0.0007356	UP
GJA3	4.686102	1.27665	0.356063	3.585457	0.000336489	0.0007365	UP
PPP3CC	433.1107	-0.36702	0.102365	-3.58537	0.000336603	0.0007367	NOT
APBA2	109.0004	0.997293	0.278168	3.58522	0.000336795	0.0007371	UP
CTSC	3372.289	0.592475	0.165258	3.585145	0.000336891	0.0007372	UP
GAPDHP4	2.545335	1.058239	0.295211	3.584682	0.00033749	0.0007385	UP
COPZ1	5644.657	0.246677	0.068824	3.584174	0.000338147	0.0007398	NOT
FRY-AS1	2.891959	1.208691	0.337231	3.584165	0.000338158	0.0007398	UP
HIST1H3A	3.151828	1.027752	0.286749	3.584149	0.000338179	0.0007398	UP
DDX21	2645.016	-0.40795	0.113836	-3.5837	0.000338768	0.000741	NOT
CSGALNA(	373.662	0.67996	0.189744	3.583563	0.000338938	0.0007414	UP
PRR4	17.79372	0.454747	0.126917	3.583015	0.000339651	0.0007429	NOT
CD247	172.3676	-0.64841	0.180986	-3.58263	0.000340153	0.0007439	DOWN
MYO7B	218.3861	0.997363	0.278406	3.582409	0.00034044	0.0007445	UP
RP11-284I	47.06926	-0.9213	0.257196	-3.5821	0.000340837	0.0007453	DOWN
RP11-524(	1.149164	1.637299	0.457225	3.580946	0.000342352	0.0007485	UP
NR1I3	3161.352	-0.90043	0.251462	-3.58078	0.000342572	0.0007489	DOWN
CEP104	1108.608	-0.32495	0.090752	-3.58066	0.000342731	0.0007492	NOT
RP4-671O	11.00723	0.732252	0.204516	3.580417	0.000343046	0.0007498	UP
FAM85B	3.581135	-1.15185	0.32171	-3.58042	0.000343047	0.0007498	DOWN
DNAJC13	1543.26	0.298203	0.083295	3.580083	0.000343485	0.0007507	NOT
C14orf105	728.3256	-0.71798	0.200574	-3.57963	0.000344081	0.000752	DOWN
RP11-56B:	4.885569	0.725459	0.202735	3.578365	0.000345751	0.0007555	UP
RP11-400I	1.911394	1.660247	0.464043	3.577791	0.00034651	0.0007571	UP
NEURL2	54.54815	0.493972	0.13807	3.5777	0.000346631	0.0007573	NOT
THUMPD3	1215.589	0.202011	0.05647	3.577309	0.00034715	0.0007584	NOT
LPCAT2	364.9572	0.709435	0.198346	3.57676	0.000347879	0.00076	UP
SLC6A4	11.85133	1.000361	0.279685	3.576735	0.000347913	0.00076	UP
RPL27	19015.09	0.459101	0.12837	3.576402	0.000348356	0.0007609	NOT
ARAP1-AS	3.739335	0.899039	0.25139	3.576273	0.000348527	0.0007612	UP
C19orf53	3365.651	0.44293	0.123856	3.576167	0.00034867	0.0007614	NOT
RP4-777O	1.338473	1.298418	0.363078	3.576141	0.000348704	0.0007615	UP
ACOT4	606.03	-0.53532	0.149707	-3.57578	0.000349187	0.0007624	NOT
GPRASP1	133.1945	-0.59775	0.167182	-3.57547	0.000349606	0.0007633	DOWN
RP11-333I	3.969963	-0.73559	0.205741	-3.57533	0.000349788	0.0007636	DOWN
RP11-10A:	8.606499	-0.96737	0.270599	-3.57491	0.000350352	0.0007648	DOWN
CTC-558C	10.23782	-0.94308	0.26381	-3.57483	0.000350461	0.000765	DOWN
KLF2	575.7238	-0.58213	0.162844	-3.57475	0.000350558	0.0007651	NOT
UBN2	685.292	0.328019	0.091761	3.5747	0.000350629	0.0007652	NOT
RP4-740C	39.96163	0.636348	0.178026	3.574464	0.000350946	0.0007659	UP
LPP-AS2	113.7442	-0.38716	0.108344	-3.57347	0.000352285	0.0007687	NOT
RP11-424I	1.158018	1.48419	0.415348	3.573368	0.000352419	0.000769	UP
CRLF3	330.2731	0.323943	0.090666	3.572919	0.000353024	0.0007702	NOT
AMN	2410.822	-1.11362	0.311693	-3.57282	0.000353163	0.0007705	DOWN
VDAC2	1890.066	0.298504	0.083556	3.572487	0.000353607	0.0007714	NOT
KIAA1324	87.53133	0.998639	0.279547	3.572352	0.00035379	0.0007717	UP
RP11-982I	1.994626	-1.22548	0.343065	-3.57214	0.000354072	0.0007723	DOWN
RP11-499I	1.732042	2.294726	0.642428	3.571959	0.000354321	0.0007727	UP
CTD-2124	51.03251	0.517246	0.144821	3.571624	0.000354774	0.0007737	NOT

NGLY1	1091.887	-0.24007	0.067218	-3.57148	0.000354964	0.000774	NOT
CRABP1	1.444574	2.763818	0.77395	3.571053	0.000355548	0.0007752	UP
GLRX5	3417.501	-0.3772	0.105637	-3.57068	0.000356062	0.0007763	NOT
IKBKGP1	1.818975	1.147627	0.321425	3.570434	0.00035639	0.0007769	UP
SIRT4	120.145	0.450985	0.126312	3.570394	0.000356445	0.000777	NOT
UHRF2	797.2234	0.298098	0.083495	3.57025	0.000356641	0.0007774	NOT
RP11-169I	4.964944	-0.89275	0.250073	-3.56996	0.000357036	0.0007782	DOWN
CEACAM2	47.12282	-0.73949	0.207148	-3.56983	0.000357209	0.0007785	DOWN
RP11-121	32.29778	0.714047	0.200032	3.569667	0.000357435	0.0007789	UP
TP63	28.19901	0.881887	0.24705	3.569665	0.000357438	0.0007789	UP
MORC3	882.5236	-0.34467	0.096557	-3.5696	0.000357521	0.000779	NOT
CA15P1	1.976675	1.446589	0.405254	3.56959	0.000357541	0.000779	UP
HSPA5	38243.13	0.345158	0.096695	3.569543	0.000357604	0.000779	NOT
GNG2	344.0772	-0.53642	0.150292	-3.56921	0.000358056	0.00078	NOT
PTPRN2	186.2205	-0.66649	0.186747	-3.56896	0.000358404	0.0007807	DOWN
ST14	2300.63	0.833457	0.233571	3.568327	0.000359267	0.0007825	UP
CDC42EP4	2755.214	0.336922	0.094426	3.568095	0.000359587	0.0007831	NOT
RP11-1E1	1.179299	1.61211	0.451826	3.567992	0.000359728	0.0007834	UP
DNM1P46	6.390295	1.265561	0.354718	3.5678	0.000359991	0.0007839	UP
TRAFD1	1031.968	0.306177	0.085817	3.567769	0.000360033	0.0007839	NOT
RP11-686	33.1702	0.560634	0.157152	3.567467	0.000360448	0.0007847	NOT
ESAM	1043.272	0.407349	0.114211	3.56662	0.000361615	0.0007872	NOT
ANXA13	1109.895	1.049001	0.294128	3.566483	0.000361804	0.0007876	UP
CCDC13-A	7.728255	0.900896	0.252604	3.566433	0.000361873	0.0007877	UP
CTD-2302	3.258448	0.889137	0.249335	3.566031	0.000362429	0.0007888	UP
KB-1410C	8.433352	0.635948	0.178339	3.565953	0.000362537	0.000789	UP
TREH	266.7114	-1.13382	0.317958	-3.56592	0.000362578	0.000789	DOWN
AC104667	68.47412	-0.75208	0.210912	-3.56586	0.000362663	0.0007891	DOWN
RP11-977	11.43022	0.593092	0.166335	3.565651	0.000362954	0.0007897	UP
RP11-15H	1.703717	-1.37647	0.386037	-3.56564	0.000362968	0.0007897	DOWN
LINC00957	87.12841	0.597367	0.167569	3.564907	0.000363985	0.0007918	UP
RP11-67K	10.6795	0.971497	0.272526	3.564786	0.000364153	0.0007921	UP
RNF2	462.3318	0.395051	0.110822	3.564737	0.000364221	0.0007922	NOT
ADARB2	17.4402	-1.2071	0.338698	-3.56393	0.000365347	0.0007946	DOWN
SRP68	3552.374	0.246977	0.0693	3.563872	0.000365424	0.0007947	NOT
RDX	4006.573	-0.36788	0.103234	-3.56351	0.000365931	0.0007957	NOT
AC108938	8.598954	0.923643	0.259247	3.562799	0.000366921	0.0007978	UP
RP11-417I	13.89242	0.664965	0.186642	3.562786	0.00036694	0.0007978	UP
CTDP1	954.2612	0.227033	0.063727	3.562564	0.00036725	0.0007984	NOT
CTA-221G	1.545537	1.331972	0.373909	3.562287	0.000367639	0.0007992	UP
CH17-431	13.42955	0.647849	0.181884	3.561873	0.000368219	0.0008004	UP
UACA	1462.85	0.336199	0.094403	3.561303	0.000369019	0.0008021	NOT
DNAJC7	2466.269	0.222101	0.062368	3.56112	0.000369277	0.0008026	NOT
DCUN1D2	1.438938	1.271543	0.357065	3.561094	0.000369313	0.0008026	UP
FBXO34	655.3363	-0.30507	0.085669	-3.56106	0.000369368	0.0008026	NOT
RSF1	1038.256	-0.26522	0.074479	-3.561	0.000369452	0.0008028	NOT
MTRNR2L1	8.230746	-0.81149	0.227902	-3.56068	0.000369898	0.0008037	DOWN
PNISR	1579.023	0.365029	0.102524	3.560419	0.000370263	0.0008044	NOT
CTD-2506	16.63898	0.569861	0.160058	3.560331	0.000370388	0.0008046	NOT
ARHGAP25	372.7969	-0.51804	0.145517	-3.55997	0.000370894	0.0008056	NOT
RP11-21L	21.43087	0.819646	0.230269	3.559523	0.000371529	0.000807	UP
ST3GAL2	1256.076	0.258043	0.072499	3.559275	0.00037188	0.0008077	NOT

EDA	255.2853	0.83757	0.235322	3.559246	0.000371921	0.0008077	UP
TMEM109	3428.353	0.28607	0.080375	3.559198	0.000371989	0.0008078	NOT
TRDC	59.3027	-0.76283	0.214341	-3.55897	0.000372319	0.0008084	DOWN
ADNP2	661.9083	0.31448	0.088365	3.558878	0.000372443	0.0008086	NOT
MRPS14	1204.154	0.270456	0.076004	3.558441	0.000373063	0.0008099	NOT
CTD-2651	1.817263	1.049435	0.294962	3.557861	0.000373888	0.0008116	UP
PGLYRP4	2.281887	1.832992	0.515216	3.557717	0.000374092	0.000812	UP
FAM117B	327.7544	0.594531	0.167128	3.55733	0.000374643	0.0008132	UP
CYP4F23P	8.048255	1.331865	0.37444	3.556956	0.000375176	0.0008142	UP
AC005487	1.203529	1.546555	0.434856	3.556476	0.000375863	0.0008157	UP
CRCP	1677.98	-0.32498	0.091408	-3.55526	0.000377603	0.0008194	NOT
SNAP91	2.4396	2.049747	0.576541	3.555247	0.000377624	0.0008194	UP
PPP1R9A	209.9518	0.903304	0.254094	3.555007	0.00037797	0.0008201	UP
CARKD	2865.563	-0.391	0.10999	-3.55491	0.000378111	0.0008203	NOT
IGHV3-53	43.15277	-1.42402	0.400588	-3.55482	0.000378235	0.0008205	DOWN
AC005306	4.539559	0.81656	0.229733	3.554381	0.00037887	0.0008218	UP
IGHV3-74	126.585	-1.38239	0.388934	-3.55431	0.000378966	0.000822	DOWN
RP11-876I	26.46434	0.539259	0.151726	3.554152	0.0003792	0.0008224	NOT
RP1-145M	2.198016	-0.73977	0.208145	-3.55408	0.000379305	0.0008225	DOWN
GPR156	2.97389	1.23139	0.346473	3.554073	0.000379314	0.0008225	UP
CTC-436P	2.025424	0.957033	0.269279	3.55406	0.000379332	0.0008225	UP
ABCG5	2222.397	-0.77873	0.21914	-3.55359	0.000380014	0.0008239	DOWN
HERC2P8	0.76077	1.825226	0.5137	3.553099	0.000380721	0.0008254	UP
CTD-3222	49.26322	0.550722	0.15501	3.552808	0.000381142	0.0008262	NOT
OR7E126P	2.867174	1.053469	0.296522	3.552753	0.000381222	0.0008263	UP
ANKRD26	213.3839	0.379842	0.106922	3.552518	0.000381563	0.000827	NOT
NAV2-AS3	1.347702	1.855422	0.522305	3.552369	0.000381779	0.0008274	UP
AP000705.	0.883432	2.142505	0.603134	3.552286	0.0003819	0.0008276	UP
GABRG1	4.425672	-1.19455	0.33629	-3.55215	0.000382095	0.000828	DOWN
TNXA	85.79124	0.882826	0.248545	3.551982	0.000382341	0.0008284	UP
CTC-268N	1.178973	1.34709	0.379261	3.551885	0.000382481	0.0008287	UP
RP1-273G.	1.418066	1.396593	0.393222	3.551662	0.000382806	0.0008293	UP
MYOZ3	10.17174	0.75356	0.212177	3.551558	0.000382957	0.0008296	UP
FGF13-AS1	2.084257	1.654077	0.465751	3.551417	0.000383162	0.0008299	UP
AC064850	1.103764	1.416356	0.398853	3.551077	0.000383659	0.000831	UP
TLE4	330.0703	-0.58024	0.163405	-3.55094	0.000383853	0.0008313	NOT
GABRB1	2.705714	2.239391	0.630683	3.550741	0.000384148	0.0008319	UP
RP11-78F1	1.076907	2.444626	0.688517	3.550566	0.000384404	0.0008324	UP
TSHZ1	1051.502	-0.32946	0.092792	-3.55048	0.000384535	0.0008326	NOT
RP4-738P1	1.244541	1.717782	0.483825	3.55042	0.000384616	0.0008327	UP
LINC0135E	5.131194	0.776149	0.218621	3.550205	0.000384931	0.0008333	UP
LETM1P2	1.139174	1.746126	0.491849	3.550122	0.000385052	0.0008335	UP
UFSP1	86.14006	0.525276	0.147963	3.550054	0.000385153	0.0008337	NOT
CYCS	5191.855	0.439566	0.123822	3.549967	0.00038528	0.0008339	NOT
GABRR2	4.210483	0.882005	0.248475	3.54967	0.000385714	0.0008348	UP
N6AMT2	272.9456	-0.39178	0.110374	-3.54953	0.000385915	0.0008351	NOT
ARMS2	2.111446	1.24299	0.350212	3.549252	0.000386328	0.000836	UP
RP11-332I	1.57743	1.206315	0.33991	3.548926	0.000386806	0.0008369	UP
TESK1	966.4534	0.278644	0.078515	3.548906	0.000386835	0.0008369	NOT
RP11-378.	1.289303	1.227687	0.345983	3.548398	0.000387582	0.0008385	UP
RP11-468I	1.279952	2.418966	0.681919	3.547295	0.000389208	0.0008419	UP
RP11-114!	18.90059	1.156772	0.326109	3.54719	0.000389364	0.0008422	UP

SLC26A7	12.51515	0.826264	0.232955	3.546887	0.000389811	0.0008431	UP
CTC-492K	2.852425	0.917082	0.258588	3.546498	0.000390387	0.0008443	UP
ZC3H11A	3862.857	0.29126	0.08213	3.546326	0.000390643	0.0008448	NOT
STAMBP	1270.231	0.211102	0.059531	3.546099	0.00039098	0.0008454	NOT
LINC00412	1.084356	1.322723	0.373035	3.545841	0.000391363	0.0008462	UP
GYG2	1234.456	-0.66232	0.186799	-3.54564	0.000391655	0.0008468	DOWN
EMCN	425.8989	0.571897	0.161298	3.545585	0.000391742	0.0008469	NOT
ICAM3	37.46308	-0.74893	0.21125	-3.54523	0.000392271	0.000848	DOWN
IGFBP2	25418.28	-1.02017	0.287779	-3.54497	0.000392661	0.0008487	DOWN
GIMAP4	925.3941	-0.54565	0.153968	-3.54392	0.000394221	0.000852	NOT
AC005795	2.865595	0.874505	0.246777	3.543712	0.000394537	0.0008527	UP
IGLV9-49	23.81128	-1.62568	0.458776	-3.54352	0.000394818	0.0008532	DOWN
ABHD16A	261.4551	0.327537	0.092433	3.543502	0.00039485	0.0008532	NOT
IGKV2D-4	8.247948	-1.62665	0.459053	-3.54349	0.000394868	0.0008532	DOWN
PEX11A	1108.883	-0.49551	0.139862	-3.54283	0.000395863	0.0008552	NOT
KRT8P26	0.938219	1.637369	0.46224	3.542252	0.000396726	0.000857	UP
SAMD9	348.048	-0.68275	0.192746	-3.54222	0.000396777	0.0008571	DOWN
CSRP1	5757.279	-0.47333	0.133628	-3.54213	0.000396904	0.0008573	NOT
CTC-451P	64.03307	-0.7028	0.198451	-3.54142	0.000397982	0.0008596	DOWN
YWHAZP2	4.000553	0.838768	0.23685	3.541354	0.00039808	0.0008597	UP
KCNJ8	2259.553	-0.76733	0.216705	-3.54088	0.000398801	0.0008612	DOWN
SCNN1D	248.7411	0.64493	0.18214	3.540845	0.000398848	0.0008612	UP
NENF	2921.727	0.478943	0.135267	3.540716	0.000399042	0.0008616	NOT
RP11-254I	3.322082	0.925013	0.26131	3.539909	0.000400265	0.0008642	UP
THADA	1218.655	0.319209	0.090187	3.539414	0.000401016	0.0008657	NOT
MANEA-A	12.55445	0.645624	0.182411	3.539382	0.000401065	0.0008657	UP
BCLAF1	2489.748	-0.21998	0.062155	-3.53922	0.000401313	0.0008662	NOT
URM1	2232.489	0.33885	0.095755	3.538706	0.000402094	0.0008678	NOT
AP001476.	3.295776	1.692844	0.478423	3.538385	0.000402582	0.0008688	UP
SLC4A10	14.36456	-1.05526	0.298267	-3.53795	0.000403242	0.0008702	DOWN
G3BP2	3218.979	-0.29555	0.083547	-3.53751	0.000403913	0.0008716	NOT
AC007064	1.122254	2.084773	0.589379	3.537237	0.000404336	0.0008724	UP
CEACAM5	9.509805	2.153561	0.608878	3.536932	0.000404804	0.0008733	UP
OFCC1	1.730204	2.676202	0.756648	3.536917	0.000404827	0.0008733	UP
LINC00997	138.9966	0.383642	0.10847	3.536831	0.000404958	0.0008735	NOT
KATNAL2	144.2085	0.611603	0.172955	3.536195	0.000405934	0.0008756	UP
RP11-412I	2.003914	2.526597	0.71451	3.536125	0.000406042	0.0008757	UP
SLC48A1	1170.11	0.334082	0.094477	3.536106	0.000406072	0.0008757	NOT
PM20D1	8.569541	-0.91056	0.25751	-3.536	0.000406237	0.000876	DOWN
RP11-44N	3.91323	0.834677	0.236059	3.535886	0.00040641	0.0008763	UP
EIF1AD	848.1861	0.205308	0.058069	3.535588	0.000406869	0.0008772	NOT
PCDHGB1	20.69896	1.005069	0.284279	3.535508	0.000406992	0.0008774	UP
DDHD1	366.8097	0.458365	0.129654	3.535293	0.000407324	0.0008781	NOT
TECRL	2.26153	3.121422	0.883	3.53502	0.000407744	0.0008789	UP
SLC47A1	5506.959	-0.74782	0.21155	-3.53497	0.000407823	0.000879	DOWN
GAPDHP7(	2.599282	0.99581	0.281731	3.53461	0.000408378	0.0008802	UP
TMEM107	136.556	0.396625	0.112236	3.533857	0.000409543	0.0008826	NOT
PDE12	1352.595	-0.25927	0.073372	-3.53368	0.000409816	0.0008831	NOT
TCEB1P2	1.587686	1.157527	0.327592	3.533436	0.000410195	0.0008839	UP
NGF	44.9786	-0.71643	0.202768	-3.53324	0.0004105	0.0008845	DOWN
CTD-3116	1.849939	1.225315	0.34682	3.533002	0.00041087	0.0008852	UP
RP11-745/	2.920361	0.958084	0.271201	3.532741	0.000411275	0.000886	UP

HSD17B8	1981.514	-0.55876	0.158169	-3.53268	0.000411375	0.0008861	NOT
S100A8	318.8054	-1.11088	0.314491	-3.5323	0.000411957	0.0008873	DOWN
RPL21P119	16.39139	-0.72078	0.204062	-3.53217	0.000412168	0.0008877	DOWN
RP11-157I	0.922822	2.082579	0.589643	3.53193	0.000412539	0.0008884	UP
EIF3CL	6.52118	0.642129	0.181822	3.531635	0.000412999	0.0008893	UP
DTHD1	7.56438	-1.04234	0.295145	-3.53162	0.000413026	0.0008893	DOWN
ORC2	585.5395	0.315312	0.08929	3.53131	0.000413507	0.0008903	NOT
USP5	3486.322	0.290398	0.082238	3.5312	0.000413678	0.0008906	NOT
GLTPD2	1531.752	-0.84306	0.238747	-3.53118	0.000413717	0.0008906	DOWN
RP11-158I	3.828162	0.88411	0.250375	3.531146	0.000413763	0.0008907	UP
AASS	1117.009	-0.95577	0.270706	-3.53065	0.000414532	0.0008922	DOWN
DCUN1D2	356.4498	0.408411	0.115679	3.530557	0.000414685	0.0008925	NOT
IL9R	3.023928	1.193775	0.338145	3.530361	0.000414992	0.0008931	UP
TMED5	5814.609	-0.33559	0.095074	-3.52976	0.000415932	0.000895	NOT
RP11-1C1.	2.811343	2.838464	0.804173	3.529666	0.000416085	0.0008953	UP
POU2F3	25.26022	1.154297	0.327055	3.529366	0.000416556	0.0008962	UP
RP11-283(	3.247609	1.411223	0.399856	3.52933	0.000416613	0.0008963	UP
HRH1	75.64489	0.59233	0.167834	3.52927	0.000416707	0.0008964	UP
RP11-361I	13.07562	1.103288	0.312635	3.528995	0.000417141	0.0008973	UP
RP11-498I	6.044649	-1.07087	0.303477	-3.52866	0.000417664	0.0008983	DOWN
GALC	644.4977	-0.59652	0.169052	-3.52866	0.000417673	0.0008983	DOWN
RING1	2330.179	0.323497	0.091693	3.528026	0.000418671	0.0009004	NOT
LPXN	415.3691	-0.36459	0.103358	-3.5274	0.000419661	0.0009024	NOT
DSCR3	1839.147	-0.25192	0.07142	-3.52726	0.000419881	0.0009028	NOT
SERF1B	7.728545	0.633222	0.179525	3.527208	0.000419967	0.000903	UP
CTD-2260	1.68407	1.259394	0.357071	3.527014	0.000420275	0.0009035	UP
AC022431	1.465659	1.734837	0.491955	3.526413	0.000421229	0.0009055	UP
RP11-345I	23.78598	-0.87276	0.247493	-3.52641	0.000421239	0.0009055	DOWN
GS1-166A	4.669148	0.86871	0.246346	3.526388	0.000421269	0.0009055	UP
CTD-3157	12.3103	-0.83482	0.23674	-3.5263	0.000421411	0.0009057	DOWN
CTD-2007	10.50174	0.66724	0.189232	3.526045	0.000421816	0.0009065	UP
RP11-164J	58.25678	0.772202	0.21905	3.525233	0.00042311	0.0009092	UP
RP11-33H	1.727931	2.398053	0.680345	3.524758	0.00042387	0.0009108	UP
ASB13	2765.617	-0.51137	0.145091	-3.52449	0.000424293	0.0009116	NOT
RPL29P33	2.339791	0.992848	0.281712	3.524334	0.000424549	0.0009121	UP
MUSTN1	5.735462	1.04835	0.297474	3.524178	0.000424799	0.0009126	UP
TP53AIP1	2.222622	1.373958	0.389871	3.524137	0.000424865	0.0009126	UP
SIRPB2	55.01963	-0.74924	0.212604	-3.5241	0.00042493	0.0009127	DOWN
RP11-379I	3.396556	0.952919	0.270418	3.523871	0.000425291	0.0009134	UP
HAT1	1076.581	0.259607	0.073682	3.523363	0.000426108	0.0009151	NOT
BRD3	869.7526	0.401754	0.114028	3.523283	0.000426236	0.0009153	NOT
RBM27	737.7441	-0.20665	0.058657	-3.52296	0.000426751	0.0009163	NOT
HNRNPA3I	4.803145	0.636697	0.180739	3.522749	0.000427096	0.000917	UP
GATB	1183.908	-0.37945	0.107728	-3.5223	0.00042782	0.0009185	NOT
ARHGDIG	2.206011	1.392007	0.395206	3.522233	0.000427928	0.0009186	UP
FAM86DP	126.3811	0.408918	0.1161	3.522129	0.000428095	0.0009189	NOT
PHF5A	798.5777	0.270916	0.076932	3.521519	0.000429082	0.000921	NOT
POLR2J3	64.48285	0.406766	0.115518	3.521245	0.000429526	0.0009219	NOT
LRRC56	62.46836	0.569171	0.161655	3.520893	0.000430097	0.000923	NOT
PPARD	1555.497	0.407775	0.115819	3.520782	0.000430276	0.0009233	NOT
ABI2	947.2714	0.372526	0.10582	3.520367	0.000430949	0.0009247	NOT
RP11-535A	3.209205	-0.89008	0.252907	-3.51941	0.000432515	0.0009279	DOWN

SMAD7	567.9796	-0.47386	0.134643	-3.5194	0.000432525	0.0009279	NOT
FMN2	5.053208	1.956679	0.556034	3.518994	0.000433186	0.0009293	UP
RP11-178(	25.82316	-0.63356	0.180044	-3.51893	0.00043329	0.0009294	DOWN
ZNF688	470.6535	-0.39902	0.113398	-3.51873	0.000433623	0.0009301	NOT
MTMR1	878.7182	0.314487	0.089376	3.518703	0.000433662	0.0009301	NOT
RP11-532I	14.83522	0.557715	0.15851	3.518479	0.000434028	0.0009308	NOT
FLVCR2	800.031	-0.56864	0.161632	-3.5181	0.000434656	0.0009321	NOT
RP4-669P1	13.94819	0.682332	0.193955	3.517996	0.000434819	0.0009324	UP
RNU6-104	1.083751	1.383153	0.393228	3.517433	0.000435743	0.0009343	UP
BTNL9	362.1011	0.768991	0.218636	3.517214	0.000436101	0.000935	UP
AC008592	31.23018	-0.60807	0.17291	-3.5167	0.000436949	0.0009367	DOWN
NR1D2	1673.088	-0.38998	0.110896	-3.51662	0.000437079	0.0009369	NOT
RP11-725(	11.04966	0.938486	0.266874	3.51659	0.000437129	0.0009369	UP
WWTR1-A	6.45602	0.664166	0.188869	3.516547	0.000437199	0.000937	UP
TRAT1	26.06719	-0.88271	0.251072	-3.51574	0.000438533	0.0009398	DOWN
PCSK6	6488.591	-0.70436	0.200364	-3.51542	0.000439067	0.0009409	DOWN
SHANK1	7.694567	0.914703	0.260208	3.515283	0.000439285	0.0009413	UP
MORN4	149.1267	-0.48581	0.138202	-3.51521	0.000439399	0.0009414	NOT
HSPA1L	79.20028	0.415646	0.118246	3.51511	0.000439572	0.0009417	NOT
ARID2	626.0998	0.288535	0.082088	3.514933	0.000439864	0.0009423	NOT
IGLV3-12	1.743269	-1.37427	0.391008	-3.51468	0.000440289	0.0009431	DOWN
ZFP69	122.8321	0.380225	0.108182	3.514673	0.000440297	0.0009431	NOT
RP11-288(	33.92142	0.545027	0.155104	3.513942	0.000441509	0.0009456	NOT
RP11-101(	3.529852	0.75474	0.214827	3.513255	0.000442653	0.000948	UP
RP11-379I	13.41045	-0.51538	0.146752	-3.5119	0.000444918	0.0009527	NOT
RPS20P33	3.609756	0.755317	0.215074	3.511893	0.000444928	0.0009527	UP
RP11-546I	2.673095	1.201793	0.342251	3.51144	0.000445686	0.0009542	UP
KIZ-AS1	2.397936	1.127653	0.321176	3.511019	0.000446393	0.0009557	UP
LYAR	509.834	0.375178	0.106878	3.510338	0.000447537	0.0009581	NOT
ZFYVE19	1331.522	0.346794	0.098795	3.510223	0.000447732	0.0009584	NOT
RP11-134I	2.643054	1.581371	0.450518	3.510116	0.000447911	0.0009587	UP
AC005498	3.36433	1.348916	0.384344	3.509662	0.000448677	0.0009603	UP
RP11-139I	13.81336	-0.60522	0.172462	-3.50928	0.000449328	0.0009616	DOWN
C2-AS1	20.63169	0.775899	0.221115	3.509023	0.000449756	0.0009624	UP
LRRC37A4I	25.22454	1.012798	0.288627	3.509018	0.000449764	0.0009624	UP
FSCN1	1883.445	0.717044	0.204379	3.508403	0.000450805	0.0009645	UP
RP1-232L2	33.06307	-0.71651	0.204254	-3.50793	0.000451605	0.0009662	DOWN
TMC4	638.9248	1.149757	0.327777	3.50774	0.000451931	0.0009668	UP
AC004951	5.023435	0.636383	0.181433	3.507534	0.000452281	0.0009675	UP
RP11-390I	0.836999	2.211266	0.630512	3.507095	0.000453027	0.000969	UP
MKLN1	1279.215	-0.34988	0.099781	-3.50648	0.000454069	0.0009711	NOT
CHST12	527.4603	0.356312	0.101653	3.505165	0.000456325	0.0009759	NOT
SMARCC2	1767.477	0.414591	0.118289	3.504891	0.000456795	0.0009768	NOT
SPESP1	51.1592	1.329461	0.379326	3.504798	0.000456954	0.0009771	UP
SVOPL	1.762551	1.579373	0.450637	3.504759	0.000457021	0.0009772	UP
KCTD6	473.3615	0.48515	0.138436	3.504494	0.000457475	0.0009781	NOT
RP11-136(	7.548724	-1.04627	0.298557	-3.50442	0.000457604	0.0009783	DOWN
RPL36AP4:	6.157879	0.768768	0.219399	3.503964	0.000458388	0.0009799	UP
CTD-3074	109.6893	0.475809	0.135798	3.503793	0.000458681	0.0009804	NOT
LINC0064!	1.315953	2.783862	0.794539	3.503746	0.000458763	0.0009805	UP
FAM19A3	1.399609	1.350428	0.385425	3.503738	0.000458776	0.0009805	UP
CTC-250I1	2.346091	1.059004	0.302274	3.503456	0.000459263	0.0009814	UP

EVA1B	292.9569	0.552027	0.157568	3.503412	0.000459338	0.0009815	NOT
HIST1H3P!	4.123612	0.666396	0.190219	3.503315	0.000459506	0.0009818	UP
MYBPC2	4.398022	1.280236	0.365532	3.502392	0.000461101	0.0009851	UP
FLT1	1673.273	0.494447	0.141186	3.502085	0.000461632	0.0009862	NOT
TMEM50A	2897.265	0.282051	0.08054	3.502014	0.000461755	0.0009864	NOT
CTC-444N	12.85191	-0.52381	0.149588	-3.50169	0.000462316	0.0009875	NOT
MYBBP1A	1700.301	0.328812	0.093902	3.501668	0.000462355	0.0009875	NOT
RP11-799I	349.6458	-0.70172	0.200396	-3.50164	0.000462407	0.0009875	DOWN
CTB-138E!	1.228834	1.907696	0.544907	3.500954	0.000463596	0.00099	UP
CPS1	96970.33	-1.10565	0.315887	-3.50015	0.000464996	0.0009929	DOWN
R3HDML	2.818305	1.433842	0.409838	3.498557	0.000467783	0.0009988	UP
RP11-588I	252.4821	-0.60669	0.17342	-3.49841	0.000468041	0.0009993	DOWN
PCDHGA6	59.03054	0.746609	0.213416	3.498368	0.000468115	0.0009993	UP
MOGAT1	19.4719	-0.88406	0.252711	-3.49829	0.000468248	0.0009995	DOWN
LINC0127٤	655.8369	0.321285	0.091845	3.498117	0.000468556	0.0010001	NOT
C15orf65	24.85293	0.516776	0.147734	3.498024	0.000468719	0.0010004	NOT
EBNA1BP2	2239.887	0.324632	0.092811	3.497767	0.00046917	0.0010013	NOT
XX-CR54.1	3.220635	2.842639	0.81284	3.497169	0.000470224	0.0010034	UP
LINC0116٤	1.483353	2.458814	0.703158	3.496815	0.000470848	0.0010047	UP
ARHGEF10	243.0233	0.631063	0.180472	3.496727	0.000471004	0.001005	UP
RP11-54C٤	1.786135	1.894352	0.541778	3.496545	0.000471326	0.0010056	UP
HSPA8P1	17.71811	0.759064	0.217112	3.496189	0.000471954	0.0010068	UP
SLC22A16	2.612688	1.273556	0.364276	3.496125	0.000472067	0.001007	UP
RP1-309F2	8.608971	0.890628	0.254771	3.495804	0.000472635	0.0010081	UP
TCEAL2	5.056003	-1.35757	0.38836	-3.49565	0.000472917	0.0010086	DOWN
RP11-66H	4.205219	1.058904	0.302936	3.495475	0.000473219	0.0010092	UP
DISC1	158.8058	0.755587	0.216167	3.495387	0.000473375	0.0010095	UP
RPL7AP6	53.38874	0.566948	0.162223	3.494857	0.000474316	0.0010114	NOT
EXOSC8	579.4302	0.298758	0.085487	3.494773	0.000474464	0.0010116	NOT
METTL10	381.2684	-0.27895	0.079826	-3.49442	0.000475089	0.0010129	NOT
MFSD8	549.8984	-0.29339	0.083964	-3.4942	0.000475482	0.0010136	NOT
FABP7	2.089732	1.837184	0.525806	3.494032	0.000475783	0.0010142	UP
PCP4	4.463624	1.848916	0.529186	3.493888	0.00047604	0.0010147	UP
OXCT1-AS	2.305506	1.67577	0.479706	3.493326	0.000477043	0.0010167	UP
RAB3IP	823.1181	0.45026	0.1289	3.493086	0.000477472	0.0010175	NOT
PMF1-BGL	63.73702	0.555689	0.159083	3.493083	0.000477479	0.0010175	NOT
TSTA3	3915.454	0.460024	0.131701	3.492943	0.000477728	0.001018	NOT
U95743.1	1.320815	2.346556	0.671834	3.492764	0.000478049	0.0010186	UP
RP11-364I	6.439836	0.779888	0.223301	3.492546	0.00047844	0.0010193	UP
INGX	1.299294	1.441635	0.412792	3.492401	0.000478698	0.0010198	UP
UCHL3	331.9224	-0.45844	0.131282	-3.492	0.000479415	0.0010212	NOT
C1QTNF3-	1.746477	1.1274	0.322863	3.49188	0.000479634	0.0010216	UP
RP4-714D	61.21281	0.508889	0.14574	3.491754	0.000479859	0.001022	NOT
CFTR	318.364	-1.54279	0.441868	-3.49152	0.000480278	0.0010229	DOWN
DNAJC19	1456.188	-0.35069	0.100449	-3.49125	0.000480763	0.0010238	NOT
CSNK1A1	1747.048	-0.30951	0.088662	-3.49092	0.000481357	0.0010249	NOT
FAM47E	107.5662	0.540115	0.15472	3.490918	0.000481363	0.0010249	NOT
NPM1P6	10.63994	0.589549	0.168916	3.490195	0.000482668	0.0010276	UP
ZNF322	127.896	0.427	0.122347	3.49008	0.000482876	0.001028	NOT
HPGD	4946.097	-1.22217	0.350191	-3.49	0.000483021	0.0010282	DOWN
EIF4BP3	37.15151	0.597282	0.171159	3.489631	0.000483687	0.0010296	UP
TGIF2	1015.692	0.451365	0.129382	3.488622	0.000485517	0.0010334	NOT

RP11-125!	63.20096	-1.3615	0.390366	-3.48775	0.000487103	0.0010367	DOWN
C2orf88	159.403	-0.67052	0.192266	-3.48747	0.00048762	0.0010377	DOWN
HEXA	3547.543	0.376498	0.107968	3.487141	0.000488214	0.0010389	NOT
RP11-710I	1.163476	1.800773	0.516411	3.487091	0.000488305	0.001039	UP
HUS1	599.5106	-0.24052	0.068985	-3.48652	0.000489352	0.0010411	NOT
C19orf43	5171.178	-0.40962	0.117493	-3.48631	0.000489727	0.0010418	NOT
FER1L6-AS	1.438131	2.848714	0.817115	3.486307	0.000489739	0.0010418	UP
IPPK	90.1823	-0.42723	0.122545	-3.4863	0.000489759	0.0010418	NOT
RP11-361I	2.375832	1.050218	0.301267	3.486006	0.00049029	0.0010428	UP
RP4-777L!	4.489031	0.835654	0.239732	3.485788	0.00049069	0.0010436	UP
AC093673	194.5421	0.521244	0.149555	3.485297	0.000491592	0.0010454	NOT
ECSCR	97.52487	0.532316	0.15274	3.485111	0.000491934	0.0010461	NOT
RP11-676J	4.000059	0.727917	0.208887	3.484743	0.000492609	0.0010474	UP
CDS1	205.4766	1.062793	0.304991	3.484666	0.000492752	0.0010476	UP
PLA2G4D	1.929939	1.900198	0.545321	3.48455	0.000492965	0.001048	UP
SORD2P	577.0802	-0.91383	0.262254	-3.48452	0.000493014	0.001048	DOWN
STX17-AS	31.19233	-0.53235	0.152778	-3.48449	0.000493075	0.0010481	NOT
SLC51B	233.7633	1.106631	0.317625	3.48408	0.000493833	0.0010496	UP
DPY19L1P	6.246228	0.615968	0.176796	3.484065	0.000493859	0.0010496	UP
CD99L2	4025.477	0.430424	0.123551	3.483783	0.000494381	0.0010506	NOT
RP3-431P	46.23328	0.717037	0.205828	3.483662	0.000494603	0.001051	UP
TEX37	2.79492	3.930164	1.12821	3.48354	0.000494829	0.0010514	UP
SOX21	1.166554	1.619348	0.46495	3.482841	0.000496122	0.0010541	UP
AC092198	2.598751	1.606517	0.461289	3.482667	0.000496446	0.0010547	UP
CCDC69	1571.877	-0.55989	0.16077	-3.48258	0.000496611	0.001055	NOT
DDX10	804.3436	0.267834	0.076909	3.482485	0.000496783	0.0010552	NOT
MTMR12	1432.963	-0.25281	0.072605	-3.48199	0.000497702	0.0010571	NOT
RP11-7M8	28.16497	1.271866	0.365275	3.481939	0.000497797	0.0010572	UP
CHORDC1	690.8094	0.417078	0.119798	3.481507	0.000498601	0.0010589	NOT
RP11-507I	8.683343	0.692198	0.198833	3.481303	0.000498981	0.0010596	UP
RRAS	1194.393	0.518196	0.148854	3.481241	0.000499095	0.0010597	NOT
ZNF253	256.7152	0.491694	0.141261	3.480755	0.000500003	0.0010616	NOT
GMFG	592.4052	-0.51023	0.146586	-3.48073	0.000500045	0.0010616	NOT
CTD-2540	1.728942	1.372785	0.394463	3.48014	0.000501152	0.0010639	UP
LINC0101	3153.377	-1.31407	0.377597	-3.48008	0.000501272	0.001064	DOWN
ANKRD24	259.5442	-0.60346	0.173428	-3.47963	0.00050211	0.0010657	DOWN
LINC0095!	47.64025	-0.51029	0.146665	-3.47928	0.000502756	0.001067	NOT
RP11-102I	16.81537	0.589015	0.169302	3.479088	0.000503123	0.0010677	UP
RARB	166.6167	-0.64203	0.18454	-3.47908	0.000503143	0.0010677	DOWN
CXorf56	659.9536	0.27785	0.079873	3.478638	0.000503968	0.0010694	NOT
AC000078	4.319847	1.064419	0.306033	3.478116	0.000504952	0.0010714	UP
RP11-843I	5.164832	0.897301	0.257991	3.478036	0.000505103	0.0010716	UP
GAS8-AS1	3.314042	0.993671	0.285788	3.476949	0.000507155	0.0010758	UP
SRP9	6181.682	0.254838	0.073294	3.476937	0.000507177	0.0010758	NOT
GOLGA7	1702.19	-0.32859	0.094514	-3.47661	0.000507798	0.0010771	NOT
SPATA2L	741.7361	-0.43722	0.125765	-3.47652	0.000507973	0.0010774	NOT
RP11-419J	1.324129	2.460593	0.707805	3.476372	0.000508247	0.0010779	UP
ASXL3	40.43693	-1.03456	0.297608	-3.47625	0.000508471	0.0010782	DOWN
RP11-368I	1.03307	2.503733	0.720246	3.476218	0.000508539	0.0010783	UP
THBS2	1889.782	0.927476	0.266816	3.476085	0.000508791	0.0010787	UP
NRGN	152.7269	0.589623	0.169623	3.476084	0.000508793	0.0010787	UP
B4GALT5	2513.509	-0.44866	0.129082	-3.47579	0.000509355	0.0010798	NOT

MRTO4	1373.569	0.348392	0.100237	3.475671	0.000509577	0.0010802	NOT
FAM107A	367.9195	-0.70901	0.204	-3.47553	0.000509839	0.0010806	DOWN
EAF1	1259.804	-0.29622	0.085237	-3.47528	0.00051032	0.0010816	NOT
CTB-47B1	3.000374	1.100442	0.316676	3.474972	0.000510907	0.0010827	UP
NPIPA3	5.838114	1.021637	0.29402	3.474723	0.000511381	0.0010837	UP
MOAP1	1056.762	-0.27589	0.079406	-3.47444	0.000511929	0.0010847	NOT
AP000476.	3.907346	-0.93786	0.269935	-3.47438	0.000512034	0.0010849	DOWN
PELI3	286.1624	0.42707	0.122935	3.473933	0.00051289	0.0010866	NOT
C20orf194	418.4441	0.454597	0.13087	3.473663	0.000513405	0.0010876	NOT
NUS1P2	1.373986	2.721397	0.783509	3.473345	0.000514014	0.0010888	UP
CD163L1	242.5668	0.70421	0.202774	3.472885	0.000514896	0.0010906	UP
XXbac-B44	40.68401	0.982642	0.283003	3.472197	0.000516218	0.0010933	UP
C15orf43	23.69594	-1.45972	0.420487	-3.4715	0.000517565	0.0010961	DOWN
RPS6KA5	145.0024	-0.4662	0.134299	-3.47136	0.000517831	0.0010966	NOT
ZMPSTE24	2728.728	0.242626	0.06991	3.470569	0.000519357	0.0010997	NOT
LINC00441	6.653023	-0.60639	0.174724	-3.47055	0.000519398	0.0010997	DOWN
AC024937	1.294669	1.209344	0.348462	3.47052	0.000519452	0.0010997	UP
RP11-727I	4.54843	0.828277	0.238662	3.470503	0.000519484	0.0010997	UP
RP11-113	8.672027	1.053633	0.303612	3.470333	0.000519813	0.0011004	UP
RP11-531I	12.27218	0.591332	0.170425	3.46975	0.000520943	0.0011027	UP
HAVCR2	312.642	-0.6387	0.184079	-3.4697	0.000521041	0.0011028	DOWN
AKR1C2	13547.72	0.961553	0.277141	3.469542	0.000521346	0.0011033	UP
RP5-1139I	5.455292	0.933243	0.269003	3.469271	0.000521872	0.0011044	UP
TRIM44	2973.088	-0.25608	0.073818	-3.46912	0.000522167	0.0011049	NOT
LMX1B	21.23371	1.881904	0.542483	3.469056	0.00052229	0.0011051	UP
LINC0093(	2.876281	1.813476	0.522776	3.468937	0.000522521	0.0011055	UP
AQP11	631.8523	-0.53303	0.153691	-3.4682	0.000523959	0.0011085	NOT
LIN28A	0.964013	1.868092	0.538645	3.468131	0.000524092	0.0011087	UP
PDE1B	122.8611	-0.50465	0.145533	-3.46759	0.000525147	0.0011108	NOT
NEAT1	12301.03	0.609913	0.175895	3.467475	0.000525372	0.0011112	UP
RP11-114I	5.749129	0.881261	0.254174	3.467156	0.000525997	0.0011124	UP
EPHA5	2.938587	2.895787	0.835246	3.466989	0.000526324	0.001113	UP
OR6E1P	1.457863	1.241416	0.358081	3.466857	0.000526583	0.0011135	UP
CTD-3010	1.83261	1.982255	0.571826	3.466537	0.000527209	0.0011147	UP
FAM120B	944.3348	-0.27781	0.080144	-3.46637	0.000527528	0.0011153	NOT
CD63	22858.38	0.394717	0.113889	3.46581	0.000528638	0.0011176	NOT
RP11-477I	44.45651	-0.49982	0.144214	-3.46578	0.000528691	0.0011176	NOT
PTPRD-AS	13.27449	-0.9834	0.283748	-3.46574	0.000528768	0.0011177	DOWN
CXXC5	4915.434	-0.32139	0.092745	-3.46526	0.000529727	0.0011196	NOT
SKA2P1	1.653327	1.019279	0.294157	3.465081	0.000530072	0.0011203	UP
CLSTN2	151.534	-0.99243	0.286413	-3.46502	0.000530183	0.0011204	DOWN
LA16c-321	1.682428	1.053847	0.304144	3.464967	0.000530297	0.0011206	UP
PTGES3L-/	1.0093	1.45343	0.419491	3.464747	0.000530731	0.0011214	UP
CTC-425C	5.035706	1.001459	0.289044	3.464732	0.000530759	0.0011214	UP
LINC0092	9.625432	1.428825	0.412458	3.464171	0.000531868	0.0011236	UP
RP11-45A	2.848664	0.869606	0.251053	3.463829	0.000532545	0.001125	UP
TPP2	1613.107	-0.33207	0.095871	-3.46376	0.000532673	0.0011252	NOT
PPIB	13423.62	0.31245	0.090209	3.463645	0.000532909	0.0011256	NOT
RPS27	19953.5	0.436199	0.125943	3.463477	0.000533243	0.0011262	NOT
PMPCB	3070.86	-0.28236	0.081528	-3.46341	0.000533368	0.0011264	NOT
PIK3IP1	901.5052	0.540468	0.156064	3.463113	0.000533965	0.0011276	NOT
INO80C	571.5664	0.418825	0.120941	3.463047	0.000534095	0.0011277	NOT

DNAJC5B	20.67794	-0.79195	0.228698	-3.46287	0.000534442	0.0011284	DOWN
LINC0100(	337.5058	0.441308	0.127444	3.462755	0.000534674	0.0011288	NOT
SLC16A10	474.0676	-0.80443	0.23235	-3.46215	0.000535888	0.0011313	DOWN
RP5-828H	1.7439	2.025504	0.585102	3.461797	0.000536582	0.0011326	UP
PDIA6	9861.82	0.356077	0.102862	3.461705	0.000536765	0.0011329	NOT
RP11-112	0.894168	2.092422	0.604558	3.461079	0.000538014	0.0011355	UP
MGAT5B	8.599769	0.883836	0.255375	3.460927	0.000538319	0.001136	UP
CTPS1	2308.048	-0.45391	0.131152	-3.46091	0.000538357	0.001136	NOT
TMEM72-	2.903005	1.060083	0.306307	3.460856	0.00053846	0.0011362	UP
OSBPL1A	894.7804	-0.47634	0.137645	-3.46064	0.000538891	0.001137	NOT
RP11-804I	10.76939	0.665302	0.192251	3.460593	0.000538987	0.0011371	UP
RP13-644I	2.222104	2.210547	0.638815	3.460389	0.000539395	0.0011379	UP
DGCR12	1.605085	1.156075	0.3341	3.460261	0.000539652	0.0011383	UP
RP11-701I	2.234233	1.45432	0.420315	3.460075	0.000540024	0.001139	UP
HNRNPA1I	2.122431	1.105121	0.319402	3.459967	0.000540243	0.0011394	UP
TMTC2	102.5234	0.720949	0.208412	3.459247	0.000541687	0.0011424	UP
DPP9-AS1	9.938385	0.608678	0.175973	3.458919	0.000542347	0.0011437	UP
RPL22	7357.403	-0.29442	0.085121	-3.45881	0.000542577	0.0011441	NOT
ENC1	1528.276	-0.63476	0.183542	-3.45841	0.000543379	0.0011457	DOWN
DLEU2L	3.740181	1.026555	0.296854	3.458115	0.000543969	0.0011468	UP
RP11-298I	21.93592	0.661191	0.191201	3.458096	0.000544008	0.0011468	UP
CTD-3020	1.503777	1.417224	0.409871	3.457733	0.000544742	0.0011483	UP
WRAP53	335.7699	0.365337	0.105659	3.457707	0.000544794	0.0011483	NOT
RP11-553/	1.431295	1.550547	0.448508	3.457122	0.000545978	0.0011507	UP
PCBP2	11863.15	-0.21882	0.063298	-3.45707	0.000546092	0.0011509	NOT
CADPS	25.9485	1.369876	0.396262	3.456993	0.000546238	0.0011511	UP
MMAB	3839.049	-0.51617	0.149319	-3.45683	0.00054656	0.0011517	NOT
TENM3	43.49491	-1.39115	0.402489	-3.45636	0.000547529	0.0011536	DOWN
RP1-65P5.	1.494534	2.377462	0.687925	3.455989	0.000548277	0.0011551	UP
HNRNPA1I	4.230882	0.700011	0.202561	3.455806	0.000548651	0.0011558	UP
YWHABP2	1.801176	0.996905	0.288477	3.45575	0.000548764	0.001156	UP
RP5-1042I	58.23795	-0.41359	0.119683	-3.45571	0.000548836	0.001156	NOT
MED1	994.2568	0.271161	0.078468	3.455671	0.000548926	0.0011561	NOT
TRAPPC5	44.74919	-0.58761	0.170055	-3.45542	0.000549439	0.0011571	DOWN
KRT8	36484.66	-0.51151	0.148032	-3.45536	0.000549553	0.0011573	NOT
ABCB7	904.431	-0.28477	0.082428	-3.45473	0.000550841	0.0011598	NOT
TMEM131	2129.102	0.266642	0.077182	3.454726	0.000550852	0.0011598	NOT
CTD-2027	7.480171	0.865788	0.25061	3.454724	0.000550856	0.0011598	UP
FAM134A	5373.498	0.241728	0.069971	3.454674	0.000550958	0.0011599	NOT
RP11-459(	1.77025	3.218557	0.931689	3.45454	0.000551232	0.0011604	UP
ACTC1	7.140582	1.430057	0.414006	3.454196	0.000551936	0.0011618	UP
ARF3	3853.216	0.239611	0.06937	3.454113	0.000552106	0.001162	NOT
RP11-366I	1.369826	1.889359	0.547038	3.453799	0.000552748	0.0011633	UP
RPLP2	23288.7	0.522747	0.151358	3.453718	0.000552915	0.0011636	NOT
PPP1R11	2866.14	0.257592	0.074584	3.453695	0.000552962	0.0011636	NOT
IPO5P1	289.2264	0.606036	0.175482	3.453548	0.000553265	0.0011641	UP
BRAP	773.6332	0.187513	0.054296	3.453525	0.000553312	0.0011641	NOT
ERICH3	18.49692	-0.93593	0.271036	-3.45315	0.000554083	0.0011656	DOWN
RAB22A	1470.352	0.222193	0.064345	3.453128	0.000554125	0.0011656	NOT
RP11-737(	2.487255	-0.95554	0.276717	-3.45313	0.000554127	0.0011656	DOWN
SEC63P1	35.32835	-0.50061	0.144977	-3.45302	0.000554349	0.001166	NOT
HEG1	1428.217	0.51057	0.147891	3.452329	0.00055577	0.0011688	NOT

MIR137HG	1.431004	2.785724	0.806915	3.452314	0.0005558	0.0011688	UP
ZNF587B	356.9899	0.377392	0.109336	3.45168	0.000557108	0.0011715	NOT
CNPPD1	2658.761	0.261934	0.075888	3.451586	0.000557302	0.0011718	NOT
1-Mar	203.7484	-0.57633	0.166981	-3.45147	0.000557538	0.0011722	NOT
RP11-566I	5.001792	1.17232	0.339665	3.451403	0.00055768	0.0011724	UP
IMPACT	1015.708	-0.32476	0.094111	-3.4508	0.000558923	0.001175	NOT
AC241377	10.1142	1.099809	0.318721	3.450697	0.000559142	0.0011753	UP
HEXB	6520.079	0.333939	0.096781	3.450448	0.000559657	0.0011763	NOT
FAS	704.1106	-0.62063	0.17987	-3.4504	0.000559746	0.0011764	DOWN
AC073283	5.46681	0.903972	0.261994	3.450346	0.000559869	0.0011766	UP
IGLC3	697.7266	-1.23189	0.357052	-3.45018	0.000560219	0.0011772	DOWN
RP11-469I	1.302818	2.172777	0.629853	3.449656	0.000561302	0.0011794	UP
RP11-162I	3.222076	1.12236	0.325358	3.449612	0.000561393	0.0011795	UP
ZNF597	157.6607	-0.38987	0.113028	-3.44933	0.000561987	0.0011807	NOT
TMEM14A	2259.281	0.498196	0.144443	3.449087	0.000562486	0.0011816	NOT
LINC0009	290.9935	0.4676	0.135588	3.448677	0.00056334	0.0011834	NOT
RP11-69E1	58.31159	0.666597	0.193301	3.448493	0.000563724	0.0011841	UP
PRKACA	5383.347	-0.34177	0.099118	-3.44807	0.000564607	0.0011858	NOT
RP11-499I	0.839526	1.735886	0.503475	3.447812	0.000565148	0.0011869	UP
DDX28	606.3045	-0.32272	0.093617	-3.44729	0.000566244	0.0011891	NOT
THAP5	1020.507	-0.30211	0.087646	-3.44689	0.000567075	0.0011907	NOT
HMGB3	2668.318	0.55047	0.159705	3.446792	0.000567285	0.0011911	NOT
SCN1A	5.883046	1.868418	0.542245	3.445711	0.00056956	0.0011958	UP
RP11-177(	1.084842	1.919315	0.557043	3.445541	0.000569917	0.0011964	UP
GRM1	2.805021	1.335585	0.387634	3.445478	0.00057005	0.0011966	UP
TXNP6	2.877906	0.972001	0.282116	3.445393	0.00057023	0.0011969	UP
RBM15	445.0094	-0.24512	0.071145	-3.44537	0.000570285	0.0011969	NOT
RNU4-62P	2.909056	-1.0532	0.305703	-3.44518	0.000570678	0.0011977	DOWN
RINT1	573.5672	0.256512	0.074458	3.445061	0.000570931	0.001198	NOT
ABO	52.96724	1.138861	0.330578	3.44506	0.000570933	0.001198	UP
FGF11	3.645733	1.150001	0.333832	3.444854	0.000571367	0.0011988	UP
HSPA2	450.1176	0.562028	0.163162	3.444608	0.000571888	0.0011999	NOT
RP11-467I	107.578	0.491866	0.142796	3.444534	0.000572044	0.0012001	NOT
AIDA	721.4629	0.308902	0.089684	3.444347	0.000572441	0.0012008	NOT
CARM1P1	1.875145	3.043511	0.88363	3.444326	0.000572485	0.0012008	UP
RP11-566I	1.303367	1.195549	0.347135	3.444047	0.000573077	0.0012019	UP
TDRG1	0.954964	2.402728	0.697649	3.444036	0.000573099	0.0012019	UP
ORAI2	423.2531	0.615456	0.178706	3.443966	0.000573247	0.0012022	UP
MTMR10	1638.749	-0.33896	0.098432	-3.44356	0.000574113	0.0012039	NOT
FAM157A	1.414653	2.024847	0.588046	3.443351	0.000574552	0.0012047	UP
ACSM2B	14027.44	-0.86478	0.25116	-3.44312	0.000575045	0.0012057	DOWN
DBF4P1	1.675038	0.985352	0.286206	3.442813	0.000575698	0.0012069	UP
COL16A1	532.6013	0.890486	0.258653	3.442786	0.000575755	0.001207	UP
MGC1627!	13.84788	0.618872	0.179764	3.442697	0.000575944	0.0012073	UP
FRMD6-A!	5.342164	0.783416	0.227567	3.442579	0.000576196	0.0012077	UP
CTD-2012	9.332848	0.693732	0.201517	3.442546	0.000576266	0.0012078	UP
EIF3K	6880.347	0.437808	0.127219	3.44136	0.000578798	0.001213	NOT
HTR2C	2.308734	2.798277	0.813183	3.441139	0.000579272	0.0012139	UP
RP11-296/	3.496019	0.823523	0.239322	3.44107	0.000579419	0.0012141	UP
ANAPC13	1962.119	-0.21893	0.063625	-3.44092	0.000579748	0.0012147	NOT
RP11-731I	1.532606	-1.07494	0.312406	-3.44084	0.000579908	0.0012149	DOWN
RADIL	18.77392	0.752043	0.218585	3.440506	0.000580629	0.0012163	UP

NFKBIE	914.6604	0.479854	0.139512	3.439525	0.000582735	0.0012207	NOT
FBXL21	35.12418	1.049858	0.305266	3.439156	0.000583532	0.0012222	UP
GPR64	170.1442	1.025378	0.298169	3.438912	0.000584058	0.0012232	UP
HDLBP	24928.87	-0.22961	0.066773	-3.43871	0.000584485	0.0012241	NOT
BRWD1-A!	27.54769	0.628147	0.182691	3.438299	0.000585381	0.0012258	UP
RP13-554I	7.561612	0.836349	0.243261	3.43808	0.000585854	0.0012267	UP
AC015849	16.09069	0.986701	0.286998	3.438007	0.000586012	0.001227	UP
MIR3176	1.864643	1.226755	0.356827	3.437956	0.000586123	0.0012271	UP
RNU4ATA(	2.307804	1.30675	0.380166	3.43732	0.000587502	0.0012299	UP
CECR5	2002.87	-0.30801	0.089627	-3.43656	0.000589161	0.0012333	NOT
SLC19A2	1520.177	-0.50995	0.1484	-3.43633	0.000589659	0.0012342	NOT
AL162151.	14.10748	0.744576	0.216695	3.436062	0.000590235	0.0012353	UP
AC114494	17.66297	0.739845	0.215346	3.435607	0.000591229	0.0012373	UP
FAM155A	19.78673	0.903461	0.262977	3.435509	0.000591443	0.0012377	UP
COLQ	88.70043	0.539945	0.157177	3.43527	0.000591963	0.0012387	NOT
CSRP2	1017.44	0.55207	0.160717	3.435048	0.00059245	0.0012396	NOT
UBA52P5	2.168784	0.944229	0.274895	3.434869	0.00059284	0.0012403	UP
CST3	15222.53	-0.47243	0.137548	-3.43463	0.000593362	0.0012413	NOT
RP11-243I	1.716353	1.893742	0.551401	3.434418	0.000593827	0.0012422	UP
MTATP8P1	17.36709	-0.73071	0.212786	-3.43403	0.000594668	0.0012439	DOWN
ZNHIT1	7138.156	-0.43708	0.12729	-3.43369	0.00059542	0.0012454	NOT
AC144833	2.978546	2.910434	0.84764	3.433575	0.000595678	0.0012458	UP
RANBP6	879.9086	-0.3632	0.105822	-3.43224	0.000598626	0.0012519	NOT
NLRP14	11.13222	-1.01255	0.295042	-3.4319	0.000599373	0.0012533	DOWN
CTC-548K	1.85522	1.113164	0.32437	3.431768	0.000599661	0.0012538	UP
PET117	202.7878	0.325626	0.094888	3.431704	0.000599802	0.0012539	NOT
USHBP1	138.357	0.453792	0.132235	3.431703	0.000599804	0.0012539	NOT
PPWD1	545.0537	0.221343	0.064502	3.431547	0.000600149	0.0012546	NOT
PLEKHD1	5.849193	1.019187	0.297032	3.431231	0.000600849	0.0012559	UP
RP11-427.	34.25804	-1.14288	0.333089	-3.43115	0.000601018	0.0012561	DOWN
SCRG1	5.536799	-0.88046	0.256608	-3.43115	0.000601018	0.0012561	DOWN
PCDHB12	30.55777	0.721429	0.210268	3.430991	0.00060138	0.0012568	UP
AC007163	0.832647	1.786569	0.520784	3.43054	0.000602381	0.0012587	UP
FAM86B3F	79.49822	-0.60573	0.17657	-3.43053	0.000602406	0.0012587	DOWN
RP13-122I	13.01055	0.738577	0.215301	3.430443	0.000602597	0.001259	UP
LY96	308.1674	0.816402	0.238013	3.430076	0.000603411	0.0012606	UP
IGHV1-24	61.39523	-1.38672	0.404296	-3.42995	0.000603682	0.0012611	DOWN
MYH15	4.917072	1.113603	0.324676	3.429886	0.000603835	0.0012613	UP
CTD-2619	10.40763	0.610105	0.17788	3.429863	0.000603886	0.0012613	UP
LRRC63	6.653544	0.952442	0.277705	3.429689	0.000604272	0.0012621	UP
C3orf38	505.9235	-0.21218	0.061868	-3.42961	0.000604446	0.0012623	NOT
CAPN1	5769.058	0.272285	0.079394	3.429529	0.000604629	0.0012626	NOT
SLC15A3	1017.353	-0.51983	0.15158	-3.42941	0.000604893	0.0012631	NOT
CNPY2	1599.994	0.388789	0.113372	3.429333	0.000605067	0.0012633	NOT
RP11-541I	1.194065	1.351618	0.394159	3.429122	0.000605538	0.0012642	UP
RP5-855D	8.783354	0.585663	0.170807	3.4288	0.000606256	0.0012656	UP
GNL3L	212.5927	0.378527	0.110422	3.428014	0.000608015	0.0012692	NOT
BNIP3P1	17.88164	-0.59131	0.172503	-3.42783	0.000608428	0.00127	DOWN
NLRP4	1.794597	2.205361	0.643425	3.427531	0.000609097	0.0012713	UP
AC002310	50.88636	0.507998	0.148216	3.427414	0.00060936	0.0012717	NOT
APOA1-AS	3.835417	0.821627	0.239749	3.427022	0.00061024	0.0012735	UP
ARL14EP	731.0016	-0.18521	0.054046	-3.42696	0.000610386	0.0012737	NOT

RBMS1	1392.026	-0.4958	0.144691	-3.42662	0.00061115	0.0012752	NOT
RPSAP4	3.033124	0.908548	0.265148	3.426572	0.000611253	0.0012753	UP
HFM1	5.65208	1.153688	0.336715	3.426308	0.000611847	0.0012764	UP
IGHV3-71	2.426105	-1.60433	0.468282	-3.42599	0.000612563	0.0012778	DOWN
NPHP3	203.2739	0.299634	0.087465	3.425743	0.000613121	0.0012789	NOT
ATRIP	2.514019	0.743345	0.216992	3.425681	0.00061326	0.0012791	UP
SERBP1	9367.891	-0.21909	0.063968	-3.42503	0.000614731	0.0012821	NOT
SLC35E1	2072.339	0.254346	0.074263	3.424954	0.000614904	0.0012823	NOT
AC006050	3.733632	3.985389	1.16365	3.424903	0.000615018	0.0012825	UP
CMTM2	8.185282	-0.85557	0.249819	-3.42475	0.000615365	0.0012831	DOWN
EIF2S2P3	4.4009	0.670555	0.195821	3.424316	0.000616348	0.001285	UP
RP11-508I	0.775411	1.620622	0.473272	3.424294	0.0006164	0.001285	UP
RP4-633O	2.07552	1.100979	0.32152	3.42429	0.000616408	0.001285	UP
CCIN	1.952808	1.363396	0.398175	3.424115	0.000616804	0.0012857	UP
RIMBP3C	1.095284	1.49466	0.436523	3.424012	0.000617039	0.0012861	UP
MDM1	208.214	0.345768	0.100987	3.423888	0.00061732	0.0012866	NOT
RP3-400B:	6.73056	0.591391	0.172744	3.423503	0.000618197	0.0012883	UP
RP11-390I	202.2398	-0.37245	0.1088	-3.42327	0.000618731	0.0012893	NOT
TUFMP1	2.961197	1.359832	0.397251	3.423103	0.000619105	0.00129	UP
CYB5D1	43.60276	-0.51508	0.150481	-3.42287	0.00061963	0.001291	NOT
IGKV6-21	13.11001	-1.54393	0.451101	-3.42257	0.000620314	0.0012923	DOWN
RP11-553I	2.440315	0.953018	0.27846	3.422461	0.00062057	0.0012928	UP
RP11-359:	3.379329	1.023674	0.299114	3.422358	0.000620805	0.0012932	UP
MAP6	96.33243	0.704604	0.205893	3.422191	0.000621186	0.0012939	UP
FAM196A	3.835671	1.013313	0.296118	3.421996	0.000621632	0.0012947	UP
RP11-435I	3.094204	0.859612	0.251218	3.421777	0.000622133	0.0012956	UP
RALBP1	2236.048	0.27959	0.081715	3.421531	0.000622695	0.0012967	NOT
RP11-74E:	16.39632	0.624864	0.182669	3.420743	0.000624503	0.0013004	UP
CLPS	3.764167	4.304559	1.2587	3.419845	0.000626569	0.0013046	UP
EMD	2241.132	0.326347	0.095428	3.41981	0.000626648	0.0013046	NOT
ELF4	316.1488	0.704869	0.206123	3.419656	0.000627005	0.0013053	UP
MINA	940.046	0.415429	0.121498	3.41922	0.00062801	0.0013073	NOT
DYX1C1	5.252899	1.041289	0.304544	3.419174	0.000628116	0.0013074	UP
LSG1	1399.166	0.199251	0.058276	3.419078	0.000628336	0.0013078	NOT
SCG3	6.291058	1.314139	0.384383	3.418827	0.000628918	0.0013089	UP
NFS1	1700.984	-0.27429	0.080231	-3.41877	0.000629047	0.0013091	NOT
HMGN3	2116.491	-0.35245	0.103098	-3.41858	0.000629482	0.0013099	NOT
SLC16A5	110.8864	-0.60524	0.177057	-3.41835	0.000630015	0.0013109	DOWN
RP11-384(	4.468279	1.457489	0.426395	3.418165	0.000630449	0.0013117	UP
SPTSSA	3427.736	0.50011	0.146313	3.418087	0.000630629	0.0013119	NOT
H3F3BP1	3.137139	0.826762	0.241884	3.418008	0.000630812	0.0013122	UP
SFTPB	2.885425	-1.09856	0.321413	-3.4179	0.000631071	0.0013127	DOWN
AQP6	48.01452	1.475536	0.431758	3.417508	0.000631972	0.0013144	UP
OLA1P1	20.96441	0.448289	0.131177	3.417438	0.000632135	0.0013147	NOT
NIPA2	878.2759	0.318803	0.093295	3.417148	0.000632808	0.001316	NOT
TFF3	295.6267	1.058554	0.309867	3.416156	0.000635119	0.0013207	UP
STK16	1509.673	-0.28994	0.084881	-3.41583	0.000635872	0.0013222	NOT
AC097374	1.065373	2.431886	0.711958	3.41577	0.000636019	0.0013224	UP
ZBTB18	1788.36	0.446431	0.130712	3.415372	0.000636949	0.0013242	NOT
PTRF	3953.134	0.514521	0.150652	3.415286	0.000637151	0.0013245	NOT
RP11-202I	2.464504	3.751508	1.098543	3.414984	0.000637857	0.0013259	UP
RP11-74C:	3.316519	0.706835	0.206986	3.414894	0.000638068	0.0013262	UP

RP5-856G:	1.610752	2.246489	0.657927	3.414494	0.000639006	0.0013281	UP
NCR3LG1	51.61927	1.013931	0.296968	3.414274	0.000639522	0.0013291	UP
CDKL5	330.8221	0.460743	0.134958	3.413983	0.000640205	0.0013304	NOT
GIMAP6	525.134	-0.54375	0.159287	-3.41367	0.000640952	0.0013318	NOT
RPS16	27102.4	0.493349	0.144523	3.413627	0.000641042	0.0013319	NOT
FAM156A	3.405986	0.694256	0.203379	3.413613	0.000641077	0.0013319	UP
LONRF3	352.2189	-0.53487	0.156691	-3.41353	0.000641265	0.0013322	NOT
RP11-115:	1.26441	1.62553	0.476231	3.413324	0.000641757	0.0013331	UP
HPRT1	1392.392	-0.391	0.114553	-3.41323	0.000641969	0.0013334	NOT
RP11-848I	8.927416	0.748389	0.219278	3.412976	0.000642576	0.0013346	UP
RASSF2	406.4729	-0.52878	0.154944	-3.41269	0.000643259	0.0013359	NOT
MBD6	2291.34	0.283953	0.083211	3.412462	0.000643788	0.0013369	NOT
U1	5.205978	0.826735	0.242279	3.412322	0.00064412	0.0013375	UP
SGMS1	1288.813	-0.31209	0.091481	-3.41156	0.000645921	0.0013411	NOT
TMEM184	1129.274	-0.33131	0.097126	-3.41118	0.000646832	0.0013429	NOT
APOLD1	1176.012	0.644632	0.188991	3.410922	0.000647437	0.0013441	UP
RP11-667I	2.192639	1.295301	0.379756	3.410873	0.000647553	0.0013442	UP
AC073072	1.16395	1.609034	0.471797	3.410439	0.000648585	0.0013463	UP
AC007308	3.326406	0.927482	0.271957	3.410394	0.00064869	0.0013464	UP
POU2F2	136.6414	-0.65536	0.192189	-3.40996	0.000649726	0.0013484	DOWN
TADA2B	1374.947	-0.24394	0.071568	-3.40845	0.000653323	0.0013558	NOT
PROSER1	597.5681	0.35616	0.104498	3.408287	0.00065372	0.0013565	NOT
IDS	1953.859	-0.36877	0.108207	-3.40797	0.000654482	0.001358	NOT
C20orf24	1371.35	0.382725	0.11231	3.407759	0.000654988	0.0013589	NOT
IER3IP1	1815.682	0.238969	0.070139	3.407091	0.000656592	0.0013622	NOT
SLC16A1	4824.002	-0.57929	0.170034	-3.40689	0.000657065	0.0013631	NOT
LINC0111(	18.84891	0.776701	0.227982	3.406852	0.000657168	0.0013632	UP
RP11-627I	15.8591	0.653927	0.191951	3.406744	0.000657426	0.0013636	UP
RP11-626(	3.059395	0.780411	0.229087	3.406618	0.000657732	0.0013641	UP
ISCA1	1472.489	-0.2693	0.079065	-3.40607	0.000659059	0.0013668	NOT
TPRN	1473.519	0.583278	0.171254	3.405922	0.000659411	0.0013674	NOT
H3F3A	879.8988	0.273467	0.080299	3.405616	0.000660149	0.0013688	NOT
EXOC5	1463.135	-0.24865	0.073028	-3.40485	0.000662004	0.0013726	NOT
PELP1	2184.266	0.333983	0.098095	3.404699	0.00066237	0.0013732	NOT
AC073254	34.1866	0.464767	0.136513	3.404561	0.000662706	0.0013738	NOT
KB-1615E4	22.86703	1.14561	0.336532	3.404162	0.000663673	0.0013757	UP
LINC00504	20.90539	0.916418	0.269208	3.404131	0.000663748	0.0013758	UP
GS1-304P	1.30091	1.411019	0.414521	3.403972	0.000664136	0.0013764	UP
KB-1507C!	90.36154	0.51498	0.151288	3.403968	0.000664146	0.0013764	NOT
CTA-253N	12.22444	0.570632	0.167673	3.403246	0.000665903	0.0013799	NOT
GLIS2-AS1	19.6734	0.944363	0.277516	3.402907	0.000666729	0.0013815	UP
DTX2P1	32.39014	0.438986	0.129007	3.402811	0.000666964	0.0013819	NOT
LL22NC03	1.291838	1.302197	0.382724	3.402442	0.000667864	0.0013837	UP
PROL1	38.70464	1.626892	0.478178	3.40227	0.000668285	0.0013845	UP
ZCCHC4	262.8662	0.240898	0.070815	3.401803	0.00066943	0.0013867	NOT
ARSI	49.72018	0.860194	0.252868	3.401754	0.000669548	0.0013869	UP
TTC39C	6331.294	-0.55001	0.161705	-3.40131	0.000670628	0.001389	NOT
RP11-676I	2.942876	3.82289	1.123965	3.401253	0.000670778	0.0013892	UP
CELF2-AS:	3.339703	1.734764	0.510073	3.401013	0.000671367	0.0013903	UP
YTHDF2	2200.185	-0.20248	0.059538	-3.40089	0.000671676	0.0013909	NOT
CTD-2600	9.580334	0.660879	0.194334	3.400741	0.000672033	0.0013915	UP
CALML3-A	15.21217	0.912793	0.268424	3.400556	0.000672489	0.0013923	UP

ZNF12	706.6239	0.289179	0.085043	3.400396	0.000672884	0.001393	NOT
RAB35	1389.896	0.221135	0.065033	3.400371	0.000672944	0.0013931	NOT
NCF1C	48.0419	-0.66891	0.196728	-3.4002	0.000673357	0.0013938	DOWN
IRF7	1659.405	-0.54352	0.159857	-3.40002	0.000673817	0.0013947	NOT
THAP6	424.8144	-0.26765	0.078721	-3.39999	0.000673875	0.0013947	NOT
RP11-80H	2.072162	-0.79723	0.234482	-3.39995	0.00067397	0.0013948	DOWN
FMNL2	607.5	0.688178	0.202409	3.399938	0.000674011	0.0013948	UP
EXTL2	426.5947	0.3314	0.097476	3.399828	0.000674283	0.0013952	NOT
CA7	1.070637	1.671278	0.491632	3.399447	0.000675223	0.0013971	UP
BTBD1	1676.225	-0.23255	0.068409	-3.39939	0.000675364	0.0013972	NOT
SMIM2-AS	163.266	-0.77843	0.229006	-3.39918	0.000675879	0.0013982	DOWN
AC019185	1.140393	1.964661	0.578007	3.399027	0.00067626	0.0013989	UP
UBALD2	1727.58	0.477808	0.14058	3.398841	0.00067672	0.0013997	NOT
RP4-758J1	68.91432	0.658527	0.193798	3.398	0.000678805	0.0014039	UP
MOK	157.9461	0.453086	0.133357	3.397552	0.000679917	0.0014061	NOT
IL1R2	392.1139	0.576419	0.169676	3.397184	0.000680833	0.0014079	NOT
PAPL	2.32246	1.378353	0.405739	3.397138	0.000680947	0.0014081	UP
TNFSF10	5106.777	-0.60496	0.17809	-3.39693	0.000681454	0.001409	DOWN
TKTL1	17.73351	1.311127	0.38603	3.396442	0.000682681	0.0014114	UP
AP000688.	10.42142	0.682812	0.201049	3.396243	0.000683177	0.0014123	UP
C7orf31	124.1674	0.664059	0.19553	3.396204	0.000683274	0.0014124	UP
SLC25A32	1011.887	-0.32296	0.095099	-3.39603	0.000683703	0.0014132	NOT
RHOD	1930.431	-0.50782	0.149539	-3.39589	0.000684061	0.0014139	NOT
RP11-864I	2.337623	1.197258	0.352586	3.395646	0.000684669	0.001415	UP
RP11-19G	1.944263	1.115491	0.328517	3.395532	0.000684953	0.0014155	UP
LRRC3	1607.625	-0.7221	0.212668	-3.39545	0.000685166	0.0014158	DOWN
RP1-266L2	7.861007	0.522257	0.153814	3.39537	0.000685358	0.0014161	NOT
SNRPB2	1626.407	0.25542	0.075234	3.395003	0.00068628	0.0014179	NOT
RP11-182J	3.766919	1.289182	0.379756	3.39476	0.000686888	0.0014191	UP
FBN1	1281.609	0.651485	0.191919	3.394578	0.000687344	0.0014199	UP
RP11-223I	256.1046	0.689875	0.203233	3.394503	0.000687533	0.0014202	UP
RP4-647C	5.272371	0.755743	0.222665	3.394088	0.000688576	0.0014222	UP
NUDT15	624.9098	-0.29602	0.087217	-3.394	0.000688796	0.0014226	NOT
RP3-425C	1.751106	0.998524	0.294217	3.393833	0.000689218	0.0014233	UP
RP11-622A	294.5976	-0.93398	0.275228	-3.39348	0.000690118	0.0014251	DOWN
RP11-286I	20.62676	0.387948	0.114323	3.393448	0.000690187	0.0014251	NOT
ZNF729	1.670658	2.49265	0.734606	3.39318	0.000690862	0.0014264	UP
RP5-912I1	1.410285	2.940834	0.866699	3.393142	0.000690957	0.0014265	UP
TRIM52-A	272.0071	0.453698	0.13373	3.392631	0.000692248	0.001429	NOT
RP11-452I	29.18558	0.469377	0.138352	3.392625	0.000692263	0.001429	NOT
RP11-69L1	1.147266	2.535207	0.747303	3.392475	0.000692641	0.0014297	UP
ADAMTSL	193.4031	0.731021	0.215515	3.39197	0.000693921	0.0014322	UP
RPS19BP1	2632.199	0.389997	0.114983	3.391784	0.000694392	0.0014331	NOT
ARMC3	2.835884	1.721706	0.507649	3.391531	0.000695033	0.0014343	UP
RETNLB	1.225152	2.536025	0.747801	3.391312	0.000695588	0.0014353	UP
DDX52	1060.079	0.231196	0.068174	3.391287	0.000695653	0.0014354	NOT
CD38	208.8948	-0.81758	0.241089	-3.39119	0.000695903	0.0014358	DOWN
RPL10AP2	7.06681	0.766143	0.225942	3.390876	0.000696695	0.0014373	UP
DNTT	1.77869	2.2681	0.668894	3.390821	0.000696836	0.0014375	UP
CTC-458G	1.476397	3.016213	0.889555	3.390699	0.000697146	0.001438	UP
TSPY26P	63.48436	0.720693	0.212571	3.39037	0.000697983	0.0014396	UP
TMEM216	183.0775	0.3866	0.114043	3.389945	0.000699066	0.0014417	NOT

LRFN3	577.1401	0.403218	0.118947	3.389914	0.000699146	0.0014417	NOT
IGKV2D-2!	22.50986	-1.40732	0.415149	-3.38991	0.000699156	0.0014417	DOWN
GRM5	1.4098	2.18116	0.643534	3.389347	0.000700593	0.0014446	UP
EPDR1	964.8479	0.626131	0.18477	3.388704	0.000702237	0.0014479	UP
ZNF598	1470.779	0.284569	0.083977	3.388641	0.000702398	0.0014481	NOT
CWC25	722.6808	-0.2089	0.061655	-3.38815	0.00070366	0.0014505	NOT
LRRC28	1478.24	-0.43562	0.128571	-3.38814	0.000703683	0.0014505	NOT
IZUMO4	128.7435	-0.68143	0.201123	-3.38811	0.000703763	0.0014506	DOWN
MT-RNR1	73242.56	-0.60058	0.177301	-3.38734	0.000705745	0.0014545	DOWN
LTB4R	285.0454	0.510201	0.150635	3.387003	0.000706607	0.0014562	NOT
LRBA	1695.143	-0.35933	0.106094	-3.38688	0.000706923	0.0014568	NOT
DUSP4	282.8149	0.879516	0.259704	3.386607	0.000707627	0.0014581	UP
ANPEP	31234.66	-0.55329	0.163382	-3.38646	0.000708005	0.0014588	NOT
MMP16	34.63711	0.94445	0.27892	3.386101	0.000708932	0.0014606	UP
SRMS	13.25682	0.856587	0.25298	3.385983	0.000709237	0.0014611	UP
USP11	1748.45	0.305349	0.090205	3.385062	0.000711622	0.0014659	NOT
PRDX6	25236.63	-0.38503	0.113766	-3.38444	0.000713232	0.0014691	NOT
PHF2P2	1.923194	3.149881	0.930878	3.383773	0.00071497	0.0014726	UP
RP11-182	3.149974	-0.79489	0.234923	-3.38362	0.000715358	0.0014733	DOWN
CDK2AP1	1141.162	0.327953	0.096927	3.383495	0.000715695	0.0014738	NOT
ADAM17	426.7148	0.47947	0.141715	3.383328	0.00071613	0.0014746	NOT
LYSMD3	1170.769	-0.344	0.101677	-3.38328	0.000716266	0.0014748	NOT
TIMM17A	3302.831	0.363061	0.107317	3.383083	0.00071677	0.0014757	NOT
RP11-304I	38.61053	0.455196	0.134565	3.382729	0.000717695	0.0014775	NOT
FAM98C	637.2118	0.303131	0.089612	3.382721	0.000717716	0.0014775	NOT
RP11-24F1	6.659979	0.895297	0.264673	3.382657	0.000717881	0.0014777	UP
ACOT9	861.7334	0.316087	0.093449	3.382467	0.00071838	0.0014786	NOT
TIMMDC1	1908.203	0.220912	0.06532	3.381995	0.000719615	0.001481	NOT
RP3-500L1	7.425781	0.991367	0.293135	3.381942	0.000719754	0.0014812	UP
RP11-94P1	0.893193	2.300693	0.680364	3.381565	0.000720743	0.0014831	UP
ZUFSP	199.9266	0.285997	0.084591	3.380954	0.000722346	0.0014863	NOT
MPHOSPH	541.751	0.357175	0.105657	3.380516	0.000723497	0.0014886	NOT
HPS3	846.2927	0.305812	0.090467	3.380377	0.000723864	0.0014892	NOT
RP11-837	2.984629	0.816476	0.241536	3.38035	0.000723935	0.0014893	UP
C8orf34	2.048637	1.653538	0.489223	3.379928	0.000725049	0.0014914	UP
RP11-107I	1.67489	1.691402	0.50044	3.379829	0.00072531	0.0014919	UP
RP4-660H	1.749373	3.134034	0.927416	3.37932	0.000726654	0.0014945	UP
NACC2	1335.075	0.361837	0.107078	3.379177	0.000727032	0.0014952	NOT
KLK10	22.2597	-1.42538	0.421849	-3.37888	0.000727806	0.0014967	DOWN
CCL19	468.0515	-1.28002	0.378871	-3.3785	0.000728812	0.0014986	DOWN
GAN	200.9635	0.313065	0.092667	3.378394	0.000729106	0.0014991	NOT
XXbac-BP(	2.389771	1.241169	0.367406	3.378194	0.000729636	0.0015001	UP
LAMTOR4	2637.513	0.424181	0.125568	3.378104	0.000729875	0.0015005	NOT
AC019117	25.77793	0.915085	0.270916	3.377749	0.000730816	0.0015023	UP
AXIN1	1107.631	0.370521	0.109714	3.377151	0.000732409	0.0015055	NOT
AKIRIN2	1616.694	-0.34463	0.102052	-3.37704	0.000732707	0.001506	NOT
PSMD12	2770.395	-0.22402	0.066347	-3.37648	0.000734203	0.0015089	NOT
ZSCAN5A	131.6664	0.357038	0.105745	3.376401	0.000734409	0.0015092	NOT
RP11-184I	3.019697	2.105215	0.623555	3.37615	0.00073508	0.0015105	UP
RP11-265I	4.964196	1.019681	0.302052	3.37585	0.000735881	0.001512	UP
FAM216A	224.3602	0.446975	0.132406	3.375787	0.00073605	0.0015123	NOT
AKNAD1	1.516536	1.480861	0.438851	3.374403	0.000739759	0.0015198	UP

AC007255	3.268325	1.502987	0.44542	3.374317	0.00073999	0.0015201	UP
ZNF562	669.2219	0.274886	0.081469	3.374112	0.000740542	0.0015212	NOT
AC006116	4.729872	0.75149	0.222732	3.37396	0.000740953	0.0015219	UP
TM9SF2	9906.978	-0.30446	0.090253	-3.37338	0.000742523	0.001525	NOT
AP006621.	1.764687	1.071477	0.31765	3.373138	0.000743166	0.0015262	UP
SYT15	15.81842	0.75816	0.224782	3.37286	0.000743917	0.0015276	UP
FP325331.:	1.716742	1.334012	0.395523	3.372784	0.000744124	0.001528	UP
TMEM205	7896.602	-0.42715	0.126662	-3.37237	0.000745241	0.0015301	NOT
PTPRC	919.3867	-0.75881	0.225019	-3.37218	0.00074576	0.0015311	DOWN
RNU12	2.307963	1.105956	0.328	3.37182	0.000746732	0.001533	UP
RP11-439I	2.384117	1.79006	0.531018	3.370995	0.000748972	0.0015375	UP
CARD10	1807.86	0.441183	0.130894	3.370542	0.000750204	0.0015399	NOT
CT83	3.053135	3.760478	1.115723	3.370442	0.000750478	0.0015403	UP
C2orf71	1.591114	2.733843	0.811241	3.369954	0.000751807	0.0015429	UP
MNDA	147.0281	-0.66532	0.197428	-3.36995	0.000751825	0.0015429	DOWN
LINC0155(	2.584009	1.428155	0.423944	3.368736	0.000755138	0.0015495	UP
DDX24	4290.548	-0.25657	0.076165	-3.3686	0.000755502	0.0015501	NOT
AC093702	1.351361	2.812756	0.834995	3.36859	0.000755539	0.0015501	UP
RP11-100I	2.936827	1.186461	0.35222	3.36852	0.000755729	0.0015504	UP
DCDC2B	2.128989	0.981183	0.291284	3.36847	0.000755867	0.0015505	UP
COL23A1	68.68727	0.861671	0.255805	3.368464	0.000755883	0.0015505	UP
C1orf43	11889.02	0.263168	0.078128	3.368428	0.00075598	0.0015506	NOT
UBE3B	1760.341	0.205986	0.061153	3.368399	0.00075606	0.0015506	NOT
GPR20	5.953104	1.003093	0.297818	3.368142	0.000756765	0.001552	UP
BACE1	2078.384	-0.31197	0.092635	-3.36771	0.000757962	0.0015543	NOT
KB-1836B!	3.103992	0.964137	0.286302	3.367551	0.000758391	0.0015551	UP
FAM26F	213.3119	-0.79808	0.236998	-3.36747	0.000758625	0.0015554	DOWN
RP1-78O1	4.322529	1.066585	0.316785	3.366908	0.000760161	0.0015585	UP
ZNF280A	1.462047	2.679512	0.795849	3.366861	0.000760291	0.0015586	UP
LINC0027:	1.61468	2.495281	0.741135	3.366837	0.000760355	0.0015586	UP
PPY2	2.462113	3.505335	1.041143	3.366814	0.00076042	0.0015586	UP
SPN	221.5608	-0.65194	0.19364	-3.36676	0.000760582	0.0015589	DOWN
PROSER2	1376.783	-0.43991	0.13067	-3.36654	0.000761178	0.00156	NOT
RP11-930I	6.836931	0.874627	0.259807	3.366454	0.000761414	0.0015603	UP
ZNF226	382.5912	0.381128	0.113235	3.365818	0.000763171	0.0015638	NOT
HOXB-AS1	16.09595	0.702857	0.208863	3.365166	0.000764975	0.0015674	UP
UTP15	524.2862	0.25468	0.075698	3.364425	0.000767034	0.0015715	NOT
RP11-13K:	1.670632	1.587694	0.471925	3.364293	0.000767399	0.0015721	UP
RP11-367I	2.674051	0.757812	0.225294	3.36366	0.000769163	0.0015756	UP
RP5-1042I	1.738755	1.100029	0.327061	3.363381	0.000769941	0.0015771	UP
LHFPL3-A!	1.323702	2.402673	0.714377	3.363312	0.000770133	0.0015774	UP
BATF	192.095	0.915757	0.272281	3.363276	0.000770232	0.0015775	UP
RP11-227(	4.014177	0.893843	0.265779	3.363106	0.000770707	0.0015783	UP
ARHGEF19	148.7552	0.616726	0.183394	3.362844	0.00077144	0.0015797	UP
RANGAP1	6289.16	0.388216	0.11546	3.362335	0.000772862	0.0015825	NOT
C6orf120	1050.654	-0.31237	0.092917	-3.36182	0.000774292	0.0015853	NOT
RBMXP2	15.53619	-0.83563	0.248595	-3.3614	0.000775477	0.0015876	DOWN
BAZ1B	3100.164	0.250683	0.074587	3.360969	0.000776696	0.00159	NOT
SLC25A46	1817.603	-0.25595	0.076176	-3.36002	0.00077937	0.0015954	NOT
AC136289	3.304997	-0.67933	0.202186	-3.35993	0.000779629	0.0015958	DOWN
RP1-117B:	10.99366	0.774001	0.230388	3.359556	0.000780679	0.0015978	UP
DLEU7-AS	3.894571	0.995698	0.29639	3.359421	0.000781059	0.0015985	UP

NOL6	1637.454	0.261459	0.077842	3.358843	0.000782695	0.0016017	NOT
ZNF204P	72.78468	0.931784	0.27742	3.35875	0.000782957	0.0016021	UP
TNFSF12	624.5978	-0.46104	0.137265	-3.35872	0.000783043	0.0016022	NOT
MIPOL1	139.4892	0.434687	0.129434	3.358374	0.000784024	0.0016041	NOT
CWC22	794.1296	-0.24127	0.071846	-3.35818	0.000784565	0.001605	NOT
C3AR1	310.9382	-0.6182	0.184133	-3.35736	0.000786908	0.0016097	DOWN
CTD-2545	4.989935	0.709986	0.211491	3.357057	0.00078777	0.0016114	UP
RP11-386I	5.503961	0.676044	0.201387	3.356944	0.00078809	0.0016119	UP
RNF113A	593.5487	0.321164	0.095686	3.356434	0.000789545	0.0016148	NOT
KCNMA1	513.5926	-0.75998	0.226443	-3.35618	0.000790285	0.0016161	DOWN
ANKRD36(	28.61493	0.655099	0.195207	3.355921	0.000791012	0.0016175	UP
AC012358	1.241724	1.311961	0.390952	3.355811	0.000791327	0.001618	UP
CA3	29.19637	-0.89364	0.266303	-3.35572	0.000791591	0.0016185	DOWN
TMEM191	118.0447	0.719392	0.214387	3.355574	0.000792005	0.0016192	UP
RNASEH2E	5.985091	0.579451	0.17269	3.355439	0.000792392	0.0016199	NOT
SPAG17	5.053508	1.204439	0.359027	3.35473	0.000794426	0.0016239	UP
DPP6	3.275661	1.068806	0.318606	3.354629	0.000794715	0.0016244	UP
SCPEP1	2160.615	0.59759	0.17816	3.354231	0.000795859	0.0016266	UP
C14orf28	202.6849	-0.34992	0.104341	-3.35367	0.000797477	0.0016298	NOT
AC110615	1.449538	1.01206	0.301788	3.353549	0.000797823	0.0016303	UP
PTPRA	1908.202	0.213233	0.063584	3.353537	0.000797857	0.0016303	NOT
RNF32	77.75245	0.419199	0.125004	3.353472	0.000798046	0.0016306	NOT
MPG	1055.056	-0.32227	0.096113	-3.35306	0.000799237	0.0016329	NOT
RP11-148I	2.724351	-1.05583	0.314933	-3.35257	0.000800648	0.0016356	DOWN
INSM1	2.611	2.102119	0.62704	3.352445	0.000801011	0.0016363	UP
ELK4	2271.034	0.361893	0.107956	3.352233	0.000801626	0.0016374	NOT
RP11-656(	2.000498	1.449287	0.432452	3.351328	0.00080425	0.0016426	UP
RP11-329I	3.97282	-0.77832	0.232259	-3.35107	0.000805002	0.0016441	DOWN
AC078942	2.573007	-1.38174	0.412364	-3.35078	0.000805848	0.0016457	DOWN
KANK1	4632.8	-0.46942	0.140099	-3.35063	0.000806268	0.0016464	NOT
A1BG-AS1	90.16815	-0.63081	0.188268	-3.35059	0.000806408	0.0016466	DOWN
LMCD1-A:	11.15879	0.584066	0.174327	3.350399	0.000806952	0.0016475	NOT
ARHGAP27	545.8986	0.46324	0.13827	3.35027	0.000807327	0.0016482	NOT
TMEM156	207.5498	1.014402	0.302789	3.350193	0.000807554	0.0016485	UP
RP11-12A:	30.74421	-1.23914	0.369885	-3.35006	0.000807933	0.0016492	DOWN
RP11-143(	6.232726	1.455482	0.434483	3.34992	0.000808349	0.0016499	UP
ARHGAP6	76.98669	0.52362	0.156312	3.349848	0.000808559	0.0016502	NOT
RP11-669I	3.247132	0.873897	0.260911	3.349405	0.000809854	0.0016527	UP
PCDHA8	1.516066	2.231179	0.666168	3.349274	0.000810237	0.0016534	UP
COTL1	2135.862	-0.59301	0.177093	-3.34857	0.0008123	0.0016575	DOWN
IKZF1	284.4534	-0.68369	0.204187	-3.34834	0.000812972	0.0016587	DOWN
RP11-347I	16.93865	1.311747	0.391769	3.34827	0.000813178	0.001659	UP
TNK2	1453.395	0.370704	0.110728	3.347867	0.000814362	0.0016613	NOT
RP11-83M	4.758841	1.642042	0.490479	3.347833	0.000814461	0.0016614	UP
RP5-1116I	1.598727	1.12863	0.337128	3.347776	0.000814629	0.0016616	UP
PBXIP1	5299.224	0.322116	0.09622	3.347706	0.000814835	0.0016619	NOT
SNX18P26	1.448545	2.252645	0.672899	3.347671	0.000814938	0.001662	UP
C11orf91	3.018782	1.085264	0.324196	3.347551	0.000815288	0.0016626	UP
RP11-291I	27.71373	0.554589	0.165675	3.34746	0.000815557	0.001663	NOT
GS1-44D2	7.384866	0.808352	0.241504	3.347164	0.000816429	0.0016647	UP
IGLV1-36	21.03958	-1.54681	0.462131	-3.34712	0.000816552	0.0016648	DOWN
CRYZP1	1.973368	1.063622	0.317811	3.346719	0.000817742	0.0016671	UP

RP11-586I	2.183403	2.413328	0.721162	3.346441	0.000818561	0.0016687	UP
RP4-568C	206.0798	-1.10647	0.330657	-3.34629	0.000819013	0.0016695	DOWN
FFAR4	26.88029	0.66572	0.198948	3.346202	0.000819267	0.0016699	UP
WBP2	8042.648	-0.30752	0.091913	-3.34575	0.000820605	0.0016725	NOT
FAM169A	200.7713	0.871307	0.260429	3.345659	0.000820872	0.0016729	UP
RSBN1	478.8182	-0.29216	0.08733	-3.34546	0.000821454	0.0016739	NOT
LAMTOR2	3586.818	0.439079	0.131275	3.344724	0.000823645	0.0016783	NOT
HEATR4	38.76689	-0.47417	0.141778	-3.34447	0.000824398	0.0016797	NOT
DLG3	349.1794	0.788589	0.235798	3.34434	0.000824787	0.0016804	UP
SP2-AS1	61.39957	0.48251	0.144283	3.344188	0.000825238	0.0016812	NOT
CNTN2	9.909635	1.036456	0.309939	3.344065	0.000825605	0.0016818	UP
PPP4R2	2921.376	-0.30177	0.090244	-3.34397	0.000825882	0.0016822	NOT
AC114730	47.8891	1.017138	0.304187	3.343787	0.000826432	0.0016832	UP
RP11-395I	10.1556	1.171923	0.350487	3.343704	0.00082668	0.0016836	UP
SH2D4A	1367.602	-0.46368	0.138678	-3.34359	0.000827019	0.0016842	NOT
PQLC2	966.2022	0.383506	0.114714	3.343147	0.00082834	0.0016867	NOT
SRF	1808.121	-0.29452	0.088099	-3.34303	0.000828698	0.0016873	NOT
ANKRD37	257.6948	-0.71749	0.214636	-3.3428	0.000829362	0.0016886	DOWN
RP11-621I	1.366435	1.561233	0.467088	3.342481	0.000830329	0.0016904	UP
C9orf43	46.57011	-0.45979	0.137584	-3.34188	0.000832126	0.0016939	NOT
AC137932	15.11756	0.661185	0.197853	3.341805	0.000832354	0.0016943	UP
RP11-359I	16.10604	-0.81843	0.24494	-3.34137	0.000833662	0.0016968	DOWN
GPR15	3.000628	-1.3122	0.392759	-3.34098	0.000834846	0.0016991	DOWN
C2CD5	877.7065	0.317175	0.094939	3.340819	0.000835317	0.0016999	NOT
CTD-2623	1.132573	1.485323	0.44462	3.34066	0.000835795	0.0017008	UP
RNFT1	623.5352	-0.37973	0.113678	-3.34038	0.000836634	0.0017024	NOT
FGFRL1	2937.785	-0.39259	0.117537	-3.34016	0.000837312	0.0017036	NOT
RHCG	82.00678	1.232901	0.36913	3.340023	0.000837713	0.0017043	UP
MRPL38	331.8882	0.285151	0.085375	3.339996	0.000837795	0.0017044	NOT
ADORA2A	81.71487	0.604029	0.180859	3.339783	0.000838439	0.0017055	UP
RP11-177	2.322065	1.053339	0.315407	3.339619	0.000838934	0.0017064	UP
ARL17B	13.35029	0.671644	0.201171	3.338676	0.000841785	0.0017121	UP
SP4	216.6406	0.398675	0.119413	3.338615	0.000841971	0.0017124	NOT
AC009299	111.6308	-0.71036	0.212802	-3.33816	0.000843363	0.0017151	DOWN
YRDC	647.2046	-0.26015	0.077936	-3.33797	0.000843926	0.0017161	NOT
SERPIND1	45644.24	-0.70176	0.210247	-3.33777	0.000844528	0.0017172	DOWN
MIR6753	1.160865	1.36176	0.408011	3.337557	0.000845184	0.0017184	UP
PSG5	2.011284	2.05544	0.615876	3.337425	0.000845587	0.0017191	UP
MOCOS	1752.888	-0.45514	0.136388	-3.33712	0.000846527	0.0017208	NOT
TREML4	2.711689	1.814171	0.543637	3.337099	0.000846578	0.0017208	UP
AL357515.	4.950769	-0.52679	0.157881	-3.33659	0.000848132	0.0017239	NOT
SZT2-AS1	1.558218	1.201495	0.360118	3.336396	0.000848722	0.0017249	UP
ARID4B	1345.789	0.33054	0.099093	3.335669	0.000850944	0.0017293	NOT
FAM217A	2.690495	1.057745	0.31711	3.335575	0.000851231	0.0017298	UP
APOC1	212562	-0.71811	0.215298	-3.33541	0.000851749	0.0017307	DOWN
DPYSL3	821.4491	0.723247	0.216848	3.335267	0.000852176	0.0017314	UP
COG6	505.5643	-0.33775	0.101274	-3.33504	0.000852861	0.0017327	NOT
C7orf43	563.7418	0.302608	0.090737	3.335015	0.000852949	0.0017328	NOT
NHLH1	4.260872	0.898484	0.269411	3.334996	0.000853006	0.0017328	UP
SFXN5	3033.356	-0.44252	0.132699	-3.33481	0.000853585	0.0017338	NOT
SVOP	30.84348	-0.86923	0.260665	-3.33467	0.000854018	0.0017346	DOWN
BBS7	175.7429	0.40601	0.121773	3.334138	0.000855641	0.0017377	NOT

CTD-2515	3.481818	0.917841	0.275288	3.334112	0.000855721	0.0017378	UP
LIMD1	2011.908	0.352856	0.105848	3.333614	0.000857255	0.0017407	NOT
TMEM144	165.9845	0.672038	0.201602	3.333496	0.000857618	0.0017414	UP
TIFAB	13.61162	0.885573	0.265664	3.333433	0.000857813	0.0017416	UP
RP3-483K:	27.48491	0.72432	0.217318	3.332991	0.000859176	0.0017443	UP
FAM222A-	18.10165	-0.73602	0.220867	-3.33243	0.000860925	0.0017476	DOWN
GRINA	19159.77	0.423618	0.12712	3.332417	0.000860952	0.0017476	NOT
RP11-295I	1.588683	1.06288	0.31899	3.332018	0.000862188	0.00175	UP
RP11-624I	14.30777	1.051358	0.315549	3.33184	0.000862738	0.001751	UP
RP11-479(	2.772377	0.906541	0.272095	3.331709	0.000863145	0.0017517	UP
RPS2P5	904.2942	0.491589	0.147567	3.331296	0.000864427	0.0017542	NOT
CR1L	7.603082	-1.09955	0.330085	-3.3311	0.000865038	0.0017553	DOWN
CTC-479C	47.10547	0.501223	0.150474	3.330951	0.000865498	0.0017561	NOT
NCAM2	71.52995	-1.1464	0.344179	-3.33083	0.00086586	0.0017567	DOWN
CTD-3162	4.512115	-0.88945	0.267098	-3.33007	0.000868255	0.0017614	DOWN
CPB2-AS1	47.46558	-0.60101	0.180494	-3.32981	0.00086906	0.0017629	DOWN
CTD-2233	20.30827	-0.42193	0.126746	-3.32892	0.000871837	0.0017684	NOT
ZSCAN22	239.1058	0.223589	0.067171	3.328642	0.000872705	0.00177	NOT
DDX50P1	4.483443	0.74597	0.224127	3.328337	0.00087366	0.0017718	UP
NKAPL	18.6026	-0.89413	0.268646	-3.3283	0.000873786	0.001772	DOWN
C11orf45	36.85056	0.711561	0.213793	3.328274	0.00087386	0.001772	UP
MTND4P1	5.781775	-1.13423	0.340801	-3.32813	0.000874305	0.0017728	DOWN
C6orf3	23.55038	0.443509	0.133309	3.326917	0.000878124	0.0017804	NOT
CTD-2529	20.03472	-1.07132	0.32202	-3.32688	0.000878238	0.0017805	DOWN
RP4-669H	3.340863	1.008457	0.30314	3.326702	0.000878804	0.0017815	UP
CLUHP3	383.1201	0.453078	0.136205	3.326436	0.000879642	0.0017831	NOT
LPCAT3	1148.912	-0.54783	0.164716	-3.32593	0.000881242	0.0017862	NOT
OAS3	1569.311	-0.63878	0.192103	-3.32518	0.000883617	0.0017909	DOWN
ZNF672	1795.752	0.26131	0.078588	3.325056	0.000884009	0.0017914	NOT
ANKRD30/	1.770232	2.013823	0.605652	3.325048	0.000884034	0.0017914	UP
MTF2	493.8947	0.325051	0.097759	3.325026	0.000884103	0.0017915	NOT
CTD-2134	2.019288	2.479302	0.7457	3.324797	0.000884828	0.0017928	UP
HNRNPF	8676.352	-0.2228	0.067015	-3.32467	0.000885221	0.0017935	NOT
RP5-1172/	3.213598	1.435374	0.43176	3.324473	0.00088586	0.0017946	UP
CCL28	206.5423	0.743362	0.223606	3.324424	0.000886013	0.0017948	UP
RP11-890I	1.667075	1.399968	0.421131	3.324302	0.000886401	0.0017954	UP
BTBD2	2734.855	0.332354	0.09999	3.323866	0.000887789	0.0017981	NOT
AQP4	65.80926	-0.98765	0.297143	-3.32381	0.000887953	0.0017983	DOWN
RP1-240K(	22.67623	-0.62986	0.189505	-3.32372	0.000888238	0.0017988	DOWN
DEXI	382.81	-0.39845	0.119882	-3.3237	0.000888318	0.0017988	NOT
GOLGA6L4	3.385524	0.939731	0.282738	3.32368	0.000888379	0.0017988	UP
GABBR1	259.9441	0.813755	0.244873	3.323174	0.000889994	0.0018019	UP
TBC1D30	189.1143	0.709838	0.213607	3.323106	0.000890212	0.0018022	UP
AC129492	4.068577	1.407619	0.42361	3.322908	0.000890842	0.0018034	UP
RP11-297I	2.479155	0.954671	0.287316	3.322715	0.00089146	0.0018045	UP
NPBWR1	38.67137	-1.40848	0.423907	-3.32263	0.000891744	0.001805	DOWN
BOLA2P2	1.49359	1.055426	0.317657	3.322532	0.000892044	0.0018054	UP
STK17A	837.8629	0.427317	0.128615	3.322452	0.000892301	0.0018058	NOT
RP11-310I	1.445307	1.744853	0.525249	3.321953	0.000893896	0.0018089	UP
SPI1	636.8633	-0.55201	0.16619	-3.32156	0.000895159	0.0018113	NOT
RP11-635I	4.392746	1.021012	0.307391	3.321546	0.000895204	0.0018113	UP
RP11-96H	79.50474	-0.5233	0.157552	-3.32142	0.0008956	0.001812	NOT

DUSP22	1305.354	0.299129	0.09007	3.321086	0.000896678	0.0018139	NOT
RP11-291I	16.88568	0.53591	0.161366	3.321081	0.000896696	0.0018139	NOT
TAL1	87.90973	-0.58142	0.175092	-3.32066	0.000898049	0.0018165	NOT
FOLR3	3.288938	-1.25556	0.378137	-3.32039	0.000898913	0.0018181	DOWN
HECTD3	2095.437	-0.2751	0.082855	-3.32025	0.000899358	0.0018189	NOT
HIST1H2A	3609.392	0.61501	0.185242	3.320033	0.00090007	0.0018202	UP
RP11-767(	3.126569	0.971197	0.292556	3.319697	0.000901152	0.0018223	UP
LRRC8D	1658.126	0.304311	0.091669	3.319667	0.00090125	0.0018223	NOT
RP11-288(	3.332582	1.463044	0.440746	3.319476	0.000901867	0.0018234	UP
MT-TC	21.59631	-0.81213	0.244657	-3.31947	0.0009019	0.0018234	DOWN
RP11-384(	1.820057	1.068868	0.322012	3.319348	0.000902279	0.001824	UP
RP11-65J3	1.388664	1.617619	0.487341	3.319278	0.000902507	0.0018243	UP
KLC1	696.3529	0.310218	0.093461	3.319218	0.0009027	0.0018246	NOT
WWOX	578.3323	-0.3912	0.117865	-3.31903	0.000903308	0.0018257	NOT
CELA3B	1.212224	2.127359	0.641028	3.31867	0.000904474	0.0018279	UP
AMPD3	164.6397	0.608713	0.183436	3.318396	0.000905361	0.0018294	UP
CTD-2231	2.643915	0.979083	0.295047	3.318395	0.000905363	0.0018294	UP
GPI	17857.31	0.45469	0.137023	3.318343	0.000905532	0.0018296	NOT
ERICH6	4.372289	0.776137	0.233897	3.318289	0.000905709	0.0018299	UP
RNF115	1824.4	0.248398	0.07486	3.318161	0.000906123	0.0018306	NOT
ABCB4	6472.582	-0.8536	0.257255	-3.31811	0.000906298	0.0018308	DOWN
RNU4-1	6.503483	-1.15304	0.347527	-3.31784	0.00090715	0.0018323	DOWN
CTD-2132	10.83279	0.686994	0.207061	3.317836	0.000907178	0.0018323	UP
AC010524	1.093221	1.461966	0.440714	3.317269	0.000909021	0.0018359	UP
CTB-35F2	1.195607	1.36467	0.411513	3.316229	0.000912409	0.0018426	UP
PPP5D1	9.000885	-0.43606	0.131494	-3.31617	0.000912617	0.0018429	NOT
DDI2	2838.064	-0.37595	0.113374	-3.31599	0.000913191	0.0018439	NOT
GATA2	123.2792	0.602104	0.181586	3.315814	0.000913765	0.0018449	UP
AC007131	2.755538	3.243421	0.978226	3.315615	0.000914418	0.0018461	UP
CASP4	1792.752	-0.4092	0.12342	-3.31551	0.00091476	0.0018467	NOT
LINC0139	4.277997	1.004558	0.303013	3.315224	0.000915697	0.0018484	UP
DLGAP1-A	269.2591	0.568793	0.171588	3.314879	0.000916829	0.0018506	NOT
NCCRP1	10.75765	1.15135	0.347337	3.314797	0.000917096	0.001851	UP
RP11-553I	1290.814	-0.43464	0.131124	-3.31469	0.000917443	0.0018516	NOT
SYBU	2661.492	-0.72994	0.220242	-3.31425	0.00091888	0.0018543	DOWN
AKAP12	1652.482	-0.80092	0.241676	-3.31403	0.000919601	0.0018556	DOWN
AC010642	6.642546	0.691235	0.208581	3.313983	0.000919771	0.0018558	UP
SYCE2	36.85913	0.525359	0.158529	3.31397	0.000919814	0.0018558	NOT
CTD-2017	7.72081	-0.58042	0.175153	-3.31379	0.00092042	0.0018569	NOT
ATP6V0A1	3583.402	0.321652	0.097073	3.313511	0.000921324	0.0018586	NOT
INPP4A	558.7794	0.342133	0.103261	3.313291	0.00092205	0.0018599	NOT
ANKRD26I	2.016513	2.780087	0.839279	3.312471	0.000924756	0.0018652	UP
CWF19L2	393.8587	-0.25039	0.075599	-3.31213	0.000925897	0.0018674	NOT
BLCAP	3729.242	-0.34382	0.10382	-3.31165	0.000927489	0.0018704	NOT
SOX7	184.8122	-0.58419	0.176406	-3.31164	0.000927513	0.0018704	NOT
LINC0114	1.373808	2.52036	0.761077	3.311573	0.000927731	0.0018707	UP
CALCRL	793.6088	0.58555	0.176845	3.311085	0.000929349	0.0018738	UP
RP11-349I	1.315971	-1.17373	0.354522	-3.31075	0.000930455	0.0018759	DOWN
CHCHD4	683.1979	0.281223	0.084946	3.310604	0.000930947	0.0018767	NOT
AC022384	5.071993	1.11371	0.336426	3.310419	0.000931564	0.0018779	UP
CCL21	1111.961	-1.11586	0.337089	-3.31028	0.000932035	0.0018787	DOWN
NDP	1.534586	1.775064	0.536373	3.309383	0.000935017	0.0018845	UP

NDUFS8	4168.214	0.441328	0.133359	3.309319	0.000935231	0.0018848	NOT
RP11-146I	1.160179	1.635357	0.494171	3.309294	0.000935315	0.0018849	UP
RP1-313L4	2.103197	1.212788	0.366485	3.309243	0.000935486	0.0018851	UP
FAM172A	467.7318	0.283259	0.085604	3.308942	0.000936492	0.001887	NOT
RP4-681N	5.154922	0.720881	0.217861	3.308908	0.000936606	0.0018871	UP
RP11-510I	18.53462	0.953051	0.288042	3.308725	0.000937219	0.0018882	UP
RP11-134I	2.444464	0.971105	0.293504	3.308656	0.000937449	0.0018884	UP
NUCB2	2932.556	0.400852	0.121153	3.308645	0.000937487	0.0018884	NOT
PAOX	744.2485	-0.44293	0.13387	-3.30863	0.00093755	0.0018884	NOT
SCART1	64.94789	0.71851	0.217168	3.308546	0.000937817	0.0018888	UP
RP11-855/	1.289715	1.403977	0.424361	3.30845	0.000938139	0.0018893	UP
RP11-243I	3.599476	1.520469	0.459664	3.307783	0.000940375	0.0018937	UP
AP000866.	4.323586	0.863043	0.260921	3.307676	0.000940737	0.0018943	UP
CTD-2033	7.548404	-0.91958	0.278021	-3.30761	0.000940957	0.0018946	DOWN
GBP1P1	57.80971	-0.6818	0.206133	-3.30758	0.00094105	0.0018946	DOWN
NUP50-AS	382.6343	0.483538	0.146201	3.307363	0.000941789	0.001896	NOT
SH3TC1	1334.846	-0.40588	0.122729	-3.30711	0.000942639	0.0018976	NOT
MYZAP	30.62941	-0.66522	0.201153	-3.30702	0.00094294	0.001898	DOWN
TMEM44-	276.2584	0.48148	0.145598	3.306911	0.000943309	0.0018986	NOT
RP11-10A1	85.24541	-0.6613	0.200009	-3.30636	0.000945172	0.0019022	DOWN
RP11-592I	16.16834	0.572503	0.173158	3.306242	0.000945565	0.0019029	NOT
RP11-11N	1.436949	2.552664	0.772093	3.306163	0.000945831	0.0019033	UP
RP11-35G!	26.51285	-0.44984	0.136063	-3.30607	0.000946144	0.0019038	NOT
LINC01428	10.9541	-0.90935	0.275086	-3.30571	0.000947363	0.0019061	DOWN
RNF103	2792.143	-0.3109	0.094056	-3.30546	0.000948191	0.0019076	NOT
NLK	965.5368	0.226647	0.068571	3.305296	0.000948762	0.0019086	NOT
XAF1	309.5042	-0.70624	0.213669	-3.30528	0.000948814	0.0019086	DOWN
CTD-2020	42.68973	0.767124	0.232131	3.304703	0.00095077	0.0019124	UP
BPTF	1619.056	0.316432	0.095755	3.304602	0.000951115	0.0019129	NOT
EML5	7.588167	1.077158	0.326018	3.303987	0.000953203	0.001917	UP
RP11-351/	2.270786	1.162003	0.351704	3.303921	0.000953425	0.0019173	UP
KIAA1109	1595.869	-0.37001	0.112	-3.30366	0.000954302	0.0019189	NOT
AC015849	1.66607	1.306396	0.395468	3.303415	0.000955148	0.0019205	UP
RP11-145I	3.033176	1.694148	0.512852	3.303383	0.000955257	0.0019205	UP
CTB-26E1!	1.655092	2.072442	0.627371	3.303374	0.000955289	0.0019205	UP
CTD-2240	6.901007	-0.75364	0.228204	-3.3025	0.000958285	0.0019264	DOWN
RP11-296(	1.192697	1.33693	0.404917	3.301737	0.000960881	0.0019314	UP
APELA	1.463445	2.541873	0.769863	3.30172	0.000960938	0.0019314	UP
CDR2L	280.3775	0.659133	0.199653	3.301398	0.000962045	0.0019335	UP
FGFBP3	45.91349	0.544326	0.16489	3.301147	0.000962903	0.0019351	NOT
ACKR4	11.77446	-0.66696	0.202112	-3.29994	0.000967057	0.0019433	DOWN
LGI4	233.0789	0.762268	0.230997	3.299904	0.000967179	0.0019434	UP
RP11-216I	3.706852	0.952823	0.288811	3.299117	0.000969896	0.0019487	UP
UBE2FP1	33.7545	-0.56843	0.172328	-3.29853	0.000971914	0.0019526	NOT
CTD-2192	2.513694	0.891101	0.270166	3.298346	0.000972561	0.0019538	UP
HOXD13	1.570901	3.034168	0.919927	3.298269	0.000972827	0.0019542	UP
OPCML	7.051597	-0.86753	0.26304	-3.29808	0.000973491	0.0019554	DOWN
CTA-268H	3.128406	1.139149	0.34542	3.297863	0.000974235	0.0019567	UP
ACAP2-IT1	2.80585	0.819225	0.248426	3.297667	0.000974918	0.001958	UP
CHMP4C	439.7878	0.448398	0.135987	3.297349	0.000976022	0.00196	NOT
CTC-231C	18.30578	0.911688	0.27651	3.297121	0.000976815	0.0019615	UP
ATP5A1P3	13.13601	-0.74709	0.22659	-3.29708	0.000976944	0.0019616	DOWN

RP11-420I	307.104	0.447025	0.135588	3.296927	0.000977488	0.0019626	NOT
RP11-1C8.	2.081947	1.188035	0.360351	3.296882	0.000977644	0.0019627	UP
SLC34A1	42.45557	-0.91622	0.277919	-3.2967	0.000978265	0.0019638	DOWN
ARPC3P5	1.009649	1.370313	0.415673	3.296613	0.000978584	0.0019643	UP
UBE2L5P	3.534601	0.649781	0.197109	3.296565	0.000978751	0.0019645	UP
GREM2	1083.924	-1.25831	0.381722	-3.29639	0.000979344	0.0019656	DOWN
TPTE2P2	1.078802	1.976272	0.599603	3.29597	0.000980826	0.0019684	UP
RP13-147I	2.669185	3.809225	1.155795	3.295761	0.000981554	0.0019697	UP
SC22CB-1	20.73949	0.651847	0.197787	3.295694	0.000981788	0.0019701	UP
EDEM2	1943.193	0.226034	0.068586	3.295645	0.000981961	0.0019703	NOT
ATAD1	1541.709	-0.25935	0.078699	-3.2955	0.000982472	0.0019711	NOT
PABPN1	2885.343	0.276751	0.083981	3.295413	0.000982771	0.0019716	NOT
RBM22	1464.999	0.201622	0.061197	3.294635	0.000985496	0.0019769	NOT
RP11-111I	32.97056	0.452263	0.137299	3.294009	0.000987693	0.0019812	NOT
LINC00858	1.732514	2.734701	0.830278	3.293716	0.000988722	0.0019831	UP
TRABD	1719.875	0.31196	0.094714	3.293684	0.000988835	0.0019832	NOT
RP11-108I	1.803431	1.176873	0.357389	3.292978	0.000991321	0.001988	UP
RP11-176I	0.952326	1.406074	0.427062	3.292437	0.000993232	0.0019917	UP
TTC1	1843.76	0.278186	0.084499	3.292161	0.000994208	0.0019935	NOT
RP11-84C.	5.018247	0.641719	0.194962	3.291509	0.000996515	0.0019979	UP
CTD-2095	18.14249	0.802839	0.243913	3.291498	0.000996552	0.0019979	UP
ZBTB20	59.82103	-0.43023	0.130712	-3.29139	0.000996922	0.0019985	NOT
ABHD12B	33.82138	0.76584	0.232685	3.291323	0.000997172	0.0019989	UP
CCNB1IP1	1643.185	-0.54508	0.165637	-3.29082	0.000998953	0.0020023	NOT
TSPYL2	821.4725	0.469016	0.142532	3.290615	0.000999685	0.0020036	NOT
APLF	107.7283	0.422158	0.128293	3.290566	0.000999861	0.0020039	NOT
RP11-104J	13.55608	-0.74239	0.225631	-3.29029	0.001000858	0.0020057	DOWN
FAM83E	10.52851	-1.19531	0.363294	-3.29019	0.001001208	0.0020063	DOWN
BTN3A1	1272.064	0.490671	0.149156	3.28966	0.001003085	0.0020099	NOT
SETD7	4345.594	-0.41597	0.126468	-3.28911	0.001005061	0.0020137	NOT
AC073130	2.800304	0.955659	0.290558	3.28905	0.001005261	0.0020139	UP
SCAF11	3130.017	-0.25232	0.076723	-3.28872	0.001006425	0.0020161	NOT
AC008440	1.528599	1.363822	0.414717	3.28856	0.001007014	0.0020172	UP
SPATA31C	1.20787	2.403893	0.731004	3.28848	0.001007298	0.0020175	UP
POLR1C	821.5751	0.301116	0.091567	3.288473	0.001007324	0.0020175	NOT
NAP1L3	40.89627	-0.80343	0.244354	-3.28797	0.001009128	0.002021	DOWN
YWHAG	6589.976	0.24982	0.075994	3.287366	0.001011294	0.0020252	NOT
HMGB3P3.	1.856569	1.312918	0.399404	3.287188	0.001011933	0.0020263	UP
TBC1D19	107.6691	0.427722	0.130148	3.28642	0.001014696	0.0020317	NOT
RP11-710I	2.033338	1.086709	0.330689	3.286201	0.001015485	0.0020331	UP
CEP57	1972.091	-0.32299	0.098291	-3.2861	0.001015835	0.0020335	NOT
RP11-883(	5.003387	0.987171	0.300408	3.286102	0.001015842	0.0020335	UP
LETM2	29.73589	0.643187	0.195756	3.28566	0.001017438	0.0020366	UP
GOLGA5	2360.088	-0.24908	0.075816	-3.28538	0.001018446	0.0020384	NOT
AC092338	3.155944	0.933345	0.284147	3.284726	0.001020818	0.002043	UP
HPN-AS1	41.39287	0.662671	0.201745	3.2847	0.001020909	0.0020431	UP
PITRM1-A	10.01491	0.671102	0.204318	3.284589	0.001021313	0.0020437	UP
LINC01374	1.29104	1.482475	0.451433	3.283933	0.001023691	0.0020483	UP
ANKRD28	1672.583	-0.25778	0.078496	-3.28392	0.001023739	0.0020483	NOT
FTH1P15	9.221405	0.610605	0.185979	3.28319	0.001026394	0.0020535	UP
RP1-27K1.	310.8417	1.170262	0.356445	3.283154	0.001026527	0.0020536	UP
PTPLAD2	95.09805	-0.53958	0.16437	-3.28273	0.001028088	0.0020565	NOT

ZNF831	38.81962	-0.84241	0.256647	-3.28238	0.001029366	0.002059	DOWN
SHISA3	61.63548	1.047552	0.319154	3.282279	0.001029716	0.0020595	UP
RP11-291I	8.807016	-1.05502	0.321457	-3.282	0.001030743	0.0020614	DOWN
WDR77	861.4571	0.290731	0.088587	3.281874	0.001031198	0.0020622	NOT
GABPA	1021.612	-0.25869	0.078831	-3.28165	0.001032023	0.0020637	NOT
QRICH1	1803.59	0.175103	0.05336	3.281532	0.001032449	0.0020644	NOT
ALKBH8	235.9468	-0.25904	0.078943	-3.28138	0.001033001	0.0020653	NOT
ZNF112	78.48882	0.530409	0.161652	3.281187	0.001033713	0.0020666	NOT
RP11-362I	6.723187	0.739628	0.225442	3.280795	0.00103515	0.0020693	UP
ATL3	2788.592	0.284284	0.086652	3.280747	0.001035325	0.0020694	NOT
RP11-296(	43.34296	0.445597	0.135822	3.280741	0.001035348	0.0020694	NOT
PMEL	278.976	-0.53329	0.162587	-3.28001	0.00103802	0.0020746	NOT
FCRL2	7.595491	-1.19582	0.364579	-3.27999	0.001038094	0.0020746	DOWN
LINC01397	1.043904	2.121117	0.646927	3.278757	0.001042652	0.0020836	UP
TNKS2	1616.885	-0.28543	0.08708	-3.27782	0.001046104	0.0020903	NOT
CDH7	1.964476	1.921831	0.586365	3.277534	0.001047183	0.0020923	UP
SLC38A11	157.997	0.824359	0.251523	3.277464	0.001047441	0.0020927	UP
CTGF	4052.144	-0.66353	0.202456	-3.27741	0.001047639	0.0020929	DOWN
CACNA1D	225.013	0.615559	0.187827	3.277265	0.001048178	0.0020939	UP
ZCCHC18	7.162389	0.809815	0.247114	3.277084	0.00104885	0.002095	UP
PTN	129.8995	-0.74934	0.228664	-3.27705	0.001048987	0.0020952	DOWN
ADCY3	315.1854	0.548999	0.16753	3.277027	0.001049065	0.0020952	NOT
RP13-93L1	1.725965	0.943347	0.287899	3.276664	0.001050414	0.0020977	UP
DUSP8P3	3.385931	0.76386	0.233163	3.276082	0.00105258	0.0021019	UP
CCDC109E	191.2438	0.680723	0.2078	3.275857	0.00105342	0.0021034	UP
RP3-441A1	1.374632	1.14343	0.349083	3.275525	0.00105466	0.0021057	UP
BLACAT1	2.034428	1.666705	0.508863	3.275353	0.001055301	0.0021068	UP
WDR87	1.468802	2.027304	0.61896	3.275336	0.001055362	0.0021068	UP
RP11-108(	80.46932	-0.73816	0.225402	-3.27488	0.001057071	0.0021101	DOWN
RP11-476I	6.811186	0.740607	0.226158	3.274734	0.001057616	0.002111	UP
TBC1D3B	2.897237	1.010281	0.308543	3.274367	0.00105899	0.0021136	UP
EGFL7	2134.016	0.558506	0.170574	3.274272	0.001059345	0.0021142	NOT
SULT1E1	499.2035	-1.10985	0.338967	-3.27421	0.001059584	0.0021145	DOWN
RP11-744I	16.87444	-0.60403	0.184485	-3.27416	0.001059765	0.0021147	DOWN
APITD1	99.19886	0.382328	0.116788	3.273696	0.001061507	0.002118	NOT
AC010907	2.950313	1.393176	0.42558	3.273591	0.001061901	0.0021187	UP
ARL13A	2.122043	0.926245	0.282965	3.273357	0.001062781	0.0021203	UP
RSRC1	654.1675	0.255041	0.077922	3.273037	0.001063986	0.0021225	NOT
RPL27AP5	2.531318	1.073758	0.328081	3.272847	0.001064702	0.0021238	UP
RGS13	7.494029	1.284968	0.39269	3.27222	0.001067067	0.0021284	UP
ZNF768	1996.13	0.300948	0.091974	3.272088	0.001067562	0.0021292	NOT
RP11-288I	6.223581	0.612834	0.187306	3.271838	0.001068509	0.0021309	UP
GTF2IP1	37.87092	0.396298	0.121135	3.271552	0.00106959	0.0021329	NOT
CASC11	1.345546	1.270345	0.388345	3.271177	0.001071009	0.0021356	UP
PPARG	871.4353	0.571853	0.174818	3.271128	0.001071194	0.0021358	NOT
RP11-468I	8.736065	0.569933	0.174239	3.270978	0.001071761	0.0021368	NOT
FAM35A	1459.337	-0.28441	0.086958	-3.27072	0.001072758	0.0021386	NOT
MAMLD1	331.3656	0.63937	0.195492	3.27057	0.001073312	0.0021396	UP
SPTLC3	1169.051	-0.57261	0.175084	-3.27047	0.001073699	0.0021402	NOT
ALG1	934.0629	0.275213	0.084152	3.270418	0.001073886	0.0021403	NOT
POTENP	1.50349	1.091026	0.333605	3.270412	0.001073909	0.0021403	UP
RP5-1033I	172.0588	-0.62304	0.190565	-3.26943	0.001077659	0.0021476	DOWN

LEPR	8866.325	-0.82051	0.250977	-3.26927	0.001078268	0.0021487	DOWN
CAV1	1579.672	0.454469	0.139024	3.268998	0.001079289	0.0021506	NOT
RP1-178F1	6.806502	0.608217	0.186104	3.268166	0.001082469	0.0021568	UP
SOD2	43358.08	-0.59569	0.182281	-3.26798	0.001083172	0.002158	DOWN
YTHDF3-A	63.82211	0.56206	0.171998	3.267834	0.001083738	0.002159	NOT
SPDYE6	8.250209	0.598579	0.183206	3.267243	0.001086003	0.0021633	UP
TMEM51	382.5344	0.706167	0.216136	3.267227	0.001086064	0.0021633	UP
TMEM115	2262.16	0.23952	0.073315	3.266997	0.001086947	0.0021649	NOT
C19orf52	505.8056	0.238537	0.073015	3.266977	0.001087025	0.0021649	NOT
COL12A1	707.4246	0.675633	0.206817	3.266817	0.001087641	0.002166	UP
RP11-659I	17.72611	-0.91045	0.278702	-3.26674	0.001087932	0.0021664	DOWN
PSORS1C2	1.08552	1.694937	0.518858	3.266671	0.001088201	0.0021668	UP
C5orf22	784.4401	0.232523	0.071182	3.266583	0.001088539	0.0021673	NOT
HSPBP1	1865.486	0.399939	0.12244	3.26641	0.001089206	0.0021685	NOT
MIR5010	1.220666	1.243124	0.380587	3.266334	0.001089496	0.0021689	UP
RP11-373I	6.939971	0.778054	0.238215	3.26619	0.001090052	0.0021698	UP
CFHR1	20119.31	-0.91894	0.28136	-3.26608	0.001090479	0.0021705	DOWN
RP4-583P1	20.39832	0.825342	0.252702	3.266066	0.001090528	0.0021705	UP
NAALAD2	56.68973	0.746496	0.228567	3.265988	0.001090827	0.0021709	UP
AC002480	4.2278	1.066281	0.326505	3.265746	0.001091761	0.0021726	UP
KB-176G8.	3.729868	0.87171	0.267023	3.264548	0.001096388	0.0021817	UP
RAB7B	148.6588	0.608703	0.186478	3.264205	0.001097716	0.0021841	UP
RP11-386(	3.133807	0.809458	0.247982	3.264174	0.001097838	0.0021842	UP
RAD51B	85.52812	0.430324	0.131835	3.264118	0.001098052	0.0021845	NOT
ST7-AS1	35.90721	0.486301	0.148989	3.264007	0.001098485	0.0021852	NOT
RP11-131(	8.606293	0.720109	0.220628	3.263913	0.001098849	0.0021858	UP
SP100	2463.306	-0.32221	0.098721	-3.26388	0.001098988	0.0021859	NOT
RP11-12J1	4.713967	0.775676	0.237676	3.263584	0.001100125	0.002188	UP
LINC01474	27.7065	0.904457	0.277162	3.263282	0.001101299	0.0021902	UP
IST1	2891.393	0.211295	0.06476	3.262743	0.001103395	0.0021942	NOT
ZFAND3	3883.254	0.236907	0.072626	3.262033	0.001106162	0.0021995	NOT
ACSL5	7169.53	-0.71444	0.219027	-3.26187	0.001106794	0.0022006	DOWN
CKMT2-AS	356.0466	0.439514	0.134747	3.261762	0.00110722	0.0022013	NOT
ZNF223	28.13992	0.474517	0.145486	3.261594	0.001107875	0.0022025	NOT
NFATC2	719.6587	-0.5184	0.158961	-3.26114	0.001109657	0.0022058	NOT
ZNF35	116.2948	0.416167	0.127615	3.261104	0.001109791	0.0022059	NOT
ZNF197-A	2.159207	1.047696	0.321372	3.260072	0.001113841	0.0022138	UP
NSFL1C	3402.419	0.24048	0.073768	3.25995	0.00111432	0.0022145	NOT
C2orf27AP	1.280555	2.147287	0.658689	3.259941	0.001114353	0.0022145	UP
NPRL3	1298.906	0.285681	0.087639	3.259762	0.001115059	0.0022158	NOT
SLC4A8	20.27925	0.668783	0.205197	3.25922	0.00111719	0.0022199	UP
RP11-114I	1.96348	1.253908	0.384768	3.258867	0.001118582	0.0022225	UP
ZNF423	91.15698	0.590979	0.18137	3.258424	0.001120328	0.0022257	UP
IRS2	4763.695	-0.57267	0.175751	-3.25842	0.001120351	0.0022257	NOT
ARGLU1	1506.854	0.320648	0.098416	3.258099	0.001121613	0.002228	NOT
TRNT1	471.9313	0.241339	0.074076	3.257992	0.001122037	0.0022287	NOT
C16orf95	75.49973	-0.42927	0.131764	-3.25789	0.001122438	0.0022293	NOT
RP13-317I	7.378073	0.593364	0.182136	3.257799	0.0011228	0.0022299	UP
CTC-281F	5.29174	0.971481	0.298247	3.257307	0.001124746	0.0022336	UP
MOV10	3498.791	0.298163	0.09154	3.257168	0.001125298	0.0022343	NOT
CSK	2685.659	-0.23888	0.073339	-3.25716	0.001125348	0.0022343	NOT
CTD-3064	20.85134	0.77616	0.238294	3.25715	0.00112537	0.0022343	UP

ITGA7	1008.937	0.47899	0.147059	3.257132	0.001125441	0.0022343	NOT
RPH3A	3.081173	-0.79531	0.24419	-3.25693	0.001126231	0.0022357	DOWN
ANKRD42	239.6196	-0.29924	0.091888	-3.25659	0.001127582	0.0022383	NOT
IGLV4-3	1.695134	-1.71707	0.527339	-3.2561	0.001129524	0.002242	DOWN
ARSG	202.074	0.42243	0.129738	3.256021	0.001129856	0.0022424	NOT
CABP4	39.78281	-0.68457	0.210256	-3.25589	0.001130358	0.0022433	DOWN
DUOXA1	7.797811	1.108185	0.34039	3.25563	0.001131412	0.0022452	UP
RFX2	222.294	-0.67318	0.206828	-3.25479	0.001134774	0.0022517	DOWN
LYPLAL1-A	5.599834	0.873675	0.268469	3.254288	0.001136768	0.0022555	UP
RP6-105D	5.165572	0.73763	0.226681	3.254048	0.001137732	0.0022571	UP
CDC42BPC	241.1383	0.845081	0.259702	3.254047	0.001137734	0.0022571	UP
OSGEPL1-	8.141416	0.502922	0.154571	3.25367	0.001139245	0.0022599	NOT
DSG2-AS1	15.6536	-0.64573	0.19847	-3.25354	0.001139769	0.0022608	DOWN
CPEB1	14.78022	1.247759	0.383518	3.25346	0.001140088	0.0022613	UP
ATP2C1	2409.091	0.244136	0.07504	3.253399	0.001140333	0.0022616	NOT
RP11-83J1	4.016721	0.813714	0.250134	3.253114	0.001141477	0.0022636	UP
IGHV3-62	1.084343	-1.71332	0.526671	-3.25311	0.001141512	0.0022636	DOWN
CH17-118	141.8014	0.481537	0.148039	3.252763	0.001142887	0.0022662	NOT
RP11-111I	16.40356	-0.56154	0.172673	-3.25204	0.001145785	0.0022716	NOT
ZNF287	36.03839	0.703469	0.216316	3.252042	0.001145792	0.0022716	UP
GALT	3535.955	-0.38469	0.118294	-3.25195	0.001146145	0.0022722	NOT
RABGGTB	2283.517	0.270436	0.083174	3.251464	0.001148123	0.0022759	NOT
RP11-203I	24.82109	0.984743	0.302902	3.251028	0.001149886	0.0022793	UP
LA16c-358	42.8364	0.548073	0.168588	3.250972	0.001150112	0.0022795	NOT
STX18	1080.752	0.292958	0.090135	3.250212	0.001153191	0.0022855	NOT
AC093106	1.919671	1.078786	0.331916	3.250174	0.001153346	0.0022856	UP
NR5A2	2507.659	-0.47854	0.147235	-3.25014	0.001153484	0.0022857	NOT
RP11-358I	4.105052	-0.67449	0.207553	-3.24971	0.001155241	0.0022891	DOWN
FAM177A1	2001.716	-0.30553	0.094021	-3.24957	0.001155789	0.00229	NOT
MRPS6	1069.805	-0.32838	0.101065	-3.2492	0.001157298	0.0022928	NOT
CPNE2	809.8341	0.366487	0.112797	3.249093	0.001157735	0.0022935	NOT
RP5-1009I	2.165282	1.133133	0.348764	3.248996	0.001158132	0.0022941	UP
PLA2G4B	8.074768	0.731374	0.225116	3.248883	0.00115859	0.0022949	UP
SNRPGP14	2.096613	0.859094	0.264437	3.248771	0.001159048	0.0022956	UP
UBFD1	1901.599	0.264374	0.08138	3.248646	0.001159557	0.0022965	NOT
RP3-423B2	5.841836	0.715184	0.22019	3.248036	0.001162045	0.0023012	UP
GRASP	298.1433	-0.52183	0.160671	-3.24783	0.001162907	0.0023028	NOT
RP4-800G	6.224057	0.666248	0.20514	3.24777	0.001163131	0.002303	UP
IKZF5	730.1261	-0.30194	0.092973	-3.24755	0.00116402	0.0023046	NOT
LINC00996	12.99748	0.773366	0.238143	3.247488	0.001164285	0.002305	UP
CXCL1	463.6437	1.233088	0.379806	3.246625	0.001167821	0.0023118	UP
SLC38A3	26437.79	-0.67708	0.208554	-3.24656	0.001168088	0.0023122	DOWN
RP11-35G	63.81901	0.362715	0.111727	3.246443	0.001168569	0.0023129	NOT
CTB-25B1	2.37765	1.007987	0.31049	3.246437	0.001168592	0.0023129	UP
GDF11	236.8612	0.632087	0.194721	3.246118	0.001169904	0.0023153	UP
SNORD83A	2.342752	0.941618	0.29014	3.245389	0.001172903	0.0023211	UP
CTD-2562	2.204964	0.737887	0.227391	3.245014	0.001174447	0.0023239	UP
RP11-756I	16.44371	-0.7995	0.246396	-3.24476	0.001175492	0.0023258	DOWN
AL135745.	3.526097	1.010383	0.311395	3.2447	0.001175745	0.0023262	UP
RLF	568.9155	-0.30635	0.094424	-3.24443	0.001176864	0.0023282	NOT
LSR	10028.5	-0.57067	0.175903	-3.24424	0.001177644	0.0023296	NOT
LIN54	526.4721	-0.2633	0.081162	-3.24413	0.001178093	0.0023303	NOT

WDR49	1.363008	1.452946	0.447875	3.244087	0.001178279	0.0023305	UP
CXorf22	9.156653	1.231343	0.379581	3.243951	0.001178841	0.0023315	UP
RP1-79C4.	1.161488	1.513735	0.466647	3.243854	0.00117924	0.0023321	UP
TMEM88	122.5835	0.554555	0.170987	3.243254	0.001181727	0.0023368	NOT
TTLL13P	6.460617	0.607591	0.187341	3.243236	0.001181801	0.0023368	UP
IGKV3-7	9.004982	-1.42422	0.439141	-3.24319	0.001181979	0.002337	DOWN
FAM153B	28.49209	1.282808	0.395554	3.243071	0.001182489	0.0023379	UP
C19orf47	535.4606	0.319932	0.098654	3.24296	0.001182948	0.0023386	NOT
RPS11P5	26.3502	0.569012	0.175474	3.242721	0.001183942	0.0023404	NOT
RPS15AP1(	2.712476	0.762067	0.235015	3.242632	0.001184309	0.002341	UP
COX6A2	104.1473	-1.26731	0.390839	-3.24255	0.001184662	0.0023415	DOWN
ZC3H4	1225.013	0.18687	0.057634	3.242334	0.001185549	0.0023431	NOT
AC019109	1.666778	1.76242	0.543576	3.242271	0.00118581	0.0023434	UP
METTL2B	854.3955	0.248681	0.0767	3.242234	0.001185964	0.0023436	NOT
DLGAP1	17.60654	0.963268	0.297113	3.242093	0.001186555	0.0023446	UP
LDB3	27.666	0.604031	0.186342	3.241519	0.001188945	0.0023491	UP
CSE1L-AS:	1.669923	1.060742	0.327247	3.241411	0.001189394	0.0023498	UP
DDX23	3397.824	0.180015	0.055548	3.240698	0.001192374	0.0023555	NOT
AC108004	1.505763	1.341488	0.413977	3.240493	0.001193231	0.0023571	UP
CD7	433.6462	0.886841	0.273696	3.240244	0.001194273	0.002359	UP
AC004967	80.90816	0.413151	0.127508	3.240189	0.001194504	0.0023592	NOT
CTB-39G8	6.684341	0.893375	0.275776	3.239496	0.001197414	0.0023648	UP
USP25	1119.606	-0.27252	0.084135	-3.23907	0.001199205	0.002368	NOT
BTBD16	162.8314	-0.696	0.214877	-3.23907	0.001199215	0.002368	DOWN
CTD-2616	5.533951	0.651183	0.201107	3.237985	0.00120377	0.0023769	UP
EFCAB13	64.70673	0.491361	0.151756	3.237842	0.001204375	0.0023779	NOT
AQP7P1	100.2853	-0.98765	0.305058	-3.23757	0.001205519	0.00238	DOWN
PLEKHA2	677.9292	-0.47301	0.146145	-3.23659	0.001209679	0.002388	NOT
CTD-2561	4.573356	1.070265	0.330706	3.236305	0.001210879	0.0023902	UP
MYH9	31276.5	0.288173	0.089045	3.236264	0.001211052	0.0023903	NOT
RP11-93I2	1.6522	1.234903	0.381584	3.236253	0.0012111	0.0023903	UP
EPB41L3	305.5366	-0.67052	0.207217	-3.23586	0.001212786	0.0023935	DOWN
TDRD10	99.91486	0.709602	0.2193	3.235754	0.001213221	0.0023942	UP
C1orf228	87.55605	-0.56454	0.174474	-3.23568	0.001213528	0.0023946	NOT
GAK	3496.745	0.21139	0.06534	3.235205	0.001215553	0.0023983	NOT
IGHV3OR1	1.953987	-1.59029	0.49156	-3.23519	0.001215619	0.0023983	DOWN
IGKV1D-2	1.229291	-1.71122	0.528941	-3.23519	0.001215639	0.0023983	DOWN
AC012499	2.701362	1.638826	0.506593	3.234997	0.001216442	0.0023997	UP
RP11-147I	41.61512	0.491845	0.152045	3.234871	0.001216977	0.0024005	NOT
RP11-426I	0.985797	1.281835	0.396285	3.234632	0.001217997	0.0024024	UP
AC092066	578.3605	0.42664	0.131902	3.234529	0.001218434	0.0024031	NOT
IFNL3P1	1.459919	1.699759	0.525519	3.234436	0.001218833	0.0024037	UP
CTD-2530	4.467707	0.706781	0.218531	3.234228	0.001219721	0.0024053	UP
ARHGAP18	497.4955	0.412305	0.127492	3.233971	0.00122082	0.0024073	NOT
RP11-187(	7.645594	0.781225	0.241589	3.233694	0.001222004	0.0024094	UP
SMURF2P1	3.102181	0.842321	0.26051	3.233352	0.001223466	0.0024121	UP
ENY2	3019.216	0.361186	0.111726	3.232789	0.001225882	0.0024167	NOT
RP11-516/	2.607717	0.923806	0.285821	3.232114	0.001228781	0.0024223	UP
TIMM23B	192.9867	-0.29987	0.092782	-3.23201	0.001229247	0.002423	NOT
RP11-517/	1.438847	1.255863	0.388576	3.231962	0.001229436	0.0024232	UP
PMEPA1	835.0567	0.818506	0.253285	3.231565	0.001231144	0.0024264	UP
KLHDC2	2200.795	-0.37032	0.114599	-3.23147	0.001231544	0.0024269	NOT

PEX6	2770.026	0.450261	0.139337	3.23145	0.001231639	0.0024269	NOT
ADAMTS7	2.851703	1.052452	0.325691	3.231447	0.001231652	0.0024269	UP
INSL6	2.274891	2.133603	0.660275	3.231383	0.001231927	0.0024273	UP
CD2AP	2075.58	0.320671	0.099254	3.230809	0.001234404	0.002432	NOT
RNA5SP38	2.113006	0.932994	0.288809	3.230481	0.001235819	0.0024346	UP
RP11-345I	24.14291	-0.65641	0.20322	-3.23006	0.001237636	0.002438	DOWN
CRELD1	1781.528	0.295499	0.091492	3.229766	0.001238918	0.0024403	NOT
RP11-68I3	4.518095	0.830282	0.2571	3.229414	0.001240441	0.0024432	UP
GCNT2	794.7609	-0.53682	0.166233	-3.22932	0.001240865	0.0024438	NOT
FAM196B	1.82552	-1.19364	0.369641	-3.2292	0.001241386	0.0024447	DOWN
NEK3	375.7746	0.558612	0.173013	3.228728	0.001243421	0.0024485	NOT
RP5-837J1	2.522468	0.871004	0.269778	3.228598	0.001243988	0.0024495	UP
RMND1	1039.381	-0.32779	0.101531	-3.22849	0.001244457	0.0024502	NOT
CD27-AS1	396.5926	0.328026	0.10163	3.227667	0.001248044	0.0024571	NOT
MRPS31P5	9.420447	0.737544	0.228522	3.227445	0.00124901	0.0024588	UP
AIFM1	5799.624	-0.36665	0.113604	-3.22741	0.00124915	0.0024589	NOT
RP11-673(	234.1766	-0.33178	0.10281	-3.22712	0.001250416	0.0024612	NOT
SCN9A	371.277	-0.72913	0.225947	-3.22699	0.001251014	0.0024622	DOWN
SLC6A15	1.74448	2.447127	0.758335	3.226972	0.001251076	0.0024622	UP
IL12A-AS1	1.403376	1.528638	0.473732	3.2268	0.00125183	0.0024635	UP
CTD-2650	13.70706	0.633802	0.196423	3.226727	0.001252149	0.002464	UP
LIMS2	1714.094	-0.49932	0.154751	-3.22663	0.001252593	0.0024647	NOT
SERPINB6	2900.963	0.328438	0.101799	3.226343	0.001253832	0.0024669	NOT
SERP1	8365.41	-0.26822	0.083141	-3.2261	0.001254898	0.0024688	NOT
DCUN1D1	1479.382	-0.27131	0.084107	-3.22581	0.001256174	0.0024712	NOT
SGMS1-AS	183.9657	-0.36195	0.112215	-3.22554	0.001257364	0.0024733	NOT
LBX2	209.0828	0.66161	0.205149	3.225026	0.001259614	0.0024776	UP
KIAA1377	32.7886	0.715971	0.22201	3.22495	0.001259947	0.0024781	UP
EIF1AX	2589.041	-0.25916	0.080362	-3.2249	0.001260184	0.0024784	NOT
RPL23P2	11.13069	0.493986	0.153184	3.224784	0.001260678	0.0024792	NOT
RP11-234(	3.916904	1.208122	0.37464	3.224756	0.001260802	0.0024792	UP
OSGIN1	8539.307	0.735315	0.228028	3.224669	0.001261185	0.0024798	UP
RP5-907C	4.884185	1.796844	0.557253	3.224468	0.001262071	0.0024814	UP
CUTC	744.2518	-0.3021	0.093695	-3.22425	0.001263044	0.0024831	NOT
CTD-2376	16.93231	-0.50816	0.157641	-3.22355	0.001266141	0.002489	NOT
RGS10	280.1245	0.563844	0.174921	3.223423	0.001266684	0.0024899	NOT
COL27A1	2471.82	0.48947	0.151909	3.222133	0.001272399	0.0025008	NOT
TPRG1	216.8247	-0.70814	0.219775	-3.22213	0.001272426	0.0025008	DOWN
RP11-640I	13.10956	-0.58075	0.180244	-3.22204	0.001272825	0.0025014	NOT
CTD-3214	3.231519	0.834343	0.258956	3.221948	0.001273223	0.002502	UP
GRB14	1402.219	-0.45596	0.14152	-3.22189	0.001273495	0.0025022	NOT
LINC00637	2.164178	1.020793	0.316831	3.221885	0.001273502	0.0025022	UP
TPST2	4229.544	-0.36274	0.112591	-3.22178	0.001273973	0.002503	NOT
BIN1	2473.656	0.397963	0.123525	3.221714	0.001274264	0.0025034	NOT
KIAA0196-	3.12901	0.759742	0.235841	3.221422	0.001275564	0.0025058	UP
NAPA	3919.879	-0.27929	0.086703	-3.22129	0.001276129	0.0025067	NOT
ADAD2	4.379626	-0.80069	0.248572	-3.22115	0.001276761	0.0025077	DOWN
TDG	515.8971	0.290849	0.090298	3.220982	0.001277521	0.0025091	NOT
RP11-806I	14.11428	1.001583	0.310971	3.220827	0.001278213	0.0025102	UP
KCTD15	500.5633	0.631408	0.196061	3.220474	0.001279787	0.0025132	UP
C16orf62	1029.032	-0.43554	0.135245	-3.22037	0.001280246	0.0025139	NOT
BCL10	523.0171	-0.25354	0.078731	-3.22033	0.00128044	0.0025141	NOT

NEGR1	17.01064	-0.93428	0.290126	-3.22024	0.00128082	0.0025146	DOWN
RDH11	5695.865	-0.39809	0.123636	-3.21982	0.001282731	0.0025182	NOT
RP11-861I	3.427611	1.013448	0.314791	3.219432	0.00128445	0.0025214	UP
PROM2	51.75751	-0.93266	0.2897	-3.21939	0.001284631	0.0025216	DOWN
LCN6	3.149925	-0.95102	0.295425	-3.21917	0.00128562	0.0025234	DOWN
ANKRD13/	1536.54	0.286382	0.088966	3.219015	0.001286319	0.0025246	NOT
RIC3	51.39551	-1.14133	0.35458	-3.21884	0.001287105	0.0025259	DOWN
RP11-5P1:	2.070654	1.777252	0.55221	3.218438	0.001288907	0.0025293	UP
RP11-234I	1.157093	1.301675	0.404486	3.218101	0.001290426	0.0025321	UP
EMBP1	117.6096	-0.56918	0.176872	-3.21805	0.00129067	0.0025324	NOT
RP11-399I	2.20207	1.028472	0.319613	3.21787	0.001291463	0.0025337	UP
ST6GALNA	98.15328	-0.56967	0.177037	-3.21778	0.001291868	0.0025344	NOT
PLEK	666.6931	-0.68018	0.211409	-3.21737	0.001293712	0.0025378	DOWN
C20orf197	5.120809	1.005166	0.312456	3.216989	0.001295435	0.002541	UP
RP11-261(	2.121512	1.078474	0.335301	3.216439	0.001297923	0.0025457	UP
CDRT15	2.097182	1.143364	0.355508	3.216142	0.001299264	0.0025481	UP
RP1-29C1:	2.833127	0.953412	0.296476	3.215813	0.001300757	0.0025509	UP
C15orf61	293.648	0.329928	0.102604	3.215542	0.001301983	0.0025531	NOT
AQP7P2	8.905941	-1.21827	0.378924	-3.21507	0.001304114	0.0025571	DOWN
RNA5SP37	1.844156	-0.96774	0.301053	-3.21453	0.001306599	0.0025618	DOWN
DOCK2	408.0318	-0.63956	0.198961	-3.21448	0.001306829	0.0025621	DOWN
MAP2K6	328.5823	0.736101	0.229017	3.214173	0.001308209	0.0025646	UP
ASB11	3.455316	1.249492	0.388774	3.213933	0.001309302	0.0025666	UP
MGAM	50.32164	1.154506	0.359226	3.213875	0.001309565	0.0025669	UP
RARRES2P(	1.960859	3.050585	0.949284	3.213564	0.001310985	0.0025695	UP
PARVA	1619.507	-0.33975	0.105725	-3.21353	0.001311159	0.0025697	NOT
RP11-728I	12.9447	0.973579	0.302973	3.213416	0.001311662	0.0025705	UP
RP11-345I	194.4158	-0.42642	0.132718	-3.21299	0.001313618	0.0025741	NOT
GRIA2	2.546806	1.850968	0.576095	3.212957	0.00131376	0.0025742	UP
ROBO2	75.29028	1.177593	0.366523	3.212874	0.00131414	0.0025748	UP
PPP2R5C	3329.933	-0.23892	0.074371	-3.21255	0.00131564	0.0025775	NOT
XPNPEP1	1373.571	0.230223	0.071675	3.212037	0.001317975	0.0025819	NOT
RPRM	1.377677	1.959709	0.610207	3.211547	0.001320223	0.0025861	UP
LINC0088(	185.0402	0.79484	0.247519	3.211228	0.00132169	0.0025888	UP
RP11-84G:	28.19845	-0.45048	0.140295	-3.21095	0.001322978	0.0025912	NOT
EPB42	2.888512	-0.81545	0.254053	-3.20976	0.00132844	0.0026017	DOWN
TMPO	3548.774	0.328044	0.102203	3.209719	0.001328647	0.0026019	NOT
RP5-888M	32.4605	-0.7232	0.225317	-3.2097	0.001328752	0.0026019	DOWN
PGAP3	2615.534	-0.35453	0.110468	-3.20937	0.001330276	0.0026047	NOT
GNL2	1963.827	0.269306	0.083921	3.209045	0.001331766	0.0026075	NOT
HSD17B7F	80.03223	0.585261	0.182412	3.208456	0.001334499	0.0026126	UP
GAGE1	2.231046	3.362667	1.048098	3.208351	0.001334983	0.0026134	UP
RP3-407E4	1.915343	-1.11527	0.347627	-3.20825	0.001335458	0.0026141	DOWN
ZNF146	2149.986	0.266803	0.08317	3.207919	0.00133699	0.0026169	NOT
ESF1	753.7573	0.308988	0.096322	3.207862	0.001337255	0.0026173	NOT
RP11-23J9	20.91568	0.658461	0.205289	3.207489	0.001338992	0.0026205	UP
RP11-263I	1.798021	2.651012	0.82653	3.207402	0.001339398	0.0026211	UP
HSPA1A	5653.867	0.563777	0.175775	3.207386	0.001339474	0.0026211	NOT
RP11-161I	1.792556	1.509564	0.470696	3.207093	0.001340838	0.0026235	UP
CTD-2595	1.226701	1.466821	0.457375	3.207045	0.001341062	0.0026237	UP
ANKRD20/	1.750161	2.565059	0.799821	3.20704	0.001341082	0.0026237	UP
C9orf153	3.50105	0.802178	0.250149	3.206804	0.001342184	0.0026256	UP

FAM83F	140.7894	-1.29862	0.404972	-3.2067	0.001342691	0.0026264	DOWN
RP11-142(	23.27971	0.696466	0.217218	3.206298	0.001344548	0.0026299	UP
ALG13	941.03	0.234915	0.073274	3.205961	0.001346123	0.0026328	NOT
RP11-89K:	7.72481	0.747637	0.23322	3.205722	0.001347239	0.0026348	UP
FTO-IT1	5.037261	0.661499	0.206364	3.205487	0.001348341	0.0026367	UP
OPA1	2527.372	-0.2367	0.073843	-3.20539	0.001348818	0.0026375	NOT
ZNF565	94.50647	0.270857	0.084508	3.205126	0.001350032	0.0026397	NOT
RP11-755I	10.35023	0.681973	0.21278	3.205068	0.001350306	0.00264	UP
LINC0134	18.07548	0.971015	0.302986	3.204812	0.001351507	0.0026422	UP
CC2D1A	1952.055	0.357271	0.111483	3.20472	0.001351939	0.0026428	NOT
SPATA5L1	424.5365	0.247656	0.07729	3.204228	0.001354251	0.0026472	NOT
SYNE4	475.6921	0.698818	0.218138	3.20356	0.001357399	0.0026531	UP
ZNF878	8.723187	-0.91163	0.284583	-3.2034	0.001358161	0.0026544	DOWN
GAPDHP4(	10.29425	0.661584	0.206535	3.203255	0.001358836	0.0026556	UP
PLAUR	377.4805	-0.68109	0.21265	-3.2029	0.001360531	0.0026587	DOWN
UBA1	10186.94	0.205197	0.064069	3.202768	0.001361136	0.0026597	NOT
NMT1	5021.661	0.218146	0.068123	3.202238	0.001363641	0.0026644	NOT
PLA2R1	100.3705	-0.7359	0.229816	-3.20213	0.001364141	0.0026652	DOWN
RP11-340I	1.37445	1.158216	0.361806	3.201202	0.001368553	0.0026736	UP
LINC0088	39.11527	0.69173	0.216086	3.201174	0.001368687	0.0026737	UP
RP11-436I	24.39432	0.825003	0.257722	3.20114	0.00136885	0.0026738	UP
RP11-876I	81.26369	0.628091	0.196213	3.201077	0.001369151	0.0026742	UP
RP11-108I	1.781241	0.969655	0.302936	3.200853	0.001370213	0.0026761	UP
AC092620	1.664683	1.181905	0.369263	3.200718	0.001370858	0.0026772	UP
IGSF9B	5.90479	0.995447	0.311038	3.200406	0.001372339	0.0026799	UP
LINC0122(	4.622727	-0.69297	0.216551	-3.20004	0.001374074	0.002683	DOWN
TMEM8C	4.278777	1.455109	0.454718	3.200028	0.001374144	0.002683	UP
RP11-482I	5.356492	0.646983	0.202193	3.199828	0.001375095	0.0026847	UP
RP11-36I1	1.604548	1.20411	0.37631	3.19978	0.001375324	0.0026849	UP
RP3-522J7	1.710349	1.090984	0.340972	3.19963	0.001376043	0.0026862	UP
LINC0047	4.219547	0.969186	0.302913	3.199558	0.001376385	0.0026866	UP
RP11-95I1	1.951449	1.182467	0.369615	3.199181	0.001378184	0.00269	UP
HEPACAM	2.410467	1.557552	0.486889	3.198988	0.001379107	0.0026916	UP
TMC3-AS:	38.466	-0.56352	0.1762	-3.19818	0.001382961	0.0026988	NOT
ZNF720	295.7104	0.314439	0.098318	3.19818	0.00138298	0.0026988	NOT
RP11-630I	1.851318	0.929031	0.290504	3.197999	0.001383848	0.0027003	UP
DLGAP3	27.04463	0.674974	0.21108	3.197723	0.001385173	0.0027026	UP
PTMAP5	66.06594	0.488721	0.152847	3.197447	0.0013865	0.002705	NOT
RP13-580I	1.688286	1.012847	0.316773	3.197389	0.001386777	0.0027054	UP
LRRC71	3.178949	0.800166	0.250263	3.197298	0.001387213	0.0027061	UP
WIPF3	195.7513	0.909965	0.284632	3.196989	0.001388701	0.0027088	UP
AC007238	17.65626	0.474692	0.148484	3.196934	0.001388968	0.0027091	NOT
SPTBN5	122.3541	0.728117	0.227766	3.196773	0.001389743	0.0027104	UP
LAMTOR3	1060.089	-0.21953	0.068674	-3.19665	0.001390336	0.0027114	NOT
RAET1G	7.994891	0.659897	0.206441	3.196534	0.001390894	0.0027123	UP
TEX30	840.0694	-0.41794	0.130755	-3.19632	0.001391939	0.0027141	NOT
HOMEZ	503.1932	0.266274	0.083308	3.196264	0.001392195	0.0027144	NOT
RP11-710(	5.867509	-0.8014	0.250788	-3.19551	0.001395816	0.0027213	DOWN
NDUFAF4	815.9156	-0.38163	0.119432	-3.19539	0.001396404	0.0027223	NOT
RP11-347I	2.66339	3.373708	1.05581	3.195374	0.0013965	0.0027223	UP
RP11-159I	2.516751	0.807995	0.252878	3.195192	0.001397377	0.0027238	UP
RP11-752I	13.89448	0.672172	0.210373	3.195148	0.001397592	0.002724	UP

MFSD9	667.2568	-0.3524	0.110311	-3.1946	0.001400259	0.002729	NOT
RP3-337H	14.31974	0.551703	0.172707	3.194452	0.001400967	0.0027302	NOT
IL24	30.83681	-0.63502	0.198794	-3.19436	0.001401423	0.0027309	DOWN
RPSAP31	1.877249	0.969023	0.303373	3.194165	0.001402361	0.0027325	UP
CCDC30	40.83503	0.488376	0.152906	3.193962	0.001403347	0.0027343	NOT
AAMP	4536.91	-0.20958	0.065621	-3.19382	0.001404053	0.0027354	NOT
FCGR3B	62.88135	-0.97757	0.306106	-3.19357	0.001405271	0.0027376	DOWN
RP11-316I	3.947613	0.619538	0.194013	3.193281	0.001406661	0.0027401	UP
RP11-196(	9.021041	0.664351	0.208051	3.193207	0.001407022	0.0027406	UP
FOXN1	2.579307	1.778299	0.556936	3.193007	0.001407998	0.0027422	UP
RP4-616B8	1.76236	1.099956	0.34449	3.192999	0.001408033	0.0027422	UP
RP11-425I	3.620526	0.668551	0.209409	3.192552	0.001410213	0.0027463	UP
BMS1P1	72.5743	0.477796	0.149669	3.192339	0.001411258	0.002748	NOT
CYP26B1	144.2417	1.035117	0.324251	3.192336	0.001411273	0.002748	UP
EIF2S3	5606.887	0.252432	0.079095	3.191517	0.00141528	0.0027556	NOT
GMPPA	2604.677	0.307195	0.09626	3.191306	0.001416314	0.0027574	NOT
RP5-882O	1.55265	1.123034	0.351909	3.191266	0.001416509	0.0027576	UP
OTUD5	2542.262	-0.19189	0.060134	-3.19102	0.001417725	0.0027597	NOT
BAG5	1087.798	-0.19006	0.059562	-3.1909	0.001418317	0.0027607	NOT
RP11-697I	3.561693	0.839786	0.263202	3.190653	0.001419517	0.0027628	UP
RP11-685I	5.933304	0.588833	0.184565	3.190388	0.001420817	0.0027652	UP
AFG3L2	2574.7	-0.25318	0.079359	-3.19028	0.001421358	0.002766	NOT
CHST2	187.8892	0.489849	0.153551	3.190139	0.001422044	0.0027672	NOT
IGKC	9792.027	-1.15084	0.360757	-3.19006	0.001422409	0.0027677	DOWN
CERS3	2.230724	1.29636	0.40639	3.189944	0.001423005	0.0027687	UP
CCDC173	12.43939	-0.68923	0.2161	-3.18942	0.001425601	0.0027735	DOWN
VPS37D	236.4045	0.546421	0.171328	3.189331	0.001426026	0.0027741	NOT
RP11-600I	2.303375	0.854334	0.267913	3.188843	0.001428436	0.0027786	UP
PAN3-AS1	16.43816	0.498773	0.156429	3.188483	0.001430215	0.0027817	NOT
CILP2	54.96689	1.0241	0.321187	3.188482	0.001430221	0.0027817	UP
RP5-1025/	1.127082	1.630851	0.511501	3.188365	0.001430799	0.0027826	UP
SMARCA5	2210.634	-0.25355	0.079534	-3.18802	0.001432517	0.0027858	NOT
BPIFA1	1.720749	2.979925	0.934884	3.187482	0.001435175	0.0027908	UP
JMJD1C-A	12.62472	0.619475	0.194375	3.187012	0.001437509	0.0027951	UP
AC074289	43.71074	0.602307	0.188992	3.186948	0.001437828	0.0027955	UP
VRK3	1726.43	-0.27719	0.086983	-3.18676	0.001438769	0.0027972	NOT
SCGB1A1	0.904419	1.965831	0.616908	3.186586	0.001439628	0.0027986	UP
RP11-343I	2.44707	0.914829	0.287091	3.186544	0.001439836	0.0027988	UP
RP11-649I	2.224983	0.905681	0.284226	3.186484	0.001440133	0.0027992	UP
PRICKLE2	224.8426	-0.66011	0.207163	-3.18644	0.001440354	0.0027995	DOWN
SLC22A17	389.4959	0.955976	0.300017	3.18641	0.001440504	0.0027996	UP
GXYLT1	1021.263	-0.26296	0.082535	-3.18601	0.001442479	0.0028032	NOT
DOK5	56.61956	0.773459	0.24277	3.185978	0.001442655	0.0028033	UP
ACVR1	1154.757	-0.33042	0.103727	-3.18552	0.001444949	0.0028076	NOT
SELPLG	484.4915	-0.56932	0.178728	-3.18542	0.001445419	0.0028083	NOT
SLIT1	18.36964	0.745959	0.234201	3.185125	0.001446913	0.002811	UP
FOXP2	178.7849	-1.06016	0.332857	-3.18503	0.0014474	0.0028118	DOWN
KAT6A	1133.518	-0.35516	0.111512	-3.1849	0.001448061	0.0028129	NOT
LMTK2	1414.12	0.321564	0.100973	3.184655	0.001449267	0.002815	NOT
PIK3CD-A:	23.98677	0.973002	0.305535	3.184585	0.001449616	0.0028155	UP
SERPINC1	140062.6	-0.836	0.262519	-3.18454	0.001449856	0.0028157	DOWN
AC026471	5.867306	0.855569	0.268755	3.183456	0.001455281	0.0028261	UP

TOP1MT	1798.993	0.407466	0.127997	3.183412	0.001455506	0.0028263	NOT
MFAP1	952.768	0.181866	0.057134	3.183118	0.001456981	0.002829	NOT
C16orf91	413.5736	0.294935	0.09267	3.18262	0.00145949	0.0028337	NOT
RP11-666/	3.2412	0.742537	0.233353	3.182026	0.001462488	0.0028393	UP
VN1R42P	0.988542	1.255748	0.394669	3.181779	0.001463734	0.0028415	UP
RP11-38L1	1.331223	1.474761	0.463504	3.181764	0.001463812	0.0028415	UP
STT3B	6400.064	0.235469	0.074016	3.18134	0.001465954	0.0028454	NOT
OR56A3	1.890085	3.241844	1.019088	3.181122	0.00146706	0.0028474	UP
PRAMEF25	1.278692	2.676952	0.841584	3.18085	0.001468438	0.0028498	UP
RRBP1	25885.21	-0.31003	0.097471	-3.18077	0.001468824	0.0028504	NOT
RP11-177I	1.514576	2.306671	0.725216	3.180667	0.001469363	0.0028512	UP
PNO1	753.2511	0.2554	0.080299	3.180615	0.001469629	0.0028515	NOT
IGHV4-55	11.06847	-1.405	0.441762	-3.18045	0.001470443	0.0028528	DOWN
TRPS1	217.2908	0.667419	0.209851	3.180448	0.001470477	0.0028528	UP
SRD5A3	978.6512	0.389039	0.122333	3.180165	0.001471912	0.0028554	NOT
CTD-2005	1.483001	1.258558	0.395768	3.180039	0.001472554	0.0028564	UP
RP11-114	90.39361	0.398604	0.12535	3.179921	0.001473153	0.0028574	NOT
RP11-491I	3.454927	1.017292	0.319938	3.179647	0.001474544	0.0028599	UP
NTSR1	16.08478	1.088921	0.342477	3.179544	0.001475068	0.0028607	UP
RP11-141I	2.201599	0.871754	0.27418	3.179493	0.001475328	0.002861	UP
SPDYE1	4.471474	0.624806	0.196512	3.179477	0.00147541	0.002861	UP
PSMD5	1181.533	-0.29132	0.091642	-3.17888	0.001478434	0.0028666	NOT
RP1-28O1	74.45129	0.77209	0.242883	3.17885	0.001478604	0.0028668	UP
TDP2	2228.165	0.282267	0.088797	3.178784	0.001478942	0.0028672	NOT
CDCP1	224.579	1.000087	0.314615	3.178768	0.001479026	0.0028672	UP
AC073869	2.627554	0.90891	0.285935	3.178729	0.001479221	0.0028673	UP
OR2H1	1.290469	2.629188	0.827282	3.178104	0.001482413	0.0028733	UP
RP11-91P2	1.464271	1.056654	0.332537	3.177547	0.001485265	0.0028787	UP
ZNF850	84.20717	0.478866	0.150714	3.177322	0.00148642	0.0028807	NOT
RP11-557I	1.029158	1.345826	0.423584	3.177232	0.001486882	0.0028814	UP
RP11-725I	2.376102	1.085663	0.341778	3.176513	0.001490572	0.0028883	UP
ATP10B	43.66005	1.525595	0.480342	3.17606	0.001492899	0.0028926	UP
PPP1R3C	1962.293	-0.63149	0.198838	-3.17588	0.001493845	0.0028943	DOWN
AKAP9	2827.085	-0.45284	0.142591	-3.1758	0.001494236	0.0028948	NOT
HNRNPA1I	3.590808	0.666185	0.209812	3.175152	0.001497581	0.0029011	UP
CTD-2538	1.583687	1.292313	0.407014	3.175105	0.001497821	0.0029014	UP
FKBP1C	13.43718	0.439679	0.138479	3.175046	0.001498125	0.0029018	NOT
DPY30	1418.222	0.278182	0.087619	3.174924	0.001498759	0.0029028	NOT
CTD-2008	7.13952	-1.53851	0.484635	-3.17456	0.001500619	0.0029062	DOWN
RP11-598I	14.0463	0.66922	0.210845	3.173993	0.001503571	0.0029117	UP
RP11-491I	1.801894	1.390593	0.438202	3.173405	0.00150662	0.0029174	UP
EDC4	961.6931	0.350723	0.11052	3.173383	0.001506738	0.0029174	NOT
TUSC2	1137.007	0.279473	0.088071	3.173284	0.001507252	0.0029182	NOT
GPC6	1892.324	-0.54369	0.171364	-3.17273	0.00151014	0.0029236	NOT
PODN	574.6484	-0.88972	0.280486	-3.17205	0.001513661	0.0029302	DOWN
LL21NC02	11.26039	0.711894	0.224432	3.17198	0.001514033	0.0029306	UP
CTA-276C	2.288031	0.783764	0.247091	3.171968	0.001514098	0.0029306	UP
CTSLP8	1.331127	2.318854	0.731189	3.171347	0.001517339	0.0029367	UP
LRRC73	36.35158	0.818187	0.257995	3.171327	0.001517442	0.0029367	UP
RP11-269(	1.280276	1.395554	0.440058	3.171295	0.001517607	0.0029368	UP
TC2N	523.0228	0.901721	0.284367	3.170982	0.001519247	0.0029398	UP
RP11-415I	8.948789	-0.52802	0.166526	-3.17081	0.001520125	0.0029413	NOT

MAL2	3405.661	0.66086	0.208426	3.170721	0.00152061	0.002942	UP
EFEMP1	1447.562	0.994382	0.31362	3.170661	0.001520923	0.0029424	UP
GNAS-AS1	5.452441	1.228649	0.387529	3.170473	0.001521909	0.0029441	UP
RP11-710I	0.995398	1.795828	0.566517	3.169945	0.00152468	0.0029493	UP
RTCA-AS1	30.99566	0.502993	0.158679	3.169882	0.001525006	0.0029497	NOT
KDM1B	629.3063	0.367536	0.115953	3.169708	0.001525921	0.0029513	NOT
RP13-766I	2.130392	1.208582	0.381341	3.169292	0.001528106	0.0029553	UP
RP11-532I	44.85596	1.127946	0.355921	3.169096	0.001529138	0.0029571	UP
GRIK1	9.083902	-0.56848	0.1794	-3.16876	0.001530913	0.0029603	NOT
C14orf37	54.15877	-0.64977	0.20506	-3.16871	0.001531179	0.0029606	DOWN
RABL2A	141.7303	0.334868	0.105684	3.168569	0.001531914	0.0029618	NOT
RFTN2	102.8287	0.526867	0.166282	3.168518	0.001532184	0.0029621	NOT
RITA1	1492.11	-0.30127	0.095099	-3.16799	0.001534982	0.0029673	NOT
TRMT112	4559.231	0.314424	0.099255	3.167852	0.001535698	0.0029685	NOT
CTD-2023	2.112936	1.063137	0.335621	3.16767	0.001536656	0.0029699	UP
HDAC2	1825.765	0.310047	0.097879	3.167653	0.00153675	0.0029699	NOT
CTD-2649	3.558412	0.92028	0.290526	3.167633	0.001536856	0.0029699	UP
RPL7AP31	1.454865	1.12531	0.355253	3.167631	0.001536863	0.0029699	UP
STON1	166.9199	0.713001	0.225118	3.167236	0.001538955	0.0029738	UP
LCP1	3341.86	-0.52912	0.167068	-3.16707	0.001539845	0.0029753	NOT
MVK	2435.604	-0.44529	0.140602	-3.16704	0.001539967	0.0029753	NOT
PTPRF	15665.46	0.388777	0.122768	3.166765	0.001541448	0.002978	NOT
RP11-624I	25.45173	-0.93526	0.295448	-3.16556	0.001547855	0.0029901	DOWN
SCNN1G	1.677643	1.380848	0.436279	3.165057	0.001550526	0.0029951	UP
HTRA2	811.1939	0.220866	0.06979	3.164716	0.001552345	0.0029984	NOT
SRPRB	4097.749	0.302874	0.095705	3.164662	0.001552635	0.0029987	NOT
RP11-305(	3.720714	0.776019	0.245219	3.164595	0.001552988	0.0029992	UP
AC079807	32.47843	0.343149	0.108458	3.16388	0.001556809	0.0030064	NOT
GYG1	816.6376	0.308222	0.09743	3.163531	0.001558679	0.0030098	NOT
LINC0151	60.63038	0.51283	0.162138	3.162926	0.001561922	0.0030158	NOT
STMN1P1	1.155151	1.165878	0.36872	3.161958	0.00156712	0.0030256	UP
SRSF11	3215.681	0.308642	0.097614	3.161858	0.00156766	0.0030265	NOT
RP11-33A	1.211077	1.941554	0.614157	3.16133	0.001570507	0.0030318	UP
RP11-186I	2.414674	0.90171	0.28526	3.161009	0.001572236	0.0030349	UP
CST5	5.34686	1.792288	0.567077	3.160574	0.001574584	0.0030392	UP
ZDBF2	168.8487	-0.80065	0.253343	-3.16035	0.001575797	0.0030413	DOWN
CALCA	37.27893	1.134077	0.358858	3.160239	0.0015764	0.0030423	UP
NCKAP5L	727.6993	0.405679	0.128377	3.160064	0.001577347	0.0030439	NOT
C11orf53	7.24503	2.072704	0.655944	3.159879	0.001578346	0.0030456	UP
HLA-K	141.9633	0.613003	0.194013	3.159596	0.001579879	0.0030484	UP
RP11-25E	4.430525	-0.9952	0.315003	-3.15933	0.001581335	0.003051	DOWN
ANKMY2	525.5696	0.281737	0.089178	3.159262	0.001581691	0.0030514	NOT
MCTS1	1470.873	0.2885	0.091323	3.1591	0.001582572	0.0030529	NOT
RP11-142	1.745569	1.804906	0.57138	3.158854	0.00158391	0.0030553	UP
VWA5B1	1.272335	1.567706	0.496309	3.158728	0.001584591	0.0030564	UP
SLC25A21	30.82642	0.684705	0.216797	3.158271	0.001587078	0.003061	UP
RP11-434I	1.394318	1.037242	0.328424	3.158235	0.001587273	0.0030611	UP
CEP85L	144.813	-0.45683	0.144653	-3.1581	0.001587988	0.0030623	NOT
IGKV2OR2	1.958987	-1.43881	0.455605	-3.15803	0.001588392	0.0030629	DOWN
HEMGN	2.552983	-1.33908	0.424028	-3.158	0.00158855	0.003063	DOWN
RAMP2	541.616	0.496282	0.157165	3.15772	0.001590081	0.0030657	NOT
HACL1	1764.972	-0.38415	0.121673	-3.15724	0.001592702	0.0030704	NOT

SPRYD7	743.7337	-0.34835	0.110334	-3.15724	0.001592719	0.0030704	NOT
RP3-388N	0.712029	1.604661	0.508351	3.156599	0.001596208	0.0030769	UP
DDX1	3776.39	0.211698	0.067068	3.156463	0.001596952	0.0030781	NOT
RP11-91H	1.125769	2.560043	0.81108	3.156339	0.001597633	0.0030792	UP
MTHFD1P:	6.763105	-0.67741	0.21463	-3.15619	0.001598446	0.0030805	DOWN
RP11-443I	0.78924	1.945836	0.616521	3.156153	0.00159865	0.0030807	UP
AC007326	1.169225	1.797374	0.569555	3.155749	0.001600866	0.0030848	UP
GUK1	8416.466	0.356764	0.113059	3.155544	0.001601991	0.0030867	NOT
RP11-100{	2.877363	1.062975	0.336863	3.155511	0.001602174	0.0030869	UP
TPT1P8	1.119551	2.219317	0.703352	3.155343	0.001603095	0.0030882	UP
WBSCR22	2713.988	0.24783	0.078543	3.155339	0.001603115	0.0030882	NOT
RP11-14C:	42.54557	-0.61297	0.194273	-3.15518	0.001603965	0.0030897	DOWN
GLB1L2	67.69842	1.022328	0.324066	3.154692	0.001606674	0.0030947	UP
MOCS1	2421.573	-0.42216	0.133827	-3.15457	0.001607364	0.0030958	NOT
RP13-15M	7.169026	0.71475	0.226594	3.154319	0.001608729	0.0030982	UP
KHDRBS3	649.26	0.460261	0.145922	3.154152	0.00160965	0.0030998	NOT
ELAC2	2578.655	-0.2536	0.080405	-3.154	0.001610497	0.0031012	NOT
CCDC171	52.00174	0.476526	0.151089	3.153949	0.001610774	0.0031015	NOT
HMP19	4.487297	1.029973	0.326568	3.153925	0.001610903	0.0031015	UP
RP11-405/	5.021393	1.278186	0.405303	3.153657	0.001612386	0.0031042	UP
PRSS35	31.11799	0.757313	0.240142	3.153612	0.001612636	0.0031044	UP
GSTA1	31704.34	-0.92825	0.294356	-3.1535	0.001613251	0.0031054	DOWN
CTD-2575	4.123621	1.285174	0.407542	3.153475	0.001613389	0.0031054	UP
TMEM134	1367.009	-0.30884	0.097945	-3.15321	0.001614863	0.0031081	NOT
GRIK5	15.37318	0.703533	0.223128	3.153046	0.001615766	0.0031096	UP
CSNK2A3	4.216782	0.674078	0.213798	3.152872	0.001616725	0.0031111	UP
EXOC6	827.5605	0.242585	0.076941	3.152866	0.001616758	0.0031111	NOT
MON2	1487.323	-0.25733	0.081638	-3.15215	0.001620725	0.0031185	NOT
TRUB1	666.7653	-0.28069	0.089049	-3.15205	0.00162126	0.0031193	NOT
NAIF1	369.4713	0.205326	0.065141	3.152007	0.001621522	0.0031196	NOT
ISY1	579.5655	0.306712	0.097311	3.151868	0.001622298	0.0031209	NOT
SF3A3	2422.042	0.219868	0.069774	3.151133	0.001626383	0.0031285	NOT
SLC7A4	16.32578	-1.16634	0.370188	-3.15066	0.001629047	0.0031334	DOWN
LINC0085:	17.92247	0.46096	0.146312	3.150526	0.001629766	0.0031346	NOT
EEF2K	1143.994	0.268141	0.085128	3.149849	0.00163355	0.0031416	NOT
B4GALNT3	104.105	0.939058	0.298153	3.149588	0.001635006	0.0031442	UP
LINC0022:	8.290882	0.950269	0.301734	3.149363	0.001636271	0.0031464	UP
PLS3	5569.337	-0.33693	0.106998	-3.14889	0.001638926	0.0031513	NOT
KB-1125A:	8.579676	-0.82116	0.260804	-3.14857	0.001640739	0.0031546	DOWN
RP11-536(	6.457049	0.520755	0.165401	3.148433	0.001641486	0.0031558	NOT
N6AMT1	291.2542	0.26309	0.083565	3.148344	0.001641984	0.0031565	NOT
FAM117A	608.1005	-0.37145	0.117988	-3.14824	0.001642593	0.0031574	NOT
RP11-2H3	3.929368	0.838251	0.266262	3.14822	0.001642683	0.0031574	UP
TMEM256	10.95292	0.476881	0.151492	3.147889	0.001644543	0.0031608	NOT
YBX1P1	21.68945	0.524016	0.166469	3.147826	0.001644898	0.0031613	NOT
RP11-293I	80.92475	-0.64575	0.205168	-3.14741	0.001647225	0.0031655	DOWN
DLX2-AS1	1.090129	2.492535	0.791949	3.147341	0.001647627	0.0031661	UP
CDH6	423.8273	0.813651	0.258567	3.146766	0.001650872	0.0031721	UP
RTCA	1077.348	0.234704	0.074589	3.146615	0.001651721	0.0031735	NOT
CTD-2270	85.62234	0.412894	0.131254	3.145769	0.001656507	0.0031825	NOT
HOXA5	45.17478	0.746373	0.237292	3.145373	0.001658752	0.0031865	UP
CYP24A1	2.668634	1.798024	0.57169	3.145105	0.00166027	0.0031891	UP

MDH1B	11.41098	0.754028	0.239747	3.145098	0.001660312	0.0031891	UP
ATRAID	2865.107	0.280343	0.089146	3.144766	0.001662197	0.0031925	NOT
FAM160B1	1404.587	-0.29659	0.094317	-3.14457	0.001663337	0.0031945	NOT
RP4-798A	38.53517	-0.67901	0.215944	-3.14436	0.001664477	0.0031964	DOWN
MMP3	6.166669	1.619504	0.515075	3.144209	0.001665365	0.0031979	UP
RP5-1065(	2.419119	-1.56923	0.499135	-3.14391	0.001667067	0.003201	DOWN
CITED1	7.531801	0.909461	0.289297	3.143695	0.001668295	0.0032031	UP
RP11-195I	0.997124	1.523313	0.484583	3.143556	0.001669083	0.0032044	UP
MIMT1	2.471206	1.738353	0.553099	3.142935	0.001672631	0.003211	UP
TCEAL1	551.3132	0.395755	0.12592	3.14291	0.001672775	0.003211	NOT
CLEC14A	768.9039	0.423324	0.134693	3.142887	0.001672906	0.0032111	NOT
ATP6V0B	5657.199	0.336314	0.10702	3.142538	0.001674901	0.0032147	NOT
AC093642	22.96502	0.816859	0.259953	3.142327	0.001676106	0.0032168	UP
RP11-472I	3.236152	0.87003	0.276877	3.142305	0.001676233	0.0032168	UP
PTPRK	3231.643	-0.29901	0.095164	-3.142	0.001677999	0.0032199	NOT
EFCAB10	11.24113	0.429294	0.136637	3.141846	0.001678864	0.0032214	NOT
C6orf52	18.55232	0.6942	0.220957	3.141789	0.001679189	0.0032218	UP
RANBP3	1848.088	0.2093	0.066619	3.141748	0.001679423	0.003222	NOT
RP5-1099I	2.745839	0.742141	0.236237	3.141511	0.001680785	0.0032244	UP
ARHGEF10	4586.73	-0.33483	0.106604	-3.14091	0.001684227	0.0032308	NOT
RP4-669P	20.09906	-0.95798	0.305015	-3.14076	0.001685075	0.0032322	DOWN
RARA	2546.299	0.308707	0.098294	3.140642	0.001685781	0.0032332	NOT
PIK3CA	699.406	-0.2779	0.088485	-3.14063	0.001685858	0.0032332	NOT
AC005152	3.034939	-1.16456	0.370865	-3.14013	0.001688741	0.0032385	DOWN
RP11-106(	3.38192	-0.63976	0.203744	-3.14004	0.001689274	0.0032393	DOWN
CTB-96E2.	1.241631	1.284483	0.409076	3.139963	0.001689691	0.0032399	UP
RP11-114I	2.109374	1.151885	0.366865	3.139807	0.001690594	0.0032414	UP
HNRNPA3I	2.435725	0.970873	0.309244	3.139504	0.001692343	0.0032445	UP
ZNF551	115.5548	0.449889	0.143311	3.13925	0.001693809	0.0032471	NOT
CTD-2330	1.393729	1.119534	0.35665	3.139024	0.001695117	0.0032494	UP
EBP	10805.22	-0.50547	0.161041	-3.13875	0.001696691	0.0032522	NOT
RP1-60O1	1.277351	1.391443	0.443342	3.138529	0.001697982	0.0032545	UP
SCN8A	306.6971	0.487988	0.155507	3.138036	0.001700842	0.0032597	NOT
CTD-2377	12.57912	0.871545	0.277737	3.138019	0.001700936	0.0032597	UP
RP3-412A	3.151935	0.790165	0.251816	3.137871	0.001701796	0.0032611	UP
RP11-140I	8.82691	-0.59129	0.188441	-3.13781	0.001702178	0.0032616	DOWN
KLRK1	9.205529	-0.90349	0.287951	-3.13763	0.001703188	0.0032633	DOWN
RP11-554	23.40511	1.128819	0.359793	3.137411	0.001704471	0.0032655	UP
SLC30A4	259.0167	-0.57951	0.184717	-3.13726	0.001705368	0.003267	NOT
RP11-544I	1.78955	1.499886	0.478122	3.137036	0.001706649	0.0032693	UP
MIR4697H	11.03247	0.898466	0.286419	3.136896	0.001707464	0.0032706	UP
RP13-20L1	56.95229	-0.4519	0.144062	-3.13682	0.001707889	0.0032712	NOT
PPL	1410.425	-0.62233	0.198409	-3.13661	0.001709126	0.0032733	DOWN
TFAP2C	19.14124	1.37425	0.438281	3.135545	0.00171535	0.003285	UP
FREM1	48.6508	-1.06322	0.339109	-3.13535	0.001716497	0.003287	DOWN
HSD17B7	841.545	0.466391	0.148778	3.134801	0.001719706	0.0032928	NOT
PAPD5	774.2257	-0.29889	0.095347	-3.13479	0.001719797	0.0032928	NOT
CHRNB1	489.3987	-0.35307	0.112632	-3.13472	0.001720172	0.0032933	NOT
FYB	439.4288	-0.65789	0.209878	-3.13463	0.001720731	0.0032942	DOWN
PTGES3P4	3.07315	1.075797	0.343219	3.134435	0.001721853	0.0032961	UP
RP11-867(	4.929355	-0.64998	0.207401	-3.13392	0.001724872	0.0033016	DOWN
ALDOAP1	1.365743	1.399736	0.446743	3.1332	0.001729117	0.0033095	UP

NEK7	1976.595	0.3318	0.105908	3.13291	0.001730825	0.0033126	NOT
RPSAP47	15.17019	0.894558	0.285563	3.132609	0.001732602	0.0033158	UP
CIDEB	40.06119	-0.6847	0.218615	-3.132	0.001736217	0.0033224	DOWN
OPN1SW	67.94058	0.509411	0.162657	3.131808	0.001737337	0.0033244	NOT
TMEM167	2931.37	0.215079	0.068678	3.131714	0.001737888	0.0033252	NOT
ZFYVE1	1005.949	-0.28004	0.089428	-3.13142	0.001739609	0.0033281	NOT
DCLRE1CP	1.562832	-1.05172	0.335862	-3.13142	0.001739636	0.0033281	DOWN
RP11-762I	8.264758	0.653953	0.208877	3.130804	0.001743286	0.0033348	UP
IRX5	17.5494	1.259043	0.402163	3.130682	0.00174401	0.003336	UP
EIF4G1	18979.68	-0.2156	0.068882	-3.13004	0.0017478	0.003343	NOT
MKRN2	906.7478	0.218491	0.069806	3.129949	0.001748366	0.0033438	NOT
MKNK1-A:	4.438371	0.727906	0.232575	3.129763	0.001749476	0.0033457	UP
CTC-444N	12.23189	0.491529	0.15707	3.129362	0.001751862	0.0033501	NOT
RP11-837.	6.593518	0.676579	0.216206	3.129323	0.001752099	0.0033503	UP
CLSTN3	3250.947	0.285346	0.091185	3.129302	0.001752223	0.0033503	NOT
INHBB	2206.458	-0.55747	0.17815	-3.12921	0.001752773	0.0033511	NOT
RP1-278E:	18.3202	0.55443	0.177185	3.12911	0.001753364	0.003352	NOT
SLITRK2	4.379401	-1.27491	0.407499	-3.12862	0.001756266	0.0033573	DOWN
RPS27A	16226.51	0.3847	0.122996	3.127739	0.001761564	0.0033672	NOT
RP11-467I	11.90727	0.472289	0.151004	3.127667	0.001761996	0.0033677	NOT
FAM174A	673.7682	-0.25355	0.081066	-3.12766	0.001762032	0.0033677	NOT
BHLHE41	169.9409	0.860365	0.275105	3.1274	0.001763598	0.0033704	UP
GAPDHP7:	2.549434	0.939555	0.300445	3.12721	0.001764735	0.0033724	UP
RP11-295I	1.712725	-1.68678	0.539431	-3.12695	0.001766285	0.0033751	DOWN
ABCB10	1294.208	0.307736	0.098424	3.126654	0.001768081	0.0033783	NOT
FIGN	240.4159	-0.61255	0.195914	-3.12662	0.00176831	0.0033785	DOWN
XX-C0071	1.375893	1.257355	0.402154	3.126551	0.001768701	0.003379	UP
RP11-408I	2.034332	3.294201	1.053631	3.126522	0.001768873	0.0033791	UP
RP11-425I	1.707091	-0.8231	0.263295	-3.12617	0.00177101	0.0033829	DOWN
GAPDHP2	3.574635	0.742479	0.237527	3.125867	0.001772818	0.0033862	UP
C9orf142	1165.644	0.428251	0.137003	3.12584	0.001772979	0.0033862	NOT
AC008391	1.244774	1.398561	0.447484	3.125388	0.001775706	0.0033911	UP
AC004837	1.959966	1.016038	0.325093	3.125376	0.001775777	0.0033911	UP
TMEM229	165.4295	0.597219	0.191134	3.124612	0.001780394	0.0033997	UP
LINC0092	63.40005	-0.62898	0.201316	-3.12432	0.001782174	0.0034029	DOWN
AP001205.	2.149712	0.926407	0.296519	3.124275	0.001782439	0.0034031	UP
UBE2R2	3737.731	-0.17874	0.057222	-3.12358	0.001786624	0.0034109	NOT
ACTN3	3.430269	1.081455	0.346229	3.123522	0.001787005	0.0034114	UP
RP11-2E1:	8.029382	0.607876	0.194628	3.123266	0.001788559	0.0034141	UP
HAPLN2	3.357568	0.889763	0.284912	3.122942	0.001790531	0.0034176	UP
MAGEB16	2.130606	3.54602	1.135495	3.122886	0.001790874	0.0034181	UP
GOPC	1051.249	-0.2474	0.079225	-3.12277	0.001791581	0.0034192	NOT
KLK13	2.426656	1.898757	0.608066	3.122616	0.001792513	0.0034206	UP
RP11-713I	1.655821	1.060064	0.339481	3.122601	0.001792605	0.0034206	UP
BTF3P4	1.585842	1.033649	0.331027	3.122553	0.0017929	0.003421	UP
PDHB	3262.109	-0.27513	0.088118	-3.1223	0.001794456	0.0034237	NOT
NLGN2	385.5841	0.585135	0.187414	3.122157	0.001795309	0.003425	UP
DNAAF1	43.44309	1.074031	0.344004	3.122144	0.001795392	0.003425	UP
TUBA3FP	10.88607	-0.50039	0.160294	-3.12172	0.001798001	0.0034298	NOT
PSMD6-A:	11.16176	0.559278	0.179184	3.121248	0.001800864	0.003435	NOT
SIGLEC10	139.483	-0.70081	0.22454	-3.12109	0.001801813	0.0034366	DOWN
RP11-60A:	1.767768	1.289636	0.413228	3.120879	0.001803121	0.0034388	UP

C17orf49	50.86232	0.3512	0.112553	3.120296	0.001806692	0.0034454	NOT
IGKV1D-3!	1.547345	-1.56943	0.503044	-3.11987	0.001809338	0.0034502	DOWN
RP11-163I	16.59186	0.524394	0.16809	3.119727	0.001810186	0.0034516	NOT
SLFN13	339.3991	0.943587	0.302464	3.119665	0.001810569	0.0034521	UP
USP8	1626.578	-0.21259	0.068147	-3.11951	0.001811518	0.0034536	NOT
APCDD1	414.656	1.030571	0.330398	3.119177	0.00181357	0.0034573	UP
RP1-266L2	40.66403	0.485073	0.155524	3.118964	0.001814883	0.0034596	NOT
RP11-275I	3.133583	0.848883	0.272178	3.11885	0.001815584	0.0034607	UP
CCDC169	2.590273	1.593424	0.510994	3.118286	0.001819063	0.0034671	UP
ZNF611	136.235	0.553735	0.177593	3.118008	0.001820779	0.0034701	NOT
BAAT	34542.2	-0.69183	0.221908	-3.11765	0.001823008	0.0034741	DOWN
SLC17A2	2290.527	-0.82935	0.266046	-3.11733	0.001824944	0.0034773	DOWN
RP3-424M	25.66687	0.446012	0.143075	3.117333	0.001824955	0.0034773	NOT
RP11-376I	3.50985	-0.76635	0.245865	-3.11696	0.001827276	0.0034815	DOWN
CCL3L3	82.34714	-0.72881	0.233838	-3.11672	0.001828726	0.003484	DOWN
FAM218A	3.684477	1.16545	0.373948	3.116611	0.001829426	0.0034851	UP
TMEM251	366.4751	0.278616	0.089408	3.11625	0.001831671	0.0034892	NOT
MFNG	366.2893	-0.39168	0.125698	-3.11602	0.001833102	0.0034917	NOT
FAM19A5	318.4191	1.216691	0.390497	3.115747	0.001834793	0.0034946	UP
KIAA0825	30.04381	-0.49642	0.159336	-3.11554	0.0018361	0.0034969	NOT
RP11-20J1	9.069322	-0.9064	0.290944	-3.11538	0.001837071	0.0034985	DOWN
RP4-737E2	2.755862	0.835989	0.268403	3.114678	0.00184146	0.0035066	UP
TRPC5OS	3.043769	1.388866	0.44592	3.114607	0.001841903	0.0035072	UP
IL12RB2	32.4233	0.850295	0.273053	3.114029	0.001845513	0.0035139	UP
PPM1E	108.0743	0.948792	0.304687	3.113993	0.001845737	0.003514	UP
UBE2CP2	1.534224	1.009789	0.324287	3.113876	0.001846468	0.0035152	UP
SLAMF6	108.1876	-0.73319	0.235503	-3.11327	0.001850236	0.0035221	DOWN
RP11-64C1	1.296003	2.287619	0.73488	3.112914	0.001852503	0.0035262	UP
RP4-539M	3.534344	-0.77307	0.248358	-3.11274	0.001853606	0.0035278	DOWN
CGB7	1.072481	1.229758	0.395073	3.112736	0.001853618	0.0035278	UP
HLCS	1133.748	-0.27206	0.087403	-3.11271	0.001853797	0.0035279	NOT
RN7SL81P	1.588332	1.072266	0.34449	3.112623	0.00185433	0.0035287	UP
BNC1	6.802629	-0.94037	0.302174	-3.11202	0.001858141	0.0035357	DOWN
C7orf55	689.8462	-0.43217	0.138871	-3.112	0.001858265	0.0035357	NOT
GPR123	14.38626	-1.55244	0.498942	-3.11147	0.001861603	0.0035418	DOWN
RP11-348I	9.582241	0.578839	0.18606	3.111032	0.001864346	0.0035468	NOT
NPR1	525.7822	-0.46284	0.148778	-3.11091	0.001865089	0.003548	NOT
MT-TL1	17.64996	0.810066	0.260409	3.110744	0.001866166	0.0035498	UP
ADCY8	20.15429	1.727896	0.555498	3.110533	0.0018675	0.0035521	UP
NUDT16P1	346.1597	-0.71114	0.228641	-3.1103	0.001868958	0.0035546	DOWN
TM9SF4	3838.485	0.193114	0.062093	3.110081	0.001870358	0.0035569	NOT
RLN3	1.141815	2.442643	0.785399	3.110067	0.001870447	0.0035569	UP
SLC26A8	9.855891	0.720987	0.231844	3.10979	0.001872206	0.00356	UP
IGHV3-60	1.368926	-1.53471	0.49357	-3.10941	0.001874589	0.0035643	DOWN
PRDM5	33.88478	0.806433	0.25939	3.108957	0.001877493	0.0035696	UP
RP11-166(	3.416133	0.644223	0.20723	3.108729	0.001878941	0.0035719	UP
RP4-570O	1.516652	2.021625	0.650306	3.108728	0.001878946	0.0035719	UP
FMO7P	0.787696	1.760428	0.566356	3.108344	0.001881389	0.0035763	UP
GAS1	109.7892	-0.80823	0.26002	-3.10832	0.001881536	0.0035763	DOWN
RP4-584D	22.11351	0.628208	0.202115	3.108167	0.001882517	0.0035779	UP
RP11-248J	11.60047	0.495324	0.159376	3.107891	0.001884275	0.003581	NOT
RP11-305I	6.106427	0.733938	0.236156	3.107851	0.00188453	0.0035812	UP

ITPR2	5489.384	-0.52466	0.16882	-3.10782	0.00188472	0.0035814	NOT
RBM44	10.59328	0.553948	0.178278	3.107212	0.00188861	0.0035884	NOT
CNTN3	118.0445	-1.15449	0.371555	-3.1072	0.001888705	0.0035884	DOWN
RP11-66D	1.242817	1.102699	0.35489	3.107159	0.001888946	0.0035886	UP
PCSK1	8.259338	0.833796	0.268367	3.106922	0.001890464	0.0035913	UP
LINC0125(	0.897725	1.769635	0.56964	3.106586	0.001892615	0.0035951	UP
GTF3C6	1469.006	0.399098	0.128478	3.106351	0.001894115	0.0035976	NOT
RP11-313I	3.936334	1.311199	0.422104	3.106341	0.00189418	0.0035976	UP
HS3ST4	5.872261	2.428897	0.781931	3.10628	0.001894573	0.0035981	UP
FIP1L1	958.4934	0.224595	0.072304	3.106262	0.001894686	0.0035981	NOT
PIH1D2	19.41083	0.47796	0.153879	3.106066	0.001895944	0.0036002	NOT
GGH	7268.164	0.604089	0.194493	3.105975	0.001896531	0.0036011	UP
LINC00987	155.6938	-0.71855	0.231449	-3.10456	0.001905602	0.0036181	DOWN
IL20RB	112.7656	-0.91239	0.293896	-3.10447	0.001906216	0.003619	DOWN
TRPM4	850.5958	0.476111	0.153365	3.104437	0.001906415	0.0036191	NOT
RP11-444I	5.733236	0.664843	0.214171	3.104255	0.001907591	0.0036211	UP
ZNF557	230.5208	0.230997	0.074415	3.104194	0.001907982	0.0036216	NOT
GGT8P	1.082448	1.800033	0.579926	3.103899	0.001909883	0.0036249	UP
RNASE2	17.92245	1.012108	0.326081	3.103858	0.00191015	0.0036252	UP
LRRTM2	7.722091	-0.66254	0.213459	-3.10383	0.001910351	0.0036253	DOWN
PEPD	8684.463	-0.42064	0.135531	-3.10362	0.001911717	0.0036275	NOT
B3GALT5-/	7.443657	-1.52886	0.492609	-3.10359	0.001911884	0.0036275	DOWN
RNU6-418	1.810675	1.035344	0.333596	3.103586	0.001911909	0.0036275	UP
PURB	1555.535	0.211927	0.068288	3.10342	0.001912979	0.0036293	NOT
PIN4P1	4.069506	0.651557	0.209963	3.103205	0.001914372	0.0036317	UP
RP11-389(	101.7874	0.429549	0.138447	3.102617	0.001918175	0.0036387	NOT
RPAP3	788.8171	0.196976	0.063491	3.102426	0.001919415	0.0036408	NOT
GSTA2	10479.01	-1.14709	0.369744	-3.10239	0.00191964	0.003641	DOWN
RP11-815I	1.520689	1.653451	0.532984	3.102252	0.001920541	0.0036424	UP
ZNF700	321.8771	0.29652	0.095584	3.102207	0.001920839	0.0036427	NOT
TSEN34	1725.414	0.290411	0.093623	3.101918	0.001922712	0.003646	NOT
LINC0092(	36.77737	0.588221	0.189634	3.101875	0.00192299	0.0036463	UP
AL117187.	1.39052	1.320059	0.425658	3.101216	0.001927276	0.0036542	UP
WDR82P1	1.401026	2.564742	0.827153	3.100687	0.001930721	0.0036605	UP
TESC-AS1	4.499046	1.376373	0.443959	3.100223	0.00193375	0.0036659	UP
WAPAL	1964.203	-0.2153	0.069448	-3.10021	0.00193384	0.0036659	NOT
RP11-16E1	36.5783	0.493727	0.159284	3.099671	0.001937359	0.0036723	NOT
MYB	30.37592	0.845881	0.272936	3.099192	0.00194049	0.003678	UP
CXorf66	5.566516	-1.18248	0.381608	-3.09867	0.00194392	0.0036842	DOWN
ZNF627	353.276	0.250278	0.080773	3.098547	0.001944723	0.0036855	NOT
KLHL5	2168.295	0.36721	0.118519	3.098319	0.001946217	0.0036881	NOT
IDH1-AS1	32.8529	0.615339	0.198639	3.097768	0.001949838	0.0036946	UP
RBM12	1609.383	0.207999	0.067145	3.097754	0.001949932	0.0036946	NOT
SFTPD	35.28311	-0.89019	0.287392	-3.09748	0.001951715	0.0036977	DOWN
MIR3653	2.003098	0.857954	0.277024	3.097036	0.00195466	0.0037031	UP
KLRC1	8.819992	-0.74806	0.241584	-3.09649	0.00195825	0.0037096	DOWN
NUCKS1	14293.68	0.260732	0.084208	3.096308	0.001959468	0.0037117	NOT
EXOC3L2	309.8066	0.432155	0.139572	3.096277	0.001959675	0.0037118	NOT
RAB3C	92.84592	1.104766	0.356925	3.095234	0.001966579	0.0037246	UP
IGLV2-34	1.45803	-1.42033	0.458921	-3.09492	0.001968628	0.0037283	DOWN
TTC29	0.967111	2.147493	0.694143	3.093733	0.001976554	0.003743	UP
RPL21P75	17.06588	-0.59225	0.191447	-3.09356	0.0019777	0.0037449	DOWN

RPL35P5	19.21337	0.639484	0.206719	3.093497	0.001978123	0.0037455	UP
EXOC3L4	1500.007	-0.71318	0.230577	-3.09303	0.00198127	0.0037512	DOWN
JMJD8	1717.071	-0.28808	0.093142	-3.09288	0.001982261	0.0037528	NOT
RP6-159A	22.04081	-0.65114	0.210568	-3.09229	0.001986204	0.00376	DOWN
SNAPC5	769.2945	-0.22832	0.073839	-3.09207	0.001987687	0.0037625	NOT
ANKRD10	2115.126	0.302906	0.097964	3.092025	0.001987964	0.0037628	NOT
KNG1	114625.1	-0.68417	0.221281	-3.09188	0.001988926	0.0037644	DOWN
RP5-1050I	1.299492	1.091586	0.353097	3.091458	0.00199176	0.0037695	UP
AMY2B	295.8735	0.567384	0.183541	3.091322	0.001992676	0.003771	NOT
F3	192.1139	-0.72643	0.234997	-3.09126	0.001993106	0.0037715	DOWN
SND1	10517.82	0.228506	0.073924	3.091108	0.00199411	0.0037732	NOT
AC244250	9.861933	-1.56441	0.506129	-3.09093	0.00199528	0.0037751	DOWN
CKM	3.922649	0.945655	0.305992	3.090458	0.00199848	0.0037809	UP
PNPLA6	2252.2	0.259792	0.084067	3.090273	0.001999723	0.003783	NOT
CASP16	99.61268	0.898455	0.290778	3.08983	0.002002711	0.0037884	UP
RP11-559I	1.762797	1.196915	0.3874	3.08961	0.002004195	0.0037909	UP
RP11-367I	7.386504	0.603102	0.195223	3.089307	0.002006243	0.0037946	UP
TRGV7	2.899141	-0.81152	0.262689	-3.08928	0.002006456	0.0037946	DOWN
GRK5	684.9421	-0.33678	0.109015	-3.08927	0.002006524	0.0037946	NOT
TCP10L2	6.296624	1.325052	0.428929	3.089214	0.002006868	0.003795	UP
RP11-162/	27.29612	-0.5057	0.163711	-3.089	0.002008319	0.0037974	NOT
RP11-883(	9.65401	0.877078	0.283941	3.088943	0.002008702	0.0037979	UP
PRMT5	1201.33	0.288457	0.093387	3.088839	0.002009401	0.003799	NOT
RP11-311I	2.284896	1.133403	0.366945	3.088756	0.002009967	0.0037998	UP
SRRD	534.1215	0.22048	0.071395	3.08817	0.002013931	0.003807	NOT
AC087884	3.118183	0.822051	0.266208	3.088009	0.002015022	0.0038088	UP
RP11-391I	7.27307	0.837478	0.271224	3.087769	0.002016649	0.0038116	UP
PNPLA2	4645.676	-0.34429	0.111504	-3.08773	0.002016888	0.0038118	NOT
TNFAIP8L3	148.3348	0.669144	0.216715	3.087674	0.002017299	0.0038121	UP
RPS3	25598.29	0.407055	0.131832	3.087673	0.002017305	0.0038121	NOT
MPHOSPH	343.3594	0.297492	0.096349	3.087655	0.002017428	0.0038121	NOT
PDGFD	421.8729	0.634438	0.205505	3.087208	0.002020459	0.0038175	UP
RP11-20B2	1.042895	1.176687	0.381226	3.086588	0.002024678	0.0038252	UP
LRP5L	204.8208	0.384054	0.124449	3.086031	0.002028475	0.0038321	NOT
RP11-181(	4.006204	0.964566	0.312565	3.085964	0.002028932	0.0038328	UP
SLC25A30	13.65105	0.612448	0.198501	3.085364	0.002033033	0.0038402	UP
CLPTM1	8746.643	0.282957	0.09171	3.085338	0.002033212	0.0038403	NOT
GLIPR1L2	27.9372	-0.64557	0.209251	-3.08515	0.002034497	0.0038425	DOWN
EIF2B5	1885.537	0.169519	0.054956	3.084612	0.002038177	0.003849	NOT
SLIT3	466.778	0.713781	0.231401	3.084605	0.002038227	0.003849	UP
GPRC5B	900.495	0.614156	0.199142	3.084004	0.002042347	0.0038565	UP
MRPL21	1687.137	0.38683	0.125435	3.083919	0.002042935	0.0038574	NOT
FAM151A	337.7016	-1.14844	0.372456	-3.08344	0.002046256	0.0038634	DOWN
RP11-149I	3.070101	0.969557	0.31449	3.082948	0.002049612	0.0038694	UP
PCYT1A	2003.62	0.164679	0.053422	3.082637	0.002051752	0.0038732	NOT
IGHV3-35	2.871691	-1.32456	0.429828	-3.0816	0.002058915	0.0038865	DOWN
PPP2R5A	4357.569	0.29385	0.095358	3.081526	0.002059427	0.0038872	NOT
AC079250	17.67135	0.601743	0.195289	3.081297	0.002061007	0.0038897	UP
COBL	2077.59	-0.48192	0.156404	-3.08129	0.002061056	0.0038897	NOT
PKIG	1412.724	-0.27732	0.090012	-3.08098	0.002063177	0.0038935	NOT
LINC0089(	360.1697	-1.52695	0.495735	-3.08018	0.002068762	0.0039037	DOWN
LMCD1	728.2122	0.510719	0.165819	3.079975	0.002070177	0.0039061	NOT

AC024937	3.630088	0.73823	0.239695	3.079869	0.002070918	0.0039073	UP
TCF7L2	1195.231	-0.27951	0.090759	-3.07969	0.00207215	0.0039093	NOT
RP11-165J	9.678637	-0.47728	0.154978	-3.07963	0.002072551	0.0039098	NOT
TMSB15B	1.983223	1.152664	0.374295	3.07956	0.002073068	0.0039105	UP
CYYR1	366.6093	0.435833	0.141542	3.079188	0.002075659	0.0039151	NOT
RPS7P3	3.83663	0.7512	0.244005	3.078626	0.002079575	0.0039223	UP
RP1-150O	1.488335	1.291844	0.419635	3.078497	0.002080476	0.0039237	UP
P2RY11	193.5105	0.403195	0.130985	3.078181	0.002082685	0.0039276	NOT
RP11-576I	7.518058	0.667036	0.216708	3.078038	0.002083683	0.0039292	UP
HIST1H1A	1.301934	1.405702	0.456716	3.077848	0.002085013	0.0039314	UP
CASP8AP2	361.2095	0.383931	0.124761	3.077325	0.002088675	0.0039381	NOT
PANK4	726.4743	-0.2257	0.073368	-3.07624	0.002096275	0.0039521	NOT
RP11-347(	93.7409	0.444257	0.144422	3.076099	0.002097279	0.0039538	NOT
RP11-305(	6.801548	-0.53141	0.172779	-3.07564	0.002100494	0.0039596	NOT
CTD-2369	8.611431	0.544075	0.1769	3.075604	0.002100766	0.0039598	NOT
CLTC	15072.02	0.225396	0.073287	3.075511	0.002101419	0.0039608	NOT
SNHG19	376.314	0.619633	0.201478	3.075432	0.002101977	0.0039614	UP
RP11-350(	3.109491	0.771625	0.250901	3.075421	0.002102053	0.0039614	UP
PIK3CD	388.6558	-0.58247	0.189418	-3.07506	0.00210459	0.0039659	NOT
RPL7P6	11.93358	0.722376	0.234935	3.07479	0.002106507	0.0039693	UP
CTD-3185	47.2288	-0.33291	0.108299	-3.07401	0.002112027	0.0039794	NOT
C4orf47	10.97006	0.659117	0.214445	3.073594	0.002114969	0.0039847	UP
LY9	69.16729	-0.66785	0.217291	-3.07353	0.002115424	0.0039853	DOWN
RP11-209I	1.989172	1.595412	0.519119	3.073307	0.002117007	0.003988	UP
C2orf72	7732.336	-0.40964	0.133306	-3.07294	0.002119612	0.0039926	NOT
RPS2P55	14.91583	0.597731	0.194522	3.072823	0.00212044	0.0039939	UP
RP11-617I	256.2273	0.623467	0.202923	3.072429	0.002123243	0.0039989	UP
CSF3R	341.8758	-0.6833	0.222418	-3.07215	0.002125212	0.0040023	DOWN
RP11-659I	1.639772	1.197477	0.389787	3.072133	0.002125348	0.0040023	UP
RP11-495I	0.815864	1.952336	0.635544	3.071914	0.002126909	0.004005	UP
BCL6	2149.838	-0.42641	0.138812	-3.07183	0.002127515	0.0040058	NOT
C5orf66-A	1.356248	2.81962	0.917962	3.071609	0.002129086	0.0040085	UP
ZC3H12D	48.44226	0.600679	0.195569	3.071446	0.00213025	0.0040105	UP
IGHV4-34	99.55943	-1.14688	0.373438	-3.07114	0.002132404	0.0040142	DOWN
TCEA1P4	3.968555	0.625001	0.203533	3.070757	0.002135169	0.0040192	UP
NFX1	1918.92	-0.22208	0.072332	-3.0703	0.002138472	0.0040251	NOT
HTR7	13.19682	-0.72224	0.235237	-3.07025	0.002138811	0.0040255	DOWN
SP110	235.7238	-0.34575	0.112616	-3.07014	0.002139591	0.0040267	NOT
CTD-3247	2.367415	-1.04498	0.34039	-3.06995	0.002140963	0.0040289	DOWN
RP11-664I	1.92931	1.880215	0.612462	3.06993	0.002141091	0.0040289	UP
LL21NC02	46.25082	0.672045	0.218928	3.069706	0.002142693	0.0040317	UP
SUB1P3	2.540158	0.752465	0.245137	3.069573	0.002143652	0.0040332	UP
ITIH6	1.902719	1.972957	0.642762	3.069497	0.002144195	0.004034	UP
DCLK2	82.49152	0.474742	0.154676	3.069266	0.002145851	0.0040368	NOT
RP11-541I	2.184742	3.514746	1.14528	3.068898	0.002148502	0.0040413	UP
ARRDC3-A	7.344109	0.570061	0.185755	3.068896	0.002148516	0.0040413	NOT
RP11-104J	41.49222	-0.70922	0.231118	-3.06864	0.002150372	0.0040445	DOWN
RP11-420I	1.940538	0.828868	0.270115	3.068581	0.002150782	0.004045	UP
ROR1-AS1	1.414716	1.694502	0.552214	3.068561	0.002150924	0.004045	UP
RP11-83B2	1.216129	1.194518	0.389382	3.06773	0.002156911	0.004056	UP
ABCG8	3090.402	-0.75921	0.247494	-3.0676	0.002157873	0.0040575	DOWN
FSIP2-AS1	2.545416	0.947963	0.309035	3.067494	0.002158617	0.0040586	UP

LSAMP	55.67599	1.073014	0.349852	3.067054	0.002161797	0.0040643	UP
HLA-DOB	77.35267	0.816126	0.266137	3.066567	0.002165324	0.0040707	UP
SEC14L4	627.8524	-0.75732	0.246968	-3.06647	0.002166019	0.0040717	DOWN
1-Dec	2.654295	1.172675	0.382458	3.066154	0.002168314	0.0040757	UP
RP11-120J	3.069063	1.133799	0.369787	3.066087	0.002168805	0.0040764	UP
PDP2	605.8903	-0.31723	0.103467	-3.06598	0.002169602	0.0040776	NOT
BIRC7	12.79665	1.108872	0.361751	3.065294	0.002174565	0.0040864	UP
SELT	2858.279	-0.1972	0.064332	-3.06529	0.002174568	0.0040864	NOT
AC079630	16.29518	-0.60757	0.198217	-3.06516	0.002175517	0.0040879	DOWN
A2M-AS1	78.87616	-0.43086	0.140571	-3.06504	0.002176437	0.0040893	NOT
CTC-367J1	12.0025	0.938284	0.306128	3.065005	0.002176665	0.0040895	UP
RP11-350I	7.64032	0.606569	0.197912	3.064849	0.002177797	0.0040913	UP
SFRP5	575.9934	-1.54965	0.505653	-3.06465	0.00217923	0.0040938	DOWN
RP11-303I	14.64283	-0.61474	0.200596	-3.06459	0.002179697	0.0040943	DOWN
CTD-2024	10.54894	-0.55771	0.182017	-3.06408	0.002183401	0.004101	NOT
RP11-127I	17.47791	0.597478	0.195002	3.06396	0.002184281	0.0041024	UP
RP11-324J	2.352304	0.892827	0.291404	3.063877	0.002184887	0.0041033	UP
MGAT4EP	1.281099	1.269463	0.414338	3.063838	0.002185173	0.0041035	UP
DPP8	843.7347	0.238929	0.077984	3.063799	0.002185459	0.0041038	NOT
NCF1B	27.42555	-0.63849	0.208403	-3.06372	0.002186034	0.0041046	DOWN
FAM225A	12.18165	0.901454	0.294242	3.063649	0.00218655	0.0041053	UP
RP11-336I	0.938479	1.982711	0.647189	3.063573	0.002187105	0.004106	UP
SHOC2	1343.094	-0.20814	0.067943	-3.06338	0.002188546	0.0041085	NOT
TIMM13	3224.638	0.408718	0.133422	3.063348	0.002188752	0.0041086	NOT
AC034228	2.184568	3.392872	1.107581	3.063318	0.002188973	0.0041087	UP
RP13-20L1	4.313202	0.901544	0.29437	3.062625	0.002194049	0.004118	UP
LA16c-349	2.201229	1.280637	0.418295	3.061564	0.002201838	0.0041323	UP
ZNF385D-	2.033532	3.477736	1.13633	3.060497	0.002209697	0.0041468	UP
MYCBP	469.6887	0.274667	0.089752	3.06028	0.002211303	0.0041495	NOT
ST6GALNA	17.94301	-0.91269	0.298255	-3.06009	0.002212703	0.0041518	DOWN
RP11-16P2	1.541317	1.105116	0.361143	3.060047	0.00221302	0.0041522	UP
LINC01376	14.32512	0.46042	0.150468	3.05991	0.002214035	0.0041537	NOT
AC009237	6.937517	0.792714	0.259066	3.059897	0.002214129	0.0041537	UP
IGLV2-14	363.2816	-1.1904	0.389188	-3.05869	0.002223055	0.0041701	DOWN
GMDS-AS	150.7822	0.272567	0.089117	3.058527	0.002224278	0.0041721	NOT
LYNX1	2485.277	0.681047	0.222681	3.058403	0.002225203	0.0041736	UP
RP11-121(	1.309744	1.083637	0.354327	3.058296	0.002225994	0.0041748	UP
CTD-2267	338.1758	0.351817	0.115039	3.058246	0.002226366	0.0041752	NOT
FAM129A	422.2785	0.576068	0.188381	3.057991	0.002228263	0.0041785	NOT
CEP120	617.9143	-0.29263	0.095707	-3.05758	0.00223131	0.0041839	NOT
HNRNPC	11059.76	0.145684	0.047648	3.057522	0.00223175	0.0041845	NOT
RP5-1059I	17.32709	0.876381	0.286684	3.056954	0.002235986	0.0041921	UP
RPS3AP26	32.55036	0.53977	0.176578	3.056836	0.002236866	0.0041935	NOT
TTC17	2931.816	-0.20781	0.067986	-3.05659	0.002238667	0.0041966	NOT
RP11-264I	1.798501	1.003749	0.328399	3.056489	0.002239454	0.0041978	UP
KRT8P11	23.44053	-0.78989	0.258454	-3.05621	0.002241526	0.0042014	DOWN
TP53BP2	2351.412	0.373194	0.122125	3.055831	0.002244378	0.0042064	NOT
HBP1	1666.996	-0.35706	0.116854	-3.05557	0.002246323	0.0042098	NOT
AC131097	8.600732	1.333516	0.436435	3.055473	0.002247056	0.0042109	UP
RP11-395I	1.808433	0.950874	0.311219	3.055321	0.002248199	0.0042127	UP
CASP9	583.6685	-0.32834	0.107484	-3.05483	0.002251866	0.0042193	NOT
CTIF	1820.078	0.332071	0.10871	3.054659	0.002253168	0.0042215	NOT

LDLRAD4	1149.817	-0.43481	0.142345	-3.0546	0.002253585	0.004222	NOT
RP11-23P1	17.99861	0.904294	0.296067	3.054357	0.002255434	0.0042251	UP
LINC01242	1.242594	2.771405	0.907375	3.05431	0.002255786	0.0042255	UP
RP11-102I	2.097662	0.812301	0.265964	3.054177	0.002256788	0.0042271	UP
AC012360	3.427867	-0.90986	0.297943	-3.0538	0.002259596	0.0042321	DOWN
FCRL1	6.171671	-1.10344	0.361359	-3.05357	0.002261376	0.0042351	DOWN
ASPHD2	48.60627	0.61686	0.202024	3.053405	0.002262604	0.0042369	UP
CLPB	1024.928	-0.3344	0.109516	-3.0534	0.002262638	0.0042369	NOT
CADM2	28.229	-1.21475	0.397918	-3.05276	0.002267479	0.0042457	DOWN
AC145676	6.241487	0.955216	0.312926	3.052528	0.002269224	0.0042487	UP
C5orf15	2908.211	-0.22405	0.073399	-3.0525	0.002269434	0.0042488	NOT
SH2D7	2.139979	1.219945	0.39968	3.052307	0.002270895	0.0042512	UP
SRGAP3-A	1.175402	2.201377	0.721226	3.052271	0.002271169	0.0042512	UP
RP11-12A1	3.798916	1.135355	0.371971	3.052263	0.002271228	0.0042512	UP
KRT18P59	6.190479	-0.64016	0.209734	-3.05225	0.002271337	0.0042512	DOWN
STK32C	226.3522	0.428947	0.140543	3.052061	0.002272762	0.0042536	NOT
HTATIP2	3879.967	0.398663	0.130622	3.05204	0.002272917	0.0042536	NOT
RP3-426I6	1.822561	0.78902	0.258566	3.051518	0.002276871	0.0042607	UP
ARVCF	785.2323	-0.37762	0.123764	-3.05118	0.002279448	0.0042652	NOT
CIITA	499.0652	0.596337	0.195484	3.050563	0.002284125	0.0042737	UP
TM9SF3	7366.531	-0.19326	0.063354	-3.05053	0.002284372	0.0042739	NOT
TNFAIP6	19.00493	0.977537	0.320512	3.049924	0.002288996	0.0042822	UP
PPTC7	1394.757	-0.27177	0.089123	-3.04935	0.002293338	0.00429	NOT
CTB-55O6	455.3446	0.585516	0.192033	3.049043	0.002295715	0.0042942	UP
RP11-89K1	6.40035	0.904496	0.296656	3.04897	0.002296276	0.004295	UP
TRIM5	1032.979	-0.33248	0.10905	-3.04886	0.002297132	0.0042963	NOT
LCT	1.251057	1.194855	0.391914	3.048772	0.00229779	0.0042972	UP
HSPA8P5	3.622193	0.711903	0.233517	3.048613	0.002299008	0.0042992	UP
GNPTAB	983.3397	0.316125	0.103702	3.048402	0.002300622	0.0043019	NOT
SNED1	557.1977	-0.48192	0.158091	-3.04839	0.002300746	0.0043019	NOT
HNRNPA1I	1.153657	1.091689	0.358161	3.04804	0.002303394	0.0043065	UP
VAMP8	3958.733	-0.38239	0.125461	-3.04786	0.002304784	0.0043088	NOT
RBMY2FP	4.336082	4.566874	1.498419	3.047796	0.002305265	0.0043094	UP
RP11-113(	5.799355	0.962795	0.315905	3.04774	0.002305695	0.00431	UP
CD93	1824.505	0.432003	0.141759	3.047439	0.002308002	0.004314	NOT
ARMC5	1102.202	-0.48537	0.15928	-3.04729	0.002309139	0.0043158	NOT
ZNF577	179.0518	0.583332	0.191434	3.047166	0.002310101	0.0043173	NOT
PCSK5	370.5951	0.641537	0.210564	3.046754	0.002313266	0.0043229	UP
LINC00844	777.6394	-1.25442	0.411753	-3.04653	0.002315003	0.0043259	DOWN
AC100830	1.558906	0.987744	0.324226	3.046462	0.002315514	0.0043266	UP
CHCHD2P	1.132983	1.751725	0.575026	3.046339	0.002316467	0.004328	UP
RP13-766I	0.806608	1.880693	0.617405	3.046125	0.002318113	0.0043308	UP
OSBPL8	1339.878	-0.28707	0.094249	-3.04591	0.002319798	0.0043337	NOT
RPL24P8	15.31524	0.633679	0.208046	3.045866	0.002320113	0.004334	UP
LGALS3	2515.285	0.696835	0.22881	3.045481	0.002323088	0.0043392	UP
DOCK8	621.1492	-0.62455	0.20509	-3.04523	0.002325059	0.0043426	DOWN
DIO1	5205.893	-0.77575	0.254756	-3.04508	0.002326207	0.0043445	DOWN
MBD4	1327.51	-0.22939	0.075341	-3.04476	0.002328676	0.0043488	NOT
RP11-85G1	1.850986	0.802834	0.26368	3.044735	0.002328851	0.0043488	UP
SNRPGP4	2.493592	1.03154	0.338805	3.044645	0.002329554	0.0043499	UP
CTD-3148	1.330003	1.154361	0.37916	3.044527	0.002330467	0.0043513	UP
SSX3	1.580764	2.786066	0.91514	3.044415	0.002331334	0.0043526	UP

DGCR8	973.226	0.194963	0.064044	3.04422	0.002332842	0.0043551	NOT
STX3	742.2414	0.442341	0.145317	3.043974	0.00233475	0.0043584	NOT
RFX7	446.7865	0.307326	0.10097	3.043724	0.002336698	0.0043617	NOT
RP11-231(	22.25129	0.465861	0.153062	3.043607	0.002337599	0.0043631	NOT
RMDN2-A	1.808058	0.978523	0.321514	3.043482	0.002338577	0.0043646	UP
FXR1	3676.802	0.193647	0.063633	3.043205	0.002340731	0.0043684	NOT
BST1	148.395	-0.5338	0.17544	-3.04261	0.002345399	0.0043768	NOT
CTD-2369	20.74394	-0.85817	0.282067	-3.04244	0.002346669	0.0043789	DOWN
TAS2R15P	4.467209	0.655506	0.215456	3.04242	0.002346838	0.0043789	UP
COX6B1	10294.98	0.483197	0.158838	3.042069	0.002349584	0.0043837	NOT
AC010967	1.478577	1.3355	0.439014	3.042043	0.002349787	0.0043838	UP
RP11-554/	3.000258	1.201183	0.394908	3.041679	0.002352624	0.0043888	UP
FAM45A	786.3287	0.337364	0.110917	3.041585	0.002353362	0.0043897	NOT
KIAA1191	3789.172	-0.25993	0.085459	-3.04157	0.002353442	0.0043897	NOT
AC006369	5.801694	-0.80461	0.264569	-3.04123	0.002356143	0.0043945	DOWN
HLA-DMA	2432.668	0.610003	0.200583	3.041147	0.002356791	0.0043954	UP
RP11-345I	3.95391	0.877337	0.288513	3.040891	0.002358791	0.0043988	UP
RSPO4	7.141127	1.071413	0.352338	3.040864	0.002359007	0.0043989	UP
CDC42EP5	86.27776	0.495175	0.162847	3.040738	0.002359991	0.0044004	NOT
RP11-320(	3.550913	1.022538	0.336302	3.040534	0.002361587	0.0044031	UP
RP1-292B1	10.89848	0.596435	0.196176	3.040302	0.002363414	0.0044062	UP
ZNF225	130.4839	0.317462	0.104427	3.040035	0.002365509	0.0044098	NOT
EGFR	5897.795	-0.47815	0.157298	-3.03979	0.002367427	0.0044131	NOT
RP11-214I	1.709999	1.010316	0.332387	3.039582	0.002369065	0.0044159	UP
NAP1L1P3	3.749995	0.687956	0.226351	3.039339	0.002370982	0.0044192	UP
ZDHHC22	1.244876	1.808975	0.59522	3.039171	0.002372298	0.0044213	UP
RP11-190J	1.520088	2.807196	0.923744	3.038931	0.002374192	0.0044245	UP
CLDN7	1600.42	0.683835	0.225065	3.038385	0.002378495	0.0044323	UP
RP11-318I	8.076359	-0.4923	0.162046	-3.03804	0.00238123	0.0044371	NOT
ADH7	8.837196	-0.93045	0.306278	-3.03791	0.00238226	0.0044387	DOWN
RP11-347(	3.411802	1.018444	0.335316	3.03727	0.002387317	0.0044478	UP
SRL	22.57922	-0.54156	0.178314	-3.03709	0.002388742	0.0044502	NOT
LGALS9B	8.109563	1.402609	0.461849	3.036944	0.002389901	0.0044517	UP
HSPB3	1.418604	2.629679	0.865897	3.036943	0.002389908	0.0044517	UP
MADCAM1	31.01499	0.560443	0.184549	3.03683	0.0023908	0.0044531	NOT
SAMD12	171.6796	-0.85682	0.282156	-3.03668	0.002391966	0.004455	DOWN
RP11-401I	7.094676	0.580896	0.1913	3.036562	0.002392926	0.0044565	NOT
RPL5P24	3.115422	0.703313	0.231634	3.03632	0.002394853	0.0044595	UP
RP11-807I	4.565585	-0.9258	0.304909	-3.03632	0.002394865	0.0044595	DOWN
ESPN	4409.625	-0.61818	0.203605	-3.03617	0.002396036	0.0044613	DOWN
TDPX2	3.245452	0.73294	0.241408	3.036104	0.002396563	0.004462	UP
GAR1	510.1192	0.257942	0.084963	3.035935	0.00239791	0.0044642	NOT
RP11-297I	1.723442	-1.25211	0.412469	-3.03565	0.002400155	0.0044679	DOWN
CORO1C	3577.403	-0.29749	0.097999	-3.03565	0.002400205	0.0044679	NOT
FAM150B	50.6792	-1.08102	0.356116	-3.03559	0.002400633	0.0044684	DOWN
AC093818	16.22893	0.447301	0.147354	3.035557	0.002400916	0.0044686	NOT
XYLB	1813.748	-0.48261	0.159013	-3.03504	0.002405057	0.004476	NOT
RABGGTA	1009.59	0.246234	0.081139	3.034708	0.002407685	0.0044806	NOT
PHF10	1927.855	-0.26186	0.086297	-3.03442	0.002409982	0.0044846	NOT
RP11-139(	19.60819	0.653116	0.215341	3.032942	0.00242182	0.0045063	UP
FLG	9.293071	1.121733	0.369882	3.032676	0.00242396	0.00451	UP
CTRB1	0.976866	2.080792	0.68617	3.032472	0.002425599	0.0045127	UP

CROCCP2	1112.057	-0.38733	0.127729	-3.03244	0.002425849	0.0045129	NOT
RP11-644I	101.0255	-0.40991	0.135177	-3.03241	0.00242609	0.0045131	NOT
IFI30	45.7613	0.51291	0.169145	3.032375	0.002426376	0.0045133	NOT
RP11-22C	5.74019	1.594107	0.525743	3.032101	0.002428576	0.0045171	UP
IGHV1-69	35.58514	-1.42649	0.4705	-3.03187	0.002430473	0.0045203	DOWN
FLCN	1128.285	0.287717	0.0949	3.031791	0.002431073	0.0045211	NOT
EXD3	407.8134	0.335024	0.110523	3.031269	0.002435279	0.0045286	NOT
C19orf18	30.03905	0.759172	0.250469	3.031008	0.002437388	0.0045322	UP
RPS27L	3635.051	-0.33528	0.11062	-3.03091	0.002438164	0.0045334	NOT
RRNAD1	1508.633	0.281923	0.093028	3.030519	0.00244134	0.004539	NOT
PCP2	13.77646	0.696774	0.229925	3.030436	0.002442005	0.0045399	UP
GPR171	38.32223	-0.78345	0.258538	-3.03031	0.002443036	0.0045415	DOWN
FAM136A	1701.277	0.278572	0.091937	3.030041	0.002445208	0.0045453	NOT
CTC-459F	1.274221	1.085164	0.35816	3.029831	0.002446909	0.0045481	UP
IREB2	1783.582	-0.23931	0.07899	-3.02966	0.002448285	0.0045504	NOT
KLHL2P1	6.527896	0.830882	0.274256	3.029584	0.002448904	0.0045512	UP
RPSAP8	2.318373	0.880136	0.290537	3.029344	0.002450852	0.0045545	UP
RP11-561I	1.938048	3.408852	1.125351	3.029145	0.002452466	0.0045572	UP
CD8BP	1.991629	1.826846	0.603227	3.028458	0.002458053	0.0045672	UP
AC004951	25.46718	0.440203	0.145356	3.028445	0.00245816	0.0045672	NOT
NUTM2G	3.749112	0.722656	0.238697	3.027501	0.002465846	0.0045812	UP
AC005077	1.715357	-1.36894	0.452184	-3.02739	0.002466738	0.0045825	DOWN
RP11-313I	1.969326	0.84484	0.279079	3.027248	0.002467917	0.0045844	UP
RP11-259I	107.2283	0.388999	0.12852	3.026766	0.002471851	0.0045911	NOT
MYPN	2.240329	1.117261	0.369127	3.026762	0.002471884	0.0045911	UP
RPSAP54	10.20036	0.550039	0.181738	3.026546	0.002473655	0.0045941	NOT
GLYATL1P	1.914383	1.685783	0.557015	3.026459	0.002474363	0.0045951	UP
POLE3	1834.194	0.212864	0.070335	3.026415	0.002474724	0.0045955	NOT
RP11-568	2.918876	0.831878	0.274903	3.026085	0.00247743	0.0046002	UP
CTC-277H	7.411175	0.733378	0.242434	3.025067	0.00248578	0.004615	UP
AIM2	23.67159	-0.78467	0.25939	-3.02506	0.002485822	0.004615	DOWN
PROX2	3.020732	0.786935	0.260139	3.025053	0.002485893	0.004615	UP
UNC5D	3.761099	2.020023	0.667909	3.0244	0.002491266	0.0046247	UP
AC079922	79.67546	0.396212	0.131011	3.024267	0.002492368	0.0046264	NOT
RP11-168	3.332436	0.711221	0.235185	3.024088	0.00249384	0.0046288	UP
ACTR8	571.3386	0.191748	0.06341	3.023934	0.002495112	0.0046309	NOT
SMAD4	2393.233	-0.22103	0.073099	-3.02372	0.002496909	0.0046339	NOT
XXbac-BP	5.997574	0.660876	0.218576	3.023551	0.002498266	0.0046361	UP
TMEM160	295.6314	0.508413	0.168159	3.023403	0.002499493	0.0046379	NOT
AC093690	2.840324	0.796283	0.263374	3.023393	0.002499576	0.0046379	UP
HLA-U	10.51906	0.826361	0.273329	3.023321	0.002500168	0.0046387	UP
LINC0047	36.2351	1.15541	0.38217	3.023285	0.002500469	0.0046389	UP
FUNDC2P	8.212384	1.184285	0.391781	3.022827	0.002504256	0.0046457	UP
ADCY2	2.749031	1.189589	0.393544	3.022763	0.002504786	0.0046463	UP
RPS14P8	12.92137	1.109441	0.367055	3.022548	0.002506565	0.0046493	UP
TMEM185	216.046	0.232702	0.076996	3.022262	0.002508936	0.0046534	NOT
SLC17A3	705.9546	-0.74399	0.246194	-3.02199	0.002511201	0.0046573	DOWN
ACSM6	2.429925	0.989604	0.327476	3.021912	0.002511833	0.0046581	UP
KCMF1	2105.68	0.171302	0.056687	3.021878	0.002512114	0.0046584	NOT
TUBA8	9.337645	0.617762	0.204452	3.021554	0.002514809	0.004663	UP
CTD-2527	1.560244	1.287522	0.426143	3.021341	0.002516577	0.004666	UP
ABCA10	134.7381	-0.6604	0.21861	-3.02092	0.002520081	0.0046722	DOWN

RP11-466I	1.831653	0.865597	0.286607	3.020157	0.002526438	0.0046837	UP
ZYX	5751.219	0.381092	0.126189	3.020004	0.002527717	0.0046857	NOT
ANXA5	7557.827	0.40202	0.133147	3.019378	0.002532941	0.0046951	NOT
TMEM30B	945.2956	-0.59785	0.198011	-3.01929	0.002533691	0.0046962	DOWN
CTD-2562	221.3911	0.583497	0.193268	3.019105	0.002535228	0.0046987	NOT
AC004775	1.910388	1.081325	0.358186	3.018891	0.002537017	0.0047017	UP
TUFM	11764.31	-0.27523	0.091173	-3.01877	0.002538072	0.0047033	NOT
RP11-464I	16.41627	0.597525	0.197963	3.018374	0.002541351	0.0047091	UP
ZNF20	13.23503	0.411589	0.136365	3.018279	0.002542144	0.0047103	NOT
PTPRO	53.21222	-0.62348	0.206572	-3.01822	0.002542644	0.0047106	DOWN
RIN3	1363.799	-0.34911	0.115667	-3.01822	0.002542682	0.0047106	NOT
MRPL40	2235.234	-0.31887	0.105651	-3.01818	0.002542967	0.0047108	NOT
HNRNPA3I	3.562356	0.627276	0.207846	3.017984	0.002544621	0.0047136	UP
RP11-230(	11.60147	0.592456	0.196317	3.017853	0.00254572	0.0047153	UP
IL15RA	915.9313	-0.46732	0.154858	-3.01775	0.002546595	0.0047166	NOT
NCOA3	1519.014	0.297292	0.098532	3.017204	0.002551183	0.0047248	NOT
RIPPLY2	1.640248	2.335125	0.773999	3.016963	0.002553212	0.0047282	UP
RP11-627(	200.2853	0.784259	0.26	3.016377	0.002558147	0.004737	UP
RP11-543(	2.869419	-0.65592	0.217456	-3.01634	0.002558492	0.0047374	DOWN
PDZD8	2062.169	-0.38232	0.126751	-3.0163	0.002558803	0.0047376	NOT
GATSL3	64.31892	0.526814	0.174675	3.015959	0.002561677	0.0047426	NOT
TBP	381.8326	0.224144	0.074325	3.015741	0.002563525	0.0047457	NOT
TP53TG1	1051.919	-0.43862	0.145446	-3.01568	0.002564061	0.0047464	NOT
VIM	11085.01	0.466647	0.154742	3.015646	0.002564328	0.0047466	NOT
LINC0048:	2.711991	1.16622	0.386799	3.01505	0.002569367	0.0047556	UP
CFHR2	451.4165	-0.72701	0.241136	-3.01496	0.002570124	0.0047567	DOWN
RP13-347I	0.956405	1.461765	0.484946	3.014283	0.002575876	0.004767	UP
RP11-210I	33.98405	1.239014	0.411122	3.013739	0.002580496	0.0047752	UP
NDNL2	578.4906	-0.21444	0.071166	-3.01327	0.002584456	0.0047822	NOT
AL590762.	2.462188	0.796685	0.264406	3.013112	0.002585837	0.0047845	UP
SACS-AS1	1.180426	2.427339	0.805676	3.012797	0.002588516	0.0047891	UP
HOOK2	837.9072	0.387081	0.12848	3.012761	0.002588829	0.0047894	NOT
RP11-458I	10.25849	0.645024	0.21411	3.012582	0.002590357	0.0047919	UP
LA16c-30(	4.057261	0.675328	0.224173	3.012528	0.002590812	0.0047924	UP
RP11-395I	3.09268	0.820474	0.272386	3.012168	0.002593886	0.0047978	UP
ELOVL2-A	27.71827	0.761168	0.252712	3.011998	0.002595339	0.0048001	UP
PANX1	892.5562	-0.39073	0.129726	-3.01195	0.002595787	0.0048006	NOT
ZNF418	77.457	0.604199	0.200606	3.011868	0.002596457	0.0048016	UP
NGFRAP1	3314.711	0.635575	0.211046	3.011551	0.002599167	0.0048063	UP
MEF2A	1355.041	-0.32728	0.108679	-3.01147	0.002599838	0.0048072	NOT
RP11-211(	18.83264	0.585733	0.194514	3.011259	0.002601666	0.0048102	UP
FAM43A	455.8452	0.61669	0.204843	3.010551	0.002607738	0.0048211	UP
POLR2J4	145.3465	0.337745	0.112193	3.010402	0.002609022	0.0048232	NOT
LINC0151(	2.209005	3.217755	1.068894	3.010359	0.002609391	0.0048235	UP
AC007278	2.936462	-1.03269	0.343076	-3.01009	0.002611703	0.0048275	DOWN
LACE1	192.9724	-0.25169	0.083616	-3.01005	0.002612034	0.0048278	NOT
TGFB1	1617.361	0.595619	0.197886	3.009907	0.002613281	0.0048296	UP
MEIS2	696.0665	0.491102	0.163162	3.009898	0.002613352	0.0048296	NOT
LINC0030(	0.765695	2.087031	0.693557	3.009168	0.002619642	0.0048409	UP
MRPL57	2420.035	-0.33899	0.112662	-3.00894	0.002621572	0.0048441	NOT
EMP1	1552.905	-0.63134	0.209875	-3.00819	0.002628048	0.0048558	DOWN
SLC12A8	630.5644	0.575496	0.191337	3.007764	0.002631774	0.0048623	NOT

RP11-119:	6.948283	0.902751	0.300158	3.007585	0.002633322	0.0048649	UP
ALG1L13P	34.82951	-0.97412	0.323906	-3.00743	0.002634641	0.004867	DOWN
RP1-137D	2.398489	1.219291	0.40546	3.007178	0.002636855	0.0048707	UP
RASSF8-A:	196.1678	-0.45551	0.151494	-3.00679	0.002640209	0.0048766	NOT
CHADL	238.8179	0.70575	0.234744	3.00646	0.002643084	0.0048816	UP
LRRC2-AS:	5.251763	0.711907	0.23681	3.006238	0.002645019	0.0048848	UP
GSTT2B	141.7024	-0.93121	0.309781	-3.00603	0.002646785	0.0048878	DOWN
SOCS1	237.0137	-0.68849	0.22905	-3.00583	0.002648536	0.0048907	DOWN
CHMP2B	1488.533	-0.23622	0.078588	-3.00575	0.00264928	0.0048917	NOT
CTA-351J:	5.098422	0.659507	0.219428	3.005567	0.002650861	0.0048943	UP
SNORA81	5.985374	0.775417	0.258003	3.005462	0.002651778	0.0048957	UP
RP11-734I	30.13531	0.344708	0.114703	3.005234	0.002653763	0.004899	NOT
RP11-187(	4.2343	0.781974	0.260227	3.004971	0.00265606	0.0049029	UP
RP11-340I	15.02759	-0.65273	0.217258	-3.00438	0.002661209	0.0049121	DOWN
RP11-521(	40.8917	-0.37245	0.124004	-3.00355	0.002668462	0.0049252	NOT
AF127577.	1.824839	2.796611	0.931111	3.003522	0.002668743	0.0049254	UP
PCGF6	370.4447	-0.25563	0.085113	-3.00339	0.002669907	0.0049272	NOT
RP11-396(	5.466462	0.657069	0.21878	3.003332	0.002670409	0.0049278	UP
RPL34P33	1.682749	1.161857	0.386873	3.003202	0.002671551	0.0049296	UP
PAXBP1	698.9382	0.266244	0.088661	3.00293	0.002673939	0.0049336	NOT
RPL23AP2	17.12132	0.516943	0.17219	3.002173	0.0026806	0.0049456	NOT
RP11-15F1	3.753015	0.933097	0.310811	3.002137	0.002680912	0.0049458	UP
PLEKHG6	383.9816	0.634033	0.211237	3.001518	0.002686375	0.0049556	UP
IGHV3-63	2.101185	-1.35908	0.452825	-3.00134	0.002687919	0.0049581	DOWN
TSPAN11	60.45893	-0.75774	0.252495	-3.001	0.002690921	0.0049633	DOWN
RP4-604A:	3.744385	0.702631	0.234173	3.000475	0.002695589	0.0049716	UP
PRUNE2	109.9211	-0.74705	0.249009	-3.00008	0.002699067	0.0049777	DOWN
NRROS	183.5356	-0.43454	0.144845	-3.00002	0.002699647	0.0049784	NOT
KIAA0391	153.9672	-0.24252	0.080845	-2.99986	0.002701032	0.0049806	NOT
RP11-478:	61.36739	0.571631	0.190568	2.999617	0.002703195	0.0049843	NOT
ZNF846	189.7065	0.3648	0.121621	2.999474	0.00270446	0.0049863	NOT
NLRP11	87.36368	0.906277	0.302228	2.998657	0.002711727	0.0049994	UP
RP11-334I	1.258635	1.364415	0.45504	2.998454	0.002713532	0.0050024	UP
RPL18A	10682.01	0.470076	0.156775	2.998403	0.002713985	0.0050029	NOT
TCP11L2	127.805	-0.43002	0.143423	-2.99827	0.002715131	0.0050046	NOT
RP11-196I	3.514414	0.656982	0.219125	2.998207	0.002715734	0.0050054	UP
CCL20	1360.588	1.070879	0.357178	2.99817	0.002716058	0.0050057	UP
OIP5-AS1	4066.852	-0.27231	0.090843	-2.99764	0.002720752	0.005014	NOT
RP11-164I	2.282654	0.93248	0.311079	2.997571	0.002721406	0.0050149	UP
RP11-167I	0.835897	1.654546	0.551968	2.997539	0.002721688	0.005015	UP
RP11-565:	3.847107	0.725531	0.242044	2.997519	0.002721871	0.0050151	UP
ZNF720P1	1.439272	2.852031	0.951538	2.997287	0.00272394	0.0050185	UP
RP11-5C2:	122.9348	-0.32604	0.10878	-2.99721	0.002724633	0.0050195	NOT
CTD-2014	1.891128	0.995464	0.332155	2.996985	0.002726642	0.0050228	UP
C5AR2	307.6544	0.74073	0.247166	2.996896	0.002727439	0.0050236	UP
SYDE1	395.6312	0.44772	0.149394	2.996896	0.002727441	0.0050236	NOT
LINC01293	1.338171	1.53297	0.511526	2.996859	0.002727771	0.0050237	UP
LINC01508	3.180761	2.38494	0.795815	2.996853	0.002727826	0.0050237	UP
UGDH-AS:	93.43742	-0.39323	0.131222	-2.99665	0.002729606	0.0050266	NOT
PLAT	596.699	0.576028	0.192267	2.995984	0.002735611	0.0050373	NOT
SLC2A3P4	1.883595	0.907053	0.302781	2.995741	0.002737786	0.005041	UP
VDAC1P1	6.028177	0.552682	0.184511	2.995381	0.002741021	0.0050466	NOT

FBXO5	193.2581	0.454964	0.151902	2.995121	0.002743364	0.0050506	NOT
RP11-39C	3.612958	0.69528	0.232154	2.994909	0.002745269	0.0050538	UP
AC124861	1.265332	1.508248	0.503609	2.994876	0.002745562	0.005054	UP
RP11-320I	2.400219	3.27544	1.093693	2.994843	0.002745857	0.0050542	UP
SEMA6D	178.482	-0.69695	0.232739	-2.99454	0.002748559	0.0050588	DOWN
RP11-473I	5.37466	0.600357	0.200501	2.994277	0.002750958	0.0050629	UP
RN7SKP29	3.322432	-0.86155	0.28774	-2.99419	0.002751761	0.0050641	DOWN
ZNF268	764.7279	-0.31137	0.103993	-2.99415	0.002752099	0.0050643	NOT
RP1-5O6.6	1.988527	0.856366	0.286028	2.99399	0.002753549	0.0050667	UP
CENPC	439.6643	-0.28602	0.095546	-2.99352	0.002757833	0.0050742	NOT
DHX32	786.5708	-0.39976	0.133592	-2.99242	0.002767768	0.0050922	NOT
KRTAP5-9	4.679887	0.844542	0.282229	2.992395	0.002767978	0.0050922	UP
PPT1	2654.931	0.367145	0.122713	2.991889	0.002772573	0.0051003	NOT
MACROD2	195.515	0.564157	0.18858	2.991605	0.002775149	0.0051047	NOT
OPTN	5240.475	0.309448	0.103441	2.991543	0.002775711	0.0051054	NOT
TCN1	13.41389	-1.47575	0.49341	-2.99092	0.00278142	0.0051156	DOWN
RPA2	1111.043	0.244422	0.08174	2.99023	0.002787675	0.0051267	NOT
ITGB5	6687.872	0.30211	0.101037	2.990092	0.002788936	0.0051287	NOT
WDR44	669.5893	-0.36255	0.121268	-2.98964	0.002793025	0.0051356	NOT
DLEC1	39.56714	-0.53513	0.178995	-2.98964	0.00279304	0.0051356	NOT
KIF12	1239.706	0.704858	0.235781	2.989466	0.002794652	0.0051382	UP
GUSBP4	6.133836	0.537811	0.17992	2.989171	0.002797359	0.0051428	NOT
RPL5P17	2.162447	0.847689	0.283606	2.988966	0.002799237	0.0051459	UP
RPS20	31245.9	0.420315	0.140625	2.988912	0.002799724	0.0051465	NOT
CTD-2002	16.43961	0.545559	0.182529	2.988891	0.002799918	0.0051465	NOT
CTC-260F	38.04383	0.43181	0.144498	2.988344	0.002804937	0.0051554	NOT
FUT5	3.01033	-0.82965	0.277631	-2.9883	0.002805329	0.0051558	DOWN
CSRP2BP	779.6652	0.257291	0.086102	2.988189	0.002806355	0.0051573	NOT
MIR762HG	31.18457	0.367111	0.122856	2.988132	0.002806885	0.0051579	NOT
RRP7A	1934.827	0.360625	0.120691	2.988003	0.002808071	0.0051598	NOT
AC068138	1.699688	3.157852	1.056881	2.987896	0.002809047	0.0051612	UP
BRI3P1	7.555677	0.663961	0.222218	2.987881	0.002809189	0.0051612	UP
AC009974	1.786857	1.080452	0.361649	2.987575	0.002812003	0.005166	UP
RBM8B	6.503658	0.673094	0.225301	2.987531	0.002812403	0.0051664	UP
IBTK	3385.868	-0.31199	0.104435	-2.98737	0.002813896	0.0051688	NOT
RPARP-AS	360.3833	0.348068	0.116536	2.986771	0.002819413	0.0051786	NOT
LL0XNC01	1.951171	0.941223	0.315144	2.986644	0.002820577	0.0051804	UP
IMMP1L	334.6989	0.260258	0.087147	2.98642	0.002822648	0.0051838	NOT
RP1-197B	47.95516	-0.42163	0.141191	-2.98623	0.002824402	0.0051867	NOT
SRR	197.0297	-0.31486	0.105461	-2.98561	0.002830112	0.0051968	NOT
MPRIPP1	3.951283	0.721007	0.241548	2.984951	0.00283624	0.0052077	UP
PPP4R1L	88.92664	0.507645	0.170091	2.984556	0.002839905	0.0052141	NOT
RP5-1172I	1.603923	1.09022	0.365294	2.984502	0.002840406	0.0052147	UP
KCNT1	4.703703	0.707464	0.237052	2.984422	0.002841143	0.0052157	UP
RP11-515	30.71295	0.57838	0.193802	2.984385	0.002841489	0.005216	NOT
TAS2R60	1.242213	-1.54956	0.519229	-2.98435	0.002841775	0.0052162	DOWN
NCAM1-A	2.400521	1.423217	0.477084	2.983159	0.002852896	0.0052362	UP
CCDC106	807.2906	-0.42686	0.143092	-2.98312	0.0028533	0.0052366	NOT
PDK3	187.573	-0.41066	0.137666	-2.98303	0.002854108	0.0052378	NOT
ATP1B2	202.3183	-0.62611	0.209928	-2.98253	0.002858782	0.0052459	DOWN
HYDIN	23.7965	-1.08572	0.36403	-2.98251	0.002858932	0.0052459	DOWN
DICER1-A	120.096	0.427422	0.143316	2.982367	0.002860286	0.0052481	NOT

ZNF652	1458.097	0.24686	0.082776	2.982284	0.00286106	0.0052491	NOT
AURKAPS1	1.477266	1.169866	0.392275	2.98226	0.002861292	0.0052492	UP
NUDT8	696.3432	-0.47305	0.158634	-2.98205	0.002863281	0.0052525	NOT
LINC00909	247.2177	0.276368	0.092692	2.981563	0.002867811	0.0052605	NOT
RP11-690I	89.3307	-0.33597	0.112685	-2.9815	0.002868384	0.0052612	NOT
HRK	2.1702	1.289548	0.432537	2.98136	0.002869716	0.0052633	UP
ADAM10	3143.581	0.2754	0.09238	2.98118	0.002871404	0.005266	NOT
MYH2	0.950923	1.90649	0.639542	2.981025	0.002872856	0.0052683	UP
NAP1L1P1	11.66352	0.47359	0.158899	2.98045	0.002878257	0.0052779	NOT
RP11-791(	3.612802	0.698043	0.234234	2.980106	0.002881482	0.0052834	UP
RBPMS2	593.2567	0.475726	0.159637	2.980051	0.002882008	0.0052841	NOT
RTN1	121.9337	0.610026	0.204713	2.979903	0.002883394	0.0052863	UP
NTHL1	1534.596	-0.45596	0.153014	-2.97985	0.002883938	0.0052869	NOT
RP11-159I	11.77378	1.19263	0.400243	2.979761	0.002884737	0.005288	UP
RP11-379I	7.591966	-1.0339	0.346985	-2.97967	0.002885634	0.0052893	DOWN
OTUB1	2733.194	0.230262	0.07728	2.979584	0.0028864	0.0052904	NOT
CD53	795.2593	-0.57001	0.191337	-2.9791	0.00289094	0.0052983	NOT
ZNF236	313.4535	0.317745	0.106664	2.978935	0.002892523	0.0053009	NOT
LYZL2	1.480031	1.529264	0.513383	2.978799	0.002893806	0.0053026	UP
TMEM200	311.2256	0.639737	0.214764	2.978796	0.002893838	0.0053026	UP
RP11-166I	20.91767	0.773754	0.259758	2.978742	0.002894341	0.0053032	UP
MEF2BNB-	14.39746	0.457944	0.15374	2.978698	0.00289476	0.0053036	NOT
PTRH1	2.560685	0.769545	0.258381	2.978339	0.002898156	0.0053094	UP
RP11-134(	1.769538	0.848534	0.284976	2.977559	0.002905533	0.0053226	UP
RP11-850/	1.546072	0.952221	0.319807	2.977485	0.002906241	0.0053236	UP
RP11-297I	160.9063	0.746769	0.250835	2.977133	0.002909578	0.0053293	UP
CHSY3	106.373	-0.58767	0.197407	-2.97696	0.002911229	0.005332	DOWN
RP11-114(	28.09997	-0.44685	0.150104	-2.9769	0.002911804	0.0053327	NOT
KIAA0020	777.2091	0.361778	0.121534	2.976759	0.00291313	0.0053348	NOT
FAM161A	108.7811	0.409953	0.137725	2.976604	0.002914601	0.0053371	NOT
PCDH19	15.14851	-0.76936	0.258492	-2.97636	0.002916949	0.005341	DOWN
HYAL3	299.1607	0.501546	0.168528	2.97604	0.002919968	0.0053462	NOT
RP4-814D	2.494229	0.713164	0.239646	2.97591	0.002921208	0.0053481	UP
PON2	6695.248	0.359597	0.120841	2.975773	0.002922514	0.0053502	NOT
CTC-453G	1.290684	0.975969	0.327979	2.975711	0.002923098	0.0053509	UP
SH2D6	21.63537	0.640467	0.215275	2.975116	0.00292878	0.0053609	UP
IGHV5-51	224.5876	-1.16358	0.391115	-2.97503	0.002929634	0.0053621	DOWN
CYP1B1	837.1726	0.646573	0.217354	2.974747	0.002932303	0.0053667	UP
RP11-545/	0.90898	1.522554	0.511865	2.974523	0.002934441	0.0053702	UP
IGHV4-61	17.61108	-1.3411	0.450895	-2.97431	0.002936526	0.0053737	DOWN
RP13-210I	1.991557	1.085961	0.365119	2.974266	0.002936904	0.005374	UP
PYHIN1	58.04816	-0.68095	0.228958	-2.97414	0.002938121	0.0053759	DOWN
RP11-158I	4.155802	0.762456	0.256366	2.97409	0.002938593	0.0053764	UP
SERBP1P3	1.890571	1.175382	0.395214	2.974038	0.002939084	0.005377	UP
RP3-337O	3.198469	0.708566	0.238267	2.973824	0.002941138	0.0053804	UP
RP11-427I	2.19025	-0.61421	0.206547	-2.9737	0.002942329	0.0053822	DOWN
RP11-305I	1.623604	3.15106	1.059718	2.973488	0.002944363	0.0053855	UP
RP11-252/	6.014518	-0.6041	0.203174	-2.97332	0.002946005	0.0053882	DOWN
RP11-162/	57.4694	-0.40298	0.135544	-2.97307	0.002948375	0.0053922	NOT
RP1-287H	1.017708	2.288838	0.769877	2.972991	0.002949131	0.0053932	UP
CTD-2619	24.83632	0.540796	0.181962	2.972033	0.002958352	0.0054096	NOT
PSMG4	641.3166	0.334626	0.112592	2.972018	0.002958497	0.0054096	NOT

RP11-443(	3.58496	1.061093	0.357033	2.971978	0.002958881	0.0054099	UP
FAM110D	49.88065	0.523639	0.176201	2.971823	0.002960373	0.0054123	NOT
RP11-566I	38.32165	0.449686	0.151327	2.971616	0.002962366	0.0054156	NOT
RP3-395M	1.908412	1.372888	0.462113	2.97089	0.002969385	0.0054281	UP
LINC0095	6.945389	0.71869	0.241941	2.970513	0.002973032	0.0054344	UP
PIK3IP1-A	4.712065	0.65201	0.219503	2.970395	0.002974168	0.0054361	UP
DSCR4-IT	1.925103	3.395001	1.14296	2.970359	0.002974522	0.0054364	UP
RP11-818(	2.670127	0.811985	0.273367	2.970313	0.002974969	0.0054368	UP
RP11-551I	1.857154	1.127088	0.379474	2.97013	0.002976737	0.0054397	UP
EFCAB14	3439.329	-0.23377	0.078713	-2.96996	0.002978409	0.0054424	NOT
RP11-469.	1.34594	1.037043	0.349238	2.969447	0.002983366	0.0054511	UP
PTPRD	501.4027	-0.84308	0.28395	-2.96913	0.00298648	0.0054564	DOWN
C17orf100	110.3956	-0.43668	0.147088	-2.96882	0.002989426	0.0054615	NOT
MAP3K5	853.6296	-0.61904	0.208531	-2.96858	0.002991826	0.0054655	DOWN
RB1CC1	2476.096	0.354281	0.119346	2.968526	0.002992315	0.005466	NOT
NOSTRIN	251.1381	-0.46345	0.156134	-2.96828	0.002994731	0.0054701	NOT
CASC16	1.395792	1.475116	0.497035	2.967832	0.002999085	0.0054777	UP
DNAJC28	73.30976	-0.35219	0.118675	-2.96772	0.003000183	0.0054793	NOT
ANXA7	6062.643	-0.20813	0.070132	-2.96765	0.003000826	0.0054801	NOT
ZCCHC11	1120.712	0.34183	0.115218	2.966817	0.003008999	0.0054947	NOT
PCDHB18I	7.217283	0.678903	0.228837	2.966756	0.003009593	0.0054954	UP
ACRBP	72.7185	0.554625	0.187012	2.965715	0.003019802	0.0055135	NOT
EIF3G	5571.128	0.313129	0.105583	2.965705	0.003019903	0.0055135	NOT
RP11-165I	2.068154	0.810328	0.273253	2.965492	0.003021993	0.005517	UP
AC106876	82.00792	-0.47518	0.160244	-2.96538	0.003023062	0.0055185	NOT
CCBL2	747.0642	-0.28426	0.09587	-2.96506	0.003026247	0.005524	NOT
OSBPL6	285.5928	-0.56654	0.191078	-2.96496	0.003027257	0.0055255	NOT
CPS1-IT1	16.59236	-1.17882	0.397622	-2.96467	0.003030103	0.0055303	DOWN
TRAPPC13	655.518	0.221465	0.074711	2.964276	0.003033957	0.005537	NOT
PHF20	1057.78	0.180155	0.060776	2.964238	0.003034332	0.0055373	NOT
SHISA5	7348.99	-0.23457	0.079135	-2.96419	0.003034786	0.0055378	NOT
PARP11	239.2489	-0.30802	0.103922	-2.96392	0.00303744	0.0055422	NOT
C16orf86	135.934	-0.43965	0.148339	-2.96384	0.003038272	0.0055434	NOT
Six3os1_1	1.039672	2.517806	0.849568	2.963631	0.003040323	0.0055468	UP
BCL2L2	831.4939	0.241302	0.081425	2.963484	0.003041782	0.0055491	NOT
9-Mar	386.7931	0.469545	0.158456	2.963247	0.003044119	0.005553	NOT
TOX2	311.0694	0.605888	0.204484	2.963003	0.003046539	0.005557	UP
CHI3L2	29.78603	0.822658	0.277654	2.962889	0.003047661	0.0055587	UP
ADAMTS3	33.03125	0.833291	0.281247	2.962846	0.00304809	0.0055591	UP
STARD4	1030.242	-0.43108	0.145505	-2.96264	0.003050107	0.0055624	NOT
FAM214B	516.6614	0.296398	0.100065	2.962048	0.003055998	0.0055728	NOT
PHB	5199.858	0.260653	0.088009	2.961667	0.003059787	0.005579	NOT
RP3-468K	4.495269	0.69751	0.235513	2.961666	0.003059793	0.005579	UP
RP11-461(	18.07229	-1.02219	0.345158	-2.96151	0.00306139	0.0055815	DOWN
LINC0158	0.769345	2.107379	0.71161	2.961423	0.003062212	0.0055826	UP
ZNF649	153.7607	-0.43293	0.146223	-2.96073	0.00306907	0.0055948	NOT
SDCCAG8	592.5492	0.231193	0.078095	2.960414	0.003072257	0.0056002	NOT
RP11-485I	1.772752	0.826737	0.279276	2.960286	0.003073537	0.0056022	UP
MICAL2	1596.511	0.388972	0.131399	2.960236	0.003074038	0.0056027	NOT
RER1	4910.404	-0.25111	0.084834	-2.96001	0.003076247	0.0056064	NOT
SPATA18	311.7476	-0.83769	0.283036	-2.95966	0.003079793	0.0056125	DOWN
RARRES2	37640.8	-0.51957	0.175583	-2.95911	0.003085285	0.0056219	NOT

RP11-405/	3.463626	1.549822	0.523748	2.959102	0.003085371	0.0056219	UP
C10orf71-	1.036974	2.485826	0.840118	2.9589	0.003087392	0.0056252	UP
RNU6-101	1.340917	0.974244	0.329311	2.958427	0.003092134	0.0056335	UP
AC108868	1.85622	2.672465	0.903364	2.958348	0.003092931	0.0056346	UP
RP11-114:	2.057627	0.909997	0.307622	2.958166	0.003094751	0.0056375	UP
FAIM2	11.9477	0.903657	0.305498	2.957978	0.003096638	0.0056406	UP
PTOV1	5356.444	0.355235	0.120097	2.957896	0.003097469	0.0056417	NOT
RP11-517I	3.499655	0.912656	0.308551	2.957874	0.003097686	0.0056417	UP
TCL1A	14.44764	-1.07535	0.363576	-2.9577	0.003099483	0.0056446	DOWN
UGT1A4	3422.105	-1.08733	0.367651	-2.95752	0.003101295	0.0056476	DOWN
WHAMM	554.1446	-0.21956	0.074242	-2.95741	0.003102304	0.005649	NOT
C10orf126	14.62543	-1.06258	0.359338	-2.95706	0.003105925	0.0056551	DOWN
RP4-724E:	15.63194	0.642798	0.217379	2.957041	0.003106068	0.0056551	UP
USP30-AS	170.0068	-0.6277	0.212289	-2.95681	0.003108407	0.005659	DOWN
RPL7P50	1.460615	1.037606	0.350939	2.956659	0.003109915	0.0056614	UP
AUTS2	1260.393	-0.55794	0.188708	-2.95664	0.003110115	0.0056614	NOT
LRRC37A1!	3.735108	0.720822	0.243821	2.956363	0.003112903	0.0056661	UP
GIPC3	203.0955	0.399271	0.135062	2.9562	0.00311455	0.0056687	NOT
RP11-247I	3.20603	0.782174	0.264594	2.956125	0.003115307	0.0056697	UP
RP11-120I	5.935489	0.619974	0.209727	2.956097	0.003115592	0.0056699	UP
EMR2	91.57073	-0.57971	0.196122	-2.95588	0.003117762	0.0056734	NOT
ANKRD19I	29.78921	0.500813	0.169455	2.955426	0.003122372	0.0056815	NOT
LYPD5	31.80924	0.690718	0.23373	2.955194	0.003124722	0.0056854	UP
C1orf56	547.5162	0.311941	0.105586	2.954372	0.003133061	0.0057002	NOT
TMA16	472.7901	0.235366	0.079682	2.953811	0.003138762	0.0057102	NOT
IGHV1OR1	3.171248	-1.34336	0.454825	-2.95359	0.003141029	0.0057139	DOWN
RP11-274I	104.7022	0.38996	0.13204	2.953344	0.003143512	0.0057181	NOT
EMC8	792.7051	0.2309	0.078186	2.95322	0.003144779	0.00572	NOT
RP13-580I	14.79893	-0.69677	0.23595	-2.95304	0.003146638	0.005723	DOWN
BZRAP1	279.6206	0.534427	0.180988	2.952839	0.003148663	0.0057263	NOT
LHFPL3	4.209169	1.567207	0.53076	2.952758	0.003149492	0.0057274	UP
RNU6-531	2.152288	-0.68012	0.230363	-2.95238	0.003153385	0.0057341	DOWN
RIPK1	1872.252	-0.19263	0.065248	-2.95224	0.00315477	0.0057363	NOT
TTI2	281.9977	-0.31225	0.105777	-2.95198	0.003157428	0.0057407	NOT
ECM2	1413.557	-0.58965	0.199758	-2.9518	0.003159238	0.0057436	DOWN
SRSF9P1	7.584371	-0.54762	0.185537	-2.95152	0.00316217	0.0057486	NOT
FAM160B2	1160.956	0.324173	0.109845	2.951174	0.003165684	0.0057546	NOT
RP11-129I	1.534766	1.117942	0.378827	2.951065	0.003166801	0.0057562	UP
GUSBP9	2.485456	0.805134	0.272851	2.950817	0.003169348	0.0057605	UP
RIN1	160.2862	0.586179	0.198669	2.950534	0.003172255	0.0057654	UP
RP11-790I	0.989931	1.711662	0.580179	2.95023	0.003175372	0.0057707	UP
AURKC	44.88256	-0.45927	0.15569	-2.94991	0.003178621	0.0057762	NOT
RP11-216I	4.268054	0.667247	0.226243	2.949249	0.003185467	0.0057883	UP
C11orf42	3.065451	0.870864	0.295296	2.949121	0.003186793	0.0057903	UP
IGKV3-15	180.6976	-1.19644	0.405786	-2.94844	0.003193805	0.0058027	DOWN
MAT2B	2526.148	-0.26955	0.091439	-2.94782	0.003200221	0.0058139	NOT
ARHGAP31	2.839795	1.17494	0.3986	2.947669	0.003201799	0.0058163	UP
SENP7	463.8454	0.291479	0.098885	2.947654	0.003201952	0.0058163	NOT
RP11-423I	309.2531	0.437206	0.148334	2.947439	0.003204183	0.00582	NOT
TACSTD2	430.8385	-1.03734	0.351976	-2.94718	0.003206892	0.0058245	DOWN
PPARA	4527.738	-0.42225	0.14328	-2.947	0.003208694	0.0058274	NOT
MXRA8	722.4509	0.639324	0.216942	2.946977	0.003208966	0.0058275	UP

RP11-1038	1.521893	2.288268	0.776528	2.946793	0.003210883	0.0058306	UP
MRPL47	1221.579	0.282712	0.095942	2.9467	0.003211848	0.005832	NOT
CCDC74A	47.85955	0.726957	0.246705	2.946665	0.003212205	0.0058323	UP
RNA5SP31	1.337111	1.180784	0.400736	2.946538	0.003213533	0.0058343	UP
RP11-101	11.78088	0.583128	0.197907	2.946471	0.003214231	0.0058352	NOT
SMG1	2497.256	-0.30335	0.102954	-2.94642	0.00321471	0.0058357	NOT
ST13P15	9.243239	-0.58219	0.197641	-2.9457	0.00322226	0.005849	NOT
DCBLD2	1158.933	-0.3267	0.110925	-2.94525	0.003226906	0.005857	NOT
LYPLAL1	1367.545	0.334979	0.113737	2.945202	0.003227443	0.0058576	NOT
RP11-3D4	9.302906	0.496139	0.168468	2.945008	0.00322946	0.0058605	NOT
SNORA53	3.046509	-0.68095	0.231223	-2.945	0.003229554	0.0058605	DOWN
AC097662	14.37714	0.415861	0.14121	2.944988	0.003229667	0.0058605	NOT
TRHDE-AS	77.56839	1.554792	0.527978	2.944805	0.003231578	0.0058634	UP
AK4P2	1.174173	2.484367	0.843646	2.944797	0.003231671	0.0058634	UP
CTD-2201	5.022981	-0.8099	0.275071	-2.94434	0.003236439	0.0058716	DOWN
NPTXR	317.2564	0.974195	0.330884	2.944222	0.003237674	0.0058735	UP
AL022393.	1.66017	1.282622	0.435652	2.944143	0.003238499	0.0058746	UP
RPL12L3	4.401636	0.589437	0.20021	2.944097	0.003238984	0.0058751	UP
SOWAHD	25.40628	0.655163	0.222584	2.943446	0.003245809	0.0058871	UP
LINC0024(	65.08736	0.524006	0.178057	2.942917	0.003251359	0.0058968	NOT
CTF1	60.09333	-0.70059	0.238072	-2.94277	0.003252858	0.005899	DOWN
HDDC2	680.427	0.34938	0.118725	2.942762	0.003252988	0.005899	NOT
SSC5D	196.9071	-0.7413	0.251922	-2.94256	0.003255094	0.0059024	DOWN
DOK4	1960.972	0.374001	0.127108	2.942377	0.003257029	0.0059055	NOT
MLLT10P1	1.716066	0.870635	0.295942	2.94191	0.003261947	0.005914	UP
RP11-430I	2.544	0.802843	0.272912	2.941763	0.003263499	0.0059165	UP
RP11-66N	19.74781	0.794361	0.270043	2.941609	0.003265118	0.005919	UP
KLK3	2.133105	3.221606	1.09521	2.941541	0.00326584	0.0059199	UP
ZNF890P	1.43318	1.153064	0.392044	2.941156	0.003269897	0.0059269	UP
DSTNP1	6.27558	0.571323	0.194257	2.941065	0.003270855	0.0059283	NOT
RASA4B	1.660693	1.026111	0.348902	2.940974	0.003271825	0.0059296	UP
TRDV1	2.286331	-0.97941	0.333036	-2.94084	0.003273211	0.0059316	DOWN
SYNE3	316.1257	0.411699	0.139994	2.940831	0.003273331	0.0059316	NOT
ZGPAT	566.4228	-0.36888	0.125447	-2.94057	0.003276088	0.0059359	NOT
ZNF805	57.68549	-0.30925	0.105168	-2.94054	0.003276427	0.0059359	NOT
LINC0091(	121.7719	0.413426	0.140596	2.940526	0.00327656	0.0059359	NOT
FEM1B	2155.19	-0.24024	0.0817	-2.94052	0.003276599	0.0059359	NOT
SMEK2	3509.338	-0.21258	0.072294	-2.94043	0.003277605	0.0059374	NOT
ENO1-AS1	5.013368	0.797929	0.271385	2.940212	0.003279876	0.0059411	UP
GUSBP5	11.27078	-0.64557	0.219586	-2.93993	0.003282851	0.0059461	DOWN
ARPC2	5175.114	0.197817	0.067302	2.939229	0.003290295	0.0059592	NOT
AC008753	2.994682	0.74239	0.2526	2.938999	0.003292742	0.0059632	UP
DOCK10	348.7643	-0.55588	0.189225	-2.93767	0.003306938	0.0059885	NOT
RHOA	13024.21	0.176649	0.060155	2.936584	0.003318494	0.0060091	NOT
UBE2E2	714.1907	-0.41154	0.140145	-2.93653	0.003319107	0.0060098	NOT
BEND5	32.16757	0.773413	0.263382	2.936466	0.003319757	0.0060106	UP
HLX	785.99	-0.40847	0.139132	-2.93581	0.003326769	0.0060229	NOT
SH3BP1	338.415	0.545245	0.185757	2.935262	0.003332661	0.0060332	NOT
RP11-110I	3.037894	0.735202	0.250535	2.934533	0.003340495	0.0060469	UP
KIAA2012	37.58208	-0.7499	0.255558	-2.93436	0.003342316	0.0060498	DOWN
LDLRAD3	190.2536	0.55692	0.189803	2.934202	0.00334406	0.0060526	NOT
PDF	137.588	-0.33913	0.115582	-2.93413	0.003344805	0.0060536	NOT

CD44	2496.85	0.698062	0.237928	2.933919	0.00334712	0.0060573	UP
SYT1	171.941	0.943125	0.321485	2.933655	0.003349964	0.0060621	UP
RP11-561I	1.137507	2.513407	0.856934	2.933022	0.003356801	0.0060741	UP
SLC39A11	2453.209	-0.3277	0.111731	-2.93292	0.003357852	0.0060754	NOT
PURG	1.625245	1.161367	0.395977	2.932914	0.003357971	0.0060754	UP
RP11-43F1	56.89543	1.085707	0.370275	2.93216	0.003366128	0.0060898	UP
GEMIN8P4	58.40353	0.387883	0.132291	2.932033	0.003367508	0.0060919	NOT
MCF2L-AS	100.5653	0.651294	0.222136	2.931961	0.003368289	0.0060929	UP
AC004067	5.330213	0.676617	0.230787	2.931782	0.00337023	0.006096	UP
RP11-327I	1.863616	1.025189	0.349686	2.931739	0.003370703	0.0060964	UP
CTC-490E	217.6215	-0.80806	0.275632	-2.93168	0.003371289	0.0060971	DOWN
DEGS2	55.22463	0.703686	0.240035	2.931597	0.003372238	0.0060984	UP
RP11-407I	2.172475	0.989911	0.337698	2.931353	0.00337489	0.0061028	UP
IGLV1-51	271.0412	-1.02811	0.350736	-2.93129	0.003375563	0.0061036	DOWN
FAM205CI	1.66937	1.500954	0.512082	2.931078	0.003377879	0.0061074	UP
RPS3AP46	1.077863	1.659952	0.566379	2.930813	0.00338076	0.0061122	UP
SPINT2	1812.124	0.962417	0.328389	2.930721	0.003381769	0.0061137	UP
UBE2F-SCI	1.459856	0.944177	0.322192	2.930481	0.003384374	0.006118	UP
RP11-731(	31.66054	0.362129	0.123597	2.929923	0.003390458	0.0061286	NOT
RP11-156I	91.99051	0.304769	0.104022	2.929859	0.003391155	0.0061294	NOT
CTD-2366	164.9053	0.496392	0.169428	2.929814	0.003391653	0.0061299	NOT
BAIAP2	3723.958	-0.43978	0.150114	-2.9296	0.003393936	0.0061336	NOT
GS1-259H	12.15894	0.714317	0.243867	2.929128	0.003399149	0.0061427	UP
USP31	664.1726	0.320255	0.109354	2.928607	0.003404843	0.0061526	NOT
RRAGC	741.2359	0.277651	0.094823	2.92811	0.003410293	0.006162	NOT
FAHD1	3132.032	-0.35611	0.121633	-2.92771	0.003414671	0.0061695	NOT
PHLDB3	950.8085	0.363351	0.124115	2.927529	0.003416669	0.0061724	NOT
ACOXL	4.975506	0.739827	0.252716	2.927509	0.003416895	0.0061724	UP
PDXDC2P	184.5521	0.434543	0.148434	2.927507	0.003416919	0.0061724	NOT
RP3-323P2	4.330196	0.510221	0.174292	2.9274	0.003418093	0.0061741	NOT
RPL35A	12941.67	0.35318	0.12065	2.927307	0.003419108	0.0061755	NOT
RP11-950(	2.639415	0.717498	0.245117	2.92716	0.003420725	0.006178	UP
CYBA	2861.005	0.721501	0.246495	2.927042	0.003422031	0.00618	UP
RP11-340I	0.744355	1.492225	0.509901	2.9265	0.003427998	0.0061904	UP
SH3RF2	588.5173	0.593811	0.202923	2.926285	0.003430368	0.0061942	UP
SPTY2D1	918.4942	-0.20394	0.069694	-2.92624	0.003430884	0.0061948	NOT
TM4SF18	367.6522	0.427416	0.14607	2.926107	0.003432324	0.0061969	NOT
AC008074	5.557115	0.572248	0.195568	2.926078	0.003432651	0.0061969	NOT
RP4-614O	2.716943	0.649287	0.221897	2.92607	0.003432739	0.0061969	UP
TAF1	912.7547	0.314181	0.107423	2.924717	0.003447691	0.0062235	NOT
RP11-793/	4.850877	-0.51889	0.177424	-2.92459	0.00344909	0.0062256	NOT
HSP90B2P	59.65826	-0.51305	0.175437	-2.92442	0.003450944	0.0062285	NOT
SCAPER	492.3229	-0.28496	0.097444	-2.92432	0.003452058	0.0062301	NOT
LHFPL2	647.9584	0.534994	0.182955	2.924192	0.003453516	0.006232	NOT
CNNM2	709.0362	0.313228	0.107116	2.924191	0.003453529	0.006232	NOT
LINGO1-A	1.400103	1.939582	0.663363	2.923863	0.003457167	0.0062381	UP
MLLT1	2197.793	0.238758	0.08166	2.923797	0.003457899	0.0062391	NOT
KRTDAP	3.394684	1.75268	0.599486	2.92364	0.003459643	0.0062418	UP
7-Mar	1819.306	-0.20178	0.069021	-2.92348	0.003461381	0.0062445	NOT
ANXA3	121.8697	-0.84928	0.290511	-2.92338	0.003462517	0.0062462	DOWN
TMEM236	11.65529	0.509901	0.174423	2.923355	0.003462814	0.0062463	NOT
RP5-1050I	6.82353	1.527363	0.522502	2.92317	0.003464874	0.0062496	UP

IFRD2	2573.159	0.267762	0.091608	2.922919	0.003467666	0.0062542	NOT
UBE2W	1145.903	-0.22946	0.078504	-2.92288	0.003468142	0.0062547	NOT
ZNF709	7.390288	0.507264	0.173556	2.922767	0.003469363	0.0062565	NOT
RP11-360I	1.915598	0.807207	0.276236	2.922165	0.003476072	0.0062682	UP
DPAGT1	1901.939	0.207607	0.071049	2.922027	0.003477611	0.0062705	NOT
TGFBRAP1	639.1164	0.225064	0.077026	2.921933	0.003478664	0.006272	NOT
UBE2V1	231.5835	0.23218	0.07947	2.9216	0.003482382	0.0062783	NOT
HK1	1012.779	0.558603	0.1912	2.921569	0.003482736	0.0062785	NOT
ABHD17AI	2.402524	0.746764	0.255615	2.921445	0.00348412	0.0062806	UP
HS3ST5	1.427958	2.0109	0.688332	2.921409	0.00348452	0.0062809	UP
NME9	7.583625	0.632046	0.216365	2.921197	0.003486896	0.0062848	UP
C17orf70	1825.096	0.23112	0.07912	2.921138	0.003487554	0.0062856	NOT
FAM114A1	3023.634	-0.27455	0.093991	-2.921	0.003489078	0.0062879	NOT
PDE6B	63.28806	0.758849	0.25983	2.920555	0.003494085	0.0062965	UP
RP11-301(	17.40364	0.501321	0.171663	2.920371	0.003496153	0.0062999	NOT
AC087350	3.973637	0.735956	0.252035	2.920059	0.003499653	0.0063058	UP
CHRM5	12.18119	0.602121	0.206213	2.919905	0.003501381	0.0063085	UP
SMAP1	361.7093	0.283875	0.097232	2.919572	0.003505124	0.0063148	NOT
CHCHD6	341.5634	0.331806	0.113654	2.919449	0.003506505	0.0063169	NOT
RAB26	715.3109	-0.63594	0.217882	-2.91873	0.003514577	0.0063309	DOWN
C10orf12	90.81481	-0.3444	0.117997	-2.91872	0.003514734	0.0063309	NOT
SH3BP5-A	162.5555	0.435439	0.149199	2.918507	0.003517121	0.0063348	NOT
CTD-2007	3.346039	0.708397	0.242729	2.91847	0.003517537	0.0063351	UP
RP11-392(	1.377327	1.460431	0.500421	2.918405	0.003518272	0.0063359	UP
IGKV1D-4:	1.832643	-1.35194	0.463248	-2.91839	0.00351844	0.0063359	DOWN
TMED1	609.346	-0.25661	0.08793	-2.91829	0.003519545	0.0063375	NOT
IGKV2D-3(	1.162124	-2.20604	0.755951	-2.91823	0.003520221	0.0063383	DOWN
RNY3P16	4.899835	0.820598	0.281208	2.918121	0.003521482	0.0063401	UP
QTRTD1	516.3071	0.22622	0.077539	2.917494	0.003528566	0.0063525	NOT
PCID2	1067.796	0.276813	0.094883	2.917409	0.003529529	0.0063538	NOT
SLC2A1-A	6.58298	0.867728	0.297433	2.917386	0.003529787	0.0063538	UP
ALDH16A1	1744.348	0.340327	0.116655	2.917369	0.003529983	0.0063538	NOT
COL18A1-	0.799027	1.379977	0.473043	2.917235	0.003531495	0.0063561	UP
RAB42	75.27488	0.641469	0.219931	2.916686	0.003537722	0.0063669	UP
MPC2	11136.43	0.319556	0.109569	2.916489	0.003539956	0.0063705	NOT
EIF2S1	2123.814	-0.2056	0.070503	-2.91623	0.003542866	0.0063753	NOT
NCBP2-AS	540.5918	0.323091	0.110804	2.915888	0.003546775	0.0063819	NOT
RPL36AL	6531.243	-0.33806	0.11594	-2.9158	0.003547749	0.0063833	NOT
AC010127	9.217433	1.341575	0.460133	2.915624	0.003549777	0.0063865	UP
RP11-95M	0.844361	1.587421	0.544467	2.915549	0.003550633	0.0063876	UP
BCL7C	1245.622	0.375391	0.128771	2.91518	0.003554835	0.0063948	NOT
RP11-179/	2.170696	0.828747	0.284325	2.914791	0.003559272	0.0064023	UP
ST8SIA1	27.68197	0.720615	0.247246	2.91457	0.003561784	0.0064064	UP
RP4-594A!	0.934307	2.287116	0.784925	2.913803	0.003570551	0.0064218	UP
SNHG18	114.0785	-0.69814	0.239602	-2.91376	0.003571053	0.0064223	DOWN
HSCB	688.3305	-0.3106	0.106612	-2.91335	0.00357577	0.0064303	NOT
KB-1043D	1.859906	0.982804	0.337365	2.913173	0.003577761	0.0064335	UP
CTD-2256	13.18221	-0.60798	0.208704	-2.91312	0.003578373	0.0064342	DOWN
BOLA3-AS	20.6875	0.623166	0.213954	2.912619	0.003584115	0.0064441	UP
POLDIP2	10049.18	-0.23599	0.081028	-2.91252	0.003585206	0.0064452	NOT
FADD	364.0192	0.317192	0.108906	2.912523	0.00358522	0.0064452	NOT
LL0XNC01	1.163363	1.291566	0.443557	2.911836	0.003593112	0.006459	UP

MAP7D3	321.7393	-0.47684	0.163786	-2.91136	0.003598553	0.0064684	NOT
LINC0087(	2.737663	3.723057	1.278842	2.911273	0.003599594	0.0064698	UP
MAGEA4	5.539372	3.471202	1.192399	2.911108	0.003601501	0.0064728	UP
RP11-207(	2.208934	0.960339	0.329914	2.910881	0.003604116	0.0064771	UP
HMGB1	5756.418	-0.21341	0.073318	-2.91079	0.003605124	0.0064785	NOT
CYGB	830.7753	-0.5005	0.171949	-2.91074	0.003605685	0.0064791	NOT
PDK1	718.9728	0.449988	0.154603	2.910597	0.00360739	0.0064813	NOT
NDUFA8	1930.941	0.330069	0.113403	2.910596	0.003607406	0.0064813	NOT
TWIST1	13.52734	0.760524	0.2613	2.910538	0.003608067	0.0064821	UP
GNAQ	2280.44	-0.26149	0.089854	-2.91016	0.003612471	0.0064896	NOT
SLC27A6	1.796789	-1.65247	0.567872	-2.90993	0.003615125	0.0064939	DOWN
RP11-501(	1.245436	2.708523	0.930796	2.909899	0.003615456	0.0064941	UP
RP11-174(	1.061232	1.049932	0.360865	2.909488	0.00362021	0.0065022	UP
IGKV3D-2(	30.73152	-1.2406	0.426419	-2.90935	0.00362179	0.0065046	DOWN
C1orf226	788.7156	0.434778	0.149448	2.909228	0.003623219	0.0065065	NOT
DMRT2	2.226697	2.03069	0.698018	2.909222	0.0036233	0.0065065	UP
FAM120A(	1107.539	0.188883	0.064938	2.908661	0.003629802	0.0065178	NOT
NCOA1	1662.239	-0.24499	0.08423	-2.9086	0.00363047	0.0065185	NOT
CTD-2574	1.370143	0.902542	0.310336	2.90827	0.003634341	0.0065251	UP
CYP2T1P	23.92407	0.705657	0.242643	2.908208	0.003635069	0.006526	UP
ADAMTS9	12.95213	-0.66581	0.229008	-2.90734	0.003645151	0.0065436	DOWN
IL17RB	2488.584	0.538635	0.185273	2.907246	0.003646269	0.0065452	NOT
RP11-312I	10.29897	0.612973	0.210847	2.907197	0.003646837	0.0065458	UP
GRK4	119.7716	0.26511	0.091198	2.906982	0.003649345	0.0065499	NOT
KCNQ2	1.964048	1.668667	0.574065	2.906757	0.003651968	0.0065542	UP
NSUN7	43.89067	1.052765	0.362184	2.906717	0.00365243	0.0065546	UP
PAK7	7.50385	-1.38956	0.478099	-2.90644	0.003655722	0.00656	DOWN
RP11-385I	0.865105	2.255348	0.776153	2.905803	0.003663121	0.0065729	UP
EGOT	10.44592	0.900984	0.310076	2.90569	0.00366444	0.0065748	UP
HSBP1L1	808.2268	-0.36891	0.126966	-2.90562	0.003665239	0.0065758	NOT
LINC0127(	177.2231	0.443755	0.152734	2.905406	0.003667768	0.00658	NOT
WDR41	853.0583	0.241725	0.083226	2.904444	0.003679061	0.0065998	NOT
JADE1	1323.727	-0.29696	0.102247	-2.90438	0.003679843	0.0066008	NOT
ASS1P12	9.366831	-0.46107	0.158753	-2.90435	0.003680128	0.0066008	NOT
FCF1	1101.151	-0.20164	0.069431	-2.90418	0.00368217	0.0066041	NOT
RP11-151I	5.726885	0.691698	0.238183	2.904058	0.003683599	0.0066062	UP
EIF1P6	3.234413	0.848543	0.2922	2.903985	0.003684457	0.0066073	UP
RP11-45M	22.70499	0.612988	0.21109	2.903913	0.0036853	0.0066084	UP
RP11-667I	1.785871	0.959048	0.330274	2.903791	0.003686737	0.0066106	UP
CCND1	7150.373	-0.56504	0.194591	-2.90373	0.0036875	0.0066115	NOT
AC003075	37.64403	-0.52386	0.180432	-2.90339	0.003691457	0.0066182	NOT
COX16	795.993	-0.22881	0.078825	-2.9027	0.003699589	0.0066323	NOT
HUWE1	6424.849	0.201574	0.069445	2.902644	0.003700267	0.0066331	NOT
RP11-182.	3.886102	0.778942	0.26839	2.90228	0.003704576	0.0066402	UP
IL10RB	1489.783	-0.22108	0.076173	-2.90227	0.003704734	0.0066402	NOT
WISP2	98.64845	-0.95895	0.330419	-2.90224	0.003705074	0.0066404	DOWN
PPP1R2	1205.807	0.226803	0.078153	2.902037	0.003707445	0.0066442	NOT
RPS2P46	114.2948	0.469351	0.161733	2.902007	0.003707799	0.0066445	NOT
AL590762.	1.681315	1.078524	0.371677	2.90178	0.003710494	0.0066488	UP
PEX11B	1452.68	0.234185	0.080705	2.901742	0.003710945	0.0066492	NOT
TRIAP1	1606.381	-0.23942	0.082514	-2.90162	0.003712431	0.0066515	NOT
RPAIN	716.3399	-0.27161	0.093609	-2.90153	0.003713403	0.0066528	NOT

AC245100	102.3466	0.424819	0.146416	2.901452	0.003714381	0.0066541	NOT
SNORD46	2.15492	0.825935	0.284708	2.900985	0.003719914	0.0066636	UP
PALM3	1295.908	-0.69135	0.238353	-2.90055	0.003725096	0.0066724	DOWN
KRTAP1-1	2.573074	-1.67234	0.576597	-2.90037	0.00372727	0.0066759	DOWN
RP11-616I	1.68022	1.095436	0.377712	2.90019	0.00372937	0.0066792	UP
SMG8	525.6479	0.283316	0.097694	2.900048	0.003731057	0.0066818	NOT
PKP2	1342.382	-0.48478	0.167186	-2.89966	0.003735619	0.0066895	NOT
CCDC67	3.881203	1.036262	0.357376	2.899641	0.003735904	0.0066896	UP
MZB1	164.9372	-0.93954	0.324045	-2.8994	0.003738766	0.0066941	DOWN
FAM160A2	1534.734	-0.22631	0.078054	-2.89939	0.003738867	0.0066941	NOT
KIAA1210	1.661524	1.11278	0.383828	2.899164	0.003741595	0.0066985	UP
HNRNPA3I	3.63496	0.63478	0.218968	2.898964	0.003743978	0.0067023	UP
CTD-2240	15.80039	-0.50412	0.173932	-2.89837	0.003751073	0.0067146	NOT
CDC73	1254.385	0.254582	0.087838	2.898332	0.003751529	0.006715	NOT
GTPBP8	239.7084	-0.21549	0.074353	-2.89815	0.003753741	0.0067182	NOT
CTD-2376	8.015848	-0.49031	0.169182	-2.89814	0.003753786	0.0067182	NOT
HEXDC	1111.49	0.316029	0.10906	2.897767	0.003758301	0.0067258	NOT
CLDN12	2673.479	-0.31159	0.107534	-2.89763	0.003759956	0.0067281	NOT
RP11-384(	4.043598	0.675951	0.233278	2.897621	0.003760044	0.0067281	UP
RNA5SP18	0.983898	1.345046	0.464195	2.89759	0.003760416	0.0067283	UP
TWF2	2064.641	0.388936	0.134258	2.89693	0.003768337	0.006742	NOT
RP11-505I	1.73679	1.333068	0.460173	2.896887	0.003768851	0.0067425	UP
NPPA	5.092206	0.867267	0.2994	2.896679	0.003771353	0.0067465	UP
FURIN	22758.37	-0.4447	0.153529	-2.8965	0.003773483	0.0067499	NOT
ZNF546	165.426	0.306561	0.105841	2.896435	0.003774293	0.0067509	NOT
ZNF585B	267.5523	0.41187	0.142206	2.896298	0.003775931	0.0067534	NOT
PTPN12	1810.813	0.281339	0.097149	2.895966	0.003779929	0.0067601	NOT
MAMDC2	40.50421	-0.76705	0.264902	-2.89561	0.003784211	0.0067674	DOWN
RP11-186I	45.6938	-0.42524	0.14687	-2.89536	0.003787247	0.0067723	NOT
MOXD1	218.7626	0.862946	0.298085	2.894969	0.003791965	0.0067801	UP
GPR110	11.53106	1.376616	0.475521	2.894961	0.003792053	0.0067801	UP
ZACN	17.35812	0.506663	0.175018	2.894926	0.003792481	0.0067801	NOT
LGALS7B	3.106186	1.608843	0.555748	2.894917	0.003792582	0.0067801	UP
LDLRAP1	1633.462	-0.29074	0.100437	-2.8947	0.003795224	0.0067844	NOT
RP11-701I	1.216419	1.382106	0.477543	2.894205	0.003801195	0.0067947	UP
RP11-350I	3.336525	1.313218	0.453765	2.894048	0.003803096	0.0067976	UP
QPRT	9792.766	-0.42924	0.148329	-2.89381	0.003805997	0.0068024	NOT
SNTA1	1169.094	0.346822	0.119853	2.893723	0.003807042	0.0068038	NOT
PRKRA	795.8412	-0.20639	0.071325	-2.89367	0.003807684	0.0068045	NOT
BTF3L4P2	23.17293	-0.30644	0.105916	-2.89321	0.003813247	0.006814	NOT
RP11-366I	13.72747	-0.44493	0.153802	-2.8929	0.003817064	0.0068204	NOT
RP13-884I	1.147463	1.310281	0.452934	2.892876	0.003817317	0.0068204	UP
TRPC3	8.768347	1.059898	0.366413	2.892635	0.003820251	0.0068252	UP
GSK3B	1793.646	0.177567	0.061393	2.892289	0.003824462	0.0068323	NOT
FOXD3-AS	2.52396	1.702099	0.588509	2.892222	0.00382528	0.0068333	UP
LGALS4	13446.3	0.865825	0.299383	2.892032	0.003827592	0.006837	UP
MIR186	2.465337	0.942994	0.326105	2.891689	0.003831766	0.006844	UP
AC012442	6.495197	0.530258	0.183377	2.891632	0.00383247	0.0068444	NOT
RP1-167A	2.813746	0.705378	0.243938	2.891631	0.003832476	0.0068444	UP
HSPE1-M(	2.418256	0.707536	0.244731	2.891076	0.00383925	0.006856	UP
CTB-167G	1.310146	1.489969	0.515541	2.890106	0.003851116	0.0068764	UP
CCNG2	548.1857	0.371615	0.128582	2.890104	0.003851147	0.0068764	NOT

ETNK2	8119.829	-0.67874	0.234857	-2.89002	0.00385217	0.0068778	DOWN
ZNF585A	144.395	0.351003	0.12146	2.889854	0.003854202	0.006881	NOT
PPIG	2257.066	-0.2005	0.069388	-2.88956	0.003857825	0.006887	NOT
RP11-47A:	4.432805	0.846333	0.292903	2.889462	0.003859022	0.0068887	UP
DNMT3L	38.23588	-1.0694	0.37011	-2.8894	0.003859779	0.0068896	DOWN
ITGAM	439.6388	0.58029	0.200885	2.88867	0.003868744	0.0069051	NOT
RP11-133I	3.196355	-0.61505	0.212938	-2.88842	0.003871809	0.0069102	DOWN
MZT2A	1542.437	0.431357	0.149357	2.888096	0.00387582	0.0069169	NOT
RP11-483I	12.17367	0.639113	0.221297	2.888028	0.003876648	0.0069179	UP
THTPA	223.1841	0.320868	0.111108	2.887903	0.003878195	0.0069202	NOT
RP11-299I	22.10143	0.427062	0.147887	2.887766	0.003879888	0.0069228	NOT
NHP2L1	3699.345	0.255056	0.088351	2.886849	0.003891203	0.0069422	NOT
ULK1	2933.282	0.292392	0.101284	2.886846	0.003891242	0.0069422	NOT
RP13-254I	2.523534	0.919235	0.318471	2.886404	0.003896713	0.006951	UP
ZFP37	48.64356	0.575341	0.199328	2.886403	0.003896727	0.006951	NOT
LAMP1	19160.88	-0.2794	0.096803	-2.88626	0.003898497	0.0069538	NOT
FLG-AS1	26.06758	0.874999	0.303197	2.885912	0.003902811	0.006961	UP
CYBRD1	1306.894	-0.55183	0.191224	-2.88579	0.003904276	0.0069627	NOT
ANKIB1	1067.798	0.290764	0.100757	2.885793	0.003904286	0.0069627	NOT
SCAF4	1319.12	-0.19736	0.068391	-2.8857	0.003905468	0.0069644	NOT
RP11-121/	9.528929	-0.57742	0.200099	-2.88568	0.003905737	0.0069644	NOT
RPAP2	482.7628	0.219346	0.076015	2.885571	0.003907045	0.0069663	NOT
RPL29P24	2.154247	0.757534	0.262531	2.885504	0.003907876	0.0069673	UP
CTC-250I1	1.737744	1.204228	0.417417	2.884951	0.003914744	0.0069791	UP
NCF1	67.7021	-0.60757	0.210602	-2.88489	0.003915464	0.0069796	DOWN
SLC38A5	53.70013	0.625288	0.216746	2.884892	0.003915483	0.0069796	UP
UBE2D2	2486.055	0.167047	0.057908	2.884711	0.003917726	0.0069831	NOT
TRIM68	402.4115	0.302376	0.104822	2.884666	0.003918286	0.0069836	NOT
RP5-1050I	3.568447	1.360075	0.471487	2.88465	0.003918487	0.0069836	UP
RP11-455J	3.807485	0.659395	0.228607	2.8844	0.003921598	0.0069887	UP
CAPN3	47.59601	0.635177	0.220217	2.884324	0.003922551	0.0069899	UP
AC090587	5.607402	0.557854	0.193437	2.883905	0.00392777	0.0069988	NOT
TOMM5	708.6279	0.258549	0.089661	2.883626	0.003931253	0.0070045	NOT
RP11-2K6.	2.429246	1.159304	0.402035	2.883589	0.00393171	0.0070049	UP
CPSF1P1	22.21981	0.976891	0.338795	2.883431	0.003933692	0.0070079	UP
RP11-863I	9.5496	-0.66324	0.230026	-2.88333	0.003934916	0.0070097	DOWN
CALM3	9671.238	0.275776	0.095648	2.883236	0.003936127	0.0070114	NOT
ITK	123.9181	-0.70836	0.24569	-2.88316	0.00393712	0.0070127	DOWN
AC067959	4.992845	-0.93174	0.323191	-2.88293	0.003939914	0.0070172	DOWN
FAM26E	113.9831	0.472024	0.163746	2.882652	0.003943426	0.007023	NOT
FLJ36000	1.09382	2.590047	0.898671	2.882085	0.003950526	0.0070352	UP
AP001469.	4.63201	0.820361	0.284646	2.882042	0.003951066	0.0070357	UP
AC114803	0.875182	2.077798	0.720953	2.882016	0.0039514	0.0070359	UP
RP11-545J	13.13888	-1.06984	0.371249	-2.88173	0.003955027	0.0070419	DOWN
RP11-206I	6.107763	-0.53306	0.185007	-2.88132	0.003960137	0.0070505	NOT
RP11-333/	3.408895	-1.20686	0.418929	-2.88084	0.003966232	0.0070609	DOWN
RPS15P5	3.885742	0.656751	0.227993	2.880577	0.003969485	0.0070663	UP
VN1R107P	1.538931	1.168084	0.405522	2.880449	0.003971097	0.0070687	UP
RP11-178I	6.991226	0.871738	0.302657	2.88028	0.00397322	0.007072	UP
ADAM33	37.19578	0.67885	0.235723	2.879867	0.003978426	0.0070808	UP
ABI3BP	277.0789	-0.71931	0.249801	-2.87954	0.00398262	0.0070878	DOWN
NKAPP1	36.45319	0.305855	0.106217	2.879515	0.003982871	0.0070878	NOT

LLNLR-304	1.276275	1.096094	0.380664	2.87943	0.003983943	0.0070893	UP
FCRL3	35.71277	-0.83525	0.290085	-2.87932	0.003985391	0.0070914	DOWN
DYRK1A	1784.917	-0.1977	0.068664	-2.87916	0.003987373	0.0070944	NOT
AC007318	75.45821	0.422887	0.146908	2.878587	0.003994613	0.0071069	NOT
ATG7	725.3736	0.194253	0.067489	2.878294	0.003998329	0.007113	NOT
GALNT5	9.828025	1.115544	0.387583	2.878207	0.003999431	0.0071145	UP
ST3GAL5	665.6057	-0.36302	0.126137	-2.87803	0.00400169	0.0071181	NOT
SDHAF1	641.4437	0.357645	0.124275	2.877848	0.004003981	0.0071217	NOT
OLR1	53.5574	0.907341	0.315295	2.877753	0.004005185	0.0071234	UP
RP11-88H	3.85428	-0.56338	0.195774	-2.87771	0.004005787	0.0071238	NOT
SUPT4H1	2580.866	0.219076	0.076129	2.877693	0.004005947	0.0071238	NOT
XPOTP1	2.392441	0.835343	0.290286	2.877656	0.004006416	0.0071242	UP
ADIPOR1	6092.724	0.218787	0.076034	2.877477	0.004008697	0.0071278	NOT
LMF2	4461.829	0.258339	0.089782	2.877402	0.004009643	0.007129	NOT
COA7	1226.815	0.257923	0.08964	2.877319	0.004010699	0.0071304	NOT
AC092415	1.28076	2.560629	0.889954	2.877261	0.004011431	0.0071313	UP
AC006116	4.115815	0.676591	0.235212	2.876517	0.004020907	0.0071477	UP
PRKAR2A	2286.829	0.233359	0.081134	2.87621	0.00402482	0.0071542	NOT
ANP32A	4323.936	0.192795	0.067034	2.876064	0.004026679	0.007157	NOT
SF3B2	6944.267	0.15285	0.053146	2.876038	0.004027016	0.0071572	NOT
CTC-487M	2.505104	0.763342	0.265429	2.875883	0.004028985	0.0071597	UP
RNA5SP40	1.180297	1.689425	0.587447	2.875878	0.004029053	0.0071597	UP
RP11-661(	2.709691	0.771907	0.268409	2.875866	0.004029204	0.0071597	UP
IGHV3-49	68.06346	-1.17999	0.41038	-2.87535	0.004035767	0.0071709	DOWN
WDR81	2286.486	-0.28021	0.097458	-2.87522	0.004037402	0.0071733	NOT
CD84	291.575	-0.57923	0.201481	-2.87486	0.004042023	0.0071811	NOT
MTX2	1002.059	0.187893	0.065358	2.874812	0.004042683	0.0071818	NOT
SETD5	2222.224	0.224865	0.078221	2.874729	0.004043748	0.0071832	NOT
RIOK3	3056.841	-0.22776	0.079229	-2.87468	0.004044315	0.0071837	NOT
CTD-3105	1.28734	1.174901	0.408736	2.874474	0.004047013	0.0071881	UP
G6PC	34498.64	-0.84016	0.292291	-2.87438	0.004048253	0.0071898	DOWN
RP11-124I	1.20379	2.52405	0.878345	2.873645	0.004057648	0.007206	UP
RP11-151/	3.842093	0.904615	0.31481	2.873531	0.004059107	0.0072077	UP
PARD3-AS	6.561116	0.636013	0.221335	2.873529	0.004059134	0.0072077	UP
CEBPA	7383.595	0.51869	0.180507	2.873513	0.004059343	0.0072077	NOT
NRIP3	16.30697	0.697912	0.242881	2.873478	0.004059794	0.007208	UP
RP11-676J	1.41267	0.959673	0.334014	2.873155	0.004063942	0.0072149	UP
MYH1	4.812017	1.736997	0.604573	2.873094	0.004064731	0.0072158	UP
RP11-365(	3.826582	0.656873	0.228689	2.872337	0.004074479	0.0072327	UP
ZFYVE9	808.7549	-0.23068	0.080315	-2.8722	0.004076254	0.0072354	NOT
AC098824	1.086222	1.037264	0.361143	2.872172	0.004076613	0.0072355	UP
SLCO3A1	583.8587	0.492031	0.17134	2.871664	0.004083168	0.0072467	NOT
FGF19	124.08	1.458165	0.507825	2.871394	0.004086657	0.0072524	UP
RCC2P6	1.248151	1.143006	0.398074	2.871343	0.004087323	0.0072532	UP
RP11-74D	6.101009	-0.55453	0.193157	-2.87088	0.004093249	0.0072632	NOT
LINC01297	1.51681	2.80086	0.97564	2.870794	0.004094424	0.0072647	UP
PTDSS1	3610.166	0.280811	0.097817	2.870777	0.00409464	0.0072647	NOT
FOXL2	2.5725	1.787307	0.622592	2.87075	0.004094996	0.0072649	UP
OXSR1	1419.662	-0.17491	0.060933	-2.87052	0.004097973	0.0072697	NOT
HPD	66090.64	-0.92636	0.322747	-2.87023	0.004101742	0.0072759	DOWN
ANGPTL4	7742.835	-0.69581	0.242426	-2.87021	0.004101964	0.0072759	DOWN
MBOAT2	170.4608	0.768138	0.267632	2.870125	0.004103094	0.0072774	UP

HIST1H2A	3.926918	0.976528	0.340292	2.869674	0.004108958	0.0072873	UP
LL0XNC01	1.37279	1.036298	0.361156	2.869391	0.004112636	0.007293	UP
SMCO4	1773.231	-0.35736	0.124544	-2.86938	0.004112805	0.007293	NOT
XG	57.03912	-0.97663	0.340364	-2.86937	0.00411292	0.007293	DOWN
PYCARD-A	2.135376	0.967787	0.3373	2.869218	0.004114879	0.007296	UP
C21orf33	138.1136	-1.00768	0.351243	-2.86889	0.004119125	0.007303	DOWN
PITPNM2	1280.093	0.42847	0.149354	2.868821	0.004120051	0.0073042	NOT
MIR202HG	1.044981	2.049436	0.714443	2.868577	0.00412323	0.0073094	UP
CISD3	4516.874	-0.36739	0.12808	-2.86845	0.004124913	0.0073119	NOT
SLC3A1	691.5916	-1.10344	0.384703	-2.86829	0.004127001	0.0073151	DOWN
SEPHS1P6	7.543431	-0.66978	0.233518	-2.86824	0.004127601	0.0073157	DOWN
GSN	7923.688	0.431091	0.15031	2.868023	0.004130453	0.0073203	NOT
ROPN1L	7.354733	-0.69272	0.241551	-2.86779	0.004133437	0.0073251	DOWN
XIRP2	1.182834	1.984676	0.692277	2.866882	0.004145381	0.0073458	UP
TGFBR2	4123.661	-0.3526	0.122997	-2.86673	0.004147393	0.0073489	NOT
PLA2G2C	2.389396	0.92896	0.324107	2.866212	0.004154163	0.0073604	UP
CTLA4	38.52678	0.775575	0.270747	2.864577	0.004175672	0.0073981	UP
CBWD1	297.4863	0.318567	0.111214	2.864444	0.004177414	0.0074007	NOT
TERF1	853.6065	0.252946	0.088309	2.864314	0.004179133	0.0074033	NOT
SUN3	1.768634	1.202473	0.419822	2.864247	0.004180019	0.0074044	UP
AL161645.	1.471433	-1.2505	0.436613	-2.86409	0.004182155	0.0074077	DOWN
RP1-241P1	3.648858	0.668721	0.233493	2.863985	0.004183476	0.0074095	UP
CCDC184	20.45042	-0.54616	0.190735	-2.86343	0.004190813	0.007422	NOT
STOX2	253.8551	0.515355	0.179989	2.863253	0.004193159	0.0074254	NOT
PACRG-AS	4.254551	-0.86259	0.301261	-2.86325	0.004193228	0.0074254	DOWN
RP11-570I	1.492298	0.917151	0.320427	2.862282	0.00420603	0.0074476	UP
HMGN2P4	9.471422	0.623744	0.217953	2.861828	0.004212055	0.0074578	UP
IGKV5-2	8.737685	-1.41995	0.496184	-2.86175	0.004213155	0.0074592	DOWN
XXyac-YX6	6.834995	0.716112	0.250289	2.861139	0.004221218	0.007473	UP
GCK	339.6238	1.403289	0.490506	2.860904	0.004224354	0.0074781	UP
EXOC3L1	226.9083	0.343015	0.119904	2.860739	0.004226542	0.0074815	NOT
SELE	186.4971	-0.82873	0.289719	-2.86047	0.004230182	0.0074875	DOWN
NFIC	7920.844	-0.34011	0.118903	-2.86042	0.004230866	0.0074882	NOT
RP13-270I	79.77966	-0.41963	0.146706	-2.86036	0.004231658	0.0074891	NOT
HSD11B1	18510.76	-0.97797	0.341958	-2.85992	0.004237496	0.007499	DOWN
RP11-138I	4.482276	1.595318	0.557913	2.85944	0.004243895	0.0075098	UP
LAG3	183.1473	-0.62077	0.217106	-2.85928	0.004246034	0.0075131	DOWN
AF064858.	170.993	-0.91275	0.319234	-2.85919	0.0042472	0.0075147	DOWN
RP1-45I4.3	21.07309	-0.64831	0.226754	-2.85911	0.004248274	0.0075161	DOWN
RP11-427I	4.904889	0.600233	0.209959	2.858806	0.00425239	0.0075229	UP
CTC-471J1	11.78114	0.550154	0.192488	2.858117	0.004261633	0.0075388	NOT
VAMP4	658.9887	0.263432	0.092176	2.857929	0.00426416	0.0075428	NOT
RP11-161I	3.49311	0.750866	0.262738	2.857851	0.0042652	0.0075441	UP
PPT2	511.1296	0.413359	0.144652	2.857605	0.004268519	0.0075495	NOT
PHAX	1388.175	0.190243	0.066587	2.857053	0.004275948	0.0075622	NOT
MUC20	379.6924	0.808008	0.282951	2.855644	0.004294968	0.0075953	UP
ZNF721	470.8863	0.254663	0.089184	2.855493	0.004297012	0.0075985	NOT
TPD52	2383.335	0.373486	0.130802	2.855355	0.004298872	0.0076013	NOT
CTD-2547	1.886286	-0.84205	0.294922	-2.85518	0.004301284	0.007605	DOWN
LINC01484	56.55676	0.586189	0.205313	2.855099	0.00430234	0.0076064	UP
NKG7	346.8331	-0.61929	0.216938	-2.85469	0.004307948	0.0076158	DOWN
AC093698	2.294239	-0.65495	0.229438	-2.85458	0.004309318	0.0076178	DOWN

MYBPH	14.60999	-1.12127	0.392817	-2.85442	0.004311495	0.0076211	DOWN
RP3-417L2	3.886782	1.854166	0.649611	2.854271	0.004313569	0.0076243	UP
BTK	153.7175	-0.51654	0.180991	-2.85393	0.004318245	0.0076321	NOT
SNCB	1.487271	1.233876	0.432428	2.853371	0.004325815	0.007645	UP
RP11-67C	4.145174	1.011463	0.3545	2.853212	0.004327976	0.0076483	UP
RP11-349	0.856367	1.721139	0.603267	2.853031	0.004330445	0.0076522	UP
NPIPB1P	2.123891	0.898273	0.314882	2.852726	0.004334592	0.007659	UP
TMBIM4	2215.013	-0.25438	0.089173	-2.85264	0.004335839	0.0076603	NOT
FTH1	24170.83	0.302472	0.106033	2.852632	0.004335886	0.0076603	NOT
PSD	132.0071	0.389107	0.136404	2.8526	0.004336316	0.0076606	NOT
RP11-44N	7.471429	0.66165	0.231959	2.852441	0.004338491	0.0076639	UP
BCL2L14	52.15941	0.82137	0.287955	2.852424	0.004338723	0.0076639	UP
SLC9C1	2.323452	1.296869	0.454779	2.851647	0.004349332	0.0076821	UP
CTAGE4	6.475445	0.846809	0.296962	2.851569	0.004350404	0.0076835	UP
FTLP2	121.3917	0.681774	0.239096	2.85147	0.004351754	0.0076854	UP
LAMA2	550.0439	-0.74021	0.259598	-2.85137	0.004353173	0.0076874	DOWN
ST13P5	15.11685	-0.484	0.169755	-2.85117	0.004355924	0.0076918	NOT
HYLS1	205.8892	0.315279	0.110585	2.851019	0.004357932	0.0076949	NOT
P4HA1	3776.119	-0.55876	0.196026	-2.85045	0.004365686	0.0077081	NOT
RP5-1126	21.87217	0.41917	0.147059	2.85035	0.004367116	0.0077101	NOT
MTRNR2L1	84.20072	-0.59535	0.208911	-2.84978	0.004374952	0.0077234	DOWN
SSH3	953.3261	0.318399	0.11173	2.849725	0.004375707	0.0077243	NOT
PDCD10	844.4579	0.193419	0.067888	2.849082	0.004384554	0.0077394	NOT
RP11-517I	6.63774	0.57924	0.203324	2.848849	0.004387765	0.0077446	NOT
SASH1	826.3079	-0.37413	0.131337	-2.84865	0.004390487	0.0077489	NOT
AC009264	1.375103	2.021066	0.709506	2.848554	0.004391841	0.0077506	UP
ATP6AP1L	124.4116	0.377621	0.132566	2.848543	0.004391997	0.0077506	NOT
RP11-459I	29.0275	-0.59085	0.207445	-2.84824	0.004396204	0.0077575	DOWN
BANCR	0.971262	1.409768	0.49499	2.848075	0.00439845	0.007761	UP
PNPLA5	3.663994	-1.37408	0.482486	-2.84793	0.004400505	0.007764	DOWN
HAUS8	235.474	0.295495	0.103759	2.847911	0.004400725	0.007764	NOT
THAP9	168.8663	-0.24557	0.086232	-2.84783	0.004401816	0.0077653	NOT
RP11-380	9.071104	0.507536	0.178219	2.847818	0.004402006	0.0077653	NOT
TRAF3IP2	566.3891	-0.36001	0.126421	-2.84772	0.004403396	0.0077672	NOT
GK-IT1	3.149229	-0.83149	0.291993	-2.84765	0.00440433	0.0077682	DOWN
SMCHD1	1131.944	0.309459	0.108672	2.847635	0.004404537	0.0077682	NOT
PCDHGB6	62.51509	-0.56273	0.197632	-2.84738	0.004408121	0.0077741	NOT
CYP4F22	952.8949	0.95458	0.335299	2.846952	0.004413998	0.0077839	UP
RP11-8L8.	6.020098	-0.57867	0.203261	-2.84691	0.004414547	0.0077844	NOT
ARHGAP3	560.179	-0.48749	0.171246	-2.84674	0.004416874	0.007788	NOT
CLIC4	3326.965	-0.306	0.107493	-2.84672	0.004417213	0.0077881	NOT
SLFNL1	16.08408	0.679052	0.238541	2.84669	0.004417632	0.0077884	UP
MUC5AC	14.31842	1.911785	0.671595	2.846633	0.004418432	0.0077893	UP
SNTG1	28.80118	1.20694	0.423995	2.846589	0.004419037	0.0077898	UP
MAN2A2	2392.737	0.370429	0.130144	2.846302	0.00442302	0.0077964	NOT
AC244230	5.527163	0.78338	0.275237	2.846205	0.004424369	0.0077982	UP
GRIK3	4.533544	-1.05631	0.37114	-2.84613	0.004425348	0.0077995	DOWN
GNA13	2254.093	-0.28346	0.099597	-2.84607	0.004426229	0.0078005	NOT
OXA1L	5402.945	0.213256	0.074933	2.84595	0.004427911	0.007803	NOT
SCOC	1970.833	-0.2074	0.072892	-2.84535	0.004436284	0.0078173	NOT
RP11-110	1.394775	1.423759	0.500472	2.844831	0.004443505	0.0078295	UP
CSN2	2.235427	3.611608	1.269581	2.844724	0.004444995	0.0078316	UP

UBC	22118.39	-0.22689	0.079768	-2.8444	0.004449461	0.007839	NOT
LRRFIP1P1	20.72518	0.628229	0.220886	2.844136	0.004453206	0.0078451	UP
MBOAT4	44.75423	0.95092	0.334369	2.843928	0.004456119	0.0078497	UP
ERN1	2016.728	-0.36518	0.128408	-2.84388	0.004456733	0.0078503	NOT
RP11-421I	5.934085	0.507295	0.178387	2.843783	0.004458142	0.0078523	NOT
HLF	4970.34	-0.70827	0.249069	-2.84367	0.004459657	0.0078544	DOWN
DHRS11	618.3422	0.31683	0.111424	2.843454	0.004462749	0.0078594	NOT
LIPH	94.88761	0.939385	0.330415	2.843044	0.004468486	0.007869	UP
RP11-34P1	2.03802	0.859265	0.302278	2.842629	0.004474315	0.0078787	UP
TRPM2-AS	4.084131	0.905127	0.318436	2.842417	0.004477284	0.0078835	UP
ANAPC1P1	1.278746	1.251065	0.440146	2.842388	0.004477694	0.0078837	UP
SNORA12	1.706331	0.896631	0.315536	2.84161	0.004488641	0.0079025	UP
CTD-2325	20.3251	0.505257	0.177841	2.841057	0.004496424	0.0079157	NOT
KRT8P3	63.34596	-0.5607	0.197371	-2.84083	0.004499608	0.0079208	NOT
PPA2	2249.427	-0.29067	0.10232	-2.84078	0.004500305	0.0079215	NOT
AQP9	23104.65	-0.88788	0.312555	-2.84072	0.004501199	0.0079226	DOWN
AC099668	8.320525	-0.60663	0.213555	-2.84065	0.00450217	0.0079238	DOWN
TMEM55A	277.361	0.567724	0.199869	2.840488	0.004504454	0.0079273	NOT
CD209	243.5092	-0.5684	0.200117	-2.84032	0.004506844	0.007931	NOT
IGHV1-46	97.79901	-1.14225	0.402206	-2.83996	0.004511927	0.0079394	DOWN
UQCC1	1423.119	0.215105	0.075745	2.839878	0.00451308	0.0079409	NOT
ARNTL2	491.0791	0.636416	0.224153	2.839208	0.00452256	0.0079571	UP
XXbac-BP(	2.082168	-0.99748	0.351345	-2.83903	0.004525066	0.007961	DOWN
FARSA	2307.106	0.231177	0.081431	2.838922	0.004526619	0.0079632	NOT
LYL1	131.3089	0.421599	0.148511	2.838833	0.004527887	0.007965	NOT
CTC-338M	32.92141	0.580717	0.204565	2.838785	0.004528571	0.0079657	NOT
RP6-159A	3.811622	-0.66633	0.234727	-2.83872	0.004529464	0.0079664	DOWN
PSD2	7.087579	0.753958	0.265598	2.838717	0.004529536	0.0079664	UP
CSTA	717.2149	0.734852	0.258937	2.837961	0.004540278	0.0079847	UP
XCL2	22.38716	-0.64275	0.226492	-2.83787	0.004541572	0.0079865	DOWN
RP11-326I	3.88339	0.752087	0.265046	2.837572	0.004545811	0.0079935	UP
ZNRF1	1284.127	-0.28892	0.101825	-2.8374	0.004548222	0.0079972	NOT
GIMAP5	61.60107	-0.52841	0.186248	-2.83712	0.00455231	0.0080039	NOT
RAD9B	20.32493	0.447205	0.157646	2.836771	0.004557223	0.008012	NOT
AC002511	1.312415	1.621153	0.571607	2.836135	0.004566315	0.0080275	UP
SYNJ2BP-(	6.574797	0.473938	0.167126	2.835815	0.004570894	0.008035	NOT
AKR7A2	2315.921	-0.30185	0.10645	-2.83561	0.004573808	0.0080396	NOT
RP11-403I	1.604351	-0.90272	0.318389	-2.83529	0.004578463	0.0080473	DOWN
MXRA5	561.3638	-0.74166	0.261588	-2.83524	0.004579109	0.0080477	DOWN
NRAP	263.5773	-0.67654	0.23862	-2.83523	0.004579257	0.0080477	DOWN
KRT36	2.117803	-0.84525	0.298133	-2.83513	0.004580725	0.0080497	DOWN
FOXL2NB	1.000418	1.682818	0.593588	2.834995	0.004582644	0.0080526	UP
AC018865	1.917279	-0.64246	0.226625	-2.83488	0.004584241	0.0080549	DOWN
GATA5	71.63992	1.354267	0.477743	2.834719	0.004586596	0.0080581	UP
SHH	863.6882	-0.54147	0.191014	-2.83471	0.004586678	0.0080581	NOT
LMAN2	14049.47	0.291576	0.102865	2.834538	0.004589195	0.0080621	NOT
COG7	735.4855	0.201812	0.071198	2.834504	0.00458969	0.0080624	NOT
RP11-108	1.378418	1.33409	0.470709	2.834215	0.004593848	0.0080692	UP
LINC0094(	5.450248	1.365906	0.481994	2.833864	0.004598885	0.0080775	UP
RP1-167O	17.92044	-1.3932	0.491689	-2.8335	0.004604141	0.0080863	DOWN
RP11-312	16.82497	0.406064	0.143314	2.833389	0.004605728	0.0080885	NOT
LINC0056	3.28729	1.062044	0.374836	2.833353	0.004606249	0.0080889	UP

ARAP3	419.8463	0.354193	0.125018	2.833133	0.004609423	0.0080939	NOT
AC058791	24.56919	-0.55533	0.196015	-2.83312	0.004609645	0.0080939	NOT
LINC0140Z	1.065568	1.240858	0.438052	2.832673	0.004616053	0.0081046	UP
RP11-159I	1.838965	0.800183	0.282496	2.832548	0.004617857	0.0081068	UP
MIA	2.544577	1.128835	0.398523	2.832545	0.0046179	0.0081068	UP
RP11-208(	9.09292	1.275736	0.450439	2.832208	0.004622781	0.0081149	UP
CCR7	69.33398	-0.65651	0.231815	-2.83205	0.00462511	0.0081184	DOWN
TUBA4A	2422.245	0.440014	0.155374	2.831969	0.004626227	0.0081199	NOT
CCDC39	46.66504	0.476247	0.168171	2.831912	0.004627058	0.0081208	NOT
PHACTR4	1861.763	-0.21885	0.077285	-2.83173	0.004629684	0.0081249	NOT
RP11-204I	13.07807	-0.77647	0.27425	-2.83123	0.004636908	0.0081371	DOWN
COPZ2	975.4712	-0.48357	0.170802	-2.8312	0.004637412	0.0081375	NOT
NOC4L	958.7647	0.292632	0.103361	2.831173	0.004637767	0.0081376	NOT
SLC22A3	1749.461	-0.60963	0.215333	-2.8311	0.004638827	0.0081389	DOWN
LINC0088Z	8.12476	0.740836	0.261685	2.831023	0.004639936	0.0081403	UP
ATF7	917.1721	0.191966	0.067812	2.830857	0.004642351	0.0081441	NOT
RP11-280(	1.014367	1.94002	0.685335	2.830764	0.004643699	0.0081459	UP
RP11-395I	23.29664	0.562736	0.198823	2.830345	0.004649785	0.0081561	NOT
B4GALT2	1905.253	0.286718	0.101306	2.830203	0.004651851	0.0081592	NOT
bP-2171C:	5.488446	1.160535	0.410217	2.829075	0.004668274	0.0081875	UP
ZDHHC19	76.57046	-0.78093	0.276079	-2.82863	0.004674795	0.0081984	DOWN
RP11-657(	1.194949	2.181131	0.771158	2.828383	0.004678379	0.0082041	UP
NHP2P1	2.02277	0.774853	0.273967	2.828269	0.00468004	0.0082065	UP
RP11-568I	1469.885	0.431777	0.152667	2.828232	0.004680583	0.008207	NOT
MNT	506.8317	0.235896	0.083412	2.828069	0.004682974	0.0082106	NOT
CTC-458I2	7.058521	0.503549	0.178061	2.827959	0.004684584	0.0082129	NOT
IKBKAP	1785.845	0.327406	0.11579	2.827577	0.004690168	0.0082222	NOT
C15orf57	289.0774	-0.22287	0.078827	-2.8273	0.004694302	0.0082289	NOT
IGHV4-4	6.686122	-1.34452	0.475617	-2.8269	0.004700169	0.0082387	DOWN
RP11-843I	8.78634	0.677538	0.239683	2.826808	0.004701448	0.0082404	UP
CTD-2184	4.093705	0.791667	0.280126	2.826105	0.004711779	0.008258	UP
ZNF292	726.4278	0.370449	0.131092	2.825864	0.004715327	0.0082637	NOT
HOXB-AS3	6.351629	0.834511	0.295331	2.825678	0.004718063	0.008268	UP
LINC0057Z	72.33327	-0.61217	0.216681	-2.82523	0.004724609	0.0082789	DOWN
ARL11	47.97274	-0.48361	0.17118	-2.82518	0.004725341	0.0082797	NOT
GVINP1	96.47683	-0.59316	0.20998	-2.82485	0.004730286	0.0082878	DOWN
HOXD11	1.497549	2.444836	0.865511	2.824731	0.004732033	0.0082903	UP
KRT87P	20.68556	1.097218	0.388469	2.824466	0.004735951	0.0082961	UP
CARD14	30.40273	0.612526	0.216865	2.824455	0.004736106	0.0082961	UP
ARHGEF28	828.2608	0.477603	0.169097	2.824433	0.004736432	0.0082961	NOT
AC007040	2.790585	-0.88887	0.314707	-2.82443	0.004736494	0.0082961	DOWN
12-Sep	1.130547	1.384697	0.490345	2.823921	0.004744009	0.0083087	UP
WAS	371.0398	-0.41933	0.14851	-2.82358	0.004749081	0.0083171	NOT
CRX	2.404657	-0.76719	0.271768	-2.82296	0.004758316	0.0083327	DOWN
MRPS18AI	1.841373	0.919505	0.325794	2.822351	0.004767301	0.0083479	UP
LGSN	124.8605	-0.86086	0.305046	-2.82208	0.004771385	0.0083545	DOWN
PRKCE	665.9173	-0.50821	0.180086	-2.82202	0.00477228	0.0083556	NOT
BHMG1	1.375987	1.256764	0.445349	2.821978	0.004772847	0.008356	UP
HOXA2	11.32677	0.738344	0.261649	2.821893	0.004774105	0.0083577	UP
RPL39	3914.979	0.378755	0.134256	2.821142	0.004785306	0.0083768	NOT
STARD10	15380.04	-0.42224	0.149682	-2.82091	0.004788742	0.0083823	NOT
RNF145	1108.824	0.34754	0.123213	2.82064	0.004792793	0.0083885	NOT

TAF1C	954.3508	0.227506	0.080658	2.820631	0.004792923	0.0083885	NOT
AC004471	4.037247	0.622477	0.220703	2.82043	0.004795936	0.0083933	UP
WFDC10A	0.775781	2.082509	0.73848	2.819994	0.004802462	0.0084037	UP
AC078899	2.669013	0.732534	0.259765	2.819982	0.004802642	0.0084037	UP
LRP10	4853.282	0.299608	0.106245	2.819969	0.004802823	0.0084037	NOT
RNA5SP11	7.76095	0.822596	0.291721	2.819803	0.004805319	0.0084076	UP
BIRC3	2622.508	0.674598	0.239254	2.819594	0.004808441	0.0084125	UP
CAMTA1	1193.717	-0.25636	0.090947	-2.81884	0.004819739	0.0084317	NOT
LCORL	351.1841	0.318553	0.113024	2.818461	0.004825441	0.0084412	NOT
LA16c-325	7.885975	0.731732	0.259633	2.818331	0.004827406	0.0084441	UP
CTD-2293	2.168555	0.85803	0.30447	2.818109	0.00483074	0.0084494	UP
UGT2B15	13371.49	-0.79494	0.282144	-2.81749	0.00483999	0.008465	DOWN
SLC35C2	2584.054	0.186684	0.066262	2.817373	0.004841822	0.0084677	NOT
LRRC4B	71.23162	0.427703	0.151815	2.817268	0.00484341	0.0084699	NOT
RP11-1024	39.76721	-0.35209	0.124985	-2.81706	0.004846544	0.0084749	NOT
CTD-2161	5.210599	0.592443	0.210308	2.81702	0.004847152	0.0084754	UP
PRSS21	11.14391	1.204431	0.427612	2.816645	0.004852806	0.0084847	UP
ZBTB8OS	706.2124	0.279681	0.099304	2.816414	0.0048563	0.0084899	NOT
AMMECR1	399.9313	0.31301	0.111138	2.81641	0.004856362	0.0084899	NOT
WI2-1896	73.67968	-0.72171	0.256259	-2.81633	0.004857648	0.0084916	DOWN
GID4	454.1427	-0.26579	0.094375	-2.8163	0.004858061	0.0084918	NOT
COPS4	900.5782	-0.19297	0.06853	-2.81588	0.004864431	0.0085024	NOT
AC124944	1.945805	0.981068	0.348507	2.815061	0.004876798	0.0085235	UP
PAK2	3734.798	-0.1883	0.066897	-2.81483	0.004880241	0.0085284	NOT
MICALCL	19.94345	-0.57394	0.2039	-2.81483	0.004880255	0.0085284	NOT
RP11-87H	109.1763	0.290955	0.103366	2.814814	0.004880553	0.0085284	NOT
AC091814	1.324971	1.798195	0.63884	2.814784	0.004881001	0.0085287	UP
CCNC	2345.819	-0.27049	0.096107	-2.81451	0.004885152	0.0085354	NOT
CYP4F32P	1.822036	1.947047	0.69182	2.814386	0.004887058	0.0085382	UP
CEACAM1	3436.995	-0.45156	0.160449	-2.81435	0.004887548	0.0085385	NOT
RP11-302I	4.180971	0.665826	0.236591	2.814248	0.004889143	0.0085407	UP
SIDT2	2143.802	-0.25616	0.091024	-2.81416	0.004890552	0.0085426	NOT
ARHGAP36	13.33931	3.510876	1.247972	2.813266	0.004904102	0.0085658	UP
RP11-493I	1.938862	1.241822	0.44142	2.813244	0.004904441	0.0085658	UP
CASP1	485.2482	-0.44096	0.156772	-2.81273	0.00491228	0.0085787	NOT
C11orf72	2.142649	0.743572	0.26436	2.812722	0.004912409	0.0085787	UP
TOMM20P	1.786039	0.868677	0.308847	2.812642	0.004913625	0.0085802	UP
IGHV3-19	2.033944	-1.24947	0.444235	-2.81262	0.004913897	0.0085802	DOWN
CALML3	26.1109	-1.13713	0.404332	-2.81237	0.004917767	0.0085864	DOWN
RP11-793I	84.55459	0.484877	0.17241	2.812344	0.00491819	0.0085866	NOT
TMEM213	1.056912	1.386241	0.492931	2.812238	0.004919803	0.0085889	UP
COASY	4035.33	0.241373	0.085836	2.812029	0.004923005	0.0085939	NOT
RP5-1170I	8.287431	0.991668	0.352683	2.811787	0.004926707	0.0085998	UP
MUC1	163.3507	0.867259	0.308453	2.811639	0.004928982	0.0086033	UP
ZNF295-A	13.84449	-0.87243	0.310312	-2.81146	0.004931779	0.0086076	DOWN
RPL23	21388.25	0.342699	0.121908	2.811115	0.004937014	0.0086157	NOT
ZDHHC20	1.330903	0.920165	0.327331	2.811114	0.004937032	0.0086157	UP
DAB1	273.2613	-0.68247	0.242785	-2.81101	0.004938553	0.0086177	DOWN
BEST3	3.163125	1.468	0.522235	2.810996	0.004938845	0.0086177	UP
TCF25	3828.929	-0.24745	0.088035	-2.81086	0.004940962	0.0086209	NOT
RPL41P5	139.4991	0.429536	0.152834	2.810478	0.004946794	0.0086305	NOT
ZNF574	593.0677	0.211116	0.075124	2.810241	0.004950437	0.0086363	NOT

PPFIA1	1648.878	0.231084	0.082247	2.809619	0.004960021	0.0086525	NOT
RP3-510H	5.019741	0.681867	0.242715	2.809335	0.004964397	0.0086596	UP
RPL7P44	1.559839	0.927014	0.329982	2.809285	0.004965159	0.0086601	UP
FOXN3	2810.858	-0.31239	0.1112	-2.80928	0.004965305	0.0086601	NOT
CTD-3220	1.556289	0.870988	0.310043	2.809251	0.004965691	0.0086602	UP
RP11-689I	17.10656	0.542332	0.193067	2.809033	0.004969058	0.0086655	NOT
HMX1	1.075963	2.344788	0.834785	2.808854	0.004971815	0.0086698	UP
TMEM232	21.20897	-0.59659	0.2125	-2.80746	0.004993327	0.0087068	DOWN
RP4-594I1	8.563487	-0.75508	0.268969	-2.80732	0.004995505	0.00871	DOWN
PLEKHA3P	3.130467	-0.68195	0.242922	-2.80728	0.004996234	0.0087107	DOWN
SUMO3	3032.59	0.214392	0.076374	2.807145	0.004998272	0.0087137	NOT
CBLN4	77.60664	1.153883	0.411096	2.806844	0.005002946	0.0087213	UP
RP11-401I	12.49469	-1.25446	0.446932	-2.80682	0.005003364	0.0087215	DOWN
RBM10	2437.073	0.180343	0.064254	2.806725	0.005004795	0.0087235	NOT
RP1-102E	47.57642	-0.48877	0.174147	-2.80664	0.00500612	0.0087252	NOT
ZG16B	57.25763	0.950973	0.338852	2.806452	0.005009031	0.0087298	UP
GPCPD1	803.2866	-0.31796	0.113327	-2.80568	0.005020987	0.00875	NOT
CTD-3203	18.73924	-0.58011	0.206781	-2.80541	0.005025253	0.0087569	NOT
CYP2G1P	5.557665	-0.89816	0.320161	-2.80533	0.005026512	0.0087586	DOWN
AC114730	1.878875	-0.82903	0.295524	-2.80529	0.005027193	0.0087592	DOWN
TUBBP5	41.80506	1.15363	0.411303	2.804814	0.005034556	0.0087715	UP
CTD-2140	0.669908	1.630051	0.581167	2.804788	0.005034967	0.0087716	UP
RP11-214I	4.013847	0.609106	0.217168	2.804768	0.005035282	0.0087716	UP
FAM95C	16.6568	1.144756	0.408207	2.804349	0.005041831	0.0087825	UP
POTEKP	2.784135	1.159407	0.413464	2.804133	0.005045206	0.0087878	UP
PTGDS	3065.112	1.081088	0.38557	2.803871	0.005049304	0.0087944	UP
RP11-175I	135.0513	-0.59764	0.213209	-2.80309	0.005061483	0.0088151	DOWN
NPY6R	100.373	1.297441	0.46287	2.803038	0.005062371	0.0088161	UP
RPS2	22394.61	0.37156	0.132559	2.802982	0.005063246	0.008817	NOT
RP11-347(	1.338153	1.09235	0.389758	2.802638	0.005068653	0.0088259	UP
CD48	337.7321	-0.59084	0.21082	-2.80259	0.005069455	0.0088267	DOWN
CBX3P9	2.681072	0.65705	0.234455	2.802457	0.005071501	0.0088297	UP
AKR1C4	10778.02	-0.72274	0.257906	-2.80233	0.005073503	0.0088327	DOWN
TP53TG3D	0.861292	1.461231	0.52148	2.802085	0.005077348	0.0088388	UP
RP11-180I	1.425145	1.265242	0.451554	2.801974	0.005079103	0.0088413	UP
TMEM14B	2336.847	0.260118	0.09284	2.801776	0.00508221	0.0088461	NOT
FRMPD2L2	1.623222	1.698204	0.606171	2.801527	0.005086139	0.0088524	UP
MCOLN1	1360.318	0.247766	0.088444	2.801397	0.00508818	0.0088554	NOT
RP11-7F17	44.06517	-0.75721	0.270304	-2.80133	0.005089213	0.0088567	DOWN
RP11-267I	47.1635	0.390581	0.139429	2.801287	0.005089916	0.0088573	NOT
HYAL4	1.750715	1.463749	0.52259	2.800952	0.005095215	0.008866	UP
LYRM7	726.6365	-0.24051	0.085886	-2.80037	0.005104408	0.0088814	NOT
RP11-165I	3.931986	0.848689	0.303067	2.800332	0.005104999	0.0088819	UP
GABPB1-A	385.996	0.403554	0.144112	2.800287	0.005105725	0.0088826	NOT
ITGB6	48.34146	0.855487	0.305509	2.800203	0.005107053	0.0088844	UP
ALPPL2	1.096588	1.979846	0.707192	2.799587	0.005116804	0.0089008	UP
TMEM248	5050.138	-0.15084	0.053883	-2.79939	0.005119912	0.0089056	NOT
FBXO48	184.5968	-0.27249	0.097347	-2.79919	0.005123065	0.0089105	NOT
NUP214	2195.512	0.171953	0.061432	2.799099	0.005124535	0.0089125	NOT
HNRNPUP	3.869269	0.527852	0.188595	2.79887	0.005128181	0.0089183	NOT
SNORD6	5.011341	0.62724	0.224116	2.798731	0.005130385	0.0089216	UP
GALNT3	73.74034	-0.71543	0.255634	-2.79864	0.005131842	0.0089236	DOWN

RP11-127	10.18587	0.738087	0.26374	2.798541	0.0051334	0.0089257	UP
RP11-53I6	2.266281	0.91447	0.326804	2.79822	0.005138513	0.008934	UP
RPL37	24376.63	0.369511	0.132064	2.797969	0.0051425	0.0089404	NOT
RP3-467K	4.814038	0.667962	0.238761	2.797621	0.005148042	0.0089495	UP
RABGAP1	1123.127	0.221111	0.079037	2.797544	0.005149273	0.0089511	NOT
PSMA1	2310.063	0.190583	0.068141	2.796893	0.005159667	0.0089686	NOT
RP11-519	2.610383	1.024714	0.366383	2.796837	0.005160554	0.0089695	UP
RAB37	718.7576	-0.49	0.175215	-2.79659	0.005164427	0.0089757	NOT
ERVV-2	0.908092	2.115395	0.75643	2.796552	0.005165105	0.008976	UP
LRRC37A5	24.96093	-0.63324	0.226437	-2.79654	0.005165226	0.008976	DOWN
LINC0052	6.362584	1.724133	0.616554	2.796404	0.005167481	0.0089793	UP
ISCA2	623.8596	0.231217	0.082689	2.796233	0.005170213	0.008983	NOT
MTRNR2L	5.386812	-0.67288	0.24064	-2.79623	0.005170262	0.008983	DOWN
SCD5	184.0228	-0.67393	0.241017	-2.7962	0.005170782	0.0089834	DOWN
IGHV1-45	5.010071	-1.2565	0.449467	-2.79553	0.005181536	0.0090015	DOWN
RP11-946	3.069258	0.625185	0.223723	2.794466	0.005198558	0.0090305	UP
SMIM17	2.657644	0.934189	0.334317	2.794322	0.005200867	0.0090339	UP
KLF3-AS1	43.51894	0.502849	0.179962	2.794198	0.005202866	0.0090368	NOT
CELSR2	515.3248	0.476973	0.170718	2.793929	0.005207187	0.0090438	NOT
FAM131A	374.8298	0.296781	0.106249	2.793264	0.005217904	0.0090618	NOT
LRRC37A7	219.656	-0.85191	0.305058	-2.79261	0.005228489	0.0090796	DOWN
WEE2	2.519575	0.751809	0.26923	2.792447	0.005231099	0.0090836	UP
ATE1	1379.993	-0.23506	0.084186	-2.79211	0.00523656	0.0090925	NOT
CH17-125	1.812654	2.146397	0.768945	2.791352	0.005248838	0.0091132	UP
SH3GL1	2741.36	0.26568	0.095183	2.791258	0.005250355	0.0091153	NOT
RP11-449	124.1915	0.665616	0.238467	2.791233	0.005250765	0.0091154	UP
AC009245	12.21256	0.494111	0.177071	2.790465	0.005263246	0.0091365	NOT
ABHD11-A	5.034957	0.934298	0.334864	2.79008	0.005269506	0.0091468	UP
MAN2B1	2935.2	0.25663	0.091984	2.789938	0.005271819	0.0091503	NOT
ZNF18	309.705	-0.25518	0.091467	-2.78982	0.005273794	0.0091526	NOT
CTD-2562	1.217967	1.604483	0.575122	2.789814	0.00527383	0.0091526	UP
CTC-510F	1.87566	0.763187	0.273577	2.789659	0.005276354	0.0091564	UP
PSMA3-AS	1062.936	0.273183	0.097932	2.789522	0.005278586	0.0091597	NOT
KLK15	1.141794	2.298984	0.824309	2.788984	0.005287374	0.0091744	UP
NRP2	747.9275	-0.41536	0.148937	-2.78881	0.005290267	0.0091788	NOT
RP11-70K	1.06247	1.550335	0.55595	2.788621	0.005293302	0.0091835	UP
NUP160	1340.655	0.220362	0.079024	2.788533	0.005294728	0.0091854	NOT
LINC0112	10.02033	0.882298	0.316482	2.787833	0.005306184	0.0092047	UP
NMU	2.310303	1.273145	0.456792	2.787142	0.005317512	0.0092238	UP
SH3GLB1	2642.284	-0.21065	0.075581	-2.78705	0.005319034	0.0092259	NOT
CD1C	68.49303	-0.63332	0.227298	-2.78631	0.005331168	0.0092463	DOWN
DDX54	3114.993	0.178874	0.064217	2.785469	0.005345041	0.0092694	NOT
TP73-AS1	488.2717	0.254452	0.09135	2.785464	0.005345113	0.0092694	NOT
CALR	59227.8	0.290208	0.104222	2.784506	0.005360941	0.0092962	NOT
PLEKHM3	96.7111	-0.39015	0.140148	-2.78388	0.005371363	0.0093137	NOT
HOMER2	2547.54	-0.51477	0.184919	-2.78374	0.005373561	0.0093169	NOT
PRR33	15.20389	0.74074	0.266103	2.783661	0.005374925	0.0093187	UP
TSC22D1	7036.582	-0.49406	0.177497	-2.78346	0.005378271	0.0093239	NOT
RP11-776	1.207704	-1.71661	0.616743	-2.78334	0.005380223	0.0093267	DOWN
BTG1	8665.002	-0.35754	0.128487	-2.78273	0.005390381	0.0093438	NOT
AC010976	26.25179	0.468196	0.168253	2.78269	0.005391035	0.0093443	NOT
UBE3D	87.75429	0.331628	0.119197	2.782181	0.005399491	0.0093583	NOT

WHAMML	72.96109	-0.31699	0.113937	-2.78216	0.005399759	0.0093583	NOT
AP000580.	7.840004	0.505836	0.181846	2.781679	0.005407853	0.0093717	NOT
MEST	1799.731	-0.40983	0.147356	-2.7812	0.005415909	0.0093851	NOT
RN7SL8P	2.267008	1.634339	0.587735	2.780742	0.005423483	0.0093976	UP
RP11-274I	9.156728	0.692889	0.249188	2.780586	0.005426084	0.0094015	UP
CNTF	13.30606	0.499132	0.179541	2.780044	0.005435145	0.0094166	NOT
RGS3	2244.711	-0.33759	0.121438	-2.77991	0.005437376	0.0094199	NOT
C17orf89	1195.25	0.442242	0.159114	2.779395	0.005446028	0.0094343	NOT
RP11-426(	15.16994	0.726635	0.261449	2.779259	0.0054483	0.0094372	UP
CD79A	93.93301	-0.84514	0.30409	-2.77925	0.005448407	0.0094372	DOWN
HABP4	622.0711	0.405558	0.145926	2.779209	0.005449147	0.0094379	NOT
XXbac-BP(	80.30435	0.4566	0.164315	2.778805	0.005455922	0.0094491	NOT
PIGCP1	33.37466	0.460955	0.165899	2.778532	0.005460507	0.0094564	NOT
CTD-2540	82.87184	0.649937	0.233918	2.778477	0.005461438	0.0094574	UP
IGHJ2	3.440789	-1.32306	0.476242	-2.77812	0.005467523	0.0094666	DOWN
SLC19A1	1677.803	0.459221	0.1653	2.778105	0.005467691	0.0094666	NOT
RP11-95P:	1.045514	2.105862	0.758022	2.778102	0.00546775	0.0094666	UP
GFRA1	2033.403	-0.85158	0.306562	-2.77783	0.005472369	0.009474	DOWN
ACTR1A	3659.33	0.197177	0.071	2.777128	0.00548415	0.0094938	NOT
RP11-124I	12.38307	-0.48197	0.173562	-2.77693	0.005487522	0.009499	NOT
RAB2B	492.8901	-0.20276	0.073038	-2.77605	0.005502396	0.0095242	NOT
KCNK15-A	2.183832	1.370207	0.493593	2.775984	0.005503499	0.0095255	UP
FMO9P	1.05068	2.314314	0.833739	2.775827	0.005506143	0.0095295	UP
NAP1L2	95.69084	0.850821	0.306516	2.775776	0.00550701	0.0095304	UP
RP11-708.	1.514841	0.928795	0.334625	2.77563	0.005509479	0.0095341	UP
CCDC42B	5.152468	0.547296	0.197182	2.775583	0.005510291	0.0095349	NOT
GAPDHP7:	1.706522	0.983544	0.354403	2.775215	0.005516515	0.0095451	UP
HIST1H2A	1.999153	1.102475	0.39733	2.774712	0.005525057	0.0095592	UP
GS1-124K!	147.7221	0.454616	0.163853	2.774538	0.00552801	0.0095637	NOT
ZNF430	95.05393	0.507248	0.182829	2.774446	0.005529586	0.0095659	NOT
KLHDC4	929.5725	0.271131	0.097728	2.774356	0.005531113	0.0095679	NOT
YARS2	501.6261	0.197045	0.07103	2.774116	0.005535197	0.0095744	NOT
NPHP3-AS	2.078629	-0.76669	0.276406	-2.77379	0.005540828	0.0095835	DOWN
DDX56	3038.115	0.238331	0.085925	2.773724	0.005541861	0.0095847	NOT
GLTP	1037.957	0.207561	0.074836	2.773539	0.005545014	0.0095896	NOT
ATP7B	1231.159	-0.4417	0.159263	-2.7734	0.005547373	0.009593	NOT
GS1-259H	1.797123	1.168533	0.42141	2.77291	0.005555748	0.0096069	UP
TNFSF8	44.68105	-0.58863	0.212284	-2.77282	0.005557339	0.0096087	DOWN
SPRR1A	2.266034	1.866198	0.673035	2.77281	0.00555746	0.0096087	UP
TMEM110	411.1502	-0.38286	0.138091	-2.77256	0.005561766	0.0096155	NOT
RP11-522I	1.479773	-0.9263	0.334123	-2.77233	0.005565732	0.0096218	DOWN
RP13-401I	14.73825	0.652233	0.235299	2.771934	0.005572437	0.0096328	UP
ZNF816	97.13282	0.551468	0.198951	2.77188	0.005573358	0.0096338	NOT
KRT18	32207.14	-0.41528	0.149823	-2.77178	0.005575153	0.0096363	NOT
SHFM1	5240.325	0.329017	0.118718	2.771426	0.005581134	0.009646	NOT
RGS11	28.57976	-0.61232	0.220965	-2.77111	0.005586571	0.0096548	DOWN
AHRR	26.83654	0.533222	0.192437	2.770891	0.005590306	0.0096606	NOT
SEC22A	527.1938	0.201218	0.072628	2.770531	0.0055965	0.0096707	NOT
CTB-131K:	211.3274	0.285067	0.103004	2.767533	0.005648237	0.0097595	NOT
LINC0029E	2.702189	1.039185	0.375501	2.767467	0.005649376	0.0097609	UP
SLC35F5	1521.791	-0.22995	0.083106	-2.76697	0.005658063	0.0097753	NOT
AC017060	7.611636	0.846607	0.306032	2.766403	0.005667843	0.0097916	UP

BTD	3086.51	-0.41161	0.148794	-2.76631	0.005669547	0.0097939	NOT
RP11-614I	1.469297	2.435367	0.880427	2.766121	0.005672744	0.0097988	UP
CTRB2	1.174173	1.474571	0.533126	2.765894	0.005676691	0.009805	UP
FOXP4-AS	16.70249	0.771569	0.278963	2.765853	0.005677418	0.0098057	UP
SUB1P1	1.89178	0.786681	0.284478	2.765347	0.005686234	0.0098203	UP
MUC19	2.220367	1.342425	0.485454	2.765299	0.005687063	0.0098211	UP
ZNF407	428.2978	0.211181	0.07637	2.765236	0.005688161	0.0098224	NOT
TXNRD3	439.0168	-0.25057	0.090639	-2.7645	0.005701053	0.009844	NOT
AC093323	507.3774	0.26919	0.097379	2.764339	0.005703821	0.0098482	NOT
PRKCH	467.5734	-0.40253	0.145617	-2.76429	0.005704736	0.0098492	NOT
AP000350.	2.353215	0.878363	0.317797	2.763911	0.005711305	0.0098599	UP
HM13-IT1	6.371428	0.494574	0.178972	2.763412	0.005720056	0.0098744	NOT
CCNYL2	1.386195	2.139702	0.774335	2.763276	0.005722429	0.0098779	UP
CRIM1	2571.958	0.597016	0.216088	2.762835	0.005730167	0.0098906	UP
AVEN	559.1789	-0.27146	0.098271	-2.76241	0.005737692	0.009903	NOT
RP11-140I	155.3719	0.440715	0.159542	2.762381	0.005738139	0.0099031	NOT
OR5H8	1.128513	2.433946	0.881249	2.761928	0.005746122	0.0099163	UP
HMGN2	4796.142	0.27171	0.098385	2.761705	0.00575004	0.0099224	NOT
DLAT	1524.915	0.324021	0.117347	2.761211	0.005758741	0.0099368	NOT
AEN	1149.493	0.314372	0.113886	2.760402	0.005773026	0.0099609	NOT
RP3-326I1	0.994634	2.329671	0.844029	2.760177	0.005776999	0.0099671	UP
GPR133	776.1121	0.847206	0.306941	2.760158	0.005777343	0.0099671	UP
CHRDL2	342.033	0.693759	0.25136	2.760022	0.005779755	0.0099706	UP
PLD6	185.9641	0.35516	0.128681	2.759998	0.005780179	0.0099707	NOT
SIGLEC17F	12.99723	-0.60558	0.219417	-2.75995	0.005781108	0.0099717	DOWN
RTN3P1	2.441847	0.740043	0.268146	2.759852	0.005782749	0.0099739	UP
RP11-400I	10.33194	0.576064	0.208748	2.759614	0.005786965	0.0099805	NOT
DACT1	152.7951	-0.58284	0.211229	-2.75929	0.005792706	0.0099898	NOT
TRAP1	3760.766	-0.30634	0.111027	-2.75914	0.005795423	0.0099939	NOT
BRF1	1076.949	0.194331	0.070437	2.758939	0.005798929	0.0099993	NOT
SEL1L	7628.496	-0.30541	0.110711	-2.7586	0.005804894	0.010009	NOT
KRR1	1056.851	-0.18412	0.066744	-2.75857	0.005805533	0.0100095	NOT
HLA-DOA	585.2712	-0.61486	0.222895	-2.75851	0.005806589	0.0100106	DOWN
DHX15	2645.96	0.203134	0.073648	2.758165	0.00581268	0.0100205	NOT
AC079807	2.038469	0.89868	0.325849	2.757967	0.005816201	0.010026	UP
IGSF8	4888.419	0.389912	0.14138	2.7579	0.005817399	0.0100274	NOT
COPG1	8473.132	0.176228	0.063902	2.757776	0.005819614	0.0100306	NOT
DSG4	4.184857	1.107073	0.401459	2.757625	0.005822293	0.0100346	UP
ZBED3	1460.005	0.394271	0.14298	2.757529	0.005824001	0.0100369	NOT
CBL	531.6336	0.333873	0.121088	2.757275	0.005828531	0.0100441	NOT
HMGN2P4	3.484424	0.579144	0.210054	2.757121	0.005831283	0.0100482	NOT
ATXN1L	1103.503	0.279757	0.101472	2.756996	0.0058335	0.0100514	NOT
LUZP4	1.194329	2.70884	0.982566	2.756905	0.005835128	0.0100536	UP
WDR78	61.37387	-0.44541	0.161576	-2.75666	0.005839435	0.0100604	NOT
GRIP1	18.06403	0.90956	0.330152	2.754978	0.005869615	0.0101117	UP
RP11-267J	11.54758	0.401563	0.145762	2.754925	0.005870562	0.0101127	NOT
RRP1B	1450.246	0.211334	0.076714	2.754829	0.005872274	0.0101151	NOT
RP11-332I	17.53089	-0.54098	0.196384	-2.75473	0.005873978	0.0101174	NOT
ALX1	1.571931	3.04257	1.104676	2.754266	0.005882389	0.0101312	UP
RP11-511I	7.540192	-0.7131	0.258913	-2.75422	0.00588323	0.010132	DOWN
DFFA	1466.829	0.205116	0.074476	2.754132	0.005884807	0.0101341	NOT
VIP	13.42636	-0.53475	0.194177	-2.75393	0.00588851	0.0101399	NOT

CTD-2527	18.98178	-0.76919	0.279339	-2.75359	0.005894475	0.0101495	DOWN
RP3-522D	5.417615	-0.7212	0.26192	-2.75352	0.005895815	0.0101512	DOWN
RP11-461I	1.598124	2.225275	0.808164	2.753493	0.005896306	0.0101514	UP
SEC11C	4757.696	0.373137	0.135519	2.75339	0.005898159	0.010154	NOT
HIST1H1T	1.028678	1.419474	0.515558	2.753278	0.005900173	0.0101568	UP
DLD	4055.536	-0.2697	0.097964	-2.75299	0.005905337	0.0101651	NOT
RP11-287I	1.653404	0.818162	0.297197	2.752924	0.005906553	0.0101665	UP
CNTN5	9.189242	1.122477	0.407778	2.752664	0.00591126	0.010174	UP
GLYATL1P4	8.999515	1.101546	0.400177	2.752644	0.005911614	0.010174	UP
SNRPD3	3925.61	0.257893	0.0937	2.75233	0.00591728	0.0101831	NOT
CHCHD7	967.94	0.312909	0.113691	2.752268	0.005918401	0.0101844	NOT
PLIN3	1672.846	0.291127	0.105788	2.75199	0.005923434	0.0101924	NOT
RP11-153I	17.19583	-0.79903	0.290358	-2.75189	0.005925296	0.010195	DOWN
RP11-274I	179.4342	0.311327	0.113154	2.751349	0.00593504	0.0102106	NOT
ARAP1-AS	1.386727	2.21819	0.80622	2.751345	0.005935115	0.0102106	UP
CTD-3126	2.786231	0.825277	0.299961	2.751278	0.005936329	0.0102121	UP
NCBP1	1212.312	-0.18397	0.066868	-2.75116	0.005938454	0.0102151	NOT
RPL6	20656.39	0.287993	0.104682	2.751135	0.005938923	0.0102152	NOT
RP11-274I	109.7919	0.464661	0.1689	2.751099	0.005939572	0.0102157	NOT
IGKV3D-1	10.74143	-1.24368	0.452073	-2.75107	0.005940148	0.0102161	DOWN
CTC-448F	18.79063	0.364157	0.132374	2.750974	0.00594183	0.0102183	NOT
RPL13AP6	6.440952	0.621759	0.226051	2.75052	0.005950076	0.0102319	UP
RGS2	913.3696	-0.6377	0.231868	-2.75026	0.005954865	0.0102395	DOWN
RP11-545I	2.931596	0.846994	0.307992	2.750052	0.00595858	0.0102452	UP
RP11-649A	2.266031	0.787545	0.28639	2.749904	0.005961274	0.0102492	UP
RP11-996I	7.256357	0.734789	0.267218	2.74977	0.005963703	0.0102528	UP
CEACAM6	46.30837	-1.45948	0.530854	-2.7493	0.005972333	0.010267	DOWN
RP11-452I	1.420371	-1.02161	0.371824	-2.74757	0.006003784	0.0103198	DOWN
TBC1D2	716.6784	-0.34722	0.126375	-2.74757	0.006003827	0.0103198	NOT
RP11-109I	1.606039	3.129412	1.139004	2.747499	0.006005171	0.0103215	UP
LDHAL6A	2.672228	0.810011	0.294839	2.747298	0.006008851	0.0103272	UP
PSMB6	4085.061	-0.29527	0.107485	-2.74713	0.006012007	0.0103319	NOT
RPL41P1	478.9427	-0.36182	0.13171	-2.74711	0.006012327	0.0103319	NOT
ZRANB1	843.9105	-0.40972	0.149147	-2.74707	0.006013054	0.0103325	NOT
SLC33A1	2463.695	0.231503	0.084289	2.74655	0.006022573	0.0103482	NOT
IGHV3-38	1.911787	-1.23895	0.451104	-2.74648	0.006023844	0.0103497	DOWN
RPP25	402.8318	0.589064	0.214503	2.746178	0.006029397	0.0103586	UP
RP11-335I	4.534582	-0.54697	0.199223	-2.74552	0.006041466	0.0103787	NOT
GEN1	510.1969	0.316838	0.115431	2.744828	0.006054255	0.0104	NOT
RP5-963E	10.16056	0.489689	0.17841	2.744737	0.006055942	0.0104018	NOT
RP11-38H	4.509033	-1.07447	0.391467	-2.74473	0.006056039	0.0104018	DOWN
DNPH1	3035.241	0.340885	0.124201	2.744625	0.006058001	0.0104045	NOT
KANK2	3430.037	0.306732	0.111763	2.744479	0.006060701	0.0104085	NOT
AC005775	4.572993	0.692241	0.252249	2.74427	0.00606456	0.0104145	UP
NOSIP	2019.219	0.37923	0.138232	2.743431	0.006080086	0.0104405	NOT
AC004449	3.81921	0.809376	0.295028	2.743389	0.006080851	0.0104412	UP
ALOX5	383.6279	0.694149	0.253037	2.74327	0.006083066	0.0104443	UP
RIBC1	28.33112	0.332255	0.121131	2.74293	0.006089365	0.0104545	NOT
FCGR2A	523.3269	0.478949	0.174631	2.742628	0.00609497	0.0104635	NOT
AC013444	1.360963	1.597147	0.582349	2.742596	0.006095565	0.0104639	UP
LRP3	2500.722	-0.36086	0.131591	-2.74233	0.00610059	0.0104718	NOT
STKLD1	45.009	0.366835	0.133781	2.742049	0.006105716	0.01048	NOT

SLC37A1	497.0636	0.404489	0.147526	2.741821	0.006109972	0.0104866	NOT
FAM188B	63.59671	0.481815	0.175744	2.741566	0.0061147	0.0104941	NOT
C16orf71	38.33656	0.411776	0.150213	2.74128	0.006120036	0.0105026	NOT
N4BP1	1188.359	-0.16859	0.061502	-2.74125	0.00612063	0.010503	NOT
GMNC	118.6889	-0.78643	0.286911	-2.74102	0.006124816	0.0105095	DOWN
PCSK7	580.4093	0.292359	0.106661	2.741005	0.006125165	0.0105095	NOT
TXLNB	36.66891	0.701825	0.256061	2.740848	0.006128085	0.0105138	UP
TXNP4	5.914425	0.738487	0.269459	2.74063	0.006132153	0.0105201	UP
RP11-114(	7.352009	1.169165	0.426632	2.740455	0.006135417	0.0105251	UP
ONECUT1	509.3503	0.557619	0.203482	2.740389	0.00613665	0.0105265	NOT
CTC-492K	1.706452	0.954039	0.34821	2.739842	0.00614687	0.0105434	UP
LA16c-313	6.217094	0.521062	0.190222	2.739231	0.006158302	0.0105624	NOT
ZNF845	143.2273	0.434635	0.158672	2.739207	0.006158762	0.0105625	NOT
SNX21	428.6585	0.240681	0.087866	2.739188	0.006159117	0.0105625	NOT
RNU4ATA(	1.864092	0.771027	0.281506	2.738936	0.006163838	0.0105699	UP
RP11-112I	14.4826	0.567144	0.20709	2.738644	0.006169314	0.0105785	NOT
KIAA0141	2721.434	-0.21061	0.076902	-2.73863	0.006169615	0.0105785	NOT
ZNF787	2221.635	0.337554	0.123264	2.738471	0.006172563	0.0105829	NOT
MIATNB	48.21282	0.356813	0.13033	2.737757	0.006185984	0.0106053	NOT
SRSF4	2678.815	-0.1642	0.059982	-2.7375	0.006190766	0.0106128	NOT
RP5-965F(	1.744165	-0.97832	0.357389	-2.73741	0.006192513	0.0106151	DOWN
AREG	70.17837	-0.82438	0.301232	-2.73669	0.006206079	0.0106377	DOWN
RAPGEF1	2635.53	0.220072	0.080426	2.736333	0.006212809	0.0106486	NOT
TTC8	426.1828	-0.30788	0.112518	-2.7363	0.006213522	0.0106492	NOT
FBP2	5.461575	0.733171	0.26796	2.736118	0.006216868	0.0106542	UP
LBH	1243.936	0.479861	0.175388	2.736004	0.006219024	0.0106573	NOT
NEFM	1.555526	1.46551	0.535686	2.735765	0.006223541	0.0106644	UP
CATSPERG	68.98094	-0.44104	0.161215	-2.73572	0.006224389	0.0106651	NOT
LINC00943	4.065251	0.92501	0.338179	2.735263	0.006233047	0.0106793	UP
CTD-3222	2.282062	0.863737	0.315792	2.73514	0.006235371	0.0106826	UP
CDK19	663.2204	0.315792	0.115463	2.735005	0.006237925	0.0106864	NOT
CCDC110	22.88127	0.638881	0.233598	2.73496	0.006238787	0.0106872	UP
MORF4L2	7668.484	0.20217	0.073928	2.7347	0.006243708	0.0106949	NOT
OXER1	1260.508	-0.55209	0.201898	-2.73452	0.006247068	0.0107	NOT
ANXA8	29.20989	-1.0028	0.366744	-2.73433	0.006250817	0.0107058	DOWN
RAC1	8857.509	0.219169	0.080166	2.733936	0.006258213	0.0107178	NOT
AC006129	3.413052	-0.83822	0.306634	-2.73363	0.006264041	0.0107271	DOWN
CFL1P2	1.760055	0.708698	0.259257	2.733569	0.006265194	0.0107284	UP
CFHR4	2441.106	-0.90014	0.329322	-2.73331	0.006270182	0.0107363	DOWN
PIM2	658.9165	-0.36019	0.131786	-2.73317	0.006272747	0.01074	NOT
RP11-941I	3.98648	0.66461	0.243196	2.732815	0.006279552	0.010751	UP
CCBL1	631.7224	0.314939	0.115267	2.732261	0.006290126	0.0107684	NOT
LPPR3	3.75327	1.127315	0.412612	2.732143	0.00629239	0.0107717	UP
ZC3HC1	789.7391	0.202876	0.074259	2.732009	0.006294945	0.0107754	NOT
FUT10	132.9307	-0.34438	0.126057	-2.73191	0.006296803	0.0107778	NOT
PCDHB17I	2.001742	1.099157	0.402342	2.731894	0.006297147	0.0107778	UP
RP11-321/	31.13767	0.50064	0.183275	2.731627	0.006302253	0.0107859	NOT
HNRNPL	5771.472	0.147048	0.053841	2.731145	0.00631147	0.010801	NOT
AC005077	277.0517	-0.73855	0.270448	-2.73083	0.006317476	0.0108106	DOWN
SUSD2	429.4413	0.624	0.228536	2.730424	0.006325282	0.0108233	UP
GDPD5	440.421	0.3604	0.132001	2.730276	0.006328128	0.0108275	NOT
RP11-709I	1.49854	0.908975	0.33302	2.729491	0.006343221	0.0108526	UP

SMIM24	1192.899	-1.15612	0.423746	-2.72834	0.006365477	0.01089	DOWN
PPP2R3C	503.8285	0.210082	0.077004	2.728215	0.00636781	0.0108934	NOT
POLR2L	4781.974	0.373576	0.13694	2.728027	0.006371438	0.0108989	NOT
AC007952	2.74886	1.333699	0.488931	2.727786	0.006376105	0.0109062	UP
MIR4482	11.903	-0.75607	0.277234	-2.72721	0.006387216	0.0109245	DOWN
RP11-927I	3.848605	0.756881	0.277542	2.727082	0.006389711	0.0109281	UP
KCTD8	1.529502	2.041305	0.748551	2.727009	0.006391129	0.0109299	UP
RP11-135/	5.958837	-0.75641	0.277393	-2.72686	0.006393987	0.0109341	DOWN
KCNK7	11.72701	0.511356	0.187528	2.726828	0.006394628	0.0109345	NOT
RP1-34L1(	1.282566	1.135271	0.416337	2.726807	0.006395049	0.0109345	UP
RP11-567(	3.113708	0.640968	0.235093	2.726442	0.006402128	0.010946	UP
RP11-467I	2.925287	0.697533	0.255844	2.726403	0.006402878	0.0109466	UP
RP11-864J	3.580228	0.568748	0.208637	2.726015	0.006410411	0.0109588	NOT
KPNA3	1574.819	-0.24304	0.08917	-2.7256	0.006418482	0.0109719	NOT
COL6A4P2	5.485308	0.76576	0.280956	2.725548	0.006419491	0.0109729	UP
AC016745	3.374741	1.015198	0.3725	2.725368	0.006422986	0.0109782	UP
RP11-247/	2.415562	1.070503	0.39281	2.725241	0.006425465	0.0109818	UP
VN1R108P	4.005187	0.602	0.220907	2.725134	0.006427538	0.0109847	UP
TTC37	2355.364	0.268143	0.098398	2.725094	0.00642832	0.0109853	NOT
CH17-353	2.35876	0.994561	0.364968	2.725064	0.006428899	0.0109856	UP
MYSM1	552.61	0.338013	0.124046	2.724905	0.006432003	0.0109901	NOT
AC139452	2.879265	0.747181	0.274206	2.724888	0.006432329	0.0109901	UP
EEF1GP5	4.130567	0.612534	0.224809	2.72469	0.006436196	0.0109961	UP
AC009950	24.82664	-0.48843	0.179305	-2.72403	0.006449137	0.0110175	NOT
RP11-375I	4.303778	-0.668	0.245279	-2.72343	0.006460815	0.0110368	DOWN
C2orf70	5.060337	1.374107	0.504629	2.723003	0.006469154	0.01105	UP
NEK1	335.1654	-0.28366	0.104171	-2.72299	0.006469359	0.01105	NOT
SLC6A13	504.7653	-0.73469	0.269854	-2.72253	0.006478407	0.0110648	DOWN
SLC7A7	509.6644	-0.49672	0.182461	-2.72233	0.006482258	0.0110707	NOT
NOMO2	323.5633	0.421396	0.154808	2.722063	0.006487583	0.0110785	NOT
NHLH2	0.884681	1.832064	0.673043	2.722059	0.00648765	0.0110785	UP
RP11-750I	247.9403	0.810601	0.29784	2.7216	0.00649667	0.0110932	UP
DES	74.41252	-0.91897	0.337689	-2.72136	0.00650141	0.0111006	DOWN
RP11-332I	211.5258	-0.28146	0.103431	-2.72119	0.006504711	0.0111056	NOT
RP11-461/	10.83802	0.531107	0.195186	2.721035	0.006507795	0.0111102	NOT
EFNB2	675.422	0.366042	0.134526	2.720984	0.00650879	0.0111109	NOT
GPRASP2	433.8208	-0.38687	0.142182	-2.72097	0.006509034	0.0111109	NOT
JTB	7547.99	0.254077	0.093384	2.720783	0.006512743	0.0111165	NOT
EPHA6	8.173473	1.39027	0.511084	2.72024	0.00652345	0.0111341	UP
SLC51A	3950.577	-0.69697	0.256249	-2.7199	0.006530156	0.0111449	DOWN
AP000350.	26.10187	0.972592	0.357831	2.718021	0.006567367	0.0112077	UP
ZNF304	255.9813	0.319761	0.117651	2.717877	0.006570232	0.0112119	NOT
SGTA	3427.81	0.214185	0.078808	2.717818	0.006571396	0.0112132	NOT
SLC9A7P1	17.25629	0.738941	0.27192	2.717495	0.006577805	0.0112234	UP
RP11-60L3	0.945676	1.7153	0.631237	2.717363	0.006580436	0.0112272	UP
FAM175B	729.429	-0.16172	0.059513	-2.71733	0.006581104	0.0112277	NOT
RP11-98J2	1.984374	0.954307	0.351202	2.717263	0.006582426	0.0112293	UP
RP5-997D	57.38527	-0.31154	0.114652	-2.71723	0.006583095	0.0112297	NOT
RP11-355I	87.95874	-0.34584	0.127278	-2.71719	0.006583874	0.0112303	NOT
SETD1B	1245.707	0.196335	0.072265	2.716887	0.006589907	0.0112399	NOT
RYR1	59.70182	0.661198	0.243398	2.716533	0.006596962	0.0112513	UP
SPG21	4225.29	-0.18817	0.069273	-2.71636	0.006600374	0.0112564	NOT

SNORD63	4.90677	0.58698	0.216107	2.716159	0.006604417	0.0112626	UP
RP11-626I	2.71699	-0.68363	0.251728	-2.71575	0.006612651	0.0112759	DOWN
SEMA6A-/	15.62389	0.458738	0.168932	2.71552	0.006617172	0.011283	NOT
LINC0099	19.67865	1.040744	0.383266	2.715462	0.00661833	0.0112842	UP
WWP2	1795.322	0.218955	0.080638	2.715285	0.006621873	0.0112894	NOT
RAI2	206.3	-0.54046	0.199043	-2.71527	0.006622148	0.0112894	NOT
MIR4740	2.237422	0.761919	0.280617	2.715156	0.006624458	0.0112926	UP
RP11-390I	18.36957	0.441276	0.162556	2.714608	0.006635429	0.0113106	NOT
FMO8P	1.098498	2.170455	0.799633	2.714314	0.006641313	0.0113194	UP
IDO1	142.1667	0.763077	0.281131	2.71431	0.006641403	0.0113194	UP
GK-AS1	16.75299	-0.66897	0.24647	-2.71423	0.006643061	0.0113215	DOWN
CACNA1C	3.239388	-0.70938	0.261379	-2.71399	0.006647861	0.011329	DOWN
LINC0084	3.69864	0.616498	0.22718	2.713693	0.006653776	0.0113379	UP
RP13-539.	1.333337	1.957512	0.721348	2.713686	0.006653927	0.0113379	UP
SYT11	230.3312	0.426199	0.157062	2.713568	0.006656287	0.0113413	NOT
CTD-2095	3.874616	0.611302	0.225313	2.71312	0.006665306	0.0113559	UP
XAB2	2343.285	0.246725	0.09094	2.713049	0.006666715	0.0113576	NOT
KCND1	55.48049	0.471371	0.173746	2.712989	0.006667936	0.011359	NOT
RP11-244I	22.90522	0.890574	0.328266	2.712963	0.006668447	0.0113592	UP
FBXW12	5.417155	0.78957	0.291088	2.712483	0.006678129	0.011375	UP
AC138472	0.905678	1.421211	0.524009	2.71219	0.006684036	0.0113843	UP
LHFP	697.9421	0.488638	0.180165	2.712163	0.006684573	0.0113846	NOT
RP6-114E	1.015736	2.348635	0.86602	2.711986	0.006688139	0.0113899	UP
KDM4D	26.09315	0.403788	0.148901	2.711791	0.006692079	0.0113959	NOT
COPS8	1250.441	0.163648	0.060348	2.71176	0.006692704	0.0113963	NOT
CYP4F26P	2.44129	1.264499	0.466324	2.711633	0.006695266	0.0114	UP
RP11-11N	39.78592	0.780677	0.287909	2.711543	0.006697091	0.0114024	UP
RP11-254I	99.49632	-0.77526	0.285921	-2.71146	0.006698827	0.0114046	DOWN
ASUN	715.1766	0.224748	0.082918	2.710489	0.006718403	0.0114372	NOT
RP11-118I	1.505949	0.89296	0.329515	2.709918	0.006729976	0.0114562	UP
RP11-342I	60.30881	-0.37002	0.136566	-2.70948	0.006738876	0.0114707	NOT
ZNF608	267.3833	0.383106	0.141399	2.709405	0.006740408	0.0114726	NOT
SLFNL1-A	41.37496	0.548386	0.202403	2.709379	0.006740938	0.0114728	NOT
SNRPG	1337.859	0.245352	0.090562	2.709215	0.006744264	0.0114777	NOT
TMC8	408.4798	-0.52056	0.192144	-2.7092	0.006744644	0.0114777	NOT
ALCAM	4572.067	0.352134	0.129987	2.709001	0.006748619	0.0114837	NOT
RP4-737E	2.52689	1.252776	0.462526	2.70855	0.006757791	0.0114986	UP
EEF1B2P2	3.918056	0.594822	0.219618	2.708438	0.006760074	0.0115018	UP
C10orf90	14.18356	1.298729	0.479523	2.708375	0.006761365	0.0115033	UP
ZBTB33	1691.096	0.318746	0.117697	2.708196	0.006765008	0.0115088	NOT
SGCD	130.5174	-0.72334	0.267113	-2.70798	0.006769497	0.0115157	DOWN
CEP44	353.1371	0.24143	0.089158	2.707885	0.006771343	0.0115182	NOT
GPR143	113.4446	0.71895	0.265505	2.707865	0.006771765	0.0115182	UP
HACE1	177.8928	0.354235	0.130824	2.707721	0.006774699	0.0115224	NOT
C7orf26	943.0715	0.19933	0.073628	2.707262	0.006784077	0.0115377	NOT
XXbac-BP	1.810361	1.400067	0.517249	2.706758	0.006794376	0.0115545	UP
CMAS	2011.962	-0.25748	0.095159	-2.70573	0.006815426	0.0115896	NOT
IGHV4-31	68.74779	-1.21245	0.448236	-2.70494	0.006831705	0.0116165	DOWN
LRRC15	39.92209	0.909316	0.336172	2.704913	0.006832236	0.0116167	UP
TNP1	1.184423	2.432486	0.899304	2.704854	0.006833434	0.0116176	UP
SH2D1A	66.79766	-0.68067	0.25165	-2.70485	0.006833586	0.0116176	DOWN
PAM	1141.743	0.459322	0.169825	2.704669	0.00683725	0.0116231	NOT

RP11-724I	7.568798	0.75149	0.277857	2.704589	0.00683889	0.0116252	UP
CASC18	2.36125	0.996561	0.368477	2.704543	0.006839842	0.0116261	UP
RP5-874C:	1.387057	1.032061	0.381628	2.704364	0.006843526	0.0116316	UP
RNF170	953.1281	-0.29156	0.107826	-2.70396	0.006851945	0.0116452	NOT
UQCRC1	13424.81	-0.2879	0.106477	-2.70388	0.006853437	0.0116471	NOT
PANX3	0.905315	2.068216	0.764988	2.703592	0.006859444	0.0116565	UP
RP11-302I	16.36303	-0.64493	0.238567	-2.70334	0.006864597	0.0116646	DOWN
TBC1D14	558.1643	0.430851	0.159389	2.703139	0.006868795	0.011671	NOT
IGHV3-33	74.40635	-1.09118	0.403709	-2.70288	0.006874203	0.0116795	DOWN
CTNND1	8177.122	-0.22263	0.082376	-2.70264	0.006879075	0.011687	NOT
TPTE2P3	1.352986	1.927182	0.713104	2.702527	0.00688146	0.0116904	UP
RP11-881I	18.96501	-0.52622	0.194727	-2.70236	0.006884827	0.0116951	NOT
ZNF254	373.1168	-0.32936	0.121879	-2.70235	0.006885064	0.0116951	NOT
ISCU	3173.591	-0.21174	0.078366	-2.70191	0.006894212	0.0117099	NOT
AP000662.	1.712672	0.761621	0.281945	2.701311	0.006906669	0.0117303	UP
SEH1L	1412.534	-0.20748	0.076813	-2.70111	0.006910825	0.0117367	NOT
AP2A1	4925.147	0.200228	0.07413	2.701038	0.006912345	0.0117385	NOT
NPC2	4683.631	0.342582	0.126849	2.700709	0.006919194	0.0117494	NOT
SLC25A42	4177.918	-0.47374	0.175413	-2.70069	0.006919588	0.0117494	NOT
LINC0027	6.898564	-0.56347	0.208708	-2.69978	0.006938536	0.0117808	NOT
MAN1B1-/	49.5783	0.405594	0.150255	2.699364	0.006947213	0.0117948	NOT
RP11-178I	1.621493	-0.8144	0.30172	-2.69921	0.006950533	0.0117997	DOWN
RP11-252I	1.019171	1.493679	0.553451	2.698846	0.006958034	0.0118117	UP
DNAJB14	771.0558	-0.24268	0.08992	-2.69883	0.006958413	0.0118117	NOT
RP11-85B	3.119421	1.024551	0.379638	2.698756	0.006959909	0.0118135	UP
RP11-535/	13.25647	0.521741	0.193356	2.698349	0.00696844	0.0118272	NOT
SNORD3B-	1.240098	1.165344	0.431892	2.69823	0.006970928	0.0118307	UP
NEDD8	2519.631	0.253935	0.094121	2.697972	0.006976336	0.0118392	NOT
GRIN3A	33.7363	0.376314	0.139497	2.697647	0.00698315	0.01185	NOT
NARFL	1201.473	0.2407	0.089234	2.69741	0.006988122	0.0118577	NOT
RPL30P4	2.503405	0.821205	0.304479	2.697086	0.006994927	0.0118686	UP
RP11-483I	1.431937	1.057272	0.392019	2.69699	0.00699694	0.0118712	UP
ZNF780B	296.8952	0.325432	0.120682	2.696619	0.007004736	0.0118837	NOT
PLTP	1623.8	-0.58131	0.215592	-2.69635	0.007010389	0.0118926	NOT
C6orf195	2.247572	1.099752	0.408061	2.695071	0.007037365	0.0119376	UP
SCARNA24	1.627679	0.761046	0.282426	2.69467	0.007045842	0.0119513	UP
TMEM177	820.7953	-0.27692	0.102772	-2.69447	0.007050105	0.0119578	NOT
FAM13A-/	31.58054	0.427371	0.158663	2.693583	0.007068847	0.0119888	NOT
RNF212	12.39228	-0.77067	0.286119	-2.69352	0.007070153	0.01199	DOWN
ZNF735	1.463487	2.813464	1.044535	2.69351	0.007070406	0.01199	UP
MFSD11	786.1667	0.236495	0.087807	2.693356	0.007073674	0.0119948	NOT
PYGL	7601.645	-0.43868	0.162892	-2.69304	0.00708039	0.0120055	NOT
CCDC37	3.034704	0.62053	0.230468	2.692474	0.007092405	0.0120251	UP
LINC0098	2.741222	0.806287	0.299532	2.691823	0.00710626	0.0120479	UP
OGFOD1	1130.213	-0.1862	0.069174	-2.69176	0.007107645	0.0120491	NOT
CTA-29F1	106.0835	0.390041	0.144902	2.691746	0.007107896	0.0120491	NOT
RP11-350	2.324555	0.76978	0.28598	2.691728	0.007108293	0.0120491	UP
UBXN7	964.6253	0.231604	0.086057	2.691303	0.007117358	0.0120637	NOT
WDR35	301.7473	0.331289	0.123107	2.691057	0.007122603	0.0120719	NOT
CTC-441N	1.002656	2.066044	0.767822	2.690786	0.007128381	0.0120809	UP
RP11-130	5.276865	-0.62373	0.231818	-2.6906	0.007132285	0.0120868	DOWN
SLC25A38	1801.779	-0.26946	0.100154	-2.69048	0.007134933	0.0120905	NOT

USP53	542.1041	-0.40574	0.15082	-2.6902	0.007140924	0.0120999	NOT
ATP5S	695.1722	-0.22041	0.081934	-2.6901	0.007143019	0.0121028	NOT
RP11-150(	5.979085	-0.62878	0.23374	-2.69007	0.007143805	0.0121033	DOWN
PRIMA1	18.39625	-1.2337	0.458809	-2.68892	0.007168305	0.0121434	DOWN
RP5-933K2	7.334798	-0.42753	0.158999	-2.68892	0.007168348	0.0121434	NOT
ANXA6	10922.01	-0.42497	0.158049	-2.68887	0.007169524	0.0121447	NOT
PIK3C3	918.0497	-0.17084	0.063539	-2.68878	0.007171443	0.0121472	NOT
IFNG-AS1	2.389931	-0.99517	0.370158	-2.6885	0.007177468	0.0121567	DOWN
ZNF280B	17.47578	0.837327	0.311467	2.688331	0.007181012	0.0121619	UP
HSPA13	983.0384	0.264144	0.098297	2.687193	0.007205531	0.0122027	NOT
GSPT1	3887.202	-0.17241	0.064167	-2.68695	0.007210787	0.0122108	NOT
CLCA2	3.541823	1.772532	0.659687	2.68693	0.0072112	0.0122108	UP
RP11-1072	3.975058	0.575465	0.214176	2.68688	0.00721228	0.0122119	NOT
HAS2	83.22273	-0.95611	0.355873	-2.68666	0.007217114	0.0122193	DOWN
GALNT9	8.191305	1.059961	0.394613	2.686074	0.007229705	0.0122399	UP
CTD-2132	12.77255	0.539337	0.200803	2.685901	0.007233452	0.0122455	NOT
UBQLN2	1725.337	-0.22405	0.083433	-2.68539	0.007244536	0.0122635	NOT
CD40	815.1648	-0.45661	0.170039	-2.68533	0.007245791	0.0122649	NOT
MAPK15	18.86354	0.793573	0.295528	2.685266	0.0072472	0.0122665	UP
AC012506	2.454496	1.190426	0.443341	2.685127	0.007250225	0.0122709	UP
BLOC1S5	671.2579	0.21765	0.081061	2.685011	0.007252732	0.0122744	NOT
RP11-291I	64.39001	0.399322	0.148724	2.684983	0.007253346	0.0122747	NOT
RP11-148(	3.564788	-1.0163	0.378586	-2.68447	0.007264576	0.0122929	DOWN
CTDSP2	5597.598	0.258845	0.096451	2.683698	0.007281292	0.0123204	NOT
NLN	1843.091	-0.35105	0.130819	-2.68349	0.007285836	0.0123274	NOT
LIPE	93.08454	0.488466	0.182049	2.683159	0.007293026	0.0123388	NOT
ENTHD1	1.036324	1.449544	0.540249	2.683105	0.007294211	0.01234	UP
RP11-478I	1.549866	2.152492	0.80226	2.683034	0.007295764	0.0123419	UP
CTD-2248	15.65156	0.609464	0.227163	2.682937	0.007297863	0.0123447	UP
STX8	839.4759	-0.28884	0.107668	-2.6827	0.007303094	0.0123528	NOT
RP11-643(	2.733387	-0.76538	0.28533	-2.68245	0.00730847	0.0123611	DOWN
RP11-108I	21.93981	0.885678	0.330187	2.682357	0.007310544	0.0123639	UP
TNNC2	14.93662	0.563464	0.210085	2.682078	0.00731665	0.0123735	NOT
SENP6	1709.46	-0.19677	0.073375	-2.68173	0.007324216	0.0123855	NOT
RP11-693J	1.298093	2.357138	0.878978	2.68168	0.007325353	0.0123867	UP
PKHD1L1	22.69894	-0.85339	0.318247	-2.68152	0.007328752	0.0123917	DOWN
RP11-815J	3.544397	0.58207	0.217077	2.681398	0.007331536	0.0123956	NOT
IQCA1	24.74754	0.680779	0.253909	2.681191	0.007336069	0.0124025	UP
UBR1	737.5722	-0.24965	0.093113	-2.68117	0.00733656	0.0124026	NOT
RP11-585I	9.274568	0.51226	0.191087	2.680767	0.007345361	0.0124167	NOT
PTMAP8	1.92579	0.708202	0.264253	2.68002	0.007361784	0.0124437	UP
SLC23A3	85.1573	-0.72416	0.270261	-2.67949	0.007373362	0.0124625	DOWN
CHKB-CPT	30.54804	0.524686	0.195844	2.679097	0.00738211	0.0124763	NOT
RP11-394I	1.741028	1.080302	0.403236	2.679081	0.007382443	0.0124763	UP
TOB2	2031.467	0.257511	0.096131	2.678767	0.007389381	0.0124873	NOT
CCDC73	27.94749	0.449733	0.167931	2.678074	0.007404683	0.0125124	NOT
LRRC74A	1.704271	1.239656	0.462916	2.67793	0.007407873	0.012517	UP
FO538757.	1.176216	1.382089	0.516115	2.677868	0.007409234	0.0125186	UP
MRPL52	1711.632	0.344944	0.128822	2.677686	0.007413263	0.0125246	NOT
SRP72	3206.357	0.158335	0.059137	2.677431	0.007418919	0.0125334	NOT
SUGCT	885.6175	-0.40756	0.152234	-2.67716	0.007424816	0.0125426	NOT
RP11-196(	14.2905	0.354646	0.132472	2.677126	0.007425677	0.0125433	NOT

CYP51A1-	7.550873	0.50652	0.189214	2.676964	0.00742927	0.0125486	NOT
RP11-16L2	2.161408	1.091683	0.407815	2.676907	0.00743053	0.0125499	UP
EYA4	19.78237	1.356872	0.506933	2.676632	0.007436636	0.0125595	UP
FOXCUT	0.867626	1.560055	0.582941	2.67618	0.007446654	0.0125756	UP
RP11-680I	2.981221	1.142505	0.426939	2.67604	0.007449768	0.0125801	UP
RP11-185I	15.42853	0.714128	0.26693	2.675334	0.007465476	0.0126059	UP
AGO3	524.4975	-0.22627	0.084579	-2.6753	0.007466162	0.0126063	NOT
TRAPPC2B	245.6539	0.371563	0.138889	2.675263	0.007467056	0.012607	NOT
FDX1P1	3.217711	-0.53181	0.198849	-2.67444	0.007485365	0.0126371	NOT
AC006126	37.50405	0.502764	0.188014	2.674085	0.007493346	0.0126499	NOT
NUDT16	3115.521	-0.28082	0.10502	-2.67402	0.007494707	0.0126514	NOT
OR7E47P	9.99731	-0.79166	0.296079	-2.67383	0.007499027	0.0126579	DOWN
TMEM171	137.3884	0.655982	0.245415	2.672943	0.007518911	0.0126907	UP
BPHL	2806.558	-0.38033	0.142307	-2.67262	0.007526056	0.012702	NOT
RPS19P1	5.012339	0.642422	0.240384	2.672484	0.007529193	0.0127065	UP
CTD-2568	1.438187	1.119815	0.41902	2.672464	0.007529656	0.0127065	UP
KIF26A	116.7169	-0.50992	0.190847	-2.67186	0.007543278	0.0127287	NOT
RXRA	9966.526	-0.33373	0.124907	-2.6718	0.007544523	0.01273	NOT
TARBP2	1024.495	0.26529	0.099301	2.671574	0.007549634	0.0127379	NOT
INAFM1	427.7214	0.430023	0.160965	2.671527	0.007550693	0.0127389	NOT
CIB1	4948.73	-0.26437	0.098965	-2.67132	0.00755536	0.012746	NOT
GPR88	1190.379	1.079046	0.403956	2.671199	0.007558093	0.0127498	UP
CSRNP2	634.6963	0.271483	0.101634	2.671172	0.007558692	0.0127501	NOT
RP11-163I	3.684321	-1.58132	0.592081	-2.67078	0.007567424	0.012764	DOWN
SMLR1	2046.821	-0.53881	0.20175	-2.67067	0.00757006	0.0127677	NOT
CRYM-AS1	1.922972	0.813038	0.304447	2.670536	0.007573024	0.0127719	UP
AC012456	1.890943	1.073636	0.402105	2.670042	0.007584166	0.0127892	UP
GLG1	4819.074	0.195966	0.073395	2.67004	0.007584213	0.0127892	NOT
LAIR1	505.5407	0.528544	0.197966	2.669866	0.007588145	0.0127951	NOT
LINC01281	1.101994	1.461704	0.547558	2.669498	0.007596469	0.0128083	UP
WNT10B	10.23696	0.734301	0.27509	2.669317	0.007600572	0.0128144	UP
RP11-700I	24.45437	0.735559	0.275643	2.668519	0.007618651	0.0128441	UP
C1orf52	402.1872	0.191122	0.071628	2.668274	0.007624198	0.0128527	NOT
AC093620	3.444835	0.651823	0.244303	2.668092	0.007628335	0.0128589	UP
TALDO1	8815.735	0.354938	0.133032	2.66806	0.007629059	0.0128593	NOT
HDHD1	654.2328	0.334394	0.125371	2.667247	0.007647547	0.0128897	NOT
U2AF1	24.62323	0.402847	0.151039	2.667168	0.007649349	0.012892	NOT
RPL23AP67	1.495471	0.952766	0.35724	2.667016	0.007652796	0.0128967	UP
RP11-384I	2.075681	0.723159	0.271151	2.666996	0.007653261	0.0128967	UP
ENOX2	439.3403	0.284994	0.10686	2.666982	0.007653576	0.0128967	NOT
C9orf139	13.58684	0.53318	0.199923	2.666925	0.007654865	0.0128981	NOT
MEPCE	2605.746	0.211706	0.079393	2.666543	0.007663587	0.012912	NOT
LINC01215	5.797433	-0.76033	0.28518	-2.66616	0.007672397	0.0129261	DOWN
OR2A9P	9.661467	0.641833	0.240736	2.666129	0.007673029	0.0129264	UP
RP11-684I	2.363026	1.022234	0.383435	2.665992	0.007676159	0.0129309	UP
RP11-378J	0.664007	1.796574	0.67398	2.665619	0.007684672	0.0129444	UP
COX20	338.2573	0.294724	0.110574	2.665404	0.007689597	0.0129519	NOT
H3F3AP4	90.24302	0.364715	0.136838	2.665298	0.00769202	0.0129552	NOT
VPS13D	1938.598	-0.25041	0.093963	-2.66496	0.007699751	0.0129669	NOT
RP11-113I	9.760645	-1.50365	0.564234	-2.66494	0.007700251	0.0129669	DOWN
BBS9	282.7155	-0.25221	0.094642	-2.66493	0.007700484	0.0129669	NOT
ARNTL	621.6694	-0.40563	0.15221	-2.66491	0.007700861	0.0129669	NOT

EYA2	101.4344	-0.85375	0.320382	-2.66478	0.007703861	0.0129712	DOWN
IER5L	503.6508	-0.58853	0.220902	-2.66422	0.007716655	0.0129919	DOWN
SRI	1280.355	0.368554	0.138343	2.664054	0.007720519	0.0129977	NOT
RP11-466/	2.554297	0.765103	0.287198	2.664025	0.007721186	0.012998	UP
OS9	16551.75	-0.23679	0.088887	-2.66398	0.007722168	0.0129989	NOT
RP11-696I	1.24629	0.930231	0.349203	2.663869	0.007724771	0.0130024	UP
FAM124A	57.45916	-0.42536	0.15968	-2.66384	0.00772552	0.0130029	NOT
AC093590	1.589719	-0.77097	0.289438	-2.66368	0.007729036	0.013008	DOWN
ZNF426	280.3089	0.428347	0.160812	2.663642	0.007729989	0.0130082	NOT
RP11-528I	3.511139	0.879835	0.330313	2.663637	0.00773009	0.0130082	UP
CTD-2022	1.494263	0.802057	0.301138	2.66342	0.007735074	0.0130158	UP
ZFP36L2	6430.808	-0.34462	0.129402	-2.66319	0.007740357	0.0130239	NOT
AC068831	1.268681	0.967568	0.363334	2.663028	0.007744092	0.0130294	UP
KIF13B	1653.704	-0.31188	0.117123	-2.66286	0.007747979	0.0130352	NOT
BPIFB6	1.262693	2.587598	0.971965	2.662234	0.0077624	0.0130586	UP
RNU6-322	2.70755	0.720662	0.270713	2.66209	0.007765721	0.0130634	UP
LANCL3	4.557908	0.689445	0.25899	2.662051	0.0077666	0.0130641	UP
ZNF341-A	1.805677	0.887145	0.333268	2.661957	0.007768789	0.013067	UP
PREX2	222.8326	0.587354	0.220686	2.661494	0.00777948	0.0130842	UP
RPSAP52	0.792361	1.423478	0.534851	2.661446	0.00778058	0.0130852	UP
LA16c-329	1.79858	0.907035	0.340891	2.660776	0.007796069	0.0131098	UP
NDUFAF5	474.4328	0.215088	0.080837	2.660773	0.007796142	0.0131098	NOT
ABTB2	979.5709	-0.35551	0.133652	-2.65995	0.007815318	0.0131413	NOT
FAM179A	22.67677	0.611341	0.229854	2.659699	0.007821062	0.0131501	UP
RP11-873I	19.99756	0.482121	0.181272	2.659658	0.007821997	0.0131509	NOT
EPHB4	4455.771	0.424425	0.159586	2.659545	0.00782463	0.0131545	NOT
SLC35B4	1220.314	0.29061	0.10928	2.659315	0.007829983	0.0131622	NOT
RP11-530/	2.588281	0.61395	0.230868	2.659306	0.007830171	0.0131622	UP
LIPE-AS1	23.37743	0.615895	0.231625	2.659022	0.007836774	0.0131725	UP
SEBOX	2.475882	-0.85002	0.319722	-2.65864	0.00784576	0.0131868	DOWN
CTA-299D	1.292239	2.413104	0.907858	2.658018	0.007860169	0.0132103	UP
RP11-314I	2.242207	1.960875	0.737758	2.657884	0.007863299	0.0132147	UP
SNCA	28.44816	-0.60342	0.227041	-2.65777	0.007866066	0.0132186	DOWN
RP11-454I	23.61311	0.348337	0.13107	2.657629	0.007869241	0.0132231	NOT
C22orf24	2.375766	0.709345	0.266925	2.657475	0.007872853	0.0132284	UP
ALDH3A2	14794.37	-0.38703	0.145658	-2.65712	0.007881147	0.0132415	NOT
CDKN1C	427.3207	0.737165	0.277435	2.657073	0.007882234	0.0132425	UP
AC002398	2.774403	0.65116	0.24508	2.656929	0.007885605	0.0132469	UP
AC003104	1.898624	0.872558	0.328409	2.656922	0.007885775	0.0132469	UP
DRG2	1012.862	0.244492	0.092044	2.656257	0.007901338	0.0132722	NOT
RP11-632I	26.22947	-0.52589	0.197997	-2.65607	0.007905785	0.0132789	NOT
TNIK	337.4648	-0.58576	0.220552	-2.6559	0.007909729	0.0132847	DOWN
AP006621.	7.737706	0.784168	0.295284	2.65564	0.007915796	0.0132941	UP
AP000351.	45.4188	-0.68336	0.257345	-2.65541	0.007921128	0.0133022	DOWN
MTMR8	13.36703	0.591299	0.222688	2.655281	0.007924241	0.0133066	UP
ASTE1	249.0174	-0.18008	0.067825	-2.65509	0.007928653	0.0133132	NOT
GPR17	28.35301	0.780604	0.294049	2.654673	0.007938529	0.013329	UP
SLC22A7	16609.75	-0.82395	0.310405	-2.65442	0.007944385	0.013338	DOWN
GCAT	2532.354	-0.5416	0.20404	-2.65439	0.00794514	0.0133385	NOT
RP11-461I	3.351881	-0.4837	0.182249	-2.65406	0.007953065	0.013351	NOT
MEI1	48.64829	-0.54091	0.20383	-2.65374	0.007960468	0.0133626	NOT
AGAP7P	5.558989	0.804359	0.303119	2.653609	0.007963604	0.013367	UP

RNGTT	614.3009	-0.22223	0.083751	-2.65342	0.007967966	0.0133736	NOT
IGKV2-30	12.75182	-1.19309	0.449664	-2.65328	0.007971294	0.0133783	DOWN
BTBD7	693.7194	0.225565	0.085036	2.652582	0.007987878	0.0134048	NOT
ZNF660	35.20396	0.52537	0.19806	2.652576	0.007988013	0.0134048	NOT
SPDYE3	293.7468	0.241654	0.091107	2.652426	0.007991563	0.0134099	NOT
CPHL1P	28.49453	0.755025	0.284666	2.652322	0.007994029	0.0134132	UP
RP11-244I	66.5455	0.387659	0.146198	2.651611	0.008010874	0.0134407	NOT
ARHGEF25	167.4767	0.517403	0.195139	2.651451	0.00801467	0.0134462	NOT
GPN2	970.6033	0.180394	0.068078	2.649798	0.008053989	0.0135114	NOT
RPL31P11	3.526934	-0.88344	0.333437	-2.64949	0.008061229	0.0135227	DOWN
RP11-297/	2.505704	0.69714	0.263141	2.649308	0.00806569	0.0135294	UP
PBX2P1	2.816083	0.665879	0.251354	2.649171	0.008068944	0.013534	UP
CEBPZOS	1645.298	0.218145	0.082356	2.648817	0.008077401	0.0135474	NOT
CD164L2	1.789476	-1.67554	0.63257	-2.64878	0.008078287	0.013548	DOWN
RP11-93O	1.703149	0.743891	0.280857	2.648652	0.008081343	0.0135523	UP
RP4-782L2	22.70384	0.595036	0.22466	2.648602	0.008082536	0.0135535	UP
IGKV3-20	659.5547	-1.00419	0.379159	-2.64847	0.008085731	0.0135581	DOWN
AC025627	11.60239	0.551377	0.208202	2.648277	0.008090324	0.0135649	NOT
UNC45B	4.158702	-0.55483	0.209539	-2.64788	0.008099752	0.0135799	NOT
NPSR1	0.605835	1.58778	0.599662	2.647792	0.008101946	0.0135828	UP
ATP6V0A4	1.842733	1.185955	0.447943	2.647559	0.008107523	0.0135913	UP
FAM60A	545.3214	0.471267	0.178016	2.647332	0.008112955	0.0135996	NOT
RBM48	313.8508	0.187842	0.070962	2.647073	0.008119178	0.0136092	NOT
PIP4K2A	1031.753	-0.28946	0.109357	-2.64693	0.008122527	0.013614	NOT
RP11-369I	5.690425	-1.03704	0.391879	-2.64633	0.00813706	0.0136375	DOWN
CDH2	4287.034	-0.39663	0.149898	-2.64599	0.008145287	0.0136505	NOT
TNKS2-AS	15.16537	-0.62327	0.235556	-2.64595	0.008146274	0.0136513	DOWN
RP11-50D	2.768392	0.675774	0.25542	2.64574	0.008151252	0.0136588	UP
TMEM255	41.45058	0.728363	0.275329	2.645425	0.008158844	0.0136707	UP
RP11-71E1	14.05091	0.775043	0.293007	2.645134	0.00816585	0.0136816	UP
C5orf38	0.846103	1.270527	0.480569	2.643796	0.008198213	0.013735	UP
AP3M1	1688.441	0.188733	0.071389	2.643705	0.008200415	0.0137379	NOT
LINC0046(	1.358461	1.993783	0.7542	2.643574	0.008203581	0.0137423	UP
MIR210	1.369499	1.031368	0.390168	2.643395	0.008207912	0.0137481	UP
IL32	15922.98	0.553976	0.20957	2.643391	0.008208018	0.0137481	NOT
SAR1A	4264.578	-0.17031	0.06443	-2.64336	0.008208688	0.0137484	NOT
CLIP4	451.6894	0.630011	0.238341	2.643324	0.008209645	0.0137492	UP
HCG11	35.97144	-0.47264	0.178813	-2.64321	0.008212327	0.0137528	NOT
RP11-465I	1.760985	0.779549	0.294941	2.643065	0.008215938	0.013758	UP
UBE2J1	2211.424	-0.20221	0.07651	-2.64289	0.008220168	0.0137643	NOT
FTLP5	3.4174	0.796785	0.301507	2.642677	0.008225349	0.0137721	UP
PRPSAP1	3140.703	0.260308	0.098515	2.642313	0.008234199	0.0137861	NOT
LINC0053(	5.87349	0.52997	0.200611	2.641787	0.008246983	0.0138067	NOT
PIH1D1	2544.746	0.2848	0.107822	2.641391	0.008256625	0.0138212	NOT
RP11-344I	4.164173	-0.60245	0.228079	-2.64139	0.008256659	0.0138212	DOWN
MSNP1	2.224481	0.763552	0.289083	2.641292	0.008259059	0.0138244	UP
BARX1	15.78982	1.113719	0.42168	2.641148	0.008262564	0.0138293	UP
RRN3P3	67.98986	0.298348	0.112962	2.64113	0.008262995	0.0138293	NOT
CSDC2	33.80109	-0.95354	0.361062	-2.64095	0.008267474	0.013836	DOWN
GEMIN5	783.2725	0.195532	0.074046	2.64069	0.008273733	0.0138456	NOT
CCL15-CC	112.2808	-0.56379	0.213502	-2.64067	0.008274284	0.0138457	NOT
MRPS10	1913.355	0.185287	0.070173	2.640419	0.008280367	0.013855	NOT

10-Mar	1.508807	1.281367	0.485352	2.640077	0.008288724	0.0138682	UP
CTD-3018	2.222755	0.922194	0.349328	2.639913	0.008292735	0.013874	UP
RP4-715N	1.006742	1.781206	0.674849	2.639416	0.00830491	0.0138936	UP
OSM	45.72604	-0.70103	0.265608	-2.63936	0.008306308	0.0138951	DOWN
AC022210	6.125798	0.535723	0.203004	2.638973	0.00831576	0.01391	NOT
EGFLAM	201.692	-0.40773	0.15453	-2.63852	0.00832678	0.0139276	NOT
RP11-25I1	3.050685	0.692327	0.2624	2.638442	0.008328795	0.0139302	UP
CFAP46	36.58645	1.01544	0.38488	2.638331	0.00833152	0.0139339	UP
RP11-839I	5.896379	0.719542	0.272771	2.637893	0.008342283	0.0139502	UP
RBBP6	1583.264	-0.1743	0.066075	-2.63789	0.008342293	0.0139502	NOT
MAK	14.3396	-0.49616	0.188107	-2.63767	0.008347788	0.0139586	NOT
LRIT3	7.580337	0.564329	0.213956	2.637593	0.008349681	0.0139609	NOT
FUT4	327.6003	0.615581	0.233434	2.637066	0.008362646	0.0139817	UP
RP11-414I	1.040013	1.257047	0.476768	2.6366	0.008374152	0.0140001	UP
NDNF	11.36186	-0.90481	0.343193	-2.63644	0.008378178	0.014006	DOWN
RAG1	52.9777	-0.61387	0.23286	-2.6362	0.008383965	0.0140144	DOWN
LINC01562	2.794681	-0.59253	0.224769	-2.63619	0.008384254	0.0140144	DOWN
RP11-575I	9.762192	0.520255	0.197355	2.636134	0.008385652	0.0140159	NOT
RPL12P8	2.791874	0.682999	0.259148	2.63556	0.008399868	0.0140389	UP
SPPL2A	4287.149	-0.23669	0.08982	-2.63519	0.00840914	0.0140535	NOT
RABGEF1	298.0784	0.320939	0.121802	2.634926	0.008415553	0.0140634	NOT
HIPK1-AS1	12.75058	-0.4151	0.157545	-2.63482	0.008418149	0.0140669	NOT
RP11-407I	28.44759	-0.28404	0.107805	-2.63477	0.008419461	0.0140682	NOT
LCMT1-AS	2.03184	0.907268	0.344364	2.634623	0.008423077	0.0140734	UP
OSCAR	74.83447	-0.45308	0.17198	-2.63448	0.008426729	0.0140786	NOT
RP11-256I	2.376786	0.95137	0.361135	2.634387	0.008428938	0.0140815	UP
TUBB4B	8488.403	0.326946	0.124118	2.634155	0.008434688	0.0140902	NOT
AC019185	0.888101	2.155585	0.81841	2.633869	0.008441793	0.0141013	UP
RP11-270(	8.964235	0.510208	0.193727	2.633639	0.008447518	0.01411	NOT
RP11-218I	508.5489	-0.32018	0.121579	-2.63353	0.00845025	0.0141137	NOT
RPL7P32	7.772014	0.586796	0.22283	2.633383	0.008453887	0.0141189	UP
DDX26B	95.82711	0.571134	0.216926	2.632856	0.008467035	0.01414	NOT
PEG3	717.849	1.005537	0.381931	2.632772	0.00846912	0.0141426	UP
FLJ41941	1.450501	1.132433	0.430188	2.632414	0.008478044	0.0141567	UP
MAGI3	628.7023	-0.31741	0.12062	-2.6315	0.008500901	0.014194	NOT
BMPR1A	654.1255	0.216264	0.082187	2.631347	0.008504703	0.0141995	NOT
RP11-106(	28.8784	0.875801	0.332882	2.630968	0.008514206	0.0142145	UP
RN7SL809I	1.666993	0.894279	0.339919	2.630862	0.008516864	0.0142181	UP
WAC	3984.478	0.168906	0.064205	2.630727	0.008520236	0.0142228	NOT
CNOT6LP1	1.886585	1.22791	0.466781	2.630593	0.008523609	0.0142276	UP
HDGFL1	1.117356	2.410304	0.916309	2.63045	0.008527182	0.0142327	UP
GRAMD4	2838.736	-0.43421	0.165104	-2.62993	0.008540233	0.0142536	NOT
RP11-973I	2.626944	0.800194	0.304345	2.62923	0.008557847	0.0142822	UP
CC2D2A	194.694	0.365767	0.139126	2.629041	0.008562605	0.0142893	NOT
MYDGF	5733.806	0.28089	0.106852	2.62877	0.00856942	0.0142998	NOT
CTD-2006	4.903698	-0.66992	0.25485	-2.62869	0.008571323	0.0143021	DOWN
IGLV1-40	273.4228	-1.05617	0.401809	-2.62854	0.008575234	0.0143077	DOWN
SPRY3	53.44779	0.333309	0.126822	2.628161	0.008584785	0.0143228	NOT
LINC0155(	15.75245	-0.80245	0.305336	-2.62808	0.00858673	0.0143252	DOWN
ERICH6B	7.105682	0.523409	0.199197	2.627592	0.008599162	0.0143451	NOT
RP11-603J	13.3955	0.452264	0.172125	2.627528	0.008600777	0.0143469	NOT
MBNL1	3283.372	-0.21256	0.080902	-2.62742	0.008603569	0.0143507	NOT

ERAL1	2393.911	0.198736	0.07566	2.62669	0.008621979	0.0143804	NOT
RP11-729I	2.792081	-0.72175	0.274776	-2.62667	0.008622395	0.0143804	DOWN
CTC-336P	9.747154	-0.51569	0.196336	-2.6266	0.008624327	0.0143827	NOT
RBFA	1008.949	-0.26878	0.102357	-2.62596	0.008640476	0.0144088	NOT
INTS9	343.3403	0.26197	0.099767	2.625811	0.008644275	0.0144142	NOT
TAS2R64P	1.331377	1.003209	0.382172	2.625021	0.008664373	0.0144469	UP
ZXDA	90.30508	-0.28975	0.110386	-2.62484	0.008668908	0.0144536	NOT
C9orf147	8.419897	0.386004	0.147088	2.624299	0.00868274	0.0144758	NOT
RP11-473(	2.598476	0.774382	0.295104	2.624101	0.008687808	0.0144833	UP
IGHV3-41	1.866541	-1.12793	0.429864	-2.62392	0.008692528	0.0144903	DOWN
GTF2F1	2452.754	0.175508	0.066891	2.623792	0.008695682	0.0144947	NOT
HSPE1P3	4.819374	0.546219	0.208193	2.623622	0.008700038	0.0145011	NOT
ZNF536	1.610421	-1.01391	0.386505	-2.62328	0.008708852	0.0145149	DOWN
OR52N4	2.130955	-0.80908	0.308433	-2.62321	0.008710659	0.0145171	DOWN
C1orf140	12.99659	1.1584	0.441604	2.623163	0.008711765	0.014518	UP
RP11-90B(	3.073747	0.61163	0.233175	2.623057	0.00871447	0.0145217	UP
CTC-529G	0.951062	2.171955	0.82814	2.622692	0.008723801	0.0145363	UP
RP11-118I	2.134005	1.053196	0.401593	2.622547	0.008727527	0.0145414	UP
RP11-179/	1.003855	2.074152	0.790897	2.622532	0.008727917	0.0145414	UP
FCER2	14.15369	-0.65576	0.250059	-2.62242	0.008730825	0.0145454	DOWN
TSPO	2250.375	0.450206	0.171682	2.622318	0.008733394	0.0145486	NOT
NPM1P37	2.265424	0.622476	0.237378	2.622303	0.008733769	0.0145486	UP
C9orf89	486.4876	0.279186	0.10647	2.622189	0.008736691	0.0145526	NOT
CRKL	2981.755	0.193599	0.073841	2.621834	0.008745795	0.0145668	NOT
RP11-47P1	0.846646	2.159912	0.824132	2.620831	0.008771564	0.0146089	UP
GADD45GI	2520.371	0.427094	0.16298	2.620527	0.008779392	0.014621	NOT
RP1-37N7	3.261152	1.153486	0.440181	2.62048	0.008780614	0.0146222	UP
PARP16	667.7275	-0.21879	0.083498	-2.62033	0.008784394	0.0146276	NOT
HIST1H4B	1.977525	1.099672	0.419687	2.620222	0.008787262	0.0146315	UP
P2RY10	24.81637	-0.74624	0.284814	-2.62012	0.008789968	0.0146351	DOWN
C8G	12171.09	-0.51958	0.198308	-2.62008	0.008790964	0.0146359	NOT
RP11-800/	9.115142	-0.60495	0.231005	-2.61878	0.008824561	0.014691	DOWN
PRKCZ	503.1905	-0.40584	0.154988	-2.61853	0.008830963	0.0147007	NOT
ICAM1	3216.497	-0.57055	0.217911	-2.61826	0.008837905	0.0147114	NOT
MLIP-AS1	9.36183	0.983874	0.375819	2.617946	0.008846091	0.0147241	UP
CTD-2145	7.553409	0.519447	0.198431	2.617779	0.00885042	0.0147305	NOT
AC026271	108.0314	0.249698	0.095386	2.617752	0.008851107	0.0147307	NOT
HLA-DPA3	2.514109	-1.00537	0.384125	-2.61729	0.008863106	0.0147498	DOWN
SUMO2P1	4.198795	0.52724	0.201454	2.617176	0.008866055	0.0147532	NOT
RP5-978I1	1.116473	2.202011	0.841371	2.617171	0.008866189	0.0147532	UP
SLC9A3R1	11168.59	0.268883	0.102754	2.616764	0.00887676	0.0147699	NOT
RP11-384I	1.366141	1.924991	0.735746	2.61638	0.008886758	0.014785	UP
WTAP	1963.4	-0.19284	0.073704	-2.61637	0.00888694	0.014785	NOT
THEMIS	40.04013	-0.68315	0.261137	-2.61605	0.00889544	0.0147983	DOWN
SEMA5A	780.5308	-0.49377	0.18876	-2.61584	0.008900902	0.0148065	NOT
PARD3B	625.002	0.423932	0.16207	2.615734	0.008903578	0.0148094	NOT
A4GNT	14.97281	1.281468	0.489909	2.615728	0.008903737	0.0148094	UP
ZSWIM4	306.578	0.407628	0.155887	2.614898	0.008925409	0.0148446	NOT
C11orf73	700.0867	0.229821	0.087906	2.614377	0.008939028	0.0148663	NOT
NPTN-IT1	8.610607	0.706468	0.270243	2.614194	0.008943812	0.0148734	UP
SVIL-AS1	460.0646	0.305266	0.116786	2.613889	0.008951814	0.0148858	NOT
CTD-2061	1.640704	0.862551	0.330028	2.613569	0.008960195	0.0148984	UP

MRFAP1	11067.03	-0.1671	0.063934	-2.61356	0.008960463	0.0148984	NOT
GUSBP3	1.739807	1.310742	0.501537	2.61345	0.008963329	0.0149023	UP
VSIG2	170.8646	-0.85195	0.32599	-2.61341	0.008964325	0.0149023	DOWN
ZNF613	123.3345	0.271103	0.103737	2.613375	0.008965281	0.0149023	NOT
ZKSCAN1	5095.223	0.309461	0.118414	2.613373	0.008965341	0.0149023	NOT
ZSWIM8-/	1.366151	0.878963	0.336335	2.613355	0.008965819	0.0149023	UP
ATOH7	14.25668	-0.58444	0.223637	-2.61335	0.008966055	0.0149023	NOT
HOXB5	43.12799	0.518978	0.1986	2.61318	0.0089704	0.0149086	NOT
TUBB8	1.782332	1.132567	0.433488	2.612681	0.008983519	0.0149295	UP
CHMP1B2I	6.332229	1.391003	0.532434	2.612537	0.008987309	0.0149349	UP
VN1R83P	5.400332	0.700447	0.268193	2.611727	0.009008606	0.0149694	UP
PALMD	1761.681	-0.4597	0.17602	-2.61165	0.009010669	0.014972	NOT
RP11-393I	10.7629	0.406422	0.155625	2.61154	0.009013539	0.0149754	NOT
LINC0110(	15.22357	0.655036	0.250825	2.611529	0.009013829	0.0149754	UP
AC008074	2.266191	0.695977	0.266527	2.611282	0.00902034	0.0149852	UP
TMEM130	27.45054	0.898347	0.344028	2.611265	0.009020804	0.0149852	UP
CEACAM2	15.86234	0.709229	0.271607	2.61123	0.00902172	0.0149858	UP
RAB43	230.4822	-0.35058	0.13426	-2.6112	0.009022428	0.0149861	NOT
SPATA5	223.5829	0.259151	0.099249	2.611132	0.009024294	0.0149883	NOT
DDIT4	2886.537	0.535695	0.20517	2.61098	0.009028326	0.0149941	NOT
PLEKHG1	476.2186	-0.35495	0.135955	-2.61083	0.009032173	0.0149996	NOT
GZMB	74.59915	-0.54972	0.210585	-2.61046	0.009042125	0.0150152	NOT
IGFBP6	172.3209	-0.50026	0.191655	-2.61022	0.009048396	0.0150247	NOT
DAAM1	1180.917	-0.35014	0.134155	-2.60995	0.009055633	0.0150353	NOT
RP11-361I	1.787741	0.892329	0.341896	2.609939	0.009055836	0.0150353	UP
RP13-608I	1.809717	0.921284	0.353042	2.609557	0.009065943	0.0150512	UP
ZNF625-ZI	1.261439	0.900893	0.345236	2.609497	0.009067551	0.0150522	UP
PPM1N	38.9013	-0.44533	0.170657	-2.60948	0.009067997	0.0150522	NOT
LBP	37578.2	-0.64195	0.246007	-2.60947	0.009068203	0.0150522	DOWN
GAS7	329.9339	0.558685	0.214106	2.609386	0.009070487	0.0150551	NOT
YEATS4	451.6062	0.227892	0.08735	2.608965	0.009081644	0.0150727	NOT
OAT	3405.193	-0.75783	0.290524	-2.60848	0.009094439	0.015093	DOWN
PGAM1	1228.954	-0.265	0.101596	-2.60841	0.009096382	0.0150953	NOT
CD79B	134.5587	-0.39466	0.151325	-2.608	0.009107216	0.0151124	NOT
LTA	18.09483	0.663602	0.25446	2.607887	0.009110291	0.0151166	UP
KIAA1033	1974.792	-0.22938	0.08796	-2.60776	0.009113717	0.0151214	NOT
DEPDC4	30.55273	0.297192	0.113994	2.607073	0.009131988	0.0151508	NOT
IL7R	514.3776	-0.76493	0.293424	-2.60691	0.009136247	0.015157	DOWN
TXLNA	3452.177	0.216259	0.082961	2.606764	0.00914022	0.0151626	NOT
TFIP11	1317.625	0.254896	0.097808	2.606094	0.009158138	0.0151915	NOT
ZDHHC11I	167.2834	0.612742	0.235134	2.605925	0.009162649	0.015198	UP
WDR37	578.4574	0.210939	0.080961	2.605439	0.009175649	0.0152187	NOT
APOBR	285.0401	-0.34441	0.132203	-2.60518	0.009182558	0.0152292	NOT
RP11-384I	120.0921	-0.35643	0.136832	-2.60484	0.009191829	0.0152437	NOT
RP11-285I	2.844691	-0.70044	0.268915	-2.60467	0.009196172	0.01525	DOWN
HPR	16888.27	-0.73091	0.28064	-2.60446	0.009201939	0.0152586	DOWN
PLBD1	466.7063	0.66217	0.254273	2.604166	0.009209815	0.0152708	UP
RP4-761J1	2.702759	0.6429	0.246891	2.60398	0.009214809	0.0152781	UP
RP11-320I	49.38969	0.369733	0.14199	2.603929	0.009216179	0.0152795	NOT
RP11-253I	36.79875	0.58733	0.225601	2.6034	0.009230431	0.0153022	UP
GLRX2	489.4234	0.280894	0.107897	2.603358	0.009231538	0.0153031	NOT
RP11-193I	1.91588	1.019543	0.391631	2.603323	0.009232497	0.0153038	UP

BAG1	2417.68	-0.26976	0.103633	-2.60302	0.009240649	0.0153164	NOT
HNRNPA1I	3.481489	0.696907	0.267736	2.60296	0.009242278	0.0153182	UP
RP11-218I	1.322808	2.729749	1.048747	2.602866	0.009244804	0.0153214	UP
UBE2B	2515.475	-0.2053	0.078902	-2.60197	0.009268937	0.0153605	NOT
PIWIL1	2.296131	0.969335	0.372552	2.601879	0.009271448	0.0153637	UP
AKT2	6004.488	-0.23324	0.089648	-2.60177	0.009274481	0.0153679	NOT
OPLAH	3446.625	0.38163	0.146687	2.601667	0.009277189	0.0153714	NOT
RP11-262I	1.960005	-0.76308	0.293319	-2.60152	0.009281268	0.0153773	DOWN
ZSCAN16-	720.8246	0.453773	0.174433	2.60142	0.00928388	0.0153807	NOT
RHOH	99.70704	-0.56367	0.216681	-2.60139	0.009284804	0.0153813	NOT
BRINP1	24.51107	-0.95405	0.366769	-2.60122	0.009289305	0.0153878	DOWN
RPUSD4	879.4743	-0.21053	0.080936	-2.60115	0.009291282	0.0153902	NOT
GNLY	153.3827	-0.50265	0.193256	-2.60096	0.00929627	0.0153975	NOT
CD36	3234.121	0.612559	0.235516	2.600927	0.009297234	0.0153978	UP
PACSIN3	1722.944	-0.44796	0.172231	-2.60092	0.009297545	0.0153978	NOT
ACTN4P1	1.554724	0.797243	0.306547	2.600724	0.009302726	0.0154054	UP
RP11-144(	4.019643	0.546785	0.210255	2.600575	0.009306779	0.0154112	NOT
RP11-296I	2.933612	-0.65723	0.25274	-2.60042	0.009310923	0.0154172	DOWN
CFAP221	131.5735	0.850413	0.327054	2.600221	0.009316373	0.0154253	UP
CTB-113I2	38.78554	0.455722	0.175267	2.600153	0.009318213	0.0154274	NOT
ZNF215	24.10185	0.77214	0.296966	2.600093	0.009319863	0.0154292	UP
PFN1P1	14.69279	0.554667	0.213365	2.599622	0.009332652	0.0154494	NOT
CTD-2186	111.6527	-0.32222	0.123952	-2.59953	0.009335204	0.0154527	NOT
AC019117	1.284494	1.074181	0.413257	2.599302	0.009341351	0.015462	UP
LLNLR-24(	8.888619	0.519979	0.200082	2.598824	0.009354365	0.0154826	NOT
CAAP1	899.6936	-0.2695	0.103711	-2.59853	0.009362462	0.0154951	NOT
DTX3L	3877.005	-0.23299	0.089663	-2.59849	0.009363433	0.0154958	NOT
LBX1	1.302562	2.614812	1.006301	2.59844	0.009364842	0.0154972	UP
RP11-680(	31.31583	-0.46549	0.179165	-2.5981	0.009374214	0.0155117	NOT
COMMD9	1555.848	0.217129	0.083585	2.597711	0.009384738	0.0155282	NOT
C14orf119	1344.367	0.18445	0.071011	2.597499	0.009390545	0.0155369	NOT
CBY3	3.463141	0.635974	0.244846	2.597448	0.00939194	0.0155383	UP
RP11-783I	2.187296	0.801296	0.308509	2.597315	0.009395563	0.0155425	UP
BST2	8123.84	-0.54789	0.210943	-2.59731	0.009395596	0.0155425	NOT
PRPF18	379.2803	-0.17085	0.065782	-2.5972	0.009398841	0.0155469	NOT
SNRPF	1589.121	0.314905	0.121273	2.596671	0.009413204	0.0155689	NOT
GAPDHP38	2.855115	0.679352	0.261625	2.596669	0.009413258	0.0155689	UP
HENMT1	115.6687	0.638839	0.246121	2.595631	0.00944174	0.0156151	UP
RP11-597I	2.027044	0.776811	0.299304	2.595386	0.00944847	0.0156253	UP
C12orf45	397.2447	0.288024	0.110977	2.595348	0.009449515	0.0156261	NOT
MIR378H	1.82315	0.866589	0.333905	2.595314	0.009450453	0.0156267	UP
RP11-455(	1.555011	1.083896	0.41765	2.595222	0.009452994	0.01563	UP
AP000442.	2.594339	0.750691	0.289282	2.595018	0.009458609	0.0156383	UP
MAP3K7	1074.045	0.197159	0.075977	2.594962	0.00946015	0.0156399	NOT
ALG8	2030.272	0.221662	0.085426	2.594798	0.009464651	0.0156464	NOT
ERVW-1	2.063845	0.963908	0.371504	2.594608	0.009469879	0.0156541	UP
RNU6-720	1.369415	-0.83769	0.322922	-2.59409	0.009484131	0.0156768	DOWN
RP11-93G	2.387558	0.926219	0.357053	2.594067	0.009484805	0.0156769	UP
RP1-118J2	1.754709	0.953114	0.367425	2.594035	0.009485676	0.0156774	UP
TUBBP9	3.504226	0.993667	0.383091	2.593812	0.009491844	0.0156867	UP
KRBOX4	358.3092	0.159339	0.061437	2.593536	0.00949945	0.0156983	NOT
LINC00092	13.93453	-0.62402	0.240661	-2.59294	0.009515969	0.0157247	DOWN

TCP1	5637.905	0.244991	0.0945	2.592506	0.009527961	0.0157436	NOT
HAUS2	533.6853	0.173185	0.066804	2.59245	0.009529499	0.0157452	NOT
IGHJ1	0.899926	-1.34024	0.516993	-2.59237	0.009531585	0.0157477	DOWN
FN1	200080.3	-0.33059	0.127537	-2.59211	0.009538908	0.015758	NOT
FKRP	462.2406	-0.18513	0.071422	-2.59211	0.009538951	0.015758	NOT
TGFB1I1	408.4702	0.342868	0.132281	2.591961	0.009543047	0.0157638	NOT
NDUFB5	3311.68	-0.19834	0.076524	-2.59188	0.009545326	0.0157666	NOT
SSBP2	230.4138	0.476286	0.183766	2.591809	0.009547285	0.0157689	NOT
SPTBN4	67.57074	0.555899	0.214529	2.591259	0.009562541	0.0157931	NOT
CTD-2235	3.307231	0.630842	0.243489	2.590845	0.009574049	0.0158112	UP
IGHV1OR1	3.532811	-1.19337	0.460649	-2.59062	0.009580327	0.0158206	DOWN
RP11-244J	4.841371	0.6387	0.246556	2.590492	0.009583894	0.0158256	UP
KRT5	95.31807	1.287574	0.497085	2.590251	0.00959061	0.0158357	UP
RP3-461F1	7.205269	0.412486	0.159261	2.589997	0.009597678	0.0158464	NOT
ZNF594	77.8933	0.442075	0.170696	2.589832	0.009602268	0.0158531	NOT
NOTCH1	1432.885	0.323174	0.124797	2.589602	0.009608682	0.0158627	NOT
EXD1	1.169557	1.062584	0.410349	2.589465	0.00961253	0.0158681	UP
CCL11	13.85221	0.893923	0.345243	2.589256	0.009618364	0.0158768	UP
AGK	797.6745	0.22266	0.085995	2.589211	0.009619603	0.0158779	NOT
PTX3	38.85797	0.780453	0.301428	2.589191	0.009620167	0.0158779	UP
LINC00900	15.00675	0.599869	0.2317	2.58899	0.009625793	0.0158862	UP
SCGB2A2	0.990105	-2.51326	0.970768	-2.58894	0.009627129	0.0158875	DOWN
KLF2P4	0.990024	2.453926	0.947859	2.588916	0.009627862	0.0158878	UP
LINC00506	4.462858	0.801011	0.309446	2.588531	0.009638622	0.0159046	UP
LHX9	90.88746	0.934905	0.361212	2.588243	0.009646696	0.0159169	UP
AC017104	10.67553	0.712591	0.275322	2.58821	0.009647627	0.0159175	UP
CTD-2357	1.921554	1.124102	0.434339	2.588078	0.009651308	0.0159218	UP
RP11-415J	1.359993	1.073418	0.414755	2.588076	0.009651364	0.0159218	UP
RP11-30L1	2.215022	0.710512	0.274541	2.588	0.0096535	0.0159244	UP
MRPS30	1547.753	-0.17538	0.067768	-2.58794	0.009655289	0.0159264	NOT
RP11-354I	0.941053	1.673114	0.646542	2.587787	0.009659463	0.0159323	UP
TCF23	7.029104	0.696864	0.269303	2.58766	0.009663028	0.0159372	UP
AC096582	2.885812	0.825344	0.318971	2.587521	0.009666943	0.0159427	UP
SLAMF7	305.4519	-0.6238	0.241119	-2.58712	0.009678272	0.0159605	DOWN
C1QTNF9E	5.509962	0.604243	0.233596	2.5867	0.009689988	0.0159788	UP
CTC-303L	1.337173	0.924663	0.357483	2.586592	0.009693035	0.0159829	UP
RP11-94B	0.912922	2.125588	0.821856	2.586326	0.009700527	0.0159943	UP
RP11-678(	3.206607	0.784112	0.303192	2.586194	0.009704238	0.0159995	UP
UQCC3	1083.84	0.365734	0.141433	2.585917	0.009712044	0.0160114	NOT
SCGB2B2	15.16208	-0.49087	0.189845	-2.58566	0.009719308	0.0160224	NOT
RNF175	19.77413	-0.58122	0.224797	-2.58555	0.009722412	0.0160266	NOT
SPIB	65.48765	0.923689	0.357313	2.585099	0.009735099	0.0160465	UP
TRAM1	11381.42	0.256237	0.099159	2.584096	0.00976347	0.0160923	NOT
MTND4P1	5199.201	-0.81018	0.313554	-2.58388	0.009769692	0.0161013	DOWN
NINL	710.7949	0.406603	0.157362	2.583863	0.009770064	0.0161013	NOT
RP13-188A	5.645797	0.61177	0.236772	2.583799	0.009771885	0.0161033	UP
RP11-248(	2.329221	0.823456	0.318706	2.583744	0.00977343	0.0161049	UP
SGOL1-AS	1.908565	0.764913	0.296055	2.583687	0.009775045	0.0161066	UP
ZNF462	107.5617	-0.53683	0.207779	-2.58365	0.009776161	0.0161075	NOT
RP11-1E2	1.299372	1.580058	0.611607	2.583452	0.009781713	0.0161157	UP
UNC5C	91.08519	-0.5411	0.209489	-2.58295	0.009795826	0.016138	NOT
IGLV2-8	89.91788	-1.10877	0.429272	-2.58291	0.009797044	0.016139	DOWN

RP11-413I	1.223382	0.95897	0.371289	2.582813	0.00979983	0.0161426	UP
ASPRV1	43.42884	-0.36523	0.141416	-2.58263	0.009804948	0.0161501	NOT
RP11-38L1	103.9407	-0.58053	0.224794	-2.58248	0.009809432	0.0161565	NOT
CTD-2666	5.183928	0.718201	0.278109	2.582445	0.009810299	0.0161565	UP
FRY	1220.573	-0.33347	0.12913	-2.58244	0.009810579	0.0161565	NOT
TULP1	1.209608	1.325657	0.513388	2.582172	0.009818057	0.0161679	UP
ZBTB46-A!	0.973113	2.141522	0.829356	2.582151	0.009818662	0.0161679	UP
IGHV4-59	206.7104	-1.08098	0.418763	-2.58137	0.009840881	0.0162035	DOWN
PTCHD4	62.51547	0.829266	0.32128	2.581132	0.009847678	0.0162137	UP
HNRNPAB	4523.462	0.204467	0.079233	2.580587	0.009863244	0.0162384	NOT
RPA1	2018.925	0.202602	0.078512	2.580515	0.00986532	0.0162409	NOT
CCNB3	34.98302	0.4121	0.159728	2.580009	0.009879771	0.0162637	NOT
LATS1	729.1004	-0.23285	0.090257	-2.5799	0.009882894	0.0162679	NOT
RP11-96P1	1.092155	1.913715	0.74179	2.579862	0.009883993	0.0162687	UP
RP1-163G!	0.960106	1.919994	0.744328	2.579502	0.0098943	0.0162847	UP
UBE2Q2L	1.559375	1.030578	0.399544	2.579384	0.009897668	0.0162893	UP
RP11-466I	255.0777	0.495852	0.192249	2.579218	0.009902428	0.0162961	NOT
AC109333	5.549382	0.537625	0.208454	2.5791	0.009905816	0.0163007	NOT
TFE3	1994.085	0.187924	0.072872	2.578828	0.009913626	0.0163126	NOT
DNAJA2	2928.607	-0.24788	0.096151	-2.57807	0.009935327	0.0163474	NOT
MRPS35	3207.73	-0.17743	0.068834	-2.57761	0.00994856	0.0163682	NOT
CCL22	37.32347	-0.66759	0.259037	-2.57721	0.009960265	0.0163864	DOWN
RSPH10B	2.434461	-0.65437	0.253943	-2.57684	0.00997069	0.0164026	DOWN
RP11-313I	3.810821	-0.50242	0.194999	-2.57656	0.009978982	0.0164153	NOT
ZNF275	1436.46	-0.34674	0.134588	-2.57634	0.009985223	0.0164246	NOT
EMX2	1.491675	1.285449	0.498988	2.576112	0.009991829	0.0164345	UP
BCAS2	1240.961	-0.18525	0.071918	-2.57588	0.009998609	0.0164446	NOT
MEIOB	2.119548	1.122753	0.43594	2.57548	0.010010112	0.0164626	UP
SULT1C3	0.961127	2.184567	0.848359	2.57505	0.010022564	0.0164821	UP
CTC-820M	3.261537	0.726539	0.282181	2.574726	0.010031957	0.0164965	UP
SNAPC1	190.5528	-0.25189	0.097858	-2.57405	0.010051575	0.0165278	NOT
SNORD11(	1.313707	1.169001	0.454278	2.573316	0.010072911	0.0165619	UP
APOBEC3H	21.67437	0.56791	0.220705	2.573163	0.010077362	0.0165682	NOT
RP11-810I	31.05457	0.383765	0.149159	2.572859	0.010086223	0.0165812	NOT
FAM186B	33.01382	-0.41474	0.161198	-2.57285	0.01008645	0.0165812	NOT
LINC00863	66.9303	0.359931	0.139902	2.572747	0.010089488	0.0165852	NOT
RP11-656I	8.550941	0.394658	0.153408	2.572599	0.010093805	0.0165913	NOT
FAM181B	2.129192	1.091209	0.424216	2.572295	0.010102675	0.0166043	UP
IGHV3-21	134.172	-1.0611	0.412511	-2.57229	0.010102885	0.0166043	DOWN
HSP90AA4	1.385449	0.885224	0.344165	2.572093	0.010108585	0.0166127	UP
AP000619.	0.888928	2.235196	0.869055	2.571986	0.010111704	0.0166162	UP
FJX1	219.9333	0.543749	0.211413	2.571978	0.010111932	0.0166162	NOT
FKBP1B	288.9361	0.73395	0.28542	2.571475	0.010126628	0.0166394	UP
RP11-195I	5.390906	0.444301	0.172788	2.571358	0.010130065	0.016644	NOT
PTGES3P2	46.01288	-0.54638	0.212491	-2.5713	0.01013168	0.0166457	NOT
CTD-2377	2.545341	1.086451	0.422553	2.571158	0.010135897	0.0166516	UP
ITIH2	99836.39	-0.51569	0.200588	-2.5709	0.010143498	0.0166631	NOT
RP11-39H	76.21339	-0.91724	0.356782	-2.57088	0.010144098	0.0166631	DOWN
PEAR1	243.3773	-0.36747	0.142941	-2.57081	0.010146232	0.0166656	NOT
CTD-2245	2.3889	0.970358	0.377486	2.570578	0.0101529	0.0166756	UP
ATRN	9256.827	0.33156	0.128995	2.570331	0.010160141	0.0166865	NOT
ZNF317	828.5767	0.172564	0.067143	2.570087	0.010167304	0.0166973	NOT

NACA2	5.981721	0.525133	0.204329	2.570038	0.010168741	0.0166986	NOT
IGHV3-48	42.91211	-1.08898	0.423745	-2.56989	0.010172952	0.0167046	DOWN
RP4-616B8	11.68216	0.628538	0.244643	2.569208	0.010193127	0.0167367	UP
AGO4	691.2178	-0.22638	0.088115	-2.56909	0.010196638	0.0167415	NOT
MSRB3	372.7657	0.430048	0.167405	2.568909	0.010201926	0.0167492	NOT
LINC00922	0.734935	1.757927	0.684373	2.568669	0.01020899	0.0167598	UP
AC093063	0.846042	2.084192	0.81158	2.568069	0.010226678	0.0167878	UP
TTLL11	139.3826	-0.27605	0.107497	-2.56802	0.010228137	0.0167892	NOT
ABCB11	2045.209	-0.85128	0.331497	-2.568	0.010228806	0.0167893	DOWN
MED11	718.6048	-0.26012	0.101296	-2.56794	0.010230508	0.0167911	NOT
IGHV1-2	80.22879	-1.10441	0.430149	-2.5675	0.010243561	0.0168115	DOWN
RP11-435I	5.402377	0.626245	0.24392	2.56742	0.010245832	0.0168143	UP
LINC00824	2.04933	1.057578	0.411996	2.566965	0.010259306	0.0168354	UP
ERMARD	751.2737	-0.26494	0.103216	-2.56684	0.010263104	0.0168406	NOT
RP5-881L2	8.303775	-0.67999	0.264925	-2.56672	0.010266456	0.0168451	DOWN
RNF133	2.304972	0.851728	0.331845	2.566649	0.010268648	0.0168477	UP
SMC1A	3269.078	0.252203	0.098298	2.565704	0.010296653	0.0168926	NOT
TTLL7	187.1239	0.558972	0.217878	2.565533	0.01030175	0.0169	NOT
SUGT1P	8.205038	0.611588	0.238416	2.565215	0.010311184	0.0169145	UP
KCNK10	5.623782	0.998728	0.389349	2.565124	0.010313882	0.0169179	UP
TMEM217	40.27927	-0.48707	0.189925	-2.56453	0.010331466	0.0169457	NOT
L3MBTL3	225.462	-0.38007	0.148224	-2.56416	0.010342563	0.0169629	NOT
RP4-775C1	1.907533	0.764153	0.29809	2.563493	0.010362467	0.0169946	UP
ANGPTL7	8.314049	1.419966	0.554018	2.563031	0.010376277	0.0170162	UP
ABCG2	1627.19	-0.64193	0.250469	-2.56289	0.01038043	0.017022	DOWN
TIFA	506.3395	-0.33238	0.129691	-2.56283	0.010382267	0.017024	NOT
SERPINE3	6.469188	0.546712	0.213343	2.562595	0.010389307	0.0170345	NOT
AC079305	0.961115	1.40282	0.547461	2.562409	0.010394884	0.0170427	UP
C17orf107	219.1709	-0.39924	0.15581	-2.56236	0.010396466	0.0170443	NOT
RP11-334A	12.38236	0.843969	0.329438	2.561846	0.010411737	0.0170683	UP
KRT8P45	40.1652	-0.52347	0.204345	-2.56171	0.010415959	0.0170742	NOT
ONECUT2	3048.65	0.434171	0.169494	2.56157	0.010420032	0.0170799	NOT
RP11-775A	1.436115	0.958147	0.374123	2.561051	0.010435612	0.0171044	UP
CHMP1B	1958.94	-0.22072	0.086188	-2.56093	0.010439285	0.0171094	NOT
SLC5A6	4440.229	0.47832	0.1868	2.560607	0.010448936	0.0171242	NOT
TNFRSF17	13.82179	-0.96036	0.37526	-2.55919	0.010491621	0.0171931	DOWN
TMEM110	34.70012	0.386073	0.150885	2.558725	0.010505682	0.0172152	NOT
DCAF10	1628.54	-0.19544	0.076393	-2.55834	0.010517195	0.017233	NOT
RBM26-AS	58.23105	-0.37151	0.145221	-2.55825	0.010520089	0.0172367	NOT
FER	358.4823	0.300313	0.117406	2.557914	0.0105302	0.0172523	NOT
EEF1A1P14	11.98913	0.528063	0.206447	2.557864	0.01053173	0.0172537	NOT
STPG2	3.958349	-0.59772	0.233681	-2.55785	0.0105323	0.0172537	DOWN
IGKV3-11	682.5479	-0.97243	0.380192	-2.55773	0.010535832	0.0172584	DOWN
AFF3	551.3198	0.54289	0.212293	2.557263	0.010549943	0.0172805	NOT
NDUFA6-A	291.1711	-0.58208	0.22763	-2.55712	0.010554151	0.0172864	NOT
NBEAL1	378.4396	-0.31277	0.122347	-2.55642	0.010575425	0.0173202	NOT
DBP	863.9469	0.404601	0.15829	2.55608	0.010585865	0.0173363	NOT
SERP2	97.44829	-0.46837	0.183247	-2.55593	0.010590518	0.0173429	NOT
CALCR	4.956454	0.90642	0.354658	2.555758	0.010595691	0.0173503	UP
SLC5A9	791.9845	0.549439	0.214985	2.555707	0.010597241	0.0173518	NOT
PFKFB3	1919.035	-0.62097	0.243015	-2.55525	0.010611115	0.0173735	DOWN
ABHD3	1659.698	0.301553	0.118017	2.555159	0.010613919	0.0173763	NOT

SLC6A1	6061.788	-0.56684	0.221841	-2.55515	0.010614066	0.0173763	NOT
RP5-994D	4.188659	0.539052	0.211014	2.554581	0.010631556	0.0174039	NOT
DUSP11	575.5191	-0.1662	0.065063	-2.55438	0.010637847	0.0174132	NOT
RP11-489(	8.69798	-0.57506	0.22517	-2.55388	0.010652848	0.0174367	NOT
ZDHHC11	127.8558	0.55761	0.218349	2.553748	0.010657036	0.0174425	NOT
AC063976	1.442676	0.929613	0.364041	2.553592	0.010661824	0.0174493	UP
EFCC1	62.8047	-0.38834	0.152103	-2.55313	0.01067589	0.0174713	NOT
MEIS3	84.20358	0.508199	0.199123	2.552195	0.010704673	0.0175174	NOT
TRIM21	771.4383	-0.26186	0.102627	-2.55162	0.010722348	0.0175453	NOT
CASR	27.75998	-1.00927	0.395562	-2.55148	0.010726527	0.0175511	DOWN
UBAP1L	96.48625	0.392108	0.153684	2.551387	0.010729516	0.0175549	NOT
HSPA8P13	0.849171	2.165495	0.848889	2.550975	0.010742208	0.0175737	UP
CCDC158	71.08174	-0.50311	0.197224	-2.55095	0.010742847	0.0175737	NOT
AC016712	5.061343	0.546604	0.214274	2.550952	0.010742924	0.0175737	NOT
RP11-342I	31.14734	0.537315	0.210635	2.550927	0.010743689	0.0175739	NOT
DUX4L27	6.079214	0.709311	0.278118	2.550392	0.010760197	0.0175999	UP
ENTPD7	405.9145	-0.34811	0.136527	-2.54972	0.010781061	0.017633	NOT
RP11-889I	49.10128	0.422643	0.165785	2.549346	0.010792529	0.0176507	NOT
KCNE2	20.88915	-0.48721	0.191116	-2.54931	0.010793729	0.0176516	NOT
TSSC4	1446.5	0.283078	0.111049	2.549129	0.010799243	0.0176596	NOT
RP11-434I	45.18932	-0.41633	0.163333	-2.54896	0.010804549	0.0176672	NOT
EID1	4464.788	-0.24866	0.097557	-2.54884	0.010808284	0.0176723	NOT
CRB1	4.868082	0.859236	0.337123	2.548731	0.01081155	0.0176766	UP
RP11-115:	3.258372	1.013	0.397465	2.548654	0.010813958	0.0176795	UP
AC009303	98.62137	0.385294	0.151198	2.548271	0.010825831	0.0176978	NOT
CH17-140	1.150675	1.225242	0.480924	2.547686	0.010844018	0.0177265	UP
ZDHHC4	1519.559	-0.26272	0.10313	-2.54748	0.01085033	0.0177358	NOT
STK33	26.49971	0.918285	0.360524	2.547084	0.010862715	0.017755	UP
CLEC5A	32.58786	0.776293	0.304805	2.546849	0.010870048	0.0177659	UP
RP11-150I	0.848715	2.21421	0.869431	2.546735	0.010873602	0.0177707	UP
AP000439.	2.218256	-1.31074	0.514704	-2.54658	0.010878353	0.0177774	DOWN
RP11-758I	70.79821	0.367713	0.144397	2.546547	0.010879449	0.0177781	NOT
PPP2R3A	166.0279	0.479603	0.188344	2.546419	0.010883437	0.0177836	NOT
WEE2-AS1	49.1876	-0.36356	0.142797	-2.54597	0.010897533	0.0178056	NOT
LINC00675	10.53823	-0.81646	0.320719	-2.54573	0.010904903	0.0178166	DOWN
RAB4B	471.545	0.320523	0.125938	2.545096	0.010924768	0.017848	NOT
RP11-367(	7.77059	0.555674	0.21835	2.544882	0.010931482	0.0178579	NOT
RP11-6L6.‹	1.744668	0.896714	0.352394	2.544633	0.010939263	0.0178689	UP
KRT79	0.680409	1.382345	0.543241	2.544625	0.010939528	0.0178689	UP
EFCAB6	32.74002	-0.46612	0.183188	-2.54449	0.010943625	0.0178746	NOT
RP11-305I	0.872245	2.262272	0.889107	2.544431	0.010945588	0.0178767	UP
RP11-316(	9.728737	0.538712	0.211733	2.544294	0.010949899	0.0178827	NOT
GZMA	193.4439	-0.5493	0.215902	-2.54422	0.010952243	0.0178855	NOT
RPL10A	16391.99	0.286895	0.112766	2.544166	0.010953888	0.0178868	NOT
RP11-709I	1.571949	0.785637	0.308801	2.544152	0.010954332	0.0178868	UP
RP11-407I	6.258655	-0.64897	0.255135	-2.54364	0.010970256	0.0179117	DOWN
RP11-4K3_	4.535088	0.595026	0.233947	2.543423	0.01097723	0.017922	UP
KIAA1161	4980.985	-0.40247	0.158245	-2.54332	0.010980306	0.017926	NOT
PGLS	2294.316	0.336659	0.132371	2.543301	0.010981043	0.0179262	NOT
PDCD1	61.62888	0.713003	0.280391	2.542891	0.010993941	0.0179462	UP
RP1-278O	1.61686	0.764461	0.300655	2.542649	0.011001569	0.0179575	UP
ORC3	817.4636	0.249191	0.09802	2.542261	0.011013802	0.0179765	NOT

ST3GAL1	7460.094	-0.44388	0.174608	-2.54218	0.011016352	0.0179794	NOT
ZNF705E	2.692404	0.627428	0.246809	2.542161	0.011016926	0.0179794	UP
IDH3B	3215.637	0.199259	0.078387	2.541987	0.011022438	0.0179874	NOT
LRRC27	218.4412	0.255493	0.10051	2.541963	0.011023195	0.0179875	NOT
RP5-881L2	15.467	0.714236	0.280986	2.541893	0.011025391	0.0179901	UP
PELI1	891.3007	-0.37803	0.148747	-2.5414	0.011040993	0.0180145	NOT
FBXL17	989.4303	-0.22861	0.089964	-2.54113	0.011049538	0.0180267	NOT
CH17-373	2.36034	0.798401	0.314193	2.541119	0.011049826	0.0180267	UP
RP4-612B1	3.350838	0.613169	0.241308	2.541027	0.011052747	0.0180304	UP
MIR3646	7.70218	0.652706	0.256938	2.540327	0.011074893	0.0180655	UP
PSMB3	6453.22	0.326028	0.128353	2.540083	0.011082614	0.018077	NOT
DHX38	1737.097	0.188639	0.074284	2.539439	0.011103049	0.0181093	NOT
RNASEH2E	538.6769	0.254236	0.100119	2.53934	0.011106198	0.0181134	NOT
ALKBH3	499.9147	-0.296	0.116585	-2.5389	0.011120062	0.0181349	NOT
CTD-2246	1.730967	0.822498	0.323961	2.53888	0.011120807	0.0181351	UP
SEPHS1	2495.794	0.176114	0.069372	2.538683	0.011127062	0.0181442	NOT
CTD-2286	9.437876	0.457482	0.180232	2.538301	0.011139227	0.018163	NOT
VN1R87P	0.689858	1.765847	0.69569	2.538267	0.011140294	0.0181636	UP
GSN-AS1	4.592515	0.568809	0.224142	2.537716	0.01115785	0.0181903	NOT
SAMD12-A	1.990391	1.31914	0.519815	2.537712	0.011157963	0.0181903	UP
IGHV3-7	6.28714	-1.13465	0.447134	-2.5376	0.011161593	0.0181951	DOWN
TGIF2-C20	3.349379	-0.54475	0.214675	-2.53756	0.011162717	0.0181959	NOT
LINC00571	4.687938	-0.6178	0.243481	-2.53739	0.011168361	0.018204	DOWN
ZNF780A	384.107	0.239844	0.094542	2.536912	0.011183522	0.0182277	NOT
AC107081	5.204491	0.554172	0.218473	2.536569	0.011194456	0.0182444	NOT
RP11-10O	2.157798	0.641278	0.252828	2.536416	0.011199357	0.0182513	UP
RP11-394A	0.865107	1.695724	0.668588	2.536278	0.011203783	0.0182575	UP
DMAP1	1448.909	0.228706	0.090181	2.536072	0.011210358	0.0182671	NOT
RP11-236I	1.301124	1.095535	0.431995	2.535993	0.011212894	0.0182701	UP
RP11-716(	1.262981	1.220512	0.481317	2.535777	0.011219826	0.0182804	UP
RP11-200A	10.6481	-0.68682	0.270855	-2.53575	0.011220546	0.0182805	DOWN
SPINK14	1.609731	1.229343	0.48487	2.535408	0.01123163	0.0182974	UP
SEMA3C	130.9376	0.817577	0.322469	2.535366	0.011232997	0.0182986	UP
KLHL32	29.67911	-0.49971	0.197126	-2.53496	0.01124612	0.0183189	NOT
RP11-490(	0.815665	1.811737	0.714707	2.534935	0.011246827	0.018319	UP
TMEM87A	1176.256	0.199033	0.078521	2.534787	0.011251587	0.0183256	NOT
GTF2H1	1163.233	0.159504	0.062927	2.53476	0.011252431	0.0183259	NOT
TRPV3	52.23025	-0.74382	0.293493	-2.53439	0.011264332	0.0183442	DOWN
C1orf132	59.93941	0.485577	0.191598	2.534351	0.011265587	0.0183447	NOT
POLR2B	2116.33	0.220282	0.086919	2.53434	0.011265921	0.0183447	NOT
RPL7AP30	17.81179	0.447755	0.17669	2.534132	0.011272617	0.018354	NOT
RP11-771I	1.9421	0.743019	0.293206	2.534121	0.011272976	0.018354	UP
PIWIL4	78.73801	0.632377	0.249567	2.533901	0.011280057	0.0183644	UP
AC104135	1.14594	1.276139	0.50382	2.532925	0.011311504	0.0184146	UP
ZFAND2A	1612.674	0.357881	0.141308	2.532635	0.01132089	0.0184288	NOT
PPIAP16	1.582935	0.772225	0.304988	2.531982	0.011341966	0.018462	UP
CCL5	1038.794	-0.6052	0.23907	-2.53147	0.01135864	0.018488	DOWN
AC062017	29.75838	0.479231	0.189332	2.531162	0.011368523	0.018503	NOT
TMEM41B	2061.842	0.2057	0.081339	2.528907	0.011441845	0.0186213	NOT
C3orf67	103.4346	0.46718	0.184741	2.528842	0.011443947	0.0186236	NOT
RP11-57H	3.131251	-0.72627	0.287266	-2.52822	0.011464347	0.0186557	DOWN
RP11-111I	6.042081	-0.60478	0.239224	-2.52811	0.011467699	0.0186601	DOWN

C15orf38-	6.566168	0.547033	0.216387	2.528034	0.011470339	0.0186632	NOT
NCKAP1	3851.066	0.216197	0.085526	2.527864	0.011475882	0.0186712	NOT
CEBPG	3988.347	0.265217	0.104926	2.527665	0.01148238	0.0186806	NOT
RP11-245(	2.129793	1.125536	0.445345	2.527334	0.011493224	0.0186972	UP
SRPX2	179.0581	0.555652	0.21986	2.527302	0.011494263	0.0186978	NOT
PCDHGA7	30.70966	0.624209	0.247013	2.52703	0.011503162	0.0187112	UP
RP11-701I	4.411615	0.871097	0.34473	2.526893	0.01150767	0.0187174	UP
MBTPS2	1290.346	-0.22875	0.090531	-2.52675	0.011512415	0.018724	NOT
RP5-1028I	14.11566	0.802001	0.317445	2.526421	0.011523126	0.0187403	UP
RP11-272(	8.629096	-0.40475	0.160222	-2.5262	0.011530228	0.0187508	NOT
CTB-133G	5.374079	-0.54083	0.21409	-2.52618	0.011531062	0.018751	NOT
KRT16	27.8771	1.499421	0.593597	2.525991	0.011537259	0.01876	UP
FYTTD1P1	48.67087	0.838248	0.331865	2.525869	0.011541236	0.0187654	UP
RP4-601P(	47.40533	1.016702	0.40254	2.525715	0.011546305	0.0187725	UP
PRSS3	384.768	1.006763	0.398644	2.52547	0.011554372	0.0187845	UP
CA11	133.7869	0.448558	0.17771	2.5241	0.011599506	0.0188568	NOT
KDR	1483.04	-0.42012	0.166481	-2.52355	0.011617611	0.0188851	NOT
GOSR1	1839.481	-0.14391	0.057043	-2.52286	0.011640535	0.0189213	NOT
MSANTD3	578.3836	0.2833	0.112311	2.522468	0.011653462	0.0189408	NOT
FBXO2	1898.637	0.678459	0.26897	2.522434	0.011654579	0.0189408	UP
PLA2G4A	52.12629	-0.57582	0.22828	-2.52243	0.01165461	0.0189408	NOT
RP11-379I	116.8835	0.572022	0.226862	2.521458	0.011686973	0.0189923	NOT
FAM162A	4253.951	-0.35275	0.139924	-2.52099	0.011702432	0.0190162	NOT
RP11-231(	3.239896	0.673831	0.26729	2.520973	0.011703075	0.0190162	UP
RP13-216I	8.408501	-0.44062	0.17482	-2.52042	0.011721496	0.019045	NOT
SLC25A11	3114.239	-0.25604	0.10159	-2.52031	0.01172528	0.0190501	NOT
LDHAP7	30.65184	-0.595	0.236101	-2.52009	0.011732358	0.0190599	DOWN
AL078471.	1.131719	2.572868	1.020946	2.520083	0.0117327	0.0190599	UP
PSG9	1.397604	2.024415	0.803365	2.519919	0.011738176	0.0190676	UP
RFESD	59.99267	0.277483	0.110117	2.519898	0.01173887	0.0190677	NOT
RP11-8H2	2.455721	0.712377	0.282714	2.519781	0.011742786	0.0190729	UP
ARL9	5.267543	0.741844	0.294441	2.519503	0.01175208	0.0190869	UP
KCNIP2	28.46992	0.423703	0.168176	2.5194	0.0117555	0.0190913	NOT
RP11-173/	3.094551	0.738147	0.292989	2.519372	0.011756423	0.0190917	UP
LINC0101(	28.69869	-0.83112	0.329902	-2.51929	0.011759069	0.0190931	DOWN
AC073850	2.516758	0.907045	0.36004	2.519288	0.011759234	0.0190931	UP
MUCL1	3.601206	1.327999	0.527134	2.519285	0.011759352	0.0190931	UP
GOLGA6L5	10.76701	0.547649	0.217424	2.518799	0.011775594	0.0191183	NOT
ST6GALNA	4.288144	0.544301	0.2161	2.518743	0.011777466	0.0191202	NOT
RPS6KA6	38.32934	-1.02711	0.407793	-2.5187	0.011778985	0.0191216	DOWN
RPL10	39586.23	0.267539	0.106229	2.518521	0.011784876	0.01913	NOT
LINC0150:	86.53945	0.581328	0.230829	2.518432	0.011787866	0.0191338	NOT
ASTN1	23.16734	-0.64116	0.254607	-2.51822	0.011795116	0.0191444	DOWN
ARHGEF38	0.79748	1.827628	0.725786	2.518137	0.011797736	0.0191475	UP
SFPQ	6320.155	0.186972	0.074256	2.51795	0.011804021	0.0191566	NOT
AF186192.	1.057606	1.049645	0.416897	2.517755	0.011810531	0.019166	UP
NCLN	4332.417	0.250498	0.099504	2.517471	0.011820074	0.0191804	NOT
TMEM135	1404.163	-0.27002	0.107272	-2.51718	0.011829809	0.0191951	NOT
ADO	610.3794	-0.1859	0.073859	-2.51699	0.011836206	0.0192043	NOT
AC004041	2.528179	0.695818	0.276544	2.516119	0.011865497	0.0192507	UP
ZRANB2-A	5.452931	-0.4763	0.189315	-2.51593	0.011871858	0.0192599	NOT
DECR2	4307.939	-0.35565	0.141369	-2.51574	0.011878237	0.0192691	NOT

RP11-505I	5.868105	-0.41646	0.165592	-2.51498	0.011903864	0.0193096	NOT
SPAST	736.5693	0.172508	0.068598	2.514764	0.011911193	0.0193203	NOT
CDH23	696.1298	-0.50471	0.200705	-2.51468	0.011913984	0.0193237	NOT
TNFAIP2	2680.22	0.449817	0.178924	2.514014	0.011936567	0.0193592	NOT
CTSW	174.3652	-0.56068	0.223133	-2.51274	0.011979629	0.0194279	NOT
RP11-660I	143.3982	-0.42795	0.170316	-2.51267	0.011982085	0.0194308	NOT
BCRP3	35.84641	0.652843	0.259854	2.512343	0.01199326	0.0194478	UP
AC114752	1.431694	0.843187	0.335653	2.512083	0.0120021	0.0194609	UP
PRKACB	1122.809	-0.25496	0.101502	-2.5119	0.012008285	0.0194698	NOT
IL11RA	813.9	0.326677	0.130058	2.511768	0.012012799	0.019476	NOT
PDE3A	295.6625	0.500383	0.199225	2.511646	0.01201695	0.0194816	NOT
GCSHP5	7.5645	-0.54193	0.215796	-2.51129	0.012029007	0.0195	NOT
RBM43	347.0202	-0.28035	0.111658	-2.51077	0.012046885	0.0195278	NOT
RP4-641G	8.717147	0.421231	0.167788	2.510498	0.012056086	0.0195416	NOT
PSMC5	6111.651	0.244829	0.097529	2.510305	0.012062696	0.0195512	NOT
CTD-2373	2.878936	0.607041	0.241825	2.510253	0.012064468	0.0195529	UP
SNORA59A	3.046951	0.517896	0.20632	2.51016	0.012067632	0.0195569	NOT
SAP25	4.74234	0.612833	0.244271	2.508827	0.012113271	0.0196297	UP
PRKXP1	7.694272	0.622379	0.248103	2.508551	0.012122745	0.0196439	UP
RP11-108	1.068842	1.333652	0.531652	2.508504	0.012124343	0.0196453	UP
FAM73B	815.9668	0.232365	0.092632	2.508472	0.012125468	0.019646	NOT
EIF4B	10798.22	-0.20448	0.081541	-2.50765	0.012153598	0.0196904	NOT
GTF3C1	2597.23	0.180317	0.071919	2.507222	0.012168427	0.0197133	NOT
MAGED1	7457.596	0.345899	0.137977	2.506922	0.012178756	0.0197289	NOT
RP4-665J2	445.8725	-0.4204	0.167705	-2.50681	0.012182657	0.0197341	NOT
LINC01342	0.975	1.193773	0.476245	2.506637	0.012188592	0.0197425	UP
PGM2L1	175.5458	0.432479	0.17254	2.50654	0.012191943	0.0197468	NOT
GLUD1P8	10.04943	-0.5546	0.221268	-2.50645	0.012195187	0.0197509	NOT
RPS19P3	5.087729	0.477271	0.190426	2.506331	0.012199125	0.0197561	NOT
CTD-2034	62.06104	-0.83387	0.332714	-2.50627	0.012201233	0.0197584	DOWN
AC005884	3.926713	0.59514	0.23748	2.506064	0.012208351	0.0197687	UP
UBE2DNL	2.301102	-1.90911	0.761805	-2.50603	0.012209446	0.0197693	DOWN
IGKV1OR2	1.116654	-1.20361	0.480295	-2.50599	0.012210907	0.0197706	DOWN
FAM174B	461.9223	0.485037	0.193559	2.505884	0.012214565	0.0197753	NOT
RP11-79P	11.12493	0.474644	0.189427	2.505677	0.012221717	0.0197857	NOT
GFI1B	2.348051	-0.93364	0.372637	-2.5055	0.012227684	0.0197942	DOWN
AC026202	27.23617	-0.46133	0.184131	-2.50545	0.012229668	0.0197963	NOT
RP11-135	1.814559	0.774151	0.30899	2.505426	0.012230411	0.0197963	UP
EVL	2167.287	-0.36435	0.145442	-2.5051	0.012241606	0.0198133	NOT
RP4-541C	6.652235	1.117719	0.44619	2.505029	0.012244122	0.0198162	UP
RN7SL381	2.509865	0.750062	0.299444	2.504847	0.012250444	0.0198249	UP
SMS	1919.205	0.251945	0.100584	2.504823	0.012251277	0.0198249	NOT
RAB15	1029.72	0.335537	0.133957	2.504813	0.012251631	0.0198249	NOT
RP11-4K1	2.188576	0.811831	0.324183	2.504235	0.012271635	0.0198559	UP
ATP5E	10478.03	0.334487	0.13357	2.504218	0.012272245	0.0198559	NOT
RP11-108I	4.018262	-0.55446	0.221427	-2.50404	0.012278549	0.019865	NOT
GS1-124K	11.84235	0.501598	0.200335	2.503795	0.012286905	0.0198773	NOT
ENPP5	136.0936	0.892185	0.356356	2.503633	0.012292549	0.0198843	UP
RP11-483I	15.48762	0.860615	0.343747	2.50363	0.012292668	0.0198843	UP
CRTAM	26.4168	-0.62076	0.247964	-2.50341	0.012300332	0.0198955	DOWN
DSCAS	3.368573	0.666243	0.266226	2.50255	0.012330233	0.0199427	UP
DIRC3	5.884898	0.53126	0.212294	2.502473	0.012332917	0.0199459	NOT

KLK6	3.648485	1.628289	0.65078	2.502056	0.012347432	0.0199682	UP
FAM120A	6754.561	-0.14852	0.059361	-2.5019	0.012352818	0.0199758	NOT
TTLL12	2316.561	0.25367	0.101404	2.501588	0.012363779	0.0199923	NOT
ANO7P1	58.39458	0.552793	0.221042	2.500845	0.012389756	0.0200332	NOT
KIRREL3	12.41852	-0.61206	0.244874	-2.49947	0.012437773	0.0201096	DOWN
GPR162	195.4865	-0.56365	0.225527	-2.49925	0.01244551	0.020121	NOT
ANKRD33E	131.8379	0.683982	0.273677	2.499231	0.012446318	0.0201211	UP
SCLT1	165.3735	0.273277	0.109359	2.498894	0.01245816	0.0201391	NOT
AC073046	6.873095	0.553972	0.221695	2.498797	0.012461583	0.0201434	NOT
CCNJ	179.6117	0.453612	0.181537	2.498729	0.012463962	0.0201461	NOT
RPL7P47	7.199098	0.549162	0.219781	2.498681	0.012465656	0.0201476	NOT
OLAH	2.479232	0.835027	0.334195	2.498623	0.012467698	0.0201498	UP
SLC37A3	650.4011	0.253697	0.101543	2.498417	0.012474938	0.0201603	NOT
AC006548	3.894456	0.657087	0.263011	2.498327	0.012478108	0.0201642	UP
GJD3	1.793304	0.905246	0.362391	2.497982	0.012490258	0.0201827	UP
KIFC3	2400.419	0.298225	0.11943	2.497082	0.012521982	0.0202328	NOT
AC006116	2.186336	0.741175	0.296821	2.497042	0.012523422	0.0202328	UP
RAB11FIP2	504.372	0.284678	0.114006	2.497038	0.01252357	0.0202328	NOT
PCDHGC3	176.48	-0.55946	0.224049	-2.49702	0.012524167	0.0202328	NOT
CTB-50E1	33.65744	-0.51851	0.207692	-2.49654	0.012541238	0.0202591	NOT
RP11-104I	8.235706	0.571603	0.228973	2.496373	0.012547047	0.0202671	NOT
CDK18	1244.818	0.523967	0.209892	2.496358	0.012547595	0.0202671	NOT
PCMTD1	1592.228	-0.21815	0.087391	-2.49629	0.012549905	0.0202696	NOT
AP5M1	1194.597	-0.22951	0.091994	-2.49481	0.012602308	0.020353	NOT
ZFHX4	538.1841	-0.45379	0.181902	-2.49471	0.012606107	0.020358	NOT
ZNF22	1123.961	-0.25017	0.100285	-2.49461	0.012609679	0.0203626	NOT
LDLRAD2	31.13998	0.408071	0.163605	2.494251	0.012622318	0.0203818	NOT
NPR2	937.6975	0.463228	0.185763	2.493657	0.012643468	0.0204148	NOT
RP11-255I	1.030114	-1.20594	0.483733	-2.49299	0.012667074	0.0204517	DOWN
TGFBI	9952.156	0.375405	0.15059	2.492897	0.012670575	0.0204561	NOT
RPL36	13797.01	0.392061	0.15728	2.492765	0.012675285	0.0204625	NOT
SLMO2	1842.212	0.195857	0.078573	2.492665	0.012678843	0.0204671	NOT
POLR1B	945.7396	-0.21167	0.084921	-2.49258	0.012681879	0.0204708	NOT
RP11-703I	36.83396	-0.37742	0.151461	-2.49186	0.012707533	0.020511	NOT
RPL13A	53198.88	0.348653	0.139921	2.491775	0.012710658	0.0205149	NOT
LIG4	487.8306	-0.29031	0.116516	-2.49158	0.012717467	0.0205247	NOT
CT55	1.121642	2.502784	1.004504	2.491563	0.012718227	0.0205247	UP
CHD5	4.445692	0.841441	0.337747	2.491335	0.012726417	0.0205367	UP
ITPK1	4468.034	0.283934	0.114014	2.490341	0.012762059	0.020593	NOT
TLR1	243.2537	-0.43618	0.175156	-2.49025	0.012765473	0.0205973	NOT
OMA1	777.6759	-0.23044	0.092554	-2.48974	0.012783549	0.0206253	NOT
IGLV6-57	128.4991	-1.00305	0.402889	-2.48964	0.012787156	0.0206299	DOWN
MSTN	4.920082	0.722782	0.290327	2.489548	0.012790569	0.0206342	UP
2-Sep	6689.132	0.235046	0.094415	2.489492	0.012792581	0.0206363	NOT
RP11-93B	19.74813	-0.65405	0.262741	-2.48933	0.012798532	0.0206446	DOWN
CYP2A7	3565.138	-1.18801	0.477278	-2.48914	0.012805259	0.0206543	DOWN
SHROOM3	1570.881	-0.41373	0.166252	-2.48856	0.012826073	0.0206867	NOT
WBP1LP2	17.59152	0.349671	0.140548	2.48791	0.012849641	0.0207235	NOT
LHFPL5	1.190472	1.466275	0.589412	2.487693	0.012857479	0.0207349	UP
RP11-62F2	1.070254	1.171711	0.471101	2.487178	0.012876096	0.0207637	UP
SLC30A5	1598.81	0.168611	0.06781	2.486529	0.012899594	0.0208004	NOT
PEBP1P2	12.68088	-0.51968	0.209003	-2.48648	0.012901395	0.0208021	NOT

RP11-857I	2.742524	0.8849	0.355907	2.486324	0.012907031	0.02081	UP
PDE8B	136.6537	-0.35824	0.144088	-2.48624	0.012910007	0.0208135	NOT
FLJ33581	6.58958	1.391246	0.559629	2.486012	0.012918355	0.0208258	UP
BAGE5	1.129456	2.565284	1.031903	2.485974	0.012919725	0.0208268	UP
FAM216B	0.853014	1.801171	0.724689	2.485441	0.012939094	0.0208568	UP
ZNF629	767.9534	0.266178	0.10711	2.4851	0.012951503	0.0208756	NOT
SPACA1	1.723476	1.569563	0.631608	2.485029	0.012954099	0.0208781	UP
ZNF383	251.7679	0.244567	0.098417	2.485016	0.012954569	0.0208781	NOT
C5orf63	130.3799	0.46484	0.1871	2.484449	0.012975204	0.0209101	NOT
RP11-344I	20.97489	-0.56519	0.227526	-2.48409	0.012988441	0.0209302	NOT
ABC7-430	1.438482	0.740741	0.298243	2.483685	0.013003076	0.0209526	UP
LRRC16A	390.7136	0.442328	0.178096	2.48365	0.01300435	0.0209526	NOT
RPS15AP1	3.452586	-0.60613	0.244047	-2.48364	0.013004589	0.0209526	DOWN
BMS1	1459.563	0.144062	0.058015	2.483187	0.013021257	0.0209782	NOT
FAM92A1	502.395	-0.27754	0.111783	-2.48281	0.01303524	0.0209995	NOT
UMODL1	9.335565	1.109938	0.447068	2.482704	0.013038956	0.0210043	UP
CH507-9B	138.3316	-0.48546	0.195554	-2.4825	0.013046344	0.021015	NOT
TMEM138	899.9811	0.208249	0.083893	2.482328	0.013052705	0.021024	NOT
OGDH	6180.456	0.290034	0.116851	2.482085	0.013061601	0.0210371	NOT
NIP7	777.8368	-0.22341	0.09001	-2.48202	0.013064044	0.0210398	NOT
UBE2D4	964.426	-0.24893	0.1003	-2.48189	0.013068867	0.0210464	NOT
RP11-686I	5.302206	0.674769	0.271939	2.481325	0.013089488	0.0210783	UP
RASA2	407.9349	-0.21906	0.088287	-2.48127	0.013091675	0.0210806	NOT
USP40	1820.02	0.208231	0.083924	2.481184	0.013094689	0.0210843	NOT
LL22NC03	1.34729	1.472101	0.593407	2.480761	0.013110216	0.021108	UP
RP11-488(	4.148471	0.613122	0.247154	2.48073	0.013111371	0.0211087	UP
CTB-58E1	129.0088	0.344236	0.138769	2.480644	0.013114522	0.0211125	NOT
ASF1A	680.8044	0.261194	0.105297	2.480547	0.013118108	0.021117	NOT
RP11-125(	6.882408	-0.67712	0.272993	-2.48035	0.013125407	0.0211276	DOWN
CTD-3224	1.320631	0.981274	0.395636	2.480241	0.013129371	0.0211327	UP
VAV2	3783.259	-0.23327	0.094051	-2.48022	0.013130263	0.0211329	NOT
CTD-2666	6.052028	0.524776	0.211635	2.479626	0.013152014	0.0211667	NOT
LINC00871	1.248776	1.886982	0.761029	2.479513	0.013156191	0.0211722	UP
SPCS2	1995.725	0.241245	0.097302	2.479335	0.013162775	0.0211816	NOT
RNPEP	3453.58	0.242113	0.097661	2.479109	0.01317111	0.0211937	NOT
TAAR3	14.29602	-0.76848	0.309984	-2.47909	0.013171941	0.0211938	DOWN
CYS1	123.5929	-0.8415	0.339468	-2.47889	0.013179214	0.0212043	DOWN
VAV1	250.1266	-0.47001	0.189616	-2.47877	0.013183724	0.0212103	NOT
HDAC9	110.3385	-0.53372	0.215333	-2.47857	0.01319107	0.0212209	NOT
AP000473.	17.05005	-0.58789	0.237323	-2.47716	0.013243306	0.0213033	DOWN
CCT6P1	101.8919	0.269036	0.108607	2.477144	0.013243843	0.0213033	NOT
C6orf229	1.525354	1.174046	0.473962	2.477089	0.013245871	0.0213054	UP
RP11-320I	12.81639	-0.6134	0.247665	-2.47674	0.013258747	0.0213248	DOWN
CCDC121	102.3137	0.275245	0.111135	2.476662	0.013261747	0.0213284	NOT
AP3S1	1960.531	0.211376	0.085348	2.476637	0.013262653	0.0213286	NOT
TXNP5	4.502888	0.762238	0.30779	2.476482	0.013268429	0.0213367	UP
RP11-806I	1.420714	0.893648	0.36087	2.476372	0.013272508	0.021342	UP
RP11-677I	4.497011	0.544295	0.219805	2.476268	0.013276371	0.021347	NOT
BLOC1S5-	12.58606	-0.68476	0.276543	-2.47613	0.013281526	0.0213531	DOWN
AC108078	63.7373	-0.6935	0.280075	-2.47612	0.013281734	0.0213531	DOWN
RPL26P37	2.618414	-0.63576	0.256783	-2.47587	0.013291019	0.0213668	DOWN
RP11-503(	16.46179	-0.67534	0.272792	-2.47566	0.013298835	0.0213781	DOWN

CTA-384D	3.148297	0.799267	0.322959	2.474827	0.013330064	0.0214271	UP
RSRC2	1727.248	0.14648	0.059199	2.474359	0.013347566	0.021454	NOT
CARD8	846.3629	0.245638	0.099296	2.4738	0.013368469	0.0214863	NOT
RP11-669I	1.514669	0.890613	0.360053	2.473565	0.013377241	0.0214983	UP
LINC0137(	242.8053	-1.10311	0.44596	-2.47356	0.013377468	0.0214983	DOWN
FRAT2	794.9708	0.324539	0.131207	2.473486	0.013380201	0.0215014	NOT
KRT18P11	15.81921	-0.55223	0.223277	-2.4733	0.013387191	0.0215114	NOT
ITGA8	104.5002	0.513828	0.20778	2.472944	0.013400519	0.0215316	NOT
POU3F4	1.289554	2.752613	1.113102	2.47292	0.013401398	0.0215317	UP
OXT	103.3511	-1.17797	0.476353	-2.4729	0.013402297	0.0215319	DOWN
ABCG1	700.4682	0.462218	0.186953	2.472382	0.013421607	0.0215617	NOT
SMIM13	986.4495	0.220696	0.089281	2.471932	0.0134385	0.0215876	NOT
GOT2P3	9.405575	-0.73217	0.296265	-2.47134	0.013460668	0.0216219	DOWN
RP11-187/	2.72524	0.632655	0.255999	2.471314	0.013461759	0.0216224	UP
AC010890	3.558653	-0.77472	0.313535	-2.47093	0.013476213	0.0216444	DOWN
ASB1	702.8758	0.277579	0.112362	2.470393	0.013496459	0.0216757	NOT
SCAF8	862.6865	-0.20005	0.080999	-2.46984	0.013517271	0.0217078	NOT
C17orf67	81.37259	0.44713	0.181046	2.469707	0.013522372	0.0217148	NOT
ZNF674	116.7722	0.201943	0.081773	2.469571	0.013527524	0.0217218	NOT
COMMD8	425.7523	0.277309	0.112299	2.469381	0.013534686	0.021732	NOT
ZNF490	25.89378	0.352414	0.142724	2.469196	0.013541686	0.021742	NOT
CD70	8.933848	0.644529	0.26103	2.469174	0.013542529	0.0217421	UP
RP3-336K2	1.347711	0.986347	0.399481	2.469073	0.013546365	0.021747	UP
IGHV1-67	2.981897	-1.19898	0.485604	-2.46905	0.013547209	0.0217471	DOWN
RP11-522I	1.246456	0.923152	0.374026	2.46815	0.013581354	0.0218006	UP
LINC0086⁄	69.99719	-0.73148	0.296382	-2.46805	0.013585163	0.0218055	DOWN
PAM16	408.5192	0.253066	0.102544	2.467887	0.013591324	0.0218141	NOT
RP11-715J	2.96393	0.705652	0.285942	2.467813	0.013594132	0.0218173	UP
HSPB6	542.3864	-0.56744	0.22994	-2.46776	0.013596205	0.0218194	NOT
CES2	27288.48	-0.52461	0.212618	-2.46739	0.013610389	0.0218397	NOT
LINC0128!	10.95171	0.522837	0.211899	2.467383	0.013610453	0.0218397	NOT
RPS7P4	7.719375	0.470078	0.190524	2.467287	0.013614134	0.0218444	NOT
THAP9-AS	499.1648	0.298411	0.12095	2.467223	0.013616554	0.021847	NOT
CCDC51	618.6922	0.221976	0.089973	2.467133	0.013619959	0.0218512	NOT
ZNF346-IT	3.419127	0.562813	0.228161	2.466735	0.013635107	0.0218737	NOT
SDR42E1	245.3989	-0.56163	0.227684	-2.46672	0.013635583	0.0218737	NOT
MRPS36	1055.468	-0.2439	0.09891	-2.46583	0.013669499	0.0219268	NOT
SH3TC2	18.95135	0.505083	0.204837	2.465783	0.013671426	0.0219287	NOT
TCEAL8	1276.026	0.423157	0.17162	2.465657	0.013676215	0.0219351	NOT
RP11-182J	1.873614	0.90615	0.367564	2.465285	0.01369044	0.0219566	UP
RP11-598I	15.64487	0.560576	0.227422	2.464915	0.013704573	0.021978	NOT
IGLV3-19	251.8533	-0.99018	0.401716	-2.46488	0.013706072	0.0219791	DOWN
DISP1	607.0778	0.406994	0.165138	2.464574	0.01371762	0.0219964	NOT
SMAD5	2282.493	0.251361	0.102002	2.464271	0.013729213	0.0220119	NOT
RP11-244I	1.151085	2.199065	0.892381	2.464267	0.013729379	0.0220119	UP
RP11-731I	1.842444	-1.11826	0.453798	-2.46423	0.013730693	0.0220119	DOWN
RP13-616I	59.39237	0.621987	0.252406	2.464228	0.013730873	0.0220119	UP
TRAK1	1399.558	0.206963	0.083987	2.464217	0.013731311	0.0220119	NOT
CTNNBL1	2745.551	0.338152	0.137232	2.464096	0.013735942	0.0220181	NOT
KRT8P13	2.406529	-0.5734	0.232723	-2.46389	0.01374381	0.0220294	NOT
RP11-753I	1.955322	0.753866	0.305998	2.463628	0.013753872	0.0220443	UP
CCDC92	1624.887	-0.21497	0.087264	-2.46343	0.013761635	0.0220554	NOT

TTC4	86.67779	0.209975	0.085243	2.463255	0.013768179	0.0220647	NOT
ZNF671	140.5059	0.402702	0.163523	2.462655	0.013791248	0.0221003	NOT
CLDN23	505.1371	-0.32534	0.13211	-2.46262	0.013792437	0.022101	NOT
RPL5P22	1.717578	0.910226	0.369622	2.462584	0.013793986	0.0221016	UP
TRGV2	2.404284	-0.79254	0.321836	-2.46257	0.013794582	0.0221016	DOWN
POLR2D	1393.898	0.154232	0.062631	2.462552	0.013795207	0.0221016	NOT
C12orf76	355.0561	0.246641	0.10017	2.462233	0.013807497	0.02212	NOT
CTD-3193	4.031092	0.711838	0.289118	2.462099	0.01381264	0.0221269	UP
AMMECR1	887.217	0.183044	0.074348	2.461996	0.013816602	0.022132	NOT
RP13-463I	2.06175	0.991729	0.402955	2.461142	0.013849547	0.0221835	UP
AC018816	18.9467	0.602773	0.244929	2.461013	0.01385455	0.0221902	UP
AKNA	962.1435	-0.35301	0.143451	-2.46084	0.013861129	0.0221991	NOT
CST4	1.396803	2.216109	0.900554	2.460827	0.013861702	0.0221991	UP
AP000695.	37.36953	-0.49496	0.20114	-2.4608	0.013862848	0.0221997	NOT
MLC1	16.26444	-0.52007	0.211357	-2.46062	0.013869892	0.0222096	NOT
MLLT4-AS	67.20935	0.377186	0.15332	2.460124	0.013888883	0.0222388	NOT
IGLL5	384.2314	-0.92732	0.376949	-2.46007	0.013890827	0.0222406	DOWN
RP11-800/	2.177382	0.690643	0.280787	2.459672	0.013906422	0.0222643	UP
MIPEPP3	12.31027	0.360617	0.14663	2.45937	0.013918105	0.0222817	NOT
RPL11	22495.05	-0.26791	0.108938	-2.45933	0.013919752	0.022283	NOT
RP11-8L2.	1.087829	2.374678	0.965773	2.458836	0.013938823	0.0223123	UP
CTC-350I8	1.686584	0.845944	0.344138	2.458154	0.01396531	0.0223534	UP
VCX3B	1.348747	1.449396	0.589714	2.457795	0.013979309	0.0223745	UP
ZBBX	0.728162	1.687503	0.686622	2.457687	0.013983489	0.0223799	UP
TNNT3	5.148511	0.717762	0.292062	2.457572	0.013987964	0.0223858	UP
DPY19L1	1299.584	-0.25935	0.105552	-2.45713	0.014005238	0.0224121	NOT
MDS2	3.816015	0.634345	0.258185	2.456937	0.014012724	0.0224228	UP
ILKAP	843.9689	0.19286	0.078509	2.45653	0.014028606	0.0224469	NOT
UBR4	5170.388	-0.2394	0.097462	-2.45631	0.014037175	0.0224593	NOT
MPST	14804.35	-0.36749	0.14963	-2.45599	0.014049607	0.0224779	NOT
RP11-144I	3.273622	0.652802	0.265804	2.455956	0.014051048	0.0224789	UP
AP000696.	1.117773	2.544928	1.036275	2.455842	0.014055502	0.0224847	UP
RP11-421I	548.7597	-0.3138	0.1278	-2.45542	0.014071927	0.0225088	NOT
SZT2	1095.428	0.192736	0.078494	2.455415	0.014072196	0.0225088	NOT
DNAJC17	698.9098	-0.24958	0.101662	-2.45496	0.014090072	0.0225361	NOT
RP11-690I	1.453034	0.783948	0.319377	2.454619	0.014103402	0.0225561	UP
GAPDHP68	1.510383	0.848712	0.345772	2.454543	0.014106393	0.0225596	UP
LRRC49	37.22495	0.5163	0.210354	2.454433	0.014110682	0.0225652	NOT
UPF3AP2	4.545755	0.477734	0.194651	2.454306	0.014115668	0.0225713	NOT
RNF25	787.246	0.215376	0.087755	2.454295	0.014116111	0.0225713	NOT
AF124730.	16.08571	-0.7175	0.292359	-2.45416	0.014121285	0.0225774	DOWN
RHOJ	250.5165	0.326326	0.132969	2.454156	0.014121554	0.0225774	NOT
PLGRKT	589.9366	0.240936	0.098177	2.454109	0.014123399	0.0225786	NOT
RP3-331H	33.94584	0.565996	0.230633	2.454094	0.014123997	0.0225786	NOT
RP3-512B.	174.2168	0.45278	0.184517	2.453872	0.014132709	0.0225913	NOT
TAF5L	1110.415	0.165908	0.067638	2.452876	0.014171903	0.0226516	NOT
RP11-638I	1.524298	0.834102	0.340051	2.452871	0.01417212	0.0226516	UP
SLC7A6OS	114.6716	0.250604	0.10217	2.452828	0.014173823	0.0226531	NOT
RP11-111.	0.79797	2.075639	0.84624	2.452778	0.014175797	0.0226549	UP
FMO6P	1.609216	0.930841	0.379516	2.452705	0.014178673	0.0226573	UP
FASTKD1	621.9592	0.263221	0.107319	2.452698	0.014178916	0.0226573	NOT
CARNS1	62.76363	0.492385	0.20076	2.452601	0.01418275	0.0226621	NOT

CACNA1H	1634.281	0.786191	0.320557	2.452574	0.014183818	0.0226625	UP
UNC93B1	1136.976	0.266492	0.10866	2.452527	0.014185663	0.0226636	NOT
C17orf78	1.237266	0.881149	0.359284	2.452515	0.014186154	0.0226636	UP
AP000350.	1.971518	-0.76773	0.313109	-2.45195	0.014208389	0.0226978	DOWN
JMJD7	2.998409	0.610797	0.249131	2.451708	0.014218012	0.0227119	UP
PCED1B	207.5649	0.461092	0.188096	2.451358	0.014231819	0.022732	NOT
ISCA1P1	1.808051	0.666326	0.27182	2.451347	0.014232263	0.022732	UP
APAF1	417.8839	0.320238	0.130639	2.451322	0.014233273	0.0227323	NOT
HIST1H2BI	1215.59	0.477815	0.194925	2.451275	0.014235111	0.0227339	NOT
ABCB8	2070.105	0.26491	0.108072	2.451226	0.014237044	0.0227357	NOT
NR2F1	930.5506	-0.44605	0.181994	-2.45089	0.014250176	0.0227554	NOT
AC010084	2.155595	-1.01478	0.414107	-2.45053	0.014264524	0.022777	DOWN
FAM9A	2.445957	1.63448	0.667023	2.450409	0.014269386	0.0227834	UP
ANKRD44	379.8069	-0.31026	0.126617	-2.45037	0.014271054	0.0227841	NOT
ZBTB20-A:	1.826498	0.815123	0.332655	2.450357	0.014271469	0.0227841	UP
PIANP	4.171681	0.712531	0.290836	2.449943	0.01428788	0.022809	UP
RP1-154K	0.807322	1.689986	0.689833	2.44985	0.014291583	0.0228136	UP
ATPIF1	4928.817	-0.26823	0.109495	-2.44967	0.014298778	0.0228238	NOT
ZNF827	92.50258	0.532664	0.217453	2.449558	0.014303186	0.0228294	NOT
KB-1552D	10.34275	-0.61299	0.250247	-2.44954	0.01430397	0.0228294	DOWN
RP11-301I	22.54586	0.633164	0.258534	2.449055	0.014323167	0.0228587	UP
RPL19	37213.77	0.268415	0.109609	2.448839	0.014331743	0.0228711	NOT
IGKV1D-1:	19.97316	-1.49259	0.609525	-2.44878	0.014334193	0.0228737	DOWN
RPS10P16	2.910184	0.59839	0.244394	2.448466	0.014346615	0.0228922	UP
MSANTD3	1.246946	0.797831	0.325988	2.447424	0.014388144	0.0229571	UP
ARSE	5006.893	-0.6038	0.246719	-2.44731	0.014392515	0.0229628	DOWN
GUCY1A3	353.879	-0.4533	0.185232	-2.44719	0.014397542	0.0229695	NOT
RP11-683I	3.363631	1.141524	0.466614	2.446398	0.014429158	0.0230186	UP
RP11-253I	6.814759	0.602825	0.246515	2.445387	0.014469688	0.0230819	UP
RP11-242(	11.82556	-0.58193	0.238075	-2.44433	0.014512071	0.0231482	NOT
UBA5	1491.499	0.176451	0.07219	2.444238	0.014515842	0.0231529	NOT
KRT34	0.828534	1.833959	0.750411	2.443939	0.014527896	0.0231708	UP
RP11-158I	0.740408	1.471745	0.602494	2.442755	0.014575619	0.0232455	UP
HERC2	1675.328	0.207828	0.08509	2.442445	0.014588138	0.0232642	NOT
AC004987	25.20137	-0.29593	0.121169	-2.4423	0.014594032	0.0232722	NOT
STEAP2	421.05	0.647889	0.265288	2.442209	0.01459769	0.0232764	UP
FGF18	13.97691	0.706496	0.289288	2.442193	0.014598359	0.0232764	UP
HMGN2P3	12.70674	0.462402	0.189352	2.44202	0.014605342	0.023286	NOT
RYK	1169.019	0.176658	0.072342	2.441995	0.014606349	0.023286	NOT
KRT8P7	5.374291	-0.56766	0.23246	-2.44198	0.014606898	0.023286	NOT
CCAT1	141.5681	-0.74514	0.305159	-2.44181	0.014613842	0.0232958	DOWN
RP11-673I	2.222199	-0.60023	0.245816	-2.44177	0.014615444	0.023297	DOWN
PLA2G12A	3.960893	0.446573	0.182953	2.440922	0.014649828	0.0233504	NOT
AC125232	59.42682	-0.35653	0.146066	-2.44089	0.014651028	0.023351	NOT
CYP2B7P	3536.669	-0.91716	0.375758	-2.44083	0.014653368	0.0233534	DOWN
LINC0112;	297.963	-0.57823	0.236906	-2.44074	0.014657018	0.0233579	NOT
RP11-983I	370.7331	0.322827	0.132293	2.440231	0.014677881	0.0233898	NOT
CAMSAP1	761.6592	0.215397	0.088274	2.440102	0.014683107	0.0233967	NOT
ARHGAP5-	71.5633	0.324303	0.132919	2.43985	0.014693376	0.0234118	NOT
CNTRL	445.583	0.320951	0.131561	2.439563	0.014705039	0.023429	NOT
RP11-54C	152.7605	0.354224	0.145202	2.439525	0.01470657	0.0234301	NOT
GOT2P2	19.40072	-0.6773	0.277674	-2.43918	0.014720659	0.0234512	DOWN

LINC01587	11.66211	0.957629	0.392622	2.43906	0.014725512	0.0234576	UP
ROCK2	2182.511	0.252095	0.10337	2.438775	0.014737138	0.0234747	NOT
ATP6V1G2	21.53656	0.475188	0.194859	2.438623	0.014743345	0.0234833	NOT
HLA-F	4002.891	0.477362	0.195766	2.438429	0.014751271	0.0234945	NOT
RP11-348I	4.21054	0.597056	0.244872	2.438238	0.014759058	0.0235056	UP
TRGV3	2.600086	-0.74525	0.305678	-2.43802	0.014768155	0.0235187	DOWN
ANGPT4	6.185553	-0.71679	0.294114	-2.43712	0.014804934	0.0235759	DOWN
RP11-356(	1.117883	1.176537	0.48285	2.436653	0.014823897	0.0236048	UP
SLC35A2	1432.184	0.182665	0.074972	2.436453	0.014832097	0.0236165	NOT
AGGF1	1217.881	0.153254	0.062905	2.43629	0.01483879	0.0236258	NOT
RP11-378/	5.5848	0.539494	0.221446	2.43623	0.014841237	0.0236283	NOT
HBG2	2.432915	-0.80623	0.330984	-2.43586	0.01485647	0.0236512	DOWN
LIPN	1.553197	-1.2402	0.509212	-2.43553	0.014870035	0.0236713	DOWN
NT5CP1	1.917521	0.755111	0.310042	2.43551	0.014870829	0.0236713	UP
IL36G	2.50663	1.034603	0.424835	2.435308	0.014879133	0.0236832	UP
PRKX	369.1146	0.490257	0.201357	2.434771	0.014901197	0.0237169	NOT
EMR3	7.264196	-0.75884	0.311711	-2.43444	0.014914906	0.0237374	DOWN
RP11-666/	0.802312	1.343414	0.551919	2.434078	0.014929766	0.0237597	UP
ATP5G2	9818.544	0.247939	0.10188	2.433649	0.014947491	0.0237865	NOT
MTND5P1	2.515951	-0.83857	0.344586	-2.43355	0.014951716	0.0237919	DOWN
RP11-798I	668.0618	0.546349	0.224516	2.433456	0.014955446	0.0237964	NOT
LRP2	113.1545	0.979647	0.402609	2.433248	0.014964036	0.0238087	UP
ALKBH7	3235.492	-0.36345	0.149371	-2.43322	0.014965337	0.0238094	NOT
ZNF777	785.8568	0.17553	0.072144	2.433043	0.014972508	0.0238182	NOT
CTD-2337	6.268021	0.749031	0.307858	2.433042	0.014972577	0.0238182	UP
ZNF484	81.21499	-0.24786	0.101879	-2.43285	0.014980576	0.0238296	NOT
TYMP	4663.424	0.450918	0.185349	2.432805	0.014982387	0.0238311	NOT
RP11-507I	7.010259	0.459166	0.188751	2.432654	0.014988613	0.0238396	NOT
DUX4L26	3.675834	0.965126	0.396778	2.432407	0.014998823	0.0238545	UP
RP11-689(	0.967578	1.779652	0.731666	2.432327	0.01500214	0.0238584	UP
FBXO25	1732.455	-0.29298	0.120483	-2.4317	0.015028222	0.0238985	NOT
PGD	5405.86	0.369353	0.151897	2.431598	0.015032364	0.0239031	NOT
RP11-366I	1.189441	2.503046	1.029388	2.431587	0.015032842	0.0239031	UP
AC005758	1.400679	-1.99514	0.820529	-2.43152	0.015035499	0.0239059	DOWN
AP000320.	3.84196	-0.55564	0.228531	-2.43134	0.015042933	0.0239164	NOT
ACOT8	1092.146	0.244466	0.100557	2.43111	0.015052646	0.0239304	NOT
ZNF567	132.7817	0.246324	0.10133	2.430912	0.015060869	0.0239421	NOT
APOPT1	631.099	0.265373	0.109175	2.43072	0.015068852	0.0239535	NOT
PDZD3	8.140506	0.844237	0.347425	2.429984	0.015099509	0.0240008	UP
TRIM13	487.8901	-0.23151	0.095294	-2.4294	0.015123813	0.0240381	NOT
RP11-500(	2.610722	0.806231	0.331891	2.429204	0.015132015	0.0240497	UP
OGFRP1	19.80876	-0.35425	0.145833	-2.42915	0.015134311	0.024052	NOT
THAP2	175.6382	0.205236	0.08449	2.42912	0.015135513	0.0240525	NOT
RP11-384I	1.58865	0.732042	0.301378	2.428986	0.015141114	0.02406	UP
TNK1	262.765	-0.28177	0.116005	-2.42893	0.015143425	0.0240623	NOT
GSTM1	3923.491	-1.23175	0.507183	-2.4286	0.01515712	0.0240827	DOWN
RP11-20O	2.39268	0.654887	0.269687	2.428318	0.015169023	0.0240985	UP
RP1-224A(	2.876458	0.648451	0.267038	2.42831	0.015169381	0.0240985	UP
RP11-757I	8.353868	1.094372	0.450674	2.428302	0.015169693	0.0240985	UP
NCALD	982.9922	-0.44666	0.183961	-2.42803	0.015180993	0.0241151	NOT
SEC23IP	1463.906	-0.17111	0.070479	-2.42781	0.01519014	0.0241282	NOT
RP11-436I	5.07751	0.571802	0.235541	2.427612	0.0151986	0.0241403	NOT

RP11-504I	12.24178	0.479443	0.197498	2.427589	0.015199559	0.0241404	NOT
TYR	0.955121	2.18908	0.901858	2.427301	0.015211646	0.0241581	UP
RP11-228I	67.46962	0.450663	0.185666	2.427281	0.015212462	0.0241581	NOT
AC023115	2.778889	0.984214	0.405488	2.427233	0.015214472	0.0241599	UP
AC147651	7.734629	1.29192	0.532281	2.427139	0.015218437	0.0241649	UP
RP11-406/	9.264045	-1.01262	0.417268	-2.42678	0.01523351	0.0241874	DOWN
OVOL2	20.97174	1.010931	0.416641	2.42638	0.015250285	0.0242127	UP
RP11-101I	2.128856	0.636184	0.262239	2.42597	0.015267512	0.0242386	UP
ZSCAN1	3.230587	0.877968	0.361921	2.425853	0.015272452	0.0242451	UP
RP11-424I	2.623061	0.726604	0.299549	2.425661	0.015280548	0.0242565	UP
CDH1	9333.556	-0.44126	0.181913	-2.42564	0.015281415	0.0242565	NOT
CES1P2	7.370877	0.711867	0.293485	2.425564	0.015284636	0.0242602	UP
VPS37B	644.9806	-0.2934	0.120969	-2.42541	0.015291166	0.0242692	NOT
HRH3	1.247426	1.212603	0.499991	2.425249	0.015297879	0.0242785	UP
BLOC1S6	1662.938	-0.15568	0.064196	-2.42501	0.015307877	0.0242929	NOT
SF3B6	2170.054	0.184988	0.076292	2.424744	0.015319175	0.0243093	NOT
CTD-2008	12.46896	-0.33307	0.137366	-2.42471	0.015320446	0.0243093	NOT
C6orf201	13.31521	0.419404	0.172971	2.424705	0.015320822	0.0243093	NOT
GPR153	332.2651	0.502903	0.207466	2.42403	0.01534932	0.0243531	NOT
RP11-352I	291.3176	0.239807	0.098965	2.423149	0.015386614	0.0244109	NOT
RP11-532I	15.46069	-0.48563	0.200414	-2.42311	0.015388193	0.024412	NOT
VAPB	2325.476	0.158528	0.065427	2.422971	0.015394145	0.02442	NOT
FANK1	10.72945	0.671666	0.277249	2.422614	0.015409278	0.0244426	UP
POC1B-G/	3.998771	0.459149	0.189531	2.422558	0.015411671	0.024445	NOT
IGKV1-27	59.64121	-1.05298	0.434713	-2.42223	0.015425423	0.0244654	DOWN
RP11-51F1	34.86093	-0.35152	0.145172	-2.42144	0.015459255	0.0245177	NOT
BMS1P2	3.029082	0.63087	0.260575	2.421073	0.015474757	0.0245409	UP
UBE2N	2140.699	0.138331	0.057139	2.420954	0.015479847	0.0245475	NOT
ATOX1	5336.639	0.372945	0.154051	2.420916	0.015481475	0.0245487	NOT
RP11-587I	3.166623	0.647394	0.267496	2.420196	0.015512122	0.0245959	UP
RP4-591C:	93.2307	0.326351	0.134876	2.419641	0.015535817	0.0246321	NOT
C2	26075.56	-0.40664	0.168092	-2.41913	0.015557793	0.0246655	NOT
SLC22A13	1.436187	0.899641	0.371904	2.419015	0.015562606	0.0246704	UP
CTC-1337	2.31784	-0.63603	0.262929	-2.41901	0.015562651	0.0246704	DOWN
C4orf32	516.0777	-0.29971	0.123902	-2.4189	0.015567324	0.0246764	NOT
TRPV2	423.4233	0.41019	0.169603	2.418523	0.015583644	0.0247008	NOT
LINC0061	7.863854	0.495381	0.204832	2.418471	0.015585908	0.024703	NOT
FAM127C	158.3778	0.518448	0.21438	2.418357	0.015590759	0.0247093	NOT
DAD1	4930.53	0.195034	0.080674	2.417559	0.015624989	0.0247621	NOT
RHEBP1	9.760604	-0.53969	0.223247	-2.41747	0.015628733	0.0247666	NOT
RPS4XP11	6.308018	0.530831	0.219609	2.417168	0.015641816	0.0247859	NOT
CXCR5	1.59965	-1.02182	0.422738	-2.41715	0.015642681	0.0247859	DOWN
LINC0152	10.23682	0.703469	0.291043	2.41706	0.015646454	0.024789	UP
RP1-253P	5.326153	0.549159	0.227201	2.417059	0.015646473	0.024789	NOT
IL7	163.7165	-0.32973	0.136429	-2.4169	0.015653416	0.0247986	NOT
AL022326.	1.378319	0.819264	0.338979	2.416859	0.015655089	0.0247998	UP
FUNDC2	2238.421	0.289224	0.119684	2.416567	0.015667657	0.0248183	NOT
CTD-2536	1.97684	-0.7493	0.310107	-2.41627	0.015680418	0.0248371	DOWN
PDILT	2.899073	-0.69998	0.289698	-2.41624	0.015681652	0.0248377	DOWN
RSPH3	344.243	-0.22485	0.093062	-2.41616	0.015685195	0.0248419	NOT
TAF13	973.313	-0.20136	0.083343	-2.41606	0.015689573	0.0248474	NOT
TRIM9	103.1937	0.868302	0.359447	2.415664	0.015706541	0.0248728	UP

TBC1D25	596.881	0.158825	0.065752	2.415516	0.015712934	0.0248815	NOT
KCND2	14.23358	0.846276	0.350386	2.41527	0.015723552	0.0248969	UP
AC010136	5.567295	0.733523	0.303776	2.414685	0.015748836	0.0249355	UP
LIN7A	1503.354	-0.34221	0.14174	-2.41435	0.015763487	0.0249573	NOT
RP11-111:	9.38797	-0.36516	0.151262	-2.4141	0.015774069	0.0249726	NOT
RP11-507I	1.803093	1.258348	0.521276	2.413974	0.015779589	0.0249799	UP
RP11-815.	3.944167	0.657179	0.272306	2.413384	0.015805174	0.025019	UP
PABPC1P3	8.983162	0.400159	0.165816	2.413276	0.015809855	0.025025	NOT
AC002519	1.351764	-0.82729	0.342813	-2.41323	0.01581195	0.0250268	DOWN
FAM131C	23.41676	0.982004	0.406998	2.412798	0.015830588	0.0250549	UP
RPL7P23	21.89009	0.436344	0.180861	2.412597	0.01583934	0.0250673	NOT
FAM81B	1.241081	1.179172	0.488776	2.412503	0.015843419	0.0250723	UP
RP11-127I	2.176043	0.936261	0.38811	2.41236	0.015849608	0.0250807	UP
NDUFB8	2030.966	-0.32068	0.132935	-2.41229	0.015852702	0.0250835	NOT
ARFGEF2	2828.302	0.244514	0.101362	2.412279	0.015853165	0.0250835	NOT
MAP3K6	407.2442	0.343869	0.142559	2.412106	0.015860661	0.0250939	NOT
RP11-210I	0.874508	1.121706	0.465091	2.411799	0.015874027	0.0251136	UP
MAGEH1	547.8733	-0.34852	0.144525	-2.41148	0.015888071	0.0251344	NOT
MAP3K1	902.3847	0.280673	0.116396	2.411363	0.015893004	0.0251408	NOT
RAD50	1875.988	0.204208	0.084697	2.411041	0.015907049	0.025161	NOT
ANGEL1	971.1848	0.29149	0.120898	2.411029	0.015907597	0.025161	NOT
P2RY14	32.24719	0.482526	0.200149	2.410831	0.015916223	0.0251732	NOT
CDC27	1896.082	0.173139	0.071824	2.410609	0.0159259	0.025187	NOT
RP11-542I	2.023095	-0.73095	0.303276	-2.41017	0.015944993	0.0252158	DOWN
TPT1P9	16.24024	-0.44526	0.184773	-2.40976	0.015962963	0.0252428	NOT
IGLV3-10	73.78556	-1.04575	0.434119	-2.40891	0.01600038	0.0253005	DOWN
LINC0052(	1.046213	1.30936	0.54358	2.40877	0.016006378	0.0253085	UP
ASCL1	192.3981	-1.30011	0.539759	-2.40869	0.016009921	0.0253127	DOWN
RPS15	9643.19	0.3528	0.146482	2.408488	0.016018747	0.0253252	NOT
KIRREL	577.5699	0.478867	0.198836	2.408355	0.016024586	0.025333	NOT
CLIP3	217.7577	0.435655	0.180895	2.408334	0.01602553	0.025333	NOT
RP11-699I	11.35538	0.513017	0.21306	2.407854	0.016046612	0.0253649	NOT
PLA2G3	1.027137	1.43153	0.594576	2.407648	0.016055636	0.0253777	UP
NAGA	2757.562	-0.26861	0.111567	-2.40758	0.01605876	0.0253799	NOT
MREG	312.0605	0.320654	0.133186	2.407575	0.016058861	0.0253799	NOT
SLC37A2	304.7303	-0.43531	0.180822	-2.40737	0.016067881	0.0253918	NOT
RP1-53C1:	1.015492	-1.3591	0.564564	-2.40734	0.016069155	0.0253918	DOWN
CTD-2186	2.59238	-0.77957	0.323831	-2.40734	0.016069163	0.0253918	DOWN
CTD-2215	1.52158	0.901095	0.374347	2.40711	0.01607933	0.0254064	UP
PTPRZ1	14.23722	0.805632	0.334695	2.407059	0.016081569	0.0254085	UP
PRR27	1.57676	1.466152	0.609119	2.407006	0.01608391	0.0254108	UP
UBAC2	3084.68	0.21867	0.090859	2.406686	0.01609802	0.0254316	NOT
RP11-281(	2.679182	0.799447	0.332274	2.405991	0.016128665	0.0254786	UP
CXCL11	128.5012	0.722511	0.300301	2.40596	0.016130022	0.0254793	UP
MORC2-A	5.405481	0.528065	0.219499	2.405776	0.016138172	0.0254907	NOT
CYB561D2	1058.679	0.238425	0.099108	2.405706	0.016141266	0.0254941	NOT
NOP9	1269.199	0.16252	0.067573	2.405108	0.016167668	0.0255344	NOT
KIF3B	2903.614	0.18969	0.078878	2.404866	0.016178403	0.0255499	NOT
RP11-737(	6.234823	-0.51253	0.213133	-2.40477	0.016182743	0.0255552	NOT
RP1-170O	0.798263	2.062585	0.857783	2.404553	0.016192266	0.0255688	UP
AGR3	8.479826	-1.27797	0.531488	-2.40451	0.016194245	0.0255705	DOWN
AKAP2	9.502276	-0.61261	0.25481	-2.4042	0.016207976	0.0255907	DOWN

FA2H	48.30476	0.95355	0.396693	2.403747	0.016228022	0.0256209	UP
ZDHHC7	1062.526	0.246532	0.102577	2.403392	0.016243768	0.0256443	NOT
ASMT	2.063255	0.85729	0.356709	2.403328	0.016246604	0.0256473	UP
ADAMTS7	3.481928	0.559306	0.232725	2.403291	0.016248232	0.0256484	NOT
PRMT6	455.271	-0.45224	0.188179	-2.40325	0.016249962	0.0256497	NOT
SMG1P1	4.983504	0.50493	0.210127	2.402978	0.016262144	0.0256674	NOT
C1orf234	1.786354	0.776476	0.323147	2.402856	0.016267613	0.0256746	UP
AC004231	3.539527	1.107273	0.460857	2.402639	0.01627726	0.0256874	UP
BZW1P2	14.00435	-0.40545	0.168751	-2.40263	0.016277562	0.0256874	NOT
TFF2	47.94487	-1.3499	0.561985	-2.40202	0.016304843	0.025729	DOWN
AC018804	5.67933	0.679784	0.283019	2.401903	0.016310052	0.0257357	UP
SDC1	40681.66	-0.33761	0.140567	-2.40179	0.016314872	0.0257419	NOT
KRT8P48	10.40708	-0.64306	0.267753	-2.4017	0.016318938	0.0257468	DOWN
HCG4P3	18.73025	0.435009	0.181129	2.401658	0.01632095	0.0257485	NOT
ZNF763	44.39428	0.392923	0.163635	2.401218	0.016340584	0.025778	NOT
RP11-20E2	9.517443	-0.44634	0.185883	-2.40117	0.016342559	0.0257797	NOT
ZNF222	79.24758	0.325813	0.135695	2.401074	0.01634704	0.0257853	NOT
RP11-4C2(	0.972329	1.208432	0.503323	2.400909	0.016354408	0.0257954	UP
POU3F1	5.802001	0.7368	0.306894	2.400832	0.016357861	0.0257994	UP
GPR89B	158.7457	0.253337	0.10553	2.400612	0.016367662	0.0258134	NOT
SH3BGRL	1507.564	-0.35385	0.147409	-2.40046	0.016374369	0.0258225	NOT
LINC0020(	1.67812	2.107947	0.878222	2.400244	0.016384145	0.0258364	UP
CHD9	1090.812	-0.25285	0.105346	-2.40021	0.016385606	0.0258373	NOT
LGMNP1	8.131039	-0.39655	0.165219	-2.40014	0.016388958	0.0258411	NOT
RP11-820I	2.340336	-0.84031	0.350181	-2.39964	0.016411075	0.0258745	DOWN
KIAA1755	130.6555	0.633621	0.264084	2.399312	0.016425934	0.0258964	UP
FAM27C	4.71127	0.968367	0.403635	2.399112	0.016434891	0.0259091	UP
RHOXF2	1.213034	2.599543	1.083559	2.399079	0.016436388	0.02591	UP
AC004510	2.591112	0.910864	0.379781	2.398396	0.016467047	0.0259568	UP
NFYC	1927.728	0.155939	0.065035	2.397763	0.016495548	0.0260002	NOT
ACER2	45.75921	-0.51066	0.213012	-2.39734	0.016514703	0.026029	NOT
MTDH	6966.23	0.236783	0.09877	2.397311	0.01651591	0.0260294	NOT
ING3	276.1779	-0.19134	0.079821	-2.39706	0.016527236	0.0260457	NOT
CCDC116	4.066449	0.531582	0.221806	2.396606	0.016547716	0.026076	NOT
HERC2P10	1.638659	0.722247	0.301364	2.396593	0.016548293	0.026076	UP
VGLL3	94.81263	0.586175	0.244676	2.395724	0.016587556	0.0261363	UP
RP11-142(	3.745589	0.832781	0.347616	2.395693	0.016588969	0.0261371	UP
CTD-3035	13.6229	0.46056	0.192261	2.395498	0.016597795	0.0261495	NOT
IGF1R	345.3888	0.590211	0.246416	2.395181	0.016612176	0.0261707	UP
RP11-129I	71.58827	-0.37822	0.157917	-2.39505	0.016618064	0.0261784	NOT
FN3KRP	1766.75	0.233777	0.097614	2.394914	0.016624283	0.0261867	NOT
PPP3CB-A	139.7084	-0.26118	0.109067	-2.39465	0.016636317	0.0262042	NOT
LSM14A	3700.322	0.156246	0.065252	2.394493	0.01664337	0.0262138	NOT
RPL9P32	3.970926	0.579791	0.242138	2.394467	0.016644533	0.0262142	NOT
CRYZ	7517.759	-0.34095	0.142463	-2.39325	0.016699642	0.0262995	NOT
RP11-203J	47.52266	-0.63648	0.265965	-2.39311	0.016706188	0.0263083	DOWN
AC016700	3.547986	0.548345	0.22916	2.39285	0.016718089	0.0263255	NOT
KLK11	17.88803	-1.304	0.545254	-2.39155	0.016777593	0.0264177	DOWN
AC000120	2.59721	0.670952	0.280623	2.39094	0.016805315	0.0264599	UP
GMCL1	943.991	0.217488	0.090967	2.39085	0.016809426	0.0264648	NOT
DNAJC14	643.8415	0.244043	0.102077	2.390768	0.016813196	0.0264693	NOT
ERGIC2	1385.109	-0.16933	0.070836	-2.39044	0.016828165	0.0264913	NOT

NDRG4	100.42	0.551123	0.230574	2.390221	0.016838247	0.0265057	NOT
SNORD15I	1.987049	0.872205	0.36493	2.390062	0.016845538	0.0265156	UP
CAMTA2	1136.323	-0.21701	0.090816	-2.38951	0.016870791	0.0265539	NOT
CHMP4B	4254.478	0.192176	0.080431	2.389322	0.016879495	0.0265661	NOT
RP11-307I	4.800713	-0.51267	0.214595	-2.38902	0.016893586	0.0265867	NOT
SRBD1	541.4311	-0.18226	0.076297	-2.38878	0.016904433	0.0266023	NOT
RP11-293/	15.6514	-0.48644	0.203644	-2.38868	0.016909236	0.0266083	NOT
STMN4	1.761882	1.028512	0.430632	2.388378	0.016922913	0.0266283	UP
RP11-894I	25.99179	0.417876	0.17497	2.388275	0.016927666	0.0266343	NOT
RP11-360I	6.453656	0.510326	0.213719	2.38784	0.016947724	0.0266644	NOT
RP11-70C	34.04205	0.917176	0.384115	2.387764	0.01695123	0.0266684	UP
USP42	470.5233	0.185361	0.077636	2.387577	0.016959866	0.0266804	NOT
RP11-582	33.54273	0.392935	0.164641	2.386622	0.01700397	0.0267483	NOT
ZDHHC9	4083.806	0.250694	0.105042	2.386595	0.017005223	0.0267487	NOT
FAM184B	9.406932	0.419039	0.175589	2.386473	0.017010869	0.0267561	NOT
TFDP1	1806.534	0.341635	0.143157	2.386445	0.017012157	0.0267566	NOT
CTC-436P	8.196004	-0.45365	0.190096	-2.3864	0.017014154	0.0267582	NOT
NUDT5	3915.18	0.219286	0.091893	2.386324	0.017017728	0.0267623	NOT
AC006116	1.683224	0.845394	0.354339	2.385833	0.017040472	0.0267966	UP
RP11-669I	26.7183	0.804645	0.337438	2.38457	0.017099081	0.0268872	UP
DPH5	716.6557	0.201833	0.084648	2.384365	0.017108618	0.0269007	NOT
RP11-358I	2.184784	-0.71958	0.301817	-2.38416	0.017118318	0.026913	DOWN
RP11-13N	1.169727	0.90423	0.379267	2.384155	0.017118406	0.026913	UP
MIR100HG	28.40655	-0.63478	0.266256	-2.38411	0.017120574	0.0269149	DOWN
GMPSP1	7.876461	0.508222	0.213178	2.38402	0.017124687	0.0269198	NOT
CLEC2D	162.1569	0.467557	0.196134	2.383864	0.017131941	0.0269297	NOT
RNA5SP82	1.633478	0.852801	0.357843	2.383171	0.017164225	0.0269789	UP
PCM1	3328.316	-0.27936	0.117225	-2.38308	0.017168668	0.0269844	NOT
ZNF880	111.2901	0.569787	0.239116	2.382885	0.017177567	0.0269968	NOT
ARID5A	1300.939	-0.30703	0.128855	-2.38278	0.017182388	0.0270028	NOT
HHLA3	428.1745	-0.3642	0.15287	-2.38243	0.017199	0.0270274	NOT
RP11-677I	2.475244	0.852015	0.357632	2.382378	0.017201236	0.0270294	UP
MGEA5	3878.955	-0.17086	0.071718	-2.38233	0.017203308	0.0270311	NOT
RP11-126(	1.760082	-0.69905	0.293433	-2.38231	0.017204447	0.0270314	DOWN
DEPTOR	1151.723	0.295265	0.123955	2.382042	0.017216938	0.0270494	NOT
DSEL	330.5912	-0.53008	0.222536	-2.38202	0.017218073	0.0270494	NOT
GIMAP1	266.7149	-0.36066	0.151409	-2.382	0.01721883	0.0270494	NOT
VCPIP1	1226.802	-0.20805	0.087345	-2.38188	0.017224384	0.0270566	NOT
RP11-150(	0.700877	1.802652	0.757028	2.381221	0.017255336	0.0271036	UP
GPRC6A	0.855184	2.232708	0.937724	2.380986	0.017266386	0.0271194	UP
GALNT2	7683.955	-0.27039	0.113585	-2.3805	0.017289082	0.0271536	NOT
HDGFRP3	237.5285	0.431835	0.181439	2.380057	0.017309941	0.0271848	NOT
RHOXF2B	1.471133	3.011605	1.265381	2.379998	0.017312716	0.0271876	UP
RP11-755I	2.920061	-0.62602	0.263039	-2.37993	0.017315853	0.027191	DOWN
WARS2	541.9689	0.218092	0.091642	2.379826	0.017320814	0.0271972	NOT
RP11-176I	22.23751	-0.36835	0.154799	-2.37956	0.017333333	0.0272153	NOT
TSPAN4	2504.838	0.304038	0.127807	2.37888	0.017365321	0.027264	NOT
POLR3E	1203.371	0.153027	0.064347	2.378166	0.017398982	0.0273153	NOT
GLB1	3479.689	0.203752	0.085684	2.377931	0.017410066	0.0273312	NOT
RP1-309F2	1.876569	0.867011	0.364658	2.377604	0.017425549	0.0273539	UP
RPL14	18395.61	0.298316	0.125472	2.377554	0.017427882	0.027356	NOT
GSTM2	227.0549	0.466361	0.196205	2.37691	0.017458363	0.0274023	NOT

UBQLN1	4993.323	-0.1558	0.065582	-2.37566	0.017517413	0.0274934	NOT
RAP2C-AS	36.94338	0.392382	0.165187	2.375386	0.017530603	0.0275126	NOT
RP11-490I	2.875543	0.60908	0.256421	2.375311	0.017534148	0.0275166	UP
CD1A	6.504542	0.730605	0.307609	2.375108	0.017543815	0.0275302	UP
RP11-55L3	1.326315	2.667714	1.123223	2.375052	0.017546493	0.0275328	UP
RARRES2P	1.544896	1.237871	0.521295	2.374608	0.017567576	0.0275643	UP
RP11-867(	222.3286	0.363752	0.153199	2.374379	0.017578518	0.02758	NOT
BDNF	11.15792	0.630019	0.265364	2.374173	0.017588318	0.0275938	UP
RP11-594I	1.00174	1.343112	0.565755	2.374016	0.017595806	0.0276039	UP
SPATA31D	1.735928	3.186286	1.342256	2.373829	0.017604708	0.0276163	UP
GRAMD2	4.90009	0.647809	0.272903	2.373775	0.017607275	0.0276188	UP
RP4-561L2	10.89875	0.333268	0.140411	2.373518	0.017619519	0.0276364	NOT
RP11-81A	227.9623	-0.36413	0.15348	-2.37248	0.017669206	0.0277128	NOT
FTLP3	377.4418	0.512005	0.215815	2.372425	0.017671751	0.0277152	NOT
AC104076	1.938809	-0.67459	0.284372	-2.37222	0.017681774	0.0277294	DOWN
SYPL1	3606.202	0.183235	0.077261	2.371632	0.017709699	0.0277716	NOT
TACR2	15.79876	0.584304	0.246411	2.371255	0.017727777	0.0277984	NOT
ABI3	365.0663	-0.33799	0.142547	-2.37109	0.01773573	0.0278093	NOT
CALM2P2	8.15765	0.461589	0.194681	2.370996	0.017740212	0.0278147	NOT
CTTNBP2N	444.7283	0.342425	0.144472	2.370185	0.017779182	0.0278742	NOT
CTD-2541	8.906946	0.767245	0.323736	2.369975	0.017789278	0.0278885	UP
AC008781	1.670484	0.630736	0.266153	2.369824	0.017796537	0.0278983	UP
RP11-180I	10.76682	0.418028	0.176479	2.368713	0.017850114	0.0279807	NOT
SNHG8	1601.304	0.362332	0.152975	2.368566	0.017857176	0.0279902	NOT
TNFRSF13(	27.03183	-0.58749	0.248042	-2.36852	0.017859613	0.0279924	DOWN
RP11-326(	27.98831	0.546672	0.230855	2.368038	0.017882688	0.028027	NOT
ZDHHC8P	13.16755	0.895014	0.378037	2.367531	0.017907241	0.0280639	UP
AC002310	1.394601	0.855767	0.361483	2.367377	0.017914661	0.0280739	UP
ZNF524	813.6201	0.322097	0.136069	2.367157	0.017925346	0.0280888	NOT
MROH5	1.266231	0.987881	0.417331	2.36714	0.017926162	0.0280888	UP
RP11-166I	4.424255	0.470897	0.198956	2.366841	0.017940644	0.0281099	NOT
PPM1B	2136.496	-0.1992	0.084165	-2.3668	0.017942651	0.0281114	NOT
PPP1CA	5910.243	0.178089	0.075253	2.366548	0.017954867	0.028129	NOT
ARL4C	1142.101	-0.44614	0.188585	-2.36571	0.017995512	0.028191	NOT
PIK3CB	1226.365	0.187398	0.079217	2.365635	0.017999156	0.0281951	NOT
RP11-311I	1.264136	0.944619	0.399382	2.365201	0.018020308	0.0282267	UP
ALG2	1855.791	-0.20886	0.088316	-2.36494	0.018032774	0.0282446	NOT
IFT74-AS1	1.838908	0.95679	0.404593	2.364819	0.018038903	0.0282526	UP
ZFAND4	308.3686	-0.21073	0.089112	-2.36472	0.018043465	0.0282582	NOT
LAX1	34.67735	-0.53727	0.227241	-2.36433	0.018062627	0.0282866	NOT
RP11-170I	0.807741	-1.35726	0.574187	-2.3638	0.018088524	0.0283255	DOWN
RP11-242(	1.981335	-0.73805	0.312249	-2.36366	0.018095502	0.0283348	DOWN
TACO1	2546.847	-0.27265	0.115353	-2.3636	0.018098114	0.0283366	NOT
ADAP1	216.5366	0.661285	0.279781	2.363578	0.018099417	0.0283366	UP
GMEB1	458.9773	0.167053	0.070678	2.363572	0.018099718	0.0283366	NOT
CTD-2313	17.44174	0.359888	0.15228	2.363333	0.018111374	0.0283533	NOT
RPL13AP3	8.829713	0.468722	0.198385	2.362684	0.018143129	0.0284014	NOT
CHCHD1	1465.35	0.241847	0.102363	2.362629	0.018145821	0.028404	NOT
NNT-AS1	553.5212	0.210014	0.08891	2.362097	0.0181719	0.0284419	NOT
AC006026	3.974384	0.668677	0.283087	2.362092	0.018172122	0.0284419	UP
TBK1	1032.723	-0.15232	0.064489	-2.36189	0.018182026	0.0284558	NOT
KIAA1731I	1.61808	0.963316	0.407909	2.361593	0.018196628	0.0284771	UP

RP11-216I	8.962449	0.854629	0.361918	2.361386	0.018206775	0.0284913	UP
MS4A1	49.23503	-0.79337	0.335999	-2.36122	0.018214833	0.0285023	DOWN
KRT17P4	10.85188	-0.96263	0.407705	-2.36108	0.018221811	0.0285116	DOWN
SLC25A24I	2.246677	1.785477	0.756228	2.361031	0.018224188	0.0285137	UP
AFMID	4849.448	-0.28847	0.122194	-2.36078	0.018236351	0.0285312	NOT
SDF2	1597.675	0.175121	0.074186	2.360574	0.018246657	0.0285457	NOT
RNF121	888.1465	0.172087	0.072903	2.360501	0.018250281	0.0285497	NOT
RP11-206I	2.692522	1.433291	0.60721	2.360455	0.018252523	0.0285516	UP
CTB-33G1	13.91912	0.399302	0.169169	2.360379	0.018256275	0.0285559	NOT
ENTPD1-A	37.29983	0.264065	0.111877	2.360316	0.018259362	0.0285591	NOT
HOXC5	0.744862	1.905489	0.807382	2.360084	0.01827081	0.0285746	UP
AP000432.	5.734002	0.426231	0.180601	2.360073	0.018271331	0.0285746	NOT
NUBPL	558.1176	-0.34012	0.144119	-2.36	0.018274892	0.0285785	NOT
RSU1	2050.022	0.207436	0.087914	2.359525	0.018298325	0.0286136	NOT
FAM84B	1347.226	0.369141	0.156516	2.358483	0.018349797	0.0286924	NOT
RBPJ	1634.842	0.180626	0.076619	2.357436	0.018401618	0.0287718	NOT
DTWD2	311.4663	-0.26915	0.114186	-2.35714	0.018416335	0.0287932	NOT
RNU6-101	1.355643	0.895663	0.380035	2.356788	0.018433777	0.0288189	UP
RP11-149I	1.72924	-1.34648	0.571391	-2.3565	0.01844816	0.0288397	DOWN
RP11-424I	1.600327	-1.328	0.56359	-2.35633	0.0184567	0.0288514	DOWN
RP11-58O	1.804715	-0.66406	0.281844	-2.35614	0.018466145	0.0288646	DOWN
MGRN1	3730.837	-0.21737	0.092289	-2.35535	0.018505254	0.0289241	NOT
FGF20	2.498695	1.239492	0.526297	2.355121	0.018516695	0.0289403	UP
ATL1	44.71726	0.405396	0.172143	2.354999	0.018522749	0.0289481	NOT
RP11-301(	1.513834	2.476356	1.051564	2.354927	0.018526355	0.028951	UP
FAM153C	2.278132	1.002452	0.425684	2.35492	0.018526695	0.028951	UP
LRRC2	470.9281	-0.46244	0.196389	-2.35474	0.018535811	0.0289636	NOT
ZNF829	88.35561	0.465765	0.197815	2.354541	0.018545606	0.028976	NOT
SLC2A11	246.5631	0.252725	0.107335	2.354537	0.018545797	0.028976	NOT
RNU6-725	0.736615	1.823266	0.774444	2.35429	0.018558136	0.0289936	UP
IGLC2	1298.692	-0.84986	0.361024	-2.35402	0.018571584	0.029013	DOWN
TEF	1694.581	0.360217	0.153027	2.353944	0.018575427	0.0290174	NOT
RPL5P9	3.295263	0.604053	0.256745	2.35274	0.018635675	0.0291098	UP
DNPEP	3058.87	-0.16752	0.071205	-2.35262	0.018641793	0.0291177	NOT
AC002519	1.46001	0.885187	0.376299	2.352348	0.018655306	0.0291357	UP
MEAF6	1974.762	-0.22978	0.097682	-2.35234	0.01865568	0.0291357	NOT
RP11-461I	1.439373	1.504119	0.639418	2.352325	0.018656444	0.0291357	UP
RP11-118I	6.241796	-0.79986	0.340106	-2.35179	0.018683093	0.0291757	DOWN
RBX1	1670.721	0.224805	0.0956	2.351522	0.018696794	0.0291954	NOT
ZHX1	2599.674	0.250199	0.106408	2.351312	0.018707326	0.0292102	NOT
DENND4C	961.7381	-0.30086	0.12796	-2.35122	0.018712036	0.0292159	NOT
AC022816	49.31325	-0.67334	0.286411	-2.35096	0.018724964	0.0292335	DOWN
RP1-90G2	1.973186	1.038892	0.441902	2.350953	0.018725413	0.0292335	UP
AC016723	4.80912	0.961125	0.408877	2.350645	0.01874092	0.0292561	UP
CTD-2303	11.39798	0.79715	0.339133	2.350556	0.018745398	0.0292614	UP
MKL2	831.7307	0.265814	0.1131	2.350258	0.018760407	0.0292832	NOT
DAPP1	66.07862	-0.51978	0.221166	-2.35019	0.018763787	0.0292868	NOT
BNC2	42.88006	0.540519	0.229994	2.350144	0.018766136	0.0292888	NOT
RP11-254I	5.677673	0.553396	0.235538	2.349493	0.018798987	0.0293384	NOT
DPM3	1751.815	0.357625	0.15222	2.349402	0.018803603	0.029344	NOT
SEC14L2	9026.146	-0.60709	0.25846	-2.34887	0.01883048	0.0293843	DOWN
AC009166	43.05465	-0.51109	0.2176	-2.34875	0.018836762	0.0293924	NOT

CLIC6	156.8114	-0.92032	0.391911	-2.34829	0.018860058	0.0294271	DOWN
TNRC18P1	6.071448	0.682955	0.290857	2.348075	0.018870723	0.0294421	UP
MYLK3	14.92325	0.523708	0.223041	2.348028	0.018873102	0.0294441	NOT
CECR1	1556.882	-0.42334	0.180319	-2.3477	0.018889676	0.0294683	NOT
SORCS3	2.490467	1.907907	0.812719	2.347561	0.018896766	0.0294777	UP
GPR135	50.93328	-0.3195	0.136104	-2.34749	0.018900187	0.0294814	NOT
RP1-292L2	28.35712	-0.38487	0.163982	-2.34699	0.018925617	0.0295194	NOT
NEK11	73.8154	0.442834	0.188689	2.346904	0.018930157	0.0295248	NOT
RP3-508I1	3.010503	0.559214	0.238296	2.34672	0.018939495	0.0295377	NOT
SPRR2D	1.977385	2.185637	0.931458	2.34647	0.018952196	0.0295558	UP
CALHM3	0.92004	1.374476	0.585768	2.34645	0.018953213	0.0295558	UP
RP5-968D	30.47379	-0.66037	0.281442	-2.34639	0.018956034	0.0295585	DOWN
ADAMTS1	571.138	0.645539	0.275124	2.346357	0.01895793	0.0295598	UP
USF2	4900.564	-0.20592	0.087764	-2.34624	0.018963869	0.0295674	NOT
RP11-116(	2.204975	-1.18074	0.503369	-2.34568	0.018992274	0.02961	DOWN
AC013460	1.026112	1.318566	0.56218	2.345452	0.019004049	0.0296267	UP
CTD-2526	4.171312	0.56152	0.239433	2.345209	0.019016416	0.0296429	NOT
FZD5	2747.865	-0.28358	0.120919	-2.34521	0.019016602	0.0296429	NOT
RP11-331I	7.987962	0.572325	0.24406	2.345015	0.019026333	0.0296564	NOT
TEX261	3304.18	0.135281	0.057701	2.344525	0.019051323	0.0296937	NOT
RPGRIP1	39.45552	-0.37709	0.160853	-2.34432	0.01906206	0.0297088	NOT
NFIB	2320.006	-0.28969	0.123573	-2.34428	0.019063836	0.0297099	NOT
RP11-360I	3.614437	-0.53306	0.227438	-2.34376	0.019090203	0.0297493	NOT
KRT18P63	1.985566	0.658857	0.281157	2.343373	0.019110267	0.0297789	UP
CTD-2587	1.597304	1.157503	0.49397	2.343265	0.019115794	0.0297858	UP
MFSD1	2487.201	-0.18851	0.08046	-2.34294	0.019132282	0.0298098	NOT
KLRC4-KLI	2.307992	-0.80755	0.344703	-2.34273	0.019143428	0.0298255	DOWN
SYN1	85.71369	-0.65335	0.278888	-2.3427	0.01914464	0.0298257	DOWN
ECT2L	5.934154	-0.3649	0.155787	-2.34232	0.019164153	0.0298544	NOT
HS3ST2	105.9584	0.667394	0.284939	2.342233	0.019168742	0.0298599	UP
ZNF100	130.3445	0.319777	0.136532	2.342138	0.019173626	0.0298658	NOT
GTF2A1	1361.039	-0.18465	0.078842	-2.34198	0.019181987	0.0298751	NOT
NCOA2	3107.178	0.274282	0.117116	2.341971	0.019182228	0.0298751	NOT
CTD-2650	3.32897	0.595209	0.25415	2.341959	0.0191828	0.0298751	UP
RP11-166I	98.96913	0.328817	0.140412	2.341793	0.019191344	0.0298867	NOT
RP4-564F2	1.421832	0.829919	0.354448	2.341441	0.019209474	0.0299132	UP
RP11-14K3	1.126296	2.494336	1.065402	2.341215	0.019221083	0.0299296	UP
MLIP-IT1	3.357642	-0.77851	0.332531	-2.34116	0.019223753	0.0299321	DOWN
ABCA2	2905.328	0.317144	0.135472	2.341024	0.019230917	0.0299416	NOT
RP11-45M	2.356495	0.663931	0.283649	2.340681	0.01924859	0.0299674	UP
B3GALT4	147.7037	0.462503	0.197623	2.340325	0.019266941	0.0299943	NOT
RP11-463(	2.214324	0.622387	0.265952	2.34022	0.019272386	0.0300011	UP
RP11-404I	2.230502	0.816241	0.348812	2.340063	0.019280495	0.030012	UP
CTAGE15	4.775261	0.963073	0.411605	2.339797	0.019294206	0.0300317	UP
MRVI1-AS	1.638456	0.887523	0.379416	2.339183	0.019325975	0.0300794	UP
RP4-631H	21.63782	0.616415	0.263521	2.339147	0.019327828	0.0300806	UP
RP4-742J2	1.717756	0.860293	0.367861	2.338636	0.019354282	0.0301201	UP
ATG4D	461.008	0.246541	0.105424	2.33856	0.019358219	0.0301245	NOT
NSDHL	2040.462	0.309519	0.132363	2.338412	0.019365873	0.0301347	NOT
LRRTM4	23.32863	-1.0077	0.431071	-2.33768	0.019404089	0.0301925	DOWN
AC128709	2.354138	0.772697	0.330562	2.337522	0.019412054	0.0302032	UP
RP11-108I	0.885059	1.385234	0.592626	2.337451	0.019415735	0.0302072	UP

IGLV2-33	1.208381	-1.37075	0.586499	-2.33717	0.019430314	0.0302278	DOWN
RP11-161I	1.720944	2.783855	1.19113	2.337154	0.01943116	0.0302278	UP
ZNF227	292.3085	0.256222	0.109633	2.337087	0.019434664	0.0302316	NOT
ECSIT	2434.629	-0.24025	0.102813	-2.33678	0.019450578	0.0302546	NOT
C9orf3	1647.689	-0.21297	0.091153	-2.33638	0.019471505	0.0302855	NOT
NANP	303.1648	0.194928	0.083433	2.336348	0.01947312	0.0302863	NOT
Z69720.3	1.346516	1.013101	0.433638	2.336283	0.019476491	0.0302898	UP
USP4	1354.354	-0.18893	0.080873	-2.33617	0.019482393	0.0302973	NOT
ASS1P5	2.596121	-0.72754	0.311436	-2.33609	0.019486352	0.0303018	DOWN
ACKR1	93.61736	0.805033	0.344615	2.336038	0.01948926	0.0303046	UP
AC009234	2.83396	1.223319	0.523679	2.336009	0.019490789	0.0303053	UP
NOP56P3	4.177175	0.749136	0.320701	2.335934	0.019494656	0.0303086	UP
LINC0096:	3073.036	-0.2567	0.109893	-2.33593	0.019495115	0.0303086	NOT
LILRB3	77.18903	-0.37157	0.159074	-2.33586	0.019498641	0.0303124	NOT
NDUFV1	9186.766	-0.2421	0.103648	-2.33576	0.019503703	0.0303185	NOT
ZNF439	49.6278	0.481744	0.206275	2.335444	0.019520259	0.0303425	NOT
RBM26	1181.717	0.184529	0.079027	2.335013	0.019542757	0.0303758	NOT
TSR3	1921.749	0.243762	0.104419	2.334459	0.019571712	0.0304191	NOT
TMX3	870.1404	0.238481	0.102163	2.33432	0.019578981	0.0304287	NOT
FADS1	4719.905	0.542948	0.232605	2.334208	0.01958481	0.030436	NOT
LMOD2	3.06617	0.903549	0.387141	2.333899	0.019601022	0.0304595	UP
HADHAP1	2.168437	0.658815	0.282305	2.333697	0.019611599	0.0304743	UP
MTND4P2	31.82729	-0.83412	0.357503	-2.33319	0.019638408	0.0305142	DOWN
EEF1A1P6	258.8248	-0.29848	0.12797	-2.3324	0.019679475	0.0305763	NOT
CTD-2619	9.275165	0.358979	0.153987	2.331229	0.019741309	0.0306706	NOT
SUCLG1	5921.349	-0.24547	0.1053	-2.33113	0.019746353	0.0306768	NOT
SLC2A13	550.7118	-0.35171	0.150884	-2.33099	0.019753953	0.0306868	NOT
RAB27B	189.0778	0.70124	0.300847	2.330886	0.019759389	0.0306936	UP
C8orf44-S	2.419706	0.539863	0.231627	2.330745	0.019766792	0.0307028	NOT
GCNT4	260.4743	0.430913	0.184883	2.330731	0.01976754	0.0307028	NOT
GNG5P2	3.963116	-0.7665	0.328896	-2.33054	0.019777836	0.030717	DOWN
SRP54	2319.128	-0.16917	0.072604	-2.33002	0.019805103	0.0307577	NOT
BAIAP3	563.9403	-0.3841	0.164889	-2.32946	0.01983489	0.0308022	NOT
PGBD4	104.5041	0.231056	0.099208	2.329013	0.019858398	0.030837	NOT
RP11-203I	1.548962	1.104865	0.474536	2.328303	0.01989601	0.0308936	UP
MIR1295A	7.0199	0.815467	0.350265	2.328146	0.019904338	0.0309048	UP
RP11-266I	129.4197	-0.46257	0.19872	-2.32778	0.019924021	0.0309337	NOT
APOM	14679.85	-0.5617	0.241316	-2.32765	0.019930594	0.0309421	NOT
RERG	124.1471	0.467609	0.200902	2.327551	0.019935971	0.0309487	NOT
TMEM230	5312.312	-0.16556	0.071135	-2.32734	0.019946916	0.030964	NOT
KLHL24	1915.569	-0.23203	0.099719	-2.32681	0.019975523	0.0310067	NOT
C9orf24	20.82134	0.519359	0.223214	2.326733	0.019979479	0.0310111	NOT
HNRNPA1I	5.744308	0.418221	0.179766	2.326468	0.01999361	0.0310313	NOT
VAV3	234.8657	0.503342	0.216391	2.326072	0.02001469	0.0310622	NOT
ANKRD12	1162.977	-0.23895	0.102747	-2.32561	0.020039521	0.031099	NOT
PANX2	904.0956	0.583375	0.250865	2.325456	0.020047594	0.0311098	NOT
TAS2R14	22.80825	0.351123	0.151036	2.324758	0.02008492	0.031166	NOT
CTA-246H	5.414343	0.537175	0.231088	2.324546	0.020096253	0.0311818	NOT
RP11-603I	1.042734	1.487658	0.640131	2.32399	0.02012602	0.0312263	UP
GPBAR1	81.06719	-0.48954	0.210676	-2.32367	0.02014319	0.0312511	NOT
RP11-257(	13.12859	-0.40446	0.174091	-2.32329	0.020163837	0.0312814	NOT
RP11-244I	1.376554	2.722559	1.17192	2.323161	0.020170525	0.03129	UP

RP13-20L1	1.768335	0.698633	0.300735	2.323087	0.020174471	0.0312944	UP
CSN3	1.340437	1.991472	0.857285	2.322999	0.020179204	0.0313	UP
HESX1	31.05496	-0.30729	0.132287	-2.32291	0.020183878	0.0313055	NOT
LINC01036	1.087029	2.445877	1.053165	2.322407	0.020211026	0.0313442	UP
INPP5K	1056.935	-0.21268	0.09158	-2.32241	0.020211111	0.0313442	NOT
RP11-684I	1.099026	1.043943	0.449587	2.322007	0.020232575	0.0313757	UP
MIR568	41.86965	-0.43632	0.187943	-2.32156	0.020256814	0.0314116	NOT
C1orf111	10.34133	0.866686	0.373336	2.321467	0.020261671	0.0314173	UP
AF121897.	0.965055	1.25826	0.542083	2.32116	0.020278207	0.0314379	UP
PRSS51	21.88518	0.795255	0.342611	2.321159	0.020278244	0.0314379	UP
C21orf62-	24.46269	-0.32703	0.140893	-2.32116	0.020278334	0.0314379	NOT
AC018892	1.240214	1.056195	0.455082	2.320891	0.020292733	0.0314585	UP
FAM182A	2.773804	1.005128	0.433102	2.320765	0.020299527	0.0314672	UP
HMX3	0.895317	1.970592	0.849147	2.320674	0.02030446	0.0314731	UP
POMC	20.48632	-0.50379	0.217106	-2.32048	0.020314815	0.0314859	NOT
SAFB	3220.667	0.135623	0.058446	2.320479	0.020314994	0.0314859	NOT
MID1IP1	3653.564	0.339474	0.146315	2.320154	0.020332558	0.0315114	NOT
LPIN1	2014.219	0.365503	0.157541	2.320047	0.020338325	0.0315185	NOT
HCG4P5	306.5202	0.691811	0.298191	2.320025	0.020339525	0.0315186	UP
PSMA4	4454.427	0.177837	0.076662	2.319754	0.020354165	0.0315396	NOT
NAIP	17.82098	-0.45291	0.195261	-2.31953	0.020366179	0.0315564	NOT
RP11-122I	3.129418	0.880768	0.3798	2.319034	0.020393166	0.0315964	UP
MRPL20	3065.913	-0.24392	0.105185	-2.31894	0.020398336	0.0316027	NOT
HOMER1	172.9199	0.533764	0.230225	2.318441	0.020425384	0.0316428	NOT
PIN1	1491.442	0.216987	0.093593	2.318403	0.020427418	0.0316442	NOT
TOMM20L	5.62028	0.424085	0.182934	2.318235	0.020436565	0.0316566	NOT
RP11-679(	2.784083	0.544791	0.235024	2.318027	0.020447871	0.0316723	NOT
RP11-468I	4.389441	0.930699	0.401574	2.317626	0.020469683	0.0317043	UP
SOS1	1581.433	0.222488	0.096005	2.317462	0.02047856	0.0317163	NOT
RP11-64K1	2.691332	0.619197	0.267238	2.31703	0.020502124	0.0317502	UP
LINC00616	0.865805	2.108875	0.910168	2.317018	0.02050275	0.0317502	UP
AP000593.	0.969749	2.275519	0.982277	2.316576	0.020526824	0.0317857	UP
AC005702	5.198516	0.376292	0.162449	2.316374	0.020537864	0.031801	NOT
GACAT3	1.022402	2.210614	0.954442	2.316133	0.020550982	0.0318196	UP
NPTN	2795.272	0.176495	0.076217	2.315697	0.020574827	0.0318543	NOT
BABAM1	1723.488	0.23283	0.100545	2.315681	0.020575693	0.0318543	NOT
BHLHB9	166.2728	0.351468	0.151794	2.315432	0.020589305	0.0318736	NOT
TIGD4	5.09836	0.539312	0.232944	2.315202	0.020601904	0.0318913	NOT
RP11-566I	0.765897	1.286251	0.555576	2.315167	0.020603809	0.0318924	UP
AC005822	2.848265	0.545543	0.235661	2.314954	0.020615452	0.0319087	NOT
ITIH4	1739.837	-0.4438	0.191718	-2.31486	0.020620507	0.0319147	NOT
AC008268	1.845591	1.206618	0.52128	2.314722	0.020628146	0.0319248	UP
UPF3AP3	3.99635	0.51831	0.223941	2.31449	0.020640835	0.0319426	NOT
RP11-138I	1.720222	0.699301	0.302216	2.313912	0.020672539	0.0319894	UP
RP11-23N	9.740421	0.555325	0.239996	2.313897	0.020673388	0.0319894	NOT
RP11-667I	3.24415	0.517717	0.223746	2.313863	0.020675248	0.0319905	NOT
SLU7	1444.545	0.144171	0.062309	2.313802	0.020678582	0.0319939	NOT
TMEM150	1493.122	0.256819	0.111001	2.313671	0.020685802	0.0320032	NOT
AC012501	0.807844	2.083568	0.900597	2.313541	0.020692894	0.0320124	UP
IGKV3OR2	4.079688	-0.95611	0.413316	-2.31327	0.020707781	0.0320337	DOWN
SSSCA1	857.5772	0.269938	0.11671	2.312894	0.020728479	0.0320639	NOT
ZNF394	649.5342	-0.14402	0.062272	-2.31282	0.020732344	0.0320681	NOT

TCEA1	2483.59	0.202824	0.087697	2.312789	0.020734256	0.0320692	NOT
DISC1FP1	5.563275	-1.01497	0.43887	-2.31268	0.020740384	0.0320769	DOWN
FAM163A	9.654818	-0.58862	0.254583	-2.31208	0.020773403	0.0321262	DOWN
LINC01079	1.266491	1.470044	0.635839	2.311973	0.020779152	0.0321333	UP
PPIAP11	16.74398	0.366012	0.158319	2.311868	0.02078498	0.0321405	NOT
SIKE1	1082.401	-0.15862	0.068614	-2.31176	0.020790952	0.0321479	NOT
NINJ2	325.4702	0.464274	0.200873	2.311279	0.020817448	0.0321871	NOT
ATF7IP	1786.189	0.209204	0.090519	2.311155	0.020824281	0.0321959	NOT
EMP2	3953.229	-0.30069	0.130115	-2.31098	0.020834154	0.0322094	NOT
SKAP2	862.0837	0.370606	0.160377	2.310842	0.020841572	0.032219	NOT
LINC01277	17.4809	-0.47337	0.204859	-2.31072	0.020848171	0.0322274	NOT
INSR	7214.29	-0.25614	0.11085	-2.3107	0.020849541	0.0322277	NOT
ENDOU	3.658175	-0.53884	0.2332	-2.31061	0.020854215	0.0322332	NOT
AC002056	1.969944	0.678753	0.293768	2.310505	0.020860216	0.0322406	UP
TAPBPL	1526.738	-0.30385	0.131512	-2.31045	0.020863328	0.0322436	NOT
ZNF385B	538.105	-0.61722	0.26715	-2.31039	0.020866604	0.0322469	DOWN
KLHDC1	72.04027	-0.3434	0.148666	-2.30989	0.020894348	0.032288	NOT
RP11-546I	10.57905	0.437372	0.189358	2.309759	0.020901527	0.0322973	NOT
CTD-3105	10.94263	0.435671	0.188666	2.309216	0.020931603	0.0323419	NOT
C6orf211	1291.08	-0.2619	0.113423	-2.3091	0.020938262	0.0323504	NOT
RP11-554J	19.53735	0.350899	0.151984	2.308785	0.020955501	0.0323752	NOT
C2orf74	128.9076	-0.27556	0.119375	-2.30836	0.020979379	0.0324103	NOT
MCAT	810.5287	-0.24026	0.104084	-2.30829	0.020982824	0.0324138	NOT
CTD-2256	112.5086	-0.34454	0.149274	-2.30808	0.020994753	0.0324305	NOT
PCAT2	2.307421	1.042941	0.451893	2.307935	0.021002768	0.032441	UP
ELANE	4.098592	-0.7683	0.332916	-2.3078	0.021010178	0.0324507	DOWN
KIAA0355	916.7606	0.221453	0.09596	2.307762	0.021012397	0.0324523	NOT
NACA	20730.01	0.181812	0.078821	2.306658	0.02107392	0.0325455	NOT
GCFC2	886.084	-0.19575	0.084873	-2.30632	0.021092606	0.0325725	NOT
C17orf47	1.81071	0.800052	0.346922	2.306142	0.021102685	0.0325863	UP
RP11-48B1	11.20528	0.354476	0.15372	2.30598	0.021111735	0.0325984	NOT
AC009095	2.543772	0.611047	0.265026	2.305613	0.021132285	0.0326283	UP
CISD2	2041.804	-0.18675	0.08101	-2.3053	0.021150002	0.0326539	NOT
ZMAT2	3093.7	0.203115	0.088111	2.305225	0.021153952	0.0326581	NOT
MBL1P	37.19214	-0.64671	0.280599	-2.30474	0.021181145	0.0326983	DOWN
RP1-200K1	1.224383	2.230441	0.967933	2.304334	0.021203906	0.0327316	UP
C22orf39	690.9337	0.186018	0.080742	2.303856	0.02123073	0.0327712	NOT
ME2	981.7595	0.246047	0.106824	2.303303	0.021261819	0.0328173	NOT
IMPA2	1799.308	-0.31884	0.138443	-2.30303	0.02127729	0.0328394	NOT
AC104654	0.580044	1.487802	0.646044	2.302944	0.021282012	0.0328448	UP
BAIAP2L1	2829.834	0.369671	0.160561	2.302377	0.021313922	0.0328923	NOT
TBL3	1987.725	0.218965	0.095109	2.302265	0.021320243	0.0329002	NOT
CARHSP1	5099.668	-0.25414	0.110387	-2.30224	0.021321639	0.0329005	NOT
ABCB6	530.1647	0.349381	0.15176	2.302189	0.021324501	0.0329031	NOT
ZNF420	220.6261	0.244228	0.106087	2.302149	0.021326763	0.0329047	NOT
RC3H1	1196.349	-0.16064	0.06979	-2.30181	0.021345987	0.0329326	NOT
ALG13-AS	2.090283	0.693149	0.301147	2.3017	0.021352072	0.0329401	UP
RPL26L1	794.6168	0.214512	0.093209	2.301407	0.021368619	0.0329638	NOT
AC073343	6.353881	-0.56496	0.245497	-2.30128	0.021375986	0.0329733	NOT
CDRT15P1	6.427628	0.547981	0.238148	2.301012	0.021390931	0.0329943	NOT
RTFDC1	3201.111	0.134747	0.05856	2.300994	0.021391963	0.0329943	NOT
EPHA4	97.95961	0.456091	0.198217	2.300965	0.02139362	0.032995	NOT

RP11-753I	1.689104	0.978131	0.425184	2.300487	0.021420645	0.0330348	UP
MLK4	456.6348	0.485953	0.211307	2.299753	0.021462223	0.0330971	NOT
CHPT1	5556.684	-0.2668	0.116014	-2.29973	0.021463708	0.0330976	NOT
TMED7	4886.558	-0.2005	0.0872	-2.29931	0.021487584	0.0331325	NOT
TM7SF3	3538.559	-0.27187	0.118246	-2.29915	0.021496256	0.0331441	NOT
RP11-400I	187.3424	0.164457	0.071535	2.298973	0.021506462	0.0331579	NOT
STAP1	9.084556	-0.62857	0.273417	-2.29895	0.021507841	0.0331582	DOWN
PCDHGB3	14.63677	0.653276	0.284169	2.298902	0.0215105	0.0331605	UP
RP11-65L3	7.557313	0.526744	0.229143	2.298756	0.021518772	0.0331714	NOT
SIAH3	1.064228	1.219831	0.530696	2.298549	0.021530537	0.0331875	UP
FAM91A1	2453.714	0.194787	0.084744	2.29853	0.021531629	0.0331875	NOT
FAM66C	10.71488	0.468686	0.203911	2.298488	0.02153403	0.0331893	NOT
DFFB	121.3804	0.23451	0.102029	2.298466	0.021535307	0.0331895	NOT
BPI	26.91986	-0.67388	0.293305	-2.29755	0.021587354	0.0332678	DOWN
ITGB2	2084.712	-0.40935	0.178199	-2.29714	0.021610929	0.0333023	NOT
ABALON	18.52448	0.438618	0.190952	2.297001	0.021618703	0.0333124	NOT
TNFRSF10A	278.0652	-0.29699	0.1293	-2.29687	0.021626154	0.0333221	NOT
CRP	144250.3	-0.95346	0.415153	-2.29665	0.021638527	0.0333393	DOWN
XPC	1647.702	-0.22183	0.096604	-2.29629	0.021659427	0.0333684	NOT
METTL17	1208.329	0.170898	0.074424	2.296281	0.021659839	0.0333684	NOT
COG3	1762.857	-0.27227	0.118588	-2.29596	0.02167828	0.0333949	NOT
VTI1BP1	1.613845	-0.78003	0.339746	-2.29592	0.021680355	0.0333963	DOWN
AC004069	3.217754	0.542027	0.236094	2.295809	0.021686811	0.0334044	NOT
DPYD-AS1	1.777842	-0.69094	0.301088	-2.2948	0.021744536	0.0334914	DOWN
XKR8	660.8453	-0.23761	0.103554	-2.29459	0.021756654	0.0335082	NOT
DNAJC27	122.5601	-0.23534	0.102566	-2.2945	0.021762039	0.0335146	NOT
DIMT1	704.035	0.167251	0.072895	2.29441	0.02176697	0.0335204	NOT
CTD-2525	1.791466	0.654671	0.285426	2.293658	0.021810147	0.033585	UP
RP11-73K9	10.24429	-0.39363	0.17163	-2.29351	0.021818833	0.0335965	NOT
CDC5L	2283.034	0.187833	0.081908	2.293228	0.021834898	0.0336194	NOT
STARD3NL	589.7698	0.231885	0.101131	2.292928	0.021852148	0.033644	NOT
NPHP1	57.90607	0.401908	0.175286	2.292874	0.02185528	0.033647	NOT
RP11-730A	9.882155	-0.3466	0.151174	-2.29275	0.021862482	0.0336562	NOT
CCDC125	1178.659	-0.21537	0.093941	-2.29264	0.021868514	0.0336636	NOT
AF131215.	1.127509	1.202178	0.524459	2.292226	0.021892633	0.0336989	UP
EFCAB6-A	1.374843	1.0014	0.436877	2.292176	0.021895462	0.0337013	UP
AP000255.	1.750118	0.662499	0.289039	2.292076	0.021901264	0.0337079	UP
RP1-151F1	214.5982	-0.36596	0.159664	-2.29206	0.021902131	0.0337079	NOT
AKR1C1	23986.13	0.539171	0.235277	2.291645	0.021926128	0.0337429	NOT
GOLGA6B	7.831681	-0.98526	0.43004	-2.2911	0.021957711	0.0337896	DOWN
TSPAN3	3499.838	0.266322	0.11625	2.290945	0.021966596	0.0338014	NOT
RP11-539I	99.10116	-0.49757	0.217196	-2.29087	0.021970783	0.033806	NOT
TAPT1-AS	44.23038	0.332546	0.145165	2.290807	0.02197457	0.0338099	NOT
KRT18P15	2.09158	0.649624	0.283612	2.290541	0.021989972	0.0338318	UP
PSMD6	2151.161	-0.14932	0.065195	-2.29037	0.021999713	0.0338449	NOT
LRRC57	516.1316	0.154579	0.067497	2.290167	0.022011664	0.0338614	NOT
NLGN4X	183.9276	0.743567	0.324699	2.29002	0.022020141	0.0338725	UP
ZNF205-A	22.78726	0.347668	0.151821	2.289985	0.022022204	0.0338738	NOT
RP11-403A	57.70232	-1.09472	0.478069	-2.28989	0.022027956	0.0338808	DOWN
FTH1P22	6.13378	-0.68078	0.297301	-2.28985	0.022030073	0.0338822	DOWN
RP11-44D	1.807611	0.790002	0.345069	2.289403	0.022055943	0.0339201	UP
BAMBI	1598.331	0.526934	0.230229	2.288735	0.022094733	0.0339778	NOT

CCDC6	1501.278	0.171289	0.074842	2.288683	0.022097764	0.0339806	NOT
LINC0116(	13.67755	0.639726	0.279527	2.288596	0.02210281	0.0339865	UP
TMEM33	3736.883	-0.19952	0.087189	-2.28835	0.022116935	0.0340063	NOT
PABPC1P1	1.471953	0.683633	0.298805	2.287891	0.022143898	0.0340449	UP
RP11-517I	45.09896	-0.34073	0.14893	-2.28788	0.022144528	0.0340449	NOT
TMEM255	209.8978	-0.28298	0.123693	-2.28779	0.022150031	0.0340515	NOT
CAMTA1-I	0.773414	1.460407	0.638519	2.287177	0.022185506	0.0341041	UP
COMMD1	947.0477	-0.22851	0.099985	-2.28541	0.022288518	0.0342606	NOT
RP11-649/	2.075782	0.640133	0.280129	2.285138	0.022304744	0.0342836	UP
AC087163	2.474652	0.605907	0.265166	2.285012	0.022312108	0.034293	UP
RP11-173/	1.332363	0.845106	0.369862	2.284923	0.022317316	0.0342991	UP
NDST4	1.214401	2.201359	0.963453	2.284863	0.022320852	0.0343013	UP
MEGF6	614.5096	0.525146	0.229838	2.284857	0.022321198	0.0343013	NOT
UTS2B	10.3405	0.56682	0.248082	2.284811	0.02232392	0.0343036	NOT
RP11-126(	2.476773	1.254355	0.549112	2.284335	0.022351862	0.0343446	UP
RP11-80H	3.598851	0.56887	0.249041	2.284245	0.022357123	0.0343508	NOT
PWRN1	5.608644	-1.10771	0.484968	-2.28409	0.022366148	0.0343627	DOWN
REST	1012.871	-0.17807	0.077994	-2.28318	0.022419602	0.034443	NOT
GKAP1	298.4818	0.210734	0.092321	2.282615	0.022453062	0.0344924	NOT
PROK1	5.858567	0.786496	0.344569	2.282552	0.02245675	0.0344962	UP
TATDN1P1	2.058041	0.690166	0.3024	2.282297	0.022471839	0.0345175	UP
MFSD12	1719.282	0.238986	0.104715	2.28225	0.022474605	0.0345198	NOT
CCDC60	1.378329	1.212052	0.531095	2.282178	0.022478825	0.0345232	UP
RP11-157.	15.143	-0.80548	0.352943	-2.28217	0.022479323	0.0345232	DOWN
RP11-813I	1.951501	0.697627	0.3057	2.282069	0.022485283	0.0345304	UP
NPHP4	225.4156	0.460205	0.20167	2.281976	0.022490735	0.0345369	NOT
ACTBL2	1.235245	1.311829	0.574918	2.281768	0.02250303	0.0345539	UP
AC022182	1.67097	0.816125	0.357696	2.281615	0.022512089	0.0345641	UP
TIMM8BP2	4.983249	-0.46475	0.203693	-2.28161	0.022512211	0.0345641	NOT
RP11-554I	7.932766	-1.31813	0.577737	-2.28154	0.022516788	0.0345692	DOWN
HERPUD2	1235.305	-0.1498	0.065671	-2.28112	0.022541113	0.0346046	NOT
ALDH1L1	29831.25	-0.68009	0.298158	-2.28096	0.022550892	0.0346166	DOWN
HRH2	13.33703	0.69933	0.306596	2.28095	0.022551428	0.0346166	UP
ANKK1	7.091694	0.628009	0.275332	2.280914	0.022553555	0.034618	UP
ZCCHC3	987.257	0.212364	0.093121	2.280528	0.022576406	0.0346511	NOT
CTD-2006	10.64255	0.4588	0.201208	2.280228	0.022594161	0.0346765	NOT
ANGPTL2	840.377	-0.34787	0.152596	-2.27966	0.02262786	0.0347263	NOT
RP11-446I	40.48195	-0.45236	0.198434	-2.27964	0.022629338	0.0347266	NOT
MTERF2	552.3058	0.209095	0.091724	2.279607	0.022630993	0.0347272	NOT
MAPK1	4044.394	0.171638	0.075304	2.279259	0.022651695	0.0347571	NOT
SEC61A2	372.8601	-0.27335	0.119945	-2.27897	0.022668882	0.0347804	NOT
FLJ16779	1.10508	1.219949	0.53531	2.27896	0.022669451	0.0347804	UP
RP1-131F1	9.037006	-0.41329	0.181364	-2.27881	0.022678495	0.0347924	NOT
RP11-434I	4.96234	0.46984	0.206186	2.278713	0.022684132	0.0347978	NOT
TMPRSS3	407.4117	0.790963	0.34711	2.278706	0.022684561	0.0347978	UP
FAM201A	46.43702	0.808067	0.354624	2.278662	0.02268719	0.0347999	UP
XXbac-BP(	64.66834	0.342828	0.150466	2.278437	0.022700529	0.0348185	NOT
RP11-44K(	2.932216	-1.06075	0.465601	-2.27823	0.022712891	0.0348345	DOWN
AC104389	2.470236	1.134545	0.497996	2.278219	0.02271353	0.0348345	UP
RP11-793I	2.465179	0.604461	0.265328	2.278169	0.022716512	0.0348372	UP
RSL24D1	2304.888	-0.15972	0.07011	-2.27806	0.022723017	0.0348452	NOT
RP11-100	8.946015	0.40139	0.1762	2.278035	0.02272447	0.0348455	NOT

ENTPD3-A	228.7456	-0.29378	0.128968	-2.27794	0.022730317	0.0348526	NOT
CDK12	1746.334	0.184119	0.080829	2.277886	0.022733365	0.0348553	NOT
ALDH3B2	6.682394	1.183463	0.51963	2.27751	0.022755784	0.0348877	UP
KANK4	126.866	-0.85865	0.377092	-2.27704	0.022783987	0.0349291	DOWN
CRB2	9.287184	0.742746	0.326197	2.276988	0.02278694	0.0349316	UP
RECK	291.3814	-0.36844	0.161861	-2.27625	0.022831342	0.0349978	NOT
C14orf1	3820.332	-0.30691	0.134851	-2.27595	0.022849282	0.0350233	NOT
SDS	29647.61	-0.87412	0.384106	-2.27572	0.022862864	0.0350422	DOWN
CDC20P1	4.592486	0.632992	0.278185	2.275438	0.022879663	0.035066	UP
ENPP4	802.9188	0.358988	0.157786	2.275164	0.022896067	0.0350892	NOT
AC005532	3.540214	0.707715	0.311069	2.275106	0.022899595	0.0350927	UP
CHMP6	1300.177	0.228874	0.100601	2.275061	0.022902256	0.0350948	NOT
DNAJB4	751.6093	-0.32781	0.144092	-2.275	0.022905931	0.0350985	NOT
FKBP3	1787.59	0.162832	0.071578	2.27488	0.022913131	0.0351076	NOT
CTD-2562	1.952035	0.944411	0.415154	2.274845	0.022915209	0.0351088	UP
RP11-187(	8.788878	0.450945	0.198235	2.274797	0.02291813	0.0351114	NOT
RP11-779(	3.012727	-0.60684	0.266779	-2.27471	0.022923528	0.0351177	DOWN
PRKCSH	16085.37	0.17692	0.077803	2.27396	0.022968385	0.0351844	NOT
DNASE1L1	526.9007	0.240367	0.105712	2.273792	0.022978516	0.035198	NOT
RPL18	23248.92	0.33395	0.146893	2.273422	0.02300078	0.0352284	NOT
RNA5SP33	1.949756	-1.30989	0.576177	-2.27342	0.0230009	0.0352284	DOWN
RP11-206I	1.592093	0.623441	0.274263	2.273152	0.023017034	0.0352504	UP
CREBZF	1286.34	0.214501	0.094363	2.273139	0.023017797	0.0352504	NOT
RPL37P23	6.351083	0.501357	0.220586	2.272843	0.023035653	0.0352758	NOT
XRCC5	9159.526	-0.14367	0.063213	-2.27281	0.023037477	0.0352759	NOT
CTB-54O9	2.229826	-0.52068	0.229091	-2.2728	0.023038257	0.0352759	NOT
POLR2E	7654.176	-0.21697	0.095475	-2.27257	0.023052238	0.0352953	NOT
RP11-428(	11.3435	0.511501	0.225113	2.272193	0.023074881	0.035328	NOT
HSPA8P4	3.988913	-0.5936	0.261334	-2.27141	0.023122145	0.0353972	DOWN
RP11-344I	4.826746	0.546601	0.240645	2.271402	0.023122641	0.0353972	NOT
RP11-50B3	1.715805	0.759152	0.334269	2.271081	0.023142094	0.035425	UP
ATF1	646.7133	-0.17093	0.075282	-2.27047	0.023179125	0.0354798	NOT
BNIP1	376.6821	0.23436	0.103227	2.270339	0.023187008	0.0354897	NOT
SPATA7	199.4494	-0.19817	0.087289	-2.27032	0.023188193	0.0354897	NOT
PCDHA11	11.5841	0.992298	0.437196	2.269687	0.023226581	0.0355465	UP
HNRNPLP2	14.7647	0.381867	0.168254	2.269592	0.023232355	0.0355534	NOT
PTMAP4	12.86781	0.298482	0.131516	2.269539	0.023235559	0.0355563	NOT
P2RX5-TA	16.72349	-0.29203	0.128696	-2.26912	0.023261115	0.0355928	NOT
RP11-524I	28.06213	-0.43999	0.193905	-2.2691	0.023261975	0.0355928	NOT
RNU6-485	2.808549	0.790823	0.348563	2.26881	0.02327986	0.0356164	UP
AC092580	15.63584	-0.5319	0.234439	-2.26881	0.023279977	0.0356164	NOT
RPL17P50	59.06106	-0.46703	0.205857	-2.26872	0.023285081	0.0356222	NOT
KRT8P37	2.150006	-0.57719	0.25442	-2.26864	0.023290403	0.0356284	NOT
RP11-93H	0.90347	1.358667	0.598968	2.268347	0.023308071	0.0356535	UP
RP3-476K8	1.942573	0.574075	0.253109	2.268093	0.023323561	0.0356752	NOT
PARD3	2100.984	0.21069	0.092901	2.267903	0.023335148	0.0356909	NOT
TMEM106	210.2661	-0.39613	0.174679	-2.26776	0.023344122	0.0357027	NOT
AC090286	0.727636	1.568082	0.691514	2.267608	0.023353136	0.0357145	UP
HNRNPA1I	1.62387	0.670319	0.295634	2.267392	0.02336626	0.0357326	UP
RNASE10	2.126263	0.804133	0.35467	2.267273	0.023373576	0.0357418	UP
INPP5A	839.4876	-0.1665	0.073452	-2.26674	0.023405861	0.0357892	NOT
GDPD4	25.49186	0.698005	0.307945	2.266659	0.023411094	0.0357952	UP

RP11-106/	2.977651	0.608759	0.268578	2.266603	0.02341447	0.0357984	UP
RPS3AP44	2.720244	0.628563	0.277318	2.266578	0.023416021	0.0357988	UP
SLC39A9	4161.868	-0.19096	0.084255	-2.26649	0.023421644	0.0358054	NOT
GNAI1	1433.004	0.332953	0.146921	2.266211	0.023438492	0.0358292	NOT
PITPNC1	896.6982	0.26829	0.11839	2.266149	0.02344226	0.035833	NOT
PTPMT1	562.7897	0.178005	0.078552	2.266079	0.023446571	0.0358376	NOT
AC004895	3.390096	0.483013	0.213164	2.26592	0.023456262	0.0358504	NOT
FTLP17	4.104012	0.633075	0.279398	2.265855	0.023460258	0.0358546	UP
KLHL13	247.2954	0.438002	0.193349	2.265342	0.023491729	0.0359007	NOT
ATF4P3	6.849857	0.447977	0.197765	2.265196	0.023500655	0.0359123	NOT
RP4-647J2	14.03202	-0.48716	0.215068	-2.26514	0.023504109	0.0359156	NOT
USP46-AS	54.6269	0.254975	0.11257	2.265032	0.023510712	0.0359237	NOT
RC3H2	1378.436	-0.1679	0.074129	-2.26493	0.023517067	0.0359315	NOT
RP11-246I	10.26274	-1.05116	0.464154	-2.26468	0.023532305	0.0359524	DOWN
SHROOM1	3223.114	0.315979	0.139526	2.264662	0.023533407	0.0359524	NOT
RP1-35C2	2.334103	1.239481	0.54732	2.264638	0.023534888	0.0359527	UP
PPP1R16B	250.6987	-0.38517	0.170114	-2.26421	0.023561441	0.0359913	NOT
RP11-261I	74.80341	1.047436	0.462662	2.263931	0.023578341	0.0360125	UP
AC016582	1.820192	1.068758	0.472084	2.263916	0.023579268	0.0360125	UP
RP11-757I	0.746642	1.572179	0.694456	2.263902	0.023580145	0.0360125	UP
ADPGK	1192.332	0.176494	0.07796	2.263896	0.02358053	0.0360125	NOT
RP11-701I	4.641095	0.713394	0.315124	2.263849	0.023583408	0.0360149	UP
NONOP2	20.41536	-0.35221	0.155596	-2.26362	0.023597287	0.0360341	NOT
OVGP1	391.9438	-0.49397	0.218255	-2.26329	0.023617888	0.0360636	NOT
EPS8L1	172.737	0.740629	0.32729	2.262911	0.023641191	0.0360972	UP
ENHO	728.3828	0.678034	0.299723	2.262201	0.023685008	0.0361621	UP
ADAMTS7	5.190054	0.583889	0.25815	2.261816	0.023708793	0.0361964	NOT
KRT2	0.971307	1.419054	0.627457	2.261596	0.023722378	0.0362151	UP
RP11-586I	1.390221	1.381488	0.61086	2.261547	0.023725416	0.0362178	UP
TAT-AS1	39.05718	0.671193	0.296796	2.261461	0.023730702	0.0362239	UP
EPC2	624.1126	-0.192	0.0849	-2.26143	0.023732847	0.0362251	NOT
SUMO4	10.95872	-0.68097	0.301138	-2.26132	0.023739544	0.0362334	DOWN
CTSS	5112.598	-0.39252	0.173667	-2.26017	0.023810622	0.0363398	NOT
RGS22	2.414376	0.823155	0.364227	2.260004	0.023820985	0.0363521	UP
LINC01001	9.626699	0.394494	0.174555	2.259999	0.023821286	0.0363521	NOT
PSMD13	4601.028	0.213661	0.094564	2.259427	0.023856813	0.0364043	NOT
WDR86	28.82044	-0.41454	0.183486	-2.25922	0.023869667	0.0364219	NOT
H3F3C	7.541617	0.37933	0.167939	2.258736	0.023899838	0.0364659	NOT
MGAT4A	1168.886	0.257169	0.113897	2.257911	0.023951235	0.0365423	NOT
GCA	469.9954	-0.32786	0.14521	-2.25785	0.023955168	0.0365463	NOT
CTC-510F	6.552082	0.717124	0.317621	2.257798	0.02395826	0.036549	UP
RP3-406A	83.65422	-0.40618	0.179927	-2.25748	0.023977915	0.036577	NOT
GIN1	191.9493	-0.1652	0.073181	-2.2574	0.023983096	0.0365829	NOT
RPS8	27680.69	0.274675	0.121703	2.256937	0.024012021	0.036625	NOT
GAPT	23.58193	-0.5021	0.222476	-2.25687	0.024015982	0.036629	NOT
PLAGL1	164.6489	0.51447	0.227971	2.256739	0.024024403	0.0366398	NOT
UVRAG	736.3002	-0.14492	0.064229	-2.25634	0.024049062	0.0366754	NOT
SSBP1	2198.873	0.17527	0.077684	2.256189	0.024058773	0.0366882	NOT
SLC2A10	2202.781	-0.41986	0.186145	-2.25557	0.024097357	0.036745	NOT
RP11-313I	2.647384	0.700241	0.3105	2.255204	0.024120507	0.0367783	UP
MDP1	43.45897	-0.29305	0.129972	-2.25475	0.024149317	0.0368202	NOT
AC012506	1.022487	1.04797	0.464815	2.254598	0.024158573	0.0368323	UP

PRPF8	7131.026	-0.17493	0.077601	-2.25426	0.024179566	0.0368622	NOT
VN2R19P	0.734431	1.319136	0.585301	2.253772	0.024210487	0.0369074	UP
RNASEK-C	15.74415	0.31554	0.140017	2.253582	0.024222453	0.0369236	NOT
ERCC5	1049.813	-0.21784	0.096676	-2.25326	0.024242516	0.0369521	NOT
FCRLB	55.95601	0.59212	0.262787	2.253235	0.024244319	0.0369528	UP
SLC13A3	2761.174	0.852389	0.378308	2.253163	0.024248852	0.0369577	UP
AGT	131499.1	-0.33785	0.149964	-2.2529	0.024265743	0.0369814	NOT
ZFP30	182.2106	0.366611	0.162731	2.252862	0.02426783	0.0369825	NOT
RP11-461(	4.009882	-0.71129	0.315764	-2.25261	0.024283719	0.0370047	DOWN
HSPA8P8	5.98436	0.691324	0.306992	2.251929	0.02432674	0.0370682	UP
RPS7P11	36.93836	0.385476	0.171214	2.251424	0.024358688	0.0371149	NOT
HCAR3	13.57865	0.855283	0.380002	2.250732	0.024402489	0.0371796	UP
HSPE1P4	2.410617	0.627231	0.278785	2.249875	0.024456863	0.0372604	UP
6-Mar	4823.798	0.169156	0.075196	2.249545	0.024477827	0.0372902	NOT
FLII	4049.426	0.194338	0.086407	2.249105	0.024505845	0.0373309	NOT
SRP14	7213.99	0.147674	0.065662	2.248994	0.024512888	0.0373395	NOT
BRD7	1536.326	0.160627	0.071423	2.248961	0.024515009	0.0373407	NOT
GRM8	94.58204	-0.88602	0.393992	-2.24883	0.024523619	0.0373518	DOWN
RP11-110I	37.19244	0.314908	0.140042	2.248669	0.024533538	0.0373648	NOT
PLEKHA5	999.2014	0.231609	0.103015	2.248303	0.024556847	0.0373983	NOT
AC009495	1.355932	-0.71771	0.319249	-2.24812	0.024568527	0.037414	DOWN
CYP51A1P	7.49127	0.445755	0.19832	2.24766	0.024597855	0.0374566	NOT
RP1-50J22	1.476848	0.793108	0.352904	2.247376	0.024616	0.0374822	UP
IGHV3-52	1.693676	-1.06133	0.472287	-2.24722	0.024625805	0.037495	DOWN
SKI	2279.247	-0.20406	0.090811	-2.2471	0.024633665	0.0375049	NOT
RP11-260(	1.177836	2.426388	1.080043	2.246566	0.024667783	0.0375548	UP
YBX1P10	87.40048	0.266604	0.118694	2.24614	0.024695063	0.0375943	NOT
SPATA13	1451.706	-0.2458	0.109436	-2.24604	0.024701335	0.0376018	NOT
ZBTB6	279.3084	0.194243	0.086496	2.245699	0.024723301	0.0376325	NOT
RP11-397I	1.505099	0.652584	0.290595	2.245684	0.024724227	0.0376325	UP
AC006994	5.209211	-0.69131	0.307845	-2.24563	0.024727829	0.0376351	DOWN
XKR6	11.49512	0.638153	0.284177	2.245615	0.024728666	0.0376351	UP
C17orf64	2.767822	0.823123	0.366575	2.24544	0.024739926	0.0376501	UP
GZMM	86.16427	-0.52219	0.232569	-2.24533	0.02474689	0.0376587	NOT
AC007246	395.352	-0.21477	0.095658	-2.24522	0.024754043	0.0376675	NOT
RBMXL2	1.542162	-0.75964	0.338359	-2.24508	0.024762804	0.0376787	DOWN
AC009948	85.57717	0.299183	0.133266	2.245005	0.024767793	0.0376843	NOT
RP11-717I	3.958706	0.634413	0.282615	2.244798	0.024781106	0.0377014	UP
UROD	4166.73	-0.22057	0.098259	-2.24479	0.024781793	0.0377014	NOT
ZNF385A	582.679	0.344818	0.153616	2.244684	0.024788407	0.0377094	NOT
SUMO2P1	12.94937	0.41462	0.18472	2.24458	0.024795088	0.0377175	NOT
TRPC5	32.11741	-0.81483	0.363034	-2.24451	0.02479959	0.0377223	DOWN
RP1-181J2	2.183993	0.699853	0.311853	2.244175	0.024821137	0.037753	UP
MGC3280	123.5774	-0.59285	0.264212	-2.24386	0.024841325	0.0377816	DOWN
RP11-619	2.204703	-0.91751	0.408934	-2.24367	0.024853941	0.0377987	DOWN
AC090602	1.557472	0.704326	0.313961	2.243356	0.024873858	0.0378269	UP
MORC1	2.127095	-0.86698	0.386478	-2.24328	0.024878546	0.037832	DOWN
EXOSC3	503.4646	0.167306	0.07464	2.241516	0.024992672	0.0380034	NOT
HNRNPK	16588.15	-0.10299	0.045956	-2.24115	0.025016615	0.0380377	NOT
SAGE1	1.030127	1.703762	0.760274	2.240985	0.025027067	0.0380515	UP
TET2	402.9415	-0.25263	0.112751	-2.24057	0.025053991	0.0380904	NOT
DDX3P1	2.030661	0.725991	0.324026	2.240532	0.025056411	0.038092	UP

RP11-491I	0.905755	1.090224	0.48663	2.240354	0.025067921	0.0381074	UP
MIER3	777.5695	-0.18987	0.084757	-2.2402	0.025077715	0.0381202	NOT
RP11-887I	4.954166	-0.48395	0.21611	-2.23936	0.025132804	0.0382018	NOT
RP11-276I	3.027634	-0.62565	0.279393	-2.23933	0.025134405	0.0382021	DOWN
RP11-756I	51.80301	-0.5053	0.225675	-2.23908	0.025150988	0.0382252	NOT
RP1-283E3	32.81776	0.3167	0.141467	2.238692	0.025175958	0.0382611	NOT
CAMKK1	82.87248	0.364058	0.162626	2.238618	0.025180755	0.0382663	NOT
RP11-34P1	3.247639	0.657867	0.293894	2.238452	0.025191626	0.0382807	UP
SH3KBP1	1325.141	0.259838	0.116095	2.238141	0.025211867	0.0383094	NOT
SNX24	453.9168	0.194837	0.087054	2.238109	0.025213931	0.0383104	NOT
RP11-686I	1.306025	0.859266	0.384095	2.237116	0.025278731	0.0384067	UP
LYPD6B	57.89874	0.996789	0.44559	2.237011	0.025285598	0.0384151	UP
RNU6-925	2.343528	0.571943	0.255743	2.236399	0.025325625	0.0384738	NOT
FOXE1	4.999115	1.433547	0.641097	2.236085	0.025346233	0.0385029	UP
TRABD2B	382.4433	-0.53854	0.240842	-2.23606	0.025347697	0.0385031	NOT
PLOD1	10574.35	0.273675	0.122397	2.235955	0.025354751	0.0385117	NOT
ZNF655	1446.548	0.175525	0.078508	2.23577	0.025366813	0.0385279	NOT
SNX25	491.3198	-0.23951	0.107128	-2.23573	0.025369408	0.0385297	NOT
PSMC2	4307.042	0.164081	0.073394	2.235605	0.025377668	0.0385401	NOT
RP11-112J	11.9422	0.44655	0.199766	2.235363	0.025393507	0.0385621	NOT
MYO7A	1297.208	-0.38205	0.170916	-2.23533	0.025395448	0.0385622	NOT
MTMR14	1867.095	0.15603	0.069802	2.235319	0.025396398	0.0385622	NOT
RP11-650I	1.771179	0.644943	0.288559	2.235052	0.025413931	0.0385867	UP
AC006116	1.158159	0.86884	0.388816	2.234579	0.025445016	0.0386318	UP
CORO7	383.8566	0.282213	0.126323	2.23406	0.025479101	0.0386814	NOT
DGKA	286.0082	0.418988	0.187569	2.233781	0.025497488	0.0387072	NOT
ZNF367	505.5212	-0.37538	0.168101	-2.23307	0.025544286	0.0387759	NOT
RP11-374A	3.073337	-0.78833	0.353027	-2.23305	0.025545553	0.0387759	DOWN
CAPN7	1084.893	-0.16437	0.073609	-2.23296	0.025551765	0.0387832	NOT
SRP19	897.2755	0.195254	0.087451	2.232735	0.025566416	0.0388033	NOT
HAS2-AS1	5.259292	0.947807	0.424545	2.232524	0.025580383	0.0388224	UP
TNPO3	2075.311	0.155273	0.069552	2.232459	0.025584642	0.0388267	NOT
REV3L	594.3984	-0.24526	0.109868	-2.23233	0.025593328	0.0388378	NOT
RP11-638I	48.22699	0.334854	0.150016	2.232118	0.025607174	0.0388567	NOT
RP11-425I	6.579984	-0.3781	0.169442	-2.23145	0.025651439	0.0389217	NOT
CADPS2	1029.865	0.303261	0.135944	2.230774	0.025696135	0.0389874	NOT
RP5-1004I	2.23265	-0.78448	0.351667	-2.23075	0.025697729	0.0389877	DOWN
PPP2R2B	52.72534	0.614472	0.275484	2.230519	0.025713026	0.0390087	UP
ERAS	3.413759	0.572039	0.256472	2.230416	0.025719851	0.0390169	NOT
MED9	824.5738	-0.21956	0.098445	-2.23028	0.025729038	0.0390287	NOT
MFSD4	75.34701	0.407768	0.182848	2.230097	0.025741033	0.0390448	NOT
SLFN5	812.4227	-0.3855	0.172871	-2.23001	0.025746793	0.0390514	NOT
RP11-258I	6.670711	0.474209	0.21266	2.229895	0.025754408	0.0390588	NOT
SLIT2	194.2053	0.575992	0.258305	2.229894	0.025754472	0.0390588	NOT
CTA-242H	1.648189	0.822253	0.368752	2.22983	0.025758731	0.0390631	UP
WFDC2	262.147	0.909441	0.407959	2.229248	0.025797414	0.0391196	UP
FABP3	211.4269	-0.46338	0.207895	-2.22891	0.025819612	0.0391492	NOT
LEP	2.114836	1.048778	0.470534	2.228912	0.025819757	0.0391492	UP
ACTR6	703.6474	0.176286	0.079103	2.228561	0.0258431	0.0391824	NOT
RP4-739H	3.050787	-0.46751	0.209794	-2.22843	0.025851608	0.0391932	NOT
RP11-10B2	16.16899	-0.5969	0.267873	-2.22829	0.025861469	0.039206	DOWN
RP11-352I	8.575542	-0.53911	0.24196	-2.22812	0.025872777	0.039221	NOT

RP1-167F1	1.302056	1.136522	0.510137	2.227876	0.025888763	0.0392431	UP
NOXA1	579.939	0.312024	0.140086	2.227383	0.025921682	0.0392899	NOT
NDUFA6	3556.164	0.248261	0.111459	2.227371	0.025922483	0.0392899	NOT
CFAP57	62.47396	-0.55058	0.247202	-2.22725	0.025930334	0.0392992	NOT
ZNF626	151.5503	0.493664	0.221649	2.227237	0.025931448	0.0392992	NOT
RP11-568J	1.048356	1.042129	0.467969	2.226918	0.025952721	0.0393292	UP
GDPGP1	248.8644	-0.21085	0.094685	-2.22683	0.025958307	0.0393356	NOT
HLA-DPA1	5512.434	-0.47301	0.212458	-2.22636	0.025989904	0.0393813	NOT
RP11-996I	30.17388	-0.25697	0.115468	-2.22551	0.026046957	0.0394656	NOT
RP5-1165I	5.0425	0.389269	0.174919	2.225427	0.026052569	0.0394719	NOT
ZNF45	307.0207	0.249994	0.11236	2.224943	0.026085062	0.039519	NOT
LINC00525	16.51109	-0.48124	0.216355	-2.22431	0.026127346	0.0395809	NOT
RERE	2359.796	0.209356	0.094127	2.22418	0.026136365	0.0395924	NOT
ATXN3	616.223	-0.16579	0.07456	-2.22358	0.026176507	0.0396495	NOT
VSIG8	1.604215	0.908278	0.408476	2.223576	0.026176974	0.0396495	UP
RP11-544A	1.814376	0.632733	0.284563	2.223525	0.026180433	0.0396526	UP
C16orf58	1985.023	-0.29796	0.134005	-2.22349	0.026182469	0.0396535	NOT
FMN1	99.95902	0.571186	0.256918	2.223227	0.026200467	0.0396786	NOT
RP11-274I	25.18731	0.483448	0.217508	2.22267	0.026238062	0.0397334	NOT
EPHA5-AS	1.414471	2.751776	1.238069	2.222635	0.02624041	0.0397347	UP
IGLV4-69	103.0668	-0.92933	0.418132	-2.22258	0.026243872	0.0397378	DOWN
RP11-66A	8.166612	-0.72532	0.326414	-2.22209	0.026277221	0.0397843	DOWN
DPY19L2P	3.315159	0.825863	0.371661	2.222087	0.02627743	0.0397843	UP
CD99P1	79.90233	0.237835	0.107042	2.221885	0.026291093	0.0398028	NOT
RP1-81D8	4.75105	-0.81687	0.367717	-2.22148	0.026318797	0.0398425	DOWN
MAMDC2-	11.66394	-0.4459	0.200741	-2.22129	0.026331641	0.0398598	NOT
ZNF33A	1346.383	0.155621	0.070065	2.221078	0.026345681	0.0398789	NOT
TAS2R19	2.308862	0.655084	0.295006	2.220575	0.026379753	0.0399283	UP
RP5-887A	2.182113	-0.92239	0.415402	-2.22048	0.026386206	0.0399358	DOWN
FLJ13224	1.820204	0.660446	0.297468	2.220229	0.026403213	0.0399594	UP
KEL	40.56976	0.724984	0.326565	2.220027	0.026416963	0.039978	UP
CTD-2514	9.541943	0.548386	0.247138	2.218944	0.026490539	0.0400853	NOT
HS6ST1	2663.331	0.305275	0.137577	2.218941	0.026490732	0.0400853	NOT
RP11-416I	0.998881	1.038341	0.468025	2.218558	0.026516806	0.0401225	UP
GJB1	17875.12	-0.37412	0.168633	-2.21854	0.026518222	0.0401225	NOT
RP3-465N	3.286523	0.652248	0.294004	2.218498	0.026520889	0.0401225	UP
AC008982	4.439379	0.515642	0.232429	2.218495	0.026521114	0.0401225	NOT
RP11-178I	14.78839	0.441461	0.19904	2.217949	0.02655829	0.0401765	NOT
CREB3	1477.197	0.191336	0.086268	2.217923	0.026560112	0.0401771	NOT
PBRM1	1469.645	-0.18507	0.083451	-2.21765	0.026578596	0.0402028	NOT
AC145124	6.005947	-0.47615	0.214712	-2.21761	0.026581741	0.0402054	NOT
RPL7P8	1.411473	0.728837	0.328673	2.217512	0.026588144	0.0402129	UP
RP1-90G2	4.99569	0.883342	0.398363	2.217428	0.026593884	0.0402175	UP
CCL15	765.2983	0.46671	0.210474	2.217424	0.026594099	0.0402175	NOT
PSENEN	1725.716	0.292362	0.131868	2.217083	0.026617414	0.0402505	NOT
RPL26	8375.281	-0.29424	0.13272	-2.21697	0.026625072	0.0402591	NOT
IGLV1-50	2.627118	-0.80514	0.363173	-2.21696	0.026625999	0.0402591	DOWN
ABR	844.7025	0.437663	0.197434	2.216754	0.026639915	0.040278	NOT
SPTSSB	28.81679	0.700473	0.316026	2.216506	0.02665688	0.0403014	UP
IER3	1652.199	-0.49522	0.223428	-2.21647	0.026659614	0.0403033	NOT
HYAL2	2009.52	0.197921	0.089302	2.216309	0.026670344	0.0403174	NOT
AC022182	1.502382	0.701106	0.316349	2.216241	0.026675033	0.0403223	UP

RP11-344I	0.930417	1.82792	0.824846	2.216074	0.026686412	0.0403372	UP
NPIPB6	1.883326	0.745905	0.336623	2.215846	0.026702048	0.0403587	UP
CTD-2194	20.35454	-0.55977	0.252639	-2.21569	0.026712446	0.0403722	NOT
DIO3OS	180.1831	-0.75903	0.342613	-2.21543	0.026730813	0.0403977	DOWN
RP11-638I	1.521061	0.671904	0.303374	2.214769	0.026775952	0.0404637	UP
RP11-845(	1.01202	0.935835	0.422569	2.214629	0.026785512	0.040476	UP
LRIG1	2205.835	-0.30808	0.139148	-2.21405	0.026825193	0.0405337	NOT
LINC0147(	0.654013	1.40394	0.634177	2.213797	0.026842723	0.040558	UP
ARHGAP5	1998.331	-0.21259	0.096034	-2.21371	0.026848912	0.0405651	NOT
RP11-110!	0.981582	1.832295	0.82783	2.213371	0.026872079	0.0405979	UP
MACF1	4994.595	-0.21139	0.095517	-2.21316	0.026886488	0.0406175	NOT
RP1-47M2	12.77996	-0.56877	0.257047	-2.2127	0.026918173	0.0406631	NOT
AC005307	0.876545	2.113457	0.955177	2.212635	0.02692284	0.040668	UP
LACC1	295.9916	-0.28027	0.126703	-2.21203	0.026964578	0.0407288	NOT
CHST3	276.0189	0.422215	0.190881	2.211932	0.026971376	0.0407368	NOT
RP5-981O	1.869571	0.58451	0.264256	2.211904	0.026973278	0.0407375	NOT
CMC1	943.8631	0.206207	0.093231	2.211796	0.026980787	0.0407466	NOT
MEF2C	596.2401	0.283615	0.128246	2.211484	0.027002332	0.0407769	NOT
CTC-204F	7.258305	0.460578	0.208282	2.211321	0.027013628	0.0407917	NOT
RPS26P11	2.798876	-0.54535	0.246643	-2.21107	0.027031039	0.0408158	NOT
RP11-333(	0.944741	1.021113	0.461866	2.210844	0.027046629	0.0408371	UP
HDAC8	691.5811	0.168914	0.076406	2.21073	0.027054509	0.0408468	NOT
RFFL	1810.318	-0.28716	0.129914	-2.21039	0.027078251	0.0408804	NOT
IGHV1-58	12.89814	-1.01155	0.457686	-2.21014	0.027095691	0.0409045	DOWN
AKAP7	783.2296	-0.40176	0.181792	-2.21001	0.027104793	0.040916	NOT
NGDN	984.6518	0.155596	0.07041	2.209859	0.027114965	0.0409291	NOT
C18orf32	336.8448	0.170164	0.077006	2.209753	0.027122336	0.040938	NOT
CCDC23	408.6999	0.294497	0.133325	2.208866	0.02718398	0.0410288	NOT
BTN1A1	1.278609	0.986282	0.446566	2.208592	0.027203047	0.041052	UP
RP11-240I	8.289496	0.614698	0.278322	2.208589	0.027203237	0.041052	UP
RP4-751H	12.94586	0.32803	0.148525	2.208581	0.027203798	0.041052	NOT
LRRC37A1	2.298532	-0.59169	0.267924	-2.20843	0.027214369	0.041065	DOWN
UNC80	6.98335	0.660253	0.298972	2.208415	0.027215361	0.041065	UP
ST7L	298.818	0.1663	0.07531	2.208207	0.027229872	0.0410846	NOT
C20orf195	3.610186	0.738863	0.334617	2.208083	0.027238499	0.0410954	UP
TRIM23	537.3492	-0.20115	0.0911	-2.20805	0.027240869	0.0410967	NOT
GEM	459.8513	-0.51557	0.233501	-2.20801	0.027243388	0.0410983	NOT
CTSF	5896.837	-0.35017	0.158621	-2.20759	0.027272993	0.0411407	NOT
SNX18P7	0.956062	1.263882	0.572607	2.207243	0.027297093	0.0411748	UP
GPER1	1001.944	-0.44121	0.199925	-2.20686	0.027323679	0.0412127	NOT
LTBR	5935.731	-0.19038	0.086268	-2.20684	0.027325394	0.041213	NOT
NPRL2	864.6533	0.181356	0.082181	2.206779	0.027329518	0.041217	NOT
GRID2	0.818006	1.832825	0.830571	2.206704	0.027334726	0.0412226	UP
GAPDHP3	1.642876	0.975987	0.442333	2.206451	0.027352472	0.0412471	UP
MINK1	2062.21	0.20992	0.09515	2.206206	0.027369607	0.0412707	NOT
MBOAT1	260.7882	0.515469	0.233694	2.205742	0.027402104	0.0413175	NOT
TPBG	160.3336	0.621252	0.281789	2.204667	0.027477438	0.0414288	UP
AC053503	16.59538	-0.4911	0.222762	-2.2046	0.027482262	0.0414338	NOT
C3orf58	1200.921	-0.37066	0.168173	-2.20404	0.027521869	0.0414913	NOT
C3orf20	4.230295	-0.58866	0.267091	-2.20396	0.027526902	0.0414956	DOWN
BTNL3	9.878527	1.242101	0.563579	2.203951	0.027527772	0.0414956	UP
SNORD3B-	1.527676	1.051626	0.477172	2.203872	0.027533343	0.0415015	UP

OR8G3P	0.880234	2.283539	1.036157	2.203853	0.027534684	0.0415015	UP
ALG1L12P	1.514294	-0.75696	0.343507	-2.20363	0.027550655	0.0415233	DOWN
GNL3	2469.197	0.197569	0.08966	2.203545	0.027556365	0.0415297	NOT
AC002128	2.992243	0.648656	0.29441	2.203238	0.027578013	0.04156	UP
AC010761	2.415532	0.623273	0.282898	2.203172	0.027582593	0.0415647	UP
AZIN1	5856.711	0.247364	0.11228	2.203102	0.027587544	0.0415699	NOT
RP11-616I	5.021678	0.765478	0.347555	2.202463	0.027632626	0.0416355	UP
NUMA1	4275.797	0.180744	0.082071	2.202289	0.02764491	0.0416518	NOT
FAM179B	409.1489	-0.21974	0.099782	-2.20223	0.027649207	0.041656	NOT
AC004893	100.0945	-0.22772	0.103407	-2.20213	0.027656303	0.0416644	NOT
VEPH1	110.9824	0.760685	0.345445	2.202047	0.027661955	0.0416707	UP
DHH	9.991745	0.54412	0.24714	2.201671	0.027688566	0.0417085	NOT
RELN	2511.185	-0.73923	0.335773	-2.20156	0.027696282	0.0417178	DOWN
RAB1B	5300.814	-0.13119	0.059599	-2.20127	0.027717149	0.041747	NOT
U6	4.097594	0.427212	0.194089	2.201111	0.027728185	0.0417613	NOT
RPL3P2	22.55191	0.361926	0.164443	2.200915	0.027742028	0.0417799	NOT
LTB4R2	54.21584	0.380318	0.172853	2.200241	0.027789819	0.0418496	NOT
RNU6-118	1.657488	0.669313	0.304209	2.200172	0.027794699	0.0418547	UP
INTS12	651.4952	-0.15483	0.070382	-2.1999	0.02781385	0.0418812	NOT
KCTD19	2.224601	0.894207	0.406545	2.199527	0.027840437	0.041919	UP
CH17-472	123.1362	0.425909	0.193681	2.199023	0.027876279	0.0419707	NOT
C14orf159	1344.2	-0.27584	0.125441	-2.19896	0.027880442	0.0419747	NOT
PAG1	952.9965	0.419783	0.190913	2.19882	0.027890754	0.0419879	NOT
HGD	19780.07	-0.48154	0.219004	-2.19879	0.027892804	0.0419887	NOT
PLA2G7	309.3784	0.510533	0.232221	2.198483	0.027914728	0.0420187	NOT
TFEC	107.4434	-0.45649	0.20764	-2.19847	0.027915743	0.0420187	NOT
RP11-118I	44.92703	-0.90048	0.409621	-2.19834	0.027925218	0.0420306	DOWN
TMEM42	593.6295	0.227471	0.103478	2.198268	0.027930035	0.0420348	NOT
CTC-360J1	2.977569	0.715215	0.325356	2.198254	0.02793104	0.0420348	UP
CAPRIN2	525.944	0.235272	0.107036	2.198057	0.027945045	0.0420536	NOT
MFAP5	17.91802	0.704434	0.32049	2.197991	0.027949744	0.0420584	UP
IFNGR2	2232.935	0.250631	0.114031	2.197925	0.027954449	0.0420632	NOT
TRPA1	7.114159	0.874058	0.397692	2.197826	0.027961505	0.0420715	UP
RP11-59C5	18.11994	0.413576	0.188177	2.197798	0.027963497	0.0420722	NOT
KIAA1467	231.3949	-0.26813	0.122036	-2.19711	0.028012817	0.0421441	NOT
KLK1	2.249442	0.854798	0.389066	2.197049	0.028016955	0.0421481	UP
RPS4XP7	2.505051	0.667808	0.304006	2.196691	0.028042539	0.042184	UP
MAATS1	86.51534	0.5249	0.238953	2.196668	0.028044184	0.042184	NOT
XPNPEP3	1126.237	-0.1807	0.082261	-2.19665	0.028045423	0.042184	NOT
Z83851.4	60.72483	-0.2906	0.132293	-2.19661	0.028048421	0.0421842	NOT
ASNSD1	1684.368	-0.13221	0.060187	-2.19661	0.028048571	0.0421842	NOT
PPIAP13	1.84464	0.617128	0.280963	2.196474	0.028058007	0.0421961	UP
IGHV3-30	132.0793	-0.89965	0.409603	-2.19641	0.028062866	0.0422011	DOWN
LINC00174	256.7155	0.324681	0.147842	2.196132	0.028082509	0.0422283	NOT
RP11-98D	2.195517	0.631673	0.287682	2.19573	0.0281113	0.0422693	UP
KIAA1147	1840.356	0.282899	0.128887	2.194934	0.028168302	0.0423527	NOT
GUCA2B	140.9087	0.771335	0.351484	2.194511	0.028198696	0.0423961	UP
DHCR24	62520.02	-0.26071	0.118815	-2.19424	0.028217948	0.0424228	NOT
RNA5SP12	1.656817	0.662409	0.301979	2.193555	0.028267393	0.0424948	UP
RP11-513(	4.756282	-0.52686	0.24023	-2.19314	0.028297168	0.0425372	NOT
TEKT2	7.07548	-0.63878	0.29129	-2.19294	0.028311372	0.0425563	DOWN
RP11-753I	9.964332	-0.66655	0.303993	-2.19264	0.028333097	0.0425866	DOWN

RP11-334/	10.53079	-0.66628	0.303917	-2.1923	0.028358143	0.0426219	DOWN
EIF6	6995.674	0.232946	0.106264	2.19215	0.028368643	0.0426354	NOT
MAGOH	1082.111	0.175954	0.080282	2.191683	0.028402419	0.0426839	NOT
GPKOW	1048.254	0.158145	0.072173	2.19118	0.028438734	0.0427361	NOT
RP11-256I	2.215076	0.726633	0.331656	2.190922	0.028457442	0.0427619	UP
RP11-425I	261.6609	0.407236	0.185877	2.19089	0.028459765	0.0427631	NOT
TSTD3	29.39039	0.265245	0.121098	2.190329	0.0285004	0.0428218	NOT
RASL10A	16.12918	-0.39853	0.181951	-2.19031	0.028501975	0.0428218	NOT
AF127936.	3.587188	-0.60181	0.274772	-2.19023	0.028507774	0.0428276	DOWN
C20orf203	1.127522	0.860943	0.393087	2.190211	0.028508921	0.0428276	UP
MED15P9	0.812979	1.962775	0.896172	2.190175	0.028511529	0.0428292	UP
PLA1A	1618.751	-0.44126	0.201513	-2.18975	0.028542142	0.0428729	NOT
SSBP3-AS:	13.44165	0.430404	0.196557	2.18972	0.028544551	0.0428742	NOT
RP5-1139I	1.200398	0.986175	0.450395	2.189577	0.028554911	0.0428874	UP
PTP4A2	5500.368	-0.16495	0.075342	-2.18933	0.028572803	0.0429119	NOT
HAR1B	4.388932	-0.73897	0.337556	-2.18916	0.028585062	0.042928	DOWN
RP11-722I	1.149193	2.525621	1.15401	2.18856	0.028628822	0.0429914	UP
RP11-536I	1.348451	0.820762	0.375068	2.1883	0.028647779	0.0430159	UP
NUTM2A-	281.0729	0.215218	0.09835	2.188294	0.028648221	0.0430159	NOT
RP11-131I	8.496546	-0.95086	0.434552	-2.18815	0.02865874	0.0430293	DOWN
CTD-2256	4.887879	-0.75198	0.343672	-2.18807	0.028664825	0.0430359	DOWN
GRB10	2639.571	0.188011	0.085926	2.188047	0.028666212	0.0430359	NOT
LARGE	1088.727	0.273066	0.12481	2.187859	0.028679875	0.043054	NOT
RP11-458I	6.494152	0.541021	0.247307	2.187652	0.028694961	0.0430744	NOT
RP11-274I	11.28924	0.484522	0.221491	2.18755	0.028702362	0.0430831	NOT
RP11-118I	1.497222	3.035535	1.388346	2.18644	0.028783459	0.0432025	UP
TUBBP1	28.46497	0.289252	0.132318	2.186028	0.028813559	0.0432453	NOT
ZNF79	155.447	0.17667	0.080825	2.185842	0.028827199	0.0432635	NOT
RP11-258(	4.016157	0.601389	0.275147	2.185698	0.028837708	0.0432769	UP
RP11-561I	2.052	0.663119	0.303418	2.185496	0.028852472	0.0432967	UP
PDLIM4	54.59292	0.575402	0.263291	2.185425	0.028857717	0.0433022	NOT
EDN3	2.806374	1.281098	0.58639	2.184718	0.02890951	0.0433776	UP
ALDOC	3209.275	-0.46518	0.212932	-2.18464	0.02891502	0.0433835	NOT
GTF2IRD2I	171.2844	-0.25755	0.117912	-2.18421	0.028946572	0.0434285	NOT
ARL15	709.1999	0.201068	0.092064	2.183999	0.028962301	0.0434497	NOT
HHATL-AS	1.78965	1.01763	0.465959	2.183948	0.028966086	0.0434531	UP
RP11-405I	2.447552	0.622401	0.285013	2.183768	0.028979306	0.0434705	UP
RP11-740(	42.03413	-0.54198	0.248236	-2.18334	0.029010842	0.0435155	NOT
STMN2	94.27481	0.995672	0.456228	2.182402	0.029079887	0.0436167	UP
NEBL-AS1	2.141789	1.229285	0.563294	2.182315	0.029086299	0.0436239	UP
RP11-356.	145.9231	-0.49242	0.225644	-2.18229	0.029088264	0.0436245	NOT
RBM25	1940.569	0.162385	0.074414	2.182188	0.029095648	0.0436332	NOT
DFNB59	50.23689	0.312575	0.143289	2.181429	0.029151675	0.0437149	NOT
AIMP2	1102.445	0.209712	0.096138	2.181363	0.029156556	0.0437198	NOT
COMMD6	2083.012	-0.25604	0.117395	-2.18098	0.029185213	0.0437604	NOT
RAB40B	847.4577	0.260421	0.11944	2.180342	0.029232107	0.0438284	NOT
RP11-332I	0.866337	2.197201	1.00793	2.179913	0.029263889	0.0438736	UP
AC002331	1.934273	0.877682	0.402643	2.179805	0.029271939	0.0438833	UP
PTPN22	70.11509	-0.46086	0.211431	-2.17971	0.029279299	0.043892	NOT
MSL3	610.9609	0.140193	0.064323	2.179515	0.029293463	0.0439108	NOT
GZMK	124.2297	-0.62814	0.288215	-2.1794	0.029301679	0.0439208	DOWN
RP11-359I	53.03473	0.297405	0.136469	2.179282	0.029310703	0.0439319	NOT

C3orf52	51.90481	0.7573	0.347509	2.17922	0.029315318	0.0439365	UP
RAB5C	3546.254	0.13687	0.062814	2.178968	0.029334079	0.0439622	NOT
AL845472.	1.49464	0.997689	0.457881	2.178929	0.029336906	0.043964	UP
LRPPRC	5973.811	0.165643	0.076024	2.178825	0.029344669	0.043972	NOT
SMC5-AS1	14.18474	-0.32304	0.148263	-2.17882	0.029345392	0.043972	NOT
CGNL1	4459.272	-0.36973	0.169711	-2.1786	0.029361358	0.0439935	NOT
TMEM184	1132.456	0.391059	0.179506	2.178532	0.02936642	0.0439987	NOT
FAM19A1	3.829138	-0.64683	0.296927	-2.1784	0.029376002	0.0440107	DOWN
RP11-429I	2.196096	0.630419	0.289413	2.178264	0.029386352	0.0440238	UP
MTIF2	1761.401	0.145686	0.066892	2.177925	0.029411625	0.044057	NOT
EMILIN1	1893.685	-0.4977	0.228522	-2.17792	0.029411688	0.044057	NOT
TTC5	267.1207	0.184015	0.084498	2.177735	0.029425802	0.0440758	NOT
ZFP90	463.2937	0.218594	0.100387	2.177518	0.029441924	0.0440975	NOT
PARVG	324.7374	-0.3789	0.174021	-2.17729	0.029458678	0.0441202	NOT
MCTS2P	14.40214	0.332557	0.152766	2.176899	0.029488088	0.0441619	NOT
AKR1E2	24.96318	0.580853	0.266868	2.176551	0.029514051	0.0441984	NOT
ARL6IP5	2767.167	-0.21046	0.096707	-2.17625	0.029536807	0.0442301	NOT
KHSRPP1	1.618265	0.853506	0.392212	2.176135	0.029545132	0.0442382	UP
RP11-824I	1.287867	0.756017	0.347413	2.176132	0.029545421	0.0442382	UP
CACNA2D	54.24065	0.451824	0.207665	2.17573	0.029575441	0.0442794	NOT
KRBOX1	8.205567	0.635282	0.291987	2.175721	0.029576136	0.0442794	UP
ITPR1	578.4743	0.353583	0.162545	2.175299	0.029607737	0.0443243	NOT
MOB2	1032.01	0.179574	0.082553	2.175254	0.029611112	0.0443269	NOT
ZCWPW2	23.81315	-0.25177	0.115746	-2.17515	0.029618612	0.0443358	NOT
EIF4BP7	111.4234	0.244367	0.112373	2.174607	0.029659573	0.0443947	NOT
MXRA7	1165.602	0.339708	0.15624	2.174275	0.029684475	0.0444296	NOT
OSMR-AS1	7.33163	0.529333	0.243459	2.17422	0.029688598	0.0444317	NOT
MTAP	1104.51	-0.21099	0.09704	-2.17421	0.029689121	0.0444317	NOT
ALS2CR12	9.732356	0.390503	0.179617	2.17408	0.02969911	0.0444442	NOT
PYROXD2	516.0427	-0.58028	0.266912	-2.17405	0.029701444	0.0444453	NOT
HAS1	21.25537	-0.89585	0.412071	-2.17401	0.029704329	0.0444473	DOWN
LINC00216	3.272688	0.542512	0.249563	2.173845	0.02971675	0.0444634	NOT
CRY2	3253.457	-0.28437	0.130839	-2.17343	0.029747953	0.0445077	NOT
NPAS3	13.12776	-0.54215	0.249468	-2.17323	0.029762716	0.0445267	NOT
RP11-500I	9.63322	-0.95151	0.437834	-2.17322	0.029763858	0.0445267	DOWN
POU6F2-A	1.259357	1.921631	0.884536	2.172473	0.02982003	0.0446083	UP
SERBP1P6	1.733053	-0.49026	0.225687	-2.17229	0.029833689	0.0446263	NOT
RP11-492I	2.160568	0.57644	0.265368	2.172228	0.029838496	0.0446311	NOT
RP11-28F1	4.096499	0.576427	0.265376	2.172115	0.029847017	0.0446414	NOT
RAB11B	4898.86	-0.18513	0.085232	-2.17207	0.029850487	0.0446442	NOT
MTPN	3457.639	0.167733	0.077231	2.171821	0.029869155	0.0446697	NOT
GOLT1A	3995.572	-0.38272	0.176233	-2.17168	0.029879526	0.0446828	NOT
PRAMENP	18.74987	-0.8553	0.39386	-2.17158	0.029887353	0.0446921	DOWN
LRRC75A-/	5957.96	0.330539	0.152214	2.171538	0.029890498	0.0446944	NOT
RP11-736I	46.26807	-0.39501	0.181927	-2.17128	0.029909921	0.044721	NOT
AGBL5-AS	1.947298	0.614063	0.282823	2.171187	0.029917059	0.0447293	UP
SGCE	865.6897	0.506655	0.233431	2.170466	0.029971561	0.0448063	NOT
ARHGAP17	1399.66	0.156018	0.071883	2.170462	0.029971845	0.0448063	NOT
PGM3	1931.98	-0.23962	0.110405	-2.17033	0.029981959	0.044819	NOT
C22orf15	9.19371	0.417776	0.192517	2.170069	0.03000163	0.044846	NOT
LINC01337	1.249427	0.858931	0.39585	2.16984	0.030019001	0.0448677	UP
NEK4	727.0245	0.250276	0.115343	2.169835	0.030019369	0.0448677	NOT

ASAP1-IT2	3.405954	0.696796	0.321153	2.169671	0.030031791	0.0448838	UP
RP11-129I	5.354672	0.482948	0.222606	2.16952	0.030043246	0.0448985	NOT
ETF1	3619.397	-0.15052	0.069383	-2.16947	0.030046808	0.0449014	NOT
APOA4	5528.566	1.024493	0.472326	2.16904	0.030079684	0.0449481	UP
RP11-158I	96.59327	-0.25824	0.119068	-2.16881	0.030096833	0.0449713	NOT
AC068831	4.493875	0.504596	0.232727	2.168183	0.030144758	0.0450405	NOT
FAM129B	2802.266	0.362681	0.167294	2.167933	0.03016382	0.0450663	NOT
KLHL6-AS1	29.85077	-0.68091	0.314088	-2.16791	0.030165311	0.0450663	DOWN
NRL	108.1671	-0.25842	0.119208	-2.16783	0.03017163	0.0450734	NOT
RP4-791C1	3.309647	-0.49401	0.227887	-2.16778	0.030175592	0.0450768	NOT
WDR24	713.4322	0.233428	0.107689	2.167618	0.030187761	0.0450926	NOT
RAB39A	9.479837	-0.57432	0.26498	-2.1674	0.03020424	0.0451148	NOT
CEP76	214.9344	-0.20759	0.095785	-2.16719	0.030220152	0.0451361	NOT
RP11-59N	3.162979	0.723189	0.333726	2.167018	0.030233476	0.0451536	UP
C11orf97	14.50262	-0.64251	0.296504	-2.16696	0.030237814	0.0451576	DOWN
RPL7AP15	2.957762	0.641308	0.295992	2.166643	0.030262121	0.0451915	UP
SMIM1	167.5551	-0.46563	0.21495	-2.1662	0.030295628	0.0452391	NOT
Vax2os1_3	0.620383	1.729007	0.798332	2.165773	0.030328504	0.0452857	UP
HNRNPH1	3.109645	0.550794	0.254451	2.164638	0.03041542	0.045413	NOT
HSPA9P1	6.509704	0.431775	0.199472	2.164589	0.030419154	0.0454162	NOT
PRSS46	2.539486	0.869595	0.401805	2.164223	0.030447251	0.0454556	UP
CTC-512J1	7.548148	0.300653	0.138965	2.163522	0.030501084	0.0455336	NOT
SLC25A53	99.05609	0.224583	0.103824	2.163116	0.030532253	0.0455776	NOT
RP5-961K1	1.084781	0.955115	0.441573	2.162984	0.030542376	0.0455903	UP
PLN	42.37787	0.573192	0.265006	2.162943	0.030545592	0.0455926	NOT
DCDC1	86.39744	-0.6919	0.319902	-2.16284	0.030553465	0.0456019	DOWN
AC007292	15.18754	0.305709	0.14135	2.162786	0.030557617	0.0456057	NOT
RP11-383I	2.223139	0.52945	0.244813	2.162675	0.030566224	0.045616	NOT
TMEM185	545.1547	0.222601	0.102942	2.162393	0.030587865	0.0456459	NOT
MAN1A2	2234.584	-0.19162	0.088629	-2.16204	0.030615063	0.045684	NOT
CTC-251I1	43.67942	0.556292	0.257381	2.161358	0.030667723	0.0457601	NOT
EIF2AK3	703.0642	-0.19599	0.090701	-2.16082	0.030709405	0.0458195	NOT
LINC01141	2.281158	0.676283	0.312978	2.1608	0.030710814	0.0458195	UP
RP11-266I	2.654295	0.610846	0.282705	2.160719	0.030717083	0.0458263	UP
VPS29	2473.933	0.136337	0.063103	2.160537	0.030731137	0.0458417	NOT
C6orf99	6.409015	-0.78756	0.364522	-2.16052	0.030732307	0.0458417	DOWN
ELP5	1377.372	-0.2276	0.105344	-2.16052	0.030732376	0.0458417	NOT
MAP6D1	47.28118	0.30834	0.142717	2.160496	0.030734307	0.0458422	NOT
NATD1	564.2132	0.249172	0.115343	2.160263	0.030752284	0.0458644	NOT
ARMCX2	191.6973	0.48444	0.224251	2.16026	0.03075255	0.0458644	NOT
RIMS1	1.993754	0.925421	0.428407	2.160143	0.030761572	0.0458754	UP
RP11-10J2	0.96427	0.951898	0.440683	2.160053	0.030768541	0.0458833	UP
RP11-355I	6.08932	0.559289	0.258964	2.159718	0.030794521	0.0459196	NOT
SOGA3	2.118594	0.609671	0.2823	2.159658	0.030799147	0.0459225	UP
EZR	4512.388	-0.30718	0.142238	-2.15965	0.030799816	0.0459225	NOT
ASB7	554.3911	-0.15605	0.07227	-2.15922	0.030832814	0.0459693	NOT
MIR659	1.815923	0.597571	0.276766	2.159115	0.03084125	0.0459794	UP
RP11-727A	2.340535	0.540622	0.250442	2.158672	0.030875612	0.0460257	NOT
AC073415	1.67101	0.699335	0.323965	2.158672	0.030875638	0.0460257	UP
RPL3P4	589.3258	-0.42444	0.196637	-2.1585	0.030888689	0.0460426	NOT
PTPRJ	2049.177	0.20487	0.094916	2.158428	0.030894596	0.046049	NOT
ZNF573	41.66648	0.223184	0.103403	2.158383	0.03089808	0.0460517	NOT

TAF7L	9.913212	0.638516	0.295878	2.158042	0.030924594	0.0460876	UP
LINC0126Ę	1.445463	1.130085	0.523665	2.15803	0.030925503	0.0460876	UP
IGHV4-28	28.16054	-0.99462	0.4609	-2.15801	0.03092723	0.0460877	DOWN
IPMK	442.4912	-0.21789	0.100979	-2.15777	0.030945784	0.0461128	NOT
IGKV3D-1!	10.25264	-1.06337	0.492962	-2.15711	0.030996816	0.0461864	DOWN
FAM184A	292.3306	0.41624	0.192964	2.157081	0.030999387	0.0461877	NOT
LURAP1	12.17469	0.494394	0.229207	2.156979	0.031007291	0.046197	NOT
SLC25A17	953.55	-0.14165	0.065675	-2.15692	0.031011867	0.0462014	NOT
RPE	946.1177	0.139008	0.06445	2.156844	0.031017789	0.0462077	NOT
TCEB2P2	2.22883	-0.52714	0.244444	-2.15649	0.031045758	0.0462469	NOT
RPL13AP5	341.4342	0.353142	0.163764	2.156411	0.031051551	0.046253	NOT
RP11-2L8.:	15.04993	-0.62106	0.288094	-2.15574	0.031103627	0.0463281	DOWN
RP11-181(	12.42041	0.418327	0.194087	2.155359	0.031133768	0.0463705	NOT
TAF15	5090.991	0.123205	0.057165	2.155257	0.031141756	0.0463799	NOT
IGHV1-12	2.868287	-1.15588	0.536342	-2.15512	0.031152792	0.0463938	DOWN
POLR2A	4374.475	-0.19944	0.092548	-2.15503	0.031159104	0.0464007	NOT
EFNB1	1225.354	0.262222	0.121686	2.154915	0.031168449	0.0464121	NOT
RAB11FIP5	863.7532	0.283508	0.131577	2.154694	0.031185757	0.0464354	NOT
PAX3	0.796365	1.851849	0.859576	2.154374	0.031210868	0.0464703	UP
SNRPCP3	3.646119	0.568814	0.264034	2.154327	0.031214567	0.0464733	NOT
CTD-2184	1.819728	0.948467	0.440305	2.154112	0.031231362	0.0464958	UP
FUK	972.9997	0.201864	0.093715	2.154014	0.031239039	0.0465047	NOT
RP11-122ﾓ	2.32043	0.634602	0.294651	2.153742	0.03126042	0.0465341	UP
PIGK	1120.597	-0.18977	0.088121	-2.15355	0.03127577	0.0465519	NOT
C1QTNF4	7.757915	0.531692	0.246891	2.153546	0.031275782	0.0465519	NOT
RAP1GDS1	1202.901	-0.22	0.102161	-2.15348	0.031280809	0.0465569	NOT
SULT1B1	1313.575	-0.62996	0.292573	-2.15316	0.031306189	0.0465922	DOWN
NDUFA3	3049.854	0.366694	0.170339	2.152728	0.031340083	0.0466401	NOT
RP11-70P:	5.007964	0.503764	0.234071	2.152186	0.031382729	0.0467011	NOT
AC006272	1.785651	0.674411	0.313433	2.151692	0.031421646	0.0467564	UP
ZNF469	101.3107	0.510111	0.237084	2.151608	0.031428268	0.0467638	NOT
TBC1D10A	568.936	-0.22163	0.103012	-2.15147	0.031439171	0.0467775	NOT
RP11-368I	46.59658	-0.32476	0.150948	-2.15145	0.031441076	0.0467778	NOT
AC009948	4.591101	0.41435	0.192595	2.151409	0.031443964	0.0467796	NOT
KHDRBS1	4073.364	0.12391	0.057597	2.151314	0.031451456	0.0467882	NOT
AGAP3	2708.829	0.189378	0.088032	2.151231	0.031457961	0.0467954	NOT
TEX9	40.92006	0.352957	0.164088	2.151024	0.031474343	0.0468172	NOT
CDC42SE2	1860.49	0.188258	0.087542	2.150493	0.031516244	0.046877	NOT
PPAN	274.0525	0.336703	0.156574	2.150446	0.031519965	0.0468801	NOT
KDELR1	7895.143	0.190288	0.088499	2.150165	0.031542132	0.0469105	NOT
RP11-184I	2.616718	1.845324	0.858303	2.149969	0.031557683	0.0469311	UP
BLVRB	7700.03	-0.30563	0.142171	-2.14972	0.031577003	0.046957	NOT
DAGLB	918.7788	0.159732	0.074304	2.149706	0.031578489	0.046957	NOT
TTC32	264.5454	0.283549	0.131905	2.149643	0.031583474	0.0469619	NOT
PREPL	1229.283	0.198053	0.092142	2.149441	0.03159944	0.0469831	NOT
FAM83A	43.2457	0.778649	0.362278	2.149312	0.0316097	0.0469958	UP
FOXA2	2228.377	-0.30858	0.143572	-2.14928	0.031612229	0.0469971	NOT
FAM169B	66.98747	-0.92074	0.428406	-2.14923	0.031616568	0.047001	DOWN
ZNF773	111.6616	0.35899	0.167041	2.149115	0.031625258	0.0470114	NOT
TFB1M	425.6582	-0.25929	0.120654	-2.14903	0.031631729	0.0470185	NOT
SDPR	1135.55	-0.46265	0.215292	-2.14895	0.031638327	0.0470258	NOT
RP11-138/	7.325985	0.544997	0.253615	2.148917	0.031640941	0.0470271	NOT

L3MBTL2	1399.552	0.140353	0.065317	2.148817	0.031648894	0.0470364	NOT
SNORA16E	1.378917	0.72993	0.339696	2.148772	0.031652447	0.0470392	UP
ZCCHC10	460.183	0.160437	0.074682	2.148268	0.031692465	0.0470961	NOT
ARHGEF7-	2.82464	0.983692	0.457956	2.148004	0.031713452	0.0471248	UP
KRT1	7.798123	1.055866	0.491565	2.147967	0.031716362	0.0471262	UP
TRIP11	1253.26	-0.19252	0.089628	-2.14795	0.031717812	0.0471262	NOT
MTOR	2899.216	0.24162	0.112495	2.147832	0.031727115	0.0471375	NOT
CLPP	2390.688	-0.2201	0.102481	-2.14775	0.031733708	0.0471447	NOT
CTSLP7	7.830064	0.558854	0.260236	2.147488	0.03175445	0.047173	NOT
RPL9P7	11.89799	0.378506	0.176267	2.147341	0.031766105	0.0471878	NOT
RNF139	1501.753	-0.16266	0.075754	-2.14724	0.031774197	0.0471973	NOT
F13B	5861.751	-0.53735	0.250269	-2.14711	0.031784674	0.0472103	NOT
RP11-159I	17.8983	0.302597	0.140943	2.146937	0.031798269	0.047228	NOT
TDRD6	69.8678	0.456406	0.212597	2.146814	0.031808117	0.0472401	NOT
RP11-602I	1.214993	0.728728	0.339467	2.14668	0.031818747	0.0472533	UP
RP11-603I	5.011382	0.984194	0.458477	2.146658	0.031820519	0.0472534	UP
IQCJ-SCHI	65.95753	-0.38047	0.177254	-2.14645	0.031836916	0.0472752	NOT
TM2D1	666.2979	0.15599	0.072678	2.146317	0.031847679	0.0472887	NOT
AC132008	3.258505	0.572579	0.26678	2.146256	0.031852553	0.0472934	NOT
RP11-696I	79.83486	0.368117	0.171549	2.145835	0.031886123	0.0473407	NOT
IKZF2	118.5877	-0.36446	0.169857	-2.14566	0.031899844	0.0473585	NOT
XXbac-BP(	1.865307	0.728798	0.339726	2.145253	0.031932639	0.0474046	UP
INF2	4733.852	-0.22836	0.10649	-2.14438	0.03200226	0.0475052	NOT
CLIC2	331.4963	-0.37166	0.17332	-2.14436	0.032003834	0.0475052	NOT
RP11-196I	2.204358	0.964066	0.449633	2.144117	0.032023527	0.0475319	UP
TAF1A-AS	94.68929	0.32131	0.149924	2.143151	0.032101007	0.0476424	NOT
RP4-539M	9.818955	-0.52396	0.244483	-2.14315	0.032101425	0.0476424	NOT
RP11-84A:	5.092357	-0.71096	0.331813	-2.14266	0.032140809	0.0476983	DOWN
C15orf56	2.752824	0.893312	0.416993	2.142272	0.032171584	0.0477414	UP
DNAJB3	6.756457	0.705391	0.329353	2.141746	0.032213916	0.0478017	UP
CFAP70	81.80918	0.391423	0.182763	2.141704	0.03221727	0.0478041	NOT
RP11-128I	1.417822	0.752136	0.351208	2.141568	0.032228254	0.0478178	UP
RP11-350I	1.440018	0.945878	0.441803	2.140951	0.032277995	0.0478891	UP
RP11-530I	8.300475	-0.51062	0.238527	-2.14072	0.032296443	0.0479118	NOT
C6orf106	10353.33	0.211388	0.098746	2.140718	0.03229676	0.0479118	NOT
RP5-828K:	2.453249	-0.57628	0.269214	-2.14061	0.032305784	0.0479207	NOT
FTH1P1	2.814483	0.542182	0.253285	2.1406	0.032306277	0.0479207	NOT
C21orf59	552.7563	0.180603	0.084374	2.14052	0.032312782	0.0479278	NOT
ZC3HAV1	1453.024	0.162019	0.075701	2.140265	0.032333378	0.0479558	NOT
SECISBP2	1774.323	-0.14748	0.068914	-2.14005	0.032350659	0.0479789	NOT
EXOC2	1153.735	0.151125	0.07062	2.139985	0.032355943	0.0479841	NOT
RP11-336/	3.319885	0.844101	0.394528	2.139521	0.032393486	0.0480372	UP
MAGEA8-/	0.811339	1.209347	0.565293	2.139327	0.032409208	0.0480557	UP
RP11-524I	7.989102	-0.50653	0.236771	-2.13932	0.032409408	0.0480557	NOT
CNTFR-AS	2.454951	1.11665	0.522087	2.138821	0.032450204	0.0481136	UP
SOD3	606.4847	0.535476	0.250413	2.138376	0.032486238	0.0481644	NOT
UBLCP1	738.6462	0.13815	0.064625	2.137706	0.03254058	0.0482424	NOT
FAM228A	5.521491	-0.53104	0.248427	-2.13762	0.032547696	0.0482504	NOT
RPS4XP23	1.181915	0.833297	0.389834	2.137566	0.032552018	0.0482542	UP
CNKSR2	139.5647	0.541045	0.253156	2.137199	0.032581781	0.0482957	NOT
RP11-17E:	53.02236	-0.37047	0.173357	-2.13705	0.032593539	0.0483106	NOT
PDE4A	382.4012	0.411828	0.192745	2.136649	0.032626528	0.0483569	NOT

UST	147.4886	0.615493	0.288184	2.135763	0.032698773	0.0484614	UP
CTB-36O1	5.98556	-0.6312	0.295562	-2.13557	0.032714075	0.0484815	DOWN
RP11-267I	9.109908	0.353327	0.165453	2.135509	0.032719421	0.0484868	NOT
AC005523	6.606583	0.545249	0.255352	2.135282	0.032738022	0.0485117	NOT
PRPF38A	1211.723	0.167356	0.078379	2.135217	0.032743283	0.0485169	NOT
FLJ45079	0.955592	0.995731	0.466352	2.135148	0.032748938	0.0485227	UP
RP11-66B2	2.16889	-0.87782	0.411159	-2.135	0.032760977	0.048538	DOWN
RP11-107	1.499119	0.664049	0.31107	2.13473	0.032783087	0.0485661	UP
CTD-2521	297.312	-0.38726	0.181412	-2.13472	0.032783489	0.0485661	NOT
RP11-777I	1.752995	-0.67454	0.316034	-2.13439	0.032810837	0.048604	DOWN
LPO	2.562687	0.835466	0.391447	2.1343	0.032818248	0.0486124	UP
RP4-612B1	9.000736	0.355126	0.166392	2.134275	0.032820268	0.0486128	NOT
SORCS1	15.15192	1.02276	0.479339	2.13369	0.032868163	0.0486811	UP
RP4-798P1	143.6685	0.443317	0.207841	2.132961	0.032927926	0.048767	NOT
LAMTOR5	2284.145	0.182461	0.085549	2.132831	0.032938561	0.0487802	NOT
C1orf122	1333.517	0.278344	0.130512	2.132704	0.032949044	0.0487931	NOT
DNM1P47	1.309738	0.867046	0.406558	2.132647	0.03295367	0.0487973	UP
RP11-73O	2.338518	-0.58551	0.274556	-2.13258	0.032959168	0.0488029	DOWN
DCAF6	5191.197	-0.19026	0.089229	-2.13222	0.032988655	0.0488439	NOT
COL9A3	197.4705	0.599553	0.281197	2.132147	0.032994768	0.0488503	UP
RP11-276I	5.204741	-0.5563	0.260955	-2.13179	0.033024497	0.0488917	NOT
RP11-500	17.34397	0.316847	0.148638	2.131668	0.033034162	0.0489034	NOT
RP11-141I	18.49299	-0.34997	0.16422	-2.13113	0.03307875	0.0489668	NOT
RNU1-138	1.798949	1.140433	0.535386	2.130114	0.03316222	0.0490877	UP
AP001271.	2.006988	0.711942	0.33425	2.129971	0.033173995	0.0491026	UP
ST18	7.157747	-0.57004	0.267661	-2.12969	0.033197294	0.0491344	NOT
KIAA0430	1830.465	-0.1658	0.077855	-2.12961	0.033203531	0.049141	NOT
KIT	114.2178	0.451697	0.212114	2.129498	0.033213079	0.0491525	NOT
UCP2	1411.282	-0.3852	0.180924	-2.12904	0.033250576	0.0492054	NOT
RP11-400I	2.349306	0.582841	0.27381	2.128636	0.033284355	0.0492527	NOT
RHCE	52.81992	-0.52487	0.246582	-2.12857	0.03329022	0.0492588	NOT
RAB3IL1	527.1082	-0.39196	0.184163	-2.12831	0.033311069	0.049287	NOT
EPB41L4A-	25.10384	-0.43146	0.20273	-2.12827	0.033314544	0.0492892	NOT
ASCC1	719.57	-0.18651	0.087634	-2.12825	0.033316117	0.0492892	NOT
MTSS1L	1773.679	-0.22327	0.104919	-2.12804	0.033334103	0.0493132	NOT
ZNF772	132.3874	0.324929	0.152693	2.127994	0.03333758	0.0493157	NOT
HSP90B1	50922.46	0.196641	0.092413	2.127852	0.033349387	0.0493305	NOT
CLLU1OS	1.519848	1.26862	0.596256	2.127644	0.033366632	0.0493534	UP
HIST1H2B	235.2853	0.472691	0.222219	2.127136	0.033408778	0.0494131	NOT
WIPI1	729.8188	0.260054	0.122281	2.126694	0.033445556	0.0494648	NOT
LINC0093	33.57884	-0.25894	0.121767	-2.12654	0.033458469	0.0494813	NOT
IGLV2-23	389.5904	-0.89279	0.41988	-2.1263	0.033478549	0.0495083	DOWN
CDK6	1565.935	0.447078	0.210274	2.126169	0.033489199	0.0495214	NOT
SP140	97.34588	0.496116	0.233356	2.126	0.033503244	0.0495395	NOT
ST13P19	3.374946	0.457613	0.215275	2.125716	0.033526944	0.0495719	NOT
RP11-902I	6.378969	0.508128	0.239064	2.125488	0.033545944	0.0495974	NOT
CDC14A	128.5705	0.291529	0.137161	2.12545	0.033549097	0.0495994	NOT
RBFOX3	2.560791	0.581088	0.27344	2.125106	0.033577809	0.0496392	NOT
AP000770.	3.285806	1.053204	0.495772	2.124372	0.033639084	0.0497271	UP
RP11-27K1	5.391498	0.415706	0.195691	2.124296	0.033645373	0.0497337	NOT
SIPA1	1952.161	0.218391	0.102813	2.124169	0.033656008	0.0497468	NOT
DNAJC9-A	4.058107	0.468789	0.220734	2.123776	0.03368888	0.0497927	NOT

ASB8	1323.247	-0.12614	0.059397	-2.12374	0.033692297	0.0497937	NOT
LINC0053:	0.662952	1.871195	0.881091	2.123725	0.033693146	0.0497937	UP
RP11-5C2:	19.53077	0.291544	0.137282	2.123687	0.033696339	0.0497958	NOT
RP11-414(	2.873765	0.811323	0.382156	2.123016	0.033752509	0.0498761	UP
GALNT12	43.53278	0.599042	0.282172	2.122968	0.033756536	0.0498794	UP
PARP10	4705.73	0.267843	0.126243	2.121638	0.033868111	0.0500416	NOT
GEMIN6	672.8538	0.183196	0.086357	2.12137	0.033890645	0.0500722	NOT
ATP5C1P1	2.25386	0.572671	0.26997	2.121235	0.033902012	0.0500863	NOT
CNN2P6	1.062703	2.064577	0.973427	2.120936	0.033927214	0.0501209	NOT
SLC38A9	739.2945	0.188795	0.089021	2.120791	0.033939412	0.0501362	NOT
AC007228	46.96411	0.351467	0.165771	2.120191	0.033989925	0.0502082	NOT
COMTD1	780.9652	0.346386	0.163379	2.120145	0.033993793	0.0502112	NOT
CTD-2192	100.512	0.317666	0.149835	2.120101	0.03399749	0.050214	NOT
PTPLA	59.77188	0.651359	0.307259	2.119907	0.034013896	0.0502355	NOT
RP11-143I	3.002496	0.505917	0.238657	2.119855	0.03401829	0.0502394	NOT
MLH1	1114.887	0.135556	0.063954	2.119562	0.034042974	0.0502731	NOT
UPK3BL	15.81818	0.947474	0.447137	2.118977	0.03409241	0.0503434	NOT
RP11-69E:	1.957146	0.62027	0.292751	2.118758	0.03411092	0.0503681	NOT
GDA	2702.889	-0.58937	0.278192	-2.11859	0.034125038	0.0503862	NOT
RP11-867(	1.123345	1.576408	0.744149	2.118405	0.034140745	0.0504067	NOT
PEX19	5649.905	-0.21568	0.101831	-2.11801	0.034174209	0.0504535	NOT
RP11-712I	67.68677	-0.19697	0.093003	-2.11791	0.034182438	0.0504629	NOT
NFATC1	321.6353	-0.39775	0.187812	-2.1178	0.034191737	0.050474	NOT
IMPDH1P4	1.226706	2.75378	1.300562	2.117377	0.034227839	0.0505245	NOT
TMEM64	1800.939	0.341516	0.161303	2.117236	0.034239851	0.0505372	NOT
CTC-444N	170.8769	0.239631	0.113181	2.117233	0.034240052	0.0505372	NOT
QTRT1	1662.706	0.235303	0.111171	2.116585	0.034295064	0.0506157	NOT
NT5C3B	1696.009	0.226986	0.107243	2.116553	0.034297816	0.050617	NOT
BASP1	524.8673	-0.57342	0.270932	-2.11649	0.034303026	0.050622	NOT
TBC1D8B	517.1663	-0.27793	0.131336	-2.1162	0.034327574	0.0506556	NOT
HSPB7	65.84492	0.590262	0.278938	2.116109	0.03433553	0.0506646	NOT
SCOC-AS1	25.82727	0.330372	0.156142	2.11584	0.034358379	0.0506956	NOT
CD81-AS1	2.534464	0.588936	0.27839	2.115506	0.034386856	0.0507349	NOT
LHX6	43.72556	0.492374	0.232784	2.115153	0.034416951	0.0507766	NOT
BET1L	2635.262	0.129545	0.061249	2.115057	0.034425089	0.0507859	NOT
ANTXR1	883.6041	0.471771	0.223077	2.114829	0.034444538	0.0508119	NOT
USP27X	171.4281	0.405669	0.191842	2.114607	0.03446349	0.0508371	NOT
NRK	20.23622	-0.69572	0.32901	-2.11458	0.034465423	0.0508373	NOT
RANGRF	295.2607	0.350672	0.165852	2.114367	0.034483953	0.0508619	NOT
CRYBA1	1.466013	0.64178	0.303574	2.114079	0.034508524	0.0508954	NOT
FAM105A	145.2843	0.428367	0.20269	2.11341	0.034565662	0.050977	NOT
TBC1D29	1.753342	0.759234	0.359255	2.11336	0.03456996	0.0509806	NOT
RP11-395I	14.62579	0.394539	0.186694	2.113298	0.034575289	0.0509857	NOT
PTPLB	4153.069	0.243048	0.115025	2.113002	0.034600606	0.0510204	NOT
MIR143HG	18.23278	0.409439	0.1938	2.112693	0.034627053	0.0510566	NOT
ACTG1	69367.98	-0.23803	0.112672	-2.11259	0.034635483	0.0510663	NOT
RP1-13D1	1.118122	0.867892	0.410888	2.112234	0.034666365	0.0511091	NOT
ZNF679	0.917096	2.195407	1.039557	2.111868	0.034697791	0.0511504	NOT
KDM5A	1462.973	-0.15452	0.073166	-2.11186	0.034698054	0.0511504	NOT
PRDM2	683.6883	-0.1798	0.085158	-2.11136	0.034741382	0.0512116	NOT
RP11-245:	1.39212	0.672606	0.318614	2.111036	0.0347692	0.0512498	NOT
CDHR1	28.45753	-0.57589	0.272806	-2.11098	0.034773971	0.0512541	NOT

RPS4XP16	10.63206	0.351164	0.166362	2.110843	0.034785783	0.0512688	NOT
RPL37A	25199.96	0.31765	0.150494	2.11072	0.034796353	0.0512817	NOT
TACC1	3052.43	0.341284	0.161695	2.110673	0.034800428	0.0512849	NOT
KLHL18	366.1979	0.142669	0.067606	2.110315	0.034831274	0.0513277	NOT
NBR2	241.2422	0.244947	0.116122	2.109393	0.034910716	0.051442	NOT
IGHV3-15	205.9055	-0.9216	0.436925	-2.10928	0.034920659	0.0514539	NOT
RP11-109	1.979893	0.864496	0.409868	2.109204	0.034926995	0.0514605	NOT
RP5-940J5	15.45693	0.325521	0.154351	2.108963	0.034947753	0.0514883	NOT
IGHV6-1	8.95826	-0.99854	0.473654	-2.10816	0.035017354	0.0515881	NOT
RP11-262	10.93465	-0.40149	0.190513	-2.10743	0.035080093	0.0516778	NOT
OTUD7B	1682.131	0.18726	0.088873	2.107038	0.03511432	0.0517255	NOT
RP11-5G9.	2.631419	0.642233	0.304849	2.106723	0.035141609	0.0517629	NOT
DUSP3	6567.085	0.169313	0.080396	2.105981	0.035205995	0.051855	NOT
ATP5F1	5508.329	-0.16822	0.079908	-2.10522	0.035272489	0.0519502	NOT
KCTD20	3464.804	0.171999	0.081705	2.105114	0.035281426	0.0519606	NOT
SMIM2	2.283595	0.788407	0.374566	2.104858	0.035303671	0.0519906	NOT
RP3-525L6	4.11542	-0.40228	0.191131	-2.10471	0.035316818	0.0520072	NOT
EPM2AIP1	1086.663	-0.20278	0.096355	-2.10454	0.035331348	0.0520258	NOT
LINC0065	92.36545	-0.73754	0.350482	-2.10437	0.035345805	0.0520443	NOT
RP11-730	2.191652	0.597442	0.283908	2.10435	0.035347933	0.0520447	NOT
SEC14L1	1796.599	-0.20945	0.099539	-2.10417	0.035363573	0.0520649	NOT
RP5-1054	5.197823	0.697335	0.331451	2.103886	0.035388412	0.0520987	NOT
EVC2	109.1666	0.550245	0.261639	2.103065	0.035460073	0.0521993	NOT
TTC12	297.7126	-0.21169	0.10066	-2.10306	0.035460538	0.0521993	NOT
MUC3A	1183.248	0.73012	0.347217	2.102779	0.035485108	0.0522327	NOT
HIRIP3	985.7263	0.256933	0.122206	2.102457	0.035513304	0.0522715	NOT
MIRLET7D	2.862403	0.604142	0.28738	2.102239	0.035532329	0.0522967	NOT
RPL7AP60	2.296179	0.520405	0.247578	2.101983	0.035554791	0.052327	NOT
RP11-321	1.906516	0.721552	0.343278	2.101948	0.035557869	0.0523287	NOT
ARID5B	796.0368	-0.30238	0.143869	-2.10176	0.035574495	0.0523504	NOT
RP11-155	3.135363	0.659173	0.313643	2.101666	0.035582561	0.0523595	NOT
MALRD1	4.924106	0.736909	0.350682	2.101361	0.035609286	0.052396	NOT
IGFL1	1.007315	1.53525	0.730638	2.101246	0.03561942	0.0524081	NOT
FOXD3	2.022907	-1.11705	0.531626	-2.10119	0.03562422	0.0524124	NOT
TRA2B	3153.451	-0.10817	0.051483	-2.1011	0.035632264	0.0524215	NOT
ZNF860	7.258952	0.661087	0.314654	2.100999	0.035641091	0.0524317	NOT
ZFP2	35.19397	0.355874	0.169415	2.100611	0.035675164	0.052479	NOT
RP11-254	1.6514	0.597478	0.284445	2.100507	0.035684297	0.0524893	NOT
MAPK1IP1	2617.043	-0.13235	0.063011	-2.10049	0.035685971	0.0524893	NOT
RP11-674	0.989432	1.032663	0.49166	2.100359	0.035697272	0.0525031	NOT
CALHM2	262.1893	0.30772	0.146522	2.100164	0.03571445	0.0525256	NOT
TMEM38A	587.6872	0.318114	0.151478	2.100068	0.03572286	0.0525352	NOT
RP11-973	3.301793	0.986512	0.469762	2.100027	0.035726427	0.0525374	NOT
LSM6	431.7159	-0.17494	0.083305	-2.10001	0.035728154	0.0525374	NOT
SERBP1P5	7.551627	0.368189	0.175336	2.099905	0.035737169	0.0525478	NOT
GS1-306C	0.813034	1.307037	0.622438	2.099868	0.035740412	0.0525498	NOT
SCARF1	441.305	-0.21989	0.104721	-2.09981	0.035745497	0.0525545	NOT
SYTL1	144.0428	0.429023	0.204319	2.099773	0.035748852	0.0525566	NOT
PSMA6	867.2584	0.184031	0.087647	2.099692	0.035755972	0.0525643	NOT
COQ7	637.8609	-0.14833	0.070645	-2.09958	0.035765574	0.0525756	NOT
PMS2P10	6.83909	-0.4129	0.19667	-2.09945	0.035777442	0.0525903	NOT
PNLIP	1.135804	2.441498	1.163021	2.099273	0.035792842	0.0526101	NOT

RBM11	3.210773	-0.7691	0.366421	-2.09896	0.035820199	0.0526475	NOT
AC083949	3.222089	-0.52946	0.252255	-2.0989	0.035825972	0.0526532	NOT
RP11-762I	11.29384	0.421018	0.200617	2.098619	0.035850543	0.0526865	NOT
RP11-279I	508.3146	0.613119	0.292166	2.098527	0.035858596	0.0526956	NOT
SLC25A36	298.8759	0.445776	0.212425	2.098506	0.035860494	0.0526956	NOT
TPT1P5	3.56924	-0.4717	0.224809	-2.09823	0.035885	0.0527288	NOT
C4orf36	75.14121	-0.24246	0.115565	-2.09801	0.035904035	0.052754	NOT
AC090181	1.202622	0.811762	0.387003	2.097559	0.035944151	0.0528101	NOT
AKAP13	3289.767	-0.20536	0.097946	-2.09671	0.036019202	0.0529175	NOT
LINC0108	1.625037	1.289029	0.614837	2.09654	0.036034332	0.052937	NOT
RP11-359I	1.692167	1.030027	0.491314	2.096474	0.036040121	0.0529427	NOT
S100A4	869.0303	0.440205	0.210019	2.096028	0.036079743	0.052998	NOT
PRR34	12.38206	0.372599	0.177775	2.095904	0.036090674	0.0530113	NOT
PTGR1	15520.56	-0.47664	0.227426	-2.09582	0.036097831	0.053019	NOT
GORASP1	1553.367	-0.11507	0.054906	-2.09577	0.036102482	0.053023	NOT
GLI3	61.57617	0.470278	0.224397	2.095742	0.036105101	0.053024	NOT
RP11-439I	2.561309	0.618972	0.295399	2.095377	0.036137466	0.0530688	NOT
RP11-576	2.556368	-0.53292	0.254391	-2.0949	0.036180085	0.0531285	NOT
RP4-765C	75.60362	0.959835	0.458195	2.094819	0.036187109	0.053136	NOT
RSPO2	29.43257	0.96654	0.461409	2.094757	0.036192635	0.0531413	NOT
DYNC1I2	1535.845	0.192772	0.092042	2.094401	0.03622423	0.0531849	NOT
FAM3C2	45.42244	-0.31815	0.151922	-2.09417	0.036244645	0.053212	NOT
RP11-394I	23.45354	0.376594	0.179863	2.093779	0.036279648	0.0532606	NOT
EXOSC10	1704.177	0.138582	0.066202	2.093324	0.03632024	0.0533173	NOT
DNAJC19F	10.33573	-0.4249	0.203026	-2.09284	0.036363719	0.0533783	NOT
RP11-178I	8.149511	-0.51695	0.247053	-2.09246	0.036396964	0.0534243	NOT
CTD-2173	17.27128	-0.41354	0.197648	-2.0923	0.036411283	0.0534425	NOT
RP1-41C2	6.423413	0.478518	0.228721	2.092146	0.036425452	0.0534599	NOT
NLRP2	48.02897	0.721754	0.344985	2.092129	0.036426983	0.0534599	NOT
MOBP	1.879062	0.712402	0.340596	2.091636	0.036471097	0.0535218	NOT
ARHGAP3	5300.964	-0.18176	0.086902	-2.09157	0.036476725	0.0535272	NOT
POLR2M	600.1343	-0.14816	0.070841	-2.09149	0.036483791	0.0535347	NOT
PMM1	2535.362	-0.28087	0.134298	-2.09135	0.036496328	0.0535503	NOT
ABT1	1358.213	0.165977	0.079386	2.090758	0.036549786	0.0536259	NOT
STIM1	3955.51	0.170942	0.081764	2.090692	0.036555688	0.0536317	NOT
OLFM1	206.2204	-0.59596	0.285099	-2.09036	0.036585303	0.0536723	NOT
GJB7	1.751417	1.078703	0.516114	2.090047	0.036613605	0.053711	NOT
AC022819	3.131113	0.524813	0.251118	2.089903	0.036626505	0.053727	NOT
ATP5F1P5	2.022559	-0.69327	0.331748	-2.08975	0.036640651	0.0537449	NOT
RP11-563I	2.166208	0.549714	0.263103	2.089349	0.036676309	0.0537944	NOT
SNX30	730.2794	0.20436	0.097833	2.088878	0.036718687	0.0538537	NOT
HEATR9	2.628445	0.666355	0.319028	2.088706	0.036734216	0.0538736	NOT
COMMD1	641.5223	0.17213	0.082412	2.088647	0.036739515	0.0538785	NOT
MAD2L2	1415.381	0.266814	0.127752	2.08853	0.036750035	0.0538911	NOT
ADPRHL2	1078.656	0.193905	0.092855	2.088271	0.036773402	0.0539212	NOT
ANKRD22	61.23302	0.689251	0.33006	2.088259	0.036774466	0.0539212	NOT
AC007279	2.014158	0.58551	0.280392	2.088179	0.036781691	0.0539289	NOT
CTD-2047	3.046291	0.636767	0.304972	2.08795	0.03680233	0.0539563	NOT
DNAJC27-	67.87481	0.346733	0.166076	2.087795	0.036816346	0.053974	NOT
DCUN1D4	1745.335	-0.1583	0.075828	-2.08767	0.036827825	0.053988	NOT
SLC7A5	1075.083	0.412166	0.197458	2.087366	0.0368551	0.0540251	NOT
TEX15	8.21208	1.089787	0.522153	2.087102	0.03687893	0.0540572	NOT

RP11-568/	1.1782	1.175924	0.563466	2.086946	0.036893022	0.054075	NOT
ZNF667	62.33289	0.554916	0.265903	2.086913	0.036896038	0.0540765	NOT
RHEBP2	52.01731	-0.54964	0.263379	-2.08688	0.036899037	0.0540781	NOT
RP11-317I	6.847351	0.573842	0.275033	2.08645	0.036937893	0.0541321	NOT
C19orf80	4636.652	0.637759	0.305697	2.086243	0.036956655	0.0541568	NOT
RP11-718(	0.841113	1.118719	0.536274	2.086095	0.036969999	0.0541722	NOT
PROB1	78.61356	0.317818	0.152352	2.086083	0.036971136	0.0541722	NOT
SNAPC4	692.9343	0.211222	0.101257	2.086013	0.03697748	0.0541787	NOT
AC096772	157.3394	-0.21796	0.104502	-2.08568	0.037007775	0.0542202	NOT
TRGC1	18.05752	-0.49703	0.238328	-2.08549	0.037024893	0.0542404	NOT
MAP1LC3E	36.29039	-0.23449	0.11244	-2.08548	0.037025471	0.0542404	NOT
WDFY3	897.4402	-0.27059	0.12977	-2.08512	0.037058581	0.054286	NOT
ACSS2	5000.357	-0.33068	0.158662	-2.08421	0.037141254	0.0544042	NOT
SLC18A2	17.50118	-0.5112	0.245282	-2.08413	0.037148767	0.0544124	NOT
OSTC	3929.69	-0.18027	0.086518	-2.08361	0.03719543	0.0544778	NOT
ODCP	2.921502	0.517171	0.248289	2.082943	0.037256457	0.0545643	NOT
RP11-534I	1.629001	0.755065	0.362604	2.082341	0.03731132	0.0546418	NOT
MED17	608.6024	0.162395	0.077997	2.082077	0.037335466	0.0546742	NOT
ANKDD1B	14.43828	0.513668	0.246743	2.081792	0.037361436	0.0547094	NOT
ZC3H11B	1.560334	0.65363	0.314046	2.081317	0.037404864	0.0547701	NOT
SLC26A5	6.327026	-0.42975	0.206513	-2.081	0.037434118	0.05481	NOT
UPF2	1379.139	0.166416	0.079976	2.080823	0.037450155	0.0548306	NOT
ZNF132	75.62669	0.296412	0.142472	2.080492	0.037480386	0.054871	NOT
NCL	16533.79	0.139484	0.067044	2.080478	0.037481745	0.054871	NOT
AC125421	1.190786	0.888571	0.427255	2.079722	0.037551032	0.0549695	NOT
CTD-2134	0.764328	2.012903	0.967989	2.079469	0.037574256	0.0550006	NOT
SNX16	231.6796	0.214552	0.103212	2.078748	0.037640547	0.0550948	NOT
AC092652	0.832665	2.034759	0.978874	2.078672	0.037647531	0.0551021	NOT
RP11-894J	10.35696	0.396357	0.190685	2.078599	0.037654177	0.0551089	NOT
TASP1	215.6381	0.21068	0.101367	2.078391	0.037673319	0.055134	NOT
UBE4A	1331.301	-0.2032	0.097779	-2.07812	0.037698277	0.0551676	NOT
SLC25A34	130.757	-0.4523	0.217703	-2.07763	0.03774373	0.0552312	NOT
RP11-204(	9.779135	0.442346	0.212941	2.077314	0.037772569	0.0552704	NOT
CTC-265F	4.363115	0.667771	0.321473	2.07722	0.0377813	0.0552803	NOT
RP11-506I	4.137799	-0.49583	0.238735	-2.07691	0.03780983	0.0553191	NOT
FXYD7	6.163444	-0.47685	0.229612	-2.07678	0.037822213	0.0553317	NOT
BID	2039.187	0.243428	0.117215	2.076774	0.03782242	0.0553317	NOT
RP11-981(	21.93618	0.642372	0.309333	2.076633	0.037835442	0.0553478	NOT
INO80B	155.0694	0.264511	0.127377	2.076593	0.037839138	0.0553503	NOT
RP1-223B	1.038784	1.023225	0.492859	2.076099	0.037884779	0.0554141	NOT
UBXN10	715.9485	-0.6163	0.296956	-2.0754	0.037949843	0.0555064	NOT
RP11-77P	9.171529	-0.42834	0.206428	-2.07501	0.037985338	0.0555553	NOT
CTD-2340	0.619382	1.418091	0.683492	2.074774	0.03800748	0.0555848	NOT
RP4-569M	22.01567	0.491506	0.236945	2.074344	0.038047334	0.0556401	NOT
GOLGA6A	7.092631	-0.81956	0.395147	-2.07405	0.038074297	0.0556766	NOT
RP11-343I	15.81565	0.731177	0.35256	2.073908	0.03808789	0.0556935	NOT
HSPA1B	2843.319	0.433882	0.209221	2.073798	0.038098049	0.055705	NOT
C2orf54	856.664	0.646284	0.311646	2.07378	0.038099763	0.055705	NOT
WDR20	569.6611	-0.13833	0.066712	-2.07359	0.03811721	0.0557276	NOT
FAM189A2	30.61808	0.438762	0.211611	2.073432	0.038132126	0.0557464	NOT
SAMD14	57.75911	0.301294	0.145316	2.073365	0.038138312	0.0557525	NOT
AC005255	12.74497	0.262442	0.126585	2.073243	0.038149666	0.0557662	NOT

RP5-864K:	34.06627	0.397691	0.191867	2.072747	0.038195838	0.0558307	NOT
HPSE	118.1928	-0.40516	0.195493	-2.07249	0.038219609	0.0558601	NOT
RIMKLB	195.1254	0.380145	0.183424	2.072488	0.038219992	0.0558601	NOT
MAGEC3	1.130199	1.401284	0.676223	2.07222	0.038244954	0.0558937	NOT
CTD-2027	4.527534	0.551723	0.266272	2.072031	0.038262585	0.0559165	NOT
IGKV1-39	4.115972	-1.15271	0.556398	-2.07173	0.038290662	0.0559546	NOT
RAP2C	2358.429	-0.23715	0.114478	-2.07159	0.038303301	0.0559701	NOT
RPL13AP7	23.32292	0.357236	0.172481	2.071167	0.038343176	0.0560254	NOT
CASP6	655.5438	-0.17994	0.086885	-2.07097	0.03836193	0.0560498	NOT
HTR2A	7.791887	-0.58248	0.28128	-2.07084	0.038373793	0.0560642	NOT
ZNF157	1.160153	1.018507	0.492111	2.06967	0.038483235	0.0562211	NOT
ARHGAP9	253.6775	-0.38922	0.18811	-2.06914	0.038533358	0.0562914	NOT
DLGAP1-A	4.146696	-0.97573	0.471611	-2.06894	0.038551863	0.0563154	NOT
RP11-274I	22.7814	0.270116	0.13057	2.068738	0.038570707	0.05634	NOT
VPS51	3207.667	-0.16454	0.079542	-2.06858	0.038585444	0.0563585	NOT
RPL4P1	2.592066	0.586768	0.28368	2.068419	0.038600616	0.0563777	NOT
RP11-53B:	4.888449	0.49663	0.240108	2.068358	0.038606386	0.0563832	NOT
RP11-20G	0.964446	0.893409	0.43195	2.068315	0.038610441	0.0563861	NOT
EPB41L4A	161.3835	-0.49222	0.237986	-2.06826	0.038615315	0.0563903	NOT
GHRL	16.49947	0.352441	0.170452	2.067685	0.03866962	0.0564666	NOT
NHSL1	1884.234	-0.30545	0.147746	-2.06743	0.038693727	0.0564988	NOT
RUFY3	712.5911	-0.19455	0.094109	-2.0673	0.038706344	0.0565142	NOT
CTB-171A	97.48343	0.346757	0.167748	2.067133	0.038721655	0.0565336	NOT
RP11-537I	2.079381	0.631443	0.305481	2.067044	0.038730023	0.0565428	NOT
ICE2	921.3918	-0.15167	0.073382	-2.06691	0.038742629	0.0565571	NOT
HMGB3P1(	6.356814	0.465425	0.22518	2.066897	0.038743839	0.0565571	NOT
AKTIP	817.2278	-0.23142	0.112014	-2.06596	0.038832027	0.0566828	NOT
CFAP36	555.6569	0.195364	0.094594	2.065301	0.038894518	0.056771	NOT
SENP2	1327.869	-0.14305	0.069266	-2.06528	0.03889672	0.0567712	NOT
NBPF15	348.2176	0.376541	0.182368	2.064732	0.03894833	0.0568436	NOT
GPR87	1.20044	-1.10457	0.535126	-2.06412	0.039006058	0.0569248	NOT
SSR4P1	61.04903	0.282905	0.13709	2.063641	0.039051773	0.0569885	NOT
GPR155	665.8288	-0.29	0.140544	-2.06342	0.039072544	0.0570158	NOT
FCRL5	37.18822	-0.76811	0.37232	-2.06304	0.039108948	0.0570659	NOT
RP11-274I	11.42181	0.492932	0.238956	2.062854	0.039126457	0.0570885	NOT
MRPS17	596.9804	0.213101	0.103317	2.06259	0.03915164	0.0571222	NOT
SNORA34	1.159009	0.686778	0.332976	2.062545	0.039155872	0.0571254	NOT
RN7SL5P	2.915949	-0.48674	0.235996	-2.06248	0.039161718	0.0571309	NOT
C12orf42	5.383243	0.657446	0.318774	2.062421	0.039167718	0.0571366	NOT
RNF150	101.5886	-0.59515	0.288601	-2.06221	0.039187947	0.0571631	NOT
ASNSP3	3.079884	0.708665	0.343682	2.061976	0.039210026	0.0571923	NOT
THRB-AS1	31.21878	-0.33539	0.162662	-2.06186	0.039221371	0.0572058	NOT
DESI1	3815.997	-0.1969	0.095506	-2.06167	0.039239125	0.0572287	NOT
RP11-400I	6.617946	0.774249	0.375618	2.061268	0.039277465	0.0572816	NOT
IGKV1OR2	10.40213	-0.72431	0.351438	-2.061	0.039303013	0.0573159	NOT
SRCIN1	544.0499	0.345251	0.167535	2.060768	0.039325133	0.0573451	NOT
RP13-487(	2.244907	0.652827	0.316803	2.060671	0.039334407	0.0573556	NOT
GTF2I	1085.319	0.332537	0.161381	2.060576	0.039343507	0.0573658	NOT
HELLPAR	54.97275	-0.37766	0.183283	-2.0605	0.039350327	0.0573728	NOT
GRHL1	360.8694	-0.35078	0.170244	-2.06045	0.039355164	0.0573768	NOT
NUTM2B	8.183423	-0.42914	0.208306	-2.06014	0.039385281	0.0574177	NOT
APOC4	28.82279	-0.53431	0.259433	-2.05955	0.039441597	0.0574967	NOT

LIMA1	1263.898	0.25654	0.124562	2.059527	0.03944381	0.0574969	NOT
SH3RF1	689.8123	-0.31872	0.154756	-2.05947	0.039449233	0.0575018	NOT
C12orf10	1446.72	0.17207	0.083556	2.059349	0.039460834	0.0575157	NOT
CCL16	4149.435	-0.58593	0.284529	-2.05929	0.039466936	0.0575216	NOT
ZNF271P	861.4336	0.135206	0.06566	2.059182	0.039476804	0.0575329	NOT
SLC25A3	16813.86	0.175435	0.085198	2.059158	0.039479097	0.0575332	NOT
LA16c-431	2.309281	0.624605	0.303334	2.059133	0.039481545	0.0575338	NOT
ZBTB5	635.2726	0.183957	0.089362	2.058564	0.039535992	0.0576101	NOT
RP1-40G4I	3.750516	0.436106	0.211867	2.058395	0.039552196	0.0576306	NOT
UBXN1	3209.119	0.192163	0.093367	2.058147	0.039576012	0.0576623	NOT
RP11-474I	2.49521	0.605169	0.294053	2.058028	0.039587416	0.0576759	NOT
RP11-1C1.	1.095582	2.32009	1.127533	2.057668	0.039621968	0.0577216	NOT
RP1-130G:	1.624533	0.656748	0.319173	2.057658	0.03962295	0.0577216	NOT
GREM1	186.3995	0.682735	0.331819	2.057554	0.039632999	0.0577332	NOT
ZNF839	226.4245	0.212534	0.103324	2.056971	0.039689049	0.0578115	NOT
SLC10A7	328.3464	-0.20337	0.098872	-2.05695	0.039690976	0.0578115	NOT
CD22	85.55141	-0.47005	0.228545	-2.05669	0.039715777	0.0578446	NOT
KRAS	1191.934	-0.20897	0.101625	-2.05627	0.039756934	0.0579015	NOT
CTD-2083	2.071876	0.575573	0.279988	2.05571	0.039810457	0.0579764	NOT
ECI1	5891.899	-0.27992	0.136179	-2.05553	0.03982744	0.0579981	NOT
RP11-419(	0.780712	2.100067	1.0217	2.055464	0.03983424	0.0580049	NOT
RP11-38G!	2.704625	0.675917	0.328846	2.055422	0.039838292	0.0580078	NOT
PTPN13	157.6689	-0.59864	0.291251	-2.0554	0.039840811	0.0580084	NOT
RNU6-510	1.709292	0.535493	0.260548	2.055256	0.039854256	0.0580249	NOT
SRA1	1937.45	0.228374	0.111137	2.05488	0.039890623	0.0580748	NOT
MUC4	29.07587	0.50613	0.246311	2.054844	0.039894112	0.0580768	NOT
AURKAIP1	4969.456	-0.26929	0.13107	-2.05455	0.039922644	0.0581153	NOT
FLJ38576	19.06238	0.403048	0.196189	2.054385	0.039938479	0.0581353	NOT
TGFA	326.0124	-0.55531	0.270309	-2.05434	0.039943008	0.0581388	NOT
RP11-449I	1.525206	0.634654	0.308949	2.054232	0.039953203	0.0581506	NOT
RP11-54O	2.300301	1.133853	0.552256	2.053129	0.040060077	0.0583031	NOT
EFHB	13.37391	0.478036	0.232848	2.05299	0.0400735	0.0583196	NOT
IGKV1-5	486.1012	-0.8244	0.401577	-2.05292	0.040080425	0.0583266	NOT
RP11-30H	0.976961	2.009019	0.97863	2.05289	0.04008323	0.0583276	NOT
RP11-10J5	1.565371	-0.95899	0.467158	-2.05281	0.040091412	0.0583364	NOT
ADORA2BI	101.9976	-0.68344	0.333124	-2.0516	0.040208961	0.0585044	NOT
EPOR	301.748	-0.24668	0.120279	-2.05093	0.040273905	0.0585931	NOT
MYO1C	6141.204	0.200647	0.097832	2.050926	0.040274158	0.0585931	NOT
BRWD3	560.6977	0.214751	0.104716	2.050804	0.040286027	0.0586073	NOT
PLCL2	592.032	-0.30076	0.146677	-2.05052	0.040313424	0.0586441	NOT
RP3-508I1	6.932974	-0.34459	0.16806	-2.05039	0.040326252	0.0586596	NOT
PSMD8	5556.472	0.181509	0.088539	2.050044	0.040360157	0.0587059	NOT
VIMP	2567.401	0.185646	0.090578	2.04957	0.040406437	0.0587701	NOT
SMDT1	1946.356	-0.24093	0.117572	-2.04922	0.040440509	0.0588166	NOT
RP11-77K:	5.334971	0.599363	0.292491	2.049166	0.040445923	0.0588213	NOT
ACSM1	1286.085	0.719435	0.351122	2.048963	0.040465757	0.0588471	NOT
LINC01014	2.058185	1.447604	0.70676	2.048225	0.040537982	0.058949	NOT
EIF4A2P1	1.671456	-0.61177	0.298692	-2.04816	0.040544023	0.0589547	NOT
LIMD1-AS	4.340535	0.416763	0.203497	2.048007	0.040559269	0.0589738	NOT
ARHGAP8	7.836533	0.779838	0.380866	2.047537	0.040605402	0.0590378	NOT
GBP4	1384.459	-0.38634	0.188722	-2.04714	0.040643905	0.0590906	NOT
TMEM19	1195.807	0.218765	0.106869	2.047036	0.040654515	0.059103	NOT

RP11-77K1	36.8897	-0.40314	0.196949	-2.04694	0.040663541	0.059113	NOT
RP11-552I	14.75016	0.339997	0.166133	2.04653	0.040704209	0.059169	NOT
RP11-342A	7.531316	0.577928	0.282439	2.046204	0.040736331	0.0592126	NOT
CHEK2P2	0.85673	1.605901	0.785079	2.045529	0.04080271	0.0593059	NOT
CALML5	2.217025	1.749198	0.855263	2.045215	0.040833652	0.0593478	NOT
RP11-775(	1.139326	2.638192	1.28999	2.045127	0.04084235	0.0593573	NOT
TDRD1	12.84884	-0.95251	0.46577	-2.04502	0.040853266	0.0593701	NOT
ACPP	12.91719	0.662153	0.323836	2.044713	0.040883151	0.0594104	NOT
KCNN4	56.23889	0.456213	0.223145	2.044472	0.040906918	0.0594418	NOT
ZNF521	96.23793	0.414588	0.202816	2.04416	0.040937776	0.0594835	NOT
BCAS3	1130.286	0.218617	0.106976	2.043604	0.040992728	0.0595602	NOT
NTAN1P2	21.81722	0.282894	0.138451	2.043284	0.041024301	0.059603	NOT
CTD-2515	1.069485	1.797447	0.879796	2.043027	0.041049757	0.0596368	NOT
DFFBP1	2.977688	0.490816	0.24027	2.042766	0.041075637	0.0596713	NOT
UQCRFS1P	7.041335	-0.38597	0.188987	-2.04233	0.041118593	0.0597306	NOT
POLR2J2	4.188949	0.6301	0.308573	2.041977	0.041153768	0.0597785	NOT
ZBTB8B	2.163026	0.652171	0.319444	2.041584	0.04119282	0.0598321	NOT
RP11-342I	258.099	0.160057	0.078399	2.041558	0.041195412	0.0598327	NOT
BATF3	53.70634	-0.37491	0.183653	-2.04139	0.041212467	0.0598543	NOT
TMEM121	49.2474	-0.51522	0.252388	-2.04136	0.041214679	0.0598544	NOT
EIF4A2P2	2.155128	0.495215	0.242606	2.041232	0.041227801	0.0598703	NOT
LINC0044(	3.136005	0.515407	0.252526	2.041009	0.041249883	0.0598993	NOT
MTG2	1841.593	0.169204	0.082911	2.040781	0.041272615	0.0599281	NOT
EPHB6	379.2492	0.576761	0.28262	2.040766	0.041274072	0.0599281	NOT
RPL15P2	2.825099	0.478888	0.234691	2.040507	0.04129985	0.0599624	NOT
FFAR2	32.4513	-0.58247	0.285497	-2.04021	0.04132951	0.0600023	NOT
IFI16	1232.368	-0.38314	0.187797	-2.04015	0.041334973	0.0600071	NOT
WWP1	5724.598	-0.22147	0.108568	-2.03996	0.041354375	0.0600321	NOT
FSD1	15.64845	-0.49518	0.24276	-2.03978	0.041371825	0.0600543	NOT
PRELID1	2306.159	0.191618	0.09396	2.039357	0.041414419	0.0601129	NOT
ZNF276	437.2534	0.175356	0.086017	2.038619	0.041488091	0.0602167	NOT
RP13-753I	5.845191	0.503152	0.246827	2.038482	0.041501722	0.0602333	NOT
LOH12CR1	323.1948	0.224281	0.110029	2.038382	0.041511723	0.060242	NOT
GLIPR1	429.1103	0.446382	0.218989	2.038379	0.041512041	0.060242	NOT
RP11-762I	3.045688	0.513195	0.251774	2.038317	0.041518239	0.0602478	NOT
PARP12	1822.28	0.219627	0.107751	2.038287	0.041521259	0.0602491	NOT
NXPE3	219.277	-0.40384	0.198142	-2.03816	0.041533918	0.0602643	NOT
FBXW4P1	16.06356	0.348424	0.170953	2.038129	0.041537004	0.0602656	NOT
LEPROTL1	1390.866	-0.20173	0.099003	-2.03761	0.041588894	0.0603377	NOT
CTD-2521	12.60494	-0.39135	0.192069	-2.03754	0.041596301	0.0603453	NOT
RP13-279I	1.898992	-0.50258	0.246682	-2.03735	0.041614771	0.0603689	NOT
RBBP9	1531.216	-0.22821	0.112027	-2.03714	0.041636497	0.0603973	NOT
RPS11	34262.34	0.274806	0.134909	2.036974	0.041652686	0.0604176	NOT
VRK2	349.0342	0.215905	0.106007	2.036699	0.041680176	0.0604543	NOT
MMP20	0.827568	1.213958	0.596048	2.036677	0.041682371	0.0604543	NOT
RP11-386I	5.752137	-0.46686	0.229233	-2.03663	0.04168669	0.0604574	NOT
C1QTNF9E	1.919416	0.770498	0.378394	2.036234	0.041726862	0.0605125	NOT
RP11-165I	4.528538	0.440604	0.216475	2.035358	0.0418149	0.060637	NOT
RP11-535I	2.523521	0.73292	0.360131	2.035149	0.041835899	0.0606642	NOT
UNC50	1030.073	0.123747	0.060813	2.034881	0.041862851	0.0607001	NOT
ACOT11	56.18308	0.52841	0.259707	2.034636	0.041887536	0.0607328	NOT
RP11-522I	128.2755	-0.27327	0.13432	-2.03449	0.041902592	0.0607514	NOT

OTUD7A	46.60418	0.304853	0.14987	2.03412	0.041939515	0.0608017	NOT
RP3-405J1	2.148003	0.524225	0.257765	2.033732	0.041978589	0.0608552	NOT
ANXA9	2112.817	0.319217	0.156975	2.033546	0.041997385	0.0608793	NOT
RP11-585I	48.0018	-0.38519	0.189451	-2.03317	0.042035678	0.0609316	NOT
RP11-126	7.381671	-0.6683	0.328735	-2.03295	0.042057884	0.0609606	NOT
AC079586	0.976414	2.220478	1.092324	2.032802	0.042072523	0.0609786	NOT
VASP	2792.523	0.235677	0.115968	2.032252	0.042128133	0.061056	NOT
CTAGE3P	4.248024	0.496025	0.244128	2.03182	0.042171871	0.0611146	NOT
PSMD8P1	2.147671	0.575273	0.283133	2.031809	0.042172985	0.0611146	NOT
RP11-120I	6.425891	-0.37604	0.185093	-2.03164	0.042189811	0.0611358	NOT
UPK1B	9.678266	-0.92769	0.456735	-2.03114	0.042240347	0.0612058	NOT
WDR3	1281.484	-0.21526	0.105983	-2.03109	0.04224557	0.0612102	NOT
FZR1	2060.446	0.176296	0.086802	2.031004	0.042254558	0.0612175	NOT
RP11-420I	8.945355	0.436788	0.21506	2.031	0.042255043	0.0612175	NOT
DAG1	6702.593	0.186382	0.091785	2.030636	0.042291983	0.0612678	NOT
NAA60	1940.497	0.201684	0.099325	2.030539	0.042301815	0.0612788	NOT
MSN	9240.33	0.28212	0.138943	2.030473	0.042308493	0.0612853	NOT
CATSPER3	29.288	0.323005	0.15913	2.02982	0.0423748	0.0613781	NOT
NDUFB3	1590.127	0.18526	0.091271	2.029785	0.042378413	0.0613801	NOT
KDM4C	460.3089	0.181941	0.089636	2.029763	0.042380639	0.0613802	NOT
P2RX5	31.50705	0.574458	0.283044	2.029574	0.042399911	0.0614049	NOT
SNHG15	287.1583	0.361098	0.177937	2.029357	0.042421916	0.0614335	NOT
FAM171A1	1890.454	0.485609	0.239301	2.029277	0.042430044	0.0614421	NOT
KCNS1	10.2037	0.918386	0.452603	2.029121	0.042445932	0.0614619	NOT
CD96	163.6853	-0.44794	0.220761	-2.02907	0.042451539	0.0614668	NOT
RNU6-882	2.34229	0.554268	0.273175	2.028984	0.042459897	0.0614756	NOT
RAB11B-A	488.4379	-0.35373	0.174399	-2.0283	0.042530095	0.0615741	NOT
RP11-655	0.938304	2.369818	1.16851	2.028067	0.042553402	0.0616033	NOT
RAB11FIP1	730.1189	0.453823	0.223773	2.028054	0.042554774	0.0616033	NOT
ADRBK1	2984.445	0.134574	0.06636	2.027938	0.042566579	0.0616168	NOT
RP11-141I	91.06949	-0.55585	0.274098	-2.02792	0.04256852	0.0616168	NOT
HSPB9	110.6539	-0.40785	0.201124	-2.02785	0.042575772	0.0616241	NOT
AC098828	7.537681	0.60955	0.300611	2.027706	0.042590273	0.0616418	NOT
LRRC58	1557.445	0.198754	0.09802	2.027679	0.042593042	0.0616426	NOT
AF131216.	1.421554	-1.24988	0.616418	-2.02766	0.04259546	0.0616429	NOT
SVIP	709.6184	0.206881	0.102055	2.027151	0.042646936	0.0617141	NOT
LHFPL1	2.196862	1.017533	0.502043	2.026785	0.042684453	0.0617652	NOT
ECE2	666.8418	0.24427	0.120523	2.02675	0.042688027	0.0617671	NOT
TYW1B	157.3768	-0.40509	0.199891	-2.02653	0.042710039	0.0617958	NOT
AP5S1	715.7997	0.170645	0.084216	2.026275	0.042736642	0.061831	NOT
MSRB2	2383.53	-0.27262	0.134546	-2.02622	0.042742114	0.0618357	NOT
EIF4A1P2	5.394575	0.395274	0.195098	2.026033	0.042761394	0.0618604	NOT
snoU13	2.173796	0.586248	0.289373	2.025927	0.042772263	0.0618729	NOT
RP11-429I	2.876216	-0.6042	0.298312	-2.02538	0.042828009	0.0619503	NOT
TTC14	622.8575	0.203535	0.100514	2.024942	0.042873311	0.0620125	NOT
GABRB2	3.237914	0.83845	0.414078	2.024864	0.04288138	0.062021	NOT
PINX1	260.4396	-0.21012	0.103787	-2.02457	0.042911351	0.0620611	NOT
ITGA4	194.258	-0.43408	0.214426	-2.0244	0.042929353	0.0620839	NOT
RP11-10K	6.020358	0.518175	0.255971	2.024356	0.042933582	0.0620867	NOT
ZHX2	1969.299	0.20487	0.10121	2.024218	0.042947725	0.0621009	NOT
SHQ1	498.3299	0.16343	0.080737	2.024216	0.042947883	0.0621009	NOT
CHMP1A	2862.818	0.166129	0.08208	2.023991	0.042971081	0.0621312	NOT

KDM6A	898.6501	-0.22734	0.112324	-2.02393	0.042977422	0.0621371	NOT
RIN2	602.1603	-0.27079	0.133801	-2.02386	0.042985043	0.0621449	NOT
RP11-367I	1.462701	0.914659	0.452043	2.023391	0.043032817	0.0622107	NOT
RP11-223/	10.57456	0.35394	0.174999	2.022523	0.043122327	0.0623369	NOT
AC022182	4.0356	0.571431	0.28254	2.022474	0.043127449	0.062341	NOT
RP11-384I	396.888	-0.38488	0.190312	-2.02237	0.04313814	0.0623532	NOT
B3GNT8	62.9868	0.496175	0.245436	2.021602	0.043217443	0.0624646	NOT
ZBTB7A	1612.011	-0.16215	0.080223	-2.02129	0.043249722	0.062508	NOT
RP11-45A:	14.83712	-0.31485	0.155776	-2.02118	0.043261318	0.0625215	NOT
KRT8P8	7.331154	-0.47728	0.236169	-2.02094	0.043285737	0.0625535	NOT
CTC-484M	4.541377	0.44578	0.22061	2.020666	0.043314352	0.0625916	NOT
MACC1	61.6355	-0.64972	0.321592	-2.02031	0.043350814	0.062641	NOT
LINC0064	197.7633	0.431953	0.213824	2.020134	0.043369483	0.0626647	NOT
SPDYE5	7.018738	0.377079	0.18669	2.019816	0.043402462	0.0627091	NOT
RP11-374I	6.73271	0.539208	0.267006	2.019465	0.04343892	0.0627585	NOT
CRYGN	1.517648	-0.63384	0.313921	-2.01909	0.043477579	0.062811	NOT
MGC4592	2.537931	0.559383	0.277099	2.018713	0.043517037	0.0628648	NOT
CT47B1	0.720667	1.91357	0.947937	2.018668	0.043521756	0.062868	NOT
CX3CL1	1906.601	-0.3282	0.162587	-2.01865	0.043523838	0.062868	NOT
NARS2	950.5682	-0.19726	0.097744	-2.01814	0.043576766	0.0629377	NOT
MPP5	1129.847	-0.18593	0.092131	-2.0181	0.043581096	0.0629377	NOT
TRBV19	8.489084	-0.55576	0.275387	-2.0181	0.043581136	0.0629377	NOT
ADSSL1	752.3504	-0.38925	0.19288	-2.0181	0.043581176	0.0629377	NOT
CDK11A	184.5119	0.197154	0.097701	2.017929	0.043598651	0.0629596	NOT
AGPAT6	3436.815	0.231469	0.114714	2.017792	0.043612955	0.062977	NOT
CTD-2555	10.24629	0.313513	0.15538	2.017726	0.043619814	0.0629836	NOT
MGAT1	9616.611	-0.17296	0.085725	-2.01762	0.043630399	0.0629956	NOT
WDFY4	205.5918	-0.43558	0.215895	-2.01757	0.043636118	0.0630006	NOT
KANSL1-A	140.9079	-0.42511	0.210764	-2.017	0.043695603	0.0630832	NOT
RAD51AP2	11.00999	0.529238	0.26243	2.01668	0.043728892	0.063128	NOT
EEF2	72912.08	-0.16878	0.083699	-2.01651	0.043746988	0.0631508	NOT
TRIM41	1352.196	0.166234	0.082448	2.016233	0.043775643	0.0631889	NOT
RP11-260/	1.35227	2.561122	1.270286	2.016178	0.043781406	0.0631939	NOT
RP11-214(	52.1392	0.560296	0.277904	2.01615	0.043784285	0.0631947	NOT
RP4-613B:	3.232294	0.453952	0.225165	2.016091	0.043790485	0.0632004	NOT
SFXN4	1330.177	0.213558	0.105929	2.016058	0.043793943	0.0632021	NOT
RP11-331I	1.300569	0.699545	0.347003	2.015962	0.043803965	0.0632132	NOT
RP11-1D1	4.747796	-0.49507	0.245594	-2.0158	0.043820785	0.0632342	NOT
AC005224	16.1975	-0.34586	0.17158	-2.01571	0.043830524	0.063245	NOT
RNF135	358.6203	-0.37478	0.185958	-2.01539	0.04386379	0.0632897	NOT
TMEM159	154.1162	0.451056	0.22383	2.015174	0.043886434	0.063319	NOT
AC097461	2.839128	-0.47748	0.236973	-2.01491	0.043914362	0.063356	NOT
AC131263	6.57557	0.355949	0.176661	2.014872	0.043918101	0.0633581	NOT
AC005042	1.050052	0.935658	0.464538	2.014168	0.04399194	0.0634613	NOT
VSTM2L	226.6036	0.698148	0.346651	2.013977	0.044011921	0.0634869	NOT
KB-1507C	3.153367	0.556363	0.276294	2.013666	0.044044635	0.0635307	NOT
FAM71D	5.322098	0.448283	0.222648	2.013414	0.044071134	0.0635656	NOT
LINC0155	215.931	-0.45351	0.22526	-2.01328	0.044085644	0.0635833	NOT
TTLL2	39.33323	-0.55777	0.277057	-2.01319	0.044094772	0.0635926	NOT
MRPL27	2590.604	0.272213	0.135216	2.01317	0.044096709	0.0635926	NOT
RINL	449.1431	0.289806	0.143963	2.013055	0.044108896	0.0636068	NOT
RP11-49K:	1.437047	0.670969	0.333328	2.012941	0.044120813	0.0636207	NOT

RP11-159(	43.39616	-0.33688	0.167381	-2.01266	0.044150283	0.0636598	NOT
RP11-54A	11.58387	0.414191	0.205795	2.01264	0.044152528	0.0636598	NOT
HMX2	0.755226	1.990452	0.989093	2.012402	0.044177607	0.0636926	NOT
RP11-76E:	2.552547	-0.65653	0.326266	-2.01227	0.044191764	0.0637097	NOT
NXF5	1.897782	0.835166	0.415111	2.01191	0.044229391	0.0637607	NOT
TRO	463.2235	-0.4196	0.208625	-2.01128	0.044296152	0.0638536	NOT
RP5-1142/	6.290907	0.399718	0.198747	2.011191	0.044305296	0.0638634	NOT
MBP	1587.334	-0.2347	0.116702	-2.01111	0.04431339	0.0638718	NOT
LINC0024:	110.0888	-0.34487	0.171508	-2.01083	0.0443438	0.0639123	NOT
SKP1	7594.347	0.139655	0.06947	2.010305	0.044398922	0.0639884	NOT
MMP21	3.222843	0.520801	0.259071	2.01026	0.044403629	0.0639918	NOT
PCBP3-OT	1.056274	1.072032	0.533307	2.010159	0.044414406	0.064004	NOT
MIR590	1.532967	0.680034	0.338314	2.010068	0.044423987	0.0640145	NOT
MAB21L3	49.71048	-0.49746	0.247502	-2.00993	0.044438435	0.064032	NOT
RP11-365I	7.824587	0.359367	0.178824	2.009611	0.044472336	0.0640748	NOT
XKR4	4.395155	-0.81136	0.403742	-2.00961	0.04447276	0.0640748	NOT
AC093388	8.807811	0.373432	0.185831	2.009519	0.044482083	0.0640849	NOT
MYO6	1824.791	0.237673	0.118294	2.009164	0.044519778	0.0641358	NOT
NDUFA7	160.2254	0.291078	0.144886	2.009018	0.044535228	0.0641529	NOT
ELOF1	1852.256	-0.19815	0.09863	-2.00901	0.044536278	0.0641529	NOT
ANO6	5214.633	-0.20193	0.100515	-2.00898	0.044538814	0.0641532	NOT
LINC0124(	1.008702	1.903372	0.947497	2.008843	0.04455377	0.0641714	NOT
PAICS	5971.503	-0.14462	0.071993	-2.00876	0.044562476	0.0641806	NOT
RP11-350:	19.16938	0.485493	0.241699	2.008665	0.04457266	0.064192	NOT
MYOM1	2087.496	-0.48579	0.241875	-2.00845	0.044595141	0.064221	NOT
RP4-809F1	1.190052	1.860542	0.926427	2.008299	0.04461151	0.0642412	NOT
CALB1	6.610757	0.556975	0.2774	2.007845	0.044659798	0.0643074	NOT
RP11-473I	6.225294	0.768359	0.3827	2.007731	0.044671839	0.0643214	NOT
ICA1	661.0665	0.420471	0.209465	2.007356	0.044711748	0.0643755	NOT
GLYCTK-A	6.099449	0.432839	0.21563	2.007324	0.044715136	0.064377	NOT
AC013271	10.8673	0.582113	0.29001	2.007218	0.044726474	0.06439	NOT
AHCYL1	5241.555	-0.16092	0.080188	-2.00684	0.044766412	0.0644414	NOT
CHRNA10	18.51869	0.289938	0.144475	2.006839	0.044766858	0.0644414	NOT
TREML2	6.91742	-0.57121	0.284677	-2.00651	0.044802236	0.064489	NOT
UNC13C	1.491263	1.017088	0.506931	2.006364	0.044817395	0.0645075	NOT
ATP12A	3.55901	-1.13963	0.568022	-2.00631	0.044823366	0.06451	NOT
NOMO3	112.891	0.471483	0.235001	2.006304	0.044823813	0.06451	NOT
RP11-552I	0.992007	1.499982	0.747653	2.006254	0.044829184	0.0645144	NOT
AP000254.	53.24384	0.230253	0.114779	2.006057	0.044850137	0.0645412	NOT
RNU6-116	0.992398	1.31003	0.653103	2.005855	0.044871713	0.0645656	NOT
AP000354.	4.128041	0.515693	0.257094	2.005855	0.044871755	0.0645656	NOT
CNN2	2655.01	-0.2676	0.133412	-2.00581	0.044876778	0.0645694	NOT
CDKN1B	2102.028	-0.18128	0.090379	-2.00574	0.044883583	0.0645759	NOT
SCML1	1187.191	0.240282	0.119806	2.005595	0.044899508	0.0645954	NOT
RPS28	11045.33	0.302916	0.151049	2.005408	0.044919459	0.0646205	NOT
NUTM2D	26.99739	0.344348	0.171711	2.005388	0.044921614	0.0646205	NOT
RPL27A	24947	0.294773	0.146998	2.005291	0.044931963	0.0646308	NOT
COMMD7	1761.717	0.174527	0.087034	2.005277	0.044933453	0.0646308	NOT
RPS24	29280.01	0.282884	0.141085	2.005058	0.044956799	0.064661	NOT
CSF2RA	157.9034	0.416511	0.207738	2.004984	0.044964755	0.0646691	NOT
EIF3M	4214.613	0.153682	0.076662	2.004663	0.04499907	0.0647129	NOT
RP13-497I	1.571227	0.831565	0.414817	2.004656	0.044999843	0.0647129	NOT

SNRNP48	653.1081	0.185023	0.092324	2.004071	0.045062481	0.0647996	NOT
SSPN	111.422	0.49001	0.244583	2.003453	0.045128649	0.0648903	NOT
TCP10L	243.6745	-0.56828	0.283654	-2.00344	0.045130284	0.0648903	NOT
RP11-351(	0.757915	1.802938	0.89995	2.003376	0.045136945	0.0648965	NOT
AC069213	62.90223	0.579904	0.289472	2.003314	0.045143584	0.0649027	NOT
AC092835	3.868202	0.496129	0.247717	2.002806	0.045198153	0.0649778	NOT
ELP4	433.6217	-0.13966	0.069753	-2.00219	0.045264378	0.0650696	NOT
MAD1L1	1546.457	0.300316	0.150033	2.001667	0.045320592	0.065147	NOT
RP11-327J	3.561599	-0.51334	0.256508	-2.00127	0.045363501	0.0652053	NOT
SNORA13	2.175498	0.503907	0.251799	2.001226	0.045368064	0.0652085	NOT
TNIP3	7.302319	-0.61951	0.309593	-2.00103	0.045388919	0.0652351	NOT
INPP4B	178.7809	0.402731	0.201286	2.000791	0.045414937	0.0652691	NOT
RP11-22L1	1.381695	1.120321	0.559986	2.000625	0.045432825	0.0652914	NOT
KATNAL1	392.0404	-0.26277	0.131346	-2.00059	0.045436927	0.0652939	NOT
SOBP	297.7105	0.47157	0.235737	2.000407	0.04545631	0.0653184	NOT
PRDM11	203.0606	0.251184	0.125573	2.000308	0.045466987	0.0653303	NOT
PSMD10P2	7.618174	0.623649	0.311789	2.000228	0.045475598	0.0653393	NOT
RP11-294J	1.329599	0.610804	0.305455	1.999655	0.045537552	0.0654249	NOT
RP11-231I	2.116599	0.634843	0.317514	1.999418	0.045563169	0.0654583	NOT
IGHV3-66	20.47327	-0.89534	0.447879	-1.99906	0.045602156	0.0655109	NOT
LINC0092(	20.18877	-0.39844	0.19932	-1.99901	0.04560764	0.0655154	NOT
CCZ1B	152.2285	0.225822	0.112972	1.998924	0.045616597	0.0655249	NOT
GSTK1	12648.58	-0.23086	0.115494	-1.99887	0.045622855	0.0655305	NOT
RP11-527I	0.544094	1.381605	0.69121	1.99882	0.045627795	0.0655341	NOT
C1orf54	217.963	-0.2633	0.131732	-1.99875	0.045635642	0.065542	NOT
AC002456	31.39671	0.432199	0.216273	1.998399	0.045673396	0.0655928	NOT
NPIPB15	142.4391	0.546884	0.273669	1.998346	0.0456792	0.0655978	NOT
HMGB1P2	3.076652	-0.60093	0.300821	-1.99763	0.045756418	0.0657052	NOT
TBX5	1.829137	1.286458	0.644071	1.997384	0.045783475	0.0657407	NOT
FCHSD1	191.1634	0.352056	0.176279	1.997153	0.045808604	0.0657733	NOT
PTPN1	1651.897	0.143554	0.071881	1.997114	0.045812828	0.065776	NOT
ZNF770	812.1479	-0.35261	0.176582	-1.99683	0.045843334	0.0658164	NOT
APH1B	277.0173	0.256177	0.128298	1.996737	0.045853754	0.0658279	NOT
THSD1	196.0717	0.406688	0.203738	1.996136	0.045919128	0.0659184	NOT
ADAM28	113.2063	0.526467	0.26379	1.995784	0.045957432	0.0659699	NOT
IFT43	872.0765	-0.20006	0.100276	-1.99511	0.046030901	0.066072	NOT
MMP7	540.7122	0.793066	0.397542	1.994927	0.046050825	0.0660971	NOT
NSUN2	2680.403	-0.13253	0.066441	-1.99463	0.046083658	0.0661408	NOT
ERCC4	272.2525	-0.21219	0.106385	-1.99459	0.046087869	0.0661423	NOT
TTC30B	183.3269	0.246905	0.123788	1.994573	0.046089487	0.0661423	NOT
RP11-295I	1.827005	0.624348	0.313041	1.994459	0.04610196	0.0661568	NOT
RP11-507I	7.239037	-0.30957	0.155238	-1.9942	0.046130477	0.0661943	NOT
AC137934	11.46237	0.445606	0.223494	1.993813	0.046172529	0.0662512	NOT
RP11-411I	2.557349	0.473023	0.237284	1.993487	0.046208177	0.0662989	NOT
AC002076	1.421069	1.1193	0.561525	1.993322	0.046226236	0.0663214	NOT
RP11-367J	30.71306	-0.38774	0.194526	-1.99323	0.046236609	0.0663328	NOT
SPG11	2133.464	-0.16665	0.083609	-1.9932	0.046239732	0.0663339	NOT
RPS23	17113.63	0.225191	0.112987	1.993068	0.046253975	0.0663479	NOT
GDAP2	511.408	-0.15128	0.075906	-1.99307	0.046254307	0.0663479	NOT
UNG	1848.464	0.181872	0.09126	1.992907	0.04627162	0.0663693	NOT
SUCLA2-A	10.20763	-0.31985	0.1605	-1.99281	0.046282437	0.0663806	NOT
ARHGEF33	6.172709	0.375971	0.188665	1.992791	0.046284348	0.0663806	NOT

CLDN14	1126.399	-0.43843	0.220021	-1.99269	0.046295795	0.0663936	NOT
LRIG3	715.9556	0.315842	0.158508	1.992592	0.046306114	0.066405	NOT
PARK7	8477.203	-0.21713	0.10897	-1.99252	0.046313803	0.0664126	NOT
RP3-486I3	3.110215	0.525211	0.263594	1.992499	0.046316282	0.0664127	NOT
GRTP1	1626.078	-0.28705	0.144106	-1.99193	0.046378422	0.0664983	NOT
TPTE2P5	8.33222	0.413634	0.207669	1.991793	0.046393756	0.0665169	NOT
DHFR	827.6956	-0.24089	0.120972	-1.99132	0.046446207	0.0665886	NOT
INIP	783.2096	-0.1493	0.074977	-1.99124	0.046454274	0.0665967	NOT
RP11-401I	4.252914	0.906756	0.455453	1.990887	0.046493265	0.0666492	NOT
RP11-107	2.497373	0.470628	0.2364	1.99081	0.046501797	0.066658	NOT
BMP1	1657.617	-0.22737	0.114219	-1.99065	0.046518905	0.066679	NOT
FP325317.	10.08948	-0.73545	0.369495	-1.99043	0.046543775	0.0667105	NOT
CTB-51J2	17.88046	-0.60699	0.304957	-1.99041	0.04654569	0.0667105	NOT
ZNF428	1188.742	0.259104	0.130182	1.99032	0.046555671	0.0667214	NOT
OCEL1	1926.238	-0.28085	0.141122	-1.99016	0.046573047	0.0667428	NOT
KRT6C	14.68841	1.314043	0.660433	1.98967	0.046627251	0.066817	NOT
ZNF569	186.0737	0.266158	0.133774	1.989602	0.046634835	0.0668244	NOT
RP11-298I	44.85493	0.255938	0.128665	1.989185	0.046680773	0.0668868	NOT
2-Mar	1802.373	0.193845	0.097461	1.988955	0.0467062	0.0669198	NOT
CPSF3L	2759.046	0.156566	0.078724	1.988786	0.04672487	0.066943	NOT
NCMAP	249.2931	-0.48234	0.242568	-1.98846	0.046760413	0.0669905	NOT
HMSD	11.73273	0.682303	0.343139	1.988414	0.04676588	0.0669949	NOT
RP13-514I	1.97613	0.826515	0.415683	1.988331	0.046775104	0.0670046	NOT
RP11-111I	1.245547	0.833172	0.419176	1.987642	0.046851264	0.0671102	NOT
CARD17	1.890964	0.751172	0.378008	1.987184	0.046902	0.0671794	NOT
ADAM1B	12.43881	-0.35944	0.180914	-1.98682	0.046942282	0.0672319	NOT
RP5-1180I	29.32633	0.503001	0.25317	1.986809	0.046943521	0.0672319	NOT
COL20A1	2.296731	-0.71234	0.35859	-1.98652	0.046976137	0.0672752	NOT
DACT3	107.8028	-0.41001	0.206421	-1.98627	0.047003025	0.0673102	NOT
RP11-21K	6.056765	0.497148	0.250297	1.986232	0.047007526	0.0673131	NOT
RP11-368I	9.952995	0.38375	0.193241	1.985866	0.047048257	0.067368	NOT
RPL24P2	36.23944	0.358531	0.180575	1.985495	0.047089397	0.0674234	NOT
ESRRA	3301.707	0.184576	0.092964	1.985455	0.047093906	0.0674264	NOT
RP4-758J2	10.84818	0.302193	0.152248	1.984869	0.047159028	0.0675133	NOT
DOCK7	977.7191	0.2071	0.10434	1.984865	0.047159498	0.0675133	NOT
ADAT1	533.894	0.176347	0.088854	1.984687	0.047179331	0.0675382	NOT
SF3B1	7467.85	0.134577	0.067813	1.984534	0.047196382	0.0675591	NOT
ANKEF1	452.8802	0.223706	0.112763	1.98386	0.047271481	0.0676631	NOT
PNPLA3	842.9901	-0.36988	0.186461	-1.9837	0.047289493	0.0676854	NOT
IGHG1	22343.26	-0.77135	0.388849	-1.98367	0.047293032	0.0676869	NOT
RP11-392I	4.532909	0.415789	0.209632	1.983427	0.047319785	0.0677217	NOT
RNA5SP28	1.44829	-0.62609	0.315758	-1.98282	0.047387242	0.0678148	NOT
SPECC1L	1487.794	0.154393	0.077881	1.982415	0.047432791	0.0678732	NOT
HSPE1	7125.966	0.244991	0.123582	1.982413	0.047433003	0.0678732	NOT
SEMA3D	53.0974	-0.52472	0.264714	-1.98221	0.047456013	0.0679026	NOT
RPL7P57	1.309852	0.604181	0.304879	1.981706	0.047512115	0.0679794	NOT
ZNF528	189.9334	0.402146	0.202943	1.981568	0.047527632	0.0679981	NOT
VPS26A	1765.709	0.118241	0.059672	1.981513	0.047533764	0.0680033	NOT
YBEY	524.6085	0.265997	0.134266	1.981119	0.047577906	0.068063	NOT
TFPI	12242.6	-0.37375	0.188679	-1.98088	0.047605048	0.0680983	NOT
SURF6	1356.985	0.157073	0.079305	1.980629	0.047632852	0.0681345	NOT
TTLL9	3.533193	0.442828	0.2236	1.980449	0.047653095	0.0681576	NOT

RASSF10	3.253669	0.990268	0.500024	1.980442	0.047653891	0.0681576	NOT
KAT6B	630.1275	-0.18623	0.09405	-1.98011	0.047691317	0.0682076	NOT
RP11-295(	7.331481	-0.44771	0.226117	-1.97997	0.047706567	0.0682225	NOT
FH	9955.994	-0.23119	0.116764	-1.97997	0.047706708	0.0682225	NOT
ING2	272.3057	-0.1835	0.092683	-1.97991	0.047714027	0.068228	NOT
CCR4	33.89312	-0.54129	0.273394	-1.97989	0.047715428	0.068228	NOT
GRID2IP	7.81357	0.603161	0.304667	1.979734	0.047733402	0.0682501	NOT
EIF3LP2	3.011201	0.491014	0.248029	1.979663	0.047741424	0.0682581	NOT
YTHDF1	2310.285	0.101281	0.051163	1.979562	0.047752744	0.0682707	NOT
CENPBD1	151.8181	0.221122	0.111733	1.979015	0.047814344	0.0683553	NOT
KPNA6	2152.179	-0.147	0.074282	-1.9789	0.047827643	0.0683707	NOT
TP53RK	584.8367	0.174889	0.088389	1.978617	0.047859138	0.0684122	NOT
AC079305	6.654875	0.410229	0.207345	1.978486	0.047873873	0.0684298	NOT
ELMO2	944.1185	0.144019	0.072794	1.978443	0.047878739	0.0684332	NOT
ILK	333.5194	0.236862	0.119727	1.97836	0.047888153	0.0684431	NOT
TMEM260	661.7126	0.20319	0.102712	1.97825	0.047900521	0.0684572	NOT
CLDN5	998.4963	0.428659	0.216693	1.978182	0.047908223	0.0684637	NOT
IGKV1OR2	1.803072	-1.08972	0.550875	-1.97816	0.047911052	0.0684637	NOT
AC015971	6.666458	0.515985	0.260843	1.978144	0.047912466	0.0684637	NOT
RP11-73E:	66.5513	0.193128	0.097646	1.977835	0.047947321	0.06851	NOT
MMGT1	736.155	0.18222	0.092148	1.977471	0.047988363	0.0685651	NOT
PGAP1	264.1519	0.245315	0.124061	1.977369	0.047999891	0.068578	NOT
HNRNPKP4	12.44411	0.322739	0.163286	1.976527	0.048095144	0.0687105	NOT
FAM209A	2.412879	0.54385	0.275179	1.976351	0.048114981	0.0687353	NOT
FGF21	1778.63	0.693142	0.350803	1.975873	0.048169148	0.0688092	NOT
FUZ	393.8694	-0.33699	0.170578	-1.9756	0.048200446	0.0688503	NOT
CCND2-A!	2.174511	0.853566	0.43207	1.975525	0.048208638	0.0688585	NOT
SDCBP2	596.9391	0.520159	0.263307	1.975488	0.048212798	0.0688608	NOT
DR1	1707.291	0.151596	0.076747	1.975274	0.048237097	0.068892	NOT
RP11-284I	7.013564	-0.36263	0.183659	-1.97449	0.048326487	0.0690161	NOT
RP11-145I	1.708804	0.906985	0.459376	1.974384	0.048338107	0.0690291	NOT
PI4KA	2605.39	0.188674	0.095579	1.974006	0.04838104	0.0690869	NOT
PAK6	1.215149	0.892242	0.452022	1.973891	0.048394109	0.069102	NOT
TIMM10B	1085.129	0.140336	0.071106	1.973632	0.048423644	0.0691406	NOT
SNW1	1763.925	0.114865	0.058204	1.973503	0.048438325	0.069158	NOT
CDKN2D	176.9731	0.239889	0.121558	1.973447	0.048444667	0.0691635	NOT
RAD21-AS	7.811504	0.402622	0.204039	1.97326	0.048465928	0.0691902	NOT
CEBPZ	1565.291	-0.13529	0.068575	-1.9729	0.048506586	0.0692447	NOT
C20orf166	2.234133	0.767616	0.389228	1.972151	0.048592381	0.0693636	NOT
NID2	308.6853	0.411523	0.208708	1.971768	0.048636149	0.0694225	NOT
SEPT4-AS:	14.14125	-0.31518	0.15989	-1.97123	0.048697273	0.0695062	NOT
RP11-108(	2.215647	1.041192	0.52828	1.97091	0.048734133	0.0695552	NOT
IGKV1-16	78.79954	-0.86965	0.441295	-1.97068	0.048760562	0.0695893	NOT
ITGAX	544.8191	0.341892	0.173499	1.97057	0.048773045	0.0696035	NOT
NUDT4	1161	-0.20329	0.103181	-1.97022	0.048813054	0.069657	NOT
CTC-278L:	0.78825	1.569999	0.796939	1.970037	0.048834141	0.0696835	NOT
FILIP1	193.6972	-0.33118	0.168136	-1.96974	0.048867957	0.0697282	NOT
RN7SKP16	3.349673	-0.53494	0.27165	-1.96924	0.048925326	0.0698065	NOT
GART	1871.643	0.144972	0.07364	1.968654	0.048992884	0.0698992	NOT
TRMT61B	345.202	0.175022	0.088956	1.967509	0.049124523	0.0700834	NOT
PSPHP1	241.0833	1.095814	0.556982	1.967413	0.049135605	0.0700956	NOT
CTDSPL	1694.728	0.21572	0.10965	1.967344	0.049143562	0.0701034	NOT

RGS6	9.915509	0.51707	0.262841	1.967232	0.04915646	0.0701182	NOT
TCP11	6.222767	0.591771	0.30083	1.967127	0.049168615	0.0701319	NOT
EID3	26.88809	0.382145	0.194277	1.967014	0.04918162	0.0701468	NOT
DHX29	1397.576	0.135154	0.068716	1.966863	0.049198988	0.070168	NOT
RP6-74O6	1.995239	0.598783	0.304483	1.966554	0.049234696	0.0702153	NOT
PPP1R12A	1075.051	0.168758	0.085821	1.966382	0.049254531	0.0702399	NOT
FIBIN	123.0353	0.573707	0.291762	1.966353	0.049257794	0.070241	NOT
NBN	1690.57	0.176838	0.089968	1.965562	0.04934926	0.0703678	NOT
STARD13	636.3661	-0.26285	0.133759	-1.96508	0.049405132	0.0704438	NOT
CSDE1	15451.88	-0.13596	0.069189	-1.96502	0.049412302	0.0704504	NOT
VCP	12776.21	0.12197	0.062092	1.964356	0.049488854	0.0705559	NOT
MTRNR2L3	4.9443	-0.47441	0.241542	-1.96407	0.049521967	0.0705995	NOT
TUBB2A	2067.594	0.338328	0.172267	1.963979	0.049532551	0.0706109	NOT
FLJ37035	16.35056	-0.30908	0.157388	-1.96381	0.049552463	0.0706347	NOT
CRYBB2	5.822473	0.549393	0.279761	1.963791	0.049554329	0.0706347	NOT
SFTA2	5.071642	-0.91336	0.46512	-1.9637	0.049564812	0.0706439	NOT
RP11-728I	2.91643	0.526499	0.268117	1.963692	0.049565859	0.0706439	NOT
FBXO4	403.9036	0.170829	0.087003	1.96348	0.049590422	0.0706752	NOT
DDX17	12153.95	0.171802	0.087509	1.963258	0.04961615	0.0707083	NOT
C3orf49	4.364768	-0.36598	0.186453	-1.96285	0.049663192	0.0707716	NOT
CTD-2026	16.52374	-0.35492	0.18082	-1.96283	0.049666231	0.0707723	NOT
MMP25	67.84566	-0.35915	0.182991	-1.96267	0.049684089	0.0707941	NOT
FTCD-AS1	5.499247	0.562779	0.286759	1.962551	0.04969839	0.0708109	NOT
ACTG1P1	7.103554	0.363606	0.185278	1.962489	0.049705552	0.0708174	NOT
IL17RA	1086.829	-0.16028	0.081701	-1.9618	0.049785822	0.0709281	NOT
LCMT2	371.6819	-0.16118	0.082162	-1.96177	0.049788932	0.0709289	NOT
EIF3C	195.648	0.258487	0.131777	1.96155	0.049814892	0.0709622	NOT
KLF1	4.174672	0.689168	0.351356	1.961451	0.049826441	0.070975	NOT
CTB-78F1.	14.01197	-0.75287	0.383867	-1.96128	0.0498462	0.0709995	NOT
HHEX	2339.616	-0.20297	0.1035	-1.9611	0.049867678	0.0710237	NOT
CAMK2N1	3942.13	-0.23516	0.119911	-1.96109	0.049868299	0.0710237	NOT
FAM32A	3248.148	-0.14494	0.073917	-1.9608	0.049901932	0.0710679	NOT
U47924.27	1.354266	1.426027	0.727374	1.960515	0.049935673	0.0711123	NOT
HLA-DQA1	2184.624	-0.41613	0.212273	-1.96037	0.049952634	0.0711328	NOT
FTH1P11	24.3927	0.312034	0.159194	1.960084	0.049985929	0.0711766	NOT
AC137695	1.493182	0.672026	0.34293	1.959662	0.050035272	0.0712432	NOT
AC009542	3.488156	0.513064	0.261844	1.959425	0.050062985	0.071279	NOT
CTGLF10P	4.892324	0.385888	0.196993	1.958897	0.050124908	0.0713634	NOT
RPL9P3	2.338316	0.561688	0.286748	1.95882	0.050133814	0.0713725	NOT
LA16c-325	1.173011	1.714664	0.87546	1.958586	0.050161245	0.0714078	NOT
YWHAZP5	15.98434	0.397773	0.203115	1.958367	0.05018698	0.0714408	NOT
MYO1E	2397.227	-0.29475	0.150525	-1.95816	0.050210792	0.071471	NOT
AC015987	2.152496	0.664427	0.339318	1.958125	0.05021536	0.0714738	NOT
CERS2	25543.23	0.222588	0.113692	1.957824	0.050250712	0.0715205	NOT
LINC0158(	0.881977	1.998081	1.020607	1.957739	0.050260692	0.071531	NOT
TBC1D9B	5596.489	0.157371	0.080407	1.957177	0.050326646	0.0716212	NOT
ZNF593	224.3028	-0.3004	0.153517	-1.95679	0.0503723	0.0716825	NOT
SLA2	69.24522	-0.3984	0.203618	-1.9566	0.050394889	0.0717109	NOT
RPL7A	29238.9	0.23816	0.12173	1.956455	0.050411632	0.0717311	NOT
CTD-2299	3.409246	0.704223	0.360004	1.956152	0.050447216	0.071778	NOT
RNF219-A	1.782265	0.572203	0.292529	1.956053	0.050458887	0.0717909	NOT
GPR98	865.8858	-0.44972	0.230009	-1.95523	0.050555408	0.0719245	NOT

RP11-6F2.5	23.72761	0.615321	0.314713	1.955181	0.050561668	0.0719297	NOT
ITGB2-AS1	46.62163	0.448947	0.229631	1.955079	0.050573691	0.0719431	NOT
RGS4	168.8442	-0.55957	0.286262	-1.95475	0.050612754	0.071995	NOT
LINC00348	16.64816	1.03707	0.530575	1.954617	0.050628347	0.0720135	NOT
AC006129	19.93695	-0.38715	0.198106	-1.95425	0.050671131	0.0720706	NOT
CHCHD10	6004.426	-0.34141	0.174709	-1.95416	0.050682355	0.0720829	NOT
NEK10	55.0376	-0.48434	0.247857	-1.9541	0.050688907	0.0720885	NOT
SPICE1	274.8302	0.198393	0.101544	1.953774	0.050727948	0.0721403	NOT
SLC10A6	7.535586	0.535198	0.273969	1.953497	0.050760762	0.0721833	NOT
SNORD62B	1.926531	0.515763	0.264054	1.953247	0.050790313	0.0722216	NOT
RP11-29G8	24.54903	-0.28528	0.146063	-1.95315	0.050802112	0.0722347	NOT
MT-TL2	1.594588	0.710841	0.364002	1.952851	0.050837311	0.072281	NOT
RLIM	1229.704	-0.17579	0.090027	-1.95261	0.050866166	0.0723183	NOT
MT-TT	6.699937	0.684872	0.350779	1.95243	0.050887147	0.0723444	NOT
TDRD3	537.744	-0.16217	0.083097	-1.95156	0.050990686	0.0724879	NOT
MROH7-T	1.315464	0.995194	0.509969	1.95148	0.050999919	0.0724973	NOT
HNRNPA3I	1.817426	0.554003	0.283899	1.95141	0.05100824	0.072504	NOT
COL17A1	16.48683	0.573438	0.29386	1.951397	0.051009862	0.072504	NOT
RP11-392I	5.389673	0.804136	0.412174	1.950965	0.051061217	0.0725732	NOT
DLEU1	181.7063	-0.22581	0.115762	-1.95061	0.05110338	0.0726286	NOT
FANCL	458.1374	0.170368	0.087342	1.950594	0.051105391	0.0726286	NOT
ZNF10	211.3563	0.216103	0.1108	1.950393	0.05112926	0.0726588	NOT
HLA-DRB6	758.8128	-0.46293	0.237401	-1.94998	0.051179037	0.0727258	NOT
C1orf189	2.709328	-0.3976	0.20392	-1.9498	0.051199885	0.0727516	NOT
CDK15	3.942128	0.72375	0.371336	1.949043	0.051290273	0.0728763	NOT
PLBD1-AS	12.78883	-0.55908	0.286859	-1.94898	0.051297819	0.0728833	NOT
MFHAS1	958.3283	0.224822	0.115366	1.948781	0.051321617	0.0729134	NOT
SLC25A4	2536.493	-0.26161	0.134246	-1.94876	0.051324649	0.0729139	NOT
RP11-332I	1.719331	0.68771	0.352998	1.948198	0.05139124	0.0730048	NOT
SCGB1B2P	22.37302	0.397229	0.203907	1.948092	0.051403899	0.0730165	NOT
RP4-605O	40.37295	-0.33324	0.171061	-1.94809	0.051404729	0.0730165	NOT
Z69890.1	1.153773	0.695722	0.357152	1.947973	0.051418254	0.0730319	NOT
RP11-430I	3.366961	0.849225	0.436031	1.947627	0.051459649	0.073087	NOT
OVCH1-AS	3.292541	-0.62936	0.323189	-1.94735	0.051493018	0.0731275	NOT
PDZD4	110.7105	-0.40263	0.206759	-1.94734	0.051493503	0.0731275	NOT
B3GALT6	933.2575	0.198493	0.101942	1.947127	0.051519496	0.0731607	NOT
INSIG1	27902.98	-0.46833	0.24057	-1.94673	0.051566539	0.0732237	NOT
RAB4A	2484.376	0.193489	0.099399	1.946586	0.051584432	0.0732454	NOT
ETFB	16420.28	-0.33112	0.170152	-1.94601	0.051653817	0.0733401	NOT
DSG1	494.78	-0.64501	0.331518	-1.94563	0.051698781	0.0734002	NOT
ITM2A	301.5688	0.406726	0.209086	1.945254	0.051744367	0.0734612	NOT
GS1-24F4.	1.77039	1.040459	0.53503	1.944675	0.051814125	0.0735564	NOT
AK4P1	30.65703	-0.40492	0.208234	-1.94453	0.051831009	0.0735766	NOT
ARHGDIB	2773.42	-0.2641	0.135822	-1.94448	0.051837107	0.0735815	NOT
RP1-111C	12.71069	-0.30989	0.159395	-1.94416	0.051876363	0.073632	NOT
IL18	227.71	-0.40815	0.20994	-1.94414	0.051878016	0.073632	NOT
MID2	267.5034	0.274352	0.141122	1.944078	0.051886001	0.0736396	NOT
TGFB3	538.5198	-0.35201	0.181159	-1.94311	0.052002368	0.0738009	NOT
VDAC1	10443.33	0.158232	0.081445	1.942814	0.052038582	0.0738486	NOT
RP11-362	1.717904	0.586165	0.301725	1.942715	0.052050652	0.0738619	NOT
ATP6V1G1	3596.572	-0.17725	0.091247	-1.94258	0.052066493	0.0738806	NOT
IL1A	2.262387	0.631221	0.324962	1.942442	0.052083561	0.073901	NOT

MAPRE3	843.7819	-0.22715	0.116968	-1.94201	0.052135977	0.0739716	NOT
PTPRU	1076.945	0.369408	0.190234	1.941862	0.05215382	0.0739931	NOT
IGLV5-45	28.55356	-0.87647	0.451462	-1.94141	0.052208311	0.0740666	NOT
GFRA3	14.96689	0.846552	0.436075	1.9413	0.052221871	0.0740821	NOT
TNFAIP1	3867.645	-0.15481	0.079756	-1.94099	0.052259148	0.0741311	NOT
EFCAB1	19.64502	-0.59393	0.306095	-1.94035	0.052336755	0.0742374	NOT
INAFM2	160.7792	-0.25086	0.129289	-1.94028	0.052346221	0.074247	NOT
ARHGEF40	3457.849	0.222469	0.114668	1.940114	0.052365779	0.0742682	NOT
RP11-428(	2.612176	-0.63425	0.326916	-1.94011	0.052366514	0.0742682	NOT
GCNT7	2.761064	0.457143	0.235681	1.93967	0.052419866	0.0743401	NOT
RP11-521I	72.69657	0.670164	0.345517	1.9396	0.052428338	0.0743483	NOT
RP11-467I	110.3489	0.714249	0.368281	1.939417	0.052450634	0.0743761	NOT
ODF3B	850.9702	-0.27705	0.142872	-1.93913	0.052485418	0.0744216	NOT
WASF3	420.8111	-0.48267	0.248929	-1.93898	0.052503589	0.0744435	NOT
HSP90AA5	2.74872	-0.46419	0.239407	-1.93893	0.052509989	0.0744488	NOT
TAF9P3	1.687422	0.497971	0.25685	1.938757	0.052530951	0.0744747	NOT
GPR137B	669.4122	-0.32261	0.166448	-1.93822	0.052596223	0.0745634	NOT
UQCRFS1	3816.633	-0.22241	0.114755	-1.93814	0.052605845	0.0745732	NOT
SFRP2	23.66424	0.950016	0.490192	1.938048	0.052617358	0.0745829	NOT
COPS2	2079.535	-0.11123	0.057392	-1.93804	0.052618036	0.0745829	NOT
RP11-373I	9.003222	-0.34166	0.176303	-1.93794	0.052630455	0.0745967	NOT
RP11-44F1	30.94408	-0.28827	0.148761	-1.93782	0.052645264	0.0746138	NOT
OLFM3	3.204319	1.56753	0.809027	1.93755	0.052678178	0.0746567	NOT
CCDC113	120.812	0.394485	0.203621	1.937352	0.052702352	0.0746871	NOT
LRRCC1	272.0031	0.328419	0.169537	1.937154	0.052726466	0.074717	NOT
TNMD	4.205365	-0.77661	0.400908	-1.93713	0.052728851	0.074717	NOT
SNRPGP2	62.90815	0.20868	0.107737	1.936943	0.052752279	0.0747464	NOT
CNP	4088.141	0.134455	0.06942	1.936835	0.052765484	0.0747612	NOT
RP11-368J	15.61654	0.440409	0.227406	1.936669	0.052785799	0.0747862	NOT
RP11-616I	1.653659	0.939977	0.485371	1.936614	0.052792501	0.0747919	NOT
AC010733	9.871126	-0.32505	0.167909	-1.93588	0.052882999	0.0749162	NOT
PNOC	7.444668	-0.73107	0.377674	-1.93573	0.052901126	0.0749381	NOT
DIS3L	1081.842	-0.17334	0.089549	-1.93566	0.052909453	0.074946	NOT
LINC00671	165.862	0.474341	0.245143	1.934954	0.052995895	0.0750646	NOT
TTN-AS1	56.10361	0.338442	0.174912	1.934926	0.052999322	0.0750656	NOT
RP11-612I	69.57622	-0.32837	0.169731	-1.93464	0.053034715	0.0751119	NOT
TMEM132	12.55548	-0.6403	0.331051	-1.93414	0.053096529	0.0751956	NOT
RP11-644I	1.434852	0.659881	0.341181	1.934107	0.053100001	0.0751967	NOT
ZNF451	1078.429	0.147984	0.076516	1.934028	0.053109643	0.0752065	NOT
ADNP-AS1	25.14019	0.21364	0.110472	1.933881	0.053127715	0.0752282	NOT
FOXA1	1580.908	-0.30257	0.156462	-1.93383	0.053134083	0.0752334	NOT
RNF181	3554.848	0.236901	0.122518	1.933608	0.053161384	0.0752682	NOT
FAM86HP	21.40205	0.300893	0.155618	1.93354	0.053169662	0.0752761	NOT
NSRP1	889.254	-0.13406	0.069349	-1.93313	0.053219815	0.0753432	NOT
LRRC8B	419.4698	-0.33854	0.175183	-1.93249	0.053298447	0.0754507	NOT
RP11-215I	4.158003	-0.36849	0.190704	-1.93228	0.053324351	0.0754835	NOT
SCRN3	617.2706	-0.16472	0.085252	-1.93215	0.053341238	0.0755035	NOT
POLR3GL	1526.022	-0.20072	0.103901	-1.93185	0.053377577	0.0755511	NOT
DUS2	437.9956	0.176911	0.091584	1.931688	0.053398028	0.0755762	NOT
PGBD2	200.6446	0.16536	0.085612	1.931516	0.053419298	0.0756024	NOT
GPR97	91.99688	0.517628	0.268005	1.93141	0.05343236	0.075617	NOT
TSPAN14	2578.85	0.183278	0.094898	1.931326	0.053442708	0.0756278	NOT

STARD8	626.2307	0.238985	0.123754	1.931121	0.053468145	0.0756599	NOT
RPL18AP7	1.423405	0.524489	0.271656	1.930708	0.05351923	0.0757284	NOT
RNU6-833	1.169018	0.704525	0.364933	1.930562	0.053537291	0.07575	NOT
BCL2L10	392.7242	0.723029	0.374636	1.929952	0.05361284	0.0758531	NOT
HMGB1P2(	1.717928	0.511796	0.265216	1.929731	0.053640223	0.0758879	NOT
ZNF614	173.63	0.316898	0.164222	1.929695	0.053644625	0.0758903	NOT
IGLL3P	1.341276	0.795525	0.412283	1.92956	0.05366135	0.0759084	NOT
LINC0031!	1.750933	0.938454	0.486359	1.929547	0.053662957	0.0759084	NOT
OR8A1	0.770301	1.959883	1.015806	1.929387	0.053682871	0.0759327	NOT
DPH3	1302.675	0.156696	0.081217	1.929352	0.053687149	0.0759349	NOT
AC068039	3.207838	0.556621	0.288526	1.929191	0.053707133	0.0759593	NOT
PTPN6	1494.328	-0.17537	0.090914	-1.92902	0.053728773	0.075986	NOT
ENPP2	2401.722	0.385447	0.199853	1.928652	0.053774065	0.0760462	NOT
ALG5	1534.533	-0.17533	0.090931	-1.92819	0.053831202	0.0761231	NOT
RP11-132I	3.722857	0.641061	0.332486	1.928086	0.053844372	0.0761378	NOT
FSTL3	1236.514	0.471337	0.244482	1.927899	0.053867747	0.076167	NOT
ADD2	23.4554	0.493744	0.256129	1.927718	0.053890249	0.0761949	NOT
CXCR3	73.77907	0.459576	0.238407	1.927691	0.053893539	0.0761956	NOT
APLNR	1095.925	-0.39104	0.202866	-1.92756	0.053909949	0.0762149	NOT
FLNB-AS1	39.37752	-0.31061	0.161159	-1.92732	0.053939773	0.0762496	NOT
ISOC2	6820.324	-0.299	0.155139	-1.92732	0.053939969	0.0762496	NOT
C10orf105	4.065477	-0.47639	0.247198	-1.92717	0.053957927	0.0762711	NOT
RP5-907C:	62.75701	-0.50529	0.26221	-1.92704	0.053974258	0.0762903	NOT
MPZL2	1203.671	0.292346	0.151723	1.926845	0.053998905	0.0763212	NOT
SERPINB2	3.171066	-0.89019	0.462016	-1.92675	0.054011017	0.0763344	NOT
SCG5	243.8278	-0.42888	0.222632	-1.92639	0.054055668	0.0763936	NOT
ABCA13	23.19404	-0.62267	0.323257	-1.92624	0.054074938	0.076417	NOT
ID3	1078.414	-0.30959	0.160738	-1.92607	0.054095212	0.0764417	NOT
ZFAND2B	1798.298	0.219362	0.113905	1.925826	0.054126091	0.0764799	NOT
CHI3L1	17591.47	0.715359	0.371458	1.925813	0.054127755	0.0764799	NOT
ASB9	445.5917	-0.34272	0.177966	-1.92574	0.054136992	0.076489	NOT
FAM86FP	48.5519	-0.36773	0.190973	-1.92557	0.054158297	0.0765152	NOT
TBC1D17	2280.409	0.168591	0.087573	1.925153	0.054210256	0.0765847	NOT
CRY1	722.3487	-0.21842	0.113457	-1.92513	0.054213392	0.0765852	NOT
RP11-123I	0.954674	1.574886	0.818101	1.925051	0.054223014	0.0765949	NOT
SP2	803.5088	0.139328	0.072398	1.924474	0.054295196	0.076693	NOT
OLFM2	3614.038	0.461029	0.239581	1.924314	0.054315293	0.0767174	NOT
SLC26A1	1228.026	-0.29866	0.155206	-1.92426	0.054321852	0.0767228	NOT
WIPI2	3379.828	0.140278	0.072907	1.924065	0.054346457	0.0767536	NOT
CTA-246H	2.7897	0.63576	0.330451	1.923914	0.054365356	0.0767764	NOT
COL13A1	49.0998	0.436104	0.22671	1.923624	0.054401745	0.0768239	NOT
CPB1	12.20198	-0.64704	0.336373	-1.92359	0.054406156	0.0768262	NOT
IGHV1-3	6.830783	-1.03249	0.536762	-1.92356	0.054410377	0.0768282	NOT
RXRG	60.07682	0.741417	0.385469	1.923416	0.054427869	0.076849	NOT
CNN2P9	3.246715	-0.35979	0.187085	-1.92312	0.054464623	0.076897	NOT
RP11-571I	1.956741	0.501727	0.260907	1.923009	0.054478905	0.0769132	NOT
YIPF6	2464.649	-0.15154	0.078809	-1.92282	0.0545021	0.076942	NOT
NUCB1	23237.63	-0.21528	0.111981	-1.92244	0.054549947	0.0770056	NOT
SV2C	10.72017	0.460541	0.239565	1.922409	0.054554284	0.0770078	NOT
ZNF415	65.57677	0.476019	0.24764	1.92222	0.054578088	0.0770375	NOT
LINC0060!	16.71662	0.657741	0.342183	1.922189	0.054581963	0.0770375	NOT
DIS3L2P1	2.009169	-0.7101	0.369428	-1.92218	0.054583673	0.0770375	NOT

NOTCH2N	9.02458	0.370256	0.192638	1.922031	0.054601822	0.0770565	NOT
TGIF1	1848.771	-0.23932	0.124516	-1.92202	0.054602683	0.0770565	NOT
GFI1	58.96007	0.438261	0.228059	1.921701	0.054643441	0.0771101	NOT
KBTBD8	53.91717	-0.31807	0.165519	-1.92165	0.054650288	0.0771158	NOT
CES1	103297.4	-0.45799	0.238349	-1.92151	0.054667237	0.0771358	NOT
SNTB2	634.6567	0.183819	0.095665	1.92148	0.054671264	0.0771375	NOT
TMEM52	202.2843	-0.4029	0.209704	-1.9213	0.054693267	0.0771644	NOT
FLOT2	5271.955	0.21139	0.110025	1.921284	0.054695893	0.0771644	NOT
RP11-266I	5.429227	0.524538	0.273019	1.921251	0.054700098	0.0771664	NOT
AP1M1	4195.9	0.169826	0.088401	1.921097	0.054719506	0.0771899	NOT
SMG1P3	20.67486	0.300441	0.15641	1.92085	0.054750668	0.0772283	NOT
B4GALT4	847.2639	0.169554	0.088272	1.920816	0.054754933	0.0772283	NOT
SLC15A5	2.016667	0.888042	0.462326	1.920814	0.05475511	0.0772283	NOT
M1AP	7.400606	0.468841	0.244104	1.920657	0.054774906	0.0772522	NOT
RP1-130H	2.622961	0.518801	0.270121	1.920625	0.054779036	0.0772541	NOT
CUX2	2438.29	-0.53948	0.280897	-1.92056	0.054787093	0.0772616	NOT
CTD-2287	275.2005	-0.36635	0.190786	-1.92024	0.054828046	0.0773154	NOT
TRGV4	3.075865	-0.58561	0.305048	-1.91974	0.054891335	0.0774007	NOT
AC008063	2.20464	0.633692	0.330162	1.919339	0.054941457	0.0774648	NOT
CH17-189	87.66927	0.329418	0.171632	1.919331	0.054942437	0.0774648	NOT
RP11-490I	1.111237	0.747167	0.389359	1.918968	0.05498842	0.0775257	NOT
ZNF121	778.9707	0.194193	0.101207	1.918769	0.05501354	0.077554	NOT
RP11-613I	4.678585	0.482297	0.251358	1.918765	0.055014068	0.077554	NOT
CXADR	2700.164	-0.24335	0.126835	-1.91865	0.055029052	0.0775711	NOT
MED28P3	2.707797	-0.4978	0.259494	-1.91836	0.055065541	0.0776186	NOT
NCF2	475.2377	0.38046	0.198372	1.917914	0.055121979	0.0776942	NOT
HIAT1	1289.907	-0.16258	0.084806	-1.91709	0.055226753	0.0778379	NOT
USP6	17.35749	0.332391	0.173396	1.916943	0.055245119	0.0778599	NOT
FBXW2	2615.647	0.180472	0.09415	1.916857	0.055256057	0.0778713	NOT
SATB1-AS	12.91018	0.573408	0.299186	1.916564	0.055293381	0.0779199	NOT
RP11-219I	1.588998	0.635615	0.331742	1.915995	0.055365742	0.0780179	NOT
MLLT4	4037.786	-0.20549	0.107269	-1.91564	0.055411373	0.0780783	NOT
RBM23	1978.436	-0.15551	0.081197	-1.91524	0.055461704	0.0781452	NOT
EIF2S2P4	8.508905	0.347598	0.181516	1.914978	0.055495336	0.0781886	NOT
ZMYM4	1257.941	0.155536	0.081225	1.914891	0.055506466	0.0782003	NOT
RP11-381I	2.634421	0.664379	0.346984	1.914727	0.055527355	0.0782202	NOT
RP11-679I	9.676085	0.376871	0.196828	1.914724	0.05552768	0.0782202	NOT
FBXO10	285.7545	0.228985	0.119592	1.914713	0.055529082	0.0782202	NOT
TRPV6	93.52777	-0.82813	0.432557	-1.91449	0.055557711	0.0782566	NOT
RP11-57H	7.866113	0.369256	0.192882	1.914417	0.055566908	0.0782655	NOT
PSMA8	1.153598	1.134783	0.592771	1.914369	0.055572981	0.0782695	NOT
ADAMTS2	941.0985	-0.44187	0.230822	-1.91435	0.055575403	0.0782695	NOT
CCDC181	11.75204	0.32548	0.170031	1.914245	0.055588853	0.0782845	NOT
TNS1	4670.642	-0.22676	0.118462	-1.91419	0.055596219	0.0782909	NOT
RPSAP53	39.66507	0.845331	0.441661	1.913982	0.055622431	0.0783219	NOT
ENDOD1	645.0342	-0.35421	0.185068	-1.91397	0.055623935	0.0783219	NOT
UBL5	4998.967	0.226516	0.118358	1.913822	0.055642886	0.0783446	NOT
RP11-797I	2.457584	0.452788	0.236607	1.913674	0.055661882	0.0783674	NOT
TOP3BP1	0.901224	1.06158	0.554765	1.913568	0.055675377	0.0783824	NOT
HLA-DQA:	584.9388	-0.5779	0.302017	-1.91346	0.055688923	0.0783975	NOT
IGLV1-47	163.7681	-0.74609	0.389991	-1.91309	0.055737019	0.0784612	NOT
RP11-53O	336.9734	0.179743	0.093956	1.913059	0.055740514	0.0784621	NOT

PXK	574.0425	0.213077	0.111383	1.91302	0.055745502	0.0784651	NOT
SLC15A4	1155.867	-0.17193	0.089881	-1.91283	0.055769519	0.0784947	NOT
VCPKMT	179.1192	-0.14971	0.078265	-1.91281	0.055772206	0.0784947	NOT
RP11-10A	17.60822	-0.43194	0.225827	-1.9127	0.055786444	0.0785108	NOT
RP11-864I	194.9313	-0.32612	0.170554	-1.91211	0.055862416	0.0786137	NOT
AC022153	13.28289	-1.30703	0.683766	-1.91152	0.055937432	0.0787153	NOT
WT1	17.9521	0.825532	0.431891	1.911437	0.055948382	0.078725	NOT
LINC0054	1.322791	-1.00092	0.523658	-1.91141	0.05595209	0.078725	NOT
TXNDC15	1825.894	-0.11937	0.062453	-1.9114	0.05595286	0.078725	NOT
RP11-192I	2.58103	0.582956	0.305012	1.91126	0.055971239	0.0787468	NOT
XRRA1	316.066	0.243079	0.127199	1.911005	0.056003879	0.0787858	NOT
RP11-149I	1.135719	0.886908	0.464107	1.911	0.056004635	0.0787858	NOT
RP1-90G2	2.411806	0.858814	0.449425	1.910916	0.05601534	0.0787968	NOT
RP5-1139I	0.85748	1.123097	0.587741	1.910871	0.056021222	0.0788011	NOT
THAP10	86.20815	0.311219	0.162885	1.910665	0.056047648	0.0788343	NOT
TMED7-TI	10.81975	0.329084	0.172257	1.910428	0.056078067	0.0788731	NOT
MGST3	3899.644	0.222549	0.116513	1.910069	0.056124332	0.0789341	NOT
AC011343	2.651888	-0.61955	0.324466	-1.90943	0.056206218	0.0790431	NOT
ITPRIPL1	42.78956	0.386596	0.202468	1.909423	0.056207557	0.0790431	NOT
GALNT1	3630.709	0.180868	0.094732	1.90927	0.056227331	0.0790635	NOT
XXbac-BP	12.94585	0.294383	0.154186	1.909266	0.056227744	0.0790635	NOT
C8orf46	179.8524	0.456822	0.239318	1.908848	0.056281713	0.0791353	NOT
RP11-138I	2.796346	1.080657	0.566278	1.90835	0.056345976	0.0792217	NOT
ERN2	8.743382	-1.01149	0.530203	-1.90773	0.05642553	0.0793295	NOT
SERPINB9I	58.42415	0.500624	0.26244	1.907577	0.056445855	0.079354	NOT
MIB1	1674.631	0.193662	0.101545	1.907144	0.056501961	0.0794289	NOT
RP11-69J7	1.73835	0.673989	0.353425	1.907025	0.056517401	0.0794465	NOT
KIF9-AS1	50.40175	0.224215	0.117578	1.906941	0.056528243	0.0794578	NOT
MCMBP	1781.335	-0.14296	0.074983	-1.90662	0.05657021	0.0795127	NOT
RN7SL689I	15.79677	0.328423	0.172285	1.906278	0.056614085	0.0795703	NOT
TSPAN18	510.0151	0.284875	0.149455	1.9061	0.056637185	0.0795988	NOT
ZIC1	299.5077	0.755261	0.39627	1.905924	0.056660094	0.0796269	NOT
PSMB10	1002.122	-0.25373	0.133135	-1.90582	0.056672953	0.0796409	NOT
LRRC48	97.42908	0.253546	0.133056	1.905558	0.056707579	0.0796855	NOT
CBY1	701.8696	0.18674	0.098005	1.905415	0.056726156	0.0797076	NOT
AC100830	3.138599	-0.36686	0.192542	-1.90537	0.056732344	0.0797122	NOT
CTC-465D	1.152428	1.345979	0.706452	1.905265	0.056745626	0.0797269	NOT
GPR55	10.39396	-0.44255	0.232292	-1.90516	0.056759748	0.0797426	NOT
AC018755	13.7176	-0.46844	0.245888	-1.9051	0.056766818	0.0797485	NOT
WNT10A	20.25285	-0.56366	0.295878	-1.90505	0.056773246	0.0797535	NOT
DEPDC5	833.9233	-0.19816	0.104054	-1.90441	0.056856525	0.0798664	NOT
SSU72	4508.685	-0.16891	0.088729	-1.90369	0.056950215	0.079994	NOT
ST20	161.6796	0.287524	0.151065	1.903312	0.056999795	0.0800596	NOT
XXbac-BP	1.844063	0.531028	0.279048	1.903	0.057040551	0.0801127	NOT
SLITRK1	1.290005	1.277761	0.671582	1.902615	0.057090792	0.0801773	NOT
STIM2	751.3016	0.142236	0.074759	1.902603	0.057092303	0.0801773	NOT
MED29	2719.615	0.133971	0.070442	1.901878	0.0571871	0.0803063	NOT
FASLG	26.93555	-0.46109	0.242443	-1.90184	0.05719144	0.0803084	NOT
AL162759.	14.30517	0.789519	0.415142	1.901805	0.057196615	0.0803115	NOT
MRPS7	2783.514	0.162898	0.085659	1.901716	0.057208292	0.0803239	NOT
RP11-944I	1.907651	0.622202	0.327201	1.901586	0.057225275	0.0803436	NOT
NUTM2A	12.05787	0.393877	0.207172	1.901212	0.057274231	0.0804083	NOT

KB-1269D	0.510708	1.298204	0.682994	1.900754	0.057334202	0.0804884	NOT
MIXL1	5.178541	0.742116	0.390455	1.900643	0.057348808	0.0805048	NOT
RP11-138I	1.110586	0.948024	0.498842	1.90045	0.05737406	0.0805362	NOT
CTD-2251	0.801007	1.981328	1.042726	1.900143	0.057414395	0.0805887	NOT
RP11-545I	0.71282	1.96603	1.034816	1.899884	0.057448332	0.0806323	NOT
RPL5P29	2.889865	0.434108	0.228535	1.899527	0.057495212	0.080693	NOT
TMA7	2860.871	0.210743	0.110946	1.89951	0.057497443	0.080693	NOT
ATG12	1477.587	0.117243	0.061749	1.898705	0.05760322	0.0808373	NOT
VPS41	1626.373	0.17652	0.092975	1.898581	0.057619526	0.0808543	NOT
RP11-244I	2.104483	0.500172	0.263447	1.898569	0.057621127	0.0808543	NOT
GPR84	13.80174	0.567297	0.298834	1.898371	0.057647223	0.0808845	NOT
C2orf49	489.1867	0.113313	0.05969	1.898361	0.057648529	0.0808845	NOT
C10orf107	5.794924	-0.516	0.271999	-1.89706	0.057820399	0.0811181	NOT
LINC0086	335.2738	0.263788	0.139051	1.897053	0.057820892	0.0811181	NOT
RNASE6	262.6519	-0.32167	0.169594	-1.89674	0.05786275	0.0811727	NOT
RPS15AP1	3.502891	0.458558	0.241824	1.896248	0.057927252	0.0812591	NOT
RP11-351I	3.48973	0.409772	0.216125	1.895996	0.057960615	0.0812979	NOT
RRAS2	1165.625	-0.20713	0.109247	-1.89599	0.057960814	0.0812979	NOT
NOS3	391.9189	-0.27646	0.145821	-1.89586	0.057978705	0.0813189	NOT
RP11-473I	21.59916	0.280739	0.148099	1.895618	0.058010589	0.0813595	NOT
UGCG	2092.539	-0.23224	0.122525	-1.89547	0.058029519	0.0813819	NOT
CTC-339C	0.982117	0.775362	0.409122	1.895186	0.058067827	0.0814315	NOT
RAC1P2	59.34989	0.209018	0.110317	1.894704	0.058131571	0.0815168	NOT
HP09025	5.585859	-0.47552	0.250979	-1.89464	0.058139827	0.0815242	NOT
ANTXRLP1	1.269362	-0.65274	0.344531	-1.89458	0.058148537	0.0815305	NOT
NRG4	55.95745	-0.48525	0.256126	-1.89456	0.058150166	0.0815305	NOT
B4GAT1	1626.143	0.205101	0.108277	1.894227	0.058194938	0.0815891	NOT
GAS2	716.3569	-0.33664	0.17777	-1.89367	0.058269388	0.0816893	NOT
NRP1	4279.592	0.270694	0.142951	1.893617	0.058275895	0.0816943	NOT
TMEM254	63.03532	0.309983	0.163735	1.8932	0.058331228	0.0817678	NOT
GIMAP7	414.5071	-0.31063	0.164085	-1.89308	0.058346631	0.0817852	NOT
PGPEP1	2832.829	0.239088	0.126327	1.892604	0.058410503	0.0818706	NOT
RP11-108I	1.889341	0.568431	0.300357	1.892518	0.058421935	0.0818825	NOT
LINC00371	1.014919	1.191248	0.629484	1.892421	0.058434966	0.0818966	NOT
RNU2-27P	4.645282	0.400052	0.21142	1.892216	0.058462146	0.0819305	NOT
AC004862	341.8206	-0.67111	0.354976	-1.89059	0.058678669	0.0822298	NOT
GOLGA8H	3.52141	0.426444	0.225593	1.890329	0.058714	0.0822751	NOT
MAP1LC3A	1366.344	-0.36365	0.192383	-1.89025	0.058723953	0.0822849	NOT
CYP51A1P	2.036866	0.622122	0.329156	1.890054	0.058750804	0.0823184	NOT
DNAJB12	2195.178	-0.12309	0.065128	-1.88998	0.058760652	0.082328	NOT
CHDH	3965.805	-0.24259	0.128363	-1.88991	0.058770412	0.0823375	NOT
ATP5HP2	1.687826	0.612337	0.324027	1.889774	0.058788164	0.0823582	NOT
GULOP	4.516254	-0.52384	0.277243	-1.88945	0.058831985	0.0824154	NOT
CTD-2619	8.149798	0.309415	0.163767	1.88936	0.058843584	0.0824275	NOT
POU5F1P3	3.504397	0.429141	0.22719	1.888911	0.05890381	0.0825075	NOT
C11orf86	5.280476	-0.61799	0.327173	-1.88889	0.05890665	0.0825075	NOT
HMBOX1	338.1626	-0.16005	0.084736	-1.88882	0.05891638	0.0825169	NOT
MED23	660.6777	0.166404	0.088115	1.888487	0.058960647	0.0825748	NOT
LINC0118	9.387733	0.719122	0.380815	1.888377	0.058975371	0.0825912	NOT
SPPL3	1738.457	0.130962	0.069355	1.8883	0.058985693	0.0826015	NOT
CTD-2383	3.180388	0.428632	0.227116	1.887287	0.059121794	0.0827879	NOT
FGF7	62.7235	0.60412	0.320112	1.887218	0.059131071	0.0827967	NOT

PTCH1	350.2246	-0.32882	0.174242	-1.88712	0.059143679	0.0828101	NOT
AC006277	5.764619	0.375643	0.199087	1.886829	0.059183394	0.0828591	NOT
ETV7	173.932	-0.46666	0.247328	-1.88682	0.059184628	0.0828591	NOT
C4BPB	12520.51	-0.37264	0.197516	-1.88664	0.059208937	0.0828889	NOT
HSD17B14	1560.366	0.502463	0.266359	1.88641	0.059239791	0.0829279	NOT
MMP2	1488.279	0.437162	0.231748	1.886368	0.059245446	0.0829316	NOT
LINC0003	1.041768	0.806769	0.427771	1.885985	0.059296973	0.0829996	NOT
RP11-416	4.446656	-0.5347	0.28358	-1.88554	0.059356617	0.0830788	NOT
CTB-50L1	4.372485	0.401551	0.213044	1.884828	0.059453042	0.0832096	NOT
TRADD	784.9415	0.197677	0.104922	1.88403	0.059560908	0.083356	NOT
ARFGAP2	4310.474	-0.13317	0.070687	-1.884	0.059565572	0.083356	NOT
LXN	161.8395	0.396411	0.210411	1.883988	0.059566662	0.083356	NOT
RP11-615	1.586498	0.567422	0.301193	1.883916	0.059576329	0.0833653	NOT
RP11-403	40.82977	0.332191	0.176362	1.883578	0.059622069	0.0834251	NOT
PCDHB1	1.747131	0.875084	0.464613	1.88347	0.059636747	0.0834414	NOT
LINC0069	3.370727	0.632127	0.335653	1.883277	0.059662862	0.0834737	NOT
TMF1	1034.312	-0.17679	0.093883	-1.88314	0.059681533	0.0834956	NOT
NRD1	5507.478	0.133883	0.071097	1.883105	0.059686156	0.0834978	NOT
ZNF644	1159.624	-0.15276	0.081133	-1.88283	0.059723359	0.0835457	NOT
RP11-462	65.02529	0.565609	0.300416	1.882752	0.059733931	0.0835562	NOT
SLC25A33	1070.866	0.291673	0.154932	1.882588	0.059756285	0.0835833	NOT
CLECL1	15.26384	-0.45522	0.24181	-1.88255	0.059761758	0.0835867	NOT
GLIPR1L1	5.728264	0.433986	0.230584	1.882118	0.059820035	0.083664	NOT
TINAGL1	1316.908	-0.28834	0.153235	-1.8817	0.059877144	0.0837396	NOT
KLHL14	4.918137	-0.70417	0.374287	-1.88138	0.059920581	0.0837949	NOT
OGFR	3321.918	-0.17178	0.091305	-1.88136	0.059922745	0.0837949	NOT
NACAP1	12.59383	-0.31511	0.167497	-1.88131	0.05992927	0.0837998	NOT
MAX	1440.18	-0.11999	0.063786	-1.88114	0.05995234	0.0838278	NOT
MRPL48	899.9181	0.165067	0.087791	1.880236	0.060075933	0.0839964	NOT
TRIP6	3019.795	0.266626	0.141815	1.880099	0.060094538	0.0840182	NOT
PKP1	23.07371	0.657606	0.349788	1.880012	0.060106485	0.0840306	NOT
NELL2	76.55018	0.583251	0.310254	1.879916	0.060119464	0.0840445	NOT
AC010872	1.454724	-0.58353	0.310461	-1.87957	0.060166523	0.0841061	NOT
RPL5	24488.62	0.208989	0.111211	1.879205	0.060216541	0.0841717	NOT
UGT1A6	1464.291	0.516591	0.274912	1.879112	0.060229164	0.0841851	NOT
SLC23A1	1254.645	-0.32303	0.171915	-1.87898	0.060246869	0.0842052	NOT
RP11-463	37.07742	-0.34283	0.182456	-1.87896	0.060249585	0.0842052	NOT
L3HYPDH	414.3482	0.228171	0.121445	1.878798	0.060272149	0.0842324	NOT
STK26	324.9645	0.365236	0.194428	1.878512	0.06031115	0.0842827	NOT
SLED1	10.79724	-0.48326	0.257311	-1.87811	0.060366449	0.0843557	NOT
KDELC2	886.8087	-0.24558	0.130791	-1.87764	0.060430728	0.0844413	NOT
ZNF474	3.363792	0.465963	0.248225	1.877182	0.060493108	0.0845242	NOT
EBLN2	14.20405	0.377998	0.201388	1.876962	0.060523321	0.0845621	NOT
CNN3	6117.356	0.219025	0.116698	1.876859	0.060537369	0.0845775	NOT
UBXN2A	685.2502	0.131208	0.06991	1.876797	0.060545889	0.0845851	NOT
ZBTB16	487.574	-0.48817	0.260256	-1.87571	0.060695114	0.0847893	NOT
RP11-812	2.009501	-1.0397	0.554341	-1.87555	0.060716921	0.0848155	NOT
GFPT1	3418.213	0.185194	0.098746	1.875455	0.060730107	0.0848296	NOT
RP11-464	1.563741	0.559586	0.298389	1.875357	0.060743639	0.0848442	NOT
RP11-260	1.143717	2.584052	1.377957	1.875277	0.0607546	0.0848552	NOT
RP11-219	30.71563	1.039128	0.554144	1.875196	0.06076572	0.0848665	NOT
RHOT1	972.9226	0.126135	0.06728	1.874763	0.060825388	0.0849455	NOT

RP13-101(	3.142488	0.657978	0.351004	1.874558	0.060853568	0.0849806	NOT
C6orf203	692.254	-0.20021	0.106806	-1.8745	0.060861564	0.0849875	NOT
MYL12A	9294.014	-0.18037	0.096239	-1.87417	0.060907121	0.0850468	NOT
RP1-224A(	2.152091	0.495758	0.264538	1.874052	0.060923269	0.0850651	NOT
RP11-589I	43.84715	-0.32856	0.175336	-1.8739	0.060944279	0.0850901	NOT
RP11-114I	1.865548	0.580462	0.309884	1.873161	0.061046224	0.0852281	NOT
RP11-115.	2.518248	-0.7752	0.413916	-1.87283	0.061091215	0.0852866	NOT
PTK6	322.0659	0.526142	0.280947	1.872749	0.061103021	0.0852988	NOT
RP11-122I	22.52682	0.483557	0.258245	1.872476	0.061140762	0.0853472	NOT
RP11-183(	1.458061	0.584328	0.312163	1.871868	0.061224842	0.0854603	NOT
AC019097	3.36727	0.396023	0.211623	1.871358	0.061295544	0.0855546	NOT
RP11-219I	14.2107	-0.42711	0.228246	-1.87128	0.061306333	0.0855654	NOT
snoZ196	1.609571	0.600909	0.321132	1.87122	0.061314662	0.0855715	NOT
MTERF4	766.9521	-0.13037	0.069672	-1.8712	0.061317476	0.0855715	NOT
GABARAPL	2343.301	-0.13927	0.074427	-1.87118	0.061320825	0.0855715	NOT
SPR	3454.058	-0.18046	0.096441	-1.87116	0.061323086	0.0855715	NOT
RP11-345I	0.779038	1.778053	0.950584	1.870485	0.061416467	0.0856975	NOT
TMEM14D	2.368879	0.488623	0.261251	1.870318	0.061439669	0.0857255	NOT
RRS1-AS1	11.16115	-0.42648	0.228041	-1.87018	0.061459163	0.0857484	NOT
RP11-196(	268.4833	-0.15915	0.085114	-1.86983	0.061507653	0.0858117	NOT
KLF8	40.35457	-0.35129	0.187887	-1.86968	0.061528106	0.0858359	NOT
EPHX3	16.65855	0.487084	0.26052	1.869658	0.061531366	0.0858361	NOT
ATXN7L3B	3655.754	0.176402	0.094359	1.86948	0.061556021	0.0858642	NOT
ZNF358	2499.878	-0.25078	0.134146	-1.86947	0.061557687	0.0858642	NOT
ACKR3	1050.141	-0.44393	0.237489	-1.86927	0.061585417	0.0858986	NOT
TRAPPC2	463.651	0.138981	0.074375	1.868656	0.061670646	0.0860131	NOT
CBLC	719.9092	-0.39579	0.211812	-1.8686	0.06167892	0.0860185	NOT
TRIM69	1101.52	-0.2251	0.120465	-1.86858	0.061680876	0.0860185	NOT
RPSAP58	292.271	0.381636	0.204241	1.868562	0.061683844	0.0860185	NOT
GLYR1	3434.506	-0.13765	0.073675	-1.86841	0.061704874	0.0860435	NOT
RP11-248I	9.112533	-0.87813	0.470028	-1.86825	0.061727486	0.0860707	NOT
RP5-882O	3.326122	0.509634	0.272793	1.868206	0.061733404	0.0860746	NOT
PTENP1	35.81028	-0.25379	0.13585	-1.86816	0.061739696	0.086079	NOT
MIR4458H	258.4785	0.363824	0.194756	1.868103	0.061747783	0.086086	NOT
AC010761	4.239123	0.497616	0.2664	1.867929	0.06177192	0.0861113	NOT
CTD-2562	2.80154	0.766712	0.410461	1.867927	0.061772193	0.0861113	NOT
DDX18	2312.655	-0.13496	0.072258	-1.86773	0.061800274	0.0861432	NOT
ASB4	124.5804	0.61365	0.328556	1.867719	0.061801286	0.0861432	NOT
RP11-650I	6.306908	-0.37955	0.203239	-1.8675	0.061831634	0.0861811	NOT
RP11-221.	1.529988	-0.59566	0.318992	-1.86733	0.061855508	0.0862101	NOT
GRAP2	114.0564	0.440308	0.235799	1.867299	0.061859834	0.0862118	NOT
ZNRF2P1	5.536725	0.346877	0.18578	1.86714	0.061882105	0.0862385	NOT
PPP1R36	12.34262	0.493434	0.26429	1.867016	0.061899288	0.0862581	NOT
ATP11AUN	1.169092	0.759961	0.40711	1.86672	0.061940745	0.0863115	NOT
CPPED1	3166.521	0.242718	0.130029	1.866642	0.061951639	0.0863223	NOT
INTS5	1194.147	-0.12516	0.067065	-1.86632	0.061996253	0.086379	NOT
SUSD1	232.0124	0.263391	0.14113	1.866306	0.061998584	0.086379	NOT
TP53I13	1426.253	0.279421	0.149789	1.865433	0.062120727	0.0865448	NOT
KIAA0753	263.6154	0.196445	0.105315	1.865302	0.062139062	0.086566	NOT
RBBP8	701.8739	0.255977	0.137241	1.86516	0.062159037	0.0865895	NOT
POU6F2-A	36.05577	-0.76903	0.412443	-1.86458	0.062239932	0.0866978	NOT
INA	5.27133	-0.63931	0.342999	-1.86389	0.062336552	0.086828	NOT

FLI1	460.8883	-0.26265	0.140945	-1.86353	0.062388257	0.0868957	NOT
USMG5	3764.691	0.252651	0.1356	1.863203	0.062433747	0.0869547	NOT
AC009299	5.435304	0.390172	0.209443	1.862903	0.062475935	0.0870064	NOT
FIS1	5524.969	-0.20888	0.112129	-1.86289	0.062477187	0.0870064	NOT
PCAT14	4.191444	0.830294	0.445741	1.862726	0.062500792	0.0870349	NOT
PCDHGA3	35.0816	0.571605	0.306915	1.862417	0.062544309	0.0870911	NOT
PATZ1	1687.334	0.165117	0.088659	1.862375	0.06255029	0.0870921	NOT
LRRN4	16.84661	-0.58339	0.31325	-1.86237	0.062551345	0.0870921	NOT
RP11-103(	51.68116	-0.84218	0.452295	-1.86201	0.062602235	0.0871586	NOT
NDN	365.7964	0.460898	0.247574	1.861658	0.062651287	0.0872225	NOT
RP11-792/	66.87826	0.320511	0.172169	1.861605	0.062658772	0.0872286	NOT
H19	22232.02	0.75354	0.404799	1.861516	0.062671376	0.0872417	NOT
CAB39	2504.371	-0.1151	0.061836	-1.86137	0.062692121	0.0872662	NOT
MS4A14	32.23397	0.382502	0.205563	1.860749	0.062779667	0.0873837	NOT
MRPL14	3206.914	0.232777	0.125113	1.860536	0.062809767	0.0874212	NOT
COL4A4	220.2602	-0.44709	0.240334	-1.8603	0.062842903	0.0874629	NOT
ABCC2	7189.906	-0.3816	0.205167	-1.85997	0.06289021	0.0875243	NOT
HMGB1P1	6.855559	0.378183	0.20339	1.8594	0.062970453	0.0876316	NOT
SNORD94	5.130817	0.415707	0.223637	1.858849	0.063048477	0.0877358	NOT
RELT	146.9426	0.303958	0.163551	1.858485	0.063100173	0.0877999	NOT
RP11-229I	4.802848	0.436748	0.235003	1.85848	0.063100935	0.0877999	NOT
RP11-359I	1.486422	0.723457	0.38929	1.858399	0.063112329	0.0878114	NOT
RP11-573I	1.387796	0.583754	0.31413	1.85832	0.063123639	0.0878227	NOT
SLC30A7	1439.167	-0.15108	0.081303	-1.85823	0.063135923	0.0878353	NOT
EAF2	75.75361	0.240958	0.129706	1.857718	0.063209052	0.0879327	NOT
CDK20	195.4594	0.214235	0.115334	1.857516	0.063237819	0.0879683	NOT
RP11-505I	1.743266	0.562716	0.302969	1.857338	0.063263125	0.087999	NOT
HSPD1P5	7.878741	-0.50524	0.27203	-1.85731	0.063267234	0.0880003	NOT
RP13-383I	1.637776	0.555752	0.299244	1.857187	0.06328453	0.08802	NOT
DYNC1LI1	1034.13	0.13669	0.073611	1.856925	0.063321785	0.0880674	NOT
MAGI2	149.8092	-0.33721	0.181607	-1.85679	0.063340998	0.0880896	NOT
KRTAP4-1	0.61685	1.698168	0.914982	1.855957	0.063459618	0.0882502	NOT
SLCO1B7	6.156533	-0.78314	0.422037	-1.85562	0.063508268	0.0883134	NOT
RP11-963I	15.84488	-0.54319	0.292827	-1.85497	0.063600592	0.0884373	NOT
RP11-996I	17.87021	0.388664	0.209569	1.854585	0.063655493	0.0885092	NOT
ZNF160	403.3904	-0.2976	0.160477	-1.85447	0.063671571	0.0885262	NOT
NME2P1	16.99583	0.369311	0.199148	1.854455	0.063674066	0.0885262	NOT
SERPINA7	11609.53	-0.56749	0.30607	-1.85411	0.063723067	0.0885898	NOT
BPIFB1	8.42618	0.805018	0.434262	1.853761	0.063773319	0.0886552	NOT
GSTT2	4.645662	0.842371	0.454468	1.85353	0.063806472	0.0886969	NOT
STRN	862.8112	-0.15128	0.081618	-1.85347	0.063814419	0.0887035	NOT
CHRM1	1.822357	0.952083	0.513697	1.853395	0.063825709	0.0887147	NOT
RHOG	1673.51	0.184539	0.099579	1.853196	0.063854337	0.08875	NOT
CHAD	976.3044	-0.44103	0.23799	-1.85315	0.063861085	0.0887549	NOT
CT45A10	1.039642	2.5129	1.356089	1.853049	0.063875369	0.0887703	NOT
FAM9B	65.4348	-0.66995	0.361623	-1.85261	0.063938491	0.0888536	NOT
SLC2A8	1494.565	-0.21957	0.118525	-1.85253	0.063949544	0.0888645	NOT
APOL3	2762.734	-0.39663	0.214116	-1.85241	0.063966432	0.0888835	NOT
PAICSP1	4.61504	0.32374	0.174771	1.852359	0.063974231	0.0888898	NOT
ENOSF1	2110.494	-0.18831	0.101662	-1.85234	0.063977415	0.0888898	NOT
LRRC43	12.27744	-0.50852	0.274536	-1.85228	0.063985393	0.0888964	NOT
ANKRD31	9.413247	0.393748	0.212597	1.852084	0.064013818	0.0889315	NOT

ZFP28	72.91067	0.370988	0.200314	1.852036	0.064020724	0.0889366	NOT
RPS23P8	41.53241	0.338951	0.183024	1.851949	0.064033217	0.0889495	NOT
RP11-136I	38.97607	-0.64953	0.350832	-1.8514	0.064112347	0.0890549	NOT
SPRR1B	3.775597	1.888109	1.019926	1.851222	0.064137621	0.0890856	NOT
IGHV3OR1	1.99021	-0.87085	0.470523	-1.85082	0.064195508	0.0891615	NOT
RP11-139I	6.936755	-0.33767	0.182446	-1.85077	0.0642027	0.089167	NOT
7-Sep	3121.05	0.122101	0.065982	1.850509	0.064240198	0.0892146	NOT
APBB1IP	1341.001	-0.39158	0.211641	-1.85022	0.06428182	0.0892667	NOT
SIN3A	1786.584	-0.13403	0.072443	-1.8502	0.064284162	0.0892667	NOT
CTSLP2	8.579046	0.546766	0.295524	1.850154	0.064291338	0.0892722	NOT
NDUFA4	9834.447	0.213948	0.115645	1.850047	0.064306846	0.0892892	NOT
RP11-215I	26.11638	-0.66659	0.360332	-1.84994	0.064322576	0.0893066	NOT
RP11-429J	1.811432	0.725453	0.392169	1.849847	0.064335586	0.0893192	NOT
NUP50	1934.68	-0.14663	0.079269	-1.84983	0.06433813	0.0893192	NOT
RP11-454I	2.025293	0.617499	0.33389	1.849409	0.064398758	0.0893989	NOT
IGF2	115643.8	0.806632	0.43617	1.849351	0.064407125	0.089406	NOT
ARL3	654.6856	0.181463	0.098127	1.849277	0.06441787	0.0894154	NOT
OR7E7P	5.921086	0.4819	0.260591	1.84926	0.064420323	0.0894154	NOT
CCDC144A	6.306929	0.834682	0.451416	1.849033	0.064453084	0.0894564	NOT
WDPCP	126.1851	0.258807	0.139977	1.848925	0.064468692	0.0894736	NOT
RPL3	70843.68	-0.19833	0.107269	-1.84888	0.064475048	0.0894779	NOT
CLK3	1303.102	0.09845	0.053258	1.848549	0.064522989	0.0895399	NOT
KCTD21-A	94.04417	0.279198	0.151039	1.848521	0.064526946	0.0895409	NOT
DNAJC8	2778.259	0.129512	0.070084	1.847971	0.064606549	0.0896469	NOT
RP11-534I	3.110655	0.438932	0.237535	1.847863	0.064622185	0.0896641	NOT
AC002463	0.71352	1.883809	1.01951	1.847758	0.06463731	0.0896806	NOT
KTN1	6797.959	-0.15719	0.085088	-1.84734	0.064698351	0.0897608	NOT
COL6A2	7942.534	0.380641	0.206052	1.847303	0.064703249	0.0897631	NOT
AC005682	1.900559	0.55625	0.301135	1.84718	0.064721002	0.0897832	NOT
VEZF1	1142.453	0.157184	0.085112	1.846781	0.064778928	0.089859	NOT
CASC21	1.240214	0.669919	0.362762	1.846717	0.064788222	0.0898674	NOT
CLEC4F	23.3254	-0.47195	0.255584	-1.84654	0.06481445	0.0898993	NOT
SIGMAR1	8894.133	-0.20789	0.112584	-1.8465	0.064818934	0.089901	NOT
RPP38	466.9768	0.139722	0.075679	1.846235	0.064858042	0.0899507	NOT
KALP	4.30994	-0.77897	0.421934	-1.84618	0.064865547	0.0899566	NOT
FRRS1	806.2668	-0.36486	0.19764	-1.8461	0.064878206	0.0899697	NOT
RP11-485(	20.45698	-0.78978	0.427829	-1.84603	0.064888008	0.0899788	NOT
FAM27E3	4.037792	0.535858	0.290296	1.845903	0.064906275	0.0899996	NOT
RP11-343I	5.486844	0.328861	0.178186	1.845608	0.064949166	0.0900546	NOT
JPH3	6.201399	0.561705	0.304375	1.845435	0.064974323	0.0900849	NOT
IGLV2-28	1.259492	-0.88323	0.478738	-1.84492	0.065048828	0.0901837	NOT
SERPINB4	1.518294	1.820415	0.986881	1.844615	0.065093514	0.0902411	NOT
MIR320E	1.329939	0.596905	0.323604	1.844552	0.0651027	0.0902493	NOT
AL928768.	3.163356	-0.78418	0.425191	-1.84431	0.065138004	0.0902914	NOT
SHROOM2	545.8484	-0.31144	0.168864	-1.8443	0.065139582	0.0902914	NOT
KLKP1	1.025115	1.960418	1.06303	1.844181	0.065156828	0.0903108	NOT
LINC00993	1.349319	2.044272	1.108737	1.843785	0.065214496	0.0903862	NOT
P2RY1	121.0086	-0.30598	0.165959	-1.84369	0.065228858	0.0904016	NOT
RP5-1021I	76.88064	-0.31343	0.17003	-1.84336	0.065276102	0.0904625	NOT
CCDC120	473.9433	0.22436	0.121758	1.842678	0.065376057	0.0905959	NOT
MTHFD2P.	1.334198	0.963418	0.522842	1.842658	0.065378888	0.0905959	NOT
RP11-69I8	5.63098	-0.40814	0.221508	-1.84254	0.065395816	0.0906148	NOT

RRP8	890.5697	-0.13018	0.070662	-1.84225	0.065438251	0.0906691	NOT
IGLV2-18	20.08309	-0.82674	0.448803	-1.84211	0.065459481	0.0906939	NOT
BRD9P2	2.554721	0.627765	0.340861	1.841704	0.065518421	0.0907711	NOT
TRGV10	6.55928	-0.49713	0.269954	-1.84152	0.065544843	0.0908031	NOT
RPS3P6	2.368389	0.4982	0.270562	1.841353	0.065569838	0.0908332	NOT
KLHL25	703.3684	-0.23725	0.128894	-1.84066	0.065671666	0.0909688	NOT
RP11-222I	2.457264	0.578907	0.314515	1.840637	0.065674832	0.0909688	NOT
GATA3-AS	1.594921	0.822238	0.446719	1.840618	0.06567762	0.0909688	NOT
CTD-2588	3.217241	-0.56791	0.308583	-1.84038	0.065712788	0.091013	NOT
RP11-664I	1.697106	-0.58757	0.319304	-1.84016	0.0657449	0.0910529	NOT
DLG4	250.4969	0.240422	0.130689	1.83965	0.065819585	0.0911518	NOT
SLC43A1	7705.503	-0.24474	0.133045	-1.83952	0.065838932	0.0911728	NOT
RP1-137D	3.458304	0.517873	0.281529	1.839502	0.065841338	0.0911728	NOT
RNU4-47P	4.443884	-0.464	0.252255	-1.8394	0.065855913	0.0911884	NOT
WNT7A	9.540516	-0.87202	0.474247	-1.83875	0.065952345	0.0913174	NOT
RP3-366N	2.510777	-0.62482	0.33989	-1.8383	0.066018109	0.0914038	NOT
PCDHB4	34.17938	0.368923	0.200697	1.83821	0.06603143	0.0914177	NOT
SYNE2	2677.152	-0.26941	0.146584	-1.83789	0.066078591	0.0914784	NOT
RDH14	749.1038	-0.12182	0.066283	-1.83783	0.066087663	0.0914864	NOT
ARPC4-TT	5.141605	0.331796	0.180543	1.837768	0.066096575	0.0914942	NOT
RP11-100I	0.579724	1.251618	0.681124	1.837578	0.066124557	0.0915283	NOT
LIMS1	1553.843	0.187309	0.101936	1.83751	0.066134685	0.0915338	NOT
ABLIM1	3213.268	0.241354	0.131348	1.837507	0.066135095	0.0915338	NOT
RP11-550/	1.529597	0.677293	0.368601	1.837468	0.066140765	0.091537	NOT
LEFTY1	50.15208	0.577933	0.314624	1.8369	0.066224695	0.0916486	NOT
CTC-398G	2.231524	0.433413	0.235967	1.836748	0.066247128	0.0916751	NOT
PARP14	3995.382	-0.19832	0.107999	-1.83632	0.066310958	0.0917588	NOT
TPI1P1	36.67225	0.300268	0.163538	1.836078	0.066346182	0.0918029	NOT
MAP3K11	6102.626	0.154753	0.084288	1.836005	0.066356977	0.0918133	NOT
RP5-906C	5.769921	0.442157	0.240866	1.835696	0.066402595	0.0918718	NOT
SPNS3	60.38423	0.44734	0.243704	1.835589	0.066418492	0.0918892	NOT
MTRF1	296.6735	-0.18228	0.099315	-1.83534	0.06645538	0.0919356	NOT
CYP17A1-	3.292743	0.57541	0.313523	1.835302	0.066461014	0.0919388	NOT
RAB43P1	42.37818	-0.24936	0.135916	-1.83465	0.066557273	0.0920674	NOT
PLB1	62.50957	-0.30663	0.167139	-1.83456	0.066571316	0.0920822	NOT
TIMM23	2142.456	-0.12671	0.069085	-1.83406	0.066644652	0.0921791	NOT
15-Sep	5035.352	-0.12769	0.069623	-1.83401	0.06665242	0.0921852	NOT
RP11-390I	79.86826	0.346747	0.189088	1.833786	0.066685702	0.0922266	NOT
NOP56P1	8.021707	0.837609	0.456798	1.833654	0.066705372	0.0922492	NOT
PLEKHB1	221.7728	0.611411	0.333445	1.833618	0.066710759	0.092252	NOT
MYL5	510.3783	0.253768	0.138404	1.833538	0.066722592	0.0922638	NOT
RP11-888I	33.77862	-0.29142	0.158962	-1.83326	0.066763686	0.092316	NOT
RPS11P6	15.72691	0.353499	0.192831	1.833203	0.06677246	0.0923235	NOT
LINC00967	1.279485	1.018415	0.55559	1.833034	0.06679756	0.0923536	NOT
CHMP5	2074.199	-0.12706	0.069329	-1.8327	0.066846979	0.0924173	NOT
NPPC	3.469958	0.678695	0.37036	1.832527	0.066872909	0.0924485	NOT
TIMM8A	596.7282	0.212531	0.115984	1.832416	0.066889498	0.0924669	NOT
DET1	195.436	0.177912	0.097105	1.832163	0.066927132	0.0925143	NOT
TOLLIP-AS	47.65252	0.272436	0.148705	1.83206	0.066942506	0.0925309	NOT
DDC	2222.104	-0.43366	0.236721	-1.83194	0.066960629	0.0925513	NOT
CTB-47B8.	2.044056	0.559862	0.305618	1.831901	0.066966189	0.0925544	NOT
OAZ1	16385.27	0.217149	0.118546	1.831765	0.066986448	0.0925778	NOT

AC006126	1.748493	0.588089	0.321102	1.831469	0.067030582	0.0926341	NOT
AL133243.	17.12663	-0.22056	0.120439	-1.83127	0.06706084	0.0926713	NOT
RP11-213(	107.1576	-0.17549	0.095832	-1.83117	0.067074606	0.0926788	NOT
ILVBL	4435.31	-0.23385	0.127706	-1.83116	0.067076134	0.0926788	NOT
EEF1A1P2!	6.391365	0.363416	0.198462	1.831162	0.067076342	0.0926788	NOT
C15orf62	79.95171	0.299357	0.163502	1.83091	0.067114042	0.0927263	NOT
CTD-2600	20.82731	-0.35914	0.196158	-1.83086	0.067121727	0.0927323	NOT
BTAF1	1094.361	0.220487	0.120438	1.830711	0.067143743	0.0927581	NOT
RP11-691I	3.537128	0.523686	0.286223	1.829646	0.067302832	0.0929732	NOT
AC019050	3.97515	0.482653	0.263943	1.82863	0.067455068	0.0931789	NOT
RP11-307I	7.446568	0.295924	0.161848	1.82841	0.067488	0.0932197	NOT
LRRC8E	23.83713	0.388114	0.212274	1.828362	0.067495182	0.093225	NOT
CCR2	88.85294	-0.42708	0.233592	-1.82832	0.06750211	0.0932299	NOT
LINC0065Z	4.110224	0.500579	0.273902	1.827584	0.067611984	0.093377	NOT
REP15	13.57815	0.437444	0.239373	1.827459	0.067630755	0.0933982	NOT
SLC27A4	2261.872	-0.20731	0.113446	-1.82742	0.067636469	0.0934015	NOT
RP11-572I	2.407111	0.544815	0.298156	1.827281	0.067657564	0.0934259	NOT
SVILP1	9.910944	0.396194	0.216861	1.82695	0.06770736	0.09349	NOT
RP11-661(	1.517087	0.627595	0.343536	1.826869	0.06771951	0.0935021	NOT
LMLN	240.2801	0.332495	0.18203	1.826588	0.067761758	0.0935512	NOT
USP33	1469.042	0.141977	0.077728	1.826588	0.067761788	0.0935512	NOT
CTC-428G	10.61196	-0.25012	0.136942	-1.8265	0.067774873	0.0935646	NOT
SLTM	2414.512	-0.10373	0.0568	-1.82626	0.067810613	0.0936092	NOT
UBA52	18692.81	0.229571	0.125724	1.825987	0.067852179	0.0936619	NOT
PAQR9	972.6448	0.309099	0.169311	1.82563	0.067906089	0.0937317	NOT
HMGN2P4	1.324443	-0.64261	0.352062	-1.82527	0.067960326	0.0938019	NOT
VTI1A	533.957	0.108622	0.059519	1.825002	0.068000771	0.0938522	NOT
DUSP8	533.0183	0.331497	0.181644	1.824976	0.068004619	0.0938522	NOT
SMNDC1	831.8328	-0.11151	0.061105	-1.82496	0.068006939	0.0938522	NOT
C4BPAP1	2.551316	0.598127	0.327785	1.824753	0.068038373	0.0938909	NOT
HLA-E	27370.9	-0.22411	0.122832	-1.82457	0.068066569	0.0939251	NOT
C15orf26	6.093196	0.445387	0.244143	1.824287	0.068108675	0.0939785	NOT
HYMAI	1.07884	0.851789	0.466929	1.824234	0.068116645	0.0939848	NOT
NMI	833.0685	-0.18262	0.100132	-1.82382	0.068179553	0.0940669	NOT
RP11-445(	2.031395	0.450545	0.247053	1.823675	0.068201261	0.0940922	NOT
MIRLET7D	2.324314	0.57675	0.316328	1.823263	0.068263574	0.0941734	NOT
FPGT-TNN	7.918205	0.411704	0.225836	1.823023	0.068299922	0.0942147	NOT
AC005593	1.645963	0.531671	0.291643	1.82302	0.06830031	0.0942147	NOT
C19orf60	1618.004	0.271314	0.148835	1.82291	0.068317051	0.0942317	NOT
TAF9B	767.0058	0.188859	0.103604	1.822894	0.06831945	0.0942317	NOT
RPS2P48	2.794768	0.470285	0.258012	1.822722	0.06834557	0.0942631	NOT
SCML2P2	1.469988	0.666897	0.36589	1.822669	0.068353534	0.0942693	NOT
TPT1P6	6.688548	-0.39241	0.215329	-1.82238	0.068398011	0.094326	NOT
RP11-407(	2.883467	0.506399	0.277883	1.82235	0.068401933	0.0943267	NOT
ARL8B	2725.245	0.116778	0.064084	1.822258	0.068415829	0.0943411	NOT
TMPRSS6	10062.04	-0.40987	0.224966	-1.82191	0.068469247	0.0944101	NOT
GTPBP1	1706.148	-0.1657	0.090982	-1.82128	0.068564434	0.0945366	NOT
RP11-576(	1.725378	0.866653	0.475897	1.821094	0.06859254	0.094569	NOT
C14orf79	216.5489	0.200657	0.110186	1.82108	0.068594722	0.094569	NOT
SELENBP1	12425.12	-0.35348	0.194154	-1.8206	0.068667908	0.0946652	NOT
ACOT6	17.14616	-0.51462	0.282673	-1.82053	0.068677966	0.0946743	NOT
RP11-977(	112.1087	0.291695	0.160273	1.819992	0.068760189	0.0947829	NOT

LINC01347	15.08706	0.342522	0.18822	1.819798	0.068789749	0.094819	NOT
RP5-834N	25.1529	-0.50814	0.279259	-1.8196	0.068820566	0.0948567	NOT
CLEC11A	271.662	-0.33174	0.182338	-1.81938	0.068854045	0.0948981	NOT
CLDN8	2.052736	-0.97309	0.534868	-1.8193	0.06886546	0.0949091	NOT
MTND4P2	529.4321	-0.56538	0.310794	-1.81916	0.068887684	0.094935	NOT
UBE2E3	1492.049	0.137969	0.075859	1.818743	0.068950593	0.095017	NOT
TNFRSF11	558.4039	-0.54973	0.30227	-1.81867	0.068961043	0.0950267	NOT
RP11-2B6.	1.676455	0.63211	0.347575	1.818627	0.068968373	0.0950289	NOT
AC011997	1.627928	0.901789	0.495865	1.818619	0.068969546	0.0950289	NOT
APCDD1L-	1.675549	0.787839	0.433223	1.818553	0.068979694	0.0950382	NOT
RPS6KB2	2007.411	0.14315	0.078722	1.818417	0.069000488	0.095061	NOT
PGAM1P7	1.530239	0.697147	0.383385	1.818399	0.069003122	0.095061	NOT
FTLP15	1.613613	0.714758	0.393085	1.81833	0.069013635	0.0950707	NOT
CTD-2651	4.934066	0.558893	0.307442	1.817879	0.069082604	0.095161	NOT
RP11-85A	6.140712	0.396813	0.218312	1.817641	0.069119001	0.0952064	NOT
FSTL1	3505.764	0.366688	0.201745	1.817577	0.069128825	0.0952152	NOT
MYNN	530.8983	-0.11102	0.061088	-1.81742	0.069152315	0.0952428	NOT
ARHGEF6	443.8095	-0.28225	0.155317	-1.81728	0.069174713	0.0952689	NOT
MATN4	6.431323	0.619395	0.340865	1.817125	0.06919798	0.0952962	NOT
RP4-800M	7.331696	-0.31203	0.171721	-1.8171	0.069202141	0.0952972	NOT
LPAR5	77.94064	-0.36312	0.199865	-1.81685	0.069240655	0.0953455	NOT
SNORD14	5.791116	0.439597	0.242052	1.816122	0.069351651	0.0954936	NOT
PRODH	645.0292	0.654115	0.36021	1.815925	0.069381889	0.0955304	NOT
RP11-107	0.857844	0.992651	0.546802	1.815376	0.069466205	0.0956418	NOT
TOR2A	587.7683	0.142886	0.078721	1.815104	0.069507869	0.0956944	NOT
PSPC1P1	3.703136	0.508436	0.280174	1.814715	0.069567724	0.095772	NOT
RP11-506	2.301796	0.526579	0.290329	1.813732	0.069719006	0.0959755	NOT
KMT2E	1459.424	0.186273	0.102708	1.813621	0.069736018	0.0959941	NOT
HVCN1	131.2934	-0.27562	0.151989	-1.81344	0.0697641	0.096028	NOT
GGT1	2853.199	-0.43739	0.241218	-1.81326	0.069791656	0.0960612	NOT
PLEKHB2	1794.722	0.219628	0.12113	1.813152	0.069808426	0.0960795	NOT
RP11-30P	367.9795	-0.5386	0.297066	-1.81306	0.069821829	0.0960931	NOT
RP11-134	69.65798	0.284313	0.156846	1.81269	0.069879581	0.0961678	NOT
PGM2	1029.075	-0.31524	0.173924	-1.81251	0.069907611	0.0962016	NOT
RP11-547	7.849875	-0.40067	0.221112	-1.81207	0.069975445	0.0962902	NOT
VPS26B	2703.779	-0.13208	0.072897	-1.81188	0.07000508	0.0963262	NOT
RNU6-516	6.778946	0.376481	0.207823	1.81155	0.070055737	0.0963911	NOT
RP11-329	7.645987	-0.3857	0.212945	-1.81127	0.070098952	0.0964458	NOT
RP11-219	1.36077	0.61939	0.342046	1.81084	0.070165555	0.0965326	NOT
C14orf142	402.9733	0.147083	0.08123	1.810706	0.070186373	0.0965564	NOT
ZNF784	451.9636	0.240121	0.132653	1.810149	0.070272712	0.0966704	NOT
RP11-513	4.460976	-0.67807	0.374621	-1.81003	0.070291723	0.0966917	NOT
MLLT10	1002.601	0.158972	0.08783	1.809994	0.070296732	0.0966938	NOT
YES1P1	2.743469	0.555608	0.306982	1.809906	0.070310423	0.0967079	NOT
PAX9	4.467061	0.71629	0.395797	1.80974	0.070336146	0.0967384	NOT
LINC00845	1.35233	1.278285	0.706357	1.809687	0.070344352	0.0967449	NOT
RP11-109	6.365568	-0.30535	0.168742	-1.80957	0.070362313	0.0967624	NOT
LGALS17A	3.660587	0.731813	0.404415	1.80956	0.070364064	0.0967624	NOT
CEP170P1	2.47583	0.490112	0.270866	1.809429	0.070384391	0.0967855	NOT
CTC-301C	24.85725	0.27341	0.151109	1.809363	0.070394597	0.0967948	NOT
MAP1LC3C	4.313575	-0.39494	0.218292	-1.80924	0.070413289	0.0968156	NOT
C11orf70	5.635016	0.521411	0.288243	1.808929	0.070462079	0.0968735	NOT

RPL21P39	2.19171	-0.39372	0.217653	-1.80893	0.070462356	0.0968735	NOT
CTD-2619	48.67528	0.365116	0.201848	1.808868	0.070471461	0.0968812	NOT
PEX12	443.0609	-0.18473	0.102129	-1.80879	0.070483765	0.0968933	NOT
STARD4-A	61.44254	0.370387	0.204773	1.808766	0.070487415	0.0968935	NOT
SLC36A2	1.787041	0.818233	0.452386	1.808707	0.070496598	0.0969013	NOT
IGHJ3	6.741287	-0.83563	0.462055	-1.80852	0.070526184	0.0969372	NOT
HSD3B7	3121.566	-0.26923	0.148905	-1.8081	0.070591101	0.0970216	NOT
TOB1	6819.576	-0.31777	0.175755	-1.80801	0.070605647	0.0970367	NOT
PCDHGA1(	97.30922	-0.3953	0.21865	-1.80793	0.070617019	0.0970403	NOT
RP11-159I	2.379983	0.444161	0.245674	1.80793	0.070617378	0.0970403	NOT
PAXIP1-AS	381.1217	0.206871	0.114425	1.807921	0.07061874	0.0970403	NOT
MKRN1	2862.531	0.105202	0.058212	1.807215	0.070728717	0.0971866	NOT
RP11-706(	167.2238	-0.28722	0.158963	-1.80685	0.070785876	0.0972603	NOT
TAS2R31	1.256712	0.681058	0.376959	1.806716	0.070806507	0.0972838	NOT
GGT7	736.5755	0.200344	0.110922	1.806167	0.070892226	0.0973967	NOT
C17orf104	11.68059	0.30715	0.170061	1.806116	0.070900248	0.0974029	NOT
KRT8P9	2.835161	-0.47186	0.261266	-1.80604	0.070911502	0.0974109	NOT
SLC26A10	6.734493	0.436819	0.241867	1.806033	0.070913138	0.0974109	NOT
SENP3-EIF	1.791988	0.550305	0.304719	1.805945	0.070926974	0.0974251	NOT
DERL1	4938.635	0.158587	0.08783	1.805617	0.070978239	0.0974907	NOT
PIK3CG	64.00556	-0.38191	0.211565	-1.80516	0.071049099	0.0975832	NOT
DNAH7	15.21267	0.456813	0.253122	1.804713	0.07111954	0.0976751	NOT
IL20RA	72.51334	0.847668	0.469717	1.804635	0.07113174	0.097687	NOT
RP11-689I	6.178489	0.6059	0.335798	1.804362	0.071174612	0.097741	NOT
CDC42BPA	2545.113	0.230092	0.127532	1.804186	0.071202101	0.0977739	NOT
APP	20636	-0.26807	0.148601	-1.80393	0.071242514	0.0978245	NOT
NFYB	1412.697	0.193341	0.107201	1.803534	0.071304366	0.0979046	NOT
VIL1	2422.826	0.543151	0.301167	1.80349	0.071311338	0.0979093	NOT
SNUPN	577.7633	0.132642	0.073564	1.80309	0.071374019	0.0979905	NOT
PARP8	582.7499	-0.26906	0.149237	-1.80294	0.071398291	0.098019	NOT
RP11-714I	126.4163	-0.65946	0.36583	-1.80265	0.071443871	0.0980767	NOT
HMGB1P3	1.447604	0.640428	0.355299	1.802506	0.071465868	0.098102	NOT
PSMD9	795.4701	0.128871	0.071506	1.802238	0.071507962	0.0981539	NOT
RP11-314I	3.367106	0.428578	0.237806	1.80222	0.071510731	0.0981539	NOT
AC144831	20.49064	0.459963	0.255254	1.801984	0.07154789	0.0982	NOT
RPSAP9	30.1463	0.239972	0.133182	1.801843	0.071570086	0.0982256	NOT
MIR3189	4.722682	0.632053	0.350817	1.801658	0.07159924	0.0982607	NOT
PNLIPRP3	0.832197	1.308975	0.726603	1.801499	0.071624328	0.0982903	NOT
SLC46A2	3.548861	0.565982	0.314204	1.80132	0.071652437	0.098324	NOT
RP11-110I	3.181175	0.369037	0.204882	1.801215	0.071668946	0.0983388	NOT
KIAA1551	1768.791	-0.24286	0.13483	-1.80121	0.07167034	0.0983388	NOT
RP1-198K1	61.01538	-0.24339	0.135142	-1.80098	0.071705442	0.0983821	NOT
LINC00551	1.934389	-0.99328	0.551702	-1.80039	0.071798864	0.0985014	NOT
HMHA1	1156.948	0.24965	0.138665	1.800387	0.071799521	0.0985014	NOT
HIST1H1D	1.417789	0.792562	0.440224	1.800362	0.071803532	0.098502	NOT
FAF2	2211.909	0.093303	0.051833	1.800069	0.071849796	0.0985606	NOT
IGKJ5	1.134163	-0.92799	0.515643	-1.79968	0.071910682	0.0986392	NOT
SKIL	1427.312	-0.2015	0.111997	-1.79916	0.071993052	0.0987473	NOT
VEGFC	229.4269	0.275429	0.153096	1.799061	0.072008958	0.0987642	NOT
SLC7A14	7.162174	0.649275	0.360949	1.798802	0.072050079	0.0988157	NOT
LINC00402	19.39868	-0.65559	0.36452	-1.7985	0.07209762	0.098876	NOT
AP001347.	2.834815	0.493818	0.27463	1.798121	0.072157822	0.0989537	NOT

CADM4	474.3151	0.334492	0.18606	1.797764	0.072214443	0.0990264	NOT
EEF1A1P1	5.938098	0.449827	0.250287	1.797242	0.072297287	0.0991351	NOT
LEO1	1144.536	0.128767	0.071666	1.796778	0.072370834	0.099231	NOT
ATG16L1	1045.619	-0.10882	0.060567	-1.79669	0.072384577	0.099245	NOT
FAM89A	413.2446	-0.28564	0.158996	-1.79655	0.072406468	0.0992701	NOT
RP11-338(	3.243497	-0.42908	0.238844	-1.79649	0.072416959	0.0992795	NOT
DDX6	3293.499	-0.11416	0.063554	-1.79632	0.072444336	0.0993121	NOT
GPS2P1	11.04935	0.472624	0.263117	1.796252	0.072454393	0.099321	NOT
SYT7	5320.944	-0.53145	0.295873	-1.79619	0.072463594	0.0993287	NOT
ARFIP1	938.4468	-0.13297	0.074033	-1.79608	0.072482261	0.0993494	NOT
CD8A	293.9554	-0.47474	0.264346	-1.79591	0.072509366	0.0993816	NOT
RPL34P34	2.59893	0.470006	0.26173	1.795764	0.072532079	0.0994078	NOT
RP11-632(	225.2611	0.414573	0.230909	1.795396	0.072590645	0.0994831	NOT
RP11-314I	1.653931	1.050214	0.585037	1.795123	0.072634065	0.0995377	NOT
RP11-468I	64.01628	0.221814	0.123569	1.795056	0.072644849	0.0995476	NOT
PPFIA2	9.274498	0.435214	0.242527	1.7945	0.072733342	0.0996639	NOT
GPM6B	33.95572	-0.32655	0.182063	-1.79359	0.072877964	0.0998571	NOT
PSMB9	1748.883	0.314038	0.175136	1.79311	0.072955379	0.0999582	NOT
EXOC8	636.6644	0.139377	0.077766	1.792256	0.073091912	0.1001403	NOT
CTC-338M	2.536775	0.579501	0.323403	1.791888	0.073150945	0.1002163	NOT
NDUFAB1	2651.981	-0.19513	0.108903	-1.79182	0.073161308	0.1002221	NOT
LTN1	875.7641	-0.17927	0.100052	-1.79182	0.073162443	0.1002221	NOT
COX14	1928.1	-0.20062	0.111968	-1.79172	0.073177817	0.1002382	NOT
ZNF217	1660.853	0.223267	0.124633	1.791399	0.073229246	0.1003037	NOT
HNRNPA0	4675.061	0.12025	0.067161	1.790475	0.073377567	0.1005018	NOT
TNS4	7.378065	0.476766	0.266305	1.790302	0.073405427	0.100535	NOT
CTD-2012	4.092693	0.467803	0.261346	1.789975	0.073457887	0.1005991	NOT
HIGD2A	3057.909	0.218926	0.122307	1.789965	0.073459474	0.1005991	NOT
SASH3	349.1296	-0.34078	0.190408	-1.78975	0.073494323	0.1006418	NOT
KLK5	0.940511	1.741625	0.97345	1.789127	0.073594319	0.1007738	NOT
EPN2	602.3818	0.174707	0.097708	1.788052	0.073767604	0.1010061	NOT
RPS12	23698.2	0.256404	0.143419	1.7878	0.073808341	0.1010568	NOT
SUGT1	1513.28	-0.15833	0.088565	-1.78778	0.073812256	0.1010572	NOT
INCA1	83.34963	0.216939	0.121363	1.787517	0.073853952	0.1011093	NOT
RP11-544I	2.577097	0.45551	0.254864	1.787267	0.073894302	0.1011595	NOT
CTD-2081	12.92664	0.294836	0.164977	1.78714	0.073914928	0.1011827	NOT
SCFD2	489.5098	0.127674	0.071465	1.786529	0.074013584	0.1013128	NOT
CTD-2555	33.94622	0.272454	0.152532	1.786215	0.074064428	0.1013774	NOT
PPAP2A	2501.045	0.244611	0.13698	1.78575	0.074139805	0.1014755	NOT
PPRC1	1260.69	-0.19501	0.109237	-1.78518	0.074232505	0.1015974	NOT
6-Sep	2230.347	0.264109	0.147954	1.785071	0.074249748	0.1016138	NOT
AC034243	18.19535	-0.53957	0.302268	-1.78506	0.074251882	0.1016138	NOT
RPS7P14	4.880097	0.346651	0.194205	1.784975	0.074265339	0.1016272	NOT
NDUFC2	3162.513	-0.23074	0.129291	-1.78465	0.074318259	0.1016946	NOT
TRAF3IP3	122.0113	-0.33507	0.18777	-1.78445	0.074350734	0.101734	NOT
RP1-206D	2.389091	0.621137	0.34809	1.784418	0.074355834	0.101736	NOT
RP11-231I	261.5641	-0.4384	0.245688	-1.78438	0.074361783	0.1017391	NOT
RP11-298J	12.02193	-0.37364	0.209402	-1.7843	0.074374927	0.101752	NOT
EDN2	31.22694	-0.58637	0.328637	-1.78424	0.074385179	0.101761	NOT
CMTR1	2081.753	0.137938	0.077319	1.784012	0.074421665	0.1018059	NOT
RP11-981(	1.187141	0.857392	0.480671	1.783739	0.074466099	0.1018616	NOT
BCL2A1	87.23287	-0.44234	0.247989	-1.7837	0.074471793	0.1018644	NOT

RPL29P14	2.005291	0.528617	0.296375	1.783609	0.07448728	0.1018805	NOT
RP11-390I	3.037594	0.562242	0.31527	1.783366	0.074526672	0.1019294	NOT
AC104809	27.96719	-0.62059	0.34806	-1.78301	0.074585408	0.1020002	NOT
SERPINB3	2.23296	1.488695	0.834937	1.783003	0.074585792	0.1020002	NOT
DGKE	249.2843	-0.28658	0.160857	-1.78158	0.074817875	0.1023125	NOT
LRRC3-AS:	8.720775	-0.44831	0.251653	-1.78145	0.07483838	0.1023355	NOT
SEC24C	1453.043	0.271045	0.152176	1.781127	0.074891775	0.1024034	NOT
PSMA7	7119.319	0.152515	0.085631	1.781087	0.074898303	0.1024073	NOT
S1PR4	70.35795	-0.3596	0.201916	-1.78092	0.074926226	0.1024404	NOT
RP4-769N	82.20419	0.173707	0.097549	1.780721	0.074958063	0.1024789	NOT
KDM2A	3821.144	0.119664	0.067224	1.780081	0.075062647	0.1026168	NOT
DPP3P2	0.673836	1.376329	0.773394	1.779595	0.07514232	0.1027206	NOT
COL6A1	7918.326	0.354212	0.199045	1.779555	0.075148845	0.1027245	NOT
C10orf32	2431.265	-0.23834	0.133961	-1.77921	0.075205941	0.1027974	NOT
ATP5G3	10726.24	-0.19186	0.107843	-1.77905	0.075230993	0.1028266	NOT
RP3-522J7	3.727273	0.467252	0.262754	1.778287	0.075356796	0.1029935	NOT
PRAMEF17	0.806247	1.213994	0.682944	1.77759	0.075471293	0.1031448	NOT
FGF1	66.65791	0.435898	0.24526	1.777291	0.0755204	0.1032069	NOT
IL17REL	2.241998	0.710791	0.399966	1.77713	0.075546783	0.1032378	NOT
RP11-567I	1.567425	0.699646	0.393884	1.776274	0.075687794	0.1034254	NOT
RN7SKP70	2.916448	0.483823	0.272403	1.776133	0.075710978	0.103452	NOT
ATPAF2	738.7862	-0.19734	0.111132	-1.77572	0.075779153	0.1035396	NOT
MTHFD2	278.1806	0.345379	0.194503	1.775699	0.075782613	0.1035396	NOT
FBXO6	821.1036	0.223251	0.125737	1.775539	0.075809009	0.1035706	NOT
RP11-398I	1.200877	1.435681	0.808798	1.77508	0.075884741	0.1036689	NOT
KANSL1L	498.6912	0.242958	0.136937	1.774226	0.07602585	0.1038566	NOT
ST3GAL1P:	1.493812	0.527408	0.297273	1.774154	0.076037659	0.1038676	NOT
AC091180	1.165676	0.644692	0.363399	1.774061	0.076053098	0.1038835	NOT
GPR179	3.423041	0.5393	0.304001	1.774009	0.07606163	0.103889	NOT
VAT1L	96.18025	0.56656	0.319373	1.773974	0.076067385	0.103889	NOT
RP11-158I	106.1468	-0.35149	0.198135	-1.77397	0.076068356	0.103889	NOT
RP11-154I	4.453444	0.564532	0.318273	1.773733	0.07610734	0.1039345	NOT
HNRNPLL	777.4481	0.099284	0.055975	1.773722	0.076109191	0.1039345	NOT
OSBPL5	270.4653	0.288619	0.162765	1.773228	0.076190856	0.1040409	NOT
RHPN2	1566.708	-0.23295	0.131373	-1.77317	0.07620004	0.1040483	NOT
ITFG3	2498.271	0.216755	0.122262	1.772868	0.076250617	0.1041122	NOT
RP11-75A!	2.009535	0.440565	0.24851	1.772825	0.076257737	0.1041168	NOT
AC016700	84.16974	-0.32386	0.182696	-1.77267	0.076283609	0.104147	NOT
UHMK1	5680.09	0.177161	0.099943	1.772619	0.076291787	0.1041498	NOT
SSTR1	547.6339	-0.50918	0.28725	-1.77261	0.076293237	0.1041498	NOT
TRAPPC12	1649.402	0.145306	0.081999	1.772048	0.076386508	0.104272	NOT
SLC2A4	290.6204	0.443072	0.250076	1.771748	0.07643636	0.1043349	NOT
CC2D2B	2.590865	0.414185	0.233781	1.771684	0.076447026	0.1043443	NOT
TOMM22	3047.989	0.157872	0.089113	1.771606	0.076459933	0.1043568	NOT
SHANK2	1155.892	0.268619	0.151641	1.771415	0.076491705	0.104395	NOT
LINC00311	1.895738	-0.61852	0.349207	-1.77121	0.076525127	0.1044355	NOT
HSPE1P2	9.207004	0.307214	0.173502	1.77066	0.076617341	0.1045562	NOT
C2orf27AP	1.706229	0.811716	0.458494	1.770397	0.076660985	0.1046106	NOT
LINC00854	18.31129	0.316388	0.178718	1.770315	0.076674746	0.1046242	NOT
REEP1	112.7253	0.473211	0.267411	1.769601	0.076793619	0.1047812	NOT
LRCH4	391.7266	0.209339	0.118314	1.769352	0.076835118	0.1048327	NOT
POLR2J	1819.881	0.205553	0.116215	1.768732	0.076938579	0.1049686	NOT

SELK	1891.634	-0.16112	0.091094	-1.76871	0.076942307	0.1049686	NOT
GCG	1.04903	1.278589	0.722913	1.768663	0.076950166	0.1049741	NOT
EYA3	624.5864	0.115369	0.065245	1.768227	0.077022917	0.1050682	NOT
RP5-1057.	44.48117	-0.30869	0.174582	-1.76819	0.077029252	0.1050717	NOT
USP43	176.0822	-0.47767	0.270225	-1.76767	0.077116289	0.1051809	NOT
PSMC1P9	2.206222	-0.38721	0.219055	-1.76766	0.077116931	0.1051809	NOT
FBXW11	1863.832	0.14473	0.081905	1.767051	0.077219719	0.1053159	NOT
AC012531	0.74153	1.327072	0.751088	1.766865	0.07725076	0.105353	NOT
FRG1	755.7144	-0.16627	0.094125	-1.76643	0.077323743	0.1054474	NOT
RP11-697I	26.33979	-0.37932	0.214758	-1.76626	0.077352703	0.1054817	NOT
NYAP2	0.650188	1.175354	0.665484	1.766164	0.077368305	0.1054977	NOT
RAPGEF6	339.4273	0.144323	0.081722	1.766019	0.077392604	0.1055257	NOT
AC004076	1.94003	0.560428	0.31742	1.765574	0.077467337	0.1056224	NOT
AC010468	8.466089	0.340107	0.192665	1.765275	0.077517581	0.1056857	NOT
RBM5-AS1	3.059973	0.457354	0.259231	1.764271	0.077686296	0.1059105	NOT
SAMD3	28.20702	-0.38766	0.219779	-1.76387	0.077753292	0.1059966	NOT
RPL12P38	3.530516	0.390824	0.221645	1.76329	0.077851571	0.1061253	NOT
OSTM1	1112.487	0.224003	0.127056	1.763025	0.077896198	0.1061809	NOT
ADIRF-AS.	37.74851	0.434771	0.24662	1.762918	0.077914271	0.1062003	NOT
MIA3	6123.966	-0.195	0.110632	-1.76263	0.077962267	0.1062556	NOT
MRPS16	3567.613	0.13575	0.077016	1.762632	0.077962531	0.1062556	NOT
TOMM70A	4652.391	-0.10969	0.062241	-1.76233	0.078014243	0.1063209	NOT
LL22NC03	7.128732	0.530236	0.300885	1.762259	0.078025609	0.1063311	NOT
IWS1	1723.423	0.096271	0.054632	1.762177	0.078039454	0.1063448	NOT
SCNN1B	24.82441	0.4972	0.282169	1.762065	0.078058258	0.1063651	NOT
RP11-230I	7.715784	0.363436	0.20626	1.762028	0.078064637	0.1063686	NOT
COX7B	6002.248	0.217258	0.123333	1.761565	0.078142882	0.10647	NOT
IL20	1.890681	0.985813	0.559829	1.760917	0.078252386	0.1066139	NOT
MED26	273.5761	0.122832	0.069771	1.760503	0.078322586	0.1067043	NOT
CTD-2013	1.900281	0.473199	0.268812	1.760338	0.078350552	0.1067371	NOT
RTN4R	427.6001	0.339111	0.192654	1.760205	0.078372981	0.1067624	NOT
RP11-702I	2.623389	0.503547	0.28612	1.759916	0.078422051	0.106824	NOT
CDRT4	6.533522	0.36564	0.207832	1.759305	0.078525655	0.1069599	NOT
RP11-25G.	7.01221	0.552973	0.314335	1.759183	0.078546424	0.1069829	NOT
RP11-440(	7.974373	0.410057	0.233106	1.759103	0.078560069	0.1069962	NOT
RTTN	321.7341	-0.1929	0.109664	-1.75904	0.078570388	0.107005	NOT
YTHDC2	1131.011	-0.15512	0.088193	-1.75888	0.078598343	0.1070378	NOT
SNORA65	6.935076	0.274865	0.156286	1.758734	0.078622622	0.1070656	NOT
JARID2-AS	3.308158	0.439571	0.249941	1.758699	0.078628688	0.1070686	NOT
ZMYND15	264.5059	0.350659	0.19941	1.758481	0.078665673	0.1071137	NOT
RP1-168L1	4.334238	-0.37433	0.212972	-1.75765	0.078807526	0.1073016	NOT
CCL8	50.00255	0.410768	0.233732	1.757431	0.078844308	0.1073464	NOT
ODC1	4521.128	0.25858	0.147161	1.757128	0.078895931	0.1074114	NOT
PUM1	2416.068	-0.11964	0.068091	-1.75704	0.078911657	0.1074275	NOT
EPHA3	304.9104	-0.4513	0.256908	-1.75666	0.078975839	0.1075096	NOT
FZD8	259.207	-0.35132	0.200004	-1.75656	0.078993266	0.107528	NOT
ZBTB48	816.3393	-0.16601	0.094516	-1.75642	0.079016811	0.1075537	NOT
CCT8P1	702.2982	-0.33	0.187884	-1.7564	0.079019888	0.1075537	NOT
RP11-371I	2.623684	0.498534	0.283878	1.756158	0.07906135	0.1076048	NOT
KCTD12	1160.598	-0.29819	0.169801	-1.75613	0.079066954	0.1076071	NOT
IGHV3-64	15.23974	-0.87695	0.499398	-1.75601	0.07908749	0.1076298	NOT
SH2B3	1701.161	-0.20586	0.117276	-1.75535	0.079198674	0.1077758	NOT

LINC01016	4.062426	-0.55756	0.317656	-1.75524	0.07921756	0.1077962	NOT
ADAM18	0.774236	1.878405	1.07025	1.755108	0.079240785	0.1078225	NOT
AKR1C6P	230.983	-0.50917	0.290236	-1.75434	0.079372549	0.1079965	NOT
ZNF680	509.5876	-0.26304	0.149946	-1.75422	0.079392877	0.1080151	NOT
TDRD15	18.99375	-0.61964	0.353231	-1.75421	0.079394041	0.1080151	NOT
CTC-248C	3.005929	0.641476	0.365689	1.754155	0.079403911	0.1080232	NOT
RP13-638	2.192683	-0.41949	0.239226	-1.75355	0.079507554	0.1081589	NOT
AF064858.	9.110046	-0.441	0.251526	-1.7533	0.079551226	0.108213	NOT
IFNLR1	404.5411	-0.39379	0.224623	-1.75311	0.079583418	0.1082514	NOT
GTF2H2B	16.39278	0.361071	0.206023	1.75258	0.079674188	0.1083696	NOT
PIGHP1	3.796815	-0.36975	0.210984	-1.75252	0.079685242	0.1083793	NOT
TBRG1	772.8825	-0.14212	0.081096	-1.75248	0.079691749	0.1083828	NOT
ADAM19	533.371	-0.2638	0.150549	-1.75224	0.079732185	0.1084273	NOT
RP4-635A	10.54725	-0.27158	0.154989	-1.75224	0.079732297	0.1084273	NOT
PAPOLG	350.695	0.127343	0.072676	1.752198	0.079739819	0.1084322	NOT
CH507-24	1.67917	0.624934	0.356672	1.752123	0.079752593	0.1084442	NOT
MIR205HG	3.259649	-1.01095	0.577389	-1.75091	0.079962028	0.1087237	NOT
UBE2Q2P1	34.24959	0.282451	0.161323	1.750842	0.079973065	0.1087333	NOT
RP11-66B	14.48637	-0.41579	0.237501	-1.75069	0.080000173	0.1087649	NOT
ZBTB42	500.5559	0.194516	0.111118	1.750533	0.080026447	0.1087952	NOT
CTC-232H	2.044765	0.507372	0.289862	1.750395	0.080050179	0.1088221	NOT
RP11-244	1.110926	2.401246	1.37218	1.74995	0.080126928	0.1089211	NOT
NKD2	124.0097	0.47124	0.269434	1.748998	0.080291363	0.1091393	NOT
RRN3P2	21.31137	0.280925	0.160666	1.748501	0.080377238	0.1092507	NOT
CASKIN2	1657.431	-0.16523	0.094517	-1.74811	0.080444904	0.1093337	NOT
GPR78	1.817937	0.960987	0.549732	1.748103	0.080446266	0.1093337	NOT
STT3A-AS	1.401588	0.540304	0.309146	1.747729	0.08051095	0.1094126	NOT
RABL2B	300.6807	0.160871	0.092046	1.747722	0.080512233	0.1094126	NOT
HIPK4	6.696168	0.346472	0.198265	1.747522	0.080546904	0.1094544	NOT
ARHGEF7	1629.562	0.164483	0.094126	1.747487	0.080552976	0.1094573	NOT
SNN	812.771	0.188128	0.107698	1.746808	0.080670579	0.1096078	NOT
TMEM70	1718.96	0.210563	0.120542	1.746802	0.080671669	0.1096078	NOT
MON1A	554.2273	0.174752	0.100045	1.746722	0.080685542	0.1096212	NOT
MRGPRF-A	8.754762	0.839747	0.480773	1.746661	0.080696182	0.1096303	NOT
RP5-1184	2.907027	0.520022	0.297776	1.746351	0.08074993	0.1096979	NOT
M6PR	1494.386	-0.19653	0.112538	-1.74631	0.080756362	0.1097013	NOT
FOXA3	3144.345	-0.41864	0.239762	-1.74604	0.080803206	0.1097595	NOT
RP11-788	1.685315	0.87934	0.503635	1.745987	0.080813207	0.1097677	NOT
TFEB	693.3304	0.219824	0.125936	1.745524	0.080893704	0.1098717	NOT
LAMC3	862.3661	-0.38117	0.218388	-1.7454	0.080915526	0.1098959	NOT
DCHS2	13.98395	0.63212	0.362179	1.745325	0.080928274	0.1099078	NOT
SH3BP5	229.7765	-0.32332	0.185256	-1.74525	0.080942118	0.1099212	NOT
NUDT3	851.1441	0.13979	0.080107	1.74505	0.080976109	0.109962	NOT
RP11-236	7.590694	-0.39868	0.228552	-1.74436	0.081096793	0.1101205	NOT
EEFSEC	1359.441	-0.16182	0.092773	-1.74429	0.081108526	0.110131	NOT
RP11-341	0.725404	1.649536	0.945778	1.744105	0.08114074	0.1101694	NOT
SYT14	1.101751	0.912829	0.523417	1.743979	0.081162835	0.1101939	NOT
SV2B	90.48135	0.532412	0.305299	1.743901	0.081176418	0.110207	NOT
RP11-159	2.723871	0.429971	0.246585	1.743706	0.081210438	0.1102478	NOT
KRT8P14	1.91637	0.515202	0.295493	1.743533	0.081240475	0.1102831	NOT
SSX2B	0.696864	1.690411	0.96957	1.743463	0.081252686	0.1102943	NOT
AC006272	2.401809	0.471572	0.270509	1.743281	0.081284625	0.1103322	NOT

LRRC37A1	4.089638	0.627973	0.360272	1.743052	0.081324463	0.1103809	NOT
KIAA1644	20.60975	-0.55986	0.321242	-1.7428	0.081367879	0.1104344	NOT
TOR1AIP2	7293.48	0.196787	0.112925	1.742633	0.081397695	0.1104694	NOT
TRPM7	1886.786	-0.21494	0.123353	-1.74248	0.081424175	0.1104959	NOT
RNF6	825.6253	-0.1541	0.088436	-1.74248	0.081425187	0.1104959	NOT
RP11-480(	1.396323	0.740668	0.425202	1.74192	0.081522392	0.1106201	NOT
ELK3	942.9146	0.231298	0.132785	1.741907	0.081524712	0.1106201	NOT
KCTD14	201.9579	-0.3751	0.215361	-1.74174	0.081553884	0.1106543	NOT
FKBP4	5900.809	0.193755	0.111259	1.741479	0.081599658	0.110711	NOT
RPS15AP3	2.715315	0.482175	0.276881	1.741453	0.081604171	0.1107116	NOT
FXYD5	1385.762	0.305144	0.175233	1.741366	0.08161944	0.1107269	NOT
CAMK1D	1403.17	0.338131	0.194198	1.741164	0.081654777	0.1107694	NOT
NEBL	397.7922	0.579362	0.332749	1.741141	0.081658901	0.1107696	NOT
LL22NC03	0.811235	1.321487	0.758988	1.741117	0.08166308	0.1107698	NOT
RP11-418I	1.58392	-0.66182	0.380123	-1.74105	0.08167405	0.1107793	NOT
MTRNR2L1	1056.898	-0.96727	0.55583	-1.74023	0.081819082	0.1109706	NOT
SOAT1	1649.512	0.181525	0.104315	1.740169	0.081829391	0.1109791	NOT
SUMF2	5958.31	0.167526	0.096274	1.740086	0.081843879	0.1109933	NOT
NEO1	1406.789	0.232271	0.13349	1.739992	0.081860428	0.1110103	NOT
SLAMF8	249.3018	0.353124	0.202957	1.739897	0.08187709	0.1110241	NOT
LYRM9	260.2072	-0.21204	0.121869	-1.73989	0.081878648	0.1110241	NOT
IL6R	5576.916	0.317568	0.182564	1.739492	0.081948229	0.111113	NOT
NLRP7	2.784732	-0.6015	0.3458	-1.73945	0.08195618	0.1111184	NOT
XXbac-BP(	78.60107	0.316738	0.182099	1.739373	0.081969225	0.1111306	NOT
SNORD19	4.418838	0.364016	0.209298	1.739224	0.081995383	0.1111606	NOT
DMXL1	1021.919	-0.1678	0.096505	-1.73875	0.082078457	0.1112678	NOT
RP3-402G	17.93632	0.309337	0.177917	1.738659	0.082094838	0.1112846	NOT
ALDH1L1-	3.464144	0.573768	0.330043	1.738465	0.082128837	0.1113252	NOT
RPS3AP25	3.937525	-0.38588	0.221979	-1.73836	0.082147016	0.1113444	NOT
TMEM167	1561.924	0.115453	0.066418	1.738274	0.082162563	0.11136	NOT
LINC0148	28.6682	-0.48402	0.278488	-1.73802	0.082207121	0.1114149	NOT
NUDT21	1816.395	-0.12683	0.07298	-1.73781	0.082244019	0.1114595	NOT
RP11-323I	1.168305	0.707334	0.407051	1.737706	0.082262684	0.1114793	NOT
FXR2	1410.102	-0.13026	0.074985	-1.73717	0.082356511	0.111601	NOT
RP11-170I	13.93815	-0.32999	0.189976	-1.73703	0.082382394	0.1116306	NOT
RP5-1153I	1.167764	0.594744	0.342437	1.736796	0.082423136	0.1116773	NOT
NEU4	1674.841	-0.57435	0.330697	-1.73679	0.082424955	0.1116773	NOT
AC006942	17.9415	0.349557	0.201271	1.736752	0.082430931	0.11168	NOT
PREX1	1177.714	0.249065	0.143417	1.736649	0.082449082	0.1116991	NOT
HR	66.71853	0.484331	0.278955	1.736233	0.082522717	0.1117934	NOT
TUSC5	1.448436	-0.64747	0.373045	-1.73563	0.082628599	0.1119239	NOT
RP11-478(	5.440495	0.391382	0.225499	1.735628	0.082629647	0.1119239	NOT
RHAG	1.176965	-0.88947	0.512482	-1.73562	0.082631212	0.1119239	NOT
CD28	92.28664	0.34693	0.199894	1.735568	0.082640181	0.1119275	NOT
HNRNPKP	6.594987	0.258826	0.149131	1.735558	0.082641979	0.1119275	NOT
TRMT112F	4.522032	0.365461	0.210652	1.734906	0.082757422	0.1120784	NOT
ZNF558	448.2098	0.246572	0.142131	1.734817	0.082773163	0.1120942	NOT
RP11-357I	2.237293	0.87295	0.503219	1.73473	0.082788611	0.1121097	NOT
KIN	640.3423	-0.15202	0.087635	-1.73468	0.082797046	0.1121124	NOT
SSR4	7714.396	0.218293	0.125841	1.734673	0.082798741	0.1121124	NOT
RP11-609I	6.383681	-0.44046	0.253942	-1.7345	0.08283004	0.1121493	NOT
CTNS	796.4611	0.140952	0.081266	1.73445	0.082838212	0.1121549	NOT

RP11-514(	7.966236	0.428545	0.247087	1.734387	0.082849521	0.1121647	NOT
CXorf38	727.8639	-0.12534	0.072278	-1.73408	0.08290412	0.1122331	NOT
RHOQP2	2.396194	0.394474	0.227512	1.733862	0.082942592	0.1122797	NOT
RP11-707(	2.404163	0.566627	0.326936	1.733142	0.08307043	0.1124465	NOT
TADA1	1034.185	0.208294	0.120185	1.733122	0.083073906	0.1124465	NOT
CUL5	1268.208	-0.11483	0.06628	-1.73253	0.083178939	0.1125831	NOT
STK11	2311.474	0.151385	0.087402	1.732056	0.083263622	0.1126922	NOT
LY6D	4.175939	1.242606	0.717606	1.7316	0.083344736	0.1127965	NOT
TCAF1	895.1825	-0.1944	0.112298	-1.73109	0.083436063	0.1129146	NOT
SNAP29	2304.608	-0.12388	0.071581	-1.7306	0.083523942	0.113028	NOT
RPA4	2.713176	0.455022	0.262993	1.730165	0.083600772	0.1131174	NOT
ART5	17.90618	-0.67686	0.391209	-1.73016	0.083601236	0.1131174	NOT
MSX2	8.583879	0.666744	0.385366	1.730157	0.083602262	0.1131174	NOT
DIP2C	1295.166	-0.25038	0.144744	-1.72982	0.08366178	0.1131924	NOT
ALG10B	276.8102	-0.2042	0.118053	-1.72969	0.083685765	0.1132193	NOT
CMTM8	1146.125	-0.21623	0.125027	-1.72949	0.083720867	0.1132559	NOT
DUS3L	978.5557	0.16899	0.097711	1.729492	0.083721037	0.1132559	NOT
RP11-294(	2.117581	-0.79983	0.462647	-1.72881	0.08384271	0.113412	NOT
RP1-317E2	37.79903	-0.25285	0.14626	-1.7288	0.083844654	0.113412	NOT
TMX1	1438.623	-0.13267	0.076745	-1.7287	0.083863235	0.1134316	NOT
CDV3	5968.192	-0.11906	0.068874	-1.72867	0.083868355	0.113433	NOT
TVP23A	27.37102	0.279947	0.161966	1.728437	0.083909857	0.1134836	NOT
AC002467	62.03984	0.245159	0.141872	1.728038	0.083981448	0.1135719	NOT
RPL9	14600.09	0.241584	0.139804	1.728027	0.083983372	0.1135719	NOT
RNU6-339	2.040685	-0.58494	0.338791	-1.72655	0.084248156	0.1139244	NOT
MAFB	1635.251	-0.30384	0.175988	-1.72649	0.084259005	0.1139335	NOT
PRSS23	1493.066	0.313112	0.18137	1.726371	0.084280682	0.1139573	NOT
RP11-25K1	39.89143	0.58098	0.336588	1.726087	0.084331744	0.1140207	NOT
RP11-326(	13.18655	0.467893	0.271093	1.72595	0.084356323	0.1140484	NOT
RP11-88H	40.66459	-0.55153	0.319568	-1.72587	0.084370464	0.1140619	NOT
IL18BP	508.7406	0.277294	0.16071	1.725431	0.084449901	0.1141583	NOT
DSG3	5.581948	1.257626	0.728877	1.72543	0.084449991	0.1141583	NOT
RP11-334I	12.11176	0.259143	0.150202	1.725296	0.084474192	0.1141854	NOT
HARS	2071.344	0.135237	0.07841	1.724737	0.084574924	0.114316	NOT
GABRA5	8.065037	-1.50419	0.872247	-1.7245	0.084617127	0.1143675	NOT
GNB2L1	32351.84	0.187136	0.10853	1.724272	0.084658816	0.1144182	NOT
SWSAP1	196.965	-0.18654	0.108184	-1.72425	0.084662996	0.1144183	NOT
RPL31P63	3.654303	0.411835	0.238865	1.724129	0.084684482	0.1144417	NOT
PDLIM1	6670.202	-0.19094	0.110767	-1.72384	0.084737491	0.1145078	NOT
SLC13A2	168.7368	0.613747	0.356072	1.723659	0.084769451	0.1145454	NOT
AC021016	3.234968	0.368734	0.213966	1.723328	0.084829317	0.1146207	NOT
PDIA3	24452.78	0.146848	0.085213	1.7233	0.084834271	0.1146218	NOT
PGM5P2	4.976491	0.379988	0.220504	1.723275	0.084838847	0.1146223	NOT
IGKV1D-1	3.137837	-0.9426	0.547087	-1.72295	0.084897567	0.1146961	NOT
GHDC	1676.165	0.170334	0.098876	1.722706	0.084941767	0.1147502	NOT
RP11-116(	2.022478	0.497014	0.288573	1.722318	0.085011931	0.1148394	NOT
MRPL51	3682.977	0.181831	0.105606	1.721777	0.085110002	0.1149662	NOT
MYO10	935.2015	-0.39972	0.232161	-1.72173	0.085118582	0.1149722	NOT
RP11-642I	20.62904	0.24791	0.144002	1.721568	0.085147883	0.1150062	NOT
RP11-888I	1.840282	0.733842	0.426292	1.721452	0.085168792	0.1150288	NOT
ZNF235	90.17944	0.170974	0.099355	1.720852	0.08527762	0.1151702	NOT
CHPF	4955.775	-0.2379	0.138267	-1.72058	0.085327661	0.1152321	NOT

AC005251	8.113329	0.336163	0.195383	1.720534	0.085335485	0.1152371	NOT
TRIB3	4030.894	0.326126	0.189611	1.719976	0.085436798	0.1153682	NOT
NYNRIN	793.6322	-0.36709	0.213438	-1.71988	0.085454592	0.1153866	NOT
AC093642	3.934488	0.641295	0.373037	1.71912	0.085592567	0.1155673	NOT
EDN1	162.9444	-0.39552	0.230091	-1.71898	0.085617899	0.1155959	NOT
LILRB4	306.2285	-0.36218	0.210765	-1.71839	0.085725391	0.1157353	NOT
RP11-448(	1.759817	0.458652	0.266991	1.717853	0.085823433	0.1158621	NOT
GMIP	433.3244	0.244397	0.142272	1.717816	0.085830201	0.1158626	NOT
FAM172BF	1.231168	0.876826	0.510434	1.717805	0.085832178	0.1158626	NOT
AP000640.	2.71277	0.40504	0.23582	1.717581	0.085873063	0.1159121	NOT
RP11-744I	1.124006	0.958911	0.558306	1.717537	0.085881153	0.1159174	NOT
PIGL	270.9982	0.1788	0.104109	1.717437	0.085899406	0.1159363	NOT
SLC25A24	312.4498	0.383261	0.223211	1.717037	0.085972347	0.1160291	NOT
AC010091	7.430346	0.837797	0.48803	1.716692	0.086035442	0.1161086	NOT
ZNF622	1479.149	0.15232	0.088747	1.716334	0.086100879	0.1161913	NOT
FLRT2	246.3552	-0.48423	0.282162	-1.71613	0.086138093	0.1162358	NOT
PTPN20A	24.16269	0.683225	0.398163	1.715944	0.086172324	0.1162763	NOT
OXCT2	20.18504	0.486851	0.283742	1.715822	0.086194543	0.1163006	NOT
GRIK1-AS1	1.667655	0.539003	0.31415	1.71575	0.08620788	0.116313	NOT
KIAA0125	17.02585	-0.5939	0.346177	-1.7156	0.086235832	0.116345	NOT
KLK14	4.877535	0.526441	0.306885	1.715433	0.086265852	0.1163798	NOT
RP11-49K2	3.948339	0.393025	0.229126	1.715326	0.086285557	0.1164007	NOT
MBD3	3516.703	0.193713	0.112996	1.714329	0.086468314	0.1166373	NOT
RN7SL752I	1.840528	-0.48458	0.282663	-1.71432	0.08646932	0.1166373	NOT
ATP5G2P4	1.534018	0.542902	0.316713	1.714177	0.086496162	0.1166678	NOT
CTD-3098	253.084	-0.7246	0.422785	-1.71388	0.086550468	0.1167311	NOT
ATP8A2P1	1.016579	2.064749	1.204725	1.713876	0.086551561	0.1167311	NOT
MTRNR2L4	2.058769	0.442385	0.25813	1.713809	0.086563738	0.1167418	NOT
PLEKHO2	1012.385	-0.23058	0.134576	-1.71341	0.0866372	0.1168352	NOT
RP11-390I	8.230707	0.412276	0.240626	1.713348	0.086648608	0.1168449	NOT
COX7A2	4905.527	0.226832	0.132401	1.713212	0.086673507	0.1168728	NOT
KTI12	310.2018	0.138113	0.080619	1.713155	0.086684024	0.1168813	NOT
NBPF25P	41.41274	-0.1871	0.10926	-1.71241	0.086820816	0.11706	NOT
ANO7	120.5396	0.286651	0.167427	1.712094	0.086879341	0.1171332	NOT
ZNF571	85.18339	0.187521	0.109533	1.71201	0.086894841	0.1171484	NOT
CTD-3162	7.941842	-0.42163	0.246295	-1.71188	0.086918827	0.117175	NOT
SRSF10	2313.462	-0.08391	0.049018	-1.71186	0.086923187	0.1171752	NOT
ARMC7	707.2947	0.182065	0.106373	1.711565	0.086976806	0.1172418	NOT
RP11-307I	1.712856	-0.61406	0.358805	-1.71142	0.087004177	0.1172715	NOT
GLRXP3	2.314959	-0.72315	0.422549	-1.7114	0.087007325	0.1172715	NOT
AC005682	22.97846	0.426423	0.249201	1.711161	0.087051476	0.1173253	NOT
METTL16	672.1872	0.150205	0.087786	1.711026	0.087076354	0.1173531	NOT
PHF11	703.926	-0.14858	0.086842	-1.71097	0.087086768	0.1173614	NOT
CCDC115	901.5843	-0.16156	0.094436	-1.71083	0.087111806	0.1173894	NOT
RP11-35N	154.1362	-0.44125	0.258019	-1.71015	0.087238227	0.1175541	NOT
PRKY	78.80764	0.714944	0.418138	1.709826	0.08729797	0.1176288	NOT
TMEM106	2825.382	-0.16179	0.094633	-1.70969	0.087322453	0.1176561	NOT
RP11-349I	1.411023	-0.57804	0.338101	-1.70966	0.08732935	0.1176597	NOT
NDUFA12	1765.079	0.154555	0.090409	1.709503	0.087357762	0.1176922	NOT
AMICA1	537.6948	0.329311	0.192693	1.708993	0.087452173	0.1178137	NOT
WNK1	5015.421	0.153777	0.089986	1.708898	0.087469884	0.1178318	NOT
TLE1	3586.449	0.169223	0.099055	1.708369	0.087567897	0.1179553	NOT

AC006978	208.79	0.150061	0.087839	1.708357	0.087570061	0.1179553	NOT
TLE3	1365.427	-0.25325	0.148288	-1.70779	0.087675429	0.1180914	NOT
CTC-559E	94.58169	0.220802	0.129299	1.707685	0.087694804	0.1181118	NOT
RP11-80I1	3.60903	-0.3756	0.219992	-1.70736	0.087756	0.1181884	NOT
PLAC1	2.618915	0.810717	0.474891	1.707166	0.087791229	0.1182301	NOT
PGAP2	1380.858	0.144242	0.084502	1.706965	0.087828567	0.1182747	NOT
RP13-977.	16.61258	0.418869	0.245425	1.706709	0.08787621	0.1183331	NOT
FGF10	2.595922	-0.73696	0.431869	-1.70645	0.087924948	0.1183929	NOT
PSMB8-AS	341.3451	0.242731	0.142249	1.706386	0.087936258	0.1184024	NOT
TRIM33	1323.185	0.140531	0.082375	1.70599	0.088009849	0.1184957	NOT
AC090616	19.02086	-0.31634	0.185477	-1.70556	0.088090737	0.1185989	NOT
MIR222HG	32.91006	0.363358	0.213078	1.705284	0.088141463	0.1186614	NOT
CTB-186H	7.710604	-0.47583	0.279076	-1.70503	0.088188814	0.1187193	NOT
C8orf37	125.8174	0.212441	0.124602	1.70496	0.088201798	0.118731	NOT
CSRNP3	48.16443	-0.42606	0.249906	-1.7049	0.088213543	0.1187411	NOT
RP11-475.	9.080409	-0.43574	0.255618	-1.70463	0.088263112	0.118802	NOT
LINC01588	128.4402	-0.31578	0.185274	-1.70441	0.088303653	0.1188508	NOT
SENP5	1089.648	0.100609	0.05905	1.703796	0.088419156	0.1190005	NOT
EDEM3	2805.838	0.202065	0.118615	1.703531	0.088468812	0.1190615	NOT
EIF4EBP3	370.2589	-0.27511	0.161499	-1.70348	0.088479187	0.1190697	NOT
HHLA2	7.544587	-0.81092	0.476163	-1.70303	0.088562782	0.1191764	NOT
RP11-139I	3.706826	-0.32839	0.192837	-1.70292	0.088583761	0.1191988	NOT
AF129075.	9.309399	0.279043	0.163887	1.702658	0.088632121	0.1192581	NOT
OR10J6P	80.27961	-0.53718	0.315575	-1.70222	0.088715023	0.1193638	NOT
PTPN11	4383.883	-0.15828	0.093005	-1.70188	0.088777166	0.1194416	NOT
MSH3	726.9518	-0.16304	0.095815	-1.70163	0.088825375	0.1195007	NOT
RP11-111.	6.212187	-0.30049	0.176646	-1.70106	0.088931756	0.119638	NOT
TOMM20P	2.885872	-0.49953	0.293743	-1.70056	0.089025337	0.1197581	NOT
GFER	930.621	0.18817	0.11067	1.700272	0.089079798	0.1198255	NOT
SNORD3A	1.326774	0.675173	0.397311	1.699355	0.089252343	0.1200477	NOT
APPBP2	1122.657	-0.13134	0.07729	-1.69935	0.089253681	0.1200477	NOT
EIF2S2P2	1.544072	0.599556	0.352903	1.698925	0.089333338	0.120149	NOT
ZNF750	3.988192	0.46596	0.274311	1.698658	0.089383715	0.1202109	NOT
TAS2R62P	1.005964	-1.15199	0.678228	-1.69853	0.089406864	0.1202321	NOT
ETNK1	2124.184	-0.14914	0.087803	-1.69853	0.089408145	0.1202321	NOT
BIRC6	2635.614	-0.15337	0.090299	-1.69845	0.08942347	0.1202469	NOT
IMPA1	1319.943	0.184181	0.108474	1.697923	0.089522283	0.1203739	NOT
IGKV1D-1	7.184575	-1.03168	0.60766	-1.69779	0.089547657	0.1203967	NOT
PCDH1	2003.788	-0.23629	0.139175	-1.69779	0.089547937	0.1203967	NOT
FAM3A	2859.028	0.189386	0.111565	1.697545	0.089593752	0.1204524	NOT
MRPL42	1629.989	0.101858	0.060009	1.697367	0.089627331	0.1204917	NOT
MADD	1548.112	0.11042	0.065102	1.696111	0.089864884	0.1208052	NOT
RP4-575N	13.96685	-0.33552	0.19782	-1.69607	0.089871879	0.1208087	NOT
AC023137	2.529383	-0.50789	0.299476	-1.69592	0.089901183	0.1208422	NOT
RP11-791(	16.26868	0.384165	0.226546	1.69575	0.089933218	0.1208794	NOT
DNM1	319.3516	0.423022	0.24952	1.695342	0.09001063	0.1209776	NOT
NDUFAF4F	3.046771	0.433948	0.256045	1.69481	0.090111448	0.1211072	NOT
RPS3AP6	30.26369	0.270757	0.159784	1.694516	0.090167183	0.1211745	NOT
ATF2	1673.283	0.129064	0.076166	1.6945	0.09017025	0.1211745	NOT
TMEM223	906.8544	0.170319	0.100516	1.694434	0.090182928	0.1211856	NOT
EMX2OS	2.037632	0.609212	0.359565	1.694304	0.090207468	0.1212127	NOT
GPHA2	2.280507	0.830815	0.490417	1.6941	0.090246256	0.121259	NOT

TSHZ2	923.2491	-0.30707	0.181277	-1.69395	0.090275434	0.1212923	NOT
CIRBP	6360.502	0.178983	0.105676	1.693697	0.090322826	0.1213501	NOT
ZNF410	45.05284	-0.17587	0.10385	-1.69349	0.090361383	0.121396	NOT
CDK2AP2	3435.48	0.188816	0.111534	1.692903	0.090474025	0.1215414	NOT
C2CD2L	557.9732	-0.13676	0.08082	-1.69216	0.090615927	0.1217261	NOT
CYP21A2	341.4715	0.405899	0.239876	1.692124	0.090622254	0.1217287	NOT
DDB2	1332.3	-0.20583	0.121652	-1.69193	0.090659577	0.1217729	NOT
IGLL4P	2.290736	1.167855	0.690487	1.691351	0.090769742	0.121915	NOT
RP3-340B1	5.618183	0.373676	0.220948	1.691237	0.090791526	0.1219383	NOT
PLD5	12.15506	0.591618	0.349821	1.691203	0.090798039	0.1219411	NOT
LINC01504	14.41814	0.385155	0.227759	1.691065	0.090824367	0.1219706	NOT
SLC25A36I	1.710291	0.555427	0.328462	1.690994	0.090837881	0.1219828	NOT
DYNC1I2P	8.468695	0.302161	0.178801	1.689932	0.091041022	0.1222497	NOT
NUP210L	3.294575	0.507485	0.300335	1.689729	0.091079842	0.1222958	NOT
C6orf141	76.74629	0.567607	0.335935	1.689634	0.091097975	0.122314	NOT
KY	1.69611	0.689456	0.408056	1.689612	0.091102223	0.122314	NOT
CTD-2528	53.28233	0.213929	0.126621	1.689522	0.091119374	0.1223311	NOT
PAX2	8.070367	0.870612	0.515349	1.689363	0.091149827	0.1223661	NOT
RP11-488I	0.731586	1.939855	1.148619	1.688859	0.091246367	0.1224897	NOT
TRI-TAT2-	4.088168	0.446934	0.264743	1.68818	0.091376693	0.1226587	NOT
TIRAP	325.0494	0.151572	0.08979	1.688072	0.091397375	0.1226805	NOT
UBE4B	1712.671	0.157874	0.093527	1.688013	0.091408643	0.1226897	NOT
RP11-260I	2.095833	0.787683	0.466664	1.687901	0.091430275	0.1227086	NOT
GCSAM	22.92774	-0.40092	0.237526	-1.68789	0.09143159	0.1227086	NOT
LINC00881	5.310778	0.491362	0.291127	1.687792	0.091451178	0.1227289	NOT
SHC4	17.35305	0.471534	0.279425	1.687512	0.091504935	0.1227951	NOT
RP11-404(	138.169	-0.5618	0.333025	-1.68696	0.091610423	0.1229307	NOT
NDUFB8P2	3.777695	-0.35206	0.208715	-1.68679	0.091643612	0.1229693	NOT
FAM91A3I	1.611711	-0.44243	0.26236	-1.68636	0.091726969	0.1230752	NOT
BTG2	1804.594	-0.28736	0.170413	-1.68629	0.09174082	0.1230878	NOT
NDUFS3	3528.1	-0.16696	0.099072	-1.68524	0.091942498	0.1233524	NOT
HNRNPH2	3298.111	-0.11572	0.068671	-1.68514	0.091962019	0.1233726	NOT
ZNF570	169.5725	0.216312	0.128374	1.68501	0.091986593	0.1233996	NOT
PSMC1P1	18.08127	0.236347	0.140291	1.684689	0.092048685	0.1234769	NOT
RP11-20G1	1.270043	0.76698	0.455301	1.684555	0.092074504	0.1235055	NOT
GPAA1P2	2.707229	0.571878	0.339673	1.683613	0.092256429	0.1237434	NOT
CTD-2240	9.967402	0.420014	0.249475	1.683591	0.092260808	0.1237434	NOT
DNAJA1P3	2.191496	0.466156	0.276901	1.683473	0.092283585	0.123768	NOT
COL6A5	1.338711	0.944682	0.561189	1.683359	0.092305571	0.1237915	NOT
DDX5	14831.74	-0.12533	0.07447	-1.68298	0.092378537	0.1238833	NOT
CTD-2636	70.28727	-0.3667	0.217912	-1.68278	0.092417952	0.1239302	NOT
RP11-596I	2.609518	0.473628	0.281486	1.682599	0.092452827	0.1239709	NOT
DUSP26	9.947261	0.457117	0.271698	1.682442	0.092483201	0.1240056	NOT
MOB4	717.6129	-0.11564	0.068734	-1.68242	0.092487606	0.1240056	NOT
RP11-286I	3.284015	0.37335	0.221924	1.682334	0.092504158	0.1240217	NOT
ACSBG1	6.079553	0.4074	0.242182	1.682203	0.092529463	0.1240458	NOT
ELOVL5	5071.504	0.200159	0.118987	1.682195	0.092531045	0.1240458	NOT
INSL3	4.346519	0.49545	0.294586	1.68185	0.092598004	0.1241295	NOT
TMEM231	105.3133	-0.31605	0.188013	-1.68099	0.092763904	0.1243459	NOT
VWA2	6.93201	-0.63638	0.378601	-1.68086	0.092789845	0.1243746	NOT
RP1-102K2	10.42861	0.60112	0.357673	1.68064	0.092832937	0.1244247	NOT
MYL6	23686.78	0.166228	0.098909	1.680623	0.092836199	0.1244247	NOT

RP11-386I	0.592562	1.049364	0.624411	1.680565	0.092847521	0.1244338	NOT
TMEM43	1693.842	0.154322	0.09185	1.680158	0.092926544	0.1245337	NOT
RP11-220I	2.089781	0.556444	0.331206	1.680052	0.092947216	0.1245554	NOT
KIF6	44.37687	0.507409	0.302154	1.679307	0.093092228	0.1247437	NOT
PDE10A	82.25011	0.348584	0.207604	1.679082	0.093136103	0.1247964	NOT
RNU6-722	5.557804	-0.27629	0.164552	-1.67902	0.093148372	0.1248068	NOT
TMPPE	40.17566	0.221878	0.132192	1.678449	0.093259448	0.1249496	NOT
MRS2	1782.691	0.179146	0.106757	1.678079	0.0933317	0.1250403	NOT
IVNS1ABP	3342.28	0.17929	0.106878	1.677528	0.093439242	0.1251784	NOT
IL2RG	783.7303	0.385624	0.229913	1.677263	0.093491091	0.1252418	NOT
SCNN1A	523.1497	0.476335	0.284007	1.677195	0.093504448	0.1252536	NOT
NBPF10	58.34263	0.245832	0.146607	1.676811	0.093579366	0.1253479	NOT
EIF3J	2336.265	-0.10915	0.06511	-1.6764	0.093659659	0.1254494	NOT
UBAC2-AS	50.87698	0.256416	0.152962	1.676341	0.093671403	0.125459	NOT
LDHAL6B	2.181648	-0.44406	0.264907	-1.6763	0.093679692	0.125464	NOT
RP11-405I	2.291659	-0.4744	0.283009	-1.67627	0.093685457	0.1254657	NOT
IGHV3-47	2.400561	-0.6577	0.392422	-1.67601	0.093737128	0.1255288	NOT
CHCHD2P	17.97574	0.248881	0.148501	1.675958	0.093746507	0.1255353	NOT
RP11-57C	4.948531	0.408496	0.243825	1.675365	0.093862705	0.1256797	NOT
IGHV7-40	0.622273	1.448862	0.864814	1.675344	0.093866742	0.1256797	NOT
RP11-632I	1.490694	0.721687	0.430771	1.675338	0.093868014	0.1256797	NOT
PRTN3	3.738862	-0.67607	0.403657	-1.67487	0.093960033	0.1257969	NOT
ANKH	4154.71	-0.18733	0.111918	-1.67378	0.094174559	0.126078	NOT
CD3E	408.9201	-0.38818	0.231926	-1.67372	0.094185164	0.1260861	NOT
SLFN12	79.7007	0.353023	0.210973	1.673311	0.09426611	0.1261883	NOT
APBA1	883.4334	-0.37954	0.226828	-1.67327	0.094273351	0.1261919	NOT
IGLV1-44	243.8293	-0.67342	0.402505	-1.67308	0.094310635	0.1262357	NOT
TDRP	538.8967	-0.30169	0.180332	-1.67295	0.094337644	0.1262658	NOT
PRH1	10.06824	-0.31362	0.187562	-1.67211	0.094503364	0.1264815	NOT
RN7SL4P	5.732094	-0.37073	0.221738	-1.67194	0.094535564	0.1265184	NOT
NPM1P46	1.994503	0.450866	0.269732	1.671532	0.094616595	0.1266207	NOT
CTD-2639	2.910102	0.45819	0.274155	1.671279	0.094666633	0.1266816	NOT
CHST13	2093.172	0.316759	0.189572	1.670917	0.094738002	0.1267701	NOT
CCR6	6.20986	0.578439	0.346185	1.670898	0.094741924	0.1267701	NOT
TNFSF13B	205.1592	-0.32832	0.196524	-1.67065	0.094791106	0.1268297	NOT
FAR1	346.7094	0.306346	0.183391	1.67045	0.094830445	0.1268762	NOT
SCAP	5547.372	0.142918	0.085563	1.670327	0.094854607	0.1269024	NOT
POGLUT1	489.0997	0.157455	0.094272	1.67022	0.09487575	0.1269246	NOT
FOXO4	701.3882	0.183478	0.10986	1.670101	0.094899285	0.1269499	NOT
HHATL	4.14941	0.627792	0.375929	1.669973	0.094924793	0.1269779	NOT
NECAP2	1856.5	-0.1189	0.071199	-1.66993	0.094932351	0.1269819	NOT
CTD-2235	15.92684	0.388355	0.232611	1.669548	0.095008881	0.1270781	NOT
LINC01554	2398.415	-0.71041	0.425539	-1.66943	0.095032645	0.1271038	NOT
LINC00167	2.700772	0.447419	0.268024	1.669326	0.095052702	0.1271244	NOT
SCYL2	1739.09	-0.14116	0.084573	-1.66905	0.095107631	0.1271917	NOT
SYNC	15.69538	0.443562	0.26577	1.668968	0.095123681	0.1272071	NOT
UBE2D3P2	3.715717	-0.33722	0.202091	-1.66863	0.095190451	0.1272902	NOT
SPATA6	119.1356	-0.29029	0.174041	-1.66791	0.095332792	0.1274744	NOT
RP11-390(	3.002223	0.409931	0.245795	1.66778	0.095359341	0.1275037	NOT
PEX10	573.2222	0.173642	0.104128	1.667582	0.095398764	0.1275503	NOT
HIST1H4J	9.961698	0.437622	0.262444	1.667486	0.095417848	0.1275696	NOT
ADAMTS5	221.9584	0.287474	0.172412	1.667364	0.095442062	0.1275958	NOT

RP11-118(	2.196004	0.485139	0.291	1.667146	0.095485429	0.1276476	NOT
SFTPA2	2.883298	0.868698	0.521147	1.666897	0.095534815	0.1277075	NOT
TBX3	2492.9	0.428054	0.256801	1.666868	0.095540614	0.1277091	NOT
LINC0099{	833.1779	-0.19506	0.117047	-1.66653	0.09560851	0.1277936	NOT
RP11-789(	1.476676	0.61198	0.367366	1.665862	0.095740951	0.1279582	NOT
STARD13-	7.08965	-0.32115	0.192783	-1.66585	0.095742762	0.1279582	NOT
STK38L	686.9034	-0.19491	0.117006	-1.66584	0.095745463	0.1279582	NOT
RP11-3L21	1.551054	0.90074	0.540845	1.66543	0.095826929	0.1280608	NOT
NET1	2456.449	-0.16647	0.09997	-1.6652	0.095871907	0.1281148	NOT
ACTG1P3	7.213415	-0.29483	0.177056	-1.66515	0.095882447	0.128122	NOT
RAI14	2043.318	0.18304	0.109925	1.665131	0.095886591	0.128122	NOT
SPHK2	965.0284	-0.21743	0.130594	-1.66497	0.095919566	0.1281599	NOT
SEPT10P1	2.678227	-0.3758	0.225717	-1.66491	0.09593055	0.1281684	NOT
ZNF747	703.945	-0.16012	0.096186	-1.6647	0.095972398	0.1282181	NOT
GGT3P	2.557604	-0.57778	0.347096	-1.66461	0.095991236	0.1282371	NOT
LRRN1	31.80593	-0.64033	0.384816	-1.66399	0.096114035	0.1283949	NOT
LINC0066;	1128.145	-0.19254	0.115732	-1.66368	0.096175777	0.1284712	NOT
RORC	5794.89	-0.35986	0.21631	-1.66361	0.096189911	0.1284839	NOT
RP11-13K:	1.419443	0.725758	0.436276	1.66353	0.096206414	0.1284997	NOT
LRRC29	114.0906	-0.21747	0.130772	-1.66296	0.096320648	0.1286461	NOT
GSTA6P	2.066484	0.498226	0.299617	1.662877	0.096337098	0.1286618	NOT
CTD-2382	4.122938	-0.49338	0.29675	-1.6626	0.096393099	0.1287304	NOT
C5orf56	180.8226	0.202355	0.121716	1.662521	0.096408442	0.1287447	NOT
B3GALTL	347.8578	0.149498	0.089924	1.662491	0.096414291	0.1287463	NOT
CLTB	2873.842	0.197189	0.118634	1.662161	0.096480466	0.1288284	NOT
VTCN1	101.106	-0.63018	0.379162	-1.66204	0.096504508	0.1288543	NOT
MYO9A	391.8592	-0.2332	0.140321	-1.66193	0.096525864	0.1288766	NOT
OGN	107.1941	0.567612	0.341654	1.661364	0.096640392	0.1290233	NOT
RP11-736I	46.28787	-0.29057	0.174917	-1.66119	0.096675338	0.1290637	NOT
RP11-339I	2.588852	0.545674	0.328491	1.661154	0.096682567	0.1290671	NOT
CPM	2496.713	-0.23184	0.139574	-1.66109	0.096695728	0.1290785	NOT
CRLS1	5065.734	-0.20479	0.123348	-1.66026	0.096862847	0.1292953	NOT
KLHL9	1026.148	-0.16026	0.096538	-1.66003	0.096908405	0.1293499	NOT
UXT-AS1	20.00689	-0.20444	0.123168	-1.65981	0.096953486	0.1294038	NOT
PSAP	60954	-0.12758	0.076871	-1.6596	0.096994441	0.1294523	NOT
LL21NC02	6.954186	0.336062	0.202525	1.65936	0.097043284	0.1295112	NOT
AKAP1	4535.797	-0.14892	0.089748	-1.65926	0.09706268	0.1295308	NOT
RP11-109I	7.433168	-0.47493	0.286263	-1.65907	0.097100817	0.1295755	NOT
RP11-685I	3.855491	0.485797	0.292839	1.658923	0.097131373	0.12961	NOT
SRSF7	2786.458	-0.10297	0.062078	-1.65873	0.097169484	0.1296546	NOT
UTP11L	895.8297	0.122962	0.074138	1.658556	0.097205238	0.1296961	NOT
RP4-635E:	7.878465	0.295097	0.177947	1.658336	0.097249697	0.1297491	NOT
PIKFYVE	1008.692	-0.15156	0.091402	-1.65816	0.097284494	0.1297893	NOT
CDK14	1169.387	0.218801	0.131998	1.657606	0.097397064	0.1299332	NOT
RGS1	978.995	-0.42082	0.253929	-1.65722	0.097475237	0.1300312	NOT
RP11-303I	4.478329	0.356162	0.21492	1.657186	0.097481814	0.1300337	NOT
GSTM3	965.1142	0.400761	0.241852	1.657049	0.097509661	0.1300646	NOT
SPRR2A	1.854106	1.368692	0.826186	1.656638	0.097592679	0.1301691	NOT
CLDN6	5.579593	0.558652	0.337319	1.656152	0.097691018	0.1302939	NOT
DOLPP1	1149.607	-0.15433	0.093189	-1.6561	0.097702343	0.1303028	NOT
PRSS8	1144.916	-0.64492	0.389491	-1.6558	0.097763048	0.1303774	NOT
PRKCQ	53.20169	-0.39827	0.240649	-1.65499	0.097926568	0.1305864	NOT

GPN3	917.6931	-0.13384	0.08087	-1.65495	0.097933685	0.1305864	NOT
USP41	1.008641	-0.65623	0.396526	-1.65495	0.097933871	0.1305864	NOT
RFXAP	80.01263	0.209191	0.126433	1.654567	0.098012442	0.1306834	NOT
CHMP2A	5909.72	0.215959	0.130524	1.654549	0.098016106	0.1306834	NOT
RP11-222I	13.31478	-0.43119	0.260651	-1.65428	0.098070966	0.1307503	NOT
AP5B1	722.7615	0.168862	0.102103	1.653839	0.098160299	0.1308631	NOT
ASTN2	186.1331	0.228276	0.138084	1.653172	0.098295899	0.1310375	NOT
BAALC	176.4427	0.449956	0.272211	1.652968	0.098337403	0.1310865	NOT
NGRN	1027.15	0.134043	0.081122	1.652348	0.098463629	0.1312485	NOT
NUS1P1	29.39174	-0.27349	0.165523	-1.65228	0.098478396	0.1312618	NOT
KCTD11	343.3951	-0.16539	0.100107	-1.65212	0.09851041	0.1312959	NOT
SLC18A1	1.944339	-0.57781	0.349741	-1.6521	0.098513432	0.1312959	NOT
PPP1R26P1	1.926896	0.612364	0.370743	1.651723	0.098591094	0.1313901	NOT
SERPINB5	5.670299	0.940286	0.56928	1.65171	0.098593618	0.1313901	NOT
TNRC6A	1845.325	-0.15608	0.094546	-1.65088	0.098763562	0.1316102	NOT
UBE2G2	2753.473	-0.10311	0.062462	-1.65084	0.098770703	0.1316134	NOT
RP11-380I	1.278965	0.782267	0.473919	1.650634	0.098813359	0.1316639	NOT
MARVELD1	422.0811	0.318431	0.192953	1.650302	0.098881199	0.1317479	NOT
RORA-AS1	4.544761	0.342803	0.207736	1.650186	0.098904857	0.1317731	NOT
RICTOR	1014.291	-0.14101	0.085456	-1.6501	0.098921611	0.1317891	NOT
RP11-102(	82.72206	0.142902	0.086608	1.649981	0.098946855	0.1318164	NOT
FDXACB1	84.05581	0.16724	0.101363	1.649911	0.098961181	0.1318291	NOT
FAM86B1	18.19131	-0.34885	0.211488	-1.64949	0.099047381	0.1319376	NOT
DZIP1	172.8152	0.360576	0.218674	1.648916	0.099164864	0.1320877	NOT
AC002539	0.885315	1.176324	0.713412	1.648871	0.099174144	0.1320937	NOT
C1QBP	2919.31	-0.16765	0.101696	-1.64858	0.099233121	0.1321659	NOT
RP11-217I	7.029904	-0.34935	0.211926	-1.64843	0.09926378	0.1322004	NOT
TBC1D2B	1975.12	-0.2293	0.139109	-1.64834	0.099283453	0.1322202	NOT
GTPBP6	2294.485	0.148602	0.090164	1.648126	0.099326744	0.132265	NOT
FBXW5	7136.564	-0.16359	0.099257	-1.64811	0.099329378	0.132265	NOT
ZNF487	101.8145	-0.15738	0.095495	-1.6481	0.099331374	0.132265	NOT
RP11-168I	46.40509	-0.55933	0.339428	-1.64787	0.09938024	0.1323236	NOT
CYP4F60P	9.366508	-0.86039	0.522229	-1.64754	0.099447123	0.1324063	NOT
PPP2R1B	4826.776	-0.237	0.143891	-1.64706	0.099544712	0.1325299	NOT
ZBTB1	656.8381	-0.15934	0.096751	-1.64687	0.099584172	0.132576	NOT
TRIT1	466.8937	0.125011	0.075912	1.646804	0.099598421	0.1325886	NOT
UBE3C	2993.434	0.144482	0.087754	1.646446	0.099671908	0.1326801	NOT
AF131215.	18.57858	0.502047	0.304934	1.646409	0.099679504	0.1326838	NOT
RP1	1.401031	-0.67994	0.412993	-1.64636	0.099689586	0.1326851	NOT
C12orf57	2452.903	0.226949	0.137849	1.646358	0.099690107	0.1326851	NOT
ZC3H18	1304.927	0.099578	0.060492	1.646135	0.099735992	0.1327398	NOT
CADM1	3965.843	0.254938	0.154889	1.645941	0.09977584	0.1327865	NOT
OGFOD1P1	1.914821	0.491519	0.298762	1.645185	0.099931669	0.1329875	NOT
KLHDC10	2693.702	0.174166	0.105912	1.644437	0.100085973	0.1331864	NOT
PLEKHG3	1712.16	-0.16068	0.097711	-1.64439	0.100095607	0.1331928	NOT
RP11-342I	3.851207	0.405397	0.246576	1.644107	0.100154172	0.1332643	NOT
RP11-96O	2.374609	0.424369	0.258127	1.644032	0.100169678	0.1332722	NOT
BRCC3P1	2.172138	0.387039	0.235421	1.644031	0.100169704	0.1332722	NOT
SUGT1P3	12.70585	-0.23379	0.142216	-1.64394	0.100188396	0.1332906	NOT
MBD2	1908.095	-0.10658	0.064874	-1.64293	0.100397617	0.1335626	NOT
NMNAT3	199.1584	0.236831	0.144154	1.642904	0.100402726	0.1335629	NOT
FBXL14	271.8528	-0.19239	0.117109	-1.64283	0.100418892	0.133578	NOT

CCDC147-	2.56858	-0.42758	0.260273	-1.6428	0.100425038	0.1335798	NOT
UNC13D	420.6134	-0.29912	0.18209	-1.64272	0.100441066	0.1335947	NOT
SMG1P5	12.95635	0.254832	0.155135	1.642643	0.100456866	0.1336093	NOT
Z83844.1	3.734542	-0.42742	0.260211	-1.6426	0.100466281	0.1336116	NOT
LINC0143!	0.831491	-0.99041	0.602958	-1.64259	0.100468259	0.1336116	NOT
DAP3P2	2.039673	0.36491	0.222162	1.642538	0.100478655	0.133619	NOT
VAMP5	2275.311	0.237129	0.144406	1.642101	0.100569053	0.1337328	NOT
RP11-273I	131.1558	-0.24941	0.151904	-1.6419	0.100610749	0.1337818	NOT
ATP2C2	28.53118	0.647247	0.394274	1.641617	0.100669426	0.1338534	NOT
AJAP1	24.78223	-0.57527	0.350471	-1.64142	0.100711283	0.1339026	NOT
RP11-863I	2.973583	0.56199	0.342386	1.641391	0.100716237	0.1339027	NOT
RNU6-113	1.584339	0.483336	0.29449	1.641262	0.100743034	0.1339319	NOT
PET100	1199.489	0.256097	0.156056	1.641062	0.100784631	0.1339808	NOT
HIRA	755.9296	0.129566	0.078973	1.640643	0.100871452	0.1340898	NOT
RP11-465I	13.32064	0.630397	0.384381	1.64003	0.100998866	0.1342527	NOT
RP11-324I	0.766519	2.073879	1.264574	1.639983	0.101008758	0.1342594	NOT
RP11-216I	3.921362	0.42503	0.259178	1.639915	0.101022818	0.1342716	NOT
ZNF717	194.5473	-0.23788	0.145069	-1.63978	0.101050895	0.1342963	NOT
GEMIN8	431.4659	0.12505	0.076261	1.639755	0.101056146	0.1342963	NOT
ZNF549	101.3347	0.332083	0.202521	1.639741	0.101058977	0.1342963	NOT
COA4	2740.031	0.150423	0.091736	1.639732	0.101060809	0.1342963	NOT
KCNB1	574.0118	0.55158	0.336401	1.639652	0.101077576	0.1343122	NOT
RALYL	9.550239	-0.7024	0.428431	-1.63948	0.101114267	0.1343545	NOT
APEH	5239.218	-0.1372	0.083703	-1.63913	0.101186686	0.1344442	NOT
ARL8A	2326.789	-0.14595	0.089047	-1.63903	0.101207261	0.1344611	NOT
RP11-96H	0.63601	1.346804	0.821713	1.63902	0.101209107	0.1344611	NOT
IDI1	6625.525	0.250177	0.152644	1.63896	0.101221529	0.1344661	NOT
SPTBN1	21864.14	-0.16586	0.101197	-1.63896	0.10122261	0.1344661	NOT
CASZ1	177.1364	0.273145	0.166726	1.638288	0.101361618	0.1346443	NOT
MYO3A	7.234066	-0.80535	0.491637	-1.6381	0.101399793	0.1346886	NOT
CTD-2210	1.715655	0.432439	0.263996	1.638047	0.101411816	0.1346981	NOT
ALG1L8P	2.006084	0.449164	0.274254	1.637769	0.101469942	0.1347688	NOT
C11orf74	347.9398	0.158062	0.09652	1.637604	0.101504225	0.1348079	NOT
TSPAN6	5100.105	-0.2038	0.124478	-1.63727	0.10157391	0.1348939	NOT
ATG4C	456.4973	-0.11862	0.072455	-1.63712	0.101605386	0.1349293	NOT
RP11-575I	10.35544	0.582441	0.355863	1.6367	0.101693222	0.1350394	NOT
TUNAR	0.941003	1.048653	0.640802	1.636469	0.101741537	0.1350956	NOT
CTD-2306	2.425631	-0.499	0.304928	-1.63645	0.101745277	0.1350956	NOT
CCDC153	28.15035	0.244129	0.149188	1.636382	0.101759708	0.1351083	NOT
PPARGC1B	174.3372	0.250687	0.15331	1.635159	0.102015729	0.1354417	NOT
FAM135A	336.2555	0.205271	0.125542	1.635071	0.102034194	0.1354597	NOT
GAPVD1	1177.747	-0.10251	0.062706	-1.63484	0.102081598	0.1355161	NOT
GNB1	9079.377	0.11744	0.071849	1.634527	0.102148225	0.135594	NOT
PRSS36	86.43604	-0.25934	0.158666	-1.63452	0.102150043	0.135594	NOT
GFOD1	518.7903	-0.20758	0.127012	-1.63436	0.102184054	0.1356326	NOT
CLCF1	209.4302	-0.35318	0.216203	-1.63357	0.102349019	0.1358451	NOT
ADAT3	79.01545	0.251927	0.154232	1.633435	0.10237756	0.1358764	NOT
ADIPOR1P	1.286932	0.707845	0.433408	1.633207	0.102425386	0.1359334	NOT
RP11-472I	2.224987	-0.59089	0.361865	-1.63291	0.102487204	0.1360089	NOT
PCDH7	59.76093	-0.4323	0.264753	-1.63285	0.102501536	0.1360214	NOT
DSCR10	0.673085	1.742456	1.067153	1.632807	0.102509493	0.1360254	NOT
CADM3-A	1.964749	0.783039	0.479625	1.632607	0.102551612	0.1360748	NOT

RNF7	2007.179	0.117682	0.072087	1.632495	0.102575304	0.1360997	NOT
RP11-195(	1.705626	0.549002	0.33632	1.632381	0.102599224	0.1361249	NOT
RPS15AP1	42.33111	-0.26205	0.160544	-1.63226	0.102625505	0.1361494	NOT
AGPS	2059.364	-0.14015	0.085863	-1.63225	0.102627525	0.1361494	NOT
AGAP10	105.1704	0.289911	0.177621	1.63219	0.102639488	0.1361587	NOT
POLD2	5015.758	-0.17202	0.105408	-1.63195	0.102690258	0.1362196	NOT
PHLDA2	301.3623	0.440095	0.269761	1.631427	0.10280029	0.136359	NOT
LINC0148!	2434.374	-0.5249	0.321758	-1.63135	0.102816962	0.1363746	NOT
HMGN2P1	35.27264	0.233244	0.142992	1.631166	0.10285523	0.1364188	NOT
RP11-316I	1.324347	0.54297	0.332963	1.63072	0.102949459	0.1365372	NOT
chr22-38_	194.9382	0.186473	0.114356	1.630645	0.102965162	0.1365515	NOT
ARFGAP3	2388.49	-0.15159	0.092973	-1.63049	0.102996935	0.1365871	NOT
METTL15P	17.58744	0.30071	0.184444	1.630363	0.10302483	0.1366141	NOT
DNTTIP1	865.5812	0.122442	0.075102	1.630352	0.103027152	0.1366141	NOT
MEIS1	222.504	0.184374	0.113103	1.630141	0.103071706	0.1366666	NOT
METTL25	131.408	0.124631	0.076456	1.63009	0.103082474	0.1366721	NOT
MCFD2	7368.317	-0.15487	0.095011	-1.63007	0.103085727	0.1366721	NOT
RP5-956O	2.771308	-0.41838	0.256707	-1.62979	0.103146563	0.1367462	NOT
RPL4P4	107.5751	0.260853	0.16006	1.629726	0.103159409	0.1367567	NOT
HERC1	1727.041	0.197058	0.120929	1.629535	0.103199794	0.1368036	NOT
RP11-115(	5.652086	0.42366	0.260012	1.629389	0.103230801	0.1368382	NOT
SLC9A8	1075.33	0.142851	0.087678	1.629267	0.103256433	0.1368656	NOT
MYO18A	4462.834	-0.20137	0.123606	-1.62912	0.103287487	0.1369002	NOT
AC011242	7.658052	0.505116	0.310091	1.628929	0.103328153	0.1369475	NOT
ZNF595	215.3174	0.189578	0.116385	1.62888	0.103338429	0.1369546	NOT
U4	1.832703	0.400966	0.246166	1.628845	0.103345892	0.1369579	NOT
RP11-95G(	5.139375	-0.36858	0.226296	-1.62876	0.103362962	0.136974	NOT
AC007000	4.143259	0.323599	0.198732	1.628321	0.103456784	0.1370918	NOT
CARD9	106.6315	0.25941	0.159463	1.626776	0.103784577	0.1375195	NOT
GS1-293C	3.398235	0.391623	0.240745	1.626714	0.103797749	0.1375304	NOT
RP4-575N	2.784998	0.490796	0.301794	1.626261	0.103894078	0.1376513	NOT
RP1-206D	21.73901	-0.35681	0.21941	-1.62624	0.10389892	0.1376513	NOT
RP11-887I	3.130399	-0.31691	0.194907	-1.62597	0.103955717	0.1377199	NOT
AC012668	13.83863	-0.46883	0.288387	-1.6257	0.104012833	0.137789	NOT
NLRC3	126.8563	-0.28353	0.174432	-1.62545	0.104066417	0.1378522	NOT
TNKS	1031.404	0.172172	0.105924	1.625432	0.104070495	0.1378522	NOT
TMEM194	241.0931	0.150374	0.092526	1.625215	0.104116656	0.1379067	NOT
SUFU	672.5362	0.11376	0.07	1.625131	0.104134622	0.1379239	NOT
MED18	696.3885	-0.13534	0.083316	-1.62438	0.104294736	0.1381294	NOT
UBE2J2	1511.1	0.140665	0.086604	1.624236	0.104325365	0.1381633	NOT
RP11-365I	5.667662	0.274295	0.168911	1.623905	0.104396141	0.1382504	NOT
NOS1AP	128.2583	0.314587	0.193726	1.623873	0.104402832	0.1382527	NOT
RP11-481(	36.22337	-0.18562	0.114313	-1.62379	0.104420644	0.1382696	NOT
GLRB	113.9002	0.534674	0.32931	1.623621	0.104456807	0.1383109	NOT
MIF-AS1	40.87396	0.354607	0.218439	1.623366	0.104511118	0.1383762	NOT
MIR3936	8.537031	0.319191	0.196655	1.623105	0.104567068	0.1384437	NOT
RP11-675I	0.779055	1.129534	0.696073	1.622725	0.104648227	0.1385445	NOT
RP13-977(	1.91679	0.495153	0.305319	1.621758	0.104855213	0.1388119	NOT
TINCR	7.596881	0.47141	0.290733	1.621455	0.104920064	0.138889	NOT
ZKSCAN2	237.4394	-0.1527	0.094176	-1.62144	0.104923523	0.138889	NOT
RP11-317(	8.890477	0.434635	0.26806	1.62141	0.10492975	0.1388906	NOT
RP4-669L(	5.795129	0.321722	0.198446	1.621207	0.104973178	0.1389414	NOT

RP11-777I	2.884529	-0.40171	0.247795	-1.62113	0.104989982	0.1389522	NOT
FTO	1193.995	-0.14005	0.086389	-1.62112	0.104991331	0.1389522	NOT
AC007364	1.681624	-0.74677	0.460665	-1.62107	0.105003194	0.1389612	NOT
IGSF22	29.63701	0.29949	0.184784	1.620756	0.105070075	0.1390431	NOT
ARPP19	3514.637	0.119188	0.073563	1.62022	0.105185122	0.1391887	NOT
ZNF625	4.326715	0.372299	0.229871	1.619599	0.105318408	0.1393581	NOT
RPL4P3	3.144169	0.360087	0.222334	1.619577	0.105323213	0.1393581	NOT
FBXO31	2890.213	0.282116	0.174238	1.619145	0.105416079	0.1394743	NOT
NUAK2	608.3964	0.338792	0.20929	1.618765	0.105497819	0.1395732	NOT
WDFY2	601.2957	-0.16289	0.100628	-1.61875	0.105500928	0.1395732	NOT
ZSCAN23	3.713833	0.638282	0.394368	1.618493	0.10555641	0.1396399	NOT
PAICSP4	2.631906	0.357846	0.221104	1.618451	0.105565474	0.1396452	NOT
CXCR4	1361.307	-0.30544	0.188747	-1.61826	0.105607137	0.1396901	NOT
SARS2	258.9668	0.195853	0.121028	1.618247	0.105609469	0.1396901	NOT
VHL	1071.702	0.15935	0.098486	1.617988	0.105665086	0.139757	NOT
PRKAG3	4.248054	0.461589	0.285329	1.617741	0.105718489	0.1398209	NOT
SCARNA7	6.238164	-0.42872	0.265046	-1.61753	0.105763359	0.1398736	NOT
COL4A3	74.64223	0.477587	0.295271	1.61745	0.105781092	0.1398903	NOT
LINC0096	2.182552	0.531725	0.328761	1.61736	0.105800591	0.1399094	NOT
TMEM37	4058.156	-0.25107	0.155258	-1.61712	0.105851943	0.1399706	NOT
PLA2G12B	1978.293	-0.32154	0.198866	-1.61688	0.105903305	0.1400319	NOT
PEX16	1848.802	-0.16204	0.100228	-1.61675	0.105931572	0.1400625	NOT
XXYLT1-A	2.153004	-0.54077	0.334597	-1.61619	0.106053411	0.1402169	NOT
BTLA	14.61207	-0.42397	0.262338	-1.61611	0.10607067	0.1402331	NOT
TAC1	1.816455	-1.05014	0.649857	-1.61596	0.106103705	0.14027	NOT
EDDM3A	0.820062	2.099389	1.299345	1.615729	0.106152991	0.1403285	NOT
RP11-12J1	4.737105	0.418602	0.259092	1.615649	0.106170139	0.1403444	NOT
TRPM1	7.865533	0.531842	0.329245	1.615339	0.106237244	0.1404201	NOT
TP53INP2	3513.061	-0.22684	0.140429	-1.61534	0.106237513	0.1404201	NOT
RP11-88E	13.05213	0.381597	0.236243	1.615275	0.106251079	0.1404313	NOT
LDHAP4	72.76559	-0.31408	0.19445	-1.61524	0.106258798	0.1404348	NOT
ESRRB	6.620098	-0.38095	0.235862	-1.61515	0.106278671	0.1404544	NOT
UBE2FP3	5.424092	0.290681	0.179993	1.614963	0.106318642	0.1404934	NOT
TMEM203	1804.053	-0.13353	0.082685	-1.61494	0.106322863	0.1404934	NOT
AC005082	5.021469	-0.39708	0.245878	-1.61494	0.106323472	0.1404934	NOT
AC073072	3.641174	0.352114	0.218043	1.614886	0.106335484	0.1405026	NOT
RP11-255I	1.988549	0.385621	0.23883	1.614626	0.106391643	0.1405701	NOT
IFITM3	47039.53	-0.21671	0.134247	-1.61426	0.106472074	0.1406696	NOT
UTRN	2227.978	0.201523	0.124852	1.614095	0.106506843	0.1407089	NOT
RNF114	2853.018	-0.09909	0.061401	-1.61377	0.106577782	0.1407959	NOT
RP11-167I	2.778484	0.450675	0.279282	1.61369	0.106594611	0.1408114	NOT
R3HDM2	1682.675	-0.12754	0.079057	-1.61321	0.106699264	0.1409397	NOT
CHD2	2059.662	-0.11992	0.074334	-1.6132	0.106701971	0.1409397	NOT
RP11-109	23.66611	-0.37645	0.233359	-1.61316	0.106708746	0.1409419	NOT
BMP3	1.880561	-0.70737	0.438506	-1.61313	0.106716369	0.1409453	NOT
RP1-244F	2.480956	0.591818	0.366923	1.612919	0.10676203	0.1409989	NOT
RP11-770	20.51143	-0.31409	0.194782	-1.61251	0.106851221	0.1411099	NOT
ZNF853	83.54509	0.345104	0.214072	1.612095	0.106941292	0.1412221	NOT
IL12RB1	121.9383	-0.31431	0.194982	-1.61197	0.106968155	0.1412509	NOT
RP11-649	4.963112	0.342995	0.212815	1.611704	0.107026308	0.1413209	NOT
MRPL15	2373.369	0.17153	0.106454	1.611306	0.107113017	0.1414286	NOT
ELFN1	1166.253	-0.46901	0.291184	-1.61071	0.107242762	0.1415932	NOT

LA16c-380	154.7486	0.333245	0.206917	1.610526	0.107283092	0.1416397	NOT
CD207	34.03082	-0.4873	0.302579	-1.61048	0.107293205	0.1416463	NOT
ROPN1B	23.37152	-0.42716	0.265253	-1.61037	0.107316082	0.1416697	NOT
PCDHGA8	12.25012	0.467297	0.290194	1.610291	0.107334437	0.1416872	NOT
PCDHGA1	22.54498	0.396939	0.246515	1.610206	0.107352795	0.1417047	NOT
RP1-102E	13.73298	0.278445	0.17296	1.60988	0.107423981	0.1417919	NOT
C2orf43	667.8106	0.120468	0.074855	1.609354	0.107539058	0.141937	NOT
RP11-43A	0.762324	0.802242	0.498577	1.609064	0.107602258	0.1420069	NOT
RP11-327	61.89038	-0.20617	0.128128	-1.60906	0.107602288	0.1420069	NOT
NIPBL	1914.841	0.124244	0.077228	1.608791	0.107661957	0.1420789	NOT
TRGV9	1.552957	-0.57066	0.354742	-1.60867	0.107688083	0.1421066	NOT
RPS4XP17	1.936005	0.387566	0.241021	1.608018	0.107831274	0.1422887	NOT
CAPZA1	3507.15	0.124283	0.077314	1.60751	0.107942474	0.1424287	NOT
P2RY2	148.3539	0.276266	0.171866	1.607448	0.107956086	0.1424398	NOT
TRPC4AP	3817.925	0.094719	0.058928	1.607365	0.107974253	0.142457	NOT
BBS12	54.56312	0.227791	0.14172	1.60733	0.107981981	0.1424604	NOT
AC021218	106.3059	0.567972	0.353454	1.60692	0.108071959	0.1425723	NOT
NF1P8	0.791258	2.049571	1.275854	1.60643	0.108179378	0.1427072	NOT
BMPR1APS	2.45419	0.451656	0.281193	1.606214	0.10822695	0.1427585	NOT
RP11-730	0.784896	1.864369	1.160728	1.606207	0.108228534	0.1427585	NOT
KRT8P10	1.566604	-0.43801	0.272704	-1.60617	0.108235578	0.142761	NOT
RP11-225	59.50813	0.184979	0.11519	1.605857	0.108305302	0.1428461	NOT
LINC0031	16.96175	0.548784	0.341775	1.605689	0.108342262	0.142888	NOT
LINC0048	3.189079	-0.51399	0.320158	-1.60542	0.108401354	0.1429592	NOT
KIAA2022	4.61516	0.594472	0.37041	1.604902	0.108515283	0.1431026	NOT
WHSC1L1	1252.544	0.181702	0.113244	1.604521	0.10859932	0.1432066	NOT
ZW10	670.7719	0.100951	0.062924	1.604321	0.108643314	0.1432578	NOT
IFT122	774.9843	-0.13358	0.083269	-1.60421	0.108668463	0.1432841	NOT
MGAT4B	10952.73	0.185012	0.115344	1.604008	0.108712326	0.1433351	NOT
RP11-445	0.641038	1.658832	1.034215	1.603953	0.10872444	0.1433442	NOT
EIF2A	2918.876	0.104229	0.064989	1.603798	0.108758551	0.1433824	NOT
SNORA7	1.553274	0.447368	0.278954	1.603736	0.108772331	0.1433912	NOT
COA3	3960.92	0.198549	0.123806	1.603714	0.108777206	0.1433912	NOT
IFT20	827.9478	0.109978	0.068579	1.603678	0.108785101	0.1433912	NOT
TEX10	638.5911	0.136057	0.084841	1.603674	0.108786001	0.1433912	NOT
RP11-366	8.234093	-0.4562	0.284484	-1.60362	0.108798087	0.1434003	NOT
AC092881	2.307166	0.601698	0.375232	1.603535	0.108816656	0.143418	NOT
DPH6-AS1	16.76617	0.431236	0.26899	1.603171	0.108896942	0.143517	NOT
RP11-399	5.869966	-1.14033	0.711316	-1.60312	0.108907762	0.1435194	NOT
ZNF211	195.7181	0.194096	0.121074	1.603115	0.108909164	0.1435194	NOT
CXorf40A	404.8072	0.141989	0.088608	1.602445	0.10905734	0.1437078	NOT
NOP16	887.4123	0.201171	0.125571	1.602047	0.10914523	0.1438168	NOT
AC008154	1.410715	-0.8072	0.503989	-1.60162	0.109239608	0.1439343	NOT
STK38	1725.472	0.143758	0.089761	1.601571	0.109250416	0.1439417	NOT
RP11-430	3.075499	0.417069	0.260438	1.601411	0.109285858	0.1439775	NOT
RP3-333H	2.154021	0.564902	0.352755	1.601401	0.109288038	0.1439775	NOT
TARS	3602.794	-0.14542	0.090812	-1.60137	0.109295369	0.1439803	NOT
RRN3P1	112.9634	-0.25095	0.156748	-1.60097	0.1093836	0.1440897	NOT
RP11-950	1.97741	0.394796	0.246666	1.600528	0.10948149	0.1442118	NOT
GNAS	26038.06	0.181217	0.113242	1.600254	0.10954219	0.1442849	NOT
KRTAP20-	1.036279	2.502822	1.564185	1.600081	0.109580675	0.1443287	NOT
CD52	417.2079	-0.33469	0.209175	-1.60004	0.109589398	0.1443333	NOT

CTD-2031	23.18794	-0.23632	0.147737	-1.59962	0.10968186	0.1444482	NOT
WNT9B	5.160386	0.565603	0.353642	1.599368	0.109738784	0.1445163	NOT
ARID1B	1472.346	-0.13248	0.082864	-1.59875	0.109875465	0.1446894	NOT
POLRMTP1	69.42957	0.366007	0.228937	1.598725	0.109881688	0.1446907	NOT
PPM1J	28.56809	0.306389	0.19165	1.598692	0.109889167	0.1446937	NOT
RP11-215I	1.598002	0.436223	0.272874	1.598624	0.109904165	0.1447065	NOT
RP11-10N	2.039024	0.562029	0.351619	1.598402	0.109953434	0.1447645	NOT
CTB-43E1!	30.32378	0.313858	0.196381	1.59821	0.109996247	0.144814	NOT
KCNK15	29.80694	0.662561	0.414572	1.598181	0.110002785	0.1448157	NOT
RPL32	26054.19	0.216224	0.135307	1.598028	0.110036673	0.1448534	NOT
UBXN4	5354.356	-0.12712	0.079554	-1.5979	0.110064742	0.1448835	NOT
RP3-461P1	8.516531	0.349321	0.218687	1.597358	0.110186028	0.1450362	NOT
KLF16	969.0016	-0.22823	0.142897	-1.59719	0.110222498	0.1450723	NOT
UBE2L3	2920.155	0.118498	0.074191	1.597188	0.110223872	0.1450723	NOT
OMP	5.341527	0.421108	0.263677	1.597059	0.11025258	0.1450973	NOT
AC005932	3.915402	0.42106	0.263648	1.597055	0.110253398	0.1450973	NOT
RP11-177(	22.5666	0.401494	0.251421	1.596902	0.110287607	0.1451354	NOT
SEC11A	3763.379	0.102244	0.064029	1.596853	0.110298544	0.1451429	NOT
LEMD3	689.0526	-0.12564	0.078683	-1.59678	0.110313739	0.145156	NOT
C5orf24	2233.766	-0.14518	0.090945	-1.59636	0.110407395	0.1452723	NOT
TMSB4XP2	1.830514	0.499158	0.31269	1.596337	0.110413611	0.1452736	NOT
RSG1	99.6419	0.223546	0.14009	1.595735	0.110547945	0.1454434	NOT
RPL7AP34	22.34679	-0.34461	0.215997	-1.59543	0.110615895	0.1455259	NOT
ZNF32	906.0042	0.148183	0.092916	1.594808	0.110755197	0.1456954	NOT
TTC21A	71.04272	0.18885	0.118417	1.59479	0.110759314	0.1456954	NOT
IGHV2-70	30.43472	-0.77544	0.486238	-1.59478	0.110760717	0.1456954	NOT
ACER3	564.9062	0.167961	0.105322	1.594743	0.110769706	0.1456954	NOT
STAG3	212.5231	-0.3064	0.192132	-1.59474	0.110771068	0.1456954	NOT
C2orf78	3.459691	-0.45701	0.28661	-1.59454	0.11081563	0.1457471	NOT
RP3-337H	3.063929	0.386856	0.242622	1.59448	0.110828664	0.1457544	NOT
U3	16.00719	0.339509	0.21293	1.594466	0.110831684	0.1457544	NOT
LEFTY2	2.385286	-0.54446	0.341489	-1.59436	0.110856352	0.1457799	NOT
IL1RL2	122.0707	-0.39561	0.248313	-1.59321	0.111113402	0.146111	NOT
PDE9A	442.0987	0.399599	0.250856	1.59294	0.111173604	0.1461832	NOT
PRMT7	1027.208	0.140262	0.088061	1.592779	0.111209838	0.1462239	NOT
ADCY1	1279.749	-0.54053	0.339372	-1.59273	0.111221165	0.1462318	NOT
DYNLRB2	9.797138	-0.40154	0.252121	-1.59265	0.111239342	0.1462464	NOT
RP11-486/	5.28511	0.418628	0.262853	1.59263	0.111243127	0.1462464	NOT
RP11-93B1	148.7201	-0.37928	0.238152	-1.59261	0.111248088	0.1462464	NOT
RP11-302I	2.675972	-0.37172	0.233451	-1.59227	0.111324652	0.1463401	NOT
MCOLN2	43.79936	0.466066	0.292718	1.592201	0.111339567	0.1463527	NOT
MAN2A1	4410.466	-0.15558	0.097732	-1.59189	0.11141024	0.1464386	NOT
AC111186	81.00889	-0.36825	0.231377	-1.59155	0.11148512	0.1465301	NOT
RP11-552I	1.809237	-0.69552	0.437045	-1.59141	0.111516396	0.1465643	NOT
RP11-3P17	2.003458	0.598752	0.376323	1.591056	0.111596999	0.1466632	NOT
RNF138P1	1.668292	0.473104	0.297365	1.590986	0.111612629	0.1466768	NOT
TRIM29	58.4939	-0.39495	0.248258	-1.59088	0.111636861	0.1467017	NOT
CREG1	11495.67	-0.22335	0.140402	-1.59081	0.111651497	0.1467139	NOT
SH3BGR	179.9209	-0.20846	0.1311	-1.59009	0.111813408	0.1469197	NOT
LGALSL	614.2624	0.15146	0.095303	1.589248	0.112004351	0.1471636	NOT
VASN	2127.426	0.20483	0.12889	1.589187	0.112018222	0.147171	NOT
AP000487.	5.424382	0.341329	0.214783	1.589176	0.112020593	0.147171	NOT

ANP32AP1	2.403292	0.354554	0.223154	1.588828	0.112099246	0.1472673	NOT
CTNNBIP1	1003.079	0.152604	0.096071	1.588441	0.112186681	0.1473752	NOT
RP13-228.	1.095268	0.660532	0.415861	1.588347	0.112207997	0.1473962	NOT
RP11-380(	3.324847	0.426594	0.268674	1.587775	0.112337261	0.147559	NOT
MINOS1	847.391	0.186415	0.11741	1.587731	0.112347251	0.1475651	NOT
RP11-345I	4.618577	-0.79519	0.500854	-1.58766	0.112362813	0.1475785	NOT
KCNK1	770.7788	-0.3835	0.241599	-1.58734	0.112436472	0.1476617	NOT
RP11-25I1	1.033766	0.784145	0.494006	1.587318	0.112440619	0.1476617	NOT
IKZF3	285.9062	-0.36984	0.232999	-1.5873	0.112443949	0.1476617	NOT
BHLHE22	33.67186	-0.48299	0.304289	-1.58729	0.112447448	0.1476617	NOT
CRYBB1	22.55343	0.362453	0.228359	1.587204	0.112466511	0.1476797	NOT
GP1BA	41.95667	-0.29852	0.188082	-1.58716	0.112476851	0.1476863	NOT
NOS1	8.462968	-0.56261	0.354508	-1.58702	0.112508218	0.1477204	NOT
PCAT1	5.120355	0.382578	0.241126	1.586628	0.112596861	0.1478298	NOT
AGRP	4.38256	0.493119	0.310808	1.586571	0.112609786	0.1478398	NOT
DCAF17	400.359	0.129758	0.081794	1.586397	0.112649172	0.1478844	NOT
NR2F6	4338.78	0.202844	0.127872	1.58631	0.112668925	0.1479034	NOT
RP11-632I	4.16457	-0.40799	0.257246	-1.58598	0.11274463	0.1479957	NOT
RP11-227(	1.850219	0.468567	0.29546	1.58589	0.112764341	0.1480146	NOT
DEFA4	2.039409	-0.86356	0.544559	-1.5858	0.112785107	0.1480348	NOT
CLASP2	943.0971	0.128119	0.080814	1.585366	0.112883133	0.1481564	NOT
RELA	3215.079	-0.08011	0.050551	-1.58483	0.113004391	0.1483085	NOT
TRIP12	4318.203	-0.09926	0.06264	-1.58468	0.113039355	0.1483474	NOT
RP4-620E.	5.988315	-0.25871	0.163299	-1.5843	0.113125552	0.1484535	NOT
GTF2H2	86.5577	0.315769	0.199318	1.584245	0.113137873	0.1484626	NOT
PRSS42	4.049087	0.400131	0.252607	1.584005	0.113192691	0.1485275	NOT
WDR45BP.	1.416026	0.64358	0.406348	1.583815	0.113235789	0.148577	NOT
UMPS	1645.599	-0.11261	0.071118	-1.58336	0.113338743	0.148705	NOT
RP1-122P.	8.520149	-0.32476	0.205133	-1.58318	0.113380986	0.1487534	NOT
SCO2	987.2277	-0.1821	0.115036	-1.58296	0.113429492	0.14881	NOT
HOXA4	21.72622	-0.29013	0.183287	-1.58292	0.113440451	0.1488173	NOT
RP11-206I	4.913128	-0.24567	0.155215	-1.58277	0.11347284	0.1488527	NOT
IGHV1-18	209.4399	-0.66933	0.422908	-1.58268	0.113494645	0.1488713	NOT
VAMP7	2328.629	0.168759	0.106631	1.582652	0.113500886	0.1488713	NOT
CACHD1	256.8625	0.312929	0.197726	1.582642	0.113503121	0.1488713	NOT
GPR3	29.55601	0.349076	0.220584	1.582511	0.113532926	0.1489033	NOT
RPS12P23	1.350937	0.568074	0.359014	1.582316	0.11357734	0.1489545	NOT
DIAPH2-A	2.434234	0.470377	0.297296	1.582184	0.113607668	0.1489872	NOT
CYP4F11	5012.4	-0.34901	0.220597	-1.58212	0.113621802	0.1489926	NOT
GPR62	16.24148	0.459971	0.290731	1.582118	0.113622534	0.1489926	NOT
ZBTB25	415.2489	-0.14129	0.089314	-1.58197	0.113655969	0.1490294	NOT
RP11-446I	10.31249	0.251783	0.159171	1.581837	0.113686809	0.1490627	NOT
RP11-166I	2.533712	0.365681	0.231188	1.581748	0.113707217	0.1490824	NOT
CYP26C1	2.488331	0.461939	0.292051	1.581706	0.113716639	0.1490877	NOT
RIPK3	71.9063	-0.30086	0.190398	-1.58016	0.114070707	0.1495408	NOT
VCAM1	1708.044	0.394979	0.249964	1.580148	0.114073044	0.1495408	NOT
CTD-2541	9.636671	0.267256	0.169139	1.580094	0.114085417	0.1495499	NOT
HS2ST1	1564.806	0.131896	0.083486	1.579857	0.114139594	0.1496139	NOT
OMD	25.88693	-0.52598	0.333081	-1.57912	0.114308235	0.1498278	NOT
HEXIM1	1784.278	-0.14871	0.094194	-1.57876	0.114390382	0.1499284	NOT
AC093901	19.71093	0.354455	0.224529	1.578661	0.11441384	0.149952	NOT
RP11-309I	3.123451	0.333507	0.211298	1.578377	0.114479098	0.1500305	NOT

MYH14	6595.527	0.224226	0.14211	1.577836	0.114603199	0.150186	NOT
UBE2V1P2	1.869613	0.395364	0.250674	1.577207	0.11474789	0.1503685	NOT
RP11-778I	5.176556	0.331998	0.210515	1.577073	0.114778839	0.1504019	NOT
ERCC1	1793.586	0.194971	0.123639	1.576931	0.114811318	0.1504374	NOT
RPL7P15	1.640515	0.474608	0.300981	1.576873	0.114824785	0.1504479	NOT
ARHGAP29	1941.867	-0.19243	0.122055	-1.57661	0.11488583	0.1505207	NOT
HIC1	279.8996	-0.26239	0.166442	-1.57649	0.114912944	0.1505491	NOT
RP11-316I	6.032401	0.323113	0.205013	1.576063	0.11501137	0.1506709	NOT
RNF217	773.361	-0.22568	0.143245	-1.57546	0.115150779	0.1508464	NOT
SP1	2715.347	0.093671	0.059463	1.575289	0.115189741	0.1508903	NOT
RP11-345J	4.785738	0.489728	0.310918	1.575101	0.11523319	0.1509401	NOT
TMX2	3347.219	0.115323	0.073223	1.574943	0.115269593	0.1509806	NOT
SYVN1	6966.202	-0.15449	0.098101	-1.57482	0.115297585	0.1510101	NOT
MAP3K8	384.5498	0.293014	0.186152	1.574058	0.115473997	0.151234	NOT
NRG3	26.00545	-0.52937	0.336324	-1.57399	0.115488874	0.1512464	NOT
TNF	23.38341	-0.44106	0.280302	-1.57351	0.115601397	0.1513866	NOT
LINC01422	5.574391	0.428039	0.27206	1.573327	0.115643082	0.151434	NOT
GPR157	560.0013	-0.20978	0.133347	-1.57316	0.115681078	0.1514766	NOT
LA16c-306	3.711337	0.374103	0.23789	1.572583	0.115815366	0.1516452	NOT
MRPS28	1014.13	-0.18332	0.116574	-1.57254	0.11582535	0.1516511	NOT
IGHV3OR1	2.631582	-0.79438	0.505187	-1.57245	0.115846885	0.1516721	NOT
RP4-550H	1.402746	0.603466	0.38393	1.571813	0.115994014	0.1518564	NOT
AC073635	2.090266	0.486884	0.309763	1.571793	0.115998574	0.1518564	NOT
TAF4B	40.83047	0.40409	0.257138	1.57149	0.11606896	0.1519413	NOT
CORO2B	96.46475	0.404369	0.257326	1.571426	0.116083665	0.1519534	NOT
ZFYVE28	357.5062	-0.23325	0.148438	-1.57134	0.1161032	0.1519718	NOT
pk	2089.629	-0.19087	0.121478	-1.57121	0.116133075	0.1520037	NOT
SRSF2	3611.45	0.093079	0.059255	1.570812	0.116226247	0.1521184	NOT
IPO8P1	1.513248	0.516652	0.328951	1.570606	0.116274095	0.1521739	NOT
SRP54-AS1	36.70867	-0.19223	0.122414	-1.57031	0.116342645	0.1522564	NOT
MRPL22	1154.506	0.144735	0.092202	1.56976	0.116470848	0.1524169	NOT
WDR93	7.017353	0.348298	0.221957	1.569213	0.116598391	0.1525766	NOT
SMUG1	1231.826	0.146719	0.093511	1.569004	0.116647046	0.1526331	NOT
ZNF564	29.49603	-0.17336	0.110497	-1.56889	0.116673605	0.1526561	NOT
DENND1A	1484.624	-0.12216	0.077862	-1.56888	0.116675702	0.1526561	NOT
AP006621.	244.1858	0.244244	0.155684	1.568845	0.1166841	0.1526599	NOT
RPL21P134	5.792271	0.363138	0.231474	1.568811	0.116691885	0.1526629	NOT
PTGER4	195.8624	-0.32314	0.206027	-1.56843	0.116780247	0.1527712	NOT
RP11-650I	1.283383	0.750986	0.47899	1.567854	0.116915262	0.1529406	NOT
IGKV4-1	710.0832	-0.62619	0.399425	-1.56772	0.116945946	0.1529636	NOT
RP5-875O	6.070325	0.543375	0.346602	1.567719	0.116946664	0.1529636	NOT
MPLKIP	2107.119	-0.17887	0.1141	-1.5677	0.116952032	0.1529636	NOT
IGHG3	2507.107	-0.57263	0.365271	-1.56768	0.116954938	0.1529636	NOT
RP11-473I	6.457531	0.318904	0.203448	1.567494	0.116999315	0.1530144	NOT
RPS20P10	1.752978	0.440765	0.281242	1.567211	0.117065488	0.1530937	NOT
RP11-849I	1.669512	0.459508	0.293215	1.567138	0.117082339	0.1531085	NOT
REC8	154.7696	-0.26427	0.168656	-1.56693	0.117131321	0.1531653	NOT
SLC52A1	10.40862	-0.39527	0.252391	-1.5661	0.117325729	0.1534123	NOT
PTPN7	183.4536	0.32822	0.209582	1.566071	0.117332085	0.1534134	NOT
GTF2H3	845.6568	0.112189	0.071649	1.565805	0.117394271	0.1534874	NOT
RP3-391O	3.727552	0.378672	0.241884	1.56551	0.117463484	0.1535707	NOT
RPL37P6	23.22646	0.387382	0.247488	1.565257	0.117522763	0.1536409	NOT

C1orf101	38.64586	0.239368	0.152929	1.565222	0.117530808	0.1536442	NOT
PCOLCE	3635.455	-0.40767	0.260481	-1.56506	0.11756865	0.1536864	NOT
RTP3	1447.972	-0.48931	0.312655	-1.56503	0.117576781	0.1536897	NOT
TUBGCP5	520.8795	0.136549	0.087278	1.564527	0.1176939	0.1538276	NOT
NKRF	374.9004	0.119648	0.076477	1.564499	0.117700351	0.1538276	NOT
AC104653	2.570823	0.523728	0.334759	1.564496	0.117701229	0.1538276	NOT
LSM1	554.4598	-0.14275	0.091243	-1.56448	0.117704486	0.1538276	NOT
GLUD1P7	20.91753	-0.26636	0.170303	-1.56402	0.1178119	0.1539607	NOT
RP11-79H	19.1422	-0.3663	0.234316	-1.56327	0.117990027	0.1541862	NOT
AGO1	981.0659	0.127617	0.081639	1.563179	0.118010401	0.1542055	NOT
ZNF586	137.2417	-0.17282	0.110575	-1.56294	0.118065799	0.1542642	NOT
PMM2	489.0084	-0.18125	0.115969	-1.56294	0.118066477	0.1542642	NOT
ABCC6P1	857.965	-0.29107	0.18628	-1.56255	0.118158606	0.1543773	NOT
TUBGCP2	2489.147	0.121656	0.077873	1.562233	0.118233224	0.1544675	NOT
RP11-247I	3.807139	0.301698	0.193149	1.561995	0.118289255	0.1545334	NOT
KDELR2	16182.23	-0.12141	0.077734	-1.56183	0.118327527	0.1545761	NOT
SGTB	207.3737	0.174433	0.111725	1.561272	0.11845956	0.1547413	NOT
IGHV3-13	25.08299	-0.74125	0.474825	-1.5611	0.118500126	0.154787	NOT
SYT17	933.5093	-0.30684	0.19656	-1.56106	0.118508547	0.1547907	NOT
SNX1	3893.087	-0.11316	0.072493	-1.56103	0.118516842	0.1547942	NOT
SRFBP1	286.04	0.146055	0.093567	1.56097	0.118530892	0.1548052	NOT
AC006160	4.418715	-0.30409	0.194856	-1.56059	0.118619926	0.1549142	NOT
HLA-J	124.3029	0.347624	0.222755	1.560567	0.118625967	0.1549148	NOT
RP1-153G	5.352573	-0.3126	0.200377	-1.56008	0.118740878	0.1550575	NOT
ITPRIPL2	882.5614	0.212891	0.136467	1.560015	0.118756223	0.1550677	NOT
POU3F3	1.147654	1.076209	0.689878	1.56	0.118759881	0.1550677	NOT
DNAJC4	1455.251	-0.20616	0.132172	-1.5598	0.118807988	0.1551232	NOT
IARS2	6169.166	0.142806	0.091557	1.559752	0.118818407	0.1551295	NOT
OLIG3	0.712625	1.915211	1.228035	1.559574	0.118860603	0.1551773	NOT
TMSB4XP1	2.326052	0.42256	0.270995	1.559293	0.118927049	0.1552567	NOT
RP11-6N1	7.554748	-0.23122	0.148298	-1.55913	0.118965125	0.1552991	NOT
SNORA75	2.545572	0.389639	0.249913	1.559098	0.118973111	0.1553021	NOT
CHRM3-A	6.897512	0.423715	0.271844	1.558671	0.119074339	0.155427	NOT
TSPAN19	0.661418	1.564321	1.003819	1.55837	0.119145554	0.1555126	NOT
AC010761	1.520489	0.483422	0.310216	1.55834	0.11915278	0.1555147	NOT
AC108488	57.47904	0.214916	0.137928	1.558167	0.11919371	0.1555608	NOT
MTATP8P2	17.99672	-0.41649	0.267302	-1.55811	0.119207928	0.155572	NOT
MORF4L1	5544.352	-0.08567	0.054985	-1.55798	0.119237403	0.1556031	NOT
FRAT1	607.3346	0.212745	0.136608	1.557342	0.119389194	0.1557904	NOT
LINC0150(	10.35599	-0.52472	0.336935	-1.55733	0.119392193	0.1557904	NOT
RP11-125	9.66705	-0.32549	0.209013	-1.55726	0.119408735	0.1558046	NOT
DNAJB7	2.49776	0.39072	0.250938	1.557036	0.119461843	0.1558666	NOT
KIAA1919	206.2735	-0.13995	0.089892	-1.55689	0.119495527	0.1559032	NOT
SERF2	22872.61	0.22432	0.144158	1.556078	0.119689562	0.156149	NOT
ATP6V0D1	3810.498	0.14083	0.090518	1.555825	0.119749735	0.1562201	NOT
RP11-107	6.01811	0.350517	0.225362	1.555349	0.119862884	0.1563603	NOT
AC115522	2.785811	0.513104	0.329907	1.555299	0.119874961	0.1563687	NOT
SERAC1	273.1525	0.152656	0.098173	1.554973	0.119952565	0.1564626	NOT
ESYT1	6144.498	0.12928	0.083148	1.554817	0.119989586	0.1565035	NOT
RP11-555I	0.794272	2.127481	1.368406	1.554715	0.120014032	0.156528	NOT
PCDH18	135.4723	-0.29913	0.192415	-1.55462	0.120037056	0.1565505	NOT
AARD	1.899611	0.630067	0.405293	1.554595	0.120042623	0.1565505	NOT

RP11-754I	2.488546	0.682368	0.438967	1.554486	0.120068467	0.1565768	NOT
AC005336	289.1464	-0.40385	0.259835	-1.55426	0.120121954	0.1566392	NOT
HSD11B1L	182.0337	0.215163	0.138463	1.553935	0.120199893	0.1567335	NOT
SPAG5-AS	45.10104	0.219931	0.14156	1.553631	0.12027246	0.1568207	NOT
ZNF830	479.0253	-0.12432	0.080032	-1.55342	0.120321717	0.1568775	NOT
LINC01067	3.16163	0.702967	0.452611	1.553136	0.120390765	0.1569589	NOT
LINC-PINT	169.7519	-0.21465	0.138206	-1.55312	0.120395496	0.1569589	NOT
CNNM3	2200.2	-0.15449	0.099472	-1.55306	0.120408405	0.1569684	NOT
HSPA8P9	2.731419	0.390779	0.251734	1.552346	0.120579405	0.1571839	NOT
TMED10P2	1.808679	0.791652	0.510087	1.551995	0.120663462	0.157286	NOT
RP11-250I	11.11542	-0.29346	0.189113	-1.55176	0.12072075	0.1573533	NOT
ING4	955.9554	0.142486	0.091826	1.551702	0.120733602	0.1573626	NOT
CTD-2526	7.577825	0.546044	0.351998	1.551272	0.120836552	0.1574894	NOT
RP11-1069	7.166309	-0.52163	0.336461	-1.55033	0.121061812	0.1577755	NOT
RP11-206I	5.760635	0.349442	0.225467	1.549863	0.121174378	0.1579148	NOT
RP11-347(	37.74936	0.234715	0.151497	1.549308	0.12130764	0.1580803	NOT
CTD-2240	2.993846	0.447178	0.288635	1.549287	0.121312769	0.1580803	NOT
TBL1XR1	3164.278	0.114616	0.073983	1.54922	0.121328859	0.1580938	NOT
ZNF280D	1084.131	0.117177	0.075638	1.549168	0.121341456	0.1581028	NOT
YBX1	11836.31	0.140639	0.090791	1.549039	0.121372411	0.1581356	NOT
PBX4	18.17728	0.380657	0.245811	1.548575	0.121483838	0.1582734	NOT
ARMC8	978.9741	0.087002	0.056192	1.548295	0.121551354	0.1583539	NOT
SKIV2L2	1720.219	0.095939	0.061973	1.548077	0.121603705	0.1584146	NOT
ALG11	178.9163	-0.16426	0.106111	-1.54799	0.12162385	0.1584334	NOT
CTA-292E	5.625833	0.303448	0.196097	1.547437	0.121757785	0.1586004	NOT
CXCL16	2731.516	-0.26105	0.168729	-1.54716	0.121825504	0.1586811	NOT
CTD-2049	2.320094	0.616288	0.398405	1.546891	0.121889487	0.158757	NOT
CTD-2260	47.47264	0.23985	0.155064	1.546781	0.121916119	0.1587842	NOT
DDX43	19.39073	0.514194	0.332508	1.546408	0.122006015	0.1588938	NOT
ATP5H	7438.304	0.162947	0.105389	1.546141	0.122070617	0.1589705	NOT
ARRDC1	1317.434	-0.13293	0.085987	-1.54593	0.122120983	0.1590286	NOT
CTB-50L17	91.99569	0.335556	0.217063	1.545889	0.122131335	0.1590346	NOT
RNF10	5987.273	0.089808	0.058096	1.545845	0.122142041	0.159041	NOT
GPR68	58.35914	0.315734	0.204272	1.545652	0.122188734	0.1590943	NOT
KRT18P10	17.09164	-0.36722	0.237644	-1.54526	0.122283753	0.1592106	NOT
DNAH9	5.52165	0.566146	0.366468	1.544871	0.12237736	0.1593249	NOT
KCTD3	5248.68	0.153178	0.099157	1.544807	0.122393021	0.1593378	NOT
TRAF4	2552.792	-0.15612	0.101074	-1.5446	0.122443761	0.1593964	NOT
CGGBP1	2576.155	-0.09879	0.063962	-1.54452	0.122462417	0.1594108	NOT
CHST9	835.1464	-0.46431	0.300624	-1.5445	0.122466376	0.1594108	NOT
RP11-354I	1.693609	0.507171	0.328386	1.544437	0.12248257	0.1594244	NOT
ARHGAP23	524.0123	0.243615	0.157776	1.544055	0.122575052	0.1595373	NOT
DPP10-AS	10.03	0.998577	0.646744	1.544006	0.122586985	0.1595453	NOT
MRPS34	2911.261	0.174606	0.113103	1.543777	0.122642271	0.1596098	NOT
RP11-109A	1.850979	0.450929	0.292133	1.543576	0.122690965	0.1596656	NOT
RP11-171I	11.48522	0.341797	0.221475	1.543278	0.122763445	0.1597471	NOT
RBBP4P1	6.81877	0.256541	0.166234	1.543252	0.122769757	0.1597471	NOT
KRR1P1	1.677862	0.480452	0.311326	1.543247	0.122770897	0.1597471	NOT
CH17-472	2.573285	-0.5463	0.354071	-1.54292	0.12285028	0.1598429	NOT
PCDH10	2.32572	-0.54488	0.353309	-1.54221	0.123021766	0.1600585	NOT
COPB1	4614.558	0.107867	0.069977	1.541466	0.123203323	0.1602871	NOT
OR7E38P	231.4759	0.269296	0.174724	1.541263	0.123252802	0.160344	NOT

RP5-832C:	23.90565	-0.22824	0.148216	-1.53992	0.123580662	0.1607629	NOT
RP11-106I	10.60253	-0.37414	0.24298	-1.53979	0.123610732	0.1607945	NOT
SIGLEC12	38.93443	0.450823	0.292877	1.539289	0.12373367	0.1609468	NOT
ACE2	419.3697	0.589194	0.382858	1.538938	0.123819443	0.1610508	NOT
AC114271	34.44803	0.213804	0.13897	1.538494	0.123927906	0.1611843	NOT
ZDHHC2	469.9081	-0.28436	0.184958	-1.53742	0.124189314	0.1615167	NOT
USMG5P1	1.74956	0.461736	0.300354	1.537309	0.124217739	0.1615461	NOT
CLASP1	1640.342	-0.11006	0.071597	-1.53718	0.124248818	0.1615789	NOT
RP11-736I	1.657998	0.602584	0.392032	1.537078	0.124274194	0.1616043	NOT
ZNF658	56.10614	0.200365	0.13037	1.536896	0.124318768	0.1616547	NOT
RP11-480I	4.844747	0.39959	0.260015	1.536795	0.124343426	0.1616792	NOT
RP11-421I	3.151201	0.359725	0.234086	1.536723	0.12436104	0.1616945	NOT
BRD2-IT1	1.61305	0.508344	0.330867	1.5364	0.124440279	0.1617899	NOT
CTD-3128	6472.949	-0.43313	0.281926	-1.53634	0.1244556	0.1618022	NOT
GSE1	1877.998	-0.15544	0.10118	-1.53631	0.124462504	0.1618036	NOT
GS1-21A4	2.966908	0.453116	0.295135	1.535282	0.124714561	0.1621237	NOT
RP11-130I	9.970409	-0.24981	0.162724	-1.53516	0.124743441	0.1621487	NOT
NECAB2	807.9957	0.50464	0.328722	1.535156	0.124745513	0.1621487	NOT
HNF4G	637.2153	-0.23343	0.152083	-1.53488	0.124813005	0.1622243	NOT
RP11-3K2	1.812098	0.486793	0.317156	1.534871	0.12481542	0.1622243	NOT
RB1	966.99	-0.19382	0.126289	-1.5347	0.124857705	0.1622716	NOT
RGPD1	5.343535	-0.43584	0.284022	-1.53452	0.124901832	0.1623213	NOT
RP11-108	2.022198	0.417626	0.272206	1.534226	0.124974082	0.1624076	NOT
RP11-555I	0.729792	2.009115	1.310049	1.533618	0.125123607	0.1625943	NOT
UBE2D1	547.6868	0.145144	0.094653	1.53344	0.125167598	0.1626386	NOT
AC007229	7.455386	0.227833	0.148577	1.533432	0.125169445	0.1626386	NOT
PNN	2384.969	0.123339	0.080454	1.533048	0.125264087	0.1627539	NOT
SIRPG	53.94694	0.397267	0.259152	1.532949	0.125288305	0.1627777	NOT
RP11-359I	3.075229	0.358948	0.234171	1.532849	0.12531313	0.1628023	NOT
CD80	19.60876	-0.3617	0.236029	-1.53246	0.125409102	0.1629194	NOT
DDX43P3	3.448326	0.718606	0.468941	1.532402	0.1254233	0.1629302	NOT
NLRP9	1.788466	0.576291	0.376159	1.53204	0.125512479	0.1630383	NOT
FLNB	9743.745	0.137368	0.089687	1.531642	0.125610684	0.1631583	NOT
RP11-325I	6.120386	0.403785	0.263635	1.531609	0.125618955	0.1631613	NOT
LINC01191	3.146678	0.572291	0.373681	1.531495	0.125646978	0.1631901	NOT
RP11-17M	29.62791	-0.19834	0.129524	-1.5313	0.12569465	0.1632443	NOT
POM121C	1156.849	0.111162	0.072608	1.530995	0.125770646	0.1633354	NOT
KCNS3	243.4291	0.37	0.24168	1.530953	0.125781028	0.1633412	NOT
RP1-153P:	3.147237	-0.50655	0.330888	-1.53087	0.125800516	0.1633588	NOT
RP11-155I	21.03527	-0.33131	0.216443	-1.53069	0.125845298	0.1634093	NOT
NPC1L1	2618.317	-0.48866	0.319367	-1.53009	0.125994764	0.1635957	NOT
EVI2A	135.6824	-0.28698	0.187568	-1.53002	0.12601161	0.1636099	NOT
ZNF583	140.4981	0.204129	0.13342	1.529968	0.126024709	0.1636192	NOT
AC011247	1.343186	0.483362	0.315978	1.529732	0.126083141	0.1636874	NOT
LINC01251	2.049899	0.513327	0.335572	1.529707	0.126089374	0.1636878	NOT
RP11-113	1.404787	0.624182	0.408093	1.52951	0.126137984	0.1637432	NOT
MANSC1	662.339	0.174168	0.113919	1.528871	0.126296473	0.1639413	NOT
CTA-292E	59.95461	-0.27729	0.181396	-1.52862	0.126358693	0.1640144	NOT
GPX2	15404.86	0.45525	0.297899	1.528201	0.126462752	0.1641417	NOT
FAM101B	478.6046	-0.20236	0.13242	-1.52816	0.126472288	0.1641464	NOT
FAM151B	32.93452	0.158868	0.103967	1.528061	0.126497408	0.1641713	NOT
NID1	7808.81	-0.20346	0.133239	-1.52706	0.126745831	0.1644808	NOT

RPUSD3	1185.944	0.143432	0.093927	1.527053	0.126747808	0.1644808	NOT
DNAJC11	991.1167	-0.15317	0.100321	-1.52675	0.12682208	0.1645695	NOT
RP11-999I	9.249037	-0.4014	0.263	-1.52624	0.126950844	0.1647289	NOT
KIAA1211	63.02728	0.476869	0.31246	1.526176	0.126966084	0.1647409	NOT
RP11-541I	45.65431	0.203588	0.1334	1.526147	0.126973324	0.1647426	NOT
RP3-432I1	1.095494	0.688308	0.451106	1.525824	0.127053631	0.1648391	NOT
CNTN1	74.78562	0.746975	0.489681	1.525433	0.127151212	0.1649579	NOT
LINC0039(	3.284832	-0.45123	0.295868	-1.5251	0.127234647	0.1650584	NOT
SNORD10(	5.243118	0.329793	0.216296	1.524729	0.127326787	0.1651702	NOT
PEF1	2891.278	-0.12844	0.084248	-1.52455	0.127372076	0.165214	NOT
PPP1R14A	249.6624	0.31022	0.203484	1.524546	0.127372476	0.165214	NOT
ID4	200.1478	-0.38109	0.249983	-1.52447	0.127390307	0.1652294	NOT
AF186192.	5.631678	0.523529	0.343508	1.524066	0.127492118	0.1653537	NOT
HAP1	13.15364	0.393456	0.258192	1.523888	0.127536635	0.1654037	NOT
ZNF433	106.3697	0.179263	0.117638	1.523845	0.127547347	0.1654098	NOT
PKD2L2	2.120133	0.419794	0.27552	1.523643	0.127597853	0.1654675	NOT
HOXB6	50.88548	0.334077	0.219275	1.523553	0.127620451	0.1654871	NOT
CR2	14.28757	0.631593	0.414558	1.523535	0.127624883	0.1654871	NOT
NBPF9	354.8127	0.224423	0.147313	1.523442	0.127648258	0.1655096	NOT
RP11-345I	1.839045	0.431096	0.28301	1.523253	0.127695404	0.165563	NOT
TM4SF5	7845.508	0.390639	0.256535	1.522751	0.127821011	0.1657181	NOT
ZNF277	978.0228	-0.13672	0.089793	-1.52261	0.127856686	0.1657566	NOT
RNU5B-2P	8.765835	0.266714	0.175218	1.522185	0.127962804	0.1658864	NOT
RP11-517I	57.71548	0.163547	0.107444	1.522157	0.127969638	0.1658875	NOT
HLA-DPB1	6050.38	-0.28729	0.188759	-1.52201	0.128007811	0.1659292	NOT
RP11-85G:	5.205294	-0.60995	0.400932	-1.52134	0.128175457	0.1661387	NOT
AC063976	5.658624	0.597799	0.393007	1.521092	0.128236728	0.1662103	NOT
RP11-65J3	2.354708	0.626559	0.412018	1.520706	0.128333585	0.1663281	NOT
DDX50	997.2097	0.110823	0.072879	1.520654	0.128346669	0.1663372	NOT
IGSF11	3.962317	0.559117	0.367716	1.520513	0.128382084	0.1663753	NOT
TMEM245	3917.136	0.18633	0.122557	1.520349	0.128423304	0.166421	NOT
LINC0063(	14.40139	0.306354	0.201523	1.520195	0.128461861	0.1664631	NOT
EPN1	8961.032	-0.14501	0.095391	-1.52012	0.128480447	0.1664794	NOT
ASCL2	36.03997	-0.40099	0.263891	-1.51954	0.128626809	0.1666613	NOT
RP11-374I	2.02346	0.515602	0.339338	1.519436	0.128652703	0.166687	NOT
SPRR3	3.182968	1.020356	0.67178	1.518885	0.128791516	0.166859	NOT
RP11-713I	1.813712	0.548461	0.36118	1.518524	0.128882373	0.1669689	NOT
ZNF776	388.5303	-0.1487	0.097932	-1.51837	0.128921848	0.1670123	NOT
UBTD1	616.4606	0.168253	0.110876	1.517488	0.129143635	0.1672859	NOT
AC034220	90.26905	0.226604	0.149329	1.517481	0.129145162	0.1672859	NOT
ATAD3A	1018.551	0.180031	0.118648	1.517357	0.129176497	0.1673186	NOT
NYAP1	19.13893	0.251546	0.165804	1.51713	0.129233859	0.1673851	NOT
ATG4A	829.3738	-0.1384	0.091238	-1.51688	0.129295803	0.1674575	NOT
TCP11L1	236.9224	0.13111	0.086438	1.516816	0.129313137	0.1674721	NOT
RP11-120I	4.645346	0.371235	0.244802	1.516471	0.129400423	0.1675773	NOT
NALCN	64.33723	-0.48033	0.316765	-1.51635	0.129431378	0.1676095	NOT
RP3-335N	1.462168	0.80781	0.532861	1.515984	0.129523284	0.1677207	NOT
NUDT13	231.9308	-0.23082	0.152412	-1.51443	0.12991703	0.1682227	NOT
RP11-539I	80.96332	-0.30221	0.199606	-1.51404	0.130014886	0.1683366	NOT
RN7SL574I	1.709266	0.449335	0.29678	1.514034	0.130017146	0.1683366	NOT
RP11-809I	4.470988	0.343695	0.227038	1.513822	0.130071079	0.1683985	NOT
CDH15	135.1844	0.616888	0.407544	1.513675	0.130108406	0.168439	NOT

SNX7	588.7545	0.252728	0.166988	1.513455	0.130164165	0.1685033	NOT
MYOZ2	2.439567	0.53385	0.352749	1.5134	0.130178035	0.1685133	NOT
CMTM7	417.7428	0.236403	0.156218	1.513292	0.130205468	0.168541	NOT
BRCC3	642.9081	0.129741	0.085738	1.51323	0.130221291	0.1685536	NOT
GNA11	2399.939	-0.09774	0.064625	-1.51249	0.130410318	0.1687903	NOT
LRRTM1	9.686103	-0.66261	0.438163	-1.51224	0.130471558	0.1688614	NOT
CTD-2342	49.20895	-0.42266	0.279502	-1.5122	0.130481929	0.1688614	NOT
CES4A	310.161	-0.40636	0.268722	-1.5122	0.130483518	0.1688614	NOT
DYNLL1P1	1.900658	0.379274	0.250828	1.512088	0.130511454	0.1688896	NOT
RP11-410I	6.862503	-0.27955	0.184906	-1.51187	0.13056743	0.1689472	NOT
SDR42E2	3.54154	0.368303	0.243609	1.511865	0.130568142	0.1689472	NOT
C4orf27	569.1099	0.108619	0.071854	1.511657	0.130621033	0.1690077	NOT
RP5-1184I	1.366862	0.501473	0.331833	1.511221	0.13073225	0.1691437	NOT
MRPL36	1060.661	0.146048	0.096662	1.510912	0.130810825	0.1692375	NOT
MCM8-AS	2.418816	-0.41953	0.27772	-1.51063	0.130883581	0.1693237	NOT
MIR155HG	22.62124	0.374263	0.247793	1.510386	0.130944837	0.1693887	NOT
RP11-677I	1.700435	0.463382	0.306798	1.510382	0.130946055	0.1693887	NOT
RP11-64K	32.97215	0.167296	0.110769	1.510321	0.130961661	0.1694009	NOT
GTF2F2	638.673	0.121694	0.080581	1.510209	0.130990078	0.1694298	NOT
PCOLCE-A	10.68312	-0.33876	0.224325	-1.51014	0.131008641	0.1694458	NOT
AC009404	60.02547	0.229345	0.151885	1.509988	0.131046504	0.1694869	NOT
CCDC88B	483.7921	0.242251	0.160446	1.509858	0.131079758	0.169522	NOT
LINC01137	281.0327	-0.20513	0.135868	-1.50976	0.131105378	0.1695472	NOT
CH17-360	50.99174	0.586061	0.388229	1.509574	0.131152216	0.1695995	NOT
RP5-854E1	2.160073	0.409631	0.27136	1.509551	0.13115812	0.1695995	NOT
BACH1-IT1	5.385521	0.327869	0.217238	1.509265	0.131231042	0.1696859	NOT
PCDHA13	9.927974	-0.70882	0.46969	-1.50913	0.131266083	0.1697233	NOT
RPS14	24153.79	0.188512	0.124918	1.50909	0.131275689	0.1697278	NOT
SPATA20	2678.331	0.176695	0.117116	1.508717	0.131371128	0.1698432	NOT
MLNR	1.506907	0.693347	0.45958	1.508652	0.131387781	0.1698568	NOT
FRMD5	3.493231	0.52718	0.349469	1.508517	0.131422225	0.1698934	NOT
STX2	521.2963	0.153024	0.101446	1.508436	0.131443096	0.1699124	NOT
WDFY1	1271.882	-0.11256	0.074625	-1.50838	0.13145735	0.1699229	NOT
CTD-2583	3.976772	0.345414	0.229009	1.508301	0.13147756	0.1699411	NOT
ADD1	6402.073	0.107754	0.071448	1.50814	0.13151861	0.1699862	NOT
SIK2	4765.757	-0.19411	0.128716	-1.50803	0.131546659	0.1700145	NOT
CD40LG	39.35712	-0.37559	0.249068	-1.50796	0.131563949	0.1700252	NOT
PTGES3P3	25.79408	0.204804	0.135816	1.507951	0.131567168	0.1700252	NOT
NOLC1	2767.779	-0.12064	0.080009	-1.50788	0.131585787	0.1700413	NOT
AC000032	2.772069	0.894611	0.593358	1.507709	0.131628989	0.1700892	NOT
ANKRD30I	1.273755	-0.69307	0.459771	-1.50743	0.13170094	0.1701742	NOT
CCDC126	326.1519	-0.14245	0.094524	-1.507	0.131809901	0.170307	NOT
TOX3	485.6791	0.373252	0.247727	1.506707	0.131885779	0.1703971	NOT
RP11-755	1.094953	0.693292	0.460149	1.506668	0.131895785	0.1704021	NOT
IPP	397.88	0.12793	0.084925	1.506394	0.131965953	0.1704848	NOT
AC090587	20.66243	0.248869	0.165245	1.506064	0.132050625	0.1705862	NOT
PPP1R26	807.9742	0.157464	0.104569	1.505839	0.13210857	0.1706531	NOT
AGAP2-AS	87.19946	0.286857	0.190551	1.505406	0.132219795	0.1707888	NOT
PPP1R18	2087.839	-0.23474	0.156034	-1.50442	0.13247274	0.1711075	NOT
TRBV7-9	8.73665	-0.46023	0.305935	-1.50435	0.132490122	0.1711162	NOT
MIR3180-	1.982623	0.553895	0.368196	1.504348	0.132491843	0.1711162	NOT
PLBD2	3764.62	0.123364	0.082007	1.504298	0.132504735	0.1711249	NOT

RP11-478I	8.185383	0.525286	0.349205	1.504234	0.132521176	0.1711381	NOT
PIGO	1560.895	0.130453	0.086737	1.504003	0.132580466	0.1712067	NOT
LMX1A	4.195661	0.685176	0.455696	1.503581	0.132689216	0.1713368	NOT
GNAQP1	3.550458	-0.37009	0.246141	-1.50356	0.132693571	0.1713368	NOT
XYLT1	95.35565	0.32745	0.217809	1.503384	0.132739991	0.1713872	NOT
RP11-231I	2.480005	0.399344	0.265633	1.503365	0.132745039	0.1713872	NOT
BBX	1162.099	0.188142	0.125195	1.502794	0.132892228	0.1715693	NOT
WIBG	1434.122	0.124055	0.082564	1.502535	0.132959024	0.1716475	NOT
RAB28	341.989	0.110358	0.073451	1.502482	0.132972591	0.171657	NOT
CYTH1	2492.37	0.11266	0.074986	1.502409	0.132991556	0.1716735	NOT
LARP6	232.4865	0.3606	0.240021	1.502369	0.133001846	0.1716787	NOT
LINC0141(	12.83968	-0.31284	0.208293	-1.50191	0.133120924	0.1718244	NOT
MBTPS1	3412.429	-0.12146	0.080873	-1.50183	0.133140887	0.1718422	NOT
KB-1836B!	3.143623	0.448607	0.298724	1.501742	0.133163707	0.1718636	NOT
TRAPPC1	2274.745	0.181901	0.121147	1.501484	0.133230365	0.1719349	NOT
RP11-286I	2.366158	-0.40181	0.267609	-1.50148	0.133231408	0.1719349	NOT
JAKMIP1	24.57231	0.381224	0.253908	1.501426	0.133245347	0.1719449	NOT
PPP1R32	103.3334	-0.27477	0.183072	-1.5009	0.133382706	0.1721141	NOT
NLGN3	16.98389	0.2756	0.183634	1.500812	0.133404288	0.172134	NOT
RP11-380I	3.347884	0.565686	0.377016	1.500427	0.133503755	0.1722543	NOT
CTD-2313	1.845526	0.445923	0.29722	1.500315	0.133532808	0.1722837	NOT
CTGLF8P	4.259314	0.395529	0.263721	1.499804	0.133665199	0.1724465	NOT
RP11-861/	2.082129	0.452613	0.301879	1.49932	0.133790626	0.1726003	NOT
TPI1P2	17.14863	0.25465	0.169862	1.499159	0.133832439	0.1726462	NOT
CTB-102L!	2.728162	0.762774	0.508815	1.499118	0.133843149	0.1726497	NOT
CTC-255N	2.157252	-0.81186	0.541562	-1.4991	0.13384763	0.1726497	NOT
RP11-497I	2.769251	-0.53033	0.353818	-1.49888	0.133903618	0.1727138	NOT
LSMEM2	2.198822	0.4169	0.27823	1.498401	0.134029085	0.1728676	NOT
RP5-875O	6.587167	-0.42036	0.280603	-1.49808	0.13411285	0.1729676	NOT
RP1-140K(	2.651105	-0.51181	0.341708	-1.4978	0.134186504	0.1730545	NOT
ZER1	1600.31	-0.15036	0.100429	-1.49717	0.13434819	0.1732549	NOT
TLDC1	203.7961	0.283086	0.189103	1.496989	0.13439619	0.1733088	NOT
NT5C3AP1	3.609139	0.399594	0.266964	1.496808	0.134443325	0.1733615	NOT
PTGS1	280.3642	-0.31834	0.212696	-1.49668	0.134476924	0.1733967	NOT
GSTA8P	3.73269	0.470335	0.314266	1.496616	0.134493331	0.1734098	NOT
CCDC58	766.0505	0.148667	0.099345	1.496468	0.13453165	0.1734511	NOT
NPL	1030.68	0.279413	0.186724	1.496398	0.134549911	0.1734666	NOT
TPP1	10978.54	0.141772	0.094759	1.49614	0.134617205	0.1735452	NOT
SNRPGP9	3.125488	-0.37172	0.248507	-1.49583	0.134699011	0.1736426	NOT
DTWD1	428.9069	0.110638	0.073979	1.495536	0.13477465	0.173732	NOT
PDE4D	222.7983	-0.26469	0.177004	-1.49539	0.134812042	0.1737721	NOT
BBIP1	337.1478	-0.11008	0.073622	-1.49522	0.134856485	0.1738213	NOT
UBE2QL1	204.6488	0.427904	0.286218	1.49503	0.134906717	0.173878	NOT
RP4-539M	5.649996	0.449576	0.300727	1.494961	0.134924512	0.1738917	NOT
COQ10A	911.4183	0.22745	0.152146	1.494941	0.134929967	0.1738917	NOT
DCAF8	3992.454	0.129206	0.086434	1.494858	0.134951389	0.1739112	NOT
TMEM50B	1129.141	0.117111	0.078347	1.494778	0.134972264	0.17393	NOT
ST13P4	6.499655	-0.25962	0.17377	-1.49401	0.135171769	0.174179	NOT
ITGB1	9736.5	0.201093	0.134612	1.493865	0.135210897	0.1742176	NOT
CAMKK2	2390.244	0.108552	0.072666	1.493852	0.135214287	0.1742176	NOT
AP000692.	11.64038	-0.33253	0.22261	-1.49377	0.135235029	0.1742362	NOT
LINC0047:	5.255337	0.732833	0.491001	1.492528	0.135560888	0.1746479	NOT

TLE2	600.7307	0.232859	0.156025	1.492449	0.135581436	0.1746662	NOT
ADAM8	309.4023	0.265519	0.177915	1.492389	0.13559717	0.1746783	NOT
GAFA3	8.185059	0.395622	0.265157	1.492031	0.135691054	0.1747876	NOT
MTRNR2L9	4.743267	-0.38989	0.261317	-1.49202	0.135694608	0.1747876	NOT
SERPINA13	1.1252	0.720661	0.483043	1.491921	0.135719865	0.174812	NOT
ZNF41	246.3092	-0.14081	0.0944	-1.49165	0.13579212	0.1748969	NOT
NBPF8	157.385	0.218586	0.146548	1.491563	0.135813654	0.1749165	NOT
ZNF180	266.88	0.135756	0.091022	1.491468	0.135838735	0.1749373	NOT
NEK5	32.72601	0.23113	0.154972	1.491432	0.13584823	0.1749373	NOT
GCC1	1060.354	-0.12656	0.084858	-1.49143	0.135848745	0.1749373	NOT
PHGR1	3.614694	0.68826	0.46159	1.491062	0.135945261	0.1750534	NOT
USP50	3.093545	-0.33218	0.222805	-1.4909	0.135987402	0.1750995	NOT
APOBEC3F	195.6389	-0.22222	0.149074	-1.49064	0.136055317	0.1751788	NOT
GMDS	1330.105	0.216139	0.145005	1.490564	0.136075961	0.1751972	NOT
SETD6	433.2006	0.105989	0.071115	1.490399	0.13611947	0.1752451	NOT
RNF223	1.624308	0.742509	0.498258	1.49021	0.136168968	0.1753007	NOT
DENND2A	248.6613	-0.21123	0.141752	-1.49012	0.136192296	0.175314	NOT
CTB-109A	1.749365	0.438608	0.294347	1.490105	0.136196737	0.175314	NOT
CLDN20	2.498904	0.429528	0.288255	1.490098	0.136198341	0.175314	NOT
CYP4F29P	5.811791	-0.59328	0.398266	-1.48966	0.136314288	0.1754551	NOT
CUL1	3220.977	-0.11201	0.07523	-1.48894	0.136503944	0.175691	NOT
JAK2	317.7406	-0.21458	0.144123	-1.48886	0.136523979	0.1757087	NOT
AC092597	1.278746	0.504299	0.338729	1.488797	0.13654078	0.1757221	NOT
ZNF136	218.6196	-0.13998	0.094024	-1.48876	0.136550748	0.1757268	NOT
RP11-209I	1.595989	0.437088	0.293612	1.488659	0.136577253	0.1757527	NOT
AP001065.	127.2759	0.431487	0.289866	1.488575	0.136599377	0.175773	NOT
RP5-1061I	1.26206	0.802849	0.53937	1.488493	0.136620963	0.1757926	NOT
NXNL2	3.966794	0.542333	0.364469	1.48801	0.136748315	0.1759483	NOT
LINC01420	370.1562	0.210808	0.141732	1.487365	0.136918306	0.1761588	NOT
RP11-15A1	10.16234	0.388862	0.261567	1.486661	0.137104288	0.1763857	NOT
AF131215.	24.48853	0.378065	0.254307	1.486649	0.137107442	0.1763857	NOT
AL136419.	16.73879	0.193098	0.129911	1.48638	0.137178612	0.1764691	NOT
BOD1L1	1050.519	0.167118	0.112463	1.485981	0.137284041	0.1765965	NOT
FAM189A1	12.08064	0.446798	0.300689	1.485914	0.137301967	0.1766113	NOT
SLC25A5	13750.59	0.154372	0.103933	1.485307	0.137462445	0.1768096	NOT
C3orf17	1038.184	0.084369	0.056804	1.485261	0.137474621	0.176817	NOT
CAMK1G	15.40716	0.383408	0.258165	1.485127	0.137510209	0.1768545	NOT
RP13-514I	2.832682	-0.47824	0.322074	-1.48486	0.137581049	0.1769374	NOT
LINC01144	61.82567	0.24675	0.166183	1.484813	0.137593306	0.176945	NOT
ENAM	73.33823	0.408679	0.275259	1.484708	0.13762114	0.1769725	NOT
LY6G5C	68.43932	0.222182	0.149663	1.484549	0.137663308	0.1770185	NOT
PIGW	310.2021	0.133769	0.090139	1.48403	0.137800848	0.1771872	NOT
TMEM125	63.00064	0.526341	0.354692	1.483936	0.137825926	0.1772106	NOT
LCN12	327.2982	0.261557	0.176262	1.483913	0.13783188	0.1772106	NOT
SNHG24	0.972003	0.802186	0.540597	1.483889	0.137838328	0.1772106	NOT
F10-AS1	8.712426	-0.32164	0.216821	-1.48343	0.137959013	0.1773524	NOT
BNIP3P5	5.526178	0.345079	0.232623	1.483426	0.137961389	0.1773524	NOT
TRAPPC3	1839.377	0.108777	0.07335	1.482981	0.138079449	0.1774959	NOT
GPR174	11.75387	-0.48164	0.324814	-1.48281	0.138125902	0.1775474	NOT
SOS2	934.4763	-0.13976	0.094257	-1.48271	0.138151828	0.1775724	NOT
RP11-835I	2.361127	-0.38393	0.259053	-1.48204	0.138329223	0.1777922	NOT
CYP3A7	7627.666	-0.52746	0.355952	-1.48183	0.138384546	0.177855	NOT

RP11-350J	3.285149	0.425325	0.287072	1.481596	0.138447841	0.1779281	NOT
ACTR3	4348.287	0.10711	0.072297	1.481538	0.138463387	0.1779398	NOT
METTL15	451.1273	-0.10998	0.074253	-1.48111	0.138577711	0.1780785	NOT
ANAPC2	1882.208	0.115647	0.078087	1.480997	0.138607305	0.1781052	NOT
EEF1A1P17	4.048203	0.327642	0.221236	1.480964	0.138616273	0.1781052	NOT
CIRH1A	1132.127	0.13368	0.090266	1.480958	0.138617844	0.1781052	NOT
CYP2D6	10671.66	-0.41232	0.278512	-1.48046	0.138751157	0.1782682	NOT
RP11-17P1	16.43504	-0.19693	0.133025	-1.48038	0.138772973	0.178288	NOT
TMEM252	43.83843	-0.66792	0.451273	-1.48008	0.138853084	0.1783826	NOT
DRD1	47.58032	0.526458	0.355868	1.479363	0.139043226	0.1786186	NOT
RP11-519(	2.735515	-0.37158	0.251281	-1.47874	0.139210303	0.1788249	NOT
LINC01013	2.699014	0.440886	0.298203	1.478475	0.1392807	0.1789071	NOT
SNX5P1	1.9043	0.461102	0.311902	1.478356	0.139312601	0.1789397	NOT
IGLJ2	0.953243	-0.74022	0.500785	-1.47811	0.139377917	0.1790153	NOT
MYLPF	7.364681	-0.39901	0.269969	-1.47798	0.139412238	0.1790511	NOT
MEIG1	6.971809	0.314307	0.212679	1.477842	0.139449948	0.1790847	NOT
MOSPD3	1080.99	0.171566	0.116093	1.477837	0.139451341	0.1790847	NOT
ROGDI	1182.319	-0.17029	0.115245	-1.47763	0.139508086	0.1791492	NOT
OST4	4820.787	0.139629	0.094504	1.47749	0.139544285	0.1791874	NOT
PPP1R3D	94.98914	0.222566	0.150653	1.477348	0.139582461	0.1792281	NOT
ACVR2B-A	48.36609	-0.19817	0.134143	-1.47731	0.139593711	0.1792342	NOT
FGD5	534.4108	0.213755	0.144711	1.477119	0.139643863	0.1792903	NOT
SLC34A2	340.601	0.683954	0.46319	1.476616	0.139778589	0.179455	NOT
Metazoa_S	23.8608	-0.25062	0.169865	-1.47542	0.140099737	0.1798589	NOT
NUDT18	215.0363	0.191872	0.130069	1.47516	0.140169661	0.1799403	NOT
RP11-68I3	1.395202	0.476194	0.323008	1.474251	0.140413932	0.1802455	NOT
TNFRSF12A	2450.765	-0.29395	0.199491	-1.47349	0.140619656	0.1805013	NOT
RPS18P13	1.821748	0.461562	0.313302	1.473215	0.140693079	0.1805871	NOT
PACRG	111.2923	-0.41238	0.279938	-1.47313	0.140715681	0.1806078	NOT
HCG14	5.397062	0.394053	0.267699	1.472002	0.141020211	0.1809902	NOT
KRTAP19-5	0.77259	1.996381	1.357242	1.47091	0.141315522	0.1813608	NOT
ZBP1	49.5579	-0.39687	0.269821	-1.47088	0.141323295	0.1813624	NOT
DEF6	283.5862	0.262001	0.17818	1.470429	0.141445733	0.1815111	NOT
NCAN	6.310476	0.450752	0.306582	1.470251	0.141493836	0.1815644	NOT
SYTL2	279.6806	-0.25138	0.171014	-1.46993	0.141581939	0.181669	NOT
GLUD2	482.1167	0.320356	0.217954	1.469829	0.141607975	0.181694	NOT
FBXO17	1821.384	0.2194	0.149277	1.469756	0.141627901	0.1817111	NOT
ZNF513	875.6484	0.121122	0.082421	1.469554	0.141682663	0.181773	NOT
GAB3	102.5657	-0.23071	0.15709	-1.46862	0.141935253	0.1820886	NOT
MAP2K7	1621.186	0.103462	0.070452	1.46854	0.141957536	0.1821087	NOT
FAM13B	538.6336	0.139555	0.095052	1.468207	0.14204806	0.1822164	NOT
DCP1A	675.6723	-0.10252	0.069845	-1.46789	0.142132991	0.1823169	NOT
DNAAF3	22.44254	-0.32728	0.222989	-1.46768	0.142191235	0.1823832	NOT
TMEM17	98.65116	0.143903	0.09807	1.467344	0.14228243	0.182484	NOT
NANS	1711.616	0.149273	0.10173	1.467342	0.142283007	0.182484	NOT
CTD-2288	8.309023	-0.26572	0.181105	-1.46721	0.142317739	0.18252	NOT
RP11-960I	2.805238	-0.41927	0.285784	-1.46707	0.142355834	0.1825604	NOT
ZNF285B	2.635346	0.406296	0.276978	1.466888	0.142406531	0.182617	NOT
C16orf89	17.89899	0.389802	0.265741	1.466847	0.142417727	0.1826229	NOT
EEF1A1P3	4.940569	0.322894	0.220144	1.466742	0.142446282	0.182651	NOT
RP11-133I	2.639808	-0.29944	0.20419	-1.46648	0.142517669	0.1827341	NOT
TMED2	13316.49	-0.09668	0.065957	-1.46586	0.142686266	0.1829418	NOT

RP11-578I	1.428687	0.462594	0.315608	1.465723	0.142723706	0.1829813	NOT
MRPL18	1933.138	0.133209	0.090931	1.464948	0.142935229	0.183244	NOT
PSTK	195.255	0.161219	0.110086	1.464482	0.14306218	0.1833983	NOT
RP11-21C4	2.959739	-0.47366	0.323478	-1.46426	0.143123716	0.1834686	NOT
FOXJ2	614.2295	0.117957	0.080561	1.464191	0.143141705	0.1834832	NOT
RP11-643A	2.726495	0.816912	0.557941	1.464153	0.14315202	0.1834879	NOT
DST	6256.495	-0.18006	0.122984	-1.46407	0.143174063	0.1835077	NOT
CTD-2530	2.699973	0.35428	0.242087	1.463441	0.14334666	0.1837204	NOT
FKSG62	12.24313	-0.52402	0.358094	-1.46337	0.143367472	0.1837386	NOT
ITGA9-AS1	86.88123	0.169471	0.115825	1.463163	0.143422798	0.1838005	NOT
IGLC7	30.57185	-0.67727	0.462886	-1.46314	0.143429058	0.1838005	NOT
LGALS12	5.051072	0.455425	0.311277	1.463086	0.143443933	0.183811	NOT
RP11-3M1	8.386909	-0.44136	0.301683	-1.463	0.143466831	0.1838318	NOT
SEC31A	6642.856	-0.10456	0.071476	-1.46293	0.143486178	0.1838481	NOT
GOLGA8R	7.888874	0.347752	0.237757	1.462638	0.143566506	0.183941	NOT
TTC3	4350.75	0.125735	0.085966	1.462618	0.143571982	0.183941	NOT
IFNGR1	3641.31	-0.22864	0.156353	-1.46233	0.143650362	0.1840327	NOT
VWA3A	2.502275	0.408302	0.279218	1.462308	0.143656817	0.1840327	NOT
C17orf97	96.89698	-0.26738	0.182875	-1.46207	0.14372285	0.1841087	NOT
WHAMMP	53.70971	-0.18569	0.127059	-1.46141	0.143902203	0.1843299	NOT
CTNNB1	10335.31	0.141446	0.096817	1.460963	0.144025622	0.1844795	NOT
DOCK9	1001.651	0.190318	0.130277	1.460872	0.144050618	0.184503	NOT
CALM2	12395.34	0.108034	0.073965	1.460599	0.1441254	0.1845902	NOT
C10orf62	3.928832	0.395727	0.270964	1.460441	0.144168815	0.1846373	NOT
NUB1	2712.5	0.105016	0.07191	1.460383	0.144184774	0.1846491	NOT
GTF3A	4720.262	-0.17097	0.117076	-1.46033	0.144198589	0.1846583	NOT
KRT80	243.5993	0.536061	0.367129	1.460143	0.144250809	0.1847166	NOT
MTND5P1	33.87786	-0.33402	0.228831	-1.45968	0.144377621	0.1848704	NOT
ZNF771	213.303	0.195608	0.134028	1.459455	0.144440048	0.1849418	NOT
GDNF	131.0799	0.592427	0.405984	1.459235	0.144500358	0.1850105	NOT
XXyac-YM	27.30044	0.520628	0.356791	1.459197	0.144510823	0.1850153	NOT
RP1-20N2	4.422121	0.286264	0.196193	1.459092	0.144539659	0.1850437	NOT
SPAG16	381.3069	-0.21436	0.146922	-1.45902	0.14455923	0.1850602	NOT
MED15P4	0.684443	1.765094	1.209844	1.458944	0.144580649	0.185079	NOT
CIDEC	172.3222	0.525576	0.360401	1.458309	0.144755343	0.1852941	NOT
RAC2	748.5155	-0.27888	0.191245	-1.45826	0.144768515	0.1853024	NOT
AL133243.	40.76776	0.176647	0.121186	1.457644	0.144938655	0.1855116	NOT
ANKRD18E	8.220749	0.586107	0.402105	1.457596	0.144952008	0.1855201	NOT
ZNF566	219.6348	0.132596	0.090972	1.457535	0.144968855	0.185533	NOT
GLT8D2	82.5957	-0.2631	0.18055	-1.45721	0.145057434	0.1856378	NOT
GSDMA	16.47543	0.432715	0.296975	1.457077	0.145095257	0.1856776	NOT
DHRS7	4615.446	0.186543	0.12803	1.457023	0.145109924	0.1856878	NOT
SCN3A	8.144081	-0.37526	0.257585	-1.45683	0.145164089	0.1857485	NOT
CTD-3092	122.4413	-0.21516	0.147691	-1.4568	0.145171658	0.1857496	NOT
SNORA2A	1.770732	0.388295	0.266595	1.456501	0.145254103	0.1858465	NOT
ATP6V0E1	6348.196	0.142403	0.097776	1.456421	0.14527636	0.1858664	NOT
CNGB1	5.56589	0.475232	0.326449	1.455764	0.145457983	0.1860902	NOT
SLC28A3	29.69414	-0.58147	0.399738	-1.45462	0.145775137	0.1864873	NOT
IRX2	1.445885	0.568064	0.390567	1.454458	0.145819243	0.1865351	NOT
DPP4	4141.238	0.306356	0.210678	1.454141	0.145907184	0.186639	NOT
AP000704.	7.104942	0.313969	0.21594	1.453966	0.145955713	0.1866924	NOT
GGTLC2	1.642167	-0.5072	0.348861	-1.45388	0.145980207	0.1867151	NOT

SMYD4	354.476	0.126126	0.086774	1.453505	0.146083668	0.1868388	NOT
RP11-154I	3.648162	0.338619	0.232978	1.453441	0.146101342	0.1868528	NOT
RP1-187B2	1.615614	-0.41716	0.287028	-1.45337	0.146120157	0.1868682	NOT
MRPS18A	1003.369	0.12956	0.089146	1.453337	0.146130195	0.1868724	NOT
MB21D2	76.77479	-0.26422	0.181807	-1.45328	0.146144877	0.1868825	NOT
LRTOMT	341.6855	-0.13362	0.091969	-1.45285	0.146265087	0.1870276	NOT
TOPORS-A	148.7837	0.189402	0.13041	1.452359	0.146401796	0.1871938	NOT
RPS4XP2	2.731918	0.362633	0.2497	1.452276	0.146424905	0.1872147	NOT
CD276	2978.629	-0.15617	0.107569	-1.45182	0.146550478	0.1873665	NOT
SLIRP	1615.285	0.198312	0.136615	1.451605	0.146611346	0.1874301	NOT
MYRIP	1418.784	-0.4114	0.283412	-1.4516	0.146613755	0.1874301	NOT
BTBD11	100.1939	-0.32122	0.221309	-1.45143	0.146659864	0.1874804	NOT
LINC00538	2.586376	0.380438	0.262181	1.451052	0.146765247	0.1876065	NOT
FCER1A	58.32518	-0.38996	0.268771	-1.4509	0.14680857	0.1876532	NOT
ZDHHC18	1402.012	-0.12303	0.084804	-1.45078	0.146840576	0.1876854	NOT
BAHD1	1156.968	-0.12872	0.088757	-1.45027	0.146982873	0.1878573	NOT
HLA-A	48050.64	0.225891	0.15576	1.450251	0.146988629	0.1878573	NOT
ITPKB	498.2654	0.219885	0.151622	1.450213	0.146999052	0.1878619	NOT
ZNF725P	0.564065	1.576196	1.086897	1.450179	0.14700858	0.1878654	NOT
PARP15	69.79172	0.333836	0.230274	1.449734	0.147132825	0.1880105	NOT
RWDD1	1550.828	-0.14004	0.096599	-1.44972	0.147137966	0.1880105	NOT
TENM1	292.4004	-0.50157	0.345988	-1.44968	0.147148863	0.1880105	NOT
RP11-488(	1.861939	0.412408	0.284483	1.449675	0.147149307	0.1880105	NOT
DSG2	2108.866	0.31771	0.219194	1.449443	0.147214013	0.1880845	NOT
RPL5P30	6.367275	-0.3159	0.217966	-1.44929	0.147256795	0.1881305	NOT
SYT13	150.8241	0.606538	0.418635	1.448849	0.14737968	0.1882788	NOT
CST8	0.647857	1.691842	1.167958	1.448547	0.147464229	0.1883781	NOT
RP11-616I	1.141909	0.756946	0.522595	1.448437	0.147494968	0.1884087	NOT
HMCN1	511.235	-0.33902	0.234068	-1.44838	0.147511683	0.1884213	NOT
THOC7	1483.013	0.125199	0.086459	1.44808	0.147594764	0.1885187	NOT
SPATA9	9.724336	0.281646	0.194568	1.447541	0.147745369	0.1887024	NOT
RP4-782L2	4.705919	0.464698	0.321172	1.446885	0.147929219	0.1889285	NOT
ZBTB20-AS	1.480201	-0.58805	0.406529	-1.44651	0.14803486	0.1890547	NOT
MAP3K4	749.5773	0.117349	0.081145	1.446169	0.148129809	0.1891672	NOT
MZT2B	3206.176	0.223673	0.15467	1.446137	0.148138648	0.1891698	NOT
STAT1	8978.022	-0.25367	0.175442	-1.44591	0.148203509	0.1892438	NOT
CHUK	1003.432	-0.11139	0.077133	-1.44415	0.148697438	0.1898658	NOT
FLJ33360	6.130045	0.426652	0.295473	1.443961	0.148749845	0.1899239	NOT
PRKCG	1.179489	0.593672	0.411237	1.443626	0.148844301	0.1900358	NOT
KIF13A	1796.104	0.14118	0.09781	1.443402	0.148907332	0.1901075	NOT
CPA3	105.2457	0.492634	0.341378	1.443077	0.148998648	0.1902153	NOT
LOXL3	84.05604	0.202138	0.140109	1.442719	0.149099662	0.1903355	NOT
DCSTAMP	11.50151	0.371979	0.257852	1.442607	0.149131074	0.1903668	NOT
IGHE	12.90955	-0.59495	0.412429	-1.44255	0.149146197	0.1903773	NOT
TSPAN1	224.1618	-0.26078	0.180826	-1.44218	0.149252574	0.1905043	NOT
SLC35D2	2186.336	-0.15214	0.105504	-1.44201	0.149298366	0.1905539	NOT
AMOTL1	1432.786	0.293539	0.203577	1.441906	0.149328773	0.190584	NOT
RP11-57H	6.381628	0.275801	0.191355	1.441307	0.149498087	0.1907913	NOT
CCDC151	26.76315	-0.2425	0.168289	-1.44099	0.149586289	0.190895	NOT
SHROOM2	2.31593	1.242225	0.862198	1.440766	0.149650888	0.1909686	NOT
ZFP91	2203.607	-0.10644	0.07389	-1.44046	0.149736661	0.1910693	NOT
RP11-452I	12.20101	0.261461	0.18155	1.440159	0.149822481	0.19117	NOT

TGFBR1	1384.038	0.143649	0.099751	1.440076	0.149845787	0.1911909	NOT
ZMYM6	344.7697	0.086513	0.060079	1.439986	0.14987125	0.1912146	NOT
CAPZA3	4.436441	-0.4797	0.333135	-1.43996	0.14987819	0.1912146	NOT
LNX1	308.8819	-0.20264	0.140752	-1.43968	0.149957688	0.1913072	NOT
AQP4-AS1	7.134068	-0.33681	0.233956	-1.43961	0.149978622	0.1913251	NOT
AL662800.	9.415115	0.222527	0.154592	1.439451	0.15002285	0.1913727	NOT
CNRIP1	173.4362	-0.21834	0.151695	-1.4393	0.150064997	0.1914176	NOT
AP002954.	1.763725	0.516897	0.359381	1.438298	0.150349603	0.1917718	NOT
RPSAP56	2.173561	0.466562	0.324455	1.437987	0.150437703	0.1918753	NOT
ZNF662	110.5231	-0.30749	0.213865	-1.43778	0.150497868	0.1919432	NOT
LTF	83.60586	0.355242	0.24716	1.437296	0.150633818	0.1921078	NOT
CTD-2290	1.451625	0.469211	0.326548	1.43688	0.15075217	0.1922498	NOT
ERO1LB	1837.553	0.238036	0.165679	1.436732	0.150794025	0.1922944	NOT
PATL2	52.69287	0.280143	0.195149	1.435532	0.151135488	0.1927209	NOT
CRPP1	5.519712	-0.67192	0.468087	-1.43547	0.151152983	0.1927343	NOT
AC005077	37.00029	-0.40096	0.2794	-1.43507	0.151267119	0.192871	NOT
MGA	914.1826	0.113373	0.07905	1.434195	0.151516644	0.1931795	NOT
RP3-508I1	3.007999	0.41719	0.290892	1.434172	0.151523069	0.1931795	NOT
RPS4XP13	2.99163	0.359755	0.250877	1.433991	0.151574799	0.1932328	NOT
AC006378	8.455726	0.217081	0.151384	1.433977	0.15157879	0.1932328	NOT
RP11-503I	5.078668	0.318686	0.222329	1.433396	0.1517447	0.1934353	NOT
XCL1	33.83547	0.374695	0.26146	1.433087	0.151832903	0.1935331	NOT
TMEM97	4788.941	0.232195	0.162025	1.433079	0.151835391	0.1935331	NOT
WDR82	2775.764	-0.09339	0.065178	-1.43291	0.151884484	0.1935868	NOT
NUTM2B-/	281.0618	0.137035	0.095658	1.432553	0.151985721	0.1937069	NOT
AC010504	8.592569	0.210914	0.147237	1.432475	0.152007815	0.1937261	NOT
RP11-933I	10.6907	-0.29196	0.203825	-1.43242	0.152024674	0.1937387	NOT
ANP32BP1	1.726298	0.388062	0.270932	1.432323	0.152051363	0.1937638	NOT
CSNK2A2	1993.625	0.094075	0.065689	1.432132	0.152106125	0.1938246	NOT
AC008592	24.47071	-0.49633	0.346597	-1.43201	0.152142184	0.1938616	NOT
UNC93B2	2.031953	0.639063	0.446387	1.431636	0.152248147	0.1939877	NOT
JAZF1-AS1	5.469321	0.418427	0.29229	1.431547	0.15227357	0.1940112	NOT
STAT5B	2566.847	-0.11432	0.079868	-1.4314	0.152315057	0.1940551	NOT
MTF1	446.0464	0.134357	0.093869	1.431336	0.152333921	0.1940702	NOT
RP1-124C	1.874026	-0.50509	0.353141	-1.43029	0.15263449	0.1944442	NOT
CNPY1	1.341342	0.784524	0.548535	1.430215	0.152655363	0.1944618	NOT
YIF1A	4971.828	-0.14904	0.104222	-1.43003	0.152708757	0.1945209	NOT
GEMIN4	623.6228	-0.13591	0.095046	-1.42998	0.15272371	0.194531	NOT
SLC4A7	329.7901	0.227303	0.158964	1.4299	0.152745801	0.1945501	NOT
RP11-265I	1.411669	0.781388	0.546521	1.429748	0.152789245	0.1945965	NOT
EPHA1	1562.159	0.344353	0.240917	1.429344	0.152905399	0.1947287	NOT
SLC17A1	892.3221	-0.35483	0.24825	-1.42934	0.152907099	0.1947287	NOT
POMK	25.99959	0.281842	0.197215	1.429112	0.152972091	0.1948025	NOT
ARPC4	3849.523	0.140418	0.098307	1.428359	0.153188633	0.1950693	NOT
LINC0015ᵹ	1.237794	0.629391	0.440649	1.428328	0.153197375	0.1950714	NOT
VILL	92.80142	0.259849	0.181939	1.42822	0.153228435	0.195102	NOT
MGARP	4.830509	-0.31296	0.219168	-1.42797	0.153301527	0.1951861	NOT
NIPSNAP3	35.62651	0.177738	0.124498	1.427638	0.153396021	0.1952974	NOT
RP11-530(	9.870312	-0.25083	0.175719	-1.42748	0.153442965	0.1953482	NOT
RAB9A	761.5749	-0.12361	0.086605	-1.42727	0.153502671	0.1954152	NOT
NIFK	1159.758	0.143114	0.100281	1.427124	0.153544152	0.195459	NOT
RPS20P22	2.015235	0.601777	0.421718	1.426964	0.153590187	0.1955086	NOT

HS3ST6	3.091082	0.66719	0.467608	1.426816	0.153632976	0.1955541	NOT
TRBV27	1.857218	0.654615	0.458889	1.42652	0.153718391	0.1956538	NOT
FBXL12	587.2807	0.107193	0.07516	1.426186	0.153814609	0.1957673	NOT
LMBRD2	1071.864	-0.16532	0.115944	-1.42582	0.15391952	0.1958918	NOT
AC044907	5.955325	0.470173	0.329787	1.425687	0.153958672	0.1959326	NOT
CFAP52	4.88831	0.342379	0.240182	1.425495	0.154014216	0.1959943	NOT
RP11-552I	5.260629	0.246528	0.172986	1.425135	0.154118125	0.1961175	NOT
RP11-567I	4.018546	0.347476	0.243836	1.42504	0.154145538	0.1961433	NOT
RP11-486I	2.927803	0.55709	0.391036	1.424651	0.154258019	0.1962774	NOT
RBSN	962.1601	0.095442	0.06701	1.4243	0.154359776	0.1963979	NOT
RP11-74M	11.37187	-0.43058	0.302327	-1.42423	0.154380367	0.196415	NOT
RPL34	13955.73	-0.18983	0.133292	-1.42419	0.154392401	0.1964213	NOT
RP11-71H	1.687909	-0.39548	0.277729	-1.42399	0.154449769	0.1964853	NOT
TTC16	8.419823	0.357369	0.250994	1.423815	0.154500015	0.1965327	NOT
CDON	231.5178	0.197371	0.138622	1.423811	0.154501221	0.1965327	NOT
SGCZ	3.918145	0.923512	0.648668	1.423706	0.154531572	0.1965622	NOT
EIF3LP3	1.83157	0.371596	0.261012	1.423675	0.154540612	0.1965647	NOT
SCYL3	682.8087	0.12032	0.084576	1.422625	0.154844764	0.1969425	NOT
LINC0127	40.34142	0.252405	0.177498	1.422018	0.155021047	0.1971576	NOT
CD1B	8.367881	0.463212	0.325748	1.421992	0.155028607	0.1971582	NOT
LITAF	6970.423	-0.17693	0.124438	-1.42184	0.155072318	0.1972047	NOT
KLHL11	36.57825	0.28046	0.197336	1.421231	0.155249744	0.1974213	NOT
BRD2	7480.877	0.110768	0.077949	1.421032	0.155307412	0.1974855	NOT
RP11-543I	14.79951	0.337835	0.237761	1.4209	0.155345695	0.1975251	NOT
RP11-84A	5.236527	0.248145	0.174652	1.420799	0.155375264	0.1975536	NOT
RP11-626I	18.4299	-0.53027	0.373283	-1.42055	0.155447711	0.1976367	NOT
CTDSPL2	784.5267	0.10783	0.075913	1.420438	0.155480315	0.1976642	NOT
CETN3	332.9068	0.104518	0.073582	1.420426	0.155483642	0.1976642	NOT
NFE2L1	14748.59	0.14157	0.099717	1.419713	0.155691149	0.1979189	NOT
TMEM92	499.1808	-0.5031	0.354396	-1.41959	0.155726973	0.1979553	NOT
RP11-15H	163.125	-0.2252	0.158659	-1.41939	0.15578509	0.1980201	NOT
RP11-603I	23.59511	0.442745	0.312094	1.418626	0.156008104	0.1982945	NOT
CTD-2001	2.295272	0.367253	0.258904	1.418493	0.156046748	0.1983272	NOT
PICK1	1124.523	0.160716	0.113301	1.418489	0.156048173	0.1983272	NOT
RPL21P3	2.148211	0.318171	0.224386	1.417959	0.156202689	0.1985145	NOT
APOBEC3D	94.31656	0.300876	0.212275	1.41739	0.15636879	0.1987164	NOT
TSSC1-IT1	6.605754	-0.26375	0.186135	-1.41696	0.156494089	0.1988665	NOT
EVA1C	267.6095	0.323806	0.228547	1.4168	0.156541399	0.1989175	NOT
CNPY3	3405.685	-0.18692	0.131938	-1.41674	0.156557792	0.1989292	NOT
OBFC1	493.7985	0.11873	0.083825	1.416407	0.156656262	0.1990452	NOT
NOM1	704.0872	0.104329	0.073687	1.415836	0.156823473	0.1992485	NOT
IFNG	8.458136	-0.46223	0.326488	-1.41577	0.15684375	0.1992651	NOT
CTC-343N	2.007069	0.427359	0.301931	1.415419	0.156945608	0.1993853	NOT
RP11-7I15	3.090964	0.49874	0.352468	1.414995	0.157069947	0.1995341	NOT
RNF40	1901.189	0.155043	0.109583	1.414843	0.157114575	0.1995817	NOT
TMEM214	4644.826	0.089162	0.063025	1.414705	0.157155149	0.1996241	NOT
ARL2BP	150.7432	0.209578	0.148153	1.414609	0.157183245	0.1996503	NOT
TMED8	482.0477	-0.13742	0.097142	-1.41458	0.157190281	0.1996503	NOT
KAL1	78.32912	0.361243	0.255383	1.414518	0.157209918	0.1996661	NOT
GGTA1P	306.8186	-0.2127	0.150387	-1.41437	0.157253099	0.1997118	NOT
IGHV5-78	2.910381	-0.52432	0.370834	-1.41388	0.157396609	0.1998849	NOT
RP1-168L1	4.523769	-0.28962	0.204947	-1.41315	0.157611207	0.2001482	NOT

LRP6	2383.314	-0.16981	0.120201	-1.4127	0.157742924	0.2003063	NOT
TMEM179	1696.334	-0.10974	0.07772	-1.41202	0.157944449	0.200553	NOT
CTC-329D	19.0465	0.168622	0.119432	1.411865	0.157989741	0.2006013	NOT
ALDH7A1F	10.75848	-0.26721	0.189275	-1.41176	0.158020991	0.2006318	NOT
TNPO1	2848.74	0.105411	0.074681	1.411474	0.15810495	0.2007292	NOT
CTC-786C	9.711317	-0.32462	0.230003	-1.41137	0.158135803	0.2007591	NOT
RNU6-312	1.943303	0.400767	0.284029	1.41101	0.15824152	0.2008841	NOT
GET4	184.682	0.155492	0.110203	1.410957	0.15825721	0.2008948	NOT
RNU6ATA(	3.340979	-0.3039	0.215417	-1.41076	0.158314576	0.2009584	NOT
AC010492	3.202596	0.507362	0.359667	1.410644	0.158349695	0.2009938	NOT
AP000295.	6.281107	-0.26592	0.188551	-1.41032	0.158443847	0.201104	NOT
PIAS2	701.1482	0.099968	0.070892	1.410138	0.158498943	0.2011647	NOT
LRIF1	488.9907	0.159471	0.113092	1.410106	0.158508281	0.2011674	NOT
PRPS1P2	14.15022	0.231114	0.163918	1.409939	0.158557661	0.2012208	NOT
DEDD2	1081.589	0.12138	0.086096	1.409821	0.158592615	0.2012559	NOT
KC6	12.22875	0.686531	0.487715	1.407648	0.159235187	0.2020621	NOT
RP11-305I	1.536198	0.380629	0.270422	1.407535	0.159268699	0.2020954	NOT
RPS17	22977.05	0.169553	0.120466	1.407472	0.159287614	0.2021101	NOT
RP11-764I	4.974179	0.321606	0.228637	1.406624	0.159539034	0.2024198	NOT
RP11-143J	6.576904	0.306415	0.217867	1.406433	0.159595485	0.2024822	NOT
PDZD9	1.911542	0.373578	0.265706	1.405985	0.159728528	0.2026417	NOT
CD97	1762.555	-0.23951	0.170369	-1.40581	0.15978121	0.2026992	NOT
SNHG22	43.08302	0.244531	0.173998	1.405369	0.159911711	0.2028554	NOT
VWA3B	9.873758	-0.42275	0.300881	-1.40504	0.160009542	0.2029702	NOT
CTD-2017	53.87882	0.24459	0.174106	1.404835	0.160070291	0.203035	NOT
EMC6	136.4895	-0.17093	0.121672	-1.40482	0.160075244	0.203035	NOT
RP11-472I	1.617302	0.421892	0.300403	1.404419	0.160194133	0.2031765	NOT
SDHDP6	3.027675	-0.30679	0.218504	-1.40403	0.16031096	0.2033153	NOT
RP11-110I	11.56982	0.327938	0.233584	1.403942	0.160336029	0.2033378	NOT
GS1-124K!	117.9378	0.149205	0.106301	1.403608	0.160435589	0.203451	NOT
OR7E102P	5.215852	0.464398	0.330864	1.403593	0.160440023	0.203451	NOT
AP000692.	5.527806	0.326402	0.232562	1.403506	0.160466146	0.2034748	NOT
EHD4	2135.793	0.156581	0.111582	1.40328	0.160533324	0.2035507	NOT
ISM1	290.8672	-0.39387	0.280727	-1.40303	0.160608141	0.2036362	NOT
TUBBP10	4.848322	-0.34359	0.244928	-1.40283	0.160667858	0.2037026	NOT
RP4-657E1	19.32469	-0.22628	0.161371	-1.40223	0.160846372	0.2039196	NOT
MDM2	3884.762	-0.162	0.115537	-1.40217	0.160863458	0.2039319	NOT
RP11-214I	5.019347	0.337244	0.24056	1.401911	0.160941835	0.2040219	NOT
TRAV5	2.205141	-0.52942	0.377653	-1.40188	0.160951776	0.2040251	NOT
TARSL2	325.4029	-0.13184	0.094079	-1.40139	0.161097169	0.2042001	NOT
HIGD1AP1	1.87399	-0.36875	0.263172	-1.40116	0.161166543	0.2042787	NOT
AC011290	1.362329	-0.53393	0.381199	-1.40067	0.161313981	0.204454	NOT
CTD-2410	2.209714	0.393168	0.280705	1.400647	0.161319691	0.204454	NOT
C1orf86	1485.373	0.150664	0.107584	1.400439	0.161381814	0.2045234	NOT
NDUFV3	2371.152	-0.14097	0.100697	-1.3999	0.161544679	0.2047204	NOT
EIF3A	7677.43	-0.1298	0.092764	-1.39923	0.161744809	0.2049579	NOT
VGLL1	3.895282	-0.69549	0.497054	-1.39922	0.1617469	0.2049579	NOT
HMGB1P1(	58.29275	-0.19283	0.137824	-1.39912	0.161778087	0.2049881	NOT
KBTBD6	362.214	-0.1328	0.094926	-1.39896	0.161823614	0.2050339	NOT
TFDP2	1238.673	0.115602	0.082635	1.398943	0.161830038	0.2050339	NOT
IGLV3-21	363.3009	-0.57898	0.413876	-1.39892	0.16183649	0.2050339	NOT
RP11-678(	14.84672	0.515266	0.368504	1.398264	0.16203394	0.2052746	NOT

AC005330	18.06404	0.39055	0.27942	1.397715	0.16219861	0.2054739	NOT
RBMS2	477.5744	0.189749	0.135784	1.397435	0.162282809	0.2055711	NOT
C1RL-AS1	341.375	0.235996	0.168883	1.397393	0.162295307	0.2055775	NOT
RNFT1P3	3.072916	-0.41042	0.293735	-1.39725	0.162338158	0.2056224	NOT
RFK	1352.638	-0.1233	0.088247	-1.39722	0.162346319	0.2056233	NOT
RP11-313I	3.988801	0.326313	0.233633	1.39669	0.162506761	0.2058171	NOT
TUBGCP3	915.258	-0.14245	0.102064	-1.39565	0.16282044	0.2062049	NOT
RARS2	1080.649	-0.11826	0.084757	-1.39525	0.162941022	0.2063482	NOT
AL132772.	10.67862	-0.29666	0.212692	-1.39479	0.16307942	0.2065122	NOT
SF3B5	3257.446	0.177855	0.127516	1.394769	0.163085436	0.2065122	NOT
ZBTB11	690.3518	0.110054	0.078931	1.394311	0.163223613	0.2066777	NOT
KB-318B8.	3.437329	0.416962	0.299223	1.393482	0.163474175	0.2069855	NOT
IP6K3	478.0492	-0.54438	0.390677	-1.39343	0.16349033	0.2069965	NOT
WFDC3	12.16909	0.323838	0.232428	1.393281	0.163534768	0.2070433	NOT
RP11-504I	1.928335	0.361614	0.259559	1.393187	0.163563321	0.2070699	NOT
ST3GAL5-/	3.84305	-0.29405	0.211067	-1.39315	0.163574314	0.2070744	NOT
NPM1P21	1.793269	0.406748	0.291994	1.392999	0.163620173	0.207123	NOT
WASL	3378.863	-0.11046	0.079302	-1.39293	0.163639593	0.2071338	NOT
RP11-122:	4.175149	0.576896	0.414163	1.392921	0.163643734	0.2071338	NOT
PLAC9	140.2825	0.278592	0.200012	1.392879	0.163656395	0.2071404	NOT
NSFP1	2.175384	0.54922	0.394433	1.392427	0.163793074	0.2073039	NOT
GSTA5	1.628791	-0.47418	0.340699	-1.3918	0.163982894	0.2075346	NOT
RP11-456(	2.766789	0.751001	0.539679	1.39157	0.164052729	0.2076135	NOT
RP11-249I	5.541014	0.559284	0.40192	1.39153	0.164064731	0.2076192	NOT
RP11-278I	118.6231	-0.34648	0.248998	-1.39148	0.164079742	0.2076287	NOT
RP11-459I	5.529128	-0.38821	0.279079	-1.39105	0.164209029	0.2077828	NOT
TSIX	4.168487	-0.46868	0.336932	-1.39102	0.164219806	0.2077869	NOT
SP140L	608.8488	0.155883	0.112079	1.390839	0.164274252	0.2078463	NOT
SON	5765.78	-0.10167	0.073109	-1.39069	0.164320355	0.2078952	NOT
BMP6	102.6456	-0.37641	0.270684	-1.39058	0.164352234	0.207926	NOT
FLJ42969	0.879559	0.698696	0.502556	1.390286	0.164442172	0.2080303	NOT
HCFC2	625.1855	-0.13502	0.097122	-1.39021	0.16446376	0.208048	NOT
SRSF9	2304.067	0.104937	0.075492	1.390048	0.164514327	0.2081025	NOT
RP11-319I	7.237668	-1.36643	0.983493	-1.38936	0.164723543	0.2083576	NOT
RPA3	984.6978	0.149841	0.107858	1.389249	0.164757052	0.2083905	NOT
NFIX	2849.983	0.18481	0.133045	1.389079	0.164808598	0.2084461	NOT
PHF21B	1.05014	0.783766	0.564306	1.388902	0.164862402	0.2085047	NOT
RP11-307(	7.180547	-0.34747	0.250204	-1.38875	0.164907983	0.2085528	NOT
RP11-212I	2.647869	0.403408	0.290547	1.388439	0.165003318	0.2086638	NOT
LSM12	281.1747	0.10293	0.074135	1.388411	0.16501195	0.2086647	NOT
CTC-340D	1.394168	-0.4509	0.324767	-1.38839	0.165019125	0.2086647	NOT
PCDHAC1	14.47632	-0.56891	0.409802	-1.38826	0.165058257	0.2087047	NOT
TCEA1P3	0.864942	0.600292	0.432528	1.387868	0.165177238	0.2088456	NOT
RPL10P1	2.668244	0.31888	0.229852	1.387329	0.165341383	0.2090436	NOT
HRASLS5	4.6377	0.514727	0.37118	1.386731	0.16552383	0.2092647	NOT
GLRA3	1.987081	0.731712	0.527664	1.386701	0.165532992	0.2092667	NOT
ZNF818P	14.45518	0.385845	0.278255	1.386661	0.165545188	0.2092717	NOT
CTD-2616	1.460773	0.452702	0.326475	1.386638	0.165552099	0.2092717	NOT
ZNF568	188.8528	0.262885	0.18971	1.385717	0.165833441	0.2096178	NOT
AC073257	14.73368	0.313923	0.226557	1.385628	0.165860536	0.2096424	NOT
WWP1P1	1.780767	0.394112	0.284448	1.385533	0.165889554	0.2096695	NOT
HSP90AA6	2.312606	0.390981	0.2824	1.384496	0.166206697	0.2100608	NOT

SNRPN	1988.694	0.265536	0.191856	1.384037	0.16634726	0.2102288	NOT
CTC-523E	2.448738	-0.41719	0.301471	-1.38384	0.166408641	0.2102968	NOT
RP11-304I	5.410861	0.35218	0.254515	1.383731	0.166440956	0.210328	NOT
TPRA1	1761.612	0.108687	0.078576	1.383208	0.166601229	0.2105209	NOT
CTD-2373	4.837036	-0.26687	0.192949	-1.3831	0.166634278	0.2105531	NOT
TMEM186	457.4177	0.131496	0.095111	1.38255	0.166802767	0.2107564	NOT
ITPKC	1490.416	-0.17804	0.128785	-1.3825	0.166818656	0.2107668	NOT
OR12D2	0.525767	1.551569	1.122469	1.382282	0.166885006	0.210841	NOT
LINC0084(	4.721671	0.451602	0.326831	1.381762	0.16704489	0.2110317	NOT
PCDH15	1.763621	0.660654	0.478132	1.381741	0.167051156	0.2110317	NOT
NOC2L	2920.875	0.136466	0.098796	1.381294	0.167188487	0.2111955	NOT
SLC16A14	199.9141	0.357342	0.258717	1.381205	0.167215991	0.2112145	NOT
CTD-2350	106.5111	-0.27596	0.199797	-1.3812	0.167218762	0.2112145	NOT
RP11-27I1	35.98707	0.206175	0.149277	1.381151	0.167232461	0.2112221	NOT
RP4-545K:	2.872094	-0.38871	0.281475	-1.38098	0.167286117	0.2112802	NOT
ZNF670	96.14382	0.195536	0.141647	1.380442	0.167450574	0.2114783	NOT
RPL12P33	1.533841	0.40173	0.291092	1.380077	0.1675628	0.2116104	NOT
RP11-390I	102.5651	-0.253	0.18335	-1.37989	0.167620517	0.2116736	NOT
BCL7B	1814.185	-0.09821	0.071217	-1.37896	0.167906639	0.2120252	NOT
RP11-770I	4.522651	0.457991	0.332162	1.378817	0.167951098	0.2120717	NOT
RP11-753/	4.559704	-0.60282	0.437426	-1.37811	0.168169096	0.2123373	NOT
SPCS1	4968.488	0.12646	0.091767	1.378055	0.168186222	0.2123492	NOT
FAM92B	1.75538	-0.54435	0.395039	-1.37798	0.168210553	0.2123702	NOT
LRPAP1	6526.498	0.129789	0.094199	1.377814	0.168260625	0.2124238	NOT
RNA5SP21	1.50813	0.544305	0.395068	1.377748	0.168281195	0.21244	NOT
SFMBT2	175.764	0.262081	0.190335	1.376942	0.168530077	0.2127445	NOT
RP11-206I	2.470447	0.381434	0.277027	1.376882	0.168548585	0.2127582	NOT
RP11-9L1{	9.696298	0.206155	0.149912	1.375175	0.169077064	0.2134155	NOT
ETNPPL	3847.234	-0.43317	0.315161	-1.37445	0.169300979	0.2136884	NOT
ASCC3	1664.717	0.149769	0.108976	1.374331	0.169339056	0.2137267	NOT
IQSEC3	48.6791	-0.35656	0.259457	-1.37424	0.169368032	0.2137536	NOT
CTD-2135	1.872647	0.343802	0.250271	1.373718	0.169529133	0.2139471	NOT
ACA59	3.256036	0.441415	0.321362	1.373575	0.169573764	0.2139937	NOT
CYB5R2	190.6823	-0.24255	0.176592	-1.37352	0.169589499	0.2140038	NOT
ACSS3	4095.53	-0.32515	0.236941	-1.37226	0.169981363	0.2144885	NOT
AP3S2	1101.726	-0.13164	0.095947	-1.37203	0.170055373	0.2145721	NOT
LRRIQ4	6.213728	0.376544	0.274466	1.371917	0.170089234	0.214605	NOT
MAGI1-IT:	3.853428	-0.25577	0.186477	-1.37159	0.170192223	0.2147225	NOT
SMYD1	1.871392	0.690143	0.503183	1.371554	0.170202389	0.2147225	NOT
PIAS1	908.8458	0.122157	0.089065	1.371543	0.170205597	0.2147225	NOT
ERICH1	368.5162	-0.1374	0.100226	-1.37086	0.170420017	0.2149832	NOT
RALGAPA2	3114.016	-0.16672	0.121625	-1.37076	0.170448654	0.2150095	NOT
SLAMF9	31.44263	0.370013	0.269943	1.370708	0.170466141	0.2150217	NOT
PCMTD2	2006.675	0.143431	0.104645	1.370639	0.170487414	0.2150388	NOT
RP11-595I	31.82472	0.305491	0.222919	1.37041	0.170558817	0.215118	NOT
RP5-1091I	11.09674	-0.29933	0.218428	-1.37039	0.170565751	0.215118	NOT
RP11-396I	10.80963	0.345686	0.252321	1.370026	0.17067885	0.2152415	NOT
RTP4	356.5234	-0.24545	0.179156	-1.37002	0.170679245	0.2152415	NOT
LEPROT	4146.506	-0.12132	0.088556	-1.36999	0.170690261	0.2152456	NOT
AC004019	29.62675	0.313565	0.228994	1.369318	0.170899931	0.2155001	NOT
LINC0156(	43.95776	0.131973	0.096383	1.369252	0.170920425	0.2155162	NOT
LRCH3	931.2914	0.08657	0.063233	1.369049	0.170983874	0.2155863	NOT

EEF1A1P2(	6.655291	0.273642	0.199898	1.368903	0.171029612	0.2156266	NOT
MIR573	1.983357	0.416927	0.304572	1.368897	0.171031431	0.2156266	NOT
WSB1	1857.937	0.152934	0.111735	1.368716	0.171087913	0.215688	NOT
PRPF40A	2626.426	0.095816	0.070008	1.368638	0.171112555	0.2157093	NOT
FTH1P20	130.3834	0.220917	0.16143	1.368503	0.171154737	0.2157526	NOT
HDX	75.06806	-0.24402	0.178336	-1.3683	0.171219073	0.2158162	NOT
RP11-627I	1.401706	0.444821	0.325092	1.368292	0.171220827	0.2158162	NOT
RP11-468I	11.48774	0.644545	0.471126	1.368096	0.171282075	0.2158836	NOT
RP11-167I	1.478414	1.040888	0.760913	1.367946	0.171328909	0.2159328	NOT
TLN1	10535.44	0.115741	0.084614	1.367878	0.171350386	0.2159338	NOT
GANAB	18397.47	-0.08826	0.064524	-1.36787	0.171352152	0.2159338	NOT
RP11-452I	5.276495	0.26438	0.193279	1.367869	0.171353127	0.2159338	NOT
RP13-258(	1.384805	0.434753	0.31784	1.367835	0.171363649	0.215934	NOT
AC068499	4.014588	0.377159	0.275737	1.367819	0.171368848	0.215934	NOT
SNX2	2331.332	-0.0967	0.070703	-1.36773	0.171396212	0.2159559	NOT
USP19	2394.125	0.089656	0.065552	1.367713	0.171401899	0.2159559	NOT
RP11-626I	7.374175	-0.32971	0.241087	-1.3676	0.171438367	0.215992	NOT
RNU1-47P	8.885786	-0.48587	0.355409	-1.36706	0.171605769	0.2161931	NOT
NME8	5.149662	0.354435	0.259286	1.366966	0.171635933	0.2162213	NOT
EPB41L4A-	494.7157	0.189008	0.138272	1.366927	0.171648194	0.2162269	NOT
LRRIQ3	31.95475	-0.26634	0.194871	-1.36674	0.171708333	0.2162928	NOT
ZNF576	563.0976	0.123635	0.090471	1.366575	0.171758424	0.216346	NOT
SDR16C5	1.873448	-0.78926	0.577967	-1.36557	0.172072792	0.2167321	NOT
ATP6V0C	1272.593	0.124397	0.091128	1.365077	0.172228816	0.2169123	NOT
TRG-AS1	35.2096	-0.27872	0.204181	-1.36504	0.172239104	0.2169123	NOT
EEF1A1P5	584.8609	0.261881	0.191848	1.365043	0.172239384	0.2169123	NOT
RP11-66B2	3.201486	0.569678	0.417501	1.364493	0.172412369	0.2171113	NOT
LINC01481	1.879406	0.431088	0.315934	1.364491	0.172413087	0.2171113	NOT
WDR31	158.8465	-0.19666	0.144135	-1.36441	0.17243796	0.2171328	NOT
ZNFX1	1938.199	-0.15338	0.112422	-1.36435	0.17245725	0.2171472	NOT
RP11-330I	3.088288	0.464506	0.340573	1.363894	0.172600964	0.2173182	NOT
RP11-203J	16.83137	0.275032	0.201667	1.363789	0.172633956	0.2173499	NOT
S100A7	5.7587	1.030702	0.755824	1.363681	0.172668105	0.217383	NOT
AL158801.	13.62717	0.175861	0.128972	1.363556	0.172707375	0.2174225	NOT
RIMBP2	15.4593	-0.73672	0.54051	-1.363	0.172881311	0.2176243	NOT
ANKRD36I	14.53804	-0.47247	0.346648	-1.36298	0.172889348	0.2176243	NOT
SMIM15	2084.219	-0.09034	0.066282	-1.36296	0.172895358	0.2176243	NOT
KCNH1	3.781136	0.475433	0.348827	1.362947	0.172899116	0.2176243	NOT
PDLIM3	509.4911	0.263585	0.193434	1.362663	0.172988593	0.217727	NOT
AC098614	21.58485	0.290508	0.213292	1.362018	0.173192054	0.2179732	NOT
EMG1	665.0715	0.150927	0.110827	1.361821	0.173254468	0.2180418	NOT
MCEMP1	10.16541	-0.4929	0.361971	-1.36172	0.173286588	0.2180723	NOT
TAGLN	3104.409	-0.24393	0.179188	-1.36132	0.173414049	0.2182228	NOT
RTF1	1806.261	-0.07135	0.052423	-1.36111	0.173479371	0.2182951	NOT
RP11-317J	53.40661	-0.26207	0.192561	-1.36099	0.173515993	0.2183312	NOT
TWF1P1	2.493683	0.36281	0.266609	1.360832	0.173566858	0.2183853	NOT
RP4-728D	7.541812	-0.27559	0.20256	-1.36054	0.173659594	0.218492	NOT
DAZAP2P1	21.42229	-0.22955	0.168749	-1.36033	0.173726894	0.2185668	NOT
NUP54	841.0921	-0.10152	0.074646	-1.36009	0.173802423	0.2186519	NOT
STK24	1427.957	0.179452	0.131979	1.3597	0.17392483	0.2187959	NOT
TSC22D3	4413.422	-0.27857	0.204949	-1.35919	0.174085489	0.2189881	NOT
CCDC150	147.5095	0.233172	0.171576	1.359002	0.174145983	0.2190542	NOT

PLCD1	494.7482	-0.12829	0.094412	-1.35885	0.174193509	0.2190952	NOT
EIF4A1P4	4.939876	0.253515	0.186566	1.358849	0.174194394	0.2190952	NOT
RP11-452I	11.06999	0.304203	0.223872	1.358821	0.174203166	0.2190962	NOT
CTD-2033	2.207311	-0.36709	0.270251	-1.35832	0.174362169	0.2192862	NOT
NBPF12	505.23	0.21415	0.157662	1.358284	0.174373712	0.2192908	NOT
PPM1L	736.5689	0.214831	0.158187	1.358087	0.174435983	0.2193591	NOT
RP11-151I	2.755655	0.31865	0.234669	1.357868	0.174505654	0.2194368	NOT
CYB5R3	12620.14	-0.12503	0.092094	-1.35759	0.174594851	0.219539	NOT
CARD16	195.4446	-0.21598	0.159104	-1.35748	0.174629063	0.219572	NOT
CGN	4944.246	-0.23586	0.173764	-1.35737	0.174663363	0.2196051	NOT
DENND2C	349.9436	0.203477	0.149934	1.357113	0.174745263	0.2196981	NOT
RP11-625I	1.421493	1.144925	0.843818	1.356838	0.174832667	0.219798	NOT
BACH1-IT:	5.426677	0.26402	0.194593	1.356781	0.174850798	0.2198108	NOT
RP11-148I	3.977847	0.405253	0.298706	1.356697	0.174877604	0.2198346	NOT
MKX	54.71729	-0.46291	0.341255	-1.35649	0.174943458	0.2199073	NOT
RP11-556I	39.06939	0.286388	0.211184	1.356106	0.175065455	0.2200507	NOT
POLE4	1082.387	-0.16228	0.119675	-1.35597	0.175109711	0.2200963	NOT
GS1-114I9	1.55063	0.372735	0.274948	1.355659	0.175207654	0.2202094	NOT
RP11-47I2	1.751349	-0.4886	0.36049	-1.35538	0.175296739	0.2203114	NOT
UMAD1	711.9973	0.123049	0.090791	1.355306	0.175320138	0.2203308	NOT
MAPKAPK:	1447.181	0.125405	0.092638	1.353718	0.175826488	0.2209571	NOT
FAM71E1	101.7754	0.245533	0.181415	1.353437	0.175916049	0.2210596	NOT
AC012368	1.412461	0.492718	0.364217	1.352814	0.176114979	0.2212995	NOT
RP11-33O	61.97553	0.203022	0.150082	1.352746	0.176136755	0.2213168	NOT
FAT3	22.26929	0.360358	0.266472	1.35233	0.176269617	0.2214737	NOT
PPP1R12B	1041.807	-0.16899	0.124984	-1.35206	0.176357052	0.2215669	NOT
NKAIN3	1.628809	0.632103	0.467515	1.352049	0.176359766	0.2215669	NOT
ATP9B	704.5939	0.124277	0.091938	1.351754	0.176454093	0.2216753	NOT
RP11-863I	3.662071	-0.42978	0.31796	-1.35167	0.176481784	0.2217	NOT
DND1P1	11.6711	0.49318	0.364983	1.351243	0.17661768	0.2218607	NOT
CDK10	2540.865	-0.12741	0.094298	-1.35118	0.176638337	0.2218765	NOT
RP13-349(	1.916747	0.45702	0.33833	1.350813	0.176755354	0.2220135	NOT
ACO2	4062.056	0.176373	0.130573	1.350761	0.176772115	0.2220244	NOT
UGDH	7568.369	0.214795	0.159034	1.35062	0.176817322	0.2220711	NOT
FCGR1B	16.90213	-0.34065	0.252222	-1.35059	0.176825981	0.2220719	NOT
RP4-530I1	6.596299	0.316128	0.23408	1.350517	0.176850359	0.2220925	NOT
RP1-65J11	7.862381	0.429612	0.318244	1.349946	0.177033237	0.222312	NOT
SEMA4C	1337.556	-0.14259	0.105638	-1.34981	0.177076695	0.2223565	NOT
MRPL30	1200.465	0.086598	0.064177	1.349371	0.17721784	0.2225236	NOT
RP11-775(	29.8057	-0.18811	0.13948	-1.34868	0.177440999	0.2227937	NOT
KCTD5	990.5389	-0.09823	0.072841	-1.34854	0.177484025	0.2228377	NOT
CTD-2135	4.462344	-0.24415	0.181143	-1.34782	0.177716244	0.2231191	NOT
MEMO1	112.2542	-0.09843	0.073032	-1.34771	0.177751383	0.2231531	NOT
USP32	1170.053	-0.12906	0.095781	-1.34746	0.177833032	0.2232455	NOT
NBPF1	372.7936	0.166421	0.123511	1.347413	0.17784711	0.223253	NOT
SPATA12	3.089945	0.523718	0.388737	1.34723	0.177906242	0.2233171	NOT
GUCA1B	20.98092	0.24336	0.180646	1.347168	0.177926041	0.2233318	NOT
CTD-2014	1.754043	-0.51196	0.380069	-1.34702	0.177973449	0.2233812	NOT
KRBA2	7.776336	0.2691	0.199835	1.346609	0.178106054	0.2235375	NOT
RNF123	1619.857	-0.14945	0.110986	-1.34657	0.178117635	0.2235419	NOT
RP11-142/	2.070646	0.381632	0.283489	1.3462	0.178238047	0.2236772	NOT
DBET	3.025815	0.48666	0.36151	1.346189	0.178241625	0.2236772	NOT

CLK1	2033.285	-0.13675	0.101591	-1.34606	0.178282419	0.2237182	NOT
PRDM1	238.699	-0.23035	0.171166	-1.34575	0.178382644	0.2238339	NOT
SYNCRIP	4125.246	-0.08761	0.065104	-1.34563	0.178422841	0.2238741	NOT
CA5BP1	192.6715	0.132561	0.09853	1.345381	0.178502228	0.2239636	NOT
UNC93A	582.847	-0.44922	0.333905	-1.34534	0.178515359	0.2239699	NOT
CTC-455F	1.451283	0.528292	0.392755	1.345092	0.178595551	0.2240604	NOT
HTR2B	108.2167	0.400568	0.297855	1.344842	0.178676286	0.2241515	NOT
RP11-578	5.320471	0.276881	0.205916	1.34463	0.178744709	0.2242272	NOT
SNX18P3	6.069923	-0.46252	0.34402	-1.34445	0.178804346	0.2242918	NOT
OPN3	507.2456	0.216112	0.160756	1.344354	0.178834	0.2243188	NOT
SNORD17	8.534664	-0.32088	0.238692	-1.34433	0.178843149	0.2243201	NOT
PKD1L3	15.4748	-0.32254	0.239987	-1.34399	0.178951889	0.2244463	NOT
CTB-140J	2.005416	0.479752	0.356973	1.343947	0.178965409	0.2244531	NOT
RP11-124	1.814629	0.413202	0.307609	1.343271	0.179184203	0.224709	NOT
CTD-2308	2.449182	0.513836	0.382529	1.343261	0.17918761	0.224709	NOT
ZBTB47	394.4332	0.184611	0.137437	1.343242	0.179193755	0.224709	NOT
FASTKD5	776.1644	-0.11148	0.083002	-1.34305	0.17925422	0.2247746	NOT
NACAP3	4.794987	-0.3575	0.266231	-1.34281	0.179332739	0.2248629	NOT
SDCBP	6336.348	0.162837	0.121331	1.342086	0.179568025	0.2251477	NOT
RP11-638	2.098767	0.409959	0.305491	1.341965	0.179607298	0.2251788	NOT
TECTB	24.40267	0.433451	0.322999	1.34196	0.179609106	0.2251788	NOT
RP13-131	1.420837	0.436863	0.325581	1.341796	0.179662199	0.2252351	NOT
EIF2AK2	1702.759	-0.12042	0.089759	-1.34154	0.179743778	0.2253272	NOT
HORMAD2	334.1416	0.535126	0.398943	1.34136	0.179803594	0.225392	NOT
COX17	1827.641	-0.14324	0.106804	-1.34114	0.179876376	0.2254722	NOT
LRRC31	203.0721	0.380112	0.28343	1.341113	0.179883908	0.2254722	NOT
RRAD	131.3283	0.344027	0.256587	1.340781	0.179991583	0.225597	NOT
EP300-AS	24.81951	-0.28618	0.213469	-1.34062	0.180045368	0.2256541	NOT
SLC44A2	1916.55	0.207859	0.155055	1.340549	0.180066811	0.2256708	NOT
PTER	722.8679	-0.16172	0.120648	-1.34041	0.180113226	0.2257187	NOT
RPL3L	25.25979	0.270111	0.201522	1.340358	0.18012902	0.2257283	NOT
KCNJ16	93.12287	-0.57029	0.425568	-1.34007	0.180223254	0.2258362	NOT
TM6SF1	34.3712	-0.25785	0.192429	-1.33999	0.180247866	0.2258568	NOT
PLIN4	1985.386	-0.2854	0.213136	-1.33903	0.180560474	0.2262382	NOT
GALNS	782.0166	-0.17099	0.127733	-1.33868	0.18067607	0.2263728	NOT
HOOK3	1247.188	0.161574	0.120706	1.338582	0.180706784	0.2264011	NOT
TCEAL3	269.6258	0.22626	0.169167	1.337491	0.181062537	0.2268365	NOT
ATG9B	34.33739	0.22005	0.164554	1.33725	0.181141157	0.2269247	NOT
FUT3	32.26744	-0.36475	0.272771	-1.3372	0.181157994	0.2269355	NOT
TCF7L1	916.3006	0.199185	0.148985	1.336949	0.181239354	0.2270272	NOT
RPS6	41698.6	0.157396	0.117772	1.33644	0.181405359	0.227222	NOT
NEURL1	87.77456	0.38604	0.288861	1.336422	0.181411301	0.227222	NOT
RPL3P1	2.619292	0.326468	0.244481	1.33535	0.181761767	0.2276506	NOT
IGHV1-69	103.2522	-0.60748	0.454979	-1.33519	0.181814111	0.2277059	NOT
LMF1	900.0365	-0.18745	0.140402	-1.33508	0.181848996	0.2277393	NOT
RNU7-49P	1.444085	0.427926	0.320574	1.334874	0.18191756	0.2278148	NOT
SNX13	993.644	0.115555	0.086575	1.334741	0.181961093	0.2278548	NOT
SNX10	1854.8	-0.22445	0.168167	-1.33471	0.18197238	0.2278548	NOT
RP5-968P	42.9007	0.23038	0.172608	1.334701	0.181974177	0.2278548	NOT
SNAPC3	734.2953	0.098856	0.074067	1.334674	0.181982956	0.2278555	NOT
JKAMP	1110.291	0.095547	0.071592	1.334604	0.182005974	0.227874	NOT
CTSH	7480.03	0.184838	0.138597	1.333629	0.182325503	0.2282637	NOT

PCOLCE2	1061.009	0.345679	0.25929	1.333176	0.182474204	0.2284395	NOT
SF3B3	3846.245	0.100265	0.075229	1.332796	0.182598745	0.2285835	NOT
KLHL41	8.862718	0.320462	0.240448	1.332775	0.182605742	0.2285835	NOT
PMP22	613.5638	0.251479	0.188718	1.332568	0.182673458	0.2286533	NOT
AEBP2	660.4529	-0.10664	0.080023	-1.33255	0.182677997	0.2286533	NOT
RP4-734G:	16.7024	0.23893	0.179359	1.332135	0.182815922	0.2288156	NOT
RP11-258I	1.596692	-0.33514	0.251634	-1.33184	0.182912349	0.2289259	NOT
RAB13	3921.228	0.117456	0.088192	1.331812	0.182921994	0.2289276	NOT
RP11-7D5	1.999431	0.367137	0.2757	1.331652	0.182974484	0.2289829	NOT
GNRH2	18.49502	-0.51557	0.387177	-1.33163	0.182982853	0.2289831	NOT
RP11-152I	1.741054	0.382397	0.287214	1.331402	0.183056831	0.2290577	NOT
EEF1DP1	22.87874	0.209083	0.157041	1.331395	0.183059073	0.2290577	NOT
RCAN2	387.8477	0.299689	0.225105	1.331328	0.183081096	0.2290749	NOT
RP11-787I	3.385307	0.289031	0.21712	1.331206	0.183121217	0.2291148	NOT
AP000350.	6.612204	-0.42507	0.319409	-1.33079	0.18325682	0.2292741	NOT
ZNF879	49.19774	0.250331	0.188162	1.330406	0.18338468	0.2294236	NOT
SAMD15	9.110346	0.288014	0.216504	1.330296	0.183420671	0.2294583	NOT
RP11-81A:	4.210051	0.69483	0.522358	1.33018	0.183458954	0.2294958	NOT
RP11-61I1	38.46229	0.280842	0.211148	1.330073	0.183494071	0.2295294	NOT
RP4-644L1	1.585928	0.531901	0.400057	1.329565	0.183661786	0.2297288	NOT
NAV2-AS5	1.449848	-0.50392	0.379022	-1.32952	0.183676785	0.2297371	NOT
GPR108	2625.732	0.095348	0.071719	1.329468	0.183693457	0.2297476	NOT
RP11-108I	169.5284	0.241959	0.182005	1.329405	0.183714279	0.2297633	NOT
BFAR	1772.172	0.0733	0.055139	1.329367	0.183726954	0.2297687	NOT
RP11-269I	2.666844	-0.38035	0.286167	-1.32912	0.183809695	0.2298618	NOT
SPSB3	88.79152	-0.17136	0.128935	-1.32904	0.18383341	0.2298707	NOT
TMEM198	457.7371	0.169383	0.127447	1.329044	0.183833427	0.2298707	NOT
RP11-94C:	46.73991	0.282989	0.213007	1.328545	0.183998247	0.2300664	NOT
RP11-358I	35.4884	-0.17283	0.1301	-1.32847	0.18402243	0.230076	NOT
TMEM233	50.29928	0.294083	0.22137	1.328471	0.18402256	0.230076	NOT
RP1-102K:	1.241631	0.51517	0.387808	1.328415	0.184041179	0.2300889	NOT
CCDC94	927.3285	0.136981	0.103138	1.328127	0.184136141	0.2301972	NOT
UBE2K	3532.346	-0.09561	0.072006	-1.32774	0.184265203	0.2303482	NOT
CH507-51	18.86676	0.231407	0.174291	1.327709	0.184274332	0.2303492	NOT
AWAT2	1.092628	-0.70829	0.533497	-1.32763	0.184299693	0.2303705	NOT
AP000560.	3.08252	0.347051	0.261582	1.326739	0.184595124	0.2307293	NOT
KIAA1683	189.6988	0.274337	0.206788	1.326659	0.184621655	0.230752	NOT
RALGAPA1	33.53351	-0.19549	0.14737	-1.32651	0.184672175	0.2308048	NOT
AHDC1	733.5558	-0.13627	0.102731	-1.32647	0.184685447	0.2308109	NOT
CD72	165.1465	-0.27846	0.210026	-1.32582	0.184899851	0.2310684	NOT
GIMAP2	318.985	-0.19634	0.148111	-1.32566	0.184952678	0.231124	NOT
RP11-109·	6.724617	-0.35199	0.265527	-1.32561	0.184968451	0.2311333	NOT
TAF12	714.9906	0.121214	0.091445	1.325543	0.184991129	0.2311512	NOT
ACTA2-AS	24.96592	0.298776	0.225446	1.325262	0.185084166	0.231257	NOT
AC241585	17.56922	0.254517	0.192078	1.32507	0.185147801	0.2313261	NOT
ARMCX3	1254.567	-0.28239	0.213131	-1.32494	0.185191501	0.2313702	NOT
SCARF2	186.456	0.230579	0.174052	1.324773	0.185246602	0.2314286	NOT
MED28	1282.631	0.104294	0.078744	1.32447	0.185347025	0.2315436	NOT
RP1-313I6	71.68102	0.187291	0.141434	1.324229	0.185426979	0.231633	NOT
ZNF366	42.74648	0.298155	0.225227	1.323795	0.185571095	0.2318026	NOT
ATP6AP2	4392.276	0.088261	0.066698	1.323288	0.18573957	0.2320026	NOT
EFHD2	2556.972	0.154138	0.116514	1.322911	0.185865109	0.2321489	NOT

UGT2B4	39076.81	-0.32216	0.24355	-1.32276	0.185915277	0.2322011	NOT
GFM2	1669.759	-0.11688	0.088386	-1.32237	0.18604424	0.2323516	NOT
CTC-325H	15.45183	-0.20438	0.154601	-1.32196	0.18618175	0.2325129	NOT
ACVR2B	476.6302	-0.19465	0.147311	-1.32134	0.186386967	0.2327587	NOT
SLC44A4	68.66482	0.370497	0.280408	1.321278	0.186408577	0.2327751	NOT
ANO5	256.7815	-0.30626	0.231846	-1.32096	0.186514533	0.2328969	NOT
TGM1	22.86727	0.252237	0.19097	1.320819	0.186561658	0.2329453	NOT
YARS	3543.61	0.142886	0.108194	1.320651	0.186617837	0.2330049	NOT
SPRY4-IT1	2.632175	-0.41585	0.314928	-1.32047	0.186678175	0.2330697	NOT
KRT27	2.757141	-0.45875	0.347462	-1.32029	0.186739747	0.2331361	NOT
RP11-761I	1.926401	0.413129	0.312938	1.320163	0.186780665	0.2331683	NOT
FAM204A	930.703	-0.07918	0.059975	-1.32016	0.186782427	0.2331683	NOT
PRICKLE1	78.76718	-0.33667	0.255028	-1.32012	0.186796277	0.2331751	NOT
LA16c-313	5.827116	0.370739	0.28085	1.320059	0.186815314	0.2331883	NOT
RP11-111	25.39583	0.28494	0.215868	1.319976	0.186843198	0.2332126	NOT
ZNRF3	15.40637	0.325289	0.246482	1.319729	0.18692567	0.233305	NOT
ESPNL	121.0036	-0.38494	0.29171	-1.31958	0.186974321	0.2333552	NOT
CLEC4A	55.97776	-0.19041	0.144324	-1.31932	0.187061167	0.2334531	NOT
RPL5P1	6.526131	0.272422	0.20652	1.319108	0.187133045	0.2335322	NOT
RP11-372I	7.880446	-0.23777	0.180298	-1.31874	0.187254584	0.2336734	NOT
RPL7AP64	1.706882	-0.401	0.304155	-1.31842	0.187363126	0.2337983	NOT
GALP	11.22817	-0.46129	0.349899	-1.31835	0.187386366	0.2338167	NOT
RP11-613I	53.86723	0.209536	0.158949	1.318259	0.187416913	0.2338443	NOT
PGAM1P5	1.329139	0.441277	0.334804	1.318014	0.187498943	0.2339361	NOT
FAM213A	8297.214	-0.17079	0.1296	-1.31779	0.187574529	0.2340199	NOT
RP11-573I	267.2085	-0.26352	0.200069	-1.31714	0.187790226	0.2342784	NOT
LA16c-306	1.898529	-0.41227	0.313057	-1.31691	0.187868243	0.2343652	NOT
BTRC	662.9428	0.096898	0.073584	1.316837	0.187893401	0.234386	NOT
SLC7A5P1	1.06382	0.570538	0.433381	1.316482	0.188012376	0.2345238	NOT
C2orf50	2.418536	0.486357	0.369461	1.316394	0.188041723	0.2345499	NOT
HSPD1P11	1.975694	0.437362	0.332333	1.316033	0.188163128	0.2346907	NOT
PHF8	1879.833	-0.16879	0.128317	-1.31542	0.188369104	0.234937	NOT
RP11-526I	8.989157	-0.18562	0.141159	-1.31494	0.188529845	0.2351269	NOT
GYPE	3.796846	-0.36113	0.274726	-1.31452	0.188672665	0.2352944	NOT
CCR5	163.0482	-0.29209	0.222248	-1.31423	0.188768871	0.2353973	NOT
TRAV25	1.209474	-0.5172	0.393541	-1.31422	0.188772171	0.2353973	NOT
RP5-968J1	1.31838	0.537857	0.409343	1.31395	0.188863177	0.2355002	NOT
FMR1	2461.031	0.129191	0.098325	1.313924	0.188871935	0.2355005	NOT
HMGN1P3	5.718573	0.215564	0.164124	1.313424	0.189039976	0.2356994	NOT
RP11-212I	7.43319	-0.48393	0.368545	-1.31308	0.189155694	0.2358272	NOT
RP11-180I	11.40289	0.482832	0.367713	1.313069	0.189159588	0.2358272	NOT
KIAA0226L	84.7084	-0.27294	0.207888	-1.31294	0.189203612	0.2358715	NOT
ZNF197	518.0654	-0.11303	0.0861	-1.31284	0.189238196	0.2358939	NOT
LRRC61	1367.64	0.18099	0.137862	1.312833	0.189239367	0.2358939	NOT
TRHDE	115.1082	0.548546	0.417841	1.312809	0.189247161	0.2358939	NOT
CTD-2298	0.954714	0.941739	0.717509	1.312511	0.189347696	0.2360086	NOT
AC007950	4.009095	0.281937	0.214827	1.312389	0.189389069	0.2360495	NOT
RBPMS	2517.766	-0.18686	0.142399	-1.3122	0.189453609	0.2361193	NOT
NCRNA00	3.291349	0.402515	0.306789	1.312024	0.189512183	0.2361817	NOT
ADRM1	5397.359	0.119561	0.09118	1.311276	0.189764638	0.2364857	NOT
PI4K2A	1208.971	0.111816	0.085294	1.310941	0.189877855	0.2366161	NOT
TP53INP1	7416.67	-0.20087	0.153247	-1.31078	0.189932693	0.2366738	NOT

TLN2	830.8036	-0.23363	0.178253	-1.31066	0.189973012	0.2367134	NOT
LRRC70	12.95779	-0.26561	0.202718	-1.31026	0.190108019	0.2368709	NOT
RP11-872I	2.431552	0.320327	0.244488	1.310195	0.190129899	0.2368875	NOT
MSL2	954.9917	-0.08463	0.064597	-1.31016	0.190142321	0.2368923	NOT
DNAH2	30.45512	-0.29763	0.22718	-1.31009	0.190166183	0.2369114	NOT
FRMPD3	4.006586	0.402963	0.307605	1.310002	0.190195307	0.2369275	NOT
FAUP1	12.95736	0.244493	0.186636	1.309999	0.190196294	0.2369275	NOT
UBASH3B	181.9782	0.290849	0.222095	1.30957	0.19034136	0.2370976	NOT
PAPD4	1271.904	-0.08138	0.062148	-1.30943	0.190389142	0.2371464	NOT
CUX1	3046.328	-0.1025	0.078304	-1.30896	0.190546969	0.2373323	NOT
TOP1	2831.835	-0.0942	0.072023	-1.30792	0.190899698	0.2377532	NOT
PAN3	657.6705	0.131521	0.100558	1.307915	0.190902091	0.2377532	NOT
KIR3DX1	3.90697	-0.30886	0.236168	-1.3078	0.190941165	0.2377912	NOT
KLHDC7A	464.394	-0.2975	0.227506	-1.30766	0.190989464	0.2378406	NOT
RPL13AP2	2.082994	0.352568	0.269655	1.307481	0.191049407	0.237899	NOT
C1orf177	15.18537	0.275216	0.210495	1.307469	0.191053541	0.237899	NOT
VENTX	20.9686	0.245842	0.188061	1.307247	0.191128692	0.2379819	NOT
RWDD3	213.6565	0.111535	0.085325	1.307178	0.19115241	0.2380007	NOT
POLD4	1680.097	-0.14263	0.109116	-1.3071	0.191179815	0.2380241	NOT
PP14571	2.586109	0.441005	0.33749	1.306721	0.191307427	0.2381723	NOT
AC073326	2.023365	0.349272	0.2673	1.30667	0.191324968	0.2381834	NOT
RP11-527I	4.836906	0.284	0.217402	1.306336	0.191438436	0.2383139	NOT
INTS4P2	1.921972	0.340094	0.260347	1.30631	0.19144718	0.2383141	NOT
CNST	1553.975	-0.13486	0.103246	-1.30623	0.191476003	0.2383393	NOT
RP11-798(	5.31156	0.296799	0.22731	1.305704	0.191653286	0.2385492	NOT
CTD-2256	1.763928	0.369996	0.283447	1.305344	0.191775619	0.2386907	NOT
MICD	2.515627	0.576581	0.441979	1.304544	0.192048042	0.239019	NOT
EIF3S5P1	1.501345	0.417978	0.320423	1.304459	0.192077176	0.2390445	NOT
RP11-342I	8.389928	-0.32148	0.246459	-1.30441	0.192093464	0.2390541	NOT
SUDS3P1	4.544535	0.229234	0.175753	1.304299	0.192131628	0.2390908	NOT
GJC2	33.31502	0.285565	0.219034	1.303749	0.192319244	0.2393135	NOT
PPIHP1	1.465164	0.426003	0.326786	1.303612	0.192366023	0.239361	NOT
FOXO3B	10.63537	0.244514	0.187637	1.303121	0.192533349	0.2395584	NOT
ST8SIA2	1.251541	0.670289	0.514388	1.30308	0.192547344	0.239565	NOT
DOCK5	1054.162	-0.1872	0.143671	-1.30296	0.192587849	0.2396046	NOT
MPND	1788.883	-0.16703	0.128198	-1.30289	0.19261386	0.2396262	NOT
SNX31	7.046771	0.367791	0.282444	1.302173	0.192857115	0.2399181	NOT
ACTB	120648.8	-0.11328	0.087015	-1.30188	0.19295584	0.2400301	NOT
NPFFR1	2.607363	0.494521	0.379918	1.301653	0.193034908	0.2401177	NOT
IGHV2-5	15.41297	-0.61787	0.474766	-1.30141	0.193118288	0.2402106	NOT
RP11-672I	2.265783	-0.62726	0.482017	-1.30132	0.193148968	0.2402379	NOT
MCTP2	159.3892	0.371815	0.285754	1.301173	0.19319909	0.2402895	NOT
TTC22	355.7856	0.253044	0.194539	1.300738	0.193348121	0.240464	NOT
RP11-30K	2.648499	-0.30598	0.235287	-1.30047	0.193438267	0.2405653	NOT
C19orf70	2314.341	0.175307	0.134871	1.29982	0.193662826	0.2408268	NOT
LY86-AS1	3.236904	0.533565	0.410495	1.29981	0.19366595	0.2408268	NOT
CDH20	2.210766	0.352896	0.271575	1.299443	0.193791774	0.2409724	NOT
RP11-106(	1.914271	0.652322	0.502097	1.299196	0.193876753	0.2410673	NOT
RP11-506I	10.30439	-0.25238	0.194272	-1.2991	0.193907934	0.2410952	NOT
RASA1	705.9181	0.142436	0.109665	1.298825	0.194004107	0.241204	NOT
MIR4442	8.863346	0.283734	0.218465	1.298764	0.194024844	0.2412189	NOT
STAG1	722.9429	0.163935	0.126303	1.297944	0.194306575	0.2415583	NOT

RP11-452(	3.212197	-0.34917	0.269031	-1.29788	0.194327208	0.2415731	NOT
ZNF267	251.9211	0.136115	0.104879	1.297824	0.194347991	0.2415881	NOT
RP11-74J1	14.01294	0.195489	0.150639	1.297727	0.194381127	0.2416184	NOT
TAPBP	13450.12	0.129935	0.100154	1.297352	0.19451009	0.2417679	NOT
KCNT2	326.5454	-0.28191	0.217356	-1.29701	0.194626598	0.2419018	NOT
TTC31	1501.811	0.115909	0.089374	1.296902	0.194664813	0.2419384	NOT
VEGFA	6765.149	0.150388	0.11598	1.296669	0.194745065	0.2420273	NOT
RPS26P31	4.297677	0.275325	0.21236	1.296504	0.194802068	0.2420873	NOT
TREM1	49.83641	-0.38634	0.298019	-1.29637	0.194848213	0.2421337	NOT
VPREB3	28.57209	0.298677	0.230411	1.296276	0.194880456	0.2421629	NOT
DCPS	1973.222	-0.22005	0.169806	-1.29591	0.195007337	0.2423097	NOT
KPRP	0.918386	1.265125	0.976319	1.295811	0.195040641	0.2423402	NOT
NRXN2	318.2145	0.362058	0.279422	1.295739	0.195065384	0.2423601	NOT
ACTR1B	2563.723	0.087927	0.067861	1.29569	0.195082471	0.2423704	NOT
SENCR	19.57179	0.245515	0.189551	1.295242	0.195236777	0.2425512	NOT
GBP3	1011.311	-0.26187	0.202259	-1.29474	0.195410561	0.2427562	NOT
TEX101	3.936085	0.777638	0.600725	1.294499	0.195493038	0.2428478	NOT
PRDX1	15105.35	0.152443	0.117791	1.294186	0.195601102	0.2429711	NOT
RNU6-137	1.744738	0.358342	0.276893	1.294155	0.195611749	0.2429734	NOT
RP11-642/	5.120419	0.314212	0.24282	1.29401	0.195661829	0.2430245	NOT
RP11-331I	4.089535	0.300777	0.232442	1.293986	0.195670421	0.2430245	NOT
ACOT7	1316.963	0.150962	0.116688	1.293723	0.195761135	0.2431262	NOT
FAM8A1	5600.044	-0.19173	0.14828	-1.29304	0.195996232	0.2434073	NOT
RASA4DP	3.858103	0.341842	0.264402	1.292887	0.196050009	0.2434632	NOT
RP11-403/	2.84423	0.447018	0.345815	1.292651	0.196131892	0.2435539	NOT
KLK7	1.556559	0.924554	0.715651	1.291906	0.196389575	0.2438629	NOT
RP11-26F2	3.293801	0.445898	0.345194	1.291731	0.196450464	0.2439276	NOT
RP11-848(	10.89952	-0.33157	0.256736	-1.29148	0.196537032	0.2440241	NOT
BCOR	1168.899	0.140355	0.108707	1.29113	0.196658747	0.2441643	NOT
ARID1A	2601.153	0.116117	0.089953	1.290868	0.19674956	0.2442661	NOT
KRT8P24	3.275888	0.41292	0.320097	1.289983	0.197056569	0.2446363	NOT
SIAH2	3247.688	-0.18113	0.140419	-1.28994	0.197071951	0.2446444	NOT
LINC01205	2.954235	-0.64789	0.502275	-1.28991	0.197081592	0.2446454	NOT
RP11-174(	3.134507	0.400079	0.310689	1.287715	0.197845138	0.2455822	NOT
LCA5	60.77718	-0.24683	0.191685	-1.28768	0.197858693	0.245588	NOT
TTC3-AS1	1.78536	0.332256	0.258076	1.287435	0.197942601	0.2456811	NOT
CRACR2A	85.49137	0.274877	0.213512	1.287408	0.197952201	0.245682	NOT
LGR6	167.2622	0.396446	0.30795	1.287372	0.197964596	0.2456864	NOT
RP11-120J	4.439274	0.42542	0.330502	1.287192	0.198027291	0.2457532	NOT
LYPLA2	3323.763	-0.11798	0.091671	-1.28701	0.198091141	0.2458214	NOT
KLB	2948.078	0.261448	0.203151	1.286962	0.198107383	0.2458305	NOT
RSAD1	2343.002	0.114861	0.089268	1.2867	0.198198963	0.2459331	NOT
AC079779	2.244857	0.496339	0.385815	1.286467	0.198280095	0.2460228	NOT
RP3-449M	1.621187	0.390031	0.303193	1.286413	0.198299101	0.2460353	NOT
RP11-48B3	127.1685	0.190872	0.148394	1.286249	0.19835601	0.2460949	NOT
NRADDP	2.14597	0.345195	0.268396	1.286141	0.198394031	0.246131	NOT
RP11-265I	6.15631	-0.17743	0.137979	-1.28591	0.198476127	0.2462218	NOT
SPATS2L	5958.994	0.115456	0.089796	1.285751	0.198529993	0.2462776	NOT
CAPN5	4153.082	-0.20248	0.157507	-1.28556	0.19859817	0.2463511	NOT
DSP	10865.13	0.165918	0.129178	1.284415	0.198996713	0.2468344	NOT
TCF4	897.2646	0.189121	0.147272	1.284161	0.199085799	0.2469339	NOT
RP1-197B1	8.26089	0.236504	0.184188	1.284038	0.199128794	0.2469761	NOT

C2CD2	733.3359	0.14807	0.115342	1.283745	0.199231052	0.2470919	NOT
NUFIP1	263.4296	-0.11084	0.086357	-1.28353	0.199306506	0.2471744	NOT
RP11-563J	4.557932	0.425544	0.33156	1.28346	0.199331093	0.2471938	NOT
PAFAH1B2	2761.764	-0.08691	0.067716	-1.28339	0.199354898	0.2472122	NOT
RP11-171I	1.854799	0.437356	0.340854	1.283119	0.199450379	0.2473195	NOT
RP1-288H	3.33045	0.526274	0.410207	1.282946	0.1995111	0.2473836	NOT
EIF5AL1	116.1198	-0.35875	0.279633	-1.28292	0.199521495	0.2473836	NOT
RP11-148I	1.96574	-0.4551	0.354744	-1.2829	0.199528842	0.2473836	NOT
RP5-857K2	217.7249	0.311298	0.242954	1.281304	0.200087064	0.2480646	NOT
EMILIN3	13.422	0.365418	0.285242	1.281079	0.200165977	0.2481513	NOT
RN7SL738I	2.760625	-0.26384	0.205957	-1.28105	0.200175358	0.2481518	NOT
GPX1P1	119.1411	0.318907	0.248981	1.280851	0.200245977	0.2482219	NOT
TRBV12-3	1.523513	-0.47888	0.373879	-1.28084	0.200249859	0.2482219	NOT
SLC25A45	669.7632	0.168903	0.131925	1.280297	0.200440542	0.2484471	NOT
TBC1D10C	286.608	0.268934	0.21009	1.280093	0.20051235	0.248525	NOT
AGFG2	4281.7	-0.13481	0.105317	-1.28007	0.20052179	0.2485256	NOT
PIGQ	1901.594	-0.13075	0.102142	-1.28003	0.200534266	0.2485299	NOT
ZDHHC16	1308.489	-0.09992	0.078087	-1.2796	0.200685272	0.2487059	NOT
CATSPER2	84.06682	0.145182	0.113489	1.279262	0.20080484	0.2488429	NOT
FRYL	927.2193	0.115432	0.090238	1.279191	0.200829911	0.2488629	NOT
KIAA1598	2357.582	0.164399	0.128539	1.278981	0.200903816	0.2489433	NOT
NADK2-AS	15.23927	-0.32982	0.257906	-1.27884	0.20095405	0.2489944	NOT
WNT16	3.435997	0.427489	0.33431	1.278723	0.200994712	0.2490336	NOT
TLE1P1	49.97721	0.18016	0.140908	1.278561	0.201051565	0.2490929	NOT
MUM1L1	114.5659	-0.51671	0.40419	-1.27839	0.201113214	0.2491581	NOT
TSG101	2406.192	0.066315	0.051888	1.278037	0.201236523	0.2492997	NOT
RPL4	39639.81	0.137029	0.107229	1.277911	0.201280708	0.2493433	NOT
CTC-512J1	2.456098	-0.27688	0.216693	-1.27774	0.201340591	0.2494063	NOT
DUXAP1	1.150555	-0.52847	0.413819	-1.27707	0.201578286	0.2496896	NOT
TMEM208	2665.418	-0.14895	0.116642	-1.27702	0.201595595	0.2496998	NOT
LINC00476	108.1012	-0.15744	0.123329	-1.27662	0.201736787	0.2498635	NOT
CTSD	65858.13	0.167721	0.131448	1.27595	0.201973184	0.2501451	NOT
RP11-44F1	3.612664	0.296083	0.232081	1.275775	0.202035046	0.2502105	NOT
PDHA1	4152.757	-0.11463	0.089859	-1.27561	0.202093525	0.2502718	NOT
KLHL4	33.94309	-0.29256	0.229473	-1.27493	0.202333213	0.2505574	NOT
GFPT2	137.9616	0.386776	0.303405	1.274784	0.202385564	0.250611	NOT
ORC4	838.3128	-0.09735	0.076375	-1.27467	0.202425177	0.2506488	NOT
RP11-686I	19.66588	0.261242	0.20498	1.274478	0.202494152	0.250723	NOT
RPL15P18	1.986161	0.311461	0.244488	1.273933	0.202687108	0.2509507	NOT
COX20P1	17.90156	0.463661	0.363968	1.273904	0.202697432	0.2509522	NOT
GALNT14	63.29395	-0.3297	0.258847	-1.27372	0.202761932	0.2510209	NOT
LINC01160	5.527492	-0.21496	0.16885	-1.27308	0.202991115	0.2512934	NOT
ZNF444	2720.872	0.166595	0.130876	1.272923	0.203045214	0.2513491	NOT
CSTF2T	1188.816	-0.10207	0.080204	-1.27259	0.203162199	0.2514826	NOT
BCL2L11	1289.63	-0.15447	0.121391	-1.2725	0.203194918	0.2515119	NOT
RP5-1177I	1.060735	0.620426	0.487587	1.272441	0.203216343	0.2515272	NOT
CACUL1	1767.691	0.087522	0.06882	1.271751	0.203461774	0.2518197	NOT
WSCD2	3.795288	-0.35022	0.27546	-1.27139	0.203591072	0.2519684	NOT
MR1	833.0536	0.166152	0.130723	1.271017	0.203722521	0.2521198	NOT
PAX6	91.0952	0.232495	0.182927	1.270974	0.203737817	0.2521275	NOT
STX19	4.716956	-0.35587	0.280025	-1.27084	0.203786111	0.2521741	NOT
RIC8B	626.5475	0.141101	0.111033	1.270808	0.203797065	0.2521741	NOT

OVCH1	2.884753	-0.36468	0.286972	-1.27079	0.203802823	0.2521741	NOT
PNPT1	981.9297	0.098161	0.077267	1.270408	0.203939183	0.2523315	NOT
RAET1L	1.100283	0.845248	0.665374	1.270335	0.203965336	0.2523526	NOT
ZNF106	1493.38	0.116784	0.09194	1.270222	0.204005517	0.252391	NOT
USP24	2178.555	0.090974	0.071633	1.269996	0.204086041	0.2524794	NOT
RP11-676I	78.25227	0.169664	0.133617	1.26978	0.20416301	0.2525633	NOT
CTD-2017	19.23318	0.268528	0.211512	1.269567	0.204239065	0.2526461	NOT
RP11-548I	6.098905	-0.25647	0.202035	-1.26945	0.204279836	0.2526852	NOT
MRAP	19.89463	0.47365	0.373255	1.26897	0.204451754	0.2528866	NOT
RP11-407I	1.91678	-0.34506	0.271937	-1.26888	0.204483033	0.2529062	NOT
TULP2	5.049017	-0.39953	0.314871	-1.26887	0.204485938	0.2529062	NOT
CTC-523E	7.202646	0.285131	0.224748	1.268671	0.204558391	0.2529845	NOT
SOCS4	517.9045	0.107411	0.084671	1.26857	0.204594505	0.2530179	NOT
S100Z	2.876996	0.324907	0.256134	1.268505	0.204617612	0.2530352	NOT
GLT8D1	2391.296	-0.12264	0.09671	-1.26816	0.204742019	0.2531777	NOT
LINC0084(	2.371855	-0.38901	0.306789	-1.268	0.204796951	0.2532343	NOT
UGT3A1	3287.207	-0.43051	0.339558	-1.26786	0.2048482	0.2532714	NOT
RHBDD2	4023.193	-0.13028	0.102756	-1.26785	0.20485055	0.2532714	NOT
RP13-554I	4.159423	-0.31136	0.245582	-1.26784	0.204854436	0.2532714	NOT
MIR4500H	3.450118	-0.51502	0.406238	-1.26778	0.204875493	0.2532861	NOT
NBPF26	41.64091	0.225373	0.177836	1.267305	0.205046157	0.2534858	NOT
DKFZP434.	1.444417	0.448492	0.353913	1.26724	0.205069559	0.2535034	NOT
RP11-613I	5.132674	-0.2396	0.189089	-1.26712	0.205111039	0.2535433	NOT
RP1-43E1:	12.56412	0.228064	0.180025	1.266848	0.205209711	0.253654	NOT
GPR132	69.49242	-0.26305	0.207697	-1.2665	0.205333501	0.2537957	NOT
DYRK4	409.4694	-0.13421	0.105971	-1.26644	0.2053565	0.2538127	NOT
HNF4A	13177.45	-0.19906	0.157319	-1.26535	0.205746164	0.254283	NOT
RP11-132I	1.309451	-0.48926	0.386707	-1.2652	0.205800651	0.254339	NOT
GNG8	2.525995	0.469084	0.370875	1.264803	0.205942118	0.2545024	NOT
CSF2	1.220557	0.438834	0.347034	1.264528	0.206040426	0.2546126	NOT
CTD-2653	1.183854	0.48521	0.383803	1.264217	0.206152118	0.2547392	NOT
FAM173A	621.3882	0.179449	0.14197	1.263992	0.206232917	0.2548277	NOT
RP11-235(	6.78053	0.545939	0.431953	1.263884	0.206271514	0.254864	NOT
OR2A7	14.48191	-0.3053	0.241641	-1.26344	0.206431257	0.25505	NOT
FAN1	545.9471	0.153266	0.121317	1.263353	0.206462208	0.2550768	NOT
RP11-686I	17.27113	0.291306	0.230672	1.262857	0.206640591	0.2552858	NOT
SNX20	135.5122	-0.2565	0.203206	-1.26224	0.20686069	0.2555463	NOT
LONRF2	150.7095	0.363995	0.28844	1.261945	0.206968662	0.2556683	NOT
CCDC13	24.85153	0.254791	0.201961	1.26158	0.207099833	0.2558189	NOT
AC016708	83.64051	-0.48399	0.383697	-1.26138	0.20717236	0.255897	NOT
RP11-234I	1.561307	-0.49881	0.395598	-1.26089	0.207346927	0.2560961	NOT
RP11-303I	3.172305	-0.28559	0.226497	-1.26088	0.207352006	0.2560961	NOT
PRSS45	16.55733	-0.37471	0.29721	-1.26076	0.207395506	0.2561384	NOT
MTA2	2337.295	0.080181	0.063604	1.260624	0.207444401	0.2561873	NOT
PVRL2	8295.7	0.173945	0.138039	1.260117	0.207627205	0.2564016	NOT
KCNA5	16.71645	0.329399	0.261496	1.259673	0.207787416	0.256588	NOT
FAM86C2I	119.0977	0.15452	0.122678	1.259562	0.207827307	0.2566258	NOT
ABCD2	19.90562	0.329713	0.261819	1.259315	0.207916516	0.2567245	NOT
UBTD2	843.9277	0.090185	0.071637	1.258923	0.208058207	0.256888	NOT
RP11-62H	3.801678	0.374815	0.297768	1.258747	0.208121894	0.2569552	NOT
PQLC3	535.1699	-0.15533	0.123418	-1.25855	0.208193316	0.2570319	NOT
RPL35	15400.28	0.188464	0.14981	1.258026	0.208382266	0.2572537	NOT

RP11-421I	138.1873	0.152984	0.121611	1.257977	0.208400037	0.2572641	NOT
RAB30	425.0726	-0.1502	0.119404	-1.25789	0.208430057	0.2572897	NOT
SNTG2	1.280051	0.68558	0.545052	1.257826	0.208454816	0.2572987	NOT
RPL29P12	4.23965	0.243687	0.193738	1.257818	0.208457684	0.2572987	NOT
RP11-417I	3.136597	0.343659	0.273223	1.257797	0.20846524	0.2572987	NOT
DGKB	12.35709	0.40699	0.323628	1.257585	0.208541891	0.2573818	NOT
AKR7A2P1	4.001663	-0.31401	0.249702	-1.25755	0.20855595	0.2573877	NOT
AC016907	3.823219	-0.29167	0.231999	-1.25721	0.208676208	0.2575246	NOT
BEX1	72.14252	-0.55037	0.437797	-1.25714	0.208702216	0.2575452	NOT
FOPNL	1204.541	-0.08991	0.07153	-1.25701	0.208749404	0.257592	NOT
LINC0061S	1.632847	-0.44352	0.352893	-1.25683	0.208816614	0.2576634	NOT
RHOU	3656.084	-0.15843	0.126097	-1.25642	0.208964753	0.2578289	NOT
RP11-303I	5.614575	0.26593	0.21166	1.256405	0.208969333	0.2578289	NOT
RP11-958I	20.8764	0.543442	0.432583	1.256274	0.209016553	0.2578756	NOT
AC024592	2.354422	0.44593	0.354972	1.256239	0.209029187	0.2578797	NOT
RP11-785I	1334.278	-0.11712	0.093246	-1.25608	0.209088483	0.2579414	NOT
TRAV6	1.440173	0.534684	0.425752	1.255857	0.209167807	0.2580277	NOT
RP11-70C	5.52932	-0.32529	0.259078	-1.25557	0.209273275	0.2581463	NOT
ATP6V1A	3105.059	0.085615	0.068201	1.255332	0.209358444	0.2582399	NOT
ADTRP	67.9523	0.406222	0.323641	1.255164	0.209419243	0.2583033	NOT
FAM136BF	1.520455	0.548339	0.436915	1.255023	0.209470503	0.258355	NOT
HOXB2	91.81159	0.206478	0.164536	1.254912	0.209510656	0.2583919	NOT
RP11-235I	2.90245	0.314117	0.250315	1.254889	0.209519042	0.2583919	NOT
COPB2	5079.37	0.080105	0.063854	1.254511	0.209656201	0.2585495	NOT
RP11-799I	17.49014	-0.31187	0.248628	-1.25438	0.209704467	0.2585975	NOT
TRAV8-1	1.616563	-0.49022	0.390944	-1.25395	0.209860022	0.2587752	NOT
AFTPH	1387.064	-0.09336	0.074454	-1.25393	0.209867284	0.2587752	NOT
HELZ2	1766.582	-0.16447	0.131276	-1.2529	0.210243699	0.2592278	NOT
LDOC1	284.5389	0.351599	0.280736	1.252421	0.210416451	0.2594292	NOT
OSTN	2.665397	0.549325	0.438667	1.252261	0.210474856	0.2594897	NOT
FPGT	514.0859	0.107301	0.085695	1.252124	0.210524752	0.2595396	NOT
MIR324	1.751902	0.416084	0.332469	1.251498	0.210752845	0.2597871	NOT
F2R	1093.095	-0.20808	0.166265	-1.25148	0.210757878	0.2597871	NOT
TMPRSS4	16.21163	0.414522	0.331228	1.25147	0.210763	0.2597871	NOT
DKFZp434I	1.592677	0.482081	0.385212	1.25147	0.210763049	0.2597871	NOT
MIR6772	3.279688	0.396304	0.316729	1.251239	0.210847226	0.2598793	NOT
ACP1	3571.077	0.078979	0.063125	1.251143	0.210882492	0.2599065	NOT
CDR2	1098.019	-0.13129	0.10494	-1.25113	0.210888137	0.2599065	NOT
ATG3	1555.355	0.082753	0.066145	1.251088	0.210902517	0.2599127	NOT
RP1-199J3	2.677319	0.348464	0.278579	1.250863	0.210984486	0.2600021	NOT
HIST1H4C	4.495181	0.303443	0.242612	1.250732	0.211032172	0.2600493	NOT
SYT16	1.601267	0.628447	0.502796	1.249905	0.211334114	0.2604072	NOT
AP000295.	2.557479	-0.39011	0.312115	-1.24989	0.211341466	0.2604072	NOT
ZNF80	3.024745	-0.42358	0.338908	-1.24983	0.211362604	0.2604217	NOT
PTPN2	900.7301	-0.13092	0.104779	-1.2495	0.211481973	0.2605572	NOT
ERICH1-AS	11.29111	-0.53002	0.424207	-1.24943	0.211506894	0.2605763	NOT
APCDD1L	4.59579	0.522776	0.418655	1.248702	0.211773959	0.2608937	NOT
RP11-101I	26.65358	-0.59889	0.479688	-1.24849	0.211850411	0.2609762	NOT
RP11-15I1	100.0022	-0.36474	0.292198	-1.24825	0.211938815	0.2610735	NOT
CDC42BPE	3638.33	0.106858	0.085638	1.247781	0.212111138	0.2612742	NOT
GLI2	44.74189	0.372335	0.298437	1.247615	0.212172039	0.2613375	NOT
LCK	222.3826	-0.27333	0.219113	-1.24746	0.212230598	0.261398	NOT

PSMC3	5976.148	0.110988	0.088986	1.247241	0.21230911	0.2614831	NOT
HERC4	1343.418	0.10008	0.08025	1.247114	0.212355824	0.261529	NOT
TMEM218	499.6031	0.089914	0.072103	1.247018	0.212391026	0.2615607	NOT
TBC1D5	1663.321	0.096135	0.077097	1.246935	0.212421173	0.2615862	NOT
TMTC1	428.8902	0.246497	0.197809	1.246135	0.212714739	0.261936	NOT
LTBP1	1468.905	-0.23865	0.191521	-1.24606	0.21274378	0.2619537	NOT
ABCC12	1.226303	0.672738	0.539899	1.246045	0.21274804	0.2619537	NOT
CLP1	447.1885	-0.07676	0.061622	-1.24566	0.212888354	0.262114	NOT
RP11-43F1	11.06644	0.396591	0.318384	1.245638	0.212897179	0.262114	NOT
CLDN10	181.4835	-0.56891	0.456907	-1.24513	0.213084901	0.2623335	NOT
ATP1A1-A	79.6289	0.138791	0.111496	1.244806	0.213203115	0.2624673	NOT
FAM221A	203.2255	-0.21314	0.171236	-1.24471	0.213238204	0.2624988	NOT
RP11-218I	1.994038	0.33971	0.272939	1.244638	0.213264825	0.2625199	NOT
PLSCR2	2.539785	0.46266	0.371814	1.24433	0.213378284	0.2626479	NOT
PPP2CA	4485.762	-0.0733	0.05892	-1.24397	0.213511722	0.2628005	NOT
CTD-2655	8.709579	0.277156	0.222841	1.24374	0.213595214	0.2628915	NOT
AC093110	36.04821	-0.28343	0.227926	-1.24353	0.21367266	0.2629752	NOT
RP11-713(	8.451642	-0.34654	0.278764	-1.24313	0.213819443	0.2631329	NOT
ILF3-AS1	588.6438	-0.14634	0.117721	-1.24313	0.213819824	0.2631329	NOT
LEKR1	24.70654	0.161438	0.12988	1.242981	0.213875004	0.2631847	NOT
RP11-505I	3.425535	0.360748	0.290232	1.242964	0.213880931	0.2631847	NOT
ADCY5	319.1702	-0.26317	0.211737	-1.24292	0.213896498	0.2631921	NOT
AHNAK	18018.78	0.135198	0.108783	1.242822	0.213933381	0.2632258	NOT
NDUFA13	2102.758	0.229558	0.184725	1.242698	0.213979112	0.2632703	NOT
RBM38	925.3078	0.157489	0.126736	1.242651	0.213996428	0.2632799	NOT
DDTL	251.1068	-0.23948	0.192726	-1.24261	0.214012291	0.2632877	NOT
RP11-53B2	2.043564	0.362548	0.291951	1.24181	0.214306781	0.2636336	NOT
KIAA1324L	124.094	0.283747	0.228498	1.241794	0.214312483	0.2636336	NOT
PLLP	545.3916	0.212215	0.170976	1.2412	0.214531966	0.2638919	NOT
KAT8	1118.188	-0.09052	0.072945	-1.24097	0.214616223	0.2639838	NOT
CASD1	614.5046	-0.11215	0.090374	-1.24094	0.214627353	0.2639857	NOT
RP4-555D	4.626522	-0.52564	0.42362	-1.24083	0.214668795	0.264025	NOT
RP3-468B3	6.474505	0.300387	0.24213	1.240599	0.214753786	0.2641126	NOT
ASAH2	138.0152	0.296014	0.238609	1.240585	0.214759157	0.2641126	NOT
BEAN1	34.31473	0.286065	0.230599	1.240527	0.214780573	0.2641208	NOT
ERGIC3	8591.982	0.102026	0.082245	1.240515	0.214784909	0.2641208	NOT
ALG1L2	3.848806	0.28655	0.230998	1.240486	0.214795834	0.2641225	NOT
AC083900	29.16467	0.302815	0.244145	1.240309	0.214861199	0.2641911	NOT
RP11-179I	1.41293	0.432451	0.348819	1.239757	0.215065208	0.2644302	NOT
RP11-386I	1.721345	-0.39415	0.317949	-1.23967	0.215097359	0.264458	NOT
PTCH2	46.931	-0.19066	0.153821	-1.23951	0.215155455	0.2645177	NOT
LMBR1L	746.6699	0.094226	0.076032	1.239283	0.215240576	0.2646106	NOT
PXT1	2.432286	0.289689	0.233775	1.239176	0.215280282	0.2646476	NOT
CRABP2	62.80555	-0.36144	0.291809	-1.23862	0.215485532	0.2648882	NOT
PHF23	1235.953	-0.11596	0.093635	-1.23842	0.215562058	0.2649705	NOT
TMX4	3308.695	-0.11625	0.093886	-1.23817	0.215654071	0.2650718	NOT
RP11-48O	1.304425	0.516454	0.417146	1.238066	0.215691575	0.2651061	NOT
LINC01465	27.79232	0.171972	0.13893	1.237834	0.215777495	0.2651999	NOT
AC007000	2.771079	0.286026	0.231089	1.237733	0.21581512	0.2652344	NOT
TMEM99	532.6458	0.168295	0.136002	1.237446	0.215921619	0.2653535	NOT
NAA50	3937.365	0.085726	0.069287	1.237257	0.215991669	0.2654278	NOT
TPGS1	206.7218	0.22208	0.17954	1.236944	0.216108058	0.265559	NOT

TOR1B	1378.904	-0.11215	0.090688	-1.23665	0.216217525	0.2656817	NOT
CNOT2	1706.486	0.077782	0.062907	1.236461	0.216287231	0.2657474	NOT
VPS13C	1508.57	-0.13741	0.111132	-1.23645	0.216290176	0.2657474	NOT
IDE	1691.06	0.097993	0.079255	1.236421	0.216301984	0.2657501	NOT
SCAND2P	92.4664	0.116961	0.094623	1.236074	0.216431112	0.265897	NOT
CORT	7.192034	0.232055	0.187789	1.235717	0.216563617	0.2660479	NOT
LINC0096(	46.36008	0.376211	0.304464	1.235652	0.216588023	0.2660661	NOT
TEKT4	2.777248	0.395607	0.320252	1.235298	0.216719446	0.2662157	NOT
SLC25A10	3298.66	-0.19139	0.154942	-1.23526	0.216733155	0.2662208	NOT
RP11-347I	1.608172	0.371049	0.300426	1.235076	0.216802189	0.2662937	NOT
RP11-355I	5.032047	-0.2785	0.225509	-1.235	0.216829379	0.2663153	NOT
CLRN2	1.135739	0.634742	0.514028	1.23484	0.216890237	0.2663733	NOT
FAM150A	12.65826	-0.58667	0.475107	-1.23482	0.216895834	0.2663733	NOT
FAM76A	310.1011	-0.09517	0.077098	-1.23441	0.217050162	0.266551	NOT
HCCS	931.4799	-0.08982	0.072798	-1.23388	0.21724818	0.2667823	NOT
RP11-497I	3.460916	-0.31934	0.258918	-1.23337	0.217437749	0.2669974	NOT
MAP3K3	947.3506	0.097612	0.079144	1.233357	0.217442599	0.2669974	NOT
LINC0118	444.5259	-0.18507	0.15006	-1.2333	0.217462557	0.2669993	NOT
SZRD1	3529.146	0.092578	0.075065	1.233301	0.217463512	0.2669993	NOT
ZSCAN4	1.552559	0.515141	0.41782	1.232925	0.21760366	0.2671595	NOT
RP11-133(	1.177478	-0.53235	0.431798	-1.23287	0.217623596	0.2671722	NOT
GCC2	1010.734	-0.1343	0.108968	-1.23244	0.21778633	0.2673601	NOT
GDF15	4610.264	0.31218	0.253472	1.231613	0.218093844	0.2677257	NOT
RPS24P8	15.36814	0.216132	0.175517	1.2314	0.218173289	0.2678114	NOT
LINC0086	44.97506	-0.28048	0.227796	-1.23129	0.218215345	0.2678511	NOT
RP11-170I	33.8566	0.233817	0.189973	1.230792	0.218400581	0.2680636	NOT
CDC16	2090.426	0.108287	0.087983	1.230773	0.218407859	0.2680636	NOT
RABL3	891.7825	-0.08652	0.070306	-1.23065	0.218455101	0.2681097	NOT
RPN1	17224.3	-0.08828	0.071741	-1.23051	0.21850726	0.2681501	NOT
TRMT10C	1000.209	-0.09432	0.076651	-1.23051	0.218507364	0.2681501	NOT
CTD-3126	1.635053	0.343466	0.279145	1.230421	0.218539553	0.2681764	NOT
RP13-870I	11.5539	0.426093	0.346305	1.230398	0.218548187	0.2681764	NOT
OR52K3P	5.685513	0.456238	0.370887	1.230127	0.218649508	0.2682888	NOT
BTN3A3	933.5201	-0.18972	0.15432	-1.22941	0.21891918	0.2686078	NOT
KB-1517D	0.787757	0.696572	0.566829	1.228892	0.219112329	0.2688329	NOT
KCTD21	952.5192	-0.12362	0.100626	-1.22855	0.21924051	0.2689782	NOT
AC000089	31.05385	0.240682	0.195936	1.228372	0.219307475	0.2690484	NOT
RP11-61A	3.63074	0.267352	0.217695	1.228103	0.219408272	0.2691601	NOT
RP11-50I1	33.60556	-0.22885	0.186391	-1.2278	0.219520892	0.2692864	NOT
RP11-256I	0.502157	1.414974	1.152656	1.227577	0.219605687	0.2693784	NOT
KRT8P33	16.43113	-0.16278	0.132637	-1.22726	0.219724993	0.2695128	NOT
IRF2BP1	1623.613	0.135659	0.110577	1.226833	0.219885506	0.2696978	NOT
AC092155	4.443645	-0.25599	0.208736	-1.22638	0.220054682	0.2698933	NOT
FUT7	11.41175	-0.2951	0.240651	-1.22628	0.220094497	0.2699302	NOT
HIF1A-AS	1.691076	0.489956	0.39964	1.225993	0.220201285	0.2700492	NOT
TMEM102	190.9678	-0.14828	0.120966	-1.22581	0.220270125	0.2701216	NOT
USP37	186.705	0.184151	0.150267	1.225496	0.220388283	0.2702545	NOT
RNASET2	1592.98	0.177233	0.14473	1.224578	0.220734417	0.270667	NOT
ASTL	1.370673	-0.47289	0.386199	-1.22446	0.220778113	0.2707086	NOT
CHRNA2	2.432698	0.459848	0.375563	1.224421	0.220793383	0.2707153	NOT
NEDD8-M	2.025916	0.345791	0.282423	1.224374	0.220811165	0.2707251	NOT
AMIGO1	348.5796	0.162506	0.132765	1.224017	0.220945768	0.2708781	NOT

SETP17	2.585852	0.289076	0.236189	1.223917	0.220983702	0.2709088	NOT
FGGY	3650.024	0.276064	0.225561	1.223899	0.220990391	0.2709088	NOT
PARD6B	403.7231	0.153942	0.125798	1.22372	0.221058022	0.2709797	NOT
PYY	6.387712	0.344274	0.281345	1.223673	0.221075437	0.2709891	NOT
LINGO2	2.090469	-0.55388	0.45281	-1.22321	0.221251311	0.2711926	NOT
ARL13B	242.9285	-0.13614	0.111319	-1.223	0.221327929	0.2712745	NOT
CCNK	191.971	0.181507	0.14842	1.222927	0.221357128	0.2712983	NOT
RP11-102I	1.652804	0.382753	0.313094	1.222484	0.221524691	0.2714916	NOT
PSEN1	2211.597	-0.07664	0.062727	-1.22174	0.221805411	0.2718236	NOT
STYXL1	1135.121	0.143237	0.117272	1.221405	0.221932651	0.2719652	NOT
CTD-2013	16.57085	0.161561	0.132277	1.221384	0.221940593	0.2719652	NOT
RP11-195I	4.41554	-0.42562	0.348493	-1.22133	0.221962496	0.2719736	NOT
ERCC6	276.4606	-0.10498	0.085953	-1.22131	0.221967121	0.2719736	NOT
RP11-166I	3.739307	-0.28854	0.236303	-1.22105	0.222065241	0.2720818	NOT
MMP23A	9.687694	-0.53132	0.435175	-1.22094	0.222109611	0.2721241	NOT
ZNF19	86.90292	0.107938	0.088415	1.22081	0.222158042	0.2721714	NOT
CLCN3	2077.694	-0.12753	0.104473	-1.22067	0.222212619	0.2722247	NOT
ANXA1	1408.12	0.243746	0.199687	1.220643	0.222221223	0.2722247	NOT
POMP	3599.217	-0.12174	0.099755	-1.22041	0.222310694	0.2723222	NOT
GTF2IRD2I	6.852534	-0.22266	0.182537	-1.21982	0.222534396	0.2725842	NOT
CTD-2086	1.296496	0.377977	0.309886	1.219729	0.22256745	0.2726126	NOT
AC093850	7.55937	0.353883	0.290246	1.219253	0.222748272	0.272822	NOT
OOEP	9.243434	0.700414	0.574523	1.219123	0.222797629	0.2728704	NOT
RP11-373I	5.695336	-0.35173	0.288542	-1.21898	0.222852903	0.272926	NOT
FUCA2	3643.335	0.135767	0.111413	1.218593	0.22299863	0.2730924	NOT
CTD-2619	64.08642	0.294849	0.24206	1.218085	0.22319182	0.2733169	NOT
AP001055.	2.909655	-0.31878	0.261719	-1.21801	0.223220658	0.2733401	NOT
SET	6909.437	0.091456	0.075089	1.217975	0.223233647	0.2733439	NOT
KDF1	193.131	-0.2679	0.220002	-1.21774	0.223323734	0.2734421	NOT
EMC3	3596.67	0.112365	0.092296	1.217436	0.223438469	0.2735678	NOT
SIGLEC6	3.471496	-0.45481	0.373589	-1.21742	0.223446204	0.2735678	NOT
RP11-472I	1.170322	0.658693	0.541121	1.217274	0.223499925	0.2736152	NOT
ACN9	533.7311	-0.14872	0.122177	-1.21726	0.223505724	0.2736152	NOT
RP4-682C.	13.77134	0.238519	0.195951	1.217236	0.223514514	0.2736152	NOT
RFPL4A	7.587526	0.4311	0.354174	1.217198	0.223528919	0.2736207	NOT
RP11-399(	21.69926	-0.22469	0.184655	-1.21679	0.223684014	0.2737984	NOT
SPAG1	356.1521	0.189907	0.156079	1.216737	0.223704278	0.2738022	NOT
RP11-389(	17.78362	-0.31777	0.26117	-1.21673	0.223706932	0.2738022	NOT
LGALS9	1310.172	0.227592	0.187064	1.216652	0.223736733	0.2738266	NOT
FCHO1	116.369	-0.28268	0.23238	-1.21646	0.223809706	0.2739038	NOT
TRAV1-2	4.827972	-0.36751	0.302239	-1.21596	0.224000166	0.2741248	NOT
RP11-706(	241.2885	0.386449	0.317898	1.21564	0.224122103	0.2742619	NOT
RP11-144I	1.383337	0.374576	0.308309	1.214936	0.22439056	0.2745782	NOT
HORMAD2	63.35529	-0.43838	0.360963	-1.21448	0.224562776	0.2747768	NOT
LLPH	731.4313	0.076712	0.063169	1.214388	0.224599494	0.2748078	NOT
MRPL45	1735.378	0.108588	0.08942	1.214366	0.224607993	0.2748078	NOT
TIMP3	165.2267	-0.24249	0.199699	-1.21429	0.224636862	0.274831	NOT
SCN2A	18.90031	0.390331	0.321512	1.214048	0.224729309	0.2749319	NOT
IGHV3-23	386.4024	-0.49058	0.404112	-1.21398	0.224756864	0.2749535	NOT
SDAD1P1	35.86423	-0.18734	0.154328	-1.21389	0.224789446	0.2749812	NOT
RP11-981(	32.01832	0.266172	0.219486	1.212708	0.225241511	0.275522	NOT
RP11-707.	2.032579	0.967148	0.797653	1.212492	0.225324091	0.2756003	NOT

PRPF19	4831.053	0.079362	0.065453	1.212488	0.225325469	0.2756003	NOT
GLIS3	543.527	0.276242	0.227908	1.212078	0.225482556	0.2757803	NOT
GRIA1	2.310358	0.519224	0.428472	1.211805	0.225586992	0.2758958	NOT
ZNF638	3119.942	-0.06987	0.057703	-1.21093	0.225924118	0.2762959	NOT
TMEM126	925.1459	0.093254	0.077025	1.210699	0.226010729	0.2763814	NOT
RP11-584I	1.919971	0.40113	0.331323	1.210691	0.226013993	0.2763814	NOT
UBA52P7	1.134246	0.55104	0.455275	1.210346	0.226146115	0.2765307	NOT
RPL17P22	3.127832	0.27898	0.230553	1.210047	0.226260877	0.2766588	NOT
TPRG1-AS	235.806	-0.3392	0.280376	-1.2098	0.226356293	0.2767633	NOT
KIF1B	2039.254	0.119867	0.099084	1.209753	0.226373709	0.2767723	NOT
RAET1E-A	6.170802	0.298623	0.246897	1.209505	0.226468708	0.2768687	NOT
SNORD10:	2.280551	0.332196	0.274656	1.209495	0.226472556	0.2768687	NOT
KB-68A7.2	8.635024	0.431102	0.356616	1.208867	0.226713974	0.2771516	NOT
EIF4EBP1	1702.928	0.190522	0.157617	1.208766	0.226752753	0.2771868	NOT
RBM22P2	2.96968	0.325084	0.268978	1.208591	0.226819892	0.2772566	NOT
RPS4X	32709.91	0.146387	0.121168	1.208132	0.226996376	0.2774601	NOT
CTD-2353	1.841	-0.4932	0.408269	-1.20802	0.227039031	0.2775	NOT
SEC14L1P:	9.428457	0.242507	0.200778	1.207836	0.227110478	0.277575	NOT
RP11-234I	2.283339	0.345578	0.286124	1.207792	0.227127187	0.2775832	NOT
ZPLD1	21.52082	0.489877	0.405654	1.207623	0.227192268	0.2776504	NOT
KIF25-AS1	2.655303	-0.49713	0.411793	-1.20724	0.227340269	0.277819	NOT
CYP2A13	120.5025	-0.53671	0.444653	-1.20704	0.227417801	0.2779015	NOT
TRAV12-1	3.216024	-0.3888	0.322178	-1.20679	0.227511643	0.2780039	NOT
KRT18P37	1.861351	0.353123	0.292641	1.206676	0.227557047	0.2780471	NOT
RP11-17A:	5.527492	-0.23116	0.191632	-1.20627	0.227712005	0.2782242	NOT
RP4-614O	5.329079	0.255714	0.21218	1.205173	0.228136402	0.2787304	NOT
RP11-338I	7.014662	0.249676	0.207185	1.205085	0.228170359	0.2787596	NOT
C1orf127	12.71485	0.22802	0.189221	1.205048	0.228184819	0.2787649	NOT
CTB-193M	1.7402	0.367472	0.305029	1.204712	0.228314529	0.2789111	NOT
TNC	844.3104	-0.25832	0.214509	-1.20426	0.228488602	0.2791114	NOT
RSPH1	28.2614	0.31544	0.261952	1.20419	0.228516099	0.2791231	NOT
AC008592	2.752527	-0.35767	0.297021	-1.20418	0.228518366	0.2791231	NOT
ZDHHC5	3802.071	-0.06688	0.055549	-1.20395	0.228610822	0.2792237	NOT
ROCK1	1525.641	-0.11139	0.092549	-1.20358	0.228750317	0.2793818	NOT
CTD-2192	13.67513	0.205514	0.170764	1.203495	0.228784811	0.2794116	NOT
CXorf65	7.408434	0.32355	0.268875	1.203347	0.228842057	0.2794691	NOT
KLF7	423.5732	-0.21052	0.174988	-1.20308	0.228943967	0.2795812	NOT
ANXA4	8828.851	0.194752	0.161899	1.202921	0.229006966	0.2796458	NOT
RP11-418I	5.947731	0.330555	0.274869	1.202592	0.229134198	0.2797889	NOT
RPS3A	8759.528	0.143855	0.119649	1.20231	0.229243317	0.2799097	NOT
RP3-467N	2.036092	0.283479	0.235791	1.202245	0.229268492	0.2799281	NOT
RP11-191I	9.34056	0.269498	0.224178	1.202161	0.229300964	0.2799554	NOT
RP11-507I	5.51676	0.251921	0.209565	1.202114	0.229319299	0.2799655	NOT
C9orf66	33.70238	-0.39504	0.328715	-1.20176	0.229455104	0.2801189	NOT
ARHGAP1:	1623.823	0.130603	0.108764	1.200788	0.229833482	0.2805624	NOT
RP11-483I	7.263035	-0.45377	0.377896	-1.20077	0.22983866	0.2805624	NOT
GPC1	2968.009	-0.20248	0.168635	-1.20067	0.22987862	0.2805988	NOT
RP4-725G:	6.77555	0.303142	0.252492	1.200602	0.229905518	0.2806192	NOT
RP1-127D	3.790389	0.575591	0.479493	1.200416	0.229977905	0.2806952	NOT
COX5A	5011.431	0.134206	0.111842	1.199964	0.23015328	0.2808969	NOT
AKR1C8P	92.97581	0.369963	0.308516	1.199171	0.230461553	0.2812559	NOT
LINC0147:	12.16352	0.209098	0.174371	1.199155	0.230467832	0.2812559	NOT

H1FX	3682.417	0.129202	0.10777	1.198863	0.230581407	0.2813821	NOT
CR848007.	4.562846	-0.64491	0.538031	-1.19864	0.230667629	0.2814749	NOT
NMT2	1359.132	0.1612	0.134495	1.198557	0.230700177	0.2815022	NOT
DENND2D	601.7797	-0.18906	0.157778	-1.19827	0.230813229	0.2816278	NOT
ARMCX1	165.0152	0.223855	0.18689	1.197791	0.230998291	0.2818411	NOT
CLPTM1L	5989.959	0.109418	0.091353	1.197757	0.23101148	0.2818448	NOT
RP11-333I	3.436095	0.294594	0.246005	1.197512	0.231107058	0.281949	NOT
RPL18AP3	268.9078	0.214187	0.178927	1.197066	0.231280849	0.2821486	NOT
CAMK1	411.0947	-0.12385	0.103465	-1.19703	0.231293902	0.282152	NOT
TMEM222	1797.384	-0.11558	0.096563	-1.19693	0.231334201	0.2821888	NOT
RP11-405I	1.659058	-0.4791	0.40035	-1.1967	0.231424343	0.2822863	NOT
TRAV12-2	4.980158	-0.41039	0.34298	-1.19655	0.231483174	0.2823456	NOT
RN7SL834I	5.537443	0.313113	0.261703	1.196448	0.231521848	0.2823803	NOT
EGLN1	4372.502	0.113041	0.094485	1.196389	0.23154479	0.2823958	NOT
MAGOH2F	13.19097	0.239751	0.200403	1.196346	0.231561707	0.282404	NOT
RP11-769(	1.954845	0.354984	0.29684	1.195879	0.2317438	0.2826136	NOT
EEF1A1P1(	8.806558	0.221584	0.185368	1.195373	0.231941202	0.2828419	NOT
PHF3	2930.9	-0.1225	0.102537	-1.19474	0.232187968	0.2831303	NOT
TOM1L1	2138.568	-0.1563	0.130907	-1.19401	0.232474496	0.2834672	NOT
RP4-800J2	2.83941	-0.28968	0.242628	-1.19391	0.232514805	0.2835036	NOT
COG8	842.5396	-0.08401	0.07037	-1.19388	0.232524773	0.2835036	NOT
RPS12P26	3.052718	0.264506	0.221618	1.193522	0.232664889	0.2836619	NOT
ANKRD20A	7.149876	-0.7308	0.612445	-1.19326	0.232768093	0.2837752	NOT
RP11-307(	8.028725	-0.27444	0.230007	-1.19317	0.232802365	0.2838045	NOT
ERC1	1300.556	-0.10034	0.084107	-1.19297	0.232881987	0.2838891	NOT
RP5-1024(	12.72994	0.149474	0.125307	1.192856	0.232925827	0.28393	NOT
TMCC1	1936.899	0.179913	0.150864	1.192554	0.233044061	0.2840616	NOT
EIF4A2P4	1.509442	0.330294	0.277093	1.191995	0.233263222	0.2843162	NOT
RP11-510J	10.50744	0.315656	0.264837	1.191887	0.233305557	0.2843553	NOT
CD300LG	68.32882	0.448885	0.376698	1.19163	0.233406172	0.2844654	NOT
RPL5P3	2.647461	0.303173	0.254444	1.191511	0.233453157	0.2845044	NOT
KRT18P34	58.9201	-0.2451	0.205711	-1.1915	0.233458742	0.2845044	NOT
AARS	7551.837	0.111233	0.093391	1.191054	0.233632408	0.2847035	NOT
PRKG1	136.638	-0.2127	0.1786	-1.1909	0.2336911	0.2847625	NOT
AP1B1	4507.462	0.085481	0.071803	1.19049	0.233853691	0.284948	NOT
CFAP58	4.60601	-0.24016	0.2018	-1.19009	0.234011277	0.2851275	NOT
SEC14L3	93.21502	-0.44932	0.377635	-1.18983	0.234112205	0.2852379	NOT
EIF2B3	1017.783	-0.10845	0.0912	-1.18911	0.234396522	0.2855717	NOT
RP11-517(	3.073493	0.248896	0.209429	1.188452	0.234655575	0.2858748	NOT
USE1	1199.448	0.158709	0.133545	1.188424	0.234666242	0.2858752	NOT
SETD8	700.7816	0.101929	0.085798	1.188005	0.234831276	0.2860636	NOT
ZNF417	134.2078	0.124025	0.104405	1.187919	0.234865346	0.2860925	NOT
RSPH4A	10.47121	-0.20858	0.175689	-1.1872	0.235148775	0.2864252	NOT
HLA-DRB5	2907.952	-0.26707	0.224996	-1.18701	0.235222943	0.2865029	NOT
SLC25A21	11.36323	-0.2979	0.250979	-1.18697	0.235240694	0.2865119	NOT
JAK3	335.0266	0.242926	0.204676	1.186879	0.235275391	0.2865415	NOT
WWC2-AS	26.54929	-0.17207	0.145009	-1.18663	0.235374149	0.2866492	NOT
MNAT1	427.2696	0.096841	0.081622	1.186456	0.235442411	0.2867197	NOT
RPL26P30	7.399288	0.260879	0.21991	1.186298	0.235504823	0.2867831	NOT
HIGD1C	1.869046	0.42263	0.35641	1.185796	0.235702822	0.2870116	NOT
RGCC	529.6599	-0.20658	0.174237	-1.18562	0.235773383	0.2870849	NOT
RPL41P2	22.09337	0.192258	0.162204	1.185282	0.23590581	0.2872261	NOT

FLJ38122	4.72768	-0.32064	0.270516	-1.18527	0.235910164	0.2872261	NOT
SPACA7	5.820599	-0.5212	0.439888	-1.18484	0.236080514	0.2874209	NOT
AC084082	4.719289	0.338839	0.286053	1.184532	0.236202499	0.2875465	NOT
SMG1P4	1.725597	0.518896	0.438062	1.184527	0.236204506	0.2875465	NOT
ZNF540	52.25691	-0.17454	0.147367	-1.18441	0.236250252	0.2875896	NOT
PRPS1	2010.198	-0.11798	0.099663	-1.18383	0.236481482	0.2878584	NOT
DOCK4	1018.206	-0.12859	0.108633	-1.18369	0.236536257	0.2879025	NOT
STAT2	4143.647	-0.11692	0.098773	-1.18368	0.236538529	0.2879025	NOT
WDR47	213.4895	0.12214	0.103251	1.182947	0.236830047	0.2882428	NOT
FAM84A	164.4885	-0.27101	0.229099	-1.18292	0.236838982	0.2882428	NOT
CECR5-AS	12.26184	0.222579	0.188256	1.182322	0.237078119	0.2885212	NOT
AC073325	1.227763	0.583978	0.494123	1.181849	0.237265479	0.2887365	NOT
TICAM2	3.714786	0.239333	0.202588	1.181376	0.237453314	0.2889524	NOT
RP11-203I	3.374621	0.274191	0.23216	1.181042	0.237585851	0.2891009	NOT
RPS6KA3	4474.06	0.142731	0.120857	1.180995	0.237604745	0.2891112	NOT
PTGR2	776.5877	-0.1195	0.101198	-1.1809	0.237641494	0.2891432	NOT
SNTB1	7384.011	-0.1678	0.142122	-1.1807	0.237721529	0.289227	NOT
PADI2	67.2057	0.294963	0.249826	1.180674	0.237732191	0.289227	NOT
RP3-342P2	22.50352	-0.37297	0.315911	-1.18063	0.237750484	0.289227	NOT
RFPL2	6.737983	-0.3457	0.292808	-1.18062	0.237752185	0.289227	NOT
ABCF3	2416.367	0.064406	0.054577	1.180083	0.237967137	0.2894758	NOT
CDC42P6	6.884619	0.225508	0.191192	1.179487	0.238204288	0.2897515	NOT
C11orf63	28.9514	-0.25297	0.214521	-1.17923	0.238304999	0.2898613	NOT
RNF149	2028.976	-0.11483	0.097431	-1.1786	0.238556719	0.2901486	NOT
YBX1P2	12.28115	0.1837	0.155865	1.178588	0.238562237	0.2901486	NOT
C5orf47	2.313133	0.318613	0.270517	1.177793	0.23887932	0.2905215	NOT
ATP5HP4	10.38476	0.245684	0.208709	1.177162	0.23913103	0.2908149	NOT
ZMAT1	340.4538	0.22438	0.190652	1.176909	0.239231838	0.2909247	NOT
ZNF75D	435.885	0.119953	0.10194	1.176696	0.239316773	0.2910152	NOT
AQR	1128.626	-0.07802	0.066323	-1.17644	0.239419745	0.2911276	NOT
ARHGAP26	595.6736	0.117046	0.099529	1.175999	0.239595403	0.2913284	NOT
AC016734	22.79291	0.205163	0.174526	1.175545	0.239776764	0.2915361	NOT
CTD-2616	2.710501	0.347086	0.295263	1.175513	0.23978956	0.2915388	NOT
MTND5P1	13.87764	0.363619	0.309342	1.175459	0.239811095	0.2915403	NOT
RP11-83A	118.2473	-0.12503	0.106371	-1.17546	0.239811872	0.2915403	NOT
ATM	1155.709	-0.11532	0.098171	-1.17465	0.240134234	0.2919194	NOT
RP11-180	3.459241	0.262699	0.22373	1.174181	0.240322551	0.292134	NOT
AC006994	2.046528	0.381472	0.32489	1.174158	0.240331891	0.292134	NOT
TMEM221	13.20084	0.257111	0.218982	1.174119	0.240347474	0.2921401	NOT
RP4-665N	2.031276	0.303171	0.258259	1.173904	0.240433538	0.2922319	NOT
ZRANB3	193.1537	0.110077	0.093824	1.173224	0.240705991	0.2925502	NOT
FAM109A	970.238	0.143373	0.122227	1.173003	0.240794442	0.2926331	NOT
TRAF3IP1	594.5684	0.080403	0.068545	1.173001	0.240795391	0.2926331	NOT
CTC-490E	3.256367	0.576034	0.491382	1.172274	0.241087139	0.2929748	NOT
RP11-815I	8.51198	0.292301	0.249378	1.172117	0.241149916	0.2930382	NOT
PPP4C	3946.836	-0.10504	0.089647	-1.17166	0.241331921	0.2932465	NOT
SDHAP3	167.9915	0.198661	0.16956	1.171626	0.24134744	0.2932525	NOT
RP11-23D	5.311414	0.422325	0.36047	1.171595	0.241359686	0.2932545	NOT
RALB	1424.965	-0.0942	0.080416	-1.1714	0.241439851	0.293339	NOT
RP11-408	8.687581	0.219327	0.187282	1.171108	0.241555495	0.2934666	NOT
RP11-513I	13.02958	0.172246	0.14713	1.170703	0.241718126	0.2936513	NOT
CH507-15	9.62142	0.477786	0.408243	1.170348	0.241861076	0.2938121	NOT

AC073410	2.273978	0.319073	0.272664	1.170207	0.241917671	0.2938633	NOT
CCDC148	19.41295	0.222802	0.190398	1.17019	0.241924453	0.2938633	NOT
ALKBH3-A	6.301216	0.190381	0.162701	1.170125	0.241950783	0.2938824	NOT
RP11-72I8	3.040864	0.259831	0.222126	1.169743	0.242104373	0.294056	NOT
SLC30A10	1893.578	0.224704	0.192173	1.169281	0.242290246	0.2942688	NOT
DYNLT3	1486.053	-0.10749	0.09195	-1.16903	0.242392997	0.2943807	NOT
EEF1DP3	5.434316	-0.26892	0.230127	-1.16857	0.242577304	0.2945916	NOT
DNAJC21	2020.611	0.078824	0.067465	1.168358	0.242662384	0.294682	NOT
PLEKHO1	1091.14	0.196309	0.168075	1.167982	0.242813922	0.2948531	NOT
RCN3	609.4854	0.164754	0.141064	1.167942	0.242830361	0.2948601	NOT
KRT4	4.913726	-0.73172	0.626556	-1.16784	0.242872192	0.2948959	NOT
IMMT	3717.571	0.08087	0.06925	1.167807	0.242884702	0.2948959	NOT
GARNL3	166.2705	-0.20955	0.179445	-1.16779	0.242891828	0.2948959	NOT
KCNK3	47.78055	-0.36893	0.315931	-1.16774	0.242911427	0.2949068	NOT
CELF2	356.054	-0.24557	0.210306	-1.16768	0.242934064	0.2949213	NOT
HAR1A	20.11426	-0.42484	0.363864	-1.16757	0.242978954	0.2949629	NOT
RNF111	743.6076	-0.10219	0.087533	-1.16745	0.243027708	0.2950091	NOT
ZNF140	356.0312	-0.11496	0.09848	-1.1673	0.24308769	0.295069	NOT
RP11-248I	5.715864	-0.5903	0.505947	-1.16672	0.24332421	0.2953431	NOT
NDUFB6	2079.414	-0.10603	0.090932	-1.16601	0.2436096	0.2956765	NOT
IGHJ3P	1.376416	-0.62602	0.537028	-1.16572	0.243728879	0.2958083	NOT
FNDC7	1.041001	0.489732	0.420187	1.165509	0.243812878	0.2958973	NOT
RP3-467L1	9.885993	0.22031	0.189036	1.165442	0.243840088	0.2959173	NOT
RP11-712I	15.32128	0.217381	0.186578	1.165093	0.243981215	0.2960756	NOT
FAM106A	1.592895	0.578017	0.496126	1.165061	0.243994369	0.2960786	NOT
ALOX5AP	179.0561	-0.26691	0.229127	-1.16491	0.244057394	0.2961421	NOT
RP11-51J9	6.884026	-0.20474	0.175765	-1.16488	0.244069332	0.2961436	NOT
ARMCX4	168.5028	0.1245	0.106919	1.164425	0.244251926	0.2963521	NOT
RPL7L1	4166.077	0.079369	0.068165	1.164375	0.244272131	0.2963637	NOT
MPP1	1632.901	-0.15521	0.133305	-1.16433	0.244291253	0.2963739	NOT
TMEM62	835.9568	0.105769	0.090857	1.164122	0.24437451	0.2964619	NOT
B9D2	144.035	0.149809	0.128693	1.164082	0.244390684	0.2964685	NOT
C11orf21	34.93159	-0.24622	0.211519	-1.16404	0.244409299	0.2964781	NOT
LINC01291	44.93004	0.507745	0.436357	1.163599	0.244586456	0.29668	NOT
HDGF	16710.12	0.090124	0.077464	1.16343	0.244654926	0.2967415	NOT
RP11-298(	11.55034	0.174607	0.15008	1.163421	0.244658602	0.2967415	NOT
CTBP2	432.4437	0.230169	0.19785	1.163351	0.244687024	0.2967629	NOT
STEAP1	689.9878	0.293509	0.25237	1.163011	0.244825022	0.2969173	NOT
RP11-253I	6.994728	0.272764	0.234626	1.162548	0.245012971	0.2971322	NOT
AC113189	266.3173	0.184364	0.158616	1.162329	0.245101983	0.2972271	NOT
RIF1	1361.238	0.104562	0.089988	1.161953	0.245254756	0.2973993	NOT
TMEM182	150.3521	-0.25122	0.216261	-1.16165	0.245376141	0.2975335	NOT
RP11-849I	19.6217	0.188637	0.162392	1.161615	0.245392064	0.2975357	NOT
RP11-74J1	12.42657	0.194544	0.16748	1.161596	0.245399481	0.2975357	NOT
CCNDBP1	1170.108	-0.09102	0.078407	-1.16088	0.24568915	0.2978718	NOT
AP000473.	7.173196	-0.33179	0.285814	-1.16086	0.245698235	0.2978718	NOT
HMG20B	2185.441	0.0974	0.083929	1.160505	0.245843191	0.2980345	NOT
RP11-1334	4.150081	0.269125	0.23192	1.16042	0.245878033	0.2980564	NOT
FBXL5	3720.207	-0.13135	0.11319	-1.16041	0.245882783	0.2980564	NOT
KIF9	155.6363	0.122712	0.105777	1.160097	0.246009227	0.2981966	NOT
KEAP1	3520.105	0.093803	0.080917	1.15925	0.246354304	0.2986018	NOT
PTAFR	337.2737	-0.24031	0.207332	-1.15905	0.246434405	0.2986858	NOT

KPNA4	2673.076	0.074298	0.064108	1.158941	0.246480281	0.2987283	NOT
NTRK1	6.40358	-0.26418	0.227985	-1.15874	0.246561655	0.2988138	NOT
C9orf92	1.564309	0.611673	0.527915	1.158659	0.246595082	0.2988413	NOT
RP11-477I	10.50416	-0.33878	0.292427	-1.15852	0.246653309	0.2988897	NOT
CTD-2378	2.826066	0.308054	0.265906	1.158508	0.246656625	0.2988897	NOT
KRT15	16.4188	0.539869	0.466156	1.15813	0.246810836	0.2990573	NOT
TUSC3	436.0153	0.296319	0.255863	1.158116	0.246816614	0.2990573	NOT
RP11-354I	2.212754	0.451114	0.38967	1.157682	0.246993806	0.2992589	NOT
RP13-895J	1.269883	0.722259	0.623931	1.157594	0.247029661	0.2992893	NOT
MPZL3	604.6694	-0.16591	0.143357	-1.15733	0.24713868	0.2994082	NOT
RP11-166I	145.703	-0.22363	0.193269	-1.1571	0.247232156	0.2995084	NOT
RP11-714(	1.716298	0.342914	0.2964	1.15693	0.247300798	0.2995784	NOT
POMT1	1423.36	-0.0913	0.078957	-1.1563	0.247559487	0.2998786	NOT
RP11-536(	2.210482	-0.6208	0.537014	-1.15603	0.247670401	0.2999999	NOT
FLJ37453	134.402	0.144337	0.124884	1.155765	0.247777175	0.3001161	NOT
COL4A2-A	1.695129	0.383844	0.332135	1.155687	0.247809184	0.3001417	NOT
AC009501	29.84433	0.205757	0.178077	1.155442	0.247909561	0.3002501	NOT
AC009473	2.58707	-0.27685	0.239619	-1.15535	0.247945294	0.3002803	NOT
MCM9	357.1655	-0.10252	0.088748	-1.15517	0.248022212	0.3003603	NOT
PLEKHH2	116.4158	0.302852	0.26221	1.154996	0.248092043	0.3004317	NOT
ALDH1A2	283.0057	-0.24008	0.207868	-1.15495	0.248110771	0.3004412	NOT
TIMP2	5329.889	0.238683	0.206674	1.154877	0.248140769	0.3004644	NOT
CTD-2192	3.592024	0.263626	0.228294	1.154766	0.248186363	0.3005064	NOT
SERTAD2	1268.143	-0.11036	0.095575	-1.15466	0.248228772	0.3005446	NOT
MAP4K1	195.5586	-0.21592	0.187037	-1.15441	0.248333265	0.300658	NOT
EIF1P7	1.603758	0.345925	0.29982	1.153778	0.248591063	0.3009569	NOT
PPP4R4	249.071	-0.2136	0.185145	-1.15369	0.248626212	0.3009863	NOT
LINC01024	53.20486	-0.13305	0.115335	-1.15361	0.248661283	0.3010156	NOT
INPP5D	582.8984	-0.17923	0.155367	-1.15358	0.248672185	0.3010156	NOT
AC003956	2.858806	0.362551	0.31439	1.15319	0.248832429	0.301188	NOT
RP11-475J	7.700503	-0.32985	0.286033	-1.15318	0.248836334	0.301188	NOT
ERVH48-1	4.136215	0.33882	0.293858	1.153007	0.248907395	0.3012608	NOT
PKD2	719.9283	-0.18593	0.16128	-1.15283	0.248978419	0.3013336	NOT
DAP	8445.014	-0.10012	0.086849	-1.15279	0.248996862	0.3013427	NOT
RP1-159A1	9.419008	0.20364	0.176704	1.152431	0.249144052	0.3015077	NOT
POLR1D	2424.802	0.098586	0.085587	1.151887	0.249367504	0.3017649	NOT
RP11-360I	2.431413	-0.35234	0.306029	-1.15132	0.249598573	0.3020313	NOT
SULF2	3253.911	-0.24773	0.21518	-1.15125	0.249627543	0.3020531	NOT
MAD2L1BI	860.6206	0.098419	0.085506	1.151015	0.249726063	0.3021591	NOT
TYW1	741.2873	-0.08191	0.071172	-1.15085	0.249795212	0.3022296	NOT
ZMAT3	1267.458	-0.14562	0.126551	-1.15068	0.249862381	0.3022976	NOT
MAMDC4	1845.604	-0.33943	0.29502	-1.15053	0.249923634	0.3023585	NOT
TPT1-AS1	160.8584	0.134687	0.117081	1.150374	0.249989712	0.3024252	NOT
TMEM9B-	51.57987	0.166502	0.144744	1.150321	0.250011802	0.3024387	NOT
GRWD1	1692.432	0.096437	0.083866	1.149885	0.250191148	0.3026425	NOT
HSPA6	222.7484	0.292407	0.254349	1.14963	0.250296413	0.3027566	NOT
ANKRD13(	574.7714	-0.09441	0.082129	-1.14958	0.250316283	0.3027674	NOT
PTAR1	1250.905	-0.11548	0.100458	-1.14954	0.250334289	0.3027759	NOT
RP11-110	1.747288	0.335093	0.2916	1.149154	0.250492591	0.3029541	NOT
FAM206A	478.7217	0.10772	0.093793	1.148489	0.25076659	0.3032722	NOT
RP11-84C	1.582344	0.374418	0.326051	1.148343	0.250827175	0.3033323	NOT
KLHL10	3.539693	-0.23849	0.207729	-1.14808	0.250936311	0.303451	NOT

RP11-168I	12.83316	-0.3113	0.271231	-1.14774	0.251075277	0.3036058	NOT
PRSS12	87.52606	0.413125	0.360064	1.147363	0.251231622	0.3037815	NOT
UBE2F	1155.458	-0.09691	0.084488	-1.14702	0.25137198	0.303938	NOT
C8orf58	116.7508	-0.14319	0.124871	-1.14673	0.251492065	0.3040699	NOT
USP32P1	9.593917	0.508746	0.443751	1.146466	0.251602288	0.3041898	NOT
PRR22	108.0435	-0.17933	0.156441	-1.14632	0.251662727	0.3042496	NOT
STXBP3	1212.568	-0.09772	0.085285	-1.14584	0.251861939	0.304473	NOT
C6orf165	5.531173	-0.19052	0.166271	-1.14582	0.251869478	0.304473	NOT
PHLPP2	402.3348	0.144973	0.126541	1.145661	0.251935592	0.3045396	NOT
AP002954.	5.697811	-0.50353	0.439569	-1.14551	0.251995949	0.3045992	NOT
AC067945	4.857701	0.290241	0.253406	1.145359	0.252060628	0.3046641	NOT
POU5F1B	8.391633	-0.24388	0.212938	-1.1453	0.252086195	0.3046817	NOT
C10orf128	278.0615	0.241359	0.210811	1.144907	0.252247883	0.3048638	NOT
TEKT3	3.920818	-0.28645	0.250233	-1.14471	0.25232791	0.3049472	NOT
EEF1A1P1(	9.210225	0.21054	0.183987	1.144317	0.25249221	0.3051324	NOT
RP11-846I	3.236709	-0.33904	0.296439	-1.14372	0.252738594	0.3054049	NOT
RP11-474I	2.526217	-0.31634	0.276588	-1.14372	0.252739786	0.3054049	NOT
ERI1	382.0004	0.127774	0.111755	1.143338	0.252898465	0.3055833	NOT
FGF2	261.0866	0.324033	0.283669	1.142292	0.253332782	0.3060948	NOT
RPL12P4	13.49992	0.240705	0.210753	1.142119	0.25340473	0.3061683	NOT
SRGAP2C	193.8086	0.136731	0.11972	1.142089	0.253416973	0.3061698	NOT
ZNF30-AS	2.229441	0.336483	0.294818	1.141325	0.25373452	0.30654	NOT
SDAD1	730.6845	0.114773	0.100582	1.141089	0.253832883	0.3066455	NOT
FRA10AC1	743.6584	0.099391	0.087133	1.140679	0.254003551	0.3068382	NOT
CYP2A7P1	5.174542	-0.64476	0.56541	-1.14033	0.254147201	0.3069909	NOT
CD1E	36.25144	-0.32994	0.289338	-1.14032	0.254152085	0.3069909	NOT
ZNF697	383.9241	0.134433	0.117894	1.14029	0.254165634	0.3069938	NOT
AC005062	1.046746	0.57863	0.507514	1.140127	0.254233362	0.3070622	NOT
KRT18P28	5.304405	-0.22075	0.193685	-1.13974	0.25439672	0.3072461	NOT
LINC0093(	47.54828	0.312688	0.274473	1.139228	0.254607896	0.3074877	NOT
BTBD6	1520.161	-0.13874	0.121794	-1.13912	0.254651994	0.3075276	NOT
AGPAT3	6479.939	-0.11365	0.099813	-1.13862	0.254862763	0.3077687	NOT
CLCN4	515.6661	0.118621	0.104218	1.138198	0.255037728	0.3079651	NOT
HNF1B	1103.001	0.248208	0.218076	1.138174	0.255047702	0.3079651	NOT
SNORD89	3.981028	-0.19944	0.17528	-1.13785	0.255184805	0.3081172	NOT
CYP46A1	42.17395	-0.20639	0.181426	-1.13759	0.255290743	0.3082317	NOT
PDCD4-AS	38.53812	-0.1467	0.129043	-1.1368	0.255623016	0.3086194	NOT
DTX2P1-U	18.84565	0.138036	0.121429	1.136759	0.255639118	0.3086254	NOT
AC093162	29.80125	0.24004	0.211171	1.136709	0.255659747	0.3086368	NOT
RPS26P6	3.544843	0.242434	0.213304	1.136563	0.255721014	0.3086973	NOT
RP3-523C.	3.080259	0.305837	0.269166	1.136239	0.255856431	0.3088473	NOT
SH3BGRL3	3893.285	0.154609	0.136075	1.136206	0.255870185	0.3088504	NOT
XXbac-BP(	2.096969	0.439755	0.387059	1.136146	0.255895579	0.3088676	NOT
ZNF334	90.89873	-0.28936	0.254779	-1.13575	0.256061395	0.3090543	NOT
EIF1AXP1	9.13467	-0.18515	0.163048	-1.13558	0.25613313	0.3091273	NOT
RPP25L	1282.145	-0.12804	0.112767	-1.1354	0.256209069	0.3092055	NOT
SLC27A3	2751.286	0.179993	0.158549	1.135248	0.256271314	0.3092671	NOT
MMRN2	1018.092	0.148619	0.130931	1.135093	0.25633643	0.3093322	NOT
CTD-3075	1.916404	-0.32069	0.282643	-1.1346	0.256544491	0.3095698	NOT
PSPN	122.5096	0.12087	0.10655	1.134392	0.256630352	0.3096599	NOT
TMEM2	5462.732	-0.15322	0.135085	-1.13426	0.256685695	0.3097132	NOT
SNORA66	4.101357	0.226611	0.199803	1.134173	0.256722108	0.3097325	NOT

ZNF575	87.38214	0.111781	0.098557	1.134168	0.256724104	0.3097325	NOT
WDR6	3143.274	0.094108	0.082986	1.134031	0.256781559	0.3097883	NOT
NRIR	8.027352	0.33213	0.292885	1.133995	0.256796609	0.309793	NOT
AZI2	516.4617	0.105889	0.093408	1.133618	0.256954976	0.3099705	NOT
RP11-211(	1.865824	0.571017	0.503946	1.133091	0.257175831	0.3102234	NOT
RP11-384I	89.64659	-0.22728	0.200626	-1.13284	0.257280349	0.3103359	NOT
TOX	113.8758	0.320244	0.282761	1.13256	0.257398899	0.3104654	NOT
C1orf21	1558.283	-0.21477	0.189661	-1.13238	0.257475453	0.3105442	NOT
NALCN-AS	2.818268	0.399054	0.352415	1.13234	0.257491324	0.3105498	NOT
CTD-3076	51.49967	-0.31028	0.274044	-1.13223	0.2575379	0.3105924	NOT
WDR83OS	3841.178	-0.11438	0.101036	-1.13206	0.257607298	0.3106626	NOT
ESPNP	11.99169	-0.28762	0.254145	-1.13171	0.257757056	0.3108296	NOT
AC099552	2.13276	0.687962	0.607956	1.131598	0.257803533	0.3108721	NOT
IGFBP7-AS	2.256251	0.338486	0.299204	1.131288	0.257933787	0.3110059	NOT
DDX10P1	2.136711	0.408536	0.361127	1.131281	0.257936948	0.3110059	NOT
CFAP97	1206.561	-0.11839	0.104715	-1.13055	0.258244094	0.3113627	NOT
RP11-680I	29.96658	-0.17965	0.158935	-1.13034	0.258332312	0.3114555	NOT
RAB8B	826.3994	-0.1374	0.121697	-1.12902	0.258889917	0.3121141	NOT
NUDT19	639.0343	0.140096	0.124115	1.128757	0.259000344	0.3122337	NOT
ZC3H6	295.5287	-0.13591	0.120463	-1.12826	0.259211478	0.3124746	NOT
PFN1P2	10.4351	-0.26708	0.236808	-1.12785	0.259382972	0.3126659	NOT
RP11-209I	1.469931	0.376259	0.333614	1.127827	0.259392806	0.3126659	NOT
RP11-286I	17.61041	0.418521	0.371116	1.127736	0.259431567	0.312699	NOT
KRT18P31	1.616562	-0.27812	0.246635	-1.12764	0.259472463	0.3127347	NOT
HNRNPCP	14.29486	-0.16231	0.143949	-1.12758	0.259498279	0.3127522	NOT
PSMA5	4731.205	-0.10079	0.089397	-1.1275	0.259533055	0.3127805	NOT
ZNF141	152.7187	-0.16842	0.149386	-1.1274	0.259572402	0.3128143	NOT
SHC2	3647.638	-0.1767	0.156742	-1.12732	0.259605593	0.3128406	NOT
ANXA8L1	20.0504	-0.45497	0.403608	-1.12727	0.259629909	0.3128563	NOT
RP5-1000I	8.048864	-0.16368	0.145235	-1.12703	0.259731194	0.3129647	NOT
BEAN1-AS	2.606706	-0.35561	0.315723	-1.12634	0.26002231	0.3133019	NOT
AC005076	24.53168	0.176739	0.15692	1.126299	0.260039068	0.3133084	NOT
PRRG2	229.3749	-0.25379	0.225448	-1.12571	0.260287965	0.3135946	NOT
ZNF708	112.2346	0.142178	0.126309	1.125638	0.260318761	0.3136181	NOT
KCNIP4	21.46875	0.142806	0.126889	1.125437	0.260403919	0.313707	NOT
TSSK1A	2.294699	0.285574	0.253759	1.125375	0.260430316	0.3137252	NOT
RRM2B	1502.796	0.110596	0.098284	1.125263	0.260477617	0.3137685	NOT
CLNK	8.438485	-0.29186	0.259407	-1.12509	0.26054906	0.3138189	NOT
PLA2G2A	18515.86	0.515377	0.458076	1.125089	0.260551154	0.3138189	NOT
PKD1P5	27.21101	0.278078	0.247163	1.12508	0.260555067	0.3138189	NOT
LINC0126(	4.479903	0.312671	0.277916	1.125057	0.260564864	0.3138189	NOT
OSER1-AS	457.9827	0.152269	0.135356	1.124951	0.260609805	0.3138594	NOT
DTX4	1389.647	0.217733	0.193624	1.124512	0.260795974	0.3140699	NOT
TM9SF1	281.1309	0.09381	0.083432	1.124391	0.260847011	0.3141177	NOT
GAS6-AS1	233.9151	0.296685	0.263897	1.124245	0.260909236	0.314179	NOT
RAB33A	16.54009	0.235231	0.209416	1.123268	0.261323492	0.3146551	NOT
CICP16	3.641944	0.305635	0.272097	1.123259	0.261327411	0.3146551	NOT
RP11-100(	8.990629	-0.22498	0.200331	-1.12302	0.261429582	0.3147614	NOT
ANO1-AS2	1.65492	0.480887	0.428217	1.122998	0.261438434	0.3147614	NOT
RP11-977(	32.71495	0.157836	0.140596	1.122622	0.261598273	0.3149401	NOT
CST6	2.650935	0.431633	0.38459	1.122322	0.26172549	0.3150796	NOT
CTD-3035	2.05945	0.413172	0.368169	1.122234	0.261762969	0.315111	NOT

ATP11B	1381.573	-0.12078	0.107642	-1.12207	0.261831647	0.3151703	NOT
NINJ1	3685.554	-0.11119	0.099092	-1.12206	0.261835029	0.3151703	NOT
TWIST2	18.66355	0.340379	0.303387	1.12193	0.261892075	0.3152253	NOT
ADPRM	303.5131	-0.13412	0.119556	-1.12181	0.261944689	0.3152749	NOT
SLC16A12	210.5235	0.361926	0.322716	1.1215	0.262074913	0.3154179	NOT
KRTAP5-2	1.785106	0.788176	0.702854	1.121393	0.262120453	0.315459	NOT
KB-1027C	1.793174	-0.56445	0.503395	-1.12129	0.262163763	0.3154974	NOT
SMARCAD	660.8311	-0.1185	0.105715	-1.12093	0.262318299	0.3156696	NOT
ARSJ	157.4554	-0.26529	0.236728	-1.12063	0.26244372	0.3158068	NOT
C20orf62	2.041862	-0.45238	0.403814	-1.12026	0.26260363	0.3159855	NOT
AC004057	1.781507	0.349015	0.311635	1.11995	0.262735215	0.3161301	NOT
PRPS2	1168.065	-0.14888	0.132945	-1.11987	0.262770661	0.316159	NOT
NXT1	486.9879	0.127416	0.11381	1.119549	0.262906094	0.3163082	NOT
RP13-585I	4.863894	0.227237	0.203006	1.119361	0.262986262	0.3163909	NOT
ERH	2803.611	0.083533	0.074681	1.118534	0.263338989	0.3168015	NOT
FTL	440352.3	0.202599	0.181149	1.118409	0.263392192	0.3168517	NOT
AES	10507.27	-0.11766	0.10521	-1.11835	0.263418897	0.31687	NOT
RP11-490I	3.363205	0.250024	0.223583	1.11826	0.263455936	0.3169008	NOT
ZNF507	759.2056	0.094206	0.084284	1.117718	0.263687283	0.3171653	NOT
LDHAP5	4.453873	-0.2109	0.188705	-1.11762	0.263729241	0.3171919	NOT
IDH3A	589.8607	-0.14623	0.130842	-1.11761	0.26373237	0.3171919	NOT
AC004166	39.5464	-0.16625	0.148777	-1.11742	0.263814796	0.3172773	NOT
NEIL1	559.928	0.148691	0.133086	1.11725	0.263887283	0.3173507	NOT
FBN2	26.11273	0.323464	0.289567	1.117059	0.263968949	0.3174244	NOT
DPY19L3	636.0168	0.096838	0.08669	1.117053	0.263971544	0.3174244	NOT
PARK2	176.0782	0.230664	0.2065	1.117017	0.263987218	0.3174295	NOT
IFITM10	574.7099	0.262887	0.235363	1.116942	0.264018985	0.3174539	NOT
RP1-100J1	5.055311	0.235764	0.211098	1.116845	0.264060794	0.3174903	NOT
RP13-452I	11.52092	-0.31193	0.279312	-1.11677	0.264092134	0.3175142	NOT
ITM2C	2547.938	0.195798	0.175343	1.116655	0.26414168	0.31756	NOT
STK17B	485.5237	-0.19967	0.178842	-1.11649	0.264212864	0.3176318	NOT
SLC22A2	7.640505	0.377457	0.338112	1.116368	0.26426473	0.3176803	NOT
RP4-706A	529.8869	0.131387	0.117721	1.116086	0.264385347	0.3178115	NOT
RP11-640I	4.357559	-0.29523	0.264535	-1.11602	0.264411819	0.3178295	NOT
SPRY1	917.2807	0.167558	0.150188	1.115658	0.264568534	0.3180041	NOT
CHST8	11.99177	0.389395	0.349253	1.114935	0.264878178	0.3183388	NOT
TARID	5.063614	-0.34247	0.307167	-1.11493	0.264879639	0.3183388	NOT
SEMA6A	845.642	0.281953	0.252889	1.114927	0.264881514	0.3183388	NOT
RP11-175I	2.071157	0.27357	0.245415	1.114725	0.264968314	0.3184293	NOT
CTC-325H	1.812688	0.374329	0.335842	1.114601	0.265021533	0.3184794	NOT
NUTM2E	3.608178	0.439683	0.39488	1.11346	0.265511035	0.3190538	NOT
ZMYM6NE	194.1975	-0.12624	0.11338	-1.11343	0.265523481	0.3190549	NOT
CRCT1	1.241022	0.811952	0.729412	1.113161	0.265639285	0.3191801	NOT
IGHV2-26	33.84913	-0.5275	0.473929	-1.11304	0.265689152	0.3192262	NOT
RP11-968(	23.97702	-0.21043	0.189103	-1.1128	0.265793756	0.319338	NOT
SDSL	3027.35	-0.23761	0.213591	-1.11244	0.265950554	0.3195125	NOT
GPLD1	1852.296	-0.33377	0.30009	-1.11223	0.266039763	0.3196058	NOT
EEF1A1P3	7.429939	0.214275	0.192707	1.111921	0.26617222	0.3197511	NOT
DHRSX	1725.805	-0.14542	0.130831	-1.11151	0.266348911	0.3199494	NOT
KLHL36	1077.975	0.11113	0.099987	1.111446	0.266376474	0.3199686	NOT
RP11-666	7.595623	0.218805	0.196896	1.111274	0.266450436	0.3200436	NOT
SLC7A9	779.2993	-0.21218	0.19098	-1.11099	0.266572579	0.3201764	NOT

GLYATL1P1	3.17208	0.292226	0.263103	1.110692	0.26670075	0.3203064	NOT
TLK2P1	6.762966	0.181201	0.163144	1.110685	0.266703948	0.3203064	NOT
POLR2F	2.772436	0.329204	0.296673	1.109655	0.267147707	0.3208205	NOT
C11orf30	387.1701	0.085154	0.076741	1.109638	0.267155245	0.3208205	NOT
RPS20P14	42.86347	-0.21214	0.191205	-1.10951	0.267210234	0.3208726	NOT
DERL2	1723.904	-0.10095	0.091002	-1.10935	0.267280587	0.3209432	NOT
LINC01232	192.1013	0.149033	0.134353	1.109264	0.267316362	0.3209722	NOT
AP1S2	450.3045	-0.13176	0.118801	-1.10909	0.267392195	0.321039	NOT
DNAH5	157.6918	-0.28492	0.256893	-1.10908	0.267395209	0.321039	NOT
GALR1	1.532836	0.534676	0.482103	1.109049	0.2674091	0.3210418	NOT
RP11-503I	4.873719	-0.27453	0.247624	-1.10867	0.267571413	0.3212227	NOT
PYGM	26.29417	-0.16465	0.148565	-1.10829	0.267738038	0.3214088	NOT
TNRC6B	1175.684	-0.0899	0.081156	-1.10781	0.267944333	0.3216425	NOT
RP11-367I	6.848267	0.200148	0.180787	1.107091	0.268254377	0.3220007	NOT
ELMOD2	1068.083	-0.07765	0.070159	-1.1067	0.268423317	0.3221895	NOT
SGCB	826.8943	-0.20148	0.182074	-1.10656	0.268482509	0.3222466	NOT
AP001625.	2.873496	0.324673	0.293586	1.105888	0.268775127	0.3225838	NOT
EPHB3	107.7051	0.284015	0.256838	1.105812	0.268807888	0.3226091	NOT
TAMM41	218.5343	0.091075	0.082413	1.105102	0.26911537	0.3229641	NOT
CTA-342B	2.496003	0.292277	0.264492	1.10505	0.269137799	0.322977	NOT
RP11-439I	1.334517	0.367462	0.3327	1.104486	0.269382472	0.3232566	NOT
RP11-204I	1.990969	-0.29866	0.270492	-1.10414	0.26953144	0.3234214	NOT
FYCO1	1605.698	0.119132	0.107904	1.104064	0.269565559	0.3234483	NOT
LINGO4	80.88359	0.335515	0.30394	1.103887	0.269642188	0.3235262	NOT
CTA-212A	13.97976	0.247632	0.224363	1.103711	0.269718661	0.3235983	NOT
RP5-1182/	18.35625	0.415256	0.376242	1.103695	0.269725654	0.3235983	NOT
INE1	19.75312	0.136692	0.123901	1.103239	0.269923485	0.3238082	NOT
NUDC	6066.537	-0.12445	0.112801	-1.10324	0.26992403	0.3238082	NOT
APRT	4114.515	0.137368	0.124593	1.102539	0.270227483	0.3241582	NOT
ATP5C1	9886.427	0.100917	0.091548	1.102335	0.270316113	0.3242504	NOT
WIPF1	1016.163	-0.19176	0.173965	-1.10231	0.270328728	0.3242515	NOT
EML1	275.5804	-0.16769	0.152137	-1.10223	0.270360164	0.3242751	NOT
RHOQP1	7.543237	0.214074	0.194229	1.10217	0.270387927	0.3242944	NOT
RP11-297I	6.818451	-0.25376	0.230268	-1.10203	0.270446551	0.3243506	NOT
PEX26	2093.847	0.072124	0.065458	1.101845	0.27052916	0.3244356	NOT
MERTK	1105.341	0.233409	0.211904	1.101483	0.270686707	0.3246105	NOT
EXOSC7	966.5356	0.092538	0.084083	1.100558	0.271088958	0.3250788	NOT
IBA57	583.145	0.109329	0.099354	1.100391	0.27116186	0.3251521	NOT
STX5	2126.482	-0.08731	0.07936	-1.10017	0.271256362	0.3252514	NOT
HIST2H3C	2.11099	-0.61758	0.561542	-1.09979	0.271421885	0.3254357	NOT
AC127904	6.322137	0.21289	0.193713	1.098994	0.271770868	0.32584	NOT
STARD7	5863.148	0.069221	0.062992	1.098898	0.271812762	0.3258761	NOT
SLFN14	1.562408	0.377167	0.343325	1.09857	0.271955737	0.3260334	NOT
FES	1395.376	-0.22091	0.201145	-1.09828	0.2720809	0.3261693	NOT
TMEM129	2817.18	-0.11124	0.101298	-1.09817	0.272130652	0.3262149	NOT
AC145343	1.417632	0.331736	0.302146	1.097933	0.272233891	0.3263245	NOT
LCLAT1	628.2188	0.11267	0.102655	1.09756	0.27239646	0.3265052	NOT
COX10	544.7554	-0.11339	0.103372	-1.09688	0.272694434	0.3268482	NOT
ARHGEF4	64.64689	-0.37666	0.343476	-1.09662	0.272807592	0.3269697	NOT
C11orf58	3749.814	-0.06918	0.063096	-1.09638	0.272912921	0.3270817	NOT
RP11-296I	7.512314	0.194384	0.177324	1.096205	0.272989102	0.3271589	NOT
COL14A1	1457.447	-0.28995	0.264533	-1.09607	0.273048804	0.3272125	NOT

RNF5	3912.924	-0.11584	0.105688	-1.09605	0.273057552	0.3272125	NOT
ACR	16.37553	-0.244	0.222636	-1.09595	0.273100681	0.3272376	NOT
RP11-173(	3.964146	-0.22682	0.206965	-1.09595	0.273102155	0.3272376	NOT
RNLS	463.4674	-0.18377	0.167687	-1.09591	0.273120224	0.3272451	NOT
HINT1	14572.66	-0.10925	0.099745	-1.09532	0.273376598	0.3275381	NOT
GJA4	611.4528	-0.15411	0.140732	-1.09507	0.273487087	0.3276563	NOT
RP11-22A:	1.305081	0.465906	0.425501	1.094961	0.273533847	0.3276981	NOT
GCLM	3280.916	-0.17619	0.160965	-1.09459	0.27369623	0.3278729	NOT
RP11-104I	2.034706	0.332975	0.304205	1.094574	0.273703399	0.3278729	NOT
CTD-2006	5.47713	0.243298	0.222318	1.09437	0.273792874	0.3279659	NOT
RP11-440I	4.663273	-0.36306	0.331763	-1.09434	0.27380807	0.3279699	NOT
PCDHGA9	19.70947	-0.24078	0.220103	-1.09394	0.273982567	0.3281647	NOT
AC017074	10.03785	-0.18724	0.171205	-1.09367	0.274100349	0.3282915	NOT
CSF2RB	205.9525	-0.21396	0.195709	-1.09324	0.274286521	0.3285003	NOT
ZDHHC20	1249.103	-0.11738	0.107378	-1.09311	0.274344475	0.3285555	NOT
CFAP99	2.084545	-0.34182	0.312798	-1.09278	0.274489349	0.3287147	NOT
KIAA0930	3274.238	0.130368	0.119319	1.0926	0.274569673	0.3287967	NOT
UPP1	774.1855	-0.16073	0.147132	-1.09244	0.274640303	0.3288671	NOT
FAR2	138.9348	0.190979	0.174924	1.091781	0.27492941	0.329199	NOT
NLRX1	845.6864	-0.08931	0.081834	-1.09136	0.275112417	0.3294039	NOT
ARMCX6	154.2029	0.211537	0.193861	1.091175	0.275195727	0.3294894	NOT
DCT	13.32863	0.370485	0.339609	1.090917	0.275309315	0.3296111	NOT
FRS2	881.9737	0.103571	0.094942	1.090884	0.275323738	0.3296141	NOT
ZNF812	22.93384	0.363314	0.333115	1.090656	0.275424231	0.329713	NOT
CIRBP-AS1	44.40676	-0.19391	0.17779	-1.09064	0.275430145	0.329713	NOT
RPL10P15	7.688268	0.252293	0.231342	1.090561	0.275466107	0.3297417	NOT
RNU6-118	13.63071	0.445941	0.409177	1.089849	0.275779642	0.3300971	NOT
AC008753	1.495748	0.359279	0.329664	1.089833	0.275786844	0.3300971	NOT
KRT17P7	1.410063	-0.50723	0.465471	-1.0897	0.275843292	0.3301504	NOT
CATIP-AS:	2.205003	0.35843	0.329009	1.089425	0.27596639	0.3302701	NOT
GOLGA4	3846.906	-0.11027	0.101217	-1.08942	0.275967209	0.3302701	NOT
CXCL13	256.9781	-0.4822	0.442688	-1.08926	0.276040403	0.3303434	NOT
LINC00892	10.98637	-0.27331	0.250929	-1.0892	0.276064692	0.3303582	NOT
KDM4A	1459.897	0.09317	0.085542	1.089175	0.276076856	0.3303585	NOT
MTATP6P2	8.249491	-0.301	0.276387	-1.08903	0.276138651	0.3304181	NOT
NHLRC4	16.06533	0.21419	0.196777	1.088493	0.276377566	0.3306897	NOT
IRF6	3988.696	0.206354	0.189695	1.087821	0.276674214	0.3310303	NOT
BMS1P10	19.79581	0.232524	0.213816	1.087497	0.276817184	0.3311714	NOT
EEF1A1P2:	10.82695	0.197462	0.181576	1.087488	0.276821253	0.3311714	NOT
B3GAT3P1	2.515267	0.224128	0.2061	1.087472	0.276828048	0.3311714	NOT
CALCOCO:	2133.948	-0.10146	0.093315	-1.08728	0.276913882	0.3312598	NOT
CRIP2	2373.687	0.165952	0.152653	1.087118	0.276984584	0.33133	NOT
ZBTB14	501.6613	-0.08045	0.074047	-1.08644	0.277283914	0.3316738	NOT
RP11-686I	15.54197	0.220632	0.203176	1.085913	0.277517539	0.3319389	NOT
MYOCD	28.9817	0.276536	0.254664	1.085885	0.277530065	0.3319395	NOT
RPL31	20407.49	0.135985	0.12524	1.085798	0.277568152	0.3319522	NOT
AGTPBP1	332.6717	0.107883	0.099359	1.085786	0.27757365	0.3319522	NOT
GRK6P1	2.054151	0.286194	0.26359	1.085755	0.277587217	0.3319522	NOT
GAA	8212.558	0.116914	0.10768	1.085752	0.277588655	0.3319522	NOT
RP11-322I	1.138567	0.422948	0.389774	1.08511	0.277873122	0.332278	NOT
HCST	164.2301	-0.19891	0.183318	-1.08503	0.277908606	0.3323061	NOT
RP11-480I	9.270359	0.254836	0.234884	1.084945	0.277945813	0.3323362	NOT

TMCC3	425.8398	0.164442	0.151631	1.084489	0.278148061	0.3325636	NOT
HCG4P7	99.60526	0.231302	0.213346	1.084163	0.278292579	0.332722	NOT
ZHX3	1465.982	0.124026	0.114412	1.084035	0.278349488	0.3327757	NOT
DSC1	3.816491	-0.29178	0.269202	-1.08385	0.278430166	0.3328578	NOT
FAM49A	393.9126	-0.1985	0.183169	-1.08372	0.278488344	0.3329129	NOT
RP11-283I	2.655094	0.378827	0.349608	1.083576	0.278553038	0.3329759	NOT
SNAI3-AS:	111.3708	0.167331	0.154437	1.083492	0.278590099	0.3330058	NOT
RP11-488(	44.92176	0.164792	0.152107	1.083396	0.278632699	0.3330423	NOT
TTN	127.2482	0.197586	0.182441	1.083012	0.278802919	0.3332314	NOT
RP11-379I	24.73789	-0.16317	0.150674	-1.08292	0.278842142	0.3332639	NOT
SEMA3E	68.80532	0.539816	0.49859	1.082686	0.278947611	0.3333725	NOT
RP3-413H	2.532508	0.505765	0.467149	1.082665	0.278957092	0.3333725	NOT
OSGIN2	1159.674	-0.12991	0.120071	-1.08194	0.279280218	0.3337442	NOT
ACER1	6.272722	-0.48668	0.449847	-1.08187	0.279310451	0.3337659	NOT
SYNPO	5983.707	-0.1351	0.124926	-1.08141	0.279514476	0.3339953	NOT
AP001065.	8.019757	0.379544	0.351019	1.081264	0.27957979	0.3340589	NOT
RP5-1159(	7.841866	-0.23049	0.213206	-1.08107	0.279664727	0.334146	NOT
CTD-2240	5.2195	0.284653	0.263378	1.080779	0.279795368	0.3342876	NOT
SEPHS2	22718.63	-0.13251	0.122635	-1.08052	0.279911357	0.3344118	NOT
ARRB2	1864.016	0.124086	0.114847	1.080446	0.279943493	0.3344357	NOT
SLPI	4882.325	0.335123	0.310187	1.080393	0.279967288	0.3344431	NOT
ZNF134	417.4477	0.133295	0.123378	1.080378	0.279973845	0.3344431	NOT
TMEM31	2.16291	0.390036	0.361118	1.08008	0.280106473	0.3345755	NOT
UBL5P2	2.166206	0.255788	0.236825	1.080075	0.280108869	0.3345755	NOT
CH507-9B	84.90892	0.183715	0.170118	1.079927	0.28017475	0.3346397	NOT
RP11-932(	1.514047	0.334766	0.31011	1.079504	0.280362938	0.33485	NOT
DLL1	272.8424	0.222694	0.206311	1.079407	0.280406468	0.3348876	NOT
DSCAML1	40.07569	0.374916	0.34741	1.079175	0.280509889	0.3349966	NOT
SLC35B3	1132.874	0.082515	0.076479	1.078924	0.280621606	0.3351156	NOT
CD3D	168.2994	0.261555	0.24247	1.078714	0.28071537	0.3352131	NOT
RP11-982I	1.617465	0.424424	0.393619	1.078261	0.280917114	0.3354395	NOT
RP11-571I	4.346169	-0.20079	0.186226	-1.07821	0.280941848	0.3354546	NOT
MAPK4	175.411	0.413956	0.383947	1.078159	0.280962665	0.335465	NOT
TSR1	1015.092	-0.09441	0.087604	-1.07765	0.281187849	0.3357193	NOT
RP11-415I	9.215596	0.171713	0.159377	1.077402	0.281300925	0.3358399	NOT
SERF1A	1.575085	0.341397	0.316893	1.077325	0.281335017	0.3358661	NOT
FAM66D	1.514066	0.412509	0.38301	1.07702	0.281471495	0.3360145	NOT
IMPAD1	5046.856	0.118678	0.110194	1.07699	0.281484691	0.3360157	NOT
RP11-49I1	2.477868	-0.29281	0.271948	-1.07672	0.281603418	0.336143	NOT
KRTCAP3	326.0105	-0.35257	0.327527	-1.07646	0.281719992	0.3362676	NOT
MST1L	430.9554	0.20668	0.192006	1.076428	0.281735874	0.3362721	NOT
ATXN7L1	335.2963	-0.14202	0.131955	-1.07625	0.281816439	0.3363537	NOT
TRAV2	2.65715	0.372341	0.346343	1.075064	0.282345956	0.3369712	NOT
CTD-2524	2.054863	-0.26274	0.244405	-1.07504	0.282358888	0.3369721	NOT
NAA16	300.5234	0.102647	0.095503	1.074807	0.28246114	0.3370796	NOT
YOD1	496.2879	0.106356	0.098978	1.074541	0.282580356	0.3372073	NOT
SERPINB1	3123.657	0.128682	0.119766	1.074448	0.28262178	0.3372422	NOT
RP11-973I	5.15904	0.444757	0.414027	1.074223	0.282722764	0.3373481	NOT
RP11-6B6.	2.128351	0.257992	0.240247	1.073864	0.282883482	0.3375253	NOT
OLFML1	430.36	-0.20362	0.189649	-1.07368	0.282967226	0.3376107	NOT
CDC34	3580.364	-0.13202	0.122986	-1.07348	0.283055225	0.3377011	NOT
POMZP3	128.0267	0.135708	0.126473	1.073015	0.283264479	0.3379362	NOT

RP11-768I	27.98248	-0.27639	0.257589	-1.07297	0.283283445	0.3379443	NOT
S1PR5	54.08607	-0.19834	0.184886	-1.07277	0.283374121	0.3380287	NOT
EID2	462.6286	0.096343	0.089808	1.07276	0.283378677	0.3380287	NOT
PSMB7	5924.26	-0.11034	0.102872	-1.07263	0.283439328	0.3380865	NOT
LINC0026	2.038621	-0.32732	0.305166	-1.07259	0.283455467	0.3380912	NOT
STK32A	11.33253	-0.31419	0.292958	-1.07249	0.283501842	0.3381216	NOT
RPS28P7	2961.103	0.31915	0.297582	1.072478	0.283505415	0.3381216	NOT
ORC5	672.2861	0.079393	0.074083	1.071683	0.283862363	0.3385327	NOT
CACFD1	3569.524	-0.12307	0.114844	-1.07164	0.283883602	0.3385434	NOT
CD6	206.8617	-0.25135	0.234729	-1.07082	0.284250196	0.338966	NOT
KIAA1715	1386.827	-0.11788	0.110165	-1.07006	0.284593541	0.3393608	NOT
ABHD8	957.2741	0.116152	0.108576	1.069778	0.284719116	0.3394821	NOT
RP11-362I	2.181192	-0.43371	0.405423	-1.06978	0.284719783	0.3394821	NOT
MMP15	4995.165	-0.14522	0.135755	-1.06973	0.284741947	0.3394939	NOT
ING1	804.3287	-0.1265	0.118286	-1.06941	0.284882994	0.3396334	NOT
NDUFA2	4140.436	0.145329	0.135896	1.069413	0.284883503	0.3396334	NOT
IGHV4-39	252.8969	-0.44844	0.419362	-1.06933	0.284921932	0.3396501	NOT
RP11-589I	1.757562	-0.35646	0.33335	-1.06933	0.284922076	0.3396501	NOT
NAPA-AS1	53.33806	-0.16849	0.157574	-1.06926	0.284952774	0.3396721	NOT
AP000355.	75.52046	0.293871	0.274855	1.069186	0.284985974	0.339697	NOT
KIAA0368	3725.021	0.07171	0.067091	1.068854	0.285135397	0.3398594	NOT
LINC0054	163.9121	0.189404	0.177207	1.068829	0.285146803	0.3398594	NOT
EFS	62.46854	-0.2411	0.225614	-1.06863	0.285235375	0.3399425	NOT
SUV420H1	1593.128	-0.08069	0.075508	-1.06862	0.285241072	0.3399425	NOT
USP16	1379.603	-0.07831	0.073306	-1.06823	0.28541822	0.3401359	NOT
GNAI3	2853.813	-0.06625	0.062021	-1.0682	0.285431811	0.3401359	NOT
GS1-124K	23.61472	0.183727	0.172	1.068178	0.285440233	0.3401359	NOT
MME	3190.867	0.46055	0.431179	1.068118	0.285467099	0.3401532	NOT
KLHL6	149.203	-0.21011	0.196732	-1.06798	0.285528267	0.3402115	NOT
FMNL1	531.0704	0.189669	0.17762	1.067839	0.285593247	0.3402643	NOT
ARHGEF5	477.4748	-0.16032	0.150134	-1.06783	0.285597219	0.3402643	NOT
RP11-20B	2.601797	0.273641	0.256375	1.067345	0.285816042	0.3405104	NOT
RNU6-796	1.580092	-0.38681	0.362429	-1.06727	0.285851978	0.3405385	NOT
RP1-78B3.	4.387598	-0.21242	0.199098	-1.06691	0.286013213	0.3407047	NOT
MET	5276.81	-0.13304	0.124698	-1.0669	0.286016106	0.3407047	NOT
RP11-283	4.735507	0.341821	0.320397	1.06687	0.286030651	0.3407074	NOT
NRDE2	735.9615	-0.10297	0.096538	-1.0666	0.286152568	0.3408269	NOT
IGLV3-1	113.5429	-0.45905	0.430388	-1.06659	0.2861556	0.3408269	NOT
MLF1	69.92774	-0.28488	0.267166	-1.06629	0.286290563	0.3409729	NOT
TNNI3K	1.614871	-0.34828	0.326661	-1.06617	0.286347713	0.3410263	NOT
S100A13	1423.629	0.199003	0.18667	1.066065	0.286394332	0.3410671	NOT
FBXO38	1292.023	-0.07431	0.069725	-1.06574	0.286542101	0.3412284	NOT
SLK	1915.352	-0.08806	0.082672	-1.06518	0.28679647	0.341516	NOT
ISPD-AS1	1.845746	-0.28604	0.268543	-1.06515	0.286808273	0.341516	NOT
RP11-327I	4.198714	-0.23943	0.224815	-1.06503	0.286861589	0.3415648	NOT
LETMD1	1962.536	0.085144	0.079962	1.064806	0.286963766	0.3416718	NOT
ERF	2583.009	0.10189	0.095702	1.064663	0.287028257	0.3417338	NOT
PLD2	672.5243	0.106104	0.099676	1.064483	0.287109964	0.3418164	NOT
PDCD6IP	4340.459	-0.06641	0.062399	-1.06432	0.287185211	0.3418913	NOT
FCGR1C	5.086834	-0.30342	0.285109	-1.06422	0.287230584	0.3419306	NOT
NPR3	467.9123	-0.26037	0.244815	-1.06355	0.287530501	0.3422729	NOT
AC130469	5.80277	-0.26024	0.244778	-1.06318	0.287698731	0.3424584	NOT

FLT3LG	68.9464	0.151651	0.142669	1.062951	0.287803952	0.3425689	NOT
KBTBD3	167.2095	-0.0972	0.091464	-1.06275	0.287895915	0.3426636	NOT
DYSF	2193.884	0.149041	0.140266	1.06256	0.287981413	0.3427507	NOT
MTIF3	1467.803	-0.09866	0.092867	-1.06235	0.288077281	0.34285	NOT
TMED6	134.3528	0.251007	0.236315	1.062169	0.288159188	0.3429327	NOT
GSAP	925.6195	-0.13729	0.129331	-1.06156	0.288434325	0.3432454	NOT
SS18L1	1494.227	-0.13255	0.124871	-1.06151	0.288458487	0.3432594	NOT
TMEM161	510.796	0.099911	0.094125	1.061475	0.288474064	0.3432632	NOT
RNF182	4.027915	0.35472	0.334249	1.061246	0.288577917	0.343372	NOT
FOXJ3	1179.596	-0.07758	0.073118	-1.06102	0.288683061	0.3434823	NOT
LGALS8	2993.425	0.116144	0.109473	1.060931	0.288721385	0.3435092	NOT
ATF6	3676.586	0.106565	0.100449	1.060887	0.288741374	0.3435092	NOT
uc_338	6.390154	0.196963	0.185659	1.060883	0.288742897	0.3435092	NOT
AP000354.	1.415345	-0.38959	0.367282	-1.06073	0.288813491	0.3435646	NOT
METTL21B	313.0404	0.095813	0.090328	1.060726	0.28881434	0.3435646	NOT
MRPS15	3604.019	0.118139	0.111454	1.059976	0.289155616	0.3439558	NOT
KBTBD4	240.8938	0.118239	0.111583	1.059656	0.289301303	0.3441143	NOT
AP2A2	3611.394	-0.06091	0.057492	-1.05953	0.289357787	0.3441667	NOT
ZZEF1	1249.329	0.098832	0.093293	1.059368	0.289432091	0.3442403	NOT
FAM96B	2865.128	-0.11667	0.110144	-1.05926	0.289480684	0.3442833	NOT
RNF148	4.140847	0.264827	0.250029	1.059187	0.289514712	0.344309	NOT
MYL9	2843.258	0.177731	0.167812	1.059109	0.289550266	0.3443364	NOT
TMOD3	2271.568	0.098017	0.092563	1.058921	0.289635942	0.3444235	NOT
IPO5	4469.181	0.108575	0.102571	1.058533	0.289812495	0.3446187	NOT
RP11-164I	2.216474	-0.35092	0.331618	-1.05821	0.289961107	0.3447806	NOT
RNF38	961.6504	0.102372	0.096755	1.058061	0.290027445	0.3448446	NOT
RP11-64D	5.458303	0.341804	0.323149	1.057731	0.290178217	0.3450091	NOT
ST13P3	4.29202	-0.2094	0.197988	-1.05763	0.290224949	0.3450498	NOT
RP11-20O	31.38606	0.169633	0.160397	1.057584	0.290245048	0.3450589	NOT
LMOD3	4.196843	0.220955	0.209001	1.057192	0.290424048	0.3452568	NOT
STAM2	983.7431	-0.07832	0.074133	-1.05651	0.290736597	0.3456135	NOT
RASA4CP	85.63885	0.201095	0.190444	1.055923	0.29100327	0.3459157	NOT
OTOA	23.43591	-0.20099	0.190408	-1.05557	0.291164326	0.3460923	NOT
GNG11	977.0225	-0.14638	0.138702	-1.05538	0.291251527	0.346181	NOT
RP11-736I	14.11958	0.281316	0.266609	1.055164	0.291350377	0.3462837	NOT
AC005037	4.47316	0.232667	0.220572	1.054832	0.291502065	0.3464491	NOT
RP11-475J	14.7772	0.281375	0.26678	1.054707	0.291559332	0.3465022	NOT
RN7SL608I	4.317965	0.226277	0.214593	1.054446	0.291678715	0.3466292	NOT
TUBB8P1	1.498011	0.334073	0.316938	1.054064	0.291853717	0.3468223	NOT
RP4-630A	1.924869	0.308828	0.293004	1.054009	0.291878855	0.3468373	NOT
CUEDC2	1778.253	0.095146	0.090286	1.053834	0.291958904	0.3469175	NOT
WBSCR16	2899.646	0.082004	0.07782	1.053766	0.291989955	0.3469292	NOT
SMG6	425.3298	-0.13891	0.131822	-1.05376	0.291993791	0.3469292	NOT
MED4-AS	1.704737	0.278286	0.264104	1.053696	0.292022046	0.3469475	NOT
RN7SL832I	16.22591	0.1809	0.171686	1.053669	0.2920343	0.3469475	NOT
DSC3	14.97506	0.456547	0.433441	1.05331	0.292199142	0.3471284	NOT
LINC00661	4.433452	0.545496	0.517911	1.053262	0.292221098	0.3471387	NOT
RP11-727I	41.99873	-0.11293	0.107219	-1.05324	0.292232863	0.3471387	NOT
NEK9	2711.989	0.073957	0.070221	1.0532	0.292249121	0.3471431	NOT
AC144652	111.942	-0.20582	0.195429	-1.05317	0.292263539	0.3471453	NOT
ATP1B3-A	1.6106	-0.35896	0.340922	-1.0529	0.292385352	0.3472751	NOT
QSOX1	4719.231	0.264122	0.250925	1.052592	0.29252794	0.3474295	NOT

RP3-454B2	1.426262	0.389447	0.370018	1.052506	0.292567415	0.3474615	NOT
SNORA71(	2.562561	0.286681	0.272389	1.052467	0.292585485	0.347468	NOT
ATP6V1D	1767.111	0.072643	0.069025	1.052422	0.29260591	0.3474774	NOT
TOB1-AS1	88.15319	0.161569	0.153557	1.052178	0.292717919	0.3475955	NOT
BANP	531.3559	0.086697	0.082403	1.052114	0.292747203	0.3476153	NOT
8-Mar	2009.119	0.112747	0.107167	1.052071	0.292766904	0.3476238	NOT
HLA-G	175.3511	0.206995	0.196791	1.051854	0.292866715	0.3477274	NOT
WDR11	1624.168	-0.08767	0.083353	-1.05173	0.292921659	0.3477777	NOT
EPS8	2109.511	-0.11464	0.109022	-1.05154	0.293012818	0.347871	NOT
FAM167A	59.89914	0.340302	0.323699	1.05129	0.293125479	0.3479775	NOT
ATP1A3	16.07362	0.289855	0.275715	1.051285	0.293127703	0.3479775	NOT
PRDX5	9922.322	0.122497	0.116529	1.05122	0.293157337	0.3479978	NOT
STON2	236.6923	0.200808	0.191035	1.051156	0.293187001	0.3480181	NOT
FARS2	780.6925	0.096353	0.091705	1.050683	0.293404284	0.348261	NOT
SLC24A1	318.0653	-0.12309	0.117162	-1.05061	0.293438383	0.3482866	NOT
DOK3	176.3905	0.164007	0.156119	1.050525	0.293476628	0.348317	NOT
RUNX1	941.4164	0.170187	0.16228	1.048723	0.294305727	0.3492861	NOT
AC003002	1.768568	0.285314	0.272086	1.048616	0.294354808	0.3493293	NOT
C17orf85	1010.393	-0.08738	0.083331	-1.04857	0.294377126	0.3493408	NOT
PAPPA	44.10205	0.271451	0.258888	1.048526	0.294396216	0.3493485	NOT
RP11-504/	12.97031	-0.18144	0.173053	-1.04847	0.294420483	0.3493623	NOT
FERMT3	689.4155	-0.17078	0.162949	-1.04807	0.294605049	0.3495663	NOT
CCL24	30.47707	-0.28356	0.270683	-1.04758	0.294833355	0.3498222	NOT
ICOSLG	71.53023	0.172404	0.164605	1.047378	0.294925334	0.3499163	NOT
AC006042	1.103494	0.386057	0.368664	1.047179	0.295017066	0.3500102	NOT
ZNF44	434.0171	0.094704	0.090447	1.047071	0.295066861	0.3500542	NOT
RNA5SP30	2.41411	0.358488	0.342488	1.046717	0.295230056	0.3502328	NOT
RP11-400(	9.561919	-0.317	0.302884	-1.04662	0.295276683	0.3502731	NOT
RP4-671O	2.564674	-0.26903	0.257106	-1.04636	0.2953943	0.3503976	NOT
IGSF9	1069.641	-0.32336	0.30918	-1.04585	0.29563052	0.3506628	NOT
TMEM141	3011.621	0.163432	0.156326	1.045458	0.295811322	0.3508622	NOT
RP11-408I	24.15782	0.297777	0.284906	1.045177	0.295940977	0.3510009	NOT
SDR39U1	297.5209	0.092576	0.088607	1.044791	0.296119651	0.3511978	NOT
MRPL23	1683.86	0.146459	0.140199	1.044653	0.296183378	0.3512583	NOT
ATP5EP2	23.55272	-0.22214	0.212676	-1.04448	0.296265609	0.3513408	NOT
C1orf123	1294.085	-0.09181	0.087903	-1.0444	0.296299466	0.3513642	NOT
HNF1A	842.9971	0.157609	0.150912	1.044378	0.296310713	0.3513642	NOT
CPSF2	1598.566	0.060121	0.057602	1.043728	0.296611382	0.3516958	NOT
CRISPLD1	58.57048	-0.24164	0.231518	-1.04372	0.296615837	0.3516958	NOT
RP5-837I2	2.661806	0.260836	0.249917	1.043689	0.296629391	0.3516968	NOT
TRPV5	1.434204	0.529312	0.507473	1.043035	0.296932034	0.3520406	NOT
TPSAB1	108.8025	0.359938	0.345105	1.042981	0.296956903	0.352055	NOT
PLIN5	2253.024	-0.19967	0.191471	-1.04281	0.297036846	0.3521346	NOT
RP11-463.	3.488905	-0.51583	0.494679	-1.04275	0.297061929	0.3521493	NOT
RP11-325I	1.961462	0.318742	0.305731	1.042556	0.297153757	0.3522431	NOT
SPOP	2117.726	0.060654	0.058196	1.042246	0.297297601	0.3523985	NOT
IL17RE	503.3662	0.209493	0.20106	1.041941	0.297438844	0.3525508	NOT
[truncated: 380,257 more chars]
