# Supplementary material for: Life span‐associated ferroptosis‐related genes identification and validation for hepatocellular carcinoma patients as hepatitis B virus carriers
Source: J Clin Lab Anal. 2023 Jul 18;37(13-14):e24930. doi: 10.1002/jcla.24930 (PMC10492458; doi:10.1002/jcla.24930)
Supplement: Supplementary file 10 — Tables S1–S14 [file JCLA-37-e24930-s009.zip › TableS5_DEG_survival.pdf]

Gene	baseMean	log2FoldCha	lfcSE	stat	pvalue	padj	reg
MAGEA4	25.73179	23.7882525	1.774845	13.40300015	5.81E-41	1.58E-36	UP
ANGPTL7	14.46279	-6.5570097	0.789892	-8.3011494	1.03E-16	1.40E-12	DOWN
AFP	29400.11	6.38325858	0.825928	7.728585522	1.09E-14	9.83E-11	UP
UPK3A	580.4999	5.40301997	0.72187	7.484759981	7.17E-14	4.86E-10	UP
TMEM233	62.98909	-2.0652455	0.286644	-7.20490231	5.81E-13	3.15E-09	DOWN
GNRH2	35.30772	-4.4681985	0.647647	-6.89912485	5.23E-12	2.37E-08	DOWN
MEP1A	760.1837	5.32165834	0.778934	6.831976512	8.38E-12	3.25E-08	UP
TNNT1	88.64128	4.75367182	0.704917	6.743589587	1.55E-11	5.24E-08	UP
LINC0094	295.1981	6.18438706	0.922993	6.700362831	2.08E-11	6.07E-08	UP
CMA1	18.37259	-3.9899353	0.59643	-6.68969884	2.24E-11	6.07E-08	DOWN
DQX1	165.634	5.11620019	0.782326	6.539730606	6.16E-11	1.52E-07	UP
MEX3A	480.739	2.32693331	0.356817	6.521364086	6.97E-11	1.58E-07	UP
SOAT2	802.44	4.18324611	0.655332	6.383401185	1.73E-10	3.61E-07	UP
MYBL2	1740.051	2.47838662	0.392271	6.318045222	2.65E-10	5.13E-07	UP
C6orf223	409.3389	4.52693285	0.722601	6.26477248	3.73E-10	6.75E-07	UP
ASB15	11.17604	-4.1538303	0.674241	-6.1607494	7.24E-10	1.23E-06	DOWN
ESRP1	253.0927	5.18366362	0.855442	6.059634166	1.36E-09	2.18E-06	UP
DSCC1	245.2502	1.37243101	0.227889	6.022362066	1.72E-09	2.43E-06	UP
CA9	704.7269	4.65709351	0.774053	6.016503907	1.78E-09	2.43E-06	UP
KDM8	980.7345	-2.10789	0.350411	-6.01548529	1.79E-09	2.43E-06	DOWN
NKAPL	18.06822	-2.562462	0.428237	-5.98374308	2.18E-09	2.82E-06	DOWN
AC090616.	19.57842	-1.6558081	0.279348	-5.92739726	3.08E-09	3.61E-06	DOWN
FAM49B	2042.883	1.147036	0.193595	5.924919915	3.12E-09	3.61E-06	UP
TRIP13	433.8213	2.00402658	0.338439	5.921386943	3.19E-09	3.61E-06	UP
NUDCD1	824.0611	1.09099776	0.185403	5.884453176	3.99E-09	4.33E-06	UP
MUC6	508.9421	4.84982722	0.827186	5.863042768	4.54E-09	4.74E-06	UP
XPO5	2093.484	0.82551017	0.141108	5.850192243	4.91E-09	4.93E-06	UP
CHP2	29.91693	6.62490308	1.135659	5.833532875	5.43E-09	5.26E-06	UP
CLEC2L	46.37317	5.17661301	0.893211	5.795509131	6.81E-09	6.19E-06	UP
C12orf56	43.74867	4.30992766	0.743766	5.794739672	6.84E-09	6.19E-06	UP
TNRC6C	728.5484	-1.1719905	0.202735	-5.78090283	7.43E-09	6.50E-06	DOWN
TEX15	55.32001	7.03263401	1.220428	5.762431418	8.29E-09	7.03E-06	UP
TMEM65	1099.046	1.26724191	0.220125	5.756916538	8.57E-09	7.04E-06	UP
HMGA1	5242.72	1.37857251	0.24031	5.736649617	9.66E-09	7.71E-06	UP
G6PD	3703.608	2.25030992	0.393853	5.713579214	1.11E-08	8.57E-06	UP
HILPDA	384.3111	1.65600869	0.291712	5.676862845	1.37E-08	1.03E-05	UP
PAK1IP1	847.8955	0.78543741	0.138617	5.666246862	1.46E-08	1.07E-05	UP
RP11-20J1	8.506029	-2.6951288	0.476571	-5.65525487	1.56E-08	1.07E-05	DOWN
CTB-3601.	52.85741	-2.8971701	0.513089	-5.64652732	1.64E-08	1.07E-05	DOWN
IGDCC3	41.42461	4.32413179	0.766773	5.639387303	1.71E-08	1.07E-05	UP
PFKFB4	314.0923	1.95011256	0.34595	5.636975507	1.73E-08	1.07E-05	UP
CDCA7	319.2244	2.87733978	0.510492	5.6364067	1.74E-08	1.07E-05	UP
GAD1	58.07107	3.48380855	0.618395	5.633627657	1.76E-08	1.07E-05	UP
TRIM73	19.32502	-2.3840972	0.423226	-5.63315666	1.77E-08	1.07E-05	DOWN
AGR2	402.8962	4.96316719	0.881135	5.632699422	1.77E-08	1.07E-05	UP
SPESP1	102.1292	3.77381898	0.670435	5.628909634	1.81E-08	1.07E-05	UP
SRL	20.36723	-1.4555168	0.259011	-5.61952677	1.91E-08	1.11E-05	DOWN
MTND1P23	1624.749	4.30958355	0.768066	5.6109541	2.01E-08	1.14E-05	UP

RP5-1154I	137.609	3.68586959	0.657772	5.603567254	2.10E-08	1.16E-05	UP
CHRAC1	1604.659	0.93673053	0.167299	5.599132508	2.15E-08	1.17E-05	UP
RP11-874J	16.68903	5.42269124	0.97483	5.562703287	2.66E-08	1.41E-05	UP
PKIB	465.6401	2.64247989	0.475529	5.55692402	2.75E-08	1.43E-05	UP
ALG9	559.1705	-1.1999746	0.216589	-5.54031801	3.02E-08	1.55E-05	DOWN
CMSS1	809.1119	0.93780128	0.169369	5.537019931	3.08E-08	1.55E-05	UP
NDRG1	18386.79	2.07700541	0.37615	5.521749677	3.36E-08	1.66E-05	UP
TPSB2	240.8826	-3.2402767	0.587161	-5.51854644	3.42E-08	1.66E-05	DOWN
PPM1K	519.5861	-1.8064449	0.329146	-5.4882812	4.06E-08	1.93E-05	DOWN
RP11-20J1	3.941293	-2.8222181	0.517242	-5.45628334	4.86E-08	2.27E-05	DOWN
DCAF13	2188.574	1.18111322	0.217395	5.433035657	5.54E-08	2.55E-05	UP
TUBA3C	83.5272	6.41557876	1.184291	5.417232577	6.05E-08	2.74E-05	UP
MAPK13	981.8445	2.30546675	0.42743	5.393787714	6.90E-08	3.07E-05	UP
VSIG2	152.2475	-2.6874151	0.502751	-5.34541524	9.02E-08	3.91E-05	DOWN
FDCSP	21.78169	4.06086935	0.760223	5.341683972	9.21E-08	3.91E-05	UP
PPP1R12B	1118.974	-0.9925418	0.185825	-5.34126745	9.23E-08	3.91E-05	DOWN
RAN	8245.812	0.82606566	0.155026	5.328571607	9.90E-08	4.13E-05	UP
LAPTM4B	7547.223	1.49805067	0.28205	5.311300578	1.09E-07	4.47E-05	UP
CYP19A1	53.59553	3.83194962	0.724925	5.285995185	1.25E-07	4.96E-05	UP
NEURL3	353.5936	2.36443897	0.447456	5.28418683	1.26E-07	4.96E-05	UP
TRIM54	118.018	3.69360071	0.699074	5.283558331	1.27E-07	4.96E-05	UP
CCT2	5095.559	0.7305359	0.138368	5.279650247	1.29E-07	4.96E-05	UP
MS4A8	22.34399	5.98691343	1.134073	5.279125131	1.30E-07	4.96E-05	UP
LYPD6B	63.96143	4.37748968	0.830173	5.272987294	1.34E-07	5.06E-05	UP
LINC0049	48.33488	4.75034894	0.901931	5.266865429	1.39E-07	5.08E-05	UP
MCM3	4298.591	1.13244193	0.215022	5.26662984	1.39E-07	5.08E-05	UP
CTSG	83.99835	-3.5536876	0.675011	-5.26463571	1.40E-07	5.08E-05	DOWN
INTS8	1345.335	0.82468826	0.157227	5.245199457	1.56E-07	5.57E-05	UP
SLC7A10	136.8946	4.42507791	0.844435	5.24028513	1.60E-07	5.65E-05	UP
RNF17	38.35881	5.67733387	1.08456	5.23468932	1.65E-07	5.75E-05	UP
UBE2C	1213.045	1.94851934	0.372863	5.225832775	1.73E-07	5.95E-05	UP
HHIPL2	345.1109	3.79252417	0.726566	5.219791963	1.79E-07	6.00E-05	UP
P2RX2	9.234581	-3.8203933	0.731991	-5.21917969	1.80E-07	6.00E-05	DOWN
CXCL5	234.0445	4.28058429	0.820411	5.217606481	1.81E-07	6.00E-05	UP
TNNC1	177.7403	3.10170847	0.594986	5.213077036	1.86E-07	6.07E-05	UP
PHYHIP	47.89453	-2.1112405	0.408389	-5.1696839	2.34E-07	7.57E-05	DOWN
CLTCL1	861.9855	-1.5277072	0.296112	-5.1592213	2.48E-07	7.89E-05	DOWN
VNN2	1003.913	2.56588563	0.49771	5.155382802	2.53E-07	7.89E-05	UP
PRAME	636.712	5.33167959	1.034533	5.153705983	2.55E-07	7.89E-05	UP
ASNSP1	41.47702	5.33655123	1.035567	5.153265591	2.56E-07	7.89E-05	UP
PFN1P11	166.4381	-4.1710258	0.811736	-5.13840269	2.77E-07	8.45E-05	DOWN
CEP55	333.8295	1.79854645	0.350775	5.127357585	2.94E-07	8.86E-05	UP
SMCO3	51.57437	-1.8781422	0.366564	-5.12363555	3.00E-07	8.94E-05	DOWN
MCM2	2223.091	1.41929804	0.277482	5.114916201	3.14E-07	9.26E-05	UP
C10orf91	38.28237	4.7621913	0.932194	5.108585247	3.25E-07	9.47E-05	UP
PAGE1	76.22305	6.10032329	1.197652	5.093569923	3.51E-07	0.000101	UP
RP11-35G9	79.94861	0.9655351	0.190066	5.08001061	3.77E-07	0.000107	UP
MTRNR2L1	109.6082	-3.5869934	0.70616	-5.07957805	3.78E-07	0.000107	DOWN
EZH2	812.4873	1.26378412	0.24943	5.066682862	4.05E-07	0.000112	UP

HTR3A	27.32115	4.69463443	0.926644	5.066278345	4.06E-07	0.000112	UP
NUP37	620.6408	0.76327909	0.150697	5.064985172	4.08E-07	0.000112	UP
TKT	19451.15	1.56751772	0.310284	5.051880407	4.37E-07	0.000119	UP
TNNI3	21.63288	4.58388301	0.909195	5.041693277	4.61E-07	0.000123	UP
PPFIA4	54.75328	2.6526456	0.526197	5.041166865	4.63E-07	0.000123	UP
PKD1L3	16.54907	-1.8561308	0.368536	-5.03650069	4.74E-07	0.000125	DOWN
RAD51	207.1233	1.41961897	0.282124	5.031901807	4.86E-07	0.000127	UP
HJURP	610.8869	1.6009461	0.318616	5.024690051	5.04E-07	0.000129	UP
RUVBL1	1680.17	0.7006823	0.13947	5.023904872	5.06E-07	0.000129	UP
RBP2	36.2678	3.96027572	0.788358	5.023451037	5.08E-07	0.000129	UP
SPC25	221.0784	1.49661061	0.29867	5.010913433	5.42E-07	0.000136	UP
GLDN	254.3595	2.91324459	0.581832	5.007022875	5.53E-07	0.000138	UP
REN	154.7257	-2.8129324	0.562429	-5.00139574	5.69E-07	0.00014	DOWN
STS	1388.936	-1.7468621	0.349394	-4.99969648	5.74E-07	0.00014	DOWN
THOC7	1763.673	0.734638	0.147067	4.995267856	5.88E-07	0.000142	UP
EEF1E1	398.8211	0.99067451	0.198349	4.994609903	5.90E-07	0.000142	UP
VPS72	2223.838	0.7780588	0.155876	4.991533663	5.99E-07	0.000143	UP
ALPI	128.4926	4.28252654	0.858981	4.985588924	6.18E-07	0.000145	UP
SFN	2565.112	2.67102802	0.535896	4.98422731	6.22E-07	0.000145	UP
ZNF239	112.1792	2.28305915	0.458757	4.976617574	6.47E-07	0.00015	UP
CTD-2207A	20.41884	5.85973234	1.179215	4.969181412	6.72E-07	0.000155	UP
RP11-362F	43.59479	3.63177516	0.732209	4.960021967	7.05E-07	0.000161	UP
PAGE5	218.339	4.26698434	0.860957	4.95609473	7.19E-07	0.000163	UP
ZNF623	1218.009	0.73018626	0.147656	4.945200538	7.61E-07	0.000171	UP
DGKK	16.80667	4.22482629	0.855832	4.936512121	7.95E-07	0.000177	UP
DLX6	27.28813	5.87577211	1.191103	4.933050404	8.10E-07	0.000179	UP
BUB1B	459.184	1.62469633	0.329564	4.929834596	8.23E-07	0.00018	UP
PPP1R14D	38.19896	3.0030726	0.610531	4.918786774	8.71E-07	0.000188	UP
ABCA9	295.7854	-1.8976431	0.385856	-4.91800603	8.74E-07	0.000188	DOWN
MPV17	1487.423	0.72963152	0.148483	4.913910151	8.93E-07	0.00019	UP
GTSE1	408.9649	1.60975834	0.327618	4.913527595	8.95E-07	0.00019	UP
PABPC1	50770.1	1.12334633	0.229003	4.905387664	9.32E-07	0.000196	UP
MTBP	176.3599	1.06703329	0.217619	4.903208049	9.43E-07	0.000197	UP
ASPA	193.2534	-1.8967288	0.387026	-4.90078142	9.55E-07	0.000198	DOWN
RP11-495F	95.65438	3.47308324	0.709097	4.89789547	9.69E-07	0.000198	UP
ABCB5	27.01018	3.53350989	0.721478	4.897601387	9.70E-07	0.000198	UP
KIFC1	1108.956	1.53912898	0.314388	4.895634464	9.80E-07	0.000198	UP
KPNA2	3909.863	1.09244699	0.223234	4.893732964	9.89E-07	0.000199	UP
IGFBP4	45189.79	-1.3226048	0.270464	-4.89012822	1.01E-06	0.000201	DOWN
LINC0109:	202.1916	-3.2041198	0.655552	-4.88766689	1.02E-06	0.000202	DOWN
GEMIN2	229.4809	0.74660788	0.152792	4.88643748	1.03E-06	0.000202	UP
KCNN2	97.84574	-2.4017696	0.491726	-4.88436682	1.04E-06	0.000203	DOWN
TRMT6	754.4997	0.7304669	0.149822	4.875574557	1.08E-06	0.000209	UP
SF3B4	4782.25	0.83391483	0.171072	4.874636403	1.09E-06	0.000209	UP
EVX1	18.69515	3.68172102	0.755393	4.873916424	1.09E-06	0.000209	UP
PRKCD	1275.115	0.99992756	0.205356	4.869245843	1.12E-06	0.000213	UP
MUC1	201.3462	2.53828605	0.521601	4.86633608	1.14E-06	0.000214	UP
C4orf29	484.1653	-0.7495353	0.15408	-4.8645823	1.15E-06	0.000215	DOWN
KIAA0101	600.346	1.43774607	0.295954	4.858002965	1.19E-06	0.000219	UP

H2AFY	5927.235	0.62273078	0.128238	4.856069502	1.20E-06	0.000219	UP
CYP4V2	4705.973	-1.3642848	0.280971	-4.8556148	1.20E-06	0.000219	DOWN
KIF2C	807.0442	1.73395215	0.357138	4.855130239	1.20E-06	0.000219	UP
UQCRFS1P1	9.16215	-1.6736276	0.345088	-4.84986029	1.24E-06	0.000223	DOWN
PBK	487.4609	1.73846205	0.359289	4.838621516	1.31E-06	0.000235	UP
SIK2	4933.133	-1.0398529	0.215289	-4.8300419	1.37E-06	0.000244	DOWN
SUSD2	763.4525	-2.0324842	0.421292	-4.82441265	1.40E-06	0.000249	DOWN
COL22A1	150.5166	3.11331782	0.645498	4.823128839	1.41E-06	0.000249	UP
RECQL4	1270.923	1.28566275	0.266824	4.818397036	1.45E-06	0.000253	UP
DDIT4L	33.72547	-1.8628566	0.386776	-4.81636688	1.46E-06	0.000254	DOWN
SSB	3192.358	0.76734364	0.159371	4.814835993	1.47E-06	0.000254	UP
RAD21	8073.759	0.75613027	0.157059	4.814319563	1.48E-06	0.000254	UP
GTPBP2	1996.751	0.87724413	0.182556	4.805354435	1.54E-06	0.000264	UP
PEG10	10516.5	3.64427436	0.758753	4.802980608	1.56E-06	0.000265	UP
SLC39A4	1672.433	2.91351975	0.606826	4.801242138	1.58E-06	0.000266	UP
NFYB	1582.745	-0.8733523	0.182141	-4.79492882	1.63E-06	0.000273	DOWN
KB-431C1.	229.3202	0.7906393	0.16508	4.789442512	1.67E-06	0.000278	UP
GARNL3	169.4905	-1.4816685	0.309612	-4.78555838	1.71E-06	0.000282	DOWN
PSMC3IP	121.1	1.19010276	0.24889	4.78163994	1.74E-06	0.000286	UP
SAPCD2	271.0912	2.08215809	0.435741	4.7784284	1.77E-06	0.000289	UP
DBF4	369.7401	0.98960586	0.207185	4.776441132	1.78E-06	0.00029	UP
NLE1	829.4974	0.92843045	0.194454	4.77455043	1.80E-06	0.00029	UP
CYP2G1P	11.06534	-3.2339821	0.677459	-4.77369096	1.81E-06	0.00029	DOWN
TMEM106C	4086.599	1.01921433	0.213644	4.770621614	1.84E-06	0.000292	UP
CENPW	367.1551	1.42687721	0.299132	4.770056649	1.84E-06	0.000292	UP
GINS1	645.0231	1.46323532	0.307171	4.763590828	1.90E-06	0.0003	UP
PRIM2	474.6154	0.7389601	0.155177	4.762051893	1.92E-06	0.000301	UP
NCAPH	454.9797	1.52767184	0.321098	4.757654364	1.96E-06	0.000305	UP
FARSB	1825.315	0.71479111	0.150297	4.755870761	1.98E-06	0.000306	UP
RP11-383J	17.41437	3.56043786	0.751837	4.73565104	2.18E-06	0.000337	UP
NCL	20266.54	0.5807152	0.122775	4.729906866	2.25E-06	0.000343	NOT
TRAIP	249.2085	1.37810063	0.291384	4.72949771	2.25E-06	0.000343	UP
ZNF695	16.60548	3.1750347	0.672487	4.721330194	2.34E-06	0.000355	UP
LINC01559	12.86684	4.07583491	0.863917	4.717855573	2.38E-06	0.000357	UP
F11-AS1	372.8195	-1.8821568	0.399016	-4.7170014	2.39E-06	0.000357	DOWN
DEPDC1B	356.8014	1.78777139	0.379015	4.71688886	2.39E-06	0.000357	UP
PLVAP	5912.717	-1.1040293	0.234541	-4.70718792	2.51E-06	0.000372	DOWN
FSD1L	144.5713	1.12860841	0.23985	4.705466815	2.53E-06	0.000373	UP
CH507-154	11.45857	3.337163	0.7095	4.703541419	2.56E-06	0.000375	UP
CNOT11	2594.156	0.73947262	0.15731	4.700734705	2.59E-06	0.000378	UP
STON1	225.9687	-1.7620654	0.375226	-4.69601581	2.65E-06	0.000385	DOWN
TKTL1	61.07217	3.64386696	0.776484	4.692779895	2.70E-06	0.000389	UP
CD3EAP	214.7351	0.82607408	0.176184	4.688704026	2.75E-06	0.000395	UP
CCT5	6992.616	0.7624773	0.162659	4.687592584	2.76E-06	0.000395	UP
SNRK	1623.424	-0.7175503	0.153235	-4.68267962	2.83E-06	0.000402	DOWN
KIAA0922	1363.763	-1.0410793	0.222372	-4.68170207	2.85E-06	0.000402	DOWN
RP5-1184F	3.67801	-1.7662689	0.377591	-4.67772536	2.90E-06	0.000408	DOWN
RTKN2	120.1739	1.81014392	0.387105	4.676100163	2.92E-06	0.000408	UP
SCRIB	5797.741	0.83726402	0.179083	4.675279287	2.94E-06	0.000408	UP

IGF2BP2	888.0101	2.05496735	0.439713	4.673433388	2.96E-06	0.00041	UP
YWHAZ	19530.22	0.87867938	0.18844	4.662922008	3.12E-06	0.000429	UP
MCM6	2012.026	1.11384061	0.238929	4.661804311	3.13E-06	0.000429	UP
BCYRN1	25.92047	1.67862256	0.360289	4.659103845	3.18E-06	0.000433	UP
RCOR2	86.3633	2.7143618	0.583072	4.655275316	3.24E-06	0.000439	UP
TLX2	9.85002	4.09613599	0.880433	4.65241154	3.28E-06	0.00044	UP
MIR663AHC	26.29292	6.22277604	1.337768	4.651610242	3.29E-06	0.00044	UP
NPTX2	829.7859	3.49402452	0.751157	4.651525748	3.29E-06	0.00044	UP
PITX2	16.00252	4.3824161	0.942472	4.649916669	3.32E-06	0.000442	UP
ADORA1	238.5679	2.79599315	0.601906	4.645229881	3.40E-06	0.000448	UP
RP11-228I	88.0996	-1.5586754	0.335577	-4.64476701	3.40E-06	0.000448	DOWN
MTFR2	105.2311	1.50540804	0.324155	4.64409867	3.42E-06	0.000448	UP
FXYD3	489.4017	3.27200223	0.704715	4.643014851	3.43E-06	0.000448	UP
ROBO4	949.3801	-1.0292385	0.22183	-4.63975247	3.49E-06	0.000451	DOWN
WBP1L	2802.968	-0.6744936	0.145394	-4.63908548	3.50E-06	0.000451	DOWN
IFT46	376.4279	-0.8707319	0.187741	-4.63794024	3.52E-06	0.000451	DOWN
PTPLA	74.50787	2.56245256	0.552534	4.637634112	3.52E-06	0.000451	UP
HNRNPA3	9028.81	0.55608609	0.119959	4.635649053	3.56E-06	0.000453	NOT
BOP1	4820.34	1.33915693	0.289334	4.628415292	3.68E-06	0.000467	UP
COL25A1	15.84257	-2.4423932	0.528113	-4.62475091	3.75E-06	0.000469	DOWN
POPDC3	24.58673	4.20408171	0.909107	4.624409806	3.76E-06	0.000469	UP
EXOC3L2	360.1675	-1.031722	0.223117	-4.62413723	3.76E-06	0.000469	DOWN
ATIC	3933.949	0.80728966	0.174606	4.623490966	3.77E-06	0.000469	UP
LINC00475	5.603904	-1.9206087	0.41545	-4.62296224	3.78E-06	0.000469	DOWN
ANXA10	798.1476	-2.4130089	0.522243	-4.62047125	3.83E-06	0.000472	DOWN
RQCD1	1990.777	0.55945626	0.121136	4.618406395	3.87E-06	0.000475	NOT
FAM225A	18.87152	2.33864138	0.506636	4.616017208	3.91E-06	0.000478	UP
PAGE2	16.16923	5.46712774	1.184927	4.613893468	3.95E-06	0.000481	UP
SMYD5	1373.41	0.74515957	0.161574	4.611883843	3.99E-06	0.000483	UP
PLEKHG4B	73.33627	3.34837834	0.727016	4.605644564	4.11E-06	0.000495	UP
RP11-43F1	51.79849	2.81512334	0.611329	4.604925134	4.13E-06	0.000495	UP
FAM24B	32.25547	1.59917716	0.347461	4.602461352	4.18E-06	0.000498	UP
PAIP2B	767.4657	-1.9834718	0.430995	-4.60207504	4.18E-06	0.000498	DOWN
HTR1D	135.5659	3.01659668	0.65569	4.600643416	4.21E-06	0.000499	UP
MCM10	314.2813	1.57647839	0.342845	4.598225947	4.26E-06	0.000503	UP
BRIX1	1130.523	0.69837643	0.151985	4.595045399	4.33E-06	0.000508	UP
EIF3H	9229.326	0.85585084	0.186309	4.593705552	4.35E-06	0.000508	UP
HSF2BP	25.60683	1.27092519	0.27669	4.593313705	4.36E-06	0.000508	UP
F11	3410.912	-1.8055589	0.39336	-4.59009659	4.43E-06	0.000514	DOWN
TTK	404.084	1.59598864	0.347917	4.587265037	4.49E-06	0.000518	UP
NR0B1	45.6144	4.66617609	1.017612	4.585419055	4.53E-06	0.000519	UP
C6orf25	6.172237	-1.8649091	0.406716	-4.5852821	4.53E-06	0.000519	DOWN
C11orf96	785.1712	-1.7054812	0.372029	-4.58426667	4.56E-06	0.000519	DOWN
NR4A3	153.4943	-1.7660125	0.385394	-4.58235701	4.60E-06	0.000521	DOWN
RP5-888M1	32.80555	-1.7696103	0.386224	-4.58182715	4.61E-06	0.000521	DOWN
CCR3	23.06445	3.42409524	0.747558	4.580371649	4.64E-06	0.000523	UP
RP11-286I	10.90818	-2.025132	0.442287	-4.57877525	4.68E-06	0.000524	DOWN
LINC00907	6.51856	-1.9403771	0.424154	-4.5747018	4.77E-06	0.000532	DOWN
RPIA	579.2002	0.68328168	0.149411	4.573172026	4.80E-06	0.000534	UP

SUPT3H	233.7213	0.99336976	0.217663	4.563787319	5.02E-06	0.000555	UP
DLK1	494.4864	5.59228543	1.225717	4.562459685	5.06E-06	0.000555	UP
PTCHD4	81.92843	-2.4697964	0.541351	-4.56228318	5.06E-06	0.000555	DOWN
CDK5R1	124.3991	0.91609107	0.200839	4.561313298	5.08E-06	0.000555	UP
SLC1A5	2068.229	1.77994094	0.390272	4.56077208	5.10E-06	0.000555	UP
AMD1	2092.626	0.58673558	0.128672	4.559923114	5.12E-06	0.000555	UP
CRHBP	139.2267	-2.4702496	0.542053	-4.55720916	5.18E-06	0.00056	DOWN
CCDC138	192.6881	1.08619495	0.238426	4.555685289	5.22E-06	0.000561	UP
FCN3	321.1961	-2.2354085	0.490721	-4.55535267	5.23E-06	0.000561	DOWN
CDCA2	204.2606	1.66649055	0.36618	4.551014966	5.34E-06	0.00057	UP
NPR1	528.6288	-1.0394403	0.228522	-4.54853228	5.40E-06	0.000573	DOWN
FRMD7	8.039559	-3.0480266	0.670125	-4.54844666	5.40E-06	0.000573	DOWN
UBE2E1	2034.849	0.58316078	0.128246	4.547213149	5.44E-06	0.000574	NOT
CBX1	1845.932	0.66730086	0.146885	4.543018858	5.55E-06	0.000583	UP
CPSF6	2294.615	0.49269043	0.108523	4.539983148	5.63E-06	0.000589	NOT
CBX3	5571.432	0.55644521	0.122773	4.532321801	5.83E-06	0.000609	NOT
GABRA2	50.71716	5.43686972	1.200588	4.528504323	5.94E-06	0.000617	UP
CSAG2	20.72088	4.64080635	1.024955	4.527814664	5.96E-06	0.000617	UP
RPL7AP34	24.24819	-1.6616994	0.367136	-4.52610997	6.01E-06	0.00062	DOWN
SCN4B	154.0172	-1.3444118	0.297219	-4.5233057	6.09E-06	0.000626	DOWN
SLC6A19	315.3238	4.40478337	0.975804	4.514004216	6.36E-06	0.000648	UP
MCM4	3101.503	1.12583085	0.249421	4.51378484	6.37E-06	0.000648	UP
NAALADL1	440.4658	2.36782251	0.524626	4.513357164	6.38E-06	0.000648	UP
IZUMO4	105.6564	-1.4378546	0.318682	-4.51188488	6.43E-06	0.00065	DOWN
AUNIP	59.15526	1.41601459	0.313898	4.511072982	6.45E-06	0.000651	UP
RIOK1	776.1072	0.71173125	0.157861	4.508595958	6.53E-06	0.000656	UP
NCAPG	767.0862	1.49460737	0.331774	4.504896069	6.64E-06	0.000661	UP
NSMCE2	1218.831	0.92420802	0.205163	4.504754399	6.64E-06	0.000661	UP
MCM7	4821.525	0.95517714	0.212065	4.504176768	6.66E-06	0.000661	UP
CACYBP	3456.925	0.88983808	0.197579	4.503706914	6.68E-06	0.000661	UP
SMG5	8183.648	0.73917068	0.164164	4.502629848	6.71E-06	0.000661	UP
LINC0106(	8.656928	4.93151596	1.095353	4.502215415	6.72E-06	0.000661	UP
DCHS2	17.3509	2.85136154	0.633619	4.500117549	6.79E-06	0.000663	UP
POLR2K	2674.517	0.78969277	0.175496	4.499766074	6.80E-06	0.000663	UP
MIR503HG	42.60141	-1.4375823	0.319506	-4.49938845	6.81E-06	0.000663	DOWN
C18orf42	4.358552	-3.3653208	0.749585	-4.48957808	7.14E-06	0.000691	DOWN
ZNF93	97.35908	1.52011343	0.338749	4.487431106	7.21E-06	0.000696	UP
MED10	1011.248	0.66425193	0.148141	4.483905727	7.33E-06	0.000705	UP
DLGAP5	465.0456	1.53999549	0.343574	4.482279018	7.39E-06	0.000707	UP
CKS2	1121.055	1.16354861	0.259607	4.481960879	7.40E-06	0.000707	UP
FER1L5	18.85533	2.16590272	0.483808	4.476785057	7.58E-06	0.000719	UP
UGT1A7	22.00167	2.65589662	0.593278	4.476648652	7.58E-06	0.000719	UP
GSR	4954.045	0.99343799	0.221995	4.475038276	7.64E-06	0.000722	UP
RPE	1136.407	0.51076578	0.114353	4.466562385	7.95E-06	0.000743	NOT
GABRA3	54.3259	5.19644457	1.163655	4.46562195	7.98E-06	0.000743	UP
METTL21A	693.2854	0.64819562	0.145175	4.464935541	8.01E-06	0.000743	UP
E2F3	846.5666	0.97285779	0.21791	4.464498874	8.03E-06	0.000743	UP
BAMBI	2155.569	1.79192464	0.401382	4.464390891	8.03E-06	0.000743	UP
LINC01197	52.80334	-1.3281834	0.29753	-4.4640344	8.04E-06	0.000743	DOWN

SLCO1A2	499.6888	-2.1992894	0.492705	-4.46370325	8.06E-06	0.000743	DOWN
HS3ST4	39.18971	7.65790464	1.716799	4.460571672	8.17E-06	0.000746	UP
ALPL	2803.778	-2.1304879	0.477652	-4.4603365	8.18E-06	0.000746	DOWN
ATAD2	2532.751	0.99081451	0.222163	4.459848332	8.20E-06	0.000746	UP
TONSL	1052.404	0.97448322	0.218512	4.45963376	8.21E-06	0.000746	UP
AURKB	648.1693	1.69061472	0.37912	4.459317385	8.22E-06	0.000746	UP
ZBTB21	691.4351	-0.8947139	0.200997	-4.45138013	8.53E-06	0.000772	DOWN
MCIDAS	5.59412	3.99615887	0.898839	4.445913556	8.75E-06	0.000786	UP
RP1-18D14	7.272718	-1.896043	0.426473	-4.44587197	8.75E-06	0.000786	DOWN
HRCT1	437.1009	2.01655465	0.453652	4.445160111	8.78E-06	0.000786	UP
AC018804.	7.105451	2.32603155	0.523403	4.444056956	8.83E-06	0.000788	UP
RP11-133K	2.303938	3.01121564	0.678764	4.436322284	9.15E-06	0.000814	UP
EFNA5	152.2125	2.69244033	0.606999	4.435655849	9.18E-06	0.000814	UP
SPATS2L	7103.726	0.70546274	0.159124	4.43342836	9.27E-06	0.00082	UP
MS4A2	27.4229	-2.6966811	0.609178	-4.42675405	9.57E-06	0.000843	DOWN
LINC00880	15.73098	-1.8939277	0.427936	-4.42572278	9.61E-06	0.000844	DOWN
NUP210	3719.775	0.91125963	0.205964	4.424355963	9.67E-06	0.000845	UP
MMP12	130.5868	2.92566334	0.66132	4.423973207	9.69E-06	0.000845	UP
RP11-115N	6.358249	3.54081429	0.801407	4.418249204	9.95E-06	0.000865	UP
TUBA1C	2646.769	0.94496048	0.213991	4.415886688	1.01E-05	0.000872	UP
SRPX	173.4317	-2.0022525	0.453599	-4.41414539	1.01E-05	0.000876	DOWN
SLC16A3	1635.144	1.76467531	0.400157	4.409955307	1.03E-05	0.00089	UP
RP11-380F	4.027163	-2.5276992	0.573389	-4.40835225	1.04E-05	0.000894	DOWN
XRCC2	171.8516	1.33463447	0.302821	4.407335754	1.05E-05	0.000896	UP
DLX6-AS1	42.06934	4.59478601	1.042719	4.406544614	1.05E-05	0.000896	UP
GTF2IRD2	97.66063	-0.927935	0.210637	-4.40538402	1.06E-05	0.000897	DOWN
C12orf75	777.1178	1.90820118	0.433249	4.404400195	1.06E-05	0.000897	UP
C18orf54	100.9123	1.36500096	0.309932	4.404193406	1.06E-05	0.000897	UP
DOK5	65.19106	-1.7579881	0.399619	-4.39915903	1.09E-05	0.000916	DOWN
RP1-228H1	103.0548	1.02428901	0.232976	4.396548179	1.10E-05	0.000924	UP
TMEM192	2171.194	-0.8589505	0.195518	-4.39319386	1.12E-05	0.000935	DOWN
HSP90AB1	58191.01	0.71217776	0.162133	4.39254006	1.12E-05	0.000935	UP
EPAS1	10535.28	-0.8073453	0.183857	-4.39115145	1.13E-05	0.000938	DOWN
TICRR	183.9507	1.41823888	0.323074	4.389831671	1.13E-05	0.000941	UP
SGMS1-AS1	203.2388	-0.8328346	0.189847	-4.38686634	1.15E-05	0.000951	DOWN
EHMT2	3157.193	0.64914628	0.148063	4.384258583	1.16E-05	0.00096	UP
RP11-495F	55.71888	3.28296815	0.749028	4.382968571	1.17E-05	0.000961	UP
MAGEA8	15.87357	4.42922144	1.010611	4.382718488	1.17E-05	0.000961	UP
MRPL9	2194.821	0.63522308	0.14505	4.379325908	1.19E-05	0.000973	UP
PPP1R32	103.6397	-1.3901745	0.317604	-4.37707228	1.20E-05	0.00098	DOWN
ATP5G1P4	12.90352	1.95593263	0.447065	4.375055536	1.21E-05	0.000986	UP
STIP1	6959.356	0.672213	0.153686	4.373944653	1.22E-05	0.000988	UP
ZCCHC24	1989.953	-0.8726144	0.199581	-4.3722261	1.23E-05	0.000993	DOWN
SGOL2	329.5357	1.23304304	0.282253	4.368569595	1.25E-05	0.001007	UP
HMGA2	23.32386	3.17881267	0.727914	4.367014762	1.26E-05	0.001008	UP
ADCY1	904.2522	-2.3969248	0.548872	-4.3670028	1.26E-05	0.001008	DOWN
WDHD1	310.7032	1.24936776	0.286304	4.363773231	1.28E-05	0.00102	UP
LINC01224	53.71287	2.90947233	0.667052	4.361689321	1.29E-05	0.001024	UP
LA16c-83F	4.890575	4.62713472	1.061137	4.360542534	1.30E-05	0.001024	UP

KIAA1524	280.7852	1.40452843	0.322134	4.360071322	1.30E-05	0.001024	UP
CDC20	1512.458	1.67164742	0.383408	4.359968983	1.30E-05	0.001024	UP
MAGEA10	9.84959	5.63742587	1.293267	4.359057051	1.31E-05	0.001024	UP
PSRC1	374.5473	1.26968867	0.291301	4.358681139	1.31E-05	0.001024	UP
AL133493.	49.12443	3.12690668	0.717433	4.3584648	1.31E-05	0.001024	UP
CTD-2550(	3.704098	-1.7039738	0.391036	-4.35758358	1.32E-05	0.001025	DOWN
RP11-442(	20.04743	-2.1549006	0.494898	-4.35422854	1.34E-05	0.001037	DOWN
FLVCR1	1012.984	1.11500671	0.256095	4.353878893	1.34E-05	0.001037	UP
OFCC1	4.84305	4.96320362	1.140304	4.352526743	1.35E-05	0.001039	UP
YARS	4427.462	0.82679697	0.189973	4.352172014	1.35E-05	0.001039	UP
RP11-451(	11.03403	5.10705245	1.17543	4.344838311	1.39E-05	0.001069	UP
LINC0066	8.449471	4.34718474	1.000645	4.344384673	1.40E-05	0.001069	UP
WDR4	522.3934	0.85662527	0.197191	4.344147413	1.40E-05	0.001069	UP
VAX2	44.08434	-2.5385232	0.584547	-4.34271626	1.41E-05	0.00107	DOWN
GNA14	85.28235	-1.4459925	0.333002	-4.34229615	1.41E-05	0.00107	DOWN
MATN3	120.7921	2.59862084	0.598482	4.342018424	1.41E-05	0.00107	UP
KIF20A	796.9371	1.35902553	0.313081	4.340809559	1.42E-05	0.001073	UP
OLA1	3137.5	0.63699928	0.146808	4.338983534	1.43E-05	0.001079	UP
BAIAP2L2	774.8247	1.89116159	0.436305	4.33449732	1.46E-05	0.001098	UP
OTOG	13.01993	3.68158516	0.849913	4.331721001	1.48E-05	0.001107	UP
CNTN4	114.7841	-1.7407119	0.401923	-4.33095984	1.48E-05	0.001107	DOWN
ENPEP	2532.588	-1.4992381	0.346179	-4.33081337	1.49E-05	0.001107	DOWN
CTC-510F	16.41723	-1.3057089	0.301636	-4.32875747	1.50E-05	0.001115	DOWN
RIPK2	931.8324	1.0768236	0.249035	4.32399076	1.53E-05	0.001136	UP
ACTL6A	1332.09	0.70888203	0.163973	4.323152434	1.54E-05	0.001137	UP
ERCC6L	122.487	1.42783663	0.330534	4.319785617	1.56E-05	0.001149	UP
PFN1P2	10.41639	-1.3737148	0.318043	-4.31926797	1.57E-05	0.001149	DOWN
NKAIN1	12.6115	3.29815703	0.763702	4.318646412	1.57E-05	0.001149	UP
RP11-432	13.98651	4.0622601	0.940679	4.318433446	1.57E-05	0.001149	UP
BDKRB2	295.1461	-1.6469089	0.381606	-4.31573128	1.59E-05	0.00116	DOWN
RP11-15N	12.84611	1.34687837	0.312262	4.313301877	1.61E-05	0.00117	UP
TRIM16L	1808.751	2.02437182	0.469887	4.308212209	1.65E-05	0.001194	UP
DNMT3B	199.1115	1.28118207	0.297678	4.303915838	1.68E-05	0.001214	UP
SOCS2	653.6329	-1.4595929	0.339219	-4.30280142	1.69E-05	0.001217	DOWN
MYCN	245.8478	2.72017053	0.633505	4.293842654	1.76E-05	0.001263	UP
CXCL14	188.2177	-2.848797	0.663521	-4.29345487	1.76E-05	0.001263	DOWN
KIF18B	518.27	1.55127138	0.361545	4.29066802	1.78E-05	0.001275	UP
RIBC2	101.1599	1.97624931	0.460925	4.287573119	1.81E-05	0.001288	UP
SLC38A1	2365.458	1.68651628	0.393371	4.287339621	1.81E-05	0.001288	UP
ATP6V1C1	4431.14	0.81207971	0.189513	4.285093658	1.83E-05	0.001297	UP
ZBTB12	200.5422	1.20434616	0.281099	4.284418479	1.83E-05	0.001298	UP
RP11-511	7.581584	-1.6026477	0.374285	-4.28189477	1.85E-05	0.001306	DOWN
PDCL3	667.3392	0.61881554	0.144522	4.281822524	1.85E-05	0.001306	UP
S100A9	1194.979	2.17627585	0.50903	4.275337764	1.91E-05	0.001341	UP
NMUR1	46.22739	-1.3777857	0.322474	-4.27254395	1.93E-05	0.001355	DOWN
ARHGAP39	281.0138	0.98983469	0.231798	4.270241285	1.95E-05	0.001365	UP
PRSS16	79.62717	3.54597085	0.830988	4.267174045	1.98E-05	0.001381	UP
EFNA3	210.6428	1.54036533	0.36104	4.266469418	1.99E-05	0.001381	UP
RP11-187	11.43465	2.43659496	0.571617	4.262639012	2.02E-05	0.001402	UP

PRRT3-AS1	63.58483	1.72645015	0.405256	4.260148344	2.04E-05	0.001411	UP
CDK4	2624.189	0.86357269	0.202714	4.260051136	2.04E-05	0.001411	UP
KCTD6	629.17	1.0557906	0.247934	4.258355344	2.06E-05	0.001418	UP
ACTBP8	16.23251	3.31828575	0.779554	4.256647358	2.08E-05	0.001425	UP
TAGLN2	23216.72	0.85373926	0.200651	4.254840267	2.09E-05	0.001427	UP
DUSP5P1	8.815863	2.94884198	0.693062	4.254799604	2.09E-05	0.001427	UP
PRSS22	40.75771	3.32404602	0.781263	4.254708113	2.09E-05	0.001427	UP
EPPK1	829.0155	2.33656108	0.549298	4.253720672	2.10E-05	0.00143	UP
ELFN1	1180.123	-2.2354465	0.525755	-4.25187511	2.12E-05	0.001435	DOWN
CDCA4	445.582	1.06122123	0.249595	4.251766879	2.12E-05	0.001435	UP
SBK3	28.93234	2.95260896	0.694652	4.250489561	2.13E-05	0.001439	UP
EPHB4	6124.057	-1.302934	0.306575	-4.24996534	2.14E-05	0.001439	DOWN
TUBB4A	665.1808	2.73589902	0.644131	4.247428585	2.16E-05	0.001449	UP
PNMA3	554.3414	2.87468843	0.676821	4.247341585	2.16E-05	0.001449	UP
TOP1MT	2400.457	0.97635534	0.229925	4.246415908	2.17E-05	0.001452	UP
GOLGA7B	281.6619	2.49475479	0.588039	4.242498217	2.21E-05	0.001473	UP
ASNS	548.7743	1.80183177	0.424799	4.241611721	2.22E-05	0.001473	UP
ARHGAP36	24.12146	-7.7212694	1.820514	-4.24125775	2.22E-05	0.001473	DOWN
NUP107	1366.342	0.64634656	0.152407	4.240921747	2.23E-05	0.001473	UP
XX-CR54.1	11.11181	5.64192767	1.332314	4.234683106	2.29E-05	0.001511	UP
PKM	12182.36	1.60180953	0.378359	4.233568376	2.30E-05	0.001511	UP
RP11-354I	3.475034	-2.5685859	0.606857	-4.23260607	2.31E-05	0.001511	DOWN
SKA3	310.2329	1.42238053	0.336098	4.232037869	2.32E-05	0.001511	UP
RPS6KA2	756.6678	-1.073149	0.253612	-4.2314597	2.32E-05	0.001511	DOWN
QPCT	171.4617	1.66702172	0.393994	4.231084682	2.33E-05	0.001511	UP
PMAIP1	62.52429	1.85823422	0.439273	4.23024785	2.33E-05	0.001511	UP
RP11-494(	4.771634	2.36608186	0.55938	4.229830185	2.34E-05	0.001511	UP
CLDN5	1163.065	-1.5243539	0.360402	-4.22959127	2.34E-05	0.001511	DOWN
CCL14	145.1338	-1.4900673	0.352299	-4.22954779	2.34E-05	0.001511	DOWN
EFHC2	13.73684	3.2227635	0.762015	4.229266014	2.34E-05	0.001511	UP
CTSC	4310.546	1.26155022	0.298513	4.226112117	2.38E-05	0.001529	UP
EIF3E	12624.14	0.94937064	0.224676	4.225516897	2.38E-05	0.001529	UP
LINC00632	8.883491	4.11947762	0.975163	4.224399626	2.40E-05	0.001533	UP
MMAA	854.8342	-0.7611933	0.180513	-4.21684476	2.48E-05	0.001581	DOWN
SPINT2	1829.397	2.40883653	0.571802	4.212712473	2.52E-05	0.001604	UP
SH2D5	20.60782	2.41851765	0.574114	4.212610424	2.52E-05	0.001604	UP
MYEOV	139.712	2.95473161	0.702162	4.208045757	2.58E-05	0.001633	UP
WDYHV1	520.4225	0.90084801	0.214282	4.204022448	2.62E-05	0.001657	UP
LINC00501	9.950753	3.40640428	0.810345	4.203645943	2.63E-05	0.001657	UP
RP11-77K1	39.67654	-1.3697965	0.325905	-4.20305471	2.63E-05	0.001658	DOWN
TPX2	2253.15	1.29472055	0.308277	4.199857749	2.67E-05	0.001677	UP
ARHGEF2	1776.873	1.02943835	0.245166	4.198949672	2.68E-05	0.00168	UP
CIDEA	10.39661	-4.1780391	0.995476	-4.19702504	2.70E-05	0.00169	DOWN
SLC39A1	9039.005	0.68845426	0.164051	4.196598068	2.71E-05	0.00169	UP
RND2	417.5551	2.52964321	0.602995	4.195131306	2.73E-05	0.001697	UP
LRRC1	601.6691	1.54889258	0.369383	4.193184831	2.75E-05	0.001707	UP
RP5-1157M	9.90266	1.33326687	0.317989	4.192811833	2.76E-05	0.001707	UP
PCNA	3896.334	0.78692352	0.18774	4.191570514	2.77E-05	0.001712	UP
YBX1	14839.8	0.67712466	0.161616	4.189723738	2.79E-05	0.001722	UP

FRAS1	699.4541	2.76584132	0.660659	4.186491436	2.83E-05	0.001743	UP
FCGBP	838.5042	2.08022605	0.497034	4.18528015	2.85E-05	0.001745	UP
CD7	660.1114	2.01475783	0.481413	4.185093569	2.85E-05	0.001745	UP
CTSE	108.3752	3.31770683	0.792918	4.184173157	2.86E-05	0.001745	UP
SPIB	101.4197	2.67440638	0.639177	4.184138245	2.86E-05	0.001745	UP
E2F5	333.6007	1.16520637	0.278609	4.182230969	2.89E-05	0.001756	UP
NBPF8P	263.1971	-1.2896106	0.308683	-4.17777856	2.94E-05	0.001787	DOWN
RP11-94C2	51.85265	-1.3923346	0.333334	-4.17700176	2.95E-05	0.001789	DOWN
DIO2	101.2095	-2.5530223	0.611536	-4.17477186	2.98E-05	0.001802	DOWN
SLC6A14	12.51839	3.75750527	0.900484	4.172759523	3.01E-05	0.001814	UP
AC019129.	4.597874	2.92386396	0.701266	4.169407436	3.05E-05	0.001837	UP
UBE2Q2	849.9606	0.88280058	0.211794	4.168200869	3.07E-05	0.001843	UP
NUDT17	101.1419	1.01706627	0.244226	4.164447821	3.12E-05	0.001869	UP
RP11-96D1	84.44226	-2.1262297	0.510912	-4.16163184	3.16E-05	0.001888	DOWN
PPARGC1A	2516.806	-1.6246431	0.390489	-4.16053595	3.18E-05	0.001893	DOWN
DGKG	162.894	1.58680576	0.381623	4.158045844	3.21E-05	0.001907	UP
SNRPD1	1752.693	0.74462825	0.179089	4.157874193	3.21E-05	0.001907	UP
TPRKB	791.4212	0.58577523	0.140997	4.154530714	3.26E-05	0.001931	UP
GPR114	565.4581	-2.2985083	0.553326	-4.15398673	3.27E-05	0.001931	DOWN
NOP58	3021.557	0.65412588	0.157645	4.149361376	3.33E-05	0.001966	UP
SLC6A3	8.489683	3.16453098	0.763108	4.146897464	3.37E-05	0.001983	UP
CTD-2555(	11.84204	2.77099155	0.668482	4.14520283	3.40E-05	0.001994	UP
TUBG1	2298.609	0.70682303	0.170556	4.144238322	3.41E-05	0.001995	UP
BASP1	347.4209	-1.5769078	0.380569	-4.14355123	3.42E-05	0.001995	DOWN
ACYP1	151.9596	0.82249652	0.1985	4.143550874	3.42E-05	0.001995	UP
RPL5P30	6.166355	-1.395788	0.336953	-4.14238369	3.44E-05	0.002001	DOWN
RP3-395M2	3.284213	-2.4954519	0.602718	-4.14033188	3.47E-05	0.002015	DOWN
FMO1	410.5855	2.52121504	0.609062	4.139503203	3.48E-05	0.002016	UP
NFYA	1306.605	0.70349911	0.169962	4.139162078	3.49E-05	0.002016	UP
PZP	124.9343	-2.046053	0.494471	-4.13786177	3.51E-05	0.002024	DOWN
PTDSS1	4616.363	0.698673	0.168922	4.136079844	3.53E-05	0.002031	UP
CENPA	271.5391	1.50887045	0.364813	4.136011671	3.53E-05	0.002031	UP
RP11-89K1	9.920385	1.70082693	0.411401	4.134229697	3.56E-05	0.00204	UP
AC016831.	53.00684	1.64680797	0.398347	4.134103728	3.56E-05	0.00204	UP
SMS	2423.464	0.68714565	0.166362	4.130421492	3.62E-05	0.002068	UP
MELK	505.554	1.30098125	0.315105	4.128726005	3.65E-05	0.002079	UP
MAGOH	1298.837	0.5878127	0.142419	4.127353415	3.67E-05	0.002087	UP
LMNB2	1862.027	1.0850987	0.263289	4.121314404	3.77E-05	0.002138	UP
NIFK	1393.537	0.69688469	0.169144	4.12007057	3.79E-05	0.002145	UP
CCT6A	8176.362	0.61088918	0.148423	4.11587203	3.86E-05	0.002178	UP
SMARCC1	3503.507	0.58287246	0.141626	4.115580213	3.86E-05	0.002178	NOT
STARD5	227.0762	-1.3337848	0.324207	-4.11398632	3.89E-05	0.002189	DOWN
CDC25A	290.2565	1.27947238	0.311075	4.11306475	3.90E-05	0.002193	UP
RP11-150(	66.63388	2.5674858	0.624517	4.111154581	3.94E-05	0.002207	UP
RCC2	2972.524	0.82651984	0.201096	4.110084576	3.96E-05	0.00221	UP
RP11-618I	57.73482	3.61671534	0.880017	4.109823875	3.96E-05	0.00221	UP
RP11-1267	78.74023	3.00314687	0.730907	4.10879684	3.98E-05	0.002216	UP
XRCC5	10242.94	0.43733042	0.106483	4.107054409	4.01E-05	0.002228	NOT
PRMT3	582.3966	0.64011546	0.155889	4.106235728	4.02E-05	0.002231	UP

WDR12	1293.723	0.56233832	0.136972	4.105485026	4.03E-05	0.002232	NOT
C19orf81	24.5447	3.57500432	0.870836	4.105257678	4.04E-05	0.002232	UP
SLC6A16	176.5906	-1.1187517	0.273068	-4.09697677	4.19E-05	0.002308	DOWN
CREG2	17.67222	1.78791136	0.4365	4.096019436	4.20E-05	0.002313	UP
RP11-567(	25.45996	-3.2647869	0.797632	-4.09310007	4.26E-05	0.002335	DOWN
SLC41A3	1926.045	0.60705776	0.148319	4.092907698	4.26E-05	0.002335	UP
FBXO5	272.9876	1.11519561	0.272544	4.09179556	4.28E-05	0.002341	UP
LINC0126(	11.02837	3.06398437	0.748914	4.091234729	4.29E-05	0.002342	UP
CENPC	484.3878	-0.6613787	0.161804	-4.087532	4.36E-05	0.002375	DOWN
ESCO2	180.753	1.44706078	0.354165	4.085837989	4.39E-05	0.002386	UP
VAT1	8320.552	0.82042352	0.200813	4.085503679	4.40E-05	0.002386	UP
PNCK	114.8525	3.38611088	0.829215	4.083514376	4.44E-05	0.002402	UP
DLGAP2	10.95538	-2.5476717	0.624028	-4.08262672	4.45E-05	0.002407	DOWN
SCNN1A	728.869	-2.0917294	0.512663	-4.08012546	4.50E-05	0.002423	DOWN
TJAP1	1528.677	0.47381563	0.116132	4.079975822	4.50E-05	0.002423	NOT
TIGD5	823.9402	1.01050761	0.247695	4.07964274	4.51E-05	0.002423	UP
UCK2	2151.452	0.8810994	0.216229	4.074848794	4.60E-05	0.002463	UP
EXOSC3	609.0492	0.52643688	0.12922	4.073953374	4.62E-05	0.002463	NOT
PIK3R3	717.2171	-0.996751	0.244675	-4.07378124	4.63E-05	0.002463	DOWN
BEND3	241.5697	1.01156383	0.248328	4.073506254	4.63E-05	0.002463	UP
CDKN3	571.5685	1.35851772	0.333526	4.073193189	4.64E-05	0.002463	UP
TRIM9	93.92477	2.24108806	0.550271	4.072700861	4.65E-05	0.002463	UP
CLDN19	66.51441	2.37511343	0.583185	4.072659876	4.65E-05	0.002463	UP
RP11-640I	12.35477	-1.1043282	0.271298	-4.07053881	4.69E-05	0.002481	DOWN
TM4SF19	22.08994	2.15877728	0.530671	4.06801477	4.74E-05	0.002503	UP
TMEM237	466.8313	0.87309492	0.214862	4.063519979	4.83E-05	0.002537	UP
SPCS3	4185.379	-0.5533849	0.136196	-4.06315544	4.84E-05	0.002537	NOT
C16orf59	312.6325	1.20802481	0.29732	4.06305189	4.84E-05	0.002537	UP
PLA2G4D	2.288284	3.32763515	0.819051	4.062795884	4.85E-05	0.002537	UP
HCG20	8.523752	1.96807173	0.484441	4.062558628	4.85E-05	0.002537	UP
SLC25A48	32.19187	-2.9229237	0.720294	-4.05795827	4.95E-05	0.002583	DOWN
FAM83H-AS	80.38249	1.60301952	0.395204	4.056179882	4.99E-05	0.002597	UP
MCM5	3355.973	0.8676491	0.214071	4.053091035	5.05E-05	0.002627	UP
SLC52A2	1926.522	1.08043531	0.266668	4.051619571	5.09E-05	0.002639	UP
PHF5A	983.0914	0.55571539	0.137201	4.050381728	5.11E-05	0.002645	NOT
C8orf33	3299.512	0.83757478	0.206799	4.050179679	5.12E-05	0.002645	UP
DDX55	821.8788	0.56182532	0.138738	4.049532807	5.13E-05	0.002647	NOT
P2RX4	1593.277	0.89461504	0.221021	4.047644558	5.17E-05	0.002663	UP
CBR4	1495.6	-0.9647517	0.238416	-4.04649983	5.20E-05	0.002668	DOWN
TCL6	27.58257	2.79407868	0.690522	4.046329652	5.20E-05	0.002668	UP
KLK6	5.08653	3.98544048	0.985761	4.043009693	5.28E-05	0.002701	UP
PLBD1	546.8642	1.65450979	0.409346	4.041839807	5.30E-05	0.00271	UP
AMOTL1	1905.37	-1.5337938	0.37968	-4.03969756	5.35E-05	0.002729	DOWN
HDAC2	2297.687	0.71593778	0.177304	4.037909759	5.39E-05	0.002745	UP
ARMC3	4.331742	3.1659233	0.784227	4.036999546	5.41E-05	0.002751	UP
RP11-347I	14.38622	-1.1453825	0.28385	-4.03516883	5.46E-05	0.002762	DOWN
RP11-81A2	19.22369	-1.8110622	0.448866	-4.03474875	5.47E-05	0.002762	DOWN
RP11-336K	13.59635	1.83053892	0.453694	4.034739391	5.47E-05	0.002762	UP
RP11-429J	4.457248	3.09278717	0.766929	4.032691026	5.51E-05	0.002781	UP

ARPC2	6337.224	0.49605053	0.123061	4.030916836	5.56E-05	0.002797	NOT
EIF5A2	431.1434	1.58844965	0.394249	4.029050398	5.60E-05	0.002814	UP
RP11-182J	6.695226	-0.9958292	0.247286	-4.02702733	5.65E-05	0.002831	DOWN
PLCH2	546.165	-2.1192895	0.526302	-4.02675489	5.66E-05	0.002831	DOWN
RP11-7F17	35.64527	-0.8313743	0.206512	-4.02578328	5.68E-05	0.002837	DOWN
CORO1C	4208.843	0.57891854	0.143865	4.024051743	5.72E-05	0.002851	NOT
RP11-617F	301.3934	-1.4299884	0.355394	-4.02366703	5.73E-05	0.002851	DOWN
CAD	2206.115	0.75439613	0.187504	4.023349921	5.74E-05	0.002851	UP
MAMDC4	1720.91	-1.9845097	0.493385	-4.02223475	5.76E-05	0.002854	DOWN
RAB44	4.302706	-2.028128	0.50423	-4.02223169	5.76E-05	0.002854	DOWN
RP11-358I	2.170117	-1.9937003	0.495804	-4.02114688	5.79E-05	0.002862	DOWN
SCGB1D2	8.846841	4.31711759	1.074259	4.01869241	5.85E-05	0.002876	UP
ZNF207	4274.497	0.33707865	0.083878	4.018654085	5.85E-05	0.002876	NOT
PPP2R1B	5236.168	-1.0146387	0.252503	-4.0183189	5.86E-05	0.002876	DOWN
PDE2A	400.0678	-1.2010757	0.298905	-4.01825679	5.86E-05	0.002876	DOWN
AVIL	253.4927	-1.3003915	0.323707	-4.01718459	5.89E-05	0.002884	DOWN
CD58	418.6756	0.7825605	0.194885	4.015501953	5.93E-05	0.0029	UP
RP11-632C	311.5783	1.65119596	0.4116	4.011652438	6.03E-05	0.002942	UP
WNT3A	26.2068	3.03366921	0.756287	4.011266539	6.04E-05	0.002942	UP
CCT3	20100.17	0.79685579	0.198798	4.008361977	6.11E-05	0.002973	UP
RNA5SP18	2.29331	3.17636834	0.792957	4.005724616	6.18E-05	0.003001	UP
RPL22L1	1890.128	1.31695878	0.3289	4.004132769	6.22E-05	0.003011	UP
DYNLL1-AS	229.7747	0.72871211	0.181993	4.004072779	6.23E-05	0.003011	UP
FBXO43	91.75372	1.26155957	0.315335	4.000690146	6.32E-05	0.003047	UP
HMCN2	499.9037	-2.0857769	0.52141	-4.00026433	6.33E-05	0.003047	DOWN
FANCE	231.549	0.9756698	0.243916	4.000017981	6.33E-05	0.003047	UP
SMARCD1	2097.161	0.55746046	0.1394	3.999009678	6.36E-05	0.003054	NOT
ZNF702P	72.68766	1.81951258	0.455036	3.998608077	6.37E-05	0.003054	UP
CRYAB	680.3556	1.85370858	0.463928	3.995682035	6.45E-05	0.003087	UP
RRP9	1102.676	0.77120802	0.193048	3.994898865	6.47E-05	0.003091	UP
GOT2	12102.61	-0.994117	0.248907	-3.99392927	6.50E-05	0.003093	DOWN
SCGB3A1	19.26942	-1.5897134	0.398034	-3.99391251	6.50E-05	0.003093	DOWN
KLF8	43.71405	-1.3430249	0.336302	-3.99350656	6.51E-05	0.003093	DOWN
RAE1	1329.186	0.51113085	0.128003	3.993115789	6.52E-05	0.003093	NOT
AHSA1	4020.872	0.61119578	0.153231	3.988713851	6.64E-05	0.003145	UP
RACGAP1	1083.659	1.05301901	0.264208	3.98556357	6.73E-05	0.003182	UP
RRS1	1482.082	0.92528476	0.232271	3.98364338	6.79E-05	0.003202	UP
CDCA3	543.0305	1.29317676	0.324793	3.98154629	6.85E-05	0.003225	UP
MZT2A	1856.059	0.99031807	0.248852	3.979543464	6.90E-05	0.003247	UP
PLA2G12A	1618.678	-0.7555001	0.190185	-3.97245396	7.11E-05	0.00333	DOWN
S100A10	8204.085	1.18927203	0.299393	3.972281399	7.12E-05	0.00333	UP
RAD54L	183.8071	1.41042352	0.355068	3.972261798	7.12E-05	0.00333	UP
IGLL4P	3.493336	4.48383533	1.129134	3.971039696	7.16E-05	0.003342	UP
NPM1	17538.8	0.74126781	0.186737	3.969579808	7.20E-05	0.003354	UP
CDH16	87.02143	2.60760498	0.656934	3.969354835	7.21E-05	0.003354	UP
PADI1	81.6752	-3.3317741	0.840487	-3.96410161	7.37E-05	0.003423	DOWN
FAM72C	19.04839	1.64924248	0.416311	3.961562412	7.45E-05	0.003445	UP
ERVFRD-1	5.040971	-2.4192871	0.610711	-3.96142868	7.45E-05	0.003445	DOWN
AC005682.	45.60143	1.39843942	0.353024	3.961310549	7.45E-05	0.003445	UP

CDC6	957.1578	1.2090081	0.305294	3.960145571	7.49E-05	0.003451	UP
C17orf53	193.891	0.99887399	0.252236	3.960073382	7.49E-05	0.003451	UP
TCF3	2041.635	0.79481287	0.200957	3.955136406	7.65E-05	0.003513	UP
POLD1	1289.288	0.71540147	0.180887	3.954965371	7.65E-05	0.003513	UP
DTYMK	1597.955	0.91995086	0.232659	3.954066955	7.68E-05	0.003513	UP
FAR2P1	52.30164	4.05535381	1.025655	3.953916851	7.69E-05	0.003513	UP
MBOAT4	48.35564	2.10459185	0.532299	3.953779885	7.69E-05	0.003513	UP
ANKFN1	59.04603	4.58500354	1.160142	3.952106808	7.75E-05	0.00353	UP
SUV39H2	390.4621	0.65262704	0.165145	3.951848089	7.75E-05	0.00353	UP
RNASEH2A	1152.466	0.93261571	0.23604	3.951092143	7.78E-05	0.003535	UP
RP11-932(	1.690283	-1.6329452	0.413389	-3.95014535	7.81E-05	0.003541	DOWN
TAT-AS1	49.6953	-1.9067665	0.482769	-3.94964889	7.83E-05	0.003541	DOWN
BOC	99.60835	-1.4561764	0.368698	-3.94950509	7.83E-05	0.003541	DOWN
NDC80	568.847	1.23691633	0.313331	3.947629349	7.89E-05	0.003563	UP
SPP1	54307.85	2.51826868	0.637986	3.947214584	7.91E-05	0.003563	UP
FAT4	359.3395	-1.5214109	0.385591	-3.94566412	7.96E-05	0.003576	DOWN
TRIM16	1092.148	1.67310246	0.424162	3.944490002	8.00E-05	0.003576	UP
AP001469.	54.38104	1.14872378	0.291241	3.944231619	8.01E-05	0.003576	UP
FAIM	395.219	0.62284131	0.157937	3.943603294	8.03E-05	0.003576	UP
B3GALT5	33.45581	2.82650694	0.716862	3.942887396	8.05E-05	0.003576	UP
CDH10	13.6713	4.33315979	1.099008	3.942793502	8.05E-05	0.003576	UP
SEMA6A	1131.604	1.76968029	0.448869	3.942527573	8.06E-05	0.003576	UP
PWRN1	4.794891	-3.2820596	0.832488	-3.94247272	8.06E-05	0.003576	DOWN
RP11-79N2	6.367896	2.10324436	0.533494	3.942395345	8.07E-05	0.003576	UP
PNPLA7	554.4749	-1.2334102	0.312886	-3.94204036	8.08E-05	0.003576	DOWN
RP11-116I	8.527097	-2.0092519	0.50976	-3.94156656	8.10E-05	0.003576	DOWN
TSPO2	42.23689	1.49384843	0.379	3.941554996	8.10E-05	0.003576	UP
CTD-3157I	26.32099	-1.3058668	0.331337	-3.94120387	8.11E-05	0.003576	DOWN
CEP19	60.83224	0.93997309	0.238605	3.939445272	8.17E-05	0.003597	UP
CASC19	25.13198	2.31600545	0.588271	3.936967991	8.25E-05	0.003628	UP
ORC1	286.0751	1.27041649	0.322858	3.934904508	8.32E-05	0.003654	UP
PPHLN1	1491.661	0.39073631	0.099317	3.934236216	8.35E-05	0.003658	NOT
CTD-2325A	22.3337	1.83813141	0.467338	3.933192571	8.38E-05	0.003664	UP
HTR2B	87.74103	-1.7304799	0.439986	-3.9330371	8.39E-05	0.003664	DOWN
IGFALS	1277.285	-2.2046132	0.560849	-3.93084954	8.46E-05	0.003689	DOWN
C11orf80	290.049	1.0726774	0.2729	3.930659072	8.47E-05	0.003689	UP
LINC0098	2.654144	-1.3818906	0.351753	-3.92857851	8.54E-05	0.003715	DOWN
DNM3OS	39.86139	-1.4366498	0.366035	-3.92489324	8.68E-05	0.003766	DOWN
GPRIN1	277.9911	1.3058862	0.332837	3.923501715	8.73E-05	0.003782	UP
RP11-317N	9.820857	-1.2361753	0.315235	-3.92144098	8.80E-05	0.003809	DOWN
ETV5	927.0566	0.98683084	0.251692	3.920789566	8.83E-05	0.003813	UP
DHX37	1244.919	0.57776816	0.147383	3.920178631	8.85E-05	0.003816	NOT
RP11-568K	6.585888	2.37887627	0.606921	3.919583813	8.87E-05	0.00382	UP
CHI3L2	40.48957	1.9469672	0.496823	3.918834355	8.90E-05	0.003826	UP
SLC25A15	1932.832	-1.5813083	0.403773	-3.91632554	8.99E-05	0.00386	DOWN
RP11-214(	74.29419	1.95245074	0.498928	3.913289494	9.10E-05	0.003902	UP
EIF2AK4	2206.313	-0.5449762	0.139332	-3.91133596	9.18E-05	0.003928	NOT
ILF2	7040.359	0.58529486	0.149659	3.91085561	9.20E-05	0.003929	UP
LINC0040	17.70679	-2.6413111	0.67588	-3.90795582	9.31E-05	0.00397	DOWN

MIR22HG	523.8264	-0.9821035	0.25134	-3.90747553	9.33E-05	0.00397	DOWN
CH17-472(	156.7744	-1.3876599	0.355168	-3.90704862	9.34E-05	0.00397	DOWN
MRPS23	2074.461	0.66080666	0.169141	3.906832464	9.35E-05	0.00397	UP
NCAPH2	2348.001	0.75283378	0.192796	3.90482225	9.43E-05	0.003997	UP
PPIAP9	6.590859	1.71821589	0.440242	3.902888424	9.51E-05	0.004023	UP
FAM65C	133.561	-1.7799238	0.456133	-3.90220091	9.53E-05	0.004028	DOWN
CST1	185.6384	4.22007952	1.081558	3.901852214	9.55E-05	0.004028	UP
FANCI	807.425	0.9919212	0.254244	3.90145751	9.56E-05	0.004028	UP
RAB42	106.8887	1.43706456	0.368518	3.89957983	9.64E-05	0.004053	UP
FEN1	1774.723	0.80184871	0.205686	3.898415116	9.68E-05	0.004066	UP
GGT1	2889.11	1.51569568	0.388838	3.898016162	9.70E-05	0.004067	UP
CDH8	18.02513	-2.0594187	0.528401	-3.8974552	9.72E-05	0.00407	DOWN
USP53	511.0946	-0.8751567	0.224887	-3.89153754	9.96E-05	0.004155	DOWN
SLC2A4	355.7942	-1.5340263	0.394214	-3.8913512	9.97E-05	0.004155	DOWN
XXyac-YM2	38.31489	2.42171836	0.622376	3.891083787	9.98E-05	0.004155	UP
ELN	1690.902	-1.8626803	0.478719	-3.89096391	9.98E-05	0.004155	DOWN
MTRNR2L3	4.537618	-1.5793579	0.406026	-3.8897961	0.0001	0.004168	DOWN
SNHG1	1217.156	0.93967427	0.241736	3.887191178	0.000101	0.004206	UP
HPS5	1553.866	-0.8821678	0.226962	-3.88685153	0.000102	0.004206	DOWN
S100A14	1290.306	1.98021516	0.509617	3.885690893	0.000102	0.004209	UP
TNFAIP1	4383.904	-0.5341909	0.137488	-3.8853669	0.000102	0.004209	NOT
PDE11A	393.6051	-1.8236998	0.46939	-3.88525372	0.000102	0.004209	DOWN
DNM1	463.4083	1.66734438	0.429149	3.885234023	0.000102	0.004209	UP
CHODL	32.35062	2.92822962	0.75426	3.882255825	0.000103	0.004254	UP
PLEKHS1	11.41168	2.01785498	0.519832	3.881745029	0.000104	0.004257	UP
ETFDH	2629.291	-0.9111476	0.234765	-3.88110996	0.000104	0.004261	DOWN
IL1R1	4453.263	-0.9180313	0.236636	-3.87950542	0.000105	0.004283	DOWN
LDB2	571.8646	-0.9814542	0.253094	-3.87782764	0.000105	0.004306	DOWN
AP000997.	5.225993	-2.3466956	0.605501	-3.8756279	0.000106	0.004339	DOWN
ANAPC7	1939.092	0.60268224	0.155548	3.874580876	0.000107	0.004351	UP
PRKAG2	1267.141	-0.8963547	0.231369	-3.87412947	0.000107	0.004352	DOWN
SNORD17	9.315506	1.36446442	0.352415	3.871754322	0.000108	0.004377	UP
MBL1P	34.91127	-2.0286737	0.523983	-3.87164086	0.000108	0.004377	DOWN
KLKB1	4510.546	-1.4630029	0.377879	-3.87161222	0.000108	0.004377	DOWN
AC079466.	1178.869	3.29990116	0.852422	3.871205019	0.000108	0.004377	UP
ZNF560	3.470413	4.29792007	1.110392	3.870634339	0.000109	0.004377	UP
NUP35	502.4581	0.56005363	0.144695	3.870567575	0.000109	0.004377	NOT
SV2A	135.0445	1.7301262	0.447044	3.870150527	0.000109	0.004378	UP
USP2	552.8467	-1.3640541	0.352637	-3.86815605	0.00011	0.004396	DOWN
ORC6	244.3682	1.24901639	0.322904	3.868076129	0.00011	0.004396	UP
STARD6	2.637342	3.19358657	0.825629	3.868064996	0.00011	0.004396	UP
CCL26	16.63984	2.06094687	0.533138	3.865693626	0.000111	0.004433	UP
TFF2	25.49665	3.31559338	0.857872	3.864904533	0.000111	0.00444	UP
GALNT9	25.65798	-2.9009676	0.750722	-3.8642368	0.000111	0.004442	DOWN
TMC7	160.0271	1.10769726	0.286665	3.864082521	0.000112	0.004442	UP
RP11-441(	65.78772	-1.3822442	0.357802	-3.86315634	0.000112	0.004453	DOWN
TSPYL2	969.7909	-0.9248209	0.239458	-3.86213555	0.000112	0.004465	DOWN
FABP5	363.5273	1.20802304	0.312932	3.860338254	0.000113	0.004491	UP
RP11-138I	7.692839	-3.5365806	0.916459	-3.85896168	0.000114	0.00451	DOWN

AATF	2874.8	0.55344683	0.143477	3.85738921	0.000115	0.004532	NOT
CHEK1	504.9719	0.88855144	0.230405	3.856473712	0.000115	0.004543	UP
RPL17	1234.863	0.67323495	0.174611	3.855635476	0.000115	0.004545	UP
TREM1	72.96473	2.10437591	0.545834	3.855337	0.000116	0.004545	UP
SRC	2040.726	1.06535323	0.276334	3.855306644	0.000116	0.004545	UP
PABPC3	22.48775	1.2958286	0.336203	3.854299809	0.000116	0.004557	UP
BLM	199.7241	1.14597366	0.297371	3.85368157	0.000116	0.004561	UP
HDC	46.79604	-1.6962999	0.440212	-3.85336617	0.000117	0.004561	DOWN
SEC16A	6640.134	-0.7039029	0.182698	-3.85281815	0.000117	0.004564	DOWN
NEIL3	156.8293	1.50186565	0.389844	3.852479867	0.000117	0.004564	UP
MMP10	30.075	2.2370379	0.580793	3.851695767	0.000117	0.004567	UP
HNRNPA3P3	4.771877	1.45951845	0.378937	3.851609411	0.000117	0.004567	UP
PVALB	7.683304	-2.1161429	0.549618	-3.85020682	0.000118	0.004587	DOWN
SLC26A3	105.0605	2.40090833	0.623767	3.849044028	0.000119	0.004602	UP
CTC-490E2	239.2539	-1.9518318	0.507378	-3.84689708	0.00012	0.004636	DOWN
RP11-122	5.099605	2.70907759	0.704329	3.846324504	0.00012	0.00464	UP
CHORDC1	865.9708	0.81273625	0.211459	3.843472716	0.000121	0.004688	UP
SEC24B	1992.733	-0.8022419	0.208797	-3.84220295	0.000122	0.004702	DOWN
LARP1B	1421.299	-0.8384377	0.218227	-3.84204716	0.000122	0.004702	DOWN
SLC50A1	4536.223	0.87593878	0.228022	3.841466304	0.000122	0.004706	UP
RP11-504	2.734547	-1.2178582	0.317074	-3.8409243	0.000123	0.00471	DOWN
UGT2B11	1108.785	2.01972652	0.525902	3.840496885	0.000123	0.004712	UP
PRSS56	17.20503	4.00711697	1.043494	3.840095605	0.000123	0.004713	UP
RAPGEF3	296.4986	-0.9381613	0.244351	-3.83939271	0.000123	0.004719	DOWN
DMBT1	40.15744	3.14546149	0.819463	3.838443158	0.000124	0.004719	UP
TFAP4	511.2271	0.60042746	0.156426	3.838413856	0.000124	0.004719	UP
TMEM206	264.07	0.72298957	0.188359	3.838361803	0.000124	0.004719	UP
SLC34A2	350.0263	3.2813549	0.85572	3.834613617	0.000126	0.004769	UP
FOXJ1	124.1428	2.6817308	0.699463	3.833987169	0.000126	0.004769	UP
COL2A1	726.1353	4.04091572	1.054076	3.833608304	0.000126	0.004769	UP
TNR	31.19957	-2.5467431	0.664328	-3.83356414	0.000126	0.004769	DOWN
LRRC37BP1	235.9358	0.67204395	0.175306	3.833549442	0.000126	0.004769	UP
FCN2	124.5385	-2.6235473	0.68438	-3.83346402	0.000126	0.004769	DOWN
GLP1R	65.57956	3.82142096	0.996881	3.833378243	0.000126	0.004769	UP
ADSL	2627.945	0.6576827	0.1718	3.828187064	0.000129	0.004862	UP
GHR	2933.08	-1.7187055	0.44899	-3.82793859	0.000129	0.004862	DOWN
FAM213B	1296.024	0.85572414	0.223641	3.826333915	0.00013	0.004887	UP
SLC2A1	733.5745	1.48730654	0.388782	3.825552389	0.00013	0.004894	UP
NME6	643.3766	0.50223388	0.131292	3.825330279	0.000131	0.004894	NOT
HES5	8.723019	-1.3669693	0.357465	-3.82406632	0.000131	0.004908	DOWN
PAEP	46.00765	3.0840052	0.806501	3.823931989	0.000131	0.004908	UP
CTD-2589H	44.49822	1.44889492	0.379008	3.82286022	0.000132	0.004923	UP
PCSK1N	168.8444	3.00117407	0.785159	3.822376481	0.000132	0.004926	UP
P2RX1	36.53342	-1.4050867	0.367631	-3.82200776	0.000132	0.004926	DOWN
KIF4A	865.2527	1.33837083	0.350267	3.821005833	0.000133	0.004939	UP
TMEM98	2380.039	1.21856966	0.318992	3.82006216	0.000133	0.004952	UP
RNF34	966.374	0.46632029	0.122094	3.819347447	0.000134	0.004955	NOT
MIR621	451.3313	-1.5930512	0.417113	-3.8192288	0.000134	0.004955	DOWN
UHRF1	481.0021	1.38348908	0.362421	3.817348591	0.000135	0.004984	UP

NCBP2	2507.812	0.53287875	0.139603	3.817104206	0.000135	0.004984	NOT
PPP1R1B	54.60067	2.8132596	0.737538	3.814392405	0.000137	0.005028	UP
EPO	286.0439	2.92663725	0.76735	3.813953544	0.000137	0.005028	UP
TPSAB1	169.2929	-2.3882851	0.626237	-3.81370987	0.000137	0.005028	DOWN
PRMT9	283.6845	-0.5925137	0.155384	-3.81323173	0.000137	0.005028	DOWN
RP11-596I	3.635735	-1.5598658	0.409078	-3.81312866	0.000137	0.005028	DOWN
TTC36	554.2026	-2.4041195	0.630563	-3.81265463	0.000137	0.005028	DOWN
CCL20	2085.474	2.0257947	0.531375	3.812365776	0.000138	0.005028	UP
UCN2	7.447166	2.64393608	0.693572	3.8120544	0.000138	0.005028	UP
LRRC32	1676.619	-1.2014794	0.315189	-3.81192976	0.000138	0.005028	DOWN
RBM17	2771.077	0.56604907	0.148511	3.811486489	0.000138	0.00503	NOT
DPH2	1216.706	0.66739891	0.175152	3.810398822	0.000139	0.005046	UP
RP11-38G5	3.752485	-1.8788216	0.493178	-3.80962442	0.000139	0.00505	DOWN
MAPKAPK5	1270.44	0.39062283	0.102539	3.809509457	0.000139	0.00505	NOT
CDCP1	233.6114	2.04033322	0.535671	3.808928506	0.00014	0.005055	UP
YEATS2	1023.744	0.7004051	0.183974	3.807085418	0.000141	0.005086	UP
SNRPC	4621.48	0.70983573	0.186511	3.805858158	0.000141	0.005105	UP
NAT8L	76.18241	2.22903583	0.585956	3.804103466	0.000142	0.005132	UP
SPG7	2873.56	-0.5783886	0.152052	-3.80387546	0.000142	0.005132	NOT
MAMDC2-AS	10.95174	-1.2894083	0.339075	-3.80271781	0.000143	0.005141	DOWN
LYAR	648.0867	0.70765038	0.186095	3.802635873	0.000143	0.005141	UP
KIF23	530.021	1.34598979	0.353978	3.80246362	0.000143	0.005141	UP
HNRNPC	13183.35	0.32540371	0.085605	3.801212323	0.000144	0.00516	NOT
TAF3	570.6107	0.51000517	0.134189	3.800644684	0.000144	0.005165	NOT
DUOX1	198.2761	1.5751056	0.414711	3.798076868	0.000146	0.005212	UP
LMNB1	1604.599	1.04611769	0.275579	3.796075688	0.000147	0.005242	UP
EDARADD	93.72697	2.03894008	0.537126	3.796016393	0.000147	0.005242	UP
PLOD2	5361.952	0.98773149	0.260269	3.795039017	0.000148	0.005256	UP
MT1JP	3.996769	-2.9646175	0.781792	-3.79208104	0.000149	0.005312	DOWN
BARD1	262.5675	1.05523119	0.27832	3.791427678	0.00015	0.005317	UP
SSTR3	14.32241	2.79784304	0.738011	3.791057646	0.00015	0.005317	UP
RP11-1072	5.218274	-1.2301225	0.324502	-3.79079927	0.00015	0.005317	DOWN
ZFP41	660.2602	0.8669287	0.228733	3.790137683	0.000151	0.005317	UP
MTERF3	994.6764	0.6624383	0.174799	3.789720111	0.000151	0.005317	UP
GAS2L3	424.9454	1.0444551	0.275639	3.789219762	0.000151	0.005317	UP
AC005682.	31.35007	1.68298717	0.444155	3.789192228	0.000151	0.005317	UP
WIF1	7.888951	4.62077749	1.219479	3.789139504	0.000151	0.005317	UP
PAK3	79.05778	-2.5166797	0.664222	-3.78891221	0.000151	0.005317	DOWN
SUB1	7813.962	0.61321966	0.161904	3.787542036	0.000152	0.00534	UP
TPRN	2019.875	1.08001178	0.285224	3.786536646	0.000153	0.005355	UP
AS3MT	136.1715	-1.4049272	0.371323	-3.78357607	0.000155	0.005412	DOWN
PCDHB15	65.19248	-1.2331835	0.325979	-3.78300976	0.000155	0.005417	DOWN
GAGE2A	7.974542	4.66694511	1.233862	3.782388081	0.000155	0.005419	UP
ZNF385C	72.02156	1.52429616	0.40303	3.782092873	0.000156	0.005419	UP
HERC1	2005.165	-0.8126598	0.214894	-3.78168156	0.000156	0.005419	DOWN
RP11-875(	27.49125	1.77456043	0.469259	3.78162295	0.000156	0.005419	UP
ARHGAP40	10.02337	2.80761588	0.74255	3.78104481	0.000156	0.005425	UP
SLC22A6	4.73556	4.05657804	1.073027	3.780499521	0.000157	0.00543	UP
EIF5AL1	116.9659	-1.6232143	0.429448	-3.77977083	0.000157	0.005439	DOWN

CCDC34	683.6961	0.81952307	0.216951	3.777465043	0.000158	0.005483	UP
KDELC1	590.2223	1.07699676	0.285197	3.77632566	0.000159	0.005495	UP
HAVCR1	65.69316	2.8029306	0.742289	3.776061762	0.000159	0.005495	UP
ATP1A1	29436.83	0.93669641	0.248069	3.775946741	0.000159	0.005495	UP
SIDT2	2374.978	-0.5877538	0.155671	-3.77560514	0.00016	0.005496	DOWN
DPCR1	5.309744	3.59232784	0.951911	3.7738074	0.000161	0.005523	UP
CD160	28.48372	-1.1697873	0.309981	-3.77374368	0.000161	0.005523	DOWN
PDE4C	87.61859	1.2115062	0.321189	3.771942391	0.000162	0.005556	UP
MAP3K19	4.154857	-1.8262779	0.484318	-3.77082656	0.000163	0.005574	DOWN
VSIG10	1711.577	0.65542617	0.173848	3.770104457	0.000163	0.005583	UP
SNRPG	1620.997	0.62511532	0.165847	3.769219836	0.000164	0.00559	UP
MAFG-AS1	192.2012	1.64094182	0.43536	3.769157565	0.000164	0.00559	UP
SRPR	10197.71	-0.5314889	0.141031	-3.76860318	0.000164	0.00559	NOT
BRDT	4.527462	3.62065538	0.960766	3.768509595	0.000164	0.00559	UP
LRRC4B	83.13511	-0.8889625	0.235967	-3.76731552	0.000165	0.005607	DOWN
PCDHGB2	87.30623	-1.790217	0.47522	-3.76713224	0.000165	0.005607	DOWN
ZMAT3	1321.912	-0.7879343	0.209193	-3.76654435	0.000166	0.005613	DOWN
NLRP2	59.74431	2.31298814	0.614436	3.76440587	0.000167	0.005652	UP
AP000473.	7.930939	-1.8249477	0.484825	-3.76413456	0.000167	0.005652	DOWN
CDK1	1095.012	1.24090118	0.329705	3.763671061	0.000167	0.005652	UP
RHPN1-AS1	51.88338	1.16328287	0.309089	3.763586727	0.000167	0.005652	UP
LINC0113	229.6689	0.97743107	0.259774	3.76262649	0.000168	0.00566	UP
CLSPN	159.093	1.39609994	0.371046	3.762602601	0.000168	0.00566	UP
COMMD2	1132.493	0.48118796	0.128066	3.757341401	0.000172	0.005771	NOT
TATDN1	1011.639	0.72874976	0.193966	3.757107736	0.000172	0.005771	UP
NOL7	2379.129	0.51720407	0.137676	3.75668884	0.000172	0.005773	NOT
SMYD2	3045.607	0.88992448	0.236931	3.756054955	0.000173	0.005773	UP
AP000925.	3.037077	-1.4430926	0.38423	-3.75580832	0.000173	0.005773	DOWN
CERS5	983.7231	0.55140999	0.14682	3.755694516	0.000173	0.005773	NOT
SORBS2	4572.056	-1.0259548	0.273203	-3.75528634	0.000173	0.005773	DOWN
GOLGA2P7	17.17938	1.21808279	0.324402	3.754860345	0.000173	0.005773	UP
TRIB3	5143.54	1.16017234	0.308983	3.75481254	0.000173	0.005773	UP
INPP4B	230.3328	-1.3180937	0.351063	-3.75457576	0.000174	0.005773	DOWN
A4GNT	13.56767	3.48962735	0.929591	3.753937338	0.000174	0.005781	UP
TMEM184C	1293.258	-0.6717599	0.179012	-3.75259047	0.000175	0.005805	DOWN
PDZD4	111.6006	-1.4236615	0.379412	-3.75228251	0.000175	0.005805	DOWN
TCF21	45.17416	-1.3322962	0.355189	-3.75094492	0.000176	0.005829	DOWN
LYSMD4	409.7159	0.7012265	0.187049	3.748900553	0.000178	0.005869	UP
EGLN3	636.4902	1.73076427	0.461856	3.747408644	0.000179	0.005897	UP
GRIN2D	97.61103	1.56778544	0.41847	3.746473533	0.000179	0.005912	UP
DKC1	2267.914	0.62497013	0.166903	3.744518454	0.000181	0.00595	UP
RP11-326	55.70263	-1.8169155	0.485285	-3.74401806	0.000181	0.00595	DOWN
ENO1	54647.99	0.9071946	0.242317	3.743826259	0.000181	0.00595	UP
WFS1	2659.753	-0.9097193	0.243004	-3.74364014	0.000181	0.00595	DOWN
VPS13D	2165.869	-0.6149649	0.164284	-3.74330556	0.000182	0.005951	DOWN
SP8	23.11068	3.98646864	1.065273	3.742204285	0.000182	0.00597	UP
KCNN3	180.1326	-0.8861845	0.236874	-3.74117079	0.000183	0.005983	DOWN
RP11-468	3.594133	-1.5132834	0.404508	-3.74104602	0.000183	0.005983	DOWN
KRT20	41.4568	2.96274745	0.792191	3.739939312	0.000184	0.006002	UP

AC114271.	40.64162	-0.8173295	0.218582	-3.7392275	0.000185	0.006012	DOWN
PAGE2B	6.950306	3.00630924	0.804079	3.738825322	0.000185	0.006014	UP
GRIN2C	32.06572	-1.014636	0.271448	-3.73786007	0.000186	0.006024	DOWN
RP11-437I	1.70301	-2.708776	0.724695	-3.73781707	0.000186	0.006024	DOWN
ITPKB	568.1847	-0.9167923	0.245376	-3.73627933	0.000187	0.006054	DOWN
GALNTL6	28.78297	-2.951775	0.79031	-3.73495636	0.000188	0.006078	DOWN
RP11-44F2	8.452534	3.37774885	0.904721	3.733468232	0.000189	0.006107	UP
C5orf34	101.9911	0.88292042	0.236522	3.732929433	0.000189	0.006113	UP
ZNF846	208.6367	-0.7399264	0.198321	-3.7309458	0.000191	0.006154	DOWN
GAS6-AS1	252.5388	-1.6954429	0.4545	-3.73034419	0.000191	0.006161	DOWN
PLK1	926.9429	1.26316376	0.338775	3.728626158	0.000193	0.006184	UP
RP11-626I	2.799951	-1.4879241	0.399093	-3.72826158	0.000193	0.006184	DOWN
RP11-517I	2.559445	-2.0868905	0.559749	-3.72825883	0.000193	0.006184	DOWN
MMACHC	1280.254	-0.6273622	0.168273	-3.72823791	0.000193	0.006184	DOWN
NUCB1	24903.83	-0.667195	0.178989	-3.7275708	0.000193	0.006193	DOWN
SLC8A3	8.329742	-1.5431965	0.414178	-3.72592719	0.000195	0.006226	DOWN
KIAA0196	2504.687	0.68974269	0.185299	3.722330674	0.000197	0.006308	UP
RBM45	373.0951	0.3544083	0.095244	3.721050557	0.000198	0.006332	NOT
TYK2	3538.455	-0.4663597	0.125405	-3.71882487	0.0002	0.006377	NOT
ZBED1	3608.612	-0.5791096	0.155739	-3.71845409	0.0002	0.006377	NOT
HAND2-AS1	50.89658	-2.2578506	0.607215	-3.71837049	0.000201	0.006377	DOWN
OLFM3	9.570785	4.16676389	1.12082	3.717604121	0.000201	0.006389	UP
OSBP	4893.955	-0.5353094	0.144061	-3.71586032	0.000203	0.006426	NOT
SKA1	395.3307	1.30749925	0.352131	3.71310815	0.000205	0.006489	UP
CYP2S1	229.7968	1.71357661	0.461565	3.712531909	0.000205	0.006491	UP
GPR1-AS	8.437296	4.55180602	1.226103	3.712418796	0.000205	0.006491	UP
UNG	2322.207	0.59927052	0.161437	3.712098352	0.000206	0.006492	UP
PUS7	677.8192	0.68147481	0.183636	3.711007945	0.000206	0.006512	UP
MYO1H	6.56474	-1.5415081	0.415616	-3.70897593	0.000208	0.006555	DOWN
CTD-2199(	7.475422	1.67136492	0.450651	3.708779501	0.000208	0.006555	UP
SNHG4	59.49284	1.50225182	0.405163	3.707772983	0.000209	0.006573	UP
RP4-640H8	13.45128	2.8022531	0.755836	3.707489216	0.000209	0.006573	UP
LINC0031	21.29964	-2.1125859	0.569862	-3.70719062	0.00021	0.006573	DOWN
ENOPH1	1286.249	0.51071077	0.137781	3.70667867	0.00021	0.006579	NOT
RP11-400I	3.146376	-1.2661076	0.341657	-3.70578293	0.000211	0.006594	DOWN
TMEM101	1941.275	0.6358745	0.171618	3.705172113	0.000211	0.006603	UP
AC006538.	22.72348	1.63153658	0.440594	3.703038453	0.000213	0.006646	UP
CCT7P2	4.171107	4.04270052	1.091759	3.702923036	0.000213	0.006646	UP
MCM8	550.3335	0.88256678	0.238414	3.701824965	0.000214	0.006658	UP
RPL36A	2452.563	0.96054696	0.25949	3.701671994	0.000214	0.006658	UP
CRYBB1	22.91281	-1.2328708	0.333063	-3.70161121	0.000214	0.006658	DOWN
CDC37L1	1303.76	-0.7662351	0.207087	-3.70005717	0.000216	0.006689	DOWN
GDF7	533.646	-1.526021	0.412456	-3.69984166	0.000216	0.006689	DOWN
RP11-332I	18.42508	-1.3789628	0.372779	-3.69914004	0.000216	0.006693	DOWN
NRM	942.3783	1.16989931	0.316265	3.69911061	0.000216	0.006693	UP
YBX1P1	28.28191	1.11181625	0.300591	3.69877166	0.000217	0.006694	UP
CXCL1	444.288	2.26682771	0.613032	3.697731978	0.000218	0.00671	UP
RGCC	513.6801	-0.7504419	0.202954	-3.69759562	0.000218	0.00671	DOWN
FNDC3A	3421.04	-0.8903098	0.240815	-3.69707295	0.000218	0.006716	DOWN

SLC25A24I	6.271623	4.31184198	1.166684	3.695810054	0.000219	0.006742	UP
KB-1980E6	2.495898	3.47208759	0.940109	3.693282614	0.000221	0.006802	UP
SQLE	6134.496	1.18876121	0.321917	3.692755896	0.000222	0.006808	UP
RP3-342P2	24.78429	-2.0557062	0.556829	-3.6918073	0.000223	0.006826	DOWN
CRLF1	75.13801	-1.2126043	0.328578	-3.69045991	0.000224	0.006854	DOWN
ZNF696	429.048	0.63824047	0.17301	3.689032699	0.000225	0.006885	UP
RP11-552C	4.825662	3.24838116	0.88074	3.688241138	0.000226	0.006896	UP
DNASE1L3	594.9786	-1.4743658	0.399767	-3.68805868	0.000226	0.006896	DOWN
PDRG1	1064.317	0.60647899	0.164509	3.686590216	0.000227	0.006928	UP
RP11-927I	4.683853	-1.4257453	0.38677	-3.68628587	0.000228	0.006929	DOWN
RRP7B	152.2838	0.74593432	0.20241	3.685264428	0.000228	0.006949	UP
ELMOD1	6.853714	-2.3412896	0.635478	-3.68429833	0.000229	0.006967	DOWN
RP11-73K9	26.13108	-1.0659334	0.289404	-3.68320388	0.00023	0.006989	DOWN
FDXR	1491.365	-0.9159141	0.248717	-3.68255487	0.000231	0.006999	DOWN
USHBP1	157.5934	-0.7641694	0.207631	-3.68041378	0.000233	0.007051	DOWN
PTPRB	878.5151	-0.9809663	0.266661	-3.67870222	0.000234	0.00709	DOWN
KIT	142.955	-1.2342024	0.335575	-3.67787314	0.000235	0.007105	DOWN
PTMA	19788.15	0.50932348	0.138539	3.676386972	0.000237	0.007139	NOT
CCNB1	1589.066	1.13959452	0.310038	3.675659711	0.000237	0.007151	UP
RP11-274I	48.58254	1.12947361	0.307331	3.675103403	0.000238	0.007159	UP
RP11-168I	54.25668	-2.1224838	0.577621	-3.67452477	0.000238	0.007167	DOWN
RP11-474I	3.637083	-1.6586531	0.451483	-3.67378883	0.000239	0.00718	DOWN
LINC00925	33.24341	2.03539195	0.554105	3.673293597	0.000239	0.007186	UP
PPIAP21	7.230165	1.51813603	0.413373	3.672558089	0.00024	0.00719	UP
RAD51AP1	283.134	1.17043641	0.318708	3.672440822	0.00024	0.00719	UP
BCL2L14	67.56567	1.84694011	0.502942	3.67227395	0.00024	0.00719	UP
RP11-863I	23.16245	-1.5408667	0.419664	-3.67166891	0.000241	0.00719	DOWN
PKMP3	10.82922	-1.1681556	0.318166	-3.6715242	0.000241	0.00719	DOWN
RP11-7F17	8.259118	-1.8017857	0.490825	-3.6709338	0.000242	0.00719	DOWN
TPT1P12	3.28401	-1.8124024	0.493718	-3.67092391	0.000242	0.00719	DOWN
KLHL8	810.6734	-0.8834974	0.240676	-3.6708999	0.000242	0.00719	DOWN
FAR2P4	13.98628	3.77931196	1.02965	3.670482331	0.000242	0.007193	UP
FHOD3	221.2647	2.23610538	0.609292	3.670007739	0.000243	0.007193	UP
ADAD2	3.70573	-1.4553785	0.396567	-3.66994387	0.000243	0.007193	DOWN
LY6K	34.53154	2.43769626	0.664644	3.667670181	0.000245	0.00725	UP
WNT4	469.9649	-2.236499	0.609854	-3.66726817	0.000245	0.007253	DOWN
RP11-44N1	39.94169	1.4285425	0.389671	3.666019572	0.000246	0.007281	UP
TCEB1	2682.109	0.76588151	0.208948	3.66542394	0.000247	0.007288	UP
RP11-337N	26.05955	0.87278913	0.238142	3.664993839	0.000247	0.007288	UP
PQLC2L	7.167432	2.62106555	0.715176	3.664922817	0.000247	0.007288	UP
C1R	52921.2	-1.1435132	0.312069	-3.66429475	0.000248	0.007298	DOWN
GTSF1	288.5403	2.87245729	0.784015	3.663780375	0.000249	0.007305	UP
DDX11-AS1	28.48243	1.09233887	0.298215	3.662918961	0.000249	0.007314	UP
CCT7	9690.263	0.48150406	0.131454	3.662898062	0.000249	0.007314	NOT
GNL3	3088.779	0.53982476	0.147497	3.659898933	0.000252	0.007392	NOT
DAXX	2748.126	0.45716498	0.124941	3.659046001	0.000253	0.007409	NOT
PRR11	495.5546	1.19493822	0.326609	3.658616074	0.000254	0.007413	UP
TPRG1	194.6335	-1.4492035	0.396145	-3.65826629	0.000254	0.007416	DOWN
RP11-236I	7.816098	-1.4879095	0.40697	-3.65606852	0.000256	0.007471	DOWN

BUB1	594.329	1.16181119	0.317815	3.655622908	0.000257	0.007476	UP
TEX19	17.67892	2.05672012	0.562686	3.655179825	0.000257	0.007481	UP
NDUFA13	2204.124	-1.0714636	0.293171	-3.65473944	0.000257	0.007486	DOWN
FHL3	687.8632	0.89042563	0.243658	3.654409102	0.000258	0.007488	UP
ENG	4261.141	-0.9740262	0.266585	-3.65371489	0.000258	0.0075	DOWN
CTD-2147F	12.34396	4.11869547	1.127441	3.653137541	0.000259	0.007509	UP
NAA20	3682.613	0.57031194	0.156148	3.652385037	0.00026	0.007523	NOT
MRPL13	2587.679	0.79293874	0.21712	3.652076435	0.00026	0.007524	UP
LINC01537	13.39674	-1.5114902	0.41398	-3.6511204	0.000261	0.007544	DOWN
CPEB3	602.7851	-1.207318	0.330766	-3.65006915	0.000262	0.007567	DOWN
CDC7	312.0984	1.03696016	0.284138	3.64949073	0.000263	0.007576	UP
AOC1	250.2296	2.39980081	0.657763	3.64842573	0.000264	0.007599	UP
CYP46A1	47.27969	-1.2221497	0.335012	-3.64807359	0.000264	0.007601	DOWN
TREML3P	54.08061	3.0870737	0.846311	3.647683553	0.000265	0.007605	UP
FBXW7	622.4611	-0.5320673	0.145876	-3.64740489	0.000265	0.007605	NOT
TRIM2	1088.443	-1.0218879	0.280211	-3.64684519	0.000265	0.007614	DOWN
CTB-51J22	17.3152	-1.8609707	0.510448	-3.64576154	0.000267	0.007638	DOWN
XRCC3	460.8651	0.76045691	0.208618	3.64521226	0.000267	0.007644	UP
RNU6-8	16.83838	1.46779786	0.402703	3.644866915	0.000268	0.007644	UP
CYP26B1	307.2261	2.24479173	0.615901	3.644727065	0.000268	0.007644	UP
WDR62	473.1868	1.01926091	0.279687	3.644291625	0.000268	0.007649	UP
MPZL1	4202.034	0.72262659	0.198354	3.643108409	0.000269	0.007676	UP
IER3IP1	2154.906	0.42542164	0.116838	3.641125182	0.000271	0.00772	NOT
CPA3	156.0817	-2.1909711	0.601733	-3.64110095	0.000271	0.00772	DOWN
NPM1P6	14.93181	1.00479971	0.275985	3.640781539	0.000272	0.007722	UP
HSPH1	3386.493	0.65871986	0.180974	3.639860389	0.000273	0.007736	UP
SLC4A10	9.833271	-1.7191673	0.472401	-3.6392111	0.000273	0.007736	DOWN
GABARAPL1	3999.2	-1.0875233	0.298843	-3.63911035	0.000274	0.007736	DOWN
RP11-92K1	19.77896	1.34248542	0.368928	3.638883778	0.000274	0.007736	UP
DBH	271.6965	-1.5962597	0.438679	-3.63878743	0.000274	0.007736	DOWN
C2orf27A	70.80142	1.4277076	0.392401	3.638392905	0.000274	0.007736	UP
RPL30	32352.77	0.80663631	0.221714	3.638189354	0.000275	0.007736	UP
UTP18	1458.715	0.51433662	0.141373	3.638143992	0.000275	0.007736	NOT
BICC1	1459.84	2.0638127	0.567409	3.637255279	0.000276	0.007754	UP
APBA1	942.3363	-1.5125042	0.41587	-3.63696079	0.000276	0.007754	DOWN
RP4-737E2	3.85724	-1.1754465	0.323214	-3.6367439	0.000276	0.007754	DOWN
FAM167B	258.6806	-0.9502109	0.261328	-3.63608898	0.000277	0.007762	DOWN
PKIA	51.41057	1.99380709	0.548356	3.635975318	0.000277	0.007762	UP
MTRNR2L10	7.296113	-1.3901406	0.382418	-3.63513707	0.000278	0.007767	DOWN
NPM1P39	10.5167	1.17841444	0.324174	3.635134668	0.000278	0.007767	UP
FSIP1	16.51074	1.59851287	0.439758	3.634980016	0.000278	0.007767	UP
PRDM15	456.8351	-0.852514	0.234586	-3.63412901	0.000279	0.007785	DOWN
AC003956.	3.052138	-1.7765311	0.488928	-3.6335204	0.00028	0.007795	DOWN
RP11-505K	24.16748	-1.2869575	0.354288	-3.6325154	0.000281	0.007818	DOWN
FLJ20021	168.8655	-0.9559461	0.263184	-3.63222933	0.000281	0.007819	DOWN
UGT8	31.05564	3.03299198	0.835224	3.631350693	0.000282	0.007831	UP
PLCXD1	2195.563	-1.3297903	0.366204	-3.63127904	0.000282	0.007831	DOWN
SASS6	243.0057	0.8472252	0.233337	3.630915645	0.000282	0.007834	UP
CCT4	6636.445	0.54199134	0.149293	3.630380759	0.000283	0.007843	NOT

MFSD8	607.8973	-0.5895661	0.162421	-3.62986004	0.000284	0.00785	DOWN
TUBA1B	5962.169	0.82856499	0.228326	3.628864926	0.000285	0.007869	UP
DYNC1LI1	1354.374	0.47493821	0.130883	3.62872855	0.000285	0.007869	NOT
OIP5	135.5385	1.10982205	0.305924	3.627772702	0.000286	0.007883	UP
SNRPGP2	82.42492	0.66965606	0.184594	3.627722311	0.000286	0.007883	UP
RP13-20L1	9.857196	-1.3041935	0.359695	-3.62582914	0.000288	0.007933	DOWN
TRNP1	1240.372	1.6982752	0.468505	3.624885259	0.000289	0.007954	UP
CLEC4G	71.22524	-2.3059394	0.636184	-3.62464257	0.000289	0.007954	DOWN
EDEM1	3904.248	-0.7477057	0.206371	-3.6231175	0.000291	0.00799	DOWN
CCDC58	941.2996	0.64968627	0.179326	3.622943225	0.000291	0.00799	UP
RP11-100(	9.26052	-1.2711722	0.351068	-3.62086708	0.000294	0.008044	DOWN
CTD-3037(	6.041392	2.65215322	0.732501	3.62068408	0.000294	0.008044	UP
SRSF3	7327.671	0.37013511	0.102264	3.619410565	0.000295	0.00807	NOT
PTK2	4444.012	0.61939237	0.171135	3.619320018	0.000295	0.00807	UP
EREG	29.22325	2.7785704	0.768074	3.617583102	0.000297	0.008116	UP
STX3	948.7877	0.81227032	0.224576	3.616908766	0.000298	0.008129	UP
GPR37L1	34.79817	1.2902359	0.356769	3.616447362	0.000299	0.008133	UP
C4BPAP1	3.120406	-1.7110208	0.473145	-3.61626933	0.000299	0.008133	DOWN
NUP62	2489.925	0.53979535	0.149331	3.614763731	0.000301	0.008172	NOT
POF1B	317.7289	2.45355044	0.678899	3.61401437	0.000301	0.008185	UP
CDIPT-AS1	10.76701	1.85937788	0.514515	3.613849228	0.000302	0.008185	UP
IL12A	17.22872	1.61442546	0.447005	3.611650325	0.000304	0.008246	UP
CENPM	444.4015	1.23663656	0.342434	3.611314779	0.000305	0.008249	UP
RINL	544.1774	0.96038068	0.26598	3.610720711	0.000305	0.008252	UP
HDAC11	1400.033	0.87093146	0.241209	3.610692163	0.000305	0.008252	UP
TRIM17	47.46456	2.14254905	0.593504	3.610001859	0.000306	0.008261	UP
CHD9	1161.052	-0.6691479	0.185365	-3.60988834	0.000306	0.008261	DOWN
GADD45A	3078.327	-0.9690827	0.268603	-3.60786448	0.000309	0.008311	DOWN
FZD4	2022.544	-0.9302638	0.257847	-3.60781941	0.000309	0.008311	DOWN
DSC2	831.1674	1.0977076	0.304303	3.607284919	0.000309	0.00832	UP
RASGEF1A	158.0818	2.04166713	0.566265	3.60550054	0.000312	0.008369	UP
CYP2U1	373.7712	-0.9151362	0.253867	-3.6047855	0.000312	0.008383	DOWN
TMEM14B	2882.37	0.62692225	0.173991	3.603179655	0.000314	0.008427	UP
LINC0054(	11.78114	2.96684543	0.823546	3.602526962	0.000315	0.00844	UP
CTA-989H1	22.22996	0.9054298	0.251392	3.601668212	0.000316	0.00846	UP
SRSF9	2875.048	0.50820497	0.141114	3.601376844	0.000317	0.008461	NOT
CNTN1	213.9622	-3.4729259	0.964648	-3.6001993	0.000318	0.008491	DOWN
CTD-2008I	16.45236	3.13989224	0.872252	3.599753048	0.000319	0.008497	UP
GFY	3.577154	3.46658143	0.963084	3.599460541	0.000319	0.008498	UP
ADAMTSL3	787.5817	-1.3293564	0.369365	-3.59903504	0.000319	0.008504	DOWN
CLEC4GP1	2.820641	-3.0204476	0.839316	-3.59870328	0.00032	0.008506	DOWN
POGK	3006.376	0.70028688	0.194627	3.598095454	0.000321	0.008518	UP
NUF2	487.2406	1.28758623	0.357911	3.5975063	0.000321	0.008529	UP
CTD-2201I	29.18197	-1.0182219	0.283352	-3.59348888	0.000326	0.008653	DOWN
KCNMA1	451.6713	-1.345818	0.374695	-3.5917731	0.000328	0.008699	DOWN
XPOT	4138.621	0.55068628	0.153326	3.591606092	0.000329	0.008699	NOT
CDCA8	667.9964	1.1528532	0.321161	3.589645063	0.000331	0.008756	UP
CTC-458G(	2.817873	4.17049236	1.161947	3.589226876	0.000332	0.00876	UP
TMEM88	146.1251	-1.0786561	0.300544	-3.58901694	0.000332	0.00876	DOWN

PARD3	2654.383	0.64426454	0.179541	3.588389647	0.000333	0.008772	UP
OGG1	1303.379	0.63492147	0.176973	3.58768153	0.000334	0.008788	UP
RP11-12J1	6.450881	-1.3810791	0.384978	-3.58742332	0.000334	0.008788	DOWN
GPRIN3	604.4041	-1.2608939	0.351549	-3.58668363	0.000335	0.008804	DOWN
SSRP1	5353.336	0.43734869	0.121985	3.585255698	0.000337	0.008833	NOT
DENR	2521.987	0.41387212	0.115441	3.58514231	0.000337	0.008833	NOT
RAP1A	2684.212	0.44700994	0.124697	3.584773438	0.000337	0.008833	NOT
SOCS2-AS1	92.34363	-1.4583981	0.406859	-3.58453337	0.000338	0.008833	DOWN
PNMA1	804.5767	0.8734157	0.24367	3.584419265	0.000338	0.008833	UP
MAMDC2	38.81781	-1.6853834	0.470211	-3.58431494	0.000338	0.008833	DOWN
AC005083.	25.32774	-2.0526941	0.57279	-3.58367536	0.000339	0.008841	DOWN
IL4I1	236.2484	1.50146251	0.418983	3.583584056	0.000339	0.008841	UP
FANCD2	503.1073	0.97371519	0.271832	3.582044515	0.000341	0.008885	UP
LRP8	138.597	1.58645127	0.442944	3.581602993	0.000341	0.008891	UP
RP11-92C4	4.732107	-1.4262784	0.398263	-3.58124466	0.000342	0.008895	DOWN
HAT1	1343.549	0.52602314	0.146941	3.579834305	0.000344	0.008935	NOT
HTRA2	1003.437	0.47640394	0.133096	3.579395971	0.000344	0.008935	NOT
SHC4	13.28846	-1.398124	0.390611	-3.57932416	0.000344	0.008935	DOWN
ABCA12	10.58684	2.00717301	0.560821	3.578991852	0.000345	0.008937	UP
KIF18A	219.9751	1.25902358	0.351803	3.57877148	0.000345	0.008937	UP
IQGAP3	1186.883	1.1272865	0.315036	3.578282669	0.000346	0.008944	UP
C19orf48	2877.397	0.91050046	0.254468	3.578053825	0.000346	0.008944	UP
AC006126.	44.1095	-1.1275001	0.31518	-3.57732448	0.000347	0.008948	DOWN
KHDRBS3	834.8537	0.96079099	0.268578	3.577322859	0.000347	0.008948	UP
YEATS4	540.4489	0.54020034	0.151017	3.57708881	0.000347	0.008948	NOT
PRAC2	5.335692	4.39047648	1.227439	3.576941839	0.000348	0.008948	UP
SPTSSB	34.99615	2.00939045	0.5618	3.576703453	0.000348	0.008948	UP
ENY2	3701.804	0.6878327	0.192331	3.576290685	0.000349	0.008954	UP
RFC4	946.5346	0.83679268	0.234032	3.575555633	0.000349	0.00897	UP
WDR76	415.5673	1.05538587	0.295242	3.574641998	0.000351	0.008993	UP
SPATS2	1054.17	0.69371546	0.194097	3.574060184	0.000351	0.008996	UP
PRTFDC1	209.5817	1.27671484	0.357217	3.574055812	0.000351	0.008996	UP
UTP23	743.1284	0.55253792	0.154686	3.572003373	0.000354	0.00905	NOT
PTTG1	1127.418	1.23687959	0.346288	3.571828016	0.000354	0.00905	UP
OXCT1	263.7455	1.60761683	0.450092	3.571753374	0.000355	0.00905	UP
MIF-AS1	57.37869	-1.2211527	0.341968	-3.57096086	0.000356	0.009069	DOWN
STAT4	231.9421	-1.1988347	0.335898	-3.56904433	0.000358	0.009124	DOWN
ANXA2P2	151.5053	1.12659749	0.315672	3.568886004	0.000359	0.009124	UP
PROM2	40.33475	1.91929167	0.538046	3.567153643	0.000361	0.009173	UP
CLVS2	15.14457	4.39302923	1.231579	3.566989341	0.000361	0.009173	UP
RBM14	1880.031	0.32371923	0.090807	3.564903086	0.000364	0.009231	NOT
ZFP69B	58.33805	0.79361582	0.222622	3.564851089	0.000364	0.009231	UP
GFRA3	15.46297	2.70166983	0.758721	3.560823125	0.00037	0.009365	UP
TOMM5	889.6938	0.57480172	0.161476	3.559665245	0.000371	0.009398	NOT
PKMYT1	408.6455	1.05476233	0.296341	3.559287369	0.000372	0.009402	UP
RP11-290F	52.92514	-1.4040383	0.394576	-3.55834658	0.000373	0.009427	DOWN
CCDC59	806.2462	0.44625571	0.125442	3.557469011	0.000374	0.009433	NOT
DCUN1D3	483.0698	-0.6183196	0.173813	-3.55738729	0.000375	0.009433	DOWN
B4GALT3	2094.993	0.54555407	0.153362	3.557288081	0.000375	0.009433	NOT

APMAP	14499.09	-0.7863573	0.221072	-3.55702389	0.000375	0.009433	DOWN
LBR	4756.372	0.62550936	0.175857	3.556930298	0.000375	0.009433	UP
TNFRSF11A	223.0804	1.46940324	0.413133	3.556731688	0.000375	0.009433	UP
SLC52A3	136.5634	1.77426611	0.498998	3.555658357	0.000377	0.009462	UP
CCDC64B	27.71137	2.56646614	0.722202	3.553670326	0.00038	0.009522	UP
SETD8	877.7471	0.49890854	0.140406	3.553339001	0.00038	0.009522	NOT
RBM5-AS1	4.017736	-1.4082954	0.396336	-3.55328291	0.00038	0.009522	DOWN
PRKRA	904.6873	0.44320683	0.124749	3.552792711	0.000381	0.009531	NOT
PDE6B	87.94977	1.6784046	0.472491	3.552245461	0.000382	0.009537	UP
HSPA14	868.1773	0.54760209	0.154161	3.552150163	0.000382	0.009537	NOT
AC093724.	23.47301	1.33154183	0.374881	3.551905677	0.000382	0.009537	UP
PNMA5	8.52599	2.39352604	0.674189	3.550228502	0.000385	0.009578	UP
LINC0101	5.079671	2.64310812	0.744526	3.550054404	0.000385	0.009578	UP
SESTD1	1377.993	0.63431163	0.178679	3.550001754	0.000385	0.009578	UP
NME2	35.60826	1.07647095	0.303256	3.549708971	0.000386	0.009578	UP
C15orf41	426.903	0.66585148	0.187598	3.549357386	0.000386	0.009578	UP
CKAP4	7131.605	0.79131664	0.222948	3.549333271	0.000386	0.009578	UP
BZW2	1646.383	0.65434127	0.184403	3.548427318	0.000388	0.009602	UP
MIR99AHG	146.8731	-1.3628477	0.384147	-3.54772617	0.000389	0.009619	DOWN
CTD-2116	4.963217	1.82485454	0.514477	3.54700574	0.00039	0.009636	UP
CASP2	1049.483	0.67888568	0.191414	3.546679381	0.00039	0.009639	UP
ATP10B	35.87946	2.58345302	0.728494	3.546292467	0.000391	0.009645	UP
LINC0129	5.51501	4.62202583	1.304212	3.543921497	0.000394	0.009723	UP
STRA6	54.4076	2.24618311	0.634279	3.541316099	0.000398	0.009811	UP
GPATCH4	1979.621	0.68145106	0.192544	3.539195815	0.000401	0.009876	UP
MED30	468.6079	0.71037077	0.200722	3.539080364	0.000402	0.009876	UP
RP11-314	3.948181	4.31525007	1.219403	3.5388219	0.000402	0.009877	UP
RP11-830	27.73094	-2.3094213	0.652652	-3.53851962	0.000402	0.009879	DOWN
GPR182	26.44174	-1.6848295	0.476256	-3.53765508	0.000404	0.009903	DOWN
DDI2	2962.998	-0.729889	0.206355	-3.53706115	0.000405	0.009916	DOWN
CDK5R2	3.512408	3.97243182	1.123482	3.53582222	0.000407	0.009954	UP
ST20-AS1	208.2006	-0.9041462	0.255739	-3.53542529	0.000407	0.00996	DOWN
RNU6ATAC	13.44872	-1.4828261	0.419484	-3.53487921	0.000408	0.009971	DOWN
RP11-448	7.081916	-1.4356213	0.406177	-3.53447096	0.000409	0.009978	DOWN
KNSTRN	644.1351	0.635696	0.179886	3.533875105	0.00041	0.009991	UP
CAPG	1875.005	1.20537784	0.341125	3.53353627	0.00041	0.009992	UP
RP5-905G	3.467835	3.22546295	0.912858	3.533367059	0.00041	0.009992	UP
XXyac-YX	2.250477	2.94205068	0.832768	3.532857779	0.000411	0.010003	UP
KCNK10	8.111014	2.8123549	0.796204	3.532202328	0.000412	0.010019	UP
REEP4	857.2316	0.72799006	0.206166	3.531089331	0.000414	0.010032	UP
VWF	4712.696	-1.152491	0.326395	-3.53097371	0.000414	0.010032	DOWN
KIAA0930	4096.483	0.73754677	0.20889	3.530789445	0.000414	0.010032	UP
IKBKE	408.9073	1.11941304	0.317048	3.530737235	0.000414	0.010032	UP
HSPD1	29713.06	0.6619995	0.1875	3.530663725	0.000415	0.010032	UP
VLDLR-AS	10.20301	2.01706627	0.571471	3.529601112	0.000416	0.010063	UP
PPARG	1084.147	0.89441388	0.253439	3.529109071	0.000417	0.010073	UP
AP000892.	62.94176	-1.2259708	0.347504	-3.52793487	0.000419	0.010109	DOWN
C11orf71	601.1066	-0.7261171	0.205874	-3.52699027	0.00042	0.010136	DOWN
RP11-208	13.12239	2.60973569	0.739987	3.526730903	0.000421	0.010137	UP

PDE6C	20.11245	-1.1158384	0.316507	-3.52547524	0.000423	0.010176	DOWN
RP11-540A	23.49044	1.17279241	0.332808	3.523926777	0.000425	0.010224	UP
METTL6	385.8367	0.44680781	0.126804	3.523597149	0.000426	0.010224	NOT
FPGT-TNNI	8.466605	-1.2122008	0.344049	-3.52333967	0.000426	0.010224	DOWN
CLEC1B	18.08576	-2.7372172	0.776892	-3.52328982	0.000426	0.010224	DOWN
HAUS1	478.0873	0.69810941	0.198174	3.522711478	0.000427	0.010237	UP
RP11-205M	22.01589	-2.5130446	0.713436	-3.52245099	0.000428	0.010238	DOWN
UPF3B	575.8465	0.56925954	0.161619	3.522226263	0.000428	0.010238	NOT
ADORA2B	72.52323	1.55619159	0.441867	3.521853518	0.000429	0.010243	UP
CTD-2184C	19.79534	-1.2246038	0.347748	-3.52152969	0.000429	0.010247	DOWN
KCTD17	834.9498	1.41143784	0.400958	3.520168118	0.000431	0.010281	UP
MAMSTR	117.1858	1.45501486	0.413342	3.520127005	0.000431	0.010281	UP
SAMD13	27.47729	1.23510477	0.350888	3.519942499	0.000432	0.010281	UP
RALGPS1	333.4474	-1.2243125	0.347883	-3.51932628	0.000433	0.010296	DOWN
TTLL10	3.420343	-1.2953455	0.36811	-3.51890819	0.000433	0.010303	DOWN
FZD7	319.2899	1.51615081	0.431022	3.517571967	0.000436	0.010346	UP
TMOD1	536.3143	-1.4583586	0.414785	-3.51593693	0.000438	0.010397	DOWN
B3GNT3	838.52	1.93944212	0.551635	3.515804928	0.000438	0.010397	UP
CTD-3025N	54.9656	0.85305246	0.242675	3.515208431	0.000439	0.010411	UP
TTC39C-AS	18.13681	-1.6787086	0.477619	-3.51474113	0.00044	0.010421	DOWN
VPS26B	3091.991	-0.4822912	0.137236	-3.51430789	0.000441	0.010421	NOT
DNAJB13	11.69729	1.97452381	0.56187	3.514199407	0.000441	0.010421	UP
GPR4	377.0744	-0.725778	0.20654	-3.51398663	0.000441	0.010421	DOWN
RBM19	1505.975	0.46310239	0.131795	3.513797761	0.000442	0.010421	NOT
CASC8	5.918481	2.42808641	0.691116	3.513285995	0.000443	0.010432	UP
HAUS5	788.2975	0.60693748	0.172818	3.511996018	0.000445	0.010474	UP
RP11-314P	13.71094	3.79909956	1.08185	3.511669231	0.000445	0.010478	UP
GAGE1	6.237142	4.63152032	1.319462	3.510157687	0.000448	0.010522	UP
COLEC10	78.12201	-1.8956147	0.540048	-3.51008323	0.000448	0.010522	DOWN
GUCY1A2	249.6574	-0.921735	0.262757	-3.50793657	0.000452	0.010598	DOWN
AP000695.	39.82999	-1.2912699	0.368261	-3.5063979	0.000454	0.010651	DOWN
GABRR1	2.207633	2.47402467	0.705647	3.506038322	0.000455	0.010656	UP
TADA2B	1505.657	-0.4628124	0.132033	-3.50527167	0.000456	0.010662	NOT
SLC26A6	1343.937	0.82184779	0.234464	3.505215091	0.000456	0.010662	UP
RP11-341D	7.05148	2.17182217	0.61966	3.504858712	0.000457	0.010662	UP
HBB	844.0912	-1.5919351	0.454213	-3.50482355	0.000457	0.010662	DOWN
PLEKHJ1	2415.053	0.69847694	0.199295	3.504737422	0.000457	0.010662	UP
MYO3B	31.58951	1.98039603	0.565298	3.503279696	0.00046	0.010711	UP
NCAPG2	847.6616	0.86748062	0.247698	3.502170835	0.000461	0.010742	UP
RP11-513C	11.24013	-1.3355111	0.38135	-3.50206539	0.000462	0.010742	DOWN
DUOXA1	9.634012	1.93503772	0.552636	3.501468508	0.000463	0.010755	UP
CNNM3	2494.487	-0.5663507	0.161773	-3.50090228	0.000464	0.010755	NOT
NAP1L1	9939.019	0.64828166	0.185179	3.500835387	0.000464	0.010755	UP
ESR1	344.9083	-1.7199574	0.491313	-3.50073727	0.000464	0.010755	DOWN
ZNF391	58.90118	1.55658565	0.444662	3.500600689	0.000464	0.010755	UP
ZNF860	9.171939	1.9487158	0.556845	3.499568894	0.000466	0.010787	UP
STEAP1B	17.28941	1.74191258	0.497825	3.499048681	0.000467	0.010799	UP
LAIR2	9.903697	1.4510393	0.414898	3.497339387	0.00047	0.010847	UP
RP11-30P6	412.5548	1.76637137	0.505072	3.497264363	0.00047	0.010847	UP

NEU1	6905.776	0.70697608	0.202155	3.497191579	0.00047	0.010847	UP
ANKRD7	4.017528	3.34708436	0.957291	3.496413487	0.000472	0.010864	UP
SNX25P1	31.65055	1.17675444	0.336569	3.496323574	0.000472	0.010864	UP
CGREF1	1625.189	1.42542951	0.407821	3.495229463	0.000474	0.010888	UP
RP11-80I1	3.845991	-1.193644	0.341521	-3.49508011	0.000474	0.010888	DOWN
PAQR4	492.0981	1.13099306	0.323598	3.49506026	0.000474	0.010888	UP
AXDND1	11.20418	1.54914985	0.443333	3.494328277	0.000475	0.010908	UP
TMPRSS4	13.16417	1.94905106	0.55788	3.493676182	0.000476	0.010918	UP
FTX	40.39737	-0.684395	0.195898	-3.49363562	0.000476	0.010918	DOWN
FLJ36000	2.745676	3.96747614	1.135786	3.493153156	0.000477	0.010929	UP
CHML	857.0604	1.01717387	0.29124	3.492565988	0.000478	0.010944	UP
PEBP4	62.12968	-2.9938652	0.857597	-3.49099296	0.000481	0.010999	DOWN
PER1	2733.078	-0.8784095	0.251673	-3.49027448	0.000483	0.011019	DOWN
MYB	45.82152	1.55807813	0.446465	3.489812925	0.000483	0.011029	UP
NMRK1	955.0811	-0.6209136	0.178105	-3.48621912	0.00049	0.011169	DOWN
GRIA2	4.020666	3.2088554	0.920609	3.485579884	0.000491	0.011186	UP
IL15RA	1063.846	0.93712161	0.268905	3.48495945	0.000492	0.011203	UP
FABP6	18.30276	2.62783516	0.7541	3.484729965	0.000493	0.011203	UP
CDC25C	281.2481	1.03066613	0.295814	3.484165947	0.000494	0.011217	UP
ADARB1	569.0657	-0.7005279	0.201096	-3.48354274	0.000495	0.011234	DOWN
ZNF527	181.2182	-0.5843397	0.167754	-3.48332279	0.000495	0.011234	NOT
HYDIN	19.89425	-2.2692914	0.651727	-3.48196804	0.000498	0.011281	DOWN
RP6-65G23	37.64237	1.01276104	0.290925	3.481174641	0.000499	0.011305	UP
TPM3	12530.54	0.46321387	0.133086	3.480559391	0.0005	0.011322	NOT
TRABD2A	297.0675	1.79151696	0.514992	3.4787273	0.000504	0.011381	UP
EXO1	346.1999	1.1065902	0.318105	3.478691586	0.000504	0.011381	UP
AC016768.	95.73593	-1.6098656	0.462804	-3.4785069	0.000504	0.011381	DOWN
NOVA2	169.0076	-0.788259	0.226679	-3.47743047	0.000506	0.011417	DOWN
bP-21201H	6.446154	2.70720097	0.778572	3.477136126	0.000507	0.01142	UP
SOCS3	1961.822	-1.2885696	0.370714	-3.47591186	0.000509	0.011463	DOWN
CDT1	789.4102	1.08386879	0.31186	3.47550193	0.00051	0.011471	UP
IGSF3	1161.588	1.35504774	0.389937	3.475040341	0.000511	0.011481	UP
FOXM1	1361.877	1.18709997	0.341683	3.474277189	0.000512	0.011504	UP
RPS2P32	21.29686	1.49118739	0.429256	3.473889381	0.000513	0.011505	UP
NINJ2	433.6189	1.20625802	0.347264	3.473607888	0.000514	0.011505	UP
PAQR8	450.0483	1.41208042	0.406519	3.473588771	0.000514	0.011505	UP
MAD2L2	1789.499	0.75615527	0.217731	3.47289098	0.000515	0.011525	UP
RP11-327J	24.6394	-1.3310177	0.38341	-3.47152823	0.000518	0.011571	DOWN
KREMEN2	24.11566	1.63041242	0.469687	3.471276815	0.000518	0.011571	UP
STK39	719.9628	1.1414516	0.328838	3.471163314	0.000518	0.011571	UP
PYGO2	3123.772	0.54124482	0.155947	3.470688733	0.000519	0.011582	NOT
GGT5	1496.62	-1.4585369	0.420348	-3.46983152	0.000521	0.011604	DOWN
TMEM14C	4981.325	0.56969163	0.164197	3.469554076	0.000521	0.011604	NOT
OLFM4	30.65803	3.25709868	0.938777	3.469512498	0.000521	0.011604	UP
G6PC	28736.9	-1.7611167	0.50767	-3.46901964	0.000522	0.011616	DOWN
ZBED4	873.238	0.58470648	0.168569	3.468647543	0.000523	0.011623	NOT
SNORA51	3.784813	1.91752148	0.552899	3.4681247	0.000524	0.011636	UP
ZNF16	483.591	0.57052411	0.164549	3.467208495	0.000526	0.01166	NOT
CSNK2B-LY	5.43676	0.99791244	0.287822	3.467120626	0.000526	0.01166	UP

C3orf52	71.09709	2.02939154	0.585457	3.466335539	0.000528	0.011685	UP
AGPS	2450.42	0.53586138	0.154652	3.464955685	0.00053	0.011735	NOT
EDC3	1164.789	0.39089341	0.112831	3.464424402	0.000531	0.011748	NOT
RP11-175E	153.6248	1.23005584	0.355073	3.464233614	0.000532	0.011748	UP
SCGB1B2P	29.58116	-1.309405	0.378062	-3.46346577	0.000533	0.011772	DOWN
RAET1K	6.201307	1.64584546	0.47529	3.462827215	0.000535	0.01179	UP
TM4SF18	401.6928	-0.7183081	0.207554	-3.46082819	0.000539	0.011868	DOWN
ENTPD2	322.0414	1.51482228	0.437824	3.459888613	0.00054	0.0119	UP
SYTL5	89.00808	-1.7609634	0.509147	-3.45865601	0.000543	0.011945	DOWN
SLC10A7	375.8363	-0.616623	0.17831	-3.45815018	0.000544	0.011946	DOWN
PARPBP	235.1481	1.00358745	0.290215	3.458078098	0.000544	0.011946	UP
CLCN2	443.8174	0.65896322	0.190563	3.457973391	0.000544	0.011946	UP
SOX2	7.085062	2.53330877	0.732716	3.457423882	0.000545	0.011961	UP
RP11-456(	3.179293	3.65174031	1.056539	3.456323185	0.000548	0.012	UP
AP000350.	40.10708	-2.0491184	0.593104	-3.45490854	0.00055	0.012054	DOWN
AACSP1	8.040691	3.60665315	1.043992	3.454674921	0.000551	0.012054	UP
CSE1L	4132.424	0.4522092	0.130954	3.453203094	0.000554	0.012111	NOT
TFF3	603.459	-2.0504455	0.593923	-3.45237621	0.000556	0.012138	DOWN
PPP1CC	5059.157	0.51336799	0.148809	3.44985636	0.000561	0.012232	NOT
MZT1	495.2835	0.67452166	0.195522	3.449850927	0.000561	0.012232	UP
SLC25A4	2825.02	-0.8793809	0.255041	-3.44799342	0.000565	0.012307	DOWN
FAM60A	658.7698	1.00238916	0.290898	3.445848045	0.000569	0.012394	UP
UBAP2	1355.723	0.60524068	0.175653	3.445652866	0.00057	0.012394	UP
ISM2	7.598249	4.07500203	1.182944	3.444797876	0.000571	0.012413	UP
VWDE	15.82282	2.98439159	0.866375	3.444688289	0.000572	0.012413	UP
FAM72D	29.27995	1.27092229	0.368962	3.444588715	0.000572	0.012413	UP
AC012065.	17.0253	0.90578015	0.262995	3.444090893	0.000573	0.012426	UP
P2RY8	422.5605	-1.214896	0.352783	-3.44374549	0.000574	0.012432	DOWN
SULT1B1	1294.025	-1.7631454	0.512093	-3.44301552	0.000575	0.012456	DOWN
SPATC1	23.65343	-1.2275016	0.35662	-3.44204281	0.000577	0.012488	DOWN
NME7	423.9976	0.70914061	0.206032	3.441893586	0.000578	0.012488	UP
AP006216.	34.61228	-1.8697219	0.543287	-3.44149778	0.000579	0.012496	DOWN
ARHGEF39	336.5107	0.94344016	0.274157	3.441239761	0.000579	0.012498	UP
DUSP13	30.83048	2.7629324	0.803118	3.440254996	0.000581	0.012533	UP
RNF152	1560.502	-0.9563685	0.278058	-3.43946218	0.000583	0.01256	DOWN
SLC12A4	1921.728	-0.5651694	0.16438	-3.43819171	0.000586	0.012599	NOT
PSMC1P10	3.033169	2.26855686	0.659811	3.438189685	0.000586	0.012599	UP
TMEM132A	1104.245	1.66904571	0.485501	3.437779934	0.000587	0.012608	UP
RP11-750E	543.2975	2.12761693	0.618928	3.437582718	0.000587	0.012608	UP
FBN3	85.06488	2.08418841	0.606436	3.436781753	0.000589	0.012635	UP
CSTF2	589.4867	0.71958307	0.209404	3.436338757	0.00059	0.012646	UP
RP11-624M	22.49592	-1.8258366	0.531448	-3.4355896	0.000591	0.012671	DOWN
CTB-193M1	153.3457	0.90943853	0.264768	3.434849787	0.000593	0.012687	UP
IL17RC	2816.508	-0.637916	0.185721	-3.43481312	0.000593	0.012687	DOWN
ANP32E	2944.616	0.59475934	0.173167	3.434606111	0.000593	0.012687	UP
CTC-487M2	26.98539	-0.9705269	0.282619	-3.43404282	0.000595	0.012703	DOWN
MMS22L	287.7839	0.99957832	0.291133	3.433404084	0.000596	0.012718	UP
RXFP4	3.174718	2.84804883	0.829537	3.43329877	0.000596	0.012718	UP
GUCA2A	20.48665	2.80327398	0.816747	3.432242278	0.000599	0.012757	UP

PUF60	11576.04	0.7655641	0.223071	3.431934295	0.000599	0.012757	UP
VCX3A	9.57553	3.60713519	1.05109	3.431802758	0.0006	0.012757	UP
LPCAT4	451.0212	0.99536899	0.290059	3.431608483	0.0006	0.012757	UP
COLCA2	208.1146	1.47552139	0.43004	3.431126043	0.000601	0.01277	UP
RP11-452I	35.83783	-0.8059389	0.234932	-3.43051934	0.000602	0.012789	DOWN
C8orf59	1491.007	0.74378091	0.216834	3.430185088	0.000603	0.012794	UP
PSMD14	3048.433	0.53729423	0.156653	3.429844025	0.000604	0.012798	NOT
GADD45G	2855.123	-1.2552828	0.366006	-3.42967674	0.000604	0.012798	DOWN
HSP90AB3F	43.85104	1.02863603	0.300028	3.428470934	0.000607	0.012842	UP
SLC45A1	41.73615	-0.9153558	0.266998	-3.42833026	0.000607	0.012842	DOWN
SRPK1	2301.26	0.60136136	0.175472	3.427107196	0.00061	0.012885	UP
RP11-316M	1.693067	-1.3612626	0.397216	-3.42700487	0.00061	0.012885	DOWN
SLC1A7	1139.236	2.35307477	0.6868	3.426142309	0.000612	0.012916	UP
SNRPA1	1143.977	0.56436114	0.164768	3.425184914	0.000614	0.012951	NOT
SCGB2B2	14.28772	-0.8530808	0.249122	-3.42435098	0.000616	0.012981	DOWN
CSRNP1	1447.476	-0.807773	0.235943	-3.4235982	0.000618	0.013007	DOWN
YWHAH	5151.974	0.53108602	0.155217	3.421566835	0.000623	0.013094	NOT
GRTP1-AS1	21.39711	-1.1774363	0.344212	-3.42067636	0.000625	0.013127	DOWN
RP11-496H	19.82909	1.50477177	0.440033	3.419682949	0.000627	0.013165	UP
MFI2	814.0323	1.70343035	0.498178	3.419323574	0.000628	0.013172	UP
KIF15	284.8842	1.28443869	0.375664	3.419113692	0.000628	0.013172	UP
LINC00964	3.10407	-1.5797468	0.462177	-3.41805772	0.000631	0.013213	DOWN
PLEKHB1	240.1132	2.0116153	0.588621	3.417505165	0.000632	0.013228	UP
ARL6IP6	377.6928	0.77470764	0.2267	3.417327274	0.000632	0.013228	UP
GAS5	6640.382	1.12930746	0.33049	3.41706492	0.000633	0.013231	UP
RP11-131I	10.38969	-1.3278924	0.388648	-3.41669817	0.000634	0.013238	DOWN
CRAT	7728.526	-0.7055473	0.206525	-3.4162789	0.000635	0.013248	DOWN
THUMPD3-A	507.4194	0.73080029	0.213934	3.416005966	0.000635	0.013252	UP
DNAJB5-AS	4.261583	-1.9802107	0.57974	-3.41568962	0.000636	0.013257	DOWN
GCDH	2829.342	-0.92823	0.271812	-3.41496951	0.000638	0.013282	DOWN
FAM220A	681.5997	0.46775481	0.137007	3.414086534	0.00064	0.013312	NOT
CR2	16.93053	2.53529601	0.742634	3.413925752	0.00064	0.013312	UP
MARCKS	5231.334	0.94474992	0.276837	3.41266333	0.000643	0.013364	UP
C6	14392.81	-1.6630235	0.487431	-3.41181521	0.000645	0.013395	DOWN
SLC6A20	15.44376	2.38738029	0.699912	3.410972592	0.000647	0.013422	UP
SFRP5	210.738	2.87016603	0.841479	3.410859121	0.000648	0.013422	UP
SCML2	220.3881	0.89735576	0.263104	3.410649097	0.000648	0.013422	UP
RASSF1	1051.936	0.48753977	0.142993	3.409538422	0.000651	0.013466	NOT
UGT1A6	2171.722	1.58115415	0.463815	3.40901819	0.000652	0.013473	UP
TPT1P5	3.988337	-1.3364755	0.39205	-3.40894543	0.000652	0.013473	DOWN
PHACTR3	4.832799	-1.61296	0.473192	-3.40868329	0.000653	0.013473	DOWN
MAP1LC3A	1547.756	-1.2361921	0.362671	-3.40857849	0.000653	0.013473	DOWN
SRSF7	3182.594	0.36444142	0.106938	3.40797798	0.000654	0.013475	NOT
COX18	877.6128	-0.5768749	0.169272	-3.40797736	0.000654	0.013475	NOT
RP11-182J	2.90865	-1.4569062	0.427507	-3.40791385	0.000655	0.013475	DOWN
BCAT2	2214.071	-0.8107043	0.237945	-3.40711042	0.000657	0.013504	DOWN
RP11-519C	10.43508	2.82249174	0.82855	3.406542265	0.000658	0.013516	UP
UNC13B	3450.734	-0.9463581	0.277827	-3.40627974	0.000659	0.013516	DOWN
SERPINE2	1779.21	1.62846069	0.478079	3.406258646	0.000659	0.013516	UP

WASF1	601.7032	0.8589971	0.252303	3.404624321	0.000663	0.013587	UP
PRELID2	115.8844	1.08071144	0.317445	3.404409514	0.000663	0.013587	UP
BIRC5	1754.937	1.20357934	0.353618	3.403613084	0.000665	0.013616	UP
EFTUD2	4491.733	0.43173639	0.126878	3.402758591	0.000667	0.013649	NOT
DBF4B	304.5262	0.70803162	0.208108	3.402229321	0.000668	0.013665	UP
RP11-757(	6.255111	-2.4675177	0.725383	-3.40167567	0.00067	0.013682	DOWN
HHLA2	9.705973	2.62510499	0.771921	3.400743617	0.000672	0.013719	UP
MAGEB1	26.31607	4.91001177	1.444067	3.400126649	0.000674	0.013723	UP
CAPN6	132.8689	2.81249449	0.827227	3.399906251	0.000674	0.013723	UP
KLHL2	927.7356	-0.7476454	0.219904	-3.39986559	0.000674	0.013723	DOWN
AC009120.	11.69328	-1.1110217	0.326795	-3.39975067	0.000674	0.013723	DOWN
MZT2B	3559.876	0.81844552	0.240745	3.399633057	0.000675	0.013723	UP
TBC1D2B	2160.204	-0.8460667	0.248927	-3.39885068	0.000677	0.013752	DOWN
SMIM22	63.94508	2.44570654	0.719709	3.398188707	0.000678	0.013775	UP
PHLDA2	449.8414	1.40202506	0.41262	3.397863079	0.000679	0.013777	UP
SNRPGP10	8.176892	1.01275807	0.298068	3.397745509	0.000679	0.013777	UP
STEAP3	5500.867	-1.1616922	0.342072	-3.39604181	0.000684	0.013852	DOWN
CSPP1	794.1877	0.84804071	0.249812	3.394720235	0.000687	0.013909	UP
KLF9	5109.025	-1.1875842	0.349972	-3.393372	0.00069	0.013967	DOWN
INPP5K	1158.819	-0.6323466	0.18638	-3.39277348	0.000692	0.01398	DOWN
IGKV2-29	9.25773	3.95476507	1.165765	3.392421473	0.000693	0.01398	UP
RP11-368I	12.27241	-1.0692365	0.31519	-3.39235771	0.000693	0.01398	DOWN
FBXW10	88.74109	2.36114549	0.696067	3.392122537	0.000694	0.01398	UP
C7orf31	181.8175	1.18097347	0.348154	3.392096752	0.000694	0.01398	UP
C6orf201	15.28963	-0.941939	0.27775	-3.39131975	0.000696	0.014009	DOWN
EIF3D	7782.327	0.55848045	0.164715	3.390577574	0.000697	0.014037	NOT
CTC-550B1	17.41175	-1.154459	0.340602	-3.38946545	0.0007	0.014084	DOWN
RP3-323A1	23.34158	2.14948017	0.634375	3.388342946	0.000703	0.014131	UP
DDX39BP2	2.729939	3.22166447	0.951	3.387659658	0.000705	0.014156	UP
CEP120	666.1393	-0.5963121	0.17605	-3.38716575	0.000706	0.014171	DOWN
CTC-490E2	20.05698	-1.1147974	0.32916	-3.38679363	0.000707	0.014179	DOWN
CCAT1	176.3083	1.83294161	0.54125	3.386495021	0.000708	0.014184	UP
RP11-729I	1.324493	-1.8308895	0.540721	-3.38601475	0.000709	NA	NA
HSPA6	274.1168	1.34149619	0.396222	3.385716613	0.00071	0.014214	UP
PTP4A1	28274.48	-0.7370767	0.217762	-3.38477861	0.000712	0.014246	DOWN
ADCY4	305.3646	-0.8245632	0.243615	-3.38469205	0.000713	0.014246	DOWN
PPIL1	1430.846	0.57209846	0.169057	3.384061758	0.000714	0.014269	NOT
TMCC1-AS1	77.41973	0.96257266	0.284483	3.38358489	0.000715	0.014283	UP
CTD-3064H	2.842487	2.86691964	0.847507	3.382769567	0.000718	0.014315	UP
PTPRN	5.858007	1.99644146	0.590286	3.382161746	0.000719	0.014334	UP
SPHK1	1328.23	1.75780849	0.519755	3.381996044	0.00072	0.014334	UP
KIAA0319	23.30451	1.46687178	0.433811	3.381360178	0.000721	0.014357	UP
CLIC1	10600.15	0.79792171	0.236029	3.380613525	0.000723	0.014385	UP
CTD-2135I	2.286467	-1.1008433	0.325684	-3.38009393	0.000725	0.014402	DOWN
CTD-2240J	6.309877	-1.3215187	0.391126	-3.37875145	0.000728	0.014454	DOWN
SYT5	14.40684	1.87416263	0.554752	3.378381508	0.000729	0.014454	UP
TNRC6B	1326.584	-0.5020266	0.1486	-3.37836994	0.000729	0.014454	NOT
SFXN1	4791.859	-0.7343266	0.217366	-3.37830029	0.000729	0.014454	DOWN
AP001626.	8.408914	2.52082292	0.746409	3.377268271	0.000732	0.014498	UP

IFT52	639.1832	0.57964441	0.171651	3.376871204	0.000733	0.014508	NOT
KDR	1398.178	-0.9096453	0.269456	-3.37586168	0.000736	0.014551	DOWN
ZPLD1	15.66515	2.37974315	0.705052	3.375270913	0.000737	0.014571	UP
GNG7	420.7985	-1.1039518	0.327122	-3.37474526	0.000739	0.014575	DOWN
RP11-407A	4.906048	3.7092694	1.099155	3.374655657	0.000739	0.014575	UP
TMEM129	3134.72	-0.6275105	0.185951	-3.37459918	0.000739	0.014575	DOWN
CAPN10	662.2641	0.56957516	0.168814	3.373977595	0.000741	0.014597	NOT
DNAAF5	1463.98	0.4604673	0.136527	3.372712304	0.000744	0.014654	NOT
C1orf132	70.22351	-1.1315439	0.335685	-3.37084949	0.000749	0.014743	DOWN
RP11-1008	4.556993	1.83771293	0.545275	3.370252179	0.000751	0.014764	UP
CENPI	166.2431	1.09988228	0.326399	3.369746401	0.000752	0.014781	UP
LUCAT1	135.4408	2.47869387	0.735809	3.368663713	0.000755	0.014828	UP
RP11-114F	4.451637	3.44333117	1.022252	3.368378449	0.000756	0.014833	UP
C1orf131	731.6405	0.61528134	0.182695	3.367810546	0.000758	0.014852	UP
RP11-428J	67.50423	0.8007204	0.237785	3.367416459	0.000759	0.014855	UP
AC093609.	9.110407	-1.4402474	0.427708	-3.36736248	0.000759	0.014855	DOWN
CORIN	45.95482	1.56873776	0.465984	3.366508129	0.000761	0.01489	UP
STAC	74.5318	2.18095289	0.647899	3.366191676	0.000762	0.014895	UP
RPS27L	3548.422	-0.6438945	0.191292	-3.36603359	0.000763	0.014895	DOWN
P2RY6	210.7469	1.51362702	0.449845	3.364776406	0.000766	0.014945	UP
HELQ	346.1715	-0.3823198	0.113627	-3.3647022	0.000766	0.014945	NOT
TEX101	10.39317	-2.9502632	0.876979	-3.36411976	0.000768	0.014966	DOWN
ACPT	31.38078	2.26200541	0.672521	3.363470242	0.00077	0.014984	UP
ITGB5	8350.923	0.61407857	0.182577	3.36339815	0.00077	0.014984	UP
ASPRV1	43.36689	-0.8141847	0.242111	-3.36285048	0.000771	0.014993	DOWN
OSBP2	305.8436	1.25797986	0.374083	3.362838243	0.000771	0.014993	UP
SIK3	1828.077	-0.5205449	0.154865	-3.3612753	0.000776	0.015067	NOT
RP11-131I	17.81302	1.06250168	0.316191	3.360318274	0.000779	0.015101	UP
MGAT5B	10.96369	-1.3100992	0.389881	-3.36025741	0.000779	0.015101	DOWN
BRWD1	1972.629	-0.558793	0.166319	-3.35976494	0.00078	0.015117	NOT
DDX1	4541.128	0.41370405	0.123154	3.359243486	0.000782	0.015127	NOT
PPIAP31	12.40932	1.21017077	0.360256	3.359196061	0.000782	0.015127	UP
LHFPL4	71.41462	2.9775324	0.886449	3.358942245	0.000782	0.01513	UP
MRPS17P1	2.75698	2.19347811	0.653139	3.35836572	0.000784	0.015141	UP
HS3ST2	134.186	-1.4914733	0.44411	-3.35834043	0.000784	0.015141	DOWN
YAE1D1	326.4227	0.50134295	0.149355	3.356722218	0.000789	0.015219	NOT
ZNF706	3708.07	0.64557796	0.192377	3.355804526	0.000791	0.015259	UP
WDR7	746.3778	-0.5634654	0.167919	-3.35557405	0.000792	0.015261	NOT
LPCAT1	2610.438	0.98074177	0.292358	3.354596527	0.000795	0.0153	UP
TIMM13	3730.837	0.73237617	0.21834	3.354299579	0.000796	0.0153	UP
PHF19	804.9711	0.87035775	0.259477	3.354276336	0.000796	0.0153	UP
CNGB3	6.771329	2.28161137	0.680284	3.353908659	0.000797	0.015309	UP
HDGF	20209.17	0.4736631	0.1413	3.352182367	0.000802	0.015394	NOT
BPIFA2	17.95352	3.14599851	0.93905	3.350194216	0.000808	0.015494	UP
TTC27	821.555	0.46469567	0.138767	3.348744129	0.000812	0.015559	NOT
RP11-408H	33.7846	-1.7335261	0.51768	-3.34864207	0.000812	0.015559	DOWN
SNX19	2462.18	-0.6434935	0.192215	-3.34777282	0.000815	0.015597	DOWN
LINC00879	10.56718	5.73708384	1.714164	3.346869285	0.000817	0.015634	UP
TSEN54	1497.981	0.62631216	0.187142	3.346722798	0.000818	0.015634	UP

MEGF8	2342.199	-0.7103112	0.212254	-3.34650962	0.000818	0.015635	DOWN
RP11-567(	13.98007	2.18793928	0.653869	3.346144954	0.000819	0.015645	UP
SLC7A14	10.34713	2.11784086	0.633013	3.345651551	0.000821	0.015662	UP
RP11-595I	38.27248	1.1324817	0.338515	3.345443981	0.000822	0.015662	UP
REPS2	747.4528	-0.9704572	0.290138	-3.34480931	0.000823	0.015687	DOWN
DLX5	21.96242	2.41240417	0.721421	3.343962038	0.000826	0.015724	UP
CCDC86	1105.826	0.5596938	0.16739	3.343658199	0.000827	0.01573	NOT
DALRD3	590.6364	0.51498753	0.154088	3.342172864	0.000831	0.015804	NOT
PKP3	121.821	2.35900824	0.705996	3.341390197	0.000834	0.015837	UP
TMIGD2	13.49859	-1.1501655	0.344238	-3.34119327	0.000834	0.015837	DOWN
RP11-178I	17.78615	-1.1653778	0.348829	-3.34083251	0.000835	0.015847	DOWN
SEC61G	2663.931	0.62009504	0.185746	3.338411742	0.000843	0.015974	UP
RP11-407I	15.22863	1.68285612	0.504125	3.338170972	0.000843	0.015977	UP
LPAR3	10.48399	2.56016874	0.76711	3.337422687	0.000846	0.016001	UP
SKP2	1280.923	0.86372883	0.258819	3.337188175	0.000846	0.016001	UP
BRCA1	511.0022	0.8130822	0.243644	3.337175242	0.000846	0.016001	UP
IGFBP7-AS	2.53144	-1.6103507	0.482674	-3.33630867	0.000849	0.01603	DOWN
HSP90AA1	41039.48	0.55721909	0.167018	3.336286672	0.000849	0.01603	NOT
COL9A2	239.5664	1.50232645	0.450446	3.335200805	0.000852	0.016071	UP
SH3D21	122.0926	0.98768098	0.296139	3.335191575	0.000852	0.016071	UP
CCM2L	144.7845	-0.7701671	0.230972	-3.33445374	0.000855	0.016083	DOWN
FRMD1	55.9802	2.26703991	0.679887	3.334437672	0.000855	0.016083	UP
SIK1	164.4686	-2.2015369	0.660251	-3.33439505	0.000855	0.016083	DOWN
ATP2B4	3664.753	-0.8412466	0.252367	-3.33342847	0.000858	0.016128	DOWN
GMPS	2612.885	0.45588076	0.136782	3.332898253	0.000859	0.016148	NOT
POLR3C	1140.178	0.58233085	0.17477	3.331987479	0.000862	0.016189	NOT
EVA1C	232.3161	-1.1405381	0.342354	-3.33145719	0.000864	0.01619	DOWN
PTHLH	85.25184	1.77892588	0.533986	3.331408882	0.000864	0.01619	UP
DDB2	1424.792	-0.7655636	0.229802	-3.33140237	0.000864	0.01619	DOWN
AC073635.	2.866996	-1.4990952	0.450239	-3.32955123	0.00087	0.016287	DOWN
C16orf47	5.924384	-1.4173005	0.425765	-3.32883321	0.000872	0.016317	DOWN
ANKRD22	73.20666	1.8435893	0.554017	3.327675506	0.000876	0.016374	UP
APOLD1	1399.657	-0.9927771	0.298373	-3.32729843	0.000877	0.016385	DOWN
MYCL	913.3812	-1.2486243	0.375338	-3.32666547	0.000879	0.016401	DOWN
ST6GALNAC	2331.263	-0.6727064	0.202218	-3.32663889	0.000879	0.016401	DOWN
HPDL	39.13243	1.6915584	0.508544	3.326277048	0.00088	0.016411	UP
NCAPD2	2145.322	0.9036408	0.271699	3.325887943	0.000881	0.016412	UP
GPR128	202.6005	-1.6571965	0.498274	-3.3258742	0.000881	0.016412	DOWN
CYP4F3	11158.19	-1.3138588	0.395101	-3.32537569	0.000883	0.01643	DOWN
ATP6V0D2	66.62748	1.68804411	0.5077	3.324884116	0.000885	0.016446	UP
UTP6	1841.749	0.42243412	0.127058	3.324733068	0.000885	0.016446	NOT
ACAT1	12868.73	-0.9406171	0.282983	-3.32393889	0.000888	0.016481	DOWN
CYBRD1	1311.501	-1.1105297	0.334118	-3.32376412	0.000888	0.016481	DOWN
PSPH	1540.151	0.92040702	0.276997	3.32280435	0.000891	0.016526	UP
ATP5A1P3	13.40478	-1.3637459	0.410462	-3.32246235	0.000892	0.016529	DOWN
RP1-168L1	4.426976	-1.1569682	0.348236	-3.32236479	0.000893	0.016529	DOWN
CTD-2529(	8.935027	1.34613057	0.40523	3.321891572	0.000894	0.016546	UP
AP004372.	2.646254	3.72195171	1.12066	3.321214737	0.000896	0.016575	UP
KANK3	253.6271	-0.8168262	0.245967	-3.32087761	0.000897	0.016584	DOWN

SNRPB	8267.196	0.71000525	0.213869	3.31981164	0.000901	0.016636	UP
MGAT1	10742.82	-0.5090389	0.153368	-3.31907543	0.000903	0.016669	NOT
C12orf49	1257.197	0.68315887	0.20597	3.316794686	0.000911	0.016787	UP
IARS	3718.298	0.55145357	0.166272	3.316579761	0.000911	0.016787	NOT
RP11-112J	7.362942	1.34276404	0.404871	3.316525958	0.000911	0.016787	UP
DIO3OS	152.0007	-2.0457537	0.616904	-3.316162	0.000913	0.016798	DOWN
RP11-435(	46.39315	0.964948	0.291014	3.315812085	0.000914	0.016807	UP
PRSS50	47.87228	1.87684549	0.566164	3.315023002	0.000916	0.016843	UP
CDH17	62.21363	2.68169933	0.809002	3.314825591	0.000917	0.016844	UP
GAL	9.347445	2.81467621	0.84965	3.312746756	0.000924	0.016958	UP
ARHGAP20	66.75062	-1.2932301	0.390444	-3.31220008	0.000926	0.01698	DOWN
RP11-432J	15.86775	0.87217684	0.26338	3.311477757	0.000928	0.017012	UP
RP11-73M7	9.330835	1.95378697	0.59005	3.311225132	0.000929	0.017016	UP
GAST	6.798414	3.5430912	1.070357	3.310195572	0.000932	0.017059	UP
STMN1	5148.367	0.92361969	0.279046	3.309923468	0.000933	0.017059	UP
FUBP1	2196.174	0.58388685	0.176414	3.309752065	0.000934	0.017059	NOT
SPINK4	7.780243	3.16645752	0.956722	3.309694434	0.000934	0.017059	UP
KIF3C	318.3608	1.2748422	0.385199	3.309568963	0.000934	0.017059	UP
RP13-644M	4.164278	3.22227344	0.973764	3.309090935	0.000936	0.017077	UP
TDRD5	22.72646	2.94507614	0.890049	3.30889224	0.000937	0.017078	UP
OR13A1	4.582864	2.58907997	0.782639	3.30814072	0.000939	0.017104	UP
RP11-90L1	41.4947	-1.4911717	0.450765	-3.30809083	0.000939	0.017104	DOWN
RP11-83J1	4.952011	1.35026591	0.408222	3.30767712	0.000941	0.017117	UP
MSI1	473.847	2.00056285	0.604968	3.3068927	0.000943	0.017154	UP
DUSP27	2.844883	-1.8608261	0.56288	-3.30590461	0.000947	0.017198	DOWN
ZBTB47	485.5123	-0.8359929	0.252888	-3.30577819	0.000947	0.017198	DOWN
NUP205	1640.547	0.61175435	0.185066	3.305606146	0.000948	0.017198	UP
MYO1E	2443.662	-0.8971528	0.271419	-3.30541476	0.000948	0.017199	DOWN
RP11-301I	30.51957	-1.5099884	0.456918	-3.30472691	0.000951	0.01722	DOWN
PAX8	211.1374	0.98090372	0.296826	3.304636934	0.000951	0.01722	UP
FAM3D	7.882227	-1.5798344	0.478085	-3.30450344	0.000951	0.01722	DOWN
RP11-328K	199.1469	-1.8107807	0.548066	-3.30394352	0.000953	0.01724	DOWN
DHDH	40.03438	1.59337301	0.482284	3.303805188	0.000954	0.01724	UP
EID3	40.51513	1.20918627	0.366163	3.302317477	0.000959	0.01732	UP
FOSB	1146.643	-1.8139679	0.549603	-3.30050422	0.000965	0.017418	DOWN
CYGB	883.8589	-1.0084178	0.305548	-3.30036124	0.000966	0.017418	DOWN
MTNR1B	6.272915	4.08550044	1.237962	3.300181707	0.000966	0.017418	UP
DNAJC27	133.5353	-0.5124778	0.155328	-3.29932866	0.000969	0.017459	NOT
GPR115	5.636337	2.68642664	0.814307	3.299034233	0.00097	0.017466	UP
VEPH1	120.6197	1.97160024	0.597675	3.298782969	0.000971	0.01747	UP
CTD-2566J	7.971542	2.63581398	0.799202	3.298056643	0.000974	0.017502	UP
POP1	329.9713	0.61089198	0.185245	3.297757059	0.000975	0.017502	UP
DDX27	2353.482	0.43168609	0.130907	3.297654403	0.000975	0.017502	NOT
LPPR2	1601.95	-0.7486346	0.227029	-3.29752875	0.000975	0.017502	DOWN
RP11-126(	2.634516	3.20083912	0.970923	3.296698373	0.000978	0.017542	UP
RP11-12J1	5.390096	-1.4235188	0.431851	-3.29631719	0.00098	0.017548	DOWN
SCG3	8.122136	2.21179137	0.671006	3.296231445	0.00098	0.017548	UP
PPM1G	5449.082	0.42608053	0.129309	3.295046314	0.000984	0.017605	NOT
MGAT3	70.09972	-1.1812227	0.358495	-3.29494806	0.000984	0.017605	DOWN

ABCF2	2014.088	0.45864614	0.139211	3.294608363	0.000986	0.017615	NOT
GLIDR	19.75453	-0.968887	0.294115	-3.29424634	0.000987	0.017626	DOWN
PXDNL	33.33698	-1.096884	0.333005	-3.2938954	0.000988	0.017636	DOWN
ASIC1	187.3898	1.6945345	0.514533	3.293344602	0.00099	0.017651	UP
EMP1	1562.817	-1.1136259	0.338151	-3.29328239	0.00099	0.017651	DOWN
RP11-372K	7.962462	-0.8763945	0.266179	-3.29250445	0.000993	0.017688	DOWN
PI15	140.4003	-2.0028575	0.608409	-3.29196059	0.000995	0.017688	DOWN
SNHG3	840.6523	1.16092449	0.352655	3.291959919	0.000995	0.017688	UP
RP11-70D2	43.87619	-0.9773596	0.296893	-3.29195548	0.000995	0.017688	DOWN
UQCRFS1	4189.929	-0.7334845	0.222873	-3.29103882	0.000998	0.017709	DOWN
RP11-680(	2.421815	-1.4905736	0.452925	-3.29099618	0.000998	0.017709	DOWN
CT83	9.610045	4.90471257	1.490445	3.290770078	0.000999	0.017709	UP
LAGE3P1	4.72907	1.10950037	0.337169	3.290631683	0.001	0.017709	UP
CTC-1337H	2.198662	-1.6082151	0.488739	-3.29053668	0.001	0.017709	DOWN
CNNM2	902.573	-0.7073348	0.214973	-3.29034798	0.001001	0.017709	DOWN
TMEM54	837.1113	1.34914876	0.410057	3.29014712	0.001001	0.017709	UP
C3orf36	11.00786	-1.6357421	0.497167	-3.29012299	0.001001	0.017709	DOWN
MSH2	1078.074	0.70030422	0.21286	3.289968572	0.001002	0.017709	UP
TBX4	64.11152	2.42421253	0.736905	3.289722365	0.001003	0.017709	UP
FOXF1	105.1363	-1.0928561	0.332221	-3.28954416	0.001003	0.017709	DOWN
MITD1	582.4666	0.47034211	0.142996	3.289194403	0.001005	0.017709	NOT
RPL10L	11.24001	2.83963946	0.863338	3.28913865	0.001005	0.017709	UP
KLK2	7.984685	3.76656448	1.145212	3.288968274	0.001006	0.017709	UP
AK4P3	2.667459	2.11686475	0.643645	3.288870775	0.001006	0.017709	UP
RP11-202K	4.717753	4.5666199	1.38872	3.288366636	0.001008	0.01773	UP
DHODH	2465.196	-1.0404979	0.316462	-3.2879108	0.001009	0.017745	DOWN
VRK1	403.8193	0.63702339	0.193756	3.287760706	0.00101	0.017745	UP
SMIM24	755.0145	2.25863973	0.687248	3.286498719	0.001014	0.017813	UP
NEK1	362.4717	-0.5567587	0.169458	-3.28552412	0.001018	0.017863	NOT
RP11-424M	1.141534	-3.1654038	0.963682	-3.28469813	0.001021	NA	NA
AGO2	1750.631	0.62709633	0.190931	3.284417388	0.001022	0.017922	UP
RP11-420I	166.5633	-1.3901407	0.423378	-3.28345062	0.001025	0.017972	DOWN
SOX4	2268.722	1.35373684	0.412334	3.283109943	0.001027	0.017982	UP
GYG2	1449.202	-1.1353756	0.34586	-3.28275729	0.001028	0.017983	DOWN
TARSL2	335.9345	-0.4954089	0.150914	-3.28272955	0.001028	0.017983	NOT
DDN	12.76143	1.9068148	0.580998	3.281962442	0.001031	0.01802	UP
CCRN4L	283.5933	-1.0368895	0.315967	-3.28163975	0.001032	0.018029	DOWN
DCLRE1C	250.2145	0.79450449	0.242164	3.280859362	0.001035	0.018064	UP
ZNF251	750.8403	0.60291861	0.183775	3.280738396	0.001035	0.018064	UP
RP11-35N6	150.913	-1.444286	0.440398	-3.27950538	0.00104	0.018105	DOWN
RPP40	391.3169	0.74609582	0.227507	3.279436673	0.00104	0.018105	UP
METTL5	1199.157	0.51531385	0.157142	3.279283384	0.001041	0.018105	NOT
IKBIP	801.3538	0.64434262	0.196495	3.279175425	0.001041	0.018105	UP
COPS7B	1305.139	0.48763196	0.148706	3.279167484	0.001041	0.018105	NOT
CUL5	1390.815	-0.379887	0.115854	-3.27900397	0.001042	0.018105	NOT
RP11-7F17	51.33057	-1.6692173	0.509094	-3.27879784	0.001043	0.018107	DOWN
DHRSX	2039.141	-0.8914454	0.271901	-3.27856453	0.001043	0.01811	DOWN
STAB2	64.20695	-1.6868861	0.51461	-3.27798869	0.001045	0.018135	DOWN
NTS	487.4693	2.94208966	0.897573	3.277826039	0.001046	0.018135	UP

LINC0017(	233.7124	1.84001895	0.56155	3.276677517	0.00105	0.018194	UP
RP11-672A	12.7079	-1.1867723	0.362203	-3.27653999	0.001051	0.018194	DOWN
ZNF7	907.4955	0.4916387	0.150059	3.276296578	0.001052	0.018198	NOT
RCAN3	404.7215	0.97365634	0.29728	3.275212802	0.001056	0.018257	UP
RP1-65P5.	3.046236	-3.1156886	0.951415	-3.27479359	0.001057	0.018272	DOWN
LINC0111(	40.68065	1.54901994	0.473066	3.27442902	0.001059	0.018279	UP
MARS	3944.898	0.47388644	0.144728	3.274330602	0.001059	0.018279	NOT
NOP56	3973.854	0.65056876	0.198711	3.273951833	0.001061	0.018292	UP
TMEM40	27.10249	1.93641498	0.591505	3.273705929	0.001061	0.018296	UP
ZEB1-AS1	168.7297	0.61802175	0.188797	3.273470079	0.001062	0.0183	UP
PRC1	1258.966	0.96032632	0.293429	3.272770442	0.001065	0.018333	UP
TSPYL5	73.48929	-0.9896759	0.302423	-3.27248778	0.001066	0.01834	DOWN
FER1L4	149.4673	1.64610909	0.503053	3.272240987	0.001067	0.018344	UP
HTATIP2	5303.73	0.78873856	0.241061	3.271948243	0.001068	0.018352	UP
PYCR2	2834.894	0.49780211	0.152168	3.271387888	0.00107	0.018376	NOT
FAM159A	39.23079	1.71516636	0.524362	3.270960871	0.001072	0.018393	UP
ZNF684	376.7782	-0.7921512	0.242259	-3.26985627	0.001076	0.018438	DOWN
CD177	25.43827	2.139985	0.654478	3.269760895	0.001076	0.018438	UP
MEF2C	672.4061	-0.6315963	0.193165	-3.26972349	0.001077	0.018438	DOWN
SLC27A1	873.011	-0.9409803	0.287818	-3.269357	0.001078	0.01845	DOWN
LINC0108(	4.173273	3.06566805	0.937757	3.269151094	0.001079	0.018452	UP
STEAP4	622.2322	-1.4338388	0.438645	-3.26879335	0.00108	0.018464	DOWN
PPAP2B	4676.954	-0.7900401	0.24175	-3.26800785	0.001083	0.018504	DOWN
TMEM79	366.6077	0.50662522	0.155038	3.267746498	0.001084	0.018509	NOT
NPC2	5421.455	0.70812372	0.216725	3.267379714	0.001085	0.018521	UP
RP11-717I	5.406134	1.64100207	0.502282	3.267093373	0.001087	0.018529	UP
LDLRAD2	38.79876	-0.9798287	0.299952	-3.26661438	0.001088	0.018548	DOWN
BRSK1	161.928	0.82046257	0.251289	3.265010982	0.001095	0.018633	UP
HHATL-AS1	1.701816	-2.0374048	0.62402	-3.26496874	0.001095	0.018633	DOWN
NUSAP1	1435.904	0.86537544	0.265097	3.264371091	0.001097	0.018661	UP
RP11-91I8	3.832056	3.40621951	1.043541	3.264095971	0.001098	0.018667	UP
ITGA9	639.1588	-0.9717896	0.297781	-3.26343708	0.001101	0.018699	DOWN
RP11-284M	6.9963	-0.9738517	0.298459	-3.26293797	0.001103	0.018714	DOWN
SHROOM4	188.9525	-0.8118604	0.248824	-3.26278695	0.001103	0.018714	DOWN
SLC23A2	4731.577	-1.0358434	0.317483	-3.26267671	0.001104	0.018714	DOWN
FAM162B	44.64595	-0.9432041	0.289172	-3.26173644	0.001107	0.018764	DOWN
NPM1P27	87.83547	0.83898785	0.257269	3.261132144	0.00111	0.018793	UP
FXYD2	642.3031	2.3693074	0.726581	3.260897952	0.001111	0.018796	UP
SNRPB2	2059.399	0.43533374	0.133531	3.260178469	0.001113	0.018832	NOT
ARHGAP11A	557.6538	0.91766963	0.281497	3.259961751	0.001114	0.018835	UP
ZNRF1	1411.52	-0.5857248	0.179743	-3.25868424	0.001119	0.018908	DOWN
SERINC5	5860.506	-0.7144853	0.219282	-3.25829802	0.001121	0.018922	DOWN
KLK13	3.060621	3.23546195	0.993064	3.258060024	0.001122	0.018926	UP
ASF1B	661.7359	1.07338776	0.329479	3.25783754	0.001123	0.018926	UP
RP11-339I	3.164125	1.84607184	0.566677	3.257711621	0.001123	0.018926	UP
RP11-863I	4.111008	-1.6890382	0.518598	-3.25692955	0.001126	0.018966	DOWN
SLC24A3	67.25609	-1.6423602	0.504392	-3.25611859	0.001129	0.019009	DOWN
RP11-158I	96.4868	-1.0847716	0.333198	-3.25563392	0.001131	0.01903	DOWN
SEL1L	8362.802	-0.715146	0.219738	-3.25453356	0.001136	0.019092	DOWN

RP11-81K1	4.465186	-2.5924297	0.796783	-3.25362086	0.001139	0.019141	DOWN
TBC1D31	357.1071	0.56609486	0.174002	3.253389192	0.00114	0.019145	NOT
RP3-395M2	3.661938	-2.2008478	0.676593	-3.25283747	0.001143	0.01917	DOWN
RP11-69E1	76.01975	-1.1111193	0.341662	-3.25210333	0.001146	0.019208	DOWN
BOD1	1595.626	0.4487803	0.138052	3.250804638	0.001151	0.019273	NOT
PIFO	23.08146	1.77929522	0.547356	3.250706995	0.001151	0.019273	UP
NXF3	6.283072	-1.6121887	0.495964	-3.25061571	0.001152	0.019273	DOWN
NONO	15023.4	0.52249954	0.160764	3.250101452	0.001154	0.019296	NOT
GPIHBP1	194.0014	-1.1287747	0.347397	-3.24923299	0.001157	0.019339	DOWN
CXorf67	15.93618	4.79182518	1.474806	3.249121947	0.001158	0.019339	UP
RP11-503C	16.13388	-1.4945248	0.460053	-3.24859438	0.00116	0.019362	DOWN
CDH9	8.05903	5.34533665	1.645515	3.248428409	0.00116	0.019362	UP
KPNB1	8470.833	0.42203572	0.129931	3.248160155	0.001162	0.019368	NOT
LGALS14	8.828286	5.13379981	1.580824	3.247546314	0.001164	0.019387	UP
RPL39L	343.5117	1.47908971	0.455448	3.247546186	0.001164	0.019387	UP
ANXA2	19268.06	0.9318887	0.286976	3.247270197	0.001165	0.019393	UP
SETD7	4710.894	-0.7787398	0.239966	-3.24521444	0.001174	0.019522	DOWN
CYP2B7P	4022.332	-2.2073746	0.680344	-3.24449981	0.001177	0.019559	DOWN
COPS5	3352.944	0.5387752	0.166085	3.243973592	0.001179	0.019583	NOT
IRF2BP2	8784.778	0.43200538	0.133221	3.242777158	0.001184	0.019648	NOT
RP11-274M	8.882718	4.0255113	1.241415	3.242680019	0.001184	0.019648	UP
CTA-384D8	4.204771	2.00760156	0.619263	3.241920953	0.001187	0.019689	UP
AP001189.	9.252811	-1.3788215	0.425352	-3.24160392	0.001189	0.019699	DOWN
SMCO2	11.30472	1.64514083	0.507571	3.241204327	0.00119	0.019714	UP
LINC00676	0.925226	-2.6593933	0.820617	-3.24072265	0.001192	NA	NA
FAM114A1	3309.12	-0.5035225	0.155425	-3.23965037	0.001197	0.01981	NOT
HIST1H2AI	18.94216	-1.6111965	0.497431	-3.23903598	0.001199	0.01984	DOWN
PRIM1	505.5942	0.77591852	0.239573	3.238755814	0.001201	0.019848	UP
RP11-679B	11.54287	-1.0149991	0.313486	-3.23777605	0.001205	0.019904	DOWN
SLC35F2	109.4284	1.46029574	0.451267	3.235989763	0.001212	0.020017	UP
NCEH1	838.6061	1.38184666	0.42714	3.235114978	0.001216	0.020066	UP
RP11-250B	16.91461	-1.0256694	0.317078	-3.234755	0.001217	0.020079	DOWN
TLCD1	719.1982	0.94560311	0.292367	3.234300778	0.001219	0.020099	UP
CLDN18	13.27252	1.79779147	0.55596	3.23366928	0.001222	0.020131	UP
RP11-113I	7.300272	-3.372184	1.043224	-3.23246514	0.001227	0.020204	DOWN
HN1	2990.263	0.88176274	0.272799	3.232282068	0.001228	0.020204	UP
RP11-44F1	31.7088	-0.831024	0.257129	-3.23192815	0.00123	0.020217	DOWN
RP11-396B	12.56674	-1.3352992	0.413265	-3.23109541	0.001233	0.020264	DOWN
ANLN	1002.385	1.17021	0.362253	3.230362193	0.001236	0.020298	UP
COL6A2	8491.593	-1.0279043	0.318214	-3.23023287	0.001237	0.020298	DOWN
CDC123	2517.418	0.5342521	0.165404	3.229988831	0.001238	0.020298	NOT
ITGB3BP	428.9796	0.60775806	0.188179	3.22968375	0.001239	0.020298	UP
UBQLN4	2481.177	0.52406173	0.162264	3.229681249	0.001239	0.020298	NOT
LINC00634	6.530347	1.46186242	0.452663	3.229473875	0.00124	0.020298	UP
RNF8	778.045	0.46476494	0.143923	3.229268397	0.001241	0.020298	NOT
RAPGEF2	1831.478	-0.7236795	0.224103	-3.22923331	0.001241	0.020298	DOWN
RP11-843B	3.1069	1.97534776	0.611822	3.228628982	0.001244	0.020329	UP
BAK1	993.68	0.70758968	0.219188	3.228231469	0.001246	0.020345	UP
HSF2	380.99	0.56203808	0.174153	3.227263675	0.00125	0.020401	NOT

GRAMD4	3073.808	-0.893162	0.276773	-3.22705888	0.001251	0.020404	DOWN
SLC16A11	1310.121	-1.7689444	0.548246	-3.22655115	0.001253	0.020427	DOWN
KNOP1	583.0462	0.45536479	0.141138	3.22638847	0.001254	0.020427	NOT
FAM227A	23.56109	1.43282009	0.444134	3.226098239	0.001255	0.020435	UP
TTLL4	1536.46	1.05690605	0.327633	3.225888238	0.001256	0.020438	UP
EWSR1	8229.581	0.31947391	0.099094	3.223956333	0.001264	0.020562	NOT
RASAL1	87.40396	1.89259696	0.587068	3.223810279	0.001265	0.020562	UP
CTA-14H9.	9.415521	-1.1603604	0.36002	-3.2230412	0.001268	0.020605	DOWN
FANCG	603.4768	0.67354462	0.20904	3.222081056	0.001273	0.020642	UP
LINC0125	31.46179	-1.4766383	0.45829	-3.22205958	0.001273	0.020642	DOWN
ZIM2-AS1	10.43641	2.11340827	0.655939	3.221958935	0.001273	0.020642	UP
GCNT1	160.5331	1.27957854	0.397156	3.221852144	0.001274	0.020642	UP
DNTTIP1	1043.457	0.43000539	0.133475	3.221606362	0.001275	0.020647	NOT
NFASC	566.1032	-1.0972685	0.340629	-3.22130151	0.001276	0.020657	DOWN
FOXD1	4.904057	2.76220129	0.857564	3.220987061	0.001277	0.020667	UP
C8orf88	24.54236	1.73982696	0.540201	3.220701353	0.001279	0.020675	UP
IGF2BP3	267.3966	1.92901816	0.598998	3.22041095	0.00128	0.020684	UP
VAMP2	1662.045	-0.5925684	0.184014	-3.22022935	0.001281	0.020685	DOWN
GRK4	148.6149	-0.5460104	0.169579	-3.21980736	0.001283	0.020703	NOT
RND3	1774.337	-0.9722261	0.302017	-3.21910673	0.001286	0.020741	DOWN
DIRAS3	69.78537	-1.6492955	0.512439	-3.21852121	0.001289	0.020771	DOWN
CTSO	3134.887	-0.9426316	0.292894	-3.21833627	0.001289	0.020772	DOWN
CDCA7L	457.0898	1.17364283	0.364724	3.217897144	0.001291	0.020781	UP
RP11-89K2	56.91292	3.02058378	0.938689	3.217875287	0.001291	0.020781	UP
POLQ	212.6337	1.04494269	0.324772	3.217461667	0.001293	0.020799	UP
C2orf44	479.6268	0.44213764	0.137445	3.216835715	0.001296	0.020832	NOT
RP11-545A	6.484073	2.78276921	0.86564	3.214694424	0.001306	0.020975	UP
DNAJB9	4237.167	-0.7925165	0.246584	-3.21398248	0.001309	0.021015	DOWN
TMEM143	650.6727	-0.6654371	0.207202	-3.21153607	0.00132	0.02118	DOWN
C1orf35	1075.785	0.61940343	0.192876	3.211401239	0.001321	0.02118	UP
FAM189B	1794.408	0.57122438	0.17792	3.210573104	0.001325	0.021206	NOT
AIM1L	159.4467	1.13399943	0.353209	3.210564509	0.001325	0.021206	UP
KCNH2	138.3647	2.16708666	0.674996	3.210515817	0.001325	0.021206	UP
UXS1	1678.372	0.49740052	0.154947	3.210129783	0.001327	0.021206	NOT
HGH1	2778.636	0.78159329	0.243481	3.210073126	0.001327	0.021206	UP
RP3-523K2	7.396718	2.90859142	0.906095	3.210028103	0.001327	0.021206	UP
GTF2H1	1423.166	0.34411947	0.107224	3.209358859	0.00133	0.021243	NOT
RAPH1	326.2833	-1.025415	0.319534	-3.20909858	0.001332	0.021249	DOWN
RP11-864N	207.6009	0.96400473	0.300431	3.208740882	0.001333	0.021263	UP
PRKACA	5466.741	-0.5337346	0.166359	-3.20833192	0.001335	0.021281	NOT
AC090945.	4.011039	1.69672746	0.528892	3.208080711	0.001336	0.021287	UP
RP13-225O	4.202737	-1.6905497	0.527097	-3.20728383	0.00134	0.021334	DOWN
S100A6	4917.926	1.38519177	0.431913	3.207108777	0.001341	0.021334	UP
RCL1	1477.807	-0.9275693	0.289261	-3.20668368	0.001343	0.021353	DOWN
RFPL4B	65.5172	3.89445298	1.214674	3.206171955	0.001345	0.021379	UP
CTA-292E1	62.49264	-0.9835534	0.306806	-3.20578789	0.001347	0.021395	DOWN
RP11-15F1	4.370025	1.71091265	0.533772	3.205327804	0.001349	0.021409	UP
UGT1A8	11.51915	2.23760515	0.698105	3.205255363	0.001349	0.021409	UP
SPINT1	1206.397	1.93304557	0.603149	3.204923977	0.001351	0.021415	UP

ISG20L2	2041.197	0.47783105	0.149097	3.204840494	0.001351	0.021415	NOT
RP3-508I1	46.90236	0.84659509	0.264181	3.204598895	0.001353	0.021421	UP
GFRA2	108.9849	-1.0848001	0.338563	-3.20412793	0.001355	0.021443	DOWN
PPAT	579.8771	0.51468816	0.160663	3.203530663	0.001358	0.021475	NOT
SMARCAL1	903.3991	0.38266941	0.119459	3.203357492	0.001358	0.021476	NOT
AF230666.	14.90805	0.87038752	0.271737	3.203054221	0.00136	0.021486	UP
SNRPEP2	32.61095	0.6712323	0.209585	3.202675191	0.001362	0.021501	UP
NPDC1	926.2854	-0.9782889	0.305545	-3.2017786	0.001366	0.021556	DOWN
DHRS2	3183.282	2.2654685	0.707737	3.201003361	0.001369	0.021601	UP
RGMA	60.37501	-0.9831935	0.307296	-3.19949971	0.001377	0.021691	DOWN
RP11-249I	8.614794	2.19942324	0.687434	3.199470436	0.001377	0.021691	UP
RP13-991F	2.297815	-1.5404333	0.48151	-3.19917358	0.001378	0.021701	DOWN
RBMXL2	1.272897	-2.0690419	0.646781	-3.19898212	0.001379	NA	NA
KLF2P1	4.897367	3.81775029	1.193493	3.19880444	0.00138	0.021716	UP
FILIP1	214.5884	-0.9298058	0.290719	-3.1983011	0.001382	0.021739	DOWN
TIPIN	220.8587	0.54644766	0.170862	3.19817674	0.001383	0.021739	NOT
BYSL	1104.563	0.56939394	0.17806	3.197765814	0.001385	0.021757	NOT
AC018892.	1.990869	2.57438306	0.805144	3.197418948	0.001387	0.02177	UP
GGT8P	2.154178	2.55352988	0.798703	3.197096311	0.001388	0.021782	UP
SEMA3B-AS	30.52791	-1.3318237	0.416613	-3.19678568	0.00139	0.021793	DOWN
TACC3	2107.757	0.96343303	0.301391	3.196616716	0.00139	0.021793	UP
KDM1A	2088.648	0.46261711	0.14473	3.196416204	0.001391	0.021796	NOT
SAE1	3882.52	0.49321634	0.154316	3.196138112	0.001393	0.021804	NOT
WDR82P1	3.007858	3.57520587	1.118728	3.195777217	0.001395	0.021808	UP
MIRLET7BH	31.16363	1.04498934	0.327018	3.195506189	0.001396	0.021808	UP
HPX	100031.4	-1.3925262	0.435804	-3.19530734	0.001397	0.021808	DOWN
S100A7	5.854537	3.96246203	1.240107	3.195258458	0.001397	0.021808	UP
CITF22-92	91.23727	0.71225305	0.222909	3.195257023	0.001397	0.021808	UP
RP11-348N	10.75325	-0.9696246	0.30351	-3.19470158	0.0014	0.021837	DOWN
SLC22A8	10.93191	2.80067455	0.876784	3.194257912	0.001402	0.021858	UP
C1orf116	215.2328	1.83989355	0.576138	3.193491784	0.001406	0.021904	UP
USP38	1314.581	-0.6011105	0.18827	-3.19281087	0.001409	0.021943	DOWN
RP11-498F	11.2512	1.10301257	0.345542	3.192127436	0.001412	0.021982	UP
RAB3A	215.3978	-0.8549516	0.267941	-3.19081737	0.001419	0.02207	DOWN
RP5-943J3	17.89409	1.05671108	0.331298	3.189605302	0.001425	0.02215	UP
AC114812.	1.471303	2.33943698	0.73353	3.189286212	0.001426	0.022162	UP
NAA40	754.652	0.58477492	0.183386	3.188770845	0.001429	0.022188	NOT
CCL23	14.68405	-1.1103954	0.3483	-3.18804079	0.001432	0.022232	DOWN
SLC1A4	2004.469	0.69848274	0.219124	3.187617833	0.001434	0.022252	UP
OVCH1-AS1	4.008847	-1.7079419	0.535866	-3.18725735	0.001436	0.022267	DOWN
CCDC24	364.6356	0.79409843	0.249165	3.187035618	0.001437	0.022271	UP
DYNLL1	6838.115	0.47663933	0.149565	3.18683748	0.001438	0.022273	NOT
PER2	835.4604	-0.6333185	0.19875	-3.18650872	0.00144	0.022286	DOWN
TTBK1	345.1657	-1.4461832	0.454094	-3.18476839	0.001449	0.022408	DOWN
TAGLN2P1	12.78306	1.03139732	0.323888	3.184421781	0.00145	0.022422	UP
GSTA12P	10.69403	-2.0132365	0.632281	-3.18408391	0.001452	0.022435	DOWN
SNX18P26	3.246066	-2.9129911	0.914928	-3.18384692	0.001453	0.02244	DOWN
SCX	49.83306	1.26985873	0.398864	3.183687964	0.001454	0.02244	UP
PTGES3	13132.03	0.37287975	0.117155	3.182785036	0.001459	0.022498	NOT

CHAF1B	437.9728	1.09922991	0.345419	3.1823094	0.001461	0.022522	UP
EGFR	7014.212	-0.9957297	0.312944	-3.18181899	0.001464	0.022547	DOWN
RP5-933K2	7.450998	-0.8300561	0.260906	-3.18144247	0.001465	0.022564	DOWN
RP11-166I	4.461706	-1.1836385	0.372089	-3.18106567	0.001467	0.02258	DOWN
RNF220	2400.229	0.40572462	0.127631	3.178897267	0.001478	0.022722	NOT
MMD	1036.617	0.82420816	0.259276	3.178884062	0.001478	0.022722	UP
RP4-570O1	2.364674	3.4092016	1.072622	3.178381158	0.001481	0.022722	UP
RP11-726(	24.74163	1.23644496	0.389025	3.178313682	0.001481	0.022722	UP
VWA2	6.65266	2.45151098	0.771328	3.178299847	0.001481	0.022722	UP
TROAP	649.7436	1.14992361	0.361809	3.178261445	0.001482	0.022722	UP
SCG5	218.9192	1.18177818	0.371924	3.177469347	0.001486	0.022772	UP
DUSP10	1657.159	-0.800255	0.251875	-3.17718672	0.001487	0.022781	DOWN
TCP10L2	8.530828	2.34568041	0.738535	3.17612764	0.001493	0.022852	UP
CRISPLD2	925.3352	-1.2605053	0.396934	-3.17560368	0.001495	0.022868	DOWN
AKAP13	3525.307	-0.5677381	0.178782	-3.17558895	0.001495	0.022868	NOT
TP53INP2	4108.236	-0.8133947	0.256205	-3.17478223	0.001499	0.022909	DOWN
WDR59	2164.813	-0.5681133	0.17895	-3.1746978	0.0015	0.022909	NOT
ERH	3325.171	0.45760626	0.144151	3.174487636	0.001501	0.022909	NOT
ZDHHC20-I	1.77793	-1.1775025	0.370934	-3.17442462	0.001501	0.022909	DOWN
LINC01518	8.230644	4.4444185	1.400289	3.173928932	0.001504	0.022935	UP
ZNF341	334.9312	0.61848779	0.194908	3.173222007	0.001508	0.022978	UP
NONOP2	22.80328	-0.7962099	0.250948	-3.172811	0.00151	0.022997	DOWN
PTPN12	2248.129	0.52951827	0.166901	3.17265461	0.001511	0.022997	NOT
RP11-1149	28.44288	1.72130092	0.542706	3.171702342	0.001515	0.023059	UP
PTMAP4	17.64139	0.66296713	0.20906	3.171184618	0.001518	0.023073	UP
DUSP1	11479.76	-1.0540988	0.33241	-3.17108166	0.001519	0.023073	DOWN
SEC24D	2821.112	-0.7138534	0.225141	-3.17068913	0.001521	0.023073	DOWN
CYB5D2	1007.43	-0.8132331	0.256495	-3.17056669	0.001521	0.023073	DOWN
C19orf45	15.26712	1.47108496	0.463988	3.170521599	0.001522	0.023073	UP
ST6GALNAC	486.8164	0.86874788	0.27401	3.170496612	0.001522	0.023073	UP
SERPINA12	98.06017	1.73859157	0.548382	3.170399519	0.001522	0.023073	UP
CCHCR1	1647.842	0.62873743	0.198339	3.170010544	0.001524	0.023091	UP
SPAG6	4.265934	2.06983555	0.653057	3.169454632	0.001527	0.023121	UP
MTHFD1L	703.1037	0.75246425	0.237427	3.169249501	0.001528	0.023121	UP
RP11-749I	7.093729	1.41905276	0.447781	3.169076934	0.001529	0.023121	UP
PIGC	1738.663	0.44776068	0.141295	3.168984797	0.00153	0.023121	NOT
FNDC5	1628.557	-2.0471651	0.646094	-3.16852284	0.001532	0.023135	DOWN
UBE3D	103.0209	0.63494031	0.200392	3.168483638	0.001532	0.023135	UP
ZFAND5	9707.12	-0.8195614	0.258685	-3.16818385	0.001534	0.023146	DOWN
RP11-933H	12.74057	-1.1927256	0.376494	-3.16798375	0.001535	0.023149	DOWN
CYP2B6	4904.334	-1.6933118	0.534663	-3.16706392	0.00154	0.023209	DOWN
ANGPTL1	246.8049	-1.5075012	0.476105	-3.16631921	0.001544	0.023256	DOWN
MCHR1	266.7421	1.50128379	0.474168	3.166139974	0.001545	0.023257	UP
IER3	1662.027	1.1045446	0.348951	3.165327413	0.001549	0.023299	UP
ZNF276	497.8064	-0.4347503	0.137349	-3.1652911	0.001549	0.023299	NOT
SYVN1	8057.782	-0.5524451	0.174605	-3.16397693	0.001556	0.023388	NOT
METTL21B	371.7067	0.48690071	0.153894	3.163861305	0.001557	0.023388	NOT
HNRNPU	14826.85	0.32389839	0.10238	3.163678386	0.001558	0.02339	NOT
CTC-246B1	20.66519	-1.3896099	0.439327	-3.1630423	0.001561	0.023428	DOWN

HMGB3P10	7.930414	-1.1435095	0.361559	-3.16272318	0.001563	0.023441	DOWN
RP11-359K	13.151	-1.2075476	0.381921	-3.16177175	0.001568	0.023505	DOWN
UBE2Q2P6	3673.346	1.12637435	0.356329	3.161055719	0.001572	0.023549	UP
B4GALNT2	313.4252	2.81416494	0.890443	3.160411111	0.001575	0.023579	UP
TAF9	2137.898	0.56203282	0.177845	3.160238455	0.001576	0.023579	NOT
TDP2	2798.013	0.55778644	0.176503	3.160205513	0.001577	0.023579	NOT
CADM1	4752.781	-0.8867381	0.280634	-3.1597684	0.001579	0.023602	DOWN
ELF4	450.1056	1.15133812	0.364406	3.159488587	0.00158	0.023611	UP
C10orf10	10498.7	-1.1606228	0.367443	-3.15864966	0.001585	0.023666	DOWN
SPDL1	427.6452	0.67217543	0.212862	3.157799655	0.00159	0.023722	UP
RNU6-339F	2.068266	-1.6057735	0.508636	-3.15702035	0.001594	0.023742	DOWN
RSPH1	37.75873	1.44831789	0.458766	3.156989285	0.001594	0.023742	UP
NWD2	24.29602	-2.3754574	0.752461	-3.15691568	0.001594	0.023742	DOWN
RSPH14	21.73709	1.40805818	0.446024	3.156913136	0.001594	0.023742	UP
SLC22A25	683.2944	-1.4806956	0.469117	-3.15634409	0.001598	0.023769	DOWN
PFDN6	2853.138	0.65672436	0.20807	3.156262517	0.001598	0.023769	UP
DUSP11	648.1104	0.37539796	0.118957	3.155751612	0.001601	0.023798	NOT
EPS8L3	1195.454	1.81672321	0.575887	3.154652389	0.001607	0.023875	UP
AIRE	6.799946	2.23983359	0.710252	3.15357675	0.001613	0.02395	UP
RP11-61L1	23.51377	0.66505106	0.21091	3.153250206	0.001615	0.023963	UP
TSPAN15	1166.7	1.25760904	0.398991	3.151972076	0.001622	0.024046	UP
KRBA1	252.4394	0.8857452	0.281017	3.15192727	0.001622	0.024046	UP
FMN2	8.140769	2.92841258	0.929143	3.151734337	0.001623	0.024049	UP
AL163953.	12.59732	2.07706423	0.659092	3.151400633	0.001625	0.024063	UP
AC019109.	2.805729	2.41980112	0.768328	3.149438098	0.001636	0.024199	UP
NVL	740.6178	0.44694002	0.141911	3.149437523	0.001636	0.024199	NOT
RANBP1	2753.356	0.53787331	0.170812	3.148926823	0.001639	0.024215	NOT
AC026202.	28.83566	-1.0734219	0.340889	-3.14889044	0.001639	0.024215	DOWN
MICB	290.6139	1.01681711	0.322932	3.148700748	0.00164	0.024215	UP
CDC14B	1771.474	-1.005551	0.319364	-3.14860138	0.001641	0.024215	DOWN
SSTR2	409.371	1.64087099	0.52133	3.147471984	0.001647	0.024296	UP
BMPER	32.29642	-1.7895397	0.568661	-3.14693491	0.00165	0.024327	DOWN
ECT2	967.7737	0.95291965	0.302841	3.146605278	0.001652	0.024335	UP
CLIP1-AS1	2.348501	-1.2247759	0.389248	-3.1465201	0.001652	0.024335	DOWN
CTD-2561E	7.506159	-1.6227037	0.515751	-3.14629224	0.001654	0.024341	DOWN
RP11-465I	23.11334	1.50564828	0.478811	3.14455766	0.001663	0.024473	UP
KNTC1	887.2637	0.92470991	0.294089	3.144322194	0.001665	0.024479	UP
TCAM1P	39.10216	2.78127368	0.884621	3.14402787	0.001666	0.024486	UP
PPAPDC2	1007.785	-0.6796968	0.216194	-3.14392493	0.001667	0.024486	DOWN
CGNL1	4707.112	-0.9972828	0.317252	-3.14349962	0.001669	0.024493	DOWN
GLO1	6531.897	0.54481093	0.173317	3.143435163	0.00167	0.024493	NOT
RP11-459(	4.127588	4.20875335	1.338935	3.143358182	0.00167	0.024493	UP
KISS1R	11.41467	2.59259972	0.824926	3.142826175	0.001673	0.024525	UP
ADAM23	190.861	1.88478331	0.599768	3.142518668	0.001675	0.024537	UP
POMC	19.02832	-1.0215375	0.325113	-3.14210123	0.001677	0.024548	DOWN
FAM126B	625.6278	-0.4950875	0.157567	-3.14207821	0.001678	0.024548	NOT
TAL1	86.33124	-0.9618006	0.306226	-3.14082116	0.001685	0.02464	DOWN
TRIM67	24.74317	1.26076751	0.401621	3.139195236	0.001694	0.024764	UP
TMEM220	1773.184	-0.9682782	0.308468	-3.13898994	0.001695	0.024768	DOWN

CXorf36	568.925	-0.5973311	0.190409	-3.1370945	0.001706	0.024915	DOWN
C10orf2	583.2598	0.59927011	0.191052	3.136680526	0.001709	0.024931	UP
LGI3	52.87258	2.1788565	0.694656	3.136597041	0.001709	0.024931	UP
PPIAP11	23.28743	0.87243571	0.278194	3.13606592	0.001712	0.024959	UP
CALCR	8.16305	2.12006024	0.676098	3.135729331	0.001714	0.024959	UP
RNF145	1364.705	0.62868851	0.200502	3.135575031	0.001715	0.024959	UP
ZNF154	65.0502	-0.8501284	0.27114	-3.13538637	0.001716	0.024959	DOWN
DOK4	2560.051	-0.7102011	0.226513	-3.13536046	0.001716	0.024959	DOWN
SAMD10	375.4585	0.53505959	0.170656	3.135317416	0.001717	0.024959	NOT
DTNBP1	605.9804	0.68785724	0.219437	3.13464035	0.001721	0.024995	UP
CACNA1H	2423.939	-1.8592036	0.593126	-3.13458678	0.001721	0.024995	DOWN
CPE	3312.75	-1.124288	0.358756	-3.13384904	0.001725	0.025044	DOWN
RBM28	1060.328	0.3696256	0.117953	3.133659146	0.001726	0.025047	NOT
BARX1	19.54133	2.23167225	0.712248	3.133280718	0.001729	0.025066	UP
AC116035.	2.80548	-1.5696973	0.501044	-3.13285224	0.001731	0.025089	DOWN
EMC3-AS1	66.86194	0.80949156	0.258415	3.13252814	0.001733	0.025102	UP
MFRP	3.032333	-1.4621141	0.466774	-3.13238166	0.001734	0.025102	DOWN
AADAT	416.7917	-1.2166794	0.388501	-3.13172639	0.001738	0.025105	DOWN
TRIM24	3348.635	0.5973941	0.190756	3.131711466	0.001738	0.025105	UP
RP11-389(	116.0003	-0.6618713	0.211356	-3.13155153	0.001739	0.025105	DOWN
RP11-81A2	230.0719	-0.851519	0.271919	-3.13151654	0.001739	0.025105	DOWN
RORB	8.749813	-1.7256026	0.551049	-3.13148409	0.001739	0.025105	DOWN
C5orf66-A	2.231564	-3.87425	1.23727	-3.13128802	0.00174	0.025105	DOWN
YWHAZP2	5.861786	1.21686747	0.388638	3.131104033	0.001742	0.025105	UP
RP13-890H	24.09742	1.15741826	0.369654	3.131085382	0.001742	0.025105	UP
SLC31A1	5703.57	-0.6309747	0.201528	-3.13094672	0.001742	0.025105	DOWN
H2AFZ	6008.162	0.61346105	0.195969	3.130391048	0.001746	0.025139	UP
RP11-15A1	22.68623	-0.7762894	0.248061	-3.12942563	0.001751	0.025208	DOWN
MTCH1	7659.154	0.45957368	0.146875	3.129010025	0.001754	0.025231	NOT
RHCG	89.91643	-2.0152273	0.644173	-3.12839241	0.001758	0.02527	DOWN
DCAF4	446.1948	0.45947803	0.146887	3.128103595	0.001759	0.025273	NOT
ALDH16A1	2125.184	-0.7052545	0.225461	-3.12805443	0.00176	0.025273	DOWN
ECM2	1241.784	-1.1198439	0.358052	-3.1276012	0.001762	0.02529	DOWN
INO80C	724.8107	0.67144838	0.214718	3.127120449	0.001765	0.02529	UP
CTD-2008I	5.852367	3.65589399	1.16914	3.126995302	0.001766	0.02529	UP
SALL4	133.9203	2.16554623	0.692536	3.126978182	0.001766	0.02529	UP
PARP12	2302.122	0.51891895	0.165951	3.126937754	0.001766	0.02529	NOT
SNHG9	262.9702	-1.1332816	0.362427	-3.12692448	0.001766	0.02529	DOWN
JADE1	1434.306	-0.5316103	0.170026	-3.12664479	0.001768	0.0253	NOT
PRKDC	6477.161	0.65458104	0.209377	3.126330995	0.00177	0.025314	UP
RBL2	2479.481	-0.8740143	0.279607	-3.1258654	0.001773	0.025341	DOWN
DLC1	1628.991	-0.7522718	0.240731	-3.12494572	0.001778	0.025404	DOWN
EGFL7	2922.078	-1.0582756	0.338668	-3.12481896	0.001779	0.025404	DOWN
RP11-108K	24.00896	-1.8347748	0.587353	-3.1238017	0.001785	0.025479	DOWN
AC105053.	0.889515	-1.8380893	0.588585	-3.12289506	0.001791	NA	NA
CBX2	431.0196	1.20095853	0.384573	3.122834683	0.001791	0.025549	UP
TTC26	158.6375	0.82293425	0.263613	3.121747382	0.001798	0.02563	UP
TNRC6A	2040.471	-0.5297973	0.169721	-3.1215829	0.001799	0.025631	NOT
RRM2	2042.839	0.98392222	0.315309	3.120496596	0.001805	0.025703	UP

HHATL	3.079111	-1.5012356	0.481097	-3.1204449	0.001806	0.025703	DOWN
KIAA1429	2887.633	0.46184305	0.148016	3.120227014	0.001807	0.025709	NOT
RP11-245(	5.42054	2.11925051	0.679278	3.119859377	0.001809	0.025728	UP
FBLN5	1102.765	-1.0874287	0.348596	-3.11945374	0.001812	0.02575	DOWN
HCAR1	13.44307	1.55082903	0.497208	3.119072134	0.001814	0.025769	UP
RPS7	22012.42	0.65269355	0.209436	3.116433244	0.001831	0.025988	UP
THA1P	4.127023	-1.3227939	0.424497	-3.11614668	0.001832	0.025988	DOWN
RP11-564I	11.3081	1.42183576	0.456284	3.11612198	0.001832	0.025988	UP
TNNT3	6.168314	-1.5475294	0.496691	-3.11567623	0.001835	0.026014	DOWN
TRPM5	5.886819	1.92295812	0.617269	3.115268401	0.001838	0.026026	UP
NNT	7940.487	-0.7969336	0.255819	-3.11522702	0.001838	0.026026	DOWN
PPIAP22	1186.516	0.51843636	0.166438	3.11489527	0.00184	0.02603	NOT
CYLD	1549.475	-0.5376431	0.172605	-3.11487359	0.00184	0.02603	NOT
OSBPL3	513.0628	0.83632343	0.268549	3.114227531	0.001844	0.026074	UP
ZNF181	497.826	-0.5221992	0.167721	-3.1135045	0.001849	0.026124	NOT
RRP15	1102.205	0.46062709	0.147973	3.112916803	0.001852	0.026151	NOT
RP11-126I	9.763602	1.36743764	0.439283	3.1128857	0.001853	0.026151	UP
AC012314.	39.64928	-0.88241	0.283528	-3.11225163	0.001857	0.026174	DOWN
SMPDL3B	138.0369	1.82275927	0.585682	3.11220155	0.001857	0.026174	UP
CTD-3037(	7.857551	2.46999107	0.793655	3.112173471	0.001857	0.026174	UP
FICD	519.8122	-0.5854328	0.188123	-3.11197418	0.001858	0.026178	DOWN
IRAK2	1213.211	0.74364864	0.238976	3.11180999	0.001859	0.026179	UP
BACE1	2244.048	-0.5289264	0.170006	-3.11121507	0.001863	0.026218	NOT
ACTA2	5373.711	-0.9079858	0.291997	-3.10957308	0.001874	0.026351	DOWN
TLE4	318.7413	-0.9261925	0.297969	-3.10834762	0.001881	0.026446	DOWN
PMFBP1	58.97156	1.15021255	0.370117	3.107702241	0.001885	0.026478	UP
RP5-1182A	13.99375	-1.9353383	0.622759	-3.10768586	0.001886	0.026478	DOWN
CD82	2266.577	-0.7039313	0.226531	-3.10744317	0.001887	0.026482	DOWN
SPTBN4	96.63944	-1.243587	0.400236	-3.10713253	0.001889	0.026482	DOWN
DVL2	1167.163	0.57207399	0.184118	3.107100688	0.001889	0.026482	NOT
TCN1	4.205682	2.34526086	0.754825	3.107027934	0.00189	0.026482	UP
RP1-232P2	33.43604	-2.5818195	0.831026	-3.10678418	0.001891	0.026491	DOWN
EEF1D	7120.882	0.62809596	0.202193	3.106413765	0.001894	0.026509	UP
SOX7	181.7593	-0.9139339	0.294222	-3.1062683	0.001895	0.026509	DOWN
RP11-589N	53.69681	0.85309278	0.274672	3.105864944	0.001897	0.026532	UP
ZNF540	55.10117	-0.8799079	0.283435	-3.10444475	0.001906	0.026646	DOWN
RP11-473M	6.275183	-1.8735417	0.603598	-3.10395768	0.00191	0.026676	DOWN
WDR75	1358.726	0.44856029	0.144531	3.103557183	0.001912	0.026692	NOT
SNAPIN	1520.877	0.4110738	0.132456	3.103474506	0.001913	0.026692	NOT
HKDC1	2490.595	1.31675734	0.424357	3.102946206	0.001916	0.026725	UP
SLC5A11	184.4193	1.59092001	0.512735	3.102809662	0.001917	0.026725	UP
KDM4A-AS1	47.59343	0.8198281	0.264289	3.102012913	0.001922	0.026768	UP
KIAA1109	1730.317	-0.6464905	0.208413	-3.10197003	0.001922	0.026768	DOWN
ST8SIA4	295.6274	-0.9793684	0.315735	-3.10186989	0.001923	0.026768	DOWN
NME1	3344.352	0.80839401	0.260637	3.10160507	0.001925	0.026779	UP
SLCO6A1	18.83999	3.28097013	1.057952	3.101247846	0.001927	0.026797	UP
ZNF791	820.0556	-0.4671676	0.150675	-3.1004909	0.001932	0.026852	NOT
RP11-274H	12.77986	1.25754766	0.405652	3.100066896	0.001935	0.026877	UP
MBNL3	6404.63	0.98945542	0.319376	3.098084948	0.001948	0.027036	UP

SNX5	3716.028	0.42216255	0.136269	3.098013836	0.001948	0.027036	NOT
MUTYH	572.0409	0.49777903	0.16069	3.097754071	0.00195	0.027046	NOT
TBL1XR1	3971.175	0.39956585	0.128997	3.09747979	0.001952	0.027057	NOT
SFT2D1	659.4109	0.58489955	0.188853	3.097123626	0.001954	0.02707	NOT
CCDC146	220.5919	-0.9537434	0.307954	-3.09702986	0.001955	0.02707	DOWN
FER1L6-AS	2.012979	3.33932081	1.07833	3.096751897	0.001957	0.027082	UP
CST2	27.19502	2.22540407	0.718761	3.096165518	0.00196	0.027122	UP
GPR116	1028.721	-0.6633153	0.214268	-3.0957233	0.001963	0.027138	DOWN
USH1C	660.312	2.5006667	0.807792	3.09568117	0.001964	0.027138	UP
SLC24A4	14.12318	-0.8205324	0.265075	-3.09546711	0.001965	0.02714	DOWN
C8A	10761.07	-1.3568545	0.438351	-3.09536407	0.001966	0.02714	DOWN
PCDHGA5	30.35342	-1.1027159	0.356434	-3.09374681	0.001976	0.027267	DOWN
RP11-1182	21.17718	-1.7176233	0.555211	-3.09363828	0.001977	0.027267	DOWN
AVPI1	998.997	-0.7278377	0.235278	-3.09352431	0.001978	0.027267	DOWN
TOP2A	3551.125	1.11595724	0.360808	3.09293786	0.001982	0.027307	UP
PGRMC2	5341.586	-0.4666455	0.150891	-3.09259418	0.001984	0.027312	NOT
RP11-736K	85.2992	-1.0079254	0.325925	-3.09250492	0.001985	0.027312	DOWN
LEMD2	2241.297	0.39960972	0.129222	3.09243178	0.001985	0.027312	NOT
GNB5	2120.809	-0.7735074	0.250191	-3.09166731	0.00199	0.027369	DOWN
GRN	19055.29	0.5952921	0.192588	3.091014113	0.001995	0.027409	UP
GIP	2.188015	2.91060459	0.941692	3.09082373	0.001996	0.027409	UP
SPATA18	263.6649	-1.6105549	0.521083	-3.09078089	0.001996	0.027409	DOWN
C12orf65	871.5922	0.37743322	0.12214	3.090159923	0.002	0.027452	NOT
GSG2	60.52317	1.05458597	0.341294	3.089959986	0.002002	0.027457	UP
SMC5-AS1	14.76962	-0.7516661	0.243379	-3.08846365	0.002012	0.027578	DOWN
RP11-736K	47.64202	-0.9883775	0.320034	-3.08835467	0.002013	0.027578	DOWN
SLC26A11	1006.619	0.48926756	0.158484	3.087170071	0.002021	0.027674	NOT
FILIP1L	738.3819	-0.9400413	0.304545	-3.08670688	0.002024	0.027695	DOWN
CHEK2	500.7453	0.69325556	0.224599	3.086641557	0.002024	0.027695	UP
NT5DC2	2214.203	1.23231465	0.399264	3.086463027	0.002026	0.027698	UP
LINC00657	10722.38	-0.3896322	0.126277	-3.08553738	0.002032	0.02777	NOT
MAST3	1472.394	-0.6078628	0.197023	-3.08523959	0.002034	0.027784	DOWN
RP11-817O	113.3616	-0.8432809	0.273344	-3.08505107	0.002035	0.027788	DOWN
TAPT1	1569.308	-0.5607506	0.181789	-3.0846278	0.002038	0.027797	NOT
CHRNE	106.4467	-1.0876844	0.352624	-3.08454539	0.002039	0.027797	DOWN
KIAA1841	283.5518	0.67457739	0.218699	3.084506988	0.002039	0.027797	UP
HHIP	54.60844	-1.9787506	0.641597	-3.08410276	0.002042	0.02782	DOWN
RBL1	390.1711	0.77787397	0.252275	3.083432585	0.002046	0.027864	UP
ZNF534	6.331617	2.55773876	0.829536	3.08333682	0.002047	0.027864	UP
RP11-120E	15.72915	-1.0224056	0.331615	-3.08311091	0.002048	0.027871	DOWN
RP11-813N	11.59072	1.76699676	0.573189	3.082745501	0.002051	0.027892	UP
RP11-680O	11.91391	-0.920903	0.298824	-3.0817592	0.002058	0.027965	DOWN
RHEBL1	47.99935	0.77117941	0.250247	3.081670362	0.002058	0.027965	UP
MTMR9LP	149.903	-0.7237579	0.234891	-3.08125348	0.002061	0.027988	DOWN
ACOT11	70.81953	1.35809614	0.440825	3.080806071	0.002064	0.027988	UP
CTC-277H1	2.530007	-1.1874836	0.385446	-3.08080322	0.002064	0.027988	DOWN
IL20RB	66.03654	-1.1395898	0.369908	-3.08073996	0.002065	0.027988	DOWN
TMTC1	544.9572	-1.0447678	0.339144	-3.08060131	0.002066	0.027988	DOWN
TBX19	105.8689	0.60136465	0.195215	3.080530495	0.002066	0.027988	UP

RP11-525A	43.95529	1.83410654	0.595423	3.080340145	0.002068	0.027992	UP
KLHDC1	71.61733	-0.8280825	0.268908	-3.07943006	0.002074	0.028053	DOWN
TCOF1	2355.818	0.55717129	0.180936	3.079386446	0.002074	0.028053	NOT
TOMM34	1659.48	0.58080721	0.188633	3.07904072	0.002077	0.028072	NOT
RP11-164(	15.24681	-1.4089433	0.45777	-3.07783857	0.002085	0.028171	DOWN
ISOC2	6985.174	-0.7759886	0.25219	-3.07699568	0.002091	0.028237	DOWN
RP11-167N	2.813045	-2.7016926	0.878201	-3.07639313	0.002095	0.028273	DOWN
DRD1	57.7674	-2.1262429	0.691203	-3.07614746	0.002097	0.028273	DOWN
MON1B	1663.386	-0.4501592	0.146347	-3.07596754	0.002098	0.028273	NOT
AC012360.	2.580257	-1.3898659	0.451863	-3.07585787	0.002099	0.028273	DOWN
NOTCH4	1134.216	-0.5970546	0.194117	-3.07574974	0.0021	0.028273	DOWN
RP11-890E	176.7968	-0.7060143	0.229544	-3.07572468	0.0021	0.028273	DOWN
HIST1H4PS	2.5595	-1.7144485	0.557477	-3.07536874	0.002102	0.028293	DOWN
CDYL	639.4851	0.38274583	0.124483	3.074691425	0.002107	0.028339	NOT
MTCL1	372.8317	1.30710865	0.425141	3.074531049	0.002108	0.028339	UP
SH3BP1	466.229	1.003597	0.326433	3.074436413	0.002109	0.028339	UP
UTS2	16.37441	1.95497053	0.636007	3.073820739	0.002113	0.028384	UP
RP11-175K	11.94558	-0.7058316	0.229647	-3.07354393	0.002115	0.028396	DOWN
GPD1L	513.5458	0.82135044	0.267258	3.073252522	0.002117	0.0284	UP
CTA-941F9	10.69104	-1.9190145	0.624451	-3.07312309	0.002118	0.0284	DOWN
RPF2	1526.859	0.59107749	0.192341	3.073063756	0.002119	0.0284	UP
PFKP	1542.137	1.41191975	0.459487	3.072816022	0.00212	0.028402	UP
LINGO4	84.19731	-1.6531868	0.538015	-3.07274973	0.002121	0.028402	DOWN
RN7SL751F	3.280263	2.36209966	0.768784	3.072513903	0.002123	0.028406	UP
RP11-373I	9.256887	-1.0307767	0.335494	-3.07241259	0.002123	0.028406	DOWN
RP11-746F	1.930638	2.30088906	0.749004	3.071931701	0.002127	0.028416	UP
VDAC1P8	92.45156	0.68065999	0.221586	3.071768886	0.002128	0.028416	UP
NDUFAF2	685.1606	0.69047767	0.224786	3.07171786	0.002128	0.028416	UP
GPR75	69.85124	-1.351653	0.440032	-3.07171425	0.002128	0.028416	DOWN
NTF3	32.13952	-1.4428491	0.469864	-3.07078322	0.002135	0.028491	DOWN
ACOX1	12383.51	-0.8159603	0.265739	-3.07053798	0.002137	0.0285	DOWN
EME1	158.1602	0.94272778	0.307051	3.070263401	0.002139	0.028513	UP
KNOP1P5	8.033084	3.72716325	1.214108	3.069877621	0.002141	0.028535	UP
RP11-778I	5.989068	-0.9803384	0.319379	-3.06951368	0.002144	0.028556	DOWN
HMGN4	1051.144	0.61780069	0.201342	3.068406801	0.002152	0.028648	UP
RP11-80H1	2.046866	-1.1951856	0.389559	-3.06804861	0.002155	0.028668	DOWN
GDPD4	23.46487	-1.4950183	0.487357	-3.06760485	0.002158	0.028689	DOWN
RBM24	326.8097	1.40225087	0.457126	3.067534702	0.002158	0.028689	UP
FBXL6	1551.182	0.7357587	0.239864	3.06739667	0.002159	0.028689	UP
ZNF707	455.9274	0.45407043	0.148043	3.067161855	0.002161	0.028693	NOT
G6PC3	1389.727	0.63336081	0.206504	3.067059049	0.002162	0.028693	UP
C8orf46	170.7672	-1.1338703	0.369787	-3.06627701	0.002167	0.028754	DOWN
PDE7A	639.4874	0.76867432	0.250701	3.066098744	0.002169	0.028756	UP
ZNF816	129.2888	1.06868894	0.348565	3.065969746	0.00217	0.028756	UP
ZNHIT3	1120.735	0.3771825	0.123063	3.064942475	0.002177	0.02884	NOT
RP11-223A	11.40792	-0.7889741	0.257442	-3.0646707	0.002179	0.028853	DOWN
RP11-242(	11.62642	-1.1637903	0.379776	-3.06441618	0.002181	0.028853	DOWN
FBXL17	1092.374	-0.5573008	0.181869	-3.0642941	0.002182	0.028853	NOT
PIGS	1293.844	0.61877149	0.201934	3.064227402	0.002182	0.028853	UP

WARS2-IT1	2.858514	-1.7169227	0.560398	-3.06375855	0.002186	0.028884	DOWN
CACYBPP2	8.269377	1.03490007	0.337885	3.062877532	0.002192	0.02895	UP
C3	498608.1	-0.9358654	0.30556	-3.06279064	0.002193	0.02895	DOWN
ENPP5	137.0696	1.96890504	0.643024	3.061948178	0.002199	0.029017	UP
PLGLB1	120.0964	-1.2717635	0.41548	-3.06094771	0.002206	0.029097	DOWN
AC006547.	11.62537	-0.8694064	0.284042	-3.06083347	0.002207	0.029097	DOWN
SPATA17	32.5753	1.95658871	0.639324	3.060401946	0.00221	0.029106	UP
RP11-264I	42.96941	-0.6879068	0.224782	-3.06032192	0.002211	0.029106	DOWN
SGOL1	227.5135	0.99844795	0.326258	3.060304507	0.002211	0.029106	UP
FAM90A1	17.14517	1.69088369	0.552557	3.060109023	0.002213	0.029111	UP
BPIFB2	229.2731	2.43970037	0.797377	3.059656306	0.002216	0.029141	UP
TPM3P9	226.5617	0.8852122	0.289393	3.058859512	0.002222	0.029186	UP
ZBED6CL	1156.906	0.82611938	0.27008	3.058793143	0.002222	0.029186	UP
RP11-374F	7.268161	-1.2358322	0.404042	-3.05867003	0.002223	0.029186	DOWN
GALNT15	183.9171	-1.1798244	0.385754	-3.05848666	0.002225	0.029186	DOWN
LRRC75A-A	7053.7	0.83474376	0.272929	3.05846786	0.002225	0.029186	UP
USP21	925.9342	0.50062682	0.163757	3.057123369	0.002235	0.029298	NOT
RP11-470F	2.386712	-2.5200233	0.824337	-3.05703045	0.002235	0.029298	DOWN
RANP1	7.651505	0.9897584	0.323788	3.056806436	0.002237	0.029306	UP
METTL18	341.7029	0.51295792	0.167818	3.056623844	0.002238	0.029308	NOT
NIF3L1	881.4709	0.35401601	0.115824	3.056495471	0.002239	0.029308	NOT
DCAF16	803.2532	0.6567313	0.214883	3.056232586	0.002241	0.029319	UP
RBM14-RBM	40.53464	-0.4956615	0.162204	-3.0558004	0.002245	0.029334	NOT
HOXA-AS3	5.820895	2.10448162	0.688701	3.055728178	0.002245	0.029334	UP
PNO1	940.1147	0.43201506	0.141382	3.055647935	0.002246	0.029334	NOT
ADCK5	820.3035	0.721184	0.236032	3.055448441	0.002247	0.02934	UP
OVOL2	28.49386	2.41459812	0.790297	3.055303248	0.002248	0.02934	UP
MAP6D1	57.21202	-0.7220608	0.236388	-3.05455759	0.002254	0.029386	DOWN
RP11-44D5	2.546936	-1.5930839	0.521546	-3.05454052	0.002254	0.029386	DOWN
NCAM2	56.04983	-1.8533419	0.606796	-3.05430879	0.002256	0.029395	DOWN
VILL	94.56332	-0.8619262	0.282233	-3.05395671	0.002258	0.029407	DOWN
RP13-650J	252.8528	-1.7696096	0.57946	-3.05389627	0.002259	0.029407	DOWN
PCDHB11	52.04993	-1.394719	0.456725	-3.05374234	0.00226	0.029408	DOWN
PRR13P5	41.86932	0.60203492	0.19717	3.053386083	0.002263	0.029429	UP
PRR34-AS1	178.3904	1.10258645	0.361211	3.052472051	0.00227	0.029491	UP
SCAND2P	104.771	-0.5043947	0.165242	-3.05246179	0.00227	0.029491	NOT
IDH2	13654.67	-0.8252771	0.270377	-3.05231496	0.002271	0.029492	DOWN
AC025335.	78.53689	0.90358557	0.29612	3.051420418	0.002278	0.029552	UP
OPN1SW	70.73769	0.60894635	0.199562	3.051410691	0.002278	0.029552	UP
COL14A1	1254.717	-1.3244838	0.434121	-3.05095206	0.002281	0.029583	DOWN
RP11-757C	5.998335	-2.6885216	0.881314	-3.05058359	0.002284	0.029605	DOWN
MIP	15.09597	-1.232721	0.404157	-3.05010439	0.002288	0.029626	DOWN
ZNF74	424.4208	0.49551125	0.162458	3.050083888	0.002288	0.029626	NOT
TMEM179	31.10231	2.99028348	0.980523	3.04968102	0.002291	0.029652	UP
AF131217.	9.270392	-1.2827946	0.420659	-3.04948465	0.002292	0.029657	DOWN
RP11-932C	2.307084	-1.2397322	0.406599	-3.04903131	0.002296	0.029676	DOWN
PTF1A	8.138372	4.01195159	1.31582	3.049013341	0.002296	0.029676	UP
FAM122A	759.1194	-0.4625833	0.151723	-3.04886666	0.002297	0.029676	NOT
TRANK1	1148.453	-0.7205421	0.23635	-3.04862453	0.002299	0.029686	DOWN

CD99L2	4962.213	-0.6920677	0.22706	-3.04794865	0.002304	0.029738	DOWN
DNAJC10	2699.374	0.54095789	0.177516	3.04738019	0.002308	0.029781	NOT
KCTD19	2.528717	2.41080321	0.791246	3.046842652	0.002313	0.02982	UP
SLC2A8	1587.557	-0.5957484	0.195541	-3.04666096	0.002314	0.029823	DOWN
PLD3	9234.314	-0.4314379	0.14163	-3.04623091	0.002317	0.029852	NOT
PRPF3	1910.416	0.51172711	0.167995	3.046084627	0.002318	0.029852	NOT
FCF1P2	160.0481	0.43014506	0.141259	3.045071372	0.002326	0.029914	NOT
CYP17A1-A	4.747477	-1.5359957	0.504426	-3.04503691	0.002327	0.029914	DOWN
MTURN	441.9968	-0.8232298	0.270351	-3.04503677	0.002327	0.029914	DOWN
SFR1	169.2483	0.53671309	0.176267	3.044886913	0.002328	0.029915	NOT
COX6B1P4	3.08512	-1.4129721	0.464075	-3.04470748	0.002329	0.029918	DOWN
HSPE1P2	11.62415	1.04058167	0.341879	3.043717432	0.002337	0.030003	UP
LINC0141	4.631506	-0.9737851	0.319953	-3.04352664	0.002338	0.030008	DOWN
TRIM40	10.91205	1.79774784	0.590827	3.042766396	0.002344	0.030069	UP
UTP11L	1094.401	0.41794775	0.137412	3.041560037	0.002354	0.030176	NOT
STK25	3834.964	0.42282094	0.13903	3.041224678	0.002356	0.030182	NOT
ZNF521	97.20465	-0.9115224	0.299734	-3.04110061	0.002357	0.030182	DOWN
RP11-417I	7.700724	-1.1102222	0.365076	-3.0410738	0.002357	0.030182	DOWN
RP4-6050	43.62299	-0.9663133	0.317805	-3.04058114	0.002361	0.030217	DOWN
VIPR1	126.3872	-1.3785203	0.453439	-3.04014419	0.002365	0.030247	DOWN
FBXO6	1046.012	0.67085395	0.220683	3.039905483	0.002367	0.030256	UP
CTC-260E	42.66899	1.58041508	0.519949	3.039557361	0.002369	0.030277	UP
TRIM59	155.5074	0.99056573	0.325947	3.039041685	0.002373	0.030315	UP
RUNX1T1	74.59019	-1.0018944	0.329692	-3.03887788	0.002375	0.030317	DOWN
VCAM1	2137.49	1.23200361	0.405577	3.037653117	0.002384	0.030412	UP
CCDC137	1235.606	0.48672331	0.16023	3.037645047	0.002384	0.030412	NOT
DSTNP2	181.3914	0.66202111	0.217959	3.037365732	0.002387	0.030426	UP
HNRNPA3P	38.05053	0.62410351	0.205535	3.036489241	0.002394	0.030501	UP
RSAD1	2750.07	-0.4714711	0.15529	-3.03606245	0.002397	0.030529	NOT
PPP1R13L	781.0339	0.77777869	0.256282	3.03485155	0.002407	0.030632	UP
RP11-315I	2.617584	-1.6520678	0.544394	-3.03469079	0.002408	0.030632	DOWN
CDH5	1761.568	-0.6598772	0.217449	-3.03462865	0.002408	0.030632	DOWN
LCN15	7.535823	2.90129237	0.956243	3.034053454	0.002413	0.030676	UP
FAM83H	5974.247	0.82829652	0.273052	3.033479955	0.002418	0.03072	UP
MAPK4	189.1407	-2.0072571	0.66177	-3.03316209	0.00242	0.030738	DOWN
CACNA1I	23.66946	-1.6644122	0.548919	-3.03216313	0.002428	0.030813	DOWN
CDX2	6.24055	2.26737348	0.747791	3.032095215	0.002429	0.030813	UP
RP11-405A	2.348077	-2.2281534	0.734947	-3.03171838	0.002432	0.030813	DOWN
TMEM220-A	206.7124	-1.0221458	0.337155	-3.03168072	0.002432	0.030813	DOWN
CTC-297N7	75.2936	-1.0464914	0.345191	-3.03162974	0.002432	0.030813	DOWN
ALDH7A1	7874.661	-0.8005468	0.264077	-3.03148799	0.002434	0.030813	DOWN
PCYOX1	8059.183	-0.64087	0.211408	-3.03143921	0.002434	0.030813	DOWN
MYO19	1749.974	0.48399212	0.1597	3.030625898	0.00244	0.030882	NOT
CASP12	7.034314	-1.0023777	0.330779	-3.03035305	0.002443	0.030895	DOWN
APH1A	11969.36	0.3619211	0.119478	3.029190912	0.002452	0.031	NOT
AC016708.	93.00615	1.8653345	0.615869	3.028786407	0.002455	0.031027	UP
ATP6V0A4	2.954925	2.41265549	0.79665	3.028502151	0.002458	0.031031	UP
CCDC158	79.63932	-1.1260332	0.371816	-3.02846669	0.002458	0.031031	DOWN
ERMARD	807.7589	-0.4945383	0.163307	-3.02826959	0.00246	0.031037	NOT

MSTN	6.714381	-1.445274	0.477402	-3.02737407	0.002467	0.031114	DOWN
KPNA4	3183.93	0.32219438	0.106433	3.027204383	0.002468	0.031115	NOT
ACP6	1072.664	0.63251919	0.208953	3.027082732	0.002469	0.031115	UP
LILRB5	330.4863	-1.0774012	0.355979	-3.0265867	0.002473	0.031142	DOWN
ERG	372.8992	-0.6531692	0.215814	-3.02654148	0.002474	0.031142	DOWN
PAMR1	170.3932	-1.0257686	0.338974	-3.02610174	0.002477	0.031173	DOWN
UPK1A	23.04552	1.72852628	0.571248	3.025878255	0.002479	0.031182	UP
MIR3682	28.16472	-1.038234	0.343144	-3.02564701	0.002481	0.031191	DOWN
AC138430.	9.822966	-1.5351881	0.507639	-3.02417214	0.002493	0.031329	DOWN
DARS	2891.954	0.46733505	0.154583	3.023207607	0.002501	0.03141	NOT
SMARCB1	2693.585	0.50412498	0.166757	3.02310828	0.002502	0.03141	NOT
SWSAP1	216.9692	-0.5998612	0.198463	-3.02252788	0.002507	0.031456	DOWN
BCAS4	233.1923	0.91909723	0.30411	3.022248768	0.002509	0.03147	UP
AC144449.	16.43698	-0.9581479	0.317112	-3.02148276	0.002515	0.031535	DOWN
FAM117B	443.1222	0.98130176	0.324865	3.020646979	0.002522	0.031608	UP
EMCN	482.8303	-0.8357987	0.276767	-3.01986411	0.002529	0.031664	DOWN
ETNPPL	3735.483	-1.6557339	0.548287	-3.01983176	0.002529	0.031664	DOWN
LPO	3.192934	2.16471811	0.716889	3.019600541	0.002531	0.031668	UP
KRT79	1.74574	2.77205763	0.918087	3.019384446	0.002533	0.031668	UP
RP11-546J	12.45813	1.01474198	0.336077	3.019371035	0.002533	0.031668	UP
GPSM2	397.1863	0.89567508	0.296659	3.019203313	0.002534	0.031669	UP
BAIAP2	3839.784	-0.8523995	0.282343	-3.01902145	0.002536	0.031669	DOWN
TUBB	30660.61	0.56566299	0.187371	3.018946898	0.002537	0.031669	NOT
RP11-295M	18.69329	-1.3527801	0.448127	-3.01873922	0.002538	0.031676	DOWN
ADAMDEC1	70.50501	1.64268212	0.544201	3.018521511	0.00254	0.031684	UP
PLN	53.17177	-1.3814765	0.457717	-3.01818896	0.002543	0.031704	DOWN
PGR	46.77404	-1.6446224	0.544946	-3.01795601	0.002545	0.031706	DOWN
LRRTM2	6.540536	-0.948681	0.314353	-3.01788477	0.002545	0.031706	DOWN
DDX52	1279.622	0.34895217	0.115633	3.017756384	0.002547	0.031706	NOT
SNORA81	7.825622	-1.1630306	0.385431	-3.01748105	0.002549	0.03172	DOWN
CNTFR	687.7979	-2.0203957	0.669778	-3.01651497	0.002557	0.031807	DOWN
RBM15B	2452.987	0.32938102	0.10923	3.015483166	0.002566	0.031901	NOT
SLC6A9	302.0232	1.11751249	0.370673	3.014820075	0.002571	0.031956	UP
CTD-2014E	1.414159	-2.1496804	0.713199	-3.01414032	0.002577	0.032003	DOWN
NOSTRIN	228.1599	-0.7364073	0.244325	-3.01404588	0.002578	0.032003	DOWN
TDG	637.0591	0.4523384	0.150081	3.013961887	0.002579	0.032003	NOT
RP11-416N	2.372003	1.85448181	0.615369	3.013607357	0.002582	0.032025	UP
BAG2	1053.82	0.75320637	0.249964	3.013264834	0.002585	0.032047	UP
PRPH	3.448846	2.17675351	0.722896	3.011158603	0.002603	0.032255	UP
CEBPZOS	2065.742	0.4494532	0.149303	3.010342194	0.00261	0.032327	NOT
GJD3	2.290183	-1.5215476	0.505487	-3.01006108	0.002612	0.032342	DOWN
SYCE1	15.9636	2.76667249	0.919438	3.009091938	0.00262	0.032417	UP
SASH1	914.5534	-0.7015651	0.233155	-3.0090066	0.002621	0.032417	DOWN
RARA	3155.014	-0.5389977	0.179131	-3.00895031	0.002622	0.032417	NOT
ZNF252P-A	20.28286	0.87174203	0.289881	3.007244657	0.002636	0.032573	UP
RP11-24F1	9.788989	1.34962751	0.448797	3.007209472	0.002637	0.032573	UP
UVSSA	345.4981	-0.5901927	0.196299	-3.00660188	0.002642	0.032623	DOWN
CCNB2	699.0316	0.98196385	0.326761	3.005148062	0.002655	0.032765	UP
MLK4	571.1338	1.1524203	0.383524	3.004819666	0.002657	0.032785	UP

RP11-386(	11.46981	1.34189441	0.446716	3.003907789	0.002665	0.032858	UP
TMEM68	778.9323	0.49207822	0.163815	3.003870076	0.002666	0.032858	NOT
RHPN1	505.5356	1.17703412	0.391903	3.003382635	0.00267	0.032896	UP
RP11-468M	13.81482	2.65462516	0.883954	3.003127504	0.002672	0.032908	UP
PUS1	1492.501	0.64867602	0.21602	3.002855016	0.002675	0.032923	UP
BCL2L2-PA	12.41666	0.88669488	0.295312	3.002569712	0.002677	0.032939	UP
RP5-1103E	17.11541	-1.2328997	0.410654	-3.00228516	0.00268	0.032955	DOWN
DUSP12	572.7082	0.48371802	0.161172	3.00126075	0.002689	0.033051	NOT
TXNRD1	12898.08	1.04266483	0.347508	3.00040334	0.002696	0.033129	UP
CFI	19025.71	-0.9624932	0.320845	-2.99987052	0.002701	0.033172	DOWN
ARPC3	7586.714	0.49255125	0.164212	2.999489792	0.002704	0.033194	NOT
RP11-50C1	66.31081	0.66027604	0.220137	2.99938984	0.002705	0.033194	UP
D4S234E	14.83333	-1.2650765	0.422093	-2.9971486	0.002725	0.033396	DOWN
LA16c-429	9.983985	-1.1557439	0.385626	-2.99706054	0.002726	0.033396	DOWN
LRRC63	8.342659	1.34960575	0.45032	2.996993743	0.002727	0.033396	UP
CRISP2	6.558404	4.52596618	1.510172	2.996987313	0.002727	0.033396	UP
UBD	4478.516	1.21132612	0.404266	2.996361056	0.002732	0.03345	UP
RP1-12G14	9.044893	-0.8451504	0.282161	-2.99528012	0.002742	0.033554	DOWN
RP11-498(	23.06804	1.79334639	0.598803	2.994887349	0.002745	0.033579	UP
TRPM3	44.55086	-1.5824448	0.528402	-2.99477461	0.002746	0.033579	DOWN
COQ9	3114.555	-0.6238784	0.208371	-2.994071	0.002753	0.033615	DOWN
ZZEF1	1439.758	-0.4932523	0.164745	-2.99404357	0.002753	0.033615	NOT
RP11-428F	4.293146	1.56349336	0.522202	2.994036712	0.002753	0.033615	UP
GALNT16	143.818	-1.1078457	0.370053	-2.99375189	0.002756	0.033631	DOWN
PHOSPHO2	93.151	0.65771675	0.219749	2.993034421	0.002762	0.033695	UP
TCF19	1228.933	0.94017903	0.314188	2.992407872	0.002768	0.033749	UP
SLC26A9	19.24429	1.86928706	0.624759	2.992011118	0.002771	0.033777	UP
LRRC70	12.50345	-0.9042147	0.302223	-2.99188012	0.002773	0.033777	DOWN
MYCNOS	9.836777	2.33956327	0.78204	2.991615687	0.002775	0.033791	UP
KIF3B	3604.703	-0.4830418	0.161519	-2.99061105	0.002784	0.033885	NOT
HSPB11	1004.755	0.47088793	0.157461	2.99049595	0.002785	0.033885	NOT
PIK3CD-AS	39.62711	1.65816571	0.554535	2.990189089	0.002788	0.0339	UP
RP11-495F	21.2514	2.98189943	0.997286	2.990014594	0.00279	0.0339	UP
RCC1	1565.444	0.58423908	0.195401	2.989946932	0.00279	0.0339	NOT
OR56A3	3.10936	4.31621331	1.443649	2.989794026	0.002792	0.033902	UP
RP11-2B6.	8.349392	-1.0628787	0.355524	-2.98960771	0.002793	0.033908	DOWN
UBE2Z	4118.992	0.39101513	0.130806	2.989273168	0.002796	0.03393	NOT
ADH4	25295.08	-1.9698471	0.659149	-2.98846895	0.002804	0.034004	DOWN
VGLL3	102.7776	-1.1811836	0.39532	-2.98791586	0.002809	0.03405	DOWN
RPL8	77327.53	0.86168269	0.288446	2.987324424	0.002814	0.034096	UP
CTD-2314F	4.229494	2.7941992	0.935379	2.987236935	0.002815	0.034096	UP
LINC01208	2.951752	2.45801294	0.822983	2.986710018	0.00282	0.034102	UP
SHOX2	21.72365	1.48144322	0.496055	2.986450067	0.002822	0.034102	UP
RP11-491F	2.263398	-1.6842029	0.563964	-2.98636802	0.002823	0.034102	DOWN
TMEM44	348.2747	0.77734483	0.260304	2.986297768	0.002824	0.034102	UP
PHF8	2215.105	-0.7079151	0.237056	-2.98627764	0.002824	0.034102	DOWN
PDX1-AS1	5.022653	2.45713286	0.822814	2.986255941	0.002824	0.034102	UP
BEST3	3.516046	2.73698534	0.916536	2.986228416	0.002824	0.034102	UP
RP11-503F	2.871306	-1.1017626	0.369001	-2.98580226	0.002828	0.034134	DOWN

ZCCHC14	2204.567	-0.5373077	0.179989	-2.98522411	0.002834	0.034183	NOT
MED8	2221.114	0.38725197	0.129748	2.98464206	0.002839	0.034233	NOT
IVD	7359.088	-0.6774971	0.227076	-2.98357193	0.002849	0.034333	DOWN
WDSUB1	368.7379	0.49607433	0.166274	2.9834818	0.00285	0.034333	NOT
DSG2	2523.982	1.08756419	0.364561	2.98321802	0.002852	0.034347	UP
INCA1	97.86586	-0.7097012	0.237931	-2.9828014	0.002856	0.034379	DOWN
MGARP	5.437549	-1.1226798	0.376404	-2.98264851	0.002858	0.034381	DOWN
LRBA	1825.767	-0.5736813	0.19238	-2.9820156	0.002864	0.034437	NOT
DPY19L2P2	35.94962	1.30544261	0.437831	2.98161488	0.002867	0.034448	UP
RP1-302G2	12.06605	1.47639	0.495182	2.981508229	0.002868	0.034448	UP
CYP2A13	98.49339	-2.3124049	0.775584	-2.98150004	0.002868	0.034448	DOWN
TGFBR3	1221.442	-0.9318297	0.312561	-2.98127255	0.002871	0.034448	DOWN
PYDC1	4.872304	2.91054996	0.976289	2.981239355	0.002871	0.034448	UP
DLX1	6.987621	1.74577489	0.585637	2.98098706	0.002873	0.034461	UP
OTOP3	4.298728	-3.3680246	1.129988	-2.98058381	0.002877	0.034491	DOWN
C6orf132	202.858	1.82923629	0.613898	2.97970822	0.002885	0.034563	UP
TSEN15	1186.032	0.40148289	0.13474	2.979675258	0.002886	0.034563	NOT
CTC-490E2	5.442568	-2.3158183	0.777258	-2.97947174	0.002887	0.03457	DOWN
CFAP45	22.71201	1.32953382	0.446291	2.979070849	0.002891	0.0346	UP
LA16c-359	2.986979	-1.1664263	0.39158	-2.97876743	0.002894	0.03462	DOWN
RP11-400N	3.282886	-1.9295263	0.648028	-2.97753339	0.002906	0.034742	DOWN
SNRPD3	4750.597	0.52124923	0.175068	2.97741032	0.002907	0.034742	NOT
SDCBP2	588.8431	1.32008964	0.443408	2.977144588	0.002909	0.034757	UP
CORO2A	664.0468	1.06660258	0.358325	2.976636504	0.002914	0.034797	UP
TRIM31	461.5829	1.47991232	0.497215	2.976405274	0.002916	0.034797	UP
CIRH1A	1377.293	0.4726126	0.158787	2.976388387	0.002917	0.034797	NOT
CTD-2013N	43.648	-0.8857735	0.29764	-2.97598928	0.00292	0.034827	DOWN
SMARCA2	2885.335	-0.7140775	0.240084	-2.97428495	0.002937	0.035006	DOWN
RAD18	590.158	0.40746602	0.137009	2.974004833	0.002939	0.035022	NOT
DNAJB5	341.8343	-0.620554	0.208688	-2.97359276	0.002943	0.035049	DOWN
RP11-347C	1.71267	-1.5667642	0.526962	-2.97320378	0.002947	0.035049	DOWN
GOLGB1	4638.609	-0.4321481	0.14535	-2.97314717	0.002948	0.035049	NOT
CALY	16.60421	1.77641942	0.597498	2.973099263	0.002948	0.035049	UP
CDRT1	25.25477	1.94117427	0.652928	2.973028029	0.002949	0.035049	UP
CFHR4	2152.745	-1.7989217	0.605093	-2.97296821	0.002949	0.035049	DOWN
FAM214A	1253.466	-0.5915783	0.199	-2.97274796	0.002951	0.035052	DOWN
AGFG1	2750.047	0.38826817	0.130618	2.972540412	0.002953	0.035052	NOT
IBSP	14.90748	2.11277567	0.710766	2.972531659	0.002954	0.035052	UP
GABRB1	2.656175	3.05927459	1.029321	2.972128348	0.002957	0.035083	UP
RP11-166I	154.3874	-1.13918	0.383379	-2.97142335	0.002964	0.035148	DOWN
IMPDH1	993.7082	1.01885299	0.342931	2.971016788	0.002968	0.035165	UP
SNHG6	2662.386	0.84881379	0.285704	2.970958183	0.002969	0.035165	UP
CTD-2008A	2.902191	-1.6277211	0.547916	-2.97074998	0.002971	0.035165	DOWN
SLC7A1	669.2065	1.22413431	0.412075	2.970662326	0.002972	0.035165	UP
RP1-317E2	41.7167	-0.7579464	0.255149	-2.9706079	0.002972	0.035165	DOWN
CKS1B	1382.291	0.65518462	0.220582	2.970256274	0.002976	0.03519	UP
CTA-384D8	22.78511	1.18273053	0.398259	2.969753119	0.00298	0.035232	UP
RP11-331F	7.93558	-1.0177027	0.342726	-2.96943576	0.002983	0.035253	DOWN
GDAP1	315.502	0.99786014	0.336072	2.969188377	0.002986	0.035265	UP

MT-RNR2	414490.9	-0.7727106	0.260254	-2.96906613	0.002987	0.035265	DOWN
FITM1	68.89155	-1.0539396	0.35505	-2.96842353	0.002993	0.035323	DOWN
LRSAM1	1295.368	-0.6408106	0.215905	-2.96802041	0.002997	0.035354	DOWN
METTL20	283.253	-0.6547949	0.220636	-2.96776222	0.003	0.035362	DOWN
RP11-260(	3.024696	4.28194483	1.442854	2.967691028	0.003	0.035362	UP
TIAM1	275.0233	-1.0727687	0.361549	-2.967146	0.003006	0.035402	DOWN
DCDC1	107.5738	1.72694845	0.582038	2.967072752	0.003006	0.035402	UP
RP11-616M	1.378846	-2.3023584	0.776001	-2.96695469	0.003008	NA	NA
LINC00635	1.827715	3.00053714	1.011496	2.966436262	0.003013	0.03546	UP
FGA	462775.5	-1.1657154	0.393011	-2.96611095	0.003016	0.035482	DOWN
PNPT1	1221.009	0.41494914	0.139923	2.96555612	0.003021	0.035531	NOT
NPAS2	1094.373	0.77405412	0.261026	2.96542387	0.003023	0.035531	UP
CCDC144NI	3.218637	3.67518804	1.239711	2.964553255	0.003031	0.035583	UP
RSPH9	38.23031	0.52053228	0.175591	2.964466554	0.003032	0.035583	NOT
LRRC29	122.344	-0.7249668	0.244554	-2.96444538	0.003032	0.035583	DOWN
FAM160B1	1518.336	-0.4808328	0.162214	-2.96418115	0.003035	0.035583	NOT
PLA2G4F	9.774408	2.20159238	0.742736	2.96416472	0.003035	0.035583	UP
DEGS1	2568.022	0.54079382	0.182446	2.964138849	0.003035	0.035583	NOT
LINC00959	41.45715	-0.7283273	0.245721	-2.96403802	0.003036	0.035583	DOWN
TRAPPC11	1137.023	-0.4797239	0.16187	-2.96363782	0.00304	0.035609	NOT
LTV1	835.7548	0.51838603	0.174921	2.963549716	0.003041	0.035609	NOT
PDSS1	383.2415	0.61683384	0.208209	2.962563823	0.003051	0.035708	UP
GNAQ	2501.544	-0.4392679	0.148329	-2.96144218	0.003062	0.035823	NOT
GDPD1	144.9853	0.82933829	0.280114	2.960720946	0.003069	0.035885	UP
SYNPO2	470.8012	-0.9330542	0.315153	-2.9606413	0.00307	0.035885	DOWN
ATMIN	1574.479	-0.4105151	0.138674	-2.96029641	0.003073	0.03591	NOT
CCL17	17.4323	-1.4145302	0.477918	-2.95977429	0.003079	0.035955	DOWN
AAMP	5110.236	0.36270688	0.122557	2.959499175	0.003081	0.035972	NOT
ANGPTL6	173.4188	-1.0411455	0.351826	-2.95926657	0.003084	0.035983	DOWN
FOXN1	3.041866	2.68717249	0.908174	2.958872518	0.003088	0.036004	UP
GMFB	2207.724	0.43320313	0.14641	2.958828514	0.003088	0.036004	NOT
TMEM67	135.7667	0.86090162	0.291009	2.958328719	0.003093	0.036047	UP
RP11-560J	18.91644	0.77476922	0.261931	2.957918431	0.003097	0.036079	UP
NDC1	1234.062	0.52764334	0.178398	2.957678294	0.0031	0.036087	NOT
MIR210HG	197.3146	1.13036781	0.382209	2.957462501	0.003102	0.036087	UP
AC099850.	20.94146	0.73868563	0.249779	2.957355136	0.003103	0.036087	UP
POLR2C	2959.415	-0.526665	0.178089	-2.95731817	0.003103	0.036087	NOT
KLK4	19.9649	3.38916774	1.146244	2.956758366	0.003109	0.036126	UP
CACNG4	306.8249	2.29442132	0.77602	2.956652208	0.00311	0.036126	UP
CTC-42502	50.10735	-0.9291551	0.314265	-2.95659355	0.003111	0.036126	DOWN
KB-1742H1	12.7695	1.52913891	0.517256	2.956253892	0.003114	0.03615	UP
PPT1	3629.997	0.68235935	0.230846	2.955907502	0.003118	0.036175	UP
MEI4	49.81717	2.43533176	0.823922	2.95578095	0.003119	0.036175	UP
RP3-44901	63.86945	-0.5983949	0.202461	-2.95560281	0.003121	0.03618	DOWN
MMP9	815.2976	1.23574726	0.418176	2.955086954	0.003126	0.036225	UP
FHL5	21.17135	-1.1982335	0.405542	-2.95464471	0.00313	0.036262	DOWN
FANCL	578.2652	0.50959944	0.172494	2.954299897	0.003134	0.036268	NOT
ADH7	7.446006	-1.7182356	0.581621	-2.9542197	0.003135	0.036268	DOWN
ALDH2	34615.96	-0.9072534	0.307107	-2.9541945	0.003135	0.036268	DOWN

RP11-241F	3.42913	-2.6168265	0.885906	-2.953843	0.003138	0.036294	DOWN
GSTM2	306.8345	-1.128117	0.381947	-2.95359289	0.003141	0.036308	DOWN
RNU6-722F	6.313506	-0.8135262	0.275512	-2.95277983	0.003149	0.036371	DOWN
ACYP2	574.726	-0.57498	0.19473	-2.95269636	0.00315	0.036371	NOT
NPM3	718.6302	0.84379928	0.285805	2.952356328	0.003154	0.036371	UP
STAMBPL1	246.4454	0.83827288	0.283936	2.952331232	0.003154	0.036371	UP
CHD7	1086.594	0.63998086	0.216777	2.9522578	0.003155	0.036371	UP
RP13-415(	2.999742	1.52435024	0.516344	2.952199895	0.003155	0.036371	UP
DPH6	287.3466	0.43966822	0.148932	2.95213768	0.003156	0.036371	NOT
MYL5	613.6262	-0.7801529	0.264319	-2.95155332	0.003162	0.036424	DOWN
GNG12-AS1	22.27022	-0.9935125	0.33673	-2.95047149	0.003173	0.036537	DOWN
TNFSF4	211.9234	0.99961093	0.338868	2.949849148	0.003179	0.036595	UP
OAS3	1704.105	0.74123249	0.251324	2.949312007	0.003185	0.036643	UP
AGR3	7.735219	3.08263547	1.045419	2.948708562	0.003191	0.03668	UP
MRPL10	2037.144	0.38676754	0.131166	2.948691785	0.003191	0.03668	NOT
SLC25A25	3869.032	-0.9843921	0.333871	-2.9484208	0.003194	0.03668	DOWN
GPR125	2737.493	-0.8488352	0.287908	-2.94828613	0.003195	0.03668	DOWN
ZNF816-ZN	36.73335	1.18391244	0.401567	2.948231766	0.003196	0.03668	UP
SRRT	4146.635	0.35543446	0.120559	2.948212735	0.003196	0.03668	NOT
TM4SF1	7444.656	1.06867227	0.362559	2.947584138	0.003203	0.036739	UP
NDUFB1	3076.087	-0.7789231	0.264273	-2.94741356	0.003204	0.036739	DOWN
CRK	3025.163	-0.4129916	0.140124	-2.94732393	0.003205	0.036739	NOT
WDR77	1125.172	0.49142018	0.166744	2.947146487	0.003207	0.036745	NOT
MRTO4	1801.102	0.55241791	0.187455	2.946939922	0.003209	0.036754	NOT
ZHX2	2422.04	0.54579999	0.185236	2.946504961	0.003214	0.03679	NOT
RN7SKP70	3.451035	-1.1867263	0.402814	-2.94608969	0.003218	0.036813	DOWN
KLF1	6.216185	-1.8164151	0.616559	-2.94605448	0.003219	0.036813	DOWN
HSPA1A	6937.669	0.80818392	0.274343	2.945891792	0.00322	0.036816	UP
RYR2	62.40054	-1.6604464	0.563758	-2.94531894	0.003226	0.036869	DOWN
GTF2IRD2F	176.9242	-0.6080044	0.206504	-2.94427685	0.003237	0.036978	DOWN
HCG11	33.40735	-0.7852735	0.266747	-2.94388416	0.003241	0.037009	DOWN
CTD-2410N	29.86375	-0.7495396	0.254738	-2.94239219	0.003257	0.037172	DOWN
TRAM1	14257.67	0.55246089	0.187775	2.942149525	0.003259	0.037186	NOT
MYH3	116.2908	-0.9885731	0.336028	-2.94193425	0.003262	0.037191	DOWN
CASC11	1.847349	1.67900905	0.570734	2.941843977	0.003263	0.037191	UP
KCMF1	2613.605	0.30492422	0.103684	2.940890259	0.003273	0.03729	NOT
SPTY2D1-A	22.11247	0.68878335	0.234228	2.940654305	0.003275	0.037303	UP
NAP1L5	154.4004	-0.9270565	0.315286	-2.94036919	0.003278	0.037322	DOWN
RP11-642F	24.01093	-0.7661766	0.260587	-2.94019606	0.00328	0.037327	DOWN
REEP1	140.832	1.40802626	0.47893	2.939940233	0.003283	0.037342	UP
POU1F1	1.975074	1.58967956	0.540757	2.939731057	0.003285	0.037352	UP
AC012512.	3.589268	2.66564953	0.906856	2.939439479	0.003288	0.03737	UP
CTD-2240J	5.704992	-1.2770614	0.434475	-2.93932353	0.003289	0.03737	DOWN
BCORL1	927.5761	0.71871428	0.244591	2.938436176	0.003299	0.037461	UP
RP11-91I8	2.0205	2.62084075	0.892059	2.937969166	0.003304	0.03749	UP
EGFR-AS1	165.9994	2.19497599	0.747115	2.937936328	0.003304	0.03749	UP
RP11-114N	10.04999	-0.9340101	0.317957	-2.93753754	0.003308	0.037515	DOWN
LINC0101(	22.66575	-1.5711883	0.534879	-2.93746733	0.003309	0.037515	DOWN
NFIC	8654.285	-0.636209	0.216612	-2.93708698	0.003313	0.037546	DOWN

WDR46	2494.091	0.4876509	0.16606	2.936599506	0.003318	0.037589	NOT
RPS18P13	2.150335	-1.4149983	0.481922	-2.93615902	0.003323	0.037623	DOWN
INA	5.702313	-1.724106	0.587217	-2.93606474	0.003324	0.037623	DOWN
DZIP1L	109.5689	1.21123677	0.412679	2.935061176	0.003335	0.037729	UP
KIF19	17.23962	-1.0459197	0.356399	-2.9346838	0.003339	0.037739	DOWN
DEDD	1935.404	0.32690752	0.111398	2.934596415	0.00334	0.037739	NOT
NDUFAF6	1021.388	0.57413977	0.195646	2.934586379	0.00334	0.037739	NOT
HEATR4	40.85489	-0.7616721	0.2596	-2.9340207	0.003346	0.037769	DOWN
GMNN	2164.274	0.73959895	0.252086	2.933915517	0.003347	0.037769	UP
RNF114	3369.075	0.34271745	0.116814	2.93387836	0.003348	0.037769	NOT
LINC0135	18.14561	-1.5969703	0.544331	-2.93382419	0.003348	0.037769	DOWN
FTH1P15	11.80241	0.91040118	0.310339	2.933573009	0.003351	0.037769	UP
TAF6	1157.365	0.58962414	0.200992	2.933567514	0.003351	0.037769	UP
LPAR2	253.6588	1.19404758	0.407057	2.933365073	0.003353	0.037769	UP
PJA2	4612.305	-0.5244744	0.178799	-2.9333111	0.003354	0.037769	NOT
CENPQ	271.9749	0.68839819	0.234782	2.932071749	0.003367	0.037899	UP
CREBRF	987.7054	-0.5136755	0.175197	-2.93198658	0.003368	0.037899	NOT
PRPF38A	1520.286	0.44760274	0.152736	2.93056233	0.003383	0.038046	NOT
COMMD5	1835.986	0.63566595	0.216912	2.930528712	0.003384	0.038046	UP
GINS4	194.2351	0.9531537	0.32528	2.930258357	0.003387	0.038063	UP
AKR1B10P1	220.3839	2.21834667	0.757239	2.92951811	0.003395	0.038129	UP
CH507-9B2	147.5179	-1.0472617	0.357493	-2.92946169	0.003395	0.038129	DOWN
PNMAL2	43.07899	-0.990172	0.338071	-2.92888898	0.003402	0.038164	DOWN
C6orf222	13.12045	2.36938574	0.80902	2.928711549	0.003404	0.038164	UP
TCTN2	248.8789	0.91367187	0.31198	2.928620282	0.003405	0.038164	UP
HEY2	290.0072	-0.8535483	0.291473	-2.92839351	0.003407	0.038164	DOWN
MORC3	939.6866	-0.5311549	0.181382	-2.9283773	0.003407	0.038164	NOT
RP11-312J	22.01164	0.70996076	0.242442	2.928373837	0.003407	0.038164	UP
KLF2P4	2.004914	3.69334166	1.261267	2.928279625	0.003408	0.038164	UP
PCAT19	124.9892	-0.7706128	0.2632	-2.92785623	0.003413	0.0382	DOWN
TCF4	1012.42	-0.7131085	0.243573	-2.92770118	0.003415	0.038203	DOWN
AC007099.	6.702035	2.07805244	0.7102	2.926009557	0.003433	0.038394	UP
MRPS10	2280.463	0.39961547	0.136586	2.925740201	0.003436	0.038394	NOT
OAS1	1911.035	0.75535806	0.258191	2.925573446	0.003438	0.038394	UP
AC114803.	1.581031	2.75559558	0.941934	2.925466917	0.003439	0.038394	UP
USP39	1839.591	0.32372284	0.110657	2.92545474	0.00344	0.038394	NOT
LINC0153	47.62689	-0.7283719	0.248984	-2.92538219	0.00344	0.038394	DOWN
RP11-326	55.84423	-1.0043795	0.34335	-2.92523744	0.003442	0.038396	DOWN
IGSF22	33.29605	-0.9373613	0.320481	-2.92485523	0.003446	0.038428	DOWN
RP11-468	6.814169	2.10638449	0.720376	2.924006128	0.003456	0.038517	UP
RP11-583	4.478974	1.61072129	0.551072	2.922887922	0.003468	0.038626	UP
PCDHB19P	2.773123	-1.1211593	0.383585	-2.92284524	0.003468	0.038626	DOWN
RP11-373	2.695806	2.49002498	0.851949	2.922740062	0.00347	0.038626	UP
UBE2T	573.1917	0.82685717	0.282982	2.921939707	0.003479	0.03871	UP
SLC35E1	2515.22	-0.4302032	0.147255	-2.92148651	0.003484	0.03875	NOT
RP11-768	25.77904	-1.0765318	0.368515	-2.92127344	0.003486	0.038756	DOWN
POM121L9	13.25055	-1.2434322	0.425667	-2.92113572	0.003488	0.038756	DOWN
RP11-498	112.2665	0.78247308	0.267873	2.921058925	0.003488	0.038756	UP
LINC0101	2.409937	3.77695587	1.29342	2.920132111	0.003499	0.038852	UP

ASS1P2	4.826757	-1.2022502	0.411741	-2.91991953	0.003501	0.038852	DOWN
DMRT2	2.283587	3.16145722	1.082726	2.919906327	0.003501	0.038852	UP
RAMP3	549.4154	-1.0424293	0.357041	-2.91963334	0.003504	0.03887	DOWN
HOXD10	38.68829	1.90778013	0.653541	2.919145849	0.00351	0.038915	UP
REXO4	1538.679	0.49480557	0.169534	2.91861846	0.003516	0.038951	NOT
RCBTB2	294.6235	-0.5149144	0.176425	-2.91860256	0.003516	0.038951	NOT
CHMP4C	548.439	0.77038262	0.264025	2.917837557	0.003525	0.03902	UP
STX1A	116.1539	0.60936636	0.208845	2.917796452	0.003525	0.03902	UP
NDUFA4L2	1859.177	1.14461445	0.392342	2.917391417	0.00353	0.039054	UP
C11orf84	590.9882	0.69634099	0.238777	2.916280157	0.003542	0.039178	UP
CHAD	835.7055	-1.1653339	0.399739	-2.91523799	0.003554	0.039266	DOWN
AVPR2	15.3574	-1.1632119	0.39905	-2.91495032	0.003557	0.039266	DOWN
RP11-60L3	1.777266	2.80042942	0.960716	2.914939819	0.003558	0.039266	UP
TM6SF2	687.0174	-1.134474	0.389195	-2.91492119	0.003558	0.039266	DOWN
VMA21	1596.312	0.44889269	0.154002	2.914853668	0.003559	0.039266	NOT
RP11-443I	47.36646	1.15736167	0.397062	2.914813733	0.003559	0.039266	UP
RHEB	3097.258	0.43535891	0.149409	2.913872334	0.00357	0.039369	NOT
DR1	2150.323	0.46354082	0.15909	2.913708431	0.003572	0.039374	NOT
LRP2BP	54.5194	-0.6711415	0.230372	-2.91329619	0.003576	0.039395	DOWN
ANKRD26P1	2.031585	3.00404922	1.031221	2.913099821	0.003579	0.039395	UP
RP11-542K	1.93648	2.25075095	0.772639	2.913070977	0.003579	0.039395	UP
CARF	189.8663	-0.5748105	0.197324	-2.91302821	0.003579	0.039395	NOT
METTL1	810.5247	0.60184351	0.206618	2.912825852	0.003582	0.039405	UP
WDR38	1.866221	1.80032078	0.618119	2.912580781	0.003585	0.03942	UP
RAC1P2	81.02599	0.58070139	0.199387	2.912434954	0.003586	0.039422	NOT
TRPC1	135.4635	1.09338838	0.375441	2.912274414	0.003588	0.039427	UP
GK-AS1	16.25498	-1.1214505	0.385107	-2.91205007	0.003591	0.039433	DOWN
EIF2B1	2337.636	0.30746072	0.105585	2.911973265	0.003592	0.039433	NOT
RP1-120G2	16.3481	-1.0782582	0.370312	-2.91175977	0.003594	0.039437	DOWN
RP4-775C1	2.696223	1.49976631	0.515086	2.911682961	0.003595	0.039437	UP
PET117	265.769	0.47309564	0.162492	2.911494135	0.003597	0.039445	NOT
CD68	98.66499	1.01227768	0.347713	2.911248835	0.0036	0.03946	UP
AGL	1826.474	-0.7820346	0.268656	-2.91091701	0.003604	0.039462	DOWN
RIC3	34.4785	-1.8395151	0.631942	-2.9108945	0.003604	0.039462	DOWN
INSR	8282.904	-0.6187019	0.212549	-2.91086022	0.003604	0.039462	DOWN
POLR2G	2113.939	0.41403674	0.142254	2.910551961	0.003608	0.039476	NOT
CDK2	1174.767	0.6345775	0.218031	2.910495538	0.003609	0.039476	UP
COX4I2	115.3064	-0.9734051	0.334547	-2.90962529	0.003619	0.039557	DOWN
CFL1	27354.16	0.4509692	0.154994	2.909599571	0.003619	0.039557	NOT
PANK3	4364.619	-0.5303279	0.182278	-2.9094382	0.003621	0.039562	NOT
ANKS1B	84.52247	1.54845205	0.53227	2.909149158	0.003624	0.039582	UP
GTF3C3	1006.53	0.37215563	0.127948	2.908650712	0.00363	0.03963	NOT
CIDEB	40.54628	-1.1611787	0.399238	-2.90848629	0.003632	0.03963	DOWN
RP11-403A	4.138916	3.15642556	1.085281	2.908393603	0.003633	0.03963	UP
RP11-250E	7.676052	-1.0653456	0.366334	-2.9081296	0.003636	0.039648	DOWN
RGS19	524.1065	0.67778866	0.233179	2.906725751	0.003652	0.03981	UP
OLA1P1	28.96841	0.65071553	0.22388	2.906539783	0.003655	0.039818	UP
RP3-439F8	17.31305	1.27738849	0.4396	2.905795239	0.003663	0.039856	UP
RP11-351J	52.99217	1.17176988	0.4033	2.90545391	0.003667	0.039856	UP

FBXL19	529.5342	0.5477668	0.188533	2.905415675	0.003668	0.039856	NOT
LGALS1	7187.575	0.94023595	0.323619	2.905379792	0.003668	0.039856	UP
RP11-407N	31.70012	-1.0139731	0.349	-2.90536563	0.003668	0.039856	DOWN
RP11-358I	4.2645	-1.0533336	0.362548	-2.90536339	0.003668	0.039856	DOWN
RP4-657D1	12.78879	-0.7134808	0.245575	-2.90535168	0.003668	0.039856	DOWN
RP11-61I1	44.89613	-1.1049917	0.380361	-2.90511295	0.003671	0.039856	DOWN
MT1A	334.7997	-1.4700065	0.506008	-2.90510777	0.003671	0.039856	DOWN
PLCD3	436.367	0.99578597	0.342785	2.904985358	0.003673	0.039856	UP
TK1	2142.035	0.84725516	0.291716	2.904384684	0.00368	0.039908	UP
TCEANC	151.427	-0.3980875	0.137067	-2.90432432	0.00368	0.039908	NOT
METTL14	932.0128	-0.4186491	0.144159	-2.90407016	0.003683	0.039922	NOT
SGK1	2905.386	-0.9731692	0.335118	-2.90395667	0.003685	0.039922	DOWN
RP11-737(	2.399713	-1.2870406	0.44322	-2.90384226	0.003686	0.039922	DOWN
ITGB4	1222.305	1.22878006	0.423189	2.903622668	0.003689	0.039934	UP
IER5L	508.916	0.99930257	0.344204	2.903224373	0.003693	0.039969	UP
AC009299.	4.612804	-0.9904287	0.341205	-2.90273672	0.003699	0.039997	DOWN
RNF219	263.6228	0.59274328	0.204207	2.902654952	0.0037	0.039997	UP
PCDH20	7.975529	-2.4945642	0.859415	-2.90262998	0.0037	0.039997	DOWN
ARHGEF12	8178.785	-0.5562369	0.191701	-2.90158626	0.003713	0.040115	NOT
FAM124A	70.03184	0.80120182	0.276195	2.900850937	0.003722	0.040187	UP
RP11-111M	162.7235	-0.8322573	0.286909	-2.90077501	0.003722	0.040187	DOWN
CKAP5	3994.592	0.40407801	0.139318	2.900410631	0.003727	0.040217	NOT
KANSL1-AS	127.7219	-0.9161742	0.31594	-2.89983538	0.003734	0.040275	DOWN
RP11-96H1	2.960933	3.71783312	1.282562	2.89875601	0.003746	0.040398	UP
ARHGEF15	467.7355	-0.5983553	0.206427	-2.89863516	0.003748	0.040398	DOWN
AC067945.	5.863996	-1.2064918	0.416434	-2.89719742	0.003765	0.040567	DOWN
RP4-706A1	630.505	0.60946755	0.210374	2.897060307	0.003767	0.040569	UP
MED22	1105.329	0.52529871	0.181356	2.896499305	0.003774	0.040625	NOT
FUNDC2	2855.28	0.65484115	0.226117	2.896025368	0.003779	0.04067	UP
RP3-449M8	3.508871	1.29798404	0.448479	2.894193582	0.003801	0.040845	UP
MRGBP	1082.386	0.47885728	0.165455	2.894191063	0.003801	0.040845	NOT
RP11-408I	3.963304	3.79706978	1.311963	2.894190731	0.003801	0.040845	UP
AC068134.	9.439493	1.12888999	0.390054	2.894185777	0.003801	0.040845	UP
DHRS4-AS1	1251.053	-0.7475364	0.258309	-2.8939602	0.003804	0.040858	DOWN
PCDH12	832.9791	-0.5334105	0.184331	-2.89375683	0.003807	0.040868	NOT
ECHDC2	7739.156	-0.7876026	0.272197	-2.89350373	0.00381	0.040885	DOWN
EMX1	105.4217	-1.9546248	0.675552	-2.89337363	0.003811	0.040886	DOWN
AC004540.	21.09343	1.37579406	0.475558	2.893011445	0.003816	0.040917	UP
AC022007.	73.50699	0.7191253	0.248592	2.892788276	0.003818	0.04093	UP
ALDOB	182753.4	-1.4508641	0.501582	-2.89257856	0.003821	0.040939	DOWN
AC083899.	57.69631	0.5464704	0.188929	2.892464608	0.003822	0.040939	NOT
FAM99B	121.5012	-2.0203017	0.698619	-2.89184874	0.00383	0.041004	DOWN
TAAR3	16.01502	1.60377325	0.554622	2.89165352	0.003832	0.041009	UP
RP11-320H	3.725252	4.4046913	1.523291	2.891562892	0.003833	0.041009	UP
PPP1R14C	11.24987	1.77527805	0.614045	2.891118639	0.003839	0.041048	UP
ZNF76	1564.915	0.4342293	0.1502	2.89101384	0.00384	0.041048	NOT
EPB41L1	3682.144	-1.3131824	0.454505	-2.88925658	0.003862	0.041253	DOWN
SLC25A19	308.5415	0.56872961	0.19685	2.889152525	0.003863	0.041253	NOT
MKRN4P	4.769561	3.01438647	1.043373	2.889078813	0.003864	0.041253	UP

RP5-1056I	11.95053	1.77946188	0.616025	2.888619092	0.003869	0.041297	UP
YWHAZP4	17.03717	0.89597944	0.310236	2.888057383	0.003876	0.041354	UP
TNFSF15	139.5615	1.26317472	0.437433	2.887699716	0.003881	0.041385	UP
NAAA	1051.492	-0.8184807	0.283486	-2.88720005	0.003887	0.041434	DOWN
HAMP	1283.818	-2.0321036	0.703962	-2.88666758	0.003893	0.041488	DOWN
HAUS6	692.7416	0.59326049	0.205542	2.886326436	0.003898	0.041505	UP
EIF4E3	528.8553	-0.974578	0.337664	-2.88623661	0.003899	0.041505	DOWN
TRAF2	1510.954	0.58330856	0.202108	2.886127913	0.0039	0.041505	NOT
RP11-327I	42.30357	-0.5662634	0.196207	-2.88605012	0.003901	0.041505	NOT
NDN	483.4625	1.30514958	0.452264	2.88581528	0.003904	0.041519	UP
MASTL	526.8463	0.56449751	0.195623	2.885632708	0.003906	0.041527	NOT
ZC3H13	1987.049	-0.595948	0.206579	-2.88484736	0.003916	0.041598	DOWN
DAGLA	338.2851	1.00544386	0.348545	2.884687998	0.003918	0.041598	UP
IQCB1	492.6069	0.48951012	0.169699	2.884576327	0.003919	0.041598	NOT
TXNDC9	1015.066	0.36574834	0.126798	2.884487186	0.003921	0.041598	NOT
AC027612.	68.60224	1.08734693	0.376964	2.884483305	0.003921	0.041598	UP
MARK1	78.81165	1.23524779	0.428283	2.884187614	0.003924	0.04162	UP
PIK3R1	4759.241	-0.7893414	0.273704	-2.88392494	0.003928	0.041639	DOWN
RP11-241I	1.931647	-2.1858928	0.758017	-2.88369737	0.00393	0.041653	DOWN
KB-68A7.1	81.05093	-1.2808423	0.444235	-2.88325213	0.003936	0.041695	DOWN
SIAE	2502.847	-0.7983231	0.276937	-2.88268696	0.003943	0.041754	DOWN
KDM4B	1994.179	-0.5273456	0.182962	-2.88226099	0.003948	0.04176	NOT
ASRGL1	502.8613	1.06559675	0.369713	2.882226861	0.003949	0.04176	UP
KRT1	8.134164	-2.2638816	0.785481	-2.88216038	0.00395	0.04176	DOWN
MIR137HG	2.669866	3.39449851	1.177767	2.882148965	0.00395	0.04176	UP
PCID2	1389.984	0.57527405	0.199619	2.881864426	0.003953	0.041781	NOT
RP11-122K	24.18874	-1.3100533	0.454646	-2.88147715	0.003958	0.041817	DOWN
RP11-772C	6.75325	-1.7989784	0.624371	-2.88126568	0.003961	0.041828	DOWN
LINC01352	11.03758	-1.1883936	0.412475	-2.88112657	0.003963	0.04183	DOWN
ZFPM1	780.4027	-0.6986419	0.242555	-2.88034938	0.003972	0.041917	DOWN
MRPL3	2612.108	0.31940962	0.110903	2.880083447	0.003976	0.041937	NOT
RP11-145A	15.52627	1.62762624	0.565312	2.879166596	0.003987	0.042042	UP
RP11-59D5	74.2538	-1.4928264	0.518572	-2.87872667	0.003993	0.042085	DOWN
RNU6ATAC1	3.863597	-0.9487309	0.329582	-2.87858932	0.003995	0.042087	DOWN
MANBAL	2160.82	0.4082423	0.14183	2.878385597	0.003997	0.042097	NOT
GS1-21A4.	3.108507	-1.1292643	0.392371	-2.87805006	0.004001	0.042126	DOWN
CIRBP	6930.656	-0.5484121	0.190575	-2.87767489	0.004006	0.042151	NOT
AC007131.	14.04364	4.24959566	1.476775	2.877619652	0.004007	0.042151	UP
RHOXF2	4.498144	4.15632614	1.444637	2.877072838	0.004014	0.042197	UP
CDK15	4.119438	-1.7174355	0.59698	-2.87687091	0.004016	0.042197	DOWN
ACAD8	1315.938	-0.5092454	0.177026	-2.8766688	0.004019	0.042197	NOT
MAF1	5668.429	0.62300797	0.216587	2.876484922	0.004021	0.042197	UP
AC145343.	29.15788	1.06432143	0.37001	2.876469058	0.004022	0.042197	UP
RP11-258C	4.972169	-1.1674747	0.405875	-2.87643977	0.004022	0.042197	DOWN
RUNDC3A	31.78751	1.29585523	0.450514	2.876389671	0.004023	0.042197	UP
TRIM74	7.963753	-1.3348765	0.464096	-2.8762957	0.004024	0.042197	DOWN
TLDC2	174.9967	1.05233023	0.365898	2.876020945	0.004027	0.042217	UP
NAT9	1485.244	0.51638126	0.179555	2.875901131	0.004029	0.042217	NOT
RP11-260I	3.468515	-2.0614657	0.716898	-2.8755341	0.004033	0.04225	DOWN

ZNF221	33.66719	-0.9792143	0.340565	-2.87526237	0.004037	0.042255	DOWN
MTA3	1174.761	0.47598281	0.165545	2.875249154	0.004037	0.042255	NOT
ELOVL1	3072.782	0.46197726	0.160739	2.874082797	0.004052	0.042395	NOT
RP11-395I	93.40377	0.59181174	0.205925	2.873925056	0.004054	0.0424	UP
SFRP1	90.23335	-1.6112776	0.56068	-2.87379199	0.004056	0.042402	DOWN
C10orf11	853.0324	-0.7483737	0.260456	-2.87332242	0.004062	0.042436	DOWN
AC005498.	3.04524	1.73171809	0.602695	2.873293032	0.004062	0.042436	UP
WBSCR17	31.74826	-1.8661087	0.649586	-2.87276825	0.004069	0.04249	DOWN
UGT1A5	18.229	2.10719774	0.733568	2.872532657	0.004072	0.042506	UP
CPEB2	1265.842	-0.6881531	0.239625	-2.87178778	0.004082	0.042568	DOWN
CCT8	6193.727	0.3773134	0.131386	2.871785486	0.004082	0.042568	NOT
FBXO2	2482.558	-1.3514507	0.47061	-2.87170225	0.004083	0.042568	DOWN
PARVA	1697.987	-0.5227456	0.182066	-2.87119476	0.004089	0.042597	NOT
PTPRG-AS1	18.92596	1.31012156	0.456306	2.871149212	0.00409	0.042597	UP
MRGPRF-AS	3.4809	2.43328063	0.847501	2.871124669	0.00409	0.042597	UP
TSPAN10	146.3107	0.8896062	0.309925	2.870394056	0.0041	0.042677	UP
NR3C2	367.3255	-0.9922533	0.345708	-2.87020889	0.004102	0.042677	DOWN
AC016735.	18.27937	1.82011334	0.634148	2.870169597	0.004103	0.042677	UP
ACP1	4211.994	0.33599815	0.11708	2.869811725	0.004107	0.042709	NOT
ZWINT	1210.057	0.86776157	0.302416	2.869432208	0.004112	0.042736	UP
HIPK2	4245.724	-0.727511	0.253544	-2.86936958	0.004113	0.042736	DOWN
GADD45B	5488.312	-0.9252468	0.322539	-2.86863539	0.004122	0.042819	DOWN
TFAP2E	22.98067	1.08627724	0.37873	2.868208366	0.004128	0.04286	UP
ALAD	9287.667	-0.7570794	0.264002	-2.86770581	0.004135	0.042892	DOWN
TIMELESS	2129.747	0.56562205	0.197243	2.867646479	0.004135	0.042892	NOT
PBDC1	813.9717	0.46290269	0.161425	2.867609536	0.004136	0.042892	NOT
NXPH4	230.374	1.51416042	0.528077	2.86731047	0.00414	0.042916	UP
HNF1B	1278.973	1.10612832	0.385823	2.866929519	0.004145	0.042952	UP
ASAP1	1746.964	0.67136287	0.234218	2.866406012	0.004152	0.043006	UP
FGB	280951.9	-1.1585756	0.40424	-2.86606096	0.004156	0.043022	DOWN
NEFH	29.458	1.10610006	0.385932	2.866046846	0.004156	0.043022	UP
TRIM66	446.7833	-0.6235008	0.217572	-2.86572029	0.004161	0.043022	DOWN
EIF4EBP1	2008.696	0.72225657	0.252034	2.865706839	0.004161	0.043022	UP
ATG2B	1531.91	-0.5235447	0.182694	-2.86568897	0.004161	0.043022	NOT
MAN1A2	2633.315	-0.4810841	0.167902	-2.86525938	0.004167	0.043048	NOT
ABAT	14966.76	-1.1288506	0.393979	-2.86525204	0.004167	0.043048	DOWN
SYT8	79.19822	1.85802606	0.648583	2.86474692	0.004173	0.0431	UP
RP11-110I	12.86878	-0.8822815	0.308	-2.86454799	0.004176	0.0431	DOWN
XAGE5	24.32789	3.94796629	1.378236	2.86450772	0.004177	0.0431	UP
DIAPH3	230.6817	1.01306971	0.353774	2.863606205	0.004188	0.043207	UP
RPL23AP82	214.6063	0.55622839	0.194262	2.863289808	0.004193	0.043233	NOT
RP11-64D2	6.255372	-1.5602567	0.5451	-2.86233039	0.004205	0.043348	DOWN
NASP	3647.79	0.51563802	0.18018	2.861792228	0.004213	0.043405	NOT
RIT1	873.9403	0.47333479	0.165423	2.861362237	0.004218	0.043448	NOT
FUT4	357.0988	1.14375709	0.399791	2.860890334	0.004225	0.043496	UP
BID	2584.603	0.63798494	0.223072	2.859995911	0.004236	0.043598	UP
RP11-403A	60.07065	2.4427268	0.854136	2.85988091	0.004238	0.043598	UP
AP006285.	11.51398	-2.0101999	0.702958	-2.85962834	0.004241	0.043598	DOWN
RP11-394H	7.449991	-1.0562067	0.369357	-2.85958516	0.004242	0.043598	DOWN

CTHRC1	514.0661	1.38173336	0.4832	2.8595462	0.004242	0.043598	UP
FAM206A	571.9975	0.49162856	0.171968	2.858845533	0.004252	0.043651	NOT
UNQ6494	22.52483	-1.169198	0.408985	-2.85877624	0.004253	0.043651	DOWN
SPOCD1	37.22756	1.28928491	0.451004	2.858699493	0.004254	0.043651	UP
CCDC43	622.6034	0.46593645	0.163	2.85851202	0.004256	0.043651	NOT
RP11-384I	414.7477	-0.9847183	0.344493	-2.85845888	0.004257	0.043651	DOWN
CNIH4	1956.749	0.50844224	0.177876	2.858406102	0.004258	0.043651	NOT
PIGU	1624.325	0.49104878	0.171796	2.858318021	0.004259	0.043651	NOT
SLC41A2	2449.588	-0.8863211	0.31016	-2.85762429	0.004268	0.04373	DOWN
ATP11C	1448.671	-0.838026	0.293299	-2.85724331	0.004273	0.043766	DOWN
PPIAP6	6.58827	1.11143908	0.389046	2.856834743	0.004279	0.043794	UP
ADH1A	16528.07	-1.4002241	0.490137	-2.85680078	0.004279	0.043794	DOWN
AGBL5	1354.75	0.50350033	0.176307	2.855818455	0.004293	0.043909	NOT
TMC1	6.486503	-1.2771388	0.447248	-2.85555154	0.004296	0.043909	DOWN
PODN	537.684	-1.4312729	0.501233	-2.85550626	0.004297	0.043909	DOWN
RP11-260M	8.54849	-1.9523108	0.683704	-2.85549196	0.004297	0.043909	DOWN
SET	8431.255	0.39546391	0.138502	2.855290741	0.0043	0.04392	NOT
RP11-262I	9.813459	-0.8759514	0.306857	-2.85459612	0.004309	0.044	DOWN
GAR1	618.33	0.43886538	0.153752	2.854363492	0.004312	0.044015	NOT
GTF3C2	1731.943	0.38474136	0.134799	2.854176466	0.004315	0.044025	NOT
RP11-251(	12.19335	1.30785183	0.45825	2.854011256	0.004317	0.044031	UP
AC147651.	37.30889	1.02030128	0.357622	2.853012957	0.004331	0.044153	UP
CHL1	42.49865	2.10155116	0.73668	2.852733585	0.004334	0.044175	UP
RP11-556H	1.520892	-2.1839665	0.76565	-2.85243275	0.004339	0.044201	DOWN
MRPL18	2301.108	0.44996463	0.15776	2.852214786	0.004342	0.044211	NOT
ERVMER61-	9.401303	3.41710438	1.198094	2.852116258	0.004343	0.044211	UP
TPD52L2	4475.324	0.48539868	0.170209	2.851784299	0.004347	0.044228	NOT
EDA2R	108.2938	-1.3698763	0.480362	-2.85175556	0.004348	0.044228	DOWN
AP1AR	957.6508	-0.546411	0.191625	-2.85145507	0.004352	0.044254	NOT
RPS9	27138.72	0.56944062	0.199719	2.851209312	0.004355	0.044267	NOT
ZNF250	350.6339	0.43274811	0.151786	2.851035656	0.004358	0.044267	NOT
KEL	50.71555	1.58513201	0.556004	2.850936561	0.004359	0.044267	UP
HNRNPA3P1	4.948144	0.90490053	0.317411	2.850883036	0.00436	0.044267	UP
LEFTY1	67.87296	1.57900185	0.553998	2.850195354	0.004369	0.044346	UP
TXNIP	11019.37	-0.9693297	0.340142	-2.84978062	0.004375	0.044387	DOWN
TLCD2	578.7222	-0.8258707	0.289896	-2.84885157	0.004388	0.044501	DOWN
MPDZ	2800.065	-1.0346925	0.363213	-2.84872235	0.00439	0.044502	DOWN
KCNK6	264.5833	-0.7899875	0.277335	-2.84849984	0.004393	0.044517	DOWN
RP11-47A8	71.78412	-0.7367122	0.258647	-2.84832991	0.004395	0.044519	DOWN
EIF5B	4950.182	0.42815362	0.150325	2.848179272	0.004397	0.044519	NOT
RP5-967N2	57.93	0.98984541	0.347542	2.848128146	0.004398	0.044519	UP
ARL15	826.4044	-0.4655793	0.163479	-2.84795265	0.0044	0.044527	NOT
GFI1	86.75958	1.15439507	0.405376	2.847713517	0.004403	0.044537	UP
RP11-462I	79.33702	1.32548256	0.465466	2.847643848	0.004404	0.044537	UP
RP11-325K	14.74831	-0.7751365	0.272229	-2.84736401	0.004408	0.044559	DOWN
U2AF2	5633.075	0.29479027	0.103557	2.846636182	0.004418	0.044645	NOT
AKT2	6082.987	-0.372807	0.130971	-2.84649562	0.00442	0.044648	NOT
PTH1R	129.2387	-1.0550026	0.370733	-2.84572324	0.004431	0.044739	DOWN
RP3-508I1	7.797727	-0.7694833	0.270421	-2.84549755	0.004434	0.044742	DOWN

bP-21264(	5.574898	-1.5242887	0.53569	-2.84546712	0.004435	0.044742	DOWN
EIF4HP2	13.38441	0.99536945	0.349838	2.845227198	0.004438	0.044759	UP
PPIA	17111.27	0.50579689	0.177795	2.844837558	0.004443	0.044781	NOT
RP11-336N	1.486502	2.53943606	0.892653	2.844818919	0.004444	0.044781	UP
TARBP1	1693.95	0.56259549	0.197771	2.844677176	0.004446	0.044781	NOT
HYAL4	1.534944	2.2250577	0.782205	2.844596796	0.004447	0.044781	UP
FEM1B	2335.486	-0.4320223	0.151906	-2.84400159	0.004455	0.044827	NOT
CAPN3	56.60461	-1.102344	0.38762	-2.84387681	0.004457	0.044827	DOWN
TWF2	2657.319	0.56507353	0.198698	2.843875005	0.004457	0.044827	NOT
TRNAU1AP	611.721	0.53018148	0.186439	2.843720847	0.004459	0.044827	NOT
RPL36	15024.26	0.7141886	0.25115	2.843678656	0.00446	0.044827	UP
RP11-159H	2.831308	1.87630889	0.65986	2.843497053	0.004462	0.044829	UP
RPL23	25679.98	0.61033075	0.21465	2.843379214	0.004464	0.044829	UP
BCRP2	2.181127	2.60166024	0.915009	2.843316144	0.004465	0.044829	UP
IRGQ	1282.21	-0.4318995	0.151913	-2.84306751	0.004468	0.044847	NOT
RP11-267J	14.9146	0.7520575	0.264594	2.842310071	0.004479	0.044937	UP
C3P1	4675.21	-1.5478252	0.544606	-2.84210118	0.004482	0.04495	DOWN
AC009961.	23.08446	0.89369688	0.31453	2.841376909	0.004492	0.045029	UP
ACSM5P1	45.94815	-1.4775198	0.520015	-2.84130272	0.004493	0.045029	DOWN
SLC35F3	18.31738	1.7795145	0.626359	2.841046669	0.004497	0.045049	UP
DPP9-AS1	12.46191	-0.7932578	0.279254	-2.84063232	0.004502	0.045075	DOWN
C11orf45	49.01651	0.94549413	0.332847	2.840623096	0.004503	0.045075	UP
CHTOP	3538.213	0.31130517	0.109599	2.840408536	0.004506	0.045089	NOT
SEH1L	1589.314	0.31166297	0.109749	2.839790473	0.004514	0.04516	NOT
C11orf49	506.591	0.7659082	0.269742	2.839410932	0.00452	0.045193	UP
CTC-459F4	25.6632	0.84705031	0.298328	2.839323746	0.004521	0.045193	UP
RP11-120K	1.959489	-1.4927511	0.525777	-2.83913607	0.004524	0.045203	DOWN
TSPAN18	584.1262	-0.6558739	0.231047	-2.83869738	0.00453	0.045245	DOWN
DCLRE1B	322.5992	0.52219443	0.183962	2.838604025	0.004531	0.045245	NOT
LINC00324	152.2838	-0.7733502	0.272462	-2.83837651	0.004534	0.045253	DOWN
RP11-474(	19.67111	-0.8263444	0.29114	-2.83830717	0.004535	0.045253	DOWN
EVPL	231.6108	1.85178372	0.652493	2.83801343	0.00454	0.045278	UP
SGPP2	251.355	1.89232862	0.666852	2.837705462	0.004544	0.045305	UP
PEAK1	989.0159	-0.4824971	0.170105	-2.83646502	0.004562	0.045465	NOT
PCSK7	713.4865	-0.4984278	0.175729	-2.8363474	0.004563	0.045465	NOT
MRPL48	1040.191	0.41195586	0.14525	2.836185137	0.004566	0.045472	NOT
GOLGA2P1(	75.90664	0.77301691	0.272592	2.835798223	0.004571	0.04551	UP
TSC22D3	4041.734	-0.9742101	0.343592	-2.83536925	0.004577	0.045554	DOWN
SRSF1	5228.909	0.23567707	0.08314	2.834687578	0.004587	0.045635	NOT
RBM7	749.3224	-0.3813612	0.134544	-2.83448158	0.00459	0.045648	NOT
SLC35B4	1495.138	-0.5181011	0.182797	-2.83429825	0.004593	0.045657	NOT
UBE2D1	642.9364	0.46373146	0.16365	2.833677711	0.004602	0.045727	NOT
PURA	1287.198	-0.4746414	0.167506	-2.83357926	0.004603	0.045727	NOT
NELL1	2.03605	2.98593601	1.053879	2.833282777	0.004607	0.045738	UP
CTD-2262E	6.730867	1.15051358	0.406074	2.833263777	0.004608	0.045738	UP
DTL	753.9765	0.8694359	0.306887	2.833081668	0.00461	0.045747	UP
ERP44	4283.953	-0.3525324	0.124461	-2.83247852	0.004619	0.045817	NOT
TUBAL3	4.395537	2.54662167	0.89925	2.831940327	0.004627	0.045878	UP
C6orf195	2.749671	2.00506105	0.70809	2.831646205	0.004631	0.045903	UP

UPK1A-AS1	14.45578	2.08484901	0.736326	2.831421528	0.004634	0.045918	UP
WHSC1	2326.457	0.59711778	0.210902	2.831254213	0.004637	0.045919	UP
TMPRSS5	17.63346	-0.8863937	0.313082	-2.83118305	0.004638	0.045919	DOWN
AC093850.	4.888463	1.85994159	0.656985	2.831027958	0.00464	0.045925	UP
L3MBTL1	151.3518	0.80714294	0.285207	2.83002886	0.004654	0.046052	UP
SLC51B	362.709	1.3871417	0.490256	2.829424175	0.004663	0.046106	UP
RIMS1	2.017536	-1.782797	0.630111	-2.82933822	0.004664	0.046106	DOWN
LYRM4	1161.599	0.58366332	0.206293	2.829286128	0.004665	0.046106	NOT
CMTM7	547.3535	0.76296024	0.269675	2.829187399	0.004667	0.046106	UP
CYP24A1	4.420102	2.53698753	0.896869	2.82871687	0.004674	0.046141	UP
FNTA	1680.62	0.49694811	0.17568	2.828711294	0.004674	0.046141	NOT
INSL6	3.887173	2.95953739	1.046298	2.82857982	0.004676	0.046143	UP
AKR1C1	35835.29	1.26249517	0.44643	2.827981164	0.004684	0.046191	UP
HTRA1	7610.107	-0.9260376	0.327456	-2.82797885	0.004684	0.046191	DOWN
NQO1	13886.99	1.87617054	0.663451	2.827898272	0.004685	0.046191	UP
MESP2	86.96223	1.11024261	0.392687	2.827296929	0.004694	0.046232	UP
ZNF680	581.8696	-0.7144513	0.252698	-2.8272932	0.004694	0.046232	DOWN
MT1F	438.3927	-1.3510727	0.477924	-2.82695925	0.004699	0.046232	DOWN
SQSTM1	51868.58	0.99406055	0.351637	2.826947317	0.004699	0.046232	UP
RP13-20L1	4.663056	-1.2055187	0.426442	-2.82692329	0.0047	0.046232	DOWN
DLX2	3.800304	2.13631873	0.755706	2.826916233	0.0047	0.046232	UP
CTC-523E2	64.05033	0.70657913	0.249958	2.826787068	0.004702	0.046234	UP
GUCY2C	307.2815	1.95979905	0.693338	2.826614397	0.004704	0.046242	UP
HIST1H4K	11.65473	-1.1472451	0.405945	-2.82611086	0.004712	0.046298	DOWN
LINC00958	6.352313	2.67372285	0.946158	2.82587309	0.004715	0.0463	UP
LY6D	2.144076	3.07455299	1.088003	2.825867303	0.004715	0.0463	UP
CENPL	318.6678	0.76087415	0.269274	2.825646746	0.004719	0.046305	UP
FAM161A	147.4511	0.70417879	0.249214	2.825600166	0.004719	0.046305	UP
MFAP2	204.9944	1.57610574	0.557882	2.825161318	0.004726	0.04635	UP
RHNO1	751.3956	0.56760005	0.200918	2.825034122	0.004728	0.04635	NOT
NUP93	1064.547	0.49122105	0.173887	2.824940445	0.004729	0.04635	NOT
RP11-149I	1.727076	-1.890888	0.669625	-2.82380071	0.004746	0.046498	DOWN
POLR1C	960.0381	0.45072098	0.159668	2.822867478	0.00476	0.046617	NOT
KIFC2	727.6446	0.74140537	0.262657	2.822711992	0.004762	0.046623	UP
RP11-108I	9.609361	1.05341385	0.373213	2.822552735	0.004764	0.046629	UP
NPY1R	89.11976	-1.1916226	0.42221	-2.8223479	0.004767	0.046642	DOWN
RP11-252I	1.611958	-2.1223376	0.752041	-2.82210452	0.004771	0.04666	DOWN
FBXO46	925.075	0.47253309	0.167464	2.821693912	0.004777	0.046703	NOT
CASQ2	57.06993	-1.1439133	0.405465	-2.8212372	0.004784	0.046744	DOWN
CTC-360J1	4.277351	1.64868487	0.584394	2.821187277	0.004785	0.046744	UP
IFITM1	1546.074	-0.9588741	0.339927	-2.82082772	0.00479	0.046779	DOWN
RP11-429F	2.761997	-1.0334182	0.366418	-2.82032881	0.004797	0.046813	DOWN
IFI44L	425.1837	1.27413808	0.451796	2.820162725	0.0048	0.046813	UP
AC005592.	9.370786	1.45058749	0.51437	2.820124394	0.004801	0.046813	UP
CBLN3	129.3391	-0.9611812	0.340835	-2.82008161	0.004801	0.046813	DOWN
TMEM199	836.1466	0.34051553	0.120755	2.819898522	0.004804	0.046813	NOT
ING5	806.2837	0.47967805	0.170107	2.819868173	0.004804	0.046813	NOT
SPTA1	17.4912	1.8035878	0.639619	2.819786216	0.004806	0.046813	UP
LOC100421	5.051845	-1.7228395	0.611029	-2.81957095	0.004809	0.046828	DOWN

RP11-283I	31.07277	1.42480886	0.505441	2.818939701	0.004818	0.046887	UP
RP11-1018	22.24074	-1.4954981	0.530519	-2.81893406	0.004818	0.046887	DOWN
FAM182B	15.42623	1.20667092	0.428094	2.818708631	0.004822	0.046903	UP
EIF4ENIF1	918.0058	0.33965521	0.120507	2.818541623	0.004824	0.046911	NOT
CD19	26.27372	1.53154428	0.543418	2.8183517	0.004827	0.046922	UP
HMGB3P32	3.396429	-1.514065	0.537249	-2.81818053	0.00483	0.04693	DOWN
ZCRB1	1752.339	0.30582593	0.108531	2.81785499	0.004835	0.046961	NOT
OPN4	2.8075	-2.1349262	0.757712	-2.81759581	0.004838	0.046982	DOWN
CES5A	122.857	-1.4400387	0.511122	-2.81740771	0.004841	0.046992	DOWN
MAP3K15	24.36714	1.41236073	0.501317	2.817300514	0.004843	0.046992	UP
ZNF638-IT	8.931673	-1.1244169	0.399171	-2.8168806	0.004849	0.046996	DOWN
FANCB	30.59733	0.85511725	0.303577	2.816809325	0.00485	0.046996	UP
RP11-108C	81.41985	-1.1769193	0.417836	-2.81669837	0.004852	0.046996	DOWN
PRPF19	5798.479	0.32519905	0.115454	2.816687439	0.004852	0.046996	NOT
WBP5	1254.66	0.94030898	0.333839	2.816655645	0.004853	0.046996	UP
RP11-167F	3.207922	-1.1648483	0.413568	-2.8165795	0.004854	0.046996	DOWN
FGG	264852.1	-1.1071016	0.393096	-2.81636783	0.004857	0.047011	DOWN
AP000997.	2.888659	-1.4446814	0.513012	-2.81607717	0.004861	0.047032	DOWN
SOX9-AS1	405.2981	1.34992878	0.47938	2.815990768	0.004863	0.047032	UP
DPY30	1766.073	0.43899644	0.155915	2.815608447	0.004868	0.047071	NOT
ZNF544	942.7789	0.56564051	0.200977	2.814452356	0.004886	0.047224	NOT
EMC2	1969.686	0.48192113	0.171274	2.813742822	0.004897	0.047305	NOT
METTL25	151.58	0.36649245	0.130254	2.813675745	0.004898	0.047305	NOT
CTH	2464.19	-1.2319371	0.43794	-2.81302794	0.004908	0.047383	DOWN
TRGC1	15.88615	-1.0250039	0.364425	-2.81266268	0.004913	0.04742	DOWN
DNAH8	10.23744	1.53666529	0.546397	2.812362066	0.004918	0.047448	UP
AL358852.	7.55147	-0.73682	0.262047	-2.81179037	0.004927	0.047515	DOWN
MTF2	643.1301	0.5743149	0.204263	2.811643802	0.004929	0.04752	NOT
SNORD3B-1	1.683178	2.00709576	0.71394	2.811294408	0.004934	0.047554	UP
CCDC159	890.7722	-0.6275813	0.223244	-2.8111857	0.004936	0.047554	DOWN
FIBCD1	31.47652	1.97952035	0.704238	2.810868095	0.004941	0.047571	UP
ASUN	875.3451	0.42258917	0.150342	2.810843409	0.004941	0.047571	NOT
RP11-374A	3.060584	2.13912038	0.761177	2.81027871	0.00495	0.047632	UP
ACLY	6054.223	0.45904067	0.163348	2.810201439	0.004951	0.047632	NOT
CTSLP8	1.465018	3.06529963	1.090963	2.809720103	0.004958	0.047671	UP
SYNE1	1158.699	-0.6655512	0.236875	-2.80971061	0.004959	0.047671	DOWN
CGRRF1	363.5031	0.40404484	0.143883	2.808143324	0.004983	0.047886	NOT
HS6ST1P1	24.84646	-1.1081051	0.394669	-2.80768403	0.00499	0.047938	DOWN
PRSS53	80.6148	-1.0616798	0.378202	-2.80717405	0.004998	0.047976	DOWN
CXCL16	2934.364	-0.8138368	0.289919	-2.80711948	0.004999	0.047976	DOWN
RP11-345F	188.45	-0.6693913	0.238465	-2.80708511	0.004999	0.047976	DOWN
OGDH	7746.041	-0.618555	0.220375	-2.80682929	0.005003	0.047997	DOWN
CTD-2510F	49.47488	1.08059635	0.385009	2.806678523	0.005006	0.048003	UP
HSPE1	8851.249	0.66398465	0.236597	2.806396471	0.00501	0.048028	UP
SHCBP1	341.9953	1.08874035	0.38801	2.805958977	0.005017	0.048076	UP
CNOT6	1003.162	0.43932477	0.156577	2.805810589	0.005019	0.048081	NOT
NAALAD2	76.68623	1.23909062	0.441653	2.805575612	0.005023	0.048099	UP
CCDC93	1200.503	0.41877864	0.149279	2.805347164	0.005026	0.0481	NOT
RASSF7	2688.312	0.64867001	0.231226	2.80534506	0.005026	0.0481	UP

NTRK1	8.813815	-1.0549791	0.37615	-2.80467474	0.005037	0.048183	DOWN
CCDC39	60.85234	-0.8775532	0.312977	-2.80388979	0.005049	0.048283	DOWN
SYNDIG1	27.21655	-2.0821653	0.742705	-2.80348838	0.005055	0.048326	DOWN
PJA1	730.5015	0.58520272	0.208785	2.802891113	0.005065	0.048386	UP
RP11-12A2	6.160018	1.8040813	0.643656	2.802864582	0.005065	0.048386	UP
RP11-680F	2.074301	-1.0851195	0.387178	-2.80263796	0.005069	0.048403	DOWN
TDP1	511.9587	0.47385201	0.169092	2.802331468	0.005073	0.048426	NOT
FAM216A	287.9771	0.65136876	0.232444	2.80225823	0.005075	0.048426	UP
RP11-321A	3.117242	-1.4087045	0.502768	-2.80190039	0.00508	0.048451	DOWN
AC006548.	5.726027	1.19514361	0.426553	2.801863124	0.005081	0.048451	UP
PAN2	2038.138	-0.6311736	0.225318	-2.80126163	0.00509	0.048524	DOWN
MAL2	4562.075	1.11324378	0.397458	2.800911558	0.005096	0.04856	UP
HSPA7	157.5497	1.33737574	0.477542	2.800542427	0.005102	0.048599	UP
RP11-973F	8.417234	1.70152181	0.607623	2.800289688	0.005106	0.04862	UP
DNAH14	370.0316	0.55156299	0.197035	2.799318232	0.005121	0.048749	NOT
TTC3P1	100.2194	-1.1408865	0.407634	-2.79880086	0.005129	0.04881	DOWN
RP11-258F	66.80496	-1.5590113	0.5573	-2.79743744	0.005151	0.048996	DOWN
CBFB	1057.534	0.42977018	0.153641	2.797237142	0.005154	0.048996	NOT
LYPD6	71.73895	1.78036263	0.636485	2.79717808	0.005155	0.048996	UP
RP11-650F	1.640922	2.32135683	0.829944	2.797003355	0.005158	0.048996	UP
RP11-640I	3.302629	-1.0218576	0.365354	-2.79690057	0.00516	0.048996	DOWN
FANCF	735.2315	0.36081183	0.129005	2.796890484	0.00516	0.048996	NOT
RP11-298C	14.78716	0.76744971	0.27441	2.796727943	0.005162	0.049004	UP
ATPAF1	3115.554	-0.4511864	0.161337	-2.79654911	0.005165	0.049013	NOT
CLPX	3059.103	-0.4958195	0.17731	-2.79634849	0.005168	0.049013	NOT
PPFIBP2	1837.576	-0.5376412	0.192267	-2.79632569	0.005169	0.049013	NOT
PCLO	69.53896	2.08533307	0.745829	2.795993601	0.005174	0.049047	UP
TAF1A	144.6446	0.53801238	0.192431	2.795873362	0.005176	0.049048	NOT
CDC25B	3357.841	0.65777151	0.235278	2.795722633	0.005178	0.049054	UP
RDH13	670.3936	-0.8319303	0.297615	-2.79532344	0.005185	0.049097	DOWN
DNER	34.42992	2.36878718	0.847519	2.794965558	0.005191	0.049134	UP
SIX3	5.350916	3.14052692	1.123756	2.794670348	0.005195	0.049151	UP
CTD-2231E	3.371913	-1.1160984	0.399374	-2.79461857	0.005196	0.049151	DOWN
CXCL12	2449.725	-1.1630842	0.416202	-2.79452034	0.005198	0.049151	DOWN
ZC3H15	2669.03	0.33720369	0.120701	2.793718868	0.005211	0.049255	NOT
RBM8B	9.132018	1.07817606	0.385958	2.79350275	0.005214	0.049271	UP
FAM84B	1645.211	0.85206819	0.305065	2.793068969	0.005221	0.04932	UP
MISP	359.6083	2.1484892	0.769387	2.792468428	0.005231	0.049395	UP
CDK2AP1	1440.533	0.4946524	0.1772	2.791491204	0.005247	0.049527	NOT
CKB	5029.778	-1.592211	0.570486	-2.790974	0.005255	0.049589	DOWN
RAP2C-AS1	45.14467	-0.8167821	0.292681	-2.79069458	0.00526	0.049614	DOWN
CLDN4	2799.657	1.72314484	0.617523	2.790413136	0.005264	0.04964	UP
RP1-40E16	9.617133	3.11334787	1.115806	2.790222296	0.005267	0.049652	UP
Y_RNA	38.34089	-0.5668381	0.203163	-2.79007132	0.00527	0.049658	NOT
RP11-478F	2.435403	3.96384715	1.420889	2.789694441	0.005276	0.049699	UP
SHBG	1288.175	1.46245162	0.524282	2.789434481	0.00528	0.049721	UP
GRPEL2	780.3279	0.35665084	0.12787	2.789156733	0.005285	0.049747	NOT
TOR4A	454.165	0.94907174	0.340348	2.788529965	0.005295	0.049817	UP
HMGB2	2918.879	0.73122986	0.262233	2.788473701	0.005296	0.049817	UP

RP3-510D1	4.658891	-0.8999181	0.322747	-2.78830747	0.005298	0.049825	DOWN
RP11-1267	1.760974	-1.7934888	0.643531	-2.78695036	0.005321	0.049989	DOWN
MGEA5	4312.238	-0.3606951	0.129423	-2.7869388	0.005321	0.049989	NOT
KAZALD1	167.4604	1.0755345	0.385924	2.786907192	0.005321	0.049989	UP
WAC	4903.805	0.35408162	0.12707	2.786504028	0.005328	0.050034	NOT
S100A11	4862.768	1.13319131	0.406699	2.786317495	0.005331	0.050046	NOT
HOXD1	12.40444	1.95563297	0.701977	2.785891761	0.005338	0.05008	NOT
BATF	301.0758	1.33189631	0.478089	2.785872615	0.005338	0.05008	NOT
MTND4P20	508.4767	-1.5458124	0.554926	-2.78562007	0.005343	0.050101	NOT
PCDHA4	6.581975	-1.4289826	0.513039	-2.78533104	0.005347	0.050129	NOT
CNTF	17.45613	-0.8346156	0.299732	-2.78454128	0.00536	0.050231	NOT
RP11-498I	5.46106	-1.539016	0.552719	-2.78444523	0.005362	0.050231	NOT
SYNJ2BP	1855.601	-0.5107778	0.183456	-2.78419852	0.005366	0.050252	NOT
LINC0113	75.5885	0.83743963	0.300806	2.783984874	0.00537	0.050266	NOT
ARHGEF26	1210.862	-1.0183783	0.365812	-2.78388516	0.005371	0.050266	NOT
SMN2	151.9803	0.98533135	0.354005	2.783383171	0.00538	0.050311	NOT
ETV4	1437.729	1.41750457	0.509303	2.783224552	0.005382	0.050311	NOT
ATP1B3	1831.453	0.78272822	0.281231	2.783218817	0.005382	0.050311	NOT
FTH1	31883.3	0.57678502	0.207242	2.783150019	0.005383	0.050311	NOT
RP11-126(	14.47237	0.95448491	0.343018	2.782608641	0.005392	0.050377	NOT
MAP6	132.2137	-1.0495482	0.377245	-2.78214196	0.0054	0.050432	NOT
AFF4	4264.697	-0.4930578	0.177265	-2.78147171	0.005411	0.050519	NOT
RP11-196(	293.4194	-0.3928416	0.141244	-2.7812936	0.005414	0.05053	NOT
ADAMTS3	42.22808	1.34045667	0.482011	2.780966442	0.00542	0.050554	NOT
CAGE1	4.471555	1.53452154	0.551805	2.780913427	0.005421	0.050554	NOT
RP11-51F1	39.69319	-0.7662106	0.275571	-2.78044701	0.005428	0.050607	NOT
APEX1	5947.072	0.38569682	0.138722	2.780352177	0.00543	0.050607	NOT
ZNF285	65.37364	1.09566649	0.394139	2.779897906	0.005438	0.05066	NOT
IQCD	94.95237	1.22103607	0.439299	2.779509934	0.005444	0.050703	NOT
ALPPL2	2.168959	3.0672348	1.103644	2.77918975	0.005449	0.050736	NOT
DBNDD2	54.24087	0.84680755	0.30472	2.778967549	0.005453	0.050753	NOT
ZC3H3	1946.397	0.49005517	0.176355	2.778794782	0.005456	0.050754	NOT
SLFN13	373.919	1.42823783	0.513988	2.778739927	0.005457	0.050754	NOT
NPM1P25	62.31999	-0.8127339	0.292527	-2.77831639	0.005464	0.050803	NOT
ZNF320	506.6002	1.12123393	0.403586	2.778179369	0.005466	0.050807	NOT
RP11-107(	15.86026	-1.2697311	0.457056	-2.77806488	0.005468	0.050807	NOT
FTH1P8	77.25628	0.81009234	0.291625	2.777859658	0.005472	0.050814	NOT
CAPZA1	4407.592	0.41795409	0.150462	2.777796243	0.005473	0.050814	NOT
ADAMTS4	921.8391	-0.7379523	0.265785	-2.77649959	0.005495	0.051	NOT
HDAC6	4153.144	-0.558849	0.201311	-2.77605069	0.005502	0.051053	NOT
TMIGD1	1.920973	2.39013904	0.861041	2.775870622	0.005505	0.051064	NOT
RP11-889I	21.60844	0.83030443	0.299151	2.775535086	0.005511	0.051099	NOT
LATS2	829.0244	-0.581783	0.209684	-2.77456748	0.005528	0.051234	NOT
EPN3	16.58108	1.26132975	0.454728	2.773810413	0.00554	0.051336	NOT
CLEC1A	65.8309	-0.7096739	0.255916	-2.77307626	0.005553	0.051434	NOT
SLBP	1936.879	0.43408055	0.156554	2.772717164	0.005559	0.051473	NOT
GDF2	24.96825	-3.8199712	1.377822	-2.77247048	0.005563	0.051495	NOT
CYS1	64.14536	1.57072046	0.566608	2.772148547	0.005569	0.051528	NOT
SLCO2A1	719.7744	-1.1452293	0.413162	-2.77186594	0.005574	0.051551	NOT

RNVU1-3	3.381543	1.51600262	0.546952	2.771731066	0.005576	0.051551	NOT
CT45A10	2.959655	4.07025762	1.46855	2.771617528	0.005578	0.051551	NOT
FAM101B	482.193	-0.5355369	0.193226	-2.77156252	0.005579	0.051551	NOT
RP1-89D4.	6.8039	1.09095474	0.393682	2.771158404	0.005586	0.051597	NOT
CKLF	354.8328	0.67945827	0.245203	2.771002516	0.005588	0.051604	NOT
TEK	286.3617	-0.9615868	0.34704	-2.77082141	0.005592	0.051615	NOT
ZNF215	29.18751	1.39952066	0.505114	2.770701288	0.005594	0.051617	NOT
POLN	121.4521	-1.1061613	0.39927	-2.77045832	0.005598	0.051638	NOT
RP11-28H5	2.201045	1.64052977	0.592257	2.769964936	0.005606	0.051691	NOT
DBT	1794.112	-0.5582507	0.201542	-2.76990386	0.005607	0.051691	NOT
LCN6	2.839193	-1.4243631	0.514334	-2.76933717	0.005617	0.05176	NOT
RP11-613I	5.565511	-1.2223617	0.441406	-2.76924377	0.005619	0.05176	NOT
RSU1	2598.208	0.46980412	0.169676	2.768825692	0.005626	0.051809	NOT
HSP90AA5I	2.896072	-1.0980227	0.396604	-2.76856039	0.00563	0.051834	NOT
ZMAT5	838.7292	0.48055831	0.173596	2.768262555	0.005636	0.051864	NOT
ZHX1-C8or	58.37162	0.71938403	0.259962	2.767267225	0.005653	0.052003	NOT
RAD54B	111.1087	0.65662781	0.237292	2.767166347	0.005655	0.052003	NOT
PFDN4	682.7035	0.60235935	0.217754	2.766238623	0.005671	0.052131	NOT
RP11-796I	11.57332	2.2454682	0.81178	2.766103664	0.005673	0.052131	NOT
RP11-157I	57.13719	-0.8812135	0.318584	-2.7660341	0.005674	0.052131	NOT
RPL34-AS1	5.483987	-0.9218401	0.333351	-2.76537394	0.005686	0.052219	NOT
PINX1	281.1636	0.50464421	0.182509	2.76504146	0.005692	0.052254	NOT
RP11-58E2	14.33133	-1.0539362	0.38124	-2.76449301	0.005701	0.052325	NOT
REEP2	70.79956	1.26435124	0.457443	2.763951704	0.005711	0.052394	NOT
TLL1	14.15348	-0.9460633	0.342301	-2.7638358	0.005713	0.052395	NOT
CD2AP	2542.83	0.47511308	0.171927	2.763459027	0.005719	0.052438	NOT
LINC01573	46.01093	-1.0201228	0.369191	-2.76313185	0.005725	0.052472	NOT
FYN	997.3197	-0.6507804	0.235572	-2.76255565	0.005735	0.052547	NOT
AP001065.	11.05939	-1.8168071	0.65782	-2.76185868	0.005747	0.052642	NOT
PRSS45	18.99622	1.47583063	0.534482	2.761236194	0.005758	0.05269	NOT
MCEE	625.1865	-0.587365	0.212725	-2.76115234	0.00576	0.05269	NOT
MT1G	2394.528	-1.7352508	0.628462	-2.76110665	0.005761	0.05269	NOT
PLA2G7	508.0936	1.16944942	0.423548	2.76108163	0.005761	0.05269	NOT
GORAB	595.9966	0.62538706	0.226507	2.761009437	0.005762	0.05269	NOT
EPHA6	14.97092	2.64408909	0.957868	2.760388447	0.005773	0.052772	NOT
RPS15	11363.71	0.72533162	0.262786	2.760162206	0.005777	0.052791	NOT
SNAP25	275.6629	1.53924527	0.557795	2.759520175	0.005789	0.052877	NOT
PYCRL	1646.383	0.74778124	0.271017	2.759165678	0.005795	0.052905	NOT
CLOCK	1246.043	-0.4518337	0.163759	-2.75913173	0.005796	0.052905	NOT
SYS1	2283.812	0.43567827	0.15793	2.758673039	0.005804	0.052961	NOT
ZNF630	39.32029	1.02136003	0.370254	2.758541893	0.005806	0.052964	NOT
RP11-295(	666.4884	1.03602556	0.375585	2.758430415	0.005808	0.052965	NOT
LINC0151(	4.503792	1.89725924	0.687849	2.758248611	0.005811	0.052976	NOT
DNAJC2	1332.178	0.41998172	0.152302	2.757553732	0.005824	0.053062	NOT
RP11-616M	10.36773	-2.1490144	0.779333	-2.75750372	0.005824	0.053062	NOT
ATP8A1	247.9204	-0.8357227	0.303113	-2.7571331	0.005831	0.053072	NOT
CCDC97	1437.631	0.39008654	0.141497	2.756861994	0.005836	0.053072	NOT
NAT10	2014.378	0.28799553	0.104468	2.756782147	0.005837	0.053072	NOT
LRRC16A	518.4826	0.91103471	0.330474	2.756755234	0.005838	0.053072	NOT

RAB15	1325.831	0.65983826	0.239356	2.756726975	0.005838	0.053072	NOT
S100A16	6892.91	0.76682769	0.278168	2.756709353	0.005839	0.053072	NOT
TES	1229.333	0.85478035	0.310077	2.756674573	0.005839	0.053072	NOT
RPS8	33814.76	0.59114882	0.214533	2.755510868	0.00586	0.053243	NOT
STK24P1	5.234976	0.97479966	0.353785	2.75534249	0.005863	0.053252	NOT
BTG2	2099.056	-0.8229402	0.298739	-2.75470977	0.005874	0.053338	NOT
GS1-21A4.	13.08085	-1.1767619	0.427221	-2.75445725	0.005879	0.053361	NOT
RP11-385I	7.045919	1.75848135	0.638571	2.753773733	0.005891	0.053443	NOT
POTEKP	4.739306	1.91086746	0.693918	2.753736141	0.005892	0.053443	NOT
GLMN	249.2041	0.52895141	0.192107	2.753416789	0.005898	0.05346	NOT
RUFY4	27.71432	1.40847947	0.51154	2.75341215	0.005898	0.05346	NOT
RPL23AP64	9.916101	-0.8866637	0.322052	-2.75317228	0.005902	0.053481	NOT
RAP2A	2215.481	0.62506025	0.227081	2.752585566	0.005913	0.053559	NOT
RP3-337H4	17.49713	0.77312127	0.280882	2.752478167	0.005915	0.053559	NOT
KLHL15	589.911	-0.5398653	0.196176	-2.75194533	0.005924	0.053629	NOT
B3GALT1	10.72158	-1.9305171	0.701582	-2.75166354	0.005929	0.053657	NOT
RP11-307(	6.958705	-1.0977336	0.399095	-2.75055763	0.005949	0.05382	NOT
E2F2	188.7926	0.87998228	0.319946	2.750405628	0.005952	0.053827	NOT
FMR1-IT1	6.50317	-1.0542992	0.383366	-2.75011079	0.005958	0.053852	NOT
SGMS2	756.1062	-0.6702096	0.243709	-2.75003986	0.005959	0.053852	NOT
HOXD13	3.713424	4.04945976	1.47281	2.749478917	0.005969	0.053908	NOT
NRAS	3239.097	0.46377497	0.168678	2.749475599	0.005969	0.053908	NOT
ODF3L2	10.0489	-0.8683403	0.315874	-2.74900929	0.005978	0.053967	NOT
CHAF1A	1008.209	0.55726078	0.202723	2.748883721	0.00598	0.05397	NOT
C1S	93994.24	-0.9046306	0.32917	-2.74822096	0.005992	0.054061	NOT
LINC00839	31.94984	1.21013278	0.440372	2.747976333	0.005996	0.054077	NOT
ENPP6	53.15059	1.76514755	0.642361	2.747907745	0.005998	0.054077	NOT
KBTBD11	272.4193	-1.2963166	0.47177	-2.74777075	0.006	0.054081	NOT
NEK2	615.6691	0.95499716	0.347573	2.747616916	0.006003	0.054089	NOT
NFE2L3	537.0811	0.97371302	0.354454	2.747078796	0.006013	0.05416	NOT
LINC00336	1.923295	2.39491814	0.87187	2.746875944	0.006017	0.054169	NOT
VPS4A	2004.756	-0.4546023	0.165507	-2.74672967	0.006019	0.054169	NOT
LUZP4	2.239646	3.66276379	1.33355	2.746625955	0.006021	0.054169	NOT
LINC01123	14.56568	1.66556376	0.606427	2.746518439	0.006023	0.054169	NOT
SCNM1	1450.944	0.6509763	0.237024	2.746456646	0.006024	0.054169	NOT
RP11-150(	1.252339	2.61831967	0.953365	2.746397538	0.006025	NA	NA
CMB9-55A1	2.388268	-1.0933201	0.398096	-2.74637048	0.006026	0.054169	NOT
CENPH	257.4134	0.65717856	0.239369	2.74546717	0.006042	0.054288	NOT
FBXL5	3885.017	-0.5085648	0.18524	-2.74543448	0.006043	0.054288	NOT
LRPPRC	7511.955	0.40277518	0.146729	2.745025381	0.006051	0.054337	NOT
PPM1L	886.566	-0.7407031	0.269898	-2.74438467	0.006062	0.054419	NOT
TNKS2-AS1	16.52408	-1.1720373	0.427104	-2.74415186	0.006067	0.054419	NOT
KIAA1671	3611.388	-0.5841146	0.212861	-2.74411743	0.006067	0.054419	NOT
TBCC	901.5534	0.38319073	0.139642	2.744098284	0.006068	0.054419	NOT
BNIP3P17	6.169373	1.88543042	0.687506	2.742421385	0.006099	0.05468	NOT
PPAP2C	1550.102	1.72496859	0.629064	2.742118497	0.006104	0.054712	NOT
RP11-963H	13.05547	-1.4441748	0.526718	-2.74183637	0.00611	0.054741	NOT
DHX34	1397.671	0.48304311	0.176245	2.740751958	0.00613	0.054904	NOT
GPD2	722.9854	0.58087546	0.211965	2.740432957	0.006136	0.054939	NOT

FLNC	2336.115	1.76822596	0.645321	2.740070973	0.006143	0.054967	NOT
PCGF5	2914.053	-0.4703088	0.171646	-2.74000038	0.006144	0.054967	NOT
ARID3A	1296.754	1.32837292	0.484819	2.739938328	0.006145	0.054967	NOT
ZKSCAN1	6105.176	-0.5635121	0.205683	-2.73971732	0.006149	0.054986	NOT
MORN2	400.1369	0.5682217	0.20742	2.73947258	0.006154	0.055009	NOT
AD000092.	13.71464	-0.7072162	0.258177	-2.73927047	0.006158	0.055025	NOT
TCERG1	1388.722	0.3785646	0.13824	2.738456056	0.006173	0.055143	NOT
SH2B3	1885.262	-0.5623307	0.205356	-2.73832219	0.006175	0.055147	NOT
LYSMD1	553.4748	0.39551303	0.144445	2.73815375	0.006179	0.055157	NOT
LINC0148	20.96927	-1.1547907	0.421804	-2.73774499	0.006186	0.055195	NOT
TTF2	423.0796	0.64838837	0.236836	2.737715221	0.006187	0.055195	NOT
INAFM2	179.7445	-0.6710047	0.245144	-2.73718758	0.006197	0.055261	NOT
PSD4	3053.69	-0.5659641	0.206775	-2.73710072	0.006198	0.055261	NOT
C15orf48	432.2343	1.56204506	0.570716	2.736993451	0.0062	0.055261	NOT
PTPRO	52.0552	0.94866344	0.346632	2.736805449	0.006204	0.055275	NOT
MMP14	4356.549	0.68973625	0.252044	2.736575392	0.006208	0.055295	NOT
NPY5R	6.353625	-1.402065	0.512393	-2.73630649	0.006213	0.055322	NOT
RP11-200	37.88568	0.73874927	0.270066	2.735440674	0.00623	0.05545	NOT
GGT3P	3.077426	1.65041337	0.603382	2.735272937	0.006233	0.05546	NOT
SRXN1	300.2229	1.00739626	0.368342	2.734950362	0.006239	0.055496	NOT
C8orf22	3.542233	4.15516976	1.519396	2.734750533	0.006243	0.055512	NOT
ANKRD20A1	4.372778	-1.921403	0.702814	-2.73387005	0.006259	0.055642	NOT
RP3-428L1	22.78963	0.80950998	0.296116	2.733756028	0.006262	0.055643	NOT
KIAA0020	889.8614	0.44546638	0.162979	2.733267923	0.006271	0.055707	NOT
RAPGEF4	1009.18	-0.8006816	0.292967	-2.73301045	0.006276	0.055733	NOT
NR4A2	378.8431	-0.9682862	0.354312	-2.73286179	0.006279	0.05574	NOT
RP11-252A	5.89174	-0.8517438	0.311742	-2.73221091	0.006291	0.055832	NOT
PBX1	837.5375	-1.1747366	0.430001	-2.73194087	0.006296	0.055859	NOT
RNU4-1	4.124594	-1.2926413	0.473196	-2.73172604	0.0063	0.055875	NOT
TRMT13	403.035	0.52184362	0.191037	2.731634372	0.006302	0.055875	NOT
RP11-216I	64.9826	1.3243421	0.48509	2.730094033	0.006332	0.056118	NOT
RORA	1663.035	-0.8161637	0.298984	-2.7297943	0.006337	0.056151	NOT
DDX31	534.242	0.37612146	0.137795	2.729580354	0.006341	0.056153	NOT
CCNJL	101.0096	1.35865752	0.497756	2.729563825	0.006342	0.056153	NOT
RP11-456K	32.08672	-0.6107416	0.223777	-2.72923601	0.006348	0.056171	NOT
NDEL1	1083.598	-0.4606975	0.168806	-2.72915923	0.00635	0.056171	NOT
KCNJ10	49.9204	1.35681259	0.497158	2.72913866	0.00635	0.056171	NOT
IDE	1954.936	-0.4102781	0.150368	-2.72849223	0.006362	0.056225	NOT
BCAT1	403.4683	0.92981398	0.340781	2.728482555	0.006363	0.056225	NOT
AC090954.	2.202888	2.91582364	1.06868	2.728434556	0.006364	0.056225	NOT
ERN2	4.581707	2.04066168	0.748006	2.728133863	0.006369	0.056225	NOT
TRPV5	1.766827	2.6120313	0.957444	2.728128208	0.006369	0.056225	NOT
TK2	1764.061	-0.4800017	0.175946	-2.72811913	0.00637	0.056225	NOT
LINC0113	15.52917	1.25776122	0.461045	2.728066783	0.006371	0.056225	NOT
RP5-836N1	2.802379	1.38381855	0.507414	2.727199046	0.006387	0.056355	NOT
SEPSECS	1696.941	-0.6424578	0.235632	-2.72652575	0.0064	0.056452	NOT
HCG18	680.5142	0.47551904	0.174425	2.726202412	0.006407	0.056489	NOT
RPSAP58	427.943	0.87831032	0.322195	2.72601783	0.00641	0.056502	NOT
POLA1	586.6829	0.61232106	0.224716	2.724868315	0.006433	0.056681	NOT

ZNHIT6	530.5742	0.48245388	0.177078	2.724530737	0.006439	0.05672	NOT
HPS4	1281.562	0.31466743	0.115511	2.724141216	0.006447	0.056769	NOT
NLRP6	224.6438	-1.5062422	0.552977	-2.72387968	0.006452	0.056795	NOT
RP1-118J2	2.193546	-1.4266775	0.523876	-2.72331068	0.006463	0.056875	NOT
RP11-261(	7.873195	1.84532984	0.677678	2.723019675	0.006469	0.056906	NOT
GNAT1	40.18835	-1.1806709	0.433607	-2.72290475	0.006471	0.056908	NOT
CFAP57	60.91064	-1.2670733	0.465441	-2.72230705	0.006483	0.056985	NOT
FAM26E	126.2192	-0.6816756	0.25041	-2.72224251	0.006484	0.056985	NOT
RGS7	3.307354	2.30334764	0.846218	2.721930803	0.00649	0.05702	NOT
CCDC74B	12.03397	1.64157037	0.603226	2.721319647	0.006502	0.057107	NOT
MAP3K2	2426.725	-0.399719	0.1469	-2.72102091	0.006508	0.057133	NOT
LRRC8C	355.3671	-0.6912028	0.254029	-2.7209549	0.006509	0.057133	NOT
C3orf70	73.10044	-0.6261492	0.230185	-2.72020475	0.006524	0.057244	NOT
TCEA1	3010.493	0.43339181	0.159329	2.720100922	0.006526	0.057244	NOT
RPL37P6	20.41684	0.98649632	0.362763	2.719394559	0.00654	0.057322	NOT
LMOD1	369.1376	-0.9023562	0.331826	-2.71936571	0.006541	0.057322	NOT
TMEM25	401.2089	-1.00329	0.368948	-2.71932783	0.006541	0.057322	NOT
BEAN1-AS1	2.945505	-1.6876233	0.620638	-2.71917661	0.006544	0.05733	NOT
MRPS12	2544.452	-0.6850143	0.251952	-2.71882943	0.006551	0.057372	NOT
ADAMTS1	1236.929	-0.8259512	0.303846	-2.71831831	0.006561	0.057442	NOT
ETS1	2139.96	-0.6515773	0.239709	-2.7181968	0.006564	0.057445	NOT
RP1-45C12	9.754217	1.53920388	0.56629	2.718049531	0.006567	0.057452	NOT
ACTR6	882.6094	0.45209955	0.166354	2.717693287	0.006574	0.057492	NOT
TNS2	4219.636	-0.6002905	0.22089	-2.71760406	0.006576	0.057492	NOT
LRRC37A	18.97219	0.82483683	0.303548	2.71731731	0.006581	0.057512	NOT
TSHR	5.934364	-1.0254624	0.377386	-2.71727701	0.006582	0.057512	NOT
YPEL1	124.4201	-0.8370524	0.308079	-2.71700803	0.006588	0.05754	NOT
FTH1P7	103.993	0.73565926	0.27086	2.716014209	0.006607	0.057694	NOT
RP11-295M	8.807028	-1.1064541	0.407409	-2.71583005	0.006611	0.057708	NOT
ZNF592	1839.769	-0.364274	0.134146	-2.7154962	0.006618	0.057747	NOT
IQCH-AS1	275.7182	-0.5854943	0.215626	-2.71532068	0.006621	0.057757	NOT
DNAJC25	963.121	-0.5679677	0.209179	-2.71522825	0.006623	0.057757	NOT
TFDP1	2497.231	0.8001309	0.294741	2.714692034	0.006634	0.057832	NOT
DGAT2L7P	9.198609	1.13319328	0.41759	2.713652439	0.006655	0.057995	NOT
CHD5	6.617053	1.77106822	0.652905	2.712597007	0.006676	0.058151	NOT
PTMAP2	27.73867	0.7616689	0.280794	2.712552822	0.006677	0.058151	NOT
RPLP0P6	258.7568	0.9519214	0.350965	2.712294712	0.006682	0.058177	NOT
TSSK5P	16.9967	0.95684213	0.3528	2.712141196	0.006685	0.058186	NOT
ATP10A	244.9846	-1.2748823	0.470167	-2.71155283	0.006697	0.05827	NOT
GRM6	6.503789	-1.3881084	0.512093	-2.71065481	0.006715	0.05841	NOT
MYBPC2	5.201152	1.46423099	0.540201	2.710528058	0.006718	0.058413	NOT
GLG1	5722.618	-0.3511133	0.129548	-2.71029121	0.006722	0.058436	NOT
SYNPO	6871.345	-0.6544339	0.241501	-2.70986497	0.006731	0.058493	NOT
FAM171A2	103.7207	1.1000826	0.406032	2.70934701	0.006742	0.058547	NOT
FOXO1	1278.313	-0.723446	0.267019	-2.70934345	0.006742	0.058547	NOT
CENPK	180.7629	0.82669273	0.305144	2.7091928	0.006745	0.058555	NOT
KB-1732A1	106.3939	0.79841742	0.294736	2.708926548	0.00675	0.058583	NOT
RPS18	47764.1	0.61539075	0.227204	2.708540158	0.006758	0.058633	NOT
TMEM255B	226.6984	-0.571089	0.210871	-2.70823283	0.006764	0.058647	NOT

HMSD	14.73443	1.69305759	0.62519	2.708067905	0.006768	0.058647	NOT
PPP4R1-AS	3.212693	-1.332505	0.492059	-2.70801857	0.006769	0.058647	NOT
EHBP1	2810.294	-0.560912	0.207131	-2.70800759	0.006769	0.058647	NOT
CALN1	2.418716	2.03770713	0.752496	2.707930944	0.00677	0.058647	NOT
RP11-93B1	21.86832	-1.2507917	0.461925	-2.70778214	0.006773	0.058654	NOT
DSC3	12.32474	2.1856671	0.807293	2.707403907	0.006781	0.058677	NOT
RP11-616M	2.309076	-1.8942645	0.699671	-2.70736503	0.006782	0.058677	NOT
SPACA6P	74.24558	1.12813746	0.416712	2.707233601	0.006785	0.058677	NOT
SLC39A10	672.2465	0.68217788	0.251984	2.707227536	0.006785	0.058677	NOT
RP11-755J	1.565127	-1.866194	0.689412	-2.70693696	0.006791	0.058703	NOT
PRDX2	10690.58	-0.5928981	0.219034	-2.70687354	0.006792	0.058703	NOT
CLEC9A	11.92551	-1.0935082	0.404152	-2.70568591	0.006816	0.058894	NOT
C1orf52	497.2385	0.3998579	0.147792	2.705546452	0.006819	0.0589	NOT
KB-176G8.	5.924029	-1.1560732	0.427338	-2.70529152	0.006824	0.058927	NOT
SLC10A3	818.5288	0.751805	0.277974	2.704583047	0.006839	0.059031	NOT
CARD10	2454.077	0.68821444	0.254482	2.704371312	0.006843	0.059031	NOT
RP11-999E	9.473739	-1.3116359	0.485028	-2.70424924	0.006846	0.059031	NOT
MIR635	18.42005	-0.7471829	0.276307	-2.70417393	0.006847	0.059031	NOT
BSDC1	4185.023	-0.4112373	0.152079	-2.70411073	0.006849	0.059031	NOT
GNMT	4015.739	-1.5957261	0.59012	-2.7040722	0.00685	0.059031	NOT
LCAT	2387.708	-1.1450829	0.423528	-2.70367936	0.006858	0.059082	NOT
REG1B	5.334798	3.59744482	1.33063	2.70356595	0.00686	0.059083	NOT
RPRM	2.316481	2.53139206	0.936467	2.703130674	0.006869	0.059142	NOT
ZNF485	100.8514	0.56565076	0.209279	2.702856163	0.006875	0.059172	NOT
CD8BP	2.783579	2.6636893	0.985626	2.702534618	0.006881	0.05921	NOT
ERO1L	3722.474	0.53071679	0.196398	2.702253105	0.006887	0.059225	NOT
TSSC1	1099.673	0.41847958	0.154864	2.702239577	0.006887	0.059225	NOT
EYS	116.8184	-0.8373017	0.309935	-2.70154168	0.006902	0.059331	NOT
SLC35G1	384.7819	0.78244723	0.289681	2.701062245	0.006912	0.059398	NOT
RP11-308D	16.77106	1.11193017	0.411757	2.700449374	0.006925	0.059458	NOT
NPIPA3	8.901791	1.33505366	0.494386	2.700425968	0.006925	0.059458	NOT
TNFRSF10C	114.4965	0.90243548	0.334185	2.700406526	0.006925	0.059458	NOT
CH17-118C	169.8405	0.73808929	0.273347	2.700195438	0.00693	0.059459	NOT
FAM53B	907.4183	-0.5708874	0.211425	-2.70019032	0.00693	0.059459	NOT
FUNDC2P2	14.22249	1.86800885	0.691847	2.700031474	0.006933	0.059469	NOT
RP11-452N	14.94252	0.88391082	0.327416	2.69965292	0.006941	0.059515	NOT
VPS53	1191.009	-0.3878596	0.143676	-2.6995503	0.006943	0.059515	NOT
RP11-909N	16.92612	2.19692093	0.813838	2.699458935	0.006945	0.059515	NOT
ZNF71	225.9973	0.76236645	0.282432	2.69929337	0.006949	0.059526	NOT
ACCS	599.7208	-0.6690781	0.247918	-2.69878297	0.006959	0.059598	NOT
TUBAP2	13.26203	0.87028769	0.322501	2.698554985	0.006964	0.05962	NOT
UBE2N	2582.826	0.28914739	0.107159	2.698301974	0.006969	0.059647	NOT
RAB33B	595.4254	-0.5812771	0.215432	-2.69819003	0.006972	0.059648	NOT
CCDC64	785.0705	0.93098679	0.345095	2.697768029	0.006981	0.059678	NOT
RAB41	22.30555	0.69294807	0.256865	2.697714084	0.006982	0.059678	NOT
GOLGA8H	4.074311	-0.8961702	0.332206	-2.69763707	0.006983	0.059678	NOT
NR2C2AP	853.0831	0.49697575	0.184228	2.697605966	0.006984	0.059678	NOT
RP11-307F	2.064102	2.04224409	0.757134	2.697335003	0.00699	0.059707	NOT
RP11-1C1.	3.775861	3.71957765	1.379155	2.696997033	0.006997	0.059749	NOT

DSG2-AS1	14.09912	0.96072474	0.356255	2.696730054	0.007002	0.059778	NOT
PLXND1	4788.466	-0.5884812	0.218245	-2.69641861	0.007009	0.059812	NOT
CSF3R	362.0451	1.07297178	0.397937	2.696334154	0.007011	0.059812	NOT
LINC0093:	16.50671	-0.7689771	0.285254	-2.69576103	0.007023	0.059896	NOT
B4GALT4	1033.463	0.38210552	0.141763	2.695378468	0.007031	0.059946	NOT
RP1-121G1	2.512084	-1.7344402	0.643749	-2.69428121	0.007054	0.060125	NOT
TRIM22	1206.111	-0.9742448	0.361613	-2.69416096	0.007057	0.060127	NOT
TMEM69	1103.397	0.46352058	0.172057	2.69399751	0.00706	0.060128	NOT
YWHAZP3	7.1364	1.03301296	0.383457	2.693948846	0.007061	0.060128	NOT
CLVS1	33.95152	-1.2887101	0.478392	-2.69383866	0.007063	0.060129	NOT
ZNF330	1070.397	-0.4246719	0.157659	-2.69360699	0.007068	0.060135	NOT
CHIT1	100.7541	1.62586828	0.603605	2.693594351	0.007069	0.060135	NOT
RP5-1112I	75.8331	0.6562973	0.243664	2.693452302	0.007072	0.060142	NOT
AC079630.	13.23596	-0.8585054	0.318801	-2.69291855	0.007083	0.06022	NOT
ERICH4	4.483947	2.02657248	0.752673	2.692499094	0.007092	0.060276	NOT
BUB3	3103.948	0.34660533	0.128736	2.69237405	0.007095	0.06028	NOT
CTC-537E7	17.78158	-1.5466541	0.574515	-2.69210237	0.0071	0.06031	NOT
SF3B6	2604.331	0.37777606	0.140346	2.691751404	0.007108	0.060355	NOT
PTBP1	10750.19	0.26681104	0.099136	2.691357119	0.007116	0.060376	NOT
ADRA1A	313.1882	-1.6893627	0.62771	-2.69130968	0.007117	0.060376	NOT
CTD-2240J	3.159446	-1.1284593	0.41931	-2.69123098	0.007119	0.060376	NOT
TRAPPC5	46.99428	-0.7624133	0.283297	-2.69121864	0.007119	0.060376	NOT
PCDHB6	16.57115	-0.9565333	0.35546	-2.6909716	0.007124	0.06039	NOT
VWA8	1633.975	-0.6877787	0.255591	-2.69093233	0.007125	0.06039	NOT
EPHB6	455.9801	1.42731897	0.530474	2.690646423	0.007131	0.060423	NOT
AC002076.	1.871699	2.50774653	0.932091	2.690451067	0.007136	0.060439	NOT
FAM214B	661.6198	-0.5371427	0.199678	-2.69003763	0.007144	0.060462	NOT
LRRN3	15.41312	-1.2309701	0.457611	-2.68999111	0.007145	0.060462	NOT
AC016722.	10.37969	-0.9115768	0.338886	-2.68991856	0.007147	0.060462	NOT
RP1-100J1	5.462151	-0.9334278	0.347011	-2.68991079	0.007147	0.060462	NOT
FENDRR	8.883001	-1.3367758	0.496999	-2.68969326	0.007152	0.060472	NOT
MBTPS1	3645.098	-0.3777629	0.140453	-2.68959501	0.007154	0.060472	NOT
TRIM61	3.815797	-1.6147275	0.600372	-2.68954332	0.007155	0.060472	NOT
SLC9A2	8.109489	1.58842725	0.590648	2.689296078	0.00716	0.060498	NOT
RP11-162(	8.133945	-1.047067	0.389451	-2.68857279	0.007176	0.060597	NOT
CTD-2553I	51.37044	-0.6752556	0.251161	-2.68853497	0.007177	0.060597	NOT
KANK2	4084.867	-0.5827381	0.216757	-2.68843648	0.007179	0.060597	NOT
ARHGAP31	500.7709	-0.7280967	0.270857	-2.68812215	0.007186	0.060631	NOT
PDLIM5	4318.095	-0.5285679	0.196637	-2.68804306	0.007187	0.060631	NOT
ESRRB	7.211801	-1.1309098	0.420769	-2.6877194	0.007194	0.060659	NOT
TIE1	1178.286	-0.607972	0.226207	-2.68768225	0.007195	0.060659	NOT
OR8G5	4.146744	2.98008383	1.10884	2.68756875	0.007197	0.060661	NOT
PPP2R3A	196.1974	0.89882562	0.334527	2.686856525	0.007213	0.060771	NOT
MCEMP1	5.756843	1.6606977	0.618171	2.686469739	0.007221	0.060817	NOT
CTC-204F2	9.332609	-0.8837431	0.328988	-2.68624387	0.007226	0.060817	NOT
MYO3A	4.596774	3.07883923	1.146193	2.68614395	0.007228	0.060817	NOT
YBX1P6	4.125927	1.0429896	0.388293	2.68609136	0.007229	0.060817	NOT
PCSK6	6283.418	-1.054472	0.392568	-2.68608455	0.007229	0.060817	NOT
UBASH3B	219.6034	1.03242668	0.384383	2.685930466	0.007233	0.060826	NOT

RTN3P1	3.602863	1.2173196	0.453308	2.685412215	0.007244	0.060902	NOT
ZNF557	275.076	-0.3841468	0.143065	-2.68512278	0.00725	0.060936	NOT
DLG2	75.14814	-0.9968378	0.371259	-2.68501735	0.007253	0.060936	NOT
KHSRPP1	2.473278	1.8319695	0.682342	2.684826742	0.007257	0.060943	NOT
RP11-415F	9.802254	-0.7624367	0.283995	-2.68468744	0.00726	0.060943	NOT
FAM83D	884.0652	0.69807874	0.260024	2.684671014	0.00726	0.060943	NOT
OR8A1	1.686026	3.45352884	1.286492	2.684454954	0.007265	0.060963	NOT
LIMK1	1092.578	0.71892783	0.267868	2.683886397	0.007277	0.061032	NOT
CWC27	730.5042	0.36614717	0.13643	2.683769094	0.00728	0.061032	NOT
TSG101	2816.943	0.26231289	0.09774	2.683769019	0.00728	0.061032	NOT
CPA6	12.42885	1.70833619	0.636581	2.683611742	0.007283	0.061041	NOT
PLXNA3	1006.944	0.8842763	0.329522	2.683514648	0.007285	0.061041	NOT
RP11-5407	7.540185	1.18207525	0.440577	2.683015521	0.007296	0.061113	NOT
ARHGAP23	630.9474	-0.7344401	0.273753	-2.68285772	0.0073	0.061123	NOT
ATP5A1	22410.92	-0.384885	0.143493	-2.68225626	0.007313	0.061214	NOT
OGN	116.4514	-1.7260945	0.643623	-2.68184138	0.007322	0.061271	NOT
ILDR1	114.9654	1.6627036	0.620134	2.681199676	0.007336	0.061352	NOT
EPCAM	2300.313	2.05845877	0.767764	2.681109924	0.007338	0.061352	NOT
GJB6	12.35085	2.22526549	0.829985	2.681089903	0.007338	0.061352	NOT
TMEM8C	2.999622	-1.6747528	0.624795	-2.68048324	0.007352	0.061444	NOT
CCNE1	266.5726	0.89507422	0.333941	2.680334562	0.007355	0.061453	NOT
HIGD1C	2.022407	-1.3529652	0.504889	-2.67972674	0.007368	0.061537	NOT
CYBA	2806.956	1.03071884	0.384644	2.679671838	0.007369	0.061537	NOT
PTK7	1214.132	1.33043448	0.496548	2.679365932	0.007376	0.061563	NOT
GS1-279B7	7.671314	0.89662206	0.334645	2.679324846	0.007377	0.061563	NOT
PAQR3	395.6197	-0.7141008	0.266537	-2.67918301	0.00738	0.06157	NOT
FTH1P16	56.56918	0.87894676	0.328095	2.678942086	0.007386	0.061595	NOT
RP13-20L1	60.74008	-0.7114429	0.265671	-2.67791272	0.007408	0.061756	NOT
ZNF667	80.12099	1.3204159	0.493086	2.677862454	0.007409	0.061756	NOT
PCNP	3306.54	0.2841249	0.106118	2.677454759	0.007418	0.061803	NOT
TMPO	4582.037	0.48658776	0.181739	2.677399592	0.00742	0.061803	NOT
AC093668.	4.824706	-0.9351015	0.34931	-2.67699279	0.007429	0.061804	NOT
SNX21	526.6127	-0.4458385	0.166545	-2.67698495	0.007429	0.061804	NOT
CYYR1	410.9442	-0.6254166	0.233627	-2.67698385	0.007429	0.061804	NOT
FAHD2B	180.2705	1.19722712	0.44724	2.676922594	0.00743	0.061804	NOT
PAK1	1648.984	0.48310645	0.180473	2.67688458	0.007431	0.061804	NOT
OAT	2184.702	-1.1965757	0.447068	-2.67649563	0.00744	0.061839	NOT
HERPUD1	6217.656	-0.5291448	0.197706	-2.67641979	0.007441	0.061839	NOT
ZNF812	19.48152	1.44066194	0.538286	2.676386189	0.007442	0.061839	NOT
FAM189A2	32.50024	-0.9426235	0.352219	-2.67623876	0.007445	0.061847	NOT
FOXP4-AS1	22.7098	1.32833383	0.496366	2.676118477	0.007448	0.06185	NOT
SERPINA13	1.347741	2.00528939	0.749508	2.675475573	0.007462	NA	NA
TRMT61A	1244.871	0.45903295	0.171599	2.675031926	0.007472	0.062032	NOT
TIMM9	1020.652	0.43953784	0.164343	2.674522016	0.007484	0.062108	NOT
RP11-203F	1.9792	-1.6137683	0.603415	-2.67439178	0.007486	0.062113	NOT
RP11-49I1	19.43434	0.86047575	0.32184	2.673612385	0.007504	0.062238	NOT
PSMD10	1610.851	0.41864511	0.156592	2.673469681	0.007507	0.062246	NOT
SPATA3-AS	7.773789	1.42674347	0.533718	2.673216469	0.007513	0.062274	NOT
HNRNPKP4	15.18574	0.83158427	0.311116	2.672906231	0.00752	0.062312	NOT

RP11-693J	1.744576	3.12239695	1.168254	2.672702701	0.007524	0.062331	NOT
LRIT3	9.984616	-1.0054644	0.376282	-2.67210274	0.007538	0.062423	NOT
MTND4P24	27.42967	1.56415803	0.585419	2.671861191	0.007543	0.062426	NOT
CENPF	1867.7	0.91174523	0.34125	2.671780924	0.007545	0.062426	NOT
RANGAP1	8000.801	0.54021574	0.202194	2.671771632	0.007545	0.062426	NOT
RP11-552I	2.002874	1.61480479	0.60442	2.67166053	0.007548	0.062426	NOT
ZNF572	115.3769	0.68801318	0.25754	2.671485942	0.007552	0.062426	NOT
METTL21EP	4.791625	-1.008114	0.377362	-2.67147588	0.007552	0.062426	NOT
CD302	1300.393	-0.7629882	0.285629	-2.67126031	0.007557	0.062447	NOT
THOC1	850.965	0.40112858	0.150195	2.670717245	0.007569	0.062529	NOT
ZC2HC1A	170.963	0.85075153	0.318593	2.670338252	0.007577	0.062581	NOT
ROGDI	1341.402	-0.5609659	0.210096	-2.6700446	0.007584	0.062616	NOT
RGS7BP	40.10758	-1.4811309	0.554814	-2.66959688	0.007594	0.062681	NOT
IGF1	385.6526	-1.310711	0.491073	-2.66907507	0.007606	0.062759	NOT
AGXT2	2301.171	-1.2570494	0.47105	-2.66861266	0.007617	0.062826	NOT
TARS	4050.894	0.39248486	0.147087	2.668386965	0.007622	0.06285	NOT
TXN	15820.03	0.70477665	0.264209	2.667495909	0.007642	0.062992	NOT
TRAK1	1661.699	-0.3445342	0.129164	-2.66742132	0.007644	0.062992	NOT
PDCD2L	281.8036	0.51488837	0.193063	2.666945748	0.007654	0.063062	NOT
TMEM105	94.81548	1.31184311	0.49194	2.666670913	0.007661	0.063095	NOT
RP11-150(	3.832538	1.76636845	0.662589	2.66585937	0.007679	0.063227	NOT
CACNA1C	169.2891	-0.8580906	0.321903	-2.66567877	0.007683	0.063227	NOT
RP11-415J	1.821942	2.0189451	0.75739	2.665660871	0.007684	0.063227	NOT
SYNJ2BP-(	8.016263	-0.7848032	0.294446	-2.66535121	0.007691	0.063243	NOT
SNX3	6082.529	0.41390369	0.155291	2.665340389	0.007691	0.063243	NOT
TAF1B	378.7082	0.43218737	0.162162	2.665153039	0.007695	0.063243	NOT
RP11-82H1	2.107926	1.27746464	0.479326	2.665129554	0.007696	0.063243	NOT
TGFB1	2263.709	0.96202412	0.360975	2.665067848	0.007697	0.063243	NOT
SMC1B	68.75931	1.50618313	0.565227	2.664740751	0.007705	0.063285	NOT
TCP11L1	285.0456	-0.4010853	0.150526	-2.66455434	0.007709	0.063301	NOT
DTD1	991.7739	0.75230098	0.282374	2.664197829	0.007717	0.063349	NOT
ZDHHC13	283.6344	0.85558064	0.321158	2.664047973	0.007721	0.063358	NOT
RAB2B	554.2458	-0.3735526	0.140232	-2.66381553	0.007726	0.063383	NOT
OLFML1	525.1493	-0.8916031	0.334722	-2.66370864	0.007728	0.063384	NOT
SVEP1	302.1598	-1.2383766	0.46493	-2.66357376	0.007732	0.06339	NOT
SMPD4	2242.502	0.36167409	0.135791	2.663467682	0.007734	0.063391	NOT
FMN1	120.7503	1.2117056	0.454993	2.663127461	0.007742	0.063432	NOT
TCTEX1D1	37.97454	1.27357619	0.478241	2.663045419	0.007744	0.063432	NOT
MXRA7	1316.303	0.63497435	0.23845	2.662923129	0.007747	0.063433	NOT
RP11-803I	5.346272	1.24554631	0.467752	2.662836101	0.007749	0.063433	NOT
TTC9C	632.0438	0.2755845	0.103504	2.662555658	0.007755	0.063454	NOT
KIF11	616.0023	0.79029678	0.296828	2.662471346	0.007757	0.063454	NOT
NAV2-AS4	5.585548	-1.864215	0.700196	-2.66241846	0.007758	0.063454	NOT
SRSF12	60.0419	1.00883539	0.378934	2.662298087	0.007761	0.063458	NOT
BFSP1	75.68	0.74217261	0.278794	2.662080273	0.007766	0.06348	NOT
ZG16B	69.10265	1.47566141	0.554349	2.661971909	0.007768	0.063481	NOT
AC068499.	4.909639	-1.2427482	0.466886	-2.66178057	0.007773	0.063498	NOT
CCDC3	720.4278	-0.9112142	0.342363	-2.66154487	0.007778	0.063524	NOT
KRT18P34	66.66568	-0.9643847	0.362367	-2.66134983	0.007783	0.063541	NOT

CTD-2267I	410.9108	-0.5189642	0.19503	-2.66094207	0.007792	0.063599	NOT
LRRC69	37.42251	0.91714694	0.344688	2.660802029	0.007795	0.063606	NOT
RP11-757F	11.38186	1.92084591	0.721989	2.660491557	0.007803	0.063646	NOT
SYT12	1433.49	1.09928416	0.413381	2.659250262	0.007831	0.063857	NOT
LINC0092	56.16411	-0.8463064	0.318259	-2.65917338	0.007833	0.063857	NOT
CCNF	597.8966	0.71906358	0.270453	2.658741041	0.007843	0.06392	NOT
CFAP61	5.89022	1.48414624	0.558265	2.658499372	0.007849	0.063947	NOT
TBRG1	821.6819	-0.3460167	0.130209	-2.6573853	0.007875	0.064139	NOT
LIN28A	1.870825	2.34343064	0.881927	2.657169774	0.00788	0.064148	NOT
ACD	624.2486	0.43989755	0.165553	2.657134207	0.007881	0.064148	NOT
RP11-70C1	23.36661	-1.5512685	0.583853	-2.65695248	0.007885	0.064149	NOT
CTB-102L5	2.999841	2.62571823	0.988253	2.656927842	0.007886	0.064149	NOT
MEIS3P1	11.52665	-0.9521797	0.358472	-2.65621712	0.007902	0.064265	NOT
SPATA5L1	509.5222	0.38541347	0.145125	2.655726537	0.007914	0.064339	NOT
TPPP2	58.99666	-1.2692891	0.478157	-2.65454306	0.007942	0.064546	NOT
BCKDHB	1332.711	-0.6747475	0.254227	-2.65411897	0.007952	0.064606	NOT
GPN1	1301.055	0.31834836	0.119949	2.654028478	0.007954	0.064606	NOT
BMP10	0.921448	-3.5132437	1.323857	-2.65379341	0.007959	NA	NA
CDC37L1-A	34.66134	-0.8023312	0.302413	-2.65309549	0.007976	0.064766	NOT
LINC00858	2.269627	3.33562434	1.257384	2.652828557	0.007982	0.064773	NOT
ALKBH2	1150.697	0.62324514	0.234939	2.652797833	0.007983	0.064773	NOT
CEP83	305.3676	0.37483964	0.141302	2.652754939	0.007984	0.064773	NOT
HNRNPK	19105.39	0.23648052	0.089167	2.652120151	0.007999	0.064875	NOT
RP11-443F	373.2406	1.63603604	0.61692	2.651942667	0.008003	0.064878	NOT
ZNF133	474.7338	0.41265201	0.155606	2.65190226	0.008004	0.064878	NOT
ABRACL	579.4676	0.77863978	0.293631	2.651766968	0.008007	0.064885	NOT
UBE4A	1543.382	-0.4719905	0.178007	-2.65152327	0.008013	0.064912	NOT
BEND3P3	9.828087	-1.2225358	0.461087	-2.65142143	0.008015	0.064913	NOT
LGSN	92.98218	-1.4145119	0.533559	-2.65108959	0.008023	0.064957	NOT
PTPN20A	31.55461	1.95526024	0.737567	2.650960699	0.008026	0.064962	NOT
METTL9	1378.805	0.48299167	0.182206	2.650797002	0.00803	0.064975	NOT
RPL27	22571.25	0.57146808	0.21569	2.649491264	0.008061	0.065207	NOT
WDR93	7.011411	0.9579908	0.361606	2.649264344	0.008067	0.065231	NOT
RP11-442(	15.78129	-1.2322547	0.465299	-2.64830615	0.00809	0.065397	NOT
CRYBB2P1	372.0143	0.57075765	0.215536	2.648080034	0.008095	0.065415	NOT
COL7A1	1139.711	1.43486071	0.541864	2.648011222	0.008097	0.065415	NOT
RP11-799F	18.09664	-1.0958519	0.413882	-2.64773978	0.008103	0.065448	NOT
RP11-661F	14.05161	1.32733475	0.501418	2.647163407	0.008117	0.065531	NOT
FDFT1	5679.22	0.59572936	0.225049	2.647107356	0.008118	0.065531	NOT
AC010733.	11.28525	-0.7069432	0.267091	-2.64682229	0.008125	0.065567	NOT
F8	681.2381	-0.759654	0.287021	-2.64668103	0.008129	0.065575	NOT
TBXA2R	161.0348	-0.7285898	0.275317	-2.6463699	0.008136	0.065616	NOT
RP11-247A	7.449464	-1.1424241	0.431898	-2.64512234	0.008166	0.065839	NOT
COL4A3BP	1517.479	-0.4014378	0.151837	-2.64387649	0.008196	0.066043	NOT
ENPP3	308.7441	1.51389549	0.572606	2.643870944	0.008196	0.066043	NOT
NEAT1	15539.64	-0.8412806	0.318261	-2.64336686	0.008209	0.066122	NOT
TMEM205	8199.722	-0.6181087	0.233847	-2.64322266	0.008212	0.066124	NOT
CFB	18117.98	-0.8884448	0.336131	-2.64315392	0.008214	0.066124	NOT
TTC22	465.9238	0.89144276	0.337295	2.642915047	0.00822	0.06615	NOT

AC129492.	6.182899	1.93272865	0.731313	2.642820985	0.008222	0.06615	NOT
PRDM2	751.9756	-0.4041479	0.152933	-2.64264076	0.008226	0.066166	NOT
SCNN1B	33.53191	1.37295275	0.519595	2.642351668	0.008233	0.066199	NOT
FAM86C1	211.0684	0.40230263	0.152256	2.642271758	0.008235	0.066199	NOT
LINC01057	41.3675	1.11292495	0.42125	2.641955962	0.008243	0.066241	NOT
NOS2	100.2305	-1.0377997	0.392841	-2.64177764	0.008247	0.066256	NOT
SLC7A7	582.5099	0.85788062	0.324793	2.641312492	0.008259	0.066328	NOT
KCNAB1	96.67689	-0.6287751	0.238076	-2.64107395	0.008264	0.066355	NOT
DUSP9	2442.613	1.73307724	0.656235	2.640940508	0.008268	0.066361	NOT
AC079776.	1.552019	-1.5385669	0.58262	-2.64077286	0.008272	0.066374	NOT
C21orf91	320.4868	-0.6203648	0.234938	-2.64054497	0.008277	0.066399	NOT
MARC2	4724.978	-0.7286215	0.27596	-2.64031225	0.008283	0.066399	NOT
S100A8	215.6495	1.41815764	0.537129	2.640255385	0.008284	0.066399	NOT
IL21-AS1	4.186657	2.28392264	0.86508	2.64012853	0.008287	0.066399	NOT
AC002056.	2.800914	1.26392102	0.478747	2.640060642	0.008289	0.066399	NOT
HTR2A	6.395705	-1.1761026	0.445485	-2.64004784	0.008289	0.066399	NOT
ZNF143	569.9879	0.27428502	0.103918	2.639445526	0.008304	0.066495	NOT
GSTZ1	1740.657	-0.8852416	0.335401	-2.63935674	0.008306	0.066495	NOT
RP11-556K	53.62158	0.87698727	0.332356	2.638698215	0.008323	0.066605	NOT
CERS1	89.84105	1.90858412	0.723399	2.638356155	0.008331	0.066652	NOT
AC084809.	2.064082	1.37405944	0.520929	2.637711832	0.008347	0.066759	NOT
TPD52	2879.529	0.62500589	0.236967	2.637521655	0.008351	0.066777	NOT
LINC00887	16.32837	1.40242678	0.531828	2.636991464	0.008364	0.066862	NOT
VOPP1	1494.266	0.48184567	0.182745	2.636716877	0.008371	0.066896	NOT
GLCCI1	377.6493	-0.6502661	0.246682	-2.63605251	0.008388	0.067008	NOT
MLYCD	1292.62	-0.6976032	0.264654	-2.63590649	0.008391	0.067017	NOT
TREM2	324.944	0.99780379	0.37862	2.635371787	0.008405	0.067093	NOT
BCL11A	88.47067	1.33841321	0.507879	2.635301351	0.008406	0.067093	NOT
C10orf35	262.2668	0.9545682	0.362235	2.635220563	0.008408	0.067093	NOT
AFF3	742.9857	1.05401588	0.400031	2.6348322	0.008418	0.06715	NOT
SEPT7-AS1	34.51967	-0.7114733	0.270073	-2.63436957	0.008429	0.067222	NOT
AACS	963.1966	0.54986097	0.208735	2.63425223	0.008432	0.067225	NOT
RP11-88E1	265.6045	0.79968996	0.303618	2.633865654	0.008442	0.067282	NOT
AC133528.	208.1083	0.63684555	0.241812	2.633636241	0.008448	0.067293	NOT
CTD-2201E	6.385782	1.23592882	0.46931	2.633502459	0.008451	0.067293	NOT
KIAA1045	11.79173	-1.2558572	0.476893	-2.63341254	0.008453	0.067293	NOT
LIN9	258.0923	0.53731272	0.204037	2.633410062	0.008453	0.067293	NOT
CXXC4	144.4519	-1.1849569	0.45013	-2.63247898	0.008476	0.067458	NOT
AC005062.	1.028795	-2.0944176	0.795783	-2.63189577	0.008491	NA	NA
SLC25A34	133.5544	-0.9219742	0.35031	-2.63188063	0.008491	0.06755	NOT
LINC00593	3.933605	-1.9776849	0.751452	-2.63181938	0.008493	0.06755	NOT
AATK-AS1	3.420477	3.40181799	1.292939	2.631073848	0.008512	0.067649	NOT
FMO8P	1.811794	3.17956274	1.208471	2.63106183	0.008512	0.067649	NOT
AC007952.	4.30369	2.18466249	0.830392	2.630882236	0.008516	0.067649	NOT
TRIM60P18	34.81496	0.8991076	0.341765	2.630776032	0.008519	0.067649	NOT
ARPC5	8425.185	0.3735174	0.141984	2.630706387	0.008521	0.067649	NOT
CDC45	392.1153	0.8178916	0.31091	2.630637842	0.008522	0.067649	NOT
SNX29P2	17.22277	-1.070889	0.407086	-2.63062235	0.008523	0.067649	NOT
RP11-696N	109.1951	-0.8070336	0.306802	-2.63046817	0.008527	0.06766	NOT

STARD3NL	726.4054	0.50417639	0.191719	2.629772508	0.008544	0.067779	NOT
SUMO2P1	5.288135	0.95350003	0.362593	2.629671588	0.008547	0.067779	NOT
GNE	3716.986	-0.7781193	0.295938	-2.62932799	0.008555	0.067828	NOT
RP11-174(	4.374059	-1.1547422	0.439293	-2.62863715	0.008573	0.067924	NOT
TNFRSF12A	2740.29	0.7742953	0.294569	2.628574572	0.008574	0.067924	NOT
SERBP1	10640.8	0.34300573	0.130493	2.628547248	0.008575	0.067924	NOT
PCDHGA3	44.6237	-1.35722	0.516386	-2.62830466	0.008581	0.06794	NOT
C10orf71-	0.847876	-2.3391767	0.890008	-2.62826369	0.008582	NA	NA
AP1G1	3380.999	-0.3979897	0.151429	-2.62822534	0.008583	0.06794	NOT
RP11-295I	33.16128	0.84831867	0.322779	2.628171254	0.008585	0.06794	NOT
ANP32B	5402.153	0.44181842	0.16814	2.627684426	0.008597	0.068004	NOT
RGMB-AS1	25.85324	-0.9731669	0.370359	-2.62763032	0.008598	0.068004	NOT
LHFPL1	2.460257	2.25160046	0.856919	2.627553967	0.0086	0.068004	NOT
UXT-AS1	21.47157	-0.5415455	0.206113	-2.62742016	0.008604	0.068011	NOT
AQP7P2	5.559957	1.61766249	0.615765	2.627078261	0.008612	0.068046	NOT
GPLD1	1908.118	-1.4620337	0.556532	-2.62704133	0.008613	0.068046	NOT
CTRB1	1.670546	2.49918478	0.951373	2.626923006	0.008616	0.068046	NOT
DCUN1D5	636.1995	0.42750517	0.162753	2.626717187	0.008621	0.068046	NOT
CTD-2562J	301.5125	-0.8436718	0.321199	-2.62663515	0.008623	0.068046	NOT
RP11-110I	8.641076	-0.8759603	0.333495	-2.62660472	0.008624	0.068046	NOT
NSUN5	1408.977	0.47009506	0.178978	2.626551891	0.008625	0.068046	NOT
KIAA0907	1650.955	0.53770731	0.204741	2.626280686	0.008632	0.06808	NOT
RP11-761N	18.31288	1.13183237	0.431029	2.62588375	0.008642	0.06814	NOT
RP11-353N	19.57713	1.77758831	0.676997	2.625697934	0.008647	0.068156	NOT
PPP5D1	9.395682	-0.6018612	0.229228	-2.62560503	0.00865	0.068156	NOT
ZFP36	5892.661	-0.6883187	0.262166	-2.62550383	0.008652	0.068157	NOT
RP5-856G1	2.053469	2.46761754	0.939932	2.6253137	0.008657	0.068163	NOT
NME1-NME2	2796.835	0.51053817	0.194471	2.625271728	0.008658	0.068163	NOT
ARMC7	858.221	0.43517939	0.165812	2.624538696	0.008677	0.068282	NOT
PTGDR	18.73933	-1.0606074	0.404121	-2.62448164	0.008678	0.068282	NOT
GTPBP4	2157.238	0.40235781	0.153317	2.624348653	0.008681	0.068289	NOT
WDR54	255.4783	0.81543457	0.310756	2.624036467	0.008689	0.068332	NOT
RP11-247(	4.968279	1.72493913	0.657484	2.623546815	0.008702	0.068396	NOT
RP11-1007	41.41566	-0.5288669	0.201587	-2.62352078	0.008703	0.068396	NOT
MMRN2	1133.303	-0.5983812	0.2281	-2.62333339	0.008707	0.068414	NOT
VGF	42.96651	1.65153604	0.62963	2.623025146	0.008715	0.068456	NOT
SHISA6	9.033471	-1.5714177	0.599189	-2.62257232	0.008727	0.068527	NOT
TRIP4	838.2388	0.26508001	0.101086	2.622327594	0.008733	0.06855	NOT
KLC2	822.9238	0.44374595	0.169226	2.622203186	0.008736	0.06855	NOT
NPEPPS	2527.866	0.39236645	0.14964	2.622067293	0.00874	0.06855	NOT
KRT8	36772.6	0.6367737	0.242852	2.622063436	0.00874	0.06855	NOT
TMCO1	8977.726	0.41241072	0.157322	2.621441325	0.008756	0.068655	NOT
RAP1GAP	3844.903	0.98505802	0.375833	2.620997292	0.008767	0.068725	NOT
RP11-724N	9.171425	-1.2455108	0.475233	-2.62084058	0.008771	0.068737	NOT
CCDC69	1747.718	-0.7924643	0.302398	-2.62060084	0.008777	0.068765	NOT
DEK	4577.401	0.53051751	0.202454	2.620435782	0.008782	0.068779	NOT
ZNF488	10.37445	1.61375418	0.615948	2.619953973	0.008794	0.068846	NOT
RP11-700H	25.02612	-1.2476864	0.476233	-2.61990492	0.008795	0.068846	NOT
CTD-3075F	1.954957	-1.3278319	0.506914	-2.61944207	0.008807	0.06892	NOT

RP11-25G1	8.394668	1.47293906	0.562334	2.619332658	0.00881	0.068922	NOT
ZFP64	724.9808	0.30268989	0.115566	2.619187679	0.008814	0.068931	NOT
CTD-2286N	12.28351	0.7730932	0.295194	2.618936804	0.00882	0.068947	NOT
U47924.31	54.36268	-0.7040647	0.268846	-2.61883879	0.008823	0.068947	NOT
FLJ38576	22.78017	-0.9208851	0.351643	-2.61880445	0.008824	0.068947	NOT
FAM127A	2643.997	0.67871501	0.259178	2.618717489	0.008826	0.068947	NOT
LINC00174	314.211	-0.7289016	0.278375	-2.61841267	0.008834	0.068989	NOT
TNXB	425.1821	-1.0361388	0.395733	-2.61827825	0.008837	0.068996	NOT
BMF	979.1863	0.94154982	0.359676	2.617769771	0.008851	0.069079	NOT
CDHR4	5.549426	1.46390389	0.55956	2.616167981	0.008892	0.069384	NOT
CHD1L	3385.986	0.50579435	0.193361	2.615804029	0.008902	0.069431	NOT
GOLGA1	1175.324	-0.4051986	0.154914	-2.61563994	0.008906	0.069431	NOT
FERMT1	310.0335	1.39305961	0.532588	2.615639783	0.008906	0.069431	NOT
RP11-250E	47.5481	-0.6566058	0.251066	-2.61526851	0.008916	0.069487	NOT
RP11-152F	1.956483	-0.9910848	0.378986	-2.61509474	0.00892	0.069494	NOT
RHOXF2B	3.828936	4.44313083	1.699071	2.615035261	0.008922	0.069494	NOT
ACSL3	4909.953	0.5579066	0.213372	2.614715091	0.00893	0.06954	NOT
RP5-940J5	3.527741	1.15151883	0.440442	2.614464313	0.008937	0.069571	NOT
CTC-529I1	11.10099	-0.9595985	0.367102	-2.61398211	0.008949	0.069649	NOT
GATAD2A	2956.221	-0.3453051	0.132114	-2.61368262	0.008957	0.06969	NOT
GRM7	14.18427	-1.7332019	0.663268	-2.61312573	0.008972	0.069784	NOT
TECTA	36.00104	-0.6943273	0.265725	-2.61295021	0.008976	0.0698	NOT
CCDC120	584.5338	0.53895814	0.206278	2.612780108	0.008981	0.069814	NOT
EEF1B2	11759.6	0.53842921	0.206083	2.612676869	0.008984	0.069815	NOT
RASSF3	1522.156	0.6991509	0.267624	2.612437187	0.00899	0.069827	NOT
ALPK1	436.6022	-0.6780992	0.259567	-2.6124254	0.00899	0.069827	NOT
PRSS46	3.937767	1.91302368	0.732464	2.611763899	0.009008	0.06993	NOT
HSD11B2	470.492	-0.7905443	0.302692	-2.61170911	0.009009	0.06993	NOT
FOXD2-AS1	203.142	0.84560893	0.323786	2.611627144	0.009011	0.06993	NOT
RP4-798P1	183.6406	-0.9969461	0.381757	-2.61146714	0.009015	0.069938	NOT
ZNF423	114.0893	-0.8293953	0.317606	-2.61139451	0.009017	0.069938	NOT
RP11-383H	311.2573	1.38704986	0.531196	2.611184052	0.009023	0.069961	NOT
GJB3	43.3629	1.5261536	0.584771	2.609831317	0.009059	0.070212	NOT
SHARPIN	4717.335	0.60370076	0.231328	2.609714062	0.009062	0.070212	NOT
LINC00668	47.26605	2.64825655	1.014789	2.609661946	0.009063	0.070212	NOT
ZIC2	355.5844	1.48295205	0.568339	2.609273364	0.009073	0.070268	NOT
MYCT1	294.4584	-0.5612842	0.215118	-2.60919644	0.009076	0.070268	NOT
FOXK2	2019.223	0.38676974	0.148239	2.609087914	0.009078	0.07027	NOT
HOXA11	22.99141	2.4041196	0.92152	2.608863261	0.009084	0.070295	NOT
CTC-575D1	256.2366	1.28341093	0.49196	2.608771239	0.009087	0.070295	NOT
C8orf76	528.5371	0.5700676	0.218568	2.608195631	0.009102	0.070393	NOT
RP3-407E4	1.252555	-1.8459681	0.707848	-2.60785944	0.009111	NA	NA
LYRM7	835.9155	-0.4321659	0.16573	-2.6076496	0.009117	0.070486	NOT
AL162759.	20.94908	1.97217662	0.756393	2.607343077	0.009125	0.070529	NOT
ARHGAP31-	4.449306	1.7326521	0.664578	2.607147782	0.00913	0.070549	NOT
SYT14	1.507698	2.34589224	0.899832	2.607032347	0.009133	0.070552	NOT
CCDC71L	1025.998	-0.7151355	0.274344	-2.60671523	0.009142	0.070598	NOT
PRR7	177.8144	0.91633106	0.35155	2.606545885	0.009146	0.070613	NOT
CTC-260E6	13.22628	1.09656794	0.420927	2.605128062	0.009184	0.070885	NOT

EFHC1	274.2022	0.55803225	0.214249	2.604598709	0.009198	0.070975	NOT
TTLL1	193.6213	0.53287444	0.204616	2.604260487	0.009207	0.071025	NOT
SELE	138.4738	-1.3178732	0.506066	-2.6041505	0.00921	0.071027	NOT
ALDH1A2	293.073	-1.0353564	0.397678	-2.60350288	0.009228	0.071141	NOT
DDIAS	151.4987	0.70702723	0.271622	2.602982434	0.009242	0.071229	NOT
RP11-432I	2.017269	1.90010943	0.730015	2.602834877	0.009246	0.07124	NOT
ZCCHC12	13.14038	1.81139096	0.696002	2.602566138	0.009253	0.071275	NOT
ZMYND15	303.3198	-0.8910114	0.342383	-2.60238429	0.009258	0.071293	NOT
RP11-39H3	55.2328	-1.6393454	0.630113	-2.60166713	0.009277	0.071397	NOT
GSTA7P	102.7699	-1.2845794	0.493759	-2.60163227	0.009278	0.071397	NOT
AC092071.	26.46377	-2.5175192	0.967715	-2.60150763	0.009282	0.071397	NOT
AC007405.	125.7403	0.65146723	0.25042	2.601493628	0.009282	0.071397	NOT
RP11-44N1	5.337288	0.99607989	0.382914	2.60131374	0.009287	0.071414	NOT
TOR1A	1813.516	-0.3377786	0.12987	-2.60089081	0.009298	0.071455	NOT
RP11-15H2	181.5656	-0.6960948	0.26764	-2.60086392	0.009299	0.071455	NOT
RP11-255M	5.725426	-1.5876778	0.610451	-2.60082649	0.0093	0.071455	NOT
RP11-384C	6.514205	1.90969172	0.734322	2.600618934	0.009306	0.071478	NOT
FMO9P	2.120168	3.58448901	1.378475	2.600329039	0.009313	0.071518	NOT
ZNF441	219.4485	-0.511571	0.196765	-2.59990668	0.009325	0.071586	NOT
JMJD1C	1954.473	-0.493511	0.18984	-2.59961377	0.009333	0.071627	NOT
AP000473.	17.50231	-1.2242439	0.47096	-2.59946553	0.009337	0.071638	NOT
ACVRL1	692.9856	-0.4896951	0.18842	-2.59895923	0.009351	0.071721	NOT
TG	32.95369	-1.0536801	0.405437	-2.5988726	0.009353	0.071721	NOT
AFMID	5216.504	-0.5820683	0.224037	-2.5980862	0.009374	0.071865	NOT
PLEKHB2	2173.217	0.49186003	0.189324	2.59797375	0.009378	0.071868	NOT
SPATA24	141.3242	0.60432919	0.232762	2.596344646	0.009422	0.07218	NOT
MYO9A	395.2881	-0.5938013	0.228711	-2.59629162	0.009424	0.07218	NOT
CCDC184	18.12751	-0.8368004	0.322413	-2.59543343	0.009447	0.07232	NOT
STX17	587.4781	-0.533405	0.205517	-2.59543202	0.009447	0.07232	NOT
CTC-30107	28.69287	-0.6414796	0.24721	-2.59487353	0.009463	0.072417	NOT
SLC10A6	9.188138	-1.1454747	0.441487	-2.59458562	0.009471	0.072452	NOT
CAPRIN1	6646.707	0.31105588	0.11989	2.594517363	0.009472	0.072452	NOT
PANX3	2.216732	2.75813205	1.063204	2.594170714	0.009482	0.072504	NOT
MGME1	920.1817	0.35971618	0.138672	2.594010481	0.009486	0.072518	NOT
RNPS1	3055.663	0.35328571	0.136222	2.593451005	0.009502	0.072599	NOT
WDR3	1492.589	0.54981163	0.212002	2.593429398	0.009502	0.072599	NOT
RP5-968P1	47.05453	-0.6781357	0.261525	-2.5930016	0.009514	0.072669	NOT
RP11-426I	1.092095	-1.3994304	0.539743	-2.59276984	0.009521	NA	NA
TDGF1	471.1485	1.70295366	0.65688	2.592488653	0.009528	0.072757	NOT
IL17RB	3312.786	0.84007955	0.324138	2.591735648	0.009549	0.072896	NOT
EPHX2	5388.079	-0.8569576	0.330675	-2.59154259	0.009555	0.072917	NOT
CPNE5	102.6426	-0.7708319	0.297526	-2.59080648	0.009575	0.073047	NOT
RP11-509A	6.646833	-1.7746663	0.685005	-2.59073571	0.009577	0.073047	NOT
ZMYND19	820.5428	0.48049178	0.185474	2.590620061	0.00958	0.073049	NOT
SMOX	671.9417	0.76044118	0.293546	2.590532451	0.009583	0.073049	NOT
TPGS2	1829.754	0.52276652	0.201859	2.589765838	0.009604	0.073191	NOT
NPTX1	16.04692	1.73853727	0.671416	2.589357686	0.009616	0.073257	NOT
TSNARE1	683.187	0.58262887	0.225039	2.589010495	0.009625	0.073311	NOT
LIMS2	1685.898	-0.7661585	0.295938	-2.58891328	0.009628	0.073311	NOT

PLCG1	2198.167	0.50114928	0.193605	2.588511077	0.009639	0.073342	NOT
TEX37	2.581531	-3.3727296	1.302967	-2.58849984	0.009639	0.073342	NOT
LRRC3-AS1	8.724638	-1.2273126	0.474145	-2.58847409	0.00964	0.073342	NOT
SFPQ	7978.82	0.32755255	0.126551	2.58831252	0.009645	0.073356	NOT
GPI	23024.63	-0.6784556	0.26214	-2.5881438	0.009649	0.073362	NOT
FAM149A	856.056	-0.8471734	0.327335	-2.58809416	0.009651	0.073362	NOT
APBB3	925.6734	-0.591875	0.228702	-2.58797897	0.009654	0.073366	NOT
HNRNPA2B1	26055.37	0.26546081	0.10259	2.587589716	0.009665	0.073428	NOT
RP11-317J	11.04993	-1.1029182	0.4263	-2.58718782	0.009676	0.073485	NOT
RP3-337H4	18.4484	0.62739538	0.242507	2.587127968	0.009678	0.073485	NOT
RHBDL2	36.85961	1.023859	0.395783	2.586920661	0.009684	0.073492	NOT
PTPN23	2829.737	0.33447561	0.129296	2.586904517	0.009684	0.073492	NOT
PAX9	5.423819	2.01071261	0.777298	2.586796545	0.009687	0.073494	NOT
AC073415.	2.311725	1.37393008	0.531178	2.586569611	0.009694	0.073497	NOT
IMPDH1P6	10.4582	0.95770467	0.370276	2.586463719	0.009697	0.073497	NOT
ECE1	5875.758	-0.4088075	0.158058	-2.5864375	0.009697	0.073497	NOT
RP11-394I	2.206549	1.65475366	0.639791	2.586398227	0.009698	0.073497	NOT
KAT2A	3077.492	0.50987517	0.19715	2.586234382	0.009703	0.073512	NOT
KRTAP5-AS	28.15848	-1.4524189	0.561637	-2.58604704	0.009708	0.073531	NOT
PINK1	1834.844	-0.7205335	0.278642	-2.58587409	0.009713	0.073546	NOT
RP11-410D	1.788555	-1.1532403	0.445998	-2.58575048	0.009717	0.073546	NOT
ZNF91	658.5788	-0.5491966	0.212399	-2.58568914	0.009718	0.073546	NOT
UGT1A9	2522.533	1.48775729	0.575451	2.585377969	0.009727	0.073592	NOT
AC023590.	4.309948	1.06737171	0.413023	2.584288417	0.009758	0.073804	NOT
DEPDC1	407.525	0.98740841	0.38212	2.584030284	0.009765	0.073822	NOT
RP11-164J	65.81249	-0.9508038	0.367958	-2.58399897	0.009766	0.073822	NOT
CTC-458I2	8.816413	-0.7138787	0.276278	-2.5839183	0.009768	0.073822	NOT
MYOM1	2184.193	-1.0431605	0.403737	-2.58376508	0.009773	0.073834	NOT
CIPC	750.8732	-0.5086378	0.196892	-2.58332739	0.009785	0.073907	NOT
RP3-399L1	9.704657	0.95177496	0.368457	2.583137775	0.009791	0.073927	NOT
RP11-426C	20.77065	-1.1729734	0.454138	-2.58285688	0.009799	0.073967	NOT
RP11-526D	9.66212	-0.5510301	0.213359	-2.58263863	0.009805	0.073977	NOT
CTD-2012J	3.850449	1.32974412	0.514894	2.582557308	0.009807	0.073977	NOT
IRF5	663.0448	0.52931613	0.204961	2.582518348	0.009808	0.073977	NOT
AJ006998.	120.6482	-1.7490629	0.677398	-2.58203192	0.009822	0.074061	NOT
RP11-474I	4.276069	-1.1856645	0.459519	-2.58023214	0.009873	0.074428	NOT
MRPL35P2	6.562535	-1.2794118	0.495888	-2.58004237	0.009879	0.074444	NOT
UBE2D3	8712.397	-0.2693833	0.104414	-2.57995721	0.009881	0.074444	NOT
HID1	887.2642	1.01871202	0.39487	2.579868894	0.009884	0.074444	NOT
SLC35D1	3914.806	-0.641557	0.248748	-2.57913979	0.009905	0.074581	NOT
CIC	3299.384	-0.3499781	0.13573	-2.57849213	0.009923	0.0747	NOT
DAAM1	1099.508	-0.6038516	0.234214	-2.57820959	0.009931	0.07474	NOT
RP11-800A	2.924758	1.10323834	0.427944	2.577997712	0.009937	0.074765	NOT
TTC39B	488.5531	-0.7905541	0.306682	-2.57776331	0.009944	0.074787	NOT
LSM7	1582.243	0.63650497	0.246932	2.577655137	0.009947	0.074787	NOT
RP11-297I	200.7204	1.168803	0.453452	2.577567427	0.00995	0.074787	NOT
ALKBH7	3577.542	-0.7298118	0.283146	-2.57751492	0.009951	0.074787	NOT
PSD3	1796.076	-0.9379156	0.363929	-2.57719319	0.009961	0.074836	NOT
LBHD1	177.7569	0.61226968	0.237626	2.576607984	0.009978	0.074942	NOT

MIR589	2.111497	1.32904044	0.515832	2.57649794	0.009981	0.074945	NOT
MFF	2502.845	0.36147745	0.140317	2.576148722	0.009991	0.074967	NOT
C19orf60	2066.345	-0.7366725	0.285964	-2.57610011	0.009992	0.074967	NOT
SRD5A1	2527.085	-0.8744658	0.339457	-2.57607152	0.009993	0.074967	NOT
RP11-303E	6.83101	-0.8268864	0.320995	-2.57601317	0.009995	0.074967	NOT
CPSF1	5009.983	0.42299472	0.164213	2.575883194	0.009998	0.074974	NOT
BANF1	5243.372	0.41084847	0.159512	2.575655912	0.010005	0.075003	NOT
DPM1	1467.932	0.36075556	0.140074	2.575463446	0.010011	0.075024	NOT
JOSD1	2475.123	0.42282318	0.164208	2.574922814	0.010026	0.075107	NOT
RPL13AP25	200.3884	0.83561862	0.324526	2.574889578	0.010027	0.075107	NOT
HNRNPL	7017.743	0.23278858	0.090421	2.574496552	0.010039	0.075139	NOT
CTC-448F2	3.586309	1.37473466	0.533989	2.574461103	0.01004	0.075139	NOT
LDB3	31.52829	-0.7803067	0.303095	-2.57445829	0.01004	0.075139	NOT
PHF21B	1.927205	2.53282418	0.983868	2.574353249	0.010043	0.075141	NOT
IQCC	120.5571	0.53441204	0.207646	2.573668945	0.010063	0.075269	NOT
SKOR1	11.23382	-0.7625794	0.296349	-2.57325134	0.010075	0.075339	NOT
BCKDHA	280.2256	-0.6780928	0.263538	-2.57303234	0.010081	0.075366	NOT
UBAP2L	6468.493	0.38983995	0.151523	2.572816483	0.010087	0.075392	NOT
FNBP1L	1780.386	0.58647361	0.227971	2.572577384	0.010094	0.075423	NOT
GDPD3	110.1665	1.11986739	0.435352	2.572324223	0.010102	0.075458	NOT
SLC38A2	10100.81	-0.599982	0.23326	-2.57215861	0.010107	0.075473	NOT
RP11-736N	2.485797	-1.4034802	0.545706	-2.57185925	0.010115	0.075518	NOT
SLC25A32	1145.548	0.45641966	0.177498	2.571410813	0.010129	0.075595	NOT
SLC28A3	19.32453	1.84960114	0.719482	2.570740065	0.010148	0.075682	NOT
PRRT1	72.6449	-0.6874964	0.267436	-2.57069951	0.010149	0.075682	NOT
RP11-471E	17.6563	-0.7871634	0.306214	-2.57063452	0.010151	0.075682	NOT
HNRNPLL	950.9494	0.25092645	0.097613	2.570628777	0.010151	0.075682	NOT
POTEF	3.695792	1.62079765	0.630548	2.570460498	0.010156	0.075685	NOT
EEF1B2P3	78.43872	0.72895235	0.2836	2.570357818	0.010159	0.075685	NOT
TMEM63C	30.21279	1.444498	0.562002	2.570273695	0.010162	0.075685	NOT
AP000318.	2.375526	-1.0563418	0.41099	-2.57023485	0.010163	0.075685	NOT
NMD3	2652.024	-0.3865064	0.150389	-2.57003772	0.010169	0.075707	NOT
OSGIN2	1419.331	0.52220311	0.203197	2.569934428	0.010172	0.075709	NOT
GPR160	188.0952	0.81936984	0.318883	2.569497216	0.010185	0.075784	NOT
C5orf30	253.2678	0.99500655	0.387269	2.569293099	0.010191	0.075808	NOT
FCHO1	121.5012	1.00426965	0.390892	2.569172514	0.010194	0.075814	NOT
SH3D19	3398.463	-0.5638363	0.219488	-2.56886797	0.010203	0.075859	NOT
LINC01127	274.2055	-1.0467062	0.407483	-2.56871138	0.010208	0.075873	NOT
PPIH	686.8525	0.49403855	0.192339	2.56858619	0.010211	0.075874	NOT
ERC1	1510.102	-0.4341044	0.16901	-2.56851471	0.010214	0.075874	NOT
EPHX4	19.34614	0.94932804	0.369616	2.568418402	0.010216	0.075875	NOT
PEAR1	243.0663	-0.5837117	0.227278	-2.56827549	0.010221	0.075885	NOT
KLRB1	134.3085	-0.8959736	0.34891	-2.56792042	0.010231	0.075896	NOT
SLC3A2	5751.078	0.55264852	0.215214	2.567896479	0.010232	0.075896	NOT
SDK2	228.8151	-1.6060739	0.625468	-2.56779683	0.010235	0.075896	NOT
RPS4XP5	6.967216	-1.325669	0.516282	-2.56772495	0.010237	0.075896	NOT
IL5RA	3.501429	-1.165495	0.453913	-2.56766299	0.010239	0.075896	NOT
DEPDC7	1122.837	-0.9031633	0.351746	-2.56765756	0.010239	0.075896	NOT
CTAGE4	8.30505	1.3712327	0.534091	2.567412574	0.010246	0.075924	NOT

RNF2	605.4277	0.53185577	0.207167	2.567285236	0.01025	0.075924	NOT
NEFM	2.906597	2.21695055	0.863552	2.567245202	0.010251	0.075924	NOT
CD34	2278.521	-0.5036354	0.196191	-2.56706814	0.010256	0.075942	NOT
RP11-25K1	35.41798	1.3379554	0.521382	2.566170875	0.010283	0.076118	NOT
HSPB1P1	80.604	0.9267605	0.36132	2.564933298	0.01032	0.076369	NOT
LSM14B	1666.659	0.40580379	0.158228	2.564683018	0.010327	0.076404	NOT
RP3-523E1	2.950587	1.48539609	0.579249	2.564348324	0.010337	0.076456	NOT
COMMD9	1934.983	0.39087585	0.152436	2.56419881	0.010341	0.076469	NOT
RP11-685M	4.783038	-1.2193001	0.475539	-2.56403967	0.010346	0.076483	NOT
ADH1B	62237.39	-1.3603819	0.530596	-2.56387762	0.010351	0.076498	NOT
ITGA1	5954.514	-0.5115239	0.199548	-2.56341525	0.010365	0.076579	NOT
LINC00665	595.1243	1.22270171	0.477165	2.562430386	0.010394	0.076775	NOT
ARMCX4	188.6635	-0.4755601	0.185609	-2.56215496	0.010402	0.076791	NOT
RAB8A	2082.488	-0.3801991	0.148393	-2.56211329	0.010404	0.076791	NOT
RP11-353M	10.57857	1.32468406	0.517036	2.562074702	0.010405	0.076791	NOT
RP11-793H	99.83822	-0.800306	0.312384	-2.56192763	0.010409	0.076803	NOT
RP6-109B7	6.295591	1.12093047	0.437602	2.561532296	0.010421	0.076869	NOT
COX4I1	18189.47	-0.7331409	0.286222	-2.56144089	0.010424	0.076869	NOT
AC002116.	14.625	0.78295669	0.305687	2.561303062	0.010428	0.076878	NOT
PDX1	220.8754	2.06449075	0.806295	2.560465246	0.010453	0.077043	NOT
P3H4	856.786	0.83267896	0.325237	2.560224498	0.01046	0.077076	NOT
RP11-37B2	149.4782	0.65273604	0.254972	2.560025656	0.010466	0.077094	NOT
ZNF347	189.9838	0.94231244	0.368098	2.559950921	0.010469	0.077094	NOT
RP11-3P17	103.0969	0.89823036	0.35091	2.55971878	0.010476	0.077125	NOT
SLC38A8	9.791249	-1.9558905	0.764164	-2.55951582	0.010482	0.07714	NOT
PWP2	39.86627	1.35580702	0.529732	2.55941908	0.010485	0.07714	NOT
PCNX	1900.399	-0.4370834	0.170784	-2.55928324	0.010489	0.07714	NOT
MACC1	54.49402	-1.3966204	0.54571	-2.55927257	0.010489	0.07714	NOT
SSBP1	2641.799	0.36456189	0.142469	2.558890725	0.010501	0.077204	NOT
TUBGCP5	605.5126	-0.3990447	0.155997	-2.5580201	0.010527	0.077377	NOT
STIL	329.5367	0.73568185	0.287619	2.557835666	0.010533	0.077397	NOT
RP11-445F	106.7993	1.64563494	0.643507	2.557290971	0.010549	0.077496	NOT
LPCAT2	492.0185	0.86209898	0.337133	2.557149741	0.010553	0.077496	NOT
CELF2-AS1	6.405965	2.23404254	0.873686	2.557031106	0.010557	0.077496	NOT
XXbac-BPC	2.655083	-1.6174268	0.632546	-2.55701152	0.010558	0.077496	NOT
TRPC4	23.34786	-0.8562201	0.334881	-2.55679072	0.010564	0.077517	NOT
GOLGA8M	21.32962	-1.1833486	0.462849	-2.55666224	0.010568	0.077517	NOT
ENPP7P13	4.200058	2.72220167	1.064759	2.556636891	0.010569	0.077517	NOT
MTDH	8715.039	0.48130498	0.188321	2.555764103	0.010595	0.077691	NOT
MYOC	1.012237	2.08747388	0.816787	2.555714024	0.010597	NA	NA
ZCCHC3	1192.905	0.46860748	0.183373	2.555486848	0.010604	0.077732	NOT
GCFC2	981.6029	0.4163169	0.162926	2.555246848	0.010611	0.077764	NOT
AC013275.	102.2205	1.41404057	0.553462	2.554898798	0.010622	0.077821	NOT
CISH	1550.378	-0.7812746	0.305884	-2.55415464	0.010645	0.077967	NOT
TCF25	4194.155	-0.4558738	0.1785	-2.55391117	0.010652	0.077995	NOT
CTD-2376I	17.22421	-0.6327815	0.247777	-2.55383733	0.010654	0.077995	NOT
AK4	6153.63	0.69302138	0.271455	2.552992464	0.01068	0.078155	NOT
RAMP2	684.6936	-0.767774	0.300745	-2.55290447	0.010683	0.078155	NOT
AC068831.	5.480705	0.97140011	0.380526	2.552784721	0.010687	0.078155	NOT

TGIF2-C2(	3.646679	-0.9155529	0.358664	-2.55267863	0.01069	0.078155	NOT
NT5C	1053.95	0.64463561	0.25254	2.552611921	0.010692	0.078155	NOT
LYPD8	60.47306	1.73567062	0.679972	2.552561755	0.010693	0.078155	NOT
KATNA1	480.7761	0.3764101	0.147478	2.552321476	0.010701	0.07817	NOT
OR2C1	2.297317	-1.2187727	0.477518	-2.55230814	0.010701	0.07817	NOT
CSAD	1262.469	-0.7707072	0.30202	-2.55184313	0.010715	0.078243	NOT
AP000692.	10.37279	-0.9514186	0.372852	-2.5517335	0.010719	0.078243	NOT
NANOGNBP3	5.856306	-0.8038799	0.315037	-2.55170101	0.01072	0.078243	NOT
PINK1-AS	139.6221	-0.4459378	0.174772	-2.55154403	0.010725	0.078257	NOT
CTD-3157E	8.878409	-0.9401815	0.368497	-2.55139779	0.010729	0.078269	NOT
SEPHS1	3103.633	0.34771581	0.136309	2.550930484	0.010744	0.078353	NOT
C19orf40	143.6164	0.55504813	0.217632	2.550396956	0.01076	0.078444	NOT
MICALCL	21.08741	-0.9485879	0.371946	-2.55033777	0.010762	0.078444	NOT
RP1-90G24	2.852313	1.86635865	0.731927	2.549924089	0.010775	0.078516	NOT
ALYREF	2317.836	0.52986421	0.207807	2.549788904	0.010779	0.078526	NOT
RP11-353N	21.36739	1.27188155	0.498872	2.549512668	0.010787	0.078567	NOT
MOB4	814.8764	0.31386675	0.123125	2.549161686	0.010798	0.078619	NOT
CABYR	743.7636	1.59654613	0.626325	2.549070242	0.010801	0.078619	NOT
LINC01087	2.529073	2.67240043	1.048412	2.548999486	0.010803	0.078619	NOT
JMJD6	1300.458	0.45945886	0.180288	2.548470467	0.01082	0.078717	NOT
LINC00882	12.24069	-1.08521	0.425873	-2.54819853	0.010828	0.078758	NOT
FKBP10	2153.52	1.18380135	0.464725	2.547316462	0.010855	0.0789	NOT
MTND5P1	18.54845	-1.4982243	0.588161	-2.54730174	0.010856	0.0789	NOT
RP11-512F	7.041789	0.84436627	0.331476	2.54728979	0.010856	0.0789	NOT
MED20	843.5568	0.31898785	0.125233	2.547153014	0.010861	0.078909	NOT
LRRC71	4.475706	-0.9121676	0.358136	-2.54698538	0.010866	0.078926	NOT
RP11-446J	5.080777	1.7629386	0.69227	2.546606999	0.010878	0.078984	NOT
ARRDC4	1178.035	-1.0596933	0.41613	-2.54654359	0.01088	0.078984	NOT
ZBBX	1.389184	2.62096485	1.029281	2.546404281	0.010884	NA	NA
SETX	2453.657	-0.3664293	0.143916	-2.54614142	0.010892	0.079038	NOT
ETS2	8317.179	-0.6264943	0.246059	-2.54611704	0.010893	0.079038	NOT
DMXL1	1185.174	-0.4908914	0.192828	-2.5457536	0.010904	0.079096	NOT
RP4-569M2	21.10401	-0.9116767	0.358128	-2.54567336	0.010907	0.079096	NOT
NARS	5925.227	0.32894864	0.129233	2.545382409	0.010916	0.079141	NOT
TMEM175	1528.032	-0.4900864	0.192548	-2.54527007	0.010919	0.079145	NOT
ELTD1	665.0206	-0.5128926	0.201532	-2.54496433	0.010929	0.079194	NOT
EIF2B4	1868.663	0.35819559	0.140767	2.544604914	0.01094	0.079254	NOT
SRI	1524.96	0.69731824	0.27415	2.543562699	0.010973	0.07946	NOT
YBX1P2	15.58028	0.7294918	0.286817	2.543406392	0.010978	0.07946	NOT
VTA1	1617.811	0.45045385	0.17711	2.543360076	0.010979	0.07946	NOT
BCRP3	46.07362	1.17947286	0.463761	2.543278992	0.010982	0.07946	NOT
INCENP	639.8944	0.58288366	0.229198	2.543149765	0.010986	0.07946	NOT
AC104653.	3.62103	-1.5393237	0.605297	-2.54308871	0.010988	0.07946	NOT
NR4A1	1182.816	-0.9161944	0.360293	-2.54291673	0.010993	0.07946	NOT
RP11-504F	101.5283	-0.6401046	0.251722	-2.54290225	0.010994	0.07946	NOT
ENTPD1-AS	43.80369	-0.4546322	0.178788	-2.54285866	0.010995	0.07946	NOT
PKDREJ	23.11541	-0.9454202	0.371825	-2.5426492	0.011002	0.079487	NOT
RP11-286E	7.089336	-1.0621264	0.417818	-2.54207838	0.01102	0.079595	NOT
RP11-537E	2.648287	-1.0472443	0.412019	-2.54173745	0.01103	0.079652	NOT

UBE2Q1	5260.879	0.34783639	0.136861	2.541527495	0.011037	0.079679	NOT
SPRY1	997.9683	-0.6048659	0.238066	-2.54074419	0.011062	0.079823	NOT
BSG	26005.07	0.63728319	0.250829	2.54070773	0.011063	0.079823	NOT
GLTPD2	1388.277	-1.0773865	0.424414	-2.53852914	0.011132	0.0803	NOT
HLCS	1227.381	-0.4399917	0.173358	-2.53804649	0.011147	0.08039	NOT
MPZ	824.3209	1.37247121	0.5408	2.537855587	0.011153	0.080407	NOT
SLC12A5	70.45402	1.02359195	0.403341	2.537784429	0.011156	0.080407	NOT
MCCC1-AS1	11.80742	-0.7924562	0.312299	-2.53749507	0.011165	0.080452	NOT
NSL1	1316.35	0.36071929	0.142161	2.537393206	0.011168	0.080455	NOT
GNG5	4308.666	0.43456806	0.171281	2.537161521	0.011176	0.080472	NOT
PFAS	1001.16	0.47768478	0.188277	2.537132456	0.011176	0.080472	NOT
FAM174B	502.9235	-0.8373573	0.330087	-2.5367783	0.011188	0.080532	NOT
RP11-599J	17.98633	1.23215016	0.485837	2.536137221	0.011208	0.080658	NOT
NAT2	358.1516	-1.4287945	0.563445	-2.5358203	0.011218	0.08071	NOT
RP11-66N1	59.75455	-0.861472	0.33975	-2.53560526	0.011225	0.080717	NOT
ATXN7L3	2510.033	0.32503945	0.128193	2.535539686	0.011227	0.080717	NOT
RP4-761J1	14.74835	-0.8410199	0.331696	-2.53551042	0.011228	0.080717	NOT
RPL14P1	153.6034	0.66145311	0.260888	2.535389646	0.011232	0.080723	NOT
DNAJC16	1406.968	-0.3851037	0.151904	-2.53517163	0.011239	0.080741	NOT
SCD	105780.7	0.99161285	0.391149	2.535129097	0.011241	0.080741	NOT
C19orf84	4.319991	1.43673987	0.566791	2.534865125	0.011249	0.08078	NOT
FAM72A	40.28849	0.77444922	0.305577	2.534384432	0.011265	0.08087	NOT
AC004988.	3.349422	1.31761114	0.519918	2.534266614	0.011268	0.080875	NOT
CA10	1.466554	-1.4704648	0.580374	-2.5336506	0.011288	0.080996	NOT
FUCA2	4675.57	0.49659754	0.196008	2.533553126	0.011291	0.080997	NOT
TEX22	21.64722	0.911785	0.359993	2.532785967	0.011316	0.081153	NOT
LAMA1	42.37488	1.58354368	0.625248	2.532663176	0.01132	0.08116	NOT
MEOX1	34.19796	-1.2761278	0.504063	-2.53168129	0.011352	0.081348	NOT
TTC3	5032.662	-0.4081255	0.161208	-2.53166981	0.011352	0.081348	NOT
RP11-98D1	3.184532	1.17703128	0.464969	2.531418183	0.01136	0.081384	NOT
KIF26B	310.7956	1.36142875	0.537861	2.531189449	0.011368	0.081416	NOT
FTH1P11	31.28693	0.76528875	0.302385	2.530843452	0.011379	0.081475	NOT
PRR13	3382.571	0.43145172	0.170508	2.530394735	0.011393	0.08153	NOT
PDLIM1P4	11.55078	-0.8857186	0.350039	-2.5303423	0.011395	0.08153	NOT
GRINA	25844.39	0.61353638	0.242473	2.530329347	0.011396	0.08153	NOT
PCSK9	3611.536	0.98284728	0.388467	2.53006623	0.011404	0.08157	NOT
SOWAHD	35.95809	0.99711294	0.394141	2.529835427	0.011412	0.081602	NOT
LSM1	627.8958	0.44865987	0.177356	2.529719785	0.011415	0.081607	NOT
GGA2	3263.856	-0.4317041	0.17068	-2.52931568	0.011429	0.08168	NOT
DGAT2	8051.426	-0.8389905	0.331747	-2.52901029	0.011438	0.081729	NOT
BCAS3	1339.31	-0.4882811	0.193082	-2.52888553	0.011443	0.081737	NOT
CIT	557.7421	0.74401643	0.29422	2.528771936	0.011446	0.08174	NOT
GGT6	92.83028	1.88500652	0.745449	2.528687548	0.011449	0.08174	NOT
NCOA5	1356.036	0.34197893	0.135279	2.527949299	0.011473	0.081856	NOT
TTC7B	695.1742	-0.572748	0.226571	-2.52789435	0.011475	0.081856	NOT
RP1-111B2	2.957943	1.19076077	0.47107	2.527778467	0.011479	0.081856	NOT
IFI27L2	395.1927	0.86150929	0.340827	2.527701364	0.011481	0.081856	NOT
RP11-342A	11.44205	-1.3465569	0.532728	-2.52766305	0.011482	0.081856	NOT
IGFL2	3.735731	2.17368483	0.859967	2.527636215	0.011483	0.081856	NOT

SEC31B	246.3009	-0.8274855	0.327454	-2.52702479	0.011503	0.081957	NOT
DFNA5	544.3216	0.91692199	0.362857	2.526952849	0.011506	0.081957	NOT
MIR126	4.773606	-1.3163143	0.520915	-2.52692486	0.011507	0.081957	NOT
NOL11	2142.335	0.34122112	0.135041	2.526791181	0.011511	0.081967	NOT
TRMT10C	1171.456	0.37993239	0.150371	2.526638791	0.011516	0.081968	NOT
OPA1	2802.888	-0.35817	0.14176	-2.52660291	0.011517	0.081968	NOT
MAD2L1BP	1004.731	0.39618815	0.156816	2.52645802	0.011522	0.081969	NOT
LINC0062Ž	24.56843	0.96277977	0.381089	2.526388576	0.011524	0.081969	NOT
CEP85	642.5811	0.6275662	0.248411	2.526319541	0.011526	0.081969	NOT
LHB	5.854638	1.80051651	0.712803	2.52596649	0.011538	0.08203	NOT
RP11-361I	14.57173	-1.3811255	0.546827	-2.5257105	0.011546	0.082068	NOT
C16orf45	815.1438	-0.8678508	0.343627	-2.52556002	0.011551	0.082082	NOT
EPM2A	250.6142	-0.5904418	0.233821	-2.52518293	0.011564	0.082149	NOT
MCCC2	6341.508	-0.5193683	0.205693	-2.52496731	0.011571	0.082178	NOT
MT-RNR1	64166.69	-0.7358862	0.291465	-2.52478823	0.011577	0.082198	NOT
BTD	3289.937	-0.6914069	0.273872	-2.52456278	0.011584	0.082229	NOT
NBEAL1	418.3072	-0.5596895	0.221712	-2.52439568	0.01159	0.082247	NOT
MIA2	78.06217	-0.7141205	0.282918	-2.52412372	0.011599	0.082289	NOT
TAF7L	11.40313	-1.4184866	0.562007	-2.52396681	0.011604	0.082304	NOT
RP11-715I	16.26275	-2.1537045	0.853451	-2.52352364	0.011619	0.082378	NOT
PRR19	98.22505	1.12942032	0.447567	2.523467615	0.01162	0.082378	NOT
CPED1	704.6682	-1.0978315	0.435087	-2.52324425	0.011628	0.082409	NOT
USP1	1436.867	0.51259282	0.203204	2.522552143	0.011651	0.082532	NOT
ZNF525	186.6656	0.96907274	0.384166	2.522535267	0.011651	0.082532	NOT
RP11-290I	338.7518	-1.2981366	0.514806	-2.52160339	0.011682	0.082729	NOT
RP11-902I	7.106704	-0.9343738	0.370564	-2.52149271	0.011686	0.082734	NOT
MC1R	91.69074	-0.7173146	0.284492	-2.52138617	0.011689	0.082736	NOT
RP11-138A	6.111931	1.32858004	0.526942	2.521301095	0.011692	0.082736	NOT
PCDHB4	40.637	-0.8961116	0.355439	-2.52114352	0.011697	0.082751	NOT
ZNF225	152.0265	-0.483883	0.191965	-2.52068087	0.011713	0.082813	NOT
SFI1	866.9748	0.71915827	0.285305	2.520661325	0.011713	0.082813	NOT
SLC38A4	13698.72	-1.0980489	0.435629	-2.52060752	0.011715	0.082813	NOT
RP11-215I	23.83306	-1.657433	0.657609	-2.52039377	0.011722	0.082841	NOT
NFKBIA	5670.431	-0.5307025	0.210591	-2.52006844	0.011733	0.082896	NOT
SPATA1	25.84184	-0.5345273	0.212126	-2.51986075	0.01174	0.082924	NOT
RP11-484I	2.960514	-0.8334749	0.330783	-2.51970668	0.011745	0.082938	NOT
MKI67	2439.599	0.86990679	0.345258	2.519588054	0.011749	0.082945	NOT
TET3	854.1069	0.47815744	0.18981	2.51913457	0.011764	0.08303	NOT
FTH1P20	174.8812	0.7515844	0.298364	2.519017775	0.011768	0.083036	NOT
TUSC3	583.2673	1.22449561	0.486154	2.518741968	0.011777	0.083079	NOT
SOX18	480.1847	-0.6165015	0.244879	-2.51757266	0.011817	0.083303	NOT
PRG2	9.714085	-1.1610281	0.461172	-2.51755878	0.011817	0.083303	NOT
IFNAR1	4163.838	-0.3602628	0.143105	-2.51747603	0.01182	0.083303	NOT
HES6	422.993	0.69930864	0.277787	2.517430484	0.011821	0.083303	NOT
RP11-1023	58.2369	0.68860478	0.273554	2.517257352	0.011827	0.083321	NOT
FAM153B	36.27327	1.74350208	0.692644	2.517170354	0.01183	0.083321	NOT
SLC22A15	214.7335	1.0880198	0.432336	2.516607438	0.011849	0.083429	NOT
CH17-302M	9.532525	1.02884331	0.408836	2.516521168	0.011852	0.083429	NOT
TUBA4A	3312.343	0.7167166	0.284814	2.516441459	0.011855	0.083429	NOT

ZC3H6	327.2048	-0.5599024	0.22253	-2.51608106	0.011867	0.083453	NOT
ADAMTS2	1017.524	-1.0289741	0.408973	-2.51599408	0.01187	0.083453	NOT
EGFLAM	204.1938	-0.591348	0.235036	-2.51598644	0.01187	0.083453	NOT
HNRNPA1P4	85.30021	0.51972742	0.206571	2.515974199	0.01187	0.083453	NOT
CMTM3	929.9846	0.72997166	0.290156	2.515787619	0.011877	0.083463	NOT
ST6GALNAC	80.27049	-0.6758933	0.268665	-2.51574912	0.011878	0.083463	NOT
RP11-1112	1.886282	-1.6582002	0.659226	-2.51537398	0.011891	0.08353	NOT
CTB-3102C	42.93192	-0.8934179	0.355269	-2.5147619	0.011911	0.083653	NOT
ATAD5	203.7986	0.62524884	0.24865	2.514570595	0.011918	0.083677	NOT
MED28	1498.913	0.35501233	0.141192	2.514402482	0.011923	0.083681	NOT
RP11-177I	5.502227	0.96349117	0.383193	2.514372787	0.011924	0.083681	NOT
RP11-565I	1.052607	2.55713141	1.017271	2.513717417	0.011947	NA	NA
NR6A1	478.6494	0.78980159	0.314199	2.513697407	0.011947	0.083819	NOT
RP4-785G1	13.90909	0.61631677	0.245326	2.512237813	0.011997	0.084145	NOT
XXbac-B44	41.89413	-1.1778359	0.468957	-2.51160906	0.012018	0.084273	NOT
RP11-641I	33.84886	0.76835281	0.305967	2.51122426	0.012031	0.084329	NOT
AC108463.	17.48189	0.82937012	0.330269	2.511195021	0.012032	0.084329	NOT
PTBP3	2867.128	0.42213276	0.168114	2.51099408	0.012039	0.084355	NOT
FAM178B	59.0905	2.14135542	0.8529	2.510674786	0.01205	0.08441	NOT
GCNT3	455.5458	1.42392486	0.567193	2.51047579	0.012057	0.084432	NOT
FLT4	761.1144	-0.590275	0.235132	-2.51039722	0.01206	0.084432	NOT
C10orf71	1.165921	-2.0689197	0.824208	-2.51019134	0.012067	NA	NA
STK24	1903.136	0.63079769	0.251299	2.510145221	0.012068	0.084464	NOT
LY6E	7994.297	1.15972178	0.462029	2.510061708	0.012071	0.084464	NOT
RHOV	37.44706	1.59188202	0.634218	2.509990514	0.012073	0.084464	NOT
RP11-959I	46.39766	-1.2185326	0.485504	-2.50983122	0.012079	0.084472	NOT
CLIC3	91.8168	1.31078139	0.522272	2.509769555	0.012081	0.084472	NOT
FTCDNL1	210.1628	-0.6360309	0.253431	-2.50968311	0.012084	0.084472	NOT
HOXB9	11.50627	1.85346412	0.738656	2.509238398	0.012099	0.084557	NOT
AC007229.	8.730024	0.60366802	0.240601	2.508999806	0.012107	0.084592	NOT
MT-CO1	807633.6	-0.6075065	0.24214	-2.50890321	0.012111	0.084594	NOT
CDKAL1	701.1861	0.31368216	0.125043	2.508592853	0.012121	0.084646	NOT
PLEKHA8	453.7892	0.51007241	0.203358	2.508245425	0.012133	0.084708	NOT
SLCO2B1	13069.57	-0.8648904	0.344925	-2.50747727	0.01216	0.08487	NOT
MTMR2	686.5593	0.51381245	0.204921	2.507364543	0.012164	0.084876	NOT
GLUD2	606.5739	0.99185347	0.395722	2.506438237	0.012195	0.085074	NOT
CTD-2536I	5.639278	-1.013129	0.404227	-2.5063344	0.012199	0.085074	NOT
TAF11	1402.823	0.32899257	0.131269	2.506253209	0.012202	0.085074	NOT
MTND6P21	4.503123	-1.548021	0.617691	-2.50614025	0.012206	0.085074	NOT
MLIP-AS1	8.784197	-1.5262587	0.609039	-2.50601038	0.01221	0.085074	NOT
SEMA3B	1101.541	-1.193496	0.476257	-2.50599357	0.012211	0.085074	NOT
KRT17	187.2029	1.43699152	0.573514	2.505591578	0.012225	0.085149	NOT
H2AFZP3	3.322686	0.98440462	0.393009	2.504791349	0.012252	0.08532	NOT
FTLP3	555.1929	1.07567254	0.429496	2.504500087	0.012262	0.085368	NOT
SLCO1C1	14.44126	-0.7469924	0.298374	-2.50353989	0.012296	0.085559	NOT
CNDP1	558.3257	-1.9163137	0.765444	-2.50353101	0.012296	0.085559	NOT
AC098614.	30.38933	0.97486205	0.38941	2.503431672	0.0123	0.085561	NOT
KNG1	103386.6	-1.040572	0.415675	-2.50332871	0.012303	0.085564	NOT
AC104134.	2.768336	-1.7410588	0.695782	-2.50230442	0.012339	0.08579	NOT

DPF3	78.57768	-0.9667075	0.386443	-2.50155388	0.012365	0.085936	NOT
ESPL1	790.218	0.86720704	0.346678	2.5014778	0.012368	0.085936	NOT
SEL1L3	1782.145	1.05059404	0.419997	2.501430316	0.012369	0.085936	NOT
ZNF728	14.41297	-2.3222232	0.928442	-2.50120334	0.012377	0.08595	NOT
C1orf140	15.11631	2.15611963	0.862065	2.501110355	0.01238	0.08595	NOT
EBF3	48.08371	-0.9101669	0.363914	-2.50104696	0.012383	0.08595	NOT
BTNL8	312.4057	1.86070004	0.744004	2.50092767	0.012387	0.08595	NOT
ATXN3	692.5404	-0.370175	0.148021	-2.50083572	0.01239	0.08595	NOT
ZNF486	85.05985	1.07442512	0.429637	2.500774354	0.012392	0.08595	NOT
TMEM55A	351.2499	0.80482389	0.321846	2.50064825	0.012397	0.08595	NOT
KRT19	560.1745	1.77609268	0.710254	2.500645358	0.012397	0.08595	NOT
C1RL	6507.286	-0.6509601	0.260333	-2.50049432	0.012402	0.085965	NOT
BANF1P2	63.31727	-1.0296676	0.411821	-2.50028199	0.012409	0.085994	NOT
HERC3	608.8361	-0.6303756	0.252135	-2.50015102	0.012414	0.086004	NOT
RP11-6B6.	2.533802	-0.8835613	0.35343	-2.49995926	0.012421	0.086008	NOT
RP11-242(	2.218755	-1.3792444	0.551707	-2.49995607	0.012421	0.086008	NOT
KRT18P4	6.886282	-0.6890233	0.275655	-2.49958179	0.012434	0.086067	NOT
CTD-2349F	22.53	0.68467939	0.273923	2.499532939	0.012436	0.086067	NOT
ANKRD9	971.7557	-0.6274603	0.251051	-2.4993321	0.012443	0.086091	NOT
ASMTL	1883.814	-0.5266033	0.210708	-2.49920684	0.012447	0.086091	NOT
AC004538.	47.72362	-1.6779555	0.671422	-2.49910824	0.012451	0.086091	NOT
THAP11	895.3	0.35586219	0.142398	2.499070032	0.012452	0.086091	NOT
GRM2	28.27932	-1.0281632	0.411442	-2.49892462	0.012457	0.086105	NOT
EYA1	6.309056	1.58431001	0.63404	2.498753027	0.012463	0.086124	NOT
C10orf105	4.945498	-0.9699178	0.388209	-2.4984415	0.012474	0.086178	NOT
WDR43	1592.564	0.32073764	0.12839	2.498144036	0.012485	0.086227	NOT
RALYL	6.177386	2.12298284	0.849852	2.498061567	0.012487	0.086227	NOT
CALB1	7.329401	-1.041484	0.416948	-2.4978731	0.012494	0.086246	NOT
DHX57	822.098	0.35746626	0.143112	2.497801858	0.012497	0.086246	NOT
CCNE2	166.6482	0.87758036	0.351372	2.497579242	0.012504	0.086253	NOT
FHDC1	94.36451	1.01067078	0.404665	2.497547502	0.012506	0.086253	NOT
ENPP1	3459.754	-0.7130782	0.285517	-2.49750161	0.012507	0.086253	NOT
CITF22-49	11.50055	-1.1012791	0.440998	-2.49724145	0.012516	0.086295	NOT
UBE2E3	1797.463	0.35896997	0.143796	2.496389278	0.012546	0.08648	NOT
TOX	107.3613	1.12955492	0.452503	2.496238975	0.012552	0.086495	NOT
SLC7A2	9304.212	-1.0473831	0.419613	-2.49606928	0.012558	0.086514	NOT
ASPHD2	52.7835	0.77296492	0.309699	2.495858413	0.012565	0.086524	NOT
CHCHD3	1999.552	0.37303772	0.149469	2.495757631	0.012569	0.086524	NOT
AC018641.	6.989316	2.19145173	0.878091	2.495700035	0.012571	0.086524	NOT
PES1	4118.388	0.44204775	0.177126	2.495670948	0.012572	0.086524	NOT
TRIM11	1206.727	0.42288467	0.169454	2.495574969	0.012575	0.086525	NOT
DACH2	5.44996	1.65734268	0.66418	2.495321709	0.012584	0.086565	NOT
MIXL1	6.750825	1.57662413	0.631885	2.495112789	0.012592	0.086594	NOT
CES4A	291.9247	-1.2487362	0.500544	-2.49475662	0.012604	0.086636	NOT
NEFL	6.125468	1.8515657	0.742204	2.494684824	0.012607	0.086636	NOT
TMEM201	642.0415	0.49188863	0.197182	2.494597907	0.01261	0.086636	NOT
DYNC1I2P1	10.56635	0.79271707	0.317776	2.494581969	0.012611	0.086636	NOT
APCDD1L-A	2.1236	1.78447222	0.715498	2.494029192	0.01263	0.086749	NOT
SMG1P3	25.00415	-0.6195315	0.24844	-2.49368193	0.012643	0.086812	NOT

DNMT1	2668.706	0.60407076	0.242292	2.49314845	0.012662	0.086919	NOT
NDST1	7278.63	-0.5169176	0.207342	-2.4930621	0.012665	0.086919	NOT
RHBDF2	1343.106	0.61776801	0.24782	2.492809814	0.012674	0.086959	NOT
SCAMP4	2588.374	0.36948131	0.148244	2.492389434	0.012689	0.087037	NOT
SLMO2	2331.085	0.36581094	0.146776	2.49231394	0.012691	0.087037	NOT
CLEC5A	41.29376	1.29716357	0.520498	2.492156433	0.012697	0.087049	NOT
AC010226.	30.98607	-0.529713	0.212558	-2.49208404	0.0127	0.087049	NOT
PHF20L1	1612.696	0.39073902	0.156801	2.491946743	0.012705	0.087061	NOT
RCAN1	2600.726	-0.7629985	0.306299	-2.49102515	0.012738	0.087265	NOT
RP11-136I	9.940974	-1.0705383	0.429883	-2.49030399	0.012763	0.08742	NOT
RP11-574K	170.5864	-0.756923	0.303977	-2.49006392	0.012772	0.087434	NOT
ZNF281	1977.949	-0.5082124	0.204098	-2.49003745	0.012773	0.087434	NOT
PPP2R2D	1106.824	-0.3610207	0.144993	-2.48991777	0.012777	0.087434	NOT
PCNPP1	6.059129	0.71510852	0.287205	2.489888948	0.012778	0.087434	NOT
USH2A	616.8182	-1.3008629	0.52252	-2.48959215	0.012789	0.087485	NOT
RP11-21B2	5.806294	-1.0456127	0.420081	-2.48907125	0.012808	0.087591	NOT
HAGLR	363.9616	1.47345447	0.592003	2.488931078	0.012813	0.087591	NOT
TNP1	2.775423	3.07005736	1.233503	2.488892492	0.012814	0.087591	NOT
MAGEB6	3.379911	2.62611331	1.055186	2.488769164	0.012819	0.087599	NOT
GLRX3	2069.71	0.38867591	0.156248	2.4875588	0.012862	0.087861	NOT
KRTAP5-2	2.197374	-2.7207164	1.093744	-2.48752553	0.012864	0.087861	NOT
CD28	117.6262	-0.8261041	0.33211	-2.48743881	0.012867	0.087861	NOT
N4BP2L1	982.5687	-0.6213704	0.24982	-2.48726738	0.012873	0.087881	NOT
H3F3A	1097.381	0.35503018	0.14276	2.486909535	0.012886	0.087948	NOT
KCNMA1-AS	2.17052	-1.1513503	0.462984	-2.48680216	0.01289	0.087952	NOT
ITM2C	2844.13	0.70970197	0.285412	2.486584434	0.012898	0.087984	NOT
ABHD5	610.3506	-0.4688389	0.18857	-2.48628693	0.012908	0.08803	NOT
RP11-192C	4.294051	1.23032801	0.494859	2.486218487	0.012911	0.08803	NOT
CTD-2184D	34.66417	1.61108598	0.648073	2.485962599	0.01292	0.088071	NOT
STAT3	9103.537	-0.4447246	0.178904	-2.48583113	0.012925	0.088082	NOT
CTD-2342N	3.203996	2.34126288	0.941999	2.485419195	0.01294	0.088162	NOT
RP11-452I	11.57054	-0.6530827	0.262811	-2.48498677	0.012956	0.088247	NOT
LTB4R2	62.43308	-0.8004124	0.322143	-2.48465046	0.012968	0.088291	NOT
ABCA8	1172.781	-1.2480177	0.502295	-2.48463051	0.012969	0.088291	NOT
RP11-472K	4.17858	1.60430485	0.645737	2.484454803	0.012975	0.088312	NOT
NBPF12	571.7692	-0.7152661	0.287931	-2.48416053	0.012986	0.088363	NOT
RP11-840I	1.837787	-1.2244516	0.492981	-2.48376892	0.013	0.088437	NOT
ZNF692	1176.989	0.6158709	0.247967	2.483683448	0.013003	0.088437	NOT
FAM157A	1.230524	2.42771638	0.977486	2.483633532	0.013005	NA	NA
CLEC4M	30.35016	-2.4595408	0.990398	-2.48338562	0.013014	0.088489	NOT
EIF2S2	3976.395	0.36874194	0.148496	2.483176864	0.013022	0.088497	NOT
LINC01152	42.07334	1.53531083	0.618289	2.483161678	0.013022	0.088497	NOT
RPS7P4	10.67918	0.79409016	0.3198	2.483084123	0.013025	0.088497	NOT
RP11-1299	6.639515	1.63544219	0.658711	2.482792841	0.013036	0.088539	NOT
GJA10	16.94768	3.61967855	1.457939	2.482736589	0.013038	0.088539	NOT
LINC01508	33.36604	3.8308083	1.543202	2.482376347	0.013051	0.088577	NOT
NOG	2.927834	2.13189109	0.858818	2.48235478	0.013052	0.088577	NOT
SLC11A1	174.8895	0.85906741	0.346075	2.482317171	0.013053	0.088577	NOT
MUS81	1185.19	0.2529407	0.101904	2.482148823	0.013059	0.088597	NOT

CLEC16A	1291.807	-0.3595933	0.144887	-2.48187957	0.013069	0.088641	NOT
AC009166.	43.58773	-0.9937905	0.400457	-2.4816435	0.013078	0.088678	NOT
CTC-55802	8.42934	-1.1455126	0.461631	-2.48144754	0.013085	0.088705	NOT
C1orf220	47.77488	-0.897165	0.361564	-2.48134401	0.013089	0.088708	NOT
TMPO-AS1	213.7303	0.69784563	0.281277	2.480992154	0.013102	0.088756	NOT
ANKRD33	32.53939	2.28889734	0.922606	2.480904422	0.013105	0.088756	NOT
RP11-465I	106.1995	1.06162391	0.427924	2.480870469	0.013106	0.088756	NOT
FP325317.	7.985956	1.54094123	0.621169	2.480713029	0.013112	0.088756	NOT
CTC-513N1	5.849563	1.90898285	0.769531	2.480708107	0.013112	0.088756	NOT
DNAJC9	593.064	0.51373955	0.20711	2.480510785	0.013119	0.088783	NOT
PPP2R5D	2498.666	0.32460072	0.130897	2.479820932	0.013145	0.088933	NOT
VDAC1	13152.52	0.36635498	0.14775	2.479557209	0.013155	0.088951	NOT
PLEKHG4	196.721	1.2812519	0.516737	2.479504562	0.013157	0.088951	NOT
NDUFV3	2580.763	-0.4522851	0.182411	-2.47948159	0.013157	0.088951	NOT
UBL3	2233.813	-0.4551129	0.183563	-2.47933221	0.013163	0.088966	NOT
RP11-120K	5.146201	1.3813677	0.557211	2.4790755	0.013172	0.089008	NOT
C6orf48	3301.719	0.59857023	0.241489	2.478666456	0.013187	0.089073	NOT
TRIM28	10269.11	0.43105461	0.173908	2.478636609	0.013189	0.089073	NOT
CTA-253N1	17.14148	0.6986775	0.281938	2.478128487	0.013207	0.089178	NOT
KB-1208A1	151.7387	0.69985966	0.282499	2.477388209	0.013235	0.089341	NOT
MAST2	1792.193	0.45541865	0.183852	2.477090271	0.013246	0.089366	NOT
LIPJ	3.068503	-1.2291711	0.496229	-2.477025	0.013248	0.089366	NOT
RP11-326C	37.84291	-1.0670954	0.430798	-2.47702202	0.013248	0.089366	NOT
SRSF8	1276.168	-0.4530427	0.182912	-2.47682759	0.013256	0.089392	NOT
SUPT16H	3512.143	0.3477283	0.140401	2.476687412	0.013261	0.089405	NOT
MYCBP2-AS	3.011986	-0.9321419	0.376396	-2.47649202	0.013268	0.089432	NOT
BMS1P8	198.1946	1.74517785	0.704778	2.476208785	0.013279	0.08948	NOT
AC145676.	6.939871	-1.1764691	0.475135	-2.47607399	0.013284	0.089492	NOT
RP11-407C	3.638885	-1.0168912	0.410715	-2.47590636	0.01329	0.089497	NOT
ANGPT4	5.73861	-1.1754819	0.474774	-2.47587803	0.013291	0.089497	NOT
ERICH1	425.8022	0.46364207	0.187273	2.475759234	0.013295	0.089504	NOT
HIST1H2AI	4.270616	-1.3616278	0.550186	-2.47484985	0.013329	0.089687	NOT
RP1-199J3	2.907426	-1.0348403	0.418159	-2.47475285	0.013333	0.089687	NOT
SORL1	5024.214	-0.8723819	0.352515	-2.47473721	0.013333	0.089687	NOT
AC007461.	3.989025	1.23586283	0.499455	2.474421937	0.013345	0.089687	NOT
TRMT12	532.7823	0.44070132	0.178103	2.474413905	0.013345	0.089687	NOT
RP11-77H9	7.370381	-1.1530558	0.465996	-2.47438884	0.013346	0.089687	NOT
HSPA8P13	1.719634	2.93101218	1.184553	2.474361688	0.013347	0.089687	NOT
GIMAP8	504.6668	-0.5676954	0.229435	-2.47432065	0.013349	0.089687	NOT
PIWIL4	89.60304	1.1082683	0.447956	2.474055582	0.013359	0.089732	NOT
ADAM9	2451.761	0.83280622	0.336649	2.473808765	0.013368	0.089772	NOT
LRRC14	1643.783	0.43971785	0.177783	2.473337736	0.013386	0.089868	NOT
POLR2D	1688.18	0.26964038	0.109025	2.473193967	0.013391	0.089882	NOT
HNRNPM	7903.846	0.27770346	0.112296	2.47295225	0.0134	0.08992	NOT
SF3A2	2935.272	0.54759839	0.221463	2.472644775	0.013412	0.089959	NOT
DIO3	29.0627	-1.7146167	0.693441	-2.47261989	0.013413	0.089959	NOT
C11orf52	5.638143	0.98108388	0.396823	2.472345142	0.013423	0.089999	NOT
ACTR1A	4526.226	-0.3624432	0.146603	-2.47228489	0.013425	0.089999	NOT
AC007773.	37.16509	0.91968902	0.372054	2.471923822	0.013439	0.090068	NOT

CTD-2574I	75.91899	0.45893931	0.185673	2.471758797	0.013445	0.090075	NOT
NUP43	1213.832	0.39268529	0.158882	2.471546554	0.013453	0.090075	NOT
LRRTM4	18.49552	1.7769613	0.718982	2.471496914	0.013455	0.090075	NOT
DEXI	399.6762	-0.5332079	0.215744	-2.47148765	0.013455	0.090075	NOT
RP11-209I	21.11708	-1.1564501	0.467937	-2.47138077	0.013459	0.090075	NOT
S100A2	64.33657	1.39555643	0.56469	2.471366735	0.01346	0.090075	NOT
RP1-200K1	2.616944	3.19204285	1.291775	2.471052378	0.013472	0.090106	NOT
NUDT11	8.798937	1.49788881	0.606192	2.470979934	0.013474	0.090106	NOT
RP11-96D1	12.35927	-0.6164327	0.249469	-2.47097831	0.013474	0.090106	NOT
ATG3	1900.562	0.32387777	0.131088	2.470686005	0.013485	0.090158	NOT
RP11-109N	6.459345	-0.7690869	0.311313	-2.47046335	0.013494	0.090191	NOT
IGIP	615.2227	-0.5040783	0.204067	-2.47015976	0.013505	0.09023	NOT
NOC2L	3568.94	0.45183202	0.182918	2.470132417	0.013506	0.09023	NOT
NELFE	3827.991	0.53350254	0.215999	2.469936128	0.013514	0.090258	NOT
BATF2	252.2905	0.58386819	0.236514	2.468641608	0.013563	0.090563	NOT
LRP6	2707.853	-0.5770349	0.233755	-2.46854394	0.013566	0.090565	NOT
CIB2	183.7272	1.07086584	0.433821	2.468449567	0.01357	0.090567	NOT
VRK2	454.4917	0.54344879	0.22018	2.468198326	0.01358	0.090608	NOT
LINC01023	76.22589	-0.7299327	0.295799	-2.4676672	0.0136	0.090718	NOT
CTD-2267I	33.89085	-0.676466	0.274141	-2.46758851	0.013603	0.090718	NOT
NT5C3B	1991.862	0.43853432	0.177726	2.467471042	0.013607	0.090725	NOT
LHFPL3	5.498208	2.13813869	0.866662	2.467096266	0.013621	0.090797	NOT
RP11-423I	372.2258	0.6263006	0.25387	2.467011772	0.013625	0.090797	NOT
CERK	2784.874	-0.48433	0.196332	-2.46688998	0.013629	0.090803	NOT
IL31RA	5.322102	1.50472683	0.610015	2.466703631	0.013636	0.090803	NOT
RP11-452I	5.892604	1.40274323	0.568674	2.466691141	0.013637	0.090803	NOT
CUZD1	19.14711	0.79469245	0.322182	2.466591499	0.013641	0.090803	NOT
CTSA	17482.6	0.47363947	0.192025	2.466550132	0.013642	0.090803	NOT
UGGT2	806.7529	0.42385347	0.171859	2.466281817	0.013652	0.090848	NOT
ANGPTL4	6991.876	-1.0312707	0.418176	-2.46611518	0.013659	0.090868	NOT
CHKA	2401.721	0.5268736	0.213661	2.465935836	0.013666	0.090892	NOT
USH1G	8.784985	1.90639875	0.77315	2.465756084	0.013672	0.090915	NOT
ACTC1	10.24905	-1.7003021	0.689614	-2.46558486	0.013679	0.09093	NOT
ZNF226	430.3741	-0.425826	0.172712	-2.46552171	0.013681	0.09093	NOT
C14orf159	1427.137	-0.571483	0.231805	-2.46536625	0.013687	0.090944	NOT
PRR7-AS1	10.04903	1.00686212	0.408415	2.465291748	0.01369	0.090944	NOT
RP11-108I	4.534714	-1.109648	0.45014	-2.46511604	0.013697	0.090966	NOT
PLA1A	1698.511	0.87988016	0.356971	2.464849809	0.013707	0.091011	NOT
PCDHGC3	168.1189	-0.9428002	0.38253	-2.46464545	0.013715	0.091041	NOT
AQP10	21.63657	2.11898772	0.859859	2.46434438	0.013726	0.091067	NOT
SEBOX	1.866461	-1.3538814	0.549425	-2.46417736	0.013733	0.091067	NOT
TMEM51	514.9317	0.93702189	0.380261	2.464154132	0.013734	0.091067	NOT
VPS26A	2110.775	0.25119591	0.101941	2.464137961	0.013734	0.091067	NOT
RNA5SP37	1.556659	-1.1876284	0.481971	-2.46410724	0.013736	0.091067	NOT
ULK1	3693.577	-0.4272892	0.173432	-2.46372987	0.01375	0.09114	NOT
CTD-2555(	38.97214	-0.6630205	0.269135	-2.46352544	0.013758	0.09117	NOT
RP11-262I	9.675266	-0.8058058	0.327151	-2.46309802	0.013774	0.091235	NOT
PRKAR1B	1086.789	0.72849579	0.295764	2.463095203	0.013774	0.091235	NOT
EXOC6B	765.9142	-0.5366469	0.217891	-2.46291203	0.013781	0.091259	NOT

SPC24	691.4369	0.75323793	0.305864	2.462656294	0.013791	0.091302	NOT
DUSP16	3688.111	-0.5581777	0.22673	-2.46186402	0.013822	0.091474	NOT
RAB32	1234.707	0.5508964	0.223777	2.461806021	0.013824	0.091474	NOT
TOE1	466.6706	0.33426148	0.135804	2.461346721	0.013842	0.091569	NOT
DRG1	2484.638	0.39387802	0.160042	2.461093274	0.013851	0.091611	NOT
RNA5SP111	9.933362	-1.2358183	0.502185	-2.46088486	0.013859	0.091642	NOT
KHDC1	13.53054	1.50909507	0.61327	2.460736398	0.013865	0.091658	NOT
GOT2P3	7.975079	-1.2572095	0.510928	-2.46063729	0.013869	0.091661	NOT
RP11-689F	20.9774	-0.8119324	0.329981	-2.46054266	0.013873	0.091663	NOT
HSP90AB2F	38.75822	0.77057466	0.313197	2.460348424	0.01388	0.09169	NOT
FEM1C	1374.271	-0.4333896	0.176178	-2.45995039	0.013896	0.091769	NOT
RP11-150C	6.74475	1.44585379	0.587944	2.459169042	0.013926	0.091947	NOT
MAFA-AS1	15.39335	2.05140667	0.83431	2.458807276	0.01394	0.092017	NOT
PCDHA12	6.45343	-1.8339855	0.745937	-2.45863434	0.013947	0.092037	NOT
AC011242.	10.36177	1.42278262	0.578707	2.458555918	0.01395	0.092037	NOT
HEPACAM2	3.744332	2.08024969	0.846267	2.458148066	0.013966	0.092119	NOT
RP11-499F	1.140387	1.92924219	0.784873	2.45803125	0.01397	NA	NA
BBOX1-AS1	27.10384	2.13807626	0.86995	2.457701176	0.013983	0.092211	NOT
BOK-AS1	27.47764	-1.6329436	0.664459	-2.45755529	0.013989	0.092226	NOT
ITPRIP	865.7668	-0.6478042	0.263609	-2.45743901	0.013993	0.092234	NOT
HPN-AS1	53.41026	-0.9428737	0.383724	-2.45716742	0.014004	0.092281	NOT
DDX54	3754.773	0.30350258	0.123542	2.456681707	0.014023	0.092384	NOT
FBXL22	34.07028	-0.543055	0.221071	-2.45647383	0.014031	0.092385	NOT
CTD-2600F	21.84752	-0.7786524	0.316987	-2.45641646	0.014033	0.092385	NOT
FYCO1	1994.973	-0.5288734	0.215308	-2.45636014	0.014035	0.092385	NOT
TMIE	107.9544	1.18624921	0.482936	2.456325908	0.014037	0.092385	NOT
RP11-426C	11.36535	-1.0142008	0.412913	-2.45621105	0.014041	0.092392	NOT
SPDEF	41.70246	1.56514988	0.637442	2.455359498	0.014074	0.092522	NOT
RP11-434F	47.03407	-0.7302411	0.297408	-2.45535285	0.014075	0.092522	NOT
RP11-16F1	15.63597	0.88503799	0.360454	2.455343365	0.014075	0.092522	NOT
FTH1P23	56.6796	0.73678027	0.300078	2.455299073	0.014077	0.092522	NOT
LMF1	987.0734	-0.6540205	0.266374	-2.45527187	0.014078	0.092522	NOT
IMPDH2	5078.095	0.49058673	0.199819	2.455154705	0.014082	0.092527	NOT
KAT6B	709.1277	-0.4401535	0.179286	-2.45503754	0.014087	0.092527	NOT
RP11-79P5	15.84127	0.82422187	0.335733	2.454990555	0.014089	0.092527	NOT
RAB25	26.45347	1.76038909	0.717126	2.454782712	0.014097	0.092558	NOT
RP11-60A2	2.470531	1.89055986	0.770198	2.454642827	0.014102	0.092572	NOT
CTB-140J7	2.568727	-1.3168218	0.536519	-2.45437855	0.014113	0.092618	NOT
RP11-254F	2.856012	1.26582249	0.515836	2.453926655	0.014131	0.092712	NOT
LINC01024	58.84817	-0.5154776	0.210085	-2.45365775	0.014141	0.092759	NOT
RP11-288C	5.530329	-1.5491311	0.631392	-2.45351791	0.014147	0.092772	NOT
B4GALT1-A	56.33183	-0.7472661	0.304652	-2.45285253	0.014173	0.092917	NOT
MINA	1269.665	0.58191516	0.237247	2.452782342	0.014176	0.092917	NOT
COL4A6	46.26137	-1.5659967	0.63855	-2.45242498	0.01419	0.092981	NOT
PHLDA1	3182.044	-0.8531795	0.347903	-2.45234546	0.014193	0.092981	NOT
LDHD	4150.376	-0.9351099	0.381323	-2.45227568	0.014196	0.092981	NOT
SLC35E4	120.5944	0.67339126	0.274614	2.45213772	0.014201	0.092994	NOT
EIF3M	4986.554	0.33792297	0.137813	2.452035836	0.014205	0.092998	NOT
ALPK3	1284.165	0.99193406	0.404565	2.45185302	0.014212	0.09301	NOT

RP11-168F	4.611184	1.55556636	0.634455	2.451816423	0.014214	0.09301	NOT
QTRTD1	639.4052	0.36745218	0.149881	2.451629886	0.014221	0.093035	NOT
RRAS2	1262.234	0.48421253	0.197537	2.451252369	0.014236	0.093098	NOT
C5	23478.14	-0.7601345	0.310108	-2.45119099	0.014238	0.093098	NOT
PARP9	3455.957	-0.5222326	0.213058	-2.45112906	0.014241	0.093098	NOT
CLDN11	193.9027	1.22030712	0.497985	2.450490981	0.014266	0.093238	NOT
MAP3K5	892.8041	-0.9240801	0.377112	-2.45041255	0.014269	0.093238	NOT
RP11-398K	18.93755	1.09888373	0.448502	2.450121341	0.014281	0.093291	NOT
SUOX	2464.767	-0.4957686	0.202359	-2.44994556	0.014288	0.093314	NOT
PITPNM2	1634.74	-0.6512131	0.265865	-2.44940915	0.014309	0.093431	NOT
RP11-154D	4.612902	-1.0759861	0.439366	-2.44894903	0.014327	0.093528	NOT
KRTAP4-1	1.350933	2.93732521	1.19953	2.448729699	0.014336	NA	NA
OSR2	84.42387	1.5074285	0.615602	2.448706126	0.014337	0.093568	NOT
DGKZP1	20.03873	0.5393885	0.220334	2.448044284	0.014363	0.093718	NOT
RP4-631H1	27.85244	-1.1457674	0.468107	-2.44765885	0.014379	0.093789	NOT
PARS2	337.7235	0.43853601	0.17917	2.447596081	0.014381	0.093789	NOT
CTD-2015C	8.180829	1.51569391	0.619279	2.447512585	0.014385	0.093789	NOT
RP11-196C	12.68938	-0.8438766	0.344841	-2.44714456	0.014399	0.093853	NOT
CTD-2561J	41.4157	1.30632215	0.533827	2.447087571	0.014402	0.093853	NOT
RP11-967K	54.81068	-0.6031139	0.246476	-2.44694828	0.014407	0.093853	NOT
FAAH2	372.1374	-0.8160615	0.33351	-2.44689049	0.014409	0.093853	NOT
FURIN	23715.98	-0.6992719	0.285788	-2.44682049	0.014412	0.093853	NOT
CCDC82	736.806	-0.4440711	0.181499	-2.44669229	0.014417	0.093853	NOT
TRGV10	6.536709	-1.1067377	0.452347	-2.44665858	0.014419	0.093853	NOT
MTMR6	994.8906	-0.4252815	0.173833	-2.44649944	0.014425	0.093872	NOT
EIF2AK3	784.3293	-0.3691981	0.150918	-2.44635103	0.014431	0.093888	NOT
SYN3	32.9962	1.13768513	0.465134	2.445929725	0.014448	0.093976	NOT
SLC16A8	17.1763	-0.795374	0.325198	-2.4458176	0.014452	0.093982	NOT
CYHR1	3826.802	0.53602973	0.219208	2.445305922	0.014473	0.094093	NOT
MAGEB2	157.0438	3.51014011	1.435668	2.444952313	0.014487	0.094163	NOT
KRT8P45	37.13029	0.79987906	0.327184	2.444741321	0.014496	0.094189	NOT
LINC0024C	71.40979	-0.8173532	0.334357	-2.44455122	0.014503	0.094189	NOT
C14orf80	429.417	0.68866308	0.281721	2.444489986	0.014506	0.094189	NOT
XXbac-B13	31.5852	-0.9071194	0.371087	-2.44448959	0.014506	0.094189	NOT
PHB	6570.013	0.41497954	0.169766	2.444421012	0.014508	0.094189	NOT
CALHM3	1.125338	2.31630579	0.947668	2.444216657	0.014517	NA	NA
EEF2K	1386.469	-0.3578907	0.146433	-2.44406183	0.014523	0.09426	NOT
Z97634.3	26.43716	0.71502365	0.292569	2.443952009	0.014527	0.094266	NOT
ANKRD12	1273.898	-0.4087504	0.167261	-2.4437821	0.014534	0.094288	NOT
TYW3	787.7267	0.34861919	0.142665	2.443625349	0.014541	0.094307	NOT
UBE2A	2376.508	0.32096737	0.131367	2.443288164	0.014554	0.094372	NOT
GLMP	5882.56	0.49091674	0.200948	2.443002933	0.014566	0.094424	NOT
TNS3	7529.979	-0.5488117	0.224672	-2.44272576	0.014577	0.094468	NOT
RP11-368I	5.189562	2.32299572	0.95101	2.442662775	0.014579	0.094468	NOT
RP11-1149	72.79175	0.69599459	0.284948	2.44253532	0.014585	0.094479	NOT
CTD-2342N	58.2959	1.26469718	0.517907	2.441938155	0.014609	0.094573	NOT
LCORL	458.8646	0.55338322	0.226617	2.441935708	0.014609	0.094573	NOT
CKAP2L	374.9444	0.80211062	0.328476	2.441916297	0.01461	0.094573	NOT
GABRR3	60.50276	1.8588707	0.761268	2.44180718	0.014614	0.094579	NOT

ESRRG	63.08086	-1.564248	0.640768	-2.44120836	0.014638	0.094702	NOT
TTC28	912.7758	-0.3549999	0.145425	-2.44111769	0.014642	0.094702	NOT
SLC35G2	261.178	0.78376387	0.321073	2.441079008	0.014643	0.094702	NOT
TMEM9	5822.323	0.40806278	0.167203	2.440519821	0.014666	0.094827	NOT
ZNF354A	264.4794	0.58338542	0.239064	2.440289277	0.014676	0.094865	NOT
LRRC36	8.916587	1.33727397	0.548049	2.440060919	0.014685	0.094902	NOT
CTD-2173I	17.07553	-0.87494	0.358588	-2.43996051	0.014689	0.094906	NOT
PHYHIPL	531.8839	1.15707448	0.474305	2.439517896	0.014707	0.094983	NOT
ANKRD24	226.3784	-0.7530831	0.308704	-2.4394958	0.014708	0.094983	NOT
FTH1P2	42.93376	0.75609798	0.309959	2.439352036	0.014714	0.094998	NOT
TEAD3	1184.242	0.39618676	0.162452	2.438788432	0.014737	0.095123	NOT
UBALD1	1296.446	-0.452287	0.185507	-2.43810762	0.014764	0.09528	NOT
DNAJC7	3021.469	0.28478171	0.116824	2.437701496	0.014781	0.095365	NOT
ABHD17C	505.1087	0.59770483	0.245214	2.437487255	0.01479	0.095388	NOT
OR7E29P	6.58448	-1.4851407	0.609303	-2.43744024	0.014792	0.095388	NOT
GK	1592.241	-0.7034049	0.288615	-2.43717365	0.014803	0.095436	NOT
SENP8	74.66617	-0.5634487	0.231249	-2.4365436	0.014828	0.095571	NOT
TXLNB	58.19644	1.16320304	0.477409	2.436490722	0.014831	0.095571	NOT
GS1-600G8	3.886235	2.41550462	0.991604	2.435955839	0.014852	0.095689	NOT
AP000619.	1.336724	2.94098137	1.207361	2.435875704	0.014856	NA	NA
HES2	26.76946	1.05168856	0.431755	2.435846046	0.014857	0.095696	NOT
RORC	5290.909	-0.9360273	0.384316	-2.43556605	0.014869	0.095747	NOT
UNC93A	692.9958	1.50342696	0.617302	2.435480399	0.014872	0.095747	NOT
ARHGEF7-A	3.940578	-1.7192472	0.706	-2.43519344	0.014884	0.095798	NOT
BMI1	2238.274	0.40919662	0.16804	2.435116879	0.014887	0.095798	NOT
SMN1	226.8076	0.36180697	0.148606	2.434671343	0.014905	0.095893	NOT
CTD-2231H	2.532994	2.72814104	1.120656	2.434415426	0.014916	0.095938	NOT
CTD-3193O	3.660372	-0.7439722	0.305617	-2.43433151	0.014919	0.095938	NOT
ZNF790	233.2915	-0.4025037	0.165366	-2.43402447	0.014932	0.095988	NOT
RELL2	76.89027	0.65826629	0.27045	2.433968644	0.014934	0.095988	NOT
RP11-544A	2.491753	-0.9081559	0.373164	-2.43366703	0.014947	0.096046	NOT
GOLGA8N	39.57073	-0.7804287	0.320715	-2.43340343	0.014958	0.096093	NOT
CTD-2192J	4.295155	-0.8708782	0.357899	-2.43330603	0.014962	0.096096	NOT
ALDH4A1	14742.9	-0.8000278	0.328818	-2.43304479	0.014972	0.096141	NOT
RPSAP8	2.759858	1.06588418	0.438101	2.432966894	0.014976	0.096141	NOT
FMO7P	1.416886	2.08573319	0.857364	2.432728413	0.014986	0.096181	NOT
VDR	270.7943	0.97295856	0.399976	2.432541605	0.014993	0.096208	NOT
LSM2	1515.598	0.46739177	0.192154	2.432378712	0.015	0.096229	NOT
DAZAP1	4382.123	0.31282893	0.128636	2.43189736	0.01502	0.096334	NOT
MAEL	61.97856	1.41101252	0.580307	2.431494146	0.015037	0.096418	NOT
NHP2	2262.244	0.45346042	0.186528	2.43105507	0.015055	0.096512	NOT
AGTRAP	1263.001	0.57802283	0.237797	2.43073746	0.015068	0.096574	NOT
PLAC1	1.634001	1.9702519	0.81068	2.430370858	0.015083	0.096618	NOT
PACRG	96.70794	-1.2419638	0.511026	-2.43033256	0.015085	0.096618	NOT
AC005540.	2.839415	1.39164407	0.572618	2.430316954	0.015086	0.096618	NOT
C8B	13786.65	-0.9441741	0.388518	-2.43019303	0.015091	0.096628	NOT
RP11-134N	3.721517	1.76939235	0.728235	2.429700197	0.015111	0.096737	NOT
PABPC1P1	1.877065	1.16949169	0.481377	2.429470443	0.015121	0.096775	NOT
CSNK2A3	5.582996	0.86615763	0.356613	2.428848061	0.015147	0.096914	NOT

ZNF286A	87.4644	0.58567757	0.241141	2.428779826	0.01515	0.096914	NOT
NHP2L1	4441.372	0.40752906	0.167818	2.428398422	0.015166	0.096993	NOT
SLC25A39	10199.59	0.43537524	0.179317	2.427966324	0.015184	0.097086	NOT
ARPC1B	6483.587	0.50297602	0.207185	2.427669322	0.015196	0.097142	NOT
MRI1	818.9353	-0.4258773	0.175435	-2.42754646	0.015201	0.097152	NOT
AK3	5879.227	-0.5336731	0.219888	-2.42702444	0.015223	0.097269	NOT
CLDN16	110.105	-0.838372	0.345475	-2.42672264	0.015236	0.097328	NOT
SLC1A3	396.4449	0.89565813	0.369128	2.426417807	0.015249	0.097386	NOT
RP11-100(	4.758737	-1.0349481	0.426549	-2.42633146	0.015252	0.097387	NOT
FAM134B	895.3153	-1.3271394	0.547012	-2.4261629	0.015259	0.097409	NOT
RP11-3P17	20.13402	0.98811173	0.407295	2.426036405	0.015265	0.09742	NOT
LINC0119]	4.482044	1.66661777	0.687108	2.425555892	0.015285	0.097507	NOT
ITPR3	899.1292	1.22361478	0.504471	2.425540015	0.015286	0.097507	NOT
ZEB2	752.1454	-0.7035539	0.290092	-2.42527647	0.015297	0.097532	NOT
AP000695.	9.186241	0.82748774	0.341194	2.425273874	0.015297	0.097532	NOT
RP11-326]	145.1491	-0.6152641	0.253697	-2.42519398	0.0153	0.097532	NOT
RP4-742C]	24.12293	-0.7699598	0.317567	-2.42456175	0.015327	0.097679	NOT
ITFG1-AS]	2.579366	-0.8319596	0.343194	-2.42416653	0.015344	0.097757	NOT
NT5E	2226.149	0.72766409	0.300179	2.424098636	0.015346	0.097757	NOT
NR2E1	5.470839	1.62102429	0.66878	2.423852816	0.015357	0.097801	NOT
RP11-359(	2.34738	1.0034581	0.41414	2.422994728	0.015393	0.098009	NOT
SLC22A17	449.4145	-1.3360679	0.551456	-2.42279926	0.015401	0.098021	NOT
RP11-172F	10.0224	1.31325254	0.542043	2.422781137	0.015402	0.098021	NOT
SLC6A1-AS	18.50179	-1.1005379	0.454285	-2.42257017	0.015411	0.098049	NOT
RP1-257A7	27.08839	0.77479746	0.319844	2.422419476	0.015418	0.098049	NOT
GUCA2B	149.397	1.5480851	0.639071	2.422400404	0.015418	0.098049	NOT
ITGB3	86.08804	-0.9625275	0.397355	-2.42233609	0.015421	0.098049	NOT
NPC1	2229.41	0.49625134	0.204874	2.422225826	0.015426	0.098056	NOT
AC137695.	1.800095	-1.0971583	0.452993	-2.42201959	0.015435	0.098088	NOT
ADA	422.0645	0.51952748	0.21453	2.42170071	0.015448	0.098151	NOT
KRT8P39	4.335628	0.8715621	0.359952	2.421328063	0.015464	0.098229	NOT
RP11-171]	15.8253	0.93399653	0.385833	2.420727595	0.015489	0.098368	NOT
LHX6	52.47325	-0.89015	0.367766	-2.42042596	0.015502	0.098427	NOT
DOC2B	191.4194	-0.8447435	0.349019	-2.42033855	0.015506	0.098428	NOT
RP11-154F	6.613075	1.09286738	0.451562	2.420192033	0.015512	0.098444	NOT
ILF3	8024.636	0.3326226	0.137451	2.419935584	0.015523	0.098491	NOT
LINC0026]	6777.661	-0.8136407	0.336263	-2.41965394	0.015535	0.098543	NOT
ASCL2	33.66783	1.08040996	0.44653	2.419570508	0.015539	0.098543	NOT
SNHG7	1507.344	0.77378036	0.319815	2.41946093	0.015544	0.09855	NOT
RAD1	921.6946	0.31516739	0.130306	2.418674101	0.015577	0.098714	NOT
RP11-342M	44.46396	0.97830743	0.404484	2.418652909	0.015578	0.098714	NOT
AP000648.	70.24494	0.6031841	0.249394	2.418600923	0.01558	0.098714	NOT
RP11-483I	13.4144	1.2869833	0.532149	2.41846347	0.015586	0.098728	NOT
SAMD8	731.363	-0.3967584	0.164062	-2.41834949	0.015591	0.098729	NOT
GOLGA8R	9.124658	-1.043728	0.431604	-2.41825596	0.015595	0.098729	NOT
BRD9	1226.726	0.33961966	0.140447	2.418135011	0.0156	0.098729	NOT
SNED1	549.8125	-0.7016447	0.290164	-2.41809833	0.015602	0.098729	NOT
GOT2P2	16.63388	-1.0941876	0.452511	-2.41803777	0.015604	0.098729	NOT
AC068831.	1.312024	-1.3011059	0.538105	-2.41793981	0.015609	NA	NA

AC010642.	8.871343	0.7878222	0.325853	2.417719235	0.015618	0.098792	NOT
NTN5	13.04136	-0.8987704	0.371809	-2.41729307	0.015636	0.09886	NOT
ENTHD1	1.461025	2.12807646	0.880373	2.41724279	0.015639	0.09886	NOT
RPL7P1	67.40888	0.80893048	0.334657	2.417190069	0.015641	0.09886	NOT
RP11-517I	8.12749	-0.8390585	0.347142	-2.41704464	0.015647	0.09886	NOT
DYNLL1P1	2.371638	0.914813	0.378484	2.417044385	0.015647	0.09886	NOT
RP11-632K	4.300358	-1.1171878	0.462228	-2.41696196	0.015651	0.09886	NOT
SETP14	8.54054	0.74198017	0.307017	2.416738206	0.01566	0.098897	NOT
PTEN	3702.599	-0.4169843	0.172577	-2.41621571	0.015683	0.09901	NOT
RP11-44M6	49.41345	-1.1261391	0.466087	-2.4161561	0.015685	0.09901	NOT
DSN1	1006.287	0.43762456	0.181137	2.415989625	0.015693	0.099032	NOT
ATAD3B	818.0569	0.57006629	0.235964	2.415901786	0.015696	0.099033	NOT
TRIM63	3.81083	-1.4110706	0.584121	-2.41571459	0.015704	0.099061	NOT
CCDC51	762.4183	0.40454133	0.16748	2.415461218	0.015715	0.099106	NOT
RPL6	24537.21	0.4581459	0.189685	2.415299406	0.015722	0.099127	NOT
RP11-326C	16.50326	-1.2255567	0.507447	-2.41514284	0.015729	0.099147	NOT
CTAGE9	2.348046	1.51460065	0.627229	2.414748623	0.015746	0.099231	NOT
RP11-517H	49.75564	-0.6781021	0.280886	-2.41415264	0.015772	0.099371	NOT
SENP1	539.0889	0.36260362	0.150212	2.413938774	0.015781	0.099406	NOT
UTP14A	914.4095	0.3495984	0.14484	2.413684951	0.015792	0.099452	NOT
TSPAN5	459.7347	1.20974626	0.501222	2.41359246	0.015796	0.099454	NOT
RP13-735I	19.34189	1.04321262	0.432247	2.413466428	0.015802	0.099466	NOT
TMEM72-AS	2.978028	-0.8853634	0.366897	-2.41311244	0.015817	0.099539	NOT
DUOX2	508.3791	1.78942971	0.741637	2.412809415	0.01583	0.099599	NOT
CBFA2T3	159.7764	-0.8440043	0.349843	-2.41252076	0.015843	0.09965	NOT
ZNF738	97.9536	0.95233009	0.39478	2.412307569	0.015852	0.09965	NOT
AP5Z1	2311.161	0.44070144	0.182694	2.412242166	0.015855	0.09965	NOT
KIAA1377	42.86693	0.91540154	0.379495	2.41215435	0.015859	0.09965	NOT
C16orf89	16.84106	-0.8252336	0.342117	-2.41214166	0.015859	0.09965	NOT
RP11-3K24	2.309618	1.1996182	0.497338	2.412080262	0.015862	0.09965	NOT
PSCA	15.89055	1.32687668	0.550135	2.411913136	0.015869	0.09965	NOT
PCAT2	3.565797	1.78787852	0.741287	2.41185876	0.015871	0.09965	NOT
SCNN1G	2.244269	1.69874868	0.704351	2.411792055	0.015874	0.09965	NOT
RP11-350J	24.61052	1.00961724	0.418619	2.411778248	0.015875	0.09965	NOT
PAK7	2.11472	2.48831675	1.031818	2.411584668	0.015883	0.09968	NOT
MIR6835	1.506919	1.71979246	0.713177	2.411453711	0.015889	0.099693	NOT
ALDH3B2	6.341214	1.95571692	0.811172	2.410977573	0.01591	0.0998	NOT
FTLP14	51.60965	-0.8387061	0.347922	-2.41061653	0.015926	0.099875	NOT
TMC3-AS1	35.83779	-0.7563267	0.313763	-2.41050424	0.01593	0.099883	NOT
KIAA1731N	1.859903	1.62533611	0.67435	2.410225741	0.015943	0.099936	NOT
SRRM4	2.564101	1.93355494	0.802358	2.409839496	0.01596	0.100019	NOT
CA15P1	2.355533	-1.3507609	0.560755	-2.40882461	0.016004	0.100274	NOT
COL6A4P1	4.775942	1.52718002	0.63407	2.408534001	0.016017	0.100331	NOT
METTL3	1120.743	0.32826886	0.136351	2.407536402	0.016061	0.100576	NOT
SCRG1	4.324037	-0.9914838	0.411836	-2.40747404	0.016063	0.100576	NOT
RP11-80A1	14.85927	-0.7463116	0.310038	-2.40715985	0.016077	0.10064	NOT
SOWAHB	763.9814	-0.8775231	0.364563	-2.40705763	0.016082	0.100645	NOT
WISP3	15.3214	0.90165574	0.374662	2.406585705	0.016102	0.100749	NOT
ZKSCAN3	409.3671	0.4946203	0.205534	2.406509066	0.016106	0.100749	NOT

RP11-22L1	2.597365	2.38711937	0.992036	2.406283951	0.016116	0.100788	NOT
LA16c-313	14.31253	0.74603887	0.310051	2.406179954	0.01612	0.100794	NOT
ERCC6L2	349.6825	-0.3874233	0.161027	-2.40595532	0.01613	0.100813	NOT
RP11-499I	42.64183	-0.6569482	0.273059	-2.40588394	0.016133	0.100813	NOT
KLC3	25.51074	1.40279879	0.583076	2.405859147	0.016134	0.100813	NOT
PANO1	16.94103	-0.745809	0.310026	-2.40563621	0.016144	0.10085	NOT
UBTF	5227.751	0.25656719	0.106656	2.405555305	0.016148	0.10085	NOT
ZFYVE28	408.5472	-0.6841904	0.284455	-2.4052671	0.016161	0.100884	NOT
RAB37	711.0183	-0.7862755	0.326908	-2.40518643	0.016164	0.100884	NOT
ATP13A2	1136.711	0.47506848	0.197522	2.405139196	0.016166	0.100884	NOT
RP11-195M	1.91605	1.69502296	0.704763	2.405094867	0.016168	0.100884	NOT
CMB9-94B1	6.828414	-0.8563967	0.356131	-2.40472639	0.016185	0.100953	NOT
CTC-350I8	2.140919	-1.0962008	0.455862	-2.40467845	0.016187	0.100953	NOT
KCNJ16	33.61324	1.66410626	0.692132	2.404320492	0.016203	0.101003	NOT
EIF6	8874.326	0.47659304	0.198228	2.404265121	0.016205	0.101003	NOT
ALG1L7P	2.17501	1.30075565	0.541025	2.404245103	0.016206	0.101003	NOT
BSND	12.73372	1.84737212	0.768511	2.403833187	0.016224	0.101094	NOT
NRBP1	5581.852	0.35338863	0.147018	2.403709414	0.01623	0.101101	NOT
DAD1	6035.302	0.37786312	0.157205	2.403639634	0.016233	0.101101	NOT
RP11-65L3	9.21001	0.85468072	0.355618	2.403365453	0.016245	0.101141	NOT
LINC01562	2.719993	-0.9543313	0.397087	-2.4033279	0.016247	0.101141	NOT
SLC35B2	3849.984	0.37737041	0.157032	2.403138528	0.016255	0.10117	NOT
KRT8P3	61.87917	0.83302817	0.346678	2.402884947	0.016266	0.101203	NOT
RP11-166(	14.54497	-0.7565381	0.31485	-2.40284928	0.016268	0.101203	NOT
GNL2	2469.41	0.35873386	0.149312	2.40257869	0.01628	0.101236	NOT
ZMYND10	44.43885	0.80158262	0.333636	2.402563815	0.016281	0.101236	NOT
LINC01296	28.18002	1.17571388	0.489504	2.401846965	0.016313	0.101411	NOT
BAZ2B	761.8937	-0.4861791	0.202435	-2.40165193	0.016321	0.101442	NOT
ADRA1B	202.6987	-1.0258277	0.427182	-2.40138431	0.016333	0.101493	NOT
HOXB13	29.00447	2.34469338	0.976733	2.400546797	0.016371	0.101702	NOT
SLC8B1	1466.208	-0.4889792	0.203733	-2.40010004	0.016391	0.101803	NOT
RP11-646I	3.814651	-0.6948036	0.289521	-2.39983599	0.016402	0.101853	NOT
KIF25-AS1	3.558846	1.80010338	0.750155	2.399640709	0.016411	0.101884	NOT
RP5-858L1	20.38916	0.99334335	0.413987	2.399454084	0.01642	0.101913	NOT
RPL5	29212.23	0.47737051	0.198966	2.399256961	0.016428	0.10193	NOT
TACR1	10.46807	-0.9166862	0.382076	-2.39922699	0.01643	0.10193	NOT
PCDHGC4	3.965361	-1.5593851	0.64998	-2.39912723	0.016434	0.101934	NOT
HNRNPAB	5504.783	0.29463309	0.12284	2.398512256	0.016462	0.102082	NOT
SCP2	19228.32	-0.720709	0.300513	-2.39826116	0.016473	0.102114	NOT
KCNC2	80.98611	-2.4221791	1.009986	-2.39822969	0.016475	0.102114	NOT
SBK2	1.462489	2.15327243	0.897987	2.397888874	0.01649	0.102186	NOT
DCXR	26975.08	-1.0105046	0.421442	-2.39772964	0.016497	0.102207	NOT
RP11-713M	802.9182	0.92040478	0.383943	2.397240769	0.016519	0.102297	NOT
WTAPP1	10.28338	1.44293438	0.601915	2.397239434	0.016519	0.102297	NOT
U47924.6	82.6038	0.54751821	0.22841	2.397089492	0.016526	0.102316	NOT
LSM8	872.6332	0.37141421	0.154954	2.396932119	0.016533	0.102336	NOT
LINC00238	31.98774	-1.8884375	0.788007	-2.39647196	0.016554	0.102396	NOT
STXBP6	627.9177	0.74503964	0.31089	2.396469984	0.016554	0.102396	NOT
NTNG2	24.98272	0.8841948	0.368958	2.396466012	0.016554	0.102396	NOT

LGALS9B	5.659744	-1.6845814	0.702992	-2.39630256	0.016561	0.102405	NOT
PSORS1C1	35.52247	1.11003048	0.463248	2.396192142	0.016566	0.102405	NOT
FFAR2	26.63781	1.11597355	0.465729	2.396186266	0.016567	0.102405	NOT
PDPR	1059.67	-0.475897	0.198665	-2.3954712	0.016599	0.102562	NOT
SUB1P3	3.494129	0.9070048	0.378636	2.3954551	0.0166	0.102562	NOT
CTSV	254.1192	1.44855748	0.604747	2.395312927	0.016606	0.102579	NOT
RP11-354K	1.361286	2.54547308	1.062705	2.395276412	0.016608	NA	NA
RP11-15E1	7.345258	0.72794054	0.303917	2.395194677	0.016612	0.102579	NOT
SCRN1	904.6309	1.13228265	0.472741	2.395143516	0.016614	0.102579	NOT
FGFRL1	3201.166	-0.487381	0.203503	-2.39496257	0.016622	0.102584	NOT
RP11-108M	65.82248	0.77459989	0.323429	2.394961998	0.016622	0.102584	NOT
NGDN	1163.398	0.31843289	0.132972	2.394745363	0.016632	0.102621	NOT
FTH1P12	17.47232	0.89708873	0.374702	2.394139456	0.016659	0.102737	NOT
RPS7P3	5.197962	0.96599989	0.403486	2.394134902	0.01666	0.102737	NOT
RPS4XP7	3.090285	1.13907347	0.475787	2.394080945	0.016662	0.102737	NOT
C10orf128	252.6021	-0.7638572	0.319117	-2.39366036	0.016681	0.102799	NOT
ACVR1C	72.92275	-0.8466408	0.353708	-2.39361586	0.016683	0.102799	NOT
ZNF296	74.85419	0.80324671	0.335581	2.393601493	0.016684	0.102799	NOT
UPF2	1653.702	0.34819336	0.145473	2.393517522	0.016688	0.102799	NOT
CD1A	10.44449	1.21659274	0.508302	2.393443042	0.016691	0.102799	NOT
JRK	543.5824	0.47576925	0.19879	2.393321748	0.016697	0.102809	NOT
TUFT1	858.0843	0.59717262	0.249553	2.392965619	0.016713	0.102873	NOT
C9orf163	17.17792	-0.7222834	0.301841	-2.39292876	0.016714	0.102873	NOT
STEAP1	944.7638	1.10960818	0.463825	2.392298028	0.016743	0.102979	NOT
UBAC2	4069.337	0.46539514	0.194551	2.392149994	0.01675	0.102979	NOT
ZNF805	62.79404	-0.4521391	0.189015	-2.39208135	0.016753	0.102979	NOT
UMPS	1911.685	0.30186994	0.126197	2.392054934	0.016754	0.102979	NOT
HBA1	4.378621	-1.2508732	0.522936	-2.39201778	0.016756	0.102979	NOT
RP11-45A1	3.642421	-0.8053027	0.336673	-2.39194541	0.016759	0.102979	NOT
RP1-197B1	10.09927	0.7064903	0.295369	2.391894031	0.016762	0.102979	NOT
SPDYE6	11.18943	0.72006921	0.301047	2.391885737	0.016762	0.102979	NOT
BICD1	230.4446	0.70362208	0.294185	2.391768641	0.016767	0.102985	NOT
KIAA0430	1971.1	-0.3267104	0.136602	-2.39169764	0.016771	0.102985	NOT
ERN1	2015.749	-0.5052251	0.211254	-2.39155151	0.016777	0.103003	NOT
RP11-555M	23.17887	-0.807693	0.337763	-2.39129836	0.016789	0.10305	NOT
SMIM3	357.1709	0.82250009	0.343974	2.39116688	0.016795	0.103064	NOT
GAPDHP74	6.368131	-1.7156172	0.717536	-2.39098406	0.016803	0.103087	NOT
FAM136A	2174.466	0.46051476	0.192611	2.390901043	0.016807	0.103087	NOT
CTD-2547I	14.62656	-0.8352399	0.349351	-2.39083635	0.01681	0.103087	NOT
RP11-324I	63.32547	0.68185783	0.285231	2.390546156	0.016823	0.103145	NOT
PRDX1	19833.24	0.55708461	0.233078	2.390116253	0.016843	0.103243	NOT
MCFD2	8598.235	-0.4542131	0.190068	-2.38974308	0.01686	0.103324	NOT
C12orf42	8.253243	1.26106613	0.52774	2.389561222	0.016869	0.103352	NOT
RP11-380N	3.716311	-1.088418	0.455508	-2.38946091	0.016873	0.103357	NOT
AATK	201.8832	1.0726217	0.448925	2.389311808	0.01688	0.103363	NOT
BTNL9	438.1889	-0.9716171	0.40667	-2.38920143	0.016885	0.103363	NOT
RP11-163E	22.72613	0.73709142	0.308517	2.389142099	0.016888	0.103363	NOT
MAGI1	1524.54	-0.4206894	0.176093	-2.38902492	0.016893	0.103363	NOT
SEMA6B	587.2479	-0.5457001	0.228423	-2.38898484	0.016895	0.103363	NOT

SRGAP3-AS	2.003081	2.36528075	0.990096	2.388941523	0.016897	0.103363	NOT
C14orf93	431.3404	0.28886098	0.120925	2.388756961	0.016905	0.103367	NOT
ZNF575	102.2203	-0.4006672	0.167733	-2.38871882	0.016907	0.103367	NOT
AC018890.	47.64334	1.87880233	0.786544	2.388679201	0.016909	0.103367	NOT
ENO2	397.3728	1.11597645	0.467224	2.388525014	0.016916	0.103387	NOT
OVOL3	2.262609	1.34484365	0.563103	2.388274263	0.016928	0.103434	NOT
GNPDA1	1732.757	0.53725487	0.225015	2.387645112	0.016957	0.103588	NOT
ZNF429	255.5487	-0.567517	0.237796	-2.38656977	0.017006	0.103868	NOT
CPSF4	1256.166	0.37958015	0.159065	2.386328228	0.017018	0.103886	NOT
PEG3	1040.569	1.66734461	0.698712	2.386311328	0.017018	0.103886	NOT
MRPS31P5	11.94722	-0.9349589	0.39181	-2.38625627	0.017021	0.103886	NOT
CHRNA7	4.586374	1.61653152	0.677476	2.386109587	0.017028	0.103886	NOT
KRT8P36	8.691826	0.89689108	0.37589	2.386046253	0.017031	0.103886	NOT
CTC-487M2	3.523223	-0.9014761	0.377817	-2.38601084	0.017032	0.103886	NOT
MAN2B2	3411.315	-0.4450379	0.186551	-2.38560737	0.017051	0.103977	NOT
HIST3H2A	184.6162	1.61864189	0.678806	2.384541823	0.0171	0.104251	NOT
C9orf3	1741.1	-0.3410669	0.143038	-2.38444511	0.017105	0.104251	NOT
LAMA2	491.1515	-1.1131379	0.466844	-2.38439008	0.017107	0.104251	NOT
RPL29	21754.95	0.53665299	0.225106	2.383998706	0.017126	0.104338	NOT
RP11-279F	6.986814	-1.3387576	0.561706	-2.3833778	0.017155	0.104478	NOT
CACNA2D1	93.25832	-0.7549619	0.316766	-2.38334325	0.017156	0.104478	NOT
AGBL2	153.4607	-0.925785	0.388504	-2.38294795	0.017175	0.104566	NOT
B3GNT4	24.97686	0.87664499	0.367902	2.382821179	0.017181	0.104579	NOT
MTMR7	336.6212	1.3102539	0.550128	2.381723857	0.017232	0.104797	NOT
RABL2B	344.8064	0.39472176	0.165734	2.381663111	0.017235	0.104797	NOT
LPIN2	8593.249	-0.6786266	0.28494	-2.38164975	0.017235	0.104797	NOT
RP5-1159(	21.18321	-0.6628114	0.278316	-2.38150285	0.017242	0.104797	NOT
B4GALT2	2414.395	0.44342455	0.186198	2.381462474	0.017244	0.104797	NOT
RP1-118J2	4.370358	1.51895945	0.63783	2.381448206	0.017245	0.104797	NOT
RHOJ	290.0468	-0.5047736	0.211961	-2.38144181	0.017245	0.104797	NOT
PLBD1-AS1	13.48385	1.26753477	0.532266	2.381391767	0.017247	0.104797	NOT
UBR5	3380.356	0.37415345	0.157125	2.381254143	0.017254	0.104813	NOT
OR2AT1P	4.412429	2.61173374	1.09683	2.381165962	0.017258	0.104815	NOT
RP11-47P1	1.545363	2.40862766	1.011622	2.380955902	0.017268	0.104851	NOT
FTO	1344.636	-0.3777227	0.158652	-2.38082715	0.017274	0.104864	NOT
ZNF609	1491.386	-0.4265072	0.179184	-2.38027824	0.0173	0.104997	NOT
ZCCHC7	654.0066	0.30046118	0.126265	2.379611948	0.017331	0.105164	NOT
LAMC3	866.6607	-0.8823199	0.370808	-2.37945382	0.017338	0.105185	NOT
RNF180	166.9907	-0.8634685	0.362953	-2.37901219	0.017359	0.105266	NOT
BAI3	9.969043	-1.2692444	0.533519	-2.37900709	0.017359	0.105266	NOT
ACAN	159.5328	1.37950996	0.579928	2.378762593	0.017371	0.105312	NOT
CTC-458A3	9.200345	1.59478064	0.670478	2.378574122	0.01738	0.105342	NOT
F13A1	552.5388	-1.0910779	0.458788	-2.37817435	0.017399	0.105433	NOT
RP11-426(	24.81532	-0.8425423	0.354303	-2.37802634	0.017406	0.105452	NOT
GOLGA2P8	2.764389	-1.3784265	0.579682	-2.37790099	0.017412	0.105464	NOT
RP11-295H	8.491139	0.87445629	0.367756	2.37781387	0.017416	0.105466	NOT
RP11-407H	3.545677	1.66214341	0.6991	2.37754657	0.017428	0.105518	NOT
RANBP3L	62.37664	-1.3329489	0.560696	-2.37731185	0.017439	0.105562	NOT
DRC1	10.67824	1.09064398	0.458798	2.377179244	0.017446	0.105576	NOT

AXIN1	1430.755	0.47698463	0.200673	2.376919786	0.017458	0.105627	NOT
C10orf62	5.177238	-1.071883	0.45102	-2.3765765	0.017474	0.105702	NOT
PCBP1-AS1	598.4912	-0.4533119	0.190769	-2.37623228	0.01749	0.105747	NOT
APOL6	5714.809	-0.6868756	0.289065	-2.37619984	0.017492	0.105747	NOT
RP11-243M	2.063459	2.0147605	0.847902	2.376172643	0.017493	0.105747	NOT
RP11-176H	23.8347	-0.6632974	0.279186	-2.37582948	0.01751	0.105822	NOT
ZNF583	167.7275	-0.579152	0.243793	-2.37558974	0.017521	0.105836	NOT
GRIP2	47.29223	1.25404975	0.527907	2.375513	0.017525	0.105836	NOT
INPP5J	81.24209	1.02475623	0.431387	2.375491524	0.017526	0.105836	NOT
AC019181.	4.313431	-0.9307768	0.391863	-2.37526053	0.017537	0.105836	NOT
PPA2	2388.219	-0.4271523	0.179835	-2.37525051	0.017537	0.105836	NOT
KDM1B	742.9744	0.46861545	0.197294	2.375218304	0.017539	0.105836	NOT
CTC-429P9	27.57761	-0.7923166	0.333578	-2.37520601	0.017539	0.105836	NOT
KRT13	7.69041	2.3448986	0.987364	2.374908404	0.017553	0.105898	NOT
C18orf21	547.1245	0.34158864	0.143843	2.374729994	0.017562	0.105901	NOT
ZADH2	1195.583	-0.4049723	0.170539	-2.37466344	0.017565	0.105901	NOT
RPS7P1	246.4999	0.69833779	0.29408	2.374650463	0.017566	0.105901	NOT
RP11-685M	7.336951	-0.580687	0.244561	-2.37440966	0.017577	0.105946	NOT
ZSCAN16	187.707	0.46407088	0.195466	2.374178784	0.017588	0.105989	NOT
ARAP1-AS1	2.296937	2.98889682	1.259012	2.374001294	0.017596	0.10599	NOT
TRIM50	407.3355	-1.7836077	0.751347	-2.37388116	0.017602	0.10599	NOT
CECR6	25.19907	0.81052892	0.341445	2.373817335	0.017605	0.10599	NOT
RN7SL381P	3.394971	-0.949915	0.400171	-2.37377442	0.017607	0.10599	NOT
BORA	114.8941	0.64409986	0.271351	2.373676508	0.017612	0.10599	NOT
GYPE	4.309087	-1.1185177	0.471224	-2.3736451	0.017613	0.10599	NOT
RXRG	70.86653	1.5847733	0.667666	2.373601259	0.017616	0.10599	NOT
NAV2-AS5	1.683438	-1.5823395	0.666663	-2.37352122	0.017619	0.10599	NOT
XXbac-BPC	16.5756	0.55398305	0.233417	2.373364977	0.017627	0.106004	NOT
RP11-146N	5.920386	0.94026218	0.396184	2.373294297	0.01763	0.106004	NOT
FKBP6	4.041206	1.76264322	0.74272	2.373226702	0.017633	0.106004	NOT
IGLON5	76.23963	1.27559453	0.537594	2.372785866	0.017654	0.106107	NOT
ARHGEF35	148.2868	0.92256396	0.388846	2.372569191	0.017665	0.106131	NOT
COQ6	625.2538	-0.3861143	0.162743	-2.37253714	0.017666	0.106131	NOT
CAP1	8897.29	0.31948736	0.134666	2.372448242	0.017671	0.106133	NOT
ZNF546	191.4528	-0.4115843	0.173516	-2.3720196	0.017691	0.106233	NOT
RP11-879F	8.044398	0.76511101	0.32257	2.371925371	0.017696	0.106237	NOT
POC5	285.0725	0.27766651	0.117071	2.371785008	0.017702	0.106253	NOT
PSMD6	2457.502	0.29930883	0.126214	2.371447755	0.017719	0.106311	NOT
AOC3	981.3302	-0.6078729	0.256337	-2.37138073	0.017722	0.106311	NOT
RPL7P23	25.98276	0.7768226	0.327588	2.371339131	0.017724	0.106311	NOT
EPHA1-AS1	12.27478	1.3363157	0.563708	2.370582627	0.01776	0.106505	NOT
RP11-298J	11.89933	-0.832981	0.351459	-2.3700687	0.017785	0.10663	NOT
FAM72B	36.62572	0.88296906	0.372568	2.36995414	0.01779	0.10664	NOT
RP11-809C	37.96429	1.24307387	0.524561	2.3697428	0.0178	0.106677	NOT
AMZ1	30.2852	1.19806028	0.505607	2.369548048	0.01781	0.1067	NOT
PLA2G16	2763.581	-0.7777718	0.328243	-2.36949807	0.017812	0.1067	NOT
UTP14C	912.4881	-0.522592	0.220569	-2.3692909	0.017822	0.106737	NOT
RTFDC1	3914.775	0.27957005	0.118032	2.368604988	0.017855	0.106911	NOT
PGAM5	2480.24	0.31972907	0.134995	2.368443627	0.017863	0.106934	NOT

MAN2A1	4870.946	-0.4736644	0.200028	-2.36798702	0.017885	0.107043	NOT
RRAGC	965.833	0.44351054	0.187315	2.367727608	0.017898	0.107094	NOT
AC108004.	2.150087	-1.2739235	0.538099	-2.36745192	0.017911	0.10715	NOT
LINC0155(	16.32383	-1.3057567	0.551602	-2.36720787	0.017923	0.107193	NOT
TIMMDC1	2338.737	0.30710156	0.129735	2.367138763	0.017926	0.107193	NOT
FOS	3753.221	-1.0224358	0.431954	-2.36700244	0.017933	0.107193	NOT
RP11-384K	127.4186	-0.5822211	0.245977	-2.36697831	0.017934	0.107193	NOT
RP11-936I	14.27489	-1.0714718	0.452704	-2.36682875	0.017941	0.107204	NOT
C16orf74	43.56515	0.96755753	0.40881	2.366763854	0.017944	0.107204	NOT
RP1-102K2	13.51738	1.43165513	0.604917	2.366694979	0.017948	0.107204	NOT
CBLN1	128.4525	-1.1685013	0.493771	-2.3664829	0.017958	0.107227	NOT
NACA	24450.71	0.33953171	0.143477	2.366454432	0.017959	0.107227	NOT
RALGAPA1	440.8429	-0.4559419	0.192686	-2.36624593	0.01797	0.107264	NOT
MPP3	145.3806	0.83077031	0.351137	2.365940201	0.017984	0.107329	NOT
TFAP2C	18.80333	1.82143419	0.769952	2.365646722	0.017999	0.10739	NOT
RP3-43804	1.419822	1.64303421	0.694599	2.365442525	0.018009	0.107426	NOT
UBL4A	2641.881	0.4053719	0.171392	2.36517361	0.018022	0.107475	NOT
PAPSS1	1038.847	0.48046097	0.203152	2.365029947	0.018029	0.107475	NOT
FAM64A	136.4106	1.12574539	0.475996	2.365029272	0.018029	0.107475	NOT
AC084082.	6.471615	-1.2040552	0.509165	-2.36476324	0.018042	0.10748	NOT
AP000688.	3.746232	2.08293102	0.880842	2.364704553	0.018044	0.10748	NOT
AC002117.	4.05097	-0.7325004	0.309767	-2.36468265	0.018046	0.10748	NOT
COL18A1	42081.49	-0.5939545	0.251182	-2.36463397	0.018048	0.10748	NOT
SV2B	108.5353	1.25878601	0.532345	2.364605603	0.018049	0.10748	NOT
SIRT1	778.3112	-0.4205599	0.177874	-2.36437182	0.018061	0.107524	NOT
MRPS18A	1216.766	0.41860091	0.177068	2.364074468	0.018075	0.107565	NOT
XXbac-BP(	76.72527	-0.632455	0.267528	-2.36406773	0.018076	0.107565	NOT
RP11-521E	35.3242	0.66761197	0.282422	2.363884193	0.018084	0.107573	NOT
TCTN3	1823.617	-0.3502776	0.148179	-2.36387634	0.018085	0.107573	NOT
CCDC71	779.8246	0.35536988	0.150343	2.363722349	0.018092	0.107594	NOT
SIRT3	1268.984	-0.3560988	0.15067	-2.3634393	0.018106	0.107653	NOT
RP11-109I	6.717487	-1.2524236	0.529961	-2.36323759	0.018116	0.107688	NOT
SULT1E1	429.4438	1.50544249	0.637174	2.362686435	0.018143	0.107802	NOT
RBM22	1822.303	0.30104719	0.127421	2.362614338	0.018147	0.107802	NOT
AP2M1	11358.47	0.34680012	0.146787	2.362602609	0.018147	0.107802	NOT
HAUS2	651.6441	0.31505123	0.133382	2.36201491	0.018176	0.107949	NOT
LDLR	4981.238	-0.5726118	0.242459	-2.36168595	0.018192	0.108021	NOT
IGDCC4	70.74855	1.24171646	0.525855	2.361327272	0.01821	0.108102	NOT
HNRNPA3P1	6.206748	0.73207568	0.310044	2.36119796	0.018216	0.108116	NOT
KCNH7	7.30131	1.57720542	0.668046	2.360922196	0.01823	0.108159	NOT
RP11-173M	102.6928	-0.7374982	0.312401	-2.36074209	0.018238	0.108159	NOT
RP11-304F	38.2889	0.78438503	0.332264	2.360727246	0.018239	0.108159	NOT
NHLRC3	1099.157	-0.450128	0.190675	-2.36070806	0.01824	0.108159	NOT
TRIM65	905.7663	0.39623728	0.167852	2.360641608	0.018243	0.108159	NOT
ZBTB16	486.5626	-1.0966419	0.464567	-2.36056623	0.018247	0.108159	NOT
HNRNPCP2	354.6798	0.40624545	0.172114	2.360334357	0.018258	0.108203	NOT
TAT	42400.74	-1.5097522	0.639667	-2.36021486	0.018264	0.108214	NOT
PWAR6	261.1126	1.21839686	0.516273	2.359983577	0.018276	0.108258	NOT
TRMT112	5755.329	0.41418575	0.175514	2.359846122	0.018283	0.10826	NOT

STXBP1	593.0758	-0.7218813	0.305916	-2.35973983	0.018288	0.10826	NOT
HSPA1B	3401.849	0.75099744	0.318255	2.359732838	0.018288	0.10826	NOT
AC124861.	2.353312	1.81436229	0.768977	2.359450179	0.018302	0.108303	NOT
RP11-104H	2.615841	-1.0563092	0.447705	-2.35938483	0.018305	0.108303	NOT
ZNF507	886.8204	-0.3335145	0.141359	-2.35934174	0.018307	0.108303	NOT
RPS3AP26	41.54459	0.81235817	0.344443	2.358468368	0.018351	0.108535	NOT
NUDC	7133.361	0.45498612	0.192933	2.358254842	0.018361	0.108574	NOT
MED15	2721.633	0.30897365	0.13104	2.357852455	0.018381	0.108652	NOT
RIMS2	10.70282	1.34554569	0.570689	2.357755648	0.018386	0.108652	NOT
CTC-559E9	42.06351	0.5500498	0.233295	2.357745813	0.018386	0.108652	NOT
GHDC	2003.3	0.41604277	0.17647	2.35757813	0.018395	0.108677	NOT
RP11-506M	9.76121	0.83707291	0.355069	2.357496608	0.018399	0.108677	NOT
PRMT7	1194.147	-0.3706549	0.157231	-2.35739478	0.018404	0.108683	NOT
PCK1	35398.32	-1.3629792	0.578195	-2.35730071	0.018408	0.108687	NOT
AP000344.	5.071325	2.51766566	1.068115	2.357111417	0.018418	0.108719	NOT
BLOC1S5	770.8516	0.35744853	0.151674	2.356693356	0.018438	0.108791	NOT
SH2D1B	16.45775	-0.8294147	0.351949	-2.35663522	0.018441	0.108791	NOT
LINC0133(	10.9198	-0.9597384	0.407262	-2.3565652	0.018445	0.108791	NOT
NEDD1	492.2283	0.36972043	0.156891	2.356541416	0.018446	0.108791	NOT
NMI	960.9356	0.47233499	0.200446	2.356420541	0.018452	0.108803	NOT
PPL	1432.414	-0.8758112	0.371767	-2.35580623	0.018483	0.10896	NOT
PPTC7	1506.738	-0.3791591	0.160963	-2.3555742	0.018494	0.108996	NOT
RP11-139I	33.90642	-0.6408133	0.272051	-2.35548821	0.018498	0.108996	NOT
RPS7P11	48.58041	0.74683647	0.317086	2.355311797	0.018507	0.108996	NOT
PNP	1809.527	0.45497924	0.193172	2.355308606	0.018507	0.108996	NOT
KLF2	527.5791	-0.6702135	0.284558	-2.35527977	0.018509	0.108996	NOT
VSNL1	1140.47	1.22072461	0.51834	2.355064287	0.01852	0.109035	NOT
SLC25A42	4036.49	-0.744268	0.316049	-2.35491094	0.018527	0.10905	NOT
AZIN1	7508.579	0.45261358	0.192205	2.354852777	0.01853	0.10905	NOT
RP11-573D	272.3243	-0.8518908	0.361821	-2.35445585	0.01855	0.109143	NOT
SMYD3	387.1454	0.66070771	0.280644	2.354254051	0.01856	0.109178	NOT
RP11-173E	4.807872	1.41142563	0.599763	2.353306575	0.018607	0.10941	NOT
MTHFD1	11999.81	-0.792154	0.336614	-2.35330343	0.018607	0.10941	NOT
FAM198B	851.1489	-0.6445344	0.273896	-2.35320687	0.018612	0.109413	NOT
ROR2	189.5084	1.30674384	0.555337	2.353064074	0.018619	0.109413	NOT
NABP2	1611.019	0.40911331	0.173865	2.353054111	0.01862	0.109413	NOT
ZNF729	3.160901	-2.5025022	1.063793	-2.35243414	0.018651	0.109572	NOT
STC2	670.6118	0.86041399	0.365776	2.35229665	0.018658	0.109588	NOT
UCHL1	180.103	1.32857334	0.564832	2.352155459	0.018665	0.109606	NOT
KB-1674E1	2.070636	-0.9674245	0.411384	-2.35163243	0.018691	0.10971	NOT
FAM46A	1156.257	-0.5882572	0.250159	-2.35153052	0.018696	0.10971	NOT
C11orf85	14.18086	1.75455244	0.746148	2.351480919	0.018699	0.10971	NOT
RP11-85B7	3.842336	1.45165657	0.617353	2.351418919	0.018702	0.10971	NOT
TBC1D17	2662.503	-0.3699305	0.157329	-2.35131237	0.018707	0.10971	NOT
ILF3-AS1	642.6457	-0.4769056	0.20283	-2.35125619	0.01871	0.10971	NOT
NRG3	27.04613	1.51025084	0.642321	2.351239204	0.018711	0.10971	NOT
RPL31P63	4.360474	1.03046706	0.43829	2.351110058	0.018717	0.109725	NOT
RP4-565E6	30.57548	1.26429913	0.537811	2.350823643	0.018732	0.109785	NOT
ABCC9	1244.392	-0.9477337	0.403241	-2.35029344	0.018759	0.109918	NOT

TYMS	1360.352	0.73923554	0.314547	2.350158171	0.018765	0.109934	NOT
STEAP3-AS	8.803706	-0.8763689	0.372959	-2.34977329	0.018785	0.110024	NOT
INMT	431.807	-1.1110254	0.472926	-2.34925846	0.018811	0.11015	NOT
PTGES3P3	32.05921	0.53970022	0.229739	2.349188944	0.018814	0.11015	NOT
RP11-152N	36.12716	0.59835727	0.254754	2.348766965	0.018836	0.110251	NOT
RP11-145M	4.625921	1.95775876	0.833584	2.348605219	0.018844	0.110275	NOT
RPL7	25469.29	0.59806212	0.254662	2.348452751	0.018852	0.110296	NOT
PARM1	423.6626	-0.9427178	0.401452	-2.34827322	0.018861	0.110326	NOT
RP11-737(	6.854165	-0.8181235	0.34846	-2.34782518	0.018883	0.110435	NOT
RPF1	1112.756	0.36225381	0.154333	2.347215163	0.018914	0.110592	NOT
RP11-184M	2.543361	1.17643198	0.50124	2.347045069	0.018923	0.110618	NOT
SRD5A3-AS	25.75261	-0.530518	0.226045	-2.34695498	0.018928	0.110621	NOT
RP11-192M	3.190181	-1.0308007	0.439243	-2.34676819	0.018937	0.110653	NOT
OTUD6B	527.4435	0.46804611	0.199456	2.346611503	0.018945	0.110676	NOT
ATP1A1-AS	97.72497	0.47215345	0.201215	2.346511084	0.01895	0.110682	NOT
ACADS	4324.434	-0.6259263	0.266758	-2.34642424	0.018955	0.110684	NOT
RTKN	4065.38	0.46650126	0.198835	2.346172715	0.018967	0.110735	NOT
RAET1E	8.586834	0.87294297	0.372115	2.345898335	0.018981	0.110792	NOT
BCL9	1278.309	0.57058461	0.243311	2.345080398	0.019023	0.111012	NOT
CENPO	485.4402	0.64456349	0.274879	2.344902537	0.019032	0.111041	NOT
NT5CP1	2.744063	-1.0262028	0.437688	-2.34460057	0.019047	0.111094	NOT
RP11-350N	4.592746	1.9941731	0.850553	2.344561956	0.019049	0.111094	NOT
QSOX2	965.6946	0.39796303	0.169755	2.344341039	0.019061	0.111132	NOT
KIAA0355	1030.249	-0.3672398	0.156654	-2.34427599	0.019064	0.111132	NOT
AJAP1	20.51175	1.41732084	0.604636	2.344089796	0.019074	0.111157	NOT
MFAP1	1115.997	0.2611662	0.111418	2.34402019	0.019077	0.111157	NOT
CTD-2517M	98.62431	-0.8021177	0.342215	-2.34390268	0.019083	0.111157	NOT
RP11-111A	6.052636	-0.6224735	0.265575	-2.3438699	0.019085	0.111157	NOT
RP11-799D	41.91834	-0.6882032	0.293636	-2.34373132	0.019092	0.111175	NOT
SLC52A1	12.79926	-1.0473983	0.446914	-2.34362482	0.019097	0.111177	NOT
RWDD4	321.984	-0.3043744	0.129877	-2.34356263	0.019101	0.111177	NOT
ST8SIA6	6.467971	-1.2736397	0.543501	-2.34339848	0.019109	0.111203	NOT
JAKMIP2-A	8.241785	-1.8414115	0.785829	-2.34327218	0.019115	0.111204	NOT
RP11-345J	157.7285	0.47201661	0.201438	2.343233968	0.019117	0.111204	NOT
DHX9	5528.717	0.30439394	0.129921	2.342913988	0.019134	0.11126	NOT
RP11-374F	2.935678	-1.1407291	0.486891	-2.34288583	0.019135	0.11126	NOT
TECPR2	763.1996	-0.418454	0.178618	-2.34273701	0.019143	0.111281	NOT
RP11-573(	3.154932	1.01542426	0.43346	2.342603206	0.01915	0.111297	NOT
BTN3A3	1044.224	-0.7045922	0.300816	-2.34227178	0.019167	0.111368	NOT
CALCOCO1	2331.819	-0.3747961	0.160019	-2.34220366	0.01917	0.111368	NOT
TMEM51-AS	27.41329	1.24802199	0.532916	2.341871909	0.019187	0.111421	NOT
TMEM130	30.24899	1.42788048	0.609719	2.341867446	0.019188	0.111421	NOT
RP1-292B1	14.40411	0.81900316	0.349768	2.341560911	0.019203	0.111488	NOT
RP11-167N	1.229633	2.04093787	0.871614	2.341560326	0.019203	NA	NA
CYP2W1	23.57004	1.23188891	0.526247	2.340893158	0.019238	0.111647	NOT
RP11-597D	22.51796	-0.8815881	0.376607	-2.34086969	0.019239	0.111647	NOT
RPS7P10	35.72613	0.67478384	0.28829	2.34064089	0.019251	0.111692	NOT
CSTL1	5.36611	1.75386813	0.749375	2.340442219	0.019261	0.111726	NOT
STX6	1137.982	0.39461306	0.168625	2.340178238	0.019275	0.111726	NOT

EEF1E1P1	5.614751	0.98834474	0.422367	2.340015428	0.019283	0.111726	NOT
HNRNPA3P5	8.548696	0.68342241	0.292062	2.339989408	0.019284	0.111726	NOT
IMMP2L	807.5649	-0.5818323	0.248655	-2.33991758	0.019288	0.111726	NOT
UBE2V2	2616.179	0.42608514	0.182105	2.339782236	0.019295	0.111726	NOT
NRP2	781.9885	-0.6181405	0.264209	-2.33958787	0.019305	0.111726	NOT
RPGRIP1L	140.5782	0.49779509	0.212773	2.339563111	0.019306	0.111726	NOT
APLNR	1072.793	-0.8157376	0.348676	-2.3395319	0.019308	0.111726	NOT
RP11-500C	21.62247	-0.5994162	0.256213	-2.33952241	0.019308	0.111726	NOT
C17orf107	214.301	-0.64228	0.274536	-2.33950841	0.019309	0.111726	NOT
RP11-675F	1.448189	2.86037592	1.222641	2.339506716	0.019309	0.111726	NOT
ZNF30-AS1	2.774278	-1.0667708	0.455984	-2.33949076	0.01931	0.111726	NOT
SMYD1	1.964181	2.04227073	0.872995	2.339384891	0.019316	0.111734	NOT
ACO1	8422.117	-0.6182966	0.264334	-2.3390763	0.019331	0.111802	NOT
FBLN1	3375.534	1.07135364	0.458047	2.338958001	0.019338	0.111814	NOT
COL10A1	65.23712	1.63479143	0.699086	2.338469765	0.019363	0.111936	NOT
RP11-435F	15.28828	0.7238097	0.309555	2.338223817	0.019376	0.11197	NOT
ESRP2	2652.246	-0.7028069	0.300577	-2.33819551	0.019377	0.11197	NOT
PLEKHG1	485.1052	-0.5607557	0.239866	-2.33778711	0.019398	0.112056	NOT
GP2	89.84296	-2.1516164	0.92038	-2.3377491	0.0194	0.112056	NOT
RAD51D	515.2692	0.3801523	0.162662	2.337063845	0.019436	0.112176	NOT
BPGM	741.2369	0.34844623	0.149096	2.337062815	0.019436	0.112176	NOT
ELK3	1097.11	-0.5185381	0.221879	-2.33702857	0.019438	0.112176	NOT
CRY2	3358.536	-0.5502258	0.235443	-2.33698247	0.01944	0.112176	NOT
MEX3D	717.1461	0.47416837	0.2029	2.336954499	0.019442	0.112176	NOT
RNPS1P1	4.308655	0.93434356	0.399834	2.33683064	0.019448	0.112183	NOT
H2AFY2	1020.605	1.03817964	0.44428	2.336770435	0.019451	0.112183	NOT
HSP90B2P	64.82492	-0.7323837	0.313458	-2.33646313	0.019467	0.112251	NOT
ARHGAP1	3974.029	0.27975517	0.11975	2.336163047	0.019483	0.112309	NOT
MRPL42	1898.102	0.26681326	0.114212	2.336113502	0.019485	0.112309	NOT
RP11-242J	240.4056	1.6571292	0.709399	2.335963162	0.019493	0.112315	NOT
TMEM127	5307.59	-0.2521432	0.107941	-2.33593216	0.019495	0.112315	NOT
CTB-41I6.	2.165038	-0.9649304	0.413128	-2.3356714	0.019508	0.11233	NOT
ZBTB11-AS	107.2158	0.46111947	0.197426	2.335660406	0.019509	0.11233	NOT
ZNF823	203.3741	-0.3268964	0.13996	-2.33564553	0.01951	0.11233	NOT
APITD1	117.3138	0.47365004	0.202799	2.335562082	0.019514	0.112331	NOT
ECSIT	2675.12	-0.439607	0.188238	-2.33537531	0.019524	0.112363	NOT
RP11-430E	37.51341	-1.1242609	0.481431	-2.33524594	0.019531	0.112378	NOT
AC018816.	26.24629	-1.010793	0.432867	-2.33511049	0.019538	0.112384	NOT
AC073283.	6.811986	1.00378461	0.429873	2.335069974	0.01954	0.112384	NOT
ACKR2	507.0238	-1.0082151	0.431861	-2.33458354	0.019565	0.112457	NOT
DNAI2	1.181206	-1.1770726	0.504193	-2.33456847	0.019566	NA	NA
AKNAD1	2.6197	1.5828877	0.678032	2.334530724	0.019568	0.112457	NOT
THOC5	1343.724	0.40553081	0.17371	2.334529162	0.019568	0.112457	NOT
RPS23	20348.85	0.48447815	0.207529	2.334508945	0.019569	0.112457	NOT
LTN1	980.3859	-0.4722685	0.202316	-2.33431574	0.019579	0.112491	NOT
RP11-182I	41.893	-0.5136588	0.220091	-2.33384657	0.019604	0.112603	NOT
CEP162	233.9525	0.45219522	0.19376	2.333787263	0.019607	0.112603	NOT
RIMBP3C	1.833209	1.51301605	0.648385	2.333514929	0.019621	0.112661	NOT
AFG3L1P	289.7143	-0.3744483	0.160482	-2.33327068	0.019634	0.112669	NOT

OR2H1	1.577058	2.96057212	1.268862	2.333249431	0.019635	0.112669	NOT
RP11-101(	4.719878	0.75334433	0.322875	2.333240664	0.019636	0.112669	NOT
DYNC1I2	1854.383	0.36974144	0.158472	2.333171738	0.019639	0.112669	NOT
4-Mar	13.3557	-0.8429045	0.361372	-2.3325148	0.019674	0.112843	NOT
RP11-285J	7.298726	0.88781921	0.380675	2.332226556	0.019689	0.112906	NOT
CTD-3065I	2.261842	-1.1354175	0.486974	-2.33157622	0.019723	0.113044	NOT
ACTL8	41.10428	2.0208722	0.866744	2.331567394	0.019723	0.113044	NOT
ZDHHC8P1	11.20221	1.65038352	0.707854	2.331530072	0.019725	0.113044	NOT
DCAF11	6822.541	-0.5489875	0.235477	-2.33138587	0.019733	0.113052	NOT
NDUFB10	4532.486	-0.5200682	0.223076	-2.33134669	0.019735	0.113052	NOT
PFN2	1107.589	1.01751667	0.436474	2.331219077	0.019742	0.113066	NOT
RP11-894I	32.1555	-0.6949044	0.298132	-2.33086031	0.019761	0.113151	NOT
MTX2	1214.458	0.30282889	0.129928	2.330752518	0.019766	0.113159	NOT
FUCA1	3439.088	-0.4836795	0.207555	-2.33037157	0.019787	0.113237	NOT
GLYATL1	3957.763	-1.1616366	0.498484	-2.3303393	0.019788	0.113237	NOT
RP11-510(	158.0822	0.68259128	0.292943	2.330115572	0.0198	0.113276	NOT
RRP12	1789.173	0.46757013	0.200669	2.330052261	0.019803	0.113276	NOT
PHLDA3	1208.884	-1.0821891	0.46447	-2.32994624	0.019809	0.113284	NOT
MAPK8IP2	563.4546	1.05393677	0.452413	2.329588357	0.019828	0.113368	NOT
COMMD7	2142.638	0.35604952	0.152856	2.329308767	0.019843	0.113429	NOT
SOX9	2117.588	0.99173143	0.425903	2.328537931	0.019884	0.113635	NOT
LA16c-38(	47.43004	1.01943479	0.437825	2.328407901	0.01989	0.113635	NOT
OSGEPL1	407.3745	0.29237281	0.125572	2.328326428	0.019895	0.113635	NOT
HSD17B12	3872.058	0.48459824	0.208133	2.328311818	0.019896	0.113635	NOT
TMEM64	2295.795	0.66129528	0.284098	2.32770178	0.019928	0.113796	NOT
FAM103A2I	2.668593	1.53058984	0.657777	2.326911614	0.01997	0.113991	NOT
HIST2H3D	2.702985	-1.0971835	0.471521	-2.32690138	0.019971	0.113991	NOT
CLRN3	674.7245	-1.4637903	0.629174	-2.32652768	0.01999	0.114081	NOT
RP1	1.776677	-1.4516939	0.624003	-2.32642262	0.019996	0.114089	NOT
B4GALT5	2805.363	0.46099994	0.198203	2.325897764	0.020024	0.114224	NOT
NR3C1	3320.386	-0.4339059	0.186568	-2.32572304	0.020033	0.114254	NOT
BSN-AS2	10.26011	1.19314487	0.513072	2.325490506	0.020046	0.1143	NOT
NIP7	878.8251	0.34958942	0.150348	2.325200432	0.020061	0.114352	NOT
RPL23A	16605.97	0.52748227	0.226858	2.325162224	0.020063	0.114352	NOT
C12orf73	433.5457	0.46218848	0.198784	2.325084705	0.020067	0.114352	NOT
C3orf33	186.6159	0.36601877	0.157431	2.324941193	0.020075	0.114363	NOT
RBMX	4261.089	0.26148376	0.112475	2.324819906	0.020082	0.114363	NOT
TMTC4	994.1037	0.53611429	0.230606	2.324810892	0.020082	0.114363	NOT
RP11-552I	3.166348	-0.9139812	0.393166	-2.3246698	0.02009	0.114382	NOT
SLC2A5	570.7842	1.37703207	0.59241	2.324456159	0.020101	0.114413	NOT
AK4P1	37.6649	0.89306509	0.384211	2.324411096	0.020103	0.114413	NOT
AAMDC	917.9845	-0.4952568	0.213099	-2.32406944	0.020122	0.114476	NOT
CFP	171.9977	-0.8874977	0.381894	-2.32393415	0.020129	0.114476	NOT
KTI12	382.0358	0.3634968	0.156418	2.323882913	0.020132	0.114476	NOT
KCNJ5	244.8246	-0.9047533	0.38934	-2.32381578	0.020135	0.114476	NOT
RP11-815M	9.94719	0.94322774	0.405897	2.323811779	0.020136	0.114476	NOT
FBN2	27.95538	1.0424549	0.44865	2.323536462	0.02015	0.114536	NOT
HIST1H2BI	6.889567	1.35831966	0.58463	2.323384593	0.020159	0.114558	NOT
APOE	358459.4	-0.7800799	0.335806	-2.32300506	0.020179	0.11465	NOT

HSPD1P11	2.544569	-1.0933281	0.470693	-2.32280714	0.02019	0.114686	NOT
RPAP3	962.0911	0.26645966	0.114725	2.32260391	0.0202	0.114715	NOT
ARMS2	2.896383	-1.1357163	0.489001	-2.32252582	0.020205	0.114715	NOT
ADAMTS9-A	9.096837	-0.7859656	0.33845	-2.32225215	0.020219	0.114715	NOT
LINC00639	16.56531	-0.7679323	0.330698	-2.32215436	0.020225	0.114715	NOT
ZNF778	226.2481	-0.4463115	0.192198	-2.3221479	0.020225	0.114715	NOT
RP11-173M	9.65973	0.7707138	0.3319	2.322129759	0.020226	0.114715	NOT
SEC62	6434.718	-0.3280053	0.141255	-2.32208001	0.020229	0.114715	NOT
ACTG1	80668.51	0.42550303	0.183256	2.321908219	0.020238	0.114715	NOT
CASP14	2.559011	2.72992382	1.17575	2.321857667	0.020241	0.114715	NOT
ACTR3	5193.08	0.31421522	0.135331	2.321819665	0.020243	0.114715	NOT
AC010761.	38.15488	0.66567161	0.286706	2.321792591	0.020244	0.114715	NOT
ITPR2	5580.198	-0.7219257	0.310938	-2.32176916	0.020245	0.114715	NOT
FBXL3	1272.74	-0.4158414	0.179145	-2.32125172	0.020273	0.114849	NOT
PCP4L1	215.2663	-1.3890649	0.598546	-2.32073402	0.020301	0.114984	NOT
ELFN1-AS1	39.05417	1.8546563	0.799271	2.320434239	0.020317	0.115051	NOT
RP5-1170K	11.83885	-1.4293027	0.616098	-2.31992575	0.020345	0.115183	NOT
INHBC	2648.451	-1.0520547	0.453534	-2.31968331	0.020358	0.115233	NOT
RP11-184E	2.721719	3.05759059	1.31838	2.319203403	0.020384	0.115356	NOT
FAM91A1	3010.166	0.36812048	0.158754	2.318810515	0.020405	0.115427	NOT
UBAC2-AS1	67.63528	0.6639179	0.286324	2.31876508	0.020408	0.115427	NOT
SCIN	65.64374	1.09809694	0.473624	2.318498944	0.020422	0.115427	NOT
CTC-820M8	4.858434	1.04985681	0.452821	2.318478895	0.020423	0.115427	NOT
MT-TP	2160.495	-0.5992018	0.258447	-2.318472	0.020424	0.115427	NOT
PCAT29	22.16495	-1.2781148	0.551278	-2.31845643	0.020425	0.115427	NOT
ZNF34	294.8013	0.41917015	0.1808	2.318425043	0.020426	0.115427	NOT
SLC22A24	4.228935	-1.3736091	0.592519	-2.31825389	0.020436	0.115455	NOT
MICALL1	1269.505	0.48305906	0.208385	2.318108767	0.020443	0.115476	NOT
RP5-1092A	11.20613	-1.748283	0.754256	-2.31789123	0.020455	0.115518	NOT
ABCC4	869.072	0.9289823	0.400818	2.31771717	0.020465	0.115548	NOT
ZNF556	11.77604	1.38614777	0.598107	2.317559848	0.020473	0.11555	NOT
MTMR10	1737.919	-0.4168358	0.179862	-2.31752775	0.020475	0.11555	NOT
RP11-797H	7.079992	-1.0532611	0.454498	-2.31741513	0.020481	0.11555	NOT
AC007365.	10.20345	0.82107934	0.354315	2.31737005	0.020484	0.11555	NOT
PCDHB12	32.79388	-0.8157767	0.352036	-2.31731154	0.020487	0.11555	NOT
RGS6	8.372528	-0.9538375	0.411627	-2.31723982	0.020491	0.11555	NOT
SYN2	9.847057	1.23886412	0.53473	2.316803817	0.020514	0.115633	NOT
AFF1	2566.223	-0.5076231	0.219113	-2.31672122	0.020519	0.115633	NOT
RP4-794I6	15.50813	-1.1037452	0.476452	-2.31659264	0.020526	0.115633	NOT
PSMD1	5032.292	0.25421093	0.109736	2.316576082	0.020527	0.115633	NOT
LINC01353	7.234443	1.10545142	0.477192	2.316574904	0.020527	0.115633	NOT
RP11-173M	7.268164	1.03894628	0.448498	2.316501519	0.020531	0.115633	NOT
RNF19A	3020.166	0.48674144	0.210164	2.316003855	0.020558	0.115762	NOT
ASPH	12597.75	0.77024486	0.332609	2.315764658	0.020571	0.115811	NOT
AC138035.	9.634072	-0.8716114	0.376426	-2.31549339	0.020586	0.115857	NOT
AC010127.	6.449028	1.8675226	0.806545	2.315460064	0.020588	0.115857	NOT
RPL15P3	160.1088	0.65450488	0.282692	2.315256417	0.020599	0.115896	NOT
DTNB	565.796	0.36855854	0.159228	2.314653748	0.020632	0.116049	NOT
RP11-153K	13.98429	-1.2535304	0.541575	-2.31460153	0.020635	0.116049	NOT

RP11-383I	5.456769	0.92378652	0.399159	2.314332537	0.020649	0.116108	NOT
PGD	7712.586	0.65980315	0.285124	2.314093082	0.020663	0.116135	NOT
HSF1	5492.959	0.50377778	0.2177	2.314087102	0.020663	0.116135	NOT
SAYSD1	948.6543	0.42655712	0.184376	2.313522592	0.020694	0.116285	NOT
RBFOX2	2747.973	-0.4215113	0.182229	-2.31308195	0.020718	0.116397	NOT
RP11-981(	36.60752	-0.8997838	0.389011	-2.31300042	0.020723	0.116398	NOT
TTYH3	4937.223	0.61261157	0.264882	2.312773298	0.020735	0.116445	NOT
SLIT3	549.7879	-0.8976629	0.388408	-2.31113363	0.020825	0.116928	NOT
KLHDC8A	23.48682	1.37944255	0.597016	2.31056207	0.020857	0.117042	NOT
TMEM159	171.9254	0.91661947	0.396713	2.310533219	0.020859	0.117042	NOT
RP11-96K1	28.02137	0.62976473	0.272563	2.310529914	0.020859	0.117042	NOT
PHYHD1	1127.353	-1.1572343	0.50091	-2.31026177	0.020874	0.117101	NOT
RP11-325K	14.57783	-0.6817433	0.295172	-2.30964895	0.020908	0.117222	NOT
HNRNPKP1	23.57704	0.5387235	0.23327	2.309441813	0.020919	0.117222	NOT
RNU6-137F	2.045155	0.99607729	0.431308	2.309431877	0.02092	0.117222	NOT
SLC4A1AP	1229.992	0.24905175	0.107842	2.309407488	0.020921	0.117222	NOT
AC093063.	1.676007	2.87491315	1.2449	2.309352392	0.020924	0.117222	NOT
ABHD2	12805.31	-0.7072107	0.306241	-2.30932723	0.020925	0.117222	NOT
SEC1P	5.839251	-0.8033903	0.347897	-2.3092732	0.020928	0.117222	NOT
DYNC1I1	166.9035	-1.167743	0.50568	-2.30925098	0.02093	0.117222	NOT
EED	622.3942	0.26807643	0.116101	2.30899461	0.020944	0.117225	NOT
RP11-701F	4.60011	-1.0014554	0.433722	-2.30898097	0.020945	0.117225	NOT
HSPE1P6	6.578172	1.0148992	0.439548	2.308963561	0.020946	0.117225	NOT
RP11-161M	24.95879	-0.8942461	0.387299	-2.30892814	0.020948	0.117225	NOT
RP11-802I	4.709966	0.76141194	0.329803	2.308690065	0.020961	0.117254	NOT
TGS1	698.9345	0.36449116	0.157879	2.308678393	0.020961	0.117254	NOT
CDPF1	478.6962	0.4540824	0.196694	2.308567301	0.020968	0.117265	NOT
RP13-270F	13.9045	-0.5354578	0.231973	-2.30827882	0.020984	0.11733	NOT
IL18R1	206.556	-0.8584586	0.371975	-2.30783795	0.021008	0.117443	NOT
GALR1	1.754344	-1.6058443	0.69589	-2.30761151	0.021021	0.117489	NOT
PRKCE	698.7165	-0.7115183	0.308355	-2.30746416	0.021029	0.117511	NOT
CLTA	6212.46	0.40689406	0.176369	2.307060622	0.021051	0.117569	NOT
LMO1	1.954652	2.56193099	1.110481	2.307045798	0.021052	0.117569	NOT
CTB-50L17	114.5363	-0.9403007	0.407578	-2.30704318	0.021052	0.117569	NOT
PTRH2	1363.599	0.45117723	0.195578	2.306888428	0.021061	0.117593	NOT
MMP3	9.509691	1.83351676	0.794892	2.306623826	0.021076	0.117652	NOT
RP11-544M	3.014277	-1.5236523	0.660659	-2.30626054	0.021096	0.117741	NOT
RP11-400K	15.14828	1.32410779	0.574202	2.305996544	0.021111	0.117799	NOT
TIAM2	245.5113	0.66607665	0.288874	2.305770439	0.021123	0.11783	NOT
AP001469.	5.958297	-0.9187128	0.398446	-2.30574167	0.021125	0.11783	NOT
GRIK3	5.589399	-1.5528055	0.67349	-2.30561121	0.021132	0.117846	NOT
GALNT5	8.809868	1.31948732	0.57233	2.305464027	0.021141	0.117862	NOT
SLC25A10	3586.765	-0.6273018	0.272101	-2.30540457	0.021144	0.117862	NOT
INS-IGF2	20.81477	-2.5806063	1.119414	-2.30531938	0.021149	0.117864	NOT
EVL	2488.161	-0.6115443	0.26533	-2.30484747	0.021175	0.117987	NOT
FAS	627.9886	-0.7416134	0.321781	-2.3047158	0.021182	0.118004	NOT
PYCARD	964.383	0.94618045	0.4106	2.304385946	0.021201	0.118068	NOT
CLCN3	2345.131	-0.4428333	0.192172	-2.3043581	0.021203	0.118068	NOT
RP11-134K	6.066076	-0.8114578	0.352177	-2.30411954	0.021216	0.118118	NOT

RP11-234I	1.394353	-1.1464362	0.497793	-2.30303719	0.021277	NA	NA
CLN3	1893.137	0.53632381	0.232884	2.302967843	0.021281	0.118454	NOT
C16orf72	1929.114	-0.342272	0.148631	-2.30282444	0.021289	0.118474	NOT
NNMT	19495.54	-1.2496157	0.542764	-2.30231875	0.021317	0.11859	NOT
CD109	942.7125	1.04421802	0.453555	2.302298678	0.021318	0.11859	NOT
RANBP10	1581.242	-0.473182	0.205533	-2.30222464	0.021323	0.11859	NOT
GACAT3	2.481976	3.27146269	1.421139	2.301999792	0.021335	0.118627	NOT
PECAM1	4591.588	-0.425989	0.185056	-2.30195019	0.021338	0.118627	NOT
RP11-83M1	4.344796	-1.7431774	0.757318	-2.30177715	0.021348	0.118657	NOT
TTBK2	358.5795	-0.3898676	0.169395	-2.30152518	0.021362	0.118712	NOT
ARL8B	3401.898	0.27085987	0.117697	2.301338983	0.021372	0.118746	NOT
RP4-737E2	4.349883	1.88993997	0.8215	2.300597786	0.021414	0.118954	NOT
RP11-67L3	10.37014	0.7855182	0.341455	2.300505921	0.02142	0.118959	NOT
ITCH	3066.414	-0.4211308	0.183093	-2.30009845	0.021443	0.119038	NOT
RP11-138I	8.726486	0.9504744	0.413232	2.300098218	0.021443	0.119038	NOT
NECAB1	61.59226	1.00095677	0.435205	2.299968622	0.02145	0.119055	NOT
FAM181B	2.256076	1.53310978	0.666644	2.299744267	0.021463	0.119101	NOT
HEATR1	1496.049	0.48294413	0.210027	2.299434303	0.02148	0.119174	NOT
AC098824.	1.520176	-1.0865613	0.47259	-2.29916402	0.021496	0.119235	NOT
RNF139-AS	61.18675	0.62636328	0.272459	2.298926274	0.021509	0.119285	NOT
C9orf40	293.4149	0.55327902	0.240712	2.298506097	0.021533	0.119393	NOT
OPRK1	1.883772	2.51870811	1.095914	2.298271882	0.021546	0.119443	NOT
SRSF2	4337.292	0.25283831	0.110026	2.297988952	0.021562	0.119507	NOT
IFT80	382.3762	0.7034381	0.306139	2.297776036	0.021575	0.119528	NOT
AC007128.	8.813609	2.03894554	0.887359	2.29776957	0.021575	0.119528	NOT
RP11-86H7	17.21641	1.14341503	0.497649	2.29763557	0.021583	0.119546	NOT
ZNF333	278.2185	-0.3678873	0.160129	-2.29744583	0.021593	0.119581	NOT
GTF3C6	1848.501	0.51922951	0.226011	2.297362779	0.021598	0.119583	NOT
PGBD4	122.6577	-0.3862522	0.168191	-2.29650336	0.021647	0.119819	NOT
CTD-2256F	5.006945	-1.4752892	0.642418	-2.29646197	0.021649	0.119819	NOT
SULT1C2	1185.266	1.16617257	0.507952	2.295830904	0.021686	0.119988	NOT
RP11-532I	2.996354	-1.071635	0.466805	-2.29568101	0.021694	0.119988	NOT
TLR3	429.9793	-0.7543097	0.328586	-2.2956237	0.021697	0.119988	NOT
MAPK3	1931.108	0.36168013	0.157558	2.295542886	0.021702	0.119988	NOT
CPEB2-AS1	4.060973	-0.8503749	0.370447	-2.29553885	0.021702	0.119988	NOT
PCDHB14	161.2495	-0.844385	0.367968	-2.29472248	0.021749	0.120213	NOT
E2F1	1231.582	0.69448855	0.302652	2.294675109	0.021752	0.120213	NOT
EAF2	89.46669	0.46977078	0.204731	2.294578436	0.021757	0.120219	NOT
RP11-505K	71.96713	-0.7765849	0.338456	-2.29449139	0.021762	0.120222	NOT
CPHL1P	45.30875	1.2642397	0.551094	2.294053979	0.021787	0.120336	NOT
CSNK2A1	3100.35	0.34473098	0.150281	2.293914798	0.021795	0.120338	NOT
FTL	568487.7	0.77564354	0.338134	2.293895552	0.021796	0.120338	NOT
TBC1D22A	1818.564	0.37270393	0.162485	2.293768282	0.021804	0.120353	NOT
RPL29P11	11.00198	0.71993287	0.313876	2.29368661	0.021809	0.120355	NOT
RP11-472E	10.95976	-0.5944967	0.259198	-2.29360153	0.021813	0.120357	NOT
RP11-449J	40.82299	1.23559301	0.538758	2.293408333	0.021825	0.120394	NOT
A2M	98584.52	-0.8250252	0.35978	-2.29313662	0.02184	0.120443	NOT
SAMD1	1905.512	0.43656272	0.190381	2.293099227	0.021842	0.120443	NOT
HEPH	238.1477	-0.711691	0.31038	-2.29297009	0.02185	0.12046	NOT

ADCY8	24.02091	2.16790362	0.945687	2.292410698	0.021882	0.120613	NOT
SLC28A2	50.19287	1.17947254	0.514559	2.292202647	0.021894	0.120655	NOT
NUDT16P1	370.3052	-0.9533005	0.415982	-2.29168841	0.021924	0.120794	NOT
RP5-890E1	30.1304	0.54883275	0.2395	2.291580775	0.02193	0.120803	NOT
TEX30	911.6693	0.59193769	0.258321	2.291481699	0.021936	0.12081	NOT
PTP4A2P1	27.24243	-0.6886148	0.300527	-2.29135783	0.021943	0.120825	NOT
GTSE1-AS1	8.920634	0.68266444	0.297941	2.291275821	0.021947	0.120827	NOT
DMGDH	4581.198	-0.9563664	0.417429	-2.29108619	0.021958	0.120863	NOT
RP11-130F	11.01163	-0.6094608	0.266063	-2.29066295	0.021983	0.120973	NOT
EDNRB	1039.687	-0.627968	0.274204	-2.29014936	0.022013	0.121112	NOT
RWDD4P2	32.55741	-0.3965081	0.173164	-2.28978903	0.022034	0.121199	NOT
FAN1	632.442	-0.5054617	0.220752	-2.28972355	0.022037	0.121199	NOT
KIN	693.3291	0.33831599	0.14776	2.289630522	0.022043	0.121204	NOT
ZNF761	454.018	0.63212292	0.276124	2.289270822	0.022064	0.121294	NOT
CAMKK1	94.78033	-0.6078747	0.265541	-2.28919397	0.022068	0.121294	NOT
RP11-950	6.661737	1.04131254	0.454908	2.289061407	0.022076	0.121312	NOT
KCNQ3	39.23326	0.85730705	0.374631	2.288405407	0.022114	0.121455	NOT
RP11-5360	7.892443	-0.5378737	0.235044	-2.28839262	0.022115	0.121455	NOT
SGSM1	103.8796	1.03912073	0.454086	2.288377627	0.022116	0.121455	NOT
ELFN2	387.6376	-1.3729462	0.60001	-2.28820723	0.022125	0.121455	NOT
WASIR2	3.749157	1.5777241	0.689519	2.288150554	0.022129	0.121455	NOT
CLEC3B	327.3061	-0.8163872	0.356796	-2.28810339	0.022132	0.121455	NOT
KCP	75.89689	0.94609071	0.413492	2.288050886	0.022135	0.121455	NOT
RP11-216I	3.30541	1.14568763	0.500738	2.28799672	0.022138	0.121455	NOT
CASC10	1074.364	0.74333011	0.324904	2.287842727	0.022147	0.12148	NOT
DNAJC12	637.1321	-1.0634308	0.464953	-2.28717707	0.022185	0.121668	NOT
OAF	10613.94	-0.5632072	0.246265	-2.28699628	0.022196	0.121696	NOT
RP11-3950	5.093914	-0.6745654	0.294965	-2.28693493	0.0222	0.121696	NOT
DSG3	3.111714	2.83032612	1.237678	2.2868033	0.022207	0.121714	NOT
FAM120B	1066.119	-0.3516938	0.153799	-2.28670322	0.022213	0.12172	NOT
NDST4	1.536397	3.12393973	1.366184	2.286617345	0.022218	0.12172	NOT
STK38	1930.979	0.35558461	0.155511	2.28655252	0.022222	0.12172	NOT
ANK3	354.4845	-0.986511	0.431455	-2.28647697	0.022226	0.12172	NOT
FAM131A	465.6104	0.38343243	0.167707	2.286316977	0.022236	0.121747	NOT
TP53I11	1957.518	-0.5606668	0.245247	-2.28613383	0.022246	0.121781	NOT
HS6ST3	4.351421	-1.4985683	0.655559	-2.28594019	0.022258	0.121816	NOT
AP000351.	40.55333	-0.9884954	0.432437	-2.28586891	0.022262	0.121816	NOT
MESP1	145.0132	0.71559396	0.313106	2.285466456	0.022285	0.121921	NOT
RP11-437F	10.99801	-0.5448526	0.238416	-2.2853017	0.022295	0.121949	NOT
RNASE2	30.83626	1.35072516	0.591277	2.284421329	0.022347	0.122207	NOT
MFSD2A	2429.605	-1.5395005	0.674033	-2.28401247	0.022371	0.122314	NOT
ATAD3A	1289.756	0.48720207	0.213326	2.283836653	0.022381	0.122345	NOT
ELOVL7	477.6052	1.23445788	0.540575	2.283599519	0.022395	0.122397	NOT
GINS3	223.5411	0.58056045	0.25424	2.283511484	0.0224	0.122401	NOT
RP11-218F	3.254417	1.40011405	0.613206	2.28326849	0.022415	0.122454	NOT
RBMS3	200.9039	-0.7250532	0.317582	-2.28304095	0.022428	0.122503	NOT
PAXBP1-AS	45.92547	-0.4366044	0.191285	-2.28248512	0.022461	0.122657	NOT
IGHE	14.28389	1.72847266	0.757335	2.282307954	0.022471	0.122689	NOT
FRMD6-AS1	6.810744	0.77399184	0.339168	2.282031794	0.022487	0.122753	NOT

HGFAC	4183.097	-1.5790428	0.691988	-2.28189426	0.022496	0.122773	NOT
EIF2B5	2293.789	0.2391316	0.104811	2.281560478	0.022515	0.122856	NOT
EBF2	76.96993	-0.742182	0.325341	-2.28123996	0.022534	0.122935	NOT
KCNE4	211.5137	-0.7834235	0.343448	-2.2810526	0.022545	0.12297	NOT
RP11-384K	102.5676	-0.8717404	0.3822	-2.28084878	0.022557	0.123011	NOT
SLC12A1	112.4617	-2.0639978	0.905139	-2.28030984	0.022589	0.12315	NOT
CDKL4	4.119168	1.44040701	0.631692	2.280234981	0.022594	0.12315	NOT
RFT1	1046.883	0.29078956	0.127529	2.280189987	0.022596	0.12315	NOT
RP1-97D16	2.840216	1.74394084	0.764913	2.279920491	0.022612	0.123182	NOT
CTIF	2175.074	-0.4835592	0.212095	-2.27991544	0.022613	0.123182	NOT
RP11-28F1	5.100716	1.04313317	0.457542	2.279861548	0.022616	0.123182	NOT
ARMC1	1955.878	0.35214747	0.154511	2.27910815	0.022661	0.123401	NOT
RP11-98D1	24.08594	0.62915926	0.276091	2.278813199	0.022678	0.123472	NOT
KIAA0895L	319.5046	-0.4916527	0.215799	-2.27828987	0.022709	0.12361	NOT
CPA2	10.05496	2.1222388	0.93155	2.278181161	0.022716	0.12361	NOT
ZBTB2	562.0921	0.30644961	0.134521	2.278083239	0.022722	0.12361	NOT
ZNRD1	806.1938	0.46370497	0.203551	2.278079164	0.022722	0.12361	NOT
AC092667.	16.77881	1.17664528	0.51656	2.277846131	0.022736	0.123647	NOT
EIF1B	1755.876	0.30304975	0.133044	2.277813195	0.022738	0.123647	NOT
CNKSR1	105.1707	1.48298938	0.651305	2.276950994	0.022789	0.123881	NOT
ZNF233	53.06824	0.88485284	0.388615	2.276937325	0.02279	0.123881	NOT
CACNA1S	9.930811	1.23722384	0.54341	2.276776745	0.0228	0.123909	NOT
RP4-725G1	8.006827	1.07170779	0.470755	2.276571069	0.022812	0.123951	NOT
SYNGR2	6840.468	0.38867149	0.170749	2.276269576	0.02283	0.124024	NOT
RNF133	2.786204	1.17731583	0.517235	2.276173405	0.022836	0.12403	NOT
RP11-810F	39.70012	0.522038	0.229376	2.275903848	0.022852	0.124093	NOT
RP11-273F	138.4765	-0.684952	0.300979	-2.27574839	0.022861	0.124119	NOT
SAA2	8117.852	-1.6339705	0.71805	-2.27556693	0.022872	0.124153	NOT
RFC5	788.9863	0.42512931	0.186844	2.275316297	0.022887	0.124201	NOT
RP11-33H1	2.366563	2.22782092	0.979147	2.275266964	0.02289	0.124201	NOT
RPSAP4	4.649035	0.94332387	0.414626	2.275118886	0.022899	0.124205	NOT
RP11-33B1	206.2973	-0.3779955	0.166149	-2.27503478	0.022904	0.124205	NOT
AL022341.	6.920956	-0.8723431	0.383443	-2.27502439	0.022904	0.124205	NOT
RP11-710C	5.906441	-1.018089	0.447661	-2.27424037	0.022952	0.124435	NOT
TNFRSF1A	6956.299	-0.3389432	0.149082	-2.27353791	0.022994	0.12464	NOT
PFKFB1	819.347	-1.0633322	0.467737	-2.27335497	0.023005	0.124674	NOT
CTD-2525F	2.579797	-1.2809156	0.563522	-2.27305242	0.023023	0.124748	NOT
RP11-120D	11.83665	0.68841931	0.302894	2.272808179	0.023038	0.124803	NOT
GALNT18	1126.34	0.65360433	0.28766	2.272141686	0.023078	0.124996	NOT
RP11-5017	71.13369	0.57080884	0.251241	2.271952957	0.023089	0.125016	NOT
YARS2	603.6854	0.30744168	0.135322	2.271927536	0.023091	0.125016	NOT
RP11-14C1	40.33281	-0.775897	0.341544	-2.2717355	0.023102	0.125034	NOT
PHYKPL	1817.03	-0.4451037	0.195932	-2.27172155	0.023103	0.125034	NOT
MYLK	4366.137	-0.6676663	0.293932	-2.27149636	0.023117	0.125055	NOT
ZBTB20-AS	1.669287	-1.6223795	0.714245	-2.27146221	0.023119	0.125055	NOT
RP9P	132.0977	0.56457964	0.248564	2.271364156	0.023125	0.125055	NOT
RP11-626C	3.306433	1.04893323	0.46181	2.271351312	0.023126	0.125055	NOT
SLC35A5	1019.466	-0.3408586	0.150081	-2.27116784	0.023137	0.12509	NOT
ORC4	970.6042	0.32213034	0.141846	2.270986593	0.023148	0.125107	NOT

PA2G4	6070.892	0.39904873	0.175723	2.27089731	0.023153	0.125107	NOT
ZFPM2	91.21711	-0.7038374	0.309939	-2.27088891	0.023154	0.125107	NOT
TMEM56	5504.626	-0.7597073	0.334582	-2.27061508	0.02317	0.125171	NOT
KRT8P8	7.198967	0.90816644	0.399993	2.270453825	0.02318	0.125172	NOT
P2RY4	3.609961	1.65786454	0.730212	2.270387791	0.023184	0.125172	NOT
SLC22A4	92.10108	0.78937055	0.347682	2.27038324	0.023184	0.125172	NOT
ADPRHL1	238.9509	1.01082702	0.445271	2.270136733	0.023199	0.125228	NOT
LINC01564	103.8168	0.95318032	0.419906	2.269986082	0.023208	0.125253	NOT
FNIP2	2321.713	-0.6928235	0.305224	-2.2698834	0.023215	0.125261	NOT
CUEDC1	1148.713	0.48878618	0.215347	2.269761134	0.023222	0.125264	NOT
NEK8	374.7944	0.49222747	0.216867	2.26972157	0.023224	0.125264	NOT
PARD6B	499.9293	0.51430013	0.22661	2.269533768	0.023236	0.125301	NOT
SLC44A4	57.431	0.77138755	0.339932	2.269242598	0.023254	0.125349	NOT
GRPEL1	2565.868	-0.4157132	0.183195	-2.26923458	0.023254	0.125349	NOT
RP11-110(	7.40628	-0.9704766	0.427753	-2.26877745	0.023282	0.125474	NOT
MT1XP1	11.53303	-1.1695699	0.515551	-2.26858191	0.023294	0.125493	NOT
NDRG2	8293.772	-0.5795935	0.2555	-2.26846982	0.023301	0.125493	NOT
CLUH	11593	-0.4292108	0.189207	-2.26846961	0.023301	0.125493	NOT
GGCX	9771.363	-0.4708774	0.207584	-2.268366	0.023307	0.125493	NOT
ZNF185	213.0063	0.63005248	0.277759	2.268340052	0.023308	0.125493	NOT
RAB11B	5273.542	-0.3356795	0.148033	-2.26760117	0.023354	0.125711	NOT
GPR84	18.86873	1.14342438	0.504268	2.267492572	0.02336	0.125721	NOT
SAP30L	1876.703	-0.3695524	0.162985	-2.26739815	0.023366	0.125727	NOT
ZEB2P1	6.016003	1.81455157	0.800311	2.267308974	0.023371	0.125732	NOT
PRR3	607.8076	0.35443396	0.156338	2.267097977	0.023384	0.125776	NOT
RP11-819(	57.8177	-0.4623656	0.203962	-2.2669189	0.023395	0.12581	NOT
GLS2	468.5706	-1.5019986	0.66275	-2.26631137	0.023432	0.125983	NOT
C22orf23	28.03148	0.66672492	0.294198	2.266242129	0.023437	0.125983	NOT
RP11-379I	570.962	-0.5273188	0.232729	-2.26580574	0.023463	0.126101	NOT
GAS6	3597.652	-0.6908438	0.304926	-2.26560889	0.023475	0.126141	NOT
RP11-365(	5.195623	0.80427421	0.355009	2.265502882	0.023482	0.126148	NOT
AC006539.	1.997854	1.72406224	0.761028	2.265437372	0.023486	0.126148	NOT
ZNF691	511.5883	0.34899986	0.15407	2.265196566	0.023501	0.126202	NOT
TCP1	7022.692	0.40257066	0.177737	2.264977482	0.023514	0.126249	NOT
KCNJ14	60.39412	0.70728178	0.312311	2.264670728	0.023533	0.126313	NOT
NDRG3	1588.818	0.45981177	0.20304	2.264632599	0.023535	0.126313	NOT
AC008063.	2.776186	1.36618803	0.603363	2.264288723	0.023556	0.126341	NOT
SMIM14	9086.626	-0.6175397	0.272736	-2.26423654	0.02356	0.126341	NOT
THBS1	4960.302	-0.8178551	0.361213	-2.2641894	0.023562	0.126341	NOT
RP5-1184I	1.483083	-1.1079152	0.489322	-2.26418368	0.023563	0.126341	NOT
LINC0046(	1.740863	2.58831791	1.143165	2.264167954	0.023564	0.126341	NOT
EPHB1	35.5281	-0.6777536	0.29939	-2.26377858	0.023588	0.126444	NOT
RP13-923(	17.93551	0.62173609	0.274716	2.263195291	0.023624	0.126612	NOT
KALRN	1349.364	-0.5226321	0.230934	-2.26311916	0.023628	0.126612	NOT
TMED3	1704.792	0.92472299	0.408629	2.262991695	0.023636	0.126612	NOT
CTC-360G5	88.77829	-0.7852831	0.347015	-2.26296861	0.023638	0.126612	NOT
RP11-278(	38.15244	0.55483363	0.24525	2.262315296	0.023678	0.126778	NOT
ZSCAN12	180.2437	0.47002235	0.207763	2.26230035	0.023679	0.126778	NOT
CCS	2843.574	-0.4966191	0.219525	-2.26223981	0.023683	0.126778	NOT

PGAP2	1647.806	0.33526417	0.148231	2.261763089	0.023712	0.12689	NOT
MFAP3L	1041.9	-1.0410713	0.460295	-2.26174769	0.023713	0.12689	NOT
C15orf27	103.5585	-0.9337144	0.412896	-2.26137971	0.023736	0.126987	NOT
RP11-368I	48.52403	-0.6189529	0.273743	-2.26107598	0.023755	0.127063	NOT
KRT5	32.65254	1.76042755	0.778718	2.26067257	0.02378	0.127171	NOT
RP11-536I	3.091836	-1.0146998	0.448868	-2.26057309	0.023786	0.127179	NOT
ZNF366	45.37224	-0.8347334	0.369315	-2.26022099	0.023808	0.127269	NOT
ADPRHL2	1350.117	0.40872624	0.180843	2.260112098	0.023814	0.127269	NOT
RP4-671O1	3.077178	-0.9358354	0.414073	-2.26007501	0.023817	0.127269	NOT
CYP2C9	16064.86	-1.1944871	0.528538	-2.25998539	0.023822	0.127274	NOT
SESN3	998.8182	-0.881575	0.390127	-2.25971386	0.023839	0.127339	NOT
FHL2	570.3422	-0.7540195	0.333695	-2.25960849	0.023846	0.127339	NOT
CENPE	420.9747	0.76281271	0.337593	2.259560141	0.023849	0.127339	NOT
PDZRN4	8.381738	-1.4814056	0.655694	-2.25929512	0.023865	0.127402	NOT
RP3-449M8	2.215736	1.08527734	0.480398	2.25912336	0.023876	0.127405	NOT
ST8SIA6-A	207.6944	1.66433832	0.736738	2.259062738	0.023879	0.127405	NOT
IGHV1-3	3.082348	2.29509896	1.015952	2.259061476	0.02388	0.127405	NOT
ANKLE2	2324.351	0.31025471	0.137358	2.258732568	0.0239	0.127489	NOT
C11orf98	154.2529	0.35598568	0.157626	2.258412996	0.02392	0.127548	NOT
RP11-649E	2.875644	-0.8510892	0.376854	-2.25840293	0.023921	0.127548	NOT
RP11-95M1	18.25946	1.42308107	0.630185	2.258194189	0.023934	0.127592	NOT
CYP2A7	1541.405	-1.8436891	0.816514	-2.25799958	0.023946	0.127632	NOT
EGR3	71.61649	-0.872458	0.386453	-2.257602	0.02397	0.127706	NOT
AP000295.	7.249621	-0.8089205	0.358315	-2.25756585	0.023973	0.127706	NOT
TEX2	3219.552	-0.4459775	0.197549	-2.25755182	0.023974	0.127706	NOT
AASS	1092.032	-1.1614101	0.514677	-2.25657949	0.024034	0.128004	NOT
SECISBP2L	1528.079	-0.445912	0.197672	-2.25581877	0.024082	0.128232	NOT
XRCC6	11310.46	0.31359948	0.139066	2.255045256	0.02413	0.128438	NOT
RP1-239B2	142.4455	1.18018707	0.523357	2.255032164	0.024131	0.128438	NOT
ZC3HC1	980.2792	0.30756983	0.136396	2.254975925	0.024135	0.128438	NOT
RP11-424C	19.72385	1.05010709	0.465731	2.254751152	0.024149	0.128488	NOT
RND1	2549.966	-0.788054	0.349577	-2.25430493	0.024177	0.128612	NOT
EPM2AIP1	1258.971	-0.4311854	0.19128	-2.25420529	0.024183	0.128619	NOT
CYP1B1-AS	25.37878	-0.6741697	0.299081	-2.25413497	0.024188	0.128619	NOT
HSP90AA2P	16.32392	0.79918866	0.354586	2.253863302	0.024205	0.128684	NOT
POU3F2	12.51226	1.50975441	0.669906	2.253681134	0.024216	0.12872	NOT
CRIPAK	417.7097	-0.40254	0.178637	-2.25339059	0.024235	0.128792	NOT
AC005550.	25.76854	-2.0134953	0.893854	-2.2525989	0.024284	0.129029	NOT
VPS33A	1142.226	0.33885958	0.150436	2.252517284	0.02429	0.129029	NOT
HOMER1	249.1246	0.90053625	0.399809	2.252417761	0.024296	0.129029	NOT
RP13-476E	3.530285	1.17145724	0.520097	2.252380977	0.024298	0.129029	NOT
AGAP2	168.754	-0.4771206	0.211839	-2.25228205	0.024304	0.129037	NOT
TRIM45	254.115	0.71595797	0.317941	2.251858599	0.024331	0.129154	NOT
THUMPD2	310.2092	0.35642452	0.158314	2.251376132	0.024362	0.129291	NOT
CTD-2006C	3.879779	-0.82477	0.366402	-2.25099476	0.024386	0.129391	NOT
RP11-426C	8.760159	-1.6360909	0.726852	-2.25092634	0.02439	0.129391	NOT
NFIA	2876.657	-0.4696491	0.20868	-2.25057106	0.024413	0.129481	NOT
RP11-689C	1.434658	2.65556733	1.179986	2.250507272	0.024417	0.129481	NOT
GEMIN6	830.1543	0.34729181	0.154322	2.250434016	0.024421	0.129481	NOT

AC079145.	5.521055	0.96536282	0.429003	2.250249789	0.024433	0.129517	NOT
CNPY4	390.6646	0.41811845	0.185834	2.249954542	0.024452	0.129591	NOT
NFKBIE	1213.397	0.54246744	0.241146	2.249539056	0.024478	0.129706	NOT
DEPDC1-AS	2.345538	1.44784878	0.643674	2.249352582	0.02449	0.129743	NOT
UBTD2	1016.604	0.26578685	0.118192	2.248770215	0.024527	0.129914	NOT
RP11-256I	3.634638	-1.2532888	0.557377	-2.24854806	0.024541	0.129919	NOT
IGHV4-39	89.67243	1.51311923	0.672934	2.248540924	0.024542	0.129919	NOT
EXOSC4	2223.961	0.72646956	0.323106	2.248392408	0.024551	0.129919	NOT
TCF7L1	1175.135	0.62957076	0.280011	2.248382535	0.024552	0.129919	NOT
LIPM	4.936691	1.88802164	0.839739	2.24834354	0.024554	0.129919	NOT
AC004112.	6.07477	-0.8425167	0.374734	-2.24830414	0.024557	0.129919	NOT
LINC01572	12.33809	0.95930141	0.426719	2.248088889	0.024571	0.129967	NOT
SLC6A1	6389.744	-0.9813212	0.436533	-2.24798628	0.024577	0.129974	NOT
LINC00704	7.580259	1.31272919	0.583976	2.247917291	0.024581	0.129974	NOT
QSER1	1094.086	0.4116714	0.183168	2.247507507	0.024608	0.130087	NOT
RP11-574F	26.94762	-0.5431196	0.241695	-2.24712946	0.024632	0.130153	NOT
KRT18P63	2.706023	1.08759916	0.483996	2.247124372	0.024632	0.130153	NOT
EFHD1	1118.366	-1.0588373	0.471205	-2.24708451	0.024635	0.130153	NOT
VPS37C	895.0051	0.27670147	0.123146	2.24693423	0.024644	0.130179	NOT
GAREML	183.1556	0.90782524	0.404091	2.246588246	0.024666	0.13027	NOT
CHRM1	1.906465	1.85779182	0.827072	2.2462265	0.02469	0.130367	NOT
VENTX	23.92079	-0.7478028	0.332941	-2.24605506	0.0247	0.1304	NOT
RP11-68L1	3.915575	1.00028731	0.445407	2.245780159	0.024718	0.130467	NOT
RP11-753H	2.267768	-1.3484211	0.600569	-2.24523896	0.024753	0.130603	NOT
IKZF2	123.18	-0.6641028	0.295785	-2.2452203	0.024754	0.130603	NOT
HMMR	641.517	0.70718371	0.314982	2.245153882	0.024758	0.130603	NOT
PTAR1	1395.743	-0.4086297	0.182015	-2.2450385	0.024766	0.130617	NOT
EFCAB7	138.5595	0.45792447	0.203995	2.244778768	0.024782	0.130679	NOT
ECH1	14327.29	-0.5579175	0.248563	-2.24457417	0.024795	0.130723	NOT
RP11-251M	11.27132	-0.8407155	0.374641	-2.24405571	0.024829	0.130873	NOT
TIPRL	2451.456	0.29918265	0.133336	2.243823131	0.024844	0.130921	NOT
C1orf61	24.23769	1.18324865	0.527352	2.243755024	0.024848	0.130921	NOT
RP11-582J	23.13173	-0.465957	0.207674	-2.24369126	0.024852	0.130921	NOT
DEPDC5	892.4351	-0.4184503	0.186526	-2.24338657	0.024872	0.130952	NOT
GRID2IP	8.038169	1.1788726	0.525488	2.243385965	0.024872	0.130952	NOT
ABI2	1214.953	0.43844728	0.195443	2.24335477	0.024874	0.130952	NOT
RP11-486A	6.76186	-1.0557546	0.470641	-2.24322727	0.024882	0.130952	NOT
ALDH6A1	10492.97	-0.8432166	0.375918	-2.24308798	0.024891	0.130952	NOT
CTD-2529O	18.43711	-1.3438357	0.599101	-2.24308574	0.024891	0.130952	NOT
AC019181.	18.40323	-1.0826636	0.482669	-2.24307527	0.024892	0.130952	NOT
SMAD1	513.2405	-0.6345932	0.282981	-2.24252809	0.024927	0.131112	NOT
LPHN3	36.07208	-0.7506301	0.334739	-2.24243407	0.024933	0.131119	NOT
FGD4	1141.682	-0.6520384	0.290864	-2.24172807	0.024979	0.131333	NOT
SNRNP40	1029.152	0.35458888	0.158221	2.241100691	0.02502	0.131521	NOT
DYRK2	1046.351	0.42469993	0.189513	2.241009109	0.025025	0.131527	NOT
RP11-152N	43.16094	0.67699218	0.302104	2.240925953	0.025031	0.13153	NOT
RPL37	30025.7	0.54414802	0.242887	2.240334262	0.025069	0.131706	NOT
RP11-108I	4.205209	-0.8856176	0.395322	-2.24024469	0.025075	0.131709	NOT
CSPG5	101.4667	0.8777304	0.391813	2.240174518	0.02508	0.131709	NOT

KRT23	1290.938	1.67927464	0.74973	2.239840419	0.025101	0.131793	NOT
RIPK4	1103.443	-0.5560574	0.248264	-2.23977984	0.025105	0.131793	NOT
CTB-193M1	2.433397	-0.949237	0.423959	-2.23898064	0.025157	0.13204	NOT
RP5-1065J	69.62374	-0.6462838	0.288693	-2.23865541	0.025178	0.132102	NOT
HNRNPA1P1	14.86222	0.70289461	0.313982	2.238643598	0.025179	0.132102	NOT
GRK6	1544.884	0.36612008	0.163551	2.238563066	0.025184	0.132102	NOT
IPO5	5619.8	0.44764045	0.199974	2.238498655	0.025189	0.132102	NOT
TRIM15	623.4795	0.90290142	0.40343	2.238064831	0.025217	0.132217	NOT
RP11-329I	58.58323	0.85438029	0.381758	2.238014187	0.02522	0.132217	NOT
LRRC61	1746.046	0.56425916	0.252136	2.237912479	0.025227	0.132226	NOT
CTD-2310F	17.81823	-0.6044082	0.270099	-2.23772756	0.025239	0.132264	NOT
KLHDC7B	132.186	0.97924537	0.437634	2.237589012	0.025248	0.132284	NOT
RP5-1159(	7.185942	-0.7938871	0.354807	-2.2375199	0.025252	0.132284	NOT
POP5	1237.376	0.41337019	0.184771	2.237205957	0.025273	0.132352	NOT
PRCC	4008.409	0.34802649	0.155565	2.237171088	0.025275	0.132352	NOT
USO1	3840.45	-0.424739	0.189869	-2.23701121	0.025286	0.132381	NOT
METRNL	865.5971	-0.8660893	0.38719	-2.23685833	0.025296	0.132408	NOT
TMEM246	556.0171	1.01247633	0.452751	2.236277697	0.025334	0.132581	NOT
UBE2S	1111.086	0.68869297	0.308009	2.235949925	0.025355	0.132664	NOT
CLMN	2790.071	-0.5830972	0.260792	-2.23586939	0.02536	0.132664	NOT
RP11-673(	280.6836	0.42847833	0.191651	2.235720436	0.02537	0.132664	NOT
ZBED8	174.5285	0.42826855	0.19156	2.235689654	0.025372	0.132664	NOT
RP11-316M	6.95006	-0.6859146	0.306813	-2.23561204	0.025377	0.132664	NOT
RP1-309F2	11.83711	1.088022	0.486683	2.235587789	0.025379	0.132664	NOT
SLC7A6	229.2131	0.62363847	0.278977	2.235447265	0.025388	0.132667	NOT
RP11-494F	2.165843	1.40161069	0.626999	2.235428962	0.025389	0.132667	NOT
RP11-430H	4.7388	1.67583919	0.749722	2.235281071	0.025399	0.132693	NOT
S100Z	3.101985	-0.792989	0.354812	-2.23495274	0.02542	0.132762	NOT
ZNF720	354.6527	-0.3905291	0.174739	-2.23492822	0.025422	0.132762	NOT
RP11-38L1	15.42419	0.88286816	0.39509	2.234602217	0.025443	0.13282	NOT
FAM69C	15.50682	1.13571517	0.508241	2.234599835	0.025444	0.13282	NOT
DESI2	1714.113	0.33811561	0.151313	2.234537025	0.025448	0.13282	NOT
RP11-903H	54.55906	-0.7209298	0.322648	-2.23441467	0.025456	0.132836	NOT
MAP3K3	1134.395	-0.322493	0.14434	-2.23426661	0.025466	0.132841	NOT
KRT18P11	15.50229	0.84355547	0.377556	2.234251256	0.025467	0.132841	NOT
LRRC37A4F	31.21296	1.12663674	0.504361	2.233789612	0.025497	0.132974	NOT
COX15	1991.581	-0.3012186	0.134853	-2.23367644	0.025504	0.132977	NOT
NELFCD	4411.318	0.3070647	0.137473	2.233632809	0.025507	0.132977	NOT
CH17-360I	14.57175	-1.9408484	0.868959	-2.23353182	0.025514	0.13298	NOT
CTC-429P9	13.9343	-0.6600876	0.295543	-2.23347381	0.025518	0.13298	NOT
RP11-642A	6.160493	0.85653305	0.38352	2.233344053	0.025526	0.132999	NOT
CTB-113P1	33.97053	-0.7220829	0.323439	-2.2325183	0.025581	0.133258	NOT
RP11-613F	5.211287	1.01606472	0.455141	2.232415076	0.025588	0.133267	NOT
BLOC1S4	675.7141	0.37112658	0.166252	2.232307176	0.025595	0.133279	NOT
LINC01511	1.883108	2.29325902	1.027388	2.232125811	0.025607	0.133302	NOT
RP1-167A1	3.802013	-0.7617078	0.341253	-2.2320908	0.025609	0.133302	NOT
HAPLN3	206.639	0.94729446	0.424456	2.231784756	0.025629	0.133382	NOT
RP13-314(	13.58167	0.88178693	0.395154	2.231500499	0.025648	0.133454	NOT
PROZ	1182.961	-1.1100811	0.497492	-2.23135579	0.025658	0.133478	NOT

TMEM164	959.0468	0.49073402	0.219948	2.231136118	0.025672	0.133528	NOT
ZCCHC17	911.1408	0.35127973	0.157472	2.230746633	0.025698	0.133634	NOT
SDHDP6	3.1998	-0.8110666	0.363596	-2.23068102	0.025702	0.133634	NOT
RIMKLB	225.5633	-0.7764937	0.348119	-2.23054194	0.025711	0.133656	NOT
TSLP	82.35932	-1.149887	0.515637	-2.2300313	0.025745	0.133764	NOT
CCDC38	59.21825	-0.9439841	0.423317	-2.22997111	0.025749	0.133764	NOT
COL15A1	1288.911	-0.7492864	0.336012	-2.22993793	0.025752	0.133764	NOT
CPSF3	1684.512	0.29119367	0.130584	2.229933688	0.025752	0.133764	NOT
RP11-114M	6.855432	1.59181249	0.713876	2.229817737	0.02576	0.133778	NOT
LL0XNC01-	2.261833	-0.8959759	0.401848	-2.22963936	0.025771	0.133814	NOT
TMEM5	634.1385	0.26843964	0.120403	2.229501763	0.025781	0.133836	NOT
RP11-627C	242.9777	-1.0434269	0.468036	-2.22937525	0.025789	0.133847	NOT
RP11-380I	43.41108	0.79017416	0.354449	2.229303025	0.025794	0.133847	NOT
ITGB1P1	19.00976	0.75007046	0.336477	2.229186568	0.025801	0.133847	NOT
ANO1	2999.79	-1.0533173	0.472515	-2.22917357	0.025802	0.133847	NOT
AD000684.	14.78918	-0.7126035	0.319727	-2.22878978	0.025828	0.133944	NOT
RPS19P3	6.274351	-0.6381198	0.286314	-2.22874176	0.025831	0.133944	NOT
ZNF814	446.3322	-0.5879729	0.263843	-2.22849818	0.025847	0.134003	NOT
ZIC5	162.4626	1.56929467	0.704273	2.22824877	0.025864	0.134064	NOT
RP4-541C2	8.769187	1.92176345	0.862537	2.228035553	0.025878	0.134112	NOT
MBD2	2087.815	0.29509368	0.13247	2.227632122	0.025905	0.134197	NOT
ROBO1	4028.502	0.88937724	0.399252	2.22760834	0.025907	0.134197	NOT
LGALSL	763.3015	0.40705742	0.18274	2.227525494	0.025912	0.134197	NOT
BCL2L12	927.6434	0.46140467	0.207141	2.227491067	0.025914	0.134197	NOT
CTD-2170C	5.937045	1.89281185	0.849825	2.227296145	0.025927	0.134239	NOT
C17orf61-	3.128405	-0.9256927	0.415687	-2.22689949	0.025954	0.134328	NOT
ITGA8	105.7931	-0.6447607	0.289534	-2.22689187	0.025955	0.134328	NOT
SLC25A24	444.8167	0.9280189	0.416758	2.226754895	0.025964	0.134341	NOT
SCN4A	109.0605	-0.8407242	0.377569	-2.22667894	0.025969	0.134341	NOT
SEC24A	2864.557	-0.485839	0.218195	-2.22663154	0.025972	0.134341	NOT
CTAGE11P	2.997302	-0.8948933	0.401921	-2.22654178	0.025978	0.134346	NOT
NACC2	1688.015	-0.3701327	0.16625	-2.22636552	0.02599	0.134376	NOT
MB21D1	133.3001	0.77489355	0.348062	2.226308077	0.025994	0.134376	NOT
TPT1P9	16.04555	-0.7725625	0.347058	-2.22603221	0.026012	0.134423	NOT
GPT2	9848.372	-0.8341146	0.374717	-2.22598403	0.026015	0.134423	NOT
PCDHGA2	149.4695	-1.0784867	0.484506	-2.22595003	0.026018	0.134423	NOT
RP11-278A	14.49175	0.68797132	0.309157	2.225313716	0.02606	0.134618	NOT
U8	11.66882	1.5019633	0.675019	2.225067226	0.026077	0.134664	NOT
TFG	6887.166	0.26403025	0.118664	2.225032287	0.026079	0.134664	NOT
SPSB3	96.14888	-0.5186647	0.233145	-2.2246458	0.026105	0.134772	NOT
BSPRY	183.9246	1.29974679	0.584278	2.224535763	0.026112	0.134785	NOT
RP11-814H	1.989605	0.98159886	0.441321	2.224226713	0.026133	0.134867	NOT
RBPMS	2607.396	0.52178914	0.23461	2.224071786	0.026144	0.134895	NOT
KRT8P7	5.319993	0.90840569	0.408596	2.223234149	0.0262	0.13516	NOT
SERPINF1	36296.51	-0.7422964	0.333893	-2.22315457	0.026205	0.135162	NOT
PAG1	1338.617	0.7672949	0.345179	2.222891532	0.026223	0.135228	NOT
UBA2	3111.742	0.32205424	0.144895	2.222666003	0.026238	0.135273	NOT
AC005077.	11.24005	-1.895805	0.85297	-2.222592	0.026243	0.135273	NOT
AC005618.	15.83889	-1.0102113	0.45453	-2.2225409	0.026247	0.135273	NOT

AQP7P1	86.5251	1.09964135	0.494811	2.222344458	0.02626	0.135307	NOT
TUBBP1	37.40007	0.53260679	0.23967	2.222253899	0.026266	0.135307	NOT
KLHDC10	3218.235	-0.4276946	0.192475	-2.2220743	0.026278	0.135307	NOT
SYNE4	587.2279	0.90580573	0.407647	2.222032087	0.026281	0.135307	NOT
SLC13A4	41.53945	0.83128039	0.374119	2.221966222	0.026286	0.135307	NOT
MMP13	2.07146	2.14726933	0.966396	2.221934241	0.026288	0.135307	NOT
SLC19A1	2260.66	-0.6530896	0.29393	-2.22192412	0.026288	0.135307	NOT
ILVBL	4736.379	-0.5605443	0.252298	-2.22175727	0.0263	0.13534	NOT
BCCIP	2328.548	0.30531325	0.137429	2.22161208	0.02631	0.135365	NOT
RP11-923I	222.9946	0.95232226	0.428712	2.221358997	0.026327	0.13541	NOT
SPRYD4	2530.367	-0.5081494	0.228761	-2.2213106	0.02633	0.13541	NOT
RP11-252A	51.60085	-0.455782	0.205193	-2.22123076	0.026335	0.13541	NOT
NFIX	3248.945	-0.5492627	0.247287	-2.2211505	0.026341	0.13541	NOT
HIP1R	3302.023	0.467015	0.210262	2.221112208	0.026343	0.13541	NOT
MYLK2	11.40217	0.89567178	0.403321	2.220741821	0.026368	0.135514	NOT
RP11-119F	9.010496	-0.8628977	0.388614	-2.22045088	0.026388	0.135578	NOT
LINC01106	18.81822	1.03927489	0.468056	2.220408841	0.026391	0.135578	NOT
CNST	1732.44	-0.4131606	0.186101	-2.22008573	0.026413	0.135665	NOT
RP11-173A	1.541083	-1.0362868	0.466815	-2.21991123	0.026425	0.1357	NOT
C19orf24	3069.291	0.61298578	0.276148	2.219772086	0.026434	0.135723	NOT
RP11-7M8.	30.83081	-1.422041	0.640747	-2.21934985	0.026463	0.135845	NOT
AC000068.	20.69384	-0.6062429	0.273198	-2.21906443	0.026482	0.135919	NOT
NPTXR	407.6348	1.26779551	0.571461	2.218517222	0.02652	0.136084	NOT
RASSF10	5.540134	2.57135793	1.159184	2.218247517	0.026538	0.136132	NOT
BCL2L15	24.89552	1.0283183	0.463575	2.218233212	0.026539	0.136132	NOT
ACSF2	3851.172	-0.5594925	0.252247	-2.21803869	0.026552	0.136157	NOT
CTA-414D7	2.165303	-0.9027667	0.407021	-2.21798545	0.026556	0.136157	NOT
ZSWIM5	455.573	0.85669946	0.386259	2.217939148	0.026559	0.136157	NOT
RP11-3D4.	17.3114	0.7082501	0.319387	2.217528634	0.026587	0.136271	NOT
RP11-672I	1.180386	-1.4936182	0.673551	-2.21752748	0.026587	NA	NA
RP11-353N	9.179246	1.11679785	0.503636	2.217468107	0.026591	0.136271	NOT
SUFU	785.9581	-0.2732542	0.123234	-2.21736633	0.026598	0.136281	NOT
PCBD2	467.9646	-0.3981556	0.179603	-2.21686355	0.026632	0.136431	NOT
ARHGEF5	563.2567	0.59945985	0.270427	2.216713294	0.026643	0.136458	NOT
ZNF142	671.7374	0.33316397	0.150304	2.216607562	0.02665	0.136469	NOT
CTB-49A3.	1.503167	1.95959355	0.884196	2.216242567	0.026675	0.136571	NOT
RP11-29H2	3.400245	0.85042476	0.383762	2.216023071	0.02669	0.136597	NOT
CRHR1-IT1	204.1773	0.73613917	0.33219	2.21602166	0.02669	0.136597	NOT
RP11-313I	3.502633	-0.7117964	0.321258	-2.21565316	0.026715	0.1367	NOT
RP13-16H1	2.574653	1.00930666	0.455601	2.215331221	0.026737	0.136787	NOT
ABCA6	3606.99	-0.9781145	0.441601	-2.21493039	0.026765	0.136902	NOT
RP11-694I	34.09626	-0.8235196	0.371819	-2.21483845	0.026771	0.136909	NOT
IL7R	416.4909	-1.0602956	0.47877	-2.21462548	0.026786	0.136958	NOT
LINC01424	4.810103	0.93314633	0.421419	2.214293997	0.026809	0.137048	NOT
BLACAT1	2.085456	1.89736233	0.856908	2.214195448	0.026815	0.137057	NOT
RP13-452N	14.64168	-1.0872317	0.491107	-2.21383888	0.02684	0.137157	NOT
RP11-313F	4.075347	-0.6576377	0.297081	-2.21366601	0.026852	0.137192	NOT
RP11-592N	18.63784	0.64858388	0.293066	2.213099197	0.026891	0.137365	NOT
CNTNAP5	2.494016	2.3157219	1.046539	2.212742631	0.026915	0.137462	NOT

RAET1L	1.532995	2.39348708	1.081715	2.21267793	0.02692	0.137462	NOT
HNRNPA1	15487.75	0.32869621	0.148561	2.212531839	0.02693	0.137474	NOT
ATAT1	408.6918	0.45353429	0.204987	2.212497935	0.026932	0.137474	NOT
EBAG9P1	2.916775	-0.8183139	0.369895	-2.21228759	0.026947	0.137522	NOT
SYT10	1.050529	-2.4486863	1.106901	-2.21220023	0.026953	NA	NA
RAP2B	1195.793	0.38886507	0.175783	2.212187647	0.026954	0.137531	NOT
CPNE9	6.140916	1.06186104	0.480041	2.212021562	0.026965	0.137564	NOT
VPS45	1639.895	0.30239669	0.136734	2.211561651	0.026997	0.1377	NOT
RP11-274I	44.31594	0.59627013	0.269636	2.211391251	0.027009	0.137734	NOT
C1orf168	492.588	-0.8610276	0.389425	-2.21102028	0.027034	0.137774	NOT
LINC0102I	93.45148	-1.5252585	0.689847	-2.21100949	0.027035	0.137774	NOT
HP	382951.3	-1.1516359	0.520867	-2.2109974	0.027036	0.137774	NOT
PYCARD-AS	3.060702	1.09521829	0.495353	2.210984526	0.027037	0.137774	NOT
GNGT1	15.94651	2.40766741	1.089093	2.210709061	0.027056	0.137824	NOT
USP47	3237.469	-0.2622707	0.118644	-2.21057195	0.027065	0.137824	NOT
MYC	2911.417	0.88117836	0.398629	2.210521883	0.027069	0.137824	NOT
PHKB	2454.531	-0.4052325	0.183322	-2.21049463	0.027071	0.137824	NOT
H2AFX	1809.934	0.58362404	0.26403	2.210446512	0.027074	0.137824	NOT
NET1	2770.191	0.40872528	0.18491	2.210403608	0.027077	0.137824	NOT
DLGAP1-AS	1.753252	2.2029747	0.996863	2.209907341	0.027112	0.137969	NOT
CD69	114.2414	-0.857436	0.388007	-2.20984772	0.027116	0.137969	NOT
CCDC144A	8.337798	-1.623775	0.734867	-2.20961709	0.027132	0.138024	NOT
JPH1	91.18889	1.14889371	0.520032	2.209274768	0.027156	0.13812	NOT
WI2-1896(	76.40101	-1.0441756	0.472675	-2.20907876	0.027169	0.138156	NOT
NHEJ1	51.02277	0.51336281	0.232393	2.209025842	0.027173	0.138156	NOT
GAK	4250.868	-0.2504048	0.113367	-2.20879437	0.027189	0.138212	NOT
SNRPF	1963.225	0.50272797	0.227625	2.208582762	0.027204	0.138261	NOT
RP11-534I	2.163776	1.07199647	0.485401	2.208474906	0.027211	0.138273	NOT
MCMDC2	100.7677	0.63205143	0.286214	2.208315387	0.027222	0.138304	NOT
TRAFD1	1272.624	0.34829876	0.157748	2.207946595	0.027248	0.138408	NOT
AC002511.	3.774903	2.17027926	0.983013	2.207783357	0.027259	0.13844	NOT
ZNF282	1605.389	0.34350621	0.155607	2.207525319	0.027277	0.13849	NOT
EGFL6	23.33657	1.14054213	0.516678	2.207452193	0.027282	0.13849	NOT
RPS15AP36	10.27337	-0.8495996	0.384883	-2.20742352	0.027284	0.13849	NOT
EMB	305.4767	-0.8977293	0.406714	-2.20727524	0.027295	0.138492	NOT
TXNDC15	2048.819	-0.2842816	0.128795	-2.20724546	0.027297	0.138492	NOT
HAS2-AS1	3.452701	1.34560164	0.609648	2.207176857	0.027302	0.138492	NOT
RP11-883A	2.403462	1.68753661	0.764615	2.207042133	0.027311	0.138492	NOT
RP11-234A	176.9062	0.70515232	0.319509	2.20698787	0.027315	0.138492	NOT
MGRN1	4113.235	-0.353485	0.160167	-2.2069784	0.027316	0.138492	NOT
RGS5	5809.188	-0.5542974	0.251171	-2.20685606	0.027324	0.13851	NOT
ERI1	490.5343	0.48772851	0.22102	2.206712807	0.027334	0.138521	NOT
MEX3C	917.7507	0.43088398	0.195264	2.206678927	0.027336	0.138521	NOT
EIF2S2P4	10.57034	0.71880803	0.325795	2.206318885	0.027362	0.138612	NOT
RP11-446I	2.948001	-1.0763098	0.487857	-2.20620158	0.02737	0.138612	NOT
AKR1D1	3535.22	-1.2923301	0.585772	-2.20620073	0.02737	0.138612	NOT
C10orf107	6.513035	-1.1578317	0.524921	-2.20572378	0.027403	0.138756	NOT
LINC01013	3.25514	-0.9439332	0.428007	-2.20541497	0.027425	0.138839	NOT
ZFAS1	2228.823	0.65499277	0.297015	2.205253297	0.027436	0.138871	NOT

ELAVL2	5.077248	-0.9427304	0.427528	-2.20507213	0.027449	0.138886	NOT
ZNF18	317.3464	-0.3761954	0.170605	-2.20506477	0.02745	0.138886	NOT
USP35	567.8863	-0.495196	0.224607	-2.2047189	0.027474	0.138983	NOT
ARPC1A	5657.512	0.32534837	0.147594	2.20435417	0.027499	0.13907	NOT
CKLF-CMTM	5.283041	0.80262039	0.364114	2.204312164	0.027502	0.13907	NOT
CCDC66	312.7493	0.32123624	0.145738	2.20420577	0.02751	0.13907	NOT
GGT2	3.891926	1.21176186	0.549756	2.204181925	0.027512	0.13907	NOT
RPL23AP3	2.193823	1.10682088	0.502319	2.203422963	0.027565	0.139314	NOT
ZNF467	704.0595	-0.7781515	0.353216	-2.20304952	0.027591	0.139421	NOT
RP11-13K1	1.3999	-1.2890003	0.585211	-2.20262631	0.027621	0.139546	NOT
ZNF780A	436.5606	-0.3689541	0.167516	-2.20250169	0.02763	0.139564	NOT
POLA2	838.0035	0.40781229	0.185172	2.202346688	0.027641	0.139593	NOT
MAFA	34.41247	1.46366804	0.664618	2.202268092	0.027646	0.139595	NOT
RP11-580S	1.714972	-1.014057	0.460516	-2.20199997	0.027665	0.139623	NOT
OIT3	621.0758	-0.713161	0.32387	-2.20199641	0.027666	0.139623	NOT
SAP30	287.1227	0.51333484	0.233135	2.201878776	0.027674	0.139623	NOT
RNASEH1	674.5175	0.29983294	0.136172	2.201875163	0.027674	0.139623	NOT
COA7	1560.466	0.34317734	0.155864	2.201767219	0.027682	0.139623	NOT
CTD-2616J	2.097019	1.13651841	0.516188	2.201752894	0.027683	0.139623	NOT
ARNT	3877.526	-0.3730662	0.169459	-2.20151095	0.0277	0.139683	NOT
FABP5P7	3.547673	1.01252291	0.459951	2.201370774	0.02771	0.139683	NOT
RP11-285F	184.3648	0.62982804	0.286115	2.201313829	0.027714	0.139683	NOT
PABPC4L	30.89261	-0.6636068	0.301465	-2.20127021	0.027717	0.139683	NOT
RP5-1120F	62.08017	1.15335554	0.523976	2.201161576	0.027725	0.139683	NOT
LINC00304	6.349717	1.43645292	0.652592	2.201149497	0.027725	0.139683	NOT
RP11-631N	72.19144	-0.6312132	0.28682	-2.20072847	0.027755	0.139807	NOT
CPSF1P1	31.55292	1.29472908	0.588435	2.200292572	0.027786	0.139937	NOT
DNMT3A	1602.703	0.53147124	0.241589	2.199899582	0.027814	0.140051	NOT
RP11-4C20	3.494852	1.21048825	0.550315	2.199626334	0.027833	0.140123	NOT
HCN1	4.036051	1.94190951	0.882975	2.199280436	0.027858	0.14022	NOT
CPNE1	4332.352	0.45583389	0.207288	2.199034448	0.027875	0.140282	NOT
SPAG4	313.8502	0.77486175	0.35239	2.198873979	0.027887	0.140314	NOT
REM2	8.036058	0.80385441	0.365626	2.198567724	0.027909	0.140374	NOT
CTD-3064M	3.666629	-1.7437588	0.793137	-2.19855984	0.027909	0.140374	NOT
RP11-46C2	115.8602	-0.5581377	0.253879	-2.19844357	0.027918	0.140382	NOT
RPL7P6	13.58087	0.85281611	0.387927	2.19839345	0.027921	0.140382	NOT
SLC25A28	1526.965	-0.3883387	0.176661	-2.19820834	0.027934	0.140422	NOT
EMG1	811.5103	0.4344436	0.197664	2.197886784	0.027957	0.140511	NOT
SHISA9	13.18561	1.58173726	0.719734	2.197668329	0.027973	0.140563	NOT
AC005307.	1.475462	3.06084463	1.392841	2.19755477	0.027981	0.140564	NOT
MFHAS1	1244.689	0.49195002	0.223866	2.197519784	0.027983	0.140564	NOT
RP11-799M	8.682661	-0.7919134	0.360381	-2.19743667	0.027989	0.140568	NOT
PPIE	1519.134	0.40855775	0.185968	2.196922474	0.028026	0.140726	NOT
SLC6A13	491.0921	-1.0852631	0.49404	-2.19671196	0.028041	0.140776	NOT
COG8	950.3521	-0.2905194	0.132272	-2.19638359	0.028064	0.140868	NOT
CFLAR-AS1	10.65636	-0.5557388	0.253045	-2.19620588	0.028077	0.140905	NOT
KCNJ8	1714.775	-0.7855071	0.35771	-2.19593074	0.028097	0.140978	NOT
C1orf127	16.59955	-0.6705264	0.305396	-2.19559718	0.028121	0.141072	NOT
RP11-177H	7.520074	-0.8116019	0.36971	-2.1952392	0.028146	0.141175	NOT

C4A	11036.82	-0.8018782	0.365396	-2.19454686	0.028196	0.141385	NOT
UTP20	901.8721	0.31810006	0.144953	2.194507903	0.028199	0.141385	NOT
FAM27C	8.096074	1.71916655	0.783625	2.193864889	0.028245	0.141514	NOT
EEF1A1P3(	4.765291	0.78729142	0.358863	2.193851767	0.028246	0.141514	NOT
HS6ST2	152.2465	1.87901716	0.856497	2.193838596	0.028247	0.141514	NOT
NUDT16	3549.496	-0.4229506	0.192795	-2.19378584	0.028251	0.141514	NOT
KB-1615E4	8.136805	1.30502035	0.594872	2.193782291	0.028251	0.141514	NOT
IGF1R	453.6829	1.00856362	0.459765	2.193652262	0.02826	0.141514	NOT
PCBP4	1406.322	0.49608854	0.226151	2.19361262	0.028263	0.141514	NOT
RP11-462(	7.75553	-0.7912918	0.360738	-2.19353506	0.028269	0.141514	NOT
CACNA2D4	109.9497	0.71168399	0.324452	2.193497583	0.028272	0.141514	NOT
RP11-350I	1.452533	-1.2741717	0.580911	-2.19340199	0.028278	0.141523	NOT
AKR1C3	20838.83	0.68884962	0.314094	2.193130108	0.028298	0.141594	NOT
RP11-187(	4.805885	-0.8144371	0.37144	-2.19264519	0.028333	0.141743	NOT
HDAC5	3183.575	0.34226125	0.156112	2.192412737	0.02835	0.141777	NOT
RP11-385I	52.00805	0.66982923	0.305525	2.192390049	0.028351	0.141777	NOT
MKL2	990.8361	-0.4525088	0.206405	-2.19233406	0.028355	0.141777	NOT
KCTD16	3.786961	-0.9328069	0.425505	-2.19223453	0.028363	0.141783	NOT
PCAT1	6.564884	0.94070275	0.429122	2.192158114	0.028368	0.141783	NOT
RNU5B-2P	9.151032	-0.5938939	0.270925	-2.19210074	0.028372	0.141783	NOT
SNRPE	2875.359	0.43284203	0.197466	2.191979457	0.028381	0.1418	NOT
HENMT1	148.0011	0.96269721	0.43921	2.19188456	0.028388	0.141809	NOT
AKR1B15	578.9102	1.63962475	0.7481	2.191718141	0.0284	0.141842	NOT
KRT18P13	4.202949	-1.0359307	0.472812	-2.19099725	0.028452	0.142077	NOT
CD4	2617.907	-0.668296	0.305056	-2.19073372	0.028471	0.142146	NOT
MTHFD1P1	6.267896	-0.8904612	0.406482	-2.19065125	0.028477	0.142149	NOT
RRP36	1907.862	0.34177162	0.156049	2.190154112	0.028513	0.142303	NOT
ADCY3	401.1123	-0.6328789	0.289015	-2.18977562	0.028541	0.142363	NOT
RP11-435I	3.493418	1.06019519	0.484178	2.189681159	0.028547	0.142363	NOT
AP001350.	18.85728	-0.6001138	0.274065	-2.1896789	0.028548	0.142363	NOT
HIST3H2BI	22.94196	1.74997353	0.799199	2.189660042	0.028549	0.142363	NOT
NUP155	1460.385	0.34715723	0.158546	2.189625746	0.028551	0.142363	NOT
TTLL12	2908.17	0.44077425	0.20132	2.189421521	0.028566	0.142375	NOT
LSM5	1271.558	0.39167949	0.178897	2.189416403	0.028567	0.142375	NOT
RP13-487(	2.946816	-1.0316366	0.471201	-2.18937574	0.02857	0.142375	NOT
ENDOG	424.3796	-0.5045161	0.230454	-2.18922303	0.028581	0.142405	NOT
RPSAP52	1.088663	2.1051624	0.961625	2.189171268	0.028584	NA	NA
AC073133.	2.908772	-1.0249294	0.468226	-2.18896514	0.028599	0.142447	NOT
RIMKLA	37.83521	1.03815272	0.474267	2.188962348	0.0286	0.142447	NOT
UNC5C	86.69952	-0.8024537	0.366608	-2.18886145	0.028607	0.142457	NOT
PYCR1	1573.819	1.18323245	0.540604	2.188724223	0.028617	0.14248	NOT
SC5D	6022.71	-0.6675099	0.305043	-2.18824828	0.028652	0.142591	NOT
RP11-29H2	15.48265	0.55961859	0.255749	2.188157646	0.028658	0.142591	NOT
BCDIN3D-A	27.71052	0.46147375	0.210907	2.188044803	0.028666	0.142591	NOT
RP11-179I	2.275864	1.57504158	0.719843	2.188034571	0.028667	0.142591	NOT
RPS6KL1	308.7792	0.74421132	0.340132	2.188010007	0.028669	0.142591	NOT
PCDHGB7	43.43818	-0.8308062	0.379713	-2.18798648	0.028671	0.142591	NOT
SSR4P1	73.85508	-0.4962937	0.226841	-2.18784662	0.028681	0.142615	NOT
PEX11G	358.8614	-0.634101	0.289909	-2.1872443	0.028725	0.142779	NOT

FAM127B	1393.356	0.6275744	0.28693	2.187200585	0.028728	0.142779	NOT
FZD2	66.36676	0.90591015	0.414191	2.187177714	0.02873	0.142779	NOT
KLK7	2.616825	2.25741736	1.032324	2.186733728	0.028762	0.142914	NOT
CTD-2562J	2.688165	-1.454196	0.665045	-2.18661453	0.028771	0.142931	NOT
SH2D3C	661.2969	-0.4498148	0.205792	-2.18577052	0.028832	0.143212	NOT
WDTC1	2626.523	-0.3434568	0.157142	-2.18564403	0.028842	0.143232	NOT
HAAO	5707.01	-0.6565417	0.300417	-2.18543736	0.028857	0.143281	NOT
PTBP2	571.0357	0.5020174	0.229721	2.185330646	0.028865	0.143293	NOT
RP5-1096J	5.856247	2.35933052	1.079836	2.184896951	0.028896	0.143425	NOT
EPRS	5799.999	0.35684316	0.163335	2.184737924	0.028908	0.143439	NOT
CCDC186	424.6176	-0.4273664	0.195618	-2.18469971	0.028911	0.143439	NOT
DHRSX-IT1	5.632123	-0.7685234	0.351785	-2.18464185	0.028915	0.143439	NOT
HBE1	2.198893	-1.9994821	0.91553	-2.18396066	0.028965	0.143661	NOT
C1orf43	14943.57	0.31921061	0.146189	2.18354667	0.028996	0.143785	NOT
SLC2A10	2359.569	-0.7700004	0.352652	-2.18345898	0.029002	0.143791	NOT
RP11-485M	2.453131	-0.7660173	0.350933	-2.18280528	0.02905	0.143977	NOT
RNU6-796F	1.709839	-1.2808777	0.586821	-2.1827397	0.029055	0.143977	NOT
TCP10	10.14788	1.82066082	0.83412	2.182733074	0.029055	0.143977	NOT
MRPL4	2900.109	-0.389129	0.178284	-2.18263684	0.029063	0.143986	NOT
TFDP3	18.22222	3.73982611	1.713659	2.182363571	0.029083	0.144044	NOT
RP11-728F	8.452305	2.06602707	0.946705	2.182334938	0.029085	0.144044	NOT
COL11A1	71.99446	1.45433698	0.666493	2.182074679	0.029104	0.144099	NOT
ZNF571-AS	10.82816	0.88177181	0.404104	2.182039526	0.029107	0.144099	NOT
B3GNT5	539.8566	0.80792517	0.370312	2.181744059	0.029128	0.144181	NOT
GK-IT1	3.258348	-1.0216891	0.468325	-2.18157977	0.029141	0.144214	NOT
IL6ST	10900.2	-0.4128859	0.18928	-2.18135291	0.029157	0.144271	NOT
ANKLE1	28.35035	0.8981692	0.411796	2.18109996	0.029176	0.144337	NOT
ERCC3	1534.778	0.23735469	0.108829	2.180994009	0.029184	0.14435	NOT
LINC00476	114.3816	-0.4741951	0.217455	-2.18065965	0.029209	0.144446	NOT
COL6A6	12.34461	-0.9394743	0.430844	-2.18054597	0.029217	0.144461	NOT
LYPD2	2.558667	-1.7942709	0.823124	-2.1798311	0.02927	0.144697	NOT
ZNF385D	108.4458	-1.2350968	0.566633	-2.17971294	0.029279	0.144706	NOT
RP11-96K1	14.95704	-0.5862448	0.268964	-2.17963819	0.029284	0.144706	NOT
RHOF	83.08534	0.73001346	0.334935	2.179569191	0.029289	0.144706	NOT
CTD-2647I	19.4131	-0.6166212	0.282916	-2.17951791	0.029293	0.144706	NOT
TMEM47	847.0977	-0.6883924	0.315894	-2.17918761	0.029318	0.144801	NOT
NSFP1	2.948045	1.38111165	0.633803	2.179087671	0.029325	0.144811	NOT
MMP7	720.5544	1.54402868	0.708623	2.178913229	0.029338	0.144849	NOT
RP4-536B2	2.01629	1.32650729	0.608835	2.178762402	0.029349	0.144858	NOT
RP11-96A1	29.35492	1.36400154	0.62605	2.178743317	0.029351	0.144858	NOT
COBLL1	3622.487	-0.6646286	0.305077	-2.1785612	0.029364	0.144873	NOT
CNIH2	38.50749	0.85500819	0.392478	2.178488533	0.02937	0.144873	NOT
KLRF1	15.66137	-0.8100906	0.371859	-2.17848647	0.02937	0.144873	NOT
CYP4F12	1705.697	-0.8029433	0.368634	-2.17815696	0.029394	0.144968	NOT
CTD-2315E	3.845888	-1.518838	0.697401	-2.17785441	0.029417	0.145042	NOT
ISY1	718.2757	0.3641882	0.167227	2.177811113	0.02942	0.145042	NOT
ZBTB26	229.3935	0.42334939	0.194423	2.177464462	0.029446	0.145143	NOT
RP11-365F	8.293906	0.78656676	0.361298	2.177059475	0.029476	0.145266	NOT
MTA2	2792.999	0.24777684	0.113839	2.17654665	0.029514	0.145428	NOT

RP11-876N	34.78032	-0.5756518	0.264533	-2.17610355	0.029548	0.145541	NOT
AC234917.	2.680462	2.04485014	0.939687	2.176097098	0.029548	0.145541	NOT
HLA-DQB2	476.1808	1.10968092	0.509993	2.175873727	0.029565	0.145596	NOT
SLC30A1	5463.95	-0.5973927	0.274572	-2.17572507	0.029576	0.145625	NOT
SLC4A7	419.0752	0.58917522	0.270815	2.175566475	0.029588	0.145657	NOT
RP11-215F	4.366961	-0.6810971	0.31308	-2.17547562	0.029594	0.145664	NOT
TAF6L	813.1573	-0.2804172	0.128908	-2.17532143	0.029606	0.145682	NOT
F12	22519.62	-0.9593825	0.441038	-2.17528285	0.029609	0.145682	NOT
CTD-2297I	31.11088	1.91145884	0.878753	2.175195503	0.029615	0.145688	NOT
FLT1	2008.01	-0.4829828	0.222058	-2.17502576	0.029628	0.145724	NOT
PLEKHA8P1	141.7969	0.394049	0.181207	2.174576025	0.029662	0.145863	NOT
TSIX	4.407852	-1.1820238	0.543612	-2.17438817	0.029676	0.145898	NOT
MED19	462.3544	0.31822301	0.146354	2.17433728	0.02968	0.145898	NOT
RP13-147I	11.24668	3.42900284	1.577146	2.174181979	0.029691	0.145929	NOT
TCEANC2	398.9521	0.31760238	0.146095	2.173951487	0.029709	0.145988	NOT
SMIM10	84.79515	0.77796487	0.357952	2.173377447	0.029752	0.146173	NOT
ARF4	8020.926	0.32563747	0.14985	2.173092865	0.029773	0.14621	NOT
ZMYM3	1827.285	0.3034625	0.139647	2.173063704	0.029776	0.14621	NOT
AASDH	355.2354	-0.3218722	0.148119	-2.1730623	0.029776	0.14621	NOT
RNF103-CH	4.989172	1.05063812	0.483499	2.172989962	0.029781	0.146211	NOT
C9orf147	11.27093	-0.5029684	0.231495	-2.17269624	0.029803	0.146276	NOT
SNHG14	1378.499	0.95280217	0.43854	2.172671061	0.029805	0.146276	NOT
SERPINH1	5796.088	0.53967048	0.248468	2.171990018	0.029856	0.146501	NOT
TRI-TAT2-	4.664517	-1.0001094	0.460512	-2.17173296	0.029876	0.14657	NOT
KRT8P48	10.34343	0.97608989	0.449511	2.171448833	0.029897	0.146621	NOT
RP11-230F	14.41276	0.76699389	0.35322	2.171431129	0.029899	0.146621	NOT
GFOD2	1323.453	-0.4195385	0.193217	-2.17133351	0.029906	0.146621	NOT
SPCS1	5827.833	0.39388298	0.181403	2.171309587	0.029908	0.146621	NOT
SLC25A41	10.66843	-1.0292426	0.474186	-2.17054425	0.029966	0.146878	NOT
PCDH17	437.4022	-0.5461468	0.25166	-2.17017828	0.029993	0.146987	NOT
LIN28B	87.18257	3.03645274	1.399285	2.170002304	0.030007	0.147026	NOT
CTA-221G9	90.56465	-0.6841256	0.315279	-2.1699065	0.030014	0.147035	NOT
LIPH	107.5558	1.21612402	0.560508	2.169683099	0.030031	0.147091	NOT
GCA	488.9943	0.61711787	0.284437	2.169608586	0.030037	0.147092	NOT
CTC-332L2	13.55958	-0.4887171	0.225266	-2.16951372	0.030044	0.147101	NOT
PRKRIR	1049.113	-0.2512136	0.1158	-2.16938013	0.030054	0.147121	NOT
AF186192.	2.637897	1.44599552	0.666567	2.169316407	0.030059	0.147121	NOT
CYC1	11903.43	0.58033904	0.267566	2.168960598	0.030086	0.147188	NOT
ACVR1	1310.782	0.40332967	0.185959	2.168919062	0.030089	0.147188	NOT
TMC3	9.384155	-0.8987145	0.414381	-2.16881071	0.030097	0.147188	NOT
RP11-59C5	19.48315	-0.6934347	0.319735	-2.16877751	0.0301	0.147188	NOT
CUL2	1711.937	0.30045594	0.138539	2.168740261	0.030102	0.147188	NOT
RP1-159A1	11.65028	0.75081684	0.346205	2.168708633	0.030105	0.147188	NOT
DIS3L	1185.066	-0.370709	0.170955	-2.16846442	0.030123	0.147252	NOT
OXNAD1	730.4261	-0.4128223	0.190439	-2.16774035	0.030178	0.147494	NOT
KIAA1522	4755.179	0.54140517	0.249859	2.166845315	0.030247	0.147777	NOT
RP11-710M	1.952894	2.20362864	1.016978	2.16683982	0.030247	0.147777	NOT
RP1-28H2(	16.063	-0.6434645	0.297032	-2.16631248	0.030287	0.147945	NOT
RP11-34P1	2.828508	1.12954131	0.521428	2.166245894	0.030292	0.147945	NOT

RP11-206I	3.185614	-1.020195	0.47097	-2.16615762	0.030299	0.147951	NOT
PRR26	285.898	-0.8338147	0.385007	-2.16571129	0.030333	0.148091	NOT
STK11IP	625.0747	0.34377277	0.158761	2.165354118	0.030361	0.148198	NOT
RP11-700H	30.44278	-1.0584078	0.488816	-2.1652466	0.030369	0.148211	NOT
UIMC1	741.1673	0.2670646	0.123346	2.165161181	0.030375	0.148216	NOT
SDK1	276.9001	-1.1313072	0.522581	-2.16484763	0.030399	0.148262	NOT
ARTN	29.34699	0.93070492	0.429925	2.164805754	0.030403	0.148262	NOT
RP3-388M5	7.639156	0.77862934	0.359681	2.164776315	0.030405	0.148262	NOT
RP11-731J	2.352184	1.21437113	0.560974	2.164754321	0.030407	0.148262	NOT
ABCA5	2127.396	-0.5757159	0.265982	-2.16448857	0.030427	0.148334	NOT
UPF1	5611.547	-0.3122996	0.144295	-2.16431831	0.03044	0.148368	NOT
CXCL13	176.4179	1.56615632	0.723667	2.164195178	0.030449	0.148368	NOT
CTD-2012K	3.465814	-0.9967197	0.460552	-2.16418363	0.03045	0.148368	NOT
C9orf78	2644.608	-0.2775761	0.128269	-2.16401938	0.030463	0.148384	NOT
SETDB2	489.8008	-0.3891842	0.179847	-2.16397608	0.030466	0.148384	NOT
CHFR	998.2777	0.39828511	0.184057	2.163927752	0.03047	0.148384	NOT
LLPH	859.6134	0.25935358	0.119861	2.16379387	0.03048	0.148408	NOT
NAP1L1P3	5.512661	0.78316271	0.361957	2.163692525	0.030488	0.148419	NOT
MAP9	64.97596	-1.0200286	0.47148	-2.16346195	0.030506	0.148465	NOT
RP11-148B	4.930039	0.99762028	0.461143	2.163366595	0.030513	0.148465	NOT
MOGAT2	1258.376	-1.2380349	0.572283	-2.16332675	0.030516	0.148465	NOT
ENTPD7	439.7094	-0.5121775	0.236762	-2.16325767	0.030521	0.148465	NOT
SNORA47	2.16009	-0.9643334	0.445819	-2.16306008	0.030537	0.148465	NOT
PIP4K2A	1191.851	0.38829448	0.179512	2.163059934	0.030537	0.148465	NOT
AGAP1	999.9097	0.42129327	0.19477	2.163025354	0.030539	0.148465	NOT
FXR1	4448.132	0.28191476	0.130335	2.162999653	0.030541	0.148465	NOT
SNAP91	2.584058	1.94721723	0.900292	2.162874081	0.030551	0.148485	NOT
TERF2IP	1998.456	-0.2998283	0.138649	-2.16249308	0.03058	0.148601	NOT
RPL19	44534.69	0.43766615	0.202409	2.162285031	0.030596	0.148648	NOT
RP5-1086K	30.49069	0.78223808	0.361775	2.162225251	0.030601	0.148648	NOT
LRRK2	298.6591	-0.7231798	0.334486	-2.1620659	0.030613	0.148681	NOT
RP11-815I	14.91685	0.58033918	0.268457	2.161761255	0.030637	0.148769	NOT
NDOR1	521.8638	0.40796538	0.188731	2.161625776	0.030647	0.148793	NOT
ATP6V1H	1813.978	0.34750147	0.160778	2.161381106	0.030666	0.148858	NOT
ACACA	3058.09	0.42854207	0.198288	2.161210419	0.030679	0.148895	NOT
TREML4	9.804601	2.00685242	0.928644	2.161056782	0.030691	0.148926	NOT
TIGD2	837.6944	-0.6837597	0.316412	-2.16098097	0.030697	0.148928	NOT
PELI2	81.58593	-0.7699774	0.356339	-2.16080297	0.030711	0.148951	NOT
IDUA	861.9999	-0.500391	0.23158	-2.16077105	0.030713	0.148951	NOT
RP11-745A	3.733935	0.91059453	0.421434	2.160706143	0.030718	0.148951	NOT
CNOT6L	1337.917	-0.3847054	0.178067	-2.1604518	0.030738	0.148979	NOT
TAGLN	3301.581	-0.7017147	0.324804	-2.16042466	0.03074	0.148979	NOT
CTD-2319I	9.492255	0.62418364	0.288924	2.160372865	0.030744	0.148979	NOT
TSTA3	4780.84	0.51832055	0.239925	2.160346138	0.030746	0.148979	NOT
AGXT	53578.15	-0.9680529	0.448196	-2.1598882	0.030781	0.149112	NOT
RPSAP53	71.29043	1.49949717	0.694266	2.159830041	0.030786	0.149112	NOT
PSPN	138.3638	-0.4181165	0.193592	-2.15978001	0.03079	0.149112	NOT
IQSEC1	5377.692	-0.3775357	0.174816	-2.15961659	0.030802	0.149147	NOT
OPA3	1640.006	-0.309506	0.143333	-2.15934387	0.030823	0.149196	NOT

RPA4	3.146448	0.94126423	0.435903	2.159343275	0.030824	0.149196	NOT
ZNF48	455.6506	0.30900144	0.143115	2.159110316	0.030842	0.149235	NOT
CTC-786C1	10.60434	-0.8175466	0.378652	-2.15909789	0.030843	0.149235	NOT
TNFAIP6	24.40558	1.14930121	0.532335	2.158982425	0.030852	0.149235	NOT
ISL2	21.31076	1.40031703	0.648619	2.158919385	0.030856	0.149235	NOT
HBG2	2.325432	-1.2045473	0.557963	-2.15883169	0.030863	0.149235	NOT
KNDC1	503.0731	-1.4376324	0.665936	-2.15881323	0.030865	0.149235	NOT
ZNF610	45.36945	0.97103624	0.449826	2.158692474	0.030874	0.149254	NOT
PFKFB3	2084.77	0.91824989	0.425511	2.157991283	0.030929	0.149461	NOT
NOP16	1088.844	0.49724747	0.230424	2.1579712	0.03093	0.149461	NOT
PDCD1	86.73905	1.08885509	0.504594	2.15788531	0.030937	0.149461	NOT
RP5-864K1	3.799026	0.80184603	0.371594	2.157856775	0.030939	0.149461	NOT
CCDC183	95.80132	0.85456737	0.396055	2.157701041	0.030951	0.149474	NOT
DDX20	556.8321	0.31503252	0.146007	2.157657908	0.030954	0.149474	NOT
MLANA	18.66617	-0.615638	0.285333	-2.15761022	0.030958	0.149474	NOT
PHOSPHO1	12.5387	-0.6255002	0.289931	-2.15740746	0.030974	0.149524	NOT
KIF5C	66.54432	-0.8215287	0.380815	-2.15729015	0.030983	0.149541	NOT
RP11-674N	1.339806	1.72142699	0.798064	2.157003975	0.031005	NA	NA
AC007278.	2.765415	-1.2700737	0.588839	-2.15691316	0.031012	0.149593	NOT
RP11-212F	20.48094	0.84609393	0.392276	2.156882251	0.031015	0.149593	NOT
SCMH1	959.4663	0.41209597	0.19107	2.156781785	0.031023	0.149593	NOT
NFATC3	851.9231	-0.4787644	0.221987	-2.15672144	0.031027	0.149593	NOT
CTD-2373H	4.577225	-0.7736879	0.358734	-2.15671686	0.031028	0.149593	NOT
CTD-3099C	30.66484	1.11865626	0.518687	2.156709077	0.031028	0.149593	NOT
TMEM97	6163.032	0.58486382	0.27119	2.156657429	0.031032	0.149593	NOT
RP11-62H7	11.0135	-0.7558098	0.350505	-2.1563479	0.031057	0.149658	NOT
EIF2D	3066.012	0.28119264	0.130403	2.156342828	0.031057	0.149658	NOT
GPT	5732.452	-1.0022946	0.464922	-2.15583315	0.031097	0.149823	NOT
RP11-1055	123.5228	-0.8884335	0.412182	-2.15543733	0.031128	0.149945	NOT
ITPA	2234.238	0.46008885	0.213471	2.1552789	0.03114	0.149978	NOT
COASY	4833.993	0.3199116	0.148442	2.155135337	0.031151	0.150006	NOT
LYZ	11702.28	1.07211401	0.497488	2.15505657	0.031157	0.150009	NOT
LYSMD2	554.6036	0.40729582	0.189026	2.154710271	0.031185	0.150113	NOT
RP11-473N	6.985453	0.75059416	0.348374	2.154564963	0.031196	0.150141	NOT
CACNA1C-A	2.982853	-1.1029366	0.51201	-2.15413012	0.03123	0.150278	NOT
AC073321.	4.266626	-1.4943704	0.693821	-2.15382621	0.031254	0.150366	NOT
RP5-1042K	2.428081	-0.9372746	0.435244	-2.15344647	0.031284	0.150483	NOT
PRSS3	396.7538	1.48571621	0.690018	2.153155459	0.031306	0.150563	NOT
PLCL1	88.47328	-0.5686028	0.264086	-2.15309434	0.031311	0.150563	NOT
PCDHGA9	19.62194	-0.7760099	0.360445	-2.15292303	0.031325	0.150601	NOT
RAB11B-AS	487.8029	-0.693569	0.322169	-2.15280864	0.031334	0.150617	NOT
PCDHGA1	46.79139	-1.065708	0.4951	-2.15251197	0.031357	0.150624	NOT
SSX2IP	1379.381	0.62587965	0.290772	2.152478039	0.03136	0.150624	NOT
ASB16	57.75923	-0.6212804	0.288635	-2.1524756	0.03136	0.150624	NOT
SPATA31D1	2.639293	4.0827173	1.896775	2.152452473	0.031362	0.150624	NOT
SNX19P2	2.267148	-0.8930093	0.414895	-2.15237299	0.031368	0.150624	NOT
RP11-545I	1.592349	2.61860635	1.216616	2.152368123	0.031368	0.150624	NOT
RP11-484K	2.802593	1.04020294	0.483363	2.152013678	0.031396	0.150731	NOT
KDELR3	1183.56	0.61280719	0.284786	2.151814081	0.031412	0.150738	NOT

TMED7	5512.017	-0.3620702	0.168267	-2.15175737	0.031416	0.150738	NOT
RP11-434I	3.766439	-1.6053879	0.746094	-2.15172196	0.031419	0.150738	NOT
LONP2	6275.079	-0.5068605	0.235561	-2.1517124	0.03142	0.150738	NOT
RDH8	10.05969	1.61044696	0.748484	2.151611792	0.031428	0.15075	NOT
SNRPGP4	3.717958	-1.070927	0.497765	-2.15147146	0.031439	0.150762	NOT
CITED1	9.209627	-0.9129349	0.424337	-2.15143778	0.031442	0.150762	NOT
THEM6	4769.044	0.54886404	0.255141	2.151219855	0.031459	0.150809	NOT
CTB-171A8	123.154	-0.6706747	0.311772	-2.15117301	0.031463	0.150809	NOT
RP11-438I	17.37394	0.63235613	0.293998	2.150883912	0.031485	0.150892	NOT
RBMX2	754.2325	0.2923577	0.135951	2.150461296	0.031519	0.151025	NOT
OR8G3P	1.753254	2.58891728	1.203949	2.150354295	0.031527	0.151025	NOT
IGFN1	13.23918	-1.2205048	0.567625	-2.15019737	0.03154	0.151025	NOT
RP11-496I	4.525372	1.26205281	0.586948	2.150194558	0.03154	0.151025	NOT
RP11-736N	1.788532	1.55391821	0.722692	2.150179904	0.031541	0.151025	NOT
RP11-445F	16.48953	1.06451654	0.495119	2.150020057	0.031554	0.151038	NOT
LSG1	1708.55	0.22898061	0.106504	2.14996206	0.031558	0.151038	NOT
AC078941.	1.644606	-1.3383797	0.622521	-2.14993368	0.03156	0.151038	NOT
SPATA31C2	0.83122	2.03586037	0.947127	2.149510438	0.031594	NA	NA
RP5-858B6	7.680377	0.72206657	0.335935	2.149423662	0.031601	0.151195	NOT
LINC00944	48.48205	1.28222224	0.596555	2.14937824	0.031604	0.151195	NOT
PPP2R3C	597.3232	0.31169297	0.145024	2.149247794	0.031615	0.151218	NOT
CDC42P6	8.479265	0.78114146	0.363621	2.148227317	0.031696	0.151578	NOT
WBSCR27	220.3416	0.74895838	0.348674	2.148020472	0.031712	0.15163	NOT
MT-CO2	370665.2	-0.5205566	0.242433	-2.14722254	0.031776	0.151907	NOT
KLHL24	2080.61	-0.3776531	0.175912	-2.14683543	0.031806	0.151964	NOT
STMND1	45.02226	-1.4049699	0.654455	-2.14677737	0.031811	0.151964	NOT
SLC38A10	12980.16	-0.3569801	0.166288	-2.14676034	0.031812	0.151964	NOT
ZNF721	584.0163	-0.3751138	0.17474	-2.14669428	0.031818	0.151964	NOT
STK3	1167.772	0.35818751	0.166858	2.146659813	0.03182	0.151964	NOT
PCDHB8	38.55674	-1.3388414	0.623689	-2.14665033	0.031821	0.151964	NOT
TAF12	863.8478	0.36998891	0.172369	2.146498771	0.031833	0.151993	NOT
CA3	19.12936	-0.9703429	0.452072	-2.14643468	0.031838	0.151993	NOT
GNG2	356.5515	-0.5486066	0.255621	-2.14617427	0.031859	0.152065	NOT
ADAT2	230.5562	0.49041952	0.228582	2.145484809	0.031914	0.152301	NOT
RP11-599B	4.068117	2.28489547	1.065058	2.145324422	0.031927	0.152335	NOT
KLHL26	392.4657	-0.5108986	0.238187	-2.1449517	0.031957	0.152446	NOT
PXYLP1	451.7448	0.5748059	0.267988	2.144893713	0.031961	0.152446	NOT
VWA3B	10.85114	-1.2298097	0.573411	-2.14472712	0.031975	0.152482	NOT
ATP4A	5.310237	1.94483981	0.907029	2.144187662	0.032018	0.152662	NOT
LTA4H	2611.152	0.23934154	0.111631	2.144044533	0.032029	0.152689	NOT
RP11-308I	4.217626	1.7929665	0.836315	2.143889536	0.032042	0.152722	NOT
CTC-429L1	5.464014	-0.7974159	0.371982	-2.1436976	0.032057	0.152729	NOT
LAG3	207.2762	0.86738101	0.40463	2.143638799	0.032062	0.152729	NOT
RP11-706F	19.50595	0.7731192	0.360669	2.143572531	0.032067	0.152729	NOT
SUSD1	288.0411	0.50039766	0.233443	2.143549725	0.032069	0.152729	NOT
RBBP8	840.1851	0.50901081	0.237465	2.14351846	0.032071	0.152729	NOT
WI2-89031	2.759857	1.15426528	0.538623	2.142992797	0.032114	0.152828	NOT
TNFRSF9	100.9397	1.07692063	0.502554	2.142893469	0.032122	0.152828	NOT
CTC-339F2	2.664567	-1.0135829	0.473001	-2.142879	0.032123	0.152828	NOT

DIRC3	7.489563	0.73250055	0.341831	2.142872177	0.032123	0.152828	NOT
CAPN11	29.27559	-0.5201071	0.242716	-2.14286489	0.032124	0.152828	NOT
ADH1C	19678.88	-1.2455407	0.58127	-2.14279315	0.03213	0.152828	NOT
KCNB1	703.4468	-1.3016271	0.607451	-2.14276986	0.032132	0.152828	NOT
MCU	887.2093	0.34202719	0.159669	2.142098167	0.032186	0.153058	NOT
PAPSS2	3911.728	-0.6507325	0.303829	-2.1417704	0.032212	0.153148	NOT
TCEB1P28	1.439521	-1.1530163	0.53837	-2.14168135	0.032219	0.153148	NOT
IL13RA1	7914.147	-0.4346335	0.202949	-2.14158674	0.032227	0.153148	NOT
FYTTD1P1	63.30931	-1.2377912	0.57798	-2.1415821	0.032227	0.153148	NOT
ERAL1	2937.802	0.28873799	0.13483	2.141500102	0.032234	0.153153	NOT
HLA-V	44.67105	1.11338526	0.520067	2.140849987	0.032286	0.153375	NOT
KRTAP10-4	1.627444	-1.3585036	0.634766	-2.14016532	0.032341	0.15361	NOT
KATNAL1	466.7972	-0.5322916	0.248724	-2.14008826	0.032348	0.153613	NOT
KRT80	198.4609	1.31756449	0.615973	2.13899851	0.032436	0.15396	NOT
NDUFS6	4865.4	0.53729818	0.251193	2.138981865	0.032437	0.15396	NOT
MMEL1	20.11987	1.10831865	0.518154	2.138975662	0.032438	0.15396	NOT
PDCD10	1033.827	0.27223994	0.127307	2.138450109	0.03248	0.154135	NOT
SFXN2	1048.358	-0.5885119	0.275221	-2.1383247	0.03249	0.154156	NOT
MNAT1	492.2795	0.34877266	0.163123	2.138099305	0.032509	0.154216	NOT
EIF3B	11272.98	0.4103899	0.191973	2.137747603	0.032537	0.15431	NOT
UPF3AP3	4.38739	-0.7749395	0.362511	-2.13769882	0.032541	0.15431	NOT
RP1-63G5.	3.907235	-1.2608092	0.589812	-2.13764503	0.032546	0.15431	NOT
PCBP2	13338.91	0.24566429	0.11493	2.137512501	0.032556	0.154334	NOT
ASS1	37629.61	-0.7869838	0.368254	-2.13706827	0.032592	0.154465	NOT
SERTAD1	640.8251	-0.4841928	0.226573	-2.1370313	0.032595	0.154465	NOT
CTA-204B4	86.45168	0.4862759	0.227576	2.136760898	0.032617	0.154543	NOT
ZNF136	250.8132	-0.390624	0.182865	-2.1361313	0.032669	0.154724	NOT
PLIN3	2083.653	0.37452063	0.175327	2.136122674	0.032669	0.154724	NOT
KIAA0825	28.97647	-0.5952979	0.278688	-2.13607257	0.032673	0.154724	NOT
LYG2	7.184121	-0.9298282	0.435311	-2.13601013	0.032679	0.154724	NOT
DLX2-AS1	1.554689	2.37962059	1.114186	2.135747878	0.0327	0.154775	NOT
CPVL	1570.713	0.72643767	0.340138	2.135713977	0.032703	0.154775	NOT
ELMO2	1184.317	0.27832786	0.130326	2.13562969	0.03271	0.154775	NOT
KLHL41	9.441962	-0.8372044	0.392023	-2.13559932	0.032712	0.154775	NOT
BMS1P4	15.90137	-0.5348591	0.250462	-2.13548709	0.032721	0.154791	NOT
LINC0084(	5.044988	-1.0556261	0.494405	-2.1351439	0.032749	0.154877	NOT
MICU1	3939.34	-0.3558634	0.166671	-2.13512606	0.032751	0.154877	NOT
DDRGK1	4995.226	-0.4432448	0.207614	-2.13495018	0.032765	0.154917	NOT
PALM2	61.56513	-0.9158026	0.428996	-2.13475808	0.032781	0.15494	NOT
CD93	2271.483	-0.4891272	0.229126	-2.13475189	0.032781	0.15494	NOT
RAC1	11405.66	0.33661456	0.157719	2.134268316	0.032821	0.1551	NOT
RP3-416H2	14.9988	-1.330857	0.623624	-2.13407012	0.032837	0.15515	NOT
CTD-2256F	13.49808	-0.7460625	0.349634	-2.13383925	0.032856	0.155212	NOT
AC138969.	13.25225	0.78848671	0.369603	2.133331345	0.032898	0.155381	NOT
RP11-543F	357.6809	0.80374088	0.376768	2.133251565	0.032904	0.155385	NOT
RP11-147I	261.0217	0.48980329	0.229664	2.132691659	0.03295	0.155554	NOT
HARS2	1492.732	0.29434051	0.138015	2.132675105	0.032951	0.155554	NOT
MX2	477.9562	0.82495238	0.386865	2.132404225	0.032974	0.155632	NOT
RP11-298(	1.721793	-0.992623	0.465529	-2.13224512	0.032987	0.155667	NOT

RPS6	48147.79	0.44440108	0.208503	2.131384981	0.033057	0.155974	NOT
KLF3	3166.039	-0.2685963	0.12603	-2.13121424	0.033071	0.156013	NOT
HIST4H4	22.73916	-0.5797085	0.272036	-2.13099672	0.033089	0.15607	NOT
SLC39A8	1614.999	-0.576913	0.27075	-2.13079848	0.033106	0.15612	NOT
FLJ42969	1.192946	1.84924798	0.867929	2.130643082	0.033119	NA	NA
HOXB7	89.07566	0.98259012	0.461217	2.130427618	0.033136	0.156237	NOT
FAM187B2I	2.274412	-0.8554224	0.401551	-2.13029701	0.033147	0.156251	NOT
FAM189A1	15.21527	1.22243796	0.573854	2.130222984	0.033153	0.156251	NOT
RP11-813I	1.582447	-1.8723569	0.878965	-2.13018266	0.033157	0.156251	NOT
STRIP2	162.1109	0.9426209	0.442533	2.130058693	0.033167	0.156272	NOT
CLU	195947.6	-0.5998237	0.281611	-2.12997596	0.033174	0.156277	NOT
TMEM251	472.5178	0.35714338	0.167737	2.129191732	0.033238	0.156534	NOT
SLC10A5	134.7905	-0.6049502	0.284124	-2.1291752	0.03324	0.156534	NOT
RSPO3	28.13755	-1.3519844	0.635073	-2.12886473	0.033265	0.156628	NOT
KDM3A	1574.907	0.32747792	0.153839	2.128712257	0.033278	0.15666	NOT
KL	45.29604	-0.7023773	0.329991	-2.12847116	0.033298	0.156727	NOT
CSPG4P12	12.08061	0.68472464	0.321719	2.128331292	0.03331	0.156755	NOT
UHRF1BP1	1109.39	0.38204282	0.179511	2.128236622	0.033317	0.156764	NOT
RP11-3P17	2.360465	-1.2389279	0.582176	-2.12809845	0.033329	0.156765	NOT
CEP131	890.5031	0.48489787	0.22786	2.128051256	0.033333	0.156765	NOT
COMMD3	1218.001	0.35950461	0.168938	2.128026388	0.033335	0.156765	NOT
NR2F1-AS1	135.0536	-0.67603	0.317714	-2.12779143	0.033354	0.156808	NOT
GPAA1	13102.41	0.50578064	0.237717	2.127662521	0.033365	0.156808	NOT
LRIG3	927.2775	0.66570735	0.312884	2.127651259	0.033366	0.156808	NOT
INTS4	729.3027	0.22754504	0.106949	2.127603761	0.03337	0.156808	NOT
RP11-138A	9.622441	0.8925529	0.419518	2.127565775	0.033373	0.156808	NOT
FAM19A4	7.262111	2.25796523	1.061527	2.127092307	0.033412	0.156944	NOT
AL662800.	10.99018	-0.5254251	0.247017	-2.12707937	0.033413	0.156944	NOT
RP11-279I	626.1906	-1.1192196	0.526219	-2.12690934	0.033428	0.156983	NOT
CD2BP2	2558.979	0.33111887	0.155691	2.12676352	0.03344	0.157013	NOT
ATP6V1E1	4548.024	0.30196397	0.141999	2.126528622	0.033459	0.157077	NOT
LLNLR-304	3.128183	1.00181787	0.47113	2.126413199	0.033469	0.157095	NOT
RCC2P6	1.843263	1.27222156	0.59834	2.12625315	0.033482	0.157131	NOT
LRRC41	3163.739	0.28892583	0.135909	2.125872169	0.033514	0.157249	NOT
ZNF792	199.1182	0.42502863	0.199937	2.125810908	0.033519	0.157249	NOT
SATB1	550.1122	-0.7057704	0.332012	-2.12573569	0.033525	0.157251	NOT
EYA2	79.13364	1.0980778	0.51666	2.125340017	0.033558	0.157379	NOT
SIX4	74.11959	0.93738287	0.44112	2.12500518	0.033586	0.157471	NOT
RP11-380M	14.95873	-0.9023915	0.424674	-2.12490297	0.033595	0.157471	NOT
SLC37A3	803.1005	0.40622613	0.191175	2.124895393	0.033595	0.157471	NOT
LZTS2	1879.856	0.47125387	0.221802	2.124659608	0.033615	0.157528	NOT
HCG14	5.774155	-0.8696202	0.409308	-2.12461121	0.033619	0.157528	NOT
PPAP2A	2774.861	-0.5427689	0.255479	-2.12451204	0.033627	0.157538	NOT
P4HA2	1981.557	0.56384346	0.265407	2.12444558	0.033633	0.157538	NOT
MDM2	4534.587	-0.4768691	0.224478	-2.12435078	0.033641	0.157548	NOT
C9orf57	10.72278	-1.525162	0.717972	-2.12426438	0.033648	0.157555	NOT
DCHS1	502.9075	-0.5269123	0.248076	-2.12399934	0.03367	0.157631	NOT
FTLP2	164.2312	0.9457673	0.445306	2.123857067	0.033682	0.15766	NOT
RP3-327A1	3.16754	0.86976549	0.409547	2.123727595	0.033693	0.157683	NOT

KCNIP2	36.77647	-0.6153671	0.289809	-2.12335102	0.033724	0.157768	NOT
C4orf47	14.49327	0.84911968	0.399902	2.123316967	0.033727	0.157768	NOT
FAM109B	287.8786	0.87153394	0.410465	2.123285591	0.03373	0.157768	NOT
MXD4	3424.092	-0.4733463	0.222936	-2.12323371	0.033734	0.157768	NOT
CSAG3	42.63405	2.40142085	1.131278	2.122750265	0.033775	0.15792	NOT
DDX51	999.0987	0.3387357	0.159577	2.122705409	0.033779	0.15792	NOT
NDUFAF1	1048.962	-0.4295297	0.202398	-2.12219829	0.033821	0.158092	NOT
RP11-900F	3.78015	1.25865815	0.593153	2.121979793	0.033839	0.15814	NOT
RP4-616B8	19.58521	0.84954041	0.400361	2.121937838	0.033843	0.15814	NOT
RP11-469H	1.397199	1.88454306	0.888244	2.12165035	0.033867	0.158225	NOT
SLFNL1-AS	51.49125	-0.7678476	0.361929	-2.12154186	0.033876	0.158229	NOT
PEX2	1867.854	0.34578242	0.162989	2.121503147	0.033879	0.158229	NOT
CTD-3222I	2.685886	-0.9023482	0.425399	-2.12118168	0.033907	0.158328	NOT
AC090602.	1.814091	1.08446581	0.511315	2.120932972	0.033927	0.158398	NOT
SLC25A11	3343.373	-0.4097099	0.193202	-2.12062921	0.033953	0.15849	NOT
DYNLT1	2068.806	0.41284036	0.194702	2.120366065	0.033975	0.158561	NOT
YBX1P10	112.3824	0.47862172	0.225732	2.12031124	0.03398	0.158561	NOT
TNS1	5255.606	-0.4772809	0.225226	-2.11912005	0.03408	0.158985	NOT
TCTEX1D4	9.956185	-0.9562856	0.451271	-2.119095	0.034082	0.158985	NOT
ZRANB1	850.7931	-0.5700401	0.269027	-2.11889503	0.034099	0.159005	NOT
RP11-276H	4.279821	-0.9446516	0.445823	-2.11889416	0.034099	0.159005	NOT
RP11-462C	2.602128	1.95897921	0.92459	2.118755497	0.034111	0.159005	NOT
CHSY3	105.0017	-0.7421761	0.350297	-2.11870492	0.034115	0.159005	NOT
RP11-561I	2.552293	2.83590586	1.338514	2.11869691	0.034116	0.159005	NOT
PNPLA4	964.2582	-0.4542427	0.214427	-2.11839916	0.034141	0.159085	NOT
RPL39P36	5.350382	0.52254239	0.246676	2.11833623	0.034147	0.159085	NOT
RP11-767I	6.310924	-1.2924371	0.610133	-2.11828723	0.034151	0.159085	NOT
MRPS28	1104.788	0.45950371	0.216933	2.118186673	0.034159	0.159097	NOT
MAFIP	12.15935	-0.9217141	0.435165	-2.11807798	0.034168	0.159113	NOT
FMO2	106.2764	-0.9347008	0.441376	-2.11769587	0.034201	0.159236	NOT
RP4-591C2	113.8211	-0.4963882	0.234475	-2.11701538	0.034259	0.159474	NOT
SIDT1	56.39833	-0.6725253	0.317685	-2.11695576	0.034264	0.159474	NOT
AC010761.	5.223299	-0.8563177	0.404528	-2.11683156	0.034274	0.159495	NOT
RNA5SP311	1.543122	-1.0598586	0.500718	-2.11667906	0.034287	0.159528	NOT
ZNF23	35.74598	-0.5931957	0.280292	-2.11635177	0.034315	0.15963	NOT
IL1RL1	31.83082	-1.0935979	0.516852	-2.11588072	0.034355	0.159789	NOT
PMEL	245.0758	-0.5644183	0.266807	-2.11545935	0.034391	0.159882	NOT
RP3-375P9	15.81741	0.82218428	0.388679	2.115328392	0.034402	0.159882	NOT
CTB-167G5	1.929275	1.71639373	0.811457	2.115200899	0.034413	0.159882	NOT
E2F7	157.9963	0.85016202	0.401937	2.115160587	0.034416	0.159882	NOT
CTDSPL	2116.723	0.45333971	0.214344	2.115013788	0.034429	0.159882	NOT
RP11-502M	2.833277	-2.0316634	0.960612	-2.11496852	0.034433	0.159882	NOT
CELF3	2.961705	1.28245884	0.606375	2.114960586	0.034433	0.159882	NOT
TRPC7	1.434558	1.92219519	0.908875	2.114916067	0.034437	0.159882	NOT
EIF3EP1	14.68114	0.76047343	0.359579	2.114898497	0.034439	0.159882	NOT
SLC4A2	7104.51	0.38547069	0.182269	2.114850172	0.034443	0.159882	NOT
AL117187.	1.86752	-1.2224984	0.578066	-2.11480892	0.034446	0.159882	NOT
GNPAT	3920.915	0.34630411	0.163753	2.114795814	0.034447	0.159882	NOT
AC009005.	70.6629	0.78737228	0.372327	2.114733485	0.034453	0.159882	NOT

ANO9	301.0125	1.09032447	0.515607	2.114642629	0.03446	0.159882	NOT
RP11-488I	267.2081	0.64741444	0.306184	2.114459687	0.034476	0.159882	NOT
FEM1A	71.09451	-0.4719573	0.223206	-2.11444431	0.034477	0.159882	NOT
RP11-1017	16.33713	-0.7339151	0.347099	-2.11442683	0.034479	0.159882	NOT
PCDHB13	99.10674	-0.9063426	0.428654	-2.11439067	0.034482	0.159882	NOT
CDH1	9036.534	-0.6813494	0.322258	-2.11430067	0.03449	0.159882	NOT
NPNT	1276.439	1.13553213	0.537082	2.114263774	0.034493	0.159882	NOT
SART3	1815.233	0.22073723	0.104411	2.11411739	0.034505	0.159912	NOT
JAKMIP2	90.2545	-0.875842	0.41432	-2.1139273	0.034521	0.15996	NOT
ZMAT1	360.1632	-0.7546906	0.357031	-2.11379634	0.034533	0.15997	NOT
PLEKHF2	947.908	0.35206734	0.166559	2.113765406	0.034535	0.15997	NOT
RPL5P9	4.168745	0.89054699	0.421365	2.113482065	0.03456	0.160055	NOT
FAM20A	3893.018	-0.631222	0.298781	-2.1126611	0.03463	0.160345	NOT
RP11-23D2	6.747655	-1.0316364	0.488328	-2.11258903	0.034636	0.160345	NOT
POLR3D	492.0099	0.38768891	0.183518	2.112542577	0.03464	0.160345	NOT
ANKRD27	1002.935	0.37216717	0.17618	2.112425691	0.03465	0.160349	NOT
TSN	3393.946	0.29991939	0.141994	2.112202228	0.034669	0.160349	NOT
LRP11	1574.325	0.40131628	0.189999	2.112201624	0.034669	0.160349	NOT
RP11-366I	2.051676	1.9589871	0.927463	2.112198968	0.034669	0.160349	NOT
KLHL30	30.15697	0.99118072	0.469277	2.112144003	0.034674	0.160349	NOT
AC084219.	6.045325	-0.9679574	0.458287	-2.11211967	0.034676	0.160349	NOT
MIR202HG	1.560731	-2.2535081	1.067074	-2.11185691	0.034699	0.160425	NOT
PCDHGA12	39.81199	-0.8918629	0.42241	-2.11137001	0.034741	0.160591	NOT
SYT15	15.37491	0.69592849	0.329637	2.111196161	0.034755	0.160611	NOT
ANP32A	5313.894	0.28384678	0.134453	2.11112163	0.034762	0.160611	NOT
RP11-121(	100.1904	0.50763607	0.240459	2.111114051	0.034763	0.160611	NOT
TNFRSF1B	2338.811	-0.4699556	0.22262	-2.11101942	0.034771	0.160621	NOT
GCN1L1	5140.512	0.27536781	0.130463	2.110696985	0.034798	0.160705	NOT
NBPF9	463.0422	-0.5681212	0.269166	-2.1106711	0.034801	0.160705	NOT
YY1AP1	2470.202	0.26590941	0.125998	2.110417978	0.034822	0.160778	NOT
15-Sep	5709.388	0.29504242	0.139808	2.110333323	0.03483	0.160784	NOT
LHFPL3-AS	33.69538	1.62021831	0.76781	2.110181702	0.034843	0.160817	NOT
UGT2B15	12036.98	-1.074051	0.509028	-2.11000186	0.034858	0.160825	NOT
ORAOV1	638.442	0.67420707	0.319532	2.109986035	0.03486	0.160825	NOT
F5	27273.37	-0.7444808	0.352842	-2.10995448	0.034862	0.160825	NOT
RP11-779(	2.005562	-0.8572322	0.406309	-2.10980221	0.034875	0.160855	NOT
CPT2	3119.41	-0.5207921	0.246851	-2.10974248	0.034881	0.160855	NOT
DICER1-AS	149.376	-0.5680938	0.269283	-2.10965547	0.034888	0.160855	NOT
SPAG1	403.1347	0.56703455	0.268787	2.109604451	0.034892	0.160855	NOT
NAGPA	1155.462	0.38192765	0.181052	2.109485805	0.034903	0.160875	NOT
MROH8	83.80599	-0.6185151	0.293227	-2.10933945	0.034915	0.160885	NOT
XDH	3443.435	-0.9596956	0.454978	-2.10932226	0.034917	0.160885	NOT
OTP	1.931686	2.13493752	1.012237	2.109127501	0.034934	0.160935	NOT
ZBTB43	534.591	-0.3597746	0.170596	-2.10892737	0.034951	0.160988	NOT
PPP1R8	1296.803	0.21722327	0.10301	2.108755303	0.034966	0.161029	NOT
CTB-13F3.	15.61345	0.63085401	0.299195	2.108503442	0.034987	0.161101	NOT
RRM1	2467.056	0.39708456	0.188355	2.108167571	0.035016	0.161208	NOT
NKAIN2	68.31119	1.54131921	0.731158	2.108051783	0.035027	0.161227	NOT
BCKDK	3526.005	-0.3669608	0.174087	-2.10791419	0.035038	0.161254	NOT

C14orf37	54.37217	-0.7825273	0.371289	-2.1075949	0.035066	0.161327	NOT
EIF5AP2	2.818545	-0.8123625	0.385449	-2.10757426	0.035068	0.161327	NOT
RP4-680D5	41.04402	-0.7096243	0.33671	-2.10752506	0.035072	0.161327	NOT
SERPING1	112113.2	-0.6956615	0.330099	-2.10743068	0.03508	0.161337	NOT
SDHD	4802.181	-0.4039432	0.191688	-2.10729522	0.035092	0.161364	NOT
EIF2B2	1090.698	0.30840609	0.146366	2.107082753	0.03511	0.161421	NOT
GSTA3	1.632471	-1.1333103	0.537936	-2.10677493	0.035137	0.161513	NOT
UBA3	1230.031	0.26836936	0.127388	2.106713472	0.035142	0.161513	NOT
HRG	96557.69	-1.1489539	0.545396	-2.1066402	0.035149	0.161515	NOT
RP11-36C2	50.9896	0.67066296	0.318376	2.1065091	0.03516	0.16152	NOT
DNMBP-AS1	65.18551	-0.9155573	0.434636	-2.10649182	0.035162	0.16152	NOT
UBR3	2144.436	-0.3451791	0.16387	-2.10641889	0.035168	0.16152	NOT
MT1X	4735.341	-1.1656097	0.553378	-2.10635352	0.035174	0.16152	NOT
GNAI3	3366.705	0.26645973	0.126525	2.10597679	0.035206	0.161643	NOT
VPS35	3683.458	0.37261609	0.176943	2.105850356	0.035217	0.161666	NOT
SEPHS1P6	7.143531	-0.810113	0.384729	-2.10567188	0.035233	0.161672	NOT
MBOAT7	3034.469	0.34157207	0.162216	2.105658135	0.035234	0.161672	NOT
TSC22D4	1962.242	0.36458415	0.173147	2.105630037	0.035237	0.161672	NOT
SERBP1P6	1.755901	-0.7758456	0.368499	-2.1054235	0.035254	0.161695	NOT
RP11-867(	12.17311	-0.512895	0.243609	-2.10539914	0.035257	0.161695	NOT
SERPINB3	1.98615	2.77689938	1.318963	2.105365982	0.035259	0.161695	NOT
SAP25	6.788503	-0.8099863	0.384748	-2.10523908	0.035271	0.161719	NOT
KMO	1051.767	-0.8562477	0.406784	-2.10492226	0.035298	0.161818	NOT
LDLRAD4	1141.87	-0.5020667	0.238569	-2.10449031	0.035336	0.161963	NOT
TBC1D9B	6770.801	-0.3167824	0.150534	-2.10439253	0.035344	0.161974	NOT
E2F8	247.9706	0.75358427	0.358171	2.103981375	0.03538	0.162084	NOT
GC	116875.7	-0.7707472	0.366328	-2.10398101	0.03538	0.162084	NOT
HSPE1P3	5.923996	0.74485029	0.354132	2.103313203	0.035438	0.162323	NOT
PNPLA1	2.077234	1.27771227	0.607495	2.103247049	0.035444	0.162323	NOT
HMGXB3	1952.03	0.25926941	0.123275	2.103172885	0.035451	0.162325	NOT
COX5BP6	5.217016	0.79769682	0.379388	2.102590354	0.035502	0.162508	NOT
AC097639.	2.646012	0.90182839	0.428923	2.102540338	0.035506	0.162508	NOT
RP11-276E	5.109066	1.25113394	0.595073	2.102486892	0.035511	0.162508	NOT
TMEM184B	2344.67	0.38675923	0.18396	2.102404978	0.035518	0.162508	NOT
ATP5O	4043.893	-0.4693639	0.223254	-2.10237377	0.035521	0.162508	NOT
IL18RAP	37.33438	-0.7481795	0.355901	-2.10221525	0.035534	0.162519	NOT
CTC-534A2	104.6854	0.38081276	0.181149	2.102208554	0.035535	0.162519	NOT
RP11-589M	1.891527	-1.1620925	0.552921	-2.10173367	0.035577	0.162682	NOT
INAFM1	469.7152	-0.5663001	0.269472	-2.10151934	0.035595	0.162741	NOT
RP11-532F	41.17901	1.28907829	0.613586	2.100892694	0.03565	0.162946	NOT
ACE2	443.493	1.48387938	0.706316	2.100871161	0.035652	0.162946	NOT
TMSB4XP4	20.78481	0.68478365	0.325988	2.100641859	0.035672	0.162995	NOT
CDC42SE1	2770.61	0.30097019	0.143277	2.100611884	0.035675	0.162995	NOT
GFRA1	1560.646	-1.1930882	0.568093	-2.1001642	0.035714	0.163131	NOT
RP11-817]	18.39869	0.6896277	0.328397	2.09997976	0.035731	0.163131	NOT
POU6F1	182.0179	-0.431918	0.205684	-2.09990787	0.035737	0.163131	NOT
GAPDHP23	9.487172	-1.4339297	0.682861	-2.09988631	0.035739	0.163131	NOT
C8orf44-S	2.891857	0.75501025	0.359553	2.099857773	0.035741	0.163131	NOT
MT2P1	26.97077	-1.0418667	0.496161	-2.09985427	0.035742	0.163131	NOT

AMIGO1	430.3074	0.51812689	0.246751	2.099794054	0.035747	0.163131	NOT
MIS18A	448.7601	0.42767961	0.203688	2.099680581	0.035757	0.163149	NOT
C9orf91	582.8202	-0.4683484	0.223075	-2.09951179	0.035772	0.163182	NOT
CHST15	1751.358	-0.5359599	0.255292	-2.09939763	0.035782	0.163182	NOT
NUDT7	584.6964	-0.6690212	0.318673	-2.09939431	0.035782	0.163182	NOT
RGN	6400.652	-0.7805413	0.371819	-2.09924849	0.035795	0.163189	NOT
RP11-513(	98.74652	-1.1996142	0.571465	-2.09918961	0.0358	0.163189	NOT
GUSBP9	3.104378	-0.7844375	0.373699	-2.09911804	0.035806	0.163189	NOT
SNHG11	746.3264	0.5521375	0.263035	2.099103975	0.035808	0.163189	NOT
MKLN1-AS	76.52082	0.49753493	0.237044	2.098916906	0.035824	0.163214	NOT
C1orf105	29.39098	0.85903202	0.409288	2.09884406	0.035831	0.163214	NOT
NCAPD2P1	40.47798	1.4179656	0.675596	2.098837033	0.035831	0.163214	NOT
RP11-298I	16.63894	-0.6260672	0.298401	-2.09807455	0.035899	0.163468	NOT
IDH1-AS1	41.56956	0.72679087	0.34642	2.098005897	0.035905	0.163468	NOT
RN7SL128F	1.729586	-0.853522	0.406836	-2.09795114	0.035909	0.163468	NOT
DLEC1	34.2541	-0.6420532	0.306041	-2.0979333	0.035911	0.163468	NOT
GPR133	1225.039	1.1209168	0.534433	2.097393182	0.035959	0.163636	NOT
PPP2R2B	81.85089	1.10710771	0.527853	2.097378443	0.03596	0.163636	NOT
EHHADH	10402.94	-0.8310209	0.396304	-2.09692574	0.036	0.163791	NOT
CLUHP3	471.6983	-0.4674976	0.222984	-2.0965525	0.036033	0.163884	NOT
SLC39A14	18024.37	-0.6407459	0.305624	-2.09651688	0.036036	0.163884	NOT
LYSMD3	1239.011	-0.404641	0.193016	-2.09640828	0.036046	0.163884	NOT
CSTF3-AS1	2.663863	1.00226157	0.478089	2.096389337	0.036048	0.163884	NOT
FXYD6	567.8308	-0.5378346	0.256568	-2.09626268	0.036059	0.163884	NOT
SPRN	273.7479	0.55483581	0.264682	2.096239019	0.036061	0.163884	NOT
CCDC14	1051.608	0.49732881	0.237251	2.09621661	0.036063	0.163884	NOT
DNTT	2.172582	2.12134376	1.012431	2.095296662	0.036145	0.164228	NOT
RP11-210M	1.227523	1.5222719	0.726548	2.095212951	0.036152	NA	NA
IRGM	5.116776	-1.3578006	0.648059	-2.09518017	0.036155	0.164244	NOT
AC253576.	5.861754	0.86308759	0.411951	2.095120768	0.03616	0.164244	NOT
ACKR4	11.49338	-0.6833374	0.326419	-2.09343405	0.03631	0.164898	NOT
RP11-10L7	0.850651	-1.3936987	0.665764	-2.09338334	0.036315	NA	NA
NEGR1	13.2194	-1.07378	0.512979	-2.09322446	0.036329	0.164951	NOT
MAFG	1315.285	0.462615	0.221012	2.093163901	0.036335	0.164951	NOT
RP4-758J1	92.64785	-0.782911	0.374048	-2.0930754	0.036342	0.164951	NOT
RP11-166F	27.00223	0.89936473	0.429718	2.092920317	0.036356	0.164951	NOT
HS1BP3	2860.59	0.375948	0.179631	2.092886987	0.036359	0.164951	NOT
ZC3HAV1L	49.54374	0.92736838	0.443108	2.092874168	0.03636	0.164951	NOT
SHOC2	1432.446	-0.2446421	0.116899	-2.09277126	0.03637	0.164951	NOT
HSPA8P1	22.63906	0.81534864	0.389605	2.092758957	0.036371	0.164951	NOT
DHX58	849.276	-0.4991427	0.238521	-2.0926561	0.03638	0.164965	NOT
RP11-366M	6.41145	-1.0433111	0.498712	-2.09201126	0.036438	0.165199	NOT
ZNF471	115.4745	0.94373377	0.451319	2.091057067	0.036523	0.165484	NOT
BLOC1S1	2519.041	-0.4754213	0.22736	-2.09105231	0.036523	0.165484	NOT
DDX10	990.1685	0.29066785	0.139007	2.091037273	0.036525	0.165484	NOT
GUF1	1299.466	-0.335032	0.160223	-2.09103634	0.036525	0.165484	NOT
UNC79	15.9529	-0.5824373	0.278579	-2.09074072	0.036551	0.165576	NOT
MYOCD	32.84787	-0.9546681	0.456652	-2.0905802	0.036566	0.165614	NOT
RP11-480Z	1.478115	-1.2122546	0.579935	-2.09032848	0.036588	0.165689	NOT

RP11-235(	7.682472	1.68727983	0.807252	2.090152731	0.036604	0.165732	NOT
C4BPA	53634.93	-1.0240303	0.489959	-2.09003284	0.036615	0.165754	NOT
NEURL1	96.76185	1.02715188	0.491512	2.089778527	0.036638	0.165811	NOT
RP4-781K5	27.2538	1.27194422	0.608661	2.089741772	0.036641	0.165811	NOT
FAM127C	179.0584	0.77118759	0.369044	2.08968812	0.036646	0.165811	NOT
ZNF75D	497.4564	-0.4147924	0.19851	-2.08953367	0.03666	0.165846	NOT
RP5-1074I	153.2166	0.61283313	0.293311	2.089365726	0.036675	0.165887	NOT
FRYL	1056.006	-0.3161183	0.151309	-2.08922406	0.036688	0.165917	NOT
CTB-133G6	2.770327	-0.6092241	0.291657	-2.08883735	0.036722	0.166046	NOT
AF127577.	1.299122	2.69445674	1.289979	2.088759921	0.036729	NA	NA
LMAN2L	1276.377	0.29883193	0.143088	2.088446967	0.036758	0.166155	NOT
RP11-381K	4.821063	1.22789414	0.587949	2.088435734	0.036759	0.166155	NOT
FBXO39	20.68479	-0.8827776	0.422804	-2.0879129	0.036806	0.166285	NOT
RP11-545I	71.75659	-0.5754863	0.275631	-2.08788555	0.036808	0.166285	NOT
B3GNTL1	327.2032	0.47118228	0.225678	2.087855084	0.036811	0.166285	NOT
RP11-21C4	3.130878	-1.3343489	0.639104	-2.08784445	0.036812	0.166285	NOT
COIL	963.6574	0.26002776	0.124553	2.087691687	0.036826	0.166319	NOT
AP4M1	539.7742	0.35239954	0.168835	2.08723789	0.036867	0.166465	NOT
CSMD3	6.40292	1.95432719	0.936362	2.08714912	0.036875	0.166465	NOT
PLAT	733.2111	-0.7200982	0.345021	-2.08711571	0.036878	0.166465	NOT
MMP24-AS1	1682.501	-0.5920849	0.283693	-2.08706243	0.036882	0.166465	NOT
EPB41L2	1622.782	0.47566654	0.227923	2.086961402	0.036892	0.166479	NOT
FCER1G	1153.407	0.73216805	0.350865	2.086749058	0.036911	0.166538	NOT
RAB29	2133.918	0.37140745	0.178005	2.086503139	0.036933	0.16661	NOT
BAGE5	2.066766	2.65237529	1.27135	2.086267459	0.036954	0.166679	NOT
AKR1B10	43780.22	1.3856815	0.664227	2.086157184	0.036964	0.166696	NOT
TPTE2P2	2.169281	1.88277572	0.902609	2.085925108	0.036985	0.166745	NOT
MFN2	5857.887	-0.3507302	0.168143	-2.08590105	0.036988	0.166745	NOT
DGCR12	2.105628	-0.9114673	0.43698	-2.0858314	0.036994	0.166746	NOT
MICAL2	2015.135	-0.5246263	0.25154	-2.08565778	0.03701	0.166789	NOT
ZDHHC22	1.799164	2.0347969	0.975649	2.085583169	0.037016	0.166792	NOT
FBXW12	7.827397	1.10122788	0.528075	2.085363924	0.037036	0.166831	NOT
RHBDL3	108.8052	1.34101869	0.643066	2.085351909	0.037037	0.166831	NOT
CDC42BPB	4394.486	-0.3559465	0.170695	-2.08528159	0.037044	0.166832	NOT
GPR146	93.83466	-0.6558004	0.31453	-2.0850166	0.037068	0.166913	NOT
CTB-50E14	28.28252	-0.681497	0.326894	-2.08476773	0.03709	0.166972	NOT
GPR137C	97.71901	0.58048672	0.278446	2.084736168	0.037093	0.166972	NOT
PRKG1	149.3181	-0.639895	0.306964	-2.08459002	0.037107	0.166979	NOT
PABPC1L	1263.078	0.64948393	0.311573	2.084532589	0.037112	0.166979	NOT
TRPM7	1994.145	-0.447818	0.214831	-2.08451643	0.037113	0.166979	NOT
SRP14	8502.494	0.28081395	0.134726	2.084333223	0.03713	0.167026	NOT
ABCF1	5132.946	0.26962446	0.129363	2.084245482	0.037138	0.167026	NOT
RPSAP54	14.23055	0.65627512	0.314881	2.084197405	0.037142	0.167026	NOT
PAQR7	617.8817	0.46751953	0.224327	2.084100575	0.037151	0.167038	NOT
MAN1C1	974.7658	-0.8051252	0.386361	-2.08386693	0.037172	0.167106	NOT
BAK1P1	6.017488	0.7341963	0.352378	2.083545772	0.037202	0.16721	NOT
TMPOP2	5.399843	0.80790544	0.38778	2.083413387	0.037214	0.167236	NOT
RIC1	930.3918	-0.372304	0.178715	-2.08323022	0.03723	0.167283	NOT
POLR2H	2018.102	0.38853237	0.186539	2.082844979	0.037265	0.167413	NOT

PLEKHG5	269.538	-0.4995565	0.239887	-2.08246417	0.0373	0.167542	NOT
SHROOM2	603.9109	-0.6865056	0.329739	-2.08196644	0.037346	0.167718	NOT
LINC0065	71.13635	0.79224281	0.380563	2.081766006	0.037364	0.167748	NOT
CTC-487M	5.642234	-0.6654739	0.319669	-2.08175788	0.037365	0.167748	NOT
CREBL2	2630.535	-0.3947973	0.189653	-2.08167987	0.037372	0.167752	NOT
CHST4	76.72706	1.64863804	0.792035	2.081520967	0.037386	0.16779	NOT
RP11-578	2.964076	2.17963671	1.047327	2.081142557	0.037421	0.167917	NOT
RP11-407	3.624337	-0.8721215	0.419124	-2.08081992	0.03745	0.168008	NOT
RYK	1373.8	0.2817784	0.135421	2.080758008	0.037456	0.168008	NOT
TMEM109	4184.494	0.33769897	0.162299	2.080718789	0.03746	0.168008	NOT
TMEM132E	29.194	-0.9414766	0.452498	-2.08062173	0.037469	0.16802	NOT
IL20RA	104.3532	1.96487988	0.944518	2.080298418	0.037498	0.168119	NOT
SCARF1	469.2939	-0.3509847	0.168723	-2.08024612	0.037503	0.168119	NOT
PLA2G12A	4.986783	-0.4959749	0.238442	-2.08006493	0.03752	0.168166	NOT
IGKV6-21	12.27581	1.97001418	0.94728	2.07965276	0.037557	0.168307	NOT
ATG2A	2611.45	-0.3050866	0.146707	-2.07956162	0.037566	0.168317	NOT
GMPPB	1472.73	-0.2987934	0.143691	-2.07941178	0.03758	0.168351	NOT
CEBPB-AS	32.90714	-0.5308423	0.255351	-2.07886942	0.037629	0.168546	NOT
RP11-467	154.7531	1.30020772	0.625595	2.078352788	0.037677	0.168711	NOT
CCDC150P	14.87998	0.88535518	0.425998	2.078309087	0.037681	0.168711	NOT
CASS4	86.1176	-0.6154636	0.296143	-2.07826652	0.037685	0.168711	NOT
RP11-586	3.605023	2.25072047	1.083115	2.078006172	0.037709	0.168791	NOT
LLGL1	710.6359	0.44724347	0.215245	2.077833947	0.037725	0.168818	NOT
ANKFY1	1608.621	-0.3959902	0.190581	-2.07780458	0.037727	0.168818	NOT
RABGGTB	2845.492	0.32476423	0.156318	2.077583053	0.037748	0.168881	NOT
AC131263.	8.032944	-0.5640166	0.271597	-2.07666872	0.037832	0.169231	NOT
ADCYAP1	10.32795	-1.3447398	0.647657	-2.0763133	0.037865	0.169324	NOT
GAB1	644.4193	-0.4558855	0.219568	-2.07628502	0.037868	0.169324	NOT
AKIP1	853.9287	0.3205962	0.154412	2.07624085	0.037872	0.169324	NOT
COL9A3	217.5889	0.93423875	0.450061	2.075803306	0.037912	0.169438	NOT
PTMAP8	2.425535	0.9107033	0.438726	2.075788756	0.037913	0.169438	NOT
SCLY	308.4778	0.54127548	0.260763	2.07573879	0.037918	0.169438	NOT
CA5A	797.9367	-0.9449868	0.455268	-2.07567035	0.037924	0.169438	NOT
SCAMP1	1358.427	-0.3709059	0.1787	-2.07557426	0.037933	0.169438	NOT
ATP13A3	7343.754	-0.3833234	0.184684	-2.07556138	0.037935	0.169438	NOT
BPIFB6	2.221187	2.72440886	1.312675	2.075464099	0.037944	0.16945	NOT
KRTAP5-7	11.64833	1.5526543	0.748349	2.074772226	0.038008	0.169703	NOT
ANKRD13B	157.667	0.65952662	0.317894	2.074671854	0.038017	0.169703	NOT
AC006272.	2.37336	-0.9310395	0.448778	-2.07461127	0.038023	0.169703	NOT
CERCAM	491.2863	0.8310164	0.40057	2.074583827	0.038025	0.169703	NOT
RP4-592A	10.44427	0.66333873	0.319769	2.074430069	0.038039	0.169732	NOT
BANP	607.9343	-0.2785875	0.134299	-2.07437671	0.038044	0.169732	NOT
RP11-761	2.446386	-0.9760988	0.470571	-2.07428531	0.038053	0.169736	NOT
C2-AS1	28.70247	-0.8035265	0.387385	-2.07423443	0.038058	0.169736	NOT
FAM47E-S	702.0094	-0.5575565	0.268834	-2.07397762	0.038081	0.169787	NOT
PAQR9	1267.309	0.69038928	0.332882	2.07397588	0.038082	0.169787	NOT
PANK1	2185.324	-0.5710925	0.275412	-2.07359479	0.038117	0.169917	NOT
DHRS3	11847.06	-0.4744825	0.228866	-2.07318684	0.038155	0.170048	NOT
AC139887.	9.350358	-0.5928357	0.285966	-2.07309762	0.038163	0.170048	NOT

PTGIR	141.8905	-0.701642	0.338455	-2.07307581	0.038165	0.170048	NOT
C1orf186	257.4254	1.31204283	0.632951	2.072897903	0.038182	0.170094	NOT
RP11-573I	10.30633	-0.8298109	0.400341	-2.07276073	0.038195	0.170095	NOT
CTD-2017F	24.01436	-0.7606586	0.366988	-2.072707	0.0382	0.170095	NOT
RP11-800A	156.9333	0.81502684	0.393221	2.072692036	0.038201	0.170095	NOT
RP11-384M	2.646613	-0.7775359	0.375191	-2.07237602	0.03823	0.170199	NOT
GREB1L	503.505	-0.6390556	0.308447	-2.07184913	0.03828	0.170359	NOT
NELFA	1184.701	0.25137213	0.121328	2.071843224	0.03828	0.170359	NOT
ZFPM2-AS1	204.9401	1.30238205	0.628627	2.071787243	0.038285	0.170359	NOT
RP11-307C	10.4775	1.09493596	0.52854	2.071622557	0.038301	0.170381	NOT
RP11-89H1	28.24668	0.88537749	0.427395	2.071566474	0.038306	0.170381	NOT
RP5-1065F	13.15159	-1.0932514	0.52775	-2.07153342	0.038309	0.170381	NOT
SLC9A7P1	29.68656	1.03167409	0.498143	2.07104199	0.038355	0.17054	NOT
LRRC6	56.44861	0.9003557	0.434746	2.070991311	0.03836	0.17054	NOT
RP11-725C	12.52898	-0.8312413	0.401382	-2.07094788	0.038364	0.17054	NOT
RPL23AP2	22.70132	0.65657732	0.317057	2.070848356	0.038373	0.170554	NOT
LIMD1	2512.852	-0.385272	0.186062	-2.07065905	0.038391	0.170604	NOT
FAM195CP	2.422801	1.168149	0.564172	2.070554632	0.0384	0.17062	NOT
LHX4-AS1	1596.538	0.36126771	0.174525	2.070005208	0.038452	0.170757	NOT
HIST1H1E	14.80329	0.76752457	0.370788	2.069983028	0.038454	0.170757	NOT
WRNIP1	2254.287	0.29940572	0.144644	2.069949351	0.038457	0.170757	NOT
MROH7	25.36057	-1.0251545	0.495269	-2.06989503	0.038462	0.170757	NOT
RP11-1259	57.06967	-1.5952083	0.770673	-2.06988906	0.038463	0.170757	NOT
RP11-366H	1.869863	2.87583229	1.389488	2.069706462	0.03848	0.170795	NOT
TMEM132D	15.1937	1.30173482	0.62896	2.069662811	0.038484	0.170795	NOT
FUS	8296.586	0.32833784	0.158663	2.069409449	0.038508	0.170848	NOT
OR7E7P	7.109925	1.01036866	0.488255	2.069346165	0.038514	0.170848	NOT
THOC3	406.093	0.348487	0.168405	2.069334342	0.038515	0.170848	NOT
RARA-AS1	146.8331	-0.4524931	0.218687	-2.0691314	0.038534	0.170884	NOT
PSPHP1	336.2562	1.92074494	0.928294	2.069111672	0.038536	0.170884	NOT
SAMD4B	3583.519	-0.2662369	0.128688	-2.068851	0.03856	0.170965	NOT
RP11-297F	1.543561	-1.4320168	0.692205	-2.06877545	0.038567	0.170968	NOT
RP11-115I	76.53691	-0.7141681	0.345295	-2.06828199	0.038614	0.171146	NOT
AC008592.	8.524962	-0.9308675	0.450094	-2.0681642	0.038625	0.17115	NOT
ECSCR	114.5679	-0.5379212	0.260106	-2.06808746	0.038632	0.17115	NOT
SEZ6	131.5089	1.69110491	0.817721	2.068070101	0.038633	0.17115	NOT
AP000350.	5.921059	-0.9493586	0.459087	-2.06792855	0.038647	0.171169	NOT
MASP1	5281.125	-0.7473938	0.361428	-2.06789037	0.03865	0.171169	NOT
CTD-2022H	2.159092	0.92868831	0.449151	2.067654023	0.038673	0.17124	NOT
POU5F1P3	3.844842	-0.681525	0.329652	-2.06740917	0.038696	0.171314	NOT
RP11-367C	7.384941	-0.9365854	0.453132	-2.06691455	0.038742	0.171483	NOT
RPS11P6	18.61809	0.68824266	0.332988	2.06686971	0.038746	0.171483	NOT
MS4A4E	10.94192	-0.676262	0.327217	-2.06670758	0.038762	0.171523	NOT
TMEM231	107.2013	-0.7226422	0.349687	-2.06654015	0.038778	0.17155	NOT
NTN3	16.49269	-0.9285149	0.449316	-2.06650707	0.038781	0.17155	NOT
NOP2	1703.558	0.32627216	0.157913	2.066156787	0.038814	0.171669	NOT
KCNE5	50.95531	1.17689825	0.569872	2.065196198	0.038904	0.172009	NOT
PCNT	1282.504	-0.3070779	0.148692	-2.06519435	0.038905	0.172009	NOT
RNF183	10.30701	1.16588593	0.564555	2.065141352	0.03891	0.172009	NOT

RP11-734I	35.94967	-0.4331401	0.209806	-2.06447649	0.038973	0.172148	NOT
RP11-347(	2.045436	-1.1343561	0.549479	-2.06442026	0.038978	0.172148	NOT
MIR3646	10.87689	-0.8902463	0.431255	-2.06431346	0.038988	0.172148	NOT
PRPF39	686.4116	0.33399688	0.161805	2.064197335	0.038999	0.172148	NOT
LGR6	190.3825	-1.1119667	0.538694	-2.06418983	0.039	0.172148	NOT
TDRD3	588.1922	-0.3416645	0.165523	-2.06415091	0.039003	0.172148	NOT
TSPO	2942.221	0.69591733	0.33715	2.064117229	0.039007	0.172148	NOT
RP11-57A1	5.893115	1.26866729	0.614652	2.064041323	0.039014	0.172148	NOT
HDDC3	551.2914	-0.3848168	0.186439	-2.0640373	0.039014	0.172148	NOT
RP11-563I	16.44156	-1.4242023	0.69001	-2.06403183	0.039015	0.172148	NOT
GRIA1	2.289221	1.70783211	0.827431	2.064016832	0.039016	0.172148	NOT
7-Mar	2047.417	0.26224299	0.127055	2.064003851	0.039017	0.172148	NOT
ACSL1	26157.95	-0.7369622	0.357071	-2.06391056	0.039026	0.17215	NOT
CTC-429P9	18.39432	-0.7260849	0.351808	-2.06386638	0.03903	0.17215	NOT
LRRC48	115.4709	0.55987753	0.271286	2.063791254	0.039038	0.172153	NOT
ZNF765	281.4088	0.3954223	0.191636	2.063406849	0.039074	0.172286	NOT
SDHA	11178.53	-0.426768	0.206844	-2.06323278	0.039091	0.172295	NOT
RP11-204(	13.6526	0.73746023	0.35743	2.06322959	0.039091	0.172295	NOT
HIC1	327.024	-0.6705026	0.324984	-2.06318579	0.039095	0.172295	NOT
HRASLS2	104.7678	1.34060479	0.649844	2.062964959	0.039116	0.172359	NOT
HUNK	260.7851	1.0747571	0.520997	2.062883492	0.039124	0.172365	NOT
USP50	2.871054	-0.7546511	0.365858	-2.06269092	0.039142	0.172418	NOT
SAT1	13601.48	-0.4767912	0.231161	-2.06259307	0.039151	0.17242	NOT
PIK3CA	767.1467	-0.3320066	0.160969	-2.06255258	0.039155	0.17242	NOT
ELK1	1565.577	-0.2387468	0.115761	-2.06241784	0.039168	0.172448	NOT
RP11-495I	39.84526	0.57766496	0.280119	2.062211635	0.039188	0.172507	NOT
CCDC88A	1431.473	0.47239768	0.229092	2.062039593	0.039204	0.172524	NOT
ZNF682	155.4946	0.72860119	0.35334	2.062037802	0.039204	0.172524	NOT
HECW1	47.91146	1.26194225	0.612071	2.061758013	0.039231	0.172613	NOT
RP11-334(	12.71196	0.77263037	0.374812	2.061379877	0.039267	0.172721	NOT
RP11-334(	26.73761	0.67750499	0.328669	2.061362621	0.039268	0.172721	NOT
RDX	4356.232	-0.3999045	0.19401	-2.06125815	0.039278	0.172721	NOT
C20orf27	1641.323	0.43173708	0.209459	2.061199726	0.039284	0.172721	NOT
HNRNPCP7	18.8349	-0.6461744	0.313505	-2.06112924	0.039291	0.172721	NOT
MYO16	121.1114	-0.8369406	0.406069	-2.06108151	0.039295	0.172721	NOT
RP11-834(	1.752717	-1.2525063	0.607744	-2.06090988	0.039312	0.172721	NOT
VTI1A	649.9239	-0.2546264	0.123551	-2.06090075	0.039313	0.172721	NOT
RP11-161I	5.658756	1.61609225	0.784214	2.060780315	0.039324	0.172721	NOT
MBTPS2	1436.351	-0.3771919	0.183042	-2.0606838	0.039333	0.172721	NOT
C19orf71	47.88378	-0.5540481	0.26887	-2.06065718	0.039336	0.172721	NOT
TRAPPC10	681.649	-0.4584246	0.222467	-2.06063993	0.039337	0.172721	NOT
CTAGE3P	5.910229	0.88450662	0.42924	2.060632468	0.039338	0.172721	NOT
PLCD4	126.8707	0.49177142	0.238666	2.060501368	0.039351	0.172748	NOT
RNF138P1	1.94117	-0.8163487	0.396247	-2.06020402	0.039379	0.172845	NOT
CCT6B	88.24833	-0.717536	0.348297	-2.06012716	0.039386	0.172849	NOT
LGI4	296.0869	0.84069766	0.408121	2.059924566	0.039406	0.172906	NOT
CLIC1P1	2.14418	1.06575031	0.51739	2.059858898	0.039412	0.172906	NOT
SLC1A2	3851.833	-1.3102118	0.636175	-2.05951436	0.039445	0.173022	NOT
RP11-260A	2.003643	3.50226899	1.700801	2.059188501	0.039476	0.173131	NOT

KANK1	4926.616	-0.4531295	0.220082	-2.05891668	0.039502	0.173217	NOT
ZNF224	299.9153	-0.388525	0.18873	-2.05863289	0.039529	0.173288	NOT
SAA1	34418.78	-1.4820834	0.719942	-2.05861583	0.039531	0.173288	NOT
RP4-564F2	13.19721	-0.8299866	0.403204	-2.05847717	0.039544	0.173303	NOT
PSMD4	13806.34	0.44769903	0.217494	2.058445375	0.039547	0.173303	NOT
ZFP1	346.4429	-0.4506072	0.218942	-2.05810769	0.03958	0.173417	NOT
RP11-568J	0.872445	1.49202247	0.724989	2.057993581	0.039591	NA	NA
RNF14	2472.696	-0.3355259	0.163059	-2.05770017	0.039619	0.173561	NOT
PTK6	351.1261	1.02651051	0.498886	2.05760433	0.039628	0.173571	NOT
MNS1	193.6785	-0.9089336	0.441757	-2.05754282	0.039634	0.173571	NOT
MLPH	2004.438	-0.8273014	0.402104	-2.05743274	0.039645	0.173589	NOT
LINC00843	4.522472	-0.7589078	0.368881	-2.0573236	0.039655	0.173594	NOT
MRPL23-AS	6.868527	1.60835312	0.781783	2.0572876	0.039659	0.173594	NOT
RP11-1960	5.607555	1.11121539	0.540224	2.056951844	0.039691	0.173698	NOT
NAA25	787.7058	0.29804655	0.1449	2.056908169	0.039695	0.173698	NOT
LSMEM1	67.18603	-0.5566417	0.270653	-2.05666305	0.039719	0.17376	NOT
PPP1CA	7012.388	0.2699073	0.131242	2.056565579	0.039728	0.17376	NOT
snoU13	2.445312	0.96727918	0.470338	2.056561449	0.039728	0.17376	NOT
RP11-286N	21.78641	-1.4187863	0.689946	-2.05637168	0.039747	0.173812	NOT
GOLGA2P5	872.0532	0.68283098	0.332119	2.055985636	0.039784	0.173895	NOT
RP11-5407	18.29345	-1.0889373	0.529646	-2.05597065	0.039785	0.173895	NOT
RP11-467I	2.116297	1.45855799	0.70945	2.055899059	0.039792	0.173895	NOT
C2CD2	861.0628	-0.4184078	0.203521	-2.05584278	0.039798	0.173895	NOT
AC006273.	34.19875	0.93189782	0.453292	2.055842736	0.039798	0.173895	NOT
LPA	1159.041	-1.0252749	0.498771	-2.05560261	0.039821	0.17395	NOT
UCA1	24.78167	1.20887998	0.588112	2.055527423	0.039828	0.17395	NOT
AKR1B1	1885.946	0.90040081	0.438042	2.055510506	0.03983	0.17395	NOT
ATP11A	2680.488	0.56190753	0.273388	2.055347205	0.039845	0.173991	NOT
ARHGEF4	71.24616	-1.2469442	0.606742	-2.0551456	0.039865	0.17402	NOT
AC080008.	6.843546	-1.0212501	0.496935	-2.05509945	0.039869	0.17402	NOT
CTD-2521M	13.21768	-0.6735379	0.327744	-2.05507011	0.039872	0.17402	NOT
NIM1K	23.08548	-0.783145	0.38109	-2.0550128	0.039878	0.17402	NOT
RP11-505K	6.075128	-0.5484017	0.266871	-2.05493517	0.039885	0.174025	NOT
CABP4	44.09646	0.76052183	0.370182	2.054452025	0.039932	0.174201	NOT
MYO1A	193.4549	0.95655092	0.465633	2.05430273	0.039946	0.174233	NOT
TBCE	1801.657	0.32412775	0.157785	2.054242894	0.039952	0.174233	NOT
PQLC2	1366.017	0.46217707	0.225006	2.054069358	0.039969	0.174274	NOT
RP11-133K	2.93975	-0.7171946	0.349168	-2.05401226	0.039975	0.174274	NOT
RAD51B	92.81318	0.41164492	0.200419	2.053923334	0.039983	0.174284	NOT
APOA1-AS	5.030685	-0.7284484	0.354698	-2.05371493	0.040003	0.174344	NOT
RP11-3990	6.606202	0.7206091	0.350898	2.053615923	0.040013	0.174357	NOT
ASF1A	879.7925	0.40170841	0.195618	2.053537166	0.040021	0.174363	NOT
FBXW11P1	24.91264	-0.8585664	0.41812	-2.05339906	0.040034	0.174393	NOT
ISG15	2759.179	0.70502958	0.343368	2.053275702	0.040046	0.174414	NOT
PRKAA2	870.6888	1.01787745	0.495748	2.053217376	0.040051	0.174414	NOT
DDX6	3763.346	-0.2459888	0.119817	-2.05302961	0.04007	0.174445	NOT
ALG1L6P	19.13318	0.46107529	0.224586	2.053001329	0.040072	0.174445	NOT
ATAD1	1653.676	-0.3119325	0.151944	-2.0529431	0.040078	0.174445	NOT
ADAMTS12	250.7815	-0.6960753	0.339083	-2.0528149	0.040091	0.174445	NOT

ECM1	436.8186	-0.6776392	0.330103	-2.05281205	0.040091	0.174445	NOT
STK36	693.0032	0.40089292	0.19531	2.052593784	0.040112	0.174509	NOT
P2RX3	27.9884	-0.9943084	0.484497	-2.05225076	0.040145	0.174579	NOT
RP11-174(	61.28913	-0.688459	0.335469	-2.0522285	0.040147	0.174579	NOT
PFDN2	2943.022	0.41788501	0.203626	2.052217661	0.040149	0.174579	NOT
MTCP1	70.64166	0.75016763	0.36555	2.052163403	0.040154	0.174579	NOT
DGAT1	4481.153	0.48613874	0.236915	2.051950572	0.040174	0.174641	NOT
TMEM156	278.6335	1.13104673	0.551265	2.051728171	0.040196	0.174706	NOT
TOMM22	3826.958	0.38551948	0.18791	2.051615338	0.040207	0.174706	NOT
RP11-286I	3.949656	1.76613913	0.860861	2.05159688	0.040209	0.174706	NOT
USP3-AS1	46.30997	-0.399323	0.194687	-2.05110239	0.040257	0.174887	NOT
HAUS8	285.0075	0.38445813	0.187477	2.050689658	0.040297	0.175034	NOT
CTC-232H1	2.635417	1.05566478	0.514897	2.050246209	0.04034	0.175194	NOT
CTC-260E6	2.71911	1.50770796	0.735489	2.049938432	0.04037	0.175296	NOT
RP11-89K1	7.425868	0.95287644	0.464926	2.049521044	0.040411	0.175445	NOT
ZDHHC3	2267.547	-0.2373571	0.115827	-2.04924678	0.040438	0.175508	NOT
AC016710.	14.37259	2.68562861	1.310548	2.049240204	0.040439	0.175508	NOT
RP5-940J5	2.36461	-0.8209338	0.400631	-2.04910349	0.040452	0.175538	NOT
ZNF329	521.2698	0.52945856	0.258421	2.04882538	0.040479	0.175612	NOT
IL9RP3	4.909856	-0.7563993	0.3692	-2.04875348	0.040486	0.175612	NOT
FLJ42393	2.138476	1.7608534	0.859512	2.048665689	0.040495	0.175612	NOT
RP11-1275	80.76792	-0.6373205	0.311091	-2.04866464	0.040495	0.175612	NOT
GAPDHP14	23.78106	1.30812726	0.638577	2.048503717	0.040511	0.175652	NOT
ANO7	149.2228	0.58386898	0.285076	2.048116937	0.040549	0.175788	NOT
P3H1	2324.549	0.36206437	0.176785	2.048051305	0.040555	0.175788	NOT
EXOC3L1	275.623	-0.4741992	0.23155	-2.04793724	0.040566	0.175808	NOT
HUS1B	4.969159	0.87184553	0.425764	2.047718037	0.040588	0.175873	NOT
ESAM	1246.19	-0.3735024	0.182418	-2.04751399	0.040608	0.175932	NOT
RPL35P2	17.06099	0.72468708	0.354044	2.046884225	0.040669	0.176172	NOT
RPL7L1P3	7.166474	-0.7874062	0.384742	-2.04658347	0.040699	0.176244	NOT
MAPRE3	967.5275	-0.4431109	0.216519	-2.04652143	0.040705	0.176244	NOT
VDAC1P2	5.178262	0.753395	0.368136	2.046514426	0.040706	0.176244	NOT
LRRC28	1482.674	-0.4501258	0.219962	-2.04638259	0.040719	0.176272	NOT
AURKAPS1	2.286212	1.28157642	0.626296	2.046277776	0.040729	0.176274	NOT
JAM3	472.4973	-0.4393129	0.214692	-2.04624592	0.040732	0.176274	NOT
RP11-535A	15.72602	-0.618029	0.302042	-2.04616838	0.04074	0.176279	NOT
NFKBIL1	1452.811	0.43023371	0.210275	2.046053278	0.040751	0.1763	NOT
RP11-338N	2.964999	1.91078509	0.933979	2.045855539	0.040771	0.176356	NOT
RP11-305I	5.23853	0.986905	0.482446	2.045626009	0.040793	0.176426	NOT
RP11-355N	5.636345	-0.7908587	0.386717	-2.0450601	0.040849	0.176639	NOT
CCDC153	33.32694	-0.5857507	0.28646	-2.04478937	0.040876	0.176726	NOT
RP11-90M2	2.82761	-0.9055058	0.442894	-2.04452273	0.040902	0.176812	NOT
BTBD6	1767.198	-0.4613	0.225646	-2.0443517	0.040919	0.176856	NOT
MS4A7	717.0772	-0.6407697	0.313478	-2.04406624	0.040947	0.17695	NOT
TMEM55B	1299.008	0.28284897	0.13839	2.043848681	0.040968	0.177015	NOT
CTD-2260A	14.91925	-0.5457995	0.267057	-2.04375286	0.040978	0.177027	NOT
PABPC4	6255.673	0.34194687	0.167331	2.043531718	0.041	0.177094	NOT
ATP10D	492.6572	-0.5471028	0.267744	-2.04338383	0.041014	0.177129	NOT
RP11-818(	3.550931	-0.8322923	0.407335	-2.04326257	0.041026	0.177152	NOT

RP11-507I	11.23387	0.67361025	0.329701	2.043096463	0.041043	0.177195	NOT
PIGN	951.1079	-0.3393978	0.166126	-2.04301925	0.041051	0.1772	NOT
HNRNPA0	5502.188	0.27189411	0.133102	2.042748218	0.041077	0.177287	NOT
LA16c-OS1	13.83279	-0.6866314	0.336164	-2.04254884	0.041097	0.177319	NOT
CAMK4	61.45836	-0.6599099	0.323083	-2.04254294	0.041098	0.177319	NOT
ZNF474	4.241396	0.85546243	0.418847	2.04242129	0.04111	0.177326	NOT
LINC0107	1.852702	-1.9574504	0.95841	-2.04239374	0.041112	0.177326	NOT
FAM150A	5.176	1.85479307	0.908282	2.042089473	0.041143	0.177424	NOT
GMEB2	1032.39	0.23011987	0.112695	2.041978978	0.041154	0.177424	NOT
TRAPPC4	1667.488	0.31176513	0.152679	2.041967868	0.041155	0.177424	NOT
ZCCHC4	326.0543	0.26486291	0.129748	2.041360139	0.041215	0.177653	NOT
TPBGL	79.0238	1.15757509	0.567077	2.041299732	0.041221	0.177653	NOT
PPP1R35	884.6729	0.47613035	0.233297	2.0408743	0.041263	0.177807	NOT
ZNF699	36.85757	-0.7049632	0.345439	-2.0407741	0.041273	0.17781	NOT
CTGF	3592.661	-0.6891871	0.337715	-2.0407369	0.041277	0.17781	NOT
CMB9-22P1	157.5218	0.95453655	0.46776	2.040653828	0.041285	0.177817	NOT
ABHD3	2072.186	0.44985865	0.22049	2.040267987	0.041324	0.177954	NOT
KDM6B	1295.163	-0.3919009	0.192096	-2.04013194	0.041337	0.177984	NOT
RP11-872I	63.80624	-1.0001718	0.490265	-2.04006538	0.041344	0.177984	NOT
RP11-147I	98.6846	0.45117002	0.221183	2.039805535	0.04137	0.178056	NOT
SERPINB5	2.597297	1.92368687	0.943104	2.039739626	0.041376	0.178056	NOT
LINC0020	2.846736	2.74443838	1.345565	2.039617755	0.041388	0.178056	NOT
TOX2	447.9968	0.80112855	0.39279	2.039587468	0.041391	0.178056	NOT
AC008592.	3.111591	-0.9893004	0.485054	-2.03956941	0.041393	0.178056	NOT
RP11-452I	295.6841	0.48673536	0.238659	2.039460248	0.041404	0.178074	NOT
HMGN2P41	9.937179	0.66834314	0.327746	2.039211119	0.041429	0.17813	NOT
SLC35F1	13.88921	-0.836682	0.410299	-2.03919929	0.04143	0.17813	NOT
FZD6	633.0634	0.69490188	0.340786	2.039112343	0.041439	0.178133	NOT
RP11-366I	13.20495	-0.5258046	0.257869	-2.03903545	0.041446	0.178133	NOT
PI4KAP2	199.9043	0.4494614	0.220433	2.038993659	0.041451	0.178133	NOT
MIA3	6614.543	-0.3912482	0.191904	-2.03877472	0.041473	0.17818	NOT
PTRH1	3.279927	-0.7628764	0.374188	-2.03875377	0.041475	0.17818	NOT
HSPE1P4	3.348499	0.95320546	0.467641	2.038325584	0.041517	0.178324	NOT
RP11-730A	2.792991	-0.9165323	0.449675	-2.03821262	0.041529	0.178324	NOT
NXT2	648.9945	-0.4739503	0.232537	-2.03817444	0.041532	0.178324	NOT
NUDT8	730.5276	-0.5823864	0.285742	-2.03815357	0.041535	0.178324	NOT
SCARF2	207.4962	-0.6504479	0.319146	-2.03809033	0.041541	0.178324	NOT
AVPR1A	1182.5	-1.4890684	0.730695	-2.03787935	0.041562	0.178386	NOT
RP11-793I	49.23033	-0.6087273	0.298727	-2.03773647	0.041576	0.178395	NOT
GATA3-AS1	2.131507	-1.4172379	0.69551	-2.03769501	0.04158	0.178395	NOT
ULBP2	37.39348	0.87211203	0.428003	2.037632662	0.041587	0.178395	NOT
STOM	14058.84	-0.504019	0.24736	-2.03759586	0.04159	0.178395	NOT
RP11-407I	123.8716	-0.8371193	0.410857	-2.03749594	0.0416	0.178409	NOT
TUBA3D	28.63697	1.09279936	0.536436	2.037147301	0.041635	0.178496	NOT
NDP	2.162986	1.7856884	0.876565	2.037143115	0.041636	0.178496	NOT
AC061992.	5.36009	-1.0711687	0.525831	-2.03709765	0.04164	0.178496	NOT
GOLGA8K	1.627096	-1.1548138	0.566952	-2.03688008	0.041662	0.178549	NOT
RP11-415I	3.419822	0.89434989	0.439101	2.036777103	0.041672	0.178549	NOT
PDXDC1	6968.38	-0.3662922	0.179841	-2.03676144	0.041674	0.178549	NOT

ARIH2	2575.579	0.2348141	0.115294	2.036648437	0.041685	0.178549	NOT
IL27	249.4849	-0.7816433	0.383789	-2.03664629	0.041686	0.178549	NOT
FOXP4	4030.814	0.38344839	0.188302	2.036343418	0.041716	0.17863	NOT
REXO1	1339.637	-0.3139078	0.154154	-2.03632597	0.041718	0.17863	NOT
CPNE6	12.28553	1.22268445	0.600469	2.036214488	0.041729	0.178634	NOT
RP3-423B2	6.553601	0.75706526	0.371806	2.036185002	0.041732	0.178634	NOT
SAV1	774.669	-0.4272817	0.209852	-2.0361068	0.04174	0.17864	NOT
APC	1036.613	-0.4082847	0.200558	-2.0357482	0.041776	0.178766	NOT
PHKG1	38.80516	-0.4902709	0.240849	-2.03559332	0.041791	0.178804	NOT
FLJ37453	148.8425	-0.3915928	0.192386	-2.03545034	0.041806	0.178837	NOT
LCLAT1	799.0661	0.42702915	0.209867	2.03475871	0.041875	0.179107	NOT
RP11-284F	13.80707	-1.6861641	0.828797	-2.03447109	0.041904	0.179202	NOT
ST3GAL6	1171.634	-0.676284	0.332475	-2.03408879	0.041943	0.179339	NOT
SLC43A3	4444.025	-0.5902542	0.290204	-2.03392887	0.041959	0.179355	NOT
CDCA5	882.4798	0.61676721	0.303249	2.033866773	0.041965	0.179355	NOT
IVNS1ABP	4093.101	0.39726936	0.195328	2.033854734	0.041966	0.179355	NOT
LINC00152	851.3979	0.71296745	0.350595	2.033592368	0.041993	0.179416	NOT
CELF4	17.60535	1.06159775	0.522034	2.033579704	0.041994	0.179416	NOT
GRM1	4.56539	1.33716542	0.657563	2.033517278	0.042	0.179416	NOT
EPHA5	1.726016	2.18317781	1.073824	2.033087051	0.042044	0.179573	NOT
SERPINB9P	71.62517	0.92036102	0.45276	2.03277962	0.042075	0.179677	NOT
RASEF	510.0222	0.97341704	0.478887	2.032666736	0.042086	0.179684	NOT
RP11-9E17	104.8803	0.52227685	0.256946	2.032633516	0.04209	0.179684	NOT
RP11-17E1	52.95118	0.65383051	0.321681	2.032545056	0.042099	0.179694	NOT
ZNF17	133.8748	-0.29402	0.144675	-2.03228051	0.042125	0.179758	NOT
DUSP8P5	27.37169	-0.5078325	0.249885	-2.03226566	0.042127	0.179758	NOT
FAM208A	2154.431	0.30552961	0.150367	2.031898891	0.042164	0.179888	NOT
ANKRD17	3384.594	-0.3624621	0.178401	-2.03173113	0.042181	0.179901	NOT
TAF2	1180.01	0.40853487	0.20109	2.031605	0.042194	0.179901	NOT
CTC-325H2	2.957758	-0.6643822	0.327032	-2.03155378	0.042199	0.179901	NOT
RAP2C	2453.273	-0.4067828	0.200237	-2.03150793	0.042203	0.179901	NOT
PI4K2A	1477.116	-0.3519728	0.173259	-2.03147844	0.042206	0.179901	NOT
TXNDC11	3852.092	-0.3129353	0.154043	-2.03147617	0.042207	0.179901	NOT
C10orf32	2672.974	-0.5433216	0.267504	-2.03107777	0.042247	0.18002	NOT
IL6R	6405.641	-0.6352065	0.312749	-2.0310446	0.04225	0.18002	NOT
SETP17	3.366553	-0.6992234	0.344275	-2.03100321	0.042255	0.18002	NOT
BHLHE40-A	42.85217	0.89900868	0.442673	2.030864052	0.042269	0.180052	NOT
HMGB1P27	3.117792	-0.9864834	0.485766	-2.03077724	0.042278	0.180061	NOT
SOCS6	1398.8	-0.3272993	0.161176	-2.03069565	0.042286	0.180068	NOT
TULP1	1.19266	1.46896074	0.723385	2.030675983	0.042288	NA	NA
MROH2A	305.519	-1.4454928	0.711885	-2.03051354	0.042304	0.180119	NOT
THEM4	1002.343	0.37772724	0.186039	2.030369311	0.042319	0.180153	NOT
C9orf173-	68.06687	-0.8910686	0.438914	-2.03016853	0.042339	0.180212	NOT
MRVI1	265.1327	-0.5725799	0.282072	-2.02990918	0.042366	0.180295	NOT
ENAH	5147.711	0.57027975	0.280957	2.029772681	0.04238	0.180326	NOT
XPOTP1	3.41274	0.93975432	0.463038	2.029542351	0.042403	0.180398	NOT
GABPB1-AS	436.4052	-0.5244265	0.258443	-2.02917476	0.04244	0.180528	NOT
NOD2	103.4268	0.5948825	0.293189	2.029007383	0.042458	0.180548	NOT
ZNF311	45.83785	-0.9829147	0.484435	-2.02899057	0.042459	0.180548	NOT

AC022819.	3.890981	-0.7744872	0.381721	-2.02893391	0.042465	0.180548	NOT
PSMG4	745.7399	0.40852233	0.201372	2.02869743	0.042489	0.180615	NOT
RP11-517I	62.00075	-0.500328	0.246631	-2.02864892	0.042494	0.180615	NOT
OPRL1	70.14895	0.70104331	0.345587	2.028556733	0.042503	0.180616	NOT
CIAO1	3483.211	0.19537362	0.096316	2.028464037	0.042513	0.180616	NOT
RBPMS-AS1	42.94241	0.63497596	0.313035	2.028449285	0.042514	0.180616	NOT
CARD14	42.5216	0.80961082	0.399142	2.028377046	0.042522	0.180619	NOT
FAM9A	2.13706	2.13110904	1.050725	2.028227333	0.042537	0.180656	NOT
AC093590.	1.52032	-1.0273628	0.506577	-2.02805062	0.042555	0.180704	NOT
RP11-76C1	5.138721	2.48828733	1.227085	2.027802912	0.04258	0.180783	NOT
HNRNPF	9755.812	0.26322539	0.129824	2.027554461	0.042606	0.180863	NOT
CTD-2171N	17.12092	1.39375529	0.687438	2.027462279	0.042615	0.180874	NOT
RP11-714M	134.657	-1.3965968	0.688948	-2.02714532	0.042648	0.180962	NOT
PDSS2	1279.684	-0.4033007	0.198955	-2.02709295	0.042653	0.180962	NOT
SUMO4	8.751863	-1.047305	0.516661	-2.02706436	0.042656	0.180962	NOT
LINC01094	29.00874	0.85639248	0.422535	2.026795721	0.042683	0.18105	NOT
KAAG1	22.27141	1.04218999	0.514227	2.026713084	0.042692	0.181058	NOT
RP11-535A	11.17222	-0.8073744	0.398414	-2.02646966	0.042717	0.181135	NOT
ACRV1	8.119915	0.80962733	0.399545	2.026374492	0.042726	0.181148	NOT
PNPO	3302.956	-0.5728805	0.282747	-2.026124	0.042752	0.181201	NOT
PRRC2A	9129.626	0.269168	0.132849	2.026122706	0.042752	0.181201	NOT
WHAMMP2	58.46068	-0.4620714	0.228094	-2.02579514	0.042786	0.181315	NOT
HEMGN	1.560741	-1.5474921	0.764244	-2.02486611	0.042881	0.181691	NOT
HMG20B	2510.684	-0.2889481	0.142713	-2.02467387	0.042901	0.181722	NOT
SLC25A5-A	83.13639	-0.4555038	0.224977	-2.02466375	0.042902	0.181722	NOT
LEO1	1383.637	0.27993818	0.138275	2.024508007	0.042918	0.181726	NOT
INPP5F	341.4468	0.38495759	0.19015	2.024495111	0.042919	0.181726	NOT
CTD-3131K	4.73701	-0.7780627	0.384334	-2.02444589	0.042924	0.181726	NOT
LINC00365	30.91467	-0.988705	0.488412	-2.02432406	0.042937	0.181726	NOT
CTD-2012K	165.4124	-0.6689759	0.330475	-2.02428333	0.042941	0.181726	NOT
AC018804.	16.44176	1.04271238	0.515107	2.024262845	0.042943	0.181726	NOT
GORASP2	4437.827	0.22902699	0.113152	2.024068174	0.042963	0.181783	NOT
CFH	62714.16	-0.7171658	0.354394	-2.02363741	0.043007	0.181942	NOT
TRAPPC12	1909.29	-0.268692	0.132786	-2.02349529	0.043022	0.181975	NOT
RP11-728F	14.55523	1.13839222	0.562626	2.023354	0.043037	0.182009	NOT
RP11-439E	90.8793	-0.625776	0.309315	-2.02310273	0.043063	0.18209	NOT
SLC35F6	4665.622	0.38242605	0.189071	2.022657768	0.043108	0.182237	NOT
TMEM42	692.2284	0.39360654	0.194601	2.022634611	0.043111	0.182237	NOT
IPO4	301.968	0.49540065	0.244948	2.022475464	0.043127	0.182258	NOT
AP006621.	284.7573	-0.5797653	0.286668	-2.0224293	0.043132	0.182258	NOT
POLDIP3	2867.893	0.29105967	0.143919	2.022391867	0.043136	0.182258	NOT
VPS54	1689.845	0.30702879	0.151832	2.02215527	0.04316	0.182332	NOT
MED15P9	1.143587	2.68278857	1.32671	2.022136762	0.043162	NA	NA
DDIT3	1764.667	0.54686082	0.270447	2.02206063	0.04317	0.182345	NOT
MIR100HG	27.4467	-1.0529917	0.520811	-2.02183229	0.043194	0.182417	NOT
PFN1P7	7.927649	-1.0106585	0.499893	-2.02174881	0.043202	0.182425	NOT
PSMD8	6437.857	-0.2996405	0.14824	-2.02132596	0.043246	0.182581	NOT
MS4A14	41.19076	-0.7268877	0.359624	-2.02124349	0.043255	0.182589	NOT
GPRIN2	16.53956	1.86236542	0.921489	2.021038669	0.043276	0.18265	NOT

RPUSD3	1449.838	0.34115358	0.168815	2.020874658	0.043293	0.182693	NOT
DGCR6	403.944	-0.7445898	0.368472	-2.02074968	0.043306	0.182703	NOT
RAB30	462.92	-0.4032124	0.199546	-2.02065125	0.043316	0.182703	NOT
CDC23	1603.409	0.25494711	0.126174	2.020602324	0.043321	0.182703	NOT
RP11-677M	1.907687	-0.8573049	0.424284	-2.02059059	0.043322	0.182703	NOT
ZNF227	326.6723	-0.3426392	0.169589	-2.02040963	0.043341	0.182754	NOT
SHISA5	8581.56	0.28974292	0.143416	2.020302644	0.043352	0.182772	NOT
RP11-191(	12.65526	-0.7206389	0.35672	-2.02018019	0.043365	0.182797	NOT
PDK2	1842.094	-0.4129215	0.204437	-2.01979448	0.043405	0.182938	NOT
LINC01436	75.96087	1.37030012	0.678512	2.019567249	0.043428	0.183009	NOT
CD81-AS1	3.262617	-0.9219353	0.456528	-2.01945069	0.04344	0.183031	NOT
C4BPB	12837.24	-0.7720868	0.382361	-2.01926178	0.04346	0.183066	NOT
RP11-754N	3.771982	1.4877265	0.736775	2.019242088	0.043462	0.183066	NOT
SERPINA1(	11328.5	-0.7117392	0.352504	-2.01909562	0.043477	0.183093	NOT
LINC01272	71.2478	0.76118407	0.377001	2.01904863	0.043482	0.183093	NOT
MOB3C	543.3444	-0.2851339	0.141238	-2.01882147	0.043506	0.183164	NOT
AC010969.	11.09962	1.39381616	0.690514	2.018521211	0.043537	0.183255	NOT
CHTF18	612.164	0.47480289	0.235235	2.018418823	0.043548	0.183255	NOT
RP11-104J	39.59993	-0.8295803	0.411007	-2.0184101	0.043549	0.183255	NOT
SLC44A3	903.1888	0.78360912	0.388245	2.01833582	0.043556	0.183255	NOT
SLC19A3	751.6535	-0.6841266	0.338973	-2.01823616	0.043567	0.183255	NOT
RP11-68I1	34.06248	0.70086645	0.347269	2.018225048	0.043568	0.183255	NOT
PSME2P2	71.56299	0.52664444	0.261011	2.017707909	0.043622	0.183453	NOT
BMP4	606.7864	0.95603802	0.473839	2.017643943	0.043628	0.183453	NOT
PWP1	1982.792	0.24811268	0.122992	2.017304795	0.043664	0.183573	NOT
GNAS-AS1	7.645744	1.45893512	0.723407	2.016756331	0.043721	0.183785	NOT
PLOD1	13319.03	0.45913435	0.227667	2.016690893	0.043728	0.183785	NOT
BIK	84.02217	0.98369186	0.487817	2.016516511	0.043746	0.183833	NOT
DOK6	104.9528	1.09492951	0.543059	2.016225925	0.043776	0.183933	NOT
USP32P1	13.03633	-1.6033373	0.795279	-2.01606984	0.043793	0.183973	NOT
PRPF38AP1	0.53112	-2.4374674	1.209043	-2.01602963	0.043797	NA	NA
GIT1	2062.927	0.37982145	0.188415	2.015874118	0.043813	0.18403	NOT
KHDRBS2	2.16334	1.50196345	0.745205	2.015503297	0.043852	0.184139	NOT
C1QTNF9	5.427749	-0.9161138	0.454535	-2.01549715	0.043853	0.184139	NOT
CHGA	30.18981	1.34950884	0.669629	2.01530736	0.043872	0.184184	NOT
RP11-67L3	17.79743	0.60299726	0.299215	2.015264679	0.043877	0.184184	NOT
XRCC4	262.9701	0.43780154	0.217283	2.014894789	0.043916	0.184294	NOT
AC241377.	11.56684	1.02944785	0.510921	2.014885812	0.043917	0.184294	NOT
RP11-185E	20.80079	0.98110655	0.486967	2.014727125	0.043933	0.184335	NOT
RP11-505E	6.295828	-0.6520879	0.323688	-2.01455555	0.043951	0.184382	NOT
ZNF230	129.0396	-0.4196512	0.208317	-2.01448103	0.043959	0.184386	NOT
AC012442.	9.169609	0.4649594	0.230843	2.014182504	0.04399	0.184489	NOT
ESF1	1001.145	0.38062053	0.188997	2.013892173	0.044021	0.184588	NOT
KB-318B8.	3.84892	-1.0041937	0.498704	-2.01360827	0.044051	0.184655	NOT
SCYL1	5038.421	-0.2489542	0.123638	-2.01358034	0.044054	0.184655	NOT
ZP1	3.476432	1.37776819	0.68425	2.013545792	0.044057	0.184655	NOT
CTD-2325A	2.52853	1.41596991	0.703375	2.013108755	0.044103	0.184796	NOT
GIPC3	240.7136	-0.4464566	0.221776	-2.01309677	0.044104	0.184796	NOT
TALDO1	12270.33	0.5245908	0.260616	2.012884796	0.044127	0.184837	NOT

RP13-392I	57.07177	0.9928528	0.493262	2.012830971	0.044132	0.184837	NOT
CHP1	9742.169	-0.4218975	0.209608	-2.01279579	0.044136	0.184837	NOT
RP11-170N	14.95823	-0.6458857	0.320898	-2.01274453	0.044142	0.184837	NOT
FBXO8	998.7702	-0.3636221	0.180673	-2.01259903	0.044157	0.184873	NOT
FDPS	12230.06	0.53310877	0.264934	2.012231312	0.044196	0.185006	NOT
AP000472.	31.15912	-1.0994674	0.546451	-2.01201503	0.044218	0.185062	NOT
BCL10	599.8431	0.31308274	0.15561	2.011975122	0.044223	0.185062	NOT
DNAJB6	2672.933	0.25588946	0.12719	2.011865058	0.044234	0.185082	NOT
NXT1	584.5909	0.39010427	0.193914	2.011740561	0.044247	0.185109	NOT
AP000240.	15.92659	0.74171204	0.368761	2.011364831	0.044287	0.185246	NOT
MPP2	64.9398	1.0164905	0.505433	2.011129346	0.044312	0.185321	NOT
SNW1	2117.305	0.23613751	0.117427	2.010937214	0.044332	0.185378	NOT
LRRC46	57.46326	0.49832186	0.247814	2.010872659	0.044339	0.185378	NOT
SLC7A8	391.358	0.68300519	0.339763	2.010238221	0.044406	0.185629	NOT
A1BG	3635.554	-0.9337787	0.464571	-2.00998067	0.044433	0.185689	NOT
CTD-2126E	2.358641	2.04222142	1.016061	2.009940608	0.044437	0.185689	NOT
GPHN	1334.334	-0.5755869	0.286389	-2.00980701	0.044452	0.185689	NOT
AC098820.	5.763082	0.85872481	0.427275	2.009771346	0.044455	0.185689	NOT
SNX13	1192.826	-0.3506263	0.174465	-2.00972057	0.044461	0.185689	NOT
RP11-465N	59.89975	0.48023069	0.238962	2.009650416	0.044468	0.185689	NOT
RP3-405J1	2.605853	-0.7008239	0.348739	-2.00959206	0.044474	0.185689	NOT
AC008440.	2.633922	1.23710474	0.615604	2.009578094	0.044476	0.185689	NOT
TCF24	4.945432	1.84143032	0.916367	2.009490349	0.044485	0.185689	NOT
RP11-45A1	2.695968	-0.7822233	0.389271	-2.00945576	0.044489	0.185689	NOT
P4HA3	48.78652	-0.5377793	0.267647	-2.00928942	0.044506	0.185713	NOT
NEDD4	2366.334	-0.6279189	0.312511	-2.00927237	0.044508	0.185713	NOT
RP3-434P1	73.19151	-1.197598	0.596093	-2.0090789	0.044529	0.185753	NOT
LINC00205	110.3255	0.53407002	0.265832	2.009053966	0.044531	0.185753	NOT
RP11-443K	1.410955	1.85891152	0.925299	2.008984956	0.044539	0.185755	NOT
PDE4DIP	3701.188	-0.5357549	0.26671	-2.0087518	0.044563	0.185819	NOT
FKRP	508.8784	-0.2900398	0.144391	-2.00871177	0.044568	0.185819	NOT
RP11-543C	2.694311	-0.7017735	0.3494	-2.0085124	0.044589	0.185878	NOT
PBLD	4473.129	-0.7268967	0.361995	-2.00803036	0.04464	0.186063	NOT
NUTM2B	9.657966	-0.7591987	0.378117	-2.00784043	0.04466	0.186118	NOT
TET1	108.812	0.79623014	0.396586	2.007712605	0.044674	0.186118	NOT
RP11-496H	4.037449	-0.7609673	0.379022	-2.00771188	0.044674	0.186118	NOT
TCN2	1343.851	0.55458783	0.276254	2.00752917	0.044693	0.186147	NOT
OSBPL11	1057.993	-0.364341	0.181493	-2.00746855	0.0447	0.186147	NOT
ZNF106	1758.689	-0.3356978	0.167231	-2.00738887	0.044708	0.186147	NOT
RPL14	23032.17	0.47869009	0.238472	2.007325564	0.044715	0.186147	NOT
RANBP6	894.7756	-0.3584721	0.178582	-2.00732506	0.044715	0.186147	NOT
CAP1P2	4.892954	0.74684146	0.372094	2.00712857	0.044736	0.186177	NOT
AUTS2	1282.113	-0.7446597	0.371008	-2.00712717	0.044736	0.186177	NOT
CCR10	27.21541	0.66403359	0.330862	2.006983489	0.044751	0.186213	NOT
RGS14	3197.913	0.53828386	0.268244	2.006697211	0.044782	0.186269	NOT
BCL6B	611.7869	-0.4249789	0.211784	-2.00666029	0.044786	0.186269	NOT
RP11-169D	3.99192	-0.7522032	0.374861	-2.006618	0.04479	0.186269	NOT
NBPF8	190.5656	-0.5189509	0.258628	-2.00655188	0.044797	0.186269	NOT
DYDC1	2.949709	1.38721456	0.691349	2.006534248	0.044799	0.186269	NOT

TNKS2	1712.323	-0.3222778	0.160624	-2.00640915	0.044813	0.186296	NOT
FAHD2A	1588.28	-0.4090128	0.203863	-2.00630866	0.044823	0.186312	NOT
PSMB9	2465.625	0.69573688	0.346786	2.006243962	0.04483	0.186312	NOT
RP11-973H	3.622149	1.73506888	0.864899	2.006093943	0.044846	0.18635	NOT
FOXD2	153.8348	0.69494342	0.346439	2.005960118	0.044861	0.186352	NOT
ZNF880	132.1498	0.8417751	0.419666	2.005820987	0.044875	0.186352	NOT
CYP4X1	220.6613	-0.8271628	0.412384	-2.00580557	0.044877	0.186352	NOT
CH507-24F	2.10298	1.29607766	0.646164	2.005802716	0.044877	0.186352	NOT
FAM170A	2.057641	-1.211555	0.604075	-2.00563813	0.044895	0.186352	NOT
GP6	3.938602	-0.9646195	0.480956	-2.00562837	0.044896	0.186352	NOT
FBXW5	8192.923	-0.3627133	0.18085	-2.0055992	0.044899	0.186352	NOT
CORO6	40.37495	0.70108595	0.349569	2.005574926	0.044902	0.186352	NOT
RP11-961A	4.940589	-0.9410416	0.469336	-2.00504956	0.044958	0.186518	NOT
SYTL2	287.7598	-0.5938125	0.296163	-2.00502206	0.044961	0.186518	NOT
CTR9	2987.675	-0.3514416	0.175282	-2.00500611	0.044962	0.186518	NOT
RP11-5C23	129.1026	-0.3630389	0.181085	-2.00480102	0.044984	0.186581	NOT
FIGNL1	486.6564	0.4682718	0.23362	2.004418833	0.045025	0.186722	NOT
HNRNPUP1	5.010352	0.56874071	0.283771	2.004225256	0.045046	0.186779	NOT
THOC2	1930.027	0.32839139	0.163856	2.004148373	0.045054	0.186785	NOT
PAXIP1	664.6654	0.34159838	0.170469	2.00387911	0.045083	0.186876	NOT
GEMIN8P4	71.91918	0.44412777	0.221677	2.003490771	0.045125	0.18702	NOT
EPB41L4A-	23.52524	-0.732457	0.365607	-2.00340159	0.045134	0.187024	NOT
MEA1	3408.534	0.34272537	0.171076	2.00335318	0.045139	0.187024	NOT
OCM	2.952599	-0.833933	0.416304	-2.00318318	0.045158	0.187071	NOT
EID1	4456.579	-0.3591474	0.179304	-2.00300798	0.045176	0.18712	NOT
GPRC5D	17.72361	0.82061653	0.409707	2.002932824	0.045184	0.187125	NOT
NEBL	457.1509	1.2166853	0.607498	2.002779182	0.045201	0.187165	NOT
TBCB	2186.107	0.48049773	0.239945	2.002536913	0.045227	0.187236	NOT
ZBTB7C	94.4589	-0.8484111	0.423678	-2.00248995	0.045232	0.187236	NOT
FAM205CP	2.095397	-1.5263586	0.762285	-2.00234754	0.045247	0.187267	NOT
HLX	772.8028	-0.4718583	0.235661	-2.00227776	0.045255	0.187267	NOT
MIR762HG	36.17823	-0.3849321	0.192261	-2.0021348	0.04527	0.187267	NOT
BPESC1	2.038551	1.64328502	0.820793	2.002071178	0.045277	0.187267	NOT
DCAF12L1	1.274656	2.86279509	1.429958	2.002013624	0.045283	NA	NA
RP11-693J	4.646786	-0.8886666	0.443891	-2.00199171	0.045286	0.187267	NOT
PHF1	2108.285	0.31771273	0.158699	2.001978567	0.045287	0.187267	NOT
ABCC6	6857.636	-0.6505857	0.324973	-2.00197032	0.045288	0.187267	NOT
KLHL20	784.5887	-0.4197189	0.209665	-2.00185457	0.0453	0.187273	NOT
C2orf70	5.75006	1.79295926	0.89566	2.001829829	0.045303	0.187273	NOT
LPHN1	851.3624	-0.9315513	0.465369	-2.00174914	0.045312	0.18728	NOT
MAOB	11609.49	-0.5364093	0.267982	-2.0016592	0.045321	0.187292	NOT
THSD4	800.0033	-0.7656339	0.382575	-2.00126252	0.045364	0.18744	NOT
AC130469.	6.719835	-0.870274	0.434919	-2.00100215	0.045392	0.187527	NOT
DONSON	802.077	0.37760164	0.188716	2.000899748	0.045403	0.187544	NOT
STAU2	1751.907	0.31155357	0.155752	2.00031203	0.045467	0.187777	NOT
RP11-341(	34.90254	0.81172147	0.405817	2.000214931	0.045477	0.187792	NOT
KRT17P7	1.229712	-1.4970972	0.748588	-1.99989386	0.045512	NA	NA
RP11-773H	5.782694	0.69067667	0.34537	1.999817126	0.04552	0.187941	NOT
RP11-344E	1.181914	2.5718475	1.286069	1.99977423	0.045525	NA	NA

BTF3L4P2	26.25067	0.39954615	0.199799	1.999739387	0.045528	0.187947	NOT
RP13-977J	21.49452	-0.8602932	0.430326	-1.99916739	0.04559	0.188138	NOT
VSIG1	174.5729	1.48103727	0.740847	1.99911221	0.045596	0.188138	NOT
SEMA3A	73.77605	1.09179243	0.546142	1.999101217	0.045597	0.188138	NOT
HPGDS	44.94024	-0.7784795	0.389424	-1.99905396	0.045603	0.188138	NOT
FAM73B	988.1939	-0.3396113	0.1699	-1.99888901	0.04562	0.188173	NOT
RP11-269F	7.607933	0.65665289	0.328516	1.998848173	0.045625	0.188173	NOT
NOS3	438.2397	-0.4689656	0.234626	-1.99877656	0.045633	0.188176	NOT
PKD1	2070.477	-0.3384287	0.169341	-1.99850901	0.045662	0.188267	NOT
FMO4	1182.984	-0.7178199	0.359402	-1.99726334	0.045797	0.188795	NOT
PDZD9	2.235436	-0.7406311	0.370835	-1.99719785	0.045804	0.188796	NOT
CEP44	423.5796	-0.3203822	0.160551	-1.99552253	0.045986	0.18948	NOT
RP13-631K	3.955623	-1.1697122	0.586175	-1.99549996	0.045988	0.18948	NOT
CTF1	63.94334	-0.9400177	0.471073	-1.99548037	0.045991	0.18948	NOT
CTD-2270F	240.9366	-0.5339617	0.267606	-1.99532637	0.046007	0.18952	NOT
RP11-530I	18.1865	0.69893106	0.35031	1.995176866	0.046024	0.189542	NOT
UBXN4	6165.7	-0.3004646	0.150602	-1.99508524	0.046034	0.189542	NOT
RP11-753D	2.909074	-1.187557	0.595251	-1.99505186	0.046037	0.189542	NOT
RP11-7807	24.67203	-0.5597621	0.280584	-1.99498698	0.046044	0.189542	NOT
CTD-2336C	345.393	-0.4449785	0.223052	-1.99495752	0.046048	0.189542	NOT
MTHFS	898.4226	-0.5473234	0.274378	-1.99477948	0.046067	0.189593	NOT
ACO2	4925.381	-0.4730535	0.237211	-1.99422863	0.046127	0.189811	NOT
UBE2SP1	17.42347	0.74882871	0.37551	1.994164804	0.046134	0.189811	NOT
CTD-2536I	2.003652	-1.1867576	0.595167	-1.99399078	0.046153	0.189861	NOT
RP11-554A	4.446463	1.27639982	0.640151	1.993903274	0.046163	0.189871	NOT
AC005336.	360.8392	-0.9229012	0.462887	-1.99379353	0.046175	0.189892	NOT
MAP7D2	194.5586	1.44137809	0.722982	1.993657825	0.046189	0.189924	NOT
KIAA1551	1897.374	-0.4677999	0.234683	-1.99332923	0.046225	0.190043	NOT
SLC4A9	3.393749	0.86270015	0.43281	1.993255631	0.046233	0.190048	NOT
ATF4	13396.18	0.39671986	0.19907	1.992867292	0.046276	0.190194	NOT
RP11-381N	3.732521	1.21113675	0.607774	1.992742093	0.04629	0.190221	NOT
YWHAZP5	23.19357	0.64217642	0.32227	1.992668659	0.046298	0.190225	NOT
CDK19	865.519	0.45831843	0.230015	1.992558422	0.04631	0.19024	NOT
RP11-92K2	27.27614	0.62417944	0.313263	1.992508493	0.046315	0.19024	NOT
ZNF566	245.8876	-0.3195642	0.160396	-1.99234598	0.046333	0.190284	NOT
TPT1P4	15.07996	-0.6020106	0.302246	-1.99178716	0.046394	0.190507	NOT
OR10J6P	86.82499	-1.1661007	0.585506	-1.99161251	0.046414	0.190527	NOT
SHPK	314.3303	-0.4777544	0.239885	-1.99159942	0.046415	0.190527	NOT
CRYZL1	522.7848	-0.2137929	0.10735	-1.99154047	0.046422	0.190527	NOT
VIPR1-AS1	2.7694	-0.9127406	0.458321	-1.99148782	0.046427	0.190527	NOT
EFNA4	558.2011	0.4507028	0.226328	1.991368746	0.04644	0.190552	NOT
TPPP3	344.3297	-0.6786132	0.340792	-1.99127984	0.04645	0.190556	NOT
VWA5A	459.8685	-0.6449865	0.323913	-1.9912311	0.046455	0.190556	NOT
RP11-297I	3.520895	0.91862373	0.461372	1.991070515	0.046473	0.1906	NOT
RP11-785I	17.03721	-1.5417557	0.774405	-1.99088976	0.046493	0.190652	NOT
COX5B	8517.708	-0.5027625	0.252562	-1.99064652	0.04652	0.190733	NOT
RP13-884E	1.52548	1.45089986	0.729021	1.990202844	0.046569	0.190905	NOT
HMGB1P10	72.29144	0.43457249	0.218487	1.98900409	0.046701	0.191417	NOT
RP11-571M	6.363592	-0.6610976	0.332432	-1.98866785	0.046738	0.191534	NOT

RP11-505I	2.135613	1.32891422	0.66826	1.988619276	0.046743	0.191534	NOT
SLC17A2	1833.296	-0.9914387	0.498618	-1.98837385	0.04677	0.191589	NOT
DLL1	317.7149	-0.7506402	0.377524	-1.9883262	0.046776	0.191589	NOT
SLC25A37	923.4651	-0.4386415	0.220611	-1.9883042	0.046778	0.191589	NOT
TGFBR1	1602.129	-0.3507944	0.176457	-1.98798712	0.046813	0.191704	NOT
SRP9	7464.32	0.27978087	0.140743	1.987880115	0.046825	0.191724	NOT
AREG	56.3812	1.00442739	0.505321	1.987702591	0.046845	0.191775	NOT
RP11-320N	2.889272	1.29017288	0.649169	1.987421807	0.046876	0.191849	NOT
RP11-122(	40.32052	1.61542221	0.812827	1.987412024	0.046877	0.191849	NOT
ULK2	416.5009	-0.6700465	0.337213	-1.9870139	0.046921	0.192	NOT
SRD5A3	1277.856	-0.4308129	0.216855	-1.98663887	0.046962	0.192134	NOT
PDE1A	164.1229	-0.7330374	0.368993	-1.98659143	0.046968	0.192134	NOT
RP13-143(	70.83415	1.20387248	0.606048	1.986430242	0.046986	0.192178	NOT
AC084219.	7.00766	-0.930175	0.468291	-1.98631818	0.046998	0.1922	NOT
ZBTB34	408.2464	0.41586875	0.209394	1.986054669	0.047027	0.192291	NOT
RPS28P7	3283.18	1.02786204	0.517561	1.985971153	0.047037	0.1923	NOT
FBXL8	209.8782	-0.5176828	0.260713	-1.98564021	0.047073	0.192421	NOT
FANK1	10.42253	0.87209026	0.439224	1.985523525	0.047086	0.192445	NOT
MSANTD3	750.6384	0.416358	0.20971	1.98539715	0.0471	0.192473	NOT
FH	11086.88	-0.4453786	0.224337	-1.98531174	0.04711	0.192474	NOT
IREB2	1992.766	-0.3171939	0.159774	-1.98526775	0.047115	0.192474	NOT
HERC2P9	156.4511	-0.4797925	0.241709	-1.98500108	0.047144	0.192566	NOT
BANF2	12.72024	1.34701543	0.678709	1.984671656	0.047181	0.192687	NOT
PTPRA	2368.286	-0.237706	0.119776	-1.98458455	0.047191	0.192698	NOT
FABP3	196.3307	0.62404135	0.314465	1.984451322	0.047206	0.192729	NOT
RP5-991G2	4.41246	0.78815038	0.397183	1.984352143	0.047217	0.192731	NOT
GGA1	2781.99	0.39357086	0.198351	1.984217868	0.047232	0.192731	NOT
ADAM15	4725.053	0.38545839	0.194264	1.984199132	0.047234	0.192731	NOT
SNHG12	320.4665	0.51310985	0.258615	1.984069816	0.047248	0.192731	NOT
HMGN1P36	6.999467	0.5421824	0.273271	1.984046527	0.047251	0.192731	NOT
TMPRSS6	9874.011	-0.8502858	0.428568	-1.98401716	0.047254	0.192731	NOT
AC017002.	2.677529	1.50197205	0.757042	1.984000852	0.047256	0.192731	NOT
APLP1	179.0429	1.00427309	0.506226	1.983842721	0.047273	0.192774	NOT
RNF139	1742.76	0.28269405	0.14252	1.983542659	0.047307	0.192881	NOT
RP11-280(	1.76587	2.4131442	1.216621	1.983481304	0.047314	0.192881	NOT
FLOT1	8972.078	0.36497113	0.18402	1.983325686	0.047331	0.192922	NOT
SPECC1L	1732.223	-0.2880779	0.145259	-1.98320673	0.047344	0.192926	NOT
RPL31	23321.14	0.45811667	0.231011	1.983096957	0.047357	0.192926	NOT
RP5-864K1	41.87733	0.66985256	0.337785	1.983074154	0.047359	0.192926	NOT
TMEM106A	262.8253	0.6173395	0.311311	1.983029613	0.047364	0.192926	NOT
RP4-781K5	3.227798	1.43134732	0.721809	1.982999469	0.047367	0.192926	NOT
GDF10	77.72505	1.74796578	0.881583	1.982759142	0.047394	0.192976	NOT
RP6-159A1	18.31314	-0.7033817	0.354752	-1.98274354	0.047396	0.192976	NOT
RP11-33B1	11.8411	-0.5983996	0.301816	-1.98266308	0.047405	0.192976	NOT
CTC-281F2	6.341703	-0.9791671	0.493883	-1.98258733	0.047414	0.192976	NOT
KCNK12	3.996449	1.14769039	0.578896	1.98254904	0.047418	0.192976	NOT
NAP1L2	146.1823	-1.139516	0.574785	-1.9825066	0.047423	0.192976	NOT
MOB3B	620.2926	0.77177891	0.389333	1.98230893	0.047445	0.193011	NOT
RP11-763I	2.127116	-1.1271639	0.568631	-1.98224032	0.047452	0.193011	NOT

ZNF746	1002.453	0.3716111	0.187473	1.98221126	0.047456	0.193011	NOT
CST4	3.310896	2.71376282	1.369083	1.982175735	0.04746	0.193011	NOT
RP11-266I	117.2263	-0.6754241	0.340788	-1.98194535	0.047485	0.193036	NOT
RP11-680(	4.377476	-0.7819179	0.394521	-1.98194446	0.047485	0.193036	NOT
SQRDL	2208.565	0.44114342	0.22259	1.981863624	0.047495	0.193036	NOT
SH3BGRL2	3212.365	-0.4846025	0.244522	-1.9818368	0.047498	0.193036	NOT
ERVV-2	1.728937	2.12512445	1.072318	1.981804657	0.047501	0.193036	NOT
RP11-802I	53.66489	0.63553428	0.320705	1.981680775	0.047515	0.193063	NOT
LINC0123	165.9269	1.68094894	0.848442	1.981219122	0.047567	0.193244	NOT
RP11-352M	333.3116	-0.3236617	0.163373	-1.9811204	0.047578	0.19326	NOT
MKKS	3291.299	0.36635369	0.184937	1.980969318	0.047595	0.1933	NOT
FAM19A1	4.561	-1.1793845	0.595404	-1.98081339	0.047612	0.193342	NOT
CHRNA3	8.673557	1.37931617	0.696371	1.980720188	0.047623	0.193356	NOT
WNT5A-AS1	16.73401	0.98811915	0.498894	1.980617952	0.047634	0.193373	NOT
NRIP1	1655.766	-0.4815299	0.243134	-1.98051429	0.047646	0.193392	NOT
TRAF5	436.183	0.56260269	0.284081	1.980430397	0.047655	0.193401	NOT
VPS9D1	833.7438	-0.3655757	0.184639	-1.97995318	0.047709	0.193566	NOT
FIRRE	17.97521	1.0500663	0.530352	1.97994129	0.04771	0.193566	NOT
IGHA1	3700.374	-1.1481357	0.579908	-1.97985754	0.04772	0.193575	NOT
CHRM4	4.178622	1.2225952	0.617563	1.979707754	0.047736	0.193615	NOT
CD24P4	2.95578	1.26291347	0.638089	1.979210756	0.047792	0.193812	NOT
SFXN5	3017.42	-0.4563543	0.230601	-1.97898089	0.047818	0.193888	NOT
BRD2-IT1	1.885619	-0.9585518	0.484464	-1.978581	0.047863	0.194042	NOT
RP1-159M	3.093105	0.76596997	0.387152	1.978470998	0.047876	0.194063	NOT
CYB561	2065.648	-0.6029808	0.304826	-1.97811291	0.047916	0.194198	NOT
KRT8P11	22.59432	0.91442942	0.462298	1.978009754	0.047928	0.194216	NOT
SDR42E1	229.6398	-0.8614422	0.435564	-1.97776025	0.047956	0.194262	NOT
MDM1	256.7849	0.38686543	0.195609	1.977744038	0.047958	0.194262	NOT
RAB3D	559.7387	-0.9175592	0.463948	-1.97771962	0.04796	0.194262	NOT
MARVELD2	1972.138	-0.5326453	0.269339	-1.97760342	0.047973	0.194265	NOT
VARS	6094.202	0.35057189	0.177273	1.977585077	0.047976	0.194265	NOT
PSPC1	1006.327	0.36472098	0.184464	1.977193124	0.04802	0.194402	NOT
TINAG	289.7807	1.69578334	0.857687	1.977158489	0.048024	0.194402	NOT
TM7SF2	9305.265	-0.7091868	0.358727	-1.97695284	0.048047	0.194419	NOT
P2RY14	36.79433	-0.5809871	0.293887	-1.97690794	0.048052	0.194419	NOT
NDUFA8	2410.03	-0.4401675	0.222659	-1.97687103	0.048056	0.194419	NOT
LIFR	613.7521	-0.7299397	0.36924	-1.97686852	0.048056	0.194419	NOT
NADK	3835.876	-0.3974666	0.201096	-1.9764994	0.048098	0.194559	NOT
LINC0127	53.85751	0.65106687	0.329448	1.976234459	0.048128	0.194651	NOT
RP11-51J	42.50159	-0.6406829	0.324231	-1.97600983	0.048154	0.194725	NOT
NRBF2	1153.951	-0.3280065	0.166014	-1.975779	0.04818	0.1948	NOT
RP11-279(	5.700221	1.46143729	0.739699	1.97571942	0.048187	0.1948	NOT
FAM134C	4727.208	-0.2888906	0.146236	-1.97551284	0.04821	0.194865	NOT
RP5-1158I	1.932786	1.3733959	0.695379	1.975032757	0.048264	0.195056	NOT
DRAXIN	12.48335	0.73391094	0.371618	1.974908645	0.048279	0.195058	NOT
RP11-434I	6.125231	-0.6169229	0.312387	-1.97486742	0.048283	0.195058	NOT
APBA2	163.8643	1.03338258	0.523274	1.974839258	0.048286	0.195058	NOT
AC004449.	4.735442	0.92381886	0.467914	1.974334547	0.048344	0.195254	NOT
ITLN2	51.63435	-1.1535161	0.58427	-1.97428484	0.048349	0.195254	NOT

NLRP14	9.826164	-1.1731411	0.594297	-1.97399868	0.048382	0.195357	NOT
NF1P8	1.496786	3.0855607	1.563255	1.973805414	0.048404	0.19539	NOT
RP11-348N	4.658003	-0.7594491	0.384786	-1.97369277	0.048417	0.19539	NOT
PTPN3	2221.739	-0.410641	0.208059	-1.97367749	0.048418	0.19539	NOT
ARAF	3981.588	-0.3130947	0.158636	-1.97367319	0.048419	0.19539	NOT
PHGR1	3.082961	-1.2916204	0.654458	-1.97357251	0.04843	0.195407	NOT
COL6A1	9811.103	-0.7390922	0.374514	-1.97346901	0.048442	0.195413	NOT
CEP41	397.7334	0.36066343	0.182759	1.973433084	0.048446	0.195413	NOT
CC2D2B	2.934952	-0.6463674	0.327563	-1.97326242	0.048466	0.195462	NOT
SMO	3393.731	-0.4965199	0.251639	-1.97314608	0.048479	0.195481	NOT
UQCR11	4578.272	-0.5109951	0.258981	-1.97309571	0.048485	0.195481	NOT
CCDC85A	9.644227	-0.7531365	0.381722	-1.97299863	0.048496	0.195496	NOT
AC099552.	3.206598	-2.158791	1.09436	-1.97265181	0.048535	0.195626	NOT
CYB5A	18486.18	-0.5690527	0.288526	-1.97227313	0.048578	0.195771	NOT
PDZD11	1620.531	0.32048429	0.162503	1.972173151	0.04859	0.195788	NOT
RP1-292L2	29.12756	-0.5745097	0.291335	-1.97199195	0.048611	0.195843	NOT
RP1-3100I	4.32544	-0.6575538	0.333464	-1.97189128	0.048622	0.19586	NOT
TMEM131	2755.015	-0.2936251	0.148914	-1.97177193	0.048636	0.195886	NOT
BNIP2	1619.383	-0.2445095	0.124017	-1.97158606	0.048657	0.195942	NOT
PPP1CB	6315.085	0.24332484	0.123429	1.971367836	0.048682	0.196013	NOT
ZNF468	519.0789	0.72866888	0.369694	1.971006897	0.048723	0.19615	NOT
AF186192.	7.970983	1.3390207	0.679415	1.970844389	0.048742	0.196176	NOT
ZNF667-AS	222.0639	0.93521064	0.474527	1.970825397	0.048744	0.196176	NOT
CUX2	2406.634	-0.9992519	0.507129	-1.9704083	0.048792	0.196293	NOT
FKBP1B	340.0963	0.94297063	0.478567	1.970402709	0.048792	0.196293	NOT
ANAPC1	531.4552	0.3095473	0.1571	1.970381183	0.048795	0.196293	NOT
PRKCH	479.1095	-0.5208359	0.264351	-1.97023994	0.048811	0.196329	NOT
RP11-474O	8.959343	0.65425528	0.332114	1.969971415	0.048842	0.196424	NOT
PPP1R3B	3605.716	-0.6063456	0.307809	-1.96987682	0.048852	0.196438	NOT
RP11-214F	6.726761	-0.7517164	0.381633	-1.96973467	0.048869	0.196475	NOT
BRPF1	741.387	0.22930468	0.116443	1.96925198	0.048924	0.196643	NOT
RP11-243A	40.22839	-0.9036402	0.458877	-1.96924383	0.048925	0.196643	NOT
PAPOLA	6456.614	0.23714034	0.120429	1.969130365	0.048938	0.196666	NOT
SOX17	120.475	-0.5029219	0.255444	-1.96881102	0.048975	0.196784	NOT
AC019185.	1.470508	-2.1585242	1.096466	-1.96861972	0.048997	0.196843	NOT
MYLK4	44.42015	-0.7061997	0.358747	-1.96851528	0.049009	0.196862	NOT
MXRA5	395.5674	-0.8204001	0.41682	-1.96823524	0.049041	0.196963	NOT
MST1P2	1264.895	-0.8430993	0.428451	-1.96778385	0.049093	0.197142	NOT
ZBTB8OS	878.9305	0.369441	0.187765	1.967566683	0.049118	0.197164	NOT
TRH	1.671633	2.20098913	1.11864	1.967557463	0.049119	0.197164	NOT
ZIM2	3.744265	1.50250017	0.76365	1.967525587	0.049123	0.197164	NOT
NOS1AP	143.492	-0.6021378	0.306045	-1.96748316	0.049128	0.197164	NOT
SESN1	1060.665	-0.4257937	0.216431	-1.96734396	0.049144	0.197171	NOT
CTD-2561J	8.557967	0.89172846	0.453266	1.967342448	0.049144	0.197171	NOT
PLXNA4	47.70858	-1.1713513	0.595435	-1.96721833	0.049158	0.197199	NOT
RBBP4	4562.737	0.28506383	0.144915	1.967105882	0.049171	0.197212	NOT
APOBEC3A	15.20193	0.90297922	0.459049	1.967063887	0.049176	0.197212	NOT
POLG2	377.293	0.3396312	0.172705	1.96654129	0.049236	0.197425	NOT
LRRN4	13.69931	1.11839948	0.568779	1.966317719	0.049262	0.197484	NOT

CAMK2B	304.3282	-1.2540913	0.637806	-1.96625825	0.049269	0.197484	NOT
RP11-179k	6.695831	-1.354104	0.688683	-1.96622364	0.049273	0.197484	NOT
ACADVL	21822.59	-0.4463074	0.227025	-1.96589781	0.04931	0.197606	NOT
IMPA1P	2.536956	-1.2802351	0.651296	-1.96567297	0.049336	0.19765	NOT
AD001527.	2.426114	-0.910263	0.463091	-1.96562609	0.049342	0.19765	NOT
XPO1	5715.788	0.23433925	0.119219	1.965612551	0.049343	0.19765	NOT
IFNG	10.55276	1.16020884	0.590319	1.965393319	0.049369	0.197723	NOT
ACA59	4.411714	-1.0977376	0.558675	-1.96489352	0.049427	0.197894	NOT
RP11-372I	10.78228	0.74912171	0.381254	1.96489077	0.049427	0.197894	NOT
PTPRS	1474.634	-1.1484933	0.584524	-1.96483519	0.049433	0.197894	NOT
ANKRD28	1900.061	-0.3091951	0.157373	-1.96472168	0.049446	0.197917	NOT
RP11-54A4	14.35164	-0.6822605	0.347274	-1.96461666	0.049459	0.197937	NOT
YAF2	594.9086	-0.2397564	0.122046	-1.96447109	0.049475	0.197975	NOT
BSN	213.8096	-0.6755843	0.343945	-1.96421968	0.049505	0.198037	NOT
LACE1	211.3979	-0.3141056	0.159914	-1.9642104	0.049506	0.198037	NOT
RP11-107(	7.315061	2.17034501	1.105056	1.964012839	0.049529	0.1981	NOT
AP000866.	6.051824	-0.7841684	0.399317	-1.96377529	0.049556	0.198181	NOT
RPRD1B	2524.292	-0.3877463	0.197458	-1.96369441	0.049566	0.198185	NOT
RP11-640M	3.441136	1.59383882	0.811676	1.963639337	0.049572	0.198185	NOT
C2orf78	4.215605	1.02932913	0.524225	1.963524472	0.049585	0.19821	NOT
C8orf44	124.9593	0.41080434	0.209276	1.962982681	0.049648	0.198432	NOT
CDH23	702.2084	-0.7087805	0.361099	-1.96284024	0.049665	0.198454	NOT
MT-CO3	446445.4	-0.4929613	0.251151	-1.96281011	0.049668	0.198454	NOT
AAGAB	1373.826	0.22844049	0.116401	1.962537218	0.0497	0.198551	NOT
NAA38	1694.727	-0.5559654	0.283301	-1.96245211	0.04971	0.198561	NOT
TAF1D	1855.375	0.37745166	0.192351	1.9623024	0.049727	0.198586	NOT
PARP4P2	4.90076	-0.9383142	0.478177	-1.96227329	0.049731	0.198586	NOT
RP11-211(	2.888437	1.65186124	0.841976	1.961886837	0.049776	0.198736	NOT
MAGEA11	4.379542	2.34347872	1.194558	1.96179554	0.049786	0.19875	NOT
FAM20B	3442.456	-0.3137386	0.159933	-1.96168544	0.049799	0.198766	NOT
HNRNPA1P4	5.927438	0.597576	0.304632	1.961633629	0.049805	0.198766	NOT
CTD-2026K	12.0313	-0.7081885	0.36105	-1.96146719	0.049825	0.198804	NOT
TIRAP	395.3978	-0.3157208	0.160968	-1.96138329	0.049834	0.198804	NOT
CHIC1	206.4895	-0.8985982	0.458149	-1.9613647	0.049836	0.198804	NOT
C11orf65	25.07272	-0.4851365	0.247365	-1.96121987	0.049853	0.198842	NOT
GJA4	628.7191	-0.486225	0.24795	-1.96098308	0.049881	0.198923	NOT
AP000320.	3.857298	-0.7590292	0.387113	-1.96074252	0.049909	0.199005	NOT
DMKN	773.9489	1.45144104	0.740325	1.960546763	0.049932	0.199067	NOT
ROS1	17.93359	-1.3330104	0.679963	-1.96041481	0.049947	0.199099	NOT
FGF5	1.238995	2.29224664	1.169271	1.960405904	0.049948	NA	NA
LTBP2	1683.689	-0.7543918	0.384852	-1.96021219	0.049971	0.199142	NOT
LRP5	8654.004	-0.3815756	0.194662	-1.96019742	0.049973	0.199142	NOT
C15orf65	28.39006	0.51502028	0.262758	1.960055161	0.049989	0.199179	NOT
METRN	942.5684	-0.6243689	0.318597	-1.9597437	0.050026	0.199266	NOT
SMUG1P1	17.97584	-2.0586948	1.050492	-1.95974325	0.050026	0.199266	NOT
LRCH3	1128.892	0.24426057	0.12466	1.959417968	0.050064	0.199388	NOT
RP11-532F	14.92752	-0.6862712	0.350263	-1.95930281	0.050077	0.199413	NOT
RP11-817(	19.56726	-0.6000552	0.30628	-1.95917201	0.050093	0.199444	NOT
GAB3	111.6833	-0.5376366	0.27449	-1.95867574	0.050151	0.199642	NOT

TTC19	1262.621	-0.4298817	0.219482	-1.95862234	0.050157	0.199642	NOT
RP11-203J	41.79844	-0.8572433	0.437723	-1.95841408	0.050181	0.19971	NOT
PLA2G3	1.258199	1.74461695	0.890876	1.958316463	0.050193	NA	NA
NYNRIN	913.3654	0.75957439	0.387918	1.958081389	0.05022	0.199809	NOT
C7orf60	223.7057	0.3571316	0.182389	1.958076718	0.050221	0.199809	NOT
ARHGEF9	616.9894	-0.407112	0.207956	-1.95768659	0.050267	0.199962	NOT
ITGB1BP1	1511.279	0.36843385	0.188216	1.95750257	0.050288	0.200018	NOT
SRRM2	15368.31	-0.3372942	0.172336	-1.95719442	0.050325	0.200133	NOT
DGKI	32.31191	-0.6782637	0.346576	-1.95704294	0.050342	0.200174	NOT
TIGD1	194.5098	0.60112032	0.307189	1.956839353	0.050366	0.20024	NOT
ACSS1	1051.249	0.63733787	0.325764	1.956440085	0.050413	0.200368	NOT
RAB14	4722.721	-0.2563527	0.131033	-1.95640355	0.050418	0.200368	NOT
EXD1	1.876878	1.28671091	0.657711	1.956348112	0.050424	0.200368	NOT
ATF7IP2	999.2816	-0.4964213	0.253755	-1.95629866	0.05043	0.200368	NOT
RP11-407(	3.424145	-0.8239246	0.421183	-1.95621382	0.05044	0.200368	NOT
TSPAN7	640.0733	0.58451509	0.298803	1.956190165	0.050443	0.200368	NOT
RP5-849H1	374.0504	-0.7834772	0.400528	-1.95611105	0.050452	0.200375	NOT
RP11-101	7.781202	1.16942595	0.597868	1.955993679	0.050466	0.200401	NOT
MYO1C	7544.291	-0.3729939	0.190776	-1.95514334	0.050566	0.20077	NOT
RPS3AP5	22.39614	0.71750609	0.367013	1.954986891	0.050585	0.200814	NOT
AC034220.	104.2513	-0.5578535	0.285383	-1.95475519	0.050612	0.200892	NOT
RP11-455F	5.621593	0.78317268	0.400662	1.954694551	0.050619	0.200892	NOT
STARD3	2568.596	0.26759312	0.136934	1.954178899	0.05068	0.201089	NOT
AC096559.	8.077084	-1.0676381	0.546344	-1.95414824	0.050684	0.201089	NOT
HTR7P1	128.6876	-0.5069673	0.259451	-1.95400099	0.050701	0.201129	NOT
TIGD4	6.797428	0.79894286	0.40889	1.953929823	0.05071	0.201133	NOT
HOXD4	12.62303	1.12726017	0.577	1.953657734	0.050742	0.201231	NOT
ZNF124	139.4856	0.50158796	0.256758	1.953542364	0.050755	0.201256	NOT
RMST	13.99115	1.39055843	0.711851	1.953441554	0.050767	0.201274	NOT
DDT	6072.999	-0.6305442	0.322802	-1.95334718	0.050778	0.201289	NOT
SON	6659.649	-0.2710718	0.13878	-1.95324356	0.050791	0.201308	NOT
TSACC	69.63762	0.66356226	0.339737	1.953162511	0.0508	0.201317	NOT
RP11-425M	4.797716	-0.7112989	0.364285	-1.95259011	0.050868	0.201556	NOT
UBXN11	714.3343	0.41468876	0.212388	1.952501264	0.050879	0.201568	NOT
RPS20	37222.09	0.48058398	0.24615	1.952402292	0.05089	0.20158	NOT
PNPLA2	4821.641	-0.3570462	0.18288	-1.95235145	0.050896	0.20158	NOT
SALL1	2153.313	-0.6091011	0.312047	-1.95195501	0.050944	0.201737	NOT
CTAGE8	5.144008	0.9662837	0.495116	1.951631268	0.050982	0.20184	NOT
SUPT7L	1666.332	0.18961835	0.09716	1.951609794	0.050985	0.20184	NOT
LINC0052	71.42524	0.58419241	0.299408	1.951156815	0.051038	0.201996	NOT
ZNF212	651.084	0.26732803	0.13701	1.951153861	0.051039	0.201996	NOT
RP11-81A1	95.88017	-0.4408904	0.225977	-1.95104154	0.051052	0.202019	NOT
RP11-729	2.476083	-1.0530872	0.53979	-1.95091943	0.051067	0.20204	NOT
TPSG1	41.67861	-1.3424663	0.688137	-1.95087117	0.051072	0.20204	NOT
MOGAT1	19.03768	-1.027649	0.526874	-1.95046574	0.051121	0.202163	NOT
DNAJB7	3.126963	0.77472766	0.397212	1.950411861	0.051127	0.202163	NOT
TRAPPC8	1778.704	-0.3565975	0.182836	-1.95036479	0.051133	0.202163	NOT
RP11-295F	37.27073	0.53163203	0.272581	1.950360808	0.051133	0.202163	NOT
MN1	201.8421	-0.8729554	0.44762	-1.95021651	0.05115	0.202201	NOT

RP11-218I	13.21082	0.60131412	0.308417	1.94967668	0.051215	0.202426	NOT
RP3-337H4	17.75682	0.66587875	0.34155	1.949580823	0.051226	0.202442	NOT
HOXA2	11.88632	0.86454827	0.443471	1.949503015	0.051235	0.202446	NOT
AP3S2	1174.831	-0.3545896	0.181894	-1.94943339	0.051244	0.202446	NOT
ARHGAP8	6.674347	1.24308059	0.637679	1.949383783	0.05125	0.202446	NOT
SSX5	11.63933	2.37355907	1.217686	1.949237052	0.051267	0.202486	NOT
CNRIP1	185.7086	-0.5452876	0.279778	-1.94899816	0.051296	0.202526	NOT
COLQ	108.6607	-0.5569047	0.285742	-1.94897551	0.051298	0.202526	NOT
DTX2	686.4743	0.32397186	0.166228	1.948964271	0.0513	0.202526	NOT
KRT4	4.504068	2.80037196	1.437139	1.948573513	0.051346	0.202651	NOT
MARK4	2280.847	-0.3154763	0.161906	-1.94852115	0.051353	0.202651	NOT
RP11-620J	135.0403	0.53960761	0.276933	1.948512854	0.051354	0.202651	NOT
SLC46A2	4.379164	-0.9374822	0.481196	-1.9482342	0.051387	0.202753	NOT
RP11-324(	29.43652	-0.7202947	0.369747	-1.94807538	0.051406	0.202786	NOT
BARX2	3.554948	1.25482749	0.644149	1.948037938	0.05141	0.202786	NOT
RP11-379I	29.50661	-0.5440002	0.27927	-1.94793959	0.051422	0.202803	NOT
GAP43	12.42599	1.08876039	0.55905	1.94751937	0.051472	0.202966	NOT
RP11-246K	7.286553	-1.7677323	0.907707	-1.9474705	0.051478	0.202966	NOT
LGALS9C	27.78879	-1.2312966	0.632307	-1.94730715	0.051498	0.202994	NOT
ARHGAP6	90.06866	-0.5055732	0.25963	-1.94728509	0.051501	0.202994	NOT
TSTD3	35.31303	0.43500255	0.22344	1.946842753	0.051554	0.203174	NOT
ABCA7	627.0683	-0.5088846	0.261442	-1.9464511	0.051601	0.20333	NOT
CTB-39G8.	9.428612	-0.8779979	0.45114	-1.94617702	0.051633	0.203418	NOT
SPDYA	22.25418	0.53772375	0.276303	1.946139317	0.051638	0.203418	NOT
NPTN-IT1	11.17847	-0.8209244	0.421858	-1.94597514	0.051658	0.203466	NOT
UBXN2A	829.2377	0.26908487	0.138313	1.945480586	0.051717	0.203668	NOT
NDUFA4	11778.07	-0.4274713	0.219735	-1.9453928	0.051728	0.203668	NOT
GYS2	2043.416	-1.2251887	0.629812	-1.94532291	0.051736	0.203668	NOT
ELL3	22.34783	0.5729553	0.294533	1.945298913	0.051739	0.203668	NOT
RP11-461(	14.46086	-1.3398686	0.688814	-1.9451832	0.051753	0.203693	NOT
EXOC3-AS1	205.608	-0.4763789	0.244916	-1.94507077	0.051766	0.203717	NOT
HMGN2P4	4.590409	0.60425012	0.31068	1.944926506	0.051784	0.203756	NOT
VTN	243878.8	-0.6462724	0.332305	-1.94481456	0.051797	0.203779	NOT
RP11-284F	781.0097	1.04206262	0.535918	1.944444242	0.051842	0.203897	NOT
SHKBP1	2096.041	0.3684748	0.189502	1.944441394	0.051842	0.203897	NOT
AKT1S1	3823.489	-0.3858104	0.198424	-1.94437593	0.05185	0.203897	NOT
SORD	9272.292	-0.6610227	0.339977	-1.94431698	0.051857	0.203897	NOT
CTC-451P1	64.06587	0.6041859	0.310804	1.94394489	0.051902	0.204043	NOT
METTL7A	25151.67	-0.547029	0.281462	-1.9435254	0.051953	0.204213	NOT
STPG1	210.9437	0.46276549	0.238116	1.943444691	0.051962	0.204222	NOT
RP11-286H	11.96556	0.55797534	0.287175	1.942981387	0.052018	0.204387	NOT
KLRD1	76.76361	-0.6506702	0.334884	-1.94297174	0.05202	0.204387	NOT
FAM91A3P	2.117574	0.88706761	0.456571	1.942889763	0.052029	0.204396	NOT
GGACT	574.2749	-0.6350746	0.326941	-1.94247376	0.05208	0.204564	NOT
IBTK	3649.422	-0.3621722	0.186465	-1.9423071	0.0521	0.204606	NOT
SYT2	16.49414	-0.6837742	0.352063	-1.94219263	0.052114	0.204606	NOT
SNORA31	6.590558	-0.6018215	0.309874	-1.94214824	0.052119	0.204606	NOT
ZNF28	402.2356	0.8048173	0.414398	1.942135881	0.052121	0.204606	NOT
RP11-12G1	669.3937	0.5282174	0.272058	1.941560357	0.05219	0.204849	NOT

DCX	12.89261	1.20811823	0.622289	1.941409463	0.052209	0.204849	NOT
PVRL4	39.08801	-0.9951706	0.512606	-1.94139331	0.052211	0.204849	NOT
CENPBD1P1	675.4086	0.26339369	0.135674	1.941375568	0.052213	0.204849	NOT
INSM1	2.556844	-1.6775404	0.864335	-1.94084465	0.052277	0.205072	NOT
LINC0052	5.740972	-1.8685556	0.962814	-1.94072371	0.052292	0.2051	NOT
OR52K3P	6.644181	1.2973809	0.668545	1.940604583	0.052306	0.205107	NOT
MCCC1	2238.76	-0.3645754	0.187869	-1.94058456	0.052309	0.205107	NOT
C1QTNF4	9.721431	-0.8303016	0.427891	-1.94045346	0.052325	0.20514	NOT
EPS8L1	127.3634	1.05260488	0.542489	1.940325598	0.05234	0.205145	NOT
MDC1	1840.852	0.34960428	0.180179	1.940317368	0.052341	0.205145	NOT
PNRC1	4282.301	-0.359429	0.185249	-1.94025079	0.052349	0.205147	NOT
OCA2	53.60889	1.16206552	0.598961	1.940134954	0.052363	0.205161	NOT
ABT1	1542.763	0.25487885	0.131376	1.940065016	0.052372	0.205161	NOT
CD300E	53.86393	-0.7019181	0.361807	-1.94003608	0.052375	0.205161	NOT
CTD-2034I	1.124244	-1.3766913	0.709627	-1.94002055	0.052377	NA	NA
RP11-528A	12.09902	1.03901482	0.535599	1.939910729	0.052391	0.205191	NOT
Z98750.1	2.570446	-0.8123111	0.418771	-1.93974971	0.05241	0.205238	NOT
GSDMD	8293.14	0.45870132	0.236482	1.939687653	0.052418	0.205238	NOT
PCDHGA6	69.94124	-0.6948277	0.358229	-1.93961718	0.052426	0.205242	NOT
SERPINA5	24560.37	-0.8034881	0.414299	-1.93939338	0.052453	0.205318	NOT
RP11-732A	8.027742	-0.6982778	0.360126	-1.93897969	0.052504	0.205486	NOT
NFXL1	485.5078	0.43620463	0.224997	1.938714533	0.052536	0.205563	NOT
VSTM4	1053.174	-0.7265192	0.374747	-1.93869395	0.052539	0.205563	NOT
HIST1H1T	0.932397	1.64163744	0.847041	1.938086075	0.052613	NA	NA
GPM6A	53.40801	-1.0498482	0.54173	-1.93795296	0.052629	0.205829	NOT
PPP1R1A	3618.085	-1.050939	0.542295	-1.93794549	0.05263	0.205829	NOT
SORCS3	5.811049	2.51955933	1.300196	1.937831089	0.052644	0.205829	NOT
KRT8P5	1.814155	1.0862953	0.560576	1.937821042	0.052645	0.205829	NOT
WSB1	2200.731	0.38293648	0.197616	1.937779194	0.05265	0.205829	NOT
RPS10P3	10.27356	0.79069834	0.408047	1.937763523	0.052652	0.205829	NOT
VSTM5	8.248182	0.94597241	0.488247	1.937489222	0.052686	0.20593	NOT
RP11-789C	8.281902	1.52769742	0.788542	1.937369439	0.0527	0.205957	NOT
CPLX1	598.6724	-0.8088121	0.417584	-1.93688318	0.05276	0.20616	NOT
DHX35	574.1479	0.24715275	0.127612	1.936751154	0.052776	0.206193	NOT
EBF4	511.529	-0.8720235	0.450296	-1.93655841	0.052799	0.206256	NOT
SLCO5A1	25.04806	0.89797449	0.463716	1.936474636	0.05281	0.206266	NOT
AC005253.	29.60842	-0.4768345	0.246266	-1.9362558	0.052836	0.206312	NOT
RP11-119I	8.128921	0.97895297	0.505602	1.936211174	0.052842	0.206312	NOT
ALDOAP1	2.284902	-1.2825167	0.662411	-1.93613492	0.052851	0.206312	NOT
TMSB4XP1	2.958624	0.87668539	0.452803	1.936130776	0.052852	0.206312	NOT
AC097523.	6.307087	0.48577118	0.250941	1.935799953	0.052892	0.206403	NOT
LINC00426	26.73256	-0.7106238	0.367103	-1.93576278	0.052897	0.206403	NOT
RP13-463N	2.757946	1.16205908	0.600314	1.935753403	0.052898	0.206403	NOT
FAM172A	569.0949	-0.3198505	0.165265	-1.9353848	0.052943	0.20655	NOT
SUPT4H1	3129.373	0.26284728	0.135831	1.935104415	0.052977	0.206654	NOT
RP11-676F	1.413101	2.97851832	1.539298	1.934984487	0.052992	0.206682	NOT
C7	3188.697	-1.1753604	0.607454	-1.93489754	0.053003	0.206694	NOT
GTF2IRD1	806.3305	0.43157247	0.22309	1.934523595	0.053049	0.206843	NOT
FADD	409.9444	0.29043656	0.150139	1.934445466	0.053058	0.206851	NOT

LINC0066[illegible]	14.36442	1.05213025	0.543932	1.934305257	0.053076	0.206888	NOT
WDPCP	142.9611	-0.4713848	0.243711	-1.93419735	0.053089	0.20691	NOT
TGM4	2.321121	0.94440651	0.488332	1.933942788	0.05312	0.207002	NOT
AC019050.	6.002218	0.88649489	0.458499	1.933469743	0.053178	0.20717	NOT
RP11-138[illegible]	4.187188	1.7196018	0.889387	1.933468697	0.053178	0.20717	NOT
AP1M2	1107.572	1.27906193	0.661629	1.933201364	0.053211	0.207243	NOT
RP11-867[illegible]	3.382502	-0.8081361	0.418035	-1.93317865	0.053214	0.207243	NOT
RP11-107[illegible]	6.773644	1.97047944	1.019321	1.933130144	0.05322	0.207243	NOT
OTUD6B-A[illegible]	1046.923	0.36046459	0.186474	1.933057882	0.053229	0.207248	NOT
EHD3	320.0705	-0.5637079	0.291645	-1.93285556	0.053254	0.207296	NOT
RP4-782L[illegible]	5.230714	-1.0693311	0.553245	-1.9328332	0.053257	0.207296	NOT
RP11-69L[illegible]	14.89621	0.81763131	0.423082	1.932559598	0.05329	0.207378	NOT
RRAGD	1903.274	0.63963957	0.330996	1.93246665	0.053302	0.207378	NOT
CTC-336P[illegible]	8.398324	-0.6413425	0.331883	-1.9324339	0.053306	0.207378	NOT
EIF4A3	3193.663	0.28854335	0.14932	1.932387387	0.053312	0.207378	NOT
MED6	656.9093	0.26147021	0.135312	1.932353574	0.053316	0.207378	NOT
A2MP1	20.64643	-0.9140006	0.473032	-1.93221582	0.053333	0.207414	NOT
RP11-434[illegible]	46.77329	1.29424368	0.669887	1.932031638	0.053356	0.207473	NOT
GRIN3A	43.39129	-0.4649256	0.240657	-1.93190386	0.053371	0.207491	NOT
RP11-83A[illegible]	142.6011	-0.4016242	0.207894	-1.93186979	0.053376	0.207491	NOT
PIGZ	371.3109	0.71579903	0.370676	1.931061515	0.053475	0.20783	NOT
OTOGL	5.195552	0.95915869	0.496706	1.931039299	0.053478	0.20783	NOT
PHACTR1	151.5535	-0.6345057	0.328627	-1.93077791	0.053511	0.207926	NOT
CIB3	2.175802	1.57635835	0.816465	1.93071185	0.053519	0.207928	NOT
QRSL1	882.2911	0.3138187	0.162548	1.930620703	0.05353	0.207942	NOT
ACTR5	468.6481	0.35249583	0.182601	1.930412097	0.053556	0.208	NOT
RP11-53I[illegible]	2.038641	-0.9289762	0.481242	-1.93037204	0.053561	0.208	NOT
RP11-93H[illegible]	1.45622	1.8659339	0.966683	1.930243961	0.053577	0.208	NOT
GATSL3	76.78327	-0.5490717	0.28446	-1.93022759	0.053579	0.208	NOT
CTSF	5865.498	-0.5501487	0.285023	-1.93019181	0.053583	0.208	NOT
DZANK1	60.08434	0.45863483	0.237658	1.92980739	0.053631	0.208155	NOT
HSPA4	7162.999	0.33500793	0.173622	1.929522549	0.053666	0.208262	NOT
SMIM17	4.301104	1.03893199	0.538461	1.929445925	0.053676	0.208269	NOT
NAE1	1387.097	0.25497193	0.132155	1.929344109	0.053688	0.208288	NOT
CALD1	13446.85	-0.3544532	0.183736	-1.92913991	0.053713	0.208356	NOT
CLDN6	6.925703	1.08530735	0.56262	1.92902426	0.053728	0.208356	NOT
GRK5	711.5228	-0.3576216	0.185391	-1.92901763	0.053729	0.208356	NOT
AF124730.	19.97645	1.08563934	0.562835	1.928878091	0.053746	0.208381	NOT
MDFI	237.6015	1.00794495	0.522575	1.928804287	0.053755	0.208381	NOT
SLC30A2	123.6797	1.33331147	0.691292	1.928723479	0.053765	0.208381	NOT
WSCD2	3.968026	-0.9301628	0.48227	-1.92871878	0.053766	0.208381	NOT
KLHL31	62.44844	-0.613587	0.318143	-1.92864999	0.053774	0.208384	NOT
GATA1	2.242743	-0.9534106	0.494399	-1.92842485	0.053802	0.208463	NOT
AC002310.	2.127653	1.02196838	0.530004	1.928227576	0.053827	0.208528	NOT
CYP8B1	18235.67	-1.316251	0.682693	-1.92802845	0.053852	0.208588	NOT
AC104654.	10.97256	1.24830412	0.647468	1.927978282	0.053858	0.208588	NOT
RP11-211[illegible]	24.54183	-0.6466458	0.335418	-1.92788153	0.05387	0.208588	NOT
EEF1A1P6	281.7339	0.46627169	0.241874	1.927745495	0.053887	0.208588	NOT
EXOSC2	890.1243	0.32643806	0.169338	1.927732673	0.053888	0.208588	NOT

YTHDC2	1298.71	-0.3502884	0.18171	-1.9277323	0.053888	0.208588	NOT
FAM110A	352.4156	0.47002999	0.243842	1.92760028	0.053905	0.208622	NOT
RP11-486I	22.45605	-0.5077715	0.263454	-1.92736023	0.053935	0.208708	NOT
LINC0062	22.68174	-0.7021629	0.364416	-1.92681477	0.054003	0.208941	NOT
HBA2	260.5401	-0.8132395	0.422092	-1.92668575	0.054019	0.208973	NOT
ZNF383	288.826	-0.3452363	0.179206	-1.92648142	0.054044	0.209042	NOT
TMEM169	156.5312	0.55629056	0.288769	1.926420981	0.054052	0.209042	NOT
RGS9	76.7457	-0.7583694	0.393705	-1.92623924	0.054075	0.209099	NOT
RP11-253M	8.942544	-0.6559112	0.340566	-1.92594329	0.054111	0.209212	NOT
KY	1.937311	1.22905801	0.63828	1.925579481	0.054157	0.209347	NOT
DOCK4	1204.214	-0.3989251	0.20718	-1.92549937	0.054167	0.209347	NOT
ATP1B3-AS	1.599543	-1.0234179	0.531513	-1.92547912	0.054169	0.209347	NOT
RGPD2	18.60875	-0.8813233	0.457731	-1.92541859	0.054177	0.209347	NOT
RP11-554F	2.279372	-0.8012401	0.41619	-1.92517771	0.054207	0.209429	NOT
RN7SKP97	2.533657	1.04278212	0.54167	1.925125046	0.054214	0.209429	NOT
RP11-727F	45.26061	-0.3417598	0.177544	-1.92493223	0.054238	0.209492	NOT
RP11-357F	4.344658	-0.9171424	0.476471	-1.92486572	0.054246	0.209494	NOT
NACC1	3149.655	-0.3350157	0.174052	-1.92480012	0.054254	0.209496	NOT
CASKIN1	33.63139	1.21617444	0.631866	1.924735474	0.054262	0.209498	NOT
CTC-338M1	66.70837	0.46410518	0.241159	1.92448093	0.054294	0.209591	NOT
CYP2E1	86859.59	-1.3635588	0.708674	-1.92409836	0.054342	0.209708	NOT
RP4-713B5	5.089158	0.67571949	0.351198	1.924039031	0.05435	0.209708	NOT
MEDAG	52.21728	-0.8881128	0.461608	-1.92395382	0.05436	0.209708	NOT
CTD-2240E	17.3051	-0.5912064	0.307293	-1.92391594	0.054365	0.209708	NOT
CRX	2.332058	-0.9444527	0.490923	-1.92382897	0.054376	0.209708	NOT
MARC1	2455.655	-0.5995521	0.311648	-1.92381275	0.054378	0.209708	NOT
CADPS	37.32146	1.44592424	0.751595	1.923807775	0.054379	0.209708	NOT
CEP164P1	5.994091	-0.7124793	0.370406	-1.92350694	0.054416	0.209804	NOT
RPL4P6	11.43795	0.52292924	0.271865	1.92348587	0.054419	0.209804	NOT
F10	13002.41	-0.7167779	0.37267	-1.92335645	0.054435	0.20983	NOT
TUBB8P7	4.053766	1.20887435	0.628539	1.923308108	0.054441	0.20983	NOT
NCKAP1	4821.164	0.28974057	0.150653	1.923225468	0.054452	0.20984	NOT
RP3-510D1	42.72526	-0.5490758	0.285522	-1.92305953	0.054473	0.209891	NOT
TMPRSS7	2.498281	1.40429241	0.730371	1.922710628	0.054516	0.21003	NOT
RP11-324E	4.52821	0.98378813	0.511711	1.922547008	0.054537	0.210079	NOT
NAP1L1P1	15.11806	0.49494046	0.257459	1.922401498	0.054555	0.21012	NOT
VMO1	122.1854	-0.4901798	0.254998	-1.92229098	0.054569	0.210144	NOT
FFAR3	2.74844	1.23554744	0.642879	1.921896166	0.054619	0.210305	NOT
CFHR3	3785.424	-1.1244435	0.585088	-1.92183545	0.054626	0.210305	NOT
UBAP1L	112.5624	-0.5396308	0.280818	-1.9216423	0.054651	0.210368	NOT
ANPEP	30588.12	-0.5847422	0.304342	-1.92133395	0.05469	0.210488	NOT
RAD51C	599.3069	0.3118067	0.162324	1.920891626	0.054745	0.210673	NOT
MLC1	14.18724	-0.6387593	0.332549	-1.9207954	0.054758	0.210689	NOT
VWFP1	5.561628	-1.0986099	0.572077	-1.92038944	0.054809	0.210857	NOT
RP11-156K	55.36392	-0.4636918	0.241467	-1.92031327	0.054818	0.210864	NOT
CREB1	977.2572	0.22369339	0.116497	1.92016861	0.054837	0.210895	NOT
SUCO	2235.108	0.37305203	0.194285	1.92012639	0.054842	0.210895	NOT
RP11-23J1	5.905408	-0.7333409	0.381943	-1.92002578	0.054855	0.210914	NOT
RP11-672I	27.81977	0.51332458	0.267386	1.919789984	0.054884	0.210998	NOT

BLNK	1016.541	-0.483239	0.251735	-1.91963536	0.054904	0.211023	NOT
SLFNL1	19.33484	-0.7480072	0.389665	-1.9196154	0.054906	0.211023	NOT
SPTB	82.39333	0.86305705	0.449639	1.919444409	0.054928	0.211076	NOT
RP11-404(	174.6088	1.17282436	0.611106	1.91918228	0.054961	0.211105	NOT
PDGFD	387.8345	-0.5749312	0.299578	-1.91913887	0.054967	0.211105	NOT
CASP8	1150.074	0.2793133	0.145542	1.919118793	0.054969	0.211105	NOT
C1orf229	33.38806	0.84875969	0.442272	1.919088083	0.054973	0.211105	NOT
APOC3	160445.5	-0.8504903	0.443185	-1.91904308	0.054979	0.211105	NOT
TRIM47	2247.722	0.55381444	0.288593	1.919016572	0.054982	0.211105	NOT
MTX1P1	155.5155	0.55546389	0.289464	1.91893641	0.054992	0.211105	NOT
JAM2	259.8693	-0.5591356	0.291384	-1.91889354	0.054998	0.211105	NOT
AC021016.	3.917176	0.73577976	0.383468	1.918750227	0.055016	0.211114	NOT
NPM1P9	3.38532	0.80001944	0.416954	1.918725706	0.055019	0.211114	NOT
AC093495.	42.64689	-0.406393	0.211808	-1.91869033	0.055024	0.211114	NOT
IER2	2862.117	-0.3805155	0.198355	-1.91835943	0.055065	0.211245	NOT
FIP1L1	1179.155	0.26123442	0.136192	1.918131409	0.055094	0.21131	NOT
DUSP3	7835.125	-0.2661511	0.138758	-1.91809693	0.055099	0.21131	NOT
AC011997.	2.309771	1.35225323	0.705034	1.91799787	0.055111	0.21131	NOT
SLC46A3	2098.092	-0.8496742	0.443005	-1.91797977	0.055114	0.21131	NOT
RP1-102E2	49.2935	-0.5997121	0.312711	-1.9177858	0.055138	0.211375	NOT
RP11-173(	4.261317	-0.6617171	0.345094	-1.91749555	0.055175	0.211486	NOT
RP5-1031I	9.397328	0.78583205	0.409876	1.917241672	0.055207	0.21158	NOT
ACIN1	4377.495	0.21317454	0.111192	1.917168244	0.055217	0.211585	NOT
PHLDB2	1702.244	-0.4900324	0.255636	-1.91691384	0.055249	0.211679	NOT
SPPL2B	1974.154	-0.3770222	0.196711	-1.9166347	0.055284	0.21178	NOT
MYO1B	11904.99	-0.5303928	0.276738	-1.91658494	0.055291	0.21178	NOT
FAM217B	341.8481	0.57131266	0.298137	1.916273348	0.05533	0.211902	NOT
ATP2B2	4540.718	-0.8056514	0.420499	-1.91594345	0.055372	0.212002	NOT
RPS18P9	50.13637	-0.3429282	0.178991	-1.91590146	0.055378	0.212002	NOT
BHLHE22	30.45045	-1.021628	0.53325	-1.915851	0.055384	0.212002	NOT
HPN	21904.05	-0.6794451	0.354649	-1.91582264	0.055388	0.212002	NOT
HAO2-IT1	4.046687	-1.3205662	0.689341	-1.91569408	0.055404	0.212034	NOT
PGLYRP4	3.79122	1.6281517	0.849934	1.915620368	0.055413	0.21204	NOT
MND1	145.5147	0.49002645	0.255859	1.915221212	0.055464	0.212184	NOT
RRN3P2	24.00023	-0.4775716	0.249358	-1.91520395	0.055466	0.212184	NOT
CTD-2341M	21.44657	-0.6087632	0.317893	-1.91499463	0.055493	0.212256	NOT
RPL5P4	11.23985	0.56770046	0.296504	1.914649732	0.055537	0.212394	NOT
BNIP3P11	14.19788	0.71155147	0.37165	1.914575931	0.055547	0.2124	NOT
R3HDM2	1922.844	-0.297613	0.155453	-1.91448747	0.055558	0.212409	NOT
FTCD	13235.43	-0.8879635	0.463825	-1.91443461	0.055565	0.212409	NOT
RPS15AP1(	3.810808	-0.6712748	0.350737	-1.91389785	0.055633	0.212641	NOT
C9orf72	390.1443	-0.4622368	0.24155	-1.9136317	0.055667	0.212742	NOT
L2HGDH	527.8427	-0.452529	0.236501	-1.91343309	0.055693	0.212809	NOT
RP11-307(	7.621809	-0.7857604	0.410782	-1.91284123	0.055768	0.213066	NOT
HNRNPA3P1	2.364152	-0.829872	0.433869	-1.91272704	0.055783	0.213066	NOT
SLC35E1P1	1.880525	-1.063758	0.556149	-1.91272292	0.055784	0.213066	NOT
SH3BGR	178.6015	-0.4497513	0.235149	-1.91262272	0.055796	0.213069	NOT
XRN2	3262.35	0.2765613	0.1446	1.912594211	0.0558	0.213069	NOT
RP11-705(	48.863	0.53480424	0.279662	1.912324958	0.055835	0.213171	NOT

SPAG9	2947.47	-0.3022936	0.158098	-1.91206676	0.055868	0.213267	NOT
KLHL11	46.58739	-0.7046597	0.368613	-1.91164954	0.055921	0.213414	NOT
RHOXF1	5.528367	-1.0642005	0.556707	-1.91159976	0.055928	0.213414	NOT
RP11-973M	3.336432	-0.8462103	0.442675	-1.91158304	0.05593	0.213414	NOT
RP11-611L	4.622971	-0.75613	0.395598	-1.91135827	0.055959	0.213494	NOT
RP11-415F	10.73508	-0.5075658	0.265563	-1.91128108	0.055968	0.213502	NOT
FAXC	12.98845	1.33748417	0.699831	1.911153369	0.055985	0.213534	NOT
AC109642.	6.305671	-0.6026011	0.31533	-1.9110182	0.056002	0.213536	NOT
HHIP-AS1	9.687617	-0.9268999	0.485041	-1.91097413	0.056008	0.213536	NOT
CCDC132	747.1603	0.30594054	0.160097	1.910965165	0.056009	0.213536	NOT
PIF1	194.0868	0.64448967	0.337277	1.9108636	0.056022	0.213549	NOT
RP11-219E	15.02034	-0.8076048	0.422649	-1.91081805	0.056028	0.213549	NOT
ELP2	2911.061	-0.2514435	0.131594	-1.91074976	0.056037	0.213552	NOT
CHST11	750.9597	0.70062236	0.366722	1.910500101	0.056069	0.213644	NOT
ECEL1	81.26038	-1.361023	0.712431	-1.91039253	0.056083	0.213667	NOT
RP11-74C1	13.39177	-1.0372463	0.542968	-1.9103275	0.056091	0.213669	NOT
PXK	710.5487	0.37938814	0.198641	1.90991661	0.056144	0.213811	NOT
LTBP4	1885.379	-0.6074627	0.318058	-1.90991418	0.056144	0.213811	NOT
MYO5A	707.6204	-0.5646597	0.295665	-1.90979422	0.05616	0.213811	NOT
ISCA1P4	4.846709	1.01885497	0.53349	1.909792978	0.05616	0.213811	NOT
ZNF781	39.18427	0.77829742	0.407543	1.909728542	0.056168	0.213813	NOT
CD14	16912.01	-0.7524801	0.394071	-1.90950552	0.056197	0.213862	NOT
THBS4	1060.548	-0.9441328	0.494439	-1.90950477	0.056197	0.213862	NOT
LYRM9	268.9838	-0.4454223	0.233299	-1.90923691	0.056232	0.213948	NOT
CEACAM7	19.52063	2.60815239	1.366091	1.909207853	0.056235	0.213948	NOT
RP11-230F	3.473778	0.95842184	0.502054	1.909000868	0.056262	0.21402	NOT
KCNQ4	52.71779	0.78686253	0.4122	1.908933096	0.056271	0.214023	NOT
IFT172	640.6656	0.37288123	0.195344	1.908843161	0.056282	0.214037	NOT
ST20-MTHF	4.584829	-0.762451	0.399508	-1.90847691	0.05633	0.214169	NOT
RP1-140K8	89.95782	0.96732046	0.506861	1.90845186	0.056333	0.214169	NOT
LA16c-316	4.830006	-0.7081448	0.371092	-1.90827447	0.056356	0.214198	NOT
NBPF26	53.81579	-0.6390849	0.334909	-1.90823587	0.056361	0.214198	NOT
ATP2A3	633.0392	-0.6259394	0.328033	-1.90815859	0.056371	0.214198	NOT
LAMP3	149.5265	0.68672579	0.359903	1.908087509	0.05638	0.214198	NOT
BLMH	1580.701	0.62717861	0.328695	1.90808705	0.05638	0.214198	NOT
LINC00856	2.769272	-1.2634072	0.662163	-1.9080009	0.056391	0.21421	NOT
RP11-17E2	4.421953	-0.7422472	0.389131	-1.90744896	0.056462	0.214422	NOT
ZKSCAN2	267.7806	-0.3449439	0.180841	-1.90744587	0.056463	0.214422	NOT
RCN2	1375.754	0.30313186	0.158925	1.907386428	0.056471	0.214422	NOT
AKAP2	9.196745	-0.8319644	0.436198	-1.90730765	0.056481	0.214431	NOT
PPP1R14BF	6.485899	0.98347087	0.51565	1.907246276	0.056489	0.214431	NOT
NRSN2-AS1	45.21723	0.49743754	0.260823	1.907186247	0.056496	0.214431	NOT
EBAG9	1574.329	0.37389443	0.196113	1.906527946	0.056582	0.214707	NOT
SEMA4G	4814.217	-0.5899621	0.309448	-1.90650125	0.056585	0.214707	NOT
RP11-386M	0.775059	-1.9909636	1.044408	-1.90630768	0.05661	NA	NA
ZNF689	490.1304	-0.2554435	0.134004	-1.90623187	0.05662	0.21481	NOT
RP11-1C8.	6.000415	0.73787524	0.387105	1.906139519	0.056632	0.214825	NOT
COQ4	2222.817	-0.3305964	0.17345	-1.90600284	0.05665	0.214862	NOT
ZNF317	998.9282	-0.2512647	0.131862	-1.90551124	0.056714	0.215042	NOT

DYNLL2	6068.275	-0.2908016	0.152614	-1.90547447	0.056718	0.215042	NOT
RP11-658F	184.1292	-0.4130506	0.216773	-1.90545543	0.056721	0.215042	NOT
GGPS1	1232.174	0.25271225	0.132638	1.905284136	0.056743	0.21508	NOT
DGKQ	1046.183	-0.2729787	0.143277	-1.90525358	0.056747	0.21508	NOT
RP11-221J	1.430094	1.14368287	0.6003	1.90518611	0.056756	0.21508	NOT
KIAA1919	238.0932	0.3459176	0.181571	1.905132955	0.056763	0.21508	NOT
SNRNP25	1499.711	-0.4398801	0.230959	-1.90458221	0.056834	0.215322	NOT
CTC-459F4	1.936555	0.95198679	0.499863	1.904494459	0.056846	0.215335	NOT
RP11-550A	2.480823	1.02226412	0.536787	1.904413621	0.056856	0.215344	NOT
TDRKH	641.8436	0.47611408	0.250014	1.904346962	0.056865	0.215347	NOT
RP11-88E1	13.16144	-0.7441568	0.390802	-1.90417841	0.056887	0.2154	NOT
ARSD	2912.689	-0.5339175	0.280422	-1.90398121	0.056913	0.215467	NOT
PARK2	205.7703	-0.700613	0.367999	-1.90384718	0.05693	0.215503	NOT
RP11-219C	7.704882	-1.1625579	0.610672	-1.90373553	0.056945	0.215528	NOT
LPIN1	2631.193	-0.5195427	0.272956	-1.90339468	0.056989	0.215667	NOT
MT1H	106.9251	-1.1592348	0.609099	-1.90319494	0.057015	0.215735	NOT
RP11-903H	24.47642	-0.7323806	0.384881	-1.90287683	0.057057	0.215854	NOT
KRT8P32	2.663735	0.82040463	0.431168	1.902748848	0.057073	0.215854	NOT
SAC3D1	918.0106	0.44474916	0.233744	1.902715872	0.057078	0.215854	NOT
HOXA1	12.69321	0.81521688	0.42845	1.902710779	0.057078	0.215854	NOT
CYTH1	2995.141	-0.2574324	0.135323	-1.90235816	0.057124	0.215998	NOT
ASIC5	5.789999	-1.0385783	0.546001	-1.90215601	0.057151	0.216067	NOT
CNN1	236.6438	-0.6973234	0.366645	-1.90190432	0.057184	0.216162	NOT
MAST4	1279.673	-0.5404399	0.28419	-1.90168769	0.057212	0.216189	NOT
RP11-588O	1.841387	-0.8256532	0.434188	-1.90160401	0.057223	0.216189	NOT
DCTPP1	1279.81	0.37298067	0.196145	1.901556701	0.057229	0.216189	NOT
LINC01465	31.75853	-0.4624449	0.243194	-1.90155075	0.05723	0.216189	NOT
AR	4171.702	-0.8979596	0.472227	-1.90154381	0.057231	0.216189	NOT
ART5	18.59303	1.32658485	0.697689	1.901398541	0.05725	0.216202	NOT
DNAJC8	3469.623	0.27418138	0.144202	1.901364274	0.057254	0.216202	NOT
FCHSD1	241.5001	0.60612599	0.3188	1.901271895	0.057266	0.216202	NOT
ACACB	4620.797	-0.5349418	0.281364	-1.90124384	0.05727	0.216202	NOT
CHRD	2037.887	-0.6721581	0.35355	-1.90117054	0.05728	0.216202	NOT
RPL7L1	4923.873	0.23963502	0.12605	1.901108015	0.057288	0.216202	NOT
SPNS2	504.7065	-0.778867	0.409706	-1.90103694	0.057297	0.216202	NOT
C19orf26	32.32381	0.72244526	0.380028	1.90103111	0.057298	0.216202	NOT
KCTD9P4	4.547593	-1.2558804	0.660652	-1.90097128	0.057306	0.216202	NOT
MT3	30.94236	1.75894822	0.925373	1.900798192	0.057328	0.216257	NOT
RSPO2	20.81292	-1.6195854	0.85213	-1.90063208	0.05735	0.216282	NOT
SLC30A3	49.55975	1.09243407	0.574776	1.900625862	0.057351	0.216282	NOT
RASL10A	17.08049	-0.5553039	0.292228	-1.90023922	0.057402	0.216443	NOT
TTF1	361.6687	0.30408913	0.160051	1.899950461	0.05744	0.216556	NOT
AC009120.	2.308634	-0.7583717	0.399167	-1.89988583	0.057448	0.216558	NOT
RP11-299H	30.03285	0.47024156	0.247534	1.899708618	0.057471	0.216603	NOT
ADAMTS7P1	3.948541	0.94138085	0.495549	1.899672743	0.057476	0.216603	NOT
RP5-837J1	3.508623	-0.7058669	0.371635	-1.89935772	0.057517	0.216729	NOT
ASPG	1592.393	-1.1835186	0.62329	-1.89882541	0.057587	0.216923	NOT
ZCWPW2	25.98834	-0.4315866	0.227296	-1.89878808	0.057592	0.216923	NOT
LARP4B	2550.737	0.28570838	0.150478	1.898674459	0.057607	0.216923	NOT

SNIP1	659.9079	-0.2561596	0.134917	-1.89863991	0.057612	0.216923	NOT
RALA	1881.926	0.23177824	0.122077	1.898617929	0.057615	0.216923	NOT
LRP10	6246.225	0.36938872	0.194558	1.898601442	0.057617	0.216923	NOT
TIMM8AP1	10.12326	0.80448905	0.42378	1.898363873	0.057648	0.217011	NOT
SERAC1	333.7863	0.37496108	0.197532	1.898225012	0.057666	0.217024	NOT
KLHL18	452.3311	0.23986387	0.126363	1.89821501	0.057668	0.217024	NOT
FUT7	12.20352	0.79561028	0.419164	1.898089143	0.057684	0.217033	NOT
RP11-440I	5.603223	-0.7012682	0.369463	-1.89807452	0.057686	0.217033	NOT
EEF1A1P11	92.39991	0.58421631	0.307855	1.897696514	0.057736	0.217191	NOT
CTD-2616J	6.213269	1.06157802	0.559424	1.897628369	0.057745	0.217194	NOT
ASB1	854.6165	0.39631393	0.20886	1.897508998	0.057761	0.217223	NOT
AC074212.	69.29015	-0.4620274	0.243517	-1.89731233	0.057787	0.217284	NOT
ERI2	590.2796	-0.3373132	0.177793	-1.89722457	0.057798	0.217284	NOT
MGC39584	71.8176	2.40167171	1.2659	1.897204298	0.057801	0.217284	NOT
KANSL2	777.1253	0.27769997	0.146386	1.897041442	0.057822	0.217335	NOT
RP11-45A1	15.32214	-0.5608675	0.295712	-1.89666923	0.057872	0.21749	NOT
SLC25A23	2965.148	-0.3878083	0.204487	-1.89649286	0.057895	0.217542	NOT
RDM1	60.93205	0.66935481	0.352972	1.896341901	0.057915	0.217542	NOT
APOBEC3G	284.8169	0.59635256	0.314478	1.896324917	0.057917	0.217542	NOT
PRR22	120.0925	-0.588955	0.310578	-1.89632049	0.057918	0.217542	NOT
HNRNPA1P2	1.954896	-0.8566508	0.451799	-1.89608933	0.057948	0.217627	NOT
MXI1	1965.847	-0.4003522	0.211192	-1.89567862	0.058003	0.21778	NOT
RP11-797A	1.836017	-0.8573865	0.452304	-1.89559682	0.058013	0.21778	NOT
APOA4	6472.377	1.50536394	0.794158	1.89554714	0.05802	0.21778	NOT
SPEF2	191.6137	0.52322839	0.276032	1.895537786	0.058021	0.21778	NOT
FAM102B	534.6169	0.51522987	0.271851	1.895268063	0.058057	0.217844	NOT
ZSCAN18	725.6884	0.6807581	0.359189	1.895266494	0.058057	0.217844	NOT
FBXL19-AS	72.98765	0.52945127	0.279367	1.895179281	0.058069	0.217844	NOT
ALAS1	11423.22	-0.5686876	0.300082	-1.89510953	0.058078	0.217844	NOT
VPS36	1507.224	-0.365214	0.192714	-1.89510513	0.058078	0.217844	NOT
CDK7	761.514	0.2878423	0.151905	1.894885117	0.058108	0.217921	NOT
MLLT3	234.0614	0.77617519	0.409628	1.894830328	0.058115	0.217921	NOT
RP11-166F	10.90082	-0.8790526	0.464072	-1.89421683	0.058196	0.218196	NOT
KIF5A	13.22754	1.03303204	0.545481	1.893799812	0.058252	0.218341	NOT
UGT1A10	132.4047	1.83833299	0.970722	1.893778204	0.058254	0.218341	NOT
CTB-147N1	9.63542	0.8603674	0.454331	1.893702292	0.058265	0.218341	NOT
RP11-621I	1.280225	1.35589863	0.716007	1.89369392	0.058266	NA	NA
AC010976.	31.35372	-0.56516	0.298445	-1.8936826	0.058267	0.218341	NOT
ST3GAL1P1	1.90431	-0.7408599	0.391264	-1.89350142	0.058291	0.218401	NOT
BOLA3-AS1	24.55852	0.69184369	0.365509	1.892823626	0.058381	0.218664	NOT
AC140912.	2.520467	-1.1261169	0.594966	-1.89274241	0.058392	0.218664	NOT
MFI2-AS1	66.68556	0.79111417	0.417976	1.892726003	0.058394	0.218664	NOT
ZNF155	70.74362	-0.5878849	0.310605	-1.89270722	0.058397	0.218664	NOT
RP11-295I	44.62207	-0.7468192	0.394585	-1.8926692	0.058402	0.218664	NOT
PER3	1098.036	-0.5456197	0.288295	-1.892573	0.058415	0.218682	NOT
RP3-473L9	6.518271	0.93016172	0.491517	1.89243052	0.058434	0.218723	NOT
MSL3P1	31.5735	0.63107299	0.333528	1.892113662	0.058476	0.218851	NOT
AC016747.	500.6556	0.3164018	0.167239	1.891918988	0.058502	0.21891	NOT
RP11-753H	2.773152	-0.8042436	0.425104	-1.89187389	0.058508	0.21891	NOT

ZNF189	1127.426	0.40566753	0.21446	1.891576414	0.058547	0.219028	NOT
RTCA	1371.037	0.27579339	0.14581	1.891454487	0.058564	0.219059	NOT
ABI3BP	234.2429	-0.8576337	0.453443	-1.89138266	0.058573	0.219064	NOT
STRBP	1032.552	0.31970804	0.16906	1.891094547	0.058612	0.219178	NOT
NARF	2422.201	-0.3929207	0.207793	-1.89092229	0.058635	0.219231	NOT
CTC-487M2	21.57302	-0.6047878	0.319847	-1.89086647	0.058642	0.219231	NOT
RASGRF2	329.4401	-0.5296715	0.280135	-1.89077257	0.058655	0.219248	NOT
CHRNA5	30.44504	0.85316643	0.451306	1.890438978	0.058699	0.219384	NOT
LINC00671	156.7864	-0.7356801	0.389172	-1.89037033	0.058708	0.219388	NOT
FOXQ1	297.9163	1.08050639	0.571603	1.890309505	0.058717	0.219389	NOT
CLPTM1	10378.75	-0.3055515	0.161646	-1.89024609	0.058725	0.21939	NOT
FGF14	162.3607	-1.1059266	0.585127	-1.89006158	0.05875	0.219452	NOT
CFL2	3335.875	-0.4182165	0.221286	-1.88993255	0.058767	0.219486	NOT
BRICD5	126.4916	-0.6414766	0.339488	-1.88954007	0.058819	0.219612	NOT
TM9SF3	8043.814	-0.2195988	0.116219	-1.88952421	0.058822	0.219612	NOT
PDE8B	139.2768	-0.4753303	0.251574	-1.88942783	0.058835	0.219612	NOT
PON2	8049.151	0.43572483	0.230614	1.88941242	0.058837	0.219612	NOT
MYH15	5.406817	-0.9266295	0.490441	-1.88937908	0.058841	0.219612	NOT
SNRPN	2663.029	0.70546389	0.373398	1.88930611	0.058851	0.219618	NOT
RP11-419C	11.09229	-0.6518165	0.345056	-1.88901462	0.05889	0.219733	NOT
NOX5	11.21016	1.02070768	0.540429	1.888699332	0.058932	0.219837	NOT
GRB7	788.7324	0.5711811	0.30243	1.888637887	0.05894	0.219837	NOT
ABALON	22.75256	0.62186907	0.32927	1.888627224	0.058942	0.219837	NOT
RP11-429K	3.325841	-0.9813958	0.519654	-1.88855796	0.058951	0.219841	NOT
NRXN3	158.9867	-1.0528004	0.557503	-1.88842154	0.058969	0.219855	NOT
RP11-190C	4.740325	0.71290352	0.377533	1.888321104	0.058983	0.219855	NOT
RP11-91H1	1.586178	-1.7401728	0.921551	-1.8883087	0.058985	0.219855	NOT
AC002480.	6.641743	1.03695888	0.549158	1.888270867	0.05899	0.219855	NOT
XXbac-BPC	5.26552	-0.6720494	0.355928	-1.8881604	0.059004	0.219855	NOT
EEFSEC	1528.008	0.29165083	0.154465	1.888130792	0.059008	0.219855	NOT
AC017002.	4.564574	0.84974225	0.450058	1.888074081	0.059016	0.219855	NOT
5-Mar	1560.08	-0.2404783	0.127369	-1.88804615	0.05902	0.219855	NOT
FTH1P3	47.1227	0.57754018	0.30591	1.887940493	0.059034	0.219878	NOT
SORBS1	2744.031	-0.4464055	0.236469	-1.88779537	0.059053	0.21992	NOT
FAM210B	5158.715	-0.4008255	0.212334	-1.88771087	0.059065	0.219932	NOT
ATL3	3439.872	-0.2826608	0.149753	-1.88750842	0.059092	0.220003	NOT
DAPL1	3.694696	1.73183072	0.917609	1.887328839	0.059116	0.220029	NOT
SPINK5	106.7677	-1.0417111	0.551953	-1.88731996	0.059117	0.220029	NOT
CLNK	8.957879	-0.7892827	0.418213	-1.8872766	0.059123	0.220029	NOT
RP11-844F	106.2213	0.97563318	0.51699	1.887141312	0.059141	0.220055	NOT
SLC17A8	5.386063	1.2834245	0.680103	1.887103536	0.059146	0.220055	NOT
TMEM87B	794.5638	0.3465276	0.183661	1.886776468	0.05919	0.220189	NOT
CD164L2	1.023974	2.13731633	1.132919	1.886556394	0.05922	NA	NA
COL4A2	14589.73	-0.4859025	0.25758	-1.88641374	0.059239	0.22031	NOT
FTH1P5	24.1121	0.60703018	0.321791	1.88641362	0.059239	0.22031	NOT
RP3-446N1	10.41613	1.61197201	0.854699	1.886012066	0.059293	0.22043	NOT
RP11-356J	128.1316	-0.63133	0.334753	-1.88595937	0.0593	0.22043	NOT
RP11-835E	2.516125	-0.8710906	0.461882	-1.88595814	0.059301	0.22043	NOT
HMGN2P17	47.13941	0.50427701	0.267395	1.885886984	0.05931	0.22043	NOT

CALML5	2.33963	2.60754866	1.382708	1.885826887	0.059318	0.22043	NOT
PDCD6	3456.769	0.25526285	0.13536	1.885812561	0.05932	0.22043	NOT
RP11-74M1	9.62345	-1.0179628	0.539861	-1.88559994	0.059349	0.220506	NOT
GNB3	10.66217	0.74818744	0.396812	1.885497266	0.059363	0.220527	NOT
NPAS1	34.94222	0.73994206	0.39255	1.884962591	0.059435	0.220765	NOT
UQCRC2	7122.167	-0.3264468	0.173192	-1.8848845	0.059445	0.220774	NOT
CH507-154	2.149086	0.97960176	0.519742	1.884786191	0.059459	0.220793	NOT
RBM12B	481.8698	0.34289856	0.181939	1.884690809	0.059472	0.220811	NOT
RP11-65L3	10.61915	0.76921617	0.408193	1.884441716	0.059505	0.220906	NOT
MT-ND5	156230.6	-0.5976178	0.317196	-1.88406708	0.059556	0.221048	NOT
MT-ND6	63564.6	-0.6471995	0.343518	-1.88403293	0.059561	0.221048	NOT
AEN	1296.707	-0.3785553	0.200935	-1.88396558	0.05957	0.221048	NOT
S100A12	5.966895	-0.9444602	0.501328	-1.88391732	0.059576	0.221048	NOT
RP11-465I	9.389643	1.22911403	0.652493	1.883720372	0.059603	0.22111	NOT
RP11-689I	1.473475	-1.2655493	0.671873	-1.88361332	0.059617	0.22111	NOT
KAT8	1266.846	-0.251595	0.13357	-1.88361204	0.059617	0.22111	NOT
SERPINA6	23339.95	-0.6987159	0.370964	-1.88351276	0.059631	0.22113	NOT
ZNF573	50.02976	-0.3258374	0.173034	-1.88308274	0.059689	0.221316	NOT
METTL7B	6737.736	0.59822048	0.317755	1.882644891	0.059749	0.221505	NOT
PDGFB	805.3379	-0.3702556	0.196684	-1.88249199	0.059769	0.221552	NOT
RP11-301(	23.17536	-0.5384215	0.286034	-1.88237021	0.059786	0.221583	NOT
RELT	190.7782	0.48334384	0.256798	1.882191726	0.05981	0.22162	NOT
LA16c-306	4.701962	-0.7023661	0.373167	-1.88217666	0.059812	0.22162	NOT
GPR17	38.50781	-1.0676926	0.567345	-1.88190923	0.059848	0.221724	NOT
NGF	41.13431	-0.6381806	0.33914	-1.88176105	0.059868	0.221768	NOT
GLB1L	399.183	0.44363043	0.235797	1.881406499	0.059917	0.221917	NOT
CTD-2306A	2.466439	-0.978181	0.520012	-1.88107406	0.059962	0.222048	NOT
USP32P3	5.779275	1.19041404	0.632854	1.881025572	0.059968	0.222048	NOT
AC226118.	4.988542	1.28915752	0.685387	1.880918791	0.059983	0.222055	NOT
CTD-2331H	2.995019	1.10949531	0.589878	1.88088986	0.059987	0.222055	NOT
RP11-981F	6.940698	-0.6607246	0.351374	-1.88040045	0.060054	0.222272	NOT
HTR2A-AS1	1.318352	-1.2494679	0.664492	-1.88033555	0.060062	NA	NA
ATXN2	1725.85	0.20682001	0.110003	1.880124545	0.060091	0.222381	NOT
F13B	6035.499	-0.8646566	0.45997	-1.87981276	0.060134	0.222507	NOT
DBP	1098.854	-0.5168251	0.274964	-1.87960794	0.060162	0.22258	NOT
CTD-2240J	10.97739	-0.7461623	0.397053	-1.87925014	0.06021	0.222731	NOT
RP11-98D1	18.96928	0.49493149	0.263449	1.87865893	0.060291	0.222975	NOT
PYROXD2	384.1239	-0.8011939	0.426482	-1.87861309	0.060297	0.222975	NOT
RP3-413H6	3.47962	-1.433888	0.763284	-1.87857763	0.060302	0.222975	NOT
RASSF4	3334.74	0.36711984	0.195435	1.878479679	0.060316	0.222975	NOT
RP11-655(	1.839966	2.85783851	1.521369	1.878465537	0.060318	0.222975	NOT
TBP	483.3268	0.27704797	0.147499	1.878298335	0.06034	0.223029	NOT
RP11-738I	1.610335	1.5722529	0.837091	1.878234355	0.060349	0.223031	NOT
PPP3CA	1124.204	-0.2955052	0.157348	-1.87804122	0.060376	0.223098	NOT
CTC-50503	356.8107	-1.1993604	0.638685	-1.87786037	0.0604	0.223139	NOT
AC098617.	4.999038	1.36915772	0.729113	1.877839871	0.060403	0.223139	NOT
AC009950.	25.51119	-0.5676951	0.302325	-1.87776546	0.060413	0.223147	NOT
LINC00963	3307.541	-0.3909551	0.208209	-1.87770132	0.060422	0.223149	NOT
TAP1	3747.422	0.51576927	0.27472	1.877436909	0.060458	0.223252	NOT

ORMDL2	1552.297	0.35306224	0.188076	1.877236582	0.060486	0.223323	NOT
TRIM36	46.68516	0.72355937	0.385469	1.877088199	0.060506	0.223366	NOT
ASPHD1	267.4081	1.01140158	0.53883	1.877032057	0.060514	0.223366	NOT
C20orf196	133.2366	0.44361379	0.23638	1.876695736	0.06056	0.22349	NOT
HIATL1	1417.807	0.33646731	0.17929	1.876666678	0.060564	0.22349	NOT
RP11-669N	6.422487	2.04129408	1.087774	1.876579658	0.060576	0.223503	NOT
RP11-165F	1.516845	1.33772925	0.712906	1.876445888	0.060594	0.223541	NOT
ZNF735	3.893915	2.807786	1.496432	1.876320838	0.060611	0.223573	NOT
BMP5	24.65177	-1.1497243	0.612831	-1.87608599	0.060643	0.223662	NOT
HADH	5643.557	-0.4198486	0.223805	-1.87595952	0.060661	0.223696	NOT
LGALS3	3620.895	0.69690289	0.371534	1.875742514	0.060691	0.223775	NOT
SPOCK3	9.008304	-1.9143781	1.020651	-1.87564503	0.060704	0.223794	NOT
C1orf189	2.876133	-0.6165287	0.328713	-1.87558568	0.060712	0.223794	NOT
VBP1	1610.853	0.28106514	0.149864	1.875469508	0.060728	0.223822	NOT
CTD-2622I	3.776899	-0.8850979	0.472102	-1.87480262	0.06082	0.224072	NOT
CYSLTR1	57.32938	-0.4884755	0.260551	-1.87477545	0.060824	0.224072	NOT
FGGY	3919.221	-0.7820161	0.417132	-1.87474458	0.060828	0.224072	NOT
MECR	953.1076	-0.3296419	0.175834	-1.87473718	0.060829	0.224072	NOT
RP11-284F	170.9299	1.16378312	0.620933	1.874249032	0.060896	0.224266	NOT
CHMP3	1665.004	0.26760356	0.14278	1.874234945	0.060898	0.224266	NOT
EPB41L4B	2538.821	-0.6250953	0.333539	-1.87412912	0.060913	0.224272	NOT
SHANK2	1474.731	-0.5456601	0.291158	-1.87410072	0.060917	0.224272	NOT
MED24	3649.96	0.21897453	0.116846	1.874041383	0.060925	0.224272	NOT
PARP2	927.6586	0.26955232	0.143845	1.873910002	0.060943	0.224308	NOT
CAMKMT	191.4535	0.3393474	0.181096	1.873850583	0.060951	0.224308	NOT
TSPEAR-AS	107.2888	-0.9438532	0.50374	-1.87369033	0.060973	0.224324	NOT
RP11-504F	15.83975	-0.6548896	0.349535	-1.87360258	0.060985	0.224324	NOT
GP1BA	47.41793	-0.692441	0.369578	-1.8735997	0.060986	0.224324	NOT
CTD-2224J	7.983878	1.11907785	0.597294	1.873579709	0.060988	0.224324	NOT
LINC00574	76.09297	-0.748412	0.399476	-1.87348629	0.061001	0.224341	NOT
RP11-15A1	7.109213	-0.8555552	0.456701	-1.87333735	0.061022	0.224351	NOT
RRP1	1667.996	0.3347739	0.178705	1.873330725	0.061023	0.224351	NOT
PROKR1	8.088717	1.39762085	0.746079	1.873287213	0.061029	0.224351	NOT
RP11-6N17	8.100117	-0.4914876	0.262377	-1.87321113	0.061039	0.224359	NOT
AP000255.	2.06471	-0.7392555	0.394672	-1.87308667	0.061056	0.224392	NOT
IGLC6	4.283128	-1.5843895	0.846006	-1.87278731	0.061098	0.224513	NOT
CTD-2008F	11.78312	2.42115319	1.292862	1.872708141	0.061109	0.224523	NOT
TNFSF12	618.1919	0.43325219	0.231364	1.872598223	0.061124	0.224549	NOT
GAPDHP65	38.41835	0.72460831	0.38697	1.872520404	0.061135	0.224558	NOT
CTD-2547I	2.111475	-0.9953842	0.531592	-1.87245728	0.061143	0.224559	NOT
FTOP1	113.5591	-0.6748352	0.360421	-1.87235061	0.061158	0.224583	NOT
TRAF3	907.157	0.32172119	0.171849	1.872110307	0.061191	0.224675	NOT
RP11-399J	58.67763	-0.8685747	0.464011	-1.87188202	0.061223	0.22476	NOT
RBM48	380.8728	0.24179503	0.129185	1.871689537	0.06125	0.224804	NOT
RP11-713M	2.504764	0.97123119	0.51891	1.871675672	0.061251	0.224804	NOT
XRCC6P1	32.35813	0.83472561	0.446109	1.87112281	0.061328	0.225031	NOT
SLC5A2	20.19658	0.7517098	0.401746	1.871109192	0.06133	0.225031	NOT
NDUFA9	2275.625	-0.3853192	0.205957	-1.87087268	0.061363	0.225121	NOT
LINC01151	78.41411	1.05211993	0.562386	1.870813035	0.061371	0.225121	NOT

RPP38	555.4718	0.26031612	0.139154	1.870703474	0.061386	0.225146	NOT
SGCD	117.1801	-0.9049408	0.483817	-1.87042028	0.061425	0.22526	NOT
FAM111B	526.6734	0.63314833	0.338534	1.870266309	0.061447	0.225286	NOT
ANKZF1	1491.945	0.30194889	0.161448	1.870250339	0.061449	0.225286	NOT
MPC1	3509.164	-0.4816387	0.257544	-1.87011884	0.061467	0.225322	NOT
AC017104.	14.10618	0.95042336	0.508369	1.869555481	0.061546	0.225578	NOT
RP11-158I	1.405889	1.88973452	1.010902	1.869355573	0.061573	0.22565	NOT
AGPAT3	7487.149	-0.3541996	0.189484	-1.86928772	0.061583	0.225654	NOT
AP000696.	1.243135	-3.2699131	1.749359	-1.86920652	0.061594	NA	NA
HNRNPA3P9	3.220256	-0.8134336	0.435205	-1.86908152	0.061611	0.225728	NOT
LMCD1	986.2272	-0.5470785	0.29274	-1.86882105	0.061648	0.225806	NOT
MAP3K1	1125.575	0.39088453	0.209162	1.868809723	0.061649	0.225806	NOT
CMYA5	213.9234	-0.7239244	0.387456	-1.86840626	0.061705	0.225981	NOT
AC012668.	16.67908	-0.9637117	0.515841	-1.86823504	0.061729	0.226016	NOT
C21orf62-	24.07064	-0.4648942	0.248844	-1.86821861	0.061732	0.226016	NOT
RP11-783K	24.84224	0.51459218	0.275469	1.868056658	0.061754	0.226064	NOT
RP11-408P	145.8414	0.3794551	0.203136	1.867985439	0.061764	0.226064	NOT
PIP5K1C	1450.012	-0.3622039	0.193905	-1.86794534	0.06177	0.226064	NOT
ATG16L2	670.9132	-0.3951234	0.211602	-1.86729578	0.06186	0.226365	NOT
ARHGAP42	456.2452	-0.4773078	0.255666	-1.86692114	0.061913	0.226512	NOT
CCNI2	14.38873	0.79182467	0.424141	1.86688873	0.061917	0.226512	NOT
RP11-267M	10.90224	0.54155611	0.290105	1.866756814	0.061936	0.226529	NOT
LRP2	154.7148	1.27910636	0.68521	1.866735038	0.061939	0.226529	NOT
ATP2C2	25.35464	1.33282256	0.71415	1.866305542	0.061999	0.226718	NOT
RP11-396O	45.54591	0.74593707	0.399712	1.866185581	0.062015	0.226749	NOT
DDX11L2	37.18695	-0.7958768	0.426549	-1.86585223	0.062062	0.226866	NOT
MRPS36	1181.281	-0.3659817	0.196149	-1.86583706	0.062064	0.226866	NOT
CTD-2033A	7.701765	1.22954877	0.659047	1.865646024	0.062091	0.226933	NOT
FAM132B	87.99937	0.71052036	0.380889	1.865427017	0.062122	0.227004	NOT
TSHZ3	107.5376	-0.5042481	0.270318	-1.86538929	0.062127	0.227004	NOT
FTH1P4	11.49025	0.71230671	0.382032	1.864518873	0.062249	0.227419	NOT
ZNF41	272.1575	-0.3068535	0.164612	-1.86409985	0.062308	0.227603	NOT
RP5-999L4	2.46486	1.12390746	0.602983	1.863913361	0.062334	0.227668	NOT
AURKA	1231.344	0.52079916	0.27943	1.863791078	0.062351	0.2277	NOT
NOX1	48.74791	0.52974987	0.284286	1.863442855	0.0624	0.227834	NOT
IRAK3	144.1211	-0.607667	0.326105	-1.86341118	0.062404	0.227834	NOT
TMEM87A	1321.204	0.23792385	0.127691	1.863277757	0.062423	0.227853	NOT
HMGN2P3	16.39188	0.56406678	0.302733	1.863250117	0.062427	0.227853	NOT
NSUN4	964.2881	0.29183921	0.156639	1.863135843	0.062443	0.227853	NOT
RPL5P1	8.077695	0.66959001	0.359389	1.86313401	0.062443	0.227853	NOT
VCX3B	1.970033	1.78854267	0.960021	1.86302529	0.062459	0.227879	NOT
ZHX3	1719.057	-0.405082	0.217452	-1.86285512	0.062483	0.22791	NOT
RP11-60A1	38.68967	-1.3503435	0.724883	-1.86284451	0.062484	0.22791	NOT
NKX2-5	1.49914	1.77929335	0.955233	1.862680297	0.062507	0.227946	NOT
PSMD10P2	9.543341	0.85420446	0.458595	1.862654907	0.062511	0.227946	NOT
RP11-123K	2.243877	-0.9916177	0.532395	-1.86255982	0.062524	0.227964	NOT
CRYGN	1.265308	1.16033117	0.623022	1.862424526	0.062543	NA	NA
TMEM255A	60.47948	0.89236678	0.479165	1.862338743	0.062555	0.228047	NOT
RP5-1172A	4.817727	1.40101416	0.752312	1.862278858	0.062564	0.228047	NOT

CTD-2666I	6.911947	-0.6482755	0.348123	-1.86220202	0.062575	0.228056	NOT
PRMT5	1518.152	0.32297568	0.173469	1.861862439	0.062622	0.228176	NOT
AC096772.	172.2983	-0.3615521	0.19419	-1.86184837	0.062624	0.228176	NOT
DHRS4L2	1315.161	-0.4675101	0.251115	-1.86173489	0.06264	0.228183	NOT
HSD17B6	19676.6	-0.8719813	0.468375	-1.86171659	0.062643	0.228183	NOT
MRC1	800.8361	-0.6972034	0.374517	-1.8616067	0.062659	0.228209	NOT
RP11-219A	3.506227	-2.0473523	1.09991	-1.86138084	0.06269	0.228294	NOT
ADAMTS7P4	6.866743	0.81555042	0.438248	1.860934313	0.062753	0.228493	NOT
HYOU1	12861.72	-0.4018917	0.216015	-1.86048067	0.062818	0.228696	NOT
CPEB4	2928.411	-0.4410006	0.237066	-1.86024141	0.062851	0.228772	NOT
PPP1R18	2482.03	0.49499393	0.266095	1.86021217	0.062856	0.228772	NOT
VDAC1P1	7.657861	0.62361769	0.335286	1.85995509	0.062892	0.228848	NOT
AC000032.	3.448299	-2.0761682	1.116252	-1.85994607	0.062893	0.228848	NOT
LIN7C	1607.279	-0.2606157	0.140158	-1.85943928	0.062965	0.229066	NOT
AL590762.	3.594947	-0.639246	0.34381	-1.85929923	0.062985	0.229066	NOT
ANO3	54.87324	-1.1583584	0.623013	-1.85928488	0.062987	0.229066	NOT
RNF5P1	134.6868	-0.8375521	0.450473	-1.85927421	0.062988	0.229066	NOT
MAGEC3	2.175786	1.87483076	1.008457	1.859108691	0.063012	0.229066	NOT
CFAP36	637.4963	0.3336679	0.179478	1.85910437	0.063012	0.229066	NOT
PP7080	583.4309	0.56524038	0.304042	1.859087154	0.063015	0.229066	NOT
LRRC3	1499.41	-0.697664	0.375281	-1.85904609	0.063021	0.229066	NOT
CEACAM6	10.12922	1.55299622	0.835402	1.858979988	0.06303	0.229069	NOT
TAF4B	44.65942	0.85101414	0.457867	1.858647236	0.063077	0.22914	NOT
C16orf96	13.94653	-0.7568694	0.40723	-1.85857817	0.063087	0.22914	NOT
HSPG2	5392.139	-0.6360845	0.342255	-1.85850975	0.063097	0.22914	NOT
STAMBP	1555.561	0.2253153	0.121235	1.858503629	0.063098	0.22914	NOT
ZBED3	1665.756	-0.4308552	0.231835	-1.85845315	0.063105	0.22914	NOT
PGBD4P3	2.288669	-0.7459154	0.401368	-1.85843482	0.063107	0.22914	NOT
THSD7B	20.91669	-0.8554807	0.460325	-1.8584264	0.063108	0.22914	NOT
HNRNPD	5603.995	0.23447288	0.126183	1.858195221	0.063141	0.229228	NOT
AC098820.	27.14857	0.59023716	0.317655	1.85810861	0.063154	0.229242	NOT
HECW2	396.4928	-0.5032519	0.270858	-1.85798904	0.063171	0.229251	NOT
C1QTNF7	33.13893	-0.7608033	0.409486	-1.85794627	0.063177	0.229251	NOT
KBTBD7	406.1777	-0.3544289	0.190767	-1.8579134	0.063181	0.229251	NOT
SH2B2	139.9956	0.41757469	0.224783	1.857682963	0.063214	0.229339	NOT
AKTIP	888.5854	-0.3922963	0.211199	-1.85746959	0.063244	0.229418	NOT
SH2D7	3.26058	1.23330155	0.664078	1.857164806	0.063288	0.229524	NOT
TRIM71	558.5312	1.55544097	0.837544	1.857145711	0.06329	0.229524	NOT
CPAMD8	85.64722	-0.627629	0.337997	-1.85690837	0.063324	0.229616	NOT
LINC00628	7.663603	0.75319433	0.405705	1.856507084	0.063381	0.229792	NOT
POLR3G	143.8416	0.48552435	0.261534	1.856445029	0.06339	0.229794	NOT
IGKV3-11	334.1358	1.15283048	0.621047	1.85626873	0.063415	0.229854	NOT
CFAP53	38.48792	0.64591999	0.348001	1.85608764	0.063441	0.229917	NOT
PTPRQ	11.69078	1.40133563	0.755101	1.855824557	0.063479	0.229994	NOT
ERGIC1	13103.86	-0.3701573	0.199458	-1.85581905	0.063479	0.229994	NOT
DPH6-AS1	16.91134	-0.9191139	0.495356	-1.85546229	0.06353	0.230148	NOT
SHC2	4320.289	-0.6239704	0.336318	-1.85529613	0.063554	0.230175	NOT
RBM8A	4358.893	0.2567344	0.13838	1.855290824	0.063555	0.230175	NOT
SMPD1	3801.646	-0.3654916	0.197042	-1.85489079	0.063612	0.230351	NOT

DHRS4	1290.24	-0.4212695	0.227165	-1.85446132	0.063673	0.230542	NOT
AC128709.	3.054051	0.9752246	0.525902	1.854385354	0.063684	0.230551	NOT
MOV10L1	64.71782	-0.8542188	0.460705	-1.85415569	0.063717	0.230632	NOT
PCDHB16	149.9273	-0.8998427	0.485335	-1.85406604	0.06373	0.230632	NOT
IRF2	1476.977	-0.2670501	0.144036	-1.85405094	0.063732	0.230632	NOT
RP11-638I	1.525253	1.46657962	0.791086	1.853880839	0.063756	0.230679	NOT
RP11-352I	8.60961	-0.7659204	0.413153	-1.85383998	0.063762	0.230679	NOT
DHRS7B	1272.381	-0.4564952	0.246259	-1.85371953	0.063779	0.230711	NOT
RP11-596(	5.355675	-0.5513151	0.297438	-1.85354723	0.063804	0.230742	NOT
ADD3	1593.859	0.50969033	0.274982	1.853540546	0.063805	0.230742	NOT
FTCD-AS1	8.026935	-0.9695703	0.523134	-1.85338738	0.063827	0.230786	NOT
RP11-1E22	3.126343	1.73605164	0.936718	1.853334268	0.063834	0.230786	NOT
PAX5	37.66939	1.05954748	0.571716	1.853277296	0.063843	0.230786	NOT
OLFM2	4565.159	-0.7568832	0.408466	-1.85299079	0.063884	0.230899	NOT
LINC0068(	179.9789	0.38266324	0.206517	1.852940694	0.063891	0.230899	NOT
GAPDHP1	696.954	0.8722236	0.470767	1.852771498	0.063915	0.230911	NOT
PAX8-AS1	440.084	0.95077598	0.513166	1.85276408	0.063916	0.230911	NOT
NHSL2	22.31619	-0.6862443	0.370395	-1.85273811	0.06392	0.230911	NOT
ATOH7	12.5466	-0.7751306	0.418386	-1.85266903	0.06393	0.230911	NOT
DNAL4	544.5185	0.32307854	0.174391	1.852611726	0.063938	0.230911	NOT
AC083843.	247.3216	0.53562327	0.289126	1.852561915	0.063945	0.230911	NOT
CTD-2623N	1.789505	1.3716708	0.740472	1.852428307	0.063964	0.230949	NOT
MT1M	200.1464	-1.0582254	0.57133	-1.85221331	0.063995	0.23103	NOT
AKR1C2	18696.15	0.92713719	0.500602	1.852045522	0.064019	0.231086	NOT
STK19B	46.90261	0.87377704	0.471809	1.851970585	0.06403	0.231094	NOT
CCDC88C	482.2205	0.55470786	0.299556	1.851768444	0.064059	0.231168	NOT
LYZL2	2.341302	1.50314322	0.811904	1.851379356	0.064115	0.231287	NOT
RP11-49K2	9.372522	0.83354725	0.450231	1.851377932	0.064115	0.231287	NOT
DAB2	3329.124	0.61846455	0.334059	1.851360914	0.064118	0.231287	NOT
CTC-338M1	3.640337	-0.971278	0.524654	-1.85127192	0.06413	0.231303	NOT
LDHAL6B	1.916189	-0.9434734	0.509661	-1.8511801	0.064144	0.23132	NOT
SECTM1	1081.631	0.65370487	0.353199	1.850813427	0.064196	0.231479	NOT
RP11-730K	2.768535	-0.9129167	0.493351	-1.8504408	0.06425	0.231642	NOT
RP11-600F	3.466703	0.75580288	0.408556	1.849939073	0.064322	0.231863	NOT
RP11-124(	93.00278	0.54764934	0.296058	1.849805416	0.064342	0.231863	NOT
ASGR1	22047.6	-0.6449703	0.34867	-1.84980396	0.064342	0.231863	NOT
ANO10	1465.479	0.39991715	0.216197	1.849777005	0.064346	0.231863	NOT
B3GALNT1	202.0547	0.59386698	0.321101	1.849472429	0.06439	0.231969	NOT
TMPRSS13	18.26418	0.95233542	0.514927	1.849455658	0.064392	0.231969	NOT
FAM133B	230.9128	0.28994903	0.156787	1.849322736	0.064411	0.232007	NOT
FOXN3	3201.173	-0.3654941	0.197644	-1.84925767	0.064421	0.23201	NOT
RP4-773N1	189.8398	0.47142036	0.254937	1.849164757	0.064434	0.232017	NOT
RP11-799I	25.81335	0.8456199	0.457308	1.849124283	0.06444	0.232017	NOT
HOGA1	943.4074	-0.7516397	0.406497	-1.84906675	0.064448	0.232017	NOT
NDUFS1	6063.739	-0.2738737	0.148126	-1.84892911	0.064468	0.232058	NOT
PODXL2	601.3469	1.12162318	0.606678	1.848796321	0.064487	0.232063	NOT
VPS29	2960.293	0.22497585	0.121689	1.848774125	0.06449	0.232063	NOT
KB-1460A1	268.877	0.52855983	0.285907	1.848711238	0.0645	0.232063	NOT
TET2	438.5213	-0.3792839	0.205171	-1.8486256	0.064512	0.232063	NOT

TRAF3IP1	704.0623	0.24412013	0.132055	1.848623523	0.064512	0.232063	NOT
TUBGCP2	2944.018	-0.2713028	0.146768	-1.84851474	0.064528	0.232088	NOT
STARD13	670.4288	-0.4246665	0.229746	-1.84841457	0.064542	0.232099	NOT
NPM1P46	2.677671	0.84296695	0.456058	1.848375124	0.064548	0.232099	NOT
KCNE2	17.8908	-0.6085853	0.32928	-1.84823199	0.064569	0.232141	NOT
CTD-2154I	1.669399	-0.9003097	0.487137	-1.84816416	0.064579	0.232141	NOT
NCOR1	3173.321	-0.4227908	0.228771	-1.84809274	0.064589	0.232141	NOT
DOCK1	1711.744	-0.4166504	0.225453	-1.84805743	0.064594	0.232141	NOT
FZR1	2473.395	-0.2864842	0.155038	-1.84783537	0.064626	0.232226	NOT
CPD	9275.431	0.4215732	0.228166	1.847658187	0.064652	0.232288	NOT
NFE2L1	17820.34	-0.3585703	0.19408	-1.84754047	0.064669	0.232305	NOT
MT2A	13481.5	-0.8310044	0.449798	-1.84750426	0.064674	0.232305	NOT
TMCO3	2675.235	0.53732864	0.290849	1.847446514	0.064682	0.232305	NOT
RP11-321(	3.136863	-1.296574	0.701923	-1.84717324	0.064722	0.232417	NOT
LARP7	1311.023	-0.209475	0.113409	-1.84707099	0.064737	0.232433	NOT
RAI1	948.9961	0.43081396	0.233248	1.84702322	0.064744	0.232433	NOT
C2CD4C	20.40968	-0.6429217	0.348122	-1.84682679	0.064772	0.23248	NOT
LGR4	4505.004	-0.4803041	0.260072	-1.84681493	0.064774	0.23248	NOT
PPP4C	4448.531	0.28860922	0.156283	1.846704982	0.06479	0.232507	NOT
RP11-54D1	3.842705	1.1319931	0.613045	1.846508009	0.064818	0.232533	NOT
RRAS	1360.978	0.46289597	0.250692	1.846474252	0.064823	0.232533	NOT
TMEM256	1682.006	-0.5014899	0.271598	-1.84644074	0.064828	0.232533	NOT
CFHR5	9829.084	-1.1854435	0.642024	-1.84641734	0.064832	0.232533	NOT
PTMAP5	79.68665	0.49913432	0.270369	1.846121984	0.064874	0.232656	NOT
SETD1A	1639.081	-0.2423867	0.131309	-1.84593319	0.064902	0.232724	NOT
AC010980.	6.829027	1.46149704	0.791883	1.845596441	0.064951	0.23284	NOT
PYGM	28.21671	-0.4692949	0.254279	-1.84559177	0.064952	0.23284	NOT
SH3PXD2A	2034.164	-0.3923784	0.212623	-1.84541853	0.064977	0.232892	NOT
RNU1-47P	9.662682	-1.1012782	0.596778	-1.84537341	0.064983	0.232892	NOT
UBTFL6	2.493768	-0.8946114	0.484842	-1.84516003	0.065014	0.232973	NOT
TRUB1	716.0231	-0.3061747	0.165949	-1.84499442	0.065038	0.233028	NOT
ARHGAP4	1845.372	0.5750703	0.311718	1.844839251	0.065061	0.233078	NOT
HIST1H2BK	1634.261	-0.5609095	0.304056	-1.84475854	0.065073	0.233083	NOT
PHC3	911.4257	-0.3289882	0.178341	-1.84471281	0.065079	0.233083	NOT
LSM11	309.4345	0.30312872	0.164334	1.844589547	0.065097	0.233116	NOT
CARNS1	63.17586	0.66886927	0.362666	1.844312874	0.065138	0.23323	NOT
TNKS1BP1	5774.209	-0.2586441	0.14025	-1.84416628	0.065159	0.23326	NOT
LRRC43	13.23865	-0.8200198	0.444663	-1.84413718	0.065163	0.23326	NOT
RBX1	1994.146	0.34402588	0.186577	1.843881431	0.0652	0.233317	NOT
DNHD1	474.3556	-0.4843864	0.2627	-1.8438735	0.065202	0.233317	NOT
AC004221.	2.588114	1.36891901	0.742446	1.843795008	0.065213	0.233317	NOT
RARS	2731.037	0.25568914	0.138676	1.843791181	0.065214	0.233317	NOT
TOM1L2	1393.215	-0.3855652	0.209151	-1.84347874	0.065259	0.233408	NOT
RP11-142(	5.276331	-1.1149613	0.604817	-1.84346801	0.065261	0.233408	NOT
ZNF888	10.03299	0.92685762	0.502802	1.843386365	0.065273	0.233408	NOT
AC011738.	5.716759	1.11634918	0.605625	1.843300234	0.065285	0.233408	NOT
EPB41L3	316.513	-0.6744828	0.365924	-1.84323416	0.065295	0.233408	NOT
AC008753.	2.130144	0.96991115	0.526203	1.843224954	0.065296	0.233408	NOT
TINCR	7.323102	-0.7828639	0.42473	-1.84320424	0.065299	0.233408	NOT

RP11-4B16	2.789873	-1.0569599	0.573465	-1.84311062	0.065313	0.233426	NOT
RP11-395I	17.02007	-0.6014154	0.326353	-1.84283511	0.065353	0.233539	NOT
RN7SL333I	3.413694	-0.7895667	0.428474	-1.84273918	0.065367	0.233558	NOT
RNU4-62P	2.402881	-0.9474117	0.514152	-1.84266744	0.065378	0.233559	NOT
SLC30A8	4.535474	1.75261809	0.951214	1.842505778	0.065401	0.233559	NOT
TOLLIP-AS	52.17771	-0.4231556	0.22967	-1.84245114	0.065409	0.233559	NOT
CDC27	2395.833	0.24452934	0.13272	1.842439387	0.065411	0.233559	NOT
RP1-276N6	16.20828	1.42918156	0.775708	1.842421785	0.065413	0.233559	NOT
NEK10	59.37292	0.72657298	0.394366	1.842384351	0.065419	0.233559	NOT
CTBS	1269.339	-0.3805577	0.206577	-1.84220452	0.065445	0.233622	NOT
NDUFS8	5500.587	-0.4886245	0.265321	-1.84163836	0.065528	0.233887	NOT
C20orf96	284.2445	0.39126639	0.212467	1.841537555	0.065543	0.233909	NOT
RP11-227C	2.327518	-0.7887904	0.428427	-1.84113093	0.065602	0.234091	NOT
RP11-864N	3.642446	-0.926177	0.503102	-1.8409312	0.065632	0.234164	NOT
RP11-123J	5.343825	-0.9025905	0.490357	-1.84067932	0.065669	0.234249	NOT
RP11-186I	3.126518	-0.8057477	0.437751	-1.84065192	0.065673	0.234249	NOT
CAMK2N2	154.111	-0.9154964	0.497556	-1.83998693	0.06577	0.234493	NOT
RP11-968C	28.39555	-0.6067733	0.329783	-1.83991536	0.065781	0.234493	NOT
uc_338	7.907435	-0.5900776	0.320719	-1.83985691	0.065789	0.234493	NOT
FAM86DP	153.6379	0.39264378	0.213412	1.839842928	0.065791	0.234493	NOT
RP1-228P1	12.18238	0.51245025	0.278531	1.839833997	0.065793	0.234493	NOT
RP11-485C	17.98935	-1.6554414	0.899779	-1.83983141	0.065793	0.234493	NOT
YDJC	800.6298	0.38729506	0.210524	1.839668821	0.065817	0.234541	NOT
RPH3AL	635.634	-0.4441216	0.24142	-1.83962238	0.065824	0.234541	NOT
PPEF1	10.67373	0.92803707	0.504592	1.839183729	0.065888	0.234726	NOT
HMGA1P4	5.852894	0.88847754	0.483106	1.83909578	0.065901	0.234726	NOT
EEF2KMT	462.887	0.31601096	0.17183	1.839087236	0.065902	0.234726	NOT
ZDHHC23	516.7991	0.53473927	0.290792	1.838905605	0.065929	0.234726	NOT
NLRX1	931.3187	-0.276765	0.150507	-1.83888448	0.065932	0.234726	NOT
ANG	18687.67	-0.7928735	0.431172	-1.83887888	0.065933	0.234726	NOT
WNK4	448.4382	-1.0758393	0.585059	-1.83885736	0.065936	0.234726	NOT
PDAP1	4485.343	0.23040939	0.125313	1.838663785	0.065965	0.234792	NOT
HSD17B13	7689.202	-1.4332199	0.779511	-1.83861476	0.065972	0.234792	NOT
LEPROTL1	1581.607	0.30505323	0.165925	1.838502132	0.065988	0.23482	NOT
RP11-129N	72.54861	-0.5467429	0.297453	-1.83808468	0.06605	0.235008	NOT
RARRES2P6	2.82369	2.22733721	1.211919	1.837860101	0.066083	0.235095	NOT
CD1D	232.4196	-0.6768453	0.368292	-1.83779498	0.066093	0.235098	NOT
AC013463.	42.63594	-0.7900285	0.429914	-1.83764414	0.066115	0.235146	NOT
FBN1	1610.177	-0.631689	0.343764	-1.83756564	0.066126	0.235157	NOT
HMGCS2	55434.88	-0.7712253	0.419725	-1.8374551	0.066143	0.235184	NOT
IGLL3P	1.582308	1.36301726	0.741912	1.837168394	0.066185	0.235303	NOT
RP11-611E	83.69368	-0.4123198	0.224468	-1.83687839	0.066228	0.235424	NOT
ADAMTS9-A	15.05566	-0.6841067	0.372441	-1.8368206	0.066236	0.235424	NOT
C2CD4D	16.14371	-0.6652799	0.362286	-1.83634032	0.066307	0.235646	NOT
GPC3	32036.08	1.01623673	0.55343	1.836251423	0.06632	0.235661	NOT
KLHL17	243.0141	0.36809306	0.200474	1.836112475	0.066341	0.235704	NOT
GPR82	14.71163	0.69674351	0.37952	1.835852402	0.066379	0.235809	NOT
RP11-506N	11.6988	-0.6448032	0.351251	-1.83573345	0.066397	0.235819	NOT
RP11-84C1	19.45689	-0.788596	0.429585	-1.835716	0.0664	0.235819	NOT

CCNL2	3980.79	-0.4257751	0.231955	-1.83559237	0.066418	0.235853	NOT
LIMS1	1925.116	0.36935316	0.201232	1.835461239	0.066437	0.235867	NOT
CD3D	214.0777	0.74425799	0.405491	1.835449315	0.066439	0.235867	NOT
LEAP2	4067.235	-0.72101	0.392955	-1.83484291	0.066529	0.236154	NOT
H3F3BP1	3.953738	0.73780571	0.402127	1.834756324	0.066542	0.236154	NOT
CTD-2517M	11.32871	0.65507696	0.357043	1.834726112	0.066546	0.236154	NOT
DSCR10	1.019879	2.70346903	1.473655	1.834532856	0.066575	NA	NA
GOLGA5	2618.134	-0.282233	0.153847	-1.83450123	0.06658	0.236235	NOT
MRO	110.1222	-0.6319044	0.344464	-1.83445519	0.066586	0.236235	NOT
ZNF280A	1.366218	2.21594968	1.208062	1.834300899	0.066609	NA	NA
SAP130	1207.903	0.23185405	0.126409	1.834163719	0.06663	0.236358	NOT
ASS1P5	2.021819	-0.9870538	0.538192	-1.83401671	0.066652	0.23638	NOT
ISPD-AS1	2.28916	-0.8692805	0.473993	-1.83395195	0.066661	0.23638	NOT
PLSCR4	967.4821	-0.5379975	0.293355	-1.8339458	0.066662	0.23638	NOT
AC005077.	42.28354	-0.9731047	0.530745	-1.83346804	0.066733	0.2366	NOT
AC104667.	69.52967	0.72843564	0.397328	1.833337664	0.066752	0.236638	NOT
BANF1P1	1.430062	-1.5120326	0.824847	-1.83310607	0.066787	0.236729	NOT
SGSM2	1312.572	-0.4052191	0.221079	-1.83291301	0.066816	0.236769	NOT
RP11-140I	8.677673	-0.6364928	0.347258	-1.83291273	0.066816	0.236769	NOT
GFOD1	537.8086	-0.4218879	0.230207	-1.83264832	0.066855	0.236871	NOT
ENPP7P4	2.962387	0.84979349	0.463708	1.832603134	0.066862	0.236871	NOT
FBLL1	136.5652	1.19095317	0.650022	1.832174348	0.066925	0.237066	NOT
RCC2P7	2.372893	-1.0082717	0.550354	-1.83204112	0.066945	0.237105	NOT
HTR1F	2.244346	-1.0395594	0.567466	-1.83193164	0.066962	0.237106	NOT
RNF125	520.3161	-0.6846148	0.373718	-1.83190285	0.066966	0.237106	NOT
PMF1	2011.261	0.43809846	0.239159	1.831827366	0.066977	0.237106	NOT
RP11-650I	8.183301	0.92453634	0.504713	1.831804726	0.066981	0.237106	NOT
RASGEF1C	7.679308	1.18946399	0.649379	1.831695599	0.066997	0.237133	NOT
NDUFV1	9926.782	-0.3498481	0.19102	-1.83147808	0.067029	0.237204	NOT
CTD-2026K	16.15607	-0.5648807	0.308435	-1.83144417	0.067034	0.237204	NOT
NPAS3	11.81297	-0.8192089	0.447322	-1.83136287	0.067046	0.237216	NOT
MARCKSL1	4311.437	0.57024261	0.311397	1.831237189	0.067065	0.237241	NOT
C1QTNF1-A	33.24875	-1.1337192	0.619114	-1.83119704	0.067071	0.237241	NOT
LA16c-431	2.807929	-0.8353151	0.456206	-1.831006	0.0671	0.237279	NOT
AQP5	6.960951	0.97209851	0.530926	1.830948808	0.067108	0.237279	NOT
ARF4P2	4.56246	1.31370082	0.717517	1.830898157	0.067116	0.237279	NOT
PRMT6	552.5431	0.59505645	0.325012	1.830877763	0.067119	0.237279	NOT
RPL23AP67	1.816117	-0.8518422	0.465276	-1.83083188	0.067126	0.237279	NOT
NLN	2171.662	0.48659339	0.265786	1.830771811	0.067135	0.23728	NOT
EIF4EP1	2.746339	-0.7755838	0.423662	-1.83066611	0.06715	0.237305	NOT
ANKHD1	229.964	-0.2569898	0.140391	-1.83052341	0.067172	0.237349	NOT
KIAA1644	11.16804	-0.8791868	0.480368	-1.83023683	0.067215	0.23747	NOT
RP11-496I	1.174812	1.58084307	0.86388	1.829934272	0.06726	NA	NA
RHOB	23398.21	-0.5615726	0.306899	-1.82982781	0.067276	0.237603	NOT
F9	10148.51	-1.0375694	0.567038	-1.82980608	0.067279	0.237603	NOT
RPS27A	19230.25	0.38646345	0.21121	1.829762916	0.067285	0.237603	NOT
UGT2A1	105.7756	1.23498951	0.67495	1.82975085	0.067287	0.237603	NOT
TSPAN9	3989.779	-0.3748469	0.20488	-1.82959317	0.067311	0.237655	NOT
SH3RF3-AS	24.22134	-0.9112088	0.49812	-1.82929487	0.067355	0.237745	NOT

EPB41L5	1919.364	-0.3384018	0.184996	-1.82923704	0.067364	0.237745	NOT
KLHDC2	2246.291	-0.3770875	0.206145	-1.82923543	0.067364	0.237745	NOT
ASL	8452.136	-0.6261086	0.342293	-1.82915887	0.067376	0.237745	NOT
POFUT1	5000.436	-0.2683033	0.146684	-1.82912969	0.06738	0.237745	NOT
POLD2P1	4.873038	1.3570009	0.741954	1.828955815	0.067406	0.237806	NOT
RP5-1148A	250.0086	-0.5041418	0.275663	-1.82883233	0.067425	0.237809	NOT
RP11-757A	7.490905	-1.332944	0.728851	-1.82882931	0.067425	0.237809	NOT
ZFYVE27	1452.067	-0.2711928	0.148292	-1.8287757	0.067433	0.237809	NOT
SARDH	4658.298	-0.5632419	0.308014	-1.82862334	0.067456	0.237858	NOT
LIF	407.9773	0.8974493	0.490802	1.828536815	0.067469	0.237873	NOT
HNRNPA1P1	33.6662	0.58497085	0.319943	1.828357271	0.067496	0.237911	NOT
NACAP1	14.80615	0.49731674	0.272003	1.828347994	0.067497	0.237911	NOT
NAP1L4P1	54.45789	0.91697058	0.50161	1.828054314	0.067541	0.238036	NOT
RP11-293M	79.83505	-0.7417377	0.405773	-1.82796394	0.067555	0.238052	NOT
SFTPB	1.906205	0.95247205	0.521108	1.827782069	0.067582	0.238118	NOT
C1GALT1	1237.82	0.32361051	0.177058	1.827707438	0.067593	0.238126	NOT
ASGR2	23554.84	-0.6605014	0.361431	-1.82745993	0.067631	0.238177	NOT
MRPL35	1921.992	-0.2594325	0.141965	-1.82744181	0.067633	0.238177	NOT
GS1-259H1	15.63377	-0.8551094	0.467939	-1.8273967	0.06764	0.238177	NOT
FCGR2C	64.58853	0.87281305	0.477631	1.827377984	0.067643	0.238177	NOT
SPINK13	2.682186	1.27767275	0.699226	1.827266411	0.06766	0.238205	NOT
RP11-400C	12.16833	-1.0776518	0.589818	-1.82709282	0.067686	0.238266	NOT
PIGF	446.8899	0.27664392	0.151423	1.826956891	0.067706	0.238307	NOT
TUBB2B	156.5145	0.7050042	0.385905	1.826887465	0.067717	0.238313	NOT
TEX29	17.0659	0.85295972	0.466929	1.826742009	0.067739	0.238359	NOT
CDC16	2655.62	0.35206298	0.19275	1.826526926	0.067771	0.238442	NOT
RP11-114M	2.117332	-0.9547928	0.522817	-1.82624628	0.067813	0.238532	NOT
OTUD7A	53.89262	-0.5005176	0.27407	-1.82624006	0.067814	0.238532	NOT
SAMD12-AS	2.634645	1.54623085	0.846796	1.825977614	0.067854	0.23864	NOT
AC009501.	34.50702	-0.5523933	0.302533	-1.8258915	0.067867	0.238655	NOT
RPL4P3	3.829783	0.73276634	0.401336	1.825816859	0.067878	0.238663	NOT
FBXO41	127.3228	0.71558238	0.391941	1.825740973	0.067889	0.238673	NOT
LINC01301	25.81858	0.61676739	0.337832	1.825663843	0.067901	0.238683	NOT
LINC00526	327.3114	-0.5553596	0.304212	-1.82556462	0.067916	0.238704	NOT
RP11-680H	14.85578	0.50848825	0.27857	1.825350349	0.067948	0.238775	NOT
ENPP2	2987.859	0.61929634	0.339282	1.825314215	0.067954	0.238775	NOT
DTWD2	353.6003	-0.4197168	0.229972	-1.8250783	0.067989	0.238869	NOT
MTPAP	950.3117	0.22271789	0.122058	1.824686177	0.068048	0.239044	NOT
PDE8A	1604.205	-0.3211924	0.176031	-1.82463141	0.068057	0.239044	NOT
ZSCAN20	55.65777	0.50376955	0.276105	1.824559279	0.068068	0.239052	NOT
RP11-73M1	3.936209	-0.5987642	0.328241	-1.82416189	0.068128	0.239211	NOT
RP11-64K7	40.67559	0.36229153	0.198609	1.824141119	0.068131	0.239211	NOT
CEP250	1441.984	0.25781015	0.141342	1.824010558	0.06815	0.23925	NOT
CTB-79E8.	29.36651	0.64475908	0.353507	1.823893876	0.068168	0.239281	NOT
DNAJC28	75.99112	-0.3758543	0.206103	-1.82362001	0.06821	0.239382	NOT
ITGB2	2457.91	0.60375414	0.331089	1.823539778	0.068222	0.239382	NOT
CCDC144NL	66.14033	0.83966793	0.460501	1.823380051	0.068246	0.239382	NOT
SYT7	5256.06	-0.9810666	0.538053	-1.82336254	0.068249	0.239382	NOT
RP11-736N	58.27857	-0.5883639	0.322689	-1.82331385	0.068256	0.239382	NOT

RP11-493E	2.613189	-0.7339902	0.402563	-1.82329081	0.068259	0.239382	NOT
RP11-100I	2.629383	0.73279744	0.401931	1.823189921	0.068275	0.239382	NOT
ELAVL4	6.508584	0.72779942	0.3992	1.823145604	0.068281	0.239382	NOT
RUSC1	1406.953	0.38352369	0.210372	1.823069808	0.068293	0.239382	NOT
MAP4K2	793.9104	0.31902841	0.174999	1.823026435	0.068299	0.239382	NOT
RAB4B	565.4546	-0.4307127	0.23627	-1.82296698	0.068308	0.239382	NOT
TBPL1	510.7052	0.36455902	0.199987	1.822916226	0.068316	0.239382	NOT
ACR	15.44717	-0.6941468	0.380793	-1.82289801	0.068319	0.239382	NOT
LRRIQ3	38.55447	0.67878376	0.372368	1.822886496	0.068321	0.239382	NOT
DENND1A	1740.475	-0.2578565	0.141467	-1.82272731	0.068345	0.239436	NOT
MAN2C1	2314.849	-0.325445	0.178556	-1.82265013	0.068356	0.239446	NOT
RFX1	662.7365	-0.2011099	0.110355	-1.822398	0.068395	0.239516	NOT
PLAC8	104.4951	0.90737008	0.497916	1.822337268	0.068404	0.239516	NOT
ENC1	1580.045	0.61410626	0.336995	1.8222988	0.06841	0.239516	NOT
HOXA6	5.683755	1.3651422	0.749137	1.82228596	0.068412	0.239516	NOT
Six3os1_1	1.694532	2.10387264	1.154817	1.821823036	0.068482	0.239724	NOT
SDR16C5	2.092167	1.76071154	0.96648	1.821777084	0.068489	0.239724	NOT
PSMG3-AS1	332.6537	-0.5279935	0.28984	-1.82167396	0.068504	0.239748	NOT
RP11-467J	3.491277	-1.061892	0.582998	-1.82143425	0.068541	0.239844	NOT
COPA	14081.8	0.30881706	0.169599	1.820867063	0.068627	0.240081	NOT
RPL21P11	5.202788	0.71476403	0.392551	1.820816576	0.068635	0.240081	NOT
LINC00264	2.341749	-0.970237	0.532868	-1.82078235	0.06864	0.240081	NOT
CDADC1	392.9993	-0.3780767	0.207656	-1.82069093	0.068654	0.240081	NOT
AC005593.	1.851392	-0.7273491	0.399499	-1.82065287	0.06866	0.240081	NOT
BAG6	13690.77	0.21758426	0.11951	1.820641078	0.068661	0.240081	NOT
MIR3176	2.554358	-0.913962	0.502044	-1.82048294	0.068685	0.240134	NOT
AC006262.	3.897259	1.71115757	0.940008	1.820365148	0.068703	0.240153	NOT
LIPT1	195.2661	0.27442098	0.150758	1.820268997	0.068718	0.240153	NOT
EXOSC10	2076.905	0.23587135	0.129584	1.82022214	0.068725	0.240153	NOT
RSL1D1	5326.784	0.25214684	0.138529	1.82017985	0.068732	0.240153	NOT
SUCLA2-AS	11.07633	-0.4577823	0.251507	-1.82015659	0.068735	0.240153	NOT
RDH5	356.0817	-0.6765704	0.371735	-1.82003252	0.068754	0.240183	NOT
RP11-823E	12.45286	-0.6139465	0.33734	-1.81996494	0.068764	0.240183	NOT
RPL7AP15	3.512624	-0.7912875	0.434791	-1.81992511	0.06877	0.240183	NOT
CTD-2619J	2.173738	1.3763004	0.756315	1.819745345	0.068798	0.240248	NOT
SFTA1P	14.96878	-0.7189034	0.395116	-1.81947599	0.068839	0.240352	NOT
SSX3	1.857403	2.17172002	1.19365	1.819394338	0.068851	0.240352	NOT
SPAG5	1893.486	0.49231145	0.2706	1.819332986	0.068861	0.240352	NOT
RP11-1124	1.465215	1.5227441	0.837	1.819287539	0.068868	0.240352	NOT
RP4-694B1	43.93473	0.49022593	0.269465	1.819258481	0.068872	0.240352	NOT
SP6	60.89282	0.75298689	0.41392	1.819158657	0.068887	0.240374	NOT
MAGOH2P	14.59347	-0.5813398	0.319591	-1.81900974	0.06891	0.240423	NOT
ZUFSP	248.6851	0.28743777	0.158046	1.818698732	0.068957	0.240529	NOT
EP300-AS1	28.20366	-0.7057008	0.388046	-1.81859983	0.068973	0.240529	NOT
DPEP1	72.87758	1.06785502	0.58721	1.818524367	0.068984	0.240529	NOT
KB-1460A1	15.02743	1.05949717	0.582614	1.818523502	0.068984	0.240529	NOT
TUBA3E	9.83343	1.25118893	0.688027	1.818518307	0.068985	0.240529	NOT
RP5-965G2	30.43093	0.74823029	0.411463	1.818461196	0.068994	0.240529	NOT
C2orf82	943.0588	0.77191034	0.42451	1.818356436	0.06901	0.240551	NOT

RP11-430I	2.9424	0.81158248	0.44634	1.818304031	0.069018	0.240551	NOT
HNRNPKP2	8.150711	0.47957151	0.263781	1.818067499	0.069054	0.240646	NOT
PRDM11	246.5866	0.4483477	0.246616	1.817999898	0.069064	0.240651	NOT
TOP2B	3911.321	0.24435378	0.134423	1.817799858	0.069095	0.240676	NOT
CD24	6993.66	0.93152444	0.512447	1.817796232	0.069095	0.240676	NOT
CHIA	1.461426	1.61096722	0.886228	1.817778671	0.069098	0.240676	NOT
XCL2	24.02129	-0.7251314	0.398945	-1.81762255	0.069122	0.240729	NOT
RP4-612B1	4.093456	0.68615799	0.377523	1.817524415	0.069137	0.24075	NOT
ZNF658	66.64548	-0.4146099	0.228133	-1.81740259	0.069155	0.240784	NOT
ZBED2	18.84443	1.14074945	0.627704	1.817337759	0.069165	0.240788	NOT
CCSER2	1317.132	-0.2889533	0.159018	-1.8171148	0.0692	0.240875	NOT
BAG3	1906.804	0.29695795	0.163445	1.81686418	0.069238	0.240978	NOT
UHRF2	971.0335	0.28246494	0.155476	1.816771218	0.069252	0.240997	NOT
BX842568.	12.8273	-1.2046473	0.663188	-1.81645036	0.069301	0.241137	NOT
ARMCX6	185.8405	0.65274763	0.359407	1.816180753	0.069343	0.241218	NOT
WNT8B	3.711747	-1.2831624	0.706523	-1.81616587	0.069345	0.241218	NOT
AC024560.	10.38522	0.89328882	0.491865	1.816124799	0.069351	0.241218	NOT
FAM161B	169.4089	0.34245803	0.188572	1.816059297	0.069361	0.241222	NOT
REL	183.0761	-0.3879318	0.21362	-1.81598753	0.069372	0.241229	NOT
RP11-415I	3.420524	0.66377973	0.365532	1.815927796	0.069381	0.24123	NOT
RHOA	15993.82	0.20780469	0.114447	1.815734883	0.069411	0.241302	NOT
CTD-2369F	21.94601	0.88293792	0.486289	1.815663607	0.069422	0.241309	NOT
ZNF439	60.59731	0.69988857	0.385605	1.815042206	0.069517	0.241597	NOT
CSTF3	1243.635	0.19762206	0.108883	1.815000338	0.069524	0.241597	NOT
C9	15337.52	-1.2772497	0.703738	-1.81495078	0.069531	0.241597	NOT
TGFA	302.2105	0.88532869	0.487835	1.814812768	0.069553	0.241614	NOT
ARMC5	962.2935	-0.4233821	0.233294	-1.81480333	0.069554	0.241614	NOT
NPL	1450.781	0.63215974	0.3484	1.814467463	0.069606	0.241735	NOT
BMX	27.40853	-0.6523776	0.359543	-1.8144612	0.069607	0.241735	NOT
SLIRP	1933.513	-0.474101	0.261309	-1.81432907	0.069627	0.241752	NOT
AC004012.	16.10966	-0.84441	0.465416	-1.81431316	0.06963	0.241752	NOT
YWHAEP5	3.272579	0.60176626	0.331701	1.814181276	0.06965	0.241791	NOT
RP11-133M	2.672812	-0.6506003	0.358661	-1.81396978	0.069682	0.241873	NOT
ZNF283	121.3316	-0.4429045	0.244188	-1.81378756	0.06971	0.24192	NOT
LINC01121	14.24018	1.29170853	0.712169	1.813767336	0.069714	0.24192	NOT
RP11-472F	2.525962	-1.1849624	0.653354	-1.81366015	0.06973	0.241946	NOT
ROBO2	56.99607	1.12602901	0.620975	1.813324196	0.069782	0.242071	NOT
CRIPT	763.8989	0.23545789	0.129853	1.813265621	0.069791	0.242071	NOT
WDR35	366.277	0.43683516	0.240925	1.81315503	0.069808	0.242071	NOT
ZNF473	437.2116	0.2657133	0.146549	1.813138093	0.069811	0.242071	NOT
ZYG11A	108.4975	0.74896538	0.413078	1.813133929	0.069811	0.242071	NOT
PPIG	2579.147	0.22500537	0.124101	1.813079273	0.06982	0.242071	NOT
RPL6P27	347.3511	0.61005031	0.336499	1.812934305	0.069842	0.242118	NOT
MMP16	49.97594	0.85334575	0.470719	1.812857305	0.069854	0.242128	NOT
LINC01583	1.070026	1.66671252	0.919618	1.812396521	0.069925	NA	NA
CENPBD1	183.0335	-0.3723315	0.20544	-1.81236533	0.06993	0.242335	NOT
WFIKKN2	4.71922	-1.2326174	0.680134	-1.81231583	0.069937	0.242335	NOT
CCDC169	3.046013	1.83993644	1.015252	1.812295858	0.06994	0.242335	NOT
FAM92B	1.399691	-1.2657039	0.698506	-1.81201459	0.069984	0.242434	NOT

RP11-190J	3.397409	2.1726009	1.19901	1.811995328	0.069987	0.242434	NOT
RBP4	211162.2	-0.7073383	0.390378	-1.81193206	0.069997	0.242437	NOT
CH17-437K	29.29815	-0.6616419	0.365181	-1.81181756	0.070014	0.242462	NOT
WWOX	559.3142	-0.305334	0.168528	-1.81176992	0.070022	0.242462	NOT
EIF2S1	2436.315	0.26129211	0.144224	1.811710708	0.070031	0.242463	NOT
ATXN2L	4239.868	0.22287123	0.123031	1.811505098	0.070063	0.242523	NOT
CAMTA2	1227.477	-0.3183679	0.17575	-1.81148177	0.070066	0.242523	NOT
SHROOM3	1664.196	-0.5968507	0.329497	-1.81139934	0.070079	0.242537	NOT
BNIP3P10	1.808516	0.85877873	0.474167	1.811131435	0.070121	0.242649	NOT
SDF2	1887.555	0.24900826	0.137493	1.811061669	0.070131	0.242656	NOT
CTD-2008I	12.8179	-0.4634762	0.255943	-1.81086027	0.070162	0.242732	NOT
RP11-213(	112.8421	-0.3076453	0.169899	-1.81075873	0.070178	0.242756	NOT
FBXW9	314.032	0.31309167	0.172934	1.810465242	0.070224	0.24288	NOT
DHRS12	675.7121	-0.450043	0.248593	-1.81036103	0.07024	0.24288	NOT
THAP9	185.8755	-0.2939447	0.162369	-1.81035413	0.070241	0.24288	NOT
RP11-243M	5.710428	1.37096179	0.75737	1.810162378	0.070271	0.24294	NOT
SCN1B	448.4401	-0.5294024	0.292467	-1.81012717	0.070276	0.24294	NOT
ARID3C	148.186	-0.7878452	0.435277	-1.80998422	0.070298	0.242985	NOT
RPS27	23754.6	0.41130067	0.227264	1.80979477	0.070328	0.243054	NOT
RP11-675I	1.053293	2.19564534	1.213238	1.809740104	0.070336	NA	NA
MAPRE1	2770.627	0.33377848	0.184435	1.809739849	0.070336	0.243054	NOT
RP11-218I	1.632965	2.83770061	1.568067	1.809680867	0.070345	0.243055	NOT
RP11-120K	7.223653	-0.5472214	0.302464	-1.80921004	0.070418	0.243275	NOT
RP11-69E1	2.558387	-0.78517	0.433998	-1.80915491	0.070427	0.243275	NOT
RP11-284N	34.03629	-0.7733034	0.427543	-1.80871695	0.070495	0.243479	NOT
MT-TM	3.307569	1.3254939	0.732895	1.80857153	0.070518	0.243526	NOT
CALCRL	909.418	-0.5465145	0.302211	-1.80838948	0.070546	0.243568	NOT
PPIAP29	125.7159	0.87207858	0.482243	1.80837854	0.070548	0.243568	NOT
CTC-444N2	873.5791	-0.3277438	0.181271	-1.80803572	0.070601	0.243721	NOT
PIGX	748.2742	0.24079726	0.133187	1.807969278	0.070611	0.243726	NOT
PRPF6	6769.422	0.28065197	0.155238	1.807880454	0.070625	0.243742	NOT
SYAP1	3907.207	-0.2885517	0.159615	-1.80779336	0.070639	0.243758	NOT
RP11-667K	2.817657	-0.9718587	0.537627	-1.80768169	0.070656	0.243787	NOT
REG4	14.95987	0.89302296	0.494036	1.80760731	0.070668	0.243796	NOT
FAM76B	539.2294	-0.3557518	0.19682	-1.80749397	0.070685	0.243826	NOT
PWAR1	2.788451	1.20917476	0.669084	1.807208608	0.07073	0.243944	NOT
CTD-3105I	13.04482	-0.6040829	0.334275	-1.80714306	0.07074	0.243944	NOT
TRIM72	9.949315	0.88774879	0.491256	1.807100617	0.070747	0.243944	NOT
FGFR3	4541.995	0.69244947	0.383224	1.806907002	0.070777	0.244018	NOT
NUP160	1661.518	0.27731509	0.153486	1.806780336	0.070797	0.244055	NOT
MPND	1854.982	-0.3687885	0.204141	-1.80654062	0.070834	0.244077	NOT
B4GALNT3	124.3992	0.97612822	0.540351	1.806469627	0.070845	0.244077	NOT
MYOZ1	10.41081	-0.7059896	0.390813	-1.80646574	0.070846	0.244077	NOT
PHGDH	4632.168	0.69284672	0.38355	1.80640689	0.070855	0.244077	NOT
GUCY1B2	46.54966	1.14709549	0.635018	1.80639891	0.070856	0.244077	NOT
TTLL6	18.42082	0.7844602	0.434276	1.806361652	0.070862	0.244077	NOT
RABAC1	2900.739	-0.4164043	0.230524	-1.80633442	0.070866	0.244077	NOT
BRD1	1113.25	0.29349378	0.162509	1.806010979	0.070917	0.244215	NOT
MEGF10	8.152798	-0.9352679	0.517877	-1.80596398	0.070924	0.244215	NOT

CCDC80	1860.294	-0.9522622	0.527333	-1.8058083	0.070948	0.244267	NOT
SPRY3	61.66766	-0.4046903	0.224127	-1.8056282	0.070976	0.24429	NOT
FTSJ1	1039.008	0.22874713	0.126687	1.805608784	0.070979	0.24429	NOT
KPNA1	2085.718	-0.218722	0.121136	-1.80559426	0.070982	0.24429	NOT
BTRC	773.2742	-0.2564309	0.142035	-1.80540533	0.071011	0.24436	NOT
ACPP	15.10774	1.03829269	0.575153	1.80524626	0.071036	0.244406	NOT
TRDC	50.63182	0.65581486	0.363294	1.805189001	0.071045	0.244406	NOT
ZNF711	156.2899	0.98710875	0.54683	1.805147627	0.071052	0.244406	NOT
RP11-536I	87.65915	0.53424759	0.295975	1.805040871	0.071068	0.244409	NOT
ARL4A	726.7779	0.48270842	0.267425	1.805026084	0.071071	0.244409	NOT
AC006460.	3.158572	-0.9744738	0.539887	-1.80495904	0.071081	0.244414	NOT
IL3RA	348.1737	-0.4159324	0.23045	-1.80486733	0.071095	0.244433	NOT
RP11-95M5	1.607043	1.4777114	0.818808	1.804711194	0.07112	0.244457	NOT
RP11-452I	1.126734	-1.1823061	0.655141	-1.80465976	0.071128	NA	NA
PACSIN2	4543.379	0.31976966	0.177192	1.804650103	0.071129	0.244457	NOT
SYT1	262.4094	-1.0184999	0.564391	-1.80460098	0.071137	0.244457	NOT
MT-TL1	20.33085	0.80654004	0.446947	1.804553611	0.071145	0.244457	NOT
AL161668.	69.24902	-0.8665115	0.480194	-1.80450275	0.071153	0.244457	NOT
C11orf58	4335.314	0.20908402	0.11587	1.804467106	0.071158	0.244457	NOT
RP11-536(	2.858062	-0.9572275	0.530495	-1.80440408	0.071168	0.244457	NOT
SRBD1	597.9916	-0.272187	0.150849	-1.80436182	0.071175	0.244457	NOT
AC005077.	276.6362	-0.9194592	0.509613	-1.80422889	0.071195	0.244498	NOT
NTRK3	13.3238	-0.7207844	0.399548	-1.80399745	0.071232	0.244575	NOT
ST8SIA3	25.95658	-1.3143421	0.728583	-1.80397107	0.071236	0.244575	NOT
BAI2	72.42258	0.92658837	0.513673	1.803848938	0.071255	0.244609	NOT
LINC0112(	16.85776	-0.6359082	0.352543	-1.80377246	0.071267	0.24462	NOT
AC025016.	6.811687	2.09205043	1.159919	1.803617543	0.071291	0.244624	NOT
DKFZP434I	11.94271	-0.8497271	0.471128	-1.80360201	0.071294	0.244624	NOT
TSSC4	1715.523	0.33744206	0.187095	1.803591078	0.071295	0.244624	NOT
AC007950.	4.501929	-0.589064	0.326646	-1.80336878	0.07133	0.244713	NOT
KCNK5	912.5514	0.85121038	0.47205	1.803221101	0.071353	0.244762	NOT
USP9X	6498.409	-0.2974971	0.164999	-1.80302405	0.071384	0.244837	NOT
CCSAP	464.7314	0.38656317	0.214431	1.802738608	0.071429	0.24496	NOT
RP11-318(	8.473594	-0.6715512	0.372542	-1.80261821	0.071448	0.244994	NOT
CTD-2184I	4.51605	-0.8742709	0.485037	-1.80248337	0.071469	0.245035	NOT
IP6K2	2149.247	0.31319718	0.173773	1.80233885	0.071492	0.245082	NOT
DOT1L	1666.53	0.34538968	0.191645	1.802234893	0.071508	0.245087	NOT
EFNA1	17183.04	0.51509614	0.285813	1.802214498	0.071512	0.245087	NOT
RP11-551I	5.43708	0.99346207	0.55127	1.802133715	0.071524	0.2451	NOT
PALM2-AKA	24.6171	-0.8052578	0.446898	-1.80188121	0.071564	0.245154	NOT
RP11-145N	6.340222	-0.5984586	0.332131	-1.80187335	0.071565	0.245154	NOT
PLD5	16.06683	1.17088965	0.649822	1.801862059	0.071567	0.245154	NOT
CCNO	73.20582	1.09195869	0.606095	1.801630646	0.071604	0.245219	NOT
TMEM170A	729.4777	-0.2242481	0.124472	-1.80159447	0.071609	0.245219	NOT
TPBG	187.8888	0.8886146	0.493245	1.801567903	0.071613	0.245219	NOT
IBA57-AS1	5.549415	-0.6774115	0.376034	-1.80146257	0.07163	0.245245	NOT
AIFM1	5955.93	-0.3717333	0.206372	-1.80128133	0.071659	0.245312	NOT
RRN3	1613.378	-0.2767886	0.153674	-1.80114286	0.07168	0.245356	NOT
CAMK2D	1797.279	-0.3336669	0.185274	-1.8009394	0.071712	0.245407	NOT

VWA5B1	1.820899	1.46556778	0.813783	1.800932065	0.071714	0.245407	NOT
PPIEL	6.436829	-0.7589281	0.421438	-1.80080684	0.071733	0.245424	NOT
RP11-180M	11.57753	-0.5466333	0.303573	-1.80066795	0.071755	0.245424	NOT
LMOD2	2.418945	1.01982191	0.56636	1.800660285	0.071756	0.245424	NOT
KLHL23	910.6854	0.47764533	0.265268	1.800616893	0.071763	0.245424	NOT
C21orf59	648.038	0.28158455	0.156384	1.800597779	0.071766	0.245424	NOT
STK26	408.4232	0.68451603	0.380169	1.80055704	0.071773	0.245424	NOT
WT1	22.03731	-1.2510689	0.694933	-1.80027241	0.071818	0.245547	NOT
PHLDB3	1160.54	-0.3775939	0.209783	-1.79992208	0.071873	0.245654	NOT
SCAMP3	6183.12	0.34747878	0.193059	1.799854251	0.071884	0.245654	NOT
IQGAP2	7668.284	-0.4967616	0.276004	-1.79983788	0.071886	0.245654	NOT
DNPEP	3382.132	0.23114179	0.128426	1.799801351	0.071892	0.245654	NOT
RP11-304I	50.35178	-0.4148808	0.230517	-1.79978652	0.071894	0.245654	NOT
RP11-113C	1.29458	-1.3668084	0.75947	-1.79968798	0.07191	NA	NA
LINC0150:	109.5881	0.71531836	0.397525	1.799429725	0.071951	0.245816	NOT
XIRP2	2.829273	1.76207843	0.979415	1.799112955	0.072001	0.245956	NOT
DDAH2	3699.71	0.47191685	0.262319	1.799019895	0.072016	0.245974	NOT
CCAR1	2190.091	0.23520521	0.130745	1.798965595	0.072024	0.245974	NOT
PCDHA10	11.9254	-0.8683583	0.482725	-1.79886745	0.07204	0.245996	NOT
RP11-540I	153.7179	-0.3203953	0.178138	-1.79857752	0.072086	0.246121	NOT
AP001628.	2.832984	0.80887253	0.449802	1.798283764	0.072132	0.246249	NOT
LINC0113S	61.71196	1.63009091	0.906545	1.798135505	0.072156	0.246298	NOT
WDR74	1301.466	0.28294053	0.157366	1.797976739	0.072181	0.246353	NOT
PEBP1P2	11.57324	-0.6913724	0.384559	-1.79783305	0.072203	0.2464	NOT
ARRDC5	5.903062	-0.7404506	0.411872	-1.7977675	0.072214	0.246404	NOT
ZNF219	1706.541	-0.3878646	0.215762	-1.79764987	0.072233	0.246405	NOT
MYH11	1151.288	-0.7830837	0.435615	-1.7976496	0.072233	0.246405	NOT
TBC1D19	117.1625	-0.397019	0.220861	-1.79759422	0.072241	0.246405	NOT
RP11-219I	5.639965	-0.7016908	0.390479	-1.79699938	0.072336	0.246686	NOT
ZNF713	72.48657	0.34377079	0.19131	1.796934775	0.072346	0.246686	NOT
THAP6	463.536	-0.2572603	0.143169	-1.79689968	0.072352	0.246686	NOT
DDR1	2278.846	0.77686441	0.432349	1.796846228	0.07236	0.246686	NOT
C20orf202	15.38877	0.64346735	0.358155	1.796619205	0.072396	0.246778	NOT
PKNOX1	722.0049	0.19949689	0.111056	1.796361455	0.072437	0.246887	NOT
FLVCR1-AS	195.6014	0.52352564	0.291451	1.796274011	0.072451	0.246903	NOT
NKPD1	7.412688	0.93184049	0.518992	1.795480019	0.072577	0.2473	NOT
VPS9D1-AS	77.57158	0.72751685	0.405205	1.79542743	0.072586	0.2473	NOT
CTC-436P1	8.07588	-0.6051385	0.33708	-1.79523757	0.072616	0.247372	NOT
TRPC5OS	4.906709	1.19199351	0.663999	1.795173868	0.072626	0.247375	NOT
RABIF	894.5001	0.31330847	0.174538	1.795073892	0.072642	0.247398	NOT
PAXIP1-AS	207.0408	-0.449937	0.250673	-1.79491909	0.072667	0.247424	NOT
ANKRD20A1	3.634458	1.69918147	0.946665	1.794912271	0.072668	0.247424	NOT
FSD2	2.353816	-0.7902352	0.440284	-1.79483068	0.072681	0.247437	NOT
MAGI2	139.7047	-0.5319592	0.296443	-1.79447444	0.072737	0.247599	NOT
ANTXR1	978.6227	-0.6787979	0.378306	-1.79430944	0.072764	0.247641	NOT
DIP2C	1448.554	-0.500293	0.278826	-1.79428232	0.072768	0.247641	NOT
TUBE1	484.6477	-0.4329881	0.241336	-1.79413167	0.072792	0.247641	NOT
AL132772.	12.55545	-0.7071868	0.394169	-1.7941198	0.072794	0.247641	NOT
GATM-AS1	8.07944	0.68587411	0.382292	1.794112643	0.072795	0.247641	NOT

RP11-1143	97.96858	0.98538202	0.549249	1.794054315	0.072804	0.247641	NOT
AC004453.	73.64024	0.59571539	0.332066	1.793966159	0.072819	0.247657	NOT
KIF26A	115.478	-0.6096261	0.339831	-1.79391153	0.072827	0.247657	NOT
N4BP2L2-I	48.20141	-0.4965471	0.276824	-1.79373078	0.072856	0.247685	NOT
LIG1	1682.49	0.33981645	0.189448	1.793716365	0.072858	0.247685	NOT
BANF1P3	7.527382	0.59370148	0.330995	1.793688604	0.072863	0.247685	NOT
RP11-626(	4.27035	0.7386669	0.411846	1.793552286	0.072885	0.247699	NOT
RP11-106M	15.02734	-0.7996433	0.445844	-1.79354807	0.072885	0.247699	NOT
CFLAR	4192.04	-0.218363	0.121777	-1.79313962	0.072951	0.247881	NOT
TNIK	346.8718	0.75248656	0.419657	1.793097564	0.072957	0.247881	NOT
CYP1A2	2203.985	-1.5335376	0.855302	-1.7929785	0.072976	0.247915	NOT
RP4-758J1	1087.639	-0.3165705	0.176573	-1.79286307	0.072995	0.24792	NOT
COX5A	5787.118	-0.3882206	0.216538	-1.79285539	0.072996	0.24792	NOT
COQ10B	1456.19	-0.2415548	0.134787	-1.79212687	0.073113	0.248285	NOT
SMCR8	1063.454	0.37686545	0.210303	1.792009268	0.073131	0.248318	NOT
RSRC2	2055.458	0.19887568	0.110986	1.791898562	0.073149	0.248347	NOT
XXbac-B44	7.03627	0.94733786	0.528741	1.791686106	0.073183	0.248409	NOT
DSCR8	91.16876	2.08843576	1.165636	1.791670294	0.073186	0.248409	NOT
CCDC125	1290.958	-0.3160512	0.176416	-1.79150807	0.073212	0.248421	NOT
MYRF	3435.592	0.57229427	0.319449	1.79150459	0.073212	0.248421	NOT
ABCB4	5846.125	-0.8596765	0.479872	-1.79147068	0.073218	0.248421	NOT
ZWILCH	443.4972	0.30717152	0.171468	1.791420116	0.073226	0.248421	NOT
TRIM44	3332.73	0.22947909	0.128114	1.791214381	0.073259	0.248501	NOT
RP11-491H	1.16995	1.4863999	0.829852	1.791163331	0.073267	NA	NA
RP11-588I	5.846567	-0.7663145	0.427866	-1.7910132	0.073291	0.24858	NOT
RP11-490(	13.60017	-0.5221908	0.291606	-1.79074023	0.073335	0.248668	NOT
THYN1	1208.029	-0.2812491	0.15706	-1.79071015	0.07334	0.248668	NOT
RFX6	9.797867	1.63059952	0.910604	1.790679212	0.073345	0.248668	NOT
EXTL2	524.4235	0.32660436	0.182419	1.790404913	0.073389	0.248785	NOT
RMI2	427.872	0.571936	0.319455	1.790351226	0.073397	0.248785	NOT
CHRNA6	2.385643	1.15286021	0.643985	1.790196877	0.073422	0.248838	NOT
GREM2	750.9504	-1.28256	0.716521	-1.78998197	0.073457	0.248924	NOT
ST20	172.4169	-0.4723725	0.263952	-1.78961681	0.073516	0.249092	NOT
RP11-10A1	39.81581	0.75062168	0.419492	1.789358857	0.073557	0.249201	NOT
RP11-31F1	15.70142	-0.5232674	0.292443	-1.78929675	0.073567	0.249204	NOT
DIRAS2	78.85018	1.25654168	0.702344	1.789068123	0.073604	0.249292	NOT
MED11	765.9176	-0.3436806	0.192105	-1.78902157	0.073611	0.249292	NOT
GTF2A1	1532.512	-0.2913793	0.162895	-1.7887603	0.073653	0.249387	NOT
RPL23P2	13.38401	-0.4360134	0.243757	-1.78872075	0.07366	0.249387	NOT
RP11-624I	22.85225	1.02512979	0.573127	1.788660562	0.073669	0.249387	NOT
TRIM23	600.6385	-0.3348098	0.187194	-1.78856978	0.073684	0.249387	NOT
RTN4	12676.88	0.33055033	0.184813	1.788562494	0.073685	0.249387	NOT
CNKSR3	1883.186	-0.423195	0.236621	-1.78849616	0.073696	0.249392	NOT
RPL23AP53	43.65909	0.39584533	0.221337	1.788424457	0.073708	0.2494	NOT
AMY2B	365.2234	-0.6264243	0.350327	-1.78811059	0.073758	0.24954	NOT
CEP72	346.944	0.43494647	0.243271	1.787909839	0.073791	0.249559	NOT
PLCG2	1620.874	-0.4977957	0.278441	-1.78779688	0.073809	0.249559	NOT
MYADML2	2.965599	1.00680041	0.563187	1.78768478	0.073827	0.249559	NOT
CENPJ	342.4155	0.42896628	0.239966	1.787612115	0.073839	0.249559	NOT

RP11-550F	16.9799	0.46531372	0.260301	1.787598298	0.073841	0.249559	NOT
RNASE7	29.46607	0.95337424	0.533327	1.787597716	0.073841	0.249559	NOT
NT5DC1	1619.12	-0.4480939	0.250671	-1.7875813	0.073844	0.249559	NOT
TOPBP1	1411.214	0.33812296	0.189162	1.787479614	0.07386	0.249559	NOT
GALR2	6.377737	0.64002841	0.358068	1.787450654	0.073865	0.249559	NOT
CYP4F23P	13.49645	1.13071857	0.632588	1.787448854	0.073865	0.249559	NOT
RP11-1275	125.3142	-0.5265782	0.294601	-1.78742834	0.073868	0.249559	NOT
RP11-345M	20.73787	-0.8152217	0.456096	-1.78739147	0.073874	0.249559	NOT
HNRNPR	3997.554	0.23130252	0.129421	1.787207701	0.073904	0.249612	NOT
GLYCTK-AS	8.037771	-0.6018491	0.336759	-1.78718077	0.073908	0.249612	NOT
CDH13	541.9188	-0.4141275	0.231736	-1.78706501	0.073927	0.249636	NOT
CTD-3076M	52.85636	-0.9275341	0.519057	-1.78696157	0.073944	0.249636	NOT
TM7SF3	3901.666	-0.3886997	0.217524	-1.78693154	0.073949	0.249636	NOT
LINC00589	2.470995	-1.5658133	0.87627	-1.78690821	0.073952	0.249636	NOT
GPATCH3	876.337	0.32195258	0.180191	1.786726712	0.073982	0.249704	NOT
CTD-3030D	1.047216	-1.3015361	0.728544	-1.78648879	0.07402	NA	NA
FAM182A	3.011629	1.26826981	0.709985	1.78633441	0.074045	0.249881	NOT
DIABLO	326.1593	0.25666431	0.143686	1.786283835	0.074053	0.249881	NOT
XXbac-BPC	8.329112	-0.6422199	0.359539	-1.78623011	0.074062	0.249881	NOT
DHRS13	226.5688	0.47077005	0.26357	1.786129861	0.074078	0.249881	NOT
TMEM249	7.305881	0.69580092	0.38956	1.786117848	0.07408	0.249881	NOT
SELM	2478.599	0.79175781	0.443379	1.785734653	0.074142	0.250054	NOT
AP000253.	9.577502	-0.7743001	0.433615	-1.78568742	0.07415	0.250054	NOT
SLC7A11	569.3319	0.90133543	0.504829	1.78542614	0.074192	0.250166	NOT
GS1-124K5	30.39469	-0.5890681	0.32995	-1.78532347	0.074209	0.250191	NOT
SCARNA13	10.42231	-0.5329117	0.298526	-1.78514243	0.074238	0.250259	NOT
CYP20A1	1222.891	-0.2279594	0.127709	-1.78499136	0.074263	0.25031	NOT
THBD	592.6987	-0.6188081	0.346715	-1.78477248	0.074298	0.250399	NOT
LINC00506	6.096829	0.95232841	0.53375	1.78422086	0.074388	0.250639	NOT
LMCD1-AS1	15.81329	0.52409973	0.293742	1.784219071	0.074388	0.250639	NOT
KEAP1	4201.696	-0.2993255	0.167779	-1.78405042	0.074415	0.25068	NOT
EFCAB1	18.73523	-0.9475724	0.531141	-1.78403041	0.074419	0.25068	NOT
CTNNA2	128.7204	-1.6394503	0.918996	-1.78395757	0.074431	0.250686	NOT
NAP1L4	4875.329	0.21471102	0.12036	1.783906822	0.074439	0.250686	NOT
RP1-81D8.	5.054881	-1.320265	0.740147	-1.78378801	0.074458	0.250706	NOT
PPME1	1510.732	0.22691067	0.127209	1.783755789	0.074463	0.250706	NOT
RP11-370A	49.41716	0.62903423	0.352686	1.783552106	0.074496	0.250787	NOT
ZDHHC11B	228.4586	-0.7718963	0.432838	-1.7833382	0.074531	0.250873	NOT
RFC2	1489.131	0.33960471	0.190447	1.783195139	0.074555	0.25092	NOT
CXCL6	380.2799	1.42416861	0.798764	1.782964881	0.074592	0.250986	NOT
LRFN3	663.3029	-0.375405	0.210555	-1.78292799	0.074598	0.250986	NOT
PPIAP13	2.692034	0.83222278	0.466779	1.782903605	0.074602	0.250986	NOT
DDOST	10128.32	0.25208089	0.141396	1.782805613	0.074618	0.251009	NOT
CYP2D7	633.5474	0.78591951	0.440891	1.782571812	0.074656	0.251052	NOT
MT-CYB	355276.7	-0.4667928	0.261866	-1.78256257	0.074658	0.251052	NOT
TMEM125	30.63403	1.01829029	0.571253	1.782555647	0.074659	0.251052	NOT
FAM151A	332.7746	-1.1827267	0.66355	-1.78242358	0.07468	0.251093	NOT
TRIM35	967.1523	-0.4917589	0.275902	-1.78236834	0.074689	0.251093	NOT
RP11-89C3	4.184474	0.91474457	0.513275	1.782173359	0.074721	0.251168	NOT

RENBP	588.0518	0.72851753	0.408807	1.782056856	0.07474	0.251198	NOT
LINC0146(	1.405668	1.08319047	0.607849	1.782006484	0.074748	0.251198	NOT
COPS6	4680.306	0.28365622	0.159198	1.781787341	0.074784	0.251287	NOT
CCPG1	1097.033	-0.3375216	0.189437	-1.78171102	0.074796	0.251297	NOT
SYCE2	46.07103	-0.4964271	0.278661	-1.78147573	0.074835	0.251395	NOT
RP4-781B1	0.563896	-1.2665854	0.711223	-1.78085453	0.074936	NA	NA
CTB-96E2.	6.419704	-0.936271	0.525777	-1.78073928	0.074955	0.251765	NOT
RP11-705(	95.83763	0.44231047	0.248401	1.780628767	0.074973	0.251765	NOT
SH2D3A	224.9576	0.70403155	0.395387	1.780615054	0.074975	0.251765	NOT
RPSA	21361.9	0.45867717	0.257601	1.780574127	0.074982	0.251765	NOT
RAET1G	9.85833	0.6057717	0.340231	1.780469622	0.074999	0.251791	NOT
GANC	542.6173	-0.3096286	0.173914	-1.78035622	0.075018	0.251823	NOT
ZDHHC24	1038.626	-0.3293357	0.185013	-1.78007154	0.075064	0.251948	NOT
CACNA1B	5.147023	1.54905794	0.87039	1.779729154	0.07512	0.252001	NOT
CTD-2562J	2.566172	1.36336412	0.766062	1.779704543	0.075124	0.252001	NOT
AC005152.	2.577755	1.44779986	0.813534	1.779641756	0.075135	0.252001	NOT
C5orf64	3.695319	0.94304651	0.529916	1.77961516	0.075139	0.252001	NOT
PRKCI	903.4485	0.27184667	0.152756	1.779610524	0.07514	0.252001	NOT
RP11-332H	2.073171	-0.6963399	0.391315	-1.77948669	0.07516	0.252001	NOT
RP11-730(	1.274297	3.02555866	1.700245	1.779483421	0.075161	NA	NA
RP11-136(	5.678818	-0.9594001	0.539147	-1.7794768	0.075162	0.252001	NOT
MYOM2	113.4558	-0.9445904	0.530828	-1.77946741	0.075163	0.252001	NOT
GPALPP1	678.1099	-0.3087143	0.173487	-1.77946384	0.075164	0.252001	NOT
CTD-2541M	11.20202	0.50134814	0.281779	1.779225115	0.075203	0.252077	NOT
YJEFN3	114.7364	0.65411307	0.367642	1.779212333	0.075205	0.252077	NOT
RP1-43E13	15.15939	-0.5512928	0.309899	-1.77894059	0.07525	0.252195	NOT
AIM2	22.22133	0.80112132	0.45037	1.778806192	0.075272	0.252228	NOT
GMEB1	567.2478	0.24112798	0.135559	1.778767072	0.075278	0.252228	NOT
RP11-576I	9.374513	0.63133328	0.354946	1.778672505	0.075293	0.252249	NOT
CTD-2256P	112.4532	-0.4434727	0.249356	-1.7784734	0.075326	0.252327	NOT
ACTG1P14	7.391986	0.55524742	0.312224	1.778359661	0.075345	0.252359	NOT
FAM185BP	18.47569	-0.4390755	0.246939	-1.77807473	0.075392	0.252484	NOT
MCOLN3	186.4499	1.31558181	0.740026	1.777751498	0.075445	0.252631	NOT
DAP3	6587.245	0.27706195	0.155883	1.77737222	0.075507	0.252808	NOT
DLK2	99.59397	0.59109918	0.332614	1.777131741	0.075547	0.252884	NOT
CWC22	895.2638	0.24700647	0.138992	1.777121724	0.075548	0.252884	NOT
RP11-251(	59.56507	-0.3214645	0.180919	-1.77684063	0.075594	0.253007	NOT
RP11-603B	29.37628	-0.9794102	0.551276	-1.77662506	0.07563	0.253084	NOT
RNASEH2B	660.4456	0.31451931	0.177039	1.776554817	0.075642	0.253084	NOT
PMF1-BGLA	86.71577	0.51562582	0.290243	1.776531007	0.075645	0.253084	NOT
RP11-288I	7.793982	-0.486301	0.27376	-1.77637702	0.075671	0.253089	NOT
CTD-2260A	57.08736	-0.520332	0.29292	-1.77636435	0.075673	0.253089	NOT
IMPDH1P4	2.277924	2.97620997	1.675478	1.776334514	0.075678	0.253089	NOT
BAAT	30615.34	-0.7277762	0.409716	-1.77629644	0.075684	0.253089	NOT
TRIP11	1388.069	-0.3081846	0.173518	-1.77609398	0.075717	0.253169	NOT
CTB-58E17	20.71122	0.56917314	0.320491	1.775943766	0.075742	0.253217	NOT
IRF2BPL	1289.692	-0.3703734	0.208556	-1.77589363	0.07575	0.253217	NOT
FAM118A	784.4815	0.4436915	0.249879	1.775626306	0.075795	0.25332	NOT
CTBP1-AS	43.01574	-0.504178	0.283955	-1.77555673	0.075806	0.25332	NOT

C1orf21	1608.973	-0.5234574	0.294835	-1.77542687	0.075827	0.25332	NOT
SLC12A6	494.0659	-0.3001993	0.169087	-1.77540873	0.07583	0.25332	NOT
IPO8	2046.373	-0.2325999	0.131013	-1.77538931	0.075834	0.25332	NOT
RN7SL336F	1.762692	-0.7236899	0.407628	-1.77536714	0.075837	0.25332	NOT
ZBTB25	470.502	-0.2955309	0.166486	-1.77511033	0.07588	0.25343	NOT
RP11-147I	6.486357	0.56612963	0.318982	1.774803613	0.07593	0.253568	NOT
SNORA2	7.542183	-0.47909	0.269952	-1.77472056	0.075944	0.253583	NOT
PPP1R16B	274.4975	-0.5329643	0.300342	-1.7745236	0.075977	0.25361	NOT
RP11-563N	9.324887	0.60079552	0.338569	1.774515328	0.075978	0.25361	NOT
ZNF180	296.6086	-0.2922193	0.164677	-1.77450132	0.07598	0.25361	NOT
FLRT1	27.04119	0.72821904	0.410393	1.774444772	0.07599	0.25361	NOT
C5orf17	18.45635	1.73741483	0.979206	1.774309309	0.076012	0.253654	NOT
FAM99A	589.2893	-1.331996	0.750748	-1.77422405	0.076026	0.25367	NOT
SYNCRIP	4753.176	0.21212824	0.119565	1.774162583	0.076036	0.253672	NOT
ADPGK-AS1	3.37107	-0.5852713	0.3299	-1.77408782	0.076049	0.253682	NOT
LAGE3	1355.691	0.44825976	0.252695	1.773915298	0.076077	0.253746	NOT
ACMSD	2978.726	-0.6858739	0.386694	-1.7736859	0.076115	0.253842	NOT
LINC00431	2.388827	-1.4815037	0.835352	-1.77350929	0.076144	0.253901	NOT
ST6GAL1	33198.79	-0.4647212	0.262041	-1.77346542	0.076152	0.253901	NOT
RP11-281(	2.712062	-0.843881	0.475901	-1.77322792	0.076191	0.254001	NOT
SNX7	770.1064	0.51454598	0.29019	1.773135613	0.076206	0.25401	NOT
GBF1	3667.66	-0.2462519	0.138882	-1.77309854	0.076212	0.25401	NOT
FGF17	15.00093	-0.9226652	0.520548	-1.77248921	0.076313	0.254288	NOT
CLEC14A	894.6606	-0.4083442	0.23038	-1.77248226	0.076315	0.254288	NOT
NR1D2	1845.591	-0.3413268	0.192583	-1.77236092	0.076335	0.25431	NOT
LUC7L3	3304.527	0.26396095	0.148934	1.77232915	0.07634	0.25431	NOT
HIVEP2	692.5023	-0.3287846	0.18552	-1.77222785	0.076357	0.254311	NOT
SFMBT2	190.0653	-0.531474	0.299892	-1.77221549	0.076359	0.254311	NOT
PDCD7	672.5178	0.18629798	0.105127	1.772123455	0.076374	0.25433	NOT
BLZF1	1015.83	0.3052572	0.172271	1.771962058	0.076401	0.254388	NOT
PDE1C	35.05557	-0.5135445	0.289839	-1.77182476	0.076424	0.254433	NOT
KLK14	5.11518	0.90947796	0.513322	1.771749082	0.076436	0.254444	NOT
CTB-96E2.	1.66502	-0.8846121	0.499333	-1.77158582	0.076463	0.254503	NOT
USP16	1556.619	-0.2686399	0.151644	-1.77151371	0.076475	0.25451	NOT
AC092614.	8.688796	0.45893475	0.259083	1.771381891	0.076497	0.25451	NOT
RP11-33B1	15.78151	-0.6163154	0.34793	-1.77137527	0.076498	0.25451	NOT
WIBG	1680.337	0.27913972	0.157587	1.771332547	0.076505	0.25451	NOT
HIST1H1C	7036.381	-0.7336058	0.414165	-1.77129033	0.076512	0.25451	NOT
ERVMER34-	55.09428	1.17281221	0.662162	1.771185166	0.07653	0.254537	NOT
GARS	5482.114	0.36452665	0.205817	1.771118943	0.076541	0.254542	NOT
RP11-125(	6.567067	-0.8377522	0.473039	-1.7709994	0.076561	0.254577	NOT
DEFA4	0.575405	-1.7856626	1.008338	-1.77089608	0.076578	NA	NA
U40455.1	3.1504	0.85548482	0.483127	1.770724581	0.076607	0.254682	NOT
KCNJ6	18.16217	-1.3070086	0.738133	-1.77069625	0.076611	0.254682	NOT
CTC-398G3	4.716158	0.81405438	0.459782	1.770523207	0.07664	0.254733	NOT
ART4	1145.824	-0.6863295	0.387657	-1.77045437	0.076651	0.254733	NOT
TUBD1	298.9608	0.27469456	0.155156	1.770436288	0.076654	0.254733	NOT
MYZAP	28.71806	0.62801825	0.354794	1.770093453	0.076712	0.254891	NOT
BDNF-AS	98.21024	-0.4841147	0.273551	-1.76974018	0.07677	0.255035	NOT

LINC0122!	11.99956	1.00484913	0.567827	1.769639998	0.076787	0.255035	NOT
RP11-775(	31.73199	-0.4383123	0.247688	-1.76961523	0.076791	0.255035	NOT
ERICH2	74.21198	0.63397269	0.358256	1.769607898	0.076792	0.255035	NOT
ZBTB37	859.9693	-0.3185909	0.180046	-1.76949217	0.076812	0.255068	NOT
RBM12	1994.999	0.20818973	0.117662	1.769385578	0.07683	0.255096	NOT
SAR1B	7435.38	-0.4385596	0.247905	-1.76906524	0.076883	0.255242	NOT
CTD-2192J	126.1347	0.52042283	0.294205	1.768910325	0.076909	0.255296	NOT
AC002519.	1.869684	-0.9527023	0.538669	-1.76862279	0.076957	0.255424	NOT
SEMA6C	1024.126	0.49723801	0.28118	1.768400205	0.076994	0.255517	NOT
SLK	2114.403	-0.2595045	0.146773	-1.76806924	0.077049	0.25564	NOT
SLC9A3	129.2758	1.01302365	0.572972	1.768014615	0.077058	0.25564	NOT
8-Mar	2345.136	-0.350368	0.198171	-1.76800762	0.07706	0.25564	NOT
TTC4	106.6605	0.28748447	0.162644	1.767564718	0.077134	0.255855	NOT
MFSD2B	30.93401	0.76517272	0.432931	1.767425042	0.077157	0.255901	NOT
SEPP1	38169.04	-0.5785083	0.32734	-1.76730041	0.077178	0.255939	NOT
P4HB	109938.7	-0.3409319	0.192919	-1.7672288	0.07719	0.255948	NOT
CRNKL1	1510.207	0.20020027	0.113301	1.766975238	0.077232	0.256057	NOT
RP11-159F	15.40545	1.11459493	0.630823	1.766889778	0.077247	0.256073	NOT
RPL30P4	3.470023	0.9894302	0.560105	1.766509824	0.07731	0.256218	NOT
RP11-557F	15.55762	-0.9338808	0.528664	-1.76649066	0.077314	0.256218	NOT
RP11-410	126.5341	-0.5262452	0.297909	-1.76646072	0.077319	0.256218	NOT
HERC2	1989.218	-0.2846472	0.161146	-1.7663943	0.07733	0.256223	NOT
ERCC2	1463.228	0.25958184	0.146976	1.766151559	0.07737	0.256312	NOT
ERP27	63.8695	0.78758001	0.445938	1.766121107	0.077376	0.256312	NOT
ADAL	395.9307	-0.3964952	0.224554	-1.76569977	0.077446	0.256514	NOT
RITA1	1696.006	0.32357609	0.183262	1.765646334	0.077455	0.256514	NOT
ZFP28	83.75442	0.64510483	0.365437	1.765295291	0.077514	0.256678	NOT
DNAJC30	832.3726	-0.325308	0.184301	-1.76509013	0.077549	0.25676	NOT
RP11-227F	1.614374	1.1723929	0.664237	1.765022583	0.07756	0.256764	NOT
GPR89B	193.9149	0.32682863	0.185178	1.764945622	0.077573	0.256764	NOT
BRI3	6351.905	0.4110436	0.232897	1.764913881	0.077578	0.256764	NOT
C19orf68	421.7925	0.23197306	0.131471	1.764443555	0.077657	0.256968	NOT
CD97	2249.985	0.53596207	0.303759	1.764434519	0.077659	0.256968	NOT
HNRNPA1P4	4.420956	-0.696012	0.394576	-1.76395031	0.07774	0.257207	NOT
AC245100.	122.5542	0.44006837	0.249502	1.763783997	0.077768	0.257268	NOT
HIST1H3H	214.6474	-0.7550062	0.428098	-1.76363043	0.077794	0.257304	NOT
P2RX5	41.55073	0.88654903	0.50272	1.763503587	0.077816	0.257304	NOT
ECI1	6041.817	-0.4425249	0.250942	-1.76345802	0.077823	0.257304	NOT
LINC0061	1.523537	-0.9708188	0.550525	-1.76344236	0.077826	0.257304	NOT
LINC0155	223.6956	-0.7399685	0.419618	-1.76343542	0.077827	0.257304	NOT
RP11-22C	4.200683	1.87105244	1.061059	1.763382295	0.077836	0.257304	NOT
PTK2B	841.9972	-0.418427	0.237303	-1.76325783	0.077857	0.257342	NOT
EIF3K	8073.244	-0.3829425	0.217199	-1.76309348	0.077885	0.257371	NOT
IYD	1013.865	-0.8803588	0.499326	-1.76309325	0.077885	0.257371	NOT
KIAA0232	2610.976	-0.2717631	0.154161	-1.76284932	0.077926	0.257475	NOT
MAT1A	41067.77	-0.7424962	0.421238	-1.76265445	0.077959	0.257553	NOT
YWHAQ	6823.776	0.27292672	0.154875	1.762243442	0.078028	0.25775	NOT
COA6	1747.949	0.38345748	0.21762	1.762054027	0.07806	0.257805	NOT
UFSP2	655.363	-0.2528891	0.143524	-1.762	0.078069	0.257805	NOT

ALOX12B	2.269774	1.45623314	0.826477	1.761976683	0.078073	0.257805	NOT
ADAMTS13	327.1341	-0.5356949	0.304094	-1.76161198	0.078135	0.257977	NOT
C2orf48	20.11477	0.84697393	0.480836	1.761461331	0.07816	0.257983	NOT
AC100830.	3.241818	-0.5200093	0.295218	-1.76144397	0.078163	0.257983	NOT
MIR4435-1	998.9675	0.52176571	0.296219	1.761419129	0.078167	0.257983	NOT
PLIN5	2478.798	-0.6700171	0.380394	-1.76137704	0.078175	0.257983	NOT
RN7SKP16	3.471836	-0.8434597	0.478914	-1.76119246	0.078206	0.258038	NOT
SUDS3	1527.01	0.22040081	0.125145	1.761165751	0.07821	0.258038	NOT
PLP2	2603.451	0.61970242	0.351964	1.760699379	0.078289	0.258249	NOT
CYP2C18	2207.528	-0.7852283	0.445981	-1.76067576	0.078293	0.258249	NOT
ROBO3	244.3708	0.64057795	0.363889	1.760368667	0.078345	0.258389	NOT
RP5-1186N	2.029515	1.35998962	0.772686	1.760079632	0.078394	0.258519	NOT
TMCC3	507.8597	0.40995067	0.232957	1.759765973	0.078447	0.258663	NOT
HLA-DPB2	49.3085	0.93808125	0.533154	1.759492611	0.078494	0.258759	NOT
NADK2	8508.718	-0.4950305	0.28135	-1.75948141	0.078496	0.258759	NOT
KCNJ11	209.4765	0.73906885	0.420099	1.759272253	0.078531	0.258845	NOT
IQSEC2	175.5381	-0.500985	0.284787	-1.75915976	0.07855	0.258846	NOT
RP11-434I	33.17894	-1.0750054	0.611091	-1.75915759	0.078551	0.258846	NOT
PPP6R1	3982.777	0.24572087	0.13971	1.758794715	0.078612	0.259003	NOT
UQCC3	1220.062	0.39057771	0.222075	1.758765706	0.078617	0.259003	NOT
ADAM12	149.2056	0.77766919	0.442215	1.75857598	0.07865	0.259051	NOT
PPP6R3	3117.873	-0.1797528	0.102215	-1.75856698	0.078651	0.259051	NOT
VPS51	3653.633	-0.2484683	0.141308	-1.75834591	0.078689	0.259143	NOT
RGPD1	6.002631	-0.8973845	0.510432	-1.75808723	0.078733	0.259257	NOT
RNPEPL1	5242.325	0.30373682	0.172784	1.757896987	0.078765	0.259332	NOT
VPS37A	1384.257	0.39479024	0.224605	1.757706602	0.078797	0.259407	NOT
SEC16B	172.1116	-0.5804007	0.33027	-1.75735071	0.078858	0.259575	NOT
RP13-582(	253.4995	0.52885604	0.301015	1.756911937	0.078933	0.25979	NOT
VMAC	191.9952	-0.306566	0.174512	-1.75670589	0.078968	0.259851	NOT
PDE4B	458.1967	-0.6494314	0.36969	-1.75669031	0.078971	0.259851	NOT
ADCK4	1289.867	-0.3083064	0.175526	-1.75646763	0.079009	0.259912	NOT
PPM1A	2998.625	-0.3006704	0.171184	-1.75641772	0.079017	0.259912	NOT
LHX4	62.59533	0.56292496	0.320497	1.75641339	0.079018	0.259912	NOT
CFHR1	15857.16	-0.8531684	0.485786	-1.75626367	0.079043	0.259965	NOT
RP11-563J	9.573552	0.89287584	0.508432	1.756136406	0.079065	0.259993	NOT
HDX	79.11981	-0.5908314	0.336445	-1.75610086	0.079071	0.259993	NOT
ZNF785	272.013	0.2804038	0.159686	1.755969027	0.079094	0.260025	NOT
RP11-818F	4.649446	1.36169479	0.775483	1.755932122	0.0791	0.260025	NOT
ERP29P1	3.677863	0.5882313	0.335032	1.755749198	0.079131	0.260096	NOT
RP11-33N1	7.376715	-0.6360427	0.362298	-1.75557933	0.07916	0.26016	NOT
ADAMTS7	247.9589	-0.355248	0.20237	-1.7554407	0.079184	0.260188	NOT
NRBP2	5757.094	0.55265039	0.314834	1.755368888	0.079196	0.260188	NOT
EVI5L	658.3328	-0.2815882	0.160416	-1.75535983	0.079198	0.260188	NOT
ANXA5	9852.082	0.38318673	0.218322	1.755144402	0.079235	0.26025	NOT
GPR161	172.897	0.59899696	0.341282	1.755137571	0.079236	0.26025	NOT
RNF113A	689.5174	0.29222548	0.166515	1.75494541	0.079269	0.260267	NOT
LINC00883	56.57144	-0.7115305	0.405456	-1.7548889	0.079278	0.260267	NOT
LINC00893	53.67227	-0.5187295	0.295596	-1.75486237	0.079283	0.260267	NOT
ITSN1	1919.048	-0.3412474	0.194463	-1.75481868	0.07929	0.260267	NOT

RP11-96P7	2.213624	2.13320381	1.215691	1.754725902	0.079306	0.260267	NOT
ATF7IP	2207.914	-0.2985004	0.170115	-1.75470021	0.079311	0.260267	NOT
RP11-263I	2.890199	-1.9708712	1.123197	-1.75469729	0.079311	0.260267	NOT
NUTM2F	1.976459	-1.048084	0.597315	-1.75465913	0.079318	0.260267	NOT
TLE2	695.5705	-0.5297753	0.30196	-1.75445247	0.079353	0.260331	NOT
RP5-966M1	1610.435	-0.6660991	0.379677	-1.75438471	0.079365	0.260331	NOT
RIMS3	134.8191	0.65262372	0.371997	1.754376749	0.079366	0.260331	NOT
HIST1H3A	4.447652	-0.8631808	0.492036	-1.75430442	0.079378	0.26034	NOT
GOT1	12907.37	-0.5482873	0.312576	-1.75409261	0.079415	0.260416	NOT
TRAF3IP2-	60.74653	0.29054864	0.165644	1.754058063	0.079421	0.260416	NOT
LINC00941	16.62937	0.98990206	0.564464	1.753703117	0.079481	0.260517	NOT
RP11-20I2	16.5301	-0.5035161	0.287117	-1.75369956	0.079482	0.260517	NOT
FOXD4	13.45093	0.86504058	0.493282	1.753644351	0.079491	0.260517	NOT
RDH12	193.7726	0.79195157	0.451604	1.753643394	0.079492	0.260517	NOT
MYH7B	147.1359	0.76839845	0.43819	1.753572891	0.079504	0.260517	NOT
LINC01036	2.204357	2.57392464	1.467843	1.753541704	0.079509	0.260517	NOT
ITFG3	3101.986	-0.3988766	0.227488	-1.75339749	0.079534	0.260538	NOT
AF131216.	5.278352	-0.6070305	0.346203	-1.75339296	0.079535	0.260538	NOT
HAGH	7696.306	-0.5064801	0.288868	-1.75332631	0.079546	0.260544	NOT
C11orf86	5.370678	0.90567916	0.516591	1.753185427	0.07957	0.260591	NOT
CCL19	420.8516	1.20368719	0.686721	1.75280267	0.079636	0.260775	NOT
RPL22	7651.844	0.25109327	0.143261	1.752692826	0.079655	0.260805	NOT
AC112497.	57.31369	0.35409518	0.202042	1.752580037	0.079674	0.260837	NOT
KRT34	1.786776	2.02564766	1.155931	1.752395296	0.079706	0.260878	NOT
SPSB2	550.9375	0.41827203	0.23869	1.752363975	0.079711	0.260878	NOT
ARX	2.352556	-1.7659128	1.007764	-1.75230777	0.079721	0.260878	NOT
CTC-278L1	1.028375	1.95151233	1.113686	1.752301017	0.079722	NA	NA
RP11-571M	36.99398	-0.4407606	0.251546	-1.75220984	0.079738	0.260878	NOT
LINC01140	16.77919	0.71939383	0.410569	1.752186323	0.079742	0.260878	NOT
NCRNA0025	4.767549	0.99988508	0.570659	1.752159359	0.079746	0.260878	NOT
RP11-266I	3.508154	-0.7804472	0.445431	-1.75211532	0.079754	0.260878	NOT
ZNF292	890.4635	0.40659735	0.232113	1.751721957	0.079822	0.261068	NOT
RP11-475J	29.18727	0.78054833	0.445614	1.751626068	0.079838	0.261072	NOT
SNHG21	36.70679	0.44454525	0.253796	1.751586267	0.079845	0.261072	NOT
POU5F1	98.32016	0.63344109	0.361647	1.751546809	0.079852	0.261072	NOT
ADAM32	9.827305	0.8553046	0.48835	1.751418356	0.079874	0.261113	NOT
NKD2	162.8275	0.86657652	0.494814	1.75131777	0.079891	0.261138	NOT
RP11-752O	14.97845	-0.555669	0.317408	-1.75064869	0.080006	0.261451	NOT
ACTBP11	13.72642	0.58885131	0.336381	1.75054709	0.080024	0.261451	NOT
AC009234.	4.076292	1.56367347	0.893275	1.750494318	0.080033	0.261451	NOT
TNFRSF25	368.8918	0.60256395	0.344227	1.75048499	0.080035	0.261451	NOT
AC004932.	3.491578	0.70637947	0.403534	1.750483175	0.080035	0.261451	NOT
RP11-867O	279.4818	-0.5036288	0.287737	-1.7503077	0.080065	0.261518	NOT
PABPC1P3	11.14014	0.47177831	0.269561	1.750170397	0.080089	0.261564	NOT
CH17-262A	6.994768	0.6766511	0.386675	1.749923227	0.080132	0.261672	NOT
RP11-126O	1.59875	-0.8680919	0.496148	-1.74966483	0.080176	0.261755	NOT
TMEM230	6098.211	0.23755245	0.13577	1.749663167	0.080176	0.261755	NOT
DCAKD	1500.58	-0.3204212	0.183151	-1.74949322	0.080206	0.26182	NOT
SPG20	454.0817	-0.682398	0.390091	-1.74932845	0.080234	0.261881	NOT

RP11-756H	16.38342	-0.8545811	0.488661	-1.74882058	0.080322	0.262044	NOT
FAM225B	3.755039	0.95885179	0.548295	1.748788568	0.080328	0.262044	NOT
B3GALT5-A	2.378259	1.83457092	1.049065	1.74876737	0.080331	0.262044	NOT
ZNF204P	76.69665	0.89449171	0.511501	1.748759294	0.080333	0.262044	NOT
KB-1836B5	3.586821	0.77669139	0.444144	1.748738785	0.080336	0.262044	NOT
ASAP1-IT2	4.147944	0.90517562	0.517639	1.748660882	0.08035	0.262044	NOT
CDK9	2515.998	-0.2314377	0.132352	-1.74864835	0.080352	0.262044	NOT
CALR4P	2.827847	-0.9710273	0.555429	-1.74824706	0.080421	0.262239	NOT
DYNC2LI1	479.1301	0.27910091	0.159663	1.748061738	0.080453	0.262312	NOT
MYH16	5.525723	0.62323286	0.356588	1.747767544	0.080504	0.262447	NOT
RP13-1016	4.244328	-0.9667848	0.5532	-1.74762115	0.08053	0.262467	NOT
FAM87A	6.315599	0.94407118	0.540204	1.74761946	0.08053	0.262467	NOT
CTD-2033A	13.47789	0.51472452	0.294545	1.747523814	0.080547	0.26249	NOT
ADK	2263.08	-0.38227	0.21879	-1.74719769	0.080603	0.262642	NOT
RP11-76E1	2.457823	-0.9280483	0.531208	-1.74705296	0.080628	0.262693	NOT
LINC01067	5.656606	1.23429036	0.706675	1.746617525	0.080704	0.262907	NOT
ARHGAP12	2014.143	0.36104624	0.206742	1.74635813	0.080749	0.262975	NOT
PGM1	7816.053	-0.4213763	0.24129	-1.74635054	0.08075	0.262975	NOT
LIX1	6.640143	1.98660841	1.1376	1.746315445	0.080756	0.262975	NOT
ZNF26	457.8858	0.29781177	0.170541	1.746273875	0.080763	0.262975	NOT
COL4A1	12868.74	-0.4398929	0.251927	-1.74611592	0.080791	0.263033	NOT
CLDN20	3.00117	-0.7678555	0.439771	-1.74603674	0.080805	0.263046	NOT
AC005754.	4.444047	-0.9475535	0.542748	-1.74584562	0.080838	0.263098	NOT
SOS1-IT1	41.1655	-0.3890499	0.222845	-1.7458339	0.08084	0.263098	NOT
RP11-20G1	7.489312	-0.9925539	0.56858	-1.74567291	0.080868	0.263157	NOT
DRAM1	818.7634	0.36795259	0.210812	1.745405436	0.080914	0.263277	NOT
PCMT1	2106.535	0.2654054	0.152086	1.745102313	0.080967	0.263417	NOT
RUFY1	2292.803	0.26180263	0.150034	1.744956485	0.080992	0.263425	NOT
NUS1P1	35.90683	-0.5373397	0.30794	-1.74494943	0.080994	0.263425	NOT
PACS1	2433.083	0.34796491	0.199416	1.74492041	0.080999	0.263425	NOT
AUH	785.6578	-0.3267825	0.187303	-1.74466907	0.081042	0.263536	NOT
RP13-210D	2.48059	-0.8671841	0.497159	-1.74427845	0.081111	0.263706	NOT
TRA2B	3586.635	0.16618676	0.095277	1.744240431	0.081117	0.263706	NOT
GYG1	1001.395	0.26958096	0.154558	1.74420255	0.081124	0.263706	NOT
LDOC1	388.2438	0.96097561	0.550978	1.744127625	0.081137	0.263717	NOT
H2AFJ	3677.603	-0.4980221	0.285554	-1.74405322	0.08115	0.263727	NOT
CPZ	7.8569	-1.3362805	0.76623	-1.74396874	0.081165	0.263743	NOT
CTA-445C9	109.8655	0.51704164	0.296515	1.743726306	0.081207	0.263849	NOT
RP11-311H	1.822927	-0.9969132	0.571772	-1.7435506	0.081237	0.263917	NOT
CTC-444N2	193.9454	-0.3285971	0.188482	-1.74338767	0.081266	0.263978	NOT
WDR25	429.0305	-0.314826	0.18059	-1.74331993	0.081278	0.263985	NOT
TIMP3	182.0009	-0.6470989	0.371202	-1.74325204	0.08129	0.263992	NOT
ZNF275	1368.715	-0.3537374	0.202932	-1.74312934	0.081311	0.26403	NOT
SLC28A1	2142.361	-0.9179909	0.526668	-1.74301482	0.081331	0.264063	NOT
MTND5P11	22.93293	-0.5066564	0.290723	-1.7427479	0.081378	0.264171	NOT
NPFFR2	68.0435	1.69050733	0.970067	1.742671361	0.081391	0.264171	NOT
HSD17B4	15509.68	-0.4266368	0.24482	-1.74265762	0.081393	0.264171	NOT
RP11-431K	8.795394	-0.5747253	0.329811	-1.74259074	0.081405	0.264177	NOT
RP11-302F	11.66871	0.63671817	0.365405	1.742498925	0.081421	0.264198	NOT

KLHDC3	6292.221	0.28260149	0.162194	1.74237166	0.081443	0.264238	NOT
SLC27A3	3127.586	-0.4835108	0.277521	-1.74225044	0.081465	0.264275	NOT
DNAAF1	33.62213	-0.9237338	0.530396	-1.7415937	0.08158	0.264617	NOT
STAT5B	2851.146	-0.2425585	0.139282	-1.74148726	0.081598	0.264645	NOT
AC018647.	13.93827	-0.552569	0.317354	-1.74117523	0.081653	0.264791	NOT
HTRA4	14.80997	-1.0169188	0.584094	-1.74102039	0.08168	0.264847	NOT
TSEN2	335.3398	0.27554531	0.158279	1.740883834	0.081704	0.264893	NOT
DUSP14	739.8146	-0.2951403	0.169547	-1.74075783	0.081726	0.264933	NOT
PKNOX2	40.46923	-0.7277963	0.418175	-1.74041221	0.081787	0.265098	NOT
TEX11	85.05445	-1.2698751	0.7297	-1.74026963	0.081812	0.265126	NOT
CECR1	1836.69	-0.595953	0.342452	-1.74025165	0.081815	0.265126	NOT
TOR1B	1623.431	-0.3230988	0.185677	-1.74011542	0.081839	0.265165	NOT
ZC3HAV1	1816.792	0.23250178	0.133616	1.740071432	0.081846	0.265165	NOT
SURF6	1586.355	0.25622568	0.147263	1.739916327	0.081874	0.265222	NOT
ANKS6	689.9404	0.68383207	0.39307	1.739718836	0.081908	0.265299	NOT
RP11-131K	5.948078	-1.529154	0.87903	-1.73959339	0.08193	0.265299	NOT
RP4-569M2	4.068954	-0.6769979	0.389177	-1.73956301	0.081936	0.265299	NOT
AC104532.	2.056344	-0.7905406	0.454449	-1.73955812	0.081937	0.265299	NOT
APOL3	2866.637	-0.6692824	0.384755	-1.73950179	0.081947	0.265299	NOT
RP11-380(	3.508763	-0.5688246	0.32703	-1.73936328	0.081971	0.265347	NOT
ZDHHC15	20.93635	-0.9217861	0.530033	-1.73910973	0.082015	0.265459	NOT
RP11-196(	16.82981	-0.4461789	0.25661	-1.73874183	0.08208	0.265637	NOT
HEATR5A	750.3539	-0.2867983	0.164968	-1.73850999	0.082121	0.265737	NOT
SCARA3	1203.097	0.68123097	0.391905	1.738254678	0.082166	0.265843	NOT
THAP2	203.1422	0.26042904	0.149826	1.738213826	0.082173	0.265843	NOT
RP11-156I	14.95078	-0.5596	0.321969	-1.7380577	0.082201	0.265885	NOT
RCAN2	376.7333	-0.6405448	0.368547	-1.73802852	0.082206	0.265885	NOT
LINC00601	1.62383	1.99520675	1.14818	1.737712813	0.082261	0.266033	NOT
GGNBP1	1.662764	0.94582194	0.544387	1.737408686	0.082315	0.266175	NOT
DUSP7	668.5902	0.26330066	0.151561	1.737263296	0.082341	0.266226	NOT
ENTPD8	517.5774	-0.7259379	0.417881	-1.73718858	0.082354	0.266234	NOT
RNF187	9585.408	0.38772008	0.223195	1.737137713	0.082363	0.266234	NOT
ST13P5	15.22008	0.55750719	0.320949	1.737061057	0.082376	0.266246	NOT
PRPF18	424.4163	0.21080872	0.121365	1.736976674	0.082391	0.266261	NOT
NCF1	70.61858	0.66428053	0.382446	1.736924407	0.082401	0.266261	NOT
RP11-817I	8.285334	0.63681735	0.366658	1.736815346	0.08242	0.266261	NOT
CHURC1	1351.671	-0.287565	0.165571	-1.73681253	0.08242	0.266261	NOT
AMER1	468.0627	-0.4876905	0.280828	-1.73661544	0.082455	0.266268	NOT
RP3-370M2	5.349161	0.63592116	0.366187	1.736602932	0.082457	0.266268	NOT
KHDRBS1	4968.257	0.17648894	0.101631	1.73655962	0.082465	0.266268	NOT
UROS	2267.741	-0.4092558	0.235682	-1.73647642	0.08248	0.266268	NOT
TBC1D3B	3.035997	0.77629553	0.447057	1.736458268	0.082483	0.266268	NOT
CATSPER2I	96.34678	-0.3660031	0.210785	-1.73638218	0.082496	0.266268	NOT
MPPED1	559.0765	-1.0326982	0.594747	-1.73636432	0.082499	0.266268	NOT
RP11-151N	3.114536	-0.641561	0.3695	-1.73629478	0.082512	0.266268	NOT
RP11-700A	3.112378	0.75354707	0.434011	1.736240559	0.082521	0.266268	NOT
KHSRP	7082.535	0.2446728	0.140922	1.736230019	0.082523	0.266268	NOT
RP11-112I	19.65378	0.62047066	0.357375	1.736189051	0.08253	0.266268	NOT
NUGGC	872.6497	-0.9082743	0.523217	-1.73594354	0.082574	0.266377	NOT

ZNF45	367.1633	-0.367732	0.211848	-1.73583275	0.082593	0.266408	NOT
PGBD5	795.7316	-0.7412916	0.427092	-1.7356709	0.082622	0.266444	NOT
PHEX	48.32051	0.8729187	0.502932	1.735659586	0.082624	0.266444	NOT
ZBTB3	190.3116	-0.2526002	0.145577	-1.73516352	0.082712	0.266695	NOT
MAPK15	22.28117	0.87499416	0.504306	1.735046776	0.082733	0.266728	NOT
C11orf24	2740.441	-0.2707617	0.156059	-1.73499417	0.082742	0.266728	NOT
PRDM1	270.716	-0.5045533	0.29082	-1.73493086	0.082753	0.266733	NOT
RP3-475N1	56.53498	-0.4884259	0.281542	-1.73482637	0.082772	0.266761	NOT
RP11-723(	8.147898	0.58929837	0.339707	1.734724663	0.08279	0.266787	NOT
CTD-2002I	20.31844	-0.5116093	0.294942	-1.73461243	0.082809	0.266794	NOT
PPP1R11	3557.811	0.25719916	0.148276	1.734601128	0.082811	0.266794	NOT
C5orf28	466.1846	0.26393639	0.152178	1.734390235	0.082849	0.266883	NOT
FHOD1	523.8463	-0.4593718	0.264889	-1.73420711	0.082881	0.266956	NOT
C16orf52	589.659	-0.3286506	0.189537	-1.7339672	0.082924	0.267061	NOT
ATM	1296.737	-0.2837038	0.163641	-1.73370024	0.082971	0.267182	NOT
DUSP15	76.37973	0.99897926	0.576346	1.733299289	0.083042	0.26735	NOT
MZF1	632.6996	-0.2980902	0.171979	-1.73329563	0.083043	0.26735	NOT
RP11-159I	3.172963	-0.5807031	0.335094	-1.73295813	0.083103	0.267512	NOT
AC021224.	3.085728	0.75546814	0.436055	1.732507614	0.083183	0.267728	NOT
RP4-794H1	35.50141	0.57074591	0.329441	1.732468647	0.08319	0.267728	NOT
CTC-463A1	54.03891	-0.4430096	0.255742	-1.73225522	0.083228	0.267819	NOT
SRR	202.616	-0.3454233	0.199418	-1.73215339	0.083246	0.267845	NOT
LINC0047	11.8596	-0.7625032	0.440266	-1.73191451	0.083289	0.267928	NOT
GLB1L3	19.62719	1.40120595	0.809058	1.73189724	0.083292	0.267928	NOT
TRAV1-2	5.082917	-0.9285542	0.536171	-1.73182441	0.083305	0.267938	NOT
CTD-2503(	5.568336	0.92967757	0.536855	1.73170927	0.083325	0.267973	NOT
SNRNP200	9408.513	0.24946372	0.144073	1.731509594	0.083361	0.268055	NOT
ADH6	10122.43	-0.7365316	0.425397	-1.73139994	0.08338	0.268082	NOT
CCDC150	170.297	0.51688819	0.298547	1.731345181	0.08339	0.268082	NOT
PLA2G2A	24904.74	-1.3562585	0.783378	-1.73129608	0.083399	0.268082	NOT
RP11-428I	66.46358	-0.6299293	0.363867	-1.73120765	0.083415	0.268101	NOT
RP11-102(	3.04327	0.71582314	0.413499	1.73113436	0.083428	0.268105	NOT
SCARB2	12426.2	-0.2704812	0.156249	-1.73109041	0.083436	0.268105	NOT
AL031587.	8.650715	0.55835432	0.322592	1.730839856	0.08348	0.268217	NOT
HDHD3	3525.207	-0.3510065	0.202812	-1.73070027	0.083505	0.268265	NOT
ACSBG2	5.177107	0.90043375	0.520313	1.730562319	0.08353	0.268298	NOT
CTC-428G	3.674563	-0.5606155	0.323956	-1.7305322	0.083535	0.268298	NOT
NUP50-AS1	488.1643	0.45115905	0.260732	1.730355381	0.083567	0.268339	NOT
STRAP	3975.579	0.23891264	0.138072	1.730349828	0.083568	0.268339	NOT
NT5DC4	6.341345	0.8586726	0.496266	1.730266855	0.083583	0.268355	NOT
RP11-85A1	7.00701	-0.6356262	0.367401	-1.73006228	0.083619	0.26844	NOT
LDLRAP1	1786.213	-0.3076934	0.177858	-1.72999297	0.083632	0.268448	NOT
ALK	4.172527	-0.7986	0.461695	-1.72971357	0.083681	0.268577	NOT
Metazoa_S	29.18177	-0.4557642	0.263527	-1.72948075	0.083723	0.268649	NOT
RP13-465I	3.729297	-0.4717034	0.272743	-1.72947742	0.083724	0.268649	NOT
GSTA2	8236.049	-1.0952389	0.633351	-1.72927635	0.08376	0.268732	NOT
CYFIP2	2739.658	-0.6685255	0.38663	-1.7291087	0.08379	0.268797	NOT
PACSIN1	255.5869	0.98360956	0.56889	1.728997812	0.083809	0.268822	NOT
RP11-196(	3.889666	-0.5751286	0.332654	-1.72890707	0.083826	0.268822	NOT

POU6F2-AS	1.546174	2.4007985	1.388636	1.728889543	0.083829	0.268822	NOT
RP11-259N	35.73206	0.97902323	0.566288	1.728843724	0.083837	0.268822	NOT
RP11-1267	7.12367	-1.0546289	0.610155	-1.72846023	0.083906	0.26901	NOT
AD000671.	2.137741	-0.7355421	0.425581	-1.72832394	0.08393	0.269044	NOT
FEZ1	227.6542	-0.5462995	0.316092	-1.72829057	0.083936	0.269044	NOT
AL163953.	12.5815	0.67497734	0.390574	1.728166946	0.083958	0.269083	NOT
RP11-143I	3.72947	0.65766644	0.380583	1.728051357	0.083979	0.269118	NOT
GJB5	11.12995	-0.8679213	0.502274	-1.72798305	0.083991	0.269125	NOT
CTC-498J1	36.98295	0.88695379	0.513372	1.727700411	0.084042	0.269256	NOT
SPCS2P4	44.13733	0.42117342	0.243788	1.727624497	0.084056	0.269258	NOT
SOSTDC1	21.12657	1.09304585	0.632701	1.727586535	0.084062	0.269258	NOT
TDRD15	14.78509	-1.0812992	0.625961	-1.72742172	0.084092	0.269321	NOT
CNTNAP2	678.1157	0.97369681	0.563719	1.727273138	0.084119	0.269349	NOT
SRSF10	2695.229	0.1539542	0.089132	1.727261875	0.084121	0.269349	NOT
RP11-146F	4.023019	0.80208075	0.464391	1.727166106	0.084138	0.269371	NOT
EEF2	78527.44	-0.2531674	0.146584	-1.72711221	0.084148	0.269371	NOT
EXT1	6198.647	0.40215768	0.232888	1.726825012	0.084199	0.269505	NOT
STAB1	3306.384	-0.4956893	0.287064	-1.72675382	0.084212	0.269514	NOT
LEF1	359.9716	0.74882245	0.43372	1.726510855	0.084256	0.269598	NOT
MAF	2294.498	-0.3571863	0.206885	-1.7264971	0.084258	0.269598	NOT
SF3A3	3048.008	0.22848583	0.132358	1.72626621	0.0843	0.269642	NOT
LGALS9	1595.206	0.55620285	0.322201	1.726262891	0.0843	0.269642	NOT
RP11-111M	10.62505	0.62972161	0.364791	1.726253735	0.084302	0.269642	NOT
RBM43	380.3474	-0.3134028	0.181608	-1.72571193	0.084399	0.269922	NOT
AOX2P	7.850284	-0.9764835	0.565873	-1.72562303	0.084415	0.269942	NOT
TMEM150B	627.7199	0.64835322	0.375759	1.725448917	0.084447	0.269996	NOT
MYCBP	582.0941	0.30998513	0.179658	1.725418702	0.084452	0.269996	NOT
RNF115	2340.551	0.24892954	0.144277	1.725357984	0.084463	0.269999	NOT
CTD-2033I	6.250491	-0.6965486	0.403755	-1.72517807	0.084495	0.270054	NOT
ZNF24	2793.726	-0.2105421	0.122043	-1.72515151	0.0845	0.270054	NOT
PDXP	175.8596	-0.6007125	0.348232	-1.72503611	0.084521	0.270073	NOT
RP11-59H7	5.005076	0.79917355	0.463287	1.725008112	0.084526	0.270073	NOT
RP11-303E	5.292226	-0.6098271	0.353553	-1.72485548	0.084554	0.270078	NOT
EEF1A1P5	770.4687	0.64314612	0.372884	1.724788487	0.084566	0.270078	NOT
RP11-159J	110.2471	0.80294744	0.465536	1.724781683	0.084567	0.270078	NOT
SLC25A46	1987.16	-0.2724084	0.157938	-1.72477893	0.084567	0.270078	NOT
RP4-657E1	22.86882	-0.4653641	0.269836	-1.72461532	0.084597	0.270131	NOT
PHF13	905.9932	0.25721121	0.149145	1.72457635	0.084604	0.270131	NOT
RP11-832A	4.868378	0.86463836	0.501392	1.724477438	0.084622	0.270149	NOT
CSGALNACT	341.6458	-0.4103509	0.237963	-1.72443368	0.08463	0.270149	NOT
TSPAN19	0.953636	2.42296949	1.405221	1.724262141	0.084661	NA	NA
EPHA5-AS1	1.889776	2.66450116	1.545315	1.724245086	0.084664	0.270226	NOT
NDST2	74.89657	-0.2547502	0.147785	-1.72378982	0.084746	0.27043	NOT
AC012501.	1.928516	1.99612844	1.157994	1.723781496	0.084747	0.27043	NOT
PRPF8	8148.385	-0.2586647	0.150079	-1.72352256	0.084794	0.270547	NOT
CTA-27603	2.717256	-0.5631784	0.326813	-1.72324339	0.084845	0.270676	NOT
CTD-2278I	3.64262	-0.5701946	0.330957	-1.7228659	0.084913	0.270837	NOT
CTD-3203F	10.67664	0.55292803	0.320939	1.72284353	0.084917	0.270837	NOT
C7orf73	2715.979	0.22955651	0.133246	1.722798525	0.084925	0.270837	NOT

CARM1P1	1.520071	2.35555871	1.367363	1.722701926	0.084942	0.270861	NOT
ELMOD2	1220.434	-0.2138069	0.124126	-1.722506	0.084978	0.270925	NOT
FBXO30	554.5998	0.32573413	0.189112	1.722440621	0.08499	0.270925	NOT
BCO1	65.38393	-1.0734871	0.623254	-1.7223922	0.084998	0.270925	NOT
LRRC59	7464.879	0.22802593	0.132391	1.722370594	0.085002	0.270925	NOT
RP11-490I	11.17912	0.77165563	0.448109	1.72202747	0.085065	0.271091	NOT
NRN1	189.8483	-0.6614735	0.384187	-1.72174725	0.085115	0.271221	NOT
LETM1	2732.127	-0.2731361	0.158652	-1.72160415	0.085141	0.271272	NOT
SIGLEC6	3.67991	-1.2127672	0.704495	-1.72147028	0.085166	0.271318	NOT
UBE2D2	3088.389	0.20330416	0.11812	1.721159977	0.085222	0.271465	NOT
RP11-983F	452.6452	-0.4096049	0.238003	-1.72100474	0.08525	0.27148	NOT
TRMT61B	421.3264	0.29957207	0.17407	1.720988915	0.085253	0.27148	NOT
LRPAP1	7602.137	-0.3244352	0.188522	-1.7209439	0.085261	0.27148	NOT
PAFAH1B1	3110.833	-0.2307192	0.134069	-1.72090262	0.085268	0.27148	NOT
WASH3P	239.877	0.32323026	0.187831	1.720857707	0.085277	0.27148	NOT
MFSD6	641.151	0.52631507	0.305865	1.720744733	0.085297	0.271514	NOT
FCGR3B	34.37151	0.82134824	0.47735	1.720639951	0.085316	0.271542	NOT
CD44	3034.64	0.74831184	0.434927	1.720546939	0.085333	0.271545	NOT
ZHX1	3186.224	0.34044971	0.197884	1.720450421	0.085351	0.271545	NOT
WDR17	6.496313	-0.8110784	0.471446	-1.72040675	0.085359	0.271545	NOT
TRMT112P6	5.785788	0.64648306	0.375779	1.720381535	0.085363	0.271545	NOT
MIR4645	1.456088	1.25200886	0.72776	1.720360024	0.085367	0.271545	NOT
CKMT2	166.2602	-0.9885418	0.574649	-1.7202522	0.085387	0.271575	NOT
FAM120C	468.9547	-0.3501822	0.203578	-1.72013786	0.085407	0.27161	NOT
TAOK2	2721.779	-0.2192986	0.127505	-1.71992496	0.085446	0.271701	NOT
RAD54L2	1264.067	-0.3242308	0.18853	-1.71977982	0.085472	0.271743	NOT
PSKH1	1560.225	-0.3108287	0.180754	-1.71962669	0.0855	0.271743	NOT
A1BG-AS1	82.11441	-0.5529253	0.321541	-1.71961267	0.085503	0.271743	NOT
TMX1	1626.875	0.28583906	0.166223	1.719607304	0.085504	0.271743	NOT
KCTD14	243.5605	0.64262317	0.37371	1.719577077	0.085509	0.271743	NOT
RP5-997D1	60.61218	-0.3713239	0.215947	-1.71951286	0.085521	0.271748	NOT
ZNF821	93.30648	0.30655429	0.178311	1.719211542	0.085576	0.271891	NOT
SPERT	7.332949	1.61392746	0.938798	1.719141771	0.085589	0.271899	NOT
SLC25A16	1427.218	-0.3610371	0.21004	-1.7188962	0.085633	0.272009	NOT
LIMCH1	872.0841	0.7082395	0.412118	1.718533786	0.085699	0.272161	NOT
SMAD5-AS1	11.19296	-0.6113681	0.355752	-1.71852348	0.085701	0.272161	NOT
ANXA2R	138.5041	0.60274506	0.350763	1.718383385	0.085727	0.27221	NOT
DYRK1A	1969.813	-0.234292	0.136354	-1.71826178	0.085749	0.272244	NOT
C16orf62	1115.222	-0.4702424	0.273681	-1.71821471	0.085757	0.272244	NOT
CLDN15	2485.524	-0.6904377	0.401853	-1.71813367	0.085772	0.272259	NOT
ZNF562	817.7054	-0.2656308	0.154621	-1.71794566	0.085807	0.272332	NOT
FAM110D	53.22663	-0.5070621	0.295188	-1.71775851	0.085841	0.272332	NOT
FOLR1	79.364	1.15894872	0.674687	1.717757492	0.085841	0.272332	NOT
ST6GAL2	40.16329	-0.9003433	0.52418	-1.71762177	0.085866	0.272332	NOT
ARL11	52.83798	0.53365981	0.310701	1.717599444	0.08587	0.272332	NOT
NCMAP	263.0745	0.81209929	0.472812	1.717596166	0.08587	0.272332	NOT
HORMAD2	81.94704	-1.2235343	0.712361	-1.71757611	0.085874	0.272332	NOT
LINC01430	9.242635	-1.0087511	0.587314	-1.71756728	0.085876	0.272332	NOT
AKNA	1056.013	-0.432514	0.251829	-1.71749325	0.085889	0.272343	NOT

AHCYL2	693.4709	-0.3239751	0.188641	-1.71741267	0.085904	0.272358	NOT
C16orf70	1690.686	-0.4249842	0.247498	-1.71712297	0.085957	0.272483	NOT
PSMA8	2.086639	2.19241183	1.276821	1.717086815	0.085963	0.272483	NOT
RP11-390F	10.25705	0.67435477	0.392751	1.7170047	0.085978	0.272496	NOT
TMEM115	2802.594	0.21926996	0.127709	1.716955055	0.085987	0.272496	NOT
ZFP82	97.08167	0.59172641	0.344665	1.716815919	0.086013	0.272545	NOT
NBPF25P	46.46211	-0.3142282	0.183051	-1.71661759	0.086049	0.272575	NOT
AC013444.	1.667829	1.42592298	0.830667	1.71660008	0.086052	0.272575	NOT
TMEM126B	1096.415	0.23704673	0.138091	1.716599352	0.086052	0.272575	NOT
PCDHB18P	8.363832	-0.6295393	0.36675	-1.71653437	0.086064	0.272578	NOT
HPS3	1084.6	0.29188459	0.170048	1.716483999	0.086073	0.272578	NOT
LA16c-60H	10.50022	0.79507878	0.463255	1.716288099	0.086109	0.272651	NOT
LRRC58	1991.05	0.29754686	0.173371	1.716247036	0.086117	0.272651	NOT
DDX19B	597.3166	-0.2773498	0.161633	-1.71591889	0.086177	0.272774	NOT
LINC0118	9.696081	0.83460335	0.486391	1.715911301	0.086178	0.272774	NOT
AC004381.	293.9215	0.49675897	0.289508	1.715870131	0.086186	0.272774	NOT
RP11-492E	53.52757	1.1144875	0.649574	1.715721262	0.086213	0.272829	NOT
ADNP	3507.981	0.25145731	0.146575	1.715555	0.086244	0.272893	NOT
MUSK	6.035867	1.74459696	1.017023	1.715396548	0.086273	0.272929	NOT
RP5-875H1	23.97788	-0.7447816	0.434178	-1.71538365	0.086275	0.272929	NOT
RP11-1105	14.78379	0.49211107	0.286893	1.715312153	0.086288	0.272939	NOT
JPH3	4.768672	0.8907936	0.519352	1.715203346	0.086308	0.27297	NOT
ZNF394	711.5632	-0.1762494	0.102761	-1.71514164	0.086319	0.272974	NOT
POC1A	583.5421	0.36453063	0.212564	1.714922653	0.086359	0.273069	NOT
COL28A1	40.58161	1.23567399	0.720577	1.71483983	0.086375	0.273083	NOT
IFI35	2434.307	0.41669296	0.243002	1.714770804	0.086387	0.273083	NOT
SIM2	68.74371	-0.5895071	0.343789	-1.71473281	0.086394	0.273083	NOT
TPPP	594.689	-0.637051	0.371534	-1.71464952	0.08641	0.2731	NOT
MOGAT3	1377.089	0.73768483	0.430272	1.714460877	0.086444	0.273147	NOT
COMMD8	522.2686	0.3706329	0.216181	1.714458531	0.086445	0.273147	NOT
NOV	184.2978	-0.6608481	0.385468	-1.7144044	0.086454	0.273147	NOT
pk	2439.63	-0.3912366	0.228217	-1.71431978	0.08647	0.273164	NOT
MANEAL	977.0084	0.51056512	0.297857	1.714129517	0.086505	0.273237	NOT
MGAT4C	17.13711	-0.9216484	0.537695	-1.71407306	0.086515	0.273237	NOT
RNF44	1855.728	0.31489891	0.183719	1.714029138	0.086523	0.273237	NOT
HMX3	1.037922	2.19570375	1.281071	1.713959455	0.086536	NA	NA
SP5	538.6657	0.86025959	0.50195	1.713834743	0.086559	0.273318	NOT
PDP2	711.3443	-0.3220688	0.187949	-1.71359937	0.086602	0.273407	NOT
POGLUT1	569.843	-0.2948346	0.172059	-1.71357082	0.086608	0.273407	NOT
MLLT10	1256.31	0.2832753	0.165325	1.713448951	0.08663	0.273437	NOT
PLEKHM1P	295.7221	-0.3231485	0.1886	-1.71341033	0.086637	0.273437	NOT
CTD-2383M	3.371723	-0.5190101	0.302991	-1.7129529	0.086721	0.273671	NOT
RP11-54C4	2.20485	-1.3240568	0.773143	-1.71256339	0.086793	0.273845	NOT
MCOLN2	59.14478	0.88486269	0.516713	1.712483965	0.086808	0.273845	NOT
FBXL2	87.71391	0.66202233	0.386597	1.712434425	0.086817	0.273845	NOT
FLAD1	2939.308	0.31245322	0.182462	1.712429907	0.086817	0.273845	NOT
PIP5KL1	16.91484	0.76094506	0.444379	1.712379358	0.086827	0.273845	NOT
MFAP4	706.5589	-0.9620646	0.561895	-1.71217774	0.086864	0.27393	NOT
CHRNB1	531.017	-0.3849701	0.224854	-1.71208962	0.08688	0.273948	NOT

CTD-3203F	17.08921	-0.604749	0.353234	-1.71203685	0.08689	0.273948	NOT
IGHV7-40	1.253748	2.08427329	1.217582	1.711814186	0.086931	NA	NA
TRIM3	487.1712	-0.2407572	0.140644	-1.71181413	0.086931	0.274043	NOT
DCN	3229.77	-0.8939201	0.522222	-1.71176371	0.08694	0.274043	NOT
TBC1D8B	561.0097	-0.4533537	0.264865	-1.71164285	0.086963	0.274059	NOT
ITGA5	6484.008	0.40678486	0.23766	1.711627134	0.086965	0.274059	NOT
RP5-1007H	1.718432	-0.7848897	0.45859	-1.71152945	0.086983	0.274063	NOT
FAM98B	834.4382	0.24842161	0.145148	1.711510758	0.086987	0.274063	NOT
MLIP-IT1	3.99151	-1.1692368	0.683208	-1.71139092	0.087009	0.27409	NOT
DYM	1508.112	0.20069882	0.117275	1.711354869	0.087016	0.27409	NOT
SEPT1	338.4649	-0.4356332	0.254567	-1.71127136	0.087031	0.274106	NOT
RP11-100I	8.429608	-0.5096513	0.297836	-1.71118116	0.087048	0.274127	NOT
PDCD1LG2	55.61735	-0.6139347	0.35881	-1.71103206	0.087075	0.274182	NOT
ZC3H12C	379.5209	-0.3534174	0.206583	-1.71077485	0.087123	0.274279	NOT
PPP4R1	2126.463	0.19478942	0.113862	1.710755728	0.087126	0.274279	NOT
RP11-284E	7.682141	-0.8202252	0.479521	-1.71050909	0.087172	0.274389	NOT
RPS15AP17	3.871069	0.73929866	0.432234	1.710412557	0.08719	0.274389	NOT
LLNLR-47C	8.364819	-0.9599668	0.561252	-1.71040136	0.087192	0.274389	NOT
RP11-310E	2.364183	1.45064756	0.848238	1.710188335	0.087231	0.274468	NOT
PIK3C3	1010.838	-0.2115242	0.123687	-1.7101573	0.087237	0.274468	NOT
RP11-815J	1.089236	-1.1471482	0.670801	-1.71011741	0.087244	NA	NA
RP11-290I	1.048566	-0.9331292	0.545704	-1.7099534	0.087274	NA	NA
SNORD7	2.965242	0.82928362	0.485087	1.709557505	0.087348	0.274727	NOT
PMS1	469.0269	0.23060578	0.134895	1.709521661	0.087354	0.274727	NOT
SDC1	44842.61	-0.4681498	0.273859	-1.70945709	0.087366	0.274727	NOT
RAP2CP1	6.642742	-1.1669425	0.682652	-1.70942561	0.087372	0.274727	NOT
PTGES3P4	3.041606	-0.8551874	0.50029	-1.70938497	0.08738	0.274727	NOT
RHO	7.345615	-0.9795696	0.573067	-1.70934676	0.087387	0.274727	NOT
FAM217A	3.189437	-0.7911507	0.462846	-1.70931916	0.087392	0.274727	NOT
ROPN1L	7.280151	0.78404115	0.458712	1.70922158	0.08741	0.274727	NOT
YKT6	2591.459	0.26749323	0.1565	1.709219069	0.08741	0.274727	NOT
RP11-307E	3.863767	-0.9315816	0.545075	-1.70908822	0.087435	0.274732	NOT
C16orf93	13.79013	0.49608191	0.290267	1.709052459	0.087441	0.274732	NOT
MPP1	1686.571	-0.4133873	0.241882	-1.70904714	0.087442	0.274732	NOT
PDCL3P5	3.381141	-0.5845162	0.342033	-1.70894733	0.087461	0.274758	NOT
RP11-109J	11.6036	1.3791221	0.807046	1.708852191	0.087478	0.274768	NOT
NCF2	714.3105	0.6204532	0.363089	1.708820321	0.087484	0.274768	NOT
RAB35	1734.309	0.18242565	0.106775	1.708501812	0.087543	0.274891	NOT
DDX5	16161.38	-0.2046456	0.119784	-1.7084576	0.087551	0.274891	NOT
QRICH1	2221.031	0.17020776	0.099627	1.708445039	0.087554	0.274891	NOT
LINC00412	1.330815	0.99113047	0.580283	1.708012276	0.087634	NA	NA
ZNF512	765.94	0.29640747	0.173559	1.707819518	0.08767	0.275224	NOT
ANKRD18A	28.09032	1.1800816	0.691014	1.707754228	0.087682	0.27523	NOT
TMEM232	20.00663	-0.7191446	0.421122	-1.70768689	0.087694	0.275237	NOT
DLG1-AS1	3.878877	0.84959679	0.497558	1.707533742	0.087723	0.275295	NOT
ZYX	7686.949	0.37781432	0.221288	1.707339517	0.087759	0.275376	NOT
MGAT2	192.1562	-0.4906118	0.287375	-1.70721569	0.087782	0.275416	NOT
ADAM17	547.1564	0.411304	0.240932	1.707138358	0.087796	0.27543	NOT
KIAA1456	173.0982	-0.9018003	0.528324	-1.70690744	0.087839	0.27552	NOT

SLC12A9	1865.36	0.28794659	0.168703	1.706830552	0.087854	0.27552	NOT
CNTFR-AS1	3.65666	-1.3556682	0.794266	-1.70681909	0.087856	0.27552	NOT
EBF1	195.597	-0.4383412	0.256853	-1.70658188	0.0879	0.275627	NOT
CHD1	1217.467	-0.2620913	0.153588	-1.70645626	0.087923	0.275668	NOT
ADCY9	2559.318	-0.3493479	0.204737	-1.70632675	0.087947	0.275712	NOT
RBP5	4891.669	-0.6695823	0.392552	-1.70571807	0.088061	0.276035	NOT
RP3-508I1	11.7477	-0.4128488	0.242064	-1.70553793	0.088094	0.276099	NOT
FN3KRP	2064.039	0.29964538	0.175694	1.705499726	0.088101	0.276099	NOT
NAGLU	4117.067	-0.4055072	0.237816	-1.70512996	0.08817	0.276283	NOT
PRPF38B	1554.516	0.24842649	0.145708	1.704965148	0.088201	0.276347	NOT
MICU3	204.099	-0.6187453	0.362934	-1.70484414	0.088223	0.276385	NOT
THRAP3	4703.396	-0.1760266	0.103256	-1.70475832	0.08824	0.276385	NOT
ZNF420	254.2489	-0.3124616	0.183294	-1.70469972	0.08825	0.276385	NOT
CTD-2515A	5.173351	-0.6797063	0.398729	-1.70468174	0.088254	0.276385	NOT
RP11-463J	3.52253	1.41888403	0.832487	1.704391263	0.088308	0.276471	NOT
TIA1	2027.685	0.36533626	0.214352	1.704371557	0.088312	0.276471	NOT
CTD-2366F	194.9227	-0.4846837	0.284377	-1.70437136	0.088312	0.276471	NOT
HMGB1P5	306.8074	0.30829593	0.180902	1.704214923	0.088341	0.27653	NOT
WNT5A	563.4166	0.77804838	0.45658	1.7040774	0.088367	0.276579	NOT
C17orf75	765.8252	0.21979766	0.128998	1.703880418	0.088403	0.276662	NOT
PDIA2	90.33904	1.20478593	0.707181	1.703646928	0.088447	0.276742	NOT
RP11-644F	299.0721	0.30547844	0.17931	1.703635236	0.088449	0.276742	NOT
RAB27B	250.9028	0.97294069	0.571233	1.703228225	0.088525	0.276892	NOT
SERPIND1	43699.96	-0.6566543	0.385542	-1.70319706	0.088531	0.276892	NOT
RP1-309I2	34.63912	-0.7663904	0.449975	-1.70318558	0.088533	0.276892	NOT
USF2	5149.819	-0.2564979	0.150601	-1.70316058	0.088538	0.276892	NOT
N4BP2L2	1863.668	-0.2815955	0.165349	-1.70303703	0.088561	0.276894	NOT
TSSK4	16.93736	-0.4883636	0.28676	-1.70303694	0.088561	0.276894	NOT
LIMD1-AS1	4.996132	-0.5242841	0.30786	-1.70299364	0.088569	0.276894	NOT
RP11-332J	10.44686	1.87744261	1.102547	1.70282367	0.088601	0.276961	NOT
AC006116.	5.524906	-0.5736407	0.33692	-1.70260188	0.088643	0.277059	NOT
RP11-109N	8.691652	0.52532052	0.308551	1.702542935	0.088654	0.277062	NOT
CRNDE	184.5066	0.75228229	0.441905	1.702361028	0.088688	0.277105	NOT
PTGES3P2	51.09071	-0.7117315	0.418085	-1.70236047	0.088688	0.277105	NOT
UNC119	1127.759	0.37696254	0.221446	1.702275406	0.088704	0.277123	NOT
ST3GAL4-A	122.4858	0.49707083	0.292017	1.702196755	0.088718	0.277137	NOT
PRG4	8884.283	-0.8596875	0.505108	-1.70198656	0.088758	0.277228	NOT
NPM1P24	3.761668	0.58426768	0.343349	1.701672787	0.088817	0.27738	NOT
UACA	1738.206	-0.2669726	0.1569	-1.70154736	0.08884	0.277421	NOT
CLCNKA	60.71816	-0.9395025	0.552166	-1.70148699	0.088852	0.277425	NOT
KAT2B	1869.205	-0.4613086	0.271154	-1.70127681	0.088891	0.277516	NOT
RP13-297E	4.058909	-0.9519055	0.559586	-1.70108971	0.088926	0.277552	NOT
AL035610.	1.845767	-1.1495056	0.675748	-1.7010863	0.088927	0.277552	NOT
HNRNPH1P1	4.30751	-0.6737333	0.396081	-1.70099876	0.088943	0.277552	NOT
RP11-488F	7.405161	-0.5256118	0.309002	-1.70099768	0.088943	0.277552	NOT
C1orf64	56.72516	1.55506744	0.914243	1.700934618	0.088955	0.277557	NOT
C14orf132	383.8477	-0.7845779	0.461283	-1.70085853	0.08897	0.277569	NOT
PTPN4	559.7806	-0.2851359	0.167655	-1.70073397	0.088993	0.277587	NOT
UGT1A3	543.8541	-1.0946629	0.643662	-1.70068014	0.089003	0.277587	NOT

BDH2	1138.636	-0.4431325	0.260572	-1.70061405	0.089015	0.277587	NOT
AP000275.	2.792017	-0.6093866	0.358334	-1.70061079	0.089016	0.277587	NOT
PSME3	3973.243	0.19227897	0.113075	1.700458353	0.089045	0.277644	NOT
RAB11FIP2	612.7232	-0.3530602	0.207644	-1.70031887	0.089071	0.277694	NOT
RP11-165F	4.575645	0.96788408	0.569269	1.700222283	0.089089	0.277719	NOT
SBK1	104.9831	0.79081922	0.465156	1.700114439	0.089109	0.27775	NOT
RP11-458F	8.404354	-0.6827692	0.401619	-1.70004146	0.089123	0.277761	NOT
TPM2	2119.944	0.50620526	0.297804	1.699791413	0.08917	0.277876	NOT
PLEK2	538.8756	0.80587983	0.474122	1.699729296	0.089182	0.27788	NOT
RAB43	267.35	-0.4165174	0.245094	-1.69942108	0.08924	0.278029	NOT
SLC37A4	7393.823	-0.4637918	0.272929	-1.69931472	0.08926	0.278032	NOT
RPL15	25158.22	0.27603434	0.162439	1.699307997	0.089261	0.278032	NOT
AC002511.	2.161688	1.73427644	1.020629	1.699223941	0.089277	0.278049	NOT
CAPN9	30.21974	-0.8007429	0.471259	-1.69915701	0.08929	0.278056	NOT
CCDC107	470.0625	-0.3603232	0.21208	-1.69899723	0.08932	0.278118	NOT
ARHGAP24	404.7111	-0.5345854	0.314687	-1.6987827	0.08936	0.278212	NOT
NDFIP1	8863.222	-0.2953728	0.173897	-1.69854606	0.089405	0.27822	NOT
AC156455.	27.15506	0.73638229	0.433538	1.69854311	0.089405	0.27822	NOT
LRP12	369.7316	0.69876952	0.411398	1.69852252	0.089409	0.27822	NOT
VPS41	1896.5	-0.2844071	0.167445	-1.69851278	0.089411	0.27822	NOT
PGGT1B	979.3262	-0.2290633	0.134862	-1.69849724	0.089414	0.27822	NOT
DSCR3	2015.779	-0.2468437	0.145338	-1.69840824	0.089431	0.278225	NOT
MIR3125	1.607046	1.85826147	1.094194	1.698292811	0.089453	0.278225	NOT
BIN1	3205.402	0.40166336	0.236511	1.698288066	0.089453	0.278225	NOT
KCNJ9	2.316364	1.17619243	0.692582	1.698271662	0.089457	0.278225	NOT
ZNF407	516.5651	-0.2509813	0.147813	-1.6979643	0.089514	0.278373	NOT
GLIS2	847.0562	0.65966398	0.388546	1.697775091	0.08955	0.278452	NOT
RP11-569(	3.5355	1.65227834	0.97339	1.69744823	0.089612	0.278612	NOT
RP11-635N	103.0571	-0.514899	0.303348	-1.69738785	0.089623	0.278616	NOT
RASSF5	830.9235	-0.469925	0.276878	-1.69722779	0.089654	0.278678	NOT
CHEK2P2	0.705832	1.82512696	1.075513	1.696982848	0.0897	NA	NA
SLIT2	230.9502	-0.7732828	0.455682	-1.69697988	0.0897	0.278751	NOT
LINC01146	676.5548	-0.8126599	0.478891	-1.69696305	0.089704	0.278751	NOT
PP14571	3.773377	0.96102765	0.56633	1.696940107	0.089708	0.278751	NOT
CNTNAP1	325.6583	0.64772667	0.381866	1.69621498	0.089845	0.279146	NOT
ALOX5AP	177.1699	0.66136988	0.389932	1.696116887	0.089864	0.279171	NOT
IL27RA	416.4351	0.46604144	0.274819	1.69580928	0.089922	0.27932	NOT
RP13-15M1	9.345726	-0.6379208	0.376201	-1.69569081	0.089944	0.279358	NOT
IGSF9	1088.368	-0.992745	0.585501	-1.69554757	0.089972	0.27941	NOT
OR7M1P	1.072034	-1.3127568	0.774404	-1.69518393	0.090041	NA	NA
CYSTM1	3864.147	0.37478505	0.221093	1.695147041	0.090048	0.279586	NOT
UGDH	10054.87	0.46504924	0.274356	1.695059534	0.090064	0.279586	NOT
2-Mar	2089.625	-0.3041807	0.179456	-1.6950116	0.090073	0.279586	NOT
SSR1	9865.425	0.23312042	0.137536	1.694978069	0.09008	0.279586	NOT
RP11-486F	2.980578	1.19023031	0.70221	1.694977653	0.09008	0.279586	NOT
ZNF257	52.53327	0.88441872	0.521816	1.694885784	0.090097	0.279608	NOT
CEP78	714.5766	0.33949777	0.200347	1.694546821	0.090161	0.279776	NOT
AC015849.	15.46542	-0.5284879	0.311902	-1.69440112	0.090189	0.27983	NOT
RP11-397F	1.581936	-0.67402	0.397808	-1.69433509	0.090202	0.279836	NOT

MAK16	505.5896	0.3461486	0.204325	1.694105386	0.090245	0.279898	NOT
RP11-574F	11.02501	-0.6269435	0.370078	-1.69408291	0.09025	0.279898	NOT
LACTB2	1855.901	0.45847827	0.270646	1.694014043	0.090263	0.279898	NOT
GIMAP1	263.3228	-0.440286	0.259907	-1.69401402	0.090263	0.279898	NOT
TRIM32	476.9246	0.30497746	0.180048	1.693871848	0.09029	0.279949	NOT
SLC2A2	17737.12	-0.8272459	0.488571	-1.69319369	0.090419	0.280317	NOT
HMGN3-AS1	59.04191	0.41440519	0.244769	1.693046211	0.090447	0.280372	NOT
NOMO3	149.4485	-0.6954736	0.410866	-1.69270045	0.090512	0.280544	NOT
TTC14	750.4543	-0.3297645	0.194847	-1.6924287	0.090564	0.280641	NOT
GNPTAB	1093.774	-0.2647932	0.156458	-1.69242857	0.090564	0.280641	NOT
TRPC6	52.89227	-0.4510133	0.2665	-1.69235459	0.090578	0.280652	NOT
DUSP8	651.9469	-0.4863954	0.287429	-1.69222872	0.090602	0.280683	NOT
FAM20C	4798.649	-0.3795657	0.224304	-1.69219487	0.090609	0.280683	NOT
TRO	517.893	-0.6716529	0.396948	-1.6920411	0.090638	0.280741	NOT
DRAP1	4095.185	0.32413125	0.191573	1.691950332	0.090655	0.280744	NOT
MPL	7.450005	-0.4926158	0.291165	-1.69187806	0.090669	0.280744	NOT
MBOAT2	207.6153	0.79494035	0.469858	1.691873574	0.09067	0.280744	NOT
ATP6V0E2-	62.92359	-0.5898425	0.348671	-1.69168932	0.090705	0.280821	NOT
EXPH5	290.7665	-0.9048323	0.534896	-1.6916033	0.090722	0.280838	NOT
SIX1	51.78757	0.84025055	0.496779	1.691397282	0.090761	0.280838	NOT
PNMA2	96.59964	0.88366544	0.522456	1.691368571	0.090766	0.280838	NOT
SPON2	10374.17	0.52183853	0.308535	1.691344025	0.090771	0.280838	NOT
TRIM6	141.0551	0.65648692	0.388147	1.691336659	0.090773	0.280838	NOT
KIF2A	493.3842	0.30788238	0.182037	1.69131751	0.090776	0.280838	NOT
TNNT2	42.95456	1.0132222	0.599098	1.69124609	0.09079	0.280838	NOT
CREB3L1	407.5971	0.89215865	0.527522	1.691226213	0.090794	0.280838	NOT
DBI	9831.882	0.34427107	0.20357	1.69116681	0.090805	0.280841	NOT
UPF3A	1068.227	0.35718501	0.211217	1.691078253	0.090822	0.280844	NOT
RP13-131K	1.497363	0.92431194	0.546589	1.691054027	0.090826	0.280844	NOT
ITGBL1	204.8814	-0.9035029	0.534355	-1.69082928	0.090869	0.280942	NOT
DDX11	703.1935	0.37411421	0.221267	1.690779306	0.090879	0.280942	NOT
AC009133.	2.858022	-0.8606997	0.509079	-1.69069925	0.090894	0.280957	NOT
ADRBK2	868.7954	-0.6923306	0.409532	-1.69053973	0.090925	0.281019	NOT
RP11-112J	11.96875	0.61392107	0.363165	1.690476481	0.090937	0.281025	NOT
PHYH	14281.9	-0.553173	0.327247	-1.69038594	0.090954	0.281046	NOT
PGRMC1	17623.39	-0.4433746	0.262338	-1.6900891	0.091011	0.28119	NOT
LHPP	2225.748	-0.5337701	0.315841	-1.68999582	0.091029	0.281213	NOT
EAF1-AS1	10.22019	-0.4884709	0.289059	-1.68986692	0.091053	0.281257	NOT
GTF2B	1089.138	0.24404542	0.144437	1.689626967	0.091099	0.281367	NOT
RP11-126C	12.64932	-0.6394993	0.378504	-1.68954419	0.091115	0.281384	NOT
GSTM5	37.22975	-0.9610211	0.5689	-1.68926089	0.091169	0.281519	NOT
TRAF6	364.5272	-0.2341449	0.138625	-1.68905637	0.091209	0.281569	NOT
AC091492.	3.071593	0.73935387	0.437733	1.689050564	0.09121	0.281569	NOT
FAM58A	771.6047	0.31822889	0.188411	1.689014367	0.091217	0.281569	NOT
BCO2	388.677	-0.9113548	0.539618	-1.68888998	0.091241	0.28161	NOT
OAZ2	3509.381	-0.2145695	0.127052	-1.68882748	0.091252	0.281615	NOT
IL17REL	3.411155	1.0029589	0.594055	1.688325767	0.091349	0.281855	NOT
HMGN2P5	21.15043	0.48875349	0.289492	1.688314042	0.091351	0.281855	NOT
IFNLR1	439.1304	-0.7264411	0.430309	-1.68818601	0.091376	0.281899	NOT

GNAO1	535.022	-0.8930103	0.529021	-1.68804305	0.091403	0.281951	NOT
SERINC3	7266.304	-0.2210121	0.130962	-1.68760432	0.091487	0.282179	NOT
PPP1R10	4528.694	-0.2403432	0.142428	-1.68746615	0.091514	0.282229	NOT
RP11-214(	4.229684	-0.9248514	0.548103	-1.6873665	0.091533	0.282256	NOT
MYLK3	23.10505	0.73374908	0.434865	1.687301292	0.091545	0.282262	NOT
CTD-2378I	3.467044	-0.7198883	0.426682	-1.68717847	0.091569	0.282275	NOT
PKN1	6077.559	0.40270279	0.238685	1.687172462	0.09157	0.282275	NOT
NCS1	641.6802	0.66342828	0.393247	1.687050651	0.091594	0.282315	NOT
DOCK6	1779.344	-0.2449338	0.14522	-1.68663548	0.091673	0.282504	NOT
CTD-2152M	16.01701	0.54675972	0.324175	1.686619034	0.091677	0.282504	NOT
NPM1P21	2.427326	0.76722642	0.454907	1.686556122	0.091689	0.282504	NOT
AC007792.	1.867577	-1.1785841	0.698828	-1.68651506	0.091697	0.282504	NOT
RP11-57H1	7.426616	-0.5180868	0.307245	-1.68623152	0.091751	0.28262	NOT
RASL11B	28.27591	0.96145114	0.5702	1.686165784	0.091764	0.28262	NOT
AOX1	38343.33	-0.8487819	0.503407	-1.68607541	0.091781	0.28262	NOT
CTA-246H3	6.76406	-0.6297851	0.373527	-1.6860483	0.091786	0.28262	NOT
ARHGEF38	52.73811	0.89263613	0.52943	1.686032592	0.09179	0.28262	NOT
GS1-124K5	12.57304	-0.5128891	0.304205	-1.68599549	0.091797	0.28262	NOT
MBNL1-AS1	41.10607	-0.3911488	0.232007	-1.6859339	0.091809	0.28262	NOT
MTFMT	455.4229	-0.2216356	0.131465	-1.68588593	0.091818	0.28262	NOT
CBLL1	1092.926	0.24995403	0.148286	1.68561674	0.09187	0.282721	NOT
RP13-36G1	52.86553	-0.4179342	0.247943	-1.68560776	0.091871	0.282721	NOT
AL158801.	17.21007	0.40324082	0.239244	1.685477382	0.091897	0.282735	NOT
F10-AS1	9.227817	-0.7042746	0.417849	-1.68547705	0.091897	0.282735	NOT
TRPV1	16.02326	-0.5480293	0.325168	-1.68537096	0.091917	0.282765	NOT
MLF1	78.14964	0.8040146	0.477297	1.684515016	0.092082	0.28321	NOT
RPP21	359.207	0.34307141	0.203662	1.684513973	0.092082	0.28321	NOT
PDCD2	1522.302	0.27051133	0.160597	1.684405979	0.092103	0.283242	NOT
LINC01018	2813.28	-1.2306341	0.730859	-1.68381932	0.092217	0.283544	NOT
TRMT1	1821.612	0.35389833	0.21018	1.683789873	0.092222	0.283544	NOT
CTD-2081(	15.31557	-0.4635062	0.275302	-1.6836273	0.092254	0.283608	NOT
RP11-690I	91.5741	-0.3470233	0.206158	-1.68329044	0.092319	0.283776	NOT
RAP1GAP2	316.2703	0.70686519	0.419945	1.683231341	0.09233	0.283779	NOT
SERHL2	64.57436	0.56476123	0.335559	1.683048033	0.092366	0.283856	NOT
NPHP1	70.3335	0.56884527	0.337996	1.682994747	0.092376	0.283856	NOT
FMO5	13845.17	-0.6241557	0.370906	-1.68278606	0.092417	0.283929	NOT
SNX1	4274.91	-0.2502304	0.148702	-1.6827646	0.092421	0.283929	NOT
AC111186.	81.21062	-0.6861777	0.407789	-1.68267645	0.092438	0.283949	NOT
PIN1	1763.35	-0.2901118	0.172437	-1.68241973	0.092487	0.284069	NOT
RP11-446I	12.49744	0.48571908	0.288737	1.682221221	0.092526	0.284104	NOT
SNORA34	1.523289	0.85442194	0.507923	1.68218667	0.092533	0.284104	NOT
NUDT1	612.3784	0.47404134	0.281811	1.682123549	0.092545	0.284104	NOT
AC009095.	3.638572	-0.6590032	0.391775	-1.6820968	0.09255	0.284104	NOT
CTC-241N9	53.44917	-0.3375017	0.200644	-1.68209177	0.092551	0.284104	NOT
C16orf95	74.56348	-0.4139764	0.246134	-1.68191496	0.092585	0.284177	NOT
ALDH7A1P1	10.86914	-0.5897923	0.350696	-1.68177687	0.092612	0.284227	NOT
RP11-141M	70.4461	-0.8298204	0.493485	-1.68155291	0.092656	0.284298	NOT
RP11-127I	13.81418	0.52836186	0.314211	1.681549414	0.092656	0.284298	NOT
TMEM43	2102.741	0.28993917	0.172432	1.681471698	0.092671	0.2843	NOT

DGKZ	1927.589	0.24129145	0.143503	1.681437531	0.092678	0.2843	NOT
FAM86FP	44.04665	-0.5645224	0.335818	-1.68103776	0.092756	0.284506	NOT
RP1-313L4	3.313268	0.93246982	0.554717	1.6809839	0.092766	0.284506	NOT
RP11-278I	5.290758	1.6572581	0.985974	1.680833825	0.092795	0.284564	NOT
AC004160.	7.184688	-1.5471643	0.920516	-1.68075715	0.09281	0.284577	NOT
LINC01426	175.0919	-0.8162029	0.485662	-1.68059781	0.092841	0.28464	NOT
TNRC18P1	8.321213	0.80570659	0.479461	1.68044099	0.092872	0.284701	NOT
HYAL1	6533.683	-0.4919189	0.292744	-1.6803698	0.092885	0.284711	NOT
RP11-161I	2.463762	0.74082803	0.440955	1.680053885	0.092947	0.284868	NOT
ABO	67.76467	-0.9265912	0.551554	-1.67996422	0.092964	0.284872	NOT
ZBED6	185.283	-0.765111	0.45544	-1.67993777	0.092969	0.284872	NOT
HIST1H2BI	6.584319	-0.8811346	0.524533	-1.67984556	0.092987	0.284895	NOT
SLC30A9	2121.206	-0.2597151	0.154627	-1.6796187	0.093032	0.284966	NOT
RP13-672I	10.07808	-0.6314274	0.375935	-1.67961814	0.093032	0.284966	NOT
E2F6	437.8748	0.25267629	0.150456	1.679401412	0.093074	0.285064	NOT
XG	48.94289	-0.9706393	0.577996	-1.67931721	0.09309	0.285075	NOT
C4orf46	242.3181	0.37848971	0.225389	1.67927447	0.093099	0.285075	NOT
RP11-1114	1.255952	1.89178185	1.126652	1.679117998	0.093129	NA	NA
PDHA1	4733.459	-0.2898081	0.1726	-1.67907652	0.093137	0.285161	NOT
IGLV3-10	42.03512	1.38470121	0.824762	1.678909199	0.09317	0.285225	NOT
PLK3	373.2469	-0.4097335	0.244054	-1.6788616	0.093179	0.285225	NOT
AL132988.	31.29564	-0.7001484	0.417094	-1.67863517	0.093223	0.285328	NOT
PLSCR2	2.833561	1.17177535	0.698234	1.678199665	0.093308	0.285555	NOT
MIR483	3.870395	2.51875827	1.500966	1.678090945	0.093329	0.285564	NOT
CNTN6	2.81721	-0.8392982	0.500155	-1.67807761	0.093332	0.285564	NOT
KIFAP3	1556.849	0.34802572	0.207412	1.677947012	0.093357	0.28561	NOT
RP1-267L1	8.318227	0.48604842	0.289738	1.677542114	0.093437	0.285807	NOT
PRSS1	7.889038	1.94498275	1.159447	1.67750841	0.093443	0.285807	NOT
XRN1	1584.304	-0.3359718	0.200287	-1.67745567	0.093453	0.285807	NOT
KBTBD3	180.991	-0.269938	0.16093	-1.67736256	0.093472	0.28583	NOT
RP11-348J	1.577928	-1.1526884	0.68724	-1.67727245	0.093489	0.285852	NOT
NDUFA5	2590.38	-0.2836929	0.169174	-1.67693098	0.093556	0.286024	NOT
LRFN5	7.774795	1.5734634	0.938338	1.676861998	0.093569	0.286033	NOT
MCRS1	2289.179	0.25077056	0.149554	1.676786904	0.093584	0.286045	NOT
MSH4	10.45788	0.77636753	0.463051	1.67663518	0.093614	0.286104	NOT
GNL1	2860.156	0.24795444	0.147904	1.676459345	0.093648	0.286168	NOT
PVT1	289.6461	0.67933266	0.405239	1.676374099	0.093665	0.286168	NOT
RP11-381E	5.92203	0.7325949	0.437014	1.676365815	0.093667	0.286168	NOT
CPM	2629.604	0.4142163	0.247106	1.676269441	0.093685	0.286194	NOT
MEF2D	3035.801	-0.2475177	0.147666	-1.67619424	0.0937	0.286198	NOT
RP11-399K	24.51727	-0.5212466	0.310978	-1.67615472	0.093708	0.286198	NOT
TAS1R1	4.703654	-0.7414206	0.442349	-1.67609805	0.093719	0.286199	NOT
EIF2S3L	16.17406	0.73228515	0.436938	1.675948039	0.093748	0.286257	NOT
DNTTIP2	2332.591	0.25974299	0.154999	1.67577101	0.093783	0.286304	NOT
SYNPO2L	2.680982	-0.6391316	0.381406	-1.6757247	0.093792	0.286304	NOT
TMEM194A	863.0487	0.38449237	0.229451	1.675707195	0.093796	0.286304	NOT
USP14	2978.583	0.18582457	0.110923	1.675262548	0.093883	0.286515	NOT
NUDT6	214.3106	-0.474304	0.283125	-1.67524773	0.093886	0.286515	NOT
SLCO1B1	5876.83	-0.7879554	0.470436	-1.67494686	0.093945	0.286663	NOT

GPHA2	3.308029	1.58563472	0.946711	1.674888431	0.093956	0.286666	NOT
PTN	84.48009	-0.5654261	0.337651	-1.67458899	0.094015	0.28679	NOT
AK9	140.8272	0.35322526	0.210941	1.674523433	0.094028	0.28679	NOT
RP4-568C1	150.2962	-0.9983863	0.59624	-1.67446933	0.094038	0.28679	NOT
CSPG4P13	2.433197	-0.762037	0.455106	-1.67441599	0.094049	0.28679	NOT
CSDC2	15.22127	-0.8937837	0.53379	-1.67441208	0.09405	0.28679	NOT
RP11-216E	18.60756	0.31973936	0.190977	1.674226071	0.094086	0.286869	NOT
HIST1H4E	9.407407	0.74234134	0.443488	1.673870463	0.094156	0.28705	NOT
MYH4	507.6239	-1.7222623	1.028992	-1.67373754	0.094182	0.287097	NOT
SNX18	1572.652	-0.280611	0.167666	-1.67363461	0.094202	0.287101	NOT
SURF1	2870.831	-0.3423833	0.204576	-1.67362285	0.094205	0.287101	NOT
GNAI2	11225.21	-0.2350543	0.140455	-1.6735171	0.094226	0.287115	NOT
CCL3L3	77.96633	0.60837198	0.363534	1.673492399	0.09423	0.287115	NOT
CUTA	9740.407	0.38555669	0.230405	1.673385099	0.094252	0.287138	NOT
RP11-338N	4.991949	1.30744768	0.781337	1.673346222	0.094259	0.287138	NOT
SLC24A1	338.4458	-0.3606901	0.215605	-1.67291763	0.094344	0.287363	NOT
FAM174A	725.0968	-0.2712565	0.162155	-1.67282496	0.094362	0.287381	NOT
ACAD11	110.565	-0.6100127	0.364681	-1.67272979	0.094381	0.287381	NOT
GRID2	1.200829	2.03990349	1.219508	1.672726678	0.094381	NA	NA
RUSC1-AS1	310.7894	0.4638274	0.27729	1.672714745	0.094383	0.287381	NOT
JARID2	703.613	0.25474882	0.152303	1.672647146	0.094397	0.287381	NOT
RGL2	1813.061	0.31049752	0.185636	1.672617414	0.094403	0.287381	NOT
AC006126.	1.756904	-0.788353	0.471378	-1.67244447	0.094437	0.287453	NOT
LA16c-306	5.013743	-0.5559184	0.33241	-1.67238512	0.094448	0.287456	NOT
ENDOV	603.8108	-0.3822739	0.228611	-1.67215986	0.094493	0.287559	NOT
C2orf16	118.6023	-0.5533998	0.330982	-1.67199601	0.094525	0.287571	NOT
CD300LG	72.32649	-1.186755	0.709787	-1.67198807	0.094527	0.287571	NOT
MID1	961.718	0.48437407	0.289701	1.671978919	0.094528	0.287571	NOT
SETBP1	504.6989	-0.40661	0.243204	-1.67188666	0.094547	0.287594	NOT
C5orf46	27.92505	0.90858045	0.5435	1.671720978	0.094579	0.287661	NOT
LINC01344	24.63149	-0.9169897	0.548575	-1.67158594	0.094606	0.28771	NOT
LINC01277	15.41237	-0.5957718	0.356445	-1.67142788	0.094637	0.287769	NOT
HDGFL1	1.588456	2.62743195	1.572069	1.671321448	0.094658	0.287769	NOT
RP11-499F	3.172195	1.72470526	1.031961	1.671288644	0.094665	0.287769	NOT
ABHD17B	704.7647	-0.2598262	0.155466	-1.67127233	0.094668	0.287769	NOT
TMEM245	4598.704	-0.3633104	0.217396	-1.67119484	0.094683	0.287783	NOT
KIAA2018	1433.919	-0.3369191	0.201626	-1.67101172	0.094719	0.287861	NOT
CDK5	950.3185	0.27879646	0.166861	1.670832534	0.094755	0.287934	NOT
RP11-671E	2.309539	0.78616934	0.470559	1.67071393	0.094778	0.287934	NOT
MPP6	1196.048	0.40944708	0.245082	1.670651054	0.094791	0.287934	NOT
NRIP2	156.8279	-0.3791426	0.226953	-1.67057807	0.094805	0.287934	NOT
NUPL2	986.0712	0.22949	0.137372	1.670572579	0.094806	0.287934	NOT
GPC2	20.94507	0.63621333	0.380837	1.670567299	0.094807	0.287934	NOT
RPL10AP6	75.37885	0.51990291	0.311311	1.670044826	0.09491	0.288216	NOT
AC005562.	50.77895	0.34161192	0.204601	1.669651333	0.094988	0.28842	NOT
MCAM	3707.409	-0.4582755	0.274522	-1.66935621	0.095047	0.288547	NOT
POLR3F	467.5925	0.19195929	0.114992	1.669333226	0.095051	0.288547	NOT
RP11-54H7	0.978665	1.67620136	1.004193	1.669203204	0.095077	NA	NA
RPTOR	2067.747	-0.2421285	0.145074	-1.66900206	0.095117	0.288714	NOT

CHMP2B	1622.113	0.26233533	0.157196	1.668839616	0.095149	0.288779	NOT
GGTA1P	356.759	-0.4471433	0.267958	-1.66870592	0.095176	0.288827	NOT
SEPT4-AS1	15.76005	-0.5195677	0.311374	-1.66863145	0.09519	0.28884	NOT
ZFAND3	4688.628	0.23739553	0.142285	1.668455717	0.095225	0.288913	NOT
ARSG	236.6219	-0.4068438	0.243858	-1.66836177	0.095244	0.288937	NOT
RPS18P12	17.83892	0.63082501	0.378219	1.667881958	0.095339	0.289194	NOT
RP5-1198(	14.37722	0.66713168	0.40004	1.667662575	0.095383	0.289287	NOT
ZNF90	36.88022	0.76950023	0.461449	1.667574059	0.0954	0.289287	NOT
PLEKHG7	20.44653	-0.7557676	0.453228	-1.66752053	0.095411	0.289287	NOT
LINC-ROR	4.742495	1.22661062	0.735593	1.667513585	0.095412	0.289287	NOT
HCG27	49.98425	-0.428852	0.257225	-1.66722553	0.09547	0.289395	NOT
MIR590	1.886606	-0.8252518	0.494993	-1.66719865	0.095475	0.289395	NOT
TREML1	7.707363	0.70958608	0.425622	1.667172821	0.09548	0.289395	NOT
SLC17A5	2410.431	0.39547318	0.237236	1.66700246	0.095514	0.289454	NOT
DBN1	2012.165	0.64865487	0.389123	1.666966937	0.095521	0.289454	NOT
SLITRK2	2.559734	1.31734922	0.79032	1.66685562	0.095543	0.289489	NOT
AC009403.	308.1051	0.36451197	0.218732	1.666475869	0.095619	0.289618	NOT
PRR14	1306.616	0.19271164	0.115642	1.666456239	0.095623	0.289618	NOT
CAMK1D	1585.627	-0.6278486	0.376763	-1.66642997	0.095628	0.289618	NOT
CNTN3	91.64484	-1.2318979	0.739245	-1.66642779	0.095628	0.289618	NOT
RP11-384F	1.426665	2.26481801	1.359299	1.666166565	0.09568	0.289743	NOT
ZNF605	613.8011	0.32264829	0.193654	1.66610574	0.095692	0.289747	NOT
TEFM	259.4819	0.22056707	0.132419	1.665676742	0.095778	0.289973	NOT
RPL5P23	6.641369	0.46941755	0.281836	1.665572671	0.095799	0.290001	NOT
PPP3CB-AS	155.1076	-0.3533023	0.212127	-1.66552393	0.095808	0.290001	NOT
KRT18P25	3.248091	-0.6327815	0.379996	-1.6652307	0.095867	0.290146	NOT
RP11-736K	50.68692	-0.5361535	0.322015	-1.66499305	0.095914	0.290208	NOT
RBP1	3427.649	-0.9134894	0.548649	-1.66497886	0.095917	0.290208	NOT
PYROXD1	616.1063	-0.2599579	0.156134	-1.6649671	0.095919	0.290208	NOT
SNHG24	1.51165	1.61144839	0.967933	1.664833947	0.095946	0.290256	NOT
RP11-415F	13.68316	-0.387132	0.232564	-1.66462337	0.095988	0.29035	NOT
SLITRK6	3.753861	-1.0513734	0.631659	-1.66446439	0.09602	0.290414	NOT
STXBP2	1574.126	0.36872774	0.22156	1.664233329	0.096066	0.290515	NOT
AC022816.	52.82013	0.85421593	0.513296	1.664179632	0.096077	0.290515	NOT
NPPA	6.518793	-0.7471845	0.448992	-1.6641364	0.096085	0.290515	NOT
C1orf145	19.28292	0.9163149	0.550673	1.663989705	0.096114	0.290546	NOT
BAG1	2506.081	-0.3152028	0.189427	-1.66397712	0.096117	0.290546	NOT
RP1-232L2	31.42205	-0.6030472	0.36246	-1.66376225	0.09616	0.290644	NOT
U3	21.31197	0.65253123	0.392238	1.663612174	0.09619	0.290702	NOT
RP11-744K	2.218629	1.50748505	0.906224	1.663479852	0.096216	0.29075	NOT
DENND5B-A	2.328532	0.75789737	0.455656	1.663310086	0.09625	0.29082	NOT
GLTP	1309.189	0.23911858	0.143793	1.66294063	0.096324	0.290992	NOT
LINC00896	66.15352	0.81249425	0.488603	1.662892132	0.096334	0.290992	NOT
WIPF3	281.2095	0.83947444	0.504836	1.66286496	0.096339	0.290992	NOT
ABI1	2110.906	0.23609364	0.142026	1.662332533	0.096446	0.291273	NOT
RP11-507E	2.461419	1.34752109	0.81064	1.662293132	0.096454	0.291273	NOT
SLC37A1	634.4687	0.4314159	0.259543	1.662215685	0.09647	0.291284	NOT
NOL10	1486.998	0.1950315	0.117346	1.662020205	0.096509	0.291284	NOT
SERPINA9	11.4801	0.8045033	0.484055	1.662009239	0.096511	0.291284	NOT

LAX1	34.38529	-0.5916461	0.355985	-1.66199554	0.096514	0.291284	NOT
MPEG1	1287.884	-0.4811555	0.289508	-1.66197649	0.096517	0.291284	NOT
NDUFC1	1951.032	-0.3009048	0.181055	-1.66195293	0.096522	0.291284	NOT
KCNMB2	3.807345	1.10140774	0.662892	1.661519104	0.096609	0.291515	NOT
HTRA3	570.3865	0.81386162	0.48986	1.661417897	0.09663	0.291544	NOT
NDUFA7	173.0449	-0.3645677	0.219456	-1.66123265	0.096667	0.291611	NOT
RP11-108M	192.2165	0.55926309	0.336672	1.661152211	0.096683	0.291611	NOT
SLC27A5	20206.22	-0.850737	0.512147	-1.66112023	0.096689	0.291611	NOT
MT-TC	15.62446	-0.7208153	0.433941	-1.66109216	0.096695	0.291611	NOT
DLD	4498.754	-0.3266839	0.196687	-1.66093224	0.096727	0.291676	NOT
NAPRT	7426.614	0.52483293	0.31608	1.660444918	0.096825	0.291938	NOT
VPS39	2212.945	-0.2048592	0.123394	-1.6601986	0.096875	0.292055	NOT
HSD17B14	2048.569	-0.7704866	0.464123	-1.66009076	0.096896	0.292077	NOT
AC137932.	5.980481	-0.559533	0.337073	-1.65997565	0.096919	0.292077	NOT
RP11-432J	9.501257	1.14391194	0.689124	1.659950514	0.096924	0.292077	NOT
CXorf57	183.8786	0.62307692	0.375359	1.65994822	0.096925	0.292077	NOT
AC064875.	4.082504	-0.6742385	0.406205	-1.65984714	0.096945	0.292079	NOT
SSSCA1-AS	53.18345	-0.2871687	0.173015	-1.65978814	0.096957	0.292079	NOT
SLN	2.78187	1.52334424	0.917796	1.659784672	0.096958	0.292079	NOT
KRT16P1	0.777018	-2.128995	1.282833	-1.65960454	0.096994	NA	NA
ITK	116.7371	-0.6786073	0.409009	-1.65914871	0.097086	0.292432	NOT
ENTPD6	4027.327	0.284507	0.171501	1.658927507	0.09713	0.292463	NOT
RPL37A	29335.47	0.45835111	0.276296	1.65891184	0.097134	0.292463	NOT
AF127936.	1.799373	0.75205378	0.453346	1.65889452	0.097137	0.292463	NOT
CITF22-1A	33.19313	0.40118894	0.241843	1.658883996	0.097139	0.292463	NOT
TNXA	114.4882	0.69232352	0.417413	1.658604941	0.097195	0.29254	NOT
C3orf14	173.41	0.79755029	0.480868	1.658563449	0.097204	0.29254	NOT
CDO1	7624.73	-0.7257134	0.437556	-1.65856276	0.097204	0.29254	NOT
ZNF776	442.2612	-0.3337368	0.201223	-1.65854347	0.097208	0.29254	NOT
RNASEH1-A	183.4089	0.32354458	0.195098	1.658365932	0.097244	0.292615	NOT
LINC00504	29.55702	-0.8123947	0.489912	-1.65824554	0.097268	0.292632	NOT
RP11-10L1	38.10112	0.35057292	0.211414	1.658231155	0.097271	0.292632	NOT
CTB-47B11	3.991857	-0.8223494	0.495965	-1.65807795	0.097302	0.292693	NOT
INE1	23.09545	-0.3668475	0.221268	-1.65793637	0.09733	0.292746	NOT
HIST1H2AC	196.132	-0.9555181	0.576383	-1.65778416	0.097361	0.292806	NOT
RPL21P135	2.052498	-0.7759258	0.468131	-1.65749786	0.097419	0.292948	NOT
MIR155HG	23.82427	0.62001046	0.374086	1.65740038	0.097439	0.292974	NOT
MAP3K4	891.9557	0.25638424	0.154697	1.657334745	0.097452	0.292982	NOT
CTA-268H5	4.731339	0.91649697	0.553018	1.65726569	0.097466	0.292991	NOT
RP11-227C	4.564704	-0.6874438	0.414869	-1.65701482	0.097516	0.293102	NOT
YWHAB	9405.757	0.17065239	0.10299	1.656976427	0.097524	0.293102	NOT
SRA1	2353.752	-0.3743685	0.225949	-1.65687032	0.097546	0.293131	NOT
PM20D2	702.7451	0.4142439	0.250023	1.656822476	0.097555	0.293131	NOT
TOP3BP1	1.443774	1.5316509	0.924495	1.656742552	0.097572	0.293147	NOT
SMUG1	1489.376	0.28823075	0.173989	1.65659972	0.0976	0.293201	NOT
CRISPLD1	56.87739	-0.6834903	0.412708	-1.65611124	0.097699	0.293428	NOT
RP11-327E	75.47889	0.38955132	0.235232	1.656030223	0.097716	0.293428	NOT
IFT20	966.7228	0.21024937	0.126961	1.656020801	0.097718	0.293428	NOT
SMARCA4	4537.66	0.28278367	0.170763	1.656004452	0.097721	0.293428	NOT

RNASE4	777.9276	-0.573076	0.346078	-1.65591516	0.097739	0.293428	NOT
RP11-101E	499.4928	0.27789785	0.167822	1.655905601	0.097741	0.293428	NOT
RP5-1028K	16.52178	0.85961311	0.519171	1.655740962	0.097774	0.293468	NOT
FLI1	493.3847	-0.4023637	0.243012	-1.65573305	0.097776	0.293468	NOT
AP5B1	908.357	-0.3090658	0.186682	-1.65557548	0.097808	0.293505	NOT
LIX1L	1855.674	0.28149413	0.170031	1.655546565	0.097814	0.293505	NOT
RP5-1177I	1.49238	-1.1622397	0.702059	-1.65547359	0.097828	0.293505	NOT
RP11-178I	7.888886	-0.8136812	0.491514	-1.65545979	0.097831	0.293505	NOT
RP11-274E	13.69403	-0.6461601	0.390336	-1.65539632	0.097844	0.293511	NOT
FDX1	2275.534	-0.3765343	0.227476	-1.65526927	0.09787	0.293556	NOT
RPL24	20461.48	0.38088241	0.230115	1.655183373	0.097887	0.293575	NOT
NDNF	7.108558	-0.8733079	0.527655	-1.65507305	0.09791	0.293603	NOT
ANKRD50	702.4768	-0.371346	0.224374	-1.65503067	0.097918	0.293603	NOT
RP11-593F	2.398	-0.9338854	0.564342	-1.65482073	0.097961	0.293699	NOT
C1orf174	875.1333	0.24067726	0.14546	1.654589423	0.098008	0.293807	NOT
ZNF619	168.2435	-0.2902258	0.175415	-1.65450606	0.098025	0.293813	NOT
INPP5D	667.5266	-0.4475201	0.270491	-1.65447321	0.098031	0.293813	NOT
ZNF213-AS	210.5956	-0.326582	0.197407	-1.65436108	0.098054	0.293831	NOT
CTD-2562J	2.803827	-0.4854696	0.293453	-1.65433572	0.098059	0.293831	NOT
ADAP1	254.4404	0.82050513	0.496004	1.654230289	0.098081	0.293863	NOT
RP3-388N1	1.077535	1.18833356	0.718407	1.654124163	0.098102	NA	NA
LINC00936	35.92412	-0.3759723	0.227364	-1.65361507	0.098206	0.294177	NOT
CHST7	309.5938	-0.5618091	0.339748	-1.65360718	0.098207	0.294177	NOT
COQ2	462.8715	-0.3036976	0.183676	-1.65344164	0.098241	0.294246	NOT
ARHGAP11E	126.4804	0.50354177	0.304565	1.65331484	0.098267	0.294291	NOT
UBE2G2	3041.388	-0.2069832	0.125217	-1.65299749	0.098331	0.294451	NOT
PTPRU	1321.135	-0.5543886	0.335416	-1.65283848	0.098364	0.294516	NOT
AC004076.	2.091343	-0.8879533	0.537304	-1.65260814	0.098411	0.294624	NOT
MCF2	5.675813	-0.7887225	0.477298	-1.65247399	0.098438	0.294673	NOT
RP11-141C	3.120262	0.79586254	0.481656	1.652345063	0.098464	0.294683	NOT
DDX50	1161.588	0.22627253	0.136944	1.652294643	0.098475	0.294683	NOT
GCGR	1851.627	-1.154348	0.698662	-1.65222769	0.098488	0.294683	NOT
RP3-325F2	4.019019	-0.8469694	0.51263	-1.65220447	0.098493	0.294683	NOT
SERPINI1	324.76	0.51342335	0.310759	1.652160823	0.098502	0.294683	NOT
B3GNT8	85.39205	-0.742644	0.449505	-1.65213799	0.098506	0.294683	NOT
CCDC155	12.23428	1.26230751	0.764091	1.652039184	0.098527	0.294697	NOT
GNB2L1	38938.6	0.32954117	0.199484	1.651968765	0.098541	0.294697	NOT
P2RY2	183.3714	0.48638398	0.294429	1.651955617	0.098544	0.294697	NOT
SNAI1	146.6089	-0.5835637	0.353289	-1.65180047	0.098575	0.294759	NOT
RP11-182J	4.714568	-0.652735	0.39519	-1.65170025	0.098596	0.294787	NOT
RP1-140A9	21.53398	0.57230924	0.346538	1.65150372	0.098636	0.294839	NOT
RP11-148E	1.691011	-0.8062	0.488174	-1.65146135	0.098644	0.294839	NOT
ME3	313.7299	0.44519409	0.269577	1.651456414	0.098645	0.294839	NOT
TEKT4	3.363855	-0.8568956	0.518917	-1.65131377	0.098675	0.294893	NOT
RP11-661A	6.350189	0.72746446	0.440552	1.651256766	0.098686	0.294895	NOT
TSHZ1	1145.43	-0.302323	0.183155	-1.65064285	0.098812	0.295238	NOT
RP11-187C	3.914884	0.62814228	0.380573	1.650515278	0.098838	0.295283	NOT
GSE1	2100.167	-0.3088418	0.187129	-1.65041762	0.098858	0.29531	NOT
ZNF135	88.72698	0.72823602	0.441286	1.650257351	0.09889	0.29533	NOT

JDP2	513.0374	-0.4156729	0.25191	-1.6500835	0.098926	0.29533	NOT
ADCY5	357.9286	-0.6671961	0.404341	-1.65008145	0.098926	0.29533	NOT
LINC0053(	7.826555	0.52768187	0.319794	1.650070645	0.098928	0.29533	NOT
PIH1D2	22.96229	0.45121632	0.273468	1.649976797	0.098948	0.29533	NOT
LY96	420.2515	0.70282635	0.425964	1.649968296	0.098949	0.29533	NOT
CYP3A5	9181.056	-0.6399309	0.387857	-1.64991655	0.09896	0.29533	NOT
AC005884.	5.001395	0.66192412	0.4012	1.649860919	0.098971	0.29533	NOT
EXOC8	782.3368	-0.2337988	0.141709	-1.64985151	0.098973	0.29533	NOT
RP11-541F	2.568727	2.58657181	1.567793	1.649816713	0.09898	0.29533	NOT
HRK	2.712861	1.06334953	0.644541	1.649779143	0.098988	0.29533	NOT
ERMAP	708.328	-0.3377845	0.204749	-1.6497456	0.098995	0.29533	NOT
SNX15	33.15511	-0.4009715	0.243061	-1.64967616	0.099009	0.29534	NOT
CWH43	3.814787	-1.1993593	0.727058	-1.64960532	0.099024	0.295351	NOT
CTD-2132N	14.43496	-0.573423	0.34764	-1.64947342	0.099051	0.295399	NOT
EIF4E2	3369.705	0.22276122	0.135068	1.649254612	0.099095	0.2955	NOT
RP11-342K	7.495663	-0.7119713	0.43171	-1.649189	0.099109	0.295508	NOT
TOB2	2338.475	-0.2999409	0.181889	-1.64903182	0.099141	0.295571	NOT
WDR45	2318.949	-0.2467723	0.149663	-1.64885561	0.099177	0.295646	NOT
RWDD2B	820.1747	-0.2656858	0.161154	-1.64864338	0.099221	0.295744	NOT
NFE4	2.544096	1.24386584	0.754512	1.648569098	0.099236	0.295756	NOT
RP3-468B3	7.732954	-0.7486553	0.45417	-1.64840468	0.09927	0.295824	NOT
CDC20P1	6.178189	0.75392932	0.457436	1.648161924	0.099319	0.295908	NOT
RP11-148E	2.297443	1.03575619	0.628445	1.648126434	0.099327	0.295908	NOT
PON1	11848.76	-0.8116604	0.492495	-1.64805936	0.09934	0.295908	NOT
ATF6	4556.142	-0.3280999	0.199089	-1.64800882	0.099351	0.295908	NOT
PRADC1P1	10.51323	-0.8567594	0.519877	-1.64800284	0.099352	0.295908	NOT
RABEP1	1878.274	-0.2917134	0.177019	-1.6479186	0.099369	0.295927	NOT
MIOS	734.7615	-0.2522417	0.153075	-1.64783612	0.099386	0.295945	NOT
RP11-2K6.	2.760941	0.96979473	0.588571	1.647711309	0.099412	0.295988	NOT
ST13P3	4.851337	0.61506496	0.373309	1.647604674	0.099434	0.296007	NOT
CLCN4	623.6564	-0.3276419	0.198863	-1.64757419	0.09944	0.296007	NOT
ARRB1	890.9426	-0.4767007	0.289383	-1.64730198	0.099496	0.296126	NOT
RP11-91J1	13.62136	-0.5082139	0.308518	-1.64727299	0.099502	0.296126	NOT
DPP6	4.773575	-0.8056738	0.489143	-1.64711188	0.099535	0.296192	NOT
ADAT1	636.1221	-0.253398	0.153862	-1.64691701	0.099575	0.296228	NOT
DRP2	7.649339	0.78543285	0.476913	1.646911335	0.099576	0.296228	NOT
HIBADH	5378.945	-0.4595471	0.279039	-1.64689487	0.09958	0.296228	NOT
CLCF1	243.6451	0.50910562	0.309154	1.64677215	0.099605	0.29627	NOT
ATRN	11719.19	-0.4199102	0.255005	-1.64667125	0.099626	0.296297	NOT
PGLYRP1	1.706868	-0.7872525	0.478101	-1.64662211	0.099636	0.296297	NOT
CPLX2	4672.14	1.59329385	0.96769	1.646492811	0.099662	0.296344	NOT
STAC2	1.996265	1.27879303	0.776708	1.6464269	0.099676	0.296351	NOT
TMCO2	1.870317	1.02530453	0.622788	1.646314354	0.099699	0.296388	NOT
SH3GL1P1	9.689617	-0.4952813	0.300921	-1.64588649	0.099787	0.296561	NOT
CFHR2	435.5591	-0.7356894	0.446992	-1.64586769	0.099791	0.296561	NOT
RP11-273(	51.59851	0.88640734	0.538578	1.645829939	0.099799	0.296561	NOT
GPX3	39375.32	0.65164689	0.395941	1.64581882	0.099801	0.296561	NOT
RAB11FIP1	977.6849	0.69544917	0.42257	1.645760741	0.099813	0.296564	NOT
PRRX2	19.90442	1.07734057	0.65468	1.645598116	0.099847	0.296604	NOT

DHRS7	5087.068	0.36360284	0.220956	1.645590204	0.099848	0.296604	NOT
NEK11	91.20059	0.58328424	0.354464	1.645536935	0.099859	0.296604	NOT
EHBP1L1	1718.673	0.28196689	0.171358	1.645480725	0.099871	0.296606	NOT
AHRR	33.99006	0.5428307	0.329925	1.645314213	0.099905	0.296675	NOT
TUBBP5	58.20834	1.25000647	0.759768	1.645247959	0.099919	0.296676	NOT
OSBPL7	232.3182	0.54475184	0.331114	1.645207122	0.099927	0.296676	NOT
FECH	1538.498	-0.3443262	0.209311	-1.64504726	0.09996	0.296707	NOT
MTL5	151.7697	0.589286	0.358236	1.644966401	0.099977	0.296707	NOT
RPS2P5	1087.74	0.40994989	0.24922	1.644931403	0.099984	0.296707	NOT
TBC1D24	1381.281	-0.3046977	0.185234	-1.64493076	0.099984	0.296707	NOT
ESM1	632.7595	-0.6099448	0.370823	-1.64484127	0.100003	0.296707	NOT
RP11-546K	3.679023	0.88714322	0.53935	1.644838387	0.100003	0.296707	NOT
IL17RD	57.08378	0.67500461	0.410418	1.644676762	0.100036	0.296773	NOT
SNORA71B	3.846492	-0.6342784	0.3857	-1.64448545	0.100076	0.296847	NOT
ICOSLG	79.75031	-0.4618896	0.280887	-1.64439627	0.100094	0.296847	NOT
JUNB	4871.677	-0.462642	0.281349	-1.64437244	0.100099	0.296847	NOT
FGF22	7.369287	0.82171604	0.499729	1.644324555	0.100109	0.296847	NOT
NCKIPSD	1580.741	0.25340165	0.15411	1.644290813	0.100116	0.296847	NOT
MAPK8IP1	390.4293	-0.4673532	0.284256	-1.6441271	0.10015	0.296915	NOT
RP11-168(	3.461111	0.92635386	0.56351	1.643900413	0.100197	0.297021	NOT
RP11-574K	16.00613	-0.4142758	0.252017	-1.64383781	0.10021	0.297027	NOT
CTA-390C1	27.48936	0.52386756	0.318739	1.643560533	0.100267	0.297133	NOT
PLIN4	2157.291	-0.6378632	0.388099	-1.64355974	0.100267	0.297133	NOT
RP11-61N2	28.74073	0.50313145	0.306148	1.643427138	0.100295	0.297181	NOT
RP1-90G24	7.673484	1.13543889	0.690925	1.643361031	0.100308	0.297189	NOT
RP5-1059I	25.95007	0.85852135	0.522446	1.643272124	0.100327	0.297211	NOT
HSPA5	47913	-0.2622579	0.159637	-1.64284389	0.100415	0.297429	NOT
AC074391.	13.37175	0.5427927	0.330405	1.642810943	0.100422	0.297429	NOT
SLC35E2	39.36767	-0.4125517	0.251141	-1.6427083	0.100443	0.29746	NOT
CCND2P1	329.7369	-0.9571051	0.582674	-1.64260941	0.100464	0.297488	NOT
CROCCP2	1148.744	-0.4004183	0.243788	-1.64248571	0.100489	0.297531	NOT
AKAP14	3.780177	1.14820066	0.699189	1.642189105	0.100551	0.29768	NOT
CDC26	276.75	0.24881989	0.151562	1.641699508	0.100652	0.297948	NOT
SSR3	8528.609	0.21466749	0.130772	1.641540255	0.100685	0.297969	NOT
OR6E1P	2.252633	0.91402205	0.55681	1.641533184	0.100687	0.297969	NOT
CCR7	74.28499	-0.6843629	0.416918	-1.64148024	0.100698	0.297969	NOT
GPCPD1	886.3446	-0.3147643	0.191759	-1.64145469	0.100703	0.297969	NOT
ACSF3	1212.704	-0.3800807	0.231579	-1.64125808	0.100744	0.298025	NOT
GSTO2	420.3181	0.67437904	0.410892	1.641256897	0.100744	0.298025	NOT
COX8A	7705.124	-0.3407975	0.207656	-1.64115991	0.100764	0.298026	NOT
TOB1	7159.716	-0.4953797	0.301849	-1.64114977	0.100766	0.298026	NOT
LINC01419	356.1211	1.80097866	1.097585	1.640856195	0.100827	0.29816	NOT
CDH15	168.2422	1.1333895	0.690744	1.640825055	0.100834	0.29816	NOT
CDK5RAP2	3352.285	0.3146186	0.191758	1.640705114	0.100859	0.298201	NOT
ANXA8	13.40644	-0.9257638	0.564276	-1.64062305	0.100876	0.298205	NOT
FBXL18	233.2869	-0.4058893	0.247411	-1.64054784	0.100891	0.298205	NOT
AC007277.	9.107951	1.10525195	0.673712	1.640540401	0.100893	0.298205	NOT
SNORD94	6.626847	-0.5758341	0.351018	-1.64046964	0.100908	0.298216	NOT
TFR2	38613.58	-0.6799671	0.414544	-1.64027716	0.100948	0.298302	NOT

TBCK	710.7714	-0.3390548	0.206722	-1.64015106	0.100974	0.298333	NOT
FLT3	17.7584	-0.6748663	0.411474	-1.64012038	0.10098	0.298333	NOT
RP11-116I	1824.789	-0.6770402	0.412822	-1.64002911	0.100999	0.298356	NOT
RP1-111C2	12.13408	-0.4513497	0.275224	-1.63993738	0.101018	0.29838	NOT
BTN2A3P	76.15621	-0.3665361	0.223571	-1.63946204	0.101117	0.29864	NOT
RP5-965G2	12.49744	0.62978637	0.384163	1.639370864	0.101136	0.298663	NOT
VPS25	2870.365	0.25045324	0.152789	1.63920484	0.101171	0.298733	NOT
KIAA1586	303.3623	0.27435606	0.167384	1.639081057	0.101196	0.298754	NOT
INTS5	1345.776	-0.1984445	0.121074	-1.63903838	0.101205	0.298754	NOT
RP4-669P1	18.87541	-0.5853998	0.357172	-1.63898463	0.101216	0.298754	NOT
VSX1	16.03734	0.86082777	0.525236	1.638933843	0.101227	0.298754	NOT
RP11-386M	2.069329	-0.9228629	0.563097	-1.63890607	0.101233	0.298754	NOT
HCFC2	690.5612	-0.314734	0.192048	-1.63883072	0.101249	0.298768	NOT
KRT18P10	12.2344	0.52016485	0.317449	1.638575477	0.101302	0.298892	NOT
C5orf47	3.181457	0.74007904	0.451707	1.638406681	0.101337	0.298964	NOT
RP11-385N	1.146123	1.9285035	1.177121	1.6383216	0.101355	NA	NA
CTD-3126E	3.182336	-0.6342848	0.387169	-1.63826396	0.101367	0.298992	NOT
AC012358.	70.70644	-0.51263	0.312914	-1.63824562	0.10137	0.298992	NOT
RN7SL268F	3.484357	-0.6088073	0.371635	-1.63818466	0.101383	0.298992	NOT
AC073150.	1.165423	-0.9610729	0.586676	-1.63816556	0.101387	NA	NA
GOLGA8VP	2.073816	1.13035557	0.690033	1.638119302	0.101397	0.298992	NOT
SBSPON	108.1794	0.68217535	0.416444	1.638095856	0.101402	0.298992	NOT
BOLA1	1082.08	0.32831266	0.20043	1.638042076	0.101413	0.298993	NOT
TBC1D30	270.6986	0.60916872	0.371918	1.637911158	0.10144	0.299012	NOT
DAAM2	462.4043	-0.566039	0.345587	-1.6379054	0.101441	0.299012	NOT
P2RX7	432.8213	0.50565357	0.308767	1.637655062	0.101494	0.299133	NOT
ZNF22	1257.69	-0.3224553	0.196914	-1.63753991	0.101518	0.299172	NOT
MFSD11	954.615	-0.2550579	0.155769	-1.63741262	0.101544	0.299187	NOT
APOA5	13507.25	-0.8653256	0.528472	-1.63740949	0.101545	0.299187	NOT
SNTG1	42.35345	-1.3548595	0.82747	-1.63735158	0.101557	0.29919	NOT
C19orf33	133.8231	1.09150882	0.666933	1.636608649	0.101712	0.299615	NOT
ALDH1L2	56.68137	-0.5390726	0.32941	-1.63647718	0.10174	0.299631	NOT
CEACAM20	168.5919	-1.2477381	0.762454	-1.63647699	0.10174	0.299631	NOT
KB-1471A8	5.904845	0.56465343	0.345055	1.636414966	0.101753	0.299637	NOT
FOXP3	190.1488	-0.610211	0.372919	-1.63630962	0.101775	0.299649	NOT
LAYN	114.0093	-0.5021064	0.306864	-1.63625044	0.101787	0.299649	NOT
ZNF341-AS	2.652543	0.86443095	0.528304	1.636237034	0.10179	0.299649	NOT
AGER	132.8828	-0.4160308	0.254334	-1.63576373	0.101889	0.299898	NOT
RP11-434I	192.9303	-1.0360368	0.63338	-1.63572608	0.101897	0.299898	NOT
NDUFB2-AS	33.01898	0.29266286	0.178966	1.635299116	0.101986	0.300129	NOT
GAS6-AS2	32.18452	-0.6106725	0.373445	-1.63524303	0.101998	0.300131	NOT
COQ7	701.6226	-0.222508	0.136076	-1.63517742	0.102012	0.300139	NOT
LINC01237	11.26629	-0.6096813	0.372911	-1.63492386	0.102065	0.300263	NOT
TMEM71	30.16287	-0.4398305	0.269045	-1.63478212	0.102095	0.300318	NOT
AP000692.	6.777916	-0.646001	0.395187	-1.63467256	0.102118	0.300339	NOT
NLGN4X	282.8669	-0.996779	0.609789	-1.63462968	0.102127	0.300339	NOT
RUNDC3A-A	4.619844	0.8325876	0.509356	1.63458865	0.102135	0.300339	NOT
TMEM30A	5448.793	-0.2441419	0.14938	-1.63436783	0.102182	0.300422	NOT
RP11-297K	3.175432	-0.8267352	0.505864	-1.63430318	0.102195	0.300422	NOT

FTLP15	2.469307	-1.0012267	0.612635	-1.63429624	0.102197	0.300422	NOT
RP11-531F	14.98903	0.48236676	0.295182	1.634131209	0.102231	0.300491	NOT
RP11-127	16.28901	-0.5940235	0.363566	-1.63387897	0.102284	0.300614	NOT
RP11-77H	3.809212	-0.601345	0.36813	-1.63351397	0.102361	0.300786	NOT
RP11-131I	4.37737	-0.5749712	0.351988	-1.63349518	0.102365	0.300786	NOT
CTD-3076(	4.6555	-0.8464939	0.518297	-1.63322218	0.102422	0.300911	NOT
CTD-2524I	1.83605	-0.7492486	0.458778	-1.63313821	0.10244	0.300911	NOT
SLC6A8	1951.635	0.85861609	0.525761	1.633090506	0.10245	0.300911	NOT
RP11-73K	11.3607	0.47957532	0.293663	1.633082698	0.102452	0.300911	NOT
ADAMTS14	77.78342	0.57695018	0.353311	1.632980459	0.102473	0.300941	NOT
RP11-126	1.176796	-1.3251625	0.811546	-1.63288557	0.102493	NA	NA
SACS-AS1	1.445368	2.30651304	1.412571	1.632847567	0.102501	0.300991	NOT
RP11-219E	2.934048	0.85703151	0.524924	1.632675936	0.102537	0.301064	NOT
RP1-117P	4.850963	1.32673457	0.812672	1.632558462	0.102562	0.301104	NOT
ZNF268	824.8948	-0.2940009	0.180095	-1.63247345	0.10258	0.301108	NOT
LILRB4	379.0083	0.64336363	0.39411	1.632446562	0.102585	0.301108	NOT
TMEM216	221.4315	0.34581043	0.211875	1.632144951	0.102649	0.301262	NOT
RP11-697N	5.10151	0.68940319	0.422456	1.631892612	0.102702	0.301355	NOT
WDR37	693.8163	-0.2457815	0.150612	-1.6318888	0.102703	0.301355	NOT
RNU1-106F	4.38022	0.83692338	0.512916	1.631695391	0.102744	0.301442	NOT
ST13P6	10.89738	0.50823048	0.311508	1.631518375	0.102781	0.301508	NOT
SPTBN1	24552.64	-0.2930713	0.179635	-1.63148395	0.102788	0.301508	NOT
AC009299.	6.710978	0.52062635	0.319137	1.631354824	0.102815	0.301555	NOT
VASP	3272.952	0.29628583	0.181628	1.631281681	0.102831	0.301568	NOT
TBC1D5	2066.48	-0.2361696	0.144788	-1.63114087	0.102861	0.30161	NOT
C22orf39	809.1302	-0.2616348	0.160407	-1.63106389	0.102877	0.30161	NOT
WBSCR22	3276.392	0.24561694	0.150588	1.631054432	0.102879	0.30161	NOT
ECHS1	23280.74	-0.4306242	0.26403	-1.63096937	0.102897	0.30163	NOT
TSNAXIP1	38.96404	-0.4917601	0.301542	-1.63081549	0.102929	0.301693	NOT
RP11-637A	14.82453	0.48199322	0.29557	1.630723274	0.102949	0.301707	NOT
NPR3	535.2517	-0.7765287	0.476201	-1.63067494	0.102959	0.301707	NOT
DNMBP	1064.017	-0.3189105	0.195574	-1.63063495	0.102967	0.301707	NOT
IL2RG	1003.58	0.64584293	0.396112	1.630454206	0.103006	0.301786	NOT
LINC0094	5.855592	0.80525245	0.493972	1.630157429	0.103068	0.301937	NOT
TCF20	1910.588	0.27065863	0.166043	1.63004969	0.103091	0.301943	NOT
PASK	321.3406	0.3902182	0.239401	1.629977901	0.103106	0.301943	NOT
RP4-739H	3.154993	-0.5565224	0.341434	-1.62995696	0.103111	0.301943	NOT
PRCD	31.04691	-0.4990862	0.306205	-1.62990822	0.103121	0.301943	NOT
RPL5P34	25.3632	0.44773099	0.274701	1.629884098	0.103126	0.301943	NOT
PA2G4P4	9.888478	0.48647545	0.298495	1.629761482	0.103152	0.301987	NOT
FAM131C	29.26458	1.05650557	0.648379	1.629457858	0.103216	0.302142	NOT
PPP1R14A	248.9718	-0.5644088	0.346406	-1.62932813	0.103244	0.302152	NOT
STARD9	212.0112	-0.4161602	0.255419	-1.62932639	0.103244	0.302152	NOT
RP11-762I	8.805989	0.39612289	0.243134	1.629234502	0.103263	0.302152	NOT
MAGEB16	3.639539	2.26754333	1.391787	1.629231474	0.103264	0.302152	NOT
BTBD1	1805.089	-0.1837091	0.112763	-1.62916341	0.103278	0.302162	NOT
RP11-395I	2.560035	0.79562985	0.488384	1.629108222	0.10329	0.302163	NOT
RP11-430K	3.430318	-0.6942991	0.426267	-1.6287885	0.103358	0.302329	NOT
RP1-240K6	23.27688	-0.5518352	0.338813	-1.62872956	0.10337	0.302332	NOT

CYB5RL	137.7204	0.35858734	0.22018	1.628607249	0.103396	0.30237	NOT
SMC4	1395.68	0.52179533	0.320402	1.628563216	0.103406	0.30237	NOT
TXNP6	4.552994	0.74161415	0.455482	1.628195764	0.103483	0.302565	NOT
ANKS1A	1051.652	0.27774808	0.1706	1.628070114	0.10351	0.302611	NOT
NMNAT1	452.0295	-0.3157795	0.193972	-1.62796589	0.103532	0.302643	NOT
TAS2R60	0.837725	1.62051698	0.995838	1.627289309	0.103676	NA	NA
RPL10A	18907.76	0.34097501	0.209548	1.627189433	0.103697	0.303092	NOT
TMUB2	1893.321	0.2118019	0.130172	1.627093023	0.103717	0.303119	NOT
RNF149	2306.277	0.28760239	0.176806	1.626659701	0.103809	0.303355	NOT
DCUN1D2	464.2201	0.40402068	0.248392	1.626545874	0.103834	0.303393	NOT
RIPPLY2	2.782027	2.09684318	1.289252	1.62640242	0.103864	0.30345	NOT
RP11-46A1	39.81953	0.4678117	0.287654	1.626298633	0.103886	0.303479	NOT
ARHGEF16	922.731	0.64958518	0.399446	1.626214308	0.103904	0.303479	NOT
AC069513.	8.04809	0.6164441	0.379079	1.626163156	0.103915	0.303479	NOT
SYNJ1	426.2673	-0.3155213	0.19403	-1.62614403	0.103919	0.303479	NOT
LINC01117	3.964975	1.1267445	0.692984	1.625931231	0.103964	0.303579	NOT
CTB-186H2	8.458247	-0.7936822	0.488211	-1.62569494	0.104015	0.30363	NOT
DBR1	506.0744	0.23782173	0.146291	1.625681132	0.104018	0.30363	NOT
CSTA	780.5242	-0.7637545	0.469815	-1.62564958	0.104024	0.30363	NOT
LEF1-AS1	8.734331	0.8224313	0.505913	1.625638055	0.104027	0.30363	NOT
UAP1	4780.245	-0.3616036	0.222454	-1.62551827	0.104052	0.303672	NOT
C11orf30	455.4033	-0.2439191	0.150064	-1.6254308	0.104071	0.303681	NOT
GATA4	2024.099	0.40577728	0.249648	1.625398282	0.104078	0.303681	NOT
PCDHGB3	19.36645	-0.7930372	0.487919	-1.62534516	0.104089	0.303682	NOT
CTB-60B18	1.709718	1.60433201	0.987113	1.625276443	0.104104	0.303692	NOT
C1orf210	319.858	0.69788595	0.429496	1.624895362	0.104185	0.303896	NOT
YTHDF1	2812.049	0.15125121	0.093108	1.624470339	0.104275	0.304127	NOT
RP11-256I	21.15348	-0.88052	0.542067	-1.62437548	0.104296	0.304154	NOT
TMEM261	982.0931	-0.4205127	0.258931	-1.62403278	0.104369	0.304334	NOT
ELANE	4.004127	-1.0289214	0.633619	-1.62388106	0.104401	0.304396	NOT
ELF5	5.87359	1.01638339	0.625978	1.623672637	0.104446	0.304466	NOT
MID1IP1	4689.621	0.38616527	0.237843	1.623614114	0.104458	0.304466	NOT
MLXIPL	14472.94	-0.5312054	0.327175	-1.6236111	0.104459	0.304466	NOT
RP11-472(	2.565187	0.99958645	0.615772	1.623306463	0.104524	0.304623	NOT
AP5M1	1292.003	-0.3025001	0.18636	-1.62320575	0.104545	0.304653	NOT
RP3-340B1	6.387524	0.62387064	0.384364	1.623122769	0.104563	0.304672	NOT
LINC00707	1.133108	1.52719879	0.940909	1.623109479	0.104566	NA	NA
BMP7	18.49482	-1.3626201	0.839573	-1.62299169	0.104591	0.304721	NOT
AP000355.	100.4639	-0.8493304	0.523353	-1.62286274	0.104619	0.304761	NOT
BCL7A	1055.333	0.26542742	0.163561	1.622800623	0.104632	0.304761	NOT
RP11-96D1	2.971458	-0.5741443	0.353808	-1.62275887	0.104641	0.304761	NOT
RP11-115(	74.77746	0.33226852	0.204763	1.622694275	0.104655	0.304761	NOT
ANK1	17.52722	-0.5913739	0.364454	-1.62263033	0.104668	0.304761	NOT
CTD-3185F	51.02739	-0.3018115	0.186004	-1.62261156	0.104672	0.304761	NOT
SERPINF2	46323.31	-0.6085632	0.375071	-1.62252739	0.10469	0.304781	NOT
AC145343.	1.681284	-0.7339124	0.452406	-1.62224299	0.104751	0.304921	NOT
GNAZ	787.2244	0.71935595	0.443449	1.622184894	0.104764	0.304921	NOT
CDKN2A	968.984	0.72744758	0.448448	1.622144318	0.104772	0.304921	NOT
AC009133.	168.7789	-0.2358422	0.14541	-1.62190913	0.104823	0.305014	NOT

CTC-548K1	2.505661	-0.6907368	0.425884	-1.62189078	0.104827	0.305014	NOT
RP11-425I	7.198388	-0.4984162	0.307357	-1.62161803	0.104885	0.305151	NOT
RP11-616K	7.519955	0.93525726	0.576851	1.621314477	0.10495	0.305264	NOT
RP1-27K12	480.3906	1.05410271	0.650159	1.621300811	0.104953	0.305264	NOT
POLD4	1947.344	-0.3511183	0.216574	-1.62124112	0.104966	0.305264	NOT
LINC0047(	52.15137	-1.1341279	0.699549	-1.6212273	0.104969	0.305264	NOT
CLK4	590.6011	-0.2782927	0.171676	-1.6210305	0.105011	0.305353	NOT
TRABD	2114.459	0.29101984	0.179539	1.620929965	0.105033	0.305353	NOT
PDE10A	86.17371	-0.5691314	0.351115	-1.62092625	0.105033	0.305353	NOT
HNRNPA1P7	2.198043	0.74898647	0.462147	1.620666819	0.105089	0.305474	NOT
CSNK1D	5978.576	0.15527793	0.095813	1.620628617	0.105097	0.305474	NOT
RP11-881M	18.38293	-0.5674338	0.350156	-1.62051767	0.105121	0.305494	NOT
TPM3P6	14.36553	0.60434272	0.372938	1.620491061	0.105127	0.305494	NOT
TNIP2	1548.946	0.26586385	0.164074	1.620387795	0.105149	0.305516	NOT
DYX1C1	6.686487	0.82383511	0.508437	1.620327524	0.105162	0.305516	NOT
MED15P4	1.425875	2.6516539	1.636547	1.620273837	0.105173	0.305516	NOT
IGKV1-17	30.62315	1.1624564	0.717456	1.620246605	0.105179	0.305516	NOT
CACTIN-AS	7.12196	-0.485368	0.299593	-1.62008984	0.105213	0.305581	NOT
UBXN10	622.4099	-0.8642468	0.533533	-1.61985663	0.105263	0.305693	NOT
CCL7	1.506394	1.62922586	1.005817	1.619803858	0.105274	0.305694	NOT
OXTR	77.23121	0.7325859	0.452308	1.61966148	0.105305	0.30575	NOT
CMTR1	2430.749	0.21110346	0.130353	1.619476962	0.105345	0.3058	NOT
RP11-693M	2.103181	-0.739415	0.456577	-1.61947565	0.105345	0.3058	NOT
RP11-789(	7.763198	-0.7059227	0.435927	-1.61936076	0.10537	0.305827	NOT
AC016700.	89.86795	0.5117162	0.316005	1.619328193	0.105377	0.305827	NOT
TMSB4X	28680.33	0.46391376	0.286544	1.618996221	0.105448	0.305986	NOT
PRR18	150.1935	-0.7005455	0.432714	-1.61895901	0.105456	0.305986	NOT
MACF1	5551.205	-0.2951273	0.182299	-1.61891561	0.105465	0.305986	NOT
HMOX2	2998.859	-0.3325314	0.205433	-1.61868819	0.105514	0.306085	NOT
chr22-38_	225.7695	0.33729097	0.208388	1.618571092	0.10554	0.306085	NOT
POU3F1	8.062135	0.85354426	0.527357	1.618530892	0.105548	0.306085	NOT
AC003002.	2.137509	-0.6737466	0.416277	-1.61850725	0.105553	0.306085	NOT
TBC1D14	729.2825	-0.4497329	0.277871	-1.61849402	0.105556	0.306085	NOT
RREB1	3406.455	-0.2700534	0.16686	-1.61844302	0.105567	0.306085	NOT
RP11-834(	2.22195	-0.9672512	0.597795	-1.61803073	0.105656	0.306302	NOT
RP11-178I	1.690692	-0.8108495	0.501146	-1.6179904	0.105665	0.306302	NOT
PRH2	15.63559	-0.9124529	0.563967	-1.6179199	0.10568	0.306314	NOT
NUP85	1915.114	0.24220395	0.149708	1.617843447	0.105696	0.306329	NOT
UBE2R2	4229.844	0.17386523	0.10748	1.617648402	0.105738	0.306418	NOT
RP11-1029	0.643503	-1.828453	1.130332	-1.61762437	0.105744	NA	NA
RP11-382J	16.60811	0.40184278	0.248441	1.617458645	0.105779	0.306504	NOT
WWC2	1265.022	-0.4058245	0.250936	-1.61724106	0.105826	0.306607	NOT
GS1-259H1	2.361788	-1.0405063	0.643431	-1.61712156	0.105852	0.306649	NOT
RP11-119F	59.08287	0.52420938	0.324234	1.616764542	0.105929	0.306792	NOT
AC002550.	8.466985	-0.4035958	0.249635	-1.61674511	0.105933	0.306792	NOT
ZNF131	810.1365	0.22895548	0.141618	1.616708267	0.105941	0.306792	NOT
KRTCAP3	291.832	1.00251597	0.620107	1.616683343	0.105947	0.306792	NOT
CTXN1	41.04823	0.79739873	0.493389	1.616167005	0.106058	0.307082	NOT
DLL4	785.3128	-0.3101984	0.191962	-1.61593708	0.106108	0.307193	NOT

CR1L	5.973326	0.99206892	0.614021	1.615692998	0.106161	0.307291	NOT
KRT18P28	5.73425	0.53705699	0.332421	1.615593157	0.106182	0.307291	NOT
CLCNKB	3.886322	0.95796923	0.592957	1.615580031	0.106185	0.307291	NOT
MTR	1878.563	-0.343018	0.21232	-1.6155708	0.106187	0.307291	NOT
RP11-260A	1.303976	3.06464658	1.897091	1.615444915	0.106214	NA	NA
RP13-270F	85.27601	-0.4641701	0.287365	-1.61526345	0.106254	0.307451	NOT
KIF21B	662.359	0.5802973	0.35932	1.614987054	0.106313	0.307591	NOT
NINJ1	4336.637	-0.3104229	0.192232	-1.61483785	0.106346	0.307652	NOT
ADAMTSL4	1901.092	-0.4839871	0.299738	-1.61470283	0.106375	0.307704	NOT
RP11-10J5	1.276661	1.60851716	0.996291	1.614504824	0.106418	NA	NA
ZBED5	828.2779	0.26924561	0.166797	1.614209054	0.106482	0.307981	NOT
RBCK1	6416.12	0.31127837	0.192849	1.614100682	0.106506	0.308016	NOT
AC010761.	3.033703	-0.7014841	0.434641	-1.61394039	0.10654	0.308068	NOT
TMEM139	506.4354	0.69614333	0.431339	1.613913107	0.106546	0.308068	NOT
TRIM46	26.60167	0.55014127	0.340926	1.613669124	0.106599	0.308113	NOT
RP5-907D1	1.652155	1.33269931	0.825904	1.613624741	0.106609	0.308113	NOT
WDFY3-AS2	42.1955	-0.524817	0.325244	-1.61360807	0.106612	0.308113	NOT
CLCN1	15.19458	-0.9240872	0.572689	-1.61359452	0.106615	0.308113	NOT
RP1-142L7	4.845783	-0.8320577	0.51566	-1.61357954	0.106619	0.308113	NOT
HMCES	2801.503	0.19306819	0.119658	1.613503946	0.106635	0.308128	NOT
CTB-63M22	106.0014	0.56761359	0.351816	1.613380535	0.106662	0.308144	NOT
RP11-396(	1.237442	-2.3798761	1.475103	-1.61336269	0.106666	NA	NA
ANKRD18B	12.36328	1.15280102	0.714548	1.613328133	0.106673	0.308144	NOT
AC009120.	2.057758	-0.9025782	0.559453	-1.61332146	0.106675	0.308144	NOT
ATP11AUN	1.759102	1.06279926	0.658862	1.613082836	0.106727	0.30826	NOT
EPHX3	16.97734	0.64939049	0.402599	1.612997028	0.106745	0.308281	NOT
IGLV1-51	132.1906	-0.8982136	0.5569	-1.61288044	0.10677	0.308322	NOT
RP11-444E	7.680858	-0.5776381	0.358175	-1.61272379	0.106805	0.308387	NOT
IFIT5	886.1965	-0.3119039	0.193413	-1.61263187	0.106825	0.308412	NOT
COL4A2-AS	1.804381	-0.7352437	0.455959	-1.61251984	0.106849	0.308422	NOT
NUCKS1	17362.37	-0.2461595	0.152662	-1.61244657	0.106865	0.308422	NOT
CPS1	78844.06	-0.9848203	0.610775	-1.61241121	0.106872	0.308422	NOT
TTC39C	6241.786	-0.45874	0.284506	-1.61240687	0.106873	0.308422	NOT
HDAC11-AS	2.116567	0.77499663	0.480805	1.611872523	0.10699	0.308725	NOT
PSMB5	5657.167	0.25479711	0.158091	1.611707359	0.107026	0.308784	NOT
RP11-422N	49.82087	-1.1266835	0.69914	-1.61152791	0.107065	0.308784	NOT
AATBC	21.32476	-0.5078015	0.315109	-1.61151231	0.107068	0.308784	NOT
AC008069.	5.544866	-0.9392375	0.582834	-1.61150221	0.10707	0.308784	NOT
MAD2L1	594.8018	0.48632463	0.301795	1.611440337	0.107084	0.308784	NOT
PLAGL2	670.6153	0.39292441	0.243835	1.611436182	0.107085	0.308784	NOT
CLEC4C	0.921318	1.26368819	0.78422	1.611394862	0.107094	NA	NA
RP4-655J1	11.27855	0.68883047	0.427488	1.611343878	0.107105	0.308784	NOT
TACO1	2813.114	-0.3624051	0.224911	-1.61132438	0.107109	0.308784	NOT
C11orf54	4428.88	-0.4357113	0.270424	-1.61121275	0.107133	0.308784	NOT
C9orf69	1985.592	0.28657237	0.177867	1.611159911	0.107145	0.308784	NOT
ZNF771	242.7152	-0.3705328	0.229991	-1.61107754	0.107163	0.308784	NOT
NAA11	54.83652	2.1970652	1.363731	1.611068986	0.107165	0.308784	NOT
CTB-131B5	22.61317	-0.53927	0.334731	-1.61105253	0.107168	0.308784	NOT
AC107983.	32.6872	-0.5644456	0.35036	-1.611047	0.107169	0.308784	NOT

C2orf40	12.22375	-0.7632576	0.473872	-1.61068279	0.107249	0.30898	NOT
RP4-669H2	4.421128	0.77181622	0.479217	1.610579289	0.107271	0.309012	NOT
RP11-106I	13.22184	-0.7050572	0.437817	-1.61039092	0.107313	0.309071	NOT
APOF	2484.607	-0.9072106	0.563352	-1.61038008	0.107315	0.309071	NOT
SLC37A2	365.9184	0.52920329	0.328635	1.610308962	0.10733	0.309083	NOT
TOR1AIP1	2564.401	-0.3055298	0.189767	-1.61002652	0.107392	0.309218	NOT
RP11-479(	269.6914	0.47383794	0.294316	1.609961606	0.107406	0.309218	NOT
UBOX5-AS1	7.32843	-0.4125054	0.256228	-1.60991266	0.107417	0.309218	NOT
C17orf10	13.20053	-0.4458787	0.276963	-1.6098854	0.107423	0.309218	NOT
GS1-257G1	8.926289	0.4693892	0.291596	1.60972349	0.107458	0.309287	NOT
AC114752.	2.232746	0.81724671	0.507718	1.609648106	0.107475	0.309302	NOT
WFDC3	14.1258	0.69287502	0.430483	1.60953027	0.1075	0.309343	NOT
RCE1	641.3988	0.25439431	0.15807	1.609375855	0.107534	0.309407	NOT
MAGEA8-AS	1.102114	1.54623684	0.960819	1.609289916	0.107553	NA	NA
SEC23A	3727.049	-0.3232656	0.200882	-1.6092309	0.107566	0.309466	NOT
HYI-AS1	12.60308	-0.5232283	0.325235	-1.6087695	0.107667	0.309642	NOT
ANKRD20A7	4.022017	1.55753873	0.968164	1.60875526	0.10767	0.309642	NOT
ATXN1L	1337.17	-0.3085028	0.191766	-1.6087443	0.107672	0.309642	NOT
STOX1	80.96777	0.80109615	0.497964	1.608741756	0.107673	0.309642	NOT
SART1	3580.302	0.22105169	0.13742	1.608581299	0.107708	0.30971	NOT
C1orf111	11.59027	-1.029043	0.639779	-1.60843402	0.10774	0.309768	NOT
RAB24	480.3439	0.28414151	0.176663	1.608378017	0.107752	0.309768	NOT
TPGS1	249.2987	-0.5190683	0.322743	-1.60830247	0.107769	0.309768	NOT
LRRC2	454.4521	-0.5960149	0.370593	-1.60827447	0.107775	0.309768	NOT
RNA5SP46	1.877414	-1.2999021	0.808282	-1.60822794	0.107785	0.309768	NOT
LRCH1	697.1834	-0.3084045	0.191812	-1.60784907	0.107868	0.309974	NOT
REP15	16.02974	0.71703169	0.44598	1.607764689	0.107887	0.309984	NOT
RP1-79C4.	1.539586	1.10996039	0.69039	1.60772841	0.107895	0.309984	NOT
RGS10	388.011	0.502921	0.312841	1.607592663	0.107924	0.310035	NOT
PSMG1	1095.463	0.26994678	0.167925	1.607543244	0.107935	0.310035	NOT
RP11-411E	3.084311	-0.6048449	0.376353	-1.60712255	0.108027	0.310267	NOT
RP1-287H1	1.206002	1.83845937	1.144131	1.606860658	0.108085	NA	NA
RP1-40E16	21.80082	-0.5133022	0.319465	-1.60675513	0.108108	0.310447	NOT
FLG-AS1	34.99331	0.88008585	0.547751	1.60672639	0.108114	0.310447	NOT
FAM229A	119.2509	-0.4393293	0.273439	-1.60668175	0.108124	0.310447	NOT
GS1-293C5	4.02298	-0.5493159	0.34194	-1.60646888	0.108171	0.310548	NOT
CDC42EP5	100.0011	-0.4582759	0.285281	-1.60639929	0.108186	0.310559	NOT
PRAMEF25	1.028773	1.96186167	1.221561	1.606027964	0.108268	NA	NA
ARMC10	1043.233	0.20036231	0.124778	1.605754431	0.108328	0.310933	NOT
ZNF564	33.85148	-0.3317013	0.20658	-1.60567581	0.108345	0.310941	NOT
ERO1LB	2450.717	-0.5622796	0.350191	-1.60563698	0.108354	0.310941	NOT
MAT2A	4410.671	0.3504268	0.218261	1.605541289	0.108375	0.310968	NOT
KLK5	0.684004	-2.6608078	1.657304	-1.60550352	0.108383	NA	NA
CCNT1	1002.21	-0.227371	0.141644	-1.60522489	0.108444	0.311135	NOT
AKAP9	3006.889	-0.4441861	0.276749	-1.6050147	0.108491	0.311235	NOT
RBM4B	573.9419	0.19359493	0.120628	1.604898259	0.108516	0.311276	NOT
CTD-2595I	1.225413	-1.0711799	0.667452	-1.60487824	0.108521	NA	NA
HMBOX1	378.4139	-0.2688184	0.167526	-1.60464007	0.108573	0.311393	NOT
MRPL52	2139.853	0.39511871	0.24624	1.604608284	0.10858	0.311393	NOT

SMG1P1	6.180564	-0.5270334	0.328506	-1.60433292	0.108641	0.311534	NOT
FAM171A1	2340.425	0.74723498	0.465812	1.604155692	0.10868	0.311595	NOT
HAX1	5623.821	0.2861966	0.178412	1.604131301	0.108685	0.311595	NOT
ZKSCAN4	206.1892	0.25080782	0.156383	1.603809536	0.108756	0.311706	NOT
PTCD2	438.3836	-0.2731954	0.170349	-1.60373695	0.108772	0.311706	NOT
HIPK3	2603.543	-0.2591713	0.161607	-1.60370944	0.108778	0.311706	NOT
APOM	14139.99	0.70473232	0.439465	1.603614247	0.108799	0.311706	NOT
MMP23A	10.7959	-1.2322579	0.768439	-1.60358635	0.108805	0.311706	NOT
AMMECR1	487.9755	0.3468997	0.216329	1.603574273	0.108808	0.311706	NOT
TMEM38B	1464.847	0.32486377	0.202595	1.603509934	0.108822	0.311706	NOT
KB-1615E4	23.38861	0.85326943	0.532151	1.603433468	0.108839	0.311706	NOT
UAP1L1	867.5653	0.78186944	0.487631	1.60340333	0.108846	0.311706	NOT
FAM35DP	51.80917	-0.6082825	0.379373	-1.60338994	0.108849	0.311706	NOT
LRP4	251.1507	0.82262652	0.513062	1.603365302	0.108854	0.311706	NOT
ADORA3	135.1787	0.55404173	0.345557	1.603331474	0.108861	0.311706	NOT
DSCAML1	32.35857	1.03760465	0.647197	1.603227317	0.108884	0.311739	NOT
NUP50	2201.474	0.21695435	0.135337	1.603067606	0.10892	0.311802	NOT
RP1-206D1	23.17524	-0.5706476	0.355982	-1.60302353	0.108929	0.311802	NOT
EGOT	18.0212	-0.8381132	0.522864	-1.60292792	0.108951	0.31182	NOT
SLC15A4	1351.472	0.26511012	0.165395	1.602890546	0.108959	0.31182	NOT
FAM168A	2400.548	-0.2337093	0.145844	-1.60245567	0.109055	0.312057	NOT
UBE3A	2539.813	-0.2173839	0.13566	-1.60241162	0.109065	0.312057	NOT
CDC42	7248.723	0.20446184	0.127616	1.602170415	0.109118	0.31212	NOT
GULOP	5.581699	-0.8303556	0.518273	-1.60215742	0.109121	0.31212	NOT
CTB-138E5	2.205846	1.31937126	0.823497	1.602156165	0.109121	0.31212	NOT
CCDC77	307.7999	0.31688849	0.197803	1.602043943	0.109146	0.312143	NOT
HTATSF1	2923.393	0.2397114	0.149631	1.602015321	0.109152	0.312143	NOT
RP11-254F	12.68408	0.56392885	0.35203	1.601933732	0.10917	0.312162	NOT
RP11-25I1	3.80026	0.66658317	0.41614	1.60182549	0.109194	0.312197	NOT
Z69720.3	1.998351	-1.02676	0.641021	-1.60175764	0.109209	0.312197	NOT
RP11-452H	3.804189	0.76126519	0.475279	1.601721599	0.109217	0.312197	NOT
TRDMT1	80.57232	0.39787934	0.248419	1.601644136	0.109234	0.312213	NOT
PPID	1773.465	-0.2170411	0.135527	-1.6014594	0.109275	0.312292	NOT
MOB1A	3489.773	0.23512412	0.146823	1.601414956	0.109285	0.312292	NOT
PTGR1	17707.21	0.65631928	0.40985	1.601364227	0.109296	0.312292	NOT
AC141928.	41.39296	1.07145208	0.66916	1.601189396	0.109335	0.312368	NOT
ZFHX4-AS1	16.02304	1.18983116	0.743153	1.601058671	0.109364	0.312368	NOT
BCOR	1505.572	0.31614097	0.197465	1.60099552	0.109378	0.312368	NOT
CPA1	5.083525	1.23227622	0.769696	1.600990842	0.109379	0.312368	NOT
ATXN10	4759.693	0.24129707	0.150718	1.600983521	0.109381	0.312368	NOT
PMS2P5	46.72183	-0.4896083	0.305883	-1.60063901	0.109457	0.312553	NOT
RP11-3M1.	8.057656	-0.8098497	0.505992	-1.60051949	0.109483	0.312596	NOT
SMAGP	542.7571	0.4273942	0.267114	1.600043612	0.109589	0.312864	NOT
PARP1	8263.705	0.25903187	0.161914	1.599806769	0.109641	0.312981	NOT
HIP1	2471.026	-0.3938986	0.246231	-1.59971064	0.109663	0.313009	NOT
SMG1P5	15.81569	-0.4205915	0.262958	-1.59945989	0.109718	0.313135	NOT
LINC00189	42.59325	1.03083485	0.644528	1.599364801	0.10974	0.313162	NOT
DNAJB2	4435.622	0.28651562	0.179156	1.599254477	0.109764	0.313167	NOT
THAP5	1078.59	-0.2713655	0.169683	-1.59925427	0.109764	0.313167	NOT

RP11-982M	1.924255	0.93315919	0.58352	1.599190689	0.109778	0.313174	NOT
RP11-407N	20.6678	-0.4325408	0.270494	-1.59907701	0.109803	0.313213	NOT
ERLIN1	3435.136	-0.3357784	0.209993	-1.59900153	0.10982	0.313228	NOT
LINC01372	15.63611	-0.483012	0.302094	-1.59887754	0.109848	0.313257	NOT
LARGE	1254.792	-0.387251	0.242206	-1.59885109	0.109854	0.313257	NOT
MLLT6	3080.109	0.30895806	0.193261	1.59865525	0.109897	0.313311	NOT
FCGR3A	1774.617	0.64566405	0.403879	1.598655166	0.109897	0.313311	NOT
LSM4	5466.751	0.35759665	0.223692	1.598611212	0.109907	0.313311	NOT
NGFR	497.5894	-0.709548	0.443917	-1.59838115	0.109958	0.313424	NOT
CLGN	845.0884	0.77756318	0.486489	1.598316428	0.109973	0.313432	NOT
RP11-467I	2.77296	-0.6539528	0.409199	-1.59812887	0.110014	0.31351	NOT
SPARC	24667.98	-0.401283	0.251113	-1.59801742	0.110039	0.31351	NOT
RP11-609N	13.74722	-0.6309131	0.394813	-1.59800342	0.110042	0.31351	NOT
ZNF600	288.3685	0.46885337	0.293403	1.597985725	0.110046	0.31351	NOT
KRT27	2.257938	-1.1547094	0.722698	-1.59777547	0.110093	0.31361	NOT
RP11-180C	15.67738	1.84131709	1.152506	1.597663946	0.110118	0.313648	NOT
ATG4A	886.2277	-0.2637523	0.165094	-1.59758502	0.110135	0.313648	NOT
RAPGEF5	1398.91	-0.3982851	0.249329	-1.59742595	0.110171	0.313648	NOT
SGIP1	80.76232	-0.6495278	0.406621	-1.59737874	0.110181	0.313648	NOT
CTD-2267I	2.720458	-0.5667298	0.354793	-1.59735401	0.110187	0.313648	NOT
AC096574.	2.201787	-0.71282	0.446258	-1.59732837	0.110193	0.313648	NOT
DNPH1	3596.058	0.36015958	0.225498	1.597175545	0.110227	0.313648	NOT
TRPV6	15.09254	0.95294991	0.596647	1.59717511	0.110227	0.313648	NOT
LPP-AS2	123.6594	-0.3209104	0.200929	-1.59712957	0.110237	0.313648	NOT
RP11-562A	2.085952	-0.618152	0.387041	-1.59712095	0.110239	0.313648	NOT
RP11-66B2	3.369121	1.12938079	0.707145	1.597098758	0.110244	0.313648	NOT
RN7SL608F	4.922597	-0.523702	0.327909	-1.59709345	0.110245	0.313648	NOT
XRCC6BP1	254.4538	0.35517405	0.222396	1.597031655	0.110259	0.313654	NOT
FAM103A1	476.463	0.20584639	0.128908	1.596842556	0.110301	0.31372	NOT
GLTSCR1	666.4028	-0.2221344	0.13911	-1.59682401	0.110305	0.31372	NOT
SEPT14	5.164885	1.98903772	1.245789	1.596608652	0.110353	0.313736	NOT
KRT36	1.968181	-0.8902117	0.557565	-1.59660636	0.110353	0.313736	NOT
HIST1H4I	714.6853	-0.4940459	0.309437	-1.59659589	0.110356	0.313736	NOT
RP13-514E	2.351006	-0.9077181	0.568535	-1.5965904	0.110357	0.313736	NOT
AL122127.	1.636205	1.54663461	0.968776	1.596482917	0.110381	0.313772	NOT
LINC01397	1.159694	1.41741287	0.887906	1.596354849	0.11041	NA	NA
F2RL3	211.4078	-0.6691338	0.4192	-1.59621713	0.11044	0.313907	NOT
LINS	311.6912	-0.2173814	0.136195	-1.59610856	0.110465	0.313941	NOT
CASR	14.95781	1.1060651	0.692997	1.596059743	0.110475	0.313941	NOT
RP11-707M	16.03668	0.5285063	0.331178	1.595839858	0.110525	0.314048	NOT
AC012074.	6.934193	0.65088681	0.407915	1.595644093	0.110568	0.314139	NOT
RP6-109B7	1.868362	0.79566876	0.49871	1.595452449	0.110611	0.314216	NOT
STX18	1260.549	0.23711228	0.148628	1.595338592	0.110637	0.314216	NOT
CARHSP1	5568.602	-0.3175481	0.19905	-1.59532012	0.110641	0.314216	NOT
RP11-722M	2.443644	2.70261665	1.694095	1.595315952	0.110642	0.314216	NOT
KCNC4	252.2147	0.50859691	0.318834	1.595175712	0.110673	0.314272	NOT
UBE2D3P2	3.890112	-0.4781472	0.299802	-1.59487562	0.11074	0.31443	NOT
C2CD5	1126.701	0.30705909	0.192573	1.594509823	0.110822	0.314629	NOT
WNT3	449.7881	-0.3884769	0.243665	-1.59430941	0.110867	0.314724	NOT

RP11-100I	6.209695	-0.6419665	0.402677	-1.59424618	0.110881	0.314731	NOT
NKIRAS1	524.678	-0.2926982	0.183605	-1.59417693	0.110896	0.314742	NOT
MANEA-AS1	16.8775	-0.517571	0.32468	-1.59409643	0.110914	0.31476	NOT
RP11-318(	5.210228	1.69889787	1.065894	1.593870786	0.110965	0.314871	NOT
AC005758.	0.55127	-3.2835121	2.060271	-1.5937279	0.110997	NA	NA
RP5-88207	4.718544	-0.7010012	0.439864	-1.59367653	0.111009	0.314951	NOT
OCLM	7.156951	-0.6131061	0.38472	-1.59364054	0.111017	0.314951	NOT
PRDM9	7.537581	1.11826316	0.701775	1.593478324	0.111053	0.314974	NOT
CBX7	1326.431	-0.3853526	0.241835	-1.59345272	0.111059	0.314974	NOT
RP11-616K	2.173452	0.93960364	0.589667	1.593449009	0.111059	0.314974	NOT
IGKV1-12	2.287555	-1.2688764	0.796398	-1.59327008	0.1111	0.315029	NOT
RPP30	882.1511	0.25139078	0.157784	1.59325931	0.111102	0.315029	NOT
CTD-2140(	1.0249	1.38924091	0.87198	1.593202882	0.111115	NA	NA
ZBTB8B	2.778815	0.82730718	0.519292	1.593143644	0.111128	0.31507	NOT
RP11-507K	7.994391	-0.4460963	0.280021	-1.59308191	0.111142	0.315076	NOT
RP11-209K	137.2479	-0.5488186	0.34457	-1.59276599	0.111213	0.315244	NOT
SPEF1	11.69338	0.65865208	0.413598	1.592493787	0.111274	0.315384	NOT
GTF3C5	2430.877	0.30161968	0.189426	1.59228503	0.111321	0.315484	NOT
SOX5	463.7889	-0.5382174	0.338058	-1.59208722	0.111365	0.315577	NOT
RP3-432I1	1.333065	1.25020412	0.785422	1.591760576	0.111439	NA	NA
C3orf17	1226.309	0.16791382	0.105493	1.591711385	0.11145	0.315783	NOT
ANKMY2	628.8408	0.23689369	0.148837	1.591629624	0.111468	0.315803	NOT
CTCFL	13.407	1.02240794	0.642424	1.591483475	0.111501	0.315863	NOT
CAP2	1600.737	0.48387668	0.304062	1.591372792	0.111526	0.315875	NOT
DST	6543.899	-0.3612021	0.226977	-1.5913608	0.111528	0.315875	NOT
GRHL1	415.309	0.49317707	0.309924	1.591281691	0.111546	0.315892	NOT
STK24-AS1	15.69002	0.49660861	0.312116	1.591103473	0.111586	0.315973	NOT
SLFN5	791.9737	-0.4451911	0.279816	-1.59101633	0.111606	0.315995	NOT
LRRC3C	2.631733	-1.0075929	0.633332	-1.59094018	0.111623	0.316011	NOT
TRAV8-4	3.079486	-0.8002091	0.503026	-1.59079198	0.111656	0.316053	NOT
EXOSC5	1224.059	0.30567638	0.192156	1.590771193	0.111661	0.316053	NOT
GMCL1P1	4.19875	1.54916877	0.973898	1.590689656	0.111679	0.316072	NOT
ZNF665	46.51337	0.65282465	0.410422	1.590619123	0.111695	0.316079	NOT
EEF1A1P13	117.141	0.38293172	0.240751	1.590574956	0.111705	0.316079	NOT
CTB-33018	17.94118	-0.5019686	0.315627	-1.59038585	0.111748	0.316161	NOT
ITGA2B	18.39964	0.67440572	0.424072	1.590307796	0.111765	0.316161	NOT
CTD-2124E	66.2851	0.43145344	0.271307	1.590278262	0.111772	0.316161	NOT
CTD-2357A	2.39137	1.11735817	0.702635	1.590239641	0.111781	0.316161	NOT
OBFC1	569.1937	-0.2642834	0.166227	-1.58989531	0.111858	0.316347	NOT
ZNF737	73.95589	0.62585072	0.393692	1.589695	0.111904	0.316413	NOT
RP11-253E	45.31422	0.62541698	0.393424	1.589678347	0.111907	0.316413	NOT
ARMC6	2330.422	-0.3610195	0.227118	-1.58956968	0.111932	0.316413	NOT
AP000442.	3.146675	-0.6887931	0.433326	-1.58955105	0.111936	0.316413	NOT
ANAPC5	4974.927	0.17809759	0.112044	1.589525843	0.111942	0.316413	NOT
RP11-563H	2.574304	0.73087227	0.459818	1.589481656	0.111952	0.316413	NOT
ZMYM4	1575.226	0.26953618	0.169607	1.589178876	0.11202	0.316573	NOT
CTD-2010I	16.05736	-0.3935111	0.247659	-1.58892421	0.112078	0.316703	NOT
AC004895.	3.973707	0.55882567	0.351715	1.588857947	0.112092	0.316712	NOT
CRYBB3	17.21828	-0.5380467	0.338665	-1.58872968	0.112121	0.316734	NOT

RS1	2.727001	0.77234217	0.48615	1.588689641	0.11213	0.316734	NOT
AC004022.	1.53388	-1.1698412	0.736366	-1.58866846	0.112135	0.316734	NOT
KCNA3	11.71475	-0.7511569	0.472847	-1.5885818	0.112155	0.316737	NOT
RBP7	551.963	-0.5885529	0.370494	-1.58856067	0.11216	0.316737	NOT
KLHL36	1217.487	-0.3035353	0.191101	-1.58835129	0.112207	0.316837	NOT
HAR1B	4.982266	-1.0432533	0.656872	-1.5882144	0.112238	0.316892	NOT
ZNF280D	1273.182	-0.2239166	0.141001	-1.58805024	0.112275	0.316964	NOT
CILP2	69.95615	0.8822842	0.555604	1.587973269	0.112292	0.31698	NOT
AC011343.	2.937422	-0.9829685	0.619051	-1.58786396	0.112317	0.316995	NOT
AC004837.	2.80341	-0.7275598	0.458206	-1.58784563	0.112321	0.316995	NOT
RNF169	365.0767	-0.4153213	0.261593	-1.58766433	0.112362	0.317067	NOT
C22orf34	46.99522	-0.4697244	0.295881	-1.58754453	0.112389	0.317067	NOT
EFCC1	67.06217	-0.484489	0.30519	-1.58750058	0.112399	0.317067	NOT
ZBTB4	1502.761	-0.3343729	0.210629	-1.58749447	0.112401	0.317067	NOT
TRMU	1325.038	0.33256087	0.209499	1.587413486	0.112419	0.317067	NOT
C11orf95	917.9347	-0.282044	0.177675	-1.58741189	0.112419	0.317067	NOT
LRRC75A	49.70567	0.57376763	0.36146	1.587359381	0.112431	0.317067	NOT
SLC9B2	1183.157	-0.4942225	0.311356	-1.58732037	0.11244	0.317067	NOT
PRRC1	3082.718	-0.2235503	0.140861	-1.58702531	0.112507	0.317211	NOT
PDZD7	26.69356	0.60950088	0.38406	1.586991891	0.112514	0.317211	NOT
PIK3IP1	1134.616	-0.4439787	0.27978	-1.58688302	0.112539	0.317247	NOT
EIF4A2P2	2.755124	0.63575558	0.400689	1.586655611	0.112591	0.317311	NOT
TIPARP-AS	78.24813	0.42110356	0.265428	1.586510387	0.112624	0.317311	NOT
PRODH	985.1191	-1.0377814	0.654129	-1.58650795	0.112624	0.317311	NOT
TANGO6	896.8625	-0.3336934	0.210334	-1.58649073	0.112628	0.317311	NOT
C7orf55	684.5932	-0.3903496	0.246049	-1.58647258	0.112632	0.317311	NOT
APOL4	221.1412	0.55749147	0.351406	1.586461049	0.112635	0.317311	NOT
GALNT12	60.23659	-0.8267879	0.521165	-1.58642215	0.112644	0.317311	NOT
ABCC5	971.7285	0.26483539	0.166945	1.586365456	0.112656	0.317314	NOT
NOL6	2059.682	0.23931859	0.150869	1.586272689	0.112677	0.31734	NOT
RP4-763G1	210.1893	-0.9346427	0.589256	-1.58614118	0.112707	0.317386	NOT
GIN1	214.4408	-0.2218012	0.139841	-1.58609828	0.112717	0.317386	NOT
FBXO33	500.3155	-0.2490598	0.157036	-1.58600536	0.112738	0.317412	NOT
PRKCG	1.188488	1.04922154	0.661587	1.585915259	0.112759	NA	NA
RP11-367(	10.0943	0.637067	0.401768	1.585659281	0.112817	0.3176	NOT
PTP4A3	1827.525	0.60433805	0.381166	1.585497641	0.112853	0.317671	NOT
RP11-497H	4.300296	0.74642164	0.470811	1.58539593	0.112876	0.317703	NOT
ZNF436	383.5442	-0.2408989	0.152033	-1.58451244	0.113077	0.318235	NOT
RPSAP31	2.419151	-0.6936482	0.437793	-1.58442099	0.113098	0.31826	NOT
CTD-2517(	39.44897	0.38669885	0.244185	1.583629677	0.113278	0.318695	NOT
RP11-136H	1.435516	-0.6375546	0.402594	-1.58361492	0.113281	0.318695	NOT
RP11-14C1	25.73234	1.13032836	0.713777	1.583587895	0.113288	0.318695	NOT
RP11-551I	2.807405	0.91734243	0.57935	1.583400375	0.11333	0.318772	NOT
MALAT1	3083.157	-0.4563884	0.28824	-1.58336438	0.113338	0.318772	NOT
TRIM5	1185.425	-0.2770019	0.174955	-1.58327152	0.11336	0.318798	NOT
POM121L4F	1.967139	1.13115806	0.714499	1.583148346	0.113388	0.318844	NOT
IL1RL2	132.7011	0.71459174	0.451576	1.582438436	0.11355	0.319256	NOT
CYP4B1	7.050142	-0.8728154	0.551584	-1.58237937	0.113563	0.319256	NOT
STAG3L4	274.267	0.37678027	0.238114	1.582351297	0.113569	0.319256	NOT

RP11-418J	22.34268	-0.8178196	0.516867	-1.582264	0.113589	0.319279	NOT
RP11-635I	6.883939	0.75221221	0.475424	1.582191222	0.113606	0.319292	NOT
LINC0017	10.8224	0.69036714	0.436425	1.581867313	0.11368	0.319467	NOT
RP11-2E11	0.906051	1.17677282	0.743964	1.581761472	0.113704	NA	NA
RP5-907C1	66.4923	-0.8174985	0.516852	-1.58168887	0.113721	0.319548	NOT
BTBD10	634.6068	0.19019219	0.120257	1.581541575	0.113754	0.31961	NOT
STX5	2349.827	-0.2046898	0.129452	-1.58119908	0.113833	0.319797	NOT
C8orf4	1682.655	-0.5545923	0.350756	-1.58113469	0.113847	0.319805	NOT
ZXDA	101.6802	-0.3504858	0.221681	-1.58103822	0.113869	0.319822	NOT
MT-ND4L	38394.84	-0.5303306	0.335449	-1.58095956	0.113887	0.319822	NOT
FNDC3B	4434.11	0.31726467	0.200685	1.580911648	0.113898	0.319822	NOT
BHLHA15	79.44597	-0.69212	0.437801	-1.5808998	0.113901	0.319822	NOT
APITD1-C(	18.26312	0.37582679	0.237737	1.580850723	0.113912	0.319822	NOT
AC133785.	1.916566	1.54587667	0.978066	1.580545072	0.113982	0.319946	NOT
SPPL3	2162.556	0.21908853	0.138617	1.580531879	0.113985	0.319946	NOT
MXD3	600.0984	0.41934277	0.265323	1.580501949	0.113992	0.319946	NOT
RNF144B	870.6009	-0.4687694	0.296656	-1.58017656	0.114066	0.320122	NOT
COPG2	1061.911	0.28595521	0.180983	1.580015704	0.114103	0.320193	NOT
ZNF763	53.60805	-0.4560385	0.2887	-1.57962658	0.114192	0.320381	NOT
ASB7	613.1901	-0.2378801	0.150593	-1.57961997	0.114194	0.320381	NOT
MAGEA3	567.7679	2.03550138	1.28866	1.57954831	0.11421	0.320394	NOT
EVC2	144.1972	0.72673362	0.460175	1.579253519	0.114278	0.320522	NOT
RP11-203J	18.26615	-0.512054	0.324239	-1.5792462	0.11428	0.320522	NOT
RP11-38L1	1.691549	1.04040749	0.658833	1.579168155	0.114297	0.320539	NOT
SIGLEC10	152.9071	0.61665389	0.390573	1.578845791	0.114371	0.320686	NOT
PKN2-AS1	2.632303	-0.7226406	0.457735	-1.57873299	0.114397	0.320686	NOT
HSPBAP1	299.0395	0.25496066	0.161505	1.578656058	0.114415	0.320686	NOT
TAS1R3	33.62477	0.61335422	0.38853	1.578654671	0.114415	0.320686	NOT
HIST1H3J	2.707939	-0.7317284	0.463519	-1.57863838	0.114419	0.320686	NOT
RP11-181(	6.118564	-0.7597842	0.481293	-1.57862971	0.114421	0.320686	NOT
CTD-3035I	17.90074	0.55533126	0.351802	1.578533887	0.114443	0.320715	NOT
FNDC1	271.4306	-0.8985487	0.569303	-1.57833051	0.11449	0.320812	NOT
RP11-69L1	1.212838	1.64993647	1.045517	1.578105924	0.114541	NA	NA
TESC	1085.741	0.81388344	0.515759	1.578030958	0.114558	0.320965	NOT
RP11-545A	1.518155	1.33034047	0.84306	1.577990631	0.114568	0.320965	NOT
MRPL17	1862.962	0.30995767	0.19644	1.577874667	0.114594	0.320985	NOT
SRPRB	4884.999	0.25101821	0.159088	1.577856041	0.114599	0.320985	NOT
CCDC110	32.18934	0.71192766	0.451267	1.57761849	0.114653	0.321105	NOT
PHF3	3367.445	-0.3092565	0.196037	-1.5775431	0.114671	0.32112	NOT
THY1	3206.295	-0.441222	0.279706	-1.57745155	0.114692	0.321146	NOT
ZNF14	110.06	0.59522302	0.377349	1.577380778	0.114708	0.321159	NOT
XBP1P1	4.418246	-0.5840596	0.370285	-1.57732614	0.11472	0.321161	NOT
RP11-554F	4.587607	0.5382324	0.341256	1.577208398	0.114748	0.321203	NOT
TSEN34	2108.41	0.25640182	0.162586	1.577019456	0.114791	0.321261	NOT
PHF6	830.1175	0.29615191	0.187793	1.577016228	0.114792	0.321261	NOT
FAXDC2	3720.709	-0.5648575	0.358212	-1.57688023	0.114823	0.321315	NOT
SMC2	870.6727	0.38773016	0.245895	1.576813376	0.114838	0.321322	NOT
ACTBL2	1.603783	1.40583975	0.891597	1.576766216	0.114849	0.321322	NOT
EML6	122.1081	0.5269243	0.3342	1.576674634	0.11487	0.321329	NOT

KRBOX4	436.3972	0.16077698	0.101974	1.576653417	0.114875	0.321329	NOT
C14orf119	1619.708	0.23019355	0.146018	1.576471603	0.114917	0.321403	NOT
FAT1	7320.748	-0.4466758	0.283345	-1.5764355	0.114925	0.321403	NOT
TRAM2	1561.278	-0.2793523	0.177229	-1.57622241	0.114975	0.321507	NOT
RP11-407I	5.633634	-0.7240045	0.459385	-1.57603017	0.115019	0.321598	NOT
MRAP2	178.5933	0.87114784	0.552846	1.575750488	0.115083	0.321725	NOT
RP4-545L1	3.468756	0.88159959	0.559494	1.575709508	0.115093	0.321725	NOT
ERGIC3	10131.34	0.21405551	0.13585	1.575678228	0.1151	0.321725	NOT
RP11-326K	24.17768	-0.3492916	0.221701	-1.57550981	0.115139	0.3218	NOT
6-Mar	5969.863	-0.2445113	0.155239	-1.57506306	0.115242	0.322003	NOT
RPL39P3	137.7004	0.55338993	0.351345	1.575059162	0.115243	0.322003	NOT
RP11-332M	243.8936	0.29187461	0.185312	1.575040644	0.115247	0.322003	NOT
CYP39A1	697.3077	-0.8609028	0.546613	-1.57497541	0.115262	0.322012	NOT
GPR63	11.62837	0.78527864	0.498619	1.574905641	0.115278	0.322024	NOT
AC068533.	9.545103	-0.7254428	0.460732	-1.5745456	0.115361	0.322223	NOT
ASCL5	4.141093	0.96574951	0.613476	1.574224526	0.115436	0.322397	NOT
CEBPA	8884.248	0.44870504	0.285045	1.574156309	0.115451	0.322408	NOT
EIF2A	3535.962	0.1997458	0.126898	1.574059793	0.115474	0.322437	NOT
VTCN1	78.76454	1.04482016	0.663918	1.573718782	0.115552	0.322623	NOT
RP11-665C	2.417842	-0.5483928	0.348492	-1.57361539	0.115576	0.322623	NOT
RP11-120I	5.960227	0.69441397	0.441296	1.573578379	0.115585	0.322623	NOT
COX7B	7088.119	-0.3724296	0.236682	-1.57354463	0.115593	0.322623	NOT
ACADSB	11318.2	-0.5135739	0.326386	-1.57351537	0.1156	0.322623	NOT
AGBL3	183.3053	0.38122005	0.242299	1.57334637	0.115639	0.322699	NOT
RP11-1094	266.7261	0.36509848	0.232104	1.57299566	0.11572	0.322892	NOT
ATP6AP1L	156.0797	-0.39022	0.248141	-1.57257619	0.115817	0.3231	NOT
RP11-225N	1.889901	-0.9374667	0.596136	-1.57257122	0.115818	0.3231	NOT
RP11-579I	31.62534	-0.5603739	0.356366	-1.57246838	0.115842	0.323113	NOT
RP11-225I	68.77092	0.3054401	0.194248	1.572420874	0.115853	0.323113	NOT
LMAN1	9263.482	-0.2961075	0.188316	-1.57239668	0.115859	0.323113	NOT
CH507-513	22.97902	-0.4873077	0.309925	-1.57234174	0.115871	0.323115	NOT
CTD-2299I	4.045257	-0.9108254	0.579348	-1.57215553	0.115914	0.323202	NOT
LCN12	392.9732	-0.5271416	0.335332	-1.57199862	0.115951	0.323271	NOT
RP11-111J	9.718715	-0.3890467	0.247503	-1.57188504	0.115977	0.323311	NOT
COX6B1	10611.45	-0.3750215	0.238589	-1.57183182	0.11599	0.323312	NOT
LTB4R	317.1881	-0.4374097	0.278303	-1.57170606	0.116019	0.32336	NOT
JMY	954.3737	-0.2922212	0.185939	-1.57159576	0.116044	0.323398	NOT
CCDC167	1370.69	0.37754706	0.24025	1.571477895	0.116072	0.323441	NOT
RNF186	26.94597	0.96692477	0.615343	1.57135902	0.116099	0.323485	NOT
AC005682.	2.13824	0.81140197	0.516419	1.571209523	0.116134	0.323538	NOT
RP11-475J	15.13089	-0.7533975	0.479512	-1.57117402	0.116142	0.323538	NOT
ARPP19	4154.618	0.20971629	0.133502	1.570880975	0.11621	0.323695	NOT
ACTN1-AS1	3.504705	-0.6710644	0.427207	-1.57081693	0.116225	0.323703	NOT
AC004775.	2.865689	0.87253628	0.555496	1.570733044	0.116245	0.323724	NOT
GATB	1229.26	-0.2680095	0.170637	-1.57064341	0.116266	0.323737	NOT
AC005740.	6.42162	-0.5324662	0.339019	-1.57060995	0.116273	0.323737	NOT
RP11-30J2	47.94798	1.40571946	0.895087	1.570483682	0.116303	0.323786	NOT
CLLU1OS	1.386601	1.35708422	0.864274	1.570200998	0.116368	NA	NA
GZMB	75.45522	0.61449469	0.391364	1.570137364	0.116383	0.323977	NOT

PTCHD3	3.397172	1.7159365	1.092902	1.570073138	0.116398	0.323982	NOT
MT-TL2	1.339093	1.01665484	0.647522	1.570070375	0.116399	NA	NA
MED18	801.1153	-0.2520121	0.160515	-1.57002576	0.116409	0.323982	NOT
ASS1P1	44.83673	-0.5367911	0.341934	-1.56986798	0.116446	0.324051	NOT
RNU4-2	8.442191	-0.7692467	0.490042	-1.56975567	0.116472	0.324091	NOT
KLF5	471.203	0.75196332	0.479107	1.569508876	0.116529	0.324157	NOT
NOC4L	1235.476	0.33206331	0.211572	1.569507431	0.11653	0.324157	NOT
CXCR2P1	122.4549	0.74915103	0.477318	1.569500032	0.116531	0.324157	NOT
SLC15A1	1506.116	0.78653026	0.501165	1.569405074	0.116554	0.324185	NOT
ACSL4	14034.52	0.78012162	0.497108	1.569321409	0.116573	0.324206	NOT
GBX2	3.529281	0.95977821	0.61164	1.569188602	0.116604	0.324259	NOT
ZNF331	808.0492	0.6025898	0.384043	1.569069767	0.116632	0.324302	NOT
APOC1	167982.8	-0.5672168	0.361551	-1.56884159	0.116685	0.324417	NOT
PSMB8-AS1	406.0592	-0.4097283	0.261213	-1.56855907	0.116751	0.324567	NOT
RP11-66N2	34.40268	-0.4905899	0.312812	-1.56832198	0.116806	0.324687	NOT
ZFR2	6.575917	0.81080153	0.517017	1.568231103	0.116827	0.324713	NOT
RP11-344F	6.736787	-0.5838902	0.37247	-1.56761476	0.116971	0.325079	NOT
RP11-777F	3.217322	-0.6721198	0.428783	-1.56750599	0.116996	0.325117	NOT
PAFAH2	984.5289	-0.296059	0.188889	-1.56737182	0.117028	0.32517	NOT
GADL1	2.024434	-1.239432	0.790948	-1.56701999	0.11711	0.325331	NOT
VPS28	9048.713	0.42960247	0.274165	1.566947689	0.117127	0.325331	NOT
RP11-468N	81.36123	-0.8813393	0.562462	-1.56693252	0.11713	0.325331	NOT
KPNA7	75.04512	0.68068674	0.434411	1.566919578	0.117133	0.325331	NOT
HMGN1	3858.75	0.25601527	0.163398	1.566822899	0.117156	0.325352	NOT
RP3-407E4	3.225613	-1.2059055	0.769669	-1.56678383	0.117165	0.325352	NOT
RP11-686I	4.318927	-0.8578322	0.547575	-1.56660137	0.117208	0.32537	NOT
KCNH8	19.73184	-1.1640397	0.743044	-1.56658303	0.117212	0.32537	NOT
AKR7A2P1	4.100757	-0.6220082	0.397077	-1.56646603	0.11724	0.32537	NOT
HAO2	2207.453	-0.957376	0.61118	-1.56643809	0.117246	0.32537	NOT
TMEM194B	299.7176	0.27000186	0.172367	1.566435776	0.117247	0.32537	NOT
KTN1-AS1	74.00698	0.33873887	0.216249	1.566427234	0.117249	0.32537	NOT
RPL5P12	4.10228	0.60815889	0.388253	1.566397033	0.117256	0.32537	NOT
RP11-483I	1.662673	1.16412777	0.743222	1.566326387	0.117272	0.325383	NOT
SSBP3	3119.078	-0.2781935	0.17764	-1.5660497	0.117337	0.325529	NOT
RP11-388M	3.416437	-0.6635707	0.42379	-1.56579918	0.117396	0.325636	NOT
U2SURP	2963.329	0.24933694	0.159242	1.565770705	0.117402	0.325636	NOT
GCNT7	3.555774	-0.5394576	0.34455	-1.5656888	0.117421	0.325636	NOT
ZNF616	338.0313	-0.2454504	0.156769	-1.56568059	0.117423	0.325636	NOT
LMO7	2943.927	-0.3983062	0.254484	-1.56515476	0.117547	0.325944	NOT
FKBP3	2122.868	0.21336444	0.136347	1.56486087	0.117616	0.326102	NOT
TMEM213	1.752681	1.11320628	0.711427	1.564751506	0.117641	0.326117	NOT
RP11-656C	3.52955	1.04462954	0.667608	1.56473533	0.117645	0.326117	NOT
EFCAB14	3812.536	-0.2402232	0.153534	-1.56462459	0.117671	0.326156	NOT
AF131215.	19.89267	0.87767862	0.560986	1.564528132	0.117694	0.326175	NOT
SLC39A7	10048.84	0.25533842	0.163208	1.564492433	0.117702	0.326175	NOT
SCN8A	393.7494	-0.4565034	0.291811	-1.56438076	0.117728	0.326214	NOT
CBR3	82.767	0.59854341	0.382663	1.564152615	0.117782	0.326288	NOT
CTC-492K1	1.911211	-0.6598946	0.421889	-1.5641431	0.117784	0.326288	NOT
CES1P1	785.8822	0.8401167	0.53712	1.564114242	0.117791	0.326288	NOT

UBE2J1	2464.37	-0.2146488	0.137242	-1.56401661	0.117814	0.326318	NOT
NOB1	1441.031	0.27577985	0.176345	1.563867853	0.117849	0.326381	NOT
MBLAC2	337.1924	-0.3268093	0.208993	-1.56373569	0.11788	0.326434	NOT
ATXN7L1	377.8631	-0.3733912	0.238805	-1.56358027	0.117916	0.326493	NOT
PSMA6P1	11.68185	0.51269788	0.327917	1.563500154	0.117935	0.326493	NOT
RP11-417I	13.50876	0.49284232	0.315222	1.563475952	0.117941	0.326493	NOT
RP11-29H2	4.344612	-0.563968	0.360723	-1.56343972	0.117949	0.326493	NOT
RP11-443I	151.4929	0.55131725	0.352683	1.563208813	0.118003	0.32659	NOT
RSL24D1	2477.213	0.19709692	0.126086	1.563188417	0.118008	0.32659	NOT
CCDC181	14.64009	0.47109343	0.301412	1.562957526	0.118063	0.326707	NOT
RP11-598I	3.010461	-0.9068397	0.580232	-1.56289195	0.118078	0.326717	NOT
CTC-265F1	5.488421	-0.8073688	0.51664	-1.56273028	0.118116	0.326767	NOT
UPB1	8170.613	-0.7381116	0.472327	-1.56271276	0.11812	0.326767	NOT
NADK2-AS1	15.32109	-0.6789406	0.434488	-1.56262119	0.118142	0.326793	NOT
SPIN4	189.5569	0.47356029	0.303073	1.562528965	0.118163	0.32682	NOT
MARK2	1596.404	0.21885864	0.140075	1.562439052	0.118185	0.326836	NOT
NBPF15	449.1165	-0.490708	0.314083	-1.56235304	0.118205	0.326836	NOT
RP11-511I	5.155011	1.47497831	0.944077	1.562350343	0.118205	0.326836	NOT
EXOSC1	922.1425	0.24130442	0.154458	1.562270413	0.118224	0.326853	NOT
KIAA0368	4532.669	-0.2062954	0.132053	-1.5622226	0.118236	0.326853	NOT
AL928768.	1.822142	-1.1086488	0.709729	-1.56207407	0.118271	0.326916	NOT
TUFMP1	4.46804	0.99389323	0.636303	1.561980355	0.118293	0.326944	NOT
RP11-390K	23.1826	-0.4668408	0.298895	-1.56188889	0.118314	0.32697	NOT
DISC1FP1	4.943162	-1.5027899	0.962347	-1.561588	0.118385	0.327125	NOT
IGHV3-41	1.421134	1.40647571	0.900693	1.561548521	0.118394	0.327125	NOT
LAIR1	686.5436	0.56318429	0.360699	1.561367972	0.118437	0.327209	NOT
RBAK	611.1389	-0.252386	0.161689	-1.5609377	0.118538	0.327456	NOT
RP11-1069	177.8012	-0.7776439	0.498223	-1.56083558	0.118563	0.327457	NOT
RP11-557I	1.623268	0.93281507	0.597639	1.560834094	0.118563	0.327457	NOT
SELP	91.90752	-0.7401586	0.474278	-1.5606006	0.118618	0.327552	NOT
C19orf70	2814.358	-0.4047721	0.259374	-1.56057426	0.118624	0.327552	NOT
CYP2F2P	15.73724	-0.4438033	0.284392	-1.56053489	0.118634	0.327552	NOT
ZNF786	283.4079	0.21882696	0.140262	1.560127858	0.11873	0.327784	NOT
DRAM2	1751.444	0.22629313	0.145078	1.559808071	0.118805	0.32796	NOT
SLC38A5	63.22008	0.54545882	0.349709	1.559749936	0.118819	0.327964	NOT
STEAP2	628.4824	0.78184128	0.501346	1.559485697	0.118881	0.328082	NOT
RNA5SP40	1.874855	-1.4571397	0.934383	-1.55946772	0.118886	0.328082	NOT
TNFSF8	40.78226	-0.5367035	0.344178	-1.55937789	0.118907	0.328092	NOT
TMA7	3434.166	0.31746213	0.20359	1.559320665	0.118921	0.328092	NOT
FAM86HP	24.31147	-0.4031507	0.258546	-1.5592977	0.118926	0.328092	NOT
RP5-1021I	78.68882	-0.4624064	0.296616	-1.55893683	0.119011	0.328288	NOT
AC108938.	11.31459	0.64730862	0.415235	1.558896044	0.119021	0.328288	NOT
RP11-495I	1.426328	1.40859393	0.903627	1.558822017	0.119039	0.328303	NOT
RP11-49I1	1.997353	-0.9700223	0.622452	-1.55838839	0.119141	0.328493	NOT
MRPL45	1970.251	0.24848392	0.159452	1.558366652	0.119146	0.328493	NOT
RP11-6L6.	3.333577	-1.0595909	0.67994	-1.55835993	0.119148	0.328493	NOT
RP11-467I	3.70339	-0.5581024	0.358142	-1.55832679	0.119156	0.328493	NOT
NAP1L3	41.29119	-0.7321917	0.469903	-1.55817629	0.119191	0.328539	NOT
MTMR12	1600.884	-0.2130903	0.13676	-1.55813073	0.119202	0.328539	NOT

KIAA1407	157.4446	0.40779506	0.261725	1.558103007	0.119209	0.328539	NOT
COLGALT2	80.03214	0.86096218	0.552676	1.557806833	0.119279	0.328646	NOT
RGAG4	276.4833	-0.6009124	0.385745	-1.55779614	0.119282	0.328646	NOT
COCH	371.9271	0.93484969	0.600131	1.557743399	0.119294	0.328646	NOT
APIP	1052.559	0.25232869	0.161984	1.557734161	0.119296	0.328646	NOT
AFG3L2	2792.804	-0.2147253	0.137851	-1.55766801	0.119312	0.328656	NOT
MEPE	3.378006	1.17343183	0.753405	1.557504424	0.119351	0.32873	NOT
PHF14	1725.032	0.20513758	0.131726	1.557306691	0.119398	0.328821	NOT
MLK7-AS1	15.82248	-0.5711068	0.366738	-1.55726267	0.119408	0.328821	NOT
RP11-142A	49.99809	-1.3030231	0.83678	-1.55718696	0.119426	0.328837	NOT
FAM118B	433.016	0.17659141	0.113411	1.55709273	0.119448	0.328847	NOT
RP11-16E1	63.85661	0.31620009	0.203078	1.557039317	0.119461	0.328847	NOT
RP11-44F1	4.514035	-0.5534203	0.355436	-1.55701841	0.119466	0.328847	NOT
RP11-45P1	14.50008	-0.4752649	0.305253	-1.55695519	0.119481	0.328855	NOT
DCUN1D1	1595.454	-0.2477068	0.159107	-1.55686123	0.119503	0.328862	NOT
MAP3K7CL	282.1323	-0.3496261	0.224576	-1.55682704	0.119512	0.328862	NOT
RP11-206I	7.343284	-0.6122461	0.393274	-1.55679137	0.11952	0.328862	NOT
DDX3X	8484.654	-0.2117064	0.136021	-1.55642377	0.119607	0.329056	NOT
GABRQ	26.07798	0.78996332	0.507586	1.556313941	0.119633	0.329056	NOT
ME2	1242.985	0.33203528	0.21335	1.556295976	0.119638	0.329056	NOT
ECI2	8758.529	-0.3655322	0.234874	-1.55628978	0.119639	0.329056	NOT
GIMAP6	509.2034	-0.4266202	0.274136	-1.55623511	0.119652	0.329059	NOT
RP11-802(	9.857548	0.4272122	0.274541	1.556098926	0.119685	0.329114	NOT
AC011747.	24.98899	1.0773541	0.692369	1.556040999	0.119698	0.329119	NOT
COX6CP1	72.27447	1.43039751	0.919348	1.555882818	0.119736	0.329181	NOT
DPF2	1419.669	0.19165778	0.123188	1.555819567	0.119751	0.329181	NOT
OXCT1-AS1	1.990983	1.07480761	0.690842	1.555793179	0.119757	0.329181	NOT
AP001055.	3.30385	-0.659555	0.423967	-1.55567583	0.119785	0.329224	NOT
RP11-513(	2.322056	-0.6326771	0.406718	-1.55556577	0.119811	0.329263	NOT
TMEM165	1582.86	0.27302176	0.175523	1.555479445	0.119832	0.329269	NOT
FRMD5	4.536911	0.97977388	0.629896	1.555454159	0.119838	0.329269	NOT
ARMC4	18.05039	-1.1858563	0.762559	-1.55510014	0.119922	0.329449	NOT
LINC0070?	31.12949	-0.5385005	0.346285	-1.5550778	0.119928	0.329449	NOT
IGSF23	582.9573	-0.7319442	0.470714	-1.55496673	0.119954	0.329471	NOT
GBP7	1254.216	-0.8382811	0.53911	-1.55493456	0.119962	0.329471	NOT
RP11-35N(	813.8754	0.67509634	0.434176	1.554890338	0.119972	0.329471	NOT
AF186192.	1.332929	1.06128627	0.682558	1.554865451	0.119978	NA	NA
HTR4	18.84816	0.79442768	0.511082	1.554404512	0.120088	0.329755	NOT
SLC1A1	2219.341	-0.7784074	0.500801	-1.55432329	0.120107	0.329755	NOT
CARD9	142.9612	0.46807716	0.301156	1.554267193	0.120121	0.329755	NOT
ZNF595	284.7044	-0.3483634	0.224136	-1.55425285	0.120124	0.329755	NOT
FAM153A	19.46956	0.97013053	0.624229	1.554126726	0.120154	0.329804	NOT
STX16-NPE	35.9139	-0.5262288	0.33862	-1.5540397	0.120175	0.329821	NOT
GS1-124K5	4.012356	-0.6480499	0.417021	-1.55399841	0.120185	0.329821	NOT
CETN2	1598.38	0.27080447	0.174278	1.553866874	0.120216	0.329874	NOT
AC010967.	1.597984	-0.8583252	0.552438	-1.55370533	0.120255	0.329946	NOT
DHFRL1	385.8082	-0.3206541	0.206418	-1.55342033	0.120323	0.3301	NOT
IRX5	31.41353	0.93832643	0.604084	1.553305264	0.12035	0.330131	NOT
RP11-325I	2.576719	-0.6531294	0.420486	-1.55327078	0.120358	0.330131	NOT

ENGASE	1122.38	-0.2678821	0.172479	-1.55312882	0.120392	0.330191	NOT
CYP1A1	1238.615	-1.1170918	0.719321	-1.55298076	0.120428	0.330201	NOT
HELZ2	2030.042	-0.3483557	0.224316	-1.55296874	0.120431	0.330201	NOT
NEURL1B	1305.422	-0.3706116	0.238649	-1.55295925	0.120433	0.330201	NOT
CRCT1	1.79877	1.9198763	1.236407	1.552786562	0.120474	0.330253	NOT
C16orf71	44.06744	-0.4086022	0.263145	-1.55276346	0.12048	0.330253	NOT
FGD5	647.776	-0.4111534	0.264794	-1.55272766	0.120488	0.330253	NOT
RP11-1348	18.25888	-0.3360402	0.216479	-1.55229787	0.120591	0.330475	NOT
BTN1A1	1.747617	0.97931483	0.630885	1.552287138	0.120594	0.330475	NOT
SFSWAP	1553.628	0.21823127	0.140594	1.552205511	0.120613	0.330495	NOT
MTMR1	1066.866	0.26800288	0.172675	1.55206969	0.120646	0.330551	NOT
RALY	6348.531	0.28160811	0.181451	1.551978116	0.120667	0.330577	NOT
PDZK1IP1	3321.141	1.01695445	0.655396	1.551664329	0.120743	0.330727	NOT
RP11-603J	16.44436	-0.4061697	0.261767	-1.55164766	0.120747	0.330727	NOT
RP11-47P1	7.571052	1.60275206	1.033035	1.551497836	0.120782	0.330792	NOT
RP11-235I	1.995315	-0.8836585	0.569604	-1.55135628	0.120816	0.330852	NOT
RP11-407N	3.373658	-0.5051992	0.32568	-1.55121153	0.120851	0.330888	NOT
RP11-549K	6.603873	1.42614124	0.91938	1.551198871	0.120854	0.330888	NOT
PRPSAP2	996.5598	0.29066383	0.187419	1.550876696	0.120931	0.331039	NOT
IGHA2	378.8958	-0.8544709	0.550963	-1.55086743	0.120933	0.331039	NOT
STAG3L3	53.68336	-0.3695757	0.238324	-1.55072927	0.120967	0.33108	NOT
CCDC23	518.1036	0.39607645	0.255417	1.550702944	0.120973	0.33108	NOT
VKORC1L1	2417.206	-0.2169498	0.139925	-1.55047359	0.121028	0.331114	NOT
GNLY	160.0296	0.56004419	0.361213	1.550455625	0.121032	0.331114	NOT
DHRS1	2694.451	-0.425977	0.274746	-1.55044211	0.121035	0.331114	NOT
C1QTNF9B	2.091265	-0.7648869	0.493347	-1.55040368	0.121045	0.331114	NOT
RP11-511H	15.37579	0.52841063	0.340823	1.55039685	0.121046	0.331114	NOT
CYP3A43	176.9963	-0.9439633	0.608876	-1.55033864	0.12106	0.331119	NOT
RP11-76C1	4.960327	1.73530216	1.119394	1.550216163	0.12109	0.331126	NOT
RP11-680F	471.1803	-0.5672235	0.365911	-1.55016959	0.121101	0.331126	NOT
PLAGL1	186.2956	0.64396675	0.415418	1.550164165	0.121102	0.331126	NOT
PLXDC2	803.2618	-0.5690381	0.367092	-1.55012445	0.121112	0.331126	NOT
CEP104	1222.812	-0.2662994	0.17182	-1.5498714	0.121172	0.33125	NOT
TATDN1P1	2.880669	0.73641422	0.475157	1.549833186	0.121182	0.33125	NOT
NAV1	484.3487	-0.4211275	0.271747	-1.54970675	0.121212	0.3313	NOT
S1PR3	734.8345	-0.4811737	0.310518	-1.54958549	0.121241	0.331306	NOT
CUL4A	3969.989	0.29764959	0.192091	1.54952346	0.121256	0.331306	NOT
MFSD10	1765.94	0.50275384	0.324475	1.549439241	0.121276	0.331306	NOT
MMP11	987.3372	0.70417098	0.454477	1.549408389	0.121284	0.331306	NOT
AC002454.	3.919969	0.93306562	0.60223	1.549351002	0.121297	0.331306	NOT
ETV2	112.9476	-0.4692188	0.302852	-1.54933442	0.121301	0.331306	NOT
GSS	3026.845	0.22761699	0.146914	1.549320117	0.121305	0.331306	NOT
SENP3-EIF	2.416812	0.78365602	0.505825	1.549262571	0.121319	0.331306	NOT
KRBOX1	8.014504	-0.7379132	0.476307	-1.54923949	0.121324	0.331306	NOT
MEAF6	2209.168	0.26602221	0.17173	1.549068766	0.121365	0.331374	NOT
AC021188.	4.262262	-0.5673649	0.36627	-1.54903346	0.121374	0.331374	NOT
AQPEP	9.958935	0.77190823	0.498354	1.548914836	0.121402	0.331419	NOT
ADAM18	1.128727	2.66980348	1.723791	1.548798071	0.12143	NA	NA
GNS	10509.35	-0.3057899	0.197466	-1.5485717	0.121485	0.331599	NOT

SPG11	2420.423	-0.2452402	0.158372	-1.54850907	0.1215	0.331599	NOT
ATP2A2	12806.09	-0.2393378	0.154562	-1.54848794	0.121505	0.331599	NOT
MPDU1	3857.45	-0.3364396	0.217333	-1.54803415	0.121614	0.331859	NOT
MYO7A	1510.027	-0.5044576	0.325879	-1.5479903	0.121625	0.331859	NOT
PF4V1	18.33318	1.2432808	0.803217	1.547877526	0.121652	0.3319	NOT
PIGR	8447.968	0.95445825	0.616702	1.547682119	0.121699	0.331974	NOT
ECT2L	6.482455	-0.4004498	0.258752	-1.5476176	0.121714	0.331974	NOT
ADIG	5.598951	1.17134298	0.756888	1.547576841	0.121724	0.331974	NOT
APOOL	1838.226	-0.3114591	0.201258	-1.54756091	0.121728	0.331974	NOT
LINC0069:	3.964552	-0.8520616	0.550662	-1.54734001	0.121781	0.332085	NOT
RP11-813I	2.49212	0.8323818	0.53797	1.547263534	0.1218	0.332085	NOT
RP11-235I	3.445154	-0.6681462	0.431831	-1.54723971	0.121805	0.332085	NOT
LINC0105	4.227042	-0.800816	0.517616	-1.54712467	0.121833	0.332128	NOT
SNORA16	2.997408	-0.7999888	0.51714	-1.54694703	0.121876	0.332208	NOT
RP11-472M	1.410899	-1.2039209	0.778279	-1.54690098	0.121887	0.332208	NOT
FLJ44511	66.39506	0.75017072	0.485146	1.546276715	0.122038	0.332585	NOT
TARID	4.029331	-0.8293538	0.536386	-1.54618835	0.122059	0.33261	NOT
AC008982.	6.25713	-0.5904584	0.381894	-1.54613291	0.122072	0.332613	NOT
RAPGEFL1	388.0224	0.37978565	0.245687	1.545813196	0.12215	0.33279	NOT
PVRL2	9441.136	-0.3663497	0.237015	-1.54568019	0.122182	0.332812	NOT
POTENP	2.068086	0.76948974	0.497833	1.545677283	0.122183	0.332812	NOT
RP11-255I	2.480295	0.60045563	0.38849	1.545612694	0.122198	0.332822	NOT
CALU	5288.491	0.28750071	0.186018	1.545552318	0.122213	0.332828	NOT
ITGAV	4301.101	0.44531015	0.288199	1.545148675	0.12231	0.333041	NOT
BRAP	950.583	0.17398746	0.112604	1.545127714	0.122315	0.333041	NOT
RP4-798A1	5.495683	0.75853903	0.490955	1.545026872	0.12234	0.333074	NOT
KCNJ4	169.9325	-0.851609	0.551354	-1.54457658	0.122449	0.333298	NOT
AP1G2	675.122	0.45292368	0.29324	1.544550792	0.122455	0.333298	NOT
ELP4	489.7878	0.18458656	0.11951	1.544534467	0.122459	0.333298	NOT
C2orf76	232.1165	0.27042195	0.175093	1.544449576	0.122479	0.333301	NOT
RP11-53B2	4.147782	0.67199722	0.435123	1.544382762	0.122496	0.333301	NOT
AC026471.	6.510796	0.73668392	0.477041	1.544277166	0.122521	0.333301	NOT
KLHL38	9.730743	0.90553419	0.586411	1.544198354	0.12254	0.333301	NOT
HADHB	10603.14	-0.2971723	0.192447	-1.54417818	0.122545	0.333301	NOT
COX20P1	18.47677	-0.8262773	0.535095	-1.54416956	0.122547	0.333301	NOT
AC005785.	5.335302	-0.609063	0.394438	-1.54412805	0.122557	0.333301	NOT
RP11-347(	3.601335	0.71817572	0.465116	1.544077998	0.122569	0.333301	NOT
MATN1	5.884369	0.46258691	0.299589	1.54407166	0.122571	0.333301	NOT
RP11-631M	28.38821	-0.5366863	0.3476	-1.54397834	0.122594	0.333301	NOT
ZBTB8OSP2	3.066036	0.94513551	0.612146	1.543971878	0.122595	0.333301	NOT
BAHCC1	761.3445	-0.3769736	0.24417	-1.54389877	0.122613	0.333315	NOT
DENND4A	1442.917	-0.2817996	0.182547	-1.54370752	0.122659	0.333408	NOT
ANXA2P1	4.628859	0.56982542	0.369152	1.543606012	0.122684	0.333436	NOT
SCARNA24	2.254013	0.64102767	0.415291	1.543564124	0.122694	0.333436	NOT
RP11-93B1	0.940277	-1.2603603	0.816648	-1.54333367	0.12275	NA	NA
RP11-169I	3.279664	-0.5853376	0.379315	-1.54314352	0.122796	0.333664	NOT
MSH3	842.7461	-0.2822147	0.182897	-1.54302205	0.122825	0.333664	NOT
RP11-506I	2.120641	-0.7037989	0.456126	-1.54299099	0.122833	0.333664	NOT
RP11-668(	1.791141	-0.7489259	0.485426	-1.54282141	0.122874	0.333664	NOT

RP11-603I	6.067558	-1.2351209	0.800584	-1.54277436	0.122886	0.333664	NOT
MAPK1IP1I	3012.19	0.19267202	0.124889	1.542747434	0.122892	0.333664	NOT
TBC1D12	424.2897	-0.2518018	0.163217	-1.54274186	0.122893	0.333664	NOT
SCN9A	355.3979	-0.6427345	0.416622	-1.54272681	0.122897	0.333664	NOT
IFI6	5251.811	0.56466483	0.366017	1.542726369	0.122897	0.333664	NOT
TRPM2-AS	5.363084	0.86457481	0.560426	1.542710956	0.122901	0.333664	NOT
PLA2G15	1101.591	-0.328903	0.213207	-1.54264679	0.122916	0.333673	NOT
RP11-2E11	10.90624	0.54756581	0.354976	1.542543745	0.122942	0.333707	NOT
COL6A3	4154.714	-0.4878352	0.31628	-1.54241603	0.122973	0.333758	NOT
MRPL1	887.8875	0.26378746	0.171051	1.5421565	0.123036	0.333896	NOT
ZNF205-AS	25.12922	-0.359754	0.233288	-1.54210461	0.123048	0.333896	NOT
KLLN	61.88652	-0.4079593	0.264559	-1.54203315	0.123066	0.33391	NOT
ZMYM6NB	218.4044	0.33330359	0.216189	1.541723282	0.123141	0.334081	NOT
TMBIM1	9587.339	0.23035031	0.149416	1.541672201	0.123153	0.334081	NOT
PSMC6	2624.484	0.2078685	0.13484	1.541592347	0.123173	0.334101	NOT
ITGA6	3501.371	0.36939752	0.239633	1.541512159	0.123192	0.33412	NOT
MLH1	1372.159	0.19003138	0.123283	1.541422686	0.123214	0.334146	NOT
RP11-420I	364.5074	-0.3661674	0.237565	-1.54133725	0.123235	0.334165	NOT
FKBP9P1	33.07593	0.64894185	0.421037	1.54129294	0.123246	0.334165	NOT
GPR135	52.77938	-0.3465514	0.22486	-1.54118677	0.123271	0.334169	NOT
FGFR4	10643.33	0.49060613	0.31833	1.541185609	0.123272	0.334169	NOT
AP000472.	1.175352	-1.2433376	0.806861	-1.54095572	0.123328	NA	NA
NPPB	3.176673	1.03290804	0.670377	1.540786363	0.123369	0.334399	NOT
MRPL47	1509.192	0.28144316	0.182717	1.540322045	0.123482	0.33464	NOT
RP3-454B2	1.93705	-0.935937	0.607625	-1.54031952	0.123482	0.33464	NOT
MAPKAPK2	6396.164	0.26435592	0.171634	1.54023146	0.123504	0.334665	NOT
WHAMML1	79.81364	-0.3214866	0.208748	-1.540074	0.123542	0.334735	NOT
IGLC2	1023.085	-0.9937019	0.645307	-1.53988984	0.123587	0.334823	NOT
FBXL15	862.7877	-0.3946287	0.25629	-1.53977585	0.123615	0.334856	NOT
RP11-463C	2.683956	-0.5954992	0.386753	-1.53973978	0.123624	0.334856	NOT
SLC5A4	11.91785	-0.5582014	0.362554	-1.53963707	0.123649	0.33489	NOT
RP11-8L2.	1.493675	2.16566883	1.406685	1.539554808	0.123669	0.334911	NOT
PDGFRA	773.4479	0.90392349	0.5872	1.539379026	0.123712	0.334973	NOT
ARL16	1217.812	0.34887012	0.226633	1.539358856	0.123717	0.334973	NOT
NRXN1	2.403155	1.84073706	1.195903	1.539202141	0.123755	0.335044	NOT
ZNF611	192.0323	0.50019341	0.324988	1.539112057	0.123777	0.335057	NOT
MT-ATP6	255274.1	-0.4084859	0.265409	-1.53908009	0.123785	0.335057	NOT
CTD-2292I	19.11578	0.53528996	0.347838	1.538904972	0.123827	0.33514	NOT
RP11-361I	3.250469	-0.6384937	0.414953	-1.5387138	0.123874	0.335233	NOT
RPS4XP22	26.9989	-1.043334	0.678142	-1.53851958	0.123922	0.335307	NOT
MMP20	1.641852	1.47004715	0.955507	1.538499889	0.123926	0.335307	NOT
HIST1H1PS	9.269837	0.81054692	0.526876	1.538401589	0.12395	0.335339	NOT
LAMTOR5	2779.773	0.24775114	0.161067	1.538184258	0.124004	0.335449	NOT
APOBEC3D	116.2705	0.5434671	0.353354	1.538025423	0.124042	0.33552	NOT
REPS1	1001.19	0.25238167	0.164108	1.537902679	0.124072	0.335568	NOT
NUMA1	5084.776	-0.2416845	0.157163	-1.53779647	0.124098	0.335605	NOT
RP1-8B1.4	14.33727	0.70851577	0.460795	1.537594804	0.124148	0.335682	NOT
DDHD1	497.1619	0.43346677	0.281915	1.53757849	0.124152	0.335682	NOT
FUT3	29.02498	0.70245254	0.457031	1.536991654	0.124295	0.336037	NOT

UBQLN2	2012.131	-0.2702161	0.175825	-1.53684662	0.124331	0.3361	NOT
PRPF40B	247.8295	0.30176082	0.196389	1.536549697	0.124404	0.336165	NOT
QRFP	7.229152	0.58490095	0.380659	1.536549256	0.124404	0.336165	NOT
PRR15	95.63337	1.0519557	0.684634	1.536523701	0.12441	0.336165	NOT
C21orf2	954.0069	-0.3291123	0.214195	-1.53650696	0.124414	0.336165	NOT
ELF3	4845.715	0.49942347	0.325069	1.536359455	0.12445	0.336165	NOT
KB-1027C1	2.076875	1.31149384	0.853645	1.53634571	0.124454	0.336165	NOT
SUCLG2	6739.351	-0.3835592	0.249662	-1.53631578	0.124461	0.336165	NOT
SPRY4-IT1	2.479672	-0.8190241	0.533116	-1.5362973	0.124465	0.336165	NOT
PRTG	41.6347	0.91723568	0.597045	1.53629262	0.124467	0.336165	NOT
RPLP0P2	25.80047	0.64129987	0.417466	1.536173673	0.124496	0.336211	NOT
RP11-452(	3.896435	0.7650168	0.498057	1.536003533	0.124538	0.33629	NOT
RASIP1	592.6506	-0.3570805	0.232482	-1.53594843	0.124551	0.336293	NOT
BRF2	237.1414	0.32237902	0.209897	1.535888039	0.124566	0.336299	NOT
SLC2A1-AS	9.61443	-0.6681664	0.435105	-1.53564407	0.124626	0.336427	NOT
ASTN2	205.6169	-0.3480612	0.226735	-1.53510064	0.124759	0.336738	NOT
SP3P	7.669133	1.36270987	0.887735	1.535040958	0.124774	0.336738	NOT
RAB26	691.0366	-0.5907196	0.384831	-1.53500929	0.124782	0.336738	NOT
GRAMD1C	482.8965	-0.564408	0.367699	-1.53497389	0.12479	0.336738	NOT
ASS1P9	5.521776	-0.7990376	0.520573	-1.53491889	0.124804	0.336741	NOT
SEMA3F	1249.778	-0.2898161	0.188827	-1.53482406	0.124827	0.336759	NOT
RNU6-1011	1.696548	0.72609726	0.473092	1.534790101	0.124835	0.336759	NOT
ROPN1	1.615059	1.26783203	0.82615	1.534626821	0.124876	0.336828	NOT
EMILIN3	19.07574	0.81546988	0.531402	1.534562832	0.124891	0.336828	NOT
ADSS	1811.435	0.24733049	0.161178	1.534513637	0.124903	0.336828	NOT
RP11-819M	2.181664	0.85347401	0.556212	1.534439172	0.124922	0.336828	NOT
ANXA9	2410.304	0.39429402	0.256964	1.534433539	0.124923	0.336828	NOT
NT5DC3	695.1415	0.35557792	0.231748	1.5343317	0.124948	0.336835	NOT
RP1-206D1	2.833303	-0.8689908	0.566376	-1.53429974	0.124956	0.336835	NOT
ZNF528	232.0761	0.56330945	0.367159	1.534236536	0.124971	0.336835	NOT
OMD	26.08114	-1.010725	0.658787	-1.53422051	0.124975	0.336835	NOT
COL18A1-A	1.071753	1.29313451	0.842871	1.534201826	0.12498	NA	NA
RP11-420A	74.90843	-0.4806481	0.313351	-1.53389934	0.125054	0.337015	NOT
LRRC15	41.67601	-0.8520281	0.555516	-1.53376069	0.125089	0.337073	NOT
RP3-420J1	36.87732	-1.2774903	0.832955	-1.53368549	0.125107	0.337089	NOT
METTL11B	2.65817	1.37614549	0.897522	1.533272823	0.125209	0.33733	NOT
BST1	148.559	-0.4817931	0.314244	-1.53318175	0.125231	0.337357	NOT
RP11-37N2	2.381393	0.89609377	0.584557	1.532944219	0.12529	0.337481	NOT
RFC3	480.7449	0.35516894	0.231728	1.532696686	0.125351	0.337612	NOT
SPACA1	2.7059	-1.6207215	1.057579	-1.53248293	0.125403	0.33772	NOT
KRTCAP2	1674.627	0.33789088	0.220498	1.532397956	0.125424	0.337743	NOT
CACFD1	4173.17	-0.3354373	0.218917	-1.53225785	0.125459	0.337795	NOT
MYL6	27241.35	-0.2912196	0.190064	-1.53221889	0.125468	0.337795	NOT
RP11-313F	4.680401	0.62273246	0.406469	1.532053241	0.125509	0.337871	NOT
TPM1	6418.762	0.40982363	0.267513	1.53197898	0.125528	0.337887	NOT
TMEM57	2856.529	-0.2581238	0.168502	-1.53187205	0.125554	0.337923	NOT
DDX3P1	2.117221	-0.6208334	0.405291	-1.53181983	0.125567	0.337923	NOT
ARAP3	495.6603	-0.3389674	0.221291	-1.53177302	0.125578	0.337923	NOT
ERBB2IP	3548.084	-0.2623427	0.171274	-1.53171651	0.125592	0.337927	NOT

LINC0116	2.310452	1.86693096	1.218957	1.53158015	0.125626	0.33798	NOT
ACTR10	1607.667	0.19391738	0.126618	1.531514872	0.125642	0.33798	NOT
TTLL5	578.933	0.27220286	0.177738	1.531486056	0.125649	0.33798	NOT
TMED7-TI(	13.68296	-0.4246738	0.277315	-1.53137935	0.125676	0.338017	NOT
DYSF	2655.829	-0.4058967	0.265066	-1.53130683	0.125694	0.338032	NOT
MAN2A2	2895.302	-0.3745233	0.244623	-1.53102033	0.125764	0.338189	NOT
CPSF4L	6.15006	0.84500212	0.552025	1.53073254	0.125836	0.338325	NOT
RPL4	44539.87	0.29168226	0.190558	1.530670849	0.125851	0.338325	NOT
TTC25	30.42146	-0.5270147	0.344311	-1.53063606	0.125859	0.338325	NOT
IGHV4-28	12.13961	1.29213353	0.844193	1.530614047	0.125865	0.338325	NOT
RFPL3S	7.650674	-0.5343538	0.349153	-1.53043074	0.12591	0.338413	NOT
PROM1	223.6718	1.2547016	0.819891	1.530327653	0.125936	0.338448	NOT
RP11-51B2	1.855156	-0.9030532	0.590174	-1.53014818	0.12598	0.338534	NOT
MMP2	1741.379	-0.6061534	0.396231	-1.5297969	0.126067	0.338734	NOT
PLOD3	7084.801	0.26206539	0.171338	1.529519979	0.126136	0.338885	NOT
ROR1-AS1	1.704459	1.25954084	0.82352	1.529459214	0.126151	0.338892	NOT
DNA2	253.9463	0.40023092	0.261714	1.529265938	0.126199	0.338979	NOT
U1	5.622454	-0.5165202	0.337781	-1.52915637	0.126226	0.338979	NOT
ERC2	5.262438	0.81157784	0.530739	1.529147079	0.126228	0.338979	NOT
PRAM1	58.13694	-0.4862652	0.318002	-1.52912575	0.126233	0.338979	NOT
EMC6	143.2081	-0.3301009	0.215884	-1.52906715	0.126248	0.338985	NOT
NAALADL2	155.3283	-0.6695038	0.437873	-1.52898948	0.126267	0.339003	NOT
C2orf43	822.7333	0.22283648	0.145754	1.528855377	0.1263	0.339037	NOT
MNT	614.3841	-0.2407237	0.157456	-1.5288287	0.126307	0.339037	NOT
RP11-9L18	11.24518	0.445185	0.291201	1.528787235	0.126317	0.339037	NOT
RP11-478I	3.383167	-0.887482	0.580534	-1.5287343	0.12633	0.339039	NOT
PMPCAP1	6.647691	1.439255	0.941508	1.528670304	0.126346	0.339048	NOT
RFX3	189.7047	0.3238009	0.211844	1.52849026	0.126391	0.339134	NOT
ASS1P12	10.05976	-0.447559	0.292859	-1.52824234	0.126452	0.339266	NOT
HLTF	2144.187	0.31727861	0.207628	1.528108088	0.126486	0.339321	NOT
AC026271.	132.304	0.25525978	0.167073	1.527833301	0.126554	0.339471	NOT
PSMA5	5646.65	0.27142398	0.177666	1.527724716	0.126581	0.33951	NOT
RP11-649A	7.242915	1.49968306	0.9817	1.527639149	0.126602	0.339533	NOT
BTF3L4	1345.782	0.23342812	0.152818	1.527488848	0.126639	0.3396	NOT
SLC16A4	176.7089	-0.6148709	0.402627	-1.52714622	0.126725	0.339777	NOT
RELL1	95.10076	-0.4552102	0.298104	-1.52701724	0.126757	0.339777	NOT
TTC39A-AS	8.181745	0.84007134	0.550156	1.526968167	0.126769	0.339777	NOT
ADORA2BP1	95.33561	-0.9604101	0.62898	-1.52693365	0.126778	0.339777	NOT
MYO9B	3538.03	-0.2359598	0.154537	-1.52688626	0.126789	0.339777	NOT
RP11-806I	1.750495	-0.762606	0.499467	-1.52683909	0.126801	0.339777	NOT
CRLF3	405.6324	0.22455648	0.147073	1.526835433	0.126802	0.339777	NOT
LTK	51.79604	-0.6413862	0.420107	-1.52672126	0.12683	0.339777	NOT
MEF2A	1481.96	-0.3146127	0.206073	-1.52670325	0.126835	0.339777	NOT
G0S2	3536.652	-0.6715963	0.4399	-1.52670289	0.126835	0.339777	NOT
FRMD4B	965.349	-0.3284355	0.215132	-1.52666837	0.126843	0.339777	NOT
RP13-516M	11.35025	-0.4680568	0.306656	-1.52632651	0.126929	0.339939	NOT
POLR2M	668.5112	-0.2013289	0.131904	-1.52632461	0.126929	0.339939	NOT
RP11-99E1	1.574321	-0.6997977	0.45852	-1.52620846	0.126958	0.339983	NOT
SETD1B	1535.775	-0.1951966	0.127907	-1.52608535	0.126989	0.340032	NOT

AP001626.	60.19034	0.85724921	0.561832	1.525809452	0.127057	0.340162	NOT
RP11-349I	1.285478	-1.1082537	0.726355	-1.52577374	0.127066	NA	NA
CCDC148	22.97869	0.51982522	0.340701	1.525752214	0.127072	0.340162	NOT
S100P	3195.069	1.16239424	0.761871	1.525709884	0.127082	0.340162	NOT
ARMC4P1	3.675096	-1.0249209	0.671776	-1.52568872	0.127087	0.340162	NOT
AFTPH	1584.428	0.21280223	0.139491	1.525567215	0.127118	0.340209	NOT
RNPC3	376.6024	-0.3566009	0.233759	-1.52550904	0.127132	0.340215	NOT
GLS	2418.654	0.48649873	0.318932	1.525398889	0.12716	0.340255	NOT
AC006369.	4.549296	-0.8111725	0.531803	-1.52532595	0.127178	0.34027	NOT
AC005532.	5.569623	0.76926008	0.504361	1.525215934	0.127205	0.340297	NOT
C1orf53	299.9734	-0.4007475	0.262753	-1.52518453	0.127213	0.340297	NOT
RP11-252I	14.89457	-0.9913592	0.650069	-1.525007	0.127257	0.340355	NOT
PCDH1	2381.753	0.39280241	0.257576	1.524996854	0.12726	0.340355	NOT
RP11-394I	12.1955	0.63663221	0.417522	1.524788734	0.127312	0.340457	NOT
IGHV5-51	112.6769	1.01142236	0.663339	1.524743913	0.127323	0.340457	NOT
RP11-855A	1.991343	0.9820267	0.644124	1.52459214	0.127361	0.340521	NOT
RP11-469N	16.8015	1.24424049	0.816154	1.524517628	0.127379	0.340521	NOT
AC087294.	5.548183	0.49946171	0.327627	1.524482328	0.127388	0.340521	NOT
RARRES2	38859.1	-0.481437	0.315819	-1.52440622	0.127407	0.340521	NOT
ARIH1	2282.83	-0.1910396	0.125322	-1.52439432	0.12741	0.340521	NOT
PODXL	2245.571	-0.4245408	0.278507	-1.52434508	0.127423	0.340521	NOT
RP11-706C	1.886382	1.77785037	1.16661	1.523945835	0.127522	0.340686	NOT
AC003104.	2.721578	-0.7207442	0.472957	-1.52391189	0.127531	0.340686	NOT
RP11-889I	64.05169	0.50335155	0.330304	1.5239027	0.127533	0.340686	NOT
RP1-60019	1.482068	-0.8960717	0.588013	-1.5238978	0.127534	0.340686	NOT
RPL35A	15761.78	0.34597557	0.227057	1.523739836	0.127574	0.340758	NOT
PPIAP16	2.27673	0.72345439	0.474809	1.523673322	0.12759	0.340768	NOT
FTH1P10	23.93034	0.59410202	0.390096	1.522962373	0.127768	0.34121	NOT
POLE4	1196.473	0.34139927	0.224203	1.52272321	0.127828	0.341336	NOT
CECR9	1.669366	-1.1869297	0.779525	-1.52263229	0.127851	0.341363	NOT
ZNF174	386.0482	0.18368863	0.120656	1.522418416	0.127904	0.341409	NOT
KLHL28	405.444	-0.2662962	0.174927	-1.52233118	0.127926	0.341409	NOT
CTC-378H2	56.05679	0.91320373	0.599886	1.522296593	0.127935	0.341409	NOT
FBXO9	2620.887	-0.279355	0.183511	-1.52227977	0.127939	0.341409	NOT
MYPN	2.805623	0.78466842	0.515459	1.522270098	0.127941	0.341409	NOT
SLC25A14	208.0678	0.24484048	0.16084	1.522262578	0.127943	0.341409	NOT
CTD-2621I	16.87074	-0.5369828	0.352833	-1.52191989	0.128029	0.341549	NOT
C17orf67	112.3206	0.52019983	0.341808	1.521907591	0.128032	0.341549	NOT
RETN	2.658691	1.0267929	0.674677	1.521901829	0.128034	0.341549	NOT
GCSAM	21.90038	0.61773182	0.406004	1.521491341	0.128137	0.341777	NOT
CTD-2006C	6.741023	-0.6030728	0.396378	-1.52146013	0.128144	0.341777	NOT
CPNE4	3.656256	0.93318895	0.613391	1.521361472	0.128169	0.341782	NOT
RP11-756C	2.182261	0.7305301	0.480185	1.521352681	0.128171	0.341782	NOT
RP11-680I	33.30815	-0.4603485	0.30261	-1.52125874	0.128195	0.341811	NOT
PLEKHN1	53.64296	0.6646952	0.437043	1.520890503	0.128287	0.342018	NOT
CTD-2523I	6.111792	0.7377866	0.485115	1.520849531	0.128298	0.342018	NOT
SPAG7	1821.981	-0.325406	0.213988	-1.5206734	0.128342	0.342102	NOT
TMA16	567.5874	0.22477114	0.147823	1.520543395	0.128374	0.342155	NOT
LL21NC02-	13.55702	-0.5072108	0.333632	-1.52027038	0.128443	0.342304	NOT

AC004076.	2.471842	-0.7408286	0.487331	-1.52017468	0.128467	0.342335	NOT
CTD-2530H	3.100168	-0.5291403	0.348104	-1.52006179	0.128495	0.342369	NOT
DPP8	1001.276	-0.2094769	0.137818	-1.51994835	0.128524	0.342369	NOT
RP5-826L7	1.52059	-0.7879693	0.518422	-1.51993762	0.128527	0.342369	NOT
NCALD	1110.652	-0.5464623	0.359535	-1.5199147	0.128532	0.342369	NOT
MCM3AP-AS	81.04347	0.32899656	0.216463	1.519872464	0.128543	0.342369	NOT
KIAA0226	695.2563	0.20487672	0.134806	1.519789508	0.128564	0.342371	NOT
RP11-459F	4.56562	-0.4177182	0.27486	-1.51975155	0.128573	0.342371	NOT
CREB5	202.4396	0.60389759	0.397375	1.519718503	0.128582	0.342371	NOT
RP11-778I	11.25462	-0.4503229	0.296335	-1.51964303	0.128601	0.342388	NOT
ZNF813	197.8149	0.64062759	0.421585	1.519567978	0.12862	0.342405	NOT
CH17-373J	2.617097	0.64896201	0.427168	1.519220993	0.128707	0.342604	NOT
OLIG3	1.366063	3.13944097	2.06652	1.519192257	0.128714	NA	NA
C8orf49	78.87409	0.49591942	0.326484	1.51896827	0.12877	0.342682	NOT
LTF	83.5506	0.65027851	0.428128	1.518888369	0.128791	0.342682	NOT
RP11-1143	2.756815	0.81641439	0.537512	1.518876151	0.128794	0.342682	NOT
RPS23P8	54.11837	0.53488899	0.352164	1.518864096	0.128797	0.342682	NOT
ANGPT2	471.8336	-0.4116842	0.271049	-1.51885374	0.128799	0.342682	NOT
HNRNPA1P8	5.165073	0.46459225	0.305895	1.518797103	0.128814	0.342686	NOT
ST7L	364.5819	0.22023327	0.145025	1.518585592	0.128867	0.342794	NOT
TMCC1	2394.311	0.39865868	0.26253	1.518525146	0.128882	0.342801	NOT
CCAR2	2126.491	0.25492695	0.16789	1.518419294	0.128909	0.342838	NOT
NR2C1	830.4948	0.24356655	0.160428	1.518230849	0.128956	0.342912	NOT
RP4-755D9	23.09488	-0.7793597	0.513356	-1.51816721	0.128972	0.342912	NOT
RP11-219E	1.775721	-0.7500137	0.494028	-1.51815941	0.128974	0.342912	NOT
HIST1H2AI	23.07966	0.66951122	0.441074	1.517911455	0.129037	0.343023	NOT
MAOA	8410.426	-0.3763825	0.247964	-1.51789301	0.129041	0.343023	NOT
RP4-539M6	8.285013	-0.6670989	0.439565	-1.51763391	0.129107	0.343163	NOT
ORAI1	1237.923	0.29743019	0.196012	1.517410187	0.129163	0.343247	NOT
GPX1P2	11.28989	0.53303821	0.351282	1.517408046	0.129164	0.343247	NOT
RP11-66D1	1.632652	-0.6499043	0.428314	-1.5173562	0.129177	0.343248	NOT
DPP4	4743.486	0.56965992	0.375463	1.517218788	0.129211	0.343307	NOT
TMEM31	2.381764	-0.9656155	0.636494	-1.51708433	0.129245	0.343322	NOT
DGUOK	1426.099	0.30019466	0.197879	1.517059112	0.129252	0.343322	NOT
STAT6	6373.858	-0.2386164	0.157294	-1.51701034	0.129264	0.343322	NOT
RPS27P25	2.346416	1.16339288	0.766906	1.516995837	0.129268	0.343322	NOT
RPL18AP3	319.282	0.49569444	0.326783	1.516890791	0.129294	0.343359	NOT
RP3-426I6	2.56663	0.66699075	0.439798	1.516585806	0.129371	0.34353	NOT
UBE2I	2982.349	0.23074746	0.152172	1.516359937	0.129428	0.343648	NOT
RP11-111M	40.82656	-0.3435135	0.226579	-1.51608695	0.129497	0.343797	NOT
DPY19L4	1389.847	0.27421332	0.180889	1.515918396	0.12954	0.343859	NOT
MTHFD2P1	1.904657	1.40273307	0.92535	1.515895107	0.129546	0.343859	NOT
RP11-16E1	41.61703	-0.4029856	0.265863	-1.51576466	0.129579	0.343888	NOT
TFP1	9.170093	-0.3366324	0.222096	-1.51570602	0.129594	0.343888	NOT
ELF2	909.7585	-0.1987491	0.131127	-1.5157016	0.129595	0.343888	NOT
CCDC109B	253.4085	0.55100083	0.363602	1.515396738	0.129672	0.344028	NOT
AMZ2	1699.344	0.2193761	0.14477	1.515343783	0.129685	0.344028	NOT
CHST6	15.93157	0.85615787	0.565005	1.515310719	0.129694	0.344028	NOT
RP11-417I	4.316031	-0.6959087	0.459268	-1.51525496	0.129708	0.344028	NOT

ZNF830	511.3641	0.21091916	0.139203	1.515194758	0.129723	0.344028	NOT
WNT6	14.16365	0.68065789	0.449222	1.51519272	0.129724	0.344028	NOT
PCDHGB1	27.43155	-0.688234	0.454287	-1.51497491	0.129779	0.344073	NOT
RP11-209I	1.955664	1.30164233	0.859188	1.51496836	0.12978	0.344073	NOT
CTD-2666I	4.59285	-0.5351721	0.353264	-1.51493599	0.129789	0.344073	NOT
ISPD	182.5586	-0.4770495	0.3149	-1.51492491	0.129791	0.344073	NOT
BMP3	1.387422	-1.223538	0.807771	-1.51470918	0.129846	NA	NA
SNTB1	8509.256	0.41973805	0.277108	1.514706566	0.129847	0.344186	NOT
ELOVL4	22.93352	0.75833988	0.500688	1.514595473	0.129875	0.344227	NOT
PXN-AS1	246.2576	0.35005383	0.231142	1.514455441	0.12991	0.344276	NOT
FLCN	1344.922	0.25037629	0.165328	1.51442234	0.129919	0.344276	NOT
NXPE3	263.225	0.60694701	0.40082	1.514264484	0.129959	0.344348	NOT
IRAK4	603.8709	-0.1959225	0.129422	-1.51382112	0.130071	0.344567	NOT
GLYATL2	2.780412	1.0205573	0.674166	1.513807057	0.130075	0.344567	NOT
GGT7	890.0999	0.33038651	0.218251	1.513788104	0.13008	0.344567	NOT
ETV1	428.2092	0.56866864	0.375693	1.513651668	0.130114	0.344603	NOT
TNFSF11	37.23518	-0.8342459	0.551154	-1.5136354	0.130118	0.344603	NOT
CHD2	2258.231	-0.1876905	0.124007	-1.51354556	0.130141	0.344629	NOT
RP11-277I	10.13301	0.42607896	0.281542	1.513375766	0.130184	0.344692	NOT
CACHD1	315.0267	0.52699857	0.348233	1.513351779	0.13019	0.344692	NOT
AC087884.	3.947582	-0.5853653	0.38682	-1.51327654	0.130209	0.344701	NOT
ZNF286B	25.42698	0.44790634	0.295992	1.513239564	0.130219	0.344701	NOT
PCDHGA7	39.27634	-0.6561625	0.43366	-1.51307946	0.130259	0.344775	NOT
RPL32	31607.73	0.37775793	0.249688	1.512920373	0.1303	0.344848	NOT
SNORA73B	10.45919	0.70509897	0.46611	1.512731613	0.130348	0.344932	NOT
RP11-494(	2.198239	0.79036476	0.522488	1.512695507	0.130357	0.344932	NOT
GDPD5	576.5831	-0.3955874	0.261546	-1.51249551	0.130408	0.345033	NOT
RP11-572I	184.2578	0.60142256	0.397651	1.51243752	0.130423	0.345038	NOT
NR2F1	936.419	-0.4803211	0.317623	-1.51223573	0.130474	0.34514	NOT
TGFBR2	4199.438	-0.3200959	0.211704	-1.51199536	0.130535	0.345268	NOT
GAPDHP62	2.553287	0.59208323	0.391615	1.511900846	0.130559	0.345278	NOT
B9D1	501.3198	0.41282944	0.273064	1.511844032	0.130574	0.345278	NOT
MUT	5448.737	-0.4030388	0.26659	-1.51183	0.130577	0.345278	NOT
AC005795.	3.660656	0.587559	0.388713	1.511549958	0.130648	0.345433	NOT
AC004166.	44.88243	-0.405512	0.268312	-1.51134483	0.130701	0.345525	NOT
ZNF804A	14.45636	0.79872414	0.528497	1.511313726	0.130709	0.345525	NOT
CCDC57	1220.398	-0.4087363	0.270512	-1.5109731	0.130795	0.345721	NOT
AC093702.	1.736993	2.01037198	1.330781	1.51067077	0.130872	0.345891	NOT
RP11-531A	27.15192	0.51352247	0.339952	1.510573422	0.130897	0.345922	NOT
MRPS18B	4969.595	0.27836478	0.18432	1.510226998	0.130986	0.346083	NOT
GPR162	208.4319	-0.6180658	0.409261	-1.51020063	0.130992	0.346083	NOT
SKI	2493.97	-0.2593631	0.171743	-1.51018524	0.130996	0.346083	NOT
MTMR4	3680.99	-0.3503965	0.232035	-1.5101048	0.131017	0.346103	NOT
BPNT1	1839.689	0.23644808	0.156584	1.510040701	0.131033	0.346113	NOT
ZFAT	486.6678	0.24985157	0.165474	1.5099185	0.131064	0.346162	NOT
PCDHA11	9.764464	1.21765662	0.806752	1.509331821	0.131214	0.346495	NOT
LIAS	561.9446	-0.2652608	0.175748	-1.50932253	0.131216	0.346495	NOT
CCSER1	9.959266	0.82875716	0.54911	1.509274156	0.131229	0.346495	NOT
PANK4	805.0897	-0.195958	0.129866	-1.50892496	0.131318	0.346697	NOT

MET	6160.304	-0.3617244	0.239733	-1.50886207	0.131334	0.346705	NOT
UNC13A	45.00948	0.79064087	0.524156	1.508406913	0.13145	0.34693	NOT
CSNK2B	3488.881	0.3073728	0.203774	1.508400929	0.131452	0.34693	NOT
WBP1LP2	22.58665	0.35931233	0.238216	1.508347961	0.131465	0.34693	NOT
MFAP3	440.9578	-0.3919275	0.259842	-1.50832992	0.13147	0.34693	NOT
ZNF345	100.8892	-0.2803326	0.185875	-1.50817487	0.13151	0.347001	NOT
ZNF837	143.4561	-0.3572272	0.236882	-1.50803953	0.131544	0.347058	NOT
C1orf159	412.0128	0.26692152	0.177045	1.507646086	0.131645	0.347204	NOT
TNFAIP2	3204.383	0.45965818	0.304897	1.507584168	0.131661	0.347204	NOT
CTD-2303H	15.83859	0.948114	0.628913	1.507544646	0.131671	0.347204	NOT
DAPK1	2575.321	-0.3566134	0.236573	-1.50741423	0.131705	0.347204	NOT
RNF19B	889.2821	0.33661182	0.223317	1.507325359	0.131727	0.347204	NOT
SLC25A35	195.3332	0.31024057	0.205822	1.507322897	0.131728	0.347204	NOT
MIR135A1	73.04516	-0.5435331	0.360604	-1.50728554	0.131737	0.347204	NOT
SLC22A9	2779.752	-0.7032299	0.466561	-1.50726391	0.131743	0.347204	NOT
PLTP	1806.576	0.60348264	0.40039	1.507235328	0.13175	0.347204	NOT
RP11-153M	6.177824	0.96998206	0.643569	1.507191953	0.131761	0.347204	NOT
TRMT2A	2047.678	0.23171672	0.153741	1.507189661	0.131762	0.347204	NOT
HIC2	498.2325	0.5237402	0.347495	1.507187835	0.131763	0.347204	NOT
PTPRH	1899.206	-0.6142104	0.407524	-1.50717468	0.131766	0.347204	NOT
LINC01556	3.532228	0.98288947	0.652194	1.507051288	0.131798	0.347246	NOT
LINC00242	118.9879	-0.4596816	0.305045	-1.50692974	0.131829	0.347246	NOT
ANKH	4537.785	-0.3303037	0.219192	-1.50691133	0.131833	0.347246	NOT
BCAS2	1418.631	0.22149306	0.146987	1.506892528	0.131838	0.347246	NOT
RP11-350C	4.19027	0.65815176	0.43677	1.506862471	0.131846	0.347246	NOT
GPR61	7.028438	-0.5375991	0.356833	-1.50658285	0.131918	0.347401	NOT
OS9	17971.82	-0.2729318	0.181168	-1.50650808	0.131937	0.347418	NOT
NAA50	4660.908	0.19215798	0.127557	1.506446717	0.131953	0.347425	NOT
RP11-267M	59.79125	0.37635274	0.249837	1.506395987	0.131966	0.347426	NOT
RP11-100E	0.846749	1.64866516	1.094476	1.506350796	0.131977	NA	NA
DNAJB1P1	1.813024	1.38070265	0.916654	1.50624152	0.132005	0.347497	NOT
PCDHGB9P	6.451407	-0.7679959	0.509901	-1.50616559	0.132025	0.347514	NOT
TXNP5	6.649438	0.75912825	0.504036	1.506097948	0.132042	0.347526	NOT
RP11-159D	324.8308	-0.363705	0.241512	-1.50595244	0.132079	0.34755	NOT
LRRC4	34.40553	-0.4743044	0.314982	-1.50581555	0.132115	0.34755	NOT
NXF5	2.444851	-1.1440632	0.759796	-1.5057514	0.132131	0.34755	NOT
FGD3	325.1265	0.51063777	0.339127	1.505741008	0.132134	0.34755	NOT
TMEM53	1434.134	-0.3219614	0.213824	-1.50572991	0.132137	0.34755	NOT
NUMB	2378.529	-0.2225639	0.147811	-1.50572878	0.132137	0.34755	NOT
RP11-206F	3.4255	1.73254508	1.150647	1.505713339	0.132141	0.34755	NOT
GPX2	23565.35	0.81064963	0.538444	1.505542271	0.132185	0.347632	NOT
CXXC1	1767.123	0.21755472	0.144521	1.505348842	0.132234	0.347729	NOT
IL1A	2.530132	0.69072303	0.458912	1.505133174	0.13229	0.347831	NOT
SH3PXD2A-	3.67497	-0.5576905	0.37055	-1.50503429	0.132315	0.347831	NOT
RP11-504P	253.6407	-0.3956019	0.262853	-1.50503341	0.132315	0.347831	NOT
RP1-46F2.	2.601743	1.59883088	1.062347	1.504998429	0.132324	0.347831	NOT
FPGS	3019.627	-0.2721938	0.180874	-1.5048773	0.132356	0.347879	NOT
UBL4B	3.794281	1.36366373	0.906199	1.504816523	0.132371	0.347886	NOT
SEC22A	623.1213	0.20518102	0.13637	1.504588072	0.13243	0.348007	NOT

TERF1	1072.021	0.25849442	0.171825	1.504403912	0.132477	0.348098	NOT
RP11-95D1	212.0627	0.32884728	0.218607	1.504285496	0.132508	0.348144	NOT
RPS27AP1	2.143373	0.71887678	0.477951	1.504079907	0.132561	0.34825	NOT
MON1A	657.0394	0.27344532	0.18184	1.503767635	0.132641	0.348427	NOT
RP11-473M	6.6785	-0.4603507	0.306154	-1.50365657	0.13267	0.348469	NOT
RP11-111M	1.9153	0.83498782	0.555331	1.503586473	0.132688	0.348482	NOT
PPP2R5C	3604.748	-0.2076458	0.138112	-1.50346153	0.13272	0.348533	NOT
AC074286.	55.30857	0.30033278	0.199781	1.503312105	0.132759	0.3486	NOT
MAGEH1	600.7213	0.3461234	0.230261	1.503179892	0.132793	0.34863	NOT
OLR1	74.56704	-0.8221283	0.54693	-1.50316922	0.132795	0.34863	NOT
LIPG	1915.729	-0.5369548	0.357306	-1.50278772	0.132894	0.348854	NOT
RP11-375I	4.359243	-0.7083039	0.471357	-1.50269004	0.132919	0.348887	NOT
PLAC9	150.7469	-0.5400833	0.359451	-1.50252175	0.132962	0.34894	NOT
STAM	762.6132	0.23006561	0.153121	1.502510899	0.132965	0.34894	NOT
ZNF417	156.7394	-0.2883939	0.191956	-1.50239738	0.132995	0.348984	NOT
RP11-533E	21.08439	0.47205154	0.31422	1.502295818	0.133021	0.349019	NOT
SAMD15	12.68801	0.5969976	0.397435	1.502124781	0.133065	0.349101	NOT
AC144530.	70.63793	0.38904556	0.259026	1.501953698	0.133109	0.349155	NOT
SH3BGRL	1518.641	-0.4173093	0.277854	-1.50190039	0.133123	0.349155	NOT
MAP3K7	1311.911	0.23354882	0.155508	1.501845797	0.133137	0.349155	NOT
CYB5D1	42.98755	-0.3857589	0.256857	-1.50184552	0.133137	0.349155	NOT
SBF2-AS1	169.9323	0.32458441	0.216189	1.501391354	0.133254	0.349424	NOT
TFRC	4878.417	0.32080281	0.21368	1.501321867	0.133272	0.349424	NOT
PIK3IP1-A	5.913883	-0.5024861	0.334701	-1.50129961	0.133278	0.349424	NOT
TWIST1	16.74575	0.67947258	0.45265	1.50109781	0.13333	0.349494	NOT
RAB33A	18.07173	0.50048339	0.333412	1.501096607	0.133331	0.349494	NOT
CCDC114	19.83963	0.65017914	0.433163	1.501003362	0.133355	0.349523	NOT
GOLGA6L5P	12.99923	-0.5228918	0.348373	-1.50095273	0.133368	0.349523	NOT
KIF24	149.5562	0.4269129	0.284437	1.500903948	0.13338	0.349523	NOT
GUCY2EP	16.44279	0.94357708	0.628697	1.500844476	0.133396	0.349529	NOT
RSPH4A	11.22675	-0.4565963	0.304245	-1.50075009	0.13342	0.34956	NOT
IFT22	551.3324	0.29510034	0.196681	1.50040454	0.13351	0.349735	NOT
PIGP	810.451	-0.2956045	0.197018	-1.50039141	0.133513	0.349735	NOT
PIGH	658.6276	0.21721924	0.144786	1.500278873	0.133542	0.349763	NOT
FAM71F1	1.60019	-0.9054536	0.603535	-1.50025074	0.133549	0.349763	NOT
MAPK7	543.1606	0.28825175	0.192162	1.500044629	0.133603	0.349869	NOT
ANKRD34B	6.972723	1.35898053	0.906013	1.499957217	0.133625	0.349895	NOT
RANBP17	152.2973	0.73843716	0.492333	1.499874283	0.133647	0.349917	NOT
ZNF826P	108.8972	0.78459497	0.523153	1.49974223	0.133681	0.349973	NOT
UNC13C	2.694816	1.20936397	0.806435	1.499641727	0.133707	0.349983	NOT
ELAC1	304.4026	-0.2056627	0.137148	-1.49956504	0.133727	0.349983	NOT
MSS51	47.6815	0.42367348	0.282539	1.499522991	0.133738	0.349983	NOT
RP13-33H1	3.046147	-0.5620202	0.374817	-1.49945182	0.133756	0.349983	NOT
AC079922.	65.29348	0.51244966	0.341759	1.499446788	0.133758	0.349983	NOT
CDKN2AIP	644.5889	-0.2386858	0.159184	-1.4994293	0.133762	0.349983	NOT
SPAG17	6.265123	0.88154763	0.587947	1.499366933	0.133778	0.349991	NOT
AC093106.	2.830238	0.81181387	0.541486	1.499233644	0.133813	0.350048	NOT
CYP4F22	1130.842	-0.8881072	0.592401	-1.49916669	0.13383	0.35006	NOT
AC144831.	8.079215	-0.8101469	0.540547	-1.49875375	0.133938	0.350306	NOT

CTD-2328I	16.51227	-0.5701287	0.380418	-1.4986906	0.133954	0.350315	NOT
RP1-278E1	24.81804	0.47767122	0.318755	1.498551243	0.13399	0.350376	NOT
CDC20B	16.12759	1.02058124	0.681124	1.498378722	0.134035	0.350443	NOT
RP11-138H	1.272161	1.12829022	0.753013	1.498367083	0.134038	NA	NA
RP5-1057I	2.406812	-0.591509	0.394773	-1.49835246	0.134042	0.350443	NOT
PYY	5.612266	0.72182488	0.481775	1.498260679	0.134066	0.350472	NOT
HFE2	10142.09	-0.6844595	0.456897	-1.49806019	0.134118	0.350563	NOT
RNASEL	286.6952	-0.4447985	0.296923	-1.49802672	0.134126	0.350563	NOT
SHMT1	12472.29	-0.5199632	0.347138	-1.49785729	0.13417	0.350645	NOT
CTD-3234F	5.570622	-0.5786125	0.386441	-1.49728687	0.134319	0.350998	NOT
TMEM107	163.5897	0.3192266	0.213211	1.497230937	0.134333	0.351003	NOT
LL22NC03-	7.479147	0.72634041	0.485153	1.497135664	0.134358	0.351031	NOT
RP11-95M1	2.978584	0.93151151	0.622223	1.497069393	0.134375	0.351031	NOT
MUC5B	897.5419	1.39519733	0.931971	1.497039227	0.134383	0.351031	NOT
RP11-386M	5.240019	-0.8529743	0.569793	-1.49698889	0.134396	0.351032	NOT
LINC01089	288.175	-0.4199119	0.280547	-1.49676111	0.134455	0.351143	NOT
RP11-777B	1.737757	-0.9004545	0.601624	-1.49670694	0.13447	0.351143	NOT
NRARP	216.2753	0.40534704	0.270831	1.496676282	0.134478	0.351143	NOT
VPS13C	1680.807	-0.307636	0.205556	-1.49660626	0.134496	0.351157	NOT
CYP2A6	33562.15	-1.1442727	0.764726	-1.49631782	0.134571	0.351319	NOT
EFCAB13	80.80037	-0.3981383	0.26609	-1.49625246	0.134588	0.35133	NOT
CTB-1I21.	3.038922	1.63450907	1.092452	1.496183484	0.134606	0.351343	NOT
ADIPOR2	6374.48	-0.2930044	0.195854	-1.49603626	0.134644	0.351409	NOT
RP4-594A5	2.293046	1.49265468	0.997917	1.495770165	0.134714	0.35153	NOT
RP11-370B	1.436398	-0.8806034	0.588744	-1.49573346	0.134723	0.35153	NOT
RUVBL2	3956.871	0.26943858	0.180141	1.495709451	0.134729	0.35153	NOT
RP11-104N	15.74671	-0.4456893	0.297994	-1.49563196	0.13475	0.351549	NOT
MAEA	2292.935	-0.1870641	0.125083	-1.49551409	0.13478	0.351595	NOT
RP11-141C	36.02234	0.83089494	0.555611	1.495460968	0.134794	0.351598	NOT
DUSP18	87.46419	0.32702486	0.218719	1.495183423	0.134867	0.351753	NOT
FAM83G	2489.683	0.38003985	0.25421	1.494980971	0.134919	0.351857	NOT
RP1-34B2C	20.18101	-0.7590716	0.507805	-1.49480969	0.134964	0.351939	NOT
RP4-61404	6.275966	-0.5540548	0.37069	-1.4946568	0.135004	0.35201	NOT
STC1	865.172	-0.4863987	0.32544	-1.49458944	0.135022	0.352022	NOT
KRTAP5-8	2.308883	-0.871679	0.583278	-1.49444941	0.135058	0.352083	NOT
GSTT2B	145.7683	0.82132111	0.549613	1.494363883	0.135081	0.352108	NOT
MAP2K7	1948.604	-0.2054954	0.137541	-1.49406304	0.135159	0.352268	NOT
ZNF727	17.54955	-0.918912	0.615074	-1.49398639	0.135179	0.352268	NOT
S100A3	27.59886	0.6727284	0.450293	1.493979355	0.135181	0.352268	NOT
LINC00961	44.14546	-0.3721902	0.249156	-1.49380258	0.135227	0.352354	NOT
TAF15	5807.14	0.15297053	0.102408	1.493733635	0.135245	0.352368	NOT
TEF	2180.967	-0.3875073	0.259447	-1.49358964	0.135283	0.352432	NOT
TRIM52	372.1044	-0.2562534	0.171604	-1.49328715	0.135362	0.352604	NOT
RP11-106C	6.929467	-0.5326105	0.356693	-1.49318841	0.135388	0.352638	NOT
PHC2	5310.54	0.24072929	0.16124	1.492988888	0.13544	0.35272	NOT
CLEC4F	22.6629	-0.6631537	0.444185	-1.49296879	0.135445	0.35272	NOT
RP11-545J	8.441441	-1.0981587	0.735638	-1.49279824	0.13549	0.352787	NOT
ZBTB40	872.1568	0.23709703	0.15883	1.492770288	0.135497	0.352787	NOT
JOSD2	1061.119	0.32782614	0.219621	1.492687635	0.135519	0.35281	NOT

RP11-386(	4.346152	0.57118429	0.382695	1.492530032	0.13556	0.352883	NOT
CST7	154.0404	-0.5055632	0.33883	-1.49208452	0.135677	0.353153	NOT
RP11-420	6.473027	0.58167618	0.389859	1.492016186	0.135695	0.353166	NOT
TDRD9	15.31774	0.79459425	0.532593	1.491936334	0.135716	0.353187	NOT
RP13-216I	8.271391	-0.4096983	0.27462	-1.49187344	0.135732	0.353194	NOT
RP11-231(	26.5718	0.37905503	0.2541	1.491755329	0.135763	0.353194	NOT
UBE2Q2P1	38.29749	-0.4103048	0.275051	-1.49173998	0.135767	0.353194	NOT
NAPA	4200.774	-0.2435368	0.163258	-1.49172711	0.135771	0.353194	NOT
ABCD1	2090.196	0.34188966	0.229218	1.491548953	0.135817	0.353264	NOT
CASC20	17.38513	1.67563614	1.123439	1.491524385	0.135824	0.353264	NOT
RP11-687I	3.719261	0.54771152	0.367257	1.491359102	0.135867	0.353343	NOT
RP3-453C1	17.70444	-0.4368921	0.293004	-1.49107989	0.135941	0.353484	NOT
PLEKHA4	957.8176	0.426295	0.285902	1.491053997	0.135947	0.353484	NOT
C9orf84	8.52877	0.75064649	0.50348	1.490916208	0.135983	0.353521	NOT
AC007285.	1.109511	1.05007509	0.704319	1.490908795	0.135985	NA	NA
CELSR1	877.0839	0.5373034	0.360389	1.490900281	0.135988	0.353521	NOT
E2F4	1759.193	0.2176896	0.146034	1.490673274	0.136047	0.353625	NOT
RP11-490I	1.617998	-0.8508445	0.570788	-1.49064817	0.136054	0.353625	NOT
RP11-390I	207.3181	-0.2851778	0.191325	-1.49053914	0.136083	0.353644	NOT
ANXA3	69.49247	0.71348794	0.478683	1.490521992	0.136087	0.353644	NOT
PVR	3360.012	-0.281226	0.188706	-1.49028339	0.13615	0.353773	NOT
ATG4D	561.7114	-0.2913016	0.195515	-1.48992273	0.136245	0.353982	NOT
CREBBP	3389.229	-0.2462451	0.165279	-1.48987766	0.136256	0.353982	NOT
RP11-278(	6.611401	-0.3883121	0.260655	-1.4897545	0.136289	0.354002	NOT
MKNK1-AS1	5.712343	-0.5270387	0.353777	-1.48974906	0.13629	0.354002	NOT
PGF	455.0025	0.40935679	0.274815	1.489570476	0.136337	0.35409	NOT
KCTD9	584.8336	0.33961049	0.228007	1.489473211	0.136363	0.354103	NOT
RP11-314(	5.568196	-0.584421	0.392373	-1.48945256	0.136368	0.354103	NOT
KRT39	5.424605	0.94882066	0.637066	1.489360943	0.136392	0.354108	NOT
ELL	846.8232	-0.1986392	0.133373	-1.48934671	0.136396	0.354108	NOT
STK31	17.97947	0.77608082	0.521178	1.489088562	0.136464	0.354232	NOT
STAG3L5P-	125.4334	-0.3707846	0.249005	-1.48906542	0.13647	0.354232	NOT
METAP1	1413.032	0.23203801	0.155836	1.488988818	0.13649	0.354251	NOT
PDK4	6596.126	-0.6866242	0.461183	-1.48883287	0.136531	0.354323	NOT
PSG8	0.594139	-2.1191198	1.423444	-1.48872695	0.136559	NA	NA
RP11-147I	4.818355	-0.6125762	0.411485	-1.48869504	0.136568	0.354384	NOT
SGCA	71.55847	-0.7256442	0.487518	-1.48844716	0.136633	0.354519	NOT
C12orf57	2706.4	0.34130791	0.229329	1.488286257	0.136675	0.354595	NOT
RARRES2P1	2.168541	1.33599801	0.897791	1.488094439	0.136726	0.354693	NOT
ZDBF2	158.5375	0.75978714	0.510624	1.487957213	0.136762	0.354753	NOT
CDRT15	2.813065	0.8484014	0.570229	1.487825749	0.136797	0.354778	NOT
LINC0129(	3.291034	0.54317536	0.365081	1.487821295	0.136798	0.354778	NOT
RP5-857K2	223.3365	0.65502031	0.440278	1.487744142	0.136818	0.354797	NOT
C10orf12(	17.59925	1.00577836	0.676074	1.487674183	0.136837	0.354811	NOT
RP11-553N	3.262291	0.68790489	0.462423	1.487608729	0.136854	0.354821	NOT
LA16c-325	1.001712	2.13532448	1.435527	1.487484869	0.136887	NA	NA
KB-1440D3	7.833637	0.78426135	0.527333	1.4872231	0.136956	0.355051	NOT
ARHGAP32	662.3622	-0.3031694	0.20388	-1.48699846	0.137015	0.35514	NOT
RP11-400I	13.03558	-0.446148	0.300033	-1.48699401	0.137016	0.35514	NOT

RP1-1500E	2.283194	1.00118814	0.67332	1.486942841	0.13703	0.355142	NOT
GMFG	594.6427	-0.3974529	0.267313	-1.48684261	0.137056	0.355176	NOT
TDRD6	105.4708	0.62120449	0.417841	1.486699129	0.137094	0.355241	NOT
AC007000.	4.678236	-0.4643751	0.312378	-1.48658244	0.137125	0.355286	NOT
CTB-161M1	3.077408	-0.5246654	0.353019	-1.48622413	0.13722	0.355498	NOT
PPIAP19	2.53455	0.67496581	0.454208	1.486027344	0.137272	0.355599	NOT
FUT2	184.3528	0.79705668	0.536398	1.4859418	0.137295	0.355623	NOT
RP11-289I	64.03125	0.35052488	0.235918	1.485792161	0.137334	0.355692	NOT
ATOH8	649.0328	-0.7422685	0.499704	-1.48541702	0.137433	0.355915	NOT
DLGAP1	13.15763	0.5774412	0.388771	1.485300788	0.137464	0.355915	NOT
RP11-413M	1.67528	0.85997556	0.579005	1.485264238	0.137474	0.355915	NOT
ANO4	20.23342	-0.7060783	0.475404	-1.48521704	0.137486	0.355915	NOT
PRPF40A	3166.258	0.19541966	0.131579	1.485192184	0.137493	0.355915	NOT
RIN3	1493.818	-0.3073676	0.206959	-1.48516251	0.137501	0.355915	NOT
FBLN2	959.7235	-0.6964106	0.468925	-1.48512057	0.137512	0.355915	NOT
GS1-24F4.	2.449565	1.55846956	1.049493	1.484973212	0.137551	0.355982	NOT
AK2	5421.175	0.21446363	0.144439	1.484807647	0.137595	0.356061	NOT
DDX18	2683.766	0.21685279	0.14607	1.484578585	0.137655	0.356151	NOT
XPC	1835.308	-0.2692371	0.181361	-1.484535	0.137667	0.356151	NOT
MMP1	123.3878	0.81737136	0.55061	1.484482045	0.137681	0.356151	NOT
RP11-54G1	3.673072	-0.4349271	0.292983	-1.48447856	0.137682	0.356151	NOT
IFI30	62.79229	0.45851414	0.30892	1.484247244	0.137743	0.356276	NOT
AC009264.	1.895472	1.57015571	1.058027	1.484041631	0.137798	0.35637	NOT
NR1I3	2980.093	-0.7036779	0.474173	-1.48401132	0.137806	0.35637	NOT
CACNG1	7.963959	1.10315882	0.74358	1.483576928	0.137921	0.356634	NOT
RCN1	5025.756	-0.4296624	0.28963	-1.48348525	0.137946	0.356639	NOT
CTA-941F9	25.18099	0.48444024	0.326559	1.483470799	0.137949	0.356639	NOT
MAP4K4	5985.379	0.26529212	0.178848	1.483338453	0.137985	0.356678	NOT
BHLHE40	7232.565	-0.370156	0.249547	-1.48331415	0.137991	0.356678	NOT
RP11-139K	2.406475	0.76745761	0.51745	1.483152835	0.138034	0.356755	NOT
CCNYL2	0.813917	2.00741982	1.353547	1.483080666	0.138053	NA	NA
RP1-39G22	178.5185	0.30612209	0.206414	1.483050502	0.138061	0.356756	NOT
MT-ND4	680987.7	-0.3820474	0.257617	-1.4830039	0.138073	0.356756	NOT
RP11-111K	4.29537	-0.4735856	0.319358	-1.48293039	0.138093	0.356756	NOT
LINC01547	149.1391	-0.3391551	0.228713	-1.48288268	0.138106	0.356756	NOT
RP11-540C	7.950421	0.61281825	0.413266	1.482865445	0.13811	0.356756	NOT
CTD-2012J	12.85862	0.4856604	0.32752	1.48284176	0.138116	0.356756	NOT
OGFOD2	99.35909	0.19673791	0.132682	1.482779921	0.138133	0.356756	NOT
TBC1D22B	506.6219	0.2363905	0.159427	1.48275527	0.138139	0.356756	NOT
DNAJC5B	21.36698	0.55975377	0.377576	1.482493886	0.138209	0.356902	NOT
PTGES2-AS	10.40282	0.66273834	0.447059	1.482439269	0.138223	0.356905	NOT
RP11-167E	2.087256	1.96134625	1.32338	1.482073308	0.138321	0.357096	NOT
FOXRED2	2369.788	0.32749611	0.220973	1.482061781	0.138324	0.357096	NOT
CERS2	31107.91	-0.3131691	0.211326	-1.48192358	0.138361	0.357157	NOT
KCNH3	47.39104	0.77165133	0.520751	1.481803599	0.138393	0.357188	NOT
RP4-545K1	2.795112	-0.7325432	0.494367	-1.4817796	0.138399	0.357188	NOT
HAVCR2	340.2692	0.49602946	0.334865	1.48128039	0.138532	0.357497	NOT
RP1-257I2	7.622673	-0.4134748	0.279147	-1.48121076	0.13855	0.357511	NOT
RP11-203M	5.862715	-0.5780989	0.390325	-1.48107175	0.138587	0.357573	NOT

GABPA	1097.497	-0.2312665	0.156172	-1.48084933	0.138647	0.35768	NOT
QPRT	10922.93	0.3933288	0.265616	1.480816946	0.138655	0.35768	NOT
RP11-13K1	1.359371	-0.8342481	0.56354	-1.48037197	0.138774	NA	NA
MGC50722	136.4789	-1.1498207	0.776751	-1.48029493	0.138795	0.358005	NOT
SSR4	8970.016	-0.3523493	0.238043	-1.48019041	0.138822	0.358043	NOT
TNPO3	2539.754	0.20863372	0.141003	1.479642454	0.138969	0.358322	NOT
RP11-261(	3.288453	0.74497745	0.503486	1.479638261	0.13897	0.358322	NOT
RPS27P29	2.489743	0.65707665	0.44408	1.479636457	0.13897	0.358322	NOT
CD47	2702.098	0.28397749	0.191998	1.479064371	0.139123	0.358682	NOT
PKHD1L1	19.98738	0.84879876	0.573947	1.478879806	0.139172	0.358775	NOT
AC022384.	8.062398	-0.8296104	0.561036	-1.47871118	0.139218	0.358857	NOT
PCSK2	0.826393	1.81005172	1.224481	1.478219312	0.139349	NA	NA
ABCC3	9757.876	0.45011455	0.304513	1.4781475	0.139368	0.359212	NOT
SOX8	11.81218	-0.6344775	0.429271	-1.47803341	0.139399	0.359256	NOT
RP11-775A	1.75403	-0.8436397	0.570828	-1.47792212	0.139429	0.359296	NOT
AP5S1	876.7269	-0.2356274	0.159438	-1.47786426	0.139444	0.359296	NOT
IL22RA2	1.48288	1.31390198	0.889077	1.477827557	0.139454	0.359296	NOT
TGFB1I1	500.6	-0.3646274	0.246763	-1.47764068	0.139504	0.359391	NOT
LRTOMT	363.2896	-0.2515107	0.170245	-1.4773448	0.139583	0.359561	NOT
RP4-758J2	13.5945	-0.3427372	0.232022	-1.47717657	0.139628	0.359643	NOT
PLGLA	108.9971	-0.9909548	0.670898	-1.47705811	0.13966	0.35969	NOT
TFDP2	1493.132	-0.2368838	0.160394	-1.47688413	0.139707	0.359776	NOT
CST8	0.92243	2.56814099	1.738927	1.476853559	0.139715	NA	NA
SH2D6	30.5294	0.56423591	0.382068	1.47679504	0.139731	0.359804	NOT
SKAP1	325.597	-0.6063332	0.410594	-1.47672355	0.13975	0.359819	NOT
PTER	796.064	0.29755067	0.201514	1.476577821	0.139789	0.359885	NOT
ADCYAP1R1	35.40065	-0.7584501	0.513757	-1.47628226	0.139868	0.360043	NOT
RP11-798(	6.242793	-0.5377466	0.364288	-1.47615831	0.139901	0.360043	NOT
CTB-1202.	2.469482	-0.9560002	0.647628	-1.47615663	0.139902	0.360043	NOT
VPREB3	31.92147	0.63696946	0.431507	1.476151462	0.139903	0.360043	NOT
FBXO16	58.66313	0.5874407	0.397969	1.476096236	0.139918	0.360047	NOT
GGTLC2	1.718273	0.82347239	0.557906	1.476005844	0.139942	0.36007	NOT
PAICSP1	6.066179	-0.4065526	0.275449	-1.47596396	0.139954	0.36007	NOT
USP20	1267.352	-0.1863316	0.12626	-1.47577241	0.140005	0.360168	NOT
ZNF285B	3.087646	-0.606905	0.411379	-1.47529411	0.140133	0.360465	NOT
AC011322.	3.509153	0.90251257	0.61186	1.475031426	0.140204	0.360612	NOT
CTGLF10P	5.703874	0.46991051	0.318605	1.474901271	0.140239	0.360668	NOT
PXMP2	3282.522	-0.4751758	0.322291	-1.47436794	0.140383	0.361003	NOT
SEC63	5148.895	-0.2500056	0.169577	-1.47429342	0.140403	0.36102	NOT
RNF135	364.9152	-0.4975075	0.337499	-1.47410175	0.140454	0.361078	NOT
SIRT6	858.0117	0.30517316	0.207027	1.474076927	0.140461	0.361078	NOT
RP11-159(	50.10558	-0.4328171	0.293644	-1.47394994	0.140495	0.361078	NOT
HM13-AS1	11.53688	-0.4736427	0.321348	-1.47392662	0.140501	0.361078	NOT
CNNM4	609.0838	0.28016935	0.190089	1.473882348	0.140513	0.361078	NOT
RP11-491F	4.46858	-0.6972879	0.473098	-1.47387544	0.140515	0.361078	NOT
Z83851.4	67.41946	0.36519325	0.247786	1.473824463	0.140529	0.361078	NOT
SNORA77	2.000861	-0.6518617	0.442296	-1.47381408	0.140532	0.361078	NOT
RP11-320(	5.555348	0.83426132	0.566125	1.47363551	0.14058	0.361167	NOT
TBX2	712.0721	-0.3866241	0.262387	-1.47348699	0.14062	0.361236	NOT

HMGN2P46	12.53435	0.59701554	0.405233	1.473266647	0.140679	0.361354	NOT
ARHGAP28	80.9236	0.63591969	0.431701	1.473056892	0.140736	0.361437	NOT
GTF3A	5572.722	0.33624129	0.228262	1.473048101	0.140738	0.361437	NOT
IGHV3-30	89.39711	1.08409853	0.736027	1.472906852	0.140776	0.361501	NOT
AP4B1-AS1	8.916325	-0.4957511	0.336652	-1.47259113	0.140861	0.361663	NOT
RP11-326(	275.8033	-0.9260197	0.628855	-1.47254792	0.140873	0.361663	NOT
AF196972.	11.0866	0.48693426	0.330693	1.472466129	0.140895	0.361663	NOT
GRP	2.46275	1.53613232	1.043274	1.472415347	0.140909	0.361663	NOT
SH3RF3	436.3093	-0.6564739	0.445853	-1.47240128	0.140913	0.361663	NOT
CCDC8	91.38558	0.71257133	0.48396	1.47237602	0.140919	0.361663	NOT
TSPEAR-AS	151.9131	-0.793547	0.539012	-1.47222442	0.14096	0.361733	NOT
ANTXR2	1404.82	-0.320451	0.217672	-1.47217611	0.140973	0.361733	NOT
RP11-706(	288.9575	0.81682844	0.554873	1.47210056	0.140994	0.361751	NOT
ANKUB1	1.547872	-0.7545512	0.512627	-1.47192967	0.14104	0.361836	NOT
RPS12	26940.78	0.36567269	0.248466	1.471722802	0.141096	0.361945	NOT
LYPD1	325.6508	0.77710076	0.528111	1.471473791	0.141163	0.36205	NOT
RP13-554M	10.44846	-0.5839042	0.396825	-1.47144081	0.141172	0.36205	NOT
RP3-465N2	6.731425	-0.4854895	0.329946	-1.47142212	0.141177	0.36205	NOT
TREML2	7.04844	0.71687	0.487213	1.471369902	0.141191	0.362052	NOT
ZSCAN9	359.8942	0.2153961	0.146404	1.471246824	0.141224	0.362104	NOT
LYPLA2	3905.681	0.26473152	0.179944	1.471191528	0.141239	0.362108	NOT
RP11-121A	8.431361	-0.5502776	0.374052	-1.47112445	0.141257	0.362118	NOT
TSPEAR	53.15577	-0.7296907	0.496039	-1.47103484	0.141282	0.362118	NOT
HRH4	3.714785	-0.6721615	0.456933	-1.47102826	0.141283	0.362118	NOT
NDUFB9	13686.97	0.42901169	0.291691	1.47077325	0.141352	0.362261	NOT
SIGLEC22F	2.305288	-0.7253163	0.493232	-1.47053878	0.141416	0.362389	NOT
RBM26-AS1	60.94056	-0.4120813	0.280253	-1.47039195	0.141456	0.362457	NOT
PPM1M	682.6351	0.3429439	0.233242	1.470337816	0.14147	0.36246	NOT
ZBTB14	564.3759	-0.1963478	0.133551	-1.47020621	0.141506	0.362492	NOT
KRT7	1294.77	-0.8443743	0.574329	-1.47019259	0.14151	0.362492	NOT
RP11-188(	14.51129	-0.4153616	0.282564	-1.46997546	0.141568	0.362609	NOT
AP000525.	1.823022	0.99840811	0.679249	1.469870686	0.141597	0.362647	NOT
RP11-388(	19.46603	-0.5033163	0.342439	-1.46979951	0.141616	0.362662	NOT
SLC12A2	1118.543	0.52860546	0.35968	1.469654162	0.141655	0.362729	NOT
BTBD2	3170.564	0.23771531	0.161755	1.469604569	0.141669	0.362729	NOT
AFM	5396.238	-0.7137843	0.485801	-1.46929403	0.141753	0.36291	NOT
RNU1-75P	2.79323	-0.9190943	0.625565	-1.46922346	0.141772	0.362925	NOT
CTC-429P9	197.5246	-0.3791549	0.258078	-1.46914859	0.141792	0.362943	NOT
EI24P2	1.538895	-0.7954671	0.541529	-1.46892677	0.141853	0.363017	NOT
MTX1	1067.454	0.22867389	0.155687	1.468805415	0.141886	0.363017	NOT
CTD-3193K	4.566416	0.64185125	0.43699	1.468802292	0.141886	0.363017	NOT
RP11-340I	1.825982	0.90925581	0.619054	1.468781975	0.141892	0.363017	NOT
INTS7	1506.364	0.20096825	0.136827	1.468778821	0.141893	0.363017	NOT
C7orf50	3131.312	0.30860494	0.210115	1.468745412	0.141902	0.363017	NOT
SMG7-AS1	20.03782	-0.3566586	0.242891	-1.46838808	0.141999	0.36321	NOT
CECR7	64.11796	1.20928124	0.823554	1.468368694	0.142004	0.36321	NOT
TRAPPC2L	1717.788	-0.3421855	0.233077	-1.46812	0.142072	0.363349	NOT
LGI1	6.281744	-1.0830932	0.737871	-1.46786227	0.142142	0.363473	NOT
KLK15	1.803071	1.92307295	1.310136	1.467842492	0.142147	0.363473	NOT

CEP57L1	265.6295	0.28955354	0.197281	1.467719155	0.142181	0.36352	NOT
HPR	15747.12	-0.7586466	0.516903	-1.46767563	0.142192	0.36352	NOT
CTD-2653I	1.16039	-0.8527536	0.581035	-1.46764495	0.142201	NA	NA
CCM2	3503.646	-0.2851721	0.194317	-1.46756239	0.142223	0.363547	NOT
ZSWIM6	412.5795	-0.3195079	0.217717	-1.46753823	0.14223	0.363547	NOT
MAPK8IP3	1383.948	-0.3378774	0.230247	-1.46745678	0.142252	0.36357	NOT
BNIP1	466.9859	0.29791779	0.203024	1.467399308	0.142267	0.363575	NOT
GAPVD1	1374.97	-0.1821291	0.124124	-1.46731257	0.142291	0.363593	NOT
MYF6	2.459045	-0.9811198	0.668668	-1.4672749	0.142301	0.363593	NOT
PCDHB7	46.34501	-0.5906716	0.402587	-1.46718828	0.142325	0.363597	NOT
AHNAK	21000.92	-0.2952072	0.201215	-1.46712278	0.142343	0.363597	NOT
RNU1-70P	996.0891	-0.7075478	0.482269	-1.46712131	0.142343	0.363597	NOT
NDRG4	86.88752	0.48842976	0.33294	1.467021666	0.14237	0.363629	NOT
UEVLD	787.2413	0.19854159	0.135345	1.466931534	0.142395	0.363629	NOT
PLAC8L1	18.08161	-0.549762	0.374771	-1.46692827	0.142396	0.363629	NOT
PCDH18	134.8952	-0.4302817	0.293402	-1.4665256	0.142505	0.363874	NOT
CTB-5506.	4.269054	1.28963814	0.879412	1.466477318	0.142518	0.363874	NOT
HDAC7	1700.686	0.33904201	0.231215	1.466349224	0.142553	0.363901	NOT
RP11-314A	19.96064	0.48825955	0.332984	1.466316186	0.142562	0.363901	NOT
CMTM4	802.3626	-0.4005856	0.273197	-1.46629029	0.142569	0.363901	NOT
AC067959.	4.128128	-0.9016895	0.615024	-1.46610522	0.14262	0.363995	NOT
AC023115.	3.206914	-0.9429635	0.643221	-1.46600219	0.142648	0.364021	NOT
HIST1H2BF	2.091589	-0.9746792	0.66487	-1.46596971	0.142657	0.364021	NOT
RP11-83B2	2.405372	-1.2995901	0.886585	-1.46583792	0.142692	0.364078	NOT
SLC26A8	13.29829	-0.576258	0.393175	-1.46565109	0.142743	0.364165	NOT
ITPRIPL1	55.31734	0.52804147	0.360287	1.465614271	0.142753	0.364165	NOT
MAPRE2	2115.282	-0.3167085	0.216132	-1.4653446	0.142827	0.364319	NOT
WFDC21P	93.16027	0.89433987	0.610407	1.465152508	0.142879	0.364418	NOT
SLC16A9	534.5732	1.04300972	0.711988	1.464925701	0.142941	0.364517	NOT
SMPD2	388.019	0.26147829	0.178498	1.464879377	0.142954	0.364517	NOT
DERL2	1851.988	-0.2455437	0.167622	-1.46486312	0.142958	0.364517	NOT
RP11-260M	23.80414	0.44936115	0.306792	1.464710163	0.143	0.364561	NOT
RP11-330M	5.102308	0.8463944	0.577862	1.464700643	0.143003	0.364561	NOT
CHST9	883.4023	0.83792447	0.572183	1.464434492	0.143075	0.364641	NOT
RP13-766I	2.388387	-0.7693492	0.525356	-1.46443384	0.143075	0.364641	NOT
NISCH	3019.653	0.19882269	0.135771	1.464396708	0.143086	0.364641	NOT
NLRP4	2.790802	1.40436943	0.95904	1.464348608	0.143099	0.364641	NOT
FAM87B	3.230656	-0.6014988	0.410787	-1.46425942	0.143123	0.364641	NOT
RP3-407E4	1.596981	-1.0163198	0.694089	-1.46424964	0.143126	0.364641	NOT
CTD-2521M	2.650744	0.74981927	0.512087	1.464241679	0.143128	0.364641	NOT
RPS10L	8.037361	0.55708195	0.380474	1.464180324	0.143145	0.364649	NOT
RP4-798A1	29.92503	-0.5543805	0.378724	-1.46381268	0.143245	0.364871	NOT
AARS2	1135.405	0.2094684	0.143108	1.463705436	0.143274	0.364911	NOT
RP4-717I2	258.6066	0.35765564	0.244362	1.463628933	0.143295	0.36493	NOT
RP11-30H9	2.481552	1.91464036	1.308236	1.463527816	0.143323	0.364967	NOT
RNU6-322F	3.386518	-0.6498397	0.444049	-1.46344281	0.143346	0.364991	NOT
PCYT2	10086.25	-0.3920107	0.267878	-1.46339344	0.14336	0.364992	NOT
PLEKHA1	2105.272	-0.2506086	0.171258	-1.46333705	0.143375	0.364997	NOT
TRNT1	568.1895	0.20099196	0.137378	1.463056952	0.143452	0.365157	NOT

RRAD	128.5033	0.58702765	0.401252	1.462990714	0.14347	0.365169	NOT
CEP152	153.6633	0.35259032	0.241029	1.462851842	0.143508	0.365232	NOT
SLC35D2	2526.88	0.28441445	0.194457	1.4626072	0.143575	0.365357	NOT
EDNRA	255.8847	-0.4408177	0.301399	-1.46257376	0.143584	0.365357	NOT
PCDH10	2.433794	-0.9908382	0.67751	-1.46247025	0.143612	0.365395	NOT
RP11-895M	1.764392	0.93655397	0.640432	1.462378185	0.143638	0.365396	NOT
FAM50A	4788.975	0.3352193	0.22923	1.462370646	0.14364	0.365396	NOT
RP11-265E	4.250458	0.57812286	0.395358	1.462278059	0.143665	0.365426	NOT
RMDN2	465.5141	-0.3486056	0.238426	-1.46211353	0.14371	0.365506	NOT
KLKP1	1.082925	2.61526898	1.789	1.46186075	0.143779	NA	NA
SYNDIG1L	3.409301	0.73016878	0.499484	1.461845094	0.143784	0.365653	NOT
AC004231.	5.058772	0.97032369	0.663785	1.46180511	0.143795	0.365653	NOT
C16orf54	68.75331	-0.5477134	0.374696	-1.46175338	0.143809	0.365654	NOT
RPL10P15	9.90685	-0.5660663	0.387272	-1.46167799	0.143829	0.365668	NOT
CTD-2162K	10.64312	0.89540235	0.612603	1.461635289	0.143841	0.365668	NOT
AC091849.	5.194203	0.71876541	0.491917	1.461153042	0.143973	0.36597	NOT
KIF20B	374.8373	0.34969809	0.239346	1.461058095	0.143999	0.366002	NOT
TEPP	1.343003	-0.9121955	0.624339	-1.46105721	0.144	NA	NA
SLC7A11-A	5.406667	0.98396256	0.673523	1.460918002	0.144038	0.366066	NOT
CYP2D8P	22.17847	0.49658524	0.339928	1.460853553	0.144056	0.366076	NOT
MEF2B	16.43576	-0.4383747	0.300095	-1.46078864	0.144073	0.366087	NOT
USP44	14.96966	0.78670483	0.538606	1.460632635	0.144116	0.366162	NOT
ZNF513	1072.658	0.22349698	0.153026	1.460514714	0.144149	0.36621	NOT
SERF1B	10.67497	0.44351091	0.303714	1.460292273	0.14421	0.366331	NOT
EMP3	556.7625	0.40410544	0.276747	1.460196632	0.144236	0.36634	NOT
DOC2GP	18.73356	-0.5645932	0.38666	-1.46018034	0.144241	0.36634	NOT
RP11-594N	1.106478	1.0261783	0.702807	1.460113641	0.144259	NA	NA
TMEM204	620.6175	-0.3614904	0.247619	-1.45986675	0.144327	0.366479	NOT
RILP	1101.569	-0.4115665	0.281922	-1.45985965	0.144329	0.366479	NOT
PHACTR4	2146.432	-0.2277543	0.156014	-1.45983341	0.144336	0.366479	NOT
CXCL8	979.4635	0.80990182	0.554933	1.459458312	0.144439	0.36665	NOT
ZSCAN26	375.3738	0.20443268	0.140076	1.459445829	0.144442	0.36665	NOT
RP11-449H	2.156378	-0.585471	0.401165	-1.45942665	0.144448	0.36665	NOT
OR2A9P	12.82176	0.62824946	0.430487	1.459392314	0.144457	0.36665	NOT
CASC3	3326.079	0.17301185	0.118561	1.459262662	0.144493	0.366652	NOT
RPL10P3	25.13213	0.54938657	0.376484	1.459257066	0.144494	0.366652	NOT
RP11-135A	5.532912	-0.7250486	0.496866	-1.45924266	0.144498	0.366652	NOT
ZNF334	111.6898	0.74367792	0.509713	1.459013396	0.144561	0.366747	NOT
KAL1	102.5981	0.6931156	0.475059	1.459008382	0.144563	0.366747	NOT
ZFP91	2584.501	-0.2161411	0.148148	-1.45895708	0.144577	0.366748	NOT
RP11-404O	3.556169	-0.5628369	0.385817	-1.45881967	0.144615	0.36681	NOT
PPIF	6473.839	-0.3345291	0.229327	-1.45873997	0.144637	0.366831	NOT
AP000439.	2.74864	0.86794671	0.595029	1.458663418	0.144658	0.366833	NOT
PSME4	3984.736	-0.1912053	0.131085	-1.4586394	0.144664	0.366833	NOT
CCDC170	404.1337	-0.7227286	0.495524	-1.45851305	0.144699	0.366887	NOT
UBE2L3	3567.827	0.20901441	0.143315	1.458424098	0.144724	0.366912	NOT
GGN	11.404	-0.4872343	0.334093	-1.45837909	0.144736	0.366912	NOT
AGAP2-AS1	93.68722	0.4808055	0.329706	1.458284979	0.144762	0.366938	NOT
RP11-22P4	5.486563	-0.5033624	0.345184	-1.4582439	0.144773	0.366938	NOT

LIPE	92.53318	-0.4403618	0.301992	-1.45818992	0.144788	0.366941	NOT
LSM12	360.5292	0.20928756	0.143547	1.457971262	0.144848	0.367003	NOT
CLIP4	549.8959	0.60105592	0.412263	1.457944638	0.144856	0.367003	NOT
AC108868.	1.522509	-2.1137387	1.449844	-1.45790785	0.144866	0.367003	NOT
LANCL1	2305.385	0.26229153	0.17991	1.457904653	0.144867	0.367003	NOT
RP11-3B7.	4.089387	0.80310949	0.550914	1.457775666	0.144902	0.367036	NOT
RP11-159F	8.142757	-0.3283351	0.225233	-1.45775986	0.144907	0.367036	NOT
ZNF25	261.9071	-0.2876604	0.197344	-1.45765885	0.144935	0.367057	NOT
RP11-106A	3.449643	0.67578111	0.463616	1.457631365	0.144942	0.367057	NOT
RP6-105D1	7.046962	0.59506507	0.408269	1.457531901	0.14497	0.367092	NOT
KRTAP5-5	6.951062	1.4442156	0.991034	1.457281749	0.145039	0.367199	NOT
PRELID1	2913.722	0.25901316	0.177737	1.457280746	0.145039	0.367199	NOT
PIR	1611.147	0.46724987	0.320732	1.456822829	0.145165	0.367485	NOT
CLDN14	1074.833	-0.5890714	0.404372	-1.45675667	0.145184	0.367497	NOT
LMLN	279.1976	-0.4766542	0.327246	-1.45656274	0.145237	0.367569	NOT
EML1	289.6251	-0.4253249	0.292007	-1.45655507	0.145239	0.367569	NOT
ZNRF2P2	3.765955	-0.5837639	0.400827	-1.45639765	0.145283	0.367641	NOT
ANP32BP1	2.234113	0.61736945	0.423915	1.456353553	0.145295	0.367641	NOT
AC112229.	5.945489	0.86214055	0.592088	1.456101208	0.145365	0.367784	NOT
RP11-302M	2.489479	-0.5953965	0.40896	-1.45587931	0.145426	0.367887	NOT
DLAT	2040.844	0.31688635	0.217677	1.455763466	0.145458	0.367887	NOT
RP11-350N	9.045704	-0.4531127	0.311261	-1.45573015	0.145467	0.367887	NOT
FAM21C	1379.654	0.19104637	0.131239	1.455713583	0.145472	0.367887	NOT
PCDHB2	53.80893	-0.7801336	0.535936	-1.45564668	0.14549	0.367887	NOT
HIGD1B	61.52217	-0.3650532	0.250794	-1.45558713	0.145507	0.367887	NOT
C2orf42	471.6455	-0.1921546	0.132012	-1.45558669	0.145507	0.367887	NOT
RP11-552I	1.599773	-0.9958602	0.684177	-1.45555844	0.145515	0.367887	NOT
DIP2A	896.4967	-0.2280087	0.156652	-1.45551266	0.145527	0.367887	NOT
JMJD8	1861.859	-0.2564045	0.176188	-1.45529339	0.145588	0.367999	NOT
RP11-367N	9.4318	0.45446752	0.312302	1.455217045	0.145609	0.367999	NOT
PPP1R9B	1920.654	0.27832327	0.191264	1.455175758	0.145621	0.367999	NOT
CSAG1	322.0881	1.38561708	0.952212	1.45515638	0.145626	0.367999	NOT
CTA-313A1	1.705915	-0.7174163	0.49314	-1.45479234	0.145727	0.368176	NOT
CTC-492K1	3.174059	-0.4823968	0.331593	-1.45478391	0.145729	0.368176	NOT
RPS14	29213.3	0.34462211	0.236901	1.454712239	0.145749	0.368176	NOT
RP13-638C	2.004993	-0.6104506	0.419638	-1.4547069	0.14575	0.368176	NOT
DHRS4L1	52.7004	-0.4965941	0.341398	-1.45459216	0.145782	0.368222	NOT
SLC22A20	18.46721	0.57881666	0.397972	1.454416055	0.145831	0.368311	NOT
WARS	3573.638	0.34290639	0.235795	1.454254187	0.145876	0.36839	NOT
AC006994.	4.855045	-0.9315242	0.640633	-1.45406939	0.145927	0.368469	NOT
CRTC1	692.2701	-0.2833651	0.194881	-1.45404372	0.145934	0.368469	NOT
FAM169B	54.60011	-1.0858922	0.747068	-1.45353871	0.146074	0.368788	NOT
RP11-615I	1.473078	1.05787602	0.727849	1.453428384	0.146105	0.368829	NOT
CIZ1	2439.566	0.24390889	0.167822	1.45338141	0.146118	0.368829	NOT
ACOT9	1039.107	0.27135342	0.186718	1.453281929	0.146145	0.368859	NOT
THRA	1266.188	-0.2901843	0.199681	-1.45324171	0.146157	0.368859	NOT
PSME2	8948.918	0.3331726	0.229292	1.453050243	0.14621	0.368958	NOT
RP4-803J1	3.108288	0.61888568	0.425956	1.452933063	0.146242	0.369005	NOT
NTPCR	2228.977	0.27775927	0.191181	1.452861496	0.146262	0.369005	NOT

ZNF197	588.5687	-0.2611573	0.179757	-1.45283613	0.146269	0.369005	NOT
RAB38	53.92785	0.66957934	0.46094	1.452639678	0.146324	0.369109	NOT
C3orf67-A	4.455672	-0.9444664	0.650321	-1.45230863	0.146416	0.369287	NOT
MAZ	2255.521	0.25497532	0.175568	1.45228771	0.146422	0.369287	NOT
NAMPT	8095.192	-0.4758645	0.327726	-1.45201903	0.146496	0.369441	NOT
SLC40A1	11657.89	-0.3168338	0.218241	-1.45176048	0.146568	0.36951	NOT
H3F3AP6	5.114314	0.44573925	0.307039	1.451733075	0.146576	0.36951	NOT
ZNF764	450.9472	0.25777292	0.177566	1.451705759	0.146583	0.36951	NOT
BDH1	7027.654	-0.5116888	0.352478	-1.45168836	0.146588	0.36951	NOT
HARBI1	158.3382	0.19360088	0.133367	1.451641008	0.146601	0.36951	NOT
RP11-114H	7.468778	-0.6307453	0.434509	-1.45162619	0.146606	0.36951	NOT
DRG2	1252.451	0.25802803	0.177779	1.45140044	0.146668	0.369609	NOT
RIC8A	4158.697	0.15072472	0.103849	1.451387199	0.146672	0.369609	NOT
METTL22	436.0246	-0.244359	0.16841	-1.45097923	0.146786	0.369832	NOT
PCDHGC5	6.918449	-0.9415761	0.648928	-1.45097118	0.146788	0.369832	NOT
RP11-6B4.	60.41871	1.14380382	0.788365	1.4508554	0.14682	0.369879	NOT
KRT2	0.84801	1.51165549	1.041985	1.450745315	0.146851	NA	NA
USP46-AS1	67.56476	-0.2994798	0.206458	-1.45056398	0.146901	0.370049	NOT
MBP	1775.336	-0.2996087	0.206599	-1.45019326	0.147005	0.370275	NOT
RP11-118E	3.818307	-0.7174025	0.494722	-1.45011216	0.147027	0.370298	NOT
RP11-1C1.	0.942687	2.40504977	1.658734	1.449930628	0.147078	NA	NA
ZNF202	327.9631	0.22189863	0.153068	1.44967195	0.14715	0.370547	NOT
MYL6B	1517.964	0.44022602	0.303675	1.449659643	0.147153	0.370547	NOT
NR2E3	5.379515	-0.6003475	0.414144	-1.44961078	0.147167	0.370547	NOT
RP11-770J	21.19713	-0.5507534	0.380061	-1.44911985	0.147304	0.370811	NOT
ACTN2	165.8062	0.82502144	0.569333	1.449102785	0.147309	0.370811	NOT
GJA1	936.7195	-0.3930124	0.271214	-1.44908562	0.147314	0.370811	NOT
TMEM185B	687.9122	0.27077589	0.186866	1.449039708	0.147326	0.370811	NOT
LTBR	6755.563	0.21522968	0.148582	1.448553516	0.147462	0.371118	NOT
RP11-109M	16.94854	-1.6046283	1.107832	-1.44844035	0.147494	0.371163	NOT
AP000593.	12.20574	0.93212508	0.643576	1.448352599	0.147518	0.37119	NOT
IGHV3-20	3.077969	1.30134416	0.898584	1.448215999	0.147557	0.371249	NOT
CLEC12B	1.922352	-0.8823979	0.609326	-1.44815403	0.147574	0.371249	NOT
RP4-530I1	8.04678	-0.5470741	0.377782	-1.44812261	0.147583	0.371249	NOT
GNA11	2716.01	-0.1817614	0.125534	-1.44790484	0.147644	0.371368	NOT
PSMB6	4556.744	-0.3064739	0.211683	-1.44779579	0.147674	0.37141	NOT
DHX30	3757.983	0.15622547	0.107921	1.447590909	0.147731	0.371492	NOT
SCARNA6	1.942278	-0.5712556	0.394647	-1.44751101	0.147754	0.371492	NOT
IRS2	4963.737	-0.4458521	0.30802	-1.44747893	0.147763	0.371492	NOT
LINC01257	2.01266	1.57373619	1.08723	1.447473127	0.147764	0.371492	NOT
RP11-40C6	25.62027	0.53850999	0.372045	1.447434084	0.147775	0.371492	NOT
YIPF6	2766.34	-0.2299486	0.158872	-1.44738042	0.14779	0.371496	NOT
ARHGAP35	6045.958	-0.2420216	0.167227	-1.44726135	0.147824	0.371522	NOT
AP006621.	2.003136	-0.5949949	0.411122	-1.44724513	0.147828	0.371522	NOT
MRPL24	6663.381	0.35845807	0.247731	1.446964901	0.147907	0.371685	NOT
CTD-3193C	16.99423	-0.6264301	0.432967	-1.44683039	0.147944	0.371745	NOT
RPS23P6	1.741711	-0.9103944	0.629351	-1.44656138	0.14802	0.371874	NOT
NOP10	3019.31	0.29719306	0.20545	1.446549026	0.148023	0.371874	NOT
ALKBH4	519.8634	0.18176404	0.125667	1.446399254	0.148065	0.371945	NOT

RP11-497(	15.77241	-0.8585158	0.593577	-1.44634362	0.148081	0.37195	NOT
RP11-425I	4.609384	-0.4203024	0.290658	-1.44603566	0.148167	0.372133	NOT
OTUD4	1329.318	-0.2745287	0.189901	-1.44563832	0.148279	0.372353	NOT
RP5-1142A	11.09552	0.52530558	0.363376	1.445625119	0.148282	0.372353	NOT
HSF4	538.0288	-0.444098	0.307214	-1.4455637	0.1483	0.372362	NOT
SLC35E3	1019.428	0.1995144	0.138031	1.445427584	0.148338	0.372423	NOT
GNG8	3.015117	-0.9740036	0.673973	-1.44516807	0.148411	0.372553	NOT
TCL1A	12.04567	0.95418455	0.660269	1.44514558	0.148417	0.372553	NOT
QRFPR	4.852955	-1.0146442	0.702133	-1.44508743	0.148433	0.37256	NOT
LINC0139:	5.188245	0.76530212	0.529809	1.444487554	0.148602	0.372948	NOT
WHSC1L1	1536.648	0.32949473	0.228184	1.443986713	0.148743	0.373263	NOT
NACA2	7.18883	0.5120875	0.354653	1.443909995	0.148764	0.373263	NOT
TEAD1	2535.63	-0.3306482	0.228998	-1.44389417	0.148769	0.373263	NOT
RAD23B	10108.98	-0.1973391	0.136687	-1.44372503	0.148816	0.373307	NOT
PDCL3P4	30.45063	-0.496616	0.343985	-1.44371591	0.148819	0.373307	NOT
AZGP1	32130.05	-0.6188309	0.428647	-1.44368505	0.148828	0.373307	NOT
SLC6A15	3.235668	1.87284908	1.29748	1.443451487	0.148893	0.373438	NOT
ZNF561	875.6221	-0.2262503	0.156748	-1.44340241	0.148907	0.373438	NOT
PRKCSH	19197.82	-0.1954737	0.135437	-1.44328261	0.148941	0.373448	NOT
CTD-2012K	3.820617	-0.4796049	0.332309	-1.44324869	0.14895	0.373448	NOT
PLXDC1	461.0877	-0.4598009	0.318597	-1.44320518	0.148963	0.373448	NOT
SLMO1	31.44379	0.63080152	0.437087	1.44319315	0.148966	0.373448	NOT
NFAT5	699.4413	-0.4017137	0.278388	-1.44299981	0.149021	0.373512	NOT
ELOF1	2075.809	-0.2751837	0.190713	-1.4429226	0.149042	0.373512	NOT
RNF157	1223.709	0.60721218	0.420834	1.442876872	0.149055	0.373512	NOT
MAFF	1346.945	0.4348848	0.301401	1.442876455	0.149055	0.373512	NOT
POLB	762.3592	0.32535051	0.22549	1.44285754	0.149061	0.373512	NOT
CCDC127	820.3714	0.22436042	0.155512	1.442719256	0.1491	0.373549	NOT
RAB13	4568	0.24317296	0.168553	1.442707564	0.149103	0.373549	NOT
RNF39	35.82496	-0.5543062	0.384238	-1.44261097	0.14913	0.373583	NOT
SPINK1	10293.99	1.02882334	0.713268	1.442407626	0.149187	0.373626	NOT
GPATCH1	514.5949	0.19909009	0.138027	1.442404376	0.149188	0.373626	NOT
IL21R	56.62031	0.63870697	0.442812	1.44238919	0.149193	0.373626	NOT
RP11-101I	29.9368	-1.2478932	0.865179	-1.44235196	0.149203	0.373626	NOT
PUS10	545.9017	-0.2830399	0.196245	-1.44227592	0.149225	0.373626	NOT
ZFYVE9	890.4093	-0.228507	0.158437	-1.44225599	0.14923	0.373626	NOT
ZNF687	2100.844	0.24072251	0.166925	1.442095842	0.149275	0.373683	NOT
TMX4	3532.138	-0.2551559	0.176936	-1.44207865	0.14928	0.373683	NOT
RP11-519(	3.635317	0.8852195	0.613888	1.441988497	0.149306	0.373712	NOT
AC016682.	3.305854	-1.2305	0.853374	-1.44192431	0.149324	0.373723	NOT
LINC0023(	12.65394	0.75164431	0.521341	1.441751362	0.149373	0.37377	NOT
CLK2	1799.706	0.26433182	0.183341	1.441749829	0.149373	0.37377	NOT
THNSL2	2478.178	0.51199175	0.355128	1.441711316	0.149384	0.37377	NOT
CAMKV	6.189483	1.32501215	0.919331	1.441277892	0.149506	0.374001	NOT
PCF11	1310.883	-0.187591	0.130162	-1.4412168	0.149523	0.374001	NOT
CD36	4090.352	-0.6107671	0.423793	-1.44119152	0.149531	0.374001	NOT
PALB2	493.464	0.23486162	0.162964	1.441187728	0.149532	0.374001	NOT
DACT3	107.1155	-0.5433041	0.37705	-1.4409348	0.149603	0.374146	NOT
PRSS51	21.17303	-0.8488859	0.589182	-1.44078736	0.149645	0.374201	NOT

CNOT2	1983.771	0.15945481	0.110674	1.440758671	0.149653	0.374201	NOT
AC093110.	36.65682	-0.5651673	0.392334	-1.44052711	0.149718	0.37433	NOT
AC009166.	4.540716	-0.6129634	0.425528	-1.44047664	0.149733	0.374331	NOT
IFT57	414.2818	0.43382134	0.301197	1.440324552	0.149776	0.374385	NOT
CCDC142	356.5787	0.23496196	0.163134	1.440303899	0.149781	0.374385	NOT
RN7SL130F	7.522517	-0.4954889	0.344039	-1.44021023	0.149808	0.374416	NOT
LINC0148(	18.06774	0.75843654	0.52667	1.440059567	0.149851	0.374488	NOT
L3HYPDH	493.7463	0.31977457	0.222118	1.439659699	0.149964	0.374612	NOT
PTENP1	40.10742	-0.3581414	0.248769	-1.43965382	0.149965	0.374612	NOT
ZNF500	464.7968	-0.1755604	0.121946	-1.43965227	0.149966	0.374612	NOT
LA16c-349	3.012197	1.03607142	0.719673	1.439641794	0.149969	0.374612	NOT
RPL7AP6	65.71524	0.45051516	0.31294	1.439621673	0.149974	0.374612	NOT
WFDC1	75.77754	-0.5367664	0.37286	-1.43959191	0.149983	0.374612	NOT
SNX17	5662.951	0.1797892	0.1249	1.439460706	0.15002	0.37467	NOT
GLB1L2	70.23695	0.84509337	0.587143	1.439330617	0.150057	0.374728	NOT
MIR4653	4.129031	0.62026717	0.430962	1.43926027	0.150077	0.374743	NOT
PSMA3	3942.41	0.22614219	0.157167	1.438863598	0.150189	0.374989	NOT
RP3-329A5	21.24767	0.47937782	0.333187	1.43876526	0.150217	0.37502	NOT
RP11-803F	4.553121	0.51747552	0.359697	1.438642464	0.150252	0.37502	NOT
SNHG10	159.7572	0.3826688	0.265999	1.438609922	0.150261	0.37502	NOT
SLC5A12	52.80471	1.02693622	0.713855	1.4385777	0.15027	0.37502	NOT
TRMT5	632.6934	0.20128847	0.139922	1.438576497	0.150271	0.37502	NOT
LINC00221	194.9344	1.81827249	1.264102	1.438390904	0.150323	0.375117	NOT
U6	5.365806	-0.4190011	0.291321	-1.43827737	0.150355	0.375162	NOT
FOXL2	2.883564	1.31360628	0.913418	1.438122429	0.150399	0.375238	NOT
NCR3LG1	67.79928	0.70021651	0.486963	1.437926698	0.150455	0.375342	NOT
MAP3K6	463.4773	0.3230487	0.2247	1.437690815	0.150522	0.375474	NOT
TPM4	8050.808	0.38195571	0.265737	1.437344039	0.15062	0.375685	NOT
PRSS30P	15.67547	0.74856586	0.520822	1.437278666	0.150639	0.375697	NOT
FHIT	631.3861	0.41216524	0.286803	1.437099983	0.15069	0.375781	NOT
CADM4	492.954	-0.5003228	0.348157	-1.43706241	0.1507	0.375781	NOT
PAPD7	1187.141	-0.2099166	0.146085	-1.43694999	0.150732	0.375803	NOT
DPP10	8.033526	-1.4937489	1.039598	-1.4368525	0.15076	0.375803	NOT
TMSB4XP8	53.17081	0.53652224	0.373403	1.436845815	0.150762	0.375803	NOT
RP11-143M	7.708284	-0.7393703	0.514592	-1.43680737	0.150773	0.375803	NOT
LINC00607	70.33835	0.73876207	0.514176	1.436787785	0.150778	0.375803	NOT
RP11-146F	39.48604	-0.3585649	0.249586	-1.43664156	0.15082	0.375872	NOT
CTD-2349F	5.428097	0.50780027	0.353494	1.436519255	0.150855	0.375924	NOT
RP11-363E	2.703895	0.76360545	0.531628	1.436353014	0.150902	0.376007	NOT
PCGF7P	2.953811	-0.555159	0.386531	-1.43626114	0.150928	0.376038	NOT
DUS4L	342.017	0.22748112	0.158426	1.435882172	0.151036	0.376263	NOT
FCGR1A	81.69145	0.59977078	0.417712	1.435846683	0.151046	0.376263	NOT
PNMA6A	778.1223	0.64015005	0.445872	1.435725993	0.15108	0.376314	NOT
EBNA1BP2	2699.109	0.22946587	0.159834	1.435654893	0.151101	0.37633	NOT
KCNJ13	1.235672	1.07273088	0.747251	1.435569572	0.151125	NA	NA
FAM193B	1449.579	-0.2856583	0.19899	-1.43554449	0.151132	0.376373	NOT
C19orf66	3735.924	-0.3464494	0.241348	-1.43547757	0.151151	0.376386	NOT
MIS18BP1	448.3687	0.31846263	0.221862	1.435406963	0.151171	0.376402	NOT
RP11-255F	3.9858	0.54802636	0.381852	1.435179075	0.151236	0.376498	NOT

AC007326.	1.542166	1.2671651	0.882962	1.435129543	0.15125	0.376498	NOT
EEF1B2P6	42.42649	0.44962007	0.313314	1.435044605	0.151274	0.376498	NOT
GNL3LP1	2.091743	0.69252071	0.482583	1.435030405	0.151278	0.376498	NOT
GPAA1P2	3.452189	0.80615948	0.561774	1.435024311	0.15128	0.376498	NOT
RP11-1007	10.7808	-0.4532144	0.315834	-1.43497873	0.151293	0.376498	NOT
RP11-1036	25.91186	1.12561289	0.784533	1.434755208	0.151357	0.376622	NOT
CYB561D2	1317.823	0.27587273	0.192293	1.434647394	0.151388	0.376664	NOT
FAM157C	13.86836	-0.6391657	0.445552	-1.43454676	0.151416	0.376673	NOT
LUZP2	14.84855	-0.974264	0.679162	-1.43450824	0.151427	0.376673	NOT
SMARCE1P6	3.103884	0.82261893	0.573458	1.434488599	0.151433	0.376673	NOT
CCBE1	64.7614	-0.9174344	0.639618	-1.43434647	0.151473	0.376739	NOT
CD99	9456.283	-0.2763472	0.192729	-1.43386457	0.151611	0.377047	NOT
HS3ST5	2.318479	1.64690564	1.148703	1.433708413	0.151655	0.3771	NOT
AF127936.	7.209595	-0.4543722	0.316926	-1.43368436	0.151662	0.3771	NOT
GCLM	4141.003	0.44417627	0.309823	1.433643522	0.151674	0.3771	NOT
ANKRD46	1245.009	0.3019569	0.210635	1.433555437	0.151699	0.377128	NOT
TBX18	23.39638	0.80393775	0.560855	1.433413795	0.15174	0.377194	NOT
AC000120.	3.338538	-0.6425601	0.448343	-1.43318722	0.151804	0.377307	NOT
RP11-535M	3.24198	-0.8584903	0.59902	-1.43315804	0.151813	0.377307	NOT
bP-21890S	4.627217	-0.7678168	0.535778	-1.43308818	0.151833	0.377322	NOT
TCP11	6.507214	-0.716329	0.499899	-1.43294658	0.151873	0.377376	NOT
AC007318.	83.84173	0.36648714	0.255768	1.432889018	0.15189	0.377376	NOT
UBE2QL1	241.6151	-0.7500172	0.523441	-1.43285988	0.151898	0.377376	NOT
UBE2D4	1072.581	-0.2660975	0.185718	-1.43280396	0.151914	0.377376	NOT
RP11-50I1	34.64897	-0.4932453	0.34426	-1.43276866	0.151924	0.377376	NOT
RNF207	513.7472	-0.3502787	0.244546	-1.43236578	0.152039	0.37761	NOT
RP11-568N	23.01921	0.43362334	0.302737	1.43234152	0.152046	0.37761	NOT
AC010761.	1.88527	0.71780684	0.501186	1.432215158	0.152082	0.377665	NOT
CHMP4B	5289.676	0.19495311	0.136127	1.432146619	0.152102	0.377679	NOT
FAM101A	57.49288	0.79761394	0.556963	1.432075786	0.152122	0.377695	NOT
PLCL2	675.4827	-0.4231035	0.295462	-1.43200731	0.152142	0.377709	NOT
COPZ2	957.5521	-0.5094426	0.355799	-1.43182632	0.152194	0.377803	NOT
BMPR2	2112.918	-0.281884	0.196909	-1.43154617	0.152274	0.377968	NOT
ZNF620	111.7246	0.29260093	0.204404	1.431485192	0.152291	0.377976	NOT
TLE6	151.6924	0.54856956	0.383232	1.431430861	0.152307	0.37798	NOT
SLC10A1	6481.635	-0.9338823	0.652468	-1.43130705	0.152342	0.378019	NOT
PCAT14	3.192666	1.25087914	0.873959	1.431278937	0.15235	0.378019	NOT
RP11-60E8	2.482547	-0.894062	0.624709	-1.43116596	0.152383	0.378065	NOT
ZNF83	882.624	0.63299435	0.442321	1.43107492	0.152409	0.378095	NOT
FARP1	4046.86	0.30906927	0.215982	1.430994371	0.152432	0.378118	NOT
F7	8207.012	-0.5501352	0.384469	-1.43089723	0.15246	0.378152	NOT
MRPL50	1096.02	0.2430126	0.169841	1.430826731	0.15248	0.378168	NOT
AC013439.	4.73987	0.87997035	0.615056	1.430716786	0.152511	0.378212	NOT
C3orf35	24.72369	0.42148778	0.294631	1.430561705	0.152556	0.378287	NOT
AC131056.	12.53414	0.67230031	0.469978	1.430491891	0.152576	0.378302	NOT
ATP8A2	31.477	0.73716147	0.515369	1.430356362	0.152615	0.378364	NOT
ZCCHC9	1370.676	0.2398384	0.1677	1.43016449	0.15267	0.378466	NOT
KCTD21	1057.703	-0.2607273	0.182343	-1.42987105	0.152754	0.37864	NOT
RP11-679C	3.526937	-0.5170105	0.361655	-1.42956782	0.152841	0.378764	NOT

CTNNA3	88.9447	-0.8785335	0.614545	-1.42956757	0.152841	0.378764	NOT
SLC9A1	581.4862	0.36124724	0.252702	1.429537152	0.15285	0.378764	NOT
PHF21A	1023.449	0.24881524	0.174057	1.42950275	0.15286	0.378764	NOT
PC	11851.71	-0.4562881	0.31932	-1.42893628	0.153023	0.379133	NOT
FAM46C	534.6011	-0.5322573	0.372511	-1.42883635	0.153051	0.379169	NOT
RP11-1C8.	2.770105	0.70384398	0.492653	1.428680801	0.153096	0.379213	NOT
DVL3	3827.454	0.21241262	0.148678	1.428677955	0.153097	0.379213	NOT
PRMT1	3416.667	0.28030085	0.196215	1.428537757	0.153137	0.379278	NOT
RP11-629I	8.153543	-0.4868102	0.340801	-1.42842877	0.153168	0.379316	NOT
RP5-984P4	2.229018	0.96245001	0.673806	1.428378944	0.153183	0.379316	NOT
RP11-676J	4.321588	-0.5696652	0.398831	-1.42833857	0.153194	0.379316	NOT
ACSM2A	9410.435	-0.6780108	0.47474	-1.42817354	0.153242	0.379399	NOT
CTD-3098H	137.5162	1.00311331	0.702551	1.427816348	0.153345	0.37956	NOT
RASGRP4	62.34678	-0.4004149	0.280453	-1.42774096	0.153366	0.37956	NOT
MICAL3	1105.422	-0.3815976	0.267276	-1.42772979	0.15337	0.37956	NOT
LINC01122	1.620797	1.09983317	0.77037	1.42766859	0.153387	0.37956	NOT
RP13-467H	13.73731	-0.6338356	0.443983	-1.42761274	0.153403	0.37956	NOT
IFRD1	1222.422	0.25084399	0.175709	1.42760823	0.153405	0.37956	NOT
ILKAP	1023.401	0.22117239	0.15493	1.427562959	0.153418	0.37956	NOT
IKZF5	787.3154	-0.2504622	0.175453	-1.42751666	0.153431	0.37956	NOT
MX1	2485.244	0.44481512	0.311602	1.427510728	0.153433	0.37956	NOT
RPS13	13113.85	0.2669341	0.187024	1.427272957	0.153501	0.379694	NOT
HUWE1	8055.233	-0.193972	0.135917	-1.42713992	0.15354	0.379755	NOT
DCP1B	510.9296	-0.2266658	0.158849	-1.42692683	0.153601	0.379872	NOT
KCND3	785.1499	-0.759614	0.532407	-1.42675398	0.153651	0.379961	NOT
RP5-1099D	3.87612	0.49012938	0.34358	1.426536595	0.153714	0.380048	NOT
CCDC154	59.41195	-0.6174523	0.432841	-1.42651079	0.153721	0.380048	NOT
HNRNPUL2	2216.961	0.16733552	0.117306	1.426485809	0.153728	0.380048	NOT
CCDC171	64.50973	-0.3907572	0.273997	-1.42613764	0.153829	0.380221	NOT
SH3PXD2B	830.6044	0.26874514	0.188444	1.426128367	0.153831	0.380221	NOT
UGT2A3	1601.122	0.65427087	0.458784	1.426096679	0.15384	0.380221	NOT
LRRN4CL	14.40238	-0.7841126	0.549959	-1.42576627	0.153936	0.380423	NOT
AP005901.	3.495661	-1.5300865	1.073285	-1.42561096	0.153981	0.380471	NOT
KRT8P9	2.79247	0.69202635	0.485428	1.425601485	0.153983	0.380471	NOT
HMHA1	1449.936	0.39333631	0.275994	1.425160248	0.154111	0.380718	NOT
NT5C3A	806.2117	0.29044298	0.203797	1.425157636	0.154112	0.380718	NOT
MRPL54	2008.306	-0.3910459	0.274398	-1.42510613	0.154127	0.38072	NOT
FRRS1L	14.12484	1.00284656	0.703732	1.425040521	0.154145	0.380733	NOT
TMEM154	91.54968	-0.6005287	0.421435	-1.4249628	0.154168	0.380753	NOT
PCED1A	1546.25	0.24513406	0.172041	1.424857994	0.154198	0.380755	NOT
DPM2	2433.763	0.32703074	0.229521	1.424840422	0.154203	0.380755	NOT
CH17-472G	131.5873	0.41744102	0.292979	1.424814637	0.154211	0.380755	NOT
LAD1	4227.262	0.68456014	0.480482	1.424735922	0.154234	0.380777	NOT
LINGO1	544.9861	-0.7004112	0.491671	-1.42455157	0.154287	0.380874	NOT
PLCB3	1080.896	0.24866997	0.174575	1.424430574	0.154322	0.380913	NOT
SRMS	15.92861	-0.628389	0.441161	-1.424399	0.154331	0.380913	NOT
EIF2AK2	1974.454	0.18419149	0.129323	1.424278106	0.154366	0.380959	NOT
REG1A	316.9767	1.46627148	1.029513	1.424238592	0.154377	0.380959	NOT
RP11-120K	40.94921	0.82232692	0.577419	1.424141931	0.154405	0.380987	NOT

RP11-114H	3.042763	-0.9375503	0.658345	-1.4241024	0.154417	0.380987	NOT
RIF1	1632.627	0.24466662	0.171815	1.424011816	0.154443	0.381017	NOT
FBXO22	1483.551	0.19347188	0.135896	1.423680516	0.154539	0.381219	NOT
SBSN	4.499416	1.04603786	0.734835	1.423500529	0.154591	0.381313	NOT
DCDC2	1732.931	0.85788105	0.602748	1.423283538	0.154654	0.381433	NOT
RP1-117B1	13.96246	-0.4517761	0.317467	-1.42306615	0.154717	0.381553	NOT
ABCC11	265.8555	-0.5777634	0.406025	-1.42297616	0.154743	0.381553	NOT
RP11-463C	37.28126	-0.4398655	0.309118	-1.42297002	0.154745	0.381553	NOT
CETP	245.4666	-0.5179212	0.363999	-1.42286538	0.154775	0.381569	NOT
ZNF337-AS	147.6517	0.30902163	0.217185	1.422850872	0.154779	0.381569	NOT
UGT2B10	8674.705	-0.7626866	0.536058	-1.42276817	0.154803	0.381593	NOT
PAQR9-AS1	314.5417	-0.5245532	0.368704	-1.42269637	0.154824	0.38161	NOT
AC010091.	11.44866	1.23757898	0.869987	1.422525181	0.154874	0.381698	NOT
IDO1	165.0617	0.65787423	0.462508	1.422405426	0.154909	0.381728	NOT
EIF5	8856.583	-0.2287061	0.16079	-1.42238625	0.154914	0.381728	NOT
CCNYL1	953.8658	0.22827422	0.160518	1.422113485	0.154993	0.381888	NOT
SLCO4A1-A	5.129674	1.17111967	0.823644	1.421875315	0.155062	0.381964	NOT
CA2	3278.568	0.57673261	0.405631	1.421814183	0.15508	0.381964	NOT
CPO	8.620635	-0.6744176	0.474345	-1.42178787	0.155088	0.381964	NOT
CTD-2651B	6.64051	-0.649936	0.457158	-1.42168794	0.155117	0.381964	NOT
RP11-389C	19.08041	0.60578247	0.426126	1.421604659	0.155141	0.381964	NOT
RP1-228P1	13.12913	0.57789246	0.406542	1.42148363	0.155176	0.381964	NOT
LINC00216	4.304251	-0.5421157	0.381383	-1.42144538	0.155187	0.381964	NOT
RP11-423B	3.902607	0.63142351	0.444214	1.421440133	0.155189	0.381964	NOT
TMEM106B	3191.756	-0.2664906	0.187479	-1.42143877	0.155189	0.381964	NOT
AC139100.	34.39641	-0.5119234	0.360179	-1.42130188	0.155229	0.381964	NOT
ACAA2	11856.46	-0.370414	0.260616	-1.42130131	0.155229	0.381964	NOT
FRMD8	1606.547	0.15824326	0.11134	1.421263432	0.15524	0.381964	NOT
RP11-627K	1.561666	0.75284482	0.529709	1.421243209	0.155246	0.381964	NOT
RGS22	2.323732	-0.7184188	0.505494	-1.42122169	0.155252	0.381964	NOT
RP11-350B	9.69035	0.92239611	0.649026	1.42120075	0.155258	0.381964	NOT
CNEP1R1	392.4214	-0.1921053	0.135171	-1.42119951	0.155259	0.381964	NOT
CTD-2619J	58.37705	-0.5406017	0.380389	-1.42118255	0.155264	0.381964	NOT
BTBD3	1007.943	0.294145	0.206986	1.421084886	0.155292	0.382	NOT
ACBD4	2443.255	-0.4174871	0.293814	-1.42092285	0.155339	0.382081	NOT
ZNF280C	140.5536	0.2958241	0.208236	1.420621395	0.155427	0.382262	NOT
ACADL	549.0949	-0.8128995	0.572252	-1.42052826	0.155454	0.382294	NOT
GOLGA6L9	70.30114	0.34721119	0.244462	1.420305196	0.155519	0.382399	NOT
FBL	5048.241	0.30429986	0.214253	1.420284065	0.155525	0.382399	NOT
RPL32P1	1.575303	-0.8069852	0.568377	-1.41980662	0.155664	0.382706	NOT
ZSCAN12P1	41.53362	-0.3995204	0.281416	-1.41968051	0.155701	0.382762	NOT
PAH	35178.68	-0.5837577	0.411236	-1.41952124	0.155747	0.382841	NOT
RP11-180M	13.09606	-0.4186849	0.294964	-1.41944263	0.15577	0.382863	NOT
RP11-74C1	4.19824	0.42336824	0.298287	1.419329869	0.155803	0.382909	NOT
PGK1	16182.63	0.30980664	0.21829	1.419245038	0.155828	0.382911	NOT
KIF5B	4520.247	0.21168611	0.149156	1.419229687	0.155832	0.382911	NOT
ZNF350-AS	4.328816	0.54750204	0.385799	1.419138239	0.155859	0.382942	NOT
RP11-533E	20.6537	0.36113772	0.254517	1.418914948	0.155924	0.383067	NOT
ACE	405.616	-0.38262	0.269679	-1.41880027	0.155957	0.383075	NOT

FOXO3B	14.09078	0.45582336	0.321279	1.418778254	0.155964	0.383075	NOT
SERBP1P5	9.79634	0.45769854	0.322605	1.418758492	0.155969	0.383075	NOT
MOK	204.0633	0.39927309	0.281492	1.418417991	0.156069	0.383261	NOT
RP11-342K	18.63018	-0.3747542	0.264209	-1.41840269	0.156073	0.383261	NOT
PAIP2	2678.029	0.19375119	0.136619	1.418189685	0.156135	0.383379	NOT
RNU4-47P	4.931375	-0.6714998	0.473517	-1.41811195	0.156158	0.3834	NOT
BTN3A2	1942.886	-0.3846064	0.27132	-1.4175398	0.156325	0.383754	NOT
ZNF205	702.0488	0.25564233	0.180345	1.417520707	0.156331	0.383754	NOT
TYRO3	659.8418	0.58413509	0.412098	1.417465526	0.156347	0.383759	NOT
SUMO2	4987.583	0.20408604	0.143999	1.417274434	0.156403	0.383862	NOT
ATXN7L3B	4477.73	0.25038196	0.176675	1.417186949	0.156428	0.38389	NOT
RP11-692I	15.36976	-0.3422422	0.241541	-1.41691118	0.156509	0.384053	NOT
DHX15	3268.91	0.19320945	0.136375	1.416748294	0.156557	0.384135	NOT
PSMA7	8906.151	0.21598054	0.152473	1.416518379	0.156624	0.384232	NOT
CBX3P9	3.4924	0.51262315	0.36189	1.416516315	0.156624	0.384232	NOT
MINOS1P3	3.919257	1.44353197	1.019131	1.416434554	0.156648	0.384256	NOT
OR12D2	0.841081	2.42977707	1.715674	1.416222992	0.15671	NA	NA
ZNF385B	421.684	-0.6956163	0.491188	-1.41619147	0.156719	0.38439	NOT
RAET1E-AS	7.529986	-0.5897372	0.416455	-1.41608802	0.15675	0.38439	NOT
TUBB3	14.66421	0.69604092	0.491541	1.416037967	0.156764	0.38439	NOT
ERBB2	4707.869	-0.454296	0.320826	-1.41601909	0.15677	0.38439	NOT
PTH2R	420.0959	-1.2732896	0.899216	-1.41599998	0.156776	0.38439	NOT
RP11-110I	4.442415	0.41282177	0.291558	1.415917671	0.1568	0.38439	NOT
SLC4A8	26.64135	0.49941053	0.352717	1.415897003	0.156806	0.38439	NOT
VSTM1	0.94054	0.97088491	0.685719	1.415864952	0.156815	NA	NA
UBXN2B	1808.573	-0.3101592	0.219061	-1.41586002	0.156816	0.38439	NOT
RASGRP3	618.7538	-0.3212355	0.226901	-1.4157546	0.156847	0.384431	NOT
ASPM	1435.087	0.48152588	0.340179	1.415508737	0.156919	0.384493	NOT
TUBG2	514.5048	0.24119895	0.170401	1.41547604	0.156929	0.384493	NOT
EMBP1	119.8986	-0.4731612	0.334287	-1.41543332	0.156941	0.384493	NOT
LINC0020	21.88009	-0.6149173	0.434442	-1.41541805	0.156946	0.384493	NOT
PQLC1	7030.873	-0.3213969	0.227075	-1.41537832	0.156958	0.384493	NOT
RP4-665N	2.388952	-0.5947444	0.420218	-1.41532289	0.156974	0.384493	NOT
CSNK1E	2928.28	0.28427948	0.200863	1.415289334	0.156984	0.384493	NOT
RP11-346C	16.7213	-0.3838288	0.271203	-1.41528139	0.156986	0.384493	NOT
PCGF2	1127.299	0.41357695	0.292234	1.41522547	0.157002	0.384499	NOT
METTL12	116.2166	0.33994654	0.240221	1.415138211	0.157028	0.384511	NOT
WTAP	2234.553	0.19580113	0.138369	1.415065825	0.157049	0.384511	NOT
ZFYVE21	1128.841	-0.272788	0.192774	-1.41506337	0.15705	0.384511	NOT
IGKV2OR22	1.148826	1.43483082	1.014238	1.414688918	0.15716	NA	NA
RP11-352I	9.897732	0.85004128	0.600891	1.414635361	0.157175	0.384751	NOT
RHOBTB3	2890.679	-0.3593405	0.254017	-1.41463181	0.157176	0.384751	NOT
OIP5-AS1	4360.949	-0.2485245	0.175706	-1.41443422	0.157234	0.384858	NOT
AC007041.	4.009516	0.50949509	0.360266	1.414218032	0.157298	0.384947	NOT
BCS1L	1139.81	0.24242	0.171417	1.414214441	0.157299	0.384947	NOT
PDGFC	790.8992	-0.4142291	0.292929	-1.41409304	0.157335	0.384999	NOT
ERCC6	315.206	-0.2433164	0.172078	-1.41398865	0.157365	0.385019	NOT
MRPL44	1573.02	0.2044693	0.144607	1.413968857	0.157371	0.385019	NOT
PXT1	3.120503	0.5130576	0.362887	1.413823199	0.157414	0.385089	NOT

ACOT2	1713.152	-0.3635146	0.257128	-1.41374932	0.157436	0.385107	NOT
C9orf16	1794.108	0.3771221	0.266812	1.413437515	0.157527	0.385276	NOT
CACNG8	11.95999	-0.5003621	0.354009	-1.41341784	0.157533	0.385276	NOT
MAP2	1427.439	0.66907679	0.473416	1.413297095	0.157568	0.385294	NOT
SEPT2	8225.469	0.26325232	0.186268	1.413295626	0.157569	0.385294	NOT
AP000580.	10.46955	0.45152746	0.319543	1.41304338	0.157643	0.385412	NOT
TAF4	1007.462	0.22460021	0.158949	1.413035854	0.157645	0.385412	NOT
UBALD2	2212.943	0.36422041	0.257796	1.412825756	0.157707	0.385528	NOT
PTGER4	221.4574	0.50709957	0.359048	1.412343636	0.157849	0.38579	NOT
CTD-2006I	3.645922	-0.5456912	0.386377	-1.4123267	0.157854	0.38579	NOT
PTPRZ1	13.70341	-0.8184058	0.579513	-1.41223033	0.157882	0.38579	NOT
C18orf32	389.4336	-0.2166118	0.153383	-1.4122291	0.157883	0.38579	NOT
TRIM7	71.97195	0.61874818	0.438139	1.412219726	0.157885	0.38579	NOT
RP5-1074I	35.80304	0.30995262	0.219553	1.411744911	0.158025	0.386071	NOT
AC078883.	4.560072	-0.5338114	0.378125	-1.4117321	0.158029	0.386071	NOT
AC083873.	9.092936	0.44538554	0.315501	1.411676458	0.158045	0.386077	NOT
RP11-314I	12.20613	0.44900489	0.318093	1.411550308	0.158082	0.386133	NOT
EPDR1	1426.659	0.46104079	0.326634	1.411491575	0.1581	0.38614	NOT
RASGRF2-A	2.023576	-0.6428569	0.455464	-1.41143156	0.158117	0.386149	NOT
AC000068.	8.227787	-0.4182053	0.296315	-1.41135614	0.15814	0.386168	NOT
RP5-881L2	20.58779	-0.7140483	0.506035	-1.41106465	0.158226	0.386313	NOT
SLC16A13	1490.928	0.44227761	0.313437	1.411057873	0.158228	0.386313	NOT
PIANP	6.113719	0.66409753	0.470731	1.410779393	0.15831	0.386479	NOT
PPFIBP1	1215.665	-0.2782846	0.197303	-1.41044191	0.158409	0.386687	NOT
IDI2-AS1	4.92566	-0.4993286	0.354042	-1.4103674	0.158431	0.386706	NOT
AC108488.	61.91475	0.28886203	0.204871	1.409967074	0.158549	0.38696	NOT
RP11-795I	23.40888	0.59912197	0.424952	1.40985672	0.158582	0.387003	NOT
APOH	177729.7	-0.5890341	0.417824	-1.40976683	0.158609	0.387003	NOT
RP11-180I	10.3173	-0.8172081	0.579706	-1.40969326	0.15863	0.387003	NOT
PCIF1	1664.551	0.16349459	0.115984	1.409629376	0.158649	0.387003	NOT
RNF24	502.959	0.34699564	0.246162	1.409625192	0.15865	0.387003	NOT
RP11-803I	7.443763	0.46152501	0.327412	1.409615704	0.158653	0.387003	NOT
ZNF607	141.1175	0.46691819	0.331259	1.409525167	0.15868	0.387003	NOT
AF131216.	3.717417	-0.5090965	0.361184	-1.40952134	0.158681	0.387003	NOT
TAC3	7.769452	0.87193014	0.618648	1.409412109	0.158713	0.387047	NOT
XXbac-B13	29.84219	-0.4452912	0.315974	-1.40926366	0.158757	0.387119	NOT
GDI2P2	2.072964	0.69003554	0.489677	1.409163841	0.158787	0.387156	NOT
IGLV1-36	6.879728	-1.16834	0.829255	-1.40890266	0.158864	0.387309	NOT
RP11-454I	28.37225	0.31767227	0.225495	1.408777799	0.158901	0.387365	NOT
ZNRF2P1	7.131963	-0.3803576	0.270001	-1.408727	0.158916	0.387366	NOT
RAB34	1056.293	0.62339118	0.442541	1.408664531	0.158934	0.38737	NOT
RP11-1228	2.914957	-0.593235	0.421144	-1.40862621	0.158946	0.38737	NOT
SPNS3	77.51406	-0.6696394	0.475417	-1.40853009	0.158974	0.387404	NOT
CTD-2541I	4.085817	-0.6468397	0.459281	-1.40837479	0.15902	0.387481	NOT
RP11-1055	16.28301	0.7258613	0.515442	1.408232043	0.159062	0.387549	NOT
FAM86JP	117.0382	0.35381783	0.251272	1.408107818	0.159099	0.387604	NOT
CDC42EP1	7910.831	0.37869916	0.269092	1.40732285	0.159332	0.388126	NOT
PSMB4	15978.48	0.25101489	0.17838	1.407191767	0.159371	0.388126	NOT
IL34	308.8435	-0.5663057	0.402441	-1.40717648	0.159375	0.388126	NOT

GLYCTK	7590.793	-0.4271436	0.303552	-1.40714945	0.159383	0.388126	NOT
RGS16	416.2691	-0.5138217	0.365152	-1.40714295	0.159385	0.388126	NOT
HMX2	0.820938	1.80878175	1.285514	1.40704944	0.159413	NA	NA
RP11-485F	9.915452	1.45995339	1.037659	1.406967798	0.159437	0.388176	NOT
SSR2	14794.2	0.30351013	0.215722	1.406953296	0.159441	0.388176	NOT
ITIH4-AS1	22.72545	-0.5650054	0.401588	-1.40692964	0.159448	0.388176	NOT
C12orf43	726.487	0.20793801	0.147804	1.40684892	0.159472	0.388188	NOT
ACAP1	319.877	-0.4507166	0.320381	-1.40681619	0.159482	0.388188	NOT
KIF16B	798.7822	-0.2504894	0.178105	-1.40641622	0.159601	0.388442	NOT
RP11-324H	1.233795	2.06146407	1.465825	1.406350649	0.15962	NA	NA
RNA5SP334	1.810084	-1.4910118	1.06041	-1.40607103	0.159703	0.388657	NOT
RP11-283I	4.053977	-0.7997866	0.568872	-1.40591628	0.159749	0.388734	NOT
CEP290	437.4563	0.24113889	0.171555	1.405605187	0.159841	0.3889	NOT
RP11-452I	196.9899	-0.3006859	0.213922	-1.40558982	0.159846	0.3889	NOT
MIR4500HC	4.128708	-1.096721	0.780365	-1.40539477	0.159904	0.389006	NOT
PHB2	9492.678	-0.2309043	0.164305	-1.40534059	0.15992	0.38901	NOT
DIMT1	854.2767	0.19112959	0.136008	1.405285697	0.159936	0.389015	NOT
EIF4EBP3	444.7289	0.43982683	0.313025	1.405083847	0.159996	0.389115	NOT
RC3H2	1531.179	-0.2115271	0.150548	-1.40505045	0.160006	0.389115	NOT
HMGB1P1	8.65887	-0.409865	0.291857	-1.40433694	0.160219	0.38957	NOT
CDKN2AIPN	1191.743	0.29309787	0.208711	1.404325178	0.160222	0.38957	NOT
ABCC1	1205.033	0.53643702	0.382004	1.404270818	0.160238	0.389575	NOT
ERAP2	1987.894	0.58753955	0.418437	1.404128735	0.160281	0.389643	NOT
AP001205.	2.999092	0.62006588	0.441732	1.403714431	0.160404	0.389908	NOT
RP11-475J	9.929146	-0.6822608	0.486074	-1.40361497	0.160434	0.389924	NOT
MEIOB	1.598044	-0.7806276	0.556163	-1.40359571	0.160439	0.389924	NOT
WNT5B	289.2481	-0.6721525	0.478948	-1.4033948	0.160499	0.390034	NOT
MTFP1	360.1657	0.38149025	0.271853	1.403297785	0.160528	0.39007	NOT
RNF212	5.864662	-0.4963925	0.353772	-1.40314153	0.160575	0.390148	NOT
IGKV1D-39	0.736159	-1.3065664	0.931296	-1.40295488	0.16063	NA	NA
XXyac-YM2	20.53455	1.09716871	0.782072	1.402899351	0.160647	0.390241	NOT
ZYG11B	1911.742	-0.2533545	0.180595	-1.40288681	0.160651	0.390241	NOT
VWA8-AS1	4.510844	-0.5918206	0.421865	-1.40286862	0.160656	0.390241	NOT
FGF14-IT1	1.026134	1.30166482	0.927892	1.402819635	0.160671	NA	NA
TH	2.864243	-0.8490193	0.605265	-1.40272336	0.160699	0.390311	NOT
ARHGEF40	4371.741	0.31681425	0.225888	1.402525972	0.160758	0.390419	NOT
IGHV1-58	9.495931	1.33894868	0.954769	1.402379006	0.160802	0.390479	NOT
RGMB	301.5444	-0.4619501	0.329412	-1.40234738	0.160812	0.390479	NOT
MCF2L-AS1	133.2353	0.63631793	0.453801	1.402196028	0.160857	0.390486	NOT
ZNF77	124.6552	0.26104031	0.186173	1.402135248	0.160875	0.390486	NOT
CCNJ	221.5478	0.48731178	0.347552	1.402128246	0.160877	0.390486	NOT
NDUFB7	5484.48	-0.3915968	0.279311	-1.40200773	0.160913	0.390486	NOT
DLX4	25.97263	0.94994694	0.67758	1.401970641	0.160924	0.390486	NOT
RP1-30M3.	22.90765	-0.3220291	0.229704	-1.40193109	0.160936	0.390486	NOT
AKAP6	224.4199	-0.5019866	0.35807	-1.40192344	0.160938	0.390486	NOT
C15orf61	350.2162	-0.2851216	0.203379	-1.40191952	0.160939	0.390486	NOT
CYP21A1P	323.7861	0.52453224	0.374157	1.401903322	0.160944	0.390486	NOT
FGL1	65054.59	-0.6713166	0.478909	-1.40176185	0.160986	0.390554	NOT
KCND1	68.13391	-0.450899	0.321727	-1.40149361	0.161067	0.390713	NOT

PAPD5	851.3587	-0.2610037	0.186242	-1.40142247	0.161088	0.390717	NOT
LYRM2	1198.201	0.20335511	0.145109	1.401391816	0.161097	0.390717	NOT
CTD-2619J	12.57852	-0.3969082	0.283288	-1.40107414	0.161192	0.390855	NOT
PEX5L	7.536476	-0.5223059	0.372792	-1.4010655	0.161194	0.390855	NOT
LINC0123	34.46506	-0.6494675	0.463605	-1.40090621	0.161242	0.390855	NOT
LPAL2	188.6979	-0.5183457	0.370008	-1.4009041	0.161243	0.390855	NOT
MPRIPP1	4.753039	0.57748595	0.412248	1.400822205	0.161267	0.390855	NOT
VHL	1356.292	0.26271	0.187542	1.400806345	0.161272	0.390855	NOT
SSUH2	381.0912	0.83544655	0.596405	1.400803159	0.161273	0.390855	NOT
RGS2	1067.791	0.6033459	0.430715	1.400801342	0.161273	0.390855	NOT
PLEKHH2	150.5759	0.69244452	0.494332	1.400767194	0.161284	0.390855	NOT
RIN1	176.0546	0.37257476	0.266013	1.400590095	0.161337	0.390886	NOT
CTB-134H2	1.665886	-0.7975553	0.569475	-1.40050857	0.161361	0.390886	NOT
IZUMO1	13.57865	0.53369348	0.38108	1.400476294	0.161371	0.390886	NOT
TSPYL1	2738.055	-0.2725729	0.19463	-1.40046546	0.161374	0.390886	NOT
CDS1	297.1126	0.77871562	0.556046	1.400451196	0.161378	0.390886	NOT
HIF1A	4225.192	0.41122428	0.293641	1.400432706	0.161384	0.390886	NOT
MAPK12	664.3659	0.5470152	0.390617	1.40038843	0.161397	0.390886	NOT
NOX4	73.62843	-0.3604599	0.257417	-1.40029437	0.161425	0.390919	NOT
CTA-342B1	2.647689	0.59117376	0.422194	1.400240617	0.161441	0.390923	NOT
TUBB8	2.211221	0.9401965	0.671494	1.400155538	0.161467	0.39095	NOT
GCK	278.8931	-1.2320421	0.880001	-1.40004673	0.161499	0.39097	NOT
CTC-512J1	2.918632	0.5147272	0.367654	1.400032071	0.161504	0.39097	NOT
YWHAZP6	3.268829	0.5379036	0.38426	1.399843916	0.16156	0.391042	NOT
SLC22A1	6956.328	-0.8972416	0.641	-1.3997523	0.161588	0.391042	NOT
AC241585.	20.70262	0.46836739	0.334621	1.399696867	0.161604	0.391042	NOT
AP001046.	22.66684	-0.4695586	0.335481	-1.39966002	0.161615	0.391042	NOT
CH17-260(	1.798566	0.63832501	0.45606	1.399650329	0.161618	0.391042	NOT
C10orf131	3.633777	-0.5288166	0.377829	-1.39961707	0.161628	0.391042	NOT
RP11-329F	3.807641	-0.5962121	0.426038	-1.39943389	0.161683	0.391042	NOT
RASGRF1	75.0143	-0.7704849	0.55057	-1.39943216	0.161683	0.391042	NOT
RP11-158M	13.34953	-0.4434271	0.316872	-1.39938938	0.161696	0.391042	NOT
RP11-188I	6.144772	0.47986823	0.342924	1.39934469	0.16171	0.391042	NOT
RP11-168A	3.997166	0.68817341	0.491798	1.39930127	0.161723	0.391042	NOT
RP5-834N1	24.19039	-0.733898	0.52448	-1.39928646	0.161727	0.391042	NOT
ZFHX3	1078.434	-0.2377951	0.169941	-1.3992818	0.161728	0.391042	NOT
RP11-689K	5.635647	-0.7801948	0.557595	-1.39921321	0.161749	0.391042	NOT
RP11-288I	2.335283	0.72345777	0.517058	1.399182	0.161758	0.391042	NOT
INSIG1	28605.19	-0.6056533	0.432868	-1.39916285	0.161764	0.391042	NOT
APEX2	1502.545	0.19106944	0.13657	1.399059948	0.161795	0.391081	NOT
PIGV	1049.553	-0.2965866	0.212001	-1.39898982	0.161816	0.391097	NOT
MRM1	452.2425	0.24971005	0.178516	1.398811471	0.16187	0.391157	NOT
PRADC1	1716.369	-0.3496409	0.249961	-1.39878088	0.161879	0.391157	NOT
GLUD1P8	10.67741	-0.6081822	0.4348	-1.39876295	0.161884	0.391157	NOT
ST13P15	9.386467	0.47719935	0.3412	1.398590618	0.161936	0.391219	NOT
C1QTNF1	980.5707	-0.5022687	0.359127	-1.3985825	0.161938	0.391219	NOT
NARF-IT1	5.654353	-0.509432	0.364285	-1.39844416	0.16198	0.391284	NOT
CXorf66	4.113792	-1.1239798	0.803874	-1.39820395	0.162052	0.391423	NOT
RP11-78F1	2.136627	1.27886719	0.915134	1.397464703	0.162274	0.391925	NOT

CENPV	1455.351	0.40494881	0.289808	1.397299962	0.162323	0.392009	NOT
HCG9	6.34862	0.7217843	0.516688	1.396945398	0.16243	0.392232	NOT
EEF1A1P9	100.04	0.33886249	0.242584	1.396886595	0.162448	0.39224	NOT
CTD-2251F	0.780709	1.92353563	1.377177	1.396723244	0.162497	NA	NA
AL390877.	10.33405	-0.7479874	0.535678	-1.39633807	0.162613	0.392551	NOT
TSC22D2	1254.977	-0.2043348	0.146337	-1.39633027	0.162615	0.392551	NOT
RP11-737(	5.149435	-0.5763275	0.412749	-1.39631344	0.16262	0.392551	NOT
SLC30A4	271.9519	-0.518601	0.371446	-1.3961662	0.162664	0.392623	NOT
CMC2	1289.792	-0.2413602	0.172934	-1.39567856	0.162811	0.392943	NOT
OTX1	81.12864	0.68845189	0.49331	1.395576571	0.162842	0.392982	NOT
FLII	5027.738	0.2421935	0.173588	1.395217825	0.16295	0.393208	NOT
ITIH3	76103.01	-0.5636288	0.404035	-1.39499935	0.163016	0.393307	NOT
HIST1H2AF	2.285525	-0.8280578	0.593596	-1.3949853	0.16302	0.393307	NOT
RP11-510M	8.773322	1.36450391	0.978448	1.394559447	0.163149	0.393551	NOT
NOP56P1	8.832973	1.12065127	0.803591	1.394554651	0.16315	0.393551	NOT
RP11-586I	2.087414	1.34596004	0.965203	1.394484579	0.163171	0.393567	NOT
CD164	10415.28	0.22757999	0.163214	1.394365915	0.163207	0.393618	NOT
C3orf58	1255.798	-0.402029	0.288336	-1.3943058	0.163225	0.393627	NOT
TMEM27	227.9807	-0.708488	0.508171	-1.39419226	0.16326	0.393674	NOT
EML4	5514.205	0.22931784	0.164492	1.394094691	0.163289	0.393708	NOT
INPP5B	543.6617	-0.2251052	0.161476	-1.39405079	0.163302	0.393708	NOT
RP3-340N1	106.2501	0.66659654	0.4783	1.393678394	0.163415	0.393891	NOT
AC006116.	3.01099	0.73259006	0.525654	1.39367241	0.163417	0.393891	NOT
CTD-2033I	2.181334	-0.6568091	0.471296	-1.39362219	0.163432	0.393891	NOT
IGFBP1	35561.95	-0.7489182	0.537396	-1.39360676	0.163436	0.393891	NOT
RP11-619A	18.4991	-0.7475785	0.536473	-1.39350613	0.163467	0.393929	NOT
RP11-152F	2.24966	-0.5542586	0.397794	-1.39332979	0.16352	0.393998	NOT
DPY19L1P2	8.263054	-0.3422683	0.24565	-1.39331589	0.163524	0.393998	NOT
TAZ	1335.771	0.26411809	0.18957	1.39325108	0.163544	0.39401	NOT
GDNF-AS1	31.70407	-1.0684074	0.766891	-1.39316803	0.163569	0.394011	NOT
RPLP0	59536.98	0.30690161	0.220293	1.393153723	0.163573	0.394011	NOT
XCR1	24.33075	-0.7164349	0.514325	-1.39296082	0.163632	0.394116	NOT
RP11-73M1	17.50204	0.31412647	0.225542	1.392764225	0.163691	0.394192	NOT
SPA17	169.3465	0.37174229	0.26691	1.392760422	0.163692	0.394192	NOT
ART3	6.937456	-0.7499853	0.538596	-1.39248333	0.163776	0.394286	NOT
CTD-2650F	4.281561	0.62094577	0.44595	1.392412492	0.163798	0.394286	NOT
GLI4	907.7073	0.39736658	0.285381	1.392406688	0.163799	0.394286	NOT
FATE1	27.51788	-0.6004659	0.431263	-1.39234354	0.163818	0.394286	NOT
EIF3FP3	172.4662	0.30993314	0.222603	1.392310832	0.163828	0.394286	NOT
RP11-738E	10.55669	-0.4377078	0.314383	-1.39227759	0.163838	0.394286	NOT
ERI3	2352.387	0.2082268	0.149559	1.392273055	0.16384	0.394286	NOT
ARID4A	856.5116	-0.2772154	0.199114	-1.39224377	0.163849	0.394286	NOT
RP11-144I	4.094994	0.64767044	0.465214	1.392199594	0.163862	0.394286	NOT
HID1-AS1	4.310894	-0.4966878	0.356812	-1.3920162	0.163917	0.394376	NOT
PDGFRB	3881.841	-0.3819858	0.274419	-1.39197953	0.163929	0.394376	NOT
SLAMF9	35.6139	-0.6917551	0.496993	-1.39188229	0.163958	0.394412	NOT
ARHGEF6	449.1313	-0.3811786	0.273915	-1.39159674	0.164045	0.394564	NOT
UMODL1	13.32286	-0.8739871	0.628076	-1.39153065	0.164065	0.394564	NOT
GBP5	507.6678	0.65471806	0.470502	1.391530217	0.164065	0.394564	NOT

RP4-575N6	14.15521	-0.5439667	0.390951	-1.39139288	0.164106	0.3946	NOT
FCHO2	1254.106	-0.2495491	0.179353	-1.39138449	0.164109	0.3946	NOT
LANCL2	1113.91	-0.1888329	0.135755	-1.39098151	0.164231	0.394859	NOT
AC003973.	40.03116	0.78348783	0.563292	1.390907962	0.164253	0.394863	NOT
RP4-710M1	212.266	-0.5742126	0.412846	-1.39086523	0.164266	0.394863	NOT
CTD-2623M	13.87117	-0.3133094	0.225268	-1.39083222	0.164276	0.394863	NOT
LINC0028	2.587017	1.05576252	0.759126	1.390760672	0.164298	0.39488	NOT
GBGT1	133.4117	0.40742279	0.29297	1.390663729	0.164327	0.394916	NOT
MED29	3196.692	-0.1483402	0.106709	-1.39013788	0.164487	0.395215	NOT
BNIP3P9	3.303504	-1.3182302	0.948295	-1.39010568	0.164497	0.395215	NOT
MASP2	8268.168	-0.6798273	0.489049	-1.39009973	0.164499	0.395215	NOT
RP5-8820	2.392706	0.73501698	0.528766	1.390061888	0.16451	0.395215	NOT
NEU3	520.4996	-0.2426104	0.174549	-1.38992762	0.164551	0.395278	NOT
PELI1	942.1755	0.33802745	0.243212	1.389844196	0.164576	0.395303	NOT
RP11-753A	3.082786	-1.1281632	0.811761	-1.38977329	0.164598	0.39532	NOT
PIK3R4	1457.315	-0.2536705	0.182556	-1.38954999	0.164666	0.395414	NOT
LINC0099	17.54703	-0.5207477	0.37476	-1.38954846	0.164666	0.395414	NOT
DROSHA	1822.42	0.21281872	0.15319	1.389242403	0.164759	0.395565	NOT
SCN3A	7.824585	-0.6351738	0.457217	-1.38921873	0.164766	0.395565	NOT
IGHV1-12	1.649516	-1.3585491	0.977937	-1.38919829	0.164772	0.395565	NOT
EEF1DP4	4.898473	0.85818382	0.617906	1.388858373	0.164876	0.395748	NOT
TRIM39	592.4219	0.18246592	0.131379	1.388851621	0.164878	0.395748	NOT
KCNE1	11.40889	-0.7200261	0.518467	-1.38876046	0.164906	0.395779	NOT
CD52	408.7278	0.45449344	0.3273	1.388612718	0.164951	0.395852	NOT
CTA-796E	6.901389	1.06890111	0.769917	1.388333163	0.165036	0.395965	NOT
ARCN1	6082.707	-0.1637622	0.117958	-1.3883062	0.165044	0.395965	NOT
VLDLR	621.1205	0.78888381	0.568245	1.388281915	0.165051	0.395965	NOT
RIC8B	667.8986	0.21138385	0.152265	1.388266529	0.165056	0.395965	NOT
TPTE2P3	1.179418	1.80995827	1.303756	1.388264836	0.165056	NA	NA
CTD-2342J	17.28147	-0.3167754	0.228198	-1.38816096	0.165088	0.395987	NOT
CNOT8	1581.391	-0.1528219	0.110092	-1.38813391	0.165096	0.395987	NOT
NKX1-2	1.888165	-1.3922671	1.003007	-1.38809263	0.165109	0.395987	NOT
TOMM20	10889.91	0.230676	0.166189	1.388036055	0.165126	0.395993	NOT
RP11-996F	19.25999	-0.4767334	0.343542	-1.38770134	0.165228	0.396203	NOT
COPS8	1500.008	0.16187484	0.116666	1.38750291	0.165288	0.396268	NOT
ZNF571	95.18636	-0.2546194	0.183511	-1.38748801	0.165293	0.396268	NOT
RPL3L	33.34365	-0.4817198	0.347194	-1.38746762	0.165299	0.396268	NOT
SIGLEC17F	16.45253	-0.6225122	0.448704	-1.3873566	0.165333	0.396314	NOT
ASAH1	4004.158	0.32226164	0.232306	1.387230141	0.165372	0.396329	NOT
LRRC55	13.89799	-0.6093814	0.43928	-1.38722799	0.165372	0.396329	NOT
ABCB6	756.7222	0.39656049	0.285873	1.387192128	0.165383	0.396329	NOT
AP3D1	7906.877	0.1909487	0.137659	1.38711694	0.165406	0.396331	NOT
TTLL2	40.48165	0.79725569	0.574767	1.387094618	0.165413	0.396331	NOT
RP11-299C	127.8269	-0.5260837	0.379295	-1.38700266	0.165441	0.396359	NOT
FAM71E1	117.6194	0.48485381	0.349598	1.386887783	0.165476	0.396359	NOT
RP11-425I	319.1788	0.46121692	0.332564	1.386850685	0.165487	0.396359	NOT
PGM5P2	5.939215	-0.5000249	0.360553	-1.38682771	0.165494	0.396359	NOT
PTGES	490.8239	0.85855758	0.619121	1.38673548	0.165522	0.396359	NOT
MKRN1	3331.191	0.17045009	0.122915	1.386726108	0.165525	0.396359	NOT

RP11-405(	1.477439	-0.7454974	0.537597	-1.38672082	0.165527	0.396359	NOT
GEM	430.3357	-0.5297784	0.382056	-1.38665066	0.165548	0.396359	NOT
AP000445.	19.81639	-0.9806789	0.707242	-1.38662448	0.165556	0.396359	NOT
RP11-156K	8.221177	-0.6965875	0.502416	-1.38647616	0.165602	0.396399	NOT
HINFP	588.5356	0.16070683	0.11591	1.386474154	0.165602	0.396399	NOT
MT-ND1	264597.1	-0.382698	0.276054	-1.38631531	0.165651	0.39648	NOT
RP11-403A	2.864767	-0.6453483	0.465599	-1.38606147	0.165728	0.396631	NOT
PVRL1	1097.98	0.33863241	0.244332	1.385952049	0.165762	0.396676	NOT
RP11-445F	9.556769	-0.4835738	0.349003	-1.38558643	0.165873	0.39689	NOT
TYRP1	13.42893	-1.1323029	0.817215	-1.38556271	0.16588	0.39689	NOT
C14orf169	513.0954	0.19126026	0.138052	1.385426081	0.165922	0.396955	NOT
ZNF436-AS	69.58213	-0.3232732	0.233376	-1.38520044	0.165991	0.397079	NOT
HECTD4	1624.242	-0.226616	0.163606	-1.38512957	0.166013	0.397079	NOT
HS2ST1	1867.512	0.21576458	0.155774	1.385112501	0.166018	0.397079	NOT
CDHR5	12304.58	-0.6055374	0.437207	-1.38501292	0.166049	0.397117	NOT
SLC33A1	3041.095	-0.2004353	0.144725	-1.38494339	0.16607	0.397133	NOT
ZNF428	1370.95	-0.3148802	0.227377	-1.38483836	0.166102	0.397175	NOT
PDIA3P1	299.2735	0.2770239	0.200072	1.38462251	0.166168	0.397298	NOT
CARD16	223.2689	-0.4245337	0.306639	-1.38447318	0.166214	0.397338	NOT
WFIKKN1	48.47754	0.59926747	0.432849	1.384471421	0.166214	0.397338	NOT
OTC	4759.792	-0.7119667	0.514278	-1.38440179	0.166235	0.397338	NOT
RP11-254F	4.474689	0.56875152	0.410836	1.384376178	0.166243	0.397338	NOT
IFITM4P	11.47228	-0.5028982	0.363311	-1.38420703	0.166295	0.397426	NOT
RPL10	44987.4	0.26487193	0.191375	1.384048532	0.166344	0.397507	NOT
LRRC73	47.69996	-0.6376507	0.460767	-1.38388921	0.166392	0.397589	NOT
RP11-20I2	113.5492	0.48513285	0.350595	1.3837433	0.166437	0.397661	NOT
RP11-473(	3.66872	0.65219952	0.471412	1.383501921	0.166511	0.397768	NOT
RP11-501(	9.610928	0.84827312	0.613165	1.383434807	0.166532	0.397768	NOT
PMEPA1	918.538	0.56930582	0.411523	1.383410499	0.166539	0.397768	NOT
OR2I1P	4146.819	0.64272821	0.464614	1.383360276	0.166554	0.397768	NOT
FAM198A	209.3709	-0.7937107	0.573793	-1.38326885	0.166582	0.397768	NOT
GPR88	1267.627	-0.9620909	0.695527	-1.38325413	0.166587	0.397768	NOT
AC073254.	44.65137	0.34239079	0.247532	1.383221052	0.166597	0.397768	NOT
CTPS2	958.2735	0.22790412	0.16477	1.383162213	0.166615	0.397768	NOT
TMEM243	239.9004	0.39471341	0.285371	1.383158814	0.166616	0.397768	NOT
RP11-372E	1160.774	-0.8490087	0.613836	-1.38311896	0.166628	0.397768	NOT
FAM177A1	2242.126	0.24392582	0.176408	1.382735213	0.166746	0.398013	NOT
TOMM20P2	2.556313	-0.6419338	0.4643	-1.38258464	0.166792	0.398067	NOT
RP11-210M	43.6228	1.14398932	0.827439	1.382566993	0.166798	0.398067	NOT
RP1-102K2	1.273555	-0.9356238	0.676756	-1.38251238	0.166814	NA	NA
PAK4	2374.02	0.26565652	0.192156	1.382503806	0.166817	0.398068	NOT
RP11-103E	7.47417	-0.4927772	0.356457	-1.38243083	0.166839	0.398068	NOT
RP11-461I	3.245004	0.46384479	0.335531	1.382421434	0.166842	0.398068	NOT
SPICE1	337.1501	0.27589654	0.199582	1.382370856	0.166858	0.39807	NOT
PLSCR1	1387.684	0.35549904	0.257218	1.382092781	0.166943	0.398231	NOT
AC000403.	6.920728	-0.4924657	0.356337	-1.38202128	0.166965	0.398231	NOT
ATP5S	761.0873	-0.2258907	0.163452	-1.38200328	0.166971	0.398231	NOT
GRIPAP1	2126.933	-0.1309032	0.094723	-1.38195953	0.166984	0.398231	NOT
RP11-767N	85.70105	-0.2031782	0.147055	-1.38164419	0.167081	0.39835	NOT

FCRL2	6.548507	0.97014115	0.702166	1.381640003	0.167082	0.39835	NOT
CTC-559E9	2.572033	-0.5950518	0.430695	-1.38160846	0.167092	0.39835	NOT
CPS1-IT1	10.76869	-0.9627135	0.696817	-1.38158638	0.167099	0.39835	NOT
AC073343.	6.750986	-0.6069899	0.439355	-1.3815471	0.167111	0.39835	NOT
ABHD10	1598.616	-0.1906336	0.137989	-1.38151124	0.167122	0.39835	NOT
ATP5J2-P1	5.926575	-0.4869293	0.3525	-1.38136155	0.167168	0.398424	NOT
LINC01128	400.9299	-0.3418276	0.247482	-1.38122189	0.167211	0.398492	NOT
BSCL2	225.9117	-0.272335	0.197186	-1.38110871	0.167246	0.398539	NOT
GUCD1	9152.358	-0.2943568	0.213176	-1.38081299	0.167336	0.398638	NOT
snoZ196	2.166434	0.80478462	0.582849	1.380776467	0.167348	0.398638	NOT
CLRN1-AS1	4.148877	-1.0323285	0.747672	-1.38072293	0.167364	0.398638	NOT
RP11-455(	2.843563	-0.5811226	0.420888	-1.38070708	0.167369	0.398638	NOT
TMEM180	569.0838	0.2739857	0.198445	1.380660689	0.167383	0.398638	NOT
ASS1P11	7.175318	-0.7906715	0.572677	-1.38065869	0.167384	0.398638	NOT
USP6NL	1125.528	0.2451773	0.177582	1.38064013	0.16739	0.398638	NOT
RP11-565F	6.535316	-0.6293728	0.455901	-1.38050416	0.167431	0.398657	NOT
SPACA7	7.239652	-1.2161171	0.880924	-1.38050113	0.167432	0.398657	NOT
TMEM88B	5.981283	1.18775686	0.860402	1.380467893	0.167443	0.398657	NOT
PLEKHG2	917.5469	0.32655571	0.236562	1.380423049	0.167456	0.398657	NOT
ZNF395	1371.225	0.32446284	0.23507	1.380281713	0.1675	0.398704	NOT
FAM19A5	289.1411	-0.9017364	0.653307	-1.38026367	0.167505	0.398704	NOT
MT-ATP8	25540.7	-0.4303969	0.311884	-1.37999129	0.167589	0.398868	NOT
RP11-124N	12.58467	-0.4436945	0.321581	-1.37973025	0.16767	0.399025	NOT
BMP8B	164.7326	0.67973355	0.492701	1.379606322	0.167708	0.39908	NOT
FLRT2	201.3537	-0.6720466	0.487161	-1.37951584	0.167736	0.399112	NOT
RP11-529F	9.949071	0.41117584	0.298099	1.379326329	0.167794	0.399216	NOT
RP1-265C2	5.18705	0.54746967	0.396944	1.379210018	0.16783	0.399266	NOT
CCDC60	0.882711	-0.9721604	0.704978	-1.37899449	0.167896	NA	NA
ORM2	29360.8	-0.5401637	0.391738	-1.37888951	0.167929	0.399466	NOT
DCLK2	98.67951	-0.3745958	0.271693	-1.37874806	0.167972	0.399535	NOT
RP1-78014	4.241326	-0.6209434	0.450441	-1.37852311	0.168042	0.399665	NOT
DUOXA2	151.6031	1.1375248	0.825358	1.378219731	0.168135	0.399843	NOT
RP11-266I	29.07734	-0.259111	0.188016	-1.3781349	0.168162	0.399843	NOT
SNX9	2973.995	-0.2254141	0.16357	-1.37808996	0.168176	0.399843	NOT
RP11-752I	19.72259	-0.4959809	0.359905	-1.37808862	0.168176	0.399843	NOT
RASSF2	410.5929	-0.3592596	0.260736	-1.37786629	0.168245	0.399972	NOT
NEIL1	617.4029	-0.3608501	0.261918	-1.37772377	0.168289	0.400024	NOT
CYP7A1	3723.5	-0.8881993	0.644698	-1.37769929	0.168296	0.400024	NOT
TMC5	460.3249	0.89134748	0.647114	1.377418546	0.168383	0.400195	NOT
RNF216	2088.629	0.19021933	0.138121	1.377194152	0.168452	0.400306	NOT
SCDP1	5.740359	0.84603994	0.614332	1.377171503	0.168459	0.400306	NOT
TMED9	12148.98	0.23328587	0.169414	1.377017711	0.168507	0.400384	NOT
WDR53	327.8555	0.16556776	0.120254	1.376822132	0.168567	0.400484	NOT
AC244230.	7.052339	0.64653216	0.469595	1.376786996	0.168578	0.400484	NOT
EI24	7871.796	-0.216932	0.157582	-1.37662499	0.168628	0.400568	NOT
AC087793.	4.939444	0.47439892	0.344632	1.376538736	0.168655	0.400596	NOT
RP11-362F	2.120358	1.03386454	0.751166	1.376345594	0.168715	0.400703	NOT
GDPGP1	268.6524	-0.2281776	0.165801	-1.37621304	0.168756	0.400765	NOT
RP11-314I	1.928037	1.46631909	1.065632	1.376008773	0.168819	0.40088	NOT

CCK	0.790459	1.21729606	0.884664	1.37599752	0.168822	NA	NA
PTCD1	466.5677	0.2372032	0.172415	1.375772811	0.168892	0.401019	NOT
ATRX	1741.905	-0.2461236	0.178943	-1.37542997	0.168998	0.401218	NOT
RFXANK	1661.59	0.34541317	0.251135	1.3754069	0.169005	0.401218	NOT
ODF3L1	34.69066	-0.6407743	0.465935	-1.3752426	0.169056	0.401285	NOT
DNAJB14	852.3866	-0.2312487	0.168154	-1.37521954	0.169063	0.401285	NOT
LINGO1-AS	1.127885	-1.2070936	0.877857	-1.37504643	0.169117	NA	NA
ZNF254	399.2997	-0.2944336	0.214128	-1.37503651	0.16912	0.401317	NOT
LEKR1	29.17528	0.30623715	0.222712	1.375035749	0.16912	0.401317	NOT
RP11-513(	15.06208	1.33075515	0.967869	1.37493343	0.169152	0.401317	NOT
RP11-206I	21.34111	-0.4039643	0.293807	-1.374929	0.169153	0.401317	NOT
FOSL2	2910.173	-0.3574155	0.259953	-1.37492572	0.169154	0.401317	NOT
RPL7P47	9.574561	0.53877228	0.39187	1.374874727	0.16917	0.401317	NOT
IPO7	4980.326	0.20169521	0.146704	1.374841661	0.169181	0.401317	NOT
CDR2L	356.2061	0.49004604	0.356508	1.37457096	0.169264	0.401482	NOT
AC012506.	1.428999	-0.8225134	0.598412	-1.37449317	0.169289	0.401504	NOT
RP11-146I	5.294697	0.94182952	0.685402	1.374126442	0.169402	0.401737	NOT
POLR1D	2851.112	0.22539989	0.164041	1.374044491	0.169428	0.401737	NOT
TBC1D10A	610.6543	-0.2457424	0.178849	-1.37402201	0.169435	0.401737	NOT
RP11-173I	5.573361	0.50729386	0.369225	1.373944104	0.169459	0.401737	NOT
UTS2B	14.82478	0.64682545	0.470782	1.373937985	0.169461	0.401737	NOT
NID1	8840.329	0.31191251	0.22705	1.373764093	0.169515	0.40183	NOT
CXCL3	28.54497	0.54588742	0.397386	1.373695678	0.169536	0.401845	NOT
CPA4	13.14877	0.8090702	0.58907	1.373471322	0.169606	0.401975	NOT
LARP6	272.2269	0.61327125	0.44655	1.373354239	0.169642	0.402026	NOT
ALX1	1.38944	2.07897765	1.513829	1.373323669	0.169652	NA	NA
FIGNL2	49.19675	0.58287331	0.424447	1.373253863	0.169673	0.402065	NOT
RP11-174(	2.674569	0.8208369	0.597767	1.373171581	0.169699	0.402076	NOT
CTD-3010I	1.927583	1.066743	0.776872	1.373126207	0.169713	0.402076	NOT
TP53TG1	1076.438	-0.371017	0.270205	-1.37309566	0.169723	0.402076	NOT
C9orf47	11.05625	-0.6367258	0.463761	-1.37296226	0.169764	0.402139	NOT
GS1-166A2	6.238657	0.51484029	0.375002	1.372899309	0.169784	0.40215	NOT
XXYLT1	377.9294	0.32183587	0.234462	1.372655622	0.169859	0.402246	NOT
GOSR1	2065.254	-0.1471626	0.107212	-1.37262899	0.169868	0.402246	NOT
LRCH4	481.8668	-0.30716	0.223775	-1.37262669	0.169868	0.402246	NOT
SRP9P1	8.903619	0.44886475	0.327028	1.372556708	0.16989	0.402262	NOT
C1RL-AS1	456.1072	-0.47628	0.347075	-1.37226704	0.16998	0.402441	NOT
RP11-10G1	19.2566	0.37701835	0.274754	1.372203341	0.17	0.402452	NOT
RP11-229I	6.045935	0.56262747	0.410068	1.372035432	0.170052	0.402541	NOT
GART	2259.957	0.20326004	0.148162	1.371880551	0.170101	0.402588	NOT
GUSBP1	161.5362	0.262582	0.191403	1.371876807	0.170102	0.402588	NOT
GRM5	1.25755	1.43860439	1.048692	1.371808746	0.170123	NA	NA
KRTAP1-1	1.391015	1.49791213	1.09229	1.371349953	0.170266	NA	NA
LIPN	0.910596	-1.4957189	1.09078	-1.37123741	0.170301	NA	NA
YY2	35.56513	-0.3384134	0.246829	-1.37104309	0.170362	0.403087	NOT
RP11-242I	1.814619	-0.5248324	0.382804	-1.37102076	0.170368	0.403087	NOT
RNU6-113(	1.888245	0.64483435	0.470333	1.371016477	0.17037	0.403087	NOT
SACM1L	1561.984	-0.1622715	0.118361	-1.37098625	0.170379	0.403087	NOT
GPS2	130.9821	-0.336849	0.245703	-1.37096169	0.170387	0.403087	NOT

CASC4	3401.552	-0.2513156	0.183335	-1.37079996	0.170437	0.403171	NOT
SNRNP48	758.4192	0.21339711	0.155687	1.370683268	0.170474	0.403212	NOT
RBBP8NL	2.459755	1.28731275	0.93923	1.370604176	0.170498	0.403212	NOT
DAO	2570.785	-0.6240781	0.455332	-1.37060032	0.1705	0.403212	NOT
CTD-2576I	2.286398	-0.6903277	0.503767	-1.37033002	0.170584	0.403286	NOT
MFSD5	1465.92	0.27247585	0.198842	1.370315115	0.170589	0.403286	NOT
QDPR	5604.773	-0.382963	0.279479	-1.37027329	0.170602	0.403286	NOT
LRRC75B	1151.479	0.41550908	0.303232	1.370268769	0.170603	0.403286	NOT
SERP1	8756.227	-0.1849736	0.134991	-1.37026212	0.170605	0.403286	NOT
TEN1-CDK3	84.20966	-0.4465785	0.325945	-1.37010213	0.170655	0.403365	NOT
RP11-1277	64.0232	-0.3212824	0.234503	-1.37005421	0.17067	0.403365	NOT
AC005042.	7.26339	-0.4731301	0.345347	-1.37001247	0.170683	0.403365	NOT
IRX3	287.7674	0.78706461	0.574514	1.369965026	0.170698	0.403365	NOT
RP11-111F	17.54694	-0.3928603	0.28681	-1.36975861	0.170762	0.403435	NOT
MEGF9	2742.6	-0.3071917	0.224271	-1.36973695	0.170769	0.403435	NOT
COX19	610.1587	0.26930596	0.196613	1.369726597	0.170772	0.403435	NOT
RP1-29C18	3.886427	0.64638815	0.471933	1.369660665	0.170793	0.403449	NOT
FAM177B	36.84747	-0.5295274	0.386638	-1.36956977	0.170821	0.403481	NOT
FBXL16	192.9627	-0.8121455	0.59306	-1.36941566	0.170869	0.403559	NOT
APOB	239407.8	-0.5035837	0.367757	-1.36933883	0.170893	0.403581	NOT
C8G	12730.26	-0.5312807	0.388007	-1.36925506	0.17092	0.403608	NOT
RP11-179E	2.159151	0.80237585	0.586098	1.369013275	0.170995	0.403751	NOT
SARNP	97.48474	0.34475441	0.251842	1.368933239	0.17102	0.403763	NOT
DCK	708.4822	0.2885488	0.210793	1.368871372	0.171039	0.403763	NOT
CTD-3128C	7136.337	-0.7122036	0.520292	-1.36885471	0.171045	0.403763	NOT
SMAD2	2504.682	0.1848851	0.135078	1.368724692	0.171085	0.403823	NOT
RAB10	6395.251	0.22718987	0.165996	1.368649459	0.171109	0.40384	NOT
RP11-845M	1.892282	-1.2311755	0.899614	-1.36855967	0.171137	0.40384	NOT
PAICSP4	3.227768	0.53535391	0.391181	1.368557616	0.171138	0.40384	NOT
PCP2	18.50428	0.55895537	0.40844	1.368511925	0.171152	0.40384	NOT
TAS2R10	2.297784	-0.6281354	0.459017	-1.36843637	0.171176	0.403861	NOT
RP11-367H	10.7289	0.55394211	0.404851	1.368262409	0.17123	0.40392	NOT
FTSJ2	1382.052	0.17628761	0.128841	1.368260452	0.171231	0.40392	NOT
CDKN1A	7922.719	-0.4059204	0.296689	-1.36816849	0.171259	0.403953	NOT
CHST1	276.9616	0.49155609	0.359368	1.367836698	0.171363	0.404163	NOT
FP236383.	0.774061	1.03806448	0.758951	1.367761864	0.171387	NA	NA
NIFK-AS1	154.3646	0.25582699	0.187049	1.367697825	0.171407	0.40423	NOT
SPI1	720.4701	0.43640982	0.319148	1.367420357	0.171494	0.4044	NOT
KIF1B	2455.303	-0.2386556	0.174543	-1.36731308	0.171527	0.404444	NOT
LL22NC03-	2.983565	0.91115743	0.666497	1.367084988	0.171599	0.404566	NOT
MINOS1	1051.975	-0.3075501	0.224978	-1.36702326	0.171618	0.404566	NOT
SYNGR3	31.63096	0.66635111	0.487453	1.367004626	0.171624	0.404566	NOT
CYP2AB1P	1.98253	1.26042205	0.922191	1.366769082	0.171698	0.404705	NOT
FOXK1	1333.599	0.25742239	0.188373	1.366554582	0.171765	0.404806	NOT
GINM1	1581.196	-0.2081968	0.152354	-1.3665376	0.17177	0.404806	NOT
PDE3B	1595.496	-0.3768696	0.275798	-1.36647056	0.171791	0.404821	NOT
SMARCE1P5	2.90748	0.50636032	0.370597	1.366337465	0.171833	0.40483	NOT
GNPDA2	281.9801	0.32337412	0.236674	1.366326012	0.171837	0.40483	NOT
NDUFS3	3953.392	-0.2538435	0.185787	-1.36631492	0.17184	0.40483	NOT

TMEM140	2808.899	-0.3303677	0.241804	-1.36626188	0.171857	0.404834	NOT
TRPA1	11.17194	0.95035035	0.695711	1.366012514	0.171935	0.404983	NOT
FGD6	737.3804	0.37022885	0.271039	1.365959325	0.171952	0.404987	NOT
IGKV1D-17	1.165553	1.31874967	0.96547	1.365914391	0.171966	NA	NA
NBN	2153.244	0.22905984	0.167725	1.36568507	0.172038	0.405145	NOT
RPL7P60	5.335184	-0.6999378	0.51253	-1.36565139	0.172048	0.405145	NOT
RP11-507K	9.061807	-0.4078233	0.29864	-1.36560014	0.172065	0.405148	NOT
ATP5B	33545.8	-0.2392872	0.175261	-1.36531771	0.172153	0.405321	NOT
RPS15A	13872.54	0.27759982	0.203337	1.365222367	0.172183	0.405357	NOT
AC090587.	25.73948	-0.3964356	0.290398	-1.36514792	0.172207	0.405376	NOT
CAPZB	7430.678	0.21751355	0.159346	1.365041581	0.17224	0.405419	NOT
AP003774.	2.036364	-0.5871654	0.430174	-1.36494752	0.17227	0.405419	NOT
HOXA9	5.822441	0.67977872	0.498047	1.36488967	0.172288	0.405419	NOT
TNNC2	18.19873	-0.5281137	0.386942	-1.36483787	0.172304	0.405419	NOT
METAP1D	345.0944	0.29199609	0.21395	1.364789518	0.172319	0.405419	NOT
ZNF195	621.7573	0.21197657	0.155319	1.364779633	0.172322	0.405419	NOT
EPHA8	1.787715	0.9149751	0.670431	1.364757278	0.172329	0.405419	NOT
RP11-788M	1.251501	-1.0390718	0.761412	-1.36466416	0.172359	NA	NA
PCNXL3	3161.369	-0.1838988	0.134769	-1.36454553	0.172396	0.405507	NOT
LINC01011	64.1669	0.32376505	0.23727	1.364543793	0.172396	0.405507	NOT
RP11-312B	11.74718	-0.4466299	0.327339	-1.36442575	0.172434	0.405559	NOT
RP11-697E	6.15775	0.35923835	0.263353	1.36409482	0.172538	0.405759	NOT
BECN1P1	1.906463	1.07234228	0.786164	1.364019418	0.172561	0.405759	NOT
CYP4F2	5280.132	-0.7118741	0.521897	-1.36401235	0.172564	0.405759	NOT
DPH5	845.378	0.22353977	0.16391	1.363791983	0.172633	0.405858	NOT
UQCC2	2359.672	0.34388522	0.252155	1.363783887	0.172636	0.405858	NOT
TMEM50A	3694.758	0.2247884	0.164833	1.36373284	0.172652	0.405861	NOT
CEMIP	182.2381	0.58926265	0.432309	1.363059811	0.172864	0.406257	NOT
RP11-490K	3.914219	-0.5184899	0.380394	-1.36303357	0.172872	0.406257	NOT
DSC1	3.615553	0.62480848	0.45841	1.362990375	0.172886	0.406257	NOT
RP11-111E	1.395952	-0.8262211	0.606185	-1.36298475	0.172887	0.406257	NOT
CNOT7	2141.625	0.26124186	0.191681	1.362898756	0.172914	0.406257	NOT
EIF1AX	2731.617	-0.1855796	0.136168	-1.36287144	0.172923	0.406257	NOT
TFAM	1535.846	0.1884455	0.138276	1.362825527	0.172938	0.406257	NOT
AP000344.	6.82432	1.07275447	0.78716	1.362817068	0.17294	0.406257	NOT
RP11-1103	12.83748	1.60548535	1.17813	1.362740262	0.172964	0.406264	NOT
FAM117A	646.6614	-0.3155199	0.231538	-1.36271296	0.172973	0.406264	NOT
PITPNA	2379.078	-0.2486103	0.182452	-1.36260417	0.173007	0.40631	NOT
SULT1C4	95.09349	0.63129754	0.463405	1.362301259	0.173103	0.406496	NOT
BTF3	13106.36	0.2362845	0.173451	1.362258016	0.173116	0.406496	NOT
CTD-2027I	4.92831	-0.5163701	0.379133	-1.3619755	0.173206	0.40667	NOT
CPEB1	11.9712	0.87349074	0.641483	1.361674132	0.173301	0.406858	NOT
SLC38A11	140.1424	-0.5577644	0.409709	-1.36136765	0.173398	0.40705	NOT
CD226	70.11808	-0.4771875	0.35054	-1.36129292	0.173421	0.40707	NOT
LINC01143	1.630133	-1.4766392	1.084804	-1.36120381	0.173449	0.407071	NOT
CTB-152G1	14.73851	-0.3101083	0.22782	-1.36119679	0.173452	0.407071	NOT
SELENBP1	12737.84	-0.4652745	0.341826	-1.36114532	0.173468	0.407074	NOT
CAPN13	62.71408	1.1591431	0.851637	1.361076663	0.173489	0.40709	NOT
MAP10	257.3265	-0.3942923	0.289708	-1.36099719	0.173515	0.407113	NOT

COLCA1	190.56	-0.5783711	0.425038	-1.36075227	0.173592	0.40726	NOT
STRA8	9.247416	0.88846673	0.652949	1.360697687	0.173609	0.407265	NOT
RPS24P8	18.22583	0.43383774	0.318894	1.360446184	0.173689	0.407416	NOT
ANO8	545.5676	-0.3200349	0.235253	-1.36038425	0.173708	0.407427	NOT
RP11-848(	11.53213	-0.5955852	0.43785	-1.3602491	0.173751	0.407468	NOT
FLJ16779	1.307002	1.19887823	0.881374	1.360237725	0.173755	NA	NA
ESPNP	12.4592	-0.6515194	0.478976	-1.36023391	0.173756	0.407468	NOT
PSMB3	8126.534	0.33888815	0.249202	1.359891947	0.173864	0.407662	NOT
EFNB2	803.1478	-0.3071497	0.225866	-1.35987808	0.173869	0.407662	NOT
DNAH2	25.43702	-0.4965672	0.365173	-1.35981349	0.173889	0.407674	NOT
bP-21890(	6.618116	1.15689846	0.850947	1.359541588	0.173975	0.407818	NOT
AC022182.	2.371696	0.70141956	0.515938	1.359504471	0.173987	0.407818	NOT
RP11-188I	5.916978	-0.4810026	0.353814	-1.35947748	0.173995	0.407818	NOT
C9orf173	55.92475	-0.6013076	0.442345	-1.35936435	0.174031	0.407867	NOT
CDKL3	28.42398	0.2582202	0.189965	1.359305154	0.17405	0.407875	NOT
RP11-389(	0.937971	1.85487722	1.364644	1.359239215	0.174071	NA	NA
RP11-98I(	44.62029	0.30534486	0.22466	1.35914451	0.174101	0.407959	NOT
RP4-738P1	6.598634	1.26528211	0.931014	1.359036876	0.174135	0.408004	NOT
LRAT	98.08165	-0.575542	0.423578	-1.35876224	0.174222	0.40816	NOT
RP11-636(	5.902546	1.2999658	0.956749	1.358731802	0.174232	0.40816	NOT
U95743.1	1.914244	1.16216247	0.855464	1.358516856	0.1743	0.408276	NOT
AKR1C5P	19.36632	-0.7556565	0.556254	-1.35847356	0.174313	0.408276	NOT
CD96	170.0324	-0.4860304	0.357817	-1.35832139	0.174362	0.408276	NOT
EFNB3	31.03878	-0.5115007	0.376577	-1.35829137	0.174371	0.408276	NOT
LINC0099(	2.700174	2.16474421	1.593738	1.358280961	0.174375	0.408276	NOT
EXOC7	2399.253	-0.2007601	0.147809	-1.35824412	0.174386	0.408276	NOT
ST6GALNA(	19.93407	0.8951143	0.659025	1.358239783	0.174388	0.408276	NOT
CATSPER3	34.52555	-0.3615888	0.266231	-1.35817734	0.174407	0.408276	NOT
HIST1H2AI	1.695688	0.8578866	0.631664	1.358137525	0.17442	0.408276	NOT
RP11-360I	7.720653	0.44403041	0.326949	1.358101955	0.174431	0.408276	NOT
MCM3AP	2484.521	-0.1861058	0.137045	-1.35799453	0.174465	0.40832	NOT
ZBTB41	1275.944	0.31114823	0.22915	1.357838664	0.174515	0.408401	NOT
PROS1	9337.68	-0.4022132	0.296228	-1.35778265	0.174533	0.408407	NOT
XAF1	302.8066	-0.50563	0.372447	-1.35758781	0.174595	0.40849	NOT
MAST1	139.1212	-0.5957896	0.438863	-1.35757595	0.174598	0.40849	NOT
AC144652.	129.2806	-0.4567814	0.336486	-1.35750662	0.17462	0.408506	NOT
SPATC1L	378.59	0.56673113	0.417573	1.35720405	0.174716	0.40867	NOT
ODF3B	909.7945	-0.3746411	0.276041	-1.35719163	0.17472	0.40867	NOT
ENDOU	3.439227	-0.5959575	0.439246	-1.35677378	0.174853	0.408945	NOT
PACSIN3	1811.22	0.42349995	0.312157	1.356689938	0.17488	0.408945	NOT
LRRC37A5I	21.99068	-0.5255912	0.38741	-1.35667982	0.174883	0.408945	NOT
RPL12	19666.22	0.3221679	0.237486	1.35657864	0.174915	0.408985	NOT
BLVRB	7993.159	-0.3421406	0.25224	-1.35640979	0.174969	0.409075	NOT
FGD1	443.5354	0.36031179	0.265673	1.356223181	0.175028	0.409178	NOT
GPN2	1188.316	0.18027412	0.132941	1.356049251	0.175083	0.409262	NOT
EGR1	5204.086	-0.5137995	0.378906	-1.35600894	0.175096	0.409262	NOT
LINC01251	2.67246	0.68856628	0.507815	1.355938139	0.175119	0.409262	NOT
WISP2	73.4872	-0.786904	0.580347	-1.35592045	0.175124	0.409262	NOT
STK4	1426.704	0.18022221	0.132933	1.355736667	0.175183	0.409262	NOT

GLRX2	602.5192	0.26658578	0.196638	1.355721044	0.175188	0.409262	NOT
AC005077.	1.792572	-1.349739	0.995616	-1.35568185	0.1752	0.409262	NOT
INSL3	5.660733	0.68647225	0.506397	1.355600609	0.175226	0.409262	NOT
ADCY10P1	153.2642	0.41303528	0.304691	1.355587277	0.175231	0.409262	NOT
LA16c-36(	12.43013	0.357003	0.26336	1.355568882	0.175236	0.409262	NOT
METTL17	1471.926	0.20822621	0.15361	1.35554834	0.175243	0.409262	NOT
RP11-170K	2.257182	-0.6536989	0.482242	-1.35554137	0.175245	0.409262	NOT
NODAL	5.055011	-0.5445589	0.401745	-1.35548376	0.175263	0.409267	NOT
10-Mar	2.115889	1.11341843	0.821444	1.355440628	0.175277	0.409267	NOT
SSBP3-AS1	16.52134	-0.3900272	0.287806	-1.35517357	0.175362	0.409395	NOT
FAT2	11.7417	0.53044685	0.391424	1.355173572	0.175362	0.409395	NOT
SCUBE2	149.9092	-0.6557349	0.483944	-1.35498052	0.175424	0.409503	NOT
GABRG1	3.330193	-0.8469324	0.625113	-1.35484712	0.175466	0.409567	NOT
RP11-347F	1.676536	-0.5446109	0.402006	-1.35473317	0.175503	0.409588	NOT
KCNIP4	24.33189	-0.2759129	0.203667	-1.35472452	0.175505	0.409588	NOT
RP11-370I	3.793429	0.68988552	0.509287	1.354609473	0.175542	0.409638	NOT
RP11-715J	109.8605	-0.5564173	0.41082	-1.35440595	0.175607	0.409754	NOT
TBX20	1.742717	-0.9895039	0.73061	-1.35435286	0.175624	0.409759	NOT
TM4SF1-AS	13.74614	0.56347299	0.416071	1.354271801	0.17565	0.409784	NOT
GNAS	31367.91	0.28383923	0.209599	1.35419909	0.175673	0.409803	NOT
RP11-379F	152.9182	-0.5547062	0.409671	-1.35402734	0.175728	0.409882	NOT
SLC17A7	15.20879	0.55939797	0.413153	1.353973065	0.175745	0.409882	NOT
LA16c-329	2.420986	0.63067507	0.465809	1.353936112	0.175757	0.409882	NOT
LINC00652	5.748149	0.68503859	0.505973	1.353902771	0.175767	0.409882	NOT
DCLRE1CP1	1.161432	0.86603507	0.639895	1.353402206	0.175927	NA	NA
TOPORS	868.6316	-0.2265265	0.167387	-1.35330941	0.175957	0.410289	NOT
GFAP	28.298	0.59863613	0.4424	1.353156748	0.176006	0.410367	NOT
ZFY-AS1	13.1818	-1.1876198	0.877736	-1.35304824	0.17604	0.410405	NOT
BTN2A2	581.2962	0.34851423	0.257587	1.352998747	0.176056	0.410405	NOT
ERRFI1	10670.73	-0.437994	0.323729	-1.35296372	0.176067	0.410405	NOT
SLC4A3	75.2259	-0.7454864	0.551111	-1.35269774	0.176152	0.410568	NOT
TBC1D25	716.7276	-0.1692751	0.125153	-1.35254974	0.1762	0.410643	NOT
HBP1	1743.66	-0.2826092	0.208957	-1.35247626	0.176223	0.41066	NOT
RP11-382F	7.415789	1.31692361	0.973745	1.352432051	0.176237	0.41066	NOT
BIRC7	15.22802	-0.7764717	0.574157	-1.35236709	0.176258	0.410673	NOT
GOLM1	5198.505	0.49108104	0.363175	1.352187419	0.176315	0.410754	NOT
COLGALT1	4600.673	0.21318486	0.157662	1.3521634	0.176323	0.410754	NOT
AC147651.	8.201396	1.18914407	0.879494	1.352076762	0.176351	0.410784	NOT
CITED4	1747.34	0.49207002	0.36399	1.351876492	0.176415	0.410898	NOT
RPL39	4565.694	0.33991009	0.251467	1.351709999	0.176468	0.410986	NOT
HMGCLL1	5.398985	-0.7968328	0.589634	-1.35140123	0.176567	0.411181	NOT
ITIH5	392.3393	0.66488054	0.492164	1.350931617	0.176717	0.411496	NOT
UGT2B28	10.87317	0.76627333	0.567308	1.350719118	0.176785	0.41157	NOT
AC087380.	4.014439	-0.6674686	0.49417	-1.3506856	0.176796	0.41157	NOT
RP11-1024	40.47098	-0.2976433	0.220371	-1.35064747	0.176808	0.41157	NOT
LOXHD1	12.02255	-0.5933527	0.439316	-1.35062748	0.176815	0.41157	NOT
COA1	1192.948	0.20157462	0.149249	1.350596422	0.176825	0.41157	NOT
RP5-1147A	1.516631	0.7631755	0.565117	1.350473509	0.176864	0.411616	NOT
UNC50	1231.931	0.14971871	0.110871	1.350387442	0.176892	0.411616	NOT

AP1S1	2531.057	0.22322798	0.165312	1.350345894	0.176905	0.411616	NOT
ZRSR2	340.786	-0.2232097	0.165298	-1.35034433	0.176906	0.411616	NOT
ZBTB48	877.2544	-0.2228912	0.16508	-1.35020192	0.176951	0.411687	NOT
ELP3	1455.2	0.26678022	0.197604	1.350078019	0.176991	0.411745	NOT
PDXDC2P	217.1869	-0.3477391	0.257585	-1.34999742	0.177017	0.411769	NOT
NAPA-AS1	54.70698	0.35881382	0.265813	1.349874592	0.177056	0.411787	NOT
SLC43A1	8560.373	-0.3399171	0.251816	-1.34986037	0.177061	0.411787	NOT
CHCHD7	1174.03	0.30818547	0.228318	1.349807384	0.177078	0.411787	NOT
RP11-23N2	11.71883	-0.5518421	0.408837	-1.3497848	0.177085	0.411787	NOT
RP3-441A1	1.91497	0.61849925	0.458257	1.349679108	0.177119	0.41183	NOT
SF3B1	9162.44	0.17571512	0.130223	1.349340312	0.177228	0.412048	NOT
KRT8P33	17.545	0.30340329	0.224882	1.349164776	0.177284	0.412144	NOT
SAMD12	178.0107	0.71277179	0.528333	1.349095243	0.177306	0.41215	NOT
MSRB2	2550.089	-0.3518909	0.260841	-1.34906125	0.177317	0.41215	NOT
RPL34P34	2.983216	0.68671754	0.509088	1.348916581	0.177364	0.412223	NOT
RP4-747G1	3.961311	0.54702863	0.405561	1.34882013	0.177395	0.41226	NOT
RP11-475E	0.618464	-1.19776	0.88803	-1.34878311	0.177407	NA	NA
NSUN6	1007.321	-0.2843397	0.21082	-1.34873415	0.177422	0.412268	NOT
RP11-493I	3.302919	0.92268404	0.684121	1.348714856	0.177429	0.412268	NOT
LHFPL5	1.463109	1.23098953	0.912779	1.34861779	0.17746	0.41229	NOT
EXOC6	985.8745	0.19278957	0.142961	1.348549395	0.177482	0.41229	NOT
YY1	4493.467	0.12472812	0.092493	1.348516303	0.177492	0.41229	NOT
RTN3	8098.537	0.19674327	0.145898	1.348495872	0.177499	0.41229	NOT
RFPL2	7.329161	0.73280942	0.543506	1.348301543	0.177561	0.412383	NOT
PRAMEF17	0.80615	1.38381463	1.026341	1.348298994	0.177562	NA	NA
ZNF576	636.772	-0.2052744	0.152249	-1.34827715	0.177569	0.412383	NOT
COX10-AS1	116.2255	-0.2919355	0.216559	-1.34806661	0.177637	0.412505	NOT
MAPK8	1252.74	-0.1950025	0.14466	-1.34800663	0.177656	0.412514	NOT
RP11-108M	34.98894	0.85730354	0.636019	1.347921182	0.177684	0.412526	NOT
PPM1B	2313.401	-0.2181459	0.161843	-1.34788574	0.177695	0.412526	NOT
EML3	1844.37	-0.2023354	0.150117	-1.34784824	0.177707	0.412526	NOT
DCAF6	5725.635	-0.22563	0.167408	-1.34778741	0.177727	0.412537	NOT
FBXW8	545.9271	0.17807479	0.132152	1.347496388	0.17782	0.412719	NOT
RP3-402G1	19.16132	-0.3700542	0.274656	-1.34733604	0.177872	0.412796	NOT
INTS10	1690.304	-0.2370649	0.175956	-1.34729887	0.177884	0.412796	NOT
NCF1B	26.61563	0.49426837	0.366915	1.34709191	0.177951	0.412884	NOT
VANGL2	156.9258	-0.768953	0.570827	-1.34708659	0.177952	0.412884	NOT
IL23R	3.813758	0.69849122	0.51855	1.347008259	0.177978	0.4129	NOT
TPI1P1	51.31821	0.43075241	0.319794	1.346969756	0.17799	0.4129	NOT
PTGER3	56.69819	-0.5212469	0.387012	-1.34685036	0.178028	0.412954	NOT
CCL4L1	145.0843	0.55001596	0.408404	1.346744594	0.178063	0.412998	NOT
RP11-544M	17.78528	-0.4362421	0.323956	-1.34660708	0.178107	0.413065	NOT
AKAP12	1524.977	-0.5580275	0.414413	-1.34654945	0.178125	0.413071	NOT
GVINP1	86.61565	-0.4501417	0.334307	-1.34649085	0.178144	0.413071	NOT
PIK3C2A	1734.903	-0.2295682	0.170498	-1.34645735	0.178155	0.413071	NOT
ATP2B3	3.400863	1.06954215	0.794389	1.346370402	0.178183	0.413083	NOT
ZFAND2B	2169.532	0.29117752	0.216272	1.346347703	0.17819	0.413083	NOT
RP11-690I	3.190954	-0.520037	0.386282	-1.34626255	0.178218	0.413111	NOT
TXNP4	8.696261	0.58052354	0.431287	1.346025461	0.178294	0.413253	NOT

CTA-217C2	7.567673	0.43659432	0.324374	1.345960049	0.178315	0.413263	NOT
RP3-395M2	85.10138	-0.4231901	0.314425	-1.3459172	0.178329	0.413263	NOT
TAF1C	1108.328	-0.2001076	0.14869	-1.34579934	0.178367	0.413284	NOT
TSTD2	296.2773	-0.4076213	0.302892	-1.34576565	0.178378	0.413284	NOT
PRSS44	2.673949	1.08117438	0.803401	1.345747622	0.178384	0.413284	NOT
AICDA	1.025988	1.18017431	0.877226	1.345347655	0.178513	NA	NA
FSCN2	8.272706	0.54504829	0.40515	1.345301507	0.178528	0.413582	NOT
AC118754.	6.604208	-0.727835	0.541067	-1.34518476	0.178566	0.413634	NOT
CTSW	192.1645	-0.5727893	0.425828	-1.34511896	0.178587	0.413648	NOT
RAVER2	403.2226	0.49889955	0.37095	1.344923409	0.17865	0.413759	NOT
CTD-2288(	2.412921	-0.6620724	0.492308	-1.3448334	0.178679	0.413791	NOT
DARS-AS1	48.38256	0.37489156	0.278811	1.344606342	0.178752	0.413923	NOT
CTC1	701.398	-0.2147289	0.159702	-1.34456197	0.178767	0.413923	NOT
TMEM217	37.11711	-0.4086949	0.304075	-1.34405815	0.17893	0.414241	NOT
ARSI	73.52127	0.66973808	0.498301	1.344042826	0.178935	0.414241	NOT
LINC0106:	6.914497	0.57975993	0.431427	1.343819457	0.179007	0.414373	NOT
SPIRE1	275.8326	-0.5481817	0.407959	-1.34371826	0.17904	0.414414	NOT
ORAOV1P1	4.255671	-0.6552066	0.487626	-1.34366646	0.179056	0.414417	NOT
TECRL	1.546905	-1.6687255	1.242141	-1.34342636	0.179134	0.414535	NOT
CEP70	883.7808	-0.2634614	0.196119	-1.34337487	0.179151	0.414535	NOT
SLC3A1	523.2069	0.88100376	0.655819	1.343363796	0.179154	0.414535	NOT
ISOC1	2437.908	-0.2911893	0.216768	-1.34331977	0.179168	0.414535	NOT
PCYT1A	2454.688	-0.133524	0.099404	-1.3432486	0.179191	0.414553	NOT
RP11-968A	1.908358	0.74117371	0.551826	1.343129771	0.17923	0.414607	NOT
URGCP	1860.021	-0.2191415	0.163173	-1.34300193	0.179271	0.414667	NOT
DBNL	4204.147	0.16152195	0.120282	1.34286494	0.179316	0.414734	NOT
RP11-303E	3.727715	-0.5160594	0.384324	-1.34277237	0.179346	0.414768	NOT
RP11-280H	6.63772	-0.7550645	0.562357	-1.34267842	0.179376	0.414791	NOT
PTPLB	5372.565	0.32856171	0.244717	1.342619493	0.179395	0.414791	NOT
PLIN1	421.2422	-0.6375016	0.474849	-1.34253425	0.179423	0.414791	NOT
GRAMD1B	512.4361	0.63165714	0.470524	1.342453999	0.179449	0.414791	NOT
EPB41L4A-	568.1401	0.34170338	0.254537	1.342449503	0.17945	0.414791	NOT
COPZ1	6805.35	0.1869814	0.139286	1.342429933	0.179457	0.414791	NOT
HMGB2P1	5.082005	0.69910154	0.520791	1.34238486	0.179471	0.414791	NOT
RP11-150(	6.050389	-0.5599955	0.417171	-1.34236411	0.179478	0.414791	NOT
MAP3K13	2123.451	-0.259657	0.193452	-1.34222813	0.179522	0.414858	NOT
ABCC10	814.0712	0.22281548	0.166014	1.342146102	0.179549	0.414884	NOT
RP11-397(	3.122986	0.90927423	0.677515	1.342073216	0.179572	0.414903	NOT
AOC4P	525.7861	-0.6164993	0.459402	-1.3419594	0.179609	0.414953	NOT
LDHA	28570.78	0.26441586	0.197073	1.341715477	0.179688	0.415077	NOT
EDN2	33.29099	-0.8094452	0.603335	-1.34161749	0.17972	0.415077	NOT
POC1B	486.0291	0.21835685	0.16276	1.341588161	0.17973	0.415077	NOT
CTC-510F1	18.00338	-0.3252356	0.242427	-1.34157901	0.179733	0.415077	NOT
SEC14L2	8026.147	-0.6436225	0.479758	-1.34155791	0.179739	0.415077	NOT
EXOSC3P1	4.739788	0.73695474	0.549507	1.341119329	0.179882	0.415344	NOT
RP11-455F	5.845923	-0.5551625	0.413958	-1.34110807	0.179885	0.415344	NOT
C8orf31	23.2816	0.66170542	0.493496	1.340853856	0.179968	0.41546	NOT
SCARNA21	2.290614	-0.7547082	0.562866	-1.34083163	0.179975	0.41546	NOT
WRN	317.7552	0.34585771	0.257946	1.340811931	0.179982	0.41546	NOT

FAM186B	32.96812	-0.3625667	0.270426	-1.34072641	0.180009	0.415487	NOT
CTD-2568A	1.784392	0.87820273	0.655042	1.340681055	0.180024	0.415487	NOT
AC005071.	2.28113	-0.5366254	0.400292	-1.34058326	0.180056	0.415487	NOT
KCTD18	601.8429	-0.2322696	0.173264	-1.34054976	0.180067	0.415487	NOT
PSMA1	2923.972	0.17723982	0.13222	1.340494187	0.180085	0.415487	NOT
KIF1A	110.8135	-0.915667	0.683084	-1.34049024	0.180086	0.415487	NOT
AP000936.	5.675632	0.48656542	0.362988	1.340444933	0.180101	0.415487	NOT
KIAA0040	1019.595	-0.342489	0.255533	-1.34029363	0.18015	0.415543	NOT
ST7	1616.149	-0.2293398	0.171128	-1.34016873	0.18019	0.415543	NOT
FCHSD2	1057.725	-0.2086063	0.155663	-1.34011883	0.180207	0.415543	NOT
MPHOSPH8	1492.936	-0.1806263	0.134788	-1.34007774	0.18022	0.415543	NOT
HIST2H2BE	893.8645	-0.4595936	0.342961	-1.34007682	0.18022	0.415543	NOT
MEOX2	73.67833	0.60751485	0.453355	1.340042971	0.180231	0.415543	NOT
ZNF790-AS	30.21165	0.54058865	0.403412	1.340040347	0.180232	0.415543	NOT
RP11-126(	37.37725	-0.5992021	0.447251	-1.33974479	0.180328	0.41573	NOT
INHBA	670.3287	-0.4803682	0.358766	-1.33894494	0.180589	0.416294	NOT
COBL	2309.862	-0.3923561	0.293067	-1.33879492	0.180637	0.416372	NOT
AC019117.	1.884152	0.85233599	0.636826	1.338413199	0.180762	0.416623	NOT
MSL1	2873.451	0.15833216	0.118312	1.338264382	0.18081	0.416628	NOT
PFDN1	1552.46	0.20942951	0.156495	1.338253026	0.180814	0.416628	NOT
NAB1	1658.83	0.179885	0.134426	1.338172729	0.18084	0.416628	NOT
RP11-102M	2.957914	0.50430626	0.376873	1.338133555	0.180853	0.416628	NOT
RP11-685F	0.291094	-1.9111938	1.42826	-1.33812692	0.180855	NA	NA
MTOR	3380.175	-0.2601394	0.194411	-1.3380891	0.180867	0.416628	NOT
RP5-1056I	35.83176	0.41788855	0.312302	1.338089056	0.180867	0.416628	NOT
IGFBP7	10885.85	-0.3455966	0.25829	-1.33801865	0.18089	0.416628	NOT
COPB1	5651.484	0.17449114	0.130411	1.338012133	0.180892	0.416628	NOT
CD9	2893.811	0.36540099	0.273098	1.337982422	0.180902	0.416628	NOT
DNAJC3	6212.74	-0.2727764	0.203883	-1.33790744	0.180927	0.416649	NOT
RGS11	25.60375	-0.5432644	0.40613	-1.33765982	0.181007	0.416799	NOT
RP11-250E	12.5964	-0.4594583	0.343519	-1.33750443	0.181058	0.41685	NOT
DUXAP8	113.011	0.60592423	0.45304	1.337462197	0.181072	0.41685	NOT
ZSCAN21	311.5671	-0.217524	0.162641	-1.33744665	0.181077	0.41685	NOT
GZMK	79.92266	-0.5715222	0.427337	-1.33740435	0.181091	0.41685	NOT
TRMT1L	1376.569	-0.2220332	0.166056	-1.33710087	0.18119	0.417042	NOT
AC004540.	27.16264	0.84042216	0.628633	1.336905243	0.181254	0.417154	NOT
APOA1	269994.8	-0.6254282	0.467834	-1.33685818	0.181269	0.417154	NOT
AC011290.	11.80668	0.47448539	0.354945	1.336784061	0.181293	0.417174	NOT
ABCG1	1001.924	-0.4635211	0.346783	-1.33663249	0.181343	0.417253	NOT
RP11-960Z	2.781432	0.49050358	0.366998	1.336527972	0.181377	0.417296	NOT
TACC2	1911.403	-0.2877749	0.215324	-1.33647641	0.181394	0.417299	NOT
CNN2P6	0.604567	1.971392	1.47509	1.336455011	0.181401	NA	NA
FTO-IT1	7.194429	0.39979362	0.299179	1.336304433	0.18145	0.417393	NOT
ATP6AP1	7293.883	0.18652133	0.139594	1.336171824	0.181493	0.417433	NOT
WAC-AS1	970.5561	0.2210546	0.165441	1.336157446	0.181498	0.417433	NOT
PDGFA	1366.798	0.33840717	0.253292	1.336037333	0.181537	0.417488	NOT
STOML2	4814.795	0.20495716	0.153438	1.335763285	0.181627	0.417658	NOT
TRIQK	1784.812	-0.2995308	0.22431	-1.33534428	0.181764	0.417938	NOT
C12orf74	5.508835	-0.6661119	0.498869	-1.33524437	0.181796	0.417978	NOT

HEXDC-IT1	4.818925	-0.4836287	0.362222	-1.33517061	0.181821	0.417998	NOT
TMEM170B	1068.308	-0.359252	0.269092	-1.33505226	0.181859	0.418051	NOT
EXOSC7	1132.753	0.22506719	0.168595	1.334957337	0.18189	0.418073	NOT
FCRL3	39.95181	0.73012278	0.546937	1.334929402	0.181899	0.418073	NOT
RP11-12A2	17.36302	-0.8898463	0.66665	-1.33480252	0.181941	0.418098	NOT
AC114730.	58.81931	0.71354028	0.534584	1.334758764	0.181955	0.418098	NOT
RP4-555D2	4.734911	1.21766518	0.912276	1.334754942	0.181957	0.418098	NOT
RP1-168L1	5.054621	-0.4818996	0.361054	-1.33470223	0.181974	0.418102	NOT
GLRX3P2	2.447462	0.61098771	0.457806	1.334600852	0.182007	0.418143	NOT
TUG1	4221.329	0.16993258	0.127335	1.334533609	0.182029	0.418143	NOT
CTD-2245E	3.269674	-0.8385559	0.628394	-1.33444256	0.182059	0.418143	NOT
OSGIN1	13257.77	0.56605542	0.424197	1.334416193	0.182067	0.418143	NOT
LRRC31	231.654	0.71674431	0.537123	1.334412598	0.182069	0.418143	NOT
OSBPL2	1830.987	-0.1469281	0.11012	-1.33425894	0.182119	0.418223	NOT
PSAPL1	5.963372	1.14919332	0.861397	1.334103551	0.18217	0.418256	NOT
TYW5	314.3852	0.16013995	0.120037	1.334084169	0.182176	0.418256	NOT
WRAP53	413.1252	0.27826321	0.208581	1.334074707	0.182179	0.418256	NOT
ERCC6-PGE	3.479795	-0.4043956	0.303142	-1.33401236	0.1822	0.418267	NOT
RP11-24M1	4.906211	0.50547656	0.379011	1.333672695	0.182311	0.418487	NOT
ZDHHC18	1763.569	0.215012	0.161237	1.333517065	0.182362	0.418569	NOT
PLBD2	4718.597	-0.2100935	0.157567	-1.33335867	0.182414	0.418653	NOT
RPL13P12	436.6087	-0.4857583	0.364329	-1.33329606	0.182435	0.418665	NOT
TLR4	463.4097	-0.436167	0.327181	-1.33310653	0.182497	0.418772	NOT
ATP8B2	852.2841	-0.3522741	0.264273	-1.3329939	0.182534	0.418821	NOT
WDR33	2630.702	0.11523362	0.086462	1.332758626	0.182611	0.418963	NOT
RP11-580I	20.09447	0.31629476	0.237345	1.332637448	0.182651	0.419019	NOT
N4BP1	1317.269	-0.1533658	0.115099	-1.33247396	0.182704	0.419107	NOT
ZSCAN32	61.56767	-0.2252465	0.169062	-1.33232718	0.182753	0.419182	NOT
POU3F4	0.8113	2.18515658	1.640123	1.332312421	0.182758	NA	NA
RPS3AP46	1.487799	1.21188531	0.909653	1.332250026	0.182778	0.419204	NOT
NUDT12	1049.848	-0.3766879	0.282768	-1.33214394	0.182813	0.419207	NOT
STAT1	10383.33	0.38915503	0.292135	1.332106276	0.182825	0.419207	NOT
C15orf56	3.472998	0.89910195	0.674948	1.332104967	0.182826	0.419207	NOT
MED13	3333.822	-0.2658174	0.199597	-1.33176889	0.182936	0.419421	NOT
NLRP1	661.2376	-0.4211981	0.316281	-1.33172254	0.182951	0.419421	NOT
KCTD8	1.593887	1.51424153	1.137091	1.331679876	0.182965	0.419421	NOT
IPCEF1	57.0703	-0.4544577	0.341297	-1.33156224	0.183004	0.419445	NOT
SCML2P2	2.279765	-0.6795214	0.510342	-1.3315027	0.183024	0.419445	NOT
RPA2	1324.512	0.19029651	0.142924	1.331456112	0.183039	0.419445	NOT
PATZ1	1997.942	0.22508639	0.169056	1.331429645	0.183048	0.419445	NOT
SRD5A2	1220.624	-0.8464986	0.63579	-1.33141321	0.183053	0.419445	NOT
MAP3K9	376.4839	0.26916565	0.202173	1.331361662	0.18307	0.419449	NOT
DLL3	3.824785	0.93492652	0.702271	1.331289662	0.183094	0.419467	NOT
RP13-279N	2.039707	-0.5876649	0.441493	-1.33108384	0.183161	0.419585	NOT
KBTBD2	1722.712	0.14244646	0.107019	1.331039407	0.183176	0.419585	NOT
TOLLIP	7228.576	-0.2165686	0.16272	-1.33093166	0.183211	0.419631	NOT
PTGS2	52.05013	-0.5606986	0.421369	-1.33066026	0.183301	0.419765	NOT
AC000123.	26.26386	0.30908983	0.232283	1.330659563	0.183301	0.419765	NOT
GDF5	1.412259	0.85032652	0.639051	1.330608496	0.183318	0.419768	NOT

IGHV4-34	53.83952	0.85145224	0.639949	1.330500862	0.183353	0.419776	NOT
HNRNPA1P2	20.38686	0.56779171	0.42676	1.330469758	0.183364	0.419776	NOT
RP11-454K	3.783582	0.51055399	0.383748	1.330442145	0.183373	0.419776	NOT
MARCO	97.36902	-0.7845504	0.589706	-1.33041033	0.183383	0.419776	NOT
OTUD5	2824.75	-0.1506801	0.113265	-1.33033766	0.183407	0.419795	NOT
NCOA1	1891.155	-0.2142894	0.161102	-1.33014701	0.18347	0.419881	NOT
RPL27AP5	3.165422	0.6762718	0.508425	1.330129675	0.183476	0.419881	NOT
RP11-388C	2.987379	-0.5101429	0.383548	-1.33006336	0.183497	0.419896	NOT
KLC1	869.3998	-0.2546795	0.191513	-1.32982512	0.183576	0.420001	NOT
FBXL12	693.5309	0.18375619	0.138183	1.329806522	0.183582	0.420001	NOT
MOSPD1	515.3336	0.30780575	0.231471	1.329782344	0.18359	0.420001	NOT
ZNF561-AS	148.597	-0.2209698	0.166215	-1.32942011	0.183709	0.420229	NOT
SNCAIP	108.967	0.52288023	0.393325	1.329384433	0.183721	0.420229	NOT
HAS3	89.09425	0.36892405	0.277524	1.329339639	0.183736	0.420229	NOT
FKBP1A	6860.739	0.23202458	0.174568	1.329133328	0.183804	0.420349	NOT
WAPAL	2212.129	-0.1780392	0.133975	-1.32889723	0.183882	0.42048	NOT
ARNTL2	661.8136	0.48066217	0.361708	1.328866318	0.183892	0.42048	NOT
GPD1	3939.193	-0.6563252	0.493989	-1.32862437	0.183972	0.420592	NOT
DNAH12	83.30403	0.77011214	0.579648	1.328586352	0.183984	0.420592	NOT
SLC25A5P5	7.685161	0.76897069	0.578808	1.328542734	0.183999	0.420592	NOT
SKP1P1	18.88228	-0.3069492	0.231048	-1.32850993	0.18401	0.420592	NOT
RP11-701P	4.514205	-0.6812518	0.512805	-1.32848243	0.184019	0.420592	NOT
CBWD1	313.4216	0.19981081	0.150426	1.328299782	0.184079	0.420694	NOT
KIAA0513	406.8302	-0.3065834	0.230819	-1.32824407	0.184097	0.420694	NOT
SRSF5	5932.484	-0.1845949	0.138999	-1.32802881	0.184169	0.420694	NOT
RP11-1136	4.686835	-0.6409638	0.482646	-1.3280213	0.184171	0.420694	NOT
ANKRD32	205.0154	0.23973427	0.180523	1.328000124	0.184178	0.420694	NOT
RP11-61L2	9.35151	0.53583344	0.403502	1.327958025	0.184192	0.420694	NOT
ING1	973.4953	-0.3089473	0.232652	-1.32793815	0.184199	0.420694	NOT
RP11-477H	11.23805	-0.7333392	0.552242	-1.3279317	0.184201	0.420694	NOT
SFXN3	852.7658	0.35911632	0.270434	1.327924859	0.184203	0.420694	NOT
IGLV1-40	192.9189	-0.9555596	0.719654	-1.32780414	0.184243	0.42075	NOT
HECTD1	5141.443	-0.247751	0.186615	-1.32760468	0.184309	0.420862	NOT
GPR171	38.70634	-0.6076847	0.457745	-1.32756165	0.184323	0.420862	NOT
RP11-849H	43.81154	0.27128598	0.204365	1.327458329	0.184357	0.420905	NOT
FLG	12.89217	0.8655388	0.652309	1.326885011	0.184547	0.421302	NOT
CTD-2334D	2.628687	-0.5795749	0.436855	-1.32669948	0.184608	0.421396	NOT
C7orf49	1394.871	0.21490807	0.161995	1.326630433	0.184631	0.421396	NOT
MAP4	9620.65	0.18139899	0.136738	1.326620076	0.184634	0.421396	NOT
LINC00871	0.68912	-1.3286846	1.00159	-1.3265757	0.184649	NA	NA
PI4K2B	1669.097	-0.2876376	0.216847	-1.32645125	0.18469	0.421433	NOT
SCT	4.754462	-0.5813886	0.438311	-1.32642817	0.184698	0.421433	NOT
RP3-339A1	5.347578	-0.4596575	0.346543	-1.32640827	0.184704	0.421433	NOT
KLF13	3520.601	-0.3159798	0.238227	-1.32638327	0.184713	0.421433	NOT
SCAPER	538.7856	-0.2480368	0.187028	-1.32620365	0.184772	0.421533	NOT
SUCLG2-AS	50.77319	-0.3965866	0.299064	-1.32609234	0.184809	0.421582	NOT
CTSZ	15456.39	0.28585835	0.2156	1.325870867	0.184882	0.421662	NOT
HMG20A	809.1544	-0.2874087	0.216782	-1.3257963	0.184907	0.421662	NOT
RNU4ATAC1	2.315942	-0.5682982	0.428647	-1.32579456	0.184908	0.421662	NOT

RP11-443I	10.34494	0.70197518	0.529491	1.325754873	0.184921	0.421662	NOT
LINC0039(	3.787608	-0.7220562	0.544639	-1.32575208	0.184922	0.421662	NOT
RP11-296I	9.69791	0.43186711	0.325765	1.325701028	0.184939	0.421663	NOT
NT5C3AP1	4.741283	0.64550008	0.486944	1.325613764	0.184968	0.421663	NOT
BX470102.	3.233022	1.02038391	0.769747	1.325609307	0.184969	0.421663	NOT
LINC0051(	3.126621	0.81644127	0.616025	1.32533881	0.185059	0.421832	NOT
RP11-132N	5.398264	0.73949718	0.558021	1.32521308	0.1851	0.421867	NOT
HK3	192.0632	0.5075489	0.383003	1.325181448	0.185111	0.421867	NOT
RP11-157(	1.663456	-0.7954959	0.600305	-1.32515198	0.185121	0.421867	NOT
FAM151B	39.43664	-0.2709648	0.204513	-1.32492728	0.185195	0.421897	NOT
RP11-343I	20.79008	0.76827253	0.579866	1.324913853	0.1852	0.421897	NOT
LRRC8A	3886.667	-0.2302211	0.173766	-1.32489424	0.185206	0.421897	NOT
CASP1P2	2.533977	-0.6752589	0.509671	-1.32489077	0.185207	0.421897	NOT
CD8B	87.19416	0.70578028	0.532714	1.324877538	0.185212	0.421897	NOT
LZIC	891.7745	0.22177	0.16741	1.324710909	0.185267	0.421988	NOT
ZNF596	90.32308	-0.3505832	0.264666	-1.32462639	0.185295	0.422016	NOT
SMIM12	1778.244	-0.2160125	0.16308	-1.32457928	0.185311	0.422016	NOT
REV3L	661.194	-0.2709172	0.20456	-1.32439271	0.185373	0.422122	NOT
C2orf71	0.979554	1.6820474	1.270073	1.324370353	0.18538	NA	NA
METTL4	310.7667	0.20801113	0.157082	1.324221426	0.18543	0.422216	NOT
AF064858.	133.2041	-0.72869	0.550339	-1.32407569	0.185478	0.422255	NOT
COL26A1	337.2499	0.75701311	0.571738	1.324056574	0.185484	0.422255	NOT
CTD-2535I	5.219167	1.21159548	0.915089	1.324019443	0.185497	0.422255	NOT
GZMA	169.945	-0.4575585	0.345593	-1.32398179	0.185509	0.422255	NOT
ZBTB6	334.0868	-0.2103524	0.15889	-1.32388477	0.185541	0.422293	NOT
KSR2	26.96884	0.77103088	0.582517	1.323619076	0.18563	0.422459	NOT
CSRP1	6098.643	-0.3098156	0.234154	-1.32312694	0.185793	0.422762	NOT
RP11-731(	39.37337	-0.294348	0.222464	-1.32312456	0.185794	0.422762	NOT
RPL17P22	3.765064	0.49998193	0.377945	1.322896091	0.18587	0.422858	NOT
UBXN6	5746.263	-0.1948925	0.147324	-1.32288552	0.185873	0.422858	NOT
GNRH1	27.95227	0.39186683	0.296228	1.322857527	0.185883	0.422858	NOT
PISD	1113.374	0.18411208	0.1392	1.322647873	0.185953	0.422912	NOT
ST14	2433.624	0.50917073	0.384965	1.322640129	0.185955	0.422912	NOT
XKR5	2.692086	0.93404078	0.706217	1.322598218	0.185969	0.422912	NOT
CTD-2337A	7.319495	0.7115233	0.537985	1.3225699	0.185978	0.422912	NOT
JUND	8849.453	-0.354111	0.267748	-1.32255183	0.185984	0.422912	NOT
SKA2	1171.295	0.25407919	0.192121	1.322497825	0.186002	0.422917	NOT
CA4	64.03972	-0.8480167	0.641274	-1.32239328	0.186037	0.422952	NOT
RP11-351I	14.79181	0.40033134	0.302741	1.322357905	0.186049	0.422952	NOT
JMJD7-PLA	99.69952	-0.424477	0.321042	-1.32218387	0.186107	0.423048	NOT
RP11-523(	1.98694	-0.6130803	0.463732	-1.32205789	0.186149	0.423052	NOT
RPS10	6506.094	0.32826333	0.248306	1.3220103	0.186165	0.423052	NOT
SLC4A1	3.353159	-0.7228921	0.546813	-1.32200853	0.186165	0.423052	NOT
SNHG18	126.5124	-0.6058687	0.4583	-1.32199099	0.186171	0.423052	NOT
RP11-216I	4.89091	0.62778122	0.474946	1.321794128	0.186237	0.423129	NOT
DVL1	3169.028	0.24516168	0.185483	1.321746719	0.186253	0.423129	NOT
ZNF585A	178.4076	-0.2913505	0.220428	-1.3217467	0.186253	0.423129	NOT
HMGCL	6892.741	-0.3449952	0.261023	-1.32170317	0.186267	0.423129	NOT
RP11-298I	29.65878	0.44257242	0.334889	1.321549712	0.186318	0.423209	NOT

HACL1	1921.537	-0.3225694	0.244093	-1.32150094	0.186334	0.423211	NOT
CSTB	8187.912	0.32536869	0.24625	1.321292908	0.186404	0.423309	NOT
BDP1	950.4701	-0.2505552	0.189631	-1.32127809	0.186409	0.423309	NOT
DUSP28	141.203	0.17601835	0.133227	1.32119356	0.186437	0.423311	NOT
MAVS	4421.111	-0.2295904	0.173777	-1.3211809	0.186441	0.423311	NOT
DDX12P	95.75034	0.37044315	0.28042	1.321032011	0.186491	0.423388	NOT
AC098973.	83.56475	1.42157128	1.076278	1.320821576	0.186561	0.423512	NOT
EHD1	2524.82	0.19100817	0.144631	1.320658103	0.186615	0.423601	NOT
ADHFE1	1333.033	-0.4546774	0.344304	-1.3205687	0.186645	0.423633	NOT
RP11-20J1	6.657216	1.08769576	0.823686	1.320522201	0.186661	0.423633	NOT
CTC-559E9	30.22705	-0.3831191	0.290156	-1.320391	0.186705	0.423697	NOT
HLA-DOB	85.70788	0.55809234	0.422738	1.3201841	0.186774	0.4238	NOT
DSCR4-IT1	2.503002	1.87576008	1.420857	1.320160745	0.186781	0.4238	NOT
LINC00847	551.8124	-0.2083756	0.157855	-1.32004816	0.186819	0.42382	NOT
RP11-317E	2.324841	-1.3131325	0.994766	-1.32004152	0.186821	0.42382	NOT
ROCK1	1751.613	-0.2354464	0.178388	-1.31985415	0.186884	0.423926	NOT
LINC0087(	91.37279	-0.531384	0.40263	-1.31978089	0.186908	0.423946	NOT
LINC00398	4.250351	-0.7358515	0.557677	-1.31949415	0.187004	0.424086	NOT
RPL35P5	20.46927	0.42678983	0.323455	1.319473413	0.187011	0.424086	NOT
POLE3	2228.523	0.18827374	0.142692	1.319443525	0.187021	0.424086	NOT
FLJ21408	13.27095	-0.373258	0.282898	-1.31940959	0.187032	0.424086	NOT
LINC01435	0.931707	-1.3538438	1.026114	-1.3193896	0.187039	NA	NA
WI2-2118(	2.619996	1.32407976	1.003608	1.319320264	0.187062	0.424118	NOT
TECR	6432.383	-0.2518676	0.190957	-1.31897347	0.187178	0.424345	NOT
PLG	60916.54	-0.6160997	0.467165	-1.31880431	0.187235	0.424395	NOT
ERP29	10632.56	0.21821726	0.165471	1.318761884	0.187249	0.424395	NOT
C2CD4B	29.69445	-0.5038204	0.382045	-1.31874484	0.187254	0.424395	NOT
PDE6A	9.029628	0.65877185	0.499554	1.318720706	0.187263	0.424395	NOT
AGAP7P	6.523322	0.61852625	0.469153	1.31838997	0.187373	0.424591	NOT
BVES	31.83401	-0.4503826	0.341621	-1.31836866	0.18738	0.424591	NOT
LY6H	53.62911	-0.6772819	0.513792	-1.31820316	0.187436	0.424681	NOT
CCDC81	17.1891	-0.3722137	0.282395	-1.31806109	0.187483	0.424753	NOT
MIR4263	5.699346	0.43968534	0.33362	1.317923614	0.187529	0.424815	NOT
PHKA2	3748.936	0.25703706	0.195037	1.317886637	0.187542	0.424815	NOT
MUC19	3.326018	-0.9667638	0.733637	-1.31776818	0.187581	0.424841	NOT
CDC37P1	2.734833	0.9034576	0.685602	1.317758437	0.187585	0.424841	NOT
AC010468.	10.36488	0.44960614	0.341233	1.31759223	0.18764	0.424903	NOT
RP11-314N	1.927647	1.60197916	1.215846	1.317583564	0.187643	0.424903	NOT
N6AMT2	280.0372	-0.263486	0.200015	-1.31733313	0.187727	0.425057	NOT
NREP	2306.546	0.39356385	0.298781	1.317230432	0.187761	0.425076	NOT
RP11-319(	6.960037	-0.538152	0.408566	-1.31717138	0.187781	0.425076	NOT
CH17-13I2	11.17247	0.77549828	0.588762	1.317168278	0.187782	0.425076	NOT
SNX6	1570.257	0.19471994	0.147848	1.317030361	0.187828	0.425145	NOT
NME8	5.944557	-0.5734668	0.435464	-1.31691048	0.187869	0.4252	NOT
NUFIP1	305.4661	0.2311555	0.175545	1.31678833	0.18791	0.425204	NOT
NFE2	21.65003	-0.6631926	0.503664	-1.31673696	0.187927	0.425204	NOT
EIF4EBP2	11697.33	-0.2523717	0.191667	-1.31672054	0.187932	0.425204	NOT
CEBPA-AS1	517.3746	0.3915536	0.297371	1.316718697	0.187933	0.425204	NOT
LL22NC03-	8.056392	0.64928169	0.493159	1.31657782	0.18798	0.425267	NOT

CASP8AP2	469.1565	0.30872659	0.234498	1.31654185	0.187992	0.425267	NOT
RP11-303E	251.1361	-0.3221037	0.244673	-1.31646738	0.188017	0.425288	NOT
CKM	5.851337	0.65971978	0.501294	1.316034399	0.188162	0.425581	NOT
CCDC136	20.71151	0.4858502	0.369247	1.315784898	0.188246	0.425724	NOT
AMBN	1.474623	1.53589029	1.167309	1.31575266	0.188257	0.425724	NOT
IGLV2-14	214.5347	-0.8720219	0.662892	-1.31548065	0.188348	0.425857	NOT
RPS23P1	2.730818	0.64725658	0.492046	1.315438211	0.188363	0.425857	NOT
RP11-727A	54.58334	0.30233741	0.229842	1.315415186	0.18837	0.425857	NOT
PPCDC	479.6567	0.27130022	0.206251	1.315391036	0.188379	0.425857	NOT
IL1RN	2087.643	-0.4716111	0.358582	-1.31521003	0.188439	0.4259	NOT
SNRPA	2463.986	0.24501197	0.186292	1.315206825	0.18844	0.4259	NOT
TJP3	947.7178	0.52362977	0.398139	1.315193847	0.188445	0.4259	NOT
LINC01232	253.9846	0.33043033	0.251303	1.314867622	0.188554	0.426105	NOT
RP11-513I	16.78559	0.41531428	0.315869	1.314830177	0.188567	0.426105	NOT
USP24	2678.522	0.17421033	0.132508	1.314715969	0.188605	0.426157	NOT
ZEB1	1006.318	-0.2095134	0.159379	-1.31456497	0.188656	0.426236	NOT
RASAL2-AS	31.20287	-0.3628302	0.276051	-1.3143593	0.188725	0.426357	NOT
RP11-669E	33.85232	-0.7480638	0.56924	-1.31414583	0.188797	0.426483	NOT
CDK20	222.752	0.27935573	0.212631	1.313808029	0.188911	0.426705	NOT
SS18L2	772.6757	0.23574189	0.179442	1.313750906	0.18893	0.426713	NOT
HINT2	2579.633	-0.3246687	0.247145	-1.31367953	0.188954	0.426731	NOT
PKD1P5	33.32459	-0.5638125	0.429227	-1.31355307	0.188997	0.426784	NOT
SECISBP2	1986.153	-0.1772411	0.134942	-1.3134628	0.189027	0.426784	NOT
LINC00824	2.990065	0.71691701	0.545826	1.313452576	0.18903	0.426784	NOT
TLR10	33.00935	0.63775877	0.485582	1.313391114	0.189051	0.426784	NOT
DSG4	6.206427	0.8950003	0.68145	1.313376583	0.189056	0.426784	NOT
TUSC2	1391.785	0.21561201	0.164174	1.313314846	0.189077	0.426796	NOT
SYNM	791.6609	-0.3820883	0.291001	-1.31301345	0.189178	0.426959	NOT
RP11-400I	7.879086	-1.2537477	0.954868	-1.31300624	0.189181	0.426959	NOT
SLITRK3	41.23607	1.17149579	0.892318	1.312868089	0.189227	0.426973	NOT
REC8	189.8159	-0.4371335	0.332965	-1.3128511	0.189233	0.426973	NOT
TCEB1P19	6.608741	-0.3231504	0.246144	-1.31284824	0.189234	0.426973	NOT
RNMTL1	781.8601	-0.2862834	0.21808	-1.31274292	0.18927	0.427017	NOT
CTD-3092A	16.68894	-0.381773	0.290859	-1.31257024	0.189328	0.427113	NOT
LINC01101	7.762687	0.95521142	0.727776	1.312507908	0.189349	0.427122	NOT
AP2A1	6044.666	-0.1814577	0.138257	-1.31246498	0.189363	0.427122	NOT
SPHK2	990.2016	-0.271199	0.206645	-1.31239369	0.189387	0.427141	NOT
RP11-399C	20.65286	-0.4421332	0.336913	-1.31230578	0.189417	0.427172	NOT
CIR1	1351.548	0.1637144	0.124764	1.312187847	0.189457	0.427226	NOT
EPHB3	141.5074	0.61233953	0.466684	1.312106497	0.189484	0.427253	NOT
DCTN5	1786.19	0.2012827	0.153419	1.311976719	0.189528	0.427316	NOT
RP11-148K	37.61737	0.33487507	0.255301	1.311688503	0.189625	0.4275	NOT
GLUL	149361.7	-0.6724839	0.512818	-1.31134913	0.18974	0.427693	NOT
RP11-230C	14.10691	0.42181309	0.321668	1.311331738	0.189746	0.427693	NOT
RP11-114H	2.624186	-0.7538528	0.57491	-1.31125322	0.189772	0.427693	NOT
LINC00319	2.597667	-1.0648915	0.812138	-1.31121937	0.189784	0.427693	NOT
RP11-302I	5.143951	0.58036145	0.442618	1.311201214	0.18979	0.427693	NOT
APOD	112.0494	-0.5611861	0.42802	-1.31112031	0.189817	0.427719	NOT
RP1-80N2.	50.35477	0.42039071	0.320656	1.311033927	0.189846	0.427749	NOT

RP11-96H1	1.117794	1.73670969	1.324918	1.310805396	0.189924	NA	NA
PERP	7253.476	0.32290846	0.246344	1.310801053	0.189925	0.427891	NOT
VDAC3	3316.276	0.24634587	0.187959	1.31063666	0.189981	0.427981	NOT
SSX2B	0.874876	1.72233638	1.314136	1.310623044	0.189985	NA	NA
UBR1	793.4029	-0.2339433	0.17855	-1.31023767	0.190115	0.428249	NOT
CICP14	93.33721	-0.3985697	0.304211	-1.31017692	0.190136	0.42826	NOT
RPS20P14	49.92022	0.41159962	0.314174	1.310102213	0.190161	0.428281	NOT
AC004870.	2.093299	1.60287853	1.223586	1.309984457	0.190201	0.428326	NOT
NECAP2	2110.256	0.18036695	0.13769	1.309950202	0.190213	0.428326	NOT
LINC01587	20.09874	0.87866574	0.670853	1.309773221	0.190273	0.428394	NOT
RP11-166F	126.3883	-0.3522098	0.26891	-1.30976784	0.190274	0.428394	NOT
NCOA4	13062.74	-0.2449765	0.187059	-1.30962289	0.190323	0.428469	NOT
KCNH5	1.960955	1.50504322	1.14935	1.309472804	0.190374	0.428533	NOT
AC003088.	1.925623	-0.8906719	0.68019	-1.30944485	0.190384	0.428533	NOT
RP11-456F	24.70659	0.38812305	0.296417	1.309382083	0.190405	0.428545	NOT
RP11-481(	38.78069	-0.233698	0.178488	-1.30932405	0.190425	0.428554	NOT
NKRF	435.0654	0.160963	0.122953	1.30914512	0.190485	0.428614	NOT
RP11-5407	54.22815	0.60445074	0.461727	1.309109359	0.190497	0.428614	NOT
ABCB11	2044.073	-0.8125203	0.620668	-1.30910505	0.190499	0.428614	NOT
RP11-1017	4.95993	0.46157107	0.352635	1.308918732	0.190562	0.428697	NOT
FAM57A	292.7746	0.4138827	0.316206	1.308903025	0.190567	0.428697	NOT
ORM1	100427.8	-0.5489111	0.419392	-1.30882587	0.190593	0.42872	NOT
DNAJB4	871.7839	0.36619954	0.279812	1.308735354	0.190624	0.428754	NOT
RNF148	4.855315	0.54898938	0.419498	1.308681983	0.190642	0.428759	NOT
MALL	36.72812	-0.5753978	0.439721	-1.30855082	0.190687	0.428823	NOT
CTD-2630F	44.59085	-0.2778168	0.212334	-1.30839705	0.190739	0.428905	NOT
AC008074.	3.100868	0.57032829	0.43596	1.30821156	0.190802	0.429008	NOT
SLC6A4	19.24807	0.69026802	0.52766	1.308168897	0.190816	0.429008	NOT
NDUFB8	2207.285	-0.3429347	0.262175	-1.30803725	0.190861	0.429073	NOT
ITGA9-AS1	107.2213	0.30450324	0.232837	1.307795681	0.190943	0.429221	NOT
HMGN1P37	9.88755	0.36268856	0.277352	1.307682481	0.190981	0.429272	NOT
EVI5	1027.129	-0.2307826	0.176493	-1.3076035	0.191008	0.429297	NOT
BIRC2	2158.462	-0.1679576	0.128455	-1.30751859	0.191037	0.429314	NOT
MNX1	22.70852	-1.1885695	0.909048	-1.30748807	0.191047	0.429314	NOT
KIAA1875	112.3643	0.45259879	0.34619	1.307371708	0.191087	0.429367	NOT
RNU2-11P	1.833463	0.68107728	0.520998	1.307254592	0.191126	0.429421	NOT
L3MBTL4-A	8.014752	0.5049714	0.386338	1.307070382	0.191189	0.429526	NOT
NRK	13.55506	-0.7587121	0.58049	-1.30701996	0.191206	0.429529	NOT
SNAI2	659.6944	0.53807947	0.411819	1.30659236	0.191351	0.429817	NOT
ZNF469	93.12746	-0.43059	0.329563	-1.30654848	0.191366	0.429817	NOT
RP11-46H1	3.765606	-0.4329806	0.331424	-1.30642398	0.191408	0.429838	NOT
C1orf158	2.694539	1.31827888	1.009076	1.306421211	0.191409	0.429838	NOT
AP000350.	3.579755	-0.620934	0.475308	-1.30638192	0.191423	0.429838	NOT
ZNF669	144.2007	0.33742687	0.258335	1.30615837	0.191499	0.429973	NOT
ZNF670	113.4835	0.35765364	0.27385	1.306021927	0.191545	0.430041	NOT
AC005355.	177.4754	-0.5122511	0.392259	-1.3058988	0.191587	0.4301	NOT
LIN7A	1702.162	0.35741241	0.273738	1.305672103	0.191664	0.430208	NOT
MRAS	1014.935	-0.4392776	0.33644	-1.30566429	0.191667	0.430208	NOT
RPS15AP1	45.33103	0.36552715	0.280037	1.30528276	0.191797	0.43045	NOT

OR2A13P	1.431857	0.92398235	0.707895	1.30525375	0.191806	0.43045	NOT
NDUFV2	1011.596	-0.2847024	0.218132	-1.30518516	0.19183	0.430467	NOT
TSPAN3	3889.53	0.26857916	0.205795	1.305080533	0.191865	0.430511	NOT
AL162151.	15.90024	0.52804334	0.404633	1.304991955	0.191896	0.430543	NOT
WNK2	960.199	0.88999429	0.682023	1.304933224	0.191916	0.43055	NOT
MIR4783	6.188911	-0.5850495	0.448369	-1.30484082	0.191947	0.43055	NOT
CCNI	6151.298	-0.2136548	0.163741	-1.30483121	0.19195	0.43055	NOT
RP11-351A	3.291568	-0.7403064	0.567383	-1.30477334	0.19197	0.43055	NOT
RP11-443C	3.836286	-0.6374763	0.488581	-1.30475042	0.191978	0.43055	NOT
RP11-2N1.	0.911529	-1.4022329	1.074991	-1.30441343	0.192093	NA	NA
MYBL1	239.499	0.28756829	0.220459	1.304407195	0.192095	0.430704	NOT
BTN2A1	821.6387	0.20287428	0.155536	1.304358856	0.192111	0.430704	NOT
RP11-156K	233.1358	-0.6274548	0.481045	-1.30435676	0.192112	0.430704	NOT
RAB3IP	925.2689	0.28160765	0.215901	1.304338659	0.192118	0.430704	NOT
RP11-317N	24.42247	1.4559155	1.11623	1.3043152	0.192126	0.430704	NOT
RP11-546I	181.9394	0.25935741	0.198854	1.30425747	0.192146	0.430713	NOT
RP11-672A	13.75524	0.81807408	0.627375	1.303963168	0.192246	0.430902	NOT
KIF9	175.6521	0.24151168	0.185228	1.303860072	0.192281	0.430945	NOT
MUM1	1892.239	-0.2186318	0.167692	-1.30377278	0.192311	0.430976	NOT
LINC0142C	468.163	0.36214491	0.277796	1.303638473	0.192357	0.431043	NOT
RP11-286E	2.536179	-0.6176893	0.473842	-1.30357567	0.192378	0.431056	NOT
NPIPB6	2.70465	-0.7254636	0.556582	-1.30342653	0.192429	0.431113	NOT
MYBBP1A	2121.06	0.24582532	0.188602	1.303407466	0.192436	0.431113	NOT
CYR61	2657.038	-0.4697674	0.360458	-1.30325221	0.192489	0.43117	NOT
SEC11A	4174.27	0.15178077	0.116464	1.303236515	0.192494	0.43117	NOT
FOXI2	3.870384	-0.6329759	0.485712	-1.30319291	0.192509	0.43117	NOT
SULT1C2P1	7.70487	0.69281525	0.531787	1.302805646	0.192641	0.431405	NOT
CEP76	244.1938	-0.2353583	0.180657	-1.30279331	0.192645	0.431405	NOT
GPR155	745.6534	-0.3699543	0.284069	-1.30233721	0.192801	0.431712	NOT
RP11-996F	12.14655	-0.3387459	0.260114	-1.30229891	0.192814	0.431712	NOT
RP11-522E	25.31004	-0.5114973	0.3928	-1.30218362	0.192854	0.431735	NOT
GATA2	150.9866	-0.4424068	0.339745	-1.30217476	0.192857	0.431735	NOT
SMAP2	4000.093	-0.1979112	0.151999	-1.3020546	0.192898	0.431792	NOT
RNU6-485F	4.078958	-0.7689449	0.590588	-1.30199956	0.192917	0.431798	NOT
RPL7P9	401.0141	0.47290675	0.363281	1.301764043	0.192997	0.431943	NOT
XXbac-B44	11.00613	-0.3890116	0.298852	-1.30168732	0.193023	0.431966	NOT
CELSR3	385.6225	0.44886629	0.344884	1.301500443	0.193087	0.432073	NOT
ZFYVE19	1620.657	0.25173423	0.193426	1.301446757	0.193106	0.432079	NOT
RP11-44N1	5.504784	0.95511134	0.733965	1.301303825	0.193154	0.432152	NOT
PIGCP1	43.05258	-0.3871893	0.297559	-1.30122036	0.193183	0.432181	NOT
KRT18P15	2.818352	-0.5319963	0.408873	-1.30112946	0.193214	0.432215	NOT
AL590762.	3.302624	0.4395719	0.337853	1.301074042	0.193233	0.432218	NOT
HS1BP3-IT	154.7989	0.60031668	0.461416	1.301032538	0.193247	0.432218	NOT
ACSM3	1262.528	-0.5276346	0.405647	-1.30072228	0.193354	0.43242	NOT
STX1B	237.2965	-0.4777659	0.36739	-1.30043297	0.193453	0.432605	NOT
SLC25A27	187.812	-0.4875622	0.374978	-1.30024371	0.193517	0.432715	NOT
ZNF688	473.9525	-0.2598689	0.199899	-1.30000235	0.1936	0.432864	NOT
RP11-532I	1.765737	0.81937167	0.630322	1.299924973	0.193627	0.432888	NOT
RP11-361M	2.375136	-0.7175726	0.552057	-1.29981701	0.193664	0.432935	NOT

FZD1	471.2412	0.42589846	0.327685	1.299720259	0.193697	0.432965	NOT
CYP4A22-A	5.941218	0.45815398	0.352516	1.299667808	0.193715	0.432965	NOT
HLA-W	10.27259	-0.506064	0.389388	-1.29963816	0.193725	0.432965	NOT
TEKT2	5.853844	0.64151608	0.493661	1.299508299	0.19377	0.433029	NOT
RP11-261F	3.958918	0.60936113	0.46896	1.299389541	0.19381	0.433084	NOT
FOXN4	182.2488	-0.8261795	0.635894	-1.29924002	0.193862	0.433129	NOT
RP11-274E	31.09577	-0.516799	0.397771	-1.29923827	0.193862	0.433129	NOT
UNC45B	3.928591	-0.4253196	0.327385	-1.2991422	0.193895	0.433159	NOT
MUSTN1	9.032367	0.68995056	0.531097	1.299105055	0.193908	0.433159	NOT
CDK17	880.6937	-0.1938383	0.149237	-1.29886215	0.193991	0.433295	NOT
VIMP	2843.898	0.21498242	0.165519	1.29883462	0.194001	0.433295	NOT
ALX3	12.26365	1.4824312	1.141417	1.298763554	0.194025	0.433314	NOT
KCNC3	309.8429	0.38999438	0.300351	1.298462392	0.194128	0.433509	NOT
ERCC1	1925.653	-0.2285321	0.176037	-1.29820279	0.194218	0.433673	NOT
FGFBP3	50.03066	-0.355941	0.274201	-1.29810409	0.194252	0.433713	NOT
CTLA4	56.22369	0.61336627	0.47253	1.298047856	0.194271	0.43372	NOT
TMEM8A	4333.771	0.18553269	0.142941	1.297967963	0.194298	0.433746	NOT
DNAJC5G	3.322459	0.78970047	0.608472	1.297842672	0.194341	0.433806	NOT
EEF1A1P19	72.72224	0.33399117	0.257361	1.29775268	0.194372	0.43384	NOT
AC009245.	15.72729	0.44133915	0.340106	1.297653384	0.194406	0.43388	NOT
NAF1	253.9172	-0.1988538	0.153255	-1.29753313	0.194448	0.43388	NOT
CTD-2527I	2.101416	0.90863664	0.7003	1.297495987	0.194461	0.43388	NOT
PRR34	14.99427	0.39229484	0.302348	1.29749587	0.194461	0.43388	NOT
OTOA	27.28059	-0.426321	0.328583	-1.29745456	0.194475	0.43388	NOT
RP11-977C	121.2442	0.29169857	0.224829	1.297421631	0.194486	0.43388	NOT
RP11-47A1	4.550055	0.57263108	0.441424	1.297235685	0.19455	0.433987	NOT
RIOK3	3410.562	-0.1965188	0.151521	-1.2969757	0.19464	0.43415	NOT
GOLGA4	4490.953	-0.2511645	0.193664	-1.29690729	0.194663	0.434167	NOT
MYL4	15.58724	0.4290564	0.330853	1.296820256	0.194693	0.434198	NOT
EDN3	3.495573	1.28596229	0.991782	1.29661757	0.194763	0.434293	NOT
CHST5	3.516881	1.00275368	0.773369	1.29660369	0.194768	0.434293	NOT
GJA3	8.384051	0.81432036	0.628209	1.296256261	0.194887	0.434524	NOT
SNAPC2	637.2085	0.21690825	0.167373	1.295957138	0.19499	0.434718	NOT
SEPT7P9	5.51604	-0.465887	0.359521	-1.29585617	0.195025	0.43476	NOT
GACAT2	1.465138	1.38043803	1.065482	1.29559925	0.195114	0.434893	NOT
RP11-227H	4.947547	0.70005594	0.54034	1.295584524	0.195119	0.434893	NOT
PPAPDC1B	1145.377	0.32239101	0.248846	1.295543684	0.195133	0.434893	NOT
MAGEA6	488.378	1.78752869	1.379969	1.295339846	0.195203	0.435014	NOT
GPC1	3496.6	0.36118126	0.278864	1.29518663	0.195256	0.435096	NOT
RP11-690I	2.356273	-0.6007579	0.463913	-1.29497822	0.195328	0.43522	NOT
RP11-269C	1.853264	1.05689938	0.816204	1.294895836	0.195356	0.435248	NOT
RNASE10	2.163198	0.63026133	0.48683	1.294623194	0.19545	0.435422	NOT
AP1S3	88.62122	0.35091322	0.271069	1.294553851	0.195474	0.435439	NOT
PI4KB	3510.006	0.19962241	0.154218	1.294413156	0.195523	0.435512	NOT
SPOCK1	157.2878	0.73124375	0.565101	1.294004354	0.195664	0.43579	NOT
HSPB3	1.764568	1.47373201	1.138972	1.293914183	0.195695	0.435824	NOT
ASB13	2798.737	0.33841281	0.261607	1.293592073	0.195806	0.436036	NOT
DNAJC1	2568.396	0.25741876	0.199013	1.293476586	0.195846	0.436063	NOT
OPTN	6531.499	0.23548439	0.182057	1.293463625	0.195851	0.436063	NOT

ZNF736	266.8362	0.36352486	0.281075	1.29333527	0.195895	0.436099	NOT
TBC1D2	727.9208	-0.2806206	0.216976	-1.2933237	0.195899	0.436099	NOT
TCHP	691.293	0.1725547	0.133427	1.293250229	0.195925	0.43612	NOT
HNF4A	14948.34	-0.386554	0.298938	-1.29309243	0.195979	0.436206	NOT
CTD-2583A	4.597173	-0.5273206	0.407827	-1.29300002	0.196011	0.436211	NOT
TDGF1P3	4.053523	1.37706362	1.065021	1.292992315	0.196014	0.436211	NOT
CLN8	692.3742	-0.299475	0.231645	-1.29281631	0.196075	0.436297	NOT
RP11-600F	44.88933	0.37289091	0.288445	1.292760918	0.196094	0.436297	NOT
STYX	1412.727	-0.2232974	0.172734	-1.29272053	0.196108	0.436297	NOT
AC015849.	23.2828	-0.6143102	0.475217	-1.29269449	0.196117	0.436297	NOT
TCEAL4	3151.974	-0.2133232	0.16503	-1.29263085	0.196139	0.436311	NOT
AC144833.	3.855495	1.63172976	1.262641	1.292315206	0.196248	0.436468	NOT
RP11-9D8.	2.2018	-0.6224547	0.481666	-1.29229557	0.196255	0.436468	NOT
CRADD	904.5566	-0.300392	0.23245	-1.29228685	0.196258	0.436468	NOT
GTF2H3	1064.265	0.18712927	0.144821	1.29214396	0.196307	0.436489	NOT
AC110615.	1.877953	-0.5041689	0.390184	-1.29212974	0.196312	0.436489	NOT
USP54	390.6973	0.41547248	0.321543	1.292120285	0.196315	0.436489	NOT
CCZ1B	191.3732	0.26706236	0.206704	1.292005413	0.196355	0.436494	NOT
FRZB	435.8976	-0.4275619	0.330935	-1.29198194	0.196363	0.436494	NOT
RSBN1	511.6254	-0.1956579	0.151448	-1.29191678	0.196386	0.436494	NOT
CDH6	508.7397	0.55491347	0.429528	1.291914398	0.196387	0.436494	NOT
AC009404.	69.82751	-0.3734384	0.289081	-1.29181071	0.196423	0.436494	NOT
C1orf100	3.604767	0.61103894	0.473019	1.291784927	0.196432	0.436494	NOT
PCCB	5540.331	-0.3112302	0.240935	-1.29175817	0.196441	0.436494	NOT
APCDD1L	5.100749	0.80105716	0.620137	1.291742511	0.196446	0.436494	NOT
TAP2	1578.013	0.35176975	0.272343	1.291641244	0.196481	0.436501	NOT
RP11-299(	237.6324	-0.498023	0.385575	-1.29163786	0.196483	0.436501	NOT
S100PBP	635.8573	0.1959092	0.15168	1.291593356	0.196498	0.436501	NOT
LINC00511	256.4662	0.63260725	0.489861	1.291400641	0.196565	0.436614	NOT
MRPL34	3074.729	-0.2670252	0.206821	-1.29109127	0.196672	0.436816	NOT
RNA5SP187	1.513015	-0.8249961	0.639037	-1.29099871	0.196704	0.436823	NOT
GDI2	11335.13	0.17396755	0.134755	1.290989228	0.196707	0.436823	NOT
LINC00243	2.611601	-0.5530369	0.428426	-1.29085876	0.196753	0.436888	NOT
RGSL1	41.75409	-1.0205916	0.790712	-1.29072468	0.196799	0.436899	NOT
GZF1	652.6608	-0.1363014	0.105603	-1.29070071	0.196807	0.436899	NOT
RP11-87H9	134.9358	0.24262402	0.187984	1.290662016	0.196821	0.436899	NOT
C16orf13	2027.992	-0.3211079	0.248794	-1.2906595	0.196822	0.436899	NOT
RPL7L1P1	2.514343	1.20382279	0.932771	1.290587417	0.196847	0.436918	NOT
AMBP	228260.2	-0.4897451	0.379503	-1.29048983	0.196881	0.436958	NOT
RP11-231I	3.18245	-0.5387012	0.417476	-1.29037563	0.19692	0.43699	NOT
MALT1	1089.008	0.23767503	0.184193	1.290355601	0.196927	0.43699	NOT
U91328.22	5.950875	0.45679171	0.354048	1.290195758	0.196983	0.437062	NOT
AC009120.	97.05482	-0.3661317	0.283795	-1.29012714	0.197007	0.437062	NOT
METAP2	3266.679	0.14748293	0.114317	1.290122269	0.197008	0.437062	NOT
HEXB	8325.341	0.25613692	0.198564	1.289944106	0.19707	0.437148	NOT
RP11-501(	1.704202	1.64710385	1.276906	1.289918239	0.197079	0.437148	NOT
RP1-225E1	1.440213	-0.6040243	0.468328	-1.28974704	0.197138	0.437244	NOT
CTD-2527I	17.80441	-0.7140765	0.55369	-1.28966736	0.197166	0.43725	NOT
RP11-864J	4.162617	-0.359115	0.27846	-1.28964606	0.197174	0.43725	NOT

RFX7	536.9699	-0.2167905	0.168107	-1.28960037	0.197189	0.43725	NOT
PCDHGB5	116.4017	0.59752103	0.463362	1.289534803	0.197212	0.437264	NOT
RYR3	34.15764	0.38381477	0.297694	1.289294303	0.197296	0.437414	NOT
UTRN	2869.131	0.29680574	0.230224	1.289201782	0.197328	0.43745	NOT
RP5-1050I	2.440552	-0.588182	0.45627	-1.28910984	0.19736	0.437485	NOT
RP11-309I	36.29496	0.45216139	0.350798	1.28894922	0.197416	0.437573	NOT
RP11-497I	2.489694	-0.783053	0.607576	-1.28881416	0.197463	0.437627	NOT
TIAF1	109.5496	-0.3637433	0.282237	-1.28878632	0.197472	0.437627	NOT
CTD-2302I	2.423139	0.5084834	0.394616	1.288550821	0.197554	0.437739	NOT
LRCOL1	220.998	-0.7896134	0.612793	-1.28854812	0.197555	0.437739	NOT
COX10	621.7275	-0.2584073	0.200562	-1.28841773	0.197601	0.437804	NOT
CCDC40	193.7475	0.37729367	0.292894	1.288159029	0.197691	0.437915	NOT
MYL12B	14706.55	0.19743092	0.153269	1.288134495	0.197699	0.437915	NOT
DCBLD1	602.8959	-0.2453793	0.190492	-1.28813335	0.1977	0.437915	NOT
ATF4P3	8.272231	0.48894442	0.37963	1.287949295	0.197764	0.438022	NOT
HYKK	219.2198	-0.2600157	0.201894	-1.28788175	0.197787	0.438038	NOT
RP1-179N1	2.186689	-0.5972494	0.463763	-1.28783341	0.197804	0.438039	NOT
RP11-23F2	14.84158	-0.6738857	0.523318	-1.28771667	0.197845	0.438094	NOT
CCKBR	1.343472	1.28243983	0.996195	1.28733795	0.197977	NA	NA
ELDR	2.459001	0.83061688	0.645254	1.287271093	0.198	0.438402	NOT
PIGHP1	4.238847	-0.4930466	0.383091	-1.28702057	0.198087	0.438538	NOT
CTD-2649C	4.154844	-0.5097114	0.396046	-1.28700131	0.198094	0.438538	NOT
MIA	2.372121	0.66707379	0.518339	1.28694621	0.198113	0.438545	NOT
KIAA2022	4.375141	0.8427551	0.654949	1.286750129	0.198181	0.438626	NOT
WDFY3	977.6749	-0.3014616	0.234282	-1.28674777	0.198182	0.438626	NOT
RPL7P8	1.725513	0.68789182	0.53465	1.286621496	0.198226	0.438688	NOT
CBX5	3248.224	-0.2756812	0.214288	-1.28649617	0.19827	0.438724	NOT
MNX1-AS2	3.60459	-1.5098024	1.17359	-1.28648203	0.198275	0.438724	NOT
DACT3-AS1	2.810308	-0.5021714	0.390388	-1.28634061	0.198324	0.438782	NOT
MSX1	126.0022	-0.4469166	0.347443	-1.28630227	0.198338	0.438782	NOT
RP11-102C	102.1691	-0.2073957	0.161238	-1.28626756	0.19835	0.438782	NOT
TAS2R4	8.992869	0.45706718	0.355363	1.286197874	0.198374	0.4388	NOT
TUNAR	0.793627	-0.9506552	0.739257	-1.28595981	0.198457	NA	NA
LHFPL2	914.5695	0.39726929	0.308931	1.285947173	0.198461	0.438958	NOT
LINC00167	3.445568	-0.5243015	0.407758	-1.28581542	0.198507	0.438989	NOT
RP11-274I	13.51193	-0.5113912	0.397718	-1.28581387	0.198508	0.438989	NOT
RP11-683I	5.119999	0.99266437	0.772153	1.285580239	0.19859	0.439134	NOT
DUSP5	1026.313	0.47725642	0.371339	1.285231309	0.198711	0.439346	NOT
BIN3	336.0721	0.27061632	0.210562	1.285212593	0.198718	0.439346	NOT
RP11-199I	30.7132	0.40890746	0.31818	1.28514491	0.198742	0.439363	NOT
RP11-5407	1.980429	1.28515296	1.000127	1.28498946	0.198796	0.439411	NOT
ERV3-1	210.6381	-0.3570956	0.277904	-1.28496076	0.198806	0.439411	NOT
CTBP1-AS2	766.6515	-0.176543	0.137394	-1.28494338	0.198812	0.439411	NOT
AC127904.	7.813753	-0.4325926	0.336695	-1.28481894	0.198856	0.439471	NOT
SNHG23	1.957287	1.00871309	0.78523	1.284609175	0.198929	0.439598	NOT
GNG4	1144.145	0.98708022	0.768637	1.284195298	0.199074	0.439882	NOT
PGPEP1	3392.814	-0.2992846	0.233069	-1.28410524	0.199105	0.439915	NOT
PPARD	1838.452	0.21914552	0.170668	1.284043155	0.199127	0.439928	NOT
CTC-524C5	78.41961	-0.3832082	0.298467	-1.28392338	0.199169	0.439984	NOT

EZR	4955.978	-0.3372904	0.262722	-1.2838325	0.199201	0.440019	NOT
SCARA5	51.1021	-0.8535915	0.664927	-1.28373688	0.199234	0.440037	NOT
SLAMF7	327.9547	0.56330486	0.438808	1.283715904	0.199241	0.440037	NOT
RNU6-1189	18.61345	-0.8276564	0.644787	-1.2836121	0.199278	0.440052	NOT
BRK1	5086.355	0.18773682	0.146257	1.283604778	0.19928	0.440052	NOT
CH17-140K	1.320835	-0.8662956	0.6749	-1.28359167	0.199285	NA	NA
RALGAPA2	3621.302	-0.295963	0.230584	-1.28353812	0.199304	0.440064	NOT
SAP30L-AS	9.054435	0.30612399	0.238508	1.283496007	0.199318	0.440064	NOT
RP11-893F	7.538707	0.41462313	0.323065	1.283406258	0.19935	0.440098	NOT
GPANK1	1383.031	0.23545698	0.183478	1.283296648	0.199388	0.440147	NOT
LINC01545	10.99742	0.37824104	0.294765	1.283195043	0.199424	0.440167	NOT
SRFBP1	350.1987	0.23268031	0.181331	1.283177856	0.19943	0.440167	NOT
CYP7B1	420.859	-0.4785458	0.372973	-1.28305771	0.199472	0.440198	NOT
LINC01091	35.26173	0.63265366	0.493123	1.282954222	0.199508	0.440198	NOT
DHX16	2296.21	0.14855596	0.115794	1.282930947	0.199516	0.440198	NOT
RPS26P6	3.886571	0.49452831	0.385468	1.282930557	0.199516	0.440198	NOT
PRSS3P2	8.22366	1.05238381	0.820312	1.282906275	0.199525	0.440198	NOT
ID3	1043.685	-0.347467	0.270859	-1.28283511	0.19955	0.440217	NOT
HPCA	4.666597	0.75796784	0.590928	1.282673267	0.199607	0.440306	NOT
HLF	4294.361	-0.5982705	0.466473	-1.28253996	0.199653	0.440373	NOT
RP11-861E	3.737678	0.67699913	0.527912	1.282409566	0.199699	0.440439	NOT
RP4-753F5	1.705188	-0.707463	0.551713	-1.28230305	0.199736	0.440485	NOT
RP11-318E	7.326496	0.39921011	0.311379	1.282072516	0.199817	0.440628	NOT
SNORA53	2.825758	-0.4545648	0.354581	-1.28197602	0.199851	0.440666	NOT
FUT1	210.2664	0.44236145	0.345115	1.281780844	0.19992	0.440782	NOT
UCKL1	1860.01	0.21448589	0.167365	1.281547672	0.200001	0.440926	NOT
CNTN2	12.50602	-0.6205468	0.484355	-1.28118183	0.20013	0.441173	NOT
TSC22D1-A	8.230696	-0.4722831	0.368672	-1.28103897	0.20018	0.441248	NOT
SMPD3	105.3321	-0.5092251	0.397607	-1.28072571	0.20029	0.441447	NOT
AC012531.	1.188432	-1.4006576	1.093673	-1.28069108	0.200302	NA	NA
RGS1	1066.473	0.55111469	0.430336	1.280660083	0.200313	0.441447	NOT
METTL23	1369.512	0.22575142	0.17628	1.280643484	0.200319	0.441447	NOT
LRRC37B	158.0004	0.24812695	0.193788	1.2804064	0.200402	0.441595	NOT
RP11-148E	3.679434	1.32123906	1.032003	1.28026694	0.200451	0.441667	NOT
GOLGA8B	1015.909	0.3938971	0.307718	1.280059421	0.200524	0.441792	NOT
RP11-638I	2.534155	-0.611338	0.477612	-1.27998747	0.20055	0.441812	NOT
RP11-598F	19.61054	-0.4483758	0.350326	-1.2798805	0.200587	0.441859	NOT
HAL	5247.335	-0.6945379	0.542695	-1.27979311	0.200618	0.44189	NOT
DNAJC24	267.2835	-0.2448867	0.191371	-1.2796408	0.200671	0.441931	NOT
GLIPR2	418.3903	0.38395593	0.300051	1.279634	0.200674	0.441931	NOT
SIT1	60.48784	0.52227787	0.408157	1.279601947	0.200685	0.441931	NOT
RP11-26J3	6.556028	0.40979435	0.320275	1.279508574	0.200718	0.441967	NOT
CDNF	116.6428	-0.3682246	0.287805	-1.27942204	0.200748	0.441983	NOT
RP11-213H	9.801144	-0.6722537	0.525458	-1.27936719	0.200768	0.441983	NOT
RP13-104F	90.44657	-0.320755	0.250723	-1.27932069	0.200784	0.441983	NOT
AC009506.	74.84883	-0.2751704	0.215094	-1.2793036	0.20079	0.441983	NOT
AC159540.	7.863651	0.63266382	0.494607	1.279124187	0.200853	0.442086	NOT
RNF213	11330.23	-0.2837484	0.221855	-1.27898329	0.200903	0.442159	NOT
CTD-2287C	273.1444	0.37700317	0.294821	1.278754521	0.200984	0.442301	NOT

HKR1	835.503	-0.2910385	0.227607	-1.27868636	0.201008	0.442318	NOT
RRN3P1	121.5568	-0.3609228	0.282277	-1.27861109	0.201034	0.44234	NOT
RP1-92014	18.27037	-0.4431854	0.346632	-1.27854724	0.201057	0.442347	NOT
CCDC92	1839.545	-0.234473	0.183396	-1.27850995	0.20107	0.442347	NOT
RPL7P32	10.06932	0.49341573	0.385944	1.278463184	0.201086	0.442347	NOT
RP11-363J	2.200511	0.64847428	0.507259	1.278387875	0.201113	0.44237	NOT
RAMP2-AS1	53.19934	0.54821362	0.428879	1.278249162	0.201162	0.442441	NOT
PRNP	2417.78	0.3621471	0.283383	1.277940834	0.20127	0.442614	NOT
ZFP92	106.4436	0.62761882	0.49112	1.277933456	0.201273	0.442614	NOT
AC005076.	27.33812	-0.3335655	0.261051	-1.27777898	0.201327	0.442698	NOT
RP11-364E	9.384899	0.81360257	0.636799	1.277645168	0.201375	0.442748	NOT
LRRC8D	2009.711	0.21911949	0.171507	1.277610371	0.201387	0.442748	NOT
AB015752.	3.424901	0.6291373	0.492461	1.277537612	0.201413	0.442748	NOT
CDC42EP3	480.5858	-0.3441399	0.269379	-1.27753029	0.201415	0.442748	NOT
RP11-555K	1.30156	2.322074	1.817688	1.277487338	0.20143	NA	NA
CHRNA4	1257.199	-0.9982392	0.78146	-1.27740313	0.20146	0.442803	NOT
CTD-2207F	4.059783	0.61718285	0.483168	1.277366288	0.201473	0.442803	NOT
RASA3	635.0305	0.39403617	0.308497	1.277278228	0.201504	0.442836	NOT
KMT2C	2237.018	-0.2660032	0.208281	-1.27713734	0.201554	0.442909	NOT
STARD4-AS	77.83929	-0.440348	0.344835	-1.27698358	0.201608	0.442987	NOT
RP11-673E	2.12013	-0.5615198	0.439747	-1.27691645	0.201632	0.442987	NOT
SLC17A1	804.4491	0.53730321	0.420788	1.276897616	0.201638	0.442987	NOT
RP3-508I1	2.745557	-0.4250979	0.332967	-1.27669618	0.20171	0.443108	NOT
RP11-369E	0.781185	-1.4900808	1.167274	-1.27654801	0.201762	NA	NA
RP11-192F	1.244141	-0.6471484	0.506996	-1.27643589	0.201801	NA	NA
SLTM	2700.501	-0.1343864	0.105292	-1.27631861	0.201843	0.443365	NOT
HS3ST3B1	1317.941	-0.4650924	0.364509	-1.27594149	0.201976	0.443612	NOT
THRB-AS1	33.17152	-0.3654911	0.286456	-1.27590723	0.201988	0.443612	NOT
IL36RN	0.886331	1.29200322	1.01265	1.275863486	0.202004	NA	NA
HCG4P7	118.0954	-0.4764215	0.373437	-1.2757746	0.202035	0.443672	NOT
RP11-744D	15.7826	-0.4328055	0.339265	-1.27571501	0.202056	0.443672	NOT
GS1-124K5	146.9271	0.22423588	0.175776	1.275692225	0.202064	0.443672	NOT
PRKRIRP7	5.148084	-0.5381962	0.421928	-1.27556365	0.20211	0.443735	NOT
GDF11	331.4571	0.49687063	0.389565	1.275450544	0.20215	0.443756	NOT
RBMS3-AS3	1.804424	-0.631249	0.494925	-1.27544449	0.202152	0.443756	NOT
URB2	528.0253	0.22463769	0.176186	1.274999787	0.202309	0.444006	NOT
HNRNPLP2	18.95084	0.40474405	0.31745	1.274984486	0.202315	0.444006	NOT
PPY2	3.407449	2.07815979	1.62995	1.2749841	0.202315	0.444006	NOT
RP1-170O1	0.892708	1.72838094	1.355757	1.274845819	0.202364	NA	NA
SCART1	75.83267	-0.4827744	0.378711	-1.27478337	0.202386	0.444126	NOT
NLRP9	2.173449	0.88474567	0.69409	1.274683986	0.202421	0.444167	NOT
PIWIL2	92.39567	-0.5094086	0.399656	-1.27461764	0.202445	0.444183	NOT
EEF1A1P16	10.12088	0.42422776	0.332851	1.274526952	0.202477	0.444218	NOT
RP13-516M	113.0031	-0.2947237	0.231275	-1.27434202	0.202542	0.444325	NOT
ARF6	4439.864	0.18138078	0.142368	1.274031938	0.202652	0.44453	NOT
RP11-343I	19.95116	0.35354661	0.277517	1.273961622	0.202677	0.444549	NOT
SERINC1	8165.582	-0.2382715	0.187054	-1.27381239	0.20273	0.444629	NOT
ZNF732	5.390876	0.65509718	0.514369	1.273593183	0.202808	0.444764	NOT
CALM2	14452.26	0.16336425	0.128293	1.273369319	0.202887	0.444902	NOT

RPSAP15	7.704974	0.44717224	0.351214	1.273218065	0.202941	0.444967	NOT
RP11-545M	6.268868	-0.3693555	0.290102	-1.2731931	0.20295	0.444967	NOT
AC002463.	1.01205	2.14442853	1.684623	1.272942484	0.203038	NA	NA
FABP2	5.007385	-1.0815325	0.849652	-1.27291245	0.203049	0.445149	NOT
NFX1	2111.351	-0.1869816	0.146952	-1.27239973	0.203231	0.445512	NOT
RNU6-833F	1.358314	-0.7001231	0.550285	-1.27229262	0.203269	NA	NA
RP11-334C	206.0346	0.35148329	0.276362	1.271821587	0.203437	0.445927	NOT
NKAP	1114.562	0.19156893	0.150639	1.271706393	0.203477	0.44598	NOT
LINC01073	2.193585	0.7096841	0.558165	1.271460281	0.203565	0.446136	NOT
LPPR3	4.967664	-0.7616907	0.599123	-1.27134306	0.203607	0.446191	NOT
RP11-293A	15.29383	-0.4456343	0.350544	-1.27126586	0.203634	0.446215	NOT
KRT18P59	5.500365	-0.4339658	0.341389	-1.2711756	0.203666	0.44625	NOT
PBRM1	1680.573	0.19500123	0.153411	1.271101761	0.203692	0.446271	NOT
RP11-3304	69.49176	0.34286729	0.269773	1.27094941	0.203747	0.446354	NOT
DNAJC9-AS	4.971026	-0.4624205	0.363875	-1.27082085	0.203792	0.446418	NOT
C11orf94	3.987923	0.45101978	0.354944	1.270679216	0.203843	0.446492	NOT
RNF10	7266.385	0.13974572	0.109988	1.270552947	0.203888	0.446549	NOT
UBP1	2810.471	0.1717014	0.135143	1.270514261	0.203902	0.446549	NOT
FBXO27	743.3479	0.53255377	0.419203	1.27039648	0.203943	0.446571	NOT
GATM	42757.39	-0.4434725	0.349083	-1.27039371	0.203944	0.446571	NOT
C2orf81	65.58029	0.38104467	0.299964	1.270300719	0.203978	0.446575	NOT
BBOX1	701.1422	-0.7500242	0.590433	-1.27029576	0.203979	0.446575	NOT
LINC01214	10.86156	0.58764247	0.46265	1.270165266	0.204026	0.446641	NOT
LRRC37A15	5.066448	-0.4833202	0.380589	-1.26992828	0.20411	0.446768	NOT
ST7-AS1	43.94365	-0.3106698	0.244639	-1.26990926	0.204117	0.446768	NOT
MMAB	4076.366	-0.3564476	0.280775	-1.26951165	0.204259	0.447042	NOT
CTSB	52327.67	0.28101949	0.221382	1.269388896	0.204302	0.447102	NOT
CLPB	1094.155	-0.2608207	0.205495	-1.269231	0.204359	0.447189	NOT
CTD-2323K	12.91458	0.30065018	0.236898	1.269112042	0.204401	0.447246	NOT
KLK3	0.875786	2.09592437	1.651508	1.269097697	0.204406	NA	NA
ZNF300P1	56.9161	0.77157465	0.607996	1.269045514	0.204425	0.447262	NOT
ZNF789	307.2372	0.2400526	0.189211	1.268702762	0.204547	0.447481	NOT
BMP1	1775.349	-0.2678114	0.211099	-1.26865541	0.204564	0.447481	NOT
RP11-583F	4.738114	0.54758993	0.43164	1.268626291	0.204574	0.447481	NOT
CT55	2.84703	1.961186	1.546038	1.268523707	0.204611	0.447525	NOT
SH2B1	2016.535	-0.185744	0.146434	-1.26844737	0.204638	0.447547	NOT
GPR20	7.467621	-0.631245	0.497669	-1.26840214	0.204654	0.447547	NOT
RP11-652I	5.644582	-0.6201758	0.48896	-1.26835658	0.204671	0.447547	NOT
TNRC6C-AS	160.9182	0.51654556	0.407352	1.26805571	0.204778	0.447746	NOT
STRIP1	978.9398	0.23235345	0.183245	1.267992055	0.204801	0.447759	NOT
CELA2B	1.889839	-0.559502	0.441321	-1.26778909	0.204873	0.447856	NOT
RPS4XP16	14.42033	-0.3663643	0.289003	-1.26768302	0.204911	0.447856	NOT
CELF6	1.754338	-0.7125884	0.562135	-1.26764624	0.204924	0.447856	NOT
ACTG1P9	3.009027	0.65553122	0.517135	1.267621978	0.204933	0.447856	NOT
SMOC1	4033.037	-0.4192309	0.330724	-1.26761642	0.204935	0.447856	NOT
MYH10	2731.826	-0.3541549	0.279392	-1.2675908	0.204944	0.447856	NOT
LYST	937.5635	-0.2647087	0.208839	-1.2675236	0.204968	0.447873	NOT
MLLT1	2705.798	-0.1851692	0.146096	-1.26744627	0.204996	0.447897	NOT
TMEM253	8.36529	-0.3989487	0.314791	-1.26734362	0.205032	0.447941	NOT

SLC39A9	4523.548	-0.2123037	0.167527	-1.26728387	0.205054	0.447952	NOT
DDX23	4077.117	0.13935364	0.109976	1.267127445	0.20511	0.448038	NOT
GRM3	4.002021	0.985801	0.778031	1.267046427	0.205139	0.448039	NOT
SEMA4D	432.0864	0.39952974	0.315327	1.267032838	0.205144	0.448039	NOT
RP11-2C24	3.77347	0.52246604	0.412467	1.266685868	0.205268	0.448239	NOT
TCEA1P2	76.88575	0.40097967	0.316558	1.266684466	0.205268	0.448239	NOT
DOCK9	1211.313	-0.313173	0.247253	-1.26660842	0.205295	0.448263	NOT
ZNF799	150.6009	-0.178218	0.140714	-1.266531	0.205323	0.448287	NOT
FOLH1	567.9779	-0.488488	0.385787	-1.26621125	0.205437	0.448467	NOT
AC005264.	3.602444	-0.4689391	0.370357	-1.26618225	0.205448	0.448467	NOT
VRK3	1822.827	-0.1971877	0.155736	-1.26616224	0.205455	0.448467	NOT
PBXIP1	6561.518	-0.2350744	0.185678	-1.26603085	0.205502	0.448497	NOT
IMMP1L	400.0018	0.19597978	0.154802	1.26600543	0.205511	0.448497	NOT
KCNC1	10.57802	0.77015868	0.608391	1.265894643	0.205551	0.448497	NOT
TMEM133	242.5429	-0.2641872	0.208702	-1.2658559	0.205565	0.448497	NOT
LLGL2	4224.885	0.26337926	0.208071	1.265817236	0.205579	0.448497	NOT
CTD-2588I	2.025861	-0.6095027	0.481513	-1.26580855	0.205582	0.448497	NOT
CALR	72281.32	-0.2172825	0.171659	-1.26577999	0.205592	0.448497	NOT
POLR1B	1066.925	0.20564585	0.162469	1.265754018	0.205601	0.448497	NOT
SZT2	1348.811	-0.1988237	0.157088	-1.26568728	0.205625	0.448513	NOT
RWDD4P1	2.255563	-0.479986	0.379257	-1.2655956	0.205658	0.448549	NOT
ALDOA	28116.92	0.37268303	0.294539	1.265311014	0.20576	0.448735	NOT
RP11-37L2	17.47859	-0.388651	0.307249	-1.26493707	0.205894	0.448972	NOT
SDHAF1	711.0128	-0.2443705	0.193191	-1.26491517	0.205902	0.448972	NOT
RP11-709I	1.967901	-0.5777442	0.456801	-1.26476239	0.205957	0.449056	NOT
CYP4F11	6018.631	-0.5200528	0.411233	-1.26461707	0.206009	0.449133	NOT
PSMF1	7546.685	0.17657376	0.139632	1.264569431	0.206026	0.449134	NOT
CDKN1C	640.5783	0.68175978	0.539155	1.264497823	0.206051	0.449154	NOT
KCNAB2	1457.507	0.49192803	0.389062	1.264396391	0.206088	0.449198	NOT
RP11-95P1	2.431191	0.82280332	0.650841	1.264216154	0.206152	0.449302	NOT
EFCAB6	27.88677	-0.419072	0.331543	-1.26400599	0.206228	0.449431	NOT
MSL2	1084.423	-0.1469472	0.116292	-1.26360381	0.206372	0.449709	NOT
STK4-AS1	23.25829	0.34111763	0.269983	1.26347675	0.206418	0.449773	NOT
RP5-1057I	1.528252	-0.6743966	0.533828	-1.26332121	0.206474	0.449858	NOT
KLF16	1013.386	0.28134046	0.222715	1.263228326	0.206507	0.449878	NOT
CTD-2015H	166.447	0.29649264	0.234715	1.263201517	0.206517	0.449878	NOT
WDR72	1036.676	0.60603649	0.479779	1.263156991	0.206533	0.449878	NOT
TMEM138	1104.681	-0.1978444	0.156675	-1.26276771	0.206673	0.450147	NOT
GPR158	199.4995	0.70431417	0.557819	1.262621288	0.206725	0.450225	NOT
GJA5	404.622	-0.4276172	0.338705	-1.26250727	0.206766	0.450275	NOT
OGFRL1	588.0874	0.29022012	0.229884	1.262465196	0.206781	0.450275	NOT
IGLV2-11	61.07207	0.78201551	0.619483	1.262368527	0.206816	0.450287	NOT
B3GALTL	395.1862	-0.1971326	0.156167	-1.26232132	0.206833	0.450287	NOT
HMGCR	4044.016	0.33152668	0.262636	1.262303631	0.20684	0.450287	NOT
HK2	894.7178	0.69467788	0.550342	1.262265545	0.206853	0.450287	NOT
ELP6	989.0625	0.20211	0.160129	1.262167131	0.206889	0.450328	NOT
RP11-356(	30.56123	-0.612753	0.485525	-1.26204136	0.206934	0.45039	NOT
PUS3	925.9598	-0.2041844	0.161834	-1.26169057	0.20706	0.450547	NOT
NRG2	81.7462	-0.6306449	0.499843	-1.26168649	0.207062	0.450547	NOT

DNAJC21	2466.785	0.1682284	0.133337	1.261680462	0.207064	0.450547	NOT
TYW1B	183.501	-0.4346327	0.344494	-1.26165667	0.207072	0.450547	NOT
SLFN12L	24.0658	-0.5296769	0.419885	-1.26147937	0.207136	0.450604	NOT
DDX56	3797.283	0.20690724	0.164029	1.26140269	0.207164	0.450604	NOT
OXER1	1248.939	-0.4340098	0.344077	-1.2613749	0.207174	0.450604	NOT
SLC6A12	2403.332	-0.5673922	0.449839	-1.26132184	0.207193	0.450604	NOT
FBXL4	1100.047	-0.2344908	0.185909	-1.26131833	0.207194	0.450604	NOT
SLC34A3	10.34954	0.67060331	0.531673	1.261307483	0.207198	0.450604	NOT
RNF130	5586.496	-0.2061705	0.163465	-1.26125482	0.207217	0.450609	NOT
CTC-526N1	22.06526	-0.3407359	0.270168	-1.26120099	0.207236	0.450615	NOT
RP11-758H	90.38106	-0.3059561	0.242633	-1.26098351	0.207315	0.450668	NOT
NUDT4	1321.53	-0.2387328	0.189329	-1.26093828	0.207331	0.450668	NOT
RP11-255H	31.90629	-0.7319309	0.580477	-1.2609132	0.20734	0.450668	NOT
RP11-474N	2.248005	-0.5795991	0.459669	-1.26090649	0.207343	0.450668	NOT
TMEM39B	581.1717	0.19151027	0.151883	1.260902959	0.207344	0.450668	NOT
LINC00571	3.668035	-0.5223484	0.414286	-1.26084032	0.207366	0.450681	NOT
UBIAD1	1275.182	0.209765	0.166379	1.260768622	0.207392	0.450701	NOT
EME2	407.7789	-0.3014002	0.239082	-1.26065577	0.207433	0.450745	NOT
XXbac-BPC	1.674662	-0.9032649	0.716524	-1.26062063	0.207446	0.450745	NOT
TTPAL	1729.838	-0.3874886	0.307417	-1.26046751	0.207501	0.45082	NOT
RP11-2B6.	2.50651	-0.6430392	0.510173	-1.26043266	0.207513	0.45082	NOT
RP11-265N	6.154595	0.63170338	0.5013	1.260131428	0.207622	0.450968	NOT
EXOC3L4	1234.21	-0.5229772	0.415023	-1.26011747	0.207627	0.450968	NOT
TTC31	1782.024	-0.2034079	0.161421	-1.26010547	0.207631	0.450968	NOT
GPRC5C	7927.236	-0.2887862	0.22919	-1.26003011	0.207659	0.450976	NOT
UCN	64.44757	0.43419198	0.344596	1.260002226	0.207669	0.450976	NOT
CDC42SE2	2275.492	0.20235716	0.160609	1.259940041	0.207691	0.450989	NOT
EPHA3	272.9723	-0.5548939	0.440449	-1.2598365	0.207728	0.451002	NOT
SLC4A5	16.93536	0.45222962	0.35896	1.259831154	0.20773	0.451002	NOT
IP6K1	2347.456	0.15730169	0.124866	1.259761048	0.207756	0.451021	NOT
ATP2C1	3011.903	0.17327963	0.137561	1.259656447	0.207793	0.451067	NOT
AC093850.	9.407157	-0.6444584	0.511683	-1.25948645	0.207855	0.451145	NOT
PPP2R4	6341.606	-0.2393794	0.190064	-1.25946467	0.207863	0.451145	NOT
RP11-458F	12.72663	-0.5006721	0.397613	-1.25919439	0.20796	0.451302	NOT
RP11-513M	40.78714	0.23681303	0.188087	1.25906167	0.208008	0.451302	NOT
KYNU	2071.841	0.51129254	0.406096	1.259043277	0.208015	0.451302	NOT
RP11-346C	8.124627	-0.3235977	0.25702	-1.2590352	0.208018	0.451302	NOT
MGC16275	18.35273	-0.426695	0.338918	-1.25899083	0.208034	0.451302	NOT
MTFR1	2162.393	0.25227344	0.200384	1.258948036	0.208049	0.451302	NOT
RANBP9	1987.598	-0.1893626	0.150414	-1.25894221	0.208051	0.451302	NOT
RP11-704M	136.4926	0.5123278	0.406999	1.258794303	0.208105	0.451381	NOT
RAC2	795.3257	0.41150468	0.326944	1.258638692	0.208161	0.451467	NOT
CTC-203F4	14.16544	-0.3204897	0.25465	-1.25855113	0.208193	0.4515	NOT
CCDC67	5.519234	0.78173526	0.621227	1.258372071	0.208257	0.451604	NOT
MYBPC3	7.760952	-0.5096532	0.405108	-1.25806742	0.208367	0.451807	NOT
PDE6D	630.0576	0.15846329	0.125971	1.25793019	0.208417	0.451846	NOT
TMEM120B	568.2649	0.18714398	0.148772	1.257925029	0.208419	0.451846	NOT
APOBEC3F	209.2466	0.31692776	0.252004	1.257628189	0.208526	0.452043	NOT
SLC16A14	231.5568	-0.5418439	0.430874	-1.25754471	0.208556	0.452072	NOT

ANXA11	7005.298	0.20632238	0.164093	1.257350035	0.208627	0.452174	NOT
RP11-268J	37.11226	0.38769883	0.308363	1.257279795	0.208652	0.452174	NOT
RP11-341N	10.26934	0.54466848	0.433226	1.257239762	0.208667	0.452174	NOT
AC097721.	17.55585	0.2850987	0.226767	1.257230354	0.20867	0.452174	NOT
PCDHA8	0.934586	-1.0593054	0.842578	-1.25721934	0.208674	NA	NA
RP11-286H	12.20283	-0.5682651	0.452067	-1.25703787	0.20874	0.452289	NOT
ALOX12-AS	123.2861	-0.234829	0.186851	-1.25677343	0.208836	0.452461	NOT
AC004623.	8.638755	-0.4036299	0.321204	-1.25661646	0.208893	0.452515	NOT
CTD-2196E	16.85138	-0.3430838	0.273043	-1.25652135	0.208927	0.452515	NOT
SNORA66	4.860655	0.44594694	0.35491	1.256506121	0.208933	0.452515	NOT
CDK5RAP1	1202.227	0.14038468	0.111729	1.256470701	0.208945	0.452515	NOT
OR2A1-AS1	87.87536	0.45856499	0.364975	1.256429952	0.20896	0.452515	NOT
COLEC12	250.8173	0.64784169	0.515622	1.256428268	0.208961	0.452515	NOT
IGSF21	98.55569	-0.428986	0.341499	-1.25618386	0.209049	0.45267	NOT
XPNPEP3	1266.542	-0.2120004	0.168799	-1.25593419	0.20914	0.45283	NOT
AC116366.	41.53119	-0.4049724	0.322461	-1.25587961	0.20916	0.452831	NOT
UBE2G1	1653.462	-0.1961715	0.156211	-1.25581428	0.209183	0.452831	NOT
RP11-664H	1.906475	-1.2166582	0.968866	-1.25575503	0.209205	0.452831	NOT
DOPEY1	443.3831	-0.2948168	0.234775	-1.25574107	0.20921	0.452831	NOT
AC007743.	9.508188	-0.5132649	0.408755	-1.25567806	0.209233	0.452831	NOT
FO538757.	1.588976	-0.9091343	0.724031	-1.2556571	0.20924	0.452831	NOT
RP11-291I	22.05496	-0.350125	0.278861	-1.25555282	0.209278	0.452855	NOT
RP11-65I1	24.98054	0.73479923	0.585248	1.255534965	0.209285	0.452855	NOT
TRBV7-6	2.22973	0.7864733	0.626452	1.255440504	0.209319	0.452893	NOT
TIGD3	56.88397	0.46232718	0.368295	1.255317845	0.209363	0.452945	NOT
DBET	2.993004	0.8302206	0.661382	1.255281455	0.209377	0.452945	NOT
DBH-AS1	761.1199	-0.4880999	0.388871	-1.25517296	0.209416	0.452994	NOT
MAP1B	898.5394	-0.3724345	0.296739	-1.25509129	0.209446	0.453022	NOT
RP1-182D1	28.32286	-0.5629474	0.44857	-1.25498242	0.209485	0.453072	NOT
CHCHD2P7	1.491246	1.05580703	0.841353	1.25489133	0.209518	0.453107	NOT
HDAC1	4170.151	0.20435136	0.162871	1.254681625	0.209594	0.453236	NOT
RP13-870H	13.40223	0.79620529	0.634682	1.254494218	0.209662	0.453337	NOT
RP11-216M	4.770407	0.58802406	0.468746	1.254461079	0.209674	0.453337	NOT
PDLIM7	1290.584	0.34788995	0.277429	1.253979929	0.209849	0.453679	NOT
S1PR1	987.0529	-0.3319365	0.264729	-1.25387311	0.209888	0.453726	NOT
GRHPR	14003.42	-0.3568508	0.284646	-1.25366345	0.209964	0.453855	NOT
LINC00863	71.54786	-0.3218401	0.256752	-1.25350502	0.210022	0.453936	NOT
DGUOK-AS1	18.65843	0.40331703	0.321762	1.253462887	0.210037	0.453936	NOT
LTA	22.01559	0.4947329	0.394706	1.253421941	0.210052	0.453936	NOT
TYW1	869.1255	-0.1740576	0.138898	-1.25313649	0.210156	0.454063	NOT
CBWD2	287.8837	0.17724177	0.14144	1.253125428	0.21016	0.454063	NOT
KIRREL2	6.113485	-0.7652706	0.610691	-1.25312293	0.210161	0.454063	NOT
HMGB1P3	1.775796	-0.6230533	0.497238	-1.25302828	0.210195	0.454084	NOT
EXO5	276.7738	0.20221182	0.161382	1.253004383	0.210204	0.454084	NOT
KIAA1549L	22.27463	0.71104077	0.567543	1.252840173	0.210264	0.454168	NOT
ATP6V0A1	4465.511	-0.2205109	0.176018	-1.25277845	0.210286	0.454168	NOT
SNX31	7.118913	-0.5194845	0.414672	-1.2527589	0.210293	0.454168	NOT
ZNF229	130.8793	0.61351849	0.489789	1.252617494	0.210345	0.454233	NOT
NAA30	1172.939	-0.1778218	0.141964	-1.25258432	0.210357	0.454233	NOT

SYCP2L	8.715993	-0.607828	0.485331	-1.25239946	0.210424	0.454298	NOT
RP4-539M6	24.8477	-0.5536735	0.442102	-1.25236617	0.210436	0.454298	NOT
RAF1	6274.748	-0.1761346	0.140642	-1.25236342	0.210437	0.454298	NOT
KRTAP5-1	6.616753	0.71087794	0.567673	1.252265731	0.210473	0.454339	NOT
RNU6-720P	1.22499	-0.711738	0.568425	-1.25212238	0.210525	NA	NA
LARP4	3910.425	-0.2264094	0.180841	-1.25198287	0.210576	0.454479	NOT
MTM1	807.8532	-0.3017756	0.24104	-1.25197505	0.210579	0.454479	NOT
FOXA3	2503.986	-0.4763614	0.380495	-1.25195028	0.210588	0.454479	NOT
LFNG	360.0332	0.44045765	0.351845	1.251851544	0.210624	0.454492	NOT
RABGAP1	1359.97	-0.2042933	0.163194	-1.25184191	0.210627	0.454492	NOT
BX842568.	49.04008	-0.6800145	0.543257	-1.25173591	0.210666	0.454539	NOT
CCDC147-A	2.898007	0.57931372	0.462857	1.251604378	0.210714	0.454606	NOT
CSAG4	9.450191	1.61671791	1.291929	1.251398315	0.210789	0.454732	NOT
CD5L	407.1998	-0.7980051	0.637748	-1.25128646	0.21083	0.454784	NOT
PDCD11	2182.143	0.19399794	0.155049	1.251200753	0.210861	0.454815	NOT
TOPORS-AS	165.2966	-0.3021125	0.241478	-1.25109856	0.210899	0.454859	NOT
RP11-436K	3.772251	0.81939426	0.654996	1.250991774	0.210937	0.454907	NOT
RP11-326A	2.84768	-0.6159879	0.492562	-1.25057948	0.211088	0.455196	NOT
GLULP4	9.322523	-0.7668689	0.613305	-1.2503873	0.211158	0.455309	NOT
RP11-148K	2.629483	-0.5179035	0.414209	-1.25034397	0.211174	0.455309	NOT
PTPN21	639.8174	-0.2894164	0.231513	-1.25010688	0.211261	0.455456	NOT
CHMP5	2333.102	0.17472524	0.139773	1.250064831	0.211276	0.455456	NOT
FMR1	2708.967	-0.2280377	0.182458	-1.24980844	0.21137	0.455587	NOT
LINC00861	53.13169	-0.4985877	0.398932	-1.24980607	0.21137	0.455587	NOT
CCDC50	4285.221	0.16443771	0.131577	1.249745745	0.211392	0.455599	NOT
MIRLET7D	2.917658	-0.6862168	0.549138	-1.24962642	0.211436	0.455648	NOT
RN7SL834P	6.284132	-0.5406899	0.4327	-1.2495725	0.211456	0.455648	NOT
RP3-337O1	4.498388	0.43271758	0.3463	1.249544648	0.211466	0.455648	NOT
FAM21A	1682.815	0.19700597	0.157668	1.249499939	0.211482	0.455648	NOT
RBBP7	4378.872	0.17689431	0.141582	1.249410229	0.211515	0.455682	NOT
RP11-569A	5.243155	-0.4630703	0.370668	-1.24928452	0.211561	0.455745	NOT
HGD	19512.96	-0.5282134	0.422965	-1.24883549	0.211725	0.45605	NOT
CTD-2410N	2.93589	-0.5215488	0.417638	-1.24880539	0.211736	0.45605	NOT
FAM84A	192.1157	0.48961837	0.392147	1.248558148	0.211827	0.45618	NOT
RP11-83M1	5.886548	-0.8129657	0.651136	-1.24853383	0.211836	0.45618	NOT
RP11-973H	6.28336	1.00094655	0.801717	1.24850309	0.211847	0.45618	NOT
RP11-104F	13.94581	-0.4805696	0.384942	-1.24841992	0.211877	0.456198	NOT
R3HDM4	2600.735	0.24710286	0.197937	1.248388369	0.211889	0.456198	NOT
CTD-2636A	71.69893	0.44353105	0.355331	1.248220878	0.21195	0.456293	NOT
R3HCC1L	496.7974	-0.1514562	0.121359	-1.24800114	0.212031	0.45643	NOT
SERBP1P3	2.194802	0.73023083	0.585165	1.247905738	0.212066	0.456444	NOT
TXNDC16	541.9483	-0.3094681	0.247993	-1.24789235	0.21207	0.456444	NOT
RP11-365C	9.284797	0.47907549	0.383944	1.247774093	0.212114	0.456501	NOT
WARS2	642.7448	0.20877801	0.167339	1.247633538	0.212165	0.456511	NOT
MMP21	4.024867	-0.5794753	0.464462	-1.24762602	0.212168	0.456511	NOT
AKAP11	1127.556	-0.2544456	0.203944	-1.24762371	0.212169	0.456511	NOT
KDM5A	1653.55	-0.1743572	0.139761	-1.24753645	0.212201	0.45652	NOT
AC104777.	1.432422	-0.8342072	0.668692	-1.24751993	0.212207	0.45652	NOT
FBXW2	3095.321	-0.2460485	0.197258	-1.24734109	0.212272	0.456625	NOT

SF3B2	8545.093	0.12585117	0.100908	1.247187073	0.212329	0.45671	NOT
PCBP3-OT1	1.082707	-1.0201562	0.818026	-1.24709529	0.212363	NA	NA
MIER1	1334.514	-0.176422	0.141472	-1.24704882	0.21238	0.456783	NOT
TOR1AIP2	8627.8	-0.2537093	0.203486	-1.2468174	0.212464	0.456929	NOT
RP11-560A	0.725367	-1.3449909	1.078813	-1.24673196	0.212496	NA	NA
DUSP19	131.1471	-0.2641837	0.211913	-1.24666168	0.212522	0.457014	NOT
KDELR2	18806.95	-0.1873425	0.150281	-1.24661809	0.212538	0.457014	NOT
RP11-350C	29.28981	-0.3444238	0.276298	-1.24656701	0.212556	0.457018	NOT
CORO1B	4502.729	0.19907263	0.159706	1.246496996	0.212582	0.457037	NOT
FAM222B	1223.453	0.20773112	0.166677	1.246312117	0.21265	0.457147	NOT
GGNBP2	2826.928	0.1223061	0.098142	1.246212446	0.212686	0.457189	NOT
CD81	24814.85	-0.3153454	0.253094	-1.24596376	0.212778	0.457294	NOT
AC008079.	20.98789	-0.3250282	0.260869	-1.2459442	0.212785	0.457294	NOT
RP5-1112D	21.0186	0.45749143	0.367185	1.245941359	0.212786	0.457294	NOT
CYP51A1-A	8.991407	0.34560019	0.277392	1.245889319	0.212805	0.457299	NOT
C1QC	5360.555	0.46323519	0.371867	1.24570161	0.212874	0.457411	NOT
CARKD	3075.743	-0.2715673	0.218014	-1.24563875	0.212897	0.457424	NOT
TCEB1P2	2.325594	0.56673561	0.45502	1.24551892	0.212941	0.457477	NOT
PPOX	989.0569	0.22827696	0.183284	1.24548012	0.212955	0.457477	NOT
GRHL2	8.839877	0.69016579	0.554203	1.245329802	0.213011	0.457523	NOT
TRIM8	5217.705	-0.2125372	0.170669	-1.2453145	0.213016	0.457523	NOT
CXCL2	1962.973	-0.5547356	0.445469	-1.24528445	0.213027	0.457523	NOT
RP3-402G1	48.48679	-0.3903883	0.313526	-1.24515542	0.213075	0.457585	NOT
TPRG1-AS1	249.6122	-0.6718265	0.53957	-1.2451142	0.21309	0.457585	NOT
RPS3AP6	36.92312	0.38769071	0.311416	1.244928288	0.213158	0.457662	NOT
PTPRVP	11.00712	0.41395736	0.332516	1.244924703	0.213159	0.457662	NOT
SLC35A4	4462.531	-0.1706008	0.13706	-1.24472068	0.213234	0.457787	NOT
RFTN1	2215.053	-0.4255351	0.341888	-1.24466377	0.213255	0.457795	NOT
C4orf33	220.5599	-0.3118633	0.250598	-1.24447696	0.213324	0.457798	NOT
WHAMM	588.4088	-0.1751849	0.140774	-1.24443827	0.213338	0.457798	NOT
MOCS2	3042.26	-0.2582465	0.207536	-1.24434404	0.213373	0.457798	NOT
NDUFS7	3995.823	-0.3164635	0.254325	-1.24432682	0.213379	0.457798	NOT
RP11-169K	53.9412	0.54685961	0.439492	1.24429867	0.21339	0.457798	NOT
GATC	1151.44	0.17179311	0.138065	1.244288506	0.213393	0.457798	NOT
PLEK	757.6473	0.48646149	0.390957	1.244284131	0.213395	0.457798	NOT
CTD-2666L	5.674471	-0.4988657	0.400933	-1.24426271	0.213403	0.457798	NOT
RP11-295D	2.340974	0.56104976	0.450915	1.244247405	0.213409	0.457798	NOT
CTC-523E2	10.09329	0.56421712	0.453528	1.244061307	0.213477	0.457846	NOT
EEA1	1263.871	-0.2371962	0.190665	-1.24404986	0.213481	0.457846	NOT
UNC119B	865.4287	0.24960531	0.200644	1.244017888	0.213493	0.457846	NOT
NSF	1754.357	0.17638832	0.141791	1.244003067	0.213498	0.457846	NOT
NPIPB5	46.90036	0.47727568	0.38377	1.243650638	0.213628	0.458026	NOT
WNK1	6366.95	0.20862451	0.167752	1.243645747	0.21363	0.458026	NOT
MIF	2722.805	0.38571022	0.310147	1.243637612	0.213633	0.458026	NOT
ASCL1	94.13988	-1.0324636	0.83031	-1.24346735	0.213696	0.458092	NOT
CHCHD6	390.07	0.21905807	0.176168	1.243461956	0.213698	0.458092	NOT
STXBP3	1399.848	0.20329981	0.163514	1.243314707	0.213752	0.458157	NOT
RP11-655M	9.08792	0.68550488	0.551365	1.243287855	0.213762	0.458157	NOT
CLCN5	1748.472	-0.3029041	0.243653	-1.24317925	0.213802	0.458176	NOT

ST8SIA5	12.84832	-0.6279042	0.505082	-1.2431722	0.213804	0.458176	NOT
AAK1	1477.947	-0.2518976	0.202647	-1.24303326	0.213856	0.458231	NOT
RPL29P14	2.871474	0.59096322	0.475429	1.243011507	0.213864	0.458231	NOT
TAB2	2791.327	-0.2461439	0.198031	-1.24295846	0.213883	0.458237	NOT
BRWD1-AS2	33.50216	-0.380337	0.30601	-1.24289222	0.213908	0.458253	NOT
ZDHHC9	5022.492	0.2366674	0.190448	1.242688273	0.213983	0.458378	NOT
KMT2E-AS1	217.273	-0.3265863	0.262833	-1.24256398	0.214029	0.45842	NOT
CTD-2135I	3.857575	-0.5546977	0.446421	-1.24254246	0.214036	0.45842	NOT
RP11-421M	8.455148	0.38473901	0.309739	1.242138099	0.214186	0.458703	NOT
HLA-DRB6	759.282	-0.4943194	0.398017	-1.24195537	0.214253	0.458805	NOT
LRRC37A11	6.927645	0.84228897	0.678233	1.241888135	0.214278	0.458805	NOT
RP11-480I	6.38758	0.59976439	0.482953	1.24186992	0.214285	0.458805	NOT
DYNC1LI2	1472.785	-0.2935954	0.236422	-1.24182648	0.214301	0.458805	NOT
RP11-873E	23.5865	0.37218293	0.299741	1.241681177	0.214354	0.458835	NOT
NBEAL2	1161.993	0.27618371	0.222432	1.241657095	0.214363	0.458835	NOT
RP11-356I	45.61401	-0.2881127	0.23204	-1.24165148	0.214365	0.458835	NOT
RP11-568A	4.083643	1.04784992	0.844106	1.241372678	0.214468	0.458922	NOT
RP11-120M	7.143091	-0.3887292	0.313146	-1.24136733	0.21447	0.458922	NOT
CTD-3076C	2.339581	1.03736289	0.835676	1.241346361	0.214478	0.458922	NOT
RP11-283C	6.790391	0.64844131	0.522378	1.241325052	0.214486	0.458922	NOT
RP11-492I	26.71366	0.79115696	0.637374	1.241275209	0.214504	0.458922	NOT
PCDHB3	25.12089	-0.587578	0.47337	-1.24126622	0.214507	0.458922	NOT
AC015849.	47.79542	-0.5775519	0.465328	-1.24117171	0.214542	0.45896	NOT
FAM201A	51.6818	0.82918242	0.668104	1.241098247	0.214569	0.458982	NOT
MEIS3P2	2.200761	-0.6529704	0.526149	-1.24103755	0.214592	0.458994	NOT
IL6	32.60949	-0.6561556	0.528805	-1.24082614	0.21467	0.459125	NOT
RP11-21K1	7.397033	-0.5231473	0.421629	-1.24077779	0.214688	0.459127	NOT
KRT18P19	2.345253	0.516515	0.416333	1.240628286	0.214743	0.459209	NOT
RBM3	6128.758	0.21323014	0.17188	1.240575054	0.214763	0.459215	NOT
FAM183A	22.41815	1.05342194	0.849194	1.240496532	0.214792	0.459218	NOT
AC002310.	60.34019	-0.3306386	0.266541	-1.24047852	0.214798	0.459218	NOT
RP11-480C	1.522623	-0.7406349	0.597079	-1.24043015	0.214816	0.45922	NOT
AC064850.	1.874562	0.71598016	0.57724	1.240350372	0.214846	0.459236	NOT
SLC25A45	843.1667	0.28433084	0.22924	1.240319556	0.214857	0.459236	NOT
CMBL	8278.143	-0.4377844	0.353021	-1.24010764	0.214936	0.459367	NOT
PRAMEF9	5.550748	-1.4695788	1.185128	-1.24001715	0.214969	0.459402	NOT
AC011747.	2.06739	0.8711453	0.702695	1.239719708	0.215079	0.459601	NOT
AP1S2	509.0009	-0.2745522	0.22149	-1.23956873	0.215135	0.459629	NOT
AGAP3	3381.397	0.2118095	0.170875	1.239557239	0.215139	0.459629	NOT
SCO1	1625.038	-0.2276817	0.183681	-1.23954754	0.215143	0.459629	NOT
RP11-541N	51.44704	-0.277356	0.223797	-1.23931963	0.215227	0.459773	NOT
BRINP1	12.29904	-0.7681118	0.619844	-1.23920247	0.215271	0.459829	NOT
TMSB10	27871.17	0.46315456	0.373826	1.238958908	0.215361	0.459986	NOT
FAM13A-AS	37.02537	-0.3505074	0.28293	-1.23885064	0.215401	0.460031	NOT
TMEM62	979.6758	0.22070996	0.178167	1.238781082	0.215427	0.460031	NOT
TYMSOS	144.7439	-0.401797	0.324353	-1.23876366	0.215433	0.460031	NOT
LINC01019	26.92705	1.92584003	1.554909	1.238554529	0.215511	0.460161	NOT
AZU1	4.284941	-0.5988566	0.483545	-1.23847062	0.215542	0.460191	NOT
ALG1L	375.3439	-0.6231747	0.503202	-1.23841766	0.215561	0.460193	NOT

HCAR3	18.52059	0.81938565	0.661671	1.238358149	0.215583	0.460193	NOT
UQCRBP1	7.41211	0.49992518	0.403709	1.238330765	0.215593	0.460193	NOT
MRPL40	2418.226	-0.2429184	0.196191	-1.23817453	0.215651	0.460267	NOT
ARID5A	1508.743	-0.3142274	0.253789	-1.23814496	0.215662	0.460267	NOT
DUXAP10	22.93796	0.58007079	0.468558	1.23799126	0.215719	0.460353	NOT
KCNN1	21.90785	-0.6294912	0.508549	-1.23781821	0.215783	0.460453	NOT
RPS20P10	2.332018	0.54120665	0.437291	1.237633619	0.215852	0.460563	NOT
LMBRD2	1249.946	-0.2887459	0.233315	-1.2375772	0.215873	0.460572	NOT
PCDHGA11	21.63019	-0.4760836	0.384736	-1.23742965	0.215928	0.46065	NOT
POU2F1	962.1385	0.17776687	0.143665	1.237373696	0.215948	0.46065	NOT
PHF7	242.3731	-0.2602298	0.210319	-1.23730821	0.215973	0.46065	NOT
ATXN7	881.8078	-0.1873527	0.151429	-1.23723149	0.216001	0.46065	NOT
KIF17	118.328	-0.4114022	0.332524	-1.23720933	0.216009	0.46065	NOT
STAR	11.26302	0.70406209	0.569075	1.237203662	0.216011	0.46065	NOT
RASA4	13.10982	-0.3760421	0.303959	-1.23714729	0.216032	0.460659	NOT
ARFIP1	1042.141	-0.1648718	0.13331	-1.23675275	0.216179	0.460935	NOT
EARS2	1541.33	-0.2047421	0.165557	-1.23668887	0.216203	0.460949	NOT
ARGLU1	1888.44	0.24069546	0.194663	1.23647398	0.216282	0.461083	NOT
AC006033.	8.884826	0.79867129	0.645978	1.236375018	0.216319	0.461125	NOT
RP11-57G1	7.766107	-0.3975842	0.321614	-1.2362159	0.216378	0.461215	NOT
RER1	5342.833	-0.1922229	0.155517	-1.23602136	0.216451	0.461322	NOT
LINC0065(	92.53251	-0.8162867	0.660432	-1.23598983	0.216462	0.461322	NOT
SSBP2	248.4226	-0.3886827	0.314497	-1.23588817	0.2165	0.461347	NOT
RMI1	550.1189	0.25416423	0.205657	1.235866845	0.216508	0.461347	NOT
MTPN	4223.202	0.17200126	0.139194	1.235692586	0.216573	0.461448	NOT
SPRED1	765.0649	0.32468209	0.262769	1.235618106	0.216601	0.461471	NOT
RPL29P12	5.332005	0.39733114	0.321578	1.235568263	0.216619	0.461474	NOT
MTMR11	756.1273	0.46852182	0.379251	1.235385546	0.216687	0.461583	NOT
SDCCAG3	2060.16	0.22571835	0.182756	1.235080706	0.2168	0.461758	NOT
MIR568	45.56881	-0.4435372	0.359124	-1.2350534	0.216811	0.461758	NOT
ABHD15	1410.919	-0.329608	0.266883	-1.23502737	0.21682	0.461758	NOT
ITFG1	2914.682	-0.2834118	0.229584	-1.23445777	0.217032	0.462173	NOT
HSP90AA6I	2.934448	0.54368138	0.440454	1.234364867	0.217067	0.462211	NOT
CTA-221G(	1.757029	-0.5789885	0.469145	-1.23413509	0.217153	0.462357	NOT
RP11-84N1	6.604279	1.07045549	0.867471	1.233995194	0.217205	0.462404	NOT
RP1-45I4.	17.53388	-0.5001294	0.405297	-1.23398385	0.217209	0.462404	NOT
RP11-697N	31.0801	-0.4827137	0.391212	-1.23389387	0.217242	0.462416	NOT
RP11-484N	135.7891	-0.6975888	0.565363	-1.23387736	0.217249	0.462416	NOT
ATP6V0E1	7730.561	0.24167645	0.195879	1.233807483	0.217275	0.462435	NOT
PLXNB3	164.252	0.69538457	0.563714	1.233576341	0.217361	0.462582	NOT
IGF2-AS	94.72162	1.18770362	0.962907	1.233456393	0.217406	0.462641	NOT
FGF1	59.38863	0.50576351	0.410077	1.233337127	0.21745	0.4627	NOT
ACTB	145203	0.20247087	0.164176	1.233257431	0.21748	0.462726	NOT
KLHDC4	1107.246	-0.2446296	0.198433	-1.23280866	0.217647	0.462994	NOT
FMO3	16009.75	-0.6433869	0.521892	-1.23279672	0.217652	0.462994	NOT
DNAH10	34.42183	0.48626222	0.394451	1.232757765	0.217666	0.462994	NOT
RP13-497I	2.375314	-0.8360321	0.678211	-1.23270211	0.217687	0.462994	NOT
ZNF582-AS	59.44547	-0.4901426	0.397648	-1.2326051	0.217723	0.462994	NOT
HOXD11	3.608606	1.68811729	1.369569	1.23259032	0.217729	0.462994	NOT

RP11-326I	3.315056	0.71771947	0.582288	1.232585683	0.21773	0.462994	NOT
RP3-331H2	1.896824	-0.7196777	0.583909	-1.23251727	0.217756	0.462994	NOT
KLHL10	3.808586	-0.3849298	0.312314	-1.23250826	0.217759	0.462994	NOT
RP11-864I	152.6969	-0.2194836	0.178122	-1.23220891	0.217871	0.463196	NOT
CTB-5506.	1.55544	-0.6296079	0.510988	-1.23213929	0.217897	0.463206	NOT
DPH3	1585.56	0.17749541	0.144059	1.232104594	0.21791	0.463206	NOT
TMEM236	15.25861	-0.3605035	0.292616	-1.2320018	0.217948	0.463247	NOT
ALG5	1639.449	-0.2232604	0.181224	-1.23196147	0.217963	0.463247	NOT
RP11-572(	5.207385	0.44743597	0.363284	1.231641872	0.218083	0.463465	NOT
RN7SL738F	3.182391	-0.4097511	0.332717	-1.23153072	0.218124	0.463465	NOT
TMTC2	126.579	0.46866713	0.380572	1.231481694	0.218143	0.463465	NOT
MCTS2P	17.98563	0.33402165	0.271248	1.231423417	0.218165	0.463465	NOT
RP11-790I	2.437673	-0.7193807	0.584187	-1.231422	0.218165	0.463465	NOT
RP3-522J7	2.457066	0.61848825	0.502259	1.231413104	0.218168	0.463465	NOT
KIF9-AS1	62.40357	-0.2612268	0.212156	-1.23129369	0.218213	0.463523	NOT
RNF144A-A	45.66187	-0.7598916	0.617198	-1.23119604	0.21825	0.463558	NOT
CSNK1G2	2247.599	0.18018463	0.146354	1.231158282	0.218264	0.463558	NOT
RP11-524(	1.370209	0.75723384	0.61511	1.231053641	0.218303	NA	NA
MANSC1	767.3055	0.26661954	0.216602	1.230918611	0.218353	0.463712	NOT
IRF3	2934.635	0.22757859	0.184897	1.23084131	0.218382	0.463737	NOT
SNX11	1028.216	0.15673501	0.127372	1.23053029	0.218499	0.463948	NOT
DLG5	1090.795	0.38625246	0.313922	1.230408055	0.218544	0.464007	NOT
ATP5J	5893.773	-0.240785	0.195702	-1.23036557	0.21856	0.464007	NOT
LRIG1	2266.86	-0.2896512	0.235436	-1.23027414	0.218594	0.46402	NOT
PRKAG1	2168.7	0.12425707	0.101002	1.230241932	0.218607	0.46402	NOT
BRCC3	751.1459	0.2066134	0.16795	1.230205245	0.21862	0.46402	NOT
OSBPL9	4278.2	-0.2282548	0.185548	-1.23016556	0.218635	0.46402	NOT
PSMD11	4893.855	0.14403835	0.117094	1.230110955	0.218656	0.464027	NOT
DGCR6L	3221.209	-0.311956	0.253613	-1.23004624	0.21868	0.464042	NOT
AP000254.	61.16121	-0.241234	0.196137	-1.22992343	0.218726	0.464071	NOT
TFAP2A	152.9262	0.72992406	0.593492	1.229881179	0.218742	0.464071	NOT
DDX53	24.2619	-1.249101	1.015634	-1.22987296	0.218745	0.464071	NOT
DEDD2	1169.203	-0.147427	0.11989	-1.22968906	0.218814	0.464181	NOT
APCS	42156.52	-0.6405211	0.520912	-1.22961387	0.218842	0.464205	NOT
MAP4K3	1401.505	0.25621954	0.208398	1.229473961	0.218894	0.464255	NOT
EMC7	3090.747	0.20724848	0.168569	1.229459155	0.2189	0.464255	NOT
RP11-47I2	1.982297	-0.7024455	0.57138	-1.22938466	0.218928	0.46426	NOT
CRYM-AS1	2.382671	0.57267062	0.465828	1.229361492	0.218936	0.46426	NOT
MRPL37	5144.848	0.2198955	0.178887	1.22924402	0.21898	0.464314	NOT
AC007292.	38.91989	-0.3628157	0.295164	-1.22920212	0.218996	0.464314	NOT
HMGB3P24	4.71334	-0.3824672	0.311163	-1.2291551	0.219014	0.464315	NOT
TARDBP	4241.377	0.09488129	0.077195	1.229108502	0.219031	0.464316	NOT
MRAP	26.37577	-0.8623802	0.701978	-1.2284996	0.219259	0.464764	NOT
ZNF10	258.2756	-0.2607738	0.212279	-1.22844714	0.219279	0.464769	NOT
ARRDC3	3216.478	-0.3109566	0.253158	-1.22831256	0.21933	0.464828	NOT
MTCH2	7674.867	-0.1851663	0.150753	-1.22827803	0.219343	0.464828	NOT
RP11-495F	6.553529	0.46539702	0.378915	1.228236578	0.219358	0.464828	NOT
MAGEA12	380.7378	1.46654912	1.194108	1.228154059	0.219389	0.464857	NOT
RXRA	10634.71	-0.2478365	0.201818	-1.22801982	0.21944	0.464927	NOT

SERPINC1	122015.5	-0.5814675	0.473621	-1.22770706	0.219557	0.46514	NOT
CXXC5	5377.279	0.22176325	0.180661	1.227507183	0.219632	0.465262	NOT
ACADM	5583.552	-0.356351	0.290328	-1.22740822	0.219669	0.465305	NOT
AIF1L	498.3699	-0.4437491	0.361569	-1.22728907	0.219714	0.465348	NOT
C8orf34	2.893969	1.02804252	0.837671	1.227262249	0.219724	0.465348	NOT
THNSL1	847.0237	-0.2918328	0.237822	-1.22710487	0.219783	0.465437	NOT
SYBU	2482.428	-0.5174311	0.421696	-1.22702341	0.219814	0.465447	NOT
HSPA12B	271.5945	-0.2939055	0.239536	-1.22697758	0.219831	0.465447	NOT
ZNF35	154.516	-0.2952749	0.240657	-1.22695553	0.219839	0.465447	NOT
PNMT	15.30274	0.99179765	0.808378	1.226898318	0.219861	0.465456	NOT
PCDH9-AS2	1.551138	1.97530073	1.610113	1.226809047	0.219894	0.465491	NOT
AC009120.	6.120776	-0.4415853	0.35996	-1.22676126	0.219912	0.465493	NOT
IGHV1-67	1.573342	-1.1506065	0.937998	-1.22666149	0.21995	0.465528	NOT
RPS10P16	3.363432	0.47318774	0.385764	1.226625187	0.219963	0.465528	NOT
CTD-2265M	8.051913	-0.3993591	0.32563	-1.22642088	0.22004	0.465633	NOT
ZNF8	231.4028	0.21600923	0.176132	1.226402322	0.220047	0.465633	NOT
RP11-546E	2.446953	-0.5560153	0.453389	-1.22635234	0.220066	0.465637	NOT
CHAC2	136.9671	0.24366029	0.198707	1.226230738	0.220112	0.465688	NOT
IDH3A	634.5088	-0.2878176	0.234724	-1.22619636	0.220125	0.465688	NOT
MYBPHL	18.19494	0.96488637	0.786968	1.226081178	0.220168	0.465744	NOT
AC096582.	4.324255	-0.6481874	0.528765	-1.22585084	0.220255	0.465891	NOT
CYP17A1	7033.121	-0.9933232	0.810413	-1.2256997	0.220312	0.465975	NOT
BUD31	1809.752	0.18695693	0.152543	1.225598986	0.22035	0.466018	NOT
AC004985.	10.85666	-0.3416615	0.278811	-1.22542431	0.220415	0.466095	NOT
PLCB1	947.9134	0.44338793	0.361828	1.225412278	0.22042	0.466095	NOT
MRPL23	1905.446	-0.3055419	0.249364	-1.225287	0.220467	0.466158	NOT
RP4-564F2	195.2228	-0.580669	0.474001	-1.22503671	0.220561	0.466321	NOT
AP006222.	17.32112	0.53143596	0.43387	1.224875098	0.220622	0.466413	NOT
IFI27	12662.94	0.70630723	0.576703	1.224733849	0.220676	0.466488	NOT
ACAD9	2538.713	0.14683433	0.119895	1.22469048	0.220692	0.466488	NOT
RP11-720L	4.97024	-1.0032038	0.819226	-1.22457581	0.220735	0.466526	NOT
RP11-338N	8.254383	1.02767536	0.839226	1.224551715	0.220744	0.466526	NOT
RP11-416N	5.041273	0.65815565	0.537538	1.224389967	0.220805	0.466618	NOT
MIMT1	5.198036	1.07318915	0.876597	1.224267679	0.220851	0.466679	NOT
HSD11B1L	213.2546	-0.3344246	0.273198	-1.22411231	0.22091	0.46676	NOT
AC093818.	19.70971	0.25124844	0.205258	1.224063249	0.220928	0.46676	NOT
RP11-121C	6.159861	0.41956759	0.342779	1.224016911	0.220946	0.46676	NOT
MIR3671	2.438858	-0.5397084	0.440969	-1.2239147	0.220984	0.46676	NOT
GTF2IRD2F	7.110315	-0.3954843	0.323136	-1.22389464	0.220992	0.46676	NOT
NFKB1	1755.434	-0.19226	0.157089	-1.22389217	0.220993	0.46676	NOT
NOC3L	606.4192	-0.179024	0.146293	-1.22373563	0.221052	0.466831	NOT
UNC93B3	2.629136	0.75907621	0.620335	1.223656282	0.221082	0.466831	NOT
AC007163.	0.928725	-0.9800141	0.800899	-1.22364192	0.221087	NA	NA
KARS	4929.894	0.19129029	0.156329	1.22363841	0.221089	0.466831	NOT
BFSP2	12.29828	0.61040073	0.498848	1.223621191	0.221095	0.466831	NOT
C19orf43	5299.106	-0.2698714	0.220564	-1.22355054	0.221122	0.466851	NOT
NUP188	1773.567	0.24127035	0.197218	1.223367402	0.221191	0.466961	NOT
SEC14L1	1956.16	-0.1967637	0.160863	-1.22317345	0.221264	0.467078	NOT
RP11-87H9	74.21255	0.31743152	0.259524	1.223128799	0.221281	0.467078	NOT

MORF4L2-A	15.45214	-0.3181735	0.260177	-1.22291332	0.221362	0.467214	NOT
LYRM5	1217.984	-0.2882785	0.235765	-1.22273589	0.221429	0.467311	NOT
ATP12A	2.550658	-1.2083688	0.988279	-1.22270003	0.221443	0.467311	NOT
TREX1	9.628403	0.24616203	0.201354	1.222534633	0.221506	0.467406	NOT
GATAD1	1668.241	0.19327203	0.158099	1.222476865	0.221527	0.467411	NOT
VPS4B	1555.306	-0.175751	0.143771	-1.22243729	0.221542	0.467411	NOT
RP11-333E	2.322818	0.49274428	0.403113	1.222347881	0.221576	0.467446	NOT
UCK1	2045.321	-0.2560623	0.209501	-1.22224859	0.221614	0.467489	NOT
KRT18P5	6.361539	0.49625667	0.406066	1.222109443	0.221666	0.467528	NOT
SRM	3356.818	0.29556847	0.241851	1.222108519	0.221667	0.467528	NOT
TRAF4	2910.433	0.19682585	0.161072	1.221974189	0.221717	0.467599	NOT
GAFA3	10.67458	-0.5881772	0.481363	-1.22190035	0.221745	0.467621	NOT
PDIK1L	611.6547	-0.2129343	0.174286	-1.2217533	0.221801	0.467698	NOT
MYCBP2	1153.523	-0.2553464	0.20901	-1.22169579	0.221823	0.467698	NOT
RP3-388E2	4.658503	-0.5392362	0.441393	-1.22166792	0.221833	0.467698	NOT
CHUK	1130.256	-0.1811508	0.148316	-1.22138553	0.22194	0.467879	NOT
IGF2BP1	922.3209	0.76939192	0.629952	1.221350218	0.221953	0.467879	NOT
NRADDP	2.548836	-0.4677703	0.383053	-1.22116306	0.222024	0.467992	NOT
MRPL11	2028.618	0.18385613	0.150565	1.221104557	0.222046	0.467994	NOT
RP5-821D1	153.8401	0.29212965	0.239241	1.221068629	0.22206	0.467994	NOT
RP11-380M	1.540038	-0.93027	0.762001	-1.22082502	0.222152	0.468152	NOT
RP11-212F	221.413	-0.22606	0.185181	-1.2207519	0.22218	0.468174	NOT
RP11-98G7	34.02423	0.49472897	0.405318	1.220595992	0.222239	0.468242	NOT
PPP4R4	259.2785	-0.4142023	0.33935	-1.22057638	0.222246	0.468242	NOT
CTSH	9203.043	-0.3172386	0.259926	-1.22049686	0.222277	0.468269	NOT
SUN2	7994.841	-0.2771333	0.227084	-1.22040098	0.222313	0.468309	NOT
CPN2	12022.43	-0.5019517	0.411396	-1.22011758	0.22242	0.468466	NOT
TNFRSF10E	2704.489	-0.2371423	0.194361	-1.22011338	0.222422	0.468466	NOT
LINC01342	1.338446	0.95375382	0.781888	1.21980836	0.222538	NA	NA
DIAPH1	10362.8	-0.2435552	0.199702	-1.21959161	0.22262	0.468832	NOT
ALDH8A1	3828.876	-0.5287892	0.433589	-1.21956382	0.22263	0.468832	NOT
LGI2	124.5273	0.53404154	0.437995	1.219285934	0.222736	0.468959	NOT
HPCAL1	2482.246	0.16908794	0.138684	1.219229227	0.222757	0.468959	NOT
GUSBP2	25.21918	-0.3946943	0.323731	-1.21920537	0.222766	0.468959	NOT
FRAT2	991.838	0.3092335	0.253635	1.21920483	0.222766	0.468959	NOT
ZNF697	485.5277	0.27579924	0.226218	1.219176849	0.222777	0.468959	NOT
FOXJ2	725.797	-0.2068737	0.16979	-1.21840725	0.223069	0.469537	NOT
RP11-150I	1.131808	-1.3442671	1.103336	-1.21836544	0.223085	NA	NA
MLEC	13932.87	-0.1964156	0.161218	-1.21832036	0.223102	0.469571	NOT
HDAC10	483.7145	0.27724923	0.227585	1.218225183	0.223138	0.469599	NOT
COL24A1	57.28384	0.72723096	0.596997	1.218147576	0.223168	0.469599	NOT
SUSD5	34.3299	0.58171941	0.477551	1.218130881	0.223174	0.469599	NOT
FAM212A	81.6597	-0.3378621	0.277367	-1.21810293	0.223185	0.469599	NOT
RP1-92014	13.8624	0.38641633	0.317248	1.218024762	0.223215	0.469625	NOT
ITGA4	197.6276	-0.4270508	0.350646	-1.21789625	0.223263	0.469691	NOT
TRAPPC3L	2.714331	-0.5265557	0.432404	-1.21774101	0.223322	0.469779	NOT
DACH1	30.95714	-0.4700734	0.38609	-1.21752177	0.223406	0.469911	NOT
PNMAL1	92.84348	-0.8310693	0.682612	-1.21748452	0.22342	0.469911	NOT
PPIAP3	3.065329	0.48116477	0.395239	1.217400936	0.223452	0.469923	NOT

RP11-131I	11.33798	0.43947532	0.361001	1.217378834	0.22346	0.469923	NOT
NUPR1	16472.13	0.45229964	0.371566	1.217279216	0.223498	0.469966	NOT
KIAA1468	901.1938	-0.1680993	0.138145	-1.21682833	0.22367	0.470267	NOT
ZFYVE1	1068.831	-0.2062592	0.169508	-1.21681119	0.223676	0.470267	NOT
CD1B	12.33132	0.65463354	0.538122	1.216514908	0.223789	0.470466	NOT
VEZF1	1405.286	0.19472315	0.160072	1.216472359	0.223805	0.470466	NOT
AC007036.	2.747773	0.79311689	0.652101	1.216248114	0.22389	0.470566	NOT
COMT	6386.779	-0.3215831	0.264421	-1.21617941	0.223917	0.470566	NOT
RP11-295I	10.97487	-0.3710958	0.305155	-1.21609103	0.22395	0.470566	NOT
FAM86EP	149.9287	0.24749051	0.203522	1.216038618	0.22397	0.470566	NOT
SS18	2582.798	-0.1699858	0.139787	-1.21603336	0.223972	0.470566	NOT
RP11-105N	8.44569	0.56532213	0.464902	1.216001734	0.223984	0.470566	NOT
CTD-2235C	20.59851	0.50523474	0.41552	1.21591093	0.224019	0.470566	NOT
AP001610.	1.827172	0.71696686	0.589682	1.21585339	0.224041	0.470566	NOT
ANAPC10	312.0667	-0.1468322	0.120767	-1.2158356	0.224048	0.470566	NOT
RP5-908M1	100.8385	-0.3688304	0.303357	-1.2158287	0.22405	0.470566	NOT
PAK2	4288.677	0.1357083	0.111618	1.215824218	0.224052	0.470566	NOT
AC016907.	3.85659	-0.4740084	0.389874	-1.21580046	0.224061	0.470566	NOT
RP11-347C	47.19633	-0.3445845	0.283451	-1.21567763	0.224108	0.470611	NOT
TMEM59L	11.45595	-0.5284746	0.434725	-1.21565366	0.224117	0.470611	NOT
RP11-365H	1.277982	1.11482358	0.917124	1.215565134	0.224151	NA	NA
SH3GL2	5.694955	-0.7332253	0.603229	-1.21550023	0.224175	0.470689	NOT
MT1CP	2.121445	-0.7041283	0.579324	-1.21543168	0.224201	0.470689	NOT
RP11-761E	2.875216	-0.5652621	0.465076	-1.21541958	0.224206	0.470689	NOT
LINC01348	753.5476	-0.6219483	0.511775	-1.21527631	0.224261	0.470767	NOT
ELOVL2	4492.693	0.47382998	0.389953	1.215095533	0.22433	0.470875	NOT
MT-TY	21.79262	-0.5130683	0.422302	-1.21493274	0.224392	0.470969	NOT
KRT17P4	5.998103	-0.9529638	0.784423	-1.21486017	0.224419	0.470991	NOT
DLGAP4-AS	18.65439	-0.346355	0.285114	-1.2147959	0.224444	0.471006	NOT
UFC1	4834.212	0.22008611	0.181192	1.214659687	0.224496	0.471049	NOT
ACAD10	2467.596	-0.2131796	0.175507	-1.21465131	0.224499	0.471049	NOT
SNHG19	432.7334	-0.4331328	0.356606	-1.21459648	0.22452	0.471056	NOT
TP53BP2	3021.205	0.21620225	0.178023	1.214459186	0.224572	0.471101	NOT
LRRC27	248.9307	-0.234306	0.192932	-1.21444923	0.224576	0.471101	NOT
RP11-701H	10.26732	0.66480079	0.547456	1.214345948	0.224616	0.47113	NOT
RP4-597N1	6.039835	0.54383995	0.447855	1.214322279	0.224625	0.47113	NOT
RP11-554I	1.932228	1.53078616	1.260745	1.214192131	0.224674	0.471198	NOT
ACTR2	7803.981	0.17843734	0.146983	1.213997358	0.224749	0.471292	NOT
AP000432.	7.176943	-0.3047131	0.251006	-1.21396751	0.22476	0.471292	NOT
RP11-155C	4.963657	0.43221898	0.356047	1.213938021	0.224771	0.471292	NOT
RP11-523H	26.8137	-0.3841603	0.316481	-1.21385126	0.224805	0.471324	NOT
TACSTD2	249.1175	0.70810756	0.583398	1.213764788	0.224838	0.471324	NOT
MTATP8P2	15.75535	0.5827977	0.480158	1.213761833	0.224839	0.471324	NOT
CLCN7	3881.935	0.2228365	0.183599	1.2137148	0.224857	0.471325	NOT
PREX2	273.5346	-0.4685432	0.386056	-1.21366498	0.224876	0.471329	NOT
CCDC122	97.36547	-0.2860914	0.235747	-1.21355079	0.224919	0.471353	NOT
KLF12	1256.753	-0.3374773	0.278095	-1.21353311	0.224926	0.471353	NOT
SEC14L5	24.83572	-0.5552317	0.457554	-1.21347871	0.224947	0.471353	NOT
GAL3ST1	1306.535	0.72417206	0.596787	1.213452449	0.224957	0.471353	NOT

LRRC37A16	212.5381	-0.2965114	0.244374	-1.21335234	0.224995	0.471397	NOT
CPB2-AS1	43.64253	-0.4136233	0.340923	-1.21324643	0.225036	0.47144	NOT
RPL26L1	968.4577	0.2099162	0.173032	1.213164074	0.225067	0.47144	NOT
NFU1	1026.112	0.17693057	0.145842	1.213162392	0.225068	0.47144	NOT
USP33	1811.943	0.19041195	0.156994	1.21286234	0.225182	0.471644	NOT
AC010458.	1.338964	-0.7145134	0.589154	-1.21277883	0.225214	NA	NA
CFAP70	92.12596	-0.4040281	0.333144	-1.21277317	0.225217	0.471658	NOT
TMEM198B	518.8107	-0.2951112	0.24334	-1.2127535	0.225224	0.471658	NOT
UCHL3	352.4165	0.3049543	0.251478	1.212646739	0.225265	0.471688	NOT
DSTN	8686.33	-0.1946577	0.160526	-1.21262541	0.225273	0.471688	NOT
XRCC6P2	9.433486	0.42749032	0.352577	1.212474696	0.225331	0.47173	NOT
RP11-155I	21.76978	-0.4879341	0.402436	-1.21245116	0.22534	0.47173	NOT
SPDYE5	9.016606	-0.3435593	0.283372	-1.21239663	0.225361	0.47173	NOT
IBA57	704.784	-0.220695	0.182033	-1.21239092	0.225363	0.47173	NOT
AC009495.	1.348142	-0.7325471	0.604256	-1.21231294	0.225393	NA	NA
RPA1	2453.995	0.19949884	0.164567	1.21226794	0.22541	0.471792	NOT
ANKAR	84.71217	-0.2101111	0.17334	-1.21212984	0.225463	0.471855	NOT
RWDD3	256.3529	0.20667904	0.170513	1.212098704	0.225475	0.471855	NOT
POLRMT	3955.077	0.25080326	0.206956	1.211866345	0.225564	0.472005	NOT
RP11-398C	34.22752	0.34542302	0.28507	1.211713171	0.225622	0.472091	NOT
YIPF3	7631.572	0.21180872	0.174809	1.211655661	0.225644	0.472098	NOT
ATP6V1B1	34.52115	-0.6526899	0.538706	-1.21158857	0.22567	0.472098	NOT
RP11-382M	5.778406	-0.5513474	0.455079	-1.21154224	0.225688	0.472098	NOT
CDC42BPG	264.1877	0.54873278	0.452928	1.211522696	0.225695	0.472098	NOT
ZFAND1	1167.467	0.20842893	0.172046	1.211475375	0.225713	0.472099	NOT
ASB11	3.825327	-0.6534973	0.539443	-1.21142995	0.225731	0.472099	NOT
MORF4L1	6024.202	0.12140159	0.10025	1.210991003	0.225899	0.472401	NOT
TNFSF13B	226.3688	0.42185982	0.348367	1.210962442	0.22591	0.472401	NOT
CTD-2555K	7.692131	0.35193144	0.290695	1.210655655	0.226027	0.472611	NOT
KLHL22	1010.091	-0.2062094	0.17035	-1.2105017	0.226086	0.472698	NOT
RP11-158I	30.22424	0.40556026	0.335071	1.210370242	0.226137	0.472752	NOT
CNGA1	633.6946	-0.5853448	0.483624	-1.21033048	0.226152	0.472752	NOT
ACER3	653.126	0.20950993	0.173107	1.210288757	0.226168	0.472752	NOT
RP11-466H	329.1062	0.43030215	0.355548	1.210252214	0.226182	0.472752	NOT
RP11-712E	16.0381	-0.3758104	0.310539	-1.21018711	0.226207	0.472755	NOT
RP11-970I	5.514851	0.35617894	0.294335	1.210113595	0.226235	0.472755	NOT
ZNF641	588.0382	-0.1328044	0.109746	-1.21011227	0.226236	0.472755	NOT
FAH	8903.753	-0.3220413	0.266155	-1.20997674	0.226288	0.472827	NOT
LDHB	1649.608	-0.3929879	0.324835	-1.20980674	0.226353	0.472927	NOT
ZNF514	390.7365	0.27477065	0.227152	1.209635794	0.226419	0.473028	NOT
UGT3A1	3598.158	-0.7189777	0.594414	-1.2095576	0.226449	0.473054	NOT
RP11-490E	4.299451	-0.4478701	0.370379	-1.20922242	0.226577	0.473286	NOT
SOHLH2	57.52266	1.51713594	1.254921	1.208949656	0.226682	0.473469	NOT
RP11-475J	3.093498	-0.751544	0.62171	-1.20883348	0.226727	0.473526	NOT
PSORS1C2	1.315327	1.03716055	0.857994	1.208820074	0.226732	NA	NA
CRYBG3	268.807	-0.4843616	0.400723	-1.20871834	0.226771	0.473582	NOT
ACAP3	1695.413	-0.2517428	0.208288	-1.20862732	0.226806	0.473618	NOT
SLC16A12	263.2951	0.67816772	0.561174	1.208480793	0.226862	0.473699	NOT
PCDHA1	14.92883	-0.848613	0.702306	-1.20832411	0.226923	0.473789	NOT

SLC38A6	527.6654	0.30774672	0.254748	1.208043133	0.227031	0.473978	NOT
SMIM19	1393.545	-0.2939923	0.243378	-1.20796482	0.227061	0.474004	NOT
ITIH2	101166.3	-0.4765687	0.394559	-1.20785039	0.227105	0.47406	NOT
CTB-35F21	1.819676	0.75821837	0.627813	1.207714657	0.227157	0.474084	NOT
LINC00473	6.025051	-1.0174482	0.842474	-1.20769103	0.227166	0.474084	NOT
RP11-510J	11.285	-0.5713515	0.473097	-1.20768442	0.227169	0.474084	NOT
STAC3	150.2435	-0.3256658	0.269676	-1.20761855	0.227194	0.4741	NOT
C6orf203	749.5517	-0.241947	0.20038	-1.20744205	0.227262	0.474195	NOT
LAS1L	2315.137	0.17323723	0.143478	1.207409153	0.227275	0.474195	NOT
RP11-16K1	5.914005	-0.6182033	0.512061	-1.20728393	0.227323	0.474259	NOT
AC072062.	8.374996	0.61633344	0.510562	1.207166587	0.227368	0.474309	NOT
TENM2	187.498	-0.8521346	0.705949	-1.20707707	0.227402	0.474309	NOT
AHI1	307.4368	-0.264988	0.219529	-1.20707521	0.227403	0.474309	NOT
AC008074.	7.202485	0.3768093	0.312176	1.207040707	0.227416	0.474309	NOT
NAPG	1130.564	-0.1528201	0.126647	-1.20666329	0.227562	0.474566	NOT
IGHG1	14418.41	-0.7985106	0.661769	-1.20662998	0.227575	0.474566	NOT
STRN4	3082.079	0.16414951	0.136069	1.206373949	0.227673	0.474706	NOT
BAATP1	45.39713	-0.6662135	0.552249	-1.20636496	0.227677	0.474706	NOT
RP5-966M1	4.417165	0.46203381	0.383037	1.206237832	0.227726	0.474771	NOT
RP11-452K	13.6821	0.48238346	0.399944	1.206127828	0.227768	0.474802	NOT
RP11-295K	27.02704	0.47231055	0.391618	1.206047782	0.227799	0.474802	NOT
STARD7-AS	115.5899	-0.1793292	0.148694	-1.20602708	0.227807	0.474802	NOT
AKAP8L	1849.671	0.17859366	0.148086	1.206011518	0.227813	0.474802	NOT
RP11-132N	5.597566	0.69049324	0.572576	1.205940822	0.22784	0.474802	NOT
GEMIN8	489.3705	0.18618088	0.154388	1.205926866	0.227846	0.474802	NOT
RP11-481H	6.117245	-0.7057021	0.585287	-1.20573689	0.227919	0.474919	NOT
SP3	2676.421	0.19758187	0.163913	1.205410167	0.228045	0.475124	NOT
ZIK1	89.03095	0.53520452	0.444009	1.205391234	0.228052	0.475124	NOT
SSTR1	525.9736	-0.671674	0.557262	-1.20531092	0.228083	0.475152	NOT
RP11-122K	4.229898	0.6940018	0.575872	1.205131366	0.228153	0.47526	NOT
PRELID1P4	4.857184	0.38554833	0.31995	1.205025005	0.228194	0.475309	NOT
RP11-196I	4.821483	0.37612179	0.31219	1.204783154	0.228287	0.475456	NOT
RP11-115H	77.56063	1.38885992	1.152839	1.204729727	0.228308	0.475456	NOT
KIAA1324	77.89929	-0.4303282	0.357206	-1.20470554	0.228317	0.475456	NOT
KDSR	3025.87	-0.1814528	0.150633	-1.20460471	0.228356	0.4755	NOT
CCDC185	6.381091	0.96753204	0.803225	1.204559876	0.228373	0.4755	NOT
ANKRA2	611.691	-0.2019376	0.167655	-1.20448414	0.228403	0.475506	NOT
DIS3L2P1	2.028814	0.87393649	0.725582	1.204462405	0.228411	0.475506	NOT
RP11-20B2	3.173561	-0.4273406	0.354821	-1.2043844	0.228441	0.475532	NOT
RP5-1098D	4.50429	-0.5466921	0.45401	-1.20414099	0.228535	0.475691	NOT
HOXC11	0.798873	1.36299165	1.132184	1.203860697	0.228643	NA	NA
RP5-1050D	5.295543	-0.4491438	0.373098	-1.20382198	0.228658	0.475911	NOT
LRRC26	11.16948	0.93622403	0.777788	1.20370137	0.228705	0.475972	NOT
SNRPGP15	5.781212	-0.4882959	0.405739	-1.20347189	0.228794	0.47612	NOT
RP4-673M1	25.65668	-0.3377725	0.280704	-1.20330639	0.228858	0.476132	NOT
AC006277.	6.881297	-0.3980023	0.330758	-1.20330359	0.228859	0.476132	NOT
EEF1A1P12	53.15419	0.33163271	0.275608	1.203277033	0.228869	0.476132	NOT
SLC9C2	6.789346	-0.6406263	0.532402	-1.20327484	0.22887	0.476132	NOT
CHAC1	330.6812	0.52443015	0.435882	1.20314771	0.228919	0.476198	NOT

NPM1P26	2.66945	-0.4179806	0.347427	-1.2030738	0.228948	0.476221	NOT
RP11-83N9	19.05817	-0.4912234	0.408342	-1.20296988	0.228988	0.476268	NOT
AE000661.	2.615369	0.65415656	0.543862	1.202799083	0.229054	0.476369	NOT
MKNK1	1005.9	0.16172014	0.13447	1.202646594	0.229113	0.476456	NOT
C19orf53	4338.348	-0.2911694	0.242158	-1.20239373	0.229211	0.476623	NOT
RP1-154K9	1.522409	1.17699596	0.978984	1.202262478	0.229262	0.476692	NOT
AC020571.	11.11382	0.68589092	0.570557	1.202142379	0.229308	0.476694	NOT
SLC25A29	1050.29	-0.2309793	0.19214	-1.20214127	0.229309	0.476694	NOT
RP11-553I	1363.27	-0.3230352	0.268721	-1.20212328	0.229316	0.476694	NOT
TENM4	33.76427	-0.6491288	0.54001	-1.20206787	0.229337	0.476702	NOT
GLOD4	2101.034	-0.2045277	0.170181	-1.20182329	0.229432	0.476863	NOT
ACAA1	9062.68	-0.3414455	0.284135	-1.20169967	0.22948	0.476926	NOT
RERGL	50.03315	0.6644388	0.553001	1.201515659	0.229551	0.477038	NOT
AK5	6.654732	-0.5517198	0.459241	-1.20137429	0.229606	0.477038	NOT
NDUFV2P1	81.55531	-0.3391209	0.282281	-1.20135867	0.229612	0.477038	NOT
CTD-2015H	10.45959	-0.8741481	0.727667	-1.20130223	0.229634	0.477038	NOT
TXK	25.49286	-0.3938089	0.327839	-1.20122769	0.229663	0.477038	NOT
RP11-958N	25.27696	-0.9263053	0.771192	-1.20113435	0.229699	0.477038	NOT
KIAA1598	2919.836	0.28180487	0.234628	1.201069976	0.229724	0.477038	NOT
RPL5P24	4.110498	0.45062814	0.37519	1.201067862	0.229725	0.477038	NOT
RP11-363N	13.56278	0.42941825	0.357534	1.201055776	0.22973	0.477038	NOT
RP11-602M	1.534229	-0.5487515	0.456918	-1.20098324	0.229758	0.477038	NOT
SH3BP5L	1972.514	0.16290857	0.135651	1.200939057	0.229775	0.477038	NOT
AC004019.	36.4969	-0.470189	0.391519	-1.20093625	0.229776	0.477038	NOT
ALDH3A2	15783.41	-0.3355614	0.279426	-1.20089418	0.229792	0.477038	NOT
RP13-254E	3.952039	-0.5816975	0.484408	-1.20084213	0.229812	0.477038	NOT
BTBD19	187.2592	-0.3169991	0.263983	-1.20083284	0.229816	0.477038	NOT
RP11-1072	5.273864	-0.3997808	0.332923	-1.20081929	0.229821	0.477038	NOT
ADAMTSL2	1210.964	-0.5321217	0.443143	-1.2007895	0.229833	0.477038	NOT
COL8A2	133.9271	0.56019681	0.466545	1.200735257	0.229854	0.477045	NOT
RP4-806M2	3.909732	0.89852584	0.748373	1.200638784	0.229891	0.477087	NOT
SFT2D2	3293.528	0.19828086	0.165169	1.200469857	0.229957	0.477186	NOT
NBR1	7643.299	-0.1938928	0.161526	-1.20038036	0.229992	0.477222	NOT
IGKV3OR2-	2.442977	-0.8362804	0.696778	-1.20021118	0.230057	0.477321	NOT
CTC-441N1	1.724405	1.56414604	1.303695	1.199778823	0.230225	0.477627	NOT
CCDC87	33.15086	0.40853925	0.340524	1.199736449	0.230242	0.477627	NOT
CCDC15	126.3481	0.23873856	0.198999	1.199696327	0.230257	0.477627	NOT
TM2D1	798.1725	0.15629399	0.130283	1.199649048	0.230276	0.477628	NOT
GALNT3	43.68361	0.47365436	0.394883	1.199479903	0.230341	0.477717	NOT
TOX3	539.7929	0.54381066	0.453385	1.199446786	0.230354	0.477717	NOT
ODAM	76.29939	1.32177018	1.102058	1.199365459	0.230386	0.477717	NOT
ZNF222	92.12254	-0.2661691	0.221935	-1.19930909	0.230408	0.477717	NOT
FAAH	1453.554	-0.3345473	0.27895	-1.19930885	0.230408	0.477717	NOT
ID2	7704.844	-0.3038759	0.253394	-1.19922249	0.230441	0.477717	NOT
STARD13-A	7.90848	0.42571012	0.354989	1.199221317	0.230442	0.477717	NOT
MMP8	0.970227	1.13982829	0.950612	1.199046937	0.23051	NA	NA
CLPSL2	2.334623	1.055342	0.880163	1.199029892	0.230516	0.477835	NOT
IMP4	2987.809	0.18877603	0.157454	1.19892782	0.230556	0.477881	NOT
RP11-676J	1.761282	-0.609372	0.508331	-1.19876973	0.230617	0.477972	NOT

CTD-3214I	4.276266	0.4718137	0.393607	1.198691033	0.230648	0.477999	NOT
CPB2	22757.87	-0.4886046	0.407677	-1.19850896	0.230719	0.478097	NOT
F2R	1240.418	-0.352175	0.293852	-1.19847921	0.230731	0.478097	NOT
GCNT4	352.6759	0.43909424	0.36641	1.198368939	0.230773	0.478135	NOT
RP11-727I	5.225922	-0.4451033	0.371433	-1.19834081	0.230784	0.478135	NOT
CYBB	1048.101	0.472685	0.394487	1.198227187	0.230829	0.47819	NOT
RP11-444I	7.240891	-0.6180887	0.515882	-1.19812075	0.23087	0.47824	NOT
RP11-299J	31.8747	-0.2214388	0.184897	-1.19763154	0.23106	0.478566	NOT
USF1	2717.177	0.17931622	0.149726	1.197625625	0.231063	0.478566	NOT
GINS2	468.6986	0.32960683	0.275342	1.197082023	0.231275	0.478922	NOT
MTHFD2L	395.8588	-0.2602359	0.217399	-1.19704467	0.231289	0.478922	NOT
ELMO1	1858.842	0.28167121	0.23531	1.197021127	0.231298	0.478922	NOT
PEX26	2461.523	0.15306343	0.127872	1.197003286	0.231305	0.478922	NOT
RP11-227I	3.370959	-0.5174884	0.432381	-1.19683537	0.231371	0.479021	NOT
CAND1	3563.693	0.16352177	0.13664	1.196734253	0.23141	0.479066	NOT
RP11-178(	25.25468	-0.4030532	0.336815	-1.19665913	0.231439	0.47909	NOT
SGCE	995.9268	0.49729657	0.415587	1.196611106	0.231458	0.479092	NOT
GRASP	314.5252	-0.3704256	0.309604	-1.19644781	0.231522	0.479187	NOT
PSMG3	1484.152	0.30110007	0.251683	1.19634801	0.231561	0.479228	NOT
NLRP11	123.648	0.66468358	0.555613	1.196306971	0.231577	0.479228	NOT
RP3-512B1	232.9221	0.44825729	0.374804	1.195977273	0.231705	0.479443	NOT
MROH5	1.373762	-0.6302799	0.527025	-1.19592078	0.231727	NA	NA
GFM2	1903.364	-0.2113887	0.176768	-1.19585099	0.231755	0.479443	NOT
GRAMD3	779.7596	-0.2124293	0.177645	-1.19580515	0.231773	0.479443	NOT
PID1	1949.841	-0.3132784	0.261995	-1.19573955	0.231798	0.479443	NOT
C17orf58	867.8268	0.24788953	0.207313	1.195726142	0.231803	0.479443	NOT
ZXDB	1063.957	-0.3385549	0.283146	-1.19569006	0.231818	0.479443	NOT
WDR5	2388.465	0.18428743	0.154131	1.195655064	0.231831	0.479443	NOT
RPL34P33	1.568031	-0.6133692	0.513006	-1.19563758	0.231838	0.479443	NOT
ICAM1	3571.698	0.42160206	0.352626	1.195608086	0.23185	0.479443	NOT
SCAMP5	1053.56	0.388848	0.325236	1.195587828	0.231857	0.479443	NOT
FUK	1211.311	-0.2113905	0.176872	-1.19515817	0.232025	0.479753	NOT
RSF1	1128.139	-0.1609991	0.134718	-1.19508628	0.232053	0.479775	NOT
AARD	2.416431	0.71407786	0.597594	1.194920663	0.232118	0.479861	NOT
RNU6-531I	2.1861	-0.5358713	0.44847	-1.19488858	0.232131	0.479861	NOT
PEX13	1776.636	-0.1689318	0.141389	-1.19480151	0.232165	0.479866	NOT
KIF21A	1833.154	0.24714577	0.206852	1.194792554	0.232168	0.479866	NOT
OSR1	65.78328	0.60198679	0.503929	1.194586993	0.232248	0.479947	NOT
STOX2	344.8621	-0.3585498	0.300147	-1.19458171	0.23225	0.479947	NOT
BEND3P1	41.67559	0.52427917	0.43889	1.19455588	0.232261	0.479947	NOT
CMAHP	287.7121	-0.4701078	0.393565	-1.19448563	0.232288	0.479968	NOT
TPT1	93087.48	-0.228902	0.191669	-1.19425388	0.232379	0.48011	NOT
RBM39	6964.057	0.16154135	0.135269	1.194219162	0.232392	0.48011	NOT
TRPM1	7.620643	-0.6412546	0.537029	-1.19407815	0.232447	0.480187	NOT
CCDC116	5.315906	-0.4068801	0.340769	-1.19400436	0.232476	0.480194	NOT
IGHV3-73	14.97283	0.90690919	0.759578	1.193964059	0.232492	0.480194	NOT
RPS7P14	6.401346	0.36597376	0.306531	1.193919286	0.23251	0.480194	NOT
RP1-253P7	6.802576	-0.4367618	0.365846	-1.19384128	0.23254	0.480194	NOT
CTC-518P1	3.382134	-0.488188	0.408943	-1.19378018	0.232564	0.480194	NOT

CRBN	1284.071	-0.1527979	0.127997	-1.19376594	0.23257	0.480194	NOT
RP11-124N	1.458844	1.66674697	1.396224	1.193753155	0.232575	0.480194	NOT
ATP5D	4920.033	-0.2850657	0.238863	-1.19342746	0.232702	0.48042	NOT
CLUAP1	467.5437	0.19557097	0.163919	1.193096612	0.232832	0.480628	NOT
ECE2	810.7646	0.26682396	0.223643	1.193080535	0.232838	0.480628	NOT
ZFP69	152.1151	0.25863838	0.216798	1.192995126	0.232871	0.48066	NOT
RP11-647(	5.66137	0.39456455	0.330786	1.192808705	0.232944	0.480773	NOT
LIME1	194.7343	-0.3661191	0.30695	-1.19276483	0.232961	0.480773	NOT
AP001107.	2.438516	-0.5706848	0.478525	-1.19259036	0.23303	0.480845	NOT
RP11-412F	5.719205	1.22094331	1.023778	1.192585799	0.233032	0.480845	NOT
EEF1DP3	6.522245	0.50310619	0.421898	1.192484339	0.233071	0.480846	NOT
EHD4-AS1	3.634236	0.53959236	0.452495	1.192483169	0.233072	0.480846	NOT
SLFN12	92.60374	-0.4213335	0.353335	-1.1924484	0.233085	0.480846	NOT
ERAS	4.579658	-0.4948455	0.415018	-1.19234596	0.233126	0.480892	NOT
CTD-2302F	5.087738	0.3645439	0.30579	1.192138698	0.233207	0.481024	NOT
TRIT1	551.1268	0.1687128	0.141533	1.192035586	0.233247	0.481047	NOT
ACTG1P10	2.062614	-0.4563462	0.382853	-1.19196146	0.233276	0.481047	NOT
GAN	241.8614	-0.1885084	0.15815	-1.19196051	0.233277	0.481047	NOT
CTD-2619J	9.255331	-0.3072956	0.257814	-1.19192875	0.233289	0.481047	NOT
RP11-526I	44.03126	-0.4521306	0.379351	-1.19185453	0.233318	0.48107	NOT
STON2	284.7192	0.39964786	0.33534	1.191767825	0.233352	0.481104	NOT
EZH1	1141.375	-0.1583034	0.13284	-1.1916874	0.233384	0.481132	NOT
RP11-339F	10.07527	-0.3809093	0.31966	-1.19160632	0.233416	0.481161	NOT
GPBAR1	87.85301	-0.4426324	0.371497	-1.19148259	0.233464	0.481209	NOT
HMGB1	6354.31	0.1765268	0.14816	1.191457431	0.233474	0.481209	NOT
ZNF580	556.502	0.27444544	0.230363	1.191358587	0.233513	0.481229	NOT
SRCAP	3982.548	-0.1659421	0.139295	-1.19130064	0.233536	0.481229	NOT
XXbac-BP(	2.654545	-0.589432	0.494782	-1.1912964	0.233537	0.481229	NOT
CAMK2N1	4539.667	0.26130639	0.219361	1.191215512	0.233569	0.481258	NOT
APOA2	518273.4	0.57575258	0.483386	1.191081549	0.233622	0.48133	NOT
RP11-102(	5.061416	0.4632639	0.388979	1.190975362	0.233663	0.481379	NOT
MUC20	409.6395	0.54649671	0.459003	1.19061797	0.233804	0.481608	NOT
RP11-798M	4.404719	-0.4653739	0.390881	-1.19057847	0.233819	0.481608	NOT
SAMD5	463.7219	0.62752775	0.527088	1.190556641	0.233828	0.481608	NOT
C10orf90	9.837731	0.9287267	0.780136	1.190467843	0.233863	0.481644	NOT
AC008592.	32.56328	-0.3764011	0.316194	-1.1904117	0.233885	0.481652	NOT
RP11-458F	277.1287	-0.2953113	0.248094	-1.19032109	0.23392	0.481689	NOT
AC006960.	0.409495	-1.7780514	1.49381	-1.19027928	0.233937	NA	NA
AC009014.	86.86733	-0.9916988	0.833249	-1.19015911	0.233984	0.481784	NOT
ZNF497	91.63318	-0.2249216	0.188999	-1.190066	0.23402	0.481822	NOT
HIST1H2BI	4.892016	-0.5848437	0.491523	-1.18986111	0.234101	0.481952	NOT
ACOT12	1494.499	-0.6057204	0.509108	-1.18976795	0.234138	0.481991	NOT
CBLB	755.4297	-0.2078374	0.174717	-1.18956703	0.234217	0.482068	NOT
MRPL55	3105.489	-0.2623616	0.220554	-1.18955856	0.23422	0.482068	NOT
PNPLA5	2.888689	-0.8685237	0.730147	-1.18951829	0.234236	0.482068	NOT
ZP3	129.9186	0.45077385	0.378964	1.189491067	0.234246	0.482068	NOT
IGLV3-1	43.98362	0.87092419	0.732312	1.189280032	0.234329	0.482203	NOT
RAB7B	203.3789	-0.376385	0.316517	-1.18914744	0.234382	0.482267	NOT
ZNF232	299.8974	0.20556508	0.172873	1.18911061	0.234396	0.482267	NOT

MCOLN1	1694.358	-0.2107893	0.177282	-1.18900624	0.234437	0.48229	NOT
RP11-680(	33.25379	0.39744252	0.334269	1.188991703	0.234443	0.48229	NOT
RP11-455(	4.929633	-0.4123237	0.346807	-1.18891408	0.234473	0.482291	NOT
UG0898H0(	1.439071	1.37326528	1.155072	1.188899724	0.234479	0.482291	NOT
LMNTD2	263.8073	-0.2856742	0.240336	-1.18864418	0.23458	0.482461	NOT
RP11-137(	11.05763	0.37121311	0.312346	1.188467066	0.234649	0.482568	NOT
RP11-360I	3.622324	-0.5177648	0.435683	-1.18839916	0.234676	0.482587	NOT
TTC32	315.2426	0.27980249	0.235463	1.188309085	0.234712	0.482623	NOT
RP11-139(	26.475	0.51609604	0.434341	1.188228184	0.234744	0.482632	NOT
ZNF260	695.3298	-0.2052097	0.172712	-1.18816301	0.234769	0.482632	NOT
TEKT1	6.896602	-0.5234145	0.440537	-1.18812773	0.234783	0.482632	NOT
FCF1	1230.904	0.19207739	0.161665	1.188117179	0.234787	0.482632	NOT
SYNE2	2660.916	-0.3095831	0.26061	-1.18791698	0.234866	0.482725	NOT
ORMDL1	1308.683	0.14884387	0.125299	1.187912039	0.234868	0.482725	NOT
DSCR4	18.88319	1.93351842	1.627863	1.187764556	0.234926	0.482808	NOT
RP11-163I	1.565998	-1.2239137	1.030618	-1.18755296	0.23501	0.482943	NOT
DPP3	2140.602	-0.1926011	0.162204	-1.18739797	0.235071	0.483032	NOT
TULP3	764.6387	0.23038128	0.194048	1.187238686	0.235133	0.483124	NOT
ALG3	4191.104	0.19681698	0.165804	1.187043558	0.23521	0.483246	NOT
NOP9	1545.271	-0.1568583	0.132158	-1.18689992	0.235267	0.483296	NOT
CCDC42B	6.081609	0.35761584	0.301305	1.186891504	0.23527	0.483296	NOT
B4GALT7	1649.506	-0.2374499	0.200091	-1.18670925	0.235342	0.483407	NOT
AC016995.	11.01096	0.5301822	0.446813	1.186586385	0.235391	0.48347	NOT
RP4-641G1	10.38844	0.35036483	0.295294	1.186495291	0.235427	0.483507	NOT
AC002075.	5.919524	0.47822288	0.403073	1.186441525	0.235448	0.483514	NOT
SDR9C7	2.401284	0.75532095	0.636691	1.18632277	0.235495	0.483574	NOT
POLR3E	1421.533	0.12847155	0.108305	1.186199565	0.235543	0.483637	NOT
RP1-224A(	2.543157	0.5459012	0.460258	1.186076171	0.235592	0.483682	NOT
EXOSC6	1122.331	-0.1863553	0.157124	-1.18603945	0.235607	0.483682	NOT
AC005224.	16.00023	-0.3361731	0.283457	-1.18597631	0.235632	0.483682	NOT
ZNF589	247.6651	0.21932091	0.184931	1.185963425	0.235637	0.483682	NOT
TRAV6	1.741807	0.74649455	0.629516	1.185822832	0.235692	0.483759	NOT
RP11-301(	3.254555	2.68616491	2.265339	1.185767108	0.235714	0.483768	NOT
C11orf63	31.03387	-0.4741798	0.399916	-1.18569866	0.235741	0.483787	NOT
SIRPG	68.22132	0.52355012	0.441614	1.185536526	0.235805	0.483882	NOT
C12orf5	288.3825	0.26894437	0.226886	1.18537116	0.235871	0.483978	NOT
RP11-315(	52.32901	0.37966674	0.320305	1.185327715	0.235888	0.483978	NOT
RAD9B	27.61524	-0.3472162	0.292967	-1.18517094	0.23595	0.484068	NOT
EIF2AK1	9635.713	0.16073621	0.135638	1.185040001	0.236002	0.484138	NOT
SLC25A12	310.1796	-0.3891251	0.328409	-1.18487987	0.236065	0.484231	NOT
ARL17B	18.41788	0.38830162	0.32774	1.18478568	0.236102	0.48424	NOT
KLF14	1.681463	1.2209589	1.030537	1.184778855	0.236105	0.48424	NOT
CLSTN1	3122.801	0.3253211	0.274603	1.184696832	0.236137	0.48427	NOT
CTD-2377(	15.95322	-0.5716701	0.482695	-1.18433087	0.236282	0.48453	NOT
MT-TT	8.639495	-0.6932686	0.585402	-1.1842602	0.23631	0.484536	NOT
RP11-403I	51.07234	0.37615185	0.31765	1.184170773	0.236345	0.484536	NOT
PEPD	8955.545	-0.2961586	0.250105	-1.184137	0.236359	0.484536	NOT
CILP	58.68051	-0.5797604	0.489614	-1.18411702	0.236367	0.484536	NOT
RLIM	1394.685	-0.174445	0.147323	-1.18409826	0.236374	0.484536	NOT

RNF212B	5.464584	-0.426104	0.359999	-1.18362552	0.236561	0.484883	NOT
EXOC2	1372.866	0.1528762	0.12917	1.183523924	0.236602	0.484929	NOT
AF127936.	159.9428	-0.2968824	0.250879	-1.18336943	0.236663	0.484997	NOT
CAST	6762.25	-0.2083301	0.176054	-1.18333306	0.236677	0.484997	NOT
AC093382.	3.760032	-0.5392517	0.455717	-1.18330416	0.236689	0.484997	NOT
PHBP11	21.99126	0.44841467	0.37901	1.183120173	0.236762	0.485105	NOT
POSTN	1205.681	0.60920244	0.514929	1.183080778	0.236777	0.485105	NOT
TWIST2	17.75568	0.60566631	0.511975	1.182999966	0.236809	0.485134	NOT
RP11-85I2	8.831183	0.85550375	0.72325	1.182859826	0.236865	0.485175	NOT
HIST1H3D	41.96709	0.54758604	0.462934	1.182859564	0.236865	0.485175	NOT
RPRML	4.641935	-0.6699764	0.566448	-1.18276788	0.236901	0.485213	NOT
RP5-1139F	1.078297	1.14998628	0.97231	1.182735643	0.236914	NA	NA
TXNL4A	2577.891	0.20517889	0.17351	1.182516606	0.237001	0.48538	NOT
ZRANB2	2146.296	0.17791366	0.150467	1.182408979	0.237043	0.485406	NOT
APOBEC3H	27.50693	-0.4101715	0.346899	-1.18239444	0.237049	0.485406	NOT
ZNF518B	261.8016	-0.4471301	0.378213	-1.18221875	0.237119	0.485477	NOT
AC068580.	12.58308	0.38348441	0.324377	1.182216456	0.23712	0.485477	NOT
CNOT1	4296.548	-0.2263559	0.191515	-1.1819236	0.237236	0.485679	NOT
RFPL1	0.389194	-1.0407423	0.880833	-1.18154367	0.237387	NA	NA
YME1L1	5929.264	0.15872637	0.134359	1.181357831	0.237461	0.486102	NOT
PTCD3	2678.267	0.18816483	0.159297	1.181220381	0.237515	0.486144	NOT
RP11-267I	2.791033	-0.7209067	0.610323	-1.1811889	0.237528	0.486144	NOT
COL9A1	32.77885	-0.5116274	0.433153	-1.18117053	0.237535	0.486144	NOT
ALMS1-IT1	21.38299	0.37644958	0.318723	1.18111944	0.237555	0.486149	NOT
SETDB1	1845.878	0.16545226	0.140086	1.181074665	0.237573	0.486149	NOT
SALL2	332.6291	0.55750918	0.472111	1.180885195	0.237648	0.486266	NOT
BRCA2	201.0516	0.32919161	0.278795	1.180765148	0.237696	0.486283	NOT
MRPL41	3828.251	-0.3347715	0.283524	-1.1807536	0.237701	0.486283	NOT
SLC17A3	679.916	0.52990537	0.448811	1.180687439	0.237727	0.486283	NOT
U73166.2	28.41477	-0.416393	0.352671	-1.18068385	0.237728	0.486283	NOT
RP11-711I	1.96173	0.53934075	0.456949	1.180308575	0.237878	0.486551	NOT
SUGCT	881.3207	-0.3418347	0.289636	-1.18022208	0.237912	0.486585	NOT
ST13P20	0.890568	1.20671506	1.022595	1.180051557	0.23798	NA	NA
RP1-181J2	2.894552	-0.5408238	0.458314	-1.18003029	0.237988	0.486704	NOT
FGF7	63.13367	-0.6669512	0.565272	-1.17987713	0.238049	0.486788	NOT
AC018766.	3.116477	-0.455556	0.386118	-1.17983665	0.238065	0.486788	NOT
AC002331.	2.880324	0.73903172	0.626453	1.179708433	0.238116	0.486856	NOT
RPS21	14588.43	0.32397704	0.274682	1.179463604	0.238214	0.487019	NOT
TMEM200C	63.92027	0.59318603	0.502981	1.179341184	0.238262	0.487053	NOT
RP11-576C	2.005838	-0.9100534	0.771669	-1.17933114	0.238266	0.487053	NOT
ZNF510	323.6701	-0.241658	0.204946	-1.17912925	0.238347	0.48718	NOT
SLC22A18	6046.526	0.39319672	0.333504	1.178987111	0.238403	0.487259	NOT
LINC00399	4.416786	-0.8744452	0.741949	-1.17857929	0.238566	0.487518	NOT
SNRPEP4	13.1316	0.3840105	0.325825	1.178578754	0.238566	0.487518	NOT
SEC13	5833.329	0.19179572	0.162743	1.178516518	0.238591	0.48753	NOT
ZNF490	32.75132	-0.3019914	0.256256	-1.17847374	0.238608	0.48753	NOT
AC074212.	70.19876	0.33041553	0.280434	1.178230674	0.238705	0.487661	NOT
RP11-686I	18.02477	-0.4320706	0.366714	-1.17822353	0.238708	0.487661	NOT
RPL5P29	3.360173	0.50612259	0.429623	1.178063299	0.238771	0.487754	NOT

ARMCX2	174.302	0.43472519	0.369091	1.177825553	0.238866	0.487911	NOT
THRB	1632.581	-0.2389077	0.202846	-1.17777676	0.238886	0.487914	NOT
ST8SIA2	2.008983	1.06109532	0.901038	1.177636501	0.238942	0.487979	NOT
TRMT112P4	22.18871	-0.5906622	0.501604	-1.17754756	0.238977	0.487979	NOT
ZNF440	236.6457	-0.2206049	0.187345	-1.17753589	0.238982	0.487979	NOT
CAPN7	1292.201	-0.177344	0.150608	-1.17751703	0.238989	0.487979	NOT
RP1-34L19	1.766155	0.67180533	0.570591	1.177385725	0.239042	0.48802	NOT
GOLGA2	3003.608	-0.1506534	0.127961	-1.17733976	0.23906	0.48802	NOT
FAM86B2	1.62227	0.7266053	0.617163	1.177331494	0.239063	0.48802	NOT
GLIPR1L2	25.6827	-0.4796473	0.407458	-1.17717025	0.239128	0.488115	NOT
RP11-438I	3.822393	0.47549522	0.403969	1.177057973	0.239172	0.488138	NOT
CTB-113D1	11.55941	0.55069957	0.467872	1.177031074	0.239183	0.488138	NOT
UGT3A2	90.80434	0.71867594	0.610597	1.177005871	0.239193	0.488138	NOT
SPATA22	1.096906	-0.6844491	0.581525	-1.17698935	0.2392	NA	NA
LINC01205	3.22651	1.04685962	0.889644	1.176717887	0.239308	0.48828	NOT
RP11-247I	4.271263	0.53523638	0.454864	1.176696524	0.239317	0.48828	NOT
CTNNAL1	1408.619	0.29839612	0.253588	1.176696293	0.239317	0.48828	NOT
RPS3	30321.02	0.27178837	0.231002	1.176564331	0.239369	0.488311	NOT
RP11-638I	2.053558	-0.4825813	0.410174	-1.17652966	0.239383	0.488311	NOT
BACE1-AS	126.2995	0.26576837	0.225893	1.176524011	0.239386	0.488311	NOT
AC013271.	14.28316	0.61295427	0.521019	1.176452142	0.239414	0.488332	NOT
NDE1	531.7828	0.25450129	0.216374	1.17621249	0.23951	0.48841	NOT
TCF7	1061.67	0.42371892	0.360247	1.176191066	0.239519	0.48841	NOT
C10orf88	310.7584	0.15052372	0.127977	1.176177769	0.239524	0.48841	NOT
LLNLR-246	12.1371	-0.4094966	0.348159	-1.17617669	0.239524	0.48841	NOT
ZNF444	2990.838	-0.2415226	0.205416	-1.17577501	0.239685	0.4887	NOT
SLC39A6	1611.439	0.26462183	0.225084	1.175659841	0.239731	0.488757	NOT
RP11-16P6	30.19671	0.37239984	0.31677	1.175616087	0.239748	0.488757	NOT
AP000770.	4.097782	-0.9769836	0.831154	-1.17545485	0.239813	0.488828	NOT
ACN9	575.6111	0.26440624	0.224943	1.17543792	0.23982	0.488828	NOT
LDLRAD4-A	6.686482	-1.023178	0.870507	-1.17538216	0.239842	0.488837	NOT
CSPG4P8	55.0897	0.32900285	0.279928	1.175314394	0.239869	0.488849	NOT
ZNF234	260.2239	-0.2368077	0.201499	-1.17523229	0.239902	0.488849	NOT
RP11-50B3	2.291101	-0.5927707	0.504402	-1.17519399	0.239917	0.488849	NOT
FAM222A-A	17.29607	0.45552211	0.387617	1.175187138	0.23992	0.488849	NOT
RP11-359I	1.9987	-0.6870584	0.5848	-1.1748613	0.24005	0.489077	NOT
ZNF565	110.0854	-0.1813644	0.154377	-1.17481699	0.240068	0.489077	NOT
RP11-661C	1.747947	-0.6144008	0.523141	-1.17444684	0.240216	0.489292	NOT
RP11-316M	15.60843	-0.3557292	0.302891	-1.17444428	0.240217	0.489292	NOT
RP11-104J	10.78584	-0.4472542	0.380835	-1.17440339	0.240233	0.489292	NOT
MED13L	1814.289	-0.2127296	0.18116	-1.17426573	0.240289	0.489292	NOT
ZNHIT1	7345.536	-0.2800666	0.238506	-1.17425434	0.240293	0.489292	NOT
POM121B	11.48972	0.48080102	0.409455	1.174247195	0.240296	0.489292	NOT
FBXO31	3366.946	-0.383208	0.326346	-1.17423838	0.2403	0.489292	NOT
CXCL9	1654.456	0.61532946	0.524074	1.174126883	0.240344	0.489346	NOT
TRBV13	1.395885	1.05522346	0.899113	1.173627134	0.240544	0.489679	NOT
RPSAP9	37.09621	0.29113672	0.24807	1.173605062	0.240553	0.489679	NOT
ATL2	2770.416	-0.1784169	0.15203	-1.17356303	0.24057	0.489679	NOT
ELL2	5290.051	-0.3057311	0.260521	-1.17353815	0.24058	0.489679	NOT

RNU6-1016	1.851704	-0.598436	0.509987	-1.17343372	0.240622	0.489714	NOT
HOXA11-AS	11.85701	0.84919348	0.7237	1.173405274	0.240633	0.489714	NOT
CTD-2530N	5.690734	0.38600328	0.328979	1.173337827	0.24066	0.489733	NOT
RP11-418J	122.3236	0.27709735	0.236171	1.173288714	0.24068	0.489735	NOT
ZNF79	180.2937	-0.1655622	0.141117	-1.17322764	0.240705	0.489735	NOT
KLHL13	309.6119	-0.4168639	0.355332	-1.173167	0.240729	0.489735	NOT
AES	12227.05	-0.260068	0.221684	-1.17314977	0.240736	0.489735	NOT
ANKRD33B	173.2128	0.59077958	0.503606	1.173098774	0.240756	0.489735	NOT
RNY3P8	9.787134	0.62197718	0.530216	1.17306422	0.24077	0.489735	NOT
SOBP	362.5333	0.51425496	0.438407	1.173007203	0.240793	0.489745	NOT
REM1	38.75675	-0.4064026	0.346483	-1.17293749	0.240821	0.489765	NOT
ATF2	2057.272	0.16423787	0.140032	1.17285744	0.240853	0.489782	NOT
LINC00311	1.782901	-0.7174259	0.611729	-1.17278458	0.240882	0.489782	NOT
AC022182.	6.252836	0.57301714	0.488597	1.172781778	0.240883	0.489782	NOT
FAF1	2368.113	0.16465864	0.140407	1.172726677	0.240905	0.48979	NOT
FRMPD2	3.399516	0.92381704	0.787938	1.172449018	0.241017	0.48998	NOT
HLX-AS1	2.260726	-0.7045934	0.601128	-1.17211827	0.24115	0.490213	NOT
RP11-156F	117.5314	-0.2071142	0.176708	-1.17207036	0.241169	0.490216	NOT
CREM	1193.261	-0.1698955	0.144993	-1.17174852	0.241298	0.490442	NOT
RN7SKP80	3.913239	0.60436478	0.515874	1.171536533	0.241383	0.490578	NOT
KCNJ2	138.8436	-0.2694881	0.230052	-1.17142232	0.241429	0.490634	NOT
RP11-118F	44.71502	-0.8768478	0.748608	-1.17130384	0.241477	0.490694	NOT
USP12-AS2	6.63453	0.54517896	0.465511	1.171139981	0.241543	0.490759	NOT
MIEN1	2460.768	0.2341295	0.19992	1.171113673	0.241553	0.490759	NOT
SULT2A1	24011.66	-0.5679821	0.485003	-1.1710892	0.241563	0.490759	NOT
TAS2R19	3.364478	0.63262657	0.540242	1.171006703	0.241596	0.49079	NOT
ANXA7	6675.59	-0.177353	0.151463	-1.17093561	0.241625	0.490799	NOT
CTC-327F1	1.973206	0.83931923	0.71687	1.170810803	0.241675	0.490799	NOT
NKX2-8	1.442372	1.75605931	1.499884	1.170796824	0.24168	0.490799	NOT
RP11-796E	25.21481	0.48466328	0.413974	1.170758816	0.241696	0.490799	NOT
VARS2	2414.345	-0.1779418	0.15199	-1.17074505	0.241701	0.490799	NOT
RP11-800A	8.61196	-0.4912292	0.419598	-1.17071264	0.241714	0.490799	NOT
NHP2P1	2.926532	0.50387465	0.430412	1.170680778	0.241727	0.490799	NOT
CFAP58	4.50708	-0.4052452	0.346196	-1.17056533	0.241774	0.490857	NOT
KIAA0586	457.2183	-0.1579063	0.134909	-1.17046915	0.241812	0.490877	NOT
RPL35P1	28.45542	0.36813254	0.314522	1.170450898	0.24182	0.490877	NOT
CTD-2215E	2.412805	0.70926905	0.606199	1.17002628	0.24199	0.491153	NOT
ZNF654	440.8557	-0.1991105	0.170177	-1.17002223	0.241992	0.491153	NOT
NT5C1B	13.09672	-0.4026676	0.344238	-1.16973727	0.242107	0.491349	NOT
WTIP	214.0392	0.43053634	0.368143	1.169480459	0.24221	0.491522	NOT
ABCA1	3885.439	-0.2701459	0.23103	-1.16931159	0.242278	0.491559	NOT
RP1-86C11	18.74992	-0.5759897	0.492594	-1.16929934	0.242283	0.491559	NOT
C1orf226	964.381	-0.2945273	0.251884	-1.16929605	0.242284	0.491559	NOT
PLA2G4C	1190.92	-0.3255245	0.278409	-1.16923182	0.24231	0.491559	NOT
TMEM167A	3502.207	-0.1579626	0.135102	-1.16920993	0.242319	0.491559	NOT
PIGM	1140.946	0.20001474	0.171081	1.169125453	0.242353	0.491592	NOT
RP5-1142A	7.980654	-0.3992274	0.341544	-1.16889162	0.242447	0.491626	NOT
APOC1P1	2158.034	0.64498658	0.551824	1.168825969	0.242474	0.491626	NOT
PNPLA8	1414.015	-0.2136335	0.182777	-1.16882284	0.242475	0.491626	NOT

AC010900.	3.06205	0.54500834	0.466294	1.16880762	0.242481	0.491626	NOT
OLFM1	146.6557	0.55690691	0.476483	1.168785828	0.24249	0.491626	NOT
ANAPC2	2187.611	-0.1737213	0.148636	-1.16877051	0.242496	0.491626	NOT
C17orf82	40.93846	-0.4258298	0.36434	-1.16876922	0.242497	0.491626	NOT
HMGN5	250.988	-0.4694591	0.401692	-1.16870453	0.242523	0.49163	NOT
MRPL2	2803.038	0.23307161	0.199432	1.16867437	0.242535	0.49163	NOT
PDF	152.4133	-0.252008	0.215645	-1.16862318	0.242555	0.491635	NOT
RPA3	1157.997	0.21942552	0.187808	1.168351601	0.242665	0.491772	NOT
LINC0085	24.17704	0.35952811	0.307725	1.168341447	0.242669	0.491772	NOT
MLLT11	266.1067	0.43107573	0.36897	1.168320669	0.242677	0.491772	NOT
MIR4482	12.59544	-0.5114636	0.437826	-1.16819043	0.24273	0.491841	NOT
COL19A1	4.715698	-0.9136307	0.78216	-1.16808713	0.242772	0.491889	NOT
PRH1	10.50797	-0.3580349	0.306542	-1.16798051	0.242815	0.491897	NOT
FCRL1	6.505082	0.8280821	0.708995	1.167966257	0.24282	0.491897	NOT
UBE3C	3605.683	-0.2191649	0.187658	-1.16789355	0.24285	0.491897	NOT
SEC22B	1967.527	-0.1734242	0.1485	-1.16783916	0.242872	0.491897	NOT
TBCEL	968.2526	-0.2057303	0.176164	-1.16783673	0.242873	0.491897	NOT
CA5B	190.1267	-0.3488749	0.298743	-1.16780818	0.242884	0.491897	NOT
FAM134A	6548.397	0.14980565	0.128306	1.167565611	0.242982	0.492023	NOT
C5AR1	439.4794	-0.3522702	0.301715	-1.16755871	0.242985	0.492023	NOT
PRKCDBP	420.988	0.34348833	0.294204	1.167519284	0.243001	0.492023	NOT
DCSTAMP	16.43257	0.49740878	0.426065	1.167448112	0.243029	0.492044	NOT
C19orf38	73.5968	0.33444559	0.286506	1.167325659	0.243079	0.492088	NOT
RP11-71E1	20.19762	-0.5902135	0.505628	-1.16728735	0.243094	0.492088	NOT
CNFN	66.03031	-0.6140509	0.526062	-1.16725968	0.243106	0.492088	NOT
RAB27A	1310.088	-0.2873101	0.246183	-1.16706063	0.243186	0.492214	NOT
NDUFB8P2	4.313885	-0.3788903	0.3247	-1.1668933	0.243253	0.492314	NOT
ALPK2	588.3392	0.56676088	0.485737	1.16680648	0.243289	0.492348	NOT
CEP135	183.8412	0.2641008	0.2264	1.166523495	0.243403	0.492543	NOT
RP3-496C2	2.247718	-0.5184464	0.44446	-1.16646338	0.243427	0.49255	NOT
BACE2	2266.727	0.6174532	0.529368	1.166396804	0.243454	0.49255	NOT
AC062017.	33.44506	-0.3955321	0.339115	-1.16636506	0.243467	0.49255	NOT
SORCS2	512.7483	-0.617047	0.529057	-1.16631438	0.243487	0.49255	NOT
RP11-968A	20.1129	0.43545876	0.373385	1.166244805	0.243515	0.49255	NOT
PLA2G6	896.4215	0.3611001	0.309626	1.166244705	0.243516	0.49255	NOT
ISLR2	34.63373	-0.4724151	0.405095	-1.16618292	0.24354	0.492564	NOT
TMEM191B	8.28447	-0.6004517	0.514915	-1.1661172	0.243567	0.492581	NOT
SMC3	2080.586	0.23375966	0.200468	1.166072448	0.243585	0.492581	NOT
RP11-108(	2.974347	-1.035531	0.888181	-1.16590024	0.243655	0.492685	NOT
TXNRD3	471.1641	-0.1930914	0.165629	-1.16580628	0.243693	0.492725	NOT
MAN1B1	4185.893	0.20457962	0.175491	1.165756306	0.243713	0.492729	NOT
USB1	1934.175	0.18296941	0.15706	1.164968216	0.244032	0.493332	NOT
PRKAB1	1799.445	0.16508361	0.141711	1.16492916	0.244048	0.493332	NOT
AC010890.	2.970584	0.64790824	0.556372	1.164524357	0.244212	0.49359	NOT
CCDC163P	145.277	0.29415538	0.2526	1.164510865	0.244217	0.49359	NOT
SPATA6	136.1197	0.38497352	0.330617	1.16441065	0.244258	0.49359	NOT
RP11-475(	2.602248	-0.7881081	0.676843	-1.16438827	0.244267	0.49359	NOT
RP11-646I	6.313493	-0.3793161	0.325769	-1.16437135	0.244274	0.49359	NOT
OST4	5849.646	0.21153539	0.181678	1.164342145	0.244285	0.49359	NOT

LINC0125(	1.780692	0.98352171	0.844751	1.164273654	0.244313	0.49359	NOT
HSPB1P2	10.41775	0.39909502	0.34279	1.164254563	0.244321	0.49359	NOT
BRE	1561.654	0.19513947	0.167626	1.16413576	0.244369	0.493651	NOT
HOXB8	5.862553	0.73068144	0.62776	1.16394941	0.244445	0.493732	NOT
FAM196A	4.826321	-0.5124066	0.440232	-1.16394704	0.244445	0.493732	NOT
RP11-426K	2.21353	0.5292598	0.454738	1.163878542	0.244473	0.493751	NOT
WDR70	987.6977	0.15133877	0.13004	1.163783515	0.244512	0.493792	NOT
ZNF614	211.8365	0.3496486	0.300474	1.163658397	0.244562	0.493858	NOT
SERPINB4	0.930327	1.79137188	1.539638	1.163502316	0.244626	NA	NA
NLRC3	128.1242	-0.3356194	0.288461	-1.16348224	0.244634	0.493952	NOT
RP11-53B2	1.621249	0.5869313	0.504479	1.163441064	0.244651	0.493952	NOT
SAMD3	26.77871	-0.4121721	0.35431	-1.1633085	0.244704	0.493952	NOT
RP11-61K9	115.6288	-0.2950345	0.253619	-1.16329897	0.244708	0.493952	NOT
RP11-34P1	4.361811	-0.5912234	0.508233	-1.16329231	0.244711	0.493952	NOT
SPAG16	396.7159	0.34358684	0.295365	1.163263062	0.244723	0.493952	NOT
RP11-192H	48.18422	0.32155809	0.276436	1.163229329	0.244736	0.493952	NOT
RP11-21K1	5.557607	0.51704143	0.444546	1.163076758	0.244798	0.49404	NOT
CTA-228A9	9.234674	0.42445519	0.364974	1.162973814	0.24484	0.49405	NOT
RP11-88H9	28.31627	0.64683056	0.556193	1.162961054	0.244845	0.49405	NOT
FAM86B1	21.83771	0.49090589	0.422129	1.162929705	0.244858	0.49405	NOT
DNM1P51	4.032407	0.52054258	0.447792	1.162465216	0.245047	0.494394	NOT
ARMC8	1157.492	0.1227548	0.105604	1.1624023	0.245072	0.494394	NOT
AC093388.	10.35217	-0.3634444	0.312674	-1.16237514	0.245083	0.494394	NOT
RP11-284(	17.74691	1.15546958	0.994227	1.162178398	0.245163	0.494518	NOT
WDR6	3869.314	0.1883788	0.162099	1.162122955	0.245186	0.494526	NOT
SLC22A14	3.640867	-0.6288578	0.541175	-1.16202363	0.245226	0.494526	NOT
ZFC3H1	935.6948	0.19370169	0.166698	1.161993048	0.245238	0.494526	NOT
RNF181	4290.476	-0.2477335	0.213198	-1.16198924	0.24524	0.494526	NOT
RP11-173F	3.890982	0.55827301	0.480593	1.161634186	0.245384	0.49478	NOT
DCAF8L2	46.11866	-1.2002154	1.03334	-1.16149086	0.245442	0.494861	NOT
MAP2K6	306.9412	-0.3545231	0.305262	-1.16137282	0.24549	0.494921	NOT
AC013460.	1.622943	1.1766823	1.013257	1.161286582	0.245525	0.494955	NOT
PTPDC1	190.4679	0.22158752	0.190841	1.161113463	0.245596	0.49506	NOT
RP11-442(	2.632142	0.43859792	0.377816	1.160878051	0.245691	0.495216	NOT
PLA2G2D	91.12459	0.75759211	0.65266	1.160776191	0.245733	0.495263	NOT
IFT74	432.5003	0.21832519	0.188102	1.160673649	0.245775	0.49531	NOT
C11orf31	3302.701	0.19163712	0.165117	1.160616661	0.245798	0.495316	NOT
SMC5	1234.682	-0.2267245	0.195355	-1.16057699	0.245814	0.495316	NOT
RP11-74E2	2.871627	0.88107974	0.759447	1.160159521	0.245984	0.495621	NOT
LINC00923	10.62057	0.87296679	0.752512	1.160070361	0.24602	0.495658	NOT
RP11-468E	20.96106	-0.2951039	0.254445	-1.15979475	0.246132	0.495847	NOT
AC131097.	4.146543	-0.7257236	0.625795	-1.15968228	0.246178	0.495902	NOT
NBPF2P	5.737334	-0.4552441	0.392581	-1.1596183	0.246204	0.495918	NOT
RTL1	4.721288	1.05556463	0.910326	1.159545894	0.246234	0.495941	NOT
DPEP2	85.48964	-0.3115337	0.268695	-1.1594324	0.24628	0.495952	NOT
RP11-602.	17.28379	-0.4131596	0.356365	-1.1593734	0.246304	0.495952	NOT
RP11-775D	4.494717	0.36305146	0.313146	1.159368678	0.246306	0.495952	NOT
CAMSAP2	1344.085	0.24893666	0.214733	1.159286892	0.246339	0.495952	NOT
SPPL2A	4596.679	-0.2065271	0.178152	-1.15927236	0.246345	0.495952	NOT

CAND2	132.2562	-0.5716665	0.493129	-1.1592626	0.246349	0.495952	NOT
SWAP70	1502.295	0.2296233	0.198098	1.159138283	0.2464	0.496017	NOT
MIR3685	11.65894	0.48264847	0.416432	1.159009314	0.246452	0.496046	NOT
CYP2C8	12868.16	-0.6457618	0.557169	-1.15900566	0.246454	0.496046	NOT
CTC-2310I	24.83127	0.57497015	0.496107	1.15896337	0.246471	0.496046	NOT
SLC4A4	1646.463	-0.4807677	0.41484	-1.15892408	0.246487	0.496046	NOT
SH3TC1	1476.968	-0.2903898	0.250589	-1.15882723	0.246527	0.496089	NOT
RP11-16N1	25.35123	-0.3430783	0.296084	-1.1587178	0.246571	0.496142	NOT
SLC30A5	1957.304	-0.1678899	0.144917	-1.15852178	0.246651	0.496266	NOT
HDGFRP2	2948.116	0.20359222	0.175752	1.158405642	0.246699	0.496301	NOT
ZNF165	176.3364	0.35943571	0.310289	1.158389273	0.246705	0.496301	NOT
AC079767.	10.45642	0.55779692	0.481616	1.158177785	0.246791	0.496417	NOT
RP11-190A	11.16605	-0.5636445	0.486673	-1.15815853	0.246799	0.496417	NOT
TM4SF4	23761.64	-0.4263852	0.368214	-1.15798093	0.246872	0.496526	NOT
CNTNAP3	82.64456	-0.6557933	0.56639	-1.15784787	0.246926	0.496598	NOT
MOCOS	1890.843	-0.3106985	0.268358	-1.15777529	0.246956	0.496621	NOT
DRD4	43.5169	0.42369545	0.366035	1.157527343	0.247057	0.496753	NOT
C9orf24	23.37285	-0.4630203	0.400009	-1.15752437	0.247058	0.496753	NOT
REG3G	3.904551	-1.727418	1.492435	-1.15744976	0.247089	0.496777	NOT
ELL2P1	6.142953	-0.433183	0.374295	-1.1573313	0.247137	0.496813	NOT
ITPKA	473.7384	0.48734098	0.421101	1.157301455	0.247149	0.496813	NOT
RP11-778I	25.76519	0.36719526	0.317294	1.157272181	0.247161	0.496813	NOT
FAM35BP	25.61705	-0.4545388	0.392804	-1.15716461	0.247205	0.496864	NOT
PIGW	356.8088	0.1920197	0.165974	1.15692861	0.247302	0.497021	NOT
RP11-427I	2.113514	-0.4377933	0.378444	-1.15682411	0.247344	0.49707	NOT
PLEKHG6	534.9603	0.43525997	0.376305	1.156668316	0.247408	0.497161	NOT
CRPP1	4.31151	0.92890969	0.803289	1.156383104	0.247524	0.497343	NOT
LINC0148	73.91557	0.45534505	0.393775	1.156357641	0.247535	0.497343	NOT
SPON1	177.1257	-0.5608956	0.485173	-1.15607344	0.247651	0.497528	NOT
AC093901.	23.17239	-0.4645436	0.40184	-1.15604271	0.247664	0.497528	NOT
PCDH19	11.77277	-0.5318837	0.460112	-1.15598868	0.247686	0.497536	NOT
CTB-31N19	10.52537	0.30156174	0.2609	1.155852538	0.247741	0.497611	NOT
CD59	11934.88	-0.2191577	0.18963	-1.15571188	0.247799	0.497616	NOT
JRKL	338.5971	0.23585244	0.20408	1.155686404	0.247809	0.497616	NOT
VN1R83P	6.594736	-0.4876651	0.422002	-1.1555982	0.247846	0.497616	NOT
RNF7	2366.882	0.15131259	0.130943	1.155564869	0.247859	0.497616	NOT
CTD-2396I	638.6316	-0.3764003	0.325729	-1.15556208	0.24786	0.497616	NOT
HEPHL1	9.786133	0.50794805	0.439578	1.155536676	0.247871	0.497616	NOT
AC073850.	3.174793	-0.6434298	0.556825	-1.15553233	0.247873	0.497616	NOT
TXN2	5233.216	-0.2316906	0.200514	-1.15548429	0.247892	0.497619	NOT
TRPC2	5.258225	-0.4751669	0.411297	-1.15529026	0.247972	0.497741	NOT
MGMT	4134.98	-0.3421584	0.296253	-1.15495515	0.248109	0.497975	NOT
AMFR	7762.62	-0.2489642	0.215569	-1.15491596	0.248125	0.497975	NOT
LBX2-AS1	1398.883	-0.2614391	0.2264	-1.154764	0.248187	0.498028	NOT
HMGN2	6189.368	0.22653403	0.196191	1.154657891	0.248231	0.498028	NOT
TMEM14D	2.976108	0.5117018	0.443177	1.154622742	0.248245	0.498028	NOT
LILRB3	92.58958	-0.333499	0.288841	-1.15461249	0.248249	0.498028	NOT
SEPT4	2002.679	-0.528716	0.457932	-1.15457212	0.248266	0.498028	NOT
FAM153C	2.87283	0.88275163	0.764588	1.154545124	0.248277	0.498028	NOT

GMDS	1480.073	0.31382177	0.271816	1.154538453	0.248279	0.498028	NOT
RP11-329A	14.56391	-0.3270807	0.283387	-1.15418315	0.248425	0.498283	NOT
RP11-37C7	11.26254	0.39372694	0.341157	1.154094106	0.248462	0.498319	NOT
MYOZ2	2.401078	-0.6357152	0.550897	-1.15396382	0.248515	0.498351	NOT
EEF1B2P2	5.055496	0.42033923	0.364257	1.153963301	0.248515	0.498351	NOT
RP11-536K	1.450972	0.67865585	0.58813	1.153920517	0.248533	0.498351	NOT
RP11-684E	1.362833	-0.6696986	0.580496	-1.15366646	0.248637	NA	NA
DDTL	235.4375	-0.3631567	0.314791	-1.15364402	0.248646	0.498542	NOT
LINC00316	1.959551	1.05164083	0.911763	1.153414698	0.24874	0.498673	NOT
CNPPD1	3234.287	0.16798721	0.145646	1.153395534	0.248748	0.498673	NOT
ZBTB20	65.871	-0.2743502	0.237904	-1.15319761	0.248829	0.498798	NOT
CAPZA2	3945.98	0.16170442	0.140252	1.15295643	0.248928	0.498922	NOT
RP11-113K	3.974852	-0.488944	0.42408	-1.15295273	0.24893	0.498922	NOT
ZNFX1	2242.597	-0.2259735	0.196002	-1.15291349	0.248946	0.498922	NOT
ATP8B4	137.4274	-0.316767	0.274771	-1.15283968	0.248976	0.498946	NOT
TNFSF10	5055.677	-0.3872396	0.335966	-1.15261487	0.249068	0.499094	NOT
ST3GAL1	8194.844	-0.3714828	0.322311	-1.15256132	0.24909	0.499101	NOT
ZCCHC6	1855.115	-0.293147	0.254396	-1.15232518	0.249187	0.499258	NOT
WRB	1194.941	-0.1948583	0.169109	-1.15226412	0.249213	0.499272	NOT
RNA5SP122	2.16583	-0.4915307	0.42662	-1.15214997	0.249259	0.499329	NOT
ZNF223	33.37667	-0.2929442	0.254281	-1.15204951	0.249301	0.499375	NOT
C1orf162	374.848	-0.3512459	0.304929	-1.1518925	0.249365	0.499427	NOT
SLC44A5	302.5909	-0.7167809	0.622266	-1.15188743	0.249367	0.499427	NOT
DLEU7-AS1	5.129636	0.57834862	0.502123	1.151806394	0.249401	0.499427	NOT
CALCOCO2	5240.004	-0.1734375	0.15058	-1.15179907	0.249404	0.499427	NOT
GHITM	11096.24	-0.2119538	0.18403	-1.15173241	0.249431	0.499427	NOT
GLDC	3729.323	-0.4363854	0.3789	-1.15171679	0.249437	0.499427	NOT
GALNT11	1655.9	-0.2049078	0.177932	-1.15160603	0.249483	0.499474	NOT
IL15	130.9636	-0.3655847	0.317469	-1.1515613	0.249501	0.499474	NOT
RP11-492M	3.271159	0.50939204	0.442378	1.151484714	0.249533	0.499474	NOT
RAB11FIP5	1076.494	0.25447381	0.221015	1.151387037	0.249573	0.499474	NOT
C14orf79	240.7809	0.22398928	0.194543	1.151362732	0.249583	0.499474	NOT
NEBL-AS1	2.529587	1.14957889	0.998454	1.151358817	0.249585	0.499474	NOT
MTSS1L	2091.899	-0.236757	0.205642	-1.15130801	0.249606	0.499474	NOT
VAMP8	4031.959	0.26560806	0.230709	1.151269886	0.249621	0.499474	NOT
RP5-875O1	6.893433	-0.620653	0.539109	-1.15125666	0.249627	0.499474	NOT
RP11-96C2	3.689782	0.7334658	0.637142	1.151181601	0.249658	0.499499	NOT
HLA-J	147.6444	-0.4540658	0.394456	-1.15111848	0.249683	0.499514	NOT
RP11-103E	6.444003	0.64609082	0.561349	1.150961701	0.249748	0.499601	NOT
PTPMT1	692.3067	0.17608984	0.152999	1.150923894	0.249764	0.499601	NOT
SDHB	5026.826	-0.2221163	0.193022	-1.15073128	0.249843	0.499722	NOT
MT1P1	3.460758	0.49943986	0.434114	1.150481052	0.249946	0.499892	NOT
TBX2-AS1	35.94378	-0.3933605	0.341935	-1.15039565	0.249981	0.499903	NOT
RP11-553K	2.518262	-0.4242462	0.368789	-1.1503775	0.249988	0.499903	NOT
KANSL1L	598.4497	0.28912854	0.251395	1.150095292	0.250105	0.500064	NOT
HSPA1L	96.37091	0.22839347	0.198587	1.150092965	0.250106	0.500064	NOT
TMED6	166.5076	-0.4956353	0.431056	-1.14981528	0.25022	0.500256	NOT
DPY19L3	739.3735	-0.1775242	0.154412	-1.14967677	0.250277	0.500333	NOT
RP11-113K	9.071576	-0.5078348	0.441747	-1.14960673	0.250306	0.500354	NOT

GFPT2	119.291	-0.550201	0.47866	-1.1494604	0.250366	0.500377	NOT
PPFIA3	523.0602	0.29684146	0.258258	1.149397761	0.250392	0.500377	NOT
TMEM161B	622.6107	-0.2076517	0.180668	-1.14935761	0.250409	0.500377	NOT
TOMM40L	2015.868	0.24647153	0.214444	1.149349991	0.250412	0.500377	NOT
SIRT2	2864.681	-0.2047438	0.178141	-1.14933512	0.250418	0.500377	NOT
FAM114A2	883.3628	-0.1749041	0.152182	-1.14930985	0.250428	0.500377	NOT
RP4-595K1	7.72793	1.06103557	0.923313	1.149160795	0.25049	0.500424	NOT
IGKV1-16	35.56007	0.88499762	0.770176	1.149084648	0.250521	0.500424	NOT
RP5-991G2	82.7778	0.33185229	0.288804	1.149055445	0.250533	0.500424	NOT
MAN1A1	8617.583	-0.2935837	0.255511	-1.14900411	0.250554	0.500424	NOT
TUBB2A	2563.585	0.34773052	0.302639	1.148995146	0.250558	0.500424	NOT
FDX1L	119.5214	0.25527202	0.222172	1.148983795	0.250563	0.500424	NOT
FEZF1-AS1	53.89642	-0.8543061	0.743584	-1.1489033	0.250596	0.500454	NOT
ICOS	36.16253	0.54030665	0.470315	1.148819795	0.25063	0.500459	NOT
MAGEE1	89.927	0.40678017	0.354089	1.14880787	0.250635	0.500459	NOT
RP11-1105	0.895375	1.71388549	1.491885	1.148805434	0.250636	NA	NA
RP11-98J2	2.507405	-0.5097312	0.443758	-1.14867027	0.250692	0.50048	NOT
KLHL4	36.11599	0.4716933	0.410644	1.14866759	0.250693	0.50048	NOT
NCSTN	7170.131	0.1626803	0.14163	1.1486297	0.250709	0.50048	NOT
RP11-107N	2.720544	0.91791753	0.79916	1.148603288	0.25072	0.50048	NOT
RP11-222K	12.06547	0.52981231	0.461304	1.148509473	0.250758	0.500486	NOT
RP11-212I	3.254124	0.54625992	0.475626	1.148506499	0.25076	0.500486	NOT
FNIP1	1442.568	-0.1995603	0.173794	-1.14825701	0.250862	0.500655	NOT
PPP2CB	2520.09	0.22202323	0.193371	1.148170265	0.250898	0.500689	NOT
PEX14	1262.917	-0.2065255	0.179905	-1.14796918	0.250981	0.500818	NOT
FRA10AC1	842.8016	-0.183052	0.159473	-1.14785452	0.251029	0.500876	NOT
LILRA4	12.31653	-0.5188438	0.452034	-1.14779739	0.251052	0.500886	NOT
TOMM20P4	2.416371	0.57813385	0.503735	1.147695296	0.251094	0.500933	NOT
RAD17	958.8844	-0.1360196	0.118545	-1.14741043	0.251212	0.501131	NOT
CRYAA	3.785452	-0.852985	0.743476	-1.14729388	0.25126	0.50119	NOT
ARHGEF19	185.7797	0.37003922	0.322583	1.147112295	0.251335	0.501274	NOT
HIST2H2BI	4.930999	-0.4076019	0.355332	-1.14710245	0.251339	0.501274	NOT
RP3-508I1	3.813372	0.45973439	0.400813	1.147004327	0.25138	0.501306	NOT
PNKD	9343.481	0.23145249	0.2018	1.146940165	0.251406	0.501306	NOT
RP11-479(	2.792545	-0.5431375	0.473567	-1.14690653	0.25142	0.501306	NOT
RP11-153I	4.025671	0.47719988	0.416084	1.146884665	0.251429	0.501306	NOT
ARHGEF17	991.8621	-0.2960047	0.258127	-1.1467389	0.25149	0.50139	NOT
AC145110.	3.017365	0.57973144	0.5056	1.146621208	0.251538	0.50145	NOT
C19orf80	4147.264	0.59021748	0.514779	1.146544686	0.25157	0.501476	NOT
TMEM41B	2589.005	0.17195239	0.149985	1.146461733	0.251604	0.501508	NOT
TRIM21	873.2802	0.20484774	0.178717	1.146210835	0.251708	0.501643	NOT
RP1-29C18	20.41197	0.70994711	0.619387	1.146208576	0.251709	0.501643	NOT
SARS2	309.813	-0.2504471	0.218557	-1.14591203	0.251832	0.50185	NOT
RP11-449F	173.9477	-0.5221417	0.455772	-1.14562085	0.251952	0.502054	NOT
MAP1LC3B	2312.705	-0.1957847	0.170912	-1.14552843	0.25199	0.50207	NOT
P4HTM	1024.409	0.3475448	0.303397	1.145510978	0.251998	0.50207	NOT
RP11-336A	8.764266	-0.5172904	0.451625	-1.14539895	0.252044	0.502108	NOT
RP11-578(	6.470314	0.38560447	0.336662	1.145375644	0.252054	0.502108	NOT
OCLN	1332.134	-0.3957639	0.345588	-1.14519149	0.25213	0.502223	NOT

DCTN2	4105.511	0.14417028	0.125911	1.145018814	0.252201	0.502304	NOT
TRAV29DV5	2.910814	-0.6129305	0.535355	-1.14490386	0.252249	0.502304	NOT
RP11-440(	10.66276	-0.4630184	0.404425	-1.14488153	0.252258	0.502304	NOT
RPL12L3	5.362598	0.36512935	0.318931	1.144853416	0.25227	0.502304	NOT
RP11-215A	22.89765	0.39526072	0.345253	1.144843173	0.252274	0.502304	NOT
RP11-287I	8.254408	0.64681717	0.564992	1.144825113	0.252282	0.502304	NOT
LRRC4C	18.38715	-0.5394363	0.47129	-1.14459464	0.252377	0.502443	NOT
SNORA71C	3.57086	0.48547873	0.424159	1.144567962	0.252388	0.502443	NOT
PRDM4	786.983	0.12832422	0.112123	1.144493131	0.252419	0.502468	NOT
CTTNBP2	166.9374	0.68208692	0.596065	1.14431654	0.252492	0.502577	NOT
RP11-139H	8.596926	-0.526859	0.460474	-1.14416606	0.252555	0.502664	NOT
CA7	1.116607	-0.6786308	0.59329	-1.14384396	0.252688	NA	NA
AC069213.	65.85694	0.50630065	0.442634	1.143835242	0.252692	0.5029	NOT
HIST2H4A	21.07853	0.46941054	0.410426	1.143715061	0.252742	0.502962	NOT
RP11-572(	23.74445	0.40626843	0.355264	1.143567774	0.252803	0.503021	NOT
TWF1P1	3.285114	0.53825656	0.470687	1.143554727	0.252808	0.503021	NOT
MIR3180-1	2.797321	-0.6843037	0.598495	-1.14337396	0.252883	0.503131	NOT
ARSEP1	53.56347	0.74753608	0.653823	1.143331445	0.252901	0.503131	NOT
AP000640.	2.022111	-0.5487223	0.479967	-1.14324923	0.252935	0.503162	NOT
APOC2	3432.717	-0.4856125	0.424831	-1.14307322	0.253008	0.503271	NOT
RET	110.4083	-0.6533878	0.571635	-1.14301616	0.253032	0.503281	NOT
RPL7AP30	23.04643	0.36667581	0.320885	1.14270032	0.253163	0.503468	NOT
NIPAL3	560.6253	-0.2083206	0.182313	-1.14265439	0.253182	0.503468	NOT
NUP153	2230.012	0.21419513	0.187455	1.142647634	0.253185	0.503468	NOT
CTA-384D8	179.1265	0.31227149	0.273297	1.142606827	0.253202	0.503468	NOT
ARRDC1	1482.591	0.18621866	0.162983	1.142566126	0.253219	0.503468	NOT
RP11-946F	3.625111	0.40407186	0.353682	1.142473501	0.253257	0.503474	NOT
RP11-804H	13.38923	0.37059161	0.324377	1.142470393	0.253259	0.503474	NOT
PTGIS	324.7513	-0.6288831	0.550599	-1.14218088	0.253379	0.503597	NOT
CTNND1	9209.496	-0.1728552	0.151351	-1.1420847	0.253419	0.503597	NOT
AF001548.	34.29895	0.33546296	0.293742	1.142033309	0.25344	0.503597	NOT
CHID1	5163.969	-0.2268739	0.198659	-1.14202445	0.253444	0.503597	NOT
RP11-568A	1.692545	1.00585681	0.880772	1.142016866	0.253447	0.503597	NOT
KBTBD6	409.8218	-0.210385	0.184223	-1.14200952	0.25345	0.503597	NOT
DOC2A	14.40181	0.51076346	0.44725	1.142008282	0.253451	0.503597	NOT
RP11-70C1	5.992504	-0.5800314	0.507959	-1.14188569	0.253502	0.503624	NOT
BCAN	164.2493	0.59548398	0.521498	1.141872522	0.253507	0.503624	NOT
COL18A1-A	2.324906	0.77543725	0.679128	1.141812906	0.253532	0.503624	NOT
SOX30	2.854799	0.53223706	0.466145	1.141783933	0.253544	0.503624	NOT
TIGD7	93.54156	-0.2538139	0.222302	-1.14175254	0.253557	0.503624	NOT
CTD-3138B	7.609397	0.39236537	0.343753	1.141417044	0.253696	0.503864	NOT
RP11-809(	0.735559	1.10500276	0.968122	1.141387743	0.253709	NA	NA
FAM227B	92.84041	-0.2865309	0.251042	-1.14136704	0.253717	0.503869	NOT
CASP7	1061.274	0.20557337	0.18018	1.14093155	0.253898	0.504178	NOT
ZNF890P	2.250429	-0.7003809	0.613883	-1.14090274	0.25391	0.504178	NOT
GSDMC	31.19706	0.58334784	0.511391	1.140707643	0.253992	0.50428	NOT
CLECL1	15.88892	0.48117636	0.421836	1.140671408	0.254007	0.50428	NOT
MTRNR2L6	3.528752	-0.4020686	0.352492	-1.14064558	0.254017	0.50428	NOT
FMNL2	757.3033	0.39958214	0.350348	1.140529275	0.254066	0.504339	NOT

CBFA2T2	1110.8	0.19510255	0.17108	1.140416609	0.254113	0.504379	NOT
MIR6772	3.332789	-0.6152405	0.539499	-1.14039191	0.254123	0.504379	NOT
PKP1	19.16894	-0.6315197	0.553799	-1.14033999	0.254145	0.504385	NOT
PLCXD3	59.59421	-0.640686	0.561893	-1.14022833	0.254191	0.504427	NOT
PECR	4427.928	-0.315497	0.276703	-1.14020031	0.254203	0.504427	NOT
GAA	9661.328	-0.2418496	0.212122	-1.14014173	0.254227	0.504438	NOT
RP11-10J2	1.297572	0.78453999	0.688182	1.140018995	0.254278	NA	NA
DYNC1H1	10907.84	0.18056316	0.158387	1.140014023	0.25428	0.504507	NOT
TECTB	25.10593	0.63429046	0.55642	1.139949276	0.254307	0.504524	NOT
RP11-755E	12.87066	0.40419793	0.354591	1.139897713	0.254329	0.504529	NOT
CCDC108	11.03736	-0.5255212	0.461063	-1.13980404	0.254368	0.50457	NOT
TTC24	5.997408	0.74137611	0.65048	1.139736786	0.254396	0.504589	NOT
STAG3L1	9.536328	-0.3263572	0.286371	-1.1396314	0.25444	0.504639	NOT
CCDC115	955.4581	0.20800818	0.182544	1.139495638	0.254496	0.504714	NOT
NEB	1117.923	0.58079462	0.509724	1.139429081	0.254524	0.504732	NOT
PTOV1-AS1	263.3688	0.20436364	0.179381	1.139273961	0.254589	0.504817	NOT
RP11-445I	1.706577	1.0929179	0.959343	1.139235741	0.254605	0.504817	NOT
COL27A1	3298.636	0.34528312	0.303094	1.139193147	0.254623	0.504817	NOT
TMEM81	176.9873	0.20711953	0.181831	1.139077612	0.254671	0.504875	NOT
ZNF30	178.0631	0.20742378	0.182131	1.138868311	0.254758	0.505011	NOT
TCAIM	2169.276	-0.1989389	0.174696	-1.13877161	0.254798	0.505055	NOT
TRIM34	11.5982	-0.2961145	0.26011	-1.1384202	0.254945	0.505285	NOT
ZNF782	133.0833	0.22563215	0.1982	1.138404097	0.254952	0.505285	NOT
HMGA1P2	3.306722	-0.4273551	0.375417	-1.13834765	0.254975	0.505294	NOT
MAGI2-AS3	534.3077	-0.5566317	0.489041	-1.13820959	0.255033	0.505372	NOT
ZNF248	389.0342	0.20710876	0.181984	1.138063353	0.255094	0.505456	NOT
VWA5B2	31.09714	0.58610467	0.515024	1.138015297	0.255114	0.505459	NOT
CHCHD5	1075.353	0.27410667	0.240884	1.137919056	0.255154	0.505501	NOT
TGIF1	2068.719	0.24839951	0.218329	1.13772958	0.255233	0.50557	NOT
TTI2	289.1984	0.18782126	0.165088	1.13770509	0.255244	0.50557	NOT
ARAP1	4222.316	0.13355662	0.117392	1.137702591	0.255245	0.50557	NOT
PIAS3	736.1927	0.24286203	0.213491	1.13757527	0.255298	0.505608	NOT
RP11-927F	6.789826	-0.3714257	0.326524	-1.13751394	0.255323	0.505608	NOT
KB-1552D7	8.77748	0.49698992	0.436922	1.137478645	0.255338	0.505608	NOT
CEL	55.97658	-0.561029	0.49323	-1.13745884	0.255347	0.505608	NOT
GDNF	152.236	-0.91444	0.803978	-1.13739458	0.255373	0.505608	NOT
BCAS1	231.3516	0.69895507	0.614526	1.137389121	0.255376	0.505608	NOT
RASGEF1B	811.323	-0.3538935	0.311173	-1.1372875	0.255418	0.505655	NOT
ZNF627	427.9229	-0.1857311	0.163366	-1.13690027	0.25558	0.505938	NOT
HERC4	1525.859	-0.1669254	0.146848	-1.13672567	0.255653	0.50597	NOT
GSTM1	1990.602	-0.9459731	0.832196	-1.13671859	0.255656	0.50597	NOT
COQ10A	1133.335	-0.3311146	0.291293	-1.13670726	0.255661	0.50597	NOT
LINC00563	5.190577	0.65296804	0.57445	1.136683854	0.25567	0.50597	NOT
HS3ST1	135.2304	0.48101133	0.423261	1.136442557	0.255771	0.506133	NOT
TMEM126A	891.5064	0.17775156	0.156419	1.136377881	0.255798	0.506149	NOT
ANP32AP1	2.680332	0.40491826	0.35635	1.13629228	0.255834	0.506161	NOT
EDEM3	3478.405	-0.242676	0.213578	-1.13624174	0.255855	0.506161	NOT
RP11-550I	39.29314	-0.2883367	0.253766	-1.13622973	0.25586	0.506161	NOT
LINC00677	2.723971	-0.5527255	0.486526	-1.13606649	0.255929	0.506259	NOT

KIF27	65.6109	-0.2475775	0.217943	-1.13597251	0.255968	0.5063	NOT
IGSF1	509.236	0.65997781	0.581029	1.135876635	0.256008	0.506337	NOT
RP4-669K1	20.75038	0.3224655	0.283901	1.135838946	0.256024	0.506337	NOT
CELA3B	1.868428	1.08392897	0.954518	1.135577719	0.256133	0.506459	NOT
RP1-130H1	3.220294	-0.4221413	0.371744	-1.13557012	0.256137	0.506459	NOT
HSBP1P2	3.586734	-0.761623	0.670704	-1.13555764	0.256142	0.506459	NOT
VWCE	1246.565	0.55080557	0.485088	1.13547492	0.256176	0.506491	NOT
RP11-376F	3.091785	0.48993221	0.431512	1.135384227	0.256214	0.506515	NOT
CTD-3035K	2.994029	0.72473153	0.638332	1.135352431	0.256228	0.506515	NOT
GJB1	19372.17	-0.3810066	0.335606	-1.13528055	0.256258	0.506515	NOT
RP11-91P2	3.386082	-0.430021	0.378786	-1.13526209	0.256266	0.506515	NOT
PHKA1P1	2.198134	-0.5031316	0.443238	-1.1351287	0.256321	0.506515	NOT
XIAP	2577.486	-0.1685353	0.148473	-1.13512092	0.256325	0.506515	NOT
GPR108	3124.708	-0.1557877	0.137246	-1.13510136	0.256333	0.506515	NOT
DCST1	22.72911	0.34445366	0.303459	1.135089485	0.256338	0.506515	NOT
THBS2	2251.41	-0.5488501	0.483574	-1.13498782	0.25638	0.506562	NOT
ZSCAN25	497.5525	0.16093209	0.141803	1.134901201	0.256417	0.506597	NOT
LINC00332	2.438993	1.02252198	0.90105	1.134812085	0.256454	0.5066	NOT
AC139452.	3.588542	0.54640255	0.481493	1.134808546	0.256456	0.5066	NOT
DPPA4	10.15514	-0.7090057	0.624861	-1.1346614	0.256517	0.506657	NOT
RP11-440I	62.51696	-0.2362195	0.208187	-1.13465011	0.256522	0.506657	NOT
RP11-31H5	13.62282	0.29975736	0.264196	1.134603073	0.256542	0.506659	NOT
CDKN2C	1141.502	0.37043969	0.326518	1.134516343	0.256578	0.506684	NOT
NCOA7-AS1	2.430264	-0.7707539	0.679387	-1.13448362	0.256592	0.506684	NOT
PON3	5489.998	-0.4485727	0.395425	-1.1344058	0.256624	0.506712	NOT
RP11-392O	2.383075	0.68226471	0.601581	1.134118593	0.256745	0.506913	NOT
ARHGEF34P	105.0771	0.40449531	0.356702	1.133985309	0.256801	0.506986	NOT
ATHL1	3498.703	-0.5011754	0.441997	-1.1338883	0.256841	0.507022	NOT
RP11-1008	32.92848	0.3822009	0.337104	1.133778071	0.256888	0.507022	NOT
ZSWIM3	187.5628	0.1571632	0.13862	1.133770973	0.256891	0.507022	NOT
RP11-128N	2.440991	0.58199186	0.513334	1.133748437	0.2569	0.507022	NOT
CYP26A1	68.92565	-0.7008925	0.618224	-1.13371939	0.256912	0.507022	NOT
STXBP5L	1.637216	0.92082512	0.812309	1.133590052	0.256967	0.507092	NOT
UBQLN4P1	5.686902	0.39434806	0.347891	1.133539002	0.256988	0.507098	NOT
NFIL3	2232.214	-0.2724078	0.240374	-1.13326537	0.257103	0.507202	NOT
CAPN5	4403.165	-0.3065179	0.270477	-1.13324854	0.25711	0.507202	NOT
USP32P2	2.087361	0.8652097	0.763478	1.133247791	0.25711	0.507202	NOT
DLST	4900.515	-0.1862913	0.164389	-1.13323499	0.257116	0.507202	NOT
NCK2	1330.49	0.38400802	0.33899	1.132799638	0.257298	0.507525	NOT
PSMG2	1841.354	0.12452075	0.109933	1.132698527	0.257341	0.50757	NOT
FTLP17	4.462842	0.49586675	0.437791	1.132656576	0.257358	0.50757	NOT
C22orf29	829.5142	0.19346104	0.170829	1.132485783	0.25743	0.507675	NOT
CHMP2A	6530.295	-0.2392746	0.211294	-1.13242388	0.257456	0.507689	NOT
GYLTL1B	370.4243	0.55097154	0.486561	1.132379752	0.257475	0.507689	NOT
SEC61A2	412.5368	-0.2389485	0.211046	-1.13221036	0.257546	0.507792	NOT
SHH	852.112	-0.4049687	0.357762	-1.13194854	0.257656	0.507972	NOT
CST6	3.300771	0.731392	0.646235	1.131774586	0.257729	0.50808	NOT
LINC01358	7.045205	-0.3743875	0.330819	-1.13169915	0.257761	0.508105	NOT
RP11-368I	1.801572	1.17267098	1.036274	1.131622448	0.257793	0.508132	NOT

ZNF80	3.439436	-0.6456967	0.570625	-1.13156122	0.257819	0.508146	NOT
THRB-IT1	2.954496	0.64029786	0.565939	1.131390408	0.257891	0.508199	NOT
NUTM2E	3.622082	-0.8181847	0.723178	-1.13137436	0.257898	0.508199	NOT
FAM21FP	19.5222	-0.2588892	0.228829	-1.13136411	0.257902	0.508199	NOT
C6orf165	6.094326	-0.345077	0.305158	-1.13081501	0.258133	0.508617	NOT
RP11-467H	1.872616	-0.5360227	0.474084	-1.13064915	0.258203	0.508718	NOT
CH507-42F	760.4265	-0.4768324	0.421768	-1.13055531	0.258242	0.508737	NOT
ATF4P4	8.122035	0.38050674	0.336572	1.130536903	0.25825	0.508737	NOT
MAGT1	7107.318	-0.2120855	0.187637	-1.13029933	0.25835	0.508877	NOT
RP11-944I	2.488229	-0.5551246	0.49114	-1.13027865	0.258359	0.508877	NOT
ZGPAT	597.2302	-0.2229918	0.197303	-1.13020058	0.258392	0.508877	NOT
BTC	68.55063	0.49760171	0.440282	1.130189454	0.258396	0.508877	NOT
ZNF287	43.21023	0.44426943	0.393119	1.130115769	0.258427	0.508902	NOT
FLJ46284	19.47121	-0.2874939	0.254412	-1.13003415	0.258462	0.508932	NOT
FCGR2A	688.1216	0.37338658	0.330466	1.129880504	0.258527	0.509023	NOT
RP11-176I	1.363901	0.73589874	0.651415	1.129692086	0.258606	NA	NA
CD83	349.9067	0.28169179	0.249353	1.129689196	0.258607	0.509145	NOT
S100A13	1632.494	0.36811906	0.326072	1.128951162	0.258918	0.50972	NOT
SCML4	69.0696	-0.4225037	0.374299	-1.12878644	0.258988	0.509791	NOT
ZFP90	529.848	-0.1775551	0.157299	-1.12877674	0.258992	0.509791	NOT
LINC01359	11.24115	-0.3348881	0.296801	-1.12832371	0.259183	0.510129	NOT
TMEM74	101.3926	0.50921407	0.451329	1.128253827	0.259213	0.510129	NOT
RP5-1057I	1.871409	0.76087535	0.674393	1.12823655	0.25922	0.510129	NOT
HAO1	7823.475	-0.5249179	0.465335	-1.12804416	0.259301	0.510252	NOT
RPH3A	3.45673	-0.4853288	0.430266	-1.12797501	0.25933	0.510272	NOT
CADM3-AS1	2.232954	-0.9091394	0.806084	-1.12784744	0.259384	0.510341	NOT
LCMT1	793.9546	0.18464221	0.163743	1.127631818	0.259475	0.510484	NOT
RGPD3	10.4674	-0.4484997	0.397761	-1.12756007	0.259506	0.510506	NOT
CTD-3035K	4.986473	0.66306981	0.588097	1.127483887	0.259538	0.510533	NOT
TBCA	4925.751	0.21144791	0.187571	1.127297911	0.259617	0.51065	NOT
TESC-AS1	4.685578	0.86462951	0.767033	1.127239264	0.259641	0.510662	NOT
CXCR3	94.70991	0.44060702	0.390893	1.12717928	0.259667	0.510675	NOT
ERCC4	304.2506	-0.2303856	0.20442	-1.12701948	0.259734	0.510771	NOT
ENPP7	1132.034	0.53240352	0.472465	1.126864091	0.2598	0.510863	NOT
SSTR5-AS1	154.9034	-1.1938455	1.059497	-1.12680365	0.259826	0.510876	NOT
MYL12A	9843.032	0.19874313	0.176396	1.126687676	0.259875	0.510935	NOT
BMS1	1771.88	0.12272178	0.108929	1.12661782	0.259904	0.510956	NOT
RP11-359E	60.50041	-0.2882353	0.255853	-1.12656729	0.259925	0.510961	NOT
RPL12P4	12.8347	0.34532901	0.306559	1.126468093	0.259967	0.511007	NOT
JSRP1	26.5692	-0.4423492	0.392751	-1.126283	0.260046	0.511122	NOT
HMGN1P38	4.277756	0.47717047	0.423684	1.126241079	0.260063	0.511122	NOT
GSTT2	5.221643	0.77722672	0.690196	1.126096336	0.260125	0.511204	NOT
PROX1	7535.864	-0.3348419	0.297359	-1.12605292	0.260143	0.511204	NOT
RP11-69L1	2.263494	-0.4658675	0.413802	-1.12582182	0.260241	0.511359	NOT
RTCB	4089.686	-0.1915507	0.170163	-1.12568941	0.260297	0.5114	NOT
DTHD1	6.61735	-0.5782577	0.513699	-1.12567476	0.260303	0.5114	NOT
TLR9	2.591485	0.48259988	0.428734	1.125639247	0.260318	0.5114	NOT
RP5-1021I	1.125917	0.89576084	0.795823	1.125578115	0.260344	NA	NA
H1FX-AS1	86.9672	-0.3417641	0.303679	-1.1254107	0.260415	0.511527	NOT

QRSL1P1	4.295587	0.74556441	0.66249	1.12539721	0.260421	0.511527	NOT
TMEM74B	267.2725	0.41190417	0.366044	1.125287205	0.260467	0.511582	NOT
ALG11	210.8726	-0.2371179	0.21075	-1.12511546	0.26054	0.511679	NOT
ITGB6	52.39932	0.55290981	0.49144	1.125081826	0.260554	0.511679	NOT
ANKRD26	278.0491	0.23149064	0.205792	1.124879104	0.26064	0.51181	NOT
TRABD2B	439.716	0.49786859	0.44268	1.124669839	0.260729	0.511947	NOT
AP1M1	5139.014	-0.1847369	0.164294	-1.12443189	0.26083	0.512108	NOT
KRT8P37	2.187527	0.56647914	0.503849	1.124302456	0.260885	0.512179	NOT
AC073325.	1.586362	0.81090819	0.721285	1.124255357	0.260905	0.512181	NOT
SLC39A13	1776.482	0.17808081	0.158427	1.124058029	0.260988	0.512308	NOT
SUPT20H	840.7672	0.21163155	0.188296	1.123932741	0.261042	0.512376	NOT
ASB16-AS1	351.895	-0.1926332	0.171407	-1.12383695	0.261082	0.512418	NOT
RPL37P23	8.3179	0.46942391	0.41774	1.123723822	0.26113	0.512476	NOT
RPL31P11	2.38814	0.71344786	0.634938	1.123650483	0.261161	0.5125	NOT
FGR	274.0093	0.33559691	0.298748	1.123345891	0.261291	0.512716	NOT
C1orf109	499.0596	0.19171729	0.17068	1.123257865	0.261328	0.512752	NOT
LL22NC03-	37.66518	0.34086472	0.303485	1.12316847	0.261366	0.512778	NOT
ZC2HC1C	163.4122	-0.2412783	0.214825	-1.12313761	0.261379	0.512778	NOT
RP11-242I	47.99352	0.3510781	0.312635	1.122966045	0.261452	0.512884	NOT
CH507-396	0.77876	-0.8878251	0.790707	-1.122824	0.261512	NA	NA
SLC25A30-	16.03378	-0.3974523	0.353982	-1.12280358	0.261521	0.512983	NOT
CTB-113P1	10.29047	-0.3683765	0.328106	-1.12273742	0.261549	0.513001	NOT
RP11-17M1	29.77818	-0.2278763	0.202982	-1.12264372	0.261589	0.51302	NOT
EP400NL	553.4701	-0.2299638	0.204856	-1.12256351	0.261623	0.51302	NOT
EIF3F	5494.023	0.15793647	0.140694	1.122556216	0.261626	0.51302	NOT
C17orf80	883.0217	0.16791102	0.149583	1.122526813	0.261639	0.51302	NOT
SMYD4	426.2884	-0.1919866	0.171036	-1.12249223	0.261653	0.51302	NOT
DNAJC4	1588.606	-0.2909585	0.25923	-1.12239685	0.261694	0.513062	NOT
SUV39H1	851.0652	0.18368844	0.163676	1.122268481	0.261748	0.513132	NOT
ALG13-AS1	2.490592	-0.4849235	0.432109	-1.12222403	0.261767	0.513132	NOT
CTA-29F11	122.1043	0.25981098	0.231549	1.122054119	0.261839	0.513213	NOT
PPP4R1L	91.12114	-0.3009812	0.268245	-1.12203766	0.261846	0.513213	NOT
KIAA1244	588.0654	0.6241838	0.556433	1.121760066	0.261964	0.513407	NOT
RP11-527N	21.2563	0.48388814	0.431445	1.121552545	0.262053	0.513543	NOT
PARP10	5989.132	0.27085747	0.241517	1.121482202	0.262083	0.513565	NOT
NR2F2-AS1	26.82426	-0.2606802	0.232508	-1.12116433	0.262218	0.513793	NOT
C6orf226	297.6623	-0.2891767	0.257949	-1.12106033	0.262262	0.513842	NOT
SNTB2	767.4646	-0.1993337	0.177821	-1.12097947	0.262297	0.513873	NOT
RP11-430C	46.15803	0.47359634	0.422579	1.12072813	0.262404	0.514045	NOT
UNC45A	3026.36	0.17235469	0.153822	1.120483717	0.262508	0.514212	NOT
IK	4455.787	0.12741498	0.11375	1.12013603	0.262656	0.514465	NOT
ZFAND2A	2010.174	0.30918038	0.276039	1.120058963	0.262689	0.51447	NOT
RP6-7406.	15.34155	0.4000144	0.357159	1.119989535	0.262718	0.51447	NOT
ACKR3	982.0493	-0.3208099	0.286443	-1.11997821	0.262723	0.51447	NOT
MYRIP	1460.771	-0.6071543	0.542126	-1.11995155	0.262734	0.51447	NOT
RPS24	34577.53	0.2885015	0.257669	1.11965902	0.262859	0.514677	NOT
TSC2	3326.42	-0.193196	0.172581	-1.11945299	0.262947	0.514749	NOT
SOCS5P4	5.029036	0.44645219	0.398816	1.119444359	0.262951	0.514749	NOT
CTD-2639I	23.42661	0.33987646	0.303613	1.119439464	0.262953	0.514749	NOT

FASN	45534.91	-0.3672325	0.328148	-1.11910691	0.263095	0.514915	NOT
AP006621.	10.12372	-0.5485919	0.490206	-1.11910526	0.263095	0.514915	NOT
RPL13P2	2.651107	-0.5483461	0.490002	-1.11906851	0.263111	0.514915	NOT
RP5-965G2	7.692326	0.43377887	0.387627	1.119062753	0.263113	0.514915	NOT
LRRN1	21.4785	0.77742367	0.694827	1.118873703	0.263194	0.515015	NOT
FAM120AOS	1367.446	0.13745914	0.122859	1.118835215	0.26321	0.515015	NOT
MIIP	1298.49	0.26555814	0.237358	1.118809791	0.263221	0.515015	NOT
RP11-323F	1.895689	0.79174011	0.707753	1.118667574	0.263282	0.515073	NOT
KDF1	195.1841	0.46418406	0.41495	1.118651081	0.263289	0.515073	NOT
BMS1P20	72.49006	0.22629344	0.20232	1.11849178	0.263357	0.515157	NOT
RP11-550F	4.763678	-0.7044759	0.629862	-1.11846113	0.26337	0.515157	NOT
SH3GL1P2	5.057699	0.42204922	0.377371	1.118392202	0.2634	0.515164	NOT
LRR1	292.5218	0.21814902	0.195061	1.118364148	0.263411	0.515164	NOT
CTD-2008F	3.25039	1.20208748	1.074977	1.118245299	0.263462	0.515192	NOT
RP11-715J	7.619156	-0.4067389	0.363747	-1.11819049	0.263486	0.515192	NOT
RP11-264I	2.819564	-0.639435	0.571852	-1.11818243	0.263489	0.515192	NOT
C20orf195	3.616084	0.63204102	0.565254	1.118153031	0.263502	0.515192	NOT
TCEA1P3	0.971464	0.73900368	0.660965	1.118067579	0.263538	NA	NA
SIRPB1	42.95887	0.43893693	0.392613	1.117988254	0.263572	0.515285	NOT
PPFIA2	12.58442	0.50067287	0.447848	1.117952461	0.263587	0.515285	NOT
CD40LG	38.09138	-0.4300392	0.384701	-1.11785347	0.26363	0.51533	NOT
ARFGEF1	2340.086	0.19033853	0.170281	1.11778923	0.263657	0.515347	NOT
CDKN2D	236.2042	0.24041542	0.215113	1.117626384	0.263727	0.515434	NOT
LNPEP	1351.747	-0.2080138	0.186131	-1.11756385	0.263753	0.515434	NOT
RP11-187C	10.7496	0.36809769	0.329382	1.117540824	0.263763	0.515434	NOT
STAT2	4759.216	-0.2105995	0.188455	-1.11750764	0.263777	0.515434	NOT
RP11-285E	10.50675	-0.5472481	0.489735	-1.11743681	0.263808	0.515456	NOT
GTF2E2	882.4979	0.19723932	0.176522	1.117362559	0.263839	0.51548	NOT
C12orf29	590.2192	0.16880086	0.15109	1.117223261	0.263899	0.51556	NOT
IFNGR1	4007.804	-0.3154677	0.282437	-1.11694722	0.264017	0.515745	NOT
RP11-666A	9.351627	-0.3807723	0.340915	-1.11691187	0.264032	0.515745	NOT
AC064853.	0.571375	1.37267477	1.229247	1.11667953	0.264131	NA	NA
HMGB1P14	2.184241	-0.4107664	0.367856	-1.11664973	0.264144	0.515924	NOT
ANKRD11	2464.178	-0.1602489	0.143521	-1.11655246	0.264186	0.515924	NOT
RP11-214K	3.642348	0.47098957	0.42183	1.116539249	0.264191	0.515924	NOT
HSP90AA4F	1.891432	0.57127626	0.511659	1.116518182	0.2642	0.515924	NOT
OCIAD1	6007.477	-0.1539453	0.137897	-1.11638149	0.264259	0.515924	NOT
FAM71F2	47.875	-0.4390894	0.393318	-1.11637156	0.264263	0.515924	NOT
IAH1	1645.167	0.16007043	0.143386	1.116356931	0.264269	0.515924	NOT
NANOS3	4.378972	0.50900239	0.455956	1.116342083	0.264276	0.515924	NOT
RMND5A	4475.784	-0.2216117	0.198529	-1.11626922	0.264307	0.515948	NOT
KCTD20	4142.723	0.16368167	0.146664	1.116030698	0.264409	0.51611	NOT
GSKIP	980.3662	0.18789238	0.168366	1.115974076	0.264433	0.51612	NOT
ZFYVE16	1351.812	-0.1886557	0.169068	-1.11585936	0.264482	0.516168	NOT
SDC2	20947.1	0.2645801	0.237116	1.115824484	0.264497	0.516168	NOT
SLC22A12	470.5504	1.03689443	0.929365	1.115701473	0.26455	0.516168	NOT
DENND6B	268.4245	0.25329131	0.22703	1.115671172	0.264563	0.516168	NOT
CTC-236F1	8.956498	0.61671907	0.552785	1.11565815	0.264568	0.516168	NOT
ZGLP1	50.23775	-0.3346592	0.299984	-1.11558989	0.264598	0.516168	NOT

UBLCP1	890.1023	0.13539903	0.12137	1.115587	0.264599	0.516168	NOT
RP11-344F	4.649479	0.42652004	0.382337	1.115561355	0.26461	0.516168	NOT
RP11-308E	6.696163	1.16264492	1.042256	1.115508536	0.264633	0.516175	NOT
NRCAM	1072.317	-0.703343	0.630556	-1.11543245	0.264665	0.516182	NOT
NAV2	2179.725	-0.2950719	0.264541	-1.11541189	0.264674	0.516182	NOT
DCP1A	812.0646	0.1338039	0.119984	1.115179374	0.264774	0.516339	NOT
TFCP2L1	78.99508	-0.5473531	0.490902	-1.11499464	0.264853	0.516456	NOT
CCDC177	53.19868	-0.8604393	0.771742	-1.11493091	0.26488	0.516472	NOT
ATP6V1D	2046.048	0.16243177	0.145714	1.114727505	0.264967	0.516605	NOT
AC079807.	3.125231	0.53211961	0.477379	1.114669903	0.264992	0.516616	NOT
RP11-505E	4.175338	0.52036027	0.466892	1.114519636	0.265056	0.516695	NOT
CTB-43E15	2.940392	0.90778074	0.814528	1.114486989	0.26507	0.516695	NOT
TMEM134	1393.57	-0.2024459	0.181675	-1.11433155	0.265137	0.516788	NOT
AC090587.	6.779438	-0.3414987	0.306473	-1.11428713	0.265156	0.516788	NOT
RP5-855D2	9.162836	-0.4095523	0.36762	-1.11406385	0.265252	0.516933	NOT
NEDD8-MDF	2.789332	0.53931157	0.484111	1.114024823	0.265269	0.516933	NOT
RP11-95P1	1.436773	1.41265028	1.268161	1.11393592	0.265307	0.51697	NOT
RP11-1348	31.94326	0.25972622	0.233184	1.113825072	0.265354	0.517025	NOT
PLEKHM1	956.934	-0.1777282	0.159585	-1.11368825	0.265413	0.517087	NOT
STAM-AS1	10.66185	0.40562336	0.364236	1.113626823	0.265439	0.517087	NOT
RRP8	997.0283	0.14100664	0.126624	1.113585947	0.265457	0.517087	NOT
KIDINS22C	2213.81	-0.2056977	0.184722	-1.11355594	0.26547	0.517087	NOT
RPL15P2	3.734854	0.45142519	0.405415	1.113489578	0.265498	0.517087	NOT
RP11-93B1	187.99	-0.5086753	0.456832	-1.11348474	0.2655	0.517087	NOT
RP11-16E1	87.32946	0.55303202	0.4967	1.113413603	0.265531	0.517109	NOT
CTD-2540E	2.494181	0.74178266	0.666287	1.113307491	0.265576	0.517137	NOT
RNU1-122F	1.968209	0.70458342	0.63291	1.113244064	0.265604	0.517137	NOT
ITPK1	5583.276	-0.2358158	0.211834	-1.11321017	0.265618	0.517137	NOT
FAM208B	1629.302	0.20265785	0.182049	1.113203266	0.265621	0.517137	NOT
UFM1	2600.026	-0.1834127	0.164778	-1.11309221	0.265669	0.517193	NOT
SNRPGP9	3.371093	0.47293903	0.424909	1.11303587	0.265693	0.517193	NOT
MOGS	3884.509	0.12290934	0.11043	1.113002464	0.265707	0.517193	NOT
IFT122	825.0782	-0.1397002	0.12553	-1.1128806	0.26576	0.517229	NOT
RP11-797A	5.670664	0.54490923	0.489643	1.112871488	0.265764	0.517229	NOT
TRHDE	164.7731	-0.8532854	0.766869	-1.1126867	0.265843	0.517346	NOT
KCTD7	402.192	0.21250511	0.191007	1.112548705	0.265902	0.517395	NOT
AC130469.	4.410056	-0.5337857	0.47979	-1.1125397	0.265906	0.517395	NOT
TBX10	48.47723	0.48182548	0.433132	1.112421811	0.265957	0.517456	NOT
DPY19L2	17.08649	0.5611263	0.504453	1.112346266	0.265989	0.517481	NOT
SH3BGRL3	5171.031	0.29378298	0.264125	1.112285764	0.266015	0.517481	NOT
RP11-638I	58.84989	-0.2737686	0.246144	-1.11223138	0.266039	0.517481	NOT
RP11-439C	7.548012	-0.9654247	0.86802	-1.1122144	0.266046	0.517481	NOT
INTS3	2712.751	-0.2363878	0.212554	-1.11213266	0.266081	0.517512	NOT
GLRB	150.8905	0.66362878	0.59675	1.112070778	0.266108	0.517527	NOT
MDH1B	15.6984	0.50054034	0.450209	1.11179584	0.266226	0.517608	NOT
GRIN1	8.023513	0.64655663	0.581561	1.111759755	0.266241	0.517608	NOT
RP11-474I	3.025367	-0.5458685	0.491015	-1.11171482	0.266261	0.517608	NOT
LNP1	155.3546	-0.2985492	0.268559	-1.11166975	0.26628	0.517608	NOT
HIST1H2BC	64.04853	-0.5437108	0.489108	-1.11163676	0.266294	0.517608	NOT

PPP1R21	967.8774	0.15207129	0.136801	1.111621929	0.266301	0.517608	NOT
NACAP3	3.925832	-0.4740609	0.426459	-1.11162056	0.266301	0.517608	NOT
RP11-334A	15.48975	0.66210888	0.595626	1.11161877	0.266302	0.517608	NOT
RP11-536C	2.495966	-1.1915089	1.072149	-1.11132743	0.266427	0.517769	NOT
FAM185A	227.9498	-0.1822359	0.163986	-1.11129042	0.266443	0.517769	NOT
ARL2BP	181.8644	-0.3083802	0.277498	-1.11128651	0.266445	0.517769	NOT
AGTR1	1829.719	0.42407977	0.381624	1.111249305	0.266461	0.517769	NOT
RP11-762I	13.7773	-0.4029069	0.3626	-1.11116161	0.266499	0.517803	NOT
DMPK	705.6018	-0.2665396	0.239884	-1.1111197	0.266517	0.517803	NOT
MROH6	749.9517	0.46858479	0.421808	1.110896422	0.266613	0.517953	NOT
CTB-39G8.	8.452328	0.44568802	0.401226	1.110816257	0.266647	0.517983	NOT
ZNF32-AS1	5.66912	0.39993126	0.360091	1.110640481	0.266723	0.518093	NOT
EEF1A2	5201.544	-0.8067045	0.726373	-1.11059292	0.266744	0.518095	NOT
PPP2CA	5243.863	0.12290505	0.110671	1.110540532	0.266766	0.518102	NOT
SLC16A7	844.6443	-0.463347	0.417251	-1.1104755	0.266794	0.518119	NOT
RP11-465N	123.3054	0.3859223	0.347563	1.110365816	0.266841	0.518174	NOT
HCST	201.6108	-0.4002643	0.360513	-1.11026214	0.266886	0.518196	NOT
U91328.19	257.0843	0.27050726	0.243648	1.110236605	0.266897	0.518196	NOT
OSBPL10	210.5019	0.3975789	0.358113	1.110205882	0.26691	0.518196	NOT
AGAP4	55.28603	0.27805588	0.250464	1.110162259	0.266929	0.518196	NOT
INTS6-AS1	61.58016	-0.2994596	0.26976	-1.11009521	0.266958	0.518215	NOT
SNORA55	2.268544	-0.5308258	0.478233	-1.10997435	0.26701	0.518279	NOT
ZNF845	179.1921	0.2984846	0.268953	1.109802488	0.267084	0.518386	NOT
OR7E126P	4.359785	0.50315378	0.453404	1.109726097	0.267117	0.518408	NOT
RP11-304I	30.86886	-0.3289837	0.296465	-1.10968683	0.267134	0.518408	NOT
KMT2D	2607.888	-0.2136794	0.192567	-1.10963728	0.267155	0.518412	NOT
LA16c-390	4.513871	-0.4756726	0.428741	-1.1094626	0.267231	0.518516	NOT
SNORA59A	3.917337	-0.3073771	0.27706	-1.10942446	0.267247	0.518516	NOT
RP11-834C	5.117328	-0.5290439	0.476925	-1.10928037	0.267309	0.5186	NOT
MUCL1	2.636188	0.99250003	0.895008	1.108928205	0.267461	0.518857	NOT
LINC00950	38.60117	-0.335164	0.302281	-1.10878388	0.267523	0.518941	NOT
SERPINA1	864025	-0.3540244	0.319314	-1.10870345	0.267558	0.518971	NOT
RP11-264E	17.94296	-0.3846199	0.346931	-1.10863458	0.267588	0.518992	NOT
CTSD	81955.15	-0.2838078	0.256048	-1.10841682	0.267682	0.519137	NOT
HPD	58006.95	-0.6635858	0.598824	-1.10814917	0.267797	0.519211	NOT
RP11-122K	26.24342	-0.314041	0.283397	-1.10813018	0.267806	0.519211	NOT
CTD-2619J	16.68015	0.36275026	0.327363	1.108096668	0.26782	0.519211	NOT
TGFBI	11108.27	0.31413542	0.283494	1.108084012	0.267826	0.519211	NOT
PAPOLG	417.6862	0.15072054	0.136021	1.1080664	0.267833	0.519211	NOT
MYO5B	1916.665	-0.2997847	0.270556	-1.10803194	0.267848	0.519211	NOT
MIR378H	2.718864	-0.5044729	0.455293	-1.10801874	0.267854	0.519211	NOT
RP11-119D	284.4645	-0.6470828	0.584027	-1.10796712	0.267876	0.519217	NOT
ZBTB8A	200.5906	-0.2114271	0.190843	-1.10785837	0.267923	0.519238	NOT
TRMT44	402.525	-0.1393483	0.125782	-1.10785279	0.267925	0.519238	NOT
KDM4D	31.60292	-0.2798075	0.252585	-1.10777444	0.267959	0.519246	NOT
LCT	1.945955	0.66030865	0.596078	1.107754837	0.267968	0.519246	NOT
PDCD6IPP2	17.73965	0.50502426	0.456018	1.10746591	0.268093	0.51942	NOT
DNAH10OS	82.47104	0.4036832	0.364513	1.107458804	0.268096	0.51942	NOT
ZNF169	57.08578	0.266383	0.240552	1.107384453	0.268128	0.519424	NOT

RP11-6L6.	2.394905	-0.6138021	0.554291	-1.10736456	0.268136	0.519424	NOT
RP11-104I	2.507815	-0.5245731	0.473773	-1.10722434	0.268197	0.519491	NOT
C10orf111	7.28728	0.31131532	0.281212	1.107047488	0.268273	0.519491	NOT
CABLES2	507.0595	0.26741969	0.241568	1.107018456	0.268286	0.519491	NOT
TM4SF19-A	17.66315	0.32757686	0.295911	1.107010183	0.26829	0.519491	NOT
GYPC	787.5828	0.33390509	0.301628	1.107010118	0.26829	0.519491	NOT
COPS3	1606.81	0.18317643	0.165489	1.106878797	0.268346	0.519491	NOT
RP11-566K	0.891072	-0.7801921	0.704858	-1.10687868	0.268346	NA	NA
AC009303.	119.8191	0.29065709	0.262593	1.106870876	0.26835	0.519491	NOT
CTD-3051I	5.519191	-0.3687465	0.333145	-1.10686341	0.268353	0.519491	NOT
RP11-736M	13.3487	-0.5348085	0.483178	-1.10685632	0.268356	0.519491	NOT
ZNF44	513.2299	-0.2016376	0.182179	-1.10681017	0.268376	0.519491	NOT
ZNF581	692.929	0.27818144	0.251339	1.106797844	0.268381	0.519491	NOT
WDR88	32.13588	-0.2908559	0.262807	-1.10673011	0.268411	0.519511	NOT
AP006621.	22.23393	-0.4722308	0.426719	-1.10665603	0.268443	0.519536	NOT
FAM96AP2	8.344277	-0.4807443	0.434522	-1.10637538	0.268564	0.519733	NOT
AC007966.	6.128061	0.63183869	0.571142	1.106273333	0.268608	0.519782	NOT
ADIPOR1	7346.898	-0.1519402	0.137369	-1.10607361	0.268695	0.519912	NOT
MAGIX	673.9958	0.36270247	0.327984	1.105855811	0.268789	0.520057	NOT
TEX41	131.9501	-0.6145968	0.555845	-1.10569902	0.268857	0.520114	NOT
UPK2	4.101275	-0.6035895	0.545903	-1.10567095	0.268869	0.520114	NOT
IFRD2	3204.374	0.18749072	0.169581	1.105610747	0.268895	0.520114	NOT
AP001469.	3.255279	0.4042279	0.365616	1.105607267	0.268897	0.520114	NOT
RNF11	2911.531	-0.1899692	0.17183	-1.10556711	0.268914	0.520114	NOT
RP11-577H	13.46317	0.32619564	0.295099	1.105378286	0.268996	0.520235	NOT
RP3-476K8	2.440899	0.41159429	0.37239	1.10527796	0.269039	0.520258	NOT
CD70	12.83998	-0.4816975	0.435827	-1.1052505	0.269051	0.520258	NOT
SNUPN	683.0438	0.14869246	0.134537	1.105217444	0.269065	0.520258	NOT
ATP8B3	150.6075	-0.3662617	0.331417	-1.10513907	0.269099	0.520275	NOT
H6PD	10885.94	-0.2850885	0.257973	-1.10510873	0.269112	0.520275	NOT
RTN4R	515.1637	0.37349993	0.338002	1.105023222	0.26915	0.520307	NOT
LMBR1L	865.9867	-0.1620835	0.146686	-1.10496776	0.269174	0.520307	NOT
RNA5SP219	1.862257	-0.7063226	0.639242	-1.10493807	0.269186	0.520307	NOT
GPR137	1827.788	-0.204052	0.184698	-1.10478581	0.269252	0.520397	NOT
USP41	1.217552	0.81051533	0.733723	1.104660505	0.269307	NA	NA
ST13P4	7.277738	0.36242362	0.328137	1.104489204	0.269381	0.520609	NOT
NDUFB2	4401.26	-0.2442628	0.221165	-1.10443487	0.269405	0.520617	NOT
VEGFA	8630.381	0.23239582	0.21045	1.104281798	0.269471	0.520686	NOT
RP11-33E1	73.95429	0.32374481	0.293177	1.10426391	0.269479	0.520686	NOT
DKFZP434A	1.83115	-0.6443355	0.583543	-1.10417758	0.269516	0.520722	NOT
SLC25A44	3836.459	-0.1979115	0.179252	-1.10409396	0.269552	0.520742	NOT
RAB6C-AS1	1.415222	-0.6985898	0.632756	-1.10404233	0.269575	0.520742	NOT
NBL1	273.6169	0.40333229	0.36533	1.104020409	0.269584	0.520742	NOT
INADL	1837.202	0.26755999	0.242388	1.1038508	0.269658	0.520847	NOT
COG3	1843.032	-0.2564912	0.232405	-1.10363854	0.26975	0.520956	NOT
EEF1A1P28	2.428436	-0.4446852	0.402929	-1.10363233	0.269753	0.520956	NOT
RP11-359E	33.64944	0.48188139	0.436667	1.103543458	0.269791	0.520993	NOT
SNORD116-	1.977043	0.71283046	0.645995	1.103461652	0.269827	0.521025	NOT
HELZ	1970.134	-0.1909742	0.173105	-1.10323034	0.269927	0.521182	NOT

ZBTB10	1615.95	0.23567538	0.21365	1.103088463	0.269989	0.52123	NOT
TOB1-AS1	103.3635	-0.3017856	0.273583	-1.10308453	0.26999	0.52123	NOT
ATG101	1032.169	0.15426605	0.139869	1.102930544	0.270057	0.521297	NOT
RP11-1084	2.580712	-0.4302583	0.39011	-1.10291618	0.270064	0.521297	NOT
ONECUT1	706.5224	-0.4059217	0.368144	-1.10261589	0.270194	0.521434	NOT
MED26	308.721	-0.1318038	0.119542	-1.10257091	0.270214	0.521434	NOT
GIMAP7	400.3273	-0.2902083	0.263214	-1.10255467	0.270221	0.521434	NOT
AC073842.	21.32036	-0.5326777	0.483161	-1.10248459	0.270251	0.521434	NOT
LINC00957	109.3958	-0.3349105	0.303787	-1.10245026	0.270266	0.521434	NOT
LINC01468	2.05603	-0.9530246	0.864466	-1.10244302	0.270269	0.521434	NOT
HMGXB4	776.93	0.17539034	0.159092	1.102442717	0.270269	0.521434	NOT
CTA-384D8	3.260203	0.58102194	0.527083	1.102335696	0.270316	0.521487	NOT
TFE3	2480.72	0.15198083	0.137903	1.10208297	0.270426	0.52164	NOT
RPL18P13	18.72468	-0.8522209	0.773295	-1.10206488	0.270433	0.52164	NOT
RP11-1348	2.084278	0.4363341	0.395994	1.101870738	0.270518	0.521755	NOT
ANXA13	1303.608	0.57292776	0.519977	1.101832212	0.270535	0.521755	NOT
RP11-362K	8.971401	-0.385787	0.350144	-1.10179426	0.270551	0.521755	NOT
ZNF676	40.55024	-0.8442681	0.766471	-1.10149973	0.270679	0.52193	NOT
RPL24P4	110.4883	0.33906641	0.307828	1.101479141	0.270688	0.52193	NOT
PDPK1	1205.969	-0.2035567	0.184807	-1.10145374	0.270699	0.52193	NOT
ZNF37BP	296.5961	0.2724755	0.247413	1.101296371	0.270768	0.522024	NOT
SUMF2	7331.028	-0.224051	0.203465	-1.10117937	0.270819	0.522085	NOT
MVB12A	1582.18	0.26766407	0.243084	1.101116109	0.270846	0.522101	NOT
ISCA1	1606.317	-0.1595794	0.144954	-1.1008981	0.270941	0.522246	NOT
BCLAF1	2791.736	-0.1390683	0.126327	-1.10085571	0.270959	0.522246	NOT
MTMR14	2305.843	0.15007267	0.136353	1.100618241	0.271063	0.522364	NOT
AC097374.	1.459809	1.0401478	0.945089	1.100581914	0.271079	0.522364	NOT
BAZ2A	3122.945	0.17752873	0.161304	1.100581672	0.271079	0.522364	NOT
MEIS2	822.8478	0.34035012	0.309311	1.100350416	0.271179	0.522521	NOT
DPP9	4780.416	-0.1529879	0.139042	-1.10030113	0.271201	0.522525	NOT
RP11-358I	38.20092	0.26352579	0.239514	1.100250673	0.271223	0.522531	NOT
RP11-757C	131.3194	-0.6553355	0.595658	-1.10018756	0.27125	0.522547	NOT
AIM1	745.1123	-0.3818813	0.347148	-1.10005204	0.271309	0.522623	NOT
CCL21	988.3718	0.69778073	0.634403	1.099901321	0.271375	0.522713	NOT
SHANK1	10.8082	0.51798614	0.470962	1.099847431	0.271399	0.522721	NOT
HNF1A-AS1	699.975	0.35700395	0.324609	1.099796103	0.271421	0.522727	NOT
PROSER1	769.0237	0.2196967	0.199791	1.099631951	0.271493	0.522827	NOT
RP13-580F	11.7391	-0.5013526	0.455952	-1.09957306	0.271518	0.52284	NOT
ZNF512B	2208.698	0.19735968	0.179515	1.099404815	0.271592	0.522897	NOT
CEACAM3	5.799892	0.60657375	0.551731	1.099401697	0.271593	0.522897	NOT
RP11-392F	171.8995	-0.2559599	0.232824	-1.0993718	0.271606	0.522897	NOT
RP11-343H	3.148032	0.46367264	0.421807	1.099252073	0.271658	0.522961	NOT
ZNF343	395.9265	0.1593556	0.144983	1.099130831	0.271711	0.523025	NOT
TMPRSS9	345.3369	-0.4860589	0.442265	-1.09902095	0.271759	0.523057	NOT
RAPGEF6	401.0078	-0.1674122	0.152334	-1.09898328	0.271775	0.523057	NOT
FKBP5	7226.982	0.3590965	0.32676	1.098960497	0.271785	0.523057	NOT
RP11-398C	11.02658	0.45294609	0.412188	1.098883478	0.271819	0.523085	NOT
RP11-49K2	2.187642	0.57262169	0.52119	1.098681811	0.271907	0.523217	NOT
WWP2	2207.327	-0.1825095	0.166139	-1.09853264	0.271972	0.52328	NOT

ABCB8	2599.83	-0.2372816	0.216004	-1.09850533	0.271984	0.52328	NOT
MAFK	1199.828	-0.3024715	0.275356	-1.09847402	0.271998	0.52328	NOT
CTD-2007I	12.81238	-0.3529935	0.321369	-1.09840662	0.272027	0.523299	NOT
IL13RA2	39.64742	0.64848949	0.590443	1.098310649	0.272069	0.52334	NOT
GPR15	2.319538	0.77951012	0.709762	1.098270266	0.272086	0.52334	NOT
TIMM23	2488.258	0.15215894	0.138575	1.09802571	0.272193	0.523492	NOT
CCDC84	504.6527	-0.2555066	0.232702	-1.09800089	0.272204	0.523492	NOT
FAM66B	4.804224	-0.6053556	0.551355	-1.09794105	0.27223	0.523505	NOT
RP11-63G1	2.730689	0.40098277	0.365287	1.097719149	0.272327	0.523654	NOT
MGAT4B	13961.31	0.23322432	0.212481	1.09762222	0.272369	0.523691	NOT
RP11-972I	6.524623	0.50778594	0.462639	1.097586276	0.272385	0.523691	NOT
IRF1	2793.161	0.28392844	0.258705	1.097498791	0.272423	0.523728	NOT
TMEM161B-	268.306	-0.2948555	0.268707	-1.0973113	0.272505	0.523815	NOT
TRPV2	641.7332	-0.3814878	0.34766	-1.09730224	0.272509	0.523815	NOT
TEX261	4018.322	0.10913444	0.099464	1.097227062	0.272542	0.523815	NOT
BPHL	2947.993	-0.3085134	0.281178	-1.09721826	0.272546	0.523815	NOT
RP5-1136(	218.0764	0.24815487	0.226213	1.096995399	0.272643	0.523965	NOT
RP11-17G1	2.554098	-0.4785802	0.436424	-1.09659447	0.272819	0.524265	NOT
FAM160A2	1666.659	-0.1592848	0.145293	-1.09630269	0.272946	0.524444	NOT
MRPS9	1270.981	0.18053526	0.164685	1.096245543	0.272971	0.524444	NOT
AC002059.	4.526265	-0.4449461	0.405906	-1.09617983	0.273	0.524444	NOT
RP5-1116I	2.150265	0.52659412	0.480392	1.096175433	0.273002	0.524444	NOT
UFSP1	101.0599	-0.2899823	0.264544	-1.09616074	0.273008	0.524444	NOT
TRBV12-3	1.416959	-0.6508775	0.593804	-1.096115	0.273028	0.524445	NOT
SPRR2D	1.074075	1.48105721	1.351237	1.096075041	0.273046	NA	NA
FER1L6	116.0217	0.95908803	0.875025	1.096068798	0.273049	0.524447	NOT
KIF1C	9232.75	-0.2114884	0.192999	-1.09579797	0.273167	0.524637	NOT
RP4-635E1	6.091693	-0.364323	0.332542	-1.09556855	0.273268	0.524793	NOT
RP11-22P(	4.356314	-0.328766	0.300108	-1.09549134	0.273301	0.524797	NOT
DENND4B	2226.555	0.16256444	0.148396	1.095475596	0.273308	0.524797	NOT
KCNRG	6.160052	-0.3455842	0.31549	-1.09538919	0.273346	0.524832	NOT
SUZ12P1	207.6802	0.25935788	0.236845	1.095051524	0.273494	0.525079	NOT
ALDH9A1	7245.327	-0.2518246	0.229983	-1.09497197	0.273529	0.525099	NOT
MIS12	598.2013	-0.1661975	0.151787	-1.09494015	0.273543	0.525099	NOT
CTD-3065J	107.3249	0.3228491	0.294872	1.094878897	0.27357	0.525113	NOT
KCNG3	4.563066	1.08436078	0.990438	1.09482914	0.273591	0.525118	NOT
CTC-448F2	22.12008	0.24453259	0.223363	1.094776713	0.273614	0.525125	NOT
ZNF777	951.2989	0.14187733	0.12966	1.094222397	0.273857	0.525486	NOT
CDH24	226.1475	0.33275984	0.304107	1.094218108	0.273859	0.525486	NOT
FAM107A	313.5613	-0.3739205	0.341739	-1.09417107	0.27388	0.525486	NOT
RP4-765C7	109.3654	0.87329267	0.798155	1.094139432	0.273894	0.525486	NOT
SLC39A2	1.413439	0.68766272	0.628504	1.094125773	0.2739	0.525486	NOT
MAML3	461.0972	-0.2419413	0.221172	-1.09390319	0.273997	0.525636	NOT
PEBP1	63071.72	-0.2718722	0.248592	-1.09364911	0.274109	0.525813	NOT
ACSBG1	7.487494	-0.4637072	0.424061	-1.09349228	0.274178	0.525894	NOT
ZNF720P1	2.299708	1.56658507	1.432681	1.093464279	0.27419	0.525894	NOT
RP13-895J	2.041205	1.3133558	1.201152	1.093413496	0.274212	0.5259	NOT
AC009237.	78.20479	0.31466944	0.287806	1.093337948	0.274245	0.525904	NOT
PSD	153.7356	-0.3024608	0.276644	-1.09331972	0.274253	0.525904	NOT

LINC0054ε	1.041597	0.94743835	0.866622	1.093254848	0.274282	NA	NA
CPNE7	164.2069	0.57577952	0.526698	1.09318746	0.274312	0.525979	NOT
RP11-610I	2.410114	-0.6483797	0.593143	-1.09312559	0.274339	0.525993	NOT
PDP1	384.0869	0.40390053	0.369514	1.093059439	0.274368	0.526012	NOT
RP11-180(	4.327091	0.43943206	0.402037	1.09301343	0.274388	0.526014	NOT
RP11-433(	1.342864	0.96486761	0.882817	1.092942285	0.274419	NA	NA
TMEM60	663.1926	0.17312377	0.158438	1.092688274	0.274531	0.52625	NOT
FCGR1B	16.08268	0.4489527	0.410948	1.092479874	0.274622	0.526277	NOT
BTBD7P1	5.996486	-0.5661511	0.518268	-1.09238982	0.274662	0.526277	NOT
AC007292.	4.093925	-0.4051356	0.370874	-1.09238049	0.274666	0.526277	NOT
DNAH17	69.72069	-0.2763056	0.25294	-1.0923761	0.274668	0.526277	NOT
SLC25A47	5258.73	-0.7354995	0.673303	-1.09237506	0.274668	0.526277	NOT
CTD-2619J	418.5302	-0.4589481	0.420145	-1.09235744	0.274676	0.526277	NOT
ATP5C1P1	2.854552	0.50280223	0.460295	1.092347658	0.27468	0.526277	NOT
RP11-522I	127.984	-0.2545252	0.23302	-1.09229126	0.274705	0.526287	NOT
SWT1	287.8612	0.17584359	0.160993	1.092243168	0.274726	0.52629	NOT
IRAK1	7114.777	0.21916401	0.200695	1.092023951	0.274823	0.526438	NOT
ST13P19	4.085633	0.41625528	0.381233	1.091866375	0.274892	0.526487	NOT
MAPK1	4972.941	0.16177227	0.148164	1.091847048	0.2749	0.526487	NOT
XXyac-YX6	8.933382	-0.4452721	0.407836	-1.09179153	0.274925	0.526487	NOT
PKD2L1	72.15812	0.49512068	0.453495	1.091788522	0.274926	0.526487	NOT
RP11-350(	3.014153	0.4620049	0.423236	1.09160087	0.275009	0.526608	NOT
MNX1-AS1	24.22548	-1.6062034	1.471542	-1.09151028	0.275048	0.526644	NOT
D2HGDH	1624.69	-0.2494139	0.228517	-1.09144405	0.275078	0.526644	NOT
AP001059.	2.878501	-0.6963394	0.638012	-1.09142103	0.275088	0.526644	NOT
NME3	1806.908	-0.2674099	0.24502	-1.09137843	0.275106	0.526644	NOT
SEPN1	3498.419	-0.1930318	0.176876	-1.09133793	0.275124	0.526644	NOT
PTOV1	6270.576	-0.2200033	0.201648	-1.09102716	0.275261	0.526846	NOT
IL12RB1	143.0817	0.39837614	0.365166	1.090946444	0.275296	0.526846	NOT
WDR92	60.75581	0.18411524	0.168773	1.090901463	0.275316	0.526846	NOT
PPP1R15A	2006.99	-0.2558687	0.234552	-1.09088413	0.275324	0.526846	NOT
UFL1	1639.843	-0.2177908	0.199648	-1.09087465	0.275328	0.526846	NOT
RP11-360I	2.287968	0.4474919	0.410244	1.090794056	0.275364	0.526846	NOT
ATP11B	1589.572	-0.2348375	0.215291	-1.0907887	0.275366	0.526846	NOT
HMGN3	2215.075	-0.2207506	0.202397	-1.0906831	0.275412	0.526898	NOT
COQ3	439.1746	0.21796113	0.199853	1.090605729	0.275446	0.526899	NOT
RP5-1125A	12.13644	-0.2846048	0.260963	-1.0905933	0.275452	0.526899	NOT
IGFL4	1.659013	0.75997683	0.696897	1.090514925	0.275486	0.526928	NOT
RP11-249(	0.971711	-0.8502887	0.779839	-1.0903383	0.275564	NA	NA
CCDC42	3.653432	-0.4223251	0.387342	-1.09031602	0.275574	0.527058	NOT
CHRNB4	18.35384	0.58126379	0.533185	1.090172545	0.275637	0.527142	NOT
RP11-95G6	5.500648	-0.4140262	0.379802	-1.09010911	0.275665	0.527158	NOT
ANKRD53	33.2904	0.47918651	0.439658	1.089907118	0.275754	0.527287	NOT
RP11-18C2	8.326464	-0.3129425	0.287138	-1.08986805	0.275771	0.527287	NOT
RP1-63M2.	26.99024	0.36655538	0.336351	1.089801461	0.275801	0.527306	NOT
CIART	394.6842	0.40507089	0.371727	1.089700286	0.275845	0.527354	NOT
RNF224	7.010143	0.62488873	0.573528	1.089552466	0.27591	0.527441	NOT
AZGP1P2	11.70994	-0.6640375	0.609525	-1.08943504	0.275962	0.527503	NOT
MRPS35	3603.006	0.15609022	0.143284	1.089379438	0.275987	0.527513	NOT

RPS12P26	3.761122	0.40519337	0.371989	1.089261619	0.276039	0.527564	NOT
LYPLA1	3433.547	0.22889455	0.210143	1.089230518	0.276052	0.527564	NOT
RP11-445(	2.594581	0.41716461	0.38308	1.088974379	0.276165	0.527743	NOT
H1FX	4369.496	0.2149118	0.19738	1.088822991	0.276232	0.527833	NOT
AC006272.	2.894651	-0.4678097	0.429692	-1.08871	0.276282	0.527839	NOT
GUCY2D	46.85249	0.57961895	0.532392	1.088706703	0.276283	0.527839	NOT
RP11-849F	2.406834	-0.4495165	0.412915	-1.08864293	0.276311	0.527839	NOT
RP13-631K	3.737714	-0.683393	0.627762	-1.08861767	0.276323	0.527839	NOT
NGRN	1155.436	0.1605474	0.147481	1.088595045	0.276333	0.527839	NOT
RP11-66H6	4.925977	0.48077633	0.441669	1.088543895	0.276355	0.527845	NOT
RP4-740C4	50.33377	-0.3831121	0.351971	-1.08847684	0.276385	0.527864	NOT
MAPKBP1	476.2184	-0.1584108	0.14554	-1.0884335	0.276404	0.527864	NOT
TRUB2	2540.252	-0.1847689	0.16981	-1.0880934	0.276554	0.528113	NOT
AC004854.	2.952037	0.48254675	0.443573	1.087863693	0.276655	0.528269	NOT
TAS2R64P	1.797903	0.60868721	0.559564	1.087788501	0.276688	0.528269	NOT
SMOC2	532.0041	-0.4407484	0.405183	-1.08777616	0.276694	0.528269	NOT
CCRL2	116.3302	0.33819292	0.310934	1.087668752	0.276741	0.528323	NOT
RP11-1365	5.238465	-0.3471765	0.319223	-1.08756616	0.276787	0.528363	NOT
RPS6KA3	5698.156	0.24711363	0.227228	1.087516176	0.276809	0.528363	NOT
RP5-856G1	2.365768	0.91277015	0.83936	1.087459271	0.276834	0.528363	NOT
TOP3A	1019.122	0.19280071	0.177298	1.087437568	0.276843	0.528363	NOT
NDUFAB1	2902.992	-0.2182618	0.200719	-1.08739991	0.27686	0.528363	NOT
IGHV3OR16	1.255663	-0.9511829	0.874832	-1.08727451	0.276916	NA	NA
RAB11FIP4	2254.302	-0.3486274	0.320681	-1.08714731	0.276972	0.528536	NOT
CEBPZ	1815.159	0.13303852	0.122378	1.087107242	0.276989	0.528536	NOT
ALDH1B1	5553.61	-0.273617	0.251708	-1.08703998	0.277019	0.528555	NOT
PRAMEF2	2.067686	-1.6378067	1.506934	-1.08684703	0.277104	0.528622	NOT
NIN	1453.742	-0.2690184	0.247523	-1.08684	0.277108	0.528622	NOT
RP11-701I	11.79013	0.35100564	0.322963	1.086828749	0.277112	0.528622	NOT
OCIAD2	2635.571	0.36234598	0.333453	1.086646957	0.277193	0.528738	NOT
AC006128.	90.79303	-0.3989189	0.367159	-1.08650225	0.277257	0.528788	NOT
NBPF11	360.1732	-0.2339743	0.215347	-1.08649993	0.277258	0.528788	NOT
RP11-727F	4.932642	-0.3446621	0.317251	-1.08640119	0.277302	0.528814	NOT
AMOT	387.501	0.43355069	0.399091	1.086344742	0.277326	0.528814	NOT
RP13-514E	2.83314	-0.7929519	0.729932	-1.08633647	0.27733	0.528814	NOT
TAF1A-AS1	107.3657	-0.2812565	0.258952	-1.08613459	0.277419	0.528911	NOT
CTD-2541J	6.675722	-0.5365267	0.493979	-1.08613294	0.27742	0.528911	NOT
LINC00998	882.254	0.26211567	0.24134	1.086083984	0.277442	0.528916	NOT
SCGN	894.3761	-0.6966325	0.641599	-1.08577516	0.277578	0.529139	NOT
KLF15	4386.13	-0.3584469	0.33017	-1.08564453	0.277636	0.529169	NOT
TNC	834.278	-0.3703419	0.341127	-1.08564259	0.277637	0.529169	NOT
CTA-292E1	473.5705	-0.2674553	0.246365	-1.0856076	0.277653	0.529169	NOT
GGA3	1454.036	0.1305104	0.120253	1.085299891	0.277789	0.529391	NOT
RP3-333H2	2.211425	-0.6247766	0.575758	-1.08513758	0.277861	0.529453	NOT
MAP3K8	475.0775	0.35145712	0.323889	1.085116809	0.27787	0.529453	NOT
ARHGEF33	7.32695	-0.3683976	0.339508	-1.0850939	0.27788	0.529453	NOT
KRT18P37	2.385533	-0.5110136	0.470961	-1.08504348	0.277902	0.529459	NOT
CHRNB2	11.49088	0.55146616	0.508295	1.084932382	0.277952	0.529515	NOT
FBXL14	301.0961	-0.2472731	0.227934	-1.08484631	0.27799	0.529546	NOT

CTC-308K2	16.52601	0.2381194	0.219504	1.084808122	0.278007	0.529546	NOT
SOHLH1	3.709157	0.91142594	0.840329	1.084606145	0.278096	0.529679	NOT
GABARAPL2	2499.098	-0.1501413	0.13844	-1.08452171	0.278134	0.529693	NOT
SACS	370.5616	0.36601847	0.337499	1.084501674	0.278142	0.529693	NOT
RP11-622A	255.572	-0.5444411	0.502049	-1.08443726	0.278171	0.529704	NOT
CELSR2	653.2391	-0.3275828	0.302087	-1.08440006	0.278187	0.529704	NOT
NOP14	2182.459	-0.1449443	0.133685	-1.08422041	0.278267	0.529773	NOT
CD209	293.2491	-0.3958575	0.365113	-1.08420512	0.278274	0.529773	NOT
CTD-2382E	4.516661	0.48526795	0.447595	1.084168352	0.27829	0.529773	NOT
PLCXD2	229.7666	-0.3299541	0.304351	-1.08412434	0.27831	0.529773	NOT
PPP1R3C	1798.044	0.34591293	0.319079	1.084098008	0.278321	0.529773	NOT
POM121	1309.985	-0.2471486	0.22799	-1.08403374	0.27835	0.52979	NOT
PITPNB	2843.228	0.16645593	0.153559	1.083984209	0.278372	0.529795	NOT
AC091729.	1.171599	0.75388942	0.695578	1.083832325	0.278439	NA	NA
NOTCH3	3026.425	-0.3129772	0.288779	-1.08379425	0.278456	0.529874	NOT
AGPAT9	759.9207	-0.4240908	0.391303	-1.08379262	0.278457	0.529874	NOT
RP11-73B2	6.445324	-0.5486361	0.506238	-1.08375087	0.278475	0.529874	NOT
YIF1A	5714.847	-0.2135467	0.197051	-1.08371436	0.278491	0.529874	NOT
RP11-145F	2.26352	-0.7340632	0.677481	-1.08351848	0.278578	0.529968	NOT
COL8A1	368.871	-0.4543344	0.419317	-1.08351023	0.278582	0.529968	NOT
FBXL21	33.67882	0.61173933	0.564611	1.083470618	0.2786	0.529968	NOT
CHCHD3P3	7.772755	0.30761096	0.283981	1.083210999	0.278715	0.53015	NOT
CRACR2A	101.6012	0.42427754	0.391705	1.08315637	0.278739	0.530159	NOT
NDUFB3	1936.077	0.20124807	0.185812	1.083070866	0.278777	0.530194	NOT
IMPA1	1591.549	-0.2344684	0.216518	-1.08290511	0.278851	0.530264	NOT
NFATC1	386.6076	-0.3803475	0.35125	-1.08283886	0.27888	0.530264	NOT
YPEL3	1830.807	-0.211688	0.195498	-1.08281415	0.278891	0.530264	NOT
RP5-1039K	108.5966	0.26486983	0.244613	1.082812525	0.278892	0.530264	NOT
GJB2	1422.882	-0.4005306	0.369985	-1.08255788	0.279005	0.530441	NOT
SPATA45	5.060014	-0.4967872	0.458996	-1.08233538	0.279104	0.530592	NOT
GLI1	72.85932	0.41110972	0.37987	1.082238792	0.279146	0.530614	NOT
RP11-15H7	1.177943	0.90021766	0.831823	1.082223242	0.279153	NA	NA
TBCD	4407.094	-0.1693815	0.156518	-1.08218376	0.279171	0.530614	NOT
CACNB1	80.1833	0.32833696	0.303404	1.082177161	0.279174	0.530614	NOT
IGHV3-38	1.183782	1.10260843	1.01906	1.081985784	0.279259	NA	NA
AC010872.	1.531084	-0.5089091	0.470414	-1.08183258	0.279327	0.530797	NOT
BTAF1	1316.562	-0.2479919	0.229252	-1.08174585	0.279365	0.530797	NOT
ABCG8	3059.264	-0.4871789	0.450369	-1.0817325	0.279371	0.530797	NOT
HSD3BP5	6.732423	-0.4598475	0.425133	-1.0816554	0.279406	0.530797	NOT
MFSD4	91.21146	0.37229002	0.344188	1.081646121	0.27941	0.530797	NOT
HSDL2	6866.098	-0.2555379	0.236264	-1.08158001	0.279439	0.530797	NOT
MSH5-SAPC	45.23212	-0.2989243	0.276385	-1.08154974	0.279453	0.530797	NOT
SH2D2A	147.1368	-0.4057538	0.375167	-1.08152797	0.279462	0.530797	NOT
KLK1	1.982218	0.59217871	0.547569	1.081467684	0.279489	0.530797	NOT
BLCAP	3987.468	-0.2279962	0.210839	-1.08137363	0.279531	0.530797	NOT
PDXK	5051.206	-0.1962234	0.181458	-1.08137007	0.279533	0.530797	NOT
CARD11	213.659	0.43743289	0.404521	1.08136006	0.279537	0.530797	NOT
LINC00271	6.666075	-0.4185051	0.387033	-1.0813175	0.279556	0.530797	NOT
RBSN	1173.42	-0.1223929	0.113193	-1.08127332	0.279576	0.530797	NOT

SLC5A3	800.0184	-0.2930945	0.271068	-1.08125984	0.279582	0.530797	NOT
CCDC173	11.32883	0.40378753	0.373468	1.081182885	0.279616	0.530797	NOT
HNRNPH3	3135.451	0.13648443	0.126243	1.081123736	0.279642	0.530797	NOT
ZNF844	502.4705	-0.3805	0.351965	-1.08107481	0.279664	0.530797	NOT
RP11-5G9.	3.233285	0.50089022	0.463334	1.081056086	0.279672	0.530797	NOT
PGLS	3062.036	-0.3061019	0.283152	-1.08105006	0.279675	0.530797	NOT
TIGIT	131.2829	0.48919181	0.452521	1.081036142	0.279681	0.530797	NOT
RP11-475(	209.5308	0.35403437	0.327532	1.080915878	0.279735	0.530862	NOT
RP4-575N(	3.811658	-0.5721833	0.529398	-1.08081912	0.279778	0.530888	NOT
RP11-367I	3.218787	0.6397337	0.591909	1.080796595	0.279788	0.530888	NOT
ATRAID	3564.811	0.17940257	0.166007	1.08069491	0.279833	0.530937	NOT
RP5-912I1	1.921256	-1.4749491	1.364875	-1.08064807	0.279854	0.530939	NOT
HIST1H3PS	5.129287	0.30356258	0.280927	1.080574957	0.279886	0.530964	NOT
DKFZp434I	1.608913	-0.6994508	0.647331	-1.08051516	0.279913	0.530977	NOT
RAB5C	4429.811	0.12575133	0.116391	1.08041801	0.279956	0.530996	NOT
CANT1	2652.67	0.15457638	0.143073	1.080405352	0.279962	0.530996	NOT
DIO1	5327.266	0.48288678	0.446975	1.080343885	0.279989	0.53101	NOT
ZNF346	432.3079	0.15986403	0.147992	1.080217874	0.280045	0.531058	NOT
LACC1	335.0189	-0.2640203	0.244418	-1.08019907	0.280054	0.531058	NOT
DPP3P2	1.114098	1.30182201	1.205317	1.080066108	0.280113	NA	NA
EXTL3-AS1	20.49889	-0.3769305	0.348998	-1.08003646	0.280126	0.531136	NOT
AC093616.	18.5956	0.34971342	0.323803	1.08001946	0.280134	0.531136	NOT
NDUFA3	3349.793	-0.3051434	0.282613	-1.07972156	0.280266	0.531333	NOT
ROM1	122.9224	-0.251519	0.232953	-1.07969763	0.280277	0.531333	NOT
PRRT2	36.5711	-0.3409463	0.315867	-1.07939801	0.28041	0.531529	NOT
TMED5	6449.59	0.19210261	0.177976	1.07937088	0.280422	0.531529	NOT
STX4	2177.331	0.16024847	0.148477	1.079282927	0.280462	0.531529	NOT
MLH3	867.557	0.17296913	0.16027	1.079237086	0.280482	0.531529	NOT
PGBD2	238.7909	0.16686911	0.154618	1.079236944	0.280482	0.531529	NOT
AKAP3	28.71289	-0.4222974	0.391305	-1.07920245	0.280497	0.531529	NOT
GAS7	421.9877	0.40880522	0.378853	1.079060711	0.280561	0.531611	NOT
PAX3	1.385705	1.45214293	1.345969	1.078882848	0.28064	NA	NA
KIR2DL4	5.728088	-0.4671282	0.433007	-1.07880069	0.280677	0.53174	NOT
RAB2A	6822.977	0.18238834	0.169068	1.078787733	0.280682	0.53174	NOT
AHR	4046.132	-0.303506	0.281343	-1.07877667	0.280687	0.53174	NOT
RP11-349I	8.835577	0.26604862	0.246642	1.078684915	0.280728	0.531769	NOT
OSBPL5	313.7937	-0.3124942	0.289708	-1.07865419	0.280742	0.531769	NOT
CHRM5	14.67923	-0.4164265	0.386077	-1.07861058	0.280761	0.531769	NOT
PRR29	61.3077	-0.2318694	0.215	-1.07846343	0.280827	0.531856	NOT
YRDC	737.3363	0.14875747	0.137962	1.078252433	0.280921	0.531997	NOT
ENO1-IT1	2.533068	0.55224464	0.512214	1.078152906	0.280966	0.532044	NOT
DEF8	1336.966	-0.1478512	0.13716	-1.077943	0.281059	0.532124	NOT
AC092881.	2.261918	-0.6245681	0.579416	-1.07792665	0.281066	0.532124	NOT
AP3M2	420.7707	0.22467493	0.208433	1.077926152	0.281067	0.532124	NOT
CTD-2553I	50.53282	-0.2963963	0.274992	-1.07783536	0.281107	0.532164	NOT
AC024560.	425.6496	0.21794057	0.202222	1.077727309	0.281155	0.532193	NOT
TMC2	4.19056	0.51621999	0.479011	1.077679652	0.281177	0.532193	NOT
RP11-490(	7.975119	0.32489216	0.301477	1.077668905	0.281182	0.532193	NOT
SEZ6L	6.013481	-0.6121009	0.568026	-1.07759348	0.281215	0.53222	NOT

P2RX6	49.38774	0.61684217	0.572458	1.077532691	0.281242	0.532234	NOT
BLK	26.00916	0.59867737	0.555663	1.077411575	0.281296	0.532299	NOT
GOLGA2P11	4.064942	-0.860611	0.798878	-1.07727501	0.281357	0.532371	NOT
TNFAIP8	505.8866	-0.3278108	0.304306	-1.07723918	0.281373	0.532371	NOT
TRIM38	1191.585	-0.2010273	0.186627	-1.07715853	0.281409	0.532379	NOT
ZSCAN16-A	812.074	-0.3312134	0.307493	-1.07714073	0.281417	0.532379	NOT
LMOD3	5.067855	-0.3120689	0.289742	-1.07705703	0.281455	0.532413	NOT
NR5A2	2757.94	-0.3061244	0.284286	-1.07681681	0.281562	0.53256	NOT
CTD-2035E	19.51847	0.44879579	0.416789	1.076794791	0.281572	0.53256	NOT
SCAI	284.5898	-0.1863292	0.173062	-1.07665996	0.281632	0.532637	NOT
GLP2R	27.20207	0.64437945	0.598525	1.07661181	0.281654	0.532641	NOT
LINC00694	40.40608	-0.5002151	0.464768	-1.07626944	0.281807	0.532893	NOT
DFFBP1	4.364256	0.4344835	0.403774	1.076057479	0.281902	0.533035	NOT
VPS52	2578.648	0.16171895	0.150308	1.075920496	0.281963	0.533114	NOT
BDKRB1	32.38369	-0.4439298	0.412701	-1.0756702	0.282075	0.533284	NOT
CTD-2270L	5.159345	-0.3326447	0.309255	-1.07563175	0.282092	0.533284	NOT
ZMIZ1-AS1	55.1309	-0.4056901	0.377184	-1.07557499	0.282117	0.533295	NOT
RP11-70P1	6.034757	-0.3877306	0.360581	-1.07529529	0.282243	0.533494	NOT
MTFR1L	2485.389	-0.1757648	0.163473	-1.07519269	0.282288	0.533544	NOT
BCDIN3D	217.0142	0.13456624	0.125167	1.075096271	0.282332	0.533588	NOT
DCAF5	2077.881	-0.1405469	0.130759	-1.07485352	0.28244	0.533756	NOT
DOLPP1	1305.409	-0.1753528	0.16317	-1.07466294	0.282526	0.533829	NOT
ZNF84	790.7009	0.16497603	0.153515	1.074660359	0.282527	0.533829	NOT
ITGA3	1543.685	0.52684534	0.490255	1.074635898	0.282538	0.533829	NOT
RP1-187B2	1.849594	-0.5692068	0.529702	-1.0745802	0.282563	0.533838	NOT
TNFSF18	15.61967	-0.4077747	0.379488	-1.07453783	0.282582	0.533838	NOT
ARL4C	1160.85	0.34253691	0.318842	1.074314996	0.282682	0.533938	NOT
STAP2	3369.631	0.30016692	0.279407	1.074299712	0.282688	0.533938	NOT
RP1-78B3.	5.426377	-0.3421366	0.318478	-1.07428747	0.282694	0.533938	NOT
ANGPTL3	15741.35	-0.4336784	0.403706	-1.07424392	0.282713	0.533938	NOT
SLC6A11	810.4668	-0.9513223	0.885795	-1.07397548	0.282834	0.534128	NOT
PLD1	1626.09	-0.3263512	0.303909	-1.07384576	0.282892	0.534189	NOT
APEH	5916.603	0.17456411	0.162564	1.073814866	0.282906	0.534189	NOT
GMDS-AS1	172.0167	-0.1842564	0.171652	-1.07342826	0.283079	0.534479	NOT
RP11-611C	102.3996	-0.5201778	0.484695	-1.07320672	0.283178	0.53463	NOT
CTB-12904	20.62658	0.41356636	0.385466	1.072900218	0.283316	0.53483	NOT
AC073869.	3.228499	0.42233154	0.393643	1.072878348	0.283326	0.53483	NOT
RPS19BP1	3321.161	0.24961742	0.232679	1.0727994	0.283361	0.53483	NOT
SSH3	1144.213	0.207039	0.19299	1.072794588	0.283363	0.53483	NOT
RP11-62F2	1.311254	0.92002511	0.857691	1.072677156	0.283416	NA	NA
ZNF773	131.1908	0.33496679	0.31228	1.072647774	0.283429	0.534917	NOT
IGKV1-8	7.519905	0.7650191	0.713386	1.072377677	0.28355	0.535074	NOT
TNNI3K	1.697673	-0.5948013	0.554658	-1.07237438	0.283552	0.535074	NOT
AKR1C7P	23.52697	0.51058798	0.476168	1.072284566	0.283592	0.535113	NOT
NXN	804.316	-0.4866035	0.453831	-1.07221258	0.283625	0.535137	NOT
RP11-108I	7.038075	-0.4240564	0.395517	-1.07215604	0.28365	0.535148	NOT
ST7-OT4	4.431684	-0.3806804	0.355129	-1.07194925	0.283743	0.535279	NOT
C18orf25	785.951	-0.1380401	0.128787	-1.07184479	0.28379	0.535279	NOT
RP11-197N	18.94737	0.26519978	0.247435	1.071796201	0.283812	0.535279	NOT

RP11-597I	2.693748	-0.4742914	0.442564	-1.07168922	0.28386	0.535279	NOT
CEND1	12.82394	0.40148608	0.374634	1.071676673	0.283865	0.535279	NOT
SLC6A17	6.983349	0.38860017	0.362613	1.071664946	0.283871	0.535279	NOT
GRAMD2	5.159654	-0.4766557	0.444785	-1.07165365	0.283876	0.535279	NOT
SHISA2	27.2684	0.58987082	0.550433	1.071649425	0.283878	0.535279	NOT
C9orf139	16.89133	-0.3878638	0.361957	-1.07157436	0.283911	0.535306	NOT
RNU6-312F	2.26045	0.52747767	0.492299	1.071458664	0.283963	0.535366	NOT
ALMS1P	10.76172	-0.3676468	0.34317	-1.0713247	0.284023	0.535427	NOT
ATE1-AS1	15.04392	0.42785451	0.399402	1.071238019	0.284062	0.535427	NOT
MERTK	1281.433	0.41098404	0.383657	1.071226423	0.284068	0.535427	NOT
MTERF4	858.7219	0.13966336	0.130379	1.071212123	0.284074	0.535427	NOT
PCK2	18568.93	-0.3827352	0.357319	-1.07113084	0.284111	0.535458	NOT
RP11-416N	1.624895	0.7906545	0.738284	1.070935514	0.284198	0.535587	NOT
RP11-575I	12.1944	-0.3703365	0.345831	-1.07085949	0.284233	0.535614	NOT
RP5-837I2	3.545411	0.40504994	0.378263	1.070814901	0.284253	0.535614	NOT
SCOC-AS1	30.28752	0.26961511	0.251814	1.07069354	0.284307	0.535663	NOT
SLC13A5	14263.03	-0.588756	0.549895	-1.07066984	0.284318	0.535663	NOT
LINC0057	5.83111	0.62182911	0.580865	1.070522602	0.284384	0.535747	NOT
NEDD8	3163.361	0.20201469	0.188714	1.070482784	0.284402	0.535747	NOT
GAPDHP32	2.573358	-0.5522919	0.515961	-1.07041343	0.284433	0.535769	NOT
AL450992.	128.7168	-0.3476105	0.324772	-1.07032021	0.284475	0.53581	NOT
UNC93B1	1342.257	0.20533165	0.191866	1.070182	0.284537	0.53589	NOT
PTCHD3P3	2.182038	0.90078454	0.84189	1.069955506	0.284639	0.536043	NOT
CTD-3220F	2.036493	-0.4604333	0.430349	-1.0699074	0.284661	0.536043	NOT
LINC0029	27.8951	-0.4972883	0.464812	-1.0698698	0.284678	0.536043	NOT
WBP2NL	35.68436	0.27619354	0.25818	1.069770843	0.284722	0.53609	NOT
NOP56P3	5.307656	0.55046439	0.514686	1.06951411	0.284838	0.53612	NOT
BEND4	5.32855	0.69123239	0.646332	1.069469336	0.284858	0.53612	NOT
FUNDC1	406.7909	0.17101864	0.15991	1.069468811	0.284858	0.53612	NOT
U52111.14	6.100962	0.48713529	0.455493	1.069467633	0.284859	0.53612	NOT
RP11-440I	263.0899	0.27511436	0.257251	1.069441125	0.284871	0.53612	NOT
SOX21	1.60997	0.70713368	0.661247	1.069393902	0.284892	0.53612	NOT
STT3A-AS1	1.524642	0.50723614	0.474341	1.069349156	0.284912	0.53612	NOT
RP11-358N	7.580924	-0.3699982	0.346017	-1.06930516	0.284932	0.53612	NOT
FADS3	1320.58	-0.2444963	0.228651	-1.06929874	0.284935	0.53612	NOT
IGLV3-21	177.5943	0.77427409	0.724097	1.069296277	0.284936	0.53612	NOT
LL22NC03-	98.74038	-0.1890626	0.176845	-1.06908915	0.28503	0.536259	NOT
RP11-677M	21.91178	-0.3939536	0.368548	-1.06893536	0.285099	0.536347	NOT
OARD1	1452.291	0.18358542	0.171752	1.068897393	0.285116	0.536347	NOT
SFTA2	2.557199	0.98161686	0.918487	1.068732396	0.28519	0.53644	NOT
RP11-700F	2.122051	0.49333892	0.461634	1.068680429	0.285214	0.53644	NOT
EEPD1	1321.303	0.22714565	0.212553	1.068655788	0.285225	0.53644	NOT
GHc-857G6	7.088903	-0.3384799	0.316813	-1.06839157	0.285344	0.536627	NOT
MED28P8	0.434377	-1.2232228	1.145034	-1.06828518	0.285392	NA	NA
RPL37P2	22.6117	0.45508872	0.426032	1.068203155	0.285429	0.536714	NOT
CTB-119C2	18.86138	-0.3633716	0.340172	-1.06820087	0.28543	0.536714	NOT
RP11-527J	17.50261	0.25964571	0.243085	1.068129032	0.285462	0.536738	NOT
MYL3	13.0622	-0.4569659	0.427844	-1.06806763	0.28549	0.536744	NOT
RP11-849F	12.28755	-0.2607681	0.244167	-1.06798939	0.285525	0.536744	NOT

CETN3	391.8264	0.14619607	0.136891	1.067975996	0.285531	0.536744	NOT
TMEM119	229.0537	-0.5207512	0.487634	-1.06791494	0.285559	0.536744	NOT
MDGA2	39.09367	1.19785396	1.121688	1.067903167	0.285564	0.536744	NOT
RP11-573(	6.547052	-0.4496092	0.421054	-1.06781725	0.285603	0.536774	NOT
HIVEP3	302.2052	0.35813746	0.335404	1.067779515	0.28562	0.536774	NOT
PRRX1	176.6534	-0.4315589	0.404213	-1.06765128	0.285678	0.536846	NOT
ABL1	2473.424	-0.1895062	0.17755	-1.06733883	0.285819	0.537063	NOT
AC125232.	60.33545	-0.2907297	0.272403	-1.06727721	0.285847	0.537063	NOT
SLC22A7	14909.93	-0.6278304	0.588262	-1.06726339	0.285853	0.537063	NOT
RP11-504I	4.626776	-0.4422914	0.41444	-1.06720245	0.28588	0.537077	NOT
CORO1A	1267.486	0.34989235	0.327873	1.067157025	0.285901	0.537079	NOT
C11orf73	819.5683	0.16090863	0.150799	1.067039536	0.285954	0.537103	NOT
DNM1P35	27.31289	-0.3966865	0.371767	-1.06703037	0.285958	0.537103	NOT
SPATA9	9.596897	-0.2942365	0.275772	-1.06695512	0.285992	0.537103	NOT
TPR	5081.347	0.17743319	0.166299	1.0669531	0.285993	0.537103	NOT
RAB3C	132.1542	0.69445599	0.650931	1.066865933	0.286032	0.537124	NOT
ERLEC1	2926.252	-0.1255901	0.117724	-1.066814	0.286056	0.537124	NOT
MRPL43	2867.877	-0.1680752	0.157558	-1.06675242	0.286084	0.537124	NOT
RABEP2	882.1632	-0.2293011	0.214958	-1.06672361	0.286097	0.537124	NOT
SARAF	9399.292	-0.1993581	0.186917	-1.06656127	0.28617	0.537124	NOT
BBS2	867.8966	0.20968473	0.19661	1.066501812	0.286197	0.537124	NOT
AC093162.	4.679895	0.41195144	0.38627	1.066484593	0.286205	0.537124	NOT
SIM1	63.78753	-0.9257684	0.868092	-1.06644063	0.286225	0.537124	NOT
TMC6	1466.959	0.44082335	0.41337	1.066414642	0.286236	0.537124	NOT
MEGF6	690.0007	-0.4633777	0.434534	-1.06637956	0.286252	0.537124	NOT
POM121C	1421.322	-0.1394999	0.130823	-1.06632644	0.286276	0.537124	NOT
MSC	1752.813	0.71042076	0.666235	1.066320816	0.286279	0.537124	NOT
AK1	551.4764	-0.2555358	0.239644	-1.06631588	0.286281	0.537124	NOT
AC008155.	3.234301	0.45898495	0.43044	1.066315059	0.286281	0.537124	NOT
PYHIN1	57.14736	-0.4309765	0.404224	-1.06618227	0.286341	0.537169	NOT
CTD-2531I	4.346593	0.70808836	0.664139	1.066174636	0.286345	0.537169	NOT
TMEM158	46.22999	0.39912586	0.374424	1.065973252	0.286436	0.537302	NOT
MAP2K3	2829.594	-0.2042624	0.191632	-1.06591142	0.286464	0.537317	NOT
B3GNT9	182.3199	0.36423936	0.341744	1.065826196	0.286502	0.537353	NOT
RP11-435(	6.34232	-0.3991077	0.374507	-1.06568693	0.286565	0.537434	NOT
LINC00562	10.20823	0.35748676	0.335495	1.065551269	0.286627	0.537466	NOT
IPO5P1	383.0388	-0.320067	0.300377	-1.06555046	0.286627	0.537466	NOT
CPT1A	9470.424	-0.2882629	0.270538	-1.0655173	0.286642	0.537466	NOT
TRRAP	2470.682	0.19518686	0.183209	1.065379378	0.286704	0.537475	NOT
PIP4K2B	2659.638	0.13570685	0.127384	1.065336675	0.286724	0.537475	NOT
IAPP	9.548596	-0.5980856	0.561406	-1.06533543	0.286724	0.537475	NOT
RP11-244I	2.756154	0.46945726	0.440672	1.065322294	0.28673	0.537475	NOT
PPIP5K2	1405.296	-0.1820031	0.170849	-1.06528749	0.286746	0.537475	NOT
RP11-143K	8.574389	0.28776006	0.270143	1.065215231	0.286779	0.537499	NOT
ZNF626	168.2422	0.38980547	0.365986	1.065081653	0.286839	0.537553	NOT
CTD-2330K	1.969192	0.55840659	0.524294	1.06506479	0.286847	0.537553	NOT
OAZ3	90.30737	0.26571938	0.249497	1.065019989	0.286867	0.537553	NOT
DGKE	261.4994	-0.3286579	0.308611	-1.06495972	0.286894	0.537567	NOT
MTND6P3	2.857376	-0.6466471	0.607254	-1.06487099	0.286934	0.537596	NOT

ACER2	46.58448	-0.3985223	0.374256	-1.0648387	0.286949	0.537596	NOT
KB-1507C5	112.1322	0.30892358	0.290204	1.064503395	0.287101	0.537843	NOT
TEX10	781.224	0.17047388	0.160174	1.06430576	0.28719	0.537974	NOT
CIB1	5182.559	-0.2058147	0.193396	-1.0642122	0.287233	0.538009	NOT
CNN3	7745.854	0.23579386	0.221574	1.064176939	0.287249	0.538009	NOT
SNX8	1937.304	0.22880921	0.215033	1.064067173	0.287298	0.538064	NOT
RP4-734G2	22.11901	0.31829932	0.29916	1.063976081	0.28734	0.538095	NOT
MORN4	154.3266	-0.2851514	0.268036	-1.06385447	0.287395	0.538095	NOT
RP11-211M	1.909198	0.52583142	0.494272	1.063850691	0.287396	0.538095	NOT
NACA3P	83.04721	0.32567205	0.306135	1.063817647	0.287411	0.538095	NOT
KCNK17	30.61778	-0.5536424	0.520432	-1.06381259	0.287414	0.538095	NOT
SEPT7P2	337.6915	-0.2188547	0.205752	-1.0636823	0.287473	0.538168	NOT
AP2A2	4197.037	-0.1170088	0.110052	-1.06321026	0.287687	0.538532	NOT
GCOM1	2.89304	0.5481462	0.515583	1.063157493	0.287711	0.538539	NOT
LMO2	443.8339	-0.2412149	0.226905	-1.06306332	0.287753	0.538582	NOT
G2E3	421.7499	0.18705813	0.175991	1.062883107	0.287835	0.538671	NOT
LACTB	1688.92	-0.2002556	0.18841	-1.06287031	0.287841	0.538671	NOT
RN7SL181F	1.894785	-0.4388286	0.412903	-1.06278858	0.287878	0.538683	NOT
HDGFP1	13.018	0.77814953	0.732191	1.062769125	0.287887	0.538683	NOT
RP11-524D	11.94962	-0.4133873	0.389026	-1.06262058	0.287954	0.538772	NOT
RPS2P48	3.803764	0.44027555	0.414494	1.062199665	0.288145	0.539075	NOT
KIZ	700.0936	-0.17806	0.167637	-1.06217556	0.288156	0.539075	NOT
RIPPLY1	115.1959	-0.5940959	0.559394	-1.06203494	0.28822	0.539158	NOT
TTC23	825.9157	0.2086328	0.196481	1.061846251	0.288305	0.539272	NOT
RP11-723C	25.21397	0.37808419	0.356074	1.061813134	0.288321	0.539272	NOT
RTN1	167.8017	0.41453286	0.39043	1.061734814	0.288356	0.539301	NOT
KPRP	1.271867	1.35083661	1.272437	1.061613683	0.288411	NA	NA
NDUFA2	5048.4	-0.285166	0.268634	-1.06154227	0.288444	0.539396	NOT
LYN	1652.46	0.2377516	0.223969	1.061535842	0.288446	0.539396	NOT
ZMIZ2	3377.454	0.16848012	0.158733	1.061408498	0.288504	0.539467	NOT
NEK4	917.1824	0.23308814	0.21968	1.06103727	0.288673	0.539745	NOT
SPIN3	118.1292	-0.1936461	0.18252	-1.06095899	0.288709	0.539774	NOT
AC096670.	3.087857	-0.4247614	0.400395	-1.06085483	0.288756	0.539785	NOT
ARNT2	389.0744	0.61034561	0.575336	1.06085024	0.288758	0.539785	NOT
AIP	2481.289	0.19883603	0.187437	1.060814473	0.288774	0.539785	NOT
AC007405.	21.87433	0.38549863	0.363441	1.060690687	0.28883	0.539826	NOT
DDX43	26.22047	0.63290056	0.596722	1.060629551	0.288858	0.539826	NOT
BTG3	781.0811	0.19632031	0.185103	1.060599233	0.288872	0.539826	NOT
GXYLT1	1156.19	-0.1850076	0.17444	-1.06058051	0.288881	0.539826	NOT
HOXD8	64.77449	-0.3973169	0.374634	-1.06054748	0.288896	0.539826	NOT
RP11-182M	2.522384	-0.474417	0.447372	-1.06045379	0.288938	0.53985	NOT
PMM1	2867.074	-0.2783272	0.262492	-1.06032695	0.288996	0.53985	NOT
IFITM10	690.4262	-0.4708342	0.444057	-1.06030215	0.289007	0.53985	NOT
PRDX3	11148.22	-0.2161797	0.20389	-1.06027674	0.289019	0.53985	NOT
DISP2	154.7381	-0.4710435	0.444276	-1.06024871	0.289031	0.53985	NOT
AC002467.	65.3355	0.24835908	0.234248	1.060241572	0.289035	0.53985	NOT
HLA-C	57607.49	0.3101455	0.292531	1.060213603	0.289047	0.53985	NOT
RP5-832C2	23.16405	-0.2755216	0.259898	-1.06011477	0.289092	0.539897	NOT
MED9	904.2738	-0.2023562	0.190893	-1.06005177	0.289121	0.539903	NOT

RPS14P8	17.46711	0.64969065	0.612904	1.060019878	0.289136	0.539903	NOT
ETHE1	1192.724	-0.2252812	0.212546	-1.05991611	0.289183	0.539929	NOT
ASIP	16.56816	0.42698883	0.402857	1.059901417	0.289189	0.539929	NOT
ZNF493	201.5531	-0.2772425	0.261586	-1.05985339	0.289211	0.539933	NOT
TRPV3	55.96083	-0.5599595	0.528369	-1.05978956	0.28924	0.539941	NOT
GMPPA	3139.774	0.18280875	0.172501	1.059756453	0.289255	0.539941	NOT
RP11-435F	3.688957	0.36246242	0.342122	1.059454122	0.289393	0.54016	NOT
NYAP2	0.816511	1.27582029	1.204382	1.059315706	0.289456	NA	NA
FLNB-AS1	41.90211	-0.319533	0.301664	-1.05923387	0.289493	0.54031	NOT
RP11-710F	2.880546	-0.5043151	0.476343	-1.05872239	0.289726	0.540708	NOT
MAP3K12	188.2926	-0.2335592	0.220643	-1.05853827	0.28981	0.540827	NOT
PPP6C	2194.992	-0.1131069	0.106863	-1.0584262	0.289861	0.540885	NOT
NECAP1	1010.227	-0.1562881	0.147681	-1.05828089	0.289927	0.540972	NOT
SMG6	464.244	-0.2543818	0.240421	-1.05806722	0.290025	0.541116	NOT
FOXRED1	2318.229	-0.1818659	0.171912	-1.05790053	0.290101	0.541212	NOT
HSPA8P8	8.939235	0.56109383	0.530401	1.057866824	0.290116	0.541212	NOT
AC005042.	1.338328	0.72395073	0.684539	1.057574441	0.29025	NA	NA
RAB6A	4314.007	-0.1306712	0.123574	-1.0574318	0.290315	0.541545	NOT
RP11-22B2	147.5713	0.25136847	0.237735	1.057345923	0.290354	0.541581	NOT
AC005307.	7.24972	0.90448805	0.855533	1.057221955	0.29041	0.541649	NOT
LINC00471	11.62104	0.28951293	0.273869	1.057123118	0.290455	0.541696	NOT
FP325331.	1.724401	0.58535002	0.553825	1.056922921	0.290547	0.541746	NOT
RP11-334E	1.76375	-0.7053266	0.667345	-1.05691518	0.29055	0.541746	NOT
MBL2	4277.033	-0.5509582	0.521294	-1.05690431	0.290555	0.541746	NOT
IGKV3D-15	3.068908	0.96929613	0.917192	1.056808205	0.290599	0.541746	NOT
IMPACT	1079.732	-0.1952664	0.184776	-1.0567728	0.290615	0.541746	NOT
AC019185.	2.148478	-0.8606615	0.814429	-1.05676724	0.290618	0.541746	NOT
SVIP	831.0996	0.19029214	0.180071	1.056758977	0.290622	0.541746	NOT
PIEZO2	1893.759	-0.3654137	0.345847	-1.0565763	0.290705	0.541864	NOT
LL22NC03-	220.5787	0.22266586	0.210766	1.056459913	0.290758	0.541908	NOT
HLA-S	2.713453	-0.5349508	0.506383	-1.05641498	0.290779	0.541908	NOT
CERS6	880.2736	0.25427002	0.240696	1.056392896	0.290789	0.541908	NOT
BMP8A	13.4354	-0.3963344	0.3752	-1.05632707	0.290819	0.541927	NOT
RP11-379F	51.56795	0.28939527	0.273982	1.056258238	0.29085	0.541948	NOT
STAG3L2	120.2805	-0.2275094	0.215412	-1.05615708	0.290896	0.541997	NOT
RP11-537H	2.953471	0.47050243	0.445514	1.056088893	0.290928	0.542018	NOT
RP11-360F	22.25682	0.2682208	0.254045	1.055799737	0.29106	0.542214	NOT
CHMP1B	2129.024	-0.1644218	0.155736	-1.05577123	0.291073	0.542214	NOT
AC044907.	7.185276	-0.5948939	0.563493	-1.05572597	0.291093	0.542215	NOT
RP11-70L8	6.584208	-0.3687072	0.349296	-1.05557264	0.291164	0.542297	NOT
AQP7P4	2.248706	0.81930709	0.776198	1.055538515	0.291179	0.542297	NOT
RP1-163G9	1.714493	1.24488296	1.17944	1.055486376	0.291203	0.542297	NOT
ACP5	2609.961	-0.4370874	0.414122	-1.0554546	0.291217	0.542297	NOT
RP11-254F	2.644188	-0.6897529	0.653601	-1.05531253	0.291282	0.542381	NOT
ULBP1	17.27597	0.54937289	0.520655	1.055156689	0.291354	0.54247	NOT
ATP5G2	11830.59	0.20069869	0.190214	1.055120197	0.29137	0.54247	NOT
WEE2	3.104291	-0.4142476	0.392672	-1.05494441	0.291451	0.542583	NOT
SPATA20	3151.469	-0.2345171	0.22232	-1.05486055	0.291489	0.542617	NOT
ARHGEF25	199.4971	-0.3901173	0.369934	-1.05456009	0.291627	0.542836	NOT

ANAPC4	911.2102	0.15526461	0.147271	1.054276592	0.291756	0.54301	NOT
MT-TE	1.739351	-0.6410009	0.608006	-1.05426782	0.29176	0.54301	NOT
RNU6-762I	2.587442	0.4335191	0.411244	1.054164927	0.291807	0.54306	NOT
RPS12P23	1.789894	0.63596907	0.603372	1.054024941	0.291871	0.543074	NOT
CTD-2024I	10.75373	-0.3679775	0.349129	-1.05398663	0.291889	0.543074	NOT
CRELD2	2821.263	-0.2309471	0.21912	-1.05397719	0.291893	0.543074	NOT
GOLGA6B	7.294074	-0.8521682	0.808529	-1.05397399	0.291895	0.543074	NOT
CBR3-AS1	47.78667	0.29557103	0.280518	1.053661458	0.292038	0.543283	NOT
SMURF2	670.2852	0.22318331	0.211821	1.053641592	0.292047	0.543283	NOT
SCARNA9	12.3302	-0.3382846	0.321105	-1.05350292	0.292111	0.543303	NOT
RP11-702I	3.586726	0.37745603	0.358288	1.053498564	0.292113	0.543303	NOT
SIRPB2	55.87604	-0.3893546	0.369587	-1.05348623	0.292118	0.543303	NOT
IGKV1OR22	0.672221	-0.957584	0.909	-1.05344775	0.292136	NA	NA
EPX	4.73946	-0.4025653	0.382156	-1.05340644	0.292155	0.543334	NOT
LINC00662	220.0637	-0.1595419	0.151467	-1.05331268	0.292198	0.543377	NOT
TMEM256-I	13.8194	-0.2982848	0.283209	-1.05323071	0.292235	0.543409	NOT
RP11-502I	45.94434	-0.2620468	0.248848	-1.05304164	0.292322	0.543533	NOT
RP11-158I	109.9957	-0.2198052	0.208755	-1.05293644	0.29237	0.543552	NOT
TROVE2	1413.012	-0.1952342	0.185419	-1.05293253	0.292372	0.543552	NOT
SLC45A4	601.8514	0.45815569	0.435155	1.052856318	0.292407	0.543579	NOT
RP11-231I	2.554977	0.57578264	0.547055	1.052513361	0.292564	0.543666	NOT
RP5-956OI	2.696728	-0.4130761	0.392467	-1.05251044	0.292565	0.543666	NOT
RP11-456I	43.71041	-0.4922867	0.46775	-1.0524562	0.29259	0.543666	NOT
SNORA40	5.469463	0.40311559	0.383025	1.05245271	0.292592	0.543666	NOT
TMTC3	826.9526	-0.1809897	0.171972	-1.05243782	0.292599	0.543666	NOT
ATF3	1861.287	-0.3346329	0.317961	-1.05243538	0.2926	0.543666	NOT
IGHV2-70	13.04864	-0.9761288	0.927586	-1.05233245	0.292647	0.543666	NOT
MICD	3.065868	0.65389803	0.621394	1.052308496	0.292658	0.543666	NOT
LINC0141(	14.90395	0.39212527	0.372638	1.052295692	0.292664	0.543666	NOT
IGLV2-18	8.585531	0.84557418	0.803574	1.052266757	0.292677	0.543666	NOT
NOL9	752.9872	0.16162433	0.153599	1.052245708	0.292687	0.543666	NOT
PDZD8	2108.543	-0.2302424	0.218814	-1.05223008	0.292694	0.543666	NOT
ADAM33	44.51013	-0.4718717	0.448493	-1.05212777	0.292741	0.543695	NOT
RPS13P2	33.50037	0.31328261	0.297778	1.052067467	0.292769	0.543695	NOT
ABCC6P1	925.5087	-0.3609261	0.343065	-1.05206473	0.29277	0.543695	NOT
RP11-95P2	12.02353	-0.3168214	0.301162	-1.05199664	0.292801	0.543695	NOT
NOD1	239.4964	-0.1730872	0.164543	-1.05192496	0.292834	0.543695	NOT
ROR1	129.6749	0.528387	0.502315	1.051903541	0.292844	0.543695	NOT
PLCE1	469.7481	0.24275984	0.230784	1.051890151	0.29285	0.543695	NOT
CEP85L	157.5359	-0.2931745	0.278753	-1.05173549	0.292921	0.543733	NOT
AQP4-AS1	7.433383	0.51031312	0.485229	1.051694929	0.29294	0.543733	NOT
PPARA	4949.028	-0.2642869	0.251302	-1.05166857	0.292952	0.543733	NOT
LCA5	54.49418	-0.329027	0.312869	-1.05164548	0.292962	0.543733	NOT
AC004987.	27.74562	-0.2309187	0.219585	-1.05161466	0.292976	0.543733	NOT
RP11-158M	4.677233	-0.4467829	0.424867	-1.05158345	0.292991	0.543733	NOT
LINC00892	11.89064	-0.3933612	0.374094	-1.05150321	0.293028	0.543765	NOT
ZNF418	91.11349	0.40617378	0.386328	1.051369692	0.293089	0.543816	NOT
RP11-466I	18.99415	0.28804912	0.273984	1.051334488	0.293105	0.543816	NOT
SNX22	855.5587	-0.4755417	0.452332	-1.05131219	0.293115	0.543816	NOT

TBC1D10B	1541.582	0.17709269	0.168463	1.051228159	0.293154	0.54385	NOT
ILK	403.2591	0.22134005	0.210581	1.051091115	0.293217	0.543897	NOT
ACTL6B	7.637728	-0.4994649	0.475209	-1.05104282	0.293239	0.543897	NOT
TBC1D8	1641.03	0.23085707	0.21966	1.05097544	0.29327	0.543897	NOT
RP11-435(	4.18697	0.55035245	0.523691	1.050910505	0.2933	0.543897	NOT
STX18-AS1	43.97585	0.26329945	0.250547	1.050896997	0.293306	0.543897	NOT
AC092415.	2.956829	1.29469663	1.232011	1.050880331	0.293314	0.543897	NOT
ZNF200	416.733	-0.1425484	0.135648	-1.0508675	0.293319	0.543897	NOT
RPS16	28808.2	0.2133913	0.203095	1.050697787	0.293397	0.544004	NOT
LONRF2	172.3404	0.54763201	0.521279	1.050555312	0.293463	0.544057	NOT
ZFAND4	344.1278	-0.1928022	0.183525	-1.05054814	0.293466	0.544057	NOT
RP11-452I	2.339184	0.62644947	0.596462	1.050274855	0.293592	0.54417	NOT
AC024995.	2.564676	0.51068541	0.486253	1.050247045	0.293605	0.54417	NOT
RP11-112J	14.38097	0.40035522	0.381217	1.050204214	0.293624	0.54417	NOT
RP11-30L1	8.0122	-0.3878565	0.369325	-1.05017777	0.293636	0.54417	NOT
RP11-75C1	5.098445	0.42257532	0.402391	1.050162132	0.293644	0.54417	NOT
SOD2	49122.03	-0.3589004	0.34177	-1.05012387	0.293661	0.54417	NOT
RP11-379K	6.923087	0.70118728	0.667727	1.05011042	0.293667	0.54417	NOT
LRP1	30280.39	-0.2362925	0.225048	-1.04996363	0.293735	0.544258	NOT
TMEM26	44.55081	-0.3423492	0.326103	-1.04981962	0.293801	0.544344	NOT
SMIM7	2310.129	-0.1962373	0.186938	-1.04974504	0.293835	0.54435	NOT
IL36G	2.916547	-0.6271222	0.597416	-1.04972453	0.293845	0.54435	NOT
TTC1	2318.999	0.18512555	0.176408	1.049414333	0.293987	0.544575	NOT
AC009948.	104.0953	0.2450088	0.233481	1.049373373	0.294006	0.544575	NOT
CTA-984G1	12.63595	0.31972846	0.304709	1.049290172	0.294045	0.544609	NOT
SNORD46	2.989626	0.4387473	0.418198	1.049136825	0.294115	0.544687	NOT
Z83844.1	4.154284	0.51213121	0.488157	1.049111692	0.294127	0.544687	NOT
FAM150B	26.24025	0.64566765	0.615477	1.049052944	0.294154	0.544699	NOT
EOGT	432.025	0.18809576	0.179308	1.049006961	0.294175	0.544701	NOT
DHDDS	1485.552	-0.1857856	0.177133	-1.04884648	0.294249	0.544801	NOT
RP11-399E	2.451458	0.49121874	0.468377	1.048768779	0.294285	0.54483	NOT
RP5-894A1	130.8732	0.29469054	0.28102	1.048645162	0.294341	0.544898	NOT
LINC00533	0.779856	-1.3386222	1.276771	-1.04844332	0.294434	NA	NA
KCNJ2-AS1	10.13277	-0.2290893	0.218518	-1.04837661	0.294465	0.545018	NOT
METTL8	471.8048	0.16802035	0.160269	1.048365846	0.29447	0.545018	NOT
MOSPD3	1335.264	-0.2377195	0.226754	-1.04835932	0.294473	0.545018	NOT
DIXDC1	459.2111	-0.2793467	0.266473	-1.0483104	0.294496	0.545018	NOT
RN7SL812P	1.402	0.65025365	0.620302	1.04828612	0.294507	0.545018	NOT
RP11-144F	1.657904	0.52187059	0.497882	1.048181989	0.294555	0.54507	NOT
RP11-159H	3.292914	0.45285686	0.4321	1.048038002	0.294621	0.545082	NOT
FBXO21	1504.918	0.22014177	0.210054	1.048025525	0.294627	0.545082	NOT
NCOA6	2301.233	0.15286938	0.145869	1.04798884	0.294644	0.545082	NOT
CTD-2574D	15.86157	0.38031012	0.362905	1.047959138	0.294657	0.545082	NOT
RP11-661(	3.323712	-0.3904251	0.372563	-1.04794461	0.294664	0.545082	NOT
WDR91	995.9837	0.1936233	0.184772	1.047906621	0.294682	0.545082	NOT
DLG3	389.7136	0.42224129	0.402974	1.047812749	0.294725	0.545123	NOT
C17orf70	2285.568	-0.1632446	0.155802	-1.04777065	0.294744	0.545123	NOT
TRAV12-2	5.025471	-0.5540784	0.528851	-1.04770194	0.294776	0.545145	NOT
PRRG1	416.3583	-0.250235	0.238873	-1.04756438	0.294839	0.545173	NOT

PSMA6	1048.391	0.17098015	0.163217	1.047562179	0.29484	0.545173	NOT
AMDHD1	3064.117	-0.4344976	0.41478	-1.04753843	0.294851	0.545173	NOT
RNF25	953.9135	0.18486907	0.176502	1.047404364	0.294913	0.545184	NOT
C2orf15	45.73919	0.37893377	0.361793	1.047377016	0.294926	0.545184	NOT
AC003090.	12.97524	0.74153671	0.708011	1.047351716	0.294937	0.545184	NOT
RP4-756H1	161.9053	0.18014989	0.172005	1.04735098	0.294938	0.545184	NOT
SIN3B	1383.608	-0.1330062	0.127	-1.04729428	0.294964	0.545195	NOT
KANSL1	1648.104	0.15050339	0.143736	1.047078602	0.295063	0.545342	NOT
FRMD3	206.8961	-0.4071485	0.388881	-1.0469754	0.295111	0.545392	NOT
INHBB	2449.669	0.34976336	0.334088	1.046919503	0.295137	0.545403	NOT
ERICH1-AS	9.479808	-0.9165048	0.875502	-1.04683382	0.295176	0.545439	NOT
KRT15	12.31897	0.73198935	0.699294	1.046754288	0.295213	0.545446	NOT
CTNNBL1	3074.424	0.23657596	0.226013	1.046737756	0.295221	0.545446	NOT
FGF11	5.595768	0.6242682	0.596493	1.046564385	0.295301	0.545466	NOT
PPM1D	625.059	-0.1703538	0.162777	-1.04655021	0.295307	0.545466	NOT
RPL17P11	9.298622	-0.6225872	0.594911	-1.04652125	0.29532	0.545466	NOT
XXbac-BP(	32.30669	-0.2696688	0.257684	-1.04650962	0.295326	0.545466	NOT
NPFFR1	3.284213	0.67125994	0.641435	1.046496656	0.295332	0.545466	NOT
IL16	367.3146	-0.2801555	0.267734	-1.04639456	0.295379	0.545516	NOT
SMIM20	1109.428	0.23939648	0.228804	1.046296015	0.295424	0.545563	NOT
HCG4P5	415.2629	-0.529139	0.505747	-1.04625188	0.295445	0.545563	NOT
FDX1P1	3.359409	-0.359523	0.343685	-1.04608163	0.295523	0.545671	NOT
HRNR	7.937638	0.65011225	0.621537	1.045974603	0.295573	0.545725	NOT
KRT6C	10.61937	1.10939676	1.06082	1.045791631	0.295657	0.545783	NOT
JAG1	2236.269	0.34399664	0.328936	1.04578718	0.295659	0.545783	NOT
RP11-718I	1.480628	-0.6201821	0.593035	-1.04577657	0.295664	0.545783	NOT
RP11-588K	244.8728	-0.3255945	0.311374	-1.04567185	0.295713	0.545835	NOT
RP11-1113	34.1895	-0.3925158	0.375393	-1.04561325	0.29574	0.545848	NOT
MICU2	1234.295	-0.174422	0.166846	-1.04540601	0.295835	0.545987	NOT
AP000662.	8.876191	-0.4576036	0.437796	-1.0452434	0.29591	0.546089	NOT
SEC24B-AS	19.45683	-0.2480066	0.237295	-1.04513983	0.295958	0.54614	NOT
RP11-121A	9.268582	-0.3430981	0.328302	-1.0450686	0.295991	0.546164	NOT
PROK1	6.994536	-0.6795138	0.650367	-1.04481559	0.296108	0.546282	NOT
LINC00857	132.5882	0.38040177	0.364087	1.044808645	0.296111	0.546282	NOT
HLA-DMB	1213.102	0.40020795	0.383048	1.044799058	0.296116	0.546282	NOT
RP11-386I	5.925156	-0.4080733	0.390598	-1.04473893	0.296144	0.546296	NOT
RP13-644M	6.119696	0.62026504	0.593794	1.044580332	0.296217	0.54639	NOT
RAB11A	3955.646	0.1042417	0.099797	1.044541491	0.296235	0.54639	NOT
RRH	4.954679	-0.4045326	0.387359	-1.04433568	0.29633	0.546505	NOT
CRACR2B	412.6342	0.43671111	0.418178	1.044319624	0.296338	0.546505	NOT
RC3H1	1334.41	-0.1311075	0.125628	-1.04361804	0.296662	0.547067	NOT
RPL21P75	20.03757	0.37486519	0.35926	1.043436276	0.296746	0.547149	NOT
RP11-262H	1.910425	-0.5591838	0.535907	-1.04343398	0.296747	0.547149	NOT
SELL	217.6165	0.3512143	0.336628	1.043330053	0.296795	0.547189	NOT
TWISTNB	714.641	0.16208495	0.155358	1.043299913	0.296809	0.547189	NOT
WWC2-AS2	29.74638	-0.2380782	0.228251	-1.04305643	0.296922	0.547339	NOT
SKA2P1	2.528062	0.38655369	0.370604	1.04303766	0.296931	0.547339	NOT
APPL1	1555.209	0.16907082	0.162105	1.042973082	0.296961	0.547357	NOT
LEPR	8515.254	-0.4984759	0.478017	-1.04279911	0.297041	0.547468	NOT

TPD52L1	1161.511	0.37892492	0.3634	1.042721105	0.297077	0.547498	NOT
MCMBP	2043.269	0.1509568	0.144787	1.042610264	0.297129	0.547555	NOT
COX6B2	4.18067	0.46895703	0.449874	1.042419481	0.297217	0.547681	NOT
RSPO1	2.373657	-0.8926318	0.856416	-1.04228812	0.297278	0.547756	NOT
ABCD2	25.06062	-0.4688863	0.449907	-1.04218607	0.297325	0.547806	NOT
CTD-2371(	54.54366	0.29757371	0.285606	1.041902871	0.297457	0.54801	NOT
GLRX5	3647.245	-0.2038814	0.195697	-1.0418238	0.297493	0.548041	NOT
C1D	670.7194	0.15942497	0.15307	1.041516345	0.297636	0.548232	NOT
RP11-787]	3.960558	-0.3937174	0.378025	-1.04151277	0.297638	0.548232	NOT
IGKV1-9	37.02341	0.73582183	0.706531	1.041457924	0.297663	0.548242	NOT
RP11-448(	46.05474	-0.3611415	0.346812	-1.04131863	0.297728	0.548323	NOT
ZDHHC1	227.2511	-0.3795571	0.364531	-1.04122083	0.297773	0.548366	NOT
NELFB	2910.995	0.14769125	0.14185	1.041182115	0.297791	0.548366	NOT
AP000704.	8.416713	0.38735121	0.372081	1.041041023	0.297857	0.548428	NOT
METTL24	24.62204	0.55403556	0.532223	1.040984054	0.297883	0.548428	NOT
RP5-1025A	1.583501	0.82179992	0.789449	1.040978777	0.297885	0.548428	NOT
BAALC	165.8374	-0.4673702	0.448997	-1.04092042	0.297912	0.54844	NOT
KMT2B	2629.141	0.21667837	0.208178	1.040830038	0.297954	0.54848	NOT
RP11-345N	5.022151	0.94292274	0.906008	1.040744326	0.297994	0.548516	NOT
POMP	4243.878	0.2119599	0.203693	1.040585465	0.298068	0.548615	NOT
SPTBN2	1459.843	-0.4311165	0.414334	-1.04050394	0.298106	0.548647	NOT
GABARAP	2239.671	-0.1632495	0.156909	-1.04040703	0.298151	0.548693	NOT
GALC	672.9754	-0.3530151	0.339355	-1.04025217	0.298223	0.548788	NOT
NSFL1C	4256.089	0.14908638	0.143338	1.040100228	0.298293	0.548851	NOT
IGFBPL1	20.64906	0.74080529	0.712252	1.040088676	0.298299	0.548851	NOT
RP11-770E	5.51988	-0.607764	0.584362	-1.04004748	0.298318	0.548851	NOT
KIAA0319I	4679.626	-0.1655136	0.159168	-1.03986828	0.298401	0.548936	NOT
RP11-143(	8.289671	-0.8049951	0.774137	-1.03986142	0.298404	0.548936	NOT
RP11-815M	2.398368	-0.8559811	0.823426	-1.03953614	0.298555	0.549177	NOT
ARL6	80.95226	0.23203513	0.223264	1.039287693	0.298671	0.549352	NOT
RP11-468N	2.107543	1.11553535	1.073541	1.039117453	0.29875	0.54946	NOT
PCDHB5	111.8874	0.50911943	0.490016	1.03898501	0.298812	0.549503	NOT
PIP5K1A	2176.8	0.16174039	0.155672	1.038980118	0.298814	0.549503	NOT
FUT8	239.7524	0.34352123	0.330684	1.03881904	0.298889	0.549604	NOT
MYT1L	5.055742	-0.8144801	0.784302	-1.03847709	0.299048	0.54984	NOT
SSH1	1653	-0.1398489	0.13467	-1.03845637	0.299058	0.54984	NOT
LINC01315	41.67646	0.35785853	0.344698	1.038179506	0.299186	0.550039	NOT
EFNB1	1543.937	0.24551818	0.236524	1.038027357	0.299257	0.550108	NOT
MDS2	5.244528	0.38968972	0.375419	1.038011901	0.299265	0.550108	NOT
FAM45A	993.4645	0.22479503	0.216574	1.037959206	0.299289	0.550116	NOT
KLRK1	9.778681	-0.5782074	0.557109	-1.03787061	0.29933	0.550155	NOT
WNK3	773.5701	-0.421293	0.405952	-1.03778956	0.299368	0.550172	NOT
MRPS21	4106.082	0.23969382	0.230972	1.037763371	0.29938	0.550172	NOT
MYO7B	315.8547	0.49305518	0.475165	1.03765078	0.299433	0.55021	NOT
ADAM8	309.5234	0.29303563	0.282408	1.037631649	0.299442	0.55021	NOT
RPL4P4	122.8934	0.30083819	0.289972	1.037473913	0.299515	0.550308	NOT
RNU6-817F	2.815772	0.44764103	0.43155	1.03728572	0.299603	0.550405	NOT
SULT1A1	4025.757	-0.3254081	0.313726	-1.03723596	0.299626	0.550405	NOT
CCDC126	371.8399	-0.1961341	0.1891	-1.03719698	0.299644	0.550405	NOT

RP11-16B1	2.507123	0.64837632	0.62513	1.037186037	0.299649	0.550405	NOT
GABRA5	5.480416	1.7196501	1.658161	1.037082837	0.299697	0.550456	NOT
ITPR1	770.9155	-0.3516836	0.339149	-1.03695757	0.299756	0.550515	NOT
RP11-417I	18.81872	-0.544576	0.525182	-1.03692735	0.29977	0.550515	NOT
ARF1	24357.43	0.1232277	0.118847	1.036856347	0.299803	0.550538	NOT
UBA52P7	1.375333	0.72807742	0.702273	1.036744811	0.299855	NA	NA
ARL6IP1	10048.64	0.184752	0.178214	1.036686856	0.299882	0.550646	NOT
WASF2	3643.078	0.17871624	0.172409	1.036581669	0.299931	0.550699	NOT
AP000487.	17.99648	0.22512155	0.217226	1.036348288	0.30004	0.550839	NOT
ICK	1800.563	-0.3202836	0.309055	-1.03633077	0.300048	0.550839	NOT
LINC00612	9.408822	-0.3752811	0.362234	-1.03601868	0.300193	0.551069	NOT
S100A1	230.7854	0.43112546	0.416175	1.035924167	0.300238	0.551113	NOT
RBM38	1144.132	0.21297469	0.20565	1.035619696	0.30038	0.551316	NOT
SEMA4A	200.9043	0.35432672	0.342159	1.035562865	0.300406	0.551316	NOT
ZNF252P	1584.788	0.17373932	0.167774	1.035555583	0.30041	0.551316	NOT
CTD-3128C	7.363778	0.41693321	0.402744	1.035230802	0.300561	0.551557	NOT
RP5-994D1	22.04703	0.19500284	0.188415	1.034962943	0.300686	0.55175	NOT
ATF5	32691.03	-0.5470652	0.528664	-1.03480655	0.300759	0.551783	NOT
COPE	7549.49	-0.2297625	0.222044	-1.03476266	0.30078	0.551783	NOT
RP1-34H18	2.123593	-0.8032225	0.776241	-1.03475949	0.300781	0.551783	NOT
RFTN2	126.5277	0.32464738	0.313745	1.034749826	0.300786	0.551783	NOT
NPRL2	1028.369	0.15500028	0.149811	1.034638735	0.300838	0.55182	NOT
KIAA0226L	93.1751	0.37464673	0.362111	1.034619229	0.300847	0.55182	NOT
RCHY1	545.0969	-0.159981	0.154636	-1.03456575	0.300872	0.551829	NOT
WDR11	1805.23	-0.1621591	0.156772	-1.03436243	0.300967	0.551966	NOT
IGHM	1979.389	0.64603248	0.624626	1.034270689	0.30101	0.552007	NOT
RP4-607I7	4.290391	0.69898617	0.675882	1.034183732	0.30105	0.552014	NOT
AC046143.	13.66255	0.26706476	0.258239	1.03417523	0.301054	0.552014	NOT
BTN3A1	1582.712	-0.2864316	0.277008	-1.03401963	0.301127	0.552094	NOT
VASN	2504.083	-0.215712	0.20862	-1.03399517	0.301138	0.552094	NOT
DFFA	1813.85	0.14736743	0.142535	1.03389993	0.301183	0.55213	NOT
ATP6V1C2	90.18712	0.26262797	0.254025	1.033866509	0.301199	0.55213	NOT
PCBP3	46.34827	-0.5237635	0.506646	-1.03378662	0.301236	0.552142	NOT
MFSD6L	2.788899	0.55358293	0.535502	1.033764806	0.301246	0.552142	NOT
SRF	2020.898	0.12696471	0.122842	1.033559882	0.301342	0.55228	NOT
RP11-90B9	3.479697	-0.3551266	0.343661	-1.03336279	0.301434	0.552412	NOT
RP11-146I	2.247749	0.74215719	0.718308	1.033202152	0.301509	0.552512	NOT
AC083884.	2.472521	-0.4264878	0.412813	-1.03312697	0.301544	0.55254	NOT
MXRA5Y	3.124879	0.76520024	0.740756	1.032999553	0.301604	0.552612	NOT
AHSP	1.319189	-0.6098893	0.59046	-1.0329045	0.301649	NA	NA
RP11-71L1	2.78919	-0.755216	0.7312	-1.03284423	0.301677	0.55266	NOT
FOLR2	782.468	-0.3805146	0.368427	-1.03280983	0.301693	0.55266	NOT
PLIN2	17171.69	0.35944152	0.348036	1.03277122	0.301711	0.55266	NOT
AC010883.	21.77014	-0.3331664	0.322603	-1.03274463	0.301723	0.55266	NOT
CRABP2	53.1532	0.4281426	0.414576	1.032724939	0.301733	0.55266	NOT
ALS2	1619.233	0.14275397	0.138253	1.032555236	0.301812	0.552769	NOT
CYP3A4	47319.57	-0.8539241	0.827163	-1.03235317	0.301907	0.552905	NOT
LENG9	257.9103	-0.2814385	0.272663	-1.03218474	0.301986	0.552986	NOT
MCC	775.376	-0.3579764	0.346819	-1.03217163	0.301992	0.552986	NOT

VPS18	1685.257	-0.2127257	0.206106	-1.03211818	0.302017	0.552994	NOT
CREB3L2	2973.501	-0.199356	0.193214	-1.03179106	0.30217	0.553227	NOT
AC016582.	3.198833	-0.8557858	0.829468	-1.03172889	0.302199	0.553227	NOT
HAUS3	471.3891	0.1268611	0.122961	1.031716248	0.302205	0.553227	NOT
JHDM1D-AS	283.9873	-0.2605024	0.252536	-1.03154469	0.302285	0.553337	NOT
BAX	2809.905	0.22151282	0.214777	1.03136034	0.302372	0.553458	NOT
CXorf40A	456.3383	-0.1719906	0.166775	-1.03127333	0.302413	0.553495	NOT
PPP1R3E	458.6538	-0.2054573	0.199256	-1.0311223	0.302483	0.553587	NOT
SERPINA11	6378.928	-0.5219667	0.506243	-1.03106032	0.302513	0.553603	NOT
ATP6V1E2	196.4697	0.26652785	0.25854	1.030896542	0.302589	0.553706	NOT
CRY1	752.4053	0.20537925	0.199232	1.030852477	0.30261	0.553707	NOT
EEF1A1P22	12.1227	0.36660381	0.355699	1.030657394	0.302702	0.553784	NOT
KIRREL	737.8627	-0.3743601	0.363233	-1.03063261	0.302713	0.553784	NOT
RP11-15I1	2.357131	0.71494423	0.693695	1.030631532	0.302714	0.553784	NOT
CMTM1	13.25729	0.321093	0.311587	1.030508121	0.302772	0.553853	NOT
NOS1	4.129645	0.58254754	0.56544	1.030254411	0.302891	0.554005	NOT
GLT8D2	77.05643	-0.3377159	0.327814	-1.03020675	0.302913	0.554005	NOT
RP11-154I	4.492304	0.38830531	0.376926	1.030188916	0.302921	0.554005	NOT
HCN3	1880.88	0.31964764	0.31029	1.030157255	0.302936	0.554005	NOT
RNF111	830.6427	-0.1694249	0.164486	-1.03002663	0.302998	0.554079	NOT
TTC16	9.651096	-0.4389682	0.426217	-1.02991686	0.303049	0.554136	NOT
MTUS1	4001.228	-0.2896421	0.281306	-1.02963506	0.303181	0.554341	NOT
MYH7	1.261529	0.7678629	0.745786	1.029601515	0.303197	NA	NA
TMEM91	222.0339	0.35420919	0.34408	1.029439887	0.303273	0.554471	NOT
EBLN3	2177.103	-0.2084868	0.202552	-1.02930004	0.303339	0.554554	NOT
LRRC19	20.96525	-0.5526584	0.537004	-1.0291505	0.303409	0.554645	NOT
RPS15P5	5.292353	0.41302708	0.401358	1.02907454	0.303445	0.554673	NOT
AC011524.	2.23127	-1.0768011	1.046484	-1.02897073	0.303493	0.554725	NOT
SEZ6L2	1858.528	0.58907783	0.572654	1.028679919	0.30363	0.554937	NOT
TBC1D7	918.7734	0.18866583	0.183422	1.02858716	0.303674	0.554979	NOT
LINC00885	32.85612	-0.7405333	0.719984	-1.02854101	0.303695	0.554982	NOT
TPP2	1891.293	0.20554308	0.199891	1.028277077	0.30382	0.555161	NOT
MRPS31P4	15.98588	-0.3548002	0.345054	-1.02824543	0.303834	0.555161	NOT
LYL1	159.7901	-0.2778308	0.270268	-1.02798074	0.303959	0.555318	NOT
RP3-508I1	29.39895	0.34876989	0.339279	1.027975062	0.303962	0.555318	NOT
ISCA2	751.7229	0.16637285	0.161864	1.027858755	0.304016	0.555381	NOT
KLHL7-AS1	22.56113	0.3976913	0.386947	1.027767225	0.304059	0.555422	NOT
PTS	858.8017	-0.1600605	0.155786	-1.02744145	0.304213	0.555629	NOT
RP11-452(	101.2186	0.29678424	0.288858	1.027439979	0.304213	0.555629	NOT
ATP6V1G1	3933.577	0.17688796	0.172175	1.027375881	0.304244	0.555646	NOT
ZNF768	2427.827	0.17158892	0.167033	1.027278291	0.304289	0.555693	NOT
RP11-352(	3.293222	0.5041281	0.490773	1.02721144	0.304321	0.555713	NOT
RP11-482M	6.8292	-0.3288204	0.320148	-1.02709008	0.304378	0.555771	NOT
TSGA10	84.82106	0.20972044	0.204195	1.027057331	0.304393	0.555771	NOT
NPAT	491.243	-0.1673396	0.162954	-1.02691486	0.304461	0.555834	NOT
OVOL1-AS1	26.26504	-0.8835285	0.860387	-1.02689626	0.304469	0.555834	NOT
RABL3	1018.307	-0.1360145	0.13246	-1.02683491	0.304498	0.55585	NOT
GPR157	652.6137	0.25593896	0.249311	1.026583385	0.304617	0.55601	NOT
RP11-265M	1.17487	0.93550889	0.911298	1.026567702	0.304624	NA	NA

GRIK2	18.97706	-0.6322565	0.615897	-1.02656147	0.304627	0.55601	NOT
MDM4	1412.49	-0.1894136	0.184591	-1.02612829	0.304831	0.556269	NOT
NOTCH2NL	11.38949	-0.3317517	0.32331	-1.0261091	0.30484	0.556269	NOT
CYB5B	2838.807	0.1974925	0.19247	1.02609704	0.304846	0.556269	NOT
AC104534.	23.52738	0.43336223	0.422357	1.026056735	0.304865	0.556269	NOT
SOD3	649.9844	0.45137454	0.439918	1.026043043	0.304871	0.556269	NOT
ADRB2	291.7151	-0.4378365	0.426784	-1.02589699	0.30494	0.556342	NOT
ABCC12	1.566556	0.86638885	0.844572	1.025832085	0.304971	0.556342	NOT
HMGN2P47	13.51018	-0.4289182	0.418136	-1.02578613	0.304992	0.556342	NOT
SYTL3	67.11746	-0.324819	0.316654	-1.0257841	0.304993	0.556342	NOT
CHL1-AS2	1.564932	-1.387343	1.353233	-1.02520621	0.305266	0.556768	NOT
SUCLA2	874.0037	-0.1810682	0.176619	-1.02519354	0.305272	0.556768	NOT
RLN3	1.829188	1.22625619	1.196231	1.025099813	0.305316	0.556768	NOT
SLC35E2B	1301.559	-0.226597	0.221066	-1.02502058	0.305353	0.556768	NOT
TNFRSF13(	29.6053	0.43564025	0.425008	1.025017317	0.305355	0.556768	NOT
SLC35A2	1722.237	0.14029114	0.136867	1.025017043	0.305355	0.556768	NOT
FAM166A	13.85103	-0.4984715	0.486321	-1.02498427	0.305371	0.556768	NOT
RP11-284F	115.0851	0.52821143	0.515419	1.024818934	0.305449	0.556873	NOT
RP11-7F18	6.773898	0.36003482	0.351356	1.024701102	0.305504	0.556937	NOT
TPH1	16.80372	0.45883338	0.447889	1.02443621	0.305629	0.557127	NOT
LL22NC03-	0.6433	1.24765634	1.217897	1.02443519	0.30563	NA	NA
CLK1	2175.454	-0.1958438	0.191205	-1.02426187	0.305712	0.557211	NOT
C5orf45	708.559	0.26968094	0.263296	1.024251564	0.305716	0.557211	NOT
MKNK2	5761.276	-0.1757746	0.17163	-1.02414694	0.305766	0.557245	NOT
PSMC2	5268.457	0.15253735	0.148944	1.024124727	0.305776	0.557245	NOT
NPR2	1218.582	0.3545692	0.346237	1.024063949	0.305805	0.55726	NOT
RP3-337H4	3.196821	-0.3832685	0.374289	-1.02399206	0.305839	0.557285	NOT
TAF7	3744.41	-0.1727376	0.168704	-1.02390721	0.305879	0.55732	NOT
RP11-21L2	29.35147	0.47236892	0.461428	1.023710302	0.305972	0.557446	NOT
IFFO2	762.9625	0.27820068	0.271769	1.023666732	0.305993	0.557446	NOT
OAS2	1366.123	0.32570144	0.318182	1.023631024	0.30601	0.557446	NOT
RGS17	23.96586	0.33740705	0.329658	1.023507171	0.306068	0.557515	NOT
ANKRD34A	35.6602	-0.2609554	0.255003	-1.02334326	0.306146	0.557543	NOT
PAPD4	1447.807	-0.1316573	0.12866	-1.02329519	0.306168	0.557543	NOT
TRIM68	498.1477	0.2190239	0.214038	1.023294979	0.306168	0.557543	NOT
FAM106A	2.248052	0.97510577	0.952937	1.023263127	0.306183	0.557543	NOT
RP11-758F	3.94546	-0.3425102	0.334726	-1.02325667	0.306187	0.557543	NOT
TELO2	1729.332	0.16707084	0.163313	1.023009354	0.306303	0.557697	NOT
SNX10	2038.75	0.30213397	0.295344	1.022991729	0.306312	0.557697	NOT
TMEM252	34.76311	-0.7934917	0.77579	-1.02281803	0.306394	0.557809	NOT
RP11-351(	7.369858	0.32818745	0.320911	1.022673903	0.306462	0.557895	NOT
UBE3B	2178.374	-0.1290092	0.126184	-1.02238745	0.306598	0.558105	NOT
RP11-666A	4.553421	0.31724167	0.310359	1.022177735	0.306697	0.558248	NOT
DGKD	1123.126	-0.2049127	0.200494	-1.02204036	0.306762	0.558329	NOT
DNAJC13	1986.569	0.1564447	0.153101	1.021838204	0.306857	0.558444	NOT
AC124789.	11.42522	-0.3115744	0.304921	-1.02181925	0.306866	0.558444	NOT
BIRC6	3049.808	-0.1792259	0.175423	-1.02167885	0.306933	0.558528	NOT
PRICKLE1	53.82866	-0.4350036	0.425853	-1.02148785	0.307023	0.558655	NOT
RP11-405M	3.151558	-0.4114394	0.402811	-1.02141917	0.307056	0.558677	NOT

RP11-239I	1.439811	-0.6305144	0.617324	-1.02136729	0.30708	0.558684	NOT
LINC01402	1.341513	-0.609653	0.596903	-1.02135986	0.307084	NA	NA
KIF14	367.8669	0.35582411	0.348418	1.021255809	0.307133	0.558736	NOT
RPL29P19	4.941293	0.45172531	0.442342	1.021211978	0.307154	0.558736	NOT
CCDC18	173.778	0.21586069	0.211384	1.021176253	0.307171	0.558736	NOT
MRPL53	171.6698	0.17750449	0.173831	1.021130673	0.307193	0.558738	NOT
RP3-414A1	66.5735	-0.3335782	0.326736	-1.02094056	0.307283	0.558836	NOT
VEZT	1802.029	0.16151888	0.158208	1.020929732	0.307288	0.558836	NOT
RASA4CP	101.6158	-0.375531	0.367877	-1.02080604	0.307346	0.558905	NOT
RP11-314A	9.583524	0.3835102	0.375749	1.020656438	0.307417	0.558993	NOT
RPL26P30	10.15506	-0.3965092	0.3885	-1.02061668	0.307436	0.558993	NOT
3-Mar	156.8642	0.33095008	0.324309	1.020477642	0.307502	0.559076	NOT
RP11-757C	4.537982	-0.3891538	0.381363	-1.02042891	0.307525	0.55908	NOT
C1QBP	3240.098	0.21586833	0.211585	1.020242674	0.307613	0.559142	NOT
RPL27A	28560.14	0.25689487	0.251808	1.020200676	0.307633	0.559142	NOT
RP11-132A	42.09787	-0.5553466	0.544373	-1.02015897	0.307653	0.559142	NOT
KDELR1	9387.477	0.15771601	0.154602	1.020140652	0.307662	0.559142	NOT
KLF6	6515.164	-0.2619859	0.256814	-1.02013945	0.307662	0.559142	NOT
SH3YL1	452.6159	0.35278911	0.345866	1.020017147	0.30772	0.55921	NOT
TTI1	1423.278	0.12674892	0.124304	1.019667318	0.307886	0.559442	NOT
PRPF4B	2318.262	0.14708266	0.144247	1.019660985	0.307889	0.559442	NOT
GTDC1	519.2359	0.1798467	0.176386	1.019618264	0.30791	0.559442	NOT
RP1-127D3	4.353368	0.90347883	0.886202	1.019494902	0.307968	0.559511	NOT
FAM129B	3385.668	0.30363411	0.29791	1.019213036	0.308102	0.559716	NOT
ZNF701	120.7315	0.35203671	0.345427	1.019134115	0.308139	0.559747	NOT
OSTF1	1265.356	-0.1386737	0.136081	-1.01905553	0.308177	0.559777	NOT
SRCIN1	683.0396	0.32669569	0.320617	1.018957758	0.308223	0.559824	NOT
MIR4292	8.242182	0.39752291	0.39015	1.018897085	0.308252	0.559839	NOT
ERMN	10.6891	0.50768583	0.498335	1.018763837	0.308315	0.559861	NOT
EIF4A1P2	6.50334	0.34111814	0.334842	1.018743658	0.308325	0.559861	NOT
MYOF	864.5062	-0.3514584	0.345002	-1.01871319	0.308339	0.559861	NOT
FAM195A	3703.875	-0.3041198	0.298538	-1.01869792	0.308346	0.559861	NOT
UQCRH	5557.525	0.23005319	0.225859	1.018568264	0.308408	0.559913	NOT
RP4-77702	1.803809	-0.4705078	0.461944	-1.01853957	0.308422	0.559913	NOT
SETMAR	466.9956	0.17130623	0.168195	1.018497567	0.308442	0.559913	NOT
RPL39P38	4.144987	0.43268709	0.424843	1.018463354	0.308458	0.559913	NOT
COA3	5095.191	0.24186629	0.237496	1.018400807	0.308488	0.55993	NOT
ETFB	16923.95	-0.3058781	0.300373	-1.01832736	0.308522	0.55993	NOT
RNF141	1332.341	-0.1481738	0.145513	-1.0182881	0.308541	0.55993	NOT
DSCAS	3.945129	0.44500361	0.437019	1.018270368	0.308549	0.55993	NOT
SBF2	1336.509	-0.2022605	0.198678	-1.01802916	0.308664	0.560081	NOT
RP4-67101	13.55579	-0.3455504	0.33945	-1.01797112	0.308692	0.560081	NOT
FAM98C	731.7874	-0.1642106	0.161313	-1.01796415	0.308695	0.560081	NOT
FAM219A	760.9562	0.13986912	0.137445	1.01763868	0.30885	0.560324	NOT
RNF166	625.754	-0.1648005	0.161952	-1.01759107	0.308872	0.560328	NOT
RHPN2	1687.862	-0.2587792	0.254361	-1.01736956	0.308978	0.560362	NOT
PRKAR2A	2874.438	0.15548576	0.152837	1.017332768	0.308995	0.560362	NOT
ZNRF2	1306.223	-0.199824	0.196425	-1.01730608	0.309008	0.560362	NOT
RP11-278C	16.72479	-0.2571773	0.252802	-1.01730579	0.309008	0.560362	NOT

CA5BP1	232.5107	0.17421289	0.171251	1.017294458	0.309013	0.560362	NOT
RP11-614F	2.824742	1.34407581	1.321231	1.017290751	0.309015	0.560362	NOT
ZMYM1	346.8563	0.20267398	0.199264	1.017113334	0.3091	0.560405	NOT
SLC16A1-A	154.1205	0.26471513	0.260263	1.017107042	0.309103	0.560405	NOT
MRPS2	2557.234	-0.1935405	0.190287	-1.01709608	0.309108	0.560405	NOT
RP11-359I	16.21847	0.46963604	0.461755	1.01706805	0.309121	0.560405	NOT
BZW1P2	14.6872	0.3342332	0.328639	1.017020845	0.309144	0.560408	NOT
NIPSNAP3E	41.78844	-0.2491908	0.245085	-1.01675442	0.30927	0.560587	NOT
RP13-638C	5.353166	0.32479624	0.319471	1.016667647	0.309312	0.560587	NOT
RP11-452I	5.645052	-0.315215	0.310048	-1.01666456	0.309313	0.560587	NOT
RP11-30K9	2.80299	0.44232259	0.435084	1.016637016	0.309326	0.560587	NOT
PRAMENP	13.51943	0.67107769	0.660122	1.016595821	0.309346	0.560587	NOT
MVK	2667.428	-0.2512455	0.24717	-1.01648986	0.309396	0.56062	NOT
CARS2	2288.964	0.23106439	0.22732	1.016470955	0.309405	0.56062	NOT
TAS2R6P	2.609014	0.51813059	0.509779	1.016383355	0.309447	0.560658	NOT
FMR1-AS1	5.188834	-0.4276879	0.420836	-1.01628128	0.309495	0.560709	NOT
RP11-384F	2.398548	-0.6691569	0.658549	-1.01610851	0.309578	0.56082	NOT
RPL7A	34518.59	0.22395751	0.220445	1.015934551	0.309661	0.560861	NOT
AC073218.	2.080389	-0.7108759	0.699734	-1.01592294	0.309666	0.560861	NOT
CTC-281F2	5.686613	-0.3041744	0.299413	-1.01590343	0.309675	0.560861	NOT
SLCO4A1	98.85021	0.50753719	0.499611	1.015864333	0.309694	0.560861	NOT
RPRD1A	1989.868	0.14856019	0.146243	1.01584346	0.309704	0.560861	NOT
RP11-115J	152.766	-0.5661345	0.557328	-1.01580047	0.309724	0.560861	NOT
LINC00899	79.67126	0.29156131	0.287116	1.015482035	0.309876	0.561098	NOT
AC006273.	9.046343	0.52507136	0.517095	1.015425129	0.309903	0.56111	NOT
GLIS1	4.046361	0.68119828	0.670886	1.015371843	0.309929	0.561119	NOT
CTA-299D3	2.0255	-1.5607565	1.537437	-1.01516785	0.310026	0.561257	NOT
RP11-488I	3.882422	0.5340535	0.526109	1.015099696	0.310058	0.561279	NOT
MZB1	118.7158	-0.5756786	0.567233	-1.01488898	0.310159	0.561353	NOT
POT1	732.8376	0.18277214	0.180108	1.014791089	0.310205	0.561353	NOT
NR5A1	4.453164	0.67820914	0.668343	1.014761894	0.310219	0.561353	NOT
RP11-236F	1.92766	0.63184933	0.622664	1.014751647	0.310224	0.561353	NOT
RP3-467K1	5.254563	0.40520032	0.399314	1.014740167	0.31023	0.561353	NOT
REEP3	1869.859	-0.1588329	0.156529	-1.01471625	0.310241	0.561353	NOT
PDZD3	14.72335	-0.6509755	0.641558	-1.01467952	0.310259	0.561353	NOT
RPAP1	1430.254	0.13533582	0.13338	1.014666302	0.310265	0.561353	NOT
AGRN	9246.7	0.23804701	0.234629	1.014568694	0.310311	0.561374	NOT
DRGX	19.39085	-0.7982283	0.786799	-1.01452633	0.310332	0.561374	NOT
MTO1	1243.774	-0.1823779	0.179769	-1.01451203	0.310338	0.561374	NOT
RP5-968D2	70.69146	0.45639731	0.449902	1.014436132	0.310375	0.561395	NOT
RP11-480I	7.459917	0.28118496	0.277201	1.014372821	0.310405	0.561395	NOT
CTD-2054N	12.7614	0.6091256	0.600518	1.014334066	0.310423	0.561395	NOT
AC090804.	2.931999	0.396427	0.390846	1.014278121	0.31045	0.561395	NOT
RP11-404F	90.00596	0.60084738	0.592429	1.014210093	0.310483	0.561395	NOT
CEPT1	954.5605	-0.1977326	0.194965	-1.01419667	0.310489	0.561395	NOT
TMEM120A	4204.286	-0.2046313	0.201769	-1.01418381	0.310495	0.561395	NOT
HAPLN2	4.323454	-0.4566975	0.450396	-1.01399162	0.310587	0.561523	NOT
FAM207BP	8.682855	0.28858758	0.284642	1.013861035	0.310649	0.56158	NOT
TPST1	1131.126	-0.2734567	0.269724	-1.01383937	0.310659	0.56158	NOT

RNU6-813F	1.88146	-1.018908	1.005067	-1.01377154	0.310692	0.561601	NOT
RMDN1	2700.555	0.18635365	0.183838	1.013684794	0.310733	0.561602	NOT
PMVK	4905.367	0.24364515	0.240365	1.013648265	0.310751	0.561602	NOT
FKBPL	385.6377	0.22097685	0.218003	1.013640269	0.310754	0.561602	NOT
HSD17B1	98.29657	0.23309613	0.230048	1.013251542	0.31094	0.5619	NOT
SNX29	677.7065	-0.2214452	0.21859	-1.01306063	0.311031	0.562027	NOT
HOXB-AS3	6.51507	-0.4603995	0.454561	-1.01284368	0.311135	0.56217	NOT
RP11-567F	4.857085	0.42922927	0.423802	1.012806288	0.311153	0.56217	NOT
RP5-884G6	32.99368	-0.2759917	0.272513	-1.01276472	0.311173	0.56217	NOT
RP11-44B1	2.1067	-0.7569656	0.747529	-1.01262386	0.31124	0.562226	NOT
MRPS5	3358.593	0.16436141	0.162314	1.012613886	0.311245	0.562226	NOT
FRY	1340.775	-0.2612296	0.258002	-1.01251126	0.311294	0.562241	NOT
KBTBD11-(	3.455414	-0.7242973	0.715349	-1.01250891	0.311295	0.562241	NOT
PF4	4.249389	0.67019455	0.661957	1.012443866	0.311326	0.56226	NOT
RHAG	0.712024	-1.0065445	0.994183	-1.01243366	0.311331	NA	NA
RP11-114F	2.732208	0.4357412	0.430423	1.012355476	0.311368	0.562283	NOT
AJ003147.	9.205532	-0.2997293	0.296088	-1.0122976	0.311396	0.562283	NOT
HLA-DQA1	2388.38	0.38772967	0.383029	1.012271908	0.311408	0.562283	NOT
CEACAM19	390.7539	-0.3016753	0.298034	-1.01221862	0.311434	0.562283	NOT
RP1-309F2	2.341954	0.72743514	0.718667	1.012200808	0.311442	0.562283	NOT
RP1-13D1(	1.746704	0.6595939	0.651772	1.012000301	0.311538	0.562418	NOT
KRT8P10	1.565215	0.51828599	0.51217	1.011942092	0.311566	0.562426	NOT
TRIM58	3.668043	-0.4850753	0.479369	-1.01190441	0.311584	0.562426	NOT
RP13-317I	8.826088	-0.2799385	0.276684	-1.01176118	0.311652	0.562513	NOT
TRIM33	1657.095	0.16118337	0.159349	1.011510303	0.311772	0.562692	NOT
DEFB1	3754.694	0.5885972	0.581934	1.011450511	0.311801	0.562706	NOT
AC007163.	1.234296	-0.7592666	0.750679	-1.01143982	0.311806	NA	NA
IGHV3-21	48.33899	-0.6847341	0.677086	-1.01129548	0.311875	0.562802	NOT
PDCL	539.318	0.15154961	0.149867	1.011227695	0.311907	0.562811	NOT
ZNF184	222.7457	0.1807491	0.178753	1.011167807	0.311936	0.562811	NOT
RP11-390F	100.0117	0.35492394	0.351008	1.011154838	0.311942	0.562811	NOT
RP11-519(	2.98973	0.49110027	0.485711	1.011095111	0.311971	0.562826	NOT
SLC38A7	1792.737	-0.1910305	0.188962	-1.01094751	0.312042	0.562916	NOT
RP11-299M	4.269661	0.49809012	0.492751	1.010835976	0.312095	0.562966	NOT
DDX24	4725.352	-0.1377359	0.13627	-1.01075909	0.312132	0.562966	NOT
CTC-398G3	2.561729	-0.3363674	0.332787	-1.01075875	0.312132	0.562966	NOT
RP11-66N2	93.04991	-0.2874726	0.284453	-1.01061501	0.312201	0.563053	NOT
FMOD	868.6976	-0.4680391	0.463216	-1.01041157	0.312298	0.563191	NOT
AP003068.	19.20076	0.29676399	0.293728	1.010337418	0.312334	0.563213	NOT
CES3	1655.642	-0.435062	0.430627	-1.01029932	0.312352	0.563213	NOT
SORCS1	6.926928	0.85132188	0.842701	1.0102299	0.312385	0.563236	NOT
MPHOSPH6	409.6061	0.15898481	0.157392	1.01011837	0.312439	0.563295	NOT
ZNF70	112.3446	0.24441007	0.241973	1.010070347	0.312462	0.563299	NOT
GPATCH2	527.8219	0.16340782	0.161812	1.009862264	0.312561	0.563441	NOT
CTD-2562(	8.580467	0.28176137	0.279036	1.009765315	0.312608	0.563476	NOT
RP11-400N	8.906539	-0.6710294	0.66456	-1.0097349	0.312622	0.563476	NOT
LURAP1	17.94215	-0.3778605	0.374255	-1.00963465	0.31267	0.563506	NOT
FAM105A	167.4894	0.34125336	0.338004	1.009614058	0.31268	0.563506	NOT
CH17-353F	3.745015	0.5720413	0.566624	1.009560698	0.312706	0.563513	NOT

RP11-395I	35.77321	0.37216718	0.368667	1.009493311	0.312738	0.563513	NOT
RP11-156(	2.43341	-0.4049255	0.401125	-1.00947597	0.312746	0.563513	NOT
ZBTB17	991.6474	0.14982328	0.148425	1.009422509	0.312772	0.563521	NOT
NCR3	22.42352	-0.340574	0.337423	-1.00933817	0.312812	0.563547	NOT
IFT81	369.7358	0.2153345	0.213349	1.009306339	0.312828	0.563547	NOT
LINC0122(	12.76874	0.49056923	0.486096	1.009202276	0.312878	0.563573	NOT
SATB2	608.266	-0.2833488	0.280769	-1.00918926	0.312884	0.563573	NOT
CH17-360I	26.84397	-0.5860153	0.580733	-1.00909556	0.312929	0.563617	NOT
FGFR1OP2	936.2563	-0.1176689	0.11665	-1.00873142	0.313103	0.563862	NOT
ITIH1	72299.1	-0.4136405	0.410063	-1.00872482	0.313107	0.563862	NOT
GIT2	778.3882	-0.1700462	0.168588	-1.00864731	0.313144	0.563892	NOT
RP11-342I	16.76014	-0.314404	0.311763	-1.00847266	0.313228	0.563985	NOT
TSPYL4	483.5987	-0.2329991	0.231046	-1.00845213	0.313237	0.563985	NOT
RP5-1042I	55.80184	-0.2215695	0.219724	-1.00839889	0.313263	0.563994	NOT
P2RY11	235.8796	-0.2531768	0.251102	-1.00826131	0.313329	0.564075	NOT
AC002456.	39.93296	0.38924008	0.386088	1.00816463	0.313375	0.564122	NOT
CTD-2283M	3.408606	-0.3316672	0.329021	-1.00804337	0.313434	0.564189	NOT
SDR42E2	3.715757	0.38205559	0.379054	1.007918184	0.313494	0.56426	NOT
RP11-855A	3.804726	-0.3323393	0.329777	-1.00776955	0.313565	0.564293	NOT
DKFZp434J	1.716513	0.75330077	0.747536	1.007711858	0.313593	0.564293	NOT
POLR3H	1664.64	-0.1646078	0.163349	-1.00770914	0.313594	0.564293	NOT
PPM1J	29.8783	0.30328603	0.300967	1.007705846	0.313596	0.564293	NOT
RP11-158H	34.42484	0.29186753	0.289664	1.007608835	0.313642	0.56434	NOT
TSPAN17	2009.751	0.18060556	0.179256	1.007528583	0.313681	0.564372	NOT
RNF144A	482.2435	0.34026385	0.33777	1.007383794	0.31375	0.564459	NOT
LCN2	7954.365	0.68316306	0.678221	1.00728712	0.313797	0.564506	NOT
ZNF784	475.5911	-0.1981092	0.196688	-1.00722512	0.313827	0.564522	NOT
SMC6	1520.1	0.18123108	0.179955	1.007090586	0.313891	0.564601	NOT
IGFL1	1.462357	1.02938628	1.022258	1.006973056	0.313948	0.564665	NOT
SLFN14	1.914697	-0.5283122	0.524681	-1.00692098	0.313973	0.564672	NOT
RP5-875H1	51.66798	-0.369733	0.367236	-1.00679995	0.314031	0.56471	NOT
RP11-758M	34.72904	1.08219857	1.074916	1.006775114	0.314043	0.56471	NOT
LINC0129(	1.62077	0.79260565	0.787329	1.006702005	0.314078	0.56471	NOT
LINC0104(	1.831865	-0.9606426	0.954277	-1.00667053	0.314093	0.56471	NOT
RP11-334J	4.530661	-0.4307239	0.427879	-1.00664806	0.314104	0.56471	NOT
SMAD9	87.83312	0.34845525	0.346164	1.006617853	0.314118	0.56471	NOT
RP11-656I	11.18604	0.24581545	0.244236	1.006465748	0.314192	0.564804	NOT
RP11-3L21	2.924124	0.88000412	0.874627	1.006147704	0.314345	0.565041	NOT
RP11-266I	7.283826	-0.3604392	0.358284	-1.00601545	0.314408	0.565118	NOT
RP11-430E	3.831379	-0.4339502	0.431425	-1.005853	0.314486	0.565206	NOT
FBP1	10714.62	-0.4208057	0.418368	-1.00582689	0.314499	0.565206	NOT
FERMT3	834.2684	0.30583738	0.304096	1.005726757	0.314547	0.565255	NOT
CTTN	7562.299	0.15858181	0.157692	1.005642553	0.314588	0.565291	NOT
1-Dec	3.285684	-0.6128485	0.609463	-1.00555462	0.31463	0.565305	NOT
DNAJC5	3502.259	0.14671231	0.145908	1.005514266	0.314649	0.565305	NOT
LINC0157(	1169.97	-0.1807653	0.179777	-1.00549493	0.314659	0.565305	NOT
RP11-390F	3.446687	0.51122072	0.508448	1.005452621	0.314679	0.565305	NOT
PRKXP1	9.116918	-0.4250263	0.422826	-1.00520279	0.314799	0.565429	NOT
ATP5L	7032.165	-0.1909091	0.189923	-1.00519254	0.314804	0.565429	NOT

RP11-214N	3.454006	-0.412366	0.410245	-1.0051708	0.314815	0.565429	NOT
RP11-109A	2.65865	-0.5695909	0.566693	-1.00511346	0.314842	0.565429	NOT
PLK4	196.4313	0.29956605	0.298048	1.005093592	0.314852	0.565429	NOT
CTD-2206N	16.1779	0.35666948	0.354935	1.004885428	0.314952	0.565561	NOT
METTL16	793.3111	-0.1767131	0.175859	-1.00485423	0.314967	0.565561	NOT
C1orf106	557.1088	0.59366107	0.590884	1.004700252	0.315041	0.56559	NOT
ZNF264	739.3045	-0.2142633	0.213262	-1.00469505	0.315044	0.56559	NOT
CNN2	2944.98	0.25953008	0.258319	1.004689856	0.315046	0.56559	NOT
RP11-535M	3.730251	-0.4985495	0.496265	-1.00460262	0.315088	0.56559	NOT
EP400	1773.441	0.14056579	0.139927	1.004561707	0.315108	0.56559	NOT
EFCAB5	6.494956	-0.3139583	0.312549	-1.00451072	0.315133	0.56559	NOT
HMGCS1	11300.91	0.33902643	0.337507	1.004500736	0.315137	0.56559	NOT
C5orf38	1.08615	-0.6033548	0.6007	-1.00441983	0.315176	NA	NA
RP11-472I	1.87568	0.50848025	0.506249	1.004407212	0.315182	0.56559	NOT
MUC13	7918.988	-0.704036	0.700951	-1.00440108	0.315185	0.56559	NOT
RASSF8	514.1711	0.31500464	0.313629	1.004387546	0.315192	0.56559	NOT
PNLIPRP3	0.810828	1.22146953	1.216263	1.004281169	0.315243	NA	NA
LINC01535	26.65586	-0.5565694	0.554308	-1.00408052	0.31534	0.565818	NOT
TCF12	1816.113	-0.1890234	0.188273	-1.00398774	0.315385	0.565857	NOT
RP11-439E	5.340882	0.58250396	0.580213	1.00394848	0.315403	0.565857	NOT
FRMPD2L2	2.520515	0.92901222	0.925465	1.003833289	0.315459	0.56592	NOT
TEDDM2P	3.256297	1.39504458	1.389882	1.003714221	0.315516	0.565927	NOT
MNDA	153.9461	0.36683844	0.365493	1.003682272	0.315532	0.565927	NOT
TXNDC12	3363.969	0.12389318	0.123442	1.003655132	0.315545	0.565927	NOT
MGAT4EP	1.658108	0.61126096	0.60906	1.00361451	0.315564	0.565927	NOT
DMBX1	9.983223	0.78023954	0.777435	1.003608061	0.315568	0.565927	NOT
AL365181.	9.332925	0.25855138	0.257654	1.003484185	0.315627	0.56597	NOT
NKIRAS2	2305.5	0.13903495	0.138554	1.003472516	0.315633	0.56597	NOT
RPL26P19	36.12161	0.38410601	0.382837	1.003315809	0.315709	0.566063	NOT
AWAT2	0.775082	0.80462569	0.801992	1.003284088	0.315724	NA	NA
CBX3P4	1.963591	0.50837455	0.506713	1.00327868	0.315726	0.566063	NOT
RNFT1P3	3.331072	0.53702934	0.535362	1.003113713	0.315806	0.566168	NOT
MAP7	1220.712	-0.2936076	0.292722	-1.00302532	0.315849	0.566207	NOT
RP11-140K	173.1058	0.25791012	0.257152	1.002946981	0.315886	0.566229	NOT
CTRC	2.804017	-0.4971129	0.495669	-1.00291319	0.315903	0.566229	NOT
SUZ12	1762.453	0.1652681	0.164797	1.002861005	0.315928	0.566237	NOT
SLFN11	418.5651	0.35189705	0.350933	1.002747608	0.315983	0.566281	NOT
LINC01278	817.2563	-0.1800022	0.179513	-1.0027228	0.315995	0.566281	NOT
MARVELD3	542.5541	0.30874687	0.307949	1.002592412	0.316058	0.566357	NOT
RP11-352D	2.527198	0.65171561	0.650364	1.002077759	0.316306	0.566735	NOT
CTD-2008F	2.952044	-0.6661522	0.664789	-1.00205097	0.316319	0.566735	NOT
RP13-1039	4.37707	0.54810951	0.547002	1.002025261	0.316331	0.566735	NOT
SMIM1	176.1132	0.43714675	0.436371	1.00177816	0.316451	0.566912	NOT
RP11-461C	3.207065	-0.5960994	0.595084	-1.0017061	0.316486	0.566936	NOT
SELT	3111.67	0.11993867	0.119765	1.001453615	0.316608	0.567118	NOT
AC003988.	0.848782	-1.2428733	1.241085	-1.00144105	0.316614	NA	NA
RP11-63A1	3.011966	-0.4414261	0.44086	-1.00128448	0.316689	0.567194	NOT
FOSL1	68.5442	-0.3777586	0.377277	-1.0012764	0.316693	0.567194	NOT
STX12	1459.762	-0.131481	0.131325	-1.00119151	0.316734	0.567194	NOT

MYH13	5.190698	0.97215713	0.971002	1.00118966	0.316735	0.567194	NOT
PARP6	756.0363	0.33502845	0.334644	1.001148854	0.316755	0.567194	NOT
CTD-2026I	2.515049	0.45261475	0.452184	1.000953021	0.31685	0.567279	NOT
SNORD104	107.6898	0.43915085	0.438743	1.000930512	0.31686	0.567279	NOT
LINC00092	14.5778	-0.468198	0.467786	-1.00088091	0.316884	0.567279	NOT
PPCS	2225.707	0.13379324	0.133676	1.00087744	0.316886	0.567279	NOT
LOXL1-AS1	38.42876	-0.505598	0.50521	-1.00076723	0.316939	0.567337	NOT
EGR4	3.883359	-0.7368423	0.736345	-1.00067534	0.316984	0.567377	NOT
FASTK	4693.127	-0.1926888	0.192567	-1.00063453	0.317004	0.567377	NOT
KDELC2	1034.352	-0.2368394	0.236714	-1.00053046	0.317054	0.56743	NOT
RP11-467I	14.42796	-0.2286976	0.228601	-1.00042129	0.317107	0.567461	NOT
RP11-278I	5.997548	-0.7240421	0.723747	-1.0004077	0.317113	0.567461	NOT
BAIAP2L1	3497.651	0.30269918	0.302624	1.000248866	0.31719	0.567561	NOT
DSG1	481.9281	-0.5988282	0.598837	-0.99998492	0.317318	0.567753	NOT
STK17A	1049.461	0.21062401	0.210646	0.999897114	0.31736	0.567791	NOT
TMPRSS11I	1.41219	0.90404447	0.904185	0.999844857	0.317386	0.567799	NOT
MEG8	2.279969	0.76216655	0.762363	0.999742277	0.317435	0.56785	NOT
VAMP3	2674.107	-0.1526335	0.152717	-0.99945383	0.317575	0.568046	NOT
AC016757.	12.19173	0.46805311	0.46832	0.999429569	0.317587	0.568046	NOT
DUX4L26	4.305671	0.62441311	0.62487	0.999268269	0.317665	0.568149	NOT
CEP350	2146.412	-0.1840531	0.184205	-0.99917311	0.317711	0.568193	NOT
EPG5	1008.699	-0.1796015	0.179762	-0.99910807	0.317742	0.568212	NOT
TAB3	2597.247	0.22219501	0.22242	0.998989624	0.3178	0.568278	NOT
RP11-562A	10.14075	-0.3116867	0.312109	-0.99864543	0.317966	0.568538	NOT
DLEU7	13.49793	0.42864911	0.429282	0.998525168	0.318025	0.568605	NOT
RP4-550H1	1.46742	0.63575335	0.636853	0.998273866	0.318147	0.568739	NOT
RP11-303C	2.492576	-0.5053346	0.506221	-0.99824821	0.318159	0.568739	NOT
MID1IP1-A	20.1865	-0.3208365	0.321402	-0.99824086	0.318163	0.568739	NOT
CTD-2651I	4.86581	-0.4509116	0.451734	-0.99817942	0.318192	0.568753	NOT
RPL29P24	2.909475	-0.3747556	0.375455	-0.99813822	0.318212	0.568753	NOT
RNF5	4329.884	-0.1970423	0.197429	-0.99803947	0.31826	0.568793	NOT
ARG1	16672.05	-0.466498	0.46743	-0.99800569	0.318277	0.568793	NOT
POP7	1609.449	0.18921755	0.189649	0.997722467	0.318414	0.569001	NOT
RNU6-611I	1.508511	-0.4828734	0.484042	-0.99758487	0.318481	0.569083	NOT
EFR3A	4438.607	0.2407389	0.24136	0.997425872	0.318558	0.569179	NOT
CAPN10-AS	68.03869	0.28768379	0.288454	0.99732901	0.318605	0.569179	NOT
ZGRF1	140.7807	-0.2652054	0.265925	-0.99729242	0.318623	0.569179	NOT
AC061961.	11.82658	-0.6686613	0.670494	-0.99726608	0.318635	0.569179	NOT
SYK	442.1396	0.34001498	0.340961	0.997224976	0.318655	0.569179	NOT
MPC2	13731.52	0.20658024	0.207157	0.997214715	0.31866	0.569179	NOT
OBSCN	584.7709	0.49381949	0.495287	0.997037063	0.318747	0.569295	NOT
IPO9	3996.103	0.1547381	0.155227	0.996852374	0.318836	0.569387	NOT
GPR153	427.4383	0.37767954	0.378875	0.996844607	0.31884	0.569387	NOT
PRTN3	3.016755	-0.6510507	0.653157	-0.99677563	0.318873	0.569409	NOT
IL1RAPL1	8.754691	0.65431151	0.656491	0.996680554	0.31892	0.569432	NOT
TNFSF13	166.1958	0.28739707	0.288363	0.99664933	0.318935	0.569432	NOT
RP11-96K1	10.34009	-0.35572	0.356927	-0.9966194	0.318949	0.569432	NOT
UGDH-AS1	94.95292	-0.2091958	0.20997	-0.99631283	0.319098	0.569623	NOT
EIF4HP1	10.5133	0.31499425	0.316178	0.996257188	0.319125	0.569623	NOT

LINC0156	144.7958	-0.2492591	0.250203	-0.99622841	0.319139	0.569623	NOT
PRPF4	1185.978	0.14253876	0.143079	0.996226233	0.31914	0.569623	NOT
CSN3	1.146157	1.33358009	1.338828	0.996079904	0.319211	NA	NA
PDE7B	165.7912	-0.3845878	0.386116	-0.99604157	0.31923	0.569743	NOT
STXBP5	481.0181	0.23008429	0.231008	0.996001536	0.319249	0.569743	NOT
RP1-283E3	41.953	-0.2024763	0.203324	-0.99583261	0.319331	0.569816	NOT
RPL3P1	2.876144	-0.4303754	0.432177	-0.9958312	0.319332	0.569816	NOT
PPFIA1	2073.349	-0.1576484	0.158317	-0.99577465	0.31936	0.569828	NOT
PDCD5	2454.962	0.17794301	0.178726	0.995621577	0.319434	0.569881	NOT
FIGN	264.6099	0.35745072	0.359027	0.995608687	0.31944	0.569881	NOT
RPS4XP14	4.142561	0.357839	0.359426	0.995583373	0.319453	0.569881	NOT
ZNF587B	428.0256	0.20762449	0.208573	0.995451717	0.319517	0.569958	NOT
CLEC18A	4.495863	0.40008845	0.401992	0.995264624	0.319608	0.570082	NOT
IHH	496.5001	0.5161102	0.5186	0.995198416	0.31964	0.570102	NOT
ZNF524	963.2068	-0.2308785	0.232055	-0.99493164	0.31977	0.570296	NOT
SIX2	43.16171	0.59789004	0.601001	0.994823224	0.319822	0.570353	NOT
ARHGDIA	9515.35	-0.1550036	0.155822	-0.99474487	0.31986	0.570383	NOT
ACTBP2	9.041871	0.34686612	0.348757	0.994577417	0.319942	0.570491	NOT
CAT	15810.26	-0.2768569	0.278386	-0.99450566	0.319977	0.570516	NOT
C17orf85	1132.907	-0.1670835	0.168029	-0.99437188	0.320042	0.570594	NOT
RP11-94A2	7.848843	0.65866788	0.662428	0.994324268	0.320065	0.570598	NOT
NWD1	7.049832	-0.6476124	0.651485	-0.99405594	0.320196	0.570757	NOT
DOCK5	1192.438	-0.2626121	0.264183	-0.99405498	0.320196	0.570757	NOT
OVAAL	1.737539	-0.8759246	0.881257	-0.99394928	0.320248	0.570811	NOT
LMO4	1586.178	0.22359465	0.224982	0.993831937	0.320305	0.570875	NOT
PLXNC1	1069.742	-0.347915	0.350123	-0.99369288	0.320372	0.570959	NOT
HAS1	15.80314	-0.6569255	0.661149	-0.99361159	0.320412	0.570992	NOT
RP11-392	17.48634	0.30660538	0.308599	0.993539844	0.320447	0.571016	NOT
RP11-626	9.081899	0.42529736	0.428114	0.993420074	0.320505	0.571066	NOT
MYO18B	247.7758	0.77073734	0.775885	0.993365188	0.320532	0.571066	NOT
RP11-85K1	17.08028	-0.2583863	0.260115	-0.9933537	0.320538	0.571066	NOT
FGF23	0.730327	-1.1961162	1.204323	-0.99318541	0.32062	NA	NA
ETNK2	8463.843	-0.4296189	0.432621	-0.99306141	0.32068	0.571269	NOT
TSGA10IP	5.784399	0.42532429	0.428309	0.993032498	0.320694	0.571269	NOT
CH17-189	110.9608	0.30705838	0.309385	0.992479275	0.320964	0.571712	NOT
GXYLT1P6	6.4787	0.85217413	0.85873	0.992366077	0.321019	0.571735	NOT
RAP1GDS1	1386.875	-0.1816543	0.183059	-0.99232503	0.321039	0.571735	NOT
RP11-372	13.21533	-0.4101575	0.41333	-0.99232355	0.32104	0.571735	NOT
CRTAC1	26.79682	-0.567526	0.571966	-0.99223732	0.321082	0.571772	NOT
RP11-478	4.088266	-0.4095439	0.412835	-0.99202798	0.321184	0.571916	NOT
RP11-107	1.102669	0.94696445	0.954629	0.991971566	0.321211	NA	NA
SPNS1	149.2525	0.19234883	0.193931	0.991843359	0.321274	0.572039	NOT
CHST10	196.4137	0.38138828	0.384555	0.991764505	0.321312	0.572064	NOT
RFX2	195.9315	0.26817582	0.270423	0.991690602	0.321348	0.572064	NOT
TSC22D1	7076.644	-0.3155583	0.318215	-0.99165147	0.321368	0.572064	NOT
ACAT2	3444.607	0.25110507	0.253229	0.991611313	0.321387	0.572064	NOT
CTB-5506.	48.72157	-0.201295	0.203	-0.99159896	0.321393	0.572064	NOT
ZNF547	74.57262	-0.2859593	0.288424	-0.99145537	0.321463	0.572104	NOT
TESK2	273.3599	-0.2390889	0.241154	-0.99143726	0.321472	0.572104	NOT

GSTA4	1478.201	-0.2887267	0.291236	-0.99138265	0.321499	0.572104	NOT
MTRNR2L4	2.456582	-0.3970937	0.400547	-0.99137938	0.3215	0.572104	NOT
MROH3P	5.656391	-0.6372525	0.642838	-0.99131193	0.321533	0.572108	NOT
NDUFA1	6191.825	-0.2266105	0.228602	-0.99128876	0.321545	0.572108	NOT
EEF1A1	121109.5	0.15941297	0.160825	0.991217333	0.321579	0.572133	NOT
LINC0152	6.04721	-0.8606661	0.868404	-0.99108917	0.321642	0.572175	NOT
YOD1	609.159	0.1909971	0.192726	0.991031218	0.32167	0.572175	NOT
CUTC	808.5878	0.17951126	0.18114	0.991005984	0.321683	0.572175	NOT
RP11-723(	0.626267	1.65655488	1.671603	0.990997817	0.321687	NA	NA
SLC25A21	13.55187	0.49615161	0.50066	0.990995743	0.321688	0.572175	NOT
RP11-84C1	6.157995	0.32474962	0.327746	0.990857328	0.321755	0.572229	NOT
PSME1	9344.595	0.16112561	0.162614	0.990847346	0.32176	0.572229	NOT
SLC48A1	1471.897	-0.1739396	0.175573	-0.99069715	0.321833	0.572322	NOT
SDHAP1	274.2705	-0.1519929	0.153458	-0.99045227	0.321953	0.572497	NOT
GAL3ST4	177.0923	0.37993671	0.383631	0.990369456	0.321994	0.572531	NOT
ATG9A	4555.295	0.13186925	0.133159	0.990314823	0.32202	0.572541	NOT
CTC-260F2	45.00474	-0.2422066	0.24462	-0.99013337	0.322109	0.572662	NOT
MPP5	1268.617	-0.1903245	0.192251	-0.98997916	0.322184	0.572758	NOT
AC114812.	5.555865	-0.6293289	0.635817	-0.98979634	0.322274	0.572879	NOT
BNC2	41.03019	-0.3235369	0.326905	-0.98969839	0.322322	0.572918	NOT
AC091654.	6.388975	0.38118829	0.385169	0.989665446	0.322338	0.572918	NOT
NSDHL	2403.75	0.21054033	0.212752	0.989602715	0.322368	0.572935	NOT
SIAH1	533.6241	-0.1280624	0.12943	-0.98943409	0.322451	0.573044	NOT
ATG13	3915.967	-0.1163877	0.117636	-0.98939056	0.322472	0.573044	NOT
LYVE1	320.184	-0.4701764	0.475278	-0.98926607	0.322533	0.573115	NOT
NCAPD3	831.9255	0.17606665	0.177994	0.989172267	0.322579	0.573159	NOT
AEBP2	767.151	0.15519995	0.15691	0.989100677	0.322614	0.57318	NOT
RP11-367J	30.36475	-0.3495244	0.35339	-0.98906174	0.322633	0.57318	NOT
UBC	24209.97	-0.1371193	0.13866	-0.98889224	0.322716	0.573274	NOT
RGS12	1857.862	0.17732297	0.179319	0.988867611	0.322728	0.573274	NOT
ACSM2B	12134.29	-0.4349383	0.439929	-0.98865621	0.322831	0.57342	NOT
TRPM6	21.86911	0.54738844	0.553751	0.988510209	0.322903	0.573509	NOT
RP5-1091N	10.90385	0.34074157	0.344733	0.988422151	0.322946	0.573524	NOT
ANKRD52	2061.371	0.17730774	0.179394	0.988370316	0.322971	0.573524	NOT
C8orf82	3121.153	0.29280367	0.296251	0.988363792	0.322975	0.573524	NOT
CCNB3	44.20246	-0.2832409	0.286591	-0.98830892	0.323001	0.573534	NOT
IDNK	657.9894	-0.2710681	0.274315	-0.98816526	0.323072	0.573621	NOT
RP11-522I	0.559943	0.92720681	0.93854	0.987925155	0.323189	NA	NA
FRG1	803.6587	-0.1523988	0.154264	-0.98791194	0.323196	0.573804	NOT
LINC0112	350.7299	0.43377591	0.439119	0.98783179	0.323235	0.573823	NOT
GSAP	982.7305	-0.2413899	0.244376	-0.98777988	0.32326	0.573823	NOT
SERTAD3	738.6266	-0.1948649	0.197279	-0.98776072	0.32327	0.573823	NOT
AANAT	5.651336	0.43828671	0.443837	0.987494227	0.3234	0.573992	NOT
C14orf10	671.0926	0.35458811	0.359084	0.987480423	0.323407	0.573992	NOT
RP11-462I	2.352805	-0.41401	0.419359	-0.98724382	0.323523	0.57416	NOT
POLL	1120.6	-0.1520774	0.154111	-0.98680237	0.32374	0.57447	NOT
DDX50P1	5.833665	-0.3057433	0.309833	-0.9868006	0.32374	0.57447	NOT
SLC36A1	561.8998	0.20936282	0.212191	0.98666938	0.323805	0.574547	NOT
LRGUK	16.63298	0.40169616	0.407156	0.986589354	0.323844	0.574579	NOT

HMGN2P28	1.915242	-0.3775378	0.38275	-0.98638292	0.323945	0.574705	NOT
CTNND2	583.3121	0.83052189	0.842008	0.986358448	0.323957	0.574705	NOT
RNASE1	3039.527	-0.3247617	0.329292	-0.98624375	0.324014	0.574736	NOT
CMIP	1790.608	-0.1470366	0.149089	-0.98623595	0.324017	0.574736	NOT
CEACAMP1(	2.657191	0.86059835	0.872695	0.986139301	0.324065	0.574783	NOT
CNTD2	226.7662	-0.5497222	0.55748	-0.98608337	0.324092	0.574794	NOT
REEP5	7265.832	-0.1749648	0.177454	-0.98597258	0.324147	0.574853	NOT
RP11-609I	6.76419	-0.3873561	0.392962	-0.98573407	0.324264	0.575023	NOT
ZNF425	82.846	-0.2508859	0.254595	-0.98543197	0.324412	0.575248	NOT
PEF1	3316.764	0.15762085	0.159981	0.985246325	0.324503	0.575353	NOT
PMP2	0.612099	1.19803635	1.216044	0.985191548	0.32453	NA	NA
MCF2L2	39.71369	0.42283052	0.429201	0.985158299	0.324546	0.575353	NOT
PPP1R14B	2925.208	0.27070325	0.274787	0.985139256	0.324556	0.575353	NOT
AP000560.	3.86298	-0.3871463	0.393011	-0.98507694	0.324586	0.575353	NOT
WNT9A	34.61324	0.52532088	0.533292	0.98505352	0.324598	0.575353	NOT
PPM1H	712.0434	0.39161997	0.397563	0.985051982	0.324599	0.575353	NOT
RP11-128A	5.718067	0.46786602	0.475004	0.984972762	0.324637	0.575385	NOT
GM2A	4044.415	0.18334969	0.186198	0.98470185	0.324771	0.575583	NOT
GPR97	120.0869	0.4471717	0.454177	0.984575252	0.324833	0.575643	NOT
TIMD4	34.07235	0.57834167	0.587437	0.984517441	0.324861	0.575643	NOT
RP11-344E	15.16912	-0.3123873	0.317304	-0.98450381	0.324868	0.575643	NOT
NCKAP5L	960.2198	0.24332303	0.247172	0.984427554	0.324905	0.575671	NOT
AGPAT6	4127.38	0.22605834	0.229647	0.98437428	0.324932	0.57568	NOT
CYB561A3	1924.884	-0.1552416	0.157713	-0.98432807	0.324954	0.575683	NOT
FAM98A	1720.396	0.1386355	0.140897	0.983947111	0.325142	0.57594	NOT
CC2D1B	1998.33	0.12820194	0.130297	0.983921955	0.325154	0.57594	NOT
C17orf105	2.103151	-0.3544323	0.360231	-0.98390385	0.325163	0.57594	NOT
LINC00853	66.16143	0.34657328	0.352264	0.983846318	0.325191	0.575952	NOT
CYP4F32P	1.648293	1.03275988	1.049982	0.983597537	0.325313	0.576101	NOT
RP11-662(	4.099875	1.08824554	1.106425	0.983569465	0.325327	0.576101	NOT
MYSM1	654.839	-0.2030383	0.206435	-0.98354608	0.325339	0.576101	NOT
APBB2	1736.931	-0.2198418	0.223591	-0.98323138	0.325494	0.576299	NOT
CTC-436P1	2.582208	-0.347967	0.353914	-0.98319508	0.325511	0.576299	NOT
RP11-48B3	154.1934	0.26142699	0.265897	0.983188613	0.325515	0.576299	NOT
IGLV3-19	125.9099	-0.6775429	0.689175	-0.98312105	0.325548	0.576321	NOT
ATG12	1791.795	-0.1247356	0.126887	-0.9830479	0.325584	0.576347	NOT
RP3-461F1	8.553938	-0.2429831	0.247185	-0.98300135	0.325607	0.57635	NOT
ARL8A	2731.66	0.17416654	0.17722	0.982768233	0.325722	0.5765	NOT
PIGG	1487.264	-0.1446136	0.147153	-0.98274193	0.325734	0.5765	NOT
VAT1L	125.4637	-0.5532887	0.56303	-0.98269923	0.325755	0.5765	NOT
SLC19A2	1485.711	-0.2138565	0.217639	-0.9826198	0.325795	0.576532	NOT
RP11-368J	18.97398	-0.3366192	0.342593	-0.98256166	0.325823	0.576545	NOT
NUDT18	255.5061	-0.2305908	0.234697	-0.98250509	0.325851	0.576556	NOT
CLASP1	1871.928	-0.1417778	0.144313	-0.98243192	0.325887	0.576583	NOT
CFAP44	238.4182	0.27563418	0.280646	0.982140501	0.326031	0.576766	NOT
MAGEF1	1251.861	0.18895676	0.192394	0.982135049	0.326033	0.576766	NOT
ZNF532	692.5108	0.26563528	0.270514	0.981965657	0.326117	0.57683	NOT
AC006116.	2.172086	-0.5351453	0.544991	-0.98193453	0.326132	0.57683	NOT
ENAM	99.36473	0.49619952	0.505329	0.981932751	0.326133	0.57683	NOT

IGHV7-81	2.600177	0.61666172	0.628106	0.981780165	0.326208	0.576873	NOT
CTC-559E9	104.2163	-0.2194227	0.223505	-0.98173467	0.326231	0.576873	NOT
LL09NC01-	6.084444	-0.3027457	0.308397	-0.98167613	0.326259	0.576873	NOT
MRPL38	404.6418	-0.1590525	0.162022	-0.98167044	0.326262	0.576873	NOT
GBA	4615.142	0.20216922	0.205945	0.981667404	0.326264	0.576873	NOT
GPR19	39.58101	-0.3912449	0.398661	-0.98139671	0.326397	0.577071	NOT
MRPL49	2946.865	0.13659106	0.139207	0.981209013	0.32649	0.577197	NOT
RP11-369K	4.201564	-0.6214854	0.633431	-0.98114209	0.326523	0.577218	NOT
TEN1	8.246893	0.25298646	0.257883	0.981012256	0.326587	0.577262	NOT
RP3-467N1	74.69922	0.27065262	0.275893	0.981004798	0.32659	0.577262	NOT
RP11-8P13	4.470672	0.45915676	0.468108	0.980877565	0.326653	0.577316	NOT
ALOX5	380.4606	0.39919578	0.406987	0.980857274	0.326663	0.577316	NOT
RNF216P1	762.9765	0.18601619	0.189669	0.980739644	0.326721	0.57738	NOT
MST1	10902.45	-0.3442949	0.35108	-0.98067257	0.326754	0.577392	NOT
POU4F1	2.844088	0.91413172	0.93221	0.980606685	0.326787	0.577392	NOT
ASTL	1.474538	0.70601564	0.720052	0.980507079	0.326836	0.577392	NOT
BACH1-IT2	6.844122	0.37954062	0.387091	0.980494655	0.326842	0.577392	NOT
SLC26A5	6.089573	-0.3510752	0.358071	-0.98046264	0.326858	0.577392	NOT
ZNF621	702.296	-0.1584709	0.16163	-0.98045735	0.32686	0.577392	NOT
RP11-297I	21.5483	0.2128342	0.217101	0.980346445	0.326915	0.577392	NOT
RP11-407N	33.14122	0.18100743	0.184637	0.980340519	0.326918	0.577392	NOT
AC073043.	3.182917	-0.4536966	0.462807	-0.98031509	0.326931	0.577392	NOT
RP11-498F	28.58116	0.51597736	0.526349	0.980294751	0.326941	0.577392	NOT
LINC00158	1.760754	0.67868532	0.692478	0.98008178	0.327046	0.57754	NOT
ZNF19	98.35788	-0.1428858	0.14581	-0.97994826	0.327112	0.577583	NOT
RPL14P3	5.548884	0.33645303	0.343345	0.979927777	0.327122	0.577583	NOT
RP11-378J	51.89797	0.21157941	0.215924	0.979878561	0.327146	0.577583	NOT
CTD-2325F	25.13848	0.5658865	0.577517	0.979860632	0.327155	0.577583	NOT
HRH3	1.496174	0.73359444	0.748858	0.979617673	0.327275	0.577757	NOT
RP11-82L1	26.35999	-0.4611579	0.470845	-0.97942688	0.327369	0.577886	NOT
NIPAL1	398.817	-0.3773884	0.385337	-0.97937289	0.327396	0.577895	NOT
RNU6-529F	8.548438	-0.4028987	0.411424	-0.97927806	0.327443	0.57794	NOT
CDK10	2701.741	-0.1739437	0.177647	-0.97915402	0.327504	0.577953	NOT
HYLS1	248.8347	0.19389307	0.198024	0.979138885	0.327511	0.577953	NOT
ANKRD18EF	129.4334	0.38219647	0.390341	0.979134092	0.327514	0.577953	NOT
DCP2	1238.917	0.13739317	0.140342	0.978985287	0.327587	0.578045	NOT
USP32	1377.9	-0.1676789	0.171311	-0.97879691	0.32768	0.578172	NOT
NUP133	1833.049	0.13290393	0.135797	0.978697699	0.327729	0.578221	NOT
PDE1B	114.0703	-0.1961999	0.2005	-0.97855442	0.3278	0.578308	NOT
DUX4L27	6.635749	-0.3791825	0.387589	-0.97831034	0.327921	0.578483	NOT
PCDHGA4	69.84155	-0.4919821	0.502914	-0.97826201	0.327945	0.578488	NOT
IFI44	742.3631	0.347105	0.354872	0.978113208	0.328018	0.578571	NOT
SOGA1	1267.897	0.23431814	0.239576	0.978052058	0.328049	0.578571	NOT
RP11-168K	13.62936	-0.4530927	0.463267	-0.97803735	0.328056	0.578571	NOT
SHROOM2P1	1.690923	-1.7799175	1.820656	-0.97762442	0.32826	0.57886	NOT
C15orf59	68.89437	-0.5470016	0.559524	-0.97762008	0.328262	0.57886	NOT
ZNF852	69.16763	-0.1538217	0.157353	-0.97756032	0.328292	0.578874	NOT
CD276	3417.13	-0.1887067	0.193062	-0.97744198	0.32835	0.57894	NOT
USP31	812.4037	-0.1931174	0.197613	-0.97724973	0.328446	0.57907	NOT

GPM6B	33.54981	-0.3158554	0.323229	-0.97718709	0.328477	0.579087	NOT
LINC0063ε	60.43964	0.29187981	0.298757	0.97697953	0.328579	0.579175	NOT
AP000487.	6.102296	-0.316759	0.324225	-0.97697372	0.328582	0.579175	NOT
SPTLC3	1252.817	0.32179268	0.329383	0.976957211	0.32859	0.579175	NOT
ACOT6	19.69619	-0.5385286	0.551273	-0.97688145	0.328628	0.579203	NOT
CDKN2A-AS	3.517087	0.55321615	0.566356	0.976799508	0.328668	0.579237	NOT
NQO2	4653.908	0.2696494	0.276074	0.976730073	0.328703	0.57926	NOT
RP4-734P1	2.191645	0.38442859	0.393664	0.97653935	0.328797	0.579389	NOT
SLC47A2	32.6119	0.36684316	0.375701	0.976421987	0.328855	0.579395	NOT
AAR2	2163.402	0.09457443	0.096859	0.976415116	0.328859	0.579395	NOT
SRRM2-AS1	70.98773	-0.3046366	0.311999	-0.97640292	0.328865	0.579395	NOT
RP11-410I	43.99604	0.28800379	0.295008	0.976256638	0.328937	0.579485	NOT
RP11-88H1	3.775825	-0.3295133	0.337607	-0.97602574	0.329052	0.579649	NOT
CISD3	4865.386	0.22493409	0.230497	0.975866548	0.329131	0.579699	NOT
AC016292.	40.55257	0.2534307	0.259704	0.975845479	0.329141	0.579699	NOT
DEPTOR	1373.908	0.23285821	0.238624	0.975838498	0.329145	0.579699	NOT
HSPB6	569.7666	-0.4154677	0.425907	-0.97548916	0.329318	0.579967	NOT
USP7	4282.557	-0.1264259	0.129633	-0.97526083	0.329431	0.580129	NOT
TRBV2	3.724201	-0.4625751	0.474377	-0.97512146	0.3295	0.580175	NOT
FUT10	139.3227	0.20240896	0.207573	0.975120861	0.3295	0.580175	NOT
RP11-181K	4.744177	0.46775382	0.479787	0.974919127	0.3296	0.580314	NOT
RP11-1149	29.5293	0.26191509	0.268715	0.97469429	0.329712	0.580443	NOT
JPH4	28.74475	0.4608178	0.472795	0.974668138	0.329725	0.580443	NOT
C2orf47	1297.188	0.15011369	0.154019	0.974642183	0.329738	0.580443	NOT
RP4-647C1	13.84003	0.36596676	0.375507	0.97459305	0.329762	0.580448	NOT
CABP7	18.14881	0.27126989	0.27837	0.974492887	0.329812	0.580473	NOT
PLK2	1665.003	-0.2422945	0.24864	-0.97447804	0.329819	0.580473	NOT
AEBP1	3388.079	-0.4340508	0.445545	-0.97420115	0.329957	0.580678	NOT
SUN1	3929.727	-0.1753032	0.179959	-0.97412943	0.329992	0.580703	NOT
RP3-425P1	2.026147	-0.4059482	0.416752	-0.97407684	0.330018	0.580711	NOT
AC006486.	14.54016	-0.2417529	0.24821	-0.97398647	0.330063	0.580752	NOT
IRF6	4907.971	-0.338776	0.347881	-0.97382855	0.330142	0.580852	NOT
PPP1R15B	4408.677	-0.1572921	0.161528	-0.97377798	0.330167	0.580859	NOT
TSSC1-IT1	7.38144	0.31066209	0.31906	0.973680368	0.330215	0.580907	NOT
CTA-351J1	6.626326	0.37560588	0.385812	0.973546533	0.330282	0.580931	NOT
AC007038.	16.98539	0.30328742	0.311537	0.973520473	0.330295	0.580931	NOT
RP11-497(	3.601743	-0.7963907	0.818086	-0.9734806	0.330314	0.580931	NOT
PRPS1P2	18.71116	-0.2860174	0.293809	-0.97348012	0.330315	0.580931	NOT
TMEM63A	3527.307	0.233517	0.239912	0.973345303	0.330382	0.581011	NOT
RP11-46J2	8.605538	-0.3355201	0.344781	-0.97314087	0.330483	0.581152	NOT
ANKRD61	11.98148	-0.2351498	0.241682	-0.9729738	0.330566	0.581218	NOT
STX16	2320.492	0.14636321	0.150431	0.972957921	0.330574	0.581218	NOT
RP11-268(	1.837887	0.86804727	0.892193	0.972936359	0.330585	0.581218	NOT
KDM3B	2549.34	0.1452669	0.149334	0.972762187	0.330672	0.581302	NOT
BBS9	310.1667	-0.1803573	0.185409	-0.97275402	0.330676	0.581302	NOT
ATP5C1	11665.29	0.17706403	0.182044	0.972646345	0.330729	0.581358	NOT
NKX3-1	117.513	-0.3800259	0.390821	-0.97237856	0.330862	0.581509	NOT
AC068039.	4.593127	0.50718938	0.521618	0.972339478	0.330882	0.581509	NOT
RP4-585I1	2.340961	0.37052309	0.381077	0.972304323	0.330899	0.581509	NOT

IGHV3-47	2.398568	0.77425945	0.796317	0.972300988	0.330901	0.581509	NOT
RP11-74D7	5.99597	-0.3505126	0.36053	-0.97221508	0.330944	0.581523	NOT
CTB-178M2	5.560681	0.48925969	0.503262	0.972176893	0.330963	0.581523	NOT
ATG4B	2266.403	0.16372709	0.168418	0.972144908	0.330978	0.581523	NOT
PCDHGA8	15.86473	0.48328847	0.497171	0.972076386	0.331013	0.581523	NOT
EMP2	4441.834	0.2271618	0.233695	0.972042243	0.33103	0.581523	NOT
GNG10	269.2679	-0.1858977	0.191248	-0.97202623	0.331037	0.581523	NOT
DGCR11	78.62279	0.22030254	0.226698	0.97178993	0.331155	0.581654	NOT
CACNB2	127.396	-0.3099919	0.319003	-0.97175207	0.331174	0.581654	NOT
RP11-235I	1.540774	-0.4681658	0.481777	-0.97174802	0.331176	0.581654	NOT
CTD-3099C	19.59192	0.37264398	0.383532	0.971612234	0.331243	0.581721	NOT
RP11-309I	3.525676	0.35481079	0.365197	0.971560639	0.331269	0.581721	NOT
RPS25	15554.71	0.16983703	0.174812	0.971541203	0.331279	0.581721	NOT
HEG1	1827.941	-0.2541082	0.261572	-0.97146599	0.331316	0.581749	NOT
EGFEM1P	12.92231	0.74680038	0.768886	0.971275577	0.331411	0.581849	NOT
RP11-196I	2.78075	-0.806568	0.83043	-0.97126582	0.331416	0.581849	NOT
MMS19	1921.846	0.10381878	0.106905	0.971128458	0.331484	0.581932	NOT
ANO5	297.3097	0.40392818	0.416018	0.970939048	0.331579	0.581988	NOT
HSPA8P9	3.569364	0.40034001	0.412345	0.970885699	0.331605	0.581988	NOT
S100B	40.93311	0.43732619	0.450449	0.970867572	0.331614	0.581988	NOT
LINC0011	4.41882	-0.5500599	0.566573	-0.97085371	0.331621	0.581988	NOT
CTC-498J1	6.49196	-0.2744118	0.282652	-0.97084826	0.331624	0.581988	NOT
MRPL21	2176.262	0.24967223	0.25723	0.97061687	0.331739	0.582151	NOT
MLLT4-AS1	90.53867	0.2915247	0.300363	0.970575814	0.33176	0.582151	NOT
RP5-1007I	2.650778	0.62111501	0.639998	0.970495821	0.331799	0.582183	NOT
TRBV5-4	3.008434	0.51073179	0.526343	0.970340781	0.331877	0.582281	NOT
RP11-395C	26.74432	0.46451517	0.478754	0.970258361	0.331918	0.582316	NOT
BUD13	697.0999	0.09570525	0.098651	0.970139182	0.331977	0.582382	NOT
ZCCHC10	551.4156	0.15018121	0.154829	0.96998241	0.332055	0.582482	NOT
CTGLF8P	5.645429	-0.4252391	0.43842	-0.96993618	0.332078	0.582484	NOT
NUBPL	600.9306	-0.2755189	0.284126	-0.96970765	0.332192	0.582646	NOT
HTT	4088.697	-0.1414283	0.145857	-0.96963596	0.332228	0.582649	NOT
ESPNL	121.9746	-0.4491885	0.463271	-0.96960187	0.332245	0.582649	NOT
CDKL1	45.47097	0.3899573	0.402195	0.969571784	0.33226	0.582649	NOT
RRP7A	2476.91	0.22599765	0.2331	0.969532693	0.332279	0.582649	NOT
HTR7	13.65433	-0.4258305	0.439298	-0.96934208	0.332375	0.582778	NOT
LANCL1-AS	6.097656	-0.3936126	0.406098	-0.9692561	0.332417	0.582804	NOT
CTD-2537I	9.163871	-0.298875	0.308365	-0.96922561	0.332433	0.582804	NOT
FADS2	12928.04	0.40999052	0.423067	0.969090556	0.3325	0.582864	NOT
POMT1	1626.209	-0.1492954	0.154063	-0.96905287	0.332519	0.582864	NOT
TCEAL8	1515.025	0.31226323	0.322247	0.969019462	0.332535	0.582864	NOT
RP11-571I	44.67316	-0.2576742	0.265922	-0.96898518	0.332553	0.582864	NOT
LRRIQ1	5.581895	0.94117	0.971345	0.968934975	0.332578	0.58287	NOT
ULK4	401.1172	-0.380432	0.392663	-0.96885093	0.33262	0.582906	NOT
LINC00987	165.2699	-0.4386728	0.452839	-0.96871635	0.332687	0.582958	NOT
RP11-59N2	3.366493	-0.5203568	0.537167	-0.96870581	0.332692	0.582958	NOT
MFAP5	23.10652	-0.5461954	0.56399	-0.96844903	0.33282	0.583107	NOT
MDP1	44.68374	-0.2230392	0.230306	-0.96844847	0.33282	0.583107	NOT
RP6-201G1	5.029057	-0.4203096	0.434046	-0.96835288	0.332868	0.583153	NOT

FASTKD5	912.1436	0.14900967	0.153901	0.968217768	0.332936	0.583234	NOT
DHX40P1	1.179738	0.82062262	0.847605	0.968166733	0.332961	NA	NA
SLC44A1	4036.262	0.16369031	0.169151	0.967718232	0.333185	0.583633	NOT
RALGAPA1I	38.12074	-0.2798652	0.28923	-0.96762186	0.333233	0.583667	NOT
RP11-179A	3.000798	0.40821176	0.42189	0.967579074	0.333255	0.583667	NOT
RP11-80H5	2.870703	0.4695379	0.485285	0.967550233	0.333269	0.583667	NOT
HFM1	8.193378	0.51197924	0.529233	0.967397736	0.333345	0.583744	NOT
HS6ST1	3110.166	-0.2402042	0.248305	-0.96737645	0.333356	0.583744	NOT
CRTC2	2758.76	0.13193827	0.136431	0.967068118	0.33351	0.583976	NOT
AC007308.	4.291807	-0.3751616	0.38796	-0.96701172	0.333538	0.583988	NOT
ARMC9	198.6544	0.32379913	0.334867	0.9669471	0.33357	0.584007	NOT
RP11-179A	2.086387	1.18246506	1.222996	0.966859307	0.333614	0.584046	NOT
LYPD3	72.32447	-0.3310673	0.342462	-0.96672831	0.33368	0.584123	NOT
KBTBD8	59.00891	0.30981752	0.320509	0.966643703	0.333722	0.58414	NOT
MEOX2-AS1	3.629959	-0.6813614	0.704904	-0.96660136	0.333743	0.58414	NOT
CCDC78	54.89632	0.45154589	0.467159	0.966579513	0.333754	0.58414	NOT
RP13-582C	37.3086	0.37124725	0.384145	0.966424538	0.333832	0.584238	NOT
TLX1	157.6811	0.47458227	0.491104	0.966357412	0.333865	0.584259	NOT
METTL13	1970.588	0.13223688	0.13686	0.966216636	0.333936	0.584316	NOT
CACUL1	2126.446	0.12131448	0.125565	0.966144995	0.333972	0.584316	NOT
GPR180	639.6923	0.28268698	0.292598	0.966126563	0.333981	0.584316	NOT
HNRNPH1	8154.178	0.14443601	0.149501	0.966119876	0.333984	0.584316	NOT
LINGO3	3.969495	-0.3841383	0.397633	-0.96606259	0.334013	0.584329	NOT
LOH12CR2	41.99073	-0.2231435	0.231095	-0.96559106	0.334249	0.584704	NOT
SNORA12	1.995812	-0.3806343	0.394224	-0.96552791	0.33428	0.584721	NOT
CD300A	277.1527	0.28935898	0.299714	0.965451499	0.334319	0.584725	NOT
TOM1L1	2373.252	-0.2393382	0.247906	-0.96543793	0.334326	0.584725	NOT
RP11-1336	1.108862	0.97327122	1.00827	0.965288295	0.3344	NA	NA
CCL5	1169.576	-0.4225402	0.437741	-0.96527452	0.334407	0.58483	NOT
RP11-333I	4.305122	-0.3558218	0.36866	-0.9651748	0.334457	0.584867	NOT
RP1-1700I	15.34775	-0.6540141	0.677632	-0.96514587	0.334472	0.584867	NOT
PSMC1P9	2.428329	-0.3455218	0.358026	-0.96507571	0.334507	0.584891	NOT
GALP	13.22879	0.64982945	0.673776	0.964458532	0.334816	0.585384	NOT
PSMD2	8802.231	0.1331834	0.138096	0.96442713	0.334832	0.585384	NOT
RP11-114H	1.556656	-0.7246244	0.75141	-0.96435274	0.334869	0.585411	NOT
TLR7	69.42194	0.36820858	0.381918	0.964103291	0.334994	0.585592	NOT
ADCK2	1608.277	0.18145715	0.188229	0.964022692	0.335035	0.585625	NOT
CEP95	927.8884	0.16187343	0.167933	0.963915579	0.335088	0.585681	NOT
HIVEP1	950.2048	-0.2178423	0.226033	-0.96376199	0.335165	0.585769	NOT
METTL2B	1045.468	0.14001617	0.145286	0.96372895	0.335182	0.585769	NOT
HSPA8P4	4.046961	-0.418861	0.434718	-0.96352385	0.335285	0.585911	NOT
CTD-2547I	11.52368	-0.3686188	0.382643	-0.96334996	0.335372	0.585997	NOT
MTG2	2196.275	0.14719643	0.152798	0.963339833	0.335377	0.585997	NOT
AC004754.	9.567791	-0.4169431	0.432853	-0.96324416	0.335425	0.586035	NOT
AC104076.	1.983318	0.51764782	0.537419	0.963211149	0.335442	0.586035	NOT
TPI1P2	20.96844	-0.28817	0.299235	-0.96302238	0.335536	0.586162	NOT
RP11-355N	3.530268	-0.4493407	0.466636	-0.96293556	0.33558	0.586185	NOT
OSMR	1681.632	0.36339566	0.377393	0.962910496	0.335592	0.586185	NOT
USP34	1464.502	-0.2548534	0.264695	-0.96281863	0.335639	0.586222	NOT

RANP4	4.746685	-0.3418741	0.35509	-0.96278221	0.335657	0.586222	NOT
FXYD7	6.502036	-0.4316012	0.448307	-0.96273508	0.33568	0.586225	NOT
KAT6A	1220.981	-0.2159296	0.224317	-0.96260784	0.335744	0.586299	NOT
IQCE	866.9622	0.19330808	0.200832	0.96253618	0.33578	0.586305	NOT
TMEM33	4226.844	-0.1573749	0.163504	-0.96251541	0.335791	0.586305	NOT
MECOM	426.898	0.32654086	0.339307	0.962375212	0.335861	0.58639	NOT
LIPC	5357.906	-0.3961574	0.411677	-0.96230082	0.335899	0.586396	NOT
TRBV4-1	3.416075	0.55577728	0.577562	0.962282211	0.335908	0.586396	NOT
AGMAT	3416.583	-0.2883402	0.299688	-0.96213385	0.335982	0.586488	NOT
NOL12	217.7659	0.2111087	0.219461	0.961941326	0.336079	0.586619	NOT
RP11-464I	21.42721	0.35222769	0.366239	0.961743759	0.336178	0.586755	NOT
CTC-471F3	36.83957	-0.3099939	0.322371	-0.96160629	0.336247	0.586789	NOT
GTF2I	1354.327	-0.2798354	0.29102	-0.96156826	0.336267	0.586789	NOT
NTMT1	950.2011	0.19268594	0.200387	0.961567858	0.336267	0.586789	NOT
RP1-47M23	12.07204	-0.4209513	0.437812	-0.96148861	0.336307	0.586789	NOT
FBXO48	194.9531	-0.1666993	0.173383	-0.96145318	0.336324	0.586789	NOT
CTC-2480I	3.127935	-0.5924625	0.616221	-0.96144495	0.336328	0.586789	NOT
PSMB2	7113.221	0.15312082	0.159268	0.961403746	0.336349	0.586789	NOT
PAIP1	2134.598	0.12567224	0.130725	0.961345798	0.336378	0.586796	NOT
ZNF358	2662.616	-0.2206273	0.229507	-0.9613092	0.336397	0.586796	NOT
MESDC1	809.7819	0.19207694	0.199835	0.961178012	0.336463	0.586874	NOT
ACRC	39.46098	-0.2223332	0.231395	-0.96083812	0.336634	0.587134	NOT
RP11-247A	2.022208	-0.5244586	0.545873	-0.96076965	0.336668	0.587156	NOT
SLC2A3	751.0361	-0.3385735	0.352444	-0.96064611	0.33673	0.587182	NOT
LAMB1	5616.7	0.3018239	0.314213	0.960570336	0.336768	0.587182	NOT
HCG4	25.00976	-0.4924963	0.512743	-0.9605131	0.336797	0.587182	NOT
RP11-254I	7.433136	0.3883206	0.404295	0.960489109	0.336809	0.587182	NOT
RP11-446H	2.116388	-0.5107164	0.531727	-0.96048552	0.336811	0.587182	NOT
ZC4H2	388.1274	0.24773872	0.257932	0.960482126	0.336813	0.587182	NOT
VN1R1	40.6717	-0.385767	0.401682	-0.96038021	0.336864	0.587234	NOT
ZNF517	935.6865	0.24399435	0.254084	0.960289289	0.33691	0.587249	NOT
GLIS3	584.1549	0.38123927	0.39701	0.960276769	0.336916	0.587249	NOT
CNTRL	508.684	0.21606978	0.225064	0.960036557	0.337037	0.587422	NOT
LINC-PINT	192.966	-0.2452788	0.255542	-0.9598376	0.337137	0.587559	NOT
RBFOX3	2.703429	-0.3474686	0.362038	-0.9597573	0.337177	0.587591	NOT
RP11-266A	2.117209	-0.4772365	0.497317	-0.95962318	0.337245	0.587671	NOT
CEP170	688.6024	0.20145743	0.209957	0.959517375	0.337298	0.587697	NOT
HOMER3	793.4422	0.33660898	0.350814	0.959507769	0.337303	0.587697	NOT
PRKAR2B	72.05866	0.39556483	0.412292	0.959428994	0.337343	0.587728	NOT
CUL7	2181.048	0.17440465	0.181842	0.959099278	0.337509	0.587953	NOT
BANK1	35.59555	0.54153033	0.564648	0.959057569	0.33753	0.587953	NOT
VN1R48P	5.302486	0.55011908	0.573612	0.959044185	0.337536	0.587953	NOT
C9orf92	2.022505	-0.7851166	0.818698	-0.95898191	0.337568	0.58797	NOT
ALOX15B	38.13821	0.47976564	0.500323	0.95891152	0.337603	0.587978	NOT
ASIC3	84.77203	-0.3495886	0.364594	-0.95884367	0.337638	0.587978	NOT
PARD3B	749.1286	0.29497035	0.307645	0.958801841	0.337659	0.587978	NOT
DMAP1	1775.797	0.16616683	0.173307	0.958800688	0.337659	0.587978	NOT
SLC22A16	3.401141	-0.5175786	0.539856	-0.95873541	0.337692	0.587997	NOT
RP11-161H	2.603846	-0.6883793	0.718044	-0.95868654	0.337717	0.588002	NOT

TMEM160	330.6281	-0.2617497	0.273045	-0.95863129	0.337745	0.588013	NOT
VIP	11.79389	-0.337466	0.35205	-0.95857491	0.337773	0.588025	NOT
HIAT1	1518.118	0.14921043	0.155671	0.958497745	0.337812	0.588055	NOT
DTX2P1	40.72801	0.22399943	0.233743	0.958313029	0.337905	0.588179	NOT
F11R	8610.347	-0.2570268	0.268261	-0.95812154	0.338001	0.588297	NOT
DCAF8	4616.466	-0.1530834	0.159779	-0.95809239	0.338016	0.588297	NOT
SUSD6	1496.545	-0.2035897	0.212519	-0.95798458	0.338071	0.588323	NOT
SGPL1	3656.38	-0.1725836	0.180154	-0.95797745	0.338074	0.588323	NOT
GALE	3717.836	0.23040097	0.240627	0.957502785	0.338314	0.588702	NOT
SUSD3	720.5253	0.3774148	0.394244	0.957312462	0.33841	0.588803	NOT
HEY1	316.7187	0.21125295	0.220675	0.957301657	0.338415	0.588803	NOT
KIRREL3	11.32607	0.38944416	0.406911	0.957074192	0.33853	0.588927	NOT
CSNK1A1P1	6.993217	-0.4343968	0.45388	-0.95707416	0.33853	0.588927	NOT
IGHV3-49	28.8543	-0.6711331	0.701309	-0.9569716	0.338582	0.58896	NOT
TMEM52B	10.10162	0.54762442	0.57226	0.956950032	0.338592	0.58896	NOT
ANGEL1	1131.785	0.19851061	0.20745	0.956907737	0.338614	0.58896	NOT
ACSM5	4483.795	-0.4912052	0.51347	-0.95663928	0.338749	0.589157	NOT
CORO2B	112.9022	0.43761259	0.457474	0.95658557	0.338776	0.589157	NOT
MED21	638.2368	0.15244553	0.159377	0.95650727	0.338816	0.589157	NOT
TRAC	583.8385	0.39008846	0.407841	0.956471104	0.338834	0.589157	NOT
RP11-10J2	2.150337	0.64016411	0.669326	0.956430374	0.338855	0.589157	NOT
TRIM27	3949.884	0.13523252	0.141394	0.956425068	0.338858	0.589157	NOT
ZNF491	53.65595	-0.2597197	0.271601	-0.95625377	0.338944	0.589208	NOT
RP5-1115A	7.634375	-0.3211987	0.335895	-0.95624754	0.338947	0.589208	NOT
THUMPD1	1413.442	-0.1395209	0.145909	-0.9562208	0.338961	0.589208	NOT
GLI3	72.98243	-0.3775187	0.394828	-0.95615874	0.338992	0.589208	NOT
GBP6	5.676116	-0.5597191	0.585429	-0.95608293	0.33903	0.589208	NOT
RP11-499C	30.45721	-0.3590245	0.375548	-0.95600089	0.339072	0.589208	NOT
C14orf28	204.3406	-0.2016866	0.210971	-0.95599006	0.339077	0.589208	NOT
IL4R	3233.462	-0.2025442	0.211869	-0.95598632	0.339079	0.589208	NOT
RP1-101A2	33.60544	-0.3023538	0.31629	-0.95593884	0.339103	0.589208	NOT
MUC5AC	8.850804	1.04079716	1.088771	0.955937433	0.339104	0.589208	NOT
RING1	2693.881	0.14712342	0.153931	0.955777507	0.339185	0.58928	NOT
RP11-392F	5.515536	0.35369053	0.370059	0.955768475	0.339189	0.58928	NOT
OPCML	6.599267	-0.4612842	0.482701	-0.95563199	0.339258	0.589282	NOT
AC105760.	18.21248	0.24538924	0.256783	0.955627189	0.339261	0.589282	NOT
RP11-439K	1.427614	0.56911154	0.595565	0.955581945	0.339283	0.589282	NOT
RP3-417L2	5.054226	1.03093256	1.078916	0.955526646	0.339311	0.589282	NOT
CTD-2616J	7.199004	-0.3495319	0.365806	-0.95551226	0.339319	0.589282	NOT
CYP3A7	7583.862	-0.5967463	0.624538	-0.95550071	0.339325	0.589282	NOT
AL035610.	10.79989	1.01290075	1.060111	0.955466433	0.339342	0.589282	NOT
PPP1R26P1	2.298036	-0.5072195	0.531129	-0.95498456	0.339586	0.589655	NOT
UGP2	13387.6	-0.2725228	0.285377	-0.95495561	0.3396	0.589655	NOT
RP11-73M1	237.2097	0.22369441	0.234262	0.954888186	0.339634	0.589659	NOT
RP11-217B	7.930312	-0.3607421	0.377808	-0.95482866	0.339664	0.589659	NOT
CTB-3204.	4.883308	-0.3153062	0.330225	-0.95482121	0.339668	0.589659	NOT
MYO15A	39.90407	-0.3495549	0.366141	-0.95470143	0.339729	0.589693	NOT
RP11-81A1	3.461235	-0.735815	0.77077	-0.95464879	0.339755	0.589693	NOT
AP2B1	5789.353	0.15282664	0.160098	0.954581921	0.339789	0.589693	NOT

RN7SL8P	2.976322	0.89701038	0.939709	0.954561428	0.3398	0.589693	NOT
SZT2-AS1	2.13515	0.43500808	0.455731	0.954527654	0.339817	0.589693	NOT
BAIAP2-AS	1217.83	-0.2457932	0.257507	-0.9545112	0.339825	0.589693	NOT
PRSS35	36.68983	0.38208757	0.400309	0.954482563	0.339839	0.589693	NOT
PARP4P1	4.8983	0.65378172	0.685152	0.954214209	0.339975	0.589827	NOT
TMEM191C	14.82718	0.34754936	0.364226	0.954213998	0.339975	0.589827	NOT
PLEKHM3	108.8869	-0.2512007	0.263258	-0.95420066	0.339982	0.589827	NOT
ERVK3-1	509.498	0.14118341	0.147969	0.954139337	0.340013	0.589843	NOT
KIR3DX1	3.841905	-0.3934103	0.412454	-0.95382901	0.34017	0.590078	NOT
GPA33	2.962116	-0.4912811	0.51513	-0.95370397	0.340234	0.59015	NOT
FUBP3	2504.837	-0.1506091	0.157945	-0.95355267	0.34031	0.590214	NOT
TSHZ2	1084.111	-0.3263525	0.342252	-0.95354522	0.340314	0.590214	NOT
AC097662.	17.92677	0.23430888	0.24575	0.953444349	0.340365	0.590265	NOT
HSPE1-MOE	3.287595	0.35423853	0.371572	0.953351195	0.340412	0.590272	NOT
OCEL1	2021.687	-0.2530111	0.2654	-0.95331999	0.340428	0.590272	NOT
NCR1	6.643815	-0.4070876	0.427027	-0.95330676	0.340435	0.590272	NOT
TWSG1	608.0344	-0.263122	0.276024	-0.95325614	0.34046	0.590279	NOT
LINC00298	3.756543	-0.5440318	0.570735	-0.95321288	0.340482	0.590279	NOT
RP11-394(	97.24898	-0.317552	0.333176	-0.95310524	0.340537	0.590336	NOT
RP11-693N	106.636	-0.2691355	0.282408	-0.95300332	0.340588	0.590366	NOT
PATL2	63.02754	-0.3418732	0.358745	-0.95296956	0.340605	0.590366	NOT
AC144831.	23.9683	-0.4352322	0.456725	-0.95294185	0.34062	0.590366	NOT
C22orf15	11.05936	0.33128853	0.347664	0.952899285	0.340641	0.590366	NOT
ZNF772	162.3249	0.25709191	0.269814	0.952848633	0.340667	0.590373	NOT
RP11-666A	5.616039	-0.38285	0.401901	-0.95259739	0.340794	0.590556	NOT
RP11-161H	1.528539	-0.5518941	0.579396	-0.95253377	0.340826	0.590574	NOT
RP11-403E	1.306031	-0.5142782	0.539952	-0.95245231	0.340868	NA	NA
ZNF831	36.28747	-0.4286746	0.450132	-0.95233123	0.340929	0.590678	NOT
RP11-424M	4.508387	-0.3827341	0.401892	-0.95232952	0.34093	0.590678	NOT
RP11-500(	49.01022	0.21705886	0.227948	0.952231361	0.34098	0.59069	NOT
PSMB8	4772.054	0.27158443	0.285209	0.952229554	0.340981	0.59069	NOT
IGHV3-74	66.88384	-0.6433845	0.675719	-0.95214767	0.341022	0.590725	NOT
PLCZ1	4.939109	-0.5703801	0.599122	-0.95202695	0.341083	0.590793	NOT
RP4-673D2	1.797118	0.61339993	0.644583	0.951623088	0.341288	0.59111	NOT
ALS2CL	1513.118	0.32788695	0.344643	0.951382814	0.34141	0.591283	NOT
SMARCAD1	790.2507	-0.1941522	0.204116	-0.95118677	0.34151	0.591382	NOT
LINC00939	64.26627	-0.4816576	0.506376	-0.95118524	0.34151	0.591382	NOT
COA4	3261.424	0.160622	0.168902	0.950978793	0.341615	0.591525	NOT
RP11-56M3	4.928932	-0.3346662	0.352009	-0.95073305	0.34174	0.591704	NOT
DDX60L	562.8939	-0.2380194	0.250393	-0.95058232	0.341816	0.591759	NOT
FLOT2	6598.679	0.18725107	0.196986	0.950581702	0.341817	0.591759	NOT
SAAL1	479.19	0.14989254	0.157692	0.950540667	0.341838	0.591759	NOT
GSTK1	13548.85	-0.2063634	0.217125	-0.95043397	0.341892	0.591815	NOT
TMEM11	948.9433	0.19640314	0.206678	0.950286678	0.341967	0.591895	NOT
MIR4697HG	12.18748	-0.4392707	0.46227	-0.95024699	0.341987	0.591895	NOT
SLC25A52	3.407762	-0.5792666	0.609616	-0.9502151	0.342003	0.591895	NOT
PSMD9	970.5987	0.12906347	0.135837	0.950138284	0.342042	0.591917	NOT
AC108479.	1.985972	0.44403453	0.467355	0.950101902	0.34206	0.591917	NOT
SRGAP2B	60.25279	0.25555243	0.268987	0.95005469	0.342084	0.591917	NOT

HSPB8	1197.822	-0.4715659	0.496376	-0.95001738	0.342103	0.591917	NOT
RP11-143J	8.095315	-0.3110615	0.327479	-0.94986806	0.342179	0.592011	NOT
TRIM69	1303.354	-0.2237382	0.235604	-0.9496366	0.342297	0.592163	NOT
VN1R107P	1.99994	-0.571427	0.60175	-0.94960873	0.342311	0.592163	NOT
PLXNA1	1524.524	0.26145014	0.275383	0.949404941	0.342415	0.592305	NOT
AC010148.	18.13435	0.44517568	0.469095	0.94900941	0.342616	0.592615	NOT
RP11-115D	17.51207	-0.7877205	0.830136	-0.94890529	0.342669	0.592669	NOT
POLD3	658.6949	0.19630504	0.206922	0.948688868	0.342779	0.592822	NOT
IQCH	76.9532	0.40314126	0.425018	0.948527378	0.342861	0.592926	NOT
PGBD1	247.6473	0.25622073	0.270171	0.948366769	0.342943	0.593002	NOT
JAGN1	2527.957	0.16402252	0.172966	0.948295614	0.342979	0.593002	NOT
FAM120A	7734.579	-0.1053084	0.111064	-0.94817878	0.343038	0.593002	NOT
SERPINA3	1012.422	-0.4119085	0.434429	-0.94816064	0.343048	0.593002	NOT
KLRC4-KLF	2.100394	-0.6094705	0.642799	-0.94815067	0.343053	0.593002	NOT
AC092155.	2.327547	-0.9175187	0.967702	-0.94814219	0.343057	0.593002	NOT
GBA3	1692.4	-0.5214887	0.550012	-0.94814054	0.343058	0.593002	NOT
UQCC1	1746.68	-0.1348702	0.142292	-0.94784285	0.343209	0.593226	NOT
HOXA13	451.0417	-0.6252138	0.659697	-0.94772853	0.343268	0.593289	NOT
ANKRD13D	875.1079	0.21441815	0.226256	0.947678359	0.343293	0.593295	NOT
CACTIN	856.7062	-0.1151632	0.121532	-0.94759595	0.343335	0.59333	NOT
TRAPPC2B	288.8121	-0.2259971	0.238523	-0.94748427	0.343392	0.593381	NOT
RUFY3	821.024	-0.1681324	0.177458	-0.94745144	0.343409	0.593381	NOT
TCEB2	7450.164	-0.2490482	0.262889	-0.94735148	0.34346	0.593432	NOT
LINC0119	31.17574	1.03902874	1.096841	0.947292209	0.34349	0.593434	NOT
SRGAP3-AS	1.691948	-1.1354405	1.19868	-0.94724267	0.343515	0.593434	NOT
MPPE1	792.4409	-0.1968101	0.207776	-0.94722013	0.343527	0.593434	NOT
RP11-547I	7.631885	0.39139463	0.41329	0.947022641	0.343627	0.593537	NOT
TJP2	3300.836	0.17369208	0.183413	0.947001119	0.343638	0.593537	NOT
CD200	194.5938	-0.2451206	0.258866	-0.94690262	0.343688	0.593537	NOT
DENND5B	1416.34	-0.198608	0.209745	-0.94690203	0.343689	0.593537	NOT
MSTO1	760.1236	0.19534461	0.206302	0.946888462	0.343696	0.593537	NOT
RP11-618I	2.254908	0.65579147	0.692649	0.946787445	0.343747	0.593588	NOT
CEBPE	2.089102	0.4926233	0.520456	0.946521713	0.343883	0.593778	NOT
RP11-752(	2.78444	-0.370266	0.391213	-0.94645605	0.343916	0.593778	NOT
SOS2	1033.18	-0.1822263	0.192539	-0.94644055	0.343924	0.593778	NOT
NOTUM	5936.584	-0.7526574	0.795285	-0.94639956	0.343945	0.593778	NOT
ZRANB3	222.4721	-0.1601484	0.169241	-0.94627682	0.344007	0.593848	NOT
RNF223	1.723939	0.78125172	0.825685	0.946185924	0.344054	0.593856	NOT
STIM1	4856.294	-0.1411429	0.149171	-0.9461826	0.344055	0.593856	NOT
NUTM2D	31.31003	-0.3169075	0.334986	-0.94603309	0.344132	0.593856	NOT
RP11-699(	6.686944	0.43640019	0.461295	0.946032846	0.344132	0.593856	NOT
COMTD1	1017.777	0.31621173	0.33425	0.946032477	0.344132	0.593856	NOT
RP11-216M	13.51115	0.52908527	0.55928	0.946010782	0.344143	0.593856	NOT
CALML4	243.5762	0.24470945	0.258695	0.945938272	0.34418	0.593882	NOT
AP002954.	4.748123	0.48548127	0.513315	0.945777164	0.344262	0.593918	NOT
RP11-287I	2.254967	0.42405982	0.448391	0.945737686	0.344282	0.593918	NOT
SLC12A8	803.7647	0.30995423	0.327749	0.945705783	0.344299	0.593918	NOT
C5orf49	66.08012	-0.5586528	0.590736	-0.94568957	0.344307	0.593918	NOT
XPO6	3098.073	0.11018854	0.116517	0.945682643	0.34431	0.593918	NOT

STRN3	1132.84	-0.1485463	0.157107	-0.94550756	0.3444	0.594021	NOT
RP11-49K2	5.11118	-0.36291	0.383868	-0.94540432	0.344452	0.594021	NOT
GPR39	204.0424	0.21052653	0.222689	0.945382625	0.344464	0.594021	NOT
FUZ	402.5192	-0.2952962	0.312361	-0.9453687	0.344471	0.594021	NOT
RP11-552M	6.083159	0.23239967	0.245834	0.945351017	0.34448	0.594021	NOT
SPAST	909.2032	0.12768988	0.135078	0.945307481	0.344502	0.594022	NOT
AC006116.	2.567144	0.44453521	0.470278	0.94526134	0.344525	0.594025	NOT
MRE11A	650.1307	0.14101961	0.1492	0.945169448	0.344572	0.59406	NOT
LRRC37A14	2.29418	-0.4179556	0.442218	-0.94513496	0.34459	0.59406	NOT
PRMT2	1536.127	0.17420647	0.184375	0.944850005	0.344735	0.594259	NOT
MEG9	6.854261	0.64466269	0.68231	0.94482349	0.344749	0.594259	NOT
ZNF503-AS	191.1995	0.25840562	0.273581	0.944531478	0.344898	0.594478	NOT
RP11-848F	11.82016	0.36711944	0.388752	0.944353077	0.344989	0.594597	NOT
C6orf141	78.42777	0.52081972	0.551555	0.944275639	0.345029	0.594619	NOT
UPP2-IT1	8.558499	-0.967839	1.02499	-0.94424229	0.345046	0.594619	NOT
RP11-191I	10.19306	-0.3190411	0.337906	-0.94417217	0.345082	0.594643	NOT
NCOA2	4034.923	0.21433664	0.227022	0.944123263	0.345107	0.594649	NOT
SNORA5A	2.23958	0.41336459	0.437856	0.944064882	0.345136	0.594657	NOT
RP11-651I	29.64161	0.28948919	0.306653	0.944028052	0.345155	0.594657	NOT
RP11-829H	6.358167	0.52108816	0.552024	0.94395924	0.34519	0.59468	NOT
CASC2	22.78185	-0.3641827	0.385892	-0.94374308	0.345301	0.594733	NOT
HSF5	2.178435	-0.367975	0.389958	-0.94362828	0.34536	0.594733	NOT
HYAL3	380.6603	0.29091908	0.308303	0.943613096	0.345367	0.594733	NOT
ZNF66	17.29647	0.40878428	0.433235	0.943562193	0.345393	0.594733	NOT
BEX2	317.2419	0.59308979	0.628602	0.943505314	0.345422	0.594733	NOT
CRYBB2	7.03149	-0.4180176	0.443062	-0.94347536	0.345438	0.594733	NOT
AGAP5	9.621406	-0.3384612	0.358753	-0.94343908	0.345456	0.594733	NOT
IGHG4	1234.422	0.65482426	0.6941	0.943415247	0.345469	0.594733	NOT
AC093838.	527.7596	0.18642445	0.197607	0.943412285	0.34547	0.594733	NOT
CASC9	233.2012	0.82572282	0.875261	0.943401414	0.345476	0.594733	NOT
PRRG2	193.8434	0.38768746	0.410948	0.943397532	0.345478	0.594733	NOT
RASSF8-AS	218.9027	-0.2692854	0.285446	-0.9433841	0.345484	0.594733	NOT
MORF4L2	8980.841	0.1315385	0.139528	0.942738794	0.345815	0.595187	NOT
ALG1L2	4.045184	0.31201581	0.330979	0.942705958	0.345831	0.595187	NOT
MAP2K4P1	3.929916	-0.3317401	0.351921	-0.94265469	0.345858	0.595187	NOT
ASTE1	278.1116	0.11691397	0.12403	0.942625274	0.345873	0.595187	NOT
RP11-43N1	47.59496	-0.2652392	0.281396	-0.94258436	0.345894	0.595187	NOT
CASP5	6.525322	0.39335969	0.417333	0.942556634	0.345908	0.595187	NOT
USP5	4313.906	-0.1454463	0.154315	-0.94252841	0.345922	0.595187	NOT
ERGIC2	1551.722	0.13475005	0.142967	0.942525822	0.345923	0.595187	NOT
RPS6KA1	1322.179	0.25671164	0.27239	0.942441802	0.345966	0.595197	NOT
C1QL4	18.33214	0.57897263	0.614346	0.942421089	0.345977	0.595197	NOT
SLC26A1	1421.738	-0.2699128	0.286448	-0.94227376	0.346052	0.595197	NOT
GCG	1.820859	1.04881324	1.113078	0.94226397	0.346058	0.595197	NOT
NUDT5	4679.022	0.15748715	0.167139	0.942253084	0.346063	0.595197	NOT
UBQLN1	5749.43	-0.1199267	0.127283	-0.94220652	0.346087	0.595197	NOT
VAMP5	2790.126	-0.2508777	0.266269	-0.94219464	0.346093	0.595197	NOT
IFNL3P1	2.020864	0.7039324	0.747139	0.942170903	0.346105	0.595197	NOT
GNRHR	2.027348	-0.4035553	0.428424	-0.94195361	0.346216	0.595319	NOT

PSMC1P1	20.74313	0.23272239	0.247065	0.941946841	0.34622	0.595319	NOT
NDUFS5	5559.283	0.20581491	0.218553	0.941717982	0.346337	0.595437	NOT
ZNF788	99.14258	-0.3524444	0.374261	-0.94170794	0.346342	0.595437	NOT
ARPC3P5	1.799376	0.57730245	0.61308	0.941643089	0.346375	0.595437	NOT
FAM13B	652.3283	-0.1709502	0.181557	-0.94157738	0.346409	0.595437	NOT
RP4-604G5	6.514259	-0.5531556	0.587489	-0.94155964	0.346418	0.595437	NOT
ZNF586	153.8796	0.18842622	0.200126	0.941539402	0.346429	0.595437	NOT
CTD-2013N	2.540285	-0.3542589	0.376266	-0.94151255	0.346442	0.595437	NOT
AL161645.	1.190781	-0.911802	0.968525	-0.9414334	0.346483	NA	NA
SNAPC5	836.698	0.14195244	0.150794	0.941367622	0.346517	0.595472	NOT
AC138472.	1.650419	0.75722414	0.804396	0.941357661	0.346522	0.595472	NOT
MOCS3	536.8896	0.13225966	0.140506	0.941309341	0.346546	0.595472	NOT
RP11-264M	27.62535	-0.325778	0.346093	-0.94130155	0.34655	0.595472	NOT
AC006994.	2.521304	-0.5011922	0.532519	-0.94117167	0.346617	0.595549	NOT
SNORD83A	2.649178	0.43534825	0.462636	0.941017808	0.346696	0.595643	NOT
TMLHE-AS1	1.83952	0.42974071	0.456695	0.940978882	0.346716	0.595643	NOT
TMEM132B	19.4624	-0.442226	0.470075	-0.94075713	0.346829	0.59577	NOT
RN7SKP160	3.157118	-0.3199831	0.340136	-0.94074944	0.346833	0.59577	NOT
RP11-154F	5.932962	-0.4898203	0.520779	-0.94055239	0.346934	0.595876	NOT
CTGLF12P	15.09056	-0.4487546	0.477144	-0.94050238	0.34696	0.595876	NOT
ANKRD49	395.3098	-0.1159463	0.123282	-0.94050017	0.346961	0.595876	NOT
RP11-393M	8.965916	0.36433953	0.387517	0.940190073	0.34712	0.596087	NOT
C1QB	6524.675	0.35944558	0.382334	0.94013506	0.347148	0.596087	NOT
SAA2-SAA4	1158.053	-0.7053547	0.750272	-0.940132	0.34715	0.596087	NOT
NFATC2	811.6355	0.25172406	0.267782	0.940034376	0.3472	0.596135	NOT
ZSWIM4	387.3806	0.2641028	0.280983	0.939923294	0.347257	0.596195	NOT
BEX4	428.8348	-0.3594625	0.382507	-0.93975392	0.347344	0.596307	NOT
CXorf40B	632.4746	0.13868653	0.147676	0.93912389	0.347667	0.596824	NOT
RP11-520H	14.34221	0.36772288	0.391591	0.9390483	0.347706	0.596853	NOT
MTRNR2L12	69.96526	-0.3487964	0.371578	-0.93868865	0.347891	0.597054	NOT
TSKS	10.01309	0.51199417	0.545452	0.938660426	0.347905	0.597054	NOT
ZNF750	4.763115	-0.3587745	0.382222	-0.93865445	0.347908	0.597054	NOT
RP11-256I	3.217185	0.61577704	0.656025	0.938649148	0.347911	0.597054	NOT
EPS15	2343.814	-0.1216582	0.129618	-0.93859041	0.347941	0.597068	NOT
TTC34	17.72532	-0.4014514	0.427762	-0.93849173	0.347992	0.597104	NOT
C4B	12882.99	-0.343103	0.365601	-0.93846391	0.348006	0.597104	NOT
CCDC180	34.65061	-0.36237	0.386202	-0.93829049	0.348095	0.597104	NOT
EEF1A1P25	7.460587	0.33691007	0.359101	0.938204714	0.348139	0.597104	NOT
RBMXP4	3.315105	-0.3486256	0.37159	-0.93820001	0.348142	0.597104	NOT
CTC-537E7	2.576656	-0.6584838	0.701876	-0.93817704	0.348153	0.597104	NOT
GREB1	939.558	0.46828357	0.499166	0.938131845	0.348177	0.597104	NOT
RP11-774O	176.9583	-0.2604655	0.277651	-0.93810361	0.348191	0.597104	NOT
RAB6B	307.8592	0.32626584	0.347797	0.938091632	0.348197	0.597104	NOT
SLITRK4	20.44428	0.46417853	0.494848	0.938023367	0.348232	0.597104	NOT
APTR	440.7992	-0.1860452	0.198342	-0.93800433	0.348242	0.597104	NOT
LOXL1	251.5267	-0.4052488	0.432035	-0.93799953	0.348245	0.597104	NOT
RNU6-564P	1.989469	-0.6041383	0.644076	-0.9379924	0.348248	0.597104	NOT
ABHD12B	42.87808	0.39723722	0.42363	0.93769872	0.348399	0.597325	NOT
AKT1	4566.516	-0.1360614	0.145126	-0.93753735	0.348482	0.597412	NOT

RP11-428(	13.89251	-0.3743705	0.399322	-0.93751406	0.348494	0.597412	NOT
FOCAD	942.8887	0.16292363	0.173792	0.93746567	0.348519	0.597417	NOT
NLRP3	77.95323	-0.3051886	0.325579	-0.93737151	0.348567	0.597433	NOT
FLYWCH1	1311.803	-0.1587643	0.169373	-0.93736269	0.348572	0.597433	NOT
MTF1	533.4896	-0.1777798	0.189683	-0.93724767	0.348631	0.597496	NOT
RASL11A	459.1222	0.43865288	0.468172	0.936947527	0.348786	0.597723	NOT
GRAP	34.70567	-0.2620478	0.2797	-0.93688931	0.348815	0.597737	NOT
RP11-686I	23.4314	-0.3364815	0.359282	-0.93653864	0.348996	0.597961	NOT
CDHR2	1567.608	0.54047076	0.577094	0.93653798	0.348996	0.597961	NOT
RP11-161I	2.810728	-1.611771	1.721047	-0.93650603	0.349013	0.597961	NOT
TNK1	272.0754	0.19538983	0.208659	0.936408423	0.349063	0.598009	NOT
SH3RF2	791.923	-0.3377293	0.360681	-0.93636608	0.349085	0.598009	NOT
COX7B2	195.0344	1.12880155	1.205692	0.936227358	0.349156	0.598094	NOT
POLR2L	5795.435	-0.230178	0.245879	-0.9361444	0.349199	0.598121	NOT
ST13P18	4.786989	0.39295876	0.419778	0.936110802	0.349216	0.598121	NOT
THG1L	458.9062	-0.1360085	0.145323	-0.93590379	0.349323	0.598253	NOT
COMP	112.7576	-0.6631478	0.708585	-0.93587544	0.349337	0.598253	NOT
PPP1R36	14.05583	-0.4213738	0.450334	-0.93569069	0.349432	0.598378	NOT
SEMA3C	138.576	0.5232804	0.559325	0.935557145	0.349501	0.598458	NOT
SLC16A6	79.00127	0.36309651	0.388125	0.935514361	0.349523	0.598458	NOT
RP11-563J	5.948529	0.57858045	0.618578	0.935338837	0.349614	0.598531	NOT
ZNF593	253.8101	0.27140805	0.290173	0.935333351	0.349617	0.598531	NOT
PRICKLE2	236.8196	-0.3801302	0.406425	-0.93530306	0.349632	0.598531	NOT
SOX2-OT	17.60691	-0.4810416	0.5144	-0.93515093	0.349711	0.598592	NOT
BHMT2	13647.63	-0.319848	0.34204	-0.93512008	0.349726	0.598592	NOT
RP11-775(	2.509002	-1.3327653	1.425262	-0.93510228	0.349736	0.598592	NOT
PARP4	2688.246	-0.1953455	0.208912	-0.93506285	0.349756	0.598592	NOT
CDH3	20.00912	0.42791083	0.457695	0.934926535	0.349826	0.598674	NOT
MKS1	429.2959	0.14884777	0.15922	0.934857021	0.349862	0.598698	NOT
HIGD1A	3478.882	-0.2120819	0.226912	-0.93464207	0.349973	0.59885	NOT
RP11-179(	17.50402	0.29937055	0.320346	0.934523037	0.350034	0.598917	NOT
MAGI3	684.9521	-0.2089927	0.223658	-0.93443112	0.350082	0.59896	NOT
KIAA1191	4245.97	-0.1542077	0.165064	-0.93422946	0.350186	0.59908	NOT
CCDC53	1032.372	-0.2385567	0.255357	-0.93420918	0.350196	0.59908	NOT
FOXO4	777.9552	-0.1945949	0.208344	-0.93400963	0.350299	0.599219	NOT
SP1	3257.64	-0.1016177	0.108812	-0.93387922	0.350366	0.599264	NOT
RP5-1172N	2.208615	-0.5002524	0.535675	-0.93387253	0.35037	0.599264	NOT
NFS1	1934.687	-0.1379939	0.147782	-0.93376452	0.350425	0.599264	NOT
ARRB2	2275.899	-0.184434	0.197548	-0.93361376	0.350503	0.599264	NOT
SOCS7	599.1157	0.20322328	0.217676	0.933603334	0.350509	0.599264	NOT
CATIP-AS1	21.81839	-0.3431169	0.367522	-0.9335958	0.350512	0.599264	NOT
URM1	2826.461	0.17163681	0.183847	0.933584923	0.350518	0.599264	NOT
KRT8P41	1.992475	0.43462146	0.465548	0.933569805	0.350526	0.599264	NOT
AC005306.	5.7642	0.36418479	0.390103	0.933560094	0.350531	0.599264	NOT
FZD3	164.2636	0.39884748	0.427246	0.933530318	0.350546	0.599264	NOT
RP11-421F	1.133261	-1.2101931	1.29658	-0.93337354	0.350627	NA	NA
RP1-244F2	2.07262	0.52124032	0.558487	0.933308064	0.350661	0.599422	NOT
ONECUT3	2.833222	0.57801694	0.619601	0.932885901	0.350879	0.599732	NOT
CTD-2373N	28.91117	-0.3200951	0.343129	-0.93287186	0.350886	0.599732	NOT

NUCB2	3703.447	-0.2155471	0.231115	-0.93263887	0.351006	0.599878	NOT
TOMM7	5168.923	-0.1685895	0.18077	-0.93262047	0.351016	0.599878	NOT
LINC0109(	2.159316	1.08108909	1.159267	0.932562242	0.351046	0.599892	NOT
GRAMD1A	2757.853	0.2532773	0.271608	0.932508744	0.351074	0.599901	NOT
RETSAT	7584.07	-0.2394792	0.256836	-0.93242112	0.351119	0.599921	NOT
ZNF770	865.2276	-0.3040623	0.326107	-0.93240042	0.35113	0.599921	NOT
CLIC4	3749.542	-0.1848414	0.198304	-0.93211092	0.351279	0.600139	NOT
STAG1	901.4301	-0.2185674	0.234508	-0.93202675	0.351323	0.600157	NOT
CCL28	306.4301	0.37497264	0.402343	0.931972248	0.351351	0.600157	NOT
CTD-2235(	4.291243	-0.3229469	0.346524	-0.93196172	0.351356	0.600157	NOT
RP1-81D8.	7.102734	0.68488354	0.734989	0.931828373	0.351425	0.600159	NOT
ABCB1	3261.596	0.43413615	0.465926	0.931770914	0.351455	0.600159	NOT
RP11-587I	3.794001	-0.3734887	0.400863	-0.93171232	0.351485	0.600159	NOT
RP11-354I	1.509948	0.42657407	0.457858	0.931673103	0.351505	0.600159	NOT
RP11-10C2	13.34247	0.21050434	0.225943	0.931668715	0.351508	0.600159	NOT
FASTKD3	474.0734	0.12447273	0.133603	0.931658209	0.351513	0.600159	NOT
AC007326.	3.406381	0.80180418	0.860627	0.931650685	0.351517	0.600159	NOT
AC020951.	1.31137	-0.5987377	0.642671	-0.931639	0.351523	NA	NA
HAUS4	983.9432	0.22012598	0.236289	0.931597148	0.351545	0.600159	NOT
AC012360.	3.25391	0.39953788	0.428884	0.931574599	0.351556	0.600159	NOT
CARM1	1572.985	0.14699265	0.157821	0.931390987	0.351651	0.600244	NOT
BRIP1	327.974	-0.2979145	0.31989	-0.93130426	0.351696	0.600244	NOT
RP11-528(	2.227013	0.82724858	0.888302	0.931269398	0.351714	0.600244	NOT
RP11-15I1	123.0107	0.50817294	0.545679	0.931267961	0.351715	0.600244	NOT
C5orf63	154.2676	0.35439085	0.380548	0.931263924	0.351717	0.600244	NOT
UBXN7	1200.703	-0.1593744	0.171172	-0.93107641	0.351814	0.600372	NOT
LDHAP7	32.16473	0.38820581	0.417053	0.930830852	0.351941	0.600551	NOT
TMEM171	151.8023	0.39616525	0.42564	0.930752513	0.351982	0.600582	NOT
C1orf147	3.385845	0.43430524	0.466643	0.930701838	0.352008	0.600589	NOT
SAGE1	2.344379	1.16870027	1.255805	0.930638182	0.352041	0.600608	NOT
RP11-707(	10.03864	-0.3578838	0.384617	-0.93049369	0.352116	0.600679	NOT
CTB-5506.	5.456421	-0.375197	0.403244	-0.93044737	0.35214	0.600679	NOT
RP11-261N	127.641	-0.8010996	0.861001	-0.93042784	0.35215	0.600679	NOT
RRN3P3	80.95518	0.19541156	0.210039	0.930356543	0.352187	0.600679	NOT
RAB18	2899.896	0.12706679	0.13659	0.930278628	0.352227	0.600679	NOT
UBOX5	573.3076	-0.1506689	0.161965	-0.93025364	0.35224	0.600679	NOT
RP11-123K	0.866946	-1.2056088	1.296001	-0.93025269	0.35224	NA	NA
SMARCE1	2460.56	0.12551803	0.134934	0.930217876	0.352258	0.600679	NOT
CTD-2553(	3.929583	-0.4169077	0.448188	-0.93020706	0.352264	0.600679	NOT
FAM132A	42.262	0.34757614	0.373669	0.930172172	0.352282	0.600679	NOT
UROC1	2185.406	-0.6386057	0.686593	-0.93010862	0.352315	0.600698	NOT
MIER2	674.6333	0.21652847	0.232817	0.930039077	0.352351	0.600709	NOT
RP11-750H	24.07471	-0.3436908	0.369565	-0.92998725	0.352378	0.600709	NOT
RIMKLBP2	8.353251	-0.3022954	0.32506	-0.92996777	0.352388	0.600709	NOT
RP5-1132H	10.23454	-0.4692561	0.504679	-0.92981078	0.352469	0.600788	NOT
GAPT	22.31451	0.37448806	0.402777	0.929764552	0.352493	0.600788	NOT
ARIH2OS	64.89204	0.16593064	0.178468	0.92974949	0.352501	0.600788	NOT
C4orf3	4250.343	-0.1596223	0.171698	-0.92966883	0.352543	0.600822	NOT
TRAPPC13	782.0936	-0.1407605	0.151447	-0.92943981	0.352661	0.600986	NOT

PDIA4	24373.49	-0.2008423	0.216106	-0.92936808	0.352698	0.600987	NOT
OVOL1	45.6363	0.67512033	0.726441	0.929353284	0.352706	0.600987	NOT
RP11-686I	1.25078	-0.5504226	0.592271	-0.92934244	0.352712	NA	NA
RNF6	917.8865	-0.166266	0.178932	-0.92921459	0.352778	0.601072	NOT
KIAA0753	305.4032	0.1949415	0.209804	0.929158593	0.352807	0.601078	NOT
CDON	297.7693	-0.2267817	0.244082	-0.92912227	0.352826	0.601078	NOT
RP11-57H1	87.68122	-0.2197097	0.236481	-0.929078	0.352849	0.601079	NOT
FAHD1	3316.953	-0.1887813	0.203201	-0.92903527	0.352871	0.601079	NOT
TRAV22	1.520011	-0.6130029	0.659869	-0.92897627	0.352901	0.601093	NOT
RP11-527I	0.96766	0.96675724	1.040742	0.928911665	0.352935	NA	NA
RP11-532M	19.45779	-0.2559888	0.275623	-0.92876302	0.353012	0.601244	NOT
RP11-387H	15.33015	-0.3458164	0.372371	-0.9286869	0.353051	0.601246	NOT
AIG1	5768.581	-0.198378	0.213633	-0.92859089	0.353101	0.601246	NOT
TMED10	11746.37	-0.1435148	0.154556	-0.92856044	0.353117	0.601246	NOT
RPL10P1	2.938825	0.37527445	0.404149	0.928554605	0.35312	0.601246	NOT
HECTD2	295.9501	0.20360078	0.219268	0.928546865	0.353124	0.601246	NOT
RP11-298I	9.14711	0.24973693	0.269033	0.928276316	0.353264	0.601447	NOT
STK11	2634.667	-0.1566773	0.168792	-0.9282279	0.353289	0.601452	NOT
CALCA	43.53594	0.59641432	0.642574	0.928163919	0.353323	0.601471	NOT
CTD-2015H	2.154349	-0.6038709	0.650725	-0.9279972	0.353409	0.601578	NOT
CTD-2265(	2.838418	0.70932219	0.764401	0.927945044	0.353436	0.601578	NOT
A2ML1	4.005739	-0.5861975	0.631737	-0.92791396	0.353452	0.601578	NOT
ATXN7L2	214.3764	0.17424077	0.187791	0.927843783	0.353489	0.601603	NOT
RP11-313F	2.555436	-0.354781	0.382493	-0.92754938	0.353641	0.601798	NOT
GLULP3	2.281869	0.68205983	0.735345	0.927537098	0.353648	0.601798	NOT
MIR659	2.469156	-0.3416699	0.368488	-0.92722013	0.353812	0.60204	NOT
GPRC5A	58.57299	-0.4708606	0.507862	-0.92714276	0.353852	0.602071	NOT
SRSF9P1	7.752689	-0.2959998	0.319382	-0.92679017	0.354035	0.602344	NOT
RP11-645(	6.737466	-0.4224	0.455809	-0.92670321	0.354081	0.602383	NOT
CD22	89.27912	0.38342528	0.41378	0.926640852	0.354113	0.602401	NOT
KRT18P1	2.373291	0.44034761	0.475234	0.926591314	0.354139	0.602407	NOT
CTNNA1	13499.75	0.11709544	0.126381	0.926527316	0.354172	0.602423	NOT
TIFAB	19.34419	0.39482613	0.426154	0.926487364	0.354193	0.602423	NOT
RP11-395A	53.7839	-0.1884462	0.203436	-0.92631699	0.354281	0.602471	NOT
CTAGE5	429.8893	-0.1871786	0.202073	-0.92629261	0.354294	0.602471	NOT
SLC18A2	17.38505	-0.4185302	0.451848	-0.92626348	0.354309	0.602471	NOT
GAPDHP40	14.80525	-0.3530561	0.381162	-0.92626215	0.35431	0.602471	NOT
C1QL3	38.9385	0.42155474	0.4552	0.926087278	0.354401	0.602588	NOT
LINC01521	28.49672	-0.2549079	0.275282	-0.92598969	0.354451	0.60262	NOT
CTD-3184A	177.051	-0.2429433	0.262368	-0.92596474	0.354464	0.60262	NOT
B3GNT7	215.6512	0.44923325	0.485257	0.925764078	0.354569	0.602706	NOT
TMEM38A	706.4344	-0.2862109	0.309165	-0.92575321	0.354574	0.602706	NOT
ALG8	2481.471	0.14443116	0.156027	0.925678663	0.354613	0.602706	NOT
GNPNAT1	1984.673	-0.199714	0.21575	-0.92567272	0.354616	0.602706	NOT
CSMD2	112.3062	0.44352059	0.479143	0.925653982	0.354626	0.602706	NOT
TSSK3	11.07907	-0.3018434	0.326102	-0.92560983	0.354649	0.602707	NOT
ANXA6	12058.76	-0.2802987	0.302845	-0.92555048	0.35468	0.602722	NOT
STX19	5.340426	-0.475613	0.513934	-0.92543527	0.35474	0.602786	NOT
GLOD5	63.35678	0.40079293	0.433161	0.925275519	0.354823	0.60289	NOT

CTA-243E7	4.232794	-0.3360627	0.363226	-0.92521675	0.354853	0.602904	NOT
VPS33B	592.8414	0.09321933	0.100782	0.924961385	0.354986	0.603092	NOT
ISG20	982.3192	-0.322577	0.348868	-0.9246401	0.355153	0.603239	NOT
SPATA12	3.354816	0.57254473	0.61923	0.924608156	0.35517	0.603239	NOT
C2CD2L	643.9182	-0.1465051	0.158464	-0.92453106	0.35521	0.603239	NOT
HIST1H4B	2.713748	-0.6141284	0.664298	-0.92447786	0.355238	0.603239	NOT
XXbac-BP(	96.23165	-0.3183349	0.344343	-0.92446991	0.355242	0.603239	NOT
WFDC2	189.637	-0.676392	0.731664	-0.92445755	0.355248	0.603239	NOT
RP4-782L2	27.31635	-0.3724902	0.402929	-0.92445513	0.355249	0.603239	NOT
PPP3R1	2279.044	-0.1017214	0.110034	-0.92445238	0.355251	0.603239	NOT
AC073326.	2.716571	0.42843248	0.463495	0.924351693	0.355303	0.603291	NOT
PHF23	1358.863	0.15964858	0.172736	0.924234469	0.355364	0.603356	NOT
LOXL4	1766.515	0.47163907	0.510351	0.924146805	0.35541	0.603362	NOT
RP5-1142J	2.017792	0.67202226	0.727214	0.924105815	0.355431	0.603362	NOT
ARPP21	49.73178	-0.7175005	0.776443	-0.92408616	0.355441	0.603362	NOT
LINC0010(	55.13707	-0.346317	0.374779	-0.92405759	0.355456	0.603362	NOT
KCNA5	19.24239	-0.4055098	0.438883	-0.9239589	0.355508	0.603411	NOT
VGLL1	2.348858	1.01283225	1.096261	0.923897394	0.35554	0.603428	NOT
OSGEPL1-A	10.05128	-0.2240373	0.242533	-0.92373834	0.355623	0.6035	NOT
LINC0068:	2.684265	-0.6468939	0.700328	-0.9237017	0.355642	0.6035	NOT
RPL13AP5	404.4058	0.27875603	0.301793	0.923666301	0.35566	0.6035	NOT
CTD-2527I	1.969831	-0.459564	0.497555	-0.92364475	0.355671	0.6035	NOT
RP13-608F	2.633272	-0.440869	0.477373	-0.92353183	0.35573	0.603525	NOT
UGT2B7	13069.67	-0.4457505	0.482673	-0.92350486	0.355744	0.603525	NOT
RP11-785H	1474.433	-0.1308707	0.141713	-0.92348874	0.355753	0.603525	NOT
ANKEF1	567.5761	0.19709797	0.213444	0.92341613	0.35579	0.603551	NOT
FKBP1C	17.40838	0.23287393	0.252218	0.92330287	0.355849	0.603607	NOT
RP11-164F	29.9559	0.40060078	0.433895	0.923267252	0.355868	0.603607	NOT
CTB-36H1(	77.91471	-0.2616198	0.283421	-0.92307776	0.355967	0.603664	NOT
ISYNA1	2137.179	-0.4526588	0.490408	-0.92302461	0.355994	0.603664	NOT
RP11-193H	2.613389	-0.5261307	0.570029	-0.92298993	0.356012	0.603664	NOT
EDAR	45.99067	-0.443612	0.480632	-0.92297601	0.35602	0.603664	NOT
GEMIN5	989.2466	0.13683273	0.148261	0.92291949	0.356049	0.603664	NOT
AC115617.	13.43669	-0.2873966	0.311406	-0.92290123	0.356059	0.603664	NOT
HEPACAM	187.608	0.88744966	0.96164	0.922850427	0.356085	0.603664	NOT
VAC14-AS1	65.92107	0.46237749	0.501037	0.922840589	0.35609	0.603664	NOT
AC005534.	3.524503	0.40738973	0.441462	0.922818822	0.356102	0.603664	NOT
MPO	8.598645	-0.4695833	0.50898	-0.92259588	0.356218	0.603823	NOT
GSTA8P	5.346207	0.50512216	0.54753	0.922547384	0.356243	0.603828	NOT
PAK6	1.168783	0.68899874	0.746895	0.922483934	0.356276	NA	NA
COPRS	2320.687	0.1757861	0.190563	0.922458702	0.356289	0.603869	NOT
THOC6	1118.715	0.17502562	0.189764	0.92233402	0.356354	0.603941	NOT
TRAV25	1.186413	-0.5605147	0.607721	-0.92232255	0.35636	NA	NA
FBXL20	839.0737	-0.1251558	0.135708	-0.92224325	0.356402	0.603984	NOT
ACY3	898.6404	-0.3512217	0.380862	-0.92217702	0.356436	0.604005	NOT
RP11-333I	8.596248	-0.3849394	0.417504	-0.92200223	0.356527	0.604121	NOT
MRPS36P4	0.725262	-1.2250194	1.328751	-0.92193329	0.356563	NA	NA
MRPL14	3793.638	0.21833768	0.236835	0.921897446	0.356582	0.604154	NOT
RPL38	17694.51	0.26711471	0.28975	0.921880017	0.356591	0.604154	NOT

RP11-142A	3.232033	0.84629513	0.918058	0.921831725	0.356616	0.604159	NOT
CHRM3-AS1	0.796342	0.91012337	0.98731	0.921821632	0.356622	NA	NA
MCCD1	81.21349	0.87166449	0.945785	0.921630325	0.356721	0.604285	NOT
NRSN2	1011.703	0.40903411	0.443829	0.921603847	0.356735	0.604285	NOT
AC005517.	5.372463	0.29413683	0.319186	0.921523023	0.356777	0.604319	NOT
ZNF385A	651.4508	0.22816956	0.247626	0.92142914	0.356826	0.604342	NOT
UNC5D	5.861606	-0.9951427	1.080021	-0.9214107	0.356836	0.604342	NOT
THEMIS2	559.6561	-0.3050212	0.331059	-0.92134941	0.356868	0.604359	NOT
LIN7B	167.3123	0.23110786	0.250889	0.921154663	0.35697	0.604493	NOT
LPGAT1	9060.899	0.18830267	0.204456	0.92099373	0.357054	0.604598	NOT
SLC26A10	7.471289	-0.3721167	0.404076	-0.92090803	0.357098	0.60463	NOT
PSG9	1.500348	1.08256126	1.175583	0.920871816	0.357117	0.60463	NOT
STARD4	1185.596	-0.2221747	0.241317	-0.92067488	0.35722	0.604719	NOT
HDAC4	589.8722	0.19906087	0.216222	0.920633717	0.357242	0.604719	NOT
PLD2	790.2525	-0.163047	0.177108	-0.92060623	0.357256	0.604719	NOT
A1CF	9630.326	-0.2847503	0.309324	-0.92055738	0.357282	0.604719	NOT
POR	31205.48	-0.2692246	0.292465	-0.92053764	0.357292	0.604719	NOT
SPRTN	424.4709	0.1076543	0.11695	0.920515151	0.357304	0.604719	NOT
PPP1R13B	1101.459	0.15382929	0.167123	0.9204566	0.357334	0.604733	NOT
CARD6	378.422	0.28968436	0.314791	0.920242077	0.357446	0.604885	NOT
PADI4	4.885207	-0.5662731	0.615412	-0.92015343	0.357493	0.604926	NOT
FAM171B	116.8378	0.30751267	0.334226	0.920074476	0.357534	0.604958	NOT
ARMC12	27.7704	0.32565831	0.353965	0.920030545	0.357557	0.604959	NOT
CTD-2246P	2.4157	0.48851881	0.531313	0.919456295	0.357857	0.605416	NOT
RAI2	173.8091	-0.3224603	0.350723	-0.91941676	0.357878	0.605416	NOT
IRX2	1.633818	0.55669208	0.605505	0.919385346	0.357894	0.605416	NOT
DAK	7597.996	-0.3496395	0.380337	-0.91928752	0.357945	0.605465	NOT
FSCN1	2135.895	0.29426114	0.320121	0.919219199	0.357981	0.605488	NOT
C17orf89	1460.312	-0.2607651	0.283715	-0.91910931	0.358038	0.605547	NOT
HIST1H4C	5.187672	0.35538688	0.386697	0.919031648	0.358079	0.605578	NOT
GBP1	2978.359	0.35991113	0.391679	0.918892724	0.358152	0.605663	NOT
ELMOD3	749.3728	-0.1166469	0.126958	-0.91878667	0.358207	0.605719	NOT
ARID4B	1565.309	-0.1418525	0.154399	-0.91874226	0.35823	0.605721	NOT
ACTN4P1	1.817557	0.43428785	0.472773	0.918597442	0.358306	0.605752	NOT
CD320	1680.834	0.24029557	0.261592	0.918590357	0.35831	0.605752	NOT
AL022476.	5.524574	0.30219191	0.328978	0.918578554	0.358316	0.605752	NOT
SHISA8	5.413249	-0.6903953	0.751629	-0.91853221	0.35834	0.605756	NOT
AC012358.	36.52158	0.20048712	0.218315	0.918340562	0.358441	0.605887	NOT
SLC13A3	3583.783	0.66099913	0.719897	0.918185937	0.358522	0.605986	NOT
CTTNBP2NL	529.5517	0.22112464	0.240855	0.918081839	0.358576	0.606041	NOT
AC020594.	8.587054	-0.3441645	0.374968	-0.91785012	0.358697	0.606208	NOT
GMPSP1	9.996418	0.30986849	0.337621	0.91779952	0.358724	0.606215	NOT
AC005534.	10.19288	0.3638088	0.396474	0.917609714	0.358823	0.606345	NOT
RP11-378J	7.961281	0.40232412	0.438472	0.917560074	0.358849	0.606351	NOT
YWHAE	12914.09	-0.1331532	0.145127	-0.9174918	0.358885	0.606374	NOT
PARP14	4467.778	-0.1888528	0.205857	-0.91739823	0.358934	0.606419	NOT
H2AFV	5663.282	-0.1338366	0.1459	-0.91731797	0.358976	0.606452	NOT
IFNG-AS1	1.85737	-0.5905459	0.643841	-0.91722309	0.359026	0.606477	NOT
UBE2K	4047.599	0.12124713	0.132192	0.917204808	0.359035	0.606477	NOT

CLSTN2	106.2434	-0.4382143	0.477825	-0.9171021	0.359089	0.60652	NOT
AC083843.	62.53544	0.34303344	0.374058	0.917058619	0.359112	0.60652	NOT
LA16c-38(	3.452778	0.43032075	0.469256	0.917028267	0.359128	0.60652	NOT
SIGLEC5	10.03417	0.38136917	0.415945	0.916874767	0.359208	0.606587	NOT
ASMTL-AS1	190.7563	-0.3189657	0.347887	-0.91686694	0.359212	0.606587	NOT
C17orf78	1.720623	0.49158406	0.536298	0.916625408	0.359339	0.606763	NOT
CYSRT1	58.7688	-0.3320819	0.362394	-0.91635567	0.35948	0.606964	NOT
SCAND1	3318.721	-0.2238309	0.244275	-0.91630666	0.359506	0.60697	NOT
RP11-341I	1.299333	1.28743859	1.405103	0.916259057	0.359531	NA	NA
RP3-323P2	5.425152	-0.2210209	0.241243	-0.91617676	0.359574	0.607041	NOT
DIP2B	2006.381	-0.1476222	0.161135	-0.91614142	0.359593	0.607041	NOT
SLC20A2	2518.112	-0.2319377	0.253184	-0.91608238	0.359624	0.607055	NOT
RP11-175K	17.56166	-0.2374625	0.259236	-0.91601039	0.359661	0.607081	NOT
RP11-826F	6.999308	0.30054464	0.328145	0.915889552	0.359725	0.607148	NOT
RP11-508N	10.20859	0.34889968	0.380968	0.915824988	0.359759	0.607148	NOT
FAM163B	30.24667	-0.6547544	0.714967	-0.91578298	0.359781	0.607148	NOT
HHEX	2583.716	0.163883	0.178958	0.915764054	0.359791	0.607148	NOT
BPIFA1	1.137026	1.2137455	1.325468	0.915711141	0.359818	NA	NA
CCDC157	68.42758	0.17575953	0.191939	0.915706626	0.359821	0.607161	NOT
SEPT7	3756.481	-0.1033849	0.112954	-0.91528445	0.360042	0.607497	NOT
ZNF431	237.5689	0.27575721	0.301318	0.915168542	0.360103	0.607509	NOT
RP11-419(	1.716606	1.19636561	1.307277	0.91515871	0.360108	0.607509	NOT
PCGF3	1601.716	-0.1366398	0.14931	-0.91513903	0.360119	0.607509	NOT
STX8	880.0934	-0.1845744	0.201706	-0.91506621	0.360157	0.607509	NOT
GPR137B	753.5123	-0.2649823	0.289604	-0.91498243	0.360201	0.607509	NOT
LINC00342	153.6146	-0.2887654	0.315605	-0.9149571	0.360214	0.607509	NOT
ST13	11973.76	0.13973845	0.152727	0.914954207	0.360216	0.607509	NOT
XXbac-BP(	2.647226	-0.6554782	0.716425	-0.91492981	0.360228	0.607509	NOT
MORC2	2013.437	0.1164544	0.127298	0.914817144	0.360288	0.607571	NOT
TRAV5	2.425267	0.59280729	0.648164	0.914594849	0.360404	0.607712	NOT
RPL4P5	33.54851	0.2893709	0.3164	0.91457215	0.360416	0.607712	NOT
CPT1C	115.4784	-0.3035263	0.331905	-0.91449811	0.360455	0.60774	NOT
FASLG	30.48938	0.39523911	0.432222	0.914435449	0.360488	0.607758	NOT
KMT2A	1706.779	-0.1442556	0.15777	-0.91434233	0.360537	0.607803	NOT
CASQ1	9.444342	-0.3831786	0.419143	-0.91419473	0.360615	0.607845	NOT
FUT6	731.4034	0.46451337	0.508124	0.914173401	0.360626	0.607845	NOT
ANKRD2	14.49683	0.43947769	0.480757	0.914137804	0.360644	0.607845	NOT
LRP5L	248.3555	-0.2034595	0.222582	-0.91408659	0.360671	0.607845	NOT
COX20	409.5519	0.19622319	0.214667	0.914080931	0.360674	0.607845	NOT
AC079613.	4.162611	0.92015172	1.006721	0.914008912	0.360712	0.607857	NOT
RETNLB	1.717645	0.92234067	1.009145	0.913982306	0.360726	0.607857	NOT
LRRC42	1655.885	0.18077673	0.197813	0.913877998	0.360781	0.607912	NOT
UQCRB	13203.01	0.23343624	0.255462	0.913780857	0.360832	0.607935	NOT
RP11-452K	5.780398	-0.3292244	0.360324	-0.91368895	0.36088	0.607935	NOT
FBXW4P1	20.19142	0.27288016	0.298659	0.91368333	0.360883	0.607935	NOT
C3orf38	570.0692	0.10763433	0.117803	0.913680866	0.360885	0.607935	NOT
HPSE	129.163	0.29715403	0.325343	0.913357169	0.361055	0.608184	NOT
RP11-44M(	218.2376	-0.2273624	0.248954	-0.91327141	0.3611	0.608222	NOT
EEF1DP1	29.64844	0.27484696	0.301008	0.913089658	0.361195	0.608278	NOT

CIDECP	422.5568	0.2058757	0.225477	0.913069379	0.361206	0.608278	NOT
AADACP1	186.6953	-0.426362	0.466956	-0.91306717	0.361207	0.608278	NOT
AC104532.	8.402283	-0.2727753	0.298756	-0.91303766	0.361223	0.608278	NOT
C16orf91	504.743	0.15964152	0.174866	0.912934891	0.361277	0.608332	NOT
FAM43B	40.37149	-0.562702	0.616518	-0.91271017	0.361395	0.608433	NOT
CTD-2537I	4.97601	-0.3595352	0.393926	-0.9126975	0.361402	0.608433	NOT
TP53BP1	636.1813	0.225865	0.247479	0.912665113	0.361419	0.608433	NOT
ZNF384	592.9902	0.1953782	0.214081	0.912635085	0.361434	0.608433	NOT
ERAP1	3385.354	-0.233331	0.255686	-0.91256792	0.36147	0.608433	NOT
SNTA1	1450.135	0.19260526	0.211059	0.912564454	0.361472	0.608433	NOT
TPK1	154.0349	-0.219854	0.240999	-0.91225993	0.361632	0.6086	NOT
VAPA	5709.096	0.12404504	0.135977	0.912250199	0.361637	0.6086	NOT
PORCN	239.8704	-0.1935522	0.212171	-0.91224799	0.361638	0.6086	NOT
TIMM23B	210.2777	-0.155391	0.17035	-0.91218554	0.361671	0.608618	NOT
HIST1H3E	41.10019	-0.3604933	0.395239	-0.91208961	0.361722	0.608665	NOT
RP11-578F	1.659802	0.45653676	0.500577	0.912021543	0.361757	0.608672	NOT
FAM184B	9.958219	-0.2580575	0.282964	-0.91197865	0.36178	0.608672	NOT
ATP6V0B	7300.133	0.18684567	0.204885	0.911954025	0.361793	0.608672	NOT
GNL3L	277.978	-0.1917936	0.210336	-0.91184388	0.361851	0.608723	NOT
MSR1	610.3301	0.29514829	0.323695	0.911811228	0.361868	0.608723	NOT
NKTR	1712.642	-0.2059375	0.225877	-0.91172583	0.361913	0.608761	NOT
CLCC1	980.3174	-0.1541369	0.16909	-0.91156442	0.361998	0.608806	NOT
NUAK1	957.241	-0.1746999	0.191651	-0.91155331	0.362004	0.608806	NOT
PPP4R2	3237.574	0.15446678	0.169457	0.911541661	0.36201	0.608806	NOT
SP2-AS1	74.07693	0.23323383	0.255884	0.911483171	0.362041	0.608806	NOT
ASS1P7	1.739472	-0.6052469	0.66404	-0.91146152	0.362052	0.608806	NOT
ANKRD40	1685.977	0.09303234	0.102094	0.91124162	0.362168	0.608952	NOT
RNF41	1211.197	-0.0980937	0.107652	-0.91121141	0.362184	0.608952	NOT
ENOX1	43.41172	0.40645411	0.446138	0.911050103	0.362269	0.609057	NOT
VIL1	3427.36	0.48393794	0.531223	0.910989157	0.362301	0.609074	NOT
SLC2A11	286.4256	-0.1825983	0.200458	-0.91090428	0.362346	0.609111	NOT
RNU12	3.269236	0.46100886	0.506167	0.91078375	0.362409	0.60918	NOT
RP11-22B2	2.254016	0.53044748	0.582462	0.910698931	0.362454	0.609199	NOT
RP11-386I	0.802673	-0.8055713	0.884569	-0.9106934	0.362457	NA	NA
EEF1A1P14	17.1602	-0.345603	0.379501	-0.91067654	0.362466	0.609199	NOT
PRR27	2.115895	-1.003867	1.1025	-0.91053665	0.36254	0.609286	NOT
RP11-413I	25.49037	0.43715685	0.48014	0.910478016	0.36257	0.6093	NOT
CTD-2017(	7.549946	0.29124905	0.319937	0.910331429	0.362648	0.609378	NOT
NKAPP1	43.89731	0.176065	0.193419	0.910279232	0.362675	0.609378	NOT
SIGLEC16	28.89259	0.35605248	0.391162	0.910242388	0.362695	0.609378	NOT
OMG	7.47754	0.3265789	0.358791	0.910219915	0.362707	0.609378	NOT
RP11-504(	2.795751	0.32576688	0.357996	0.909974123	0.362836	0.609477	NOT
LL0XNC01-	13.02995	-0.3254	0.357604	-0.90994595	0.362851	0.609477	NOT
RPL10AP2	9.122716	0.39187878	0.430663	0.909943697	0.362852	0.609477	NOT
CRYBA2	2.469918	-0.6935339	0.76218	-0.90993461	0.362857	0.609477	NOT
ADI1	21146.05	-0.2855319	0.313808	-0.90989501	0.362878	0.609477	NOT
THOP1	2851.848	0.21210192	0.233139	0.909767297	0.362945	0.609539	NOT
CHMP1B2P	10.36859	0.90746115	0.997495	0.909739904	0.36296	0.609539	NOT
MED7	475.6658	0.14048834	0.154464	0.909521396	0.363075	0.609657	NOT

ACTR8	693.3031	0.10869488	0.119508	0.90952085	0.363075	0.609657	NOT
NSMCE4A	1222.509	-0.142215	0.156376	-0.909443	0.363116	0.609688	NOT
RP11-355I	3.507807	-0.3536307	0.388888	-0.90933733	0.363172	0.609744	NOT
CTC-327F1	1.490923	0.62131952	0.683299	0.909293711	0.363195	0.609745	NOT
PAOX	762.4476	-0.2232605	0.245573	-0.9091417	0.363275	0.609842	NOT
SERBP1P1	6.681251	0.32560152	0.358158	0.909099189	0.363298	0.609842	NOT
CCDC130	1000.887	-0.1551245	0.170646	-0.90904485	0.363326	0.609853	NOT
INTS9	421.1759	0.17935944	0.19735	0.908838447	0.363435	0.609975	NOT
TMEM116	445.2917	0.14963803	0.164651	0.908821599	0.363444	0.609975	NOT
CTD-2005I	43.83016	0.56456905	0.621323	0.908656088	0.363532	0.61003	NOT
TTC9B	6.281396	-0.3645557	0.401231	-0.90859394	0.363565	0.61003	NOT
CICP16	4.537004	-0.4089039	0.450045	-0.90858511	0.363569	0.61003	NOT
CACNA1C-A	2.72307	-0.4527393	0.4983	-0.90856821	0.363578	0.61003	NOT
MTATP6P1	32005.11	-0.2672819	0.294186	-0.90854658	0.36359	0.61003	NOT
GEMIN7	746.8351	0.17437869	0.191952	0.90844895	0.363641	0.610079	NOT
C15orf43	14.20856	-0.7464862	0.821782	-0.90837486	0.36368	0.610103	NOT
SIGLEC7	64.25735	0.31371168	0.345369	0.908336578	0.3637	0.610103	NOT
RPL24P2	41.29479	0.30614586	0.337085	0.908214484	0.363765	0.61016	NOT
APAF1	534.8945	0.22364314	0.246252	0.908186543	0.36378	0.61016	NOT
CTD-2192J	2.922279	0.3783837	0.416716	0.908012987	0.363871	0.610276	NOT
CTD-3162I	9.607806	-0.4550112	0.501159	-0.90791827	0.363921	0.610323	NOT
RP1-151F1	36.74551	-0.3094688	0.340876	-0.90786362	0.36395	0.610333	NOT
C21orf58	234.7627	0.25205832	0.277671	0.907758988	0.364006	0.610364	NOT
RP11-285(	6.398472	0.32995809	0.363492	0.90774417	0.364013	0.610364	NOT
TAF1	1127.948	-0.1869577	0.205989	-0.90760831	0.364085	0.610446	NOT
PLS3-AS1	25.39568	-0.2998297	0.330484	-0.90724405	0.364278	0.610731	NOT
SENP3	713.2606	0.1603611	0.176765	0.907199369	0.364301	0.610733	NOT
TST	17068.17	-0.3028638	0.333862	-0.90715164	0.364327	0.610738	NOT
ALOX15	13.27789	-0.4117293	0.453905	-0.90708229	0.364363	0.610762	NOT
LRRC47	2463.143	0.11340785	0.125031	0.907037508	0.364387	0.610764	NOT
CTD-2292M	24.56985	0.31552166	0.347943	0.906820498	0.364502	0.610864	NOT
RPS26P11	3.033736	0.4258333	0.469591	0.906817729	0.364503	0.610864	NOT
AC002310.	1.823006	0.43028465	0.474511	0.906796357	0.364514	0.610864	NOT
RP11-15A1	31.1682	-0.3425276	0.377768	-0.90671353	0.364558	0.610882	NOT
GRIN3B	10.19565	0.42833936	0.472455	0.906625343	0.364605	0.610882	NOT
RP11-162(	4.822825	0.40290597	0.444407	0.90661574	0.36461	0.610882	NOT
ZFP3	113.6137	-0.3829691	0.422431	-0.90658381	0.364627	0.610882	NOT
KCNT2	389.628	-0.3839389	0.42351	-0.90656343	0.364638	0.610882	NOT
RP11-478(	4.144347	0.35730673	0.394153	0.906518365	0.364662	0.610884	NOT
STMN3	374.9134	0.42184342	0.465407	0.906397746	0.364725	0.610953	NOT
RP11-568A	3.798637	0.74268665	0.819604	0.906152582	0.364855	0.611133	NOT
TMEM30B	867.1009	-0.3676579	0.405771	-0.90607229	0.364898	0.611166	NOT
RP11-454I	2.507009	-0.4677243	0.516243	-0.90601547	0.364928	0.611171	NOT
FAM129A	509.9573	0.28583189	0.315497	0.905972729	0.36495	0.611171	NOT
RP11-279(	10.58154	-0.3908873	0.431483	-0.90591633	0.36498	0.611171	NOT
HOXC4	28.87603	0.5200104	0.574028	0.905896941	0.36499	0.611171	NOT
TPTE2P1	4.365704	0.39733124	0.438712	0.905676713	0.365107	0.611328	NOT
SMCHD1	1365.017	0.17260878	0.190605	0.905584161	0.365156	0.611373	NOT
ALDH1L1-A	94.87097	-0.4509052	0.497995	-0.90544083	0.365232	0.611462	NOT

RP11-164(	1.459163	-0.5556058	0.613684	-0.9053618	0.365274	0.611494	NOT
TERF2	1216.324	-0.1138212	0.125735	-0.90524931	0.365333	0.611504	NOT
PDIA5	2770.137	-0.1742298	0.192472	-0.90521983	0.365349	0.611504	NOT
C4orf32	581.2264	-0.220554	0.243665	-0.90515321	0.365384	0.611504	NOT
FAM13A	967.4468	-0.3570594	0.394475	-0.90515183	0.365385	0.611504	NOT
RASAL3	337.9896	-0.2667797	0.294739	-0.90513822	0.365392	0.611504	NOT
RP11-94I2	7.481216	0.5134081	0.567251	0.905081746	0.365422	0.611516	NOT
RP11-481J	38.77631	-0.1797545	0.198619	-0.90502187	0.365454	0.611532	NOT
MAPT	541.1391	0.36436225	0.402637	0.904940024	0.365497	0.611559	NOT
OTUD1	519.4936	0.15563795	0.171993	0.904906168	0.365515	0.611559	NOT
GTF3C1	3130.851	-0.1069255	0.118195	-0.90465432	0.365649	0.611744	NOT
OGFOD3	1923.384	-0.1838464	0.203263	-0.90447755	0.365742	0.611863	NOT
NCAM1	74.68567	0.45053613	0.498158	0.904404212	0.365781	0.611891	NOT
RP11-274E	219.262	-0.1885993	0.208555	-0.90431267	0.36583	0.611934	NOT
MKRN9P	1.865897	0.88688598	0.980862	0.904190654	0.365894	0.612004	NOT
BNIP3P1	18.21471	0.2934667	0.324644	0.903965409	0.366014	0.612167	NOT
INPP5A	891.4768	-0.1238723	0.137046	-0.90387702	0.366061	0.612198	NOT
SENP2	1462.629	-0.1193221	0.132016	-0.9038446	0.366078	0.612198	NOT
AGRP	4.663312	-0.4346503	0.480941	-0.90374893	0.366129	0.612239	NOT
DPH1	672.1684	-0.1502783	0.16629	-0.90371325	0.366147	0.612239	NOT
MIR4740	3.251623	0.36148855	0.400087	0.903525148	0.366247	0.612339	NOT
APLN	482.1098	-0.2988406	0.330753	-0.90351525	0.366252	0.612339	NOT
SNX12	1903.487	0.14620978	0.161849	0.903373081	0.366328	0.612428	NOT
LINC0024	2.335699	0.4931146	0.545885	0.903329975	0.366351	0.612428	NOT
CCDC101	929.7272	0.19201519	0.212604	0.903159286	0.366441	0.612542	NOT
CHRM3	225.7641	-0.4550282	0.503855	-0.90309352	0.366476	0.612562	NOT
GDF9	33.229	-0.2335901	0.258724	-0.90285392	0.366603	0.612737	NOT
SNORD56	2.5136	-0.3537616	0.391856	-0.90278387	0.366641	0.612741	NOT
AP001476.	5.480419	0.67762084	0.750617	0.902751892	0.366658	0.612741	NOT
RP11-426(	12.3352	-0.5996487	0.664291	-0.90268964	0.366691	0.612741	NOT
HCK	502.4859	0.30087522	0.333321	0.902659325	0.366707	0.612741	NOT
GPRASP2	431.436	-0.247457	0.274149	-0.90263666	0.366719	0.612741	NOT
ITGB1BP2	38.43256	0.29465681	0.326471	0.902551346	0.366764	0.612779	NOT
DENND1C	839.7196	-0.2112585	0.234084	-0.90249128	0.366796	0.612795	NOT
DXO	985.0605	0.16713075	0.185255	0.902164322	0.36697	0.61304	NOT
DPH7	686.4784	0.19237503	0.213245	0.902129719	0.366988	0.61304	NOT
TMEM9B-AS	60.33269	-0.2229606	0.247183	-0.90200488	0.367054	0.613113	NOT
ADAMTS15	79.24614	-0.3444079	0.381859	-0.90192352	0.367097	0.61313	NOT
CTD-2033I	2.901599	-0.4815398	0.53394	-0.90186068	0.367131	0.61313	NOT
ZNF2	177.1227	0.11846068	0.131352	0.901857811	0.367132	0.61313	NOT
ADAMTS7P3	4.60557	-0.3081696	0.341747	-0.90174841	0.367191	0.61319	NOT
AP000223.	5.617709	-0.3245135	0.359916	-0.901637	0.36725	0.613251	NOT
ZNF767P	256.4091	-0.1778457	0.197332	-0.90124962	0.367456	0.613557	NOT
MIPOL1	181.1631	0.22705181	0.251968	0.901113207	0.367528	0.61364	NOT
RP11-76P2	2.156645	-0.3749017	0.416164	-0.90085083	0.367668	0.613789	NOT
LAMC2	264.1014	0.54767407	0.607964	0.900833706	0.367677	0.613789	NOT
ZNF99	31.49677	-0.6296741	0.699033	-0.9007791	0.367706	0.613789	NOT
HDDC2	852.9406	0.20366787	0.226103	0.900775329	0.367708	0.613789	NOT
SEMA3F-AS	33.38525	-0.235922	0.261934	-0.9006942	0.367751	0.613813	NOT

FLJ43681	2.322066	-0.6062467	0.673111	-0.90066364	0.367767	0.613813	NOT
RP1-136B1	6.744224	0.32441205	0.36024	0.900544344	0.367831	0.613881	NOT
CTD-2376I	7.48652	-0.2518395	0.279696	-0.90040589	0.367904	0.613904	NOT
XXbac-B44	8.179466	-0.3442092	0.382285	-0.90039869	0.367908	0.613904	NOT
ESPN	4062.187	-0.3384687	0.375913	-0.90039088	0.367912	0.613904	NOT
KLHDC8B	1282.93	0.24574998	0.272964	0.900303372	0.367959	0.613944	NOT
TDRD7	802.1537	0.15914164	0.176782	0.900212016	0.368007	0.613987	NOT
CCL11	18.79693	0.58893519	0.654301	0.900098002	0.368068	0.61405	NOT
FOXCUT	1.374779	-0.7661316	0.851206	-0.90005422	0.368091	NA	NA
SKAP2	1168.323	0.23796777	0.264417	0.899972185	0.368135	0.614108	NOT
FKBP11	3269.668	0.27884445	0.309845	0.899947485	0.368148	0.614108	NOT
SLC22A18A	350.7499	-0.364051	0.404557	-0.89987496	0.368187	0.614133	NOT
ASTN1	25.13303	-0.4429455	0.492266	-0.89980851	0.368222	0.614133	NOT
LINC00441	6.987943	-0.2788719	0.309929	-0.89979282	0.368231	0.614133	NOT
KIAA0895	372.1291	0.29263754	0.325264	0.899692762	0.368284	0.614151	NOT
RP11-748I	1.670127	-0.4988783	0.554502	-0.89968731	0.368287	0.614151	NOT
SEPT6	2824.03	-0.2430552	0.270191	-0.89956724	0.368351	0.614179	NOT
SLC29A3	674.2942	0.15361705	0.170777	0.899520358	0.368376	0.614179	NOT
RNA5SP301	3.007138	0.48280468	0.536737	0.899518135	0.368377	0.614179	NOT
SLC29A1	6197.573	0.24754826	0.275211	0.899484784	0.368394	0.614179	NOT
HOOK1	1717.98	-0.2563897	0.285084	-0.89934911	0.368467	0.61424	NOT
KCNG2	7.860134	-0.3232905	0.359479	-0.89933146	0.368476	0.61424	NOT
TRAPPC6B	1012.703	-0.120348	0.133833	-0.8992437	0.368523	0.614246	NOT
C1orf101	41.88242	-0.2419823	0.269097	-0.89923933	0.368525	0.614246	NOT
RP11-424M	2.014986	0.52482382	0.583666	0.899185709	0.368554	0.614256	NOT
NF1	2434.871	-0.1446778	0.160931	-0.89900666	0.368649	0.61436	NOT
RP3-393E1	2.751617	0.44941526	0.499937	0.89894356	0.368683	0.61436	NOT
RP11-378C	4.274202	0.41342579	0.459903	0.898941457	0.368684	0.61436	NOT
RSAD2	238.2597	0.30287229	0.336992	0.898753003	0.368784	0.614485	NOT
RP11-632I	26.31918	-0.2945462	0.327742	-0.89871503	0.368804	0.614485	NOT
RP11-723C	36.57667	0.3544054	0.394464	0.898447545	0.368947	0.614647	NOT
CPXM1	203.2218	0.38219248	0.425399	0.898432693	0.368955	0.614647	NOT
RBM34	78.93492	0.17289432	0.192446	0.898405757	0.368969	0.614647	NOT
C11orf74	395.8316	0.14712346	0.163796	0.898212638	0.369072	0.614759	NOT
KRTAP5-6	24.81921	-0.4516571	0.50285	-0.89819461	0.369082	0.614759	NOT
AB019441.	107.7953	0.32538096	0.362348	0.897978346	0.369197	0.61486	NOT
RP11-659E	15.06501	-0.477609	0.531877	-0.89796936	0.369202	0.61486	NOT
FOXF2	21.80244	0.34463201	0.383797	0.897953091	0.369211	0.61486	NOT
PIGQ	2101.43	-0.1770965	0.197282	-0.89768195	0.369355	0.615052	NOT
NAV2-AS3	2.279934	-0.6488405	0.72282	-0.89765162	0.369371	0.615052	NOT
NOL4	22.69001	0.82575185	0.919995	0.8975616	0.369419	0.61508	NOT
RP11-212I	8.248719	0.58934705	0.65664	0.897519687	0.369442	0.61508	NOT
RP11-496N	1.855114	0.42838949	0.477324	0.897481287	0.369462	0.61508	NOT
VANGL1	683.6289	0.20347884	0.22673	0.897450075	0.369479	0.61508	NOT
SAMD9L	473.0311	0.3137835	0.349701	0.897290365	0.369564	0.615156	NOT
AC000089.	39.81077	-0.3403114	0.379278	-0.8972616	0.369579	0.615156	NOT
RP11-259K	143.0254	-0.2192438	0.244358	-0.89722219	0.3696	0.615156	NOT
TEP1	918.418	0.16167247	0.180198	0.897194526	0.369615	0.615156	NOT
MPG	1136.589	-0.1553108	0.173139	-0.89703116	0.369702	0.615263	NOT

COX14	2085.752	-0.1901512	0.212037	-0.89678163	0.369835	0.615437	NOT
IGBP1	1831.713	0.13886602	0.15486	0.89671762	0.36987	0.615437	NOT
PGM3	2158.226	0.19756435	0.220322	0.896708272	0.369875	0.615437	NOT
DHX8	2076.06	0.11506801	0.128334	0.896629947	0.369916	0.615469	NOT
SSFA2	4140.879	-0.1802919	0.201136	-0.89636949	0.370055	0.615662	NOT
GSN-AS1	5.327786	-0.2939495	0.327979	-0.89624443	0.370122	0.615716	NOT
AC102953.	7.514878	0.34516453	0.385144	0.89619689	0.370148	0.615716	NOT
FOXE1	3.553446	0.94314642	1.052405	0.896181874	0.370156	0.615716	NOT
DICER1	1984.52	-0.1826402	0.203826	-0.8960592	0.370221	0.615757	NOT
RNF2P1	11.93378	0.33234451	0.370915	0.896013741	0.370245	0.615757	NOT
RPS15AP24	1.973602	-0.3440817	0.384033	-0.89596833	0.37027	0.615757	NOT
AC004156.	8.004513	-0.3187689	0.355782	-0.89596587	0.370271	0.615757	NOT
CTD-2555A	1.783402	-0.3834745	0.428038	-0.89588927	0.370312	0.615787	NOT
TRIM14	1878.756	-0.1742236	0.19448	-0.89584544	0.370335	0.615788	NOT
MRPS34	3651.764	-0.1968893	0.219813	-0.89571467	0.370405	0.615866	NOT
RP11-23J9	26.39047	0.33847356	0.37791	0.895645498	0.370442	0.61589	NOT
RP11-215F	1.858387	-0.3545062	0.395949	-0.89533405	0.370609	0.61611	NOT
RP11-514O	9.597759	0.33536829	0.374582	0.895312823	0.37062	0.61611	NOT
FZD9	32.95914	0.59346923	0.662987	0.895144704	0.37071	0.616222	NOT
SYDE1	493.2789	-0.2521983	0.281774	-0.89503791	0.370767	0.616249	NOT
ZNF718	176.7212	0.22120724	0.247151	0.895029293	0.370771	0.616249	NOT
LINC00278	6.657843	-0.5800189	0.648163	-0.89486527	0.370859	0.61632	NOT
RP11-48B3	50.16432	0.20818006	0.232639	0.894863923	0.37086	0.61632	NOT
RP11-27N2	17.96183	0.35583306	0.397764	0.894582575	0.37101	0.616532	NOT
RP11-417I	18.92901	0.29333009	0.327912	0.894537727	0.371034	0.616535	NOT
FANCC	880.8032	-0.2325212	0.259959	-0.89445392	0.371079	0.616551	NOT
NKX2-2	2.045128	-0.946679	1.058519	-0.89434317	0.371138	0.616551	NOT
RP11-43F1	15.18458	0.53050977	0.593195	0.894326096	0.371147	0.616551	NOT
DBF4P1	2.608949	0.32713787	0.365811	0.894281271	0.371171	0.616551	NOT
CTB-40H15	3.542174	0.45216573	0.505623	0.894273953	0.371175	0.616551	NOT
RP11-66B2	1.946008	0.68386692	0.764774	0.894207357	0.371211	0.616551	NOT
CTC-510F1	2.446651	-0.3688494	0.412499	-0.89418227	0.371224	0.616551	NOT
RP11-296O	57.8275	0.21557928	0.241092	0.894179785	0.371226	0.616551	NOT
GRK6P1	2.52768	0.34748153	0.388654	0.894063142	0.371288	0.616616	NOT
GATA2-AS1	54.14568	0.45339869	0.507178	0.893963008	0.371342	0.616668	NOT
TMOD2	312.4581	-0.2598398	0.290713	-0.893803	0.371427	0.616772	NOT
RP11-161M	4.8488	0.31494187	0.352479	0.893505495	0.371587	0.616977	NOT
PRAMEF8	2.265465	-0.7257099	0.812222	-0.89348655	0.371597	0.616977	NOT
FAM83B	3.990189	0.9482874	1.061412	0.89342044	0.371632	0.616977	NOT
NUS1P2	1.96906	1.05230683	1.177895	0.893379461	0.371654	0.616977	NOT
ATP5HP4	12.28528	0.32659595	0.365596	0.893325761	0.371683	0.616977	NOT
RP4-545C2	13.48582	0.35759978	0.400305	0.893317488	0.371687	0.616977	NOT
GSTM4	1917.448	-0.2487019	0.278427	-0.89323966	0.371729	0.616982	NOT
RP11-428J	9.216466	-0.215219	0.240946	-0.89322446	0.371737	0.616982	NOT
C3orf22	2.878353	-0.4076222	0.456369	-0.89318473	0.371758	0.616982	NOT
RP11-798K	781.3014	-0.3514556	0.393525	-0.89309618	0.371806	0.617023	NOT
FBXW4	2323.584	-0.1669266	0.186924	-0.89301827	0.371847	0.617054	NOT
RP11-932O	8.742912	0.34168499	0.382703	0.89281931	0.371954	0.617193	NOT
FARP2	852.9841	0.14982702	0.167857	0.892586527	0.372079	0.617348	NOT

GJB4	3.977043	-0.624818	0.700029	-0.89256054	0.372093	0.617348	NOT
TRAF3IP2	617.2602	0.20769457	0.232711	0.892499522	0.372125	0.617364	NOT
CTD-2325M	23.51036	-0.2726601	0.305523	-0.89243592	0.372159	0.617383	NOT
GAS5-AS1	48.72972	-0.199673	0.223774	-0.89229629	0.372234	0.617442	NOT
ZNF639	985.8005	0.10687877	0.119786	0.892245618	0.372261	0.617442	NOT
CLYBL	1542.71	-0.2583692	0.28958	-0.89222087	0.372275	0.617442	NOT
AP003900.	6.077276	-0.9877526	1.107099	-0.89219926	0.372286	0.617442	NOT
ZNF835	42.83558	0.39733906	0.445394	0.892106281	0.372336	0.617445	NOT
ARHGEF10L	5070.882	-0.1765067	0.197857	-0.89209296	0.372343	0.617445	NOT
RP13-349(	2.187111	0.48341013	0.541898	0.892069205	0.372356	0.617445	NOT
RP11-767N	6.922389	-0.3214149	0.360374	-0.89189234	0.372451	0.617564	NOT
AC005037.	5.680107	0.35889752	0.402471	0.891735579	0.372535	0.617619	NOT
ATP6V0C	1564.239	0.13280044	0.14893	0.891695636	0.372556	0.617619	NOT
LA16c-361	3.059426	-0.3661858	0.410697	-0.891621	0.372596	0.617619	NOT
BDH2P1	2.196625	0.46713335	0.523948	0.891564012	0.372627	0.617619	NOT
TPRXL	17.45475	0.62776385	0.704117	0.891561363	0.372628	0.617619	NOT
RP11-680H	4.47077	0.6110757	0.685401	0.891559282	0.372629	0.617619	NOT
CDH7	2.540754	-0.7709106	0.864702	-0.89153376	0.372643	0.617619	NOT
PET100	1355.604	-0.2637857	0.295934	-0.89136748	0.372732	0.617719	NOT
ID2-AS1	56.80662	-0.3137154	0.351961	-0.89133624	0.372749	0.617719	NOT
ZNF92P3	4.950305	-0.4640519	0.520702	-0.89120403	0.37282	0.617799	NOT
PPBP	2.659674	-0.472437	0.530137	-0.89116103	0.372843	0.617799	NOT
RP11-119E	15.44527	-0.2256366	0.253225	-0.89105073	0.372902	0.617845	NOT
RP11-710F	1.519235	0.79128144	0.888058	0.891024274	0.372916	0.617845	NOT
FOXH1	5.839576	0.41722907	0.468379	0.890793023	0.37304	0.618012	NOT
CREB3L4	871.4525	0.19232472	0.215916	0.890739252	0.373069	0.618012	NOT
LMF2	5417.458	0.15316809	0.171962	0.890709141	0.373085	0.618012	NOT
HEXA	4476.373	-0.1993885	0.223877	-0.89061656	0.373135	0.618057	NOT
CPN1	2250.81	-0.374148	0.420152	-0.89050646	0.373194	0.618085	NOT
RP5-901A4	8.687483	0.28194063	0.316609	0.890500295	0.373197	0.618085	NOT
USP3	1237.199	-0.1162778	0.1306	-0.89033415	0.373286	0.618103	NOT
CLEC2B	250.136	-0.2594719	0.291439	-0.89031291	0.373298	0.618103	NOT
SLAIN2	1925.933	-0.1165297	0.130889	-0.8902944	0.373308	0.618103	NOT
FBXO18	2121.364	0.13504036	0.151683	0.890282786	0.373314	0.618103	NOT
SHISA4	864.326	0.34812191	0.391048	0.890227826	0.373344	0.618103	NOT
AC010084.	1.815148	-0.6206321	0.697163	-0.89022494	0.373345	0.618103	NOT
GPR124	726.6273	-0.3028505	0.340315	-0.88991176	0.373513	0.618344	NOT
NAGA	3076.449	0.19012667	0.213665	0.889834403	0.373555	0.618375	NOT
AGPAT4	251.6701	0.31456712	0.353562	0.889708791	0.373622	0.618424	NOT
UBA52P5	3.053725	-0.2999499	0.337139	-0.88969089	0.373632	0.618424	NOT
CYP4F29P	4.363699	0.59227055	0.665733	0.889651951	0.373653	0.618424	NOT
CCDC12	1654.144	0.18657723	0.209754	0.88950668	0.373731	0.618452	NOT
TUBBP10	5.042228	-0.3756931	0.422377	-0.8894725	0.373749	0.618452	NOT
ING4	1086.049	0.14306822	0.160855	0.889426091	0.373774	0.618452	NOT
OSM	47.78756	0.40016209	0.449937	0.88937315	0.373803	0.618452	NOT
PIK3R2	78.96627	-0.3074218	0.345668	-0.88935572	0.373812	0.618452	NOT
HRASLS5	5.091736	-0.5263873	0.591879	-0.88935007	0.373815	0.618452	NOT
RP11-714(	2.169497	-0.3829449	0.430603	-0.88932326	0.373829	0.618452	NOT
RP11-345K	1.681231	1.19680906	1.345839	0.889266125	0.37386	0.618465	NOT

GNG11	936.104	-0.1880636	0.211528	-0.8890713	0.373965	0.6186	NOT
BNIP3P27	1.484748	0.60072447	0.675737	0.888992038	0.374007	0.618633	NOT
MAPK14	3441.542	0.13254345	0.14914	0.888720551	0.374153	0.618837	NOT
RP11-177J	3.325106	-0.4090225	0.460286	-0.888627	0.374204	0.618865	NOT
ASB14	27.37487	0.21367449	0.240461	0.888604031	0.374216	0.618865	NOT
TMEM182	130.2081	0.25235264	0.284035	0.888457201	0.374295	0.618957	NOT
ATRIP	3.247118	-0.2596658	0.292284	-0.8884026	0.374324	0.618957	NOT
CLDN3	2020.523	0.34433824	0.387606	0.88837279	0.37434	0.618957	NOT
EMR3	6.680315	0.46684701	0.52555	0.888301907	0.374378	0.618969	NOT
RP11-282I	1.661272	0.7072884	0.796274	0.888247625	0.374408	0.618969	NOT
PSMD8P1	2.546513	-0.358803	0.403952	-0.88823275	0.374416	0.618969	NOT
RP11-85I1	3.228632	0.44711438	0.503512	0.887991871	0.374545	0.619145	NOT
BBS1	46.98184	-0.1611015	0.181435	-0.88793033	0.374578	0.619148	NOT
DCAF4L1	23.57436	0.34929001	0.393387	0.887904308	0.374592	0.619148	NOT
FOXS1	178.128	0.3780165	0.425806	0.887767413	0.374666	0.619197	NOT
LRRCC1	353.9081	0.2596292	0.292453	0.887763868	0.374668	0.619197	NOT
CCL2	786.3688	-0.3777252	0.425531	-0.88765658	0.374726	0.619253	NOT
NUTM2HP	2.117327	-0.3634527	0.409472	-0.887614	0.374748	0.619253	NOT
FAM156A	4.499763	0.28844967	0.324987	0.887573466	0.37477	0.619253	NOT
ST3GAL4	1341.285	-0.231775	0.261165	-0.88746675	0.374828	0.619266	NOT
DHX29	1694.049	-0.1293388	0.14574	-0.88746049	0.374831	0.619266	NOT
KLHL9	1141.77	-0.1796464	0.20244	-0.88740759	0.37486	0.619266	NOT
ARL14	34.0055	0.53025166	0.597554	0.887369772	0.37488	0.619266	NOT
CSF1	2219.084	0.28747611	0.323972	0.887347276	0.374892	0.619266	NOT
INPP1	967.8582	0.20508408	0.231207	0.887016665	0.37507	0.619522	NOT
RP1-267D1	63.08702	0.21195779	0.238989	0.886893761	0.375136	0.619582	NOT
AC000123.	74.80681	-0.1959512	0.220949	-0.88686383	0.375152	0.619582	NOT
TTLL9	4.050154	0.31353311	0.353604	0.886679398	0.375252	0.619709	NOT
CTC-359D2	47.68272	0.27645297	0.311812	0.88660172	0.375293	0.619725	NOT
RP11-884K	184.2539	0.15751202	0.177663	0.886576724	0.375307	0.619725	NOT
H3F3AP4	116.0922	0.21005108	0.236971	0.88639906	0.375403	0.619845	NOT
XX-C2158C	9.963791	0.64620772	0.729101	0.886307459	0.375452	0.619889	NOT
RP11-516A	3.757958	0.40797822	0.460407	0.886125215	0.37555	0.620013	NOT
ATP5J2	6524.507	-0.2145475	0.242141	-0.88604519	0.375593	0.62003	NOT
PTP4A2	6396.251	0.1170466	0.132104	0.886021429	0.375606	0.62003	NOT
RP11-676J	16.72477	0.54290821	0.612811	0.885930556	0.375655	0.620073	NOT
FOXP2	135.5691	-0.5733549	0.647218	-0.88587653	0.375684	0.620083	NOT
RP11-26J3	53.80041	0.2298425	0.259471	0.885812011	0.375719	0.620103	NOT
RP1-193H1	207.0849	-0.286828	0.323864	-0.88564386	0.375809	0.620185	NOT
CES1P2	10.44285	0.46831823	0.528797	0.885630218	0.375817	0.620185	NOT
TNPO1	3544.531	0.13004961	0.14685	0.885592752	0.375837	0.620185	NOT
RP11-753E	9.046972	-0.5105916	0.576636	-0.88546677	0.375905	0.620259	NOT
HIST1H2BN	123.3041	-0.2935166	0.331592	-0.88517293	0.376063	0.620483	NOT
RP11-1094	7.13042	-0.409868	0.463118	-0.88501923	0.376146	0.620573	NOT
AC091729.	305.7295	0.22266264	0.2516	0.884987163	0.376164	0.620573	NOT
IFIT2	1118.475	-0.3006056	0.339699	-0.88491666	0.376202	0.620598	NOT
CTD-2154I	2.676793	-0.3727433	0.42125	-0.88485078	0.376237	0.620619	NOT
LRRN2	316.8033	-0.4233371	0.478466	-0.88478045	0.376275	0.620643	NOT
LAP3	6260.068	-0.2368167	0.267696	-0.88464866	0.376346	0.620723	NOT

NFKBIB	1687.477	-0.1655204	0.187113	-0.88460247	0.376371	0.620726	NOT
PIGT	7587.379	0.13964825	0.157892	0.884455073	0.376451	0.620786	NOT
RP11-422I	66.31423	-0.1833628	0.207318	-0.88445068	0.376453	0.620786	NOT
HIST1H2AI	132.6053	-0.4067413	0.459926	-0.88436326	0.3765	0.620804	NOT
RP11-121	42.92643	-0.3218008	0.363892	-0.88432969	0.376518	0.620804	NOT
PAF1	3456.311	-0.1259894	0.142473	-0.88430276	0.376533	0.620804	NOT
GDAP2	593.4352	0.1424854	0.161154	0.8841554	0.376612	0.620898	NOT
MAK	13.91187	0.26340322	0.297977	0.883971396	0.376712	0.620996	NOT
CBX6	1423.47	0.29837339	0.337564	0.883900497	0.37675	0.620996	NOT
PHLPP2	501.9325	-0.2193085	0.24812	-0.88387947	0.376761	0.620996	NOT
AGA	1017.936	-0.1922445	0.217508	-0.88384918	0.376778	0.620996	NOT
ABHD12	3926.44	0.15187509	0.171849	0.883772743	0.376819	0.620996	NOT
MIR4635	4.372773	-0.3930437	0.444756	-0.88372932	0.376842	0.620996	NOT
RP11-151	5.244248	-0.4238479	0.479615	-0.88372584	0.376844	0.620996	NOT
BHLHA9	1.752193	0.93717252	1.060546	0.883669643	0.376875	0.620996	NOT
LILRA5	62.32501	-0.3143847	0.355774	-0.88366371	0.376878	0.620996	NOT
UGT2A3P7	7.961907	-0.4486555	0.507847	-0.88344638	0.376995	0.621151	NOT
MUC21	1.801747	0.89877212	1.017496	0.883317432	0.377065	0.621228	NOT
RP11-583I	3.194406	0.39338281	0.445374	0.883263992	0.377094	0.621232	NOT
RP3-426I6	2.373688	0.31151475	0.352703	0.883221699	0.377116	0.621232	NOT
ACTRT3	96.17411	-0.2017408	0.22843	-0.88316055	0.37715	0.621232	NOT
CTD-3131	33.19611	-0.2200645	0.249183	-0.8831438	0.377159	0.621232	NOT
ERICH6	5.568015	-0.2761421	0.312874	-0.88259887	0.377453	0.621679	NOT
WNT1	1.349471	-0.539368	0.611171	-0.88251549	0.377498	NA	NA
PLEKHG3	1856.078	-0.1467267	0.166273	-0.88244455	0.377536	0.621779	NOT
ZNF608	329.9322	0.24636896	0.27922	0.882347374	0.377589	0.621828	NOT
CXCR1	13.21101	0.4059952	0.4602	0.882214883	0.377661	0.621908	NOT
CA14	372.1736	-0.4346347	0.492985	-0.88163871	0.377972	0.622339	NOT
POLK	724.0467	-0.1763383	0.200018	-0.88161376	0.377986	0.622339	NOT
RP11-619	2.321363	-0.6641871	0.753385	-0.88160344	0.377991	0.622339	NOT
RP11-667	17.23938	-0.2263177	0.256822	-0.88122486	0.378196	0.622589	NOT
LRRC56	73.60477	0.25258398	0.286638	0.881196036	0.378212	0.622589	NOT
RP11-552	1.930823	0.96835378	1.098919	0.881187604	0.378216	0.622589	NOT
SULF2	2987.947	-0.3262926	0.370302	-0.88115351	0.378235	0.622589	NOT
KPNA6	2519.019	-0.1283472	0.145682	-0.88100713	0.378314	0.622681	NOT
ANKS3	604.3099	0.1881651	0.213621	0.880835071	0.378407	0.622797	NOT
RPS5	23772.3	0.21622388	0.245554	0.880554006	0.378559	0.62301	NOT
CEACAM1	3828.369	-0.2684434	0.304925	-0.88035865	0.378665	0.623085	NOT
FREM1	47.18385	0.59750651	0.678716	0.880348711	0.37867	0.623085	NOT
DOCK11	305.254	-0.2972758	0.337693	-0.88031291	0.37869	0.623085	NOT
ZNF433	123.1888	-0.2165747	0.246041	-0.88023775	0.378731	0.623085	NOT
IPO9-AS1	7.64038	0.29810246	0.338662	0.880236017	0.378731	0.623085	NOT
SLCO1B7	4.052484	-0.718917	0.816797	-0.88016556	0.37877	0.623085	NOT
RPL12P8	3.10564	0.34518625	0.392189	0.880152302	0.378777	0.623085	NOT
RNF185	2286.29	-0.1298181	0.147499	-0.88012972	0.378789	0.623085	NOT
RP1-93H1	2.579645	-0.3947495	0.448684	-0.87979468	0.378971	0.623346	NOT
TCTE3	70.86861	0.21534376	0.244813	0.879626751	0.379062	0.623457	NOT
RCCD1	632.9835	0.13330127	0.151553	0.879570358	0.379092	0.623457	NOT
CXorf56	809.4919	0.13464932	0.15309	0.879543232	0.379107	0.623457	NOT

MMP24	228.3579	0.40393645	0.45934	0.87938415	0.379193	0.623535	NOT
RP11-368N	9.357199	-0.2030933	0.230962	-0.87933789	0.379218	0.623535	NOT
CTDSPL2	924.6837	0.13088599	0.148856	0.879279488	0.37925	0.623535	NOT
LINC0052(	1.58395	0.79141613	0.900128	0.879226166	0.379279	0.623535	NOT
AKR1E2	25.53079	0.41750114	0.474886	0.879160653	0.379314	0.623535	NOT
YTHDF2	2475.636	0.09079761	0.103279	0.879151635	0.379319	0.623535	NOT
CCDC183-A	62.61575	-0.2540247	0.288946	-0.87914199	0.379324	0.623535	NOT
SNRNP27	908.2317	0.10264477	0.116763	0.879083105	0.379356	0.623535	NOT
SLC25A13	5679.256	-0.2078011	0.236393	-0.87904795	0.379375	0.623535	NOT
RP5-827C2	3.918895	0.56773024	0.645858	0.879032128	0.379384	0.623535	NOT
HERC2P3	75.00151	0.41285053	0.469697	0.878971842	0.379417	0.62355	NOT
RP11-598F	39.48013	-0.2942734	0.334857	-0.87880306	0.379508	0.623637	NOT
ZNF43	238.1185	0.32932042	0.374758	0.878754318	0.379535	0.623637	NOT
FAR2P2	6.535207	-0.5909802	0.672525	-0.87874804	0.379538	0.623637	NOT
POU2F3	30.58654	-0.4867703	0.553966	-0.87870017	0.379564	0.623641	NOT
RP11-359M	1.775734	-0.6593689	0.750469	-0.87860884	0.379613	0.623685	NOT
HNRNPUL1	8392.103	0.12783511	0.145509	0.878536	0.379653	0.623712	NOT
ALCAM	5677.721	0.20451368	0.232825	0.878401487	0.379726	0.623794	NOT
LINC00371	1.211994	0.68973612	0.785275	0.878337167	0.379761	NA	NA
CRIP2	2799.561	-0.2552821	0.290696	-0.87817657	0.379848	0.623957	NOT
SCN1A	8.316663	0.88431716	1.007174	0.878018178	0.379934	0.62406	NOT
MTHFD2	344.7648	0.29237241	0.333091	0.877754487	0.380077	0.624195	NOT
DDO	548.9576	0.22632457	0.257852	0.877730115	0.38009	0.624195	NOT
CTD-2270N	19.28542	0.29839098	0.339961	0.877722305	0.380094	0.624195	NOT
RP11-229D	6.630413	0.32823011	0.373967	0.877697645	0.380108	0.624195	NOT
RP11-467D	30.16893	-0.1984294	0.2261	-0.8776186	0.380151	0.624228	NOT
HABP2	17204.23	-0.3969906	0.452408	-0.87750542	0.380212	0.624291	NOT
RP11-177(	30.09357	-0.4085867	0.465657	-0.87744154	0.380247	0.62431	NOT
RP11-256I	0.755842	-1.4845327	1.692085	-0.87733951	0.380302	NA	NA
ARPC4	4939.873	0.17629181	0.200972	0.877193735	0.380381	0.624493	NOT
TOB2P1	36.75851	-0.3059329	0.348802	-0.87709521	0.380435	0.624543	NOT
RP13-104F	110.1878	-0.224706	0.256209	-0.87704184	0.380464	0.624553	NOT
DLG5-AS1	35.24855	0.32250068	0.367792	0.876855618	0.380565	0.624681	NOT
MSX2	5.404378	0.52887562	0.603198	0.876786047	0.380603	0.624705	NOT
AC007279.	2.610004	0.3789151	0.432257	0.876597143	0.380705	0.624822	NOT
TOR2A	723.3444	0.12832473	0.146394	0.876570582	0.38072	0.624822	NOT
NPSR1-AS1	67.3565	0.59037667	0.673626	0.87641588	0.380804	0.624922	NOT
IL18	246.9912	0.33017555	0.376792	0.876280102	0.380878	0.625005	NOT
LINC00616	1.131161	1.01045577	1.153203	0.876216496	0.380912	NA	NA
RANGRF	388.8425	0.28752464	0.328247	0.87594017	0.381063	0.625251	NOT
PFKM	857.759	-0.2344588	0.267682	-0.87588443	0.381093	0.625251	NOT
LINC01515	78.49728	0.26073314	0.297682	0.875877408	0.381097	0.625251	NOT
TLE1	4134.307	0.1427379	0.162998	0.875703604	0.381191	0.625368	NOT
PGAM1P5	1.519853	0.46850281	0.535155	0.875453349	0.381327	0.625554	NOT
CTA-280A3	1.804738	0.78088558	0.892105	0.875328788	0.381395	0.625572	NOT
GRIK1	10.30098	-0.2839936	0.32445	-0.87530795	0.381406	0.625572	NOT
HDGFRP3	301.5491	0.2999722	0.342708	0.875299791	0.381411	0.625572	NOT
TMEM178A	98.64046	-0.3636971	0.415529	-0.87526381	0.38143	0.625572	NOT
ZNF570	197.0142	-0.1960952	0.224076	-0.87512753	0.381505	0.625655	NOT

LINC00184	1.55593	0.56377897	0.644319	0.875000429	0.381574	0.625724	NOT
SAMD4A	971.2571	-0.2167346	0.247706	-0.87496588	0.381592	0.625724	NOT
HIST1H2BC	13.55041	-0.3969817	0.453854	-0.87469025	0.381742	0.625932	NOT
ATF1	714.6524	0.11909418	0.136189	0.874478234	0.381858	0.626084	NOT
SEPT5	490.1471	0.28944884	0.331016	0.87442665	0.381886	0.626092	NOT
MAGEA1	569.8566	0.80672144	0.92296	0.874059218	0.382086	0.626382	NOT
HYAL2	2515.021	0.14711719	0.16835	0.87387771	0.382185	0.626496	NOT
ZNF461	127.3865	-0.1581134	0.18094	-0.87384617	0.382202	0.626496	NOT
MIR143HG	20.65725	-0.2598681	0.297415	-0.87375433	0.382252	0.62654	NOT
PGAM1P7	2.162921	0.55111984	0.630819	0.873657609	0.382305	0.626552	NOT
NTRK2	74.84981	-0.4873589	0.557838	-0.8736562	0.382306	0.626552	NOT
FAM26F	172.7685	0.33307971	0.381411	0.873282429	0.382509	0.62683	NOT
RP11-305M	20.36108	0.39629627	0.453812	0.87326003	0.382521	0.62683	NOT
SYF2	1770.002	0.1129438	0.129343	0.873212349	0.382547	0.626835	NOT
GABRR2	5.924002	-0.3426806	0.392507	-0.87305668	0.382632	0.626913	NOT
RP11-572M	2.952962	-0.3919315	0.44896	-0.87297737	0.382675	0.626913	NOT
CFDP1	2202.413	0.14443624	0.165456	0.872956854	0.382687	0.626913	NOT
CDKN2B-AS	45.11965	0.34562972	0.395936	0.872943642	0.382694	0.626913	NOT
CTD-2591A	13.70071	0.77303888	0.885611	0.8728878	0.382724	0.626913	NOT
DNASE2B	9.082312	0.37538129	0.430053	0.872871229	0.382733	0.626913	NOT
IGHGP	218.7352	-0.5509517	0.631291	-0.87273769	0.382806	0.626994	NOT
CTD-2600C	11.42951	-0.2937197	0.336604	-0.87259657	0.382883	0.627082	NOT
RP11-345J	6.421578	-0.4259456	0.488198	-0.87248512	0.382944	0.62711	NOT
RGAG1	68.01125	-0.5491346	0.629411	-0.87245749	0.382959	0.62711	NOT
CCDC25	1645.622	-0.1952471	0.223795	-0.87243793	0.38297	0.62711	NOT
ADPRM	294.751	-0.1781723	0.204264	-0.87226549	0.383064	0.627226	NOT
PRDM7	8.330637	0.43897833	0.503309	0.872184832	0.383108	0.627245	NOT
C5orf58	59.22342	0.58956397	0.675999	0.872137923	0.383133	0.627245	NOT
CAV1	1949.453	-0.2277453	0.261141	-0.87211611	0.383145	0.627245	NOT
TJP1	3164.391	-0.1658385	0.19017	-0.87205467	0.383179	0.627245	NOT
BTG1	8704.79	0.20962122	0.240382	0.872032885	0.38319	0.627245	NOT
CTD-2161E	6.286846	0.32970242	0.378292	0.871555751	0.383451	0.627605	NOT
POLE	2069.137	-0.1607775	0.184484	-0.87149911	0.383482	0.627605	NOT
RP11-345F	2.158942	-0.3599162	0.412999	-0.87147078	0.383497	0.627605	NOT
THSD1	232.2221	-0.3139803	0.360298	-0.87144593	0.383511	0.627605	NOT
RP11-887F	3.441985	0.32164978	0.369111	0.871417426	0.383526	0.627605	NOT
EIF2S2P3	6.125393	-0.2559477	0.293754	-0.87129857	0.383591	0.627645	NOT
TBC1D29	2.292749	-0.4408224	0.505943	-0.87128862	0.383597	0.627645	NOT
QRICH2	295.1276	0.25626392	0.29419	0.871081748	0.38371	0.627792	NOT
CBL	672.0667	0.18753376	0.215365	0.87077172	0.383879	0.628009	NOT
HEXDC	1275.071	-0.1720589	0.197598	-0.87075391	0.383889	0.628009	NOT
AC093326.	1.295026	-0.6859033	0.787743	-0.87071918	0.383908	NA	NA
RP11-530C	9.970791	-0.2776682	0.318932	-0.87061837	0.383963	0.628074	NOT
AC005281.	2.030405	0.67511019	0.775457	0.870596158	0.383975	0.628074	NOT
RP11-977C	40.22602	-0.2340508	0.268875	-0.8704827	0.384037	0.628097	NOT
SMG1P2	35.75988	0.20624591	0.236936	0.870471795	0.384043	0.628097	NOT
KAZN	120.7443	-0.3419909	0.392893	-0.87044309	0.384058	0.628097	NOT
FBXW11	2184.438	-0.1397601	0.160578	-0.87035607	0.384106	0.628133	NOT
FNBP1	1859.251	-0.1306848	0.150158	-0.87031797	0.384127	0.628133	NOT

CTAGE15	7.353812	0.58539511	0.672744	0.870160894	0.384212	0.628236	NOT
H1F0	18367.04	0.19697503	0.226391	0.870067538	0.384263	0.62826	NOT
PADI3	11.27088	-0.7045268	0.809769	-0.8700345	0.384282	0.62826	NOT
ATP6V1G2	27.71133	0.32002195	0.367839	0.870006214	0.384297	0.62826	NOT
IGLC7	12.14411	-0.7343489	0.844206	-0.86986927	0.384372	0.628323	NOT
HRASLS	3.165787	0.54890274	0.63103	0.86985137	0.384382	0.628323	NOT
TRDN	1.118399	0.5836871	0.671106	0.869739403	0.384443	NA	NA
TLN1	12990.39	-0.1553255	0.178596	-0.86970432	0.384462	0.62835	NOT
EVPLL	13.09784	0.60780845	0.698868	0.869703718	0.384462	0.62835	NOT
ACSM1	1917.862	0.52330212	0.601708	0.869694136	0.384468	0.62835	NOT
AC078899.	3.432894	0.37059228	0.426144	0.869641009	0.384497	0.628359	NOT
MCUR1	2086.954	0.15736161	0.180971	0.869538434	0.384553	0.628412	NOT
RP11-461A	13.46747	0.28229523	0.324684	0.869445436	0.384604	0.628412	NOT
CMB9-55F2	128.2121	-0.3128516	0.359836	-0.86942933	0.384612	0.628412	NOT
NFYC	2367.99	0.10615533	0.1221	0.869412958	0.384621	0.628412	NOT
RP11-347E	18.68257	0.55456289	0.637922	0.869326778	0.384668	0.628451	NOT
FXYD5	1726.188	0.28108812	0.323405	0.869152112	0.384764	0.628569	NOT
RP11-474I	14.20811	-0.7593181	0.873931	-0.86885368	0.384927	0.628679	NOT
RP11-219I	5.436363	-0.2829096	0.325618	-0.86883942	0.384935	0.628679	NOT
FITM2	1039.429	-0.1923015	0.221335	-0.86882569	0.384942	0.628679	NOT
MADCAM1	37.02943	-0.2958509	0.34052	-0.86882185	0.384945	0.628679	NOT
RRNAD1	1805.862	0.1501192	0.172786	0.868817424	0.384947	0.628679	NOT
RNFT2	137.8695	0.33148814	0.381578	0.868728567	0.384996	0.62872	NOT
C19orf54	594.3518	-0.1567414	0.180438	-0.86867284	0.385026	0.628732	NOT
VCX	10.75109	0.76857763	0.884872	0.868575381	0.385079	0.628782	NOT
JPX	712.6854	0.14590421	0.168021	0.868368271	0.385193	0.628886	NOT
CABIN1	2094.056	-0.1310558	0.150922	-0.86836628	0.385194	0.628886	NOT
RP11-110I	47.59761	-0.2082418	0.239824	-0.86831165	0.385224	0.628886	NOT
DDX39A	3202.86	0.19574597	0.225439	0.868289291	0.385236	0.628886	NOT
RAB20	1526.484	0.2654121	0.305687	0.868246924	0.385259	0.628886	NOT
SETD5	2822.765	0.1265038	0.145724	0.868107122	0.385336	0.628973	NOT
RPL11	23940.49	0.16882837	0.194498	0.868021935	0.385382	0.629011	NOT
IGLV7-46	14.96308	0.54405403	0.627011	0.867694821	0.385561	0.629266	NOT
MTND6P4	28.541	-0.3801357	0.438145	-0.86760306	0.385612	0.629302	NOT
GPX1	17432.84	0.22604456	0.260549	0.867569592	0.38563	0.629302	NOT
HNF1A	1083.122	-0.248922	0.286938	-0.86751027	0.385662	0.629317	NOT
GALNT10	1247.775	0.21358806	0.246229	0.867435641	0.385703	0.629346	NOT
KCNT1	5.134914	-0.304245	0.350783	-0.86733155	0.38576	0.629401	NOT
RP1-145M2	1.991179	-0.3284819	0.378762	-0.86725147	0.385804	0.629435	NOT
RP11-332H	57.67695	0.20210413	0.233067	0.867151279	0.385859	0.629486	NOT
LINC01338	1.928635	-0.8441647	0.973611	-0.86704472	0.385918	0.629521	NOT
SLC38A3	26165.33	-0.3465508	0.399704	-0.86701912	0.385932	0.629521	NOT
RP5-1171I	43.27157	-0.3957352	0.45645	-0.86698557	0.38595	0.629521	NOT
RPSAP55	3.034512	-0.3331723	0.384327	-0.86689832	0.385998	0.629561	NOT
RP11-843F	11.144	-0.3495389	0.403309	-0.8666783	0.386118	0.629683	NOT
NFYC-AS1	33.03798	-0.2370706	0.273552	-0.86663796	0.38614	0.629683	NOT
RAB30-AS1	300.7176	0.16358691	0.188761	0.866634557	0.386142	0.629683	NOT
WBSCR16	3444.46	0.12665633	0.146181	0.866432305	0.386253	0.629826	NOT
LINC01016	4.146324	-0.5258593	0.606963	-0.86637763	0.386283	0.629831	NOT

GPR25	1.895813	0.50302799	0.580641	0.866331463	0.386308	0.629831	NOT
RP11-542M	1.957445	-0.4673076	0.539429	-0.86629976	0.386326	0.629831	NOT
RP11-83M1	22.11581	-0.6827888	0.788227	-0.86623428	0.386362	0.629852	NOT
RP11-4C20	1.192101	0.65291976	0.753803	0.866167585	0.386398	NA	NA
RP11-796(	4.295388	-0.3719469	0.429497	-0.86600584	0.386487	0.630018	NOT
RWDD2A	325.8157	-0.1484106	0.171429	-0.86572854	0.386639	0.630228	NOT
LA16c-313	7.846347	-0.4414495	0.50997	-0.86563849	0.386688	0.630245	NOT
MYO10	750.4031	-0.3461075	0.399835	-0.86562553	0.386696	0.630245	NOT
RAB17	3866.481	-0.28647	0.330998	-0.86547333	0.386779	0.630343	NOT
DHCR7	12120.64	0.25919018	0.299524	0.865340934	0.386852	0.63038	NOT
SMARCD2	4030.996	-0.1471625	0.170063	-0.86534035	0.386852	0.63038	NOT
FNTB	417.2131	0.12754867	0.147403	0.865304554	0.386872	0.63038	NOT
CHAMP1	946.943	0.19012861	0.219764	0.865147593	0.386958	0.630483	NOT
RP5-994D1	3.201488	-0.4035634	0.466545	-0.8650047	0.387036	0.630572	NOT
SETD6	512.3981	-0.1166873	0.134919	-0.86486811	0.387111	0.630657	NOT
WRAP73	675.4306	0.11222509	0.129775	0.864766408	0.387167	0.63071	NOT
MROH7-TTC	1.702597	-0.7077209	0.81851	-0.86464481	0.387234	0.630781	NOT
CALML6	25.60455	-0.3863733	0.446917	-0.86453104	0.387296	0.630834	NOT
AC093323.	606.2761	0.13856293	0.160281	0.864500305	0.387313	0.630834	NOT
SYTL4	950.9995	-0.2815085	0.325677	-0.86438071	0.387379	0.630889	NOT
ASXL2	1361.596	-0.1540658	0.178244	-0.86435405	0.387393	0.630889	NOT
RP13-554M	52.56447	0.2975839	0.344337	0.864222246	0.387466	0.630969	NOT
PTAFR	357.1191	0.3013552	0.348799	0.863979955	0.387599	0.631148	NOT
AC005355.	2.735724	0.42300034	0.489688	0.863815227	0.387689	0.631258	NOT
FAM32A	3571.673	-0.1243274	0.143941	-0.86373576	0.387733	0.631286	NOT
BLOC1S5-1	9.236573	-0.4054489	0.469433	-0.86369875	0.387753	0.631286	NOT
TMEM136	187.4185	0.25880222	0.29966	0.863653588	0.387778	0.631289	NOT
F2	59445.97	-0.3220471	0.372923	-0.86357515	0.387821	0.631305	NOT
RP3-394A1	258.6047	-0.3308031	0.383087	-0.86351966	0.387852	0.631305	NOT
TTC39A	770.3776	0.39178488	0.453728	0.863480417	0.387873	0.631305	NOT
RP11-530A	3.545409	0.27005773	0.31276	0.863465674	0.387881	0.631305	NOT
SPAG5-AS1	53.52883	-0.2246539	0.260225	-0.86330507	0.38797	0.631411	NOT
PAAF1	840.9449	-0.1109802	0.12856	-0.86325386	0.387998	0.631419	NOT
GS1-124K5	222.7381	-0.1665792	0.192988	-0.86315989	0.38805	0.631423	NOT
ALG10	109.8648	0.18225258	0.211147	0.863153452	0.388053	0.631423	NOT
MORF4L1P1	185.7074	0.17360033	0.201131	0.863122288	0.38807	0.631423	NOT
FAM104A	618.483	0.11838452	0.137172	0.863038968	0.388116	0.631452	NOT
IL11	7.469382	0.43835906	0.507956	0.862985875	0.388145	0.631452	NOT
MRC2	939.7408	0.36092243	0.418236	0.86296291	0.388158	0.631452	NOT
PIK3CG	70.70932	-0.3044562	0.35291	-0.86270233	0.388301	0.631647	NOT
PXDN	1689.295	-0.2649551	0.307214	-0.86244506	0.388443	0.631839	NOT
LINC00467	624.8792	0.17358089	0.201351	0.862079195	0.388644	0.631988	NOT
GRM4	6.887536	0.54108334	0.627663	0.862059959	0.388655	0.631988	NOT
WDR31	178.1733	-0.2377572	0.27581	-0.86203234	0.38867	0.631988	NOT
RP4-612B1	2.493296	-0.4756227	0.551853	-0.86186513	0.388762	0.631988	NOT
DYRK4	425.7536	-0.1649275	0.191372	-0.86181797	0.388788	0.631988	NOT
TRAPPC1	2774.041	-0.2198347	0.255091	-0.86179089	0.388803	0.631988	NOT
AOX3P	27.16999	-0.4909325	0.569681	-0.86176723	0.388816	0.631988	NOT
RP11-384K	2.003017	-0.3611953	0.419142	-0.86174869	0.388826	0.631988	NOT

RP11-366M	2.258634	0.39362888	0.456789	0.861730351	0.388836	0.631988	NOT
KIAA2012	41.9438	-0.4503708	0.522642	-0.86171966	0.388842	0.631988	NOT
C12orf76	441.3331	0.16722217	0.194066	0.861675965	0.388866	0.631988	NOT
FAM81A	83.83119	0.38974005	0.452315	0.861656374	0.388877	0.631988	NOT
RP11-353N	57.73047	0.28241626	0.327771	0.861627283	0.388893	0.631988	NOT
RP11-158M	4.643162	-0.305528	0.354602	-0.86160754	0.388904	0.631988	NOT
RBMY2FP	10.76442	1.85957979	2.158271	0.861606149	0.388904	0.631988	NOT
RP11-1023	1.667696	-0.4882231	0.566646	-0.86160222	0.388906	0.631988	NOT
FAM131B	21.08911	-0.2755469	0.319913	-0.86131742	0.389063	0.632176	NOT
DUSP23	5023.847	0.24356039	0.28278	0.861307351	0.389069	0.632176	NOT
SNHG20	207.0722	0.1688893	0.196107	0.861208603	0.389123	0.632194	NOT
NPBWR1	24.28094	-0.6262312	0.727159	-0.86120241	0.389127	0.632194	NOT
ZNF862	452.0258	-0.2286775	0.265565	-0.86109783	0.389184	0.632249	NOT
RP11-118N	4.040431	1.37436578	1.596342	0.86094714	0.389267	0.632333	NOT
C5orf22	986.7586	0.12928162	0.150167	0.86091983	0.389282	0.632333	NOT
C21orf33	97.23168	-0.5349821	0.621452	-0.86085784	0.389316	0.632351	NOT
RAB1B	5991.881	-0.0928138	0.107843	-0.86063466	0.389439	0.632475	NOT
SRPK2P	2.092314	0.63831603	0.741681	0.860634174	0.38944	0.632475	NOT
GSN	9698.171	-0.2141327	0.248832	-0.86055002	0.389486	0.632512	NOT
CTD-2095E	26.23179	0.39695842	0.461423	0.860290994	0.389629	0.632706	NOT
SNAP29	2654.467	-0.1288986	0.149857	-0.86014583	0.389709	0.632798	NOT
RP4-69502	4.331945	0.36980718	0.43001	0.859997631	0.38979	0.632885	NOT
RPL41P2	24.94764	-0.2612154	0.303751	-0.85996447	0.389809	0.632885	NOT
CHN2	1746.88	-0.2384766	0.277371	-0.85977451	0.389913	0.632986	NOT
C1orf216	562.5934	0.17409575	0.202492	0.859766814	0.389918	0.632986	NOT
COL5A2	2924.628	0.29657957	0.34497	0.85972483	0.389941	0.632986	NOT
PIGO	1949.797	-0.1469689	0.170979	-0.85957326	0.390024	0.633084	NOT
HSP90B1	61380.01	-0.1485354	0.172837	-0.85939383	0.390123	0.633182	NOT
BIRC3	3472.623	0.36091704	0.419974	0.859379238	0.390131	0.633182	NOT
MFGE8	1605.401	-0.2493793	0.290228	-0.85925335	0.390201	0.633235	NOT
DAND5	10.61332	0.43863992	0.510501	0.859234899	0.390211	0.633235	NOT
AARSD1	188.1668	0.1484914	0.17286	0.859025158	0.390327	0.633352	NOT
ANAPC13	2140.719	0.10461871	0.121788	0.859019704	0.39033	0.633352	NOT
CBX4	1954.312	0.15489182	0.180369	0.858750305	0.390478	0.633491	NOT
ATP1A2	119.7609	0.41471998	0.482939	0.858742601	0.390483	0.633491	NOT
RP4-724E1	7.125351	-0.359836	0.419029	-0.85873742	0.390485	0.633491	NOT
RP11-255M	1.211344	-0.7029288	0.818623	-0.85867215	0.390521	NA	NA
RP11-129H	2.053503	0.49632487	0.57802	0.85866444	0.390526	0.633509	NOT
SLC46A1	1692.39	0.19113421	0.222603	0.858632262	0.390543	0.633509	NOT
LA16c-306	2.291719	0.45177888	0.526193	0.858580584	0.390572	0.633518	NOT
RP11-524C	2.677469	-0.3256271	0.379343	-0.85839781	0.390673	0.633593	NOT
TOX4	2628.24	0.08878158	0.103431	0.858365582	0.390691	0.633593	NOT
RP11-326C	28.78779	-0.226719	0.264134	-0.85834923	0.3907	0.633593	NOT
PSMC3	7143.144	0.14371835	0.16744	0.858327452	0.390712	0.633593	NOT
PHKA1	197.7136	-0.3317294	0.386563	-0.85815123	0.390809	0.633713	NOT
RAPGEF1	3289.94	-0.1372489	0.159969	-0.85797434	0.390907	0.63383	NOT
ABCG5	2108.661	-0.341735	0.398339	-0.85790087	0.390947	0.63383	NOT
C1QL1	278.8605	-0.5276907	0.6151	-0.85789369	0.390951	0.63383	NOT
RP1-102E2	8.442692	0.32414661	0.377875	0.857814688	0.390995	0.633855	NOT

RP11-283I	199.8095	-0.1627192	0.189698	-0.85778125	0.391013	0.633855	NOT
ZNF516	951.8378	-0.1471501	0.171561	-0.85771424	0.39105	0.633877	NOT
RPL9P7	14.39369	0.26765853	0.312084	0.857648414	0.391087	0.633898	NOT
SBF1	2984.898	0.16942219	0.197587	0.857455229	0.391193	0.634003	NOT
PTCH1	373.4867	-0.273148	0.31856	-0.85744618	0.391198	0.634003	NOT
RP13-401N	18.36061	0.32969107	0.384588	0.857257906	0.391302	0.634134	NOT
DPYD-AS1	2.038958	0.50985915	0.594884	0.857072695	0.391405	0.634262	NOT
COL17A1	18.49024	-0.4024643	0.469738	-0.85678531	0.391564	0.634445	NOT
OR7E102P	4.281565	-0.481066	0.561507	-0.85674076	0.391588	0.634445	NOT
KCTD12	1197.97	-0.2565525	0.299481	-0.85665557	0.391635	0.634445	NOT
UBR7	1353.098	0.13373214	0.156118	0.856611149	0.39166	0.634445	NOT
RP11-65J2	40.24511	-0.286252	0.334192	-0.85654848	0.391694	0.634445	NOT
WWTR1	2236.523	0.25688766	0.299912	0.856544647	0.391697	0.634445	NOT
CTD-2373J	3.635514	-0.2767177	0.323067	-0.85653452	0.391702	0.634445	NOT
SMAD3	2694.057	0.17082161	0.199436	0.856523208	0.391708	0.634445	NOT
DAGLB	1098.665	-0.1295489	0.151256	-0.85648686	0.391729	0.634445	NOT
KIAA1147	2180.917	-0.20863	0.243624	-0.85635973	0.391799	0.634521	NOT
VWA3A	3.584482	0.40014101	0.467294	0.856293997	0.391835	0.634534	NOT
CAPS2	68.96885	-0.2592532	0.302773	-0.85626107	0.391853	0.634534	NOT
CTD-2206(	2.425231	-0.4462898	0.521248	-0.85619437	0.39189	0.634535	NOT
TRAM2-AS1	398.6743	-0.2020718	0.236017	-0.85617493	0.391901	0.634535	NOT
UBE2O	1648.817	0.12053462	0.140797	0.85608927	0.391948	0.634574	NOT
FCGRT	12036.72	-0.1973099	0.23049	-0.85604625	0.391972	0.634575	NOT
FABP1	51459.41	0.47567042	0.555708	0.855971222	0.392014	0.634604	NOT
RP11-390F	93.03902	-0.2633883	0.307746	-0.85586131	0.392074	0.634665	NOT
CDH19	45.54092	-0.5855884	0.68443	-0.85558488	0.392227	0.634874	NOT
ZBTB24	349.8378	0.12702631	0.148516	0.855301067	0.392384	0.63494	NOT
SCAANT1	5.594351	0.35626158	0.416541	0.855285765	0.392393	0.63494	NOT
TMEM240	21.45923	0.32928495	0.385012	0.855257819	0.392408	0.63494	NOT
LAMB2	8871.707	-0.1789964	0.209295	-0.85523598	0.392421	0.63494	NOT
XXbac-BP(	2.006575	0.41982419	0.490908	0.855199394	0.392441	0.63494	NOT
AC069363.	3.925005	-0.4853529	0.567534	-0.85519604	0.392443	0.63494	NOT
CCDC6	1788.518	0.10790354	0.126175	0.855188264	0.392447	0.63494	NOT
TAX1BP3	756.856	-0.2712598	0.317199	-0.85517326	0.392455	0.63494	NOT
AKR7L	298.4849	-0.2994096	0.350168	-0.85504504	0.392526	0.634991	NOT
RP11-411K	8.853285	-0.4365578	0.510581	-0.85502109	0.392539	0.634991	NOT
DEFB132	74.80438	-0.7310842	0.855079	-0.85498994	0.392557	0.634991	NOT
MYOT	4.384289	-0.482423	0.564283	-0.85493087	0.392589	0.635006	NOT
TAS2R62P	0.659594	1.3051646	1.527042	0.854701306	0.392717	NA	NA
WDFY2	675.003	0.16646955	0.194783	0.854639141	0.392751	0.635229	NOT
TMEM35	4.272759	0.36906846	0.432018	0.85428921	0.392945	0.635467	NOT
WDR83OS	4248.012	-0.1693681	0.198262	-0.85426494	0.392958	0.635467	NOT
DKFZP434I	86.716	-0.1943254	0.227482	-0.85424619	0.392969	0.635467	NOT
PACRGL	212.141	0.13115854	0.153554	0.854150042	0.393022	0.635516	NOT
EPB41L4A	159.4443	-0.3867198	0.452781	-0.85409862	0.39305	0.635524	NOT
IGLV1-44	103.3001	-0.5914882	0.692604	-0.85400648	0.393101	0.635541	NOT
VEGFC	286.8871	-0.2487827	0.291316	-0.85399536	0.393108	0.635541	NOT
RP11-74E2	2.075287	-0.4958841	0.58073	-0.85389733	0.393162	0.635591	NOT
DPM3	2173.868	-0.2431891	0.284859	-0.85371649	0.393262	0.635715	NOT

AC091633.	3.529279	-0.3849173	0.450924	-0.85361928	0.393316	0.635764	NOT
BTNL10	3.518159	-0.3596729	0.4214	-0.85351819	0.393372	0.635779	NOT
RP11-472N	4.42321	-0.3279664	0.384253	-0.85351768	0.393372	0.635779	NOT
CTD-2319I	16.15498	-0.2569013	0.301065	-0.85330824	0.393488	0.635891	NOT
AC000078.	5.445293	-0.4346624	0.509397	-0.85328728	0.3935	0.635891	NOT
RP11-424I	3.261142	0.44905861	0.526282	0.853265862	0.393512	0.635891	NOT
BTBD9	638.3099	-0.1537504	0.180208	-0.85318364	0.393557	0.635927	NOT
CYB5R2	214.8504	0.27819393	0.326105	0.853081265	0.393614	0.635981	NOT
LINC0011	27.06408	-0.2162635	0.253543	-0.85296534	0.393679	0.636043	NOT
TSSK1A	2.588675	-0.3263989	0.382689	-0.85290869	0.39371	0.636043	NOT
RP11-160H	11.7528	0.33047136	0.387475	0.852884823	0.393723	0.636043	NOT
RP11-458I	68.56484	0.31130729	0.365092	0.852682512	0.393835	0.636187	NOT
RN7SL541F	1.513342	-0.4979394	0.584065	-0.85254074	0.393914	0.636276	NOT
CLEC10A	213.4733	-0.2694188	0.316058	-0.85243351	0.393974	0.636316	NOT
TP53TG5	13.14729	-0.2039067	0.239212	-0.8524111	0.393986	0.636316	NOT
ARHGAP27	645.0853	0.20025068	0.234986	0.852181418	0.394113	0.636484	NOT
CRP	124167	0.61005336	0.715952	0.852087232	0.394166	0.636531	NOT
SIGLEC8	34.17712	-0.3752849	0.440465	-0.85201946	0.394203	0.636546	NOT
TCF7L2	1325.675	-0.1385317	0.162599	-0.85198558	0.394222	0.636546	NOT
IGHV3OR16	1.124707	-1.1190974	1.313685	-0.85187625	0.394283	NA	NA
RP11-632K	57.09702	-0.232538	0.272974	-0.85186885	0.394287	0.636582	NOT
MAGED2	6587.538	0.1448763	0.17007	0.851860501	0.394292	0.636582	NOT
CACNA1E	21.39038	0.5761123	0.67647	0.851645414	0.394411	0.636737	NOT
TBC1D1	2122.038	-0.1642435	0.192871	-0.85157079	0.394452	0.636766	NOT
CYP2D6	8925.449	0.39368	0.462345	0.851485897	0.394499	0.636804	NOT
ZBTB44	1708.653	-0.1527945	0.179454	-0.85144056	0.394525	0.636807	NOT
LINC01215	5.997247	0.45033143	0.528961	0.851351066	0.394574	0.636849	NOT
FAM3A	3315.962	0.15827004	0.185926	0.851251961	0.394629	0.6369	NOT
ARSK	322.8693	-0.1766446	0.207556	-0.85106792	0.394732	0.637005	NOT
CCDC22	1162.448	0.13894894	0.163271	0.851030999	0.394752	0.637005	NOT
CACNB4	42.55818	-0.410962	0.482912	-0.85100798	0.394765	0.637005	NOT
SSU72	5044.394	0.14291221	0.167963	0.850853007	0.394851	0.63709	NOT
DNAI1	11.97556	-0.479452	0.563511	-0.85082944	0.394864	0.63709	NOT
NR1I2	1601.09	-0.4529901	0.532494	-0.85069526	0.394939	0.63717	NOT
RP11-439H	10.14403	-0.6926545	0.81426	-0.85065564	0.394961	0.63717	NOT
ANKK1	9.2318	0.44774145	0.52644	0.85050846	0.395042	0.637264	NOT
TAS2R5	8.82405	0.36043643	0.423849	0.850388199	0.395109	0.637299	NOT
CTD-3074C	139.2826	-0.1992191	0.234269	-0.85038427	0.395111	0.637299	NOT
RP11-291E	7.732241	0.45348952	0.533309	0.850331525	0.395141	0.637309	NOT
NRXN2	390.3866	-0.3969919	0.466939	-0.85020037	0.395214	0.637388	NOT
DAPK2	613.3593	-0.2512431	0.295586	-0.84998309	0.395334	0.637539	NOT
FCAMR	550.6683	-0.5819346	0.68468	-0.84993625	0.395361	0.637539	NOT
RP11-774I	61.01404	-0.8472178	0.996838	-0.84990537	0.395378	0.637539	NOT
GREM1	162.8281	0.4610985	0.542585	0.849817439	0.395427	0.63758	NOT
CDH12	21.01556	-0.9349907	1.100313	-0.84974986	0.395464	0.637603	NOT
SIL1	7624.072	-0.2399276	0.282373	-0.84968285	0.395501	0.637611	NOT
MACROD2	279.09	0.31588797	0.371791	0.849637692	0.395527	0.637611	NOT
AC022431.	1.649413	-0.5574432	0.656116	-0.84961083	0.395541	0.637611	NOT
RP11-522I	0.430666	1.15626869	1.360944	0.849607971	0.395543	NA	NA

SLC35A3	1761.174	-0.2036126	0.239684	-0.84950571	0.3956	0.637611	NOT
AFAP1-AS1	304.2962	-0.6330581	0.745215	-0.84949709	0.395605	0.637611	NOT
KRT6B	11.4903	-0.7811283	0.91953	-0.84948669	0.395611	0.637611	NOT
RCN1P2	120.8796	-0.3059022	0.360178	-0.849308	0.39571	0.637734	NOT
RGS20	12.75793	0.44531441	0.524361	0.84925153	0.395741	0.637735	NOT
CCDC36	11.91546	0.41164945	0.484737	0.84922188	0.395758	0.637735	NOT
RP11-686I	20.42482	-0.3569422	0.420356	-0.84914261	0.395802	0.637768	NOT
RP11-427J	28.50558	-0.4930072	0.580858	-0.84875656	0.396017	0.63807	NOT
RRS1-AS1	11.77638	-0.3652792	0.430445	-0.84860784	0.3961	0.63807	NOT
AKAP5	91.07727	0.29422061	0.346716	0.848591761	0.396108	0.63807	NOT
AAED1	462.6554	-0.1577972	0.185977	-0.84847472	0.396174	0.63807	NOT
IGLV3-25	123.2377	0.60441862	0.71236	0.848473713	0.396174	0.63807	NOT
RP11-102K	6.18816	0.35801057	0.421951	0.848464225	0.396179	0.63807	NOT
AQP7	479.1364	-0.2879212	0.339379	-0.84837569	0.396229	0.63807	NOT
ATP8A2P1	1.664862	1.52847218	1.801647	0.848375139	0.396229	0.63807	NOT
SFRP2	26.86572	0.6808194	0.802513	0.84835977	0.396238	0.63807	NOT
MIR616	3.202201	-0.3807328	0.448799	-0.84833666	0.396251	0.63807	NOT
AP003774.	8.145942	0.39270372	0.462911	0.84833604	0.396251	0.63807	NOT
RP11-582E	228.0367	-0.1495866	0.176342	-0.8482751	0.396285	0.63807	NOT
ANKRD16	391.6282	0.14964411	0.176429	0.848184191	0.396335	0.63807	NOT
AC027601.	41.28374	-0.1962494	0.231385	-0.84814926	0.396355	0.63807	NOT
RNF138	724.8141	0.1416763	0.167042	0.848145875	0.396357	0.63807	NOT
GET4	232.9111	-0.164103	0.193488	-0.8481308	0.396365	0.63807	NOT
PHF2P2	2.986671	1.27227689	1.500619	0.847834689	0.39653	0.638297	NOT
C14orf142	494.7002	0.13149652	0.155116	0.847728793	0.396589	0.63834	NOT
GOLT1A	4433.949	-0.2982517	0.351835	-0.84770268	0.396604	0.63834	NOT
NOTCH2	4090.503	-0.2154932	0.254229	-0.84763478	0.396641	0.638363	NOT
CCDC7	104.7187	-0.2031722	0.239709	-0.84757701	0.396674	0.638367	NOT
RP1-241P1	4.590621	0.32145688	0.37928	0.847545522	0.396691	0.638367	NOT
ZNF519	77.85964	-0.2740057	0.323544	-0.84688821	0.397057	0.638919	NOT
RP3-354N1	1.986221	-0.3674179	0.433934	-0.8467129	0.397155	0.639038	NOT
FAM45B	6.48632	0.27003477	0.318973	0.846575878	0.397232	0.639123	NOT
RP11-1006	1.68973	-0.4471235	0.528213	-0.84648323	0.397283	0.639139	NOT
RP11-399K	3.113314	0.50995555	0.602535	0.846349903	0.397358	0.639139	NOT
LINC01509	1.848987	-0.7027072	0.830286	-0.84634363	0.397361	0.639139	NOT
KCTD11	376.0059	-0.1617784	0.191156	-0.84631482	0.397377	0.639139	NOT
PTPRT	24.02269	0.47884964	0.565808	0.846311927	0.397379	0.639139	NOT
CTC-459F4	109.6425	-0.1712447	0.202362	-0.84623052	0.397424	0.639139	NOT
EPHX1	130771.7	-0.2908749	0.343745	-0.84619459	0.397444	0.639139	NOT
ADAMTS8	16.25504	-0.3182087	0.376048	-0.8461915	0.397446	0.639139	NOT
HBEGF	403.5643	-0.2351544	0.277902	-0.84617811	0.397453	0.639139	NOT
MRPL40P1	4.475935	0.4232982	0.500311	0.846069514	0.397514	0.639198	NOT
TRGV9	1.560855	-0.528438	0.624649	-0.84597516	0.397567	0.639233	NOT
SLC41A1	947.2816	0.26414656	0.31225	0.845946834	0.397582	0.639233	NOT
CROCC	702.5008	-0.1855052	0.219315	-0.84583934	0.397642	0.639291	NOT
DHFR	959.1276	0.19191322	0.226915	0.845750652	0.397692	0.639333	NOT
KB-1125A3	7.561162	-0.4187042	0.495133	-0.84564044	0.397753	0.639385	NOT
FLNA	15431.31	0.33246012	0.393161	0.845607815	0.397772	0.639385	NOT
PTCH2	49.69859	-0.2100593	0.248453	-0.84546775	0.39785	0.639473	NOT

MIR325HG	21.92259	-0.5726192	0.677366	-0.84536125	0.397909	0.639476	NOT
UBXN8	704.192	0.19640544	0.232336	0.845352049	0.397914	0.639476	NOT
PEX11B	1806.224	0.1278802	0.151277	0.845338138	0.397922	0.639476	NOT
EGLN1	5279.162	0.15184528	0.179644	0.845258812	0.397966	0.639484	NOT
SLC25A5	16551.71	0.17612624	0.208373	0.845244319	0.397974	0.639484	NOT
RP1-315G1	6.758158	-0.3630083	0.429626	-0.84494112	0.398144	0.639689	NOT
IGHV3-23	185.5121	-0.5700331	0.674678	-0.84489647	0.398169	0.639689	NOT
LINC01229	3.762803	-0.3066564	0.362957	-0.84488366	0.398176	0.639689	NOT
RAD9A	710.449	0.14954373	0.177012	0.844821816	0.39821	0.639689	NOT
CCDC68	331.8901	-0.281364	0.333057	-0.84479143	0.398227	0.639689	NOT
ADM2	1202.152	0.35562145	0.420972	0.844762479	0.398243	0.639689	NOT
RFFL	2088.075	0.19530436	0.23124	0.844597679	0.398336	0.63974	NOT
C2orf72	8104.942	0.20149591	0.23857	0.844596961	0.398336	0.63974	NOT
CST5	7.076677	0.74895589	0.886781	0.844578476	0.398346	0.63974	NOT
RP11-715F	6.172904	0.28488675	0.337332	0.844529337	0.398374	0.639747	NOT
ZNF365	5.473781	-0.3759423	0.445187	-0.84445988	0.398412	0.639771	NOT
CTD-2331H	30.04915	0.41920567	0.496456	0.844396012	0.398448	0.639778	NOT
CCNT2-AS1	21.81528	-0.1975797	0.234016	-0.8443001	0.398502	0.639778	NOT
RP11-702H	3.079893	0.44374548	0.525606	0.844254863	0.398527	0.639778	NOT
RP11-274E	136.7404	-0.2675755	0.316942	-0.84424214	0.398534	0.639778	NOT
HSPB1	29958.6	0.25065785	0.296903	0.844241355	0.398535	0.639778	NOT
UST	108.8661	-0.394211	0.467073	-0.84400253	0.398668	0.639954	NOT
NUDT14	1137.037	-0.2995466	0.354931	-0.84395817	0.398693	0.639956	NOT
RP11-373N	9.163395	-0.2461273	0.291747	-0.84363355	0.398874	0.640192	NOT
RP5-1024N	8.021527	0.31148195	0.369225	0.843610272	0.398887	0.640192	NOT
COL4A4	207.4969	-0.3739142	0.443274	-0.84352841	0.398933	0.640228	NOT
ACBD5	3429.682	-0.1895104	0.224709	-0.84335764	0.399028	0.640307	NOT
ZNF214	51.2391	-0.2995121	0.355143	-0.8433558	0.399029	0.640307	NOT
MOXD1	243.6565	-0.4358656	0.516894	-0.84324039	0.399094	0.640373	NOT
EPHA1	1863.488	-0.3738941	0.443494	-0.84306513	0.399192	0.640492	NOT
NGLY1	1244.854	0.11637708	0.138059	0.842949538	0.399257	0.640526	NOT
RP11-815J	5.082826	-0.3687725	0.437482	-0.84294276	0.39926	0.640526	NOT
LINC01448	2.266509	-0.9934564	1.178652	-0.84287535	0.399298	0.640537	NOT
RP11-467I	140.8785	-0.2197066	0.260672	-0.84284571	0.399315	0.640537	NOT
SCAF4	1479.521	-0.1199344	0.142314	-0.84274764	0.39937	0.640588	NOT
RP11-6F2.	27.57974	-0.4751271	0.563927	-0.84253299	0.39949	0.640742	NOT
PDE9A	536.8562	0.38825603	0.460901	0.842384018	0.399573	0.640742	NOT
RAD51AP2	14.12855	0.41458734	0.492164	0.842376974	0.399577	0.640742	NOT
CEP83-AS1	11.07361	0.25163204	0.298722	0.842362424	0.399585	0.640742	NOT
CHRND	14.22392	0.60583183	0.719222	0.842343069	0.399596	0.640742	NOT
RP11-158H	5.339578	-0.341378	0.405282	-0.84232244	0.399607	0.640742	NOT
THAP7	1482.218	-0.1540575	0.18292	-0.84221293	0.399669	0.640761	NOT
PACRG-AS1	3.390958	-0.5695153	0.676218	-0.84220717	0.399672	0.640761	NOT
OR5BA1P	1.37509	0.72607909	0.862122	0.842199981	0.399676	NA	NA
MYT1	26.4199	-0.4764887	0.565784	-0.84217441	0.39969	0.640761	NOT
RBM46	4.901101	0.55347258	0.657269	0.842078976	0.399744	0.640779	NOT
RP4-583P1	57.44687	-0.2891596	0.343392	-0.84206956	0.399749	0.640779	NOT
RIMBP2	4.18337	0.7096845	0.842889	0.841967202	0.399806	0.640823	NOT
TMEM241	267.4265	0.17810988	0.211548	0.841936521	0.399823	0.640823	NOT

FBXO7	5847.239	-0.1673926	0.198832	-0.84188056	0.399855	0.640832	NOT
CLIP1	3534.134	-0.1607746	0.19098	-0.84184196	0.399876	0.640832	NOT
CST3	17149.67	-0.224436	0.266644	-0.84170526	0.399953	0.640917	NOT
NPSR1	1.223193	0.68343533	0.812049	0.841618674	0.400001	NA	NA
MBOAT1	328.4956	-0.3427936	0.407329	-0.84156348	0.400032	0.641003	NOT
FAM173A	736.4975	-0.2349356	0.279183	-0.84151148	0.400061	0.641003	NOT
SMG1P7	81.16995	-0.2145979	0.255024	-0.84148271	0.400078	0.641003	NOT
FGFR1	1082.184	0.3664516	0.435537	0.841378609	0.400136	0.641059	NOT
XPNPEP1	1665.927	0.11026455	0.13106	0.841329	0.400164	0.641065	NOT
BCORP1	4.48881	0.5820163	0.69185	0.841246093	0.40021	0.641102	NOT
ANKRD10	2672.684	0.17134427	0.203693	0.841189211	0.400242	0.641115	NOT
RP11-343N	2.165221	0.47032923	0.559206	0.84106552	0.400311	0.641188	NOT
EIF2S3	7005.15	0.12958154	0.154098	0.840901933	0.400403	0.641293	NOT
SNORD100	6.748897	0.31710547	0.377119	0.840863714	0.400424	0.641293	NOT
SRP19	1082.676	0.14718901	0.175056	0.840810844	0.400454	0.641303	NOT
ADRA2B	102.718	-0.3142365	0.373804	-0.84064619	0.400546	0.641343	NOT
HIST1H2AM	13.21715	-0.3301778	0.392774	-0.84062999	0.400555	0.641343	NOT
IKBKAP	2283.612	0.19148007	0.227785	0.840619049	0.400561	0.641343	NOT
POLE2	256.8868	0.22936652	0.272866	0.840583877	0.400581	0.641343	NOT
MCAT	881.8876	0.1774196	0.211079	0.840535407	0.400608	0.641343	NOT
COX7A1	126.7878	-0.2489516	0.29619	-0.84051355	0.400621	0.641343	NOT
HR	103.0469	0.45194234	0.537753	0.840427078	0.400669	0.641382	NOT
ATP2B1	1986.112	0.19814072	0.235779	0.840365669	0.400703	0.6414	NOT
SERF2	24640.63	0.21103583	0.251157	0.840255569	0.400765	0.641451	NOT
GLDCP1	21.75452	-0.3685975	0.438689	-0.84022458	0.400782	0.641451	NOT
RNY3P16	5.941497	0.3519431	0.41894	0.840080508	0.400863	0.641498	NOT
GLUD1P3	37.6991	-0.1960807	0.233426	-0.84001112	0.400902	0.641498	NOT
BMS1P2	3.655817	-0.367659	0.437709	-0.83996192	0.40093	0.641498	NOT
RP11-118M	2.195409	-0.4019416	0.478542	-0.83992955	0.400948	0.641498	NOT
NCOA3	1805.548	0.13927241	0.165816	0.839920604	0.400953	0.641498	NOT
DNAJA1P3	2.766133	0.38612027	0.459712	0.839918513	0.400954	0.641498	NOT
FUT8-AS1	2.833878	-0.3812658	0.454147	-0.83952141	0.401177	0.641797	NOT
N6AMT1	349.7717	0.14318794	0.170563	0.83949938	0.401189	0.641797	NOT
RP11-96B2	10.89807	0.51818131	0.61728	0.839459104	0.401212	0.641797	NOT
PRAP1	26031.96	0.40907351	0.48737	0.839348431	0.401274	0.641858	NOT
RPS17	25127.72	0.18202334	0.216895	0.839223616	0.401344	0.641897	NOT
RP11-91K8	3.406258	-0.4605028	0.548726	-0.83922148	0.401345	0.641897	NOT
XX-C00717	1.676041	0.52090892	0.620849	0.839026629	0.401454	0.642034	NOT
RP11-25I1	1.286294	-0.7423512	0.884948	-0.83886391	0.401546	NA	NA
RP11-401.	116.2031	0.21215649	0.252957	0.838706347	0.401634	0.642236	NOT
C1orf74	229.2964	0.1508233	0.179846	0.838625068	0.40168	0.642236	NOT
LEFTY2	2.194879	0.57465044	0.685235	0.838618591	0.401683	0.642236	NOT
MVB12B	784.9654	-0.1544614	0.184188	-0.83860722	0.40169	0.642236	NOT
PRSS42	5.488307	0.36285354	0.432698	0.838584038	0.401703	0.642236	NOT
MUC12	32.56234	0.44590513	0.531761	0.838544539	0.401725	0.642236	NOT
MATN2	1996.012	-0.3190238	0.380467	-0.83850579	0.401747	0.642236	NOT
RP11-809F	5.648746	0.29630152	0.353395	0.838442807	0.401782	0.642255	NOT
SREBF1	10455.04	0.25174719	0.300276	0.838385765	0.401814	0.642263	NOT
HAUS7	114.4461	-0.2576269	0.307303	-0.8383493	0.401835	0.642263	NOT

GPR87	0.840859	0.83491642	0.995951	0.838311082	0.401856	NA	NA
UBA1	12678.37	-0.1050276	0.12529	-0.83827342	0.401877	0.642282	NOT
THAP4	2296.749	0.13433727	0.16026	0.838243899	0.401894	0.642282	NOT
TMEM121	52.60644	0.40350419	0.481402	0.838186318	0.401926	0.642296	NOT
ADRA2C	380.8561	0.47997963	0.572712	0.838081711	0.401985	0.642322	NOT
KTN1	7774.352	0.13822595	0.164933	0.838073114	0.40199	0.642322	NOT
RPL17P50	61.42052	0.30473928	0.363826	0.837596935	0.402257	0.642711	NOT
MIB2	1273.103	0.14268023	0.170386	0.837394279	0.402371	0.642842	NOT
RPL21P12(	3.469979	0.37336872	0.445884	0.837366646	0.402387	0.642842	NOT
RP11-270(	11.17826	-0.2831138	0.338282	-0.83691562	0.40264	0.643209	NOT
MAFB	1611.59	-0.2540323	0.303576	-0.83679908	0.402706	0.643276	NOT
ZNF530	122.264	0.21117331	0.252381	0.836725897	0.402747	0.643304	NOT
AC004980.	2.206158	-0.455834	0.544901	-0.8365445	0.402849	0.643429	NOT
RP11-666A	14.06725	-0.2210025	0.264205	-0.8364814	0.402884	0.643448	NOT
IGKV1D-16	2.843512	0.61134249	0.731114	0.836179175	0.403054	0.643634	NOT
ENSAP2	3.573769	0.3252863	0.389026	0.836155769	0.403067	0.643634	NOT
EMILIN1	1714.116	-0.3319103	0.396963	-0.8361232	0.403086	0.643634	NOT
FERP1	2.830645	-0.3020314	0.361251	-0.8360713	0.403115	0.643634	NOT
SLC25A40	689.6175	0.15357905	0.183693	0.836063157	0.403119	0.643634	NOT
CYSLTR2	54.26961	-0.2881592	0.344691	-0.83599346	0.403159	0.643659	NOT
AC005624.	2.387097	0.38023924	0.45487	0.835929123	0.403195	0.643676	NOT
MED17	716.517	0.11677003	0.139695	0.835890231	0.403217	0.643676	NOT
AKIRIN1	2092.786	0.13306989	0.159228	0.835717855	0.403314	0.643747	NOT
EDF1	15141.46	-0.2070587	0.247763	-0.83571225	0.403317	0.643747	NOT
RP5-1039K	49.37957	0.23882414	0.285783	0.835684669	0.403332	0.643747	NOT
HSPD1P1	30.31623	0.27772412	0.332434	0.835425424	0.403478	0.643935	NOT
RP11-319(	6.206949	0.37593177	0.450007	0.835391202	0.403497	0.643935	NOT
RP11-676J	5.106046	0.25880373	0.30985	0.835253559	0.403575	0.644011	NOT
SEPT12	1.759913	-0.5554769	0.665067	-0.83521929	0.403594	0.644011	NOT
PSG5	1.5644	0.77410399	0.926914	0.83514094	0.403638	0.644011	NOT
ADAT3	92.23716	0.21593347	0.25856	0.835137393	0.40364	0.644011	NOT
RGS9BP	5.281862	0.37176199	0.445288	0.834880661	0.403785	0.644179	NOT
HEXIM1	1941.972	0.14566504	0.174481	0.834849321	0.403803	0.644179	NOT
RNU4ATAC	2.503073	-0.4578321	0.548433	-0.83479973	0.40383	0.644179	NOT
WDR34	2257.679	0.21635442	0.259183	0.834753942	0.403856	0.644179	NOT
ABLIM2	315.2818	0.33297039	0.398891	0.834739305	0.403865	0.644179	NOT
RFXAP	96.22567	0.19752539	0.236688	0.83453969	0.403977	0.644289	NOT
UBA6-AS1	297.0277	-0.133475	0.15994	-0.83453273	0.403981	0.644289	NOT
LNX2	1136.709	-0.1934052	0.231783	-0.8344227	0.404043	0.644319	NOT
RP11-156E	409.9327	0.11424457	0.136916	0.834415327	0.404047	0.644319	NOT
CTD-2033(	10.88134	-0.1891396	0.226697	-0.83432723	0.404097	0.64433	NOT
RP11-280(	3.448733	-0.3229267	0.387067	-0.83429238	0.404116	0.64433	NOT
RPS29	9977.945	0.21871955	0.262173	0.83425503	0.404137	0.64433	NOT
HSD3BP2	1.784083	-0.7100113	0.851094	-0.8342337	0.404149	0.64433	NOT
SOGA3	2.726705	-0.3743634	0.448846	-0.83405846	0.404248	0.64445	NOT
GS1-124K5	171.8995	-0.2172505	0.260533	-0.8338699	0.404354	0.644581	NOT
ABCB7	967.4525	-0.1259243	0.151027	-0.83378493	0.404402	0.64462	NOT
SPATA6L	121.7311	-0.2584168	0.310008	-0.8335821	0.404517	0.644764	NOT
RP4-714D9	81.62838	0.22404939	0.268816	0.833467691	0.404581	0.644829	NOT

RP4-635A2	2.19694	0.36970042	0.443651	0.833313946	0.404668	0.644929	NOT
LINC01341	120.9113	-0.3250911	0.390155	-0.83323536	0.404712	0.644934	NOT
EXOSC8	699.391	0.14069507	0.168856	0.833224171	0.404718	0.644934	NOT
ZNF542P	548.2821	0.2503757	0.300579	0.832978649	0.404857	0.645117	NOT
AC093159.	3.62077	-0.3896964	0.467919	-0.83282831	0.404942	0.645214	NOT
RP11-867(	1.21459	0.8803089	1.057253	0.832637733	0.405049	NA	NA
RP11-651I	13.30221	0.18818716	0.226037	0.832550857	0.405098	0.645426	NOT
GBE1	3730.414	-0.228319	0.274261	-0.8324868	0.405134	0.645446	NOT
LINC00222	12.43045	0.45125821	0.542121	0.832394323	0.405186	0.645491	NOT
IQCK	130.8162	0.24217589	0.290966	0.832317102	0.40523	0.645522	NOT
SOAT1	2147.32	0.15903857	0.191141	0.832049674	0.405381	0.645725	NOT
CSNK1A1	1956.982	-0.1440644	0.173171	-0.83192144	0.405453	0.645781	NOT
CCND1	8195.971	0.31892666	0.38337	0.831902886	0.405464	0.645781	NOT
MGC45922	3.042387	0.37540092	0.451285	0.831849401	0.405494	0.645791	NOT
RHD	17.88003	-0.2351666	0.282721	-0.83179769	0.405523	0.6458	NOT
UBXN1	3834.656	0.14026263	0.168639	0.831732341	0.40556	0.645821	NOT
RP11-585I	57.90933	-0.3125618	0.375823	-0.83167341	0.405593	0.645836	NOT
RP11-534I	4.330886	0.32253035	0.387907	0.831462284	0.405713	0.645937	NOT
CTD-2047I	1.581829	-0.4155048	0.499757	-0.83141308	0.40574	0.645937	NOT
RP11-160(	106.1578	0.27635762	0.332402	0.831395041	0.40575	0.645937	NOT
BAI1	40.15722	-0.4200314	0.505223	-0.83137851	0.40576	0.645937	NOT
KIR3DL2	1.435226	-0.4166568	0.501181	-0.83134939	0.405776	0.645937	NOT
CBLN4	33.13391	0.49639962	0.597183	0.831234784	0.405841	0.646002	NOT
WNT7A	2.969011	0.57025659	0.686198	0.831037692	0.405952	0.646142	NOT
LINC01287	564.591	0.92327372	1.11109	0.830962459	0.405995	0.646144	NOT
TTC38	7612.326	-0.2171097	0.261279	-0.83095114	0.406001	0.646144	NOT
USMG5	4466.194	-0.2199155	0.264674	-0.83089297	0.406034	0.646158	NOT
SMIM13	1132.018	0.12617958	0.151872	0.830826955	0.406071	0.646177	NOT
MAGI1-IT1	3.900325	-0.2543919	0.306206	-0.83078746	0.406094	0.646177	NOT
RPL18A	12331.15	0.22974498	0.276567	0.83070155	0.406142	0.646217	NOT
GAS2L1P2	3.684417	-0.7346382	0.88452	-0.83055025	0.406228	0.646315	NOT
MIR4489	4.827088	-0.3304302	0.397876	-0.83048552	0.406264	0.646335	NOT
BCL2L1	5959.553	0.1807986	0.217733	0.830366939	0.406331	0.646404	NOT
GPX7	454.81	0.33012457	0.397688	0.830110171	0.406476	0.646597	NOT
PRCP	3768.257	-0.1959914	0.236141	-0.8299778	0.406551	0.646678	NOT
MAGOHB	587.9291	0.12699021	0.153023	0.829876994	0.406608	0.646719	NOT
IGSF11	3.821496	0.45828717	0.552269	0.829826083	0.406637	0.646719	NOT
BNIP3P28	2.653623	-0.5723748	0.68977	-0.82980577	0.406649	0.646719	NOT
SNAPC1	218.2157	0.16805121	0.20253	0.829760183	0.406674	0.646719	NOT
AGMO	2830.424	-0.29099	0.350708	-0.82972159	0.406696	0.646719	NOT
GAPDHP70	3.605957	0.35832617	0.431927	0.829597996	0.406766	0.646792	NOT
INTS6	1217.199	-0.1461069	0.176188	-0.82926773	0.406953	0.647051	NOT
SLC25A26	928.956	0.14206732	0.171368	0.829018064	0.407094	0.647238	NOT
LINC00982	78.77984	-0.5367356	0.647519	-0.82891148	0.407155	0.647278	NOT
USP12	1370.457	-0.1672035	0.20172	-0.82888863	0.407167	0.647278	NOT
TYROBP	1768.722	0.26447738	0.319091	0.828845574	0.407192	0.647279	NOT
TIPARP	1099.522	-0.1765585	0.213087	-0.82857493	0.407345	0.647476	NOT
AGAP1-IT1	31.03373	-0.3903523	0.471136	-0.82853499	0.407368	0.647476	NOT
RP11-175I	24.51164	-0.2589125	0.312507	-0.82850032	0.407387	0.647476	NOT

MYH2	0.903527	0.76901043	0.928225	0.828474095	0.407402	NA	NA
ADO	681.5399	0.11419628	0.137842	0.828457293	0.407412	0.647477	NOT
LINC0092	0.896085	0.89951381	1.085903	0.828355878	0.407469	NA	NA
UBQLNL	22.06176	-0.3453088	0.41687	-0.8283366	0.40748	0.647547	NOT
SLC25A1	10943.52	-0.181527	0.21916	-0.82828404	0.40751	0.647557	NOT
C1orf177	18.18799	0.28909332	0.349059	0.828207221	0.407553	0.647588	NOT
PITX1	439.8787	0.57499093	0.694304	0.82815397	0.407583	0.647598	NOT
RP11-12M	2.195149	0.34634582	0.41829	0.828003911	0.407668	0.647695	NOT
BOD1L1	1248.104	-0.1661596	0.200685	-0.82796059	0.407693	0.647696	NOT
SPRR1B	2.798465	1.34610527	1.62617	0.827776426	0.407797	0.647824	NOT
ZFPL1	365.8124	0.10110922	0.122152	0.827733884	0.407821	0.647824	NOT
ANKS4B	1224.954	-0.3033703	0.36661	-0.82750166	0.407953	0.647995	NOT
CRYBA1	1.88239	0.36265307	0.438463	0.827101358	0.40818	0.648318	NOT
AP001258.	98.85643	0.18802858	0.227386	0.826911828	0.408287	0.64845	NOT
UHRF1BP1	1483.726	-0.1665929	0.201478	-0.82685405	0.40832	0.648464	NOT
BCL2L11	1420.85	-0.1862988	0.225383	-0.8265859	0.408472	0.648668	NOT
MIR3131	3.060916	0.5641254	0.682513	0.826541062	0.408497	0.64867	NOT
MIR8071-	1.604674	0.68587506	0.829858	0.826496555	0.408522	0.648672	NOT
CTD-2313	2.271342	0.37899945	0.45859	0.826444719	0.408552	0.648681	NOT
MT1HL1	0.276611	-1.4427041	1.745925	-0.8263264	0.408619	NA	NA
RP11-573	1.790872	0.37207999	0.450301	0.826291234	0.408639	0.648772	NOT
ACTN3	5.64943	0.48471058	0.586632	0.826259703	0.408657	0.648772	NOT
APOPT1	791.704	0.17148503	0.207579	0.826118761	0.408737	0.648801	NOT
SGOL1-AS	2.572254	0.31430119	0.38046	0.826107489	0.408743	0.648801	NOT
FASTKD1	763.687	-0.1738745	0.210476	-0.82610039	0.408747	0.648801	NOT
RP11-81H	316.5308	0.57686281	0.69837	0.826012796	0.408797	0.648842	NOT
TXLNA	4239.105	-0.1246147	0.150904	-0.82578625	0.408925	0.648977	NOT
ZNF112	92.53808	-0.2470399	0.29916	-0.82577823	0.40893	0.648977	NOT
LINC0137	17.00275	-0.206011	0.249543	-0.82555401	0.409057	0.649141	NOT
SELPLG	485.3414	-0.2505598	0.30366	-0.82513161	0.409297	0.649484	NOT
RFX3-AS1	17.5157	0.32933784	0.399174	0.825047999	0.409344	0.649484	NOT
IDI1	8386.239	0.22301017	0.2703	0.825046954	0.409345	0.649484	NOT
CTD-2571	5.522206	0.24259561	0.294127	0.824799276	0.409486	0.649669	NOT
RP11-10A	6.788789	-0.4069899	0.493556	-0.82460778	0.409594	0.649804	NOT
HEXIM2	135.7751	0.14624133	0.17737	0.824497606	0.409657	0.649851	NOT
RP11-64C	3.758968	0.53171353	0.644921	0.824463228	0.409676	0.649851	NOT
A4GALT	409.4223	-0.2687449	0.325977	-0.82442931	0.409696	0.649851	NOT
ASPSCR1	5730.755	0.31395424	0.380873	0.824300568	0.409769	0.649929	NOT
LINC0158	109.5139	-0.2535652	0.307632	-0.82424743	0.409799	0.649938	NOT
RP11-649	2.389782	0.34443463	0.417916	0.824172185	0.409842	0.649968	NOT
IGHV3-19	1.122227	-0.6490741	0.787681	-0.82403191	0.409921	NA	NA
GRIA3	148.387	-0.5225813	0.634229	-0.82396246	0.409961	0.650119	NOT
PI4KAP1	131.2813	0.29432948	0.357239	0.823900185	0.409996	0.650137	NOT
FABP7	1.614514	0.51964728	0.630805	0.823784499	0.410062	0.650166	NOT
CDK2AP2	4033.039	-0.1510275	0.183334	-0.8237845	0.410062	0.650166	NOT
USP28	782.4676	0.12704337	0.154248	0.823632928	0.410148	0.650236	NOT
PTOV1-AS	83.05982	0.2481341	0.301273	0.823617967	0.410157	0.650236	NOT
ACTG1P3	7.866609	-0.2613148	0.317291	-0.82358002	0.410178	0.650236	NOT
MS4A1	41.2634	0.46850589	0.568925	0.82349288	0.410228	0.650276	NOT

RCN3	687.4021	-0.217488	0.264127	-0.82342362	0.410267	0.650301	NOT
RABGGTA	1221.065	-0.1326308	0.161087	-0.82334964	0.410309	0.650329	NOT
COPG1	10515.93	0.08291869	0.100718	0.823279238	0.410349	0.650342	NOT
RSPH3	375.4369	-0.1445815	0.175623	-0.82325126	0.410365	0.650342	NOT
CAMP	1.535614	0.5425044	0.659071	0.823135529	0.410431	0.650408	NOT
RPS4XP13	3.137542	0.36161021	0.439357	0.823043696	0.410483	0.650453	NOT
XXbac-B46	7.319795	-0.3483525	0.423306	-0.82293251	0.410546	0.650515	NOT
WNT11	309.2775	-0.4305847	0.523273	-0.82286795	0.410583	0.650535	NOT
RPS6KB1	1215.804	-0.1142429	0.138842	-0.8228267	0.410607	0.650535	NOT
PVRL3-AS1	7.44309	0.28005795	0.340468	0.822568637	0.410753	0.650729	NOT
WSCD1	311.8146	-0.32275	0.392433	-0.82243265	0.410831	0.650814	NOT
AQP1	3306.839	-0.3225809	0.392292	-0.82229739	0.410908	0.650898	NOT
RP4-760C5	3.978167	-0.3912894	0.476001	-0.82203542	0.411057	0.651059	NOT
PIGBOS1	369.4662	-0.1383212	0.168267	-0.82203362	0.411058	0.651059	NOT
RP11-1094	24.84554	-0.3268613	0.397649	-0.8219846	0.411086	0.651066	NOT
POGZ	3282.067	0.14513561	0.176584	0.821907454	0.41113	0.651097	NOT
EIF1P3	4.377557	-0.3200568	0.389448	-0.82182191	0.411178	0.651136	NOT
NRIP3	23.22579	0.31347375	0.381606	0.821459798	0.411384	0.651409	NOT
F2RL1	768.3206	0.35800387	0.435827	0.821435413	0.411398	0.651409	NOT
LA16c-385	2.265809	0.37738452	0.459469	0.821349973	0.411447	0.651441	NOT
CCL24	39.31148	-0.4008023	0.488001	-0.82131532	0.411467	0.651441	NOT
SPOCK2	804.299	-0.2432658	0.296226	-0.82121577	0.411523	0.651493	NOT
ENTPD5	10807.49	-0.2751287	0.335057	-0.82113954	0.411567	0.651493	NOT
RP11-556I	162.5367	0.5719068	0.696487	0.821131088	0.411572	0.651493	NOT
AGBL5-IT1	9.207875	-0.2517809	0.306668	-0.82102109	0.411634	0.651554	NOT
USP36	2004.44	-0.1037462	0.126399	-0.82078294	0.41177	0.65173	NOT
NR0B2	2973.605	0.32846879	0.400216	0.820727768	0.411801	0.65173	NOT
PPP1R1C	112.8123	-0.4220711	0.514282	-0.82069928	0.411818	0.65173	NOT
JPH2	33.05187	-0.2957907	0.360482	-0.82054183	0.411907	0.651833	NOT
USP40	2169.768	-0.1221495	0.148872	-0.82050109	0.411931	0.651833	NOT
BTNL3	13.04124	0.75714191	0.922899	0.820395132	0.411991	0.65189	NOT
RP11-27I1	4.809735	-0.2975419	0.362716	-0.82031688	0.412035	0.651906	NOT
TBC1D13	1864.528	0.15236071	0.185742	0.820279886	0.412057	0.651906	NOT
AC010504.	9.510856	-0.182544	0.222546	-0.82025145	0.412073	0.651906	NOT
RP11-506H	4.138821	-0.3792426	0.462396	-0.82016834	0.41212	0.651943	NOT
CCBL1	805.8392	-0.1742493	0.21249	-0.82003494	0.412196	0.652025	NOT
MIR217HG	10.69353	-0.7562697	0.922776	-0.8195593	0.412467	0.652382	NOT
USP4	1548.444	0.11035519	0.134653	0.819555397	0.41247	0.652382	NOT
KRR1P1	2.192035	-0.3823315	0.466558	-0.81947285	0.412517	0.652418	NOT
SLC51A	3951.674	-0.3727699	0.45498	-0.81931054	0.412609	0.652526	NOT
PLCE1-AS1	1.292029	0.56710244	0.692221	0.819250358	0.412644	NA	NA
RP11-718(	0.856777	-0.6697367	0.817627	-0.81912207	0.412717	NA	NA
TBC1D4	1174.572	0.24711653	0.301739	0.81897357	0.412801	0.652724	NOT
APOC4	23.05729	-0.3945968	0.481825	-0.81896289	0.412808	0.652724	NOT
TTC7A	1856.616	-0.1734889	0.211841	-0.8189576	0.412811	0.652724	NOT
IL10RA	762.2807	-0.2716752	0.331747	-0.81892239	0.412831	0.652724	NOT
SOWAHA	655.2237	-0.3253349	0.397301	-0.81886233	0.412865	0.65274	NOT
RP11-806I	6.276514	-0.330044	0.40324	-0.81848005	0.413083	0.653047	NOT
CYP11A1	426.8212	-0.4950141	0.604886	-0.81835952	0.413152	0.653075	NOT

WNT7B	31.8861	0.55614858	0.679591	0.818357888	0.413153	0.653075	NOT
CALML3-AS	15.76744	-0.3598096	0.439692	-0.81832252	0.413173	0.653075	NOT
RP11-197M	2.21818	0.35913221	0.438899	0.81825622	0.413211	0.653097	NOT
DNAH11	176.98	-0.4661671	0.569756	-0.81818708	0.41325	0.653121	NOT
BCAR3	1050.798	0.16477852	0.20147	0.817881746	0.413425	0.653359	NOT
CTC-205M6	49.58213	-0.1884474	0.230453	-0.81772683	0.413513	0.65343	NOT
KIAA1024	14.7509	-0.3666382	0.448409	-0.81764314	0.413561	0.65343	NOT
GPRC6A	1.430894	1.0078974	1.232751	0.817600421	0.413585	0.65343	NOT
AC005702.	6.692151	0.19141687	0.234126	0.817581411	0.413596	0.65343	NOT
RPS19P1	5.86255	-0.3377982	0.413176	-0.81756465	0.413606	0.65343	NOT
RP11-378A	6.786548	0.29877895	0.365457	0.817549489	0.413614	0.65343	NOT
RP11-218C	11.26281	0.24750054	0.302784	0.817417457	0.41369	0.653512	NOT
RPS4XP23	1.169761	0.50789942	0.621372	0.81738441	0.413709	NA	NA
MIEF2	600.714	0.14979761	0.183276	0.817334846	0.413737	0.653548	NOT
RP11-20B2	1.314974	-0.4668381	0.57129	-0.81716439	0.413834	NA	NA
bP-2171C2	7.345211	0.56029507	0.685765	0.817036992	0.413907	0.653755	NOT
MYO18A	4978.307	-0.1972124	0.24138	-0.81702131	0.413916	0.653755	NOT
CCNL1	2648.042	-0.1495425	0.183061	-0.81690073	0.413985	0.653803	NOT
RP11-566F	50.25684	0.24360092	0.298207	0.81688415	0.413995	0.653803	NOT
XKR6	10.13811	0.37160045	0.45493	0.816829485	0.414026	0.653814	NOT
AP000442.	20.65706	-0.164954	0.20196	-0.81676392	0.414063	0.653821	NOT
WBSCR28	13.58393	-0.4659603	0.570514	-0.81673745	0.414078	0.653821	NOT
LZTS3	971.5405	-0.1888331	0.231246	-0.81658974	0.414163	0.653893	NOT
RAB40B	1040.67	0.19293402	0.236277	0.816560018	0.41418	0.653893	NOT
CDKN1B	2336.547	-0.150999	0.184943	-0.81646109	0.414236	0.653893	NOT
TRPM2	297.6509	0.28919868	0.354214	0.816451345	0.414242	0.653893	NOT
RP5-874C2	4.636607	-0.5045353	0.617965	-0.81644613	0.414245	0.653893	NOT
RP3-412A9	18.034	0.28163845	0.344975	0.816403443	0.414269	0.653894	NOT
EEF1GP1	16.83449	-0.4908601	0.601288	-0.81634785	0.414301	0.653906	NOT
APOL1	11318.27	-0.2688482	0.329395	-0.8161879	0.414393	0.654012	NOT
CKAP2	821.8955	0.21308716	0.26115	0.815956312	0.414525	0.654125	NOT
FLJ13224	2.191814	-0.3430922	0.420485	-0.81594325	0.414533	0.654125	NOT
WEE1	1743.795	0.18424873	0.225814	0.815933138	0.414538	0.654125	NOT
TAL2	11.06142	0.39649215	0.485971	0.815876757	0.414571	0.654125	NOT
IPP	496.8803	-0.1311779	0.160794	-0.815812	0.414608	0.654125	NOT
STPG2	4.255183	-0.3877789	0.47533	-0.81580972	0.414609	0.654125	NOT
SCGB2A2	0.393047	1.20620643	1.478655	0.815745792	0.414646	NA	NA
GTF2E1	493.7232	0.13188807	0.161686	0.815705808	0.414668	0.654181	NOT
RP11-299H	1.917107	-0.5232593	0.641622	-0.81552638	0.414771	0.654292	NOT
HNF4G	743.1914	0.25856776	0.317067	0.815498194	0.414787	0.654292	NOT
MZF1-AS1	96.86503	-0.2041593	0.250396	-0.81534547	0.414875	0.654385	NOT
TRBV5-6	2.08781	-0.4889691	0.599733	-0.81531145	0.414894	0.654385	NOT
ZNF496	981.5336	0.25299707	0.310504	0.814795619	0.415189	0.654813	NOT
CTC-465D4	1.298181	-0.9128892	1.120404	-0.81478595	0.415195	NA	NA
CYCSP10	2.270027	0.38458777	0.472079	0.814669053	0.415262	0.654889	NOT
DOCK3	28.34912	-0.3141306	0.385729	-0.81438196	0.415426	0.65511	NOT
PEX1	1020.333	-0.187719	0.230539	-0.81426203	0.415495	0.65518	NOT
MIR1295A	9.272233	-0.5338521	0.655669	-0.81420965	0.415525	0.655189	NOT
TMEM259	6684.212	-0.1262323	0.155062	-0.81407585	0.415601	0.655221	NOT

CROT	1701.605	-0.2356797	0.289512	-0.81405795	0.415612	0.655221	NOT
ZNF318	1523.807	0.13822057	0.169794	0.814048126	0.415617	0.655221	NOT
SS18L1	1701.525	-0.1934496	0.237668	-0.81394811	0.415675	0.655247	NOT
AF011889.	9.344857	0.33726049	0.414358	0.813935382	0.415682	0.655247	NOT
AC016999.	1.889286	-0.4027746	0.494894	-0.8138601	0.415725	0.655262	NOT
LYPLAL1-A	6.994471	-0.331527	0.407364	-0.8138345	0.41574	0.655262	NOT
OSBPL6	300.5342	0.27421962	0.336978	0.813761837	0.415781	0.655289	NOT
OGDHL	4793.423	-0.3923802	0.482418	-0.81336119	0.416011	0.655613	NOT
MTRF1	327.4958	-0.1518197	0.186714	-0.81311161	0.416154	0.6558	NOT
GATA3	79.11898	-0.3420049	0.420675	-0.81299104	0.416223	0.655871	NOT
MFN1	1199.801	0.10062743	0.123786	0.812913198	0.416268	0.655871	NOT
TMEM135	1656.113	-0.1536907	0.189063	-0.81290668	0.416272	0.655871	NOT
RP11-1152	7.639113	0.3886632	0.478219	0.812731142	0.416372	0.655985	NOT
CRABP1	1.447687	0.89041982	1.095636	0.812696738	0.416392	0.655985	NOT
MATK	149.9486	0.24213084	0.297963	0.812620518	0.416436	0.655994	NOT
CTD-2302E	3.875769	-0.299304	0.368328	-0.81260247	0.416446	0.655994	NOT
GRIK5	21.74095	-0.3505416	0.431489	-0.81239932	0.416563	0.656107	NOT
FBXO36	107.7117	-0.2357237	0.29016	-0.81239311	0.416566	0.656107	NOT
RP11-722E	23.25606	0.23053603	0.28383	0.812232542	0.416658	0.656211	NOT
HCG17	8.091265	-0.2963501	0.364876	-0.81219383	0.41668	0.656211	NOT
CTD-3092A	137.3666	-0.2182077	0.26872	-0.8120263	0.416777	0.656291	NOT
GTF2F1	2939.174	-0.1082739	0.133339	-0.81202028	0.41678	0.656291	NOT
AKAP17A	1743.327	-0.1335436	0.164489	-0.81186862	0.416867	0.65639	NOT
RP11-286N	24.64731	-0.153759	0.189401	-0.81181689	0.416897	0.656399	NOT
C2orf88	160.9268	-0.2907398	0.358167	-0.81174385	0.416939	0.656419	NOT
NTAN1	1043.566	0.14920698	0.183818	0.811710708	0.416958	0.656419	NOT
RP11-517A	1.555352	-0.3860906	0.475693	-0.81163882	0.416999	0.656445	NOT
ABCC8	27.48836	0.48217842	0.594119	0.811586189	0.417029	0.656455	NOT
ZNF597	169.9901	-0.1678365	0.206828	-0.81147992	0.41709	0.656464	NOT
PPAN	301.8113	-0.1893938	0.233401	-0.81145314	0.417105	0.656464	NOT
RP11-148I	1.966954	-0.448086	0.552204	-0.81144961	0.417108	0.656464	NOT
CKMT2-AS1	404.6314	-0.1934493	0.238426	-0.81136101	0.417158	0.656506	NOT
AC073257.	19.08381	0.31676178	0.390443	0.811287668	0.4172	0.656534	NOT
RP11-275F	2.575508	-0.2860991	0.352674	-0.81122835	0.417235	0.65655	NOT
ZNF716	44.40085	-0.9094269	1.121726	-0.81073855	0.417516	0.656954	NOT
CTD-2201(	4.471737	-0.4460556	0.550292	-0.81058048	0.417607	0.657059	NOT
IL12B	2.822904	-0.4387184	0.541305	-0.8104823	0.417663	0.65711	NOT
RP11-412I	86.98337	0.17820866	0.219893	0.810433648	0.417691	0.657113	NOT
KRT8P14	2.240602	0.40137199	0.49528	0.810393938	0.417714	0.657113	NOT
ADAM11	56.68151	-0.3771037	0.465414	-0.81025396	0.417794	0.657161	NOT
RP4-568B1	13.63503	-0.3495386	0.431412	-0.81022024	0.417814	0.657161	NOT
GABRP	27.29986	0.48441199	0.597881	0.810214155	0.417817	0.657161	NOT
GPSM1	681.0436	0.27181826	0.335654	0.809817054	0.418045	0.657482	NOT
ZNF302	971.7357	-0.1483099	0.183151	-0.80976963	0.418073	0.657487	NOT
RP5-968J1	1.53113	0.51022678	0.630456	0.80929736	0.418344	0.657876	NOT
ALG1L12P	1.584295	-0.5199766	0.642582	-0.8091986	0.418401	0.657901	NOT
RP11-440I	5.859702	-0.4971577	0.614393	-0.80918573	0.418408	0.657901	NOT
NFATC2IP	1276.997	0.1180732	0.145928	0.809118122	0.418447	0.657907	NOT
RP3-368A4	86.74119	-0.2284412	0.28236	-0.80904307	0.41849	0.657907	NOT

CHTF8	3132.059	-0.1038364	0.128345	-0.80903925	0.418493	0.657907	NOT
ZMYND12	108.1706	-0.315538	0.390049	-0.80896994	0.418532	0.657907	NOT
C9orf114	1430.945	0.12428328	0.153646	0.808895591	0.418575	0.657907	NOT
RP11-504(	2.640381	0.29780636	0.368177	0.808866863	0.418592	0.657907	NOT
OXSM	708.763	-0.1125396	0.139135	-0.80884912	0.418602	0.657907	NOT
ANXA4	9963.469	0.23548566	0.291157	0.808792727	0.418634	0.657907	NOT
AC006077.	20.85838	-0.3070232	0.379623	-0.80875821	0.418654	0.657907	NOT
RP11-276H	3.161716	0.34940572	0.432028	0.808757132	0.418655	0.657907	NOT
FAM27E3	6.310252	0.42606894	0.526997	0.808483922	0.418812	0.658116	NOT
FAM172BP	1.184128	-0.5905319	0.730461	-0.80843763	0.418839	NA	NA
AF277315.	9.64265	-1.3057017	1.615826	-0.80807064	0.41905	0.658452	NOT
C11orf21	31.5854	-0.2825235	0.34966	-0.80799507	0.419093	0.658455	NOT
TIMM44	2104.776	-0.1562297	0.193358	-0.80798275	0.419101	0.658455	NOT
SBDSP1	467.6769	-0.1823105	0.225698	-0.80776281	0.419227	0.658527	NOT
PLA2G4E-A	2.866642	-0.3362063	0.416223	-0.80775476	0.419232	0.658527	NOT
PPP1R2	1403.368	0.1197111	0.148203	0.807751577	0.419234	0.658527	NOT
CTA-212A2	2.92097	-0.3554165	0.440017	-0.80773396	0.419244	0.658527	NOT
LINC01265	1.346002	0.65144852	0.806888	0.807359238	0.41946	NA	NA
AC006942.	20.43911	-0.2765701	0.342577	-0.80732325	0.41948	0.658861	NOT
ZNF85	76.09564	0.26448576	0.327656	0.807206353	0.419548	0.658928	NOT
MREG	374.7227	-0.1843968	0.228456	-0.80714408	0.419583	0.658946	NOT
NSMAF	633.5075	0.17400603	0.215657	0.806865972	0.419744	0.659143	NOT
S1PR2	832.1051	-0.2505436	0.310527	-0.80683237	0.419763	0.659143	NOT
BNIP3P30	3.354626	-0.696487	0.863288	-0.80678438	0.419791	0.659143	NOT
CBY3	4.082313	-0.3213988	0.398388	-0.80674791	0.419812	0.659143	NOT
GID8	3511.631	0.1108143	0.137365	0.806716462	0.41983	0.659143	NOT
PRELP	938.2296	-0.4061357	0.503662	-0.80636604	0.420032	0.659413	NOT
JMJD4	1159.244	0.16372932	0.203068	0.806276843	0.420083	0.659413	NOT
CTDNEP1	2465.062	0.1565274	0.194147	0.80623096	0.42011	0.659413	NOT
ALMS1	781.5989	0.13491743	0.167358	0.806162022	0.420149	0.659413	NOT
ERICH6B	8.844924	-0.2889183	0.358392	-0.80615268	0.420155	0.659413	NOT
RP13-516M	8.115961	-0.3019686	0.374586	-0.80613902	0.420163	0.659413	NOT
RP11-480N	2.921525	0.36426223	0.451879	0.80610499	0.420182	0.659413	NOT
LSM10	1261.589	0.15343782	0.19035	0.806080607	0.420196	0.659413	NOT
RP11-405M	7.860491	-0.2980597	0.369784	-0.80603691	0.420222	0.659414	NOT
RP11-760D	4.8045	-0.8518535	1.057041	-0.80588532	0.420309	0.659513	NOT
RP11-118K	2.886773	0.47321432	0.58732	0.805717499	0.420406	0.659602	NOT
ODF2	1268.263	0.15465404	0.191949	0.80570243	0.420414	0.659602	NOT
TBX15	856.2181	-0.4267685	0.529744	-0.8056124	0.420466	0.659626	NOT
CTC-756D1	2.997876	0.40211258	0.499152	0.805591961	0.420478	0.659626	NOT
KCNK15	23.23504	-0.6199685	0.769881	-0.80527791	0.420659	0.659872	NOT
BRAF	926.1302	0.12597773	0.156485	0.805044408	0.420794	0.660045	NOT
UBR2	2337.833	-0.1256719	0.15613	-0.80492111	0.420865	0.660119	NOT
MIR25	10.9912	0.2797198	0.347612	0.804690211	0.420998	0.660261	NOT
LENG1	490.6673	-0.1650259	0.205083	-0.80467918	0.421005	0.660261	NOT
RCVRN	1.329783	-0.4460078	0.554287	-0.80465186	0.421021	NA	NA
AC007191.	56.38204	0.25366946	0.315263	0.804626838	0.421035	0.660271	NOT
LGALS4	19416.17	0.40355772	0.501613	0.804519484	0.421097	0.66033	NOT
RP1-37N7.	4.127636	0.57388655	0.713469	0.804360416	0.421189	0.660435	NOT

CTC-484M2	5.595383	-0.3108585	0.386543	-0.80420249	0.42128	0.66054	NOT
MAT2B	2836.75	-0.15336	0.190713	-0.80413932	0.421317	0.660559	NOT
RP11-63K6	23.2738	1.2260228	1.52513	0.80388108	0.421466	0.660755	NOT
PREB	5804.213	-0.1670186	0.207782	-0.80381464	0.421504	0.660777	NOT
RP11-138I	2.530209	-0.3139132	0.390667	-0.80353218	0.421667	0.660954	NOT
RP5-839B4	5.258188	0.59511328	0.740667	0.803483395	0.421695	0.660954	NOT
ZNRD1-AS1	140.3124	-0.208248	0.259196	-0.8034374	0.421722	0.660954	NOT
GS1-44D2(	9.660028	0.288807	0.359499	0.803358706	0.421767	0.660954	NOT
C4orf36	83.96252	-0.1632583	0.203223	-0.80334764	0.421774	0.660954	NOT
ILDR2	527.4246	0.43265047	0.538601	0.803285547	0.42181	0.660954	NOT
ASB2	58.7186	0.29452989	0.366668	0.803259422	0.421825	0.660954	NOT
PRICKLE3	262.8443	-0.1057952	0.131711	-0.80324005	0.421836	0.660954	NOT
ANKDD1A	107.2477	-0.188325	0.234463	-0.80321987	0.421848	0.660954	NOT
GLT8D1	2653.938	0.14454011	0.179961	0.803176546	0.421873	0.660954	NOT
CTC-1337H	11.17907	0.33178116	0.413102	0.803145924	0.42189	0.660954	NOT
TRAPPC3	2171.878	0.11384757	0.14176	0.803099934	0.421917	0.660954	NOT
LINC01202	5.166339	0.96955275	1.207307	0.803070777	0.421934	0.660954	NOT
BRI3BP	1387.462	0.14465636	0.180154	0.802957947	0.421999	0.661018	NOT
TNFRSF21	2080.454	0.2942494	0.366501	0.802860391	0.422055	0.661068	NOT
KCNN4	67.97513	0.29207795	0.363816	0.802817582	0.42208	0.661069	NOT
ZNF574	678.1949	-0.0954297	0.118885	-0.80270639	0.422144	0.661131	NOT
UHMK1	6810.708	-0.1470172	0.18317	-0.80262578	0.422191	0.661166	NOT
RP11-390K	9.974768	-0.3018257	0.376138	-0.8024339	0.422302	0.661302	NOT
TRAV39	1.184156	0.51610259	0.643229	0.802362012	0.422344	NA	NA
JMJD1C-AS	17.00507	0.30140977	0.375723	0.802212081	0.42243	0.661464	NOT
CTB-191K2	2.487491	-0.3153022	0.393087	-0.80211905	0.422484	0.661473	NOT
RP11-383(	2.401353	-0.3663354	0.45671	-0.80211866	0.422484	0.661473	NOT
TMEM173	667.5041	-0.2171315	0.270765	-0.80191755	0.422601	0.661617	NOT
ZNF663P	1.203618	0.7770973	0.969108	0.801868931	0.422629	NA	NA
DCTN6	667.6177	0.1346911	0.168001	0.801729592	0.422709	0.661749	NOT
HNRNPA1P5	2.660323	0.36552878	0.455978	0.801637045	0.422763	0.661794	NOT
CLPS	1.842186	1.22017893	1.523791	0.800751993	0.423275	0.662558	NOT
ATP13A1	3681.623	-0.114126	0.142538	-0.8006717	0.423322	0.662593	NOT
RP11-981(	1.332136	-0.6278313	0.784459	-0.80033635	0.423516	NA	NA
LTBP1	1677.627	-0.2940088	0.36743	-0.80017743	0.423608	0.663002	NOT
RP11-1109	22.45942	0.27530291	0.344115	0.800032162	0.423692	0.66308	NOT
IGLV1-50	1.555104	-0.4391118	0.548977	-0.79987324	0.423784	0.66308	NOT
RP11-305(	4.736442	-0.3076109	0.384635	-0.79974763	0.423857	0.66308	NOT
AC004593.	15.93614	0.4877596	0.609971	0.799644165	0.423917	0.66308	NOT
AC106869.	12.69486	-0.6024765	0.753444	-0.7996298	0.423925	0.66308	NOT
VAMP4	792.0839	-0.1477236	0.184743	-0.79961476	0.423934	0.66308	NOT
RP11-559N	2.359986	0.39634393	0.495674	0.799606051	0.423939	0.66308	NOT
SGCZ	2.875179	1.07061069	1.338936	0.799598139	0.423944	0.66308	NOT
RP11-832N	13.50289	0.28966912	0.362277	0.799578008	0.423955	0.66308	NOT
FCRLB	56.22032	0.3460599	0.432829	0.799530869	0.423983	0.66308	NOT
BCL2	302.9784	-0.2567159	0.321086	-0.79952418	0.423987	0.66308	NOT
RP11-347I	35.40797	-0.253284	0.316794	-0.79952236	0.423988	0.66308	NOT
LEP	2.759714	0.61892255	0.774132	0.799505412	0.423997	0.66308	NOT
URI1	2310.566	-0.0854026	0.106821	-0.79949507	0.424003	0.66308	NOT

ZDHHC14	1291.162	-0.1760222	0.220176	-0.79945979	0.424024	0.66308	NOT
RP11-66A2	6.892309	-0.5083914	0.636081	-0.79925551	0.424142	0.66319	NOT
WDR44	710.2147	-0.1932718	0.24182	-0.79923877	0.424152	0.66319	NOT
RP11-66N2	2.738665	-0.2648614	0.331428	-0.79915313	0.424202	0.66319	NOT
CH507-9B2	71.62749	-0.157273	0.196803	-0.79913999	0.424209	0.66319	NOT
YIPF5	1824.736	-0.0995336	0.124553	-0.79912723	0.424217	0.66319	NOT
ACP2	4640.742	-0.1439011	0.180119	-0.79892272	0.424335	0.663337	NOT
SPATA5	300.3311	0.15596453	0.195263	0.798741588	0.42444	0.663463	NOT
THBS3	777.3477	-0.1812952	0.227045	-0.79849967	0.424581	0.663602	NOT
DDX60	967.4239	-0.2568146	0.32163	-0.79847924	0.424592	0.663602	NOT
RP11-426I	8.687935	-0.2609348	0.32681	-0.79842927	0.424621	0.663602	NOT
RHOU	3813.983	-0.1825887	0.228696	-0.79838868	0.424645	0.663602	NOT
RP13-942N	42.74011	-0.2365899	0.296338	-0.79837732	0.424652	0.663602	NOT
RP13-128C	15.80709	-0.297582	0.372829	-0.79817278	0.42477	0.663749	NOT
CNPY2	2017.898	0.17812873	0.223224	0.797982973	0.42488	0.663883	NOT
BMP2	605.2157	0.24223459	0.303824	0.797284919	0.425286	0.664417	NOT
DENND6A	813.7925	0.0961229	0.120564	0.797276328	0.425291	0.664417	NOT
RP11-669E	4.220023	-0.3126793	0.392189	-0.79726749	0.425296	0.664417	NOT
NCDN	1000.046	0.17498315	0.219504	0.797174778	0.42535	0.664419	NOT
AC007000.	2.887797	-0.256303	0.321518	-0.79716512	0.425355	0.664419	NOT
RP11-405A	6.649144	-0.5352611	0.671478	-0.79713914	0.42537	0.664419	NOT
BEST1	108.477	0.23737551	0.297836	0.797000703	0.425451	0.664506	NOT
FAM109A	1225.162	0.17162979	0.215383	0.7968586	0.425533	0.664579	NOT
N4BP3	134.8941	0.23873668	0.299606	0.796836135	0.425546	0.664579	NOT
CHRM2	2.717614	-0.6866493	0.861904	-0.7966659	0.425645	0.664684	NOT
AC006042.	15.0383	-0.2987784	0.375053	-0.79663045	0.425666	0.664684	NOT
RHOG	2094.436	0.15720923	0.197352	0.79659366	0.425687	0.664684	NOT
RP4-785G1	8.616841	-0.3524401	0.442585	-0.79632109	0.425845	0.664893	NOT
PRIMPOL	364.885	-0.1381562	0.173534	-0.79613394	0.425954	0.665008	NOT
DCUN1D4	1997.896	-0.1064511	0.133716	-0.79610004	0.425974	0.665008	NOT
NUDT9	1820.72	0.15684565	0.197035	0.796029297	0.426015	0.665008	NOT
PABPN1	3636.78	0.13175449	0.165532	0.795946489	0.426063	0.665008	NOT
MTIF3	1654.169	-0.1446365	0.181723	-0.79591962	0.426079	0.665008	NOT
COX6A1P2	72.38291	0.21736353	0.273104	0.795901034	0.42609	0.665008	NOT
PPIHP1	1.943338	-0.4111333	0.516564	-0.79589985	0.42609	0.665008	NOT
GPR89A	281.1184	0.16197801	0.203537	0.795817119	0.426138	0.665044	NOT
RP11-274E	27.10731	-0.1940458	0.243854	-0.79574726	0.426179	0.66507	NOT
PPP2R1A	11589.39	-0.1568756	0.197171	-0.79563195	0.426246	0.665086	NOT
HMGN1P4	1.81521	0.56085822	0.70494	0.795610834	0.426258	0.665086	NOT
AC091814.	0.76333	-0.6645468	0.835309	-0.79557037	0.426282	NA	NA
TPSD1	9.222939	-0.5632989	0.708047	-0.79556678	0.426284	0.665086	NOT
RP11-843F	6.695684	-0.3175967	0.399211	-0.79556075	0.426287	0.665086	NOT
RP5-828H9	3.588959	0.66789104	0.839594	0.795493287	0.426327	0.665109	NOT
RP4-613B2	3.925002	-0.2931821	0.368586	-0.79542418	0.426367	0.665126	NOT
PVRL3	994.157	0.21395803	0.269028	0.795299168	0.426439	0.665126	NOT
TRPV4	419.327	0.35447776	0.44574	0.795256181	0.426464	0.665126	NOT
IGHV1-45	1.695034	-0.6016383	0.75655	-0.79523925	0.426474	0.665126	NOT
DNM1P46	8.030382	-0.4613749	0.580185	-0.79522007	0.426486	0.665126	NOT
RP11-415J	45.27971	-0.2294322	0.288522	-0.7951984	0.426498	0.665126	NOT

DNM1L	2037.445	0.11488229	0.144473	0.795179309	0.426509	0.665126	NOT
ZCCHC11	1447.988	0.17889022	0.225008	0.7950405	0.42659	0.665214	NOT
RPSAP14	4.407018	0.2571393	0.323506	0.794851905	0.4267	0.665304	NOT
RP4-816N1	10.28629	-0.2160024	0.27177	-0.79480011	0.42673	0.665304	NOT
SGTA	4183.624	0.11800382	0.148482	0.79473547	0.426767	0.665304	NOT
IWS1	1960.107	0.07621255	0.095898	0.794727171	0.426772	0.665304	NOT
CMTM8	1329.365	-0.1854534	0.233367	-0.79468545	0.426796	0.665304	NOT
WBP2	9217.487	-0.1463669	0.18419	-0.79465083	0.426817	0.665304	NOT
RP4-601P9	34.10174	0.55137726	0.693871	0.794639867	0.426823	0.665304	NOT
RP11-483I	10.03064	-0.553122	0.696098	-0.79460364	0.426844	0.665304	NOT
CRCP	1856.581	-0.1177767	0.148236	-0.79452338	0.426891	0.665339	NOT
PRICKLE4	310.4715	0.15831603	0.199339	0.794203242	0.427077	0.665591	NOT
NRTN	365.9167	-0.2715228	0.341944	-0.79405575	0.427163	0.665686	NOT
RP11-351I	4.637815	-0.255399	0.321747	-0.7937885	0.427319	0.66586	NOT
CCDC117	167.1545	-0.2326701	0.293117	-0.79377979	0.427324	0.66586	NOT
G3BP2	3562.441	-0.1244828	0.156838	-0.79370137	0.427369	0.665893	NOT
ZNF37A	776.9452	0.14262624	0.179719	0.793608395	0.427423	0.665939	NOT
IL1B	72.48233	0.33208898	0.418501	0.793520856	0.427474	0.66598	NOT
ACSL5	7164.785	-0.3304213	0.416438	-0.79344588	0.427518	0.66601	NOT
BAG5	1235.374	-0.097363	0.12273	-0.79331096	0.427597	0.666094	NOT
KHNYN	1615.287	-0.1724751	0.217468	-0.79310684	0.427716	0.666238	NOT
FCGR1C	5.721396	0.40119485	0.505877	0.793068368	0.427738	0.666238	NOT
CREB3L3	12406.41	-0.3106399	0.391722	-0.79301032	0.427772	0.666252	NOT
GLIPR1L1	6.496263	-0.3423824	0.431846	-0.79283437	0.427874	0.666342	NOT
ELF1	1383.888	-0.1686339	0.212699	-0.79282712	0.427879	0.666342	NOT
AC005082.	4.914569	-0.3280578	0.413883	-0.79263469	0.427991	0.666478	NOT
TMEM229B	222.9725	0.29512351	0.372463	0.792356028	0.428153	0.666693	NOT
XPNPEP2	3100.603	0.53777432	0.678777	0.792269235	0.428204	0.666734	NOT
POLR3A	1196.534	0.10157513	0.128242	0.792059147	0.428326	0.666838	NOT
SLC43A2	1215.599	-0.2471884	0.312103	-0.79200875	0.428356	0.666838	NOT
EIF4BP3	45.70125	-0.2396438	0.302597	-0.79195741	0.428386	0.666838	NOT
DDX41	3730.776	0.13297214	0.167904	0.791951052	0.428389	0.666838	NOT
MAD1L1	1756.146	-0.1868196	0.235914	-0.79189645	0.428421	0.666838	NOT
FSIP2	26.36579	0.36021687	0.454891	0.791875753	0.428433	0.666838	NOT
OR7E38P	287.9647	0.2579476	0.325749	0.791858849	0.428443	0.666838	NOT
MTMR8	18.65705	0.31818	0.40184	0.791808271	0.428472	0.666846	NOT
AC008060.	2.868128	-0.8907301	1.12516	-0.7916473	0.428566	0.666954	NOT
SLC9A6	807.7144	0.13736676	0.173561	0.791462978	0.428674	0.667083	NOT
PAX6	106.7216	0.27303136	0.345056	0.791266387	0.428789	0.667223	NOT
CD53	831.9499	0.26739516	0.338098	0.790880001	0.429014	0.667513	NOT
ORMDL3	12122.49	-0.1942661	0.245638	-0.79086265	0.429024	0.667513	NOT
LENG8	3694	-0.1658424	0.20972	-0.79078002	0.429072	0.66755	NOT
BNC1	3.3378	-0.3680727	0.465589	-0.7905531	0.429205	0.667718	NOT
AC108676.	12.03501	0.30120208	0.381066	0.790419464	0.429283	0.66778	NOT
QARS	7522.194	0.10224952	0.129364	0.790400124	0.429294	0.66778	NOT
NLRP7	2.139283	-0.4684554	0.592752	-0.79030526	0.42935	0.66779	NOT
PPIL2	2212.537	0.09359451	0.118428	0.790304301	0.42935	0.66779	NOT
AC007383.	86.25774	0.199221	0.252137	0.790129447	0.429452	0.667911	NOT
RP11-338I	7.802463	-0.3009167	0.38087	-0.79007628	0.429483	0.667921	NOT

RBMS2	541.0824	-0.1811688	0.229344	-0.78994187	0.429562	0.668005	NOT
MT1E	2434.361	-0.4249875	0.538087	-0.78981194	0.429638	0.668084	NOT
POLR1E	795.6544	0.126768	0.16059	0.789387063	0.429886	0.668432	NOT
CCDC85B	743.8924	-0.2303212	0.291915	-0.78900134	0.430111	0.668692	NOT
LAMTOR3	1183.747	0.09782612	0.124002	0.7889083	0.430166	0.668692	NOT
WDR81	2505.412	-0.1386575	0.175761	-0.78889921	0.430171	0.668692	NOT
ULK3	1556.652	-0.1360975	0.172521	-0.78887622	0.430184	0.668692	NOT
RP11-380I	12.31606	0.22296735	0.282641	0.788871251	0.430187	0.668692	NOT
LINC01447	11.63917	0.5172092	0.655682	0.78881103	0.430222	0.668692	NOT
RP11-754I	1.928008	-0.3639881	0.461442	-0.78880534	0.430226	0.668692	NOT
RP11-320I	17.58045	-0.2014149	0.255375	-0.78870335	0.430285	0.668747	NOT
ABHD17A	1175.283	-0.1376082	0.174514	-0.78852201	0.430391	0.668823	NOT
VTI1B	2677.514	-0.1148086	0.145609	-0.78847164	0.430421	0.668823	NOT
SRRD	657.5019	0.11620986	0.147392	0.788441263	0.430439	0.668823	NOT
TWF1	1877.272	0.12346151	0.156593	0.78842411	0.430449	0.668823	NOT
AGGF1P2	1.287323	0.61819324	0.78409	0.788421142	0.43045	NA	NA
AP000783.	3.768364	0.42981707	0.545171	0.788408306	0.430458	0.668823	NOT
TMEM8B	634.1822	0.1577933	0.200168	0.788304055	0.430519	0.66888	NOT
H3F3B	16946.29	-0.1282244	0.162688	-0.7881626	0.430602	0.668945	NOT
RP11-264I	2.405116	0.36501182	0.463126	0.788147716	0.43061	0.668945	NOT
TPI1	20570.52	0.151907	0.192753	0.78809023	0.430644	0.668959	NOT
RD3L	0.206141	-1.249094	1.585095	-0.78802454	0.430682	NA	NA
CTC-479C5	38.97523	-0.1574101	0.199758	-0.78800278	0.430695	0.669	NOT
RNASEK-C1	18.26748	-0.207056	0.262835	-0.78777797	0.430827	0.669131	NOT
SLC9A3R2	9000.486	-0.2031041	0.25782	-0.78777447	0.430829	0.669131	NOT
LINC01532	22.06018	0.50642602	0.643269	0.78726942	0.431124	0.669552	NOT
SDC3	2856.191	0.19557581	0.24849	0.787055727	0.431249	0.669682	NOT
ZNF638	3634.887	-0.0888024	0.112831	-0.78704181	0.431257	0.669682	NOT
CD207	25.31745	0.39509367	0.502104	0.786876284	0.431354	0.66977	NOT
RP11-127I	4.09772	0.32790379	0.416744	0.786822818	0.431386	0.66977	NOT
KRT12	7.743857	0.72365649	0.919725	0.786818283	0.431388	0.66977	NOT
TPP1	14044.65	0.1391577	0.176923	0.786544058	0.431549	0.669981	NOT
RP11-45M2	26.30717	-0.2834213	0.360416	-0.78637201	0.43165	0.670067	NOT
OVCH1	3.391147	-0.4258084	0.541489	-0.78636518	0.431654	0.670067	NOT
GDF15	6000.455	0.36158306	0.459844	0.786317128	0.431682	0.670072	NOT
NAT16	7.818101	-0.3948993	0.502297	-0.78618745	0.431758	0.670126	NOT
TVP23A	35.05447	0.2434211	0.309627	0.786174166	0.431765	0.670126	NOT
NHLRC2	1206.539	-0.1218457	0.155004	-0.78608349	0.431819	0.670159	NOT
ABCG2	1580.002	-0.3613708	0.45974	-0.78603303	0.431848	0.670159	NOT
RP11-413I	3.89613	-0.3156009	0.401522	-0.7860109	0.431861	0.670159	NOT
NNT-AS1	685.8963	-0.1213872	0.154464	-0.78586057	0.431949	0.670257	NOT
NDUFA6	4329.963	-0.181451	0.230943	-0.78569768	0.432045	0.670366	NOT
CFAP99	2.339442	0.50137423	0.63816	0.785656476	0.432069	0.670366	NOT
TTC37	2934.514	-0.1638607	0.208588	-0.78557044	0.432119	0.670406	NOT
ZCCHC16	53.54379	0.63927451	0.813816	0.785527424	0.432144	0.670407	NOT
TRBV10-3	2.706375	-0.4430117	0.564037	-0.78543065	0.432201	0.670457	NOT
ESCO1	805.9878	0.10786019	0.137354	0.785270929	0.432295	0.670523	NOT
RP11-350N	1.395588	1.02482044	1.30514	0.785218784	0.432325	0.670523	NOT
DGKB	14.50818	0.42301395	0.53873	0.785205255	0.432333	0.670523	NOT

TNFRSF11E	463.3089	0.40050907	0.510144	0.785090058	0.432401	0.670523	NOT
MRPS31	749.9451	-0.146781	0.186963	-0.78507933	0.432407	0.670523	NOT
RP1-60019	1293.795	-0.2577687	0.32834	-0.78506631	0.432415	0.670523	NOT
AF131215.	23.377	0.35109876	0.447224	0.78506232	0.432417	0.670523	NOT
PALM3	1087.93	-0.3055446	0.389256	-0.78494513	0.432486	0.670527	NOT
CTD-2270F	31.15728	-0.2536231	0.323111	-0.78494102	0.432488	0.670527	NOT
RP13-977J	2.559305	-0.376438	0.47958	-0.78493196	0.432493	0.670527	NOT
RP11-488I	1.65167	1.12057428	1.427713	0.784873623	0.432528	0.670541	NOT
CBLN2	1.924354	-0.740797	0.943973	-0.78476476	0.432592	0.670567	NOT
FAM21EP	18.2122	-0.2378932	0.303141	-0.78476137	0.432593	0.670567	NOT
AC096574.	8.181816	0.38726177	0.493519	0.784694492	0.432633	0.670572	NOT
TAF5	245.3874	-0.1279131	0.163015	-0.78467195	0.432646	0.670572	NOT
CTC-42502	7.380195	-0.3527967	0.44973	-0.78446339	0.432768	0.670698	NOT
LRRC52	6.943692	0.78678027	1.002973	0.784447999	0.432777	0.670698	NOT
FEZF1	7.533336	-0.6822254	0.86988	-0.784275	0.432879	0.670817	NOT
AC022182.	2.158499	0.3689712	0.470508	0.784198173	0.432924	0.670849	NOT
INO80E	1643.872	0.1130052	0.144144	0.783972176	0.433056	0.671016	NOT
AC097461.	3.284426	0.32833529	0.418912	0.78378172	0.433168	0.671151	NOT
GATA6-AS1	94.86102	0.30963032	0.395079	0.783717382	0.433206	0.671171	NOT
AC090154.	5.495654	0.28600753	0.365056	0.783463142	0.433355	0.671326	NOT
SLC14A2	8.404365	-0.4494882	0.57372	-0.7834627	0.433355	0.671326	NOT
AC012066.	1.834906	0.38123222	0.486713	0.783280069	0.433463	0.671454	NOT
NATD1	644.2222	-0.16671	0.212887	-0.78309314	0.433572	0.671585	NOT
RP11-615I	1.957354	-0.335874	0.428984	-0.78295215	0.433655	0.671675	NOT
MDH2	15747.56	-0.16617	0.212273	-0.78281376	0.433736	0.671763	NOT
TANC1	781.938	-0.1731388	0.221191	-0.78275681	0.43377	0.671765	NOT
ANO6	6030.436	-0.1559184	0.199212	-0.78267449	0.433818	0.671765	NOT
PLCB2	428.7948	-0.2341465	0.299167	-0.78266065	0.433826	0.671765	NOT
COG6	533.1581	-0.1534173	0.196025	-0.7826424	0.433837	0.671765	NOT
CTD-2021H	6.635048	0.61368925	0.78434	0.782427957	0.433963	0.671905	NOT
HOXB2	115.551	0.26742176	0.341849	0.782280716	0.43405	0.671905	NOT
RP11-44N2	10.04455	-0.2999894	0.383487	-0.78226822	0.434057	0.671905	NOT
DYRK1B	720.555	-0.1988488	0.254215	-0.78220572	0.434094	0.671905	NOT
FN3K	4607.737	-0.2052928	0.26247	-0.78215764	0.434122	0.671905	NOT
ZNF749	128.4615	-0.1821058	0.232831	-0.78213691	0.434134	0.671905	NOT
TMEM234	346.9489	0.1460872	0.186783	0.782121944	0.434143	0.671905	NOT
LINC00272	1.735579	0.81699959	1.044597	0.78211916	0.434145	0.671905	NOT
HSD17B10	6067.254	-0.1638569	0.209516	-0.78207351	0.434171	0.671905	NOT
AC022210.	8.145383	0.25660294	0.328109	0.782067026	0.434175	0.671905	NOT
RP1-273G1	2.255158	0.39889456	0.510181	0.78186823	0.434292	0.672048	NOT
PROL1	36.80715	-0.6213374	0.794736	-0.78181658	0.434322	0.672056	NOT
RP3-525L6	4.110631	0.26238159	0.335699	0.781597403	0.434451	0.672187	NOT
POPDC2	61.30726	-0.1969039	0.251928	-0.78158823	0.434457	0.672187	NOT
COL6A5	1.571516	-0.7845171	1.003872	-0.78149113	0.434514	0.672229	NOT
RP11-399H	2.844727	-1.2431346	1.590788	-0.78145826	0.434533	0.672229	NOT
ARPC3P1	4.847535	0.22251472	0.284778	0.781361626	0.43459	0.672236	NOT
F2RL2	91.71511	0.39558606	0.506291	0.781340776	0.434602	0.672236	NOT
APPBP2	1298.069	-0.1107879	0.141795	-0.78132445	0.434612	0.672236	NOT
SPTBN5	147.3825	0.2932716	0.375474	0.781070675	0.434761	0.672428	NOT

CTD-2313J	19.4967	0.20572366	0.263487	0.780774155	0.434935	0.67266	NOT
XPR1	1694.074	0.17557469	0.22489	0.780714322	0.434971	0.672676	NOT
FAM163A	9.017583	0.32254733	0.41319	0.780627804	0.435021	0.672716	NOT
TMUB1	3235.773	0.16260691	0.208336	0.780503991	0.435094	0.672739	NOT
CTPS1	2401.165	-0.159813	0.204762	-0.78048158	0.435107	0.672739	NOT
MICE	31.2136	0.26560647	0.340313	0.780475873	0.435111	0.672739	NOT
FAM83C-AS	5.160019	0.30082461	0.385472	0.780406826	0.435151	0.672764	NOT
C5orf24	2513.339	-0.1271278	0.162929	-0.78026487	0.435235	0.672855	NOT
ZNF800	655.0032	-0.1278733	0.164002	-0.77970724	0.435563	0.673274	NOT
PCNXL4	1087.044	0.14549611	0.186605	0.779699804	0.435568	0.673274	NOT
AF121897.	1.552538	-0.6341425	0.81334	-0.7796771	0.435581	0.673274	NOT
RP11-613I	4.786598	-0.2847488	0.365259	-0.77958103	0.435638	0.673289	NOT
MUC3A	1473.834	-0.4532585	0.581416	-0.77957711	0.43564	0.673289	NOT
GPC5	139.3306	0.54708645	0.701847	0.77949559	0.435688	0.673324	NOT
FAM210A	815.4257	0.10984953	0.140956	0.779315823	0.435794	0.67345	NOT
KDM7A	812.8081	-0.1505654	0.193238	-0.7791725	0.435878	0.673498	NOT
RP11-663F	1.558624	-0.4803508	0.616515	-0.77913857	0.435898	0.673498	NOT
RSPO4	11.36913	0.49340574	0.633273	0.77913587	0.4359	0.673498	NOT
CP	41391.59	-0.3651689	0.468787	-0.7789647	0.436001	0.6736	NOT
HOXB4	58.34871	-0.2651988	0.340461	-0.77894009	0.436015	0.6736	NOT
RASD1	2327.363	-0.3661905	0.470152	-0.77887673	0.436052	0.673619	NOT
RP11-678C	20.98246	0.55595735	0.713878	0.77878443	0.436107	0.673665	NOT
DSP	12867.6	0.18436457	0.236749	0.778734788	0.436136	0.673672	NOT
PNLIP	0.799498	-1.0775745	1.383963	-0.77861524	0.436206	NA	NA
CMTM2	6.149072	-0.2691454	0.345715	-0.77851866	0.436263	0.673823	NOT
API5	3243.04	-0.0993901	0.127671	-0.77848385	0.436284	0.673823	NOT
ABC7-4304	1.858618	0.30072425	0.386431	0.77820945	0.436446	0.674035	NOT
KIF28P	30.57865	-0.3171969	0.407639	-0.77813221	0.436491	0.674067	NOT
C12orf4	463.4597	0.10687305	0.137362	0.778040904	0.436545	0.674107	NOT
EWSAT1	22.98422	0.39914569	0.513039	0.778003318	0.436567	0.674107	NOT
C2orf68	1604.332	0.11288184	0.145118	0.777862038	0.43665	0.674168	NOT
SIPA1L1	1973.717	-0.1434692	0.184458	-0.77778631	0.436695	0.674168	NOT
RTBDN	15.39855	0.51618269	0.663658	0.777783789	0.436696	0.674168	NOT
CTD-3051I	2.948886	-0.3814414	0.490454	-0.77773094	0.436728	0.674168	NOT
SNAPC4	882.3831	0.16123569	0.207317	0.777725699	0.436731	0.674168	NOT
TMEM187	599.3048	-0.18077	0.232473	-0.77759725	0.436806	0.674247	NOT
PPIB	16045.29	-0.1336849	0.171934	-0.7775352	0.436843	0.674262	NOT
CTC-325H2	1.733534	-0.4305353	0.553746	-0.77749668	0.436866	0.674262	NOT
AC092159.	2.774466	0.32653202	0.420181	0.777122974	0.437086	0.674478	NOT
RP11-573I	3.428401	0.3931373	0.505909	0.77709132	0.437105	0.674478	NOT
RP11-644K	1.942227	-0.3613635	0.465065	-0.77701774	0.437148	0.674478	NOT
TYSND1	1537.744	0.17064194	0.219613	0.777013319	0.437151	0.674478	NOT
MIB1	1973.866	-0.150202	0.193317	-0.77697191	0.437175	0.674478	NOT
RP11-245J	1.879981	0.4094845	0.527035	0.776958439	0.437183	0.674478	NOT
NLRC5	1276.967	-0.2435975	0.313534	-0.7769399	0.437194	0.674478	NOT
CTD-2554C	5.5211	0.44699674	0.575343	0.776921817	0.437205	0.674478	NOT
COL4A5	606.8773	-0.4784618	0.616045	-0.77666713	0.437355	0.674671	NOT
TTC12	318.8771	-0.1431182	0.184322	-0.77645803	0.437479	0.674823	NOT
DNAH7	17.28558	0.36326041	0.467926	0.776319552	0.43756	0.674884	NOT

CFAP221	129.0674	-0.4497651	0.579396	-0.77626556	0.437592	0.674884	NOT
NAA15	1458.384	0.10139557	0.13062	0.776265456	0.437592	0.674884	NOT
PPIL6	25.38588	0.22847616	0.294353	0.776198733	0.437632	0.674895	NOT
OSTN	2.701716	-0.5556371	0.715871	-0.77616924	0.437649	0.674895	NOT
JUN	7241.98	-0.1998099	0.257449	-0.77611554	0.437681	0.674905	NOT
NAV3	401.4175	0.43324437	0.558303	0.776002332	0.437748	0.674945	NOT
NEDD9	1926.319	0.22394263	0.288591	0.775986885	0.437757	0.674945	NOT
ANKRD39	726.3163	0.14194267	0.182938	0.775907713	0.437803	0.674979	NOT
RP11-930I	8.86682	0.3198219	0.412266	0.77576516	0.437888	0.67504	NOT
BRWD3	676.3348	-0.1701724	0.219363	-0.77575698	0.437892	0.67504	NOT
CDH18	5.852132	0.88415083	1.139835	0.775683069	0.437936	0.675069	NOT
RP11-110S	4.285932	0.33640501	0.433797	0.775490022	0.43805	0.675206	NOT
ERBB4	2.897951	0.61862261	0.797847	0.775364757	0.438124	0.675282	NOT
RP11-406A	5.592798	-0.6129632	0.790732	-0.77518465	0.438231	0.675367	NOT
LAMP1	21463.58	0.14427045	0.186131	0.775101307	0.43828	0.675367	NOT
TEX21P	6.569502	-0.2956958	0.381507	-0.77507354	0.438296	0.675367	NOT
GOSR2	1013.515	0.08927332	0.115182	0.775060207	0.438304	0.675367	NOT
RP11-347C	13.96564	-0.289368	0.373355	-0.7750486	0.438311	0.675367	NOT
CMTR2	695.1301	-0.1595792	0.205904	-0.77501783	0.438329	0.675367	NOT
CPB1	9.34303	-0.4319928	0.55748	-0.77490333	0.438397	0.675433	NOT
TLE1P1	58.44956	0.18936022	0.24438	0.774860629	0.438422	0.675434	NOT
TMC8	427.6645	-0.2685162	0.346676	-0.77454594	0.438608	0.675659	NOT
NEK3	463.3789	-0.2494311	0.32205	-0.77451023	0.438629	0.675659	NOT
RARB	153.8814	-0.2603445	0.336176	-0.77443001	0.438677	0.675659	NOT
XKR3	3.171128	-0.7439589	0.960659	-0.77442548	0.438679	0.675659	NOT
DLX3	5.965242	0.71551339	0.923956	0.774402313	0.438693	0.675659	NOT
CDC34	4244.938	0.19574214	0.252799	0.774299226	0.438754	0.675715	NOT
SLITRK5	9.597492	-0.476342	0.615242	-0.77423482	0.438792	0.675735	NOT
MAPKAP1	3347.556	-0.0884038	0.114208	-0.77406192	0.438894	0.675807	NOT
FHP1	5.79742	0.37182372	0.480386	0.774010012	0.438925	0.675807	NOT
ARG2	164.0936	0.3142975	0.406074	0.773989885	0.438937	0.675807	NOT
CTD-2186M	2.843478	0.45215031	0.584188	0.773980884	0.438942	0.675807	NOT
RP11-790S	2.535372	0.35804648	0.462625	0.773945732	0.438963	0.675807	NOT
RP11-680A	27.31019	0.22037457	0.284767	0.77387586	0.439004	0.675832	NOT
SUCLG2P2	20.34637	-0.2526151	0.326481	-0.77375138	0.439078	0.675869	NOT
CTD-2083E	4.443203	-0.2692312	0.347956	-0.77375121	0.439078	0.675869	NOT
THAP10	95.09623	-0.2460413	0.318029	-0.77364397	0.439141	0.675895	NOT
RPL17P43	1.907219	0.38659846	0.499715	0.773637881	0.439145	0.675895	NOT
LUM	2018.294	-0.4431337	0.57291	-0.77347826	0.439239	0.675984	NOT
CTD-2526A	4.887467	-0.3026741	0.391327	-0.7734555	0.439253	0.675984	NOT
RP11-522N	1.689303	-0.4219833	0.54562	-0.77340211	0.439284	0.675995	NOT
PRSS27	29.71191	0.22047729	0.285097	0.773340766	0.439321	0.676008	NOT
NOVA1-AS1	16.3924	0.65634308	0.848753	0.773303115	0.439343	0.676008	NOT
PLD4	76.41805	0.30794893	0.398278	0.773201596	0.439403	0.676062	NOT
CFAP97	1392.707	-0.1455983	0.188343	-0.77304754	0.439494	0.676164	NOT
AP003068.	40.28381	-0.2281405	0.295155	-0.77295232	0.439551	0.676213	NOT
PACS2	1940.805	-0.1216133	0.157349	-0.77288706	0.439589	0.676233	NOT
RP11-575F	35.62485	-0.407296	0.527008	-0.77284581	0.439614	0.676233	NOT
AC005753.	2.139775	-0.3712411	0.480416	-0.77274974	0.439671	0.676249	NOT

PPP1R26-A	45.87353	0.24140946	0.312416	0.772718918	0.439689	0.676249	NOT
MAP2K5	713.9492	-0.1278589	0.16547	-0.77270219	0.439699	0.676249	NOT
TRIB2	1593.403	-0.2589511	0.335183	-0.77256675	0.439779	0.676334	NOT
IL12A-AS1	1.761834	0.56309751	0.728965	0.772461217	0.439841	0.676391	NOT
PLEKHH1	230.0643	0.21162331	0.273982	0.772398866	0.439878	0.676404	NOT
CPXM2	191.9649	-0.3408539	0.441313	-0.77236341	0.439899	0.676404	NOT
RP3-3910	4.803597	-0.3190034	0.413108	-0.77220324	0.439994	0.676511	NOT
RP4-669P1	22.2861	-0.395258	0.511899	-0.77214015	0.440031	0.676517	NOT
RP11-728K	4.062861	-0.3127387	0.405043	-0.77211289	0.440048	0.676517	NOT
UBE4B	2058.935	-0.1389615	0.179988	-0.77205824	0.44008	0.676528	NOT
TBC1D3D	0.622436	1.23531	1.600634	0.771762833	0.440255	NA	NA
LINC0115(	4.152682	0.34472166	0.446704	0.77170005	0.440292	0.676816	NOT
RBM33	1727.317	-0.0900366	0.116718	-0.77139993	0.44047	0.677051	NOT
SEC14L3	67.82906	-0.5160511	0.669242	-0.77109738	0.440649	0.677285	NOT
CD247	166.7924	-0.2444476	0.317036	-0.77104069	0.440683	0.677285	NOT
LRRC37A2	88.3136	-0.1663357	0.215735	-0.77101682	0.440697	0.677285	NOT
GALNT7	189.2098	0.3000051	0.389168	0.770889034	0.440773	0.677358	NOT
CTD-3252(	219.5569	-0.3114859	0.40408	-0.77085256	0.440794	0.677358	NOT
RP5-1033H	197.8282	0.29522515	0.383008	0.770806283	0.440822	0.677361	NOT
RP11-690F	1.724536	-0.350738	0.455165	-0.77057244	0.44096	0.677508	NOT
RP11-452I	172.7974	-0.2218108	0.287856	-0.7705608	0.440967	0.677508	NOT
AARS	9077.868	0.13183408	0.171102	0.770497833	0.441005	0.677527	NOT
AMZ2P1	272.6646	-0.1385406	0.179844	-0.77033995	0.441098	0.677633	NOT
RP11-477I	35.11048	-0.1835224	0.238252	-0.77028799	0.441129	0.677642	NOT
GALNT2	8438.422	-0.1582106	0.205441	-0.77010359	0.441238	0.677759	NOT
SIX5	510.298	0.15156392	0.196817	0.770075322	0.441255	0.677759	NOT
EMC4	3098.992	0.10351973	0.134442	0.769993047	0.441304	0.677795	NOT
EMILIN2	402.0728	0.25513827	0.331398	0.769883862	0.441369	0.677841	NOT
AC084125.	21.07262	-0.2236731	0.290538	-0.76985863	0.441384	0.677841	NOT
AC107081.	6.765804	0.28273678	0.367289	0.76979438	0.441422	0.677861	NOT
SPATA32	6.067475	0.37555229	0.487942	0.769665106	0.441499	0.677911	NOT
RP11-303E	13.52433	-0.2746876	0.356897	-0.76965583	0.441504	0.677911	NOT
ETV7	164.4272	0.2915987	0.378924	0.769543727	0.441571	0.677975	NOT
RP11-322I	1.484857	0.52842902	0.686738	0.76947668	0.44161	0.677992	NOT
AC005330.	28.27644	0.36798124	0.478245	0.76944026	0.441632	0.677992	NOT
RP4-635E1	9.846311	-0.2462763	0.320134	-0.76929223	0.44172	0.678089	NOT
PDGFRL	118.0853	0.3292492	0.428072	0.769144594	0.441807	0.678185	NOT
POLR2A	5082.256	-0.1371068	0.17828	-0.769054	0.441861	0.67822	NOT
HCG4B	60.12885	0.28105121	0.365466	0.769021548	0.441881	0.67822	NOT
JARID2-AS	3.391472	-0.2962122	0.385308	-0.76876696	0.442032	0.678375	NOT
IGHV3-11	54.91849	-0.5754019	0.748484	-0.7687569	0.442038	0.678375	NOT
C1orf233	247.911	0.39657653	0.515888	0.76872545	0.442056	0.678375	NOT
RNF175	17.52975	-0.2748014	0.3575	-0.76867617	0.442086	0.678381	NOT
APOO	666.1518	-0.1545384	0.201066	-0.76859607	0.442133	0.678401	NOT
SLC2A6	647.0598	0.27186514	0.353747	0.768529776	0.442173	0.678401	NOT
PANX1	977.0315	-0.1903923	0.247736	-0.76852847	0.442173	0.678401	NOT
DDAH1	4849.583	0.20316174	0.264414	0.768347354	0.442281	0.6785	NOT
RP3-455J7	3.177705	-0.4147704	0.539882	-0.7682619	0.442332	0.6785	NOT
RBM20	33.87034	-0.3246675	0.422615	-0.76823466	0.442348	0.6785	NOT

PCDH9	97.44303	0.44137241	0.57454	0.76821911	0.442357	0.6785	NOT
CHCHD4	867.5481	-0.1349994	0.175741	-0.76817173	0.442385	0.6785	NOT
TMEM37	4541.237	0.21650887	0.281851	0.768166565	0.442388	0.6785	NOT
PURG	2.53126	0.42997785	0.559823	0.768060991	0.442451	0.678558	NOT
CCDC96	58.65266	-0.1663166	0.216555	-0.76800976	0.442481	0.678567	NOT
RP11-549J	16.03013	-0.210866	0.274586	-0.76794223	0.442522	0.67859	NOT
ULBP3	7.262409	-0.3804947	0.495581	-0.76777501	0.442621	0.678704	NOT
CCNA2	804.6867	0.25483518	0.332132	0.767271935	0.44292	0.679124	NOT
RNU6-925F	3.015324	0.31280061	0.407793	0.767056623	0.443048	0.679268	NOT
DOCK7	1204.557	0.15316428	0.199685	0.76702947	0.443064	0.679268	NOT
SREK1IP1	1099.881	0.15040569	0.196122	0.766898668	0.443142	0.679308	NOT
CTC-479C5	63.25257	-0.2161519	0.28186	-0.76687656	0.443155	0.679308	NOT
ITGA10	63.76251	-0.2313526	0.301688	-0.76685978	0.443165	0.679308	NOT
DAPK3	1983.821	-0.1383347	0.180414	-0.76676232	0.443223	0.679358	NOT
ZNF117	370.6362	0.23674648	0.308818	0.766621927	0.443306	0.67941	NOT
SLC8A1	188.2546	-0.2472092	0.322466	-0.76662084	0.443307	0.67941	NOT
RP11-48B3	9.452224	-0.5908207	0.770819	-0.76648471	0.443388	0.679496	NOT
SSC4D	446.4736	0.34836795	0.454638	0.766254088	0.443525	0.679668	NOT
GPR107	3285.672	0.12200704	0.159235	0.766207985	0.443553	0.679671	NOT
ZNF121	961.3427	-0.149574	0.195241	-0.76609971	0.443617	0.679732	NOT
MRPL33	1445.401	0.15162672	0.197999	0.765797032	0.443797	0.679969	NOT
TP73-AS1	563.4587	-0.128844	0.16829	-0.76560683	0.44391	0.680068	NOT
AP000569.	3.44184	0.50920776	0.665106	0.765603933	0.443912	0.680068	NOT
CTD-3001H	18.1329	-0.2534755	0.33115	-0.7654405	0.444009	0.680179	NOT
SPRR3	2.926153	0.72604419	0.948589	0.765393474	0.444037	0.680184	NOT
TSPAN6	5192.892	0.16136014	0.210859	0.765250237	0.444123	0.68027	NOT
SLC18B1	618.5756	-0.1899243	0.24821	-0.76517605	0.444167	0.68027	NOT
SRPX2	218.1713	0.30283133	0.395769	0.765172039	0.444169	0.68027	NOT
PSMD12	3164.069	-0.0898223	0.117433	-0.76488189	0.444342	0.680428	NOT
NAT8	2181.736	0.35134366	0.459349	0.764872859	0.444347	0.680428	NOT
C6orf136	1175.299	0.14105214	0.184413	0.764872656	0.444347	0.680428	NOT
RP11-96D1	10.08343	-0.2840743	0.371519	-0.76463015	0.444492	0.680611	NOT
FAM63A	1654.168	-0.1567485	0.205057	-0.76441407	0.444621	0.680756	NOT
RILPL2	561.5016	-0.1427302	0.186725	-0.76438629	0.444637	0.680756	NOT
GDA	2802.746	-0.3639857	0.476241	-0.76428854	0.444695	0.680807	NOT
SEPT10	1964.805	0.14285816	0.186958	0.764117406	0.444797	0.680888	NOT
MUC4	39.07957	-0.3255428	0.426039	-0.76411516	0.444799	0.680888	NOT
CRLF2	13.94925	0.47627032	0.6234	0.76398853	0.444874	0.680937	NOT
RP11-162A	60.10568	-0.2091636	0.273782	-0.76397808	0.44488	0.680937	NOT
NMT1	6226.726	0.09144133	0.11972	0.76379173	0.444991	0.681068	NOT
GPC6	2099.049	-0.2443223	0.319968	-0.76358403	0.445115	0.681219	NOT
RP11-142C	31.10824	-0.3226709	0.422614	-0.76351241	0.445158	0.681246	NOT
RP11-195F	40.39724	-0.205971	0.269793	-0.76344119	0.4452	0.681273	NOT
ZNF653	237.9414	-0.1199921	0.1572	-0.76330816	0.44528	0.681356	NOT
B4GALT1	8242.264	-0.1318983	0.172852	-0.76307082	0.445421	0.681522	NOT
MON2	1644.898	-0.1301474	0.170575	-0.76299233	0.445468	0.681522	NOT
RP11-344F	49.89588	-0.2684596	0.35186	-0.76297268	0.44548	0.681522	NOT
CEP164	787.1324	0.11736564	0.15383	0.762957215	0.445489	0.681522	NOT
RP11-248E	5.960951	0.77068927	1.010223	0.762889993	0.445529	0.681545	NOT

RP5-1112I	66.36304	0.22820641	0.299155	0.762835583	0.445561	0.681556	NOT
EIF3J	2634.77	0.10085834	0.132239	0.762698378	0.445643	0.681643	NOT
ZNF273	110.0038	-0.1973223	0.258771	-0.76253647	0.44574	0.681693	NOT
MPZL2	1376.349	0.20098599	0.263576	0.762534734	0.445741	0.681693	NOT
RP11-93B1	1.427623	-0.5761318	0.755565	-0.76251777	0.445751	0.681693	NOT
RP11-46F1	10.27473	0.24225953	0.317739	0.762448767	0.445792	0.681717	NOT
SHFM1	6139.057	0.17038631	0.223509	0.76232573	0.445866	0.681791	NOT
GLUD1P7	23.65048	-0.2557192	0.335472	-0.76226658	0.445901	0.681806	NOT
MIR646HG	25.47517	0.45597622	0.598307	0.762111284	0.445994	0.681849	NOT
ALG13	1068.336	0.10228062	0.134213	0.762074273	0.446016	0.681849	NOT
RP11-109I	21.29106	-0.4472922	0.586944	-0.76206963	0.446018	0.681849	NOT
PCNXL2	276.1255	0.32613801	0.427991	0.762021463	0.446047	0.681849	NOT
ORC2	736.7467	0.12291241	0.161308	0.761971666	0.446077	0.681849	NOT
ASPN	757.2816	-0.2898069	0.38034	-0.76196702	0.44608	0.681849	NOT
CFAP20	1120.888	-0.1015437	0.133278	-0.76189386	0.446123	0.681878	NOT
CMTM6	4297.423	0.13888606	0.182313	0.761798811	0.44618	0.681926	NOT
RP11-553A	2.264437	-0.5225094	0.685961	-0.76171904	0.446228	0.68196	NOT
LRP3	2663.096	-0.1813154	0.238073	-0.76159421	0.446302	0.682036	NOT
SIAH3	1.167646	0.65792435	0.864166	0.761340172	0.446454	NA	NA
HSP90B3P	3.984829	-0.2924267	0.3841	-0.76132963	0.44646	0.682239	NOT
SRRM5	27.91474	0.22815563	0.299731	0.761200721	0.446537	0.682318	NOT
COL12A1	823.7996	-0.2675015	0.351458	-0.76111902	0.446586	0.682323	NOT
RP1-130G2	1.632289	-0.3151669	0.414108	-0.7610746	0.446613	0.682323	NOT
RP11-227C	8.262214	-0.253819	0.333503	-0.76106905	0.446616	0.682323	NOT
ITGA2	600.2416	0.30709859	0.403656	0.760792732	0.446781	0.682536	NOT
RP11-80H5	6.291033	0.27560286	0.362366	0.760564477	0.446917	0.682632	NOT
PYY2	18.77118	-0.335044	0.44054	-0.76053059	0.446937	0.682632	NOT
GLRA3	2.48607	0.62545121	0.822394	0.760525254	0.446941	0.682632	NOT
RP4-621B1	6.058844	-0.2873119	0.377784	-0.7605195	0.446944	0.682632	NOT
SLC29A4	1998.563	-0.4401856	0.578861	-0.76043439	0.446995	0.682671	NOT
AC093698.	2.263609	-0.3184417	0.418795	-0.76037622	0.44703	0.682686	NOT
RP11-110C	76.49193	-0.1656808	0.217926	-0.76026264	0.447098	0.682719	NOT
RFWD3	871.9555	0.13602437	0.178919	0.760255873	0.447102	0.682719	NOT
GAREM	539.3423	0.21586647	0.283978	0.760153416	0.447163	0.682765	NOT
SNORD99	8.135556	0.29881559	0.393116	0.760121233	0.447182	0.682765	NOT
LGALS7B	2.050025	-0.5553525	0.730674	-0.76005458	0.447222	0.682787	NOT
RP11-884K	9.339489	-0.2875385	0.378333	-0.76001334	0.447247	0.682787	NOT
SHD	378.125	-0.4120792	0.542312	-0.75985697	0.44734	0.682891	NOT
CTB-50L17	32.09939	0.23189321	0.305231	0.759729972	0.447416	0.682968	NOT
RP11-271K	2.616078	-0.2935997	0.386562	-0.75951457	0.447545	0.6831	NOT
NLGN2	459.3975	-0.2724941	0.358834	-0.7593882	0.44762	0.6831	NOT
ITM2B	21908.08	-0.1500252	0.197566	-0.75936611	0.447634	0.6831	NOT
SGSM3	2495.543	0.1140724	0.150221	0.759362091	0.447636	0.6831	NOT
KB-1043D8	2.602873	0.35884962	0.472605	0.759300897	0.447673	0.6831	NOT
RP11-325N	2.103377	0.40147223	0.528765	0.759263396	0.447695	0.6831	NOT
RP11-100N	9.087819	0.25308657	0.333332	0.75926323	0.447695	0.6831	NOT
NOL3	1161.863	-0.2108807	0.277761	-0.75921619	0.447723	0.6831	NOT
SH3GLB2	2046.768	0.14204852	0.187101	0.759206816	0.447729	0.6831	NOT
CCDC102A	222.5708	-0.2331285	0.307099	-0.75913047	0.447775	0.683125	NOT

CLMP	49.05562	0.3180036	0.418924	0.759095286	0.447796	0.683125	NOT
KCNJ1	1.818208	-0.40156	0.529064	-0.75900055	0.447852	0.683161	NOT
GABRB2	3.918864	0.50505846	0.665451	0.758971242	0.44787	0.683161	NOT
SPEG	61.90604	0.36591496	0.482279	0.758719799	0.44802	0.683344	NOT
LURAP1L	1302.455	-0.2695236	0.35525	-0.75868664	0.44804	0.683344	NOT
SAG	2.364244	-0.3907076	0.515012	-0.75863848	0.448069	0.68335	NOT
CMPK1	8904.873	-0.11547	0.152224	-0.75855264	0.44812	0.683365	NOT
RP11-554A	13.7449	0.44568915	0.587578	0.758519705	0.44814	0.683365	NOT
HMP19	7.126561	0.44741659	0.589874	0.758494918	0.448155	0.683365	NOT
CTB-25B13	6.470028	-0.2436056	0.321193	-0.75844077	0.448187	0.683368	NOT
CNR1	49.8938	0.47778609	0.629987	0.758405776	0.448208	0.683368	NOT
RP11-3L10	2.542132	-0.3683401	0.485703	-0.75836546	0.448232	0.683368	NOT
FAM155A	19.03704	-0.2831909	0.37348	-0.75824924	0.448302	0.683436	NOT
ZNF175	194.5637	-0.2172214	0.286515	-0.75815066	0.448361	0.683488	NOT
AP3B2	11.47877	0.50409953	0.665176	0.757843802	0.448544	0.683703	NOT
RP11-505F	2.283232	-0.3513993	0.4637	-0.75781568	0.448561	0.683703	NOT
ZKSCAN7	63.69427	-0.2664572	0.351625	-0.75778886	0.448577	0.683703	NOT
AC005785.	24.89023	-0.2220033	0.292988	-0.75772221	0.448617	0.683704	NOT
CTD-2020K	24.44687	-0.2791903	0.368469	-0.75770333	0.448629	0.683704	NOT
HCN2	93.93095	0.40150561	0.529975	0.75759388	0.448694	0.683765	NOT
HIST2H2AC	50.99548	0.26615905	0.351348	0.757536462	0.448729	0.683779	NOT
UBE2J2	1826.107	0.12900154	0.170302	0.75748753	0.448758	0.683785	NOT
NPM1P37	2.76372	0.29168161	0.385144	0.757331984	0.448851	0.683889	NOT
RP1-27K12	13.27923	0.30750882	0.406141	0.757147921	0.448961	0.683931	NOT
RP11-612E	66.61151	-0.2285195	0.301833	-0.75710466	0.448987	0.683931	NOT
FXYD1	785.0425	-0.4439852	0.586426	-0.75710378	0.448988	0.683931	NOT
C1orf56	707.9059	0.15174507	0.20043	0.757096674	0.448992	0.683931	NOT
RP11-186N	23.31519	0.20646504	0.272714	0.757075189	0.449005	0.683931	NOT
AC073052.	1.877978	0.36601713	0.483592	0.756871276	0.449127	0.684046	NOT
MIR186	2.961785	-0.3812092	0.503674	-0.75685711	0.449135	0.684046	NOT
COMMD10	764.4787	0.12433849	0.16429	0.756823062	0.449156	0.684046	NOT
RP11-126O	18.76447	-0.3176953	0.419903	-0.75659147	0.449295	0.684189	NOT
ZNF419	260.9159	0.15998319	0.211472	0.756521758	0.449336	0.684189	NOT
MEF2BNB	624.2186	-0.1380449	0.182476	-0.75651061	0.449343	0.684189	NOT
TTTY10	1.608926	-0.4735885	0.626027	-0.75649878	0.44935	0.684189	NOT
ARL13A	2.399667	-0.2730774	0.361059	-0.75632416	0.449455	0.684309	NOT
IFITM5	3.624066	-0.6938826	0.917521	-0.75625801	0.449495	0.684331	NOT
TANGO2	865.7567	0.13173253	0.174207	0.756183601	0.449539	0.684361	NOT
SLC22A31	1063.291	-0.6926514	0.916205	-0.75600058	0.449649	0.68449	NOT
ZDHHC8	1062.54	-0.1846041	0.244286	-0.75568972	0.449835	0.684655	NOT
RAPSN	18.98969	-0.2677504	0.354314	-0.75568743	0.449837	0.684655	NOT
CTC-529G1	1.028203	0.87070084	1.152212	0.755677507	0.449843	NA	NA
RP3-406P2	19.95846	0.23213904	0.307246	0.755548659	0.44992	0.684655	NOT
EFEMP1	2026.486	-0.4547343	0.601863	-0.75554439	0.449922	0.684655	NOT
GOLGA6A	6.12318	-0.534547	0.707524	-0.75551729	0.449939	0.684655	NOT
LRRC74A	1.84711	0.50517614	0.66869	0.755471439	0.449966	0.684655	NOT
SCN7A	34.15946	-0.4715653	0.624213	-0.75545566	0.449976	0.684655	NOT
RPL21P4	2.154646	-0.350528	0.463998	-0.75545078	0.449979	0.684655	NOT
RIPK1	2033.955	-0.0926911	0.122698	-0.75544041	0.449985	0.684655	NOT

CTD-2528I	1.812849	0.47370013	0.627125	0.755352519	0.450037	0.684685	NOT
KIR3DL1	2.996494	0.4284104	0.567188	0.755323751	0.450055	0.684685	NOT
GTF2A2	1310.816	-0.0976602	0.129309	-0.75524595	0.450101	0.684692	NOT
RP11-739N	25.68448	0.26747602	0.354164	0.755232339	0.45011	0.684692	NOT
VEGFB	2860.959	-0.2876281	0.380979	-0.75497066	0.450267	0.684876	NOT
TUBGCP3	1111.9	0.1662	0.220148	0.754946328	0.450281	0.684876	NOT
RAB39A	9.27075	0.3316287	0.43931	0.754885495	0.450318	0.684893	NOT
TLR1	282.9293	-0.268499	0.355729	-0.75478604	0.450377	0.684945	NOT
CD80	25.4826	-0.308096	0.408297	-0.75458864	0.450496	0.684993	NOT
CTB-131K1	259.0672	0.13708158	0.181666	0.754581979	0.4505	0.684993	NOT
RP11-244N	31.40117	0.42919092	0.56878	0.754581882	0.4505	0.684993	NOT
CPTP	2570.583	-0.1690418	0.224026	-0.75456501	0.45051	0.684993	NOT
CDK16	3056.868	0.13682243	0.181364	0.754409697	0.450603	0.685051	NOT
PCSK1	11.30608	-0.3489649	0.462578	-0.75439118	0.450614	0.685051	NOT
COX17P1	8.427034	0.22600468	0.299592	0.754375263	0.450624	0.685051	NOT
PSPC1P1	5.580316	0.33574267	0.445103	0.754303537	0.450667	0.685078	NOT
ARHGDIG	3.250308	-0.4626733	0.61343	-0.75423998	0.450705	0.685098	NOT
XIST	1159.455	-0.8446584	1.120006	-0.75415509	0.450756	0.685137	NOT
NRSN1	1.999432	0.67935215	0.900896	0.754085125	0.450798	0.68515	NOT
IGLV1-41	2.276877	-0.7342855	0.973779	-0.75405735	0.450815	0.68515	NOT
RP11-6N17	216.5881	-0.2036303	0.27016	-0.75374051	0.451005	0.6854	NOT
ACOX2	4962.986	-0.2481612	0.3293	-0.75360212	0.451088	0.685459	NOT
IDS	2016.524	-0.158609	0.210471	-0.75359195	0.451094	0.685459	NOT
ATP8B5P	14.26854	0.2529775	0.335781	0.753399778	0.45121	0.685534	NOT
RP11-10N2	20.47494	-0.2130377	0.282771	-0.75339294	0.451214	0.685534	NOT
CLEC2D	208.4039	0.23639742	0.313783	0.753377615	0.451223	0.685534	NOT
HRSP12	12901.27	-0.2873195	0.381404	-0.75332065	0.451257	0.685534	NOT
PFN4	34.7896	0.23552335	0.312658	0.753292837	0.451274	0.685534	NOT
RPS10P2	10.56072	0.25795173	0.342448	0.753257482	0.451295	0.685534	NOT
DGCR2	5174.61	-0.1057256	0.140394	-0.75306577	0.45141	0.685671	NOT
RP11-158I	9.253026	-0.3158855	0.419568	-0.75288216	0.451521	0.6858	NOT
NDUFB11	5356.964	0.14691821	0.195177	0.752744469	0.451603	0.685887	NOT
TRPC4AP	4719.725	0.08455287	0.112333	0.752695312	0.451633	0.685894	NOT
RP1-95L4.	150.2605	-0.1740965	0.231392	-0.75238736	0.451818	0.686136	NOT
RP11-27K1	7.51205	-0.2260902	0.300514	-0.75234404	0.451844	0.686138	NOT
TRIM41	1621.424	-0.1283956	0.170678	-0.75226795	0.45189	0.686169	NOT
RFWD2	2735.913	0.11804126	0.156957	0.752061797	0.452014	0.686318	NOT
CSF3	1.915024	-0.5741352	0.76348	-0.75199784	0.452052	0.686324	NOT
ANKRD36C	36.4484	-0.240662	0.320047	-0.75195794	0.452076	0.686324	NOT
TTTY14	56.67176	0.59152394	0.786675	0.751929054	0.452094	0.686324	NOT
TMEM145	69.5084	-0.3965949	0.527473	-0.75187737	0.452125	0.686333	NOT
TTPA	2798.127	-0.3421746	0.455159	-0.75176987	0.452189	0.686393	NOT
RGL4	28.50452	-0.2203259	0.293099	-0.75171206	0.452224	0.686407	NOT
AC139100.	34.1851	0.29947584	0.398464	0.751576453	0.452306	0.686477	NOT
TEAD2	1244.539	0.22975503	0.305708	0.751551496	0.452321	0.686477	NOT
RTN4RL1	393.245	-0.3524018	0.468973	-0.75143286	0.452392	0.686545	NOT
CBWD5	170.1626	0.14665097	0.195172	0.751392552	0.452416	0.686545	NOT
RP11-478C	6.431114	0.29304301	0.390112	0.751176795	0.452546	0.686704	NOT
RP11-426J	5.786253	0.38670883	0.514998	0.750894027	0.452716	0.686924	NOT

RP11-333(	0.917429	-0.4733751	0.630418	-0.75089137	0.452718	NA	NA
CUX1	3466.134	-0.1237004	0.164789	-0.75065716	0.452859	0.68708	NOT
AC015987.	2.572209	-0.3951219	0.526381	-0.75063878	0.45287	0.68708	NOT
TPCN1	3248.817	-0.148033	0.197307	-0.75026704	0.453094	0.687381	NOT
KRT87P	23.9041	-0.5299578	0.70669	-0.7499155	0.453306	0.687624	NOT
ZNF300	154.053	0.31599719	0.421404	0.749866915	0.453335	0.687624	NOT
RP11-466F	2.93123	-0.2861501	0.381616	-0.74983683	0.453353	0.687624	NOT
EMR1	35.62009	-0.3209674	0.428052	-0.74983268	0.453355	0.687624	NOT
RP11-535A	2.766654	-0.3856943	0.514545	-0.74958286	0.453506	0.687814	NOT
RP4-580N2	15.65905	-0.4125299	0.550526	-0.7493383	0.453653	0.687999	NOT
NIPAL2	1407.048	0.2141197	0.285769	0.749275178	0.453691	0.688018	NOT
PIWIL1	2.612363	0.35862162	0.478708	0.749145555	0.453769	0.688098	NOT
RP11-2H3.	5.549258	-0.3159362	0.421834	-0.74895898	0.453882	0.688176	NOT
RPS2P35	3.269594	0.30792676	0.411165	0.748913099	0.45391	0.688176	NOT
RP1-313I6	74.92874	0.18548275	0.24767	0.748912279	0.45391	0.688176	NOT
H3F3C	9.500434	-0.2108202	0.281509	-0.74889212	0.453922	0.688176	NOT
VN1R81P	7.207215	0.39262304	0.524372	0.748749607	0.454008	0.688268	NOT
TRAK2	1748.557	0.12317527	0.164581	0.748415721	0.454209	0.688502	NOT
FAM221A	240.9464	-0.2367113	0.316286	-0.74840916	0.454213	0.688502	NOT
MED4	1235.49	0.12101143	0.161754	0.748121283	0.454387	0.688727	NOT
PDHB	3529.03	0.12037795	0.160918	0.748068571	0.454419	0.688736	NOT
KCNQ5	5.169822	-0.3639866	0.486622	-0.74798687	0.454468	0.688768	NOT
UROD	4821.973	-0.1377042	0.184109	-0.7479502	0.45449	0.688768	NOT
SEC24C	1831.012	-0.201316	0.269303	-0.74754467	0.454735	0.6891	NOT
AC092580.	15.46972	-0.3101253	0.414991	-0.74730657	0.454879	0.689279	NOT
STRCP1	16.32028	0.42377084	0.567172	0.747165132	0.454964	0.689331	NOT
FGFBP1	2.063168	0.72411534	0.969169	0.747150512	0.454973	0.689331	NOT
SHC1	8260.591	0.12453429	0.166687	0.747116172	0.454993	0.689331	NOT
RBMS1P1	4.436646	-0.2739209	0.366655	-0.74708175	0.455014	0.689331	NOT
SULT1A2	749.3228	0.29579586	0.39597	0.747016018	0.455054	0.689352	NOT
ATP6V1B2	2670.674	-0.1418972	0.189978	-0.74691475	0.455115	0.689363	NOT
RP11-2C24	15.84705	-0.1889008	0.25292	-0.7468793	0.455136	0.689363	NOT
BBS12	66.22394	0.194946	0.261015	0.746878018	0.455137	0.689363	NOT
RP4-715N1	1.341527	-0.7875123	1.054615	-0.74672944	0.455227	NA	NA
CAMK2G	1222.194	-0.1201152	0.160863	-0.74669204	0.455249	0.689447	NOT
CBY1	835.1455	0.15550709	0.208266	0.746673859	0.45526	0.689447	NOT
NPTN	3255.272	-0.1144584	0.153296	-0.74665104	0.455274	0.689447	NOT
MYPOP	414.0788	-0.1251696	0.167649	-0.74661784	0.455294	0.689447	NOT
AP001271.	2.675372	-0.3365348	0.450844	-0.74645477	0.455393	0.68953	NOT
1-Mar	210.8989	0.20874056	0.279647	0.746443111	0.4554	0.68953	NOT
RP11-104(	1.688333	-0.3995465	0.535426	-0.74622148	0.455534	0.689658	NOT
RP11-1084	2.297557	0.47450849	0.635964	0.746125347	0.455592	0.689658	NOT
CTB-26E19	4.116988	-0.8017909	1.074614	-0.7461197	0.455595	0.689658	NOT
RP11-285F	7.886312	-0.354127	0.474652	-0.74607696	0.455621	0.689658	NOT
EIF3LP2	3.910706	-0.306661	0.411034	-0.74607284	0.455623	0.689658	NOT
PSD2	8.296621	-0.3179607	0.426245	-0.7459575	0.455693	0.689658	NOT
RP11-488(	52.19948	0.20286771	0.271963	0.745938086	0.455705	0.689658	NOT
RP11-404F	2.235231	0.39938646	0.535433	0.745913544	0.45572	0.689658	NOT
AC073046.	8.84438	0.2722076	0.364936	0.745904824	0.455725	0.689658	NOT

IFNA20P	3.5484	-0.3334546	0.447105	-0.74580763	0.455784	0.689658	NOT
MBD1	2143.196	0.08125627	0.108957	0.745765104	0.455809	0.689658	NOT
RPS26P13	2.427964	0.34716384	0.465514	0.745765081	0.455809	0.689658	NOT
AC005154.	3.014372	0.38097652	0.51086	0.745755711	0.455815	0.689658	NOT
CSRP2	1132.844	0.22368745	0.300032	0.745544792	0.455942	0.689794	NOT
MDN1	1743.042	-0.1353098	0.181496	-0.74552302	0.455956	0.689794	NOT
B2M	109449.9	-0.2027383	0.271976	-0.74542862	0.456013	0.68982	NOT
PIPSL	26.47441	0.17531317	0.235198	0.745385194	0.456039	0.68982	NOT
SIPA1	2386.472	-0.1414896	0.189825	-0.74536844	0.456049	0.68982	NOT
CFAP54	14.34895	-0.3116427	0.418135	-0.74531526	0.456081	0.68983	NOT
C2orf50	2.644418	0.39399965	0.528707	0.745213146	0.456143	0.689865	NOT
AC092574.	3.084183	-0.2452419	0.329109	-0.74517036	0.456169	0.689865	NOT
TNK2-AS1	6.356378	-0.3235294	0.43418	-0.74515038	0.456181	0.689865	NOT
TNIP1	10309.54	0.14685878	0.197133	0.744973102	0.456288	0.68992	NOT
SEPT10P1	3.024443	0.29375123	0.394313	0.744969167	0.45629	0.68992	NOT
ALG1L10P	2.687721	0.35885803	0.481711	0.744964959	0.456293	0.68992	NOT
RP13-131K	9.421521	-0.3317204	0.445312	-0.74491695	0.456322	0.689925	NOT
LL09NC01-	11.05713	0.23181512	0.311294	0.744682727	0.456464	0.690059	NOT
ITGAX	702.1495	0.21732098	0.291833	0.74467549	0.456468	0.690059	NOT
FAM179B	442.2886	-0.149891	0.201292	-0.74464415	0.456487	0.690059	NOT
GSTP1	1330.889	0.25030431	0.336175	0.744565591	0.456534	0.690082	NOT
TGFBRAP1	763.6742	-0.1024098	0.137549	-0.74453431	0.456553	0.690082	NOT
TRAPPC12-	20.0288	0.23308047	0.3131	0.744429296	0.456617	0.69014	NOT
CDK6	1818.717	0.29797216	0.400309	0.744355869	0.456661	0.690169	NOT
NAPEPLD	742.7461	-0.1292416	0.173647	-0.74427922	0.456708	0.6902	NOT
CAPNS1	11536.85	0.11381086	0.152924	0.744229792	0.456737	0.690207	NOT
RP11-343E	6.654948	0.22513956	0.302554	0.744130023	0.456798	0.69026	NOT
RP11-351(	1.285592	0.98370043	1.322052	0.744070807	0.456834	NA	NA
RP11-483E	7.016653	0.25981741	0.349268	0.743891672	0.456942	0.690422	NOT
IGLV9-49	12.13974	0.66077023	0.88829	0.743868097	0.456956	0.690422	NOT
C9orf152	175.7493	0.46356854	0.623225	0.743822342	0.456984	0.690426	NOT
RP4-621N1	1.893353	0.37819125	0.50851	0.743724346	0.457043	0.690477	NOT
AC009237.	8.991479	-0.3130432	0.421003	-0.74356452	0.45714	0.690585	NOT
RP4-644L1	1.780429	0.47181723	0.634862	0.743180906	0.457372	0.690897	NOT
ZNF324B	141.3893	0.10711162	0.144136	0.743129119	0.457404	0.690906	NOT
SLC26A2	551.8797	0.16543171	0.222635	0.743063181	0.457443	0.690928	NOT
NYAP1	23.282	0.20329619	0.27364	0.742931803	0.457523	0.691009	NOT
AC092620.	2.250234	0.37471806	0.504436	0.742845355	0.457575	0.69105	NOT
NTSR1	20.89418	0.45137867	0.607912	0.742506898	0.45778	0.691286	NOT
CTSLP7	10.23501	-0.3605773	0.485624	-0.74250298	0.457783	0.691286	NOT
ALDH5A1	6114.131	-0.2253584	0.303538	-0.74243897	0.457821	0.691306	NOT
DNAJA1	9684.259	0.12021152	0.16196	0.74222899	0.457949	0.691384	NOT
RNF168	815.6028	-0.1155084	0.155628	-0.74220624	0.457962	0.691384	NOT
ENHO	704.8759	0.35028159	0.471952	0.742197044	0.457968	0.691384	NOT
MESDC2	2941.467	0.09562396	0.128841	0.742185479	0.457975	0.691384	NOT
DIS3	1357.417	-0.1338857	0.180423	-0.74206528	0.458048	0.69144	NOT
PPP2R2A	1073.741	0.13142847	0.177118	0.742040072	0.458063	0.69144	NOT
RP3-424M6	30.51171	-0.1782298	0.240207	-0.74198477	0.458097	0.691452	NOT
RPL7P57	1.639434	-0.3086239	0.415986	-0.74190862	0.458143	0.691483	NOT

CALM3	12218.38	-0.1399606	0.188683	-0.74177462	0.458224	0.691536	NOT
RP5-1061I	1.493629	-0.5593918	0.754135	-0.74176644	0.458229	0.691536	NOT
SEMA3E	36.78874	-0.6729867	0.907449	-0.74162482	0.458315	0.691627	NOT
RP11-981(	2.086642	-0.35043	0.472622	-0.7414594	0.458415	0.691711	NOT
CTD-2233I	21.12712	0.15304004	0.206407	0.741449367	0.458421	0.691711	NOT
CYFIP1	4265.384	-0.1006024	0.135693	-0.74139611	0.458453	0.691721	NOT
ZNF662	107.3512	-0.3046887	0.411025	-0.7412903	0.458517	0.691779	NOT
EBLN2	18.31508	-0.2577537	0.347744	-0.7412166	0.458562	0.691808	NOT
RP11-338I	3.841436	-0.2473544	0.333862	-0.74088874	0.458761	0.69207	NOT
NFIB	2490.288	-0.1850552	0.249818	-0.74075962	0.458839	0.692138	NOT
SDS	23759.87	-0.5034669	0.67969	-0.74072971	0.458857	0.692138	NOT
CTD-2210I	2.192026	0.28543141	0.385389	0.740632043	0.458917	0.692187	NOT
RPP25	560.0754	0.31370216	0.423601	0.740560823	0.45896	0.692187	NOT
ZBTB7A	1841.499	-0.1203538	0.162519	-0.74055048	0.458966	0.692187	NOT
EP300	2941.197	-0.1342199	0.181263	-0.74047224	0.459013	0.69222	NOT
SHANK3	2119.212	-0.1550379	0.209397	-0.74040297	0.459056	0.692221	NOT
ZNF549	119.8508	0.28329442	0.38263	0.740386841	0.459065	0.692221	NOT
SERINC2	12051.22	0.20125507	0.271872	0.740256562	0.459144	0.692302	NOT
NDUFS4	2401.647	-0.1647433	0.222584	-0.74014159	0.459214	0.692336	NOT
MBNL1	3633.516	-0.1065673	0.143993	-0.74008886	0.459246	0.692336	NOT
ANKRD65	505.2683	0.40362834	0.545385	0.740079205	0.459252	0.692336	NOT
PCDH15	1.839047	-0.5885186	0.795283	-0.74001167	0.459293	0.692336	NOT
PLAUR	349.7115	0.23125031	0.312499	0.740003018	0.459298	0.692336	NOT
RNU6-1035	1.477667	-0.3880741	0.524455	-0.73995659	0.459326	0.692336	NOT
STK32A	9.992319	0.38973788	0.526727	0.739924468	0.459346	0.692336	NOT
RP11-493I	1.813425	0.67325482	0.910217	0.73966403	0.459504	0.692388	NOT
RPSAP56	2.90626	0.38707267	0.52336	0.739592305	0.459547	0.692388	NOT
SDF4	8012.887	-0.1120846	0.151557	-0.73955565	0.45957	0.692388	NOT
OTUB1	3375.001	0.10483617	0.141765	0.739505646	0.4596	0.692388	NOT
MEN1	2014.681	0.07125642	0.096365	0.739440114	0.45964	0.692388	NOT
SH3BP4	3553.126	-0.1927634	0.260699	-0.73941022	0.459658	0.692388	NOT
IFT27	1117.867	-0.1401245	0.189509	-0.73940736	0.45966	0.692388	NOT
RP5-1085I	229.3054	0.2107225	0.284996	0.739388709	0.459671	0.692388	NOT
RNLS	513.4881	0.24421102	0.330302	0.73935595	0.459691	0.692388	NOT
LZTS1	310.8043	-0.1965324	0.265816	-0.73935538	0.459691	0.692388	NOT
FAM65B	159.0893	-0.2431433	0.328865	-0.73934136	0.4597	0.692388	NOT
ATP7A	341.0666	0.17477435	0.236393	0.739338097	0.459702	0.692388	NOT
KIAA0556	671.8082	0.1501127	0.203043	0.739313961	0.459716	0.692388	NOT
ZNF408	924.4918	0.13496651	0.182576	0.739236635	0.459763	0.692388	NOT
GABRD	221.5278	-0.211908	0.286685	-0.73916607	0.459806	0.692388	NOT
RP11-497I	5.095204	-0.3132855	0.423842	-0.73915615	0.459812	0.692388	NOT
FAM111A	1241.512	-0.1420468	0.192175	-0.73915326	0.459814	0.692388	NOT
H19	22047.85	0.52474254	0.710061	0.739010949	0.4599	0.692442	NOT
HHAT	465.4397	-0.1938087	0.262254	-0.73900999	0.459901	0.692442	NOT
ZNF787	2685.7	0.15363222	0.207905	0.73895363	0.459935	0.692455	NOT
SNRPGP14	2.75325	0.28264861	0.382601	0.73875529	0.460056	0.692547	NOT
RP11-807I	4.271847	-0.3784173	0.512253	-0.73873075	0.460071	0.692547	NOT
CTD-2353I	3.643358	0.33166175	0.448964	0.73872709	0.460073	0.692547	NOT
LMF1-AS1	2.915628	-0.2682432	0.363214	-0.73852558	0.460195	0.692693	NOT

CAPRIN2	644.0678	-0.1559439	0.211213	-0.7383261	0.460316	0.692796	NOT
IRF2BP1	1792.475	-0.1298754	0.175906	-0.73832437	0.460317	0.692796	NOT
HIBCH	2396.86	-0.1636975	0.221726	-0.73828647	0.46034	0.692796	NOT
GCH1	1592.749	-0.2313837	0.313517	-0.73802659	0.460498	0.692959	NOT
TVP23C-CI	2.893095	0.28832723	0.390675	0.738023748	0.4605	0.692959	NOT
ATG10	396.2309	-0.1356186	0.183827	-0.73775322	0.460664	0.693141	NOT
STK10	1145.781	-0.1373266	0.186148	-0.73772782	0.46068	0.693141	NOT
LINC0137(	242.4244	-0.6062968	0.821905	-0.73767297	0.460713	0.693141	NOT
GPR37	1037.979	-0.3883275	0.526433	-0.73765733	0.460723	0.693141	NOT
SCEL	1.893382	-0.7915196	1.073083	-0.73761255	0.46075	0.693143	NOT
DMTN	1425.829	0.23063025	0.312699	0.737547807	0.460789	0.693164	NOT
ZEB2-AS1	5.443577	-0.2731706	0.370401	-0.73750031	0.460818	0.693169	NOT
SHMT2	10276.85	0.13319727	0.18062	0.737445831	0.460851	0.69318	NOT
RP1-167F1	1.084003	0.63329213	0.858792	0.737422259	0.460866	NA	NA
PPARGC1B	230.7666	-0.1866125	0.25308	-0.73736613	0.4609	0.693207	NOT
ZBTB32	16.00977	-0.2438691	0.330745	-0.73733229	0.46092	0.693207	NOT
RBM23	2204.282	-0.1156765	0.1569	-0.73726061	0.460964	0.693234	NOT
DCAF12	2083.029	0.11114606	0.15077	0.737190238	0.461007	0.693241	NOT
HELLPAR	60.54433	-0.2214099	0.300351	-0.73716929	0.461019	0.693241	NOT
BRD8	1716.131	0.10458816	0.141896	0.737078497	0.461075	0.693286	NOT
RP11-145I	1.457656	0.63220822	0.857797	0.73701369	0.461114	0.693307	NOT
PROC	12965.88	-0.2474983	0.335867	-0.73689403	0.461187	0.693343	NOT
IL2RB	420.1641	-0.2686622	0.364589	-0.7368902	0.461189	0.693343	NOT
ZDHHC6	1559.801	-0.1225833	0.166369	-0.73681724	0.461233	0.693371	NOT
TRPS1	270.7449	0.26443275	0.358912	0.736763024	0.461266	0.693382	NOT
AL135745.	4.479916	-0.3543448	0.481086	-0.73655189	0.461395	0.693537	NOT
TM9SF1	341.1016	-0.1199386	0.162866	-0.73642635	0.461471	0.693547	NOT
LINC00487	3.528896	-0.3885309	0.527591	-0.73642455	0.461472	0.693547	NOT
RP11-225I	1.986027	-0.3796153	0.515491	-0.73641493	0.461478	0.693547	NOT
PPM1F	1684.631	-0.1250328	0.169815	-0.73629048	0.461554	0.693622	NOT
RIMBP3	4.81126	0.32609907	0.442995	0.736123758	0.461655	0.693702	NOT
RP11-846I	3.666659	-0.4016639	0.545707	-0.73604372	0.461704	0.693702	NOT
ESYT1	7458.926	-0.1251336	0.170013	-0.73602452	0.461716	0.693702	NOT
PGM5-AS1	2.994202	0.52056151	0.707264	0.736021826	0.461717	0.693702	NOT
ESYT3	64.40323	0.35565047	0.483225	0.735992814	0.461735	0.693702	NOT
VGLL4	823.7615	0.16378857	0.222565	0.735913313	0.461783	0.693727	NOT
PLA2G10	2.901881	0.34554211	0.469562	0.73588195	0.461803	0.693727	NOT
ZDHHC16	1458.729	0.09592196	0.130367	0.735786832	0.46186	0.693775	NOT
ASB8	1461.733	-0.0846125	0.115004	-0.7357372	0.461891	0.693782	NOT
PLEKHO2	1193.774	-0.1844263	0.250687	-0.73568241	0.461924	0.693794	NOT
XK	216.0017	0.37461852	0.509293	0.735566481	0.461995	0.693826	NOT
LINC00909	286.6211	0.11518112	0.156594	0.735539303	0.462011	0.693826	NOT
UBE2L6	3804.3	0.19604654	0.266541	0.735521424	0.462022	0.693826	NOT
TRIP12	4931.367	0.08659332	0.117741	0.735453723	0.462063	0.693849	NOT
HEXA-AS1	14.82886	0.2729444	0.371282	0.735140868	0.462254	0.694096	NOT
KB-1125A3	5.677762	0.24634669	0.335141	0.735053411	0.462307	0.694096	NOT
B3GALNT2	285.8843	-0.1589004	0.216176	-0.73504986	0.462309	0.694096	NOT
AC133644.	26.82541	0.26780487	0.364352	0.735016109	0.46233	0.694096	NOT
BDNF	11.85135	-0.2721294	0.370266	-0.73495561	0.462367	0.694103	NOT

NACAD	32.21866	-0.3210933	0.43693	-0.73488494	0.46241	0.694103	NOT
RP11-677M	14.5721	-0.2203826	0.299888	-0.73488203	0.462411	0.694103	NOT
EIF3CL	8.945693	0.20311272	0.276407	0.734833081	0.462441	0.694109	NOT
AC005822.	3.89372	0.254103	0.345909	0.734595392	0.462586	0.694183	NOT
PTGES3P1	146.3992	0.18844851	0.256536	0.734588494	0.46259	0.694183	NOT
RP1-209A6	5.154627	-0.6206518	0.844899	-0.73458706	0.462591	0.694183	NOT
RP11-483H	1.864319	0.38595181	0.525401	0.734585168	0.462592	0.694183	NOT
MDK	10645.83	0.32611205	0.444028	0.734440954	0.46268	0.694276	NOT
RP11-234K	5.981039	-0.415259	0.565527	-0.73428635	0.462774	0.694379	NOT
DNM1P47	1.615911	-0.4315973	0.587888	-0.73414869	0.462858	0.694439	NOT
DDX4	2.829641	0.50905494	0.693412	0.734130154	0.462869	0.694439	NOT
TRERF1	143.9002	-0.2155949	0.293688	-0.73409439	0.462891	0.694439	NOT
PRUNE	1686.667	0.12921423	0.176046	0.7339818	0.46296	0.694494	NOT
RP11-661A	70.60141	0.21197535	0.288823	0.733928488	0.462992	0.694494	NOT
NDUFB6	2266.357	-0.1211029	0.165016	-0.73388685	0.463018	0.694494	NOT
RP11-394(	1.122665	-0.6976518	0.950637	-0.73387863	0.463023	NA	NA
C6orf99	5.928115	-0.5642051	0.768811	-0.73386697	0.46303	0.694494	NOT
CDYL2	325.9674	-0.2073525	0.282579	-0.73378632	0.463079	0.694529	NOT
CD72	191.777	0.27913501	0.380443	0.733710367	0.463125	0.694551	NOT
ZSCAN31	707.2627	0.27542095	0.375421	0.733631936	0.463173	0.694551	NOT
CTC-457E2	1.415092	-0.7749483	1.056384	-0.7335861	0.463201	0.694551	NOT
CTA-292E1	6.80289	0.22713252	0.309646	0.733523883	0.463239	0.694551	NOT
ALG12	975.2453	0.15762366	0.214886	0.733522479	0.46324	0.694551	NOT
HINT3	1578.526	-0.1159015	0.158009	-0.73351045	0.463247	0.694551	NOT
PARP16	714.8629	-0.1187529	0.161933	-0.73334377	0.463349	0.694611	NOT
GRPR	38.21288	-0.5018622	0.68436	-0.73333013	0.463357	0.694611	NOT
TMEM14A	2677.963	0.18747628	0.255655	0.733318119	0.463364	0.694611	NOT
XXbac-BP(	8.713082	-0.3195658	0.435851	-0.73320004	0.463436	0.694681	NOT
POMT2	671.1419	0.11927437	0.162696	0.733112747	0.46349	0.694722	NOT
AC093642.	31.06314	-0.3660974	0.499463	-0.73298239	0.463569	0.694803	NOT
KHK	9858.159	-0.2626145	0.358316	-0.73291411	0.463611	0.694827	NOT
RP11-31F1	1.66098	-0.4980531	0.679665	-0.73279242	0.463685	0.6949	NOT
SATB1-AS1	17.67277	0.41483646	0.566325	0.732505443	0.46386	0.695103	NOT
RP5-828K2	2.415072	0.36940815	0.504329	0.732474315	0.463879	0.695103	NOT
RP11-23J9	7.080295	-0.3216333	0.439123	-0.73244448	0.463897	0.695103	NOT
CDIP1	2631.601	-0.1471728	0.200976	-0.73229173	0.463991	0.695196	NOT
RP11-464I	1.921797	-0.3569184	0.487421	-0.73225844	0.464011	0.695196	NOT
RP11-43F1	388.9122	0.21691746	0.296265	0.732172704	0.464063	0.695206	NOT
HMX1	1.533072	1.0173914	1.389567	0.732164359	0.464068	0.695206	NOT
KIAA1549	334.6755	0.33075087	0.451799	0.732074736	0.464123	0.695249	NOT
TRAV35	1.111293	0.6500426	0.888017	0.73201613	0.464159	NA	NA
CSGALNACT	771.8377	-0.1209526	0.165242	-0.73197018	0.464187	0.695272	NOT
IGKV1-27	36.22775	0.61193147	0.836011	0.731965603	0.46419	0.695272	NOT
PREX1	1411.094	-0.1857161	0.25377	-0.73182921	0.464273	0.695358	NOT
CLEC11A	296.6876	-0.2471523	0.337748	-0.73176562	0.464312	0.695378	NOT
COX11	2211.539	-0.1009258	0.137953	-0.73159448	0.464416	0.695496	NOT
SPRR1A	2.330152	0.8113727	1.109362	0.731386743	0.464543	0.695583	NOT
SHQ1	616.2178	0.12154827	0.166191	0.73137565	0.46455	0.695583	NOT
HMBS	1420.042	0.13252073	0.181194	0.731373056	0.464551	0.695583	NOT

SNORD15B	1.790131	0.39676946	0.542626	0.731202288	0.464656	0.695701	NOT
NBPF10	74.80918	-0.1969737	0.269402	-0.73115289	0.464686	0.695708	NOT
LINC00308	1.568756	-0.6843769	0.936203	-0.73101307	0.464771	0.695797	NOT
IGLV2-33	1.381566	-0.7587407	1.037975	-0.7309818	0.46479	NA	NA
RP11-373I	6.478618	-0.3483255	0.476669	-0.73074845	0.464933	0.695988	NOT
HSPB7	61.0508	-0.3238313	0.443188	-0.73068538	0.464971	0.695988	NOT
RP11-1148	12.39244	-0.1605698	0.219766	-0.73063999	0.464999	0.695988	NOT
RP11-219(	1.481557	0.36677715	0.501996	0.730636979	0.465001	0.695988	NOT
PLEKHA3P1	2.931427	0.30653777	0.419628	0.730498101	0.465086	0.696045	NOT
DARS2	2055.637	0.15093268	0.206625	0.730467245	0.465105	0.696045	NOT
EFCAB2	501.6435	-0.1688844	0.231206	-0.73044842	0.465116	0.696045	NOT
RP11-713(	1.371788	-0.505025	0.691392	-0.73044673	0.465117	NA	NA
RP11-798M	34.12279	0.18378075	0.25163	0.730361453	0.465169	0.696086	NOT
TMEM262	42.21302	-0.1472233	0.201589	-0.73031401	0.465198	0.696089	NOT
C6orf106	12435.58	0.13204409	0.180814	0.730274733	0.465222	0.696089	NOT
AC023283.	1.640707	0.46600286	0.6382	0.730183449	0.465278	0.696095	NOT
KIAA1217	1943.703	0.12725727	0.174281	0.730183351	0.465278	0.696095	NOT
RP11-142A	2.827092	0.33708072	0.461667	0.730138315	0.465306	0.696098	NOT
GJC3	30.32937	-0.3153143	0.431927	-0.73001829	0.465379	0.69614	NOT
DTX4	1617.195	-0.2528491	0.346365	-0.7300084	0.465385	0.69614	NOT
RP11-1379	10.28321	-0.2234919	0.306221	-0.72983887	0.465489	0.696247	NOT
LINC00881	7.44013	-0.3875953	0.531105	-0.72979019	0.465518	0.696247	NOT
NOP14-AS1	436.235	-0.1327952	0.18197	-0.72976566	0.465533	0.696247	NOT
TMPRSS15	10.05532	0.80264606	1.100065	0.72963498	0.465613	0.696328	NOT
LINC0156(	53.24704	-0.1223132	0.167685	-0.72942065	0.465744	0.696456	NOT
SLC22A3	1843.238	-0.2991933	0.410185	-0.72941074	0.46575	0.696456	NOT
RP11-769(	2.532294	0.35846507	0.491515	0.729306973	0.465814	0.696513	NOT
HES7	2.037576	0.57233054	0.784991	0.729091504	0.465946	0.696664	NOT
KB-1269D1	0.504854	0.89009382	1.220866	0.729067767	0.46596	NA	NA
COL21A1	151.1841	-0.2820706	0.386921	-0.7290127	0.465994	0.696664	NOT
TBC1D27	4.494459	-0.3694071	0.506749	-0.72897428	0.466017	0.696664	NOT
PRR15L	332.7896	0.41352767	0.567274	0.728973748	0.466018	0.696664	NOT
LRRC23	166.4818	0.18238557	0.25021	0.728931178	0.466044	0.696665	NOT
RP11-816J	5.023582	0.25037784	0.343515	0.728871141	0.46608	0.696681	NOT
LECT2	2981.23	-0.4422371	0.60694	-0.7286337	0.466226	0.696811	NOT
RP11-434H	106.0384	-0.2230531	0.306159	-0.72855264	0.466275	0.696811	NOT
IGHV4-59	98.8054	0.51313343	0.704322	0.728549633	0.466277	0.696811	NOT
FAM89B	718.6278	0.13994276	0.19211	0.728450835	0.466338	0.696811	NOT
RP11-335I	4.618759	-0.2576984	0.353763	-0.72845011	0.466338	0.696811	NOT
RP5-1014I	81.06005	-0.1641437	0.225346	-0.72840684	0.466365	0.696811	NOT
RP1-124C6	2.211299	0.4948576	0.679372	0.728404111	0.466366	0.696811	NOT
BMS1P1	86.7752	-0.1920023	0.263612	-0.7283522	0.466398	0.696811	NOT
RP11-521E	22.42179	-0.2090349	0.286997	-0.72835153	0.466398	0.696811	NOT
RP11-57H1	2.598194	-0.3413787	0.468814	-0.72817458	0.466507	0.696901	NOT
PSMA2	1032.427	-0.135722	0.186388	-0.72816951	0.46651	0.696901	NOT
CDK8	615.8324	0.10710373	0.147107	0.728065904	0.466573	0.696957	NOT
RP11-622(	3.309685	0.32803656	0.450619	0.727969494	0.466632	0.696974	NOT
CNNM1	276.5745	0.44995043	0.618095	0.727963402	0.466636	0.696974	NOT
RP11-296A	4.871481	-0.2752126	0.378139	-0.72780731	0.466732	0.69705	NOT

CGA	7.531381	0.54063069	0.742832	0.727796549	0.466738	0.69705	NOT
DHX36	1364.346	-0.1009065	0.138674	-0.7276538	0.466826	0.697142	NOT
PLEKHH3	1214.608	-0.1326546	0.182359	-0.72743633	0.466959	0.697239	NOT
KCNF1	105.7677	0.47435914	0.65211	0.727422268	0.466967	0.697239	NOT
PUSL1	672.5195	0.20556253	0.282591	0.727421182	0.466968	0.697239	NOT
SNRPD2	5565.615	0.1657789	0.227913	0.727378496	0.466994	0.69724	NOT
CORO7	499.7806	0.17204142	0.236548	0.727300994	0.467042	0.697273	NOT
NHLRC1	207.9526	0.19357209	0.266167	0.727258328	0.467068	0.697273	NOT
RP11-313I	3.464565	0.27358861	0.376259	0.727127626	0.467148	0.697322	NOT
SRP14-AS1	221.3497	-0.1751687	0.240907	-0.72712057	0.467152	0.697322	NOT
GOLT1B	1874.731	0.09144038	0.125785	0.726959878	0.467251	0.697367	NOT
LRRC25	296.8214	0.22884299	0.314799	0.726950253	0.467256	0.697367	NOT
KRT18P16	2.932628	0.35802938	0.492531	0.726916769	0.467277	0.697367	NOT
C8orf58	139.6456	-0.1813214	0.249443	-0.72690363	0.467285	0.697367	NOT
RP11-791(	4.047041	-0.2567182	0.353193	-0.72684892	0.467319	0.697379	NOT
RP11-338K	3.836821	-0.3136232	0.431587	-0.72667384	0.467426	0.697492	NOT
ZNF502	107.1327	-0.2924324	0.402444	-0.72664194	0.467445	0.697492	NOT
ANGPTL2	861.0844	-0.1789108	0.246253	-0.72653121	0.467513	0.697554	NOT
AF129075.	10.44776	-0.2154189	0.296549	-0.72641878	0.467582	0.697619	NOT
MYD88	2287.637	-0.1201712	0.165451	-0.72632324	0.467641	0.697668	NOT
ACOT8	1374.313	0.13758786	0.189444	0.726271768	0.467672	0.697677	NOT
SKIL	1628.358	0.1493885	0.20577	0.725997338	0.46784	0.697879	NOT
RP5-857K2	5959.79	-0.2336222	0.321817	-0.72594784	0.467871	0.697879	NOT
LINC0031(	6.786836	0.22037419	0.303577	0.725924848	0.467885	0.697879	NOT
EPHB2	573.0897	0.37285648	0.513698	0.725828537	0.467944	0.697928	NOT
OPHN1	109.8189	-0.2311899	0.318575	-0.72570058	0.468022	0.697987	NOT
TPTE2P5	9.165837	-0.2740529	0.377649	-0.72568081	0.468034	0.697987	NOT
RP3-336K2	2.273235	-0.4239266	0.584322	-0.72550146	0.468144	0.698001	NOT
RP1-137D1	3.010555	0.28017748	0.386185	0.725501368	0.468145	0.698001	NOT
ZSWIM8	2776.389	-0.1484557	0.204625	-0.72550079	0.468145	0.698001	NOT
CTD-3187F	3.484061	0.39673024	0.546839	0.725497616	0.468147	0.698001	NOT
RP11-863K	8.763315	-0.316504	0.436291	-0.72544182	0.468181	0.698013	NOT
LINC0008(	20.64705	0.28493554	0.392946	0.725126834	0.468374	0.698237	NOT
ITGAM	607.1314	0.26742676	0.368818	0.72509069	0.468396	0.698237	NOT
RP3-508I1	2.909536	0.29550335	0.407569	0.725038945	0.468428	0.698237	NOT
GBP2	5017.144	0.23325433	0.321717	0.725029893	0.468434	0.698237	NOT
PLA2G4B	9.401271	-0.274701	0.378919	-0.7249594	0.468477	0.698263	NOT
AMPD3	201.9893	0.21870937	0.301718	0.724879494	0.468526	0.698297	NOT
ZBED5-AS1	512.2594	0.17006611	0.234653	0.724757075	0.468601	0.698371	NOT
CTD-2013N	19.51384	0.16588855	0.228937	0.724603907	0.468695	0.698435	NOT
LPXN	441.2849	-0.1339169	0.184814	-0.72460287	0.468696	0.698435	NOT
CTD-2184I	2.448193	0.51220213	0.706993	0.724479337	0.468772	0.698478	NOT
SNX24	563.9628	-0.1182091	0.163168	-0.72446383	0.468781	0.698478	NOT
ZNF335	1111.239	0.09178403	0.126698	0.724430485	0.468801	0.698478	NOT
HIST1H4H	78.39683	0.31426143	0.433885	0.724296544	0.468884	0.698562	NOT
CTD-2047H	3.553573	0.34935935	0.48241	0.724195422	0.468946	0.698607	NOT
RP11-435(	7.522596	0.30125815	0.416009	0.724163151	0.468966	0.698607	NOT
ST3GAL2	1548.618	-0.09556	0.132011	-0.72387926	0.46914	0.698791	NOT
COX17	1960.044	0.1420361	0.196215	0.723878819	0.46914	0.698791	NOT

RP11-14N7	14.64034	0.34198236	0.472542	0.723707601	0.469245	0.698909	NOT
DLEU2	135.5538	0.20308423	0.280714	0.723456934	0.469399	0.6991	NOT
TRAV13-1	4.512117	-0.4073207	0.563069	-0.7233934	0.469438	0.699106	NOT
DYDC2	84.03341	0.4227217	0.584381	0.723366148	0.469455	0.699106	NOT
PSMB7	6679.788	-0.1367108	0.189008	-0.72330554	0.469492	0.699123	NOT
RP11-565A	6.100978	0.76446283	1.05726	0.723060697	0.469643	0.699274	NOT
FLT3LG	80.93926	-0.1816137	0.251184	-0.72303128	0.469661	0.699274	NOT
RP11-278C	32.64325	0.14183725	0.196175	0.723014308	0.469671	0.699274	NOT
PYGL	7381.891	-0.2054797	0.284244	-0.72289829	0.469742	0.699327	NOT
TTC6	115.2493	-0.2252129	0.311553	-0.72287253	0.469758	0.699327	NOT
HIATL2	74.48881	0.11840746	0.16385	0.72265948	0.469889	0.699484	NOT
FBXO38	1446.124	-0.1006527	0.139312	-0.72249874	0.469988	0.699527	NOT
RP1-90G24	3.492678	0.48702466	0.674092	0.722489941	0.469993	0.699527	NOT
TMEM254	1823.827	-0.157378	0.217828	-0.72248586	0.469996	0.699527	NOT
TRAT1	26.47718	-0.3353014	0.46415	-0.7223994	0.470049	0.699562	NOT
RP11-16L2	3.55089	0.41920302	0.580321	0.722364426	0.47007	0.699562	NOT
VN1R85P	4.951924	-0.6821851	0.944588	-0.72220355	0.470169	0.699671	NOT
SATB2-AS1	12.55722	-0.2821547	0.390757	-0.72207259	0.47025	0.699723	NOT
RP11-181C	16.69202	0.25459853	0.352605	0.722049971	0.470264	0.699723	NOT
MAN2B1	3616.665	-0.1258976	0.174373	-0.72200093	0.470294	0.699723	NOT
RP11-416I	4.917155	0.39165857	0.542485	0.721971492	0.470312	0.699723	NOT
ETNK1	2373.657	0.12521022	0.173437	0.721936832	0.470333	0.699723	NOT
CCNC	2517.29	0.14636583	0.202794	0.721745105	0.470451	0.69986	NOT
IGKV1D-43	1.082992	0.73010134	1.011651	0.721692935	0.470483	NA	NA
LINC00449	4.359238	-0.2813737	0.38992	-0.7216194	0.470529	0.699872	NOT
ZNF679	2.183384	-1.0930662	1.514816	-0.72158368	0.47055	0.699872	NOT
RP11-51F1	36.02064	0.25129471	0.348269	0.721553092	0.470569	0.699872	NOT
LRRC66	21.97574	0.34367947	0.476319	0.7215328	0.470582	0.699872	NOT
ZNF624	91.80499	0.17129169	0.237403	0.721522348	0.470588	0.699872	NOT
ABCD3	5315.65	-0.1449224	0.20087	-0.72147485	0.470617	0.699877	NOT
STK38L	723.825	-0.1476442	0.204801	-0.72091539	0.470962	0.700327	NOT
DDX43P3	4.753402	0.58906288	0.817165	0.720861891	0.470994	0.700327	NOT
HNMT	4864.877	-0.1774481	0.246185	-0.720791	0.471038	0.700327	NOT
RP11-402I	4.880995	0.31007167	0.430217	0.720733445	0.471074	0.700327	NOT
LINC01266	1.527876	-0.4404748	0.611167	-0.72071165	0.471087	0.700327	NOT
SETD9	316.9982	-0.1565469	0.217231	-0.72064694	0.471127	0.700327	NOT
CYP4Z1	7.512379	-0.3861306	0.535813	-0.72064462	0.471128	0.700327	NOT
GABBR2	82.80672	0.56841442	0.788777	0.720627227	0.471139	0.700327	NOT
PARP15	77.68593	-0.2745832	0.381045	-0.72060525	0.471152	0.700327	NOT
L3MBTL4	533.4641	-0.1481682	0.205633	-0.72054519	0.471189	0.700343	NOT
HIST1H2AJ	1.700519	0.4160234	0.577492	0.720396202	0.471281	0.700441	NOT
CTB-25B13	380.0444	0.17299978	0.240195	0.720247124	0.471373	0.700529	NOT
FAM63B	569.9728	-0.138344	0.192099	-0.72017112	0.47142	0.700529	NOT
CTD-2501E	2.281381	0.36600381	0.508245	0.720133147	0.471443	0.700529	NOT
SLC34A1	34.98585	-0.364033	0.505534	-0.7200958	0.471466	0.700529	NOT
RP11-191I	9.429073	0.62112044	0.862558	0.720091073	0.471469	0.700529	NOT
TNFAIP8L1	3245.679	-0.1544628	0.214528	-0.72001271	0.471517	0.700562	NOT
ENTPD4	982.3134	-0.1575611	0.218857	-0.71992725	0.47157	0.700595	NOT
ETFA	8537.5	-0.1453178	0.201862	-0.71988732	0.471594	0.700595	NOT

MICALL2	1243.213	0.1784864	0.247949	0.719851265	0.471617	0.700595	NOT
RP11-32D1	2.698423	0.34826136	0.483906	0.71968802	0.471717	0.700659	NOT
MIPEPP3	15.08469	0.15990834	0.222197	0.719669612	0.471728	0.700659	NOT
TEAD4	364.3149	0.25928613	0.360299	0.719641643	0.471746	0.700659	NOT
NMD3P1	1.805197	-0.3640911	0.506	-0.71954715	0.471804	0.700659	NOT
CSNK1G3	1075.659	-0.1147368	0.159475	-0.71946556	0.471854	0.700659	NOT
RP13-122E	17.87005	-0.2726242	0.378937	-0.71944418	0.471867	0.700659	NOT
RP11-632F	1.431586	0.52712512	0.732691	0.719436971	0.471872	0.700659	NOT
CTC-296K1	21.46587	-0.548749	0.762764	-0.71942162	0.471881	0.700659	NOT
AC009487.	19.87721	0.22630463	0.314573	0.719403528	0.471892	0.700659	NOT
SEC14L4	648.3773	0.33586543	0.466965	0.719251792	0.471986	0.700759	NOT
KCNC4-AS1	2.910553	-0.3520085	0.489604	-0.71896573	0.472162	0.700983	NOT
YLPM1	1775.833	-0.1219451	0.169658	-0.71876955	0.472283	0.701082	NOT
LTB	429.1848	0.28126062	0.39132	0.71874918	0.472295	0.701082	NOT
RP11-747H	41.36653	0.38508327	0.535782	0.718731803	0.472306	0.701082	NOT
GPR110	4.81546	0.43982797	0.611989	0.718686525	0.472334	0.701085	NOT
SVIL	2086.337	-0.1803027	0.250927	-0.71854747	0.47242	0.701116	NOT
TMEFF2	3.171599	0.52130855	0.725531	0.718520013	0.472437	0.701116	NOT
SH3GL1	3321.087	0.12625371	0.175719	0.718496394	0.472451	0.701116	NOT
RP11-482I	2.84493	0.35371191	0.492303	0.718484517	0.472459	0.701116	NOT
PRR5L	295.3555	0.29634367	0.412497	0.718414165	0.472502	0.701142	NOT
VSIG10L	672.1086	0.32679116	0.455097	0.718069794	0.472714	0.701419	NOT
CDHR3	260.5315	-0.1916538	0.266927	-0.71799991	0.472757	0.701444	NOT
ELK4	2820.405	-0.1494608	0.208273	-0.71761969	0.472992	0.701754	NOT
RPL4P2	3.910187	0.31734646	0.442323	0.717454189	0.473094	0.701825	NOT
RTN2	416.1879	0.24631082	0.343331	0.717415982	0.473117	0.701825	NOT
CTD-2005H	2.095555	-0.4012638	0.559373	-0.71734567	0.473161	0.701825	NOT
C20orf24	1905.568	0.15644592	0.218103	0.717304411	0.473186	0.701825	NOT
CTC-378H2	2.532897	0.33963038	0.473509	0.717262046	0.473212	0.701825	NOT
RPL7AP11	10.21971	0.24527479	0.341962	0.717256665	0.473216	0.701825	NOT
RP11-397E	12.50553	0.26323829	0.367012	0.717247695	0.473221	0.701825	NOT
ADPRH	304.7024	-0.1547556	0.2158	-0.71712423	0.473297	0.7019	NOT
TDO2	9662.249	-0.379925	0.529883	-0.71699727	0.473376	0.701978	NOT
ZNF815P	59.49264	0.16056856	0.223998	0.71683023	0.473479	0.702092	NOT
RN7SK	163.1826	-0.2782327	0.388177	-0.71676797	0.473517	0.702111	NOT
RP11-685E	5.597698	-0.5220205	0.728346	-0.71672055	0.473547	0.702114	NOT
UBE2V1	289.0464	0.10659944	0.14874	0.716680843	0.473571	0.702114	NOT
ZDHHC5	4452.297	-0.0762257	0.10639	-0.71647644	0.473697	0.702197	NOT
TNNI2	61.7677	0.32233777	0.449908	0.716452753	0.473712	0.702197	NOT
RP11-495F	3.204183	0.38098976	0.531801	0.71641406	0.473736	0.702197	NOT
CTD-2201E	117.4312	0.24724612	0.345127	0.716392265	0.473749	0.702197	NOT
AC114498.	14.95209	-0.3514031	0.490529	-0.71637518	0.47376	0.702197	NOT
FNBP4	1588.623	0.12076374	0.168601	0.716270488	0.473824	0.702197	NOT
RPL15P18	2.248477	0.29497116	0.411831	0.716243458	0.473841	0.702197	NOT
RP4-630A1	2.409723	-0.3185912	0.444826	-0.71621546	0.473858	0.702197	NOT
VCAN	1917.626	0.36317537	0.507078	0.716212739	0.47386	0.702197	NOT
CTD-2047H	18.64128	-0.2096157	0.292707	-0.71612703	0.473913	0.70221	NOT
RP11-513M	15.67542	-0.19039	0.265865	-0.71611458	0.473921	0.70221	NOT
IGLV4-60	8.364343	0.61842855	0.863648	0.716065546	0.473951	0.702217	NOT

SBDS	3632.294	-0.1143857	0.159758	-0.71599569	0.473994	0.702242	NOT
IGHV3OR16	1.012863	-0.6224264	0.869402	-0.71592498	0.474038	NA	NA
RNF219-AS	2.31426	0.32115534	0.448725	0.715705613	0.474173	0.702469	NOT
EYA4	22.44739	0.6175355	0.863039	0.715536368	0.474278	0.702571	NOT
TMEM50B	1320.133	-0.1195017	0.167018	-0.71550024	0.4743	0.702571	NOT
SPTLC2	1396.973	0.10558567	0.147576	0.715468482	0.47432	0.702571	NOT
EGR2	163.4109	-0.2947935	0.412164	-0.71523286	0.474465	0.702747	NOT
CASK	2032.477	-0.1451184	0.202908	-0.71519227	0.47449	0.702747	NOT
PXN	2940.213	0.08607787	0.120384	0.715029995	0.474591	0.702857	NOT
RP11-174(	1.453432	0.38979872	0.545225	0.714931515	0.474651	0.702886	NOT
CTD-2545(	6.149185	0.25141118	0.351678	0.714891162	0.474676	0.702886	NOT
HIRA	894.347	-0.1155239	0.161601	-0.71487236	0.474688	0.702886	NOT
EEF1A1P1	6.217029	0.3353087	0.469078	0.71482488	0.474717	0.702891	NOT
XXbac-BP(	2.025852	0.42923106	0.600539	0.714742487	0.474768	0.702928	NOT
ITLN1	15.29221	-0.444379	0.621777	-0.71469206	0.474799	0.702936	NOT
TRAP1	4163.051	0.14842371	0.207732	0.714495524	0.474921	0.703046	NOT
C17orf59	428.4657	0.14278716	0.199845	0.714487788	0.474926	0.703046	NOT
RPUSD4	990.4782	0.11093639	0.155289	0.714388172	0.474987	0.703099	NOT
CHST8	18.00245	-0.4698594	0.658011	-0.71405985	0.47519	0.703361	NOT
TUBB8P1	1.860239	-0.3363818	0.471138	-0.71397798	0.475241	0.703398	NOT
FZD5	3157.57	0.1628239	0.228172	0.713602828	0.475473	0.703703	NOT
RP11-165F	2.860151	0.30752599	0.430988	0.713536953	0.475514	0.703725	NOT
CDC14A	166.839	0.16915225	0.237092	0.713445839	0.47557	0.70377	NOT
RP11-148E	2.431528	0.45931755	0.644037	0.713185038	0.475731	0.70397	NOT
LRRC3B	6.1702	0.34989393	0.490674	0.713087694	0.475791	0.703975	NOT
DEPDC4	36.64863	0.14532779	0.203805	0.713073358	0.4758	0.703975	NOT
ASMT	2.899991	-0.4347216	0.609662	-0.71305371	0.475813	0.703975	NOT
PTRHD1	797.0273	-0.1539418	0.215936	-0.71290512	0.475904	0.704073	NOT
RP11-776A	0.626728	-0.8353539	1.172065	-0.71271949	0.476019	NA	NA
DEGS2	76.50073	-0.348127	0.48853	-0.71260097	0.476093	0.704313	NOT
FGF20	1.265358	0.54590178	0.766153	0.712522913	0.476141	NA	NA
RP11-295F	6.986189	-0.2686607	0.377058	-0.71251868	0.476144	0.70435	NOT
SPEN	2758.512	-0.1292083	0.18139	-0.71232385	0.476264	0.70449	NOT
LENG8-AS1	57.71224	0.15232018	0.213888	0.712149848	0.476372	0.704552	NOT
CTD-2384A	9.085516	0.82059929	1.152339	0.712116356	0.476393	0.704552	NOT
GSC	3.831987	0.4727753	0.663949	0.712065682	0.476424	0.704552	NOT
HAND2	77.05628	-0.3921824	0.550789	-0.71203754	0.476442	0.704552	NOT
PFN1	24165.76	0.14588025	0.204881	0.712024589	0.47645	0.704552	NOT
RPL7P26	2.346951	0.30785051	0.432372	0.712003939	0.476462	0.704552	NOT
TMEM150A	1855.349	-0.1394292	0.195845	-0.71193464	0.476505	0.704573	NOT
PNRC2	1506.016	0.09635911	0.135355	0.711897908	0.476528	0.704573	NOT
GALNT1	4283.707	-0.1328856	0.186717	-0.71169595	0.476653	0.704685	NOT
SMNDC1	950.7749	0.0781529	0.109813	0.711690927	0.476656	0.704685	NOT
RP11-254F	1.994354	-0.3140185	0.441263	-0.71163499	0.476691	0.704698	NOT
BROX	2240.568	0.10268316	0.14432	0.711497175	0.476776	0.704786	NOT
AL022393.	2.079895	0.52830368	0.74262	0.711405364	0.476833	0.704817	NOT
PKP4	3205.829	0.13367248	0.187906	0.711379268	0.476849	0.704817	NOT
RP11-466A	2.843236	-0.3256931	0.457975	-0.71115843	0.476986	0.70495	NOT
IMPG1	5.92675	-0.2625465	0.369185	-0.71115089	0.476991	0.70495	NOT

CHPF	5738.223	-0.1982689	0.278855	-0.71101095	0.477077	0.705039	NOT
CCDC62	4.526285	0.32112351	0.45191	0.710592128	0.477337	0.705383	NOT
C9orf9	215.5197	-0.2075029	0.292031	-0.71055173	0.477362	0.705383	NOT
ALDH1L1	29105.02	0.38161497	0.537145	0.710451227	0.477424	0.705437	NOT
ATG5	1161.164	0.10329075	0.145406	0.710363271	0.477479	0.705452	NOT
RPN1	19683.29	0.08612795	0.12125	0.710333269	0.477497	0.705452	NOT
MARS2	478.763	0.13793572	0.194191	0.71030862	0.477513	0.705452	NOT
LINC0111９	21.35743	0.2539341	0.357581	0.710144759	0.477614	0.705564	NOT
FIS1	6117.898	-0.1581777	0.222762	-0.71007613	0.477657	0.70557	NOT
C9orf106	16.63727	-0.3681642	0.518513	-0.71003872	0.47768	0.70557	NOT
TARS2	2686.944	0.13621626	0.191851	0.710011713	0.477697	0.70557	NOT
PIGB	339.1828	-0.1133821	0.159705	-0.70994731	0.477737	0.70559	NOT
RNF121	1040.937	-0.0955991	0.134664	-0.70990691	0.477762	0.70559	NOT
RP11-766F	11.37553	0.52626525	0.74169	0.709548961	0.477984	0.705879	NOT
SH2D4A	1460.281	-0.1910663	0.26932	-0.70943887	0.478052	0.705941	NOT
STAU1	9625.695	0.10449294	0.147322	0.709283666	0.478148	0.706045	NOT
AC098828.	2.078116	0.43228891	0.609532	0.709214469	0.478191	0.70607	NOT
POLR2F	3.112355	-0.3506648	0.494558	-0.70904624	0.478296	0.706186	NOT
NUBP1	690.9731	-0.0933697	0.13173	-0.70879772	0.47845	0.706356	NOT
IDH3G	2785.396	-0.1427055	0.201341	-0.70877694	0.478463	0.706356	NOT
RP11-73M7	3.072734	0.31157025	0.439647	0.70868223	0.478522	0.706404	NOT
RP11-783K	36.67106	-0.2544356	0.359057	-0.70862074	0.47856	0.706422	NOT
LINC00087	77.48104	-0.2510872	0.354383	-0.70851828	0.478623	0.706475	NOT
RAB5B	6828.677	-0.0931664	0.131506	-0.7084571	0.478661	0.706475	NOT
BCAP31	15624.23	0.13703021	0.193426	0.708436531	0.478674	0.706475	NOT
BCAR1	3151.994	-0.1141536	0.161152	-0.70835741	0.478723	0.706509	NOT
AK8	25.92687	0.34384888	0.48549	0.708251777	0.478789	0.706568	NOT
TRAV24	1.017055	-0.56224	0.793864	-0.70823216	0.478801	NA	NA
CDC37	6347.125	-0.0867566	0.122533	-0.70802917	0.478927	0.706701	NOT
RP11-128(	9.663181	0.25002831	0.353136	0.708022502	0.478931	0.706701	NOT
CNBD2	17.05811	-0.1940344	0.274112	-0.70786532	0.479029	0.706783	NOT
MAP1S	1530.447	0.12240732	0.172928	0.707849421	0.479039	0.706783	NOT
FAM228B	174.9834	0.20462964	0.289147	0.707700577	0.479131	0.706881	NOT
FERMT2	2591.815	-0.1420316	0.200724	-0.70759631	0.479196	0.706938	NOT
MSRB1	6398.086	-0.1657247	0.234353	-0.70715933	0.479467	0.7073	NOT
DNAH5	198.676	-0.3329471	0.470907	-0.7070337	0.479546	0.70732	NOT
FKBP4	7179.605	0.14380312	0.203396	0.707010312	0.47956	0.70732	NOT
BLOC1S3	793.8964	0.10696623	0.151303	0.706967267	0.479587	0.70732	NOT
MRPL46	1051.686	-0.1650781	0.233513	-0.70693468	0.479607	0.70732	NOT
CHST14	719.132	0.13079356	0.185024	0.70689994	0.479629	0.70732	NOT
RP11-294J	1.728311	-0.2999827	0.424373	-0.70688533	0.479638	0.70732	NOT
RP11-87H9	3.907893	-0.3331703	0.471411	-0.70675154	0.479721	0.707373	NOT
CTA-223H9	4.96914	0.2846435	0.402753	0.706744232	0.479725	0.707373	NOT
WDR27	355.893	0.18164032	0.257081	0.706549741	0.479846	0.707513	NOT
RPS3AP47	23.82027	0.23518041	0.332906	0.70644637	0.479911	0.707569	NOT
ESRRAP2	1.999737	-0.2685611	0.380251	-0.70627356	0.480018	0.707689	NOT
DNAJC27-A	80.97781	-0.2188746	0.309946	-0.70617015	0.480082	0.707735	NOT
VMP1	7566.084	0.11849999	0.167831	0.706065366	0.480147	0.707735	NOT
FAM212B	325.6476	-0.2031903	0.287792	-0.7060315	0.480169	0.707735	NOT

DNMT3L	26.14648	-0.4573017	0.647719	-0.70601831	0.480177	0.707735	NOT
RP4-561L2	13.17617	-0.1701417	0.240989	-0.70601342	0.48018	0.707735	NOT
KLF7	475.4911	-0.2142959	0.30357	-0.70591998	0.480238	0.70777	NOT
USP8	1750.269	-0.1002457	0.142013	-0.70589078	0.480256	0.70777	NOT
DIAPH2	1276.397	-0.1393117	0.197377	-0.70581565	0.480303	0.707801	NOT
CTD-2526M	8.055579	-0.4379536	0.620651	-0.70563591	0.480415	0.707927	NOT
FAM200B	829.2402	-0.1102955	0.15635	-0.70543846	0.480537	0.70807	NOT
AC017074.	10.72175	-0.2050515	0.290695	-0.70538269	0.480572	0.708082	NOT
PPP1R3D	114.5005	0.1834946	0.260228	0.705128935	0.48073	0.708277	NOT
EPHA4	98.82236	-0.2367036	0.335741	-0.70501761	0.480799	0.70834	NOT
COQ5	1951.845	0.11542776	0.163737	0.704957981	0.480836	0.708356	NOT
RP11-74J1	16.72056	-0.1896486	0.269042	-0.70490277	0.480871	0.708369	NOT
RP4-604A2	4.817301	0.30247792	0.429217	0.704720539	0.480984	0.708497	NOT
PRR33	17.08023	-0.322789	0.458299	-0.7043193	0.481234	0.708827	NOT
RBBP9	1660.676	-0.1428619	0.202863	-0.70422761	0.481291	0.708861	NOT
SPECC1	632.7886	0.32166983	0.456789	0.704197353	0.48131	0.708861	NOT
RP11-553A	16.39162	0.39309343	0.558296	0.704094918	0.481374	0.708917	NOT
ZFP36L2	6560.023	-0.1495309	0.212427	-0.70391636	0.481485	0.709042	NOT
C1orf112	438.16	-0.1966349	0.279361	-0.70387409	0.481511	0.709042	NOT
RP11-545I	2.09864	-0.2602392	0.369749	-0.70382683	0.481541	0.709047	NOT
SPTSSA	4602.911	0.20002211	0.284212	0.703776838	0.481572	0.709055	NOT
GDAP1L1	4.426445	-0.3775308	0.536501	-0.70369127	0.481625	0.709095	NOT
RP11-89B1	1.039628	-0.444572	0.632057	-0.70337322	0.481823	NA	NA
ZSCAN5B	2.168763	-0.3037158	0.43185	-0.70329071	0.481875	0.709395	NOT
PIAS2	831.9293	0.1003205	0.142647	0.703279667	0.481881	0.709395	NOT
CFAP74	20.80393	0.27629906	0.392906	0.703219772	0.481919	0.709412	NOT
RBBP6	1798.061	-0.0885458	0.125928	-0.70314467	0.481966	0.709442	NOT
TRPT1	1315.374	-0.1470781	0.209188	-0.70309179	0.481999	0.709452	NOT
RP11-5C23	22.73801	-0.1663066	0.236621	-0.7028398	0.482156	0.709607	NOT
LRRC37A7P	202.8116	-0.3948804	0.561836	-0.70283913	0.482156	0.709607	NOT
LINC00485	3.707409	0.39967783	0.568775	0.702699591	0.482243	0.709683	NOT
RP11-792A	80.08315	-0.1973879	0.280925	-0.70263648	0.482282	0.709683	NOT
TPCN2	1236.468	-0.1764693	0.251155	-0.70263023	0.482286	0.709683	NOT
EPHA10	99.7427	0.51150154	0.728117	0.702498964	0.482368	0.709739	NOT
RP11-305O	6.998453	0.21770433	0.309906	0.702485479	0.482376	0.709739	NOT
LIN54	600.3759	-0.1055353	0.150242	-0.70243711	0.482407	0.709745	NOT
RP11-317N	3.238968	0.29717646	0.423096	0.702385807	0.482439	0.709753	NOT
POT1-AS1	15.32977	0.22291715	0.317415	0.702290162	0.482498	0.709759	NOT
LYPLA2P1	3.066511	0.28052855	0.399483	0.702228222	0.482537	0.709759	NOT
CENPP	172.8648	0.1354422	0.192882	0.702203692	0.482552	0.709759	NOT
TTC30B	218.3324	0.16584024	0.236181	0.70217545	0.48257	0.709759	NOT
TUBA3FP	10.16483	-0.1902482	0.270946	-0.702164	0.482577	0.709759	NOT
FLJ38122	4.185111	-0.3034604	0.432201	-0.70212824	0.482599	0.709759	NOT
AC093620.	4.649246	-0.288558	0.411052	-0.70199888	0.48268	0.709817	NOT
LRG1	26659.11	-0.2896563	0.412627	-0.70198095	0.482691	0.709817	NOT
CTD-2373J	4.941023	-0.2443371	0.348202	-0.70171039	0.48286	0.709986	NOT
IGLV5-45	10.24021	-0.5572617	0.794158	-0.70170118	0.482866	0.709986	NOT
USP42	593.0124	-0.1031616	0.147028	-0.70164569	0.4829	0.709986	NOT
RP11-256K	2.989708	0.40607376	0.578759	0.701628181	0.482911	0.709986	NOT

SLC2A14	55.26692	-0.4208791	0.599958	-0.70151396	0.482982	0.710044	NOT
RP5-994D1	5.489587	-0.2288898	0.32631	-0.70144969	0.483022	0.710044	NOT
IGHV3-64	4.392721	-0.5825887	0.830593	-0.70141311	0.483045	0.710044	NOT
RNF32	106.2371	0.17114522	0.244006	0.70139749	0.483055	0.710044	NOT
RP11-101E	2.515442	0.30117047	0.429433	0.701320568	0.483103	0.710063	NOT
RP11-132F	32.03452	0.31246933	0.445578	0.701268113	0.483136	0.710063	NOT
MB	75.75354	-0.3566239	0.508554	-0.70125066	0.483147	0.710063	NOT
SLC7A6OS	145.6314	0.1278578	0.182348	0.701176599	0.483193	0.710093	NOT
TAS2R14	28.92116	-0.2186368	0.311867	-0.70105851	0.483267	0.71013	NOT
RP11-180I	2.222284	0.54824859	0.782036	0.701052468	0.48327	0.71013	NOT
RP11-430H	4.755446	-0.3986942	0.568882	-0.7008382	0.483404	0.710261	NOT
ZNF501	69.66382	-0.2194072	0.31307	-0.70082518	0.483412	0.710261	NOT
IL33	511.407	-0.2987136	0.426342	-0.70064376	0.483525	0.710389	NOT
FAAHP1	11.31235	0.2819106	0.402431	0.700519605	0.483603	0.710464	NOT
INPP4A	691.632	-0.121508	0.173478	-0.70042359	0.483663	0.710514	NOT
HOXC13	3.361859	-0.650689	0.929161	-0.70029711	0.483742	0.710591	NOT
RP11-455C	7.99403	0.27723985	0.395921	0.700239802	0.483778	0.710606	NOT
SENCR	22.76596	-0.2298382	0.328254	-0.70018453	0.483812	0.710607	NOT
LINC0127C	217.454	-0.1882144	0.268829	-0.70012648	0.483848	0.710607	NOT
CRYZP1	2.693311	-0.3144276	0.44911	-0.70011274	0.483857	0.710607	NOT
POU6F2	20.56039	-0.5980856	0.854348	-0.7000488	0.483897	0.710627	NOT
C9orf142	1560.286	0.19733141	0.281946	0.699889755	0.483996	0.710734	NOT
ZNF75A	649.6443	-0.1226844	0.175302	-0.69984725	0.484023	0.710735	NOT
AQP8	202.2599	0.52369532	0.74837	0.699781636	0.484064	0.710756	NOT
AC019186.	8.02402	0.19936792	0.284977	0.699593472	0.484181	0.710891	NOT
EXD3	483.0253	-0.1563801	0.223563	-0.69948969	0.484246	0.71093	NOT
SERGEF	683.9529	0.15110453	0.216028	0.699466573	0.48426	0.71093	NOT
KIAA1614-	5.121851	0.28704015	0.410421	0.699379059	0.484315	0.710972	NOT
RP11-13N1	1.598441	0.37676958	0.538993	0.699024631	0.484537	0.711222	NOT
TAB1	1550.383	0.09132619	0.130648	0.69902281	0.484538	0.711222	NOT
KB-208E9.	10.99631	0.33056594	0.472964	0.698923923	0.4846	0.711274	NOT
CYP4F26P	3.081638	0.53011617	0.75865	0.698762886	0.4847	0.711383	NOT
AC109826.	9.845316	0.31681882	0.453485	0.698630677	0.484783	0.711422	NOT
C2	25282.5	-0.2053375	0.293929	-0.69859541	0.484805	0.711422	NOT
AP003068.	293.85	-0.2470775	0.353709	-0.69853241	0.484844	0.711422	NOT
G3BP1	6601.901	0.09093822	0.130187	0.698520757	0.484852	0.711422	NOT
AC113189.	292.7755	-0.2101378	0.300841	-0.69850233	0.484863	0.711422	NOT
PANK2	1006.397	0.07772106	0.111273	0.698469057	0.484884	0.711422	NOT
MRPL16	2228.82	-0.094862	0.135823	-0.69842557	0.484911	0.711423	NOT
MRPL39	833.5024	-0.1045248	0.149678	-0.69833121	0.48497	0.711445	NOT
PLGLB2	139.3116	-0.3132322	0.448553	-0.69831751	0.484979	0.711445	NOT
BRAT1	3163.328	0.11737917	0.168115	0.69820623	0.485048	0.711509	NOT
RP11-429J	12.65926	0.19519178	0.279597	0.698119535	0.485102	0.711514	NOT
AC005251.	10.38078	0.25648071	0.367428	0.698044002	0.48515	0.711514	NOT
UGT1A1	3434.599	0.38661288	0.553897	0.697986949	0.485185	0.711514	NOT
RP11-139I	5.804222	-0.4440847	0.636266	-0.69795489	0.485205	0.711514	NOT
RP1-148H1	4.669718	-0.3363518	0.48194	-0.6979118	0.485232	0.711514	NOT
PTGR2	856.0423	-0.1441934	0.206607	-0.69791174	0.485232	0.711514	NOT
RTN4IP1	425.4419	0.12634941	0.18104	0.697907027	0.485235	0.711514	NOT

TBX5	0.863722	0.57910094	0.830081	0.697643593	0.4854	NA	NA
CA13	168.9182	-0.2348787	0.336705	-0.69758028	0.48544	0.711775	NOT
IGKV2D-3(	0.305631	-0.9915524	1.421453	-0.6975623	0.485451	NA	NA
RP11-261I	11.97005	-0.2526099	0.362175	-0.69747928	0.485503	0.71183	NOT
CYP2C58P	0.98554	-0.4762271	0.682816	-0.69744586	0.485524	NA	NA
CCBL2	817.4804	-0.1248626	0.17904	-0.69739926	0.485553	0.711839	NOT
CSRP2BP	943.934	-0.1179806	0.169176	-0.6973847	0.485562	0.711839	NOT
KRT18P55	5.035535	-0.3253062	0.466524	-0.69729814	0.485616	0.71188	NOT
RAB3IL1	655.867	0.23977623	0.343914	0.697197389	0.485679	0.711934	NOT
ZIC4	149.5183	-0.4664363	0.669211	-0.69699427	0.485806	0.712082	NOT
RP11-923I	3.628361	-0.3811672	0.546942	-0.6969057	0.485862	0.712125	NOT
RP11-173I	3.570743	0.29371832	0.421536	0.696780593	0.48594	0.712201	NOT
C12orf45	470.269	-0.1368452	0.196433	-0.69664941	0.486022	0.712283	NOT
FXN	626.348	-0.1518211	0.218021	-0.69635924	0.486204	0.712511	NOT
RTEL1-TNI	76.52721	-0.1889149	0.27133	-0.69625559	0.486269	0.712555	NOT
YWHAEP7	12.59403	0.49106708	0.705326	0.696226698	0.486287	0.712555	NOT
DKK4	98.42089	0.61923716	0.889475	0.696182794	0.486314	0.712557	NOT
NUDCD3	3702.206	-0.0978869	0.14063	-0.69606095	0.486391	0.71263	NOT
CWF19L2	417.7342	-0.0901605	0.12955	-0.69595095	0.48646	0.712693	NOT
RRP1B	1729.792	-0.0986719	0.141798	-0.69586226	0.486515	0.712703	NOT
MKL1	1439.307	0.10015255	0.143927	0.69585606	0.486519	0.712703	NOT
WDR66	54.98569	0.25157706	0.361569	0.695792613	0.486559	0.712705	NOT
ZBTB42	600.5417	-0.1343263	0.193061	-0.69576962	0.486573	0.712705	NOT
CTC-251D1	9.694627	0.2240395	0.322022	0.695727499	0.4866	0.712706	NOT
AURKAIP1	5291.589	-0.1631153	0.234495	-0.69560205	0.486678	0.712782	NOT
RP13-465I	3.682467	-0.3541525	0.509273	-0.69540847	0.486799	0.712864	NOT
HOMEZ	617.1683	0.10594085	0.152346	0.695396795	0.486807	0.712864	NOT
TRAV12-3	4.297916	0.39629232	0.569887	0.695387238	0.486813	0.712864	NOT
SPTLC1	991.0951	0.13631241	0.196053	0.695282276	0.486878	0.712922	NOT
FGL2	837.7838	-0.221891	0.319198	-0.69515098	0.486961	0.712942	NOT
ZNF480	622.8621	-0.1240942	0.178523	-0.69511648	0.486982	0.712942	NOT
RP11-16F1	5.521697	0.25992664	0.373939	0.69510445	0.48699	0.712942	NOT
C1QTNF9B-	7.155629	-0.2691588	0.387227	-0.69509231	0.486997	0.712942	NOT
INTS6P1	2.053667	0.30169882	0.434127	0.69495474	0.487084	0.71303	NOT
SNORA14A	2.155079	-0.3960205	0.569953	-0.69482951	0.487162	0.713081	NOT
MYH14	8107.352	-0.1679724	0.241759	-0.69479153	0.487186	0.713081	NOT
IGHV2-5	4.508463	0.58676452	0.844558	0.694758932	0.487206	0.713081	NOT
CPQ	4855.164	0.1584796	0.228116	0.694731593	0.487224	0.713081	NOT
RP11-313I	3.586161	0.38459163	0.553637	0.694664441	0.487266	0.713086	NOT
RP11-1334	9.374778	0.28463597	0.409766	0.694629949	0.487287	0.713086	NOT
ZSCAN2	451.9645	0.11902868	0.171363	0.694600069	0.487306	0.713086	NOT
IGHJ1	0.371738	0.89253975	1.285185	0.694483649	0.487379	NA	NA
RPL18AP7	1.794173	-0.2897903	0.417324	-0.69440127	0.487431	0.71323	NOT
TCEAL1	596.1055	-0.1453371	0.20934	-0.69426304	0.487517	0.713318	NOT
RP11-169I	63.57417	0.34978908	0.503932	0.694119187	0.487607	0.713402	NOT
RP11-187(	9.731909	0.31144879	0.448726	0.694073489	0.487636	0.713402	NOT
UQCRQ	14276.16	-0.2144108	0.308932	-0.69403866	0.487658	0.713402	NOT
GS1-304P7	3.536559	-0.3819537	0.550362	-0.69400397	0.48768	0.713402	NOT
CTB-109A1	2.043668	0.3135744	0.451934	0.693849257	0.487777	0.713492	NOT

AMDHD2	1207.813	0.16138546	0.232604	0.693821439	0.487794	0.713492	NOT
DGCR9	29.72342	0.38408169	0.553655	0.693720753	0.487857	0.713546	NOT
VCP	15476.65	-0.0815494	0.117576	-0.69358703	0.487941	0.71363	NOT
HSD17B7P2	105.5312	0.20784997	0.299727	0.693465269	0.488018	0.713704	NOT
GMPR	341.3541	0.33101201	0.477392	0.693375783	0.488074	0.713747	NOT
GPNMB	2527.841	0.23532924	0.339426	0.693315387	0.488112	0.713764	NOT
RP11-367(	4.465698	-0.3402107	0.49089	-0.69304807	0.488279	0.713971	NOT
UTP3	1130.507	-0.0865	0.124823	-0.69298342	0.48832	0.713992	NOT
CD3G	94.04055	0.30696562	0.442997	0.692928973	0.488354	0.714003	NOT
RP11-489I	3.2714	-0.3177342	0.458613	-0.69281559	0.488425	0.714034	NOT
RP11-643(	2.605408	0.41692173	0.60186	0.69272231	0.488484	0.714034	NOT
LINC0108	5.341487	0.41148487	0.594016	0.692717136	0.488487	0.714034	NOT
CCDC65	62.78494	0.19493395	0.281433	0.692648506	0.48853	0.714034	NOT
HPS6	764.0055	-0.0958456	0.138376	-0.69264487	0.488532	0.714034	NOT
ZNF584	315.2694	-0.1004963	0.145092	-0.6926388	0.488536	0.714034	NOT
LA16c-358	54.70774	-0.2028892	0.292938	-0.69260213	0.488559	0.714034	NOT
ZNF410	49.00596	0.12322059	0.177931	0.692517377	0.488612	0.714055	NOT
PTPN7	233.4421	0.25243209	0.364525	0.692495368	0.488626	0.714055	NOT
RP11-332K	1.352736	0.98029782	1.41594	0.692329814	0.48873	NA	NA
RNASE13	5.873009	-0.3181707	0.459583	-0.69230299	0.488747	0.714193	NOT
IGKV3D-2(	14.96925	0.54279057	0.784104	0.692242707	0.488785	0.71421	NOT
DENND1B	729.7815	-0.1306054	0.188686	-0.69218221	0.488823	0.714227	NOT
RP11-624I	6.53824	0.30074175	0.434606	0.691987772	0.488945	0.714367	NOT
RP11-72M1	8.459159	-0.2471433	0.357196	-0.69189766	0.489002	0.714411	NOT
TBL1X	2257.509	-0.1316783	0.190367	-0.69170588	0.489122	0.714526	NOT
LINC0112	75.49409	-0.1946904	0.281471	-0.69168825	0.489133	0.714526	NOT
RP11-115I	3.41622	-0.2896499	0.418852	-0.69153359	0.48923	0.71463	NOT
TPTE	7.038746	0.9241079	1.336908	0.691227873	0.489422	0.714839	NOT
FIBP	2116.466	0.10005741	0.144754	0.6912217	0.489426	0.714839	NOT
FOXD3-AS1	1.829948	0.67992094	0.983843	0.691086975	0.489511	0.714924	NOT
WDR63	3.352415	0.37026777	0.535958	0.690852387	0.489658	0.715083	NOT
RP11-85G2	4.071241	0.51452358	0.744794	0.690826463	0.489675	0.715083	NOT
NDUFAF7	595.6808	0.08654558	0.125285	0.690788024	0.489699	0.715083	NOT
AC024937.	10.10958	0.30240928	0.437838	0.690688326	0.489761	0.715113	NOT
LA16c-32	9.942897	-0.3180142	0.460442	-0.6906716	0.489772	0.715113	NOT
RP11-521I	71.55996	0.18563719	0.268808	0.690594134	0.489821	0.715146	NOT
GLRXP3	2.320471	0.55727401	0.80705	0.690507708	0.489875	0.715186	NOT
CTC-471J1	35.67694	-0.1973876	0.285941	-0.69030828	0.49	0.715331	NOT
GSTA1	29088.2	-0.3653069	0.52925	-0.69023431	0.490047	0.71536	NOT
TNFRSF8	25.99332	0.26849146	0.389137	0.689967022	0.490215	0.715501	NOT
RP11-570I	1.951615	0.35266075	0.511136	0.689954998	0.490222	0.715501	NOT
SNX33	2162.707	-0.10425	0.151097	-0.68995483	0.490223	0.715501	NOT
DECR1	9580.079	-0.1683866	0.244114	-0.68978673	0.490328	0.715617	NOT
ANXA1	1448.357	0.23082885	0.334751	0.689554372	0.490474	0.715792	NOT
APH1B	322.2213	-0.1582579	0.229587	-0.68931596	0.490624	0.715939	NOT
SMG9	1237.044	0.13052736	0.189359	0.6893107	0.490628	0.715939	NOT
NCLP1	2.692534	0.30582085	0.443698	0.689254739	0.490663	0.715952	NOT
SMTNL2	6.690873	0.29649164	0.430231	0.689145543	0.490732	0.716013	NOT
NNAT	17.30891	-0.3127721	0.454147	-0.68870313	0.49101	0.716381	NOT

SLAMF8	327.5965	0.23368996	0.339418	0.688501764	0.491137	0.716505	NOT
PDLIM3	656.7255	0.23420341	0.340172	0.688484423	0.491148	0.716505	NOT
RABGEF1	358.6464	0.13439783	0.195222	0.688434925	0.491179	0.716506	NOT
TAS2R15P	6.157572	-0.2440229	0.354479	-0.68839894	0.491202	0.716506	NOT
MEIG1	8.576192	0.26180841	0.38034	0.688353783	0.49123	0.716509	NOT
WASH4P	32.7104	-0.1758269	0.255456	-0.68828613	0.491273	0.716533	NOT
SPATA25	58.65921	0.21341219	0.310087	0.688233608	0.491306	0.716542	NOT
RP11-629(	46.73193	0.23580142	0.342717	0.688035125	0.491431	0.716686	NOT
FMO6P	1.919516	0.4387516	0.637828	0.687883703	0.491526	0.716787	NOT
MUC2	3.010143	0.49160121	0.714734	0.687810012	0.491572	0.716809	NOT
GSDMA	22.98498	0.36860265	0.535958	0.687745265	0.491613	0.716809	NOT
EIF1P6	3.576594	-0.2579692	0.375101	-0.68773331	0.491621	0.716809	NOT
AL133243.	50.66911	0.15575854	0.226579	0.687434529	0.491809	0.717027	NOT
PCP4	8.182066	-0.6587964	0.958404	-0.68738879	0.491838	0.717027	NOT
PHF10	2207.63	-0.1145446	0.166654	-0.68732063	0.491881	0.717027	NOT
AC099552.	1.476751	0.74741483	1.087461	0.687302897	0.491892	0.717027	NOT
RP11-305I	1.933857	0.7791007	1.133589	0.687286843	0.491902	0.717027	NOT
ZNF683	45.23741	0.30308386	0.441092	0.687121447	0.492006	0.71714	NOT
RP1-234P1	19.60274	-0.1508799	0.219673	-0.68683766	0.492185	0.717362	NOT
RP11-798K	22.01999	0.26253507	0.382342	0.686649696	0.492304	0.717496	NOT
RP11-486A	77.05435	0.24820395	0.361509	0.686577049	0.492349	0.717514	NOT
FCRLA	28.87595	0.37303992	0.543357	0.68654618	0.492369	0.717514	NOT
NAA60	2416.803	-0.1271074	0.185154	-0.68649431	0.492401	0.717523	NOT
URAHP	185.4404	0.22104406	0.322079	0.686304021	0.492521	0.71766	NOT
HAGHL	140.233	-0.3210094	0.467802	-0.68620807	0.492582	0.717709	NOT
AC108448.	1.281449	-0.4151073	0.605152	-0.68595526	0.492741	NA	NA
ABR	862.9598	0.20128478	0.29344	0.685949322	0.492745	0.717877	NOT
AGAP10	125.5587	-0.2155291	0.314228	-0.68590074	0.492776	0.717877	NOT
ANKRD42	249.9314	-0.1258521	0.183494	-0.68586625	0.492797	0.717877	NOT
CBWD3	19.17184	0.19134613	0.278988	0.685857646	0.492803	0.717877	NOT
GPR31	1.676197	-0.5056779	0.737352	-0.68580226	0.492838	0.717889	NOT
RP11-390F	231.8973	-0.3285102	0.479075	-0.68571727	0.492891	0.717929	NOT
RP5-855D2	11.37922	0.20562134	0.299883	0.685672822	0.492919	0.717931	NOT
RP5-915N1	2.745607	-0.2819407	0.411237	-0.68559148	0.492971	0.717936	NOT
TMEM80	631.2015	0.11252236	0.164126	0.685583937	0.492976	0.717936	NOT
TEDDM1	2.573596	0.5851657	0.85374	0.685414722	0.493082	0.718019	NOT
ZNF211	233.7533	-0.154383	0.225242	-0.68540903	0.493086	0.718019	NOT
AC012613.	2.02491	-0.4446161	0.648761	-0.68533149	0.493135	0.718052	NOT
ZNF529-AS	93.20838	-0.1657418	0.241938	-0.68505834	0.493307	0.718214	NOT
SPATA2	783.5829	0.08158332	0.119099	0.685003956	0.493341	0.718214	NOT
ISCU	3464.178	-0.1111999	0.162336	-0.68499708	0.493346	0.718214	NOT
VKORC1	3001.069	-0.1536952	0.224377	-0.68498772	0.493352	0.718214	NOT
PHF2	1139.151	-0.1279615	0.186824	-0.68493082	0.493388	0.718222	NOT
PARP3	959.8852	-0.1321705	0.192979	-0.68489458	0.49341	0.718222	NOT
NEU4	1817.01	-0.4159871	0.607452	-0.68480674	0.493466	0.718264	NOT
SP4	287.26	0.16704861	0.24396	0.6847386	0.493509	0.718288	NOT
SGMS1	1426.297	-0.1163122	0.169875	-0.68469127	0.493539	0.718293	NOT
RP11-159F	2.949108	-0.2678068	0.391164	-0.68464087	0.493571	0.718301	NOT
CASC18	2.296216	0.37776972	0.551884	0.684508762	0.493654	0.718346	NOT

CPSF3L	3301.479	0.10260052	0.149889	0.684508204	0.493654	0.718346	NOT
NFYAP1	2.692476	-0.6110223	0.892721	-0.68444972	0.493691	0.718361	NOT
WIPF2	2025.884	0.09739464	0.142326	0.684307183	0.493781	0.718454	NOT
RP11-1293	51.39021	0.23487114	0.343256	0.684243849	0.493821	0.718473	NOT
SHFM1P1	3.823679	0.31802073	0.465013	0.683896819	0.49404	0.718753	NOT
RALY-AS1	109.949	-0.16488	0.241143	-0.68374319	0.494137	0.718838	NOT
CCDC124	3113.146	0.1480279	0.216503	0.683721029	0.494151	0.718838	NOT
MPP7	418.029	0.2225172	0.325483	0.683652315	0.494195	0.718862	NOT
PEX7	328.3424	-0.1346481	0.197019	-0.68342804	0.494336	0.718996	NOT
LINC01483	1.474212	0.44378565	0.649357	0.683423505	0.494339	0.718996	NOT
NUP54	969.4614	0.10478033	0.153339	0.683322616	0.494403	0.71905	NOT
CTSL	7377.085	-0.1204857	0.176339	-0.6832632	0.494441	0.719066	NOT
RP11-460N	8.90064	-0.2181291	0.319317	-0.68311183	0.494536	0.719166	NOT
RP11-513(	4.752944	-0.3100024	0.453917	-0.68294917	0.494639	0.719277	NOT
LINC00884	186.407	-0.1812085	0.265365	-0.68286607	0.494691	0.719315	NOT
RPS20P22	2.627441	0.50737524	0.743083	0.682797648	0.494735	0.719339	NOT
MEP1B	12.78641	-0.4575876	0.670305	-0.6826556	0.494824	0.719397	NOT
SNCB	2.277913	0.48005575	0.703233	0.682641504	0.494833	0.719397	NOT
DKKL1	14.2033	-0.3098939	0.453984	-0.68260898	0.494854	0.719397	NOT
IGSF5	2.055995	-0.4652119	0.681573	-0.6825558	0.494888	0.719407	NOT
HCAR2	45.91653	0.38122942	0.558603	0.682469649	0.494942	0.719448	NOT
DOHH	696.0854	0.12603734	0.184709	0.682357591	0.495013	0.719512	NOT
RNVU1-6	5.54905	0.27173881	0.398278	0.682284954	0.495059	0.719541	NOT
RP11-73E1	76.52845	-0.1224906	0.179576	-0.68210919	0.49517	0.719664	NOT
RP11-325F	3.614757	-0.3326764	0.487759	-0.68205001	0.495207	0.719679	NOT
RP11-459I	6.410551	-0.3629401	0.532277	-0.68186277	0.495326	0.719782	NOT
MTHFSD	515.9418	-0.1029395	0.150971	-0.68184861	0.495335	0.719782	NOT
NUDT22	1127.663	-0.14866	0.218037	-0.68181217	0.495358	0.719782	NOT
MYHAS	1.9359	-0.6585956	0.96608	-0.68171975	0.495416	0.719829	NOT
TRG-AS1	37.59744	-0.2343648	0.34386	-0.68157092	0.49551	0.719899	NOT
NDUFB4	4556.155	0.1017289	0.149259	0.681559721	0.495517	0.719899	NOT
THEM5	176.5479	-0.1989257	0.291906	-0.68147212	0.495573	0.719902	NOT
LINC00346	182.8913	0.29544697	0.433597	0.681386613	0.495627	0.719902	NOT
RP11-122(	6.534928	-0.2277134	0.334201	-0.68136631	0.49564	0.719902	NOT
RP11-458J	177.9205	-0.1071072	0.157198	-0.68135299	0.495648	0.719902	NOT
DHTKD1	7399.174	-0.1967892	0.288824	-0.68134665	0.495652	0.719902	NOT
PHLPP1	1708.762	-0.1461018	0.214447	-0.68129444	0.495685	0.719904	NOT
CASC5	767.3442	0.16764833	0.246085	0.681260756	0.495707	0.719904	NOT
REV1	849.3565	-0.1132878	0.166306	-0.68119976	0.495745	0.719921	NOT
CAPN8	65.5843	0.32950579	0.483836	0.681028251	0.495854	0.72004	NOT
RP11-134I	88.00072	-0.19474	0.285974	-0.68097081	0.49589	0.720051	NOT
PIP5K1B	18.58051	-0.240637	0.353393	-0.68093292	0.495914	0.720051	NOT
POMGNT2	794.6762	0.11368044	0.166968	0.68085056	0.495966	0.720064	NOT
DIS3L2	1047.81	0.08328075	0.122322	0.680834611	0.495976	0.720064	NOT
CS	4978.723	0.11482821	0.168694	0.68068853	0.496069	0.72016	NOT
CTA-293F1	6.823896	-0.4579609	0.672852	-0.68062681	0.496108	0.720178	NOT
PTGFRN	2552.937	0.18775899	0.275899	0.680534401	0.496166	0.720223	NOT
PSENEN	2113.025	0.16918422	0.24862	0.680494034	0.496192	0.720223	NOT
NHLH2	1.86143	0.70674523	1.038666	0.680435567	0.496229	0.720238	NOT

RP11-131M	6.760538	-0.2063017	0.303225	-0.68035913	0.496277	0.720258	NOT
RP11-297H	16.24191	-0.566933	0.833321	-0.68032981	0.496296	0.720258	NOT
GBA2	2718.169	-0.1103265	0.162184	-0.68025406	0.496344	0.720289	NOT
RP11-481F	16.16268	0.77927476	1.145774	0.680129551	0.496422	0.720314	NOT
BRD4	4530.776	0.10663728	0.156793	0.680116591	0.496431	0.720314	NOT
FGF21	1872.236	0.42110179	0.619186	0.680088919	0.496448	0.720314	NOT
BCL6	2377.001	-0.1532283	0.225316	-0.68005883	0.496467	0.720314	NOT
GPR176	424.5061	0.2398158	0.35279	0.679768358	0.496651	0.720469	NOT
SMG8	665.3013	0.1201106	0.176698	0.679749831	0.496663	0.720469	NOT
MYBPH	13.48147	-0.4532063	0.666726	-0.67974923	0.496663	0.720469	NOT
COL5A3	3632.841	-0.2566929	0.377652	-0.67970719	0.49669	0.720469	NOT
LINC0084	405.0029	-0.5046891	0.742539	-0.67968052	0.496707	0.720469	NOT
LL0XNC01-	46.80609	-0.1775449	0.261261	-0.67956922	0.496777	0.720533	NOT
RP11-42I1	21.77081	0.1813218	0.266846	0.679498559	0.496822	0.720559	NOT
SFTPD	37.91919	-0.4387207	0.645732	-0.67941638	0.496874	0.720596	NOT
RP4-67101	1.757384	-0.4137457	0.609053	-0.6793262	0.496931	0.720611	NOT
GADD45GIF	2564.199	0.16737875	0.246393	0.6793159	0.496938	0.720611	NOT
AC074117.	260.5659	0.1482245	0.218228	0.679218171	0.497	0.720663	NOT
CTD-2514(	13.3887	-0.295388	0.43494	-0.67914733	0.497045	0.720687	NOT
MOV10	4492.393	0.11438054	0.168428	0.679107674	0.49707	0.720687	NOT
ATOX1	6566.068	-0.1992647	0.293449	-0.67904294	0.497111	0.720708	NOT
SRP54-AS1	40.04131	-0.1522464	0.22435	-0.67861017	0.497385	0.721008	NOT
DENND4C	1064.396	-0.1634836	0.24091	-0.67860883	0.497386	0.721008	NOT
ZNF426	345.656	-0.2082848	0.306958	-0.67854489	0.497426	0.721008	NOT
MSMB	14.29522	0.43161209	0.636094	0.678534613	0.497433	0.721008	NOT
HERC2P2	718.4818	-0.191508	0.282249	-0.67850706	0.49745	0.721008	NOT
PTCHD1	2.722459	-0.3518046	0.518567	-0.67841747	0.497507	0.721052	NOT
MTND2P28	13071.63	-0.2647814	0.390465	-0.67811801	0.497697	0.721278	NOT
PTGES2	2697.551	0.1422063	0.209717	0.678086996	0.497717	0.721278	NOT
SRGAP2	1271.116	0.1337678	0.197289	0.678029158	0.497753	0.721293	NOT
AC012506.	3.682955	-0.452452	0.667392	-0.67794013	0.49781	0.721336	NOT
LINC0094(	4.910397	-0.4806875	0.709196	-0.67779258	0.497903	0.721377	NOT
ASH2L	1265.262	0.12611168	0.186068	0.677771702	0.497916	0.721377	NOT
TMEM86B	1247.598	-0.2938022	0.433483	-0.67777036	0.497917	0.721377	NOT
RP11-355F	6.783793	0.32626913	0.481617	0.677445477	0.498123	0.721636	NOT
HNRNPA1P1	1.427534	0.33438322	0.493643	0.677378755	0.498166	0.721637	NOT
AC007016.	1.905179	-0.3882864	0.573234	-0.67736065	0.498177	0.721637	NOT
LAMA5-AS1	244.8101	-0.4237624	0.625665	-0.67729949	0.498216	0.721649	NOT
TTC9	1347.5	0.32471674	0.479454	0.677264013	0.498238	0.721649	NOT
FGFR2	2982.047	0.36748347	0.542688	0.677154201	0.498308	0.721711	NOT
C11orf53	4.397517	-0.7870868	1.162802	-0.67688799	0.498477	0.721892	NOT
IFIT1	1534.172	-0.2320994	0.342931	-0.67681062	0.498526	0.721892	NOT
CASC21	1.433975	0.38328737	0.566322	0.676801168	0.498532	0.721892	NOT
CLTB	3526.956	-0.1588885	0.234768	-0.67679019	0.498539	0.721892	NOT
PRKG2	4.820449	0.50044974	0.739546	0.676698873	0.498597	0.721937	NOT
DCPS	2235.816	0.20547133	0.303728	0.676497668	0.498725	0.722084	NOT
MESTIT1	3.543163	0.30290161	0.448036	0.676065765	0.498999	0.722431	NOT
ANTXRLP1	1.314403	-0.3997096	0.591238	-0.67605507	0.499006	NA	NA
IGLV3-16	1.549165	0.7149574	1.057574	0.676035413	0.499018	0.722431	NOT

SULF1	1087.912	0.33275835	0.492321	0.675896758	0.499106	0.722495	NOT
PPAPDC3	31.63672	0.24480356	0.362198	0.675882746	0.499115	0.722495	NOT
RP11-848I	38.68917	0.19574897	0.289645	0.675823355	0.499153	0.722511	NOT
C15orf38-	8.018518	-0.2616735	0.387268	-0.67569052	0.499237	0.722594	NOT
RHOC	9720.974	-0.1557889	0.230626	-0.6755049	0.499355	0.722726	NOT
RP11-109(	14.99963	-0.1906971	0.282331	-0.67543716	0.499398	0.722748	NOT
CHRFAM7A	3.069846	0.37254944	0.551601	0.675397201	0.499423	0.722748	NOT
CCDC47	7177.172	-0.12224	0.181002	-0.67535325	0.499451	0.72275	NOT
RASSF6	126.5196	-0.27679	0.409946	-0.67518596	0.499558	0.722865	NOT
WDR36	1354.258	-0.0942689	0.139637	-0.67509849	0.499613	0.722907	NOT
MLLT10P1	2.249386	0.26733059	0.396115	0.674881525	0.499751	0.723039	NOT
HELLS	574.6519	-0.2515025	0.372668	-0.6748709	0.499758	0.723039	NOT
PRPS1	2167.229	-0.1213609	0.179853	-0.67477812	0.499817	0.723086	NOT
IL17D	102.1921	-0.3687056	0.546566	-0.67458578	0.499939	0.723183	NOT
IQGAP1	2346.333	0.17640971	0.261536	0.674513204	0.499985	0.723183	NOT
AIMP1	2060.966	0.09218858	0.13668	0.674484873	0.500003	0.723183	NOT
NRBF2P5	3.668176	-0.2616389	0.387914	-0.67447724	0.500008	0.723183	NOT
LRRIQ4	7.028644	0.35174458	0.521519	0.674462251	0.500017	0.723183	NOT
ANKDD1B	16.02285	-0.3005137	0.445622	-0.67436991	0.500076	0.723217	NOT
RP11-154J	13.15193	-0.2363454	0.350491	-0.67432571	0.500104	0.723217	NOT
ZNF316	1389.607	0.09380586	0.139116	0.674300228	0.50012	0.723217	NOT
SEC14L6	66.31019	0.37118061	0.550617	0.67411743	0.500237	0.723319	NOT
LL22NC03-	1.616168	0.36901323	0.547412	0.674105448	0.500244	0.723319	NOT
RP11-307I	3.168434	-0.268566	0.398472	-0.67399025	0.500318	0.723386	NOT
CTB-58E17	6.326698	-0.1969082	0.292187	-0.67391084	0.500368	0.72342	NOT
ATP5F1P5	1.901259	-0.4059282	0.602436	-0.67381153	0.500431	0.723473	NOT
RP13-714J	10.49215	0.47432044	0.704052	0.673700799	0.500502	0.723536	NOT
RSPRY1	1244.904	-0.0968168	0.143724	-0.67363066	0.500546	0.723542	NOT
LINC0089(	138.5326	-0.524451	0.778566	-0.67361106	0.500559	0.723542	NOT
LOXL3	102.9242	0.15226983	0.226076	0.673534161	0.500608	0.723574	NOT
LINC0089	63.64588	-0.2018258	0.299732	-0.67335495	0.500722	0.7237	NOT
CTA-228A(	36.61564	0.24821497	0.368671	0.673269534	0.500776	0.723708	NOT
SNORA33	27.12569	0.23635364	0.351057	0.673262139	0.500781	0.723708	NOT
HCG4P11	9.419141	0.29855971	0.443522	0.673156678	0.500848	0.723767	NOT
STX11	135.3648	-0.2462823	0.365941	-0.67301013	0.500941	0.723863	NOT
RSL24D1P(	5.116356	0.22144028	0.329103	0.67285988	0.501036	0.723962	NOT
RP11-436I	32.29931	-0.3049363	0.453286	-0.67272453	0.501123	0.723999	NOT
ITM2A	327.1737	-0.2517879	0.374299	-0.67269234	0.501143	0.723999	NOT
AC011899.	58.99657	-0.2438466	0.362503	-0.67267374	0.501155	0.723999	NOT
RP11-122(	5.491424	0.51876871	0.771236	0.67264578	0.501173	0.723999	NOT
EPS15L1	1162.575	-0.1049948	0.1561	-0.67261035	0.501195	0.723999	NOT
RP1-65J11	7.345652	-0.3536361	0.525808	-0.6725577	0.501229	0.724001	NOT
RP11-135I	24.76001	-0.2131721	0.316973	-0.67252459	0.50125	0.724001	NOT
ZSWIM8-AS	1.839499	0.37020746	0.550754	0.672182827	0.501467	0.724147	NOT
C17orf51	155.1845	-0.1964655	0.292286	-0.67216766	0.501477	0.724147	NOT
TMEM108	33.74089	0.2787125	0.414648	0.672166141	0.501478	0.724147	NOT
LRMP	125.2825	-0.171456	0.255085	-0.67215176	0.501487	0.724147	NOT
CXorf65	9.090365	-0.3064348	0.455901	-0.67215163	0.501487	0.724147	NOT
C22orf24	2.673245	0.25567789	0.38045	0.672039922	0.501558	0.724147	NOT

EXOC3	2564.159	0.10472472	0.155831	0.672039522	0.501559	0.724147	NOT
LRRC49	41.0502	0.24483444	0.364321	0.672030263	0.501564	0.724147	NOT
RP11-184A	2.750891	0.52946626	0.787982	0.67192681	0.50163	0.724199	NOT
RP11-338K	6.537981	0.17182686	0.255737	0.671890105	0.501654	0.724199	NOT
RPS4X	36972.74	0.14214302	0.211581	0.671812698	0.501703	0.724231	NOT
PDZRN3	70.58325	-0.345036	0.513647	-0.67173788	0.501751	0.724261	NOT
KRT72	1.749681	-0.5488691	0.81723	-0.67162156	0.501825	0.724302	NOT
AC093673.	254.4941	0.18492271	0.275343	0.671609384	0.501832	0.724302	NOT
MGST1	31412.54	0.21529433	0.320585	0.671566557	0.50186	0.724303	NOT
RICTOR	1203.695	-0.1123769	0.167404	-0.67129126	0.502035	0.724518	NOT
AC092066.	654.0847	-0.1348185	0.200852	-0.67123374	0.502072	0.724522	NOT
ABCE1	2120.126	0.09577454	0.142691	0.671202446	0.502092	0.724522	NOT
NSA2	3036.685	0.10108513	0.150619	0.671129642	0.502138	0.724551	NOT
RP11-65J3	3.947411	-0.4782319	0.712671	-0.67104203	0.502194	0.724593	NOT
KIAA1279	739.0717	-0.1040739	0.155112	-0.67096035	0.502246	0.724629	NOT
CTD-2281M	1.465886	-0.4429832	0.66037	-0.67081038	0.502341	0.724729	NOT
CTD-2583A	124.4572	-0.1823286	0.271833	-0.67073646	0.502388	0.724758	NOT
DNASE1L2	37.22716	-0.2769646	0.413153	-0.67036836	0.502623	0.725041	NOT
ARL13B	281.1293	0.14136263	0.210881	0.670344274	0.502638	0.725041	NOT
TRPC7-AS1	2.126342	0.56687786	0.845768	0.67025196	0.502697	0.725088	NOT
LINC01422	7.053636	-0.3465955	0.517216	-0.670117	0.502783	0.725173	NOT
AP004782.	2.039589	-0.4249393	0.634177	-0.67006384	0.502817	0.725184	NOT
SDCBP2-AS	141.0919	-0.1451633	0.216661	-0.670003	0.502856	0.725201	NOT
UQCR10	5077.059	-0.1651983	0.246622	-0.66984354	0.502958	0.725309	NOT
PTPRF	20240.6	-0.1396747	0.208559	-0.66971339	0.503041	0.72539	NOT
CTAG2	418.3227	0.93549712	1.397661	0.669330688	0.503285	0.725684	NOT
RP11-333E	3.79149	-0.206288	0.30821	-0.66930995	0.503298	0.725684	NOT
GNB2	8173.961	0.11942854	0.178552	0.668870767	0.503578	0.726049	NOT
WASH1	263.1157	-0.168563	0.252098	-0.66864154	0.503724	0.726188	NOT
ARHGEF3	694.3035	0.22652321	0.338784	0.668636283	0.503728	0.726188	NOT
EIF4B	11889.26	0.099036	0.148133	0.668560286	0.503776	0.726211	NOT
SLC22A10	982.2673	-0.3598767	0.538313	-0.66852681	0.503797	0.726211	NOT
RP5-1071N	3.044816	-0.2816955	0.421432	-0.66842435	0.503863	0.726267	NOT
YPEL2	1333.755	-0.144927	0.216851	-0.66832615	0.503925	0.726303	NOT
RP11-613M	59.80832	-0.1715917	0.256767	-0.6682776	0.503956	0.726303	NOT
RPL21	6959.415	0.16345358	0.244596	0.668259164	0.503968	0.726303	NOT
LINC01108	19.36912	-0.3653123	0.546823	-0.66806281	0.504093	0.726445	NOT
RP11-349A	1.02367	-0.5496454	0.822758	-0.66805268	0.5041	NA	NA
RP11-110H	3.7665	-0.2279873	0.341335	-0.66792795	0.50418	0.726531	NOT
RP3-486I3	5.312161	-0.2256796	0.337974	-0.66774283	0.504298	0.726625	NOT
ECD	1235.848	0.0683195	0.102314	0.667741276	0.504299	0.726625	NOT
RP11-543E	21.765	-0.2792054	0.418197	-0.66764028	0.504363	0.726679	NOT
AC002451.	12.51728	0.25788893	0.38631	0.667569569	0.504408	0.726706	NOT
PITRM1	3183.966	-0.1280893	0.191904	-0.66746706	0.504474	0.726762	NOT
KDM2A	4645.08	-0.084979	0.127342	-0.66732712	0.504563	0.726848	NOT
CCNY	4479.2	0.08986339	0.134669	0.667288815	0.504588	0.726848	NOT
HCG15	15.11339	-0.2584269	0.387544	-0.66683264	0.504879	0.72723	NOT
DPYD	2655.091	-0.1974906	0.296187	-0.66677594	0.504915	0.727243	NOT
SPCS2	2126.596	0.0774307	0.116139	0.666706137	0.50496	0.727269	NOT

TMEM150C	898.8351	-0.2197996	0.329743	-0.66657785	0.505042	0.727348	NOT
GLYATL1P3	3.332594	-0.5283607	0.792702	-0.6665315	0.505071	0.727352	NOT
PDE6G	55.14191	-0.2149313	0.322489	-0.66647539	0.505107	0.727365	NOT
IZUMO1R	1.301067	0.51673623	0.775515	0.666313326	0.505211	NA	NA
AC002066.	5.18933	-0.3235577	0.485729	-0.66612761	0.50533	0.72761	NOT
RP4-756H1	30.0366	-0.1879627	0.282173	-0.66612564	0.505331	0.72761	NOT
ADAMTSL4-	16.10369	-0.2154292	0.323438	-0.66606034	0.505373	0.727631	NOT
FBLN7	738.8721	-0.2241291	0.33654	-0.66598137	0.505423	0.727641	NOT
FGD5P1	2.84532	0.57909052	0.869549	0.665966317	0.505433	0.727641	NOT
AC005537.	9.858474	-0.6133519	0.921535	-0.66557608	0.505682	0.727932	NOT
TXNDC17	2091.459	-0.1616895	0.242936	-0.66556545	0.505689	0.727932	NOT
RP5-875O1	7.304011	0.41350602	0.621428	0.665412941	0.505786	0.728034	NOT
AP001063.	3.886858	-0.5912212	0.888746	-0.66523062	0.505903	0.728163	NOT
TPT1P6	5.865992	0.25099536	0.377358	0.665138751	0.505962	0.728209	NOT
SNORA59B	9.137177	0.28907883	0.434734	0.664955316	0.506079	0.728339	NOT
RFK	1526.333	-0.1157844	0.174135	-0.66491212	0.506107	0.72834	NOT
DDX21	2874.394	0.12101547	0.182026	0.664826844	0.506161	0.72838	NOT
AC007364.	0.941989	-0.5286738	0.795425	-0.66464328	0.506279	NA	NA
RP11-982M	2.180631	0.42152262	0.634597	0.664236601	0.506539	0.728885	NOT
MLIP	694.2726	-0.3172875	0.477728	-0.66415885	0.506589	0.728918	NOT
TIMM22	633.0214	-0.1226051	0.184729	-0.66370276	0.506881	0.729299	NOT
PPIL4	667.2683	0.10938568	0.164852	0.663539769	0.506985	0.729366	NOT
ACER1	2.113784	-0.5097983	0.76832	-0.66352342	0.506995	0.729366	NOT
SLC15A5	2.836193	-0.4712763	0.710307	-0.66348211	0.507022	0.729366	NOT
DMRTA1	459.986	-0.259194	0.390669	-0.66346218	0.507035	0.729366	NOT
LDHAP4	80.84123	0.2275459	0.34312	0.663166522	0.507224	0.7296	NOT
RP3-431P2	2.17438	-0.362343	0.546433	-0.6631063	0.507262	0.729617	NOT
CAMTA1	1310.349	0.11639689	0.175555	0.663023364	0.507316	0.729654	NOT
PHBP9	9.079372	-0.2178557	0.328707	-0.66276633	0.50748	0.729815	NOT
CCDC85C	1381.124	-0.1417893	0.213936	-0.66276456	0.507481	0.729815	NOT
LINC01281	1.947102	0.51977247	0.784302	0.662719563	0.50751	0.729818	NOT
PTGS1	361.5723	0.2750125	0.415006	0.662670845	0.507541	0.729824	NOT
NXPH3	38.08763	0.21278381	0.321171	0.662524904	0.507635	0.72992	NOT
TESPA1	55.66817	-0.2616631	0.395058	-0.66234094	0.507753	0.730051	NOT
PPP3CC	442.3351	-0.1119138	0.16899	-0.66224946	0.507811	0.730065	NOT
RP4-682C2	17.21609	-0.2379664	0.359335	-0.66224196	0.507816	0.730065	NOT
RAB39B	15.76702	0.25523539	0.385498	0.662092732	0.507912	0.73016	NOT
KIR2DS4	2.738887	-0.3122416	0.471626	-0.66205414	0.507937	0.73016	NOT
APOA1BP	7003.617	0.15064409	0.22763	0.661793107	0.508104	0.730242	NOT
KLHL6-AS1	32.26794	0.39155502	0.591753	0.661686884	0.508172	0.730242	NOT
SCUBE3	27.25101	-0.2145664	0.324275	-0.66167973	0.508176	0.730242	NOT
RNU6-516P	7.799984	0.24038761	0.363336	0.661612787	0.508219	0.730242	NOT
HAPLN1	15.83833	0.48421513	0.731874	0.661610344	0.508221	0.730242	NOT
RSRP1	1269.367	-0.149071	0.225321	-0.66159409	0.508231	0.730242	NOT
CTD-2006C	80.63272	0.17490151	0.26437	0.661579167	0.508241	0.730242	NOT
RP11-567M	2.544509	0.43009679	0.650112	0.661573353	0.508245	0.730242	NOT
DECR2	4664.37	-0.1768263	0.267291	-0.66155086	0.508259	0.730242	NOT
PRKACB	1259.302	-0.1312098	0.198338	-0.66154565	0.508262	0.730242	NOT
RP11-92G1	8.500677	-0.3544716	0.535904	-0.66144585	0.508326	0.730295	NOT

RP11-157E	1.99991	-0.5798713	0.876856	-0.66130735	0.508415	0.730384	NOT
C15orf52	550.3806	0.25090123	0.379438	0.661244616	0.508455	0.730403	NOT
IPPK	98.66602	-0.1467713	0.221999	-0.66113588	0.508525	0.730465	NOT
PPM1E	148.3383	-0.3649617	0.5522	-0.66092356	0.508661	0.730622	NOT
EDRF1	378.7349	0.10699061	0.161908	0.660810429	0.508734	0.730662	NOT
VSIG8	2.182229	-0.3690937	0.558621	-0.66072323	0.50879	0.730662	NOT
RP11-67L2	180.0821	-0.1407273	0.212991	-0.66072005	0.508792	0.730662	NOT
LPCAT3	1296.77	0.20243085	0.306383	0.660711883	0.508797	0.730662	NOT
CLSTN3	4184.708	-0.1212719	0.183567	-0.66064276	0.508841	0.730666	NOT
CENPT	1064.623	-0.1047184	0.158515	-0.66062303	0.508854	0.730666	NOT
C1orf95	49.47136	0.27259797	0.412744	0.660452864	0.508963	0.730769	NOT
PMS2CL	172.1993	-0.1224499	0.18541	-0.66042732	0.50898	0.730769	NOT
TMEM17	124.0777	0.12998376	0.196877	0.66022978	0.509106	0.730895	NOT
CTD-2619J	12.59696	-0.1584567	0.240011	-0.66020722	0.509121	0.730895	NOT
MCTP1	410.0661	-0.2513849	0.380811	-0.66013081	0.50917	0.730926	NOT
KIAA1683	254.0887	-0.2589729	0.392373	-0.66001738	0.509243	0.730955	NOT
RP11-661A	8.91095	-0.2803259	0.424726	-0.66001581	0.509244	0.730955	NOT
NCKAP5	162.8781	0.24439131	0.370331	0.659926106	0.509301	0.73097	NOT
NUDT10	3.96619	-0.4231149	0.641227	-0.65985157	0.509349	0.73097	NOT
MATR3	122.8594	-0.143552	0.217553	-0.65984909	0.509351	0.73097	NOT
FLVCR2	835.8541	-0.1995451	0.302418	-0.65983145	0.509362	0.73097	NOT
SLC25A22	1994.482	0.12654888	0.19184	0.659658467	0.509473	0.731064	NOT
ZNF879	60.10586	-0.2257192	0.342183	-0.65964515	0.509482	0.731064	NOT
FOXC1	217.2548	0.28334564	0.42964	0.65949511	0.509578	0.731164	NOT
NUTM2A	13.79458	-0.2489391	0.377533	-0.65938375	0.509649	0.731178	NOT
CTD-2547(	61.68203	-0.1750458	0.265493	-0.6593227	0.509689	0.731178	NOT
ACAP2	1342.03	-0.0977334	0.148234	-0.65931769	0.509692	0.731178	NOT
RP1-313L4	4.610553	0.21393641	0.324485	0.659311401	0.509696	0.731178	NOT
DEFA3	0.666807	-0.504572	0.765367	-0.6592549	0.509732	NA	NA
TRIO	2091.993	-0.1077479	0.163455	-0.65919051	0.509773	0.731251	NOT
CTD-2583A	104.9156	-0.1551463	0.23539	-0.65910411	0.509829	0.731292	NOT
AC010907.	4.566384	0.46349083	0.703308	0.659015736	0.509886	0.731296	NOT
RPS2	28550.9	0.16925667	0.256838	0.659001795	0.509895	0.731296	NOT
RNU6-1161	1.332385	0.61906001	0.939409	0.658988814	0.509903	NA	NA
RP11-661A	47.75695	-0.3452358	0.523899	-0.65897344	0.509913	0.731296	NOT
OR51E2	6.535069	-0.3253907	0.493872	-0.65885606	0.509988	0.731354	NOT
RP11-304I	1.628223	-0.426019	0.646633	-0.65882649	0.510007	0.731354	NOT
GKAP1	365.4245	0.1069431	0.162413	0.658465195	0.510239	0.731604	NOT
CNTROB	1031.291	0.11651904	0.176972	0.658404065	0.510279	0.731604	NOT
PKIG	1499.783	-0.1109178	0.168466	-0.65839974	0.510281	0.731604	NOT
ALKBH3-AS	7.532206	0.18247662	0.277157	0.658388171	0.510289	0.731604	NOT
AXL	687.6459	-0.2079767	0.315914	-0.65833385	0.510324	0.731611	NOT
TM6SF1	37.27254	-0.2223456	0.337759	-0.65829638	0.510348	0.731611	NOT
CYP27A1	22859.1	-0.2357234	0.358106	-0.6582502	0.510377	0.731615	NOT
FAM162A	4525.645	-0.1756371	0.266876	-0.65812263	0.510459	0.731693	NOT
CDV3	7187.923	0.08315143	0.126366	0.6580192	0.510526	0.73175	NOT
JADE2	1100.56	0.12332444	0.1875	0.657731092	0.510711	0.731911	NOT
SSPN	112.5303	-0.2562554	0.389635	-0.65768022	0.510744	0.731911	NOT
LINC01374	2.179128	0.4482032	0.681492	0.657679738	0.510744	0.731911	NOT

TIAL1	2574.165	0.07833471	0.119115	0.657637252	0.510771	0.731911	NOT
ZSWIM7	524.6615	-0.1296359	0.197124	-0.65763478	0.510773	0.731911	NOT
IGLC3	470.7616	-0.4227791	0.643127	-0.65738008	0.510937	0.732107	NOT
CRLS1	5370.473	0.15508396	0.235985	0.657175917	0.511068	0.732188	NOT
RP11-384(	2.346722	-0.3002763	0.456921	-0.65717411	0.511069	0.732188	NOT
RP11-77K1	4.550019	0.29560862	0.449824	0.657164829	0.511075	0.732188	NOT
SCARNA15	11.84677	0.22836624	0.347533	0.657106678	0.511112	0.732188	NOT
RP11-209M	1.819549	0.3565192	0.5426	0.657057625	0.511144	0.732188	NOT
SHPRH	218.8819	-0.2005765	0.305334	-0.65690823	0.51124	0.732188	NOT
YEATS2-AS	15.76192	0.19555739	0.297721	0.656847508	0.511279	0.732188	NOT
DCTN4	2797.214	-0.0844579	0.12859	-0.65679975	0.51131	0.732188	NOT
RP11-627K	18.77039	0.22572048	0.34367	0.656793459	0.511314	0.732188	NOT
MORC1	1.866138	0.48871506	0.744161	0.656732673	0.511353	0.732188	NOT
EXT2	2568.836	-0.0735518	0.112001	-0.6567074	0.511369	0.732188	NOT
MCTS1	1760.463	0.11344717	0.172759	0.656678923	0.511387	0.732188	NOT
EDA	378.5675	-0.282734	0.430552	-0.656678	0.511388	0.732188	NOT
RHEBP1	10.15349	0.26220463	0.399309	0.656646205	0.511408	0.732188	NOT
LINC01481	2.031038	-0.274918	0.418683	-0.65662594	0.511421	0.732188	NOT
CTB-92J24	45.62459	-0.2861508	0.435793	-0.65662041	0.511425	0.732188	NOT
UVRAG	847.8364	-0.0881856	0.134329	-0.65649008	0.511509	0.732249	NOT
FRK	1533.352	0.19021779	0.289764	0.656457613	0.51153	0.732249	NOT
ATPIF1	5412.185	-0.1326424	0.202073	-0.65640935	0.511561	0.732249	NOT
AL590226.	5.067248	0.48427032	0.737808	0.656363225	0.51159	0.732249	NOT
RPLP2	28542.24	0.17343657	0.264261	0.656307831	0.511626	0.732249	NOT
SLAMF6	95.99866	-0.256654	0.39106	-0.65630264	0.511629	0.732249	NOT
AC073072.	1.824254	-0.4734049	0.721402	-0.65622924	0.511677	0.732278	NOT
AC002539.	1.47479	0.82316494	1.254502	0.656168957	0.511715	0.732295	NOT
DDA1	1673.636	-0.1063504	0.162102	-0.65606964	0.511779	0.732347	NOT
CT47B1	1.556753	0.89420771	1.363481	0.655827272	0.511935	0.732508	NOT
GFM1	2932.073	-0.1008007	0.153711	-0.65578019	0.511966	0.732508	NOT
RPUSD2	501.4173	0.09897759	0.150934	0.655769414	0.511972	0.732508	NOT
PTTG1IP	10160.24	-0.0879731	0.134199	-0.65554393	0.512118	0.732677	NOT
RP11-131I	5.312169	0.26042897	0.397382	0.655362124	0.512235	0.732795	NOT
RP11-114(	12.29956	0.45744618	0.698038	0.655331552	0.512254	0.732795	NOT
CYP2R1	521.6596	0.08419093	0.128486	0.655254898	0.512304	0.732827	NOT
SLC20A1	2300.45	0.11151499	0.170202	0.655193829	0.512343	0.732845	NOT
AP001065.	184.3481	-0.3514319	0.536444	-0.65511382	0.512394	0.73288	NOT
C15orf40	731.6871	0.09767421	0.149124	0.654985473	0.512477	0.732959	NOT
RPS19	34108.28	-0.1784329	0.272468	-0.6548764	0.512547	0.732983	NOT
TSPAN13	2133.019	0.27385875	0.418184	0.654875677	0.512548	0.732983	NOT
PKP2	1464.964	-0.1996657	0.304932	-0.65478815	0.512604	0.733025	NOT
RP11-421I	7.8654	0.1699552	0.259623	0.654622107	0.512711	0.733139	NOT
RP11-1055	2.4675	-0.3817277	0.583316	-0.65441003	0.512848	0.733296	NOT
UBE2CP2	2.294865	0.27355775	0.418228	0.654087878	0.513055	0.733554	NOT
RP3-325F2	18.23305	-0.3323241	0.508169	-0.65396317	0.513136	0.73363	NOT
RP11-972F	5.065799	0.21174832	0.323853	0.65384167	0.513214	0.733676	NOT
DNAJB3	7.603575	0.32998889	0.504702	0.65382955	0.513222	0.733676	NOT
PCDH7	54.30333	-0.3179432	0.486379	-0.65369379	0.513309	0.733745	NOT
AKR7A2	2546.24	-0.1284414	0.196507	-0.65362415	0.513354	0.733745	NOT

ZC3H11B	1.743681	-0.3122343	0.477723	-0.65358824	0.513377	0.733745	NOT
YBX3	1873.086	0.24545387	0.37556	0.65356682	0.513391	0.733745	NOT
HIF1AN	1579.928	0.09725924	0.148818	0.653545007	0.513405	0.733745	NOT
ZBTB45	545.1328	0.08693366	0.133033	0.653473255	0.513451	0.733754	NOT
RP11-359F	3.549489	-0.2360259	0.361221	-0.6534115	0.513491	0.733754	NOT
RP11-837J	3.650901	-0.2209858	0.338204	-0.65340964	0.513492	0.733754	NOT
GIPC2	671.071	-0.2356849	0.360751	-0.6533168	0.513552	0.733766	NOT
PPP1R14BF	526.4997	0.19683044	0.301281	0.653312584	0.513555	0.733766	NOT
QKI	3404.636	-0.1121313	0.171692	-0.65309758	0.513693	0.733898	NOT
VCPKMT	185.7465	-0.0884198	0.135388	-0.65308442	0.513702	0.733898	NOT
KCNMB3	84.9355	0.23059877	0.353125	0.653023812	0.513741	0.733916	NOT
ZCCHC8	1034.916	0.10087583	0.154553	0.652696205	0.513952	0.734108	NOT
FAM53C	1268.814	0.08690948	0.133159	0.652672864	0.513967	0.734108	NOT
CTC-428H1	11.03358	-0.1879919	0.288042	-0.6526554	0.513978	0.734108	NOT
SRGAP1	397.1659	-0.2352889	0.360515	-0.65264732	0.513984	0.734108	NOT
DDC	2601.238	0.28688037	0.439694	0.6524547	0.514108	0.734227	NOT
RAB1A	7592.003	0.08076381	0.123795	0.6523995	0.514143	0.734227	NOT
MMADHC	3313.382	-0.096572	0.148028	-0.65238871	0.51415	0.734227	NOT
PRIMA1	3.009565	-0.510443	0.782468	-0.6523499	0.514175	0.734227	NOT
ZNF20	15.97396	-0.1434875	0.220024	-0.65214551	0.514307	0.734348	NOT
RP11-286N	4.040889	0.22075823	0.338522	0.652123887	0.514321	0.734348	NOT
RP11-584F	2.494526	-0.3613125	0.554081	-0.65209308	0.514341	0.734348	NOT
RP11-328J	40.90101	0.58774885	0.901403	0.652037843	0.514377	0.73436	NOT
RP11-57H1	3.320963	0.2236129	0.342985	0.651961982	0.514426	0.734391	NOT
KCNH4	34.05325	0.27654417	0.424229	0.651874555	0.514482	0.734433	NOT
RP11-561F	2.644162	-0.2974923	0.45643	-0.65178052	0.514543	0.734481	NOT
REG3A	1717.185	-0.7678699	1.17822	-0.65172016	0.514582	0.734498	NOT
RP11-307F	4.612432	-0.2678892	0.411149	-0.65156205	0.514684	0.734605	NOT
IGF2	165354	0.54878461	0.842411	0.651445347	0.514759	0.734674	NOT
LINC01549	35.65878	0.44972094	0.690702	0.651107251	0.514977	0.734912	NOT
RP11-248F	2.784618	0.64381226	0.988838	0.651079446	0.514995	0.734912	NOT
ZNF652	1752.927	-0.1091788	0.167708	-0.65100566	0.515043	0.734912	NOT
APOBR	296.7996	0.15282376	0.234754	0.650996037	0.515049	0.734912	NOT
PICALM	5995.808	-0.0727718	0.111789	-0.65097726	0.515061	0.734912	NOT
DOK1	231.9909	0.17797833	0.273472	0.650810452	0.515169	0.735024	NOT
RASD2	86.00451	-0.1837003	0.282281	-0.65077142	0.515194	0.735024	NOT
RP11-69I8	6.240177	0.27693124	0.425753	0.650449792	0.515402	0.735282	NOT
KCNK2	15.63	0.56861707	0.874447	0.650258972	0.515525	0.735419	NOT
ZNF841	304.5809	-0.1657376	0.254904	-0.65019646	0.515565	0.735436	NOT
CCDC102B	157.323	-0.1342784	0.206533	-0.6501557	0.515592	0.735436	NOT
RP1-506.6	2.352453	0.26119701	0.4018	0.65006799	0.515648	0.73545	NOT
OXT	44.89053	-0.5295268	0.814585	-0.6500572	0.515655	0.73545	NOT
TMX2P1	50.97832	0.16476994	0.253576	0.64978598	0.51583	0.735593	NOT
CNTNAP3B	117.3284	-0.3537971	0.544494	-0.64977207	0.515839	0.735593	NOT
RNU1-138F	2.596999	-0.56807	0.874305	-0.64973866	0.515861	0.735593	NOT
SNORD69	2.963562	-0.2356552	0.362695	-0.649734	0.515864	0.735593	NOT
DNASE1	361.277	0.14071434	0.216596	0.649662077	0.515911	0.735619	NOT
RBM26	1414.473	0.10214748	0.157241	0.649621859	0.515937	0.735619	NOT
RP11-475C	13.49978	-0.354702	0.546281	-0.64930294	0.516143	0.735874	NOT

ATG16L1	1163.251	0.07190601	0.110758	0.649215363	0.516199	0.735916	NOT
NUTM2G	4.824454	0.24935709	0.384186	0.649052599	0.516304	0.735941	NOT
CCDC13	24.71136	-0.1909377	0.294194	-0.64901985	0.516326	0.735941	NOT
CHCHD2	9360.348	0.11448098	0.176397	0.648997495	0.51634	0.735941	NOT
RP11-552F	13.64015	-0.189532	0.29204	-0.64899335	0.516343	0.735941	NOT
ZNF146	2511.55	-0.1016545	0.156638	-0.64897904	0.516352	0.735941	NOT
RP11-427M	1.621685	0.50117509	0.772378	0.648872997	0.51642	0.736	NOT
RN7SL832F	18.53387	-0.1897105	0.292397	-0.64881189	0.51646	0.736017	NOT
CBLC	710.8519	0.24394658	0.376058	0.648694292	0.516536	0.736063	NOT
TMPPE	55.53856	0.15814128	0.243814	0.648613632	0.516588	0.736063	NOT
ZNF81	110.3231	-0.1414745	0.218129	-0.64858239	0.516608	0.736063	NOT
CCL16	4357.151	-0.3405235	0.525042	-0.64856434	0.51662	0.736063	NOT
NRAP	235.7116	-0.2881926	0.444363	-0.64855213	0.516628	0.736063	NOT
RBM10	2922.68	0.07132565	0.109985	0.648500665	0.516661	0.736072	NOT
EIF1AD	1051.088	0.06927081	0.106826	0.648442389	0.516699	0.736087	NOT
TP53	1700.747	0.17904285	0.276186	0.648269942	0.51681	0.736171	NOT
HLA-K	198.7082	0.21849302	0.337069	0.64821368	0.516847	0.736171	NOT
IGKV4-1	455.3695	0.45491755	0.701865	0.648155246	0.516885	0.736171	NOT
MED1	1230.709	0.09282344	0.143218	0.648126408	0.516903	0.736171	NOT
RP11-151A	11.15216	-0.2680215	0.413546	-0.64810588	0.516916	0.736171	NOT
SERPINE1	8970.811	0.30600634	0.472192	0.648055204	0.516949	0.736171	NOT
RP11-283C	53.57768	-0.186476	0.287753	-0.64804096	0.516958	0.736171	NOT
ANGPT1	194.8803	-0.2344024	0.361723	-0.64801576	0.516975	0.736171	NOT
RP11-684E	2.648428	-0.3563808	0.550088	-0.64786114	0.517075	0.736251	NOT
GUCY1A3	364.6655	-0.2108969	0.325536	-0.64784507	0.517085	0.736251	NOT
RP13-93L1	2.388013	-0.2336952	0.360837	-0.64764742	0.517213	0.736394	NOT
RP1-199J3	5.909936	0.21843623	0.337352	0.647502945	0.517306	0.736489	NOT
RNF170	986.2276	-0.137114	0.211815	-0.64733046	0.517418	0.73656	NOT
ALG2	2032.929	-0.1052854	0.16266	-0.64727243	0.517456	0.73656	NOT
LSM12P1	45.67642	0.1492792	0.230636	0.64724948	0.51747	0.73656	NOT
C2orf49	571.7915	0.0715391	0.110533	0.647218551	0.517491	0.73656	NOT
RP11-850A	2.007898	0.30388015	0.469519	0.647215197	0.517493	0.73656	NOT
CACNA2D2	67.79235	-0.2634333	0.407118	-0.64706812	0.517588	0.73665	NOT
SRSF11	4039.016	0.12957952	0.200267	0.647033926	0.51761	0.73665	NOT
RP1-40G4F	4.879887	0.21830481	0.337468	0.646891221	0.517702	0.736743	NOT
LAMP5	29.27444	0.36493824	0.564234	0.646785635	0.517771	0.736759	NOT
NEK7	2489.107	-0.1308755	0.202355	-0.64676275	0.517786	0.736759	NOT
AC006277.	20.73587	0.17207032	0.266055	0.646748152	0.517795	0.736759	NOT
PRRG4	1299.619	-0.1984529	0.306885	-0.64666968	0.517846	0.736792	NOT
TMEM161A	2758.072	-0.1324806	0.204891	-0.64659038	0.517897	0.736827	NOT
XRRA1	391.7749	0.15140108	0.234241	0.646346606	0.518055	0.737013	NOT
RAB8B	933.0123	0.14270154	0.220827	0.646214286	0.518141	0.737096	NOT
SI	5.018985	0.79755223	1.234312	0.646151232	0.518181	0.737115	NOT
EMC9	725.982	-0.1441271	0.223069	-0.64610944	0.518208	0.737115	NOT
OSMR-AS1	8.95732	0.29278722	0.453307	0.645892161	0.518349	0.737199	NOT
RP11-80H5	5.040116	-0.2823916	0.437218	-0.64588229	0.518356	0.737199	NOT
RP11-91H1	9.441987	-0.5369161	0.831302	-0.64587398	0.518361	0.737199	NOT
Z69890.1	1.500604	-0.355011	0.54968	-0.64585022	0.518376	0.737199	NOT
IFITM3	52850.4	0.16740486	0.259263	0.645695053	0.518477	0.73727	NOT

KRT83	1.608048	0.62230578	0.963841	0.645652106	0.518505	0.73727	NOT
UTS2R	2.361925	-0.5293309	0.819938	-0.64557432	0.518555	0.73727	NOT
RRAGA	3089.499	-0.0869178	0.13464	-0.645559	0.518565	0.73727	NOT
THAP7-AS1	137.2374	-0.1703805	0.26393	-0.64555093	0.51857	0.73727	NOT
ZNF284	74.01349	0.15465989	0.239593	0.645511822	0.518596	0.73727	NOT
SMIM2	3.063102	0.39230622	0.607802	0.645450343	0.518635	0.73727	NOT
ZNF793	102.771	0.27432631	0.425024	0.645437297	0.518644	0.73727	NOT
SCARNA7	5.706144	-0.2865045	0.443971	-0.64532199	0.518719	0.737285	NOT
LRRC45	2095.834	-0.16288	0.252402	-0.64531977	0.51872	0.737285	NOT
ATF6B	4139.295	0.08680771	0.134524	0.645295868	0.518735	0.737285	NOT
RPL19P21	7.526778	0.22767707	0.353021	0.644939231	0.518967	0.737575	NOT
GRIP1	14.32183	0.34639349	0.537139	0.644885951	0.519001	0.737585	NOT
PCBP1	8302.079	0.11293548	0.175169	0.644724272	0.519106	0.737647	NOT
IL17B	4.53457	0.33262608	0.515929	0.644713354	0.519113	0.737647	NOT
ODC1	5651.399	0.16363897	0.253859	0.644604836	0.519183	0.737647	NOT
ADCK3	7435.491	0.16370563	0.253966	0.644596148	0.519189	0.737647	NOT
RP11-488(	2.209437	0.31326135	0.486037	0.644521292	0.519237	0.737647	NOT
ZNF850	105.397	-0.1686804	0.261722	-0.64450313	0.519249	0.737647	NOT
TLL2	21.26734	0.2594093	0.402534	0.644440137	0.51929	0.737647	NOT
MEIS3	83.00976	0.18152705	0.281684	0.644435126	0.519293	0.737647	NOT
PRKD3	1020.38	0.11632321	0.180511	0.644409136	0.51931	0.737647	NOT
AC004383.	1.233878	-0.3674765	0.570281	-0.64437837	0.51933	NA	NA
LIG3	1092.767	0.08408801	0.130501	0.644348367	0.51935	0.737647	NOT
C11orf42	3.854275	-0.3220636	0.499852	-0.64431798	0.519369	0.737647	NOT
RP11-299I	30.96086	-0.4347823	0.674797	-0.64431556	0.519371	0.737647	NOT
RPL13A	59271.18	0.14709978	0.228351	0.644181606	0.519458	0.737732	NOT
LINC0158(	1.364007	-0.9857623	1.53142	-0.64369165	0.519775	NA	NA
RP13-638(	2.402777	-0.2373654	0.368767	-0.64367233	0.519788	0.738115	NOT
STYXL1	1418.007	0.14129331	0.219534	0.64360469	0.519832	0.738115	NOT
SEPW1	4080.716	-0.2077963	0.322869	-0.64359366	0.519839	0.738115	NOT
RP5-940J5	20.16767	0.17874959	0.277739	0.643588587	0.519842	0.738115	NOT
RP11-216I	5.675028	-0.2415491	0.37534	-0.64354823	0.519868	0.738115	NOT
CHD8	2171.787	0.08696365	0.135148	0.643472101	0.519918	0.738115	NOT
WAS	402.1854	-0.1859012	0.288903	-0.64347163	0.519918	0.738115	NOT
JAZF1-AS1	4.044859	0.3025769	0.470305	0.643363226	0.519988	0.738176	NOT
PCCA	2676.928	-0.1597985	0.248447	-0.64318881	0.520102	0.738259	NOT
GALNT6	144.7788	0.18871752	0.293439	0.643124247	0.520143	0.738259	NOT
RXFP1	19.72952	0.2191825	0.340852	0.643042643	0.520196	0.738259	NOT
PROCA1	78.18423	0.19294073	0.30005	0.643028492	0.520206	0.738259	NOT
LOH12CR1	369.9122	-0.1271011	0.197667	-0.64300726	0.520219	0.738259	NOT
RP11-435I	7.621786	-0.2788109	0.433609	-0.64300054	0.520224	0.738259	NOT
KIF3A	297.9474	0.11186403	0.173986	0.64294935	0.520257	0.738259	NOT
LA16c-313	6.951656	-0.1770343	0.275352	-0.64293796	0.520264	0.738259	NOT
DHPS	2354.287	-0.1093855	0.1702	-0.64268826	0.520426	0.738433	NOT
LZTR1	2410.825	0.08521317	0.132594	0.642661029	0.520444	0.738433	NOT
RHBDF1	696.9563	0.15209935	0.236685	0.642623485	0.520468	0.738433	NOT
KRTAP20-4	1.698099	1.67376519	2.605022	0.642514847	0.520539	0.738494	NOT
L3MBTL2	1646.355	0.07961643	0.12393	0.642432254	0.520593	0.738531	NOT
RP11-104I	18.40681	-0.2238764	0.348512	-0.6423779	0.520628	0.738543	NOT

LINC0131]	33.34557	0.17802537	0.277237	0.642140768	0.520782	0.738685	NOT
NME4	5525.217	-0.1858062	0.289365	-0.64211638	0.520798	0.738685	NOT
EBI3	125.5695	0.2019786	0.314567	0.642084317	0.520818	0.738685	NOT
FAM69A	1028.631	0.15720975	0.24486	0.642038562	0.520848	0.738685	NOT
LINC0129]	56.50171	-0.4745288	0.739126	-0.64201346	0.520864	0.738685	NOT
SH3BP2	2293.935	-0.1396785	0.217584	-0.64195331	0.520903	0.738702	NOT
U2AF1	28.75651	-0.1692016	0.26365	-0.64176581	0.521025	0.738784	NOT
TOMM20L	6.266725	0.18463275	0.287706	0.641740122	0.521042	0.738784	NOT
RPL21P39	2.235448	0.24029118	0.374438	0.641738856	0.521043	0.738784	NOT
RP11-259(	16.86313	0.26151857	0.407569	0.641655381	0.521097	0.738784	NOT
RTEL1	39.12912	-0.1764216	0.274948	-0.64165475	0.521097	0.738784	NOT
DHH	11.83343	-0.2813897	0.438598	-0.6415667	0.521155	0.738827	NOT
AC006037.	3.213815	-0.4591195	0.715774	-0.64143077	0.521243	0.738913	NOT
RP11-214K	4.938739	0.22425184	0.349674	0.641316667	0.521317	0.738922	NOT
AC079807.	39.92342	-0.1236398	0.192791	-0.64131402	0.521319	0.738922	NOT
RP11-305I	23.66381	0.21897897	0.341464	0.641294404	0.521331	0.738922	NOT
NUDT21	2058.863	0.09013976	0.140574	0.641228415	0.521374	0.738922	NOT
RP11-475I	5.580877	0.29273758	0.456538	0.641211522	0.521385	0.738922	NOT
CD248	758.214	-0.1762664	0.274941	-0.64110664	0.521453	0.738969	NOT
RP11-135J	2.278431	0.29887542	0.466238	0.6410367	0.521499	0.738969	NOT
BAZ1B	3816.93	-0.0947206	0.147762	-0.64103431	0.5215	0.738969	NOT
RNA5SP163	3.81931	-0.3185775	0.497101	-0.64087086	0.521607	0.739081	NOT
IGHV1-18	105.7625	0.47699261	0.74455	0.640645776	0.521753	0.73924	NOT
C3orf67	125.5969	0.20520163	0.32032	0.640614452	0.521773	0.73924	NOT
RP11-33A1	1.293692	0.65317271	1.01969	0.640560345	0.521808	NA	NA
VAMP7	2781.024	-0.1362687	0.212761	-0.64047821	0.521862	0.739327	NOT
IGHV3-71	1.339981	0.73619937	1.149783	0.640294202	0.521981	NA	NA
ZNF725P	0.483906	-0.9647107	1.506814	-0.64023198	0.522022	NA	NA
CAB39	2764.826	0.07377388	0.115237	0.640194169	0.522046	0.73955	NOT
LA16c-312	24.77367	0.4236093	0.661984	0.639908368	0.522232	0.739755	NOT
LYG1	42.93126	-0.1820589	0.284517	-0.63988738	0.522246	0.739755	NOT
KDM6A	1021.471	-0.1371493	0.21436	-0.63980724	0.522298	0.739777	NOT
PTPN1	2099.629	0.08477614	0.132508	0.639779359	0.522316	0.739777	NOT
RP11-102N	22.8925	-0.2075274	0.324406	-0.63971596	0.522357	0.739781	NOT
HSPB2	19.37478	0.24977837	0.390491	0.639652065	0.522399	0.739781	NOT
TRMT10A	305.9937	-0.1090201	0.170437	-0.63964953	0.5224	0.739781	NOT
RP11-259(	9.087284	-0.5687677	0.889345	-0.63953574	0.522474	0.739847	NOT
RP3-406A7	82.88819	0.22076588	0.345234	0.639467881	0.522519	0.739871	NOT
RP11-134K	3.057955	0.3089653	0.483365	0.639196158	0.522695	0.740083	NOT
NBPF13P	193.1191	0.34170237	0.534646	0.639118917	0.522746	0.740087	NOT
PRKG1-AS1	3.32715	0.57446653	0.898857	0.639108026	0.522753	0.740087	NOT
TDRD1	6.360279	0.50068898	0.783505	0.639037017	0.522799	0.740113	NOT
RBMY1E	0.868482	1.26649535	1.982048	0.638983136	0.522834	NA	NA
DMC1	22.15859	0.28450526	0.445269	0.638950745	0.522855	0.740154	NOT
KRT16P3	0.178391	-0.9302042	1.456391	-0.63870478	0.523015	NA	NA
ALX4	2.625997	0.42773387	0.669711	0.638684026	0.523029	0.740361	NOT
RP11-268(	12.27818	-0.4408761	0.690374	-0.63860457	0.52308	0.740396	NOT
NAT1	238.7269	-0.1591082	0.249183	-0.6385207	0.523135	0.740434	NOT
RP11-46D(	187.846	0.12337714	0.193242	0.63846004	0.523174	0.740452	NOT

CTD-3162I	4.280668	-0.2935756	0.460023	-0.6381765	0.523359	0.740648	NOT
PIP	1.775591	0.54693612	0.857083	0.638136454	0.523385	0.740648	NOT
SNX25	539.6656	-0.127776	0.200238	-0.63812049	0.523395	0.740648	NOT
GPR1	11.58828	0.37719531	0.591151	0.638068944	0.523429	0.740648	NOT
TTC21B	560.6583	-0.0983887	0.154205	-0.63803766	0.523449	0.740648	NOT
MMP28	56.84313	0.28835753	0.452172	0.637716317	0.523658	0.740905	NOT
SPG21	4682.583	-0.0861281	0.135087	-0.63757313	0.523752	0.740993	NOT
TUSC8	157.612	-0.3227502	0.506257	-0.63752308	0.523784	0.740993	NOT
ATP9B	875.5807	-0.1186115	0.186064	-0.63747677	0.523814	0.740993	NOT
DDX10P1	2.247578	0.35654168	0.559331	0.637443478	0.523836	0.740993	NOT
U47924.32	4.479253	0.26573241	0.416893	0.637411423	0.523857	0.740993	NOT
SLC39A11	2687.683	-0.1346017	0.21127	-0.63710889	0.524054	0.741233	NOT
VN1R28P	1.312683	0.94982715	1.491114	0.636991791	0.52413	NA	NA
GPAM	9396.236	-0.2265726	0.355696	-0.63698329	0.524136	0.741243	NOT
NAIF1	450.4162	0.07538073	0.11834	0.63698275	0.524136	0.741243	NOT
RP11-100(	2.335321	-0.239947	0.376709	-0.63695578	0.524154	0.741243	NOT
RP13-539J	2.170355	0.74785671	1.174158	0.636930163	0.52417	0.741243	NOT
RPS11P5	33.8899	0.20660782	0.324464	0.636766757	0.524277	0.741355	NOT
RP11-285(	2.403044	-0.3088901	0.485253	-0.63655414	0.524415	0.741512	NOT
RP11-394I	31.43201	-0.2159142	0.339218	-0.63650548	0.524447	0.741518	NOT
CUBN	34.23056	-0.1555827	0.244472	-0.63640417	0.524513	0.741535	NOT
MT1DP	24.87493	0.25895805	0.406909	0.636403037	0.524514	0.741535	NOT
ATP8B1	2260.831	-0.175981	0.276587	-0.63625987	0.524607	0.741595	NOT
RP11-443I	4.157355	-0.2486429	0.390826	-0.63619911	0.524647	0.741595	NOT
PLCD1	556.5885	-0.1072093	0.168521	-0.63617651	0.524661	0.741595	NOT
RP11-157I	1.650761	0.56206693	0.883516	0.636170523	0.524665	0.741595	NOT
RP11-214K	2.67375	0.31010763	0.487555	0.63604643	0.524746	0.741616	NOT
BCHE	1703.952	-0.3519438	0.553382	-0.63598714	0.524785	0.741616	NOT
RP3-395M2	12.98584	0.23921449	0.376132	0.63598541	0.524786	0.741616	NOT
RP11-267(	8.888836	-0.5772038	0.907582	-0.63597945	0.52479	0.741616	NOT
PROSER2	1365.831	0.16427925	0.258415	0.635718723	0.52496	0.741803	NOT
RP11-706(	19.34579	-0.3784107	0.595273	-0.63569285	0.524977	0.741803	NOT
RP5-1052I	4.000697	-0.3483154	0.547998	-0.63561444	0.525028	0.741837	NOT
RP11-181(	88.28486	-0.2621195	0.4125	-0.63544189	0.52514	0.741956	NOT
TRAM1L1	76.74425	0.3863326	0.608014	0.635401263	0.525167	0.741956	NOT
CFAP46	38.14231	-0.4559328	0.717752	-0.63522337	0.525283	0.742081	NOT
CLDN2	4066.227	-0.4021452	0.633179	-0.63512103	0.525349	0.742137	NOT
AC006042.	1.311612	-0.2964121	0.466929	-0.63481129	0.525551	NA	NA
LY6G5B	64.60852	0.20144048	0.317327	0.634803585	0.525557	0.742368	NOT
SLC22A23	2237.968	-0.1534374	0.241715	-0.63478566	0.525568	0.742368	NOT
C14orf166	3603.882	0.09963425	0.156971	0.634729936	0.525605	0.742381	NOT
DDX47	45.32703	0.14713338	0.231869	0.634553911	0.525719	0.742468	NOT
GABRE	1173.166	0.30936844	0.487616	0.634450899	0.525787	0.742468	NOT
RP11-506K	9.116479	-0.2313537	0.364658	-0.63443949	0.525794	0.742468	NOT
BMPR1B	16.27709	-0.4152621	0.654545	-0.63442862	0.525801	0.742468	NOT
RP11-545I	92.88714	-0.1650089	0.260096	-0.63441466	0.52581	0.742468	NOT
PPP1R37	1671.091	0.11131183	0.175473	0.634354212	0.52585	0.742468	NOT
CTC-573N1	26.44538	0.49699373	0.783517	0.634311198	0.525878	0.742468	NOT
PLEKHA5	1183.453	-0.1247004	0.196607	-0.63426186	0.52591	0.742468	NOT

GSTCD	295.064	0.14502812	0.228676	0.634207784	0.525945	0.742468	NOT
SENP5	1289.29	-0.0648672	0.102288	-0.63416273	0.525975	0.742468	NOT
RP11-474I	1.270436	0.45676031	0.720302	0.634123698	0.526	NA	NA
ZNF397	776.8889	-0.0835149	0.131709	-0.63408533	0.526025	0.742468	NOT
ADRB1	17.20062	0.29951036	0.472357	0.634076122	0.526031	0.742468	NOT
HS3ST3A1	15.42536	0.2843409	0.448448	0.634055441	0.526045	0.742468	NOT
RP11-341(	1.875144	0.46582344	0.734681	0.634048371	0.526049	0.742468	NOT
AC003003.	2.434729	0.31032836	0.489555	0.633898486	0.526147	0.742548	NOT
CELF1	5107.453	-0.0563981	0.088982	-0.63381756	0.5262	0.742548	NOT
POLR1A	2147.205	0.09028005	0.14244	0.633809988	0.526205	0.742548	NOT
bP-2171C2	5.083234	0.287853	0.454175	0.633793292	0.526216	0.742548	NOT
ST8SIA1	32.43074	0.24770845	0.390874	0.633729579	0.526257	0.742568	NOT
SELO	3153.444	-0.155502	0.245393	-0.63368514	0.526286	0.742571	NOT
AKR1C8P	118.1097	-0.3595565	0.567536	-0.63353986	0.526381	0.742626	NOT
ADAMTS20	4.383292	0.54819842	0.865357	0.633494038	0.526411	0.742626	NOT
TSNAX	1823.303	0.08919112	0.140813	0.633400601	0.526472	0.742626	NOT
GTPBP1	1825.572	0.07287619	0.115058	0.63338886	0.52648	0.742626	NOT
NBEAP1	3.981672	-0.3568023	0.56333	-0.63338114	0.526485	0.742626	NOT
RP11-120J	4.123804	0.4022768	0.635157	0.633350539	0.526505	0.742626	NOT
RP3-417G1	24.07966	0.22991574	0.363026	0.633331984	0.526517	0.742626	NOT
HACE1	230.6916	0.1707539	0.269658	0.633224476	0.526587	0.742668	NOT
ARVCF	777.3949	0.15111212	0.238647	0.63320242	0.526601	0.742668	NOT
ATPAF2	844.2748	0.14400342	0.227454	0.633108777	0.526663	0.742715	NOT
AUP1	8179.688	0.0969934	0.153222	0.633023799	0.526718	0.74274	NOT
THRSP	2518.675	-0.4797526	0.757905	-0.63299819	0.526735	0.74274	NOT
LINC00582	0.654099	-0.3894688	0.615328	-0.63294505	0.52677	NA	NA
TFPI	14009.21	0.23515827	0.37159	0.632843145	0.526836	0.742844	NOT
GGCT	1485.139	0.12820531	0.202612	0.632763664	0.526888	0.742853	NOT
ZNF827	116.9748	-0.2493453	0.394066	-0.63274965	0.526897	0.742853	NOT
RP13-1032	159.5348	-0.1584328	0.250439	-0.63261989	0.526982	0.742896	NOT
ASAH2B	235.513	0.12789567	0.202168	0.632619569	0.526982	0.742896	NOT
SPAG8	27.1111	0.16231454	0.256614	0.632522878	0.527045	0.742946	NOT
CSN2	0.886262	-1.1155051	1.763948	-0.632391	0.527131	NA	NA
RP5-858B6	6.254081	0.38675445	0.611612	0.632352519	0.527157	0.743044	NOT
AC092171.	2.226059	0.3045587	0.481643	0.632332401	0.52717	0.743044	NOT
CES2	27335.15	-0.2502045	0.395727	-0.63226509	0.527214	0.743068	NOT
TRMT10B	260.7114	0.09146229	0.144674	0.632197272	0.527258	0.743086	NOT
CTD-2017I	60.96609	0.17394767	0.275164	0.632160669	0.527282	0.743086	NOT
UCKL1-AS1	20.99795	-0.2315292	0.366284	-0.63210363	0.527319	0.743093	NOT
GIMAP5	62.40086	-0.2165711	0.34265	-0.6320481	0.527355	0.743093	NOT
ZDHHC20	1487.248	-0.1349444	0.213518	-0.63200527	0.527383	0.743093	NOT
HOXD3	7.173083	0.29370792	0.464738	0.631985942	0.527396	0.743093	NOT
ZNF528-AS	85.88472	0.24423693	0.386495	0.631927624	0.527434	0.743108	NOT
HCG4P3	21.74821	0.19068156	0.30184	0.631730294	0.527563	0.743233	NOT
DGCR14	800.3452	0.09491324	0.150248	0.631708628	0.527577	0.743233	NOT
RP4-800O1	5.705541	0.40083889	0.634758	0.631482597	0.527725	0.743402	NOT
WDFY1	1453.781	0.09313704	0.147514	0.631379721	0.527792	0.743434	NOT
RP11-274I	130.017	-0.1469442	0.232741	-0.63136457	0.527802	0.743434	NOT
AQP9	19038.49	-0.3750713	0.594106	-0.63132032	0.527831	0.743436	NOT

CSNK1G1	511.9385	0.09636592	0.152674	0.631189496	0.527917	0.743518	NOT
ATAD3C	141.8443	-0.312755	0.495651	-0.63099865	0.528041	0.743629	NOT
EBPL	2513.068	-0.1842625	0.292024	-0.63098448	0.528051	0.743629	NOT
RP11-523H	8.407657	0.18404561	0.291715	0.630908893	0.5281	0.74366	NOT
RP11-64K1	3.371113	-0.2758215	0.437508	-0.63043798	0.528408	0.744055	NOT
PRDM5	38.43612	0.2771677	0.439695	0.630363232	0.528457	0.744085	NOT
IDSP1	1.854858	0.34755537	0.551448	0.630260114	0.528524	0.744142	NOT
COG2	1380.702	0.09659547	0.153296	0.630123607	0.528614	0.744193	NOT
IGKV5-2	1.937825	-0.5833668	0.925802	-0.63012054	0.528616	0.744193	NOT
RP11-476H	7.379294	0.20865722	0.33118	0.63004172	0.528667	0.744199	NOT
RHOA-IT1	2.179404	-0.27645	0.438788	-0.63003069	0.528675	0.744199	NOT
HDHD1	820.9656	-0.1471463	0.233575	-0.6299733	0.528712	0.744213	NOT
CELF5	112.1079	0.43923276	0.697329	0.629878642	0.528774	0.744261	NOT
RP1-278C1	7.884613	-0.2495514	0.396233	-0.62980905	0.52882	0.744273	NOT
RP11-666A	1.087305	-0.6138655	0.974828	-0.62971676	0.52888	NA	NA
RP11-848F	23.32382	-0.1832765	0.291056	-0.62969552	0.528894	0.744273	NOT
CTD-2061E	2.034658	0.32803074	0.520972	0.629651851	0.528922	0.744273	NOT
RNF123	1844.476	-0.1227966	0.195034	-0.62961771	0.528945	0.744273	NOT
RIOK2	835.8405	-0.0841635	0.133674	-0.62961692	0.528945	0.744273	NOT
CTD-2532M	3.065691	0.29918682	0.475191	0.629614023	0.528947	0.744273	NOT
PCOLCE2	1293.638	0.29501607	0.468757	0.629358654	0.529114	0.744408	NOT
KB-431C1.	12.91835	0.20292186	0.322434	0.629342908	0.529125	0.744408	NOT
RP11-78J2	5.964064	0.23019545	0.365772	0.62934191	0.529125	0.744408	NOT
CCDC141	28.81638	0.34307684	0.545232	0.629231223	0.529198	0.744408	NOT
C10orf76	1145.029	-0.0723451	0.114976	-0.62921824	0.529206	0.744408	NOT
RGS3	2383.228	0.12296125	0.19542	0.629215857	0.529208	0.744408	NOT
APBB1	778.9301	-0.1551373	0.246585	-0.62914257	0.529256	0.744437	NOT
AC007349.	1.164644	-0.4319262	0.686702	-0.62898637	0.529358	NA	NA
ZNF780B	365.0074	-0.1493077	0.237444	-0.62881189	0.529472	0.744643	NOT
PDCD4-AS1	40.23789	0.13020419	0.207066	0.628805619	0.529476	0.744643	NOT
CFTR	120.3412	-0.5048916	0.802953	-0.62879384	0.529484	0.744643	NOT
CCR5	178.9731	0.24999204	0.397623	0.628716636	0.529535	0.744675	NOT
OLFML2A	634.4647	-0.1874755	0.298267	-0.62854958	0.529644	0.744717	NOT
RP11-63P1	11.65137	0.37871736	0.602547	0.628527016	0.529659	0.744717	NOT
CTB-75G16	6.737075	0.29915953	0.475979	0.628513809	0.529667	0.744717	NOT
CCDC152	702.2015	-0.1928821	0.306891	-0.62850309	0.529674	0.744717	NOT
OMP	7.340855	-0.2954387	0.470184	-0.62834678	0.529777	0.744762	NOT
ARHGEF11	3287.273	-0.1130752	0.179959	-0.62833987	0.529781	0.744762	NOT
MIAT	77.7489	0.29678791	0.472345	0.628328572	0.529789	0.744762	NOT
SLC25A6	13692.84	-0.1466184	0.23342	-0.62813042	0.529919	0.74487	NOT
DIRC2	511.8671	-0.1077889	0.171603	-0.62812775	0.52992	0.74487	NOT
CACNA1A	20.99572	0.24635716	0.392251	0.628059276	0.529965	0.744894	NOT
YAP1	3001.861	-0.1197982	0.190776	-0.62795305	0.530035	0.744954	NOT
ANKRD55	25.04405	0.27681984	0.441024	0.627675527	0.530217	0.745059	NOT
IFT140	451.4201	0.15038866	0.239602	0.627659687	0.530227	0.745059	NOT
RPL3P6	3.056793	-0.281033	0.447763	-0.62763759	0.530241	0.745059	NOT
SNHG16	856.9251	-0.1126535	0.179498	-0.6276049	0.530263	0.745059	NOT
ALOXE3	2.225865	0.49878878	0.794781	0.627580191	0.530279	0.745059	NOT
ATP6V0D1	4593.284	-0.1152181	0.183599	-0.6275524	0.530297	0.745059	NOT

HIST1H2AE	2.088072	0.3419229	0.544877	0.627523421	0.530316	0.745059	NOT
ZNF484	87.39511	-0.1165095	0.18569	-0.62744257	0.530369	0.745059	NOT
SNAI3-AS1	136.4305	-0.192508	0.306827	-0.62741602	0.530387	0.745059	NOT
RP11-295(	7.875145	0.26988186	0.430168	0.627386733	0.530406	0.745059	NOT
CNIH3	113.2282	-0.1866541	0.297515	-0.62737772	0.530412	0.745059	NOT
TSPAN14	3151.058	-0.120008	0.191306	-0.62730853	0.530457	0.745068	NOT
CABLES1	958.767	0.14420806	0.229893	0.627283648	0.530473	0.745068	NOT
RP11-244E	1.891751	0.90568737	1.444347	0.627056557	0.530622	0.745239	NOT
RP4-575N6	4.379975	-0.2273171	0.362607	-0.62689682	0.530727	0.745347	NOT
BMP6	73.67607	0.28458526	0.453994	0.626848312	0.530759	0.745353	NOT
DLG4	318.3687	-0.168128	0.268277	-0.62669504	0.530859	0.745435	NOT
GABPB1	1115.623	-0.0601321	0.095954	-0.626676	0.530872	0.745435	NOT
TMSB15B	2.393418	0.37134746	0.592686	0.626549613	0.530955	0.745513	NOT
NEXN-AS1	5.353576	-0.2909234	0.464396	-0.62645547	0.531016	0.745556	NOT
GALNT14	47.56402	-0.2485646	0.396803	-0.62641842	0.531041	0.745556	NOT
ZNF217	2263.927	0.1488381	0.237626	0.62635405	0.531083	0.745556	NOT
SIGLEC9	99.46952	0.21228325	0.338929	0.626335359	0.531095	0.745556	NOT
IGFBP5	3883.578	-0.2177079	0.347645	-0.6262371	0.531159	0.745568	NOT
TRBV5-1	7.891842	0.2927483	0.467472	0.626236832	0.53116	0.745568	NOT
FAM218A	4.515779	-0.3982702	0.636033	-0.62617857	0.531198	0.745568	NOT
RP11-638I	1.805917	0.32626031	0.521054	0.62615476	0.531213	0.745568	NOT
RP11-29B2	4.927966	0.23385953	0.373604	0.625955118	0.531344	0.745713	NOT
NOVA1	124.0395	-0.3098256	0.495099	-0.62578569	0.531456	0.745812	NOT
AC010524.	92.64507	0.36263646	0.579562	0.625707623	0.531507	0.745812	NOT
MAP2K1	2015.738	-0.0934286	0.149323	-0.62568318	0.531523	0.745812	NOT
IL20	1.923089	-0.5546172	0.886423	-0.62568	0.531525	0.745812	NOT
RPS6KB2	2398.701	-0.0872929	0.139537	-0.62559116	0.531583	0.745823	NOT
RAMP1	5133.698	-0.2808588	0.448981	-0.62554763	0.531612	0.745823	NOT
RP11-475J	7.395993	0.32764498	0.523777	0.625542317	0.531615	0.745823	NOT
ZNF559	277.8423	-0.1463035	0.233924	-0.6254328	0.531687	0.745885	NOT
UQCRHL	116.2895	0.148433	0.237351	0.62537259	0.531727	0.745902	NOT
ALG14	815.8055	-0.103739	0.165934	-0.62518244	0.531851	0.746015	NOT
RECQL5	1540.729	0.09837116	0.157352	0.625166201	0.531862	0.746015	NOT
TUSC5	1.055478	-0.3384846	0.541434	-0.62516296	0.531864	NA	NA
HLA-U	12.85434	0.29275224	0.468317	0.625115939	0.531895	0.746022	NOT
RP11-248M	12.51312	0.17118724	0.273887	0.625029805	0.531951	0.746035	NOT
RPS6KA4	1322.386	0.09055923	0.14489	0.625018768	0.531959	0.746035	NOT
BAD	2049.079	-0.1432704	0.229251	-0.62494909	0.532004	0.74606	NOT
KRT222	61.33696	0.32073298	0.513273	0.624877403	0.532052	0.746062	NOT
PAX2	5.945291	0.5442532	0.870995	0.624863771	0.53206	0.746062	NOT
AIFM2	2874.739	0.14235086	0.227841	0.624781126	0.532115	0.746071	NOT
LONP1	7871.931	0.12287013	0.196665	0.624770087	0.532122	0.746071	NOT
RP11-34P1	50.33557	0.29146838	0.466616	0.624642326	0.532206	0.746134	NOT
SLC36A4	421.1047	-0.1331995	0.213249	-0.62461792	0.532222	0.746134	NOT
RPL35	18288.66	0.16947944	0.271395	0.62447483	0.532316	0.746199	NOT
TBL1Y	17.38	0.64747352	1.036849	0.624462986	0.532324	0.746199	NOT
RP11-4K3_	6.694551	0.26103343	0.418064	0.624386085	0.532374	0.746231	NOT
RP3-355L5	6.848694	-0.2247529	0.359985	-0.62433891	0.532405	0.746236	NOT
TGM1	29.16975	0.22775489	0.364921	0.624121908	0.532548	0.746397	NOT

RP11-149I	0.49105	-0.6719026	1.076577	-0.62410995	0.532555	NA	NA
SPSB4	4.737344	0.35787746	0.573496	0.624028123	0.532609	0.746445	NOT
PCDHAC2	21.93628	-0.3887022	0.62308	-0.62383956	0.532733	0.746518	NOT
CTD-2035I	9.118616	0.2208135	0.353964	0.623829542	0.53274	0.746518	NOT
SMIM5	17.19229	-0.2304826	0.369468	-0.62382345	0.532744	0.746518	NOT
BEND7	771.8663	0.16881818	0.270676	0.623690201	0.532831	0.746602	NOT
AC004069.	4.009955	0.2470706	0.396188	0.623618975	0.532878	0.746629	NOT
RP11-697I	2.081501	0.77296174	1.239658	0.623528152	0.532938	0.746674	NOT
RP11-383I	5.251659	-0.2567014	0.411907	-0.62320186	0.533152	0.746899	NOT
TBX6	59.21731	-0.1666167	0.267357	-0.62319932	0.533154	0.746899	NOT
AC092198.	3.565308	0.47799511	0.767124	0.62310002	0.533219	0.746918	NOT
KCNMB2-AS	67.75786	0.39087626	0.627348	0.623061517	0.533244	0.746918	NOT
WASH6P	270.7309	-0.1154813	0.185347	-0.62305406	0.533249	0.746918	NOT
STAP1	9.353553	0.33566544	0.538833	0.622948626	0.533318	0.746958	NOT
RP11-465(	1.958326	0.54318161	0.872025	0.622897017	0.533352	0.746958	NOT
BRF1	1294.653	0.07667265	0.123093	0.622884347	0.533361	0.746958	NOT
SLC4A1APF	2.649303	0.25873476	0.415445	0.622788846	0.533423	0.747007	NOT
HAGLROS	11.5242	0.24776756	0.397901	0.622686905	0.53349	0.747038	NOT
OSTM1	1351.321	-0.1497605	0.240526	-0.62263822	0.533522	0.747038	NOT
RP11-90B9	1.914596	-0.2681566	0.430684	-0.62263017	0.533528	0.747038	NOT
LINC0031	5.222846	0.2212088	0.355315	0.622570403	0.533567	0.747054	NOT
SARM1	203.1058	-0.1679634	0.269899	-0.6223196	0.533732	0.747218	NOT
RNFT1	691.2943	0.13649194	0.219336	0.622295298	0.533748	0.747218	NOT
SNRNP35	1199.789	0.14337653	0.23041	0.622267094	0.533766	0.747218	NOT
CTA-963H5	40.05626	0.16832438	0.270532	0.622198261	0.533812	0.747242	NOT
NEK9	3144.967	-0.0899514	0.144587	-0.62212605	0.533859	0.74726	NOT
ZNF432	310.9669	-0.1500283	0.241166	-0.62209584	0.533879	0.74726	NOT
C12orf66	406.5203	-0.1186762	0.190837	-0.62187197	0.534026	0.747427	NOT
AP001505.	83.64272	0.19994027	0.32163	0.621647073	0.534174	0.747596	NOT
TPRA1	2089.2	0.0882798	0.142039	0.621519391	0.534258	0.74766	NOT
LAPTM4A	12866.28	0.08149973	0.131135	0.6214934	0.534275	0.74766	NOT
ACTR1B	2958.471	-0.0772536	0.124327	-0.62137539	0.534353	0.74773	NOT
CRIM1	2989.567	0.23212518	0.373695	0.621161865	0.534493	0.747801	NOT
KIF13A	2141.493	0.10954217	0.176358	0.621134647	0.534511	0.747801	NOT
SERPINA7	10733.34	-0.3457433	0.556705	-0.62105258	0.534565	0.747801	NOT
STK33	28.65857	0.42730711	0.688039	0.621050564	0.534566	0.747801	NOT
RP11-490(	1.329839	0.66326061	1.067983	0.621040434	0.534573	NA	NA
RHOBTB1	1068.912	0.28731708	0.462642	0.621035738	0.534576	0.747801	NOT
WDR24	845.0838	-0.1221199	0.196689	-0.62087903	0.534679	0.747801	NOT
LASP1	15016.49	0.10793987	0.173852	0.62087291	0.534683	0.747801	NOT
KCTD2	1505.781	-0.0991541	0.159705	-0.62085932	0.534692	0.747801	NOT
ASB6	898.284	0.10467824	0.168614	0.620816427	0.53472	0.747801	NOT
SNORD19	6.010334	0.22491413	0.362318	0.620764758	0.534754	0.747801	NOT
GALR3	20.8669	-0.3953473	0.636896	-0.62074041	0.53477	0.747801	NOT
AC142528.	23.36729	0.23722231	0.382163	0.620735597	0.534774	0.747801	NOT
IP6K3	429.253	-0.4153377	0.669112	-0.62073005	0.534777	0.747801	NOT
RP11-289I	1.562774	-0.7477037	1.20459	-0.62071196	0.534789	0.747801	NOT
MED31	199.158	-0.1120388	0.180513	-0.62066961	0.534817	0.747801	NOT
RPS11	38496.66	0.13781237	0.222093	0.620517347	0.534917	0.747903	NOT

RP11-53B2	2.492987	0.28676521	0.462191	0.620448019	0.534963	0.747928	NOT
CNOT3	1792.742	0.07609713	0.12267	0.62033805	0.535035	0.747978	NOT
TP53I3	1789.821	0.18707292	0.30158	0.620310045	0.535054	0.747978	NOT
KDM5B	1805.674	0.14106543	0.227491	0.620091517	0.535198	0.748126	NOT
PNKP	1400.541	0.11272594	0.181798	0.620059815	0.535218	0.748126	NOT
LMTK2	1820.242	0.12355567	0.199276	0.620023357	0.535242	0.748126	NOT
SYDE2	121.3992	0.19819621	0.319683	0.619976391	0.535273	0.748131	NOT
FNDC7	1.529163	0.45627351	0.736247	0.619729147	0.535436	0.748286	NOT
RP1-102E2	16.64778	0.18038361	0.291071	0.619724362	0.535439	0.748286	NOT
INTS1	6781.402	-0.1048687	0.169322	-0.61934488	0.535689	0.748596	NOT
RP11-132N	1.45784	-0.3872588	0.625324	-0.61929262	0.535724	0.748606	NOT
HAR1A	28.85974	0.47664201	0.769722	0.619238775	0.535759	0.748617	NOT
PLA2G2C	2.875424	0.29958772	0.483915	0.619091668	0.535856	0.748714	NOT
GRB14	1453.53	0.16499234	0.266558	0.618973672	0.535934	0.748784	NOT
BGN	13351.32	-0.2519676	0.407111	-0.6189162	0.535972	0.748785	NOT
COMMD4	3881.143	0.12200824	0.197149	0.618864252	0.536006	0.748785	NOT
HSPA9P1	8.103788	0.22709335	0.366963	0.618846025	0.536018	0.748785	NOT
SPATA13	1609.291	-0.1301162	0.210282	-0.6187706	0.536067	0.748816	NOT
TDRD10	121.9605	0.24898427	0.402418	0.618719835	0.536101	0.748825	NOT
KIZ-AS1	3.191634	0.32130082	0.519395	0.618605931	0.536176	0.748875	NOT
RP11-329F	8.42617	-0.2516229	0.406779	-0.6185743	0.536197	0.748875	NOT
AFAP1	609.4142	-0.2068136	0.334358	-0.6185398	0.53622	0.748875	NOT
ABCD4	1675.716	-0.0971088	0.157015	-0.61846879	0.536266	0.748901	NOT
CELA3A	3.145087	0.54331775	0.878568	0.618412997	0.536303	0.748914	NOT
CASP16	130.9777	0.2994637	0.484316	0.618322445	0.536363	0.748959	NOT
ANKRD54	1218.592	0.12474739	0.201787	0.618211675	0.536436	0.749019	NOT
EFTUD1	815.9685	0.09054203	0.146467	0.618173703	0.536461	0.749019	NOT
ARFRP1	2387.034	0.11813513	0.191132	0.618080741	0.536522	0.749055	NOT
HERPUD2	1397.638	-0.0870371	0.14083	-0.61802786	0.536557	0.749055	NOT
RP11-196C	17.66687	-0.1338628	0.216604	-0.61800813	0.53657	0.749055	NOT
SURF2	794.6059	0.16695224	0.270165	0.61796414	0.536599	0.749057	NOT
SNORA11	7.307735	0.17367152	0.281062	0.617912492	0.536633	0.749066	NOT
RAB28	391.7442	0.08508308	0.137705	0.617863204	0.536666	0.749073	NOT
RP1-68D18	1.463003	0.39832181	0.644752	0.617790516	0.536713	0.749102	NOT
LINC01561	1.128367	-0.5715204	0.925159	-0.61775389	0.536738	NA	NA
ZCCHC18	8.240979	-0.2437397	0.394588	-0.61770626	0.536769	0.749141	NOT
FAM96A	4028.434	-0.1342614	0.217389	-0.61760876	0.536833	0.749192	NOT
CISD2	2331.727	-0.0905005	0.146555	-0.61752067	0.536891	0.749234	NOT
RP11-19D2	62.83634	-0.3291826	0.533109	-0.61747665	0.53692	0.749236	NOT
NR1D1	1916.442	0.16367817	0.265133	0.617344496	0.537008	0.749318	NOT
RP11-206I	6.9584	-0.1959775	0.317479	-0.61729307	0.537041	0.749318	NOT
TRIM37	969.9715	0.09955821	0.161293	0.617251933	0.537069	0.749318	NOT
RB1CC1	3103.302	0.14088816	0.22827	0.617198425	0.537104	0.749318	NOT
AC079354.	2.751403	0.25507477	0.413292	0.617177836	0.537117	0.749318	NOT
USP51	104.6303	0.22844438	0.370205	0.617075125	0.537185	0.749323	NOT
CTC-273B1	3.097274	-0.3439281	0.557353	-0.61707374	0.537186	0.749323	NOT
TCFL5	540.2366	0.11215012	0.181753	0.617046555	0.537204	0.749323	NOT
HERC2P4	11.14994	-0.3833593	0.621323	-0.61700521	0.537231	0.749323	NOT
HGS	3404.136	0.10460685	0.169598	0.616792947	0.537371	0.74948	NOT

RP4-584D1	31.02546	0.23018998	0.373281	0.616666412	0.537455	0.749535	NOT
RP11-478J	76.85211	0.22773608	0.369312	0.616649541	0.537466	0.749535	NOT
RP5-1009E	3.17964	0.32088207	0.520454	0.616542649	0.537536	0.749595	NOT
CRTC3-AS1	9.174186	0.15352513	0.249034	0.616481386	0.537577	0.749612	NOT
RP11-172E	2.871476	-0.4564147	0.740419	-0.61642715	0.537613	0.749624	NOT
BANCR	1.02442	-0.5061137	0.821148	-0.61634906	0.537664	NA	NA
GLYAT	3202.187	-0.3852431	0.625126	-0.61626452	0.53772	0.749735	NOT
CYCS	6915.402	-0.1574045	0.255443	-0.61620195	0.537761	0.749754	NOT
MSRB3	454.0313	-0.1862124	0.302224	-0.61614122	0.537801	0.749761	NOT
SHF	1064.174	0.21627992	0.351041	0.616110995	0.537821	0.749761	NOT
AC142472.	56.5993	-0.14813	0.240476	-0.61598779	0.537903	0.749835	NOT
C1orf228	89.59705	-0.1892434	0.30733	-0.61576672	0.538048	0.749939	NOT
GP5	7.277202	-0.386936	0.628382	-0.61576594	0.538049	0.749939	NOT
ARNTL	598.597	-0.1476145	0.239731	-0.6157494	0.53806	0.749939	NOT
ZNF558	573.6083	0.16447677	0.267146	0.615681769	0.538105	0.749949	NOT
TMEM141	3754.854	0.18714544	0.304005	0.61560076	0.538158	0.749949	NOT
OLMALINC	563.3723	-0.1995611	0.32419	-0.61556781	0.53818	0.749949	NOT
PRDM6	47.26891	-0.2769144	0.449858	-0.61555984	0.538185	0.749949	NOT
SERPINB1	3733.528	0.1199919	0.194963	0.61546116	0.53825	0.749949	NOT
MEMO1	131.881	0.08565772	0.139186	0.615418677	0.538278	0.749949	NOT
RP3-462C1	3.075311	0.46906453	0.762201	0.615408304	0.538285	0.749949	NOT
RP5-1009E	8.895909	0.2784237	0.452424	0.615404177	0.538288	0.749949	NOT
ZFHX2	70.83062	-0.2213554	0.359738	-0.61532434	0.538341	0.749983	NOT
TRAPPC13F	15.52927	-0.4606002	0.748599	-0.61528326	0.538368	0.749983	NOT
AC092597.	1.749043	-0.3209919	0.521888	-0.6150585	0.538516	0.750151	NOT
UBE2W	1319.881	0.09539183	0.155112	0.614986563	0.538564	0.750179	NOT
LINC00997	163.5337	0.12078541	0.19645	0.614840442	0.53866	0.750223	NOT
RP11-30K9	13.72867	-0.4680725	0.761302	-0.61483197	0.538666	0.750223	NOT
MAP4K1	213.1688	-0.2020874	0.328704	-0.61479985	0.538687	0.750223	NOT
PIK3C2B	1315.796	-0.1269495	0.206499	-0.61477099	0.538706	0.750223	NOT
RP11-504A	15.56385	-0.1859177	0.302456	-0.6146932	0.538757	0.750256	NOT
KIAA1614	132.0154	0.20034881	0.32603	0.614511148	0.538878	0.750309	NOT
IPO11	781.8757	-0.0806773	0.131287	-0.61451084	0.538878	0.750309	NOT
PHTF1	471.5657	-0.1052915	0.171349	-0.61448644	0.538894	0.750309	NOT
AD000864.	15.75446	0.20099007	0.327096	0.614468183	0.538906	0.750309	NOT
CRB1	6.526645	-0.3723662	0.606079	-0.61438565	0.538961	0.750346	NOT
SAT2	6275.907	-0.1672102	0.272189	-0.61431703	0.539006	0.750347	NOT
S1PR4	71.05333	-0.2199767	0.358116	-0.61426083	0.539043	0.750347	NOT
CHMP1A	3408.795	-0.1002799	0.163255	-0.61425193	0.539049	0.750347	NOT
CNTN5	9.434173	-0.4094046	0.666551	-0.61421347	0.539074	0.750347	NOT
IFT74-AS1	1.798063	-0.3228024	0.52559	-0.61417169	0.539102	0.750347	NOT
RPL35P6	1.843108	0.3251431	0.529434	0.61413335	0.539127	0.750347	NOT
PAM	1351.959	-0.1914091	0.311723	-0.61403605	0.539192	0.750398	NOT
HCCS	1071.794	0.08305973	0.135286	0.613956906	0.539244	0.750433	NOT
MTUS2	26.08176	-0.4096391	0.667292	-0.61388268	0.539293	0.750451	NOT
ZNF319	478.6266	0.09453092	0.154001	0.613834025	0.539325	0.750451	NOT
HCG23	2.233806	-0.3750101	0.610953	-0.61381176	0.53934	0.750451	NOT
LGALS2	165.5095	0.2906249	0.473575	0.613682585	0.539425	0.750458	NOT
CEACAM5	3.043187	0.57167844	0.931558	0.613680084	0.539427	0.750458	NOT

MED14OS	20.89296	-0.1484579	0.241915	-0.6136786	0.539428	0.750458	NOT
HRC	111.3191	0.21735493	0.354233	0.613593546	0.539484	0.750497	NOT
LIG4	543.9059	-0.1456447	0.237385	-0.61353748	0.539521	0.75051	NOT
RP11-1114	6.024381	0.24825451	0.404703	0.613424412	0.539596	0.750575	NOT
SCAMP2	4081.773	0.06948291	0.113278	0.613383119	0.539623	0.750575	NOT
VPS16	1771.942	-0.0863979	0.140869	-0.61332137	0.539664	0.750585	NOT
STK35	1490.352	-0.1116348	0.182037	-0.61325431	0.539708	0.750585	NOT
ADRBK1	3509.964	0.07006347	0.114256	0.613214128	0.539735	0.750585	NOT
NKG7	273.8399	0.21932524	0.35767	0.61320538	0.539741	0.750585	NOT
RP11-480I	10.8247	0.25802489	0.420891	0.61304446	0.539847	0.750693	NOT
MBNL2	2683.604	-0.164009	0.267564	-0.61297219	0.539895	0.750693	NOT
AC006116.	1.760133	-0.3803758	0.620566	-0.61294943	0.53991	0.750693	NOT
APP	21252.2	0.18055678	0.294584	0.612920434	0.539929	0.750693	NOT
DNM3	94.36254	-0.1747697	0.28521	-0.61277548	0.540025	0.750788	NOT
ST6GALNAC	5.471391	0.2203743	0.359735	0.612601141	0.54014	0.75091	NOT
RP11-1102	2.171672	0.24281848	0.396502	0.61240225	0.540272	0.751041	NOT
POFUT2	1038.086	0.10139399	0.165575	0.612374603	0.54029	0.751041	NOT
AC009236.	1.43698	0.5568872	0.909507	0.612295496	0.540342	0.751049	NOT
VAV3	323.3752	0.24809171	0.405192	0.612282061	0.540351	0.751049	NOT
OR1N2	0.995428	1.58908645	2.596295	0.612059303	0.540499	NA	NA
CBS	367.303	-0.2353666	0.384637	-0.6119188	0.540591	0.751345	NOT
PIM3	2793.577	-0.1236531	0.202165	-0.61164402	0.540773	0.75146	NOT
OSCAR	81.13584	0.2017435	0.329841	0.611638775	0.540777	0.75146	NOT
TIMP4	21.83987	0.20941092	0.34238	0.611632199	0.540781	0.75146	NOT
RP11-530N	7.827089	-0.2933758	0.479666	-0.6116257	0.540785	0.75146	NOT
BEST4	23.88258	0.25335934	0.41442	0.611358128	0.540963	0.751668	NOT
OTUD7B	2129.935	0.09993319	0.163487	0.611259892	0.541028	0.75172	NOT
RP11-195I	5.730479	0.15790819	0.258355	0.611205372	0.541064	0.751731	NOT
R3HDM1	1083.957	0.09050763	0.148092	0.61115633	0.541096	0.751738	NOT
RP1-30M3.	80.14839	-0.1341919	0.219622	-0.61101372	0.541191	0.751811	NOT
FADS6	351.9479	0.3644731	0.596576	0.610941398	0.541238	0.751811	NOT
TF	344187.5	-0.2440693	0.399508	-0.61092502	0.541249	0.751811	NOT
ARSA	3611.125	0.16364728	0.267875	0.610908835	0.54126	0.751811	NOT
VWA9	1263.328	-0.0637699	0.1044	-0.61082214	0.541317	0.751812	NOT
RASSF1-AS	18.66559	-0.1878867	0.307643	-0.61072908	0.541379	0.751812	NOT
NANP	395.0177	0.09790415	0.160313	0.610704947	0.541395	0.751812	NOT
IGHV1-69-	36.20465	-0.445137	0.728924	-0.61067708	0.541413	0.751812	NOT
OACYLP	5.925427	0.25032792	0.409924	0.610669346	0.541419	0.751812	NOT
GAPDHP2	4.724788	-0.2234944	0.36601	-0.61062448	0.541448	0.751812	NOT
GUSBP5	12.14421	-0.2743694	0.449361	-0.61057654	0.54148	0.751812	NOT
APOL2	4104.715	-0.1457078	0.238641	-0.61057393	0.541482	0.751812	NOT
ATAD2B	519.5945	-0.0971488	0.159184	-0.61029261	0.541668	0.752032	NOT
SIRPAP1	5.191494	0.24169441	0.396084	0.61021042	0.541722	0.752069	NOT
MPRIP	3554.471	-0.1114324	0.182642	-0.6101134	0.541787	0.752099	NOT
RP4-717I2	2.907531	0.23459402	0.384549	0.610050319	0.541828	0.752099	NOT
AC011551.	2.53655	0.26292885	0.431039	0.609987899	0.54187	0.752099	NOT
TYMP	6319.707	0.20441321	0.335112	0.60998535	0.541872	0.752099	NOT
PRKRIRP3	2.160715	0.50324061	0.825028	0.609968056	0.541883	0.752099	NOT
R3HDML	4.909196	-0.3982843	0.653161	-0.60977988	0.542008	0.752208	NOT

DCAF8L1	44.34856	0.81425131	1.33535	0.609766056	0.542017	0.752208	NOT
DTD2	505.2615	0.09187448	0.150684	0.609717456	0.542049	0.752214	NOT
MYO1G	246.0292	0.20603153	0.337937	0.609674507	0.542077	0.752215	NOT
CTD-2026I	81.83305	-0.1307056	0.214442	-0.60951501	0.542183	0.752323	NOT
FOCAD-AS1	2.093483	-0.2850688	0.467746	-0.60945139	0.542225	0.752343	NOT
KRT86	40.19575	-0.282755	0.464022	-0.60935635	0.542288	0.752392	NOT
RP11-327J	2.695515	0.25540446	0.419219	0.609238807	0.542366	0.752462	NOT
KIAA2026	645.8794	-0.1138907	0.186993	-0.60906474	0.542482	0.752537	NOT
BRINP3	18.0359	-0.5869204	0.963771	-0.60898302	0.542536	0.752537	NOT
MYCBPAP	15.24717	0.23921647	0.392829	0.608958462	0.542552	0.752537	NOT
CLEC12A	34.69512	-0.228178	0.374711	-0.60894392	0.542562	0.752537	NOT
SRPK2	1145.977	0.1082255	0.177736	0.608910052	0.542584	0.752537	NOT
SNORA75	3.280659	-0.2387794	0.392153	-0.60889342	0.542595	0.752537	NOT
TOM1	5937.278	0.1259533	0.206866	0.608864394	0.542614	0.752537	NOT
RP11-137H	13.86296	0.25557468	0.419862	0.608710703	0.542716	0.75264	NOT
L1TD1	2.65223	-0.2606442	0.42831	-0.60854119	0.542829	0.752757	NOT
SMURF2P1	4.415436	-0.2547534	0.418751	-0.60836429	0.542946	0.752829	NOT
DCAF15	972.911	0.0880664	0.144772	0.608311146	0.542981	0.752829	NOT
KIAA1462	1291.976	0.17045519	0.280214	0.608303995	0.542986	0.752829	NOT
RP4-616B8	11.23069	-0.2387298	0.392457	-0.60829581	0.542991	0.752829	NOT
APLF	126.3617	0.14364942	0.236238	0.608070829	0.54314	0.752997	NOT
RP11-302F	5.735367	-0.2291472	0.376933	-0.60792598	0.543237	0.753004	NOT
RP11-701H	1.928538	0.44406606	0.730473	0.607916191	0.543243	0.753004	NOT
RP11-134C	19.43308	-0.2139839	0.352	-0.60790819	0.543248	0.753004	NOT
RP11-307I	8.636836	0.16254829	0.267395	0.60789639	0.543256	0.753004	NOT
CPSF7	3101.164	0.07110364	0.116978	0.607838729	0.543294	0.753018	NOT
GABPB2	606.6856	-0.1037621	0.170725	-0.60777363	0.543338	0.75304	NOT
BAZ1A	1093.402	0.11335834	0.18655	0.607657684	0.543415	0.753091	NOT
FETUB	3692.695	-0.3439071	0.565977	-0.60763437	0.54343	0.753091	NOT
HARS	2517.687	0.0957856	0.15765	0.607582781	0.543464	0.7531	NOT
RPAP2	608.324	0.09260158	0.152427	0.607512874	0.543511	0.753125	NOT
AP2S1	4253.23	-0.1250956	0.205965	-0.60736235	0.54361	0.753183	NOT
CNN2P1	14.49102	-0.2384409	0.392629	-0.60729341	0.543656	0.753183	NOT
MTMR3	221.4376	0.10191103	0.167813	0.607288027	0.54366	0.753183	NOT
RP11-430C	4.368484	0.28562807	0.470365	0.607248254	0.543686	0.753183	NOT
CRIP3	370.5543	0.2288776	0.376928	0.60721817	0.543706	0.753183	NOT
KLRC2	2.001235	-0.3215506	0.529563	-0.60719951	0.543719	0.753183	NOT
RORA-AS1	5.11021	-0.2088197	0.343945	-0.60713158	0.543764	0.753207	NOT
CTD-2114J	88.22566	-0.3114581	0.51305	-0.60707186	0.543803	0.753223	NOT
GSTM3	1270.642	0.26823655	0.441921	0.606978346	0.543865	0.75324	NOT
ARHGAP33	368.0318	0.16249728	0.267719	0.606969589	0.543871	0.75324	NOT
LINC00535	45.01833	0.28150849	0.463908	0.606820261	0.54397	0.753286	NOT
CDIPT	5552.73	-0.1101993	0.181602	-0.60681881	0.543971	0.753286	NOT
PDIA6	13084.89	0.12879773	0.212259	0.606794638	0.543987	0.753286	NOT
PSMA3-AS1	1222.576	-0.1128171	0.18597	-0.60664212	0.544088	0.753352	NOT
RP11-169K	3.668144	0.32158457	0.530109	0.606638306	0.544091	0.753352	NOT
PCBD1	8570.441	-0.1459327	0.240621	-0.60648365	0.544194	0.753456	NOT
BACH1-AS1	3.038448	0.23359089	0.385238	0.606354399	0.544279	0.753537	NOT
TANK	1513.285	0.10215179	0.168487	0.606287746	0.544324	0.753559	NOT

SUGP2	1528.163	-0.1029892	0.169889	-0.60621301	0.544373	0.75359	NOT
AC007319.	32.35093	-0.2041541	0.336935	-0.60591508	0.544571	0.753825	NOT
DCAF7	3672.504	-0.0882934	0.14574	-0.60582784	0.544629	0.753867	NOT
RP1-261D1	48.986	-0.1622864	0.267918	-0.60573066	0.544694	0.753906	NOT
YWHABP2	2.607622	0.23726079	0.391712	0.605701888	0.544713	0.753906	NOT
TENM1	217.8616	-0.369647	0.610325	-0.60565557	0.544743	0.75391	NOT
ZNF618	944.3044	-0.1569011	0.259079	-0.60561179	0.544773	0.753911	NOT
PFKL	9100.708	-0.1054713	0.174177	-0.60554026	0.54482	0.753939	NOT
RP11-321F	8.03525	0.19315616	0.319219	0.605089403	0.54512	0.754275	NOT
CTD-2132N	14.6025	-0.2099888	0.347045	-0.60507686	0.545128	0.754275	NOT
TULP4	952.8071	-0.1004544	0.166027	-0.60504935	0.545146	0.754275	NOT
RSPH10B	2.465642	0.27482064	0.454311	0.604917088	0.545234	0.754335	NOT
RP11-332H	1.772233	0.27783333	0.459304	0.604900411	0.545245	0.754335	NOT
DHX33	845.7506	-0.0991564	0.163962	-0.60475231	0.545344	0.754356	NOT
ZBTB46	614.1906	0.13483199	0.222972	0.6047024	0.545377	0.754356	NOT
NFRKB	1340.088	0.08146691	0.134728	0.604677937	0.545393	0.754356	NOT
ZBTB38	2663.315	-0.1615273	0.267134	-0.60466867	0.545399	0.754356	NOT
ADIRF-AS1	45.93126	-0.2728068	0.451168	-0.60466798	0.5454	0.754356	NOT
PHLDB1	961.6904	0.19116089	0.316205	0.604548202	0.545479	0.754377	NOT
NELL2	86.35285	-0.3220058	0.532668	-0.60451523	0.545501	0.754377	NOT
RP11-996F	31.49076	-0.1345615	0.222597	-0.60450825	0.545506	0.754377	NOT
RP1-140K8	2.580406	0.36124669	0.597618	0.60447759	0.545526	0.754377	NOT
SVIL-AS1	530.8136	0.14916097	0.246828	0.604311207	0.545637	0.754492	NOT
RP11-486C	46.32943	0.19944505	0.33014	0.604123448	0.545762	0.754626	NOT
KCTD1	297.4559	-0.1771435	0.293273	-0.60402146	0.545829	0.754681	NOT
RP11-344F	57.085	-0.2543737	0.421291	-0.60379503	0.54598	0.754846	NOT
RP11-84A1	1.787567	0.32891814	0.544806	0.60373409	0.54602	0.754846	NOT
PCDHGB6	56.47689	-0.2053114	0.34008	-0.60371604	0.546032	0.754846	NOT
TNFRSF10D	656.3454	-0.1758507	0.29135	-0.60357093	0.546129	0.754941	NOT
CTNS	977.9911	0.09204834	0.152537	0.603449445	0.54621	0.755006	NOT
RP11-554J	22.44013	-0.1391533	0.230609	-0.60341676	0.546231	0.755006	NOT
RBM27	832.4612	-0.0665957	0.11039	-0.60327824	0.546324	0.755095	NOT
BHLHE41	158.3781	0.26054923	0.431949	0.603194209	0.546379	0.755134	NOT
ATG4C	509.1215	-0.0817666	0.135584	-0.60307063	0.546462	0.755209	NOT
RP11-129B	6.969388	0.23587845	0.391366	0.60270505	0.546705	0.755507	NOT
SPATA41	155.8252	-0.2694471	0.447143	-0.60259739	0.546777	0.755567	NOT
TRBV19	9.621932	-0.2980863	0.49478	-0.60246266	0.546866	0.755652	NOT
HINT1	17107.17	0.12507188	0.207628	0.602384788	0.546918	0.755686	NOT
NKD1	1764.844	0.43213132	0.717614	0.602178029	0.547056	0.755837	NOT
HRH1	100.295	0.17295414	0.287285	0.602029695	0.547154	0.755885	NOT
RBM12B-AS	10.48305	-0.1652941	0.274595	-0.6019567	0.547203	0.755885	NOT
RP11-410E	7.487509	0.22582822	0.375172	0.60193304	0.547219	0.755885	NOT
RP11-316C	11.23379	-0.2104113	0.349569	-0.60191706	0.547229	0.755885	NOT
ST3GAL3	741.1352	-0.1059434	0.17601	-0.6019169	0.547229	0.755885	NOT
CXorf22	16.72829	-0.400118	0.664923	-0.60175105	0.54734	0.755982	NOT
ZBP1	46.54322	0.26836695	0.445994	0.601728081	0.547355	0.755982	NOT
PIGA	421.0463	-0.1096159	0.182354	-0.60111709	0.547762	0.756505	NOT
C20orf144	8.204648	0.20872287	0.347422	0.600775623	0.547989	0.756781	NOT
ATP5F1	6227.841	0.08934117	0.14878	0.600489999	0.54818	0.757005	NOT

AC003075.	34.95434	0.2014635	0.335596	0.600315566	0.548296	0.757127	NOT
RP11-414H	1.6779	0.44341832	0.738716	0.600255191	0.548336	0.757144	NOT
SPATA21	108.006	-0.3720983	0.619952	-0.60020484	0.54837	0.757152	NOT
GTF2F2	739.846	0.0947496	0.157882	0.600128243	0.548421	0.757183	NOT
LINC00265	40.72677	0.18023067	0.300405	0.599959158	0.548533	0.757294	NOT
SMTNL1	14.56774	0.28174435	0.469633	0.599924858	0.548556	0.757294	NOT
TRAV20	1.582296	-0.3476337	0.579601	-0.5997815	0.548652	0.757352	NOT
POLR2I	1188.557	0.1335915	0.222739	0.599768007	0.548661	0.757352	NOT
CYB5R1	4070.255	0.12486618	0.208206	0.59972305	0.548691	0.757352	NOT
RP4-639F2	517.2595	0.18361431	0.306214	0.599626583	0.548755	0.757352	NOT
WEE2-AS1	50.68965	-0.1673086	0.279035	-0.5995968	0.548775	0.757352	NOT
KIF7	176.6613	0.20970638	0.34976	0.599572097	0.548791	0.757352	NOT
ADH5P4	11.66462	-0.2207108	0.368128	-0.59954869	0.548807	0.757352	NOT
TC2N	536.2354	0.29995033	0.500334	0.599499833	0.54884	0.757352	NOT
NUAK2	783.3919	0.20645092	0.344381	0.599483932	0.54885	0.757352	NOT
PRND	51.22734	-0.3324903	0.554672	-0.59943639	0.548882	0.757358	NOT
BST2	9533.74	0.24176471	0.403366	0.599368178	0.548927	0.757382	NOT
CCR1	281.3288	0.22132884	0.369317	0.599292618	0.548978	0.757392	NOT
MGST2	5129.611	-0.1464067	0.244307	-0.59927295	0.548991	0.757392	NOT
CUL3	2036.226	0.07271835	0.121382	0.599087748	0.549114	0.757524	NOT
VAV1	258.8584	0.19469211	0.325027	0.59900287	0.549171	0.757564	NOT
LHFP	713.4325	-0.1575999	0.263131	-0.59894108	0.549212	0.757566	NOT
TM4SF20	411.2196	0.5262356	0.878683	0.598891573	0.549245	0.757566	NOT
FRS3	240.4792	0.08429632	0.140758	0.598875205	0.549256	0.757566	NOT
LCMT2	392.5362	-0.0933568	0.155922	-0.59873973	0.549346	0.757652	NOT
SLA	410.8465	-0.2059791	0.344094	-0.59861279	0.549431	0.75773	NOT
CTD-2555(	12.53578	-0.1429572	0.238874	-0.59846343	0.549531	0.757829	NOT
CNGA4	4.780282	-0.2102125	0.351284	-0.59841264	0.549565	0.757837	NOT
IGHV4-4	3.048076	-0.5614772	0.938908	-0.59801093	0.549833	0.758128	NOT
RP11-417E	7.315543	0.32647739	0.545964	0.597982829	0.549851	0.758128	NOT
KCNJ3	304.923	-0.397435	0.66464	-0.59797063	0.54986	0.758128	NOT
UBA7	2370.741	-0.1238741	0.207173	-0.59792542	0.54989	0.758131	NOT
DISC1	191.9664	-0.2211216	0.369888	-0.59780689	0.549969	0.758184	NOT
EDN1	170.9051	0.25510133	0.426744	0.59778479	0.549984	0.758184	NOT
HMOX1	3859.626	0.2148088	0.359425	0.597644927	0.550077	0.758274	NOT
KCNK3	49.91354	0.33289995	0.557074	0.597586644	0.550116	0.758289	NOT
FHL1	1036.122	0.20323379	0.340117	0.597540136	0.550147	0.758293	NOT
NPAS4	1.0873	-0.3930585	0.658067	-0.59729276	0.550312	NA	NA
SEC23B	2979.507	-0.0940461	0.157456	-0.59728662	0.550316	0.758488	NOT
NFE2L2	4156.162	-0.1082309	0.181245	-0.59715203	0.550406	0.758573	NOT
RLTPR	88.30639	-0.2266031	0.379604	-0.59694644	0.550543	0.75871	NOT
RP11-395E	14.50476	-0.3817203	0.639484	-0.59691934	0.550561	0.75871	NOT
FLJ45079	1.192316	-0.4147709	0.694997	-0.59679513	0.550644	NA	NA
ITGB1	11693.29	0.14074245	0.235902	0.59661327	0.550766	0.758866	NOT
CTD-2350(	108.878	-0.223672	0.374911	-0.59659982	0.550775	0.758866	NOT
RP11-517E	67.68254	0.11078677	0.185699	0.596594286	0.550778	0.758866	NOT
IGKC	6639.394	0.37952782	0.63617	0.596582829	0.550786	0.758866	NOT
ATL1	46.59807	-0.1670733	0.280089	-0.59649988	0.550841	0.758903	NOT
IGKV2-28	0.508811	0.92955238	1.559002	0.596248413	0.551009	NA	NA

RNU2-27P	5.507749	0.19382219	0.325081	0.596226731	0.551024	0.759048	NOT
RP11-21L2	562.4536	-0.2981808	0.500129	-0.59620816	0.551036	0.759048	NOT
N4BP2	747.6113	-0.1463558	0.245496	-0.59616251	0.551067	0.759048	NOT
LINC00323	3.526074	-0.3629973	0.608904	-0.59614865	0.551076	0.759048	NOT
RP1-122P2	8.262483	0.23044242	0.386583	0.596101019	0.551108	0.759048	NOT
IGKV1D-42	0.992685	0.63290251	1.061746	0.596096019	0.551111	NA	NA
KATNBL1	551.7274	0.0686282	0.11513	0.59609128	0.551114	0.759048	NOT
CTRB2	1.731129	0.51110164	0.857894	0.595763294	0.551333	0.75928	NOT
STAG3	210.4602	-0.199975	0.335686	-0.59571948	0.551363	0.75928	NOT
GPER1	1084.563	-0.2049353	0.344021	-0.59570562	0.551372	0.75928	NOT
DYNC2H1	118.046	0.22336728	0.374984	0.595672222	0.551394	0.75928	NOT
ADAM1B	12.28513	-0.1894956	0.318164	-0.59559187	0.551448	0.759315	NOT
HOOK3	1416.948	-0.1319205	0.221559	-0.59541892	0.551563	0.759414	NOT
MAX	1621.779	0.08177969	0.137353	0.595399911	0.551576	0.759414	NOT
ZNF385D-A	4.272369	-0.878127	1.474995	-0.59534217	0.551615	0.759429	NOT
WNT10B	14.52571	0.28843454	0.484759	0.595006339	0.551839	0.759685	NOT
CTD-2203K	22.81139	-0.1722793	0.289564	-0.59496012	0.55187	0.759685	NOT
TICAM2	4.616822	-0.2043154	0.343423	-0.59493799	0.551885	0.759685	NOT
RP5-857K2	177.6387	-0.194649	0.327231	-0.59483748	0.551952	0.759739	NOT
LA16c-358	3.976032	-0.2220481	0.373372	-0.59470974	0.552038	0.759818	NOT
ARRDC1-AS	471.7236	-0.1155842	0.194413	-0.594529	0.552158	0.759926	NOT
LINC00674	95.07099	0.11366633	0.191206	0.59447053	0.552197	0.759926	NOT
SSC5D	203.7562	-0.2844416	0.47851	-0.59443229	0.552223	0.759926	NOT
LDOC1L	485.1588	0.19102966	0.321369	0.594424749	0.552228	0.759926	NOT
CTD-2515C	2.823605	0.71231369	1.198681	0.59424805	0.552346	0.76005	NOT
CTD-2515H	17.15931	0.23777519	0.400215	0.594118432	0.552433	0.760131	NOT
NDUFAF5	583.1925	-0.0960046	0.161635	-0.59395974	0.552539	0.760174	NOT
ZNF664	5018.058	0.08813441	0.148387	0.593947889	0.552547	0.760174	NOT
LINC00969	785.0896	-0.1243208	0.209327	-0.59390661	0.552575	0.760174	NOT
TLX1NB	2.964219	0.42215135	0.710807	0.593904098	0.552576	0.760174	NOT
RP4-730K3	11.07542	0.19954712	0.336032	0.593833852	0.552623	0.7602	NOT
RRM2B	1827.542	-0.1133004	0.19084	-0.59369321	0.552717	0.760289	NOT
RPL21P28	48.03779	0.21279403	0.358457	0.593638805	0.552754	0.760289	NOT
OTOF	17.46764	0.26365085	0.444181	0.593566039	0.552802	0.760289	NOT
RP11-327F	4.82879	-0.2274245	0.383154	-0.59355902	0.552807	0.760289	NOT
NOMO1	2017.43	-0.0940613	0.158489	-0.59348712	0.552855	0.760289	NOT
HDHD2	897.0487	-0.1037793	0.174864	-0.59348567	0.552856	0.760289	NOT
RNASEK	292.4773	-0.1078666	0.181819	-0.59326185	0.553006	0.760425	NOT
RP3-395M2	349.6696	0.22109001	0.372673	0.593254136	0.553011	0.760425	NOT
UGT2B17	966.1972	-0.4780216	0.805844	-0.5931935	0.553052	0.760443	NOT
NID2	381.3566	-0.2187332	0.368939	-0.59287107	0.553267	0.760652	NOT
PCOLCE	4316.054	-0.2872341	0.48454	-0.59279782	0.553316	0.760652	NOT
NPIPB15	174.1279	-0.2661419	0.448978	-0.59277269	0.553333	0.760652	NOT
ARHGAP29	2155.127	-0.1422747	0.24002	-0.59276301	0.55334	0.760652	NOT
ATP6AP2	5132.395	0.07482066	0.12623	0.592733716	0.553359	0.760652	NOT
MPPED2	68.95913	0.27268981	0.460069	0.592714712	0.553372	0.760652	NOT
SNX4	2232.49	0.08773894	0.148047	0.592642744	0.55342	0.760665	NOT
PRDX6	27518.52	0.11947738	0.201623	0.592577792	0.553464	0.760665	NOT
EEF1A1P38	9.226214	-0.2041045	0.344442	-0.59256491	0.553472	0.760665	NOT

ZACN	21.45291	0.16773811	0.283087	0.59253277	0.553494	0.760665	NOT
AC106786.	5.425393	0.35823089	0.604736	0.592375365	0.553599	0.760771	NOT
FER	415.3869	-0.1245258	0.210256	-0.59225861	0.553677	0.760793	NOT
IKBKG	540.5938	0.16852383	0.284552	0.592243236	0.553688	0.760793	NOT
POLR2J	2219.369	-0.1309547	0.221134	-0.59219607	0.553719	0.760793	NOT
ST3GAL5-A	3.931331	0.22300998	0.376589	0.592184267	0.553727	0.760793	NOT
PPP1R3F	321.5957	-0.205982	0.347928	-0.59202507	0.553834	0.760901	NOT
AC079610.	1.346825	0.39159809	0.661618	0.59187949	0.553931	NA	NA
HMGB1P8	4.069876	0.18417156	0.311175	0.591858066	0.553946	0.761016	NOT
OSBPL8	1463.236	-0.1033076	0.174573	-0.59177368	0.554002	0.761055	NOT
RP11-276H	19.10883	0.57626444	0.973904	0.591705351	0.554048	0.761079	NOT
PIN4	1081.801	-0.1023726	0.173102	-0.59139933	0.554253	0.761323	NOT
PSMD7P1	2.499044	-0.3202359	0.541539	-0.59134363	0.55429	0.761335	NOT
SOX12	1886.549	0.19590397	0.331439	0.591070537	0.554473	0.761548	NOT
PSEN1	2548.99	0.07475647	0.126511	0.590909289	0.554581	0.761658	NOT
WDR11-AS1	3.532879	0.24742413	0.418828	0.590753346	0.554686	0.761763	NOT
ST6GALNAC	12.22594	0.29491097	0.499259	0.590697402	0.554723	0.761776	NOT
DCST2	80.79849	0.19326638	0.327227	0.590619134	0.554776	0.761809	NOT
RP11-573C	1.798004	-0.2632438	0.445802	-0.5904952	0.554859	0.761859	NOT
AC079305.	1.502793	0.46410794	0.785983	0.5904808	0.554868	0.761859	NOT
RP11-503E	6.153252	-0.2141848	0.362845	-0.59029283	0.554994	0.761954	NOT
COX6C	13607.95	0.18780742	0.318173	0.590268795	0.55501	0.761954	NOT
PTPN11	4931.153	-0.0993175	0.168263	-0.59025219	0.555022	0.761954	NOT
CTA-126B4	1.187917	0.34482539	0.584297	0.590154046	0.555087	NA	NA
DMD	1788.271	-0.1792682	0.303831	-0.59002644	0.555173	0.762123	NOT
TCAF1P1	32.1087	-0.2506809	0.424922	-0.58994572	0.555227	0.762152	NOT
RP11-63E9	1.782773	0.5261036	0.891835	0.589911666	0.55525	0.762152	NOT
NT5C2	684.5909	-0.1439579	0.244139	-0.58965428	0.555422	0.762346	NOT
CTD-2368P	98.35928	0.19754155	0.335059	0.589572895	0.555477	0.762346	NOT
RP11-337C	152.696	0.12213039	0.207152	0.589568333	0.55548	0.762346	NOT
KRIT1	907.3819	0.10730369	0.182015	0.589533036	0.555504	0.762346	NOT
ZNF703	927.2207	0.22657845	0.384402	0.589431115	0.555572	0.762396	NOT
KRBA2	9.137238	-0.1997511	0.338909	-0.58939436	0.555597	0.762396	NOT
NUDT2	885.2791	0.11684467	0.198313	0.589194548	0.555731	0.762499	NOT
B3GALT4	169.4173	-0.2046257	0.347315	-0.58916386	0.555751	0.762499	NOT
TRAV14DV4	2.441508	0.38379176	0.651497	0.589091949	0.5558	0.762499	NOT
HCFC1R1	1719.802	-0.1504209	0.255349	-0.58907841	0.555809	0.762499	NOT
PAGR1	343.0329	0.094282	0.160051	0.589073671	0.555812	0.762499	NOT
FAM76A	343.05	-0.0772519	0.13117	-0.58894604	0.555897	0.762578	NOT
KLHL35	17.83275	-0.2793849	0.474455	-0.58885408	0.555959	0.762604	NOT
TRAV8-2	4.938587	0.30725037	0.521795	0.588833416	0.555973	0.762604	NOT
AZI2	644.6863	0.0986981	0.167656	0.588694732	0.556066	0.762688	NOT
FAM83A	29.48889	0.32723196	0.555897	0.588655971	0.556092	0.762688	NOT
DUSP22	1484.222	0.09245326	0.157069	0.588617127	0.556118	0.762688	NOT
TMED4	4106.962	-0.0764965	0.129985	-0.58850394	0.556194	0.762753	NOT
DEF6	337.8403	0.18308676	0.311155	0.588409845	0.556257	0.762781	NOT
CNDP2	10249.26	-0.1159485	0.197061	-0.58839005	0.556271	0.762781	NOT
CD163L1	305.6748	-0.2130594	0.362219	-0.58820657	0.556394	0.762835	NOT
EMX2OS	2.226396	-0.3029085	0.514973	-0.58820257	0.556396	0.762835	NOT

AC016700.	4.221126	0.22280239	0.378854	0.588095707	0.556468	0.762835	NOT
GMIP	527.306	0.14407212	0.24499	0.588072817	0.556483	0.762835	NOT
AC021087.	2.707876	-0.3567317	0.606615	-0.588069	0.556486	0.762835	NOT
AC010518.	0.815905	0.52013771	0.884497	0.588060381	0.556492	NA	NA
PCDHGA10	89.9635	-0.2168939	0.368843	-0.58803887	0.556506	0.762835	NOT
RAB9A	828.1671	0.09374805	0.159425	0.588037675	0.556507	0.762835	NOT
KISS1	107.2969	-0.280369	0.47697	-0.58781309	0.556658	0.763003	NOT
RP11-11N5	1.453558	0.53074867	0.903489	0.587443254	0.556906	0.763288	NOT
LPIN3	887.5415	0.1380677	0.235041	0.587419394	0.556922	0.763288	NOT
TUBBP9	3.813908	0.35347877	0.601899	0.587272651	0.557021	0.763385	NOT
PLAA	1604.346	-0.0865971	0.147509	-0.58706484	0.55716	0.763521	NOT
RXRB	2210.537	0.07873665	0.134125	0.587040806	0.557176	0.763521	NOT
SLC5A1	20.62387	-0.3813976	0.649933	-0.58682623	0.55732	0.763664	NOT
RP1-197B1	15.58797	-0.2079693	0.354411	-0.58680215	0.557337	0.763664	NOT
AP4S1	249.8271	-0.1088047	0.185479	-0.58661341	0.557463	0.763799	NOT
OSER1	1525.062	0.08032148	0.136956	0.586476773	0.557555	0.763855	NOT
LINC00493	1724.299	0.09556453	0.162949	0.586468502	0.557561	0.763855	NOT
TTC21A	88.69443	-0.1377105	0.234864	-0.58634129	0.557646	0.763934	NOT
RP11-513(	3.724351	0.42730704	0.729113	0.586064575	0.557832	0.764095	NOT
GFPT1	4080.301	-0.0895882	0.152865	-0.58606155	0.557834	0.764095	NOT
MEMO1P1	42.03567	0.14428274	0.246199	0.586040361	0.557848	0.764095	NOT
CTB-3601.	6.438807	0.3273034	0.558791	0.585734773	0.558054	0.764308	NOT
PCMTD1	1709.769	-0.0984549	0.1681	-0.58569149	0.558083	0.764308	NOT
RP11-545I	3.670872	0.28102708	0.479866	0.585635992	0.55812	0.764308	NOT
CTD-2619J	80.80665	-0.2735629	0.467134	-0.58561921	0.558131	0.764308	NOT
SOX11	17.35825	0.37592098	0.642056	0.585495353	0.558215	0.764308	NOT
CTD-3148I	1.996154	-0.3195392	0.545824	-0.58542527	0.558262	0.764308	NOT
ARHGAP5	2275.743	-0.1132897	0.193519	-0.58541891	0.558266	0.764308	NOT
CHRDL2	494.1309	-0.2775478	0.474118	-0.58539852	0.55828	0.764308	NOT
NINL	883.8556	0.16502915	0.281922	0.585372392	0.558297	0.764308	NOT
SPP2	2430.827	-0.3424897	0.585082	-0.58537051	0.558299	0.764308	NOT
SSX1	580.7787	0.72369389	1.236349	0.585347464	0.558314	0.764308	NOT
RP11-172I	3.498973	-0.2021124	0.345316	-0.58529748	0.558348	0.764316	NOT
SNX32	20.23168	0.17284735	0.29537	0.585188342	0.558421	0.764378	NOT
ARL14EP	817.8861	0.0585299	0.100067	0.584908393	0.558609	0.764596	NOT
ZBTB49	149.8008	-0.0790621	0.135179	-0.58486765	0.558637	0.764596	NOT
MTHFR	1180.866	-0.115359	0.197277	-0.58475595	0.558712	0.764657	NOT
UGT1A2P	122.0678	-0.4854725	0.830269	-0.5847169	0.558738	0.764657	NOT
BPIFB4	9.908578	0.41450726	0.709042	0.584601914	0.558815	0.764705	NOT
ACTBP7	2.751479	0.25818732	0.441681	0.58455564	0.558847	0.764705	NOT
FDPSP1	2.474301	0.26247681	0.44906	0.584502198	0.558882	0.764705	NOT
ATP2A1	45.42091	-0.1834948	0.313936	-0.58449733	0.558886	0.764705	NOT
CTB-75G16	6.7339	0.14206832	0.243108	0.58438472	0.558962	0.764747	NOT
RP11-789(	1.681904	-0.3504635	0.59973	-0.58436841	0.558972	0.764747	NOT
PARP11	252.8669	0.12508169	0.214061	0.584326419	0.559001	0.764747	NOT
ADD2	26.81384	-0.2520768	0.431528	-0.58414937	0.55912	0.764871	NOT
TTC13	895.5562	0.10567189	0.180923	0.584072205	0.559172	0.764903	NOT
ALS2CR11	8.487498	-0.2196606	0.376178	-0.58392684	0.55927	0.764999	NOT
SNURF	10.44911	0.26248186	0.449548	0.583879001	0.559302	0.765004	NOT

EEF1A1P4	16.05344	0.20430679	0.350036	0.583674257	0.559439	0.765154	NOT
KAT5	956.2712	0.06953018	0.119178	0.583412908	0.559615	0.765349	NOT
ICE2	996.4475	-0.0803905	0.137802	-0.58337838	0.559639	0.765349	NOT
GOLGA6L1(	13.28951	0.16084024	0.275894	0.5829786	0.559908	0.765679	NOT
TMEM223	1147.378	0.10932436	0.187587	0.582792932	0.560033	0.765811	NOT
KIAA1328	161.239	0.08533237	0.146433	0.582739793	0.560068	0.765821	NOT
RP11-182J	2.745939	-0.1769557	0.303773	-0.58252692	0.560212	0.76597	NOT
GYG2P1	27.16997	0.54470272	0.935122	0.582493889	0.560234	0.76597	NOT
RP11-410I	1.726652	0.26829959	0.460684	0.582394221	0.560301	0.766024	NOT
DDX39B-AS	5.443478	0.24150525	0.414707	0.58235147	0.56033	0.766024	NOT
AC010136.	8.056894	-0.330511	0.567944	-0.58194322	0.560605	0.766362	NOT
MDGA1	398.0068	-0.3059452	0.525782	-0.58188639	0.560643	0.766375	NOT
RP11-691N	3.850853	-0.2699064	0.464105	-0.58156322	0.560861	0.766634	NOT
AHDC1	839.1306	-0.1053976	0.181279	-0.58140993	0.560964	0.766705	NOT
CTD-2256F	2.17749	-0.2521341	0.433677	-0.58138703	0.56098	0.766705	NOT
HOMER2P1	11.01478	0.74215415	1.276582	0.581360505	0.560998	0.766705	NOT
SREBF2	9207.118	0.11813501	0.20322	0.581317136	0.561027	0.766706	NOT
WDR86-AS1	11.67233	-0.2648472	0.455769	-0.58109971	0.561173	0.766864	NOT
FAT3	24.1352	0.27311525	0.470028	0.581062106	0.561199	0.766864	NOT
RP11-539I	19.04291	-0.1508411	0.25968	-0.580873	0.561326	0.766965	NOT
RP11-466F	10.24389	-0.2065975	0.35567	-0.58086828	0.561329	0.766965	NOT
CHCHD10	6879.081	-0.2070374	0.356496	-0.58075694	0.561404	0.767029	NOT
HORMAD1	3.818012	-0.3277396	0.564393	-0.58069359	0.561447	0.767049	NOT
MTAP	1237.909	0.12560927	0.216355	0.580570313	0.56153	0.767102	NOT
RP11-100E	3.282084	0.29354555	0.505631	0.580552463	0.561542	0.767102	NOT
CCNH	881.7591	-0.0850442	0.146515	-0.58044892	0.561612	0.767158	NOT
FBXO28	1486.925	-0.0823598	0.141937	-0.58025495	0.561743	0.767298	NOT
TTC5	320.5292	0.09085002	0.156596	0.580153984	0.561811	0.767316	NOT
RPL24P8	19.14531	0.22614202	0.389798	0.580151952	0.561812	0.767316	NOT
RP11-168J	4.732661	0.19267751	0.332218	0.579972708	0.561933	0.767419	NOT
NSUN3	316.2137	-0.0937852	0.161711	-0.57995592	0.561944	0.767419	NOT
NUP214	2712.092	-0.0776394	0.133882	-0.57991101	0.561975	0.767422	NOT
GALNT13	11.29033	-0.3199525	0.551829	-0.57980387	0.562047	0.767482	NOT
PHC1	112.0044	0.15605154	0.269266	0.579544017	0.562222	0.767619	NOT
MFSD12	2120.377	0.1107776	0.191155	0.579518218	0.56224	0.767619	NOT
RP11-396M	10.41553	-0.1767738	0.305055	-0.57948126	0.562264	0.767619	NOT
LRRFIP1	3617.629	-0.0855677	0.147664	-0.57947471	0.562269	0.767619	NOT
NOMO2	431.6108	-0.1621052	0.279759	-0.57944553	0.562289	0.767619	NOT
SLC10A4	3.533657	0.31595362	0.545411	0.579294705	0.56239	0.76771	NOT
CBWD7	63.92203	0.13912567	0.240177	0.579262814	0.562412	0.76771	NOT
URB1-AS1	180.0961	-0.1269529	0.219252	-0.57902781	0.56257	0.767881	NOT
AGBL5-AS1	2.306695	0.27250934	0.47066	0.578993608	0.562593	0.767881	NOT
PPP5C	2495.905	0.06811987	0.117667	0.578921727	0.562642	0.767908	NOT
CTC-459F4	13.30719	-0.2138217	0.369413	-0.57881478	0.562714	0.767964	NOT
CTD-2516F	54.95022	-0.1459954	0.252248	-0.57877688	0.56274	0.767964	NOT
DMTF1	1064.926	0.09950432	0.171969	0.578616765	0.562848	0.768073	NOT
UPP1	918.4213	-0.1478645	0.255664	-0.57835421	0.563025	0.768276	NOT
RP11-177(	0.716859	-0.4240316	0.733259	-0.57828354	0.563073	NA	NA
RP11-186F	2.082948	0.42756971	0.739442	0.578233135	0.563107	0.768349	NOT

AHCYP2	7.68198	-0.2692712	0.465775	-0.57811384	0.563187	0.768363	NOT
LHX9	77.37188	-0.3553266	0.614667	-0.57807957	0.56321	0.768363	NOT
ZNF454	13.83702	-0.2425543	0.419608	-0.57804992	0.56323	0.768363	NOT
NKAIN4	3.644394	-0.3652119	0.631803	-0.57804711	0.563232	0.768363	NOT
MIR210	1.625207	0.3095308	0.535559	0.577958609	0.563292	0.768363	NOT
BFAR	2127.028	0.0626632	0.108422	0.577954662	0.563295	0.768363	NOT
PAIP1P1	10.98254	-0.1890382	0.327099	-0.57792389	0.563316	0.768363	NOT
SEC22C	1130.99	0.06739184	0.11663	0.57782374	0.563383	0.768417	NOT
TSPAN11	58.27945	-0.2691417	0.465824	-0.57777607	0.563415	0.768422	NOT
RP11-508N	1.369969	0.41351741	0.715918	0.577604461	0.563531	NA	NA
ZFP57	8.425435	0.42766621	0.740455	0.577572486	0.563553	0.768537	NOT
BGLAP	31.64404	0.19871174	0.344049	0.577567971	0.563556	0.768537	NOT
MSTO2P	105.9877	-0.1775294	0.307416	-0.57748882	0.563609	0.768571	NOT
TMEM128	553.3185	0.07878853	0.136531	0.577074054	0.563889	0.768877	NOT
RP11-462I	1.658654	0.40659303	0.704607	0.577049152	0.563906	0.768877	NOT
RP11-677I	5.584148	-0.1902307	0.329672	-0.57703031	0.563919	0.768877	NOT
RGL3	781.722	0.17109469	0.29655	0.57695095	0.563973	0.768912	NOT
RBAK-RBAK	3.482415	0.29494649	0.511291	0.576866648	0.56403	0.768951	NOT
ZNF793-AS	38.27453	0.28913618	0.501279	0.57679687	0.564077	0.768976	NOT
IRF7	1760.16	-0.1732991	0.300502	-0.57669872	0.564143	0.769028	NOT
LMO7-AS1	11.50627	-0.3115565	0.540504	-0.57641848	0.564332	0.769221	NOT
PTPN18	2366.228	-0.072106	0.125101	-0.57638364	0.564356	0.769221	NOT
SLC22A2	5.849404	-0.310544	0.538799	-0.57636317	0.56437	0.769221	NOT
UBA6	1353.904	-0.0826247	0.143385	-0.57624217	0.564452	0.769294	NOT
RNF208	515.9718	0.16583239	0.287905	0.575997145	0.564617	0.769481	NOT
RP1-257C2	10.14936	0.33226421	0.577004	0.575843459	0.564721	0.769571	NOT
POLR2E	8659.7	0.09939664	0.172633	0.575768778	0.564771	0.769571	NOT
RP13-152C	6.09491	0.23878423	0.414745	0.575737765	0.564792	0.769571	NOT
ZNF615	207.8055	-0.1392815	0.241921	-0.57573158	0.564797	0.769571	NOT
HEATR5B	970.0665	-0.0983454	0.170866	-0.57556845	0.564907	0.769683	NOT
ANKRD37	215.923	-0.1979836	0.34403	-0.57548381	0.564964	0.769693	NOT
PGS1	751.7925	0.06902157	0.119939	0.575473953	0.564971	0.769693	NOT
TMEM168	891.6797	0.10089837	0.175353	0.575402462	0.565019	0.76972	NOT
C10orf25	186.4288	-0.1184553	0.205907	-0.57528478	0.565099	0.76979	NOT
AC012485.	8.095047	-0.2398823	0.417049	-0.57519036	0.565163	0.769824	NOT
SIGLEC1	617.8847	-0.2048639	0.35621	-0.57512144	0.565209	0.769824	NOT
SYS1-DBNI	5.390147	0.15336587	0.266683	0.575087669	0.565232	0.769824	NOT
RP11-351J	61.04279	-0.2263944	0.393727	-0.57500321	0.565289	0.769824	NOT
CASP6	757.2908	-0.099852	0.173669	-0.57495729	0.56532	0.769824	NOT
NRL	116.3263	0.12982374	0.225814	0.574914042	0.565349	0.769824	NOT
MMP23B	6.198654	-0.2738774	0.476404	-0.5748852	0.565369	0.769824	NOT
USP45	243.4643	-0.1151397	0.200284	-0.57488078	0.565372	0.769824	NOT
PGM2L1	186.8448	0.13345793	0.232153	0.574869426	0.56538	0.769824	NOT
XXyac-YX1	4.123192	0.35556222	0.618686	0.574705799	0.56549	0.769936	NOT
RP11-712I	79.4388	-0.0870427	0.151477	-0.57462629	0.565544	0.769965	NOT
RP11-650I	2.344495	-0.2484645	0.432422	-0.57458784	0.56557	0.769965	NOT
CAMSAP1	957.7709	-0.0852798	0.14843	-0.57454611	0.565598	0.769965	NOT
CHKB-CPT1	35.44829	0.19927008	0.346868	0.574483694	0.565641	0.769965	NOT
TSTD1	1750.384	0.18882713	0.328701	0.574464397	0.565654	0.769965	NOT

RP11-424(	13.38524	-0.3928357	0.683916	-0.57439156	0.565703	0.769994	NOT
GHRHR	38.49002	-0.3683116	0.641342	-0.57428254	0.565777	0.770056	NOT
HNRNPA1P3	7.728001	0.15644065	0.272434	0.574233415	0.56581	0.770062	NOT
UBA5	1769.258	-0.078132	0.136079	-0.57416451	0.565856	0.770067	NOT
EIF4H	7629.236	-0.0737018	0.128368	-0.57414444	0.56587	0.770067	NOT
RNF217-AS	13.3167	-0.2381557	0.414836	-0.57409562	0.565903	0.770068	NOT
FAM110B	499.2726	0.18055088	0.314516	0.574059948	0.565927	0.770068	NOT
RP11-285A	2.425474	0.24449184	0.426053	0.573852565	0.566068	0.77022	NOT
ANKRD20A1	2.305323	0.66677695	1.162271	0.573684435	0.566181	0.770336	NOT
HMGB3	3227.979	-0.1532887	0.267263	-0.57355012	0.566272	0.770421	NOT
LINC01192	1.716148	0.59311661	1.034556	0.57330545	0.566438	0.770558	NOT
FEZ2	1524.817	0.11713084	0.204316	0.573283914	0.566452	0.770558	NOT
SULT2B1	44.94901	0.36139692	0.630407	0.573275814	0.566458	0.770558	NOT
POU2F2	117.1412	-0.1650809	0.288035	-0.57312775	0.566558	0.770655	NOT
SYMPK	4922.136	0.08181657	0.142773	0.57305492	0.566608	0.770684	NOT
PNPLA6	2707.585	-0.0883066	0.154118	-0.57298065	0.566658	0.770714	NOT
PTPN2	970.328	0.0920009	0.160599	0.57286071	0.566739	0.770786	NOT
TPRG1L	4070.829	-0.1312301	0.229098	-0.57281162	0.566772	0.770786	NOT
BATF3	57.73575	-0.1811162	0.316224	-0.57274697	0.566816	0.770786	NOT
FAM66D	1.936085	0.36076373	0.629897	0.572734525	0.566824	0.770786	NOT
ANKRD1	179.8626	0.34657716	0.605234	0.572632885	0.566893	0.770841	NOT
RP11-263K	18.37778	-0.1825169	0.318895	-0.57234162	0.567091	0.77107	NOT
GSTA6P	2.604109	0.25032996	0.437472	0.572219348	0.567173	0.771111	NOT
CD2	326.2386	0.22599155	0.394973	0.572169953	0.567207	0.771111	NOT
KRT85	9.870459	0.39752437	0.694812	0.572132154	0.567232	0.771111	NOT
RP11-206I	5.632253	-0.1564331	0.273423	-0.5721293	0.567234	0.771111	NOT
RP11-362J	2.270115	-0.2589359	0.45275	-0.57191857	0.567377	0.771267	NOT
TCEA2	2461.168	-0.1707736	0.298665	-0.57179045	0.567464	0.771344	NOT
RP11-970I	23.24382	-0.1473001	0.257671	-0.57165892	0.567553	0.771344	NOT
ABLIM3	3355.719	-0.1607642	0.28124	-0.5716263	0.567575	0.771344	NOT
TSPAN32	41.39776	-0.1975729	0.345634	-0.57162404	0.567577	0.771344	NOT
RP11-1148	109.34	0.12346443	0.215991	0.571617791	0.567581	0.771344	NOT
BCAM	9397.241	-0.1756368	0.307281	-0.57158306	0.567604	0.771344	NOT
SLC16A2	3462.736	-0.2182663	0.381897	-0.57153138	0.567639	0.771347	NOT
SLC22A11	1139.316	0.4454252	0.779421	0.571482345	0.567673	0.771347	NOT
SEC11C	5598.87	-0.1464372	0.256254	-0.57145349	0.567692	0.771347	NOT
SYTL1	174.7136	0.20151283	0.352678	0.571379511	0.567742	0.771377	NOT
TSPAN8	4153.622	-0.2917906	0.510783	-0.57126133	0.567823	0.771401	NOT
PROB1	90.38239	0.14893914	0.260727	0.571245338	0.567833	0.771401	NOT
GPR34	147.5361	0.18908846	0.331021	0.571227624	0.567845	0.771401	NOT
RP11-455J	4.890626	0.2337048	0.409204	0.571120092	0.567918	0.771405	NOT
RP11-565F	33.88707	0.14688003	0.257202	0.571067853	0.567954	0.771405	NOT
DDX28	662.587	-0.1027965	0.180011	-0.57105774	0.567961	0.771405	NOT
OSTN-AS1	22.79517	-0.4222433	0.739416	-0.57105004	0.567966	0.771405	NOT
TGM3	1354.065	0.37137304	0.650375	0.571013445	0.567991	0.771405	NOT
CTD-2213F	12.89273	0.1255417	0.219877	0.57096315	0.568025	0.771413	NOT
PLAG1	124.4762	0.23197771	0.406327	0.57091426	0.568058	0.771419	NOT
CASC22	3.271245	-0.4576099	0.8017	-0.57079975	0.568135	0.771486	NOT
CTD-2506J	1.153312	0.41011362	0.718593	0.570717788	0.568191	NA	NA

LINC0092(	46.93009	-0.1959651	0.343436	-0.57060176	0.56827	0.771629	NOT
PIK3R6	86.9009	0.16027661	0.280972	0.570435766	0.568382	0.771706	NOT
GTF2IRD1I	7.034313	-0.2280944	0.39986	-0.57043494	0.568383	0.771706	NOT
RP11-488I	426.3367	0.17818122	0.312435	0.570299342	0.568475	0.771792	NOT
HK1	1305.069	-0.2082067	0.36538	-0.56983617	0.568789	0.77218	NOT
LINC0100I	12.34025	-0.1762365	0.309342	-0.56971481	0.568871	0.772253	NOT
PEX3	671.4246	-0.1310912	0.230141	-0.56961135	0.568941	0.772268	NOT
ST5	1896.669	-0.1316828	0.231211	-0.56953513	0.568993	0.772268	NOT
HSD17B8	1803.181	-0.1628436	0.285934	-0.56951448	0.569007	0.772268	NOT
HES4	252.1981	-0.2218296	0.389517	-0.56949892	0.569018	0.772268	NOT
NFATC4	358.5088	0.19057153	0.334636	0.569488948	0.569024	0.772268	NOT
TTLL7	275.9665	-0.229066	0.402331	-0.56934746	0.56912	0.772359	NOT
RPL7AP64	1.872817	0.33499371	0.588485	0.569247975	0.569188	0.772412	NOT
MRPS22	1727.377	-0.070421	0.123729	-0.56915718	0.569249	0.772433	NOT
VSIG4	412.6675	0.22591186	0.396934	0.569141649	0.56926	0.772433	NOT
RP11-110I	13.41143	-0.1968011	0.345843	-0.56904754	0.569324	0.772481	NOT
KIAA1211	89.87052	0.32587043	0.572701	0.569005784	0.569352	0.772481	NOT
SKIDA1	253.9405	0.17522084	0.307968	0.568958255	0.569384	0.772486	NOT
RP11-2C24	1.933009	-0.2706878	0.475894	-0.56879809	0.569493	0.772587	NOT
HOXA3	156.3118	0.2947396	0.518232	0.568740417	0.569532	0.772587	NOT
RP11-403I	16.1231	-0.1574129	0.276783	-0.5687229	0.569544	0.772587	NOT
RP11-552M	17.76508	0.16111531	0.283325	0.568659283	0.569587	0.772592	NOT
RP11-159K	5.151554	0.66795079	1.174738	0.568595635	0.569631	0.772592	NOT
INTS4P1	10.54776	-0.2467183	0.433911	-0.56859155	0.569633	0.772592	NOT
RP11-401I	8.902586	0.20116579	0.353908	0.568412366	0.569755	0.772718	NOT
ZC3H12B	77.43892	0.21227132	0.373688	0.568044822	0.570005	0.772969	NOT
RP11-139I	13.03108	0.16704253	0.294068	0.568040671	0.570007	0.772969	NOT
RPL29P33	3.261856	0.2385617	0.419993	0.568013629	0.570026	0.772969	NOT
NIPAL4	3.355789	-0.2922226	0.514641	-0.56781848	0.570158	0.77311	NOT
BOLA3	911.1999	-0.1313577	0.231393	-0.56768269	0.57025	0.773197	NOT
NRG1	300.0603	-0.3397239	0.598758	-0.56738087	0.570455	0.773418	NOT
FOXP1	2639.341	-0.099236	0.174909	-0.56735878	0.57047	0.773418	NOT
COMMD6	2161.473	0.12302904	0.216888	0.567246849	0.570546	0.773482	NOT
GNAI1	1714.187	0.1564508	0.275881	0.567094932	0.57065	0.773583	NOT
RP11-5P18	3.352051	0.44772088	0.789632	0.566999597	0.570714	0.773633	NOT
PKD2L2	2.903333	-0.2461052	0.434117	-0.56690964	0.570776	0.773647	NOT
HOXC9	56.07672	-0.4918597	0.86763	-0.56690035	0.570782	0.773647	NOT
CD180	108.8606	0.18479092	0.326087	0.566692498	0.570923	0.773744	NOT
RP11-4K16	2.932543	0.29998978	0.529437	0.566619977	0.570972	0.773744	NOT
RP11-1055	5.691702	-0.2516082	0.444066	-0.56660057	0.570986	0.773744	NOT
EIF4A1P4	5.782055	0.19447897	0.343253	0.566576383	0.571002	0.773744	NOT
ZNF134	500.7837	-0.1222973	0.215862	-0.56655296	0.571018	0.773744	NOT
ZNF818P	15.66764	0.26410892	0.466177	0.566542628	0.571025	0.773744	NOT
LL22NC03-	8.368123	-0.3184057	0.562125	-0.56643251	0.5711	0.773807	NOT
AP002954.	6.722813	0.47366613	0.836314	0.566373384	0.57114	0.773823	NOT
SFXN4	1645.464	0.11709991	0.206852	0.566105897	0.571322	0.773953	NOT
MTRF1L	307.0554	-0.0843474	0.149001	-0.56608563	0.571336	0.773953	NOT
LRRC8B	464.9152	-0.1716912	0.3033	-0.56607716	0.571341	0.773953	NOT
AP000347.	32.87025	-0.1795762	0.317259	-0.56602451	0.571377	0.773953	NOT

RP3-500L1	7.563347	-0.2618186	0.462567	-0.56601234	0.571385	0.773953	NOT
CIDEC	212.2562	0.31522187	0.556948	0.565980437	0.571407	0.773953	NOT
RP11-108M	2.348667	0.27686511	0.489449	0.565667479	0.57162	0.774169	NOT
RABEPK	2118.298	-0.1454076	0.257108	-0.56555071	0.571699	0.774169	NOT
FAM122B	1013.274	0.11669079	0.206332	0.565547915	0.571701	0.774169	NOT
RBBP4P1	8.69996	0.16888869	0.298632	0.565541889	0.571705	0.774169	NOT
NCK1-AS1	108.7546	0.08920698	0.157742	0.565524399	0.571717	0.774169	NOT
DNAJC15	1347.597	-0.165425	0.292559	-0.56544192	0.571773	0.774169	NOT
C4orf27	665.6509	0.07301656	0.129137	0.565417297	0.57179	0.774169	NOT
SMC1A	4221.384	-0.1090381	0.192848	-0.56540943	0.571795	0.774169	NOT
KB-226F1.	45.42156	-0.24134	0.426907	-0.56532266	0.571854	0.774211	NOT
RP11-460N	2.170525	-0.2947388	0.521456	-0.56522291	0.571922	0.774236	NOT
SUV420H2	514.5199	-0.1318586	0.233291	-0.565211	0.57193	0.774236	NOT
LURAP1L-A	2.160291	-0.2887902	0.511026	-0.56511846	0.571993	0.774262	NOT
RP11-330H	2.182468	0.25771942	0.456102	0.565048179	0.572041	0.774262	NOT
STOML1	971.7086	0.11399365	0.201748	0.565030478	0.572053	0.774262	NOT
RP11-115I	1.433405	0.34933673	0.618338	0.564960851	0.5721	0.774262	NOT
TMEM179B	1905.323	0.08138535	0.144058	0.56495019	0.572108	0.774262	NOT
SMCO4	1870.847	-0.1296015	0.229411	-0.56493088	0.572121	0.774262	NOT
GOLGA7	1831.406	0.10650546	0.188567	0.564816391	0.572199	0.774302	NOT
AC000067.	1.928579	-0.2881763	0.510223	-0.56480412	0.572207	0.774302	NOT
CA12	1344.705	-0.346481	0.613522	-0.56474076	0.57225	0.774321	NOT
FOXL2NB	1.643146	0.55076562	0.975696	0.564484588	0.572424	0.77447	NOT
RP11-458I	2.618457	-0.2216789	0.392715	-0.56447711	0.572429	0.77447	NOT
RP3-395C1	3.582396	0.25154387	0.445641	0.564453458	0.572446	0.77447	NOT
RP11-305F	3.654656	0.67260775	1.191748	0.564387476	0.57249	0.77447	NOT
RP11-440I	18.20168	0.17213361	0.305002	0.564369253	0.572503	0.77447	NOT
NLGN1	9.27611	0.40971355	0.726158	0.564221079	0.572604	0.774568	NOT
CXorf23	330.1481	-0.0812361	0.144018	-0.56406812	0.572708	0.77466	NOT
RP11-367E	3.903202	0.18950428	0.336006	0.563990406	0.572761	0.77466	NOT
SPATA2L	783.6044	-0.1340035	0.237605	-0.56397576	0.572771	0.77466	NOT
TRBV3-1	4.337037	-0.3044435	0.539838	-0.56395382	0.572786	0.77466	NOT
LINC00641	127.5868	-0.1471943	0.261062	-0.56382985	0.57287	0.774718	NOT
ZNF649	152.9017	0.14532785	0.257762	0.563806813	0.572886	0.774718	NOT
UPP2	581.0519	-0.4057308	0.719913	-0.56358306	0.573038	0.774885	NOT
CTNNBIP1	1158.922	0.09977378	0.177098	0.563382314	0.573175	0.775031	NOT
RP11-566K	7.666099	-0.3313193	0.588318	-0.56316314	0.573324	0.77517	NOT
AVP	2.857525	0.55863771	0.992028	0.563127188	0.573348	0.77517	NOT
RP11-656D	25.69573	-0.1963067	0.34865	-0.5630487	0.573402	0.77517	NOT
RP11-872D	3.120375	-0.200913	0.356844	-0.56302785	0.573416	0.77517	NOT
AC012487.	11.89198	0.20097417	0.357015	0.562928946	0.573483	0.77517	NOT
AC012368.	1.544935	0.29744118	0.528388	0.562922431	0.573488	0.77517	NOT
MIR7111	2.8629	0.30737691	0.546053	0.562906783	0.573498	0.77517	NOT
RP11-760H	180.3037	0.12976658	0.230535	0.562892353	0.573508	0.77517	NOT
APOBEC3B	238.5436	0.28726437	0.510406	0.562815388	0.573561	0.77517	NOT
CTD-2616J	2.912099	-0.2961074	0.526121	-0.56281207	0.573563	0.77517	NOT
RP11-142C	2.284162	-0.266954	0.474413	-0.56270351	0.573637	0.775231	NOT
SKIV2L	3388.788	0.07871889	0.139958	0.562447717	0.573811	0.775428	NOT
CCER2	28.53425	-0.2387401	0.424513	-0.56238638	0.573853	0.775446	NOT

RPS26P31	4.853011	0.20859609	0.371032	0.562204665	0.573977	0.775574	NOT
HNRNPH2	3711.194	-0.0720731	0.128218	-0.56211415	0.574038	0.775619	NOT
RP5-1050E	5.229483	-0.4454927	0.792642	-0.56203489	0.574092	0.775654	NOT
HLA-DRB1	11671.06	0.21864733	0.389082	0.561956945	0.574145	0.775683	NOT
RP11-800A	15.10547	0.43138865	0.767706	0.561919145	0.574171	0.775683	NOT
RP11-503N	4.790335	0.23554593	0.419229	0.561855669	0.574214	0.775703	NOT
STAG3L5P	212.49	-0.1599691	0.284741	-0.56180512	0.574249	0.775704	NOT
ESRRA	3973.496	-0.0974915	0.173549	-0.5617523	0.574285	0.775704	NOT
RP11-872J	49.63949	0.16424177	0.292387	0.561728071	0.574301	0.775704	NOT
DPP7	4937.341	-0.17633	0.313956	-0.56163857	0.574362	0.775748	NOT
LINC01337	1.730158	-0.3002189	0.534732	-0.56143802	0.574499	0.775853	NOT
RP5-1050D	1.766253	-0.2672656	0.476055	-0.56141778	0.574513	0.775853	NOT
OR1F1	5.35199	0.45558766	0.811523	0.561398208	0.574526	0.775853	NOT
RP11-390C	3.717984	-0.2136488	0.380643	-0.56128418	0.574604	0.775855	NOT
INSIG2	1996.984	0.134888	0.240327	0.56126798	0.574615	0.775855	NOT
CTD-2036F	4.72245	-0.1666016	0.296847	-0.56123674	0.574636	0.775855	NOT
TADA1	1365.109	-0.1247074	0.222226	-0.56117374	0.574679	0.775855	NOT
FAM66C	13.1404	0.21222934	0.378194	0.561165404	0.574685	0.775855	NOT
SP140L	764.4297	0.12269534	0.218652	0.561144695	0.574699	0.775855	NOT
CTAGE7P	12.19795	-0.2012158	0.35862	-0.56108361	0.574741	0.775871	NOT
MYEF2	244.4872	-0.3187938	0.568216	-0.56104304	0.574768	0.775871	NOT
RN7SL809F	2.416344	-0.2353094	0.41948	-0.56095482	0.574828	0.77588	NOT
TP63	39.01823	0.26287349	0.468641	0.560926674	0.574848	0.77588	NOT
FSTL1	3851.889	0.196096	0.349697	0.560760283	0.574961	0.77588	NOT
AL023806.	6.657821	0.18808441	0.335421	0.560741901	0.574973	0.77588	NOT
ALDH3B1	874.6555	-0.164304	0.293016	-0.56073372	0.574979	0.77588	NOT
C12orf79	19.57413	0.21211138	0.378326	0.560658041	0.575031	0.77588	NOT
LY86-AS1	2.234849	-0.3447014	0.614817	-0.56065667	0.575032	0.77588	NOT
RTCA-AS1	38.5238	-0.1478145	0.263671	-0.56060134	0.575069	0.77588	NOT
ST18	5.649523	0.28442756	0.507379	0.560582398	0.575082	0.77588	NOT
RNH1	7049.736	0.09221516	0.164502	0.56057187	0.575089	0.77588	NOT
TRDV1	1.789702	-0.3052037	0.544501	-0.56051952	0.575125	0.77588	NOT
RPAIN	737.4619	-0.1023196	0.182564	-0.56045742	0.575167	0.77588	NOT
MROH1	2255.614	0.11971825	0.213611	0.560450615	0.575172	0.77588	NOT
AC006978.	251.4929	0.08933882	0.159406	0.560446561	0.575175	0.77588	NOT
XXbac-B56	15.90658	-0.2074175	0.370147	-0.56036583	0.57523	0.775888	NOT
SOCS4	602.217	0.08952223	0.159761	0.560349494	0.575241	0.775888	NOT
RUNX2	61.68831	0.20453387	0.365036	0.560311633	0.575267	0.775888	NOT
ESYT2	3522.165	-0.095151	0.169838	-0.56024654	0.575311	0.775904	NOT
RP11-120J	5.354761	-0.2976053	0.531239	-0.56021017	0.575336	0.775904	NOT
UBE2E2	778.4855	0.1597638	0.285363	0.559861138	0.575574	0.776187	NOT
CCDC176	151.8981	-0.11923	0.213008	-0.55974462	0.575654	0.776255	NOT
GPR113	14.03823	-0.1316745	0.235308	-0.55958356	0.575764	0.776308	NOT
RARG	360.2695	-0.1476547	0.263868	-0.55957865	0.575767	0.776308	NOT
CTD-2587H	1.83508	-0.4568221	0.816421	-0.55954227	0.575792	0.776308	NOT
RP11-752C	3.281013	-0.2692867	0.481319	-0.55947617	0.575837	0.776308	NOT
RP11-77P1	9.313761	0.20997498	0.375314	0.559464386	0.575845	0.776308	NOT
RPS6KA6	41.58911	0.48449318	0.866039	0.559435445	0.575865	0.776308	NOT
HORMAD2-A	410.8038	0.41138907	0.735434	0.55938276	0.575901	0.776318	NOT

AC005150.	11.29441	0.76922851	1.375486	0.559241381	0.575997	0.776382	NOT
LINC0126(	5.8607	0.25951082	0.464097	0.55917365	0.576043	0.776382	NOT
PRR36	146.3947	0.28310864	0.506345	0.559122568	0.576078	0.776382	NOT
JUP	8830.318	0.09541101	0.170644	0.559122113	0.576078	0.776382	NOT
FLJ12825	7.24618	-0.2770527	0.49553	-0.55910328	0.576091	0.776382	NOT
PCAT6	99.15116	0.18655749	0.33372	0.559024051	0.576145	0.776407	NOT
RP13-88F2	1.970479	-0.2086433	0.373249	-0.55899252	0.576167	0.776407	NOT
GNG5P2	3.509479	0.34640517	0.619836	0.558866106	0.576253	0.776485	NOT
THTPA	265.1923	-0.111324	0.199225	-0.55878661	0.576307	0.776519	NOT
KRTDAP	3.885722	0.51888423	0.928659	0.558745416	0.576335	0.776519	NOT
CTD-2086I	1.486171	-0.2645071	0.473588	-0.55851705	0.576491	0.776674	NOT
PBX3	1230.2	0.15301106	0.273972	0.558492413	0.576508	0.776674	NOT
C1orf204	20.40096	0.23165662	0.414829	0.558438899	0.576545	0.776685	NOT
HHIPL1	146.982	0.1986257	0.355729	0.558361662	0.576597	0.776717	NOT
NBPF3	96.94826	-0.1931163	0.345964	-0.55819752	0.57671	0.77683	NOT
IGHV2-26	12.13013	0.46118181	0.826413	0.558052193	0.576809	0.776925	NOT
TAF10	3133.153	0.10831783	0.19412	0.557993344	0.576849	0.77694	NOT
LINC0124(	0.788591	-0.8074838	1.447283	-0.5579307	0.576892	NA	NA
RP5-874C2	3.803333	-0.3393355	0.608319	-0.55782514	0.576964	0.777057	NOT
CTD-3199J	18.30666	0.14950358	0.268075	0.557693142	0.577054	0.777139	NOT
BBS7	233.7033	-0.1331711	0.238857	-0.55753599	0.577161	0.777188	NOT
EIF3I	7954.036	0.09690364	0.17382	0.557492779	0.577191	0.777188	NOT
CTD-2583F	2.546026	-0.3179005	0.570303	-0.55742366	0.577238	0.777188	NOT
RP11-701H	3.490018	0.37160379	0.666699	0.557378916	0.577269	0.777188	NOT
CTD-2210F	2.815789	-0.1982303	0.355652	-0.55737156	0.577274	0.777188	NOT
POLR2J4	168.6244	0.10356213	0.185805	0.557370195	0.577275	0.777188	NOT
CTD-3035I	25.58771	0.23539838	0.422355	0.557346779	0.577291	0.777188	NOT
KRT8P13	2.373678	0.25567784	0.459022	0.557005359	0.577524	0.777452	NOT
RP11-625I	1.647484	0.82879474	1.488031	0.556974114	0.577545	0.777452	NOT
GFER	1145.091	0.12221647	0.219445	0.556933666	0.577573	0.777452	NOT
GRIN2B	31.18144	-0.368457	0.66172	-0.55681708	0.577652	0.777502	NOT
HLA-DRB9	4.85704	0.36437193	0.654409	0.556795204	0.577667	0.777502	NOT
TMEM190	1.335659	0.42927514	0.771009	0.556770918	0.577684	NA	NA
LINC0084(	1.71412	0.62004768	1.1137	0.556745602	0.577701	0.777509	NOT
PPP1R16A	6070.433	0.18677392	0.33553	0.556654045	0.577764	0.777532	NOT
LAPTM5	4635.402	0.17302979	0.310865	0.556608147	0.577795	0.777532	NOT
LPP	3681.926	-0.0836392	0.150269	-0.55659511	0.577804	0.777532	NOT
AC006050.	3.228499	0.85476507	1.535998	0.556488579	0.577877	0.777592	NOT
LINC00667	1259.5	-0.1208403	0.217179	-0.55640811	0.577932	0.777612	NOT
SLC9A8	1305.664	-0.0925209	0.16629	-0.55638271	0.577949	0.777612	NOT
ZNF518A	898.0729	-0.1306242	0.234817	-0.55628171	0.578018	0.777639	NOT
ALKBH6	143.1295	-0.1243053	0.223462	-0.55626936	0.578027	0.777639	NOT
PAICS	7005.898	0.07595563	0.136577	0.55613927	0.578116	0.77772	NOT
POU3F3	0.932578	0.59989727	1.078871	0.556041605	0.578182	NA	NA
TMEM185A	257.6772	-0.080436	0.144698	-0.55589	0.578286	0.777911	NOT
RP11-326I	4.368822	0.2278082	0.409862	0.555816587	0.578336	0.77794	NOT
TLDC1	249.7936	0.17535884	0.315535	0.55575113	0.578381	0.777961	NOT
FBXO44	1350.555	-0.130474	0.23481	-0.55565669	0.578446	0.778009	NOT
RP11-355(	42.98667	0.14744683	0.265388	0.555589341	0.578492	0.778033	NOT

TDRG1	1.423894	-0.5012909	0.902373	-0.5555252	0.578535	0.778053	NOT
PDPK2P	13.14624	0.21587545	0.388677	0.555410287	0.578614	0.77812	NOT
CNTD1	38.00638	0.11146726	0.200797	0.555123674	0.57881	0.778313	NOT
ADM5	26.23152	-0.1959949	0.353091	-0.55508246	0.578838	0.778313	NOT
MYRFL	13.96711	-0.2630362	0.473876	-0.55507444	0.578844	0.778313	NOT
MOSPD2	635.9601	0.10812908	0.19491	0.554764513	0.579056	0.778437	NOT
RP11-977I	9.239085	0.20739936	0.373852	0.554762625	0.579057	0.778437	NOT
RP11-454I	1.707004	-0.3733378	0.67301	-0.55472827	0.579081	0.778437	NOT
ZNF775	875.9793	-0.1357174	0.244658	-0.55472287	0.579084	0.778437	NOT
FRRS1	945.679	-0.2044299	0.368548	-0.55468929	0.579107	0.778437	NOT
RP11-166N	4.75515	0.29583979	0.533349	0.554682912	0.579112	0.778437	NOT
FAM228A	4.494441	-0.2330625	0.420219	-0.55462095	0.579154	0.778437	NOT
FAM49A	450.1458	-0.1811901	0.326701	-0.5546047	0.579165	0.778437	NOT
ZNF555	195.9568	-0.1151127	0.207604	-0.55448334	0.579248	0.77851	NOT
CRMP1	369.6976	-0.2253966	0.406531	-0.55443891	0.579279	0.778512	NOT
MMP25-AS1	244.8883	-0.1701982	0.307	-0.55439202	0.579311	0.778517	NOT
RP11-43A1	1.043914	0.44864341	0.809888	0.553957051	0.579608	NA	NA
ZNF354C	108.832	-0.2177176	0.393066	-0.55389538	0.57965	0.778909	NOT
TEX38	2.605256	0.23050856	0.416192	0.553851925	0.57968	0.778909	NOT
PDE4D	226.0271	-0.1715732	0.309789	-0.55383958	0.579689	0.778909	NOT
RP11-93I2	2.33901	0.3159558	0.570569	0.553755297	0.579746	0.778948	NOT
TAF13	1066.115	0.08595913	0.155295	0.553520607	0.579907	0.779125	NOT
SUMO2P17	14.37613	-0.1662234	0.3005	-0.55315647	0.580156	0.779422	NOT
FAM86C2P	127.905	0.12572534	0.227353	0.552995327	0.580267	0.779531	NOT
RP11-553I	9.519894	0.19738459	0.356992	0.552909798	0.580325	0.779533	NOT
EIF4A1P7	1.865274	0.27100581	0.490182	0.552867417	0.580354	0.779533	NOT
BCL9L	1342.565	-0.1506038	0.272405	-0.55286719	0.580354	0.779533	NOT
NMRAL1	2414.16	-0.164348	0.297304	-0.55279462	0.580404	0.779561	NOT
RASA4DP	5.193997	0.24137807	0.436686	0.552749974	0.580435	0.779564	NOT
AC011247.	1.624834	0.27923871	0.505225	0.552701278	0.580468	0.77957	NOT
FIZ1	669.0185	-0.081846	0.148114	-0.55258631	0.580547	0.779618	NOT
PYGB	4373.388	0.11682655	0.211426	0.552565687	0.580561	0.779618	NOT
ADAMTS9	738.1626	0.15684871	0.283889	0.552499583	0.580606	0.77964	NOT
RASA1	856.8096	-0.1185817	0.21466	-0.55241693	0.580663	0.779677	NOT
TMEM39A	1108.748	0.07075131	0.128161	0.552052175	0.580913	0.779928	NOT
STK32B	37.85259	-0.2140057	0.387684	-0.55201062	0.580941	0.779928	NOT
PRRC2C	6778.123	0.10709533	0.194018	0.551985993	0.580958	0.779928	NOT
RP11-244J	5.857175	-0.241875	0.438198	-0.55197632	0.580965	0.779928	NOT
DHX32	810.2827	0.14305179	0.259218	0.551858111	0.581046	0.779986	NOT
GAS8	622.1629	-0.1158218	0.209887	-0.55183007	0.581065	0.779986	NOT
CH17-125A	1.288688	0.63768597	1.155927	0.551666414	0.581177	NA	NA
DAP	9749.503	0.09445576	0.171228	0.551636671	0.581197	0.780125	NOT
WLS	1455.128	0.2511471	0.455454	0.551421081	0.581345	0.78019	NOT
TP73	203.6126	0.25391536	0.460495	0.551396472	0.581362	0.78019	NOT
GPR35	449.1516	0.3247884	0.58908	0.551348828	0.581395	0.78019	NOT
AC125421.	1.639602	-0.3369174	0.611148	-0.55128628	0.581437	0.78019	NOT
GAMT	13562.15	0.15746483	0.285642	0.551266378	0.581451	0.78019	NOT
FLJ41941	1.892872	-0.2917095	0.529187	-0.55124086	0.581469	0.78019	NOT
GLIPR1	370.7372	0.16172975	0.293394	0.551237503	0.581471	0.78019	NOT

NUTF2	3619.117	0.11735208	0.212891	0.551230015	0.581476	0.78019	NOT
CECR2	592.0362	-0.2055904	0.373031	-0.55113515	0.581541	0.780239	NOT
AC073410.	3.137152	0.23716768	0.43055	0.550847814	0.581738	0.780431	NOT
SEPSECS-A	153.2021	0.12890697	0.234018	0.550842098	0.581742	0.780431	NOT
RP11-295F	291.382	0.12226366	0.222064	0.550578672	0.581923	0.780635	NOT
C2orf27AF	2.471487	-0.5471595	0.993915	-0.55050949	0.58197	0.78066	NOT
UCP2	1551.287	0.17583372	0.319499	0.550342184	0.582085	0.780775	NOT
RP2	602.6275	-0.1088923	0.197902	-0.5502342	0.582159	0.780836	NOT
RNF43	1794.908	0.22812613	0.414684	0.550119924	0.582237	0.780888	NOT
RP11-9N2(	2.41028	-0.2668653	0.485156	-0.55006078	0.582278	0.780888	NOT
RP4-798A1	18.82526	0.13210148	0.240165	0.550043888	0.582289	0.780888	NOT
HPRT1	1477.816	-0.1100318	0.200094	-0.54990109	0.582387	0.780888	NOT
SCNN1D	299.4344	-0.1897696	0.345098	-0.54990084	0.582387	0.780888	NOT
TMEM102	204.6783	0.12918982	0.234976	0.549799867	0.582457	0.780888	NOT
RPL3P2	26.1192	0.17404974	0.316585	0.549773366	0.582475	0.780888	NOT
ZC3H14	1644.045	-0.089138	0.162139	-0.54976381	0.582481	0.780888	NOT
RTTN	342.2864	-0.1018122	0.1852	-0.54974128	0.582497	0.780888	NOT
ZNF529	404.8088	-0.1023352	0.186156	-0.54972722	0.582506	0.780888	NOT
CTC-338M1	41.26153	-0.1977417	0.359722	-0.54970736	0.58252	0.780888	NOT
FA2H	34.79202	0.33485017	0.609187	0.549667721	0.582547	0.780888	NOT
SCN2A	16.34184	-0.2870794	0.522312	-0.54963244	0.582572	0.780888	NOT
UBA52P6	6.832696	0.18306681	0.333113	0.549562935	0.582619	0.780913	NOT
LINC01446	48.44003	-0.508487	0.925347	-0.54950973	0.582656	0.780918	NOT
EPHA2	1163.852	-0.158887	0.289162	-0.5494737	0.58268	0.780918	NOT
FBXO11	846.7435	-0.1221327	0.222339	-0.54930917	0.582793	0.780995	NOT
RAB21	2502.755	-0.0616629	0.112256	-0.54930606	0.582795	0.780995	NOT
TRHDE-AS1	111.1063	0.52416947	0.954485	0.549164759	0.582892	0.781086	NOT
GVQW1	25.27976	0.13446473	0.244883	0.549098848	0.582938	0.781108	NOT
RP11-255F	5.938513	-0.4622751	0.841944	-0.54905671	0.582967	0.781108	NOT
ADAM19	607.8382	0.13206472	0.240576	0.548951675	0.583039	0.781157	NOT
CDH4	19.65107	0.22643485	0.412526	0.548898691	0.583075	0.781157	NOT
DYNLT3	1659.62	-0.1014889	0.184909	-0.54885764	0.583103	0.781157	NOT
CTD-2575K	3.535129	-0.3293336	0.600058	-0.54883648	0.583118	0.781157	NOT
RANBP2	2885.192	-0.0780141	0.142177	-0.54870967	0.583205	0.781174	NOT
PPIC	1914.541	0.1104885	0.201376	0.548668484	0.583233	0.781174	NOT
CDK5RAP3	5445.86	-0.0984262	0.179401	-0.54863859	0.583253	0.781174	NOT
RAD50	2343.044	-0.0911096	0.166073	-0.5486117	0.583272	0.781174	NOT
RP11-318A	7.769372	0.17020272	0.310245	0.54860806	0.583274	0.781174	NOT
PRKD1	230.3124	-0.2220006	0.404715	-0.54853529	0.583324	0.781195	NOT
IL18BP	679.1474	0.16674431	0.304005	0.54849281	0.583354	0.781195	NOT
ABCA2	3856.223	-0.1373707	0.250467	-0.5484593	0.583377	0.781195	NOT
RP1-153G1	5.902867	-0.1896928	0.34591	-0.54838802	0.583426	0.781222	NOT
DDR2	570.8025	-0.2005476	0.365933	-0.5480442	0.583662	0.781499	NOT
BAALC-AS2	1.394359	0.44869853	0.819097	0.547796337	0.583832	NA	NA
RP11-36I1	2.414192	0.30368432	0.554477	0.547694983	0.583901	0.781731	NOT
IVL	1.502968	-0.6565709	1.198922	-0.54763423	0.583943	0.781731	NOT
EDIL3	331.2404	-0.1830984	0.33435	-0.54762538	0.583949	0.781731	NOT
RP3-400B1	8.722227	-0.1444221	0.263727	-0.54762038	0.583953	0.781731	NOT
ERMP1	1732.605	0.12716583	0.232232	0.547582122	0.583979	0.781731	NOT

C19orf47	630.159	0.07436914	0.135865	0.547374667	0.584121	0.781883	NOT
CHRNA1	7.447079	0.31964035	0.584035	0.547296983	0.584175	0.781886	NOT
NR1H2	3674.221	-0.0841109	0.1537	-0.54724013	0.584214	0.781886	NOT
TM2D3	1774.863	0.08039906	0.146922	0.547221464	0.584227	0.781886	NOT
CCDC73	34.15493	-0.1611149	0.294433	-0.54720391	0.584239	0.781886	NOT
TUBA8	11.50622	0.16479868	0.301234	0.547079041	0.584324	0.781952	NOT
C9orf66	31.70798	-0.3106879	0.567967	-0.54701719	0.584367	0.781952	NOT
RP11-420K	2.486832	-0.2081947	0.380608	-0.54700554	0.584375	0.781952	NOT
SYCE3	30.22651	0.24681464	0.451245	0.546964217	0.584403	0.781952	NOT
RP11-234(	5.329755	0.34622677	0.633058	0.546911691	0.584439	0.781962	NOT
IL32	20701.84	0.19098097	0.349245	0.546839275	0.584489	0.78199	NOT
RPL21P3	2.455118	-0.1969835	0.360283	-0.54674656	0.584553	0.782037	NOT
APPL2	1288.579	-0.0805612	0.147389	-0.54659063	0.58466	0.782139	NOT
RP11-468E	12.65426	-0.1480997	0.270972	-0.54655075	0.584687	0.782139	NOT
MBTD1	836.9025	-0.0967084	0.176969	-0.5464702	0.584743	0.782175	NOT
PTPRR	13.92481	-0.2550186	0.466731	-0.546393	0.584796	0.782207	NOT
MTTP	8724.765	-0.2271136	0.415727	-0.54630513	0.584856	0.782213	NOT
ITPR1-AS1	10.47623	0.16685378	0.305423	0.546303242	0.584858	0.782213	NOT
UBN2	845.5668	-0.0937116	0.17156	-0.54623104	0.584907	0.782223	NOT
ATP2A1-AS	15.47251	0.22054873	0.403787	0.546201038	0.584928	0.782223	NOT
BHMT	15977.07	-0.3170172	0.580467	-0.54614155	0.584969	0.782223	NOT
HAS2	33.9558	-0.2826812	0.517613	-0.54612461	0.58498	0.782223	NOT
DUTP6	19.24169	0.1884056	0.345065	0.546000025	0.585066	0.782299	NOT
OR7D2	3.520649	0.39283752	0.719547	0.545950961	0.5851	0.782305	NOT
GCM1	5.105173	0.29880362	0.547411	0.545848459	0.58517	0.782361	NOT
HPCAL4	3.071113	-0.3098543	0.567789	-0.54572066	0.585258	0.78244	NOT
KDM4A	1781.164	-0.088255	0.161753	-0.5456157	0.58533	0.782441	NOT
RP11-46H1	8.837715	-0.2168895	0.397535	-0.54558619	0.58535	0.782441	NOT
ZNF526	499.4682	-0.0666077	0.122088	-0.54557012	0.585361	0.782441	NOT
ZNF446	320.8625	-0.0861368	0.157907	-0.54549044	0.585416	0.782441	NOT
PAPPA	46.13447	-0.2584031	0.473722	-0.5454744	0.585427	0.782441	NOT
DNAJC19	1416.041	-0.0870226	0.159538	-0.54546748	0.585432	0.782441	NOT
OR52E8	0.413569	0.62536505	1.146926	0.545253266	0.585579	NA	NA
GPX4	20457.48	-0.1328264	0.243622	-0.54521606	0.585605	0.782611	NOT
ZNF582	105.0527	-0.1852799	0.339839	-0.54519884	0.585617	0.782611	NOT
HOMER2	2512.244	-0.2089931	0.383452	-0.54503105	0.585732	0.782727	NOT
SUSD4	977.919	0.31590827	0.579694	0.544956876	0.585783	0.782756	NOT
RP5-836J3	2.336816	-0.3200694	0.587537	-0.54476502	0.585915	0.782894	NOT
IGHJ3P	1.131226	-0.5650708	1.037539	-0.54462584	0.586011	NA	NA
CNN2P3	2.894563	0.55100141	1.011768	0.544592563	0.586034	0.783003	NOT
CASP10	763.8959	0.10107446	0.185607	0.544562899	0.586054	0.783003	NOT
CYTH2	2220.425	0.08820829	0.162038	0.544368383	0.586188	0.783068	NOT
MCPH1	467.0718	0.0967066	0.177655	0.544351423	0.5862	0.783068	NOT
UBL7-AS1	67.67403	-0.0970359	0.178274	-0.54430739	0.58623	0.783068	NOT
CHI3L1	25172.1	0.38279881	0.703284	0.544302124	0.586234	0.783068	NOT
CTC-303L1	1.751471	0.27464285	0.504623	0.544253894	0.586267	0.783068	NOT
PTPN6	1711.334	0.0906697	0.166604	0.54422225	0.586289	0.783068	NOT
ZFHX4	579.6521	-0.1871541	0.343908	-0.54419824	0.586305	0.783068	NOT
SH3GLB1	2933.29	-0.0768409	0.141245	-0.5440275	0.586423	0.783186	NOT

GDI1	4766.927	0.08716646	0.16029	0.543805402	0.586575	0.783352	NOT
TOMM70A	5466.281	0.06699586	0.123219	0.543712229	0.58664	0.783384	NOT
SFMBT1	659.2738	0.09115475	0.167667	0.543665177	0.586672	0.783384	NOT
RP11-320M	63.02764	0.12367598	0.227494	0.543644752	0.586686	0.783384	NOT
YWHAG	8033.256	0.07297948	0.134274	0.543511068	0.586778	0.783463	NOT
DOCK9-AS2	20.80702	0.18812943	0.34616	0.543474936	0.586803	0.783463	NOT
CTD-3220F	10.44458	-0.1852673	0.341086	-0.54316936	0.587013	0.783626	NOT
MILR1	90.11622	0.16253303	0.299248	0.543137916	0.587035	0.783626	NOT
POLR3B	763.8729	0.08170007	0.150424	0.54313095	0.58704	0.783626	NOT
FAM213A	10018.67	-0.1305573	0.240381	-0.54312695	0.587042	0.783626	NOT
INVS	516.3858	-0.0901073	0.165917	-0.54308709	0.58707	0.783626	NOT
PEX5	2829.505	-0.0894866	0.164823	-0.54292427	0.587182	0.783658	NOT
RHBG	1623.945	-0.3893036	0.717058	-0.54291758	0.587187	0.783658	NOT
RP11-401F	22.53008	-0.2773083	0.510803	-0.54288701	0.587208	0.783658	NOT
PLEKHD1	6.579243	-0.2795087	0.514858	-0.54288526	0.587209	0.783658	NOT
RP11-296O	1.849715	0.28246331	0.520372	0.5428108	0.58726	0.783668	NOT
CAMK1G	14.63799	0.19918714	0.366978	0.542777221	0.587283	0.783668	NOT
ARID2	784.4139	-0.0842783	0.155292	-0.5427095	0.58733	0.783668	NOT
IKBKB	1120.003	0.09272784	0.170862	0.542706413	0.587332	0.783668	NOT
PTGES3L	15.44956	0.21748812	0.400823	0.542603763	0.587403	0.783724	NOT
GLI2	43.20108	-0.2648567	0.488211	-0.54250469	0.587471	0.783776	NOT
RP4-800J2	2.911558	-0.2194322	0.404562	-0.54239477	0.587547	0.783838	NOT
RP11-393I	12.22876	-0.1308288	0.241247	-0.54230288	0.58761	0.783884	NOT
SAP30BP	2531.65	0.08113566	0.149631	0.542238269	0.587654	0.783905	NOT
RNA5SP383	2.92861	0.22960993	0.42359	0.542056955	0.587779	0.784033	NOT
RP11-530I	411.1561	-0.1046291	0.193055	-0.54196401	0.587843	0.784037	NOT
THAP8	450.9761	0.10927868	0.201639	0.541952319	0.587851	0.784037	NOT
CH25H	72.73601	-0.249767	0.460887	-0.54192676	0.587869	0.784037	NOT
ZZZ3	1431.441	0.08156278	0.150529	0.54183931	0.587929	0.784042	NOT
PAFAH1B3	1175.72	0.20138658	0.371688	0.541816919	0.587945	0.784042	NOT
HOXA5	74.53713	0.25269785	0.466438	0.541761368	0.587983	0.784042	NOT
DNAJB11	5748.882	0.08129796	0.150064	0.541754239	0.587988	0.784042	NOT
METTL2A	747.0369	0.07856161	0.145058	0.54158664	0.588103	0.784157	NOT
RHOQP2	2.919016	0.17710944	0.327072	0.541500132	0.588163	0.784177	NOT
POLM	847.9791	-0.077095	0.142388	-0.54144357	0.588202	0.784177	NOT
ALG6	749.1889	0.0836321	0.154467	0.54142438	0.588215	0.784177	NOT
KANTR	21.21994	-0.1847787	0.3413	-0.54139708	0.588234	0.784177	NOT
AGPAT4-IT	2.940457	-0.2697163	0.498262	-0.54131424	0.588291	0.784196	NOT
ZFP30	221.7237	-0.1652085	0.305211	-0.54129223	0.588306	0.784196	NOT
SEPT11	3307.334	-0.0899861	0.166257	-0.5412467	0.588338	0.7842	NOT
RP11-298H	0.84237	-0.4468178	0.825567	-0.54122507	0.588352	NA	NA
GLT1D1	1266.885	-0.2549402	0.471183	-0.54106375	0.588464	0.784329	NOT
F3	180.2031	-0.2187388	0.404636	-0.54058143	0.588796	0.784667	NOT
C17orf99	4.282253	0.36529	0.675739	0.540578819	0.588798	0.784667	NOT
SNORD101	2.89109	0.23705515	0.438528	0.54057031	0.588804	0.784667	NOT
PSTPIP1	168.2262	-0.2134738	0.395056	-0.54036323	0.588947	0.784807	NOT
NPPA-AS1	33.46284	0.2136614	0.395425	0.540333287	0.588967	0.784807	NOT
AC136289.	2.951762	0.221005	0.409073	0.540258041	0.589019	0.784838	NOT
AC110781.	1.76349	-0.3672222	0.680082	-0.53996744	0.589219	0.785067	NOT

SCN3B	10.90053	-0.2485481	0.460404	-0.53984756	0.589302	0.785137	NOT
RP11-18B1	9.249042	-0.2934903	0.5437	-0.53980196	0.589334	0.785137	NOT
RP11-248J	14.10467	-0.1374017	0.254558	-0.53976494	0.589359	0.785137	NOT
CYB561D1	332.4538	0.11154131	0.206705	0.539616957	0.589461	0.785207	NOT
RP11-278J	13.14481	-0.171713	0.31822	-0.53960471	0.58947	0.785207	NOT
RP11-375N	8.410714	0.20895254	0.387344	0.539449689	0.589577	0.785311	NOT
NRGN	207.9259	0.16435779	0.304705	0.539400374	0.589611	0.785318	NOT
CDK14	1455.255	0.13232903	0.245398	0.539243208	0.589719	0.785424	NOT
PRKCQ	46.05891	-0.2112729	0.391872	-0.53913747	0.589792	0.785442	NOT
APBB1IP	1554.311	0.20715002	0.384236	0.539121174	0.589803	0.785442	NOT
KRI1	1338.363	0.06963579	0.129171	0.539096782	0.58982	0.785442	NOT
FAM209B	5.450513	-0.2184269	0.405248	-0.53899589	0.58989	0.785497	NOT
RP11-385F	8.8217	-0.1683303	0.312506	-0.53864573	0.590131	0.785749	NOT
PCDHGB8P	4.19412	-0.3153072	0.58538	-0.53863689	0.590137	0.785749	NOT
RP4-594I1	6.948234	0.28685618	0.53262	0.538575749	0.59018	0.785767	NOT
CTC-255N2	1.018141	-0.4598612	0.853924	-0.53852728	0.590213	NA	NA
NCBP2-AS1	3.685965	0.20425381	0.379369	0.538403999	0.590298	0.785886	NOT
FOXN2	1365.229	0.113663	0.21114	0.538330348	0.590349	0.785915	NOT
CTD-2104F	4.575367	-0.2634981	0.489539	-0.53825724	0.590399	0.785944	NOT
TRIM52-AS	345.6984	0.12470436	0.231728	0.538148815	0.590474	0.786005	NOT
LARS	3658.466	0.07637607	0.141998	0.537867825	0.590668	0.786102	NOT
HOXB3	180.2372	-0.2036967	0.378715	-0.53786262	0.590672	0.786102	NOT
UBL7	1787.57	0.07828868	0.145566	0.537821544	0.5907	0.786102	NOT
AC007292.	17.86737	-0.1179032	0.219226	-0.53781579	0.590704	0.786102	NOT
SIGIRR	2720.19	-0.1761864	0.327613	-0.53778743	0.590724	0.786102	NOT
LINC00598	6.183121	-0.1989079	0.369865	-0.5377847	0.590726	0.786102	NOT
RP5-827C2	19.26063	0.19897976	0.370023	0.537749938	0.59075	0.786102	NOT
RP11-377I	5.843419	-0.165513	0.307888	-0.53757503	0.59087	0.786203	NOT
ABCC6P2	197.4602	-0.1717193	0.319448	-0.53755085	0.590887	0.786203	NOT
TRAPPC9	1924.538	0.10403543	0.193549	0.537513574	0.590913	0.786203	NOT
CROCCP3	65.68917	0.13006366	0.242004	0.537445121	0.59096	0.786227	NOT
RP11-404F	2.818149	-0.2733299	0.50865	-0.53736347	0.591017	0.786264	NOT
DANCR	1565.395	-0.1512593	0.281578	-0.53718489	0.59114	0.786358	NOT
ZNF714	97.01384	0.22966743	0.427545	0.53717762	0.591145	0.786358	NOT
MEI1	44.78925	-0.1773675	0.330262	-0.53705019	0.591233	0.786417	NOT
STIM2	905.2171	-0.0809965	0.150823	-0.53702885	0.591248	0.786417	NOT
RP11-93H2	21.91799	0.1739755	0.324036	0.536901859	0.591335	0.786495	NOT
HOXC-AS2	8.890607	-0.4522334	0.842421	-0.53682565	0.591388	0.786527	NOT
RP11-391H	10.10296	0.25325765	0.471807	0.536782124	0.591418	0.786528	NOT
TSSC2	27.49287	-0.2717683	0.506351	-0.5367195	0.591461	0.786547	NOT
CDK3	31.01213	-0.1806337	0.336649	-0.53656436	0.591569	0.786651	NOT
PCYOX1L	171.0312	0.14823104	0.276321	0.536445211	0.591651	0.786722	NOT
VPRBP	1358.849	-0.0712917	0.132928	-0.53631987	0.591737	0.786796	NOT
RP11-258C	173.66	-0.1202413	0.224213	-0.53628077	0.591765	0.786796	NOT
COL5A1	3550.191	-0.2352751	0.438796	-0.53618306	0.591832	0.786841	NOT
MED23	815.8413	0.10219743	0.190617	0.536140161	0.591862	0.786841	NOT
CLPTM1L	6878.155	0.07621667	0.142167	0.536105905	0.591885	0.786841	NOT
RP11-875C	11.87926	-0.1386988	0.258765	-0.53600341	0.591956	0.786897	NOT
PTPN5	7.047725	-0.3026712	0.564827	-0.53586525	0.592052	0.786985	NOT

MYO6	2276.988	0.12936814	0.241441	0.535817024	0.592085	0.786991	NOT
RP11-46B1	3.4785	0.44561231	0.831813	0.535712403	0.592157	0.787026	NOT
RAB36	49.78793	0.2515267	0.469534	0.535694842	0.592169	0.787026	NOT
RP11-864I	4.19747	-0.2040564	0.38098	-0.53560922	0.592229	0.787037	NOT
MAMLD1	432.1089	0.20756551	0.38754	0.535598332	0.592236	0.787037	NOT
DPRXP4	2.092127	0.26063401	0.486702	0.535510363	0.592297	0.787064	NOT
CD300LF	77.83626	0.16905435	0.315703	0.535485209	0.592314	0.787064	NOT
AC005329.	26.72387	-0.2483678	0.463908	-0.53538155	0.592386	0.787121	NOT
RP11-442M	17.35415	0.17732202	0.331334	0.535175891	0.592528	0.78726	NOT
AREL1	1292.885	-0.0804446	0.150323	-0.53514651	0.592549	0.78726	NOT
CEP57	2112.696	-0.0949093	0.177382	-0.53505663	0.592611	0.787265	NOT
RP11-75A9	2.193467	-0.1968743	0.367994	-0.53499313	0.592655	0.787265	NOT
RP3-402G1	67.82412	-0.1472215	0.275188	-0.53498594	0.59266	0.787265	NOT
ZNF266	778.2337	-0.1416468	0.264788	-0.53494479	0.592688	0.787265	NOT
KCNH6	20.08595	-0.3733182	0.697883	-0.53492977	0.592698	0.787265	NOT
RP11-254F	2.758491	0.34854622	0.651624	0.534888872	0.592727	0.787265	NOT
CTC-325J2	1.606554	-0.2516891	0.47071	-0.53470067	0.592857	0.787399	NOT
NMU	3.254455	0.3622424	0.677605	0.534592562	0.592932	0.787423	NOT
PGAP1	327.8328	-0.130636	0.244378	-0.53456502	0.592951	0.787423	NOT
SPG20-AS1	1.617579	0.4474612	0.837081	0.534549406	0.592961	0.787423	NOT
FYTTD1	1851.907	-0.0651848	0.121985	-0.53436569	0.593089	0.787553	NOT
MED28P7	2.674426	0.25196346	0.471574	0.534303024	0.593132	0.787558	NOT
NSD1	2378.936	-0.0869366	0.162718	-0.53427661	0.59315	0.787558	NOT
CNOT4	882.2291	0.06706341	0.125541	0.534197241	0.593205	0.787592	NOT
IGKV1D-12	1.488318	-0.6219923	1.164459	-0.53414676	0.59324	0.7876	NOT
RP13-347D	1.393721	0.35273519	0.660433	0.534096585	0.593275	NA	NA
AC004471.	4.031437	-0.194917	0.365016	-0.53399563	0.593345	0.7877	NOT
CPNE2	1023.721	-0.1199886	0.224725	-0.53393414	0.593387	0.787718	NOT
RP11-124N	2.07827	0.27675442	0.518428	0.533833955	0.593456	0.787771	NOT
RP11-278A	1.550853	-0.3163142	0.592678	-0.53370346	0.593547	0.787853	NOT
TCIRG1	4855.587	-0.1251903	0.234634	-0.53355502	0.593649	0.787951	NOT
NMB	208.8647	0.18531043	0.347373	0.533462751	0.593713	0.787991	NOT
RP4-742C1	4.70398	-0.2309143	0.432958	-0.53334087	0.593798	0.787991	NOT
GGTLC1	3.40322	0.2969332	0.556753	0.533330522	0.593805	0.787991	NOT
RP11-99L1	2.437049	0.46539955	0.872667	0.533307017	0.593821	0.787991	NOT
RP4-789D1	5.079627	0.20139581	0.377665	0.533265787	0.59385	0.787991	NOT
MAB21L1	2.812165	-0.3362395	0.630537	-0.5332587	0.593855	0.787991	NOT
RP11-111F	6.024664	-0.2517201	0.472126	-0.53316345	0.59392	0.788028	NOT
CTD-2260A	2.703797	0.29251094	0.548662	0.533134966	0.59394	0.788028	NOT
LPL	212.4069	-0.1570614	0.294643	-0.53305629	0.593995	0.788062	NOT
NAA10	1972.992	0.1184018	0.222206	0.532847755	0.594139	0.788204	NOT
RP11-845C	1.508472	0.32957922	0.61856	0.532817285	0.59416	0.788204	NOT
AC015971.	8.52191	-0.220441	0.413836	-0.53267699	0.594257	0.788294	NOT
RPL3P3	2.010943	-0.2810223	0.527637	-0.53260513	0.594307	0.788322	NOT
AC079250.	20.86216	0.18544756	0.348268	0.532485169	0.59439	0.788372	NOT
P2RY12	11.33217	-0.2443509	0.458904	-0.53246611	0.594403	0.788372	NOT
AP000593.	1.323396	0.72031549	1.353086	0.532350249	0.594483	NA	NA
TLK1	2605.395	0.08737982	0.164174	0.53223925	0.59456	0.788446	NOT
TTYH2	251.8329	-0.172395	0.32392	-0.53221444	0.594577	0.788446	NOT

RP11-22N1	10.67216	-0.165479	0.310926	-0.5322135	0.594578	0.788446	NOT
AF064858.	10.43686	0.25532909	0.479755	0.53220767	0.594582	0.788446	NOT
RP11-14K3	1.17257	0.71845703	1.349999	0.532190847	0.594594	NA	NA
CPA5	8.447596	0.32578869	0.612195	0.532164472	0.594612	0.788446	NOT
DCBLD2	1355.486	-0.1248178	0.234564	-0.53212693	0.594638	0.788446	NOT
SMTN	1700.698	0.10888894	0.204643	0.532091806	0.594662	0.788446	NOT
RP11-38M8	58.35895	0.27560717	0.518144	0.53191198	0.594787	0.788573	NOT
LRRC57	605.7415	0.07414943	0.139469	0.531655697	0.594964	0.78877	NOT
TRAV26-1	3.085236	0.31270186	0.588353	0.531486686	0.595082	0.788887	NOT
RP11-206I	1.832088	0.2091141	0.393521	0.531391975	0.595147	0.78891	NOT
UNK	1275.122	0.08170607	0.153763	0.531376862	0.595158	0.78891	NOT
FAM149B1	1214.94	0.07769001	0.146274	0.531127683	0.59533	0.789064	NOT
AC114730.	2.007908	-0.2959274	0.55717	-0.53112579	0.595332	0.789064	NOT
HLA-G	210.9487	0.19436111	0.36599	0.531055234	0.595381	0.78909	NOT
CHRNA2	2.465601	-0.344462	0.648755	-0.53095837	0.595448	0.78914	NOT
PLXNB2	17333.65	0.0955432	0.179961	0.530911959	0.59548	0.789145	NOT
IFT88	676.6942	0.13209151	0.248826	0.530859185	0.595516	0.789154	NOT
RP11-428(	2.706368	-0.3110826	0.586213	-0.53066484	0.595651	0.789294	NOT
RDH16	7270.9	-0.2997854	0.565011	-0.53058318	0.595708	0.789328	NOT
SLC9B1	19.08199	-0.1080356	0.203632	-0.53054443	0.595735	0.789328	NOT
IGKJ5	0.631076	-0.4470701	0.843024	-0.53031689	0.595892	NA	NA
CLP1	509.6793	0.05710938	0.107691	0.530306778	0.595899	0.78934	NOT
FAM96B	3243.637	0.1065331	0.200899	0.530282229	0.595916	0.78934	NOT
TECPR1	1526.557	-0.0992434	0.18716	-0.53026015	0.595932	0.78934	NOT
PRF1	299.3554	-0.1792272	0.338001	-0.53025612	0.595934	0.78934	NOT
MRRF	1160.75	-0.0734432	0.138509	-0.53024224	0.595944	0.78934	NOT
PFKFB2	767.1575	0.13406751	0.252855	0.530214817	0.595963	0.78934	NOT
LZTFL1	444.2438	0.13573196	0.25601	0.530182332	0.595986	0.78934	NOT
AC004947.	1.180083	-0.3829083	0.722308	-0.53011742	0.596031	NA	NA
BEGAIN	48.08576	0.31416586	0.592668	0.530087581	0.596051	0.78934	NOT
CEACAM21	40.89533	-0.177965	0.335731	-0.53008278	0.596055	0.78934	NOT
AC068282.	143.8335	0.17227761	0.325002	0.530081141	0.596056	0.78934	NOT
RP11-640I	4.520788	0.24741896	0.466835	0.529992287	0.596117	0.78934	NOT
HSPA8	40305.56	0.10629355	0.200558	0.52998957	0.596119	0.78934	NOT
COL4A3	88.92456	0.28883993	0.545032	0.529950789	0.596146	0.78934	NOT
AC092835.	4.732845	0.23432317	0.442166	0.52994417	0.596151	0.78934	NOT
GPR173	22.08596	0.20478856	0.386484	0.529876524	0.596198	0.789363	NOT
LY6G6C	0.969399	0.33925509	0.640276	0.529857379	0.596211	NA	NA
RP13-39P1	4.18924	0.22978497	0.433755	0.529757062	0.59628	0.789434	NOT
HOXC5	1.260259	0.67340745	1.271417	0.529651006	0.596354	NA	NA
IGLV10-54	9.472093	0.36460822	0.688712	0.529405812	0.596524	0.789718	NOT
DOCK8	571.8194	-0.182221	0.344284	-0.52927565	0.596614	0.789798	NOT
RP11-262I	28.24572	0.42509089	0.803217	0.529235334	0.596642	0.789798	NOT
RP11-6N17	1.510144	-0.2335617	0.441432	-0.52910067	0.596736	0.789828	NOT
NUP88	1610.239	-0.0872606	0.164947	-0.52902265	0.59679	0.789828	NOT
RP4-756G2	18.88417	-0.1360065	0.257107	-0.52898814	0.596814	0.789828	NOT
CTD-2287(	51.53373	-0.1337282	0.252801	-0.52898689	0.596815	0.789828	NOT
KIR2DL1	1.694598	-0.3208934	0.606633	-0.52897487	0.596823	0.789828	NOT
CEP170B	1972.35	0.0872303	0.164912	0.528950925	0.596839	0.789828	NOT

RP11-276I	3.327155	0.2106923	0.398405	0.528840047	0.596916	0.789891	NOT
NPB	4.581324	-0.215948	0.408505	-0.52862969	0.597062	0.790026	NOT
BACH1	1887.28	0.10258489	0.194066	0.528609152	0.597077	0.790026	NOT
AC018799.	0.969818	0.66367081	1.255577	0.528578387	0.597098	NA	NA
RP11-188I	11.52591	0.19705987	0.37286	0.528509518	0.597146	0.790029	NOT
THEMIS	36.23383	-0.2320736	0.439125	-0.52849163	0.597158	0.790029	NOT
AC009473.	2.589466	-0.2244235	0.424659	-0.52847963	0.597166	0.790029	NOT
LIMK2	1810.758	-0.1414664	0.267766	-0.52832179	0.597276	0.790135	NOT
COX7C	13092.66	-0.1135447	0.214942	-0.52825645	0.597321	0.790157	NOT
TMC4	510.294	0.28527555	0.540087	0.528203199	0.597358	0.790167	NOT
POLI	400.1532	0.10285003	0.194762	0.528081189	0.597443	0.790241	NOT
CXCL10	1373.451	0.2633519	0.498736	0.528038471	0.597473	0.790241	NOT
RP11-379I	3.597787	0.18979869	0.359512	0.52793456	0.597545	0.790277	NOT
PIK3R5	213.1142	-0.1534858	0.290765	-0.52786964	0.59759	0.790277	NOT
PPP1R27	7.529316	0.22004957	0.416887	0.527839402	0.597611	0.790277	NOT
TRAV1-1	1.476336	-0.3442248	0.652148	-0.52783195	0.597616	0.790277	NOT
SNHG17	880.7341	-0.1419872	0.269119	-0.52760023	0.597777	0.790377	NOT
RSRC1	801.3898	0.07468324	0.141565	0.527556055	0.597808	0.790377	NOT
FLJ31356	6.561029	0.30907009	0.585877	0.527534282	0.597823	0.790377	NOT
RP11-791(	18.79405	0.21685045	0.411074	0.527521073	0.597832	0.790377	NOT
BPTF	2028.025	-0.0958968	0.18179	-0.52751303	0.597837	0.790377	NOT
RP11-489(	8.856803	-0.2127089	0.4033	-0.52742087	0.597901	0.790423	NOT
LINC0090(	21.02799	-0.2067381	0.392016	-0.52737172	0.597935	0.79043	NOT
CHST3	287.3278	0.16259691	0.308343	0.527325343	0.597968	0.790434	NOT
SLC12A7	7753.966	0.09647421	0.182973	0.52726043	0.598013	0.790434	NOT
FLJ22763	80.78097	-0.3784383	0.717775	-0.52723818	0.598028	0.790434	NOT
MRPL27	3174.404	-0.1371758	0.260211	-0.52717124	0.598075	0.790434	NOT
MRPL19	2112.496	0.06840232	0.129757	0.527157015	0.598085	0.790434	NOT
TNFSF14	997.3608	-0.2438778	0.462681	-0.52709663	0.598126	0.79044	NOT
HMGN2P15	20.55737	0.23615615	0.448058	0.527066345	0.598148	0.79044	NOT
RP11-258I	8.472075	-0.1705266	0.323664	-0.52686247	0.598289	0.790586	NOT
RP11-359I	54.90324	-0.1204078	0.228574	-0.52677828	0.598348	0.790586	NOT
PROSER2-A	19.64571	-0.1528363	0.290149	-0.52675131	0.598366	0.790586	NOT
IQUB	6.763557	0.21837528	0.414579	0.526740195	0.598374	0.790586	NOT
RAB9B	19.327	-0.2359294	0.448091	-0.52652065	0.598527	0.790749	NOT
RP11-449J	55.5547	-0.197299	0.374772	-0.52645027	0.598575	0.790775	NOT
RP11-355I	89.92671	0.11101183	0.210941	0.526269248	0.598701	0.790902	NOT
TLR5	236.7534	0.18566706	0.352867	0.526167993	0.598771	0.790942	NOT
WDR49	1.965486	0.35479434	0.674332	0.52614166	0.59879	0.790942	NOT
MIATNB	57.50264	-0.111257	0.211526	-0.5259744	0.598906	0.791021	NOT
SAMD11	85.69911	0.27829898	0.529114	0.525971763	0.598908	0.791021	NOT
MPP4	4.816579	0.21404652	0.4072	0.525654491	0.599128	0.791179	NOT
PNPLA3	942.6253	-0.1734058	0.329912	-0.52561229	0.599158	0.791179	NOT
SPDYC	95.32176	-0.2507016	0.476976	-0.52560632	0.599162	0.791179	NOT
TMEM248	5746.832	-0.0568974	0.108252	-0.52559943	0.599167	0.791179	NOT
RYBP	1601.695	0.08221757	0.156429	0.525590406	0.599173	0.791179	NOT
GPKOW	1273.246	0.07750902	0.147564	0.525257712	0.599404	0.791446	NOT
IGHV3-53	14.37028	0.35534213	0.67673	0.525086818	0.599523	0.791564	NOT
TMED10P2	1.413001	0.40708512	0.775387	0.525009089	0.599577	0.791597	NOT

RP11-325I	8.674869	-0.4058691	0.773444	-0.52475584	0.599753	0.791773	NOT
CYP21A2	441.1462	0.21644278	0.412482	0.524732577	0.599769	0.791773	NOT
UBA52P8	5.584951	0.18075105	0.344519	0.524647759	0.599828	0.791811	NOT
TCEB3	2384.566	-0.0873446	0.166495	-0.52460778	0.599856	0.791811	NOT
PCDHA3	11.27186	0.33586101	0.640408	0.524448806	0.599966	0.791918	NOT
MORC2-AS1	6.989269	0.19180995	0.365835	0.524307676	0.600065	0.792009	NOT
PTPLAD2	99.31481	-0.1594752	0.304215	-0.52421906	0.600126	0.792014	NOT
SMEK1	2537.401	-0.0626181	0.11945	-0.52421826	0.600127	0.792014	NOT
ZSCAN22	284.6837	-0.0602726	0.114988	-0.5241652	0.600164	0.792016	NOT
AL672183.	2.898257	-0.2177601	0.415484	-0.52411184	0.600201	0.792016	NOT
HSBP1L1	890.3607	0.12058991	0.230093	0.524091033	0.600215	0.792016	NOT
ITGA7	1194.606	-0.1354078	0.258398	-0.52402833	0.600259	0.792035	NOT
COX7CP1	6.267556	0.17502016	0.334074	0.523896891	0.60035	0.792117	NOT
CTD-2530I	2.686178	-0.2210106	0.421944	-0.52379182	0.600423	0.792168	NOT
PPIAP2	3.729978	0.21313081	0.406927	0.523756568	0.600448	0.792168	NOT
RP11-15L1	6.978053	0.15756769	0.301012	0.52346014	0.600654	0.792402	NOT
HNRNPU-AS	252.7942	0.15726092	0.300453	0.523412047	0.600688	0.792408	NOT
RP11-171I	2.572722	-0.2827441	0.540505	-0.52311062	0.600897	0.792646	NOT
ABLIM1	3653.104	-0.1118116	0.213773	-0.52304002	0.600946	0.792672	NOT
MFSD1	2786.891	-0.0845076	0.161592	-0.52296873	0.600996	0.792678	NOT
KCNK13	76.41414	0.27459725	0.525104	0.52293875	0.601017	0.792678	NOT
FBXO32	677.9634	-0.1847304	0.353276	-0.52290697	0.601039	0.792678	NOT
SCARNA10	1.42208	0.28514224	0.545434	0.522780225	0.601127	0.792756	NOT
MT1P3	1.293753	0.49660355	0.950188	0.522637046	0.601227	NA	NA
PHIP	1269.141	0.09115133	0.174419	0.522600432	0.601252	0.792851	NOT
DDX25	9.295058	0.24865467	0.475809	0.522593226	0.601257	0.792851	NOT
RP11-806I	16.27559	0.23803866	0.455569	0.522508483	0.601316	0.792888	NOT
RP11-96H1	74.36159	-0.1412723	0.270394	-0.52246862	0.601344	0.792888	NOT
XXbac-BP(	240.1387	0.13337197	0.255358	0.522293929	0.601466	0.79298	NOT
CYP26C1	3.099204	-0.2605942	0.498981	-0.52225318	0.601494	0.79298	NOT
SLC17A9	4739.131	0.18038112	0.345397	0.522242443	0.601502	0.79298	NOT
BRPF3	2501.459	0.10727277	0.205436	0.522172464	0.60155	0.793006	NOT
TPST2	4300.896	-0.1136185	0.217701	-0.52190116	0.601739	0.793216	NOT
CTU2	670.0087	-0.1016235	0.194755	-0.52180304	0.601807	0.793246	NOT
MIR103A2	3.844113	-0.16079	0.308183	-0.52173516	0.601855	0.793246	NOT
C1orf122	1560.937	-0.1198992	0.229811	-0.52172951	0.601859	0.793246	NOT
RP11-175I	2.361729	-0.1949471	0.373699	-0.52166941	0.601901	0.793246	NOT
VIPR2	7.929327	0.29811644	0.571477	0.521659306	0.601908	0.793246	NOT
RP11-739I	2.879996	0.21202181	0.406471	0.521615654	0.601938	0.793247	NOT
RP5-1139I	6.175078	0.22046301	0.422839	0.521388108	0.602096	0.793417	NOT
PLEKHA2	757.0861	0.13710212	0.263006	0.521289032	0.602165	0.793421	NOT
ORAI2	525.9502	0.16424528	0.315134	0.521192509	0.602233	0.793421	NOT
LILRB1	201.9731	0.18418937	0.353458	0.521107136	0.602292	0.793421	NOT
RP4-761J1	3.553098	-0.1952107	0.374615	-0.52109678	0.602299	0.793421	NOT
OSER1-AS1	560.9928	0.12796082	0.245562	0.521094128	0.602301	0.793421	NOT
RP11-16P2	1.810517	0.26906149	0.516371	0.521062414	0.602323	0.793421	NOT
AC002398.	19.30647	0.14962555	0.287177	0.521021407	0.602352	0.793421	NOT
IPMK	508.5253	0.10441473	0.200407	0.521012575	0.602358	0.793421	NOT
AC005387.	5.999215	-0.200658	0.385136	-0.52100609	0.602363	0.793421	NOT

ADAP2	445.2516	0.13208644	0.253543	0.520963048	0.602393	0.793422	NOT
KSR1	409.4454	0.11080161	0.212707	0.52091084	0.602429	0.793432	NOT
CTD-2006I	61.55305	0.12264368	0.23546	0.520868384	0.602458	0.793432	NOT
RARRES3	2482.886	-0.1951986	0.374979	-0.52055914	0.602674	0.793654	NOT
CGN	5586.306	-0.1832477	0.352053	-0.5205125	0.602706	0.793654	NOT
DPY19L2P1	2.001052	0.27217485	0.522951	0.520459119	0.602744	0.793654	NOT
ATP6V1F	3339.284	0.10830131	0.2081	0.520428683	0.602765	0.793654	NOT
TCTEX1D2	68.89501	-0.1706195	0.327854	-0.52041311	0.602776	0.793654	NOT
ACVR2B	603.2823	0.14375975	0.276273	0.520353279	0.602817	0.793654	NOT
SLC25A36	389.5622	0.21059405	0.404793	0.520251467	0.602888	0.793654	NOT
CCL15-CCI	128.4264	-0.2085091	0.400786	-0.52025045	0.602889	0.793654	NOT
RMND1	1157.472	0.09897573	0.190256	0.520223751	0.602908	0.793654	NOT
MBD6	2787.605	-0.0768704	0.147769	-0.52020672	0.60292	0.793654	NOT
LINC0048?	4.859519	-0.3013091	0.579335	-0.52009438	0.602998	0.793718	NOT
KIF22	2362.339	0.11507021	0.221319	0.519928542	0.603113	0.793787	NOT
CTD-2031I	26.18551	0.14074738	0.270718	0.519904491	0.60313	0.793787	NOT
RP11-485(	3.133432	0.29625033	0.569836	0.519887074	0.603142	0.793787	NOT
RP5-894A1	56.26189	0.12975262	0.249595	0.519851793	0.603167	0.793787	NOT
OR2A20P	4.322199	-0.3964095	0.762789	-0.51968422	0.603284	0.793894	NOT
MDFIC	1017.223	0.1832237	0.35259	0.51965086	0.603307	0.793894	NOT
LINC0029/	356.0755	-0.0891678	0.171611	-0.51959315	0.603347	0.793909	NOT
FBRSL1	2545.141	0.07785028	0.149854	0.519507375	0.603407	0.793923	NOT
RP11-348I	27.90738	-0.175885	0.338584	-0.51947256	0.603431	0.793923	NOT
KRT18P38	2.148443	0.27343552	0.526393	0.519451295	0.603446	0.793923	NOT
VAPB	2825.151	-0.0618971	0.119192	-0.51930555	0.603548	0.794018	NOT
RP11-264I	10.78445	-0.1150903	0.22165	-0.51924369	0.603591	0.794033	NOT
AMOTL2	1579.082	-0.167658	0.322925	-0.51918593	0.603631	0.794033	NOT
RPL18	26303.32	0.12838834	0.247308	0.519143325	0.603661	0.794033	NOT
MGST3	4701.42	-0.1214449	0.233943	-0.5191222	0.603676	0.794033	NOT
SERPINB6	3305.343	0.09471253	0.18248	0.519028759	0.603741	0.794063	NOT
PURB	1911.905	0.06744998	0.12996	0.519005601	0.603757	0.794063	NOT
RP11-10A1	19.76077	0.23398805	0.451	0.518820373	0.603886	0.794194	NOT
IGLV5-37	1.405703	-0.5022479	0.968188	-0.5187501	0.603935	0.79422	NOT
NETO1	2.144453	-0.3349862	0.645828	-0.51869228	0.603975	0.794228	NOT
FAM83F	45.86593	0.32885596	0.634066	0.518646033	0.604008	0.794228	NOT
RP11-242I	2.220838	-0.2082026	0.401462	-0.51861126	0.604032	0.794228	NOT
LINC0090(	4.02289	-0.3739743	0.721226	-0.51852585	0.604091	0.794228	NOT
MSANTD2	213.5523	0.10178099	0.196292	0.51851835	0.604097	0.794228	NOT
TXNL1	3405.827	0.0898983	0.173385	0.518489394	0.604117	0.794228	NOT
HOXC6	58.87482	-0.4330122	0.835238	-0.51842958	0.604159	0.794244	NOT
FAM92A1P1	5.770994	0.19966909	0.385252	0.518281455	0.604262	0.794342	NOT
RFPL4AL1	1.528173	0.37552111	0.724651	0.518209638	0.604312	0.794369	NOT
DOK7	185.9323	-0.2635021	0.508552	-0.51814209	0.604359	0.794393	NOT
RP11-471I	4.348599	-0.2182888	0.421349	-0.51807175	0.604408	0.794393	NOT
KLHL2P1	8.639509	-0.2247173	0.433768	-0.51805808	0.604418	0.794393	NOT
FLJ31104	25.2532	-0.1907449	0.368286	-0.51792592	0.60451	0.794438	NOT
RABGAP1L	1253.157	0.08607119	0.166204	0.517865579	0.604552	0.794438	NOT
CTB-161C1	3.94184	-0.2861785	0.552629	-0.51784907	0.604564	0.794438	NOT
PRKAG3	4.913255	-0.232112	0.448237	-0.51783295	0.604575	0.794438	NOT

IGHV1-2	32.09452	-0.3794902	0.732891	-0.51779922	0.604598	0.794438	NOT
RP11-449(	3.976116	0.18786757	0.362858	0.517744596	0.604636	0.794449	NOT
IGHV3-52	0.989179	-0.5070676	0.979601	-0.51762657	0.604719	NA	NA
RP11-455(	1.871672	-0.2788699	0.538805	-0.5175709	0.604758	0.79457	NOT
FLJ27354	9.28595	-0.1448664	0.279943	-0.51748478	0.604818	0.79461	NOT
C5orf51	1446.814	-0.0799989	0.154625	-0.51737308	0.604896	0.794674	NOT
RP11-799I	304.6103	-0.1741274	0.336615	-0.51729026	0.604954	0.794712	NOT
WISP1	106.9265	-0.2170734	0.419689	-0.51722401	0.605	0.794714	NOT
MIER3	885.176	-0.0863524	0.166968	-0.51717872	0.605031	0.794714	NOT
NAGS	1856.517	-0.2359586	0.456257	-0.51716169	0.605043	0.794714	NOT
TRAV8-3	3.640168	0.32189177	0.622472	0.517118754	0.605073	0.794715	NOT
BABAM1	2128.554	-0.1010563	0.195465	-0.5170039	0.605153	0.794782	NOT
CELF2	322.5198	-0.1673182	0.323688	-0.51691161	0.605218	0.794828	NOT
TRAV21	4.381715	0.324553	0.628027	0.516781799	0.605308	0.794909	NOT
C2orf74	138.5038	0.11476072	0.222175	0.516533447	0.605482	0.795061	NOT
RALB	1608.128	-0.0689309	0.133449	-0.51653205	0.605483	0.795061	NOT
PRSS12	106.5276	0.33513389	0.648927	0.516442767	0.605545	0.79509	NOT
ARSF	108.5495	-0.4246419	0.822286	-0.51641613	0.605564	0.79509	NOT
TRGV4	2.979049	0.28278878	0.54767	0.516349026	0.605611	0.795113	NOT
CERS3	3.157563	0.30534181	0.591496	0.516219447	0.605701	0.795142	NOT
MAP7D3	368.8779	0.15930636	0.308615	0.516197286	0.605717	0.795142	NOT
EMR4P	14.76598	-0.2120824	0.41086	-0.51619109	0.605721	0.795142	NOT
MYLIP	435.165	-0.1175001	0.227661	-0.51611958	0.605771	0.79517	NOT
CTB-179K2	11.3773	-0.133456	0.258597	-0.5160773	0.6058	0.79517	NOT
GCNT2	807.2888	0.16082489	0.311674	0.516003804	0.605852	0.795199	NOT
EFCAB10	14.08218	-0.1198016	0.232197	-0.51594743	0.605891	0.795201	NOT
CSNK2A2	2353.192	0.06435247	0.124742	0.515885676	0.605934	0.795201	NOT
EIF5A	15262.2	0.105111	0.203777	0.515813166	0.605985	0.795201	NOT
HSD11B1	14995.99	-0.3336008	0.646776	-0.51579041	0.606001	0.795201	NOT
RP4-680D5	2.608505	-0.2094298	0.40604	-0.51578606	0.606004	0.795201	NOT
RCBTB1	846.7794	-0.1111978	0.215612	-0.51573049	0.606043	0.795201	NOT
INF2	5593.544	0.09817195	0.190369	0.515691706	0.60607	0.795201	NOT
OLFML3	525.7419	-0.2095088	0.406288	-0.51566555	0.606088	0.795201	NOT
XPA	578.8308	-0.0680452	0.132005	-0.51547249	0.606223	0.795318	NOT
CDAN1	651.859	0.06862355	0.133132	0.515454499	0.606235	0.795318	NOT
AAAS	1887.002	0.06893349	0.133762	0.515346041	0.606311	0.795344	NOT
TUBA1A	1473.755	-0.1471268	0.285498	-0.51533296	0.60632	0.795344	NOT
MYOZ3	12.28858	-0.15871	0.307995	-0.51530014	0.606343	0.795344	NOT
RP11-195I	7.912733	-0.1670284	0.324206	-0.51519185	0.606419	0.795404	NOT
NEIL2	548.9969	0.10120816	0.196469	0.515134949	0.606459	0.795418	NOT
CDH26	16.49904	-0.2200018	0.427258	-0.5149156	0.606612	0.795581	NOT
AC022153.	1.568137	-0.4843939	0.940906	-0.51481635	0.606681	0.795618	NOT
CC2D1A	2168.242	-0.0849684	0.165058	-0.51477831	0.606708	0.795618	NOT
UBL5P2	2.983463	-0.1923812	0.373738	-0.51474906	0.606728	0.795618	NOT
RP3-368A4	191.0852	0.14272763	0.277329	0.514650861	0.606797	0.795642	NOT
FAM221B	10.48004	-0.1919363	0.372976	-0.51460741	0.606827	0.795642	NOT
SNORA26	4.954473	0.1789262	0.347701	0.514597189	0.606835	0.795642	NOT
OFD1	798.5272	0.07988999	0.155292	0.51445076	0.606937	0.795738	NOT
IFNAR2	751.0454	0.07903363	0.153644	0.51439607	0.606975	0.795749	NOT

RP11-11N9	54.61156	0.26489067	0.515045	0.514305636	0.607038	0.795794	NOT
DISP1	730.88	-0.1394512	0.271208	-0.51418481	0.607123	0.795847	NOT
HOXB6	61.63099	0.21265232	0.413589	0.51416308	0.607138	0.795847	NOT
LINC00960	59.14299	0.26207669	0.509788	0.514089961	0.607189	0.795853	NOT
ACTL10	44.23108	0.23382143	0.454878	0.514031337	0.60723	0.795853	NOT
IGLL5	278.8481	0.35091626	0.682699	0.514013301	0.607243	0.795853	NOT
AC104088.	5.58187	0.56357396	1.096471	0.513989012	0.60726	0.795853	NOT
LY6G5C	79.7682	-0.1520372	0.295844	-0.51391042	0.607315	0.795887	NOT
ACSS3	4497.585	-0.2333245	0.454059	-0.51386328	0.607348	0.795891	NOT
LIPE-AS1	19.77551	0.18888539	0.36766	0.51375032	0.607427	0.795956	NOT
NRF1	579.5361	0.06303161	0.122728	0.513588763	0.60754	0.796056	NOT
AC008746.	16.812	-0.1693943	0.329845	-0.51355797	0.607561	0.796056	NOT
RNF217	848.7922	0.14895905	0.290095	0.513483512	0.607613	0.796064	NOT
ASCC2	3996.597	0.07491913	0.14591	0.513459785	0.60763	0.796064	NOT
RP11-10C2	46.09807	0.09250736	0.180182	0.513409338	0.607665	0.796064	NOT
PHF12	1483.551	0.06974949	0.135892	0.513269906	0.607763	0.796064	NOT
RP11-135A	15.96576	0.20321263	0.39594	0.513241295	0.607783	0.796064	NOT
RP11-87C1	5.714543	0.17735224	0.345558	0.513234221	0.607787	0.796064	NOT
CCDC162P	152.5826	-0.3259262	0.635093	-0.51319402	0.607816	0.796064	NOT
TSKU	10842.59	-0.203438	0.396424	-0.51318327	0.607823	0.796064	NOT
PCDHA5	3.238189	-0.3183678	0.620392	-0.51317159	0.607831	0.796064	NOT
LINC00886	185.9608	-0.2025928	0.39489	-0.51303619	0.607926	0.796132	NOT
HSPBP1	2235.092	0.10646797	0.207535	0.513013248	0.607942	0.796132	NOT
ODCP	3.694465	0.20766879	0.404835	0.512971447	0.607971	0.796132	NOT
RPL26	8773.933	0.13153677	0.256477	0.512859616	0.60805	0.79618	NOT
FAR2	149.5557	0.15203475	0.296465	0.512826094	0.608073	0.79618	NOT
TMEM41A	1384.77	0.08206604	0.160048	0.512759387	0.60812	0.79618	NOT
RP1-151F1	214.4107	-0.1611462	0.314278	-0.5127511	0.608125	0.79618	NOT
RPL13AP20	89.51428	0.16638937	0.32453	0.512709221	0.608155	0.79618	NOT
CSMD1	167.6338	0.47467132	0.926155	0.512518113	0.608288	0.796317	NOT
TACR2	21.6494	0.22250706	0.434289	0.512348002	0.608407	0.796402	NOT
AL513327.	1.862348	0.29086247	0.567713	0.512340988	0.608412	0.796402	NOT
RP11-1134	2.102905	0.37072857	0.7237	0.512268224	0.608463	0.796428	NOT
SNORA45B	2.896901	0.26531543	0.517963	0.512228636	0.608491	0.796428	NOT
BIVM	1015.957	0.11396463	0.222557	0.512069169	0.608603	0.796501	NOT
MRPL22	1397.461	0.09709849	0.189621	0.512065078	0.608605	0.796501	NOT
STK19	510.3378	0.07728618	0.151017	0.511772286	0.60881	0.796731	NOT
ANKIB1	1340.074	0.10306874	0.201418	0.511714993	0.60885	0.796745	NOT
LSM3	2107.345	0.08763909	0.171288	0.511646175	0.608899	0.796769	NOT
TMEM110-M	41.58526	-0.135688	0.265243	-0.51156064	0.608959	0.796779	NOT
RP4-625H1	1.6479	-0.374011	0.731131	-0.51155132	0.608965	0.796779	NOT
TMEM209	1068.834	0.09358119	0.182955	0.511497047	0.609003	0.796791	NOT
TADA3	3587.888	0.08015295	0.156719	0.511445268	0.609039	0.7968	NOT
KIF25	38.26416	-0.2510507	0.490937	-0.51137044	0.609092	0.79683	NOT
U47924.27	0.958	0.59868039	1.170829	0.511330151	0.60912	NA	NA
NICN1	735.1704	-0.0924707	0.180858	-0.51128849	0.609149	0.796846	NOT
AC090587.	22.81542	-0.1549092	0.302989	-0.51126943	0.609162	0.796846	NOT
CD200R1	45.29303	-0.1745452	0.341437	-0.51120703	0.609206	0.796864	NOT
CYP4F35P	2.549215	0.37306421	0.729841	0.511158226	0.60924	0.796871	NOT

ZNF157	1.832852	0.40797196	0.798309	0.511045477	0.609319	0.796935	NOT
ZNF382	101.6171	0.16162756	0.316322	0.510959317	0.60938	0.796976	NOT
PTPRD-AS1	10.88791	0.30679041	0.60054	0.510857359	0.609451	0.797031	NOT
CAMTA1-IT	1.264091	0.46927112	0.91909	0.510582191	0.609644	NA	NA
CTD-2026K	9.370992	-0.2082281	0.40783	-0.51057584	0.609648	0.797149	NOT
ZNF865	798.8492	0.08139201	0.159417	0.510560032	0.609659	0.797149	NOT
DZIP1	235.0437	0.21546642	0.422045	0.510530095	0.60968	0.797149	NOT
PLCG1-AS1	5.522646	0.16304617	0.31937	0.51052362	0.609685	0.797149	NOT
RP11-481J	3.08885	-0.2009301	0.393609	-0.51048163	0.609714	0.797149	NOT
DACT2	442.0865	-0.3443748	0.674614	-0.51047668	0.609718	0.797149	NOT
RP11-136K	34.96787	-0.3591084	0.703737	-0.51028792	0.60985	0.797283	NOT
SPATA33	179.6843	-0.085476	0.167548	-0.51015688	0.609942	0.797338	NOT
DPYSL2	2119.427	-0.1344803	0.263612	-0.51014385	0.609951	0.797338	NOT
RP11-365M	9.813567	-0.166067	0.325575	-0.51007243	0.610001	0.797365	NOT
RHOQ	1168.436	-0.1227946	0.24077	-0.51000748	0.610046	0.797386	NOT
ELMSAN1	975.146	-0.0992766	0.194706	-0.50987963	0.610136	0.797452	NOT
RP11-812E	0.788961	0.61658901	1.209381	0.509838482	0.610165	NA	NA
HSD3BP4	3.197558	-0.3192852	0.626296	-0.50979898	0.610192	0.797452	NOT
TRPC5	31.62577	0.37517095	0.735929	0.509792136	0.610197	0.797452	NOT
VAV2	4183.381	-0.0974585	0.191182	-0.50976821	0.610214	0.797452	NOT
CTC-343N3	2.521251	0.25213031	0.494756	0.509605572	0.610328	0.797562	NOT
INHA	47.87547	-0.3591309	0.704819	-0.50953657	0.610376	0.797587	NOT
RP11-15A1	10.11338	-0.2021455	0.396839	-0.50938966	0.610479	0.797648	NOT
SFRP4	280.0888	-0.2597168	0.509863	-0.5093859	0.610482	0.797648	NOT
THAP9-AS1	607.1756	-0.1137959	0.223429	-0.50931535	0.610531	0.797675	NOT
C11orf68	1559.786	-0.0717728	0.140992	-0.50905503	0.610714	0.797839	NOT
CTD-2554C	4.509494	0.17579721	0.345342	0.50905213	0.610716	0.797839	NOT
AC016745.	3.596313	-0.2983608	0.586171	-0.50899949	0.610753	0.797839	NOT
CTA-363E6	2.766669	0.36571296	0.718546	0.508962817	0.610778	0.797839	NOT
GTPBP3	802.7322	0.08352764	0.164129	0.508915399	0.610812	0.797839	NOT
RPL19P20	1.935853	-0.2779486	0.546193	-0.508884	0.610834	0.797839	NOT
C3orf80	38.78306	0.21672457	0.426023	0.508715139	0.610952	0.797935	NOT
RNA5SP203	1.576988	-0.25243	0.496265	-0.5086595	0.610991	0.797935	NOT
AC093375.	16.27582	0.26706711	0.525047	0.508653546	0.610995	0.797935	NOT
PPP2R5B	774.9948	-0.0815382	0.160328	-0.50857198	0.611052	0.797971	NOT
ADCY6	1807.223	-0.0856555	0.168458	-0.5084671	0.611126	0.798029	NOT
SP140	120.148	0.21780719	0.428417	0.508399443	0.611173	0.798039	NOT
LUZP1	722.8207	-0.0850409	0.167295	-0.50832959	0.611222	0.798039	NOT
MGC27382	36.6431	-0.2244514	0.441553	-0.50832218	0.611227	0.798039	NOT
C21orf62	4.031662	-0.2826744	0.556161	-0.50825993	0.611271	0.798039	NOT
EPB41	2620.144	-0.127426	0.250717	-0.50824638	0.611281	0.798039	NOT
C1QTNF3	270.5479	0.21182489	0.416815	0.508198826	0.611314	0.798042	NOT
CNBP	11527.93	0.05390103	0.106071	0.508158459	0.611342	0.798042	NOT
CD86	258.9465	0.16152962	0.317975	0.507995157	0.611457	0.798141	NOT
IGHG2	2135.579	-0.3277169	0.645154	-0.50796713	0.611476	0.798141	NOT
RP4-633O1	2.999163	0.2268304	0.446647	0.507851285	0.611558	0.798202	NOT
KRT17P8	381.3508	-0.2864877	0.564157	-0.50781594	0.611582	0.798202	NOT
TMOD3	2699.748	0.09462821	0.186419	0.507610158	0.611727	0.798352	NOT
TBX3	3106.106	-0.2353771	0.46392	-0.50736578	0.611898	0.798478	NOT

RP11-356I	17.33633	-0.1588474	0.31309	-0.50735341	0.611907	0.798478	NOT
CCDC88B	624.6327	0.14961238	0.294911	0.507314172	0.611934	0.798478	NOT
JAK1	6161.562	-0.0934046	0.184119	-0.5073049	0.611941	0.798478	NOT
PIK3AP1	5400.843	0.12752272	0.251427	0.507195178	0.612018	0.79854	NOT
PDE5A	251.7543	0.14947185	0.294734	0.507141691	0.612055	0.798551	NOT
LRRC40	663.8147	0.11404397	0.224928	0.507024589	0.612138	0.798586	NOT
RP11-204I	2.311586	0.23171898	0.457022	0.507018936	0.612142	0.798586	NOT
XXbac-BP(	2.131534	-0.2152325	0.424557	-0.50695733	0.612185	0.798604	NOT
FGF13	433.715	0.22666953	0.447241	0.506817926	0.612283	0.798693	NOT
RP11-380(	11.65918	0.15349436	0.302895	0.506756999	0.612325	0.798711	NOT
TGIF2	1273.61	0.11927773	0.235409	0.506684021	0.612377	0.798714	NOT
ABHD8	1127.874	0.1009047	0.199156	0.506662168	0.612392	0.798714	NOT
C20orf194	514.2147	0.11501518	0.227054	0.506553871	0.612468	0.798714	NOT
RNF122	198.1128	0.10385716	0.205037	0.506528572	0.612486	0.798714	NOT
OGFR-AS1	18.82387	-0.1593099	0.314517	-0.50652205	0.61249	0.798714	NOT
GPC4	478.3656	-0.2634556	0.520148	-0.50650138	0.612505	0.798714	NOT
NUP210P3	2.167483	-0.2657322	0.524695	-0.50645085	0.61254	0.798722	NOT
RP11-325I	7.8736	0.22585796	0.446096	0.506298696	0.612647	0.798823	NOT
LINC01322	5.43373	-0.3521983	0.695752	-0.50621236	0.612708	0.798825	NOT
IGKV1-6	27.00082	-0.3476045	0.686677	-0.50621223	0.612708	0.798825	NOT
GPR75-ASE	92.49728	0.07358648	0.145421	0.506025456	0.612839	0.798934	NOT
RP11-266K	8.402007	0.21506377	0.425044	0.505980581	0.61287	0.798934	NOT
GK5	562.9153	-0.0840638	0.166144	-0.50596802	0.612879	0.798934	NOT
WDR19	731.2482	-0.0807746	0.159684	-0.50583887	0.61297	0.798991	NOT
RPL7AP66	19.10555	0.17082309	0.337739	0.505784356	0.613008	0.798991	NOT
PTPLAD1	8432.057	-0.098569	0.194885	-0.50578005	0.613011	0.798991	NOT
RP5-902P8	7.497319	-0.1854549	0.366797	-0.50560622	0.613133	0.79911	NOT
P2RY10	23.07985	-0.2527965	0.500027	-0.50556611	0.613161	0.79911	NOT
RP11-7D5.	2.541172	-0.2311031	0.45722	-0.50545287	0.613241	0.799175	NOT
AC068831.	8.445055	-0.1315677	0.260369	-0.50531279	0.613339	0.79922	NOT
FAM104B	450.0419	0.07967448	0.157683	0.50528406	0.613359	0.79922	NOT
EID2	488.533	0.0560873	0.111015	0.505221456	0.613403	0.79922	NOT
MTMR9	325.6138	-0.0959861	0.18999	-0.50521689	0.613407	0.79922	NOT
RP11-175I	4.192754	-0.1867328	0.369635	-0.50518188	0.613431	0.79922	NOT
HSH2D	223.4173	0.22883094	0.452995	0.505151482	0.613452	0.79922	NOT
BRD9P2	3.627591	-0.3042321	0.602449	-0.50499212	0.613564	0.799328	NOT
PRORSD1P	56.69951	0.11459483	0.227025	0.5047671	0.613722	0.799495	NOT
SOCS5	767.3851	0.07391501	0.146459	0.504680059	0.613784	0.799522	NOT
NUB1	3231.507	0.07247964	0.143622	0.504653906	0.613802	0.799522	NOT
AMH	35.4701	-0.2848937	0.564633	-0.50456449	0.613865	0.799566	NOT
EID2B	56.65468	0.10731733	0.212735	0.504464516	0.613935	0.799605	NOT
C16orf46	37.98088	-0.1269711	0.251709	-0.50443709	0.613954	0.799605	NOT
HIST1H2BJ	77.24914	-0.2442368	0.484313	-0.50429561	0.614054	0.799696	NOT
GNB4	360.1667	-0.1229489	0.243826	-0.50424895	0.614086	0.799701	NOT
PDHX	1508.462	-0.0691381	0.137152	-0.50409757	0.614193	0.799801	NOT
PLEC	15711.34	0.09166359	0.181888	0.503956731	0.614292	0.799891	NOT
AC016739.	105.2322	0.15702274	0.311618	0.503894591	0.614335	0.79991	NOT
KXD1	2665.49	0.07216967	0.143318	0.503564747	0.614567	0.800173	NOT
LPAR5	83.32651	0.17850721	0.354647	0.503337537	0.614727	0.800343	NOT

DRICH1	9.404434	-0.1977504	0.392971	-0.5032187	0.614811	0.800413	NOT
ENPP4	956.7731	-0.1409687	0.280175	-0.5031447	0.614863	0.800426	NOT
CD27-AS1	473.1086	0.09069729	0.180269	0.503120628	0.614879	0.800426	NOT
EMC10	6967.077	-0.1014881	0.201754	-0.50302923	0.614944	0.800471	NOT
FZD8	284.3045	-0.1916885	0.381247	-0.50279344	0.61511	0.800649	NOT
CEP63	904.3411	-0.0511641	0.101773	-0.50272935	0.615155	0.800669	NOT
XXbac-BP(	97.59004	0.15179919	0.302023	0.502608671	0.615239	0.800741	NOT
RP11-322I	11.65839	-0.167714	0.333718	-0.50256158	0.615273	0.800746	NOT
LINC0122(	4.652313	-0.2104291	0.418868	-0.5023754	0.615403	0.800878	NOT
AC007387.	3.475377	-0.2121456	0.422403	-0.50223454	0.615503	0.800955	NOT
ARID3B	239.1124	0.10054287	0.200213	0.502180307	0.615541	0.800955	NOT
ZNF807	2.022159	-0.2298062	0.457631	-0.5021654	0.615551	0.800955	NOT
USPL1	596.8711	-0.0822391	0.163802	-0.50206332	0.615623	0.80101	NOT
LINC00327	5.107267	0.32924436	0.655855	0.502007505	0.615662	0.801022	NOT
GTF2H4	126.2797	0.08895033	0.177211	0.5019464	0.615705	0.80104	NOT
SSH2	818.9812	0.07403765	0.147522	0.501876498	0.615754	0.801051	NOT
NIPA1	1024.629	0.12528279	0.249642	0.501850229	0.615773	0.801051	NOT
RP9	298.4476	0.0934237	0.1862	0.501737741	0.615852	0.801116	NOT
DPYSL4	78.72026	-0.2832273	0.564596	-0.50164578	0.615917	0.801161	NOT
TMEM218	584.7438	0.06429958	0.128189	0.501600644	0.615948	0.801164	NOT
RN7SL4P	6.008071	-0.1785304	0.356002	-0.50148683	0.616029	0.80123	NOT
RP11-401I	27.57276	-0.1985805	0.396102	-0.50133676	0.616134	0.801301	NOT
FBXO10	349.209	0.11453847	0.228471	0.501325647	0.616142	0.801301	NOT
AP4B1	543.4834	0.07698969	0.15359	0.501268947	0.616182	0.801314	NOT
CEBPD	4703.449	-0.147979	0.295236	-0.50122184	0.616215	0.801319	NOT
TTC17	3302.181	-0.0650806	0.129881	-0.50108052	0.616314	0.801409	NOT
RP3-406P2	2.311119	-0.1826267	0.364496	-0.50103875	0.616344	0.801409	NOT
WDR89	508.4164	0.06960444	0.138998	0.500758346	0.616541	0.801567	NOT
BBS10	553.3057	-0.1014842	0.202666	-0.50074634	0.61655	0.801567	NOT
GMPR2	2350.289	0.06814205	0.136082	0.500741173	0.616553	0.801567	NOT
RP1-167O2	8.382087	0.43964164	0.878505	0.50044269	0.616763	0.801757	NOT
RP5-906C1	6.888286	-0.1950582	0.389779	-0.50043342	0.61677	0.801757	NOT
RP4-800G7	7.054188	0.17011253	0.339948	0.500407171	0.616788	0.801757	NOT
NUP210L	3.864862	-0.2151277	0.429974	-0.50032663	0.616845	0.801792	NOT
AC005154.	625.0789	0.11289428	0.225665	0.500273991	0.616882	0.801802	NOT
RGS13	6.744054	0.3277328	0.655301	0.500125497	0.616987	0.801818	NOT
FDXACB1	99.11027	-0.0876457	0.175256	-0.50010225	0.617003	0.801818	NOT
RUNX1	1130.236	-0.1320958	0.264156	-0.50006668	0.617028	0.801818	NOT
RP4-724E1	22.96621	-0.1907854	0.381535	-0.50004634	0.617042	0.801818	NOT
PIK3CB	1517.37	-0.0806704	0.16133	-0.50003401	0.617051	0.801818	NOT
NECAB2	1000.442	0.30047286	0.60094	0.500004393	0.617072	0.801818	NOT
PRPS2	1359.596	-0.1228707	0.245768	-0.49994631	0.617113	0.801833	NOT
CTD-2083I	13.58931	-0.1392316	0.278556	-0.49983437	0.617192	0.801897	NOT
ZNF280B	22.60312	0.28602987	0.572594	0.499533866	0.617403	0.802055	NOT
SRPK3	37.99117	0.22540052	0.451232	0.499522609	0.617411	0.802055	NOT
FTH1P1	3.575582	0.18961217	0.379601	0.499503474	0.617425	0.802055	NOT
FSIP2-AS1	3.640752	0.23195889	0.464417	0.499462774	0.617453	0.802055	NOT
RP11-47I2	6.559412	0.28846668	0.577575	0.499444328	0.617466	0.802055	NOT
TOP1	3334.569	0.06812508	0.136416	0.499393363	0.617502	0.802055	NOT

SCARNA5	1.505382	0.28427778	0.569294	0.499351516	0.617532	0.802055	NOT
LINC00675	8.028686	0.29043165	0.581674	0.49930335	0.617566	0.802055	NOT
GAPDHP71	3.718885	0.23327609	0.467236	0.49926861	0.61759	0.802055	NOT
RP11-641J	2.109938	0.23982519	0.480378	0.499242229	0.617609	0.802055	NOT
SLC23A1	1364.246	0.15268273	0.305956	0.4990349	0.617755	0.802182	NOT
MT-ND2	281752.3	-0.1388387	0.278237	-0.49899476	0.617783	0.802182	NOT
CHCHD2P6	22.19976	-0.1303725	0.261292	-0.49895342	0.617812	0.802182	NOT
LINC0064(	2.422048	-0.296789	0.594845	-0.49893509	0.617825	0.802182	NOT
AC007064.	1.861737	-0.4207389	0.843348	-0.49889115	0.617856	0.802184	NOT
CORT	8.183199	-0.1669156	0.334615	-0.49882911	0.6179	0.802187	NOT
ZNF876P	19.10536	-0.1375319	0.275723	-0.49880403	0.617917	0.802187	NOT
CYP2A7P1	2.301713	-0.5418695	1.086557	-0.49870315	0.617989	0.802241	NOT
INSRR	7.780742	-0.2739701	0.54942	-0.49865346	0.618024	0.802248	NOT
LINC0100(	444.3986	0.12088878	0.242493	0.498523855	0.618115	0.802328	NOT
AKT3	484.8753	-0.1569775	0.314912	-0.49848089	0.618145	0.802329	NOT
AQP4	56.40173	0.28902935	0.579897	0.498415384	0.618191	0.80235	NOT
GPR64	250.5241	0.25199387	0.505844	0.498165186	0.618368	0.802529	NOT
RP3-4770	6.699776	-0.1592671	0.31974	-0.49811488	0.618403	0.802529	NOT
ZNF337	75.5968	0.13645664	0.273971	0.498069788	0.618435	0.802529	NOT
RP11-459I	28.79133	0.18630716	0.374072	0.498052019	0.618447	0.802529	NOT
HPS1	3010.978	0.0814876	0.163708	0.497760811	0.618653	0.802733	NOT
RTP3	1298.327	-0.2918249	0.586293	-0.49774576	0.618663	0.802733	NOT
CDC5L	2715.505	0.07753889	0.155825	0.497602764	0.618764	0.80281	NOT
SP100	2642.589	-0.0911573	0.183204	-0.4975738	0.618784	0.80281	NOT
ZBTB20-AS	2.737502	-0.2549324	0.51239	-0.4975355	0.618811	0.80281	NOT
RP5-942I1	2.685831	0.27468886	0.552487	0.497185733	0.619058	0.803051	NOT
RP11-510N	23.1055	0.22754683	0.457709	0.497143327	0.619088	0.803051	NOT
RP11-110I	7.959641	-0.1572329	0.316293	-0.49711157	0.61911	0.803051	NOT
AC005387.	3.506996	-0.1777563	0.357584	-0.49710362	0.619116	0.803051	NOT
TLE3	1559.675	-0.1134431	0.228263	-0.49698411	0.6192	0.803122	NOT
KRT18P8	6.889143	-0.2123163	0.427309	-0.4968677	0.619282	0.80319	NOT
RP11-500(	3.000912	0.26436129	0.532188	0.496743994	0.61937	0.803265	NOT
RP11-100(	3.014005	-0.1811537	0.364852	-0.49651288	0.619533	0.803337	NOT
MAGEL2	5.581396	-0.2806858	0.56534	-0.49649034	0.619549	0.803337	NOT
RP4-800G7	89.4401	-0.1683937	0.339174	-0.49648121	0.619555	0.803337	NOT
CDKN2B	522.2757	0.15903392	0.320333	0.496463749	0.619567	0.803337	NOT
C19orf35	11.61871	-0.1678622	0.338135	-0.49643599	0.619587	0.803337	NOT
PARG	752.8576	0.0716809	0.144398	0.496413673	0.619603	0.803337	NOT
IGKV1D-27	0.635025	0.65058776	1.311089	0.496219327	0.61974	NA	NA
RP11-626H	12.59603	0.34551435	0.696311	0.496207134	0.619748	0.803449	NOT
POU6F2-AS	31.35948	0.37977965	0.765366	0.496206673	0.619749	0.803449	NOT
PLS1	1623.425	0.14777134	0.298022	0.4958399	0.620007	0.803746	NOT
GEN1	647.2894	-0.099181	0.200057	-0.49576304	0.620062	0.803778	NOT
DOPEY2	1474.119	-0.1379784	0.278378	-0.49565043	0.620141	0.803838	NOT
AC093627.	28.09918	0.12937865	0.261063	0.495583085	0.620189	0.803838	NOT
AC023347.	4.03258	0.47433541	0.957148	0.49557175	0.620197	0.803838	NOT
RASL10B	494.6459	-0.3068071	0.619201	-0.49548869	0.620255	0.803846	NOT
NFIA-AS2	4.324815	-0.2814116	0.567959	-0.49547885	0.620262	0.803846	NOT
RP11-356(	1.003875	-0.4046667	0.816722	-0.49547678	0.620264	NA	NA

RP11-510M	2.845845	0.17675914	0.35683	0.495359243	0.620347	0.803909	NOT
CTD-2369F	11.17675	-0.1584907	0.319973	-0.49532574	0.62037	0.803909	NOT
EGF	117.8851	0.3998964	0.807495	0.495230698	0.620437	0.803958	NOT
PLXNB1	6382.037	0.10435984	0.210821	0.495016343	0.620589	0.804028	NOT
DNALI1	605.3896	0.20432831	0.412788	0.494995788	0.620603	0.804028	NOT
C15orf62	92.0848	0.15153035	0.306139	0.494972814	0.620619	0.804028	NOT
FUOM	3716.786	0.14854169	0.30012	0.49494124	0.620642	0.804028	NOT
NCBP2-AS2	663.4272	0.10295994	0.208038	0.494910048	0.620664	0.804028	NOT
C19orf52	599.9719	-0.0740501	0.149626	-0.49490232	0.620669	0.804028	NOT
LINC0086	22.02691	0.20375753	0.411909	0.49466631	0.620836	0.804177	NOT
RAB11FIP1	26.22249	-0.1990104	0.402322	-0.4946546	0.620844	0.804177	NOT
BAALC-AS1	34.4321	-0.200565	0.405696	-0.49437315	0.621043	0.804396	NOT
FAM135B	36.63946	-0.2772762	0.561055	-0.49420472	0.621162	0.804512	NOT
SAP18	4619.563	0.07859662	0.159095	0.494023183	0.62129	0.80463	NOT
OXSR1	1649.937	0.06235043	0.126225	0.493964472	0.621331	0.80463	NOT
C9orf85	331.2814	-0.0657016	0.133013	-0.4939495	0.621342	0.80463	NOT
RP1-53C1	0.670604	0.55522315	1.124115	0.493920164	0.621363	NA	NA
AP006285.	19.50705	-0.2428169	0.491661	-0.49387106	0.621397	0.804664	NOT
TMEM176B	53809.72	-0.1781191	0.36075	-0.49374665	0.621485	0.80469	NOT
RP11-725F	3.487305	-0.2533341	0.513088	-0.49374425	0.621487	0.80469	NOT
BRD2	8678.175	0.06661952	0.134939	0.493701401	0.621517	0.80469	NOT
CHMP4A	102.9938	0.16919715	0.342755	0.493638631	0.621561	0.80469	NOT
EFHB	15.03771	0.18028673	0.365225	0.493632461	0.621566	0.80469	NOT
RP11-184F	4.910004	-0.4383654	0.888167	-0.4935617	0.621616	0.804716	NOT
MAP2K4	967.485	-0.0825765	0.167369	-0.49337975	0.621744	0.804812	NOT
RP11-297A	2.976017	0.2022161	0.409897	0.493333831	0.621777	0.804812	NOT
CTNNAP1	2.163191	0.23647131	0.479361	0.493305087	0.621797	0.804812	NOT
IGLV4-3	0.585224	0.53510657	1.08475	0.493299441	0.621801	NA	NA
DPCD	344.8464	0.12373234	0.250832	0.493288469	0.621809	0.804812	NOT
snoU109	6.610065	0.12771524	0.259046	0.493021566	0.621997	0.805018	NOT
RP11-120M	5.783097	-0.2101091	0.426295	-0.492872	0.622103	0.805033	NOT
CHPF2	2487.796	-0.0784122	0.159104	-0.49283507	0.622129	0.805033	NOT
RP11-394A	1.275863	0.50284979	1.020401	0.492796203	0.622157	NA	NA
RP11-138J	29.94446	0.37216605	0.755237	0.492780769	0.622168	0.805033	NOT
MRPL12	2657.619	0.14556728	0.295408	0.492766529	0.622178	0.805033	NOT
SYT16	1.613499	0.40854164	0.829089	0.492759889	0.622182	0.805033	NOT
RFC1	3182.624	0.07589593	0.154024	0.492752878	0.622187	0.805033	NOT
CCDC112	154.8408	0.14950024	0.303456	0.492658735	0.622254	0.805056	NOT
BRD7	1857.009	0.06616953	0.134315	0.49264442	0.622264	0.805056	NOT
HEATR6	532.1983	0.07889343	0.160194	0.492487685	0.622375	0.805161	NOT
FAM73A	1002.052	-0.0902618	0.183305	-0.49241413	0.622427	0.805189	NOT
LINC0118	20.90492	0.23373696	0.474733	0.492354537	0.622469	0.805206	NOT
LETM1P2	2.002228	0.34952846	0.710134	0.492200677	0.622577	0.805308	NOT
RP4-665J2	494.5273	-0.1563753	0.31777	-0.49210301	0.622647	0.80532	NOT
OVGP1	377.7068	0.1831597	0.372213	0.492082966	0.622661	0.80532	NOT
HOXB-AS1	17.94202	0.17977101	0.365358	0.492041072	0.62269	0.80532	NOT
AHCTF1	2231.716	0.08313104	0.168959	0.49201936	0.622706	0.80532	NOT
CRIP1	43.54291	0.18269065	0.371497	0.491769508	0.622882	0.80551	NOT
FAM110C	761.3821	-0.2079383	0.42298	-0.49160276	0.623	0.805561	NOT

RP11-806(	25.42952	-0.2136665	0.43464	-0.49159385	0.623006	0.805561	NOT
HIST1H1D	1.678923	-0.2997209	0.609699	-0.49158834	0.62301	0.805561	NOT
TOR3A	2764.012	0.11521852	0.234407	0.491530928	0.623051	0.805575	NOT
CTD-2540I	1.543166	0.36553343	0.743819	0.49142795	0.623124	0.80563	NOT
GAPDHP72	7.495361	0.21121958	0.4299	0.491322979	0.623198	0.805688	NOT
MXD1	576.4961	-0.1096646	0.22323	-0.49126333	0.62324	0.805704	NOT
ITGAE	394.39	0.13929919	0.28372	0.490974713	0.623444	0.805898	NOT
RP11-297M	7.592521	-0.1924113	0.391907	-0.49096183	0.623453	0.805898	NOT
PSAP	70330.83	-0.0714534	0.145549	-0.49092494	0.62348	0.805898	NOT
ATP1A3	15.25122	-0.2217092	0.451705	-0.49082702	0.623549	0.805931	NOT
WASH2P	130.8224	-0.1041749	0.212253	-0.49080488	0.623564	0.805931	NOT
AC007246.	423.6365	0.08015826	0.163337	0.490753919	0.623601	0.805939	NOT
FAM78A	264.6262	-0.1417875	0.288958	-0.49068492	0.623649	0.805942	NOT
CTD-3126E	1.859437	0.22692192	0.462477	0.490666684	0.623662	0.805942	NOT
LINC0154(	31.88898	-0.2337242	0.476389	-0.49061605	0.623698	0.80595	NOT
DOK2	353.3751	0.16431782	0.335058	0.49041645	0.623839	0.806093	NOT
WWTR1-AS1	7.664979	0.14852169	0.302873	0.490376541	0.623867	0.806093	NOT
RERE	2892.226	0.08194072	0.167173	0.490154018	0.624025	0.806213	NOT
WT1-AS	6.251506	-0.3616525	0.737855	-0.49014054	0.624034	0.806213	NOT
RP11-15J1	10.85227	-0.2189413	0.446711	-0.49011881	0.62405	0.806213	NOT
CERKL	183.6337	0.13872781	0.283142	0.489958051	0.624164	0.806303	NOT
NRD1	6956.749	-0.0695385	0.141958	-0.48985103	0.624239	0.806303	NOT
BX322557.	46.4847	-0.1266333	0.258526	-0.48982825	0.624255	0.806303	NOT
HTR1B	3.25949	-0.2945222	0.601289	-0.48981823	0.624263	0.806303	NOT
AC114730.	5.186856	-0.3706061	0.756641	-0.48980457	0.624272	0.806303	NOT
RP11-69M1	32.17095	-0.1287589	0.262898	-0.48976853	0.624298	0.806303	NOT
LINC00551	0.686868	0.46753974	0.954778	0.489684329	0.624357	NA	NA
GLA	1407.922	0.13310487	0.271825	0.489670439	0.624367	0.806354	NOT
ATP7B	1348.331	-0.1580941	0.322899	-0.48960785	0.624411	0.806373	NOT
LRRC8E	31.26068	-0.1903593	0.388892	-0.48949116	0.624494	0.806441	NOT
DKFZp779M	137.509	0.22297299	0.455694	0.489303714	0.624627	0.806574	NOT
PAN3-AS1	18.7083	-0.1291996	0.264119	-0.48917256	0.62472	0.806641	NOT
MTND5P10	2.146969	-0.3081722	0.63002	-0.48914638	0.624738	0.806641	NOT
PTGFR	457.9009	0.31822445	0.650681	0.489063791	0.624797	0.806663	NOT
LINC00383	4.172448	0.54312943	1.110656	0.489016991	0.62483	0.806663	NOT
CTD-2587H	25.93878	0.15331041	0.31352	0.488996702	0.624844	0.806663	NOT
SYT9	18.28862	-0.433346	0.886404	-0.48888116	0.624926	0.806724	NOT
SOWAHC	2120.587	0.11257982	0.230305	0.488828907	0.624963	0.806724	NOT
DKK1	612.969	0.39412207	0.806392	0.488747549	0.62502	0.806724	NOT
MDH1	9648.905	-0.0905932	0.185359	-0.48874414	0.625023	0.806724	NOT
RP11-794(	7.478113	0.2562379	0.524372	0.48865704	0.625085	0.806724	NOT
AC098830.	3.66054	-0.4646268	0.950872	-0.48863243	0.625102	0.806724	NOT
RPL3P4	614.2102	-0.1732776	0.354664	-0.48856815	0.625147	0.806724	NOT
RP11-255(	9.454871	-0.137294	0.281022	-0.48855303	0.625158	0.806724	NOT
VPS11	1906.299	-0.0574992	0.117693	-0.48855166	0.625159	0.806724	NOT
LARP1	9445.863	0.07132839	0.146029	0.488452169	0.62523	0.806777	NOT
RP11-21G1	2.024905	-0.2434704	0.49859	-0.48831792	0.625325	0.806861	NOT
CTBP2P8	1.263969	-0.301105	0.616673	-0.48827345	0.625356	NA	NA
RP11-703I	40.01962	-0.1183178	0.242404	-0.48810094	0.625478	0.807002	NOT

AC099668.	8.638366	-0.196134	0.401901	-0.48801571	0.625539	0.807002	NOT
MALRD1	8.022257	-0.3097148	0.634658	-0.48800242	0.625548	0.807002	NOT
LMNA	22534.95	0.10141386	0.207817	0.487995655	0.625553	0.807002	NOT
ARFGAP3	2715.756	0.0804547	0.164925	0.487827426	0.625672	0.807112	NOT
IRX6	3.799905	-0.3699838	0.758488	-0.48779157	0.625697	0.807112	NOT
ZNF843	33.93412	-0.1510363	0.309722	-0.48765093	0.625797	0.807202	NOT
IGHV3-15	82.8812	-0.3621451	0.7429	-0.48747498	0.625922	0.807324	NOT
ZNF655	1748.34	0.07918922	0.162465	0.487423685	0.625958	0.807333	NOT
ZNF236	387.2932	-0.1040256	0.213493	-0.48725605	0.626077	0.807448	NOT
ZNF98	6.143887	-0.4148574	0.851538	-0.48718589	0.626127	0.807473	NOT
CXCR5	1.27039	0.4303299	0.883438	0.487108472	0.626181	NA	NA
GPR85	32.29223	0.16522214	0.339237	0.487039976	0.62623	0.807568	NOT
TBRG4	3529.529	0.07912332	0.162533	0.486812591	0.626391	0.807724	NOT
CTD-2091N	2.583945	0.43629833	0.896474	0.486682849	0.626483	0.807724	NOT
RP11-139H	7.883009	0.15620997	0.320971	0.486679323	0.626486	0.807724	NOT
VWA7	282.8696	-0.1625957	0.334098	-0.48667135	0.626491	0.807724	NOT
HYPK	68.86544	-0.0914023	0.187816	-0.48665909	0.6265	0.807724	NOT
RARS2	1185.715	-0.0766769	0.157596	-0.48654157	0.626583	0.807733	NOT
PFN1P3	2.233946	-0.3090817	0.635277	-0.48653052	0.626591	0.807733	NOT
ACRBP	86.23656	0.15652361	0.321718	0.486523679	0.626596	0.807733	NOT
ANKRD36BF	10.06383	-0.2747261	0.564881	-0.48634359	0.626724	0.807823	NOT
RP11-26H1	3.351828	-0.1645089	0.338313	-0.48626301	0.626781	0.807823	NOT
LGALS8-AS	15.21354	-0.164391	0.338111	-0.48620419	0.626822	0.807823	NOT
TMEM151A	73.0413	-0.2590945	0.532912	-0.48618625	0.626835	0.807823	NOT
WI2-1528C	7.649424	0.1643279	0.33803	0.486133603	0.626872	0.807823	NOT
HIST2H2BF	40.28544	-0.2057026	0.423144	-0.48612927	0.626876	0.807823	NOT
MBD5	438.6617	-0.0889868	0.183062	-0.4861008	0.626896	0.807823	NOT
SLC25A30	2266.055	-0.1568128	0.322601	-0.48608918	0.626904	0.807823	NOT
CTD-2291D	7.4188	-0.3639525	0.749	-0.48591772	0.627026	0.807911	NOT
CYTH3	688.5533	-0.1198973	0.246749	-0.48590847	0.627032	0.807911	NOT
RP11-733C	10.43588	-0.1807749	0.372282	-0.48558537	0.627261	0.808133	NOT
WASF3	442.4128	-0.2368143	0.487692	-0.48558125	0.627264	0.808133	NOT
LSP1	1229.76	0.15189721	0.312871	0.485494256	0.627326	0.808175	NOT
RP11-178H	42.19954	0.14177979	0.292161	0.485279064	0.627478	0.808333	NOT
KATNB1	911.3846	-0.0802221	0.165339	-0.48519649	0.627537	0.80837	NOT
SNHG15	351.1749	0.1644339	0.339101	0.484911901	0.627739	0.808568	NOT
ZNF677	72.36319	-0.159158	0.328242	-0.4848799	0.627762	0.808568	NOT
RP11-795F	47.76922	0.17147411	0.353662	0.484853427	0.62778	0.808568	NOT
RP11-77H9	49.81883	0.09833088	0.202869	0.48470136	0.627888	0.808655	NOT
VCPIP1	1404.965	0.07445378	0.15362	0.48466208	0.627916	0.808655	NOT
P2RY13	91.18189	-0.1560942	0.322088	-0.48463194	0.627937	0.808655	NOT
UBE2F	1312.046	0.07848185	0.16203	0.484364752	0.628127	0.808861	NOT
AC022154.	6.681491	-0.1518093	0.313504	-0.48423366	0.62822	0.808943	NOT
SNORD89	4.271763	0.14422392	0.297918	0.484105985	0.628311	0.808997	NOT
NAA35	544.6805	0.06993222	0.14447	0.484060339	0.628343	0.808997	NOT
C17orf47	2.206219	-0.2457066	0.507608	-0.48404795	0.628352	0.808997	NOT
RP11-159D	32.67171	-0.0995391	0.205664	-0.48399023	0.628393	0.809005	NOT
GPR150	20.98743	-0.2319098	0.479241	-0.48391022	0.62845	0.809005	NOT
CHRM3-AS2	7.169176	-0.2012594	0.415906	-0.4839053	0.628453	0.809005	NOT

HDAC9	88.91914	-0.1612646	0.333299	-0.48384374	0.628497	0.809005	NOT
WDR61	1565.246	-0.0589854	0.121914	-0.48382934	0.628507	0.809005	NOT
POP4	1531.587	-0.0673991	0.139321	-0.48376727	0.628551	0.809023	NOT
CLPP	2621.72	-0.0896336	0.185312	-0.48368974	0.628606	0.809056	NOT
ZNF263	1159.266	-0.0543118	0.112308	-0.48359556	0.628673	0.809066	NOT
USP22	6012.719	0.09275115	0.191795	0.483594632	0.628674	0.809066	NOT
ELMO3	563.0613	-0.2131017	0.440787	-0.48345693	0.628771	0.809153	NOT
AC092338.	4.060859	-0.2055805	0.425483	-0.48317	0.628975	0.809364	NOT
C15orf39	1323.647	0.10766973	0.222853	0.483141615	0.628995	0.809364	NOT
RP11-96L1	5.541396	0.15457586	0.319985	0.483072112	0.629045	0.80939	NOT
SPIRE2	970.4274	0.1367641	0.283291	0.48276965	0.629259	0.809612	NOT
LRRC9	3.758492	-0.2118325	0.438809	-0.48274426	0.629277	0.809612	NOT
TEKT3	4.290218	-0.2278784	0.472204	-0.48258435	0.629391	0.809685	NOT
FAM188A	511.003	-0.0683271	0.141597	-0.48254504	0.629419	0.809685	NOT
AP1B1	5482.724	0.07048501	0.146084	0.482498047	0.629452	0.809685	NOT
UNC13D	406.8129	0.1465383	0.303709	0.482496158	0.629454	0.809685	NOT
PPT2-EGFI	44.31328	-0.0996582	0.206577	-0.48242641	0.629503	0.809711	NOT
TM4SF5	7263.292	0.20364835	0.422247	0.482296229	0.629596	0.809791	NOT
ZNF253	309.6291	-0.1125586	0.233426	-0.48220295	0.629662	0.809838	NOT
IGFBP2	19911.89	0.24422288	0.506632	0.482052223	0.629769	0.809901	NOT
ZNF552	291.244	-0.1028587	0.213378	-0.48205037	0.62977	0.809901	NOT
ARC	15.06685	0.21420089	0.444512	0.481879082	0.629892	0.809952	NOT
RP4-791C1	3.989917	-0.1925758	0.399639	-0.48187395	0.629895	0.809952	NOT
GS1-304P7	1.673821	-0.2814605	0.584102	-0.48186862	0.629899	0.809952	NOT
RP11-398I	0.749425	0.5854175	1.214902	0.481863868	0.629903	NA	NA
CEP192	696.3104	-0.0991911	0.205893	-0.48176053	0.629976	0.810012	NOT
RP11-118I	1.685144	-0.3050611	0.633478	-0.48156565	0.630115	0.810152	NOT
RP11-776H	25.58512	0.40478906	0.840829	0.481416794	0.63022	0.810249	NOT
TNFRSF4	235.0548	-0.1360287	0.282645	-0.48127113	0.630324	0.810332	NOT
ZNF585B	323.3475	-0.1269791	0.263872	-0.48121404	0.630364	0.810332	NOT
HIRIP3	1124.11	-0.1043019	0.216764	-0.48117609	0.630391	0.810332	NOT
AC010894.	85.0081	-0.1334105	0.2773	-0.481105	0.630442	0.810332	NOT
GPSM3	565.8491	0.13073111	0.271753	0.481065863	0.63047	0.810332	NOT
MRPS18AP1	2.469461	-0.2383957	0.495561	-0.48106257	0.630472	0.810332	NOT
PRR4	24.11445	-0.1081861	0.224904	-0.48103211	0.630494	0.810332	NOT
MCM8-AS1	2.453598	-0.2426579	0.504636	-0.48085753	0.630618	0.810453	NOT
ARL5B	2502.905	0.09182821	0.19102	0.480724716	0.630712	0.810536	NOT
RP11-839I	7.245097	0.22354478	0.465065	0.480674208	0.630748	0.810544	NOT
SH3KBP1	1685.026	-0.0982243	0.204393	-0.48056474	0.630826	0.810561	NOT
MFSD7	456.926	0.19917541	0.414483	0.480539688	0.630844	0.810561	NOT
RPS15AP38	3.184487	0.21075006	0.438579	0.480529481	0.630851	0.810561	NOT
SMG1P4	2.321317	-0.350021	0.72863	-0.48038239	0.630956	0.810621	NOT
MAST4-AS1	6.783221	-0.1685155	0.350797	-0.48037917	0.630958	0.810621	NOT
C14orf2	3244.712	-0.101914	0.212178	-0.48032239	0.630998	0.810635	NOT
ZNF32	1078.739	-0.0933097	0.194349	-0.48011316	0.631147	0.810775	NOT
PGC	795.5259	0.47135897	0.981825	0.480084491	0.631167	0.810775	NOT
B3GAT1	189.7224	0.32137334	0.66971	0.479869757	0.63132	0.810904	NOT
RP11-561C	2.052269	0.23418236	0.488023	0.479858994	0.631328	0.810904	NOT
RP11-81H1	28.68688	0.3605992	0.751592	0.47978079	0.631383	0.810923	NOT

FSTL3	1829.539	-0.213252	0.444547	-0.47970634	0.631436	0.810923	NOT
RP11-305K	100.5029	-0.1653879	0.344791	-0.47967593	0.631458	0.810923	NOT
FHAD1	52.65874	-0.2096588	0.437123	-0.47963384	0.631488	0.810923	NOT
CBX8	609.1195	0.10270806	0.214141	0.479628393	0.631492	0.810923	NOT
RP4-809F1	0.695925	0.67193331	1.401561	0.479417908	0.631641	NA	NA
SLC29A2	918.0311	0.13835995	0.288644	0.479345179	0.631693	0.811143	NOT
TMSB15A	6.693256	0.23676692	0.494131	0.479157999	0.631826	0.811276	NOT
C6orf1	1065.946	0.1304634	0.272358	0.479015171	0.631928	0.811335	NOT
GZMM	77.65402	-0.1898457	0.396334	-0.4790038	0.631936	0.811335	NOT
GS1-124K5	15.61134	-0.1840953	0.384359	-0.47896667	0.631962	0.811335	NOT
CTB-129P6	6.157134	-0.1549205	0.32349	-0.47890339	0.632007	0.811355	NOT
REXO2	1459.353	0.08563736	0.178864	0.478785038	0.632092	0.81139	NOT
PDCD4	2145.222	-0.0811597	0.169513	-0.47878059	0.632095	0.81139	NOT
ZNF444P1	2.969328	-0.3866891	0.808051	-0.47854563	0.632262	0.811566	NOT
MOBP	2.592717	-0.2601286	0.543703	-0.47843905	0.632338	0.811625	NOT
LHFPL3-AS	1.30167	-0.4834357	1.010537	-0.47839485	0.632369	NA	NA
RP5-881L2	9.07859	-0.2617182	0.547097	-0.47837591	0.632383	0.811645	NOT
RP11-708J	27.74972	-0.1340265	0.280226	-0.47827921	0.632451	0.811681	NOT
AC010492.	5.228976	-0.2992179	0.625663	-0.47824162	0.632478	0.811681	NOT
POLR2J2	4.374224	-0.2263608	0.473351	-0.47820953	0.632501	0.811681	NOT
CCNA1	1.283579	0.34956608	0.731176	0.478087199	0.632588	NA	NA
GRIK1-AS1	2.036145	-0.2270055	0.474953	-0.477954	0.632683	0.811876	NOT
BMS1P10	27.64088	-0.1801561	0.377056	-0.47779666	0.632795	0.811982	NOT
PCDHA6	5.254875	0.31362919	0.656544	0.477697193	0.632866	0.812034	NOT
PRRC2B	3228.78	-0.1056226	0.221214	-0.4774669	0.63303	0.812206	NOT
CTD-2256P	2.234765	-0.2093726	0.438932	-0.47700447	0.633359	0.81259	NOT
IFNGR2	2742.12	0.09676751	0.202921	0.476873515	0.633452	0.812671	NOT
RP11-109A	2.629389	-0.215655	0.452354	-0.4767396	0.633548	0.812755	NOT
EML5	9.837747	0.29061124	0.609704	0.476643173	0.633616	0.812805	NOT
BAHD1	1272.844	0.0763897	0.16031	0.476513389	0.633709	0.812885	NOT
AC004447.	495.9514	-0.1075538	0.225746	-0.47643695	0.633763	0.812916	NOT
RLF	649.0072	0.09019107	0.189349	0.476320665	0.633846	0.812984	NOT
CD1C	67.16391	-0.1966619	0.412963	-0.47622172	0.633916	0.813036	NOT
CTD-2554C	3.871942	0.31124882	0.653713	0.476124245	0.633986	0.813052	NOT
C5orf56	210.6809	-0.112832	0.236982	-0.47612025	0.633989	0.813052	NOT
FAM3C	984.6449	0.09102311	0.191213	0.476029428	0.634053	0.813096	NOT
SNORD6	6.165473	0.17397925	0.365607	0.475863792	0.634171	0.813162	NOT
SYCE1L	28.35077	0.20090006	0.422207	0.47583303	0.634193	0.813162	NOT
AC093162.	34.60644	0.1819025	0.382309	0.475799648	0.634217	0.813162	NOT
MIF4GD	1031.404	0.0848451	0.178336	0.475759738	0.634246	0.813162	NOT
MT-ND3	97586.73	-0.1490601	0.313336	-0.47571984	0.634274	0.813162	NOT
TRGV5	8.885946	-0.2145282	0.451005	-0.47566718	0.634312	0.813162	NOT
RP11-182J	2.374065	-0.2558137	0.537804	-0.4756637	0.634314	0.813162	NOT
CEBPB	5342.841	-0.1210877	0.254806	-0.47521518	0.634634	0.813481	NOT
RP11-94P1	1.710525	0.42468844	0.893722	0.475190776	0.634651	0.813481	NOT
CTD-2006C	14.35032	-0.1652659	0.34779	-0.47518814	0.634653	0.813481	NOT
ANXA8L1	11.35626	0.35857506	0.754833	0.475039145	0.634759	0.813578	NOT
WIPI2	4095.486	-0.0697416	0.146833	-0.47497209	0.634807	0.813601	NOT
RP11-336N	3.964904	-0.2364915	0.498306	-0.47459069	0.635079	0.813867	NOT

RP11-146I	1.85317	-0.2138698	0.450665	-0.47456459	0.635097	0.813867	NOT
RP11-386I	4.730685	0.19216585	0.404939	0.474555281	0.635104	0.813867	NOT
RP11-121(	1.505778	0.24059986	0.507072	0.474488184	0.635152	0.81389	NOT
TRBV29-1	8.054741	-0.2347318	0.494881	-0.47432014	0.635272	0.813992	NOT
RP4-742J2	2.06428	0.2794783	0.589271	0.474278161	0.635302	0.813992	NOT
RP11-7I15	4.060356	0.27754527	0.585254	0.474230295	0.635336	0.813992	NOT
UGGT1	5639.27	0.09645735	0.203407	0.47420751	0.635352	0.813992	NOT
MIR497HG	31.75609	0.15145197	0.319507	0.474017072	0.635488	0.814119	NOT
RP11-493I	4.896378	0.21566089	0.454996	0.473984154	0.635511	0.814119	NOT
RP11-686(	40.68392	-0.1285288	0.271207	-0.47391445	0.635561	0.814145	NOT
RP11-34P1	17.15402	0.21535192	0.454516	0.473805367	0.635639	0.814193	NOT
SNX16	286.2492	0.0941484	0.198719	0.473776963	0.635659	0.814193	NOT
AC004893.	105.9292	0.09187991	0.193948	0.47373527	0.635689	0.814193	NOT
CTD-2547I	1.554952	-0.2422651	0.511516	-0.47362142	0.63577	0.814258	NOT
RP11-192I	17.52603	0.16562929	0.349787	0.473514403	0.635846	0.814318	NOT
SMU1	2308.164	0.05656719	0.119485	0.473425637	0.63591	0.81436	NOT
TSPAN31	1623.525	-0.1067231	0.225514	-0.47324346	0.636039	0.814416	NOT
TMEM198	363.9383	-0.173201	0.366011	-0.47321196	0.636062	0.814416	NOT
CYTH4	507.2961	0.14181951	0.299713	0.473184035	0.636082	0.814416	NOT
BRSK2	102.3213	0.29801974	0.629856	0.473155276	0.636102	0.814416	NOT
C1orf50	386.8595	-0.0942593	0.199215	-0.47315399	0.636103	0.814416	NOT
GHRL	20.42488	-0.1349921	0.28541	-0.47297589	0.63623	0.814541	NOT
ZNF304	310.2289	-0.1108224	0.234476	-0.47263773	0.636472	0.814743	NOT
HOXD-AS2	9.66752	0.27652195	0.585094	0.472611445	0.63649	0.814743	NOT
RP1-197B1	48.15471	0.11801362	0.249707	0.472607604	0.636493	0.814743	NOT
RBFA	1127.045	0.0871033	0.184334	0.472529591	0.636549	0.814743	NOT
ZNF672	2141.679	-0.0702592	0.148689	-0.47252401	0.636553	0.814743	NOT
RP11-379I	25.6727	0.15579729	0.329777	0.472432851	0.636618	0.814743	NOT
MAP2K2	7348.227	0.11465057	0.242682	0.472430959	0.636619	0.814743	NOT
SCRN2	2505.122	-0.110048	0.232953	-0.47240407	0.636638	0.814743	NOT
PAXBP1	837.7045	-0.0798073	0.168954	-0.47236234	0.636668	0.814743	NOT
RP11-39K2	2.638213	-0.2073618	0.439016	-0.47233326	0.636689	0.814743	NOT
MFSD9	703.4385	-0.0947432	0.200724	-0.47200679	0.636922	0.814974	NOT
CYP4A22	3529.182	-0.2420962	0.51292	-0.47199601	0.63693	0.814974	NOT
KLHL21	2500.089	0.11139615	0.236035	0.471947599	0.636964	0.81498	NOT
XYLT2	1711.745	0.07209617	0.152818	0.471777404	0.637086	0.815044	NOT
DCDC2B	2.79862	-0.1788732	0.37916	-0.47176114	0.637097	0.815044	NOT
CASKIN2	1890.483	0.07507186	0.159134	0.471751242	0.637104	0.815044	NOT
LCE2D	1.062009	-0.5086771	1.078275	-0.47175064	0.637105	NA	NA
ZNF536	1.450591	0.44113306	0.935625	0.47148486	0.637295	0.815174	NOT
IGHV1-24	41.20988	-0.3784662	0.802885	-0.47138272	0.637367	0.815174	NOT
DERL3	619.3874	-0.2342205	0.496957	-0.47130978	0.63742	0.815174	NOT
NPIPB4	47.96518	0.175749	0.372895	0.471309193	0.63742	0.815174	NOT
LAMB3	1390.606	-0.1820347	0.386233	-0.471308	0.637421	0.815174	NOT
CTD-3247I	2.127729	-0.3074447	0.652345	-0.47129163	0.637432	0.815174	NOT
RP11-359J	4.573274	-0.2319176	0.492103	-0.47127887	0.637442	0.815174	NOT
GOLGA6L4	3.608584	-0.1911905	0.40569	-0.47127233	0.637446	0.815174	NOT
AC087650.	2.75449	-0.185398	0.393456	-0.47120382	0.637495	0.815198	NOT
SPRR2E	0.57046	1.07650052	2.285205	0.47107399	0.637588	NA	NA

CCDC91	755.9752	-0.059489	0.126293	-0.47103951	0.637613	0.81531	NOT
SPRED2	1584.107	0.08632882	0.183378	0.470770949	0.637804	0.815425	NOT
RP11-29G8	26.50279	0.12704323	0.269867	0.47076232	0.63781	0.815425	NOT
AC104809.	21.79628	-0.299228	0.635625	-0.47076184	0.637811	0.815425	NOT
RP11-755F	33.77782	0.10043459	0.213352	0.470745341	0.637823	0.815425	NOT
TIMM21	913.3275	0.06593115	0.14007	0.470701935	0.637854	0.815426	NOT
PDS5A	4130.167	0.07049737	0.149805	0.47059554	0.63793	0.815485	NOT
BOLA2P2	1.945811	0.20263472	0.430739	0.470435324	0.638044	0.815592	NOT
LA16c-431	4.259525	-0.2193614	0.466364	-0.47036477	0.638094	0.815618	NOT
IGLV2-23	130.2706	-0.3197369	0.679911	-0.47026313	0.638167	0.815645	NOT
ZNF511	664.148	-0.1021785	0.2173	-0.4702181	0.638199	0.815645	NOT
CTA-246H3	2.312061	0.38253511	0.813626	0.47016112	0.63824	0.815645	NOT
ANKRD30A	3.62343	-0.4203157	0.894098	-0.47010025	0.638283	0.815645	NOT
PFN1P1	19.80521	0.19054904	0.405412	0.470013165	0.638346	0.815645	NOT
PTGER1	20.60077	-0.2737855	0.582584	-0.46995012	0.638391	0.815645	NOT
CERS4	4004.812	-0.1425521	0.303356	-0.46991659	0.638415	0.815645	NOT
RP11-727A	2.596886	-0.1654464	0.352126	-0.46985008	0.638462	0.815645	NOT
USP15	1529.718	0.0548257	0.1167	0.469802359	0.638496	0.815645	NOT
RP11-396C	6.448099	0.15436053	0.328573	0.469791048	0.638504	0.815645	NOT
RP11-218M	544.0463	0.09876459	0.210232	0.469788201	0.638506	0.815645	NOT
RP13-516M	20.45816	-0.1253847	0.266898	-0.46978598	0.638508	0.815645	NOT
RAB43P1	50.36605	-0.1212547	0.258124	-0.46975423	0.638531	0.815645	NOT
ARPC5L	1812.998	0.0880851	0.187522	0.469732672	0.638546	0.815645	NOT
RP11-97C1	68.31728	0.11300072	0.240578	0.46970486	0.638566	0.815645	NOT
HTR3B	3.532293	0.38842426	0.82716	0.469587831	0.63865	0.815713	NOT
GNGT2	48.62775	-0.14728	0.313718	-0.46946554	0.638737	0.815769	NOT
CFAP69	92.9743	-0.1315065	0.280134	-0.46944193	0.638754	0.815769	NOT
CNOT6LP1	1.680401	-0.3116148	0.66398	-0.46931325	0.638846	0.815848	NOT
C16orf58	2174.703	-0.1154847	0.246175	-0.46911611	0.638987	0.81598	NOT
SMIM8	279.7235	0.08513687	0.181496	0.469084535	0.639009	0.81598	NOT
ARHGEF10	285.5462	-0.1574136	0.335694	-0.46891922	0.639127	0.816093	NOT
SMARCD3	571.4269	0.19324567	0.41218	0.468837771	0.639186	0.816129	NOT
SERTAD4-A	20.95655	-0.2283851	0.487198	-0.46877267	0.639232	0.81615	NOT
MRPS17	721.0637	0.09076339	0.193647	0.468705674	0.63928	0.816173	NOT
PP2D1	8.545691	-0.1082802	0.231096	-0.46855108	0.639391	0.816236	NOT
RP11-10K1	6.803002	-0.2086382	0.445322	-0.46851077	0.639419	0.816236	NOT
SYP	143.431	-0.183841	0.39241	-0.46849231	0.639433	0.816236	NOT
LRIG2	484.0617	-0.0732259	0.156309	-0.46846757	0.63945	0.816236	NOT
LCN10	2.882884	-0.4266654	0.911164	-0.46826439	0.639596	0.816376	NOT
OXA1L	6559.009	0.06776035	0.144724	0.468205157	0.639638	0.816376	NOT
FGF10	1.531482	-0.3625659	0.774441	-0.46816495	0.639667	0.816376	NOT
RNF38	1157.209	0.08834194	0.188706	0.468146416	0.63968	0.816376	NOT
CTD-2007I	4.546196	0.19934462	0.42591	0.468043586	0.639753	0.816431	NOT
AC021218.	176.0169	-0.2563766	0.548106	-0.46775034	0.639963	0.81666	NOT
AC008781.	2.068515	-0.1784954	0.381664	-0.46767655	0.640016	0.816689	NOT
CNR2	7.433679	-0.216645	0.463454	-0.46745702	0.640173	0.816843	NOT
PPP1R12C	2936.638	-0.0583538	0.124841	-0.46742437	0.640196	0.816843	NOT
MSN	11520.58	0.11451847	0.245085	0.46726031	0.640314	0.816949	NOT
FADS1	6934.687	0.17914727	0.383466	0.467179508	0.640371	0.816949	NOT

NTM	117.5376	-0.2612854	0.559305	-0.4671608	0.640385	0.816949	NOT
CTCF	1919.29	-0.0542584	0.11615	-0.46713988	0.6404	0.816949	NOT
ABHD4	2559.024	0.10928026	0.233969	0.467070559	0.640449	0.816955	NOT
AC074289.	55.08946	-0.1688845	0.361599	-0.46704886	0.640465	0.816955	NOT
GAPDH	113857.6	0.10744649	0.23013	0.466895381	0.640575	0.817057	NOT
RP11-594N	1.465431	0.42600394	0.912515	0.466846152	0.64061	0.817063	NOT
PLGRKT	668.2193	-0.0733985	0.157244	-0.46678141	0.640656	0.817084	NOT
TMLHE	461.1653	-0.0829895	0.177891	-0.46651978	0.640843	0.817284	NOT
RP11-360N	1.933472	-0.2353715	0.504609	-0.46644338	0.640898	0.81731	NOT
EIF3KP1	1.343795	0.34201965	0.733273	0.466429013	0.640908	NA	NA
NDUFA12	2084.272	0.08298135	0.177916	0.466406942	0.640924	0.81731	NOT
GAPDHP44	4.073053	0.20324489	0.436148	0.465999579	0.641216	0.817644	NOT
C16orf87	676.1979	-0.1124644	0.241385	-0.46591249	0.641278	0.817685	NOT
AC106876.	88.58883	0.14196078	0.304839	0.4656906	0.641437	0.817849	NOT
RARRES2P8	24.64505	-0.6615224	1.421395	-0.46540347	0.641643	0.818062	NOT
NMRK2	2.326781	0.42068769	0.903981	0.46537236	0.641665	0.818062	NOT
AC019097.	3.49135	-0.1684733	0.362109	-0.46525567	0.641748	0.818085	NOT
CCDC13-AS	11.23698	0.20737857	0.44576	0.465225054	0.64177	0.818085	NOT
C9orf64	1096.983	-0.0850326	0.182779	-0.4652215	0.641773	0.818085	NOT
RP5-1061H	48.23145	0.12308889	0.264673	0.465061119	0.641888	0.818193	NOT
ADCY2	3.920491	-0.29691	0.638663	-0.464893	0.642008	0.818308	NOT
CH17-385C	2.804658	-0.2055593	0.442313	-0.46473688	0.64212	0.818412	NOT
TMEM86A	312.1284	0.1171635	0.25215	0.46465804	0.642176	0.818445	NOT
NUDT3	1033.662	0.06401501	0.137794	0.464570947	0.642239	0.818445	NOT
CD79B	136.5313	-0.1138756	0.245141	-0.46453151	0.642267	0.818445	NOT
RP11-141C	5.551465	0.2193397	0.472201	0.46450485	0.642286	0.818445	NOT
PCAT7	70.56999	-0.1589497	0.342203	-0.46448937	0.642297	0.818445	NOT
IL10	14.44357	-0.1905476	0.410315	-0.46439393	0.642366	0.818494	NOT
LDHAP2	2.585922	-0.168502	0.363099	-0.46406609	0.6426	0.818755	NOT
CEP68	800.9125	0.07961756	0.171611	0.463941519	0.64269	0.818822	NOT
TFEC	115.4028	0.17075285	0.368107	0.46386802	0.642742	0.818822	NOT
SGPP1	1090.833	0.09140675	0.197054	0.463865855	0.642744	0.818822	NOT
SURF4	17067.76	-0.0612274	0.132143	-0.46334179	0.643119	0.819262	NOT
RP11-274H	4.802957	0.2191	0.472994	0.463218962	0.643207	0.819336	NOT
FOXO3	1854.007	0.08526422	0.184199	0.462891306	0.643442	0.819548	NOT
ZSCAN30	416.0329	-0.0799774	0.172788	-0.46286475	0.643461	0.819548	NOT
PSG4	1.834269	-0.4615383	0.997229	-0.46282098	0.643493	0.819548	NOT
PLEKHA7	913.4625	0.12120431	0.261883	0.462817774	0.643495	0.819548	NOT
ATP1B2	179.629	0.160436	0.34673	0.462711267	0.643571	0.819607	NOT
FOXC2	29.55995	0.16206473	0.350328	0.462608267	0.643645	0.819663	NOT
CTC-548K1	2.641825	0.23921709	0.517228	0.462498033	0.643724	0.819722	NOT
AL109763.	19.02096	-0.610209	1.319488	-0.462459	0.643752	0.819722	NOT
LINC00888	219.1076	0.13335847	0.288429	0.462362111	0.643822	0.819723	NOT
CTC-444N2	3.157434	0.17567429	0.379977	0.462329094	0.643845	0.819723	NOT
RP11-529K	24.57657	-0.1221032	0.264117	-0.46230772	0.643861	0.819723	NOT
IL10RB	1655.102	0.06010179	0.130013	0.462273693	0.643885	0.819723	NOT
BOLA2B	27.42819	-0.1665498	0.360307	-0.4622448	0.643906	0.819723	NOT
PHPT1	4419.828	0.13608506	0.294425	0.462205536	0.643934	0.819723	NOT
CTB-89H12	360.1257	-0.0870753	0.188468	-0.46201555	0.64407	0.819823	NOT

CTD-2284J	190.757	-0.1409226	0.30502	-0.46201101	0.644073	0.819823	NOT
RAD21-AS1	9.376417	-0.1409294	0.305131	-0.46186608	0.644177	0.819917	NOT
RP11-113K	5.099289	0.18317658	0.396649	0.461809756	0.644218	0.819924	NOT
IGLV2-8	47.96964	0.36609534	0.792921	0.461704482	0.644293	0.819924	NOT
AIF1	839.1607	0.14804108	0.320647	0.461695292	0.6443	0.819924	NOT
LRCH2	34.27773	0.17883038	0.387362	0.461661899	0.644324	0.819924	NOT
HIF1A-AS2	1.65552	0.25886978	0.560752	0.461647692	0.644334	0.819924	NOT
TP53INP1	8316.259	0.132105	0.286207	0.461571789	0.644388	0.819955	NOT
SERPINA2	2.762477	-0.2226941	0.482664	-0.46138512	0.644522	0.820087	NOT
RP11-575F	15.41437	0.30171172	0.654067	0.46128592	0.644593	0.820139	NOT
UBE2FP3	6.38389	-0.1339925	0.290529	-0.46120124	0.644654	0.820178	NOT
EIF4G2	23888.19	0.061672	0.133771	0.461027002	0.644779	0.820286	NOT
SDHAP3	190.2715	-0.1222728	0.265235	-0.46099795	0.6448	0.820286	NOT
RP11-589F	54.06264	-0.1387382	0.301094	-0.46077961	0.644957	0.820384	NOT
LPAR6	619.0246	0.11348577	0.246295	0.460771855	0.644962	0.820384	NOT
RP11-10C2	18.77879	0.10571219	0.229441	0.460738464	0.644986	0.820384	NOT
KCTD5	1159.994	0.0651404	0.141388	0.460722067	0.644998	0.820384	NOT
RP11-218E	2.118623	-0.205327	0.445895	-0.46048275	0.64517	0.820564	NOT
RP1-223B1	1.538388	0.42786744	0.929378	0.460380241	0.645243	0.820619	NOT
RP11-468E	11.91601	0.13193857	0.286717	0.460170451	0.645394	0.820772	NOT
IL1R2	472.7064	0.14152527	0.307642	0.460032377	0.645493	0.82086	NOT
TRAV17	3.482539	0.25727568	0.559417	0.459899833	0.645588	0.820915	NOT
ZNF3	1291.391	-0.0624374	0.135767	-0.45988731	0.645597	0.820915	NOT
RP11-52L5	4.416827	-0.4448968	0.967629	-0.45978046	0.645674	0.820974	NOT
SDPR	1134.921	-0.1922134	0.41818	-0.45964246	0.645773	0.821062	NOT
C12orf60	142.0624	0.12416955	0.270226	0.459502571	0.645873	0.821151	NOT
PRRT3	104.5704	-0.103516	0.225301	-0.45945699	0.645906	0.821154	NOT
GS1-358P8	597.0651	-0.1242141	0.27038	-0.45940508	0.645943	0.821163	NOT
RP11-730A	10.78432	0.12641949	0.275222	0.459336638	0.645992	0.821187	NOT
SDR39U1	341.0277	-0.0712168	0.155067	-0.4592656	0.646043	0.821213	NOT
QSOX1	5710.995	0.21169969	0.461045	0.459173189	0.64611	0.821237	NOT
RP11-290M	2.927443	-0.2278994	0.496344	-0.45915587	0.646122	0.821237	NOT
SHB	1086.104	-0.0937245	0.204203	-0.45897767	0.64625	0.821344	NOT
FAM126A	742.5402	0.11442674	0.249321	0.458953683	0.646267	0.821344	NOT
UBE2DNL	0.545401	0.84683794	1.845755	0.458802937	0.646376	NA	NA
PCDHB10	36.14914	0.18934148	0.412728	0.45875591	0.646409	0.821397	NOT
AGAP6	219.7076	0.11129645	0.24261	0.458745419	0.646417	0.821397	NOT
GAS2	817.256	-0.155296	0.338531	-0.45873536	0.646424	0.821397	NOT
CEP89	604.3119	0.07233921	0.157718	0.458662813	0.646476	0.821397	NOT
RP11-402F	38.65276	0.60060921	1.309486	0.458660146	0.646478	0.821397	NOT
HLA-L	288.3576	0.16899481	0.368484	0.458621556	0.646506	0.821397	NOT
UFD1L	3171.888	-0.0715873	0.156104	-0.45858787	0.64653	0.821397	NOT
RIIAD1	3.975937	-0.2564795	0.559384	-0.45850312	0.646591	0.821397	NOT
MIPEP	529.7118	-0.0847637	0.184912	-0.45840017	0.646665	0.821397	NOT
RP11-13A1	1.886445	0.23921894	0.521873	0.458385764	0.646675	0.821397	NOT
ZDHHC2	498.7822	0.16240115	0.354313	0.458355116	0.646697	0.821397	NOT
NCOR2	4998.111	-0.0631722	0.137827	-0.45834256	0.646706	0.821397	NOT
HECTD3	2404.211	0.07547904	0.164684	0.458327119	0.646717	0.821397	NOT
FKBP14	414.5579	0.09930311	0.216674	0.458305585	0.646733	0.821397	NOT

IL13	1.931945	0.31481135	0.687192	0.458112468	0.646872	0.821535	NOT
PRDM12	9.612917	0.15233912	0.332624	0.457991818	0.646958	0.821591	NOT
CTD-2591A	3.389988	-0.2210208	0.482613	-0.45796647	0.646977	0.821591	NOT
PBX4	20.54448	-0.1719202	0.375467	-0.45788334	0.647036	0.821629	NOT
SRGAP3	211.6031	-0.1508108	0.329426	-0.45779866	0.647097	0.821667	NOT
SYNGR4	10.6459	0.19296625	0.421639	0.457657912	0.647198	0.821757	NOT
RP11-132A	78.87662	0.19129606	0.418062	0.457578711	0.647255	0.821791	NOT
GPATCH11	442.7656	-0.0710615	0.15533	-0.45748655	0.647321	0.821837	NOT
NUDCD2	1406.579	-0.0718886	0.157157	-0.4574331	0.64736	0.821847	NOT
NDUFAF3	3253.099	-0.0951512	0.208056	-0.45733381	0.647431	0.821899	NOT
COPS2	2300.304	0.04882067	0.106775	0.457228492	0.647507	0.821956	NOT
RP11-649A	3.315562	0.19909503	0.435518	0.457145634	0.647566	0.821956	NOT
AC012354.	1.841463	-0.2144797	0.469172	-0.45714532	0.647567	0.821956	NOT
ANKRD31	11.4676	-0.1739884	0.380674	-0.45705301	0.647633	0.822001	NOT
AC007238.	23.89935	0.13311441	0.291305	0.456958476	0.647701	0.822005	NOT
UBAP1	2633.234	-0.0534718	0.117025	-0.45692618	0.647724	0.822005	NOT
SMC2-AS1	5.22052	0.16739822	0.366376	0.456902577	0.647741	0.822005	NOT
TMEM178B	164.1213	-0.2688256	0.588445	-0.45684065	0.647786	0.822005	NOT
SLC25A33	1241.056	0.11736787	0.256913	0.456838268	0.647787	0.822005	NOT
STARD10	15759.04	-0.1293127	0.283107	-0.45676301	0.647841	0.822035	NOT
RP11-460M	2.243163	-0.2196293	0.480902	-0.45670268	0.647885	0.822052	NOT
NUBP2	3035.2	-0.079291	0.173672	-0.45655608	0.64799	0.822147	NOT
RP11-365(	6.750936	0.17570074	0.384912	0.456469903	0.648052	0.822184	NOT
FKBP2	5654.476	-0.121399	0.265974	-0.45643184	0.648079	0.822184	NOT
AC104820.	3.728991	-0.2118342	0.46433	-0.4562148	0.648236	0.822321	NOT
AC015849.	2.562616	-0.2545718	0.558031	-0.45619683	0.648248	0.822321	NOT
STX10	2010.243	0.08945038	0.196175	0.455971577	0.64841	0.822463	NOT
KLF10	2457.97	-0.1094867	0.240195	-0.45582482	0.648516	0.822463	NOT
CLEC17A	2.773363	-0.2712779	0.595137	-0.45582443	0.648516	0.822463	NOT
STRADA	264.9052	-0.0742957	0.16301	-0.45577531	0.648552	0.822463	NOT
NUDT13	237.8944	-0.1304797	0.28631	-0.45572853	0.648585	0.822463	NOT
CSF2RA	201.5351	-0.1679974	0.368653	-0.45570609	0.648601	0.822463	NOT
RP11-820I	2.056842	0.28814549	0.632433	0.455613924	0.648668	0.822463	NOT
PTMS	25391.44	-0.1122578	0.24639	-0.45561079	0.64867	0.822463	NOT
TM9SF2	11524.22	0.08952415	0.196498	0.455598734	0.648679	0.822463	NOT
RP5-884C9	2.707012	0.34519777	0.757725	0.455571129	0.648698	0.822463	NOT
KLHL7	820.6082	0.06511014	0.14292	0.45556904	0.6487	0.822463	NOT
ETV3	958.572	0.07452125	0.163601	0.455505469	0.648746	0.822463	NOT
BRDTP1	2.295617	-0.4117014	0.903878	-0.45548352	0.648761	0.822463	NOT
U4	2.356677	0.16642722	0.365412	0.455451102	0.648785	0.822463	NOT
MTA1	1553.331	0.06891027	0.151429	0.455066876	0.649061	0.822731	NOT
RP11-640M	11.55595	0.11072695	0.243334	0.455040332	0.64908	0.822731	NOT
TEX9	44.82229	-0.1349661	0.296609	-0.45503064	0.649087	0.822731	NOT
PTPRJ	2474.023	-0.0805265	0.177028	-0.45487935	0.649196	0.82279	NOT
RP1-96H9.	2.438674	0.21637812	0.475719	0.454843988	0.649221	0.82279	NOT
FAM209A	3.100742	0.2120788	0.466271	0.454840072	0.649224	0.82279	NOT
RP1-20C7.	11.80911	0.12314822	0.270796	0.454764244	0.649279	0.822814	NOT
RP11-876M	111.5662	-0.1665409	0.366324	-0.45462687	0.649378	0.822814	NOT
APOBEC2	7.104133	0.16689888	0.367169	0.454556258	0.649429	0.822814	NOT

RP11-248J	24.55484	0.12855887	0.282836	0.454535565	0.649443	0.822814	NOT
MYH1	6.49163	-0.3947811	0.868598	-0.45450392	0.649466	0.822814	NOT
CHCHD2P2	55.23764	-0.1234273	0.271571	-0.45449449	0.649473	0.822814	NOT
RAB23	169.152	0.1255144	0.276176	0.45447335	0.649488	0.822814	NOT
WDR45B	3889.159	0.06779053	0.149166	0.454462916	0.649496	0.822814	NOT
RPL4P1	3.411869	0.1943592	0.427695	0.454433961	0.649517	0.822814	NOT
RP11-420K	6.490827	0.53507744	1.177733	0.454328136	0.649593	0.822872	NOT
TMEM258	3341.414	0.08680092	0.191118	0.454174484	0.649703	0.822956	NOT
FAM43A	577.46	-0.1652867	0.363946	-0.45415147	0.64972	0.822956	NOT
GOPC	1180.011	-0.0717362	0.157978	-0.45408913	0.649765	0.822975	NOT
CTC-367J1	14.78041	0.25221554	0.555486	0.454044793	0.649797	0.822977	NOT
MRPL32	1774.807	-0.0627384	0.13821	-0.45393515	0.649876	0.823038	NOT
WDR13	6128.872	0.11231643	0.247487	0.453827791	0.649953	0.823042	NOT
ZNF92	250.0718	0.0877598	0.193386	0.453806544	0.649968	0.823042	NOT
ZBTB46-AS	1.626697	0.55586077	1.22489	0.453804738	0.649969	0.823042	NOT
MIR148A	4.347769	0.19526709	0.430444	0.453641007	0.650087	0.82308	NOT
RP11-307C	82.35245	0.14315909	0.315587	0.453628323	0.650096	0.82308	NOT
LA16c-321	2.075217	-0.1755216	0.386959	-0.45359197	0.650123	0.82308	NOT
RP11-357H	1.528848	0.33254027	0.733232	0.453526437	0.65017	0.82308	NOT
RN7SL574F	2.181031	-0.2038872	0.449625	-0.45346003	0.650218	0.82308	NOT
EHD2	2279.782	0.11806802	0.26039	0.453427791	0.650241	0.82308	NOT
PTPRC	851.4749	-0.1746583	0.385202	-0.45341986	0.650246	0.82308	NOT
RP11-74J1	14.58837	0.13588363	0.2997	0.453399217	0.650261	0.82308	NOT
RP11-709D	1.948918	0.2017075	0.444921	0.453356249	0.650292	0.82308	NOT
RPARP-AS1	410.1371	0.09636643	0.212569	0.453341154	0.650303	0.82308	NOT
TULP2	4.367009	0.2346481	0.518045	0.452949033	0.650585	0.823399	NOT
LINC00689	24.03841	0.40880307	0.902875	0.452779026	0.650708	0.823516	NOT
LDHAP3	26.8847	0.17915504	0.395787	0.452655669	0.650797	0.823554	NOT
CDH11	405.253	0.18588349	0.410654	0.452652101	0.650799	0.823554	NOT
EIF4EP2	5.2336	0.15967669	0.352793	0.452607522	0.650831	0.823557	NOT
RP11-395E	5.270376	-0.2453572	0.542398	-0.45235675	0.651012	0.823747	NOT
CTD-3080F	164.6609	0.25946342	0.573738	0.452233273	0.651101	0.823821	NOT
HEBP1	3208.39	0.08266029	0.182827	0.452123979	0.65118	0.823838	NOT
NAMPTP1	261.5299	0.20167215	0.446057	0.452121821	0.651181	0.823838	NOT
RALGDS	1337.453	-0.0843623	0.186684	-0.45189894	0.651342	0.823838	NOT
FUT5	3.219645	-0.2301196	0.509312	-0.45182466	0.651395	0.823838	NOT
EIF3G	6810.345	-0.0921554	0.203968	-0.45181413	0.651403	0.823838	NOT
DNAAF2	645.1012	-0.074452	0.164785	-0.45181288	0.651404	0.823838	NOT
INSC	25.85775	0.24033907	0.531973	0.451788336	0.651421	0.823838	NOT
AC092301.	4.429753	-0.1558916	0.345064	-0.45177545	0.651431	0.823838	NOT
ANO7P1	74.81838	-0.1826236	0.404304	-0.45169887	0.651486	0.823838	NOT
DOK3	216.2974	0.12500828	0.276756	0.451691806	0.651491	0.823838	NOT
KLHL33	2.031484	0.22109644	0.489491	0.451686302	0.651495	0.823838	NOT
RP11-626C	3.818382	0.12836358	0.28419	0.451682494	0.651498	0.823838	NOT
9-Mar	484.5067	0.14848936	0.328759	0.451666904	0.651509	0.823838	NOT
CALR3	3.507085	-0.2170591	0.480674	-0.45157249	0.651577	0.823885	NOT
CTC-471J1	13.16643	0.13642595	0.302538	0.450937712	0.652034	0.824425	NOT
SLED1	12.70325	0.19952931	0.442603	0.450809271	0.652127	0.824454	NOT
CDX1	6.029445	-0.1979879	0.439239	-0.45075249	0.652168	0.824454	NOT

CECR5-AS1	13.75384	-0.1539997	0.341695	-0.45069362	0.65221	0.824454	NOT
PLRG1	2029.661	0.04958294	0.110017	0.450683029	0.652218	0.824454	NOT
RP4-800G7	2.095165	-0.1819297	0.403763	-0.45058544	0.652288	0.824454	NOT
RP11-64K1	2.090678	-0.1969494	0.4372	-0.45047933	0.652365	0.824454	NOT
TMEM242	652.8667	-0.0855247	0.189859	-0.45046554	0.652375	0.824454	NOT
EPOR	339.9485	-0.1094228	0.242927	-0.45043422	0.652397	0.824454	NOT
FPR1	108.596	-0.1888237	0.419213	-0.45042448	0.652404	0.824454	NOT
CICP27	2.005451	0.25333199	0.562521	0.45035151	0.652457	0.824454	NOT
ZXDC	1344.216	-0.0554992	0.123236	-0.45034991	0.652458	0.824454	NOT
RPS20P33	4.874903	-0.1439593	0.31967	-0.45033773	0.652467	0.824454	NOT
TAF8	1160.37	-0.0684856	0.152076	-0.45033713	0.652467	0.824454	NOT
STX7	1527.632	0.07596631	0.168695	0.450316605	0.652482	0.824454	NOT
C19orf18	28.98433	-0.1891556	0.420115	-0.45024669	0.652533	0.824463	NOT
TRIM13	527.6908	-0.0813327	0.180666	-0.45018309	0.652578	0.824463	NOT
EHMT1	2020.824	-0.0705791	0.15678	-0.45017971	0.652581	0.824463	NOT
WDR82	3207.039	0.05762136	0.128011	0.450128845	0.652618	0.824471	NOT
LINC00565	2.993015	-0.228224	0.507081	-0.45007396	0.652657	0.824483	NOT
RP11-229F	9.778103	0.15007441	0.333573	0.449899818	0.652783	0.824567	NOT
SLC14A1	72.44158	0.22316371	0.496034	0.449896427	0.652785	0.824567	NOT
MRPS30	1779.817	0.06050091	0.134534	0.449706517	0.652922	0.824702	NOT
RP11-394F	3.939423	-0.1578101	0.351055	-0.44953102	0.653049	0.824824	NOT
RP11-155C	4.441556	0.2236576	0.497946	0.449159957	0.653316	0.825123	NOT
MARVELD1	569.1939	-0.1626361	0.362181	-0.44904674	0.653398	0.825188	NOT
ORC5	770.7315	0.06282742	0.139938	0.448964621	0.653457	0.825224	NOT
SNX27	2555.028	0.0768989	0.171313	0.448879811	0.653518	0.825263	NOT
TRBV12-4	3.081454	-0.2655823	0.591783	-0.44878324	0.653588	0.825313	NOT
CTD-2588C	2.81944	-0.1921732	0.42825	-0.44874122	0.653618	0.825313	NOT
XXbac-B47	46.03673	-0.1070519	0.238667	-0.44854192	0.653762	0.825456	NOT
RP11-115C	147.9513	-0.3434694	0.765902	-0.44845078	0.653828	0.8255	NOT
GXYLT2	78.81769	0.24613205	0.548912	0.448399605	0.653865	0.825509	NOT
LINC00160	1.742716	-0.3587173	0.800203	-0.44828285	0.653949	0.825576	NOT
AMIGO2	287.297	-0.1831989	0.408716	-0.44823013	0.653987	0.825586	NOT
DCLK1	70.71028	-0.1820006	0.406296	-0.44795044	0.654189	0.825802	NOT
EIF4A2P1	1.663558	0.23417211	0.522824	0.447898848	0.654226	0.825811	NOT
FRMPD1	144.2755	0.23528014	0.525416	0.447798185	0.654299	0.825831	NOT
C20orf203	1.475529	-0.2878086	0.642728	-0.44779224	0.654303	0.825831	NOT
TLR8	70.1442	0.1983004	0.443198	0.447431139	0.654564	0.826064	NOT
AL136419.	20.81432	0.09875275	0.220744	0.447362721	0.654613	0.826064	NOT
SWI5	1043.546	0.09036167	0.20202	0.447289678	0.654666	0.826064	NOT
AF146191.	4.75871	0.17534506	0.392021	0.447285299	0.654669	0.826064	NOT
MEF2BNB-M	17.50469	0.10910872	0.243936	0.447284058	0.65467	0.826064	NOT
RP11-327I	2.600246	-0.2276534	0.508969	-0.44728394	0.65467	0.826064	NOT
RFX8	23.13819	-0.1470092	0.328767	-0.44715343	0.654764	0.826144	NOT
IGFBP3	8727.645	-0.1440772	0.322247	-0.44710156	0.654802	0.826153	NOT
RP11-295K	6.776796	-0.1363425	0.305055	-0.44694346	0.654916	0.826259	NOT
PELO	1158.044	-0.0938988	0.210132	-0.44685546	0.654979	0.8263	NOT
TMEM117	224.3912	0.10982707	0.245806	0.4468033	0.655017	0.82631	NOT
IGHV3-66	8.285113	0.36577754	0.818783	0.446732963	0.655068	0.826335	NOT
CHD4	8138.875	0.05540672	0.124075	0.446559572	0.655193	0.826455	NOT

PAM16	485.4435	-0.0822953	0.184364	-0.44637423	0.655327	0.826585	NOT
MIOX	62.98548	0.28587352	0.640701	0.446188352	0.655461	0.826665	NOT
GPR55	9.81186	-0.1606866	0.360154	-0.44616035	0.655481	0.826665	NOT
AKAP1	5252.87	-0.0738249	0.165469	-0.4461545	0.655486	0.826665	NOT
TMEM147	3428.576	0.10323059	0.231398	0.446117771	0.655512	0.826665	NOT
MORN3	15.8342	0.16571639	0.371616	0.445933889	0.655645	0.826757	NOT
FAM81B	1.587486	0.30582803	0.685817	0.445932667	0.655646	0.826757	NOT
RP11-888I	35.37376	0.11930274	0.267605	0.445815908	0.65573	0.826792	NOT
DENND5A	2164.513	0.06691985	0.150109	0.445809194	0.655735	0.826792	NOT
CNKSR2	195.6769	-0.2048256	0.459497	-0.44576049	0.65577	0.826798	NOT
RP11-630I	2.313303	0.20111462	0.451461	0.445474723	0.655977	0.826998	NOT
C14orf18(	10.66317	0.34462173	0.773727	0.445404914	0.656027	0.826998	NOT
IST1	3425.731	0.0529643	0.11894	0.445301719	0.656102	0.826998	NOT
LDLRAD1	150.2084	0.28236182	0.634102	0.445294229	0.656107	0.826998	NOT
RP1-179N1	24.57529	0.15061422	0.338238	0.445290951	0.65611	0.826998	NOT
MIR570	18.7395	-0.1254173	0.281654	-0.44528822	0.656111	0.826998	NOT
RPS4Y1	4271.856	0.34562486	0.776307	0.445216797	0.656163	0.827015	NOT
ZNF568	208.466	-0.1447867	0.325228	-0.44518544	0.656186	0.827015	NOT
MEX3B	87.78061	0.14072842	0.316186	0.445080709	0.656261	0.827019	NOT
TRBV25-1	11.16274	-0.2060194	0.462902	-0.44506088	0.656276	0.827019	NOT
RP11-930I	2.488161	-0.1687637	0.379261	-0.4449807	0.656334	0.827019	NOT
RP11-54A9	2.760303	-0.246794	0.55467	-0.44493852	0.656364	0.827019	NOT
FCRL6	35.7175	-0.1493802	0.335782	-0.4448729	0.656412	0.827019	NOT
AC062029.	95.6172	0.10148296	0.228127	0.444853182	0.656426	0.827019	NOT
FRS2	1059.765	0.08327887	0.187206	0.444850429	0.656428	0.827019	NOT
RP4-616B8	2.666858	0.22848517	0.513631	0.444842923	0.656433	0.827019	NOT
RAB3GAP1	1794.752	-0.0491843	0.110586	-0.44476028	0.656493	0.827056	NOT
RP1-288H2	4.715648	0.33760989	0.759168	0.444710261	0.656529	0.827063	NOT
ARHGAP22	109.7256	0.14363647	0.323076	0.444590482	0.656616	0.827134	NOT
CDC73	1554.407	-0.073309	0.164954	-0.44442211	0.656737	0.827231	NOT
PPRC1	1387.919	0.06747234	0.151843	0.444356628	0.656785	0.827231	NOT
LY75	99.81671	-0.1486755	0.334601	-0.44433711	0.656799	0.827231	NOT
RP11-797I	3.083977	0.16009021	0.360308	0.444314875	0.656815	0.827231	NOT
MAPK6	3065.402	0.07943487	0.178829	0.444193558	0.656903	0.827266	NOT
MB21D2	86.84062	-0.1489212	0.335316	-0.4441218	0.656955	0.827266	NOT
UTY	296.3213	-0.3563715	0.802471	-0.44409274	0.656976	0.827266	NOT
CAPN1	7304.793	0.06635582	0.149422	0.444084717	0.656981	0.827266	NOT
LPAR1	121.4638	-0.2376618	0.53524	-0.4440284	0.657022	0.827266	NOT
PRPF31	2725.562	0.07606478	0.171308	0.444024021	0.657025	0.827266	NOT
ABCA4	150.0767	-0.1670998	0.376496	-0.44382879	0.657166	0.827382	NOT
IL2RA	77.71719	0.20909673	0.471141	0.443808958	0.657181	0.827382	NOT
ALKBH8	253.0769	-0.0661899	0.149154	-0.44376953	0.657209	0.827382	NOT
NSUN5P2	58.64453	0.10188164	0.229605	0.443726581	0.65724	0.827383	NOT
GTF2IP1	48.74755	0.09474005	0.213604	0.443530953	0.657382	0.8275	NOT
RP11-247I	4.84552	-0.1447748	0.326427	-0.44351315	0.657395	0.8275	NOT
AGO3	571.238	-0.0695966	0.156988	-0.44332373	0.657532	0.827634	NOT
C2CD3	640.2058	0.06638428	0.149803	0.443143702	0.657662	0.827758	NOT
RP13-580F	2.486122	-0.201551	0.454862	-0.44310406	0.65769	0.827758	NOT
JTB	9195.743	0.07729475	0.174479	0.443001895	0.657764	0.827812	NOT

BMPR1APS1	2.934082	0.19614698	0.44284	0.442930152	0.657816	0.827839	NOT
PI16	5.636062	0.26942094	0.608552	0.44272441	0.657965	0.827988	NOT
PLA2G5	103.996	-0.149279	0.337231	-0.44266085	0.658011	0.828007	NOT
ZNF839	259.4469	-0.0834785	0.188668	-0.44246214	0.658155	0.828129	NOT
AC084117.	4.072094	0.18309541	0.413829	0.442442543	0.658169	0.828129	NOT
AC008592.	23.27625	0.28528059	0.64492	0.442350547	0.658236	0.828138	NOT
PARVB	1824.202	0.15554466	0.351633	0.442349012	0.658237	0.828138	NOT
RPS26P3	6.289136	0.12262091	0.277233	0.442302524	0.65827	0.828142	NOT
PRR14L	1349.402	0.06712824	0.151809	0.44218741	0.658354	0.828208	NOT
HIST2H3C	1.260558	-0.3411382	0.771596	-0.44212029	0.658402	NA	NA
NARFL	1441.094	-0.0757269	0.171326	-0.44200546	0.658485	0.828335	NOT
GLYR1	3871.287	-0.0614202	0.138979	-0.44193869	0.658534	0.828358	NOT
CTD-2525I	1.958489	0.18058621	0.408804	0.441742544	0.658676	0.828498	NOT
AC015933.	3.303916	0.19443337	0.440509	0.441383826	0.658935	0.82875	NOT
MRS2	2028.245	0.09073461	0.20557	0.441380831	0.658937	0.82875	NOT
RP11-517(	1.984679	0.29821534	0.675719	0.441330268	0.658974	0.828758	NOT
SCO2	1131.62	-0.0985424	0.22332	-0.4412617	0.659024	0.828769	NOT
RP11-223I	309.682	-0.168388	0.381674	-0.44118242	0.659081	0.828769	NOT
FIG4	503.8138	0.075171	0.170398	0.441150084	0.659104	0.828769	NOT
PI4KA	3302.355	0.08509129	0.192886	0.441149208	0.659105	0.828769	NOT
RP3-395M2	7.613322	-0.1960326	0.444566	-0.44095258	0.659247	0.828875	NOT
QTRT1	1895.036	-0.0910714	0.206535	-0.44094851	0.65925	0.828875	NOT
TRPC3	11.10968	-0.2912429	0.660907	-0.44067181	0.659451	0.829082	NOT
MYDGF	6920.965	-0.086694	0.196777	-0.44057028	0.659524	0.829082	NOT
DFNB59	58.44452	-0.1202454	0.272936	-0.44056323	0.659529	0.829082	NOT
HIST1H4J	10.45223	-0.1835442	0.416623	-0.44055198	0.659537	0.829082	NOT
PUS7L	681.7936	-0.068807	0.156288	-0.44025674	0.659751	0.82931	NOT
COTL1	2271.987	0.14728816	0.334581	0.440216904	0.65978	0.82931	NOT
LINC00488	28.76889	0.35454953	0.806476	0.439628106	0.660206	0.829757	NOT
ZNF853	88.30431	0.16278796	0.370311	0.439598361	0.660228	0.829757	NOT
LMO3	25.15281	0.17062824	0.38817	0.439571399	0.660248	0.829757	NOT
ALLC	9.033362	0.3174336	0.722166	0.439557564	0.660258	0.829757	NOT
TFB1M	444.3204	-0.0929716	0.211562	-0.43945382	0.660333	0.829806	NOT
SARS	6874.611	0.08993905	0.204677	0.439419095	0.660358	0.829806	NOT
ARMCX1	184.4233	0.15438689	0.351381	0.439372373	0.660392	0.82981	NOT
BHLHB9	204.6899	-0.1366375	0.311046	-0.43928358	0.660456	0.829849	NOT
CTC-431G1	4.892479	0.31370073	0.714181	0.43924524	0.660484	0.829849	NOT
ASH1L	2454.891	-0.0966565	0.220148	-0.43905268	0.660623	0.829953	NOT
LCP1	3406.406	0.1303215	0.296829	0.439046309	0.660628	0.829953	NOT
MRPS18C	553.1658	-0.0632236	0.144026	-0.43897333	0.660681	0.829981	NOT
CTB-129P6	171.9817	0.14862385	0.338772	0.438713689	0.660869	0.830179	NOT
CTB-129P6	4.395298	0.14628237	0.33357	0.438535246	0.660998	0.830227	NOT
AC011330.	3.709914	0.16480476	0.375813	0.438528706	0.661003	0.830227	NOT
DPY19L1	1468.869	-0.0901352	0.205554	-0.43849907	0.661025	0.830227	NOT
RP3-425C1	247.8076	0.09413083	0.21467	0.438491769	0.66103	0.830227	NOT
KCNK1	728.9638	-0.1946013	0.443949	-0.43834146	0.661139	0.830271	NOT
RAB22A	1837.959	-0.0510471	0.116464	-0.43830793	0.661163	0.830271	NOT
SNHG8	1829.462	-0.1279759	0.292005	-0.43826576	0.661194	0.830271	NOT
IFI16	1248.603	-0.145551	0.332128	-0.43823796	0.661214	0.830271	NOT

FCRL5	24.98936	-0.2911321	0.664332	-0.43823257	0.661218	0.830271	NOT
C17orf97	94.61156	-0.1476133	0.337048	-0.43795902	0.661416	0.830482	NOT
ABHD14A-A	26.90863	-0.1164644	0.266015	-0.43781186	0.661523	0.830556	NOT
RP13-228J	1.399096	0.29811434	0.680948	0.437792888	0.661536	0.830556	NOT
CCDC74A	56.11124	0.18134167	0.41438	0.437621502	0.661661	0.830637	NOT
RP11-480I	75.37819	0.16848907	0.385022	0.437608632	0.66167	0.830637	NOT
MRPL20	3283.107	0.08185022	0.187053	0.437577327	0.661693	0.830637	NOT
TSR1	1168.118	0.0760252	0.173791	0.437452423	0.661783	0.830688	NOT
SIRT5	1597.158	-0.0924003	0.211232	-0.43743627	0.661795	0.830688	NOT
GTPBP6	2822.212	-0.0729742	0.166848	-0.43736944	0.661843	0.830691	NOT
SMAD4	2549.846	-0.0654164	0.149592	-0.43729935	0.661894	0.830691	NOT
PROCR	281.5959	-0.1371397	0.313666	-0.43721525	0.661955	0.830691	NOT
SPINK14	1.872776	0.30581311	0.699566	0.437147208	0.662005	0.830691	NOT
RFNG	3980.438	0.09939476	0.227387	0.437117459	0.662026	0.830691	NOT
PPP1R7	2696.862	0.0742592	0.169889	0.437105267	0.662035	0.830691	NOT
UPK1B	6.229386	-0.3305903	0.756348	-0.43708772	0.662048	0.830691	NOT
TMSB4Y	31.56961	-0.3057894	0.69976	-0.43699189	0.662117	0.830691	NOT
ADAMTS16	144.2023	0.28683195	0.656391	0.436983061	0.662124	0.830691	NOT
PIK3C2G	529.4929	-0.2437925	0.557915	-0.43697093	0.662132	0.830691	NOT
RP11-431K	6.640113	-0.2389525	0.546841	-0.43696865	0.662134	0.830691	NOT
RP11-177F	3.46659	-0.4052545	0.927952	-0.43671918	0.662315	0.830845	NOT
CEP112	246.3153	0.08885073	0.203453	0.43671474	0.662318	0.830845	NOT
CCND2	455.2765	-0.1199886	0.274781	-0.43667004	0.662351	0.830848	NOT
RP11-247I	2.298027	-0.2319597	0.531445	-0.43646967	0.662496	0.830976	NOT
CCNDBP1	1274.313	-0.0679129	0.155605	-0.43644426	0.662514	0.830976	NOT
RHBDL1	172.0162	-0.1584459	0.363382	-0.4360314	0.662814	0.831312	NOT
CLN5	529.0026	-0.0795104	0.182367	-0.43599084	0.662843	0.831312	NOT
NAPSB	163.1696	0.16142868	0.370343	0.435889833	0.662917	0.831354	NOT
FAM180B	2.66426	-0.2548095	0.584613	-0.43585988	0.662938	0.831354	NOT
ADAR	15340.51	0.06510933	0.149425	0.435733787	0.66303	0.831369	NOT
MTRNR2L8	24.21364	-0.147635	0.338824	-0.43572733	0.663035	0.831369	NOT
RP11-277F	4.859472	0.23163277	0.531662	0.435676997	0.663071	0.831369	NOT
NLK	1166.92	-0.0540832	0.124137	-0.43567512	0.663072	0.831369	NOT
ANKRD36B	20.61929	0.15808405	0.362958	0.435544127	0.663168	0.831428	NOT
NABP1	658.119	0.12316057	0.282786	0.435525405	0.663181	0.831428	NOT
JAZF1	978.0984	0.11710134	0.268941	0.435416458	0.66326	0.831489	NOT
RP11-464F	13.11891	0.18120891	0.416339	0.435244131	0.663385	0.831607	NOT
ROCK2	2768.146	-0.0875818	0.201252	-0.43518399	0.663429	0.831623	NOT
RP11-241F	2.408618	-0.3839243	0.882398	-0.43509218	0.663496	0.831669	NOT
RNU6-1048	1.497498	-0.2148166	0.493976	-0.43487253	0.663655	0.831754	NOT
AP001625.	3.51693	0.20739331	0.476908	0.434871002	0.663656	0.831754	NOT
KIAA1958	663.9272	-0.0965784	0.222085	-0.4348702	0.663657	0.831754	NOT
SGSH	1388.838	-0.0729294	0.16772	-0.43482955	0.663686	0.831754	NOT
RPUSD1	969.8209	0.07209081	0.165812	0.434775642	0.663725	0.831764	NOT
HSBP1	2935.572	-0.0647957	0.14906	-0.43469603	0.663783	0.831798	NOT
RP11-757F	0.923196	0.39166902	0.901058	0.434676869	0.663797	NA	NA
PGAM4	1.97071	-0.1888227	0.4346	-0.43447475	0.663944	0.831906	NOT
OPLAH	4640.833	0.12746759	0.293385	0.434471783	0.663946	0.831906	NOT
DIEXF	1439.887	0.0771796	0.177656	0.434432267	0.663975	0.831906	NOT

SLC2A12	307.3264	-0.220819	0.50832	-0.43440919	0.663991	0.831906	NOT
ARHGAP18	617.4514	0.09899294	0.227909	0.434352683	0.664032	0.831919	NOT
ZNF506	310.2531	0.12776086	0.294388	0.433987399	0.664298	0.832196	NOT
RP11-500I	8.810109	-0.4026426	0.927826	-0.43396361	0.664315	0.832196	NOT
RP1-266L2	51.37784	0.11348401	0.261535	0.433914467	0.664351	0.832202	NOT
NDNL2	611.5453	0.05792974	0.133545	0.433785913	0.664444	0.832275	NOT
RILPL1	333.6858	-0.0835725	0.192689	-0.43371654	0.664494	0.832275	NOT
DTX3L	4243.042	-0.0696102	0.160515	-0.4336668	0.66453	0.832275	NOT
NKX3-2	17.25057	0.29630526	0.683281	0.433650615	0.664542	0.832275	NOT
RP11-36B1	4.401825	-0.1390488	0.320729	-0.43353984	0.664623	0.832275	NOT
LINC01314	793.0981	-0.1861896	0.429484	-0.43351892	0.664638	0.832275	NOT
CWC25	787.1914	0.04207138	0.097047	0.433517583	0.664639	0.832275	NOT
RP11-528I	2.834821	-0.1984449	0.457778	-0.43349633	0.664654	0.832275	NOT
EPC2	688.7591	-0.0684874	0.158031	-0.43337853	0.66474	0.832286	NOT
CLASRP	1530.777	-0.0725605	0.167432	-0.43337404	0.664743	0.832286	NOT
STT3B	8057.929	0.06182157	0.14267	0.433318284	0.664784	0.832286	NOT
ABCF3	2835.976	0.04332739	0.1	0.433274841	0.664815	0.832286	NOT
GABRG2	20.11634	-0.5714458	1.319025	-0.43323365	0.664845	0.832286	NOT
NUPL1	1243.753	0.08552932	0.197422	0.433230949	0.664847	0.832286	NOT
RP1-16A9.	2.990944	0.22073891	0.509833	0.432963299	0.665041	0.832451	NOT
GLTSCR1L	740.8885	-0.0825238	0.190625	-0.43291257	0.665078	0.832451	NOT
RP11-263(	15.32157	-0.2814872	0.65025	-0.43289087	0.665094	0.832451	NOT
AC011290.	1.265591	0.2848131	0.657946	0.432882449	0.6651	NA	NA
RP11-21L2	28.72082	0.16153344	0.373249	0.432776232	0.665177	0.832451	NOT
CCDC144B	15.71853	-0.2370997	0.547875	-0.4327621	0.665188	0.832451	NOT
NEXN	218.549	-0.1588148	0.366985	-0.4327552	0.665193	0.832451	NOT
GCC1	1137.452	-0.0720989	0.166615	-0.43272663	0.665213	0.832451	NOT
RP13-258(	1.705065	-0.2126429	0.491419	-0.43271169	0.665224	0.832451	NOT
TMEM18	940.9205	0.06066646	0.140295	0.432419882	0.665436	0.832628	NOT
SPDYE2	3.535705	-0.2181793	0.504649	-0.43233891	0.665495	0.832628	NOT
PRAMEF10	80.32169	-0.3864629	0.893952	-0.43230855	0.665517	0.832628	NOT
RBMXL1	708.7187	0.08861435	0.204981	0.432304926	0.66552	0.832628	NOT
RAB31	828.9092	-0.1296034	0.299823	-0.43226699	0.665547	0.832628	NOT
TRBV27	2.217325	0.32876233	0.760559	0.432263963	0.66555	0.832628	NOT
DYRK3	209.1313	0.12683383	0.293473	0.432182598	0.665609	0.832639	NOT
FAM129C	16.38029	0.1942558	0.449503	0.432156784	0.665627	0.832639	NOT
RP11-13N1	4.387967	0.18273836	0.422884	0.432124508	0.665651	0.832639	NOT
RP5-978I1	1.727017	-0.5709455	1.321648	-0.43199522	0.665745	0.83271	NOT
SRSF6	5347.008	0.04725521	0.109397	0.431962368	0.665769	0.83271	NOT
SNORD12B	2.513018	0.20515626	0.475022	0.431887696	0.665823	0.832739	NOT
RPL32P3	217.0744	0.08964536	0.207625	0.431764734	0.665912	0.832813	NOT
GAPDHP37	2.539097	-0.3109325	0.720372	-0.43162799	0.666012	0.832872	NOT
LINC00921	52.45446	-0.0986579	0.228579	-0.43161491	0.666021	0.832872	NOT
CERS3-AS1	2.584243	-0.4057166	0.940257	-0.43149542	0.666108	0.832942	NOT
HSD3B2	9.236342	-0.2812306	0.65187	-0.43142111	0.666162	0.832959	NOT
PTCHD2	7.532498	-0.2125744	0.492764	-0.43139208	0.666183	0.832959	NOT
CR1	55.21368	-0.2174034	0.504095	-0.43127505	0.666268	0.833027	NOT
TCEAL2	5.084093	0.38604908	0.895228	0.43122988	0.666301	0.83303	NOT
AC008268.	2.354666	-0.3710928	0.860814	-0.43109522	0.666399	0.833114	NOT

TCHH	16.02972	0.19726747	0.45775	0.430950665	0.666504	0.833207	NOT
LINC0118[illegible]	511.5699	-0.1325836	0.307737	-0.43083394	0.666589	0.833255	NOT
SEMA4C	1501.208	0.08396404	0.194912	0.430780059	0.666628	0.833255	NOT
COPB2	6086.278	0.04856119	0.112743	0.430723611	0.666669	0.833255	NOT
KLF4	693.2705	-0.1685968	0.391453	-0.43069459	0.66669	0.833255	NOT
STEAP2-AS	3.1296	-0.2503095	0.58119	-0.43068449	0.666698	0.833255	NOT
POLR2J3	80.07248	0.0786399	0.18261	0.430644109	0.666727	0.833255	NOT
CLCA2	2.801171	-0.4813457	1.117973	-0.43055214	0.666794	0.833301	NOT
CEP170P1	3.267398	0.18100506	0.420506	0.430446321	0.666871	0.833305	NOT
HCG25	32.03217	0.10239736	0.237891	0.43043848	0.666877	0.833305	NOT
SPIDR	1679.225	-0.0739484	0.171805	-0.43042053	0.66689	0.833305	NOT
OSBPL1A	948.8872	0.11926957	0.277161	0.430325863	0.666959	0.833339	NOT
MICAL1	868.4321	0.13082429	0.304031	0.430298751	0.666978	0.833339	NOT
CRB3	727.3842	-0.1196347	0.278096	-0.4301918	0.667056	0.833398	NOT
RP3-483K1	33.98904	-0.1572269	0.365538	-0.43012488	0.667105	0.83342	NOT
AOC2	73.46403	0.11154195	0.259373	0.430044652	0.667163	0.833427	NOT
PGP	1301.495	0.11448878	0.266299	0.429925023	0.66725	0.833427	NOT
TBK1	1199.891	0.05557449	0.129278	0.429882923	0.667281	0.833427	NOT
SCRN3	698.786	-0.0640612	0.149024	-0.42987259	0.667288	0.833427	NOT
CYP1B1	869.8	0.15701316	0.365262	0.429864218	0.667294	0.833427	NOT
TNFRSF10A	306.8204	0.10512674	0.244558	0.429863899	0.667295	0.833427	NOT
CYP2T1P	24.75087	0.16861718	0.392377	0.429733087	0.66739	0.833479	NOT
RP4-635A2	11.64554	-0.1142823	0.265945	-0.42972183	0.667398	0.833479	NOT
PDLIM2	845.0265	-0.0902525	0.210059	-0.42965328	0.667448	0.833503	NOT
RP11-453E	12.70746	0.09136081	0.212724	0.429479887	0.667574	0.833608	NOT
PPP2R2C	374.1684	-0.3330475	0.775515	-0.42945323	0.667593	0.833608	NOT
IL7	186.8864	-0.116725	0.2719	-0.42929437	0.667709	0.833714	NOT
PDCD6IP	5290.607	0.05440788	0.12684	0.428949905	0.66796	0.833987	NOT
PES1P1	8.824821	-0.4304471	1.003585	-0.42890949	0.667989	0.833987	NOT
SERPINB8	465.6161	-0.1429659	0.333402	-0.42880958	0.668062	0.834007	NOT
NOA1	922.9658	-0.0601962	0.140396	-0.42876052	0.668098	0.834007	NOT
LAMTOR1	3889.254	-0.0657961	0.153473	-0.42871384	0.668131	0.834007	NOT
RP11-17P1	19.09512	-0.099327	0.231728	-0.42863689	0.668187	0.834007	NOT
RIPPLY3	51.48574	0.24274394	0.566328	0.428628175	0.668194	0.834007	NOT
RP11-266K	6.747509	0.21174078	0.494009	0.428617354	0.668202	0.834007	NOT
ASLP1	10.03267	-0.1636452	0.381821	-0.42859187	0.66822	0.834007	NOT
LMX1A	4.368192	-0.3055155	0.713254	-0.42834045	0.668403	0.834197	NOT
TAOK3	1726.979	-0.0711666	0.166181	-0.42824706	0.668471	0.834209	NOT
ARFGEF2	3594.927	-0.0834154	0.194785	-0.42824343	0.668474	0.834209	NOT
ZNF559-ZN	8.436228	0.1608788	0.375757	0.428146309	0.668545	0.834259	NOT
PDDC1	2563.278	-0.0742719	0.173497	-0.42808664	0.668588	0.834274	NOT
GBP1P1	60.45361	-0.1708657	0.399203	-0.42801745	0.668638	0.83429	NOT
CCL3	185.903	-0.1528747	0.357197	-0.42798384	0.668663	0.83429	NOT
CTB-92J24	29.27278	0.33305949	0.778281	0.427942368	0.668693	0.83429	NOT
MRGPRF	347.1293	0.23996065	0.56097	0.42775991	0.668826	0.834418	NOT
RP1-131F1	9.572959	0.14458498	0.338085	0.427659275	0.668899	0.834471	NOT
BRCC3P1	2.950155	-0.1450464	0.33922	-0.42758778	0.668951	0.834497	NOT
MCL1	16210.25	-0.0736071	0.172185	-0.42748829	0.669024	0.834549	NOT
GCNT1P3	1.123024	0.32039457	0.749547	0.427450952	0.669051	NA	NA

PCOLCE-AS	12.5473	0.19788638	0.463042	0.427361541	0.669116	0.834586	NOT
SYT13	98.66428	0.30494384	0.713573	0.427347661	0.669126	0.834586	NOT
SLCO1B3	792.6878	0.34737239	0.812906	0.427321654	0.669145	0.834586	NOT
PIH1D1	2939.734	0.07735148	0.181041	0.427260029	0.66919	0.834603	NOT
GALNT8	7.331247	-0.1842103	0.431214	-0.42718994	0.669241	0.834628	NOT
TUBGCP4	585.0404	0.07366417	0.172493	0.427057012	0.669338	0.834711	NOT
CTD-2561E	27.43021	-0.1086811	0.254568	-0.42692372	0.669435	0.834769	NOT
MIRLET7DH	3.091467	0.18738566	0.438936	0.426908863	0.669446	0.834769	NOT
RP5-1057J	45.40042	0.12566046	0.294524	0.426655956	0.66963	0.834923	NOT
CTC-480C2	8.710989	-0.4034737	0.945668	-0.42665481	0.669631	0.834923	NOT
LANCL3	5.984375	0.17326297	0.40621	0.426535594	0.669718	0.834966	NOT
RP11-182J	4.24833	0.2610424	0.612077	0.42648613	0.669754	0.834966	NOT
UBE2SP2	2.708335	0.21958938	0.514887	0.426480669	0.669758	0.834966	NOT
ZNF674-AS	120.6576	0.08361187	0.196103	0.426366215	0.669841	0.835031	NOT
ICE1	1439.72	0.07346139	0.172377	0.426167897	0.669986	0.835106	NOT
DPT	139.5234	-0.2550317	0.598459	-0.42614747	0.67	0.835106	NOT
CHERP	2362.999	-0.069161	0.162327	-0.42606055	0.670064	0.835106	NOT
CRHR2	19.92101	0.15689085	0.368237	0.426059582	0.670064	0.835106	NOT
MTATP8P1	15.95918	-0.1895891	0.444993	-0.42604948	0.670072	0.835106	NOT
YTHDF3	3574.054	0.06876688	0.161413	0.426030535	0.670086	0.835106	NOT
EIF4A2	11171.06	0.07899228	0.185433	0.42598714	0.670117	0.835107	NOT
PRMT5-AS1	7.557248	0.11368737	0.266991	0.425810143	0.670246	0.835229	NOT
LGALS17A	4.333791	0.24288756	0.570516	0.425732927	0.670302	0.835261	NOT
AGBL4	12.92671	-0.2499802	0.587283	-0.42565535	0.670359	0.835289	NOT
ZPR1	2183.586	0.06854035	0.161052	0.425578517	0.670415	0.835289	NOT
RPS4XP3	2.2709	-0.1807941	0.424822	-0.42557567	0.670417	0.835289	NOT
PRR5-ARHG	2.230302	0.29736011	0.699254	0.42525309	0.670652	0.835543	NOT
PNN	2817.891	0.06634435	0.156036	0.425185588	0.670701	0.835557	NOT
RP11-445N	11.46769	0.1784705	0.419779	0.42515357	0.670725	0.835557	NOT
OLIG1	1.925024	-0.2982844	0.701733	-0.42506817	0.670787	0.835596	NOT
RP11-375H	6.836178	0.21660496	0.509632	0.425022337	0.67082	0.835597	NOT
PWAR5	17.14533	0.20902666	0.491847	0.424982815	0.670849	0.835597	NOT
AQR	1286.458	-0.0546222	0.128552	-0.42490241	0.670908	0.835632	NOT
CUEDC2	2076.765	-0.0741513	0.174632	-0.42461404	0.671118	0.835822	NOT
RP5-1092A	79.42659	-0.1449597	0.341396	-0.42460849	0.671122	0.835822	NOT
ADCY10	253.6111	-0.1742284	0.410527	-0.42440225	0.671272	0.835942	NOT
RP11-527F	5.726328	-0.1597043	0.376313	-0.42439177	0.67128	0.835942	NOT
PGAP3	2683.157	0.0860668	0.202839	0.42431041	0.671339	0.835977	NOT
RP11-347H	4.523596	0.66333154	1.563533	0.424251802	0.671382	0.835992	NOT
TGOLN2	15071.69	0.07801446	0.183952	0.424101385	0.671492	0.83609	NOT
ZNF668	135.5941	0.06351394	0.149782	0.42404184	0.671535	0.836096	NOT
AK4P2	1.516195	0.5147348	1.213967	0.424010531	0.671558	0.836096	NOT
SIGLEC12	50.08682	0.20096895	0.474069	0.423923765	0.671621	0.836122	NOT
PTPRM	1859.815	-0.1227131	0.289488	-0.42389734	0.671641	0.836122	NOT
RP11-434H	1.829674	0.18733016	0.442079	0.423747811	0.67175	0.836213	NOT
RP11-315C	2.236532	-0.310957	0.733886	-0.42371312	0.671775	0.836213	NOT
C3AR1	322.8756	0.13672901	0.322771	0.423610003	0.67185	0.836258	NOT
RP11-549H	34.86878	0.10956055	0.258654	0.42357903	0.671873	0.836258	NOT
CTD-3224K	1.659102	0.24621603	0.581406	0.423483653	0.671942	0.836306	NOT

RP11-367F	1.561634	-0.2813228	0.664404	-0.423421	0.671988	0.836325	NOT
M6PR	1726.453	0.09178278	0.216816	0.423321028	0.672061	0.836377	NOT
RPLP1	51809.5	0.09776098	0.231025	0.423162537	0.672177	0.836482	NOT
RP11-340E	1.059712	0.29879253	0.70616	0.423122842	0.672206	NA	NA
UBL5	5970.537	-0.0979066	0.231433	-0.42304544	0.672262	0.83655	NOT
RP11-656I	3.348192	0.21650585	0.511837	0.42299765	0.672297	0.836555	NOT
RP11-173A	3.395251	0.18010154	0.425846	0.422926377	0.672349	0.836582	NOT
IL22RA1	813.6311	-0.1686955	0.399251	-0.42252993	0.672638	0.836838	NOT
RP1-283E3	89.70224	-0.1158076	0.274105	-0.42249334	0.672665	0.836838	NOT
RP11-329N	24.73672	-0.1123261	0.265882	-0.4224667	0.672684	0.836838	NOT
TRBV20-1	15.23622	0.1864986	0.441461	0.422457601	0.672691	0.836838	NOT
BBS5	117.4956	0.13155454	0.311421	0.422432876	0.672709	0.836838	NOT
AC018738.	280.4892	-0.1334768	0.316092	-0.4222715	0.672827	0.836946	NOT
ZNF730	11.90253	-0.2954737	0.700162	-0.42200729	0.67302	0.837148	NOT
AC006946.	8.320862	0.35134429	0.832839	0.421863263	0.673125	0.83724	NOT
RP11-585F	9.737857	-0.1347918	0.319595	-0.42175822	0.673202	0.837297	NOT
BTF3P5	2.0612	0.21444687	0.508594	0.421646458	0.673283	0.837331	NOT
RP11-626(	4.886513	0.14783681	0.350627	0.42163608	0.673291	0.837331	NOT
RP3-525N1	2.323206	-0.2363441	0.560664	-0.42154303	0.673359	0.837377	NOT
Vax2os1_3	0.693982	-0.515119	1.222139	-0.4214895	0.673398	NA	NA
SEPT8	1833.55	-0.0692135	0.164233	-0.42143458	0.673438	0.837437	NOT
INTU	160.5141	0.13203272	0.313377	0.421322356	0.67352	0.837475	NOT
PITPNC1	1035.581	-0.0949037	0.225259	-0.42130815	0.67353	0.837475	NOT
ABHD14B	6445.795	-0.1096698	0.260375	-0.4211988	0.67361	0.83751	NOT
TTC33	535.9785	-0.0807696	0.191793	-0.4211291	0.673661	0.83751	NOT
FAM184A	381.788	0.1512081	0.359058	0.421124004	0.673665	0.83751	NOT
LKAAEAR1	5.603118	0.3530976	0.838512	0.421100219	0.673682	0.83751	NOT
RP11-203I	37.49928	-0.2235286	0.531075	-0.42089813	0.673829	0.837636	NOT
ARF5	5278.839	0.07115695	0.169091	0.420819767	0.673887	0.837636	NOT
DESI1	4372.044	-0.0865957	0.20579	-0.4207956	0.673904	0.837636	NOT
RP11-485(	5.926009	-0.1655464	0.393443	-0.42076373	0.673928	0.837636	NOT
RBM15	509.9734	-0.0564681	0.134208	-0.42075064	0.673937	0.837636	NOT
IGHG3	1716.635	0.2644259	0.628572	0.420677092	0.673991	0.837664	NOT
ACVR2A	484.4538	0.06509422	0.154772	0.420580252	0.674062	0.837711	NOT
RP5-1126H	29.35176	-0.1112744	0.264598	-0.42054138	0.67409	0.837711	NOT
RP11-473M	19.40298	-0.1380489	0.328592	-0.4201227	0.674396	0.838019	NOT
ZC3H11A	4780.145	-0.0622953	0.148282	-0.42011473	0.674402	0.838019	NOT
AZIN2	102.9412	0.11485734	0.273421	0.420074502	0.674431	0.838019	NOT
RNF165	68.76218	-0.183145	0.436074	-0.41998636	0.674495	0.838061	NOT
DTX3	405.5393	-0.1500063	0.357234	-0.41991015	0.674551	0.838075	NOT
KLHL29	642.2571	-0.1596554	0.380296	-0.41981906	0.674618	0.838075	NOT
B3GAT2	18.39156	0.14611847	0.348087	0.419775723	0.674649	0.838075	NOT
GAPDHP68	2.493613	-0.2178	0.51886	-0.41976639	0.674656	0.838075	NOT
RP11-557H	8.586621	-0.2988223	0.711959	-0.4197184	0.674691	0.838075	NOT
OXLD1	1159.176	0.10338182	0.246313	0.419716923	0.674692	0.838075	NOT
RP11-459(	1.306633	0.3675182	0.875786	0.419643667	0.674746	NA	NA
TTC23L	12.48171	0.12061854	0.287441	0.41962843	0.674757	0.838117	NOT
CTD-3162I	8.490182	-0.1855993	0.442434	-0.41949638	0.674853	0.838199	NOT
EIF3S5P1	2.333704	0.21007368	0.500922	0.419374034	0.674943	0.838244	NOT

RGP1	1414.827	-0.0677074	0.161469	-0.41932228	0.674981	0.838244	NOT
AC008391.	2.275533	-0.2531804	0.60379	-0.41931903	0.674983	0.838244	NOT
IGHV4-31	33.7345	0.34963106	0.834498	0.418971919	0.675237	0.83852	NOT
CTD-3064M	24.99201	-0.1782257	0.425429	-0.41893132	0.675266	0.83852	NOT
PKLR	7290.499	0.18559653	0.443079	0.418879451	0.675304	0.838528	NOT
RP11-10E1	6.289009	-0.1744436	0.416883	-0.41844718	0.67562	0.838882	NOT
RPS4XP6	6.252261	0.13941962	0.333302	0.418297637	0.67573	0.838953	NOT
CTD-2228K	714.6325	-0.182625	0.436605	-0.41828433	0.675739	0.838953	NOT
PLEKHO1	1418.904	0.13864045	0.331513	0.418205087	0.675797	0.838987	NOT
MPHOSPH9	706.2748	-0.0809239	0.193528	-0.41815106	0.675837	0.838994	NOT
NOXRED1	17.52321	0.10916895	0.261136	0.418054741	0.675907	0.838994	NOT
EIF4A2P4	1.876581	-0.1716344	0.410583	-0.41802587	0.675928	0.838994	NOT
MAP1LC3B2	40.24517	-0.082035	0.196252	-0.4180094	0.67594	0.838994	NOT
HIST1H1B	4.537552	0.18900225	0.452174	0.417985731	0.675958	0.838994	NOT
SLC25A15P	17.74272	-0.3440393	0.823941	-0.41755318	0.676274	0.839325	NOT
AMICA1	661.9882	-0.1408673	0.337379	-0.41753461	0.676287	0.839325	NOT
TUBB1	10.8731	-0.1587197	0.380173	-0.41749352	0.676317	0.839325	NOT
FTH1P22	6.194493	0.22742125	0.545084	0.417222212	0.676516	0.839496	NOT
RP11-84C1	1.829334	0.21025956	0.50399	0.417189732	0.67654	0.839496	NOT
KCND2	16.14761	-0.2563809	0.614559	-0.41717862	0.676548	0.839496	NOT
C12orf10	1726.248	0.06565405	0.157395	0.417129796	0.676583	0.839502	NOT
CDKL2	1.536615	-0.231023	0.55405	-0.41697156	0.676699	0.839574	NOT
PHTF2	656.9402	0.07700785	0.184686	0.41696598	0.676703	0.839574	NOT
RP11-649(	5.418486	0.14713791	0.352973	0.41685375	0.676785	0.839637	NOT
RP11-79P5	19.22823	0.12439004	0.298438	0.416804224	0.676822	0.839644	NOT
THEM7P	1.989824	-0.3473189	0.833388	-0.41675531	0.676857	0.83965	NOT
BACH2	139.666	-0.1527908	0.3667	-0.41666473	0.676924	0.839694	NOT
RNU6-30P	1.84949	0.23427222	0.562402	0.416556818	0.677003	0.839753	NOT
TRBV15	1.24636	0.29870217	0.71743	0.416350323	0.677154	NA	NA
LINC01285	14.4811	0.14696274	0.352983	0.416345102	0.677157	0.839872	NOT
C5orf60	3.032377	-0.1993926	0.478918	-0.4163396	0.677162	0.839872	NOT
KIAA1161	5311.979	-0.1193016	0.286581	-0.41629238	0.677196	0.839872	NOT
CLCN6	716.2688	-0.0707918	0.170068	-0.41625605	0.677223	0.839872	NOT
SNTG2	1.646868	-0.3628949	0.872139	-0.4160976	0.677339	0.839942	NOT
RP4-622L5	4.911907	0.18137855	0.435907	0.416095037	0.67734	0.839942	NOT
RPS2P46	150.781	0.12787385	0.307352	0.416049638	0.677374	0.839945	NOT
PDS5B	1049.136	-0.0683833	0.164438	-0.41586145	0.677511	0.840077	NOT
AC012146.	156.6396	-0.1405828	0.338135	-0.41575985	0.677586	0.840131	NOT
RNF31	593.8747	-0.0804961	0.193637	-0.41570515	0.677626	0.840142	NOT
RP11-501(	5.201194	0.2952645	0.710524	0.415558834	0.677733	0.840209	NOT
CALM2P2	10.48505	-0.1331722	0.320475	-0.41554625	0.677742	0.840209	NOT
TRAV27	1.386573	-0.265017	0.6378	-0.41551734	0.677763	NA	NA
RP4-545L1	12.01776	-0.108472	0.261067	-0.41549458	0.67778	0.840218	NOT
SH2D4B	4.293188	0.18384793	0.442597	0.415384096	0.677861	0.840258	NOT
ELAVL1	3012.269	-0.0445539	0.107264	-0.41536599	0.677874	0.840258	NOT
SRP68	4357.836	0.05443151	0.13107	0.41528654	0.677932	0.840291	NOT
RP5-1054A	6.890113	-0.2277342	0.54844	-0.41523995	0.677966	0.840295	NOT
SMAD6	326.5144	-0.1257764	0.303035	-0.41505544	0.678101	0.840411	NOT
FBP2	5.842612	-0.1662099	0.400502	-0.4150038	0.678139	0.840411	NOT

RP11-159F	2.431031	0.16390197	0.394958	0.414985316	0.678153	0.840411	NOT
CTD-2288F	9.005877	-0.133779	0.322465	-0.41486345	0.678242	0.840483	NOT
ZFP2	38.89511	-0.1255492	0.302682	-0.41478854	0.678297	0.840513	NOT
RALGAPB	2068.746	0.05517443	0.133039	0.414722652	0.678345	0.840534	NOT
RP3-467L1	12.59369	-0.1367428	0.329818	-0.41460057	0.678434	0.840579	NOT
ENO3	1099.706	-0.1626673	0.392446	-0.41449602	0.678511	0.840579	NOT
FAM155B	117.5786	0.23471537	0.566276	0.414489627	0.678516	0.840579	NOT
MAPK9	1779.407	0.05330335	0.128602	0.414483214	0.67852	0.840579	NOT
WDR60	391.3112	-0.0795106	0.191841	-0.41446187	0.678536	0.840579	NOT
ZBTB7B	4093.807	0.0808378	0.195097	0.414346118	0.678621	0.840646	NOT
ERCC8	240.5411	0.05306296	0.128085	0.414279376	0.67867	0.840668	NOT
RP11-731F	1.473714	0.39569766	0.95528	0.414221555	0.678712	0.840682	NOT
HSD17B11	8859.919	-0.104684	0.252761	-0.41416228	0.678755	0.840697	NOT
STKLD1	55.88258	0.10406942	0.251392	0.413972938	0.678894	0.840756	NOT
RP11-382I	7.750084	0.17857277	0.431388	0.413949538	0.678911	0.840756	NOT
DUSP4	288.1938	-0.1742611	0.420979	-0.41394256	0.678916	0.840756	NOT
CLDN1	15655.26	0.11906785	0.287653	0.413928536	0.678926	0.840756	NOT
RP11-304I	6.457643	0.16965734	0.409943	0.413856177	0.678979	0.840783	NOT
PALM	1228.061	0.18957516	0.458121	0.413810064	0.679013	0.840786	NOT
LINC00032	1.429146	-0.2667241	0.644754	-0.41368374	0.679106	0.840863	NOT
FAM199X	1759.527	0.06241066	0.15096	0.413425259	0.679295	0.841048	NOT
FAM124B	108.602	-0.1666933	0.403231	-0.41339442	0.679318	0.841048	NOT
CXCL11	206.3005	0.22380693	0.541495	0.413313376	0.679377	0.841083	NOT
VPS13B	1267.667	0.07381978	0.178662	0.413182194	0.679473	0.841144	NOT
RP11-863F	1.736696	-0.2885578	0.698413	-0.41316204	0.679488	0.841144	NOT
TFF1	19.01012	0.35380061	0.856535	0.413060255	0.679562	0.841198	NOT
RP11-369E	0.808411	-0.4510279	1.092869	-0.41270074	0.679826	NA	NA
SLC5A10	32.15993	0.17935091	0.434667	0.412616987	0.679887	0.841535	NOT
IKZF4	186.2995	-0.0698093	0.169192	-0.41260372	0.679897	0.841535	NOT
NAA16	363.5437	-0.0791311	0.191874	-0.41241088	0.680038	0.841606	NOT
RP5-1125A	63.19359	-0.1005365	0.243784	-0.41239944	0.680047	0.841606	NOT
SLAMF1	63.60782	-0.1474428	0.357525	-0.41239859	0.680047	0.841606	NOT
NCLN	5393.386	0.0782131	0.189677	0.412347918	0.680084	0.841613	NOT
SYCP3	9.853017	0.12496784	0.303201	0.412162244	0.680221	0.841736	NOT
NENF	3549.992	0.09831666	0.238558	0.412128188	0.680245	0.841736	NOT
ANAPC15	410.5745	-0.0630737	0.153088	-0.41201	0.680332	0.841805	NOT
CRKL	3631.437	0.05761289	0.139852	0.411954993	0.680372	0.841816	NOT
MIS18A-AS	5.024098	0.13738788	0.333637	0.411788998	0.680494	0.841867	NOT
RP11-734J	10.70431	0.13505949	0.327987	0.411783664	0.680498	0.841867	NOT
ASB9	455.3951	-0.1288425	0.312897	-0.41177241	0.680506	0.841867	NOT
TENM3	10.7946	-0.2431459	0.590586	-0.41170287	0.680557	0.841891	NOT
ATP1B1P1	1.629144	0.19895735	0.483472	0.411517841	0.680693	0.842021	NOT
C6orf163	15.49002	0.15934117	0.387312	0.411402461	0.680777	0.842087	NOT
ZNF629	901.3736	-0.0814534	0.198038	-0.41130279	0.680851	0.842139	NOT
DNAJC11	1148.911	-0.0769444	0.187132	-0.4111762	0.680943	0.842176	NOT
NUS1	1234.943	-0.0690519	0.167973	-0.41109015	0.681006	0.842176	NOT
RIMS4	1.364211	-0.3763348	0.915494	-0.41107315	0.681019	NA	NA
LINC01474	24.76234	-0.1740078	0.423327	-0.41104853	0.681037	0.842176	NOT
RP11-1319	10.83316	0.15343914	0.373296	0.411038427	0.681044	0.842176	NOT

OR11Q1P	20.62884	-0.4115676	1.001385	-0.41099856	0.681074	0.842176	NOT
PALD1	401.3434	-0.0935533	0.227627	-0.4109936	0.681077	0.842176	NOT
CHSY1	836.3402	-0.1018664	0.247871	-0.41096605	0.681097	0.842176	NOT
RP1-47A17	2.16631	0.19479346	0.474092	0.410877197	0.681163	0.842189	NOT
RP11-325I	6.717356	0.15395418	0.374706	0.410866603	0.68117	0.842189	NOT
HNRNPA1P1	4.702302	0.12343405	0.300486	0.41078145	0.681233	0.842195	NOT
SLC11A2	2127.816	0.0694502	0.169071	0.410774831	0.681238	0.842195	NOT
CUL1	3747.748	0.05939471	0.144607	0.410732244	0.681269	0.842196	NOT
MUL1	1623.574	-0.0607463	0.147932	-0.41063526	0.68134	0.842226	NOT
AC008746.	5.940013	-0.1517934	0.369675	-0.41061359	0.681356	0.842226	NOT
SAR1A	4829.485	-0.0540177	0.131645	-0.41032993	0.681564	0.842445	NOT
RPL9P18	4.706325	0.16469902	0.401635	0.410071315	0.681754	0.842641	NOT
RP11-817J	23.45529	0.33854698	0.825784	0.409970392	0.681828	0.842694	NOT
WWP1	6173.623	0.08878319	0.216613	0.409870834	0.681901	0.84271	NOT
CYP4F8	2.032525	0.37083312	0.904781	0.409859709	0.681909	0.84271	NOT
RP5-1024C	25.75832	-0.1349354	0.329251	-0.40982585	0.681934	0.84271	NOT
TBCCD1	578.4815	0.06033952	0.147281	0.409689555	0.682034	0.842773	NOT
LRP4-AS1	5.337978	0.1837722	0.448646	0.409614711	0.682089	0.842773	NOT
USP46	452.5852	0.06508965	0.158924	0.409564065	0.682126	0.842773	NOT
RP5-854E1	2.803817	-0.1853919	0.452733	-0.40949542	0.682176	0.842773	NOT
ANKRD44	408.0286	-0.0900174	0.219829	-0.40948897	0.682181	0.842773	NOT
PSMA4	5292.481	0.063027	0.15392	0.409478996	0.682188	0.842773	NOT
SNF8	3607.697	0.07608158	0.185823	0.409429654	0.682224	0.842773	NOT
RP11-685I	12.84539	-0.2164326	0.528663	-0.40939622	0.682249	0.842773	NOT
AURKC	47.24741	-0.1274746	0.311411	-0.40934489	0.682287	0.842773	NOT
RP11-34A1	4.746547	0.28853448	0.704953	0.409296328	0.682322	0.842773	NOT
AP000620.	3.817092	-0.1575438	0.384947	-0.4092609	0.682348	0.842773	NOT
GRAMD4P8	1.565266	0.26130334	0.638533	0.409224255	0.682375	0.842773	NOT
CTD-3220I	4.072697	-0.2201195	0.537942	-0.40918812	0.682402	0.842773	NOT
ATF7	1102.453	-0.0513258	0.125441	-0.40916349	0.68242	0.842773	NOT
TUBB6	852.3915	-0.1270571	0.310683	-0.40896092	0.682568	0.842919	NOT
NSUN5P1	409.0618	-0.0991549	0.242495	-0.4088947	0.682617	0.84294	NOT
RP11-568J	3.482536	-0.1659944	0.40602	-0.40883323	0.682662	0.842958	NOT
NIPSNAP1	11358.16	0.09411774	0.230411	0.40847712	0.682923	0.843242	NOT
RP4-754E2	25.05202	0.34382862	0.842115	0.408291607	0.68306	0.84337	NOT
TMEM167B	1820.24	0.04874314	0.119395	0.408251378	0.683089	0.84337	NOT
ENTPD3-AS	241.6161	0.09580969	0.234744	0.408144575	0.683168	0.843411	NOT
HIGD2A	3947.766	0.09906465	0.242772	0.408056566	0.683232	0.843411	NOT
TMEM186	539.0013	-0.0664565	0.162875	-0.40802108	0.683258	0.843411	NOT
C2orf69	745.5042	-0.0686606	0.168293	-0.40798188	0.683287	0.843411	NOT
RBMXP2	15.16653	0.16314685	0.39989	0.407979057	0.683289	0.843411	NOT
CTD-2194A	19.4943	-0.1917195	0.469956	-0.40795225	0.683309	0.843411	NOT
RP11-547I	10.6859	-0.1233972	0.302514	-0.40790525	0.683343	0.843415	NOT
RP11-11N5	0.950703	0.36785415	0.902183	0.407737736	0.683466	NA	NA
SUGT1P	9.655	-0.1787451	0.438419	-0.40770345	0.683491	0.843559	NOT
IGFBP6	171.4193	0.13796783	0.338475	0.407616518	0.683555	0.843594	NOT
TRA2A	1788.909	0.04922553	0.120784	0.407550529	0.683604	0.843594	NOT
CLCN3P1	4.902071	0.37095787	0.910241	0.407537929	0.683613	0.843594	NOT
LINC0057C	14.08725	0.25956594	0.637314	0.407280899	0.683802	0.843752	NOT

AC016738.	12.13599	0.1620557	0.39795	0.407225849	0.683842	0.843752	NOT
NRIR	10.69165	0.19643801	0.482433	0.407181768	0.683874	0.843752	NOT
RP11-57A1	22.77576	0.25198999	0.618903	0.407155623	0.683894	0.843752	NOT
DPEP3	4.182814	-0.1997105	0.490506	-0.40715221	0.683896	0.843752	NOT
RP11-524F	6.767231	0.17008939	0.41801	0.406902445	0.68408	0.84394	NOT
SLC7A4	7.250504	-0.2599451	0.638922	-0.40684946	0.684119	0.843949	NOT
NDUFA6-AS	296.1538	-0.1686261	0.414621	-0.40669946	0.684229	0.844047	NOT
PKN2	1525.821	-0.0762889	0.187646	-0.40655718	0.684333	0.844138	NOT
LLNLR-304	1.751551	0.22079279	0.543209	0.406460373	0.684404	0.844187	NOT
CTD-2245F	10.69436	0.1482024	0.364794	0.40626338	0.684549	0.844327	NOT
MFSD3	2987.119	0.13729574	0.338033	0.406160997	0.684624	0.844374	NOT
ABHD6	1708.972	-0.1166039	0.28714	-0.40608672	0.684679	0.844374	NOT
RHOQP1	8.59103	-0.1324578	0.326183	-0.406084	0.684681	0.844374	NOT
ZNF808	201.8337	-0.1172388	0.288759	-0.40600894	0.684736	0.844404	NOT
ACOX3	1146.247	-0.0785152	0.193442	-0.40588434	0.684828	0.844479	NOT
FCER1A	54.6323	0.19005846	0.46837	0.405786617	0.684899	0.844517	NOT
CD8A	259.8525	-0.176442	0.434858	-0.40574629	0.684929	0.844517	NOT
NAP1L6	23.57259	0.40414673	0.996136	0.405714343	0.684953	0.844517	NOT
SAMHD1	3580.694	-0.0972914	0.239886	-0.40557379	0.685056	0.844526	NOT
FAM136BP	1.84423	0.26181488	0.645563	0.405560563	0.685066	0.844526	NOT
FAM193A	1200.715	-0.0505285	0.12459	-0.40555881	0.685067	0.844526	NOT
ARHGAP15	206.6594	-0.1013492	0.249914	-0.40553595	0.685084	0.844526	NOT
RP11-848F	5.871236	0.18454216	0.455187	0.405420481	0.685168	0.844592	NOT
ZNF324	294.9731	-0.0511684	0.126287	-0.40517609	0.685348	0.844755	NOT
BECN1	1756.051	-0.0758321	0.187192	-0.40510416	0.685401	0.844755	NOT
ZNF577	224.6225	0.14829007	0.366076	0.405079654	0.685419	0.844755	NOT
ASB4	106.4387	-0.2239166	0.552806	-0.40505418	0.685438	0.844755	NOT
RP3-412A9	4.034825	0.13705933	0.338394	0.405028284	0.685457	0.844755	NOT
HESX1	33.04783	-0.1018659	0.251558	-0.40493959	0.685522	0.844797	NOT
LINC01176	54.61827	-0.1210537	0.29898	-0.40488912	0.685559	0.844805	NOT
GCHFR	748.2917	-0.1158359	0.286191	-0.4047505	0.685661	0.844834	NOT
LCMT1-AS1	5.194879	-0.1247139	0.308174	-0.40468722	0.685707	0.844834	NOT
NR2F6	5186.85	-0.099944	0.246968	-0.40468433	0.68571	0.844834	NOT
RPS28	12264.58	-0.1059231	0.261752	-0.40467029	0.68572	0.844834	NOT
HSPA8P15	4.003498	-0.154999	0.383095	-0.40459654	0.685774	0.844834	NOT
HIST2H2BC	7.993104	-0.1297821	0.320787	-0.40457368	0.685791	0.844834	NOT
MYH8	2.409721	-0.4257058	1.05227	-0.40455968	0.685801	0.844834	NOT
RP11-165I	5.042897	-0.1341575	0.331722	-0.40442691	0.685899	0.844916	NOT
SLC7A9	894.9068	0.13812053	0.341585	0.404352134	0.685954	0.844946	NOT
C6orf183	52.47146	-0.2500496	0.618566	-0.40424084	0.686036	0.845008	NOT
ARL10	169.7999	0.11279938	0.279119	0.404126678	0.68612	0.845073	NOT
LINC01451	167.9551	0.19671617	0.486911	0.404008119	0.686207	0.845117	NOT
PI3	121.929	-0.2519744	0.623865	-0.40389252	0.686292	0.845117	NOT
CAPN2	5426.389	-0.0947552	0.23461	-0.40388404	0.686298	0.845117	NOT
EMC1	2510.104	0.06289726	0.155734	0.403874954	0.686305	0.845117	NOT
RP11-383C	2.410034	0.17041623	0.421962	0.403866152	0.686311	0.845117	NOT
RP11-764K	5.953115	-0.1498134	0.371019	-0.40378879	0.686368	0.845139	NOT
C1GALT1C1	1296.791	0.06985721	0.17302	0.403751357	0.686396	0.845139	NOT
PROX2	4.152757	-0.1657866	0.410652	-0.40371518	0.686422	0.845139	NOT

C9orf116	86.22549	-0.1353045	0.335329	-0.40349772	0.686582	0.845297	NOT
TRIM10	184.2862	0.16020119	0.397145	0.403381702	0.686667	0.845364	NOT
SNAP23	1478.623	-0.0510715	0.126648	-0.40325476	0.686761	0.845413	NOT
AC018766.	28.6212	0.11518429	0.285663	0.403217332	0.686788	0.845413	NOT
RP11-326(	11.15392	-0.1442175	0.357682	-0.40320002	0.686801	0.845413	NOT
TMX2	4032	0.05788151	0.143613	0.403039037	0.68692	0.845489	NOT
ARID5B	896.4503	0.10588246	0.262715	0.403031933	0.686925	0.845489	NOT
RP11-407F	2.081584	0.20324534	0.504508	0.402858352	0.687052	0.845608	NOT
RP11-421F	558.3763	0.09187121	0.228147	0.402683424	0.687181	0.845681	NOT
AC006116.	5.139585	-0.146683	0.364272	-0.40267458	0.687188	0.845681	NOT
ABHD1	186.3263	-0.1970203	0.489308	-0.40265053	0.687205	0.845681	NOT
AP001372.	162.095	-0.0758441	0.188446	-0.40247068	0.687338	0.845805	NOT
RP11-371I	3.102138	0.21449576	0.533153	0.402315605	0.687452	0.845907	NOT
ABCA13	29.24197	-0.2402585	0.597359	-0.40220133	0.687536	0.845938	NOT
FCAR	4.688762	-0.2141233	0.532498	-0.40211121	0.687602	0.845938	NOT
PRKRIP1	1057.31	-0.0600579	0.149359	-0.40210429	0.687607	0.845938	NOT
AQP6	28.6755	0.2895648	0.720138	0.402096423	0.687613	0.845938	NOT
SDSL	3289.033	-0.1616471	0.402037	-0.40207047	0.687632	0.845938	NOT
CCDC9	1129.394	0.05873451	0.146133	0.401925761	0.687739	0.845992	NOT
ANAPC11	5663.134	0.10608113	0.263948	0.401901812	0.687756	0.845992	NOT
TMED8	557.0327	-0.080342	0.199914	-0.40188291	0.68777	0.845992	NOT
SULT4A1	127.1417	-0.363959	0.905927	-0.4017531	0.687866	0.846052	NOT
ICAM3	34.21096	-0.1493806	0.371846	-0.40172679	0.687885	0.846052	NOT
FMR1NB	2.265147	0.31304584	0.779322	0.401689764	0.687912	0.846052	NOT
AC011294.	41.55206	0.22725006	0.566038	0.401475031	0.68807	0.846188	NOT
RELA	3807.472	-0.0336124	0.083726	-0.40145523	0.688085	0.846188	NOT
C6orf229	2.391651	0.29656299	0.738941	0.401335355	0.688173	0.8462	NOT
LRFN1	154.6659	0.16757471	0.417547	0.401331243	0.688176	0.8462	NOT
RP11-114H	12.98333	-0.404276	1.007381	-0.40131394	0.688189	0.8462	NOT
IGKV1-33	0.934011	-0.468418	1.167426	-0.40123983	0.688244	NA	NA
RP11-396K	294.2397	-0.1013256	0.252586	-0.40115349	0.688307	0.846246	NOT
AC018462.	4.279805	0.14656816	0.365423	0.401092193	0.688352	0.846246	NOT
KLHL32	30.31708	-0.155116	0.386737	-0.40108867	0.688355	0.846246	NOT
RP11-657(	1.791323	-0.4176801	1.041438	-0.40106106	0.688375	0.846246	NOT
MRFAP1L1	2624.354	-0.0463289	0.115527	-0.40102359	0.688403	0.846246	NOT
HERC2P5	3.277121	-0.3325752	0.829344	-0.40100978	0.688413	0.846246	NOT
CASC1	5.678569	0.16912455	0.421908	0.400856527	0.688526	0.846346	NOT
AC000123.	23.36627	-0.0959289	0.239342	-0.40080247	0.688566	0.846357	NOT
ARL2	1414.348	-0.1297489	0.323793	-0.40071602	0.688629	0.846395	NOT
NAT8B	218.978	-0.2202915	0.5498	-0.40067543	0.688659	0.846395	NOT
EIF4A1	165.5424	0.10871522	0.271452	0.400494747	0.688792	0.846498	NOT
AC007255.	4.493521	0.26593559	0.664047	0.400476921	0.688805	0.846498	NOT
AP000354.	1.64544	-0.2258744	0.564179	-0.40035913	0.688892	0.846565	NOT
AKAP8	1194.699	-0.0515565	0.128789	-0.4003182	0.688922	0.846565	NOT
AC104135.	1.297384	-0.3090397	0.772165	-0.40022496	0.688991	NA	NA
IGKV1D-8	4.190036	0.33009885	0.824826	0.400204384	0.689006	0.846629	NOT
LRRC39	32.89743	-0.1016508	0.254057	-0.40010965	0.689076	0.846677	NOT
CTD-2561E	7.364739	-0.1390312	0.347759	-0.39979119	0.68931	0.846927	NOT
AC012358.	1.669246	0.21776998	0.544843	0.399693014	0.689383	0.846955	NOT

AC099684.	41.75612	-0.2071018	0.518175	-0.3996753	0.689396	0.846955	NOT
TMEM183A	1487.681	0.04855422	0.121548	0.399465964	0.68955	0.847106	NOT
IGHV1-69	12.87512	0.32744032	0.819787	0.399421254	0.689583	0.847108	NOT
RNU6-418I	2.385158	0.17668518	0.442597	0.399201227	0.689745	0.847269	NOT
MRPS33	1978.911	-0.0747727	0.187393	-0.39901523	0.689882	0.847399	NOT
PRAF2	706.4661	-0.0963243	0.241442	-0.39895437	0.689927	0.847416	NOT
RP11-215I	9.426888	-0.1107348	0.277684	-0.39877977	0.690055	0.847535	NOT
RP11-44N2	49.49197	-0.207511	0.520458	-0.39870815	0.690108	0.847562	NOT
TBX21	40.12308	-0.1284378	0.322218	-0.39860572	0.690184	0.847565	NOT
GOLGA8IP	4.485207	0.23602514	0.592155	0.398586469	0.690198	0.847565	NOT
AP001437.	5.534883	-0.1324151	0.33222	-0.3985768	0.690205	0.847565	NOT
RAB7A	10899.66	0.04387617	0.110115	0.39845708	0.690293	0.847615	NOT
WDR86	27.6901	0.11688058	0.293349	0.398434944	0.69031	0.847615	NOT
CTD-2311M	8.240801	0.17618367	0.442292	0.398342721	0.690378	0.847615	NOT
DNAJA4	266.9586	0.15661185	0.393161	0.398340543	0.690379	0.847615	NOT
SIGLEC11	38.17324	0.16042356	0.402761	0.398309764	0.690402	0.847615	NOT
PEX12	475.9339	-0.0792564	0.199078	-0.39811822	0.690543	0.847711	NOT
PRSS23	1674.317	0.1163462	0.292298	0.398039004	0.690601	0.847711	NOT
[truncated: 459,076 more chars]
