# Supplementary material for: Life span‐associated ferroptosis‐related genes identification and validation for hepatocellular carcinoma patients as hepatitis B virus carriers
Source: J Clin Lab Anal. 2023 Jul 18;37(13-14):e24930. doi: 10.1002/jcla.24930 (PMC10492458; doi:10.1002/jcla.24930)
Supplement: Supplementary file 10 — Tables S1–S14 [file JCLA-37-e24930-s009.zip › TableS6_Ferr_deg.docx]

TableS6_Ferr_deg

| TICRR |
| --- |
| RRM2 |
| POLQ |
| MCM10 |
| FANCD2 |
| STMN1 |
| RAD51AP1 |
| NQO1 |
| G6PD |
| HAMP |
| DUOX1 |
| STEAP3 |
| DUSP1 |
| MT1F |
| TXNRD1 |
| EPAS1 |
| SQLE |
| SRC |
| ASNS |
| ZFP69B |
| GABARAPL1 |
| CAPG |
| SQSTM1 |
| MAF1 |
| RPL8 |
| CA9 |
| SLC1A4 |
| SLC2A1 |
| SLC38A1 |
| SLC1A5 |
| TXNIP |
| CRYAB |
| PROM2 |
| MYB |
| MUC1 |
| ANGPTL7 |
