# Supplementary material for: Life span‐associated ferroptosis‐related genes identification and validation for hepatocellular carcinoma patients as hepatitis B virus carriers
Source: J Clin Lab Anal. 2023 Jul 18;37(13-14):e24930. doi: 10.1002/jcla.24930 (PMC10492458; doi:10.1002/jcla.24930)
Supplement: Supplementary file 10 — Tables S1–S14 [file JCLA-37-e24930-s009.zip › TableS7_cox.docx]

TableS7_cox

| Characteristics | HR | CI5 | CI95 | HR (95% CI for HR) | beta | wald.test | p.value |
| --- | --- | --- | --- | --- | --- | --- | --- |
| G6PD | 1.33 | 1.14 | 1.55 | 1.33 (1.14-1.55) | 0.28 | 13.26 | 0 |
| GABARAPL1 | 0.65 | 0.52 | 0.8 | 0.65 (0.52-0.8) | -0.44 | 16.87 | 0 |
| RRM2 | 1.45 | 1.18 | 1.79 | 1.45 (1.18-1.79) | 0.37 | 12.14 | 0 |
| FANCD2 | 1.59 | 1.23 | 2.06 | 1.59 (1.23-2.06) | 0.46 | 12.43 | 0 |
| MCM10 | 1.55 | 1.24 | 1.93 | 1.55 (1.24-1.93) | 0.44 | 14.7 | 0 |
| RAD51AP1 | 1.65 | 1.29 | 2.11 | 1.65 (1.29-2.11) | 0.5 | 16.22 | 0 |
| TICRR | 1.67 | 1.28 | 2.17 | 1.67 (1.28-2.17) | 0.51 | 14.25 | 0 |
| SLC38A1 | 1.34 | 1.13 | 1.58 | 1.34 (1.13-1.58) | 0.29 | 11.78 | 0.001 |
| DUSP1 | 0.7 | 0.57 | 0.86 | 0.7 (0.57-0.86) | -0.36 | 10.87 | 0.001 |
| ZFP69B | 2.16 | 1.34 | 3.47 | 2.16 (1.34-3.47) | 0.77 | 10.13 | 0.001 |
| SLC2A1 | 1.4 | 1.15 | 1.7 | 1.4 (1.15-1.7) | 0.33 | 11.35 | 0.001 |
| STMN1 | 1.46 | 1.17 | 1.84 | 1.46 (1.17-1.84) | 0.38 | 10.82 | 0.001 |
| SRC | 1.56 | 1.19 | 2.03 | 1.56 (1.19-2.03) | 0.44 | 10.58 | 0.001 |
| CA9 | 1.17 | 1.06 | 1.29 | 1.17 (1.06-1.29) | 0.16 | 10.11 | 0.001 |
| POLQ | 1.54 | 1.2 | 1.98 | 1.54 (1.2-1.98) | 0.43 | 11.6 | 0.001 |
| SLC1A5 | 1.28 | 1.1 | 1.5 | 1.28 (1.1-1.5) | 0.25 | 9.99 | 0.002 |
| MYB | 1.52 | 1.15 | 2 | 1.52 (1.15-2) | 0.42 | 8.92 | 0.003 |
| ASNS | 1.28 | 1.09 | 1.51 | 1.28 (1.09-1.51) | 0.25 | 9.12 | 0.003 |
| CAPG | 1.3 | 1.09 | 1.54 | 1.3 (1.09-1.54) | 0.26 | 8.98 | 0.003 |
| SQLE | 1.34 | 1.09 | 1.65 | 1.34 (1.09-1.65) | 0.3 | 7.99 | 0.005 |
| EPAS1 | 0.67 | 0.49 | 0.91 | 0.67 (0.49-0.91) | -0.4 | 6.54 | 0.011 |
| SLC1A4 | 1.4 | 1.06 | 1.86 | 1.4 (1.06-1.86) | 0.34 | 5.43 | 0.02 |
| RPL8 | 1.25 | 1.02 | 1.52 | 1.25 (1.02-1.52) | 0.22 | 4.57 | 0.033 |
| STEAP3 | 0.85 | 0.73 | 0.99 | 0.85 (0.73-0.99) | -0.16 | 4.21 | 0.04 |
| ANGPTL7 | 0.74 | 0.55 | 1 | 0.74 (0.55-1) | -0.3 | 3.96 | 0.046 |
| MUC1 | 1.14 | 1 | 1.3 | 1.14 (1-1.3) | 0.13 | 3.56 | 0.059 |
| HAMP | 0.91 | 0.83 | 1.01 | 0.91 (0.83-1.01) | -0.09 | 3.39 | 0.066 |
| TXNIP | 0.84 | 0.69 | 1.02 | 0.84 (0.69-1.02) | -0.18 | 3.24 | 0.072 |
| DUOX1 | 1.18 | 0.98 | 1.42 | 1.18 (0.98-1.42) | 0.17 | 3.21 | 0.073 |
| MAF1 | 1.3 | 0.97 | 1.72 | 1.3 (0.97-1.72) | 0.26 | 3.18 | 0.075 |
| TXNRD1 | 1.19 | 0.98 | 1.45 | 1.19 (0.98-1.45) | 0.18 | 3.05 | 0.081 |
| NQO1 | 1.06 | 0.98 | 1.15 | 1.06 (0.98-1.15) | 0.06 | 2.23 | 0.135 |
| PROM2 | 1.12 | 0.96 | 1.31 | 1.12 (0.96-1.31) | 0.11 | 1.93 | 0.165 |
| SQSTM1 | 1.16 | 0.94 | 1.43 | 1.16 (0.94-1.43) | 0.15 | 1.85 | 0.173 |
| MT1F | 0.92 | 0.8 | 1.07 | 0.92 (0.8-1.07) | -0.08 | 1.09 | 0.297 |
| CRYAB | 1.04 | 0.94 | 1.15 | 1.04 (0.94-1.15) | 0.04 | 0.63 | 0.429 |
