# Supplementary material for: Life span‐associated ferroptosis‐related genes identification and validation for hepatocellular carcinoma patients as hepatitis B virus carriers
Source: J Clin Lab Anal. 2023 Jul 18;37(13-14):e24930. doi: 10.1002/jcla.24930 (PMC10492458; doi:10.1002/jcla.24930)
Supplement: Supplementary file 10 — Tables S1–S14 [file JCLA-37-e24930-s009.zip › TableS8_comparison_of_models.docx]

TableS8_comparison_of_models

| No. | Reference | Object of study | Gene list | AUC |
| --- | --- | --- | --- | --- |
| 1 | This manuscript | Ferroptosis genes on HBV+ HCC samples | "EPAS1","GABARAPL1","SQLE","RAD51AP1","RPL8","CAPG","RRM2","SLC1A5","SLC38A1","SRC" | 0.851 |
| 2 | Screening and Identification of Potential Biomarkers in Hepatitis B Virus-Related Hepatocellular Carcinoma by Bioinformatics Analysis | HBV+ HCC samples | "KIF11","TPX2","KIF20A","CCNB2" | 0.816 |
| 3 | Construction and Validation of a Combined Ferroptosis and Hypoxia Prognostic Signature for Hepatocellular Carcinoma | Ferroptosis genes on HCC samples | "PPARGC1A","EIF2AK4","UGP2","ILVBL","SLC2A1","LDHA","SLC7A11","STC2","TXNRD1","STMN1" | 0.8 |
| 4 | The Detection and Verification of Two Heterogeneous Subgroups and a Risk Model Based on Ferroptosis-Related Genes in Hepatocellular Carcinoma | Ferroptosis genes on HCC samples | "CDCA8","SPP1","S100A9","EPO","FTCD","CFHR3" | 0.837 |
| 5 | Identification and validation of three core genes in p53 signaling pathway in hepatitis B virus-related hepatocellular carcinoma | P53 genes on HBV+ HCC samples | "CCNB1","CDK1","RRM2" | 0.8 |
