# Supplementary material for: Life span‐associated ferroptosis‐related genes identification and validation for hepatocellular carcinoma patients as hepatitis B virus carriers
Source: J Clin Lab Anal. 2023 Jul 18;37(13-14):e24930. doi: 10.1002/jcla.24930 (PMC10492458; doi:10.1002/jcla.24930)
Supplement: Supplementary file 10 — Tables S1–S14 [file JCLA-37-e24930-s009.zip › TableS9_GO_BP.docx]

TableS9_GO_BP

| ID | Description | BgRatio | pvalue | p.adjust | qvalue | geneID | Count |
| --- | --- | --- | --- | --- | --- | --- | --- |
| GO:0003018 | vascular process in circulatory system | 263/18723 | 0.000146 | 0.045631 | 0.024829 | SLC1A5/SLC38A1/SRC | 3 |
| GO:0015804 | neutral amino acid transport | 48/18723 | 0.000178 | 0.045631 | 0.024829 | SLC1A5/SLC38A1 | 2 |
| GO:1902475 | L-alpha-amino acid transmembrane transport | 65/18723 | 0.000328 | 0.045631 | 0.024829 | SLC1A5/SLC38A1 | 2 |
| GO:0015807 | L-amino acid transport | 69/18723 | 0.000369 | 0.045631 | 0.024829 | SLC1A5/SLC38A1 | 2 |
| GO:2000243 | positive regulation of reproductive process | 81/18723 | 0.000509 | 0.045631 | 0.024829 | RAD51AP1/SRC | 2 |
| GO:0150104 | transport across blood-brain barrier | 87/18723 | 0.000587 | 0.045631 | 0.024829 | SLC1A5/SLC38A1 | 2 |
| GO:0010232 | vascular transport | 88/18723 | 0.0006 | 0.045631 | 0.024829 | SLC1A5/SLC38A1 | 2 |
| GO:0003333 | amino acid transmembrane transport | 98/18723 | 0.000744 | 0.049469 | 0.026917 | SLC1A5/SLC38A1 | 2 |
| GO:0031333 | negative regulation of protein-containing complex assembly | 141/18723 | 0.001531 | 0.066661 | 0.036272 | CAPG/SRC | 2 |
| GO:0006865 | amino acid transport | 143/18723 | 0.001574 | 0.066661 | 0.036272 | SLC1A5/SLC38A1 | 2 |
| GO:1905039 | carboxylic acid transmembrane transport | 149/18723 | 0.001707 | 0.066661 | 0.036272 | SLC1A5/SLC38A1 | 2 |
| GO:1903825 | organic acid transmembrane transport | 150/18723 | 0.00173 | 0.066661 | 0.036272 | SLC1A5/SLC38A1 | 2 |
| GO:0044409 | entry into host | 151/18723 | 0.001752 | 0.066661 | 0.036272 | SLC1A5/SRC | 2 |
| GO:2000241 | regulation of reproductive process | 168/18723 | 0.002163 | 0.066661 | 0.036272 | RAD51AP1/SRC | 2 |
| GO:0052126 | movement in host environment | 175/18723 | 0.002344 | 0.066661 | 0.036272 | SLC1A5/SRC | 2 |
| GO:0051054 | positive regulation of DNA metabolic process | 201/18723 | 0.003077 | 0.066661 | 0.036272 | RAD51AP1/SRC | 2 |
| GO:0051701 | biological process involved in interaction with host | 203/18723 | 0.003138 | 0.066661 | 0.036272 | SLC1A5/SRC | 2 |
| GO:0098657 | import into cell | 227/18723 | 0.003905 | 0.066661 | 0.036272 | SLC1A5/SLC38A1 | 2 |
| GO:0051974 | negative regulation of telomerase activity | 10/18723 | 0.004266 | 0.066661 | 0.036272 | SRC | 1 |
| GO:0071803 | positive regulation of podosome assembly | 10/18723 | 0.004266 | 0.066661 | 0.036272 | SRC | 1 |
| GO:0051259 | protein complex oligomerization | 238/18723 | 0.004284 | 0.066661 | 0.036272 | RRM2/SLC1A5 | 2 |
| GO:0032329 | serine transport | 11/18723 | 0.004691 | 0.066661 | 0.036272 | SLC1A5 | 1 |
| GO:0033625 | positive regulation of integrin activation | 11/18723 | 0.004691 | 0.066661 | 0.036272 | SRC | 1 |
| GO:0070778 | L-aspartate transmembrane transport | 11/18723 | 0.004691 | 0.066661 | 0.036272 | SLC1A5 | 1 |
| GO:0140042 | lipid droplet formation | 11/18723 | 0.004691 | 0.066661 | 0.036272 | SQLE | 1 |
| GO:2000392 | regulation of lamellipodium morphogenesis | 11/18723 | 0.004691 | 0.066661 | 0.036272 | SRC | 1 |
| GO:0072584 | caveolin-mediated endocytosis | 12/18723 | 0.005117 | 0.066661 | 0.036272 | SRC | 1 |
| GO:0071801 | regulation of podosome assembly | 13/18723 | 0.005542 | 0.066661 | 0.036272 | SRC | 1 |
| GO:0046942 | carboxylic acid transport | 273/18723 | 0.005597 | 0.066661 | 0.036272 | SLC1A5/SLC38A1 | 2 |
| GO:0035791 | platelet-derived growth factor receptor-beta signaling pathway | 14/18723 | 0.005967 | 0.066661 | 0.036272 | SRC | 1 |
| GO:0038166 | angiotensin-activated signaling pathway | 14/18723 | 0.005967 | 0.066661 | 0.036272 | SRC | 1 |
| GO:0060576 | intestinal epithelial cell development | 14/18723 | 0.005967 | 0.066661 | 0.036272 | SRC | 1 |
| GO:0086103 | G protein-coupled receptor signaling pathway involved in heart process | 14/18723 | 0.005967 | 0.066661 | 0.036272 | SRC | 1 |
| GO:0044403 | biological process involved in symbiotic interaction | 290/18723 | 0.006294 | 0.066661 | 0.036272 | SLC1A5/SRC | 2 |
| GO:0009263 | deoxyribonucleotide biosynthetic process | 15/18723 | 0.006392 | 0.066661 | 0.036272 | RRM2 | 1 |
| GO:0045836 | positive regulation of meiotic nuclear division | 15/18723 | 0.006392 | 0.066661 | 0.036272 | RAD51AP1 | 1 |
| GO:0051290 | protein heterotetramerization | 15/18723 | 0.006392 | 0.066661 | 0.036272 | RRM2 | 1 |
| GO:0070207 | protein homotrimerization | 15/18723 | 0.006392 | 0.066661 | 0.036272 | SLC1A5 | 1 |
| GO:1905168 | positive regulation of double-strand break repair via homologous recombination | 15/18723 | 0.006392 | 0.066661 | 0.036272 | RAD51AP1 | 1 |
| GO:0051014 | actin filament severing | 16/18723 | 0.006817 | 0.066661 | 0.036272 | CAPG | 1 |
| GO:0015849 | organic acid transport | 303/18723 | 0.006853 | 0.066661 | 0.036272 | SLC1A5/SLC38A1 | 2 |
| GO:0033623 | regulation of integrin activation | 17/18723 | 0.007242 | 0.066661 | 0.036272 | SRC | 1 |
| GO:0036035 | osteoclast development | 17/18723 | 0.007242 | 0.066661 | 0.036272 | SRC | 1 |
| GO:0070206 | protein trimerization | 17/18723 | 0.007242 | 0.066661 | 0.036272 | SLC1A5 | 1 |
| GO:0072673 | lamellipodium morphogenesis | 17/18723 | 0.007242 | 0.066661 | 0.036272 | SRC | 1 |
| GO:0098962 | regulation of postsynaptic neurotransmitter receptor activity | 17/18723 | 0.007242 | 0.066661 | 0.036272 | SRC | 1 |
| GO:2000811 | negative regulation of anoikis | 17/18723 | 0.007242 | 0.066661 | 0.036272 | SRC | 1 |
| GO:0045787 | positive regulation of cell cycle | 313/18723 | 0.007298 | 0.066661 | 0.036272 | RAD51AP1/SRC | 2 |
| GO:0002223 | stimulatory C-type lectin receptor signaling pathway | 18/18723 | 0.007667 | 0.066661 | 0.036272 | SRC | 1 |
| GO:0051895 | negative regulation of focal adhesion assembly | 18/18723 | 0.007667 | 0.066661 | 0.036272 | SRC | 1 |
| GO:0071800 | podosome assembly | 18/18723 | 0.007667 | 0.066661 | 0.036272 | SRC | 1 |
| GO:0150118 | negative regulation of cell-substrate junction organization | 18/18723 | 0.007667 | 0.066661 | 0.036272 | SRC | 1 |
| GO:1990840 | response to lectin | 18/18723 | 0.007667 | 0.066661 | 0.036272 | SRC | 1 |
| GO:1990858 | cellular response to lectin | 18/18723 | 0.007667 | 0.066661 | 0.036272 | SRC | 1 |
| GO:2000641 | regulation of early endosome to late endosome transport | 18/18723 | 0.007667 | 0.066661 | 0.036272 | SRC | 1 |
| GO:0060065 | uterus development | 19/18723 | 0.008091 | 0.066661 | 0.036272 | SRC | 1 |
| GO:0032211 | negative regulation of telomere maintenance via telomerase | 20/18723 | 0.008515 | 0.066661 | 0.036272 | SRC | 1 |
| GO:0051900 | regulation of mitochondrial depolarization | 20/18723 | 0.008515 | 0.066661 | 0.036272 | SRC | 1 |
| GO:0038083 | peptidyl-tyrosine autophosphorylation | 21/18723 | 0.008939 | 0.066661 | 0.036272 | SRC | 1 |
| GO:0045056 | transcytosis | 21/18723 | 0.008939 | 0.066661 | 0.036272 | SRC | 1 |
| GO:0071498 | cellular response to fluid shear stress | 21/18723 | 0.008939 | 0.066661 | 0.036272 | SRC | 1 |
| GO:0010639 | negative regulation of organelle organization | 348/18723 | 0.008957 | 0.066661 | 0.036272 | CAPG/SRC | 2 |
| GO:0015711 | organic anion transport | 354/18723 | 0.009257 | 0.066661 | 0.036272 | SLC1A5/SLC38A1 | 2 |
| GO:0002220 | innate immune response activating cell surface receptor signaling pathway | 22/18723 | 0.009363 | 0.066661 | 0.036272 | SRC | 1 |
| GO:0036120 | cellular response to platelet-derived growth factor stimulus | 22/18723 | 0.009363 | 0.066661 | 0.036272 | SRC | 1 |
| GO:0046628 | positive regulation of insulin receptor signaling pathway | 22/18723 | 0.009363 | 0.066661 | 0.036272 | SRC | 1 |
| GO:0060444 | branching involved in mammary gland duct morphogenesis | 22/18723 | 0.009363 | 0.066661 | 0.036272 | SRC | 1 |
| GO:0051052 | regulation of DNA metabolic process | 359/18723 | 0.00951 | 0.066661 | 0.036272 | RAD51AP1/SRC | 2 |
| GO:0002758 | innate immune response-activating signal transduction | 23/18723 | 0.009787 | 0.066661 | 0.036272 | SRC | 1 |
| GO:0010640 | regulation of platelet-derived growth factor receptor signaling pathway | 23/18723 | 0.009787 | 0.066661 | 0.036272 | SRC | 1 |
| GO:0036119 | response to platelet-derived growth factor | 23/18723 | 0.009787 | 0.066661 | 0.036272 | SRC | 1 |
| GO:0051446 | positive regulation of meiotic cell cycle | 23/18723 | 0.009787 | 0.066661 | 0.036272 | RAD51AP1 | 1 |
| GO:0051882 | mitochondrial depolarization | 23/18723 | 0.009787 | 0.066661 | 0.036272 | SRC | 1 |
| GO:0060575 | intestinal epithelial cell differentiation | 23/18723 | 0.009787 | 0.066661 | 0.036272 | SRC | 1 |
| GO:0140694 | non-membrane-bounded organelle assembly | 367/18723 | 0.009923 | 0.066661 | 0.036272 | SQLE/SRC | 2 |
| GO:0048011 | neurotrophin TRK receptor signaling pathway | 24/18723 | 0.010211 | 0.066661 | 0.036272 | SRC | 1 |
| GO:2000209 | regulation of anoikis | 24/18723 | 0.010211 | 0.066661 | 0.036272 | SRC | 1 |
| GO:0010447 | response to acidic pH | 25/18723 | 0.010634 | 0.066661 | 0.036272 | SRC | 1 |
| GO:0010954 | positive regulation of protein processing | 25/18723 | 0.010634 | 0.066661 | 0.036272 | SRC | 1 |
| GO:0033622 | integrin activation | 25/18723 | 0.010634 | 0.066661 | 0.036272 | SRC | 1 |
| GO:0034389 | lipid droplet organization | 25/18723 | 0.010634 | 0.066661 | 0.036272 | SQLE | 1 |
| GO:0051291 | protein heterooligomerization | 25/18723 | 0.010634 | 0.066661 | 0.036272 | RRM2 | 1 |
| GO:1900078 | positive regulation of cellular response to insulin stimulus | 25/18723 | 0.010634 | 0.066661 | 0.036272 | SRC | 1 |
| GO:1904385 | cellular response to angiotensin | 25/18723 | 0.010634 | 0.066661 | 0.036272 | SRC | 1 |
| GO:0051016 | barbed-end actin filament capping | 26/18723 | 0.011058 | 0.066661 | 0.036272 | CAPG | 1 |
| GO:0070102 | interleukin-6-mediated signaling pathway | 26/18723 | 0.011058 | 0.066661 | 0.036272 | SRC | 1 |
| GO:0002433 | immune response-regulating cell surface receptor signaling pathway involved in phagocytosis | 27/18723 | 0.011481 | 0.066661 | 0.036272 | SRC | 1 |
| GO:0031954 | positive regulation of protein autophosphorylation | 27/18723 | 0.011481 | 0.066661 | 0.036272 | SRC | 1 |
| GO:0038096 | Fc-gamma receptor signaling pathway involved in phagocytosis | 27/18723 | 0.011481 | 0.066661 | 0.036272 | SRC | 1 |
| GO:1903319 | positive regulation of protein maturation | 27/18723 | 0.011481 | 0.066661 | 0.036272 | SRC | 1 |
| GO:1904357 | negative regulation of telomere maintenance via telomere lengthening | 27/18723 | 0.011481 | 0.066661 | 0.036272 | SRC | 1 |
| GO:0015740 | C4-dicarboxylate transport | 28/18723 | 0.011904 | 0.066661 | 0.036272 | SLC1A5 | 1 |
| GO:0038094 | Fc-gamma receptor signaling pathway | 28/18723 | 0.011904 | 0.066661 | 0.036272 | SRC | 1 |
| GO:1903649 | regulation of cytoplasmic transport | 28/18723 | 0.011904 | 0.066661 | 0.036272 | SRC | 1 |
| GO:1990776 | response to angiotensin | 28/18723 | 0.011904 | 0.066661 | 0.036272 | SRC | 1 |
| GO:0002862 | negative regulation of inflammatory response to antigenic stimulus | 29/18723 | 0.012327 | 0.067605 | 0.036786 | SRC | 1 |
| GO:0045737 | positive regulation of cyclin-dependent protein serine/threonine kinase activity | 29/18723 | 0.012327 | 0.067605 | 0.036786 | SRC | 1 |
| GO:0060603 | mammary gland duct morphogenesis | 30/18723 | 0.012749 | 0.06921 | 0.037659 | SRC | 1 |
| GO:0032148 | activation of protein kinase B activity | 31/18723 | 0.013172 | 0.070781 | 0.038514 | SRC | 1 |
| GO:0043254 | regulation of protein-containing complex assembly | 428/18723 | 0.013325 | 0.070888 | 0.038572 | CAPG/SRC | 2 |
| GO:1901889 | negative regulation of cell junction assembly | 32/18723 | 0.013594 | 0.071016 | 0.038641 | SRC | 1 |
| GO:0002431 | Fc receptor mediated stimulatory signaling pathway | 33/18723 | 0.014016 | 0.071016 | 0.038641 | SRC | 1 |
| GO:0040020 | regulation of meiotic nuclear division | 33/18723 | 0.014016 | 0.071016 | 0.038641 | RAD51AP1 | 1 |
| GO:0043552 | positive regulation of phosphatidylinositol 3-kinase activity | 33/18723 | 0.014016 | 0.071016 | 0.038641 | SRC | 1 |
| GO:1904031 | positive regulation of cyclin-dependent protein kinase activity | 33/18723 | 0.014016 | 0.071016 | 0.038641 | SRC | 1 |
| GO:0007015 | actin filament organization | 442/18723 | 0.014169 | 0.071113 | 0.038694 | CAPG/SRC | 2 |
| GO:0043276 | anoikis | 34/18723 | 0.014438 | 0.071122 | 0.038699 | SRC | 1 |
| GO:0098751 | bone cell development | 34/18723 | 0.014438 | 0.071122 | 0.038699 | SRC | 1 |
| GO:0032205 | negative regulation of telomere maintenance | 35/18723 | 0.01486 | 0.071222 | 0.038753 | SRC | 1 |
| GO:0033146 | regulation of intracellular estrogen receptor signaling pathway | 35/18723 | 0.01486 | 0.071222 | 0.038753 | SRC | 1 |
| GO:0034405 | response to fluid shear stress | 35/18723 | 0.01486 | 0.071222 | 0.038753 | SRC | 1 |
| GO:0038179 | neurotrophin signaling pathway | 36/18723 | 0.015282 | 0.071946 | 0.039148 | SRC | 1 |
| GO:0051385 | response to mineralocorticoid | 36/18723 | 0.015282 | 0.071946 | 0.039148 | SRC | 1 |
| GO:0032570 | response to progesterone | 37/18723 | 0.015703 | 0.072019 | 0.039187 | SRC | 1 |
| GO:0090218 | positive regulation of lipid kinase activity | 37/18723 | 0.015703 | 0.072019 | 0.039187 | SRC | 1 |
| GO:1902745 | positive regulation of lamellipodium organization | 37/18723 | 0.015703 | 0.072019 | 0.039187 | SRC | 1 |
| GO:0071398 | cellular response to fatty acid | 38/18723 | 0.016125 | 0.072057 | 0.039208 | SRC | 1 |
| GO:2000279 | negative regulation of DNA biosynthetic process | 38/18723 | 0.016125 | 0.072057 | 0.039208 | SRC | 1 |
| GO:0036297 | interstrand cross-link repair | 39/18723 | 0.016546 | 0.072057 | 0.039208 | RAD51AP1 | 1 |
| GO:0045022 | early endosome to late endosome transport | 40/18723 | 0.016967 | 0.072057 | 0.039208 | SRC | 1 |
| GO:2000781 | positive regulation of double-strand break repair | 40/18723 | 0.016967 | 0.072057 | 0.039208 | RAD51AP1 | 1 |
| GO:0001953 | negative regulation of cell-matrix adhesion | 41/18723 | 0.017388 | 0.072057 | 0.039208 | SRC | 1 |
| GO:0010907 | positive regulation of glucose metabolic process | 41/18723 | 0.017388 | 0.072057 | 0.039208 | SRC | 1 |
| GO:0051693 | actin filament capping | 41/18723 | 0.017388 | 0.072057 | 0.039208 | CAPG | 1 |
| GO:0051602 | response to electrical stimulus | 42/18723 | 0.017809 | 0.072057 | 0.039208 | SRC | 1 |
| GO:0001504 | neurotransmitter uptake | 43/18723 | 0.018229 | 0.072057 | 0.039208 | SLC38A1 | 1 |
| GO:0002861 | regulation of inflammatory response to antigenic stimulus | 43/18723 | 0.018229 | 0.072057 | 0.039208 | SRC | 1 |
| GO:0009268 | response to pH | 43/18723 | 0.018229 | 0.072057 | 0.039208 | SRC | 1 |
| GO:0031952 | regulation of protein autophosphorylation | 43/18723 | 0.018229 | 0.072057 | 0.039208 | SRC | 1 |
| GO:0060443 | mammary gland morphogenesis | 43/18723 | 0.018229 | 0.072057 | 0.039208 | SRC | 1 |
| GO:0098927 | vesicle-mediated transport between endosomal compartments | 43/18723 | 0.018229 | 0.072057 | 0.039208 | SRC | 1 |
| GO:0002066 | columnar/cuboidal epithelial cell development | 44/18723 | 0.01865 | 0.072057 | 0.039208 | SRC | 1 |
| GO:0009262 | deoxyribonucleotide metabolic process | 44/18723 | 0.01865 | 0.072057 | 0.039208 | RRM2 | 1 |
| GO:0031295 | T cell costimulation | 44/18723 | 0.01865 | 0.072057 | 0.039208 | SRC | 1 |
| GO:0045124 | regulation of bone resorption | 44/18723 | 0.01865 | 0.072057 | 0.039208 | SRC | 1 |
| GO:0089718 | amino acid import across plasma membrane | 44/18723 | 0.01865 | 0.072057 | 0.039208 | SLC1A5 | 1 |
| GO:0003254 | regulation of membrane depolarization | 45/18723 | 0.01907 | 0.072057 | 0.039208 | SRC | 1 |
| GO:0022602 | ovulation cycle process | 45/18723 | 0.01907 | 0.072057 | 0.039208 | SRC | 1 |
| GO:0030835 | negative regulation of actin filament depolymerization | 45/18723 | 0.01907 | 0.072057 | 0.039208 | CAPG | 1 |
| GO:0071354 | cellular response to interleukin-6 | 45/18723 | 0.01907 | 0.072057 | 0.039208 | SRC | 1 |
| GO:0031294 | lymphocyte costimulation | 46/18723 | 0.01949 | 0.072057 | 0.039208 | SRC | 1 |
| GO:0031648 | protein destabilization | 46/18723 | 0.01949 | 0.072057 | 0.039208 | SRC | 1 |
| GO:0014911 | positive regulation of smooth muscle cell migration | 47/18723 | 0.01991 | 0.072057 | 0.039208 | SRC | 1 |
| GO:0043114 | regulation of vascular permeability | 47/18723 | 0.01991 | 0.072057 | 0.039208 | SRC | 1 |
| GO:0045747 | positive regulation of Notch signaling pathway | 47/18723 | 0.01991 | 0.072057 | 0.039208 | SRC | 1 |
| GO:0045911 | positive regulation of DNA recombination | 47/18723 | 0.01991 | 0.072057 | 0.039208 | RAD51AP1 | 1 |
| GO:0051972 | regulation of telomerase activity | 47/18723 | 0.01991 | 0.072057 | 0.039208 | SRC | 1 |
| GO:0010569 | regulation of double-strand break repair via homologous recombination | 48/18723 | 0.02033 | 0.073079 | 0.039764 | RAD51AP1 | 1 |
| GO:0034332 | adherens junction organization | 49/18723 | 0.02075 | 0.073106 | 0.039778 | SRC | 1 |
| GO:0051445 | regulation of meiotic cell cycle | 49/18723 | 0.02075 | 0.073106 | 0.039778 | RAD51AP1 | 1 |
| GO:0070741 | response to interleukin-6 | 49/18723 | 0.02075 | 0.073106 | 0.039778 | SRC | 1 |
| GO:0038093 | Fc receptor signaling pathway | 50/18723 | 0.021169 | 0.074093 | 0.040316 | SRC | 1 |
| GO:0046850 | regulation of bone remodeling | 51/18723 | 0.021589 | 0.074579 | 0.04058 | SRC | 1 |
| GO:0048013 | ephrin receptor signaling pathway | 51/18723 | 0.021589 | 0.074579 | 0.04058 | SRC | 1 |
| GO:0002218 | activation of innate immune response | 52/18723 | 0.022008 | 0.075053 | 0.040838 | SRC | 1 |
| GO:0043090 | amino acid import | 52/18723 | 0.022008 | 0.075053 | 0.040838 | SLC1A5 | 1 |
| GO:0001541 | ovarian follicle development | 53/18723 | 0.022427 | 0.075491 | 0.041076 | SRC | 1 |
| GO:0032210 | regulation of telomere maintenance via telomerase | 53/18723 | 0.022427 | 0.075491 | 0.041076 | SRC | 1 |
| GO:0030520 | intracellular estrogen receptor signaling pathway | 54/18723 | 0.022846 | 0.075491 | 0.041076 | SRC | 1 |
| GO:0030834 | regulation of actin filament depolymerization | 54/18723 | 0.022846 | 0.075491 | 0.041076 | CAPG | 1 |
| GO:1902743 | regulation of lamellipodium organization | 54/18723 | 0.022846 | 0.075491 | 0.041076 | SRC | 1 |
| GO:0010676 | positive regulation of cellular carbohydrate metabolic process | 55/18723 | 0.023265 | 0.075931 | 0.041316 | SRC | 1 |
| GO:0061098 | positive regulation of protein tyrosine kinase activity | 55/18723 | 0.023265 | 0.075931 | 0.041316 | SRC | 1 |
| GO:0048008 | platelet-derived growth factor receptor signaling pathway | 56/18723 | 0.023683 | 0.076826 | 0.041803 | SRC | 1 |
| GO:0051785 | positive regulation of nuclear division | 57/18723 | 0.024102 | 0.077585 | 0.042216 | RAD51AP1 | 1 |
| GO:0007131 | reciprocal meiotic recombination | 58/18723 | 0.02452 | 0.077585 | 0.042216 | RAD51AP1 | 1 |
| GO:0140527 | reciprocal homologous recombination | 58/18723 | 0.02452 | 0.077585 | 0.042216 | RAD51AP1 | 1 |
| GO:0030042 | actin filament depolymerization | 59/18723 | 0.024938 | 0.077585 | 0.042216 | CAPG | 1 |
| GO:0035306 | positive regulation of dephosphorylation | 59/18723 | 0.024938 | 0.077585 | 0.042216 | SRC | 1 |
| GO:0043551 | regulation of phosphatidylinositol 3-kinase activity | 59/18723 | 0.024938 | 0.077585 | 0.042216 | SRC | 1 |
| GO:0048010 | vascular endothelial growth factor receptor signaling pathway | 59/18723 | 0.024938 | 0.077585 | 0.042216 | SRC | 1 |
| GO:0035825 | homologous recombination | 60/18723 | 0.025356 | 0.078426 | 0.042674 | RAD51AP1 | 1 |
| GO:1904356 | regulation of telomere maintenance via telomere lengthening | 61/18723 | 0.025774 | 0.079258 | 0.043126 | SRC | 1 |
| GO:0099601 | regulation of neurotransmitter receptor activity | 63/18723 | 0.026609 | 0.080446 | 0.043773 | SRC | 1 |
| GO:0016126 | sterol biosynthetic process | 64/18723 | 0.027026 | 0.080446 | 0.043773 | SQLE | 1 |
| GO:0070542 | response to fatty acid | 64/18723 | 0.027026 | 0.080446 | 0.043773 | SRC | 1 |
| GO:1900076 | regulation of cellular response to insulin stimulus | 64/18723 | 0.027026 | 0.080446 | 0.043773 | SRC | 1 |
| GO:0030837 | negative regulation of actin filament polymerization | 65/18723 | 0.027443 | 0.080446 | 0.043773 | CAPG | 1 |
| GO:0045453 | bone resorption | 65/18723 | 0.027443 | 0.080446 | 0.043773 | SRC | 1 |
| GO:0070613 | regulation of protein processing | 65/18723 | 0.027443 | 0.080446 | 0.043773 | SRC | 1 |
| GO:0046626 | regulation of insulin receptor signaling pathway | 66/18723 | 0.02786 | 0.080446 | 0.043773 | SRC | 1 |
| GO:0051893 | regulation of focal adhesion assembly | 66/18723 | 0.02786 | 0.080446 | 0.043773 | SRC | 1 |
| GO:0090109 | regulation of cell-substrate junction assembly | 66/18723 | 0.02786 | 0.080446 | 0.043773 | SRC | 1 |
| GO:2000573 | positive regulation of DNA biosynthetic process | 66/18723 | 0.02786 | 0.080446 | 0.043773 | SRC | 1 |
| GO:0061180 | mammary gland epithelium development | 67/18723 | 0.028277 | 0.080446 | 0.043773 | SRC | 1 |
| GO:1902117 | positive regulation of organelle assembly | 67/18723 | 0.028277 | 0.080446 | 0.043773 | SRC | 1 |
| GO:1903317 | regulation of protein maturation | 67/18723 | 0.028277 | 0.080446 | 0.043773 | SRC | 1 |
| GO:0010812 | negative regulation of cell-substrate adhesion | 68/18723 | 0.028694 | 0.080768 | 0.043948 | SRC | 1 |
| GO:0042698 | ovulation cycle | 68/18723 | 0.028694 | 0.080768 | 0.043948 | SRC | 1 |
| GO:0007004 | telomere maintenance via telomerase | 69/18723 | 0.02911 | 0.081509 | 0.044351 | SRC | 1 |
| GO:0043550 | regulation of lipid kinase activity | 71/18723 | 0.029943 | 0.082403 | 0.044837 | SRC | 1 |
| GO:0061515 | myeloid cell development | 71/18723 | 0.029943 | 0.082403 | 0.044837 | SRC | 1 |
| GO:0150116 | regulation of cell-substrate junction organization | 71/18723 | 0.029943 | 0.082403 | 0.044837 | SRC | 1 |
| GO:0051057 | positive regulation of small GTPase mediated signal transduction | 72/18723 | 0.030359 | 0.082403 | 0.044837 | SRC | 1 |
| GO:0071479 | cellular response to ionizing radiation | 72/18723 | 0.030359 | 0.082403 | 0.044837 | RAD51AP1 | 1 |
| GO:1901880 | negative regulation of protein depolymerization | 72/18723 | 0.030359 | 0.082403 | 0.044837 | CAPG | 1 |
| GO:0045739 | positive regulation of DNA repair | 73/18723 | 0.030775 | 0.082967 | 0.045144 | RAD51AP1 | 1 |
| GO:0002437 | inflammatory response to antigenic stimulus | 74/18723 | 0.031191 | 0.082967 | 0.045144 | SRC | 1 |
| GO:0033143 | regulation of intracellular steroid hormone receptor signaling pathway | 74/18723 | 0.031191 | 0.082967 | 0.045144 | SRC | 1 |
| GO:0051881 | regulation of mitochondrial membrane potential | 74/18723 | 0.031191 | 0.082967 | 0.045144 | SRC | 1 |
| GO:0006278 | RNA-dependent DNA biosynthetic process | 75/18723 | 0.031606 | 0.08324 | 0.045293 | SRC | 1 |
| GO:0045913 | positive regulation of carbohydrate metabolic process | 75/18723 | 0.031606 | 0.08324 | 0.045293 | SRC | 1 |
| GO:0043154 | negative regulation of cysteine-type endopeptidase activity involved in apoptotic process | 78/18723 | 0.032852 | 0.086095 | 0.046846 | SRC | 1 |
| GO:0014068 | positive regulation of phosphatidylinositol 3-kinase signaling | 79/18723 | 0.033267 | 0.086755 | 0.047206 | SRC | 1 |
| GO:0032204 | regulation of telomere maintenance | 80/18723 | 0.033682 | 0.086984 | 0.04733 | SRC | 1 |
| GO:0032272 | negative regulation of protein polymerization | 80/18723 | 0.033682 | 0.086984 | 0.04733 | CAPG | 1 |
| GO:0010833 | telomere maintenance via telomere lengthening | 81/18723 | 0.034097 | 0.08763 | 0.047681 | SRC | 1 |
| GO:0043242 | negative regulation of protein-containing complex disassembly | 82/18723 | 0.034511 | 0.088269 | 0.048029 | CAPG | 1 |
| GO:0051899 | membrane depolarization | 83/18723 | 0.034925 | 0.088864 | 0.048353 | SRC | 1 |
| GO:0006835 | dicarboxylic acid transport | 84/18723 | 0.03534 | 0.088864 | 0.048353 | SLC1A5 | 1 |
| GO:2000779 | regulation of double-strand break repair | 85/18723 | 0.035754 | 0.088864 | 0.048353 | RAD51AP1 | 1 |
| GO:0034103 | regulation of tissue remodeling | 86/18723 | 0.036168 | 0.088864 | 0.048353 | SRC | 1 |
| GO:2000117 | negative regulation of cysteine-type endopeptidase activity | 86/18723 | 0.036168 | 0.088864 | 0.048353 | SRC | 1 |
| GO:2001251 | negative regulation of chromosome organization | 86/18723 | 0.036168 | 0.088864 | 0.048353 | SRC | 1 |
| GO:0019915 | lipid storage | 87/18723 | 0.036581 | 0.088864 | 0.048353 | SQLE | 1 |
| GO:0048041 | focal adhesion assembly | 87/18723 | 0.036581 | 0.088864 | 0.048353 | SRC | 1 |
| GO:0051262 | protein tetramerization | 87/18723 | 0.036581 | 0.088864 | 0.048353 | RRM2 | 1 |
| GO:1900182 | positive regulation of protein localization to nucleus | 87/18723 | 0.036581 | 0.088864 | 0.048353 | SRC | 1 |
| GO:1901879 | regulation of protein depolymerization | 87/18723 | 0.036581 | 0.088864 | 0.048353 | CAPG | 1 |
| GO:0014910 | regulation of smooth muscle cell migration | 89/18723 | 0.037408 | 0.089826 | 0.048877 | SRC | 1 |
| GO:0046849 | bone remodeling | 90/18723 | 0.037822 | 0.089826 | 0.048877 | SRC | 1 |
| GO:0048477 | oogenesis | 90/18723 | 0.037822 | 0.089826 | 0.048877 | SRC | 1 |
| GO:0061097 | regulation of protein tyrosine kinase activity | 90/18723 | 0.037822 | 0.089826 | 0.048877 | SRC | 1 |
| GO:0097581 | lamellipodium organization | 90/18723 | 0.037822 | 0.089826 | 0.048877 | SRC | 1 |
| GO:0002065 | columnar/cuboidal epithelial cell differentiation | 91/18723 | 0.038235 | 0.090404 | 0.049191 | SRC | 1 |
| GO:0000079 | regulation of cyclin-dependent protein serine/threonine kinase activity | 94/18723 | 0.039473 | 0.092257 | 0.050199 | SRC | 1 |
| GO:0030316 | osteoclast differentiation | 94/18723 | 0.039473 | 0.092257 | 0.050199 | SRC | 1 |
| GO:0007044 | cell-substrate junction assembly | 95/18723 | 0.039886 | 0.092257 | 0.050199 | SRC | 1 |
| GO:0008585 | female gonad development | 95/18723 | 0.039886 | 0.092257 | 0.050199 | SRC | 1 |
| GO:0008593 | regulation of Notch signaling pathway | 95/18723 | 0.039886 | 0.092257 | 0.050199 | SRC | 1 |
| GO:1901655 | cellular response to ketone | 96/18723 | 0.040298 | 0.092699 | 0.05044 | SRC | 1 |
| GO:0014909 | smooth muscle cell migration | 97/18723 | 0.04071 | 0.092699 | 0.05044 | SRC | 1 |
| GO:2001237 | negative regulation of extrinsic apoptotic signaling pathway | 97/18723 | 0.04071 | 0.092699 | 0.05044 | SRC | 1 |
| GO:0070301 | cellular response to hydrogen peroxide | 98/18723 | 0.041122 | 0.092699 | 0.05044 | SRC | 1 |
| GO:1904029 | regulation of cyclin-dependent protein kinase activity | 98/18723 | 0.041122 | 0.092699 | 0.05044 | SRC | 1 |
| GO:2001243 | negative regulation of intrinsic apoptotic signaling pathway | 98/18723 | 0.041122 | 0.092699 | 0.05044 | SRC | 1 |
| GO:0046545 | development of primary female sexual characteristics | 100/18723 | 0.041946 | 0.094156 | 0.051233 | SRC | 1 |
| GO:0150115 | cell-substrate junction organization | 101/18723 | 0.042357 | 0.094681 | 0.051518 | SRC | 1 |
| GO:2001022 | positive regulation of response to DNA damage stimulus | 105/18723 | 0.044002 | 0.096834 | 0.05269 | RAD51AP1 | 1 |
| GO:0001824 | blastocyst development | 106/18723 | 0.044413 | 0.096834 | 0.05269 | RRM2 | 1 |
| GO:0030038 | contractile actin filament bundle assembly | 106/18723 | 0.044413 | 0.096834 | 0.05269 | SRC | 1 |
| GO:0043149 | stress fiber assembly | 106/18723 | 0.044413 | 0.096834 | 0.05269 | SRC | 1 |
| GO:0090263 | positive regulation of canonical Wnt signaling pathway | 106/18723 | 0.044413 | 0.096834 | 0.05269 | SRC | 1 |
| GO:2000278 | regulation of DNA biosynthetic process | 106/18723 | 0.044413 | 0.096834 | 0.05269 | SRC | 1 |
| GO:0000018 | regulation of DNA recombination | 107/18723 | 0.044823 | 0.096935 | 0.052745 | RAD51AP1 | 1 |
| GO:0007229 | integrin-mediated signaling pathway | 107/18723 | 0.044823 | 0.096935 | 0.052745 | SRC | 1 |
| GO:0007173 | epidermal growth factor receptor signaling pathway | 108/18723 | 0.045234 | 0.097034 | 0.052798 | SRC | 1 |
| GO:0034446 | substrate adhesion-dependent cell spreading | 108/18723 | 0.045234 | 0.097034 | 0.052798 | SRC | 1 |
| GO:0014812 | muscle cell migration | 110/18723 | 0.046054 | 0.098397 | 0.05354 | SRC | 1 |
| GO:0014066 | regulation of phosphatidylinositol 3-kinase signaling | 111/18723 | 0.046464 | 0.098876 | 0.053801 | SRC | 1 |
| GO:0043406 | positive regulation of MAP kinase activity | 112/18723 | 0.046874 | 0.099351 | 0.054059 | SRC | 1 |
| GO:0046660 | female sex differentiation | 114/18723 | 0.047693 | 0.100288 | 0.054569 | SRC | 1 |
| GO:0051261 | protein depolymerization | 114/18723 | 0.047693 | 0.100288 | 0.054569 | CAPG | 1 |
| GO:0008286 | insulin receptor signaling pathway | 116/18723 | 0.048512 | 0.101209 | 0.05507 | SRC | 1 |
| GO:0030518 | intracellular steroid hormone receptor signaling pathway | 116/18723 | 0.048512 | 0.101209 | 0.05507 | SRC | 1 |
| GO:0022612 | gland morphogenesis | 118/18723 | 0.04933 | 0.102514 | 0.05578 | SRC | 1 |
| GO:0010906 | regulation of glucose metabolic process | 119/18723 | 0.049739 | 0.102961 | 0.056024 | SRC | 1 |
| GO:0051897 | positive regulation of protein kinase B signaling | 120/18723 | 0.050147 | 0.103405 | 0.056265 | SRC | 1 |
| GO:0038127 | ERBB signaling pathway | 121/18723 | 0.050556 | 0.103445 | 0.056287 | SRC | 1 |
| GO:0043244 | regulation of protein-containing complex disassembly | 121/18723 | 0.050556 | 0.103445 | 0.056287 | CAPG | 1 |
| GO:0030168 | platelet activation | 123/18723 | 0.051372 | 0.104713 | 0.056977 | SRC | 1 |
| GO:0051053 | negative regulation of DNA metabolic process | 125/18723 | 0.052188 | 0.10597 | 0.057661 | SRC | 1 |
| GO:0048565 | digestive tract development | 127/18723 | 0.053004 | 0.107216 | 0.058339 | SRC | 1 |
| GO:0001952 | regulation of cell-matrix adhesion | 128/18723 | 0.053411 | 0.107225 | 0.058344 | SRC | 1 |
| GO:0035303 | regulation of dephosphorylation | 128/18723 | 0.053411 | 0.107225 | 0.058344 | SRC | 1 |
| GO:0007127 | meiosis I | 129/18723 | 0.053818 | 0.107636 | 0.058567 | RAD51AP1 | 1 |
| GO:0006282 | regulation of DNA repair | 130/18723 | 0.054225 | 0.107641 | 0.05857 | RAD51AP1 | 1 |
| GO:0042476 | odontogenesis | 130/18723 | 0.054225 | 0.107641 | 0.05857 | SRC | 1 |
| GO:0000723 | telomere maintenance | 131/18723 | 0.054632 | 0.107646 | 0.058573 | SRC | 1 |
| GO:0045089 | positive regulation of innate immune response | 131/18723 | 0.054632 | 0.107646 | 0.058573 | SRC | 1 |
| GO:0032147 | activation of protein kinase activity | 134/18723 | 0.055852 | 0.109214 | 0.059426 | SRC | 1 |
| GO:0061982 | meiosis I cell cycle process | 135/18723 | 0.056258 | 0.109214 | 0.059426 | RAD51AP1 | 1 |
| GO:0043401 | steroid hormone mediated signaling pathway | 136/18723 | 0.056665 | 0.109214 | 0.059426 | SRC | 1 |
| GO:0043624 | cellular protein complex disassembly | 136/18723 | 0.056665 | 0.109214 | 0.059426 | CAPG | 1 |
| GO:1900180 | regulation of protein localization to nucleus | 136/18723 | 0.056665 | 0.109214 | 0.059426 | SRC | 1 |
| GO:0008203 | cholesterol metabolic process | 137/18723 | 0.057071 | 0.109214 | 0.059426 | SQLE | 1 |
| GO:0030879 | mammary gland development | 137/18723 | 0.057071 | 0.109214 | 0.059426 | SRC | 1 |
| GO:0055123 | digestive system development | 137/18723 | 0.057071 | 0.109214 | 0.059426 | SRC | 1 |
| GO:0000724 | double-strand break repair via homologous recombination | 138/18723 | 0.057476 | 0.109573 | 0.059621 | RAD51AP1 | 1 |
| GO:0051783 | regulation of nuclear division | 139/18723 | 0.057882 | 0.109573 | 0.059621 | RAD51AP1 | 1 |
| GO:0000725 | recombinational repair | 140/18723 | 0.058288 | 0.109573 | 0.059621 | RAD51AP1 | 1 |
| GO:0007292 | female gamete generation | 140/18723 | 0.058288 | 0.109573 | 0.059621 | SRC | 1 |
| GO:0030177 | positive regulation of Wnt signaling pathway | 140/18723 | 0.058288 | 0.109573 | 0.059621 | SRC | 1 |
| GO:0062013 | positive regulation of small molecule metabolic process | 143/18723 | 0.059504 | 0.110664 | 0.060215 | SRC | 1 |
| GO:0070555 | response to interleukin-1 | 143/18723 | 0.059504 | 0.110664 | 0.060215 | SRC | 1 |
| GO:0014065 | phosphatidylinositol 3-kinase signaling | 144/18723 | 0.059908 | 0.110664 | 0.060215 | SRC | 1 |
| GO:0031644 | regulation of nervous system process | 144/18723 | 0.059908 | 0.110664 | 0.060215 | SRC | 1 |
| GO:0046718 | viral entry into host cell | 144/18723 | 0.059908 | 0.110664 | 0.060215 | SLC1A5 | 1 |
| GO:0010675 | regulation of cellular carbohydrate metabolic process | 146/18723 | 0.060718 | 0.111386 | 0.060608 | SRC | 1 |
| GO:0042542 | response to hydrogen peroxide | 146/18723 | 0.060718 | 0.111386 | 0.060608 | SRC | 1 |
| GO:1902652 | secondary alcohol metabolic process | 147/18723 | 0.061122 | 0.111714 | 0.060786 | SQLE | 1 |
| GO:0002181 | cytoplasmic translation | 148/18723 | 0.061527 | 0.111714 | 0.060786 | RPL8 | 1 |
| GO:0010212 | response to ionizing radiation | 148/18723 | 0.061527 | 0.111714 | 0.060786 | RAD51AP1 | 1 |
| GO:0045834 | positive regulation of lipid metabolic process | 149/18723 | 0.061931 | 0.112065 | 0.060977 | SRC | 1 |
| GO:0048754 | branching morphogenesis of an epithelial tube | 151/18723 | 0.062739 | 0.112381 | 0.061149 | SRC | 1 |
| GO:0071456 | cellular response to hypoxia | 151/18723 | 0.062739 | 0.112381 | 0.061149 | SRC | 1 |
| GO:2001236 | regulation of extrinsic apoptotic signaling pathway | 151/18723 | 0.062739 | 0.112381 | 0.061149 | SRC | 1 |
| GO:0016125 | sterol metabolic process | 152/18723 | 0.063143 | 0.112724 | 0.061336 | SQLE | 1 |
| GO:0034614 | cellular response to reactive oxygen species | 155/18723 | 0.064353 | 0.114501 | 0.062303 | SRC | 1 |
| GO:0051017 | actin filament bundle assembly | 157/18723 | 0.065159 | 0.115549 | 0.062873 | SRC | 1 |
| GO:0032200 | telomere organization | 159/18723 | 0.065964 | 0.116588 | 0.063438 | SRC | 1 |
| GO:0036294 | cellular response to decreased oxygen levels | 161/18723 | 0.066769 | 0.117232 | 0.063789 | SRC | 1 |
| GO:0061572 | actin filament bundle organization | 161/18723 | 0.066769 | 0.117232 | 0.063789 | SRC | 1 |
| GO:0051494 | negative regulation of cytoskeleton organization | 163/18723 | 0.067574 | 0.118254 | 0.064345 | CAPG | 1 |
| GO:2001242 | regulation of intrinsic apoptotic signaling pathway | 164/18723 | 0.067975 | 0.118567 | 0.064515 | SRC | 1 |
| GO:0031960 | response to corticosteroid | 167/18723 | 0.06918 | 0.119797 | 0.065185 | SRC | 1 |
| GO:1902904 | negative regulation of supramolecular fiber organization | 167/18723 | 0.06918 | 0.119797 | 0.065185 | CAPG | 1 |
| GO:0002833 | positive regulation of response to biotic stimulus | 168/18723 | 0.069582 | 0.119797 | 0.065185 | SRC | 1 |
| GO:0016482 | cytosolic transport | 168/18723 | 0.069582 | 0.119797 | 0.065185 | SRC | 1 |
| GO:0007219 | Notch signaling pathway | 172/18723 | 0.071185 | 0.121673 | 0.066205 | SRC | 1 |
| GO:0030833 | regulation of actin filament polymerization | 172/18723 | 0.071185 | 0.121673 | 0.066205 | CAPG | 1 |
| GO:0006694 | steroid biosynthetic process | 173/18723 | 0.071586 | 0.121673 | 0.066205 | SQLE | 1 |
| GO:0010469 | regulation of signaling receptor activity | 173/18723 | 0.071586 | 0.121673 | 0.066205 | SRC | 1 |
| GO:0048771 | tissue remodeling | 175/18723 | 0.072386 | 0.121956 | 0.066359 | SRC | 1 |
| GO:0010634 | positive regulation of epithelial cell migration | 176/18723 | 0.072787 | 0.121956 | 0.066359 | SRC | 1 |
| GO:0050728 | negative regulation of inflammatory response | 176/18723 | 0.072787 | 0.121956 | 0.066359 | SRC | 1 |
| GO:0043405 | regulation of MAP kinase activity | 177/18723 | 0.073187 | 0.121956 | 0.066359 | SRC | 1 |
| GO:0071453 | cellular response to oxygen levels | 177/18723 | 0.073187 | 0.121956 | 0.066359 | SRC | 1 |
| GO:0098739 | import across plasma membrane | 177/18723 | 0.073187 | 0.121956 | 0.066359 | SLC1A5 | 1 |
| GO:0006109 | regulation of carbohydrate metabolic process | 178/18723 | 0.073586 | 0.121956 | 0.066359 | SRC | 1 |
| GO:0048015 | phosphatidylinositol-mediated signaling | 178/18723 | 0.073586 | 0.121956 | 0.066359 | SRC | 1 |
| GO:0071897 | DNA biosynthetic process | 180/18723 | 0.074385 | 0.122898 | 0.066872 | SRC | 1 |
| GO:0048017 | inositol lipid-mediated signaling | 182/18723 | 0.075184 | 0.12307 | 0.066965 | SRC | 1 |
| GO:0051260 | protein homooligomerization | 182/18723 | 0.075184 | 0.12307 | 0.066965 | SLC1A5 | 1 |
| GO:0061138 | morphogenesis of a branching epithelium | 182/18723 | 0.075184 | 0.12307 | 0.066965 | SRC | 1 |
| GO:0051896 | regulation of protein kinase B signaling | 185/18723 | 0.076381 | 0.124154 | 0.067555 | SRC | 1 |
| GO:0140013 | meiotic nuclear division | 185/18723 | 0.076381 | 0.124154 | 0.067555 | RAD51AP1 | 1 |
| GO:0071478 | cellular response to radiation | 186/18723 | 0.076779 | 0.124154 | 0.067555 | RAD51AP1 | 1 |
| GO:1902115 | regulation of organelle assembly | 186/18723 | 0.076779 | 0.124154 | 0.067555 | SRC | 1 |
| GO:0033044 | regulation of chromosome organization | 187/18723 | 0.077178 | 0.124308 | 0.067639 | SRC | 1 |
| GO:0008064 | regulation of actin polymerization or depolymerization | 188/18723 | 0.077576 | 0.124308 | 0.067639 | CAPG | 1 |
| GO:0060491 | regulation of cell projection assembly | 188/18723 | 0.077576 | 0.124308 | 0.067639 | SRC | 1 |
| GO:0030832 | regulation of actin filament length | 189/18723 | 0.077974 | 0.124571 | 0.067782 | CAPG | 1 |
| GO:0009755 | hormone-mediated signaling pathway | 190/18723 | 0.078372 | 0.124832 | 0.067924 | SRC | 1 |
| GO:0030041 | actin filament polymerization | 191/18723 | 0.07877 | 0.125091 | 0.068065 | CAPG | 1 |
| GO:0007565 | female pregnancy | 193/18723 | 0.079565 | 0.125255 | 0.068154 | SLC38A1 | 1 |
| GO:0050731 | positive regulation of peptidyl-tyrosine phosphorylation | 193/18723 | 0.079565 | 0.125255 | 0.068154 | SRC | 1 |
| GO:0050777 | negative regulation of immune response | 194/18723 | 0.079962 | 0.125255 | 0.068154 | SRC | 1 |
| GO:1901654 | response to ketone | 194/18723 | 0.079962 | 0.125255 | 0.068154 | SRC | 1 |
| GO:0001763 | morphogenesis of a branching structure | 196/18723 | 0.080757 | 0.125255 | 0.068154 | SRC | 1 |
| GO:0006006 | glucose metabolic process | 196/18723 | 0.080757 | 0.125255 | 0.068154 | SRC | 1 |
| GO:0031032 | actomyosin structure organization | 196/18723 | 0.080757 | 0.125255 | 0.068154 | SRC | 1 |
| GO:0043393 | regulation of protein binding | 196/18723 | 0.080757 | 0.125255 | 0.068154 | SRC | 1 |
| GO:0007179 | transforming growth factor beta receptor signaling pathway | 198/18723 | 0.08155 | 0.126119 | 0.068624 | SRC | 1 |
| GO:0045216 | cell-cell junction organization | 200/18723 | 0.082344 | 0.126609 | 0.068891 | SRC | 1 |
| GO:0071902 | positive regulation of protein serine/threonine kinase activity | 200/18723 | 0.082344 | 0.126609 | 0.068891 | SRC | 1 |
| GO:1903046 | meiotic cell cycle process | 202/18723 | 0.083136 | 0.127459 | 0.069354 | RAD51AP1 | 1 |
| GO:0032869 | cellular response to insulin stimulus | 203/18723 | 0.083532 | 0.127571 | 0.069414 | SRC | 1 |
| GO:0071383 | cellular response to steroid hormone stimulus | 204/18723 | 0.083928 | 0.127571 | 0.069414 | SRC | 1 |
| GO:1901888 | regulation of cell junction assembly | 204/18723 | 0.083928 | 0.127571 | 0.069414 | SRC | 1 |
| GO:0060348 | bone development | 205/18723 | 0.084324 | 0.127807 | 0.069543 | SRC | 1 |
| GO:0002573 | myeloid leukocyte differentiation | 208/18723 | 0.08551 | 0.128737 | 0.070049 | SRC | 1 |
| GO:0006836 | neurotransmitter transport | 208/18723 | 0.08551 | 0.128737 | 0.070049 | SLC38A1 | 1 |
| GO:0043281 | regulation of cysteine-type endopeptidase activity involved in apoptotic process | 209/18723 | 0.085905 | 0.128737 | 0.070049 | SRC | 1 |
| GO:0071222 | cellular response to lipopolysaccharide | 209/18723 | 0.085905 | 0.128737 | 0.070049 | SRC | 1 |
| GO:0030100 | regulation of endocytosis | 211/18723 | 0.086695 | 0.129193 | 0.070297 | SRC | 1 |
| GO:0043491 | protein kinase B signaling | 211/18723 | 0.086695 | 0.129193 | 0.070297 | SRC | 1 |
| GO:0001505 | regulation of neurotransmitter levels | 213/18723 | 0.087484 | 0.129484 | 0.070455 | SLC38A1 | 1 |
| GO:0009612 | response to mechanical stimulus | 216/18723 | 0.088667 | 0.129484 | 0.070455 | SRC | 1 |
| GO:0050870 | positive regulation of T cell activation | 216/18723 | 0.088667 | 0.129484 | 0.070455 | SRC | 1 |
| GO:0007596 | blood coagulation | 217/18723 | 0.089061 | 0.129484 | 0.070455 | SRC | 1 |
| GO:0070374 | positive regulation of ERK1 and ERK2 cascade | 217/18723 | 0.089061 | 0.129484 | 0.070455 | SRC | 1 |
| GO:0008154 | actin polymerization or depolymerization | 218/18723 | 0.089455 | 0.129484 | 0.070455 | CAPG | 1 |
| GO:0045088 | regulation of innate immune response | 218/18723 | 0.089455 | 0.129484 | 0.070455 | SRC | 1 |
| GO:0097191 | extrinsic apoptotic signaling pathway | 219/18723 | 0.089848 | 0.129484 | 0.070455 | SRC | 1 |
| GO:2001020 | regulation of response to DNA damage stimulus | 219/18723 | 0.089848 | 0.129484 | 0.070455 | RAD51AP1 | 1 |
| GO:0002064 | epithelial cell development | 220/18723 | 0.090242 | 0.129484 | 0.070455 | SRC | 1 |
| GO:0044706 | multi-multicellular organism process | 220/18723 | 0.090242 | 0.129484 | 0.070455 | SLC38A1 | 1 |
| GO:0008406 | gonad development | 221/18723 | 0.090635 | 0.129484 | 0.070455 | SRC | 1 |
| GO:0010810 | regulation of cell-substrate adhesion | 221/18723 | 0.090635 | 0.129484 | 0.070455 | SRC | 1 |
| GO:0071219 | cellular response to molecule of bacterial origin | 221/18723 | 0.090635 | 0.129484 | 0.070455 | SRC | 1 |
| GO:0000302 | response to reactive oxygen species | 222/18723 | 0.091028 | 0.129484 | 0.070455 | SRC | 1 |
| GO:0007599 | hemostasis | 222/18723 | 0.091028 | 0.129484 | 0.070455 | SRC | 1 |
| GO:0050817 | coagulation | 222/18723 | 0.091028 | 0.129484 | 0.070455 | SRC | 1 |
| GO:0032984 | protein-containing complex disassembly | 224/18723 | 0.091814 | 0.129907 | 0.070686 | CAPG | 1 |
| GO:2001234 | negative regulation of apoptotic signaling pathway | 224/18723 | 0.091814 | 0.129907 | 0.070686 | SRC | 1 |
| GO:0016485 | protein processing | 225/18723 | 0.092207 | 0.130117 | 0.0708 | SRC | 1 |
| GO:0045137 | development of primary sexual characteristics | 227/18723 | 0.092992 | 0.130532 | 0.071026 | SRC | 1 |
| GO:0046777 | protein autophosphorylation | 227/18723 | 0.092992 | 0.130532 | 0.071026 | SRC | 1 |
| GO:0007160 | cell-matrix adhesion | 233/18723 | 0.095344 | 0.133131 | 0.07244 | SRC | 1 |
| GO:0032271 | regulation of protein polymerization | 233/18723 | 0.095344 | 0.133131 | 0.07244 | CAPG | 1 |
| GO:2000116 | regulation of cysteine-type endopeptidase activity | 235/18723 | 0.096126 | 0.133563 | 0.072675 | SRC | 1 |
| GO:0090068 | positive regulation of cell cycle process | 236/18723 | 0.096518 | 0.133563 | 0.072675 | RAD51AP1 | 1 |
| GO:0019318 | hexose metabolic process | 237/18723 | 0.096908 | 0.133563 | 0.072675 | SRC | 1 |
| GO:0031334 | positive regulation of protein-containing complex assembly | 237/18723 | 0.096908 | 0.133563 | 0.072675 | SRC | 1 |
| GO:1901617 | organic hydroxy compound biosynthetic process | 237/18723 | 0.096908 | 0.133563 | 0.072675 | SQLE | 1 |
| GO:1903039 | positive regulation of leukocyte cell-cell adhesion | 239/18723 | 0.09769 | 0.134292 | 0.073072 | SRC | 1 |
| GO:0006814 | sodium ion transport | 245/18723 | 0.100031 | 0.136803 | 0.074438 | SLC38A1 | 1 |
| GO:0098656 | anion transmembrane transport | 245/18723 | 0.100031 | 0.136803 | 0.074438 | SLC1A5 | 1 |
| GO:0071216 | cellular response to biotic stimulus | 246/18723 | 0.100421 | 0.136984 | 0.074536 | SRC | 1 |
| GO:0071560 | cellular response to transforming growth factor beta stimulus | 250/18723 | 0.101978 | 0.138573 | 0.075401 | SRC | 1 |
| GO:0003015 | heart process | 251/18723 | 0.102367 | 0.138573 | 0.075401 | SRC | 1 |
| GO:0006302 | double-strand break repair | 251/18723 | 0.102367 | 0.138573 | 0.075401 | RAD51AP1 | 1 |
| GO:0010951 | negative regulation of endopeptidase activity | 252/18723 | 0.102755 | 0.138746 | 0.075495 | SRC | 1 |
| GO:0060828 | regulation of canonical Wnt signaling pathway | 253/18723 | 0.103144 | 0.138918 | 0.075588 | SRC | 1 |
| GO:0009165 | nucleotide biosynthetic process | 254/18723 | 0.103532 | 0.139089 | 0.075682 | RRM2 | 1 |
| GO:0071559 | response to transforming growth factor beta | 256/18723 | 0.104309 | 0.139428 | 0.075866 | SRC | 1 |
| GO:1901293 | nucleoside phosphate biosynthetic process | 256/18723 | 0.104309 | 0.139428 | 0.075866 | RRM2 | 1 |
| GO:0005996 | monosaccharide metabolic process | 257/18723 | 0.104697 | 0.139596 | 0.075957 | SRC | 1 |
| GO:0031348 | negative regulation of defense response | 258/18723 | 0.105085 | 0.139763 | 0.076048 | SRC | 1 |
| GO:0006260 | DNA replication | 260/18723 | 0.10586 | 0.140443 | 0.076418 | RRM2 | 1 |
| GO:0051321 | meiotic cell cycle | 261/18723 | 0.106247 | 0.140606 | 0.076507 | RAD51AP1 | 1 |
| GO:0010466 | negative regulation of peptidase activity | 262/18723 | 0.106635 | 0.140768 | 0.076595 | SRC | 1 |
| GO:0032868 | response to insulin | 264/18723 | 0.107409 | 0.14109 | 0.07677 | SRC | 1 |
| GO:0050730 | regulation of peptidyl-tyrosine phosphorylation | 264/18723 | 0.107409 | 0.14109 | 0.07677 | SRC | 1 |
| GO:0030522 | intracellular receptor signaling pathway | 265/18723 | 0.107796 | 0.14125 | 0.076857 | SRC | 1 |
| GO:0001894 | tissue homeostasis | 268/18723 | 0.108955 | 0.142069 | 0.077303 | SRC | 1 |
| GO:0051348 | negative regulation of transferase activity | 268/18723 | 0.108955 | 0.142069 | 0.077303 | SRC | 1 |
| GO:0007548 | sex differentiation | 276/18723 | 0.112041 | 0.145316 | 0.07907 | SRC | 1 |
| GO:1903829 | positive regulation of cellular protein localization | 276/18723 | 0.112041 | 0.145316 | 0.07907 | SRC | 1 |
| GO:0007281 | germ cell development | 278/18723 | 0.112811 | 0.145316 | 0.07907 | SRC | 1 |
| GO:0031349 | positive regulation of defense response | 278/18723 | 0.112811 | 0.145316 | 0.07907 | SRC | 1 |
| GO:0110053 | regulation of actin filament organization | 278/18723 | 0.112811 | 0.145316 | 0.07907 | CAPG | 1 |
| GO:0044262 | cellular carbohydrate metabolic process | 283/18723 | 0.114734 | 0.147436 | 0.080223 | SRC | 1 |
| GO:0022409 | positive regulation of cell-cell adhesion | 284/18723 | 0.115118 | 0.147573 | 0.080298 | SRC | 1 |
| GO:0034599 | cellular response to oxidative stress | 288/18723 | 0.116653 | 0.148509 | 0.080807 | SRC | 1 |
| GO:0097193 | intrinsic apoptotic signaling pathway | 288/18723 | 0.116653 | 0.148509 | 0.080807 | SRC | 1 |
| GO:0034504 | protein localization to nucleus | 290/18723 | 0.117419 | 0.148509 | 0.080807 | SRC | 1 |
| GO:0071375 | cellular response to peptide hormone stimulus | 290/18723 | 0.117419 | 0.148509 | 0.080807 | SRC | 1 |
| GO:0002429 | immune response-activating cell surface receptor signaling pathway | 291/18723 | 0.117802 | 0.148509 | 0.080807 | SRC | 1 |
| GO:0002757 | immune response-activating signal transduction | 291/18723 | 0.117802 | 0.148509 | 0.080807 | SRC | 1 |
| GO:0016236 | macroautophagy | 291/18723 | 0.117802 | 0.148509 | 0.080807 | SRC | 1 |
| GO:0010632 | regulation of epithelial cell migration | 292/18723 | 0.118185 | 0.148639 | 0.080878 | SRC | 1 |
| GO:0051604 | protein maturation | 294/18723 | 0.118951 | 0.149249 | 0.08121 | SRC | 1 |
| GO:0051258 | protein polymerization | 297/18723 | 0.120097 | 0.150106 | 0.081676 | CAPG | 1 |
| GO:0031647 | regulation of protein stability | 298/18723 | 0.12048 | 0.150106 | 0.081676 | SRC | 1 |
| GO:0048511 | rhythmic process | 298/18723 | 0.12048 | 0.150106 | 0.081676 | SRC | 1 |
| GO:0051056 | regulation of small GTPase mediated signal transduction | 302/18723 | 0.122006 | 0.151068 | 0.082199 | SRC | 1 |
| GO:0007162 | negative regulation of cell adhesion | 303/18723 | 0.122387 | 0.151068 | 0.082199 | SRC | 1 |
| GO:0051222 | positive regulation of protein transport | 303/18723 | 0.122387 | 0.151068 | 0.082199 | SRC | 1 |
| GO:0060070 | canonical Wnt signaling pathway | 303/18723 | 0.122387 | 0.151068 | 0.082199 | SRC | 1 |
| GO:0006310 | DNA recombination | 305/18723 | 0.12315 | 0.151656 | 0.08252 | RAD51AP1 | 1 |
| GO:0001666 | response to hypoxia | 307/18723 | 0.123911 | 0.152123 | 0.082774 | SRC | 1 |
| GO:0006909 | phagocytosis | 308/18723 | 0.124292 | 0.152123 | 0.082774 | SRC | 1 |
| GO:0022604 | regulation of cell morphogenesis | 309/18723 | 0.124672 | 0.152123 | 0.082774 | SRC | 1 |
| GO:0070372 | regulation of ERK1 and ERK2 cascade | 309/18723 | 0.124672 | 0.152123 | 0.082774 | SRC | 1 |
| GO:0060249 | anatomical structure homeostasis | 314/18723 | 0.126572 | 0.153846 | 0.083711 | SRC | 1 |
| GO:0002768 | immune response-regulating cell surface receptor signaling pathway | 315/18723 | 0.126952 | 0.153846 | 0.083711 | SRC | 1 |
| GO:0018105 | peptidyl-serine phosphorylation | 315/18723 | 0.126952 | 0.153846 | 0.083711 | SRC | 1 |
| GO:0019058 | viral life cycle | 317/18723 | 0.12771 | 0.154414 | 0.08402 | SLC1A5 | 1 |
| GO:0008202 | steroid metabolic process | 319/18723 | 0.128469 | 0.154627 | 0.084136 | SQLE | 1 |
| GO:1904951 | positive regulation of establishment of protein localization | 319/18723 | 0.128469 | 0.154627 | 0.084136 | SRC | 1 |
| GO:0036293 | response to decreased oxygen levels | 322/18723 | 0.129605 | 0.155643 | 0.084689 | SRC | 1 |
| GO:0060562 | epithelial tube morphogenesis | 325/18723 | 0.13074 | 0.156546 | 0.08518 | SRC | 1 |
| GO:0002831 | regulation of response to biotic stimulus | 327/18723 | 0.131495 | 0.156546 | 0.08518 | SRC | 1 |
| GO:0051235 | maintenance of location | 327/18723 | 0.131495 | 0.156546 | 0.08518 | SQLE | 1 |
| GO:0030111 | regulation of Wnt signaling pathway | 328/18723 | 0.131873 | 0.156546 | 0.08518 | SRC | 1 |
| GO:0050863 | regulation of T cell activation | 329/18723 | 0.132251 | 0.156546 | 0.08518 | SRC | 1 |
| GO:0070371 | ERK1 and ERK2 cascade | 330/18723 | 0.132628 | 0.156546 | 0.08518 | SRC | 1 |
| GO:0019216 | regulation of lipid metabolic process | 331/18723 | 0.133005 | 0.156546 | 0.08518 | SRC | 1 |
| GO:0071214 | cellular response to abiotic stimulus | 331/18723 | 0.133005 | 0.156546 | 0.08518 | RAD51AP1 | 1 |
| GO:0104004 | cellular response to environmental stimulus | 331/18723 | 0.133005 | 0.156546 | 0.08518 | RAD51AP1 | 1 |
| GO:0062012 | regulation of small molecule metabolic process | 334/18723 | 0.134136 | 0.157529 | 0.085715 | SRC | 1 |
| GO:1903037 | regulation of leukocyte cell-cell adhesion | 336/18723 | 0.134889 | 0.15781 | 0.085868 | SRC | 1 |
| GO:0032386 | regulation of intracellular transport | 337/18723 | 0.135266 | 0.15781 | 0.085868 | SRC | 1 |
| GO:0062197 | cellular response to chemical stress | 337/18723 | 0.135266 | 0.15781 | 0.085868 | SRC | 1 |
| GO:0018209 | peptidyl-serine modification | 338/18723 | 0.135642 | 0.157903 | 0.085919 | SRC | 1 |
| GO:0048545 | response to steroid hormone | 339/18723 | 0.136018 | 0.157995 | 0.085969 | SRC | 1 |
| GO:0032496 | response to lipopolysaccharide | 343/18723 | 0.137521 | 0.159393 | 0.086729 | SRC | 1 |
| GO:0070482 | response to oxygen levels | 347/18723 | 0.139022 | 0.160782 | 0.087485 | SRC | 1 |
| GO:0045861 | negative regulation of proteolysis | 351/18723 | 0.14052 | 0.162162 | 0.088236 | SRC | 1 |
| GO:0006066 | alcohol metabolic process | 353/18723 | 0.141269 | 0.162322 | 0.088323 | SQLE | 1 |
| GO:0031346 | positive regulation of cell projection organization | 353/18723 | 0.141269 | 0.162322 | 0.088323 | SRC | 1 |
| GO:0007178 | transmembrane receptor protein serine/threonine kinase signaling pathway | 355/18723 | 0.142017 | 0.162745 | 0.088553 | SRC | 1 |
| GO:2001233 | regulation of apoptotic signaling pathway | 356/18723 | 0.14239 | 0.162745 | 0.088553 | SRC | 1 |
| GO:0010631 | epithelial cell migration | 357/18723 | 0.142764 | 0.162745 | 0.088553 | SRC | 1 |
| GO:0032956 | regulation of actin cytoskeleton organization | 358/18723 | 0.143137 | 0.162745 | 0.088553 | CAPG | 1 |
| GO:0071900 | regulation of protein serine/threonine kinase activity | 359/18723 | 0.14351 | 0.162745 | 0.088553 | SRC | 1 |
| GO:1901653 | cellular response to peptide | 359/18723 | 0.14351 | 0.162745 | 0.088553 | SRC | 1 |
| GO:0090132 | epithelium migration | 360/18723 | 0.143884 | 0.162745 | 0.088553 | SRC | 1 |
| GO:0051251 | positive regulation of lymphocyte activation | 362/18723 | 0.144629 | 0.162745 | 0.088553 | SRC | 1 |
| GO:0002237 | response to molecule of bacterial origin | 363/18723 | 0.145002 | 0.162745 | 0.088553 | SRC | 1 |
| GO:0031589 | cell-substrate adhesion | 363/18723 | 0.145002 | 0.162745 | 0.088553 | SRC | 1 |
| GO:0051098 | regulation of binding | 363/18723 | 0.145002 | 0.162745 | 0.088553 | SRC | 1 |
| GO:0090130 | tissue migration | 365/18723 | 0.145747 | 0.163237 | 0.088821 | SRC | 1 |
| GO:0001701 | in utero embryonic development | 367/18723 | 0.146491 | 0.163382 | 0.0889 | RRM2 | 1 |
| GO:0009615 | response to virus | 367/18723 | 0.146491 | 0.163382 | 0.0889 | SRC | 1 |
| GO:0050900 | leukocyte migration | 369/18723 | 0.147235 | 0.163868 | 0.089165 | SRC | 1 |
| GO:0007159 | leukocyte cell-cell adhesion | 371/18723 | 0.147978 | 0.164352 | 0.089428 | SRC | 1 |
| GO:0045862 | positive regulation of proteolysis | 372/18723 | 0.14835 | 0.164421 | 0.089465 | SRC | 1 |
| GO:0002253 | activation of immune response | 375/18723 | 0.149463 | 0.164968 | 0.089763 | SRC | 1 |
| GO:0018108 | peptidyl-tyrosine phosphorylation | 375/18723 | 0.149463 | 0.164968 | 0.089763 | SRC | 1 |
| GO:0018212 | peptidyl-tyrosine modification | 378/18723 | 0.150575 | 0.165233 | 0.089907 | SRC | 1 |
| GO:0030900 | forebrain development | 379/18723 | 0.150946 | 0.165233 | 0.089907 | SRC | 1 |
| GO:0050878 | regulation of body fluid levels | 379/18723 | 0.150946 | 0.165233 | 0.089907 | SRC | 1 |
| GO:0051346 | negative regulation of hydrolase activity | 379/18723 | 0.150946 | 0.165233 | 0.089907 | SRC | 1 |
| GO:0030099 | myeloid cell differentiation | 381/18723 | 0.151686 | 0.165702 | 0.090163 | SRC | 1 |
| GO:0032535 | regulation of cellular component size | 383/18723 | 0.152426 | 0.16583 | 0.090232 | CAPG | 1 |
| GO:1902903 | regulation of supramolecular fiber organization | 383/18723 | 0.152426 | 0.16583 | 0.090232 | CAPG | 1 |
| GO:0022412 | cellular process involved in reproduction in multicellular organism | 384/18723 | 0.152796 | 0.165893 | 0.090266 | SRC | 1 |
| GO:0045860 | positive regulation of protein kinase activity | 386/18723 | 0.153535 | 0.166017 | 0.090334 | SRC | 1 |
| GO:0050727 | regulation of inflammatory response | 386/18723 | 0.153535 | 0.166017 | 0.090334 | SRC | 1 |
| GO:0032970 | regulation of actin filament-based process | 397/18723 | 0.157589 | 0.170056 | 0.092531 | CAPG | 1 |
| GO:0002696 | positive regulation of leukocyte activation | 409/18723 | 0.161993 | 0.174454 | 0.094924 | SRC | 1 |
| GO:0043434 | response to peptide hormone | 414/18723 | 0.163822 | 0.176067 | 0.095802 | SRC | 1 |
| GO:0016032 | viral process | 415/18723 | 0.164187 | 0.176104 | 0.095822 | SLC1A5 | 1 |
| GO:0016311 | dephosphorylation | 417/18723 | 0.164918 | 0.176531 | 0.096055 | SRC | 1 |
| GO:0032102 | negative regulation of response to external stimulus | 420/18723 | 0.166012 | 0.176637 | 0.096112 | SRC | 1 |
| GO:0034329 | cell junction assembly | 420/18723 | 0.166012 | 0.176637 | 0.096112 | SRC | 1 |
| GO:0050867 | positive regulation of cell activation | 420/18723 | 0.166012 | 0.176637 | 0.096112 | SRC | 1 |
| GO:0042060 | wound healing | 422/18723 | 0.166741 | 0.177058 | 0.096341 | SRC | 1 |
| GO:0048608 | reproductive structure development | 424/18723 | 0.167469 | 0.177477 | 0.09657 | SRC | 1 |
| GO:0032103 | positive regulation of response to external stimulus | 427/18723 | 0.168561 | 0.177925 | 0.096813 | SRC | 1 |
| GO:0061458 | reproductive system development | 427/18723 | 0.168561 | 0.177925 | 0.096813 | SRC | 1 |
| GO:0052548 | regulation of endopeptidase activity | 432/18723 | 0.170377 | 0.179486 | 0.097663 | SRC | 1 |
| GO:0002683 | negative regulation of immune system process | 434/18723 | 0.171103 | 0.17954 | 0.097692 | SRC | 1 |
| GO:0042391 | regulation of membrane potential | 434/18723 | 0.171103 | 0.17954 | 0.097692 | SRC | 1 |
| GO:0048732 | gland development | 436/18723 | 0.171828 | 0.179945 | 0.097913 | SRC | 1 |
| GO:0045785 | positive regulation of cell adhesion | 437/18723 | 0.17219 | 0.179971 | 0.097926 | SRC | 1 |
| GO:0000280 | nuclear division | 439/18723 | 0.172914 | 0.18002 | 0.097953 | RAD51AP1 | 1 |
| GO:0050804 | modulation of chemical synaptic transmission | 439/18723 | 0.172914 | 0.18002 | 0.097953 | SRC | 1 |
| GO:0099177 | regulation of trans-synaptic signaling | 440/18723 | 0.173276 | 0.180045 | 0.097966 | SRC | 1 |
| GO:0022411 | cellular component disassembly | 443/18723 | 0.174361 | 0.180819 | 0.098388 | CAPG | 1 |
| GO:0016055 | Wnt signaling pathway | 444/18723 | 0.174722 | 0.180841 | 0.0984 | SRC | 1 |
| GO:0006979 | response to oxidative stress | 446/18723 | 0.175444 | 0.180885 | 0.098424 | SRC | 1 |
| GO:0198738 | cell-cell signaling by wnt | 446/18723 | 0.175444 | 0.180885 | 0.098424 | SRC | 1 |
| GO:0010876 | lipid localization | 448/18723 | 0.176166 | 0.180927 | 0.098447 | SQLE | 1 |
| GO:0022407 | regulation of cell-cell adhesion | 448/18723 | 0.176166 | 0.180927 | 0.098447 | SRC | 1 |
| GO:0009314 | response to radiation | 456/18723 | 0.179047 | 0.183532 | 0.099864 | RAD51AP1 | 1 |
| GO:0052547 | regulation of peptidase activity | 461/18723 | 0.180844 | 0.185017 | 0.100672 | SRC | 1 |
| GO:0009410 | response to xenobiotic stimulus | 462/18723 | 0.181202 | 0.185028 | 0.100678 | SRC | 1 |
| GO:0001819 | positive regulation of cytokine production | 467/18723 | 0.182995 | 0.186144 | 0.101285 | SRC | 1 |
| GO:0033674 | positive regulation of kinase activity | 467/18723 | 0.182995 | 0.186144 | 0.101285 | SRC | 1 |
| GO:0002764 | immune response-regulating signaling pathway | 468/18723 | 0.183353 | 0.186152 | 0.10129 | SRC | 1 |
| GO:0019221 | cytokine-mediated signaling pathway | 472/18723 | 0.184783 | 0.187247 | 0.101886 | SRC | 1 |
| GO:0031667 | response to nutrient levels | 474/18723 | 0.185498 | 0.187614 | 0.102085 | SRC | 1 |
| GO:0001667 | ameboidal-type cell migration | 475/18723 | 0.185855 | 0.187618 | 0.102088 | SRC | 1 |
| GO:0043410 | positive regulation of MAPK cascade | 480/18723 | 0.187638 | 0.18906 | 0.102872 | SRC | 1 |
| GO:0042110 | T cell activation | 487/18723 | 0.190129 | 0.191199 | 0.104036 | SRC | 1 |
| GO:0048285 | organelle fission | 488/18723 | 0.190484 | 0.191199 | 0.104036 | RAD51AP1 | 1 |
| GO:0009117 | nucleotide metabolic process | 489/18723 | 0.190839 | 0.191199 | 0.104036 | RRM2 | 1 |
| GO:0006753 | nucleoside phosphate metabolic process | 497/18723 | 0.193676 | 0.193676 | 0.105384 | RRM2 | 1 |
